# Entropy, complexity, and spectral features of EEG signals in autism and typical development: a quantitative approach

**DOI:** 10.3389/fpsyt.2025.1505297

**Published:** 2025-02-04

**Authors:** Aleksandar Tenev, Silvana Markovska-Simoska, Andreas Müller, Igor Mishkovski

**Affiliations:** ^1^ Faculty of Computer Science and Engineering, St Cyril and Methodius University of Skopje, Skopje, North Macedonia; ^2^ Department of Neurophysiology, Macedonian Academy of Sciences and Arts, Skopje, North Macedonia; ^3^ Brain and Trauma Foundation Grison/Switzerland, Chur, Switzerland

**Keywords:** entropy, complexity, brain-rate, autism, quantitative EEG

## Abstract

Autism spectrum disorder (ASD) is a neurodevelopmental condition that affects the brain’s function. Electroencephalography (EEG) is a non-invasive technique that measures the electrical activity of the brain and can reveal its dynamics and information processing. This study explores an eyes-opened resting state quantitative EEG analysis of 49 children with ASD and 39 typically developing (TD or Control) children, using various features of entropy and complexity. Time and frequency domain features were applied for all EEG channels, such as the power spectra, brain rate, sample entropy, permutation entropy, spectral entropy, Tsallis entropy, Rényi entropy, Lempel–Ziv complexity, and Higuchi fractal dimension. The features were compared between the ASD and TD groups and tested for statistical significance. The results showed that the ASD group had a lower brain rate, higher Tsallis entropy and Rényi entropy, and lower Lempel–Ziv complexity than the TD group. The entropy results show impaired neural synchronization, increased randomness, and noise in ASD. The Lempel–Ziv complexity results showed that it is a potential indicator of the existence of focal spikes in the EEG signals of ASD. The brain-rate results show a low level of arousal in ASD. The findings suggest that entropy and complexity measures can be useful tools for characterizing the EEG features of ASD and provide insights into the neurophysiological mechanisms of the disorder.

## Introduction

Autism Spectrum Disorder (ASD) represents a complex and heterogeneous neurodevelopmental condition characterized by challenges in social interaction, communication deficits, and repetitive behaviors. While behavioral and clinical observations have traditionally guided our understanding of ASD, advancements in neuroimaging techniques, particularly electroencephalography (EEG), have provided a window into the intricate neural dynamics associated with this disorder.

EEG, as a non-invasive tool, enables the recording of electrical activity generated by the brain’s neural networks. In recent years, a growing body of research has sought to unravel the unique EEG signatures associated with ASD (1–3), to enhance our understanding of the underlying neurological mechanisms that contribute to its diverse clinical manifestations. This scientific inquiry into the findings of EEG in the context of ASD has revealed a spectrum of aberrations in neural processing. Researchers have investigated patterns of connectivity, alterations in frequency bands, and event-related potentials (ERPs) to decipher the complex interplay of neural circuits involved in the pathology of ASD.

Analyses of frequency bands, such as delta, theta, alpha, and beta, have provided information on the unique neural signatures associated with ASD. Previous studies found that in resting-state EEG analysis, children with typical development have high alpha activity during rest (4), while children with autism showed mixed results (5–12). Deviations from typical spectral patterns have been observed, suggesting disruptions in fundamental neurophysiological processes, including attention regulation, sensory integration, and information processing.

Event-related potentials (ERPs), which capture the brain’s response to specific stimuli, have also been scrutinized in the context of ASD. Variations in the timing and magnitude of ERP components, such as the P300 and N400, have been identified, offering valuable clues about the neurocognitive processes involved in social and cognitive functions affected by ASD (13–19).

A prevalent theme in the literature involves the investigation of atypical brain connectivity in individuals with ASD. Studies have reported both over-connectivity and under-connectivity in various brain regions (20–24), shedding light on the intricate balance of neural networks governing social cognition, communication, and sensory processing in ASD individuals.

Furthermore, focal spikes are abnormal brain signals that occur more often in ASD (25, 26). They can be measured by EEG, which is the main method for studying epilepsy and other epileptic brain signals. Some people with ASD have these spikes even when they have no seizures (27, 28).

Despite significant strides in unraveling the neurobiological underpinnings of ASD through EEG analysis, challenges remain. The heterogeneity inherent in ASD presents a formidable obstacle that requires researchers to navigate the complex landscape of individual differences. In a recent review of EEG signal research related to mental disorders (29), caution was advised in relying too heavily on spectral analysis. The reason is that increases in band-specific power tend to overlap significantly within and between different disorders. Additionally, spectral analysis fails to account for the inherent non-linearity in brain dynamics, resulting in a distorted understanding of brain functioning. Since EEG signals are non-stationary in nature, non-linear features should be used in their analysis (30).

One approach involves non-linear dynamics and chaos theory, which examines the complex, seemingly random, and irregular patterns exhibited by systems governed by deterministic rules and influenced by initial conditions. Researchers have used complexity-based measures to explore brain dynamics, aiming to quantify neural processes with intricate and irregular behavior. The Lyapunov coefficient, a measure of the chaotic behavior of a system, has been used in studies that discriminate between ASD and TD (31–35). While the chaos-related methodology is valuable, its implementation can be challenging, and the resulting outcomes are often abstract.

An alternative, less abstract approach for investigating brain signal structure involves using entropy measures inspired by Shannon’s ideas. In this context, complexity is quantified through entropy, a concept borrowed from thermodynamics and adapted to describe probabilistic processes. The concept of entropy proved to be one of the pillars and fundamental concepts of modern-day physics. Furthermore, some researchers have even tied the concept of entropy to consciousness (36). It is a measure that quantifies the uncertainty, complexity, and information that a physical system possesses and the human brain is a complex physical system that generates and processes information. High entropy values indicate greater uncertainty, resulting in increased complexity, irregularity, and unpredictability. Studies have detected a higher EEG entropy in autism and suggest that it is related to impaired social cognition, communication, and behavior (37–39). Neural noise (40) may explain this elevated entropy. That perfectly aligns with the very definition of entropy. Researchers have linked the excess of neural noise in the autistic brain with cognitive, social, and behavioral difficulties (41, 42), and even further, there are authors who argue that, due to stochastic resonance, it could be an asset in certain occasions (43, 44) and should be pointed out loudly since autistic people face ignorance, prejudice, and discrimination (45). Different types of entropy measures, such as sample entropy, multiscale entropy (46–49), and Rényi entropy (39) have been explored as potential biomarkers for ASD, as has Lempel–Ziv complexity (LZC) (50, 51), another interesting metric that gives different perspective of complexity and is not based on entropy but originates from a family of techniques that maps time series into sequences of symbolic representations. Simply put, the regularity of the signal is determined by scanning the symbolic sequences for new patterns, increasing the complexity count every time a new sequence is detected. A recent review stated that while the concept of entropy has been investigated in the domain of ASD, studies that explore Lempel–Ziv complexity in ASD EEG signals are scarce (52).

This paper provides an extensive quantitative analysis of different EEG spectral characteristics and different entropy and complexity features extracted from the time domain of the EEG signal, and an interpretation of the results is given in the discussion section.

### Methods

### Data

#### Participants

EEG recordings and analyses were made on 88 children of which 39 had typical development (TD or Control) and 49 had autism (ASD). The mean age of the ASD group was 6.18 years with a standard deviation of 1.98 years, and the TD group had a mean age of 5.35 years and a standard deviation of 2.31 years. Since the TD children were all boys, gender was excluded in the further analysis because of the lack of a significant gender ratio between the two groups. The diagnosis of autism was made by a psychiatrist and a psychologist and both had to agree on the diagnosis for the participant to be included in the study. All participants met the DSM-V criteria for autism disorder. To ensure diagnostic validity, additional information was collected from the parents. All the recordings were conducted in Skopje, North Macedonia and written consent and approval for the research was obtained from the parents of the children. This work was approved by the Ethics Committee at the Faculty of Medicine, St’s Cyril and Methodius University in Skopje under reference number 03-4953/2.

#### Procedure

Because of the nature of the disorder, the EEG signals were obtained in the resting state condition, with opened eyes. The placement of EEG electrodes was in accordance with the international 10/20 system using an electro-cap produced by Electro-cap International. Activity in 19 channels was recorded: Fp1, Fp2, F3, F4, F7, F8, Fz, C3, C4, Cz, T3, T4, T5, T6, P3, P4, Pz, O1, and O2, referenced to linked ears. The ground electrode was placed between Fpz and Fz. To control for eye movement artifacts, the electrooculogram (EOG) was recorded, using two 9 mm tin electrodes, above and under the right eye, referenced to Fpz and Oz. The EOG rejection was set at 50 *µ*V. The bandwidth of the amplifiers was set at 0.53 Hz low-frequency filter, 50 Hz high-frequency filter, and 45-55 Hz notch filter. The impedance levels for all electrodes were set to 5 kΩ. The EEG digitization frequency was 250 Hz. EEG was continuously recorded on the hard disc for offline analysis. The processing was conducted using Mitsar WinEEG software. We obtained a time series of 3 minutes for the TD and variable lengths in the range of 7 to 30 minutes for the ASD group. It is important to note that the length of the signal in the ASD group was different because of the challenging environment during the recording. The second author took notes in real-time during the recording and also visually checked every signal and decided whether to accept or reject it based on the absence or presence of artifacts. Each of the EEG signal recordings was processed in an identical manner. First, the signals were manually checked and artifacts were corrected. For the time-domain analysis, a continuous segment of the time-series signal without artifacts was needed. The continuous segment without artifacts with the maximum length was selected from the EEG recording for each participant when the child was sitting quietly. We cut the segments to be the same length for each participant, therefore choosing the minimum length of all the maximum lengths. The minimum length was 43 seconds. Since our sampling frequency was 250Hz, we had 10,750 time point amplitude values for each of the 19 electrodes, totaling 204,250 amplitude values (43 ∗ 250 ∗ 19 = 204,250) for each participant. For spectral analysis, fast Fourier transform was carried out for five frequency bands, namely, Delta (0.5–4 Hz), Theta (4–8 Hz), Alpha (8–12 Hz), SMR (12-15 Hz), and Beta (15–20 Hz), for relative power.

### Features

A diverse set of features is derived from EEG signals to capture different dimensions of brain dynamics and complexity. Power spectral analysis was chosen to quantify the distribution of power between the frequency bands, providing information on patterns often associated with ASD. Brain rate, as a measure of dominant brain activity and level of arousal, aids in identifying shifts in cognitive and behavioral states. Measures of complexity, such as Lempel–Ziv complexity and Higuchi fractal dimension, were included to reflect the inherent irregularity and fractal nature of EEG signals. Similarly, Rényi and Tsallis entropies were selected as generalized entropy measures to assess signal diversity and statistical order. Permutation entropy (PE), spectral entropy, and sample entropy (SampEn) provide additional perspectives on signal unpredictability and regularity, each tailored to capture specific nuances in the time-series data. Collectively, these features were chosen to offer a comprehensive view of the underlying neural mechanisms, facilitating a quantitative differentiation between children with ASD and typically developing children.

#### Relative power spectra

Relative power spectra are the proportion of the power in a specific frequency band relative to the total power summed across all frequency bands.

#### Brain rate

Brain rate is defined as the weighted mean frequency of the EEG spectrum. This parameter is used as an indicator of the level of consciousness, characterizing mental arousal irrespective of its content. In other words, the brain rate expresses the mean frequency of brain rhythms (53). It is a metric that we calculated in the frequency domain of the signal with the following formula:


(1) $$BR=\frac{\sum_{i=1}^{n}\;f_{i}\cdot P_{i}}{\sum_{i=1}^{n}P_{i}}$$

where *f_i_* is the frequency of the i-th EEG band, and *P_i_* is the power of the i-th EEG band.

#### Permutation entropy

PE is a robust time series tool that provides a measure of the complexity of a dynamic system. It captures the order relations between values of a time series and extracts a probability distribution of the ordinal patterns (54). The formula for the calculation of PE is:


(2) $$H_{PE}(D,\tau )=-\sum_{\pi \epsilon S_{D}}p(\pi )\log\;p(\pi )$$

where *D* is the embedding dimension, *τ* is the embedding delay, *S_D_* is the set of all possible permutations of length *D*, and p(*π*) is the probability of the permutation *π* in the partitioned time series. In this study, the entropy was calculated with *D* = 3 and *τ* = 1.

PE is non-parametric and is free of restrictive parametric model assumptions. It is robust with respect to noise, computationally efficient, flexible, and invariant with respect to non-linear monotonic transformations of the data (55).

#### Sample entropy

SampEn is a modification of approximate entropy (ApEn), used for assessing the complexity of physiological time-series signals. It has two advantages over ApEn: data length independence and a relatively trouble-free implementation (56). It is a measure of complexity that does not include self-similar patterns as ApEn does. For a given embedding dimension, tolerance, and number of data points, SampEn is the negative natural logarithm of the probability so that if two sets of simultaneous data points of length *m* have distance *r* then two sets of simultaneous data points of length *m* + 1 also have distance *r* (56). It is a measure of the regularity or predictability of a time series. A smaller value indicates more self-similarity in the data set or less noise (56). It can be calculated using the following formula:


(3) $$SampEn\left(m,r,n\right)=-\text{ln }\frac{A\left(m+1,r\right)}{B\left(m,r\right)}$$

where *m* is the length of the template vector, *r* is the tolerance for accepting matches, *n* is the number of data points, *A*(*m* + 1*,r*) is the number of template vector pairs of length *m* + 1 that have distance less than *r*, and *B*(*m,r*) is the number of template vector pairs of length *m* that have distance less than *r*. In this study, this feature is calculated with *m* = 2 and *r* = 20% of the signal standard deviation.

#### Spectral entropy

Spectral entropy is defined as the Shannon entropy of the power spectral density (PSD) of the data (57) with the following formula:


(4) $$SE=-\sum_{i=1}^{n}p_{i}\text{ ln }p_{i}$$

where *n* is the number of bins or frequency points, and *p_i_* is the normalized PSD value at the i-th bin or frequency point. In the context of time series analysis, spectral entropy measures the “forecast” of a time series, where low values indicate a high signal-to-noise ratio and large values occur when a series is difficult to forecast (58–61).

#### Rényi entropy

Rényi entropy is a quantity in information theory that generalizes various notions of entropy. It depends on a parameter *α* that can be varied to obtain different entropy measures. For example, when *α* = 1, Rényi entropy reduces to Shannon entropy, and when *α* = *∞*, Rényi entropy becomes the min-entropy, which is the smallest entropy measure in the family of Rényi entropies (62).

Rényi entropy has been used in various EEG analyses (63–66) to characterize the complexity and predictability of an EEG signal. However, Rényi entropy is not a simple function of the length of the EEG signal but rather depends on how the EEG signal is partitioned into segments or symbols. Different partitioning methods can lead to different values of Rényi entropy, even for the same EEG signal and the same *α*. Therefore, Rényi entropy is not a universal measure of the information content of an EEG signal, but rather a relative measure that depends on the choice of partitioning scheme.

We used kernel density estimation to estimate the probability density function of the EEG signal. Then we applied the Rényi entropy formula to the resulting distribution.


(5) $$H_{\alpha }\left(f\right)=\frac{1}{1-\alpha }\text{log }\int_{-\infty }^{\infty }f{\left(x\right)}^{\alpha }dx$$

where *f*(*x*) is the probability density function. In this study, *α* = 2.

#### Higuchi fractal dimension

In fractal geometry, the Higuchi fractal dimension (HFD) is an approximate value for the box-counting dimension of the graph of a real-valued function or time series (67). This value is obtained via an algorithmic approximation, so one also talks about the Higuchi method. The feature is calculated with the formula


(6) $$D_{Higuchi}=\frac{\text{log}(L/k)}{\text{log}(k)}$$

where *L* is the total length of the curve and *k* is the interval size. We used *k* = 10. The HFD is based on the idea that the length of a curve can be measured by dividing it into smaller segments of equal size and summing their lengths. The smaller the segment size, the longer the curve length. The HFD is the slope of the log-log plot of curve length versus the segment size. The HFD can be calculated for a time series by projecting it into different sub-series with different starting points and averaging the lengths of these sub-series. The Higuchi method has been used in many EEG analyses as a feature for machine learning and to analyze the complexity and irregularity of EEG signals and reflect different states of the brain, such as wakefulness, sleep, or seizure (68–70).

#### Tsallis entropy

Tsallis entropy is a generalization of the standard Boltzmann–Gibbs entropy. It was introduced in 1988 by Constantino Tsallis as a basis for generalizing the standard statistical mechanics (71) as the following equation:


(7) $$S_{q}=\frac{1}{q-1}\left(1-\sum_{i=1}^{n}p_{i}^{q}\right)$$

where *n* is the number of possible outcomes, *p_i_* is the probability of the i-th outcome, and *q* is the entropic index. In this study, *q* = 1.5. Tsallis entropy depends on a parameter *q*, which determines the degree of non-extensivity or non-additivity of the entropy. When *q* = 1, Tsallis entropy reduces to Boltzmann–Gibbs entropy. When *q >* 1, Tsallis entropy is sub-extensive, meaning that the entropy of the whole system is less than the sum of the entropies of its parts. When *q <* 1, Tsallis entropy is super-extensive, meaning that the entropy of the whole system is greater than the sum of the entropies of its parts. Tsallis entropy can be applied to quantify the changes in the EEG signals that correspond to different states, and can also be used for machine learning and classification purposes (72). Some of the advantages of Tsallis entropy over other entropy measures are that it can capture the non-Gaussianity, non-stationarity, and multi-scale nature of the EEG signals, and it can be computed efficiently and robustly (73–75).

#### Lempel–Ziv complexity

LZC is a measure that was first introduced by two Israeli computer scientists, Abraham Lempel and Jacob Ziv (76). LZC can be used to measure the repetitiveness of binary sequences and text. It is based on an elementary principle of copying words. This complexity measure is not too restrictive in the sense that it satisfies the main qualities expected by such a measure: sequences with a certain regularity do not have a too large complexity, and the complexity grows as the sequence grows in length and irregularity. LZC corresponds to the number of different substrings (or sub-words) encountered as the binary sequence is viewed as a stream (from left to right) (76). For example, the binary string 010101010101 has low LZC because it only has two sub-sequences: 01 and 10. The binary string 011001011110 has a higher LZC because it has six sub-sequences: 0, 1, 01, 10, 100, and 111. In our study, we first binarized the signal using a threshold value. The threshold value was the mean amplitude of the signal. The segments with an amplitude value above the threshold were given a value of 1 and the segments below the threshold were given a value of 0. The advantage of using LZC is that it does not require any prior knowledge or assumptions about the structure or dynamics of the EEG signals and is robust to noise and artifacts.

### Analysis

**Figure 1:**
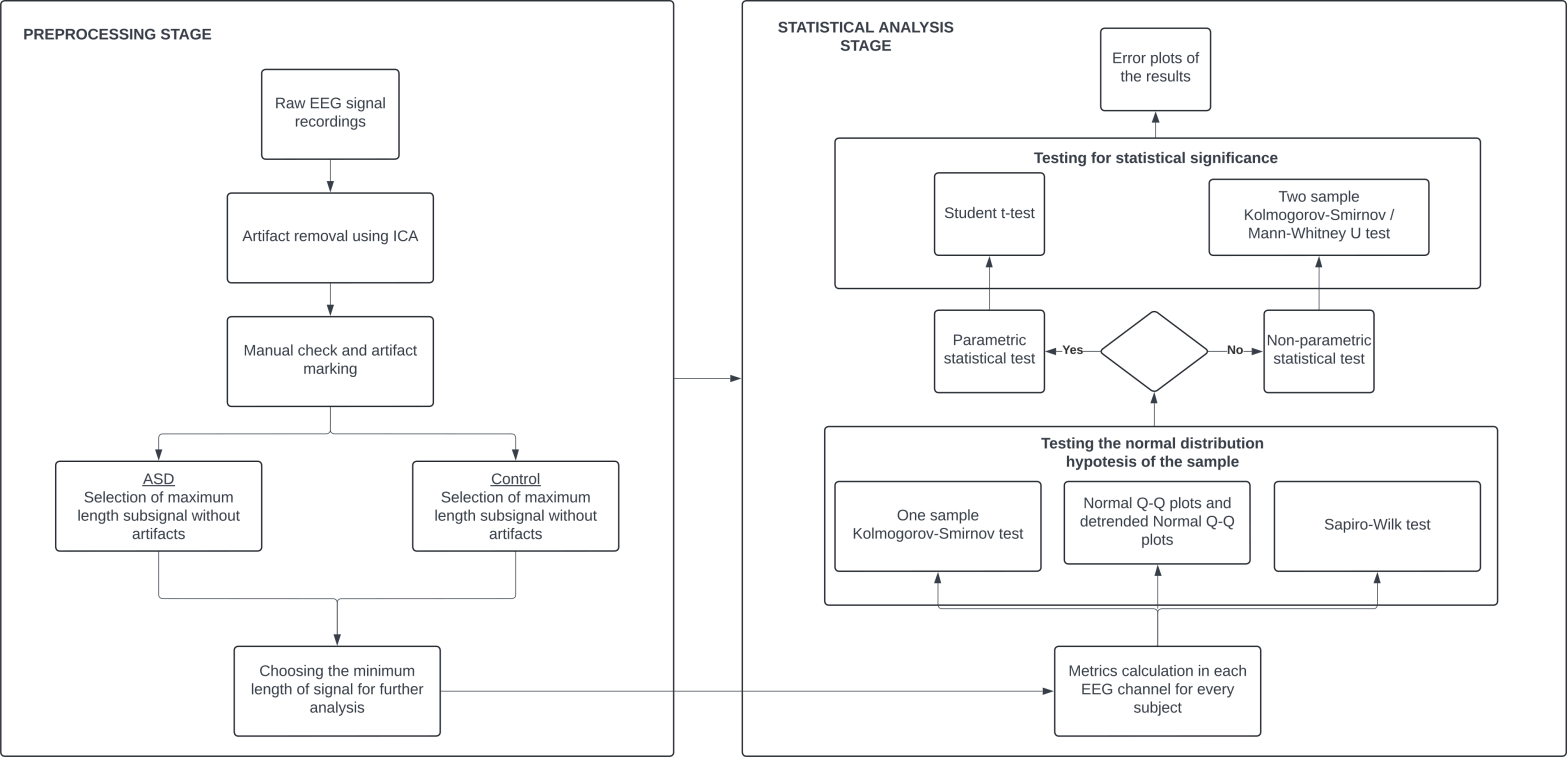
Analysis flowchart.

**Figure 2:**
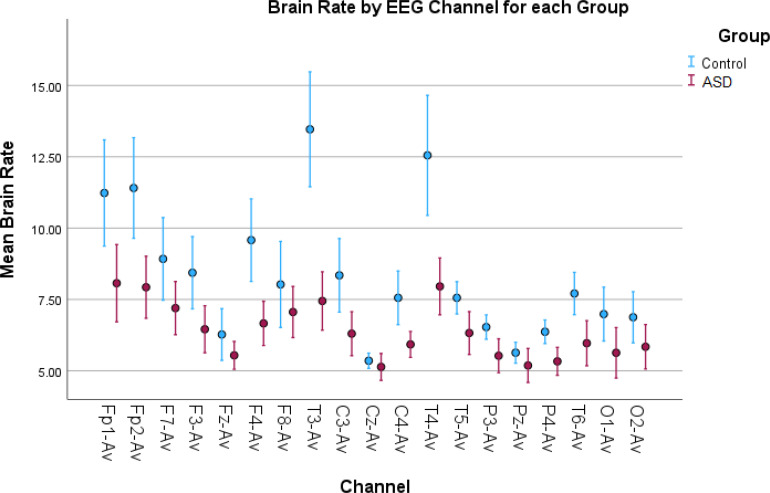
Error bar plots for brain rate in all EEG channels. Brain rate results depict increased spectral activity for the control children in most of the EEG channels compared to the children with autism. Since brain rate is an average metric, the increased values are mostly due to the increased alpha activity found in the control children.

**Figure 3:**
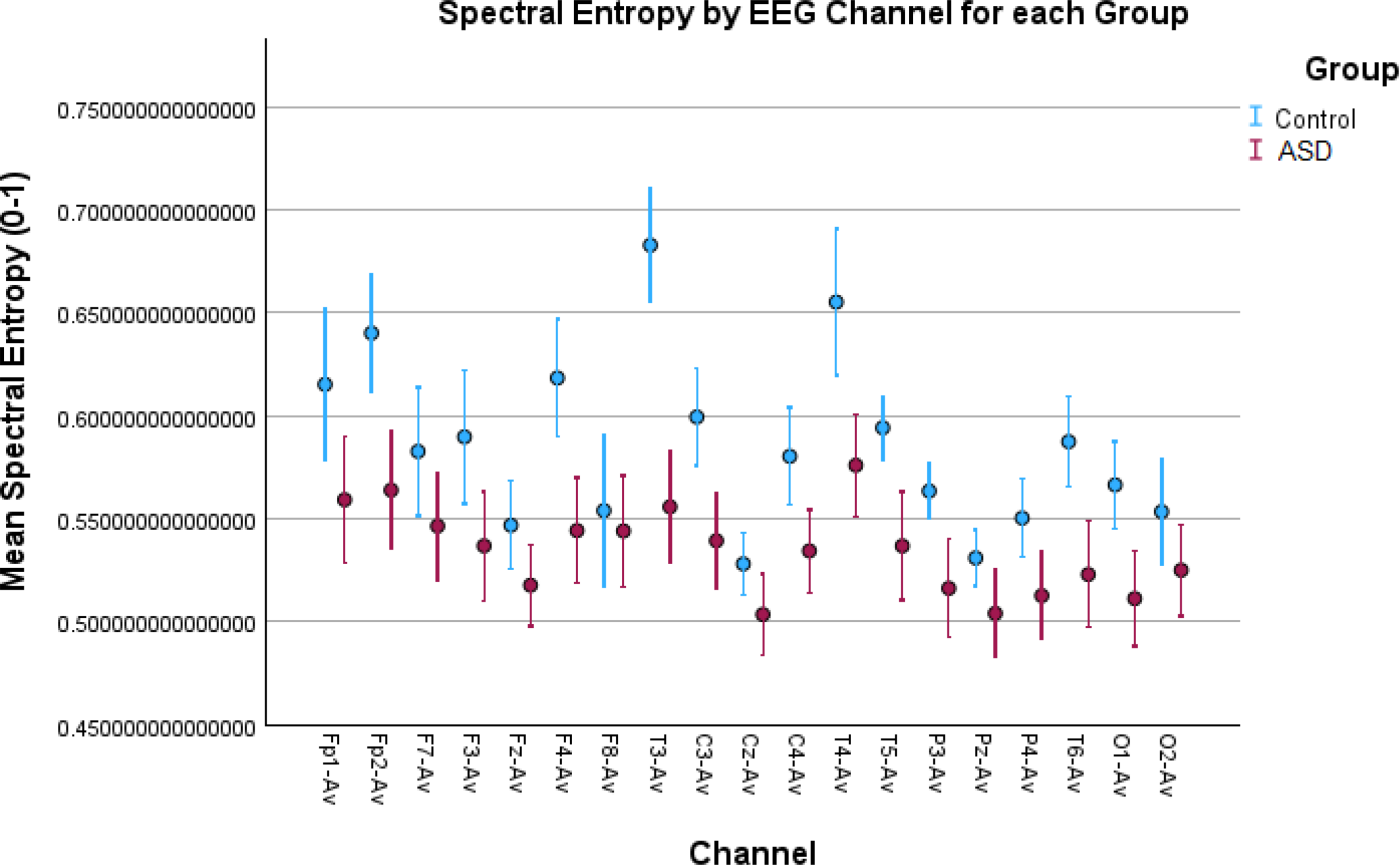
Error bar plots for spectral entropy in all EEG channels. The results depict increased spectral activity for the control children in most of the EEG channels compared to the children with autism. Spectral entropy, similar to the brain rate, is obtained in the spectral domain and the increased values are due to increased alpha activity in the control children.

**Figure 4:**
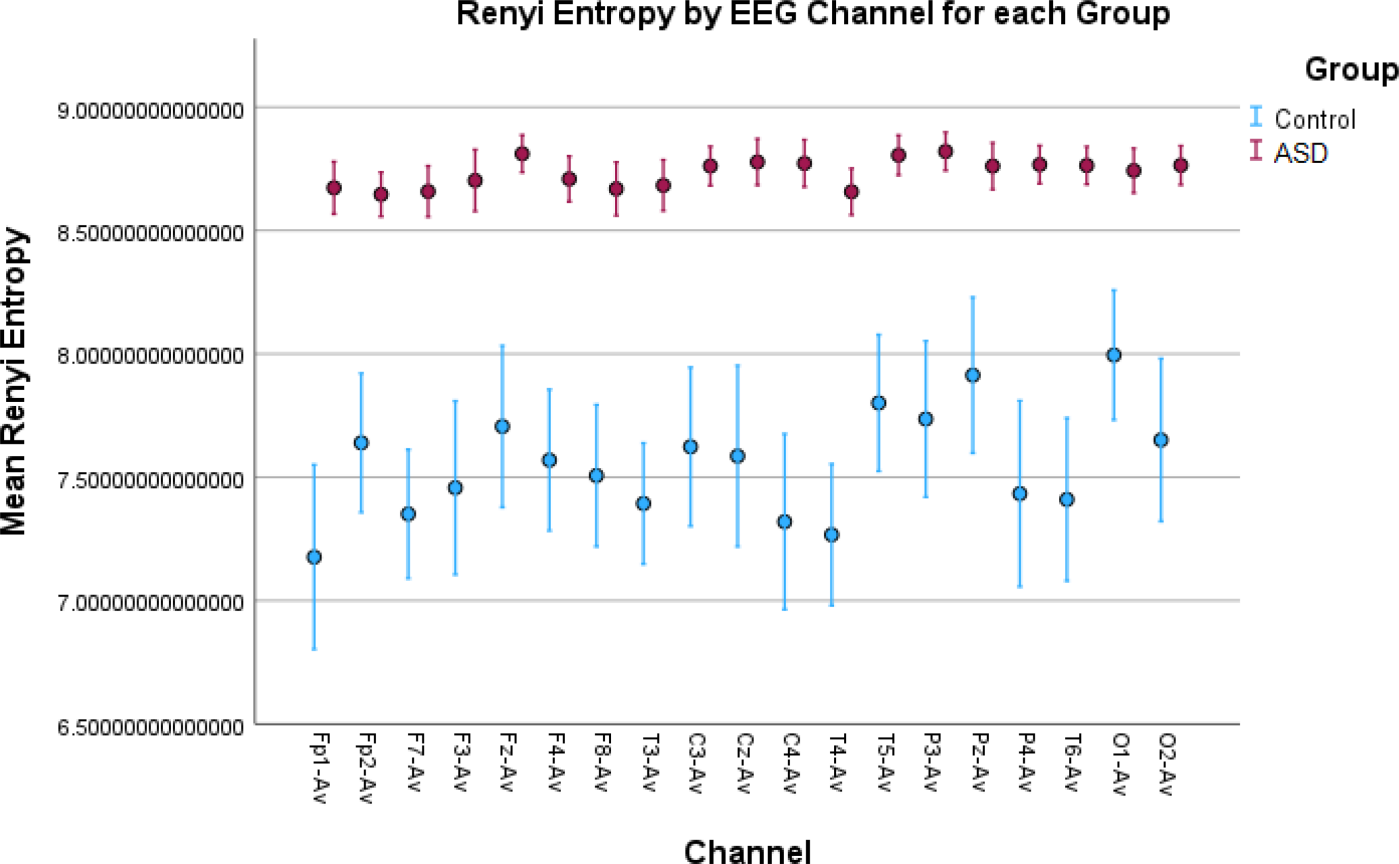
Error bar plots for Rényi entropy in all EEG channels. The results show significantly elevated entropy values for the children with autism compared to the control children. This indicates a more “noisy” signal due to increased neural noise in the ASD group and a more “smooth” signal in the Control group. This noise is captured by the metric as more amplitude variations in the signal.

**Figure 5:**
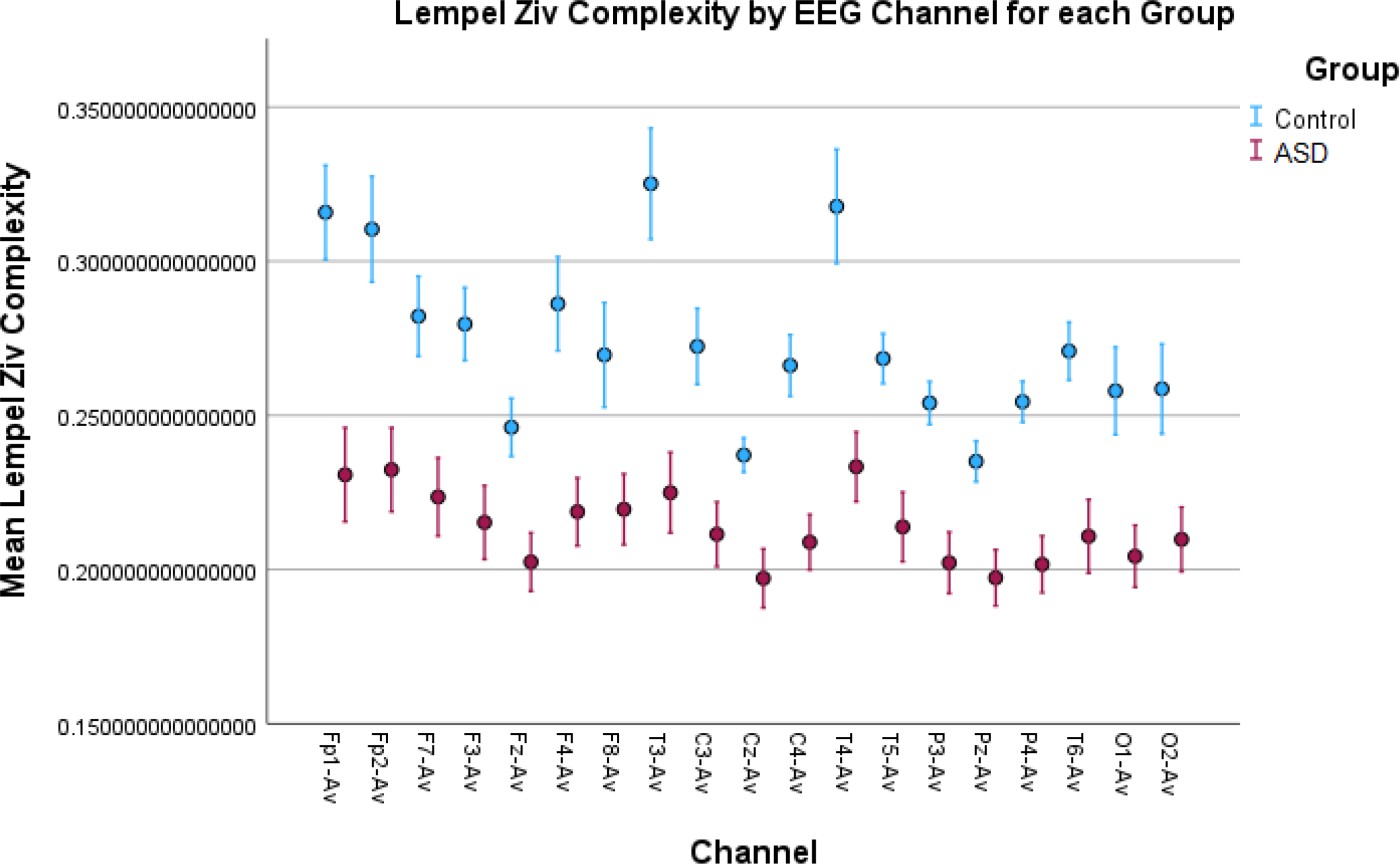
Lempel–Ziv complexity (LZC) results in all EEG channels. The ASD group shows lower complexity than the control group. After the binarization of the signal, many of the amplitude variations above or below the binarization threshold value are discarded. The lower LZC values indicate frequent and repetitive sharp oscillations in the signal between the two sides of the threshold value, which can be an indicator of more focal spikes found in ASD EEG signals.

**Figure 6:**
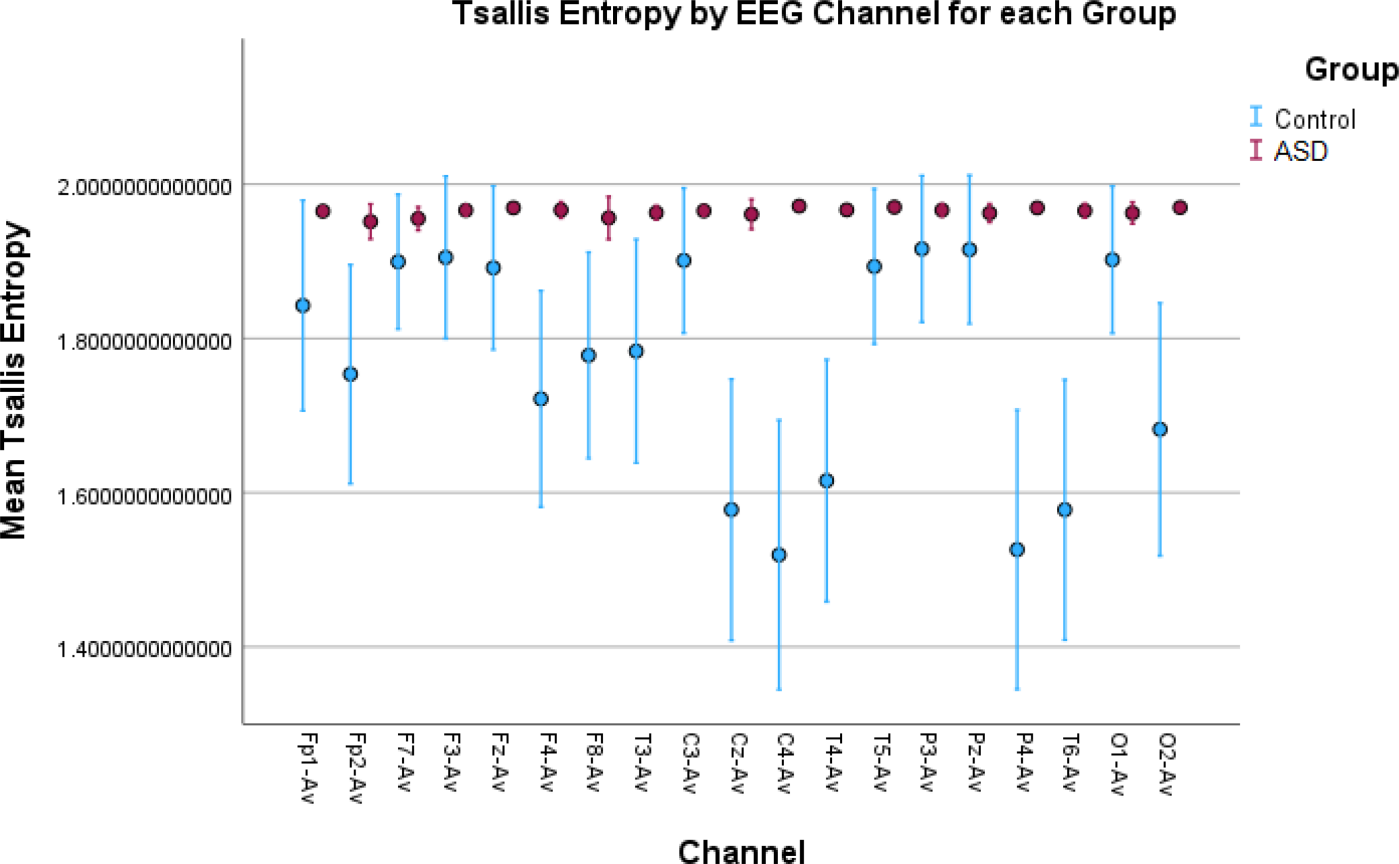
Error bar plots for Tsallis entropy in all EEG channels. The results show significantly elevated entropy values for the children with autism compared to the control children. The explanation is the same as for Rényi entropy. However, we found that there was no in-between variation of entropy values in the ASD group since the entropy for all the children with autism reaches the asymptotic maximum value for Tsallis entropy.

We calculated the relative power spectra for both groups and the previously mentioned features in all the EEG channels for each participant and made a comparative statistical analysis between the groups. The flowchart diagram of the analysis can be seen in **Figure 1**. For every metric, we calculated 19 values for 88 participants, representing 39 TD and 49 ASD children. First, we ran both the one-sample Kolmogorov–Smirnov and Shapiro–Wilk tests, together with Normal Q-Q plots and detrended Normal Q-Q plots to check whether the normality condition of the sample was satisfied. This was an important step that told us what statistical test we should choose. Since our data was not normally distributed (Appendix), we cannot run parametric tests of significance such as t-tests. Instead, we ran two non-parametric tests to check for statistical significance: two-sample Kolmogorov–Smirnov and Mann–Whitney U tests. We also plotted error bars with 95% confidence intervals for every metric so we could visually confirm and interpret the tests. The bars (**Figures 2**–**6**) show us with a probability of 95%, that if we obtain a new data point, its measures will fall somewhere across the blue line if it is a control data point, or across the red line if it is an autism data point. We also performed a Bonferroni correction to address the multiple comparison problem. Since we analyzed 19 hypotheses corresponding to 19 EEG channels, the corrected significance level *α* can be calculated by dividing 0.05 by 19, giving a value of 0.0026.

## Results

**Figure 7:**
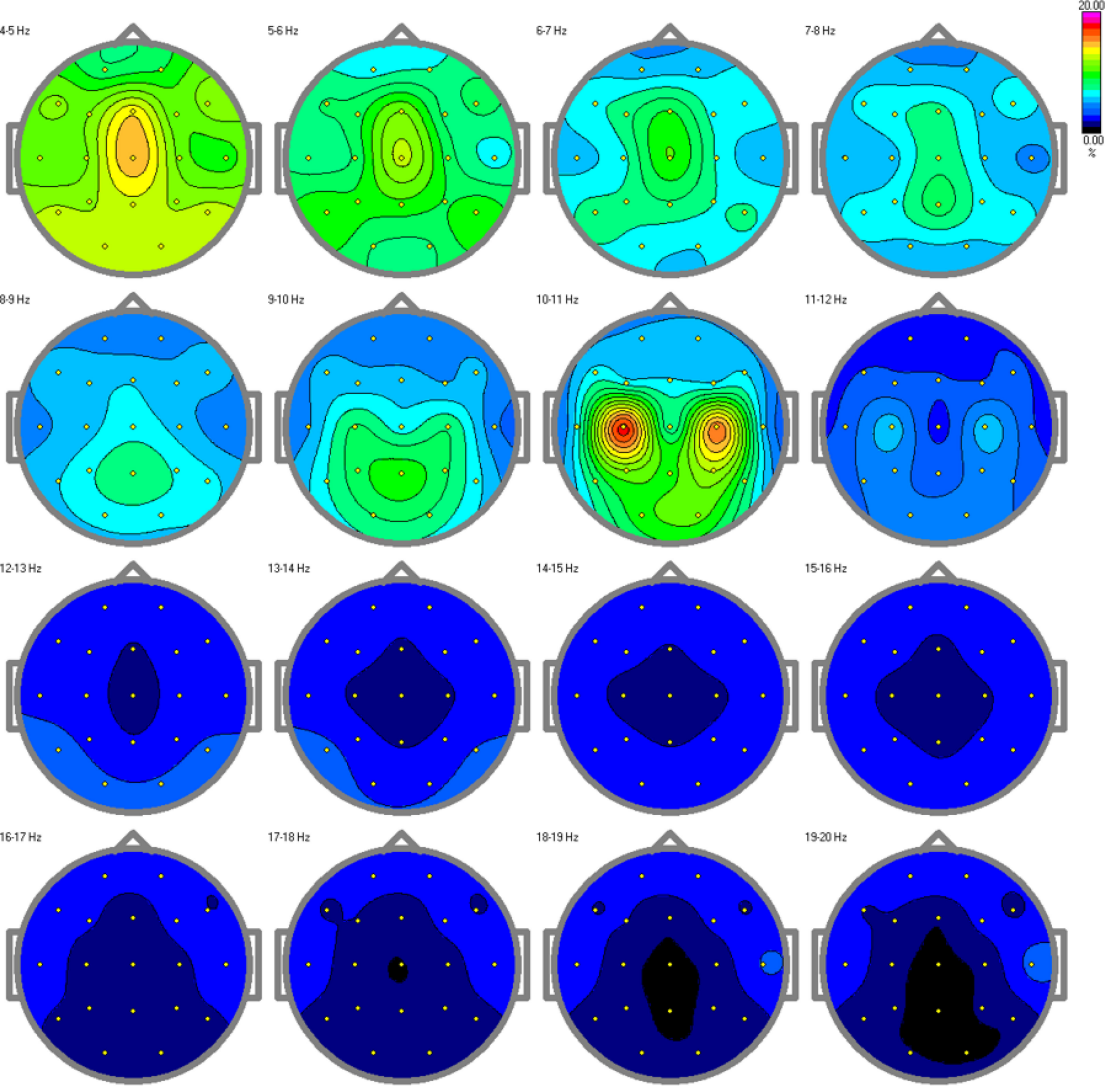
Relative power spectra for the control group. Increased alpha activity can be found in the alpha band, specifically in the 10-11 Hz range, indicating arousal and awareness in the children with typical development.

**Figure 8:**
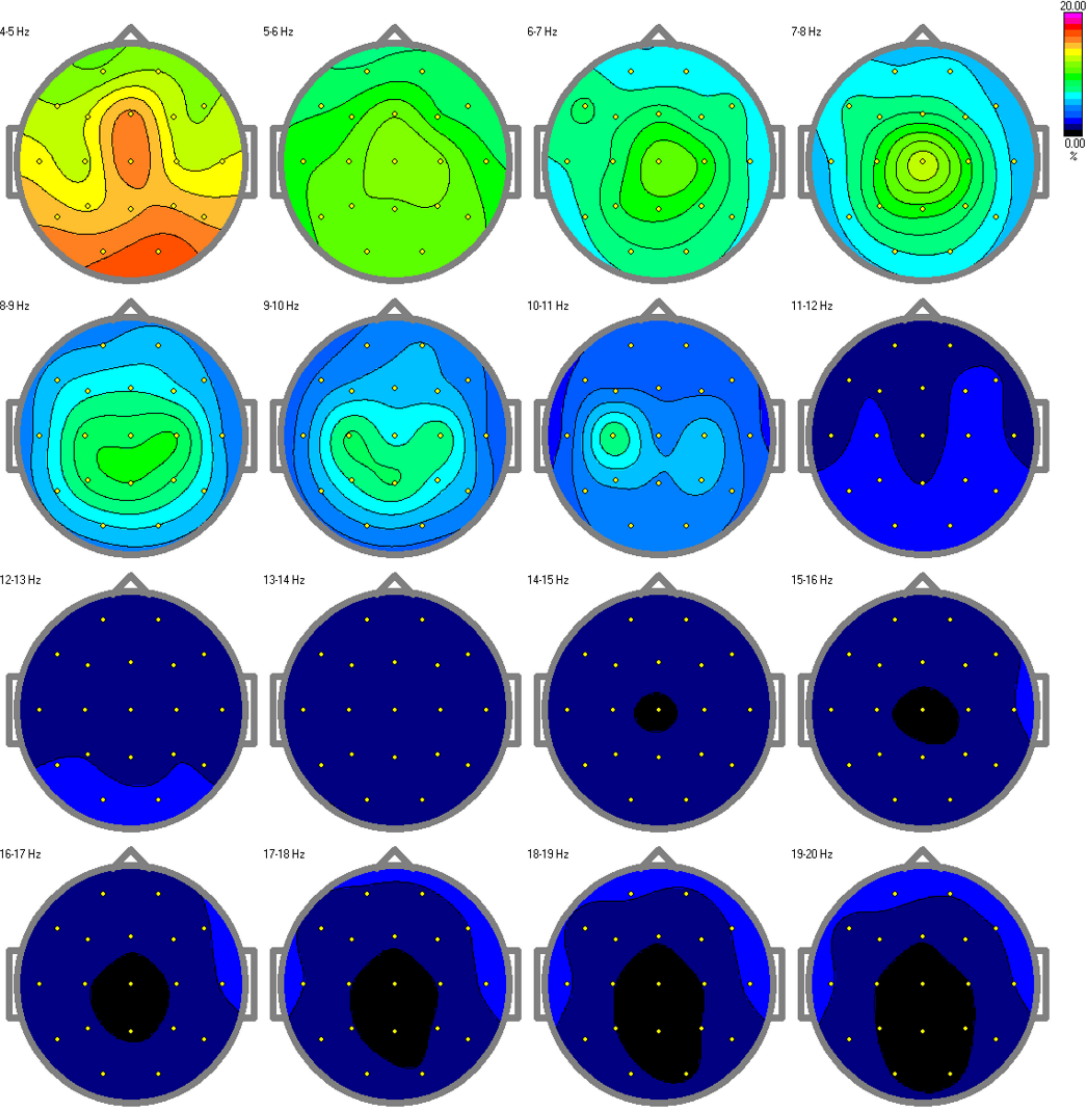
Relative power spectra for the autism group. Decreased or no activity can be found in the alpha band, specifically in the 10-11 Hz range, indicating a low level of arousal in the children with autism.

The results from the relative power spectra are shown in **Figures 7**, **8** for the TD and ASD children respectively. The spectrum is depicted hertz by hertz to avoid over-averaging of the results across different frequency bands. The results show that in the alpha band (10-11Hz), which is characteristic of a resting state, we found increased brain activity in the typical development group compared to the autism group. Elevated alpha activity is associated with a state of cortical idling, where the brain is not engaged in active processing but is still ready to respond to stimuli. This shows that the TD children were in a state of wakefulness while resting, having thoughts or fantasizing about something, completely aware of the surroundings around them. Children with ASD on the other hand, showed very little or no brain activity at all. This indicates an absence of awareness of their surroundings.

The results from both statistical tests for significance are shown in **Tables 1**, **2** for the features in each of the EEG channels. We can see that there is a significant difference between the TD and ASD groups in many features and channels.

**Table 1:** P-values for brain rate, permutation entropy, sample entropy, and spectral entropy.

EEG channel	Brain rate		Permutation entropy		Sample entropy		Spectral entropy	
	K–S	Mann–Whitney	K–S	Mann–Whitney	K–S	Mann–Whitney	K–S	Mann–Whitney
Fp1-Av	0.013	0.001*	0.001*	<0.001*	0.138	0.288	0.053	0.021
Fp2-Av	0.007	0.001*	0.011	0.003	0.135	0.076	0.009	0.001*
F7-Av	0.096	0.034	0.087	0.014	0.914	0.943	0.068	0.083
F3-Av	0.007	0.002*	0.026	0.016	0.024	0.162	<0.001*	0.002*
Fz-Av	0.188	0.292	0.275	0.798	0.536	0.817	0.060	0.086
F4-Av	0.012	0.001*	0.190	0.115	0.317	0.183	0.018	0.001*
F8-Av	0.736	0.373	0.068	0.031	0.410	0.571	0.790	0.702
T3-Av	<0.001*	<0.001*	0.004	0.001*	0.008	0.002*	<0.001*	<0.001*
C3-Av	0.004	<0.001*	0.982	0.916	0.052	0.215	0.002*	<0.001*
Cz-Av	0.076	0.095	0.184	0.367	0.396	0.753	0.028	0.009
C4-Av	0.007	<0.001*	0.329	0.520	0.396	0.684	0.002*	0.001*
T4-Av	0.001*	<0.001*	<0.001*	0.003	0.102	0.170	0.002*	0.001*
T5-Av	0.002*	0.002*	0.980	0.936	0.621	0.422	0.001*	<0.001*
P3-Av	0.007	0.001*	0.650	0.571	0.431	0.394	0.007	0.001*
Pz-Av	0.067	0.015	0.149	0.494	0.363	0.576	0.053	0.022
P4-Av	0.002*	<0.001*	0.275	0.641	0.400	0.857	0.016	0.003
T6-Av	<0.001*	<0.001*	0.572	0.582	0.167	0.418	<0.001*	<0.001*
O1-Av	0.003	<0.001*	0.929	0.997	0.149	0.078	<0.001*	<0.001*
O2-Av	0.111	0.015	0.370	0.778	0.877	0.883	0.089	0.021

The p-values marked with * are statistically significant after the Bonferroni correction to address the multiple comparison problem.

**Table 2:** P-values for Higuchi fractal dimension (HFD), Lempel–Ziv complexity (LZC), Rényi entropy, and Tsallis entropy.

EEG channel	HFD		LZC		Rényi entropy		Tsallis entropy	
	K–S	Mann–Whitney	K-S	Mann–Whitney	K–S	Mann–Whitney	K–S	Mann–Whitney
Fp1-Av	0.001*	0.002*	*<*0.001*	*<*0.001*	*<*0.001*	*<*0.001*	*<*0.001*	*<*0.001*
Fp2-Av	0.194	0.068	*<*0.001*	*<*0.001*	*<*0.001*	*<*0.001*	*<*0.001*	*<*0.001*
F7-Av	0.938	0.494	*<*0.001*	*<*0.001*	*<*0.001*	*<*0.001*	*<*0.001*	0.009
F3-Av	0.084	0.125	*<*0.001*	*<*0.001*	*<*0.001*	*<*0.001*	*<*0.001*	*<*0.001*
Fz-Av	0.120	0.647	*<*0.001*	*<*0.001*	*<*0.001*	*<*0.001*	*<*0.001*	*<*0.001*
F4-Av	0.407	0.097	*<*0.001*	*<*0.001*	*<*0.001*	*<*0.001*	*<*0.001*	0.113
F8-Av	0.650	0.635	*<*0.001*	*<*0.001*	*<*0.001*	*<*0.001*	0.004	0.950
T3-Av	0.027	0.003	*<*0.001*	*<*0.001*	*<*0.001*	*<*0.001*	*<*0.001*	0.143
C3-Av	0.498	0.427	*<*0.001*	*<*0.001*	*<*0.001*	*<*0.001*	*<*0.001*	*<*0.001*
Cz-Av	0.066	0.594	*<*0.001*	*<*0.001*	*<*0.001*	*<*0.001*	*<*0.001*	0.172
C4-Av	0.204	0.432	*<*0.001*	*<*0.001*	*<*0.001*	*<*0.001*	*<*0.001*	0.005
T4-Av	0.063	0.035	*<*0.001*	*<*0.001*	*<*0.001*	*<*0.001*	*<*0.001*	*<*0.001*
T5-Av	0.617	0.371	*<*0.001*	*<*0.001*	*<*0.001*	*<*0.001*	*<*0.001*	*<*0.001*
P3-Av	0.805	0.936	*<*0.001*	*<*0.001*	*<*0.001*	*<*0.001*	*<*0.001*	*<*0.001*
Pz-Av	0.390	0.684	*<*0.001*	*<*0.001*	*<*0.001*	*<*0.001*	*<*0.001*	*<*0.001*
P4-Av	0.460	0.520	*<*0.001*	*<*0.001*	*<*0.001*	*<*0.001*	*<*0.001*	*<*0.001*
T6-Av	0.987	0.923	*<*0.001*	*<*0.001*	*<*0.001*	*<*0.001*	*<*0.001*	0.016
O1-Av	0.629	0.721	*<*0.001*	*<*0.001*	*<*0.001*	*<*0.001*	*<*0.001*	*<*0.001*
O2-Av	0.403	0.582	*<*0.001*	*<*0.001*	*<*0.001*	*<*0.001*	*<*0.001*	0.296

The p-values marked with * are statistically significant after the Bonferroni correction to address the multiple comparison problem.

For example, as seen in **Figure 2**, the brain rate results overall show larger values for the control group compared to the autism group. This is shown in **Table 1**, as we found significant statistical differences in 14 channels confirmed by both tests. This confirms the higher level of arousal and consciousness during a resting state in the TD group because of the wakefulness in this group thanks to elevated alpha activity during rest.

Similar results can be noticed from spectral entropy (**Figure 3**), where in 13 channels there were significant differences confirmed by both tests (**Table 1**). This entropy, the same as the brain rate, is a metric computed in the frequency domain, and the results from both of these features were correlated, therefore they can be interpreted in the same way.

While permutation entropy, HFD, and sample entropy did not show significant results overall, showing significant differences in a small number of EEG channels, other features such as LZC, Rényi entropy, and Tsallis entropy had very significant results (**Tables 1**, **2**). These results were visualized by the error-bar plots shown in **Figures 4**–**6**. There were higher values in the ASD group across all of the EEG channels for both entropy measures, meaning there was a high degree of disorder in the EEG signals of the children with autism. LZC, however, had lower values in the ASD group, meaning the EEG signals in ASD were more predictable and followed a pattern.

## Discussion

The results of this paper for the spectral features and entropy confirmed the previous results obtained by other authors mentioned in the Introduction.

The spectral features analysis showed that the resting-state EEG of TD children had high alpha activity during rest because of the wakefulness and higher level of arousal, while children with autism showed low or no activity at all. This elevated alpha activity influenced the higher values of brain rate and spectral entropy.

Higher values for Rényi entropy and Tsallis entropy were due to the increased neural noise captured by the EEG signals in the children with autism, which was also mentioned in the Introduction. Since entropy is a measure of the degree of disorder in a system, and the noise itself elevates this disorder, these results were expected and showed impaired neural synchronization, increased randomness, uncertainty, and complexity in ASD. Furthermore, Tsallis entropy showed interesting results since there was no variability among the children with autism across all the channels. This was because it reached an asymptotic maximum value of entropy (77) and the properties of sub-extensive Tsallis entropy (entropic index *q* = 1.5) (78). For the TD children, the in-between variability could be because of the different states of awareness, wakefulness, and thoughtfulness during rest in each child with typical development. Future research should be conducted to optimize the entropic index *q* and achieve better false positive rates in all channels (**Figure 6**). In general, entropy features capture the amplitude variations and distribution across the signal. The higher entropy values in the ASD group indicated a more “noisy” signal with more variations in the amplitude, while lower entropy in TD children indicated a more “smooth” signal for this group.

LZC, however, showed lower values in the ASD children than in the TD children. However, it is a complexity measure that differs from entropy complexity and should not be confused with it. Entropy is a measure of complexity that considers the probability distribution of the signal amplitude values, capturing the noise and randomness in the brain dynamics that manifest as cognitive and behavioral dysfunction in autism. LZC is calculated after binarizing the signal, and binarizing a time series can affect its complexity by reducing the amount of information and diversity in the signal. By using a threshold to assign binary values to the data points, one is essentially discarding some of the amplitude variations on both sides of the threshold value. This can make the signal more regular and predictable, and therefore less complex. However, the binarization affects the signals from both groups, hence the lower LZC in the autism group could be because of the repetitive sharp oscillations around the threshold value and alternating between 0 and 1 more regularly and periodically. That could explain the increased focal spikes and epileptic discharges found in ASD (25–28, 79), and should be investigated further as a potential biomarker. The higher LZC in the TD children was because of more random oscillations around the threshold value and could indicate the state of wakefulness and thoughtfulness during rest.

## Conclusion

In conclusion, we want to mention that all non-linear features are relative and depend on the input parameters such as embedding dimensions, window lengths, *α* values, and *q* values, meaning that further research should be conducted to optimize the results based on different parameter values. Nevertheless, our results showed that the ASD group had a lower brain rate, higher Tsallis entropy and Rényi entropy, and lower Lempel–Ziv complexity than the TD group, capturing a low level of arousal in the ASD children, increased randomness and noise, and the existence of focal spikes in the EEG signals of children with autism. The results from this study can be used as a feature selection to build future machine learning models on the topic.

## Data Availability

The raw data supporting the conclusions of this article will be made available by the authors, without undue reservation.

## References

[1] Boutros NN Lajiness-O’Neill R Zillgitt A Richard AE Bowyer SM. Eeg changes associated with autistic spectrum disorders. Neuropsychiatr Electrophysiology. (2015) 1:1–20. doi: 10.1186/s40810-014-0001-5

[2] Hashemian M Pourghassem H. Diagnosing autism spectrum disorders based on eeg analysis: A survey. Neurophysiology. (2014) 46:183–95. doi: 10.1007/s11062-014-9427-4

[3] Milovanovic M Grujicic R. Electroencephalography in assessment of autism spectrum disorders: A review. Front Psychiatry. (2021) 12:686021. doi: 10.3389/fpsyt.2021.686021 34658944 PMC8511396

[4] Verbeke WJ Pozharliev R Van Strien JW Belschak F Bagozzi RP. i am resting but rest less well with you.“ the moderating effect of anxious attachment style on alpha power during eeg resting state in a social context. Front Hum Neurosci. (2014) 8:486. doi: 10.3389/fnhum.2014.00486 25071516 PMC4092365

[5] Chan AS Leung WW. Differentiating autistic children with quantitative encephalography: A 3-month longitudinal study. J Child Neurol. (2006) 21:391–9. doi: 10.1177/08830738060210050501 16901444

[6] Coben R Clarke AR Hudspeth W Barry RJ. Eeg power and coherence in autistic spectrum disorder. Clin Neurophysiol. (2008) 119:1002–9. doi: 10.1016/j.clinph.2008.01.013 18331812

[7] Cornew L Roberts TP Blaskey L Edgar JC. Resting-state oscillatory activity in autism spectrum disorders. J Autism Dev Disord. (2012) 42:1884–94. doi: 10.1007/s10803-011-1431-6 PMC3638261 22207057

[8] Elhabashy H Raafat O Afifi L Raafat H Abdullah K. Quantitative eeg in autistic children. Egyptian J Neurology Psychiatry Neurosurg. (2015) 52:176. doi: 10.4103/1110-1083.162031

[9] Orekhova EV Elsabbagh M Jones EJ Dawson G Charman T Johnson MH. Eeg hyper-connectivity in high-risk infants is associated with later autism. J Neurodev Disord. (2014) 6:1–11. doi: 10.1186/1866-1955-6-40 25400705 PMC4232695

[10] Sheikhani A Behnam H Mohammadi MR Noroozian M Mohammadi M. Detection of abnormalities for diagnosing of children with autism disorders using of quantitative electroencephalography analysis. J Med Syst. (2012) 36:957–63. doi: 10.1007/s10916-010-9560-6 20711644

[11] Sutton SK Burnette CP Mundy PC Meyer J Vaughan A Sanders C. Resting cortical brain activity and social behavior in higher functioning children with autism. J Child Psychol Psychiatry. (2005) 46:211–22. doi: 10.1111/j.1469-7610.2004.00341.x 15679529

[12] Takagaki K Russell J Lippert MT Motamedi GK. Development of the posterior basic rhythm in children with autism. Clin Neurophysiol. (2015) 126:297–303. doi: 10.1016/j.clinph.2014.04.022 24913702

[13] Donchin E Coles MG. Is the p300 component a manifestation of context updating? Behav Brain Sci. (1988) 11:357–74.

[14] Farwell LA Donchin E. Talking off the top of your head: Toward a mental prosthesis utilizing event-related brain potentials. Electroencephalography Clin Neurophysiol. (1988) 70:510–23. doi: 10.1016/0013-4694(88)90149-6 2461285

[15] Krusienski DJ Sellers EW Cabestaing F Bayoudh S McFarland DJ Vaughan TM. A comparison of classification techniques for the p300 speller. J Neural Eng. (2006) 3:299. doi: 10.1088/1741-2560/3/4/007 17124334

[16] Kutas M Hillyard SA. Event-related brain potentials to semantically inappropriate and surprisingly large words. Biol Psychol. (1980) 11:99–116. doi: 10.1016/0301-0511(80)90046-0 7272388

[17] Kutas M Hillyard SA. Reading senseless sentences: Brain potentials reflect semantic incongruity. Science. (1980) 207:203–5. doi: 10.1126/science.7350657 7350657

[18] Kutas M Hillyard SA. Brain potentials during reading reflect word expectancy and semantic association. Nature. (1984) 307:161–3. doi: 10.1038/307161a0 6690995

[19] Polich J. Updating p300: An integrative theory of p3a and p3b. Clin Neurophysiol. (2007) 118:2128–48. doi: 10.1016/j.clinph.2007.04.019 PMC2715154 17573239

[20] Anderson JS. Cortical underconnectivity hypothesis in autism: Evidence from functional connectivity mri. Compr guide to Autism. (2014), 1457–71.

[21] Leisman G Melillo R Melillo T. Prefrontal functional connectivities in autism spectrum disorders: A connectopathic disorder affecting movement, interoception, and cognition. Brain Res Bull. (2023) 198:65–76. doi: 10.1016/j.brainresbull.2023.04.004 37087061

[22] Maximo JO Kana RK. Aberrant “deep connectivity“ in autism: A cortico–subcortical functional connectivity magnetic resonance imaging study. Autism Res. (2019) 12:384–400. doi: 10.1002/aur.2019.12.issue-3 30624021

[23] Maximo JO Cadena EJ Kana RK. The implications of brain connectivity in the neuropsychology of autism. Neuropsychol Rev. (2014) 24:16–31. doi: 10.1007/s11065-014-9250-0 24496901 PMC4059500

[24] Tomasi D Volkow ND. Reduced local and increased long-range functional connectivity of the thalamus in autism spectrum disorder. Cereb Cortex. (2019) 29:573–85. doi: 10.1093/cercor/bhx340 PMC6319176 29300843

[25] Tuchman R Rapin I. Epilepsy in autism. Lancet Neurol. (2002) 1:352–8. doi: 10.1016/S1474-4422(02)00160-6 12849396

[26] Tuchman RF Rapin I. Regression in pervasive developmental disorders: Seizures and epileptiform electroencephalogram correlates. Pediatrics. (1997) 99:560–6. doi: 10.1542/peds.99.4.560 9093299

[27] Hughes JR Melyn M. Eeg and seizures in autistic children and adolescents: Further findings with therapeutic implications. Clin EEG Neurosci. (2005) 36:15–20. doi: 10.1177/155005940503600105 15683193

[28] Rossi PG Parmeggiani A Bach V Santucci M Visconti P. Eeg features and epilepsy in patients with autism. Brain Dev. (1995) 17:169–74. doi: 10.1016/0387-7604(95)00019-8 7573755

[29] Newson JJ Thiagarajan TC. Eeg frequency bands in psychiatric disorders: A review of resting state studies. Front Hum Neurosci. (2019) 12:521. doi: 10.3389/fnhum.2018.00521 30687041 PMC6333694

[30] Lo M-T Tsai P-H Lin P-F Lin C Hsin YL. The nonlinear and nonstationary properties in eeg signals: Probing the complex fluctuations by hilbert–huang transform. Adv Adaptive Data Anal. (2009) 1:461–82.

[31] Abdolzadegan D Moattar MH Ghoshuni M. A robust method for early diagnosis of autism spectrum disorder from eeg signals based on feature selection and dbscan method. Biocybernetics Biomed Eng. (2020) 40:482–93. doi: 10.1016/j.bbe.2020.01.008

[32] Oh SL Jahmunah V Arunkumar N Abdulhay EW Gururajan R Adib N. A novel automated autism spectrum disorder detection system. Complex Intelligent Syst. (2021) 7:2399–413. doi: 10.1007/s40747-021-00408-8

[33] Peck FC Gabard-Durnam LJ Wilkinson CL Bosl W Tager-Flusberg H Nelson CA. Prediction of autism spectrum disorder diagnosis using nonlinear measures of language-related eeg at 6 and 12 months. J Neurodev Disord. (2021) 13:1–13. doi: 10.1186/s11689-021-09405-x 34847887 PMC8903497

[34] Wang H Zhao X Yu D. Nonlinear features of gaze behavior during joint attention in children with autism spectrum disorder. Autism Res. (2023) 16:1786–98. doi: 10.1002/aur.v16.9 37530201

[35] Xu J Yang W. Autism diagnosis using linear and nonlinear analysis of resting-state eeg and self-organizing map. Int J Advanced Comput Sci Appl. (2023) 14. doi: 10.14569/IJACSA.2023.01409123

[36] Erra RG Mateos D Wennberg R Velazquez J. Towards a statistical mechanics of consciousness: Maximization of number of connections is associated with conscious awareness. arXiv preprint arXiv:1606.00821. (2016). 10.1103/PhysRevE.94.052402 27967157

[37] Bosl W Tierney A Tager-Flusberg H Nelson C. Eeg complexity as a biomarker for autism spectrum disorder risk. BMC Med. (2011) 9:1–16. doi: 10.1186/1741-7015-9-18 21342500 PMC3050760

[38] Djemal R AlSharabi K Ibrahim S Alsuwailem A. Eeg-based computer aided diagnosis of autism spectrum disorder using wavelet, entropy, and ann. BioMed Res Int. (2017) 2017. doi: 10.1155/2017/9816591 PMC5412163 28484720

[39] Han J Li Y Kang J Cai E Tong Z Ouyang G. Global synchronization of multichannel eeg based on rényi entropy in children with autism spectrum disorder. Appl Sci. (2017) 7:257. doi: 10.3390/app7030257

[40] Milne E. Increased intra-participant variability in children with autistic spectrum disorders: Evidence from single-trial analysis of evoked eeg. Front Psychol. (2011) 2:51. doi: 10.3389/fpsyg.2011.00051 21716921 PMC3110871

[41] Dinstein I Heeger DJ Behrmann M. Neural variability: Friend or foe? Trends Cogn Sci. (2015) 19:322–8. 10.1016/j.tics.2015.04.005 25979849

[42] Simmons DR Robertson AE McKay LS Toal E McAleer P Pollick FE. Vision in autism spectrum disorders. Vision Res. (2009) 49:2705–39. doi: 10.1016/j.visres.2009.08.005 19682485

[43] Moss F Ward LM Sannita WG. Stochastic resonance and sensory information processing: A tutorial and review of application. Clin Neurophysiol. (2004) 115:267–81. doi: 10.1016/j.clinph.2003.09.014 14744566

[44] Raul P McNally K Ward LM van Boxtel JJ. Does stochastic resonance improve performance for individuals with higher autism-spectrum quotient? Front Neurosci. (2023) 17:1110714. 37123379 10.3389/fnins.2023.1110714 PMC10140507

[45] Turnock A Langley K Jones CR. Understanding stigma in autism: A narrative review and theoretical model. Autism Adulthood. (2022) 4:76–91. doi: 10.1089/aut.2021.0005 36605561 PMC8992913

[46] Bosl WJ Tager-Flusberg H Nelson CA. Eeg analytics for early detection of autism spectrum disorder: A data-driven approach. Sci Rep. (2018) 8:6828. doi: 10.1038/s41598-018-24318-x 29717196 PMC5931530

[47] Catarino A Churches O Baron-Cohen S Andrade A Ring H. Atypical eeg complexity in autism spectrum conditions: A multiscale entropy analysis. Clin Neurophysiol. (2011) 122:2375–83. doi: 10.1016/j.clinph.2011.05.004 21641861

[48] Hadoush H Alafeef M Abdulhay E. Brain complexity in children with mild and severe autism spectrum disorders: Analysis of multiscale entropy in eeg. Brain topography. (2019) 32:914–21. doi: 10.1007/s10548-019-00711-1 31006838

[49] Papaioannou AG Kalantzi E Papageorgiou CC Korombili K Bokou A Pehlivanidis A. Complexity analysis of the brain activity in autism spectrum disorder (asd) and attention deficit hyperactivity disorder (adhd) due to cognitive loads/demands induced by aristotle’s type of syllogism/reasoning. a power spectral density and multiscale entropy (mse) analysis. Heliyon. (2021) 7. doi: 10.1016/j.heliyon.2021.e07984 PMC8477216 34611558

[50] Aksentijevic A Mihailović A Mihailović DT. A novel approach to the study of spatio-temporal brain dynamics using change-based complexity. Appl Mathematics Comput. (2021) 410:126432. doi: 10.1016/j.amc.2021.126432

[51] Sheikhani A Behnam H Mohammadi MR Noroozian M Golabi P. Analysis of quantitative electroencephalogram background activity in autism disease patients with lempel-ziv complexity and short time fourier transform measure. In: 2007 4th IEEE/EMBS international summer school and symposium on medical devices and biosensors. IEEE. (2007). p. 111–4.

[52] Lau ZJ Pham T Chen SA Makowski D. Brain entropy, fractal dimensions and predictability: A review of complexity measures for eeg in healthy and neuropsychiatric populations. Eur J Neurosci. (2022) 56:5047–69. doi: 10.1111/ejn.15800 PMC9826422 35985344

[53] Pop-Jordanova N Pop-Jordanov J. Spectrum-weighted eeg frequency (“brain-rate”) as a quantitative indicator of mental arousal. Prilozi. (2005) 26:35–42. 16400227

[54] Henry M Judge G. Permutation entropy and information recovery in nonlinear dynamic economic time series. Econometrics. (2019) 7:10. doi: 10.3390/econometrics7010010

[55] Bandt C Pompe B. Permutation entropy: A natural complexity measure for time series. Phys Rev Lett. (2002) 88:174102. doi: 10.1103/PhysRevLett.88.174102 12005759

[56] Delgado-Bonal A Marshak A. Approximate entropy and sample entropy: A comprehensive tutorial. Entropy. (2019) 21:541. doi: 10.3390/e21060541 33267255 PMC7515030

[57] Powell G Percival I. A spectral entropy method for distinguishing regular and irregular motion of hamiltonian systems. J Phys A: Math Gen. (1979) 12:2053. doi: 10.1088/0305-4470/12/11/017

[58] Pan Y Chen J Li X. Spectral entropy: A complementary index for rolling element bearing performance degradation assessment. Proc Institution Mechanical Engineers Part C: J Mechanical Eng Sci. (2009) 223:1223–31. doi: 10.1243/09544062JMES1224

[59] Sharma V Parey A. A review of gear fault diagnosis using various condition indicators. Proc Eng. (2016) 144:253–63. doi: 10.1016/j.proeng.2016.05.131

[60] Shen J-l Hung J-W Lee L-S. Robust entropy-based endpoint detection for speech recognition in noisy environments. Fifth Int Conf spoken Lang Process. (1998). doi: 10.21437/ICSLP.1998

[61] Vakkuri A Yli-Hankala A Talja P Mustola S Tolvanen-Laakso H Sampson T. Time-frequency balanced spectral entropy as a measure of anesthetic drug effect in central nervous system during sevoflurane, propofol, and thiopental anesthesia. Acta Anaesthesiologica Scandinavica. (2004) 48:145–53. doi: 10.1111/j.0001-5172.2004.00323.x 14995935

[62] Rényi A. On measures of entropy and information. In: Proceedings of the Fourth Berkeley Symposium on Mathematical Statistics and Probability, Volume 1: Contributions to the Theory of Statistics. project Euclid. vol. 4. (1961). p. 547–62.

[63] Hadiyoso S Aulia S Rizal A. Quantitative eeg based on renyi entropy for epileptic classification. J Electrical Electron Eng. (2019) 12:15–20.

[64] Inuso G La Foresta F Mammone N Morabito FC. Brain activity investigation by eeg processing: Wavelet analysis, kurtosis and renyi’s entropy for artifact detection. 2007 Int Conf Inf Acquisition. (2007), 195–200. doi: 10.1109/ICIA.2007.4295725

[65] Rahman MA Khanam F Ahmad M Uddin MS. Multiclass eeg signal classification utilizing rényi min-entropy-based feature selection from wavelet packet transformation. Brain Inf. (2020) 7:1–11. doi: 10.1186/s40708-020-00108-y PMC7297893 32548772

[66] Yin Y Sun K He S. Multiscale permutation rényi entropy and its application for eeg signals. PloS One. (2018) 13:e0202558. doi: 10.1371/journal.pone.0202558 30180194 PMC6122795

[67] Higuchi T. Approach to an irregular time series on the basis of the fractal theory. Physica D: Nonlinear Phenomena. (1988) 31:277–83. doi: 10.1016/0167-2789(88)90081-4

[68] Anderson K Chirion C Fraser M Purcell M Stein S Vuckovic A. Markers of central neuropathic pain in higuchi fractal analysis of eeg signals from people with spinal cord injury. Front Neurosci. (2021) 15:705652. doi: 10.3389/fnins.2021.705652 34512243 PMC8427815

[69] Cukic M Pokrajac D Stokic M Radivojevic V Ljubisavljevic M. Eeg machine learning with higuchi fractal dimension and sample entropy as features for successful detection of depression. arXiv preprint arXiv:1803.05985. (2018).

[70] Klonowski W. Fractal analysis of electroencephalographic time series (eeg signals). fractal geometry Brain. (2016), 413–29.

[71] Tsallis C. Possible generalization of boltzmann-gibbs statistics. J Stat Phys. (1988) 52:479–87. doi: 10.1007/BF01016429

[72] Thilagaraj M Pallikonda Rajasekaran M Arun Kumar N. Tsallis entropy: As a new single feature with the least computation time for classification of epileptic seizures. Cluster Computing. (2019) 22:15213–21. doi: 10.1007/s10586-018-2549-5

[73] Anastasiadis A. Tsallis entropy. Entropy. (2012) 14:174–6. doi: 10.3390/e14020174

[74] Capurro A Diambra L Lorenzo D Macadar O Martín MT Mostaccio C. Tsallis entropy and cortical dynamics: The analysis of eeg signals. Physica A: Stat mechanics its Appl. (1998) 257:149–55. doi: 10.1016/S0378-4371(98)00137-X

[75] Zhang D Jia X Ding H Ye D Thakor NV. Application of tsallis entropy to eeg: Quantifying the presence of burst suppression after asphyxial cardiac arrest in rats. IEEE Trans Biomed Eng. (2009) 57:867–74. 10.1109/TBME.2009.2029082 PMC3050535 19695982

[76] Lempel A Ziv J. On the complexity of finite sequences. IEEE Trans Inf Theory. (1976) 22:75–81. doi: 10.1109/TIT.1976.1055501

[77] Ramírez-Reyes A Hernández-Montoya AR Herrera-Corral G Domínguez-Jiménez I. Determining the entropic index q of tsallis entropy in images through redundancy. Entropy. (2016) 18:299. doi: 10.3390/e18080299

[78] Gell-Mann M Tsallis C. Nonextensive entropy: Interdisciplinary applications. Oxford University Press (2004).

[79] Wang J Barstein J Ethridge LE Mosconi MW Takarae Y Sweeney JA. Resting state eeg abnormalities in autism spectrum disorders. J Neurodev Disord. (2013) 5:1–14. doi: 10.1186/1866-1955-5-24 24040879 PMC3847481

