# Supplementary material for: Entropy, complexity, and spectral features of EEG signals in autism and typical development: a quantitative approach
Source: Front Psychiatry. 2025 Feb 4;16:1505297. doi: 10.3389/fpsyt.2025.1505297 (PMC11832502; doi:10.3389/fpsyt.2025.1505297)
Supplement: Supplementary file 1 [file DataSheet1.pdf]

Explore

Case Processing Summary

	Cases					
	Valid		Missing		Total	
	N	Percent	N	Percent	N	Percent
BrainRate	39	100.0%	0	0.0%	39	100.0%
PermutationEntropy	39	100.0%	0	0.0%	39	100.0%
PermutationEntropy_01	39	100.0%	0	0.0%	39	100.0%
SampleEntropy	39	100.0%	0	0.0%	39	100.0%
SpectralEntropy_bits	39	100.0%	0	0.0%	39	100.0%
SpectralEntropy_01	39	100.0%	0	0.0%	39	100.0%
HiguchiFractalDimension	39	100.0%	0	0.0%	39	100.0%
LempelZivComplexity	39	100.0%	0	0.0%	39	100.0%
RenyiEntropy	39	100.0%	0	0.0%	39	100.0%
TsallisEntropy	39	100.0%	0	0.0%	39	100.0%
PS_4_5	39	100.0%	0	0.0%	39	100.0%
PS_5_6	39	100.0%	0	0.0%	39	100.0%
PS_6_7	39	100.0%	0	0.0%	39	100.0%
PS_7_8	39	100.0%	0	0.0%	39	100.0%
PS_8_9	39	100.0%	0	0.0%	39	100.0%
PS_9_10	39	100.0%	0	0.0%	39	100.0%
PS_10_11	39	100.0%	0	0.0%	39	100.0%
PS_11_12	39	100.0%	0	0.0%	39	100.0%
PS_12_13	39	100.0%	0	0.0%	39	100.0%
PS_13_14	39	100.0%	0	0.0%	39	100.0%
PS_14_15	39	100.0%	0	0.0%	39	100.0%
PS_15_16	39	100.0%	0	0.0%	39	100.0%
PS_16_17	39	100.0%	0	0.0%	39	100.0%
PS_17_18	39	100.0%	0	0.0%	39	100.0%
PS_18_19	39	100.0%	0	0.0%	39	100.0%
PS_19_20	39	100.0%	0	0.0%	39	100.0%

Descriptives

			Statistic	Std. Error
BrainRate	Mean		11.2303	.91890
	95% Confidence Interval for Mean	Lower Bound	9.3700	
		Upper Bound	13.0905	
	5% Trimmed Mean		10.6997	
	Median		10.0400	
	Variance		32.931	
	Std. Deviation		5.73854	
	Minimum		4.57	
	Maximum		30.28	
	Range		25.71	
	Interquartile Range		7.58	
	Skewness		1.360	.378
	Kurtosis		2.121	.741
PermutationEntropy	Mean		1.5608478296	.00491638544
	95% Confidence Interval for Mean	Lower Bound	1.5508951276	
		Upper Bound	1.5708005316	
	5% Trimmed Mean		1.5626399415	
	Median		1.5640314949	
	Variance		.001	
	Std. Deviation		.03070281726	
	Minimum		1.4585155426	
	Maximum		1.6130314857	
	Range		.15451594306	
	Interquartile Range		.04864835445	
	Skewness		-.976	.378
	Kurtosis		1.885	.741
PermutationEntropy_01	Mean		.87112576012	.00274388696
	95% Confidence Interval for Mean	Lower Bound	.86557105137	
		Upper Bound	.87668046887	
	5% Trimmed Mean		.87212595682	
	Median		.87290259759	
	Variance		.000	
	Std. Deviation		.01713556858	
	Minimum		.81401302332	
	Maximum		.90025001312	
	Range		.08623698979	
	Interquartile Range		.02715116358	
	Skewness		-.976	.378
	Kurtosis		1.885	.741

Descriptives

			Statistic	Std. Error
SampleEntropy	Mean		.82961234934	.04618430401
	95% Confidence Interval for Mean	Lower Bound	.73611711384	
		Upper Bound	.92310758485	
	5% Trimmed Mean		.83570015742	
	Median		.89430336577	
	Variance		.083	
	Std. Deviation		.28842088611	
	Minimum		.24540291659	
	Maximum		1.3085076761	
	Range		1.0631047595	
	Interquartile Range		.45839366914	
	Skewness		-.425	.378
	Kurtosis		-.719	.741
SpectralEntropy_bits	Mean		4.3143203387	.12653021514
	95% Confidence Interval for Mean	Lower Bound	4.0581733096	
		Upper Bound	4.5704673678	
	5% Trimmed Mean		4.3304310638	
	Median		4.3299151048	
	Variance		.624	
	Std. Deviation		.79018094031	
	Minimum		2.6090914877	
	Maximum		5.5803574014	
	Range		2.9712659136	
	Interquartile Range		1.2600275532	
	Skewness		-.299	.378
	Kurtosis		-.696	.741
SpectralEntropy_01	Mean		.61534452978	.01804679987
	95% Confidence Interval for Mean	Lower Bound	.57881069344	
		Upper Bound	.65187836612	
	5% Trimmed Mean		.61764237643	
	Median		.61756878604	
	Variance		.013	
	Std. Deviation		.11270222909	
	Minimum		.37213049765	
	Maximum		.79591734771	
	Range		.42378685006	
	Interquartile Range		.17971569132	
	Skewness		-.299	.378
	Kurtosis		-.696	.741

Descriptives

			Statistic	Std. Error
HiguchiFractalDimension	Mean		.36761831763	.01739535106
	95% Confidence Interval for Mean	Lower Bound	.33240327046	
		Upper Bound	.40283336480	
	5% Trimmed Mean		.36667485886	
	Median		.37095361707	
	Variance		.012	
	Std. Deviation		.10863393257	
	Minimum		.14561988793	
	Maximum		.60569823977	
	Range		.46007835184	
	Interquartile Range		.12719266814	
	Skewness		.215	.378
	Kurtosis		.237	.741
LempelZivComplexity	Mean		.31583709354	.00754738563
	95% Confidence Interval for Mean	Lower Bound	.30055821013	
		Upper Bound	.33111597696	
	5% Trimmed Mean		.31442207594	
	Median		.30414947870	
	Variance		.002	
	Std. Deviation		.04713340813	
	Minimum		.23510023334	
	Maximum		.42617528481	
	Range		.19107505147	
	Interquartile Range		.06516883605	
	Skewness		.430	.378
	Kurtosis		-.253	.741
RenyiEntropy	Mean		7.1762502992	.18460306643
	95% Confidence Interval for Mean	Lower Bound	6.8025409289	
		Upper Bound	7.5499596695	
	5% Trimmed Mean		7.2324040983	
	Median		7.5232298881	
	Variance		1.329	
	Std. Deviation		1.1528457804	
	Minimum		4.4784558973	
	Maximum		8.7728302947	
	Range		4.2943743974	
	Interquartile Range		1.9732748092	
	Skewness		-.792	.378
	Kurtosis		-.480	.741

Descriptives

			Statistic	Std. Error
TsallisEntropy	Mean		1.8426561361	.06740343974
	95% Confidence Interval for Mean	Lower Bound	1.7062050060	
		Upper Bound	1.9791072661	
	5% Trimmed Mean		1.9175043542	
	Median		1.9841421550	
	Variance		.177	
	Std. Deviation		.42093434623	
	Minimum		.17044907143	
	Maximum		1.9889123594	
	Range		1.8184632880	
	Interquartile Range		.02185908256	
	Skewness		-3.247	.378
	Kurtosis		9.590	.741
PS_4_5	Mean		1.73295	.393014
	95% Confidence Interval for Mean	Lower Bound	.93733	
		Upper Bound	2.52856	
	5% Trimmed Mean		1.35432	
	Median		1.13800	
	Variance		6.024	
	Std. Deviation		2.454371	
	Minimum		.294	
	Maximum		15.850	
	Range		15.556	
	Interquartile Range		1.137	
	Skewness		5.260	.378
	Kurtosis		30.518	.741
PS_5_6	Mean		1.38397	.331507
	95% Confidence Interval for Mean	Lower Bound	.71287	
		Upper Bound	2.05508	
	5% Trimmed Mean		1.07071	
	Median		.95000	
	Variance		4.286	
	Std. Deviation		2.070262	
	Minimum		.210	
	Maximum		13.410	
	Range		13.200	
	Interquartile Range		.601	
	Skewness		5.419	.378
	Kurtosis		31.867	.741

Descriptives

			Statistic	Std. Error
PS_6_7	Mean		1.02123	.163588
	95% Confidence Interval for Mean	Lower Bound	.69006	
		Upper Bound	1.35240	
	5% Trimmed Mean		.88093	
	Median		.81100	
	Variance		1.044	
	Std. Deviation		1.021608	
	Minimum		.189	
	Maximum		6.480	
	Range		6.291	
	Interquartile Range		.530	
	Skewness		4.242	.378
	Kurtosis		22.050	.741
PS_7_8	Mean		.92462	.124291
	95% Confidence Interval for Mean	Lower Bound	.67300	
		Upper Bound	1.17623	
	5% Trimmed Mean		.81562	
	Median		.70400	
	Variance		.602	
	Std. Deviation		.776199	
	Minimum		.156	
	Maximum		4.210	
	Range		4.054	
	Interquartile Range		.549	
	Skewness		2.636	.378
	Kurtosis		8.690	.741
PS_8_9	Mean		.81708	.122856
	95% Confidence Interval for Mean	Lower Bound	.56837	
		Upper Bound	1.06579	
	5% Trimmed Mean		.70268	
	Median		.58500	
	Variance		.589	
	Std. Deviation		.767236	
	Minimum		.125	
	Maximum		3.940	
	Range		3.815	
	Interquartile Range		.454	
	Skewness		2.700	.378
	Kurtosis		8.283	.741

Descriptives

			Statistic	Std. Error
PS_9_10	Mean		.87728	.180539
	95% Confidence Interval for Mean	Lower Bound	.51180	
		Upper Bound	1.24276	
	5% Trimmed Mean		.70640	
	Median		.66400	
	Variance		1.271	
	Std. Deviation		1.127466	
	Minimum		.097	
	Maximum		7.200	
	Range		7.103	
	Interquartile Range		.429	
	Skewness		4.898	.378
	Kurtosis		27.333	.741
PS_10_11	Mean		.98195	.169932
	95% Confidence Interval for Mean	Lower Bound	.63794	
		Upper Bound	1.32596	
	5% Trimmed Mean		.83277	
	Median		.75100	
	Variance		1.126	
	Std. Deviation		1.061228	
	Minimum		.104	
	Maximum		6.560	
	Range		6.456	
	Interquartile Range		.612	
	Skewness		4.092	.378
	Kurtosis		20.538	.741
PS_11_12	Mean		.50695	.078225
	95% Confidence Interval for Mean	Lower Bound	.34859	
		Upper Bound	.66531	
	5% Trimmed Mean		.43404	
	Median		.36700	
	Variance		.239	
	Std. Deviation		.488513	
	Minimum		.077	
	Maximum		2.373	
	Range		2.296	
	Interquartile Range		.264	
	Skewness		2.786	.378
	Kurtosis		7.808	.741

Descriptives

			Statistic	Std. Error
PS_12_13	Mean		.40354	.062304
	95% Confidence Interval for Mean	Lower Bound	.27741	
		Upper Bound	.52967	
	5% Trimmed Mean		.34903	
	Median		.32400	
	Variance		.151	
	Std. Deviation		.389091	
	Minimum		.059	
	Maximum		2.310	
	Range		2.251	
	Interquartile Range		.239	
	Skewness		3.450	.378
	Kurtosis		15.112	.741
PS_13_14	Mean		.36767	.045302
	95% Confidence Interval for Mean	Lower Bound	.27596	
		Upper Bound	.45938	
	5% Trimmed Mean		.33618	
	Median		.30100	
	Variance		.080	
	Std. Deviation		.282912	
	Minimum		.057	
	Maximum		1.440	
	Range		1.383	
	Interquartile Range		.259	
	Skewness		1.909	.378
	Kurtosis		4.683	.741
PS_14_15	Mean		.39262	.053528
	95% Confidence Interval for Mean	Lower Bound	.28425	
		Upper Bound	.50098	
	5% Trimmed Mean		.35044	
	Median		.27200	
	Variance		.112	
	Std. Deviation		.334280	
	Minimum		.055	
	Maximum		1.750	
	Range		1.695	
	Interquartile Range		.292	
	Skewness		2.221	.378
	Kurtosis		6.587	.741

Descriptives

			Statistic	Std. Error
PS_15_16	Mean		.38728	.048876
	95% Confidence Interval for Mean	Lower Bound	.28834	
		Upper Bound	.48623	
	5% Trimmed Mean		.35785	
	Median		.30000	
	Variance		.093	
	Std. Deviation		.305229	
	Minimum		.049	
	Maximum		1.420	
	Range		1.371	
	Interquartile Range		.307	
	Skewness		1.525	.378
	Kurtosis		2.460	.741
PS_16_17	Mean		.39562	.049868
	95% Confidence Interval for Mean	Lower Bound	.29466	
		Upper Bound	.49657	
	5% Trimmed Mean		.36905	
	Median		.29800	
	Variance		.097	
	Std. Deviation		.311423	
	Minimum		.050	
	Maximum		1.460	
	Range		1.410	
	Interquartile Range		.341	
	Skewness		1.475	.378
	Kurtosis		2.376	.741
PS_17_18	Mean		.42638	.059222
	95% Confidence Interval for Mean	Lower Bound	.30650	
		Upper Bound	.54627	
	5% Trimmed Mean		.38826	
	Median		.32700	
	Variance		.137	
	Std. Deviation		.369839	
	Minimum		.038	
	Maximum		1.560	
	Range		1.522	
	Interquartile Range		.359	
	Skewness		1.545	.378
	Kurtosis		2.113	.741

Descriptives

			Statistic	Std. Error
PS_18_19	Mean		.43015	.058786
	95% Confidence Interval for Mean	Lower Bound	.31115	
		Upper Bound	.54916	
	5% Trimmed Mean		.39598	
	Median		.31400	
	Variance		.135	
	Std. Deviation		.367117	
	Minimum		.040	
	Maximum		1.490	
	Range		1.450	
	Interquartile Range		.384	
	Skewness		1.404	.378
	Kurtosis		1.552	.741
PS_19_20	Mean		.48767	.077494
	95% Confidence Interval for Mean	Lower Bound	.33079	
		Upper Bound	.64454	
	5% Trimmed Mean		.42479	
	Median		.33700	
	Variance		.234	
	Std. Deviation		.483948	
	Minimum		.034	
	Maximum		2.270	
	Range		2.236	
	Interquartile Range		.506	
	Skewness		2.103	.378
	Kurtosis		5.039	.741

Tests of Normality

	Kolmogorov-Smirnov[a]			Shapiro-Wilk		
	Statistic	df	Sig.	Statistic	df	Sig.
BrainRate	.123	39	.142	.883	39	<.001
PermutationEntropy	.095	39	.200*	.945	39	.057
PermutationEntropy_01	.095	39	.200*	.945	39	.057
SampleEntropy	.108	39	.200*	.959	39	.168
SpectralEntropy_bits	.074	39	.200*	.971	39	.415
SpectralEntropy_01	.074	39	.200*	.971	39	.415
HiguchiFractalDimension	.071	39	.200*	.975	39	.525
LempelZivComplexity	.122	39	.152	.973	39	.454
RenyiEntropy	.139	39	.055	.908	39	.004
TsallisEntropy	.414	39	<.001	.386	39	<.001
PS_4_5	.279	39	<.001	.418	39	<.001
PS_5_6	.294	39	<.001	.391	39	<.001
PS_6_7	.240	39	<.001	.570	39	<.001
PS_7_8	.233	39	<.001	.733	39	<.001
PS_8_9	.251	39	<.001	.696	39	<.001
PS_9_10	.287	39	<.001	.472	39	<.001
PS_10_11	.267	39	<.001	.576	39	<.001
PS_11_12	.281	39	<.001	.627	39	<.001
PS_12_13	.274	39	<.001	.643	39	<.001
PS_13_14	.172	39	.005	.828	39	<.001
PS_14_15	.163	39	.011	.789	39	<.001
PS_15_16	.160	39	.014	.858	39	<.001
PS_16_17	.158	39	.016	.862	39	<.001
PS_17_18	.176	39	.004	.835	39	<.001
PS_18_19	.159	39	.014	.851	39	<.001
PS_19_20	.202	39	<.001	.778	39	<.001

*. This is a lower bound of the true significance.

a. Lilliefors Significance Correction

BrainRate

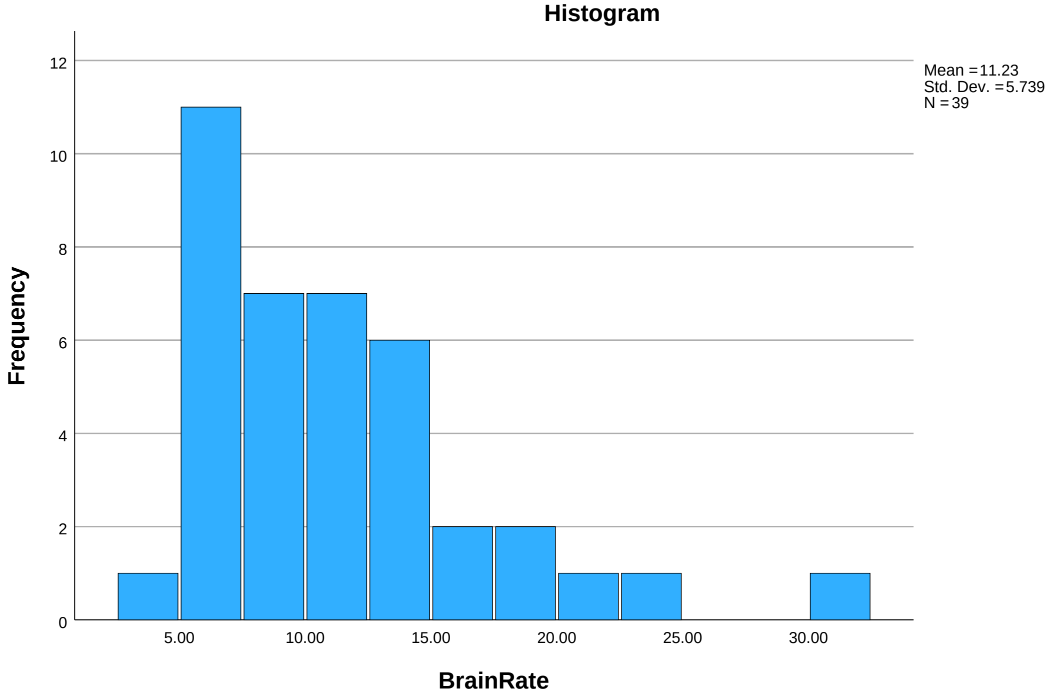


```
BrainRate Stem-and-Leaf Plot

 Frequency    Stem &  Leaf

     1.00        0 .  4
    18.00        0 .  555556666667788999
    13.00        1 .  0011222333334
     4.00        1 .  6699
     2.00        2 .  13
     1.00 Extremes    (>=30)

 Stem width:      10.00
 Each leaf:        1 case(s)
```

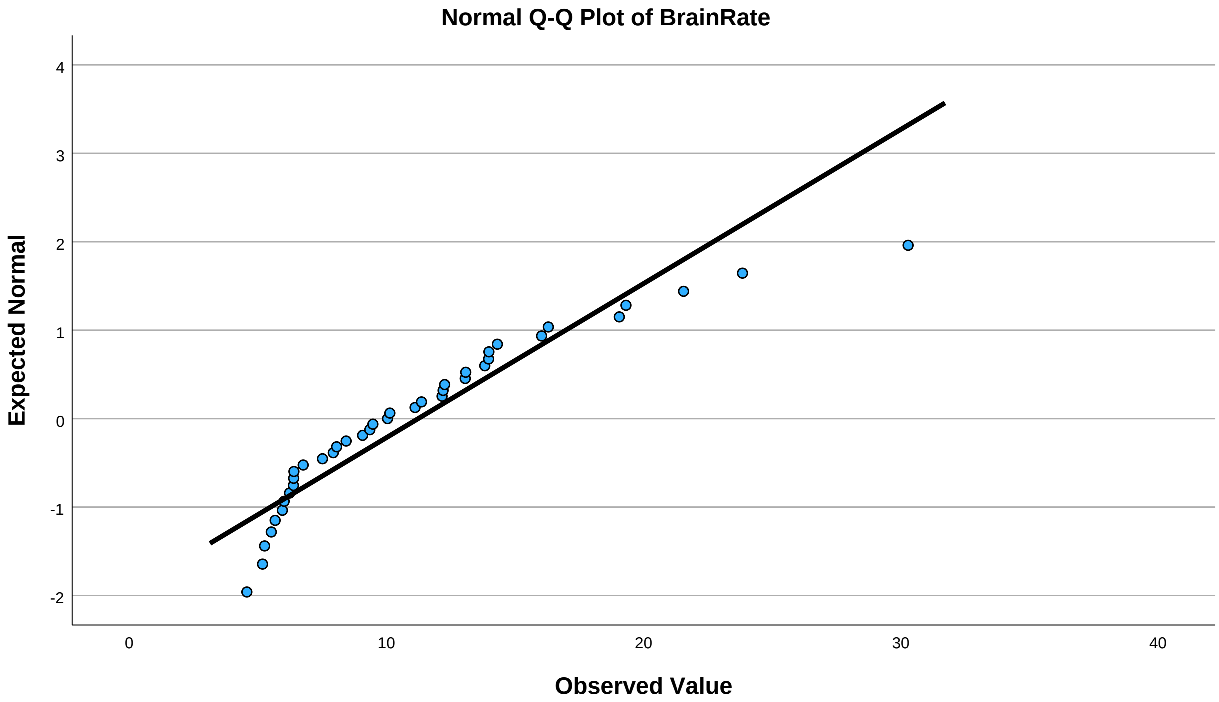
Normal Q-Q Plot of BrainRate
Expected Normal
4
3
2
1
0
-1
-2
0
10
20
30
40
Observed Value

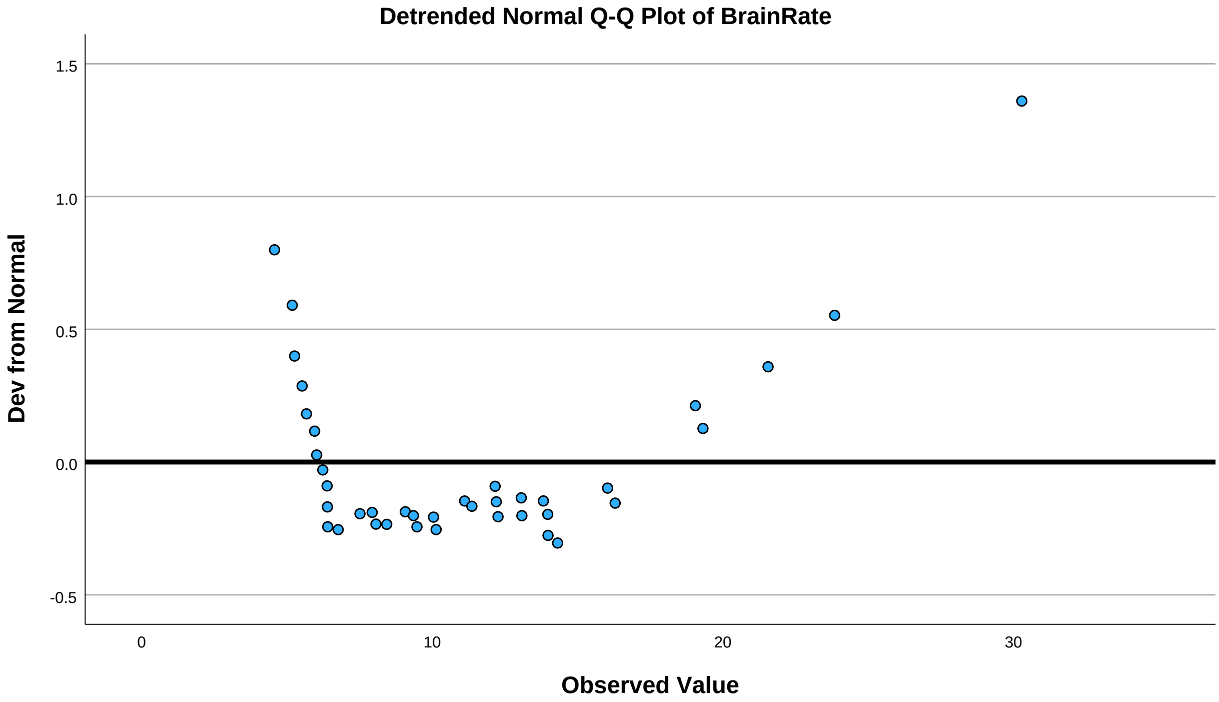
Detrended Normal Q-Q Plot of BrainRate
Dev from Normal
1.5
1.0
0.5
0.0
-0.5
0
10
20
30
Observed Value

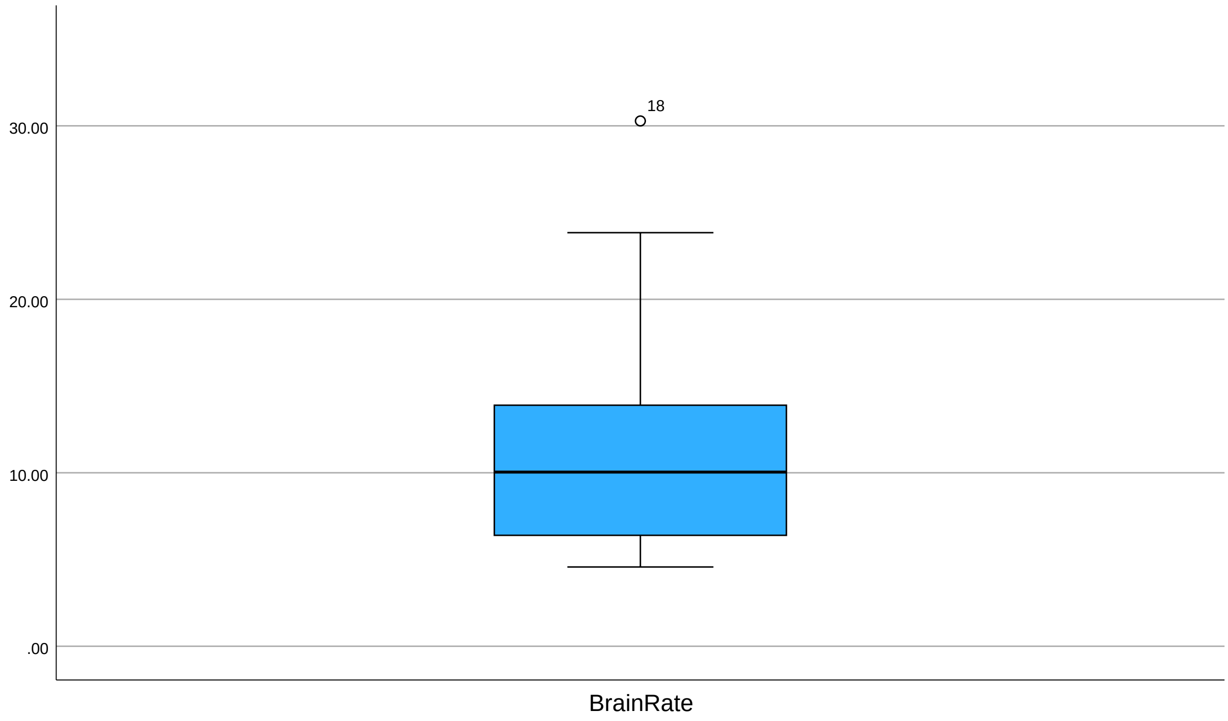

PermutationEntropy

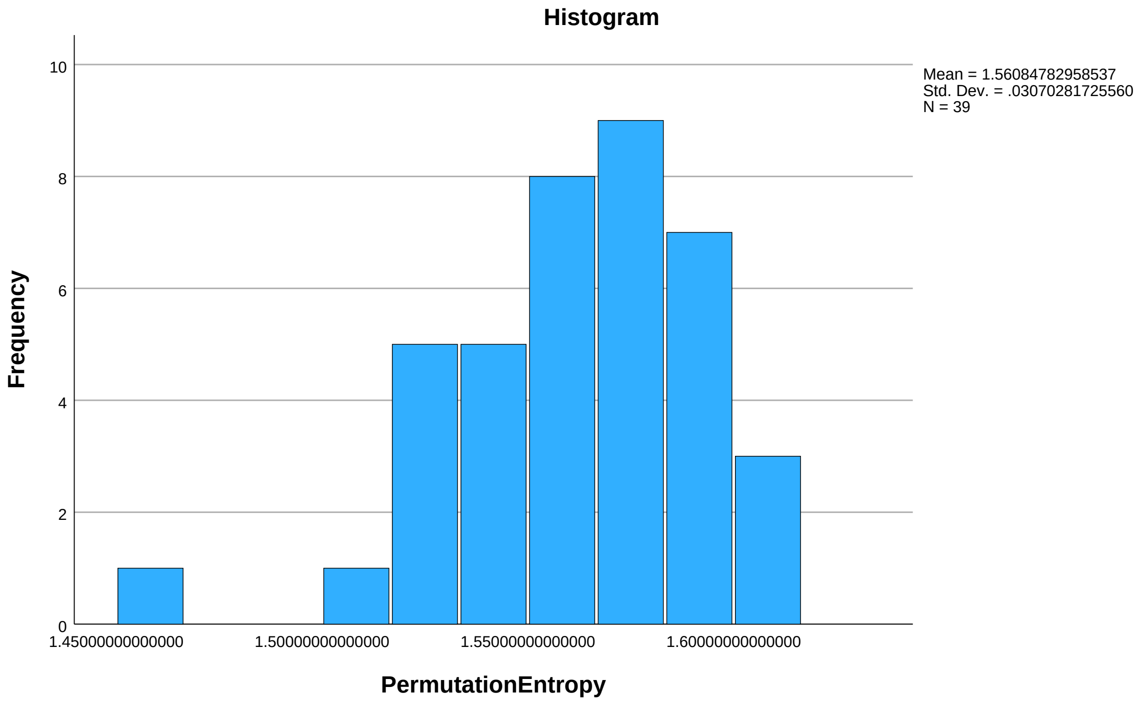


```
PermutationEntropy Stem-and-Leaf Plot

 Frequency    Stem &  Leaf
```

```
 1.00 Extremes    (=<1.459)
 1.00     150 .  3
 1.00     151 .  8
 2.00     152 .  89
 6.00     153 .  333459
 1.00     154 .  9
 5.00     155 .  22478
 4.00     156 .  1249
 6.00     157 .  001239
 7.00     158 .  2245567
 2.00     159 .  37
 2.00     160 .  14
 1.00     161 .  3

Stem width:   .0100000
Each leaf:        1 case(s)
```

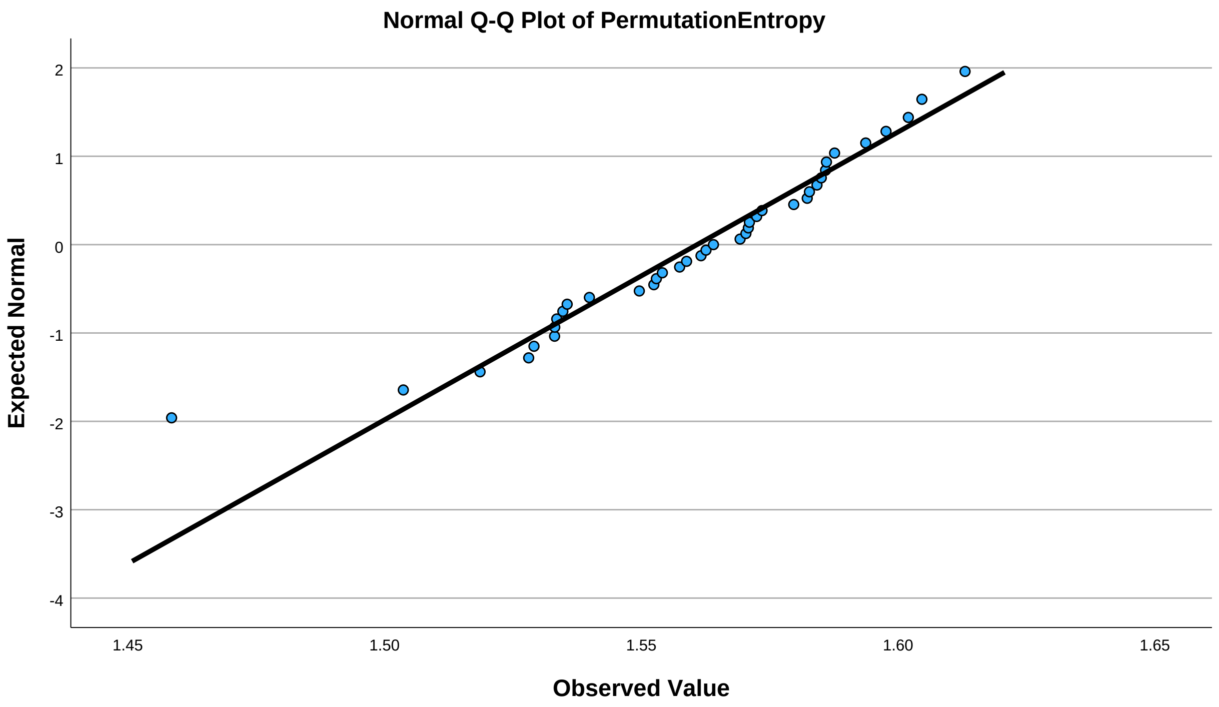

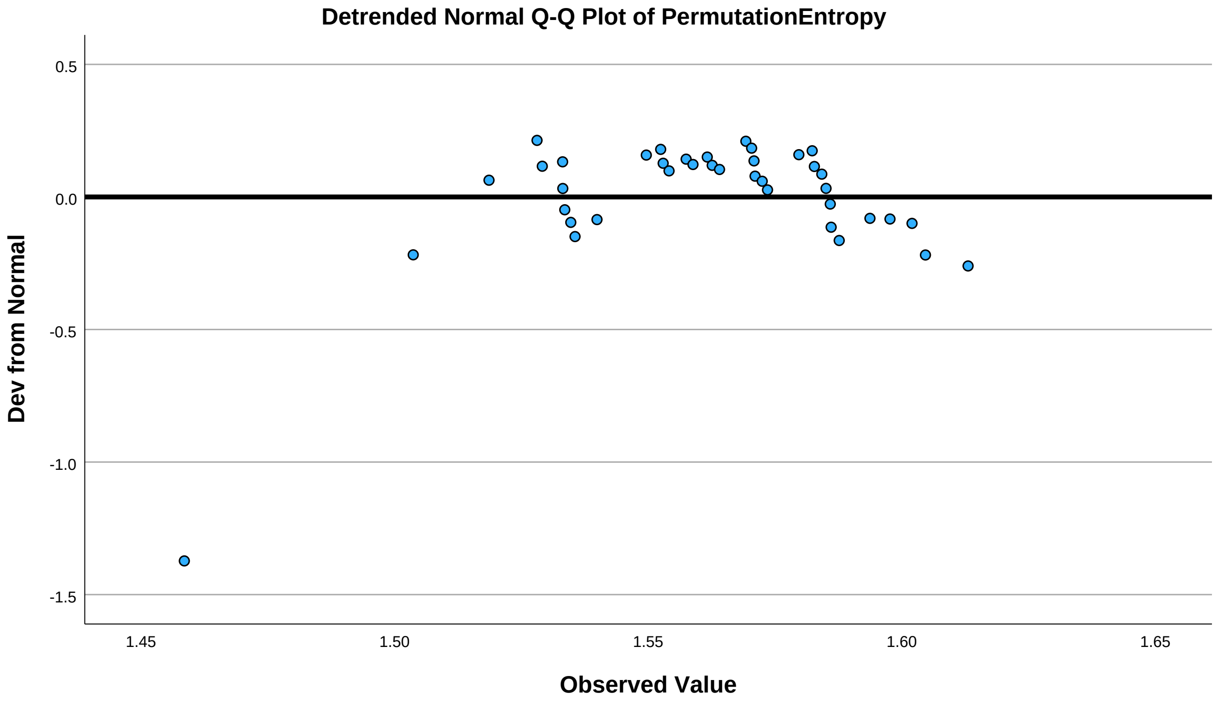

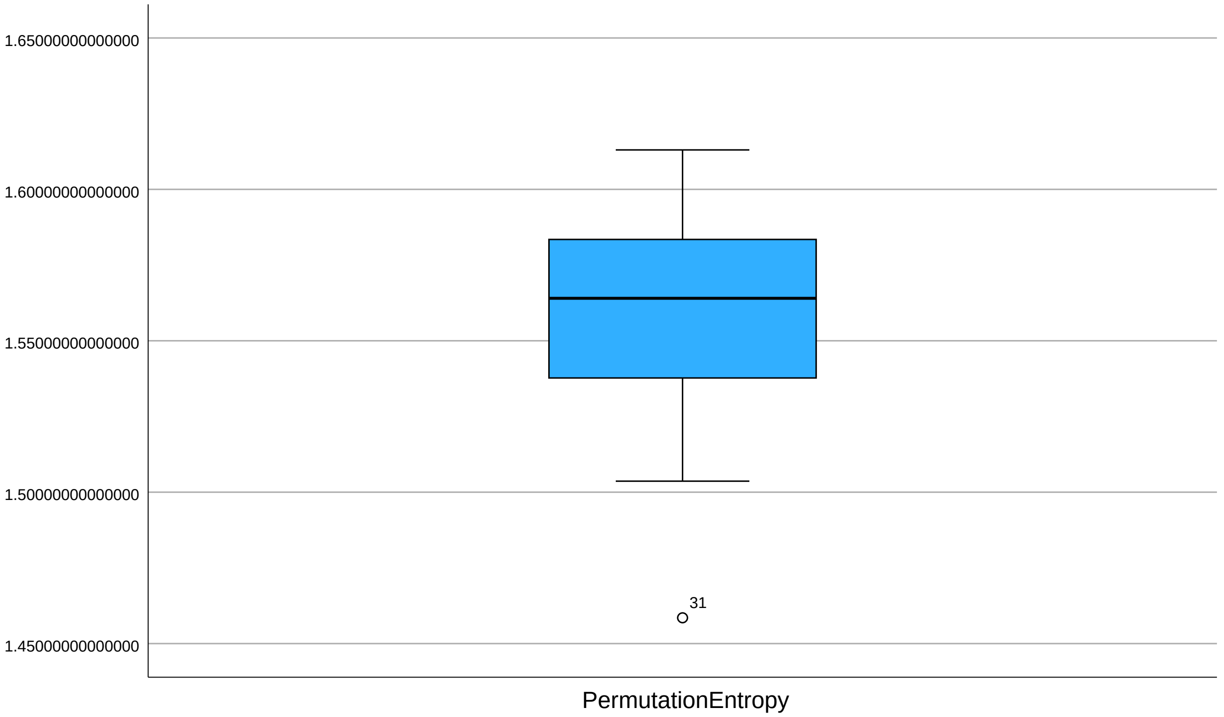

PermutationEntropy_01

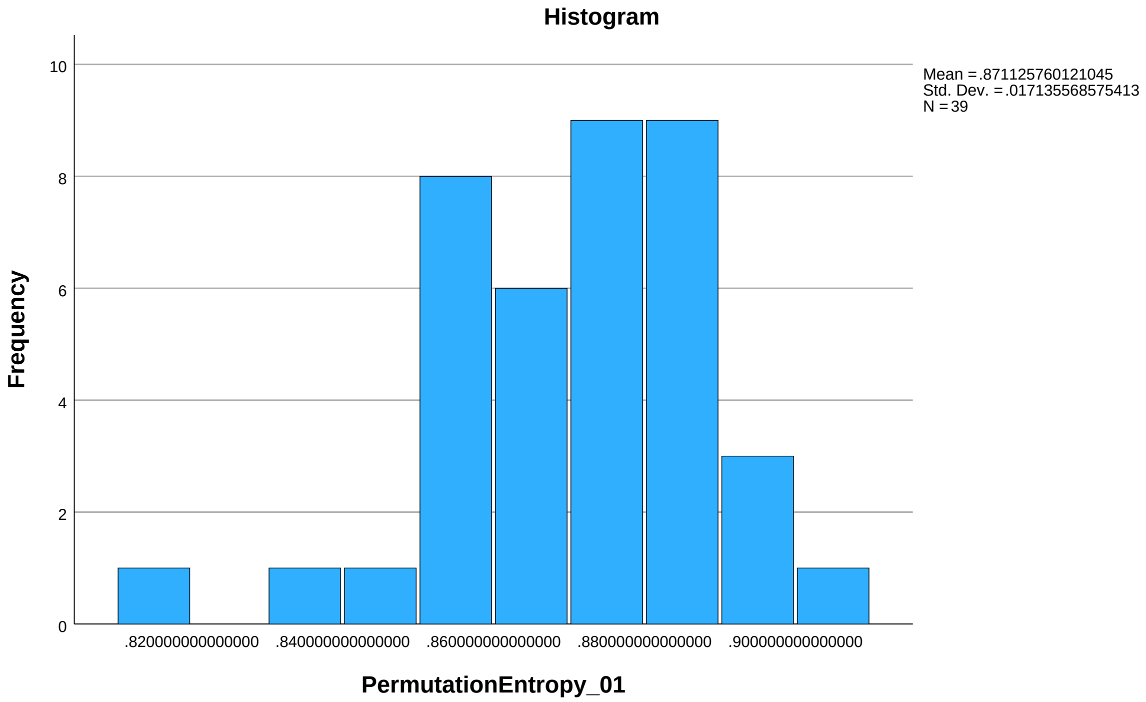


```
PermutationEntropy_01 Stem-and-Leaf Plot

 Frequency    Stem &  Leaf

     1.00 Extremes    (=<.814)
     1.00       83 .  9
     1.00       84 .  7
     8.00       85 .  23555679
     6.00       86 .  466799
     9.00       87 .  122566678
     9.00       88 .  133445569
     3.00       89 .  145
     1.00       90 .  0

 Stem width:   .0100000
 Each leaf:       1 case(s)
```

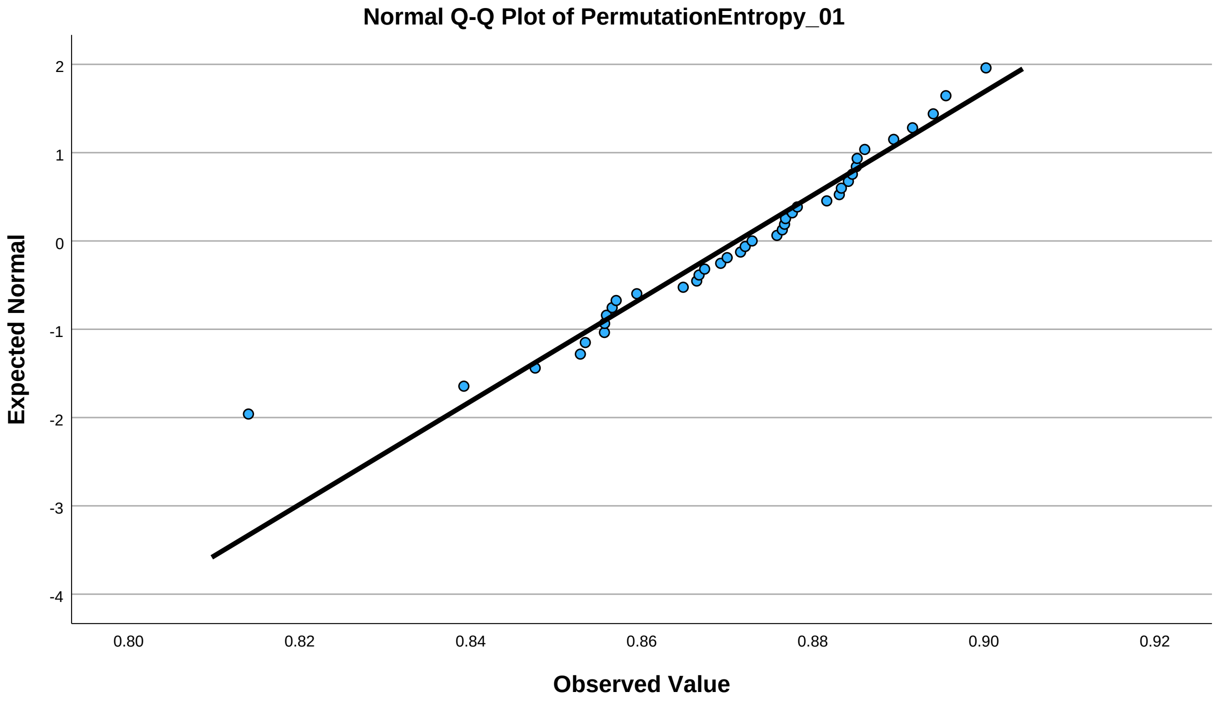
Normal Q-Q Plot of PermutationEntropy_01
Expected Normal
2
1
0
-1
-2
-3
-4
0.80
0.82
0.84
0.86
0.88
0.90
0.92
Observed Value

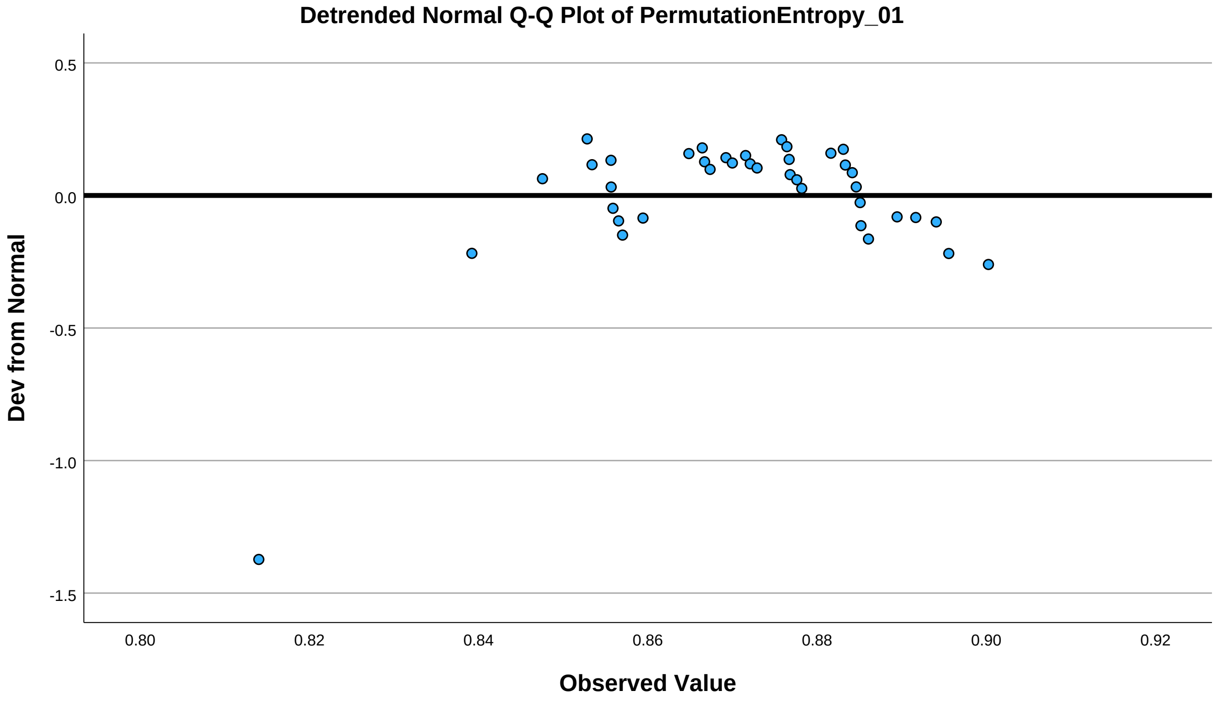
Detrended Normal Q-Q Plot of PermutationEntropy_01
Dev from Normal
0.5
0.0
-0.5
-1.0
-1.5
0.80
0.82
0.84
0.86
0.88
0.90
0.92
Observed Value

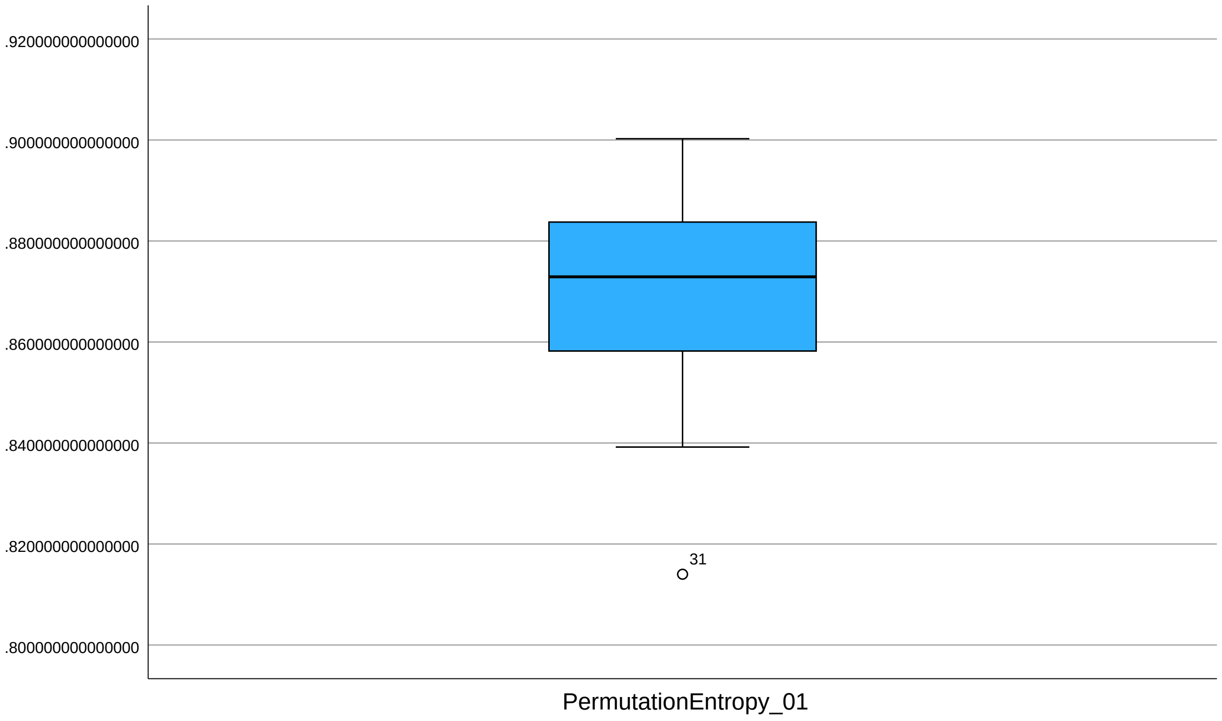

SampleEntropy

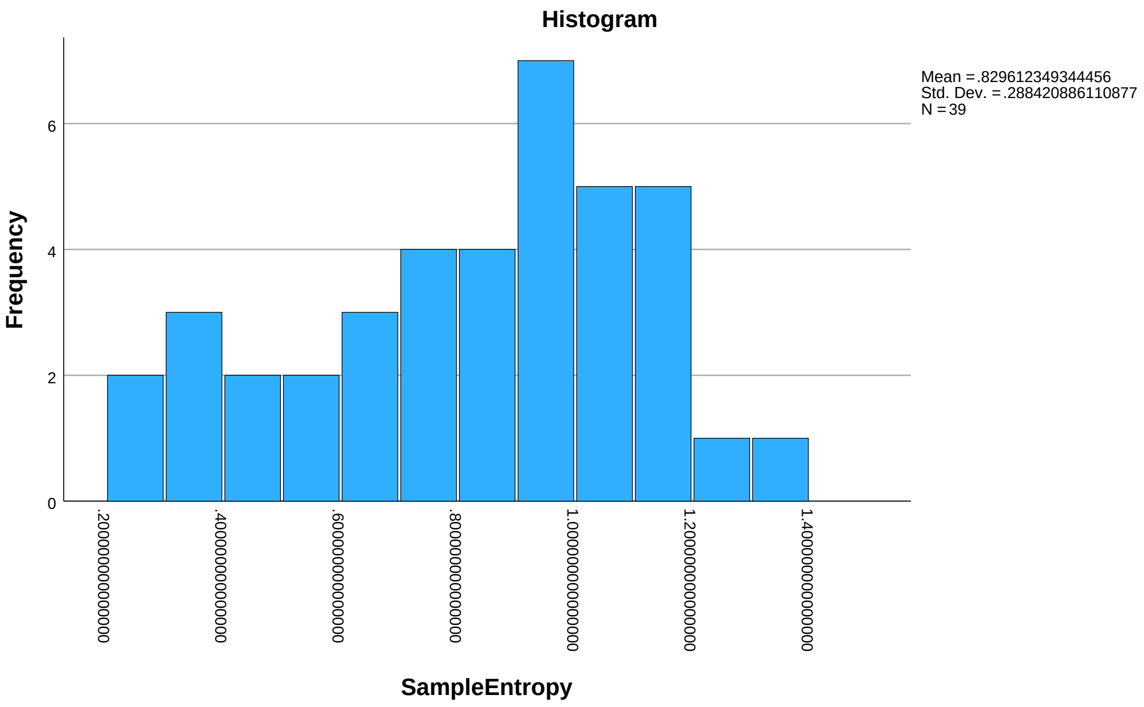


```
SampleEntropy Stem-and-Leaf Plot

 Frequency    Stem &  Leaf
```

```
 2.00        2 .  47
 3.00        3 .  379
 2.00        4 .  78
 2.00        5 .  33
 3.00        6 .  067
 4.00        7 .  4569
 4.00        8 .  4449
 7.00        9 .  0124556
 5.00       10 .  44668
 5.00       11 .  33679
 1.00       12 .  7
 1.00       13 .  0

Stem width:   .1000000
Each leaf:        1 case(s)
```

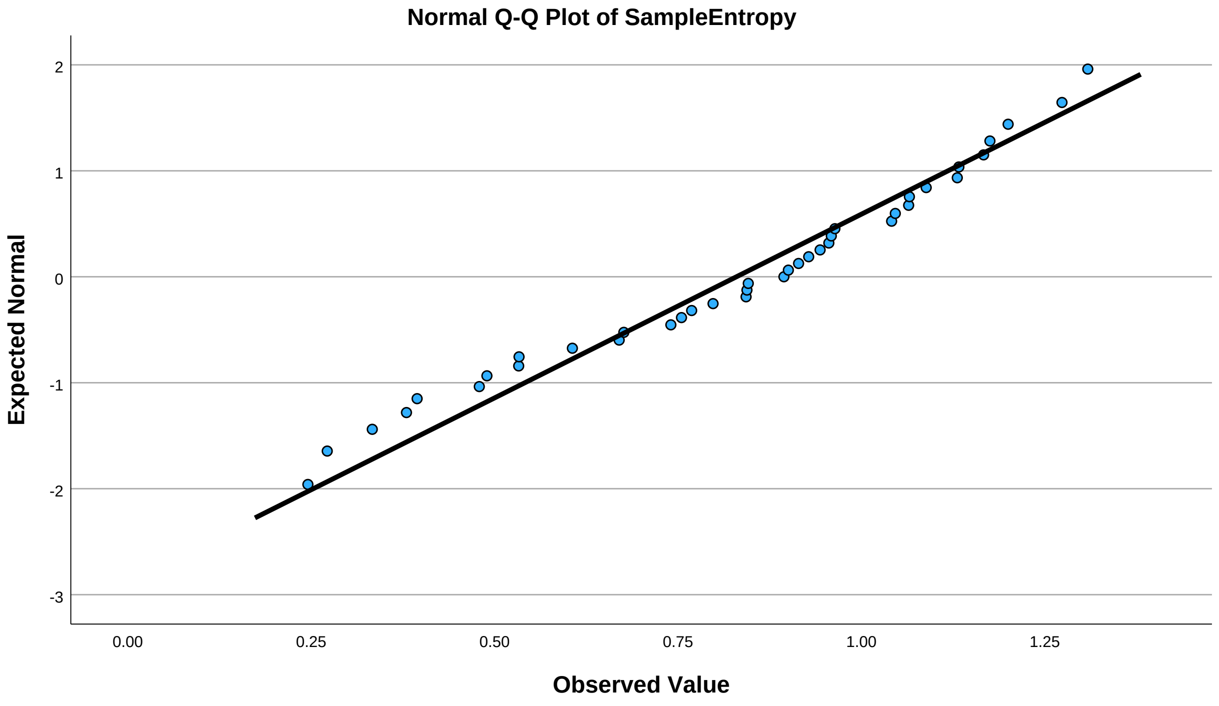

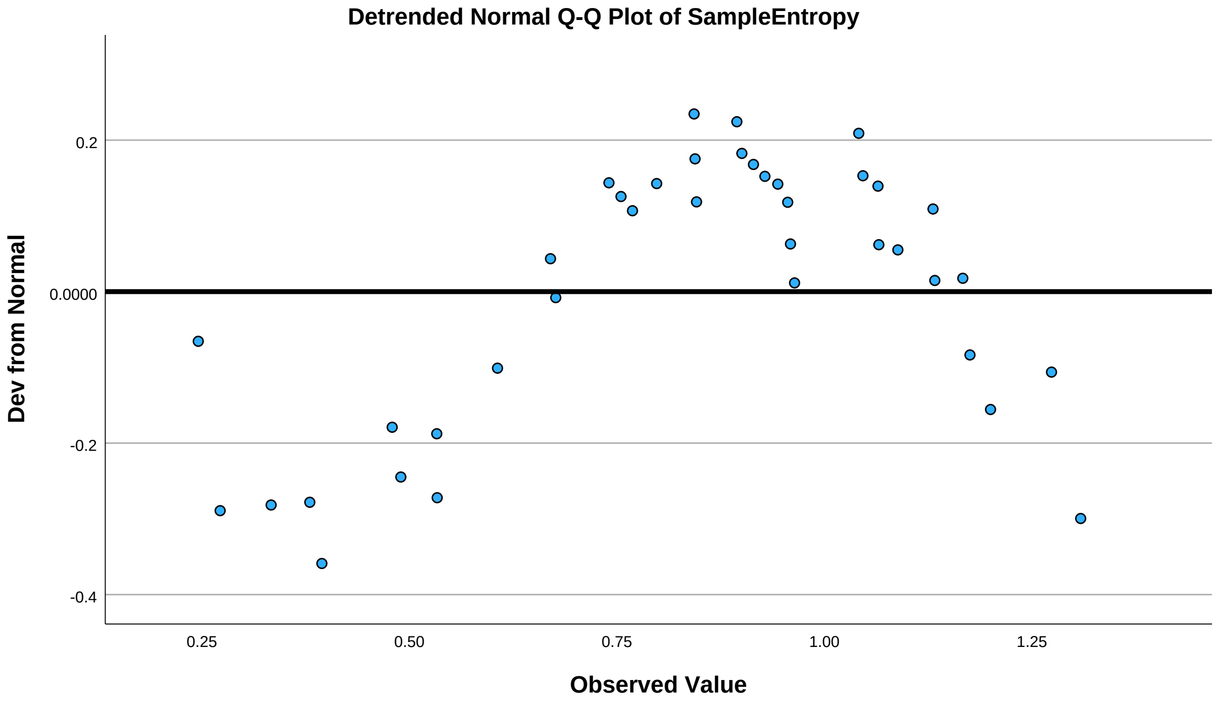

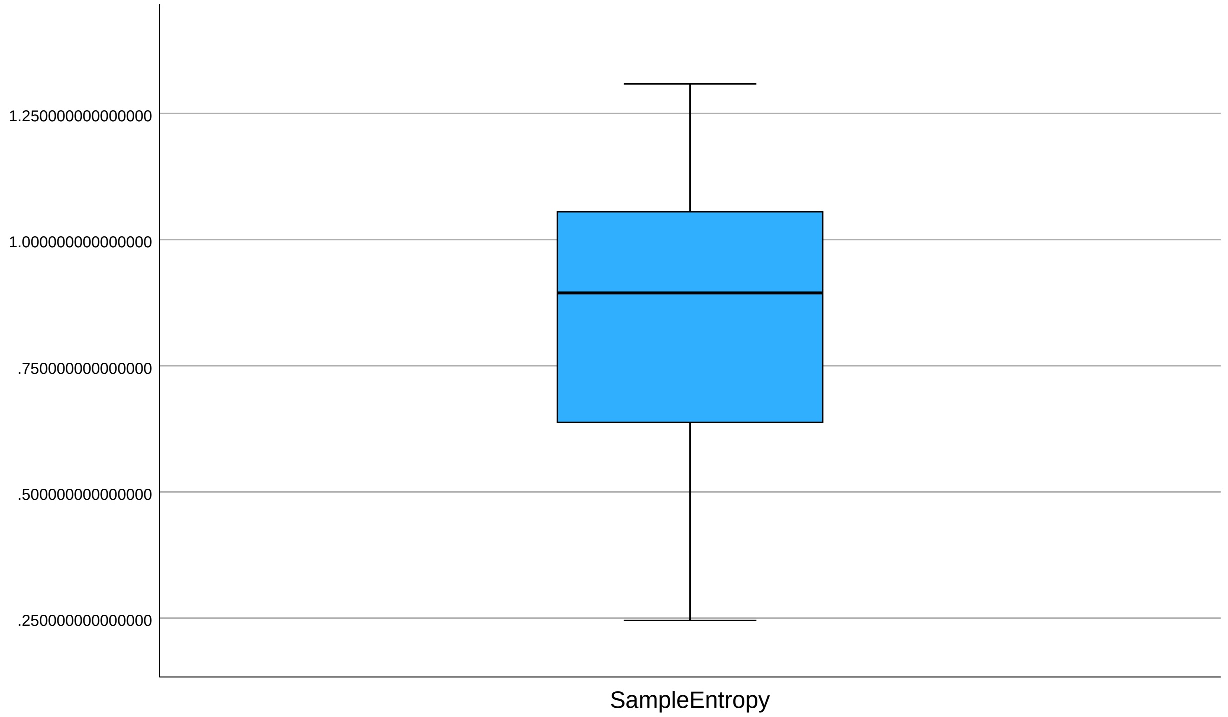

SpectralEntropy_bits

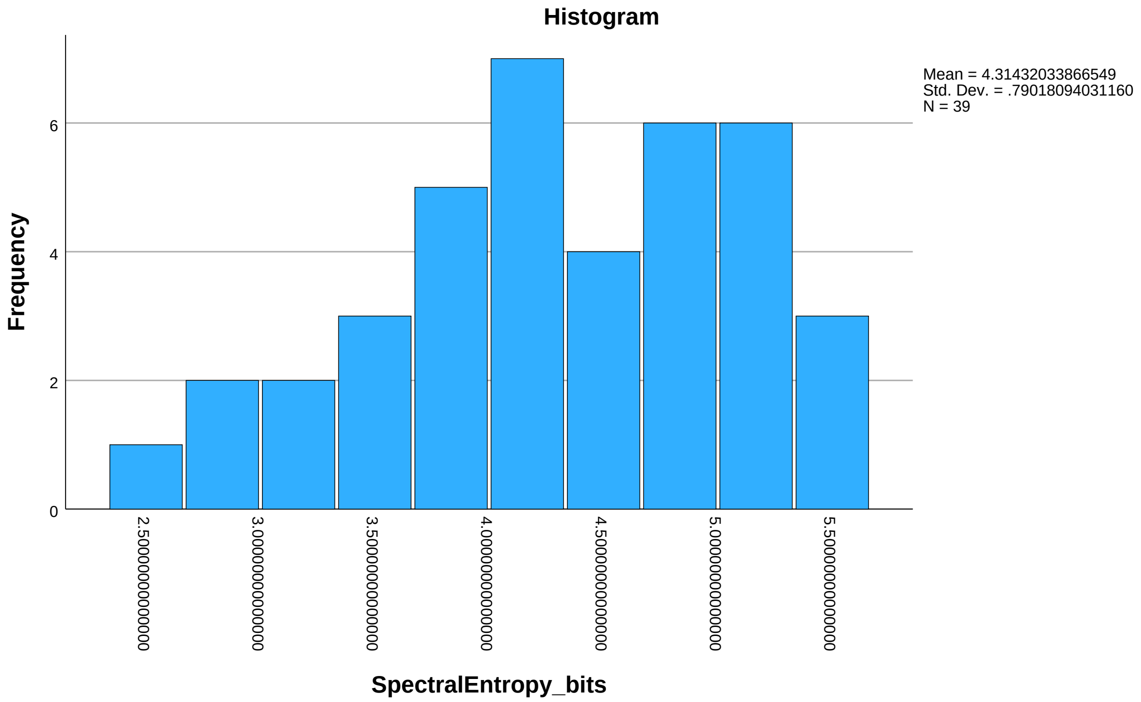

SpectralEntropy_bits Stem-and-Leaf Plot

```
 Frequency    Stem &  Leaf

     3.00        2 .  699
     4.00        3 .  0144
     6.00        3 .  567899
     8.00        4 .  00012334
     9.00        4 .  556677899
     6.00        5 .  000113
     3.00        5 .  555

 Stem width:  1.000000
 Each leaf:       1 case(s)
```

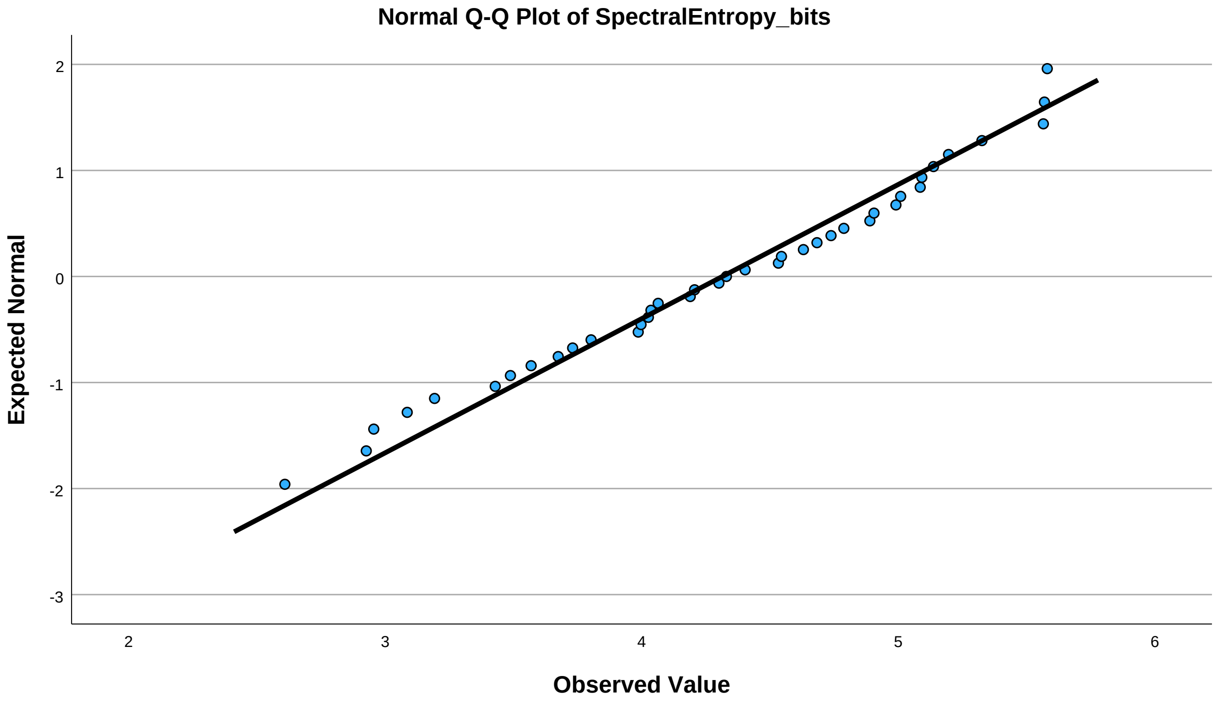
Normal Q-Q Plot of SpectralEntropy_bits
Expected Normal
Observed Value
2
1
0
-1
-2
-3
2
3
4
5
6

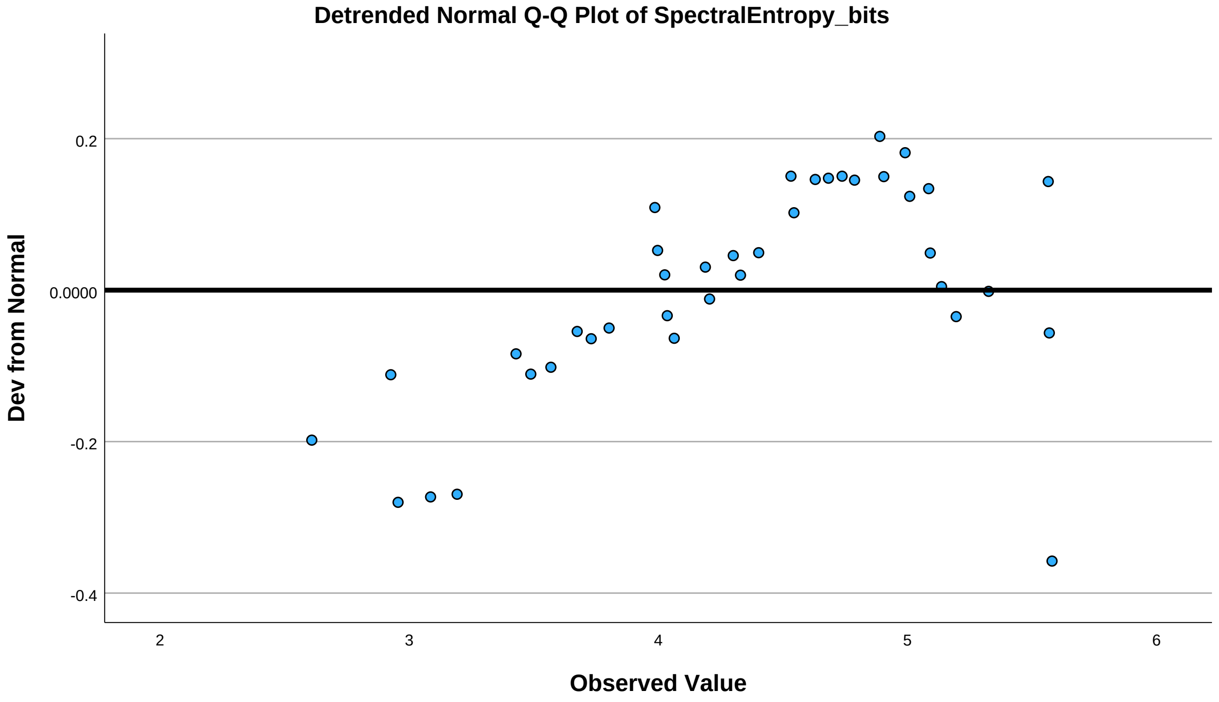
Detrended Normal Q-Q Plot of SpectralEntropy_bits
Dev from Normal
Observed Value
0.2
0.0000
-0.2
-0.4
2
3
4
5
6

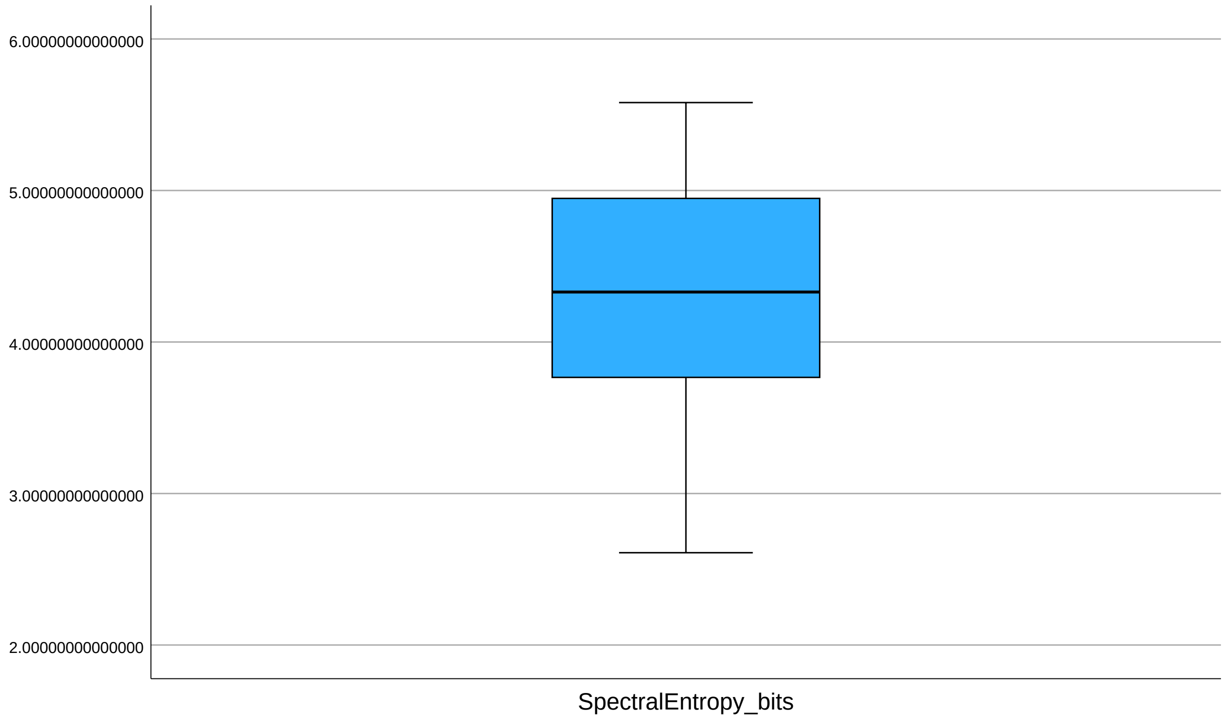

SpectralEntropy_01

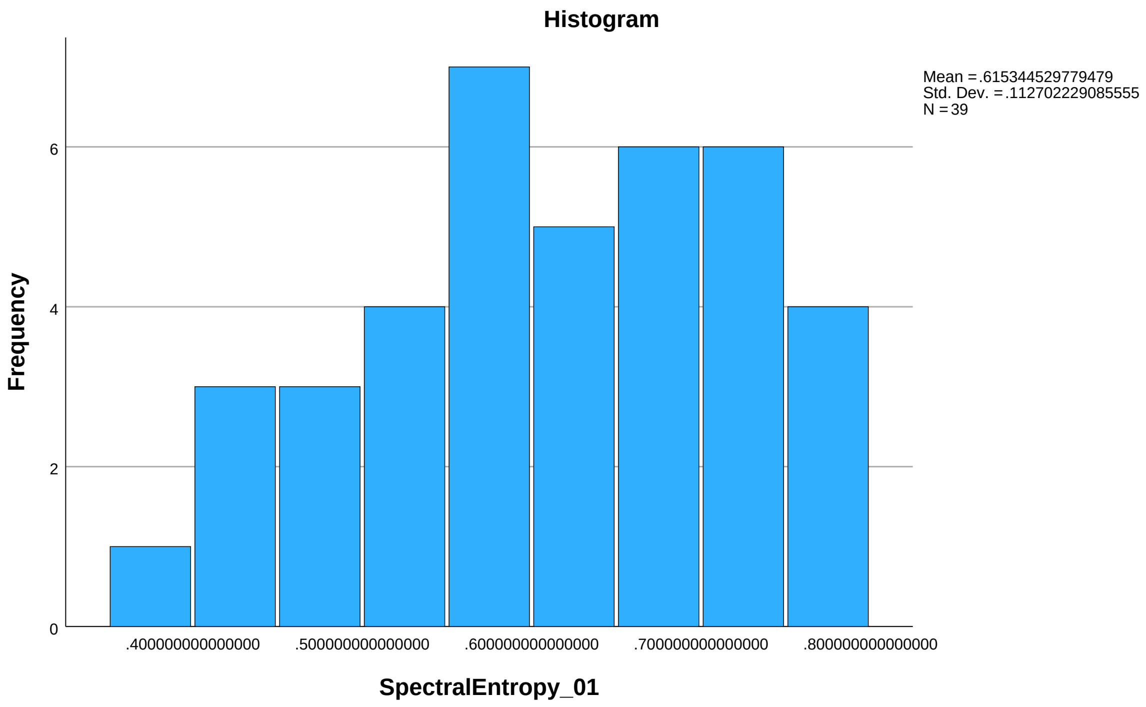


```
SpectralEntropy_01 Stem-and-Leaf Plot

 Frequency    Stem &  Leaf
```

```
 1.00      3 .  7
 3.00      4 .  124
 3.00      4 .  589
 4.00      5 .  0234
 7.00      5 .  6777799
 5.00      6 .  11244
 6.00      6 .  667899
 6.00      7 .  112234
 4.00      7 .  5999

Stem width:   .1000000
Each leaf:        1 case(s)
```

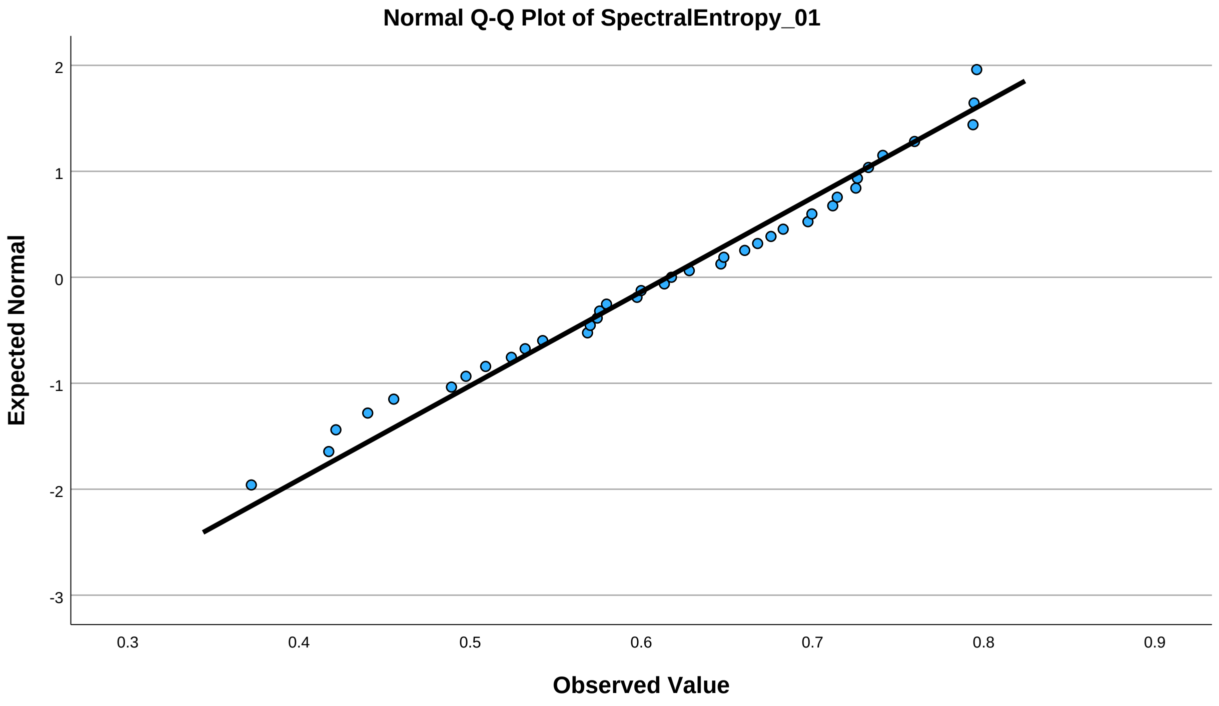

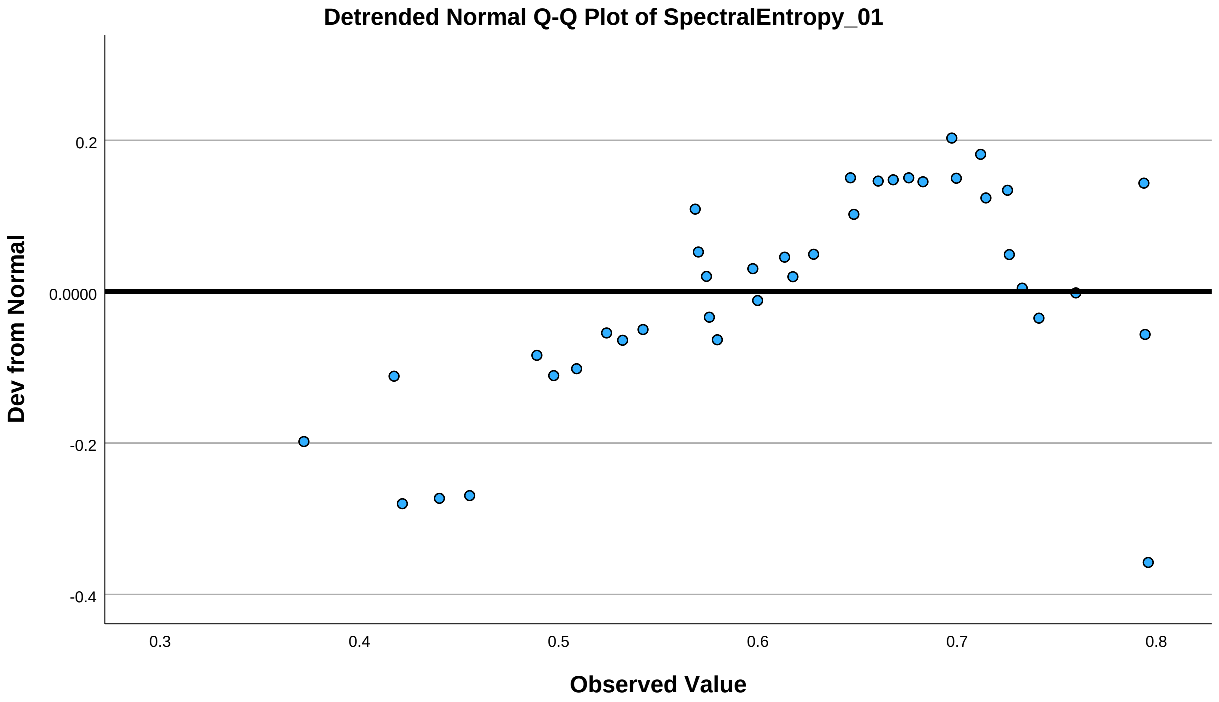

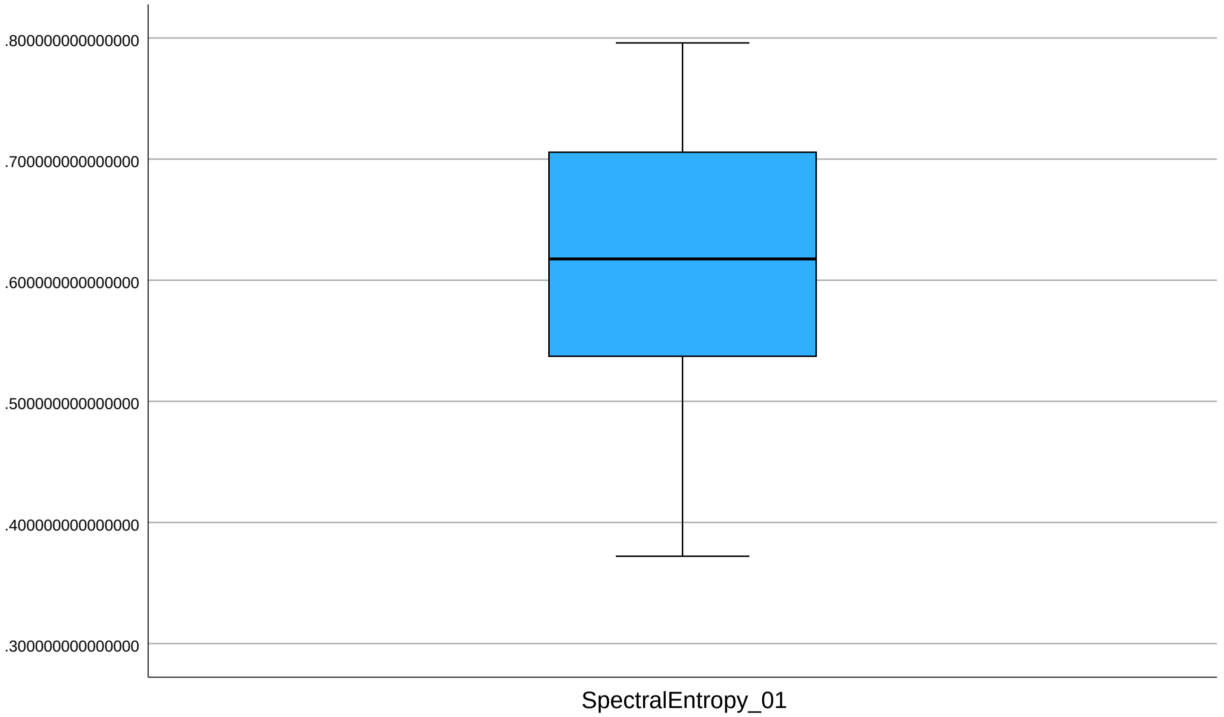

HiguchiFractalDimension

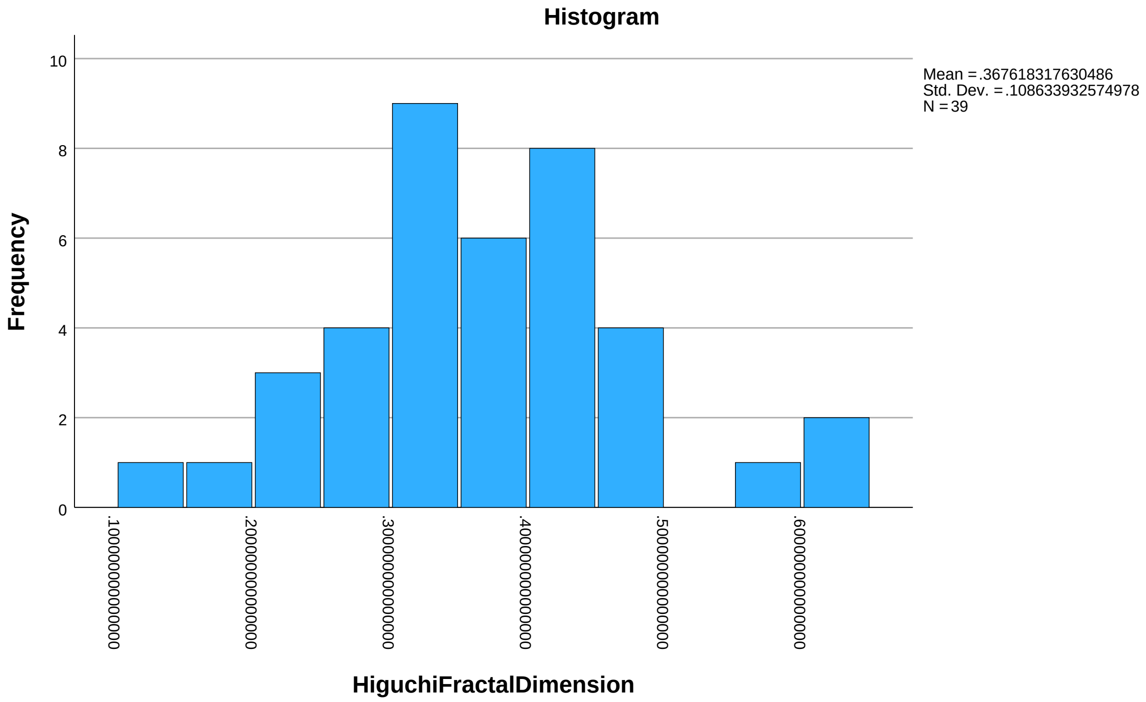


```
HiguchiFractalDimension Stem-and-Leaf Plot

 Frequency    Stem &  Leaf

     1.00        1 .  4
     1.00        1 .  5
     3.00        2 .  134
     4.00        2 .  5567
     9.00        3 .  000333444
     6.00        3 .  578899
     8.00        4 .  00112234
     4.00        4 .  5689
      .00        5 .
     1.00        5 .  9
     2.00        6 .  00

 Stem width:   .1000000
 Each leaf:        1 case(s)
```

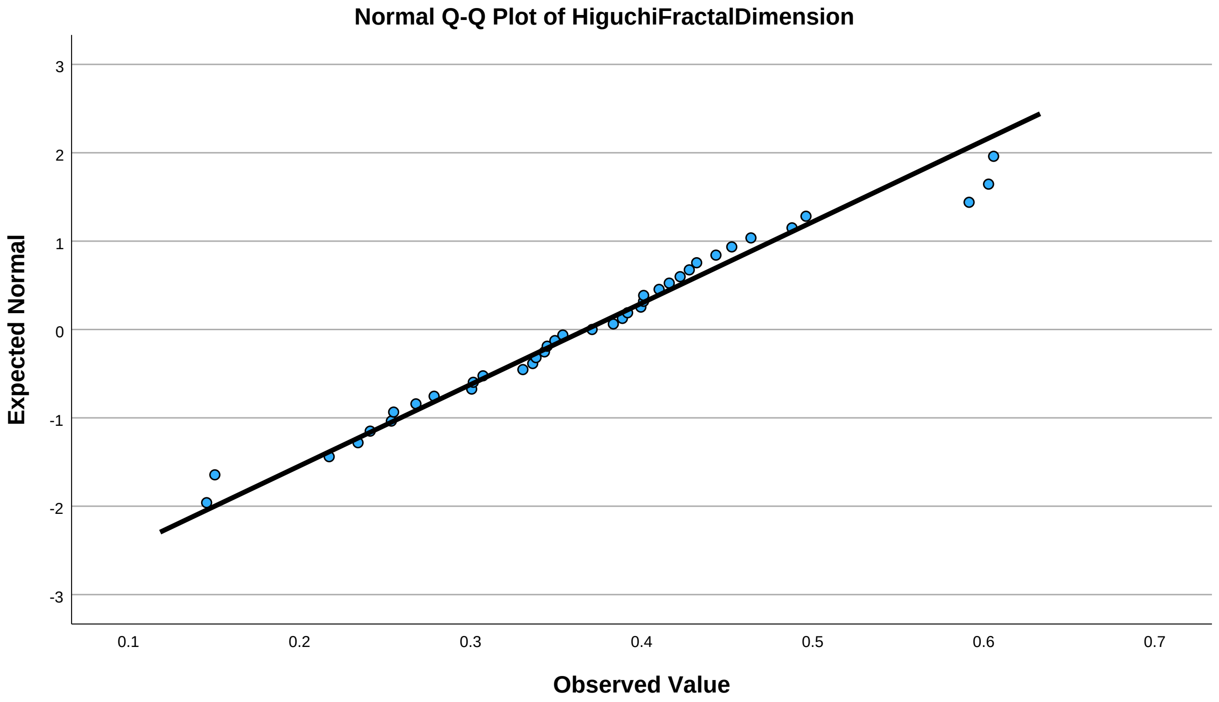
Normal Q-Q Plot of HiguchiFractalDimension
Expected Normal
3
2
1
0
-1
-2
-3
0.1
0.2
0.3
0.4
0.5
0.6
0.7
Observed Value

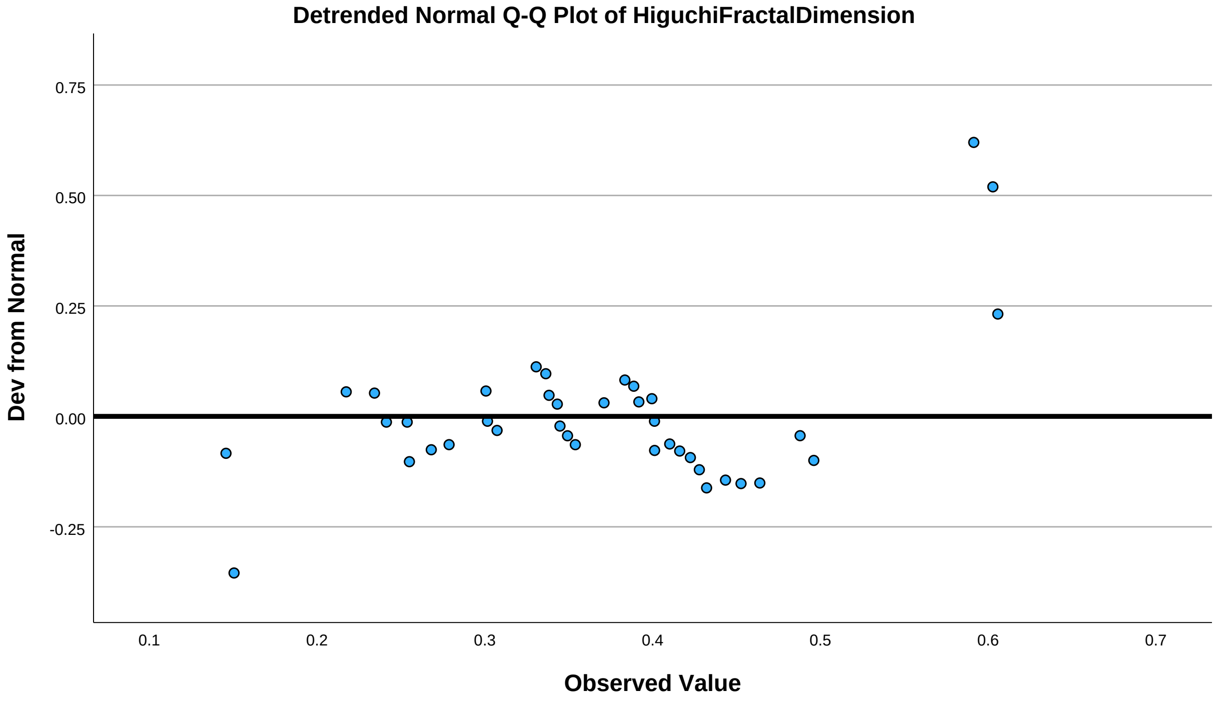
Detrended Normal Q-Q Plot of HiguchiFractalDimension
Dev from Normal
0.75
0.50
0.25
0.00
-0.25
0.1
0.2
0.3
0.4
0.5
0.6
0.7
Observed Value

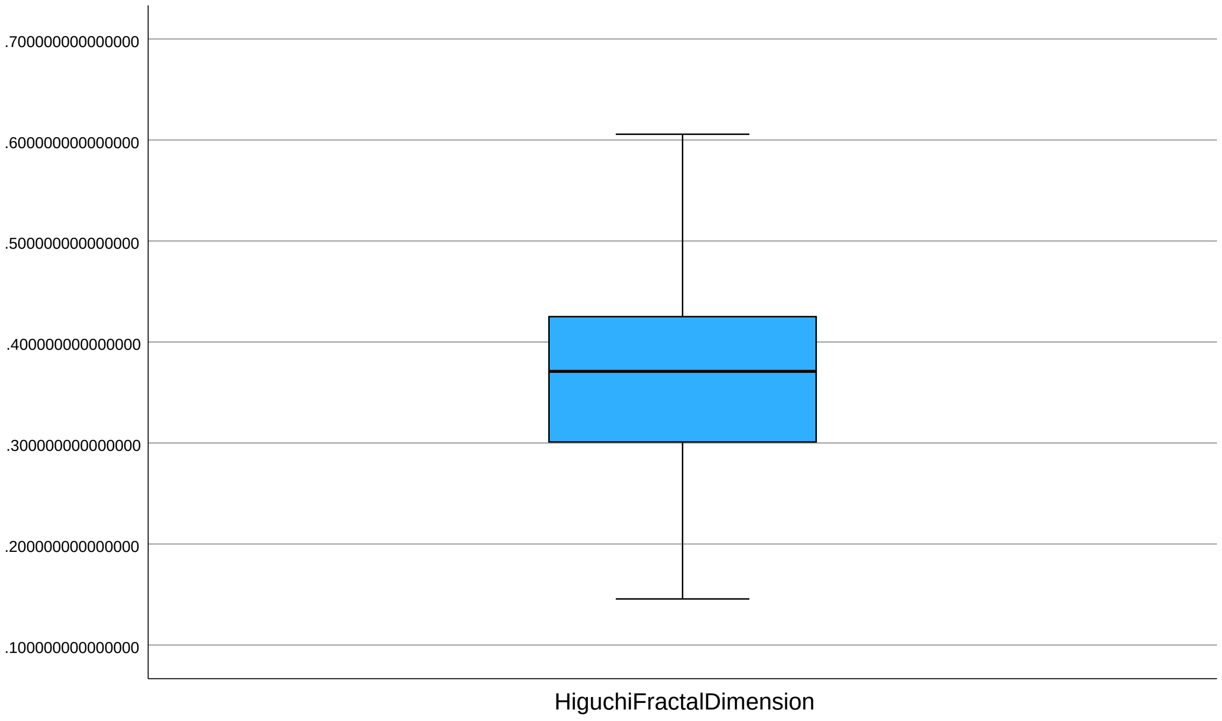

LempelZivComplexity

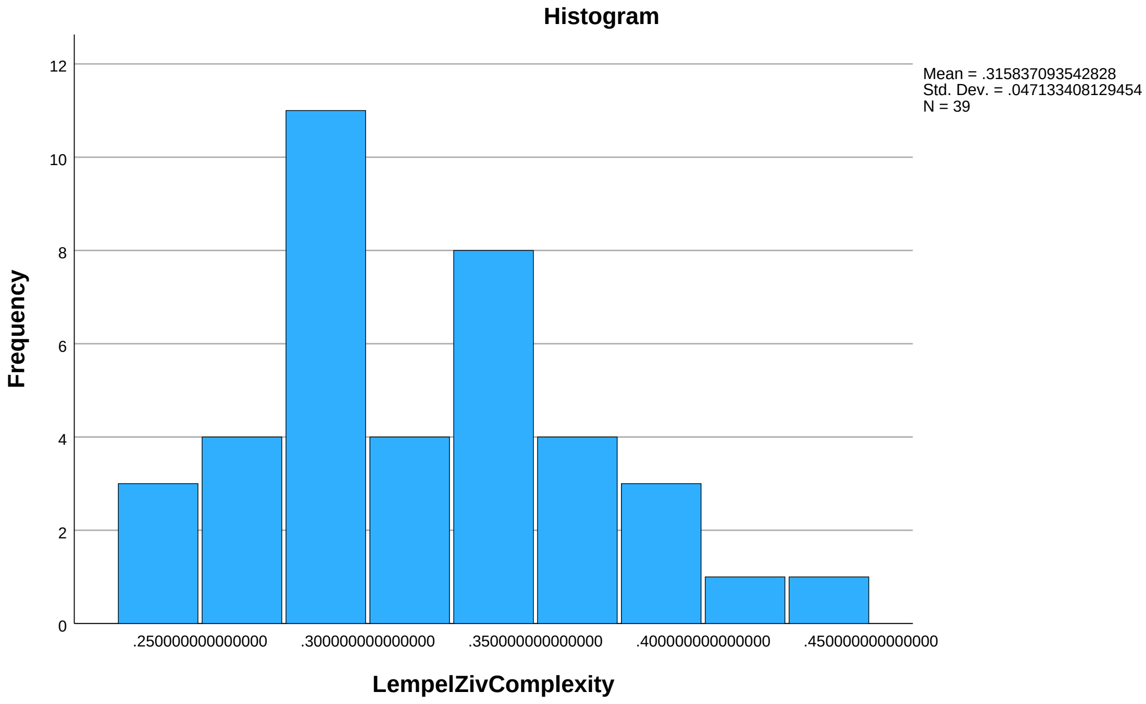


```
LempelZivComplexity Stem-and-Leaf Plot

 Frequency    Stem &  Leaf
```

```
 3.00      2 . 334
15.00      2 . 667777889999999
12.00      3 . 000122333344
 7.00      3 . 5557788
 2.00      4 . 12

Stem width:   .1000000
Each leaf:       1 case(s)
```

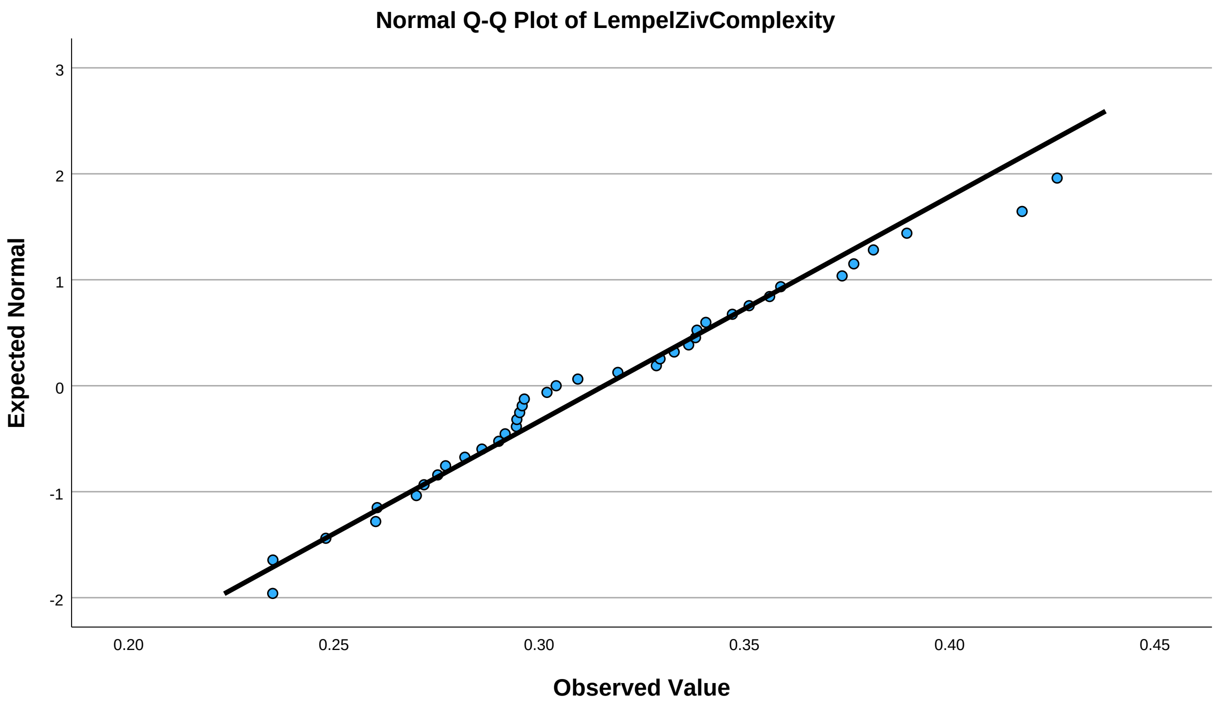

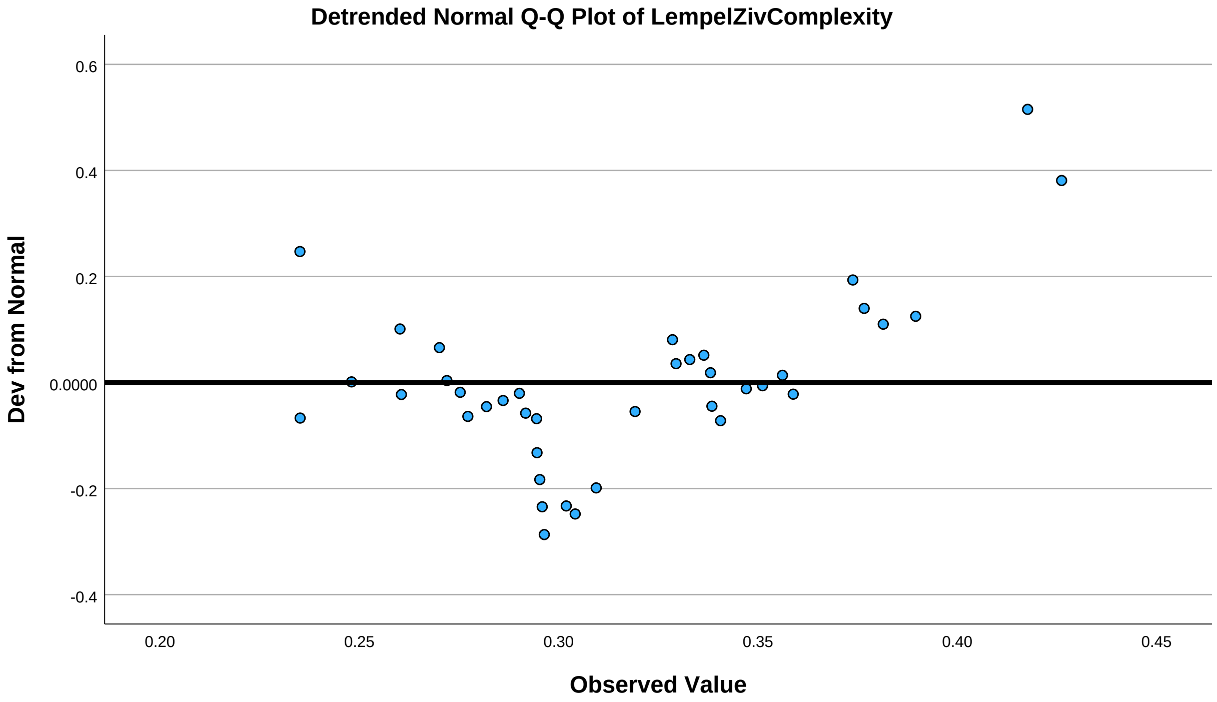

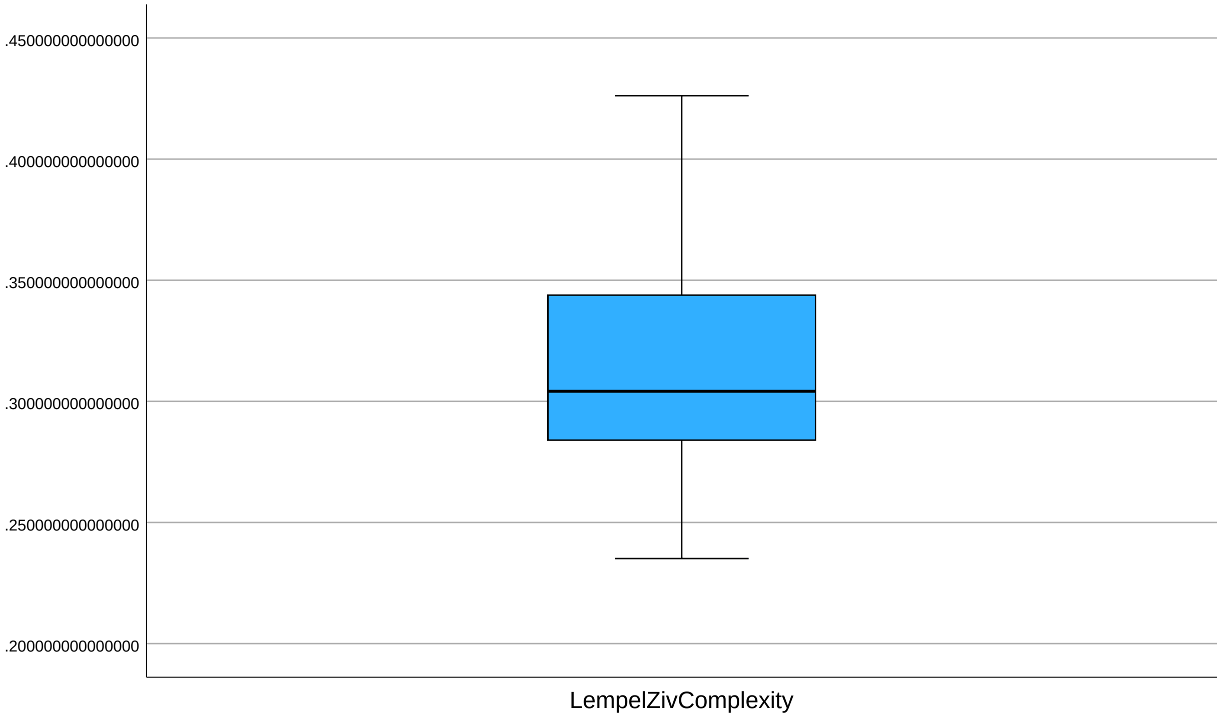

RenyiEntropy

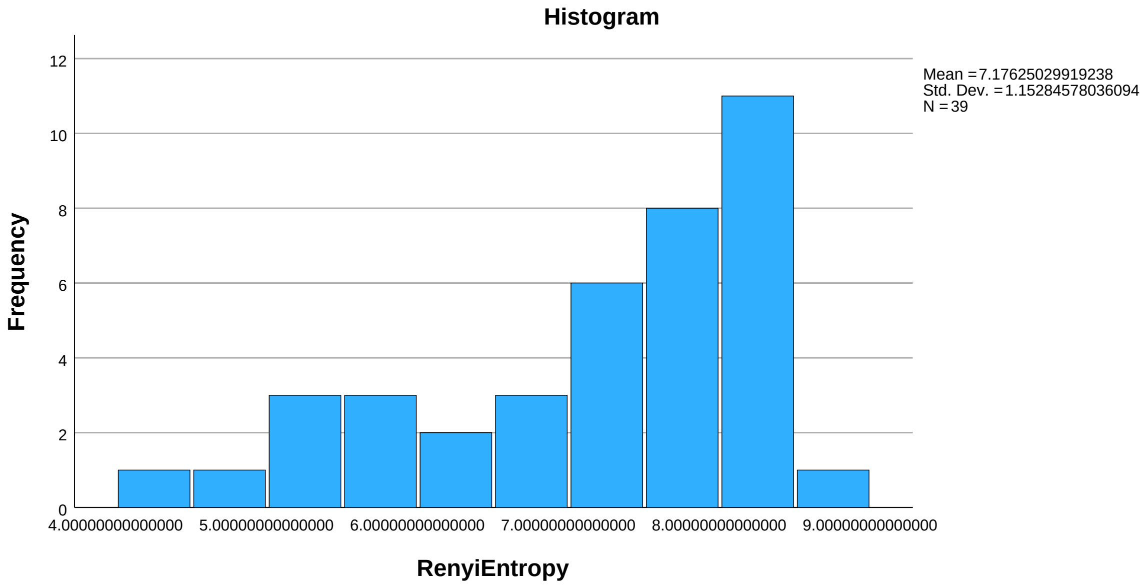


```
RenyiEntropy Stem-and-Leaf Plot

 Frequency    Stem &  Leaf

     1.00        4 .  4
     1.00        4 .  9
     3.00        5 .  134
     3.00        5 .  568
     2.00        6 .  11
     3.00        6 .  889
     6.00        7 .  012234
     8.00        7 .  55567899
    11.00        8 .  00011223444
     1.00        8 .  7

 Stem width:  1.000000
 Each leaf:       1 case(s)
```

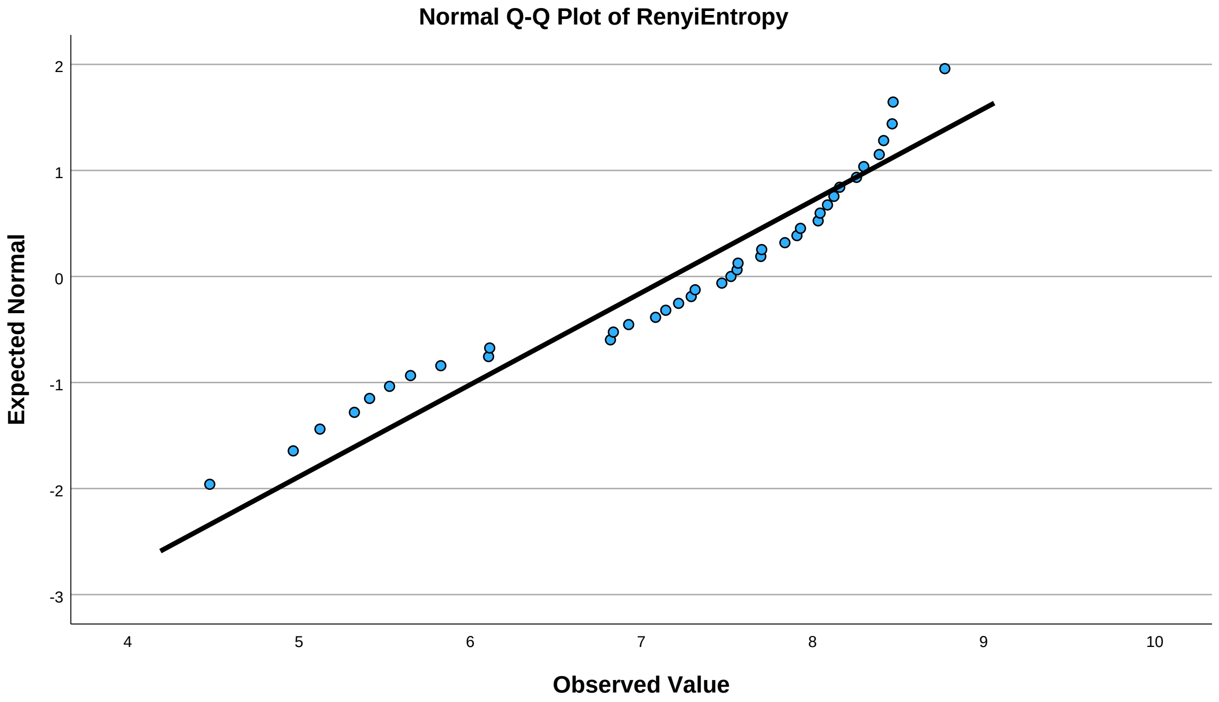
Normal Q-Q Plot of RenyiEntropy
Expected Normal
2
1
0
-1
-2
-3
4
5
6
7
8
9
10
Observed Value

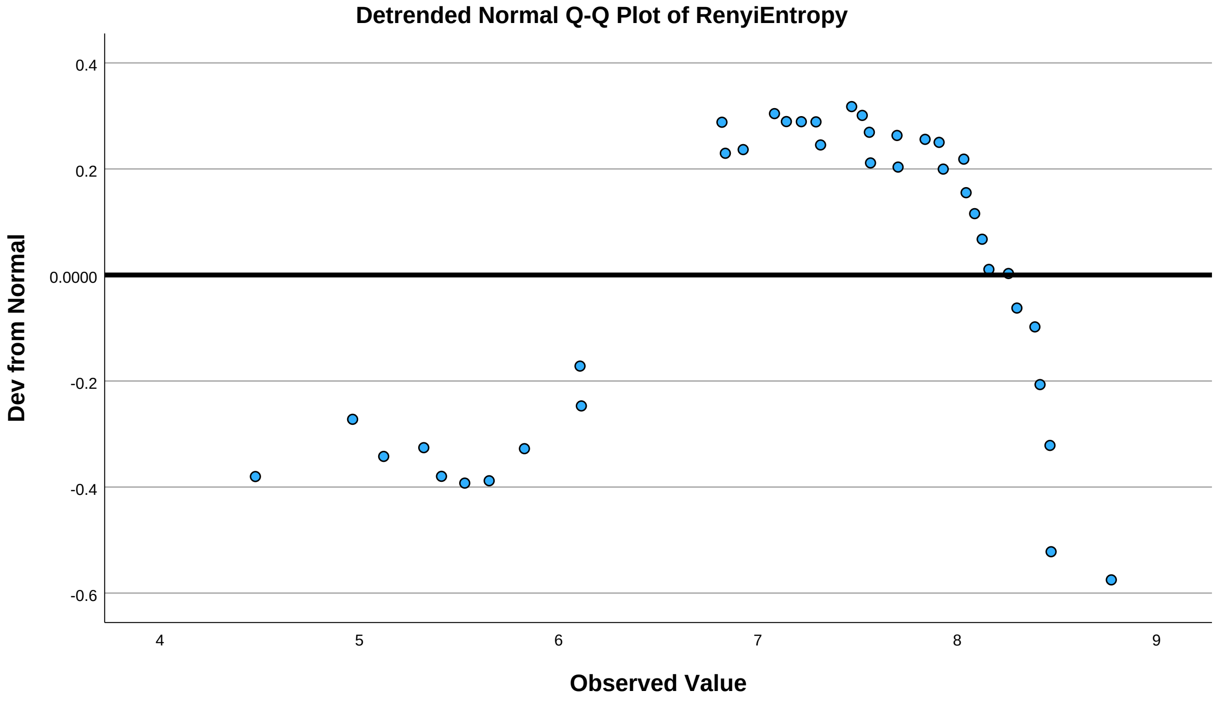
Detrended Normal Q-Q Plot of RenyiEntropy
Dev from Normal
0.4
0.2
0.0000
-0.2
-0.4
-0.6
4
5
6
7
8
9
Observed Value

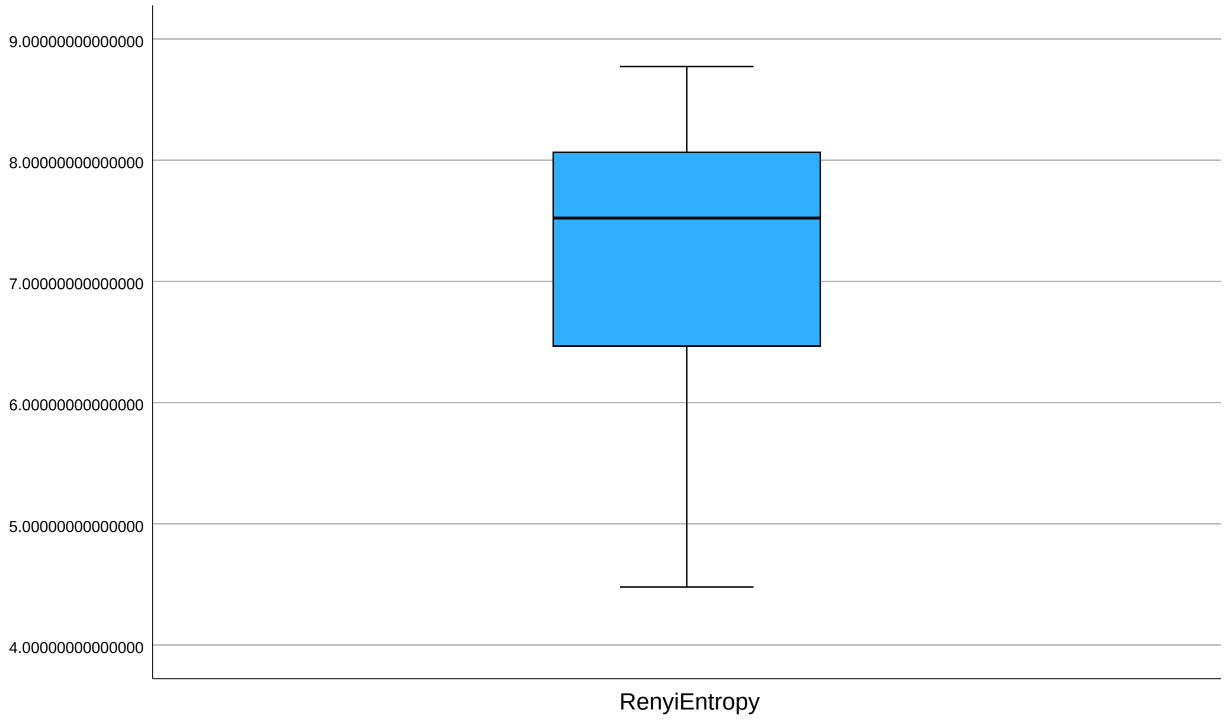

TsallisEntropy

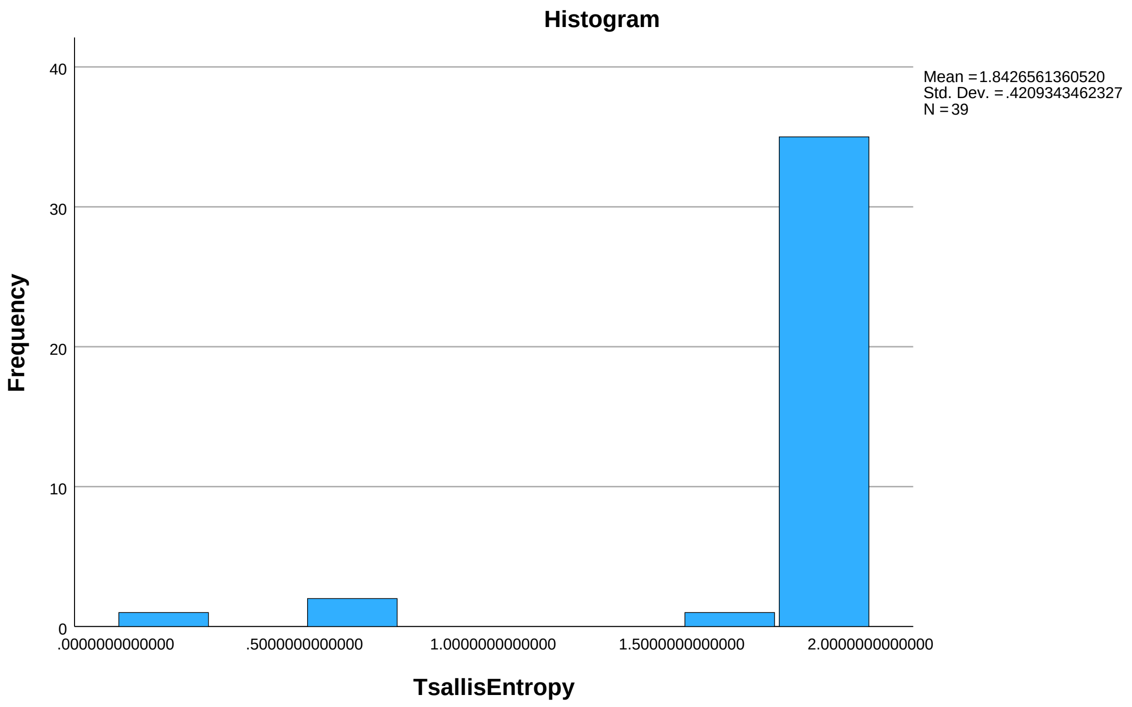


```
TsallisEntropy Stem-and-Leaf Plot

 Frequency    Stem &  Leaf
```

```
 7.00 Extremes    (=<1.904)
 1.00     194 .  2
  .00     194 .
 1.00     195 .  1
  .00     195 .
 1.00     196 .  4
 1.00     196 .  8
 1.00     197 .  4
 4.00     197 .  6899
 7.00     198 .  0224444
16.00     198 .  5555555666778888

Stem width:   .0100000
Each leaf:        1 case(s)
```

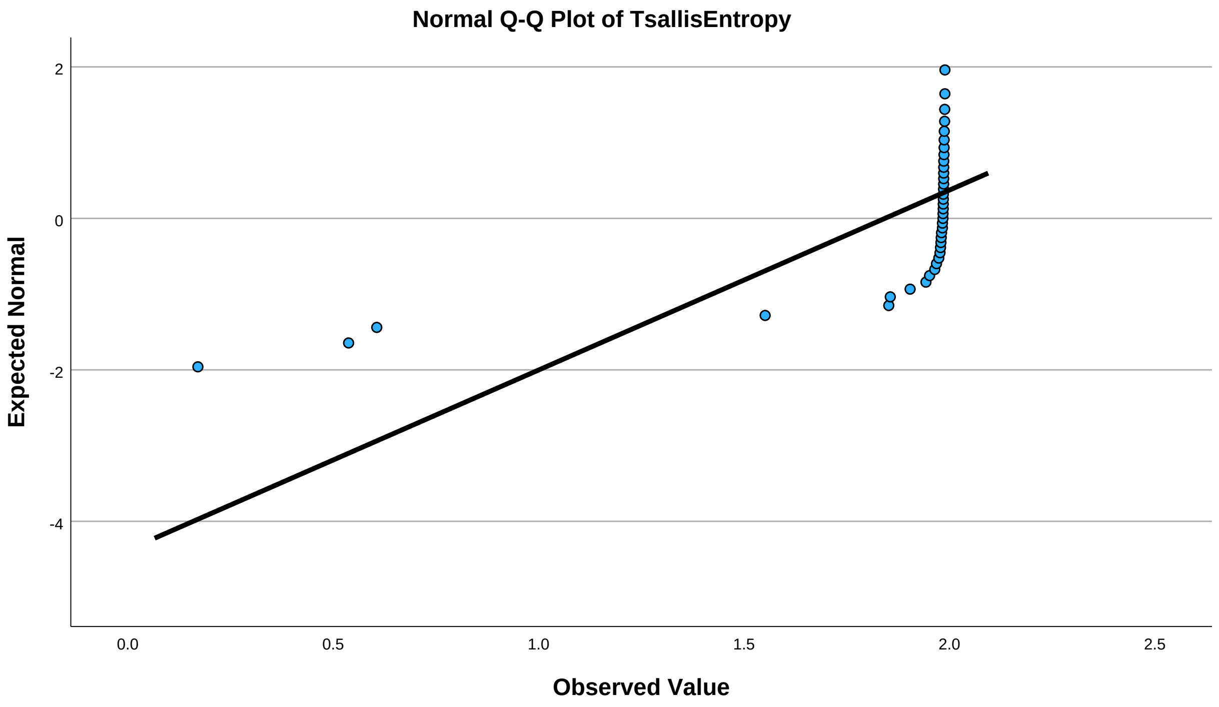

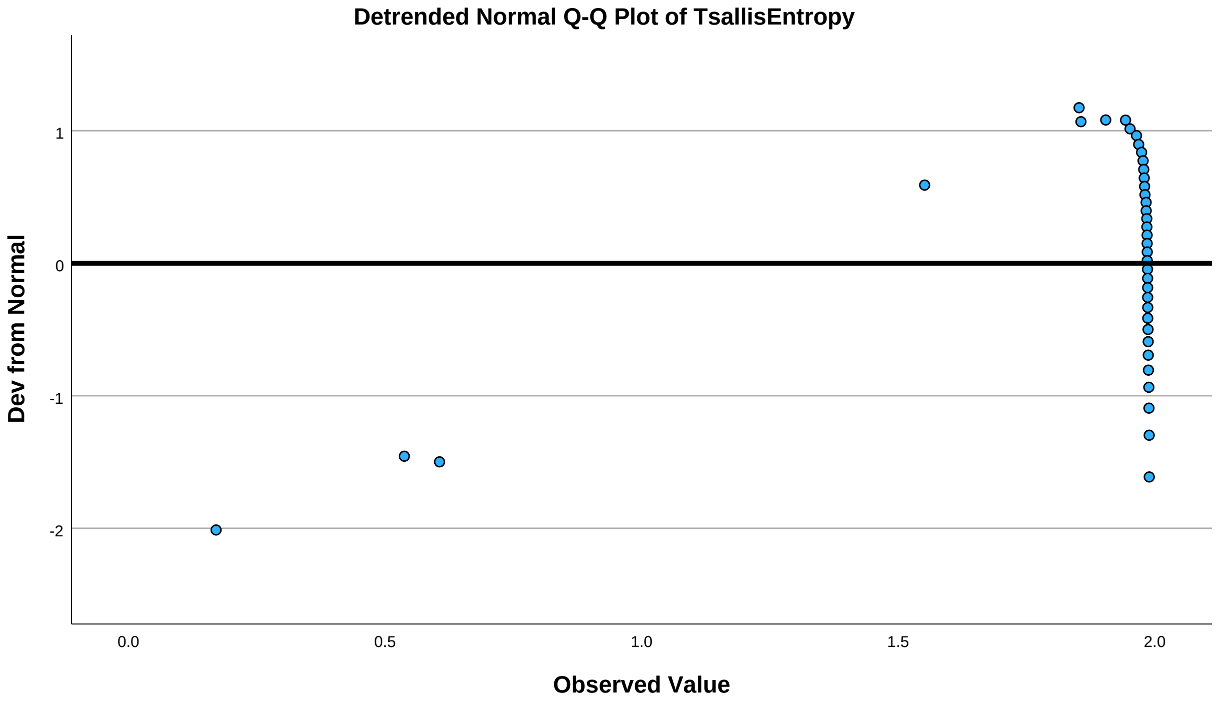
Detrended Normal Q-Q Plot of TsallisEntropy
Dev from Normal
1
0
-1
-2
0.0
0.5
1.0
1.5
2.0
Observed Value

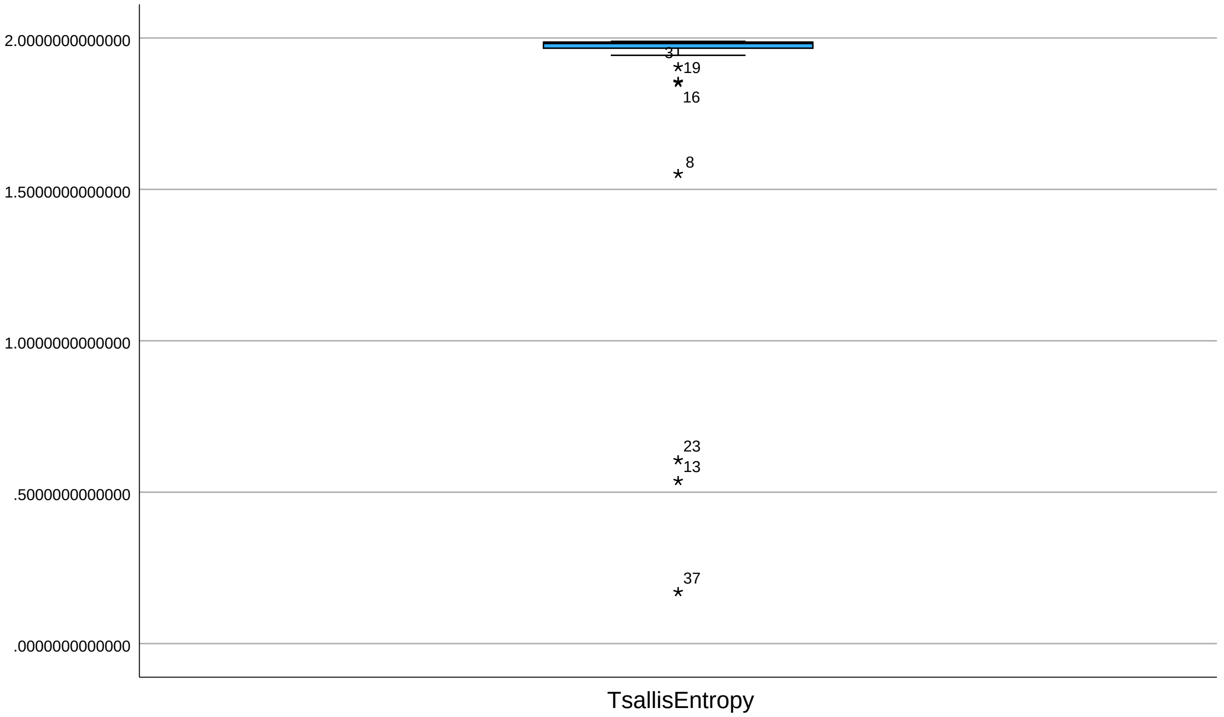
2.0000000000000
1.5000000000000
1.0000000000000
.5000000000000
.0000000000000
31
19
16
8
23
13
37
TsallisEntropy

PS_4_5

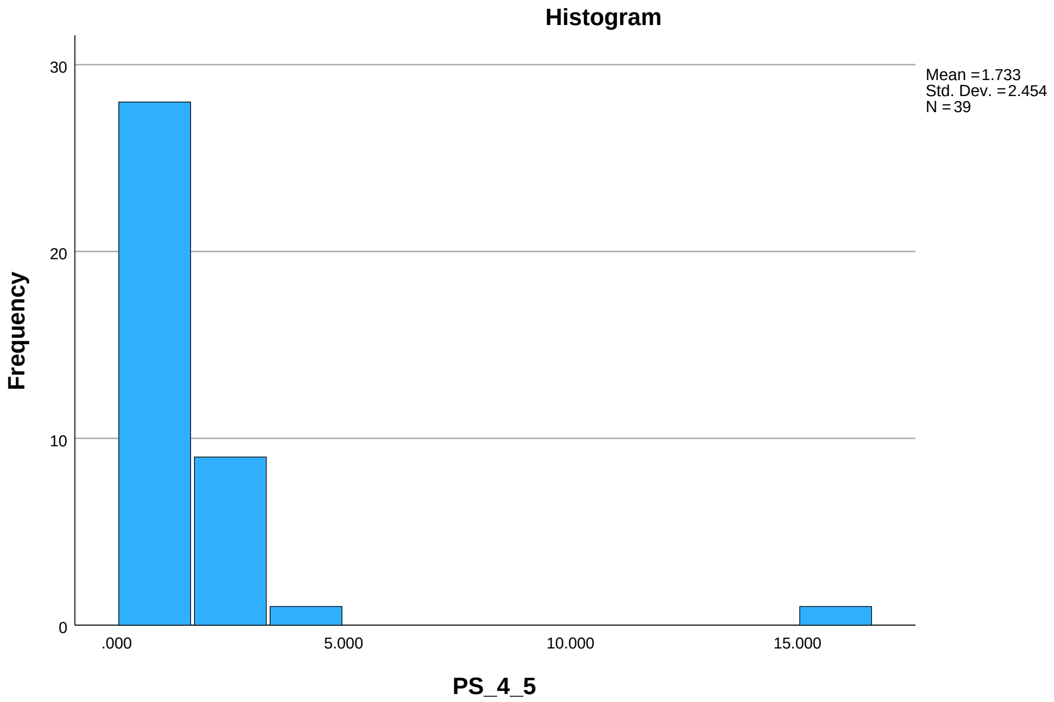


```
PS_4_5 Stem-and-Leaf Plot

 Frequency    Stem &  Leaf

     5.00        0 .  24444
     9.00        0 .  556778999
    12.00        1 .  000111223334
     4.00        1 .  5678
     3.00        2 .  034
     4.00        2 .  5789
     2.00 Extremes    (>=3.7)

 Stem width:      1.000
 Each leaf:       1 case(s)
```

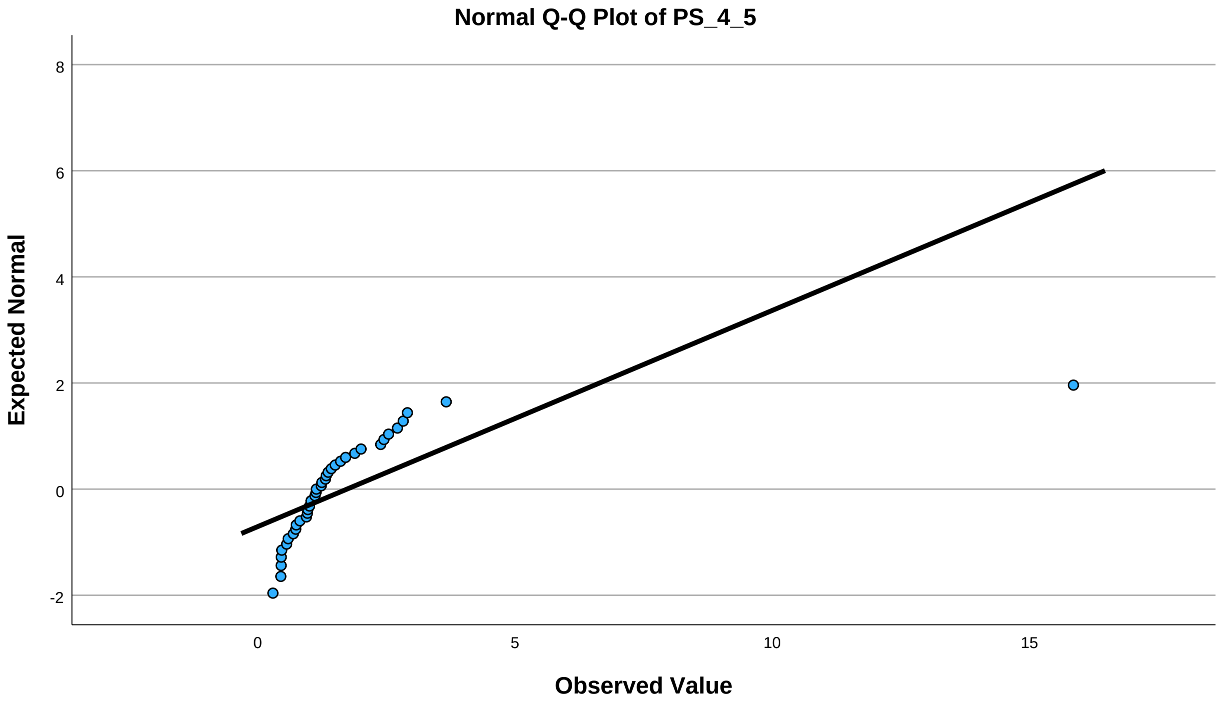
Normal Q-Q Plot of PS_4_5
Expected Normal
8
6
4
2
0
-2
0
5
10
15
Observed Value

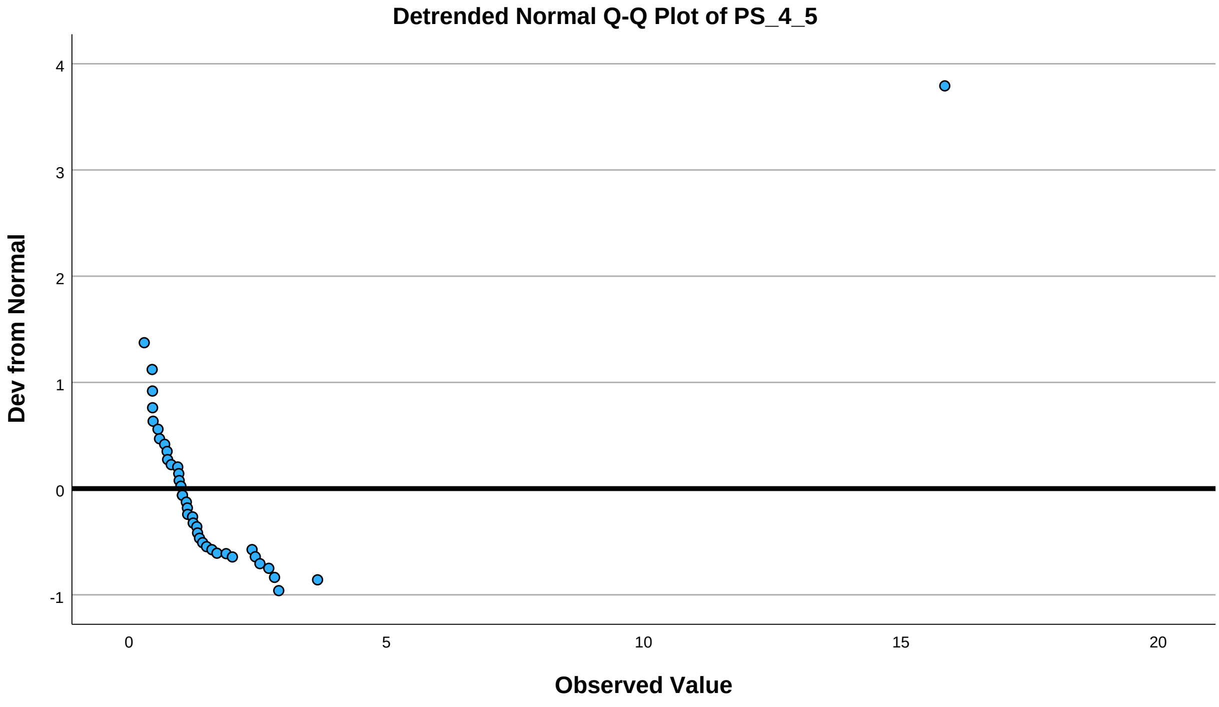
Detrended Normal Q-Q Plot of PS_4_5
Dev from Normal
4
3
2
1
0
-1
0
5
10
15
20
Observed Value

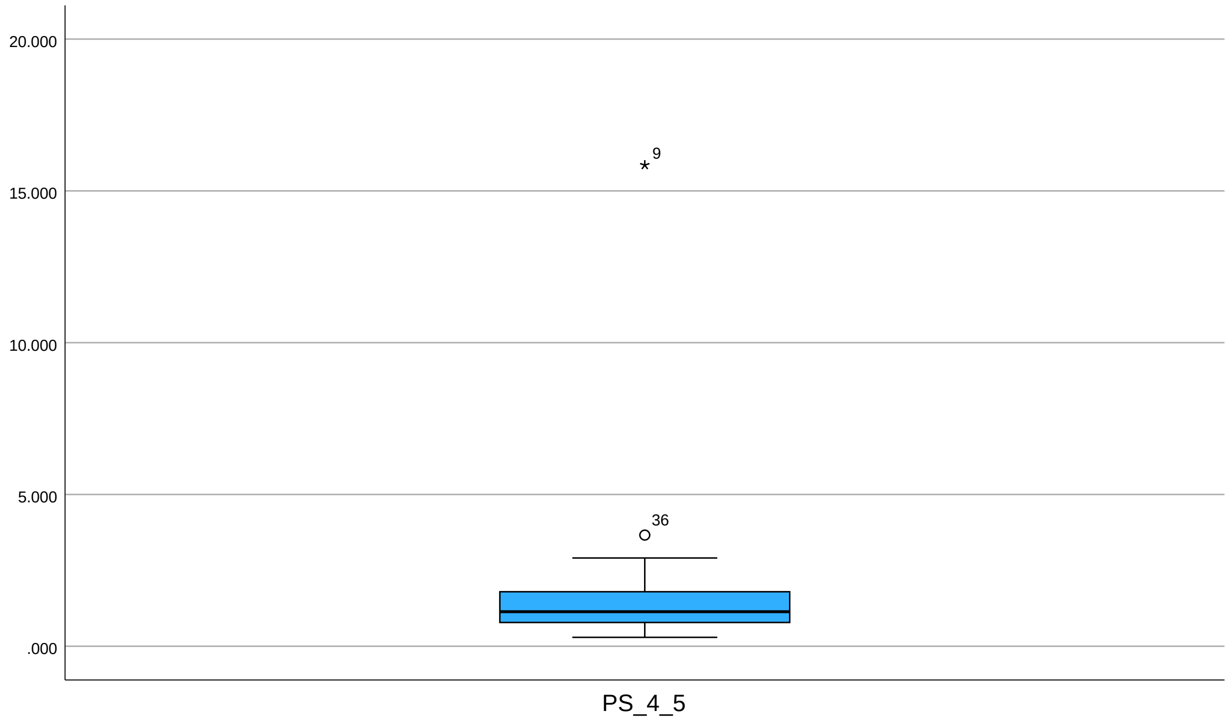

PS_5_6

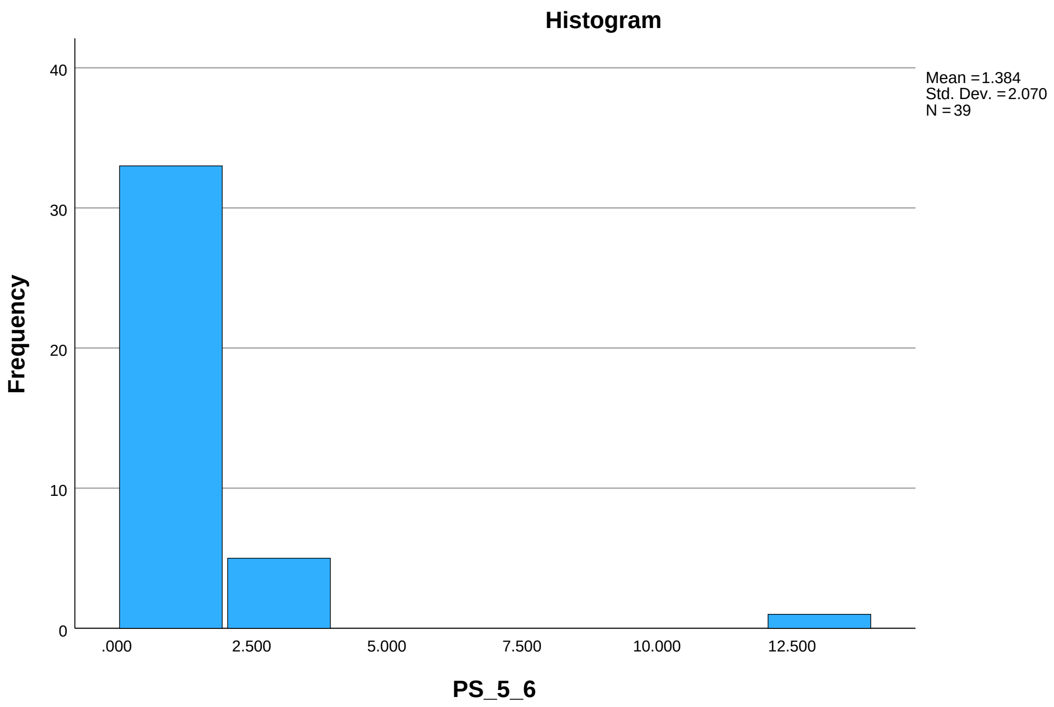


```
PS_5_6 Stem-and-Leaf Plot

 Frequency    Stem &  Leaf
```

```
     5.00        0 .  22333
    20.00        0 .  55566777788889999999
     5.00        1 .  12222
     3.00        1 .  588
     2.00        2 .  00
     4.00 Extremes    (>=2.3)

 Stem width:      1.000
 Each leaf:       1 case(s)
```

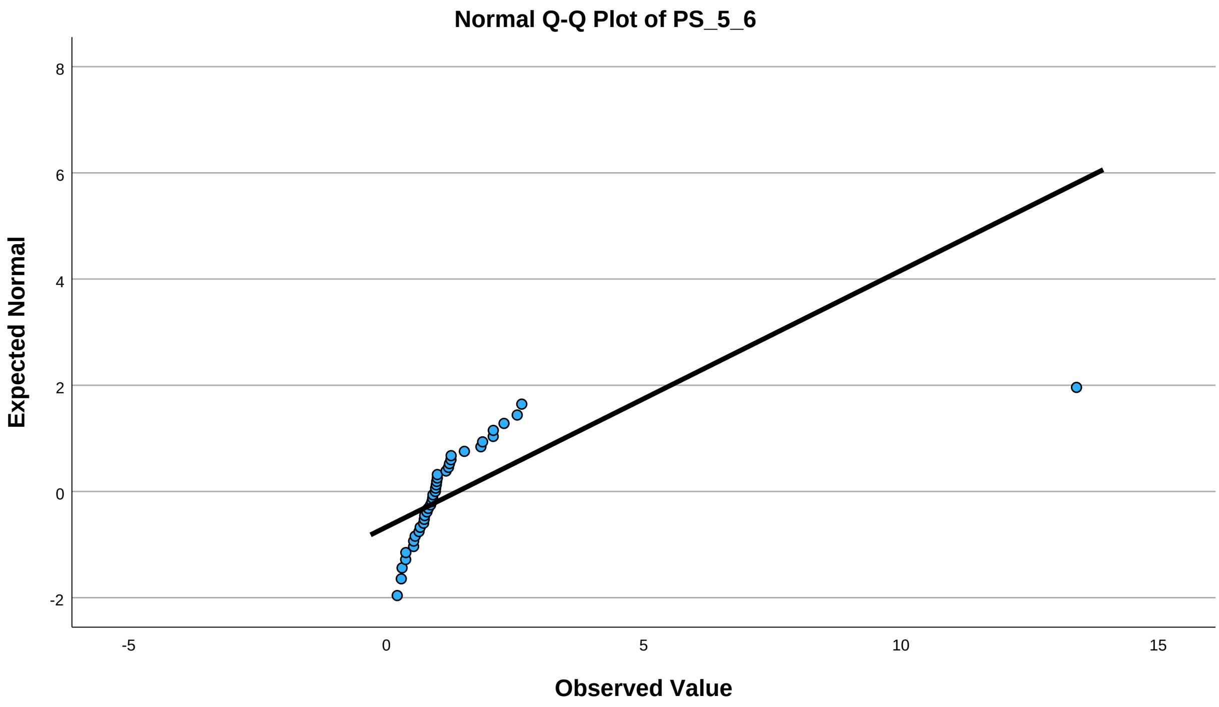

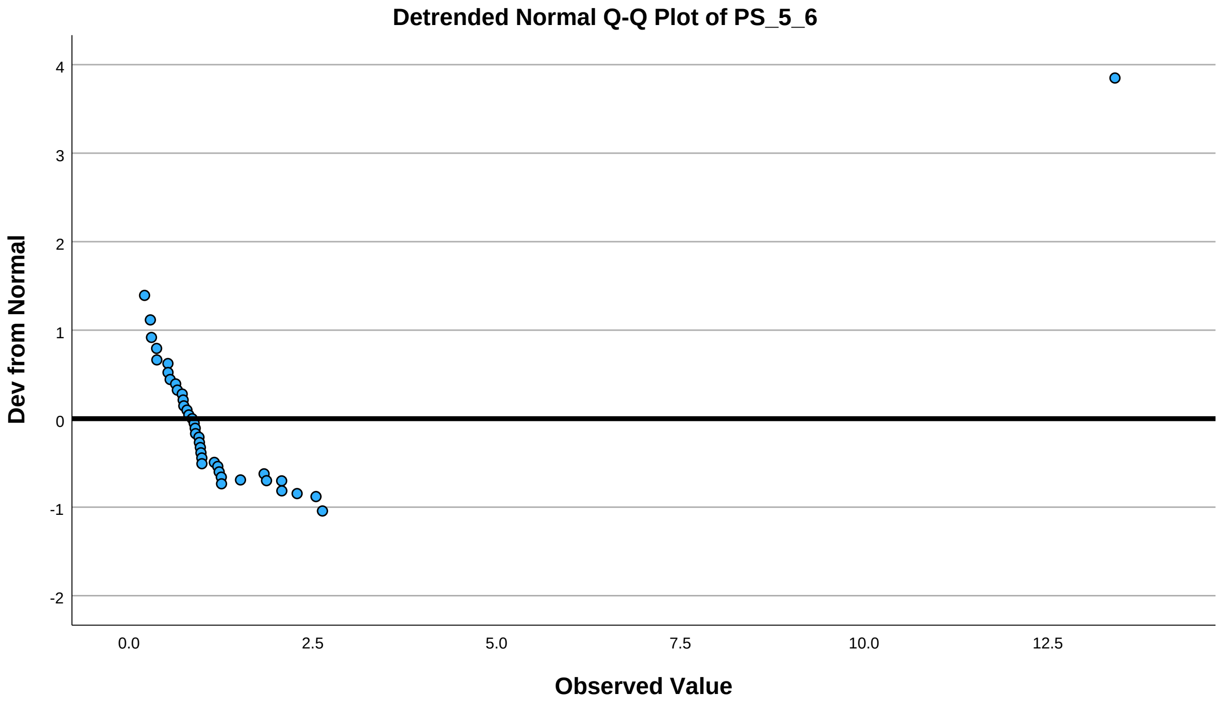
Detrended Normal Q-Q Plot of PS_5_6
Dev from Normal
4
3
2
1
0
-1
-2
0.0
2.5
5.0
7.5
10.0
12.5
Observed Value

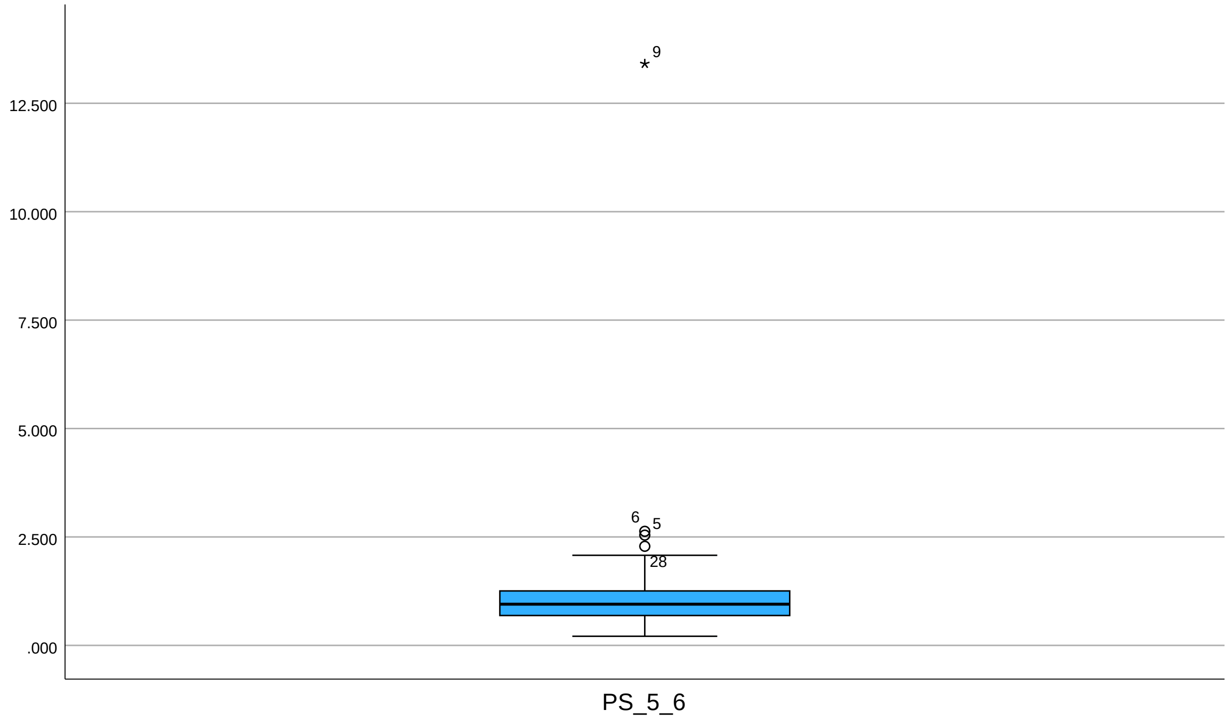
12.500
10.000
7.500
5.000
2.500
.000
9
6
5
28
PS_5_6

PS_6_7

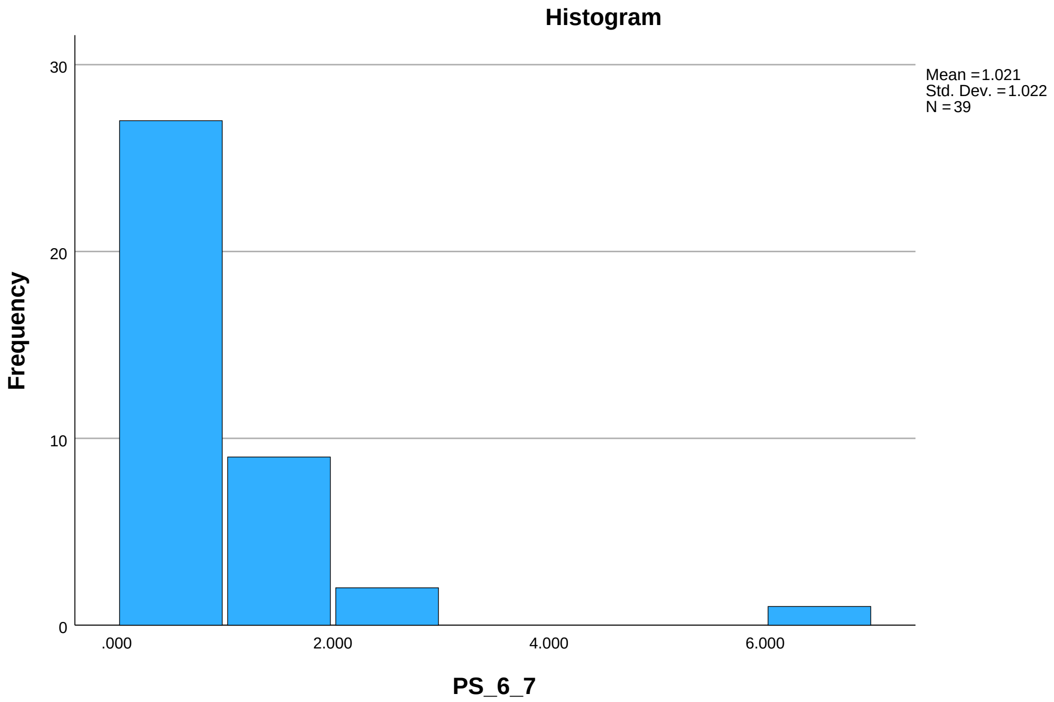


```
PS_6_7 Stem-and-Leaf Plot

 Frequency    Stem &  Leaf

     1.00        0 .  1
     4.00        0 .  2223
     7.00        0 .  4445555
     7.00        0 .  6666777
     8.00        0 .  88889999
     3.00        1 .  000
     3.00        1 .  233
     1.00        1 .  4
     1.00        1 .  7
     4.00 Extremes    (>=1.9)

 Stem width:      1.000
 Each leaf:       1 case(s)
```

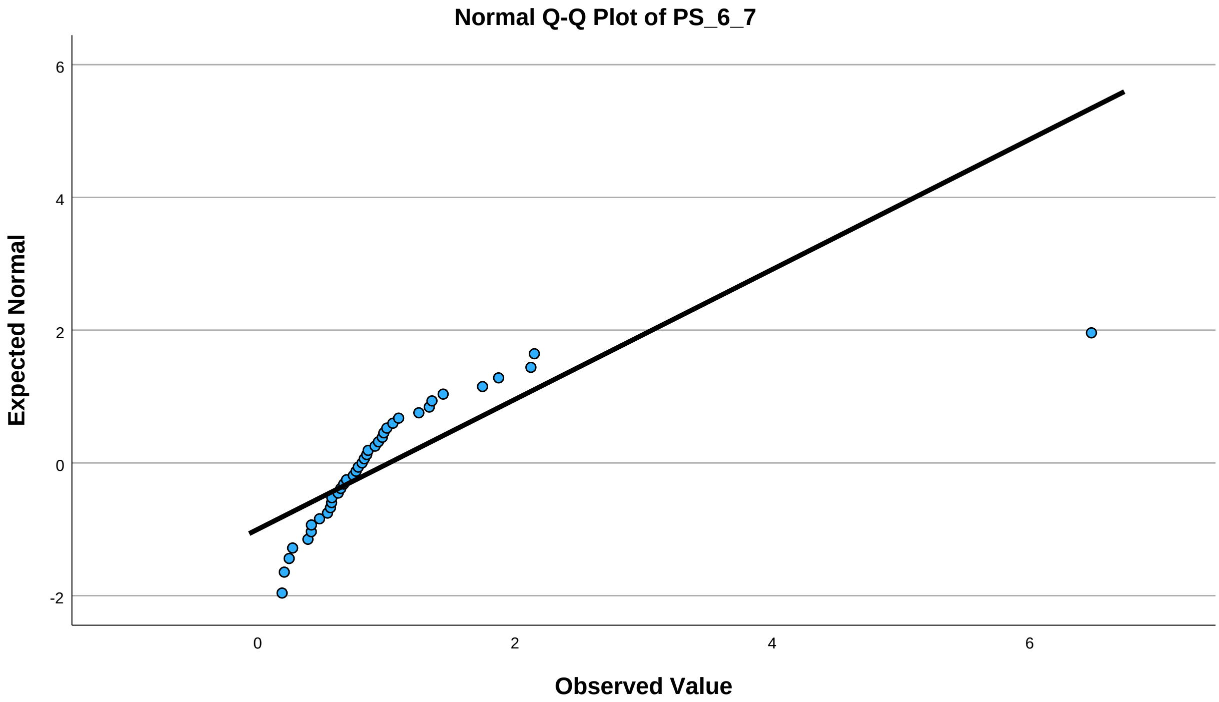
Normal Q-Q Plot of PS_6_7
Expected Normal
6
4
2
0
-2
0
2
4
6
Observed Value

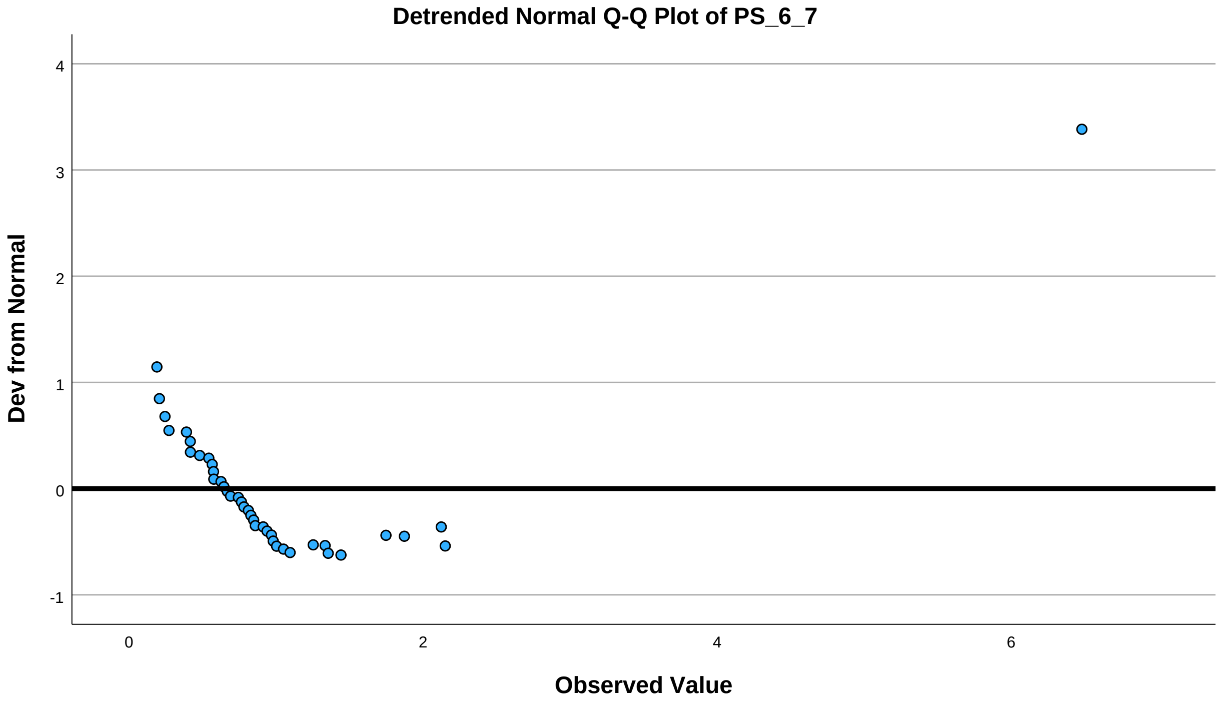
Detrended Normal Q-Q Plot of PS_6_7
Dev from Normal
4
3
2
1
0
-1
0
2
4
6
Observed Value

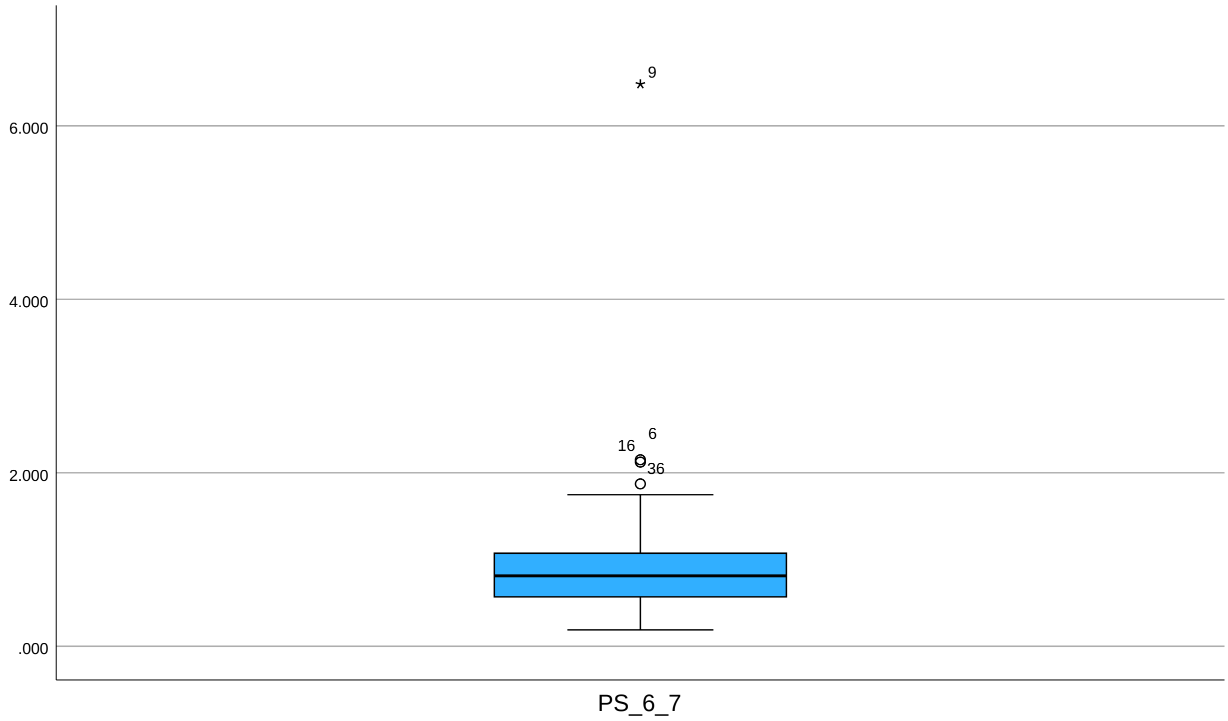

PS_7_8

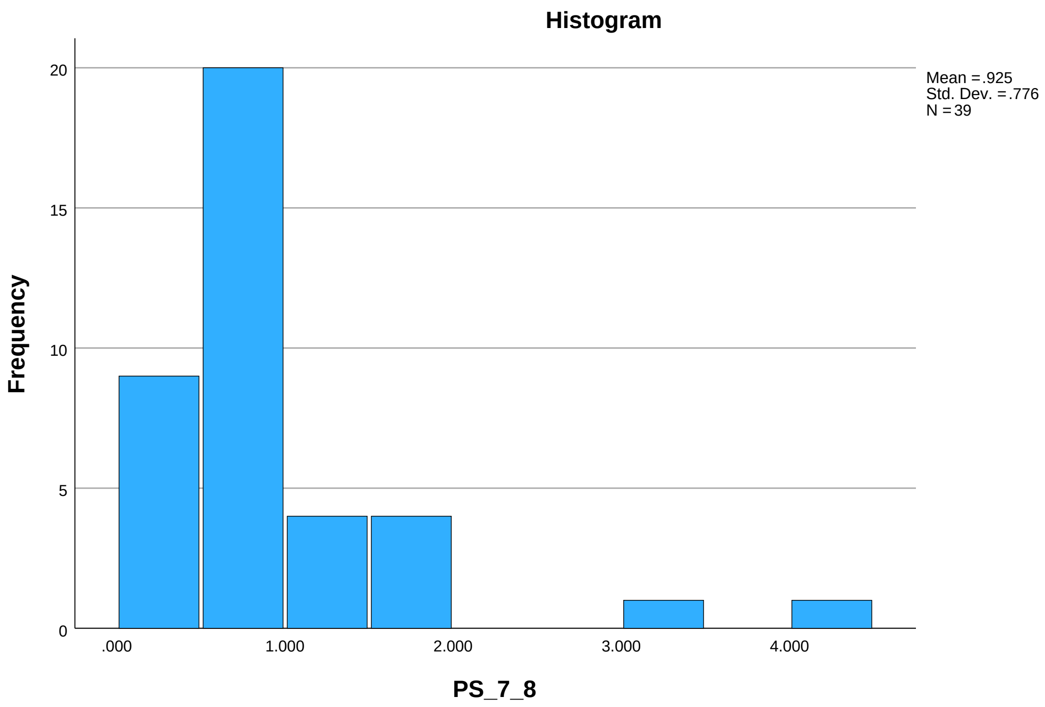


```
PS_7_8 Stem-and-Leaf Plot

 Frequency    Stem &  Leaf
```

```
 2.00     0 . 11
 5.00     0 . 22333
 7.00     0 . 4455555
 9.00     0 . 666677777
 6.00     0 . 888899
 2.00     1 . 01
 1.00     1 . 3
 3.00     1 . 455
 4.00 Extremes    (>=1.7)

Stem width:    1.000
Each leaf:      1 case(s)
```

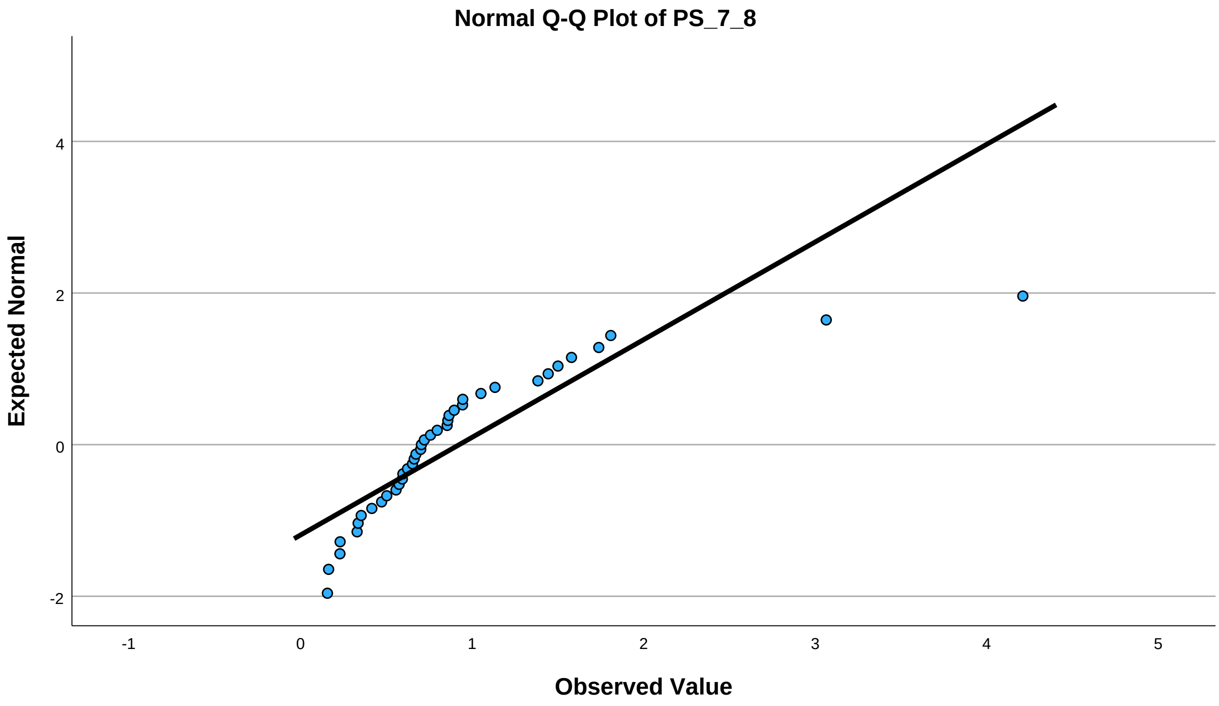

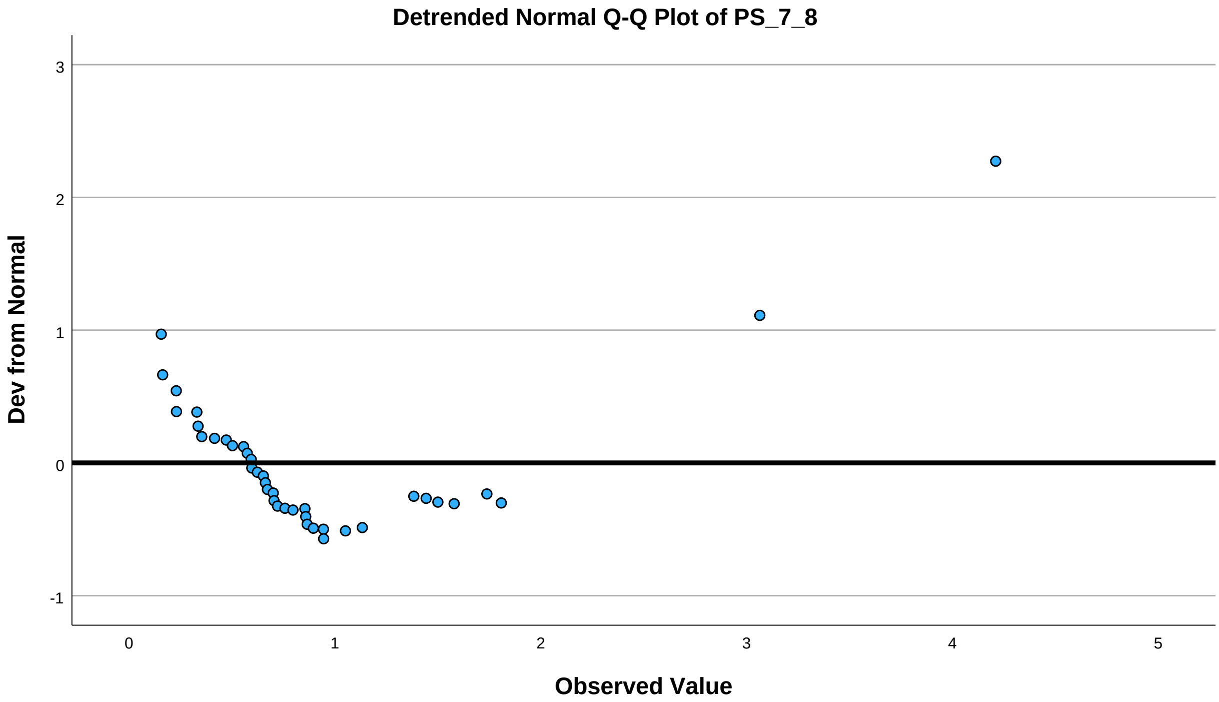

Detrended Normal Q-Q Plot of PS_7_8
Dev from Normal
3
2
1
0
-1
0
1
2
3
4
5
Observed Value

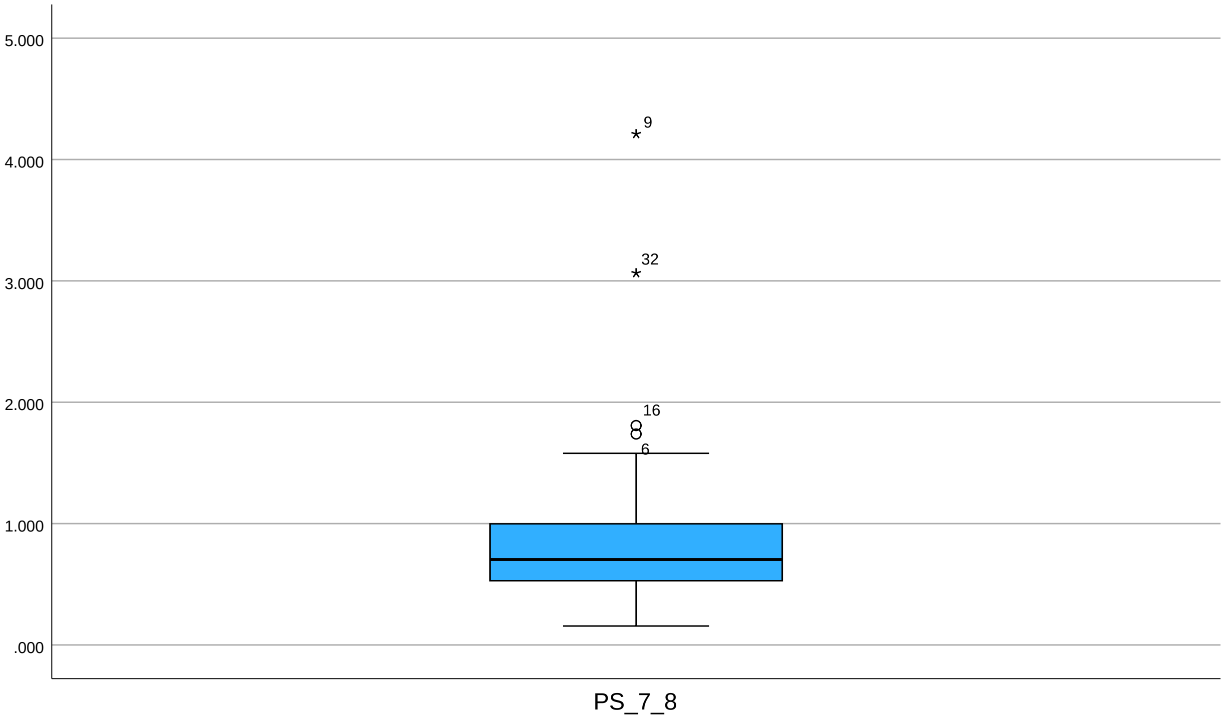

5.000
4.000
3.000
2.000
1.000
.000
9
32
16
6
PS_7_8

PS_8_9

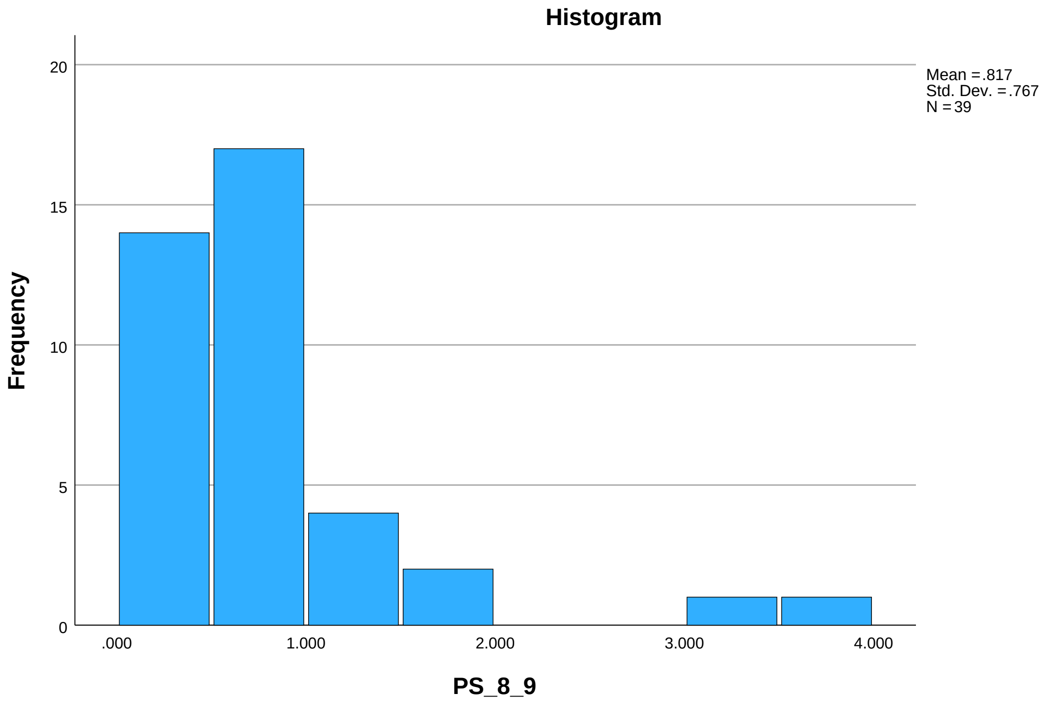

PS_8_9 Stem-and-Leaf Plot

```
 Frequency    Stem &  Leaf

     3.00        0 .  111
     7.00        0 .  2223333
    10.00        0 .  4444555555
     6.00        0 .  666677
     5.00        0 .  88889
     2.00        1 .  00
     1.00        1 .  2
     1.00        1 .  4
     4.00 Extremes    (>=1.8)

 Stem width:      1.000
 Each leaf:       1 case(s)
```

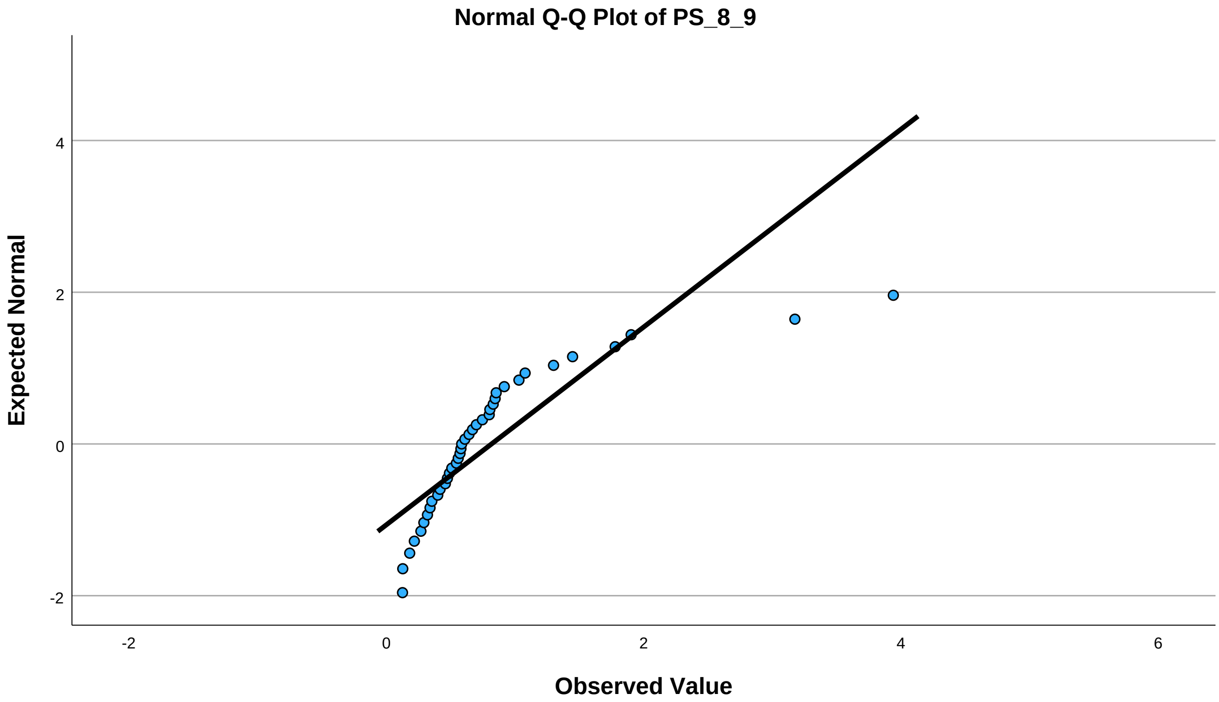
Normal Q-Q Plot of PS_8_9
Expected Normal
Observed Value
4
2
0
-2
-2
0
2
4
6

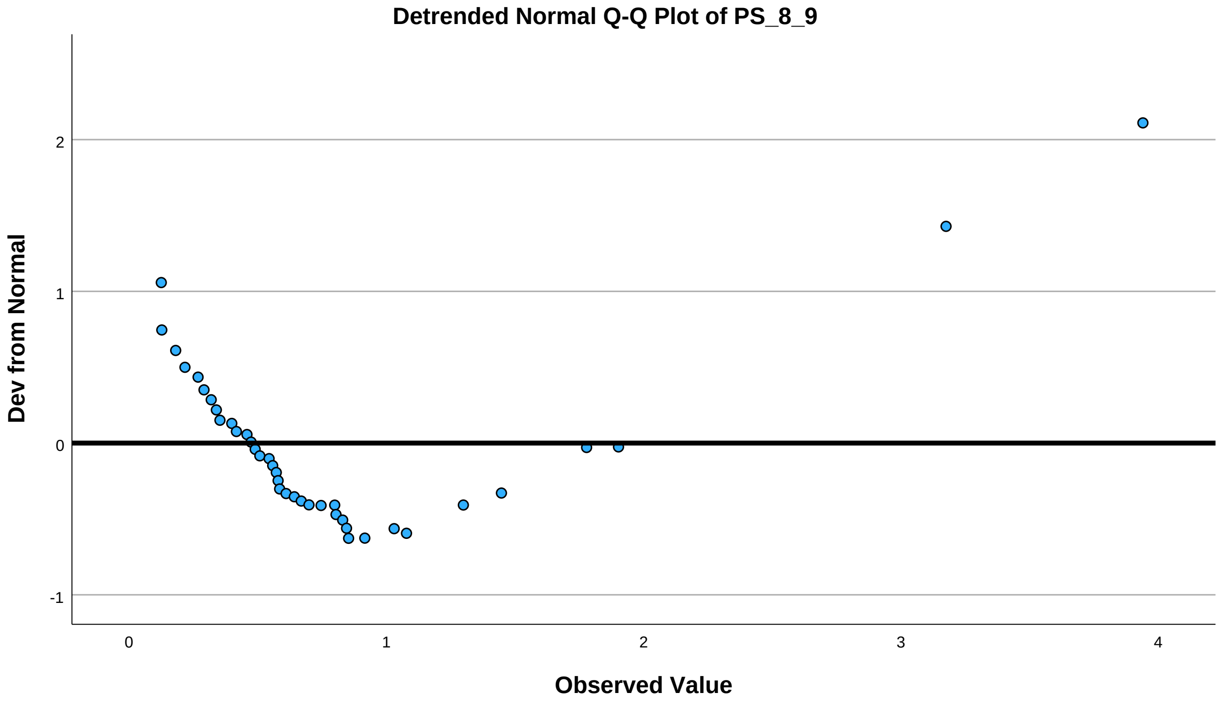
Detrended Normal Q-Q Plot of PS_8_9
Dev from Normal
Observed Value
2
1
0
-1
0
1
2
3
4

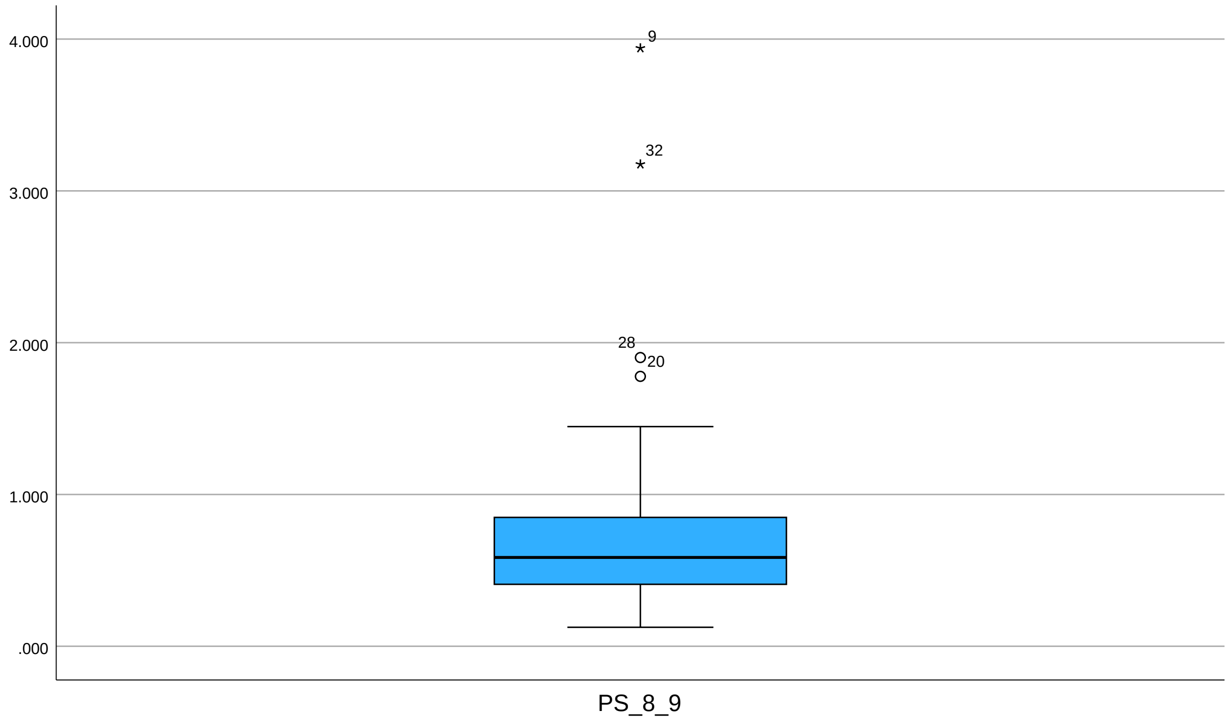

PS_9_10

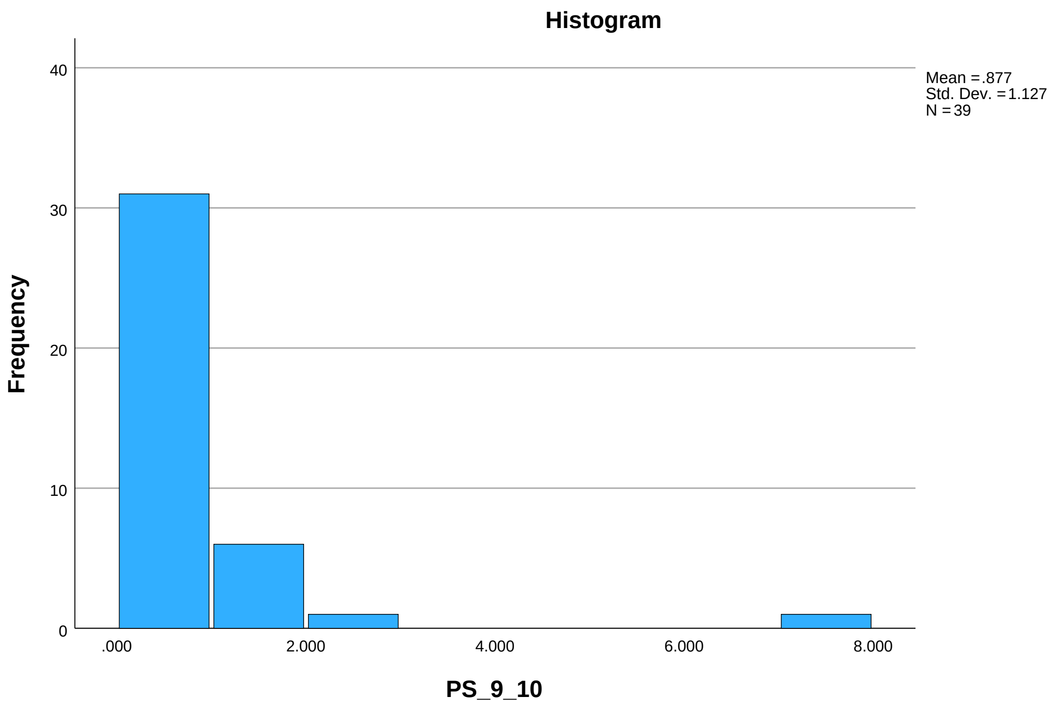

PS_9_10 Stem-and-Leaf Plot

Frequency   Stem & Leaf

```
 1.00      0 . 9
 3.00      1 . 049
 2.00      2 . 67
 2.00      3 . 14
 3.00      4 . 339
 6.00      5 . 014569
 5.00      6 . 56667
 6.00      7 . 045999
 2.00      8 . 46
 1.00      9 . 0
 1.00     10 . 8
 3.00     11 . 169
 4.00 Extremes    (>=1.63)

Stem width:      .100
Each leaf:       1 case(s)
```

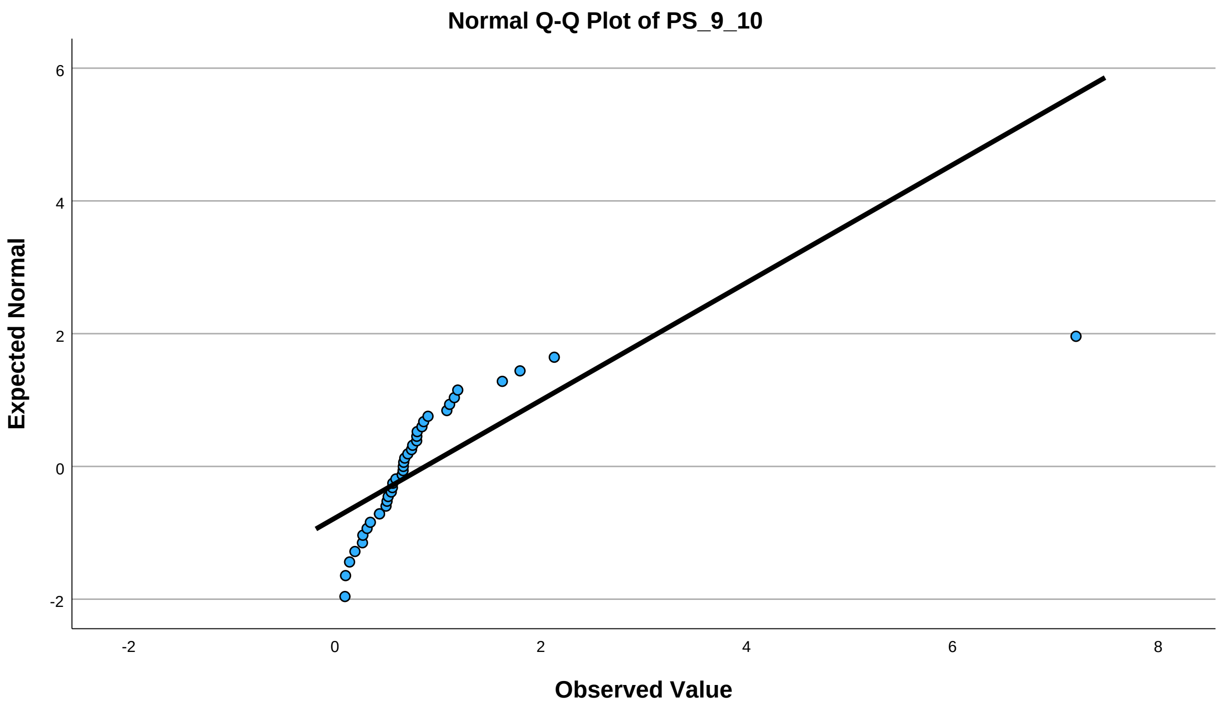

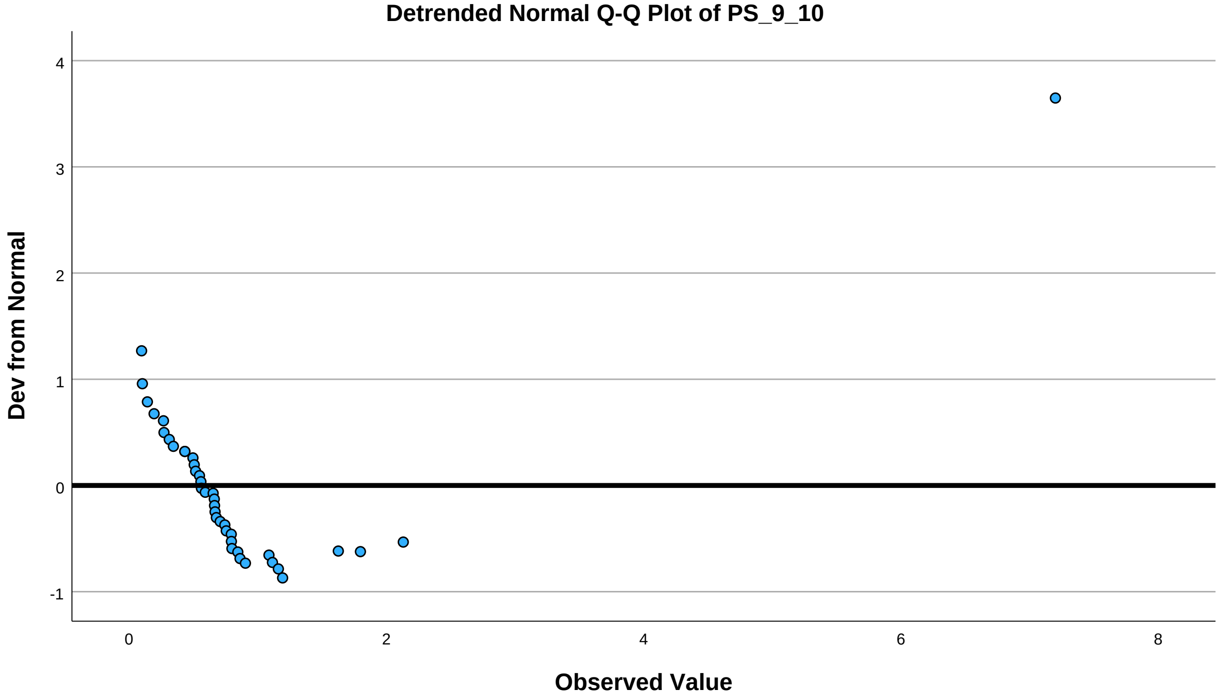
Detrended Normal Q-Q Plot of PS_9_10
Dev from Normal
Observed Value
4
3
2
1
0
-1
0
2
4
6
8

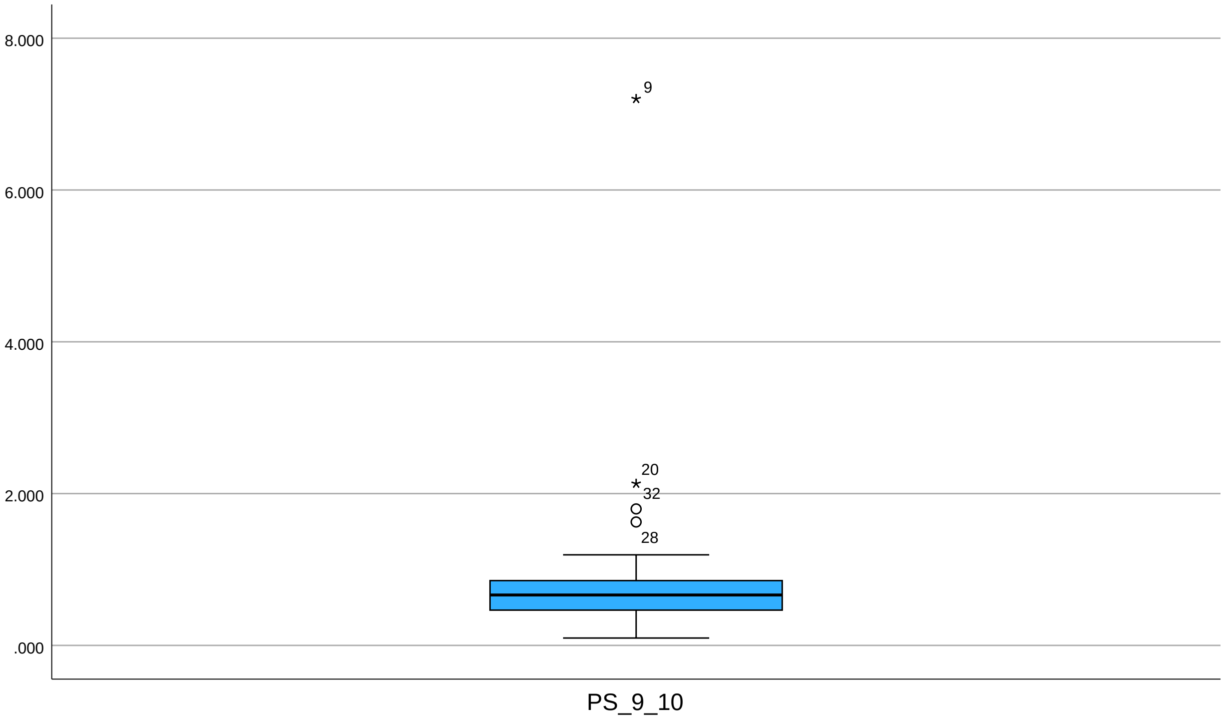
8.000
6.000
4.000
2.000
.000
9
20
32
28
PS_9_10

PS_10_11

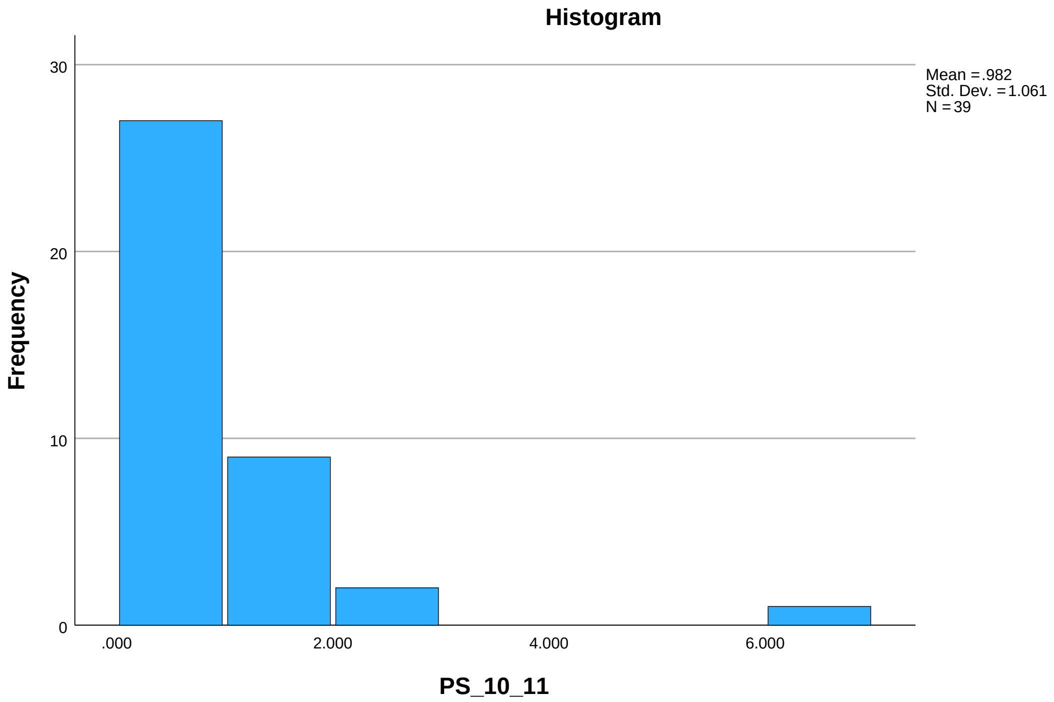

PS_10_11 Stem-and-Leaf Plot

```
 Frequency    Stem &  Leaf

     2.00        0 .  11
     7.00        0 .  2333333
     4.00        0 .  4455
    11.00        0 .  66677777777
     3.00        0 .  899
     5.00        1 .  00001
     1.00        1 .  2
     1.00        1 .  5
      .00        1 .
     2.00        1 .  88
     3.00 Extremes    (>=2.3)

 Stem width:      1.000
 Each leaf:       1 case(s)
```

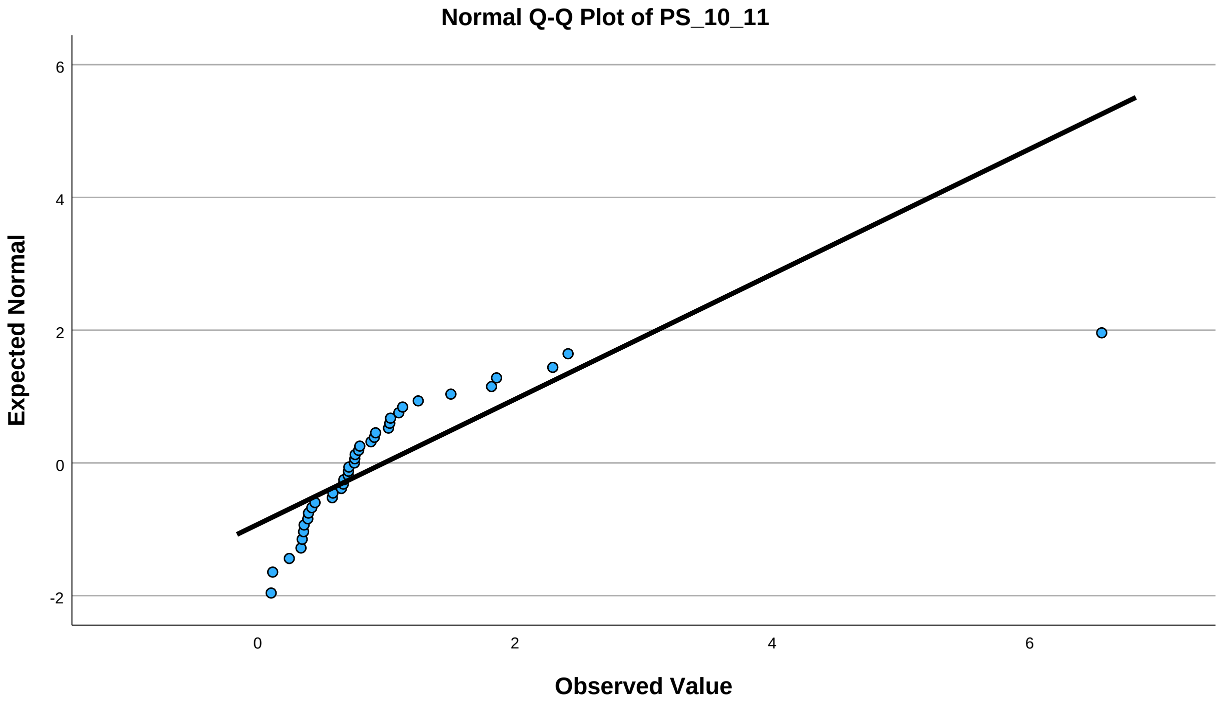
Normal Q-Q Plot of PS_10_11
Expected Normal
Observed Value
6
4
2
0
-2
0
2
4
6

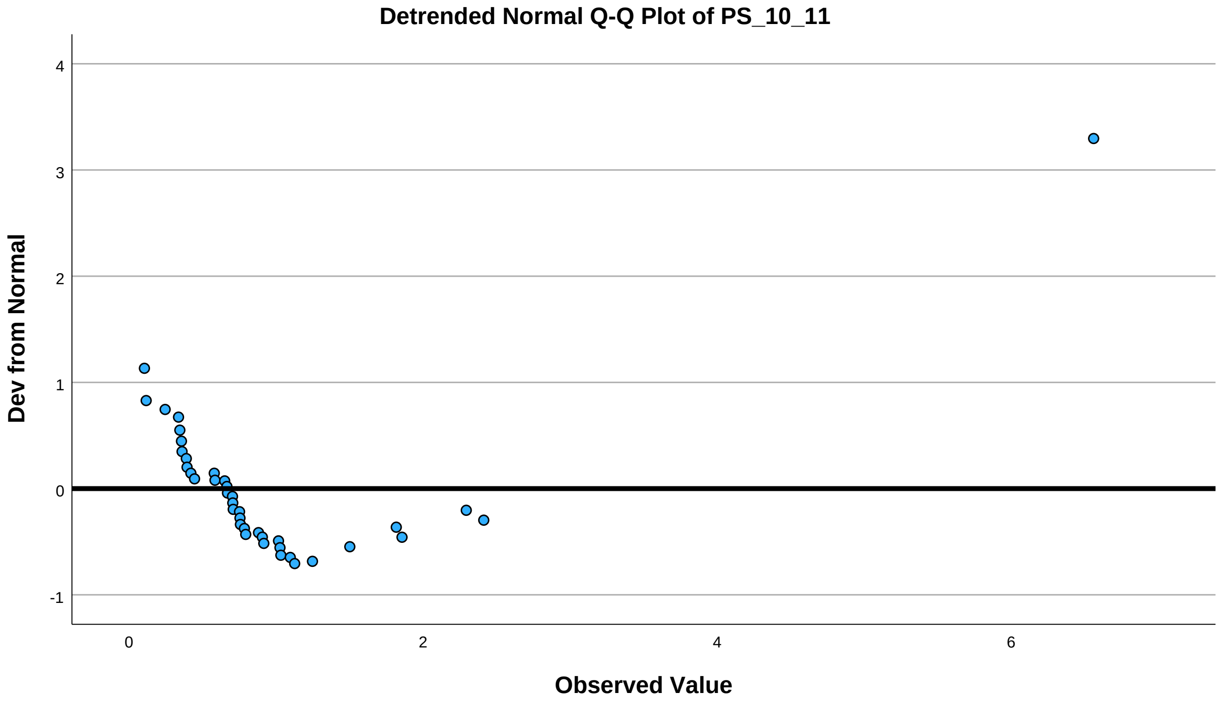
Detrended Normal Q-Q Plot of PS_10_11
Dev from Normal
Observed Value
4
3
2
1
0
-1
0
2
4
6

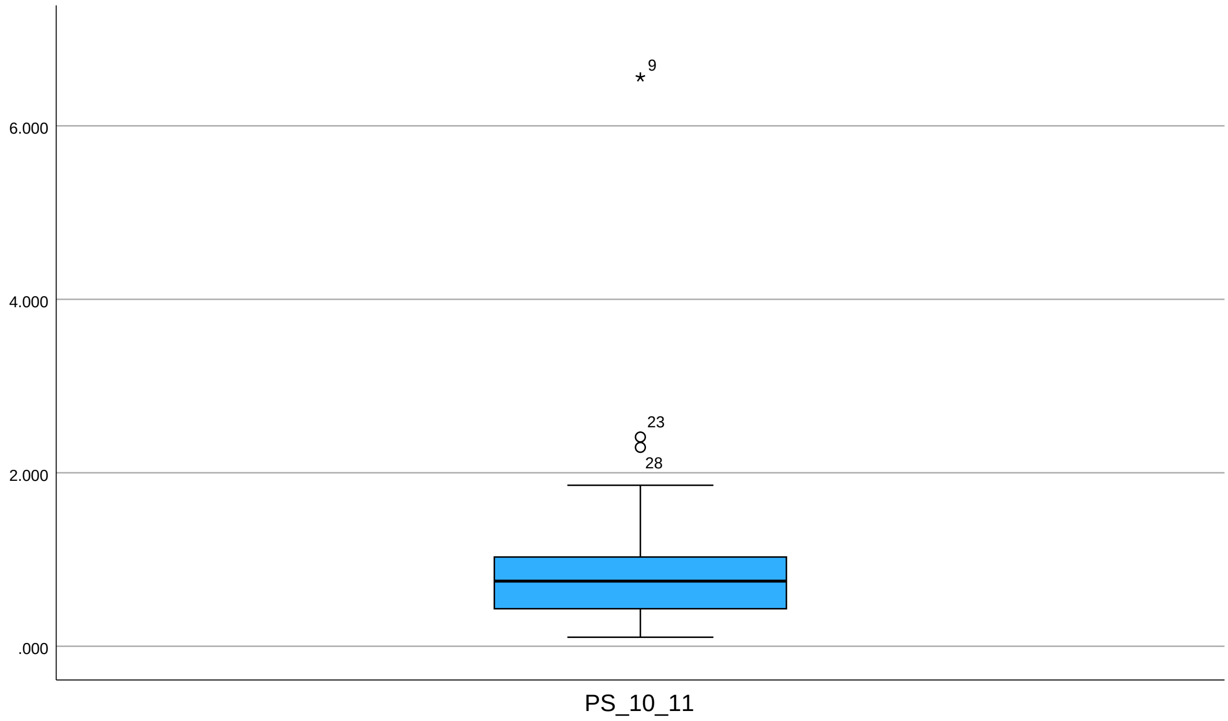

PS_11_12

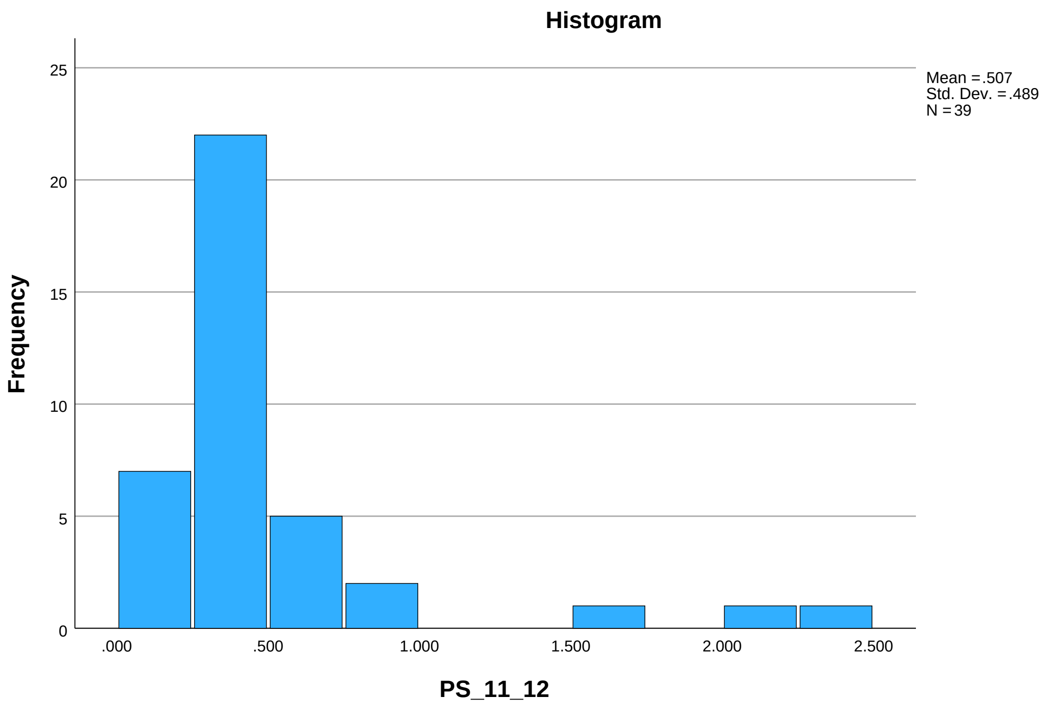

PS_11_12 Stem-and-Leaf Plot

Frequency   Stem & Leaf

```
 2.00      0 . 78
 1.00      1 . 4
 9.00      2 . 123455577
12.00      3 . 022225667789
 5.00      4 . 02467
 4.00      5 . 2289
 1.00      6 . 6
 5.00 Extremes    (>=.87)

Stem width:      .100
Each leaf:       1 case(s)
```

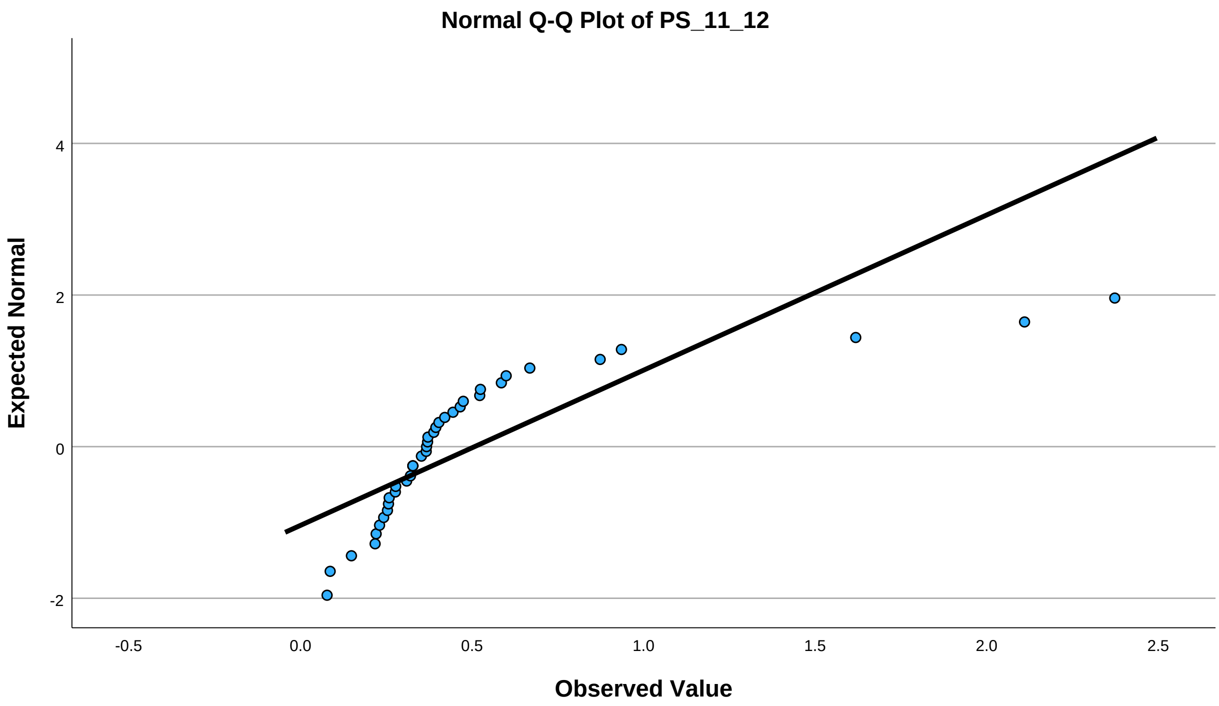

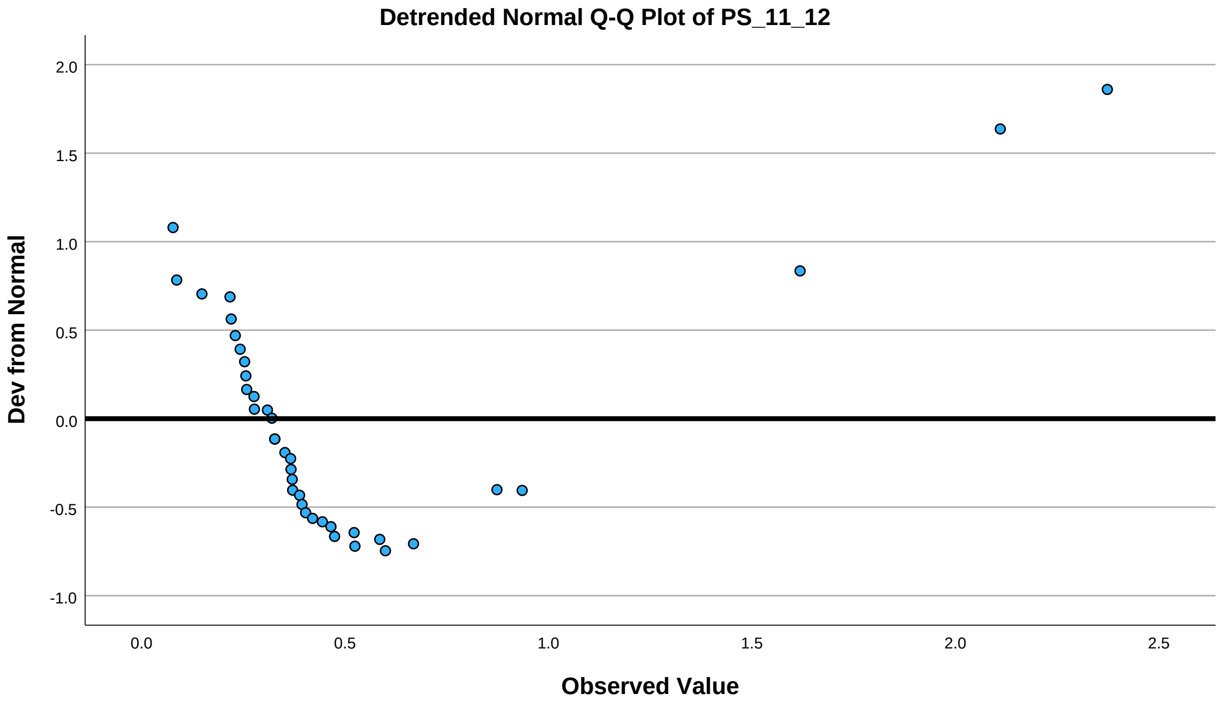
Detrended Normal Q-Q Plot of PS_11_12
Dev from Normal
2.0
1.5
1.0
0.5
0.0
-0.5
-1.0
0.0
0.5
1.0
1.5
2.0
2.5
Observed Value

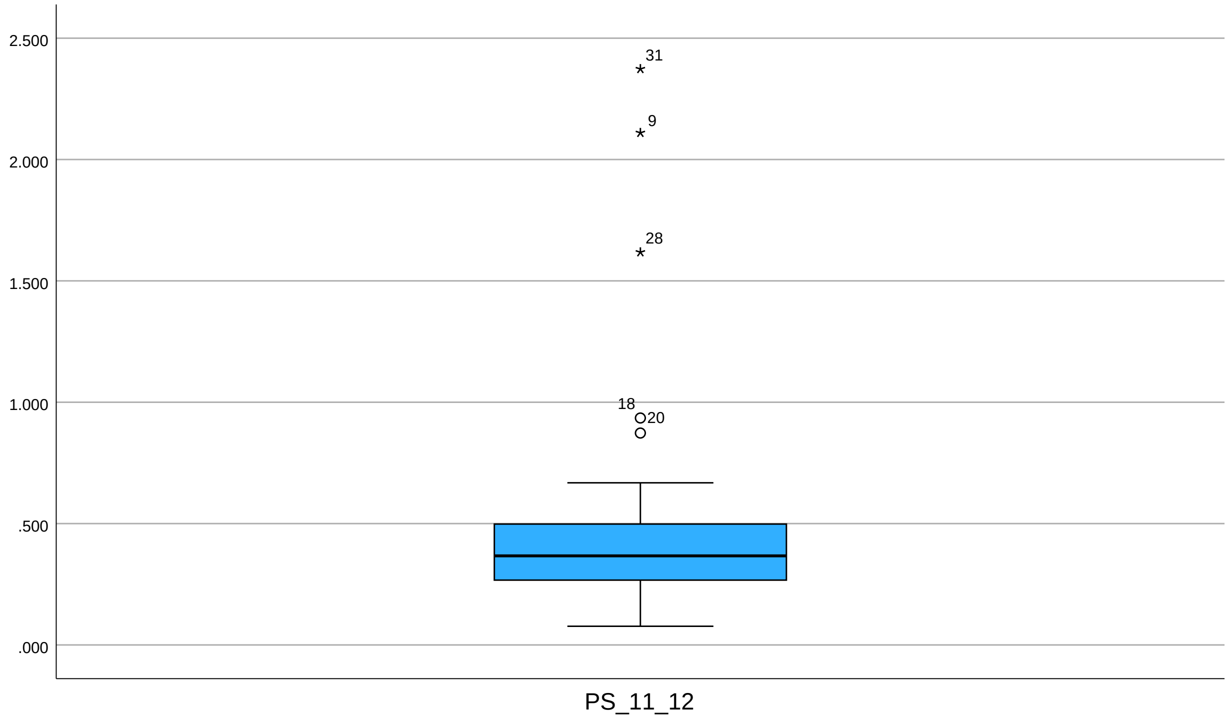
2.500
2.000
1.500
1.000
.500
.000
31
9
28
18
20
PS_11_12

PS_12_13

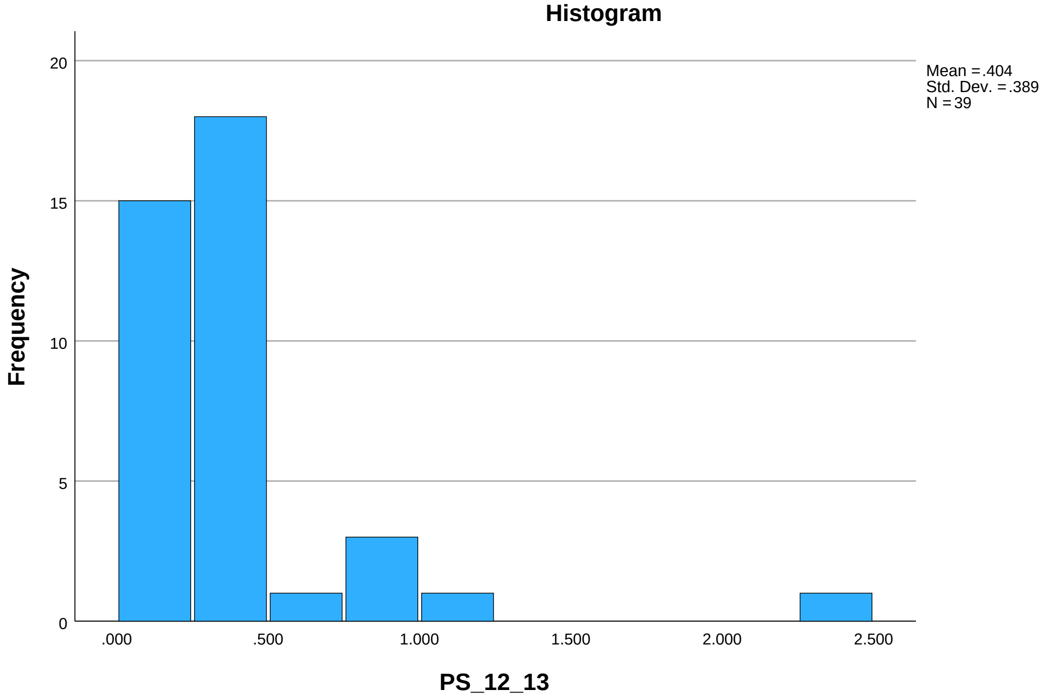


```
PS_12_13 Stem-and-Leaf Plot

 Frequency    Stem &  Leaf

     2.00        0 .  56
     4.00        1 .  1233
     3.00        1 .  679
     6.00        2 .  011134
     4.00        2 .  7888
     2.00        3 .  24
     5.00        3 .  55589
     4.00        4 .  0113
     3.00        4 .  679
     1.00        5 .  0
     5.00 Extremes    (>=.83)

 Stem width:      .100
 Each leaf:       1 case(s)
```

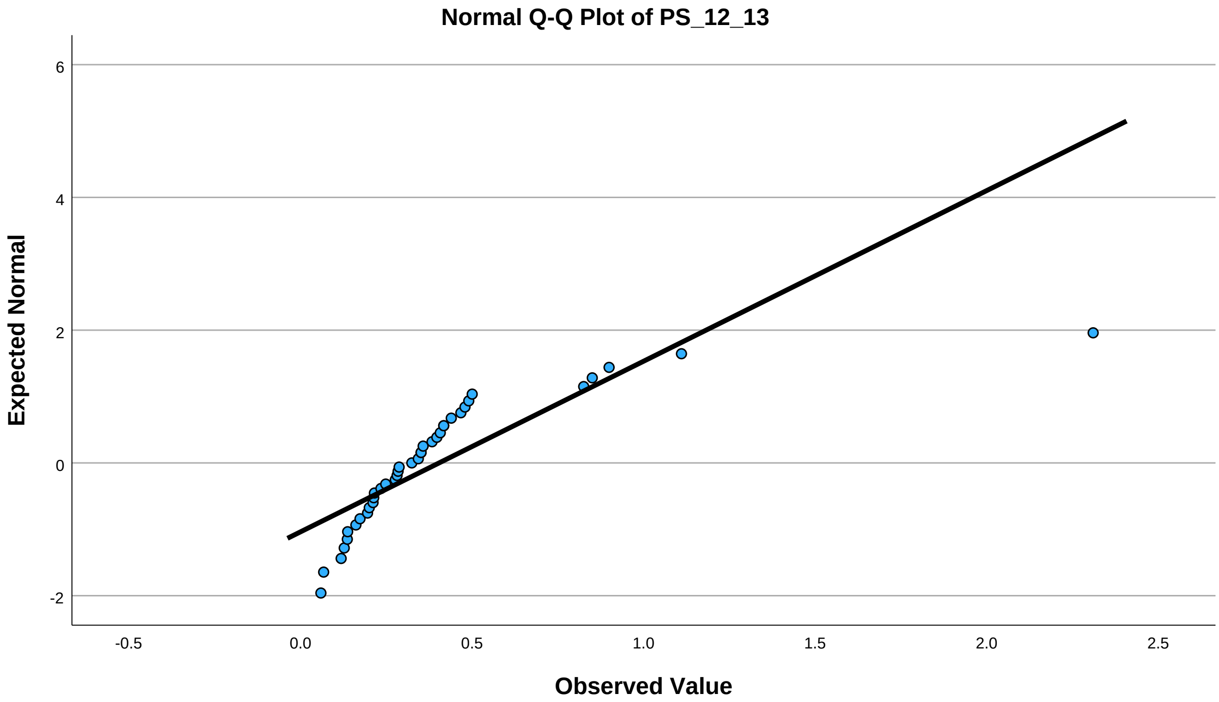
Normal Q-Q Plot of PS_12_13
Expected Normal
6
4
2
0
-2
-0.5
0.0
0.5
1.0
1.5
2.0
2.5
Observed Value

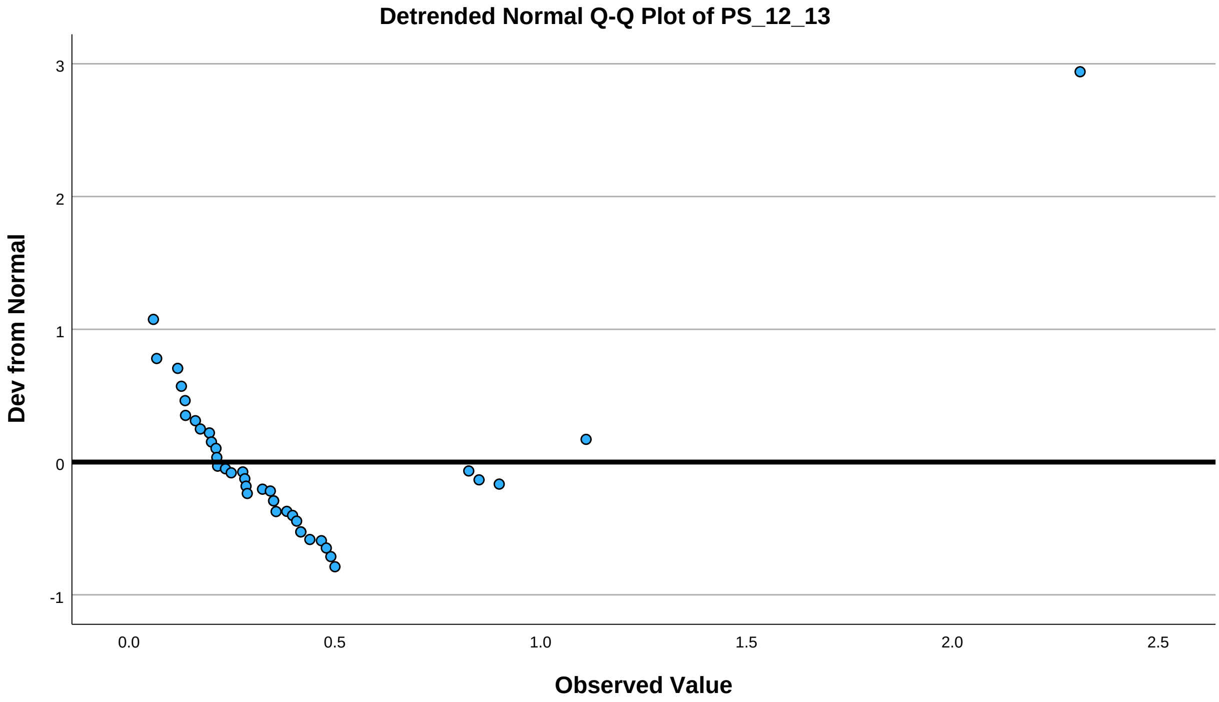
Detrended Normal Q-Q Plot of PS_12_13
Dev from Normal
3
2
1
0
-1
0.0
0.5
1.0
1.5
2.0
2.5
Observed Value

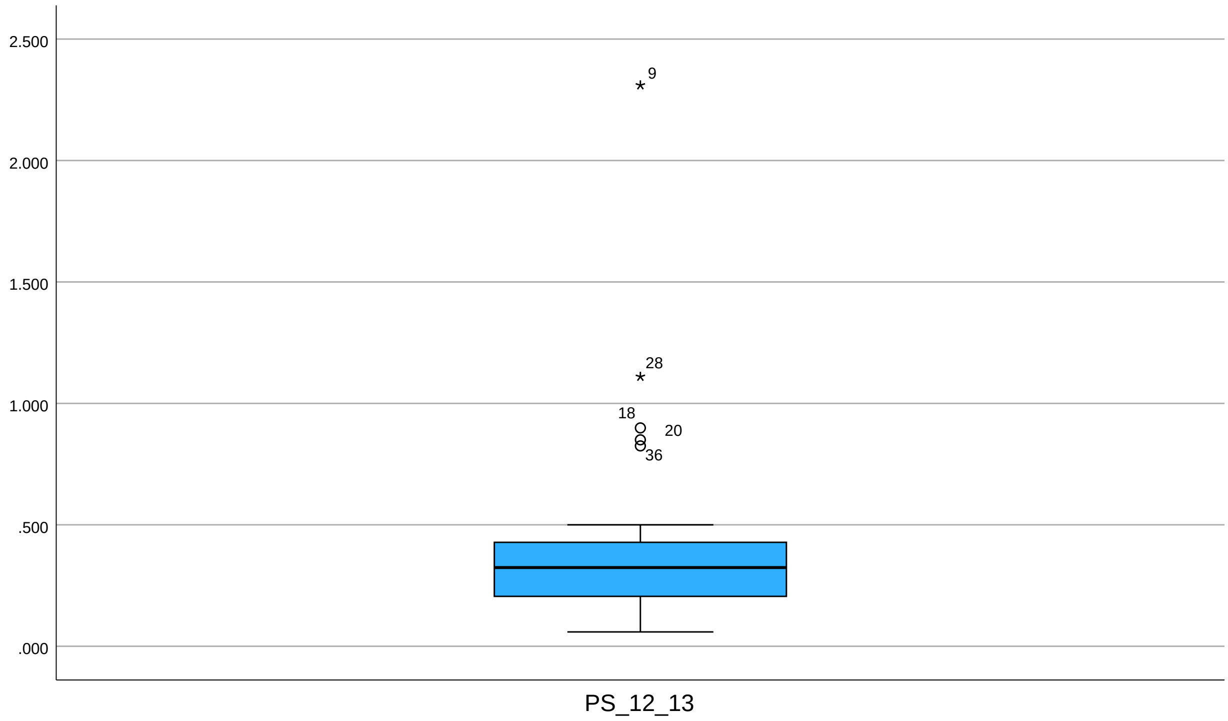

PS_13_14

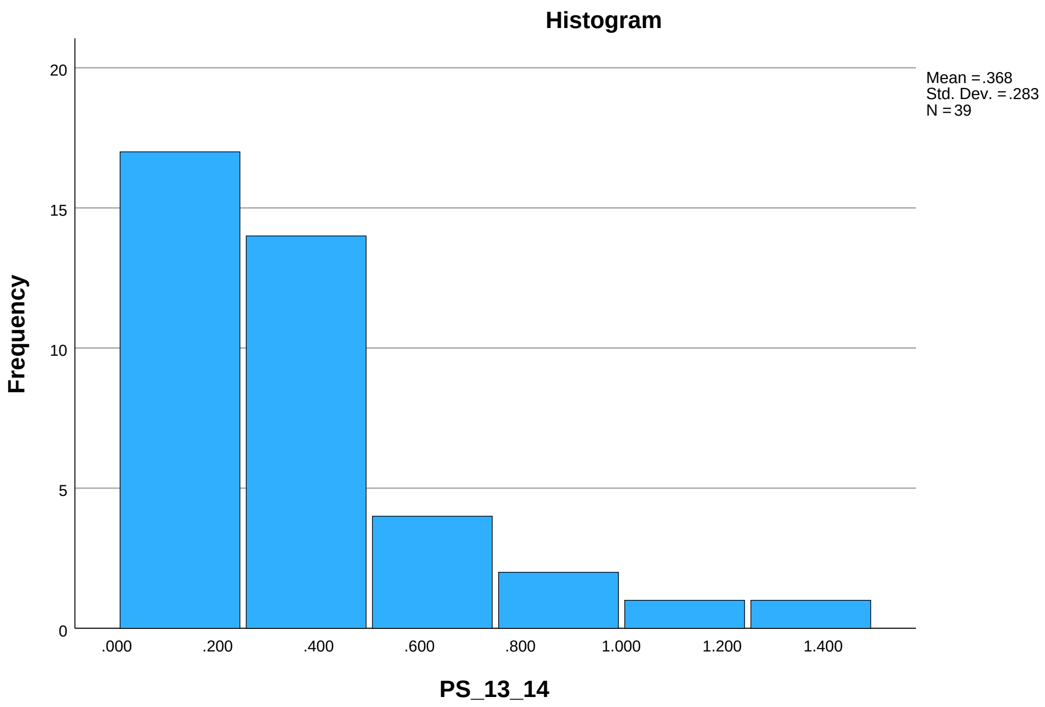

PS_13_14 Stem-and-Leaf Plot

Frequency Stem & Leaf

```
4.00      0 . 5678
7.00      1 . 0267778
8.00      2 . 12223358
8.00      3 . 03455588
4.00      4 . 2336
2.00      5 . 05
1.00      6 . 7
2.00      7 . 25
1.00      8 . 1
2.00 Extremes    (>=1.03)

Stem width:      .100
Each leaf:       1 case(s)
```

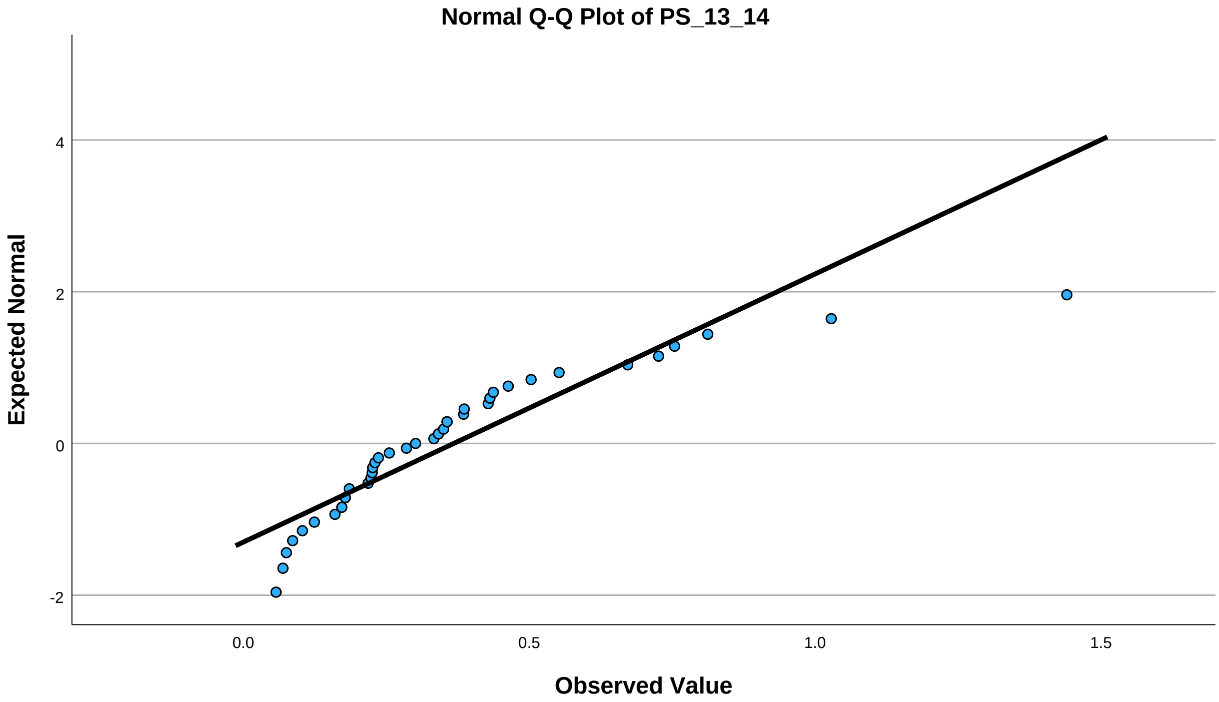

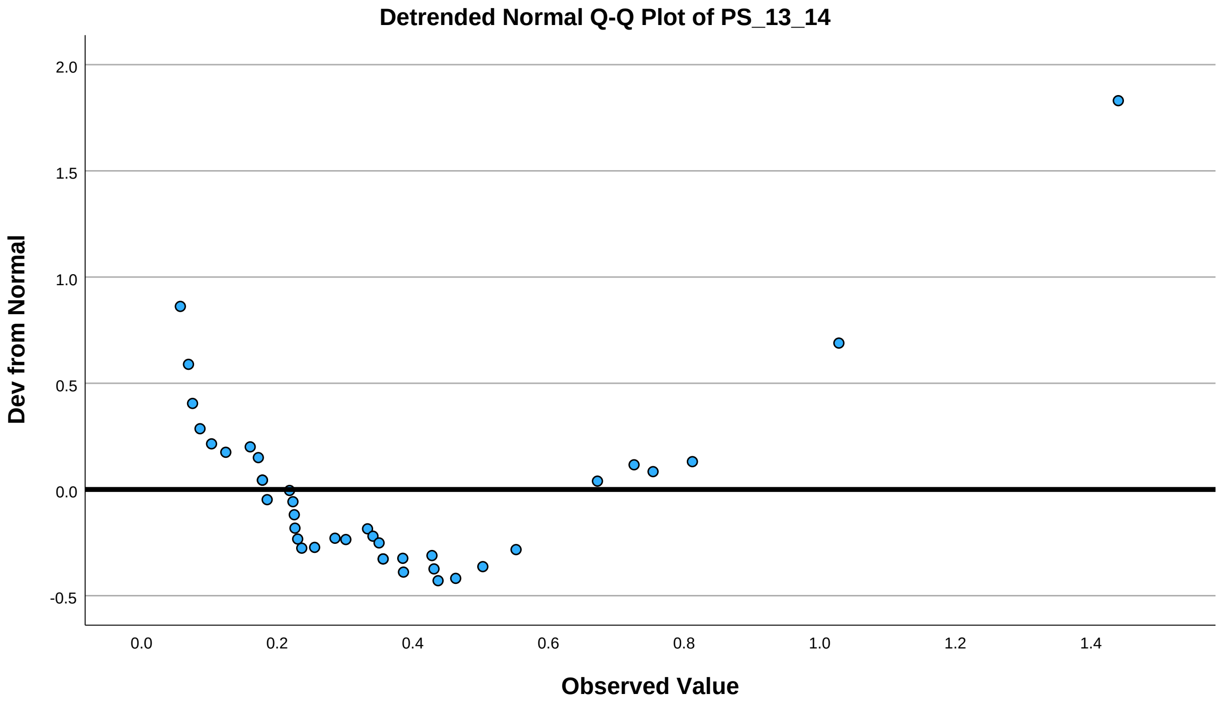
Detrended Normal Q-Q Plot of PS_13_14
Dev from Normal
2.0
1.5
1.0
0.5
0.0
-0.5
0.0
0.2
0.4
0.6
0.8
1.0
1.2
1.4
Observed Value

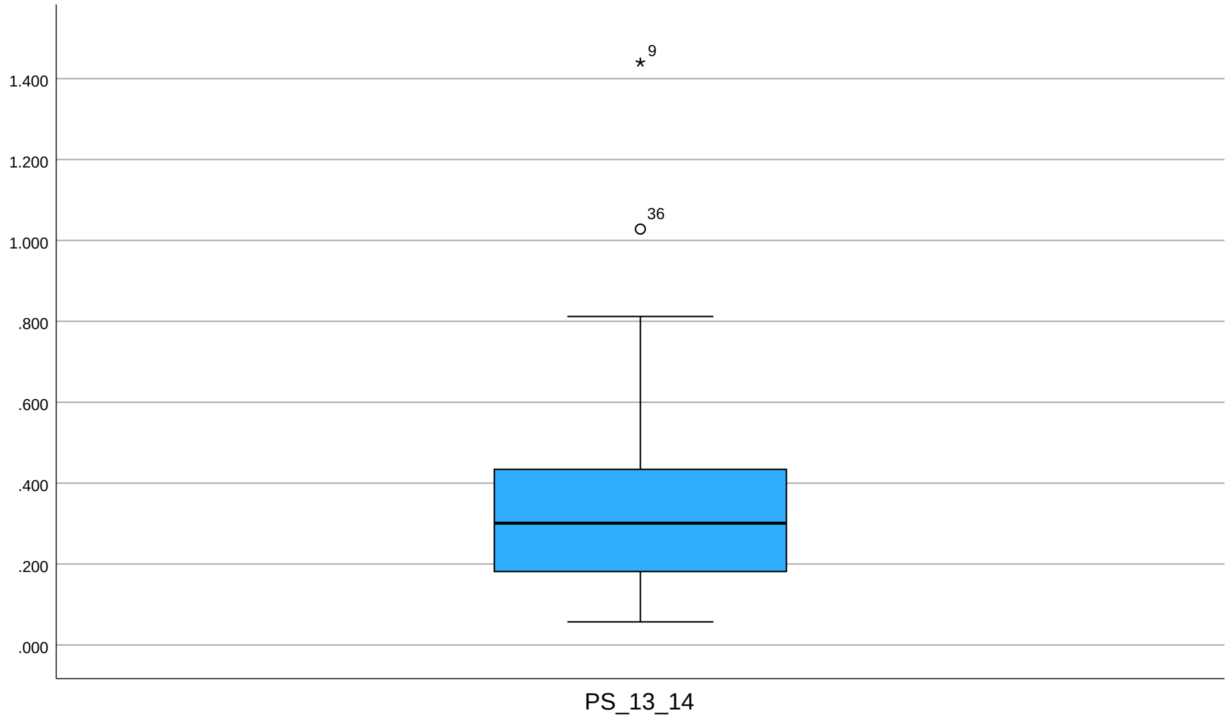
9
36
1.400
1.200
1.000
.800
.600
.400
.200
.000
PS_13_14

PS_14_15

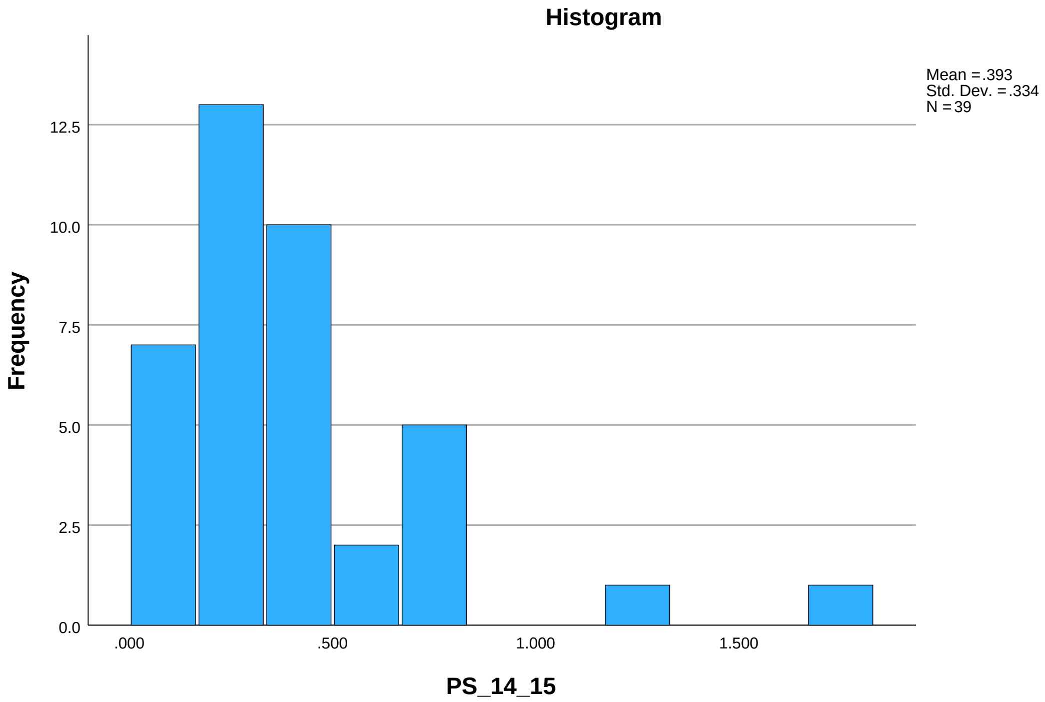


```
PS_14_15 Stem-and-Leaf Plot

 Frequency    Stem &  Leaf

     4.00        0 .  5669
     8.00        1 .  03366999
     8.00        2 .  12233347
     5.00        3 .  56778
     5.00        4 .  01578
     2.00        5 .  39
      .00        6 .
     5.00        7 .  12258
     2.00 Extremes    (>=1.21)

 Stem width:      .100
 Each leaf:       1 case(s)
```

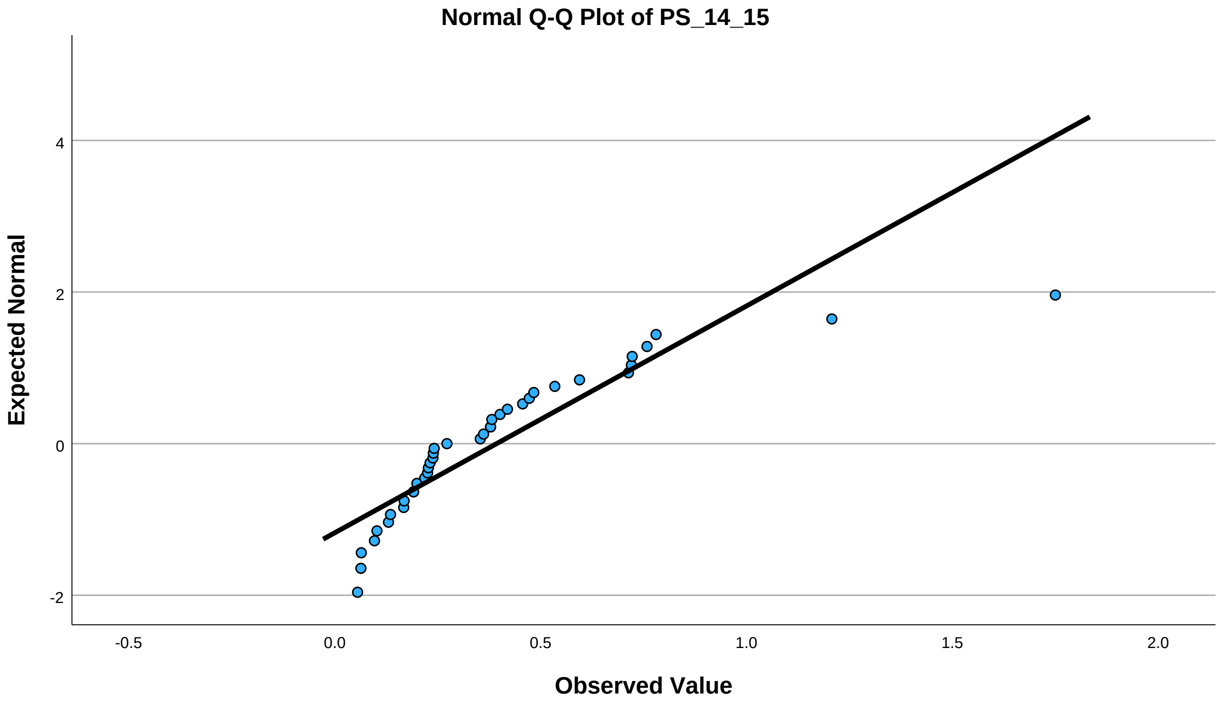
Normal Q-Q Plot of PS_14_15
Expected Normal
4
2
0
-2
-0.5
0.0
0.5
1.0
1.5
2.0
Observed Value

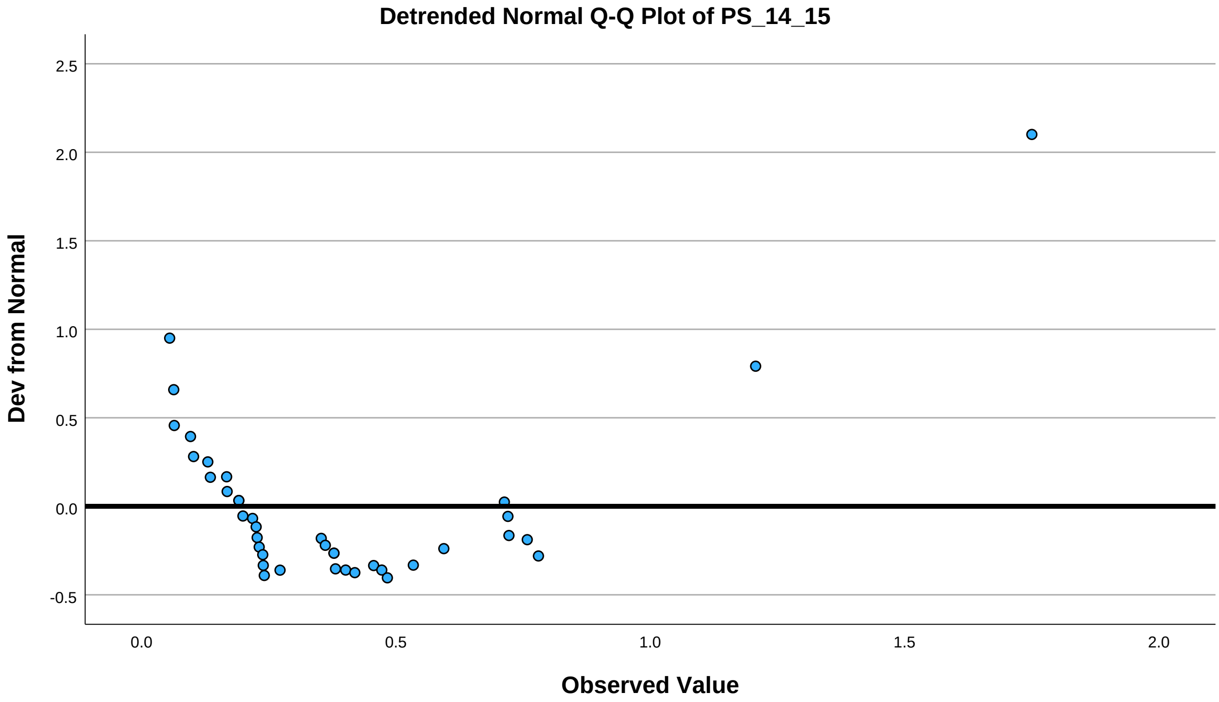
Detrended Normal Q-Q Plot of PS_14_15
Dev from Normal
2.5
2.0
1.5
1.0
0.5
0.0
-0.5
0.0
0.5
1.0
1.5
2.0
Observed Value

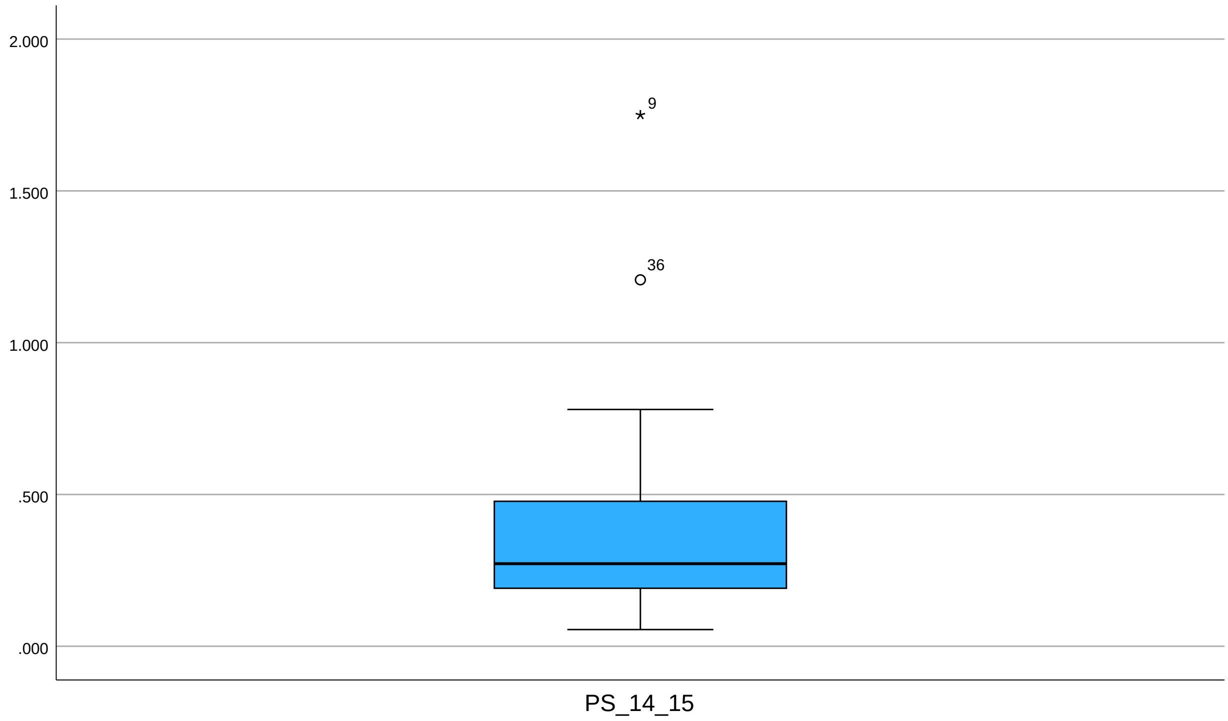

PS_15_16

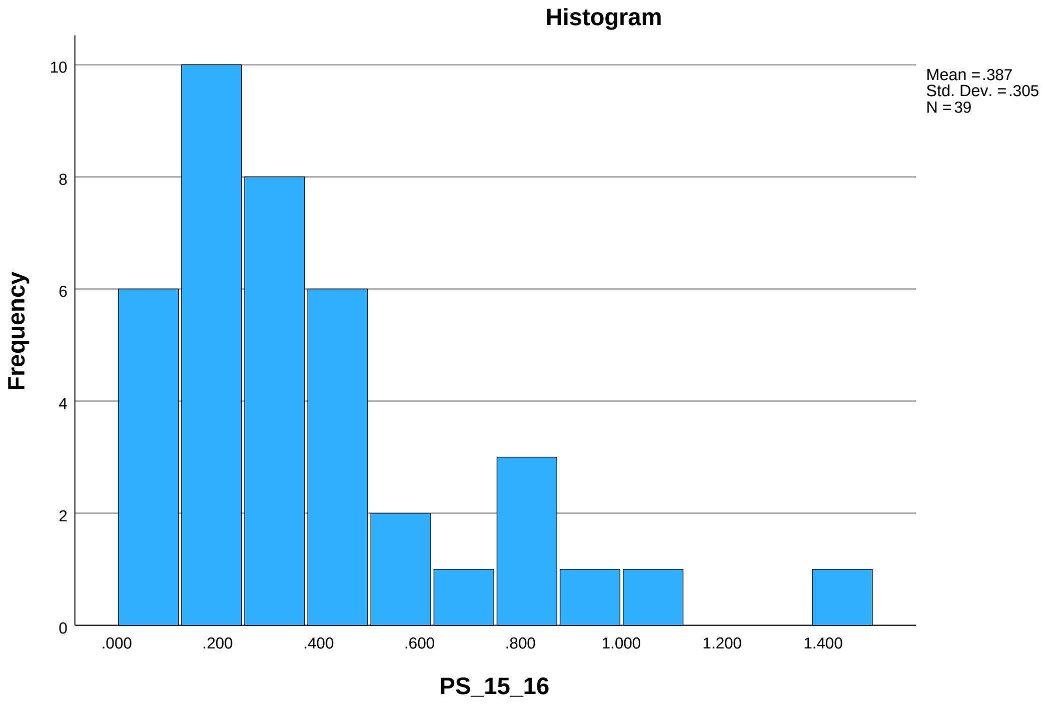

PS_15_16 Stem-and-Leaf Plot

Frequency   Stem & Leaf

```
    4.00        0 .  4569
    8.00        1 .  11446779
    7.00        2 .  1222579
    6.00        3 .  004568
    5.00        4 .  02368
    2.00        5 .  49
     .00        6 .
    3.00        7 .  369
    1.00        8 .  2
    1.00        9 .  0
    2.00 Extremes    (>=1.08)

 Stem width:      .100
 Each leaf:       1 case(s)
```

Normal Q-Q Plot of PS_15_16

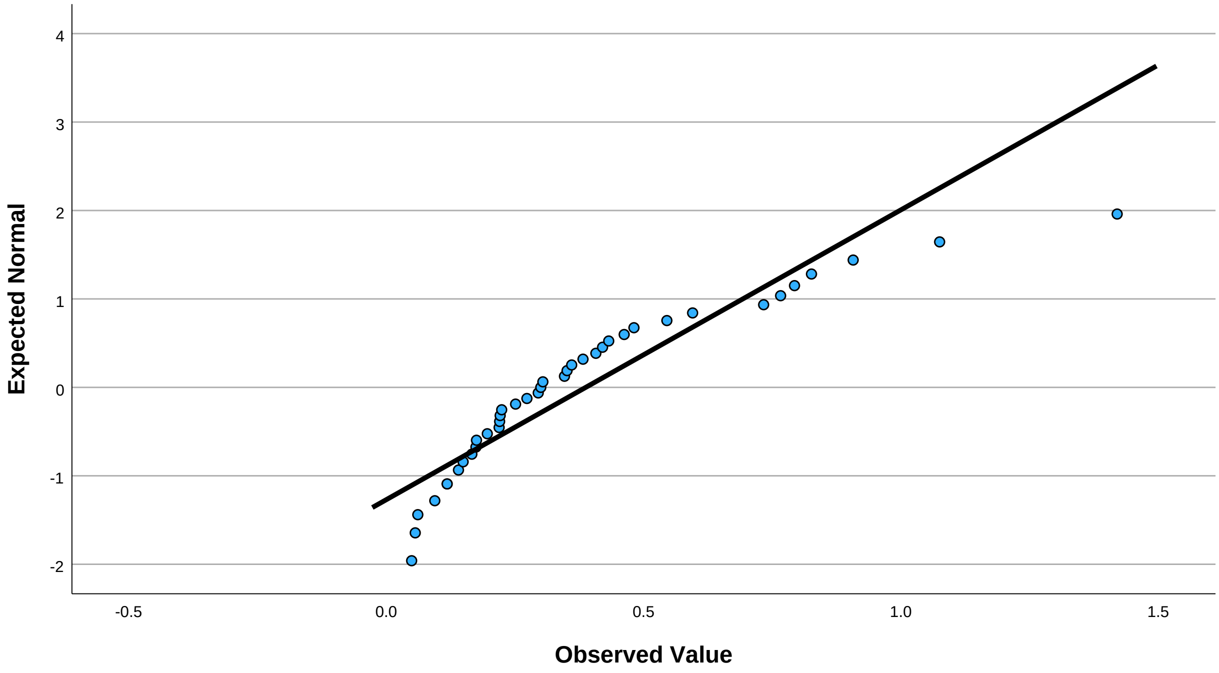

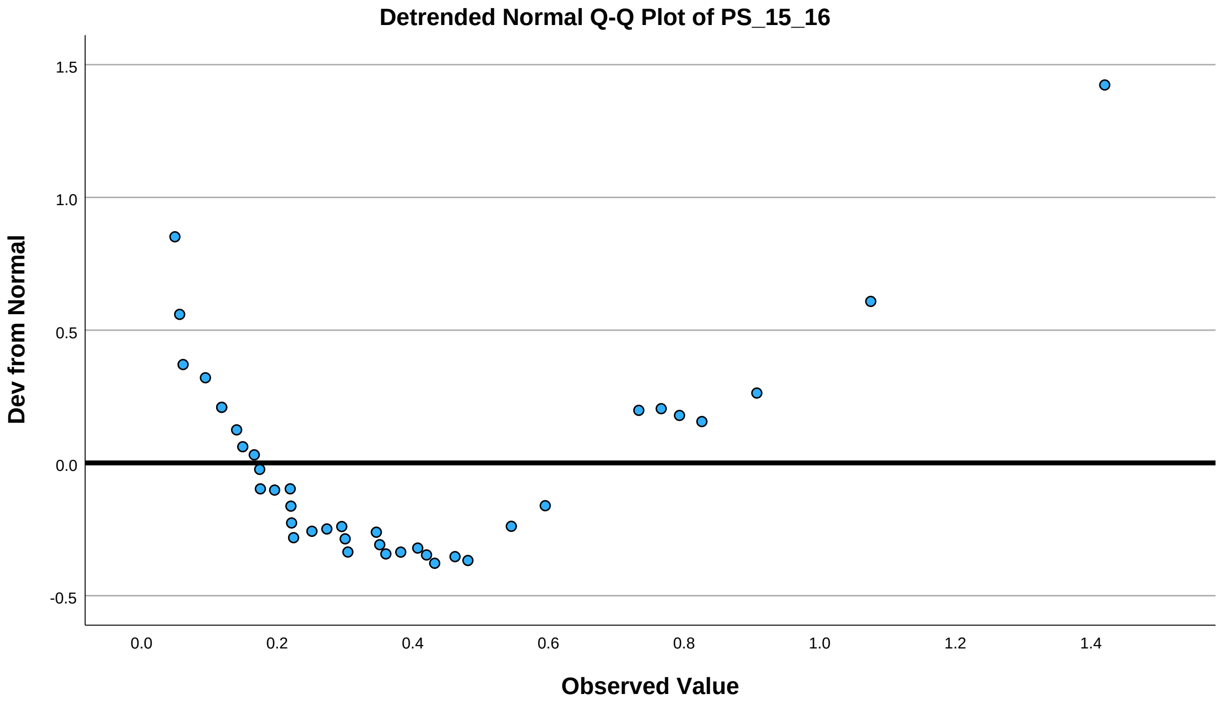
Detrended Normal Q-Q Plot of PS_15_16
Dev from Normal
1.5
1.0
0.5
0.0
-0.5
0.0
0.2
0.4
0.6
0.8
1.0
1.2
1.4
Observed Value

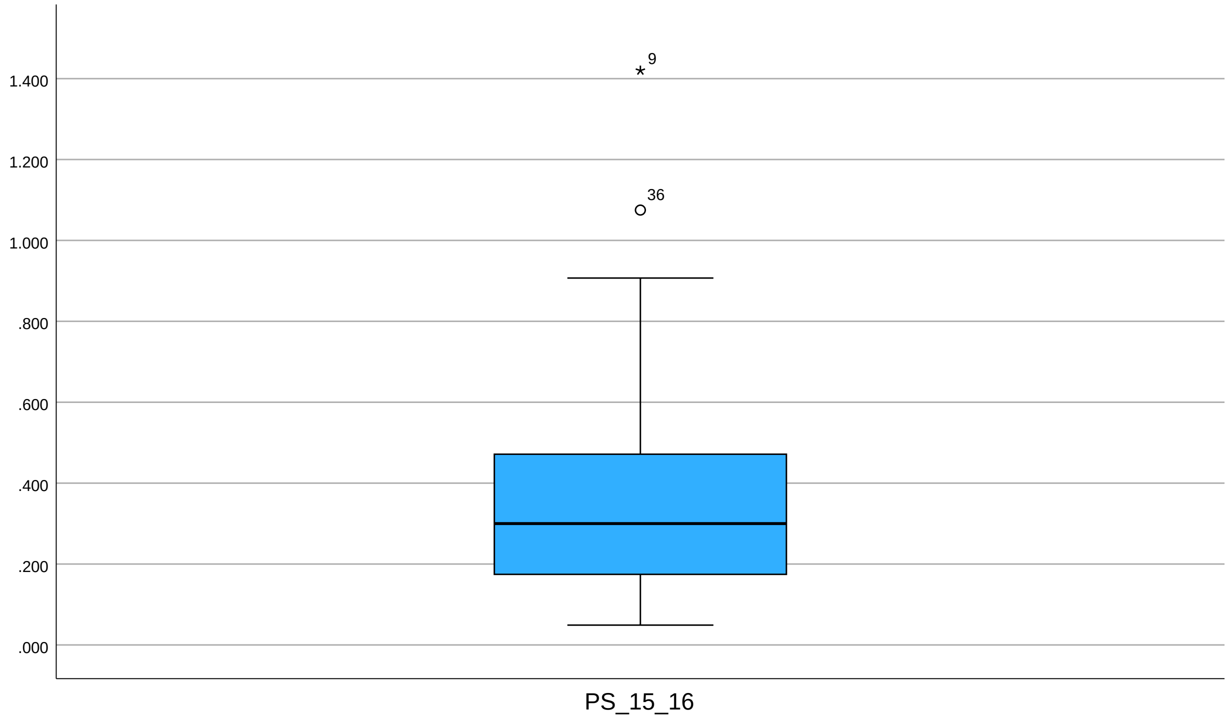
1.400
1.200
1.000
.800
.600
.400
.200
.000
9
36
PS_15_16

PS_16_17

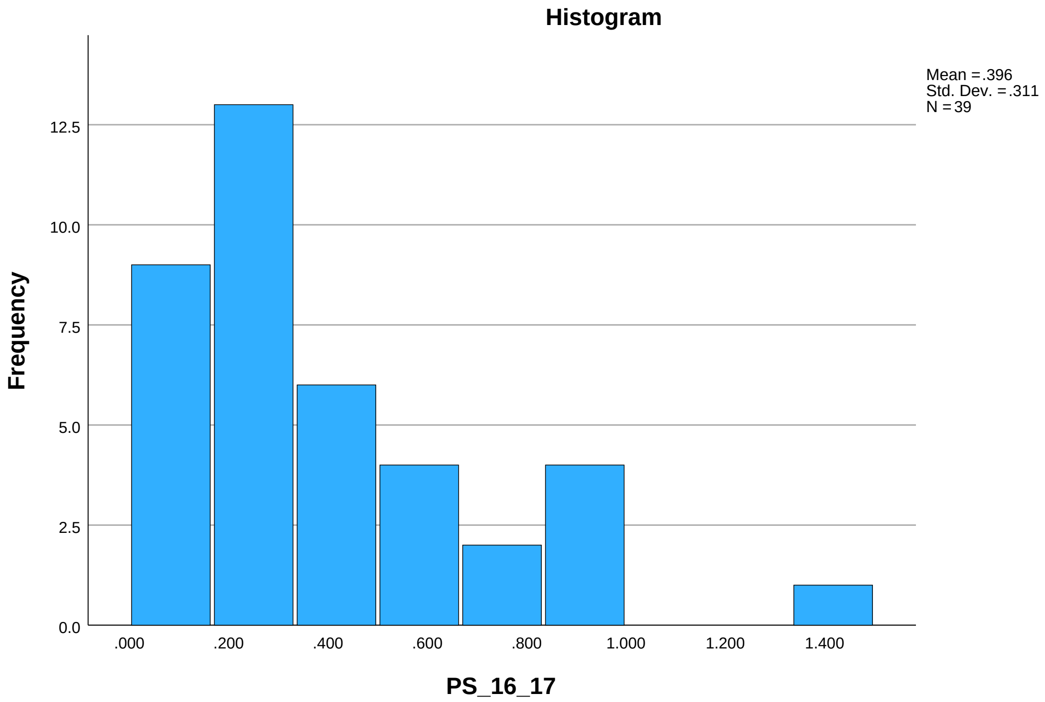


```
PS_16_17 Stem-and-Leaf Plot

 Frequency    Stem &  Leaf

     4.00        0 .  5569
     9.00        1 .  233667788
     7.00        2 .  0245999
     4.00        3 .  0269
     4.00        4 .  0278
     4.00        5 .  0147
      .00        6 .
     2.00        7 .  22
     1.00        8 .  9
     3.00        9 .  046
     1.00 Extremes    (>=1.46)

 Stem width:      .100
 Each leaf:       1 case(s)
```

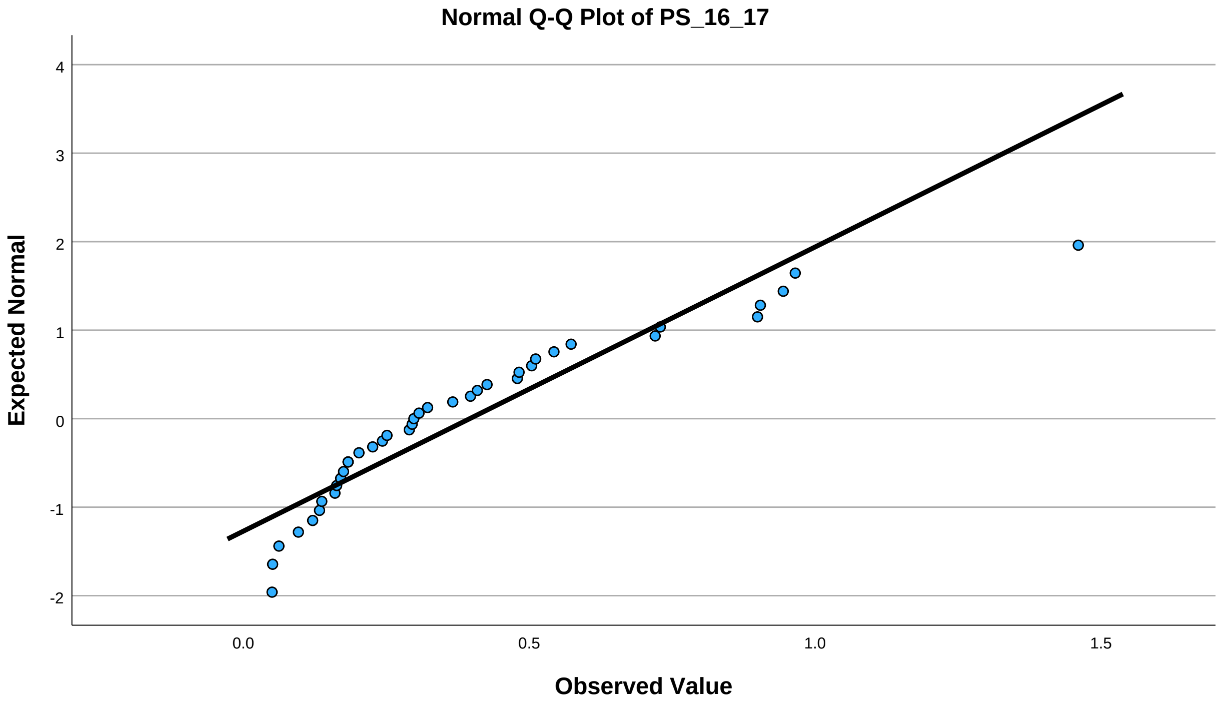
Normal Q-Q Plot of PS_16_17
Expected Normal
4
3
2
1
0
-1
-2
0.0
0.5
1.0
1.5
Observed Value

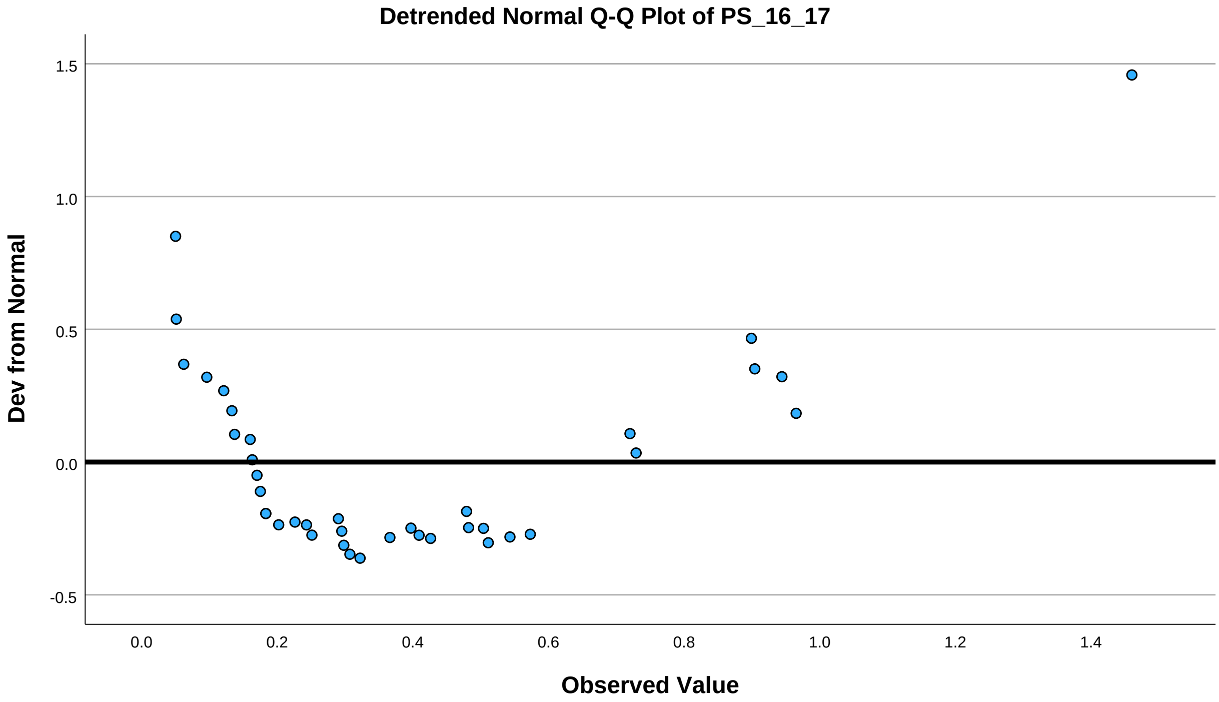
Detrended Normal Q-Q Plot of PS_16_17
Dev from Normal
1.5
1.0
0.5
0.0
-0.5
0.0
0.2
0.4
0.6
0.8
1.0
1.2
1.4
Observed Value

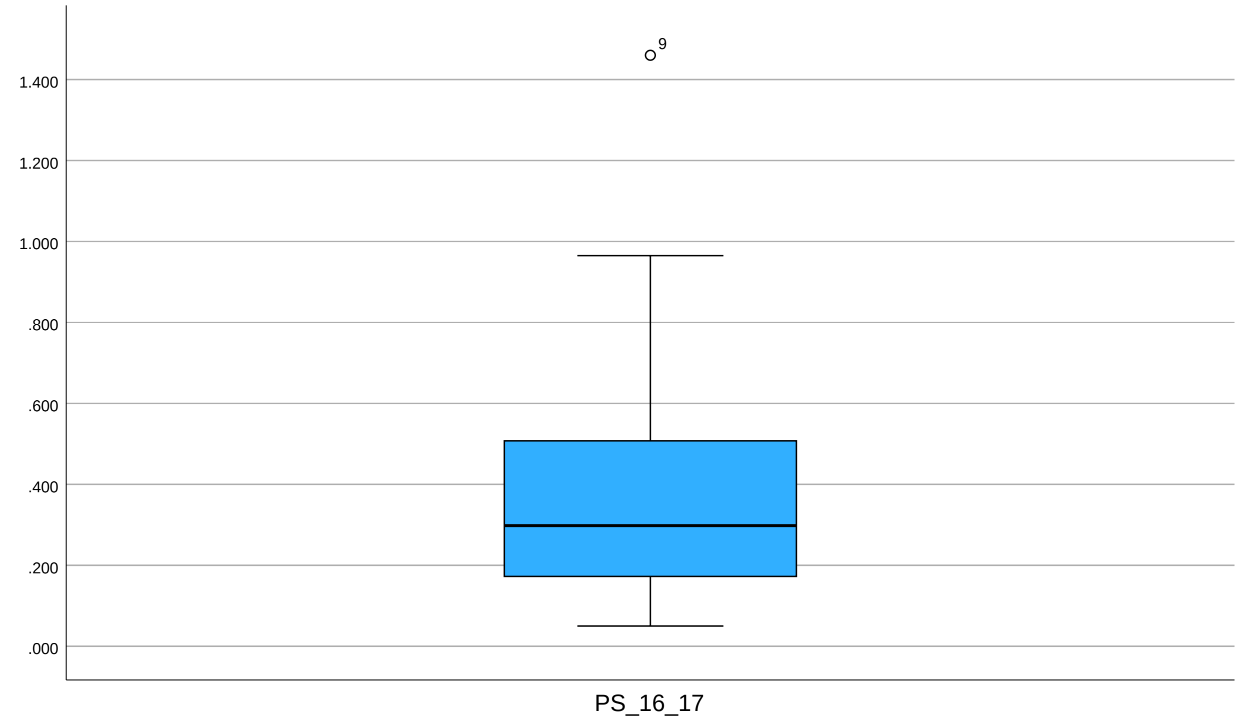

PS_17_18

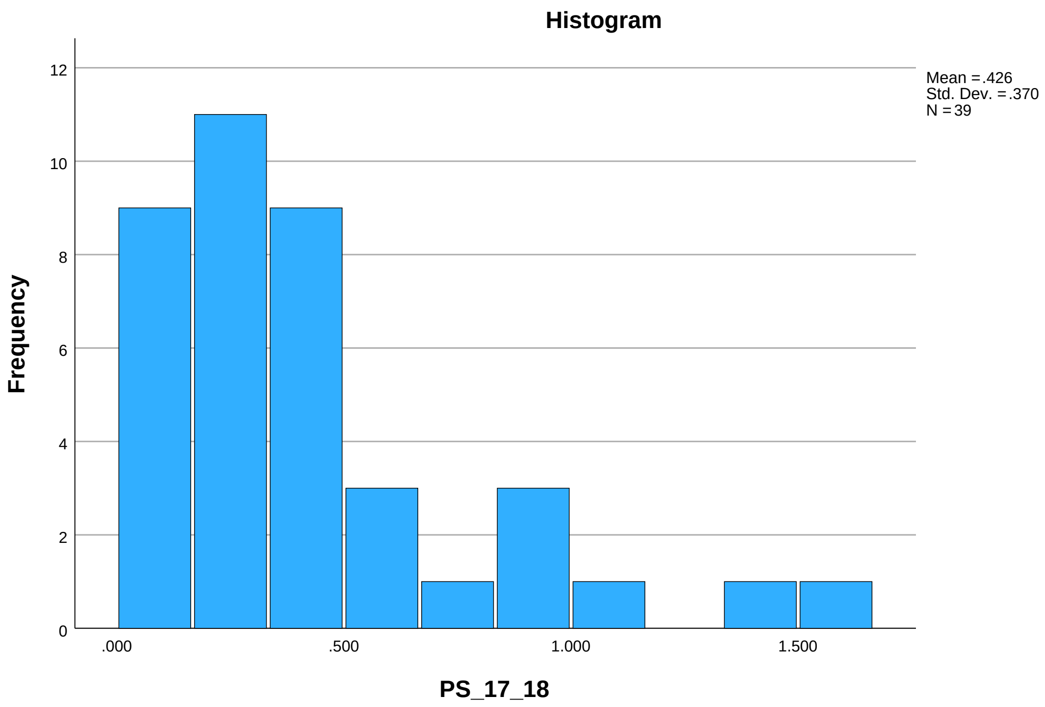

PS_17_18 Stem-and-Leaf Plot

Frequency   Stem & Leaf

```
    4.00        0 .  3457
   10.00        1 .  0335577888
    4.00        2 .  3344
    4.00        3 .  0237
    7.00        4 .  1234688
    2.00        5 .  36
    1.00        6 .  2
    1.00        7 .  3
     .00        8 .
    3.00        9 .  138
    3.00 Extremes    (>=1.11)

 Stem width:      .100
 Each leaf:       1 case(s)
```

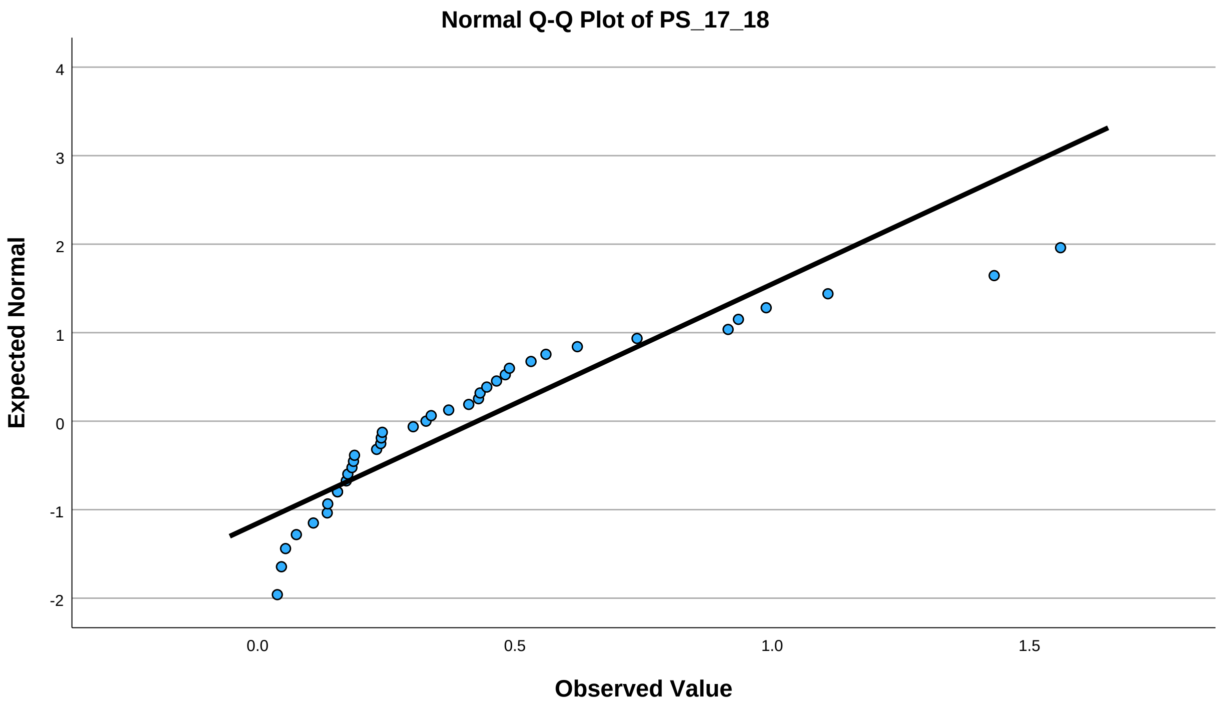

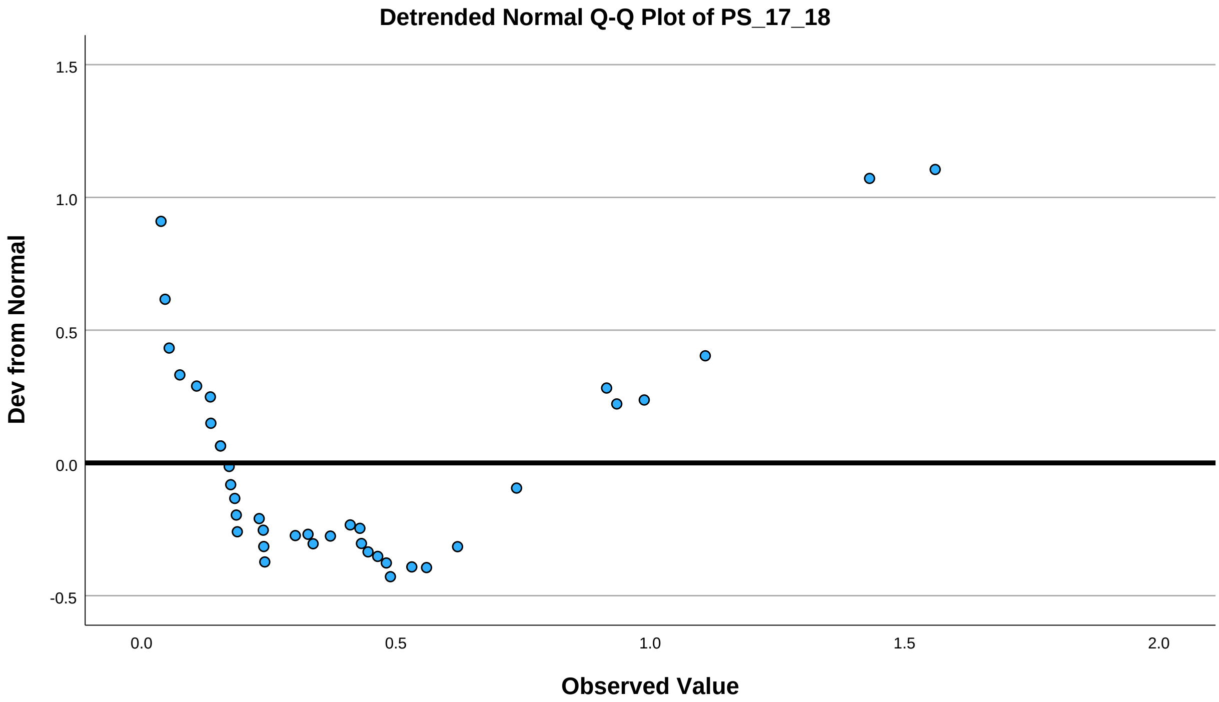
Detrended Normal Q-Q Plot of PS_17_18
Dev from Normal
1.5
1.0
0.5
0.0
-0.5
0.0
0.5
1.0
1.5
2.0
Observed Value

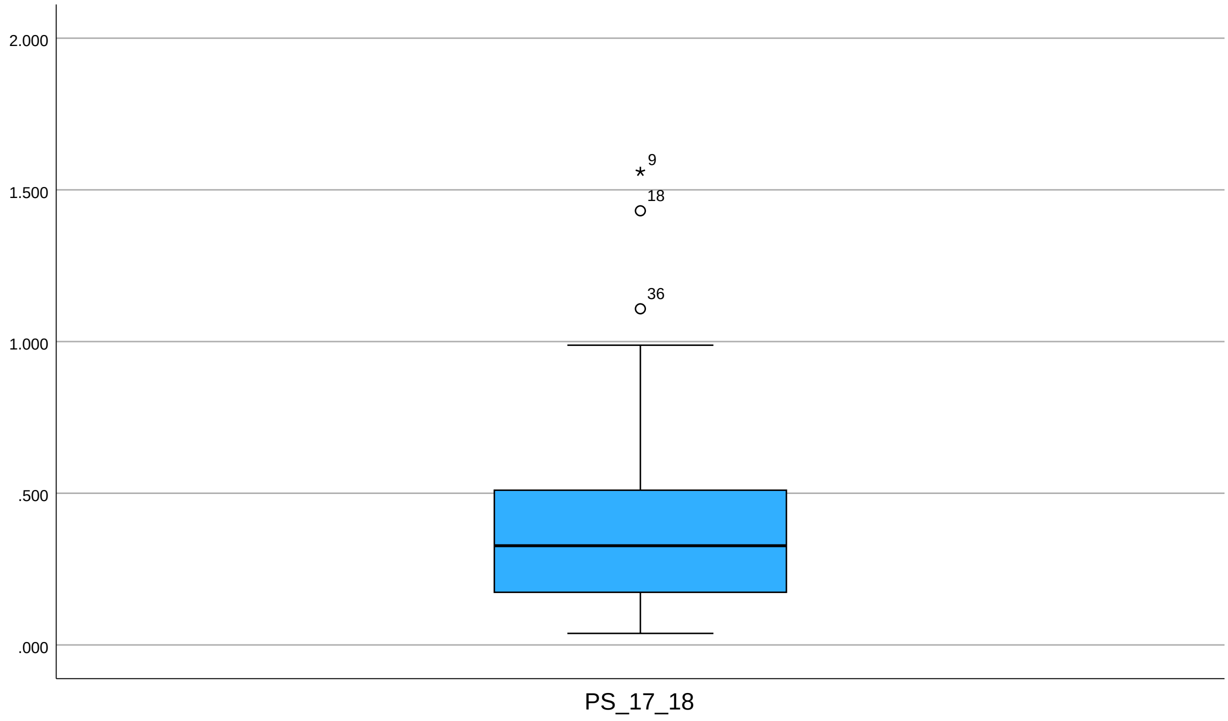
2.000
1.500
1.000
.500
.000
9
18
36
PS_17_18

PS_18_19

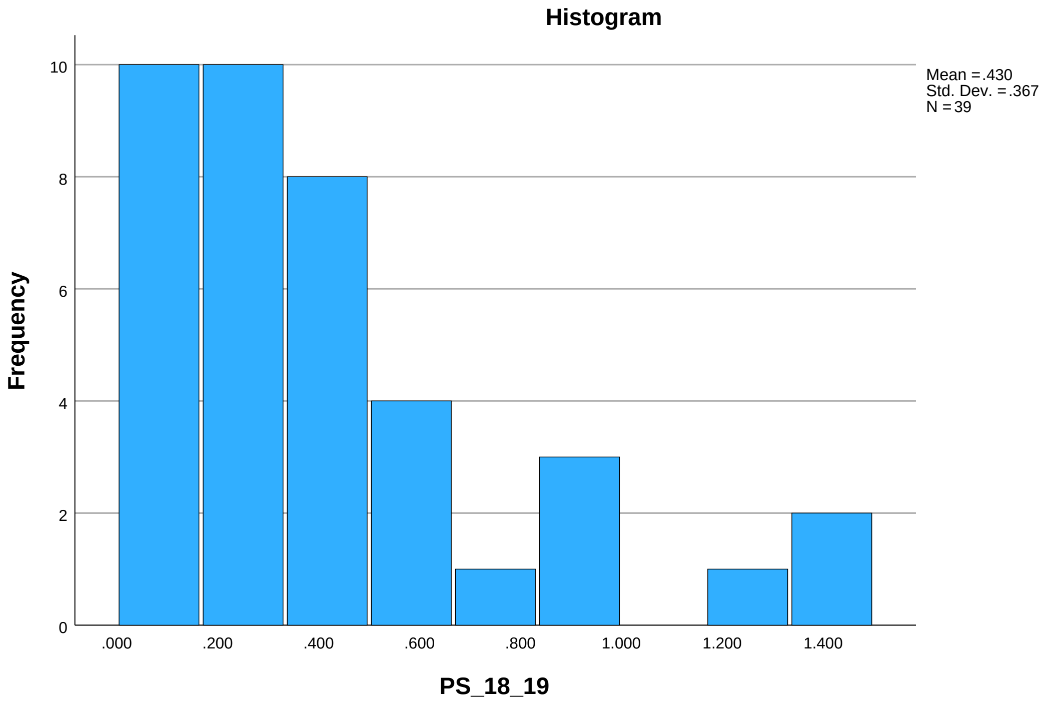

PS_18_19 Stem-and-Leaf Plot

```
 Frequency    Stem &  Leaf

     4.00        0 .  4467
     9.00        1 .  003466778
     6.00        2 .  013558
     3.00        3 .  134
     6.00        4 .  367789
     2.00        5 .  44
     2.00        6 .  13
     1.00        7 .  8
     2.00        8 .  36
     1.00        9 .  8
     3.00 Extremes    (>=1.24)

 Stem width:      .100
 Each leaf:       1 case(s)
```

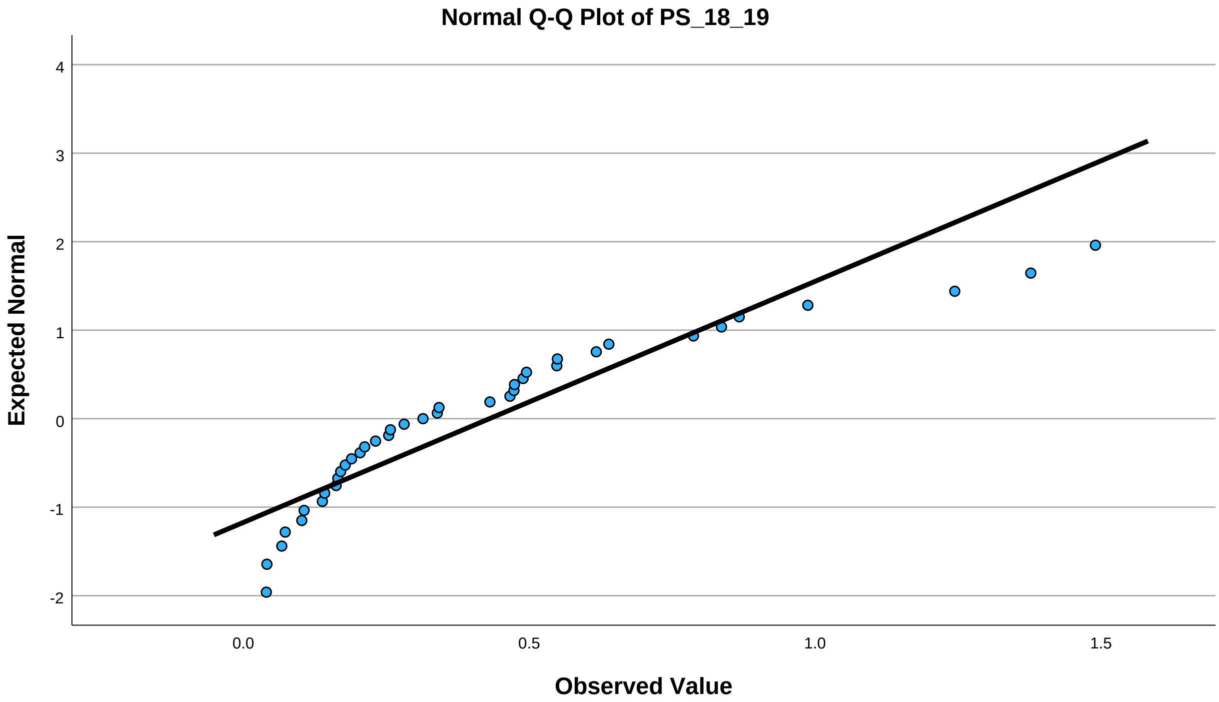
Normal Q-Q Plot of PS_18_19
Expected Normal
Observed Value
4
3
2
1
0
-1
-2
0.0
0.5
1.0
1.5

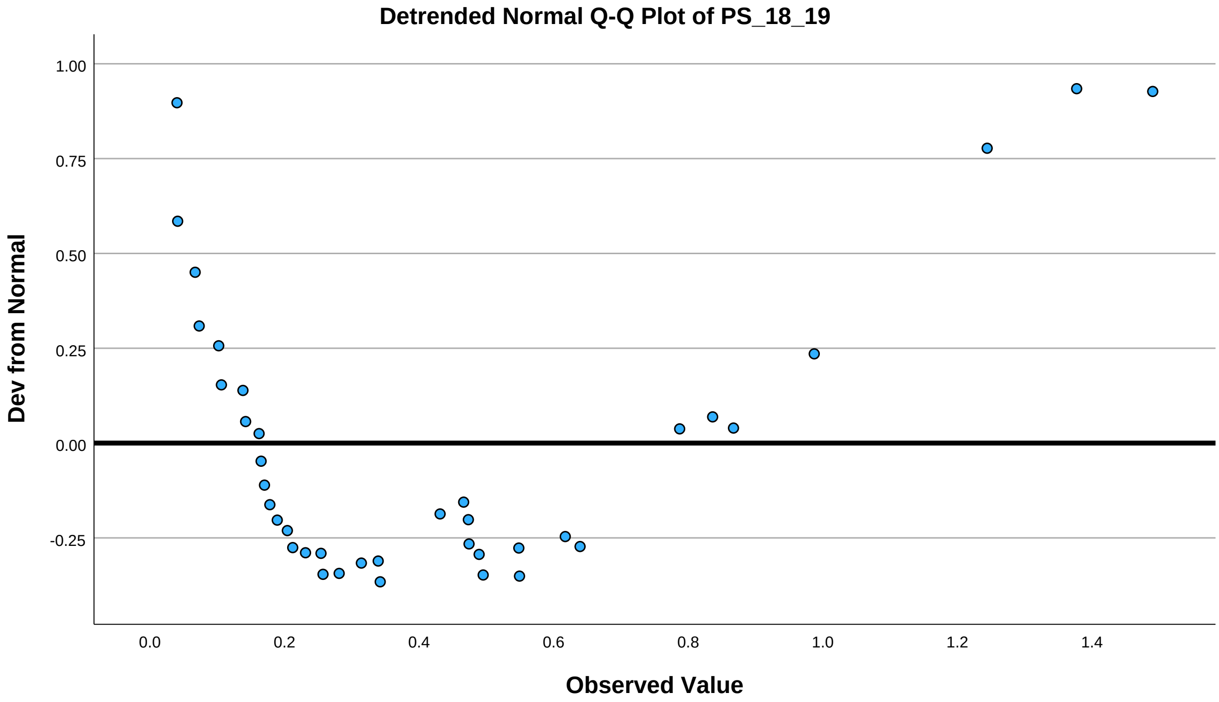
Detrended Normal Q-Q Plot of PS_18_19
Dev from Normal
Observed Value
1.00
0.75
0.50
0.25
0.00
-0.25
0.0
0.2
0.4
0.6
0.8
1.0
1.2
1.4

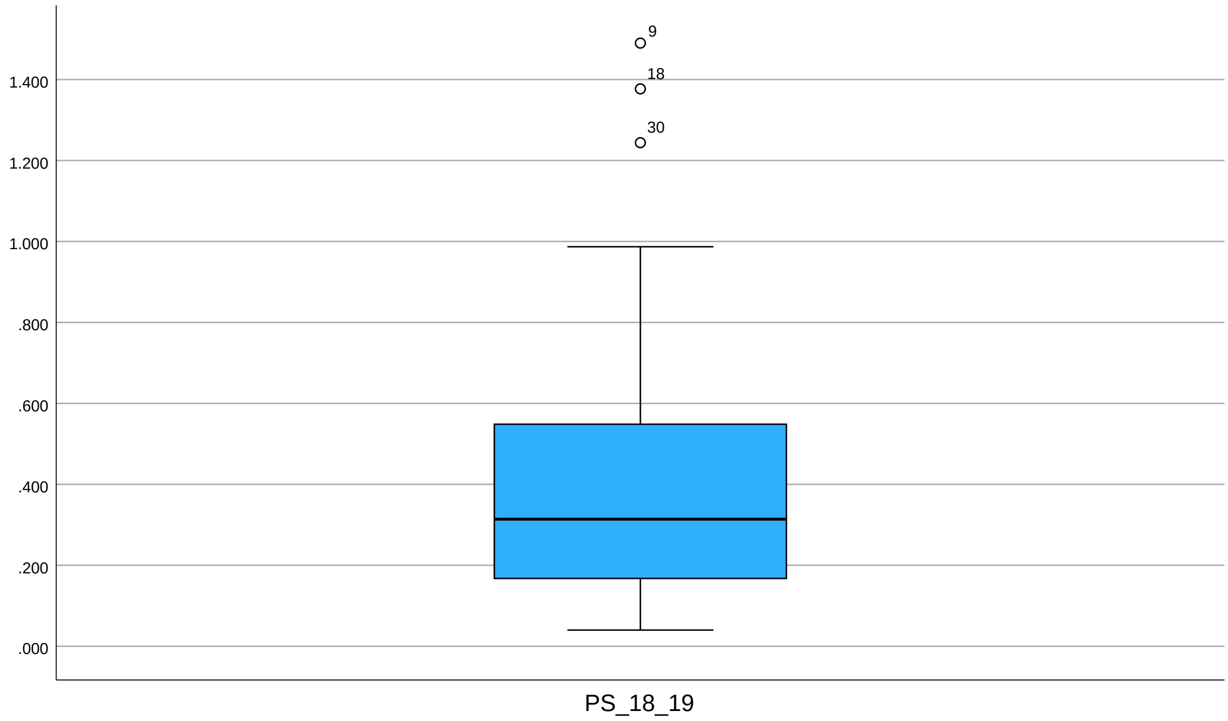

PS_19_20

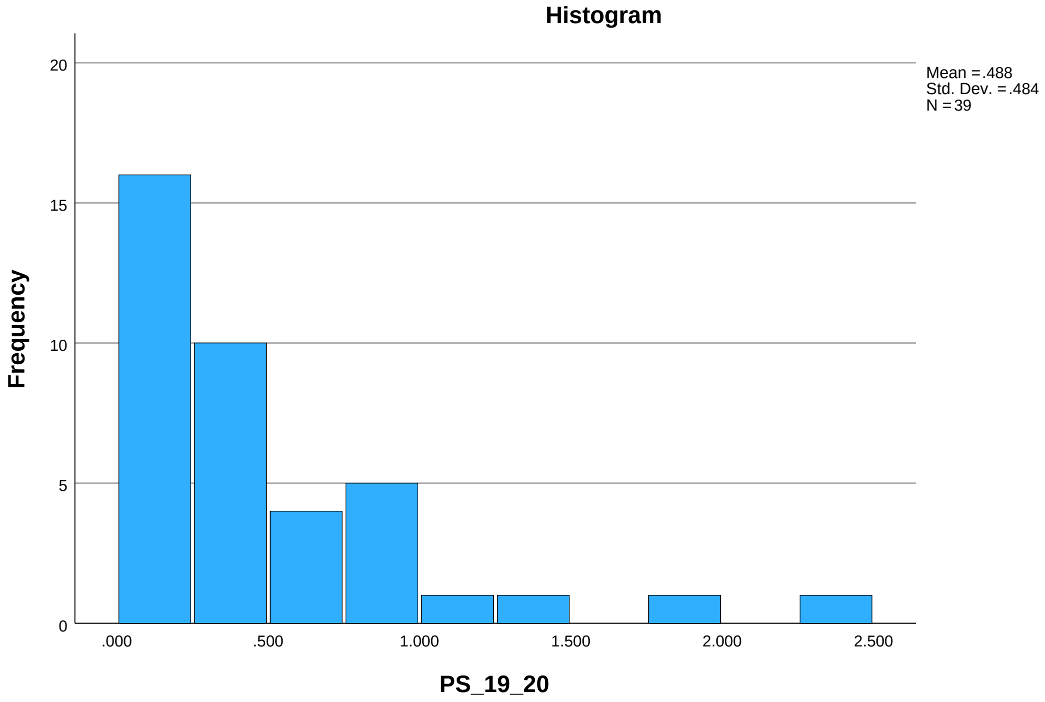


```
PS_19_20 Stem-and-Leaf Plot

 Frequency    Stem &  Leaf
```

```
 4.00      0 . 3448
 8.00      1 . 01246778
 6.00      2 . 013389
 4.00      3 . 3366
 4.00      4 . 3668
 3.00      5 . 123
 1.00      6 . 8
 2.00      7 . 58
 3.00      8 . 268
  .00      9 .
 1.00     10 . 0
 3.00 Extremes    (>=1.40)

Stem width:      .100
Each leaf:       1 case(s)
```

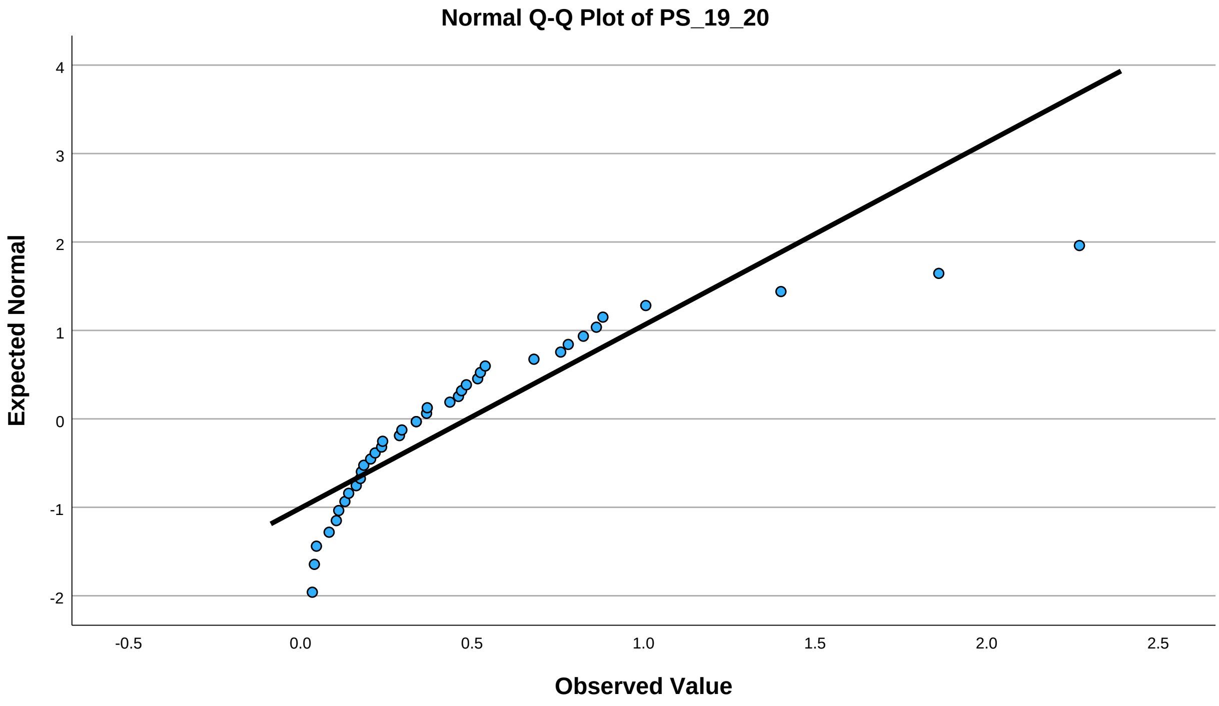

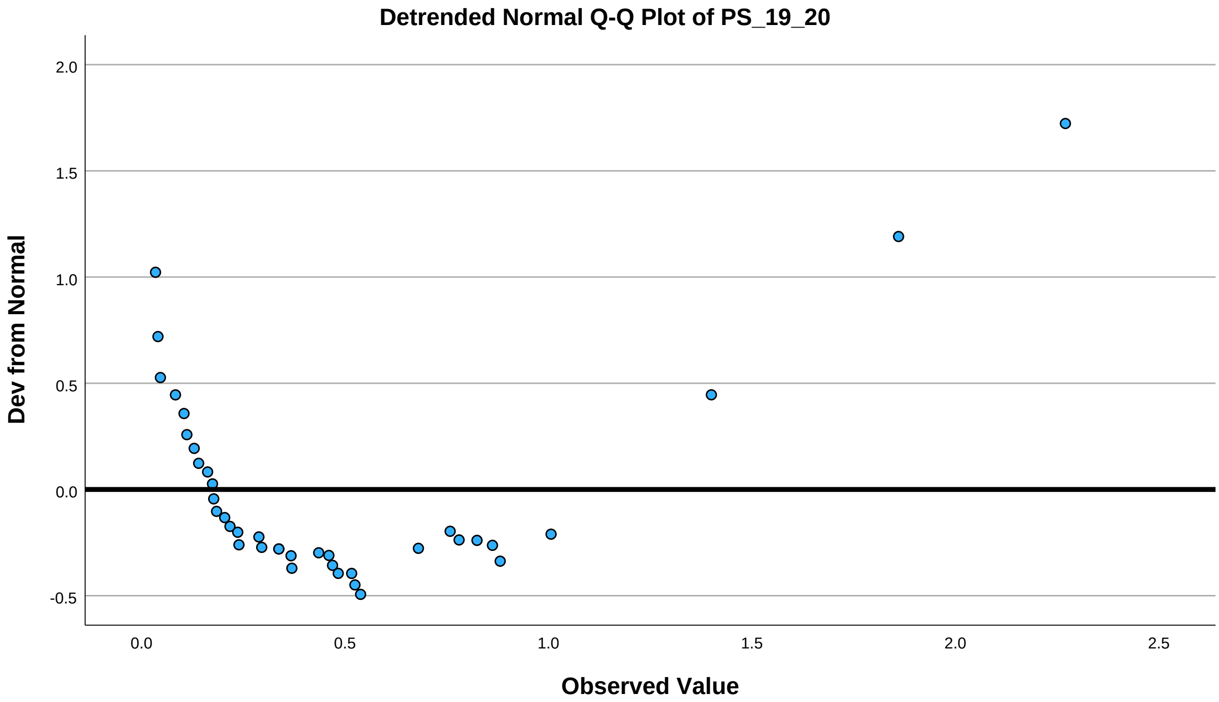
Detrended Normal Q-Q Plot of PS_19_20
Dev from Normal
2.0
1.5
1.0
0.5
0.0
-0.5
0.0
0.5
1.0
1.5
2.0
2.5
Observed Value

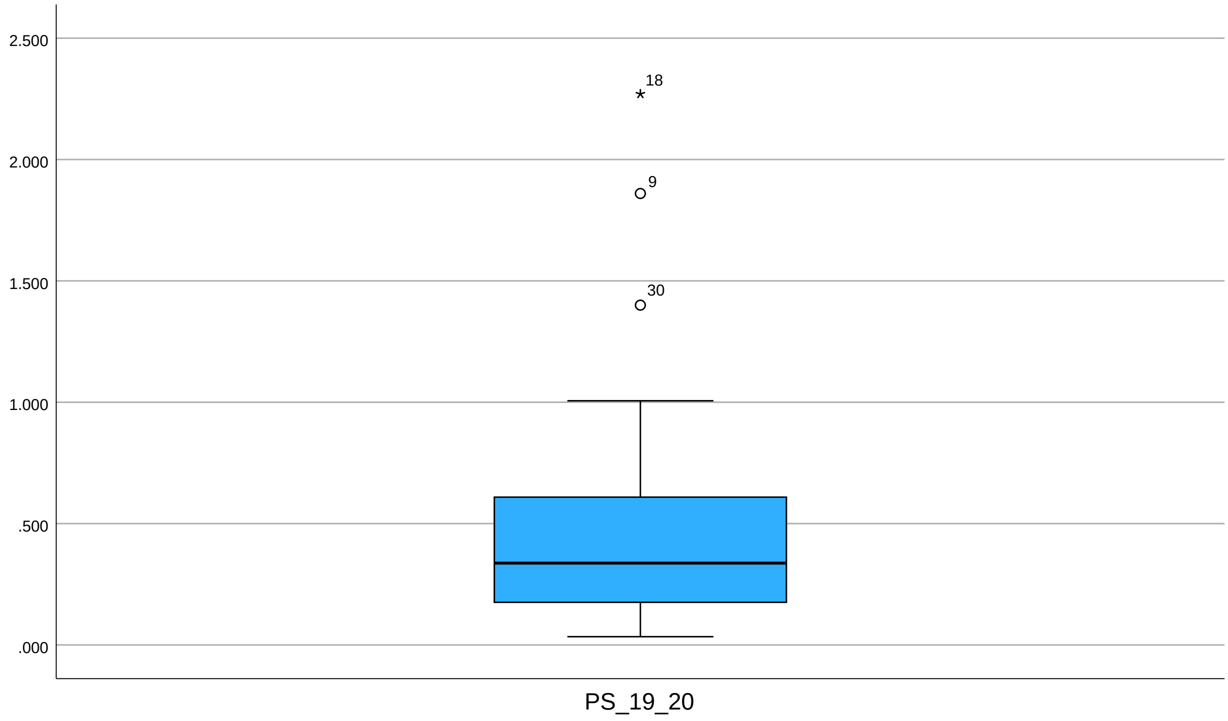
2.500
2.000
1.500
1.000
.500
.000
18
9
30
PS_19_20

Explore

Case Processing Summary

	Cases					
	Valid		Missing		Total	
	N	Percent	N	Percent	N	Percent
BrainRate	39	100.0%	0	0.0%	39	100.0%
PermutationEntropy	39	100.0%	0	0.0%	39	100.0%
PermutationEntropy_01	39	100.0%	0	0.0%	39	100.0%
SampleEntropy	39	100.0%	0	0.0%	39	100.0%
SpectralEntropy_bits	39	100.0%	0	0.0%	39	100.0%
SpectralEntropy_01	39	100.0%	0	0.0%	39	100.0%
HiguchiFractalDimension	39	100.0%	0	0.0%	39	100.0%
LempelZivComplexity	39	100.0%	0	0.0%	39	100.0%
RenyiEntropy	39	100.0%	0	0.0%	39	100.0%
TsallisEntropy	39	100.0%	0	0.0%	39	100.0%
PS_4_5	39	100.0%	0	0.0%	39	100.0%
PS_5_6	39	100.0%	0	0.0%	39	100.0%
PS_6_7	39	100.0%	0	0.0%	39	100.0%
PS_7_8	39	100.0%	0	0.0%	39	100.0%
PS_8_9	39	100.0%	0	0.0%	39	100.0%
PS_9_10	39	100.0%	0	0.0%	39	100.0%
PS_10_11	39	100.0%	0	0.0%	39	100.0%
PS_11_12	39	100.0%	0	0.0%	39	100.0%
PS_12_13	39	100.0%	0	0.0%	39	100.0%
PS_13_14	39	100.0%	0	0.0%	39	100.0%
PS_14_15	39	100.0%	0	0.0%	39	100.0%
PS_15_16	39	100.0%	0	0.0%	39	100.0%
PS_16_17	39	100.0%	0	0.0%	39	100.0%
PS_17_18	39	100.0%	0	0.0%	39	100.0%
PS_18_19	39	100.0%	0	0.0%	39	100.0%
PS_19_20	39	100.0%	0	0.0%	39	100.0%

Descriptives

			Statistic	Std. Error
BrainRate	Mean		11.2303	.91890
	95% Confidence Interval for Mean	Lower Bound	9.3700	
		Upper Bound	13.0905	
	5% Trimmed Mean		10.6997	
	Median		10.0400	
	Variance		32.931	
	Std. Deviation		5.73854	
	Minimum		4.57	
	Maximum		30.28	
	Range		25.71	
	Interquartile Range		7.58	
	Skewness		1.360	.378
	Kurtosis		2.121	.741
PermutationEntropy	Mean		1.5608478296	.00491638544
	95% Confidence Interval for Mean	Lower Bound	1.5508951276	
		Upper Bound	1.5708005316	
	5% Trimmed Mean		1.5626399415	
	Median		1.5640314949	
	Variance		.001	
	Std. Deviation		.03070281726	
	Minimum		1.4585155426	
	Maximum		1.6130314857	
	Range		.15451594306	
	Interquartile Range		.04864835445	
	Skewness		-.976	.378
	Kurtosis		1.885	.741
PermutationEntropy_01	Mean		.87112576012	.00274388696
	95% Confidence Interval for Mean	Lower Bound	.86557105137	
		Upper Bound	.87668046887	
	5% Trimmed Mean		.87212595682	
	Median		.87290259759	
	Variance		.000	
	Std. Deviation		.01713556858	
	Minimum		.81401302332	
	Maximum		.90025001312	
	Range		.08623698979	
	Interquartile Range		.02715116358	
	Skewness		-.976	.378
	Kurtosis		1.885	.741

Descriptives

			Statistic	Std. Error
SampleEntropy	Mean		.82961234934	.04618430401
	95% Confidence Interval for Mean	Lower Bound	.73611711384	
		Upper Bound	.92310758485	
	5% Trimmed Mean		.83570015742	
	Median		.89430336577	
	Variance		.083	
	Std. Deviation		.28842088611	
	Minimum		.24540291659	
	Maximum		1.3085076761	
	Range		1.0631047595	
	Interquartile Range		.45839366914	
	Skewness		-.425	.378
	Kurtosis		-.719	.741
SpectralEntropy_bits	Mean		4.3143203387	.12653021514
	95% Confidence Interval for Mean	Lower Bound	4.0581733096	
		Upper Bound	4.5704673678	
	5% Trimmed Mean		4.3304310638	
	Median		4.3299151048	
	Variance		.624	
	Std. Deviation		.79018094031	
	Minimum		2.6090914877	
	Maximum		5.5803574014	
	Range		2.9712659136	
	Interquartile Range		1.2600275532	
	Skewness		-.299	.378
	Kurtosis		-.696	.741
SpectralEntropy_01	Mean		.61534452978	.01804679987
	95% Confidence Interval for Mean	Lower Bound	.57881069344	
		Upper Bound	.65187836612	
	5% Trimmed Mean		.61764237643	
	Median		.61756878604	
	Variance		.013	
	Std. Deviation		.11270222909	
	Minimum		.37213049765	
	Maximum		.79591734771	
	Range		.42378685006	
	Interquartile Range		.17971569132	
	Skewness		-.299	.378
	Kurtosis		-.696	.741

Descriptives

			Statistic	Std. Error
HiguchiFractalDimension	Mean		.36761831763	.01739535106
	95% Confidence Interval for Mean	Lower Bound	.33240327046	
		Upper Bound	.40283336480	
	5% Trimmed Mean		.36667485886	
	Median		.37095361707	
	Variance		.012	
	Std. Deviation		.10863393257	
	Minimum		.14561988793	
	Maximum		.60569823977	
	Range		.46007835184	
	Interquartile Range		.12719266814	
	Skewness		.215	.378
	Kurtosis		.237	.741
LempelZivComplexity	Mean		.31583709354	.00754738563
	95% Confidence Interval for Mean	Lower Bound	.30055821013	
		Upper Bound	.33111597696	
	5% Trimmed Mean		.31442207594	
	Median		.30414947870	
	Variance		.002	
	Std. Deviation		.04713340813	
	Minimum		.23510023334	
	Maximum		.42617528481	
	Range		.19107505147	
	Interquartile Range		.06516883605	
	Skewness		.430	.378
	Kurtosis		-.253	.741
RenyiEntropy	Mean		7.1762502992	.18460306643
	95% Confidence Interval for Mean	Lower Bound	6.8025409289	
		Upper Bound	7.5499596695	
	5% Trimmed Mean		7.2324040983	
	Median		7.5232298881	
	Variance		1.329	
	Std. Deviation		1.1528457804	
	Minimum		4.4784558973	
	Maximum		8.7728302947	
	Range		4.2943743974	
	Interquartile Range		1.9732748092	
	Skewness		-.792	.378
	Kurtosis		-.480	.741

Descriptives

			Statistic	Std. Error
TsallisEntropy	Mean		1.8426561361	.06740343974
	95% Confidence Interval for Mean	Lower Bound	1.7062050060	
		Upper Bound	1.9791072661	
	5% Trimmed Mean		1.9175043542	
	Median		1.9841421550	
	Variance		.177	
	Std. Deviation		.42093434623	
	Minimum		.17044907143	
	Maximum		1.9889123594	
	Range		1.8184632880	
	Interquartile Range		.02185908256	
	Skewness		-3.247	.378
	Kurtosis		9.590	.741
PS_4_5	Mean		1.73295	.393014
	95% Confidence Interval for Mean	Lower Bound	.93733	
		Upper Bound	2.52856	
	5% Trimmed Mean		1.35432	
	Median		1.13800	
	Variance		6.024	
	Std. Deviation		2.454371	
	Minimum		.294	
	Maximum		15.850	
	Range		15.556	
	Interquartile Range		1.137	
	Skewness		5.260	.378
	Kurtosis		30.518	.741
PS_5_6	Mean		1.38397	.331507
	95% Confidence Interval for Mean	Lower Bound	.71287	
		Upper Bound	2.05508	
	5% Trimmed Mean		1.07071	
	Median		.95000	
	Variance		4.286	
	Std. Deviation		2.070262	
	Minimum		.210	
	Maximum		13.410	
	Range		13.200	
	Interquartile Range		.601	
	Skewness		5.419	.378
	Kurtosis		31.867	.741

Descriptives

			Statistic	Std. Error
PS_6_7	Mean		1.02123	.163588
	95% Confidence Interval for Mean	Lower Bound	.69006	
		Upper Bound	1.35240	
	5% Trimmed Mean		.88093	
	Median		.81100	
	Variance		1.044	
	Std. Deviation		1.021608	
	Minimum		.189	
	Maximum		6.480	
	Range		6.291	
	Interquartile Range		.530	
	Skewness		4.242	.378
	Kurtosis		22.050	.741
PS_7_8	Mean		.92462	.124291
	95% Confidence Interval for Mean	Lower Bound	.67300	
		Upper Bound	1.17623	
	5% Trimmed Mean		.81562	
	Median		.70400	
	Variance		.602	
	Std. Deviation		.776199	
	Minimum		.156	
	Maximum		4.210	
	Range		4.054	
	Interquartile Range		.549	
	Skewness		2.636	.378
	Kurtosis		8.690	.741
PS_8_9	Mean		.81708	.122856
	95% Confidence Interval for Mean	Lower Bound	.56837	
		Upper Bound	1.06579	
	5% Trimmed Mean		.70268	
	Median		.58500	
	Variance		.589	
	Std. Deviation		.767236	
	Minimum		.125	
	Maximum		3.940	
	Range		3.815	
	Interquartile Range		.454	
	Skewness		2.700	.378
	Kurtosis		8.283	.741

Descriptives

			Statistic	Std. Error
PS_9_10	Mean		.87728	.180539
	95% Confidence Interval for Mean	Lower Bound	.51180	
		Upper Bound	1.24276	
	5% Trimmed Mean		.70640	
	Median		.66400	
	Variance		1.271	
	Std. Deviation		1.127466	
	Minimum		.097	
	Maximum		7.200	
	Range		7.103	
	Interquartile Range		.429	
	Skewness		4.898	.378
	Kurtosis		27.333	.741
PS_10_11	Mean		.98195	.169932
	95% Confidence Interval for Mean	Lower Bound	.63794	
		Upper Bound	1.32596	
	5% Trimmed Mean		.83277	
	Median		.75100	
	Variance		1.126	
	Std. Deviation		1.061228	
	Minimum		.104	
	Maximum		6.560	
	Range		6.456	
	Interquartile Range		.612	
	Skewness		4.092	.378
	Kurtosis		20.538	.741
PS_11_12	Mean		.50695	.078225
	95% Confidence Interval for Mean	Lower Bound	.34859	
		Upper Bound	.66531	
	5% Trimmed Mean		.43404	
	Median		.36700	
	Variance		.239	
	Std. Deviation		.488513	
	Minimum		.077	
	Maximum		2.373	
	Range		2.296	
	Interquartile Range		.264	
	Skewness		2.786	.378
	Kurtosis		7.808	.741

Descriptives

			Statistic	Std. Error
PS_12_13	Mean		.40354	.062304
	95% Confidence Interval for Mean	Lower Bound	.27741	
		Upper Bound	.52967	
	5% Trimmed Mean		.34903	
	Median		.32400	
	Variance		.151	
	Std. Deviation		.389091	
	Minimum		.059	
	Maximum		2.310	
	Range		2.251	
	Interquartile Range		.239	
	Skewness		3.450	.378
	Kurtosis		15.112	.741
PS_13_14	Mean		.36767	.045302
	95% Confidence Interval for Mean	Lower Bound	.27596	
		Upper Bound	.45938	
	5% Trimmed Mean		.33618	
	Median		.30100	
	Variance		.080	
	Std. Deviation		.282912	
	Minimum		.057	
	Maximum		1.440	
	Range		1.383	
	Interquartile Range		.259	
	Skewness		1.909	.378
	Kurtosis		4.683	.741
PS_14_15	Mean		.39262	.053528
	95% Confidence Interval for Mean	Lower Bound	.28425	
		Upper Bound	.50098	
	5% Trimmed Mean		.35044	
	Median		.27200	
	Variance		.112	
	Std. Deviation		.334280	
	Minimum		.055	
	Maximum		1.750	
	Range		1.695	
	Interquartile Range		.292	
	Skewness		2.221	.378
	Kurtosis		6.587	.741

Descriptives

			Statistic	Std. Error
PS_15_16	Mean		.38728	.048876
	95% Confidence Interval for Mean	Lower Bound	.28834	
		Upper Bound	.48623	
	5% Trimmed Mean		.35785	
	Median		.30000	
	Variance		.093	
	Std. Deviation		.305229	
	Minimum		.049	
	Maximum		1.420	
	Range		1.371	
	Interquartile Range		.307	
	Skewness		1.525	.378
	Kurtosis		2.460	.741
PS_16_17	Mean		.39562	.049868
	95% Confidence Interval for Mean	Lower Bound	.29466	
		Upper Bound	.49657	
	5% Trimmed Mean		.36905	
	Median		.29800	
	Variance		.097	
	Std. Deviation		.311423	
	Minimum		.050	
	Maximum		1.460	
	Range		1.410	
	Interquartile Range		.341	
	Skewness		1.475	.378
	Kurtosis		2.376	.741
PS_17_18	Mean		.42638	.059222
	95% Confidence Interval for Mean	Lower Bound	.30650	
		Upper Bound	.54627	
	5% Trimmed Mean		.38826	
	Median		.32700	
	Variance		.137	
	Std. Deviation		.369839	
	Minimum		.038	
	Maximum		1.560	
	Range		1.522	
	Interquartile Range		.359	
	Skewness		1.545	.378
	Kurtosis		2.113	.741

Descriptives

			Statistic	Std. Error
PS_18_19	Mean		.43015	.058786
	95% Confidence Interval for Mean	Lower Bound	.31115	
		Upper Bound	.54916	
	5% Trimmed Mean		.39598	
	Median		.31400	
	Variance		.135	
	Std. Deviation		.367117	
	Minimum		.040	
	Maximum		1.490	
	Range		1.450	
	Interquartile Range		.384	
	Skewness		1.404	.378
	Kurtosis		1.552	.741
PS_19_20	Mean		.48767	.077494
	95% Confidence Interval for Mean	Lower Bound	.33079	
		Upper Bound	.64454	
	5% Trimmed Mean		.42479	
	Median		.33700	
	Variance		.234	
	Std. Deviation		.483948	
	Minimum		.034	
	Maximum		2.270	
	Range		2.236	
	Interquartile Range		.506	
	Skewness		2.103	.378
	Kurtosis		5.039	.741

Tests of Normality

	Kolmogorov-Smirnov[a]			Shapiro-Wilk		
	Statistic	df	Sig.	Statistic	df	Sig.
BrainRate	.123	39	.142	.883	39	<.001
PermutationEntropy	.095	39	.200*	.945	39	.057
PermutationEntropy_01	.095	39	.200*	.945	39	.057
SampleEntropy	.108	39	.200*	.959	39	.168
SpectralEntropy_bits	.074	39	.200*	.971	39	.415
SpectralEntropy_01	.074	39	.200*	.971	39	.415
HiguchiFractalDimension	.071	39	.200*	.975	39	.525
LempelZivComplexity	.122	39	.152	.973	39	.454
RenyiEntropy	.139	39	.055	.908	39	.004
TsallisEntropy	.414	39	<.001	.386	39	<.001
PS_4_5	.279	39	<.001	.418	39	<.001
PS_5_6	.294	39	<.001	.391	39	<.001
PS_6_7	.240	39	<.001	.570	39	<.001
PS_7_8	.233	39	<.001	.733	39	<.001
PS_8_9	.251	39	<.001	.696	39	<.001
PS_9_10	.287	39	<.001	.472	39	<.001
PS_10_11	.267	39	<.001	.576	39	<.001
PS_11_12	.281	39	<.001	.627	39	<.001
PS_12_13	.274	39	<.001	.643	39	<.001
PS_13_14	.172	39	.005	.828	39	<.001
PS_14_15	.163	39	.011	.789	39	<.001
PS_15_16	.160	39	.014	.858	39	<.001
PS_16_17	.158	39	.016	.862	39	<.001
PS_17_18	.176	39	.004	.835	39	<.001
PS_18_19	.159	39	.014	.851	39	<.001
PS_19_20	.202	39	<.001	.778	39	<.001

*. This is a lower bound of the true significance.

a. Lilliefors Significance Correction

BrainRate

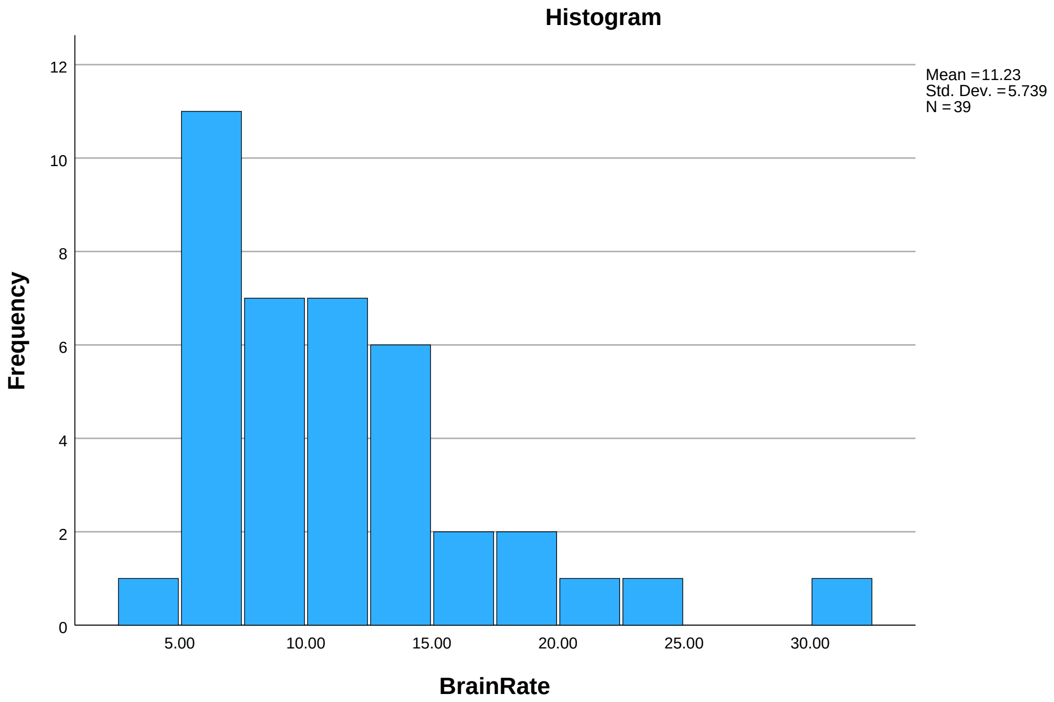


```
BrainRate Stem-and-Leaf Plot

 Frequency    Stem &  Leaf

     1.00        0 .  4
    18.00        0 .  555556666667788999
    13.00        1 .  0011222333334
     4.00        1 .  6699
     2.00        2 .  13
     1.00 Extremes    (>=30)

 Stem width:      10.00
 Each leaf:        1 case(s)
```

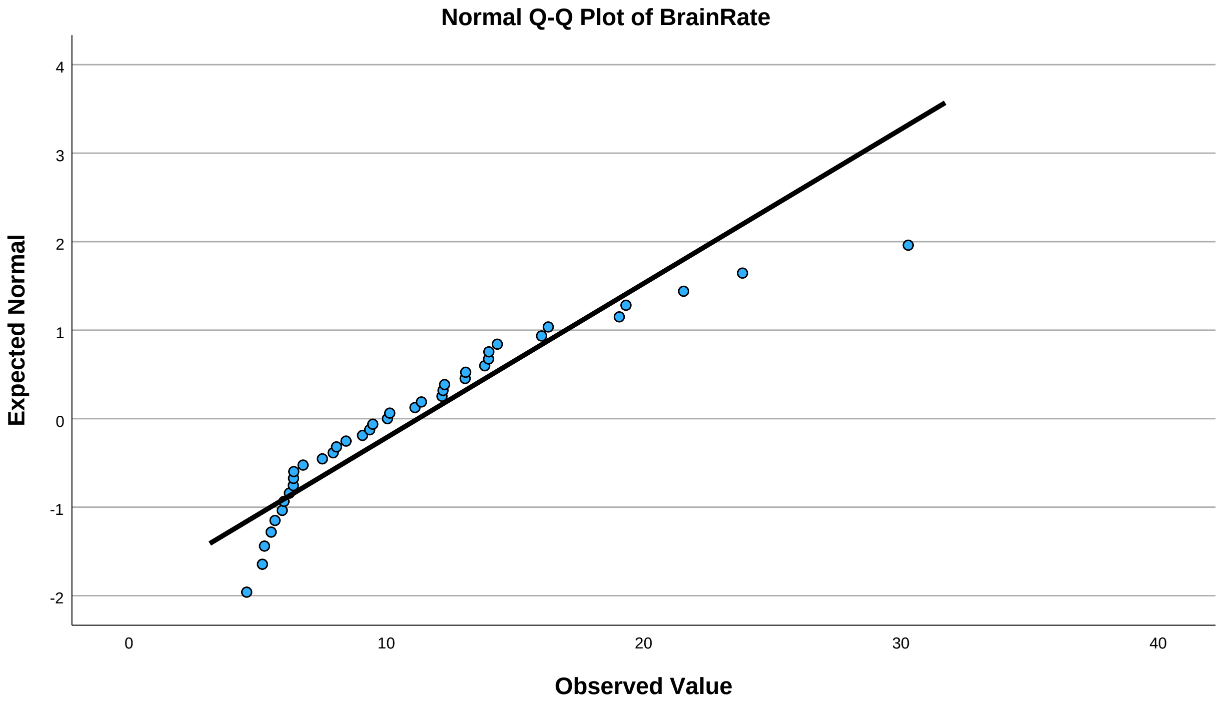

Normal Q-Q Plot of BrainRate
Expected Normal
4
3
2
1
0
-1
-2
0
10
20
30
40
Observed Value

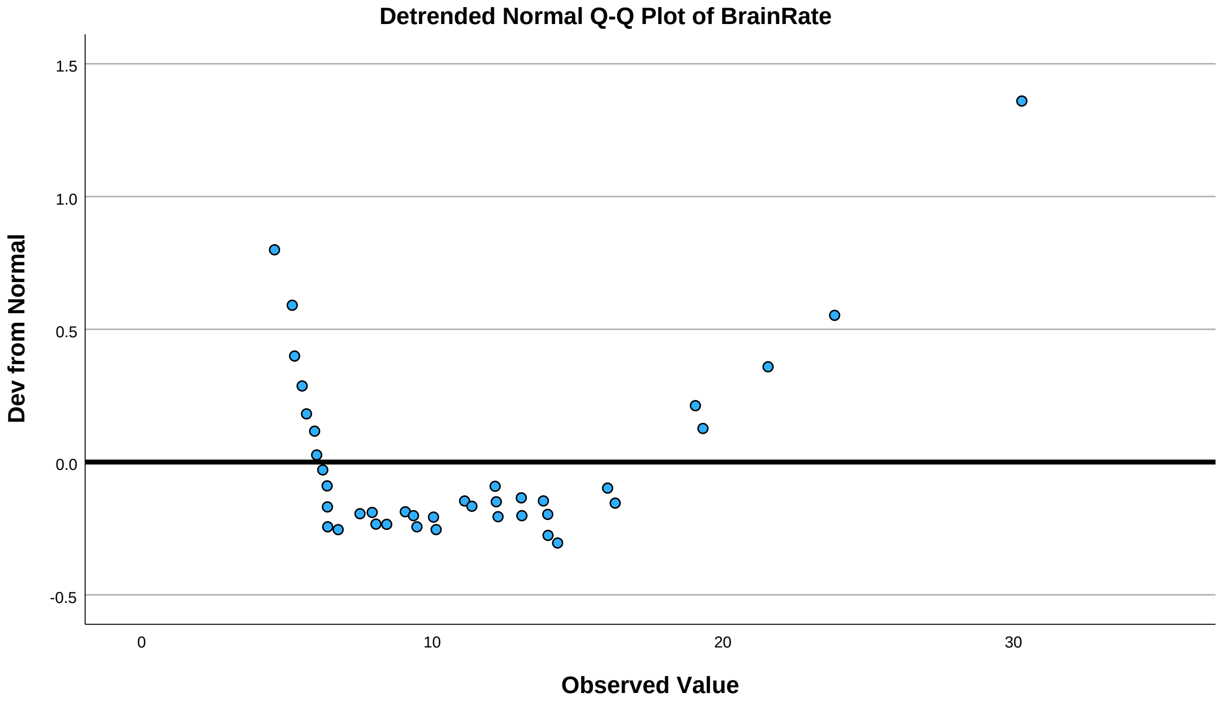

Detrended Normal Q-Q Plot of BrainRate
Dev from Normal
1.5
1.0
0.5
0.0
-0.5
0
10
20
30
Observed Value

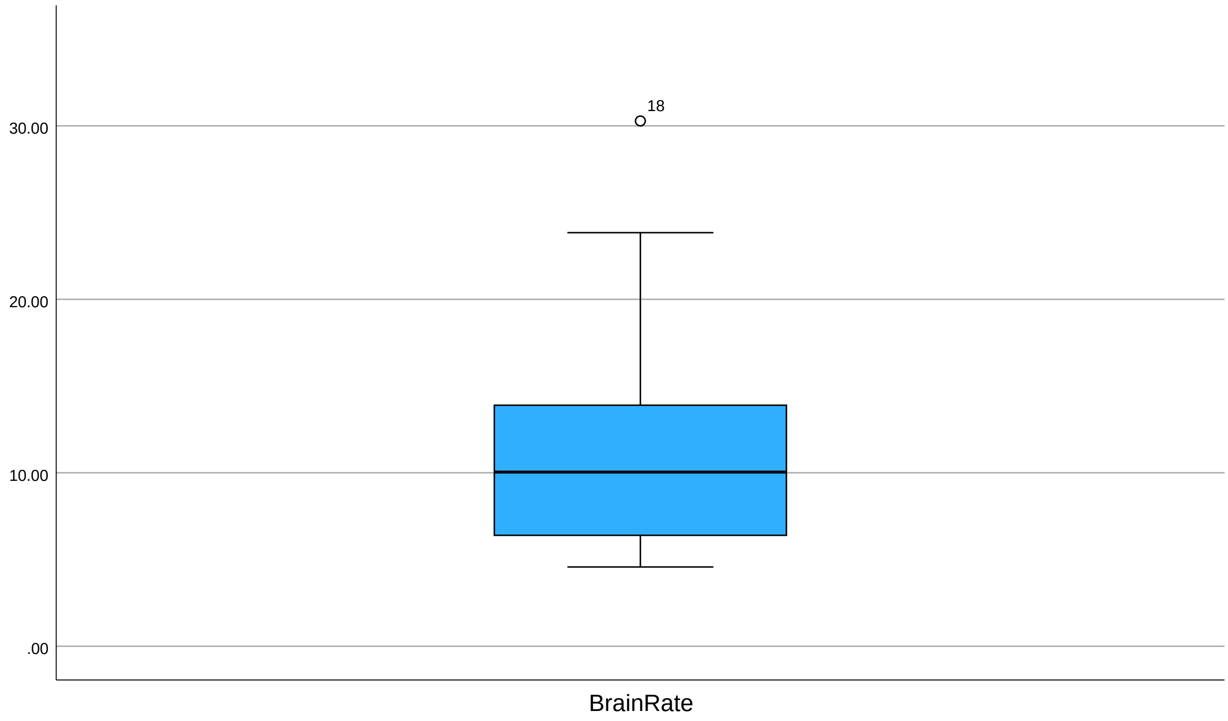

PermutationEntropy

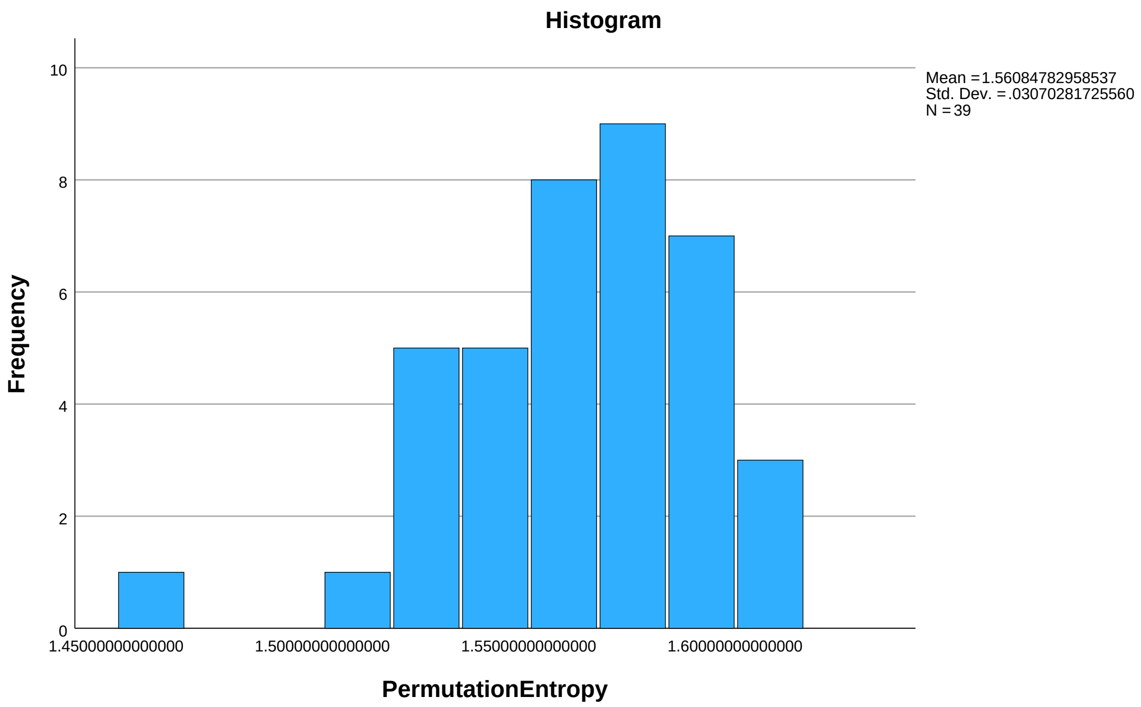

PermutationEntropy Stem-and-Leaf Plot

Frequency   Stem & Leaf

```
 1.00 Extremes    (=<1.459)
 1.00     150 .  3
 1.00     151 .  8
 2.00     152 .  89
 6.00     153 .  333459
 1.00     154 .  9
 5.00     155 .  22478
 4.00     156 .  1249
 6.00     157 .  001239
 7.00     158 .  2245567
 2.00     159 .  37
 2.00     160 .  14
 1.00     161 .  3

Stem width:   .0100000
Each leaf:        1 case(s)
```

Normal Q-Q Plot of PermutationEntropy

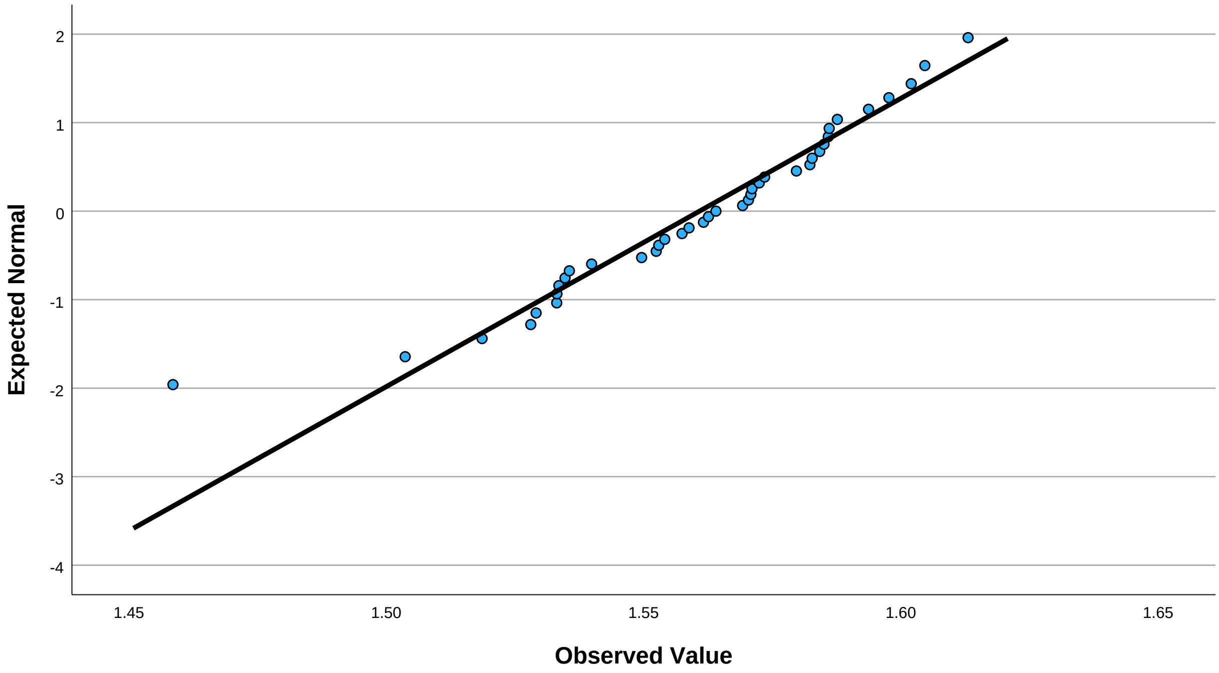

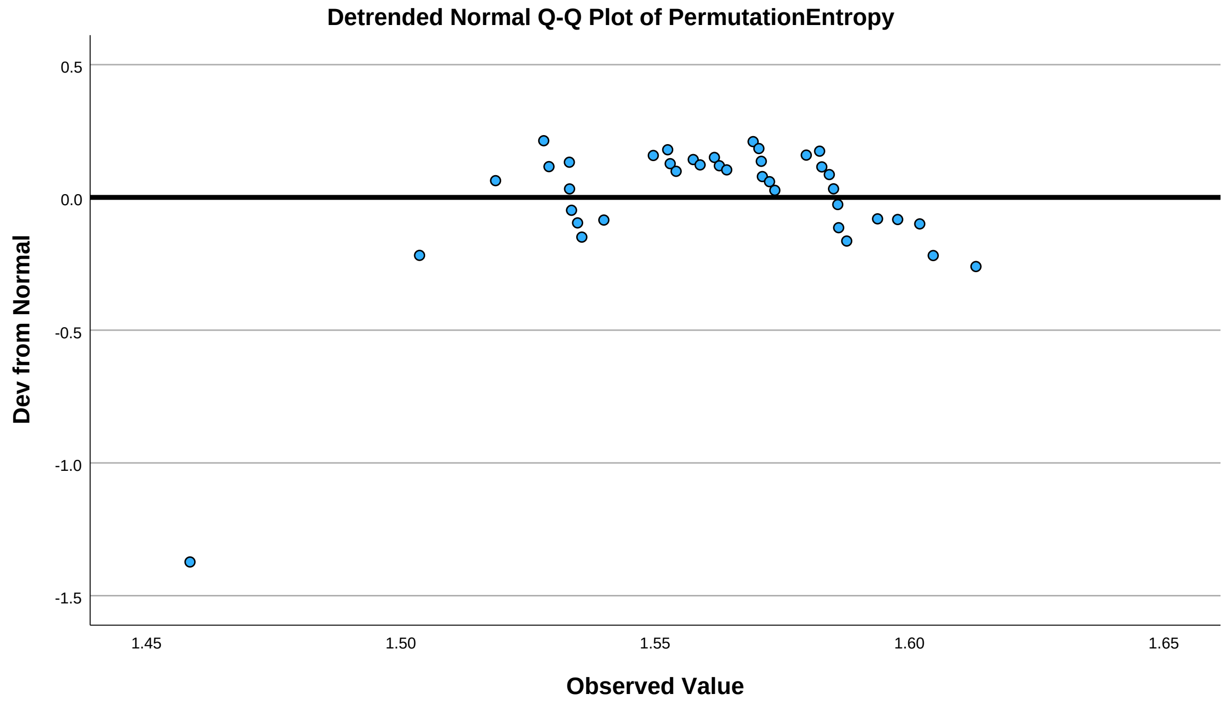

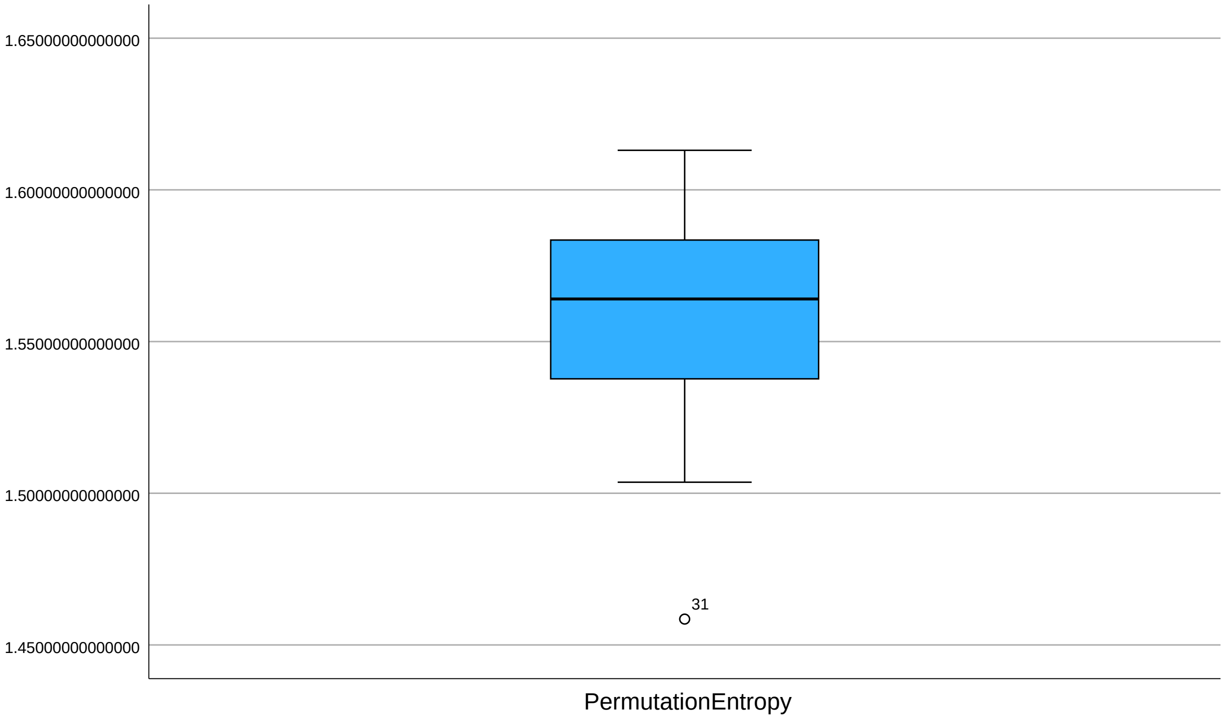

PermutationEntropy_01

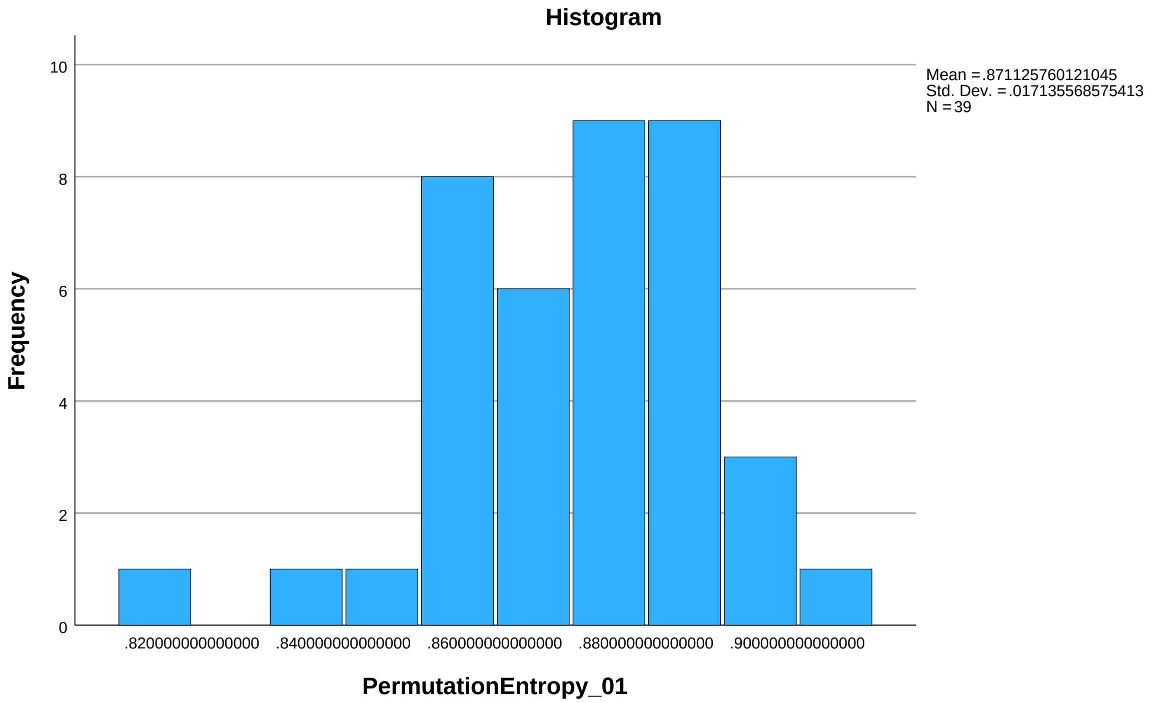


```
PermutationEntropy_01 Stem-and-Leaf Plot

 Frequency    Stem &  Leaf

     1.00 Extremes    (=<.814)
     1.00       83 .  9
     1.00       84 .  7
     8.00       85 .  23555679
     6.00       86 .  466799
     9.00       87 .  122566678
     9.00       88 .  133445569
     3.00       89 .  145
     1.00       90 .  0

 Stem width:   .0100000
 Each leaf:       1 case(s)
```

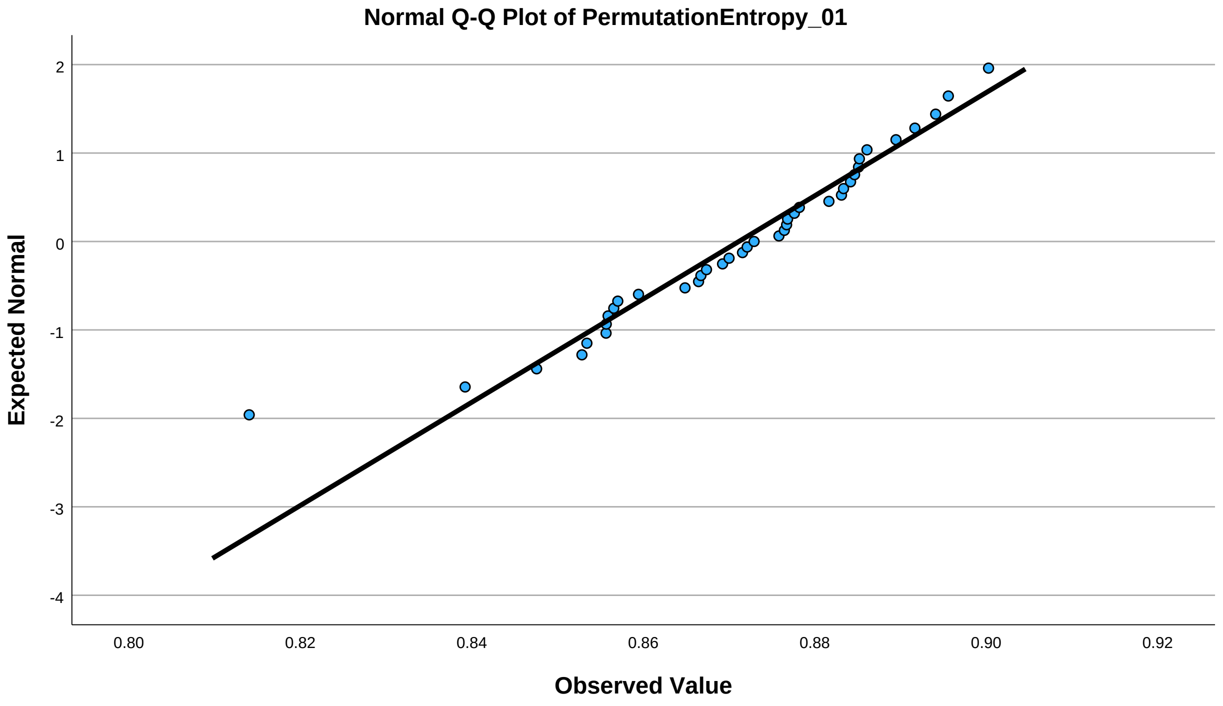
Normal Q-Q Plot of PermutationEntropy_01
Expected Normal
2
1
0
-1
-2
-3
-4
0.80
0.82
0.84
0.86
0.88
0.90
0.92
Observed Value

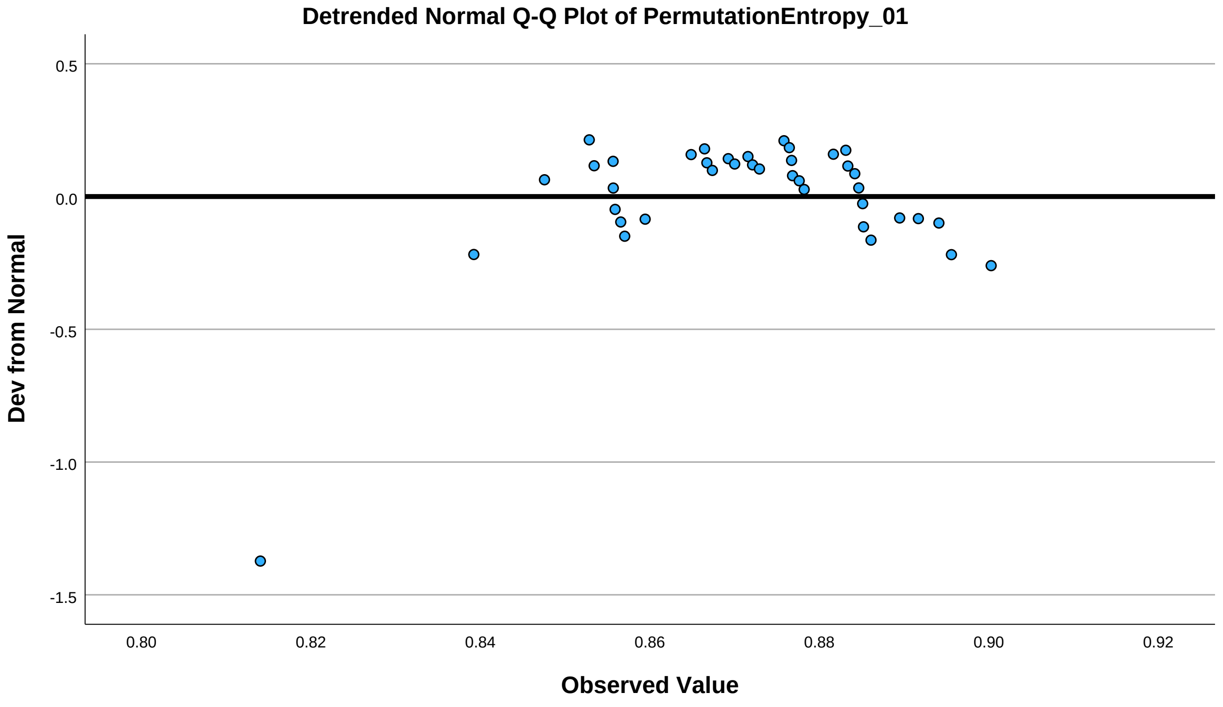
Detrended Normal Q-Q Plot of PermutationEntropy_01
Dev from Normal
0.5
0.0
-0.5
-1.0
-1.5
0.80
0.82
0.84
0.86
0.88
0.90
0.92
Observed Value

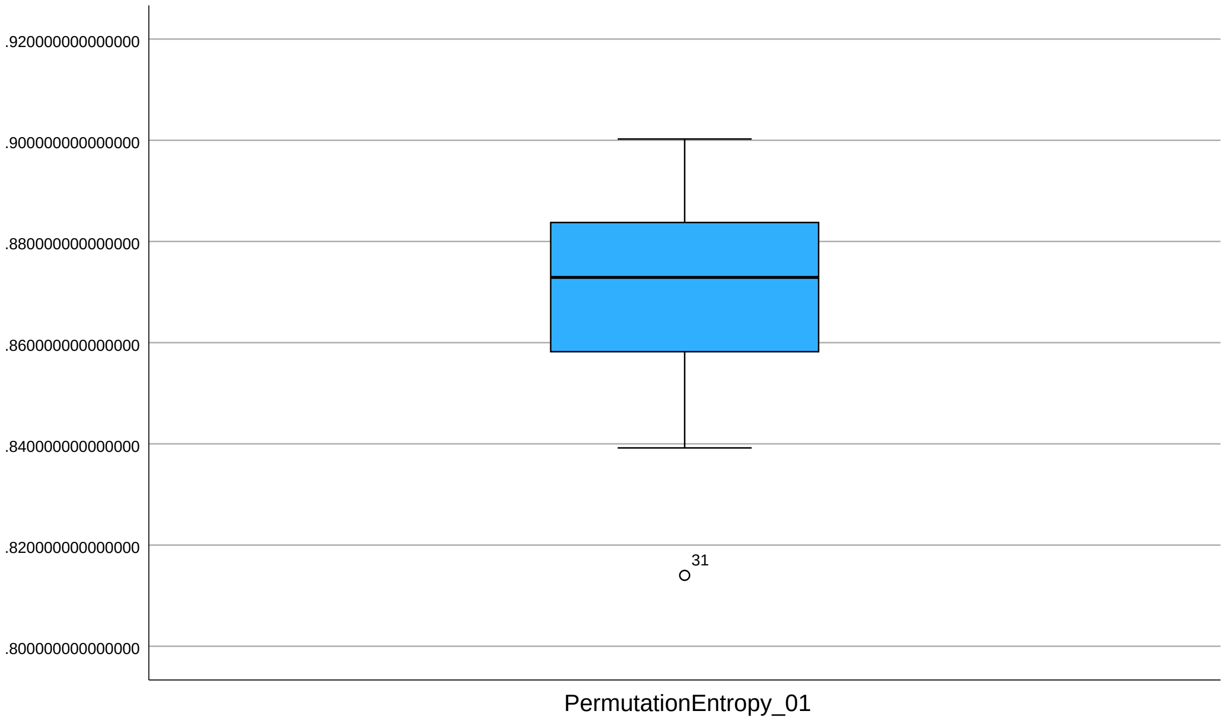

SampleEntropy

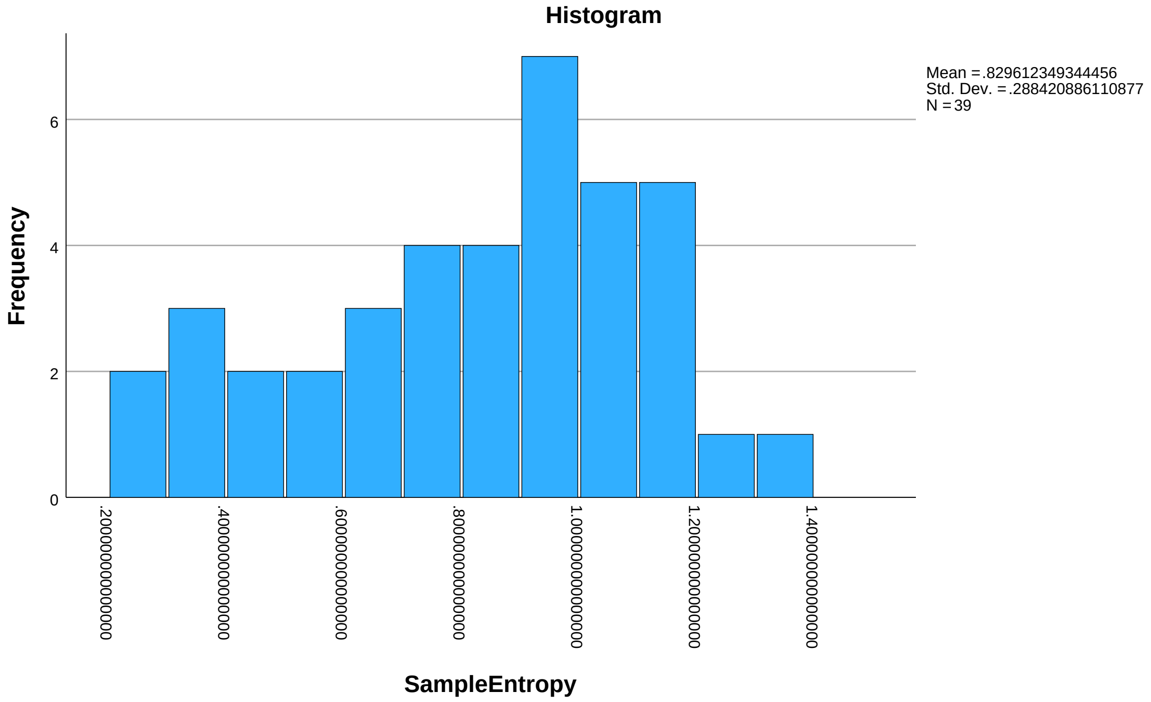


```
SampleEntropy Stem-and-Leaf Plot

 Frequency    Stem &  Leaf
```

```
     2.00        2 .  47
     3.00        3 .  379
     2.00        4 .  78
     2.00        5 .  33
     3.00        6 .  067
     4.00        7 .  4569
     4.00        8 .  4449
     7.00        9 .  0124556
     5.00       10 .  44668
     5.00       11 .  33679
     1.00       12 .  7
     1.00       13 .  0

 Stem width:   .1000000
 Each leaf:       1 case(s)
```

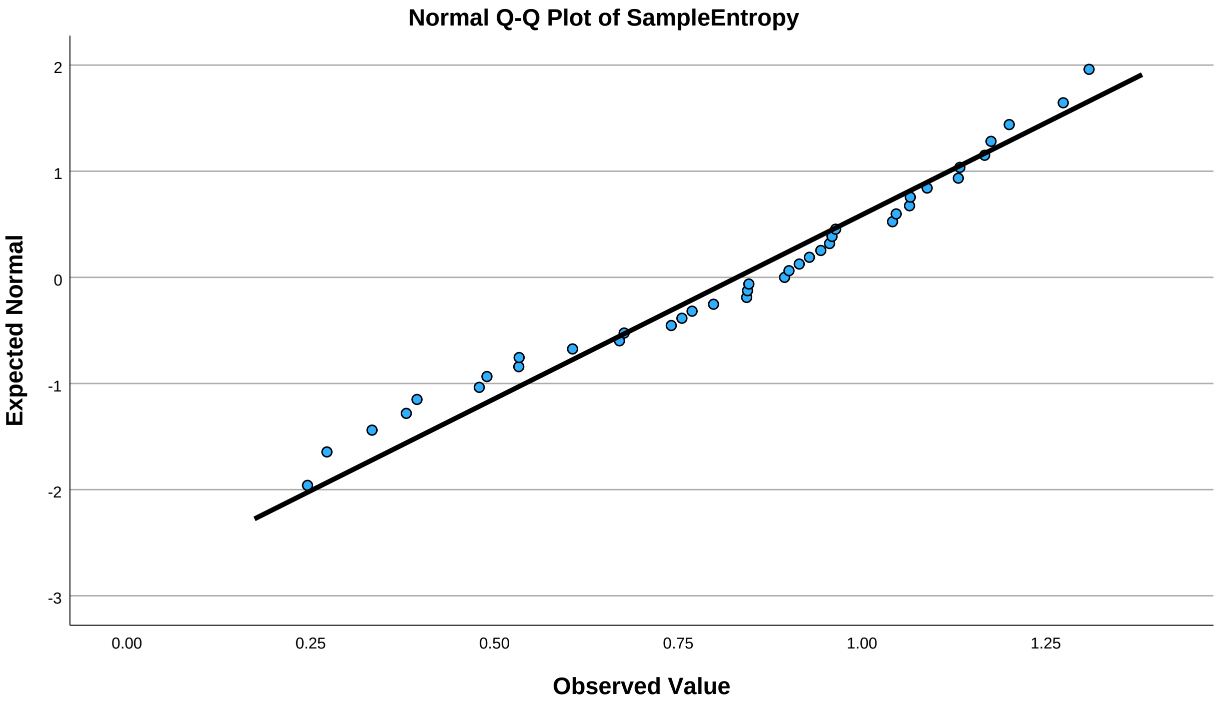

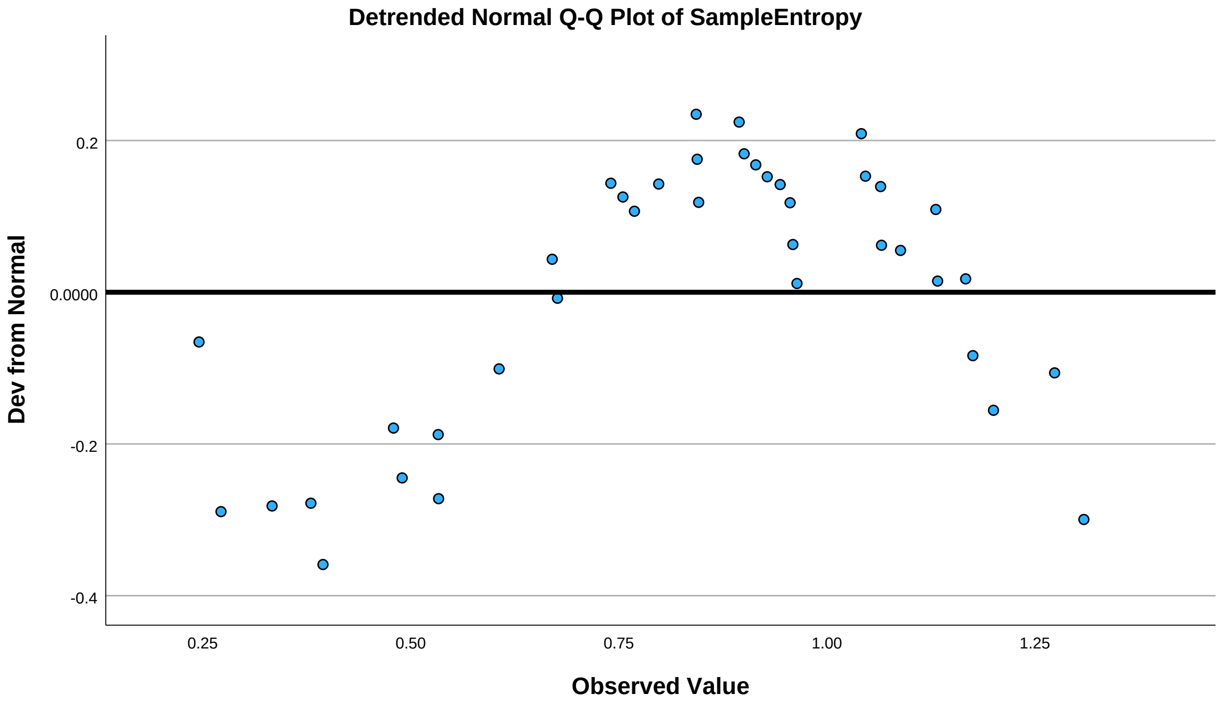

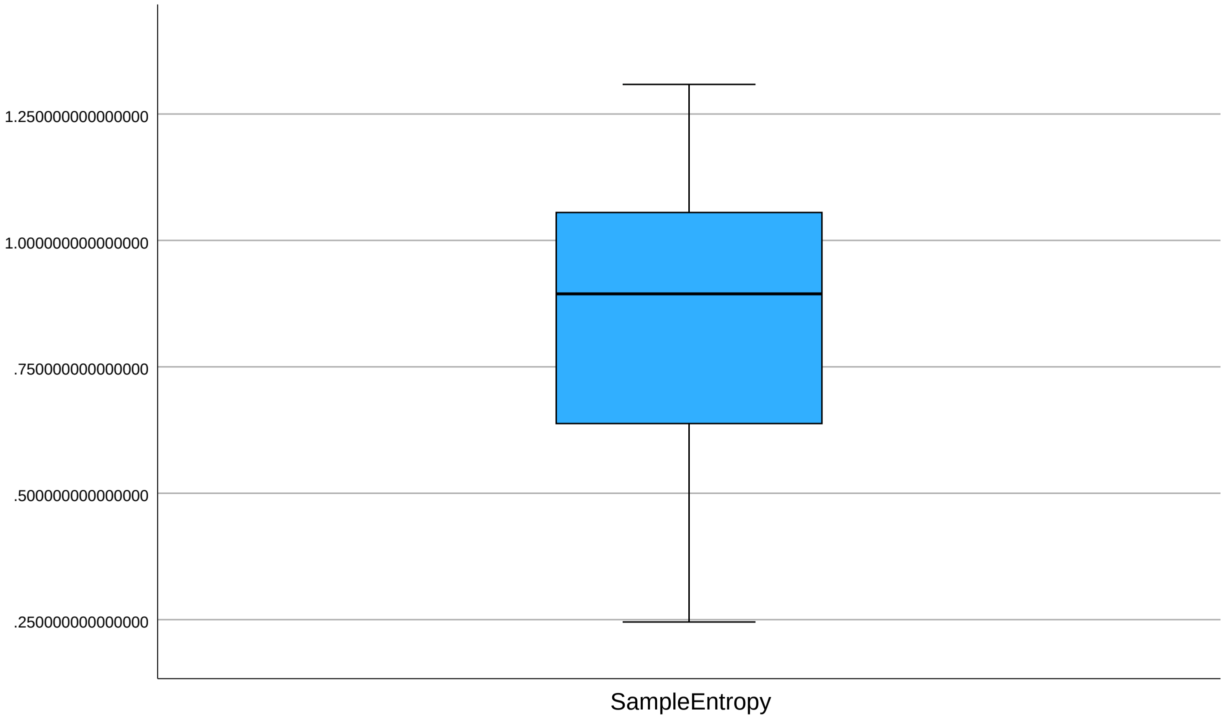

SpectralEntropy_bits

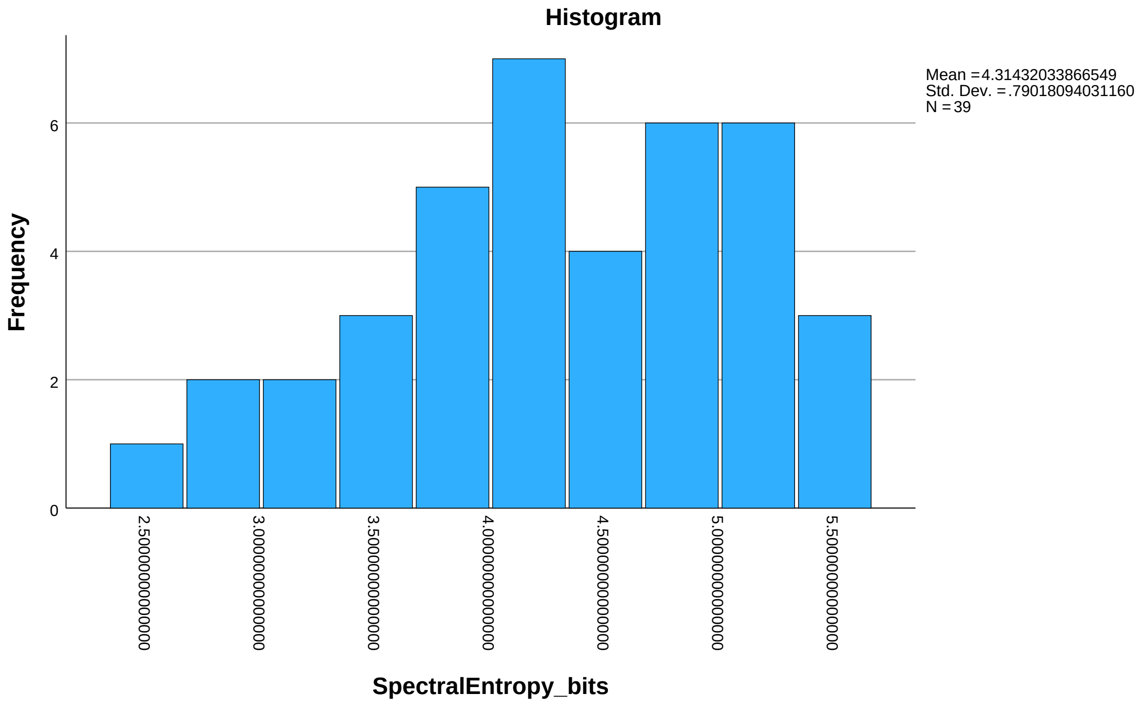


```
SpectralEntropy_bits Stem-and-Leaf Plot

 Frequency    Stem &  Leaf

     3.00        2 .  699
     4.00        3 .  0144
     6.00        3 .  567899
     8.00        4 .  00012334
     9.00        4 .  556677899
     6.00        5 .  000113
     3.00        5 .  555

 Stem width:  1.000000
 Each leaf:       1 case(s)
```

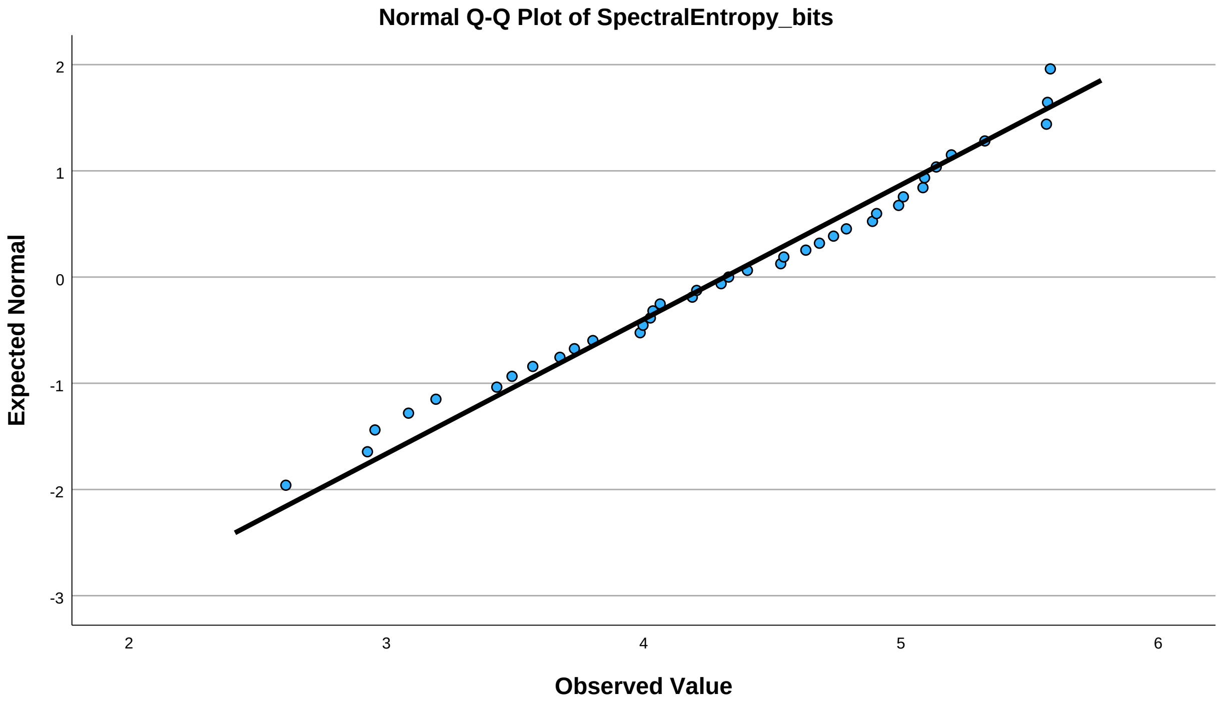
Normal Q-Q Plot of SpectralEntropy_bits
Expected Normal
Observed Value
2
1
0
-1
-2
-3
2
3
4
5
6

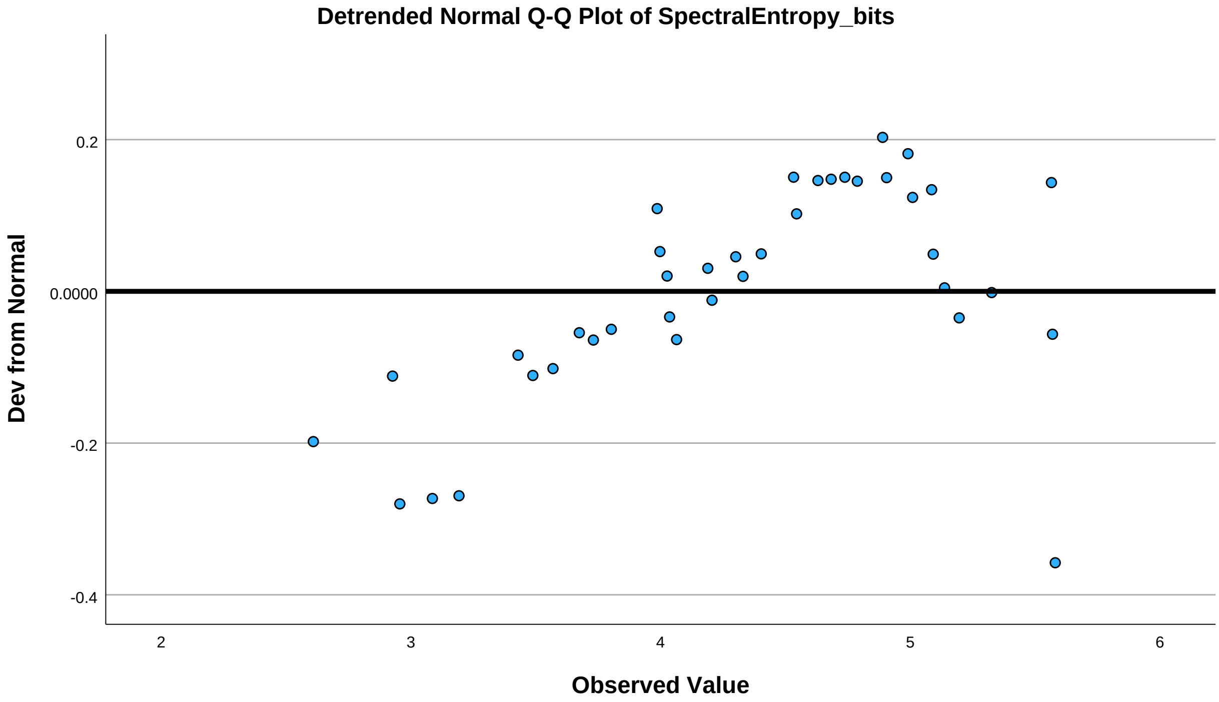
Detrended Normal Q-Q Plot of SpectralEntropy_bits
Dev from Normal
Observed Value
0.2
0.0000
-0.2
-0.4
2
3
4
5
6

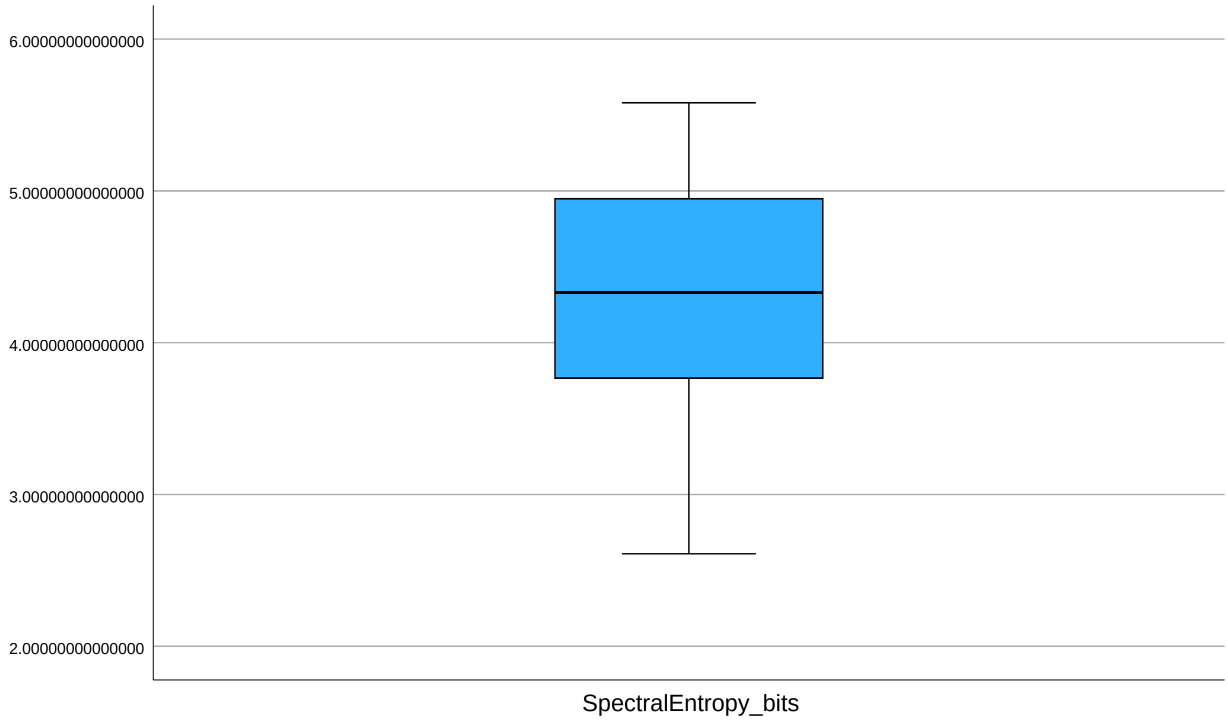

SpectralEntropy_01

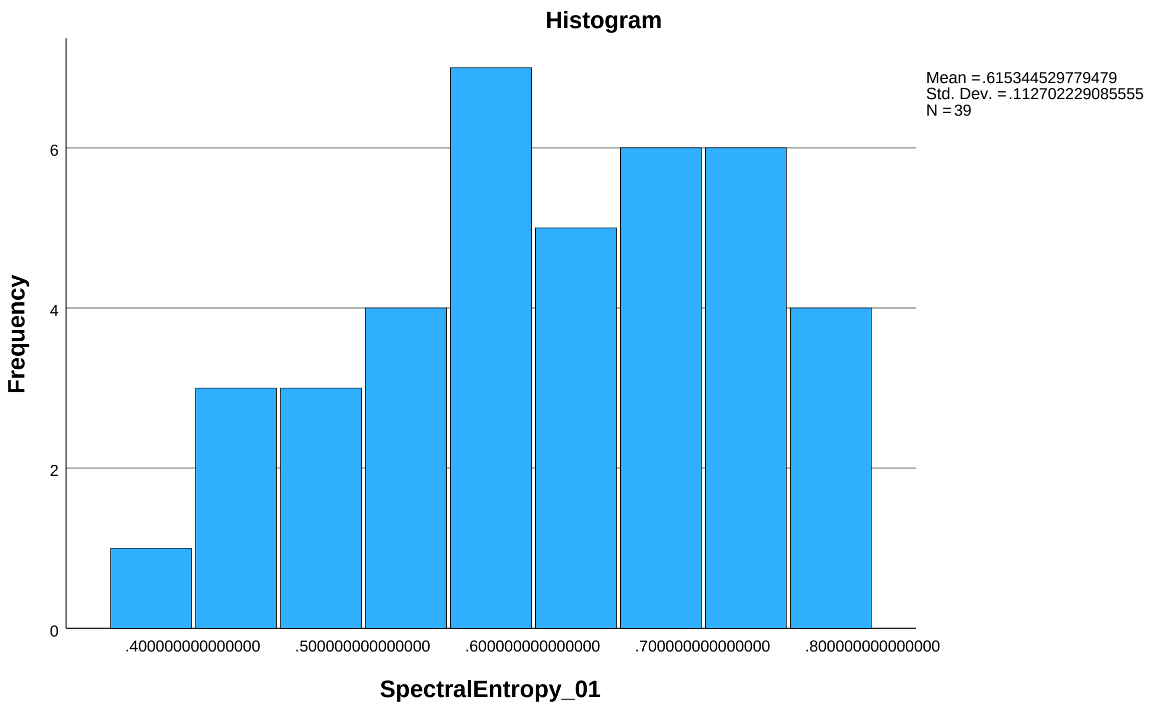


```
SpectralEntropy_01 Stem-and-Leaf Plot

 Frequency    Stem &  Leaf
```

```
 1.00     3 . 7
 3.00     4 . 124
 3.00     4 . 589
 4.00     5 . 0234
 7.00     5 . 6777799
 5.00     6 . 11244
 6.00     6 . 667899
 6.00     7 . 112234
 4.00     7 . 5999

Stem width:   .1000000
Each leaf:       1 case(s)
```

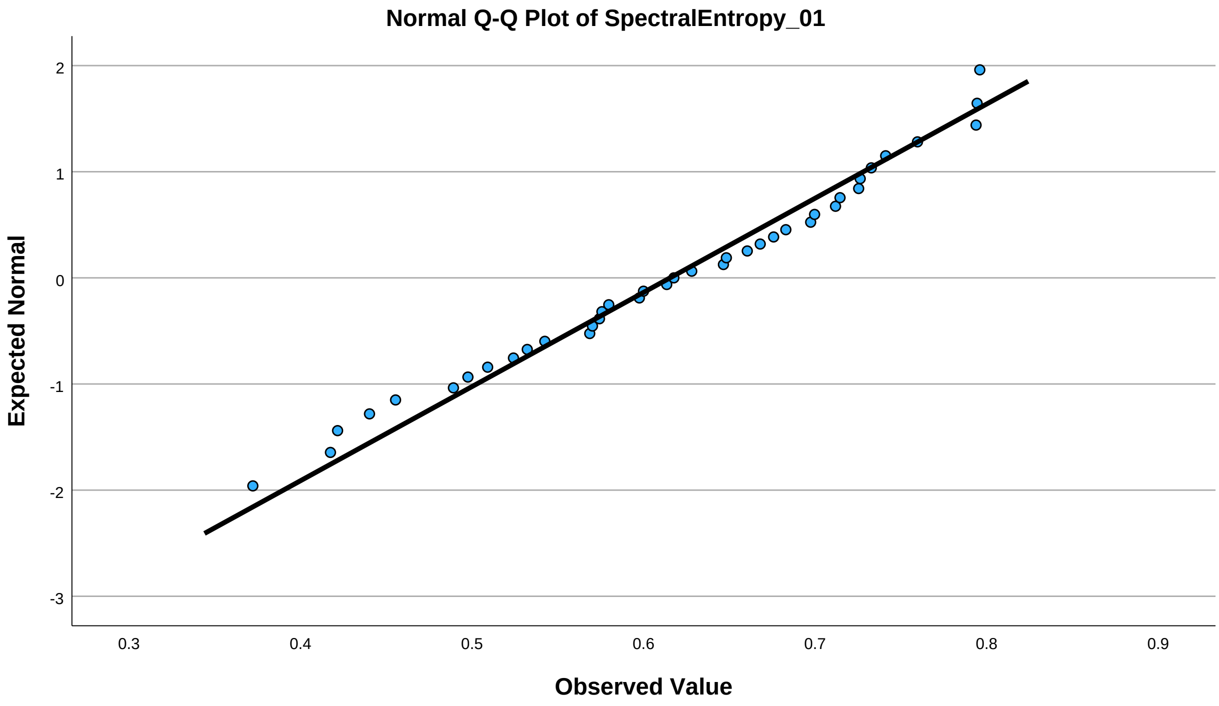

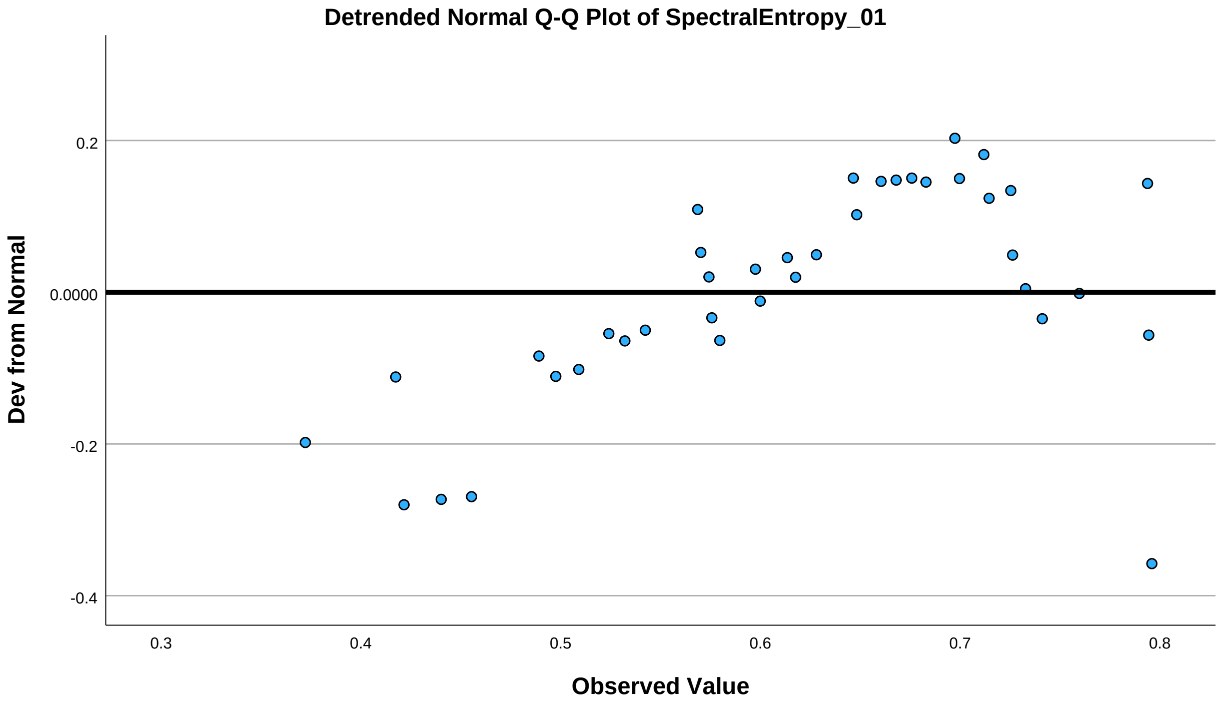

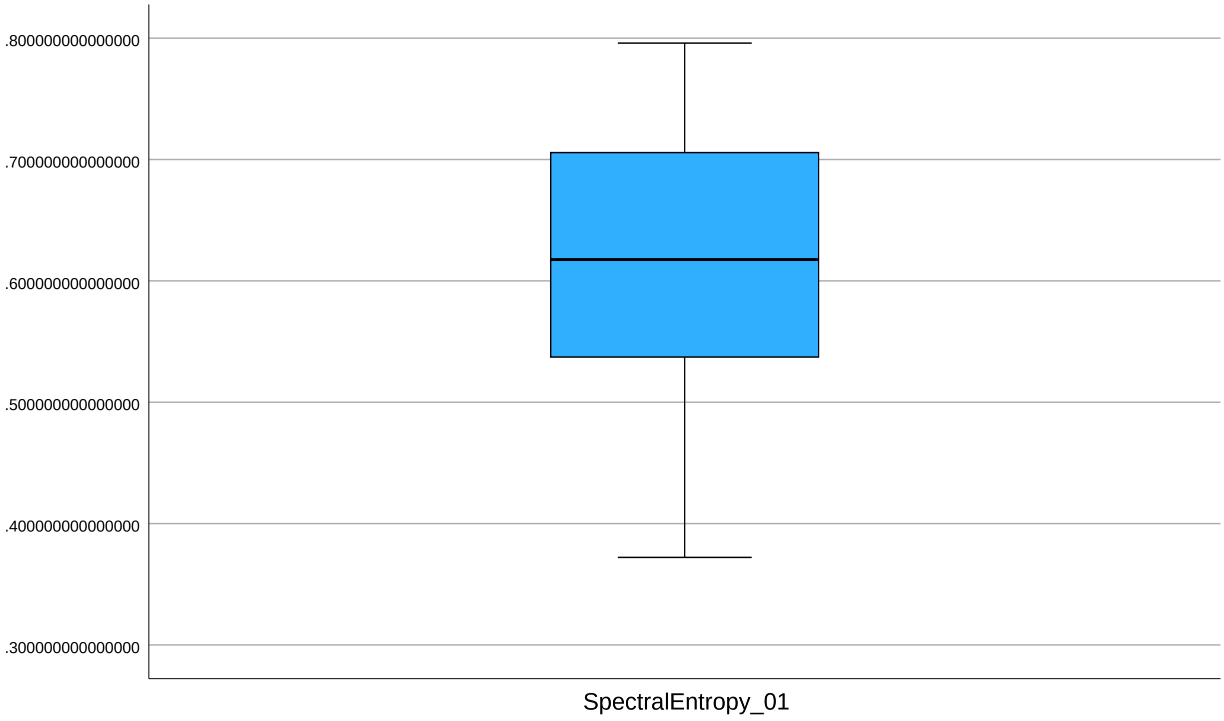

HiguchiFractalDimension

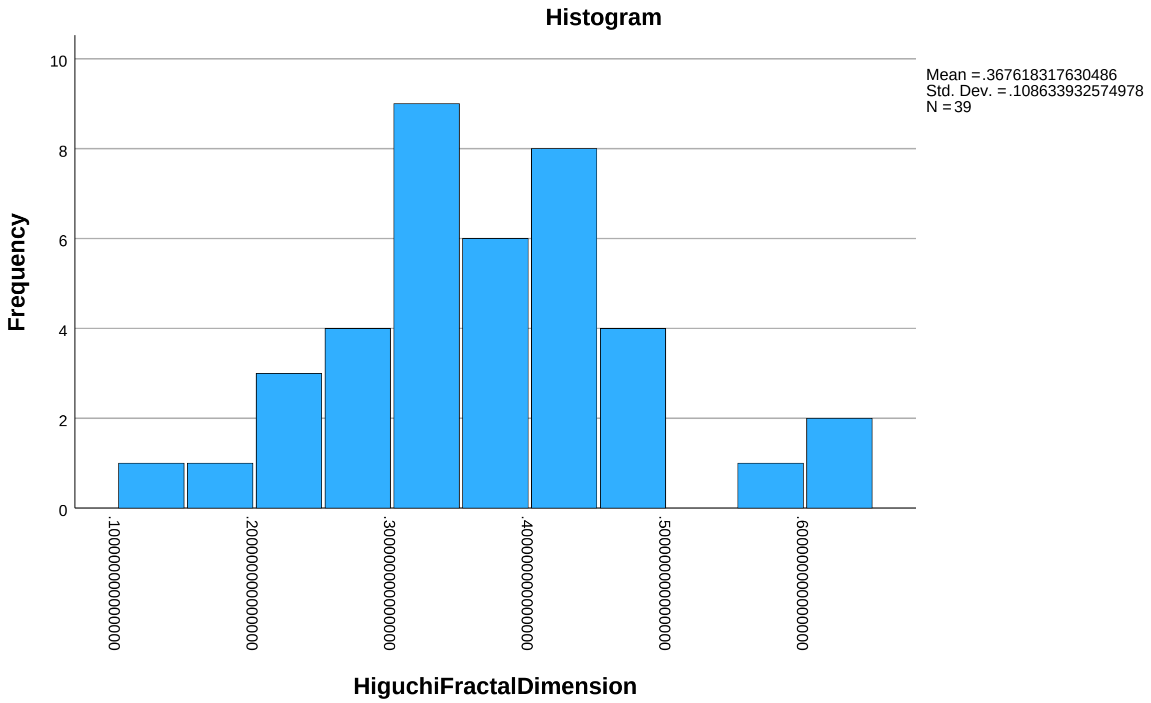


```
HiguchiFractalDimension Stem-and-Leaf Plot

 Frequency    Stem &  Leaf

     1.00        1 .  4
     1.00        1 .  5
     3.00        2 .  134
     4.00        2 .  5567
     9.00        3 .  000333444
     6.00        3 .  578899
     8.00        4 .  00112234
     4.00        4 .  5689
      .00        5 .
     1.00        5 .  9
     2.00        6 .  00

 Stem width:   .1000000
 Each leaf:       1 case(s)
```

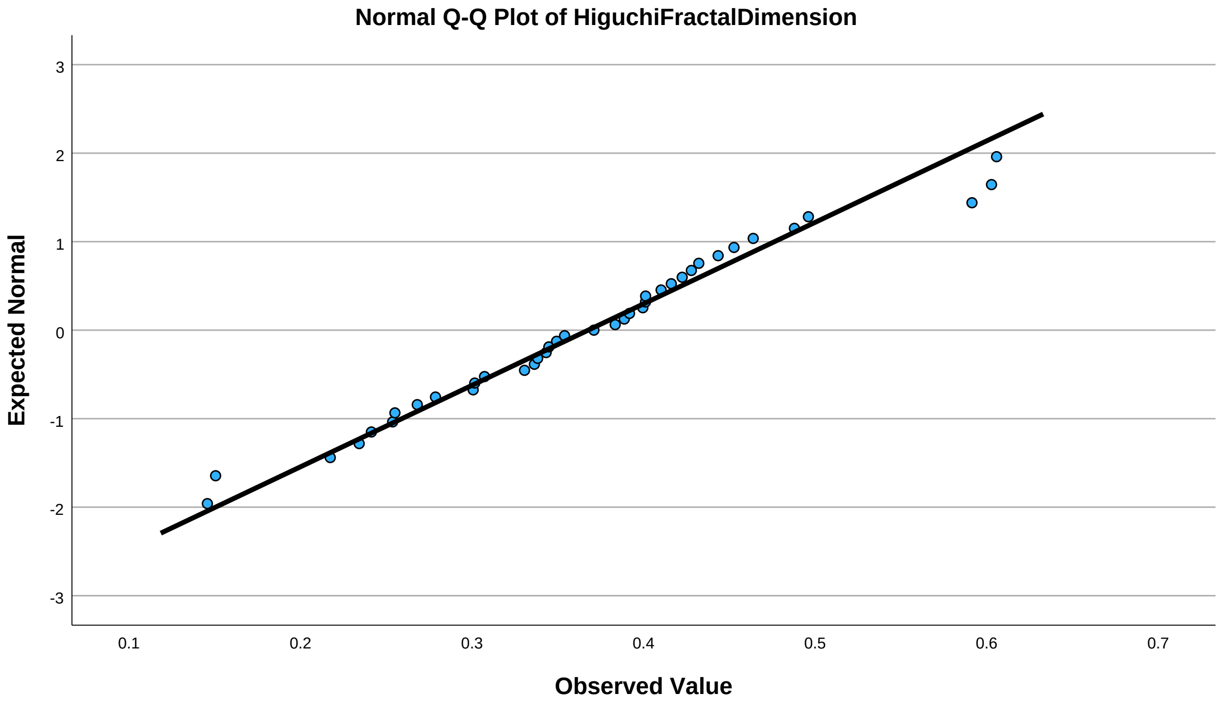

Normal Q-Q Plot of HiguchiFractalDimension
Expected Normal
3
2
1
0
-1
-2
-3
0.1
0.2
0.3
0.4
0.5
0.6
0.7
Observed Value

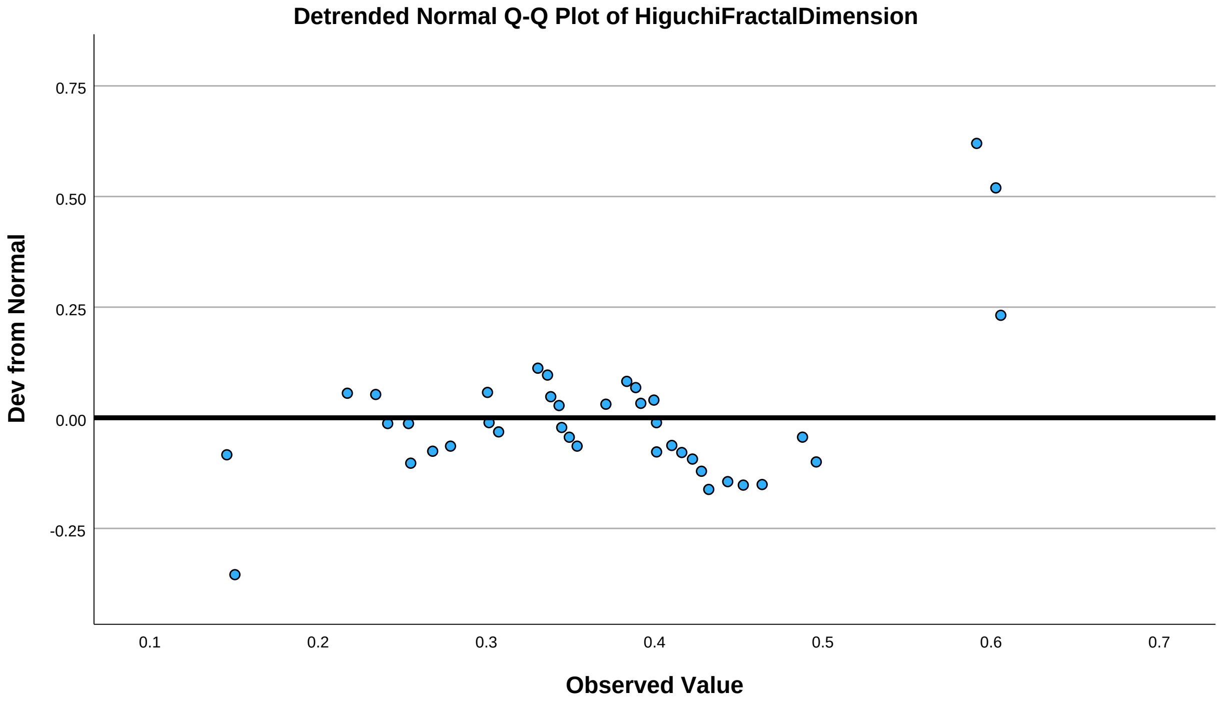

Detrended Normal Q-Q Plot of HiguchiFractalDimension
Dev from Normal
0.75
0.50
0.25
0.00
-0.25
0.1
0.2
0.3
0.4
0.5
0.6
0.7
Observed Value

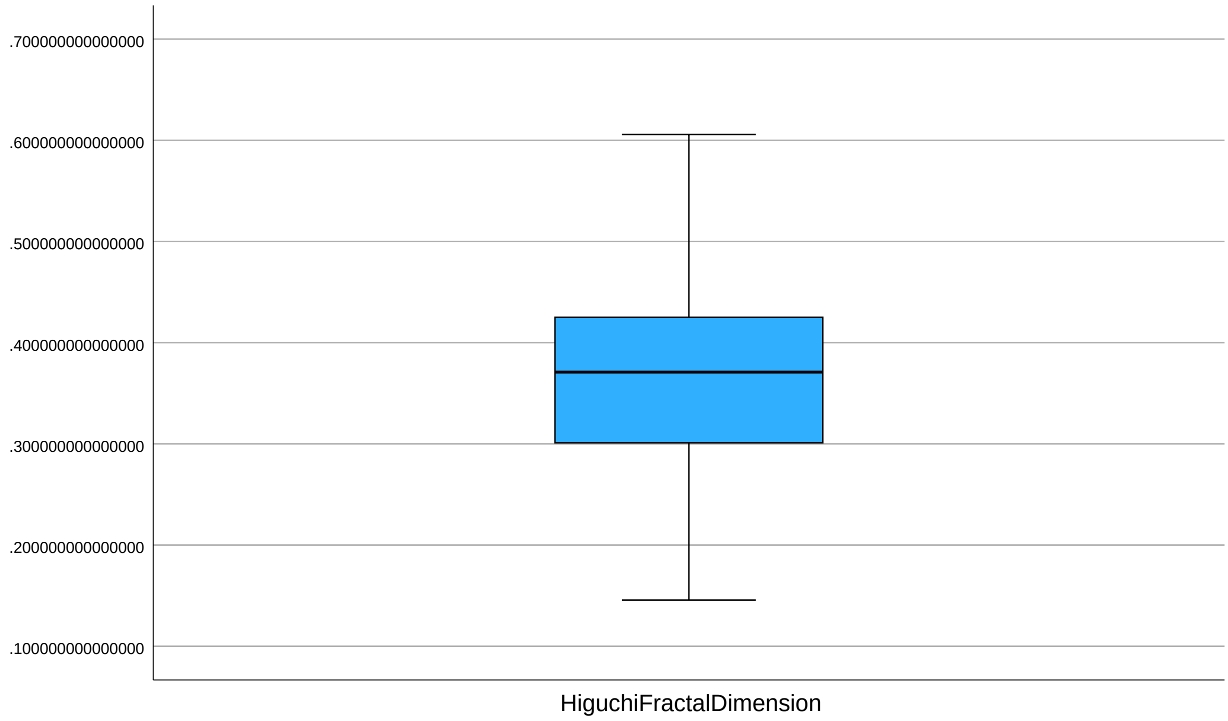

LempelZivComplexity

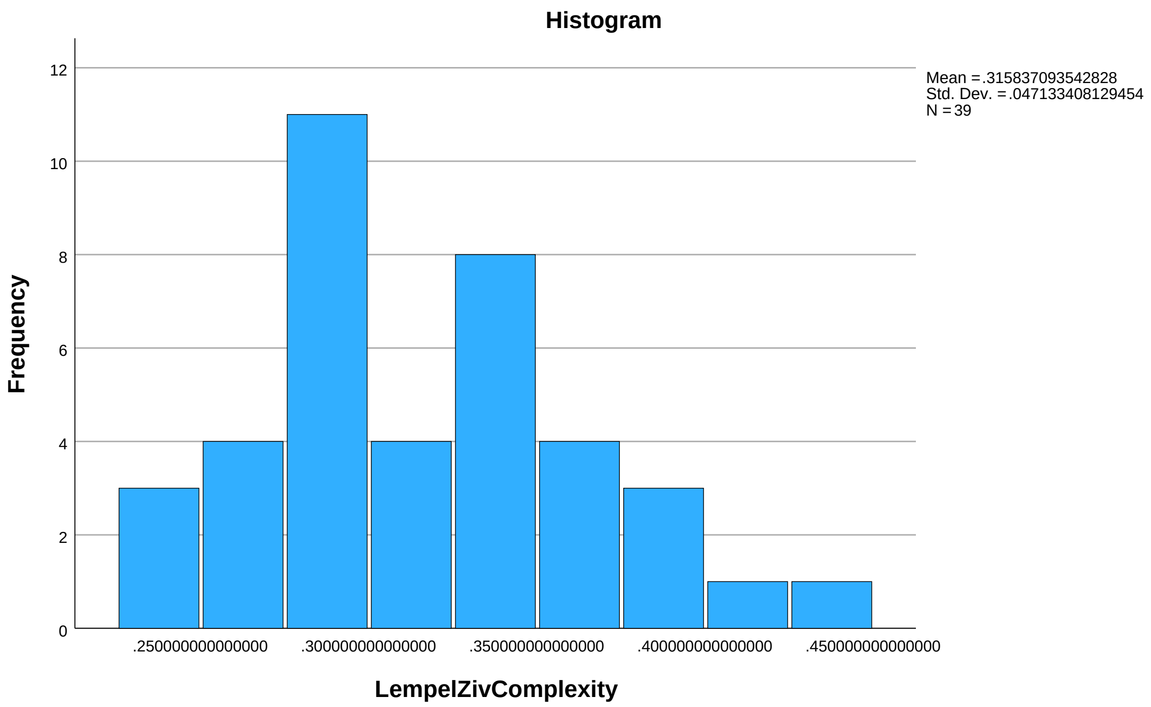


```
LempelZivComplexity Stem-and-Leaf Plot

 Frequency    Stem &  Leaf
```

```
   3.00        2 .  334
  15.00        2 .  667777889999999
  12.00        3 .  000122333344
   7.00        3 .  5557788
   2.00        4 .  12

Stem width:   .1000000
Each leaf:       1 case(s)
```

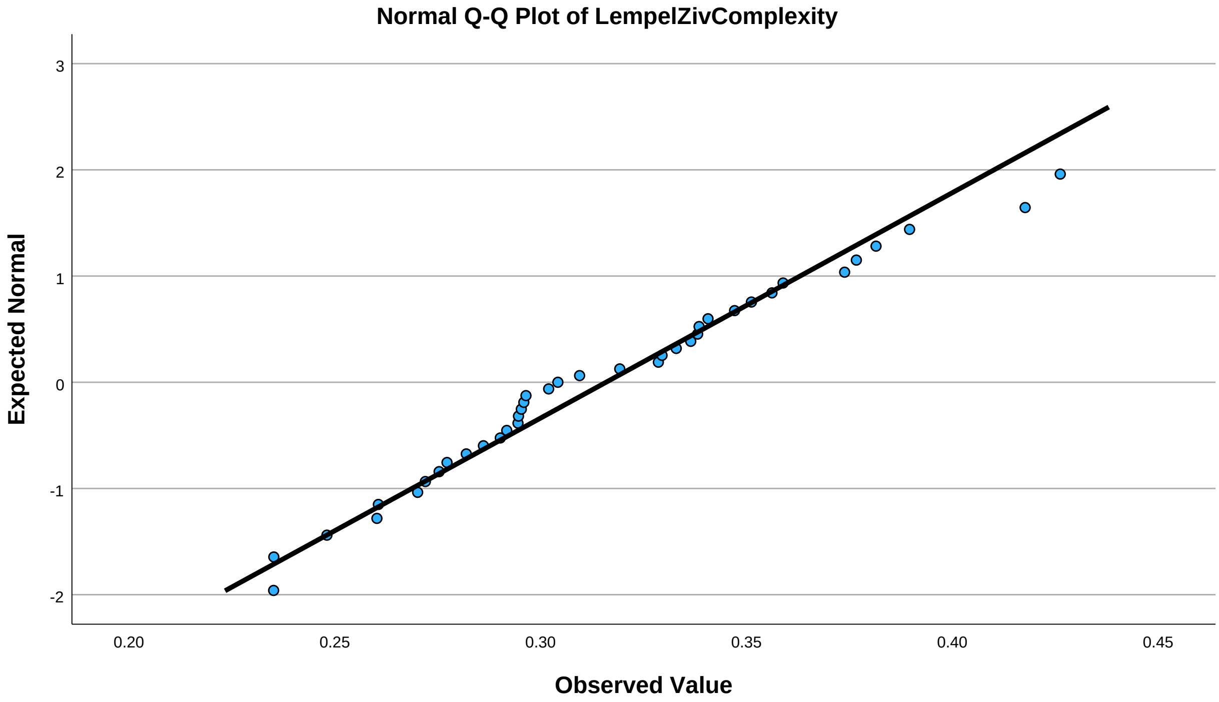

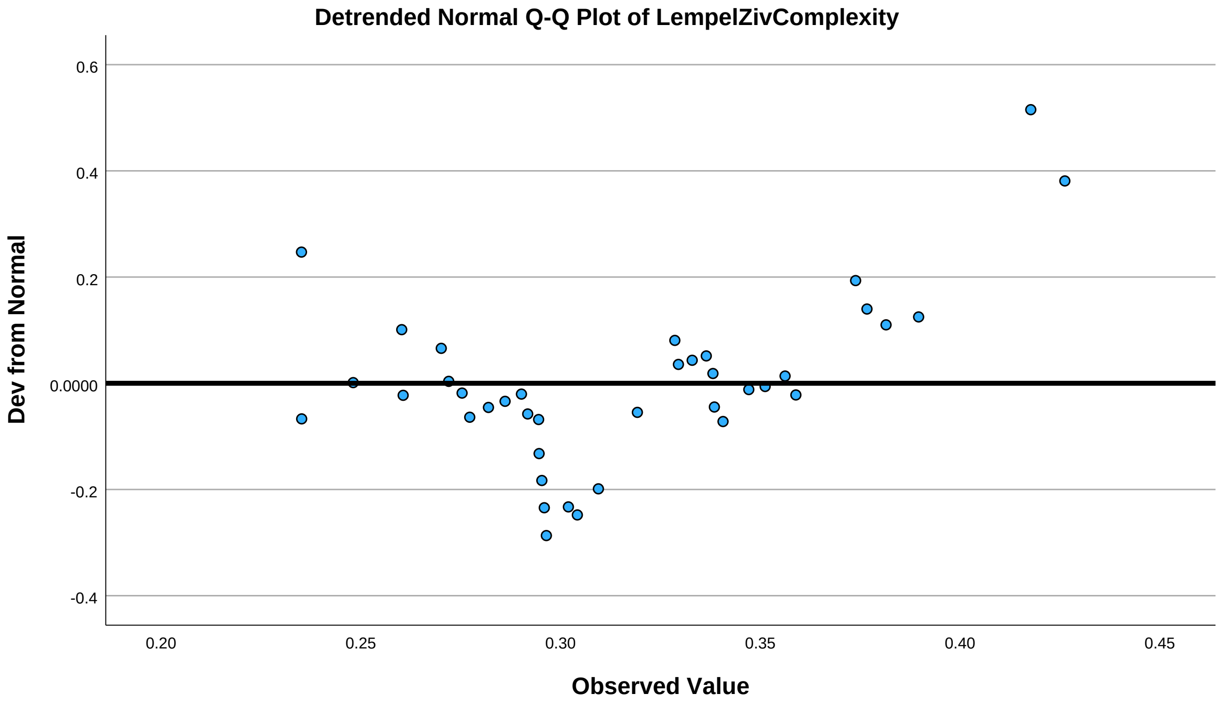

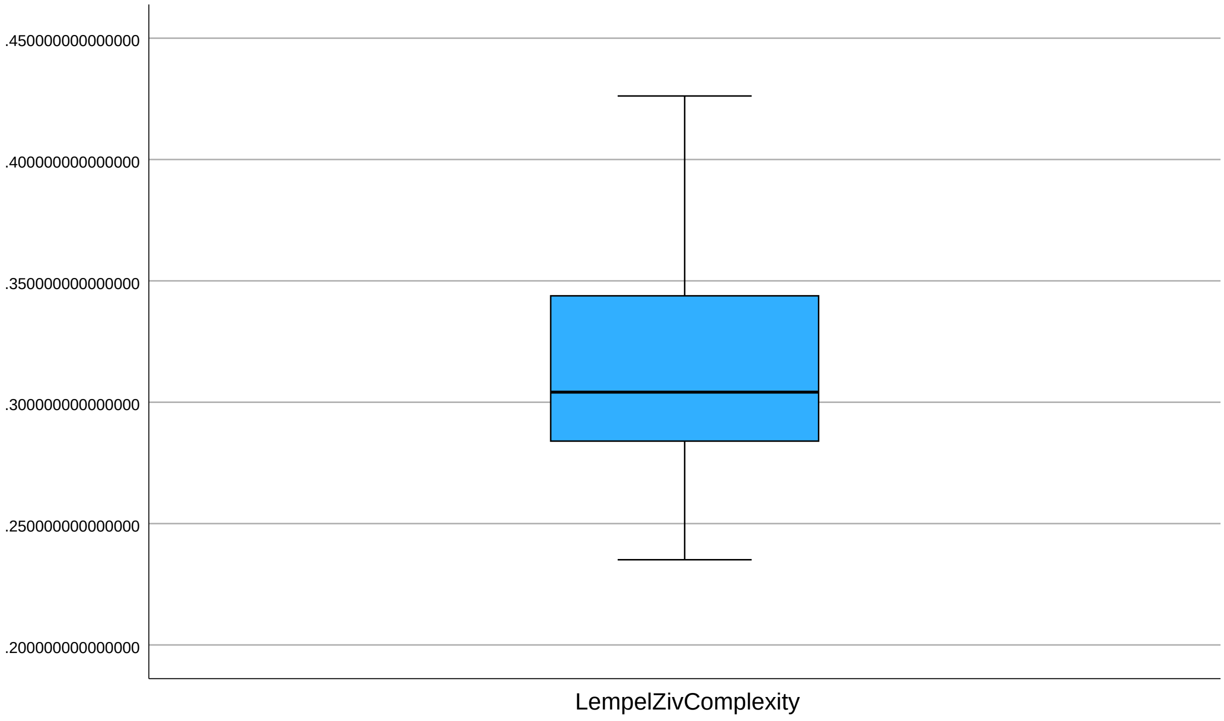

RenyiEntropy

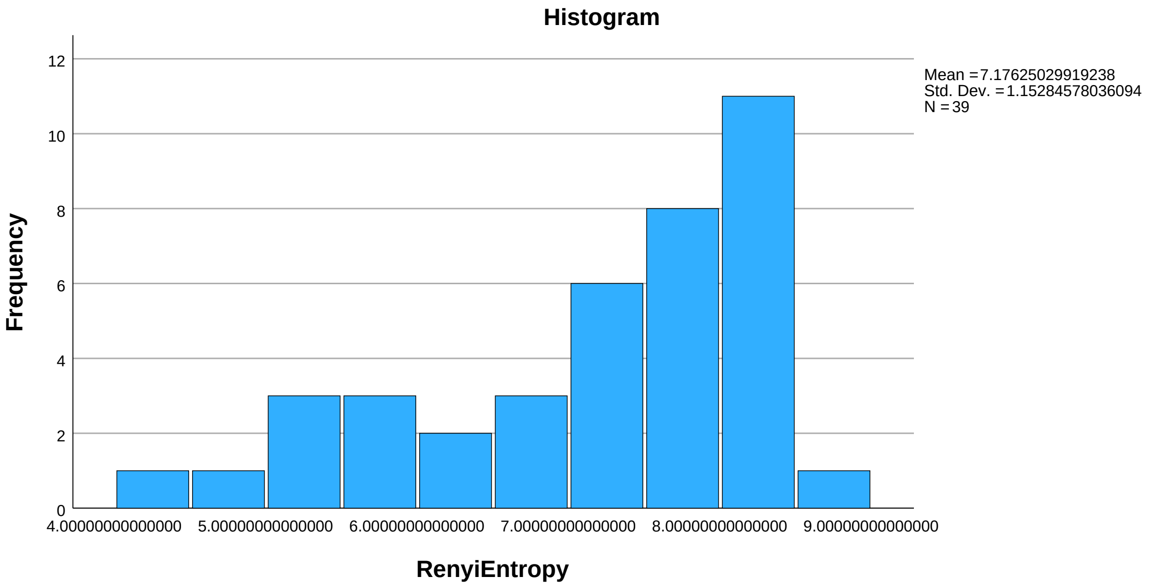


```
RenyiEntropy Stem-and-Leaf Plot

 Frequency    Stem &  Leaf

     1.00        4 .  4
     1.00        4 .  9
     3.00        5 .  134
     3.00        5 .  568
     2.00        6 .  11
     3.00        6 .  889
     6.00        7 .  012234
     8.00        7 .  55567899
    11.00        8 .  00011223444
     1.00        8 .  7

 Stem width:   1.000000
 Each leaf:        1 case(s)
```

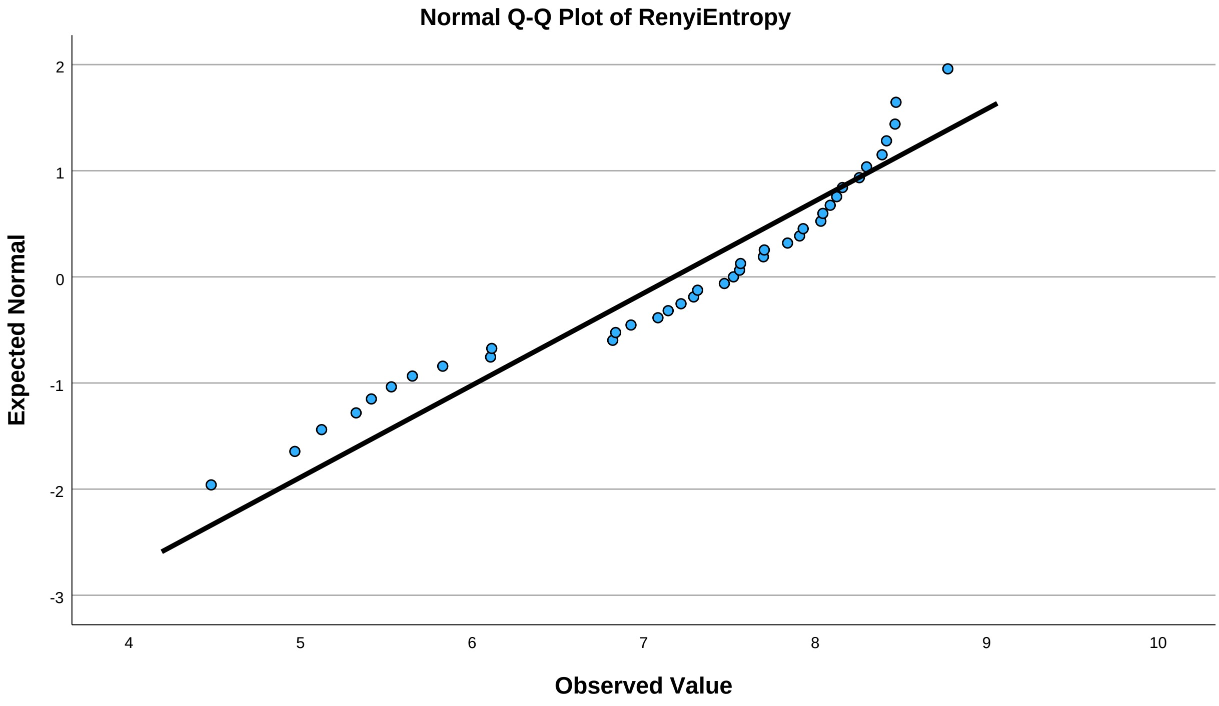

Normal Q-Q Plot of RenyiEntropy
Expected Normal
Observed Value
2
1
0
-1
-2
-3
4
5
6
7
8
9
10

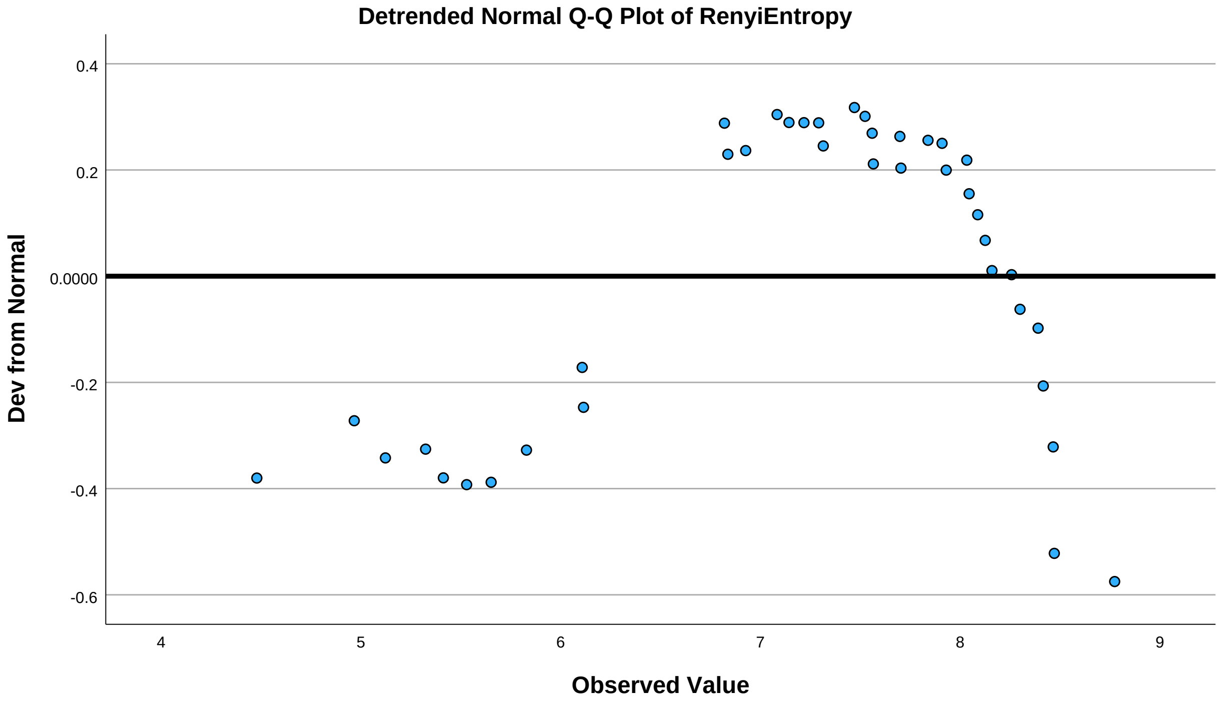

Detrended Normal Q-Q Plot of RenyiEntropy
Dev from Normal
Observed Value
0.4
0.2
0.0000
-0.2
-0.4
-0.6
4
5
6
7
8
9

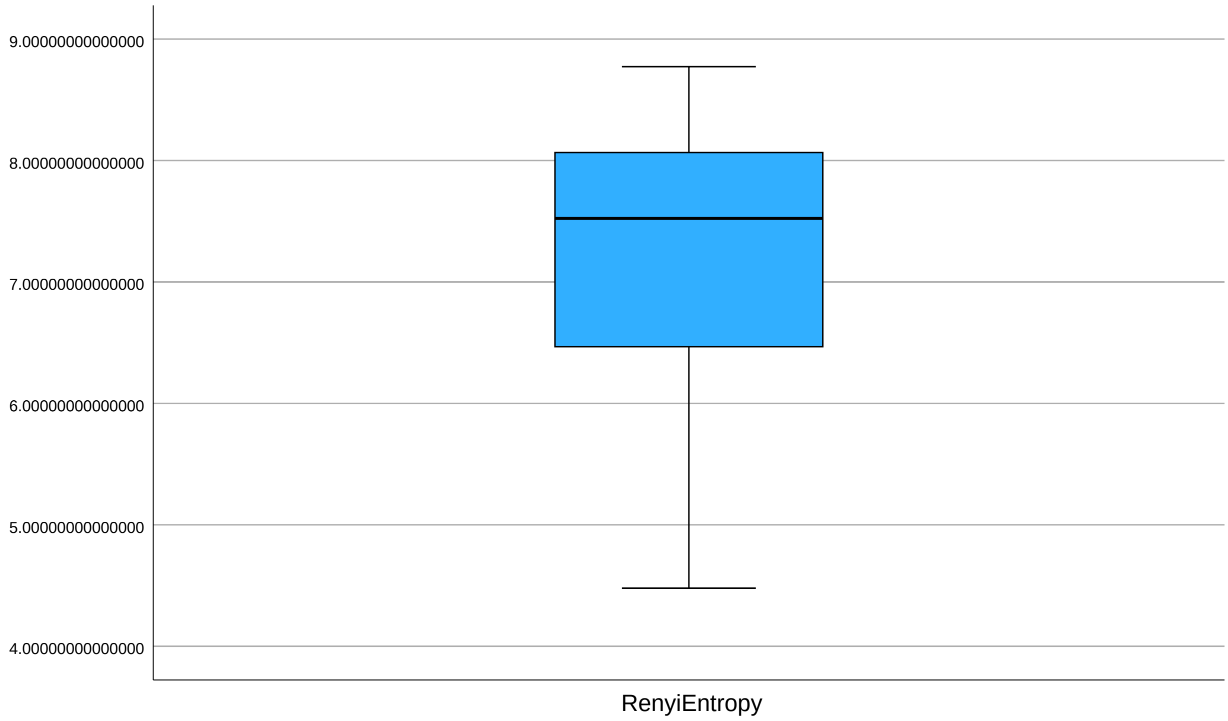

TsallisEntropy

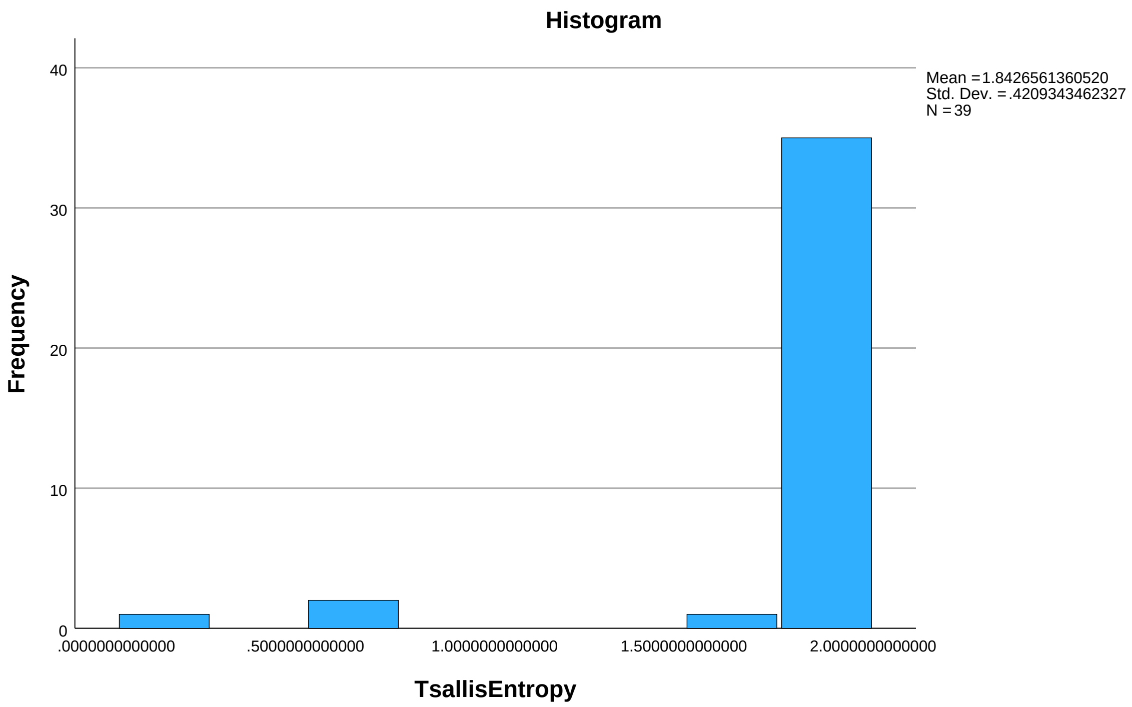

TsallisEntropy Stem-and-Leaf Plot

Frequency   Stem & Leaf

```
  7.00 Extremes    (=<1.904)
 1.00     194 .  2
  .00    194 .
 1.00     195 .  1
  .00    195 .
 1.00     196 .  4
 1.00     196 .  8
 1.00     197 .  4
 4.00     197 .  6899
 7.00     198 .  0224444
 16.00     198 .  5555555666778888

Stem width:   .0100000
Each leaf:       1 case(s)
```

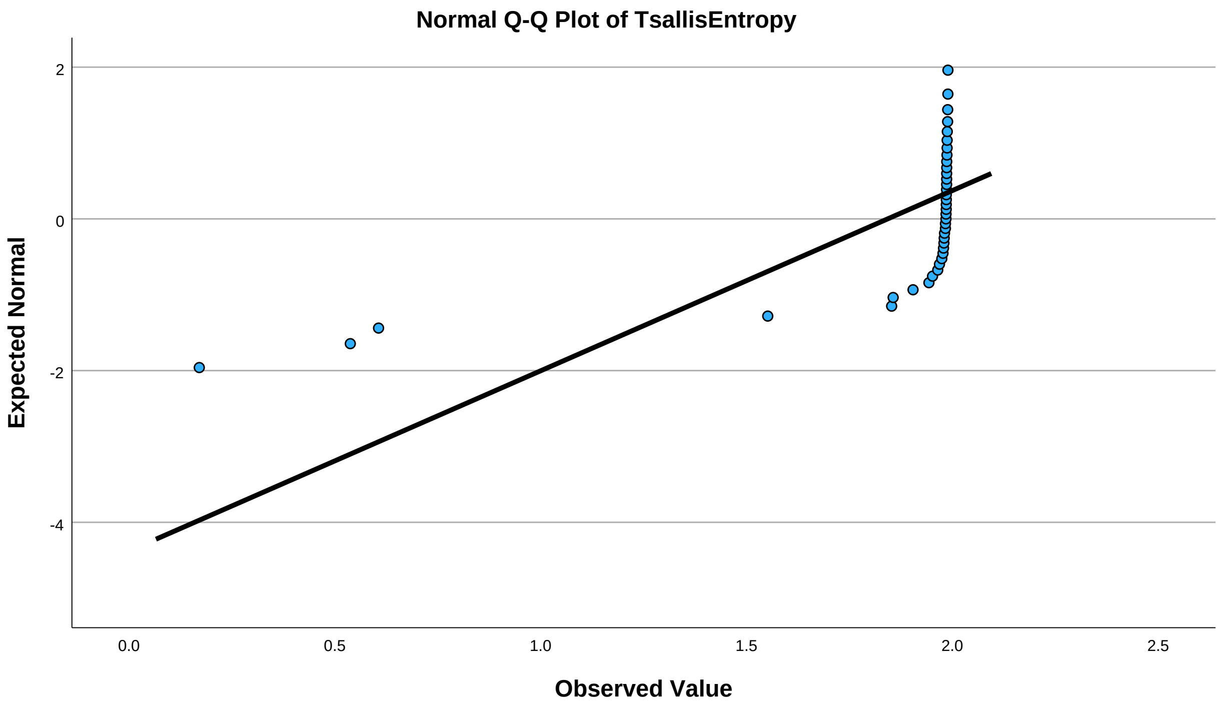

Detrended Normal Q-Q Plot of TsallisEntropy

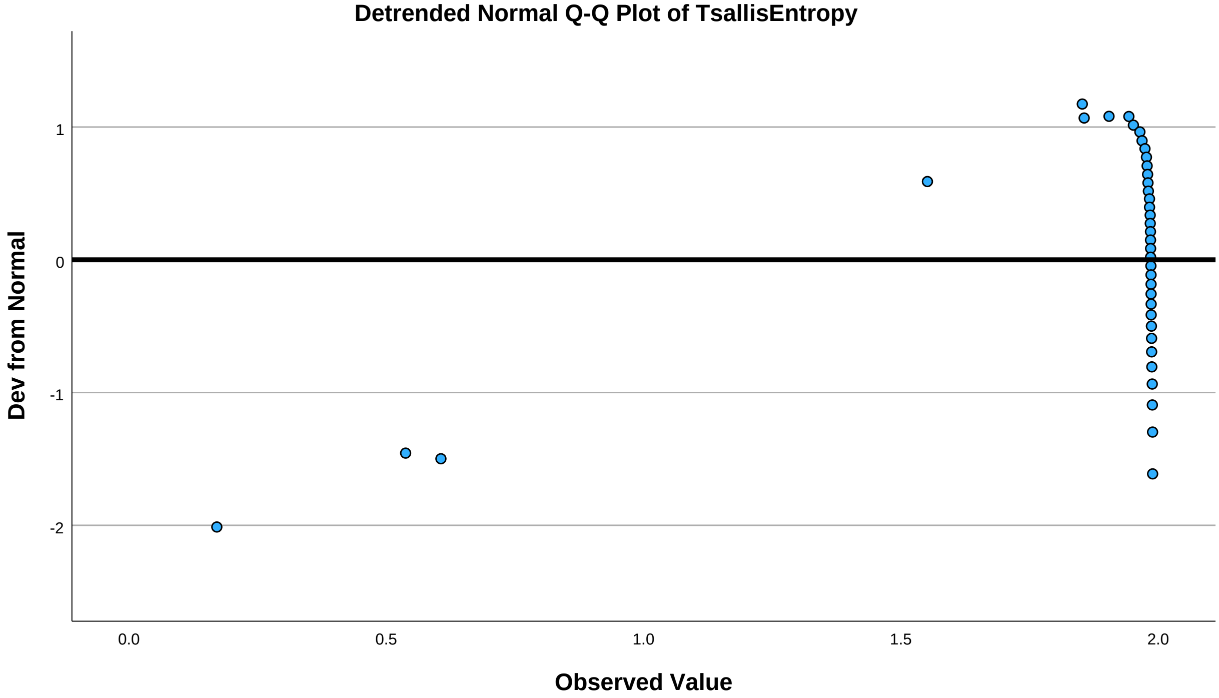

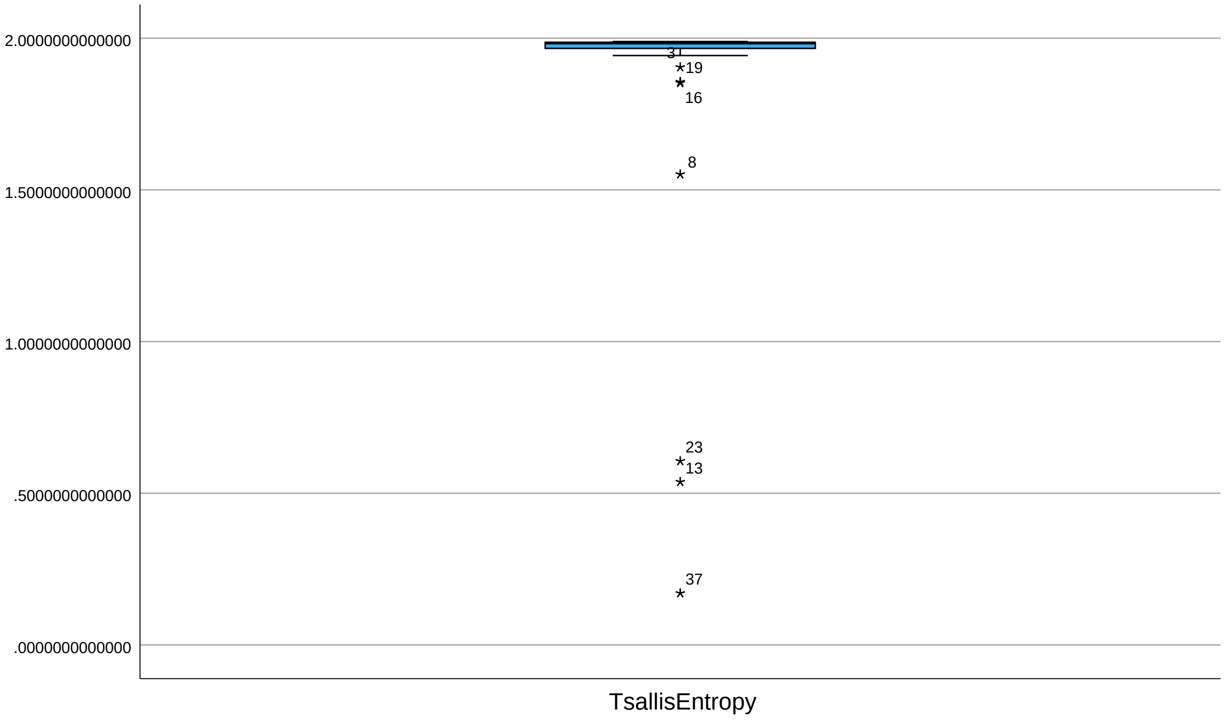

PS_4_5

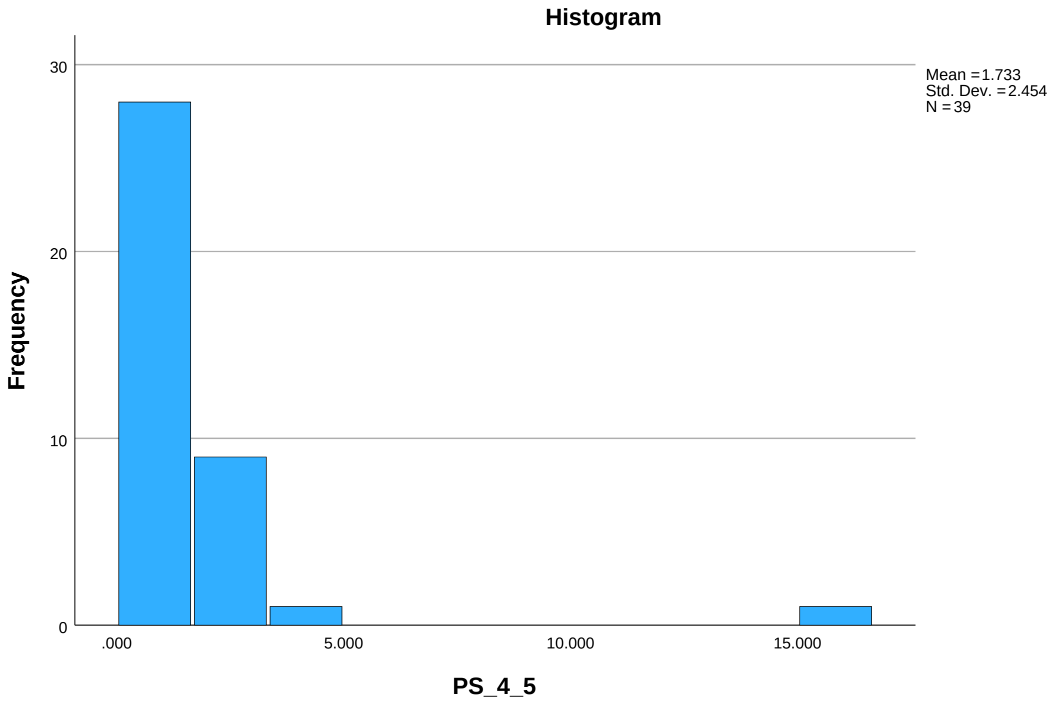

PS_4_5 Stem-and-Leaf Plot

```
 Frequency    Stem &  Leaf

     5.00        0 .  24444
     9.00        0 .  556778999
    12.00        1 .  000111223334
     4.00        1 .  5678
     3.00        2 .  034
     4.00        2 .  5789
     2.00 Extremes    (>=3.7)

 Stem width:      1.000
 Each leaf:       1 case(s)
```

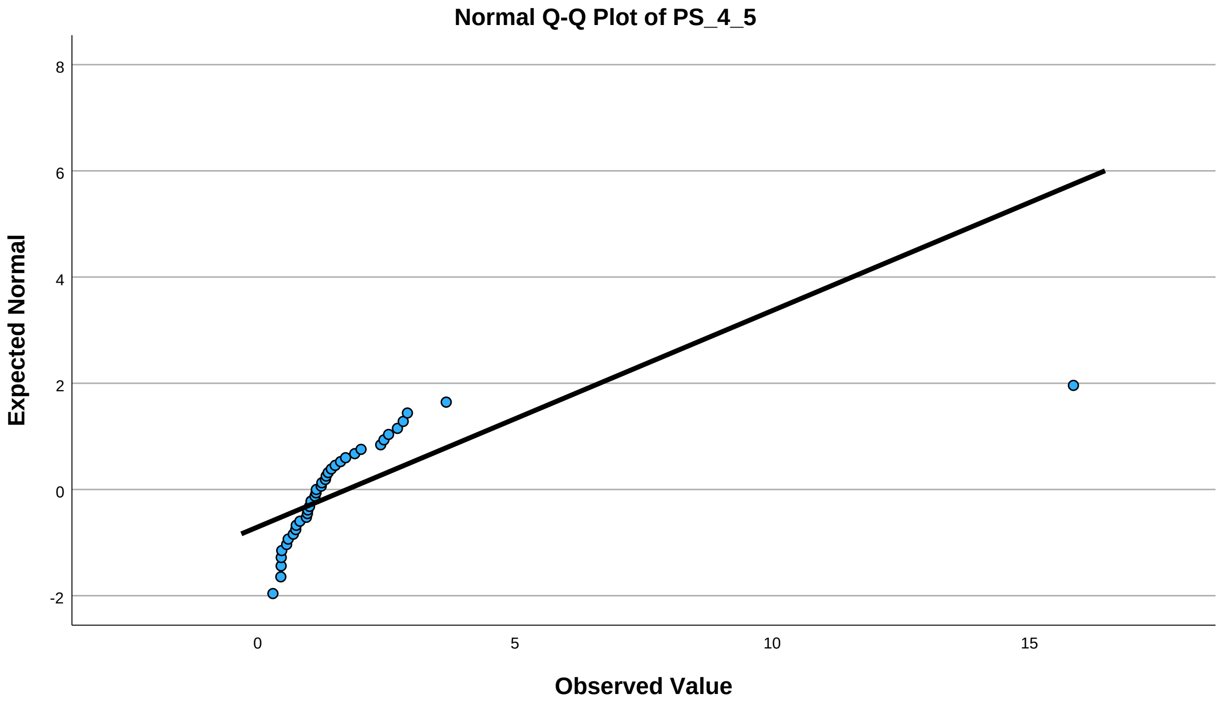
Normal Q-Q Plot of PS_4_5
Expected Normal
8
6
4
2
0
-2
0
5
10
15
Observed Value

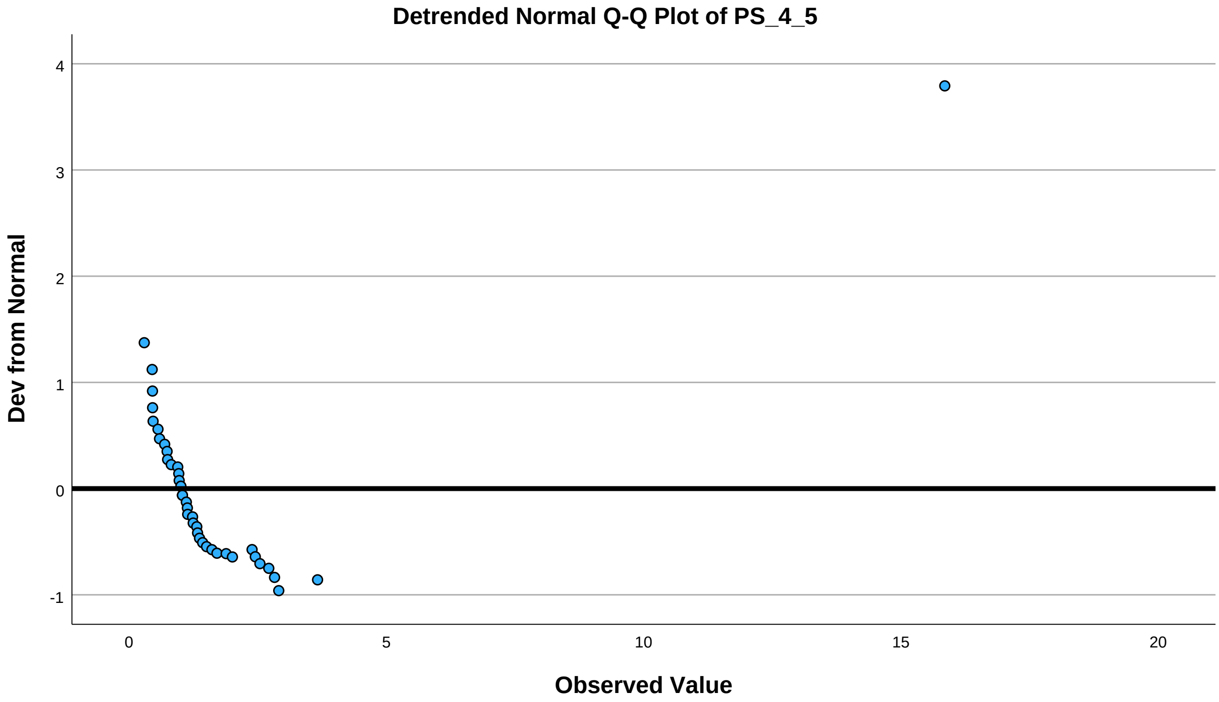
Detrended Normal Q-Q Plot of PS_4_5
Dev from Normal
4
3
2
1
0
-1
0
5
10
15
20
Observed Value

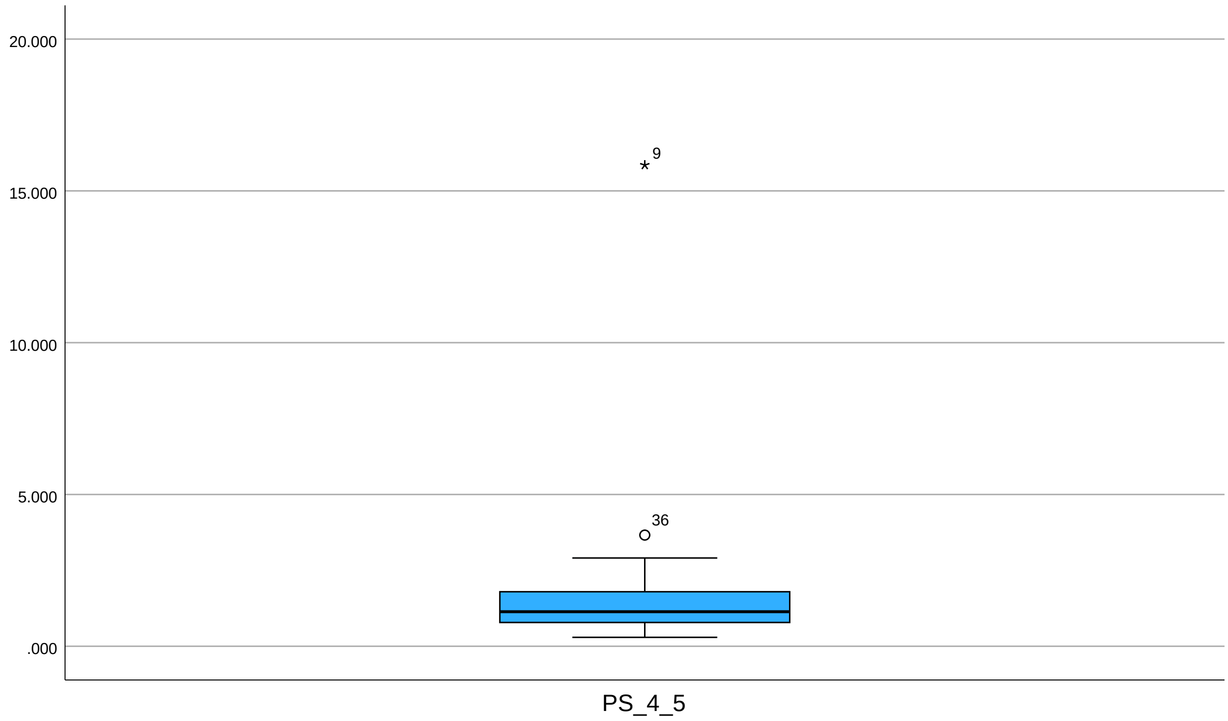

PS_5_6

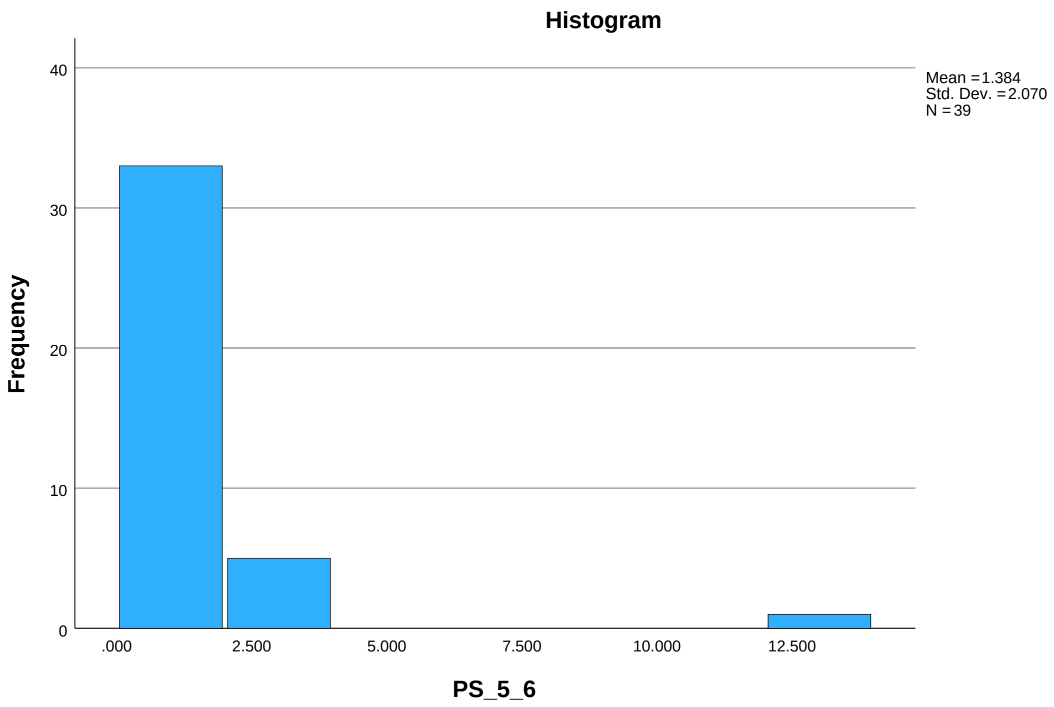

PS_5_6 Stem-and-Leaf Plot

Frequency   Stem & Leaf

```
 5.00        0 .  22333
 20.00       0 .  55566777788889999999
 5.00        1 .  12222
 3.00        1 .  588
 2.00        2 .  00
 4.00 Extremes    (>=2.3)

Stem width:      1.000
Each leaf:       1 case(s)
```

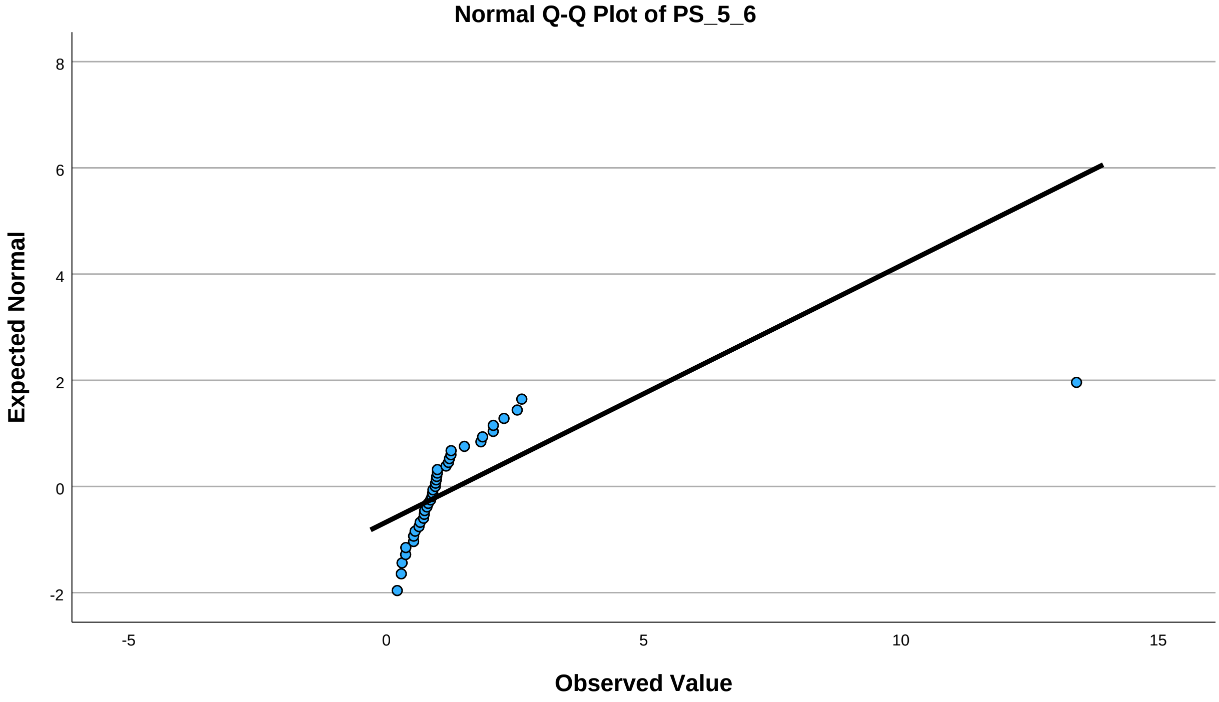

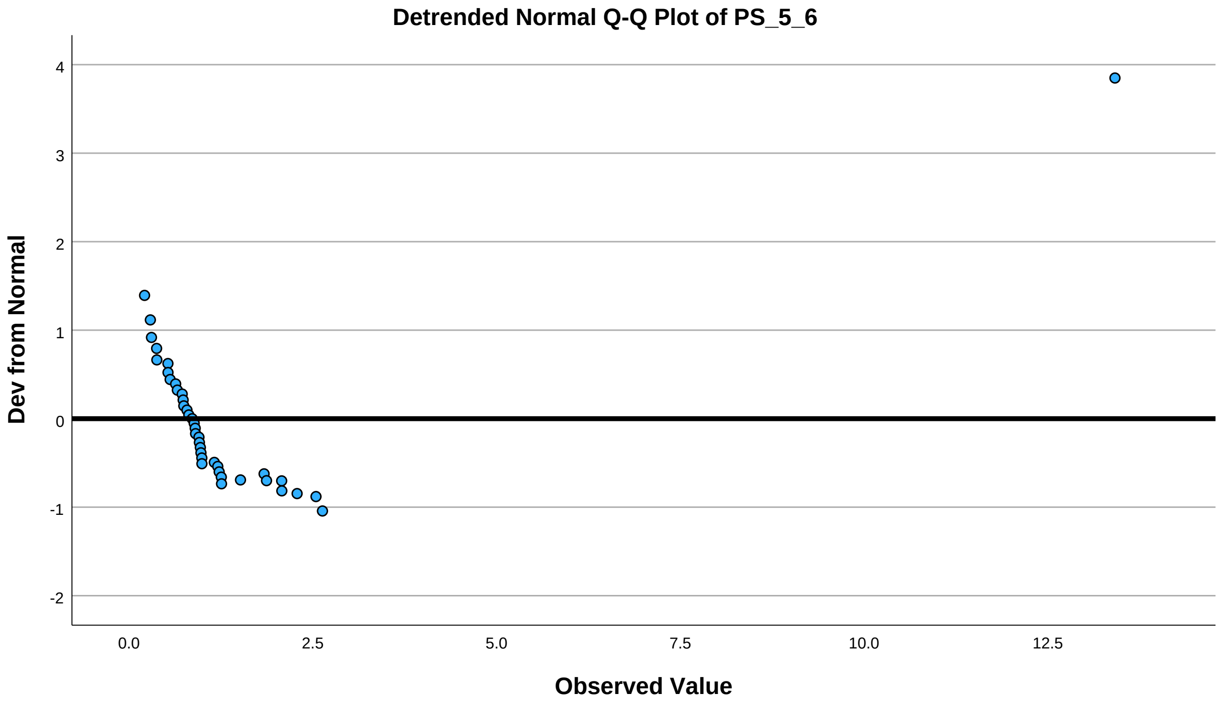
Detrended Normal Q-Q Plot of PS_5_6
Dev from Normal
4
3
2
1
0
-1
-2
0.0
2.5
5.0
7.5
10.0
12.5
Observed Value

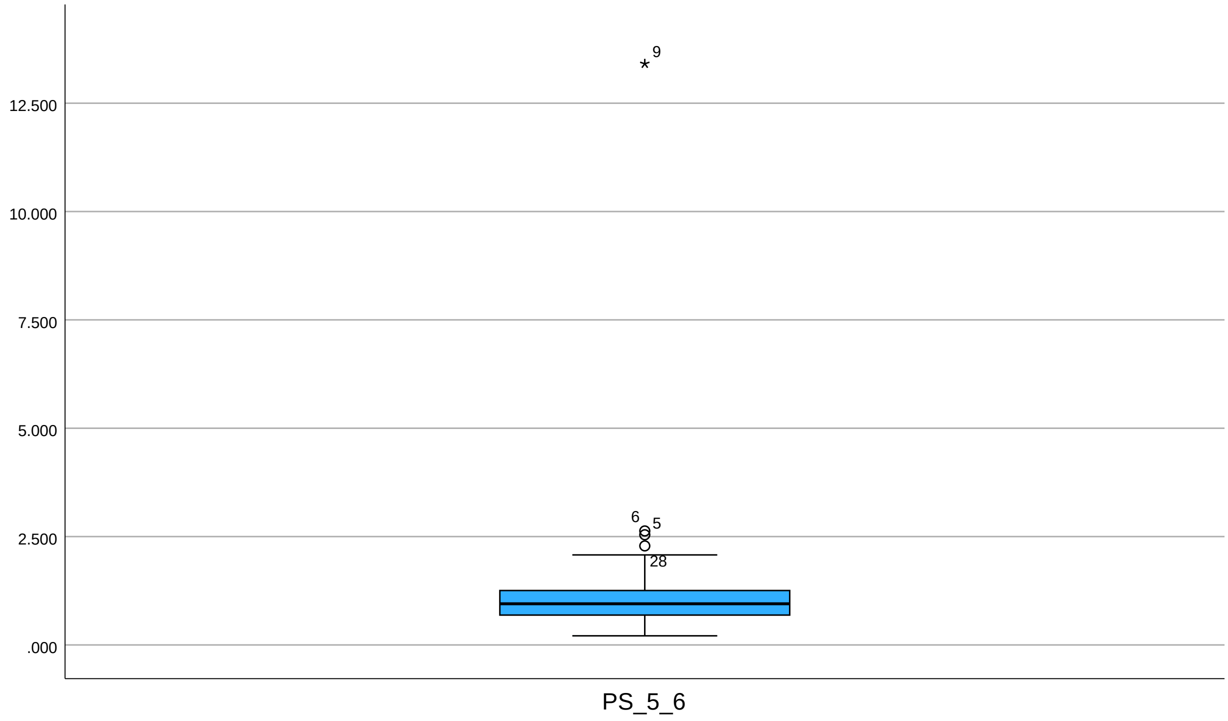
12.500
10.000
7.500
5.000
2.500
.000
9
6
5
28
PS_5_6

PS_6_7

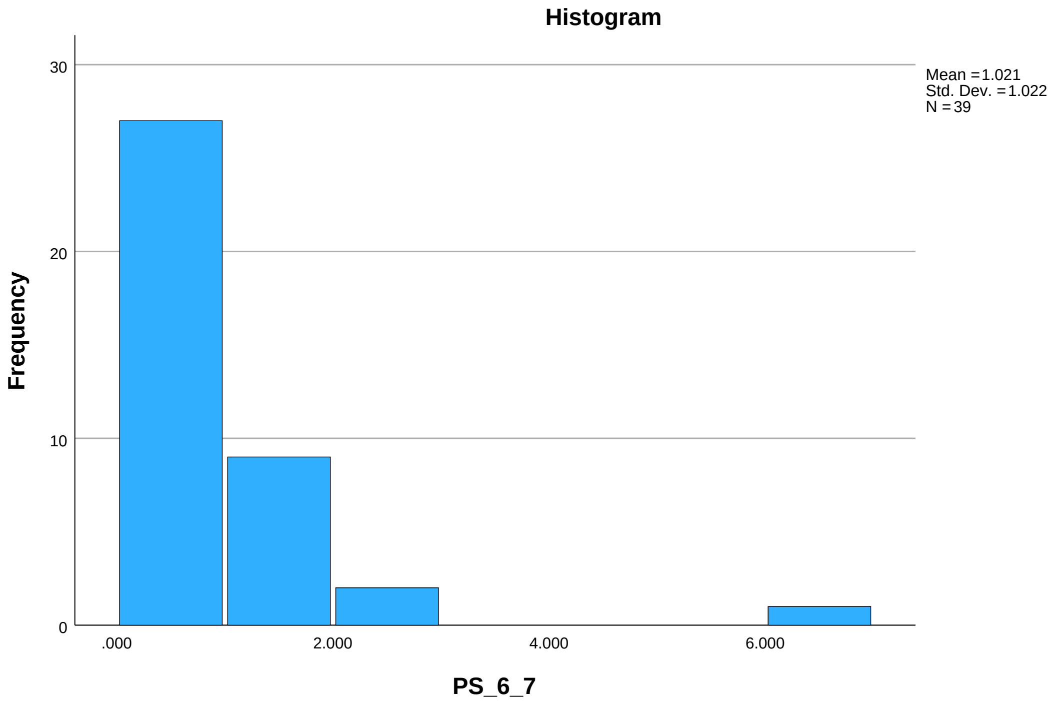


```
PS_6_7 Stem-and-Leaf Plot

 Frequency    Stem &  Leaf

     1.00        0 .  1
     4.00        0 .  2223
     7.00        0 .  4445555
     7.00        0 .  6666777
     8.00        0 .  88889999
     3.00        1 .  000
     3.00        1 .  233
     1.00        1 .  4
     1.00        1 .  7
     4.00 Extremes    (>=1.9)

 Stem width:      1.000
 Each leaf:        1 case(s)
```

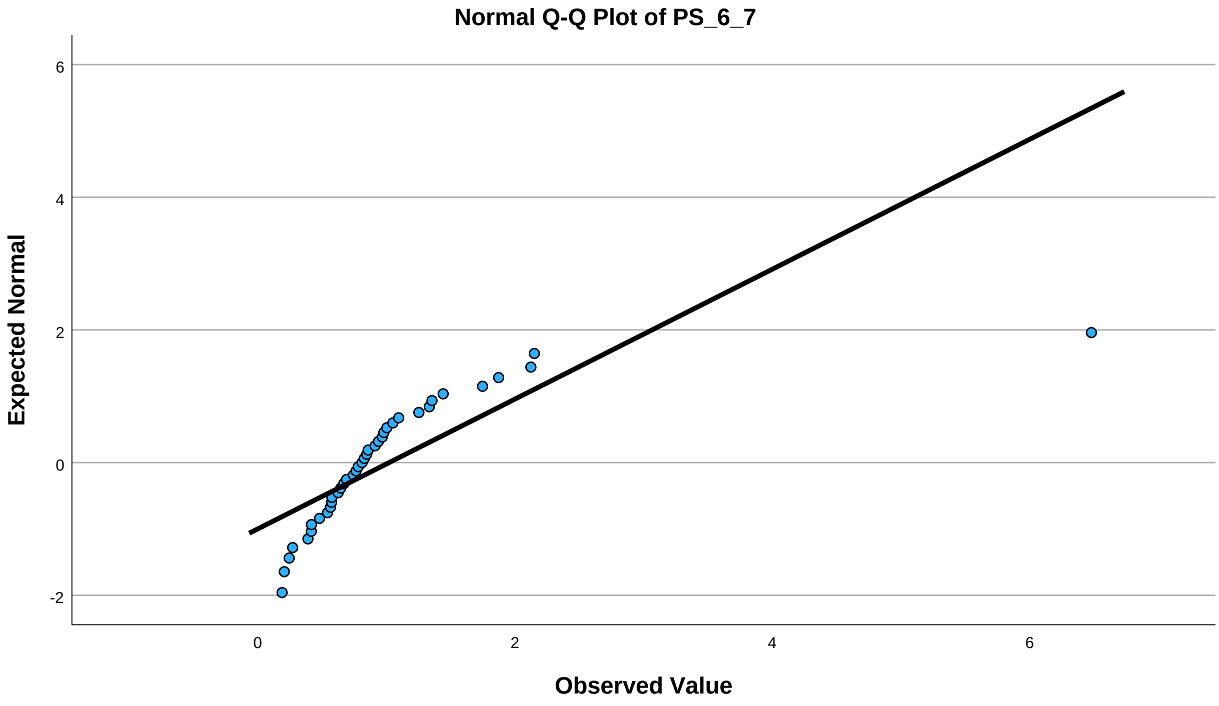
Normal Q-Q Plot of PS_6_7
Expected Normal
Observed Value
6
4
2
0
-2
0
2
4
6

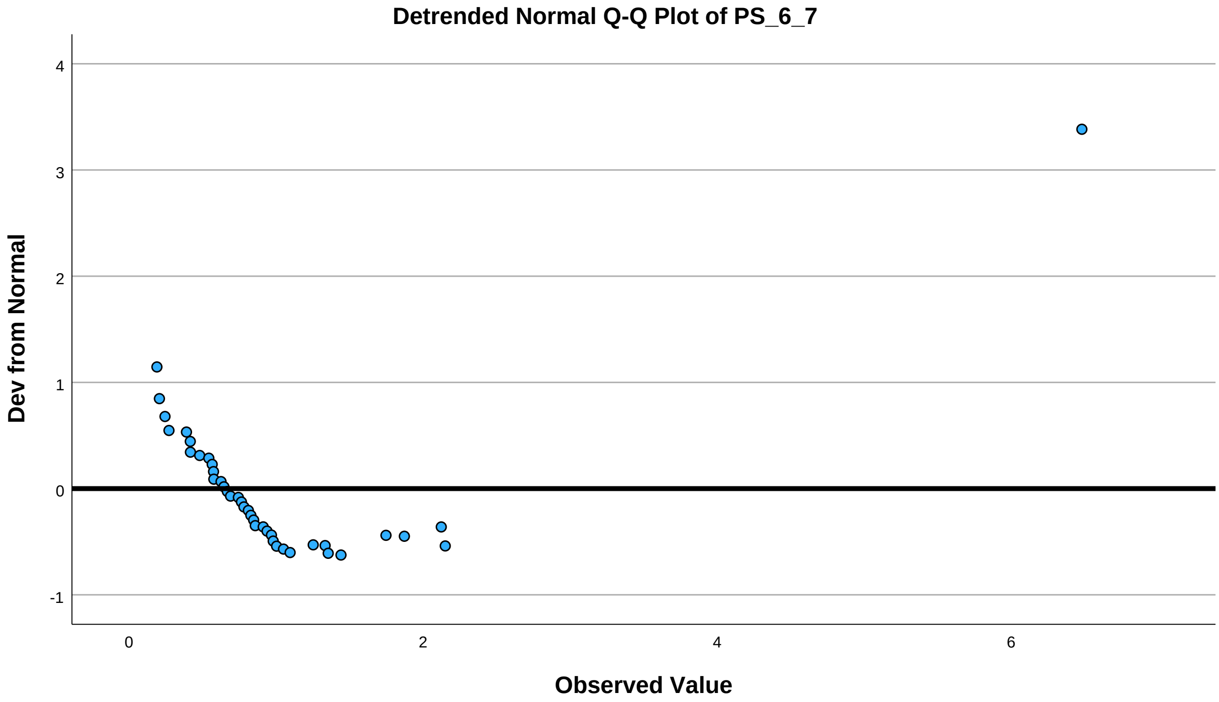
Detrended Normal Q-Q Plot of PS_6_7
Dev from Normal
Observed Value
4
3
2
1
0
-1
0
2
4
6

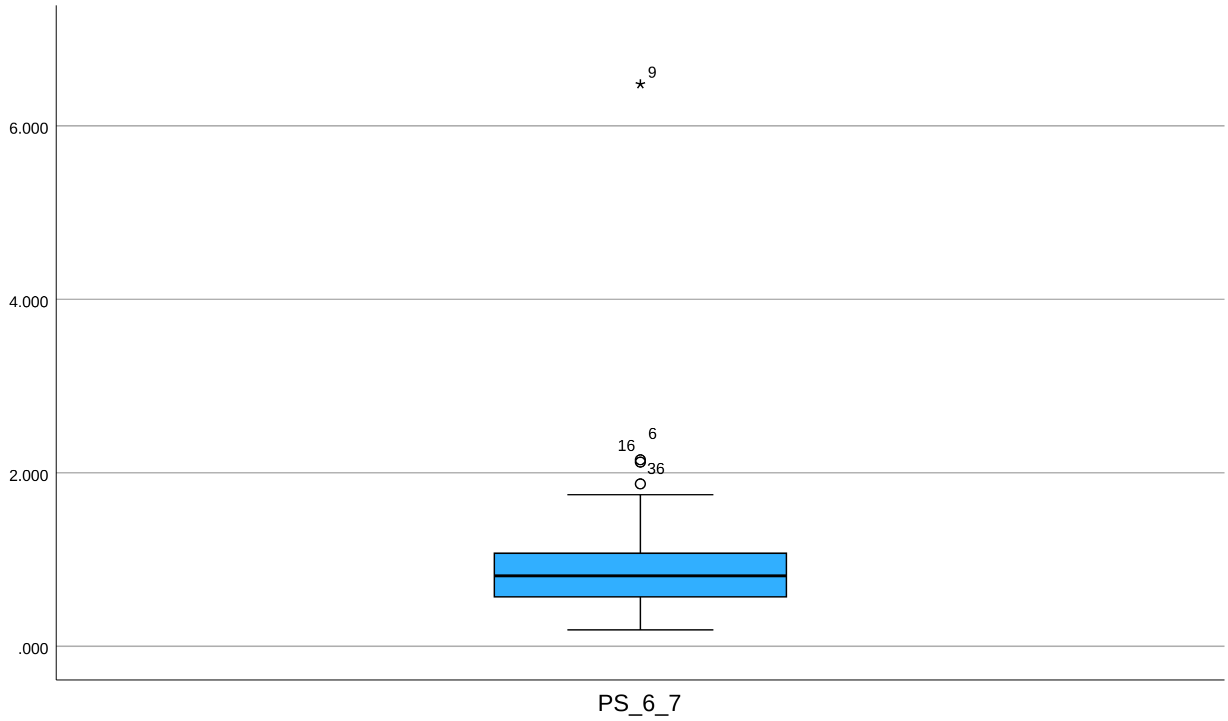

PS_7_8

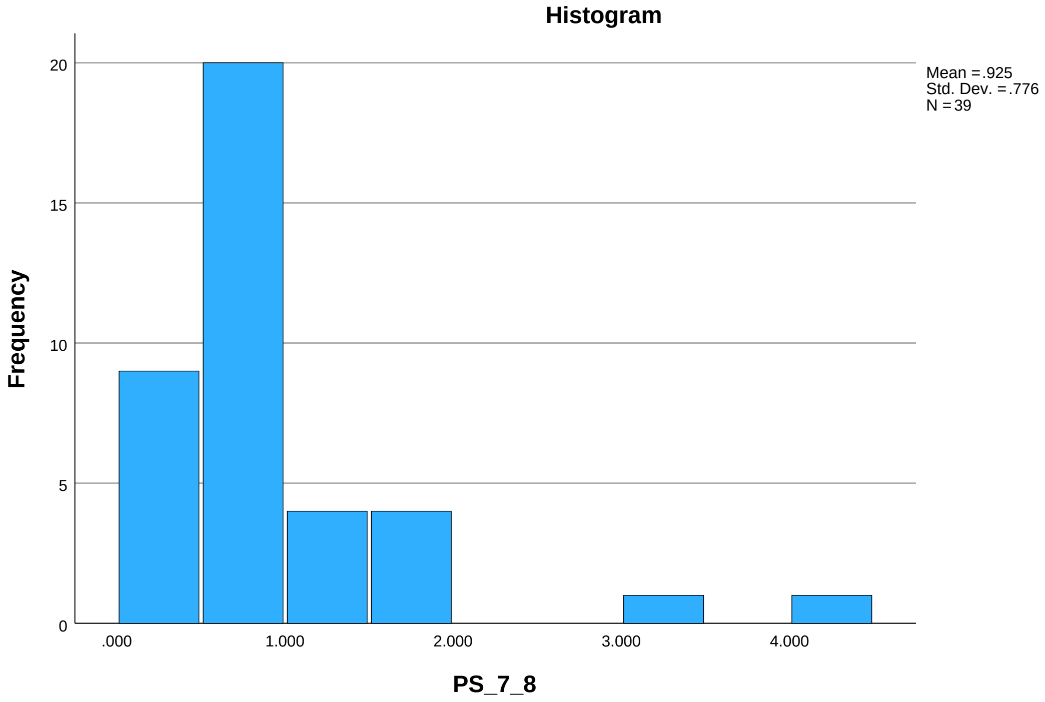

PS_7_8 Stem-and-Leaf Plot

Frequency   Stem & Leaf

```
 2.00     0 . 11
 5.00     0 . 22333
 7.00     0 . 4455555
 9.00     0 . 666677777
 6.00     0 . 888899
 2.00     1 . 01
 1.00     1 . 3
 3.00     1 . 455
 4.00 Extremes    (>=1.7)

Stem width:     1.000
Each leaf:       1 case(s)
```

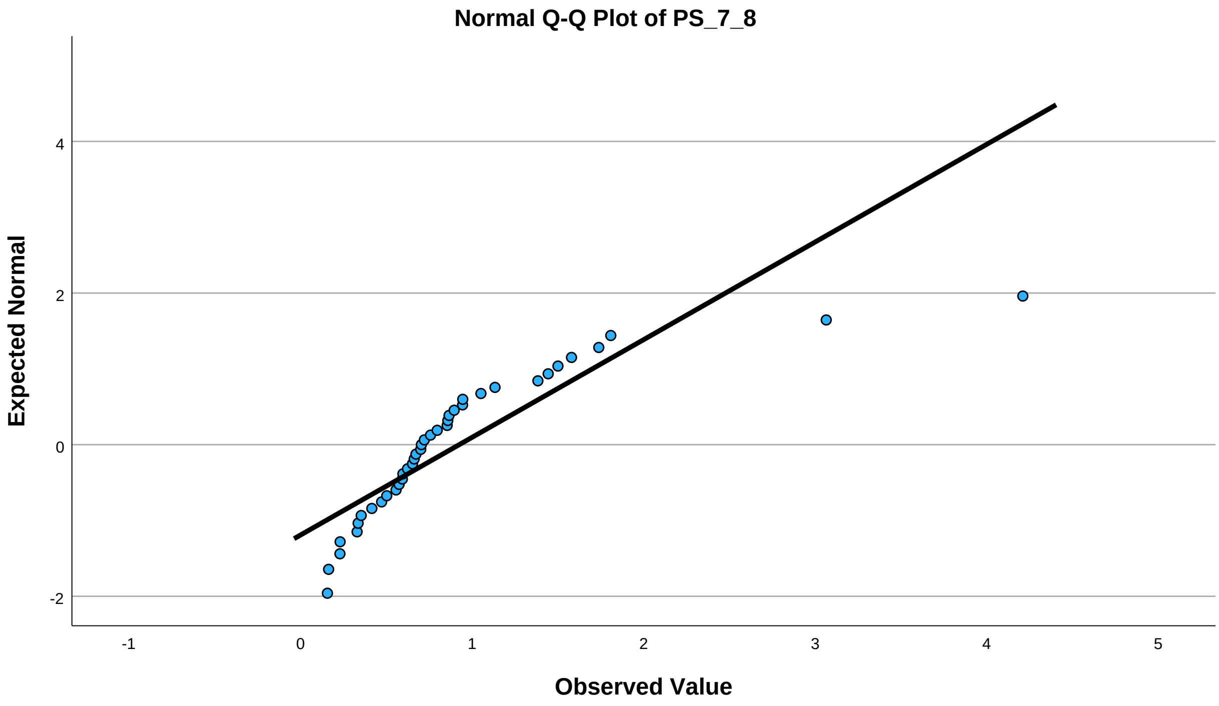

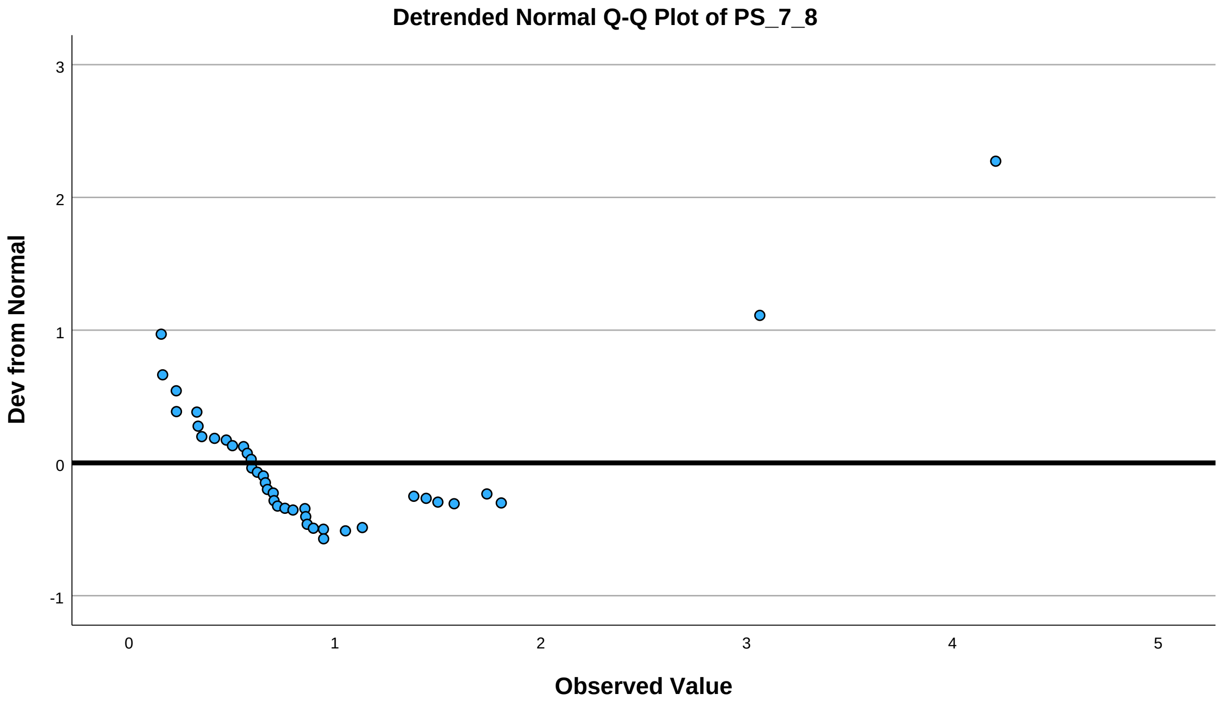

Detrended Normal Q-Q Plot of PS_7_8
Dev from Normal
3
2
1
0
-1
0
1
2
3
4
5
Observed Value

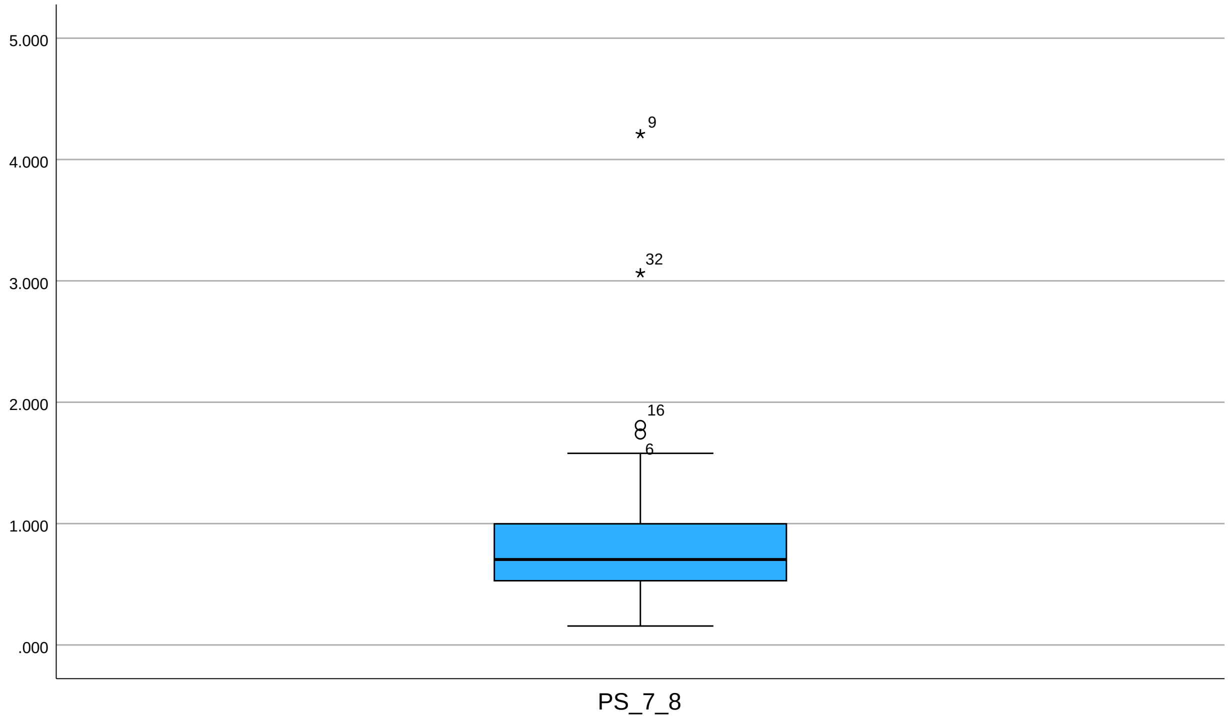

5.000
4.000
3.000
2.000
1.000
.000
9
32
16
6
PS_7_8

PS_8_9

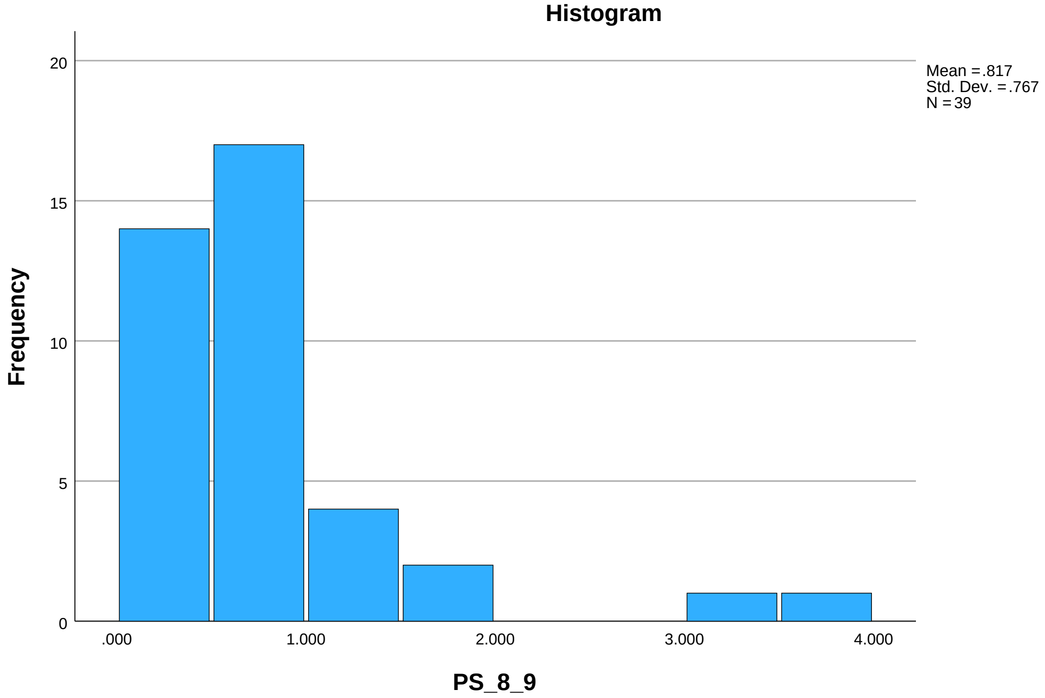


```
PS_8_9 Stem-and-Leaf Plot

 Frequency    Stem &  Leaf

     3.00        0 .  111
     7.00        0 .  2223333
    10.00        0 .  4444555555
     6.00        0 .  666677
     5.00        0 .  88889
     2.00        1 .  00
     1.00        1 .  2
     1.00        1 .  4
     4.00 Extremes    (>=1.8)

 Stem width:      1.000
 Each leaf:       1 case(s)
```

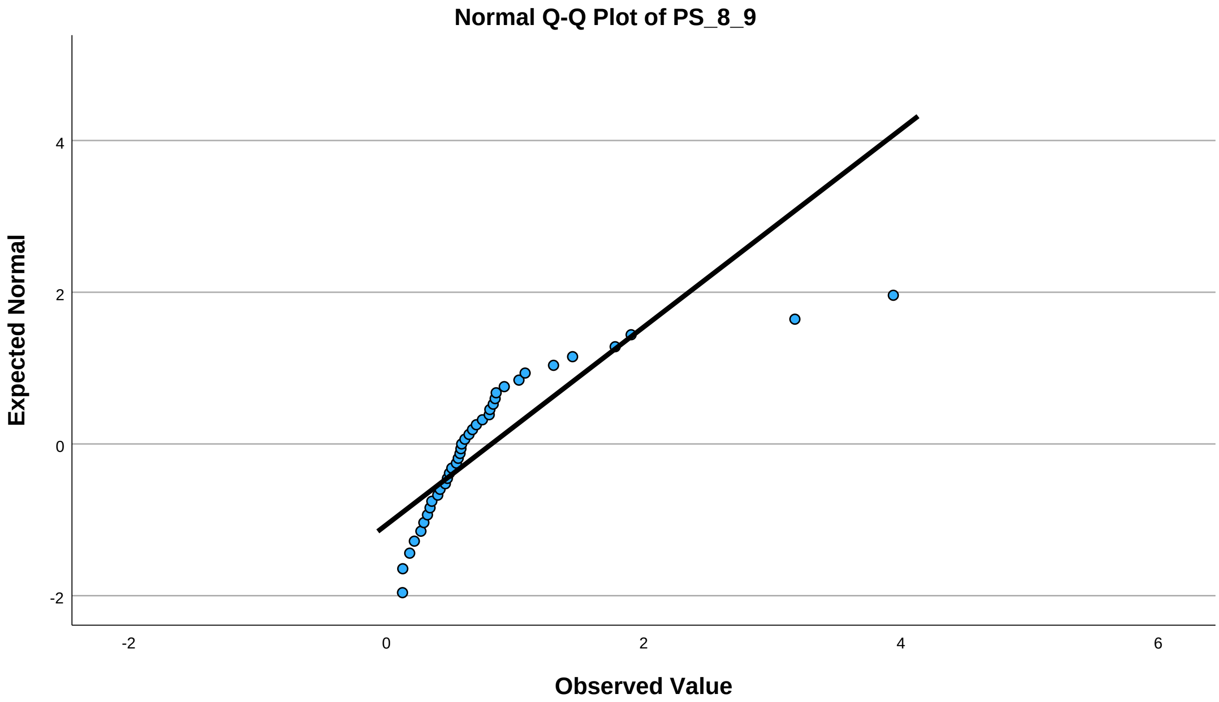
Normal Q-Q Plot of PS_8_9
Expected Normal
Observed Value
4
2
0
-2
6

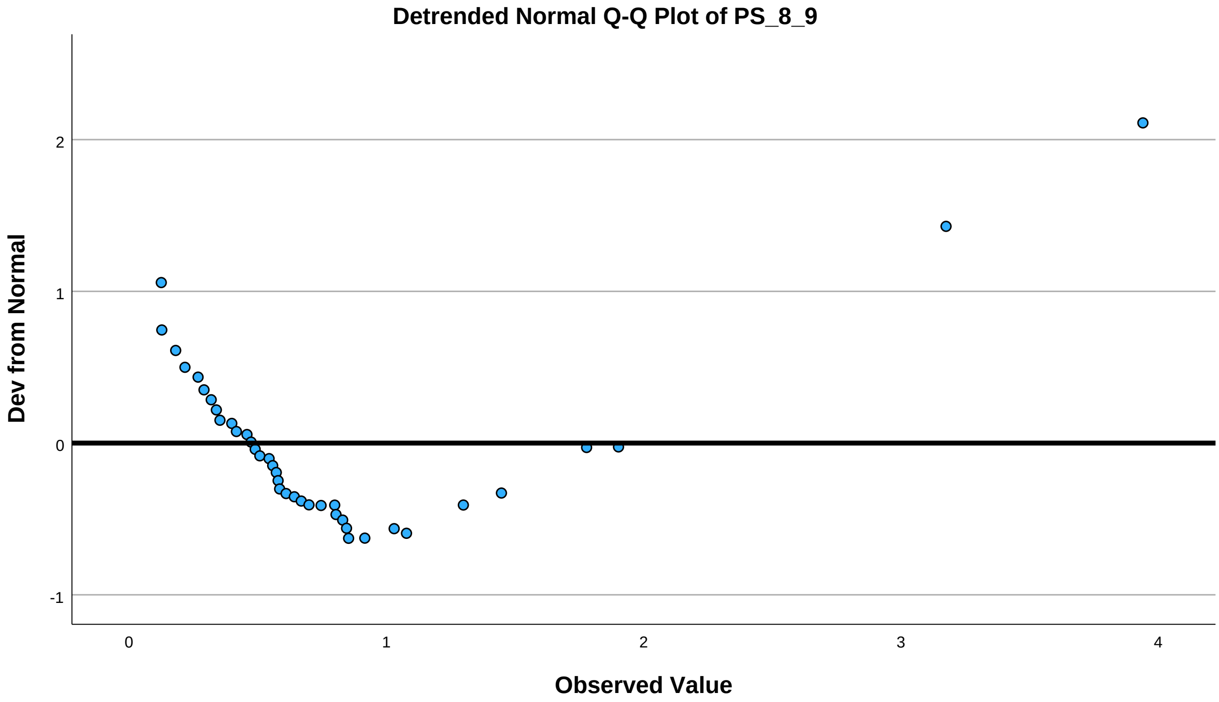
Detrended Normal Q-Q Plot of PS_8_9
Dev from Normal
Observed Value
2
1
0
-1
3
4

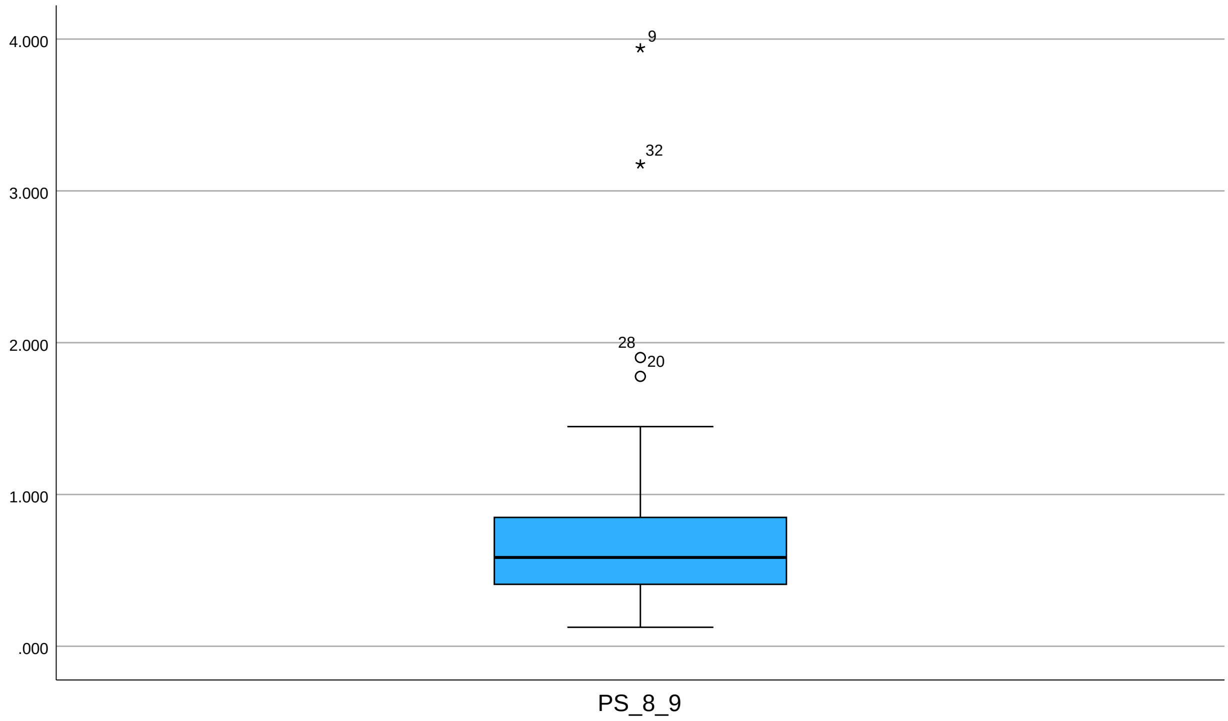

PS_9_10

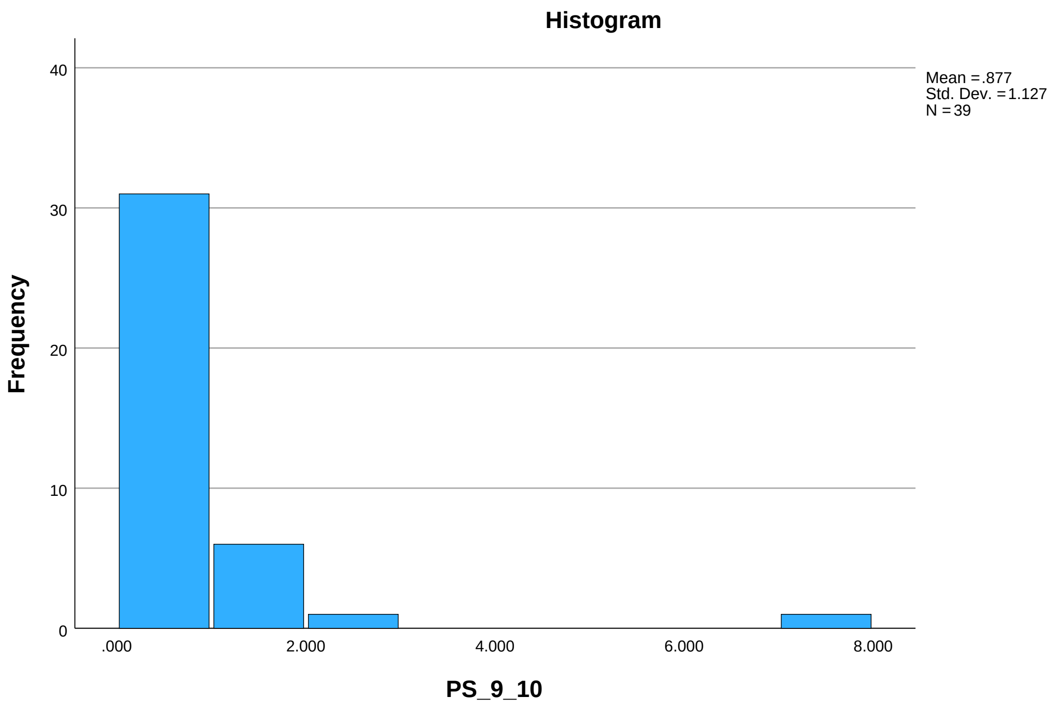

PS_9_10 Stem-and-Leaf Plot

Frequency   Stem & Leaf

```
     1.00        0 .  9
     3.00        1 .  049
     2.00        2 .  67
     2.00        3 .  14
     3.00        4 .  339
     6.00        5 .  014569
     5.00        6 .  56667
     6.00        7 .  045999
     2.00        8 .  46
     1.00        9 .  0
     1.00       10 .  8
     3.00       11 .  169
     4.00 Extremes    (>=1.63)

 Stem width:      .100
 Each leaf:       1 case(s)
```

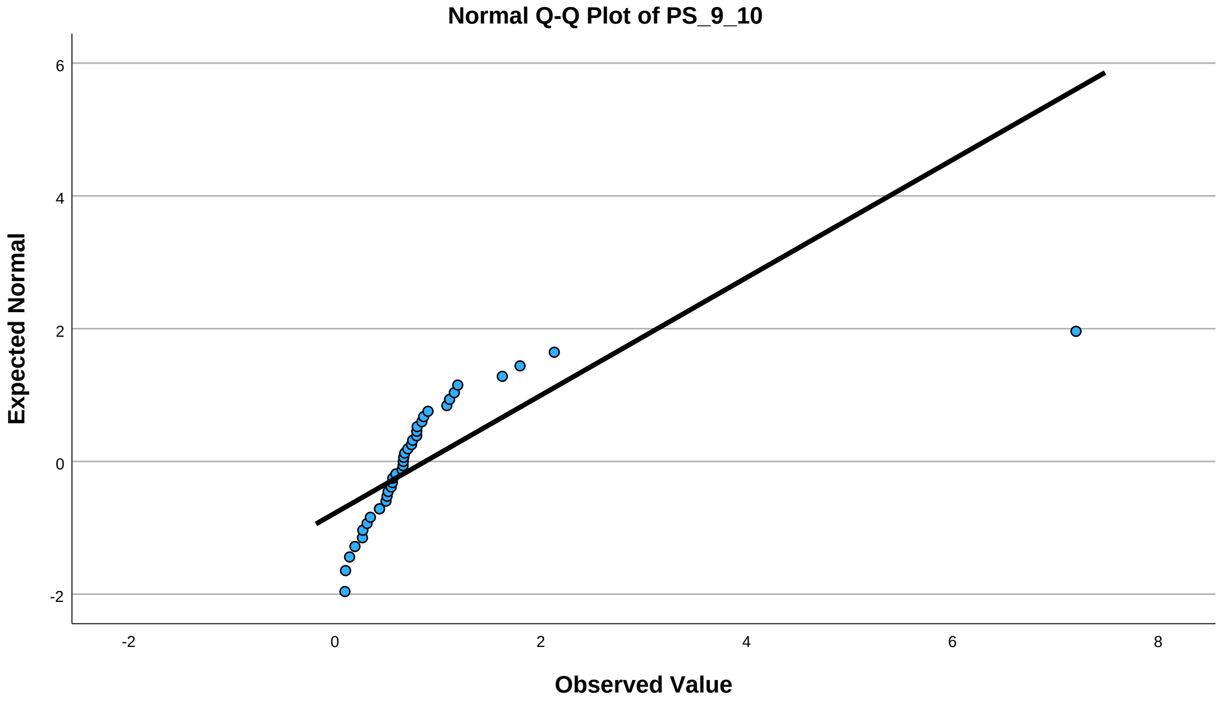

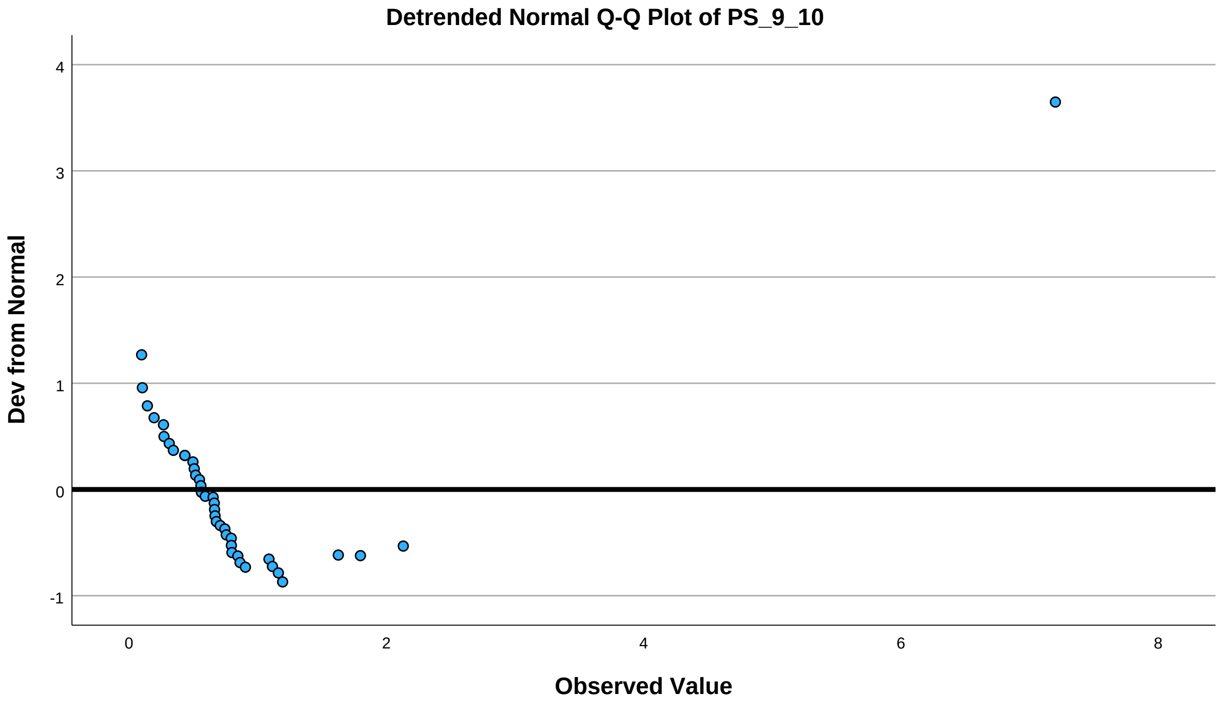
Detrended Normal Q-Q Plot of PS_9_10
Dev from Normal
Observed Value
4
3
2
1
0
-1
0
2
4
6
8

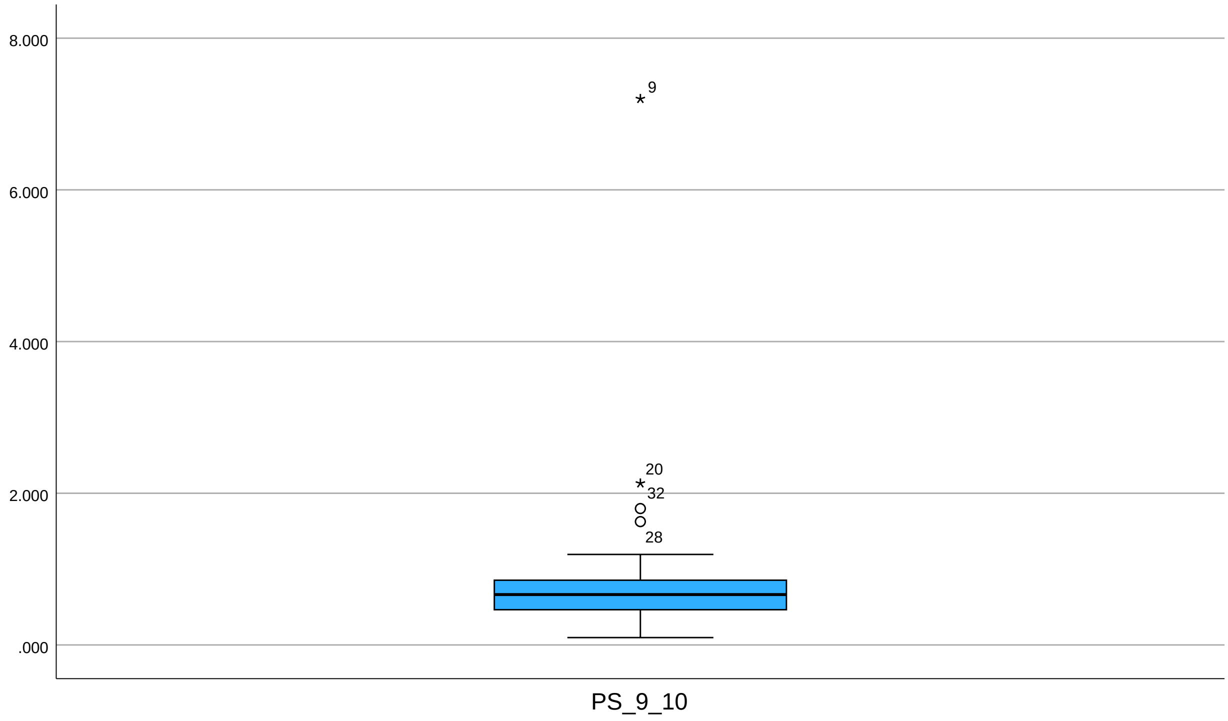
8.000
6.000
4.000
2.000
.000
9
20
32
28
PS_9_10

PS_10_11

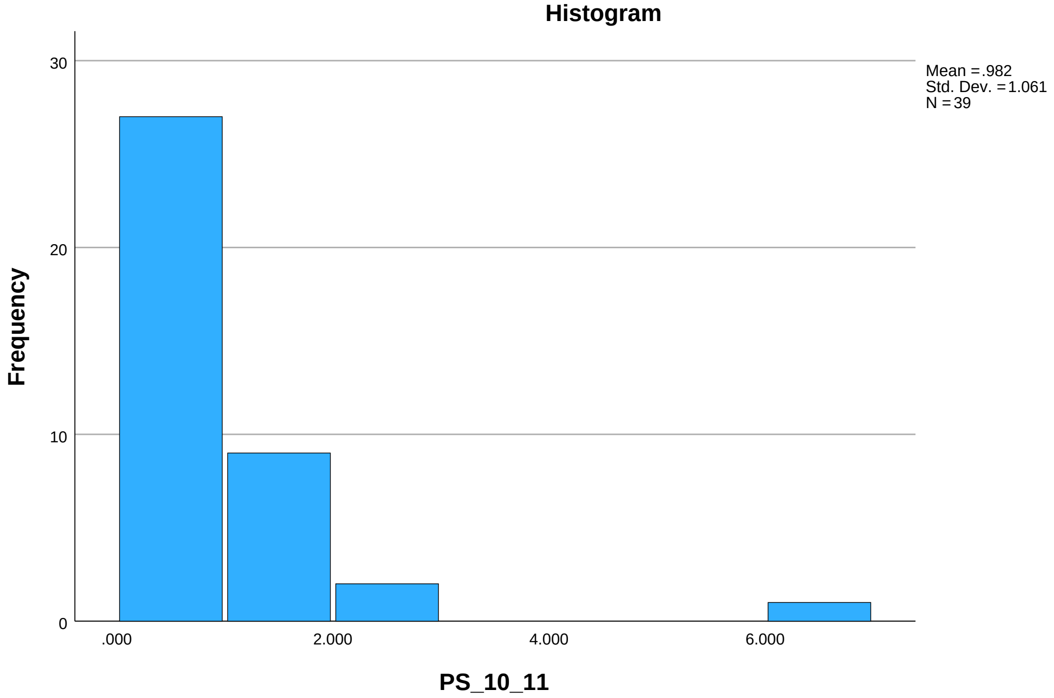


```
PS_10_11 Stem-and-Leaf Plot

 Frequency    Stem &  Leaf

     2.00        0 .  11
     7.00        0 .  2333333
     4.00        0 .  4455
    11.00        0 .  66677777777
     3.00        0 .  899
     5.00        1 .  00001
     1.00        1 .  2
     1.00        1 .  5
      .00        1 .
     2.00        1 .  88
     3.00 Extremes    (>=2.3)

 Stem width:      1.000
 Each leaf:        1 case(s)
```

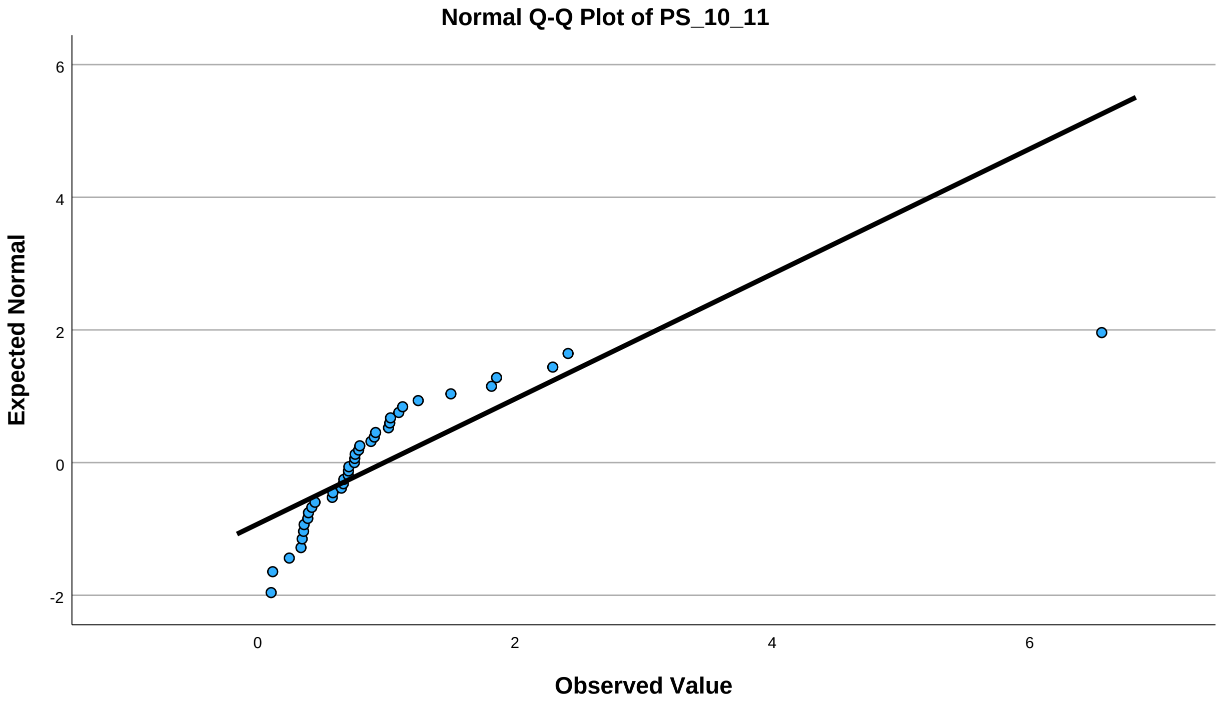

Normal Q-Q Plot of PS_10_11
Expected Normal
Observed Value
6
4
2
0
-2
0
2
4
6

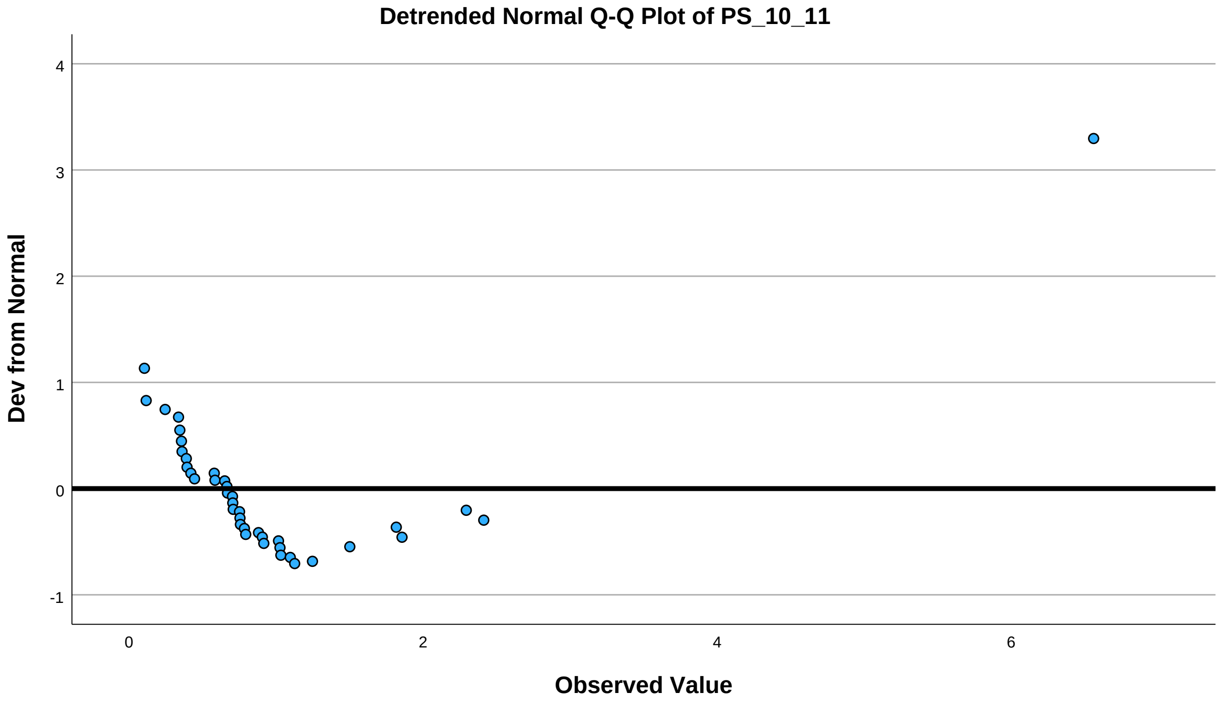

Detrended Normal Q-Q Plot of PS_10_11
Dev from Normal
Observed Value
4
3
2
1
0
-1
0
2
4
6

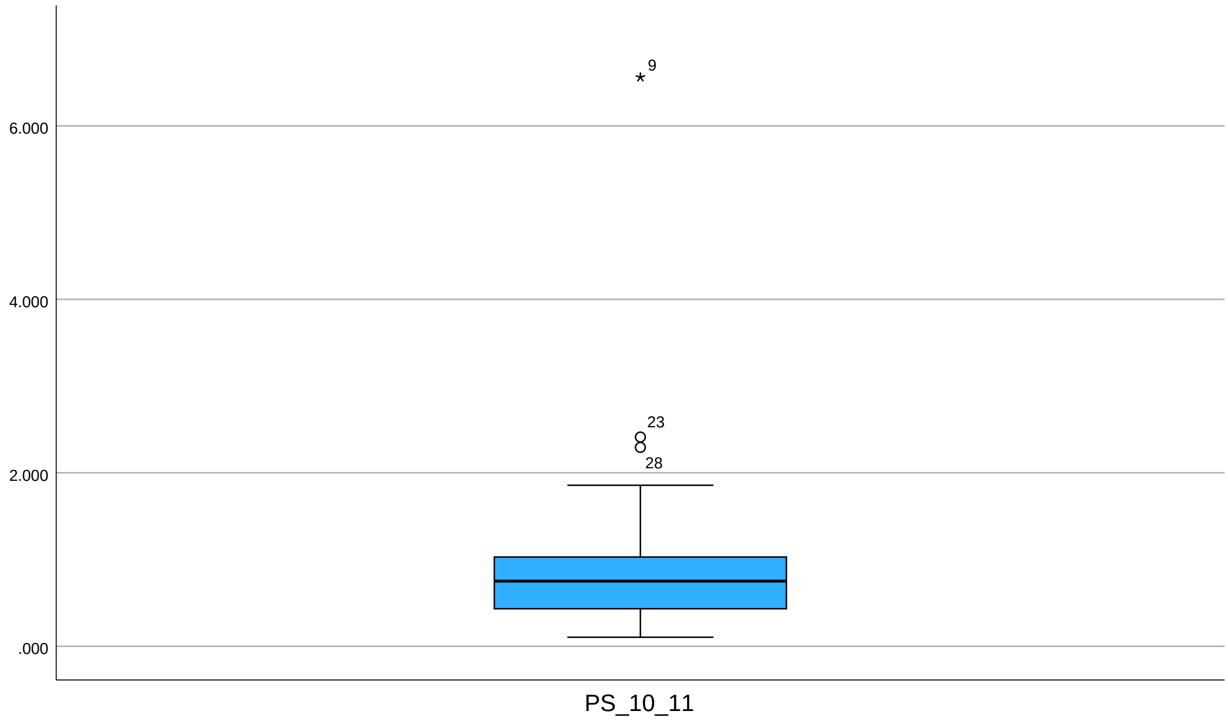

PS_11_12

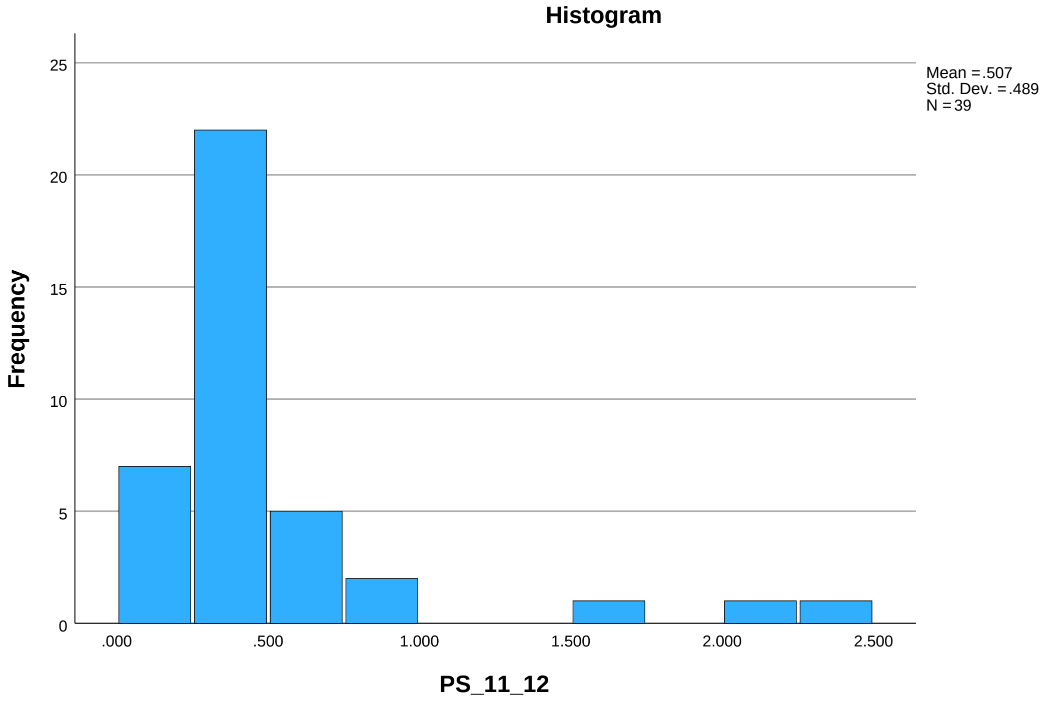

PS_11_12 Stem-and-Leaf Plot

Frequency   Stem & Leaf

```
 2.00      0 .  78
 1.00      1 .  4
 9.00      2 .  123455577
12.00      3 .  022225667789
 5.00      4 .  02467
 4.00      5 .  2289
 1.00      6 .  6
 5.00 Extremes    (>=.87)

Stem width:      .100
Each leaf:       1 case(s)
```

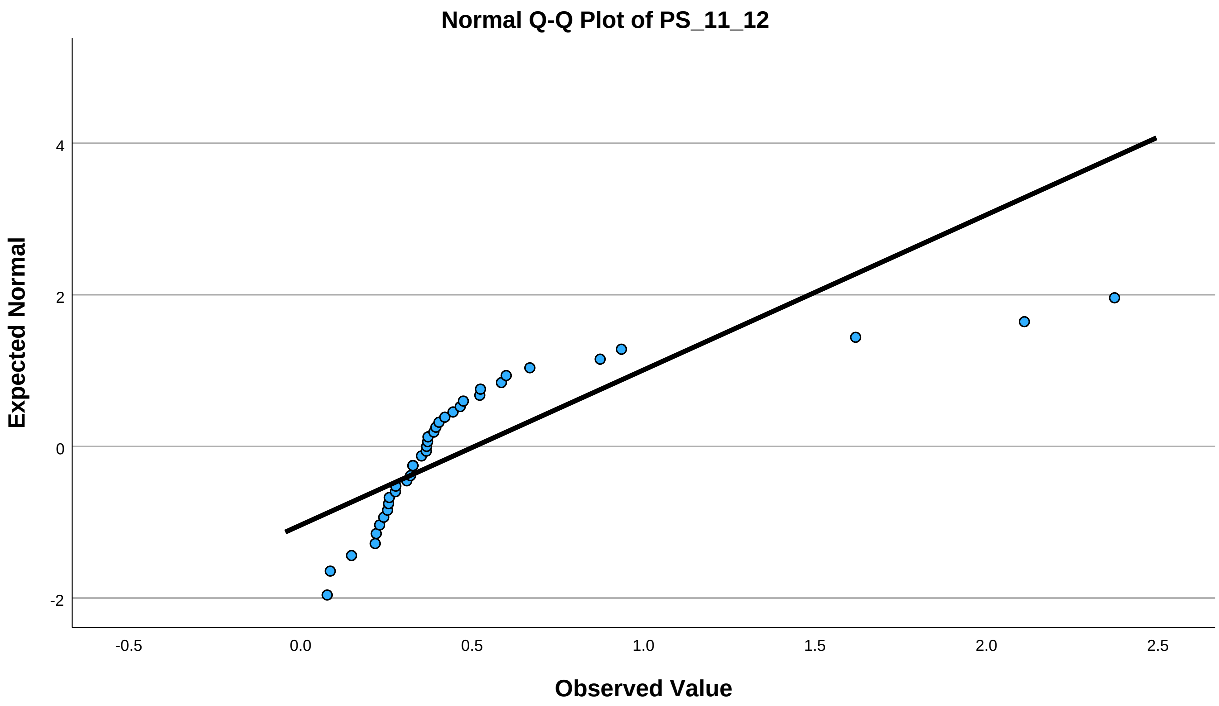

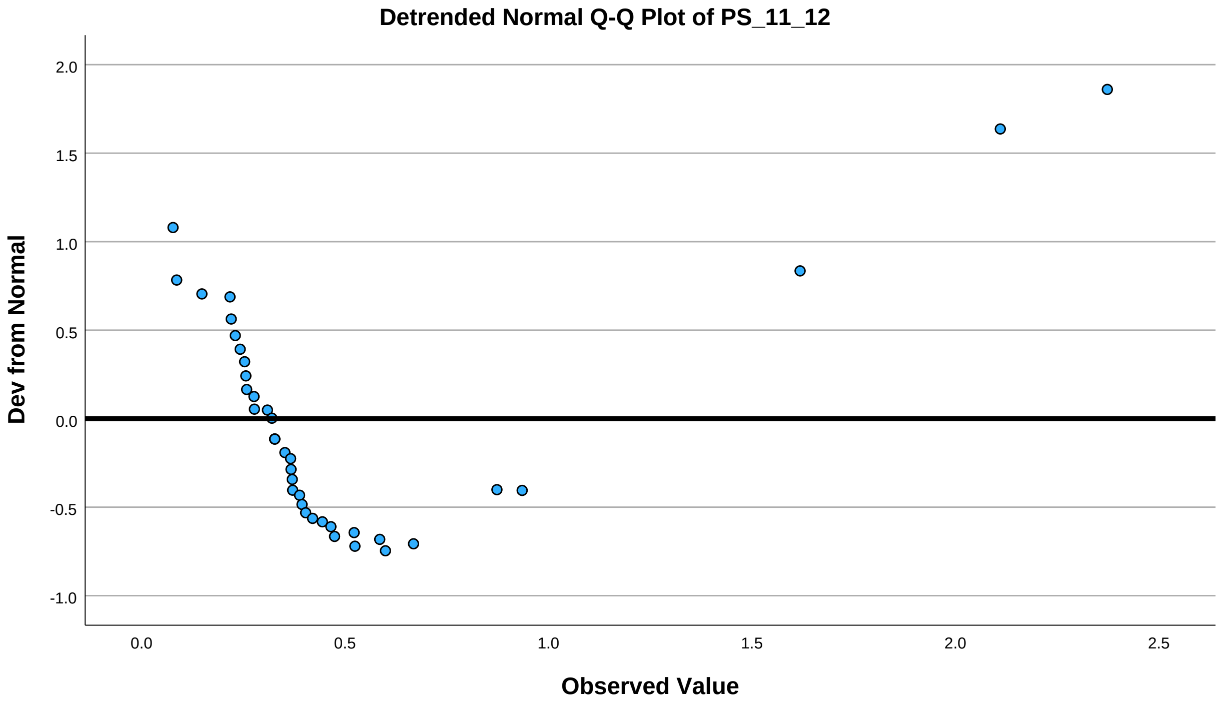
Detrended Normal Q-Q Plot of PS_11_12
Dev from Normal
Observed Value
2.0
1.5
1.0
0.5
0.0
-0.5
-1.0
0.0
0.5
1.0
1.5
2.0
2.5

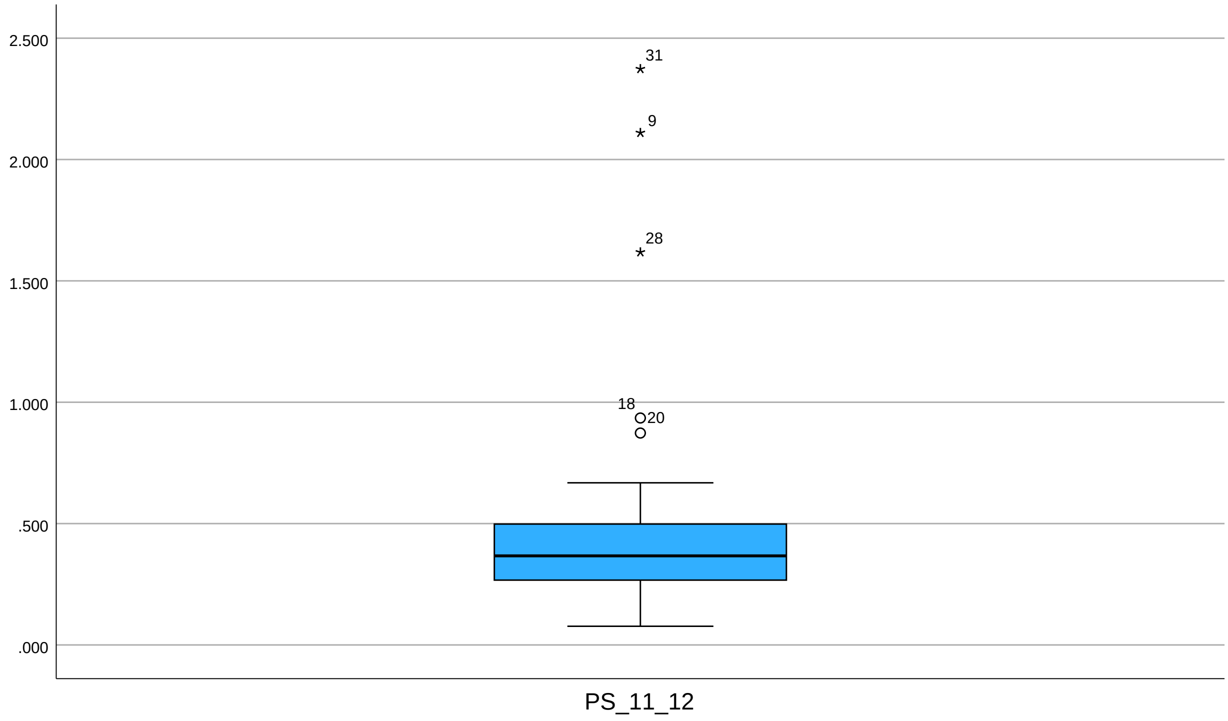
2.500
2.000
1.500
1.000
.500
.000
31
9
28
18
20
PS_11_12

PS_12_13

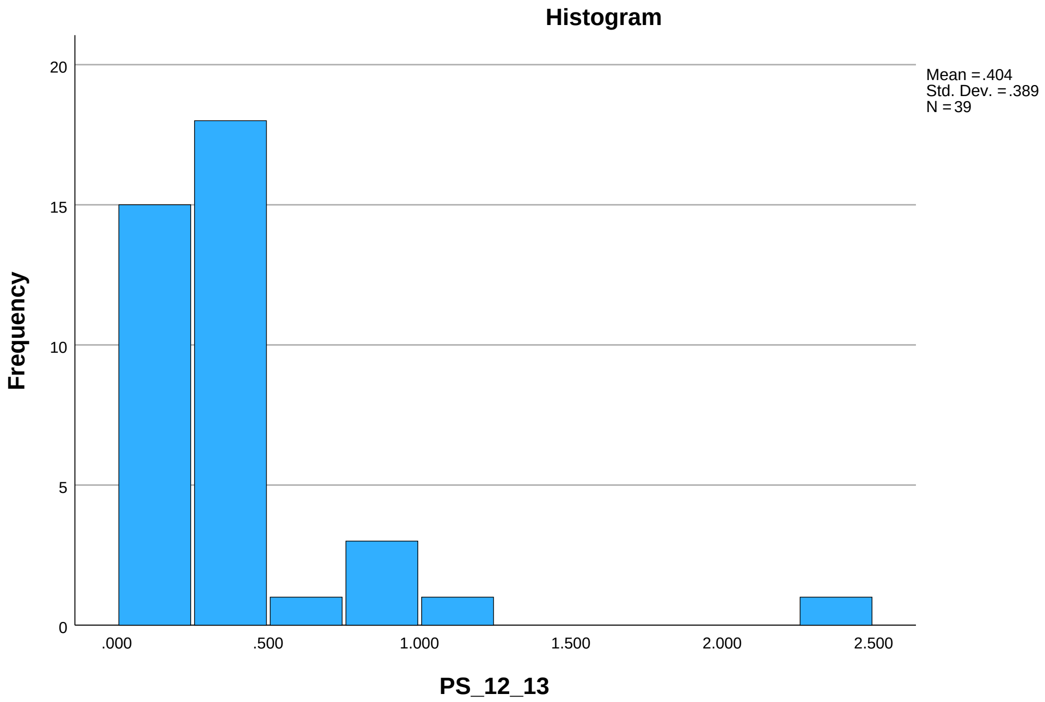


```
PS_12_13 Stem-and-Leaf Plot

 Frequency    Stem &  Leaf

     2.00        0 .  56
     4.00        1 .  1233
     3.00        1 .  679
     6.00        2 .  011134
     4.00        2 .  7888
     2.00        3 .  24
     5.00        3 .  55589
     4.00        4 .  0113
     3.00        4 .  679
     1.00        5 .  0
     5.00 Extremes    (>=.83)

 Stem width:      .100
 Each leaf:       1 case(s)
```

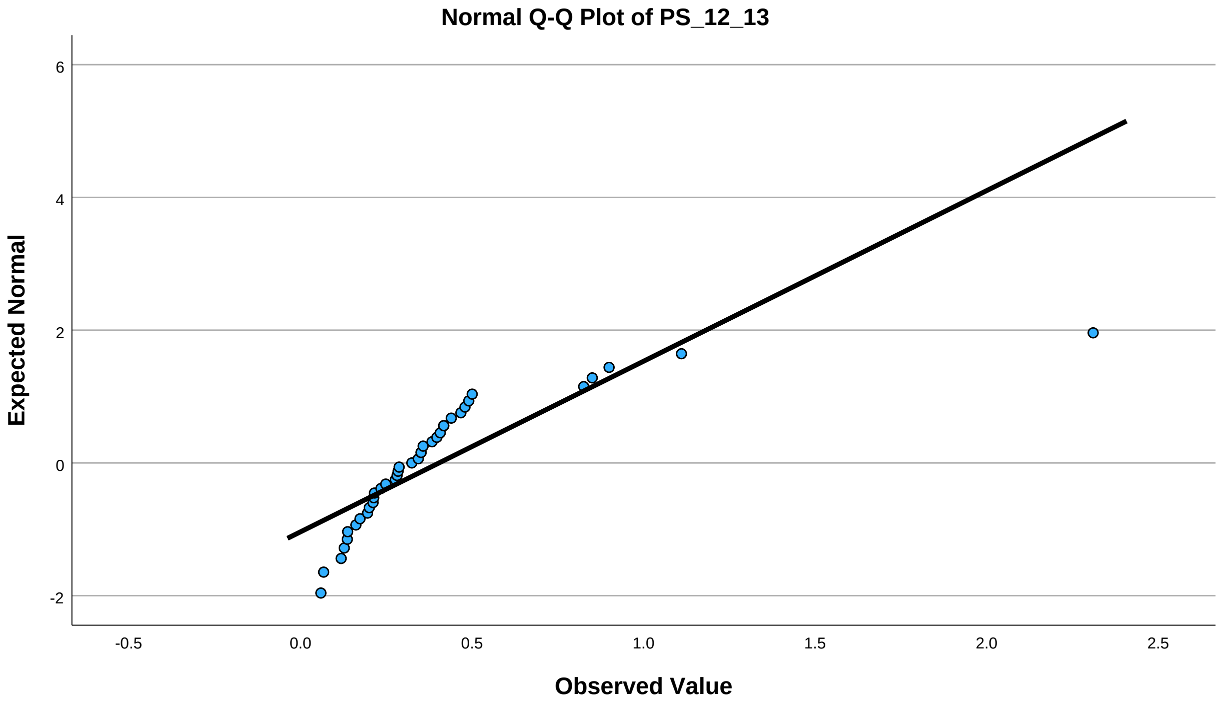
Normal Q-Q Plot of PS_12_13
Expected Normal
6
4
2
0
-2
-0.5
0.0
0.5
1.0
1.5
2.0
2.5
Observed Value

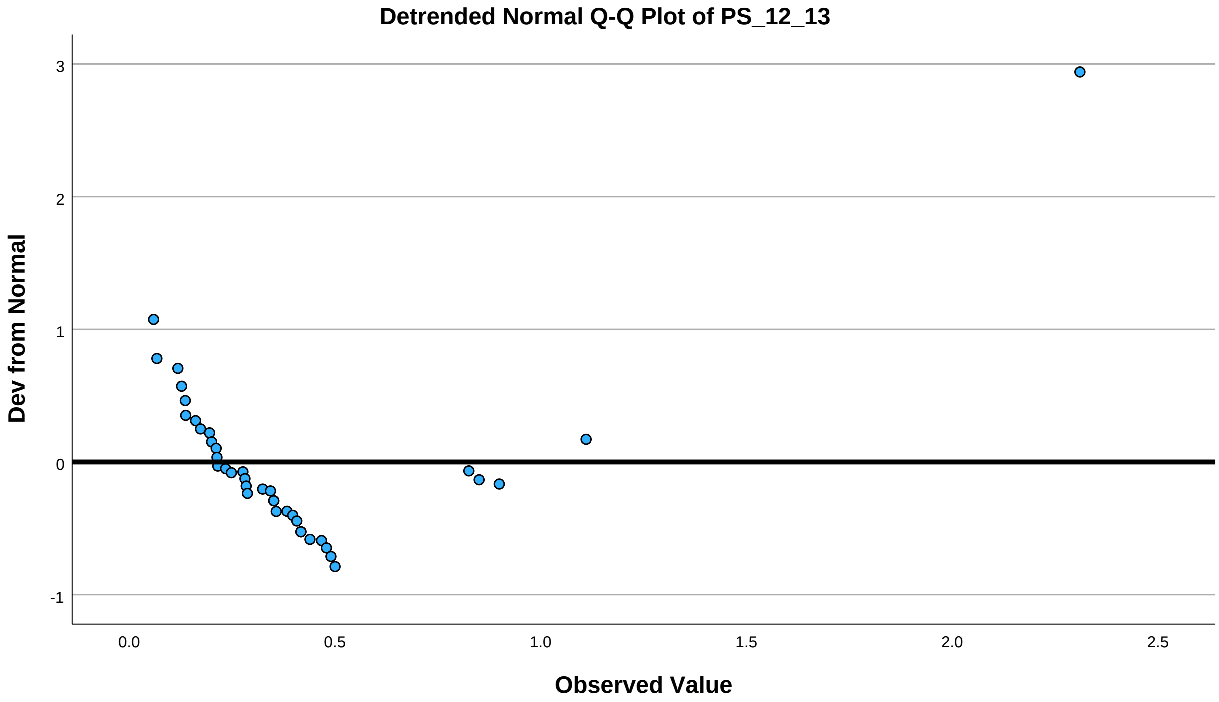
Detrended Normal Q-Q Plot of PS_12_13
Dev from Normal
3
2
1
0
-1
0.0
0.5
1.0
1.5
2.0
2.5
Observed Value

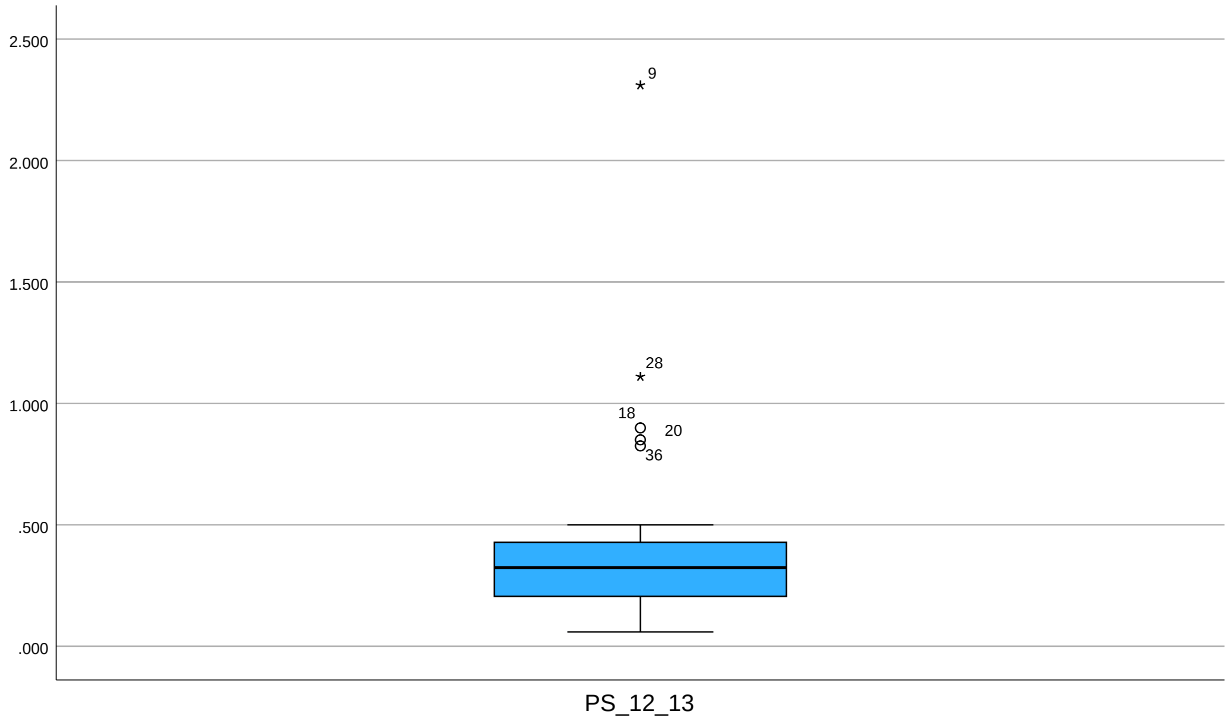

PS_13_14

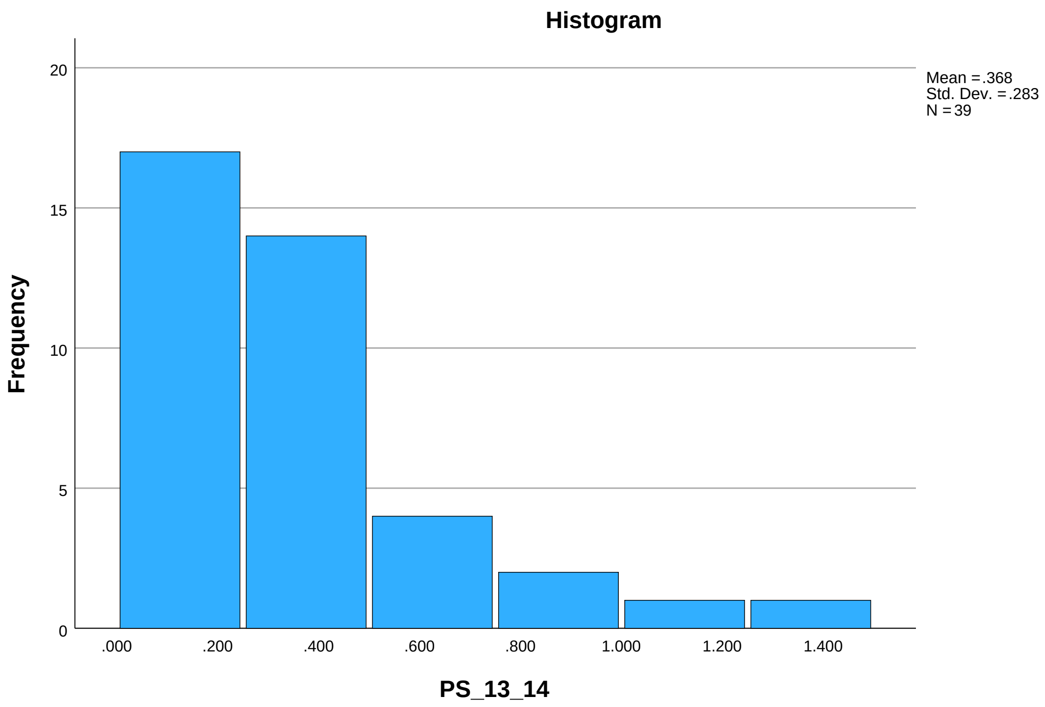

PS_13_14 Stem-and-Leaf Plot

Frequency Stem & Leaf

```
    4.00        0 .  5678
    7.00        1 .  0267778
    8.00        2 .  12223358
    8.00        3 .  03455588
    4.00        4 .  2336
    2.00        5 .  05
    1.00        6 .  7
    2.00        7 .  25
    1.00        8 .  1
    2.00 Extremes    (>=1.03)

 Stem width:      .100
 Each leaf:       1 case(s)
```

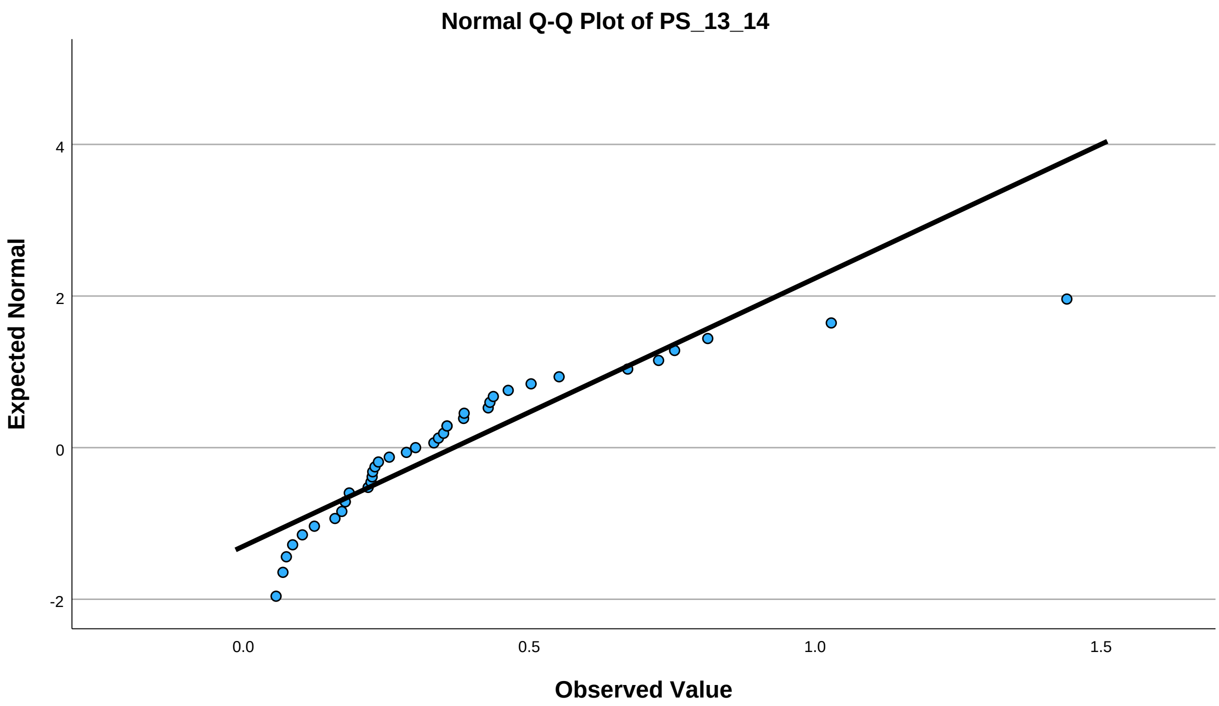

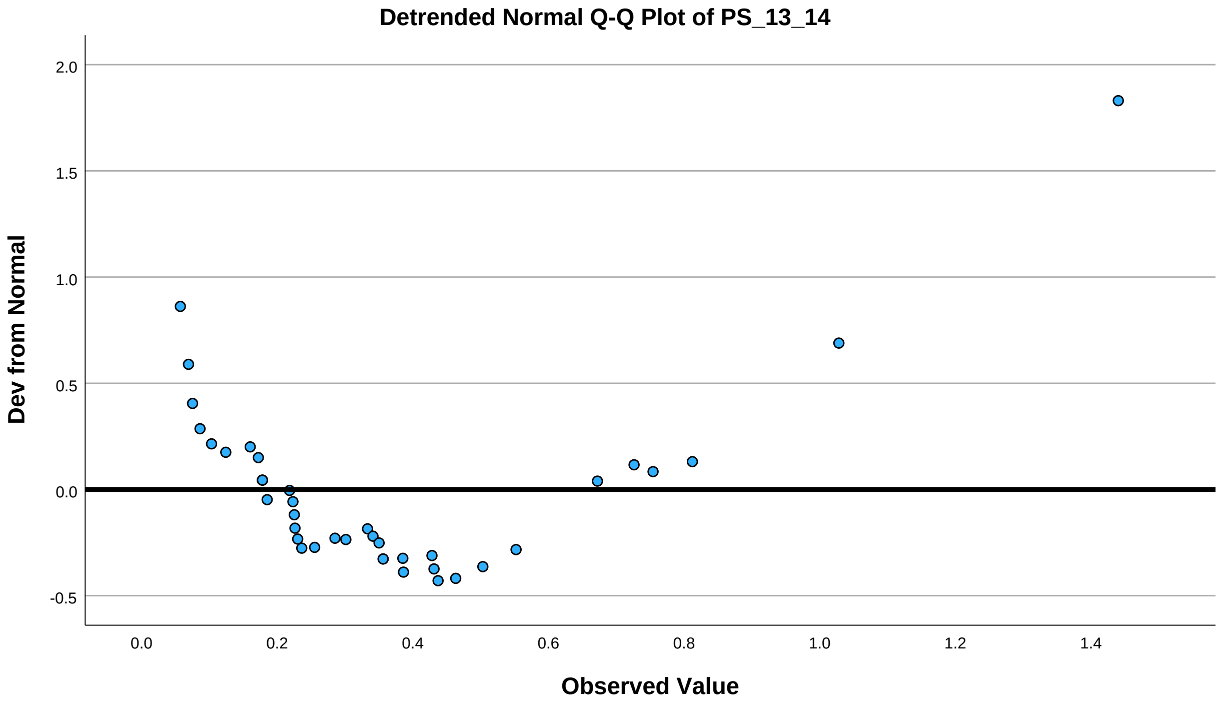
Detrended Normal Q-Q Plot of PS_13_14
Dev from Normal
2.0
1.5
1.0
0.5
0.0
-0.5
0.0
0.2
0.4
0.6
0.8
1.0
1.2
1.4
Observed Value

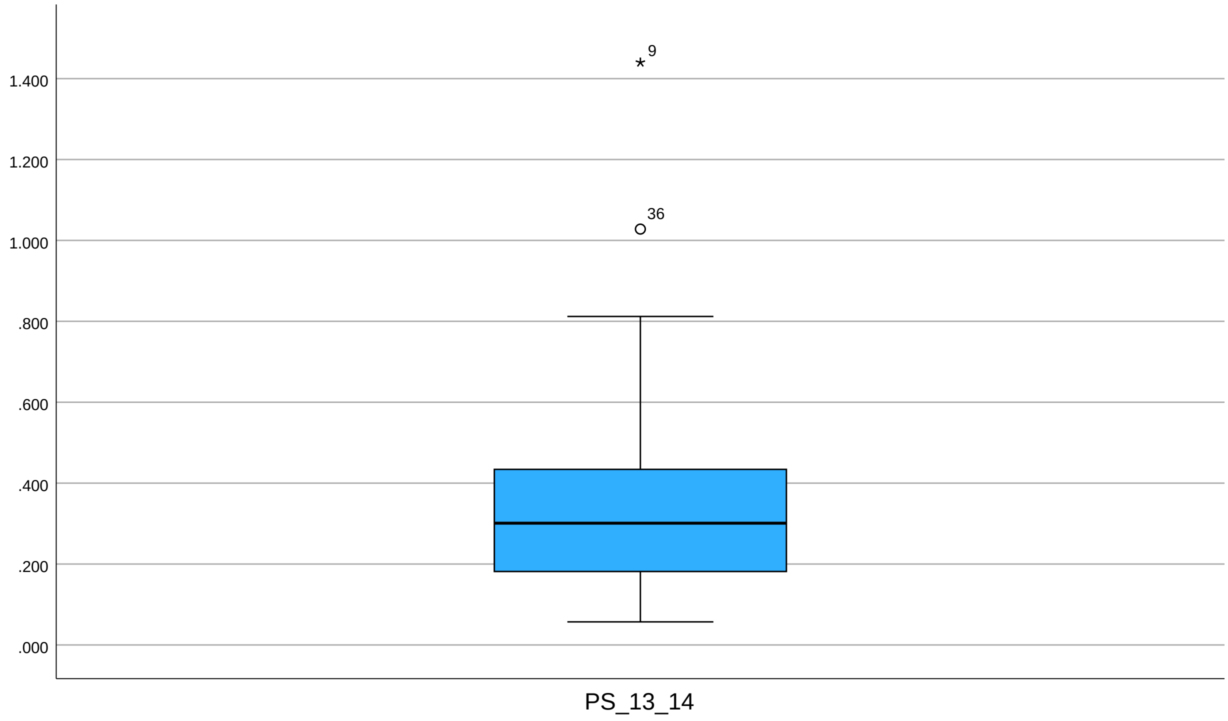
9
36
1.400
1.200
1.000
.800
.600
.400
.200
.000
PS_13_14

PS_14_15

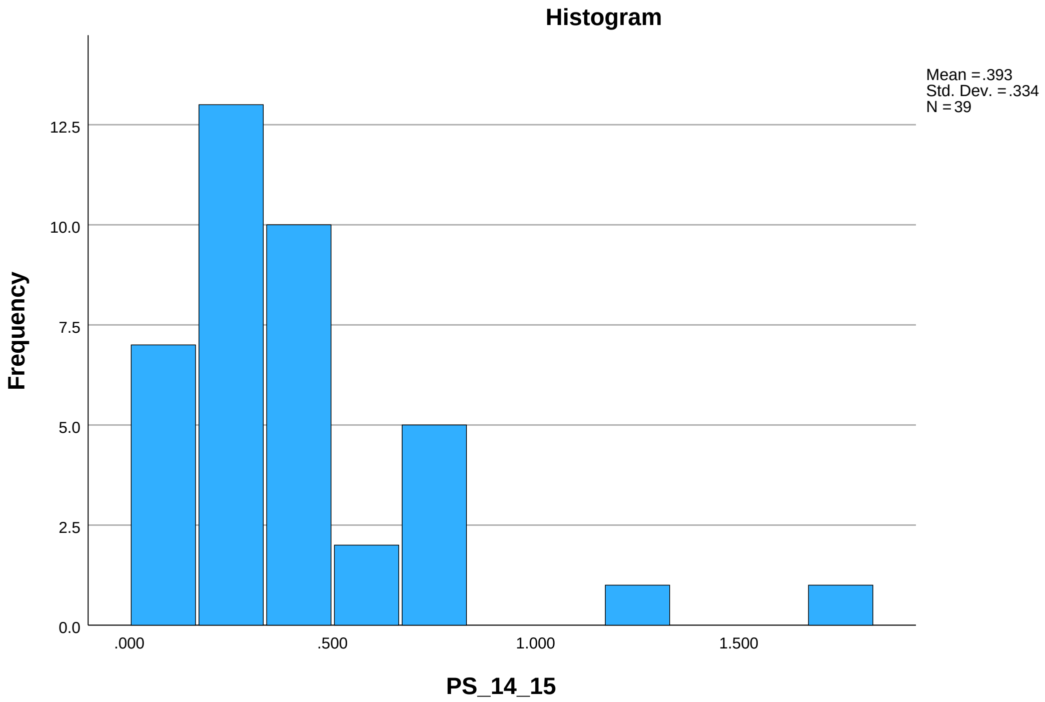


```
PS_14_15 Stem-and-Leaf Plot

 Frequency    Stem &  Leaf

     4.00        0 .  5669
     8.00        1 .  03366999
     8.00        2 .  12233347
     5.00        3 .  56778
     5.00        4 .  01578
     2.00        5 .  39
      .00        6 .
     5.00        7 .  12258
     2.00 Extremes    (>=1.21)

 Stem width:      .100
 Each leaf:       1 case(s)
```

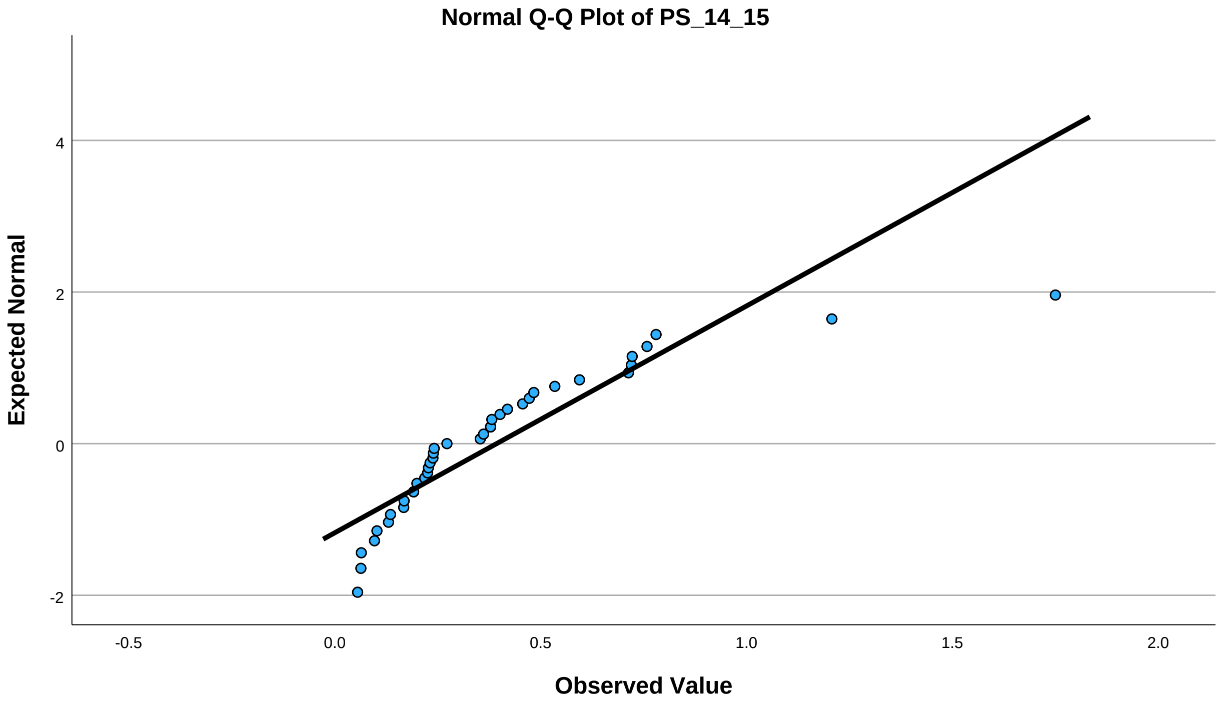
Normal Q-Q Plot of PS_14_15
Expected Normal
4
2
0
-2
-0.5
0.0
0.5
1.0
1.5
2.0
Observed Value

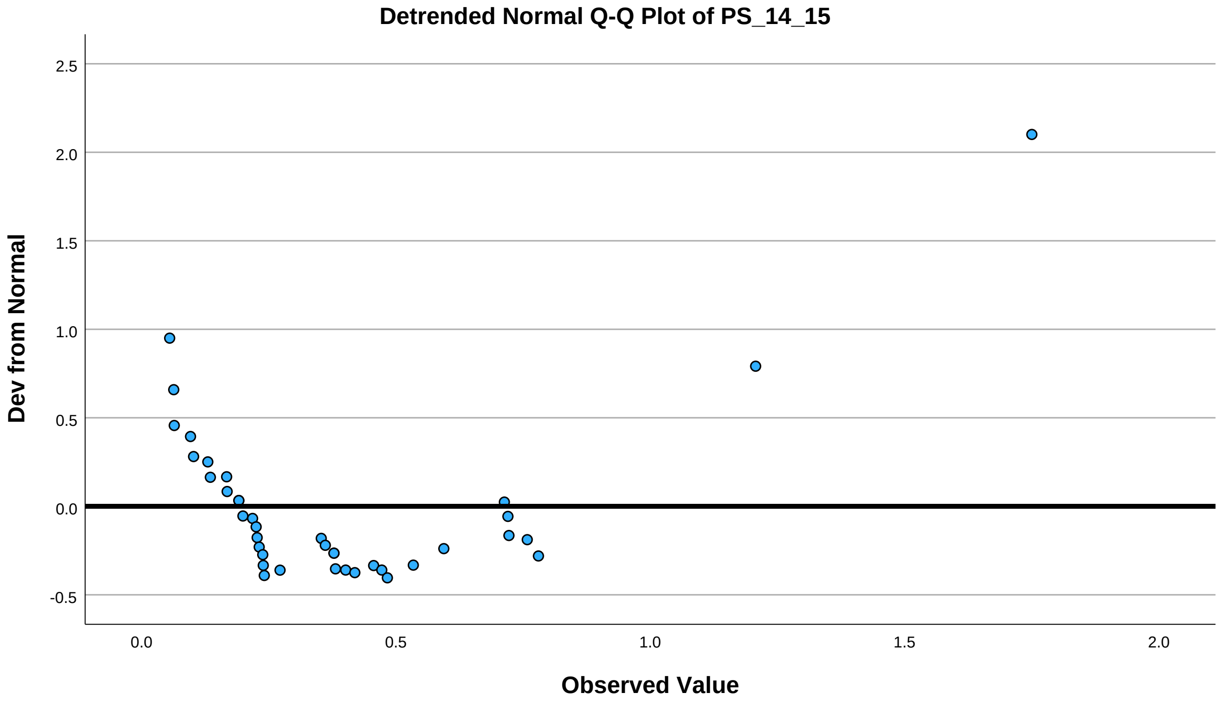
Detrended Normal Q-Q Plot of PS_14_15
Dev from Normal
2.5
2.0
1.5
1.0
0.5
0.0
-0.5
0.0
0.5
1.0
1.5
2.0
Observed Value

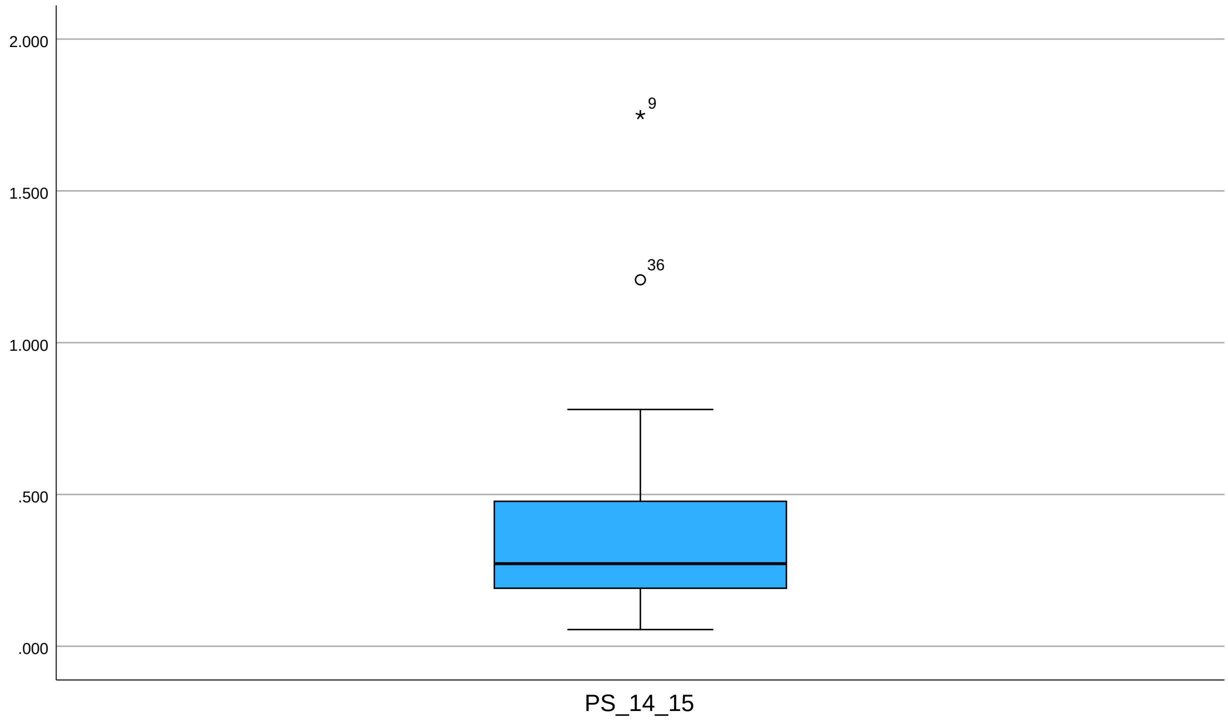

PS_15_16

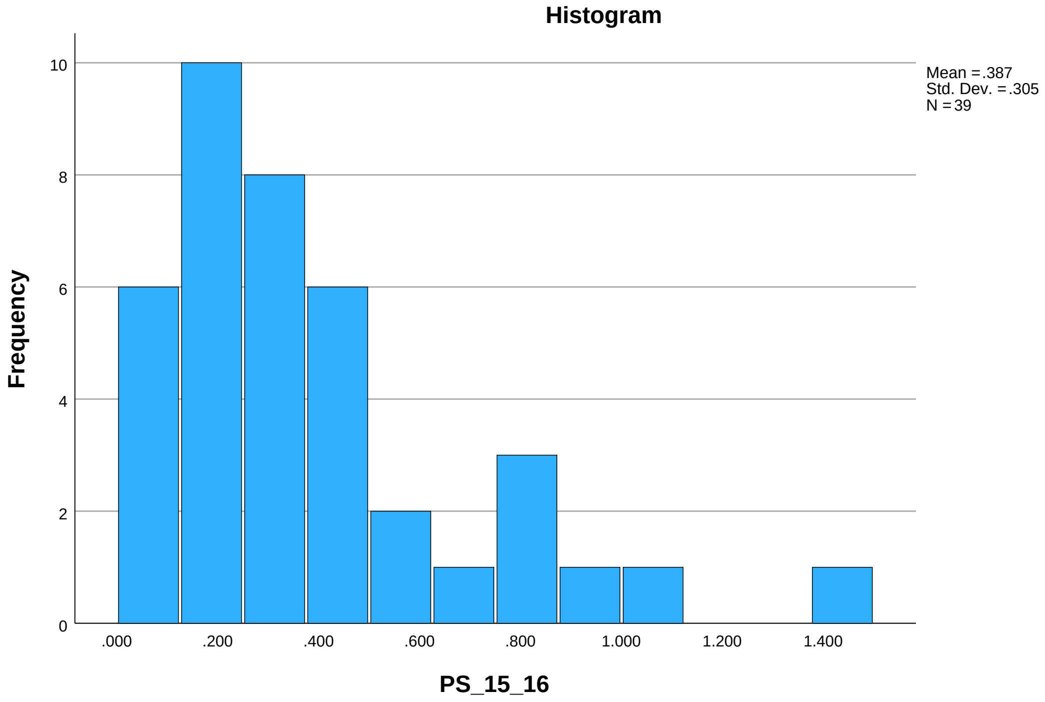

PS_15_16 Stem-and-Leaf Plot

Frequency   Stem & Leaf

```
   4.00      0 . 4569
   8.00      1 . 11446779
   7.00      2 . 1222579
   6.00      3 . 004568
   5.00      4 . 02368
   2.00      5 . 49
    .00      6 .
   3.00      7 . 369
   1.00      8 . 2
   1.00      9 . 0
   2.00 Extremes    (>=1.08)

 Stem width:      .100
 Each leaf:       1 case(s)
```

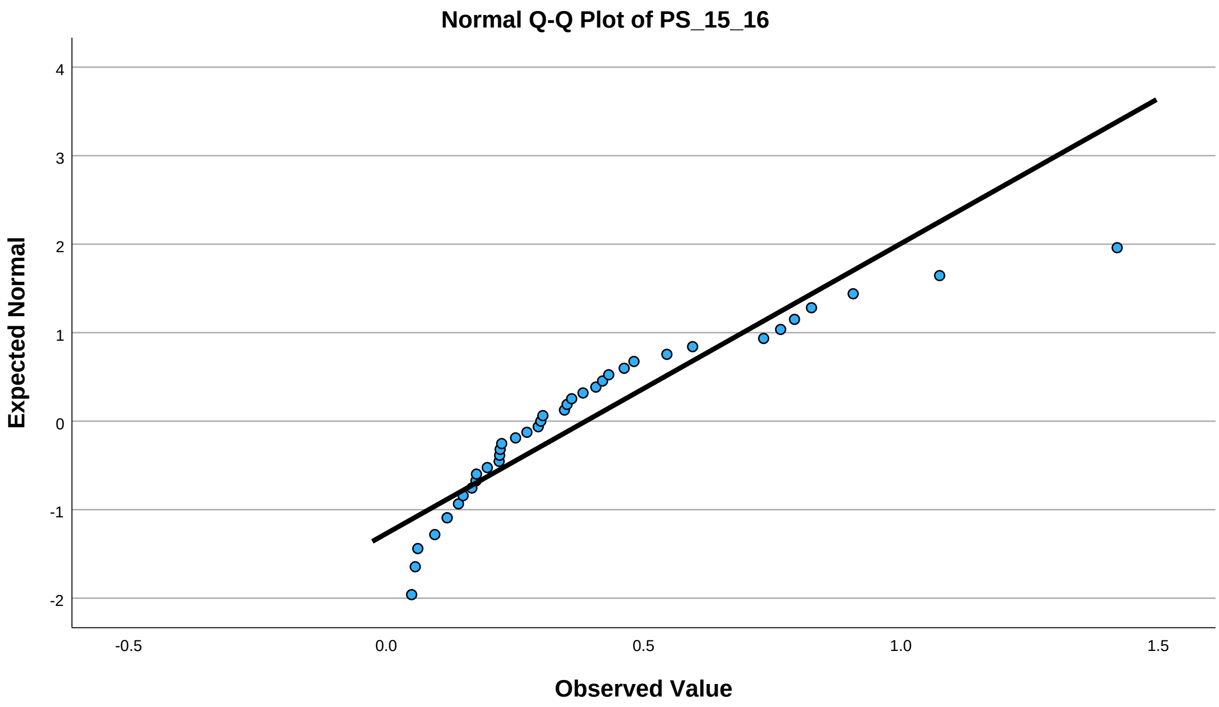

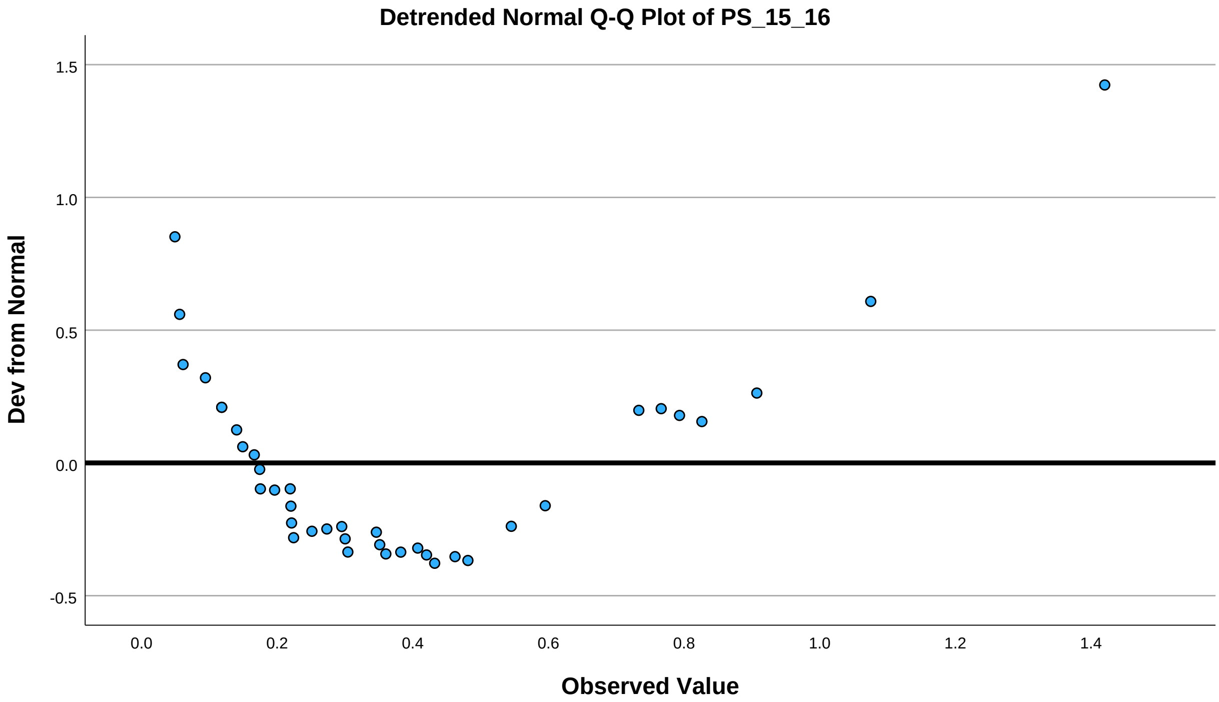
Detrended Normal Q-Q Plot of PS_15_16
Dev from Normal
1.5
1.0
0.5
0.0
-0.5
0.0
0.2
0.4
0.6
0.8
1.0
1.2
1.4
Observed Value

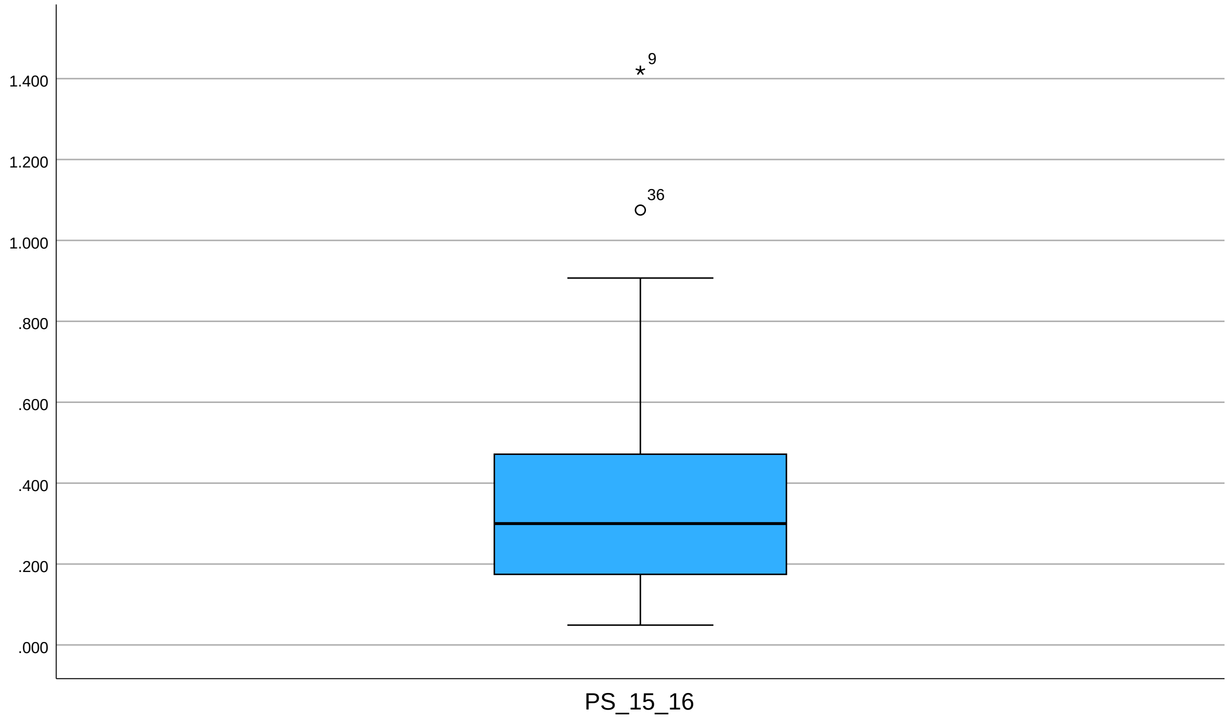
1.400
1.200
1.000
.800
.600
.400
.200
.000
9
36
PS_15_16

PS_16_17

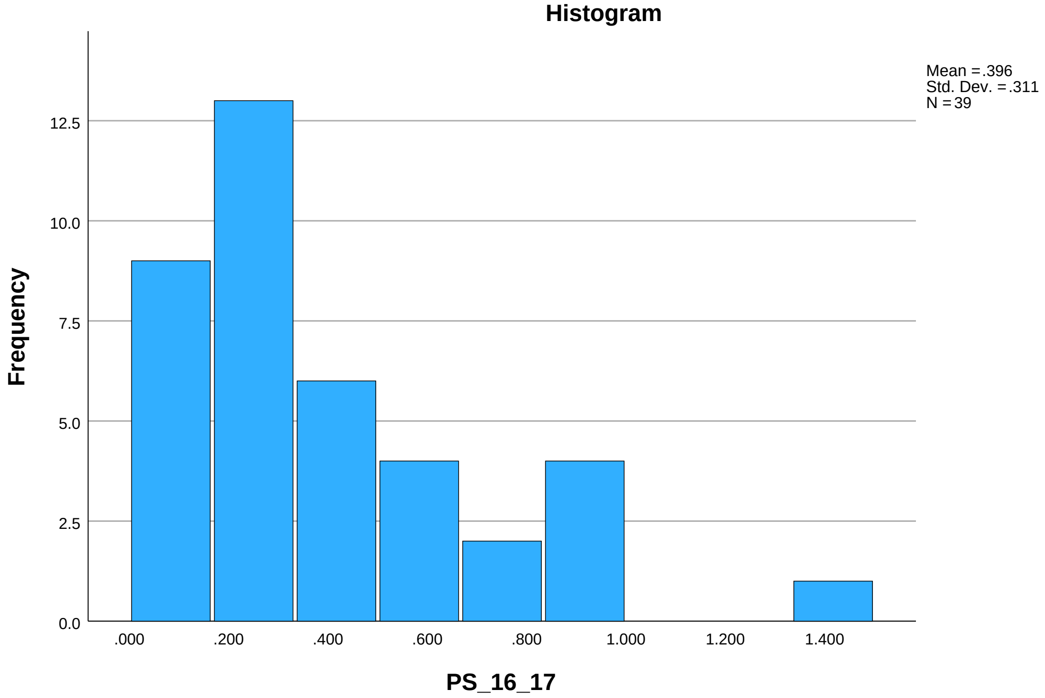


```
PS_16_17 Stem-and-Leaf Plot

 Frequency    Stem &  Leaf

     4.00        0 .  5569
     9.00        1 .  233667788
     7.00        2 .  0245999
     4.00        3 .  0269
     4.00        4 .  0278
     4.00        5 .  0147
      .00        6 .
     2.00        7 .  22
     1.00        8 .  9
     3.00        9 .  046
     1.00 Extremes    (>=1.46)

 Stem width:      .100
 Each leaf:       1 case(s)
```

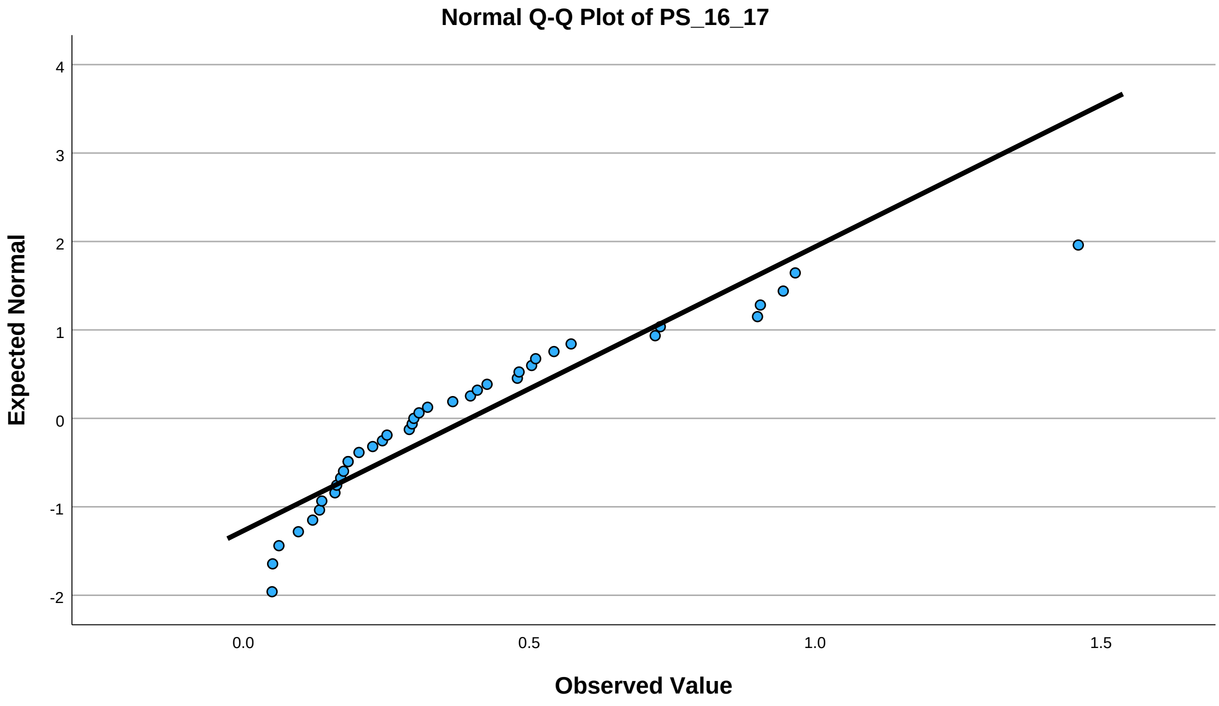
Normal Q-Q Plot of PS_16_17
Expected Normal
Observed Value
4
3
2
1
0
-1
-2
0.0
0.5
1.0
1.5

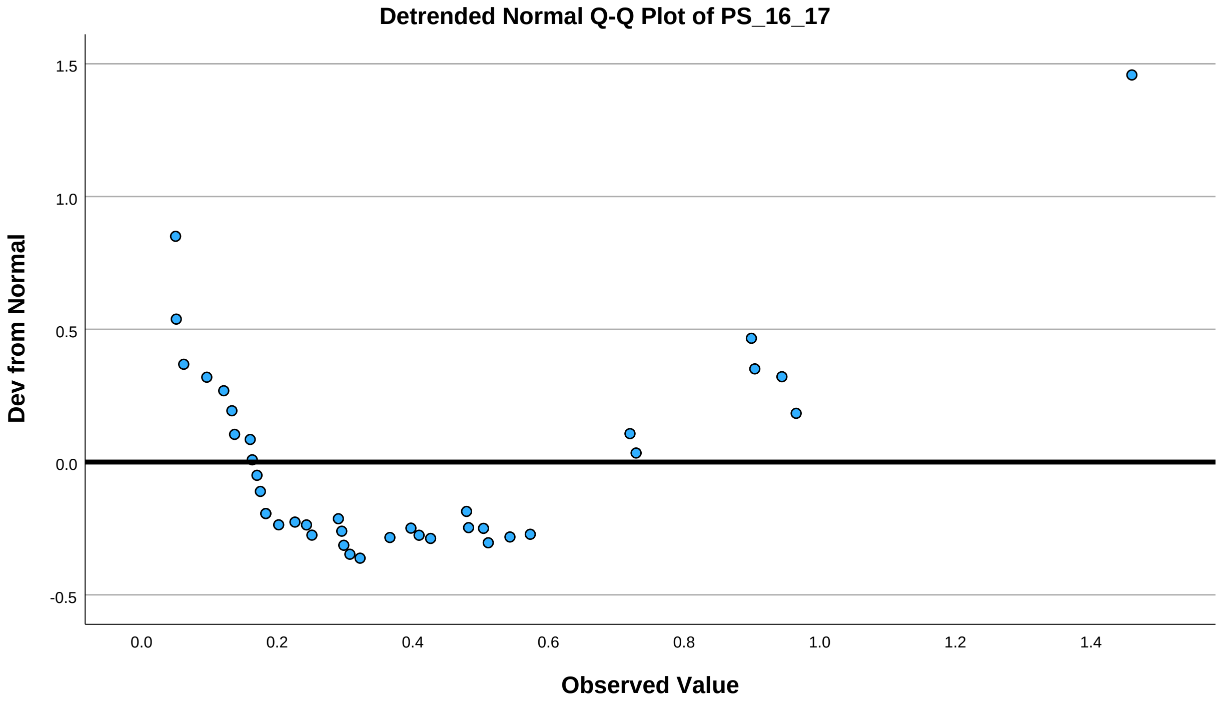
Detrended Normal Q-Q Plot of PS_16_17
Dev from Normal
Observed Value
1.5
1.0
0.5
0.0
-0.5
0.0
0.2
0.4
0.6
0.8
1.0
1.2
1.4

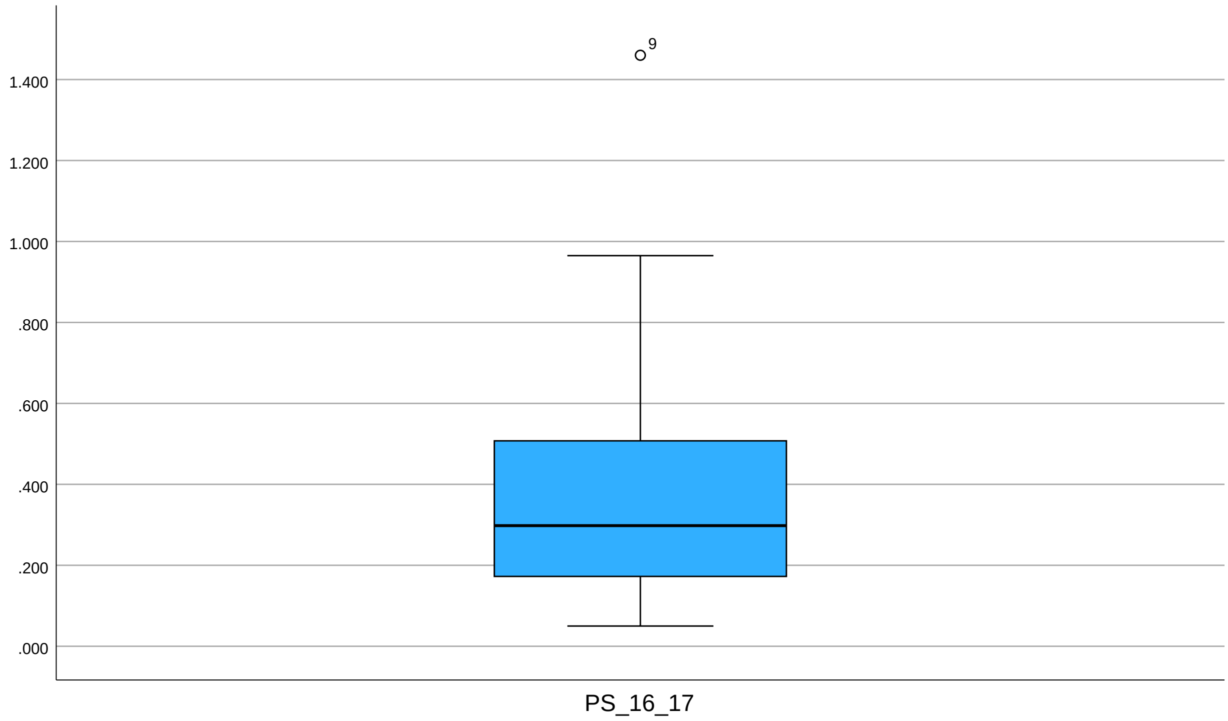

PS_17_18

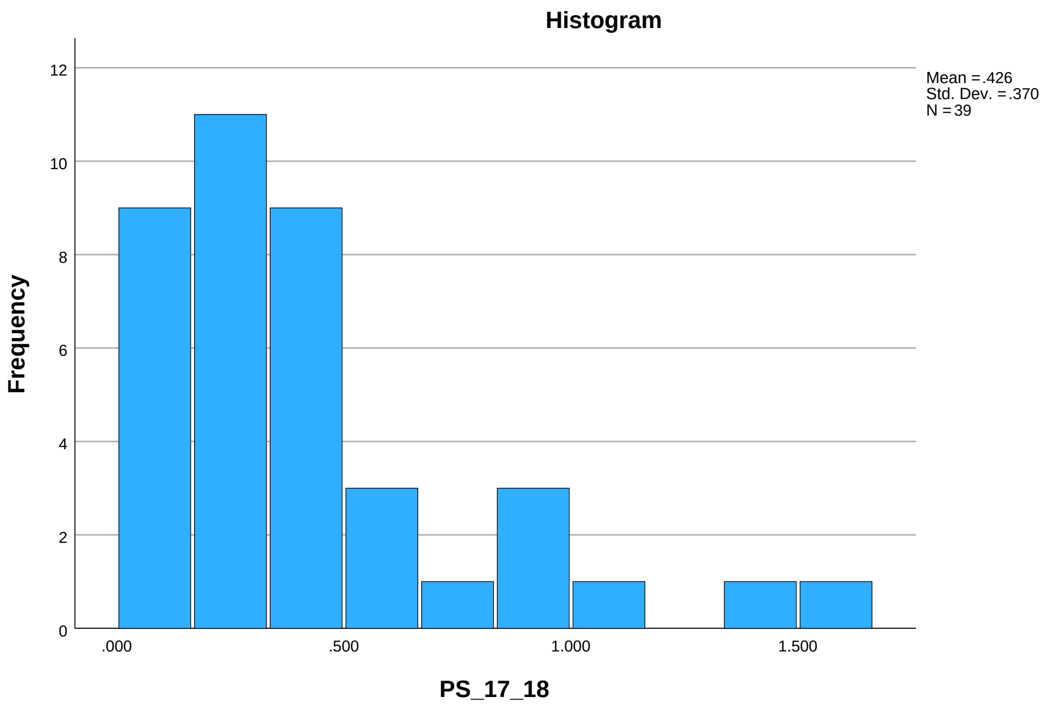

PS_17_18 Stem-and-Leaf Plot

Frequency   Stem & Leaf

```
    4.00        0 .  3457
   10.00         1 .  0335577888
    4.00        2 .  3344
    4.00        3 .  0237
    7.00        4 .  1234688
    2.00       5 .  36
    1.00       6 .  2
    1.00       7 .  3
     .00      8 .
    3.00       9 .  138
    3.00 Extremes    (>=1.11)

Stem width:      .100
Each leaf:       1 case(s)
```

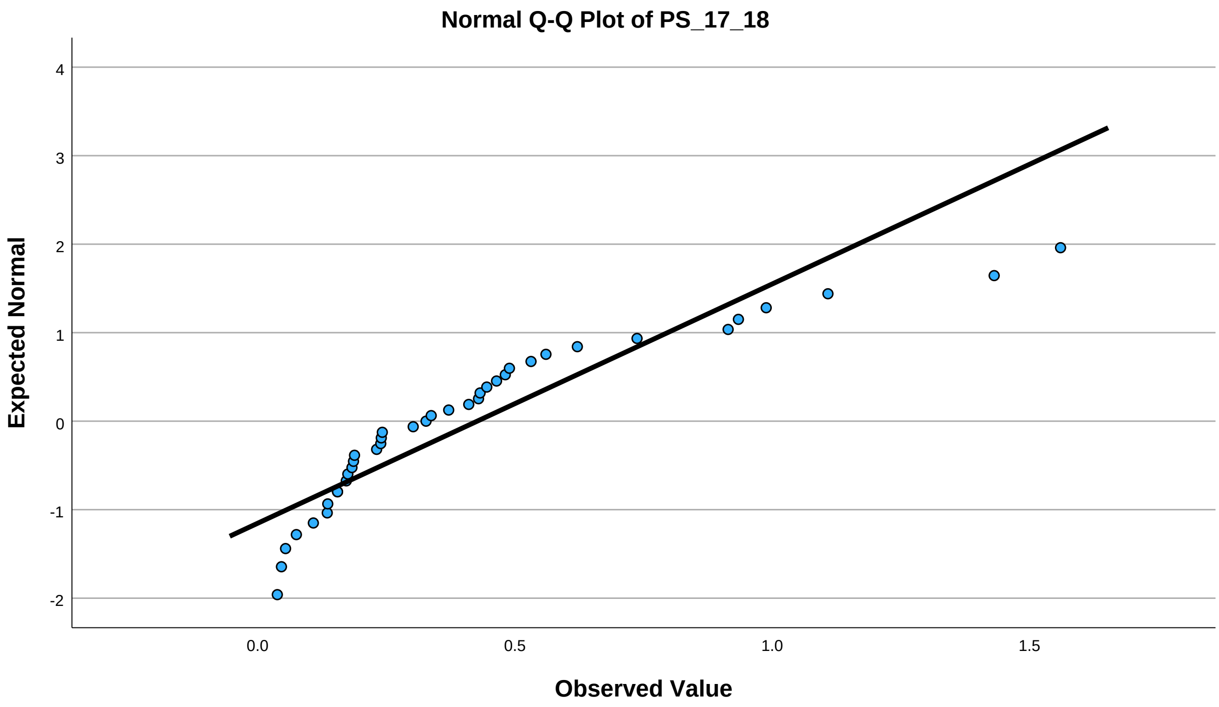

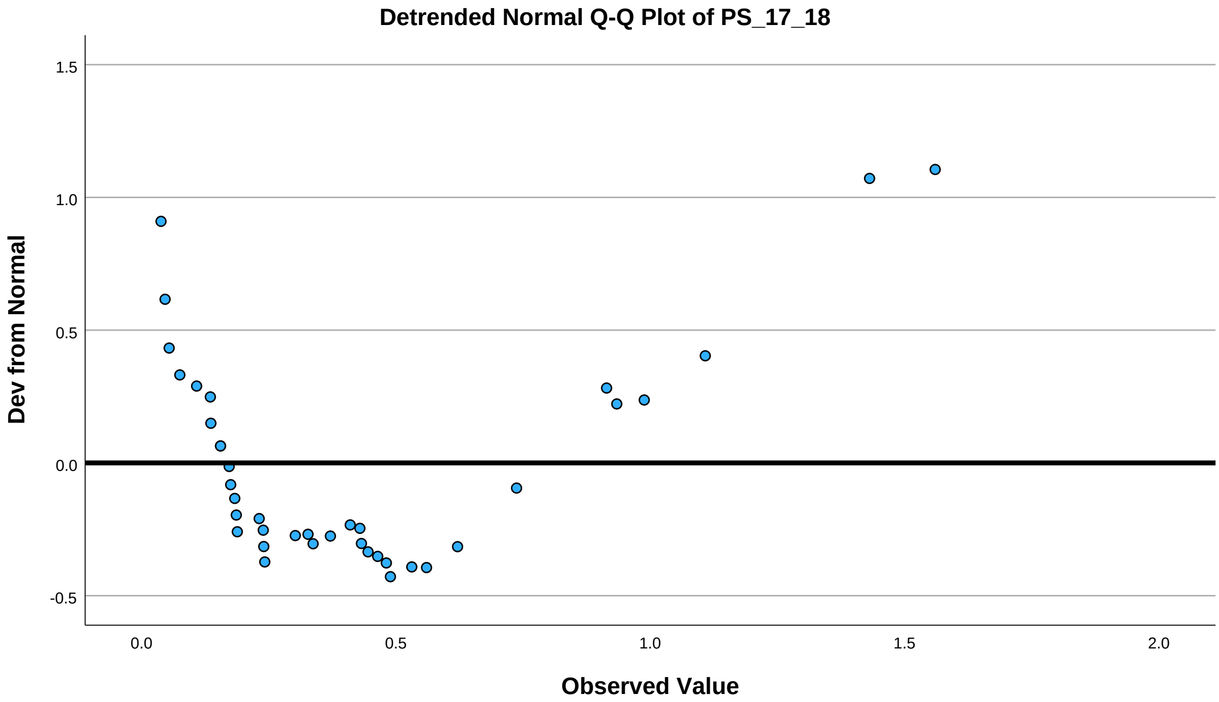
Detrended Normal Q-Q Plot of PS_17_18
Dev from Normal
1.5
1.0
0.5
0.0
-0.5
0.0
0.5
1.0
1.5
2.0
Observed Value

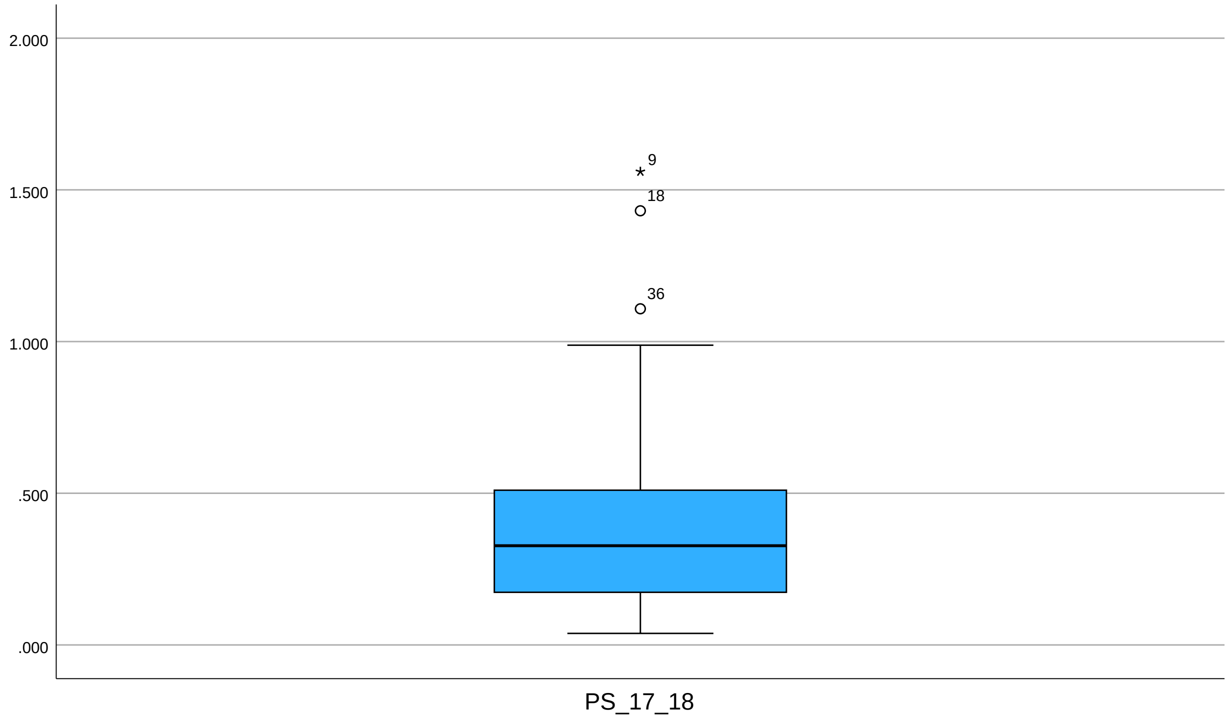
2.000
1.500
1.000
.500
.000
9
18
36
PS_17_18

PS_18_19

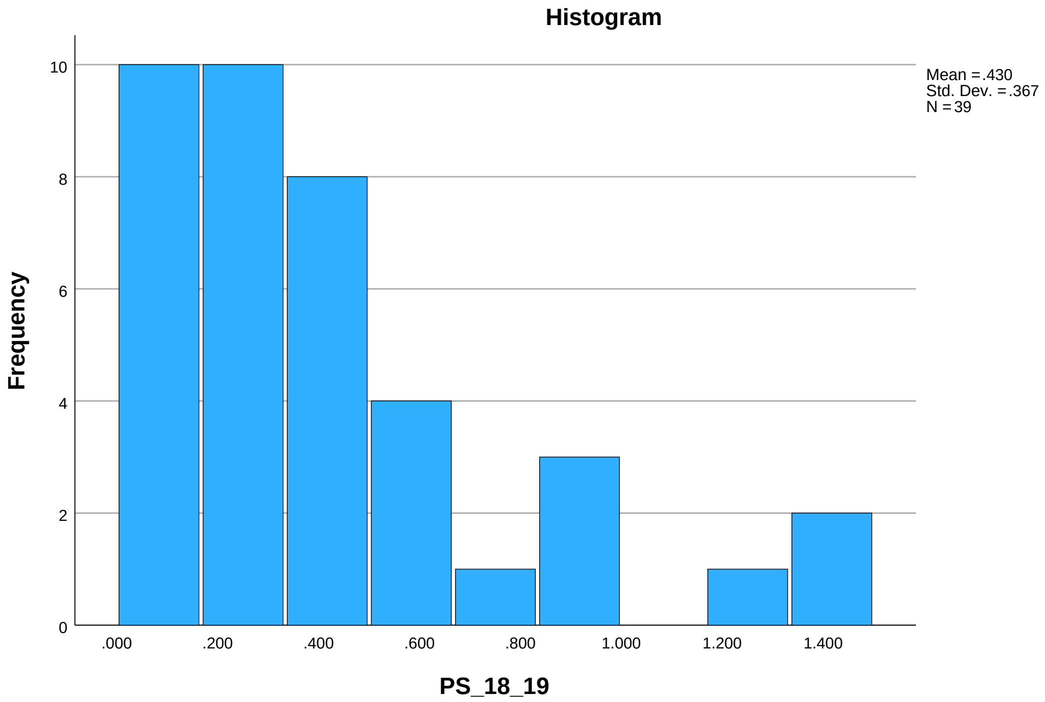


```
PS_18_19 Stem-and-Leaf Plot

 Frequency    Stem &  Leaf

     4.00        0 .  4467
     9.00        1 .  003466778
     6.00        2 .  013558
     3.00        3 .  134
     6.00        4 .  367789
     2.00        5 .  44
     2.00        6 .  13
     1.00        7 .  8
     2.00        8 .  36
     1.00        9 .  8
     3.00 Extremes    (>=1.24)

 Stem width:      .100
 Each leaf:       1 case(s)
```

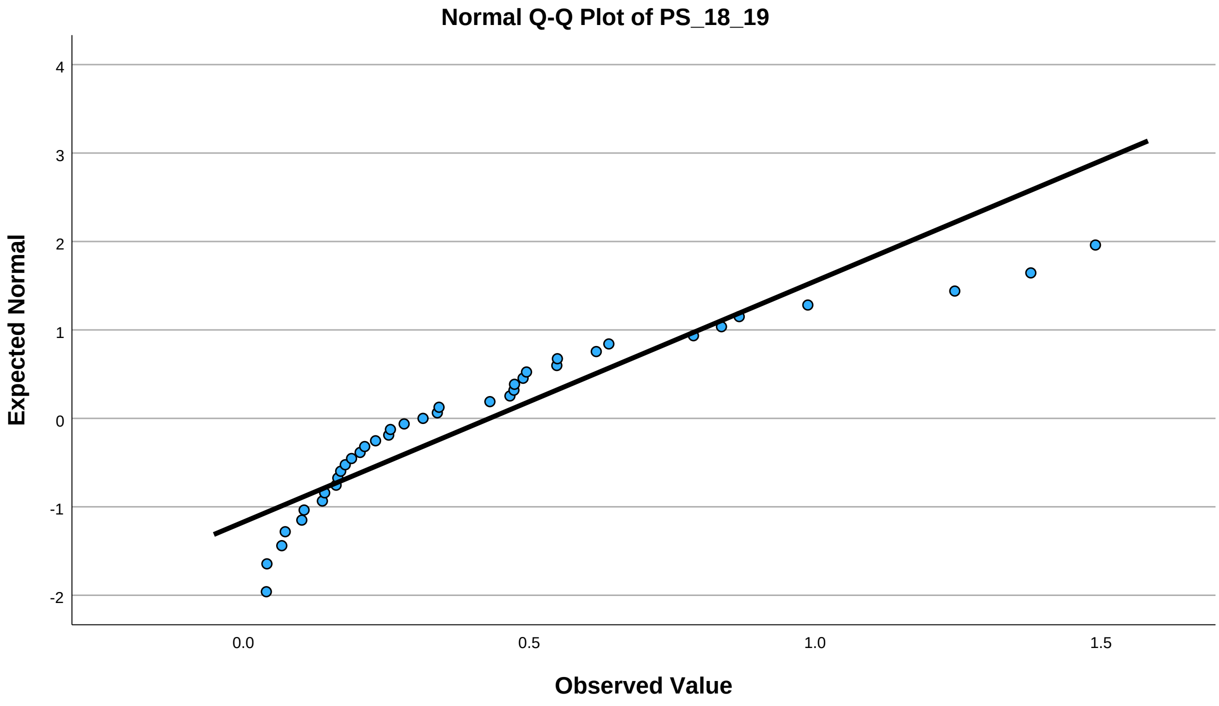
Normal Q-Q Plot of PS_18_19
Expected Normal
4
3
2
1
0
-1
-2
0.0
0.5
1.0
1.5
Observed Value

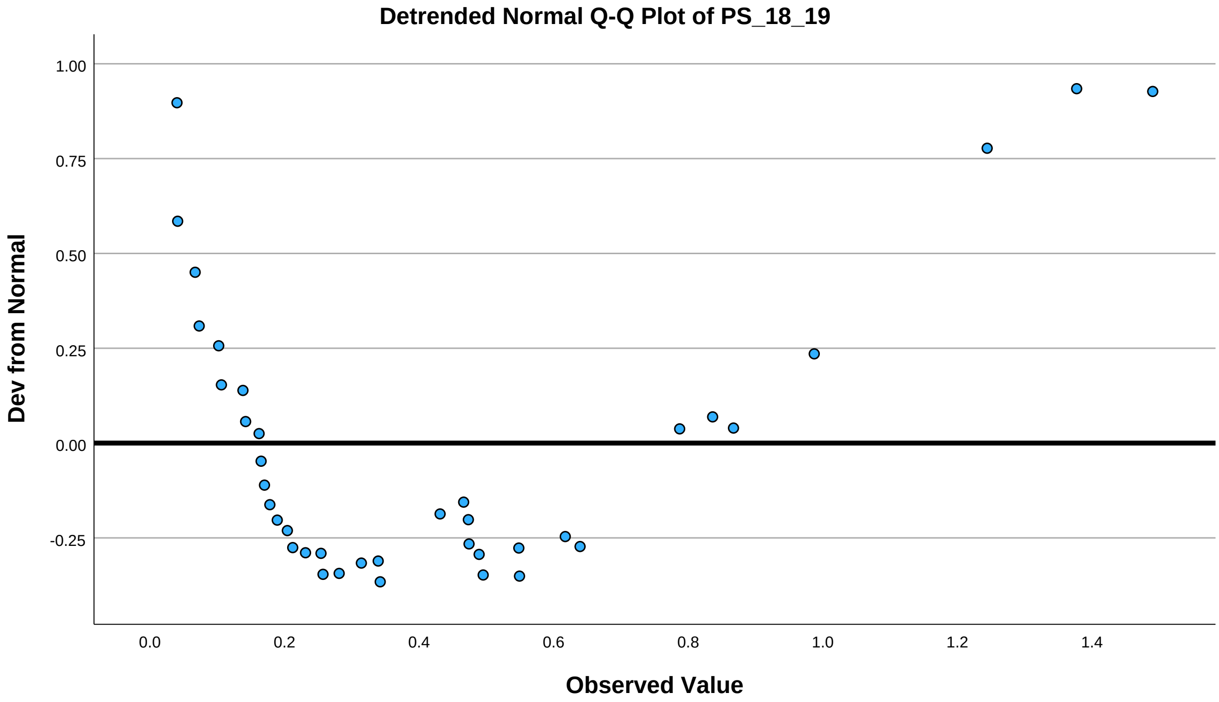
Detrended Normal Q-Q Plot of PS_18_19
Dev from Normal
1.00
0.75
0.50
0.25
0.00
-0.25
0.0
0.2
0.4
0.6
0.8
1.0
1.2
1.4
Observed Value

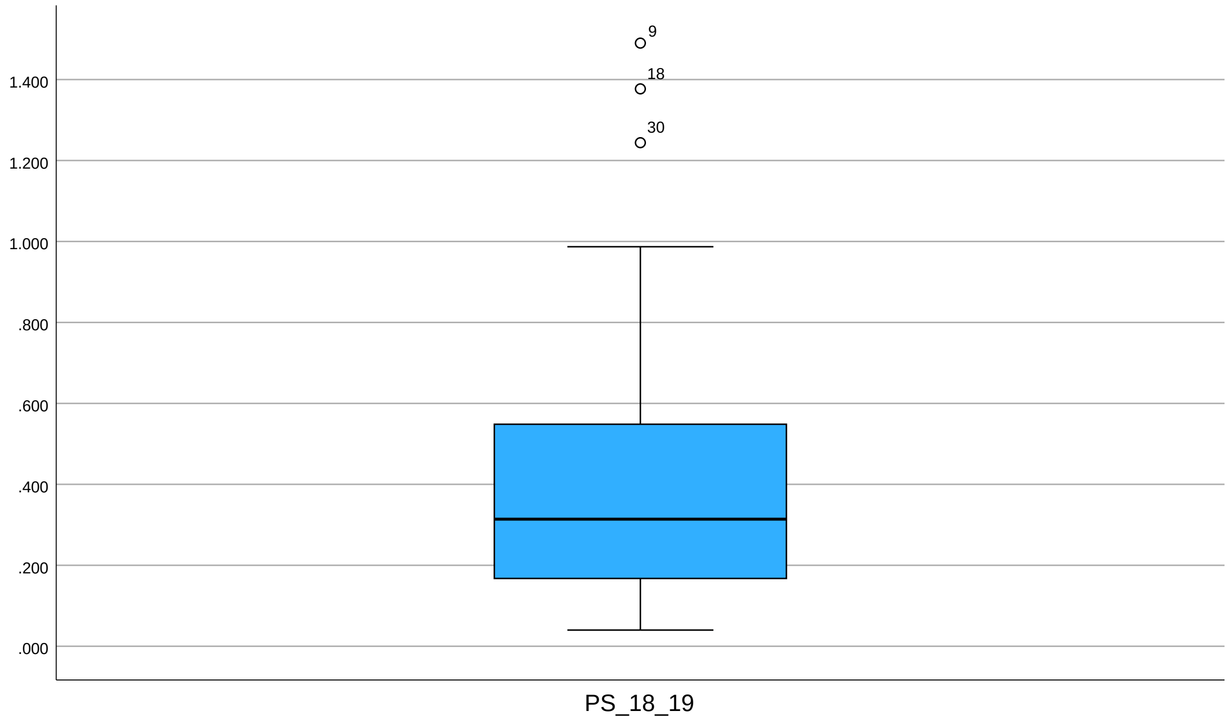

PS_19_20

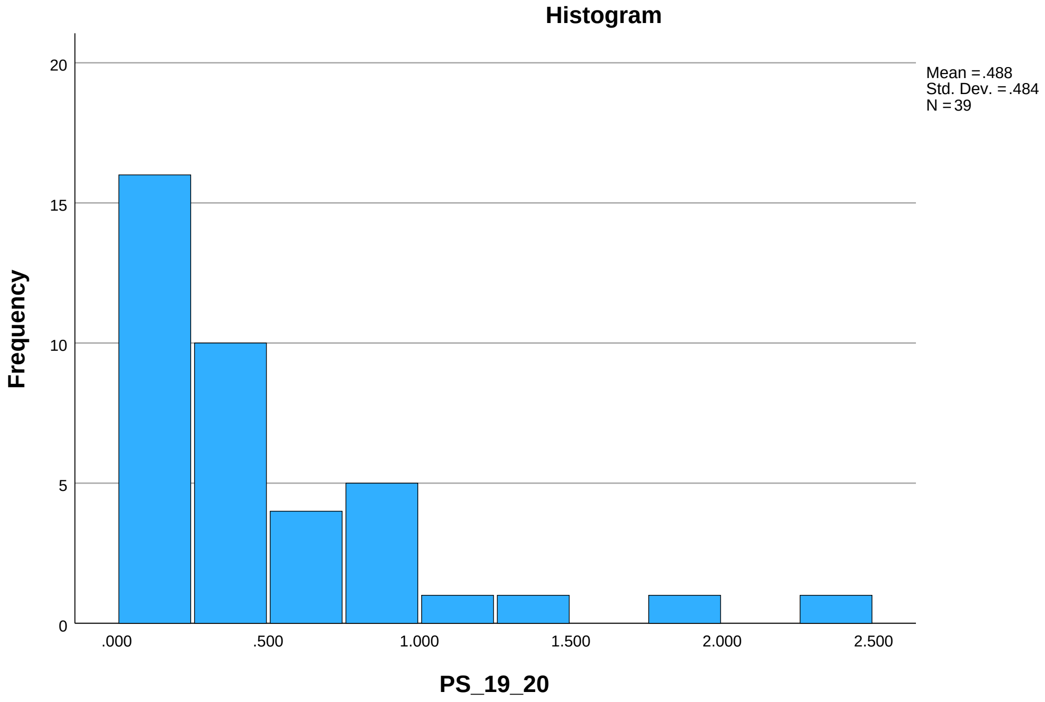

PS_19_20 Stem-and-Leaf Plot

Frequency   Stem & Leaf

```
4.00      0 . 3448
 8.00      1 . 01246778
 6.00      2 . 013389
 4.00      3 . 3366
 4.00      4 . 3668
 3.00      5 . 123
1.00      6 . 8
 2.00      7 . 58
 3.00      8 . 268
  .00     9 .
 1.00     10 . 0
  3.00 Extremes    (>=1.40)

Stem width:      .100
Each leaf:       1 case(s)
```

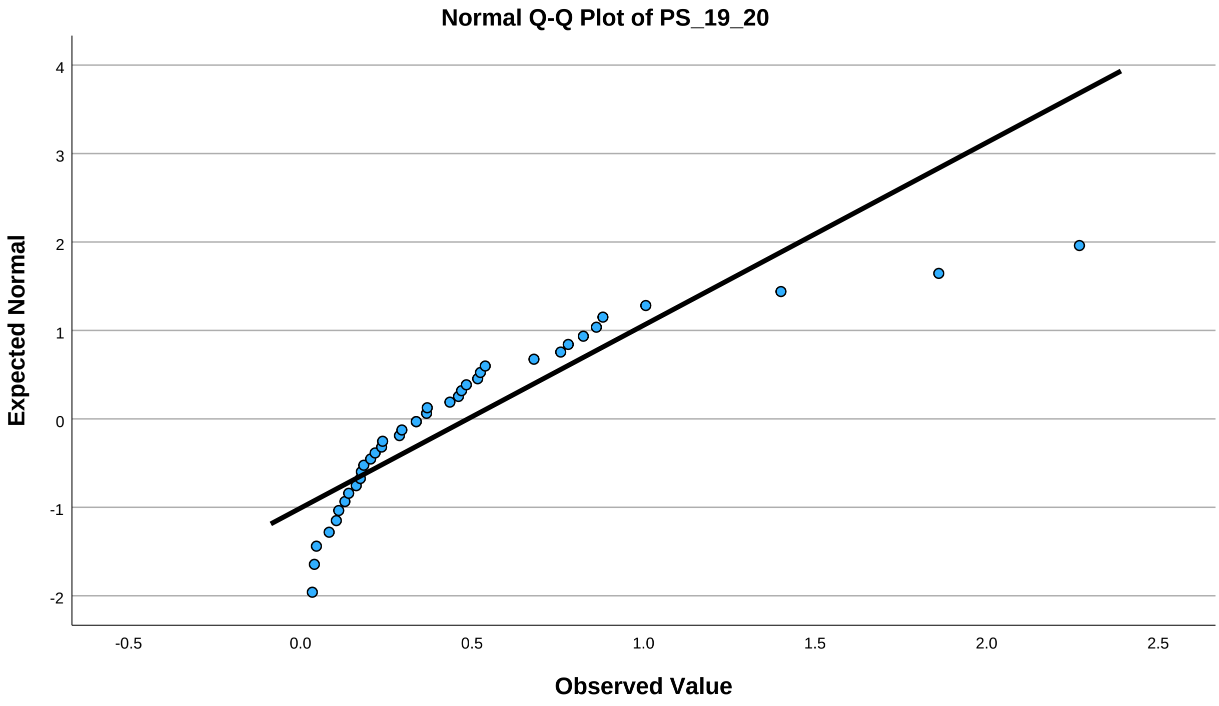

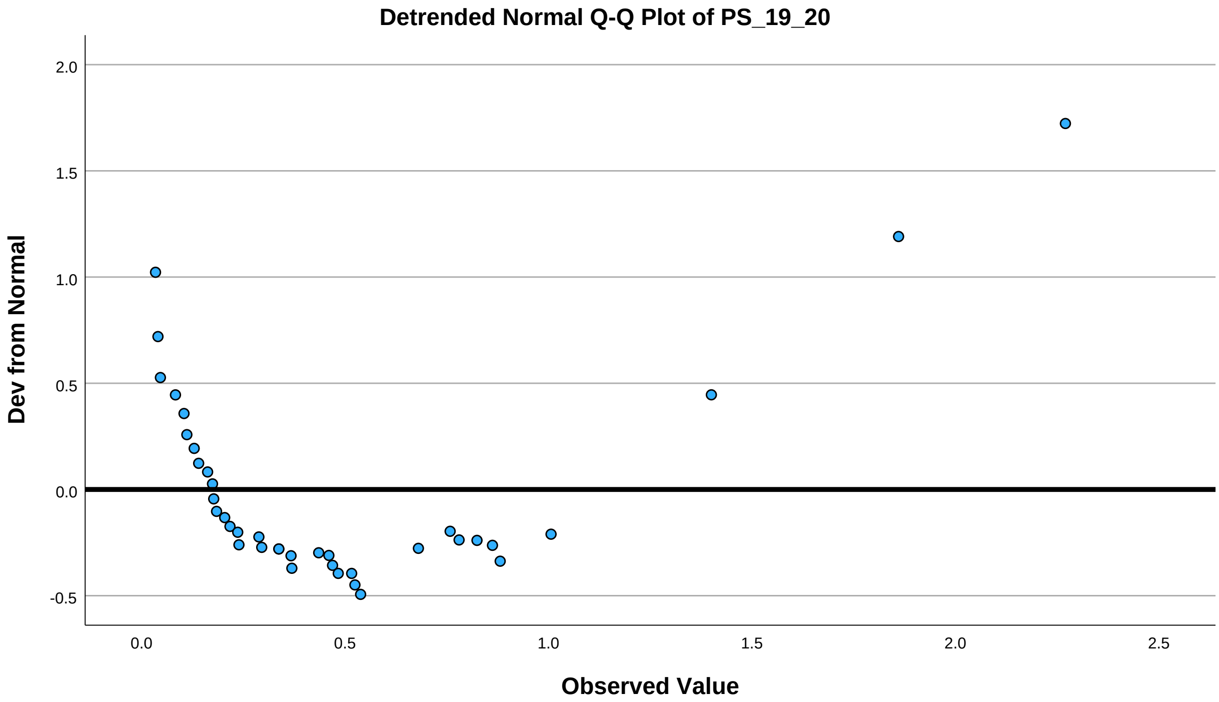

Detrended Normal Q-Q Plot of PS_19_20
Dev from Normal
2.0
1.5
1.0
0.5
0.0
-0.5
0.0
0.5
1.0
1.5
2.0
2.5
Observed Value

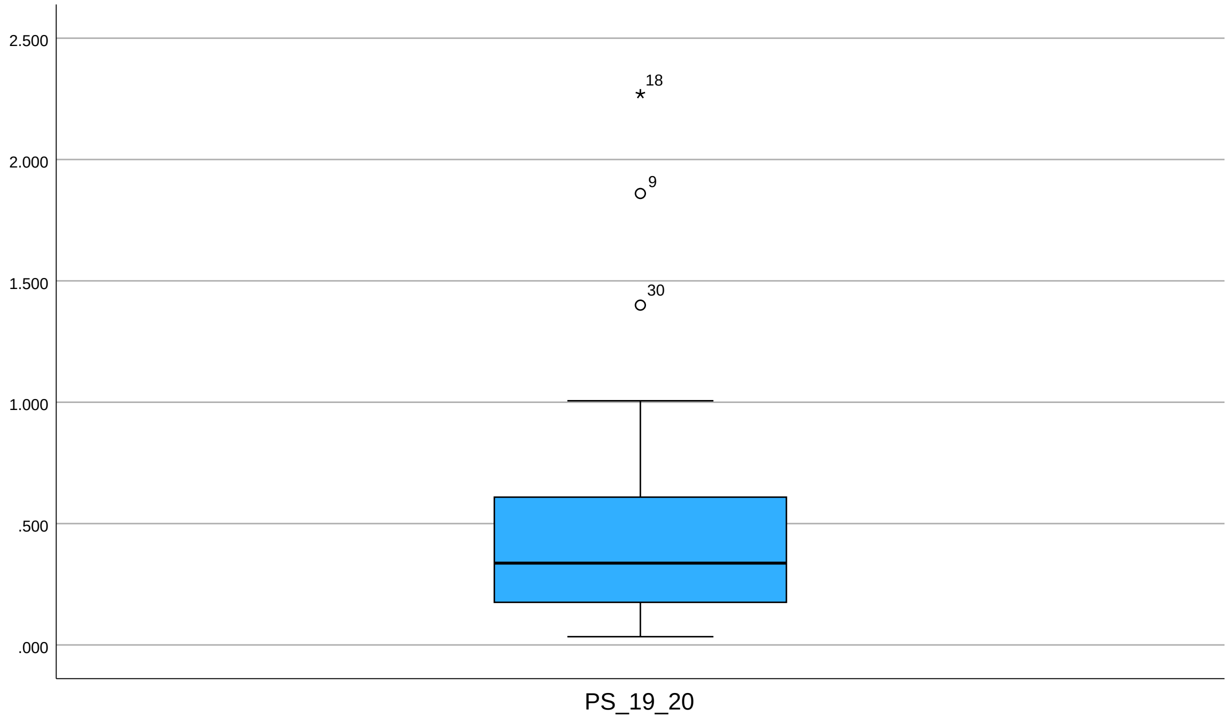

2.500
2.000
1.500
1.000
.500
.000
18
9
30
PS_19_20

Explore

Case Processing Summary

	Cases					
	Valid		Missing		Total	
	N	Percent	N	Percent	N	Percent
BrainRate	39	100.0%	0	0.0%	39	100.0%
PermutationEntropy	39	100.0%	0	0.0%	39	100.0%
PermutationEntropy_01	39	100.0%	0	0.0%	39	100.0%
SampleEntropy	39	100.0%	0	0.0%	39	100.0%
SpectralEntropy_bits	39	100.0%	0	0.0%	39	100.0%
SpectralEntropy_01	39	100.0%	0	0.0%	39	100.0%
HiguchiFractalDimension	39	100.0%	0	0.0%	39	100.0%
LempelZivComplexity	39	100.0%	0	0.0%	39	100.0%
RenyiEntropy	39	100.0%	0	0.0%	39	100.0%
TsallisEntropy	39	100.0%	0	0.0%	39	100.0%
PS_4_5	39	100.0%	0	0.0%	39	100.0%
PS_5_6	39	100.0%	0	0.0%	39	100.0%
PS_6_7	39	100.0%	0	0.0%	39	100.0%
PS_7_8	39	100.0%	0	0.0%	39	100.0%
PS_8_9	39	100.0%	0	0.0%	39	100.0%
PS_9_10	39	100.0%	0	0.0%	39	100.0%
PS_10_11	39	100.0%	0	0.0%	39	100.0%
PS_11_12	39	100.0%	0	0.0%	39	100.0%
PS_12_13	39	100.0%	0	0.0%	39	100.0%
PS_13_14	39	100.0%	0	0.0%	39	100.0%
PS_14_15	39	100.0%	0	0.0%	39	100.0%
PS_15_16	39	100.0%	0	0.0%	39	100.0%
PS_16_17	39	100.0%	0	0.0%	39	100.0%
PS_17_18	39	100.0%	0	0.0%	39	100.0%
PS_18_19	39	100.0%	0	0.0%	39	100.0%
PS_19_20	39	100.0%	0	0.0%	39	100.0%

Descriptives

			Statistic	Std. Error
BrainRate	Mean		8.9210	.71500
	95% Confidence Interval for Mean	Lower Bound	7.4736	
		Upper Bound	10.3685	
	5% Trimmed Mean		8.5227	
	Median		8.0600	
	Variance		19.938	
	Std. Deviation		4.46518	
	Minimum		3.35	
	Maximum		26.31	
	Range		22.96	
	Interquartile Range		5.15	
	Skewness		1.805	.378
	Kurtosis		4.825	.741
PermutationEntropy	Mean		1.5294273935	.00745911374
	95% Confidence Interval for Mean	Lower Bound	1.5143272071	
		Upper Bound	1.5445275798	
	5% Trimmed Mean		1.5299576091	
	Median		1.5318096939	
	Variance		.002	
	Std. Deviation		.04658215039	
	Minimum		1.4137657588	
	Maximum		1.6511770684	
	Range		.23741130957	
	Interquartile Range		.05148162149	
	Skewness		-.195	.378
	Kurtosis		.762	.741
PermutationEntropy_01	Mean		.85358968083	.00416301064
	95% Confidence Interval for Mean	Lower Bound	.84516210638	
		Upper Bound	.86201725528	
	5% Trimmed Mean		.85388559982	
	Median		.85491926800	
	Variance		.001	
	Std. Deviation		.02599799314	
	Minimum		.78903769346	
	Maximum		.92153946819	
	Range		.13250177473	
	Interquartile Range		.02873244002	
	Skewness		-.195	.378
	Kurtosis		.762	.741

Descriptives

			Statistic	Std. Error
SampleEntropy	Mean		.73026039951	.03729526968
	95% Confidence Interval for Mean	Lower Bound	.65476007324	
		Upper Bound	.80576072579	
	5% Trimmed Mean		.72851289468	
	Median		.70977634311	
	Variance		.054	
	Std. Deviation		.23290888449	
	Minimum		.28065071047	
	Maximum		1.2347827055	
	Range		.95413199502	
	Interquartile Range		.33950595191	
	Skewness		-.033	.378
	Kurtosis		-.563	.741
SpectralEntropy_bits	Mean		4.0862986464	.10801476486
	95% Confidence Interval for Mean	Lower Bound	3.8676341868	
		Upper Bound	4.3049631059	
	5% Trimmed Mean		4.0728126153	
	Median		4.1367502114	
	Variance		.455	
	Std. Deviation		.67455199033	
	Minimum		2.8127135991	
	Maximum		5.8614541728	
	Range		3.0487405737	
	Interquartile Range		.97731250336	
	Skewness		.197	.378
	Kurtosis		.124	.741
SpectralEntropy_01	Mean		.58282216472	.01540597115
	95% Confidence Interval for Mean	Lower Bound	.55163440663	
		Upper Bound	.61400992282	
	5% Trimmed Mean		.58089867393	
	Median		.59001798982	
	Variance		.009	
	Std. Deviation		.09621025903	
	Minimum		.40117278996	
	Maximum		.83600972545	
	Range		.43483693548	
	Interquartile Range		.13939250116	
	Skewness		.197	.378
	Kurtosis		.124	.741

Descriptives

			Statistic	Std. Error
HiguchiFractalDimension	Mean		.45250886111	.01744748127
	95% Confidence Interval for Mean	Lower Bound	.41718828185	
		Upper Bound	.48782944037	
	5% Trimmed Mean		.45386407729	
	Median		.44765545123	
	Variance		.012	
	Std. Deviation		.10895948561	
	Minimum		.20596779040	
	Maximum		.68886371181	
	Range		.48289592141	
	Interquartile Range		.15474350726	
	Skewness		-.219	.378
	Kurtosis		-.236	.741
LempelZivComplexity	Mean		.28215300419	.00640814317
	95% Confidence Interval for Mean	Lower Bound	.26918039656	
		Upper Bound	.29512561181	
	5% Trimmed Mean		.27952346974	
	Median		.27265908019	
	Variance		.002	
	Std. Deviation		.04001884124	
	Minimum		.22775669604	
	Maximum		.38885192736	
	Range		.16109523132	
	Interquartile Range		.04274361619	
	Skewness		.890	.378
	Kurtosis		.476	.741
RenyiEntropy	Mean		7.3508800398	.12911485879
	95% Confidence Interval for Mean	Lower Bound	7.0895006732	
		Upper Bound	7.6122594064	
	5% Trimmed Mean		7.3764928440	
	Median		7.4904371559	
	Variance		.650	
	Std. Deviation		.80632203472	
	Minimum		5.5510054320	
	Maximum		8.6675754666	
	Range		3.1165700346	
	Interquartile Range		.96080640268	
	Skewness		-.530	.378
	Kurtosis		-.188	.741

Descriptives

			Statistic	Std. Error
TsallisEntropy	Mean		1.8994397453	.04317987227
	95% Confidence Interval for Mean	Lower Bound	1.8120266639	
		Upper Bound	1.9868528268	
	5% Trimmed Mean		1.9520628787	
	Median		1.9803470809	
	Variance		.073	
	Std. Deviation		.26965821589	
	Minimum		.42723117022	
	Maximum		1.9882127318	
	Range		1.5609815616	
	Interquartile Range		.03857156722	
	Skewness		-4.781	.378
	Kurtosis		24.799	.741
PS_4_5	Mean		2.32862	.368756
	95% Confidence Interval for Mean	Lower Bound	1.58211	
		Upper Bound	3.07512	
	5% Trimmed Mean		2.02662	
	Median		1.69700	
	Variance		5.303	
	Std. Deviation		2.302882	
	Minimum		.278	
	Maximum		14.720	
	Range		14.442	
	Interquartile Range		1.419	
	Skewness		4.300	.378
	Kurtosis		22.714	.741
PS_5_6	Mean		1.72349	.309423
	95% Confidence Interval for Mean	Lower Bound	1.09709	
		Upper Bound	2.34988	
	5% Trimmed Mean		1.44945	
	Median		1.18600	
	Variance		3.734	
	Std. Deviation		1.932347	
	Minimum		.259	
	Maximum		12.590	
	Range		12.331	
	Interquartile Range		1.280	
	Skewness		4.895	.378
	Kurtosis		27.630	.741

Descriptives

			Statistic	Std. Error
PS_6_7	Mean		1.36090	.197043
	95% Confidence Interval for Mean	Lower Bound	.96201	
		Upper Bound	1.75979	
	5% Trimmed Mean		1.20600	
	Median		1.06100	
	Variance		1.514	
	Std. Deviation		1.230530	
	Minimum		.204	
	Maximum		7.830	
	Range		7.626	
	Interquartile Range		.919	
	Skewness		3.996	.378
	Kurtosis		20.461	.741
PS_7_8	Mean		1.18454	.148605
	95% Confidence Interval for Mean	Lower Bound	.88370	
		Upper Bound	1.48537	
	5% Trimmed Mean		1.05113	
	Median		.96600	
	Variance		.861	
	Std. Deviation		.928037	
	Minimum		.210	
	Maximum		4.898	
	Range		4.688	
	Interquartile Range		.864	
	Skewness		2.589	.378
	Kurtosis		8.095	.741
PS_8_9	Mean		1.04844	.132503
	95% Confidence Interval for Mean	Lower Bound	.78020	
		Upper Bound	1.31667	
	5% Trimmed Mean		.93695	
	Median		.83200	
	Variance		.685	
	Std. Deviation		.827482	
	Minimum		.196	
	Maximum		4.214	
	Range		4.018	
	Interquartile Range		.806	
	Skewness		2.283	.378
	Kurtosis		6.359	.741

Descriptives

			Statistic	Std. Error
PS_9_10	Mean		1.03895	.123341
	95% Confidence Interval for Mean	Lower Bound	.78926	
		Upper Bound	1.28864	
	5% Trimmed Mean		.95769	
	Median		.92800	
	Variance		.593	
	Std. Deviation		.770265	
	Minimum		.193	
	Maximum		3.860	
	Range		3.667	
	Interquartile Range		1.056	
	Skewness		1.733	.378
	Kurtosis		3.829	.741
PS_10_11	Mean		1.20631	.151003
	95% Confidence Interval for Mean	Lower Bound	.90062	
		Upper Bound	1.51200	
	5% Trimmed Mean		1.09524	
	Median		.96000	
	Variance		.889	
	Std. Deviation		.943011	
	Minimum		.217	
	Maximum		4.340	
	Range		4.123	
	Interquartile Range		.989	
	Skewness		1.816	.378
	Kurtosis		3.678	.741
PS_11_12	Mean		.60733	.083207
	95% Confidence Interval for Mean	Lower Bound	.43889	
		Upper Bound	.77578	
	5% Trimmed Mean		.53414	
	Median		.50000	
	Variance		.270	
	Std. Deviation		.519627	
	Minimum		.147	
	Maximum		2.920	
	Range		2.773	
	Interquartile Range		.441	
	Skewness		2.810	.378
	Kurtosis		10.066	.741

Descriptives

			Statistic	Std. Error
PS_12_13	Mean		.48000	.065579
	95% Confidence Interval for Mean	Lower Bound	.34724	
		Upper Bound	.61276	
	5% Trimmed Mean		.42292	
	Median		.35700	
	Variance		.168	
	Std. Deviation		.409540	
	Minimum		.095	
	Maximum		2.440	
	Range		2.345	
	Interquartile Range		.325	
	Skewness		3.187	.378
	Kurtosis		13.572	.741
PS_13_14	Mean		.42492	.051533
	95% Confidence Interval for Mean	Lower Bound	.32060	
		Upper Bound	.52925	
	5% Trimmed Mean		.38730	
	Median		.31500	
	Variance		.104	
	Std. Deviation		.321821	
	Minimum		.079	
	Maximum		1.490	
	Range		1.411	
	Interquartile Range		.281	
	Skewness		1.854	.378
	Kurtosis		3.528	.741
PS_14_15	Mean		.43526	.064594
	95% Confidence Interval for Mean	Lower Bound	.30449	
		Upper Bound	.56602	
	5% Trimmed Mean		.38034	
	Median		.32200	
	Variance		.163	
	Std. Deviation		.403389	
	Minimum		.081	
	Maximum		2.180	
	Range		2.099	
	Interquartile Range		.296	
	Skewness		2.697	.378
	Kurtosis		8.889	.741

Descriptives

			Statistic	Std. Error
PS_15_16	Mean		.40464	.054132
	95% Confidence Interval for Mean	Lower Bound	.29506	
		Upper Bound	.51422	
	5% Trimmed Mean		.36676	
	Median		.27800	
	Variance		.114	
	Std. Deviation		.338052	
	Minimum		.083	
	Maximum		1.510	
	Range		1.427	
	Interquartile Range		.224	
	Skewness		1.906	.378
	Kurtosis		3.150	.741
PS_16_17	Mean		.39405	.050826
	95% Confidence Interval for Mean	Lower Bound	.29116	
		Upper Bound	.49694	
	5% Trimmed Mean		.36206	
	Median		.28600	
	Variance		.101	
	Std. Deviation		.317405	
	Minimum		.092	
	Maximum		1.310	
	Range		1.218	
	Interquartile Range		.269	
	Skewness		1.756	.378
	Kurtosis		2.381	.741
PS_17_18	Mean		.37538	.050317
	95% Confidence Interval for Mean	Lower Bound	.27352	
		Upper Bound	.47725	
	5% Trimmed Mean		.33906	
	Median		.27000	
	Variance		.099	
	Std. Deviation		.314231	
	Minimum		.091	
	Maximum		1.397	
	Range		1.306	
	Interquartile Range		.196	
	Skewness		1.937	.378
	Kurtosis		3.450	.741

Descriptives

			Statistic	Std. Error
PS_18_19	Mean		.38164	.056597
	95% Confidence Interval for Mean	Lower Bound	.26707	
		Upper Bound	.49622	
	5% Trimmed Mean		.33429	
	Median		.25000	
	Variance		.125	
	Std. Deviation		.353448	
	Minimum		.088	
	Maximum		1.667	
	Range		1.579	
	Interquartile Range		.219	
	Skewness		2.309	.378
	Kurtosis		5.374	.741
PS_19_20	Mean		.37644	.056591
	95% Confidence Interval for Mean	Lower Bound	.26187	
		Upper Bound	.49100	
	5% Trimmed Mean		.32568	
	Median		.24300	
	Variance		.125	
	Std. Deviation		.353408	
	Minimum		.080	
	Maximum		1.849	
	Range		1.769	
	Interquartile Range		.240	
	Skewness		2.600	.378
	Kurtosis		7.942	.741

Tests of Normality

	Kolmogorov-Smirnov[a]			Shapiro-Wilk		
	Statistic	df	Sig.	Statistic	df	Sig.
BrainRate	.160	39	.013	.854	39	<.001
PermutationEntropy	.094	39	.200*	.980	39	.688
PermutationEntropy_01	.094	39	.200*	.980	39	.688
SampleEntropy	.094	39	.200*	.984	39	.835
SpectralEntropy_bits	.054	39	.200*	.986	39	.906
SpectralEntropy_01	.054	39	.200*	.986	39	.906
HiguchiFractalDimension	.083	39	.200*	.984	39	.857
LempelZivComplexity	.128	39	.107	.933	39	.023
RenyiEntropy	.089	39	.200*	.957	39	.142
TsallisEntropy	.371	39	<.001	.355	39	<.001
PS_4_5	.221	39	<.001	.566	39	<.001
PS_5_6	.271	39	<.001	.481	39	<.001
PS_6_7	.188	39	.001	.612	39	<.001
PS_7_8	.180	39	.003	.727	39	<.001
PS_8_9	.168	39	.007	.768	39	<.001
PS_9_10	.185	39	.002	.842	39	<.001
PS_10_11	.155	39	.019	.821	39	<.001
PS_11_12	.216	39	<.001	.710	39	<.001
PS_12_13	.187	39	.001	.692	39	<.001
PS_13_14	.174	39	.004	.803	39	<.001
PS_14_15	.233	39	<.001	.707	39	<.001
PS_15_16	.252	39	<.001	.756	39	<.001
PS_16_17	.236	39	<.001	.768	39	<.001
PS_17_18	.258	39	<.001	.760	39	<.001
PS_18_19	.278	39	<.001	.708	39	<.001
PS_19_20	.227	39	<.001	.704	39	<.001

*. This is a lower bound of the true significance.

a. Lilliefors Significance Correction

BrainRate

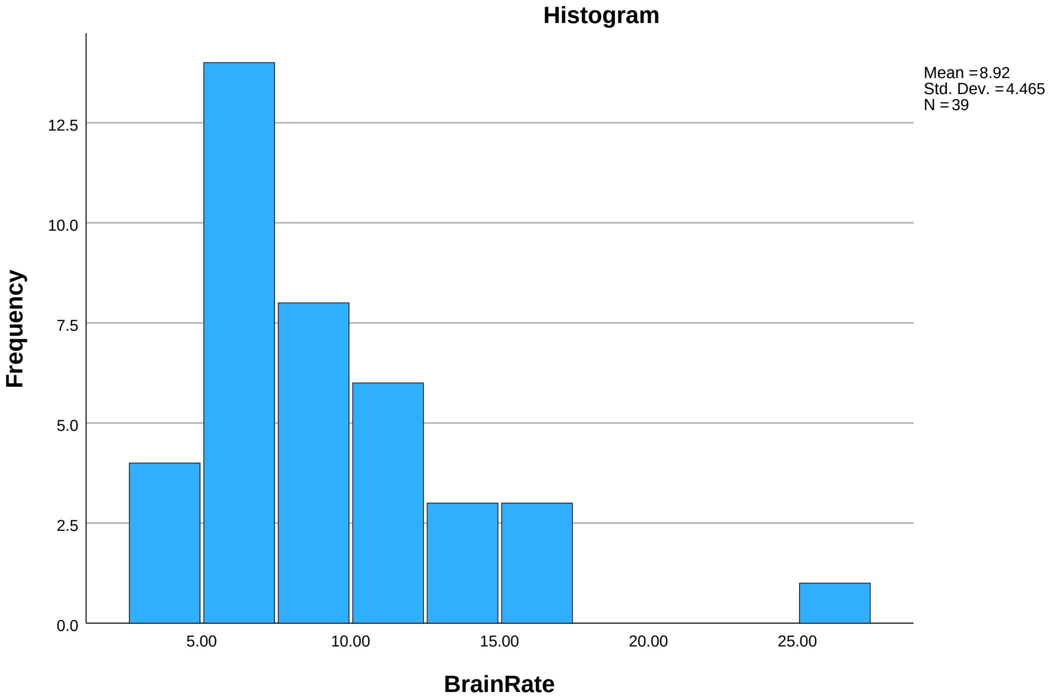

BrainRate Stem-and-Leaf Plot

```
 Frequency    Stem &  Leaf

     2.00        0 .  33
     7.00        0 .  4455555
    10.00        0 .  6666666667
     7.00        0 .  8888889
     5.00        1 .  00011
     3.00        1 .  222
     2.00        1 .  45
     2.00        1 .  66
     1.00 Extremes    (>=26)

 Stem width:      10.00
 Each leaf:        1 case(s)
```

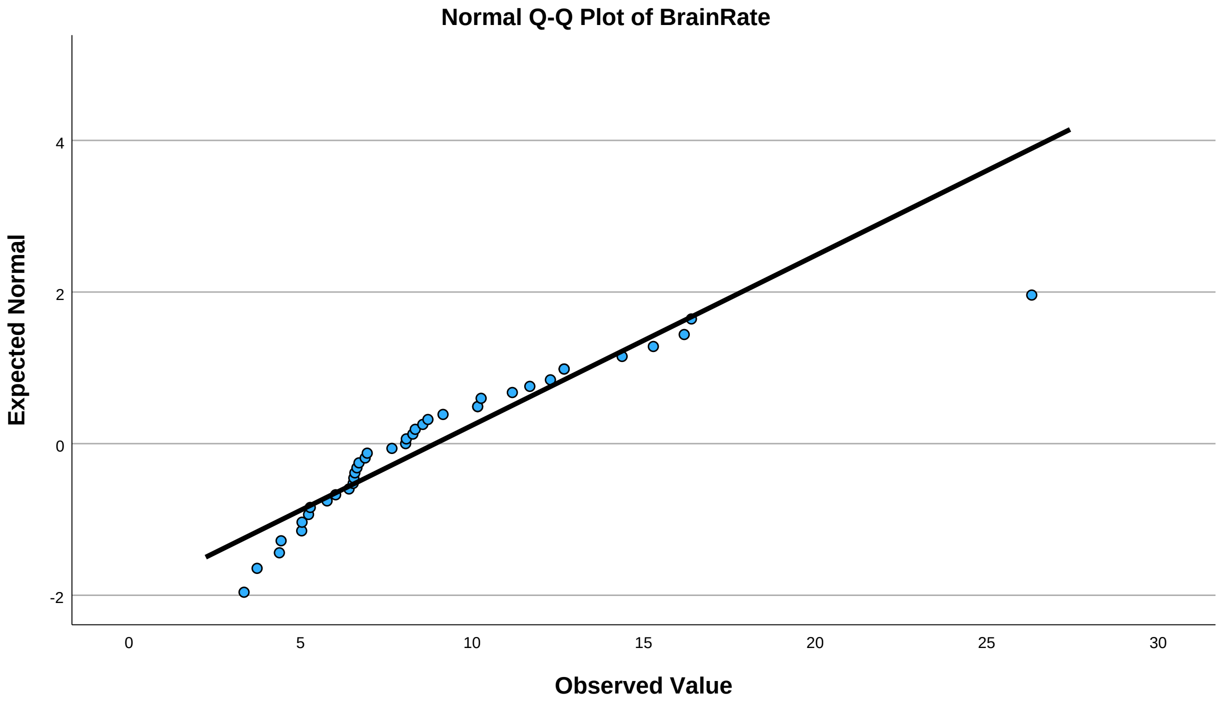

Normal Q-Q Plot of BrainRate
Expected Normal
4
2
0
-2
0
5
10
15
20
25
30
Observed Value

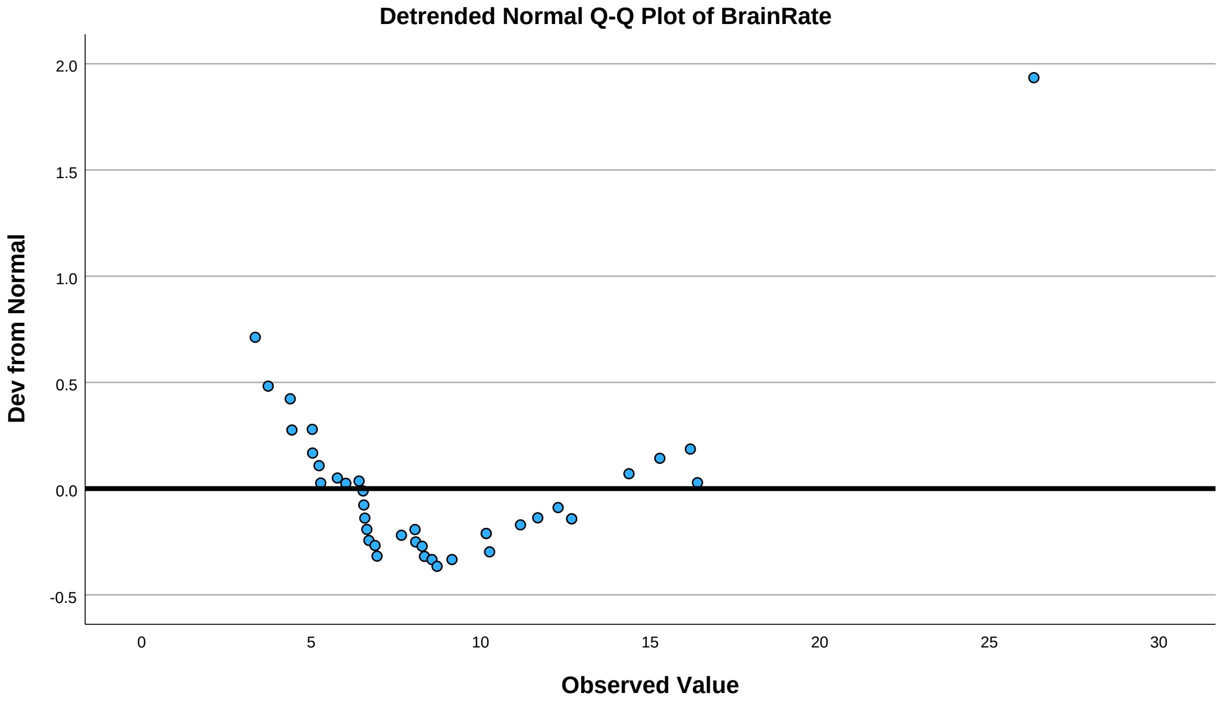

Detrended Normal Q-Q Plot of BrainRate
Dev from Normal
2.0
1.5
1.0
0.5
0.0
-0.5
0
5
10
15
20
25
30
Observed Value

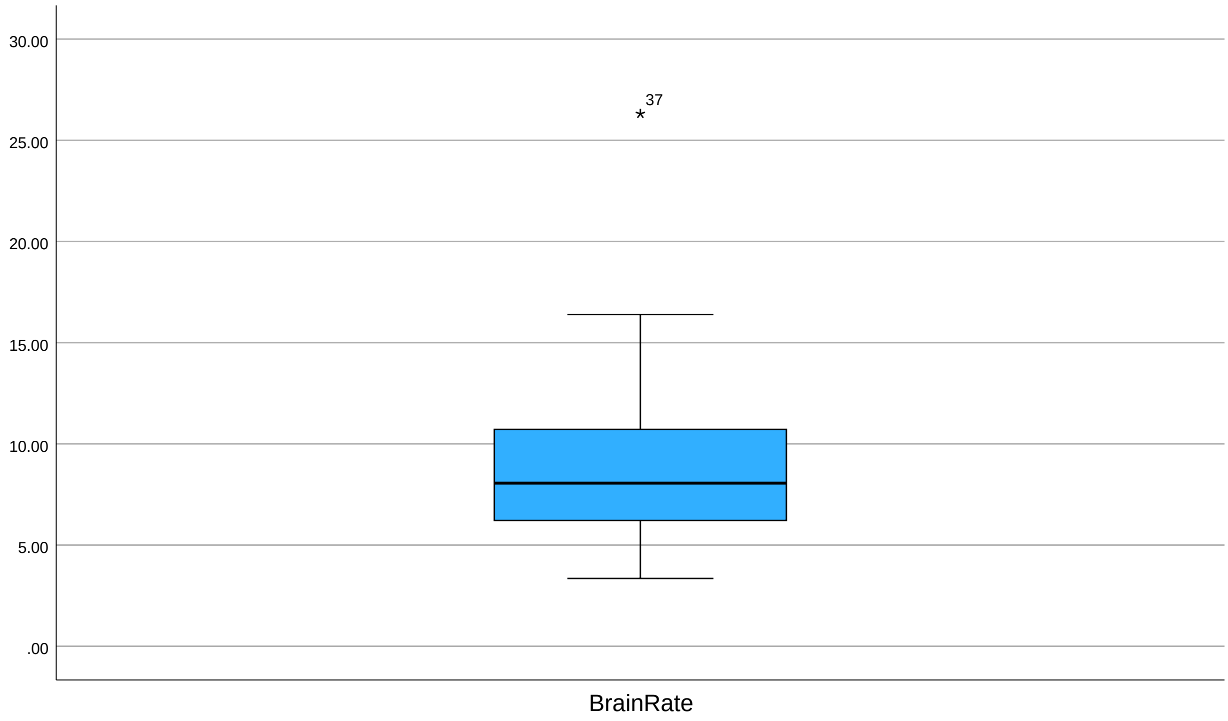

PermutationEntropy

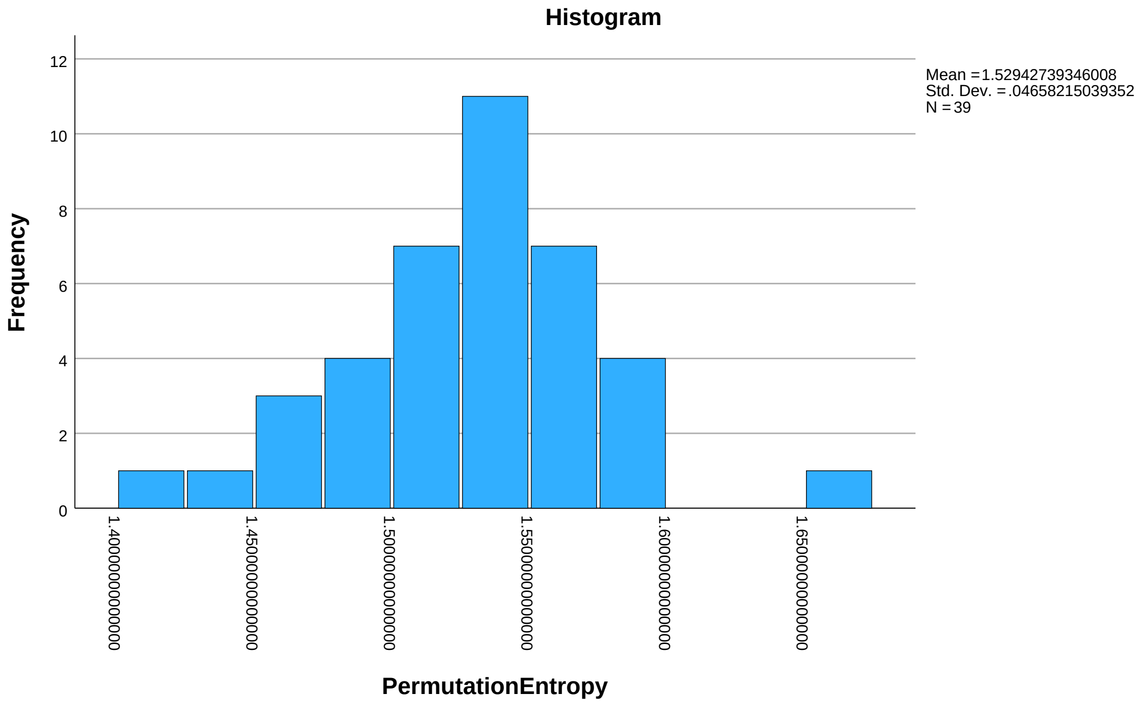

PermutationEntropy Stem-and-Leaf Plot

Frequency   Stem & Leaf

```
 1.00 Extremes    (=<1.41)
1.00     14 . 3
2.00     14 . 55
1.00     14 . 7
4.00     14 . 8899
6.00     15 . 011111
9.00     15 . 222333333
6.00     15 . 444555
5.00     15 . 67777
3.00     15 . 889
 1.00 Extremes    (>=1.65)

Stem width:   .1000000
Each leaf:       1 case(s)
```

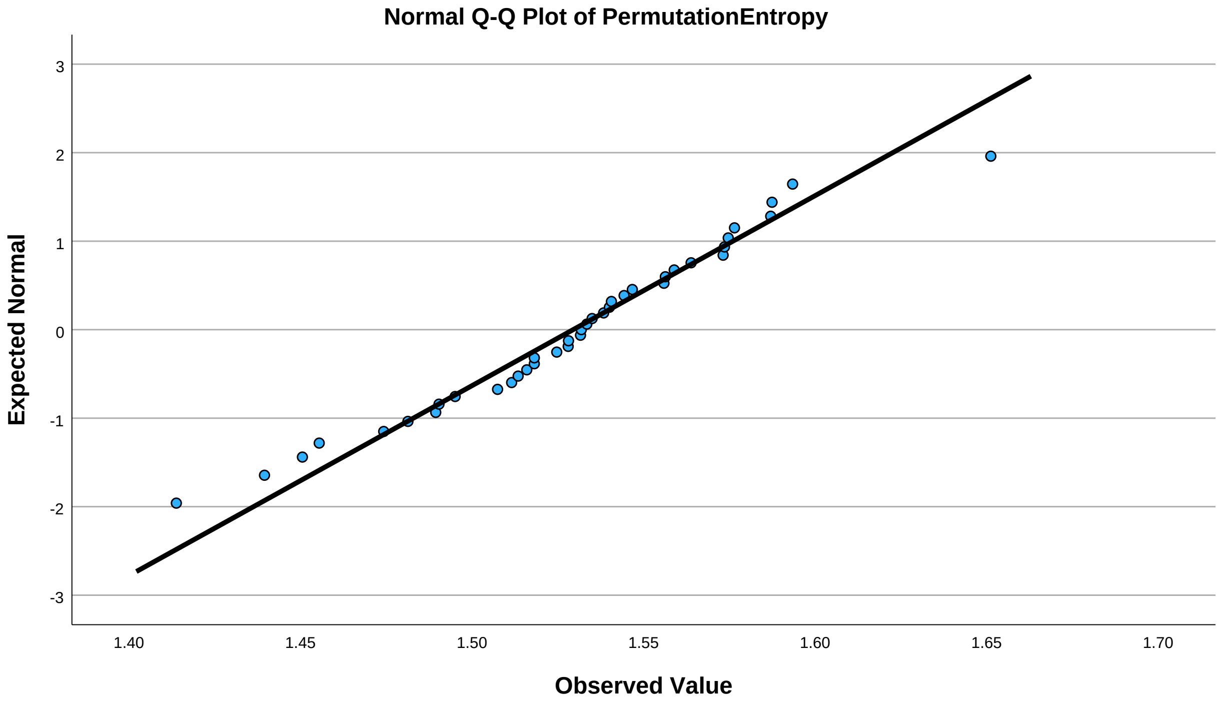

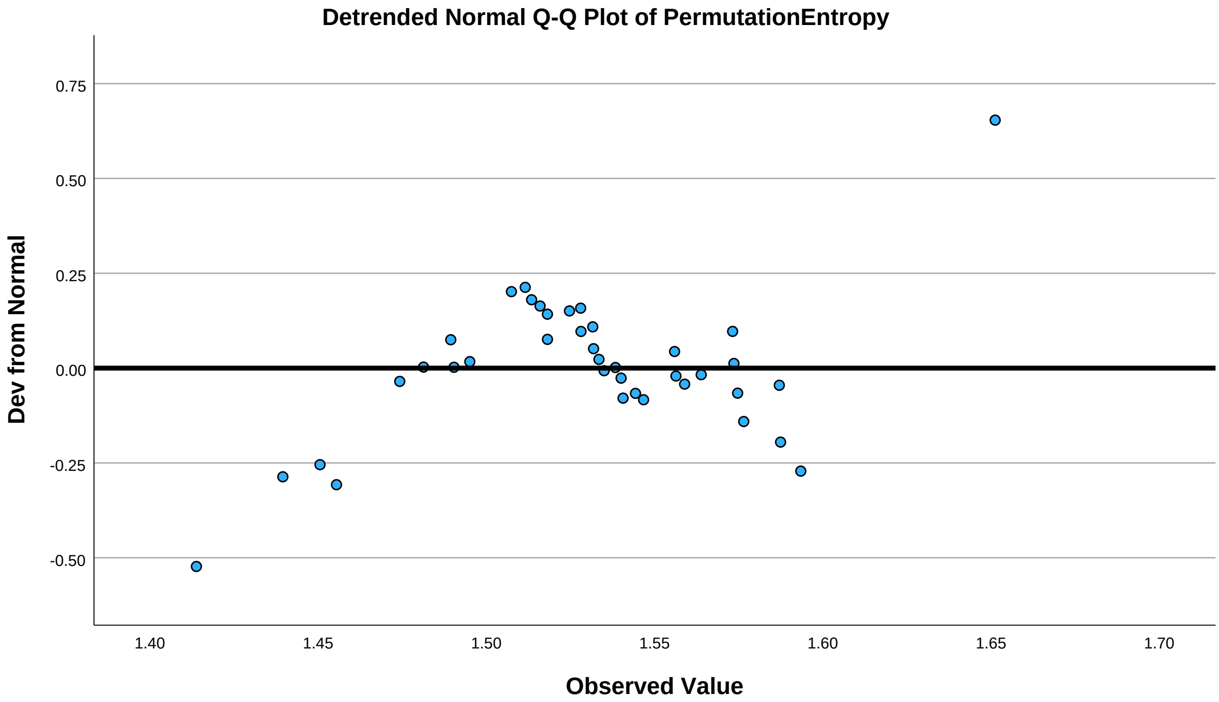

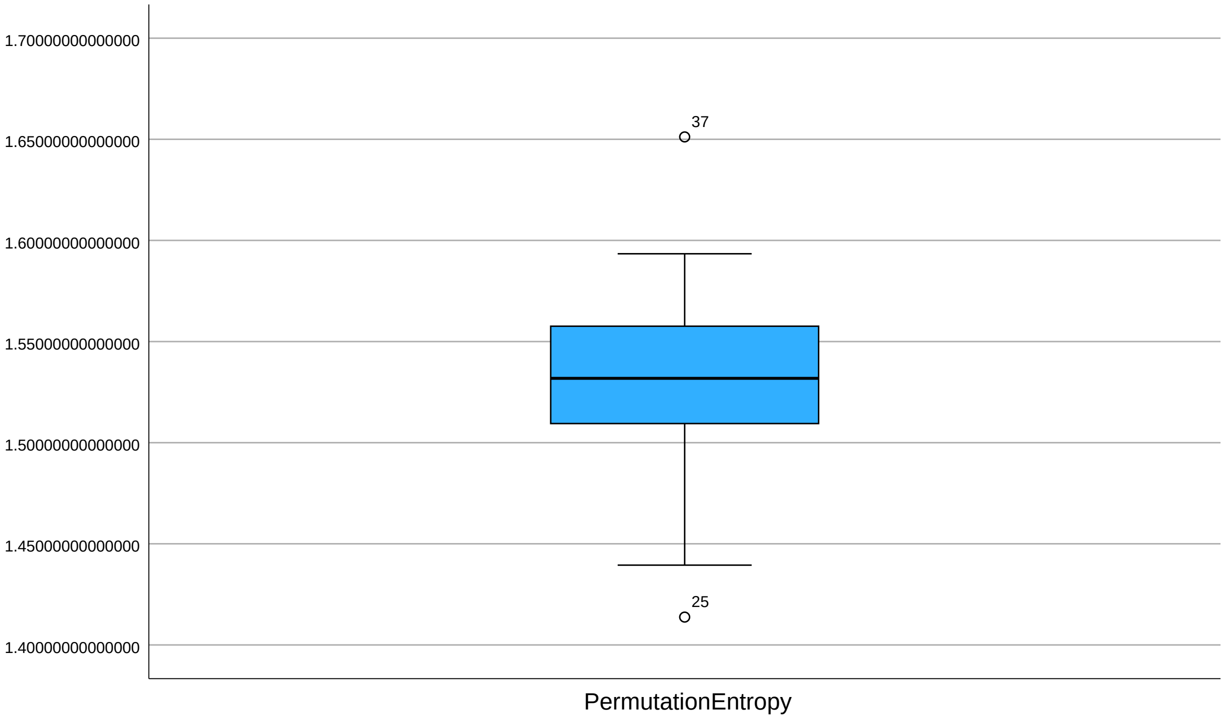

PermutationEntropy_01

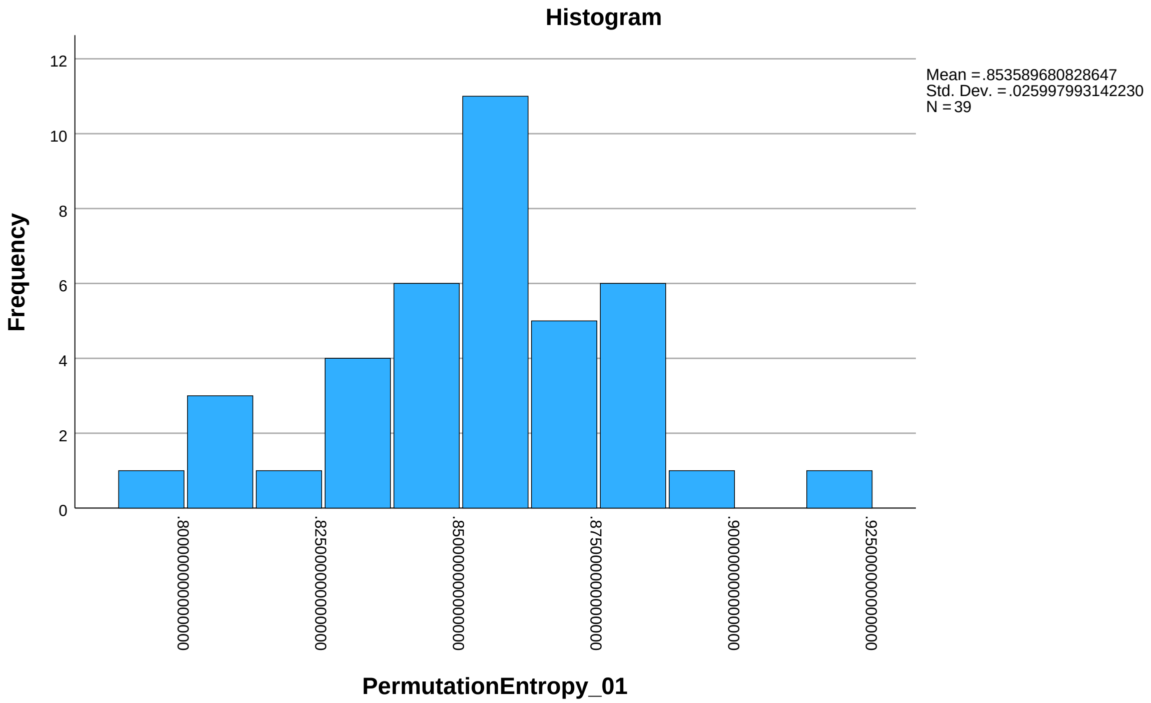


```
PermutationEntropy_01 Stem-and-Leaf Plot

 Frequency    Stem &  Leaf

     1.00 Extremes    (=<.789)
     2.00       80 .  39
     1.00       81 .  2
     2.00       82 .  26
     3.00       83 .  114
     6.00       84 .  134677
    10.00       85 .  0224456899
     4.00       86 .  1388
     6.00       87 .  027889
     3.00       88 .  559
     1.00 Extremes    (>=.922)

 Stem width:   .0100000
 Each leaf:       1 case(s)
```

Normal Q-Q Plot of PermutationEntropy_01

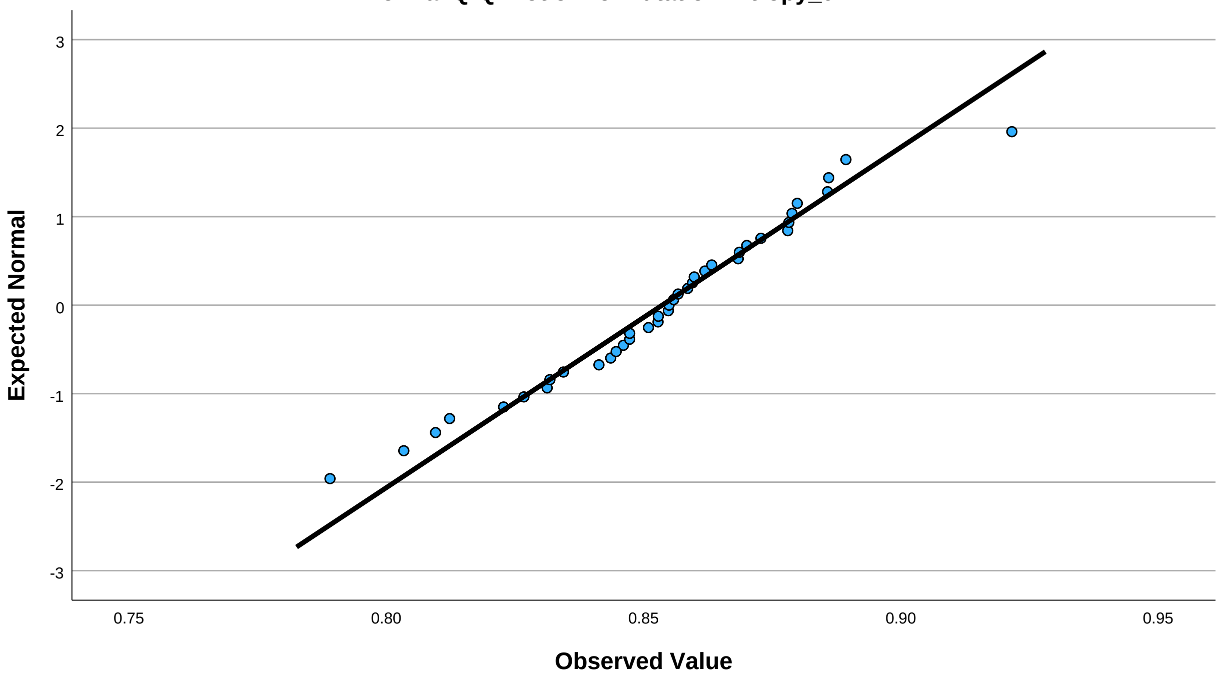

Detrended Normal Q-Q Plot of PermutationEntropy_01

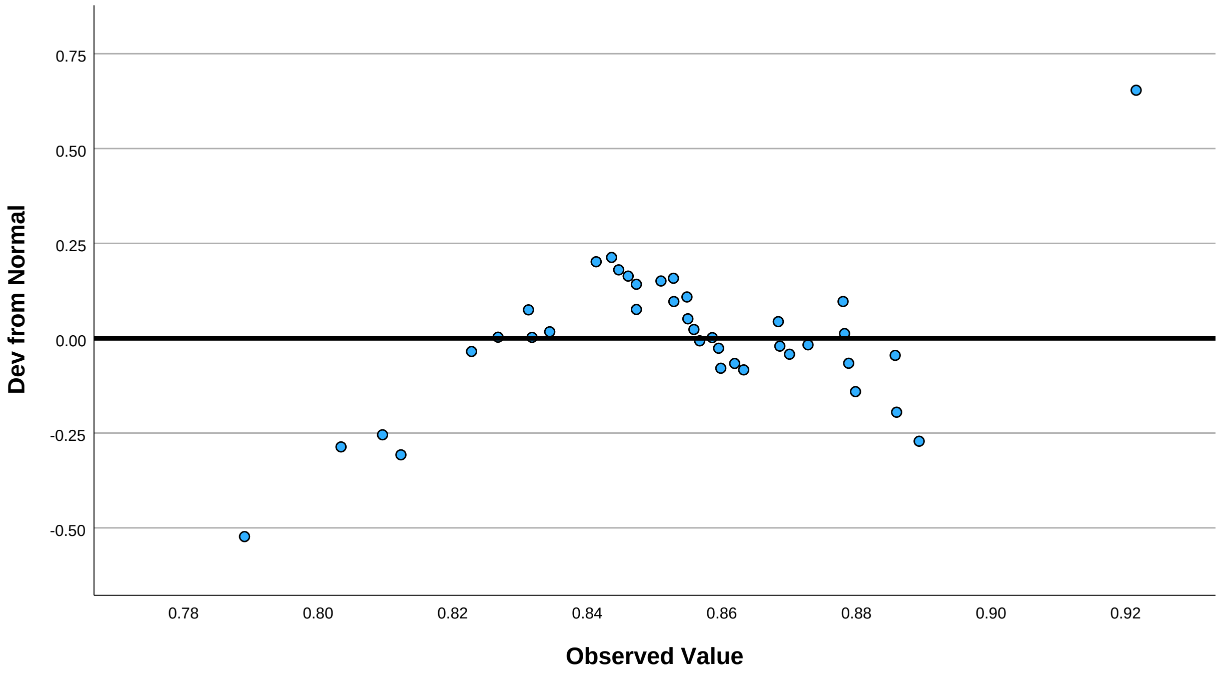

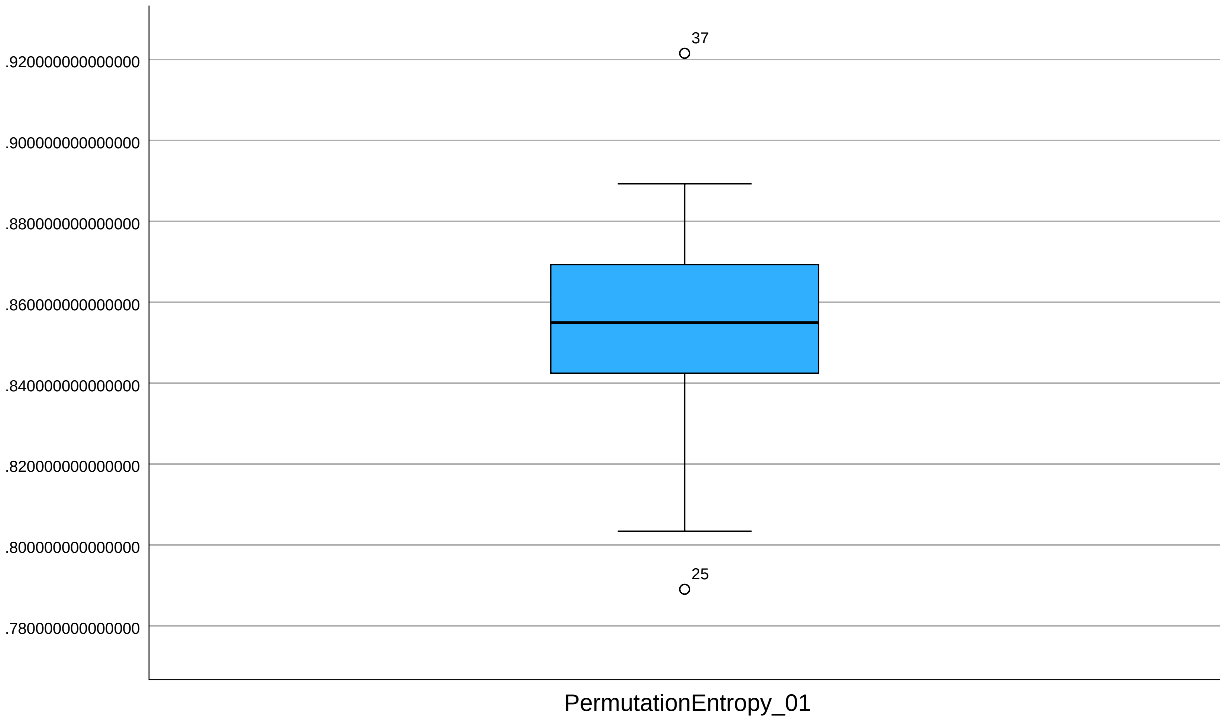

SampleEntropy

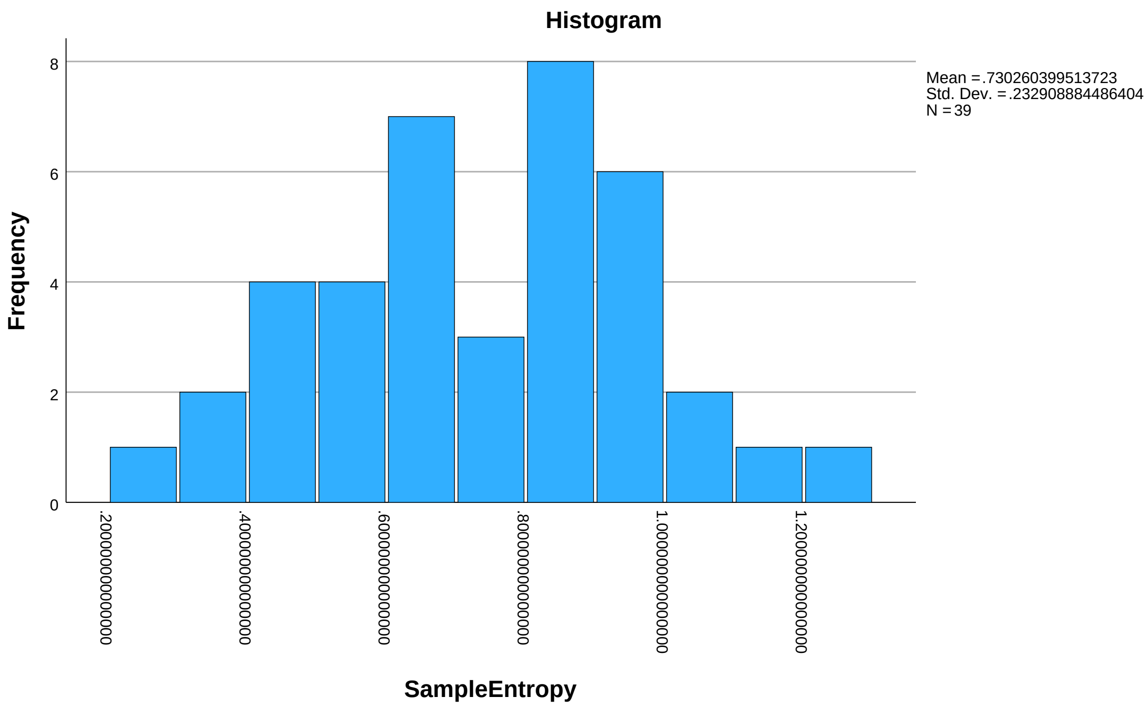


```
SampleEntropy Stem-and-Leaf Plot

 Frequency    Stem &  Leaf
```

```
   1.00        2 .  8
   2.00        3 .  15
   4.00        4 .  0347
   4.00        5 .  0378
   7.00        6 .  1133468
   3.00        7 .  006
   8.00        8 .  02344788
   6.00        9 .  113468
   2.00       10 .  05
   1.00       11 .  5
   1.00       12 .  3

 Stem width:   .1000000
 Each leaf:        1 case(s)
```

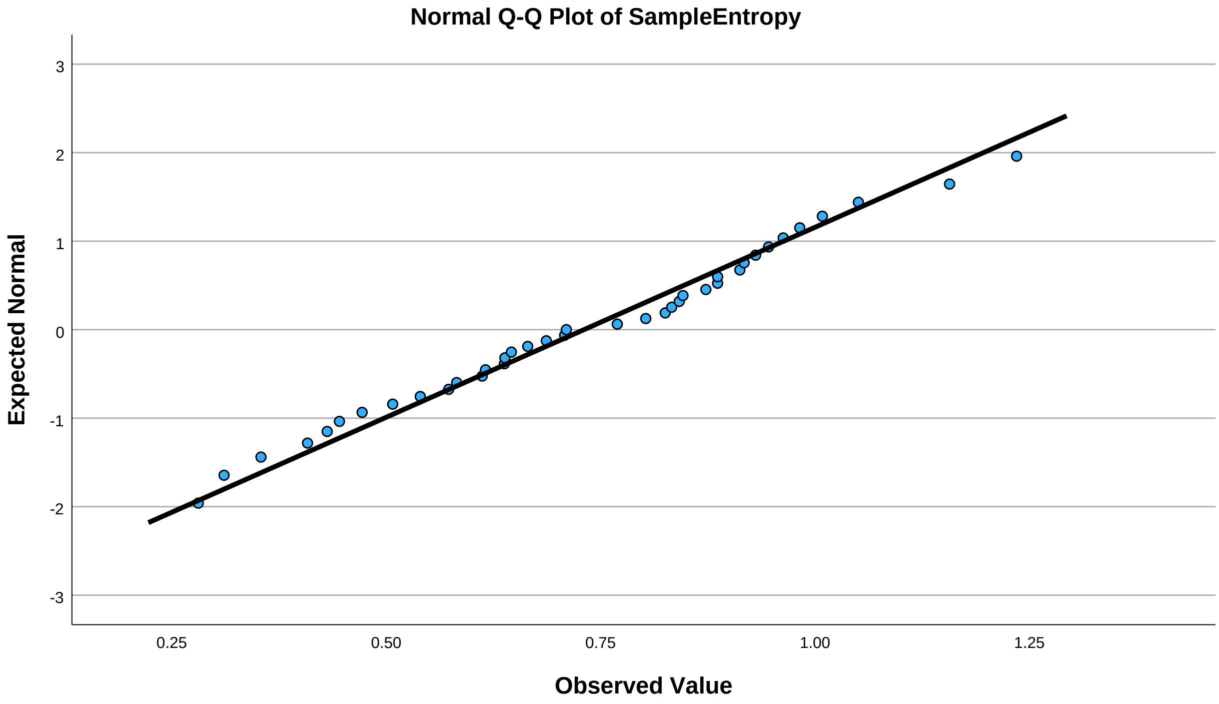

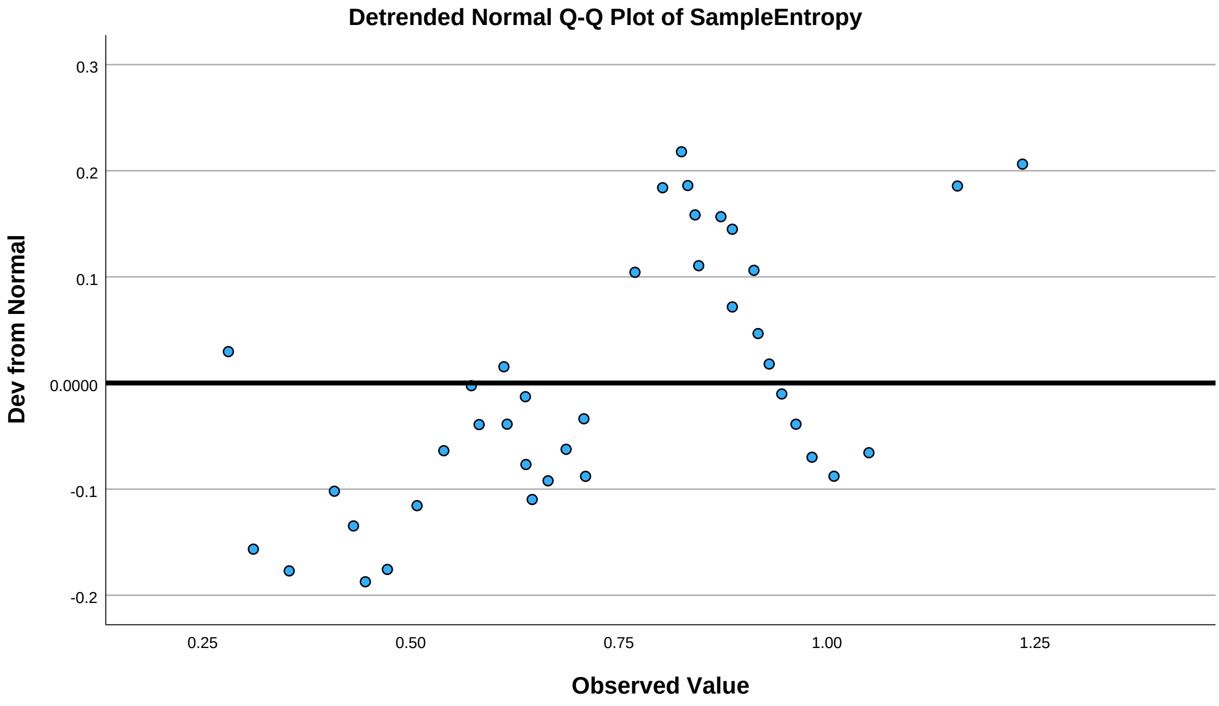

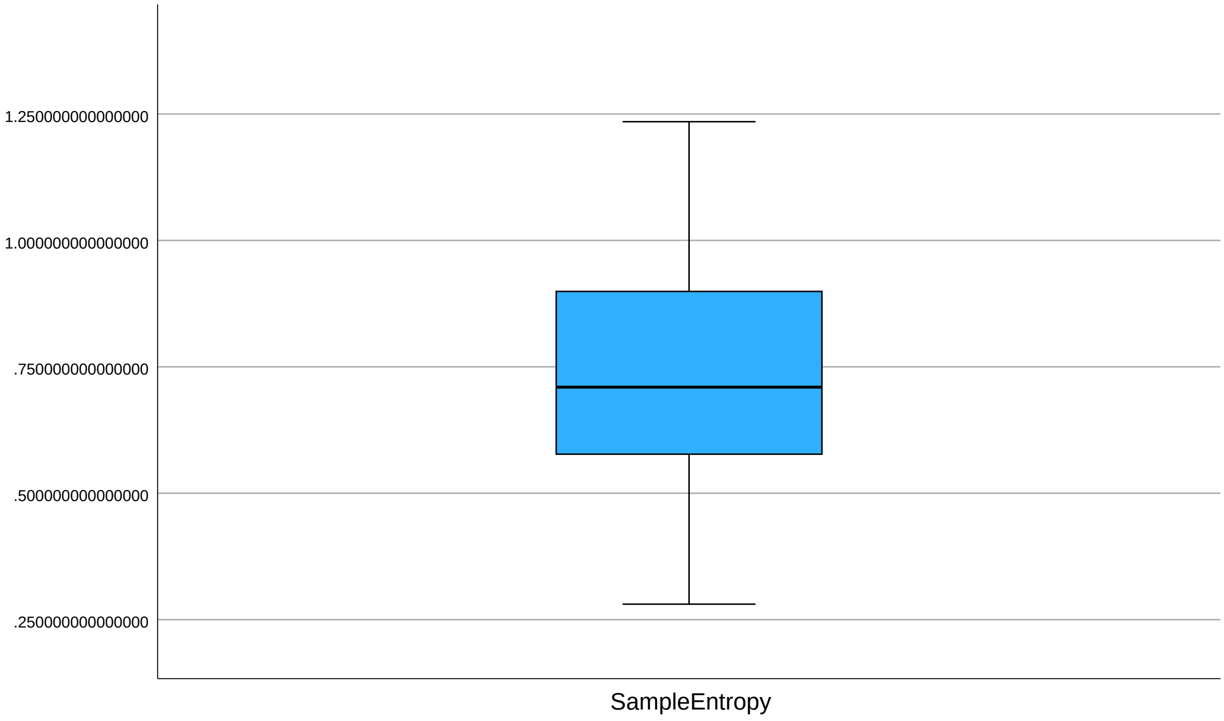

SpectralEntropy_bits

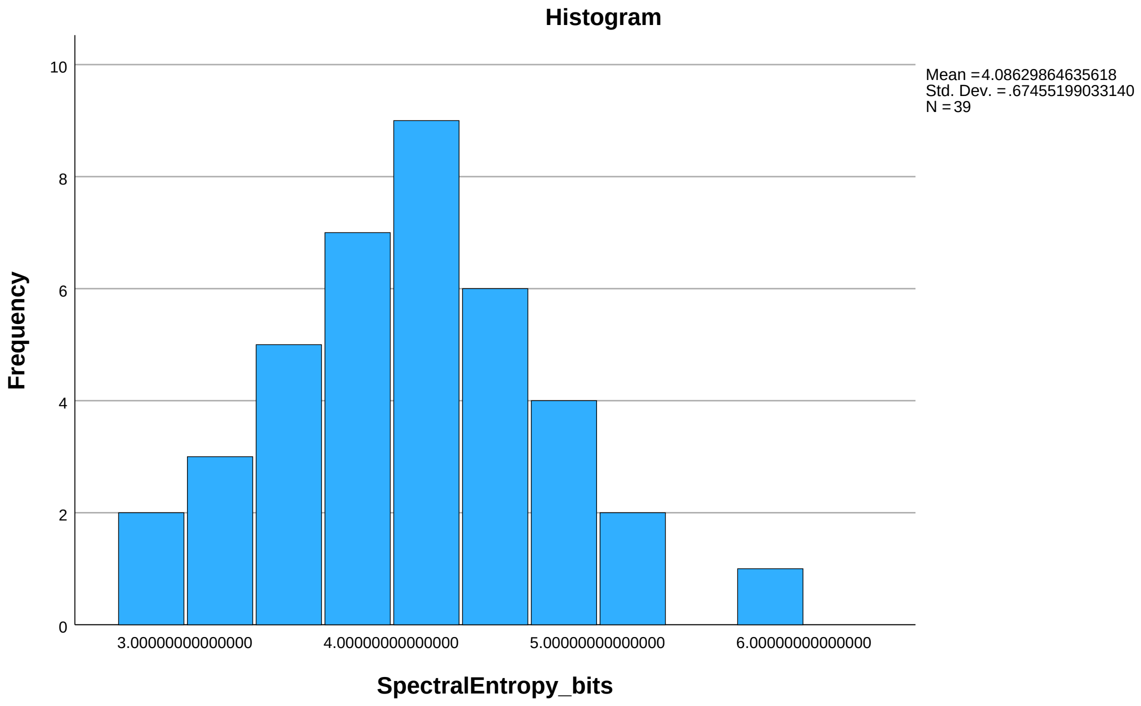


```
SpectralEntropy_bits Stem-and-Leaf Plot

 Frequency    Stem &  Leaf

    2.00        2 .  89
    6.00        3 .  001344
    9.00        3 .  566788999
   12.00        4 .  001111222444
    7.00        4 .  5666779
    2.00        5 .  12
    1.00 Extremes    (>=5.9)

 Stem width:   1.000000
 Each leaf:        1 case(s)
```

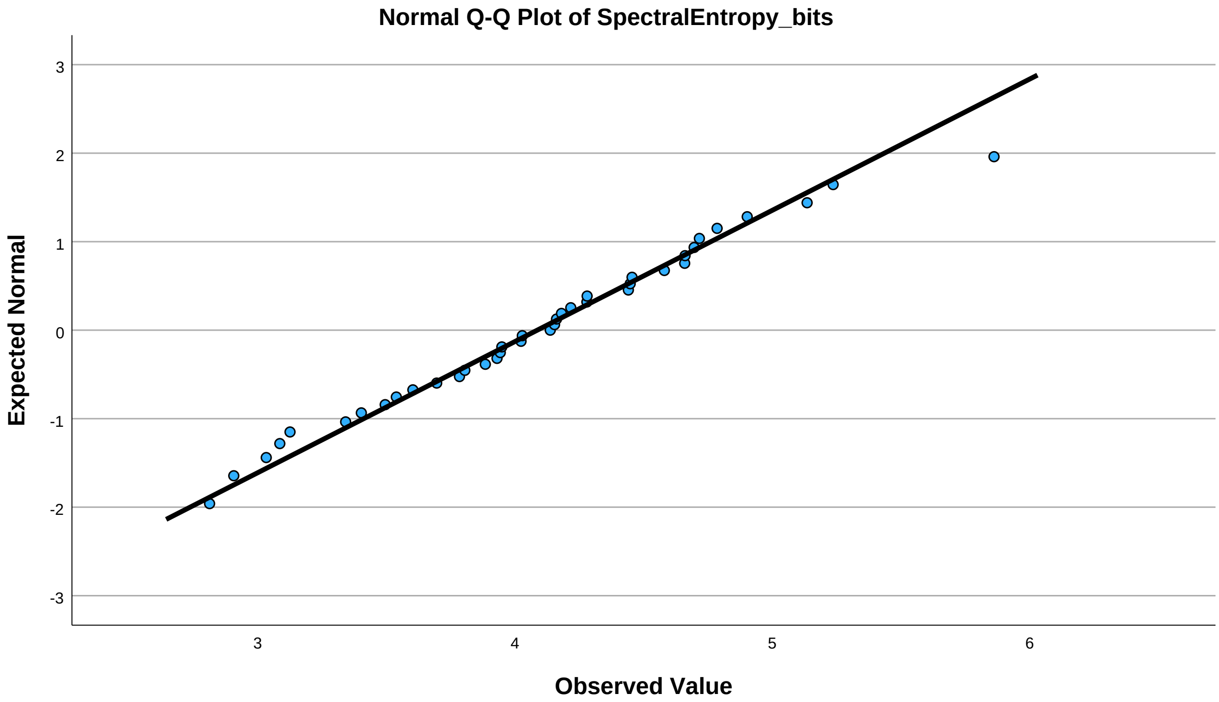
Normal Q-Q Plot of SpectralEntropy_bits
Expected Normal
Observed Value
3
2
1
0
-1
-2
-3
3
4
5
6

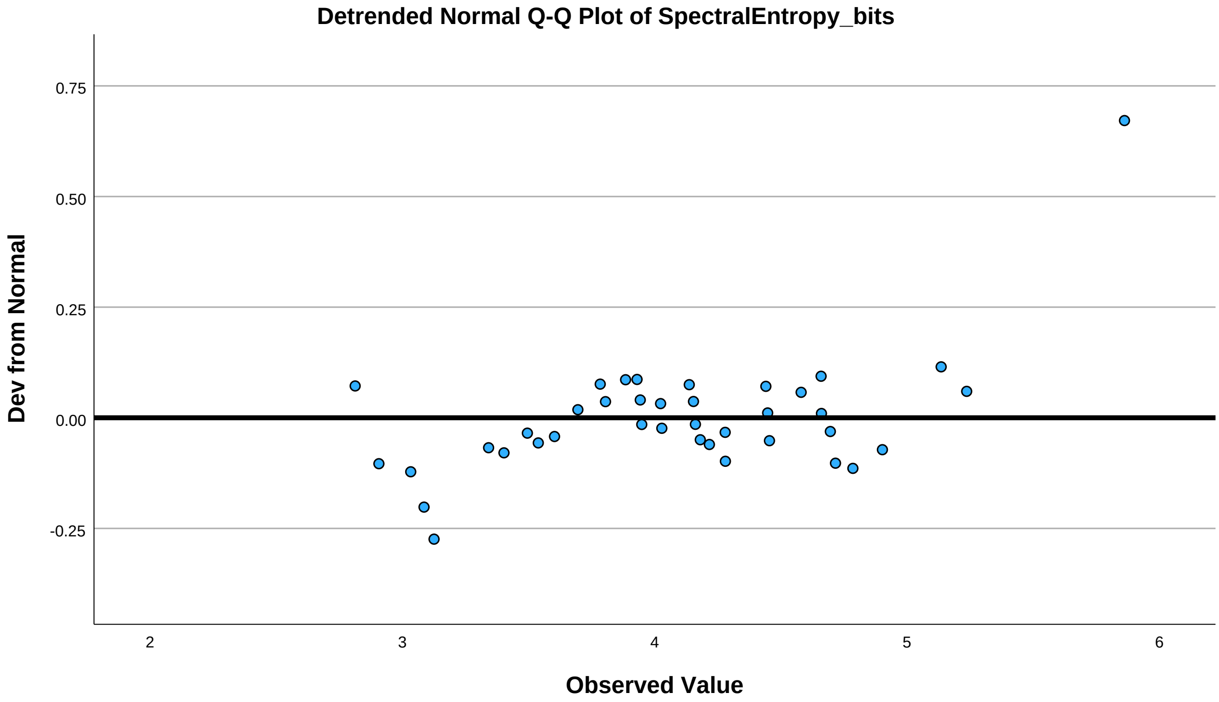
Detrended Normal Q-Q Plot of SpectralEntropy_bits
Dev from Normal
Observed Value
0.75
0.50
0.25
0.00
-0.25
2
3
4
5
6

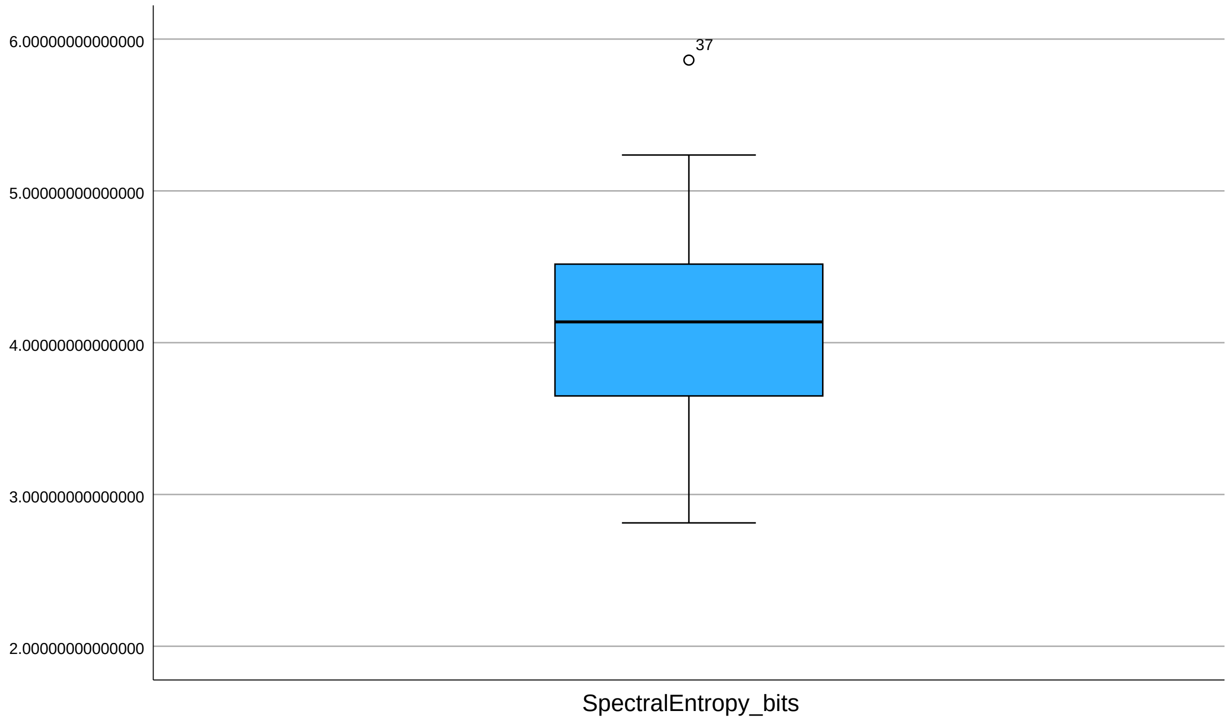

SpectralEntropy_01

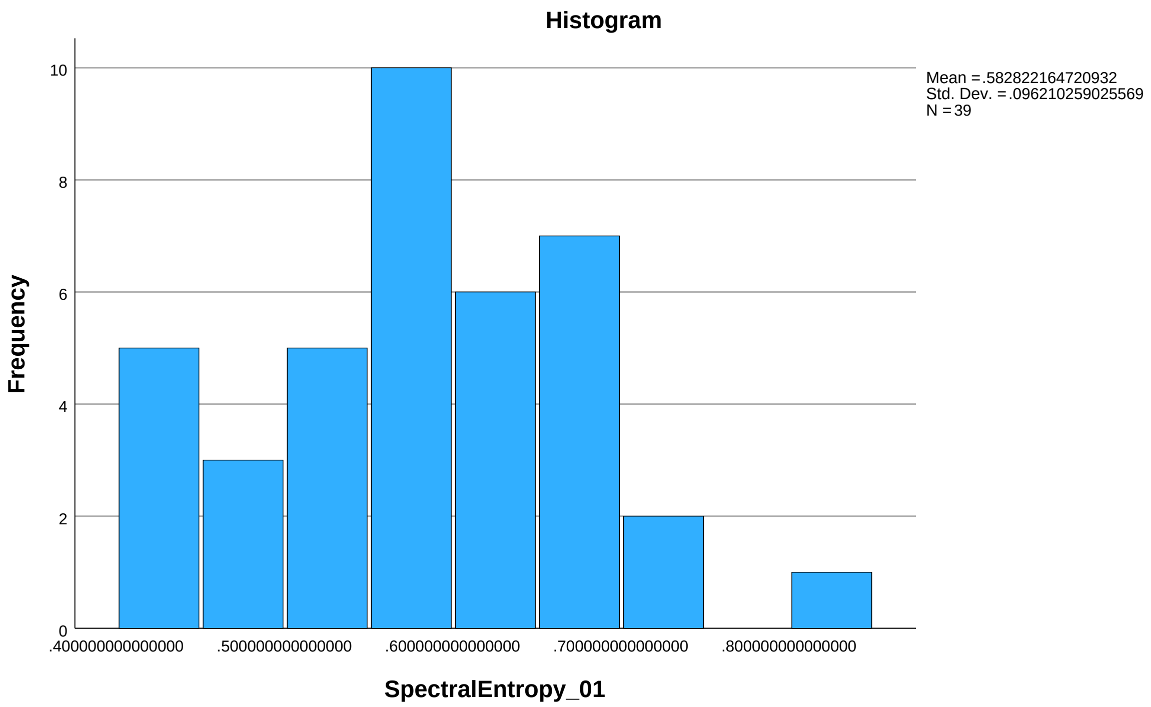

SpectralEntropy_01 Stem-and-Leaf Plot

Frequency   Stem & Leaf

```
    5.00       4 .  01344
    3.00       4 .  789
    5.00       5 .  01234
   10.00       5 .  5666779999
    6.00       6 .  011333
    7.00       6 .  5666789
    2.00       7 .  34
    1.00 Extremes    (>=.84)

 Stem width:   .1000000
 Each leaf:       1 case(s)
```

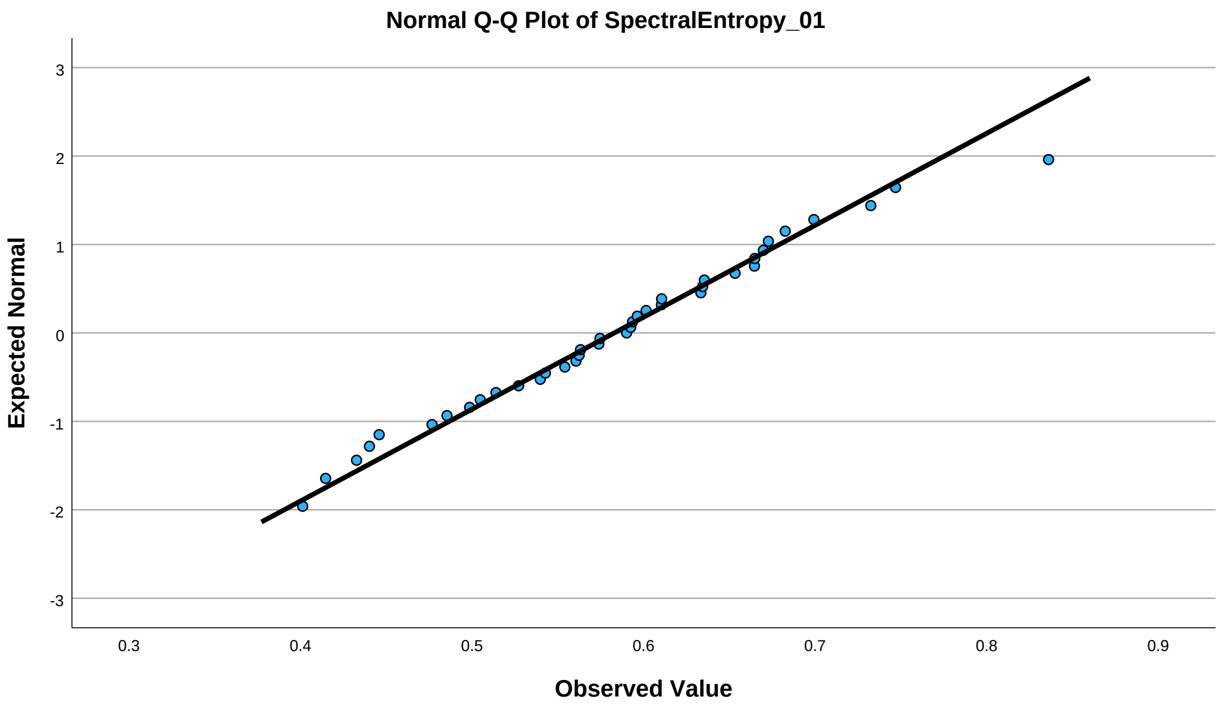

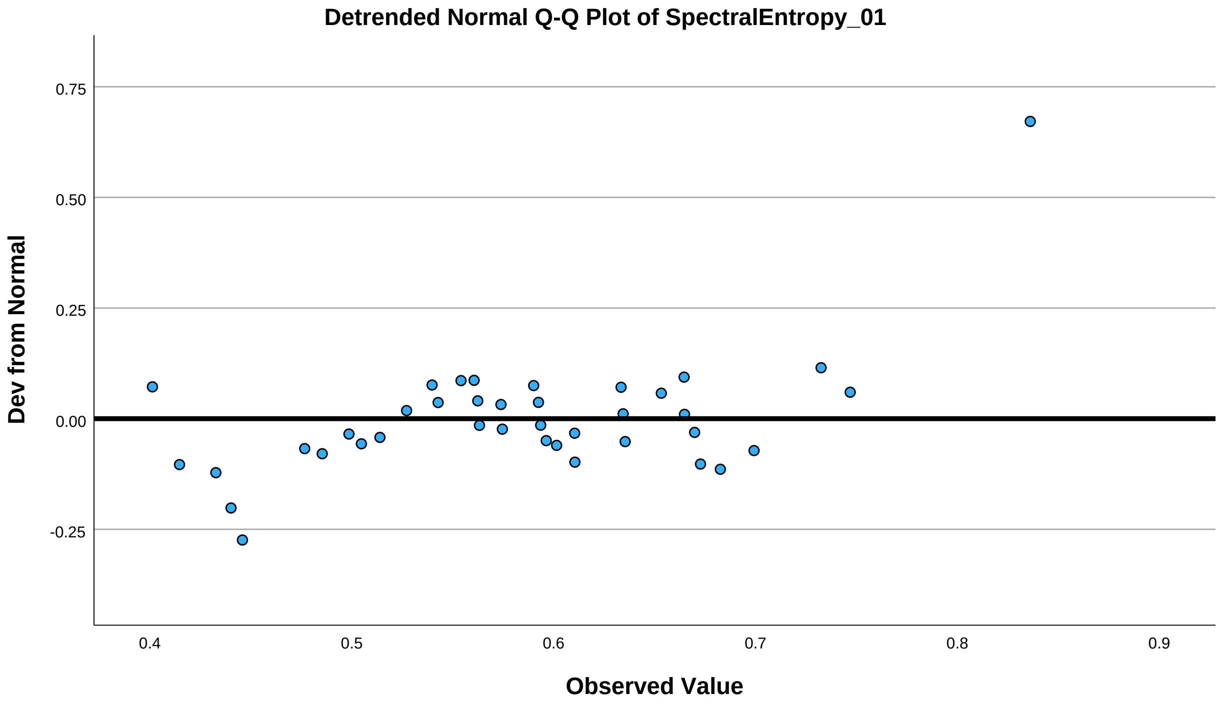

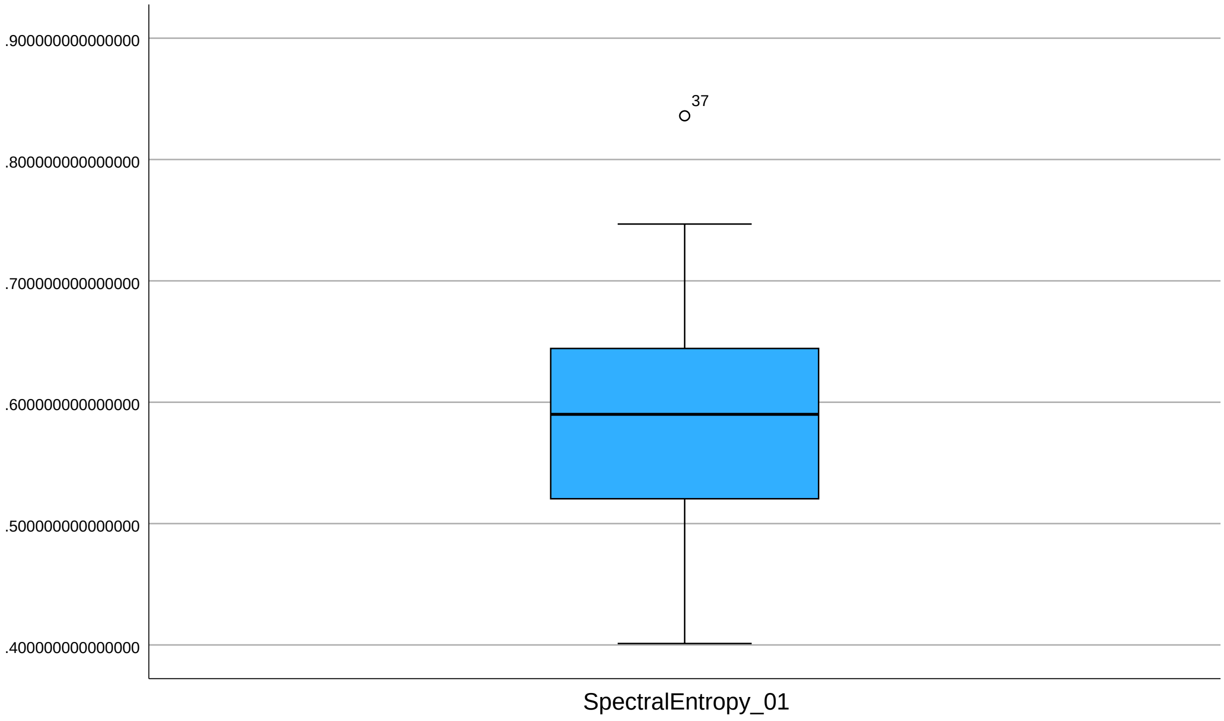

HiguchiFractalDimension

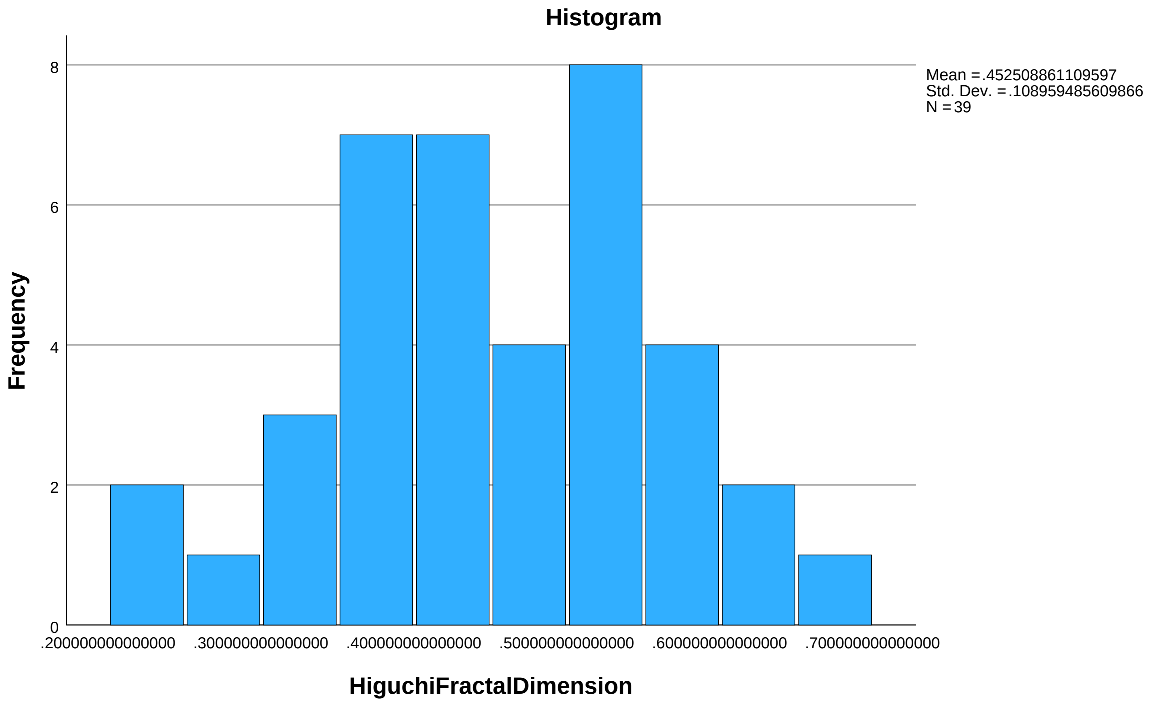


```
HiguchiFractalDimension Stem-and-Leaf Plot

 Frequency    Stem &  Leaf

     2.00        2 .  04
     1.00        2 .  6
     3.00        3 .  012
     7.00        3 .  6778899
     7.00        4 .  0023334
     4.00        4 .  5889
     8.00        5 .  01233334
     4.00        5 .  5567
     2.00        6 .  02
     1.00        6 .  8

 Stem width:   .1000000
 Each leaf:       1 case(s)
```

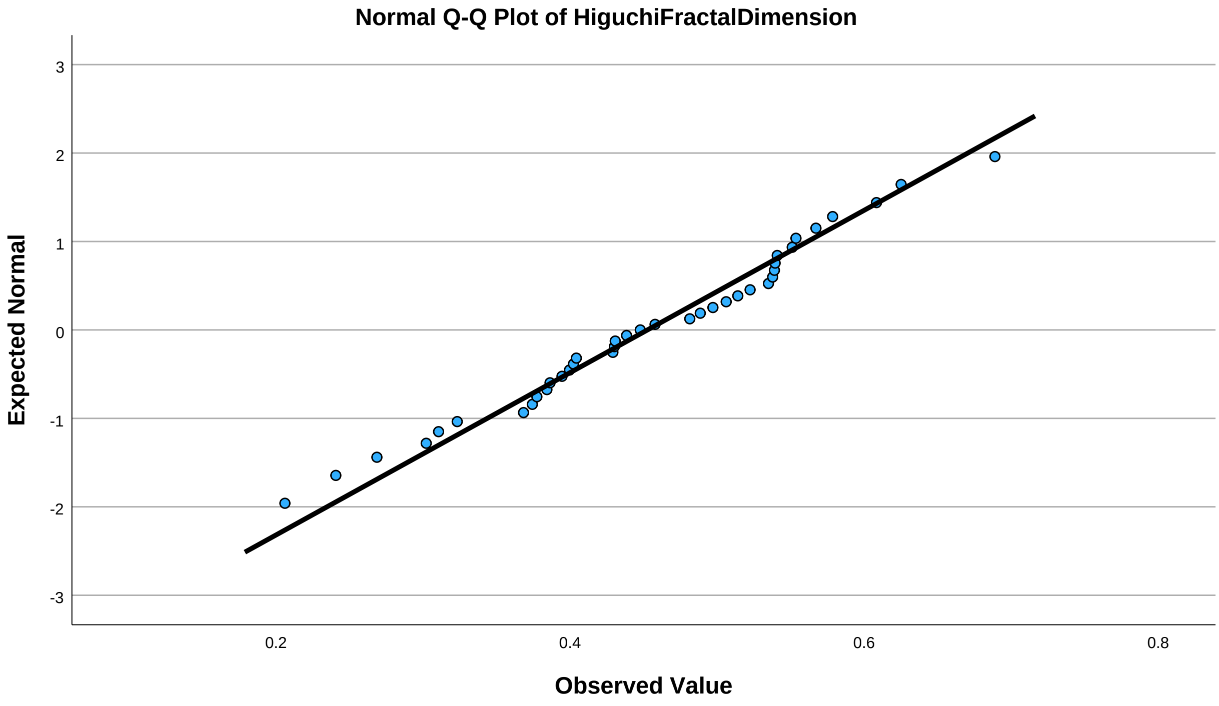
Normal Q-Q Plot of HiguchiFractalDimension
Expected Normal
Observed Value
3
2
1
0
-1
-2
-3
0.2
0.4
0.6
0.8

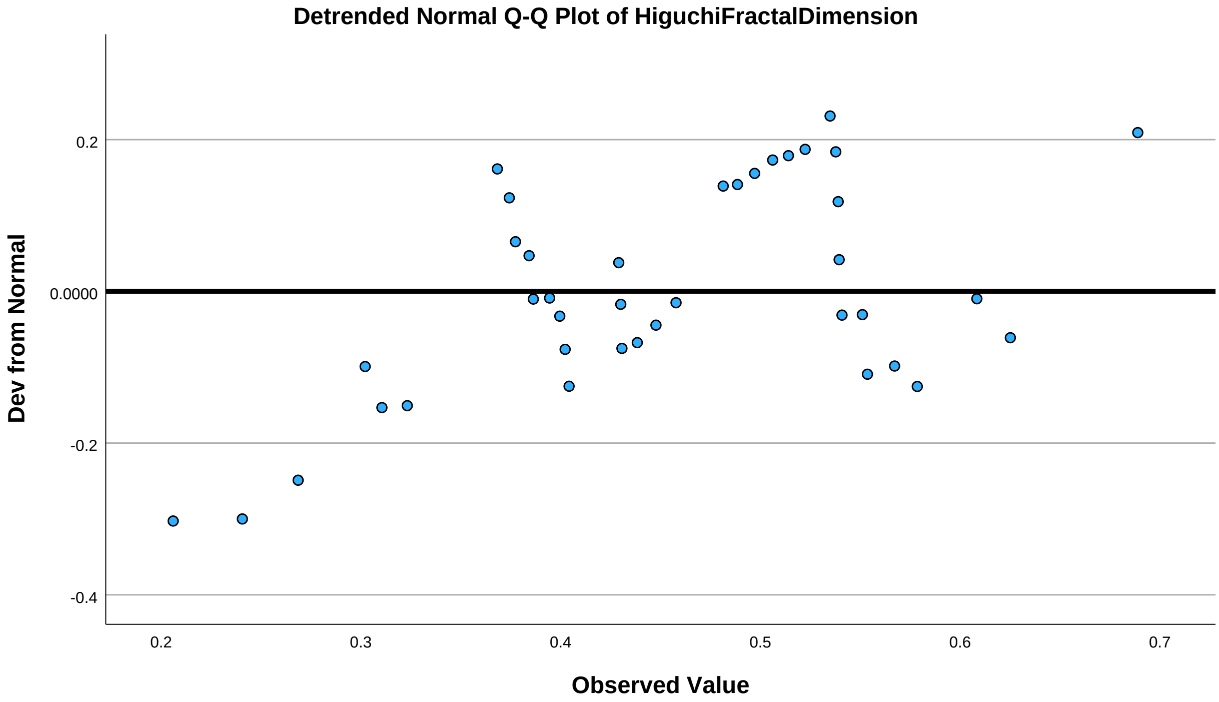
Detrended Normal Q-Q Plot of HiguchiFractalDimension
Dev from Normal
Observed Value
0.2
0.0000
-0.2
-0.4
0.2
0.3
0.4
0.5
0.6
0.7

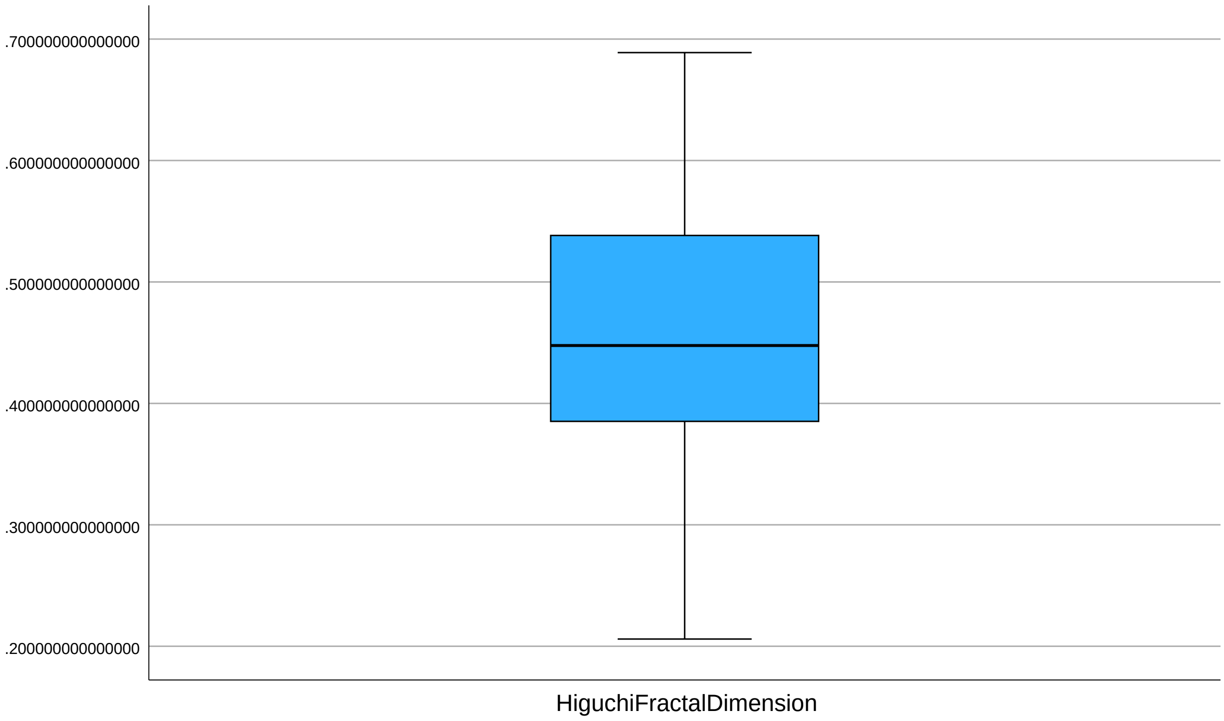

LempelZivComplexity

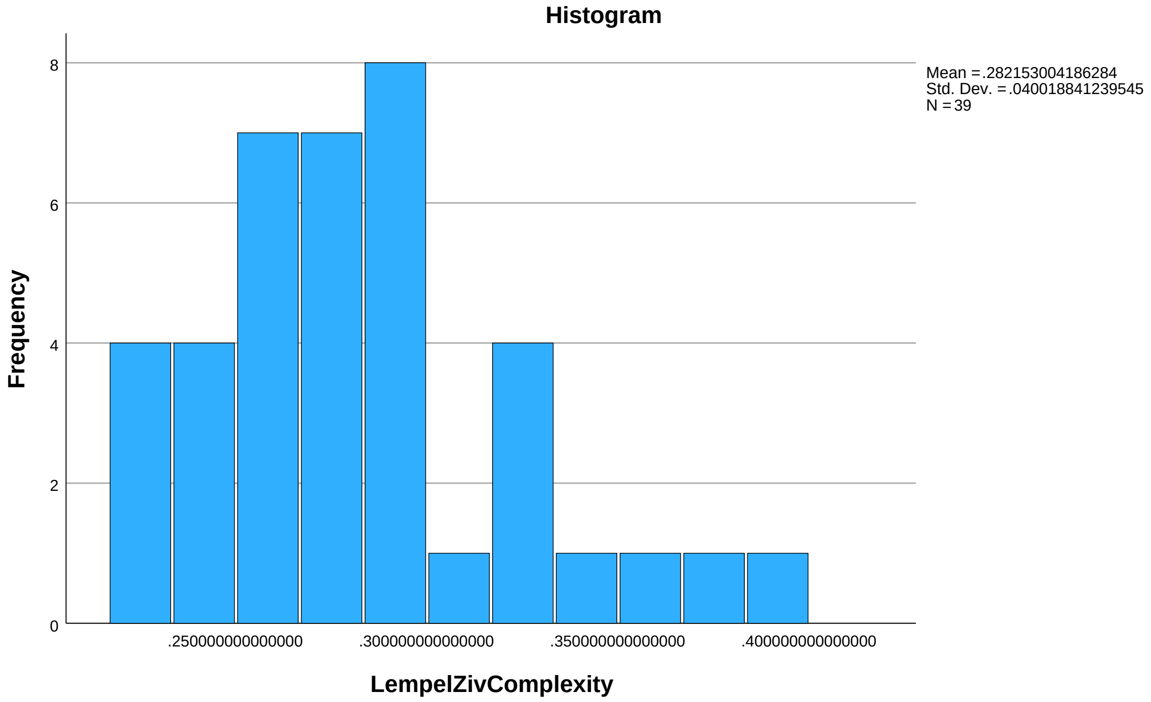


```
LempelZivComplexity Stem-and-Leaf Plot

 Frequency    Stem &  Leaf
```

```
   5.00      2 .  22333
   8.00      2 .  44455555
   8.00      2 .  66667777
   9.00      2 .  888999999
   2.00      3 .  01
   4.00      3 .  2233
   1.00      3 .  5
   2.00 Extremes    (>=.38)

 Stem width:   .1000000
 Each leaf:       1 case(s)
```

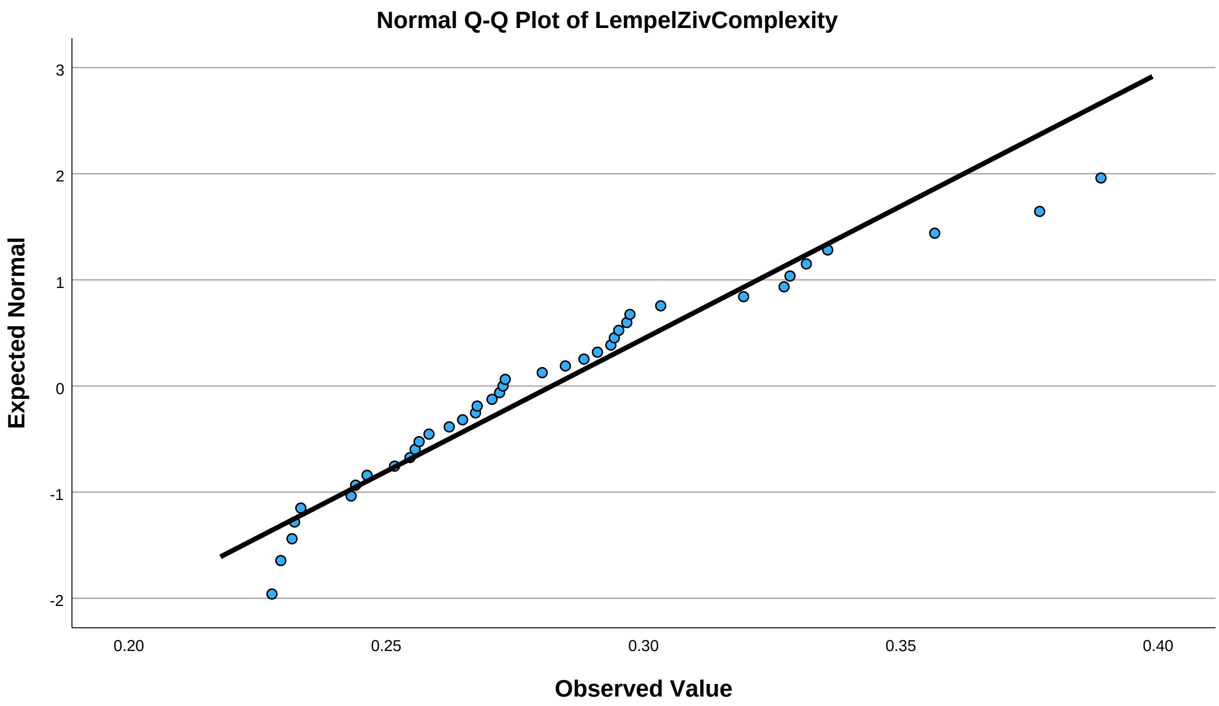

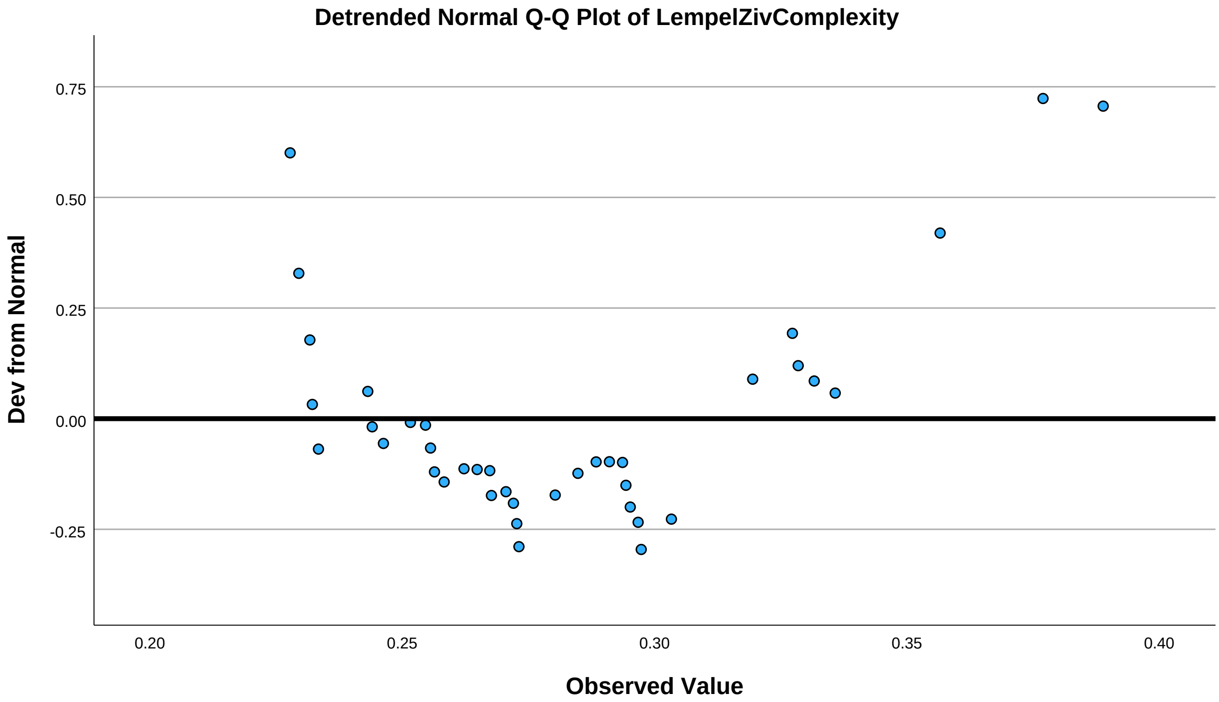

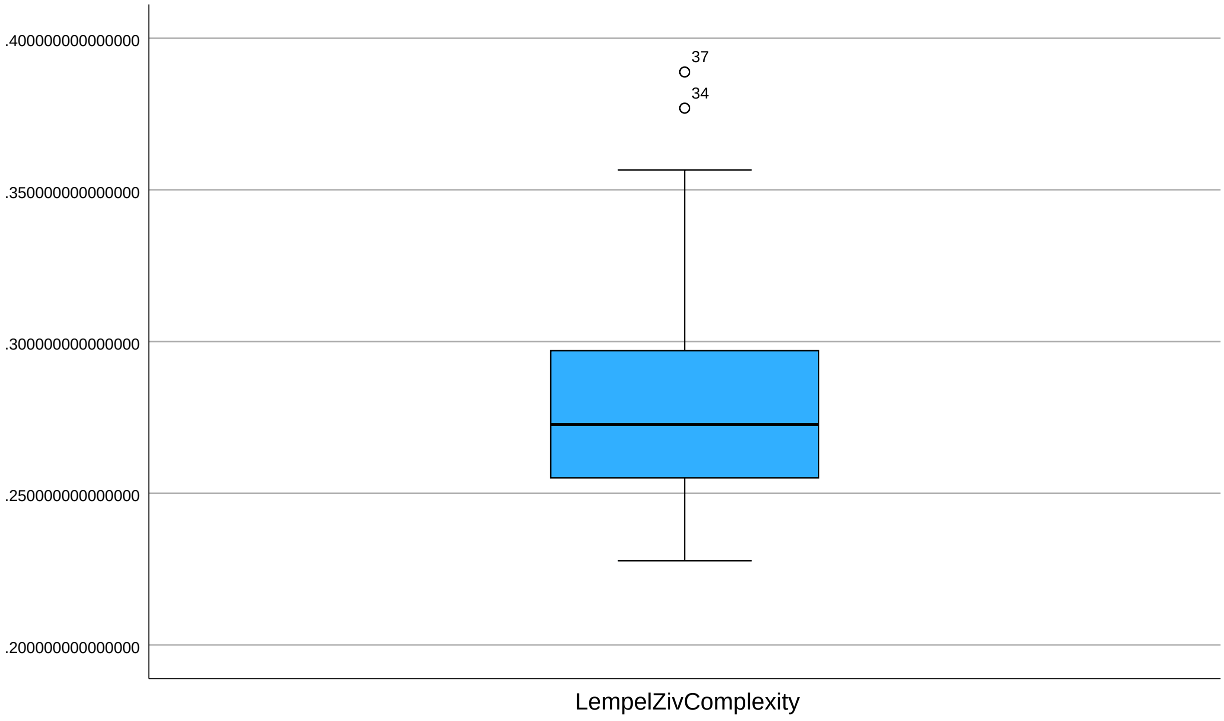

RenyiEntropy

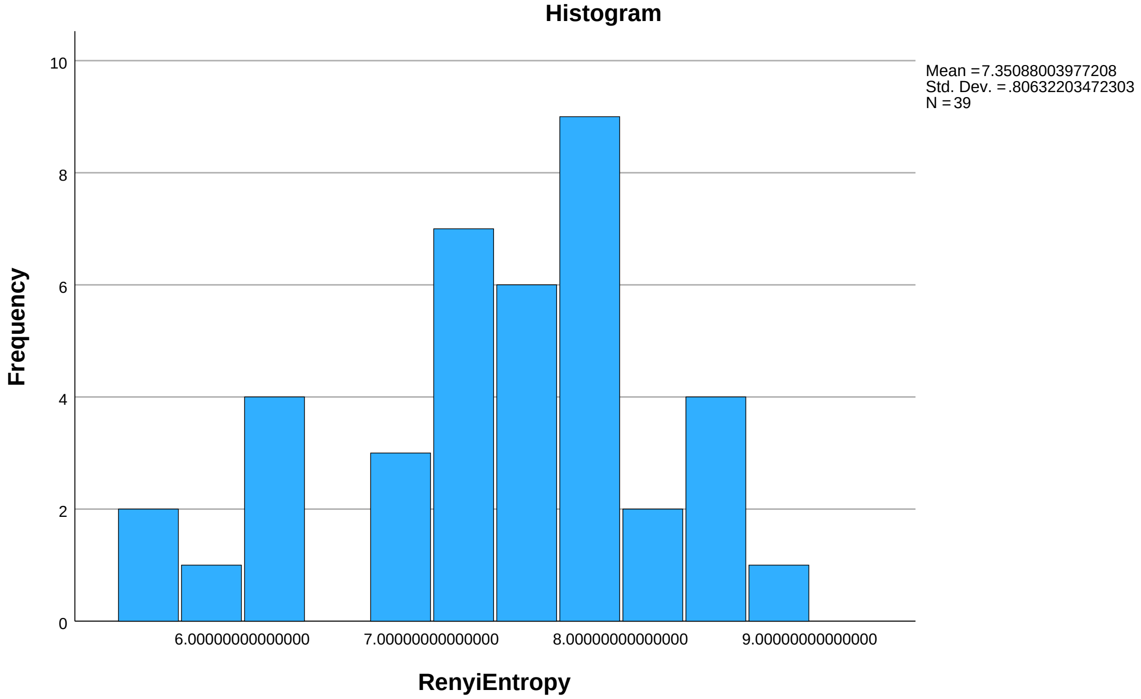


```
RenyiEntropy Stem-and-Leaf Plot

 Frequency    Stem &  Leaf

     2.00 Extremes    (=<5.6)
     1.00        5 .  9
     4.00        6 .  0223
     3.00        6 .  799
    10.00        7 .  0011222344
    12.00        7 .  556667778899
     4.00        8 .  0044
     3.00        8 .  566

 Stem width:  1.000000
 Each leaf:       1 case(s)
```

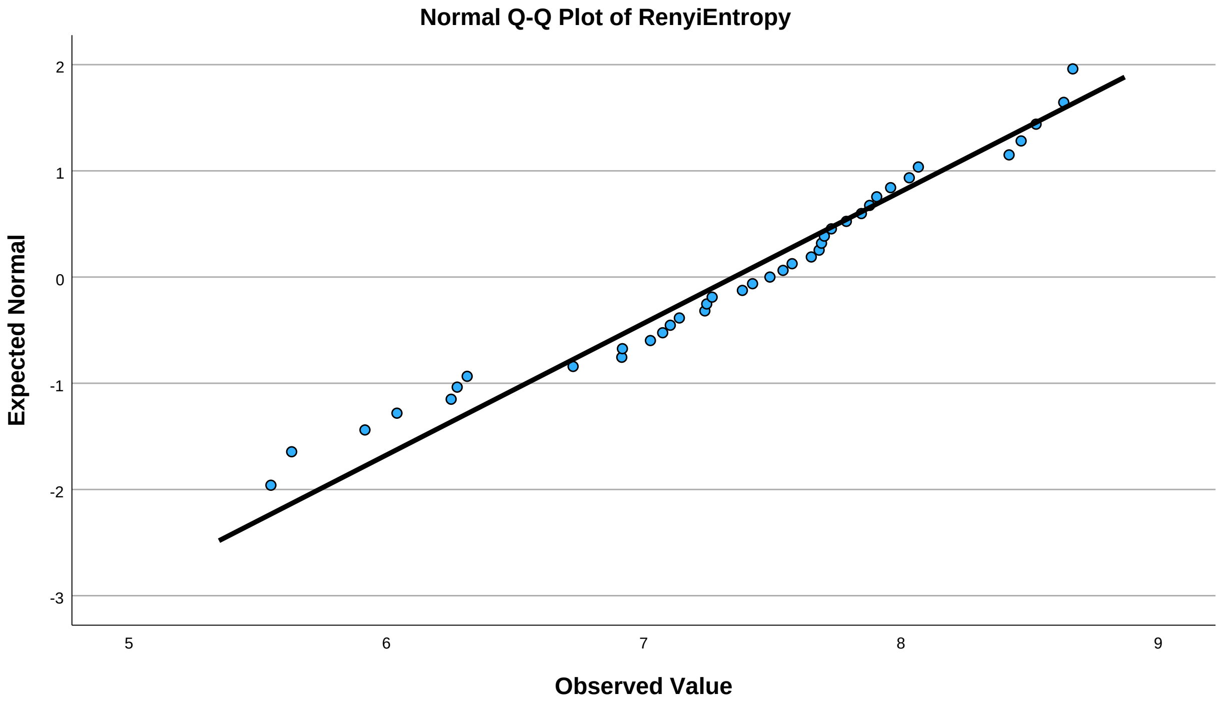
Normal Q-Q Plot of RenyiEntropy
Expected Normal
2
1
0
-1
-2
-3
5
6
7
8
9
Observed Value

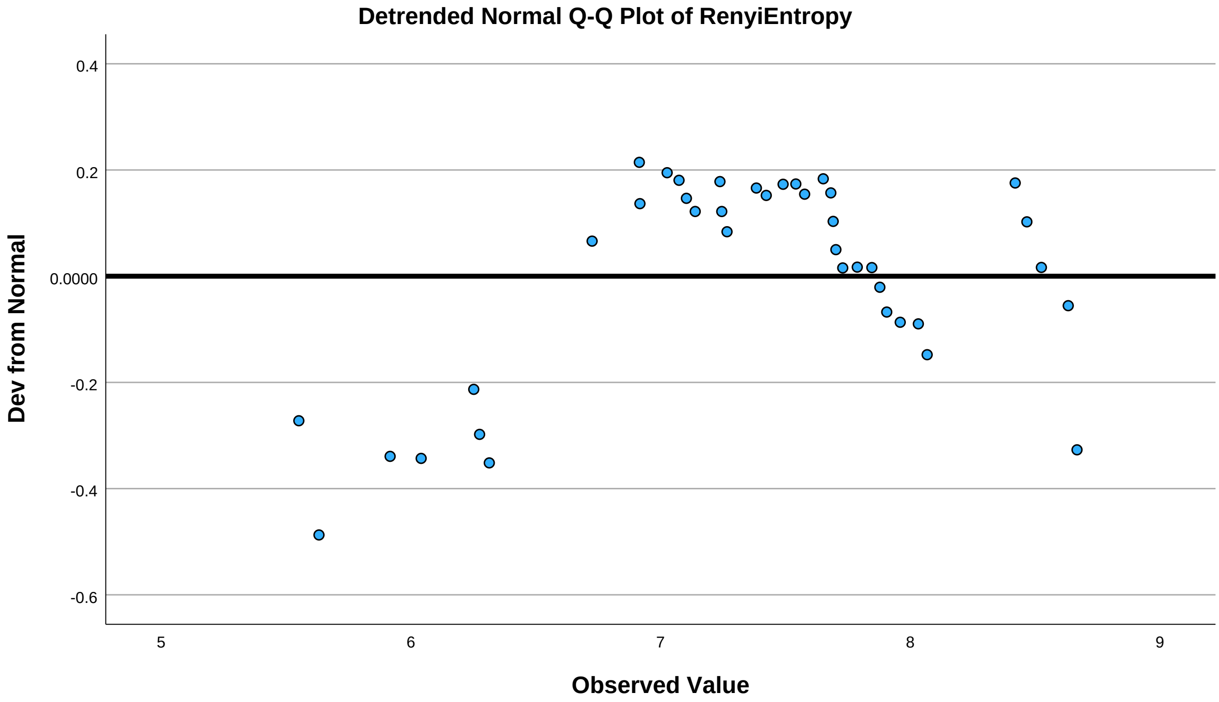
Detrended Normal Q-Q Plot of RenyiEntropy
Dev from Normal
0.4
0.2
0.0000
-0.2
-0.4
-0.6
5
6
7
8
9
Observed Value

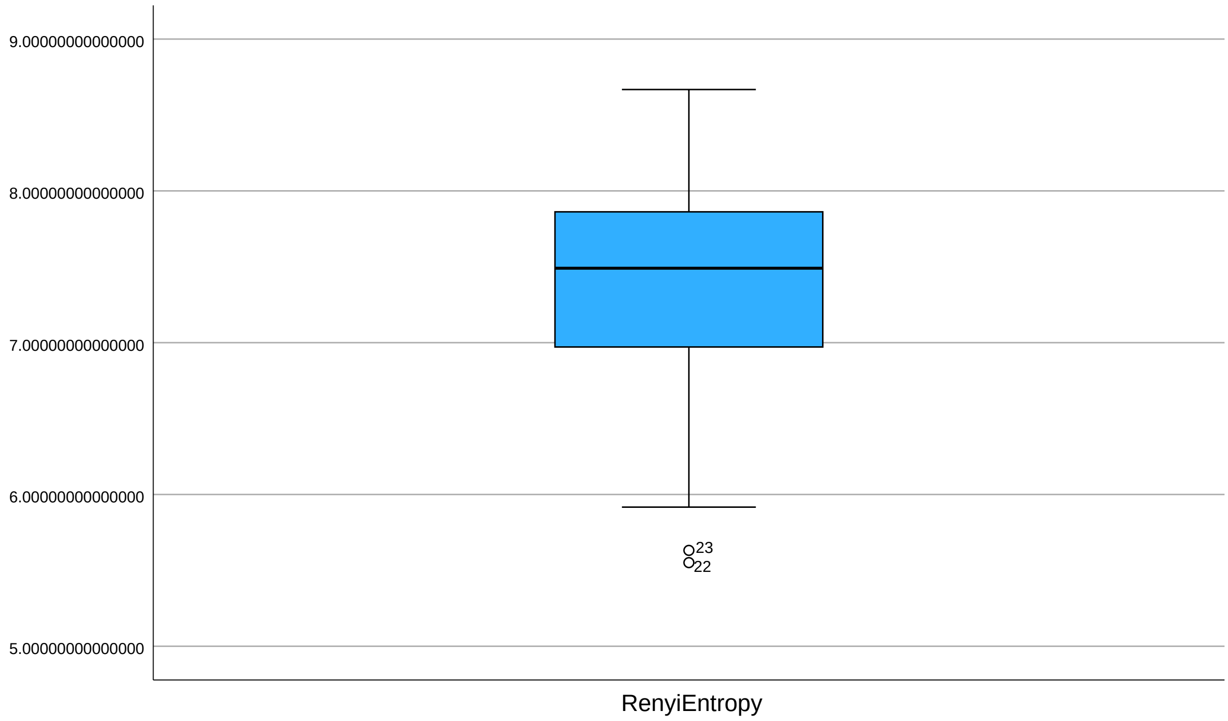

TsallisEntropy

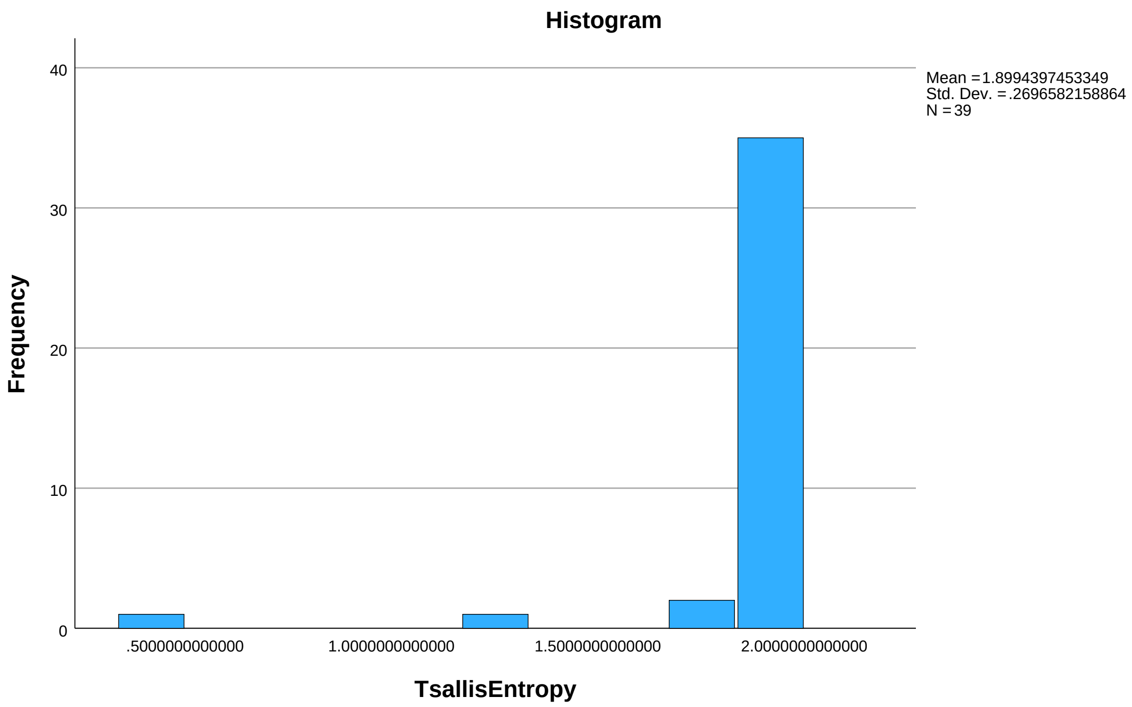

TsallisEntropy Stem-and-Leaf Plot

Frequency   Stem & Leaf

```
 8.00 Extremes    (=<1.897)
1.00    194 . 1
1.00    194 . 7
 .00    195 .
1.00    195 . 5
2.00    196 . 33
1.00    196 . 5
1.00    197 . 4
4.00    197 . 5679
5.00    198 . 00344
15.00     198 . 555566666777788

Stem width:  .0100000
Each leaf:      1 case(s)
```

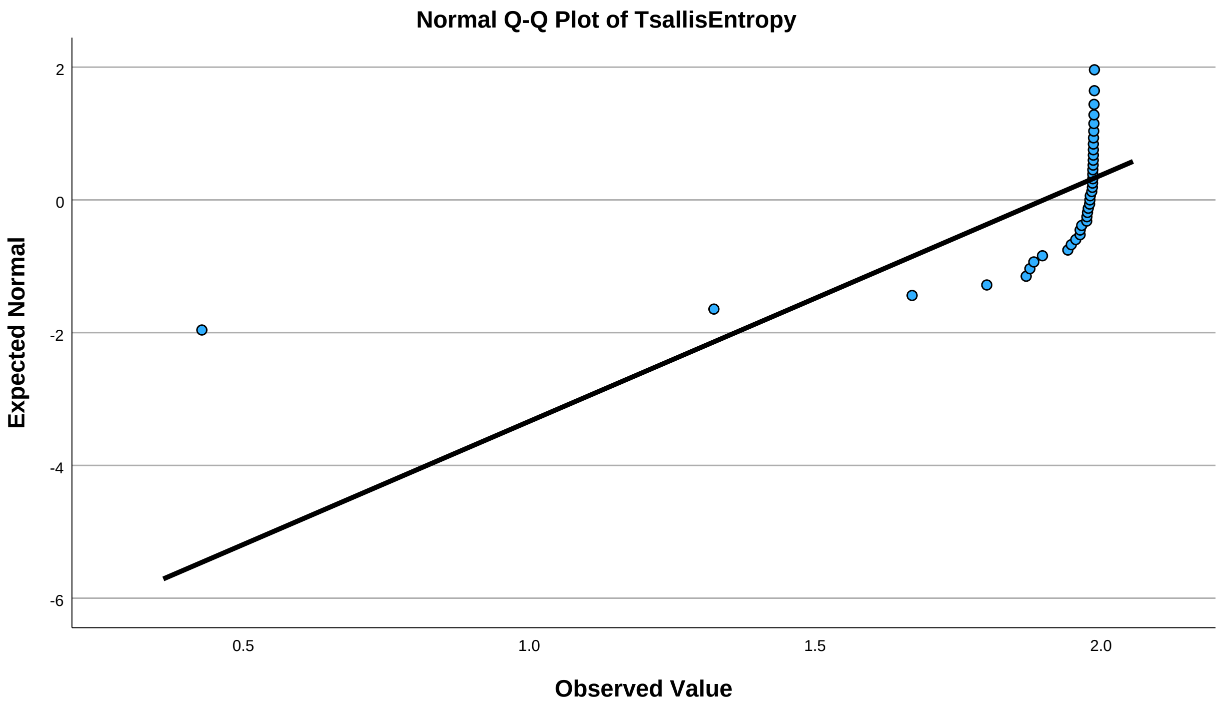

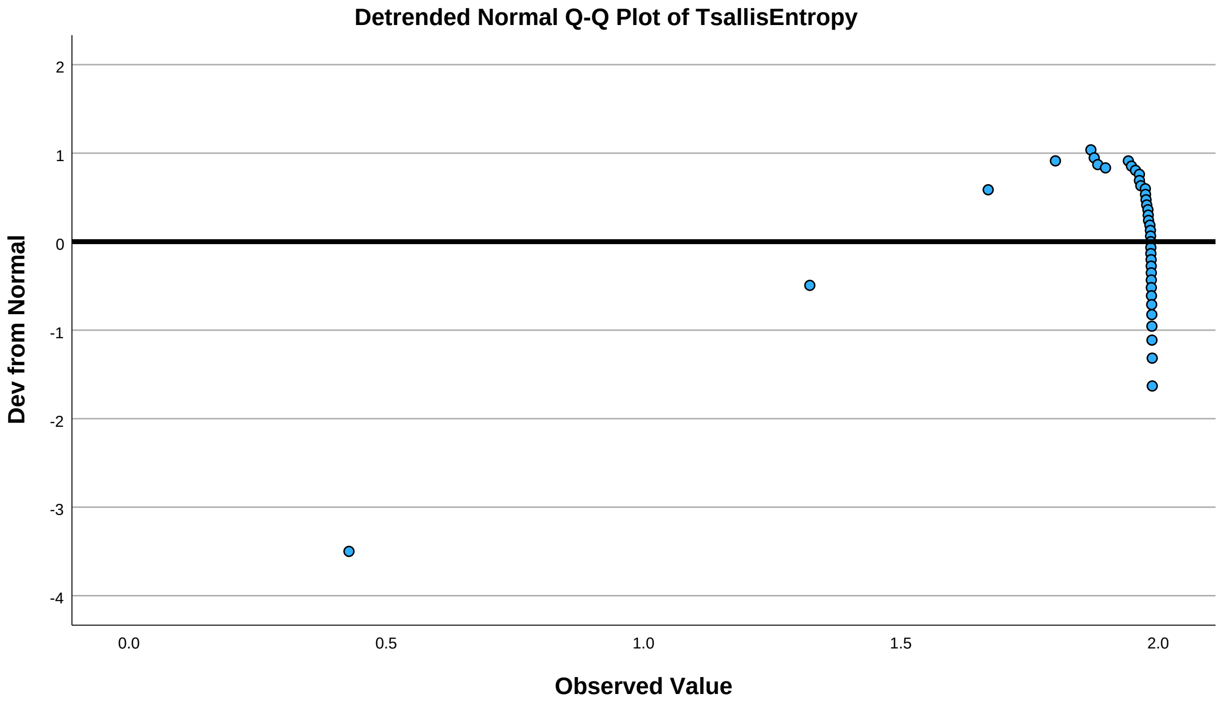
Detrended Normal Q-Q Plot of TsallisEntropy
Dev from Normal
2
1
0
-1
-2
-3
-4
0.0
0.5
1.0
1.5
2.0
Observed Value

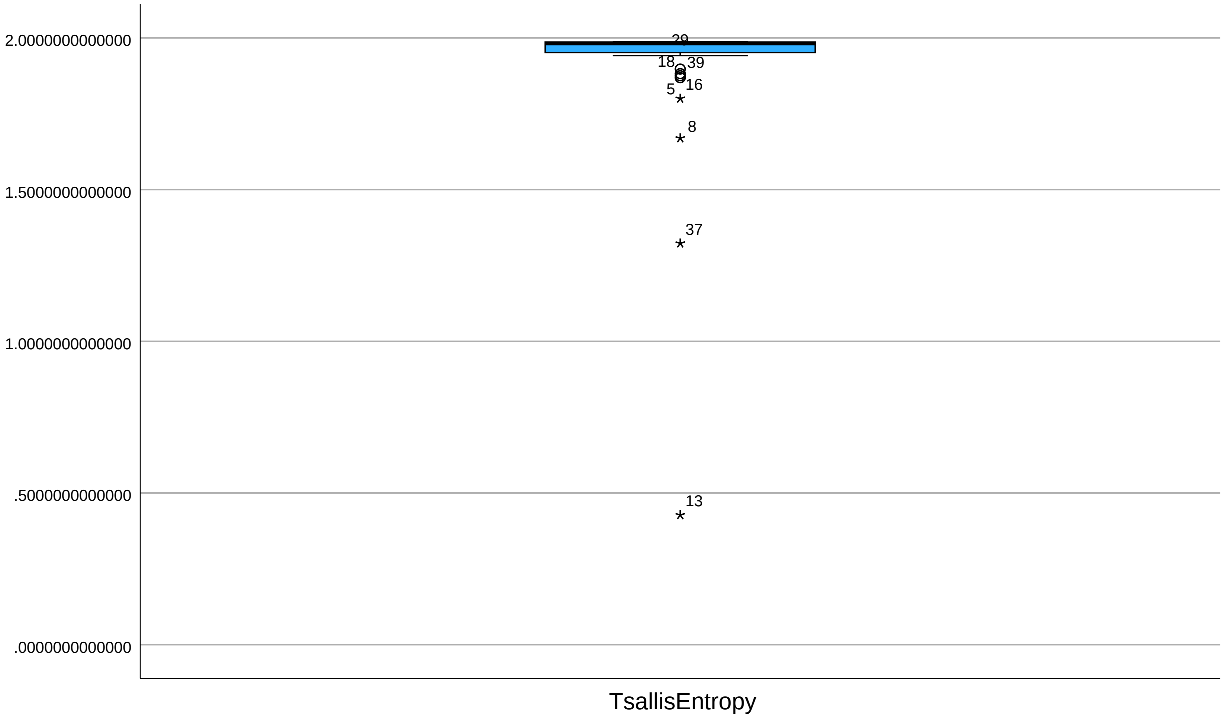
2.0000000000000
1.5000000000000
1.0000000000000
.5000000000000
.0000000000000
29
18
39
5
16
8
37
13
TsallisEntropy

PS_4_5

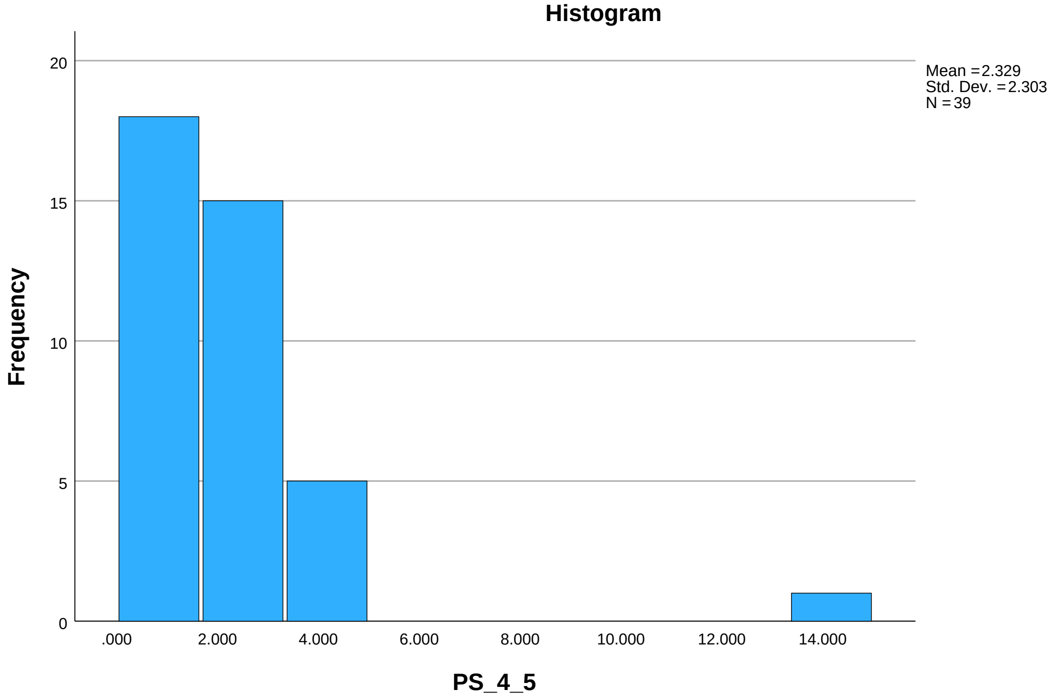

PS_4_5 Stem-and-Leaf Plot

```
 Frequency    Stem &  Leaf

    1.00        0 .  2
    4.00        0 .  5789
   11.00        1 .  00112222223
    5.00        1 .  66669
    5.00        2 .  00124
    6.00        2 .  556679
    1.00        3 .  1
    1.00        3 .  9
    4.00        4 .  0033
    1.00 Extremes    (>=14.7)

 Stem width:      1.000
 Each leaf:       1 case(s)
```

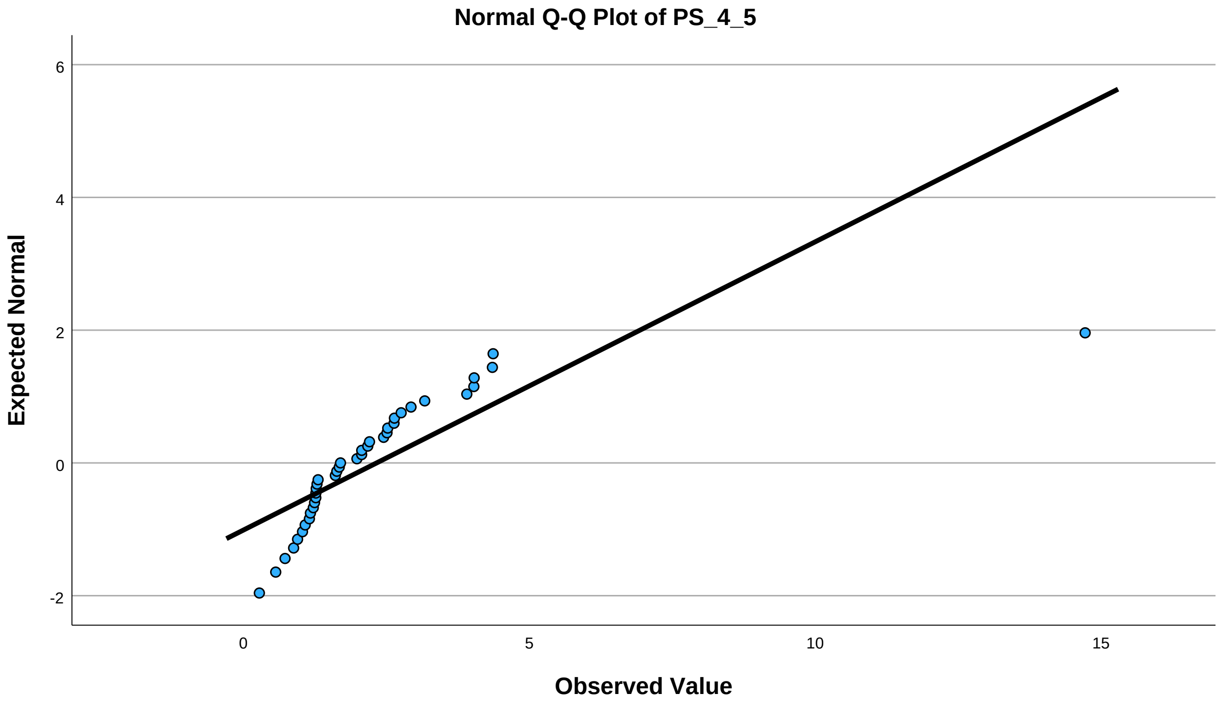

Normal Q-Q Plot of PS_4_5
Expected Normal
Observed Value
6
4
2
0
-2
0
5
10
15

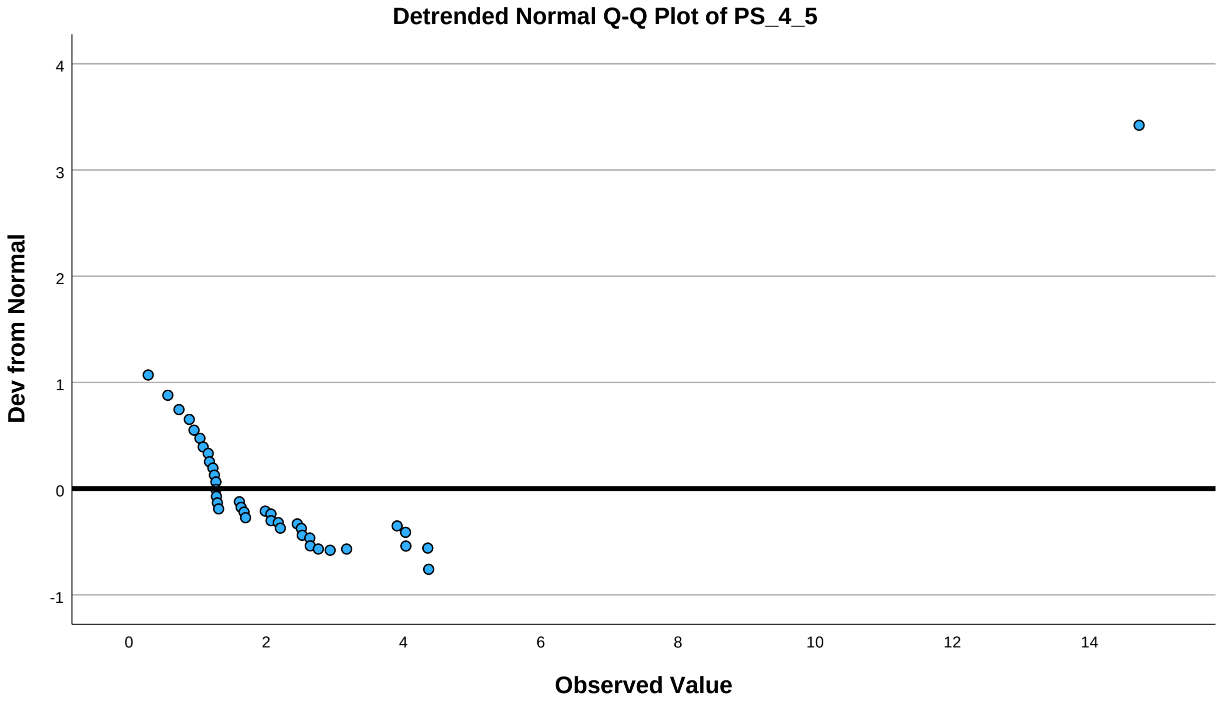

Detrended Normal Q-Q Plot of PS_4_5
Dev from Normal
Observed Value
4
3
2
1
0
-1
0
2
4
6
8
10
12
14

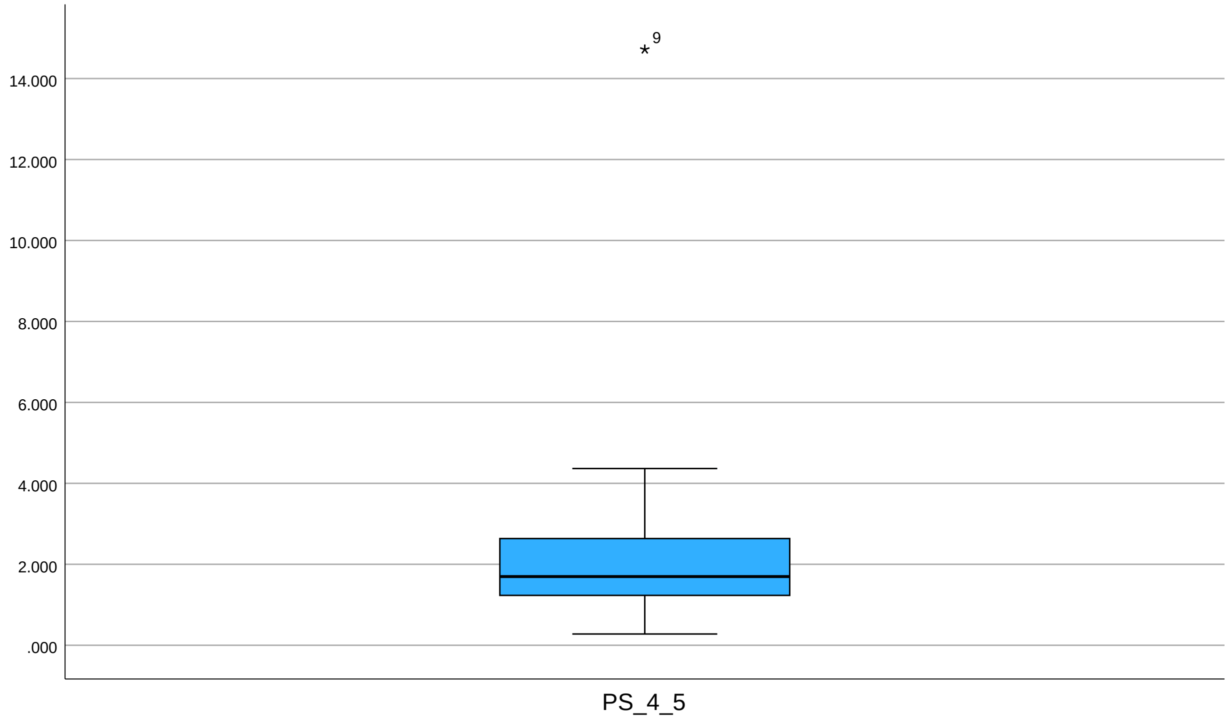

PS_5_6

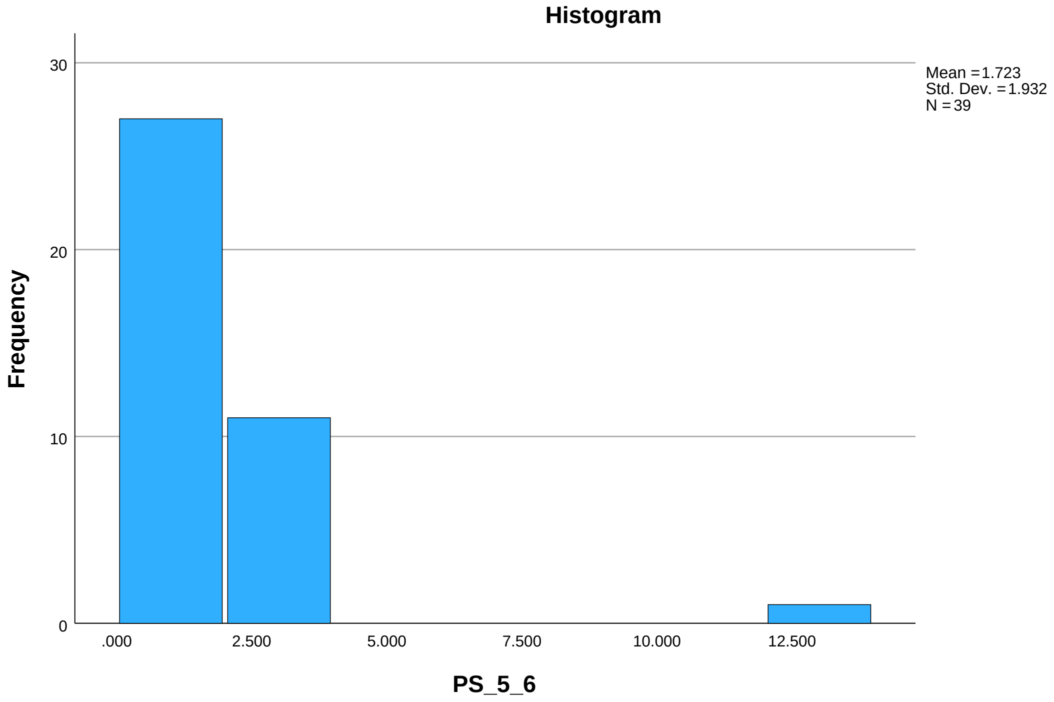

PS_5_6 Stem-and-Leaf Plot

Frequency Stem & Leaf

```
    2.00        0 .  23
    14.00          0 .  56778888899999
     7.00         1 .  0111224
     4.00        1 .  6899
     9.00         2 .  011122344
    1.00       2 .  9
    1.00       3 .  2
     1.00 Extremes     (>=12.6)

Stem width:      1.000
Each leaf:        1 case(s)
```

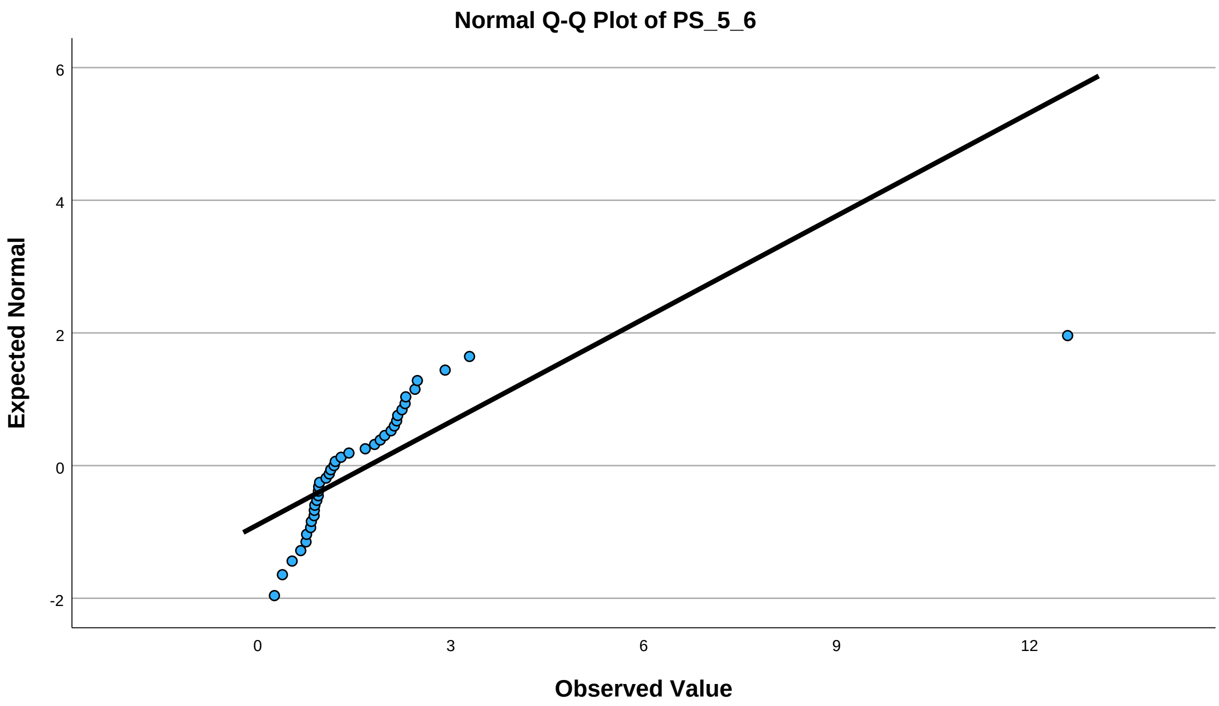

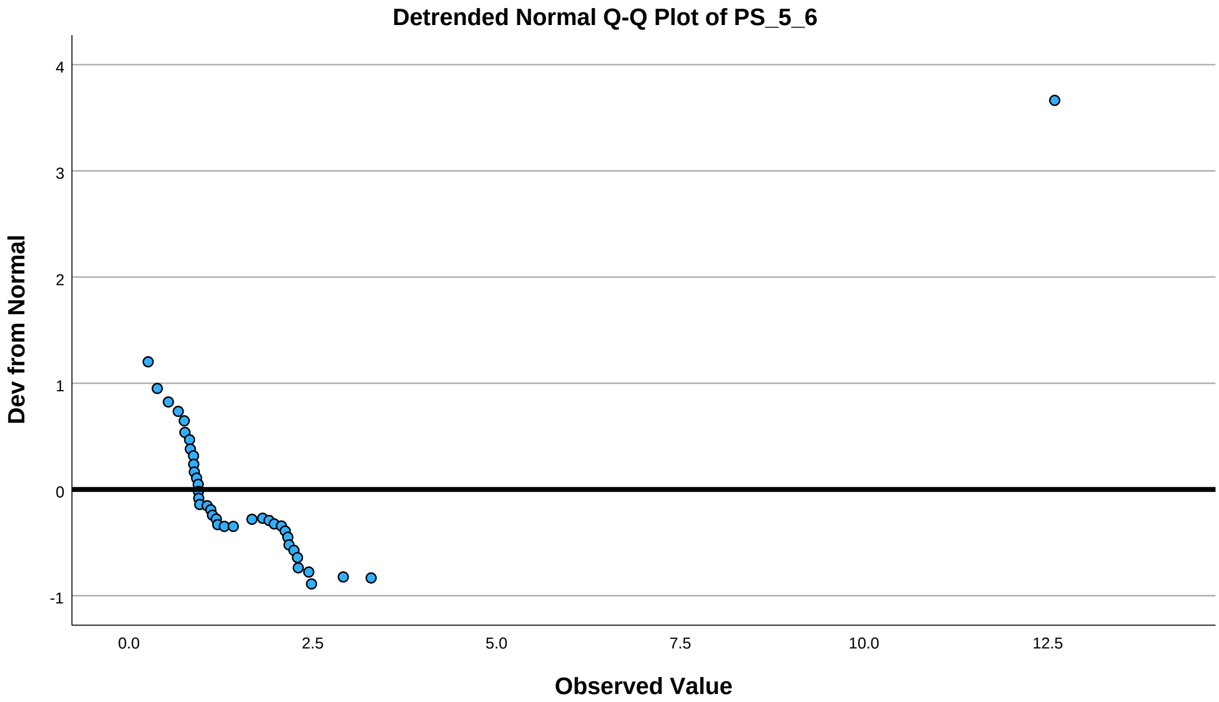

Detrended Normal Q-Q Plot of PS_5_6
Dev from Normal
4
3
2
1
0
-1
0.0
2.5
5.0
7.5
10.0
12.5
Observed Value

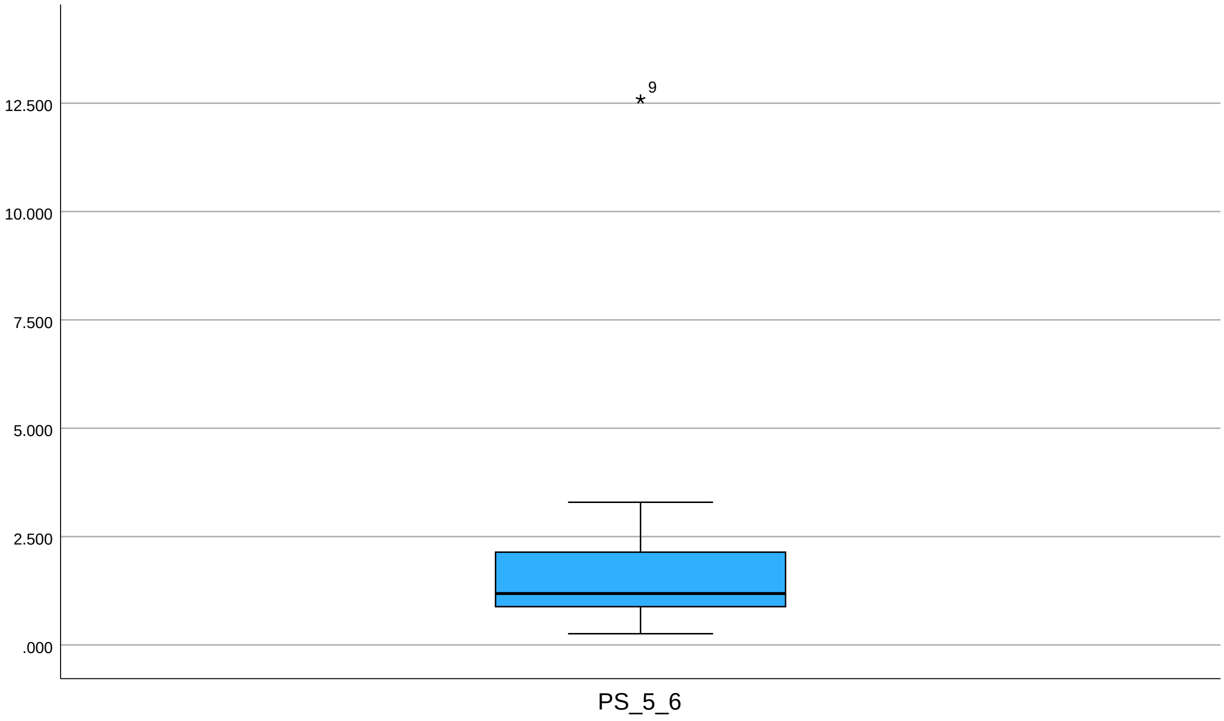

12.500
10.000
7.500
5.000
2.500
.000
9
PS_5_6

PS_6_7

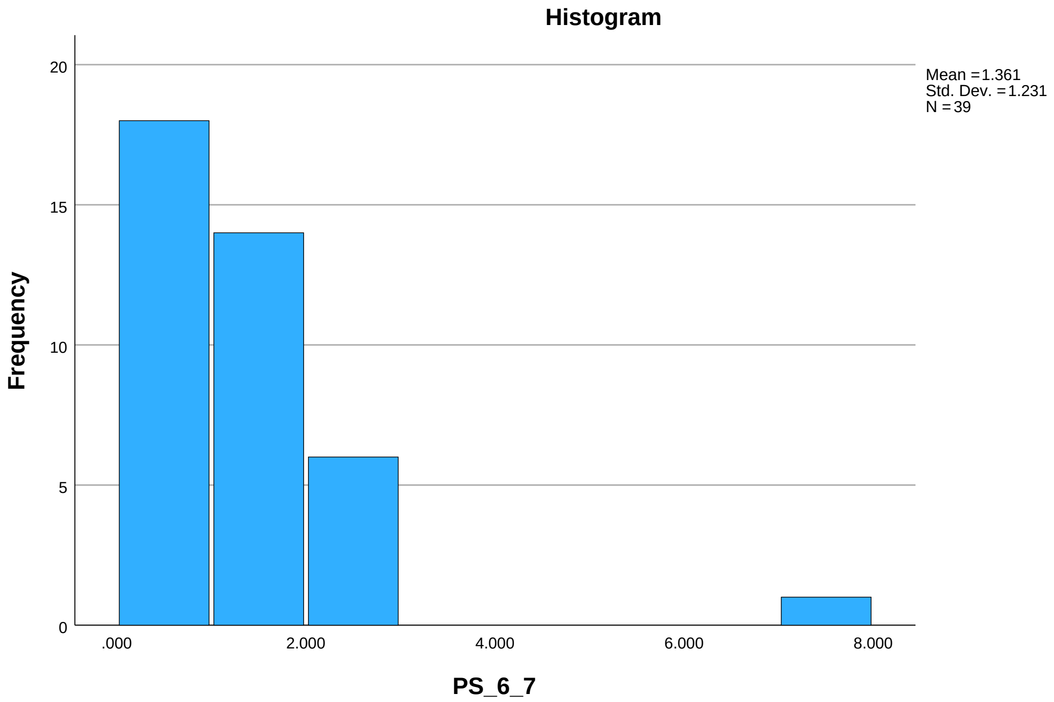


```
PS_6_7 Stem-and-Leaf Plot

 Frequency    Stem &  Leaf

     4.00        0 .  2234
    14.00        0 .  56667777788999
     8.00        1 .  00111334
     6.00        1 .  555669
     4.00        2 .  0003
     2.00        2 .  55
     1.00 Extremes    (>=7.8)

 Stem width:      1.000
 Each leaf:       1 case(s)
```

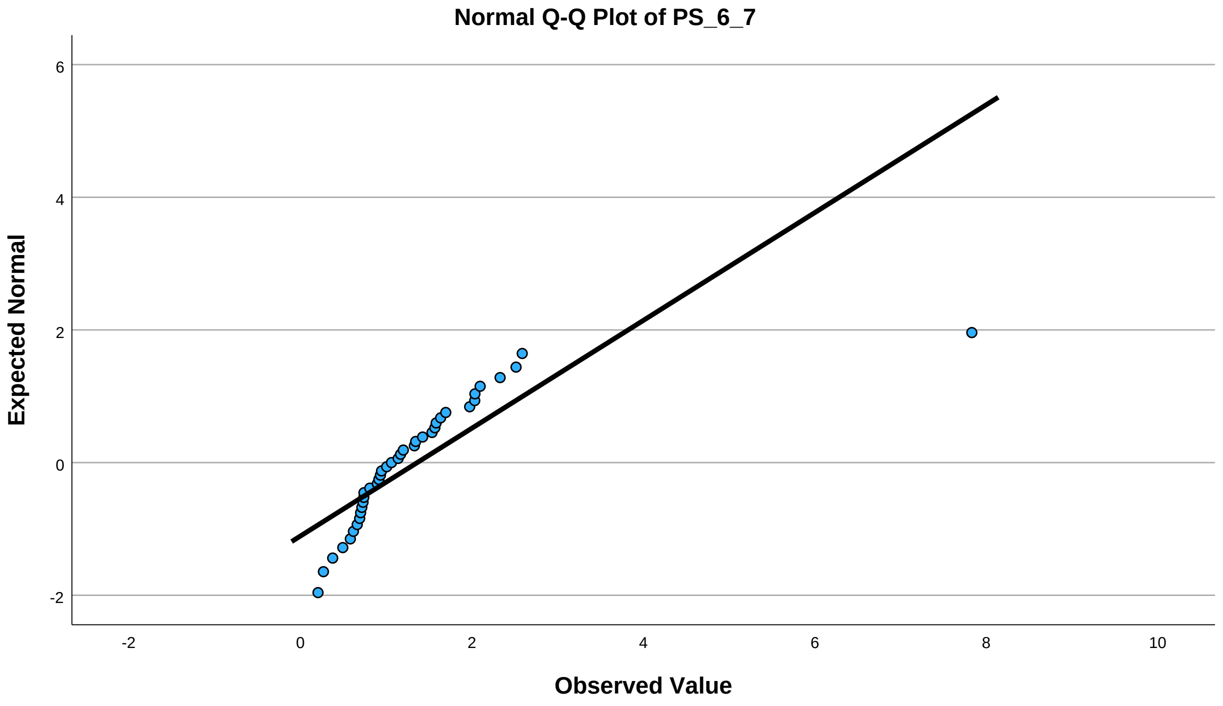
Normal Q-Q Plot of PS_6_7
Expected Normal
Observed Value
6
4
2
0
-2
-2
0
2
4
6
8
10

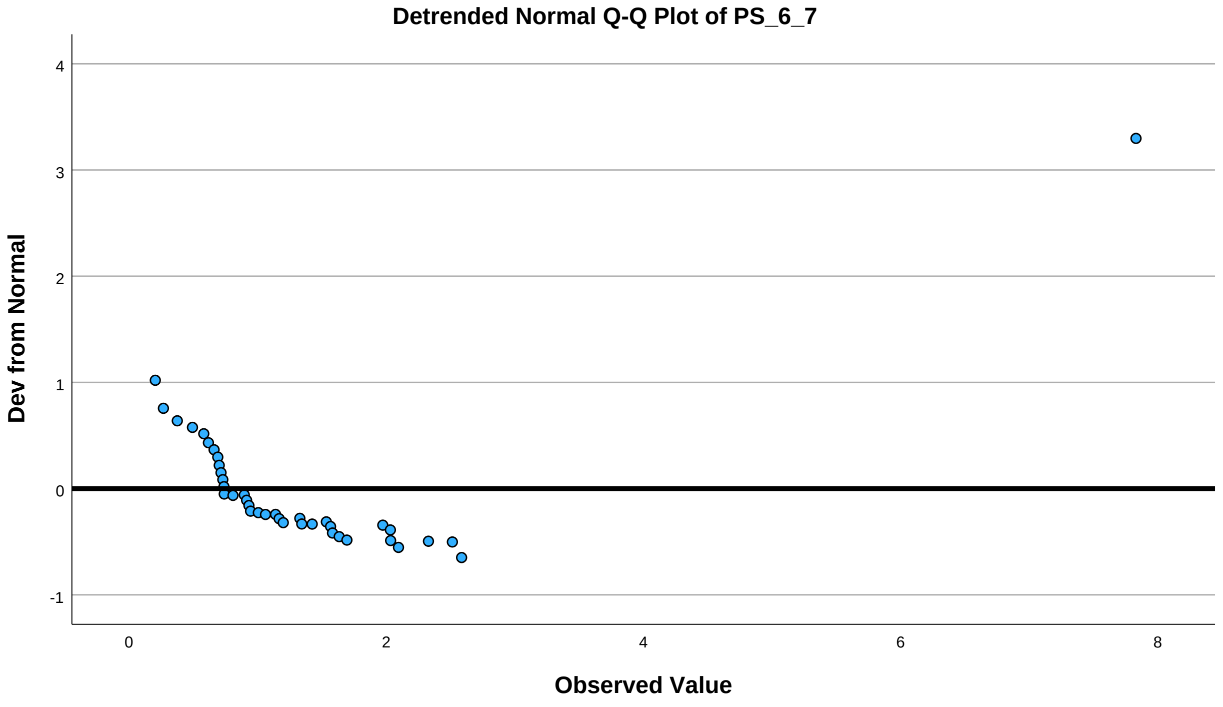
Detrended Normal Q-Q Plot of PS_6_7
Dev from Normal
Observed Value
4
3
2
1
0
-1
0
2
4
6
8

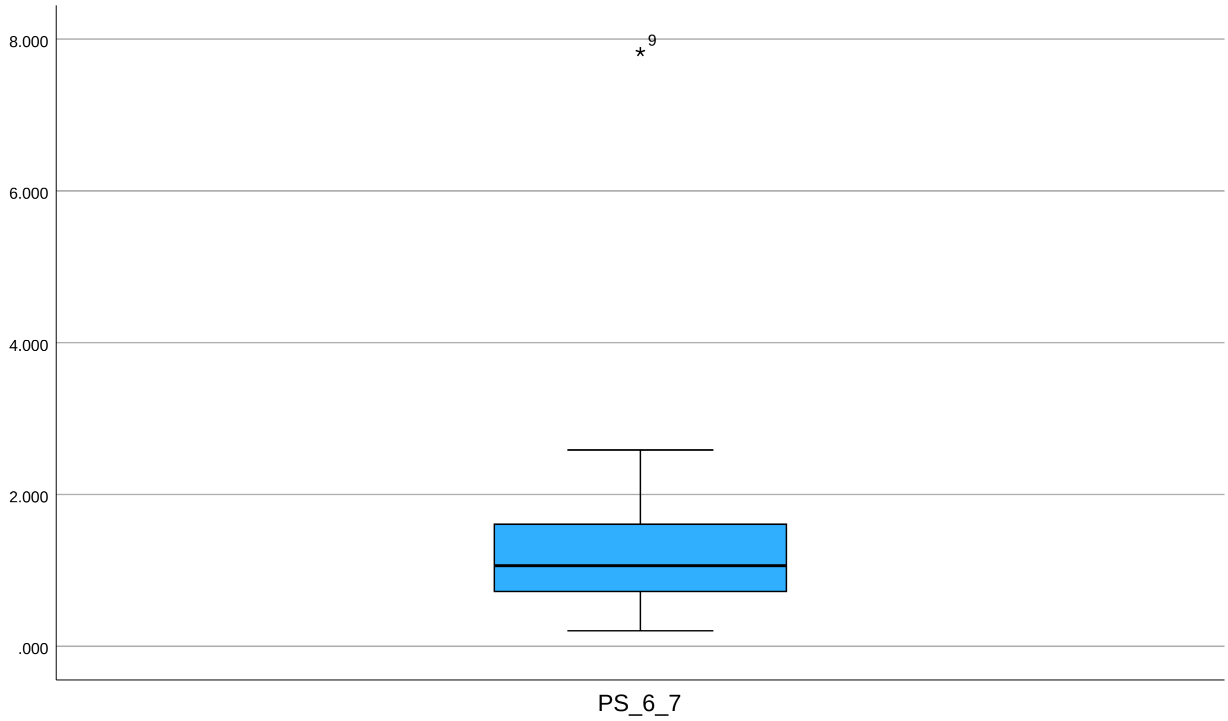

PS_7_8

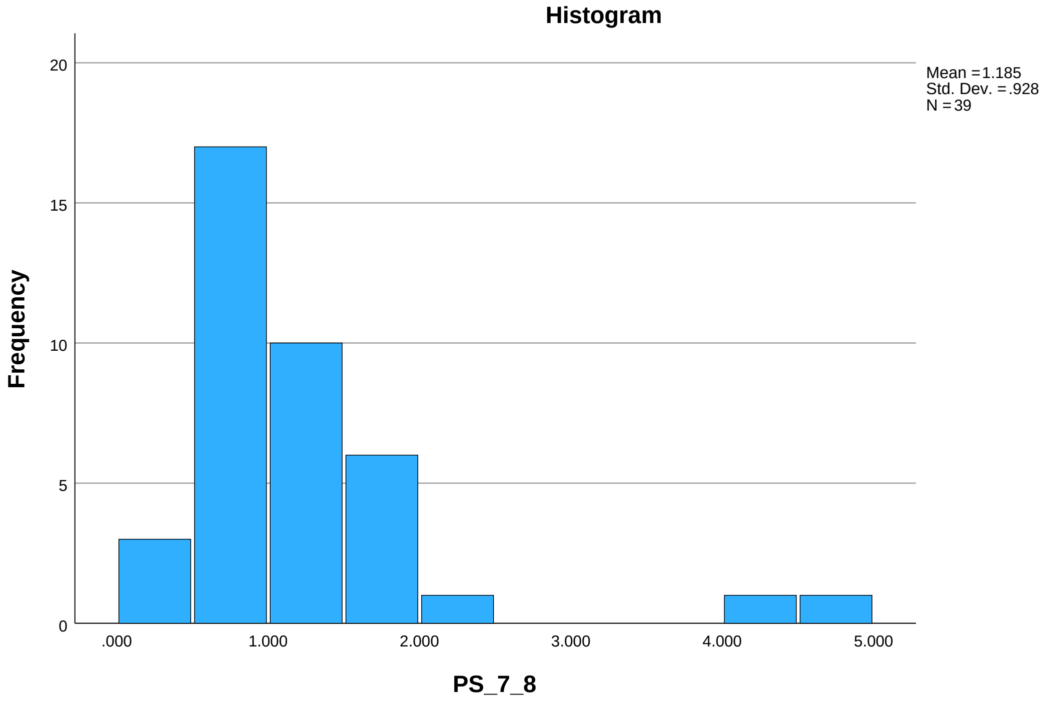


```
PS_7_8 Stem-and-Leaf Plot

 Frequency    Stem &  Leaf
```

```
    3.00        0 .  223
   17.00        0 .  55556666666777899
   10.00        1 .  0012223344
    6.00        1 .  556678
    1.00        2 .  2
    2.00 Extremes    (>=4.2)

Stem width:      1.000
Each leaf:        1 case(s)
```

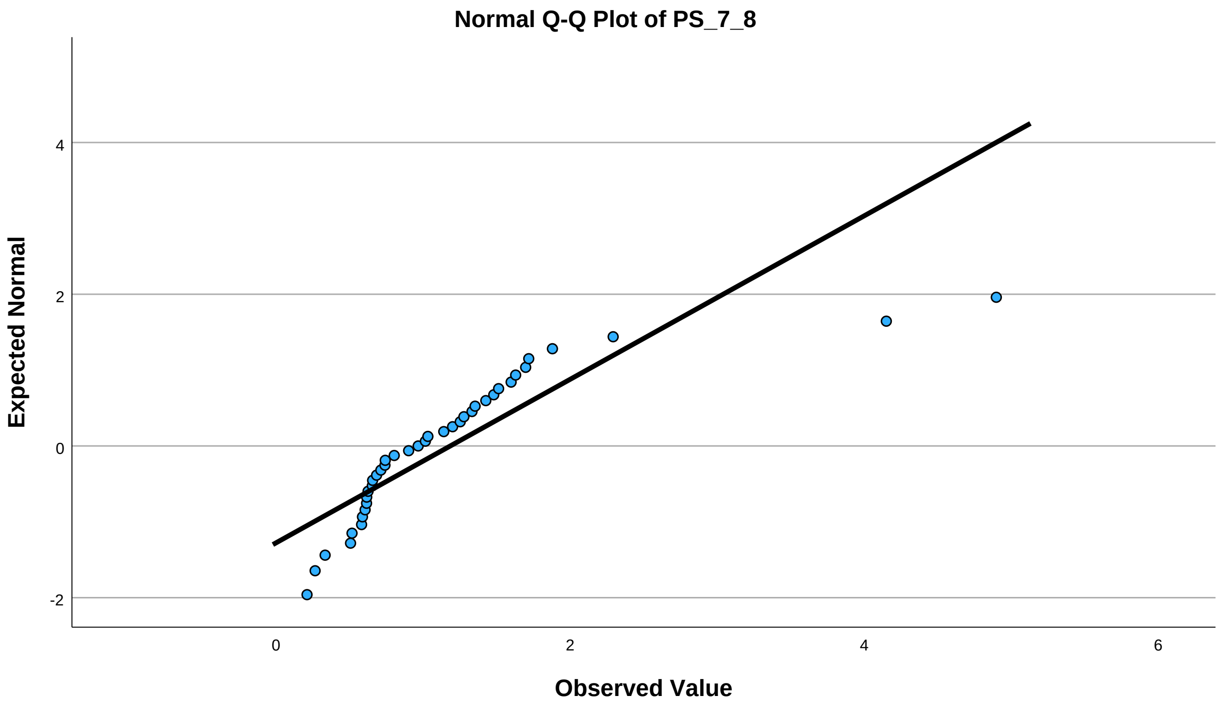

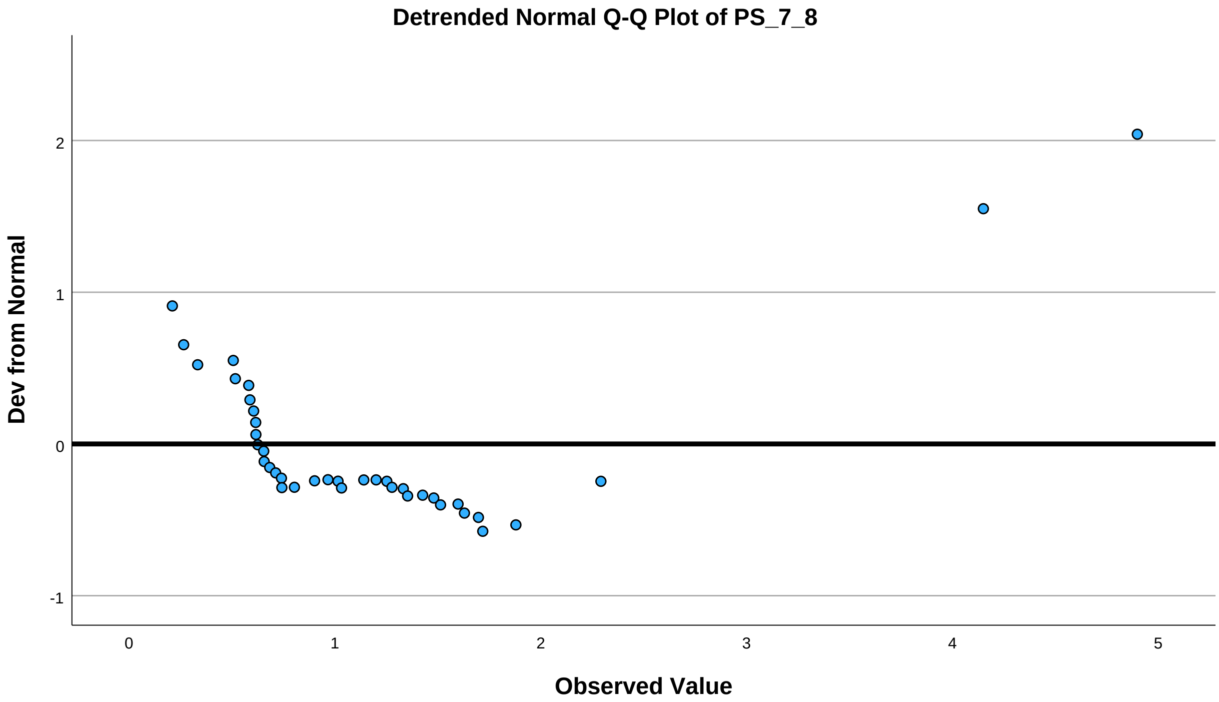
Detrended Normal Q-Q Plot of PS_7_8
Dev from Normal
Observed Value
2
1
0
-1
0
1
2
3
4
5

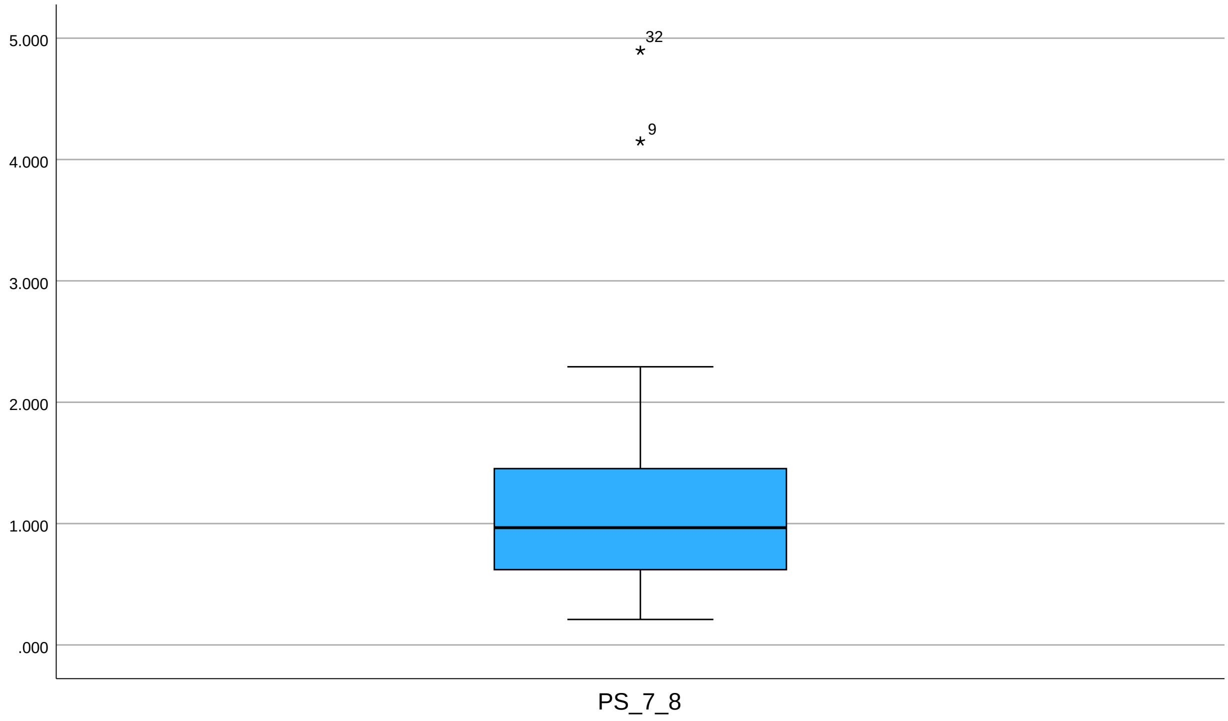
5.000
4.000
3.000
2.000
1.000
.000
32
9
PS_7_8

PS_8_9

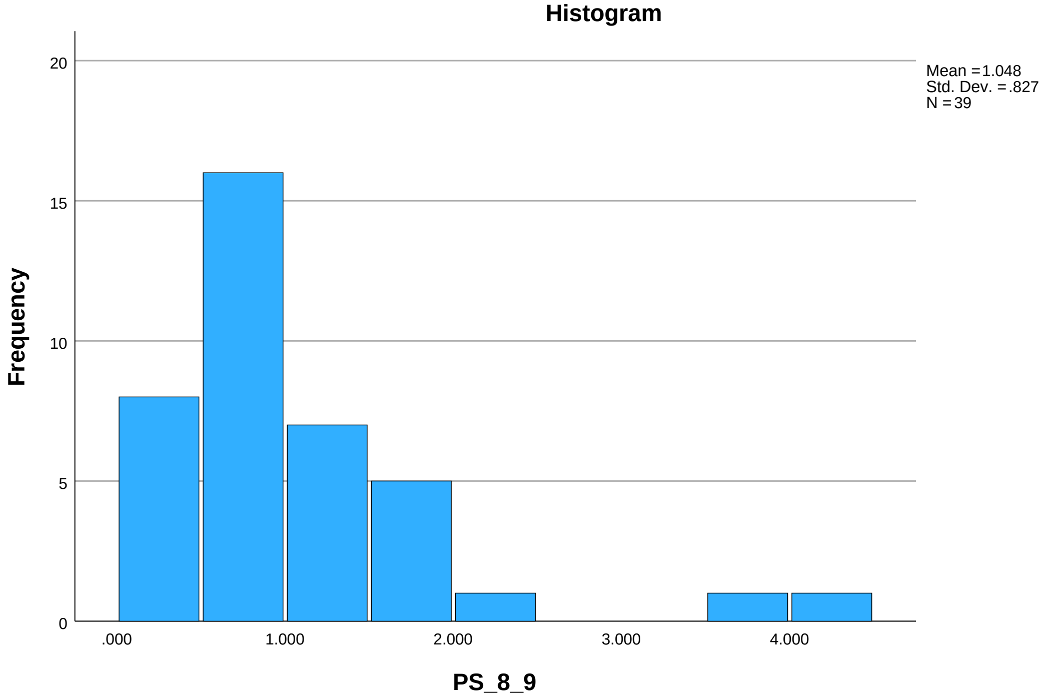


```
PS_8_9 Stem-and-Leaf Plot

 Frequency    Stem &  Leaf

     8.00        0 .  12244444
    16.00        0 .  5555556666888899
     7.00        1 .  0112334
     5.00        1 .  55578
     1.00        2 .  1
     2.00 Extremes    (>=3.6)

 Stem width:      1.000
 Each leaf:       1 case(s)
```

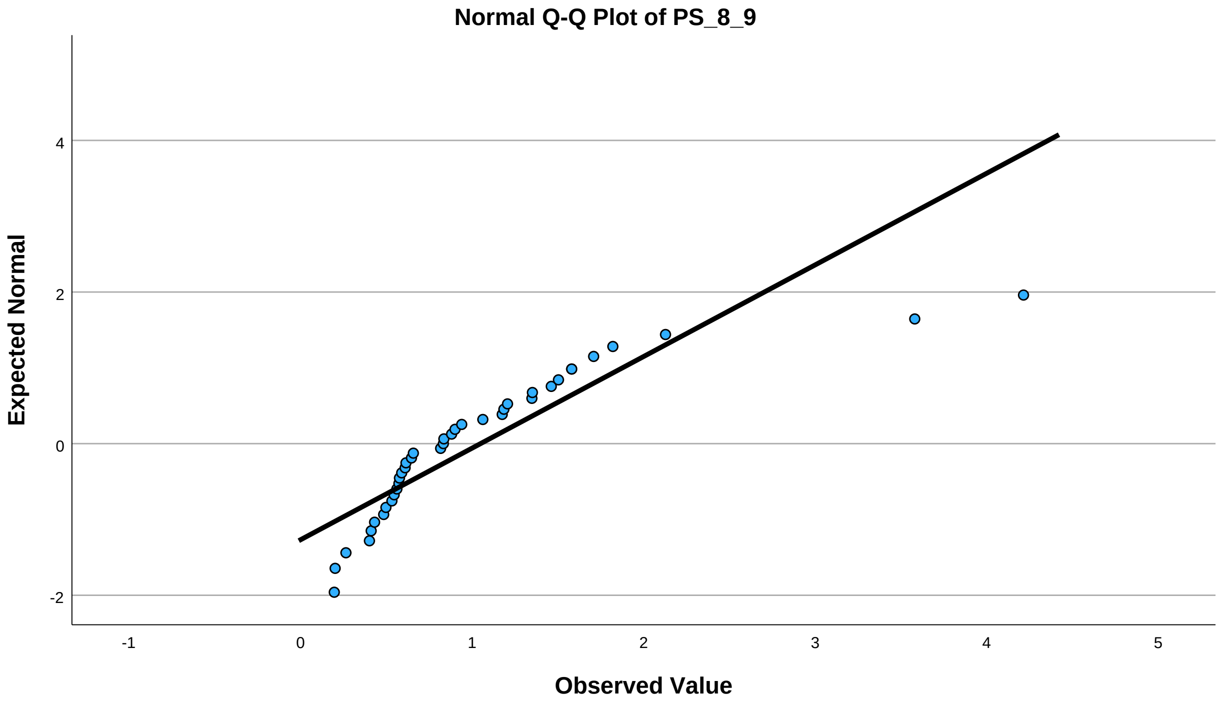
Normal Q-Q Plot of PS_8_9
Expected Normal
Observed Value
4
2
0
-2
-1
0
1
2
3
4
5

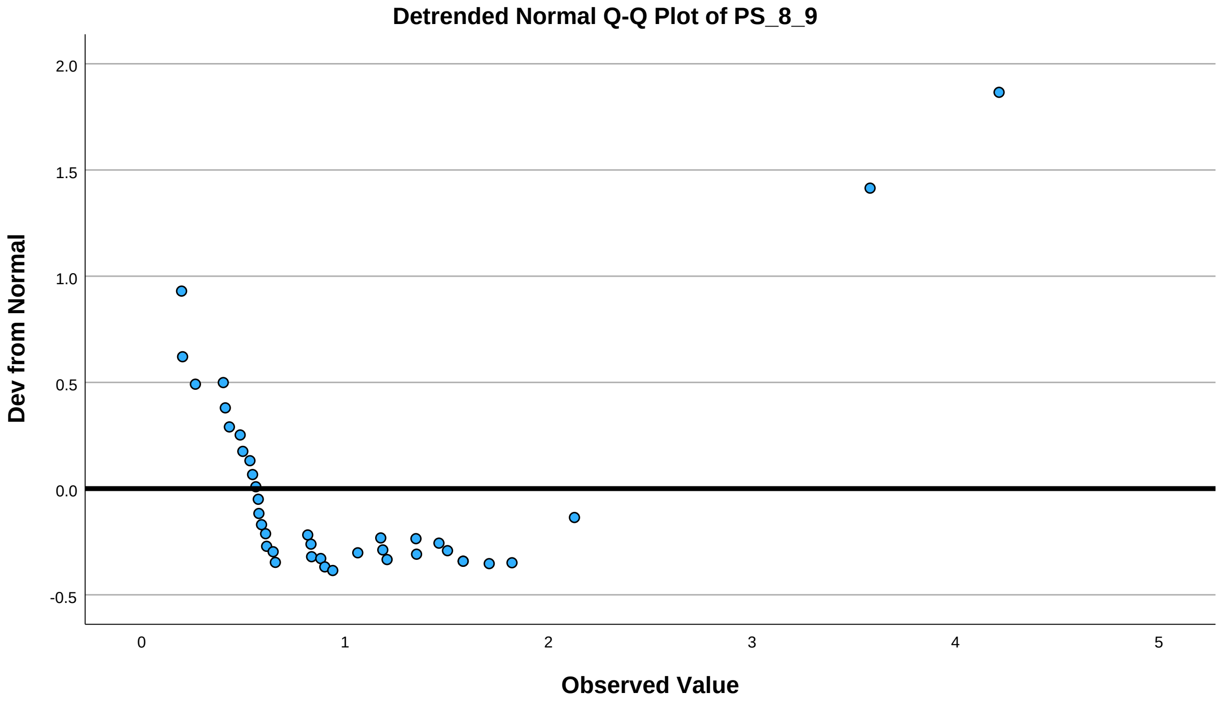
Detrended Normal Q-Q Plot of PS_8_9
Dev from Normal
Observed Value
2.0
1.5
1.0
0.5
0.0
-0.5
0
1
2
3
4
5

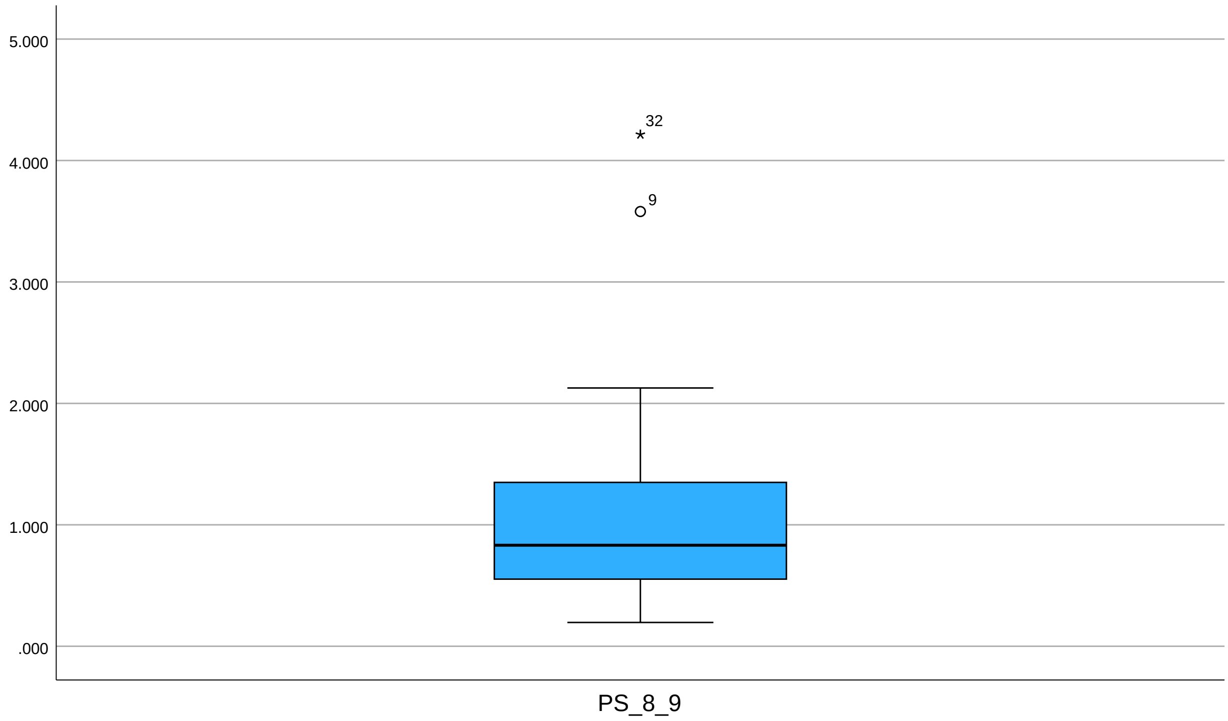

PS_9_10

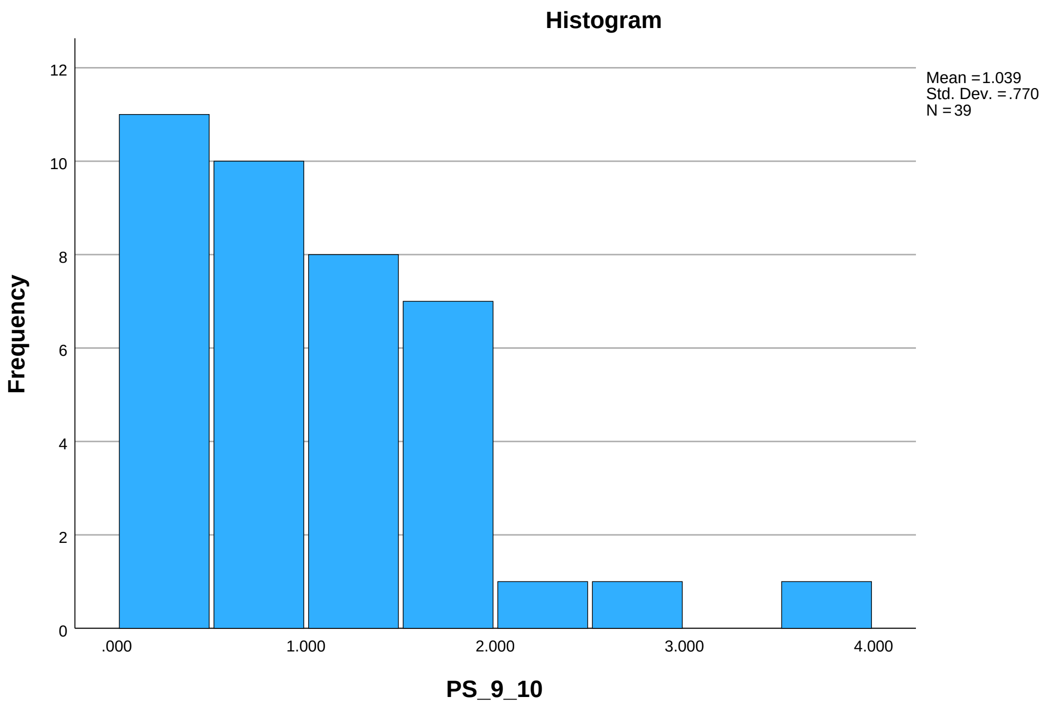

PS_9_10 Stem-and-Leaf Plot

Frequency   Stem & Leaf

```
   11.00        0 .  12233334444
   10.00        0 .  5555668899
    8.00        1 .  00000011
    7.00        1 .  5555888
   1.00     2 . 4
    2.00 Extremes    (>=2.8)

Stem width:      1.000
Each leaf:        1 case(s)
```

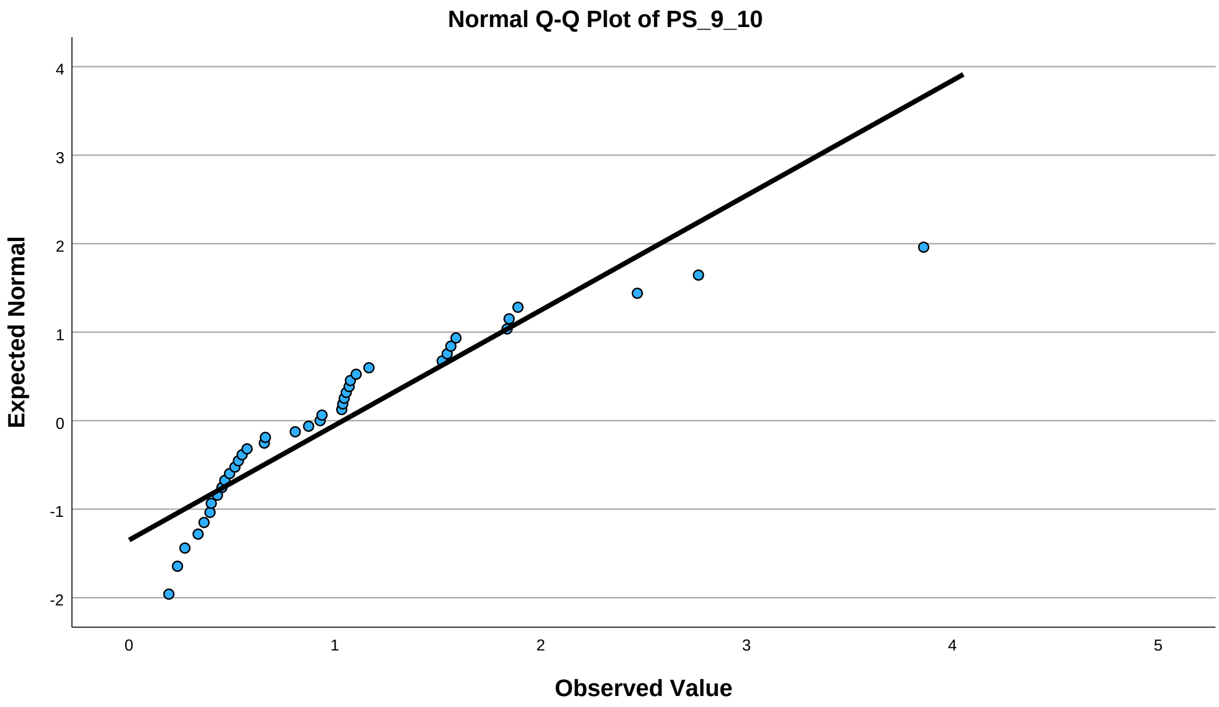

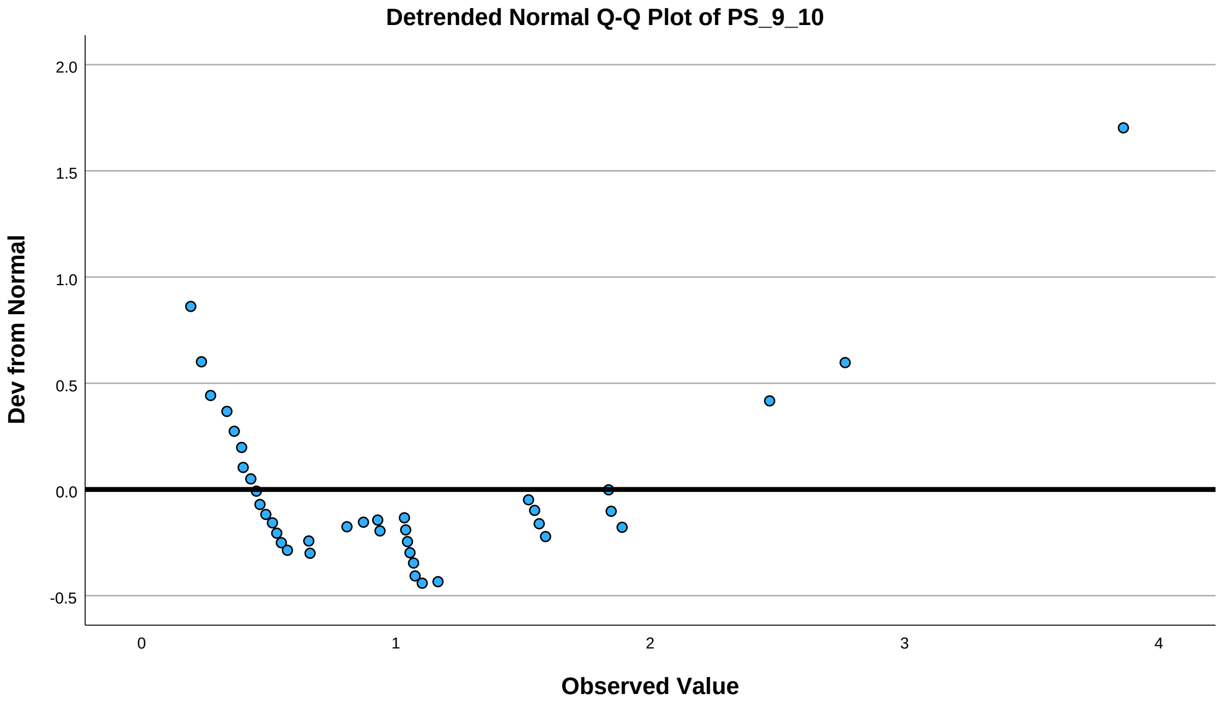

Detrended Normal Q-Q Plot of PS_9_10
Dev from Normal
2.0
1.5
1.0
0.5
0.0
-0.5
0
1
2
3
4
Observed Value

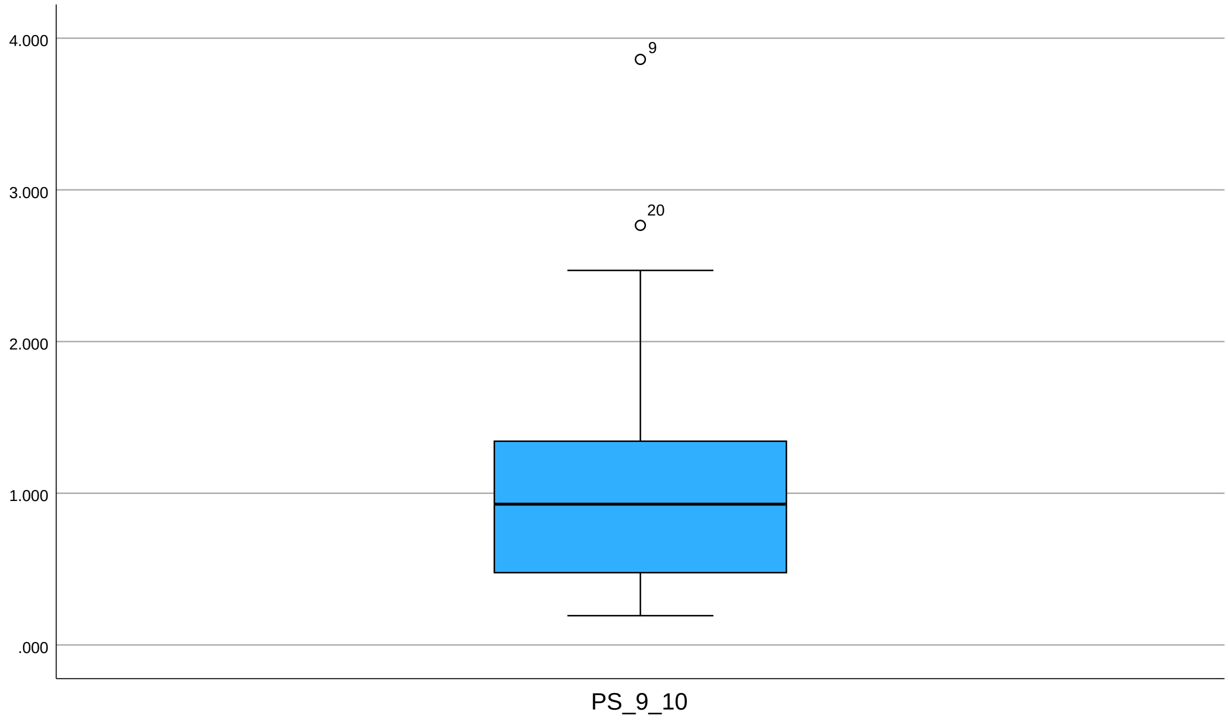

4.000
3.000
2.000
1.000
.000
9
20
PS_9_10

PS_10_11

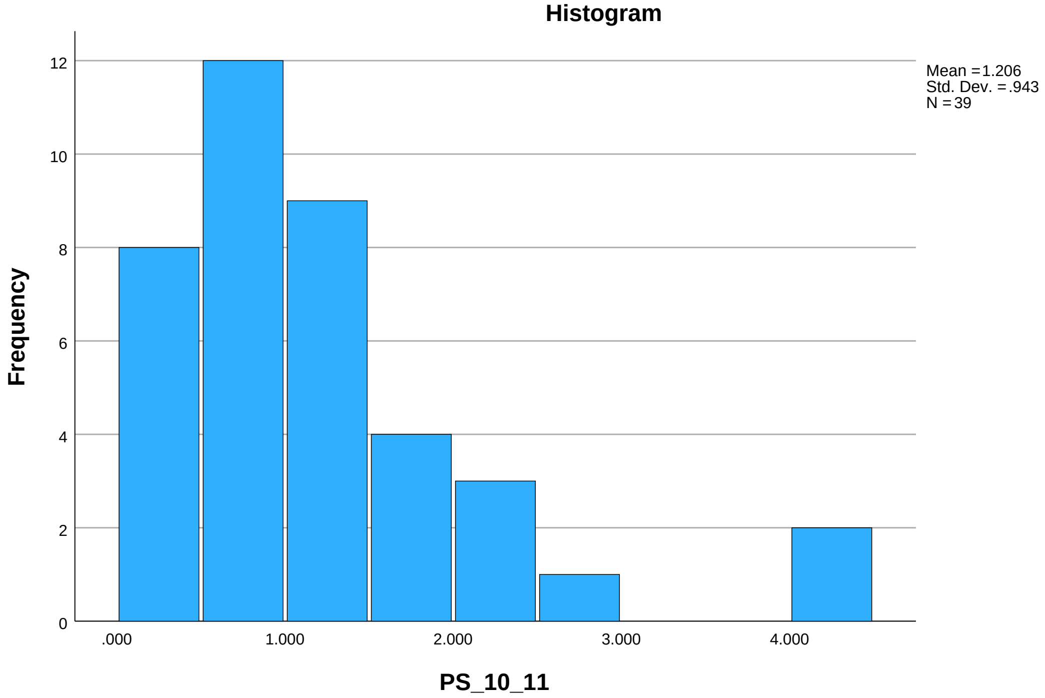


```
PS_10_11 Stem-and-Leaf Plot

 Frequency    Stem &  Leaf

     8.00        0 .  22233444
    12.00        0 .  555666778899
     9.00        1 .  000123444
     4.00        1 .  5577
     3.00        2 .  022
     3.00 Extremes    (>=3.0)

 Stem width:      1.000
 Each leaf:        1 case(s)
```

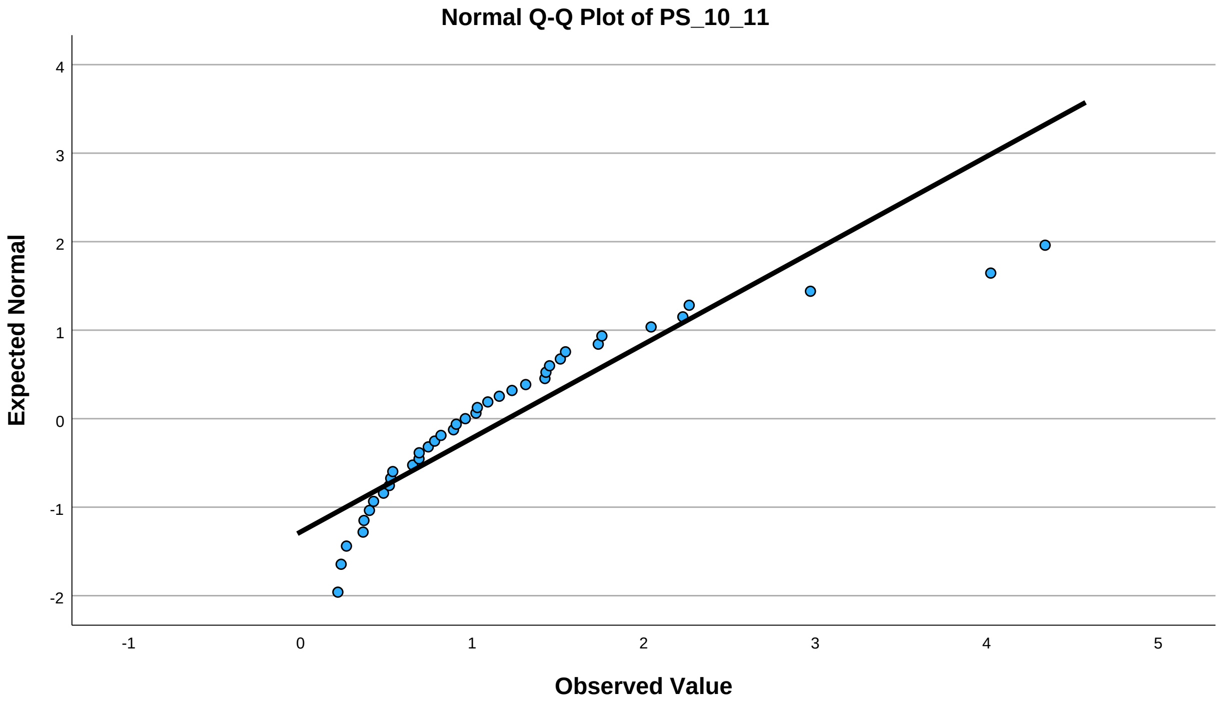
Normal Q-Q Plot of PS_10_11
Expected Normal
4
3
2
1
0
-1
-2
-1
0
1
2
3
4
5
Observed Value

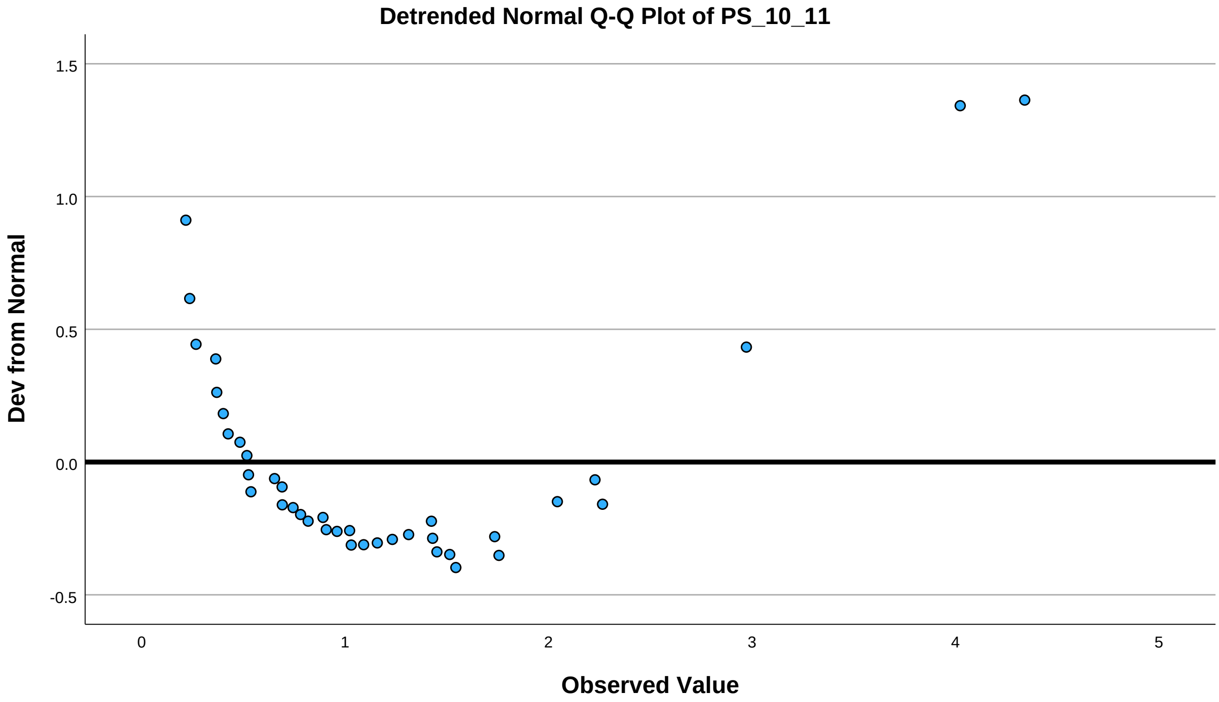
Detrended Normal Q-Q Plot of PS_10_11
Dev from Normal
1.5
1.0
0.5
0.0
-0.5
0
1
2
3
4
5
Observed Value

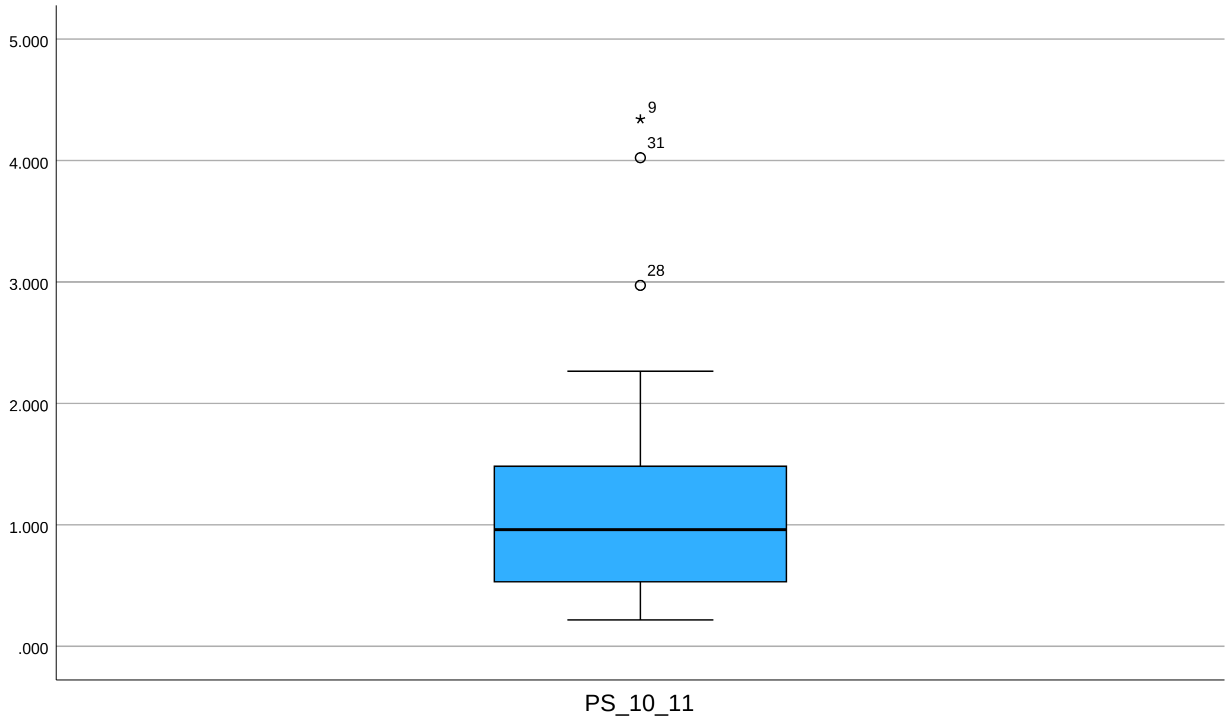

PS_11_12

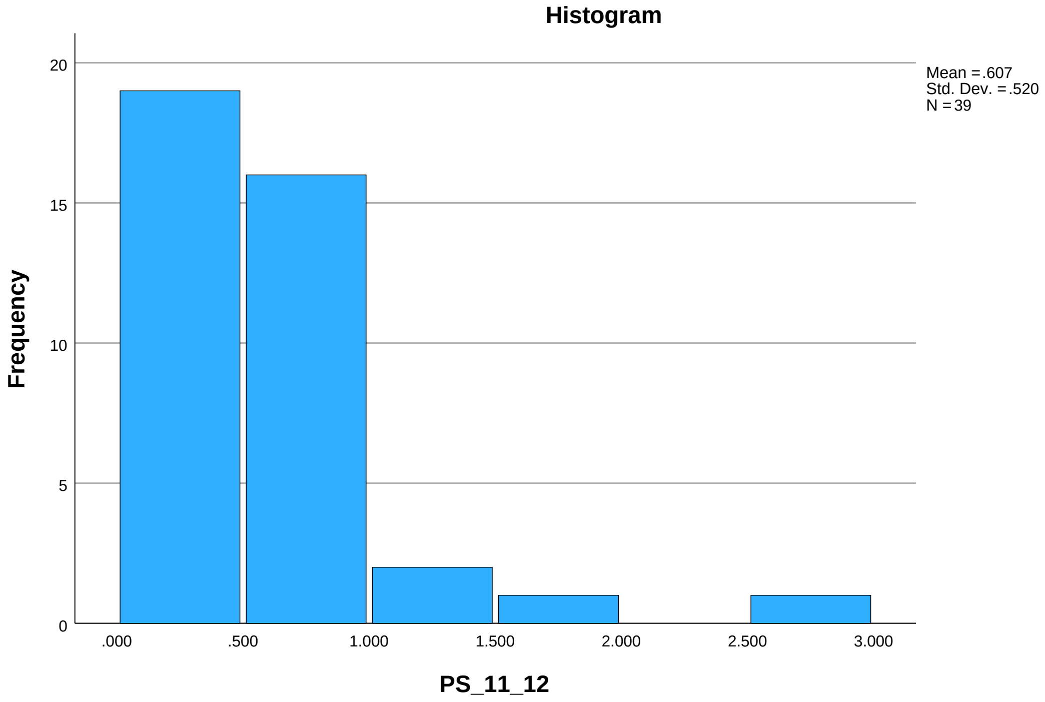


```
PS_11_12 Stem-and-Leaf Plot

 Frequency    Stem &  Leaf
```

```
  3.00      1 . 488
  9.00      2 . 124557999
  5.00      3 . 22479
  2.00      4 . 14
  5.00      5 . 00012
  3.00      6 . 346
  6.00      7 . 133368
  1.00      8 . 9
  1.00      9 . 5
   .00     10 .
  1.00     11 . 9
  3.00 Extremes    (>=1.48)

Stem width:      .100
Each leaf:       1 case(s)
```

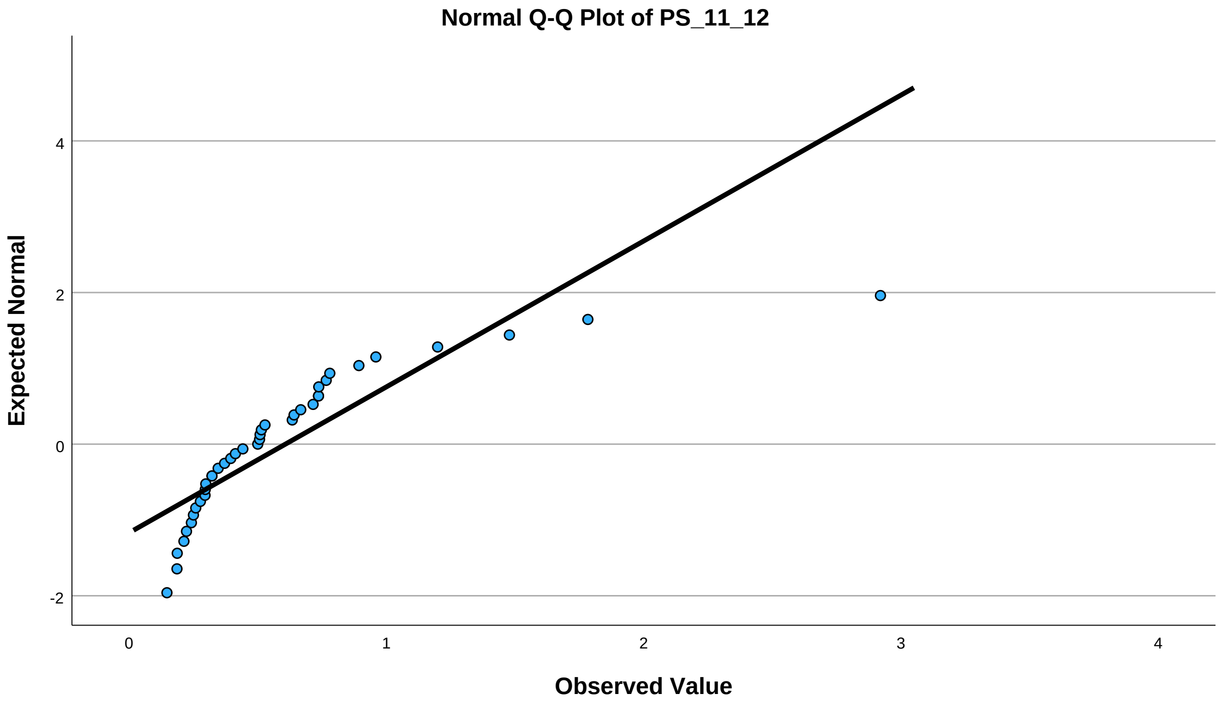

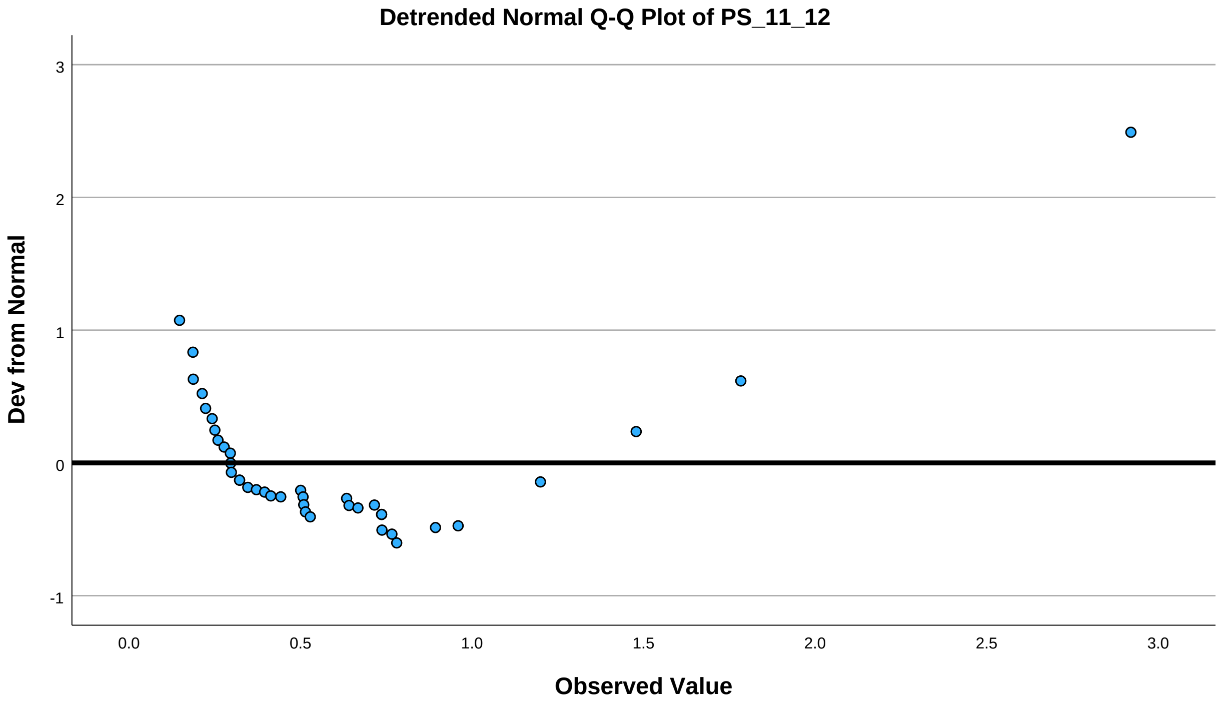
Detrended Normal Q-Q Plot of PS_11_12
Dev from Normal
3
2
1
0
-1
0.0
0.5
1.0
1.5
2.0
2.5
3.0
Observed Value

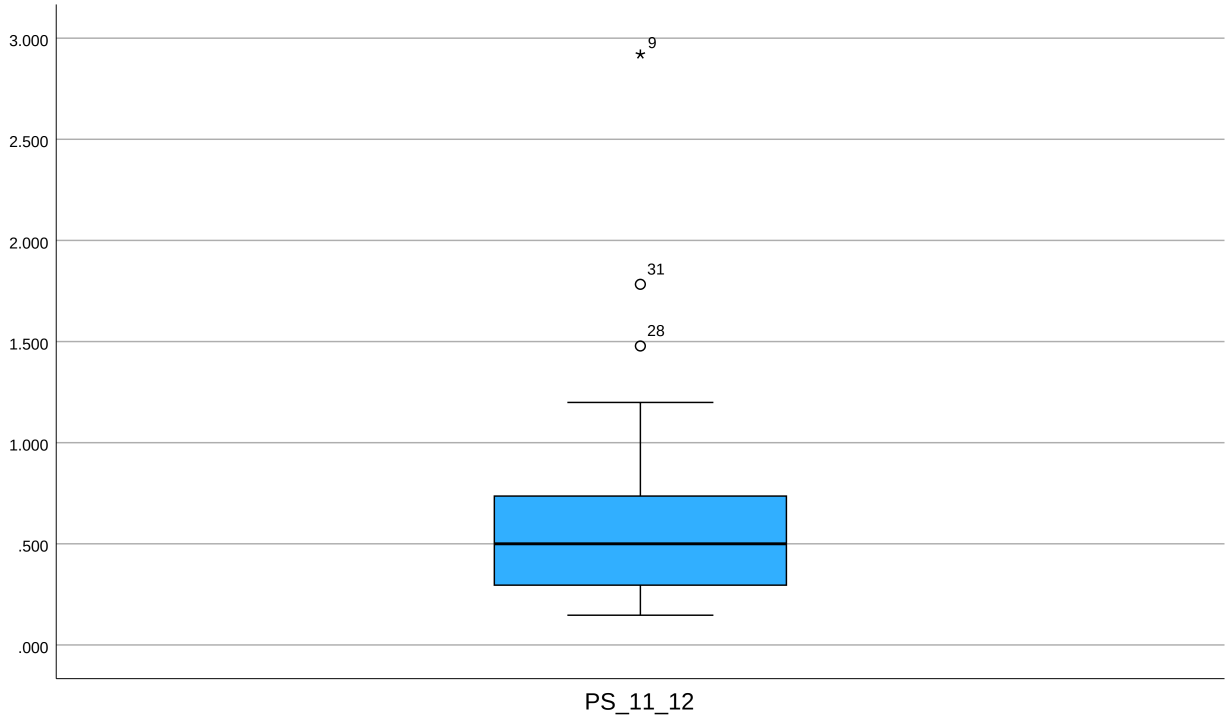
3.000
2.500
2.000
1.500
1.000
.500
.000
9
31
28
PS_11_12

PS_12_13

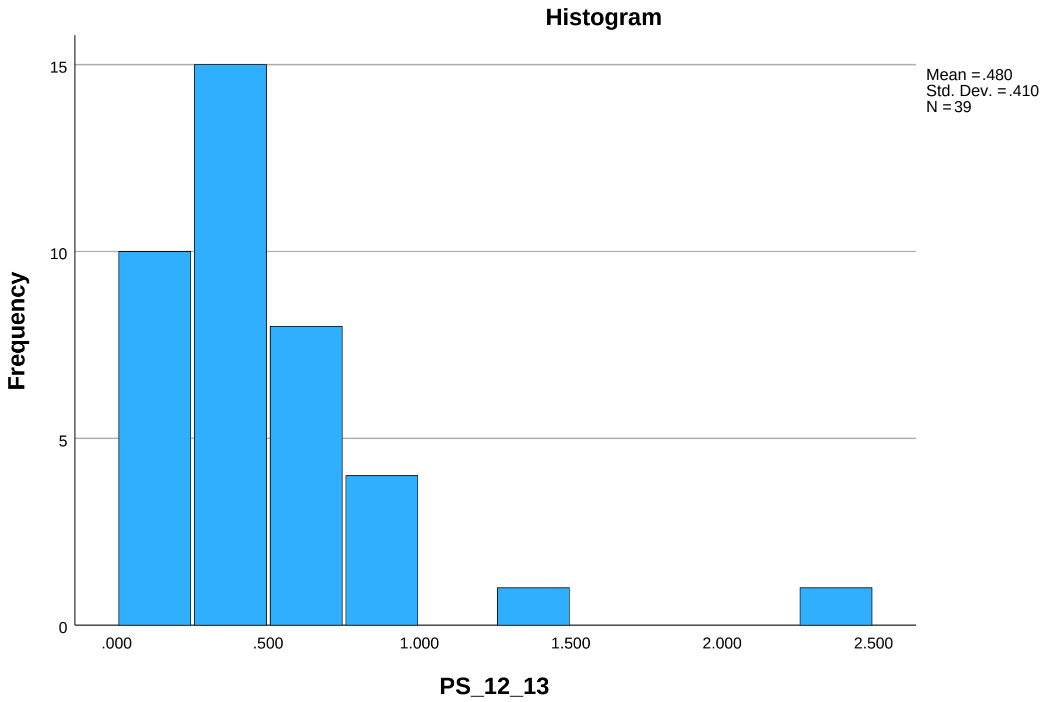


```
PS_12_13 Stem-and-Leaf Plot

 Frequency    Stem &  Leaf

     1.00        0 .  9
     5.00        1 .  23679
     9.00        2 .  223467799
     6.00        3 .  001157
     4.00        4 .  1156
     5.00        5 .  35566
     3.00        6 .  566
     2.00        7 .  89
     2.00        8 .  29
     2.00 Extremes    (>=1.29)

 Stem width:      .100
 Each leaf:       1 case(s)
```

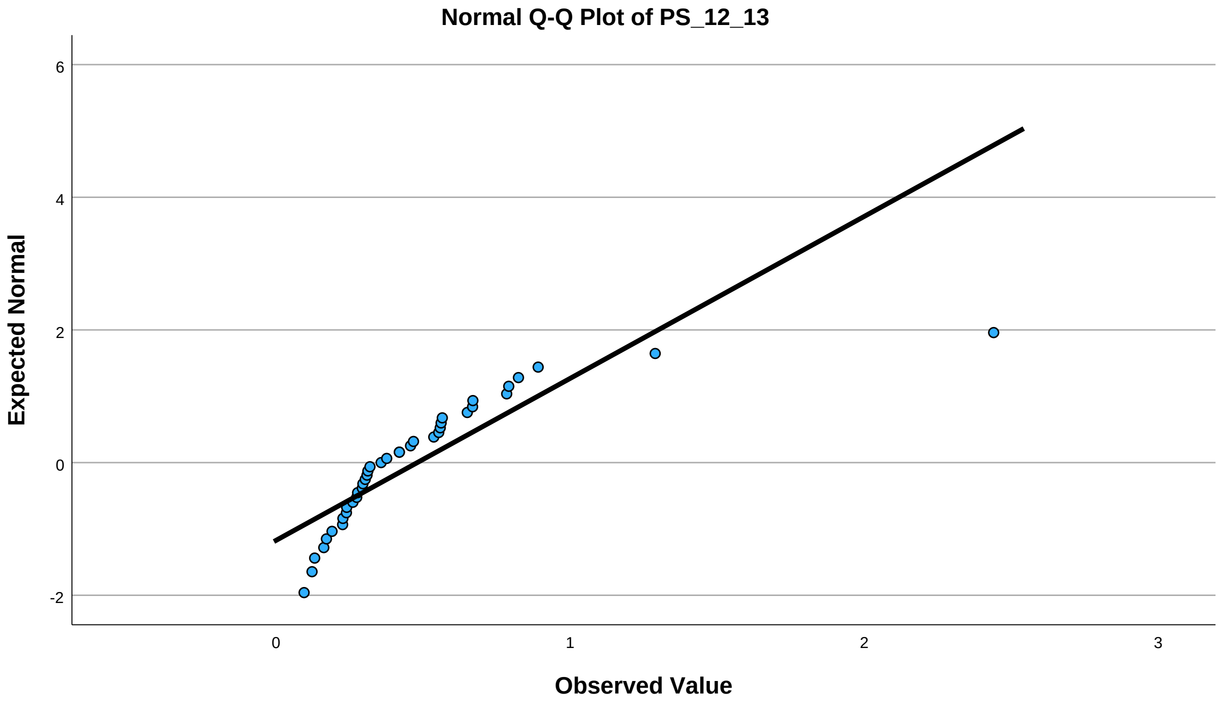

Normal Q-Q Plot of PS_12_13
Expected Normal
Observed Value
6
4
2
0
-2
0
1
2
3

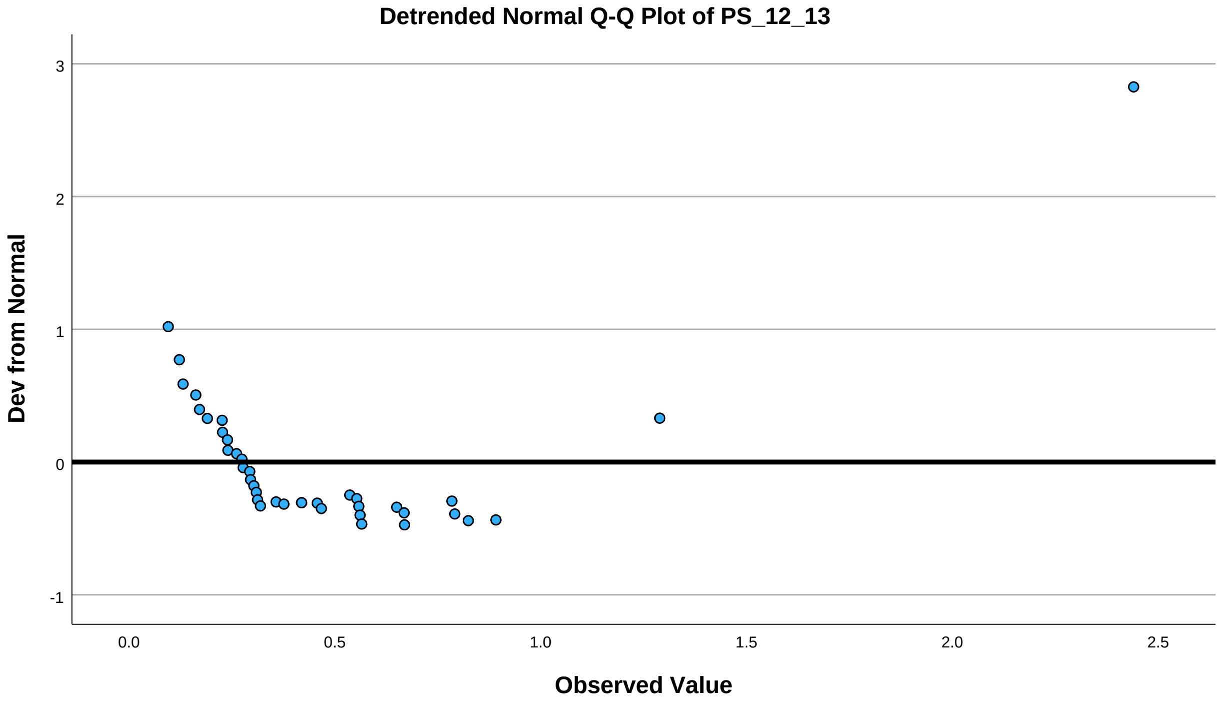

Detrended Normal Q-Q Plot of PS_12_13
Dev from Normal
Observed Value
3
2
1
0
-1
0.0
0.5
1.0
1.5
2.0
2.5

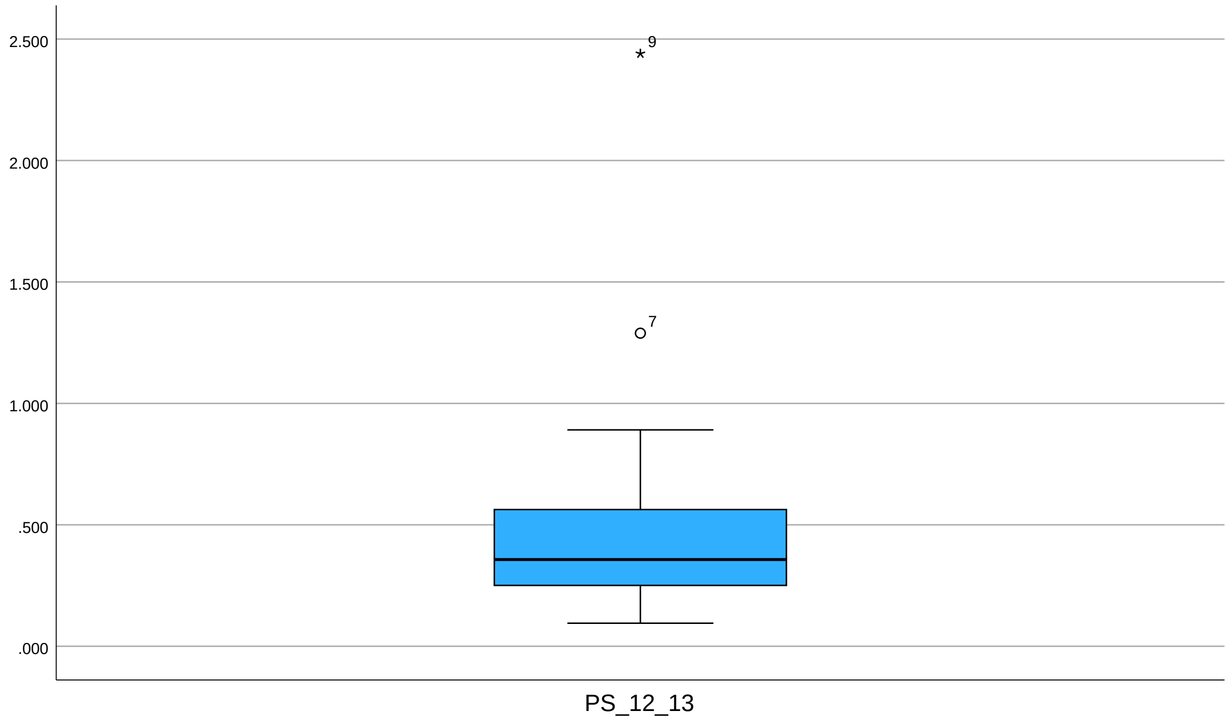

PS_13_14

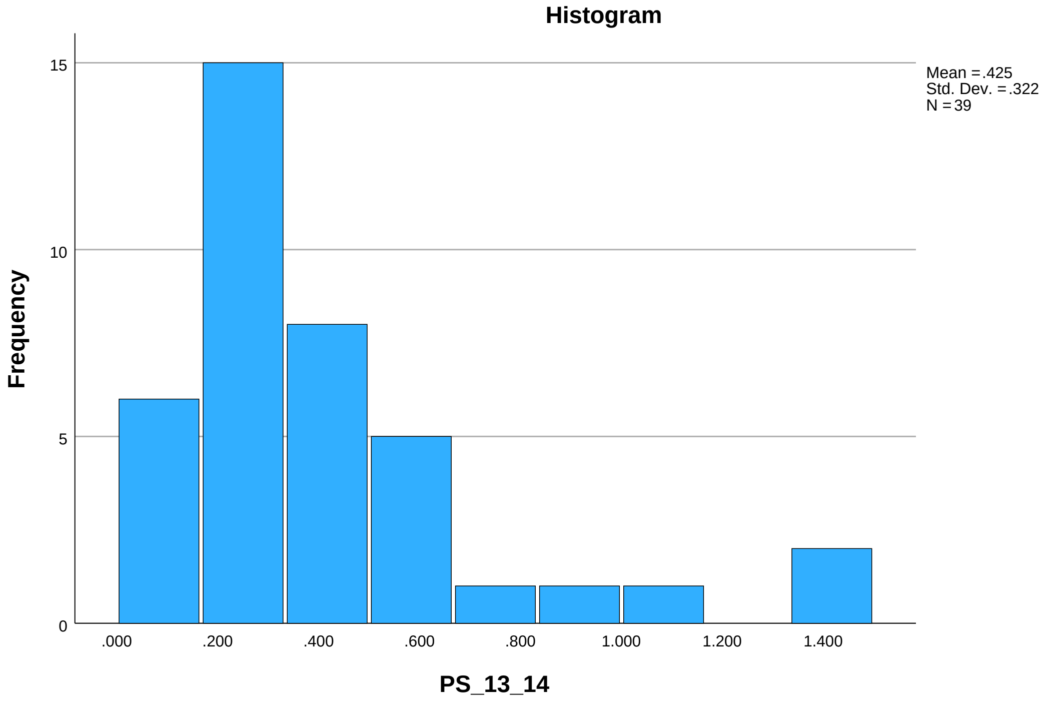


```
PS_13_14 Stem-and-Leaf Plot

 Frequency    Stem &  Leaf
```

```
1.00      0 . 7
5.00      1 . 14445
13.00     2 . 0002334556789
3.00      3 . 124
7.00      4 . 0112578
2.00      5 . 04
3.00      6 . 245
1.00      7 . 9
4.00 Extremes    (>=.95)

Stem width:     .100
Each leaf:      1 case(s)
```

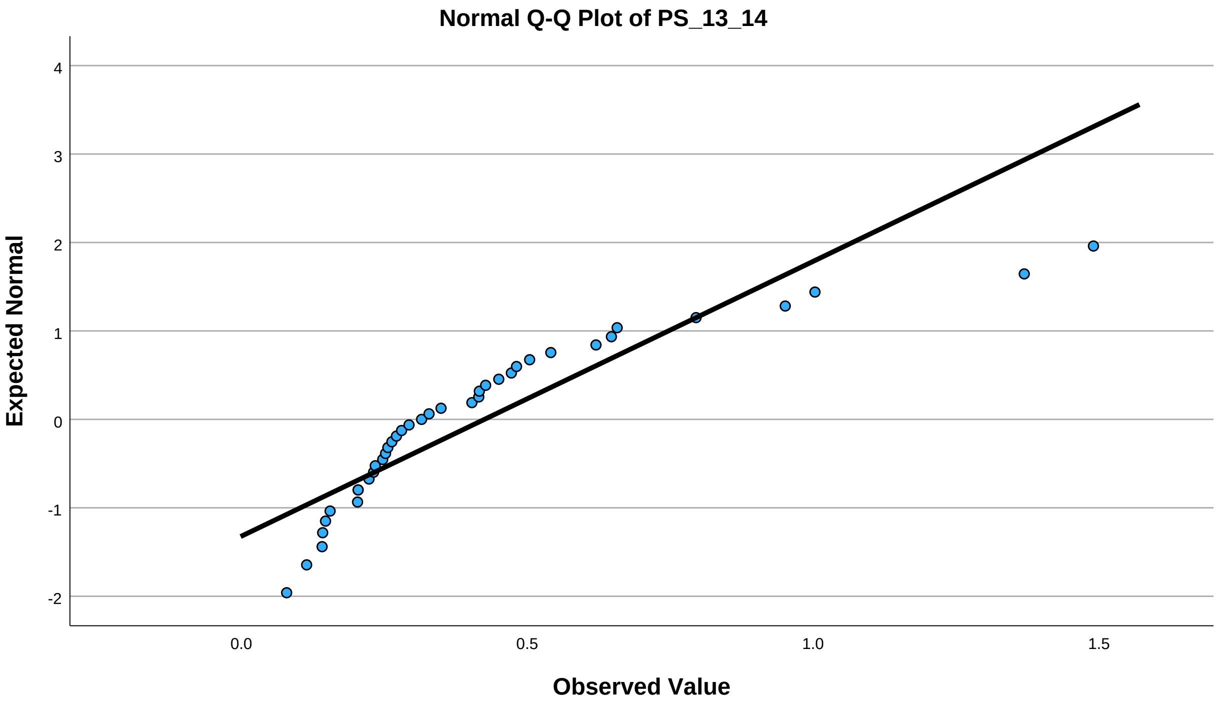

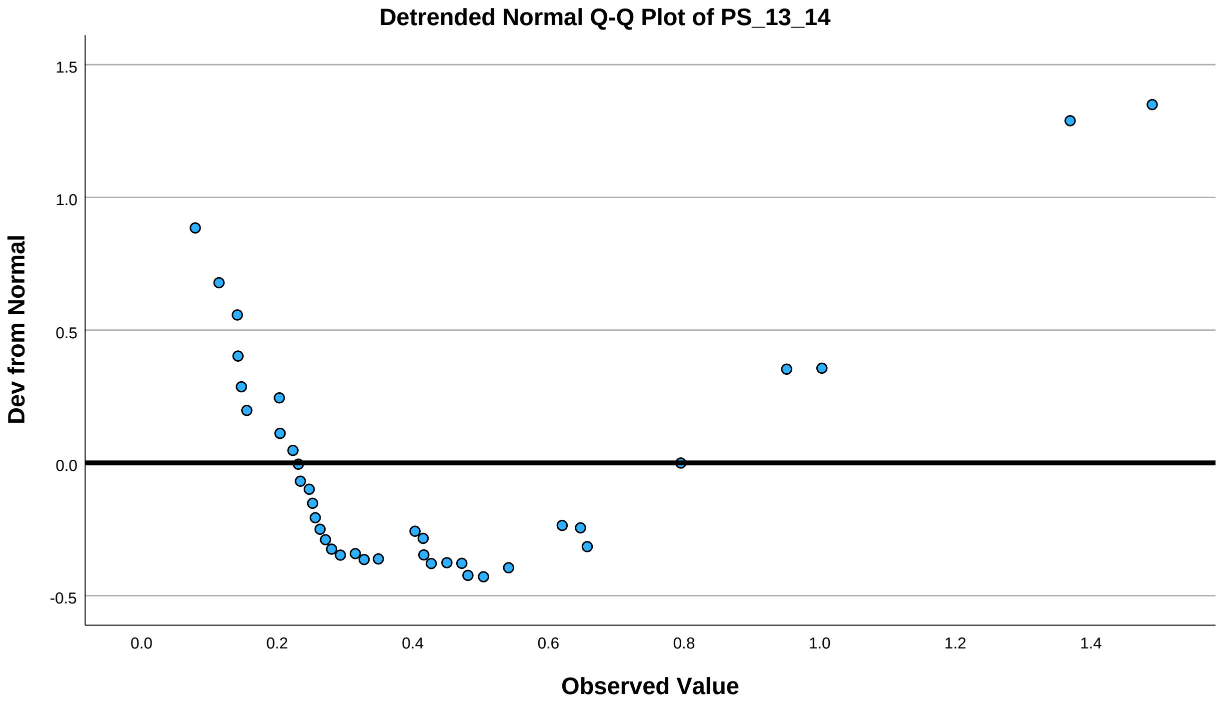
Detrended Normal Q-Q Plot of PS_13_14
Dev from Normal
1.5
1.0
0.5
0.0
-0.5
0.0
0.2
0.4
0.6
0.8
1.0
1.2
1.4
Observed Value

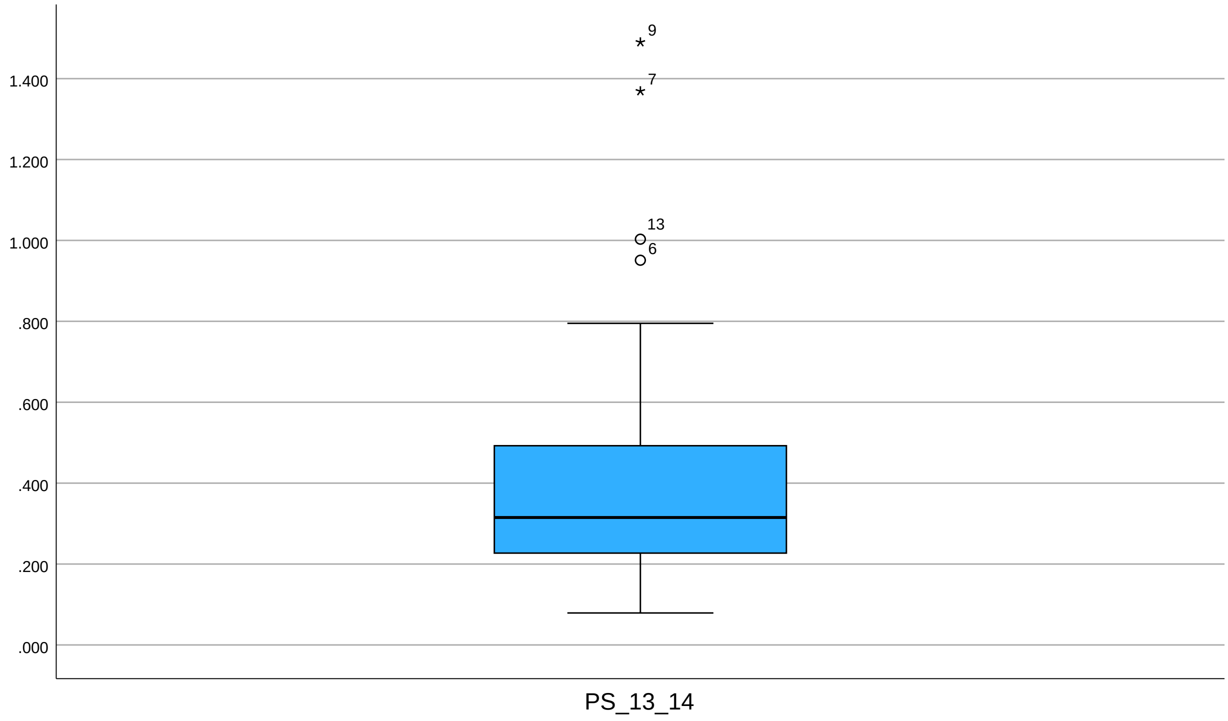
1.400
1.200
1.000
.800
.600
.400
.200
.000
9
7
13
6
PS_13_14

PS_14_15

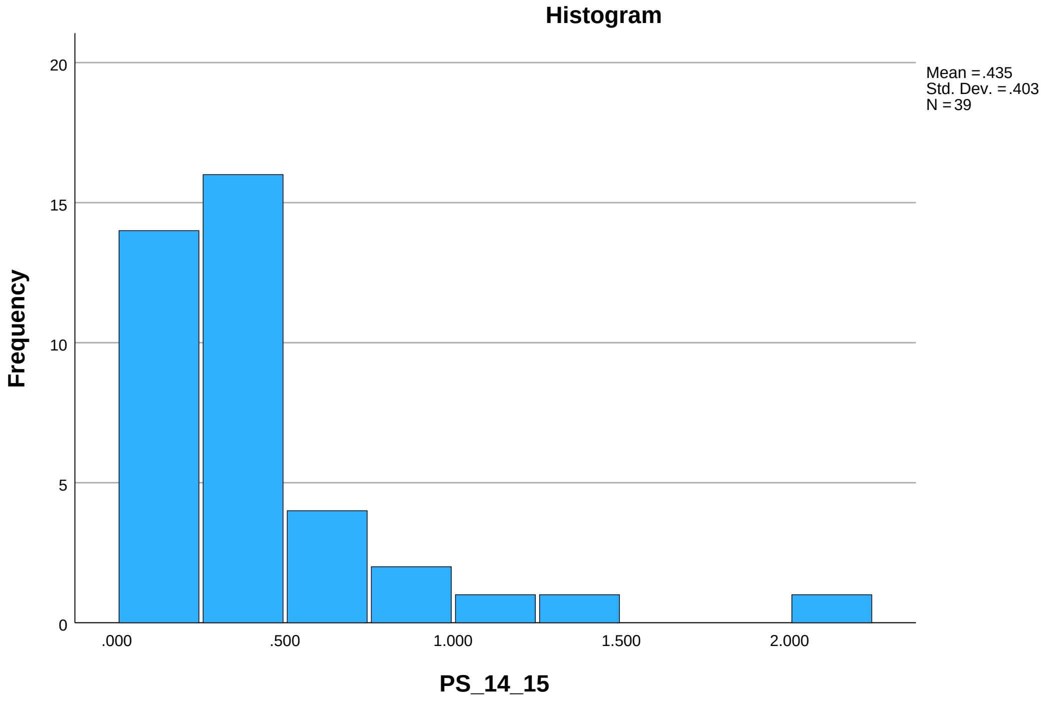


```
PS_14_15 Stem-and-Leaf Plot

 Frequency    Stem &  Leaf

     2.00        0 .  88
     7.00        1 .  1246799
    10.00        2 .  0013477779
     7.00        3 .  2444799
     4.00        4 .  0489
     2.00        5 .  12
     2.00        6 .  34
      .00        7 .
      .00        8 .
     1.00        9 .  1
     4.00 Extremes    (>=.97)

 Stem width:      .100
 Each leaf:       1 case(s)
```

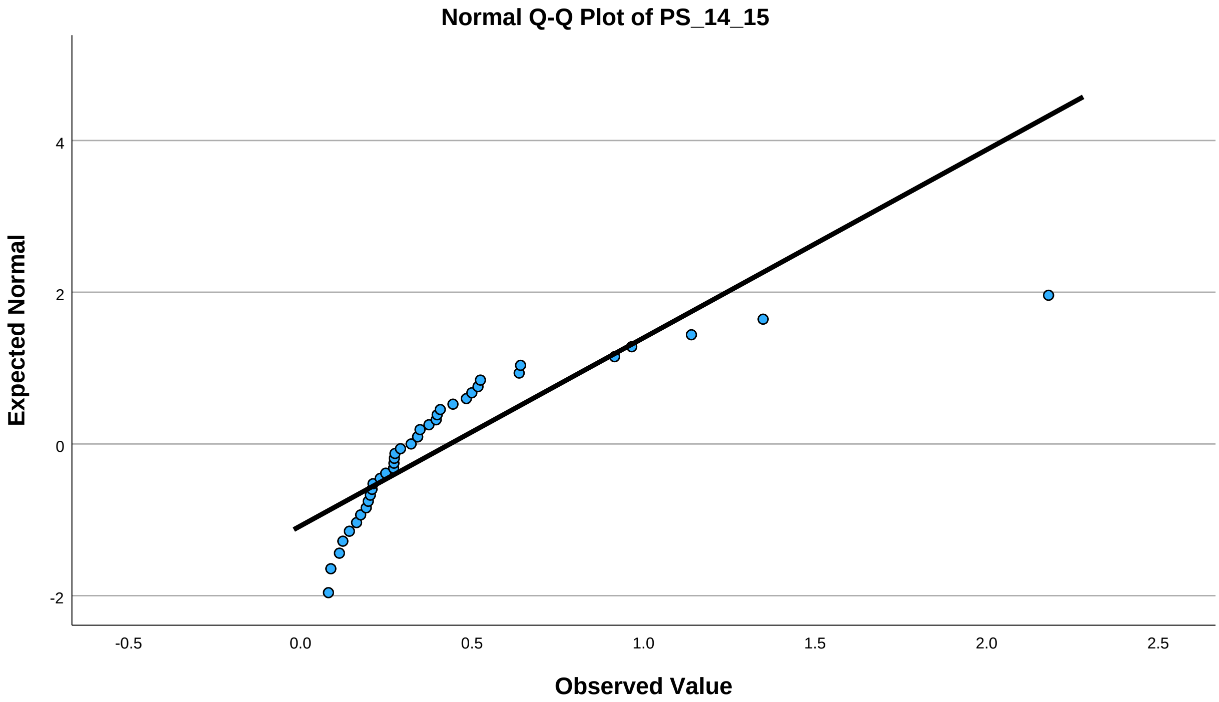
Normal Q-Q Plot of PS_14_15
Expected Normal
Observed Value
4
2
0
-2
-0.5
0.0
0.5
1.0
1.5
2.0
2.5

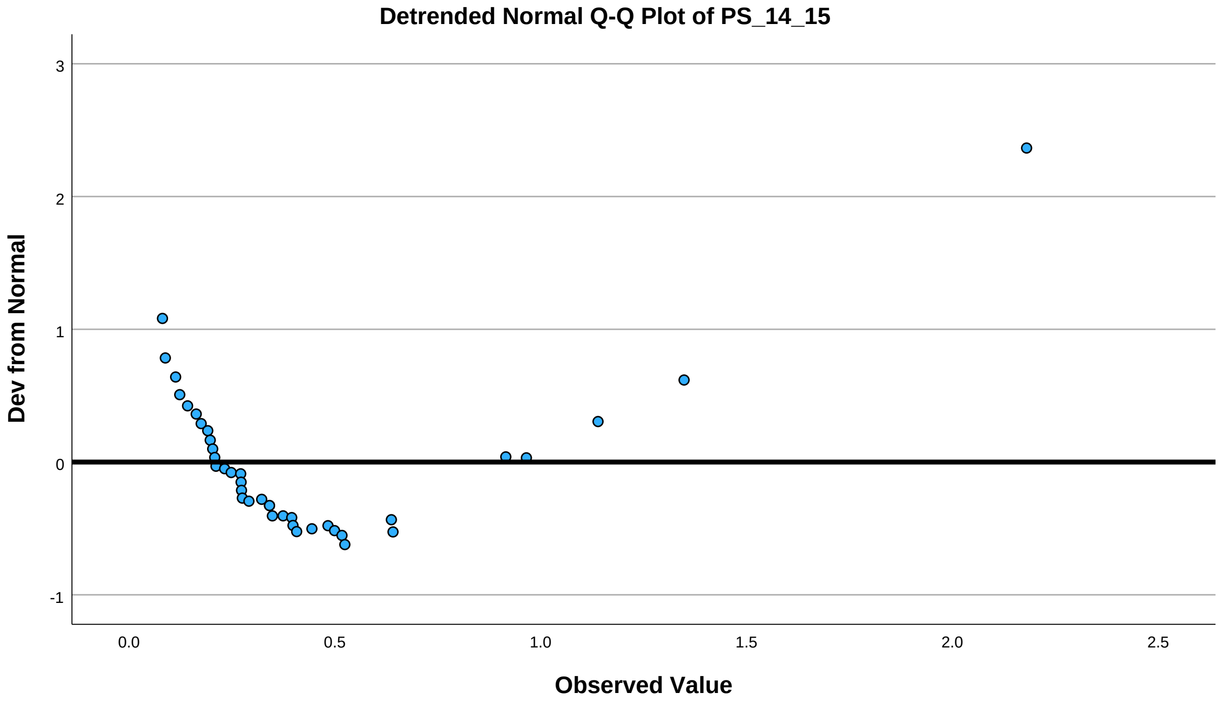
Detrended Normal Q-Q Plot of PS_14_15
Dev from Normal
Observed Value
3
2
1
0
-1
0.0
0.5
1.0
1.5
2.0
2.5

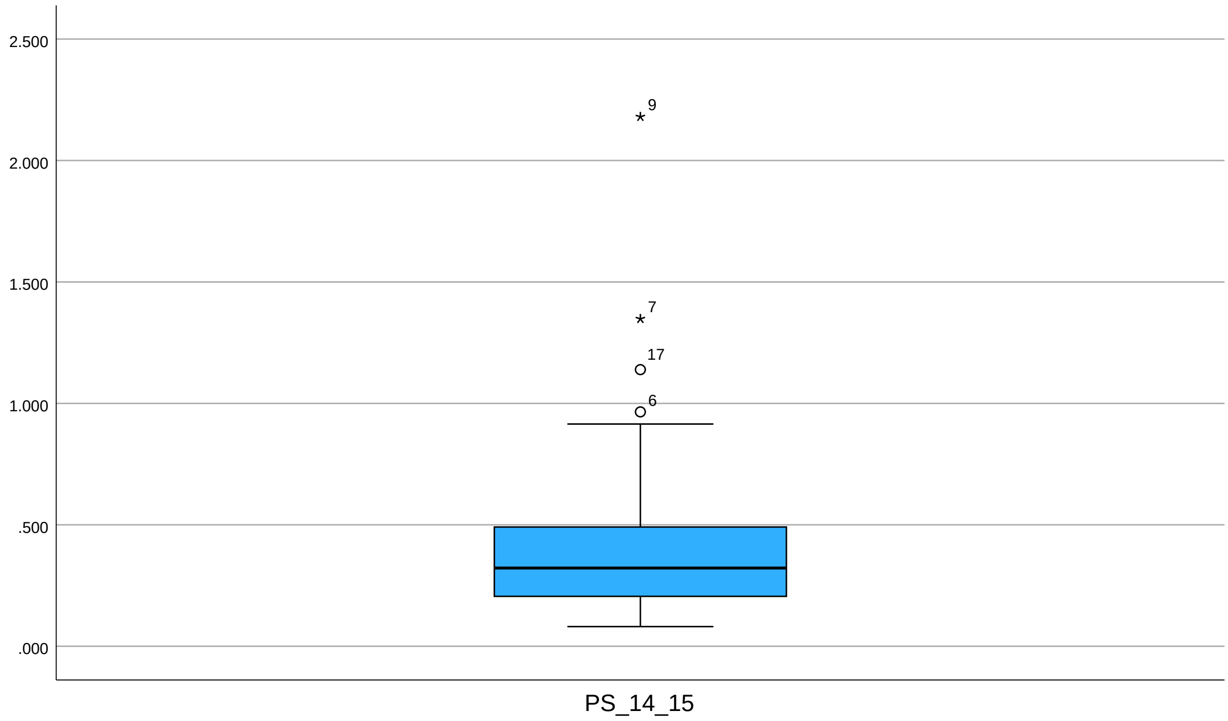

PS_15_16

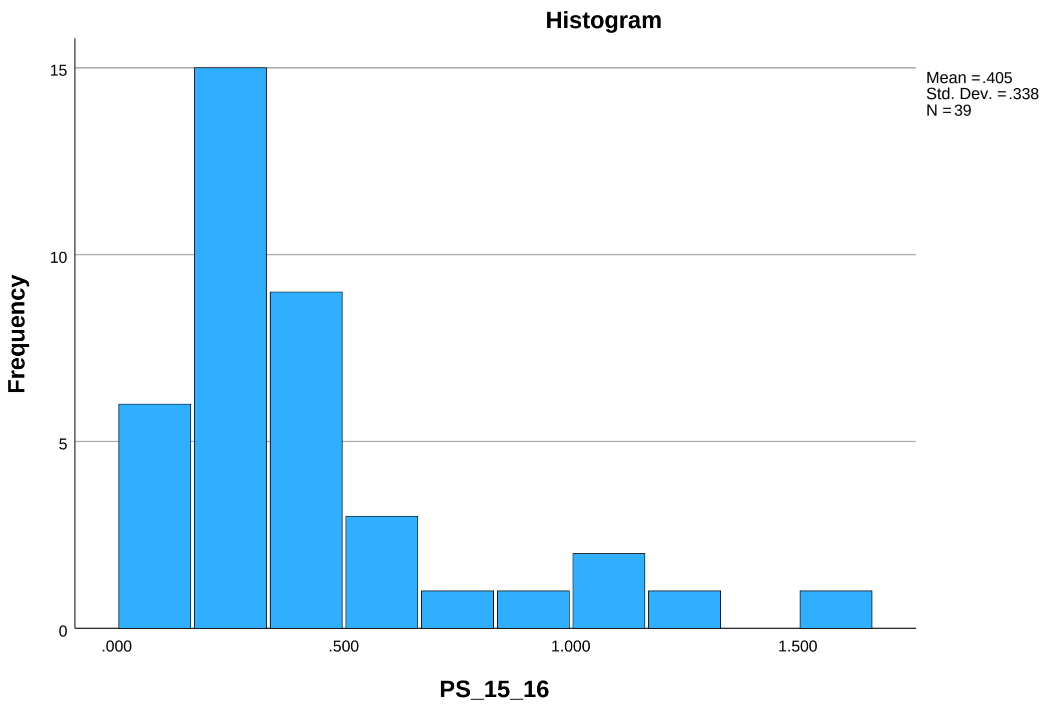

PS_15_16 Stem-and-Leaf Plot

Frequency   Stem & Leaf

```
  1.00      0 . 8
 10.00      1 . 0133588899
  9.00      2 . 023445577
  8.00      3 . 15566678
  2.00      4 . 01
  3.00      5 . 019
   .00      6 .
  1.00      7 . 0
  5.00 Extremes    (>=.86)

Stem width:     .100
Each leaf:      1 case(s)
```

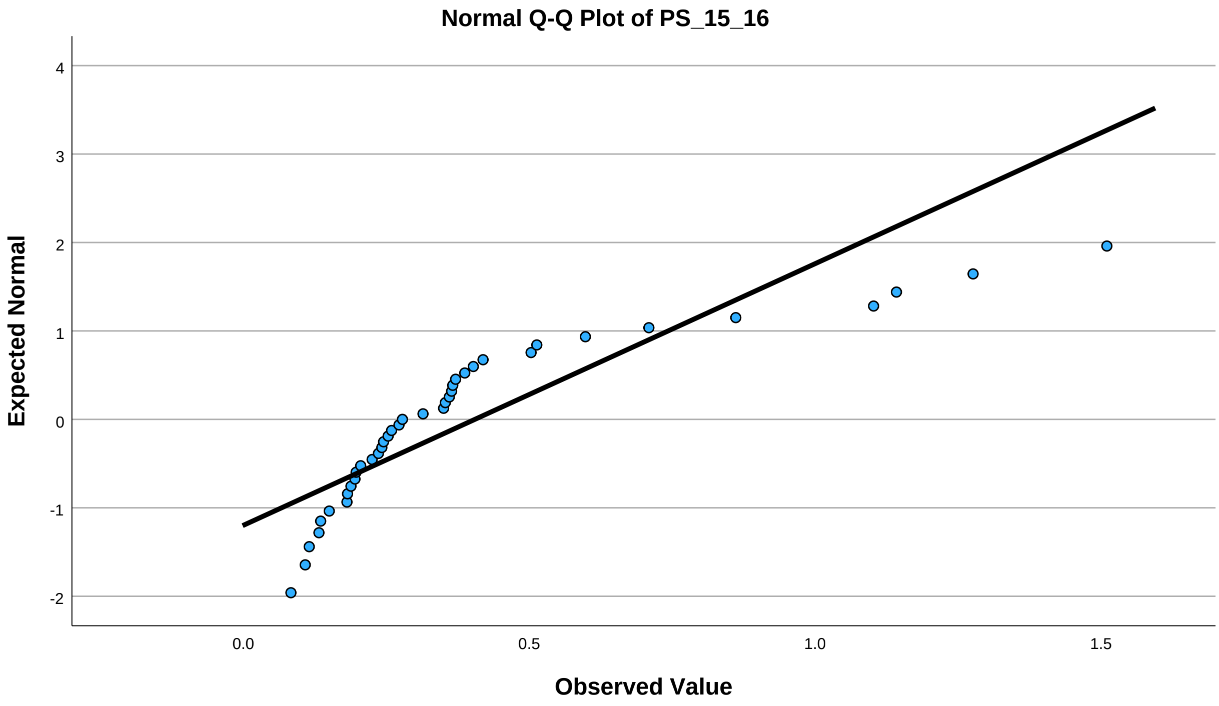

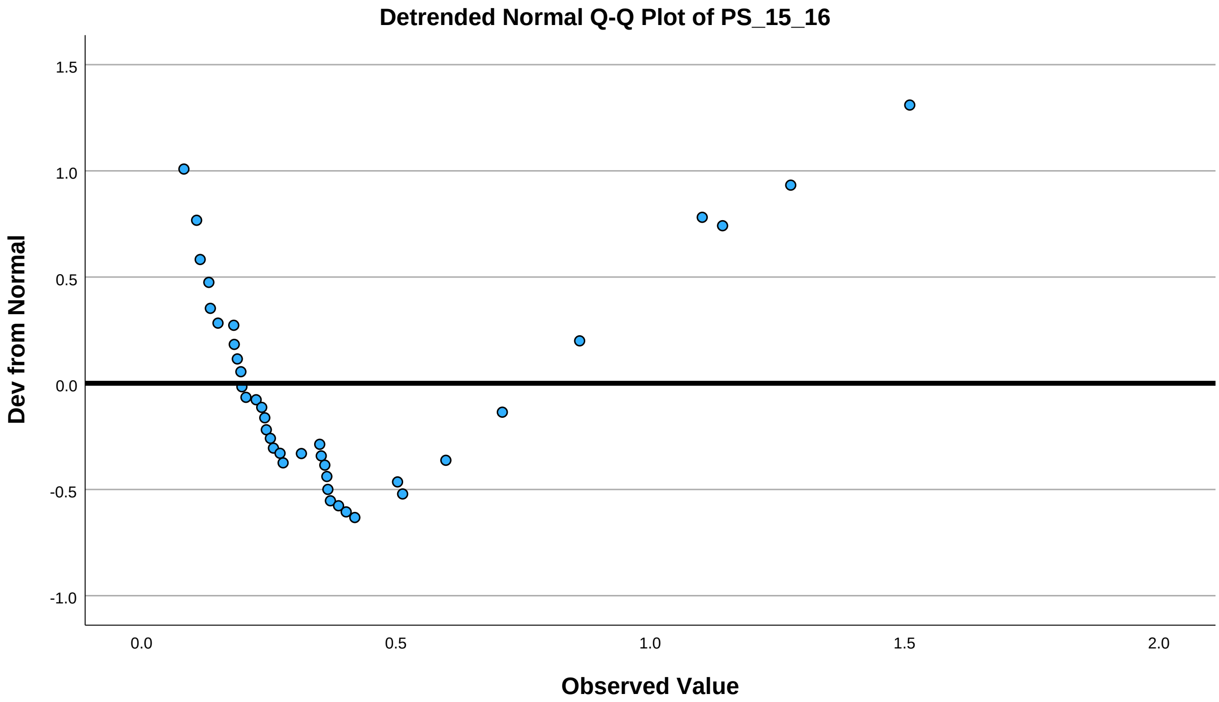

Detrended Normal Q-Q Plot of PS_15_16
Dev from Normal
1.5
1.0
0.5
0.0
-0.5
-1.0
0.0
0.5
1.0
1.5
2.0
Observed Value

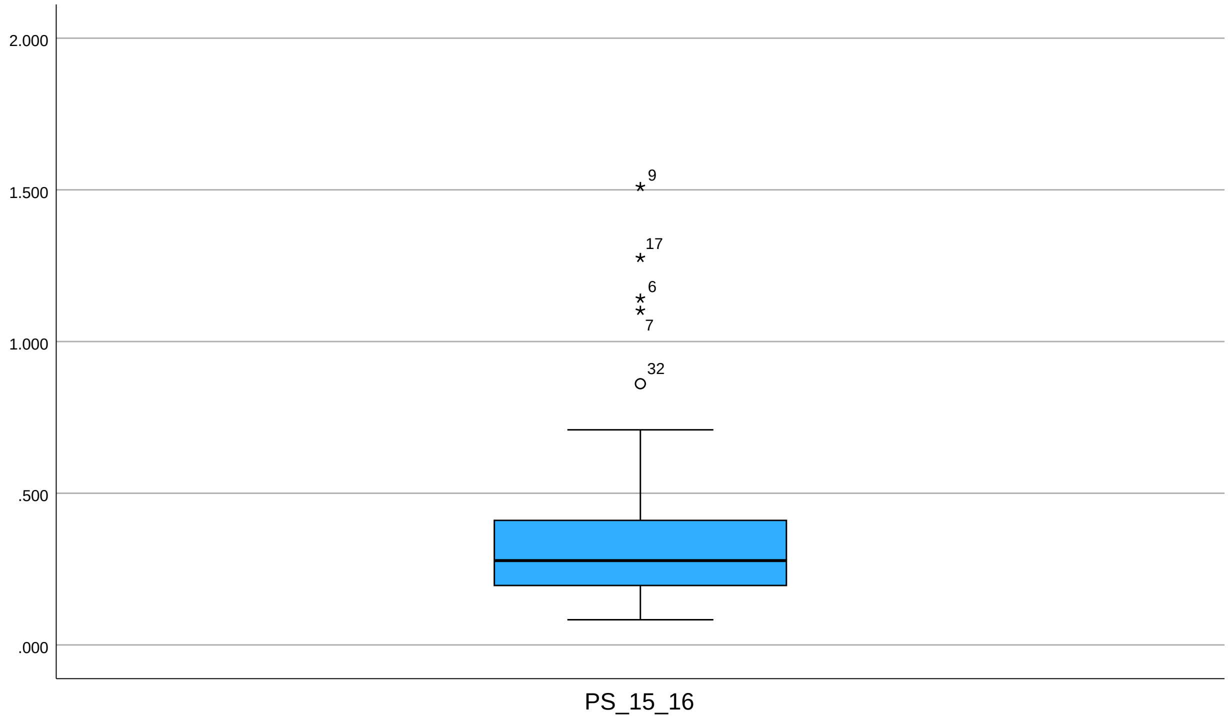

2.000
1.500
1.000
.500
.000
9
17
6
7
32
PS_15_16

PS_16_17

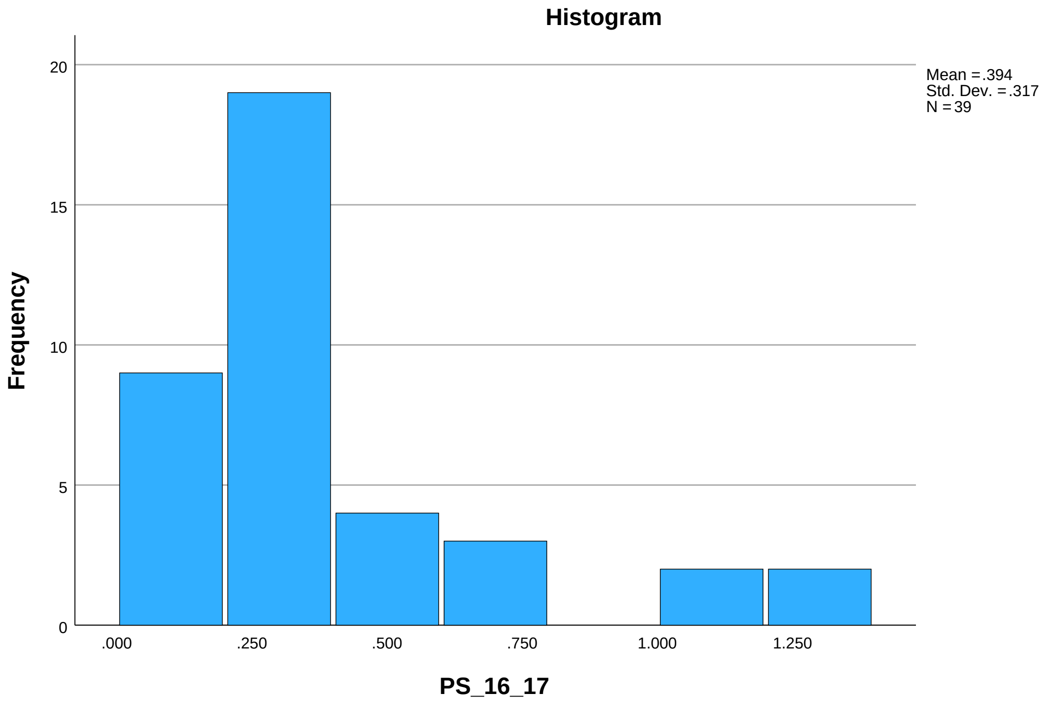


```
PS_16_17 Stem-and-Leaf Plot

 Frequency    Stem &  Leaf

     1.00        0 .  9
     8.00        1 .  01125889
    11.00        2 .  00011234458
     8.00        3 .  11444568
     2.00        4 .  67
     2.00        5 .  03
     2.00        6 .  22
     1.00        7 .  9
     4.00 Extremes    (>=1.06)

 Stem width:      .100
 Each leaf:       1 case(s)
```

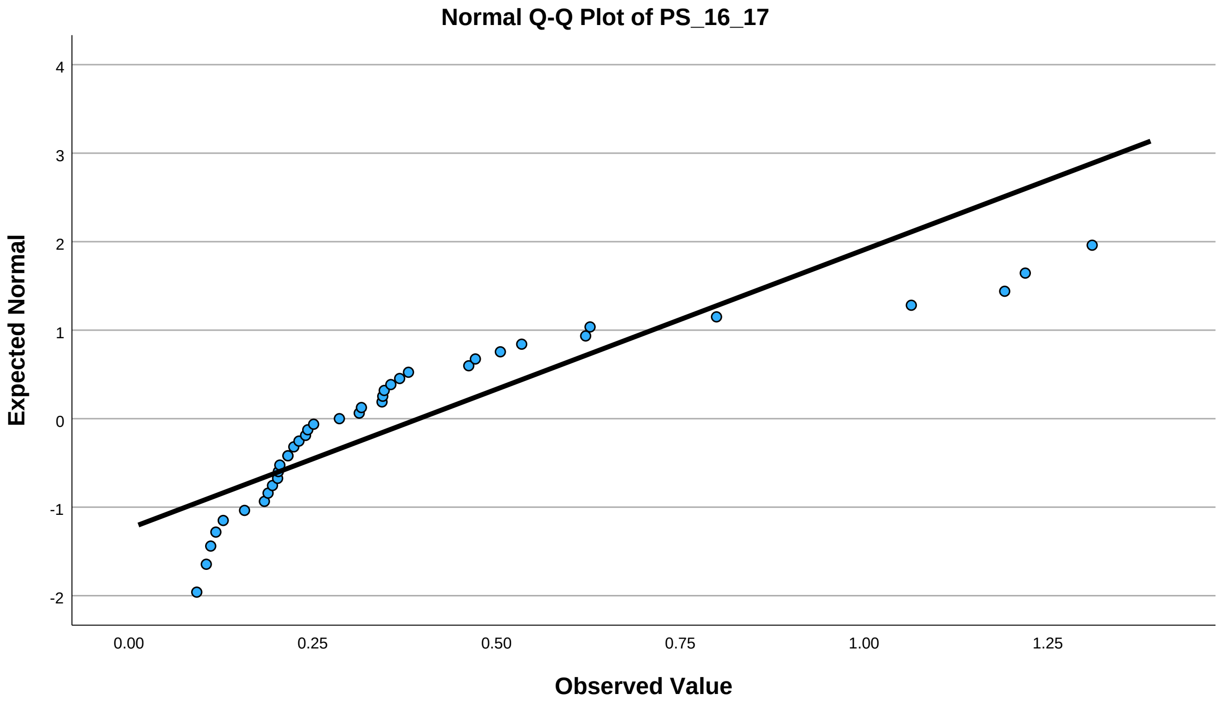
Normal Q-Q Plot of PS_16_17
Expected Normal
Observed Value
4
3
2
1
0
-1
-2
0.00
0.25
0.50
0.75
1.00
1.25

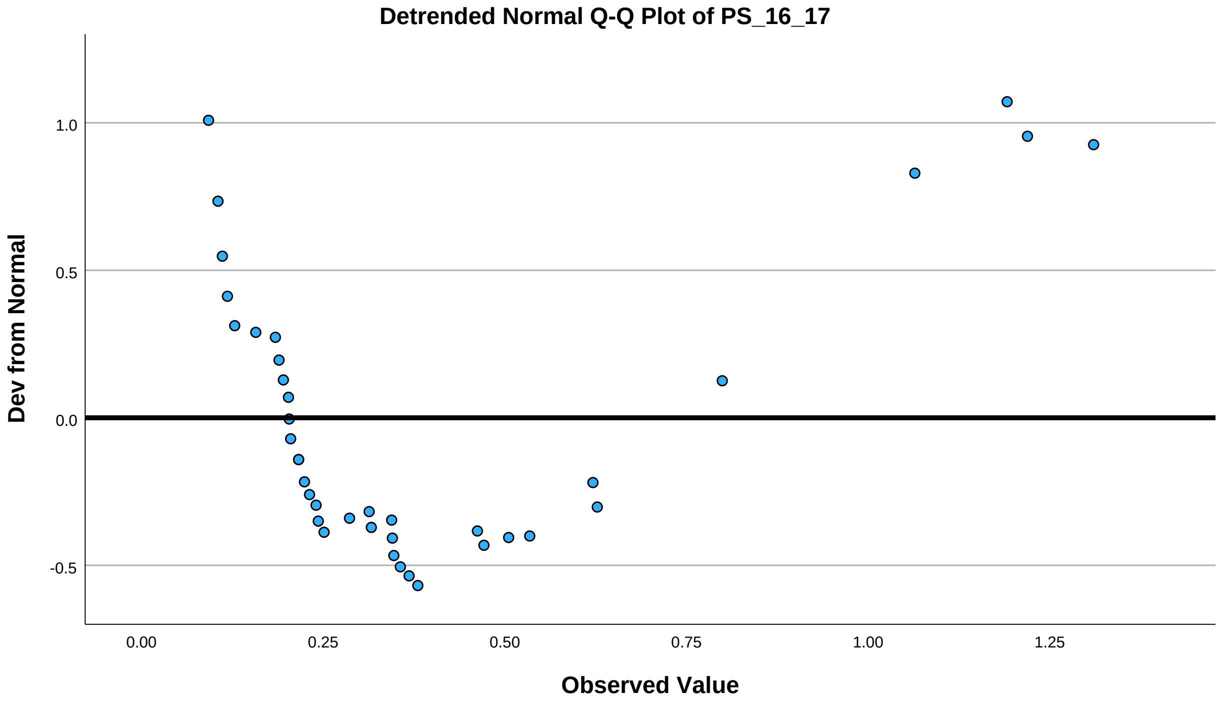
Detrended Normal Q-Q Plot of PS_16_17
Dev from Normal
Observed Value
1.0
0.5
0.0
-0.5
0.00
0.25
0.50
0.75
1.00
1.25

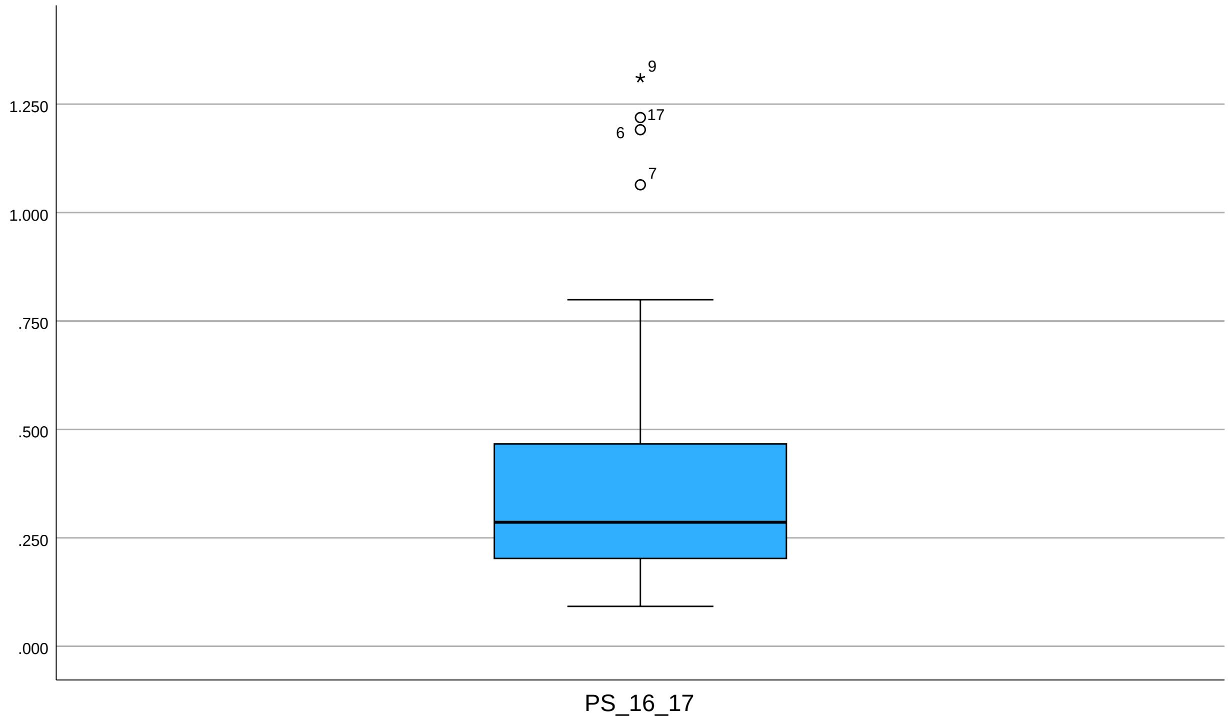

PS_17_18

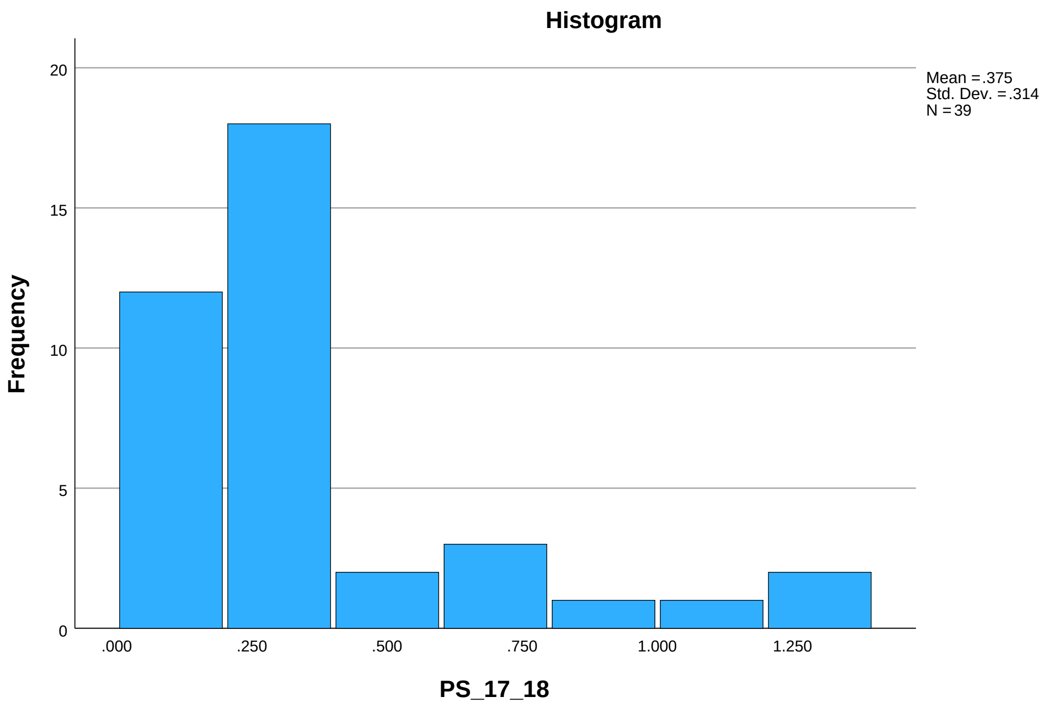


```
PS_17_18 Stem-and-Leaf Plot

 Frequency    Stem &  Leaf
```

```
 2.00      0 . 99
10.00      1 . 0113478889
 9.00      2 . 000024578
 9.00      3 . 122357788
 1.00      4 . 7
 1.00      5 . 3
 2.00      6 . 56
 5.00 Extremes    (>=.69)

Stem width:     .100
Each leaf:      1 case(s)
```

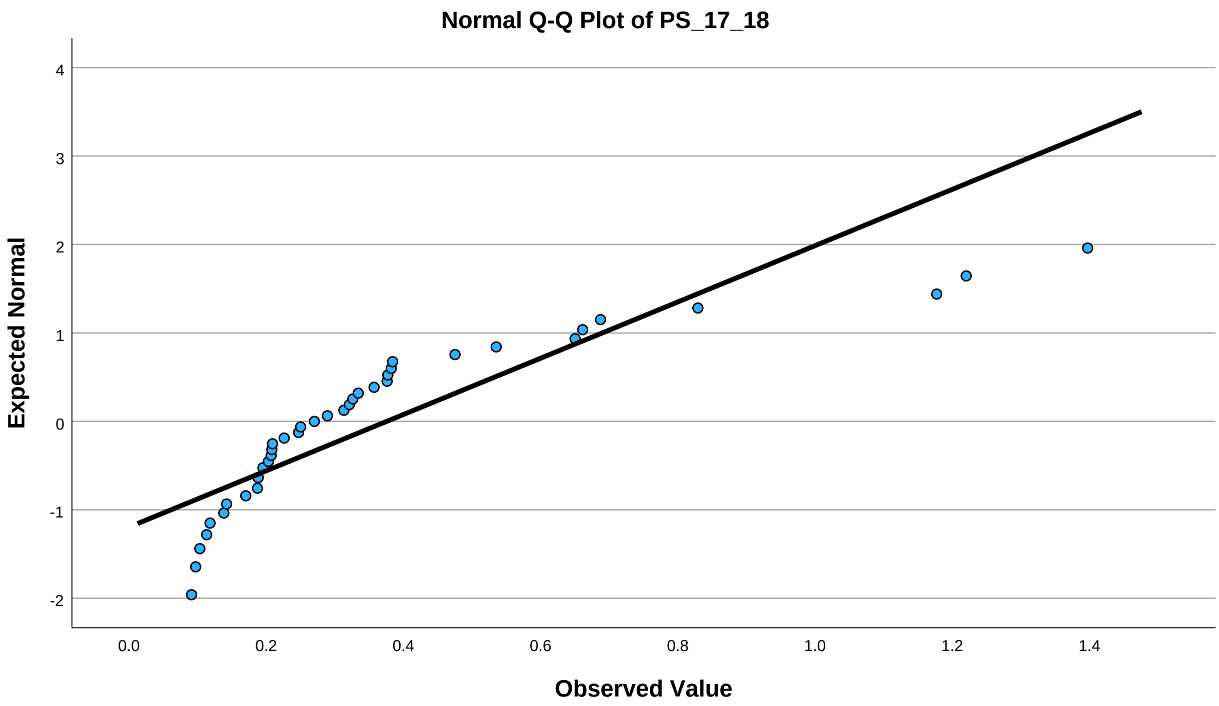

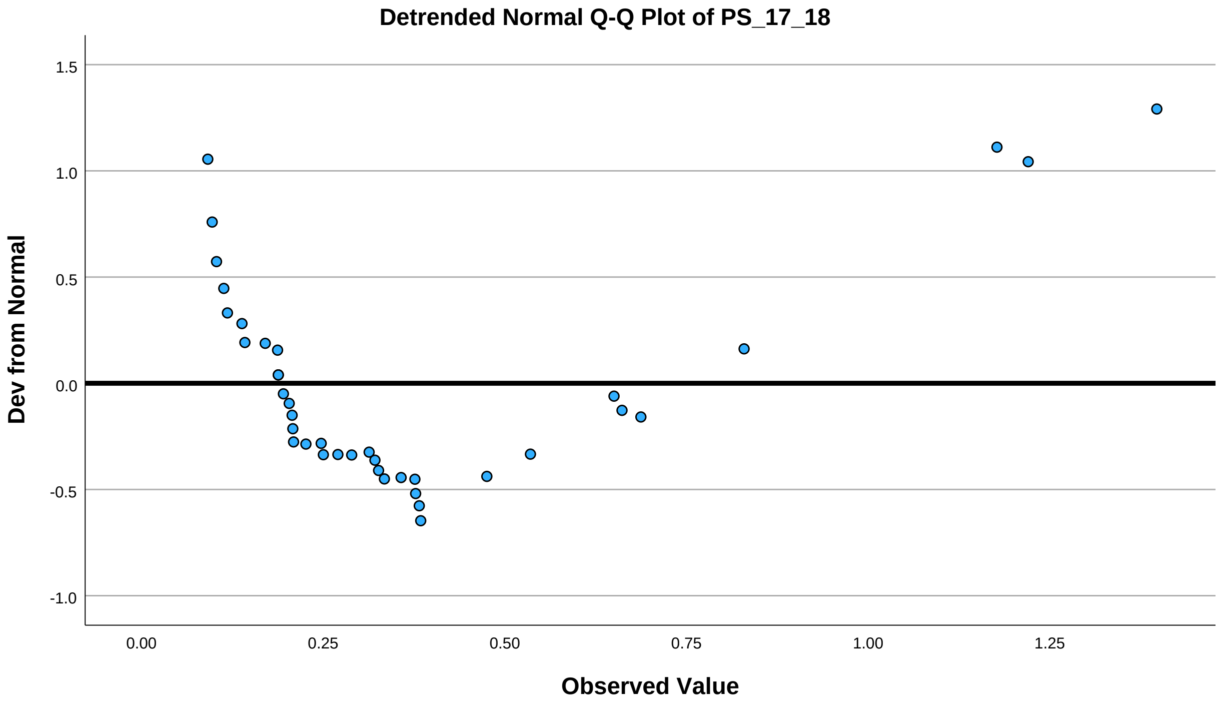
Detrended Normal Q-Q Plot of PS_17_18
Dev from Normal
1.5
1.0
0.5
0.0
-0.5
-1.0
0.00
0.25
0.50
0.75
1.00
1.25
Observed Value

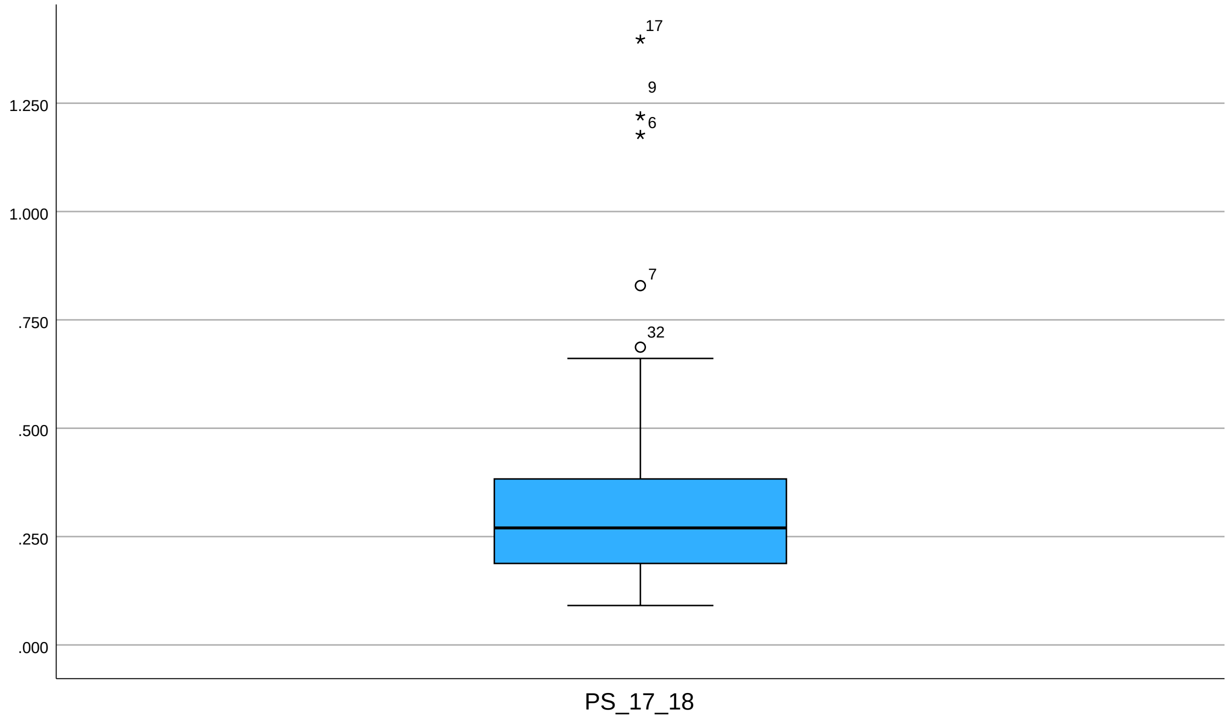
17
9
6
7
32
1.250
1.000
.750
.500
.250
.000
PS_17_18

PS_18_19

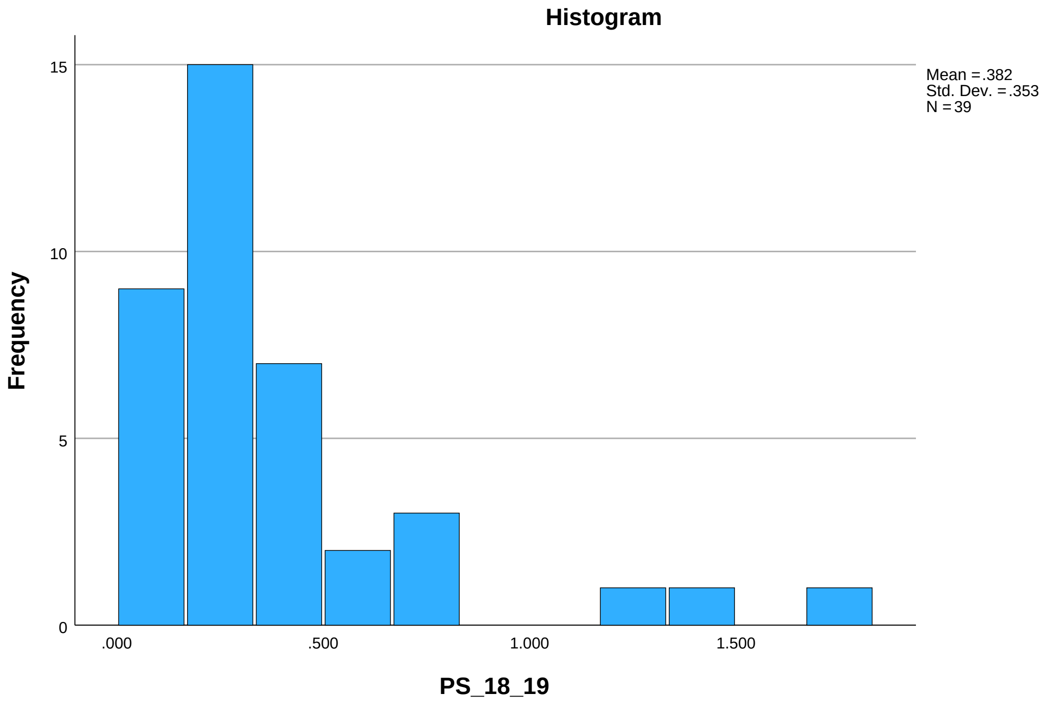


```
PS_18_19 Stem-and-Leaf Plot

 Frequency    Stem &  Leaf

     2.00        0 .  89
     9.00        1 .  113344478
    10.00        2 .  0011234456
    10.00        3 .  0004577899
      .00        4 .
     1.00        5 .  3
     2.00        6 .  09
     5.00 Extremes    (>=.73)

 Stem width:      .100
 Each leaf:       1 case(s)
```

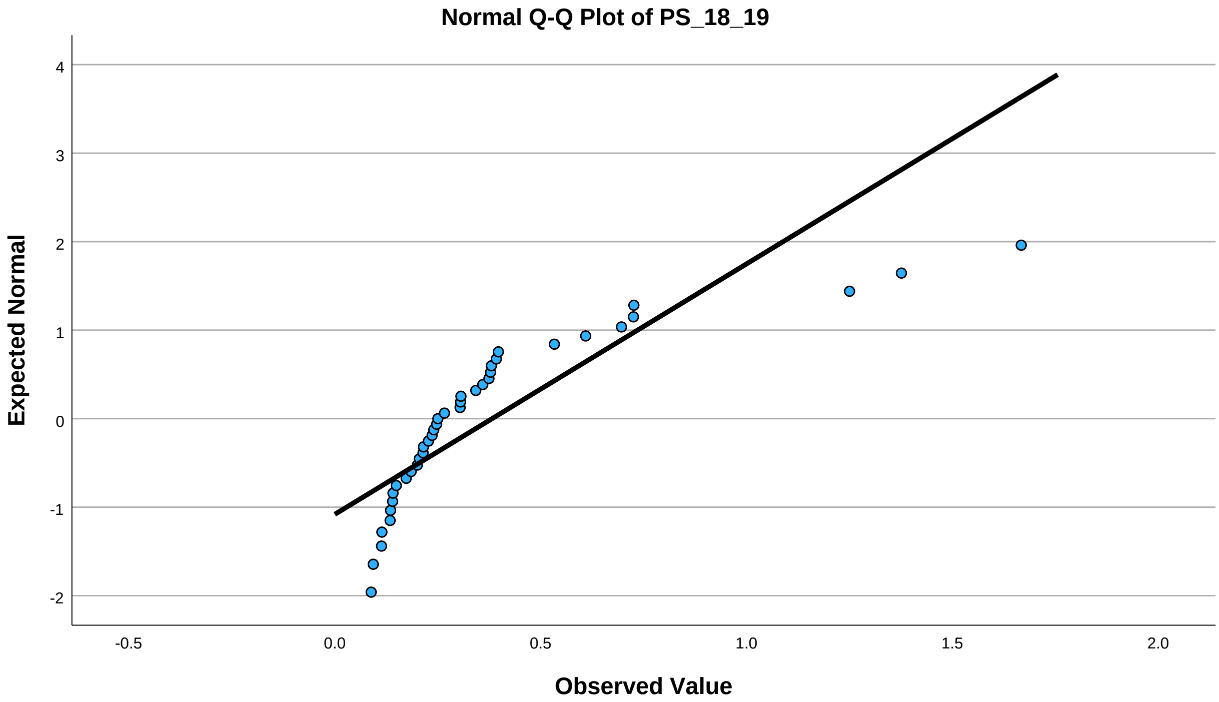
Normal Q-Q Plot of PS_18_19
Expected Normal
Observed Value
4
3
2
1
0
-1
-2
-0.5
0.0
0.5
1.0
1.5
2.0

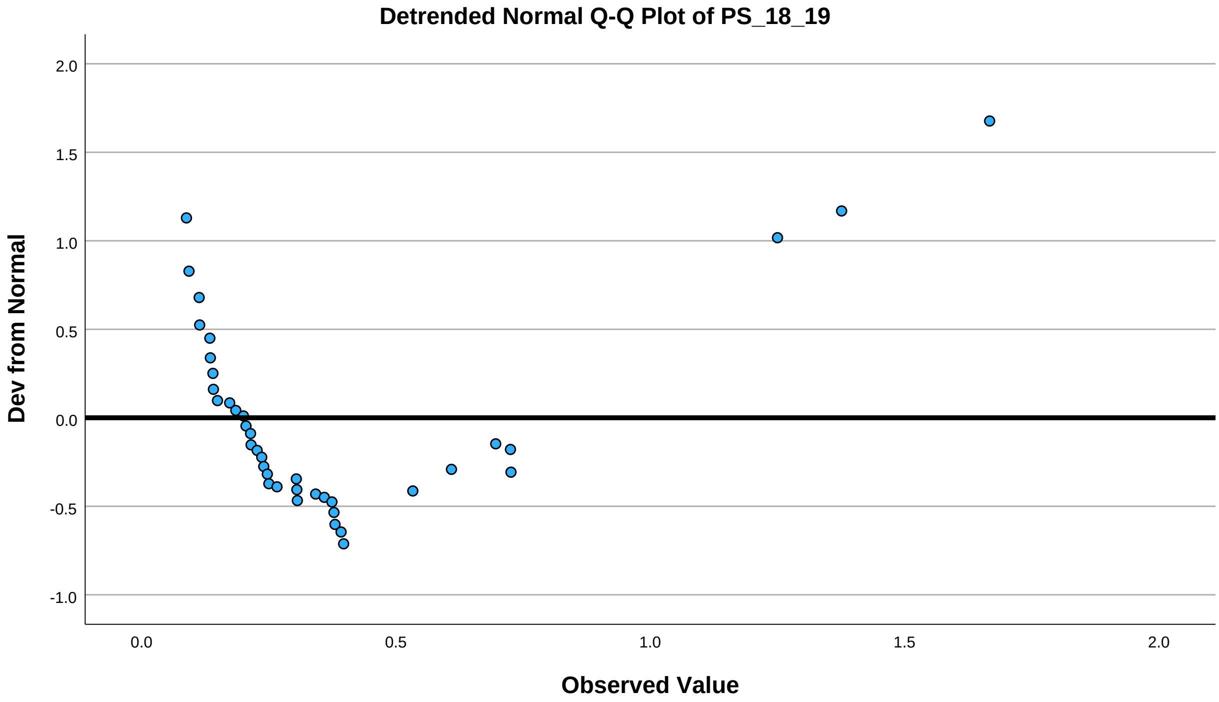
Detrended Normal Q-Q Plot of PS_18_19
Dev from Normal
Observed Value
2.0
1.5
1.0
0.5
0.0
-0.5
-1.0
0.0
0.5
1.0
1.5
2.0

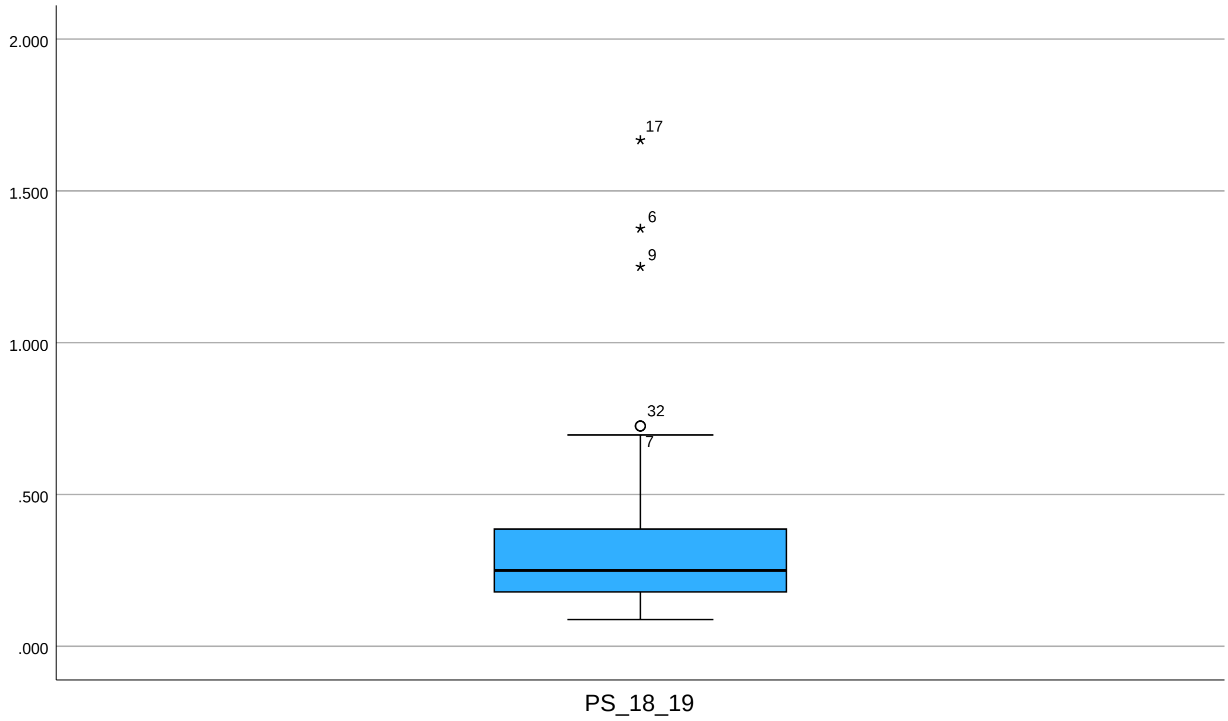

PS_19_20

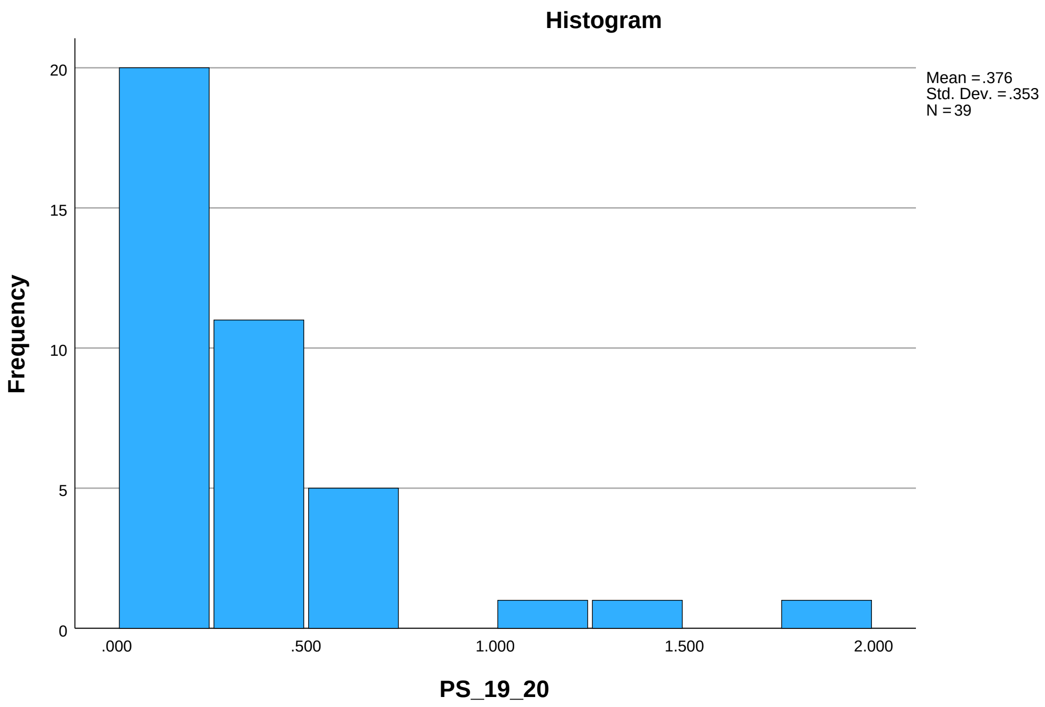

PS_19_20 Stem-and-Leaf Plot

Frequency   Stem & Leaf

```
    2.00      0 .  89
   11.00        1 .  12224457899
    9.00       2 .  001113455
    6.00      3 .  155588
    3.00      4 .  013
    1.00      5 .  0
    3.00      6 .  468
    1.00      7 .  1
     3.00 Extremes    (>=1.02)

 Stem width:      .100
 Each leaf:       1 case(s)
```

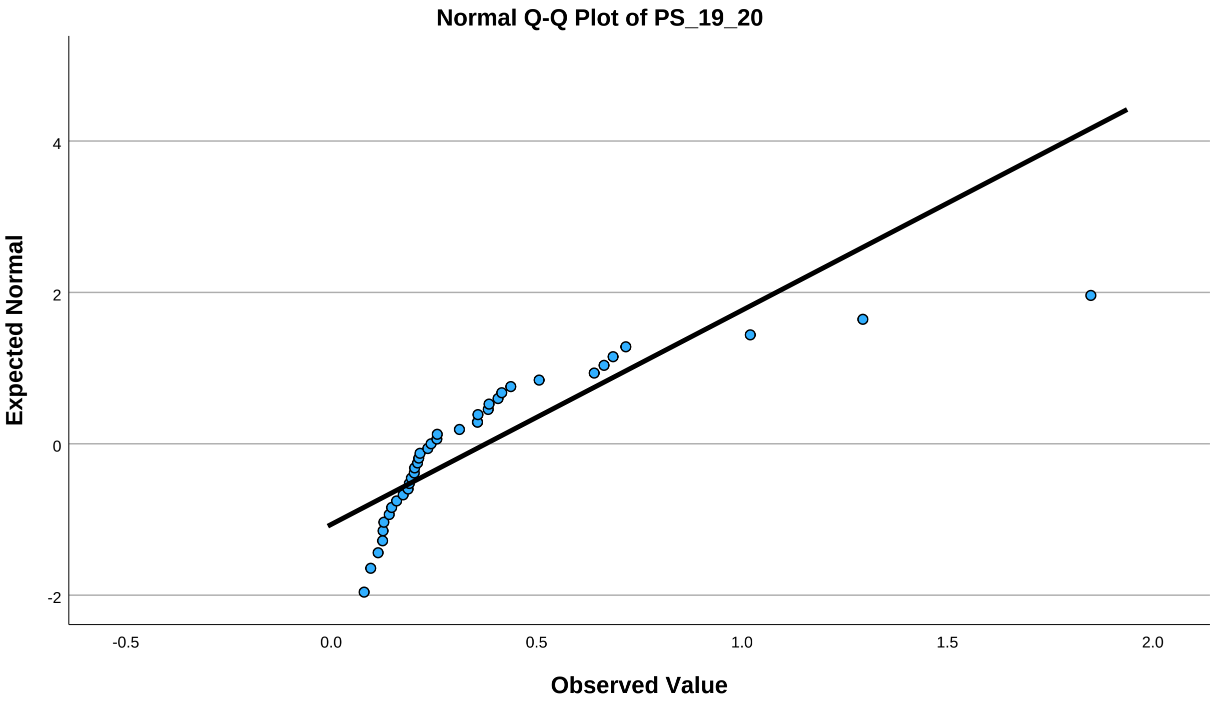

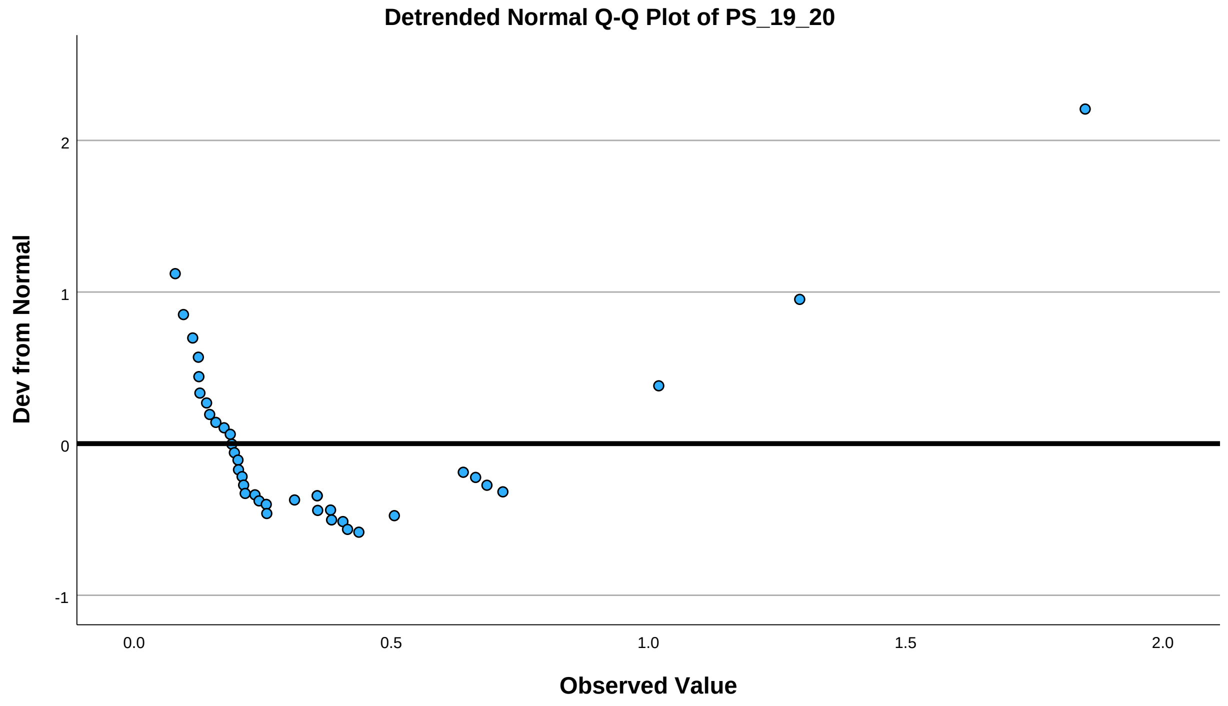
Detrended Normal Q-Q Plot of PS_19_20
Dev from Normal
2
1
0
-1
0.0
0.5
1.0
1.5
2.0
Observed Value

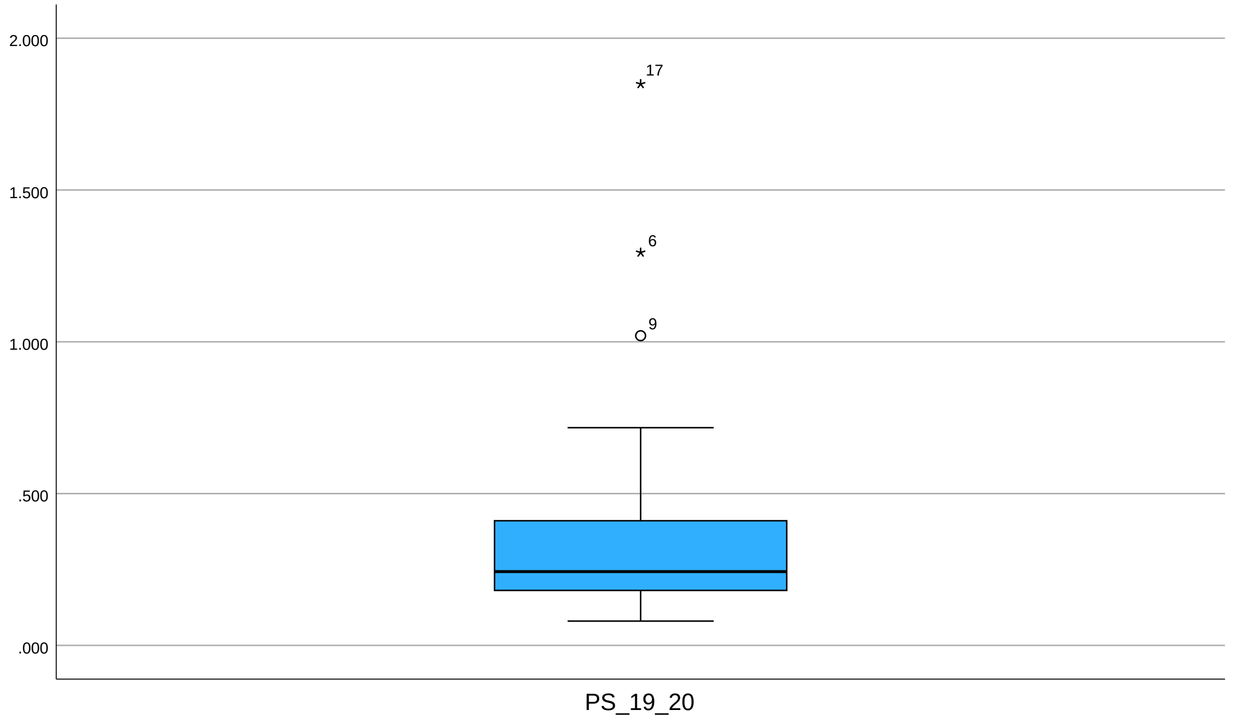
2.000
1.500
1.000
.500
.000
17
6
9
PS_19_20

Explore

Case Processing Summary

	Cases					
	Valid		Missing		Total	
	N	Percent	N	Percent	N	Percent
BrainRate	39	100.0%	0	0.0%	39	100.0%
PermutationEntropy	39	100.0%	0	0.0%	39	100.0%
PermutationEntropy_01	39	100.0%	0	0.0%	39	100.0%
SampleEntropy	39	100.0%	0	0.0%	39	100.0%
SpectralEntropy_bits	39	100.0%	0	0.0%	39	100.0%
SpectralEntropy_01	39	100.0%	0	0.0%	39	100.0%
HiguchiFractalDimension	39	100.0%	0	0.0%	39	100.0%
LempelZivComplexity	39	100.0%	0	0.0%	39	100.0%
RenyiEntropy	39	100.0%	0	0.0%	39	100.0%
TsallisEntropy	39	100.0%	0	0.0%	39	100.0%
PS_4_5	39	100.0%	0	0.0%	39	100.0%
PS_5_6	39	100.0%	0	0.0%	39	100.0%
PS_6_7	39	100.0%	0	0.0%	39	100.0%
PS_7_8	39	100.0%	0	0.0%	39	100.0%
PS_8_9	39	100.0%	0	0.0%	39	100.0%
PS_9_10	39	100.0%	0	0.0%	39	100.0%
PS_10_11	39	100.0%	0	0.0%	39	100.0%
PS_11_12	39	100.0%	0	0.0%	39	100.0%
PS_12_13	39	100.0%	0	0.0%	39	100.0%
PS_13_14	39	100.0%	0	0.0%	39	100.0%
PS_14_15	39	100.0%	0	0.0%	39	100.0%
PS_15_16	39	100.0%	0	0.0%	39	100.0%
PS_16_17	39	100.0%	0	0.0%	39	100.0%
PS_17_18	39	100.0%	0	0.0%	39	100.0%
PS_18_19	39	100.0%	0	0.0%	39	100.0%
PS_19_20	39	100.0%	0	0.0%	39	100.0%

Descriptives

			Statistic	Std. Error
BrainRate	Mean		8.4354	.62585
	95% Confidence Interval for Mean	Lower Bound	7.1684	
		Upper Bound	9.7024	
	5% Trimmed Mean		8.0565	
	Median		7.5400	
	Variance		15.276	
	Std. Deviation		3.90845	
	Minimum		2.24	
	Maximum		24.55	
	Range		22.31	
	Interquartile Range		3.28	
	Skewness		2.133	.378
	Kurtosis		6.905	.741
PermutationEntropy	Mean		1.4950417020	.00855137101
	95% Confidence Interval for Mean	Lower Bound	1.4777303564	
		Upper Bound	1.5123530475	
	5% Trimmed Mean		1.4968382944	
	Median		1.5010977319	
	Variance		.003	
	Std. Deviation		.05340329484	
	Minimum		1.3541335434	
	Maximum		1.6301072159	
	Range		.27597367244	
	Interquartile Range		.05631048757	
	Skewness		-.531	.378
	Kurtosis		1.479	.741
PermutationEntropy_01	Mean		.83439866101	.00477261103
	95% Confidence Interval for Mean	Lower Bound	.82473701509	
		Upper Bound	.84406030693	
	5% Trimmed Mean		.83540135834	
	Median		.83777859566	
	Variance		.001	
	Std. Deviation		.02980494634	
	Minimum		.75575632037	
	Maximum		.90978015960	
	Range		.15402383924	
	Interquartile Range		.03142748150	
	Skewness		-.531	.378
	Kurtosis		1.479	.741

Descriptives

			Statistic	Std. Error
SampleEntropy	Mean		.71943244294	.03391049298
	95% Confidence Interval for Mean	Lower Bound	.65078423886	
		Upper Bound	.78808064701	
	5% Trimmed Mean		.73262743624	
	Median		.75805007482	
	Variance		.045	
	Std. Deviation		.21177096077	
	Minimum		.07404590328	
	Maximum		1.0632982141	
	Range		.98925231086	
	Interquartile Range		.19643025588	
	Skewness		-1.092	.378
	Kurtosis		1.436	.741
SpectralEntropy_bits	Mean		4.1357579694	.11216378012
	95% Confidence Interval for Mean	Lower Bound	3.9086942675	
		Upper Bound	4.3628216713	
	5% Trimmed Mean		4.1749780915	
	Median		4.1660221033	
	Variance		.491	
	Std. Deviation		.70046258232	
	Minimum		1.4337093252	
	Maximum		5.6455927148	
	Range		4.2118833896	
	Interquartile Range		.50776559740	
	Skewness		-1.370	.378
	Kurtosis		5.269	.741
SpectralEntropy_01	Mean		.58987646795	.01599773849
	95% Confidence Interval for Mean	Lower Bound	.55749073952	
		Upper Bound	.62226219638	
	5% Trimmed Mean		.59547037051	
	Median		.59419299240	
	Variance		.010	
	Std. Deviation		.09990584484	
	Minimum		.20448764146	
	Maximum		.80522175493	
	Range		.60073411347	
	Interquartile Range		.07242178564	
	Skewness		-1.370	.378
	Kurtosis		5.269	.741

Descriptives

			Statistic	Std. Error
HiguchiFractalDimension	Mean		.50850014347	.01649774813
	95% Confidence Interval for Mean	Lower Bound	.47510219844	
		Upper Bound	.54189808849	
	5% Trimmed Mean		.51258831405	
	Median		.53606937088	
	Variance		.011	
	Std. Deviation		.10302840403	
	Minimum		.21610553796	
	Maximum		.71968650434	
	Range		.50358096637	
	Interquartile Range		.14991124122	
	Skewness		-.749	.378
	Kurtosis		.703	.741
LempelZivComplexity	Mean		.27960601176	.00584129909
	95% Confidence Interval for Mean	Lower Bound	.26778091997	
		Upper Bound	.29143110355	
	5% Trimmed Mean		.27764727852	
	Median		.27140052687	
	Variance		.001	
	Std. Deviation		.03647890113	
	Minimum		.20042888580	
	Maximum		.38466287459	
	Range		.18423398879	
	Interquartile Range		.03081805196	
	Skewness		.945	.378
	Kurtosis		1.416	.741
RenyiEntropy	Mean		7.4571747442	.17362526525
	95% Confidence Interval for Mean	Lower Bound	7.1056887706	
		Upper Bound	7.8086607179	
	5% Trimmed Mean		7.5336130545	
	Median		7.7808446529	
	Variance		1.176	
	Std. Deviation		1.0842894339	
	Minimum		4.3203590256	
	Maximum		8.7567122422	
	Range		4.4363532165	
	Interquartile Range		1.3667566322	
	Skewness		-1.104	.378
	Kurtosis		.471	.741

Descriptives

			Statistic	Std. Error
TsallisEntropy	Mean		1.9050746771	.05201773967
	95% Confidence Interval for Mean	Lower Bound	1.7997702685	
		Upper Bound	2.0103790857	
	5% Trimmed Mean		1.9754617969	
	Median		1.9856246218	
	Variance		.106	
	Std. Deviation		.32485068013	
	Minimum		.25855771453	
	Maximum		1.9882695896	
	Range		1.7297118751	
	Interquartile Range		.00364984985	
	Skewness		-4.503	.378
	Kurtosis		20.306	.741
PS_4_5	Mean		2.43362	.428350
	95% Confidence Interval for Mean	Lower Bound	1.56647	
		Upper Bound	3.30076	
	5% Trimmed Mean		2.02227	
	Median		1.72600	
	Variance		7.156	
	Std. Deviation		2.675042	
	Minimum		.700	
	Maximum		17.400	
	Range		16.700	
	Interquartile Range		1.546	
	Skewness		4.857	.378
	Kurtosis		27.031	.741
PS_5_6	Mean		1.83672	.275825
	95% Confidence Interval for Mean	Lower Bound	1.27834	
		Upper Bound	2.39510	
	5% Trimmed Mean		1.59409	
	Median		1.40700	
	Variance		2.967	
	Std. Deviation		1.722526	
	Minimum		.556	
	Maximum		11.350	
	Range		10.794	
	Interquartile Range		1.205	
	Skewness		4.650	.378
	Kurtosis		25.513	.741

Descriptives

			Statistic	Std. Error
PS_6_7	Mean		1.51951	.196286
	95% Confidence Interval for Mean	Lower Bound	1.12215	
		Upper Bound	1.91687	
	5% Trimmed Mean		1.35071	
	Median		1.29200	
	Variance		1.503	
	Std. Deviation		1.225805	
	Minimum		.329	
	Maximum		7.950	
	Range		7.621	
	Interquartile Range		.932	
	Skewness		4.012	.378
	Kurtosis		20.337	.741
PS_7_8	Mean		1.29159	.166765
	95% Confidence Interval for Mean	Lower Bound	.95399	
		Upper Bound	1.62919	
	5% Trimmed Mean		1.13156	
	Median		.93700	
	Variance		1.085	
	Std. Deviation		1.041448	
	Minimum		.263	
	Maximum		6.080	
	Range		5.817	
	Interquartile Range		.625	
	Skewness		3.220	.378
	Kurtosis		12.337	.741
PS_8_9	Mean		1.09887	.126396
	95% Confidence Interval for Mean	Lower Bound	.84300	
		Upper Bound	1.35475	
	5% Trimmed Mean		.99764	
	Median		.82200	
	Variance		.623	
	Std. Deviation		.789345	
	Minimum		.211	
	Maximum		4.227	
	Range		4.016	
	Interquartile Range		.722	
	Skewness		2.262	.378
	Kurtosis		6.403	.741

Descriptives

			Statistic	Std. Error
PS_9_10	Mean		1.15444	.166764
	95% Confidence Interval for Mean	Lower Bound	.81684	
		Upper Bound	1.49203	
	5% Trimmed Mean		1.00781	
	Median		.99800	
	Variance		1.085	
	Std. Deviation		1.041441	
	Minimum		.183	
	Maximum		6.350	
	Range		6.167	
	Interquartile Range		.803	
	Skewness		3.535	.378
	Kurtosis		16.331	.741
PS_10_11	Mean		1.36236	.193326
	95% Confidence Interval for Mean	Lower Bound	.97099	
		Upper Bound	1.75373	
	5% Trimmed Mean		1.20345	
	Median		1.11000	
	Variance		1.458	
	Std. Deviation		1.207318	
	Minimum		.223	
	Maximum		6.920	
	Range		6.697	
	Interquartile Range		1.030	
	Skewness		2.891	.378
	Kurtosis		11.347	.741
PS_11_12	Mean		.63544	.094396
	95% Confidence Interval for Mean	Lower Bound	.44434	
		Upper Bound	.82653	
	5% Trimmed Mean		.54716	
	Median		.43100	
	Variance		.348	
	Std. Deviation		.589506	
	Minimum		.132	
	Maximum		3.520	
	Range		3.388	
	Interquartile Range		.324	
	Skewness		3.482	.378
	Kurtosis		15.124	.741

Descriptives

			Statistic	Std. Error
PS_12_13	Mean		.46826	.054507
	95% Confidence Interval for Mean	Lower Bound	.35791	
		Upper Bound	.57860	
	5% Trimmed Mean		.42682	
	Median		.35200	
	Variance		.116	
	Std. Deviation		.340397	
	Minimum		.114	
	Maximum		1.770	
	Range		1.656	
	Interquartile Range		.294	
	Skewness		2.217	.378
	Kurtosis		5.404	.741
PS_13_14	Mean		.43367	.051558
	95% Confidence Interval for Mean	Lower Bound	.32929	
		Upper Bound	.53804	
	5% Trimmed Mean		.39774	
	Median		.35600	
	Variance		.104	
	Std. Deviation		.321980	
	Minimum		.117	
	Maximum		1.397	
	Range		1.280	
	Interquartile Range		.231	
	Skewness		1.964	.378
	Kurtosis		3.419	.741
PS_14_15	Mean		.45321	.059627
	95% Confidence Interval for Mean	Lower Bound	.33250	
		Upper Bound	.57391	
	5% Trimmed Mean		.40729	
	Median		.32700	
	Variance		.139	
	Std. Deviation		.372368	
	Minimum		.116	
	Maximum		1.724	
	Range		1.608	
	Interquartile Range		.274	
	Skewness		2.084	.378
	Kurtosis		4.086	.741

Descriptives

			Statistic	Std. Error
PS_15_16	Mean		.44736	.061575
	95% Confidence Interval for Mean	Lower Bound	.32271	
		Upper Bound	.57201	
	5% Trimmed Mean		.39954	
	Median		.31900	
	Variance		.148	
	Std. Deviation		.384534	
	Minimum		.112	
	Maximum		1.644	
	Range		1.532	
	Interquartile Range		.263	
	Skewness		2.125	.378
	Kurtosis		4.015	.741
PS_16_17	Mean		.41400	.053987
	95% Confidence Interval for Mean	Lower Bound	.30471	
		Upper Bound	.52329	
	5% Trimmed Mean		.37818	
	Median		.31100	
	Variance		.114	
	Std. Deviation		.337151	
	Minimum		.109	
	Maximum		1.377	
	Range		1.268	
	Interquartile Range		.168	
	Skewness		1.817	.378
	Kurtosis		2.500	.741
PS_17_18	Mean		.38128	.049484
	95% Confidence Interval for Mean	Lower Bound	.28111	
		Upper Bound	.48146	
	5% Trimmed Mean		.34304	
	Median		.28100	
	Variance		.095	
	Std. Deviation		.309030	
	Minimum		.110	
	Maximum		1.360	
	Range		1.250	
	Interquartile Range		.217	
	Skewness		2.197	.378
	Kurtosis		4.452	.741

Descriptives

			Statistic	Std. Error
PS_18_19	Mean		.38462	.052102
	95% Confidence Interval for Mean	Lower Bound	.27914	
		Upper Bound	.49009	
	5% Trimmed Mean		.33960	
	Median		.25800	
	Variance		.106	
	Std. Deviation		.325379	
	Minimum		.105	
	Maximum		1.549	
	Range		1.444	
	Interquartile Range		.306	
	Skewness		2.223	.378
	Kurtosis		5.223	.741
PS_19_20	Mean		.35233	.044807
	95% Confidence Interval for Mean	Lower Bound	.26163	
		Upper Bound	.44304	
	5% Trimmed Mean		.31332	
	Median		.27000	
	Variance		.078	
	Std. Deviation		.279822	
	Minimum		.110	
	Maximum		1.351	
	Range		1.241	
	Interquartile Range		.315	
	Skewness		2.170	.378
	Kurtosis		5.278	.741

Tests of Normality

	Kolmogorov-Smirnov[a]			Shapiro-Wilk		
	Statistic	df	Sig.	Statistic	df	Sig.
BrainRate	.176	39	.004	.825	39	<.001
PermutationEntropy	.107	39	.200*	.950	39	.084
PermutationEntropy_01	.107	39	.200*	.950	39	.084
SampleEntropy	.179	39	.003	.924	39	.011
SpectralEntropy_bits	.181	39	.003	.884	39	<.001
SpectralEntropy_01	.181	39	.003	.884	39	<.001
HiguchiFractalDimension	.144	39	.041	.950	39	.085
LempelZivComplexity	.187	39	.001	.924	39	.012
RenyiEntropy	.158	39	.015	.874	39	<.001
TsallisEntropy	.451	39	<.001	.274	39	<.001
PS_4_5	.262	39	<.001	.478	39	<.001
PS_5_6	.242	39	<.001	.518	39	<.001
PS_6_7	.207	39	<.001	.609	39	<.001
PS_7_8	.232	39	<.001	.645	39	<.001
PS_8_9	.182	39	.002	.780	39	<.001
PS_9_10	.216	39	<.001	.659	39	<.001
PS_10_11	.197	39	<.001	.727	39	<.001
PS_11_12	.242	39	<.001	.629	39	<.001
PS_12_13	.260	39	<.001	.748	39	<.001
PS_13_14	.248	39	<.001	.749	39	<.001
PS_14_15	.232	39	<.001	.739	39	<.001
PS_15_16	.271	39	<.001	.711	39	<.001
PS_16_17	.291	39	<.001	.743	39	<.001
PS_17_18	.259	39	<.001	.709	39	<.001
PS_18_19	.243	39	<.001	.735	39	<.001
PS_19_20	.206	39	<.001	.758	39	<.001

*. This is a lower bound of the true significance.

a. Lilliefors Significance Correction

BrainRate

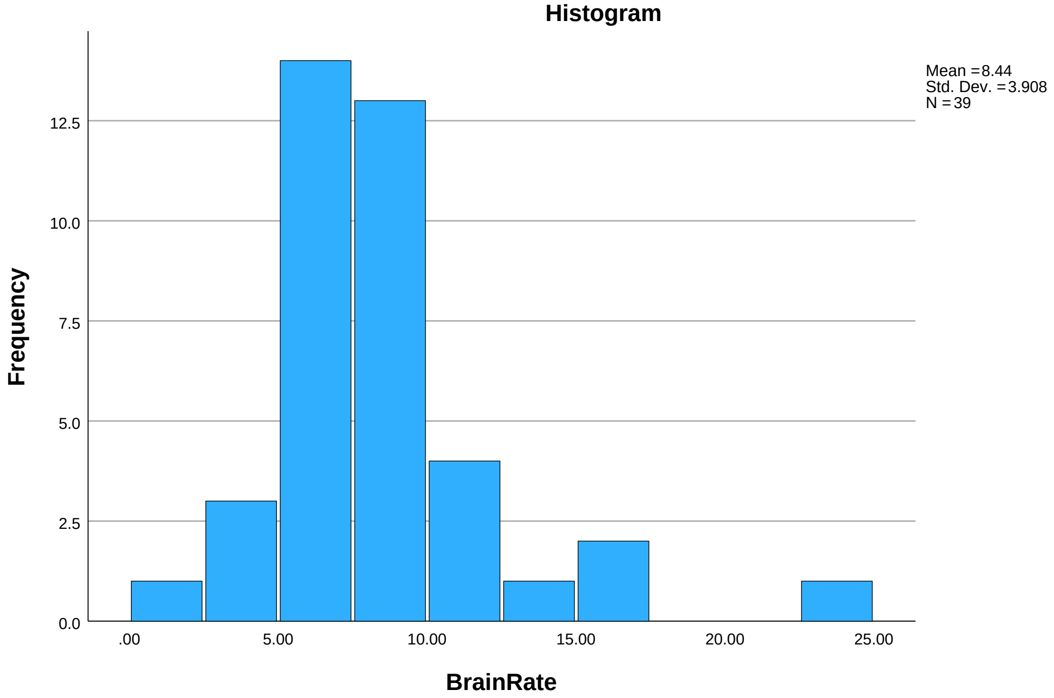


```
BrainRate Stem-and-Leaf Plot

 Frequency    Stem &  Leaf

     1.00        2 .  2
      .00        3 .
     3.00        4 .  017
     3.00        5 .  479
    10.00        6 .  0123456688
     4.00        7 .  2559
     5.00        8 .  00124
     5.00        9 .  33356
     2.00       10 .  04
     1.00       11 .  8
     2.00       12 .  38
     3.00 Extremes    (>=15.1)

 Stem width:      1.00
 Each leaf:       1 case(s)
```

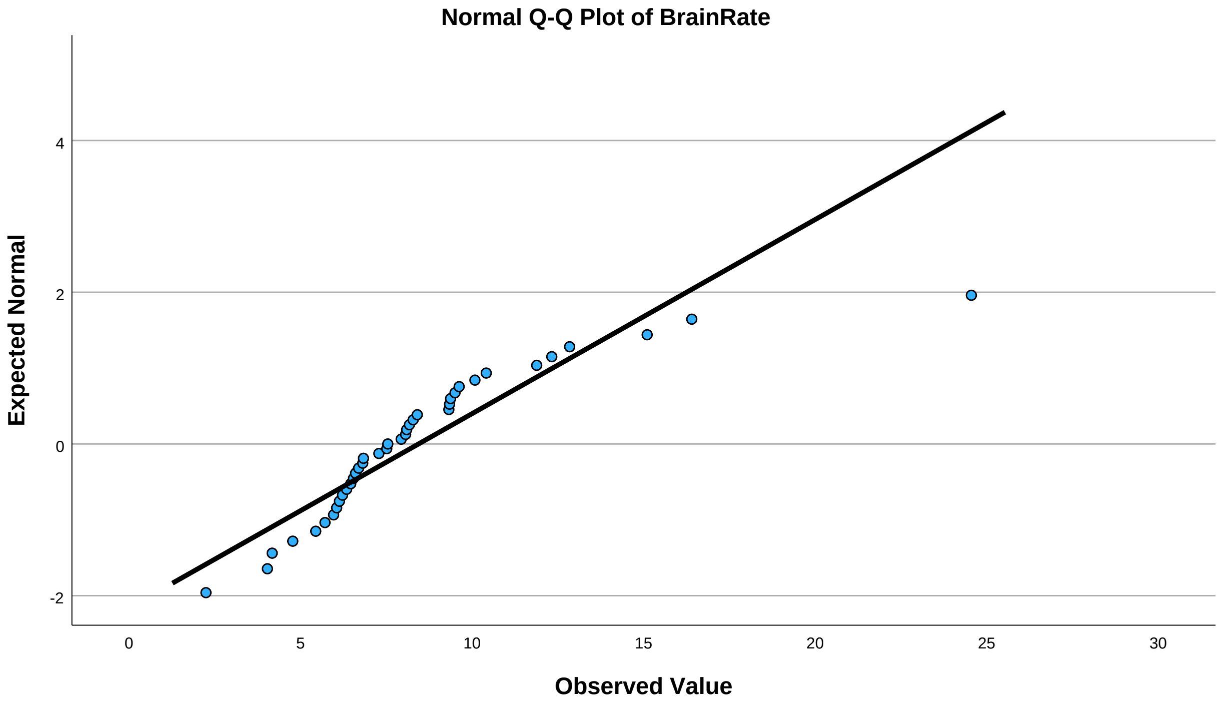
Normal Q-Q Plot of BrainRate
Expected Normal
4
2
0
-2
0
5
10
15
20
25
30
Observed Value

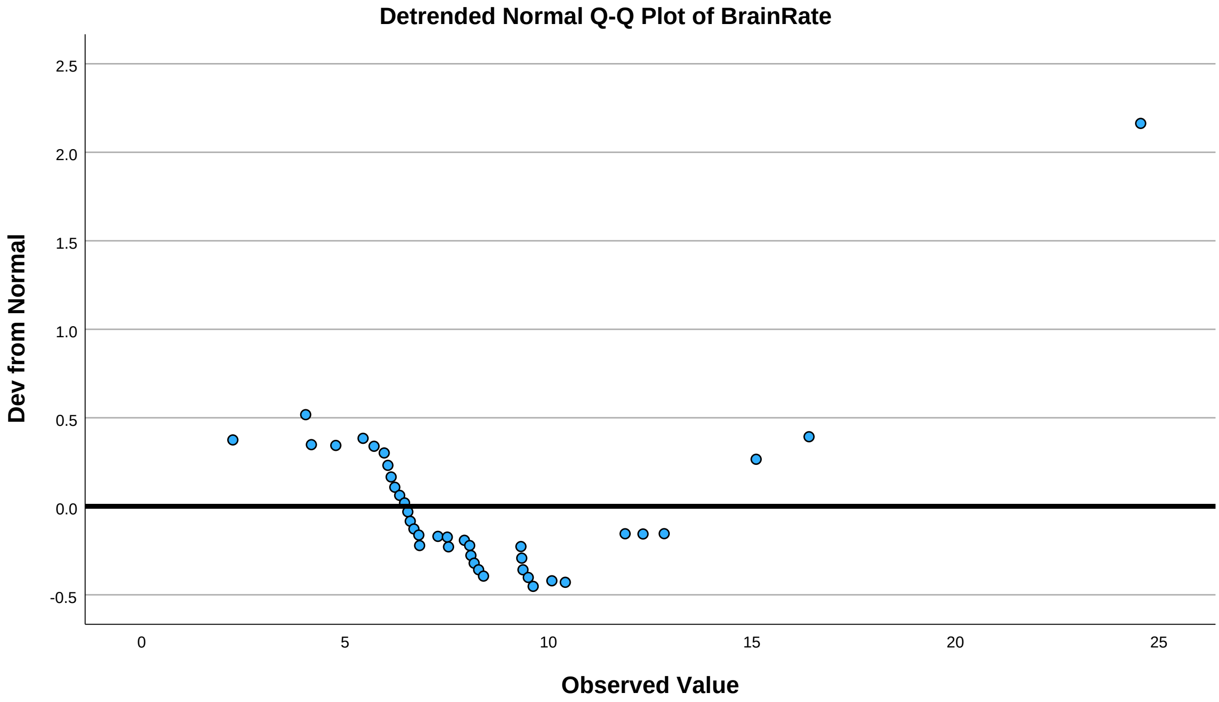
Detrended Normal Q-Q Plot of BrainRate
Dev from Normal
2.5
2.0
1.5
1.0
0.5
0.0
-0.5
0
5
10
15
20
25
Observed Value

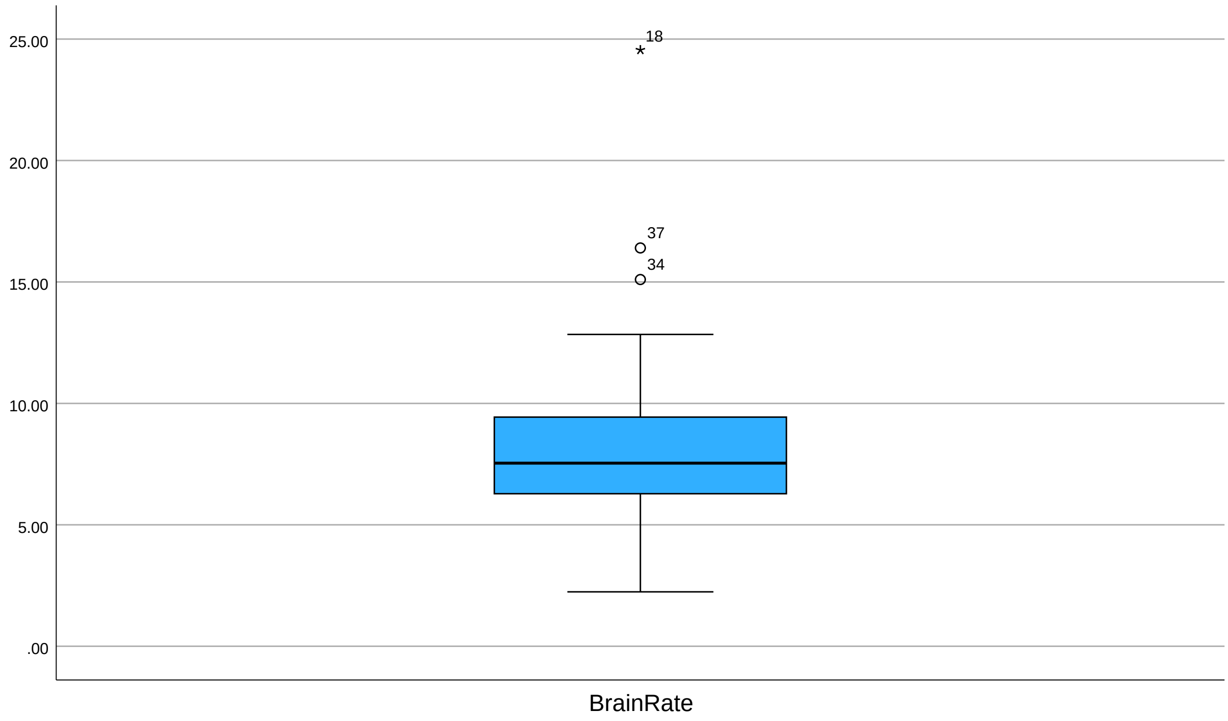

PermutationEntropy

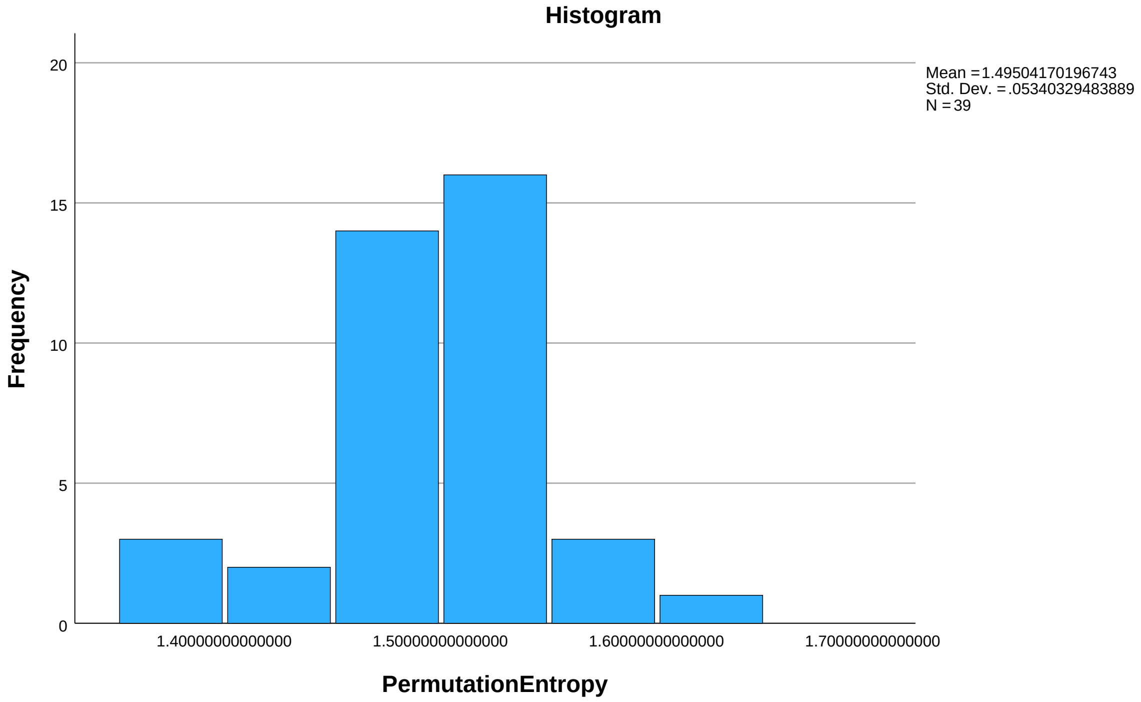


```
PermutationEntropy Stem-and-Leaf Plot

 Frequency    Stem &  Leaf
```

```
  3.00 Extremes    (=<1.39)
 1.00      14 .  2
 2.00      14 .  45
 8.00      14 .  66677777
 5.00      14 .  88889
 7.00      15 .  0001111
 8.00      15 .  22223333
 1.00      15 .  4
 3.00      15 .  666
  1.00 Extremes    (>=1.63)

Stem width:   .1000000
Each leaf:       1 case(s)
```

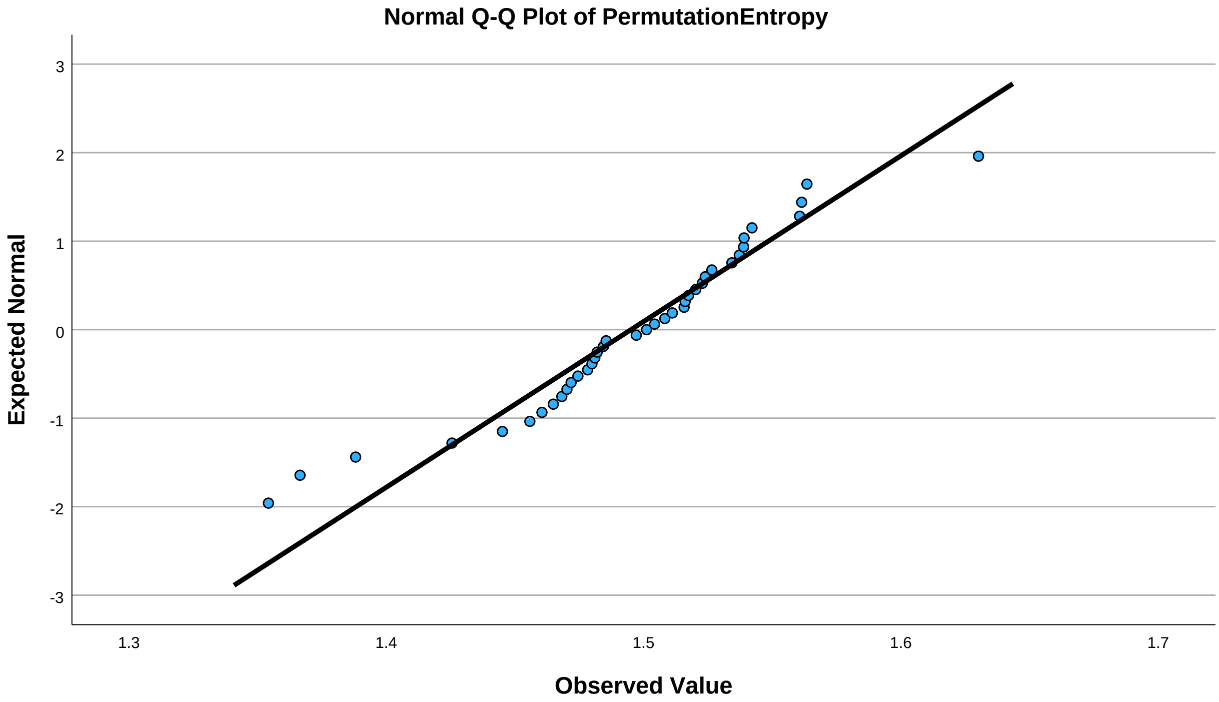

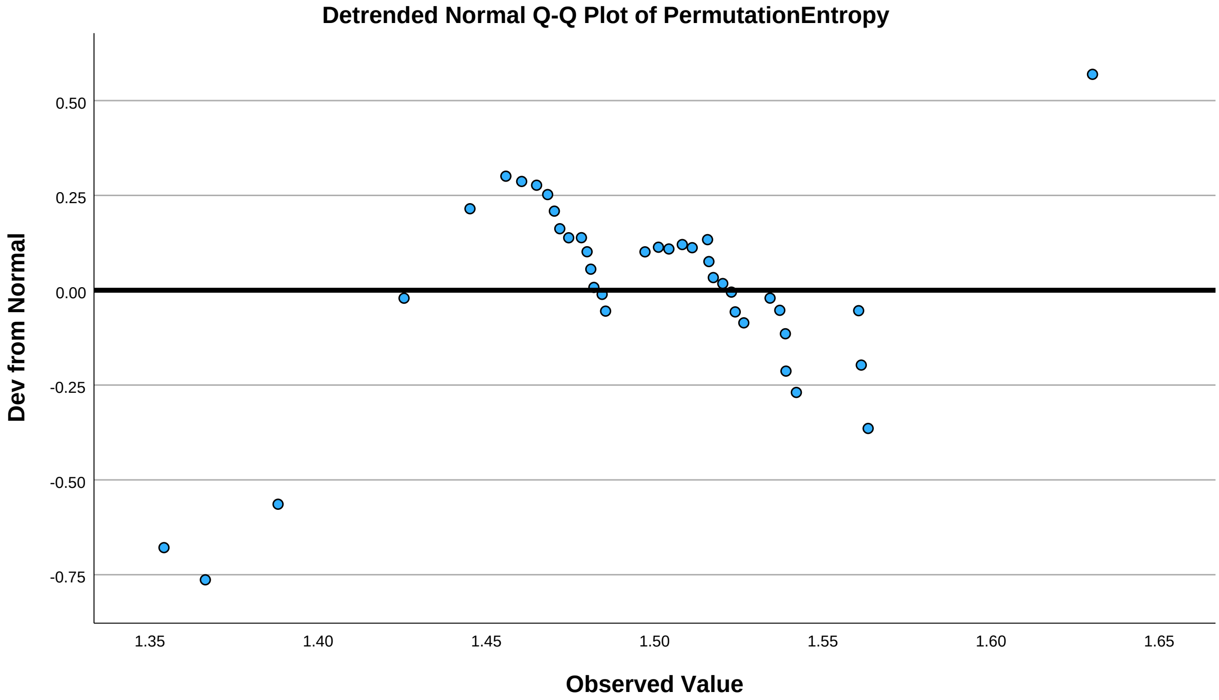

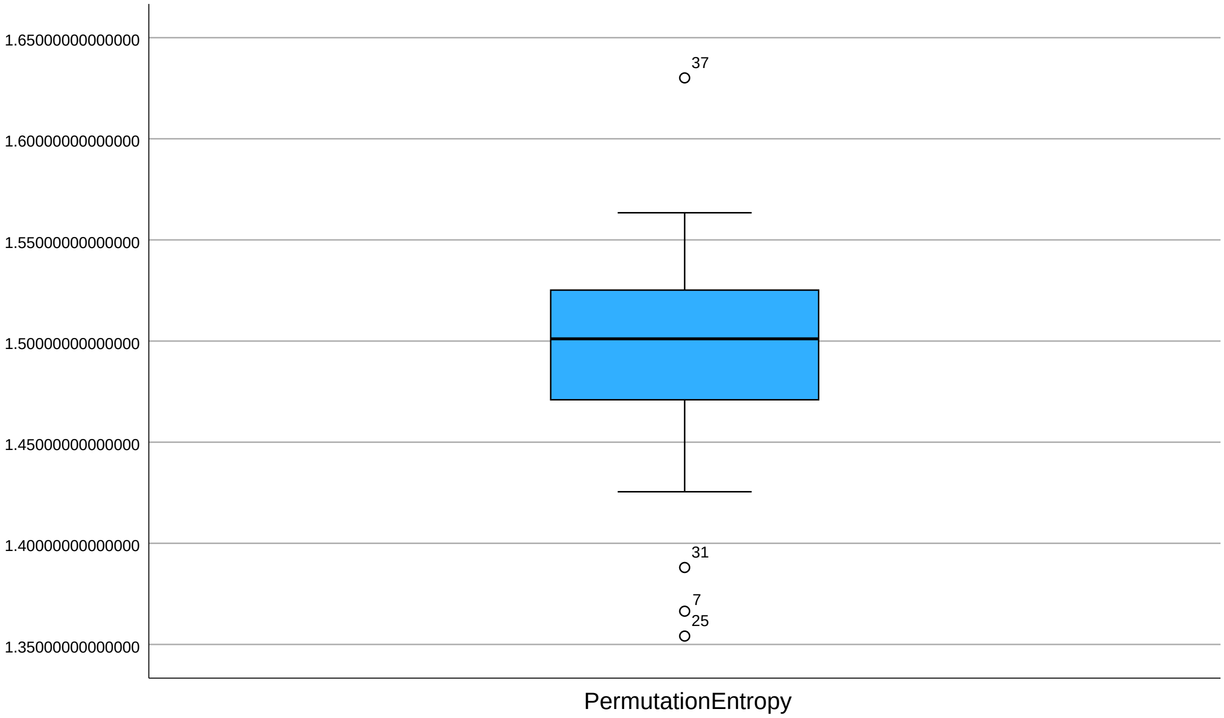

PermutationEntropy_01

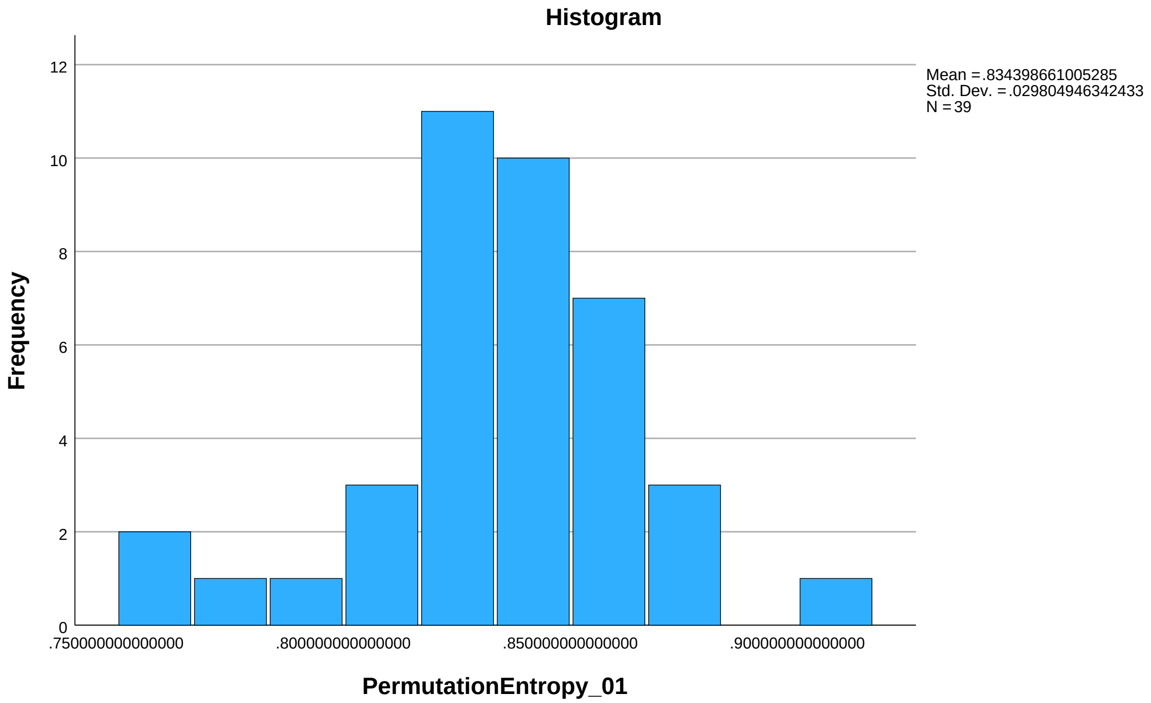

PermutationEntropy_01 Stem-and-Leaf Plot

```
 Frequency    Stem &  Leaf

     3.00 Extremes    (=<.775)
     1.00       79 .  5
     1.00       80 .  6
     4.00       81 .  2579
     9.00       82 .  012556789
     3.00       83 .  579
     7.00       84 .  1356689
     6.00       85 .  016788
     1.00       86 .  0
     3.00       87 .  012
     1.00 Extremes    (>=.910)

 Stem width:   .0100000
 Each leaf:        1 case(s)
```

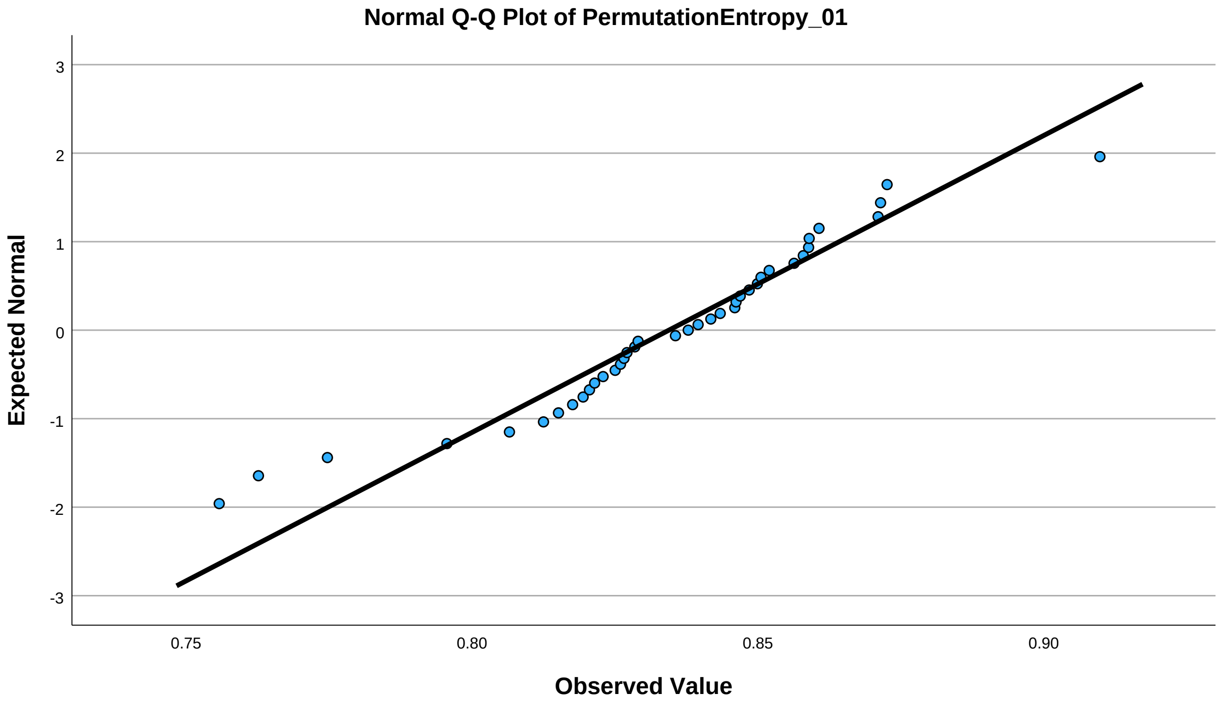
Normal Q-Q Plot of PermutationEntropy_01
Expected Normal
3
2
1
0
-1
-2
-3
0.75
0.80
0.85
0.90
Observed Value

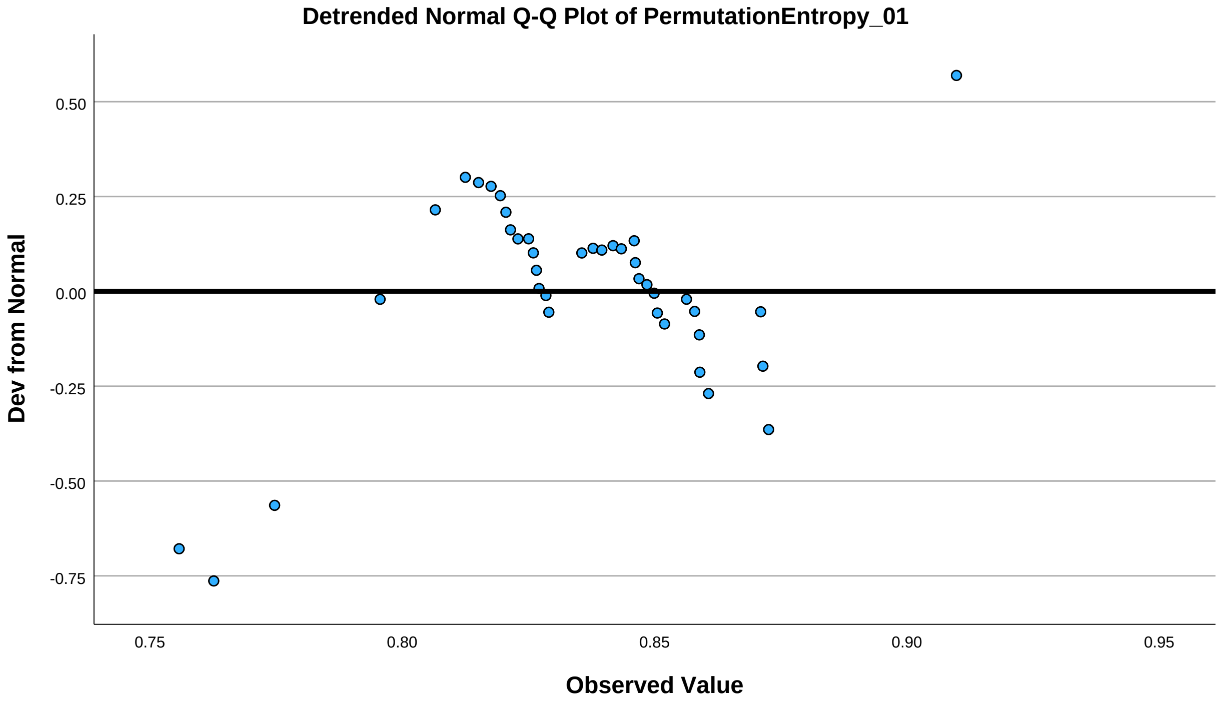
Detrended Normal Q-Q Plot of PermutationEntropy_01
Dev from Normal
0.50
0.25
0.00
-0.25
-0.50
-0.75
0.75
0.80
0.85
0.90
0.95
Observed Value

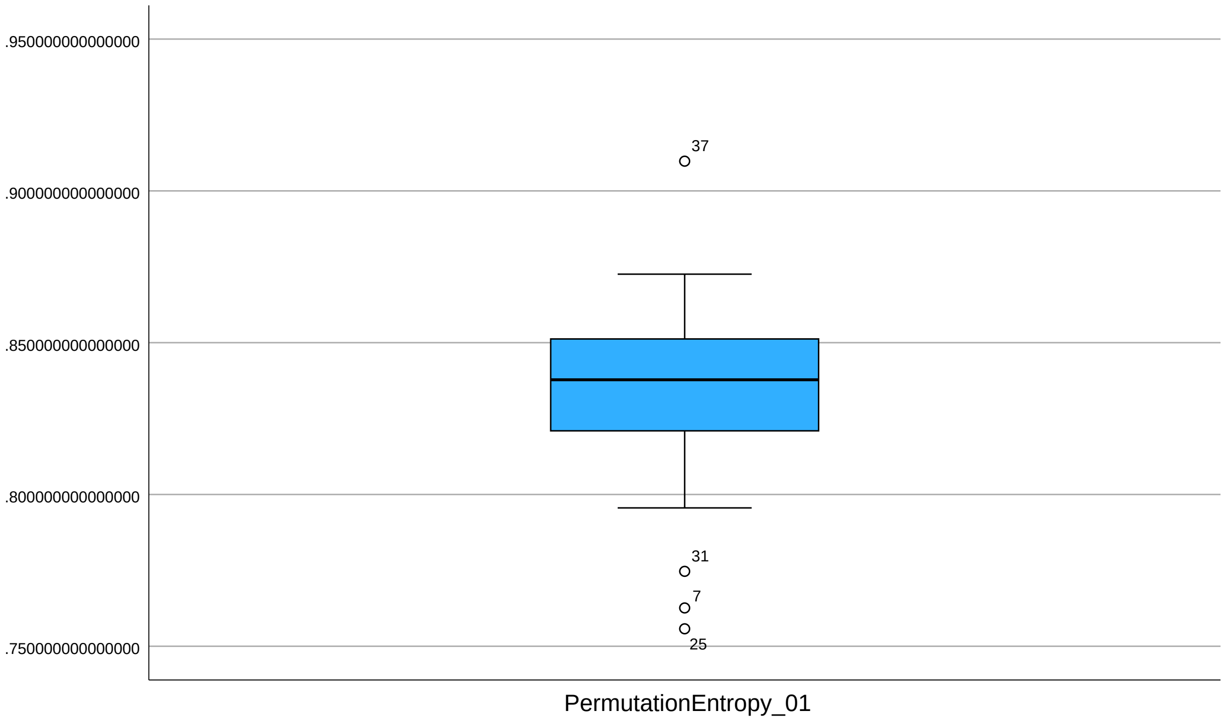

SampleEntropy

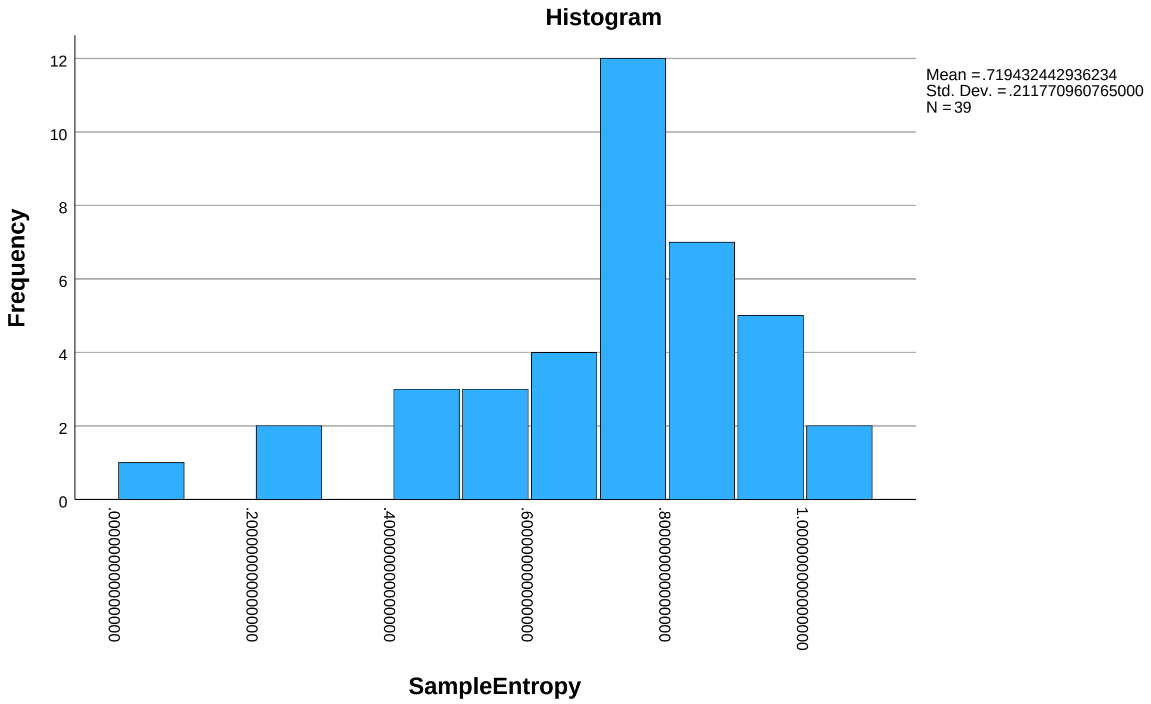


```
SampleEntropy Stem-and-Leaf Plot

 Frequency    Stem &  Leaf
```

```
 3.00 Extremes    (=<.29)
 3.00      4 . 447
 3.00      5 . 168
 4.00      6 . 5689
12.00      7 . 233334556677
 7.00      8 . 0134458
 5.00      9 . 23458
 2.00     10 . 06

Stem width:   .1000000
Each leaf:        1 case(s)
```

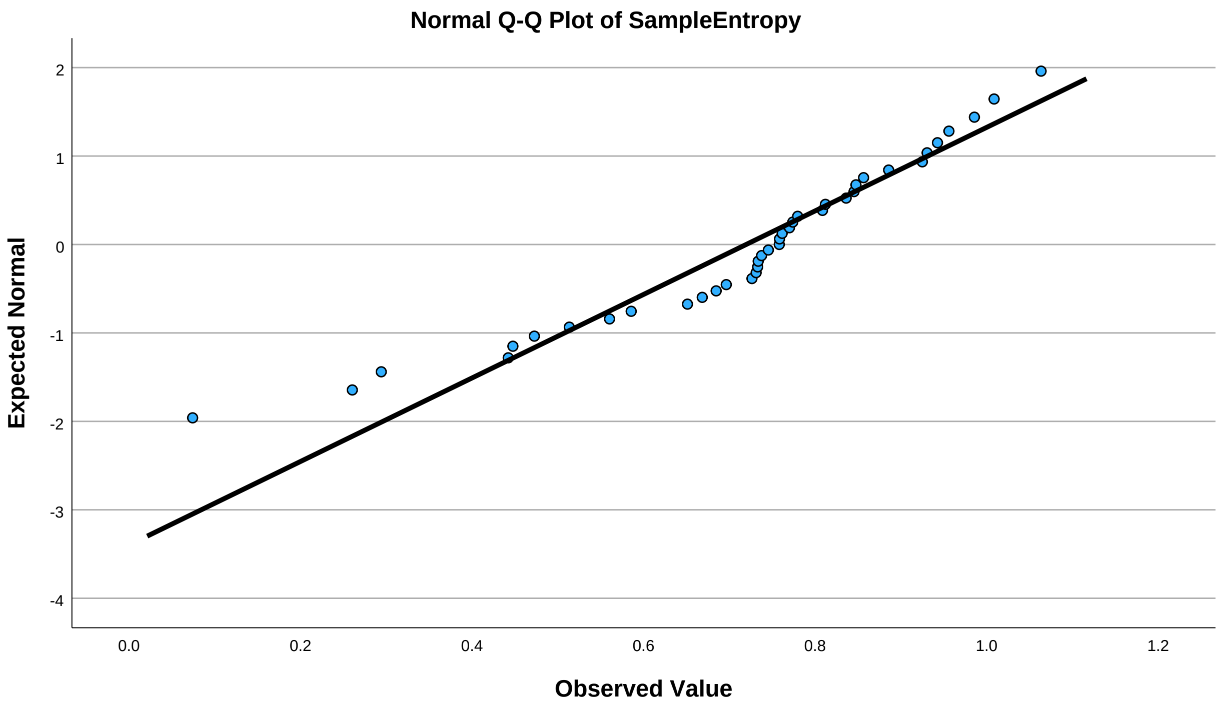

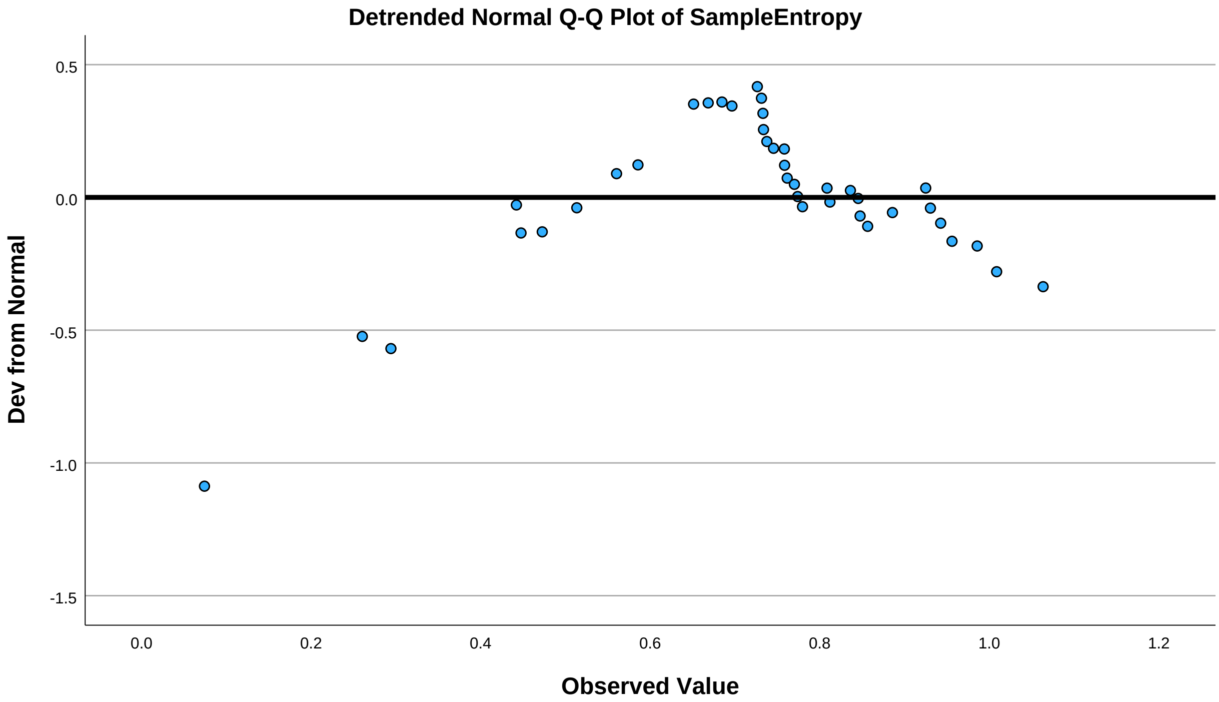

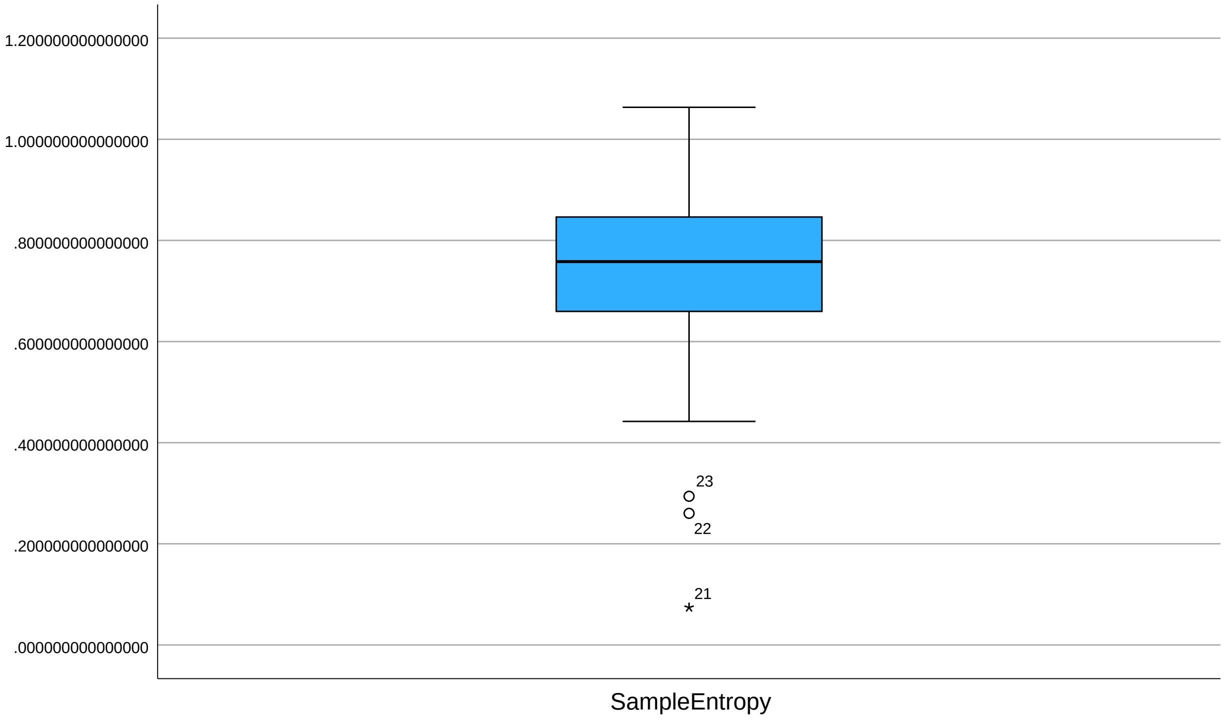

SpectralEntropy_bits

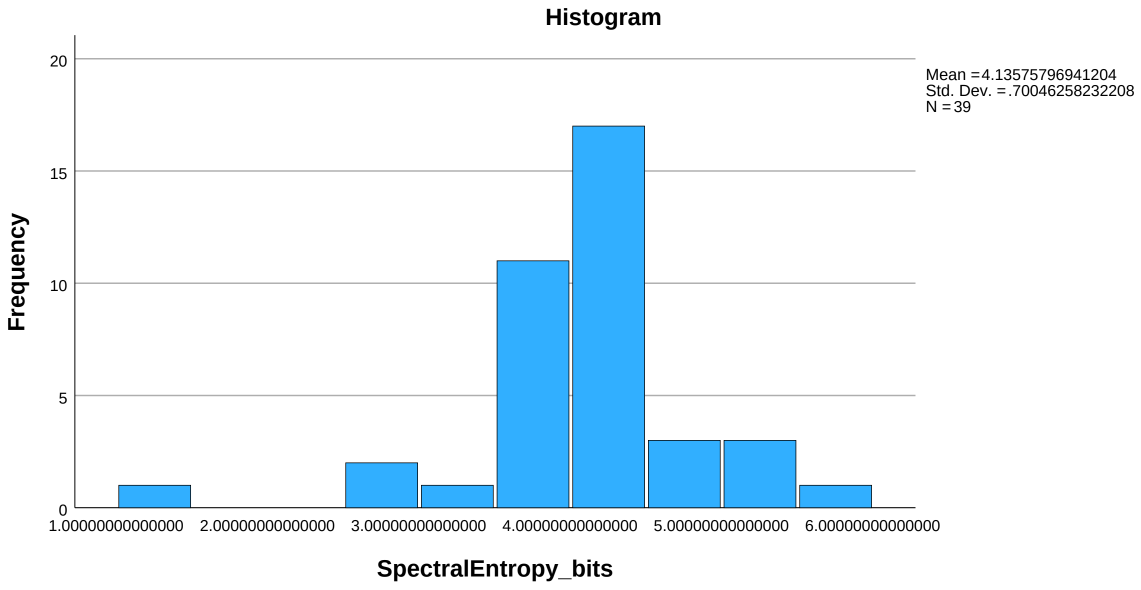


```
SpectralEntropy_bits Stem-and-Leaf Plot

 Frequency    Stem &  Leaf

     3.00 Extremes    (=<3.0)
     1.00        3 .  4
    11.00        3 .  55888999999
    17.00        4 .  01111222333334444
     3.00        4 .  578
     2.00        5 .  00
     2.00 Extremes    (>=5.2)

 Stem width:   1.000000
 Each leaf:        1 case(s)
```

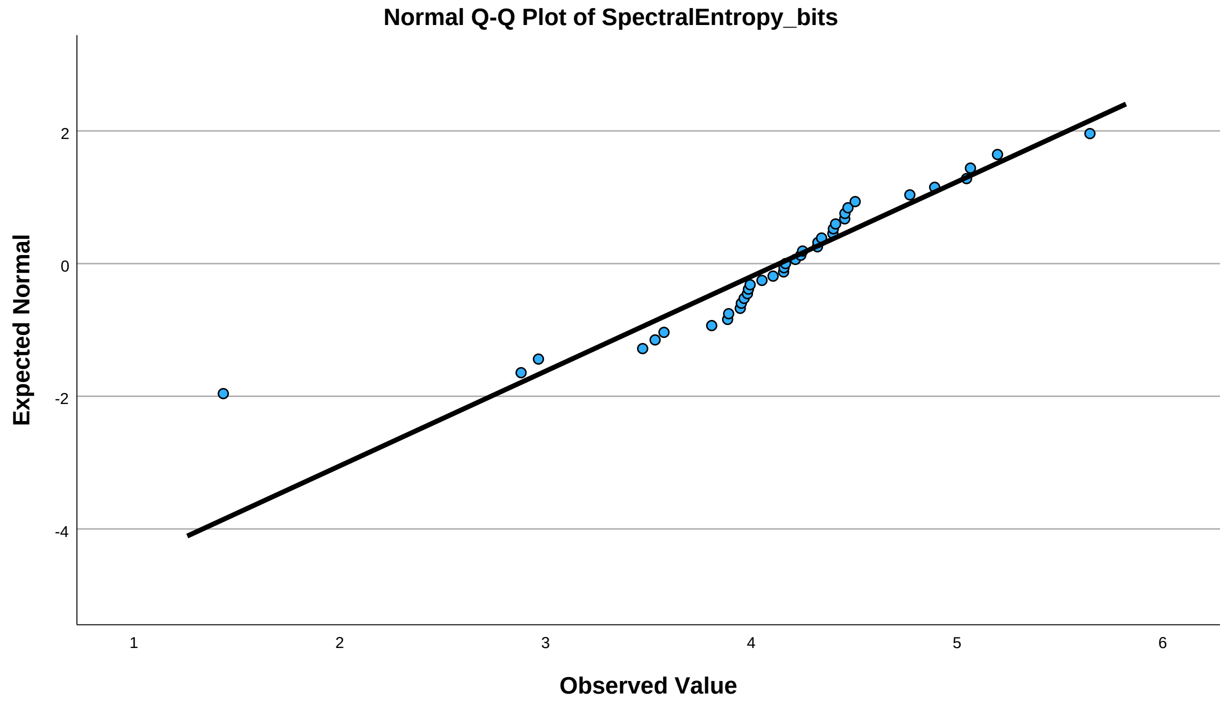
Normal Q-Q Plot of SpectralEntropy_bits
Expected Normal
2
0
-2
-4
1
2
3
4
5
6
Observed Value

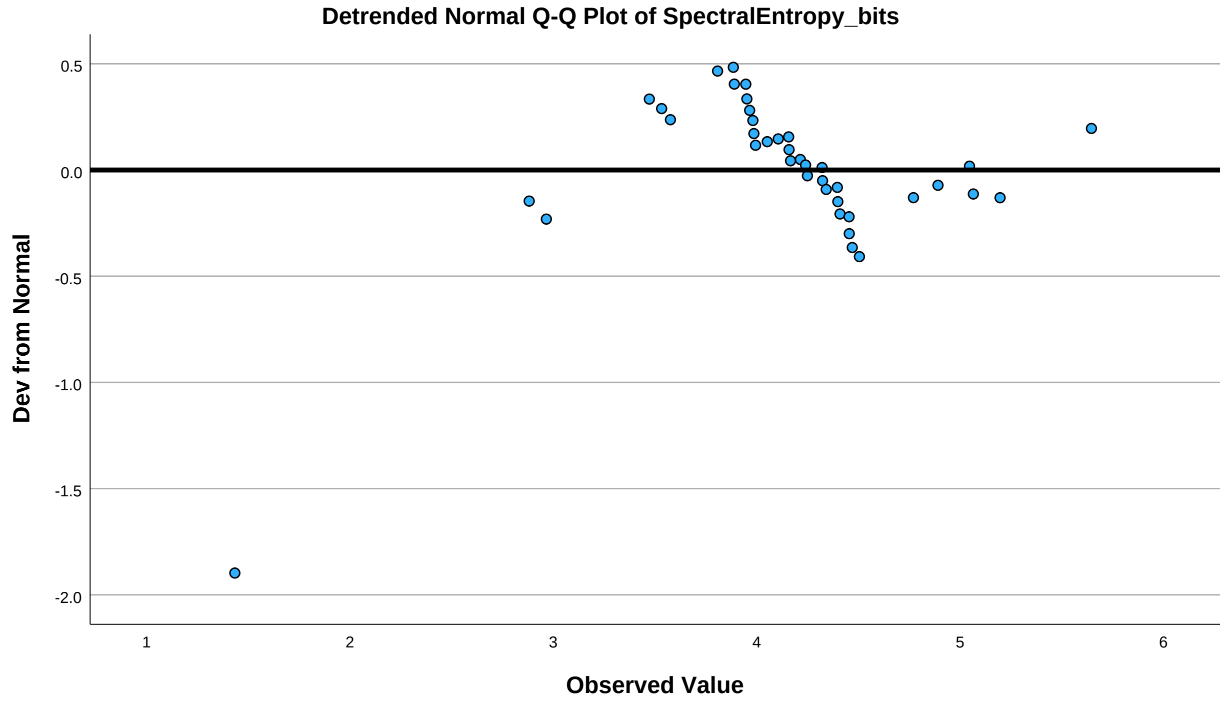
Detrended Normal Q-Q Plot of SpectralEntropy_bits
Dev from Normal
0.5
0.0
-0.5
-1.0
-1.5
-2.0
1
2
3
4
5
6
Observed Value

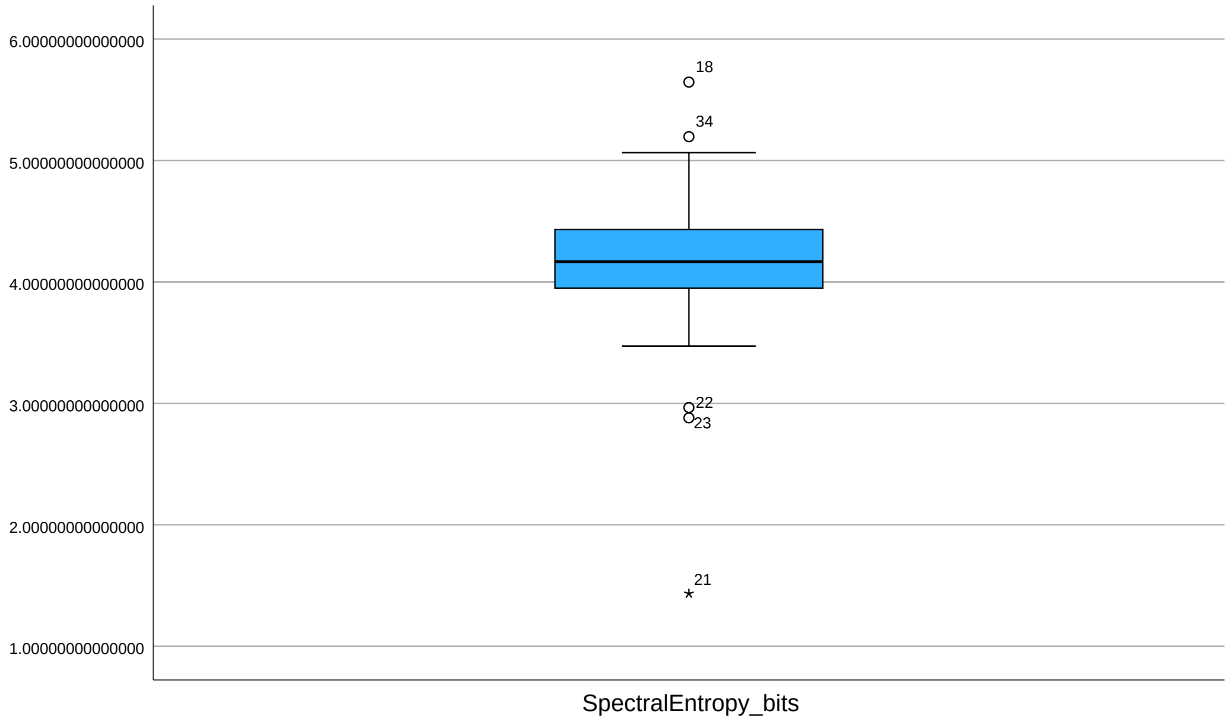

SpectralEntropy_01

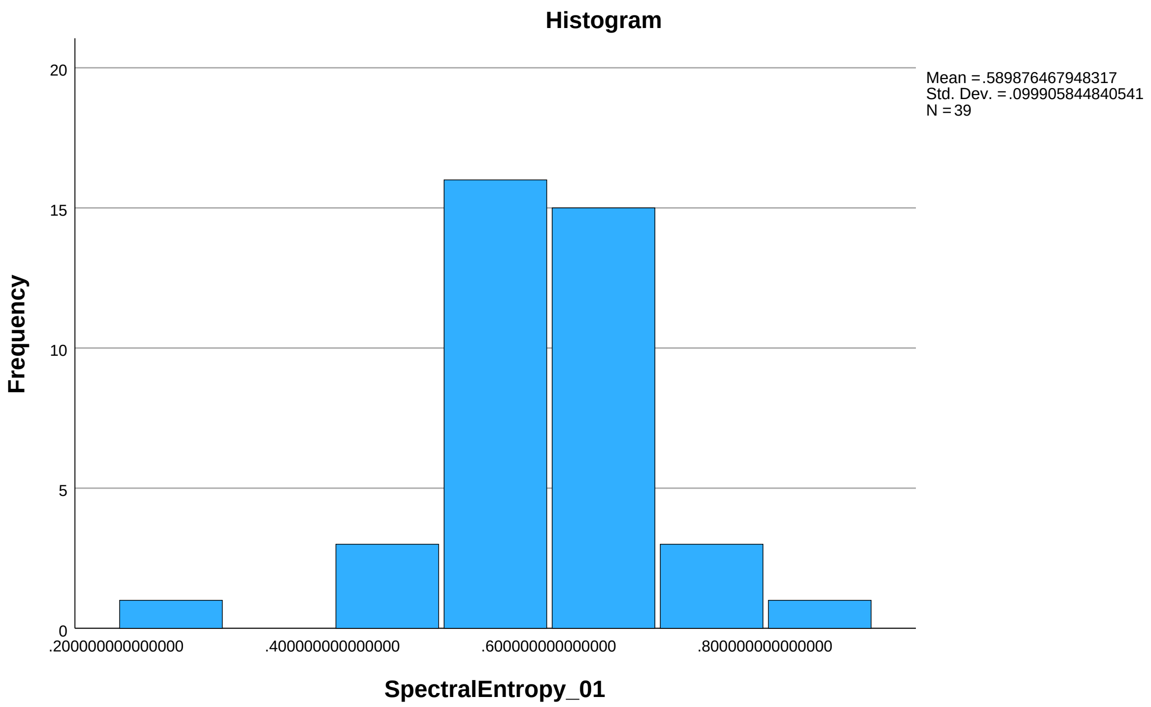


```
SpectralEntropy_01 Stem-and-Leaf Plot

 Frequency    Stem &  Leaf
```

```
 3.00 Extremes    (=<.42)
 1.00      4 . 9
 3.00      5 . 004
13.00      5 . 5566666678999
13.00      6 . 0001112223334
 2.00      6 . 89
 2.00      7 . 12
 2.00 Extremes    (>=.74)

Stem width:   .1000000
Each leaf:       1 case(s)
```

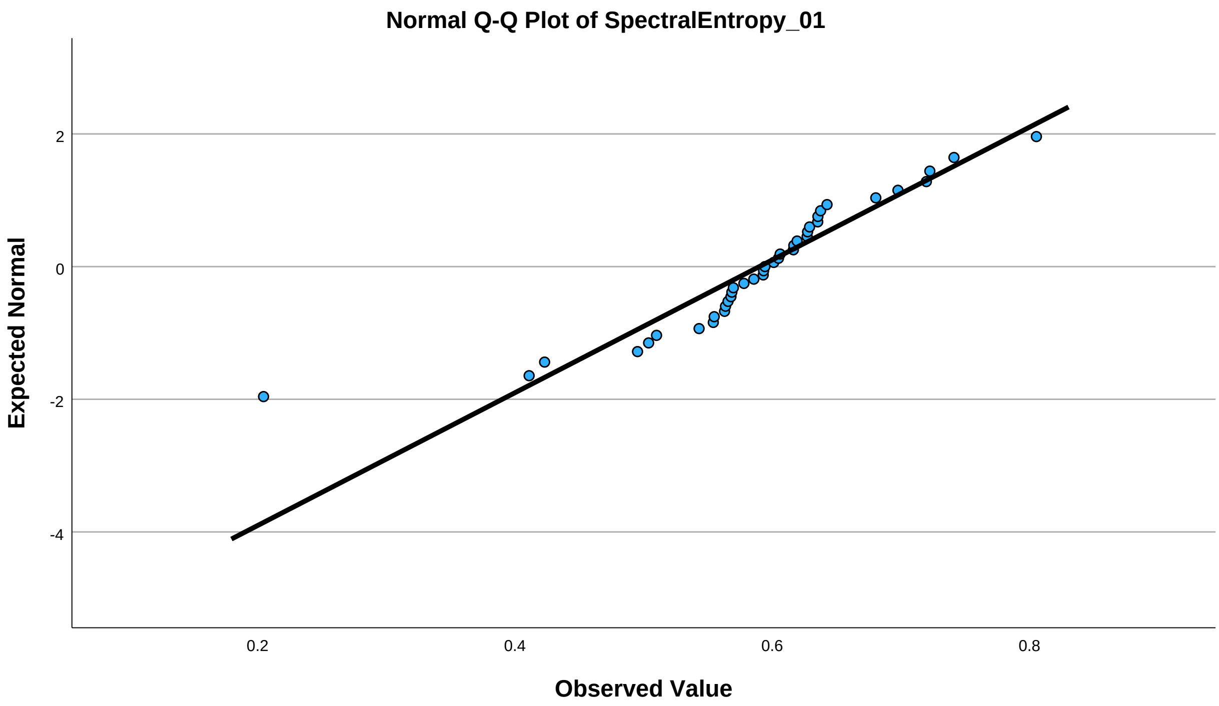

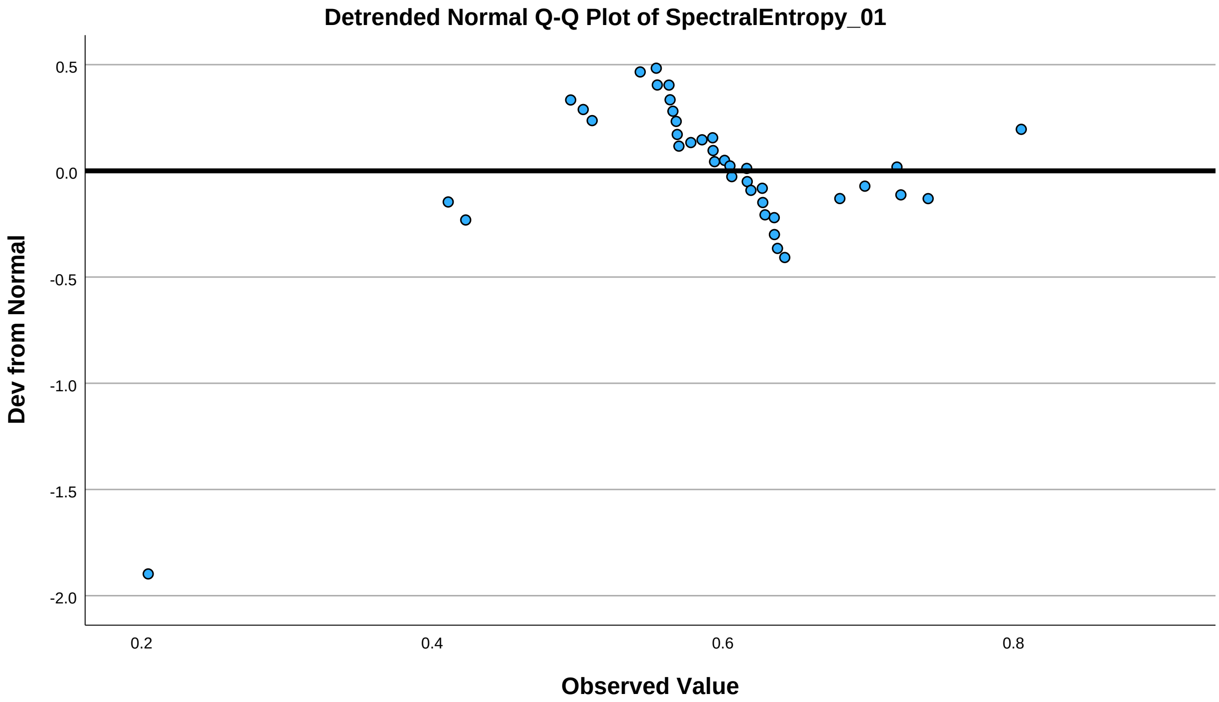

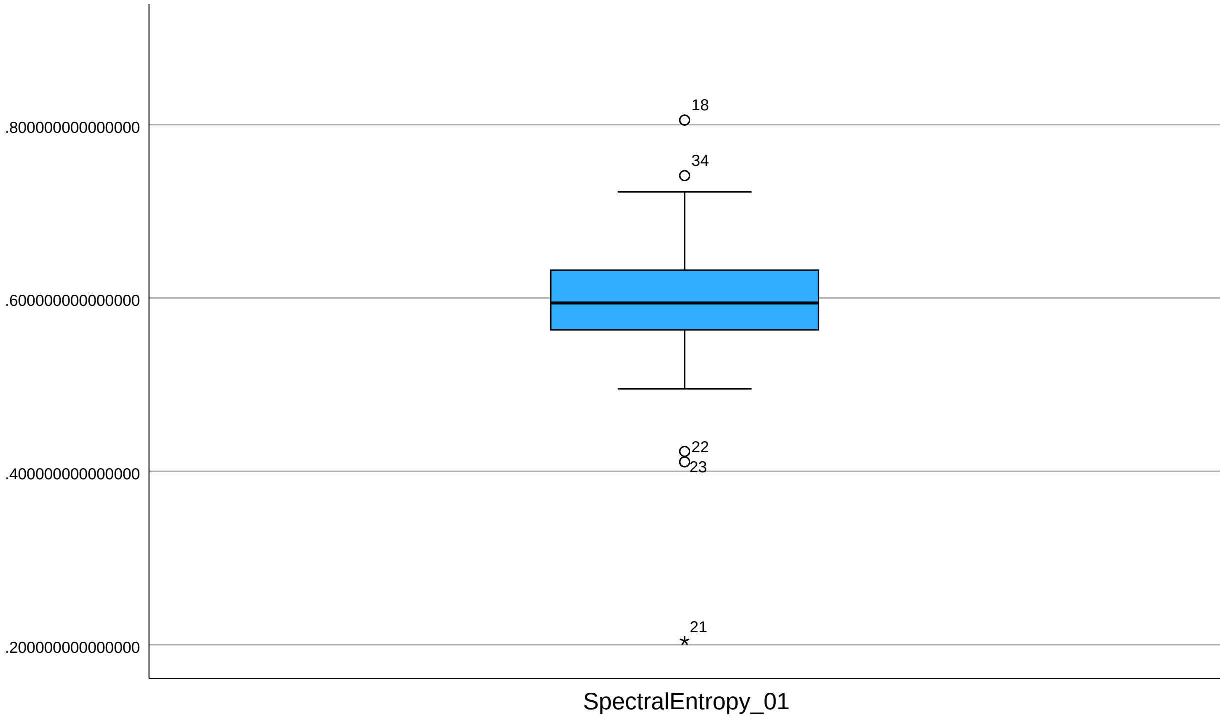

HiguchiFractalDimension

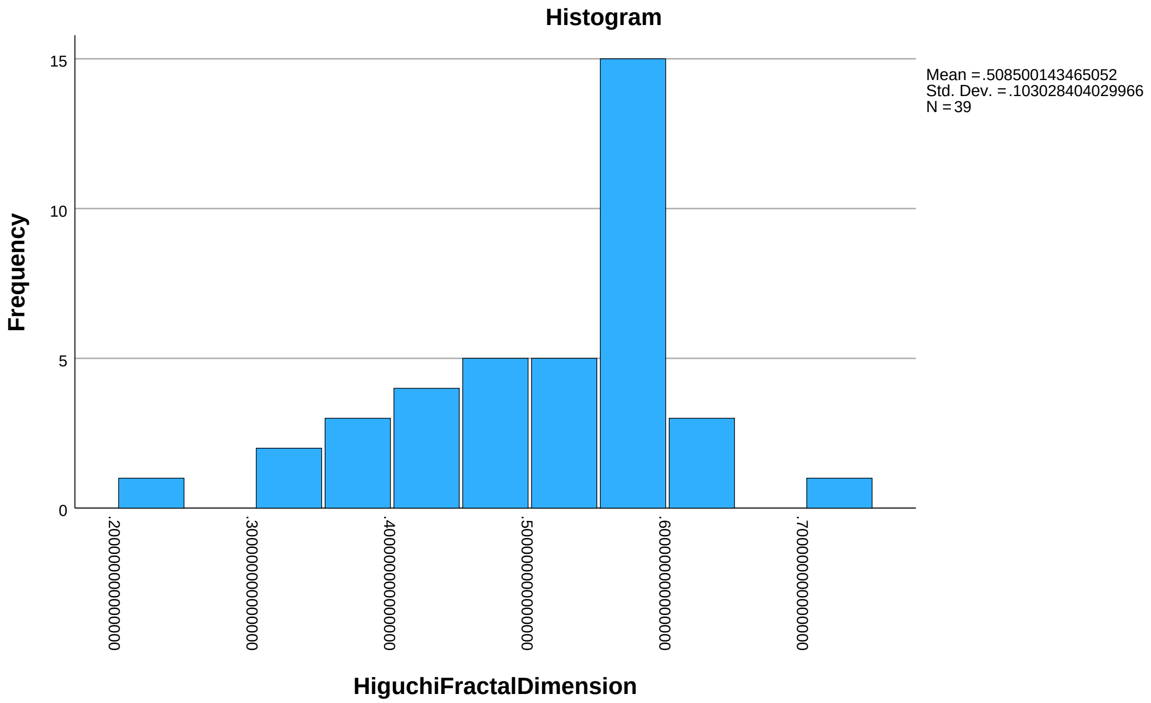


```
HiguchiFractalDimension Stem-and-Leaf Plot

 Frequency    Stem &  Leaf

     1.00 Extremes    (=<.22)
     2.00        3 .  02
     3.00        3 .  689
     4.00        4 .  0022
     5.00        4 .  67789
     5.00        5 .  00233
    15.00        5 .  555555567788899
     3.00        6 .  044
      .00        6 .
     1.00        7 .  1

 Stem width:   .1000000
 Each leaf:       1 case(s)
```

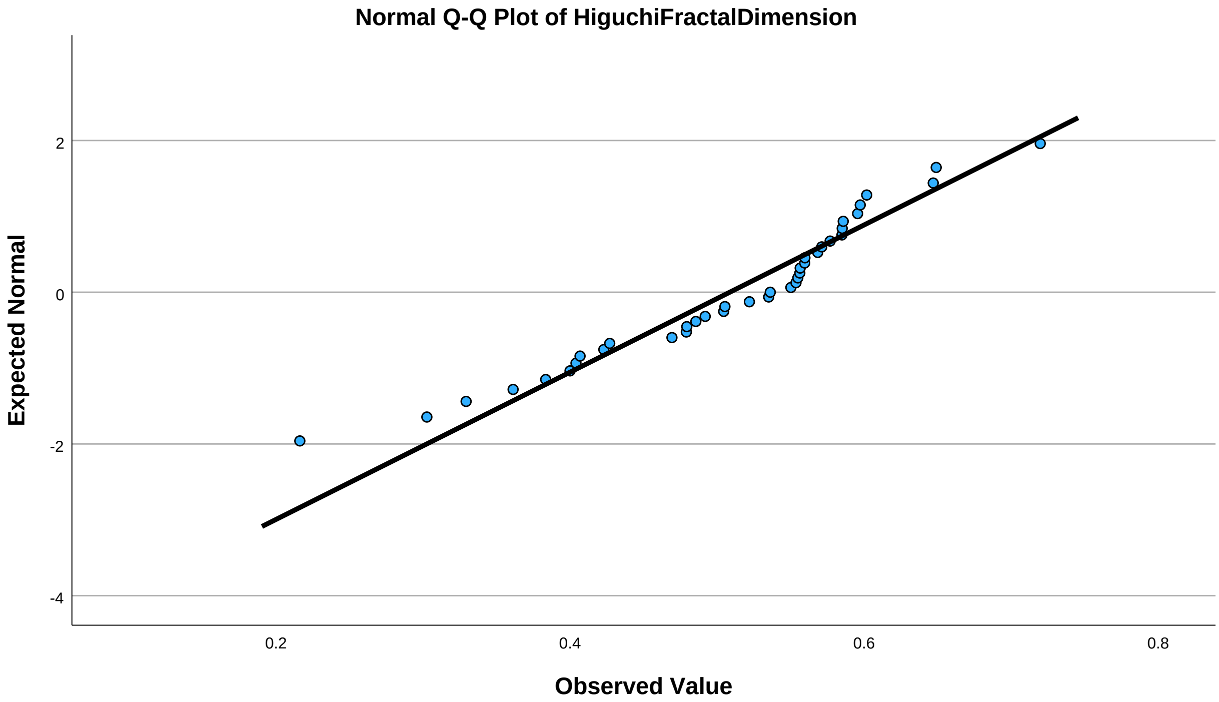
Normal Q-Q Plot of HiguchiFractalDimension
Expected Normal
Observed Value
2
0
-2
-4
0.2
0.4
0.6
0.8

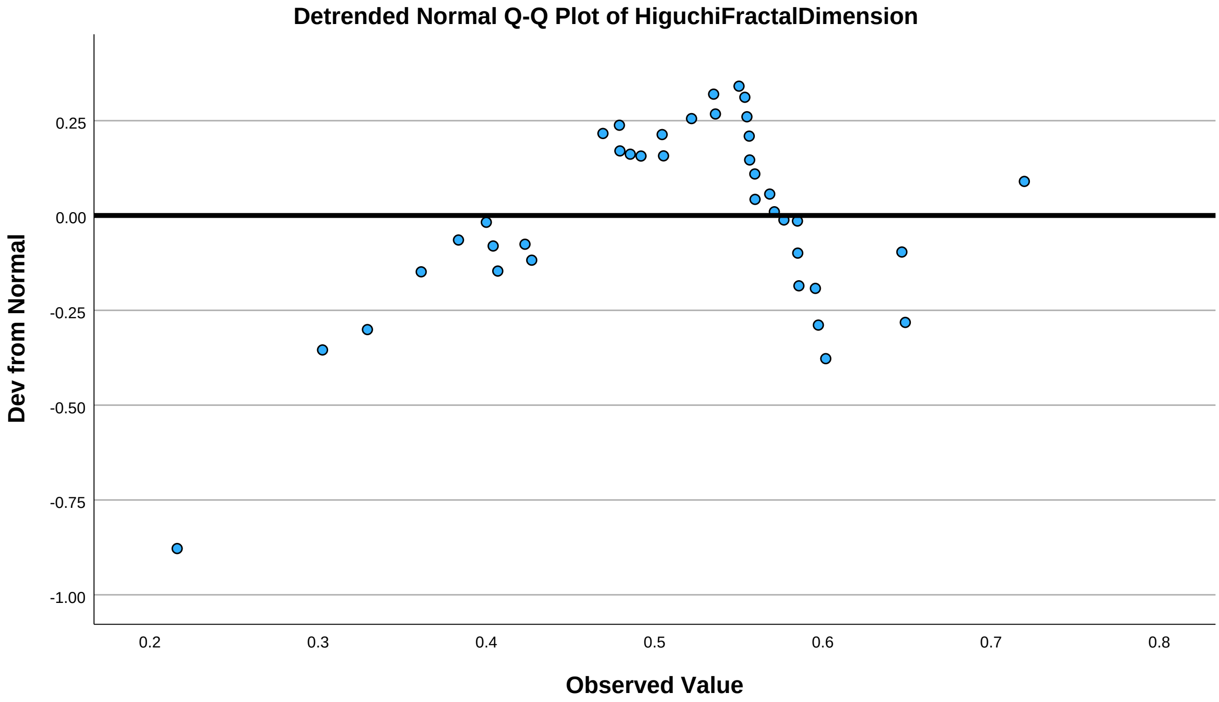
Detrended Normal Q-Q Plot of HiguchiFractalDimension
Dev from Normal
Observed Value
0.25
0.00
-0.25
-0.50
-0.75
-1.00
0.2
0.3
0.4
0.5
0.6
0.7
0.8

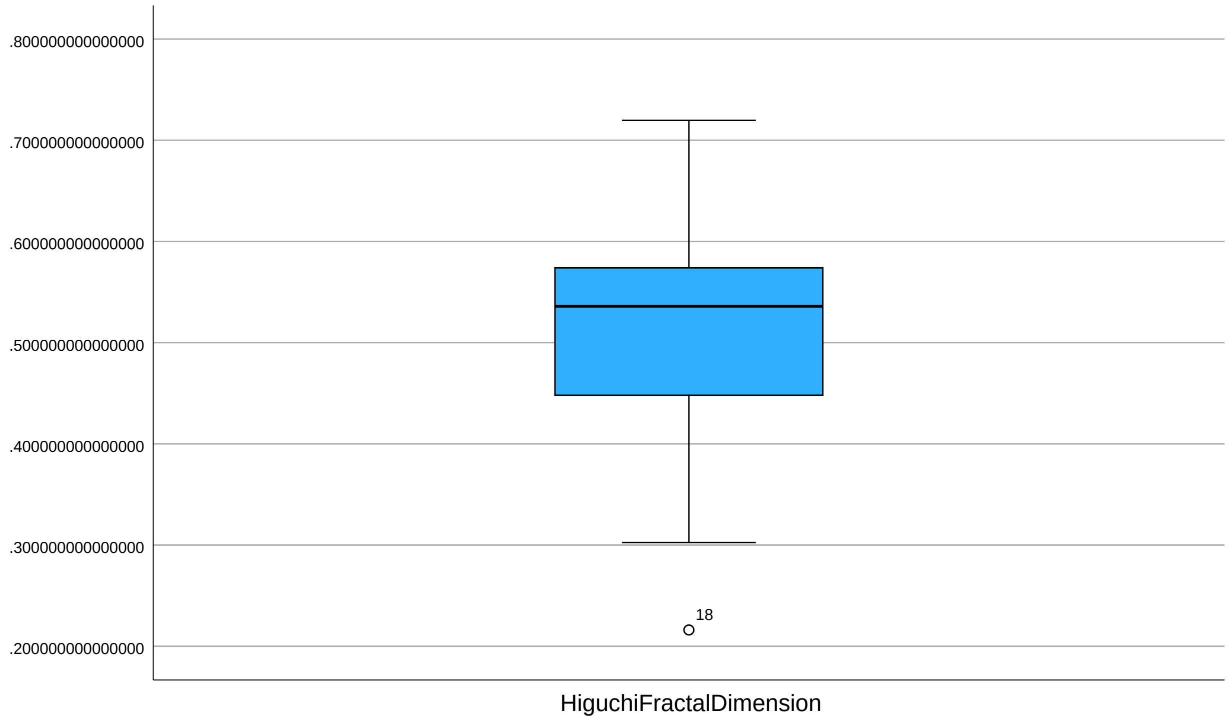

LempelZivComplexity

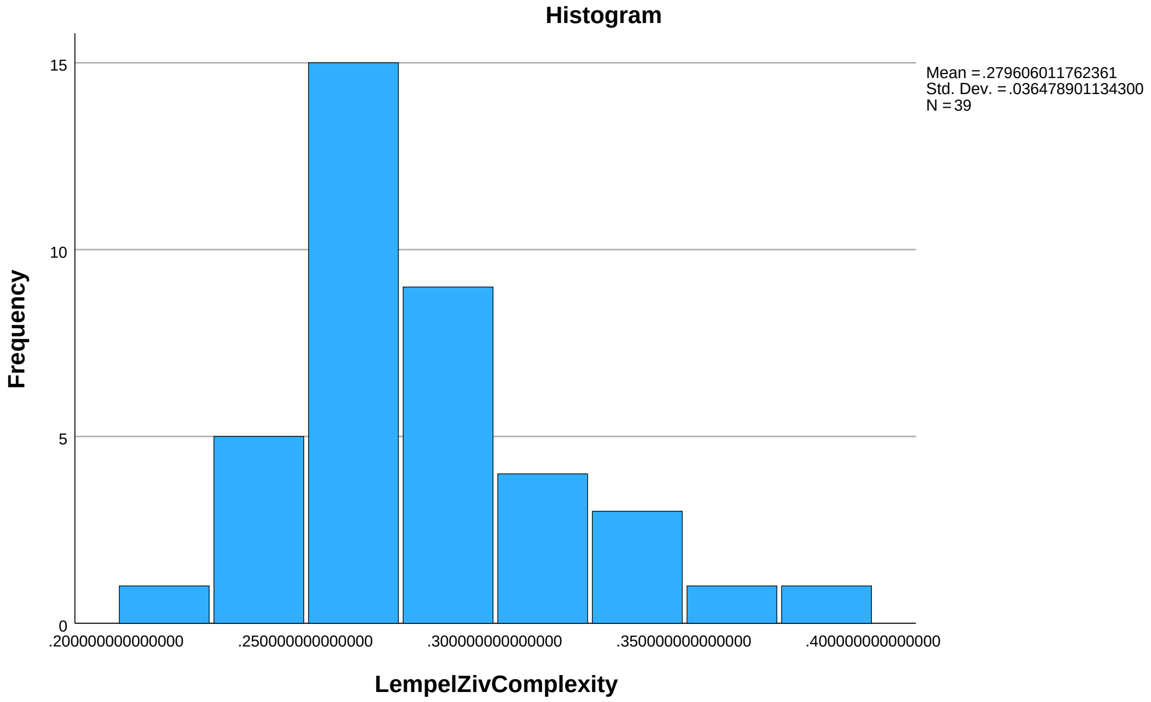


```
LempelZivComplexity Stem-and-Leaf Plot

 Frequency    Stem &  Leaf
```

```
 1.00 Extremes    (=<.200)
2.00      23 .  79
3.00      24 .  258
6.00      25 .  144669
6.00      26 .  335889
6.00      27 .  013689
6.00      28 .  113467
 .00     29 .
2.00      30 .  02
1.00      31 .  9
1.00      32 .  1
 5.00 Extremes    (>=.335)

Stem width:   .0100000
Each leaf:       1 case(s)
```

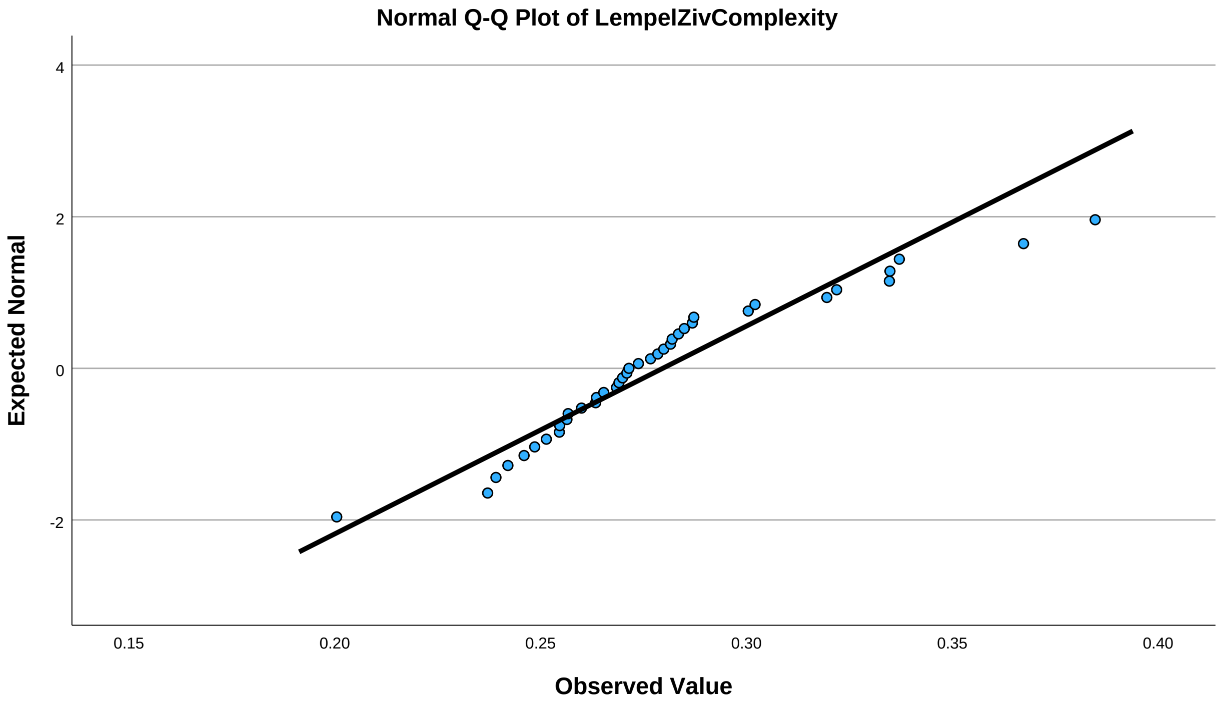

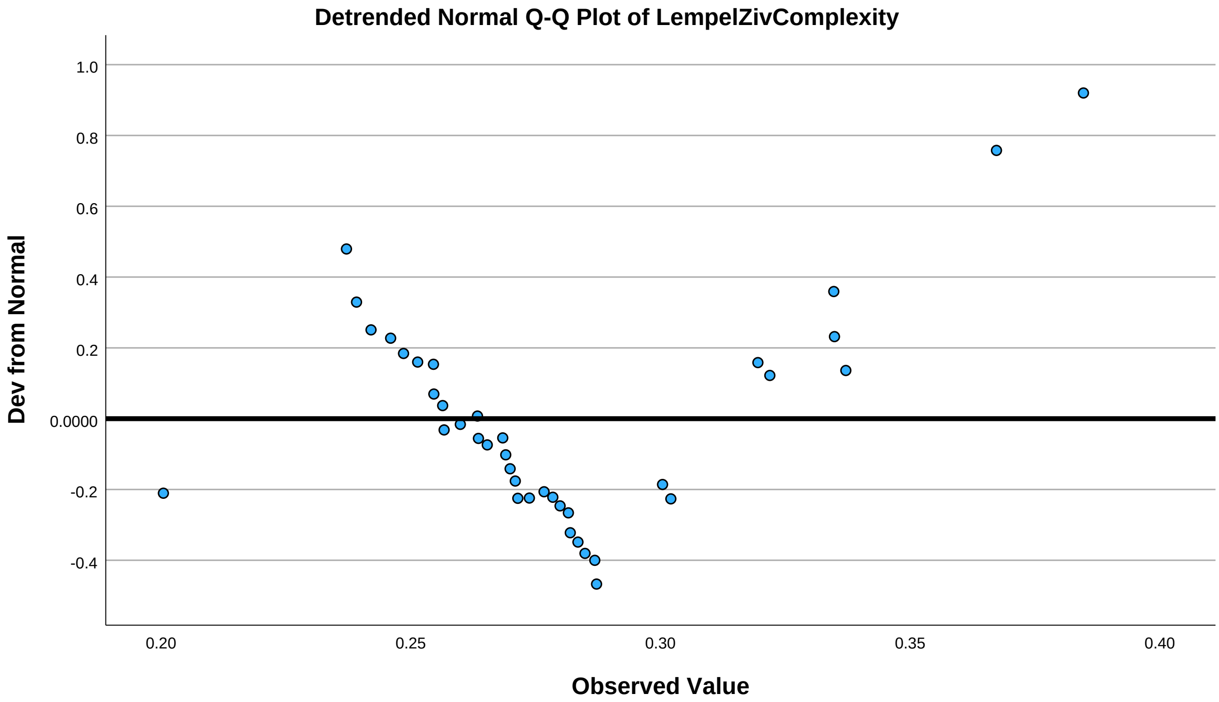

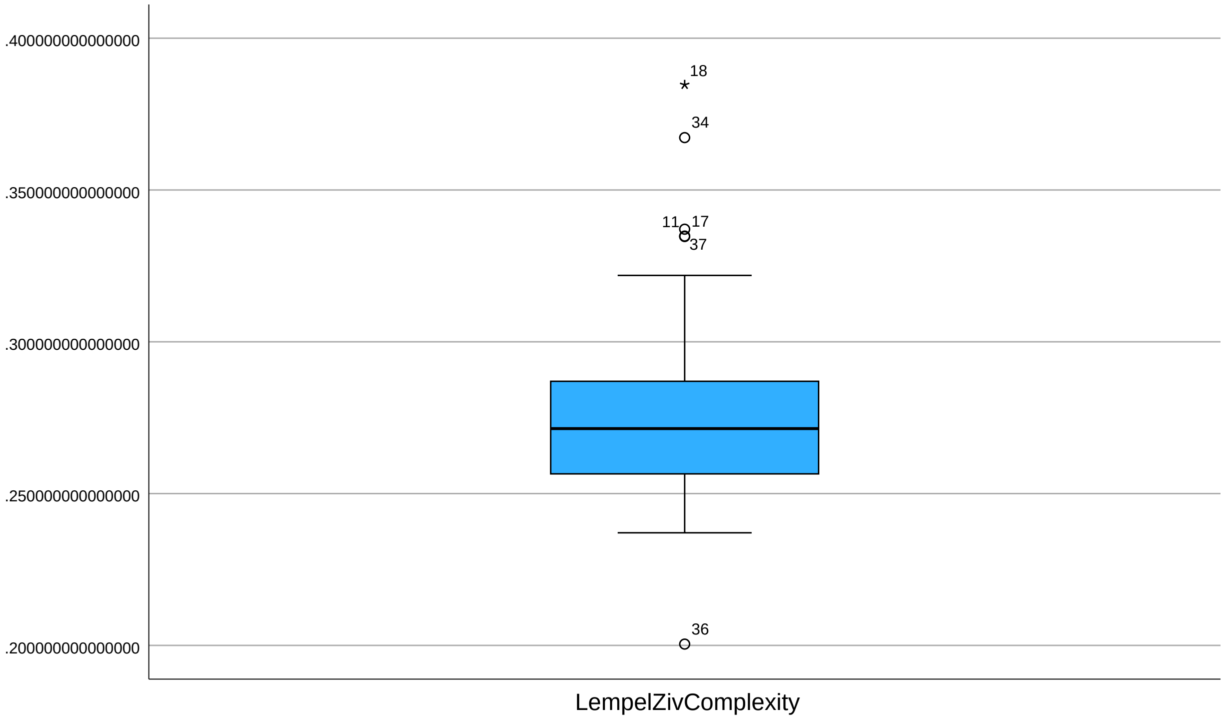

RenyiEntropy

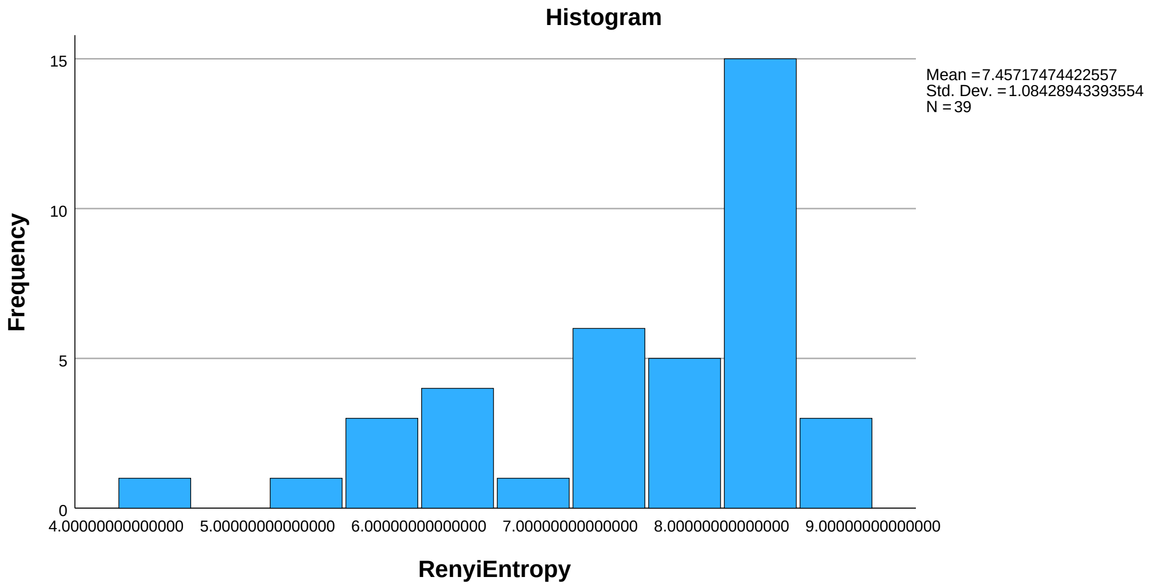


```
RenyiEntropy Stem-and-Leaf Plot

 Frequency    Stem &  Leaf

     1.00 Extremes    (=<4.3)
     1.00        5 .  4
     3.00        5 .  588
     4.00        6 .  0022
     1.00        6 .  9
     6.00        7 .  023444
     5.00        7 .  56779
    15.00        8 .  000001233333444
     3.00        8 .  557

 Stem width:   1.000000
 Each leaf:        1 case(s)
```

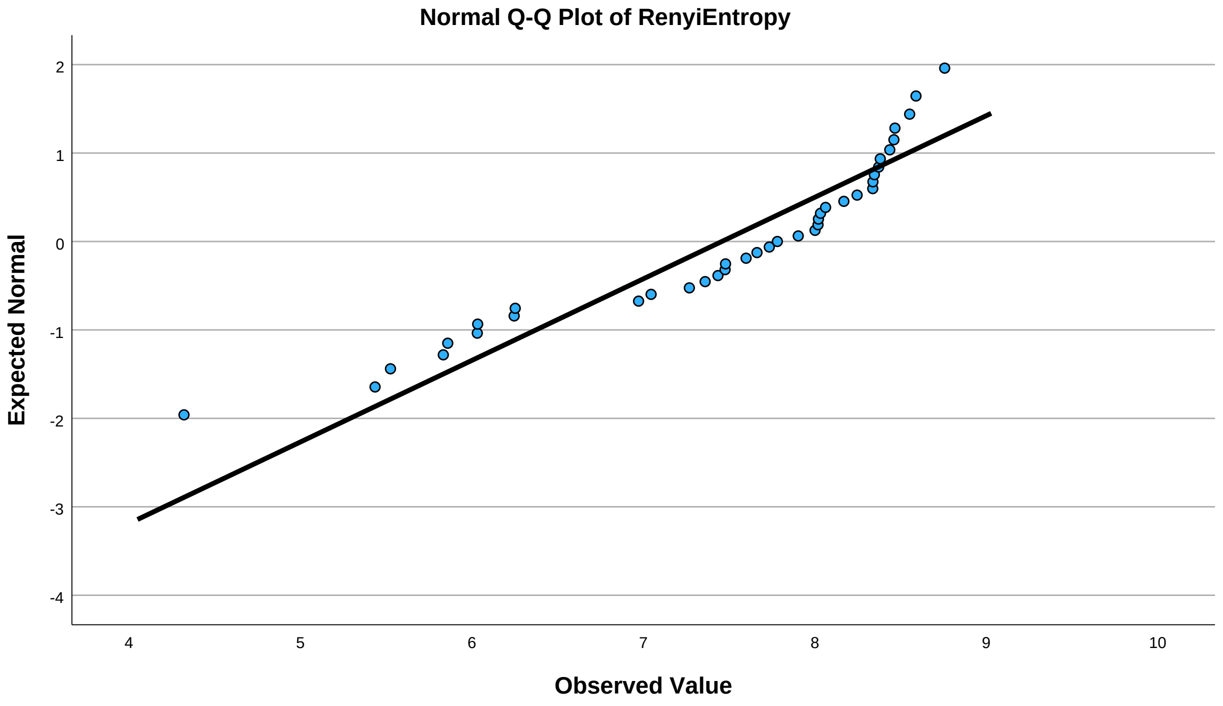
Normal Q-Q Plot of RenyiEntropy
Expected Normal
2
1
0
-1
-2
-3
-4
4
5
6
7
8
9
10
Observed Value

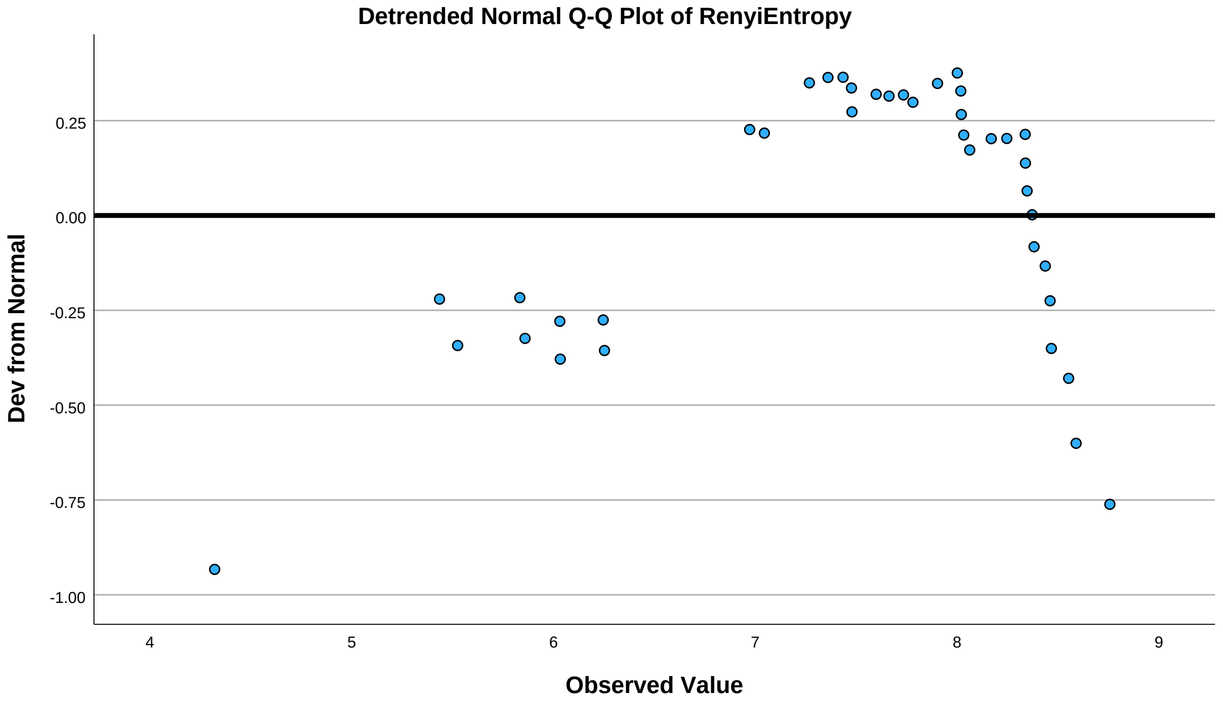
Detrended Normal Q-Q Plot of RenyiEntropy
Dev from Normal
0.25
0.00
-0.25
-0.50
-0.75
-1.00
4
5
6
7
8
9
Observed Value

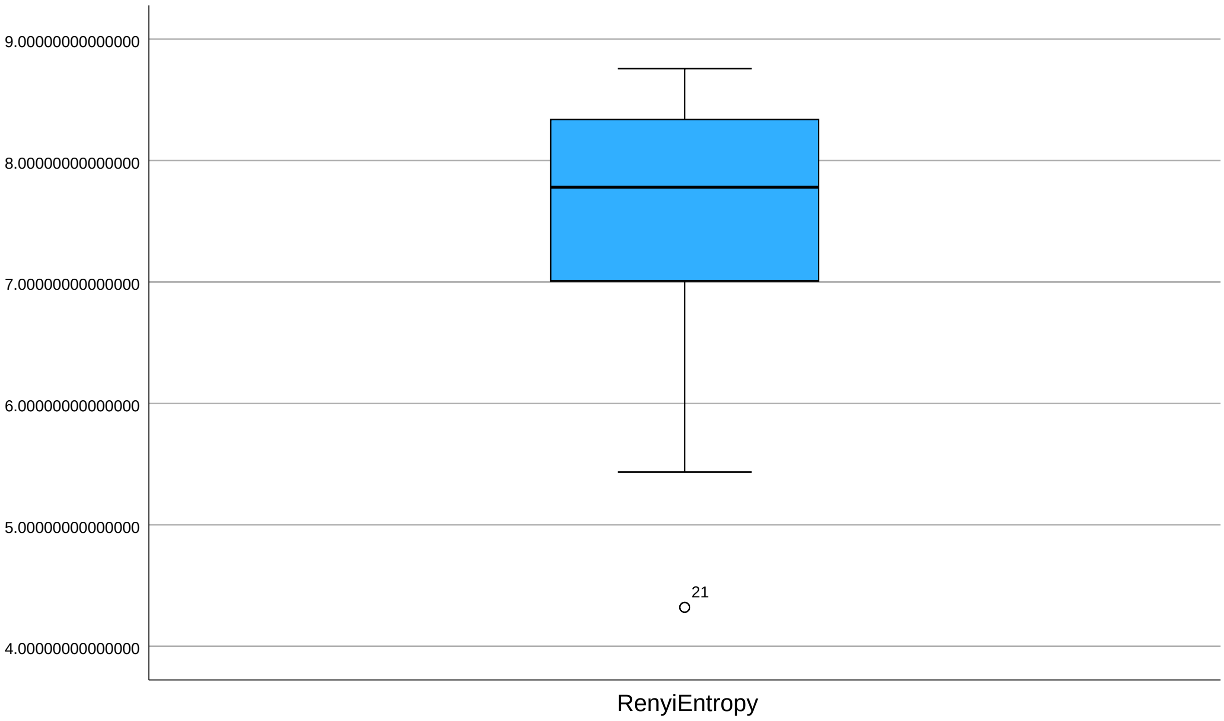

TsallisEntropy

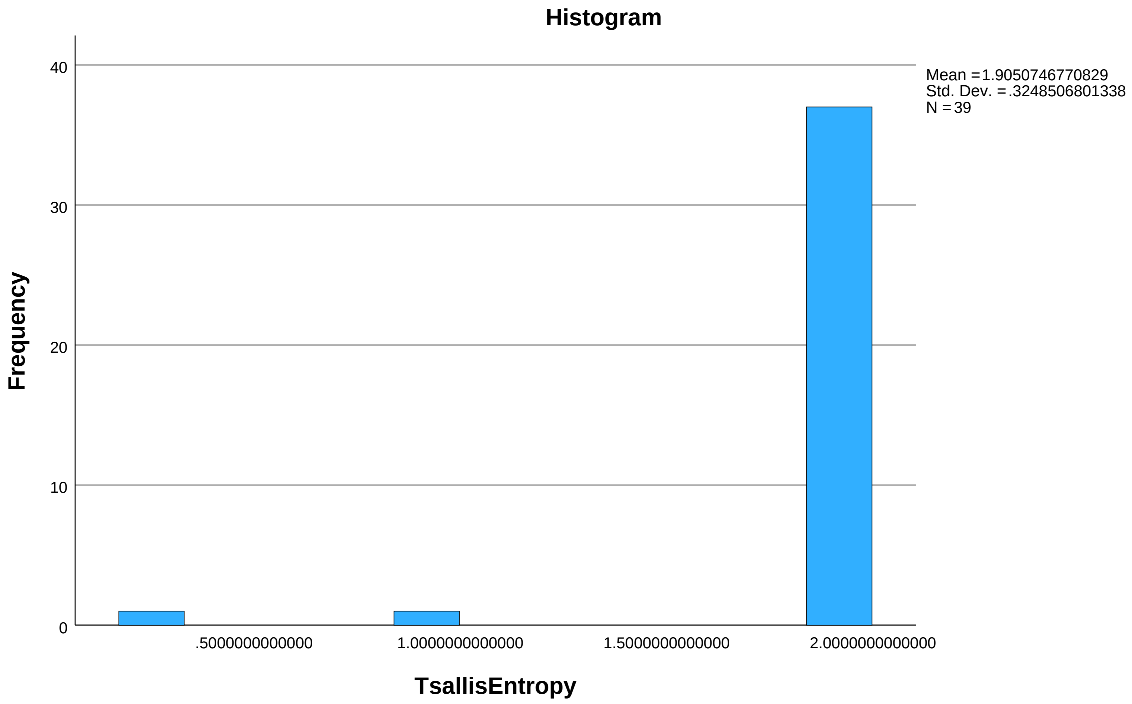


```
TsallisEntropy Stem-and-Leaf Plot

 Frequency    Stem &  Leaf
```

```
  8.00 Extremes    (=<1.9767)
 1.00    1982 .  6
 4.00    1983 .  2589
 3.00    1984 .  349
 7.00    1985 .  1226678
 8.00    1986 .  01125899
 6.00    1987 .  124458
 2.00    1988 .  12

Stem width:   .0010000
Each leaf:       1 case(s)
```

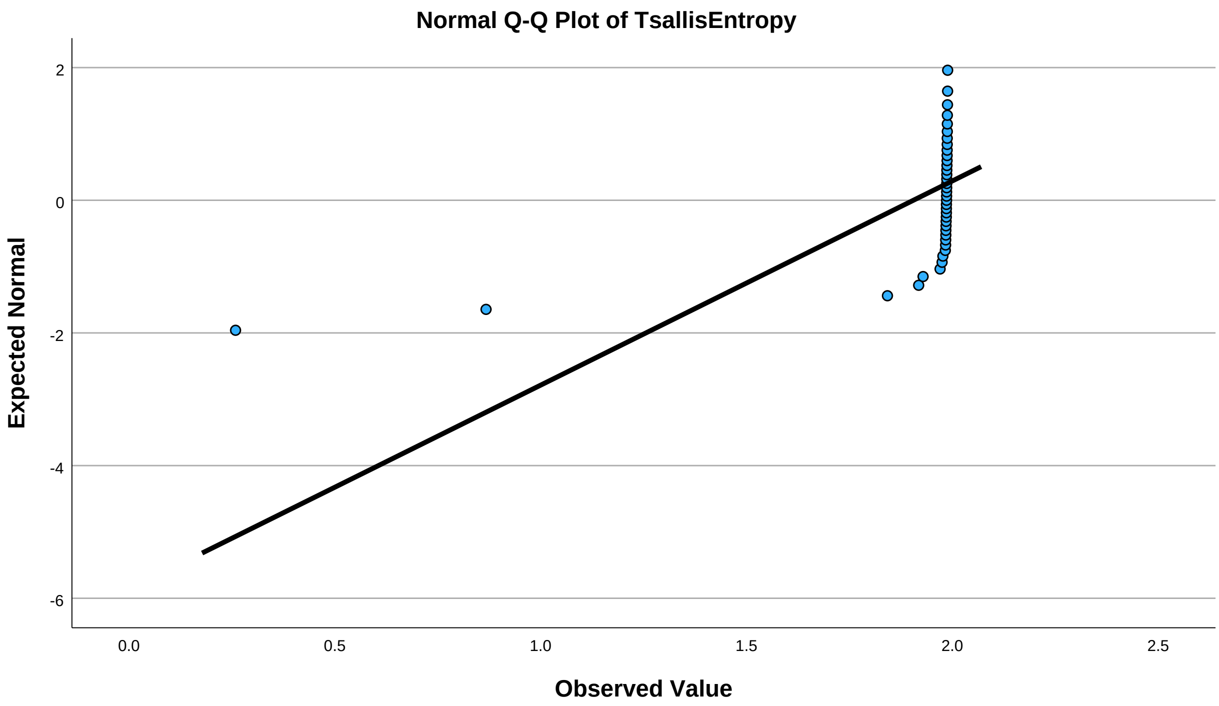

Detrended Normal Q-Q Plot of TsallisEntropy

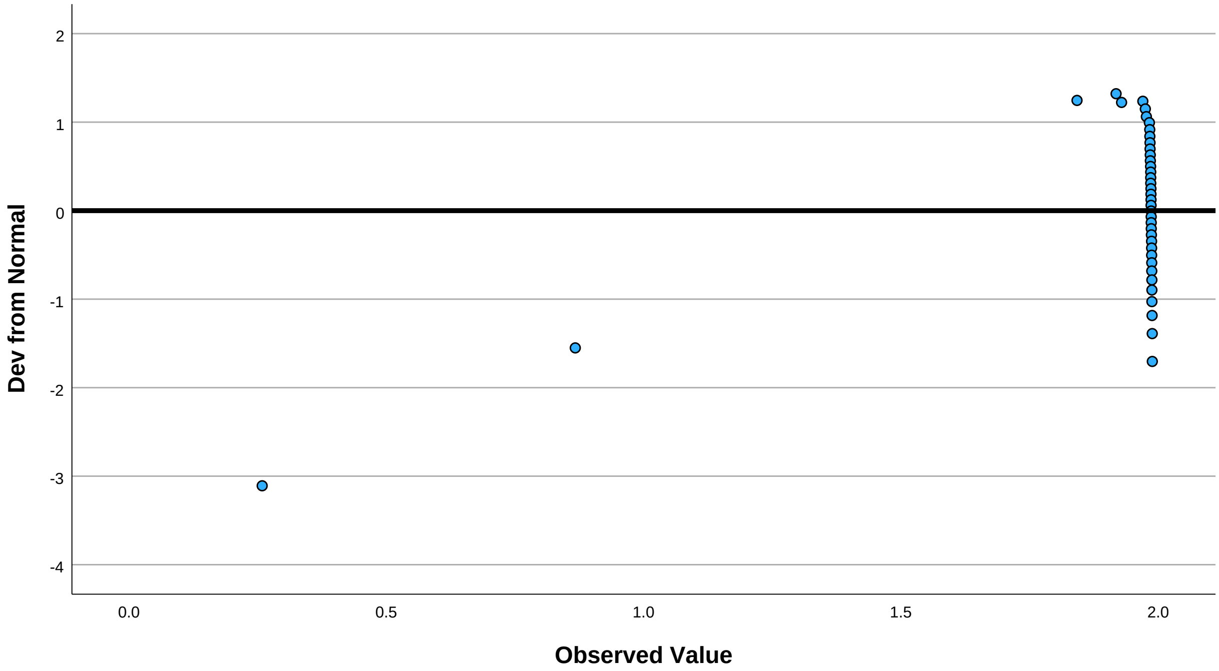

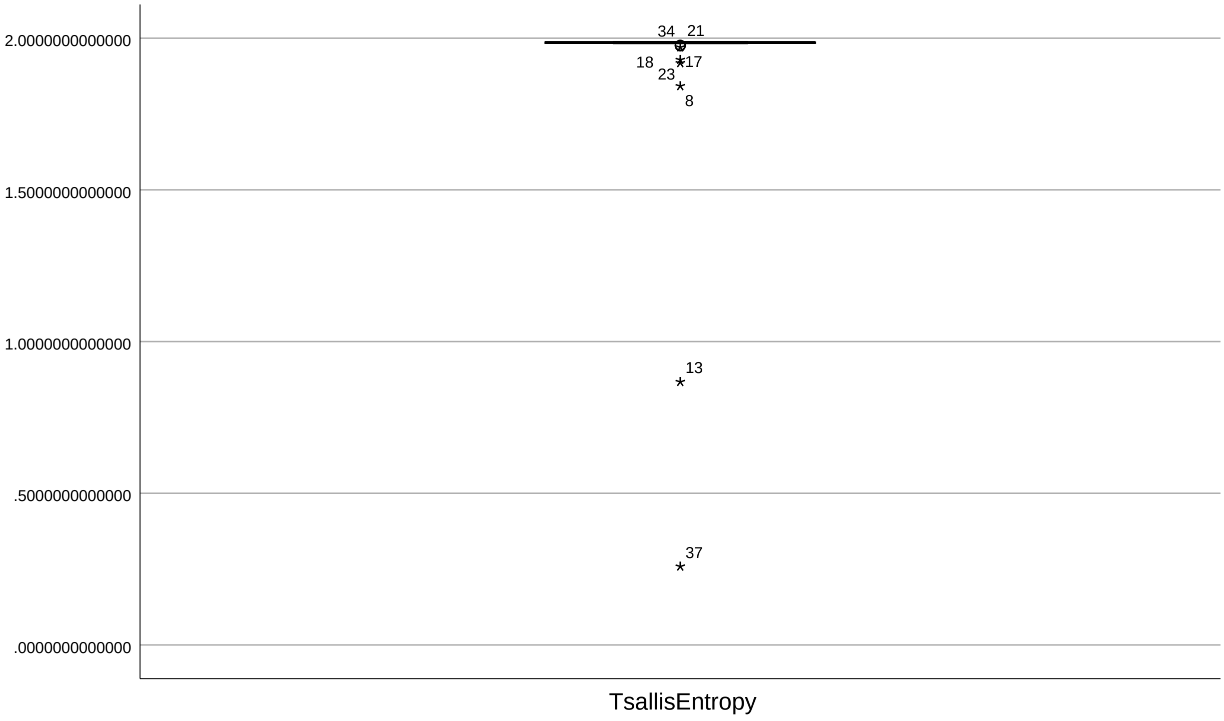

PS_4_5

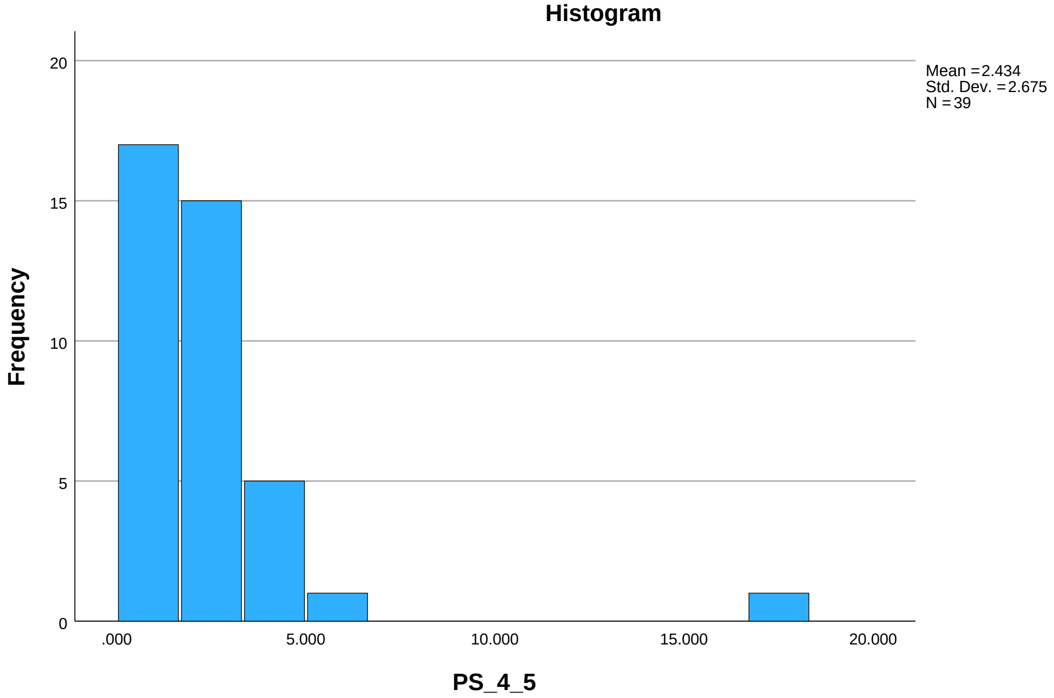

PS_4_5 Stem-and-Leaf Plot

```
 Frequency    Stem &  Leaf

     3.00        0 .  789
    10.00        1 .  0000012333
    10.00        1 .  5556777799
     6.00        2 .  012223
     2.00        2 .  79
     2.00        3 .  14
     3.00        3 .  568
     1.00        4 .  2
     2.00 Extremes    (>=5.3)

 Stem width:      1.000
 Each leaf:       1 case(s)
```

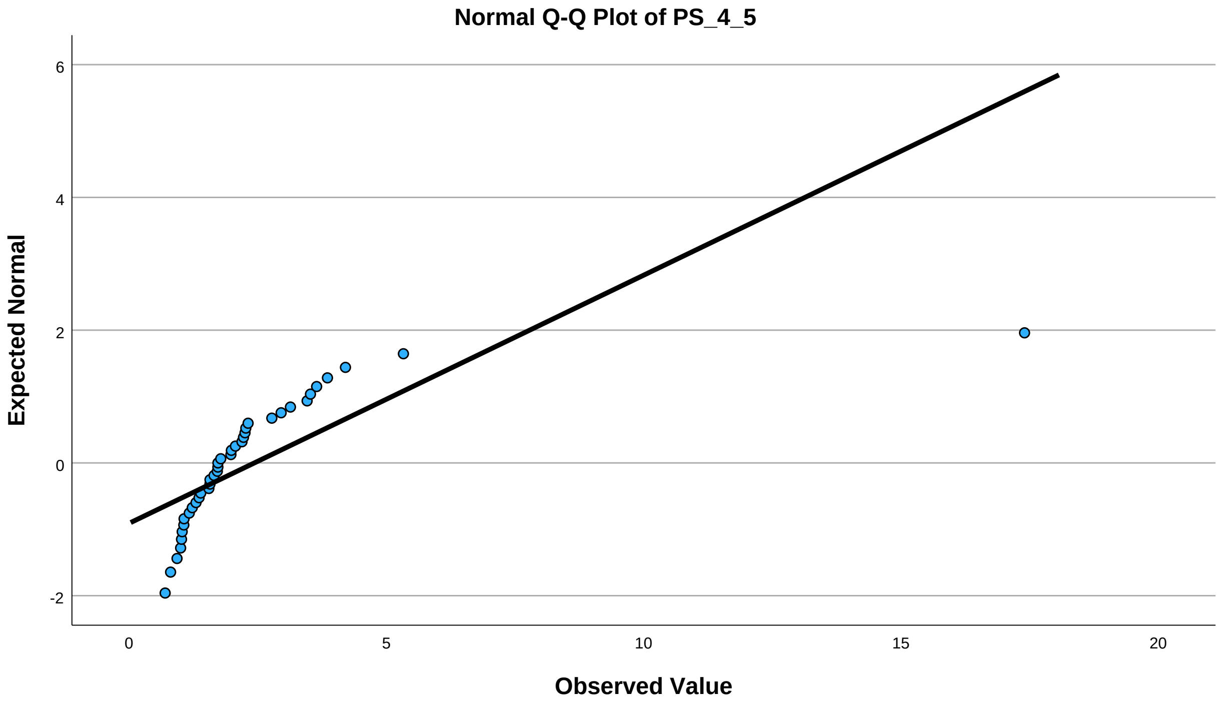
Normal Q-Q Plot of PS_4_5
Expected Normal
Observed Value
6
4
2
0
-2
0
5
10
15
20

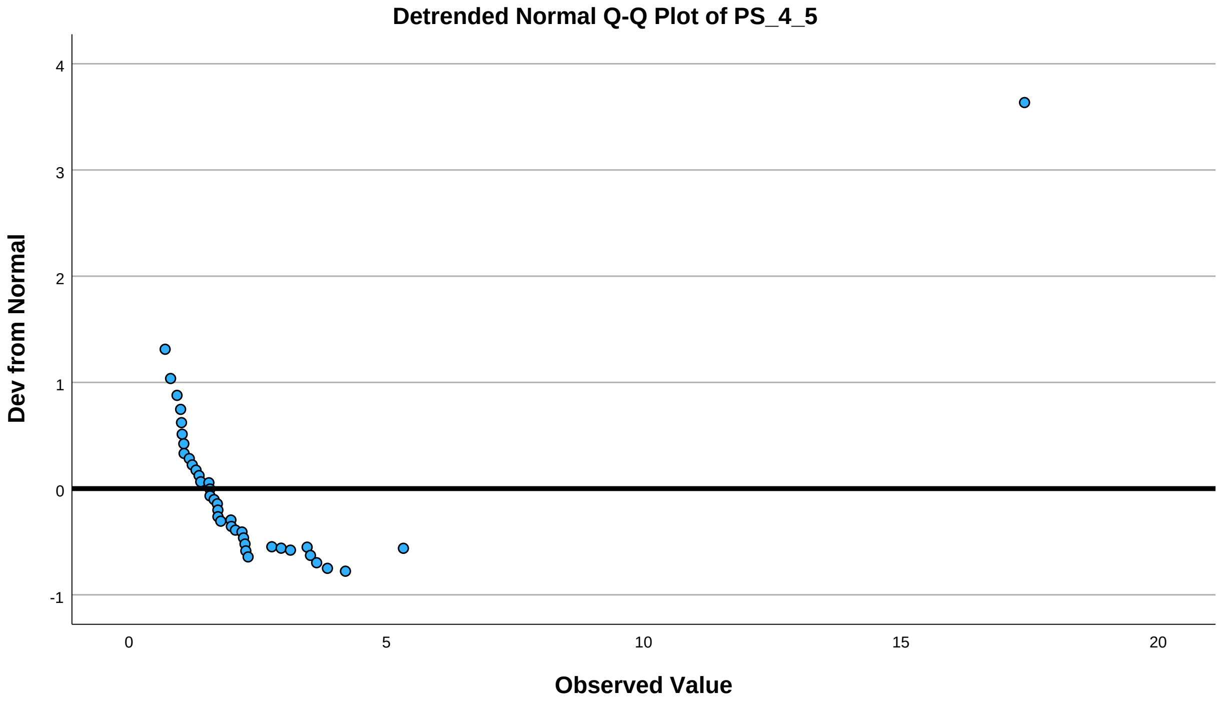
Detrended Normal Q-Q Plot of PS_4_5
Dev from Normal
Observed Value
4
3
2
1
0
-1
0
5
10
15
20

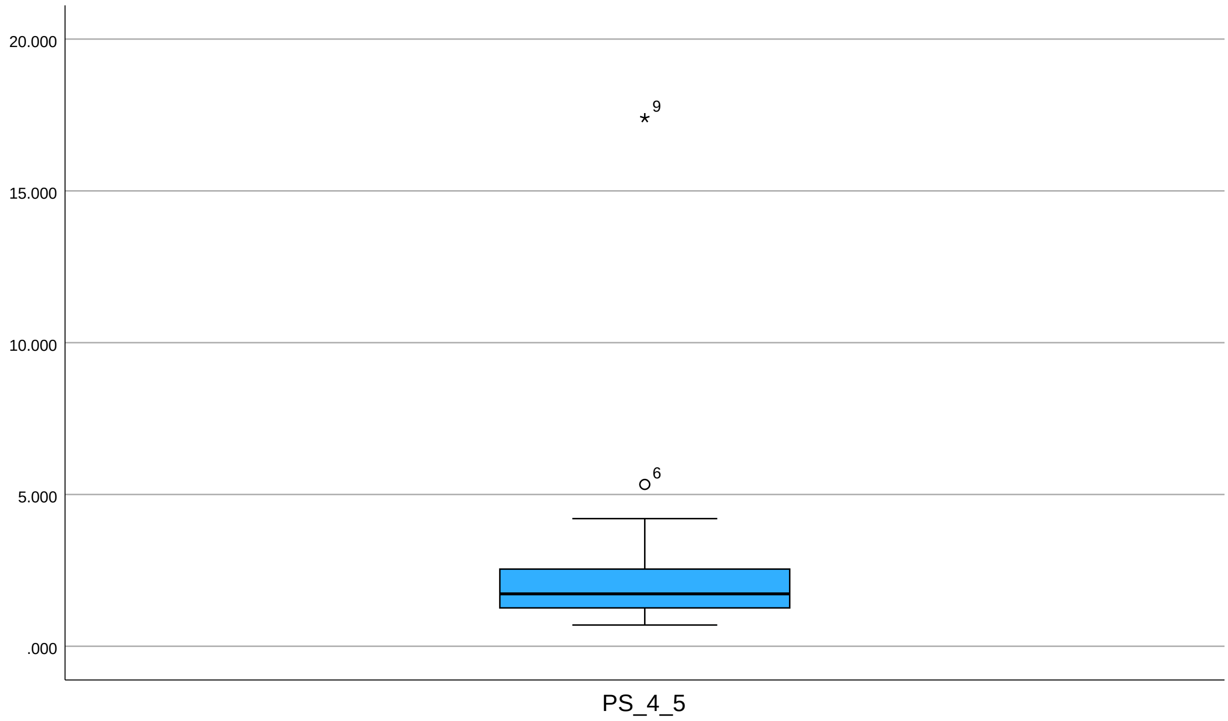

PS_5_6

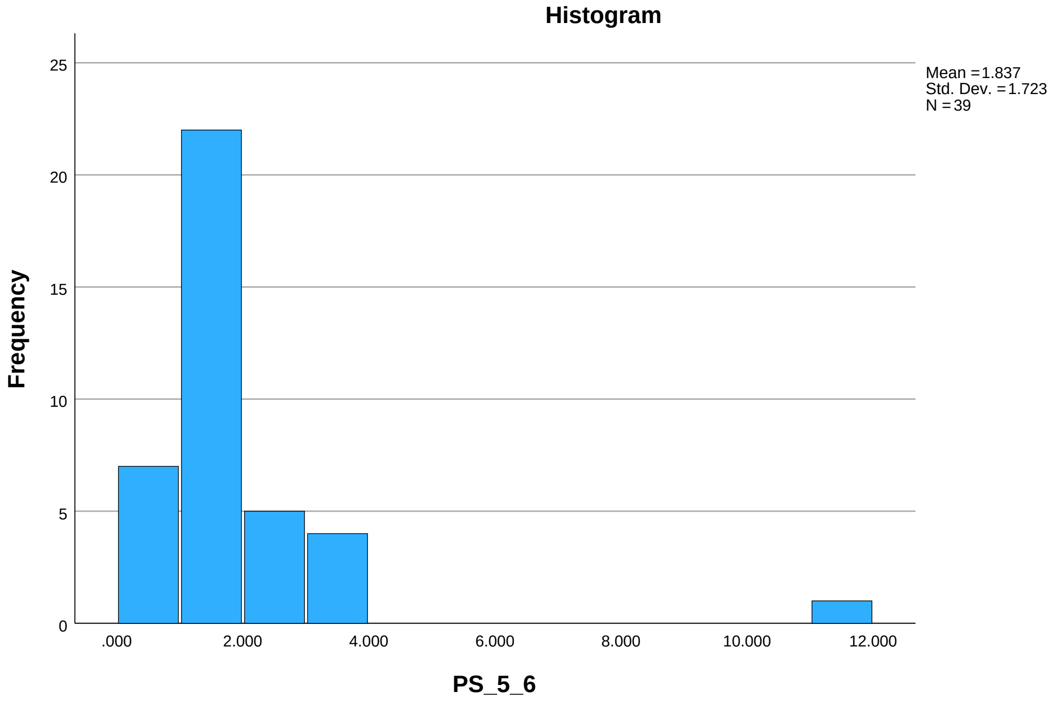

PS_5_6 Stem-and-Leaf Plot

Frequency   Stem & Leaf

```
  7.00       0 .  5567899
 13.00        1 .  0000111223334
  9.00       1 .  556688889
  5.00      2 .  22224
  .00     2 .
  4.00      3 .  0003
  1.00 Extremes    (>=11.4)

Stem width:     1.000
Each leaf:       1 case(s)
```

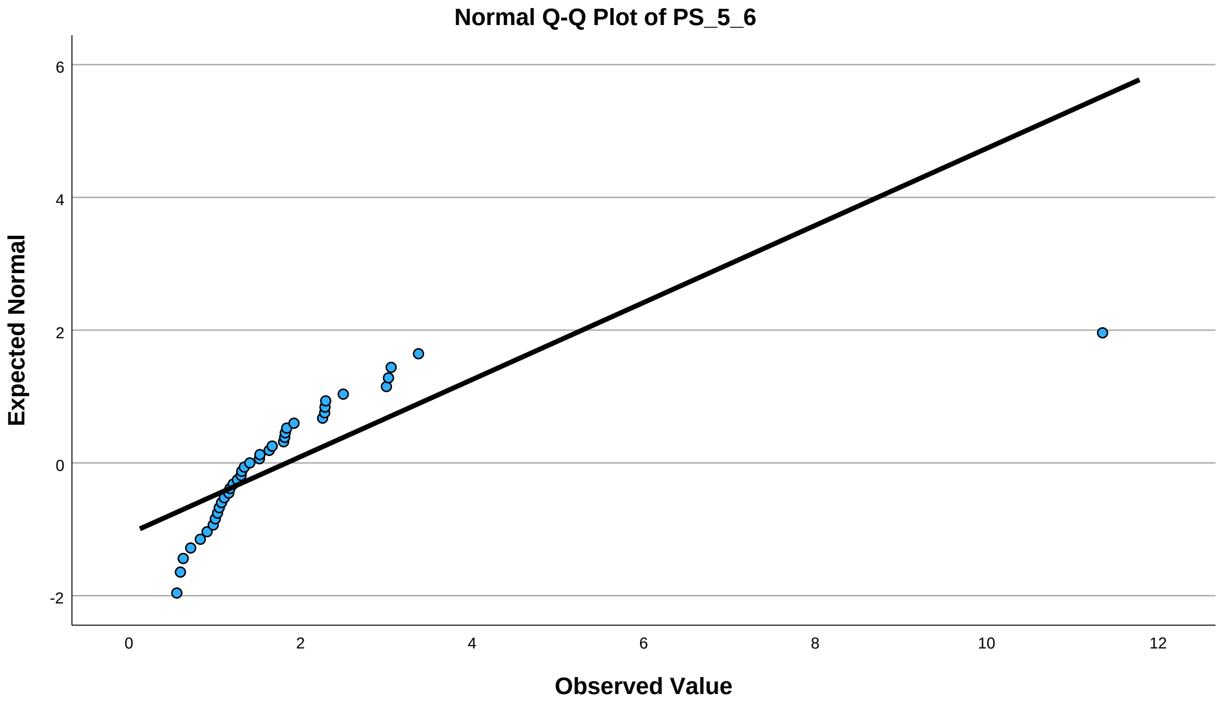

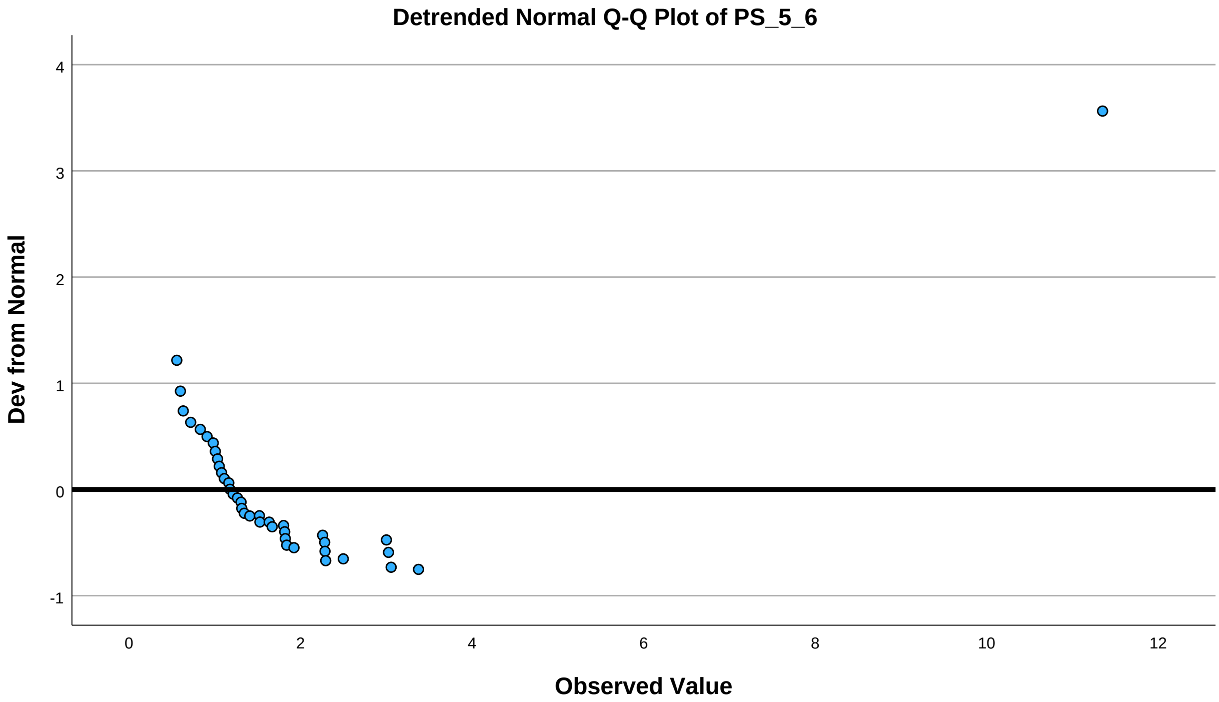

Detrended Normal Q-Q Plot of PS_5_6
Dev from Normal
4
3
2
1
0
-1
0
2
4
6
8
10
12
Observed Value

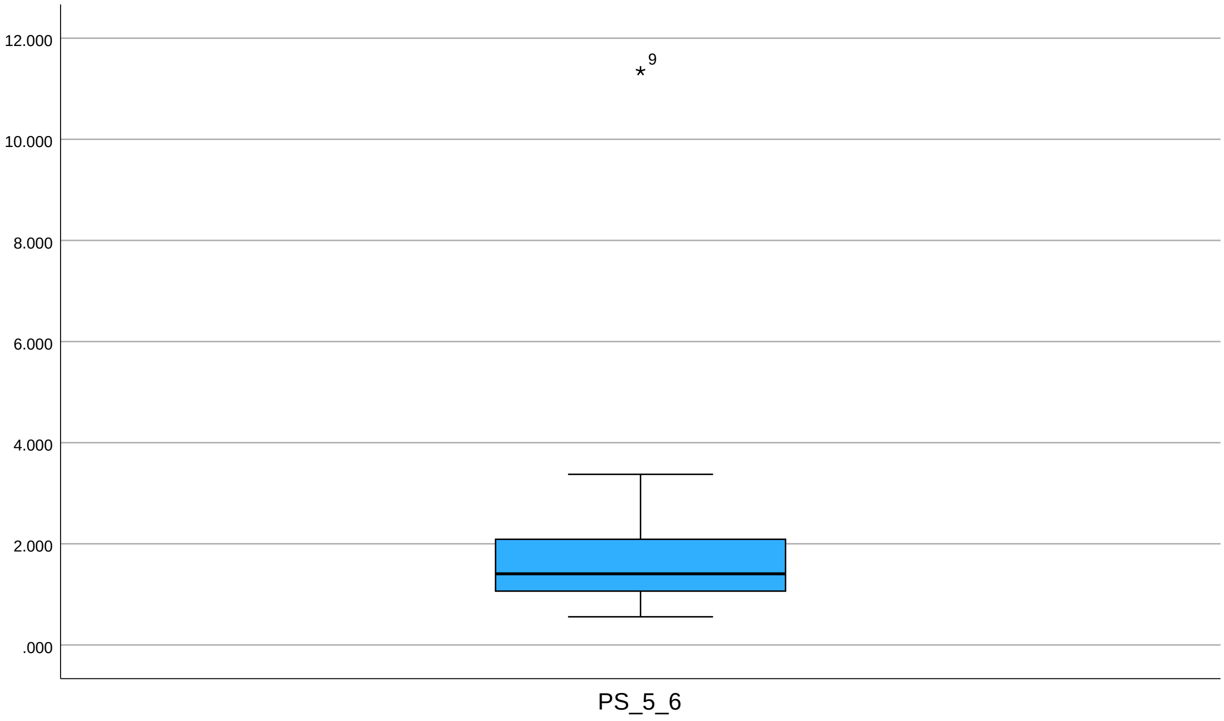

12.000
10.000
8.000
6.000
4.000
2.000
.000
9
PS_5_6

PS_6_7

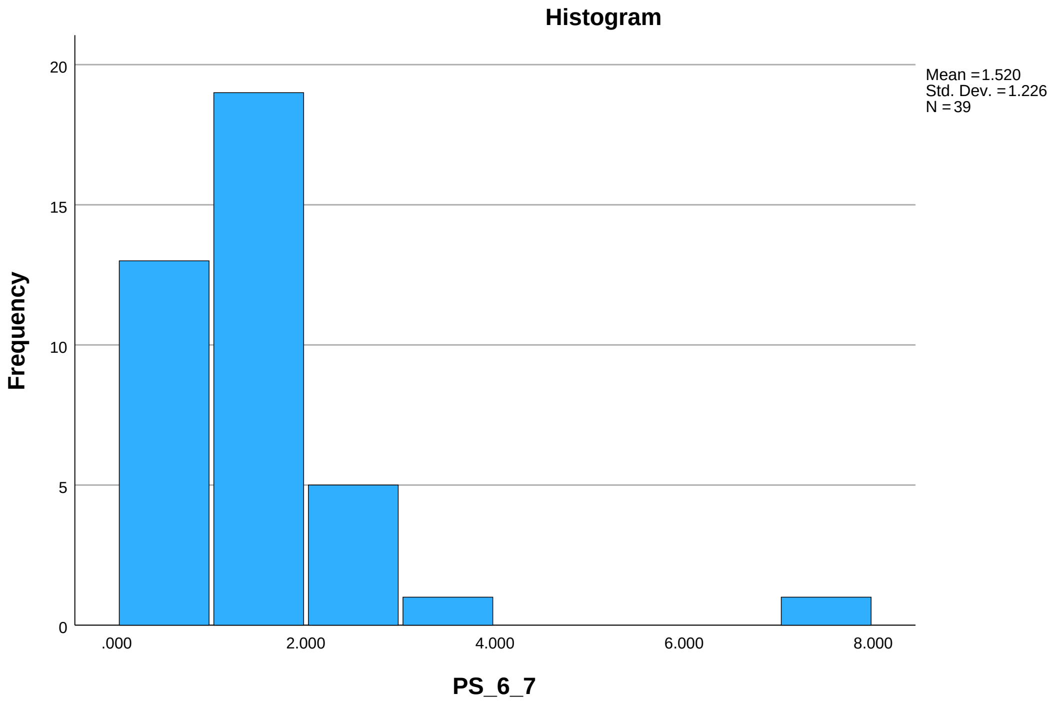

PS_6_7 Stem-and-Leaf Plot

```
 Frequency    Stem &  Leaf

     1.00        0 .  3
    12.00        0 .  556677888999
    11.00        1 .  00011123334
     8.00        1 .  55567889
     4.00        2 .  0112
     1.00        2 .  7
     2.00 Extremes    (>=3.2)

 Stem width:      1.000
 Each leaf:       1 case(s)
```

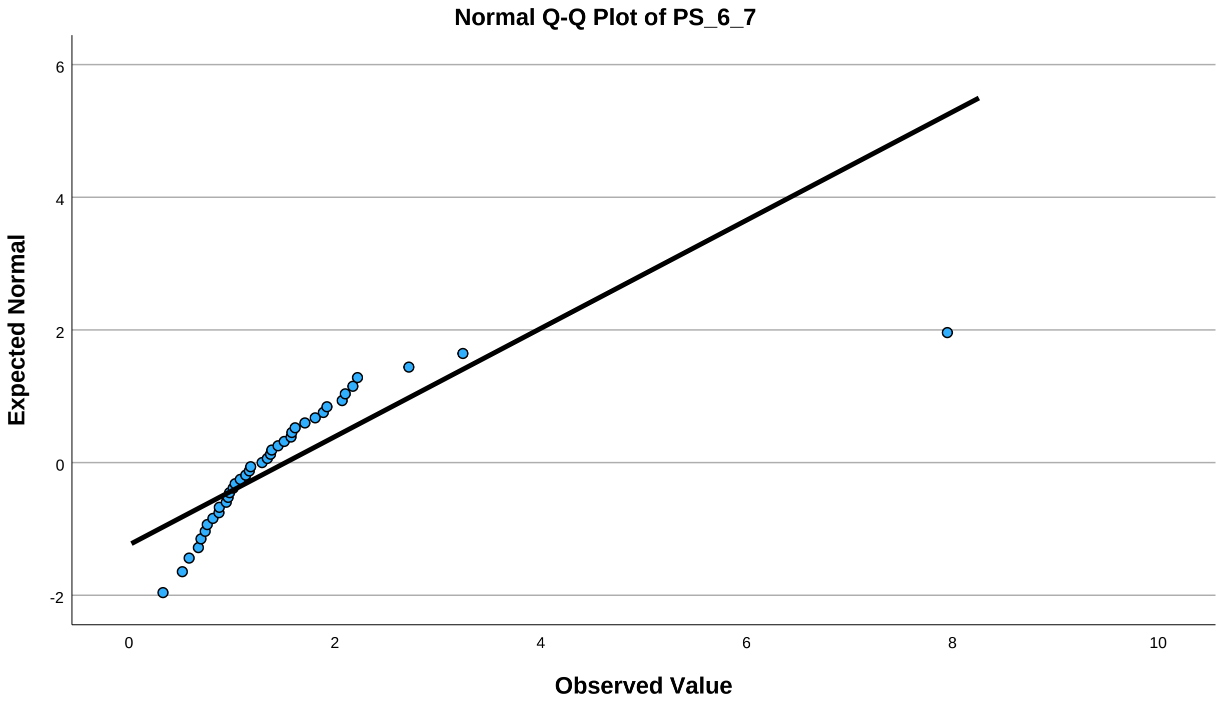

Normal Q-Q Plot of PS_6_7
Expected Normal
Observed Value
6
4
2
0
-2
0
2
4
6
8
10

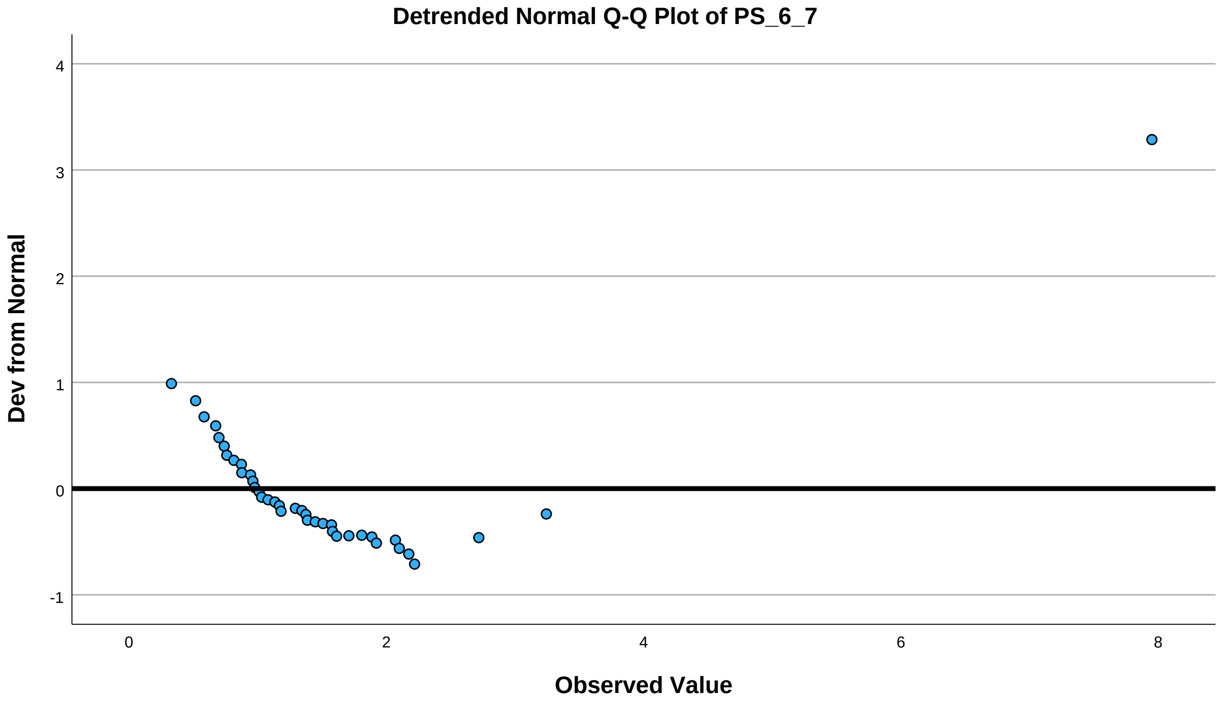

Detrended Normal Q-Q Plot of PS_6_7
Dev from Normal
Observed Value
4
3
2
1
0
-1
0
2
4
6
8

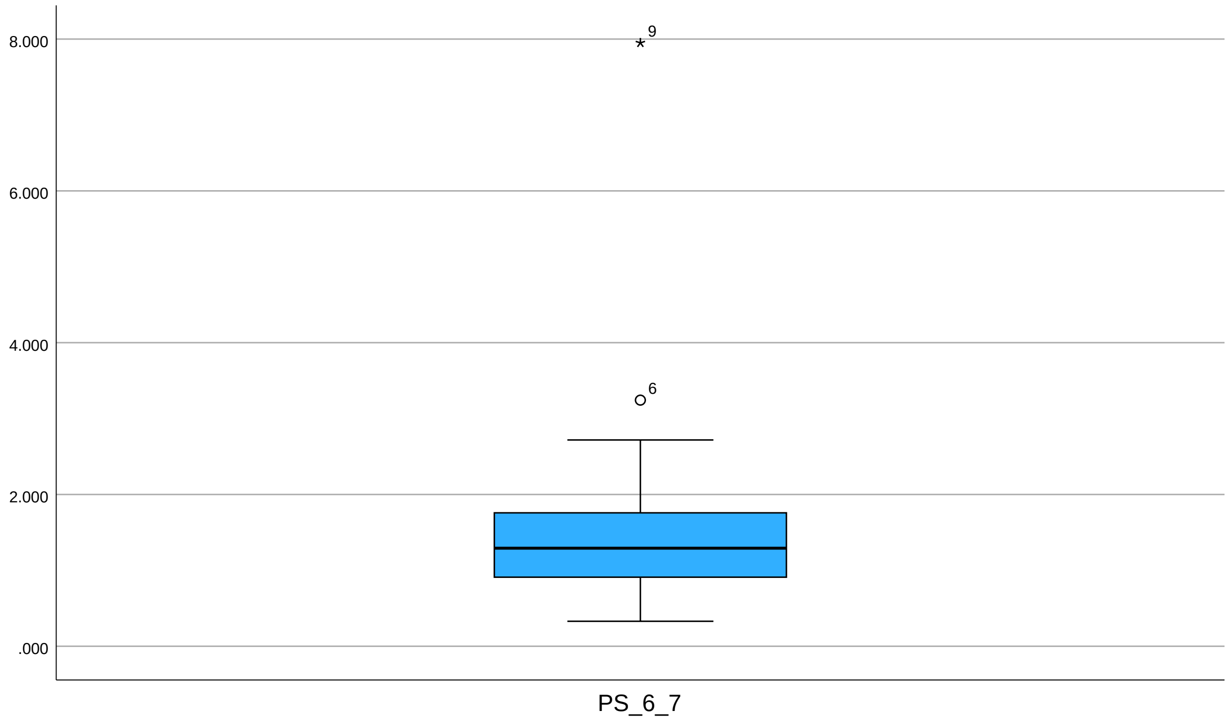

PS_7_8

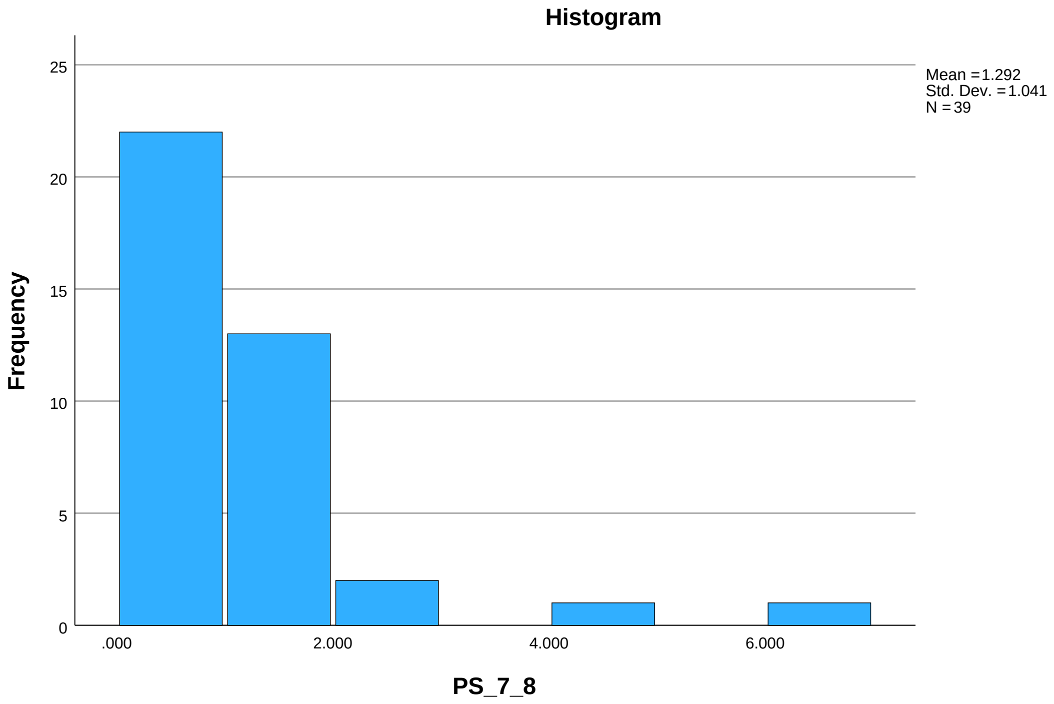

PS_7_8 Stem-and-Leaf Plot

Frequency   Stem & Leaf

```
 3.00      0 . 244
 19.00       0 .  5667777888888899999
  8.00      1 . 11222234
  5.00      1 . 56778
 1.00     2 . 0
  3.00 Extremes    (>=2.8)

Stem width:     1.000
Each leaf:       1 case(s)
```

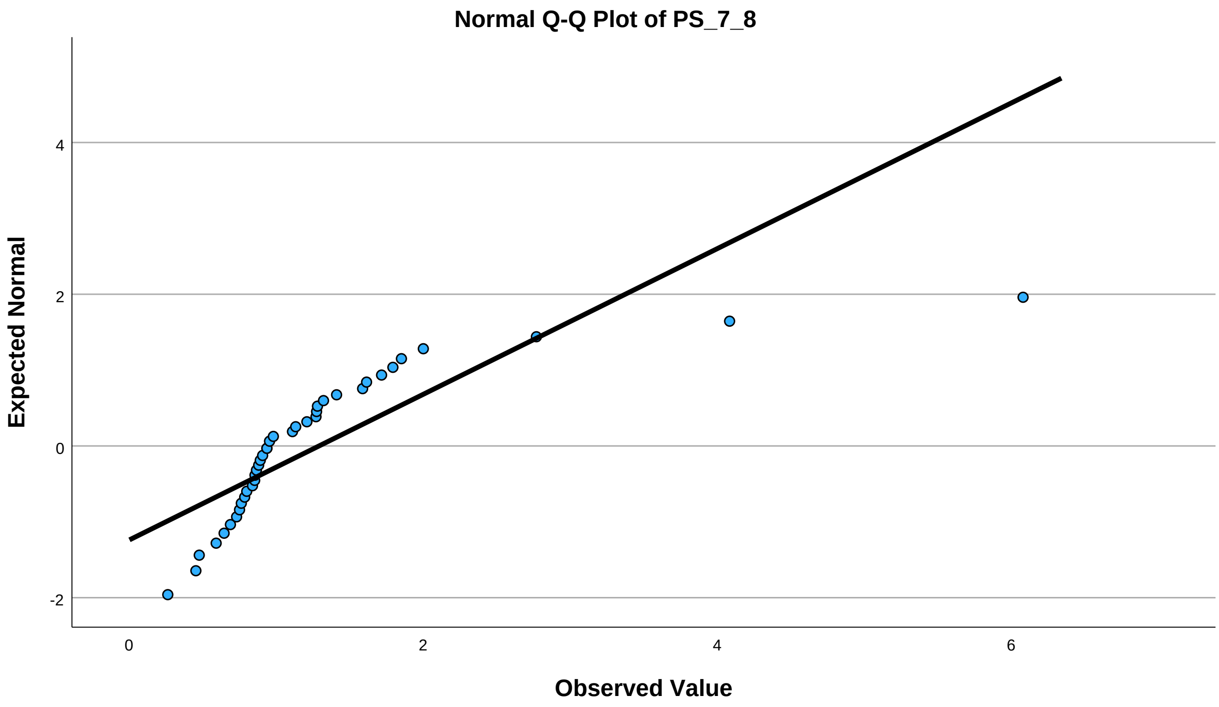

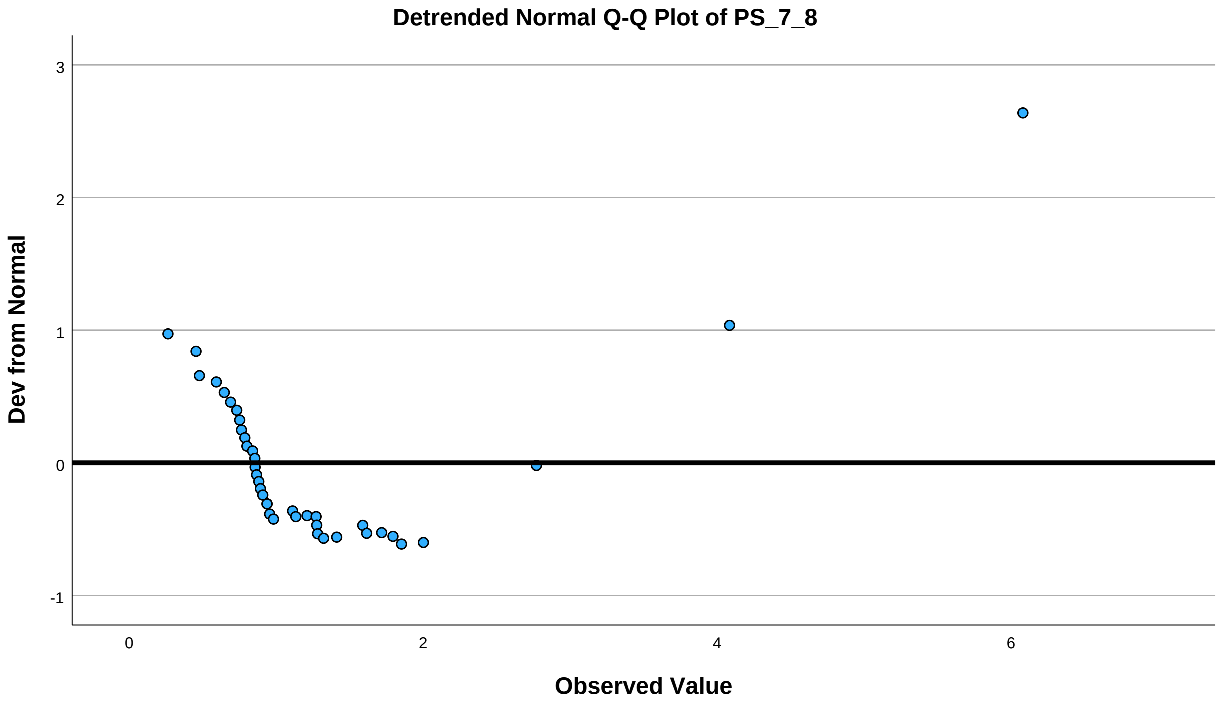

Detrended Normal Q-Q Plot of PS_7_8
Dev from Normal
Observed Value
3
2
1
0
-1
0
2
4
6

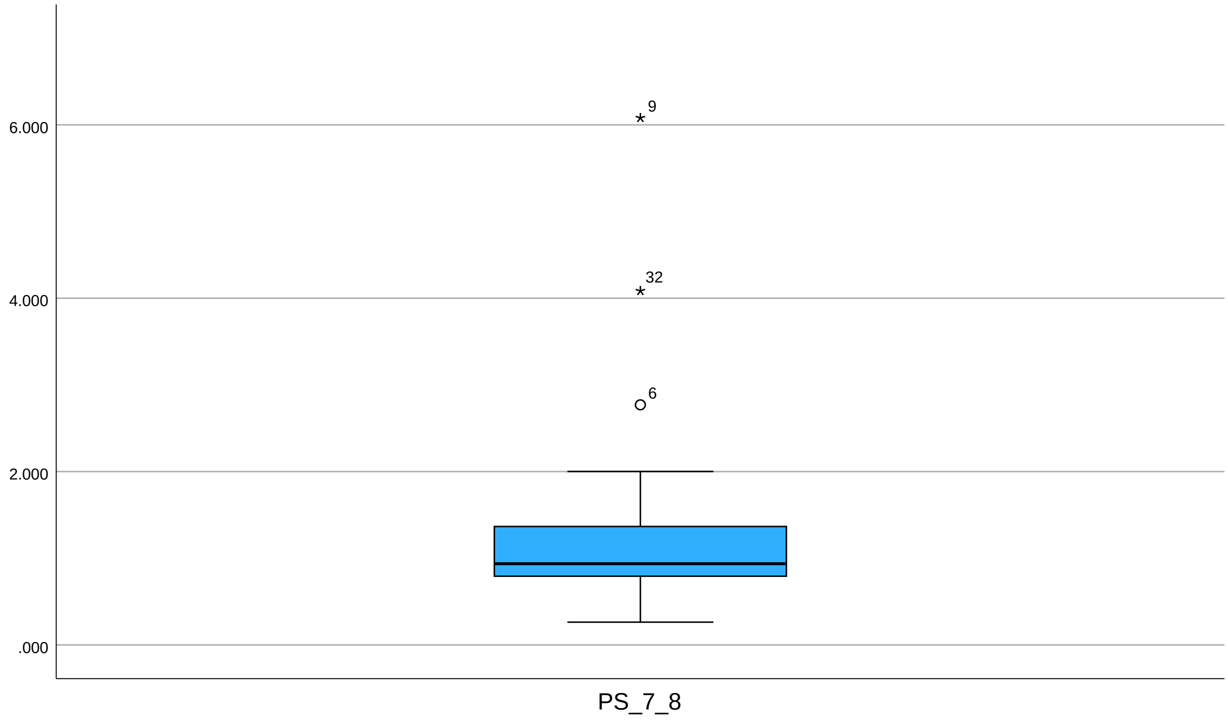

6.000
4.000
2.000
.000
9
32
6
PS_7_8

PS_8_9

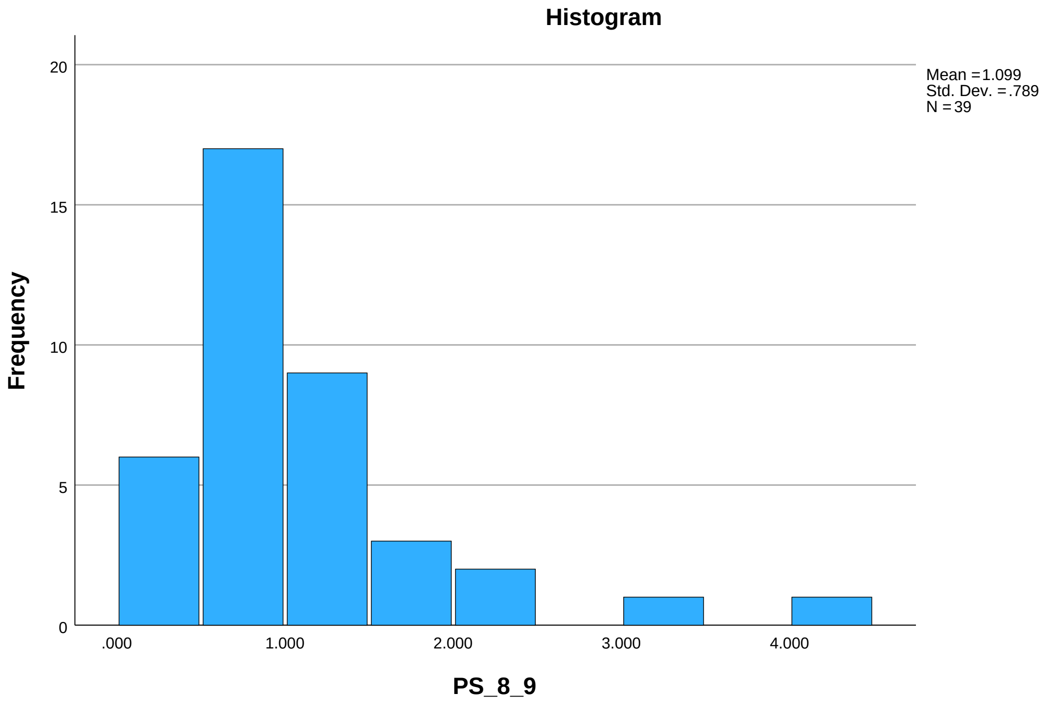

PS_8_9 Stem-and-Leaf Plot

```
 Frequency    Stem &  Leaf

     6.00        0 .  233444
    17.00        0 .  55566677777778899
     9.00        1 .  001222344
     3.00        1 .  678
     2.00        2 .  01
     2.00 Extremes    (>=3.2)

 Stem width:      1.000
 Each leaf:       1 case(s)
```

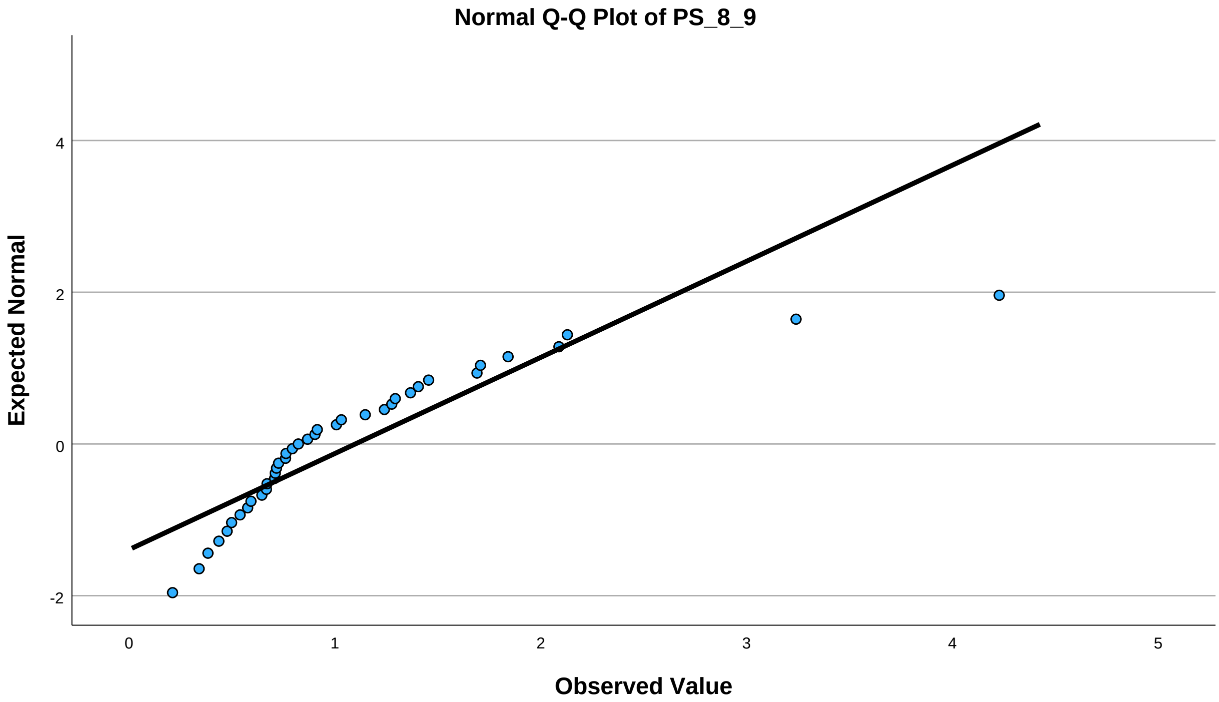
Normal Q-Q Plot of PS_8_9
Expected Normal
4
2
0
-2
0
1
2
3
4
5
Observed Value

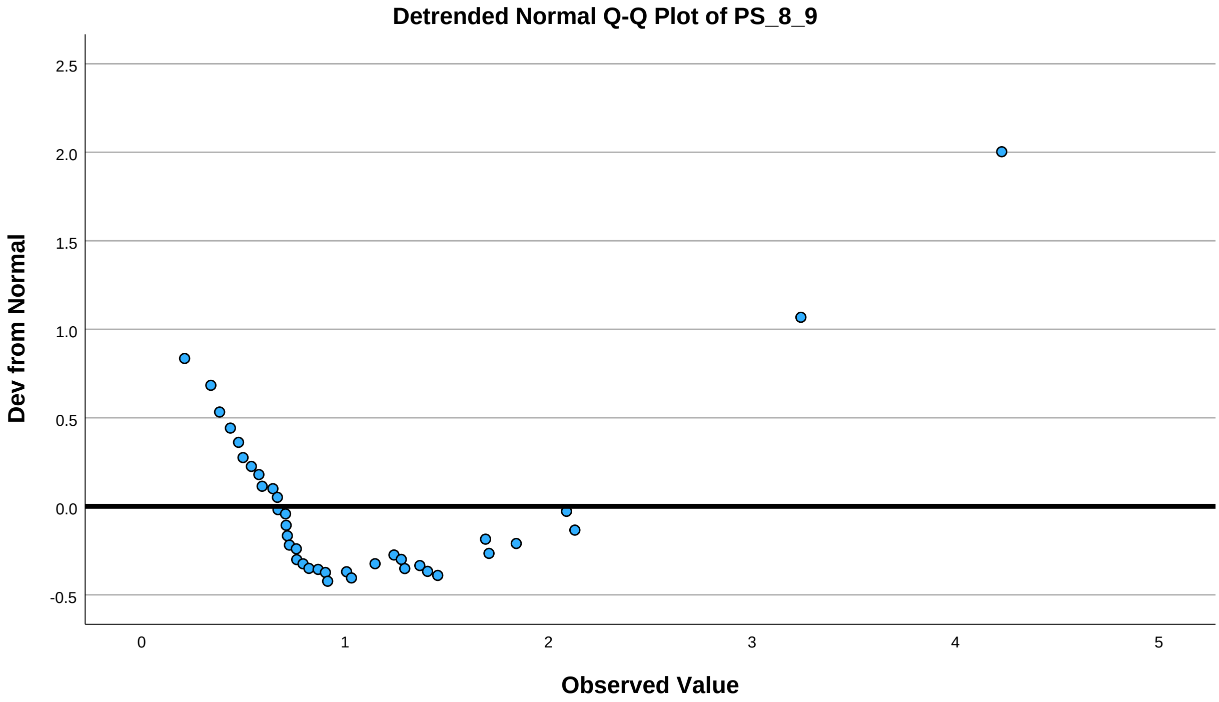
Detrended Normal Q-Q Plot of PS_8_9
Dev from Normal
2.5
2.0
1.5
1.0
0.5
0.0
-0.5
0
1
2
3
4
5
Observed Value

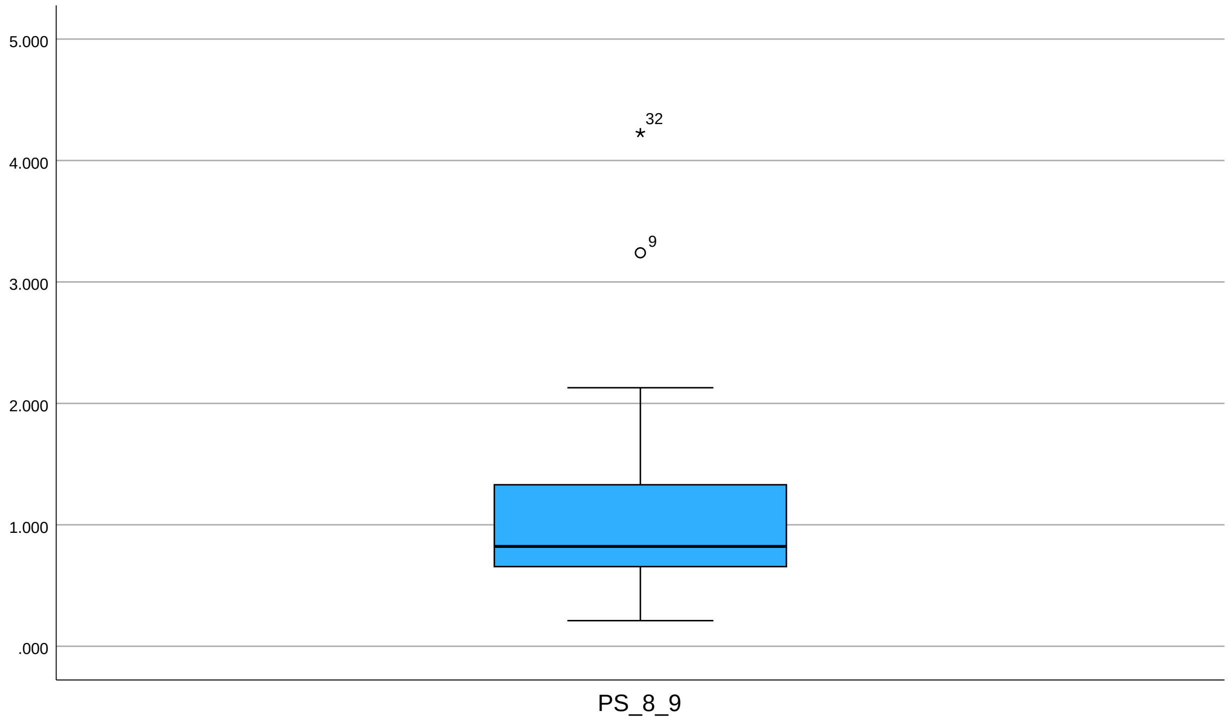

PS_9_10

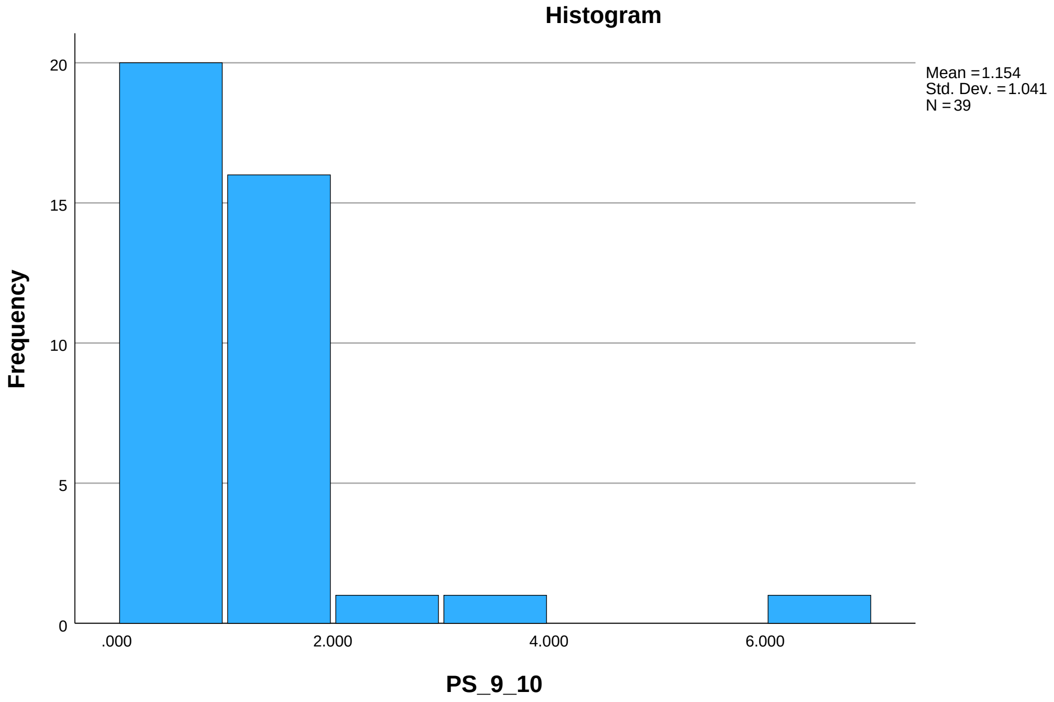

PS_9_10 Stem-and-Leaf Plot

Frequency   Stem & Leaf

```
 8.00      0 . 12234444
12.00      0 . 555577788899
12.00      1 . 000011122334
 4.00      1 . 6789
 1.00      2 . 2
 2.00 Extremes    (>=3.0)

Stem width:     1.000
Each leaf:       1 case(s)
```

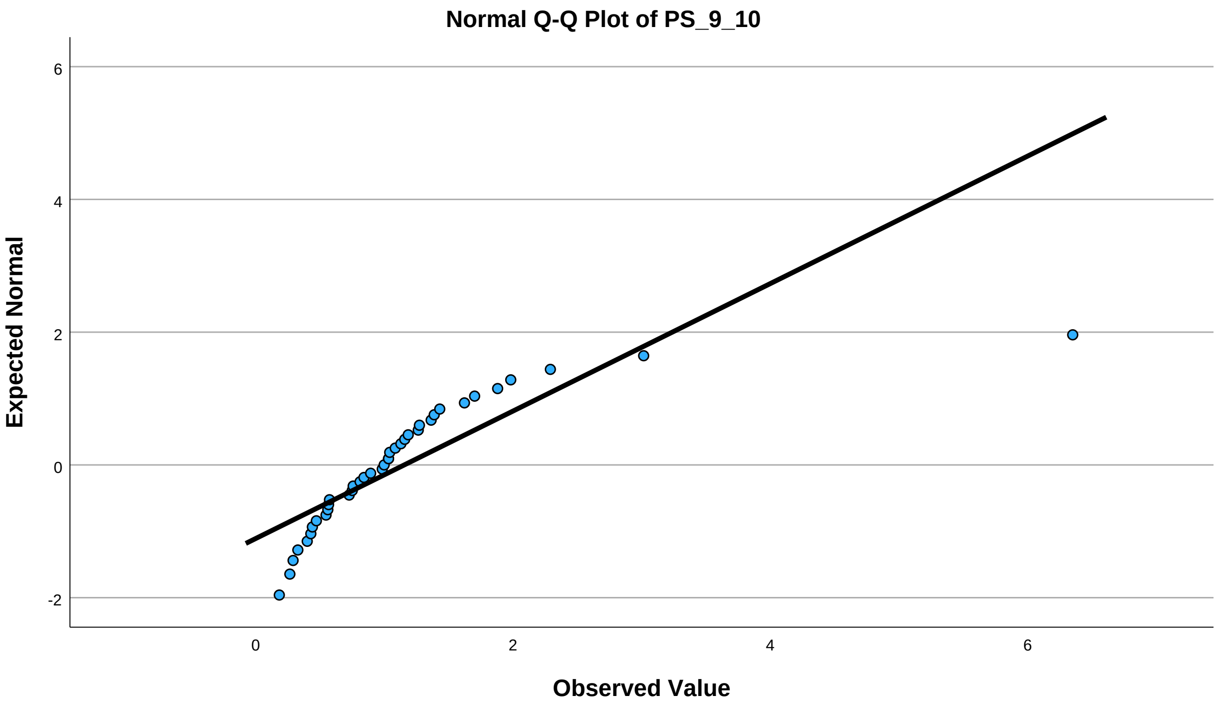

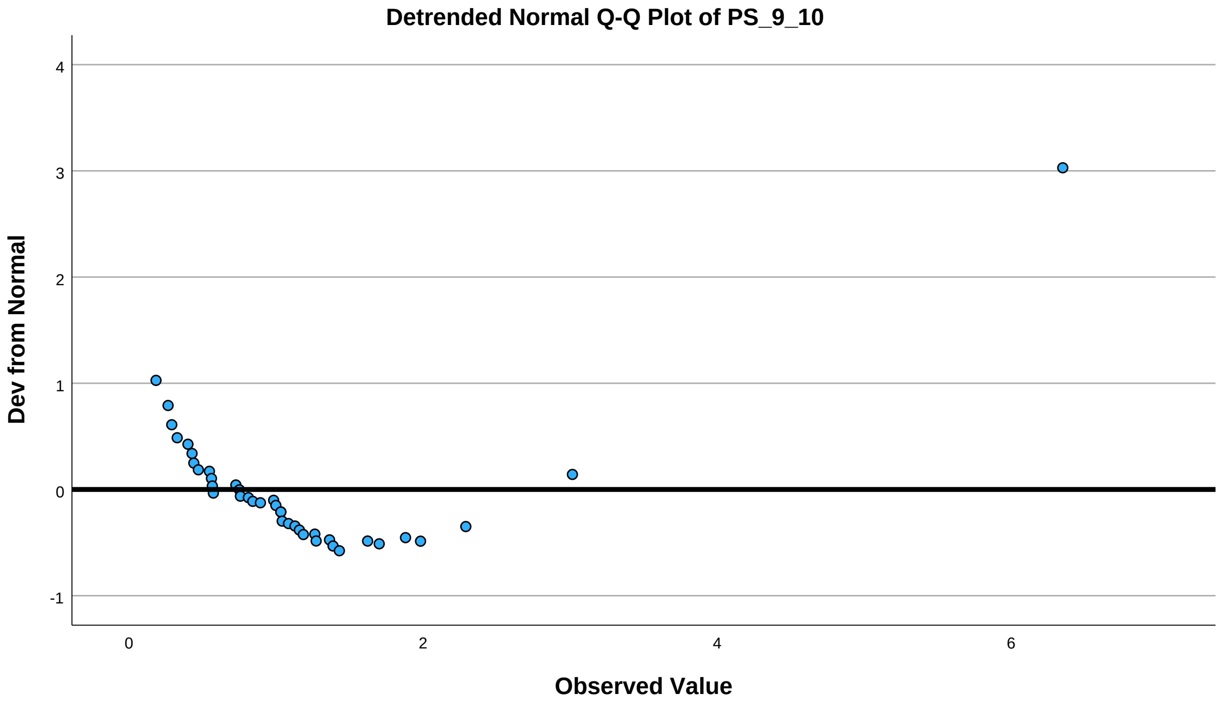

Detrended Normal Q-Q Plot of PS_9_10
Dev from Normal
4
3
2
1
0
-1
0
2
4
6
Observed Value

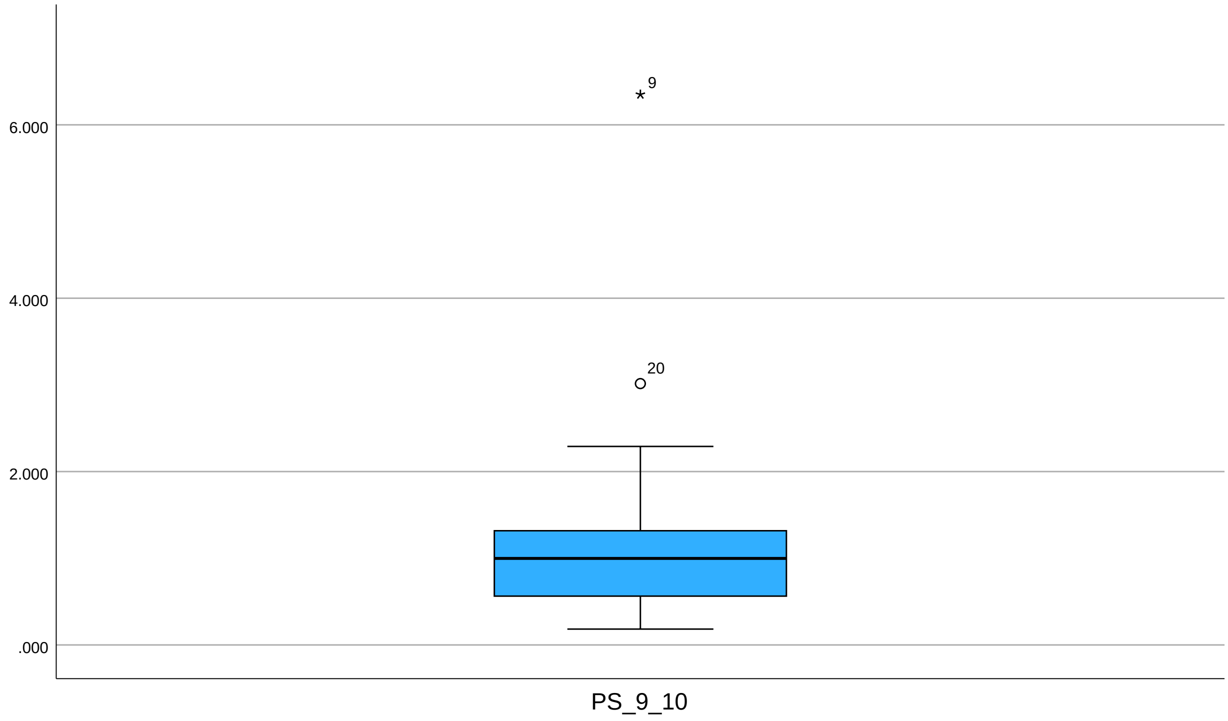

6.000
4.000
2.000
.000
9
20
PS_9_10

PS_10_11

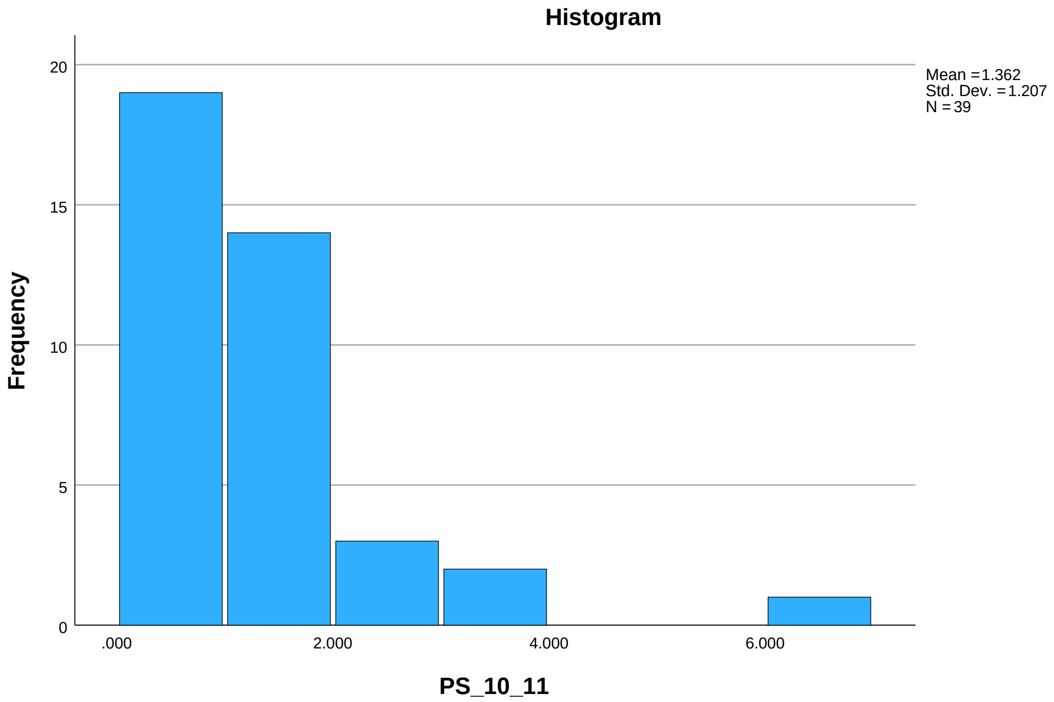

PS_10_11 Stem-and-Leaf Plot

```
 Frequency    Stem &  Leaf

     7.00        0 .  2223444
    12.00        0 .  566677788999
     9.00        1 .  112223344
     5.00        1 .  56889
     2.00        2 .  12
     1.00        2 .  8
     3.00 Extremes    (>=3.1)

 Stem width:      1.000
 Each leaf:        1 case(s)
```

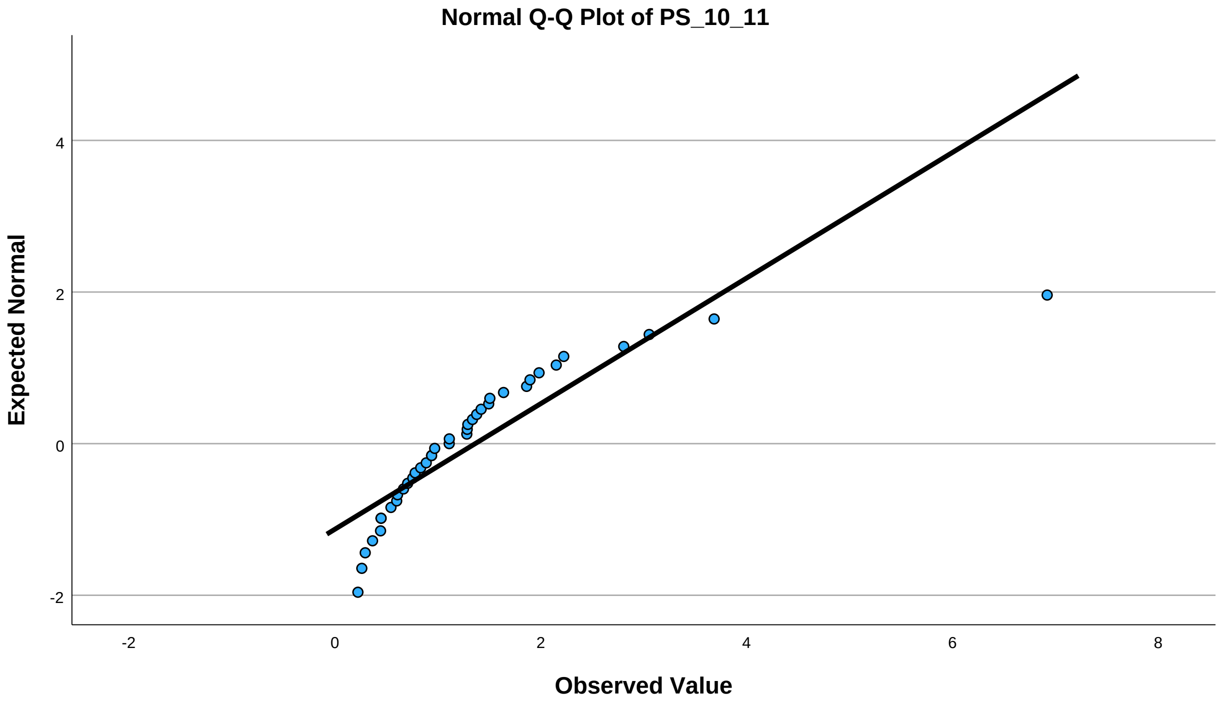
Normal Q-Q Plot of PS_10_11
Expected Normal
Observed Value
4
2
0
-2
-2
0
2
4
6
8

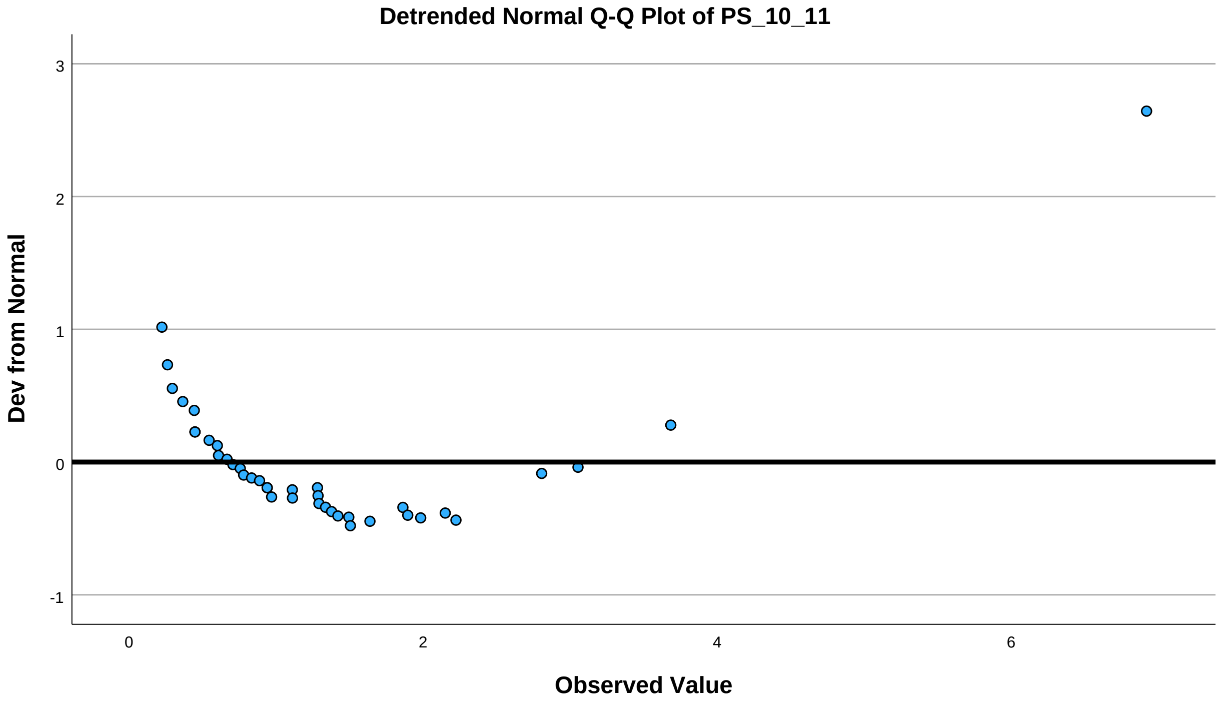
Detrended Normal Q-Q Plot of PS_10_11
Dev from Normal
Observed Value
3
2
1
0
-1
0
2
4
6

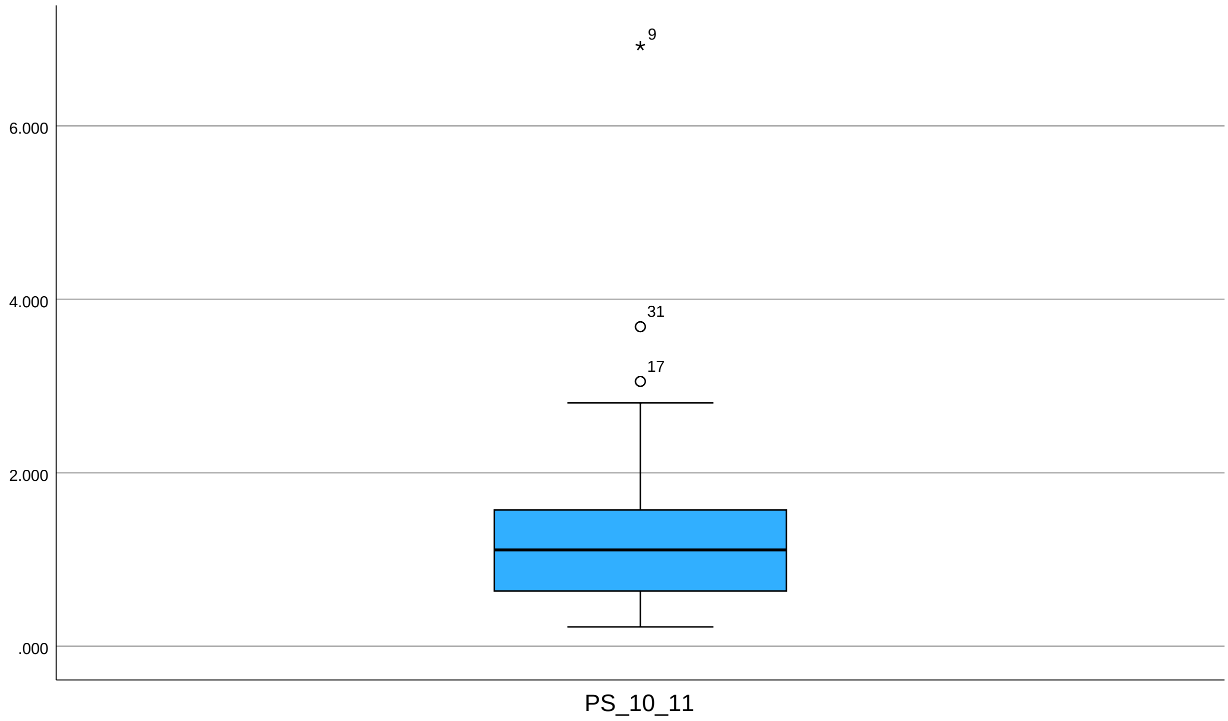

PS_11_12

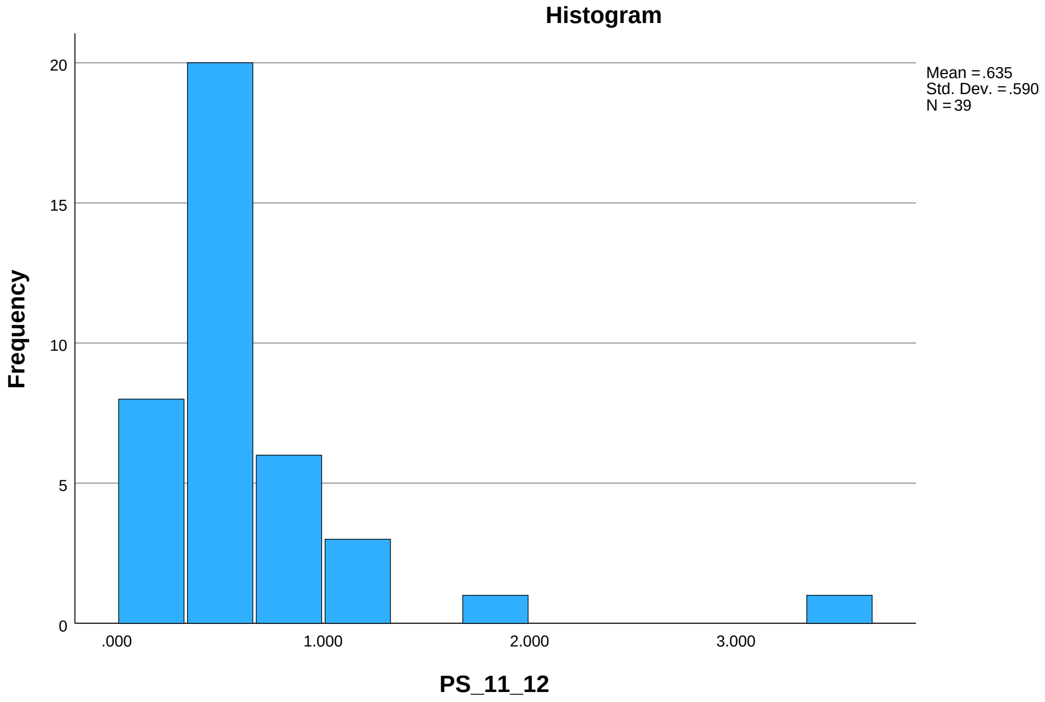

PS_11_12 Stem-and-Leaf Plot

Frequency   Stem & Leaf

```
 2.00      1 . 38
 5.00      2 . 15678
 8.00      3 . 24566679
 8.00      4 . 02223467
 3.00      5 . 138
 4.00      6 . 0477
 1.00      7 . 7
 1.00      8 . 6
 2.00      9 . 48
 5.00 Extremes    (>=1.15)

Stem width:      .100
Each leaf:       1 case(s)
```

Normal Q-Q Plot of PS_11_12

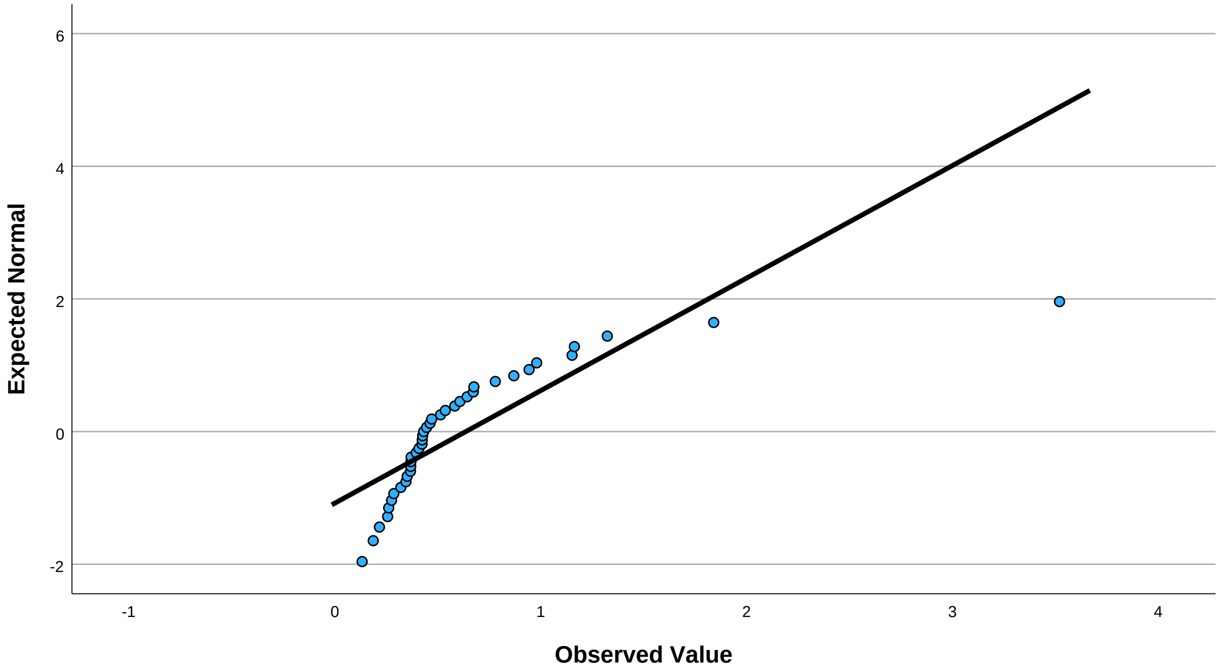

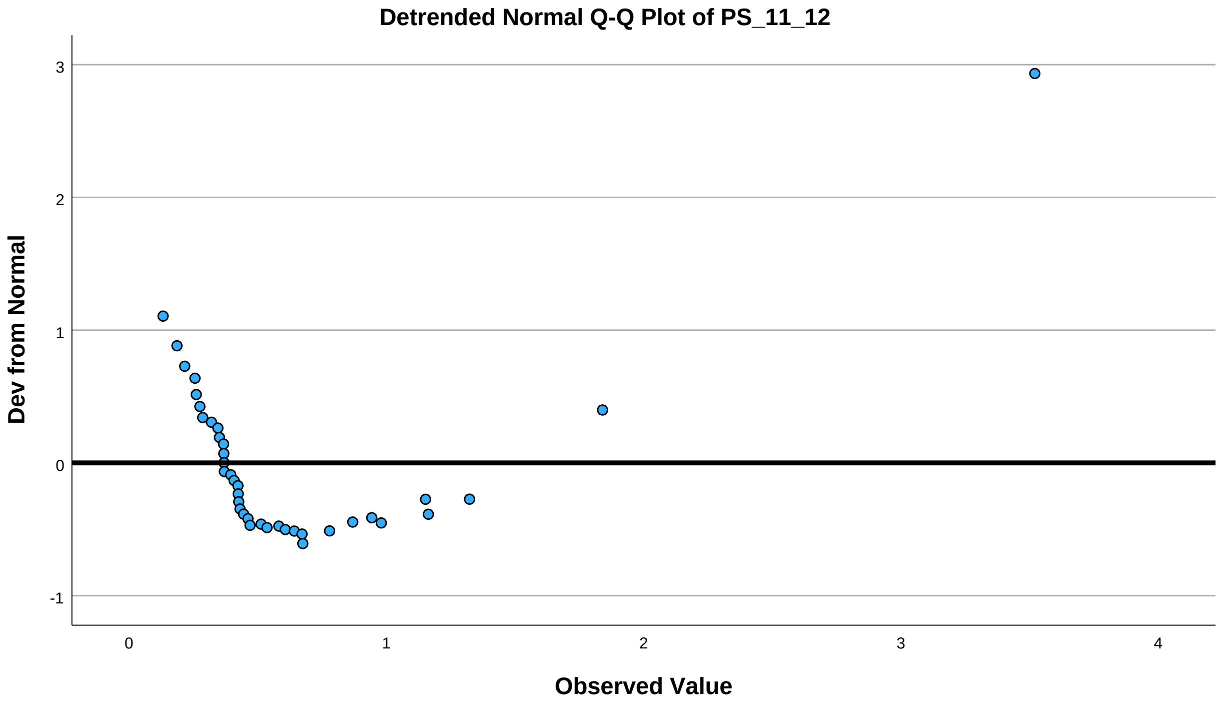

Detrended Normal Q-Q Plot of PS_11_12
Dev from Normal
3
2
1
0
-1
0
1
2
3
4
Observed Value

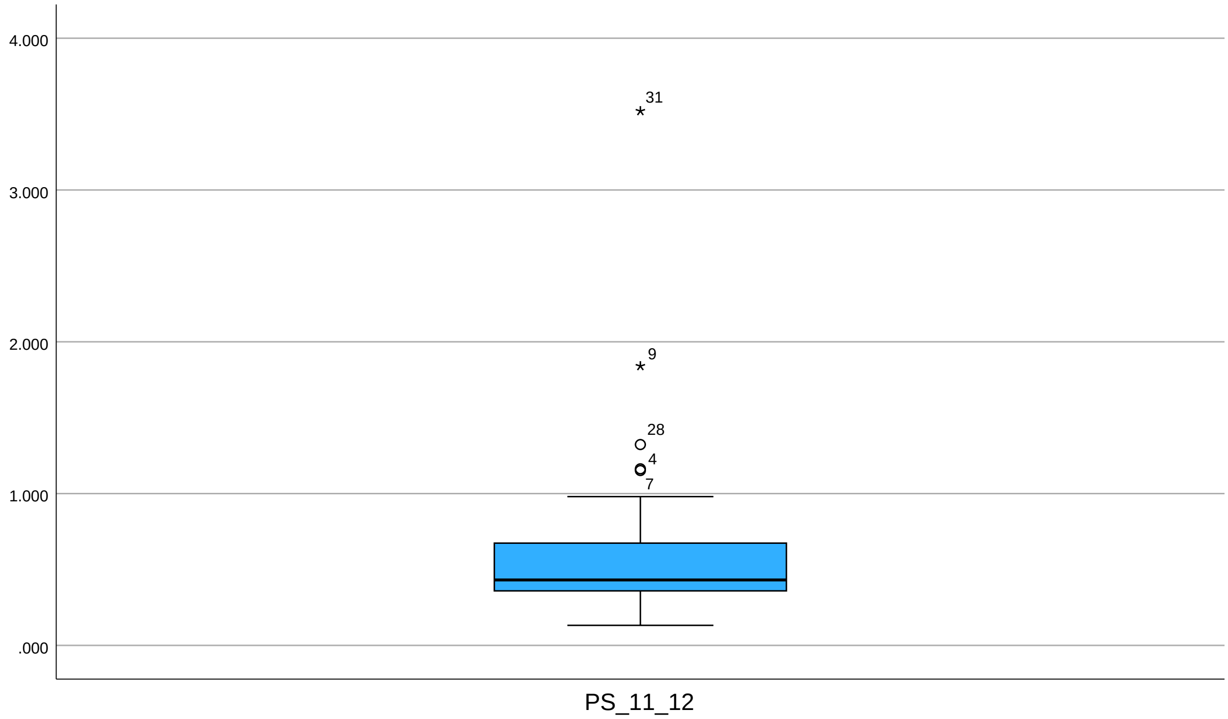

4.000
3.000
2.000
1.000
.000
31
9
28
4
7
PS_11_12

PS_12_13

Histogram

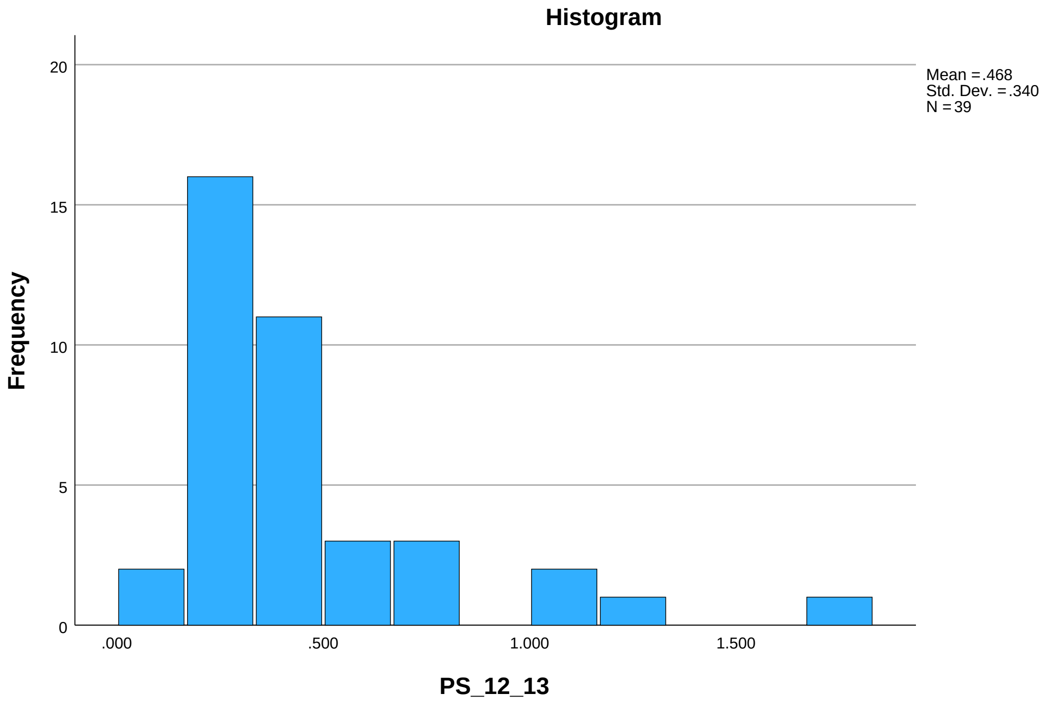

PS_12_13 Stem-and-Leaf Plot

```
 Frequency    Stem &  Leaf

     4.00        1 .  1578
     8.00        2 .  33458899
    12.00        3 .  001123557778
     5.00        4 .  00345
     2.00        5 .  77
     2.00        6 .  09
     2.00        7 .  58
     4.00 Extremes    (>=1.08)

 Stem width:      .100
 Each leaf:       1 case(s)
```

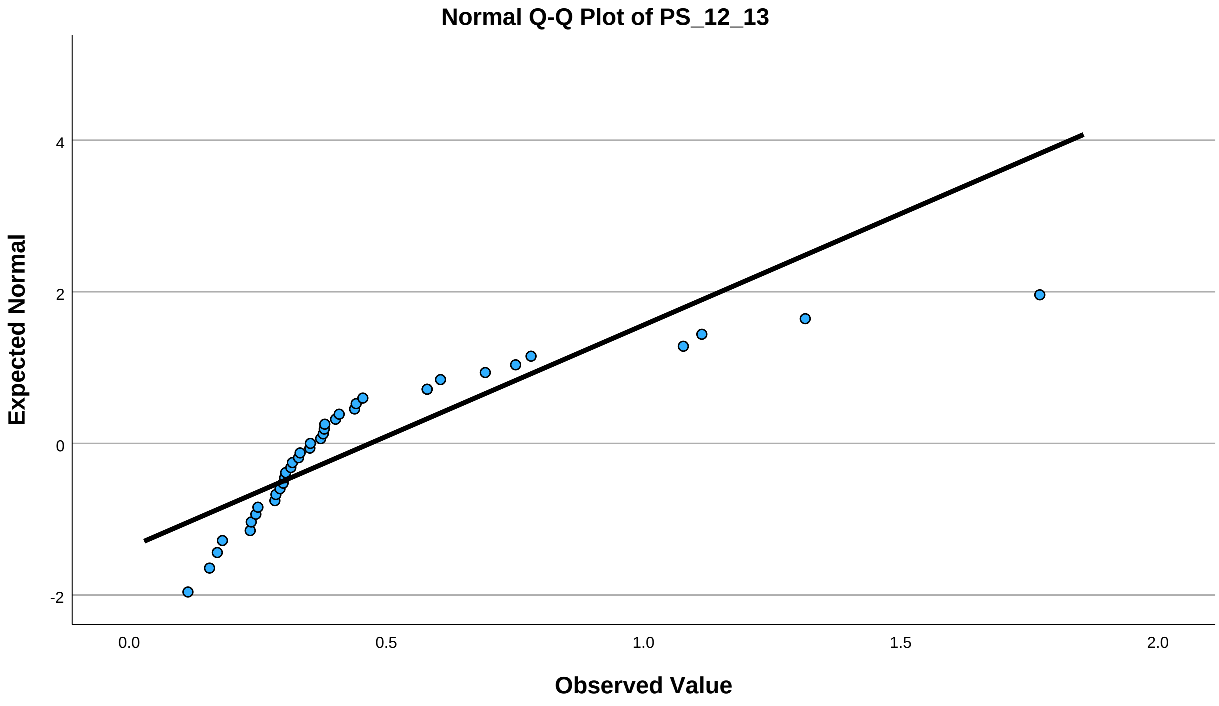
Normal Q-Q Plot of PS_12_13
Expected Normal
Observed Value
4
2
0
-2
0.0
0.5
1.0
1.5
2.0

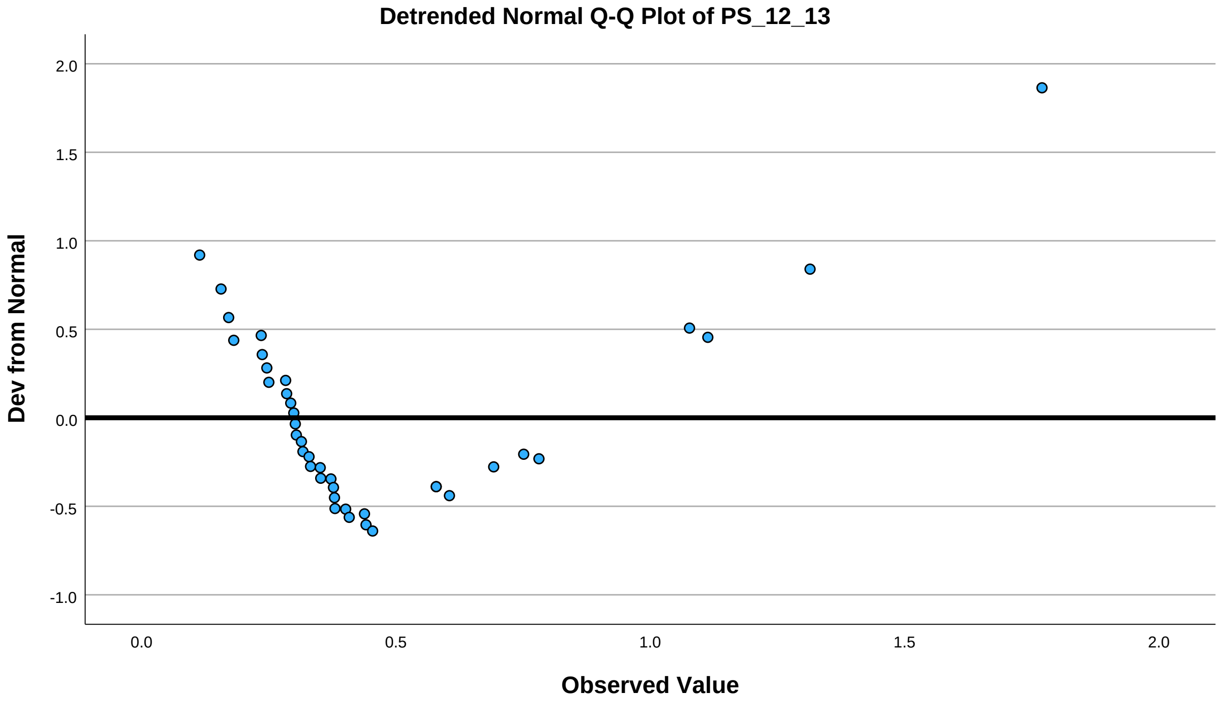
Detrended Normal Q-Q Plot of PS_12_13
Dev from Normal
Observed Value
2.0
1.5
1.0
0.5
0.0
-0.5
-1.0
0.0
0.5
1.0
1.5
2.0

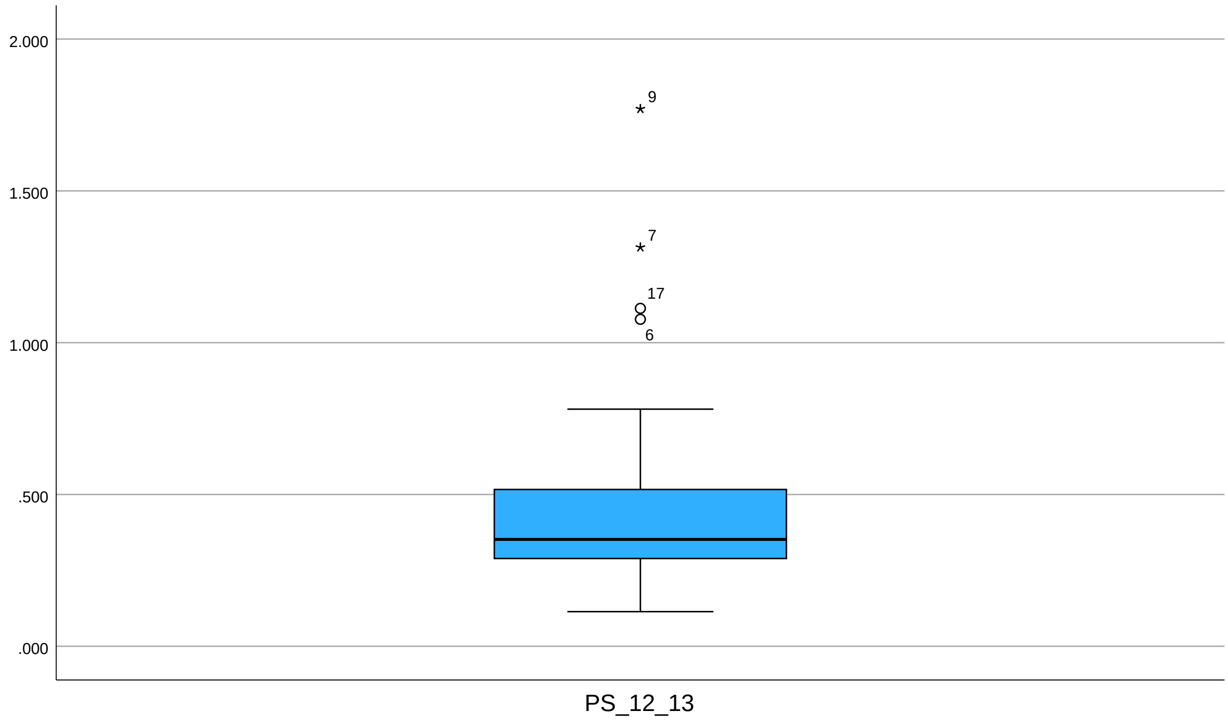

PS_13_14

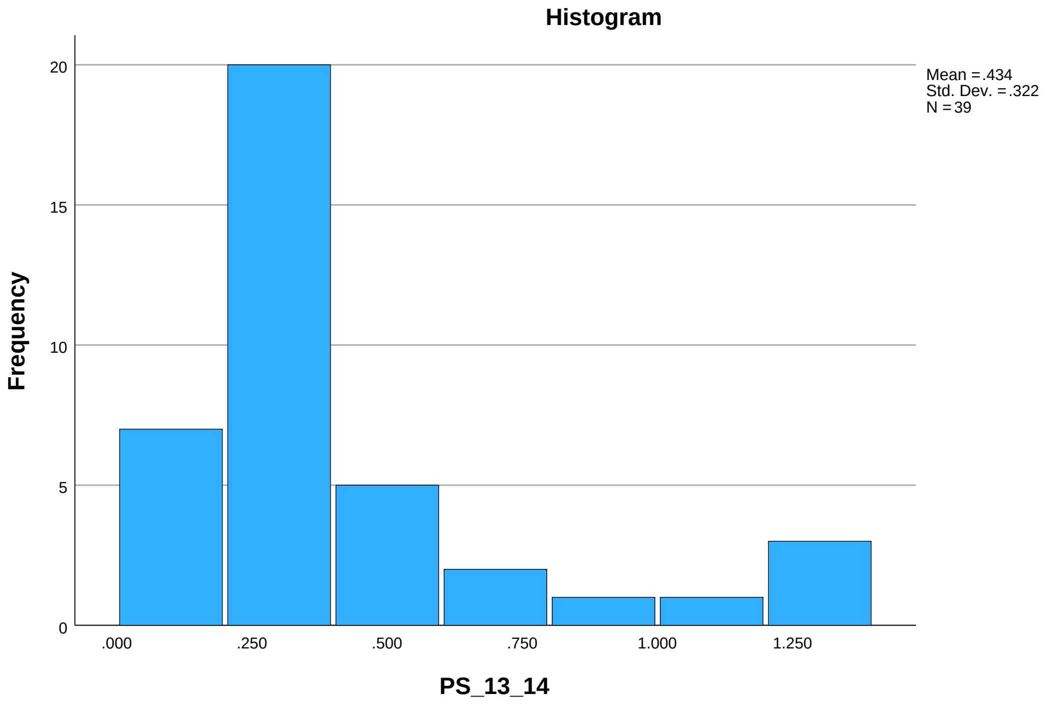

PS_13_14 Stem-and-Leaf Plot

Frequency   Stem & Leaf

```
  7.00        1 .  1356999
  8.00        2 .  01344467
 12.00        3 .  124457888999
  4.00        4 .  0679
  1.00        5 .  1
  2.00        6 .  12
   .00        7 .
  1.00        8 .  0
  4.00 Extremes    (>=1.00)

Stem width:      .100
Each leaf:       1 case(s)
```

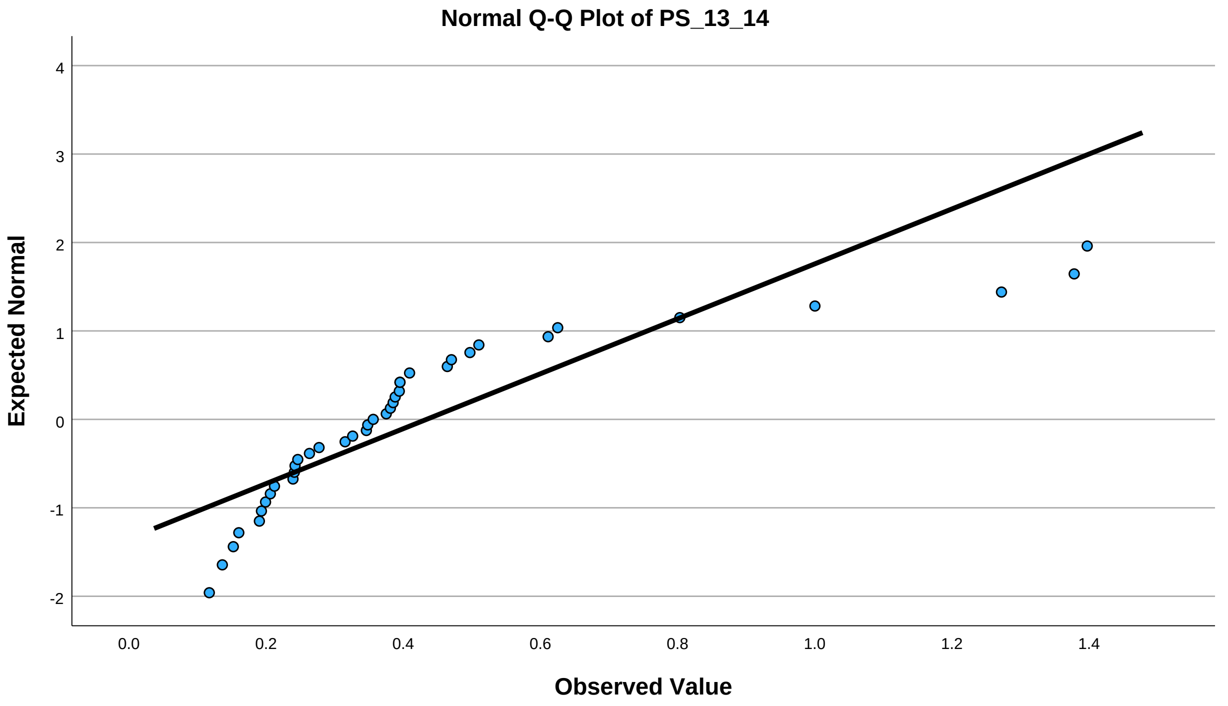

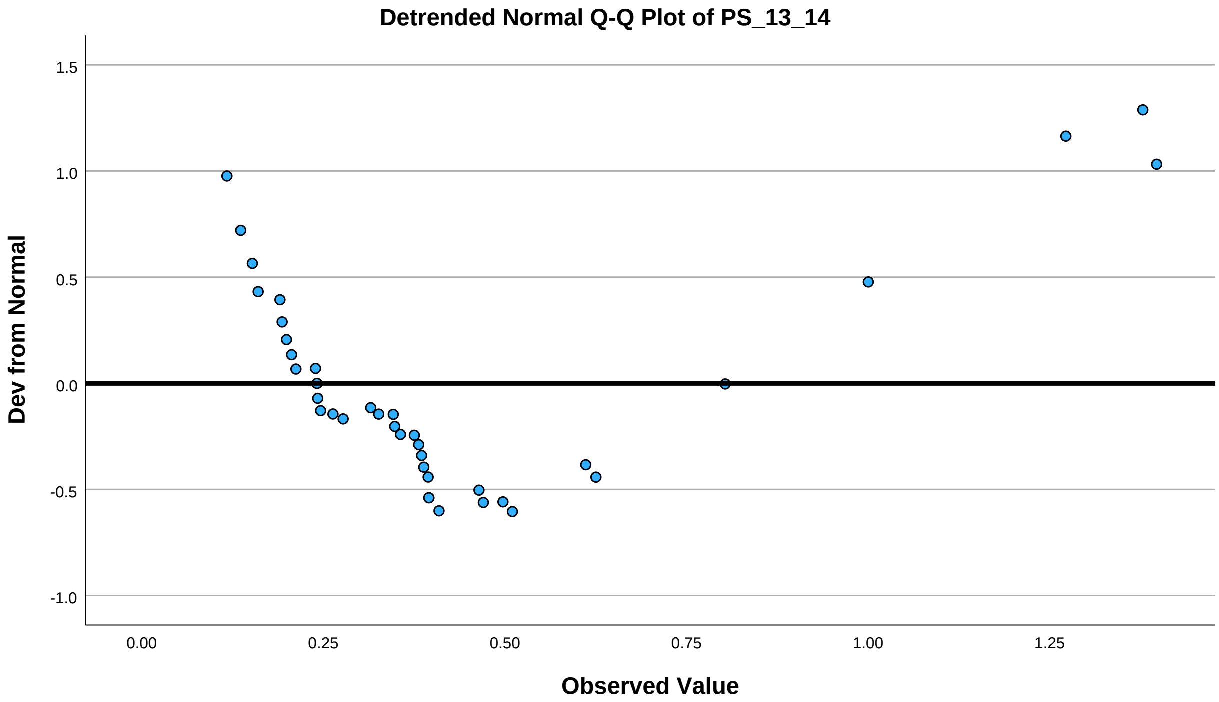
Detrended Normal Q-Q Plot of PS_13_14
Dev from Normal
1.5
1.0
0.5
0.0
-0.5
-1.0
0.00
0.25
0.50
0.75
1.00
1.25
Observed Value

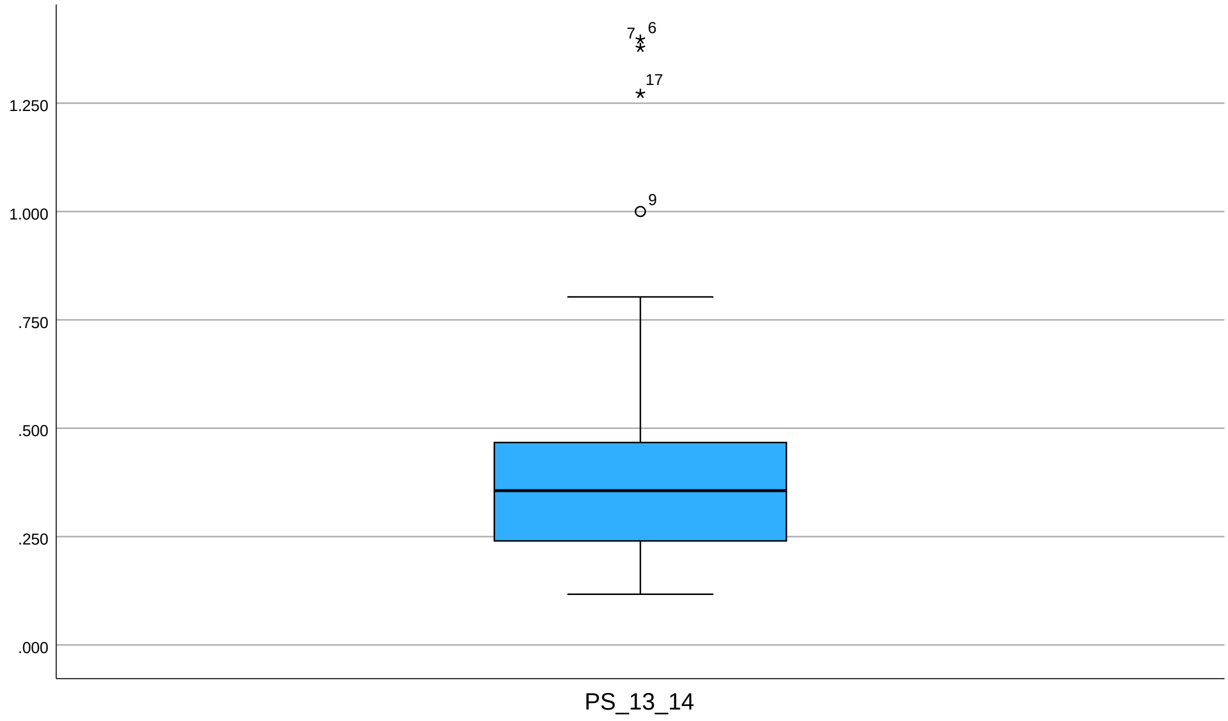
7
6
17
9
1.250
1.000
.750
.500
.250
.000
PS_13_14

PS_14_15

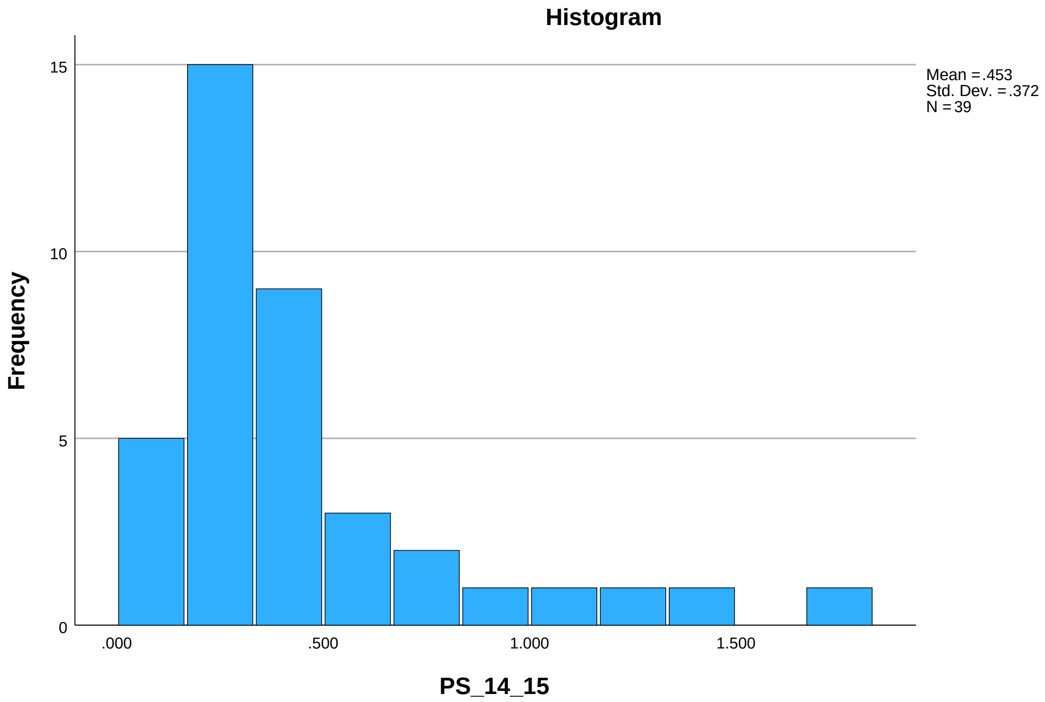


```
PS_14_15 Stem-and-Leaf Plot

 Frequency    Stem &  Leaf

     7.00        1 .  1346689
    10.00         2 .  0134566789
     8.00        3 .  12234789
     4.00       4 .  3457
     3.00       5 .  015
     1.00      6 .  7
     1.00      7 .  0
     1.00      8 .  7
     4.00 Extremes    (>=1.11)

 Stem width:      .100
 Each leaf:       1 case(s)
```

Normal Q-Q Plot of PS_14_15

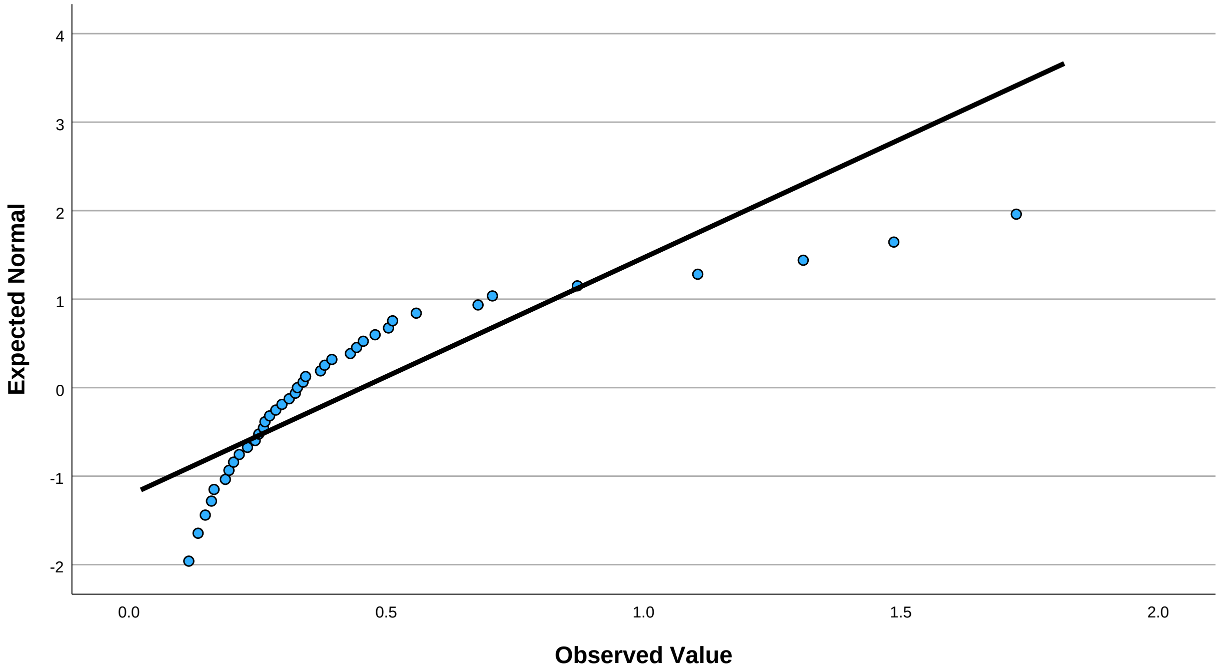

Detrended Normal Q-Q Plot of PS_14_15

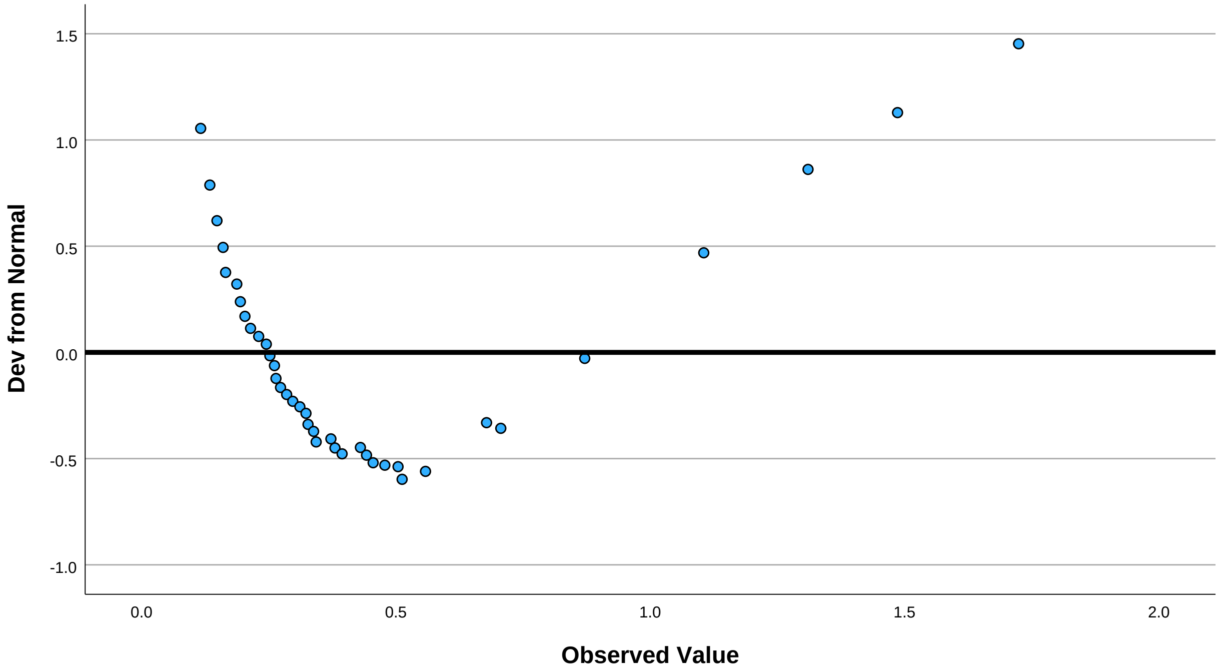

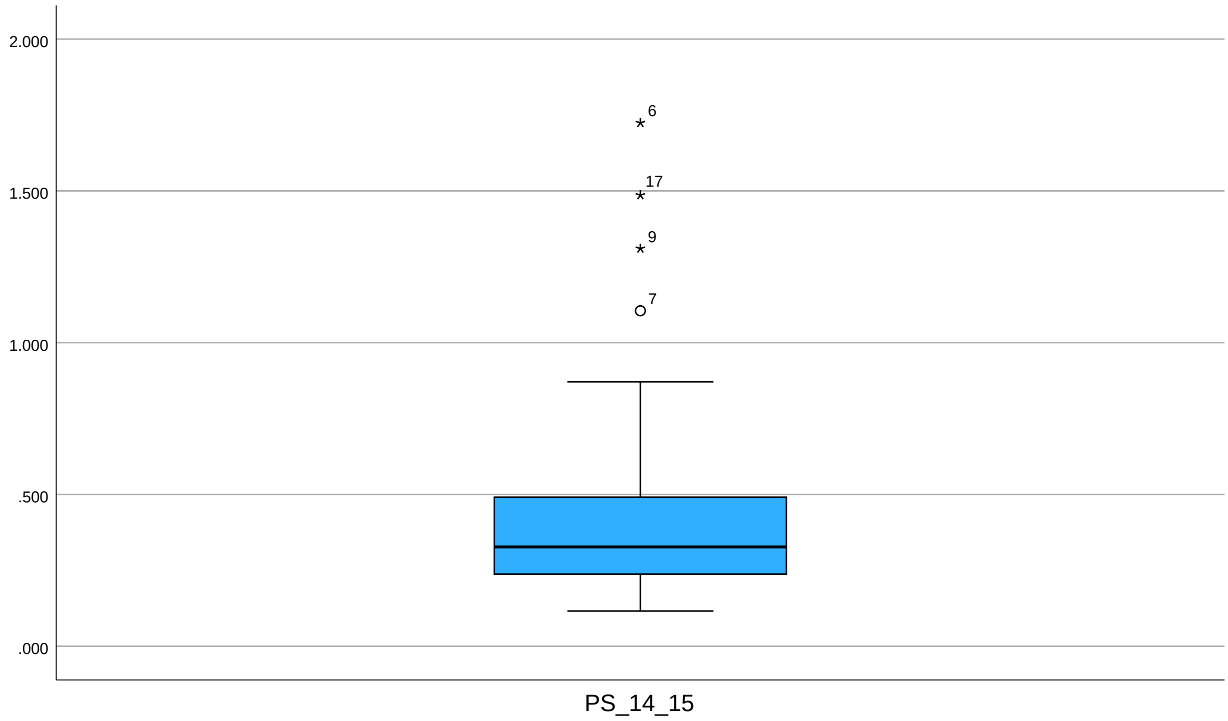

PS_15_16

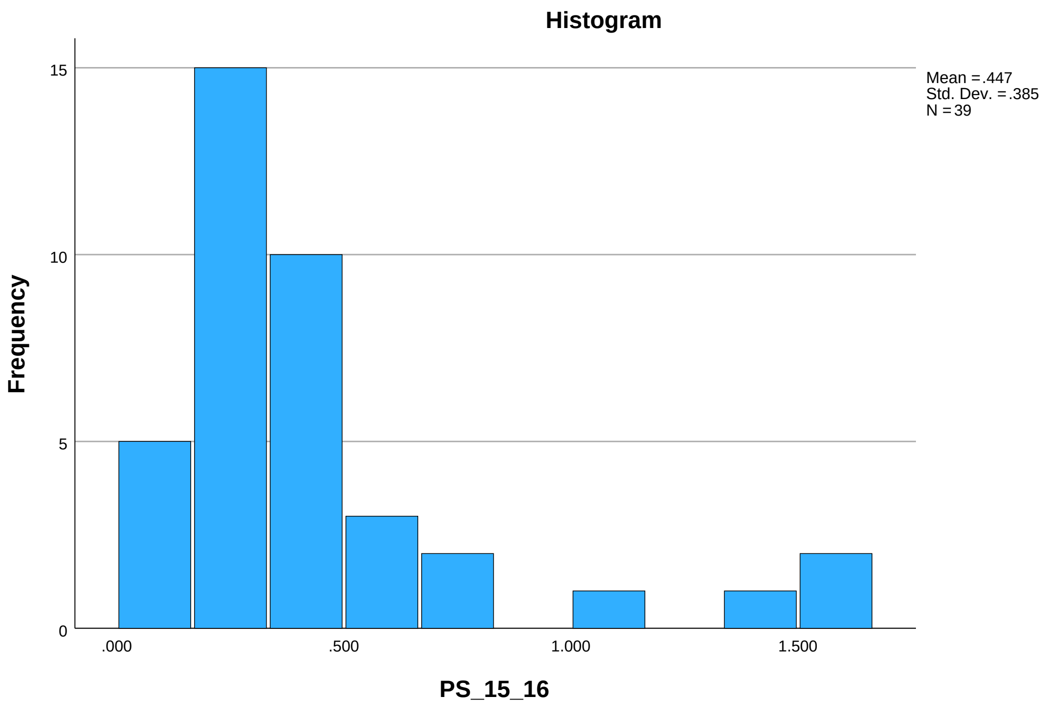

PS_15_16 Stem-and-Leaf Plot

Frequency   Stem & Leaf

```
 8.00      1 .  12346889
 8.00      2 .  33333569
10.00      3 .  0111345677
 4.00      4 .  1129
 2.00      5 .  24
 1.00      6 .  5
 2.00      7 .  56
 4.00 Extremes    (>=1.16)

Stem width:      .100
Each leaf:       1 case(s)
```

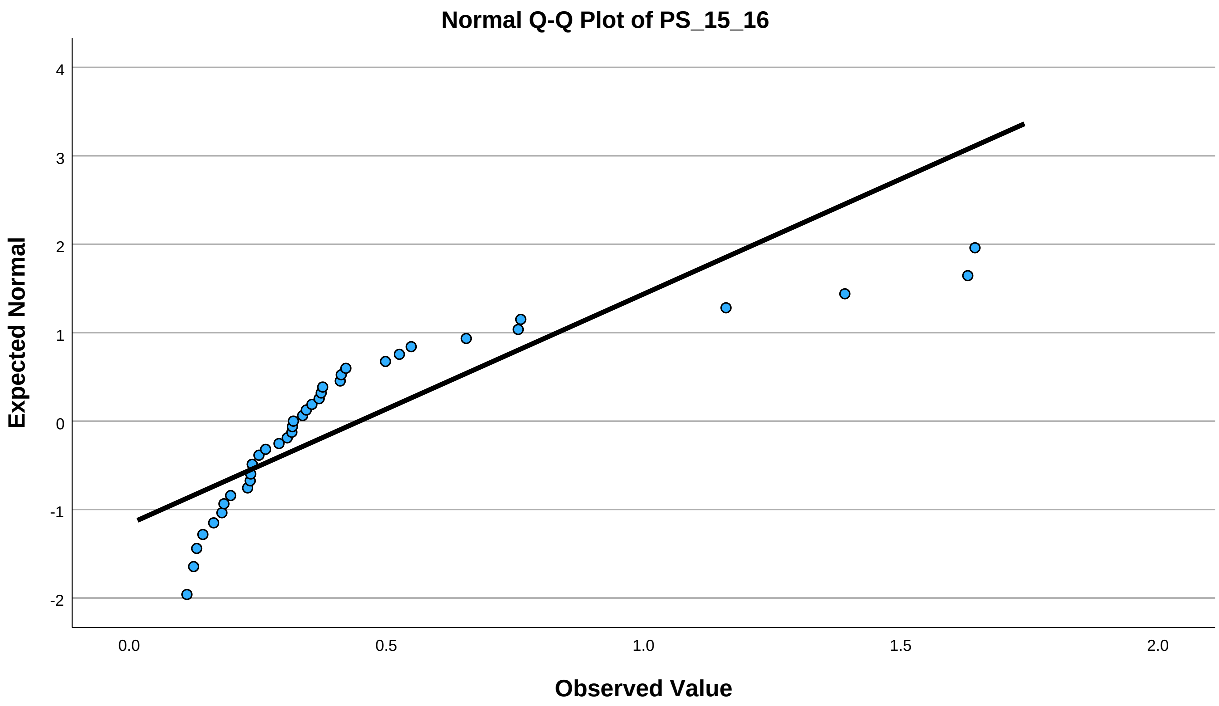

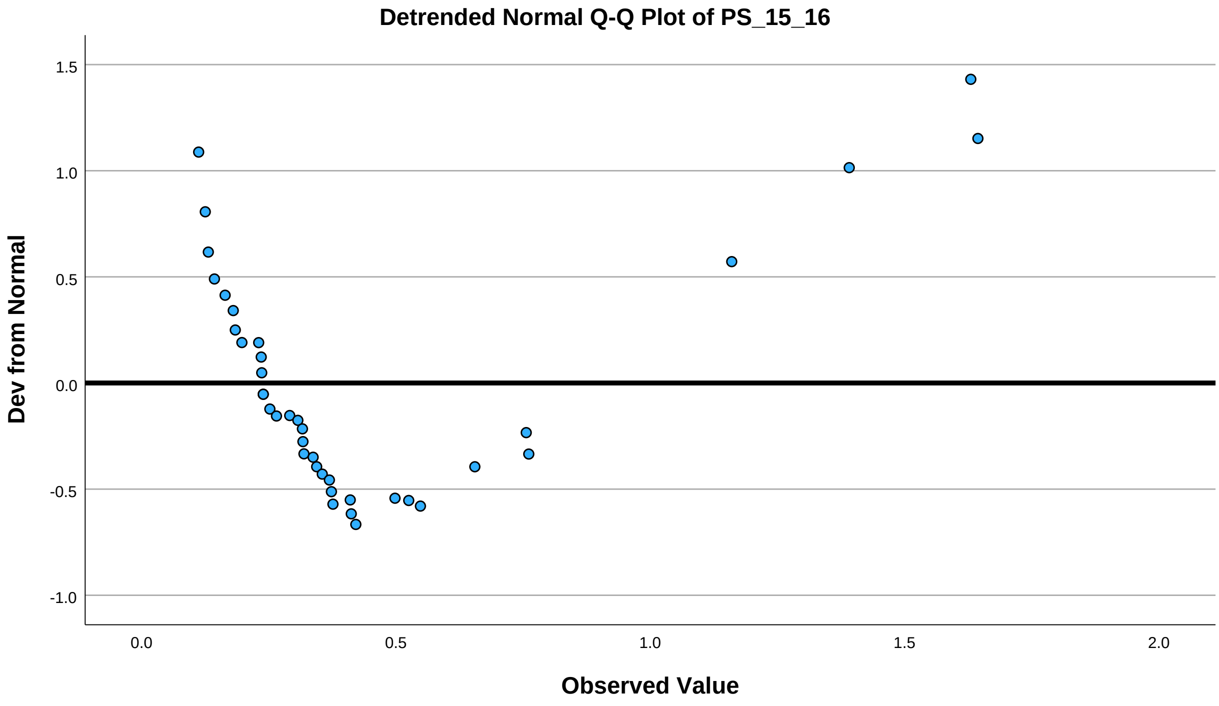
Detrended Normal Q-Q Plot of PS_15_16
Dev from Normal
1.5
1.0
0.5
0.0
-0.5
-1.0
0.0
0.5
1.0
1.5
2.0
Observed Value

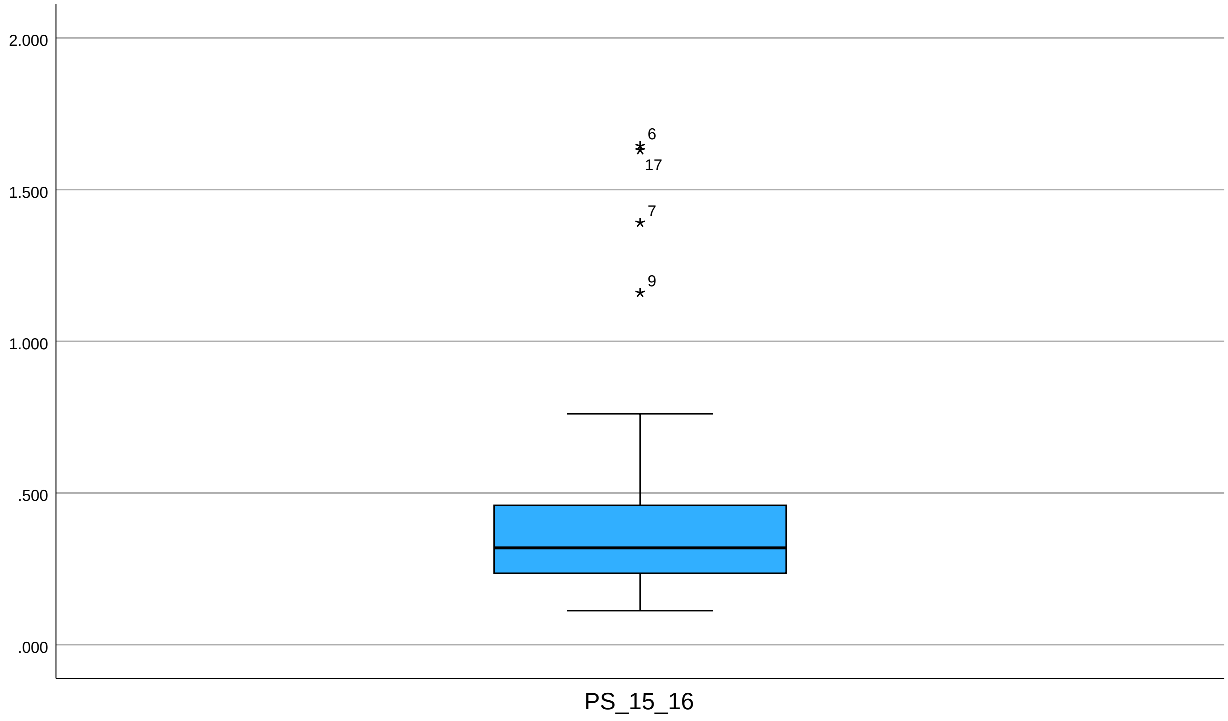
2.000
1.500
1.000
.500
.000
6
17
7
9
PS_15_16

PS_16_17

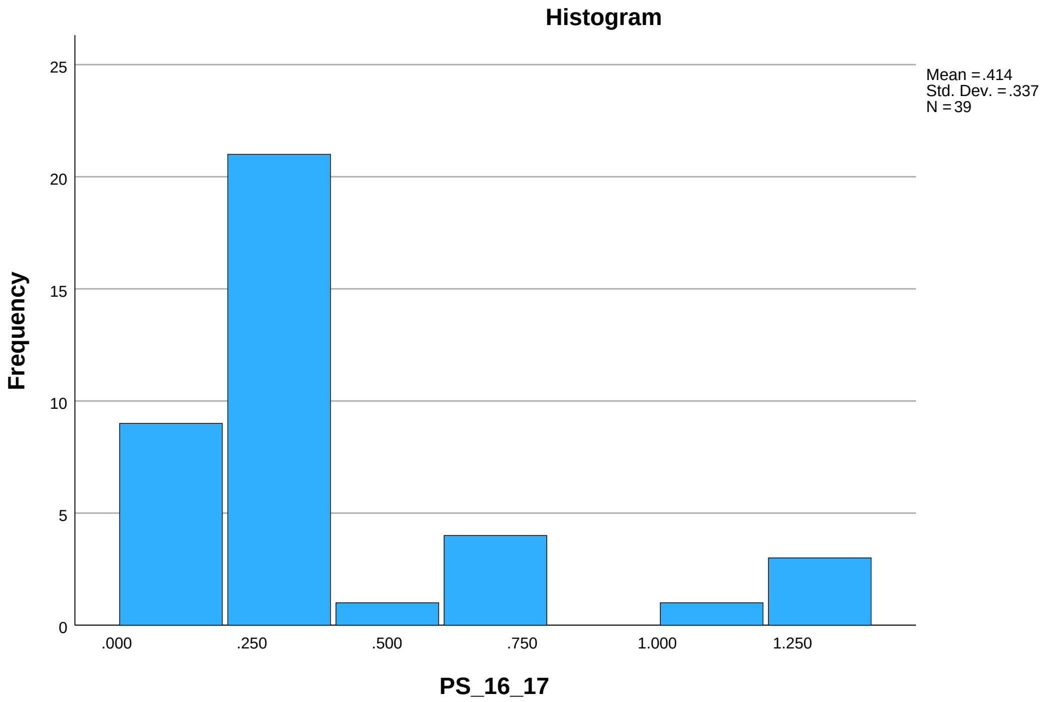

PS_16_17 Stem-and-Leaf Plot

```
 Frequency    Stem &  Leaf

     5.00        1 .  01234
     4.00        1 .  6779
     8.00        2 .  23444444
     2.00        2 .  59
     4.00        3 .  1123
     7.00        3 .  5778899
      .00        4 .
     1.00        4 .  6
      .00        5 .
      .00        5 .
     1.00        6 .  1
     7.00 Extremes    (>=.68)

 Stem width:      .100
 Each leaf:       1 case(s)
```

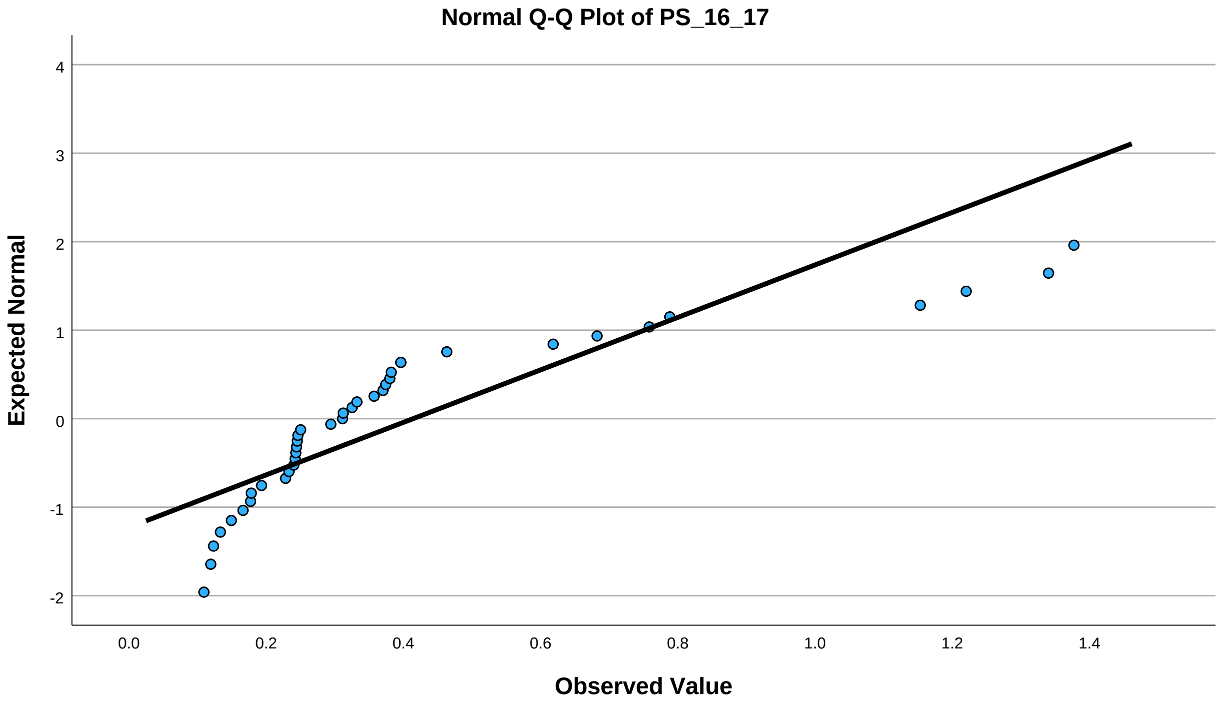

Normal Q-Q Plot of PS_16_17
Expected Normal
Observed Value
4
3
2
1
0
-1
-2
0.0
0.2
0.4
0.6
0.8
1.0
1.2
1.4

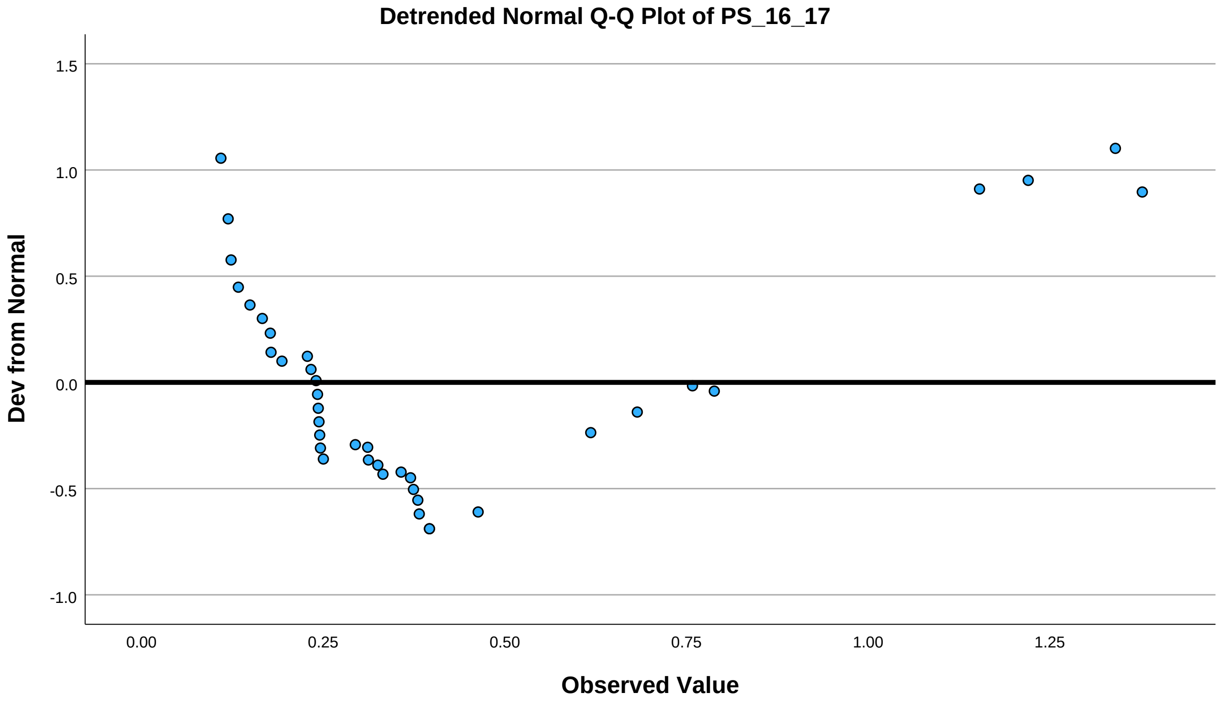

Detrended Normal Q-Q Plot of PS_16_17
Dev from Normal
Observed Value
1.5
1.0
0.5
0.0
-0.5
-1.0
0.00
0.25
0.50
0.75
1.00
1.25

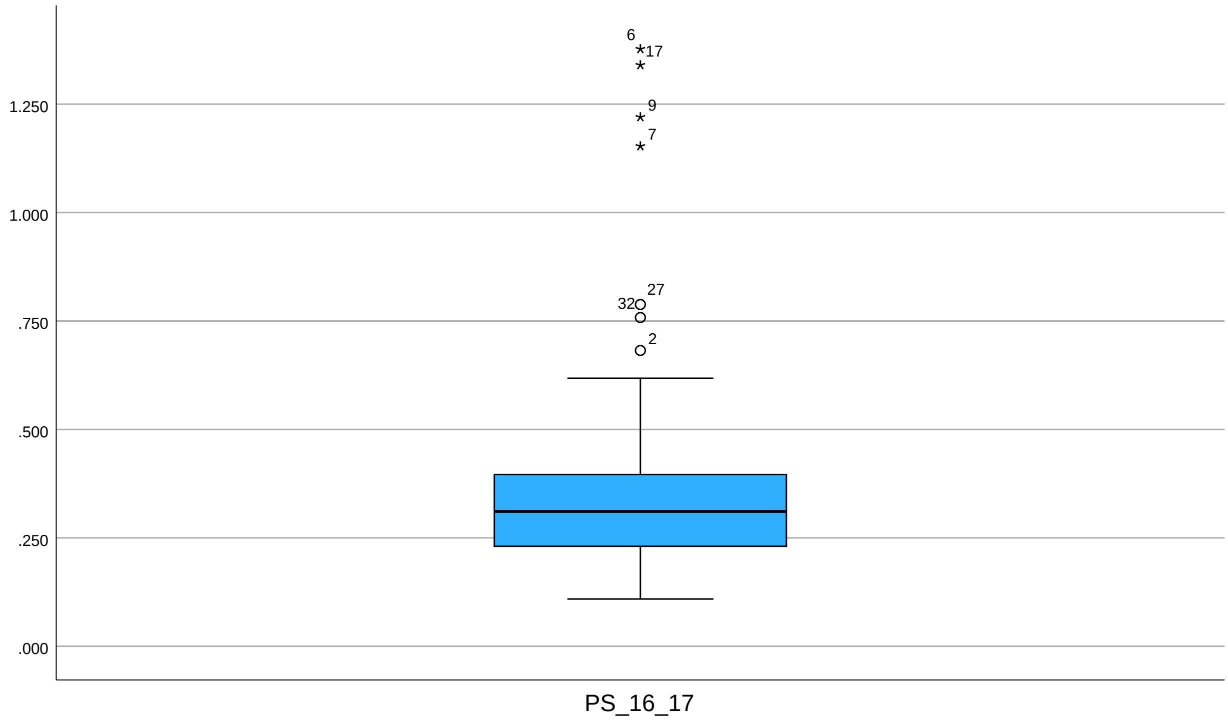

PS_17_18

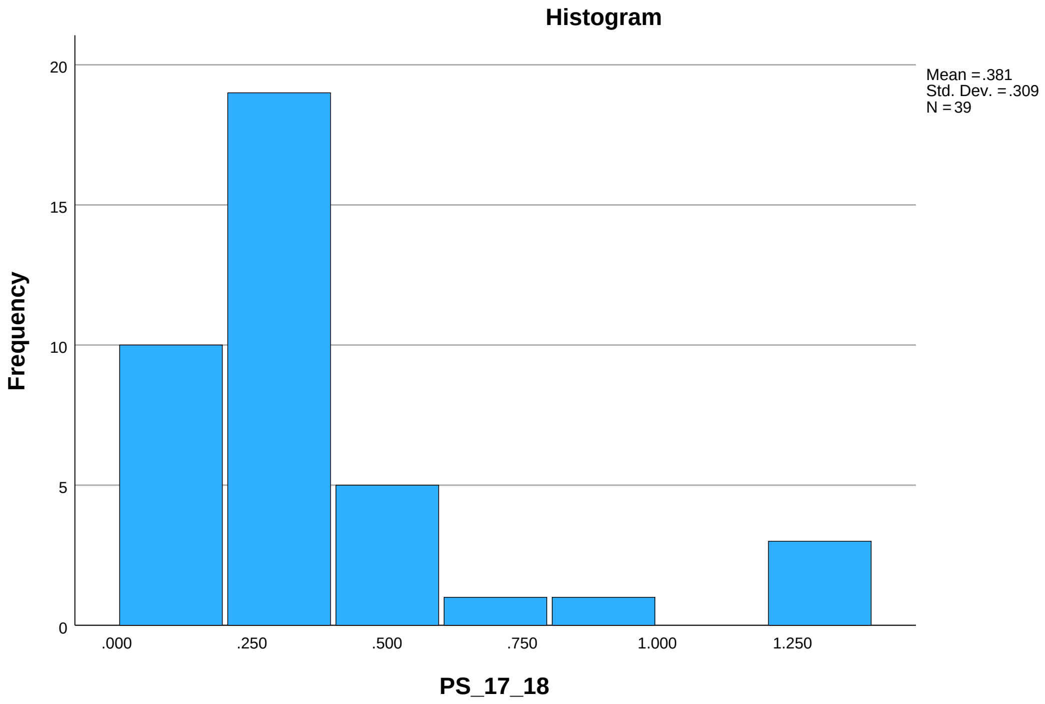

PS_17_18 Stem-and-Leaf Plot

Frequency   Stem & Leaf

```
    4.00        1 .  1124
    6.00        1 .  555789
    4.00        2 .  0334
    7.00        2 .  5557789
    4.00        3 .  1234
    4.00        3 .  6789
    2.00        4 .  00
    2.00        4 .  79
    1.00        5 .  2
    5.00 Extremes    (>=.71)

 Stem width:      .100
 Each leaf:       1 case(s)
```

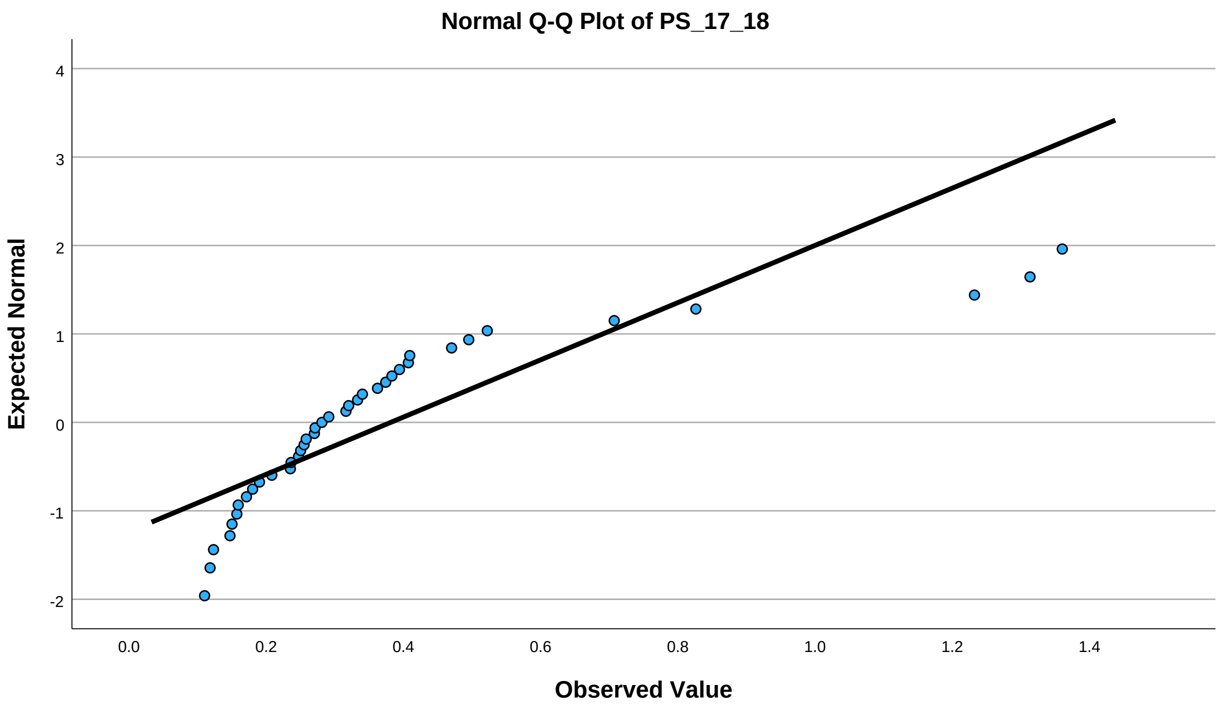

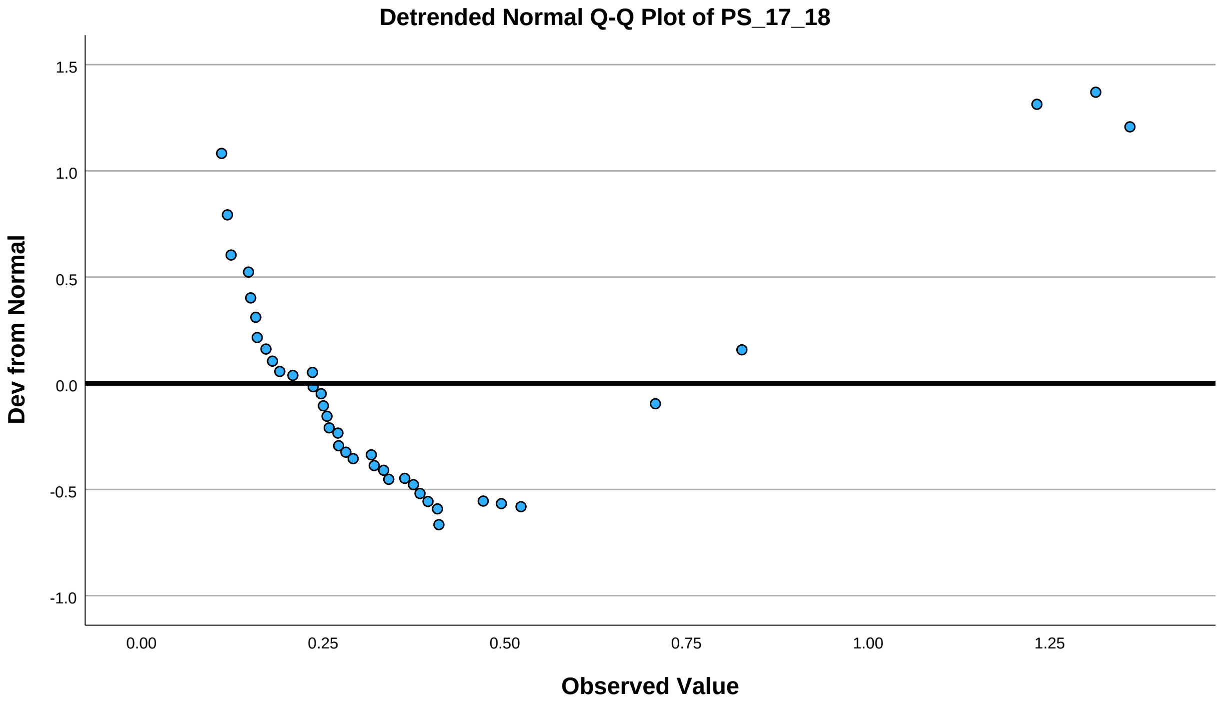
Detrended Normal Q-Q Plot of PS_17_18
Dev from Normal
1.5
1.0
0.5
0.0
-0.5
-1.0
0.00
0.25
0.50
0.75
1.00
1.25
Observed Value

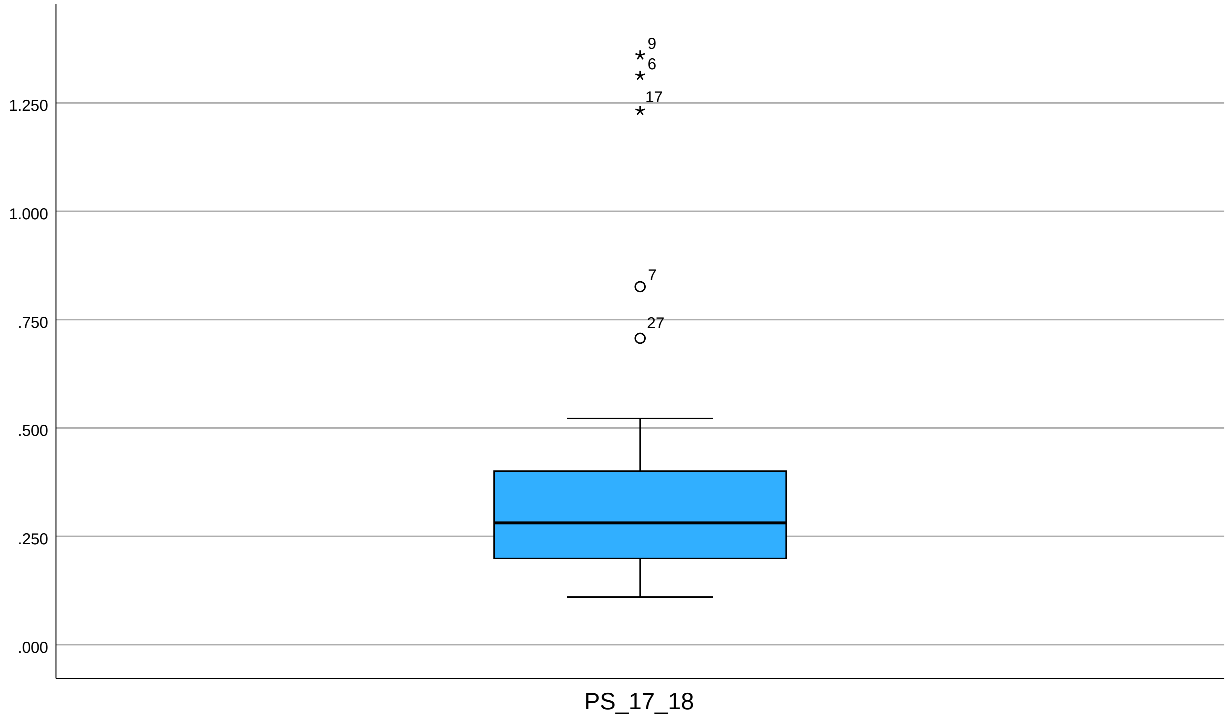
9
6
17
7
27
1.250
1.000
.750
.500
.250
.000
PS_17_18

PS_18_19

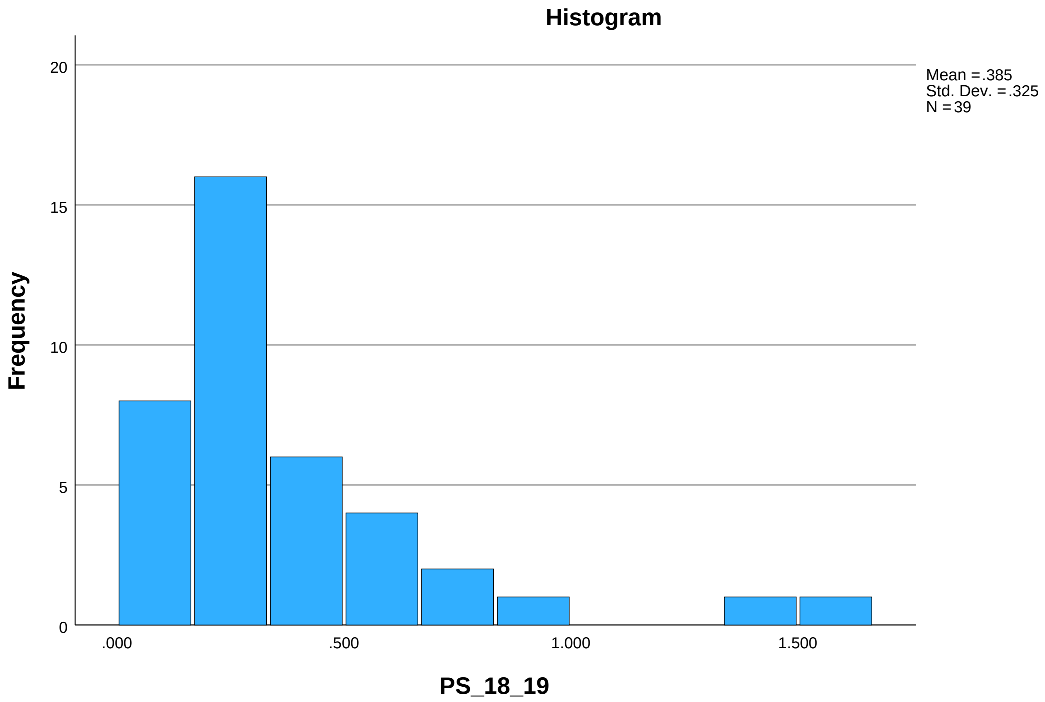


```
PS_18_19 Stem-and-Leaf Plot

 Frequency    Stem &  Leaf

    11.00        1 .  00244455789
    11.00        2 .  01334444568
     6.00        3 .  033349
     2.00        4 .  38
     2.00        5 .  07
     3.00        6 .  169
     1.00        7 .  5
     3.00 Extremes    (>=.99)

 Stem width:      .100
 Each leaf:        1 case(s)
```

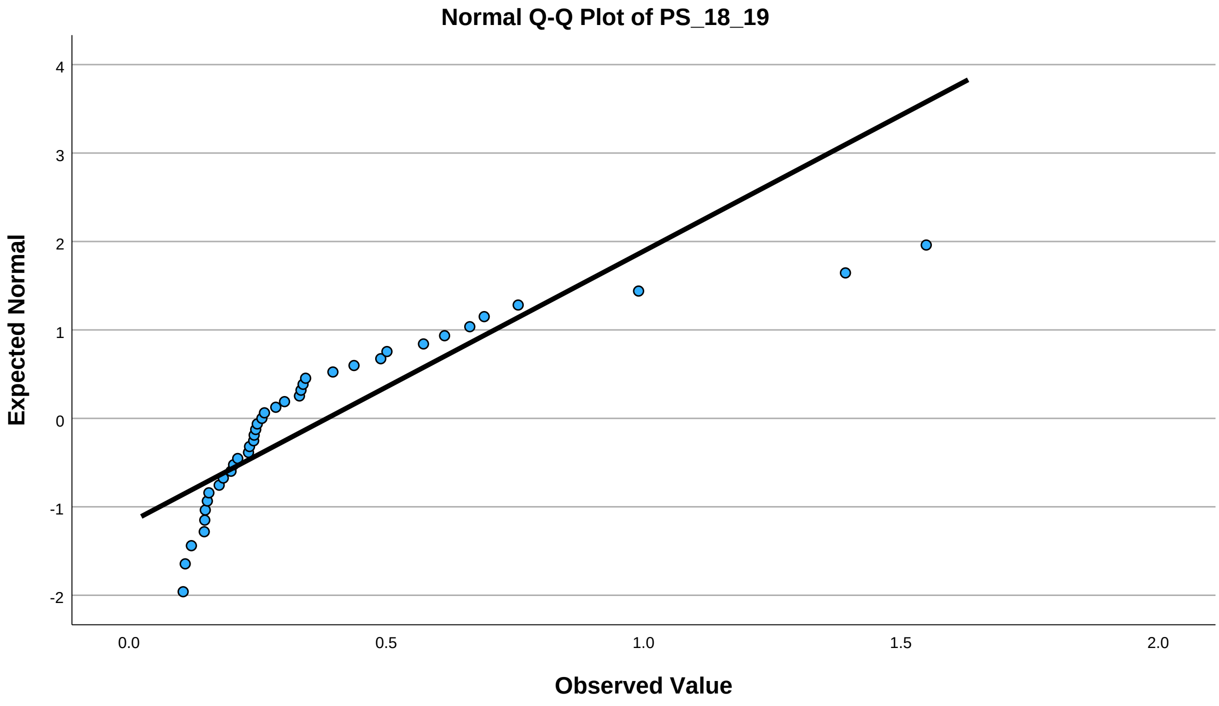

Normal Q-Q Plot of PS_18_19
Expected Normal
4
3
2
1
0
-1
-2
0.0
0.5
1.0
1.5
2.0
Observed Value

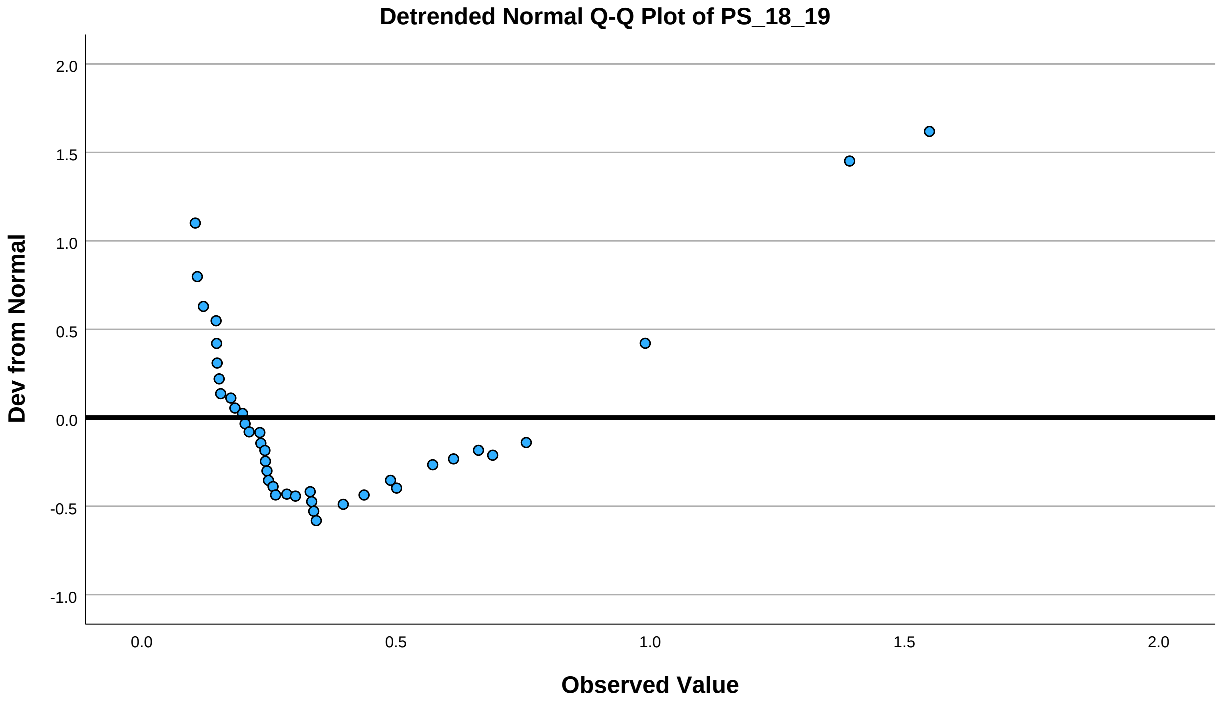

Detrended Normal Q-Q Plot of PS_18_19
Dev from Normal
2.0
1.5
1.0
0.5
0.0
-0.5
-1.0
0.0
0.5
1.0
1.5
2.0
Observed Value

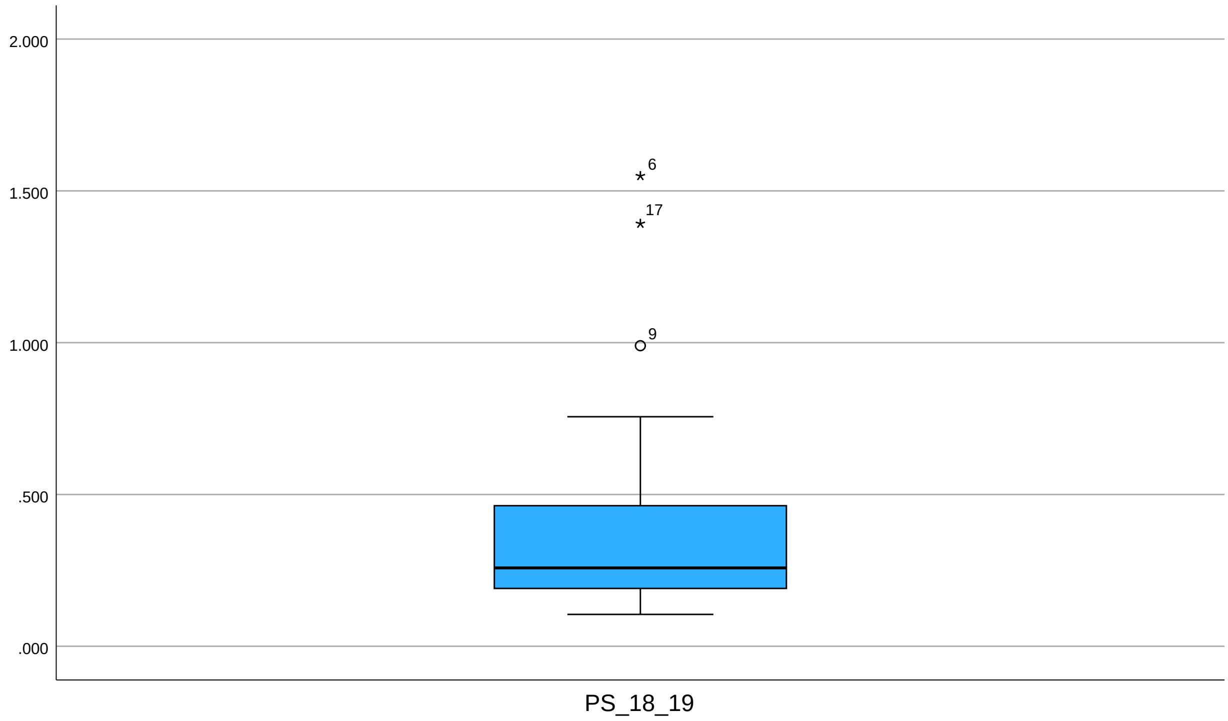

PS_19_20

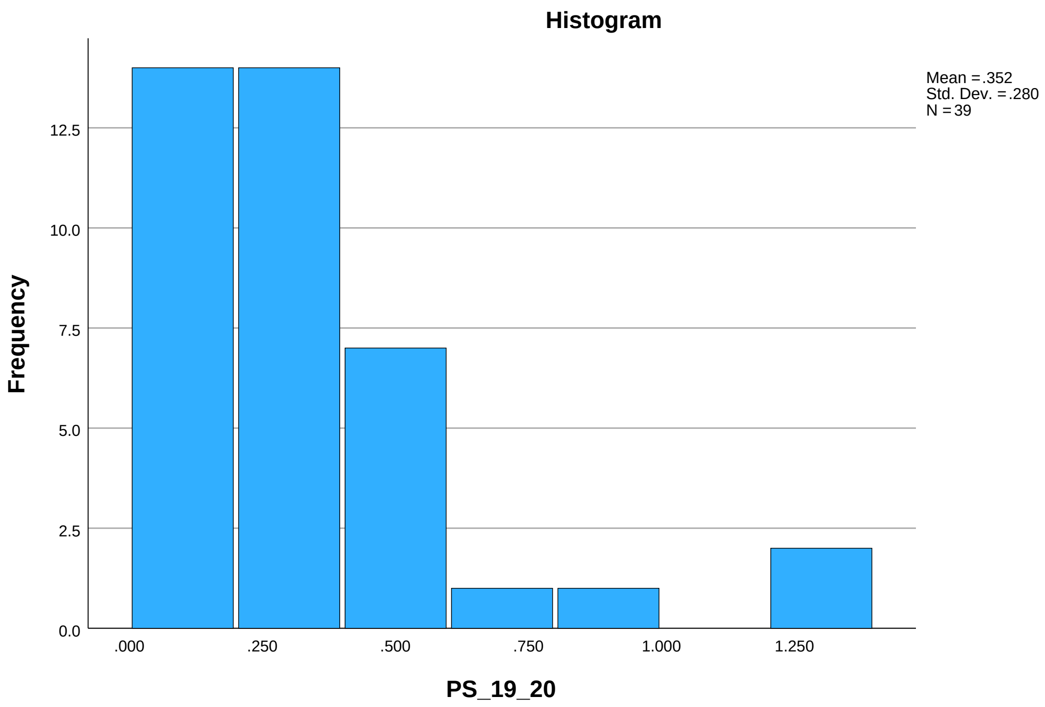

PS_19_20 Stem-and-Leaf Plot

Frequency   Stem & Leaf

```
 14.00       1 .  11111345567799
  9.00      2 .  23356779
  5.00      3 .  02347
  3.00      4 .  378
  4.00      5 .  1558
  1.00      6 .  7
   .00      7 .
  1.00      8 .  0
  2.00 Extremes    (>=1.24)

Stem width:      .100
Each leaf:       1 case(s)
```

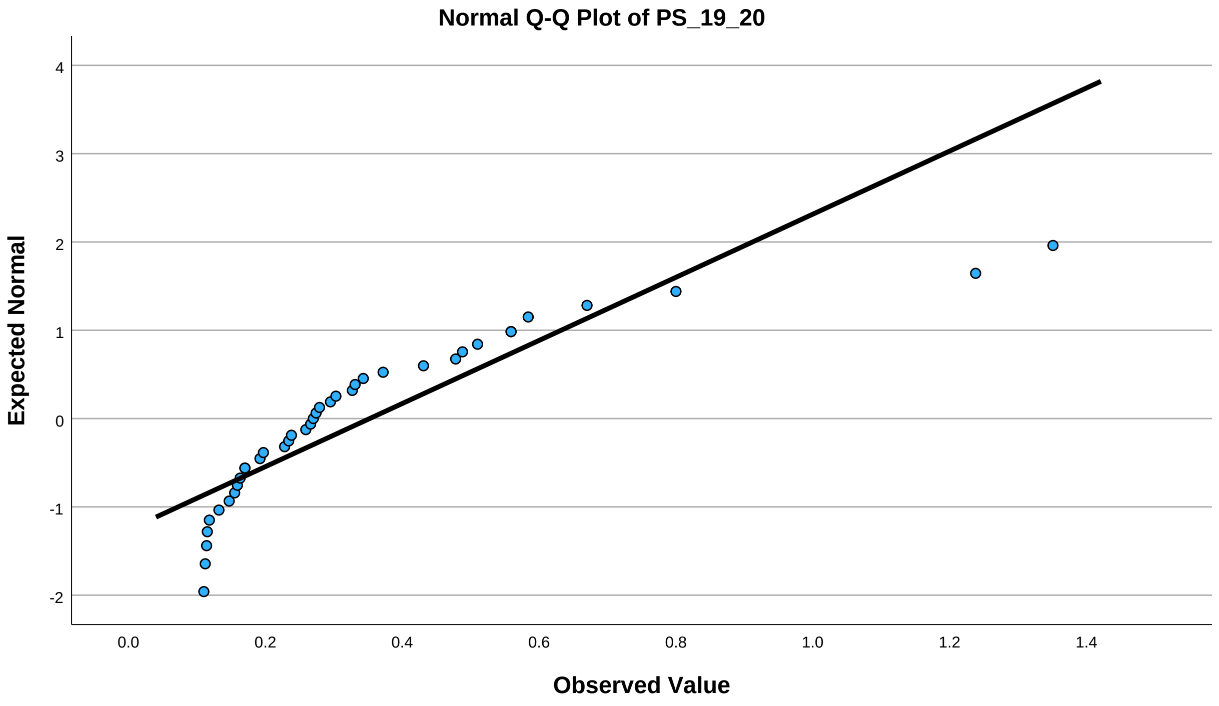

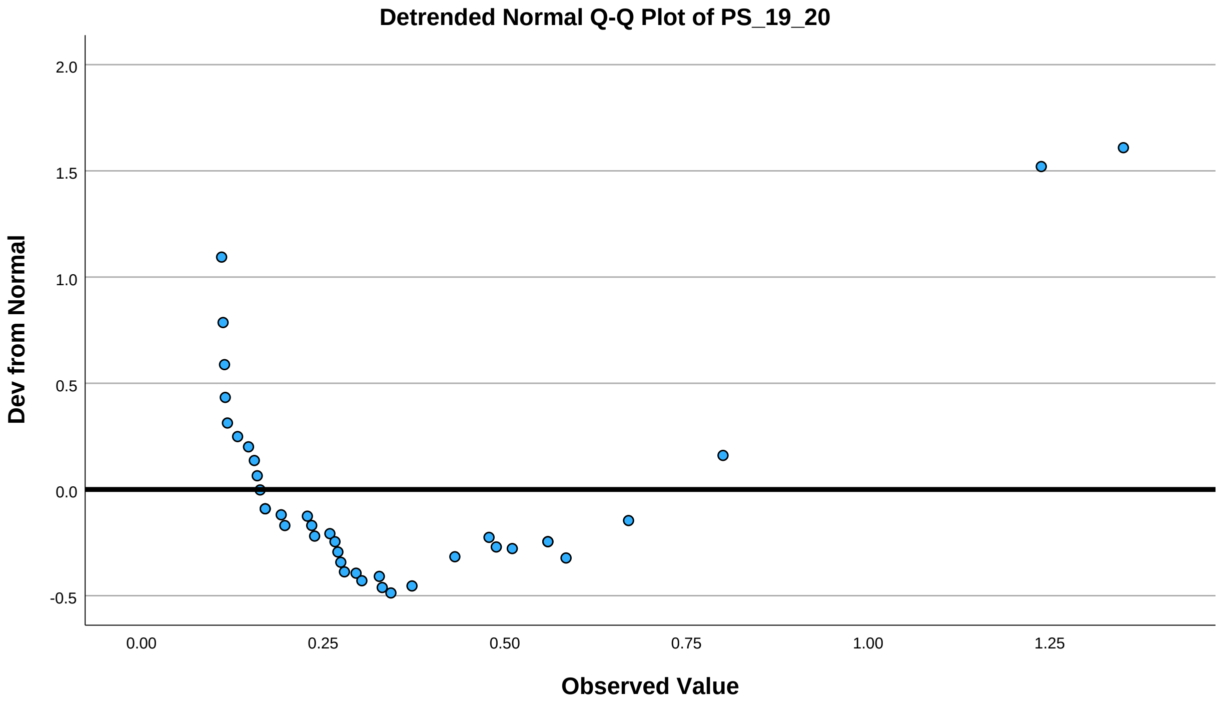

Detrended Normal Q-Q Plot of PS_19_20
Dev from Normal
2.0
1.5
1.0
0.5
0.0
-0.5
0.00
0.25
0.50
0.75
1.00
1.25
Observed Value

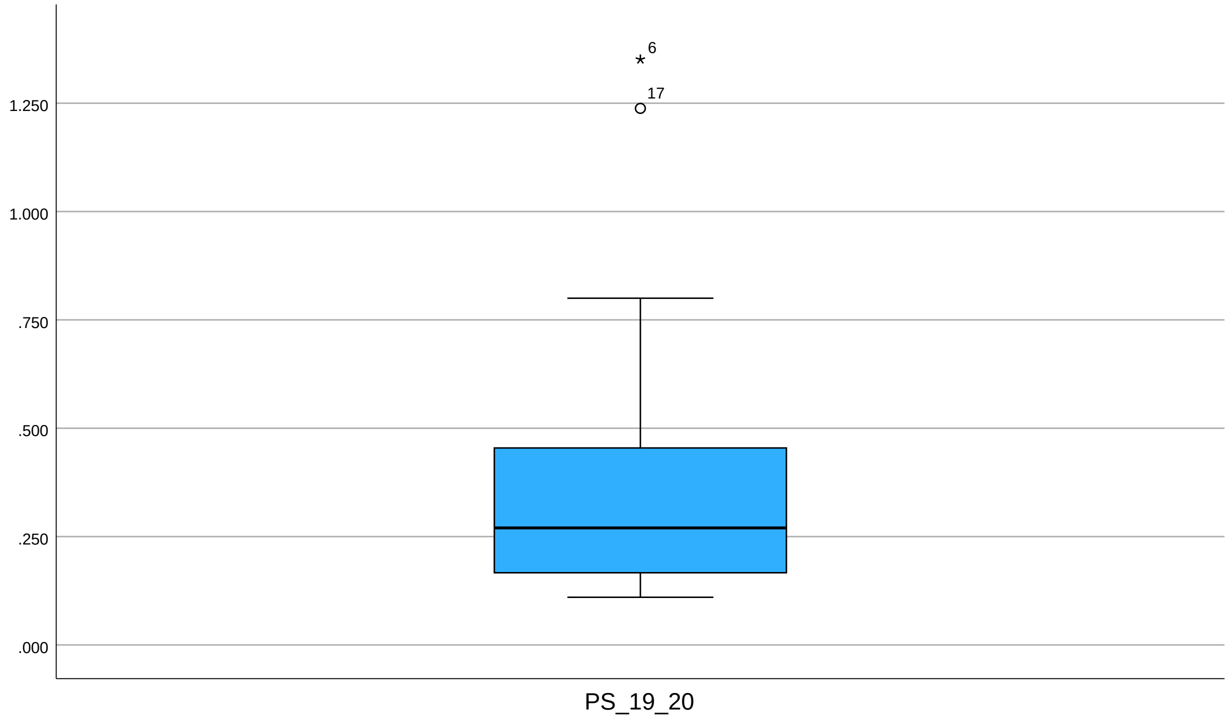

6
17
1.250
1.000
.750
.500
.250
.000
PS_19_20

Explore

Case Processing Summary

	Cases					
	Valid		Missing		Total	
	N	Percent	N	Percent	N	Percent
BrainRate	39	100.0%	0	0.0%	39	100.0%
PermutationEntropy	39	100.0%	0	0.0%	39	100.0%
PermutationEntropy_01	39	100.0%	0	0.0%	39	100.0%
SampleEntropy	39	100.0%	0	0.0%	39	100.0%
SpectralEntropy_bits	39	100.0%	0	0.0%	39	100.0%
SpectralEntropy_01	39	100.0%	0	0.0%	39	100.0%
HiguchiFractalDimension	39	100.0%	0	0.0%	39	100.0%
LempelZivComplexity	39	100.0%	0	0.0%	39	100.0%
RenyiEntropy	39	100.0%	0	0.0%	39	100.0%
TsallisEntropy	39	100.0%	0	0.0%	39	100.0%
PS_4_5	39	100.0%	0	0.0%	39	100.0%
PS_5_6	39	100.0%	0	0.0%	39	100.0%
PS_6_7	39	100.0%	0	0.0%	39	100.0%
PS_7_8	39	100.0%	0	0.0%	39	100.0%
PS_8_9	39	100.0%	0	0.0%	39	100.0%
PS_9_10	39	100.0%	0	0.0%	39	100.0%
PS_10_11	39	100.0%	0	0.0%	39	100.0%
PS_11_12	39	100.0%	0	0.0%	39	100.0%
PS_12_13	39	100.0%	0	0.0%	39	100.0%
PS_13_14	39	100.0%	0	0.0%	39	100.0%
PS_14_15	39	100.0%	0	0.0%	39	100.0%
PS_15_16	39	100.0%	0	0.0%	39	100.0%
PS_16_17	39	100.0%	0	0.0%	39	100.0%
PS_17_18	39	100.0%	0	0.0%	39	100.0%
PS_18_19	39	100.0%	0	0.0%	39	100.0%
PS_19_20	39	100.0%	0	0.0%	39	100.0%

Descriptives

			Statistic	Std. Error
BrainRate	Mean		6.2708	.44704
	95% Confidence Interval for Mean	Lower Bound	5.3658	
		Upper Bound	7.1757	
	5% Trimmed Mean		5.8317	
	Median		5.5800	
	Variance		7.794	
	Std. Deviation		2.79174	
	Minimum		3.39	
	Maximum		19.70	
	Range		16.31	
	Interquartile Range		1.88	
	Skewness		3.558	.378
	Kurtosis		14.978	.741
PermutationEntropy	Mean		1.4552717713	.00816909624
	95% Confidence Interval for Mean	Lower Bound	1.4387343005	
		Upper Bound	1.4718092420	
	5% Trimmed Mean		1.4563821678	
	Median		1.4535044643	
	Variance		.003	
	Std. Deviation		.05101598966	
	Minimum		1.3262360079	
	Maximum		1.5711143969	
	Range		.24487838896	
	Interquartile Range		.05503630404	
	Skewness		-.367	.378
	Kurtosis		.512	.741
PermutationEntropy_01	Mean		.81220264008	.00455925942
	95% Confidence Interval for Mean	Lower Bound	.80297290191	
		Upper Bound	.82143237824	
	5% Trimmed Mean		.81282236416	
	Median		.81121628728	
	Variance		.001	
	Std. Deviation		.02847256595	
	Minimum		.74018640934	
	Maximum		.87685564044	
	Range		.13666923109	
	Interquartile Range		.03071634613	
	Skewness		-.367	.378
	Kurtosis		.512	.741

Descriptives

			Statistic	Std. Error
SampleEntropy	Mean		.61015753163	.02443301397
	95% Confidence Interval for Mean	Lower Bound	.56069548075	
		Upper Bound	.65961958251	
	5% Trimmed Mean		.61061137303	
	Median		.62305795523	
	Variance		.023	
	Std. Deviation		.15258412332	
	Minimum		.22782605507	
	Maximum		.97838267084	
	Range		.75055661577	
	Interquartile Range		.13248706841	
	Skewness		-.206	.378
	Kurtosis		1.575	.741
SpectralEntropy_bits	Mean		3.8347430682	.07407000491
	95% Confidence Interval for Mean	Lower Bound	3.6847961825	
		Upper Bound	3.9846899539	
	5% Trimmed Mean		3.8167329243	
	Median		3.7860256815	
	Variance		.214	
	Std. Deviation		.46256703241	
	Minimum		2.6741356121	
	Maximum		5.3932909478	
	Range		2.7191553357	
	Interquartile Range		.37641715718	
	Skewness		.872	.378
	Kurtosis		3.540	.741
SpectralEntropy_01	Mean		.54694319960	.01056448496
	95% Confidence Interval for Mean	Lower Bound	.52555651790	
		Upper Bound	.56832988130	
	5% Trimmed Mean		.54437444192	
	Median		.53999471755	
	Variance		.004	
	Std. Deviation		.06597518745	
	Minimum		.38140763589	
	Maximum		.76923636210	
	Range		.38782872621	
	Interquartile Range		.05368777012	
	Skewness		.872	.378
	Kurtosis		3.540	.741

Descriptives

			Statistic	Std. Error
HiguchiFractalDimension	Mean		.60444305196	.01556606862
	95% Confidence Interval for Mean	Lower Bound	.57293119350	
		Upper Bound	.63595491043	
	5% Trimmed Mean		.61425953318	
	Median		.63116598219	
	Variance		.009	
	Std. Deviation		.09721006737	
	Minimum		.25111111411	
	Maximum		.76122126457	
	Range		.51011015046	
	Interquartile Range		.08201424815	
	Skewness		-1.867	.378
	Kurtosis		4.833	.741
LempelZivComplexity	Mean		.24611332224	.00464225147
	95% Confidence Interval for Mean	Lower Bound	.23671557546	
		Upper Bound	.25551106903	
	5% Trimmed Mean		.24316716113	
	Median		.24025040883	
	Variance		.001	
	Std. Deviation		.02899085115	
	Minimum		.20650917916	
	Maximum		.34918656382	
	Range		.14267738466	
	Interquartile Range		.02625856358	
	Skewness		1.775	.378
	Kurtosis		4.129	.741
RenyiEntropy	Mean		7.7057647747	.16169930362
	95% Confidence Interval for Mean	Lower Bound	7.3784216482	
		Upper Bound	8.0331079013	
	5% Trimmed Mean		7.7672993458	
	Median		8.0783002277	
	Variance		1.020	
	Std. Deviation		1.0098118274	
	Minimum		5.4008226377	
	Maximum		8.8389778159	
	Range		3.4381551781	
	Interquartile Range		1.0309541081	
	Skewness		-1.066	.378
	Kurtosis		-.115	.741

Descriptives

			Statistic	Std. Error
TsallisEntropy	Mean		1.8915363352	.05244047363
	95% Confidence Interval for Mean	Lower Bound	1.7853761464	
		Upper Bound	1.9976965240	
	5% Trimmed Mean		1.9514939652	
	Median		1.9861626555	
	Variance		.107	
	Std. Deviation		.32749065288	
	Minimum		.18405382988	
	Maximum		1.9889399128	
	Range		1.8048860829	
	Interquartile Range		.00179005420	
	Skewness		-4.286	.378
	Kurtosis		20.264	.741
PS_4_5	Mean		3.04315	.461154
	95% Confidence Interval for Mean	Lower Bound	2.10960	
		Upper Bound	3.97671	
	5% Trimmed Mean		2.65558	
	Median		2.42100	
	Variance		8.294	
	Std. Deviation		2.879905	
	Minimum		.925	
	Maximum		18.700	
	Range		17.775	
	Interquartile Range		1.957	
	Skewness		4.423	.378
	Kurtosis		23.768	.741
PS_5_6	Mean		2.40141	.337443
	95% Confidence Interval for Mean	Lower Bound	1.71829	
		Upper Bound	3.08453	
	5% Trimmed Mean		2.08848	
	Median		2.00800	
	Variance		4.441	
	Std. Deviation		2.107329	
	Minimum		.569	
	Maximum		13.520	
	Range		12.951	
	Interquartile Range		1.344	
	Skewness		4.128	.378
	Kurtosis		20.954	.741

Descriptives

			Statistic	Std. Error
PS_6_7	Mean		1.90097	.220963
	95% Confidence Interval for Mean	Lower Bound	1.45366	
		Upper Bound	2.34829	
	5% Trimmed Mean		1.72484	
	Median		1.58000	
	Variance		1.904	
	Std. Deviation		1.379916	
	Minimum		.467	
	Maximum		8.570	
	Range		8.103	
	Interquartile Range		1.055	
	Skewness		3.245	.378
	Kurtosis		14.143	.741
PS_7_8	Mean		1.46087	.137886
	95% Confidence Interval for Mean	Lower Bound	1.18174	
		Upper Bound	1.74001	
	5% Trimmed Mean		1.36486	
	Median		1.21800	
	Variance		.741	
	Std. Deviation		.861097	
	Minimum		.358	
	Maximum		4.650	
	Range		4.292	
	Interquartile Range		.877	
	Skewness		1.864	.378
	Kurtosis		4.679	.741
PS_8_9	Mean		1.19903	.132220
	95% Confidence Interval for Mean	Lower Bound	.93136	
		Upper Bound	1.46669	
	5% Trimmed Mean		1.11150	
	Median		.90600	
	Variance		.682	
	Std. Deviation		.825715	
	Minimum		.264	
	Maximum		3.690	
	Range		3.426	
	Interquartile Range		.817	
	Skewness		1.693	.378
	Kurtosis		2.680	.741

Descriptives

			Statistic	Std. Error
PS_9_10	Mean		1.24105	.210294
	95% Confidence Interval for Mean	Lower Bound	.81533	
		Upper Bound	1.66677	
	5% Trimmed Mean		1.04607	
	Median		.99900	
	Variance		1.725	
	Std. Deviation		1.313283	
	Minimum		.220	
	Maximum		8.080	
	Range		7.860	
	Interquartile Range		.835	
	Skewness		3.979	.378
	Kurtosis		19.712	.741
PS_10_11	Mean		1.31072	.190066
	95% Confidence Interval for Mean	Lower Bound	.92595	
		Upper Bound	1.69549	
	5% Trimmed Mean		1.16248	
	Median		.88800	
	Variance		1.409	
	Std. Deviation		1.186965	
	Minimum		.232	
	Maximum		6.300	
	Range		6.068	
	Interquartile Range		1.060	
	Skewness		2.419	.378
	Kurtosis		7.471	.741
PS_11_12	Mean		.60587	.111580
	95% Confidence Interval for Mean	Lower Bound	.37999	
		Upper Bound	.83175	
	5% Trimmed Mean		.50275	
	Median		.42900	
	Variance		.486	
	Std. Deviation		.696817	
	Minimum		.156	
	Maximum		4.504	
	Range		4.348	
	Interquartile Range		.295	
	Skewness		4.855	.378
	Kurtosis		27.008	.741

Descriptives

			Statistic	Std. Error
PS_12_13	Mean		.39444	.034254
	95% Confidence Interval for Mean	Lower Bound	.32509	
		Upper Bound	.46378	
	5% Trimmed Mean		.37931	
	Median		.31600	
	Variance		.046	
	Std. Deviation		.213917	
	Minimum		.113	
	Maximum		1.050	
	Range		.937	
	Interquartile Range		.196	
	Skewness		1.325	.378
	Kurtosis		1.275	.741
PS_13_14	Mean		.37882	.037280
	95% Confidence Interval for Mean	Lower Bound	.30335	
		Upper Bound	.45429	
	5% Trimmed Mean		.36134	
	Median		.30400	
	Variance		.054	
	Std. Deviation		.232816	
	Minimum		.107	
	Maximum		.955	
	Range		.848	
	Interquartile Range		.275	
	Skewness		1.329	.378
	Kurtosis		1.042	.741
PS_14_15	Mean		.39241	.042903
	95% Confidence Interval for Mean	Lower Bound	.30556	
		Upper Bound	.47926	
	5% Trimmed Mean		.36864	
	Median		.27600	
	Variance		.072	
	Std. Deviation		.267932	
	Minimum		.111	
	Maximum		1.150	
	Range		1.039	
	Interquartile Range		.225	
	Skewness		1.487	.378
	Kurtosis		1.393	.741

Descriptives

			Statistic	Std. Error
PS_15_16	Mean		.35136	.036848
	95% Confidence Interval for Mean	Lower Bound	.27676	
		Upper Bound	.42595	
	5% Trimmed Mean		.32718	
	Median		.26500	
	Variance		.053	
	Std. Deviation		.230114	
	Minimum		.102	
	Maximum		1.053	
	Range		.951	
	Interquartile Range		.254	
	Skewness		1.550	.378
	Kurtosis		2.132	.741
PS_16_17	Mean		.30677	.033750
	95% Confidence Interval for Mean	Lower Bound	.23845	
		Upper Bound	.37509	
	5% Trimmed Mean		.28137	
	Median		.23200	
	Variance		.044	
	Std. Deviation		.210766	
	Minimum		.092	
	Maximum		1.180	
	Range		1.088	
	Interquartile Range		.149	
	Skewness		2.406	.378
	Kurtosis		7.217	.741
PS_17_18	Mean		.26756	.027123
	95% Confidence Interval for Mean	Lower Bound	.21266	
		Upper Bound	.32247	
	5% Trimmed Mean		.25043	
	Median		.20200	
	Variance		.029	
	Std. Deviation		.169385	
	Minimum		.094	
	Maximum		.790	
	Range		.696	
	Interquartile Range		.136	
	Skewness		1.826	.378
	Kurtosis		2.756	.741

Descriptives

			Statistic	Std. Error
PS_18_19	Mean		.25685	.028919
	95% Confidence Interval for Mean	Lower Bound	.19830	
		Upper Bound	.31539	
	5% Trimmed Mean		.23217	
	Median		.19700	
	Variance		.033	
	Std. Deviation		.180601	
	Minimum		.084	
	Maximum		.980	
	Range		.896	
	Interquartile Range		.181	
	Skewness		2.390	.378
	Kurtosis		6.733	.741
PS_19_20	Mean		.22938	.019289
	95% Confidence Interval for Mean	Lower Bound	.19034	
		Upper Bound	.26843	
	5% Trimmed Mean		.22187	
	Median		.18600	
	Variance		.015	
	Std. Deviation		.120462	
	Minimum		.084	
	Maximum		.546	
	Range		.462	
	Interquartile Range		.154	
	Skewness		1.007	.378
	Kurtosis		.045	.741

Tests of Normality

	Kolmogorov-Smirnov[a]			Shapiro-Wilk		
	Statistic	df	Sig.	Statistic	df	Sig.
BrainRate	.265	39	<.001	.603	39	<.001
PermutationEntropy	.120	39	.168	.975	39	.510
PermutationEntropy_01	.120	39	.168	.975	39	.510
SampleEntropy	.137	39	.062	.943	39	.048
SpectralEntropy_bits	.175	39	.004	.908	39	.004
SpectralEntropy_01	.175	39	.004	.908	39	.004
HiguchiFractalDimension	.174	39	.004	.839	39	<.001
LempelZivComplexity	.163	39	.010	.850	39	<.001
RenyiEntropy	.214	39	<.001	.843	39	<.001
TsallisEntropy	.477	39	<.001	.333	39	<.001
PS_4_5	.231	39	<.001	.545	39	<.001
PS_5_6	.240	39	<.001	.583	39	<.001
PS_6_7	.207	39	<.001	.695	39	<.001
PS_7_8	.138	39	.058	.845	39	<.001
PS_8_9	.221	39	<.001	.813	39	<.001
PS_9_10	.243	39	<.001	.593	39	<.001
PS_10_11	.201	39	<.001	.748	39	<.001
PS_11_12	.259	39	<.001	.474	39	<.001
PS_12_13	.236	39	<.001	.857	39	<.001
PS_13_14	.192	39	<.001	.844	39	<.001
PS_14_15	.222	39	<.001	.808	39	<.001
PS_15_16	.207	39	<.001	.831	39	<.001
PS_16_17	.205	39	<.001	.751	39	<.001
PS_17_18	.240	39	<.001	.763	39	<.001
PS_18_19	.245	39	<.001	.738	39	<.001
PS_19_20	.204	39	<.001	.879	39	<.001

a. Lilliefors Significance Correction

BrainRate

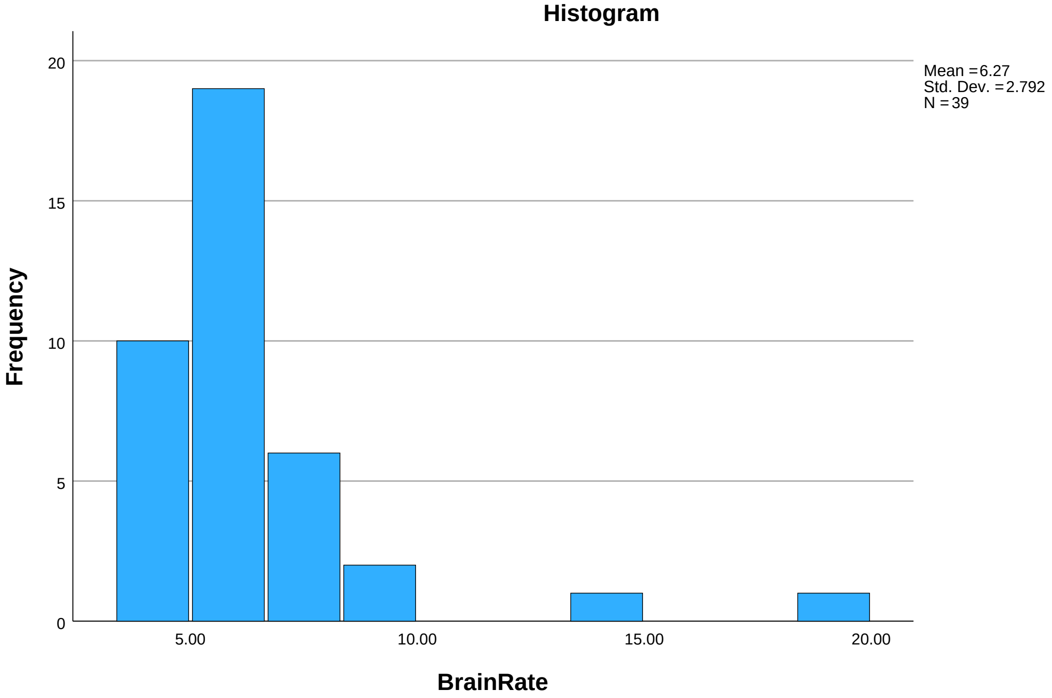


```
BrainRate Stem-and-Leaf Plot

 Frequency    Stem &  Leaf

     1.00        3 .  3
     1.00        3 .  8
     3.00        4 .  244
     5.00        4 .  67888
     4.00        5 .  3444
    14.00        5 .  55555556667789
     1.00        6 .  1
     3.00        6 .  789
     2.00        7 .  23
     1.00        7 .  6
     1.00        8 .  3
     3.00 Extremes    (>=8.6)

 Stem width:      1.00
 Each leaf:       1 case(s)
```

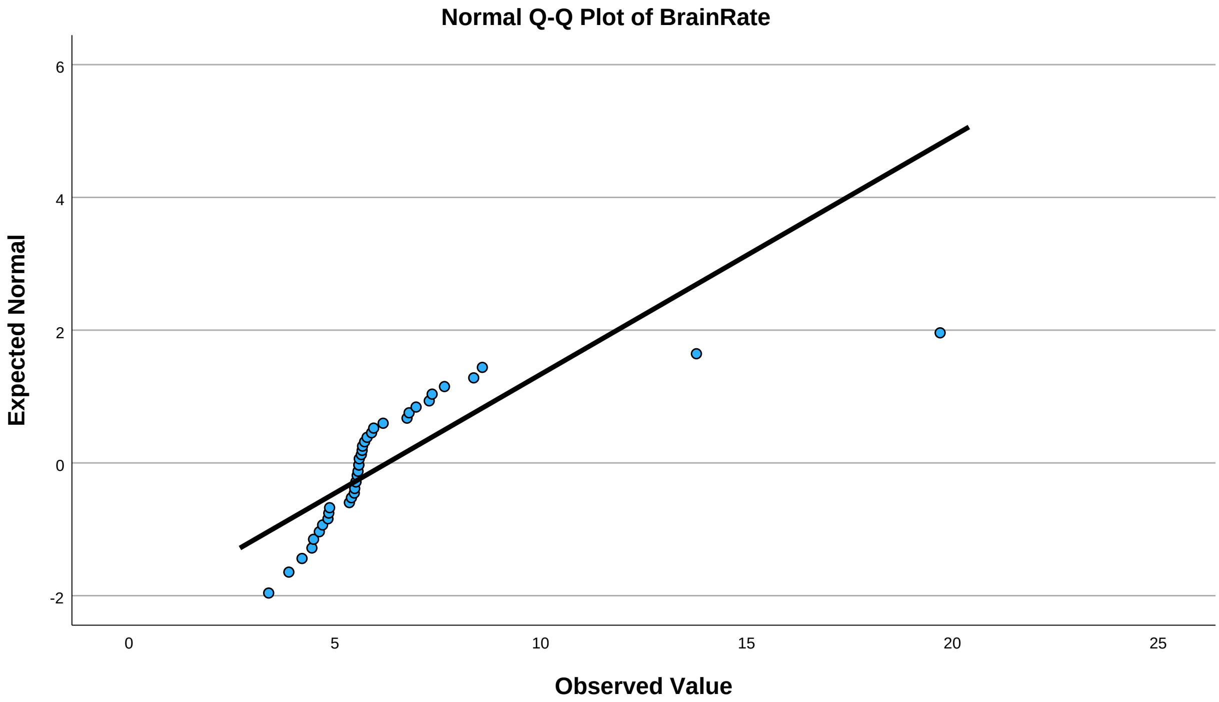
Normal Q-Q Plot of BrainRate
Expected Normal
6
4
2
0
-2
0
5
10
15
20
25
Observed Value

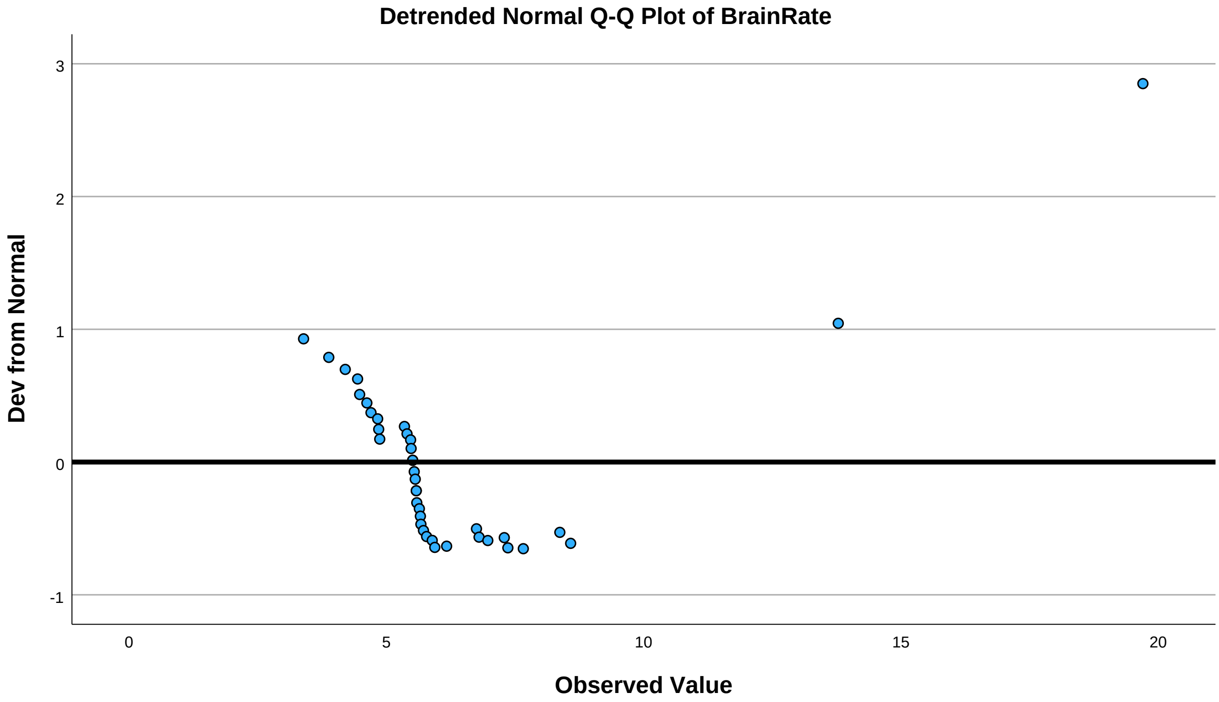
Detrended Normal Q-Q Plot of BrainRate
Dev from Normal
3
2
1
0
-1
0
5
10
15
20
Observed Value

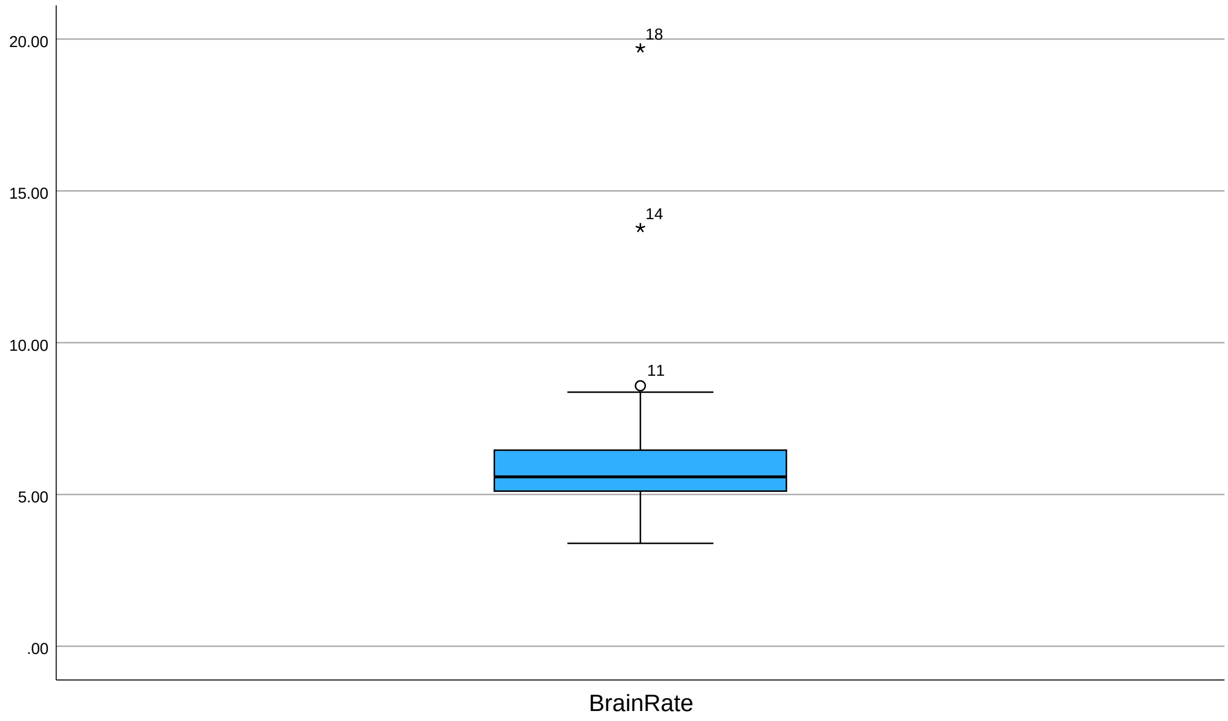

PermutationEntropy

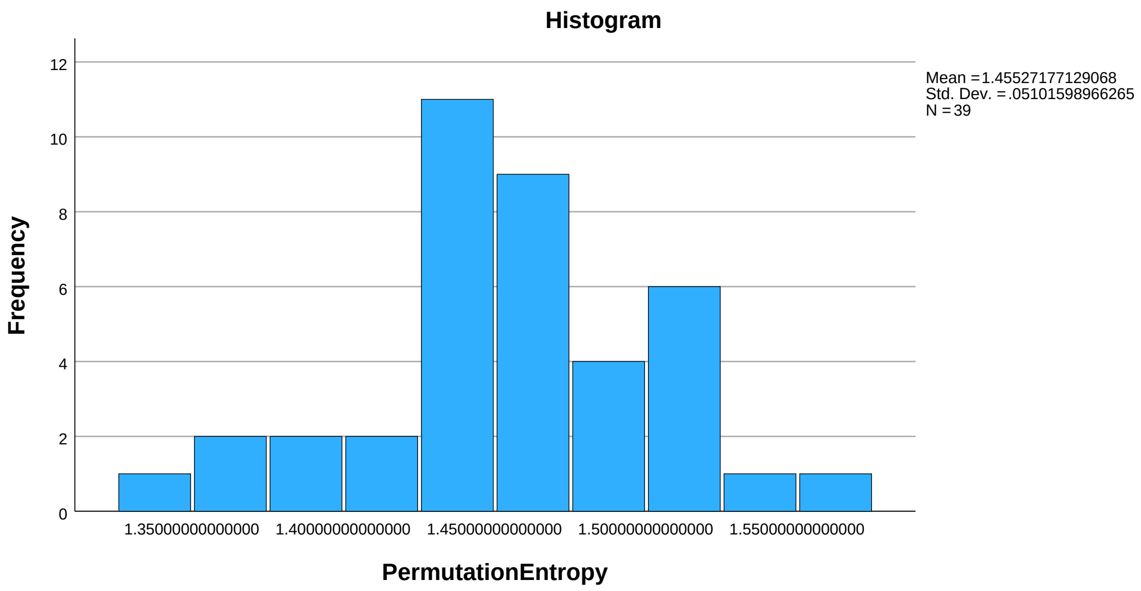

PermutationEntropy Stem-and-Leaf Plot

Frequency   Stem & Leaf

```
  2.00 Extremes    (=<1.35)
  3.00     13 .  578
 13.00      14 .  0223333344444
 13.00      14 .  5555666778889
  7.00     15 .  0001222
  1.00 Extremes    (>=1.57)

Stem width:  .1000000
Each leaf:       1 case(s)
```

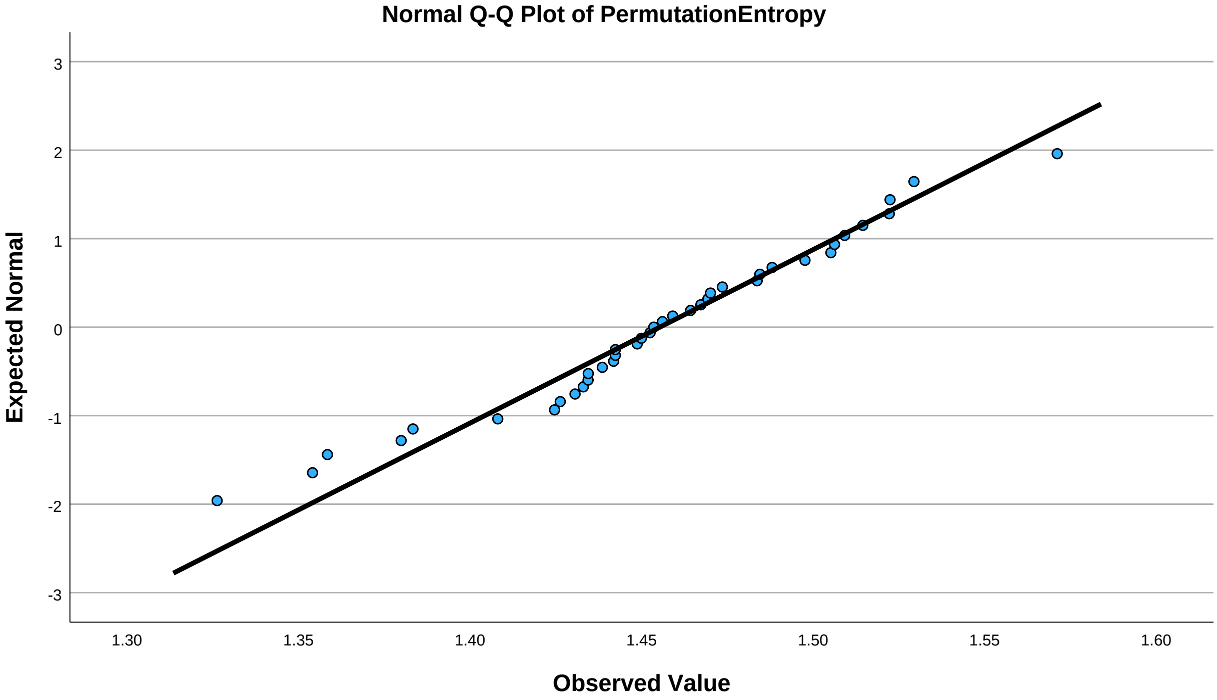

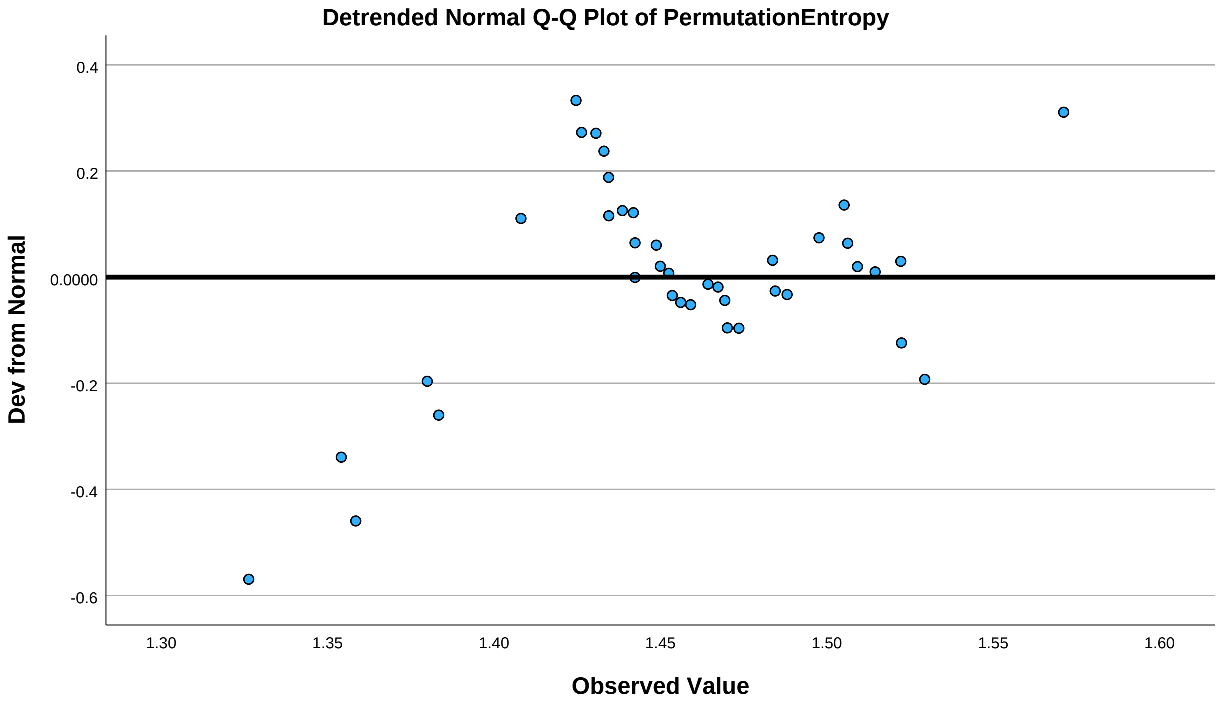

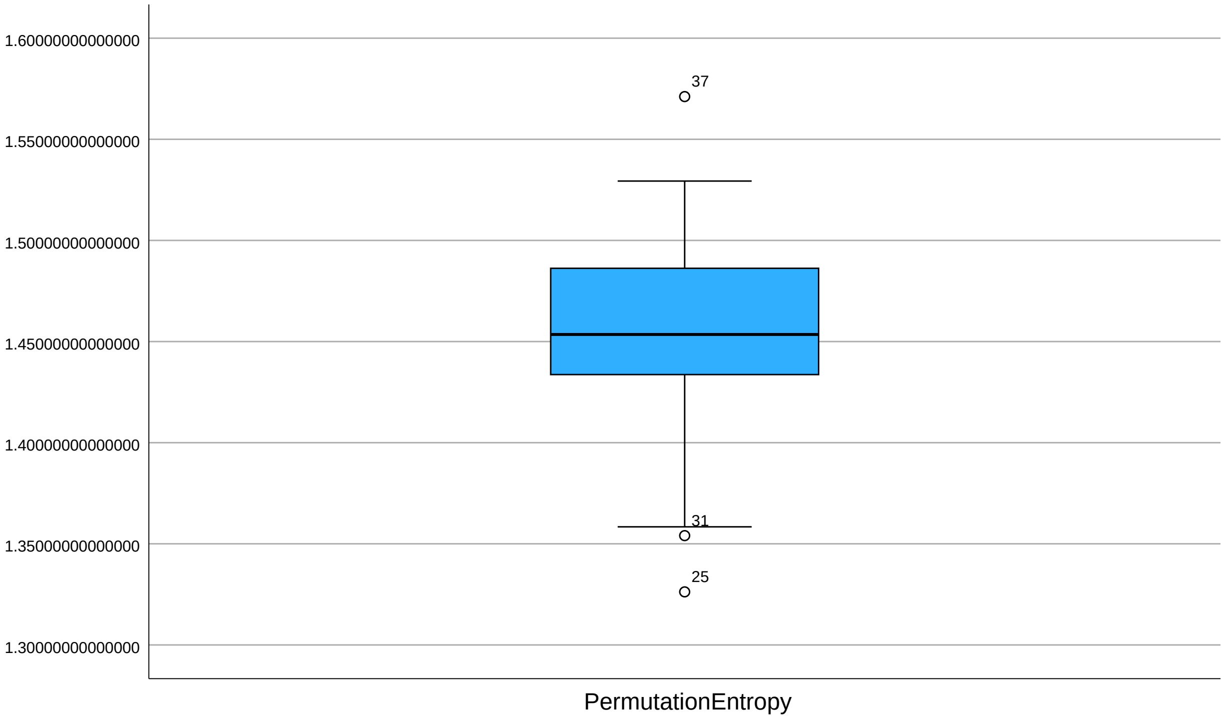

PermutationEntropy_01

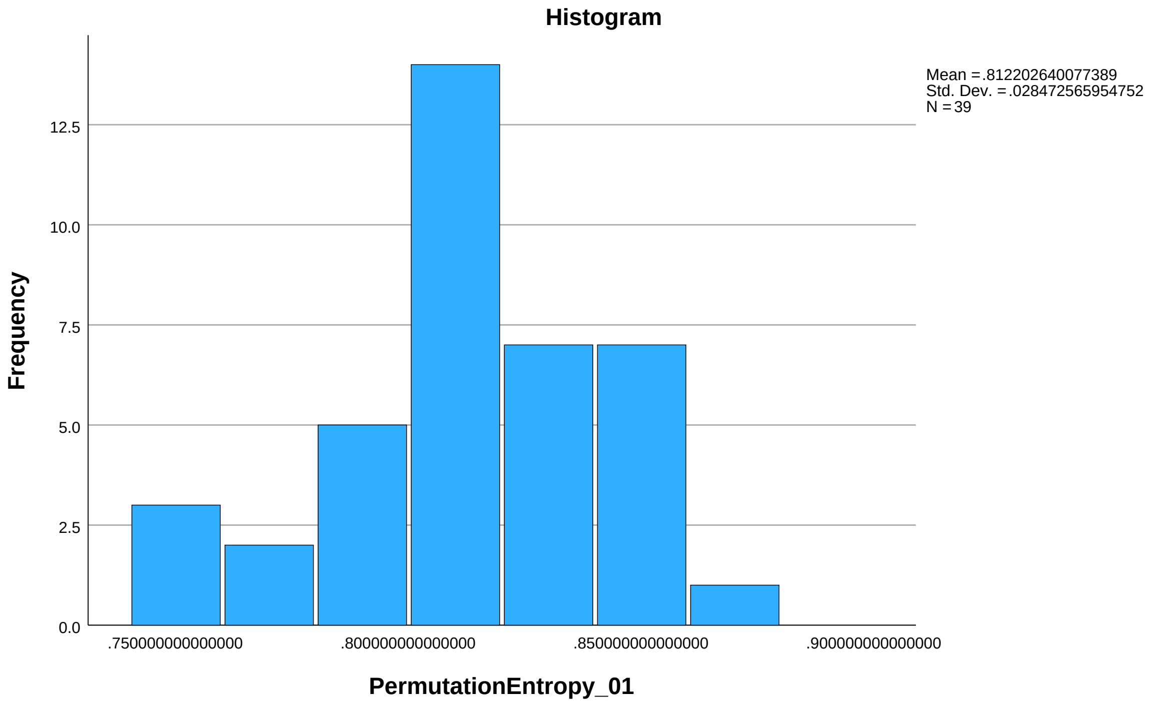


```
PermutationEntropy_01 Stem-and-Leaf Plot

 Frequency    Stem &  Leaf

     2.00 Extremes    (=<.756)
     1.00       75 .  8
      .00       76 .
     2.00       77 .  02
     1.00       78 .  5
     4.00       79 .  5689
     8.00       80 .  00244489
     6.00       81 .  012478
     5.00       82 .  00288
     2.00       83 .  05
     6.00       84 .  002599
     1.00       85 .  3
     1.00 Extremes    (>=.877)

 Stem width:   .0100000
 Each leaf:       1 case(s)
```

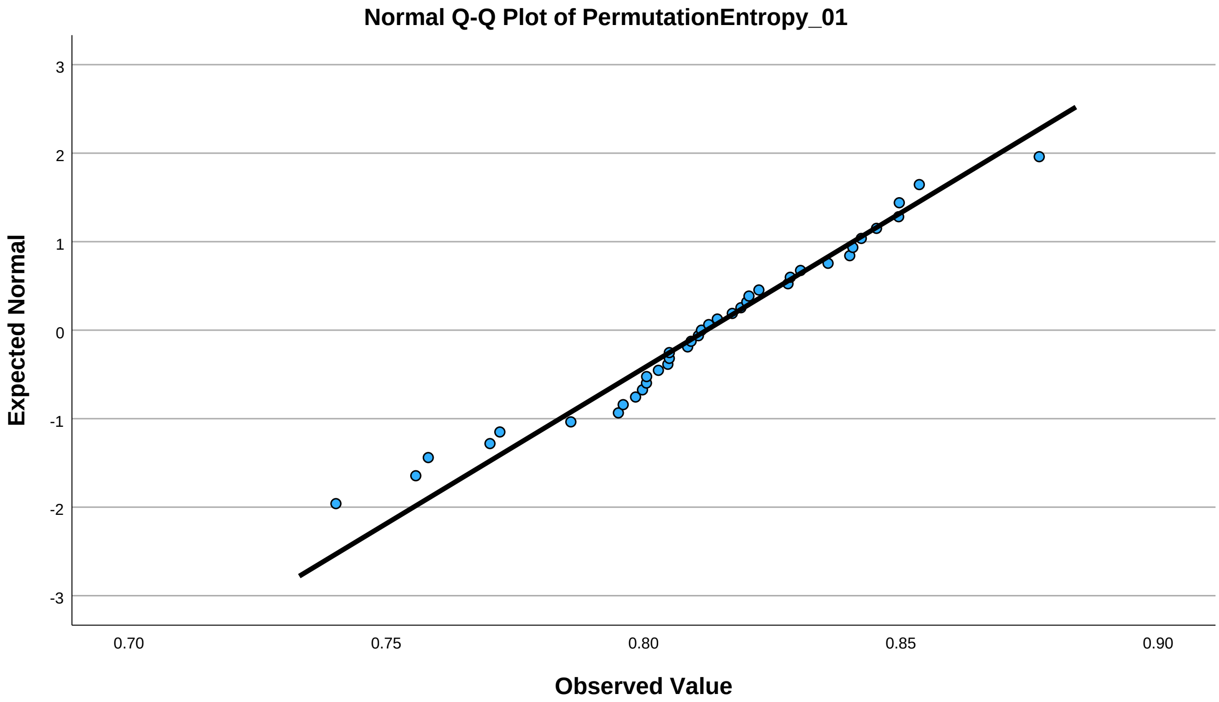
Normal Q-Q Plot of PermutationEntropy_01
Expected Normal
3
2
1
0
-1
-2
-3
0.70
0.75
0.80
0.85
0.90
Observed Value

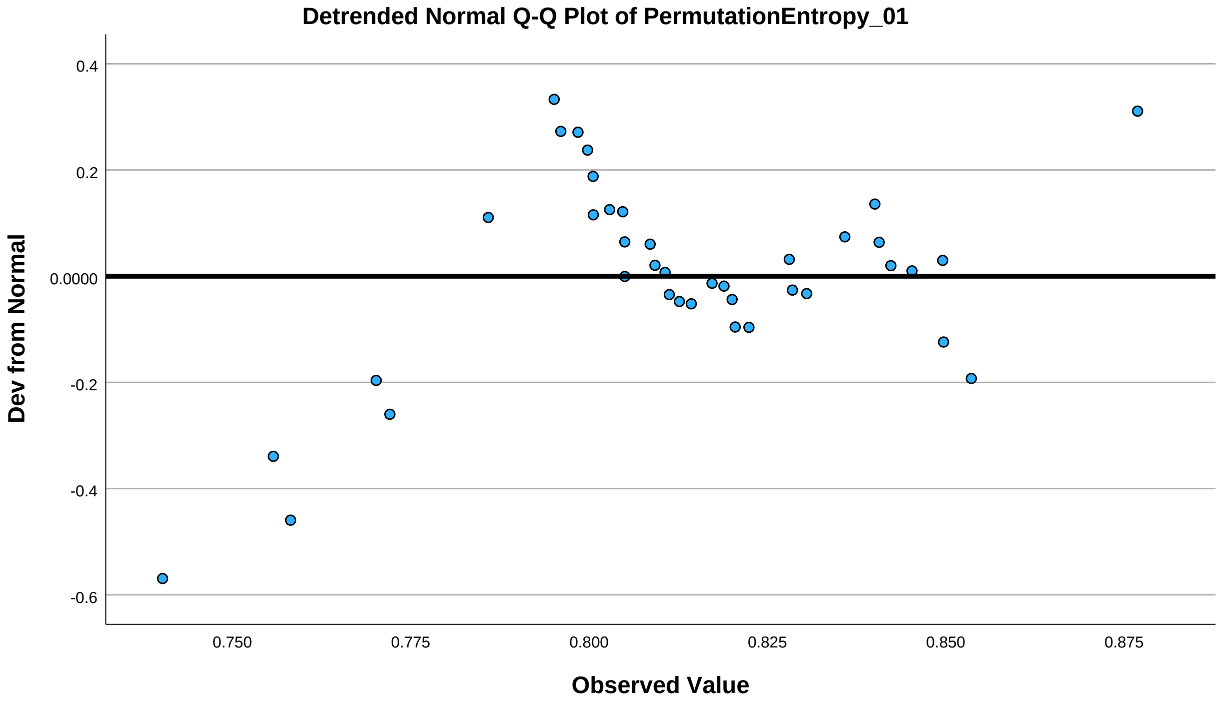
Detrended Normal Q-Q Plot of PermutationEntropy_01
Dev from Normal
0.4
0.2
0.0000
-0.2
-0.4
-0.6
0.750
0.775
0.800
0.825
0.850
0.875
Observed Value

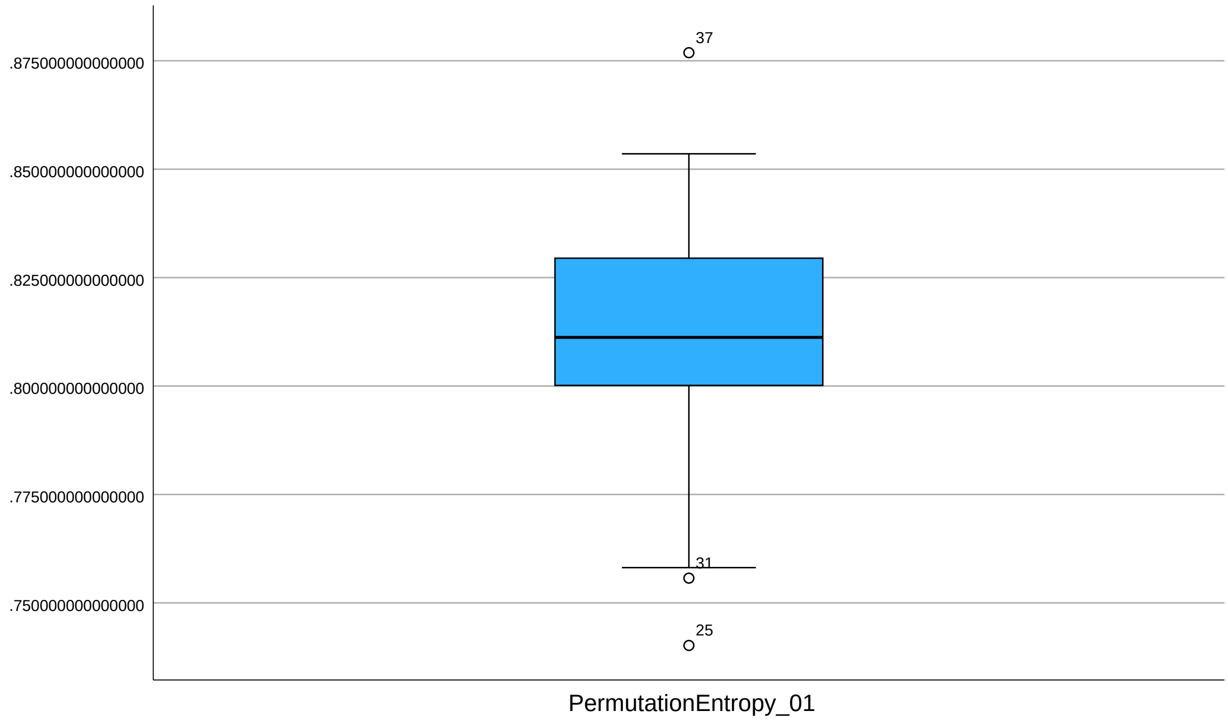

SampleEntropy

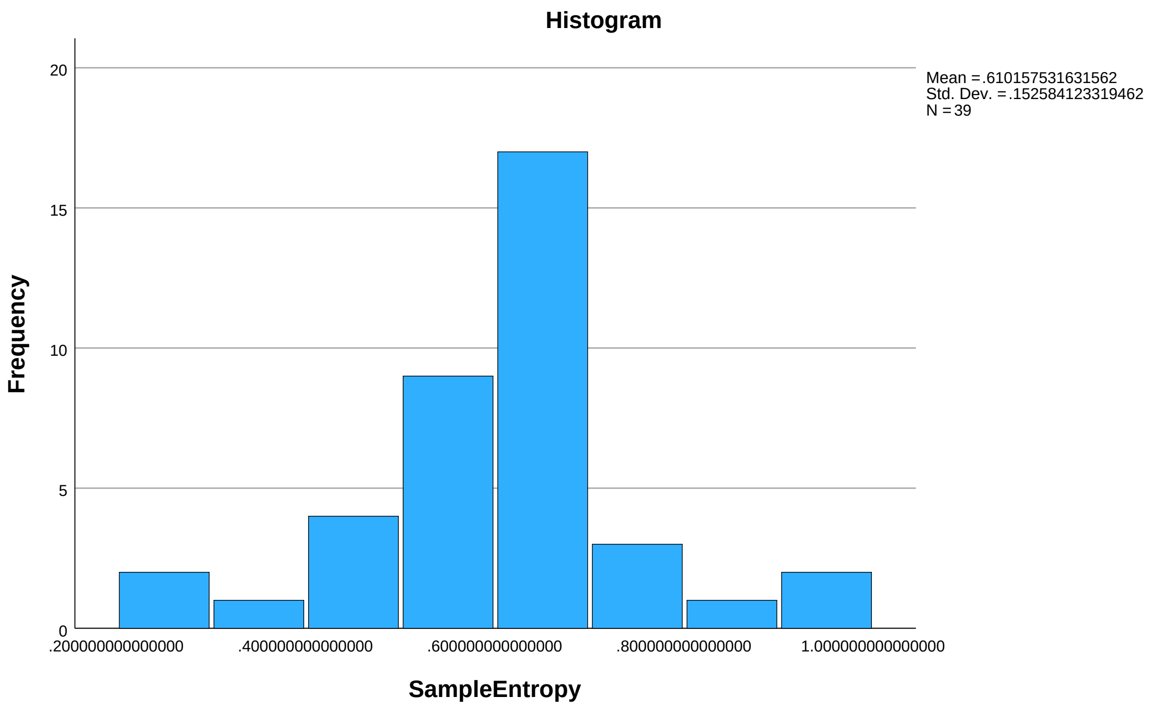


```
SampleEntropy Stem-and-Leaf Plot

 Frequency    Stem &  Leaf
```

```
 3.00 Extremes    (=<.33)
 1.00      4 . 3
 3.00      4 . 559
 3.00      5 . 044
 6.00      5 . 555789
 8.00      6 . 01222344
 9.00      6 . 567778889
 2.00      7 . 44
 1.00      7 . 5
 1.00      8 . 0
 2.00 Extremes    (>=.97)

Stem width:   .1000000
Each leaf:       1 case(s)
```

Normal Q-Q Plot of SampleEntropy

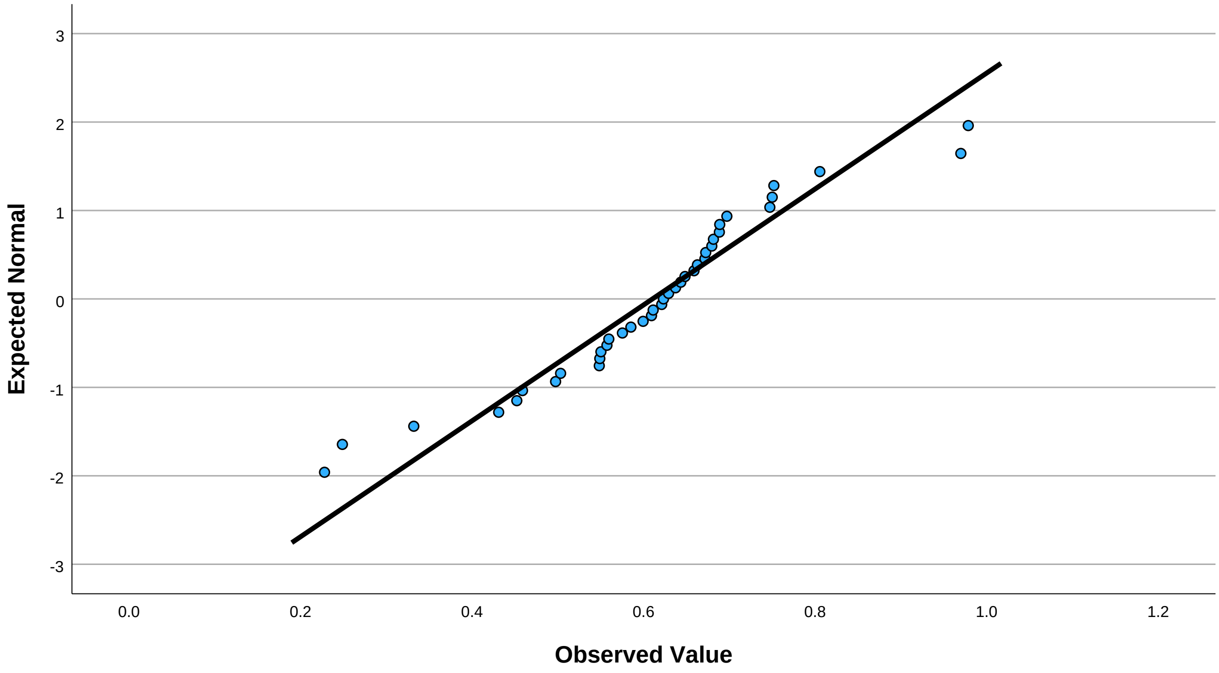

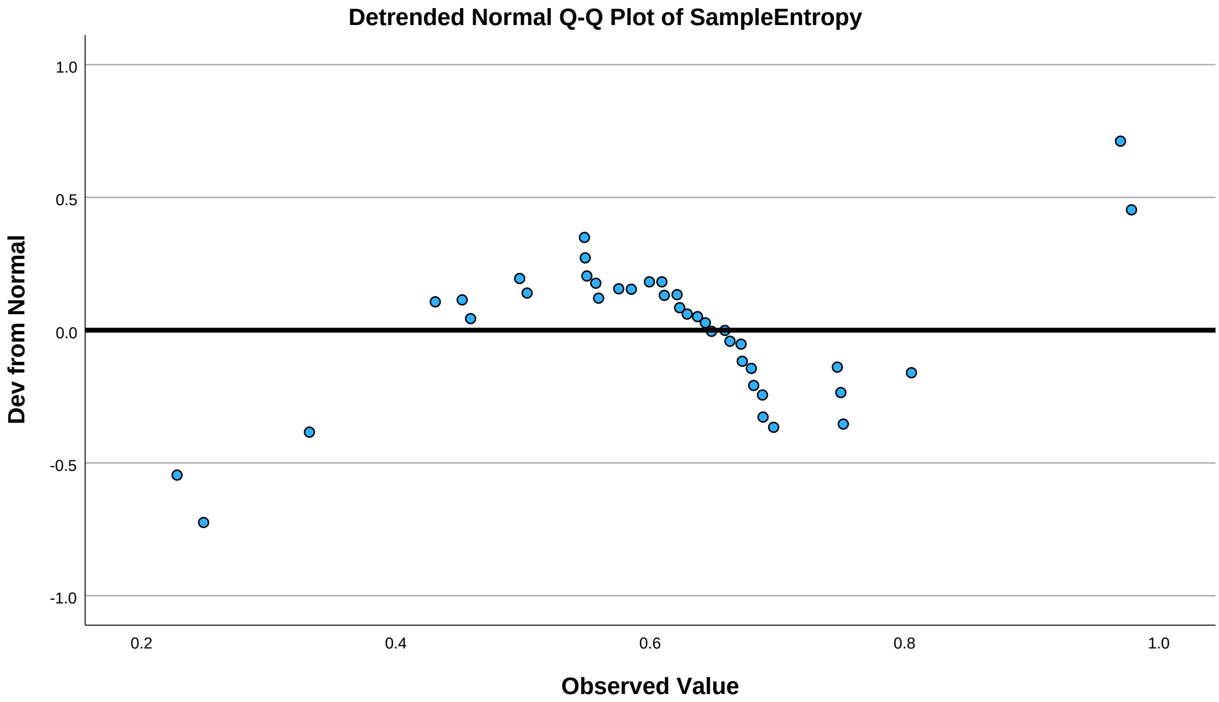

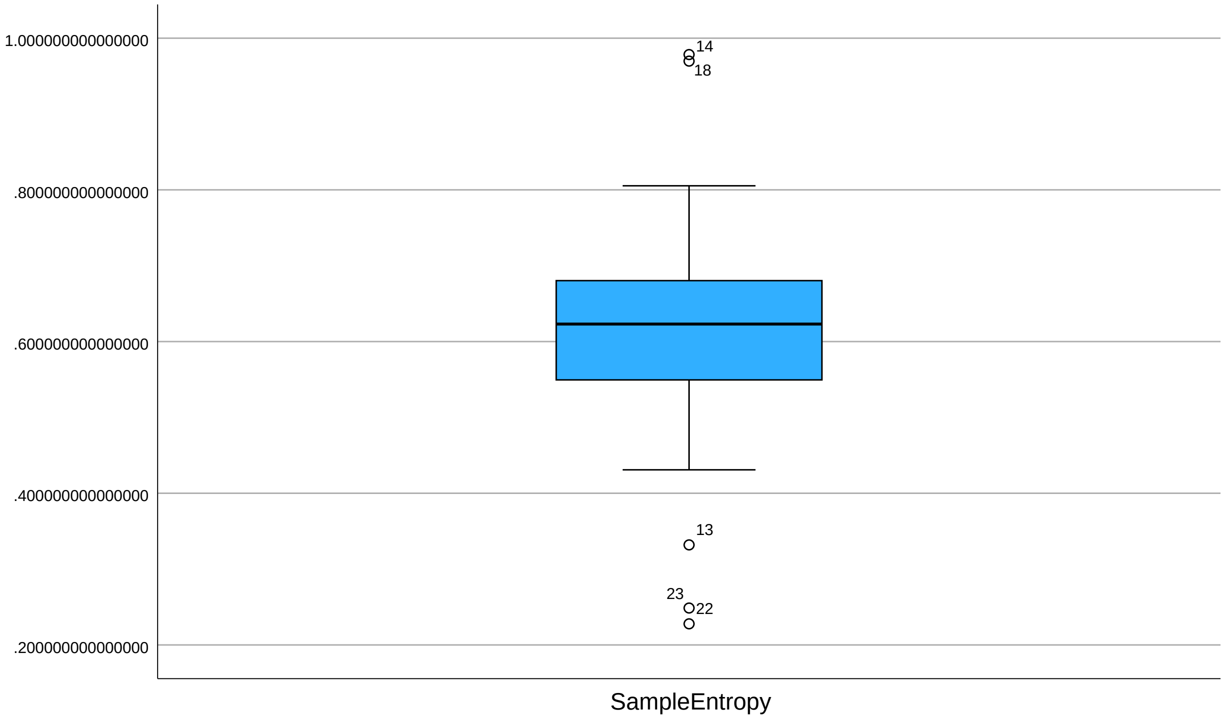

SpectralEntropy_bits

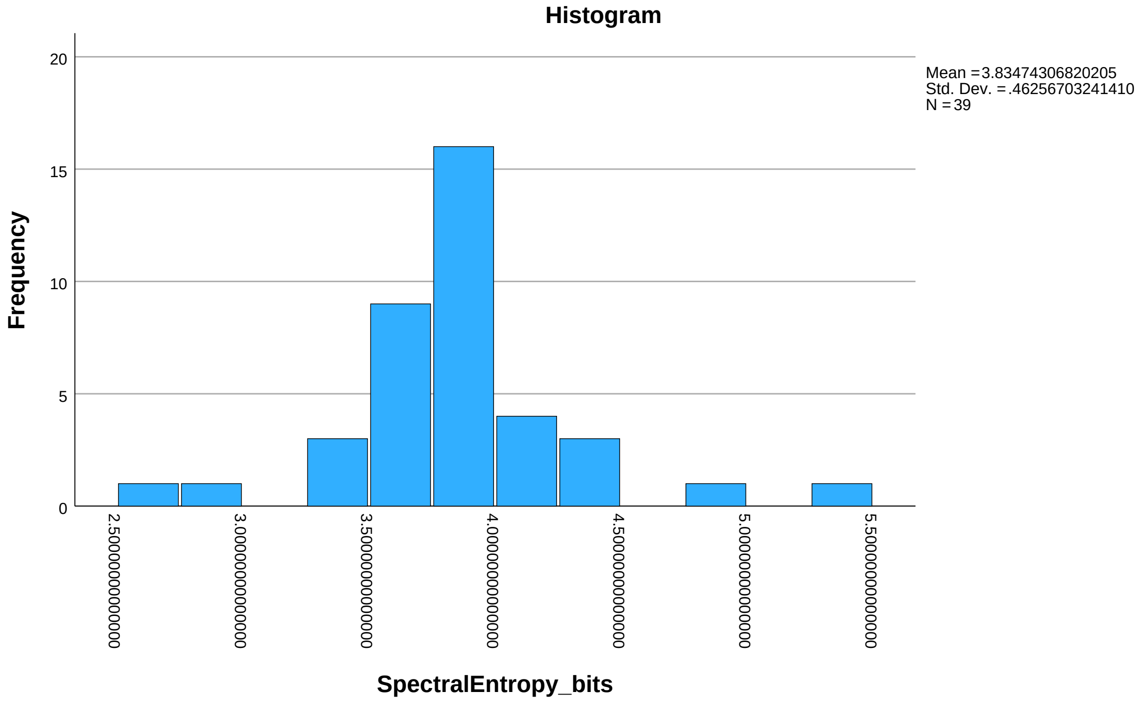


```
SpectralEntropy_bits Stem-and-Leaf Plot

 Frequency    Stem &  Leaf

     2.00 Extremes    (=<2.99)
     1.00       33 .  6
     2.00       34 .  01
     6.00       35 .  011667
     1.00       36 .  4
     8.00       37 .  34567788
     8.00       38 .  00457789
     2.00       39 .  24
     1.00       40 .  4
     2.00       41 .  17
     2.00       42 .  45
     1.00       43 .  5
     1.00       44 .  7
     2.00 Extremes    (>=4.92)

 Stem width:   .1000000
 Each leaf:        1 case(s)
```

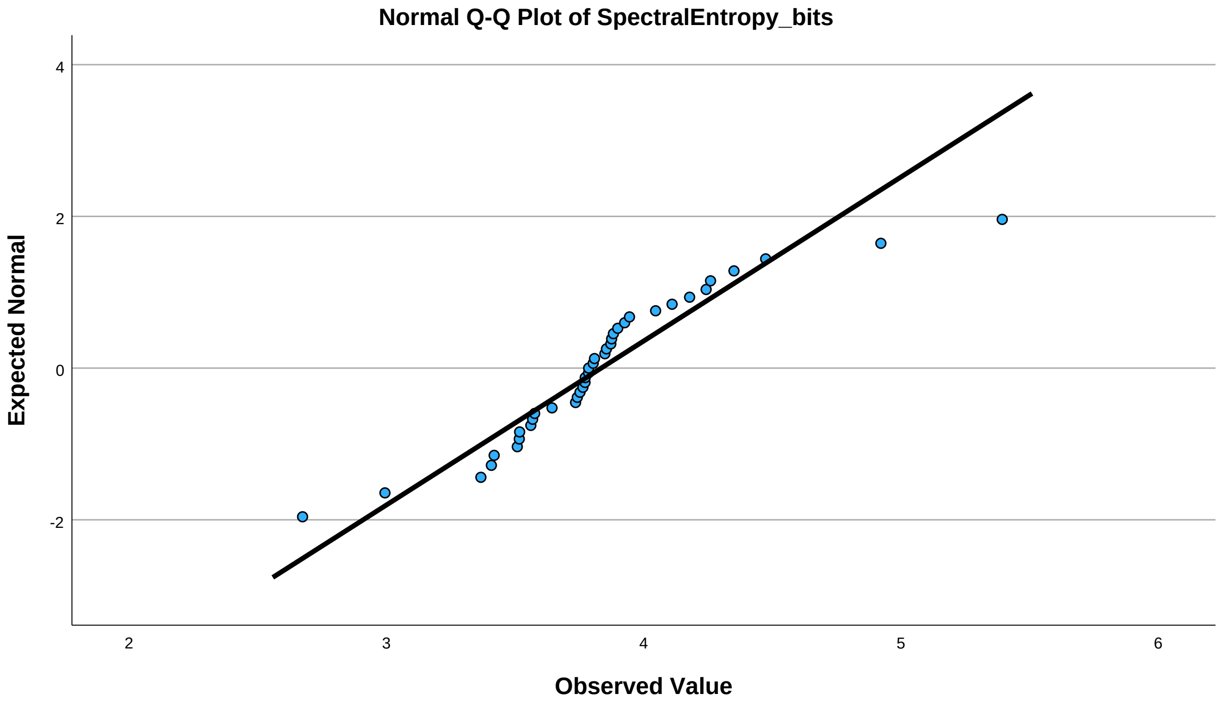
Normal Q-Q Plot of SpectralEntropy_bits
Expected Normal
4
2
0
-2
2
3
4
5
6
Observed Value

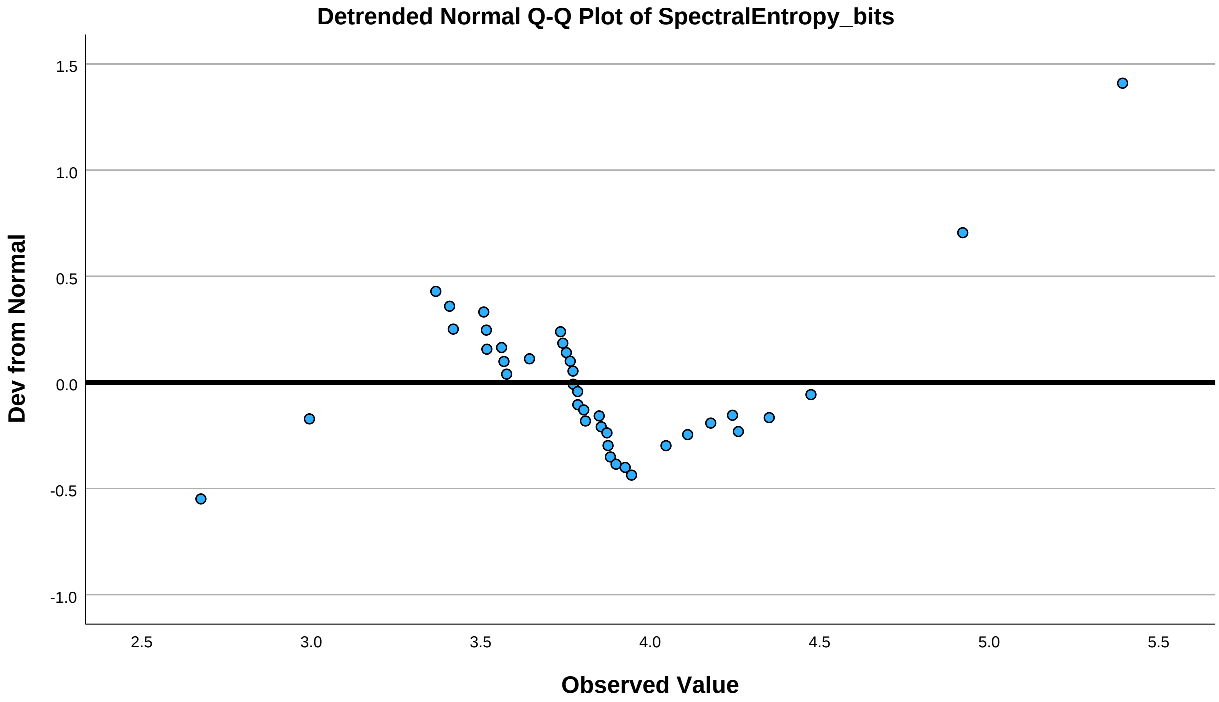
Detrended Normal Q-Q Plot of SpectralEntropy_bits
Dev from Normal
1.5
1.0
0.5
0.0
-0.5
-1.0
2.5
3.0
3.5
4.0
4.5
5.0
5.5
Observed Value

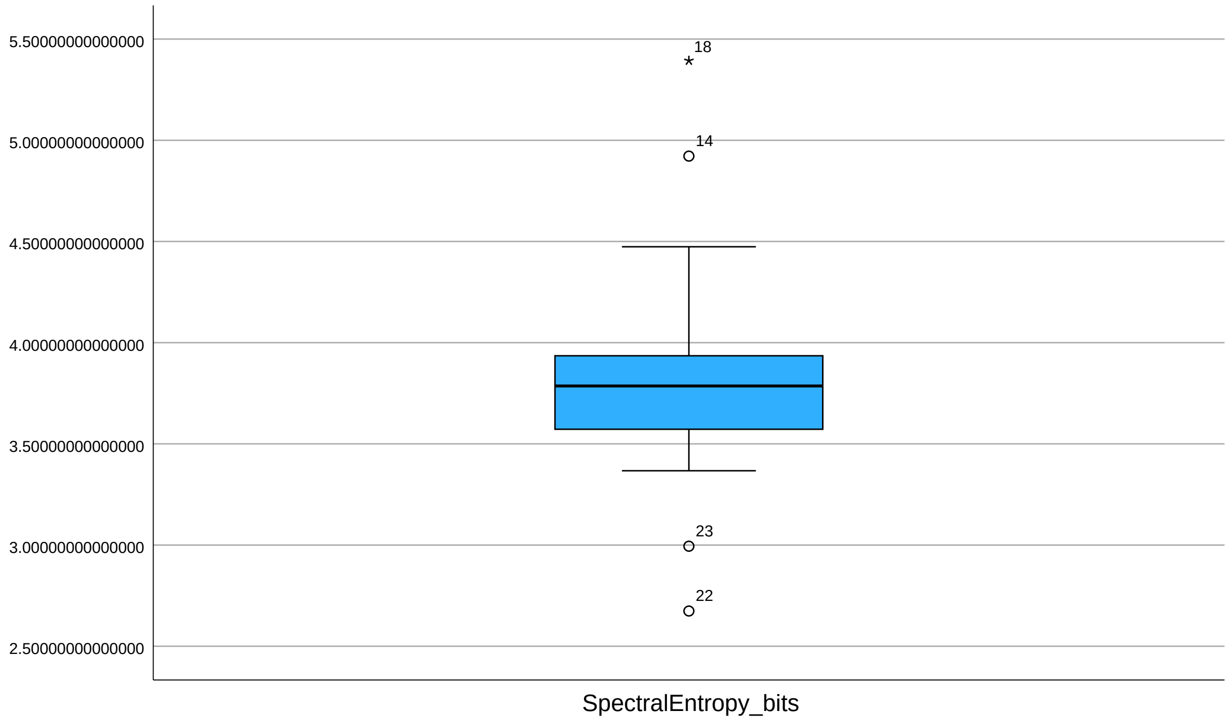

SpectralEntropy_01

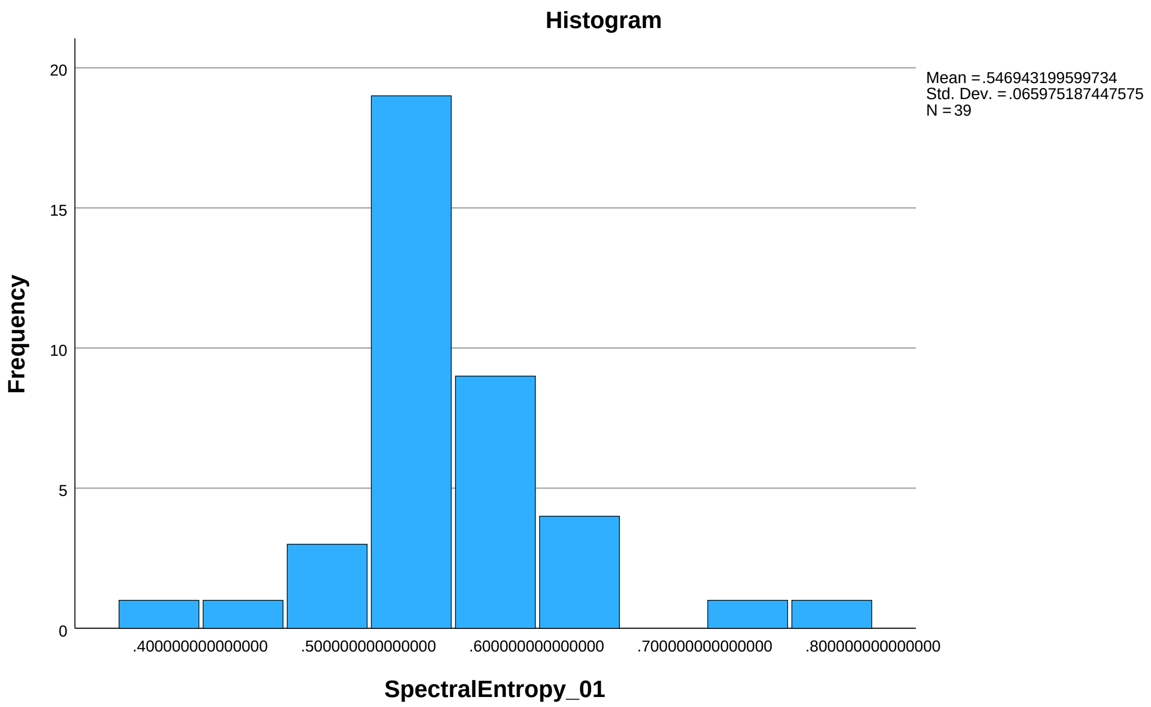

SpectralEntropy_01 Stem-and-Leaf Plot

Frequency   Stem & Leaf

```
  2.00 Extremes    (=<.43)
 3.00      4 .  888
 19.00        5 .  0000011333333334444
  9.00       5 .  555556789
 4.00      6 .  0023
  2.00 Extremes    (>=.70)

Stem width:   .1000000
Each leaf:       1 case(s)
```

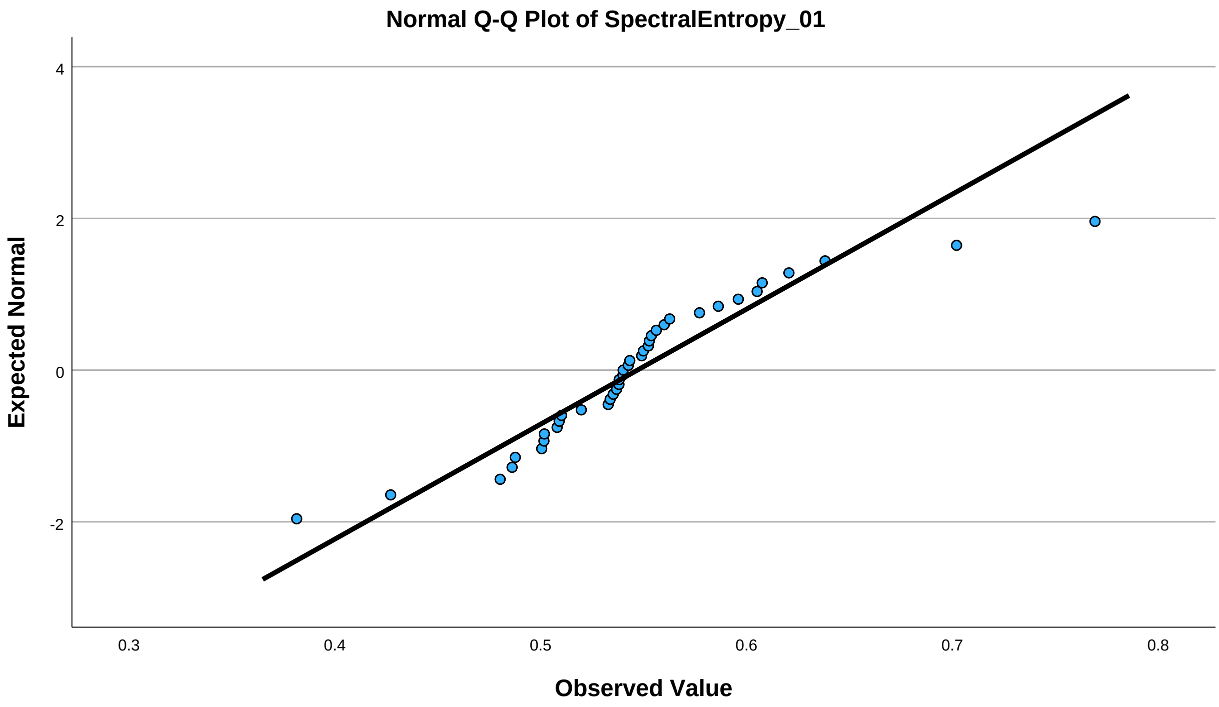

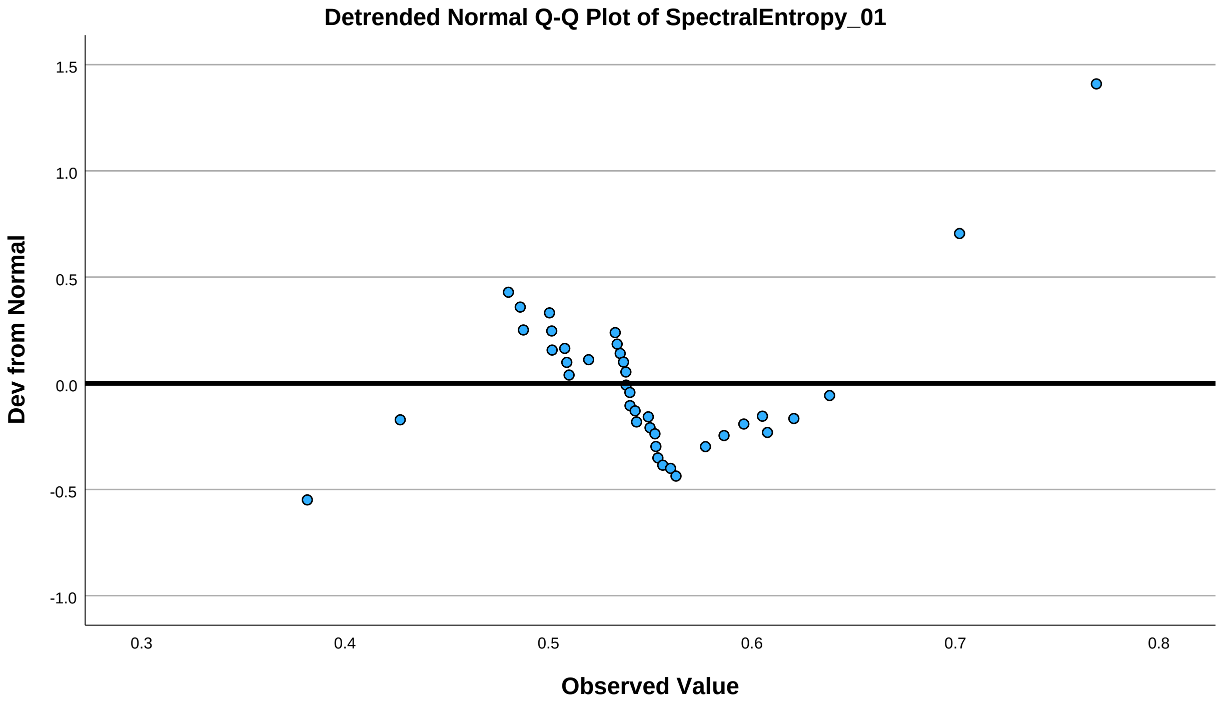

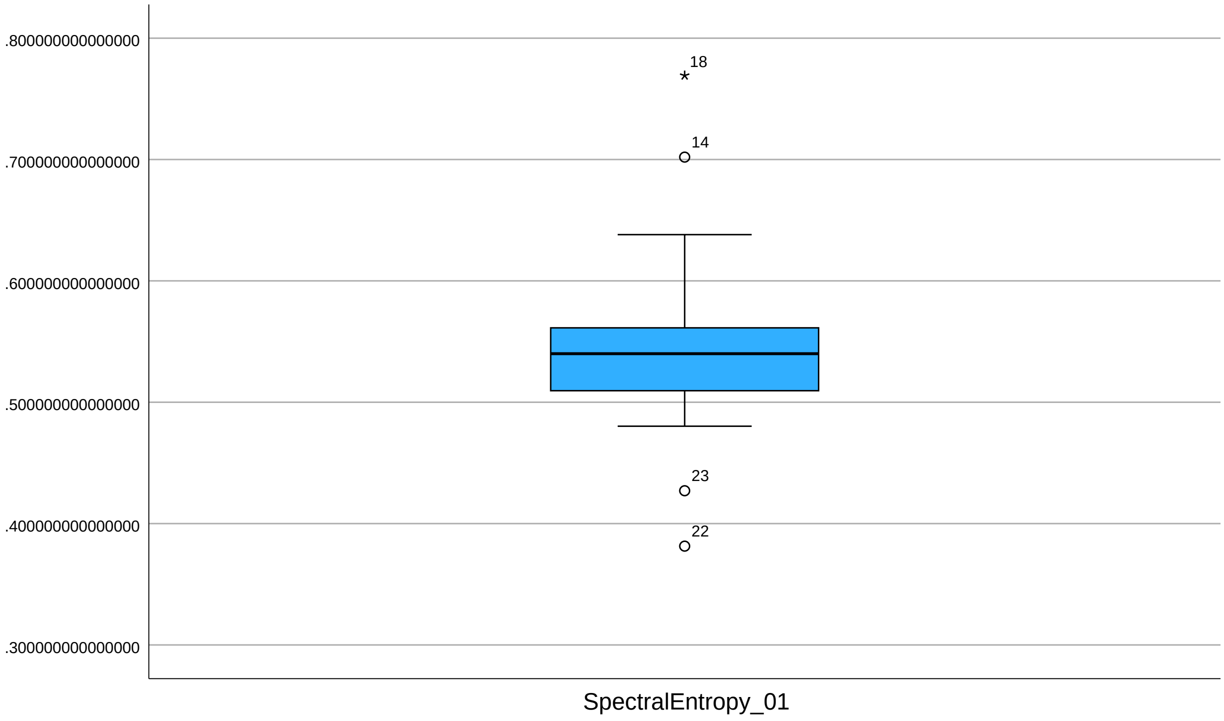

HiguchiFractalDimension

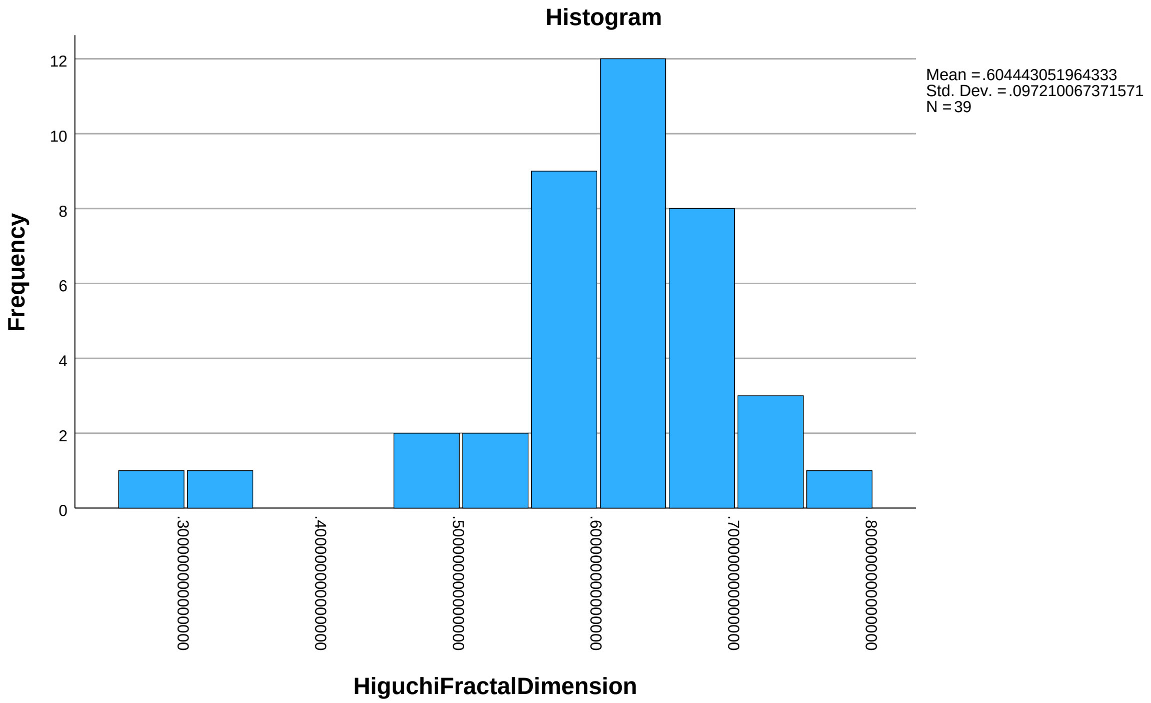


```
HiguchiFractalDimension Stem-and-Leaf Plot

 Frequency    Stem &  Leaf

     2.00 Extremes    (=<.33)
     2.00        4 .  66
     2.00        5 .  12
     9.00        5 .  667788999
    12.00        6 .  001133334444
     8.00        6 .  55666777
     3.00        7 .  012
     1.00        7 .  6

 Stem width:   .1000000
 Each leaf:        1 case(s)
```

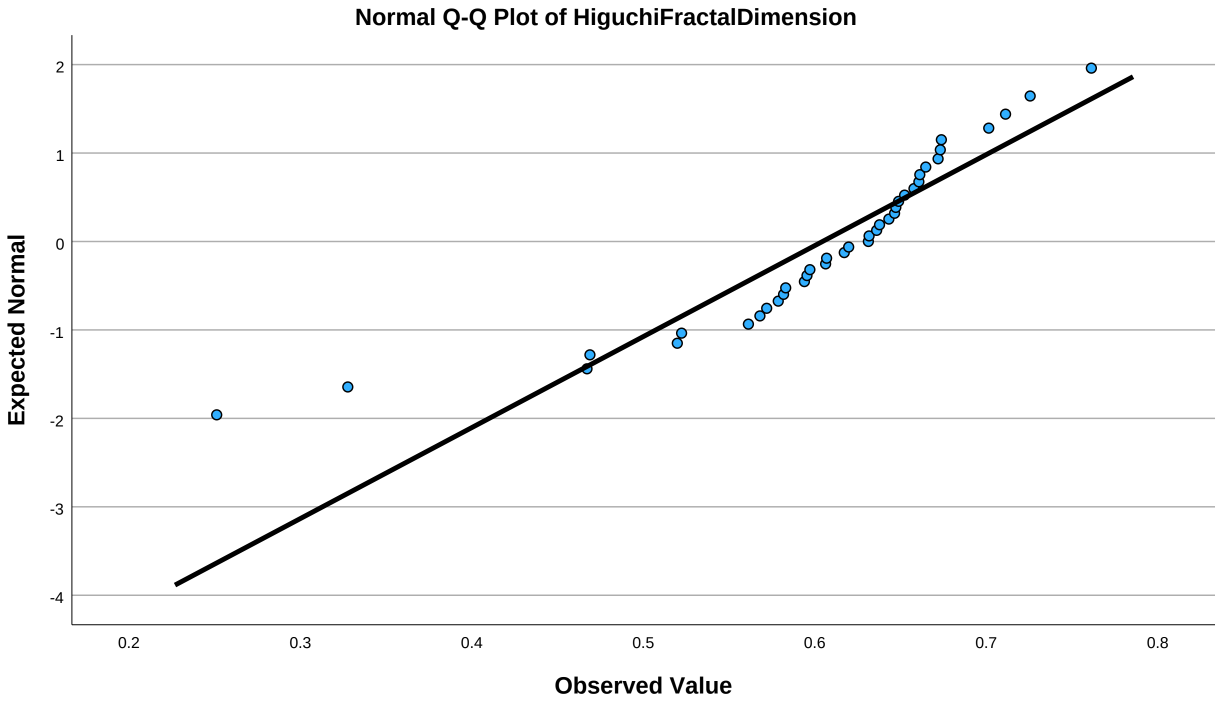
Normal Q-Q Plot of HiguchiFractalDimension
Expected Normal
2
1
0
-1
-2
-3
-4
0.2
0.3
0.4
0.5
0.6
0.7
0.8
Observed Value

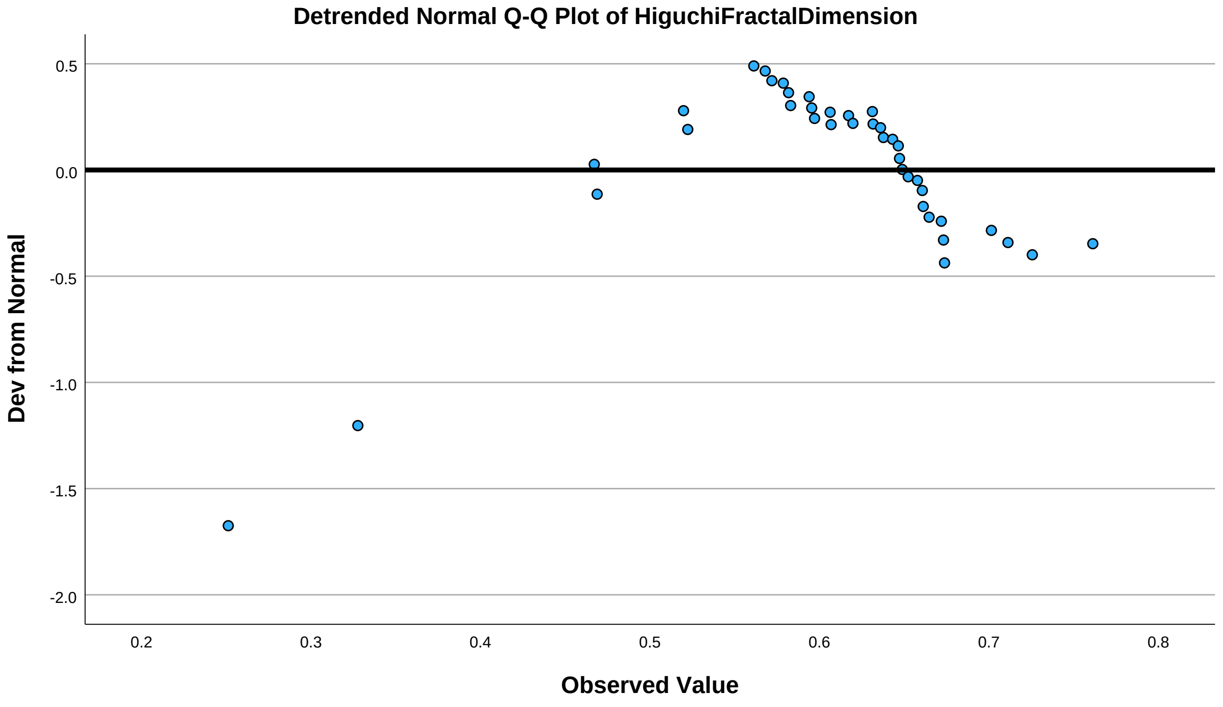
Detrended Normal Q-Q Plot of HiguchiFractalDimension
Dev from Normal
0.5
0.0
-0.5
-1.0
-1.5
-2.0
0.2
0.3
0.4
0.5
0.6
0.7
0.8
Observed Value

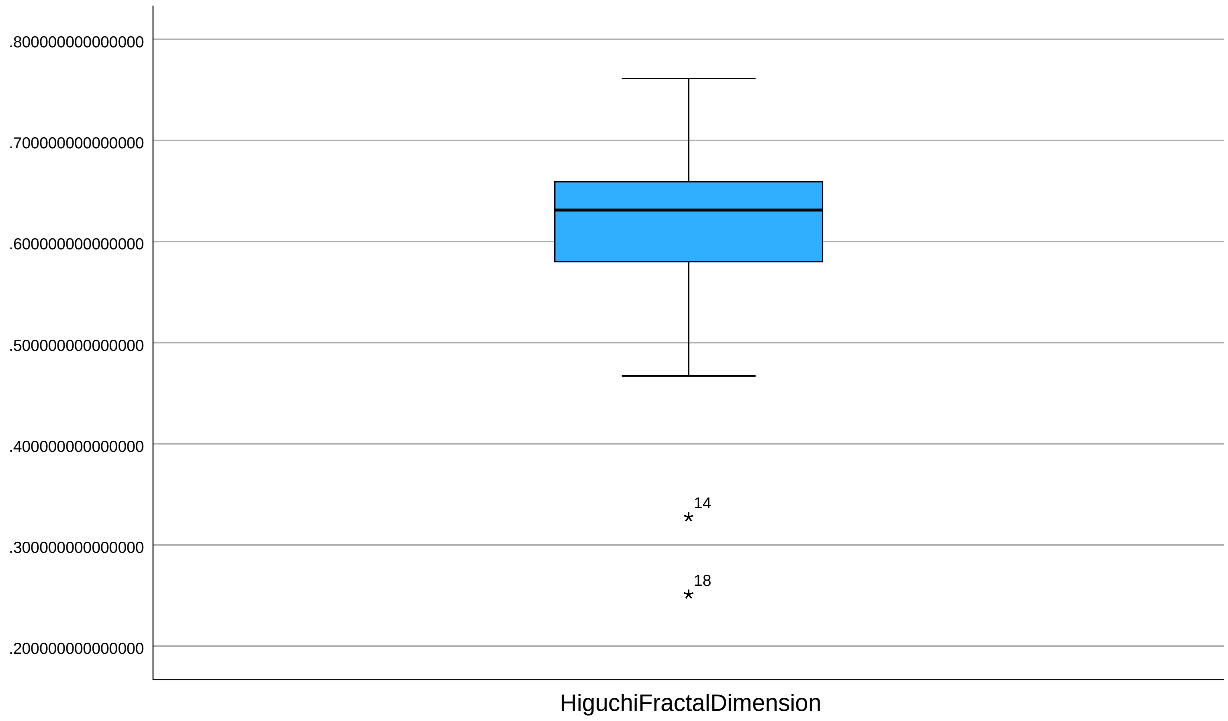

LempelZivComplexity

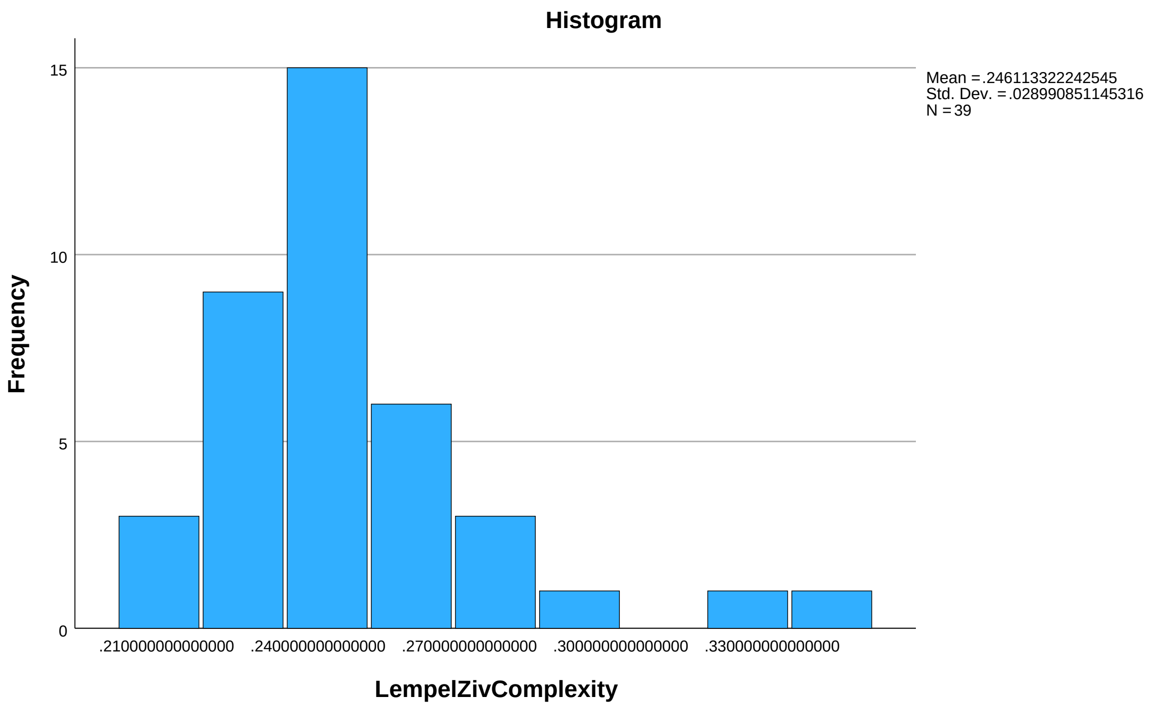


```
LempelZivComplexity Stem-and-Leaf Plot

 Frequency    Stem &  Leaf
```

```
     2.00        20 .  69
     3.00        21 .  579
     7.00        22 .  1117889
     6.00        23 .  467899
     9.00        24 .  000144469
     5.00        25 .  04479
     2.00        26 .  08
     1.00        27 .  7
     1.00        28 .  3
     3.00 Extremes    (>=.296)

 Stem width:   .0100000
 Each leaf:       1 case(s)
```

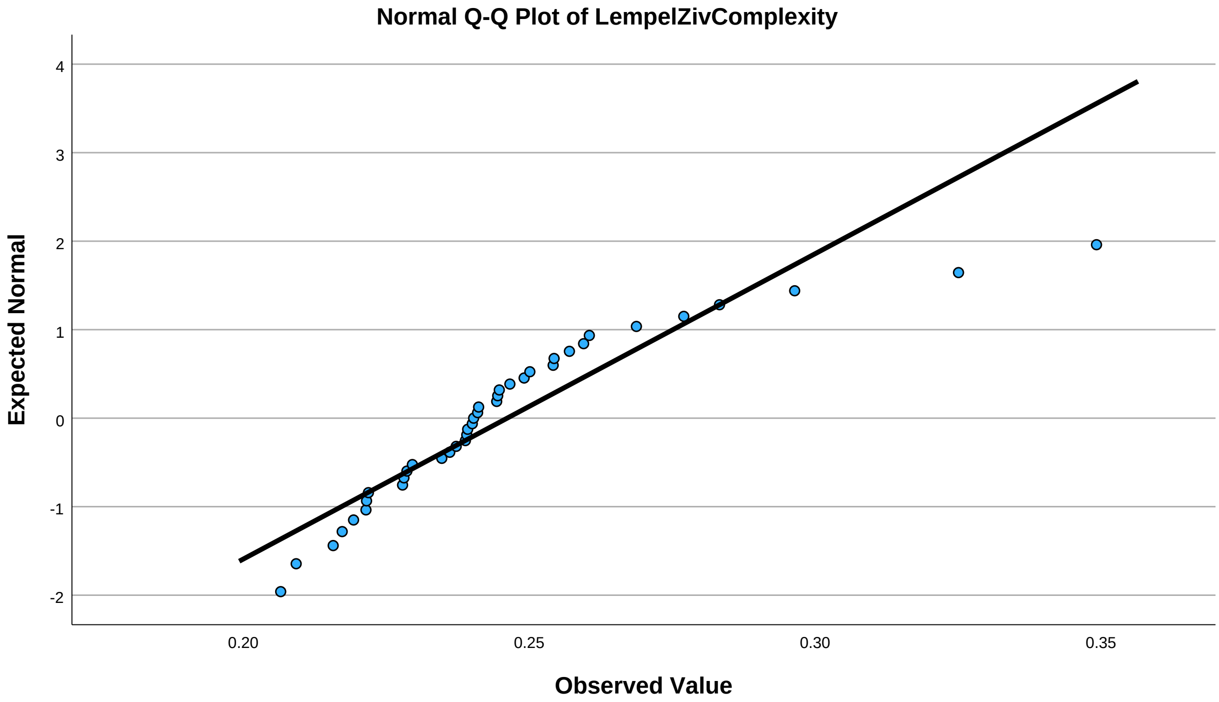

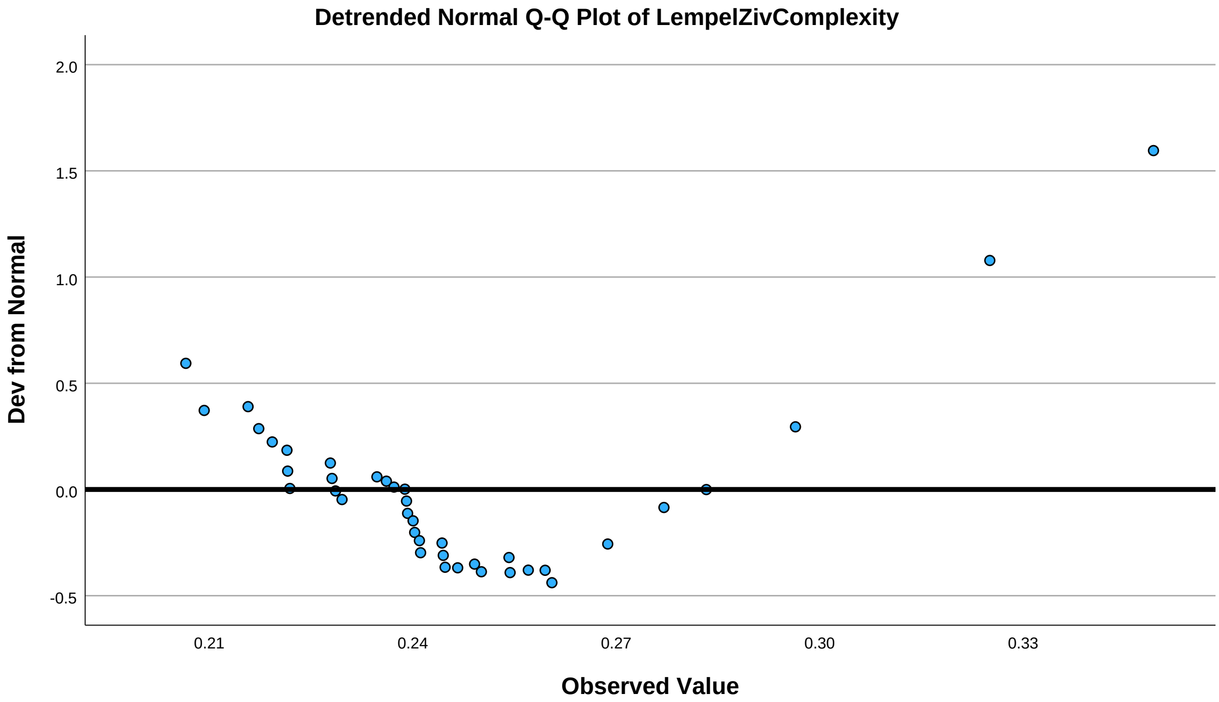

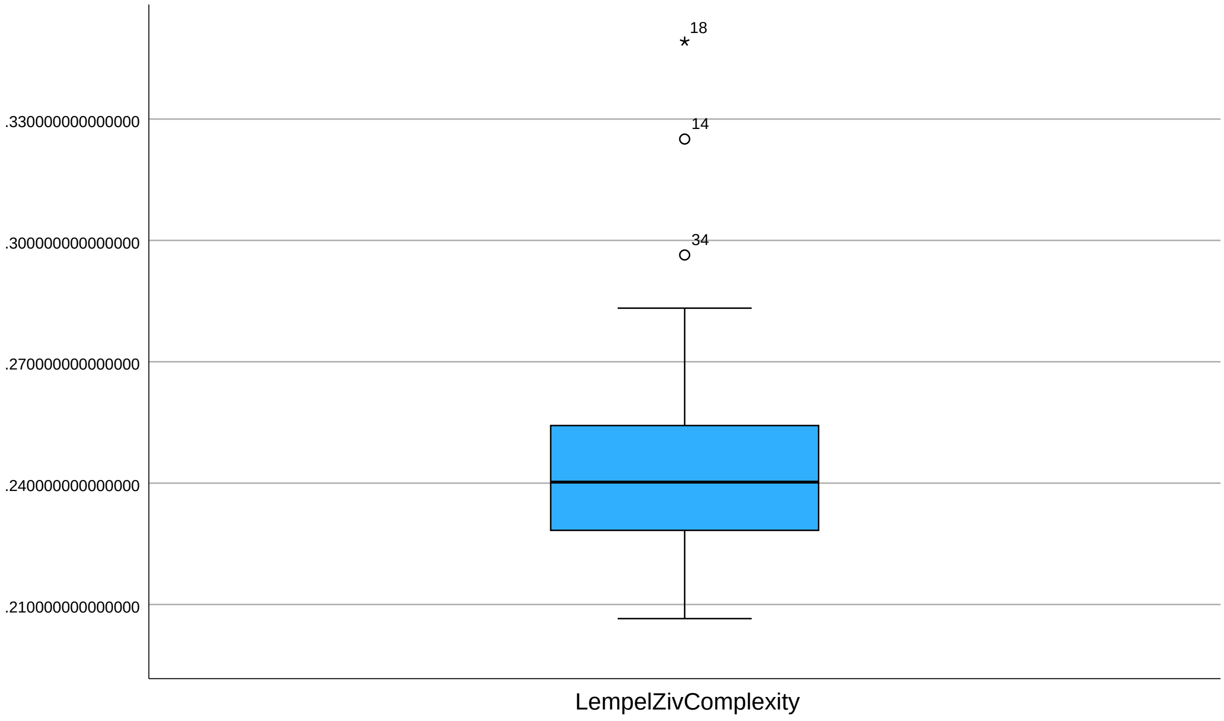

RenyiEntropy

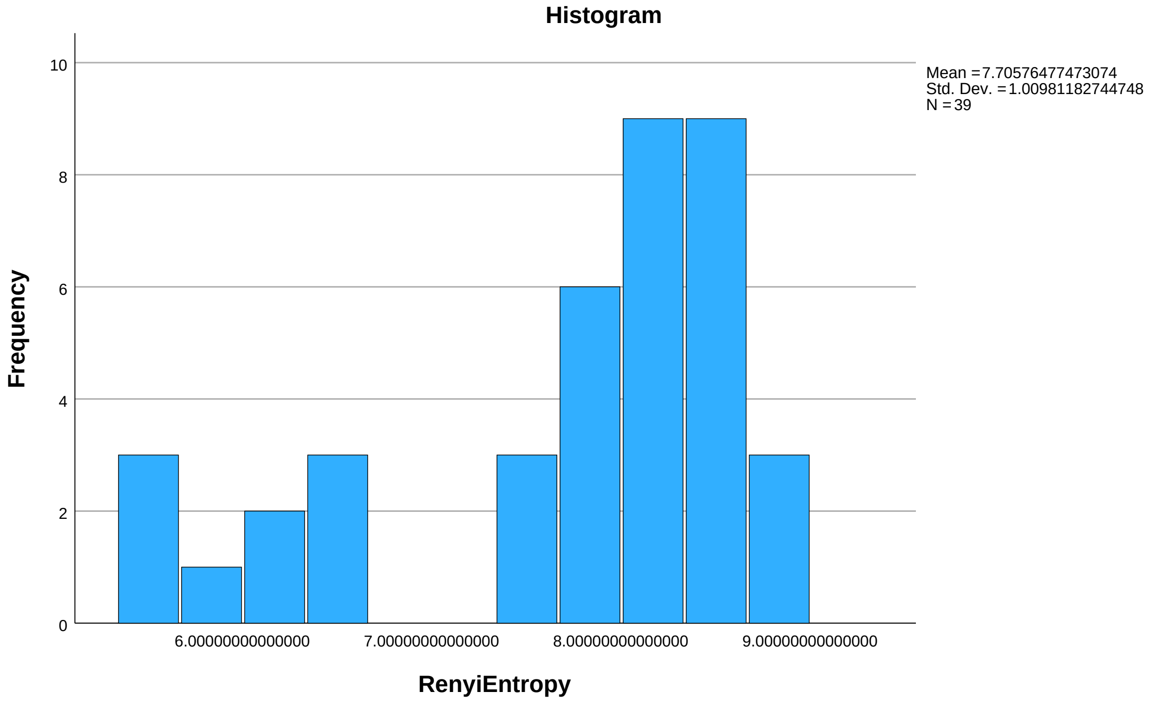


```
RenyiEntropy Stem-and-Leaf Plot

 Frequency    Stem &  Leaf

     4.00 Extremes    (=<5.9)
     5.00        6 .  13344
      .00        6 .
     1.00        7 .  4
     8.00        7 .  56777899
    12.00        8 .  000011222344
     9.00        8 .  555566788

 Stem width:  1.000000
 Each leaf:       1 case(s)
```

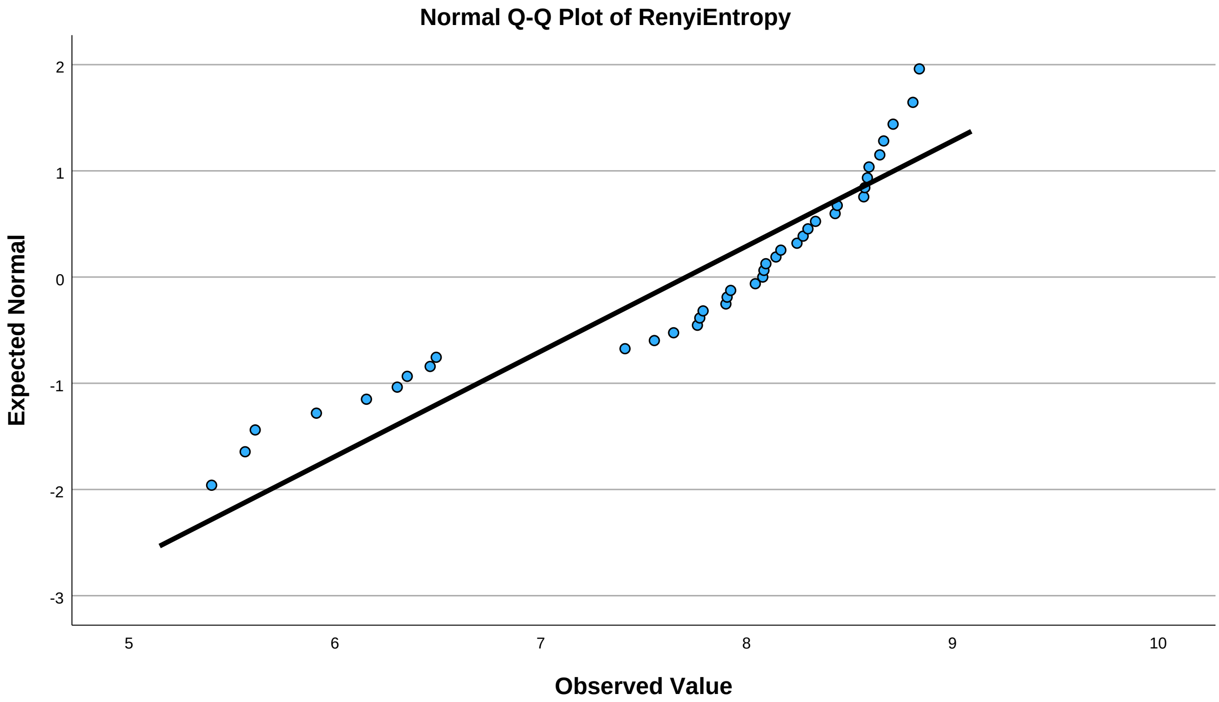

Normal Q-Q Plot of RenyiEntropy
Expected Normal
2
1
0
-1
-2
-3
5
6
7
8
9
10
Observed Value

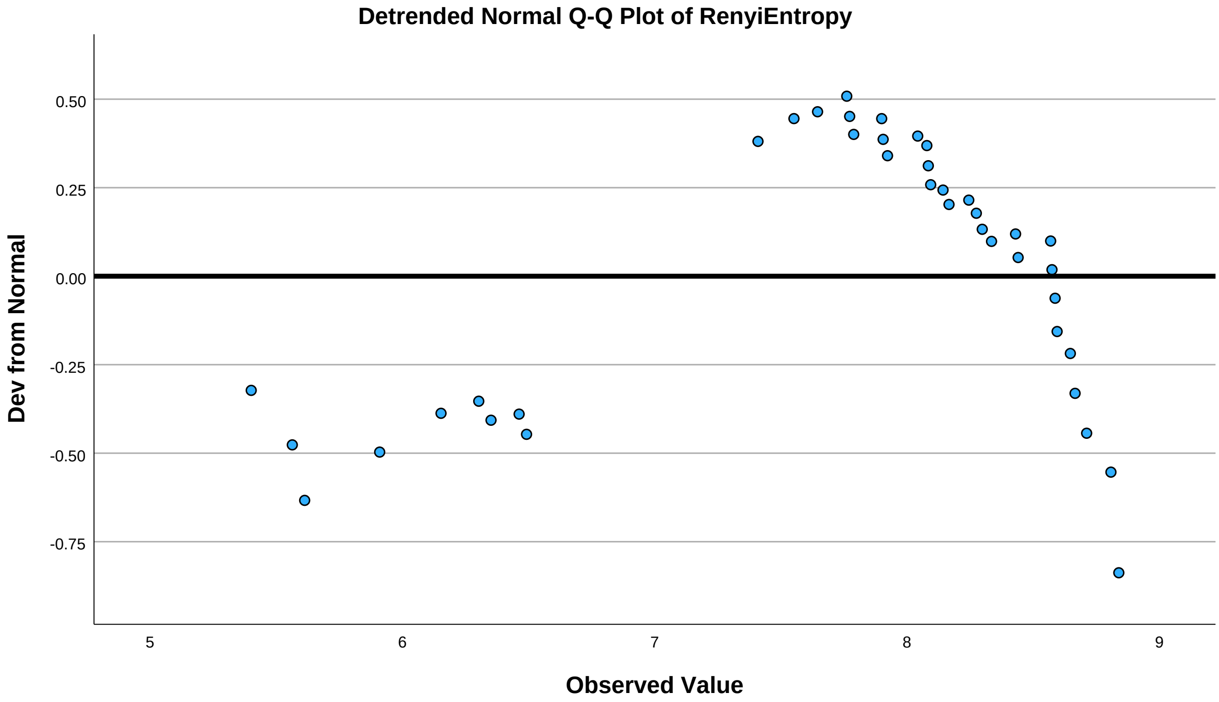

Detrended Normal Q-Q Plot of RenyiEntropy
Dev from Normal
0.50
0.25
0.00
-0.25
-0.50
-0.75
5
6
7
8
9
Observed Value

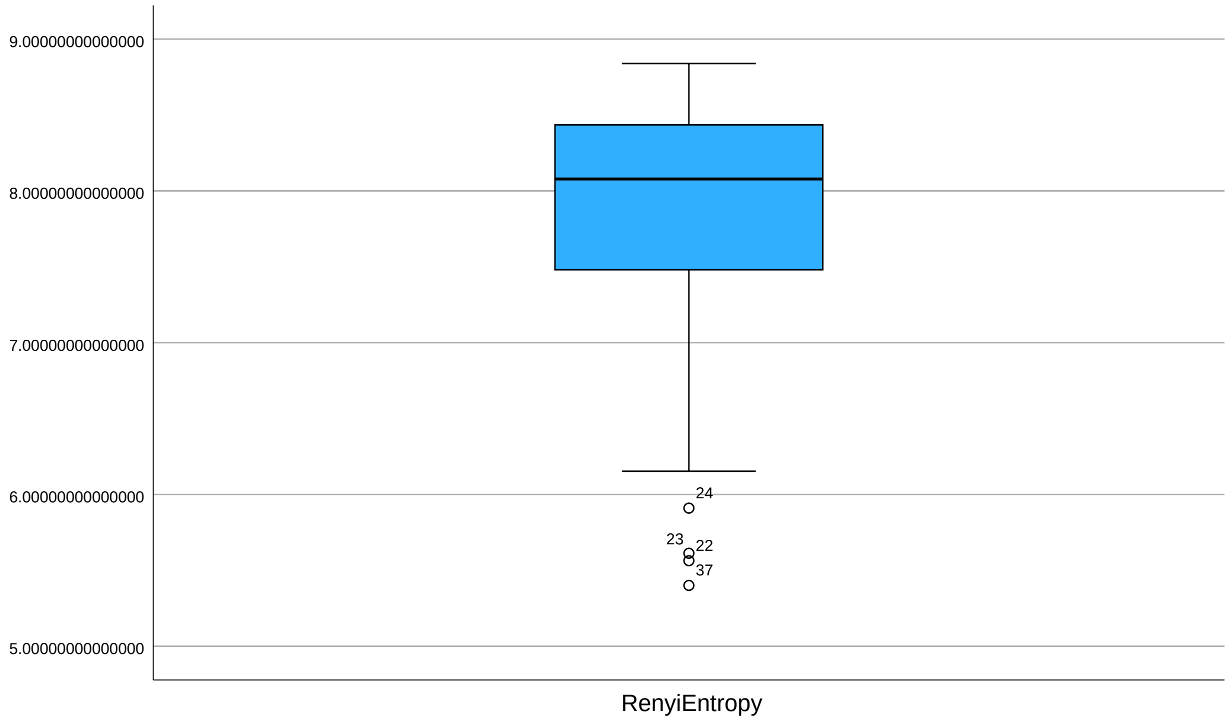

TsallisEntropy

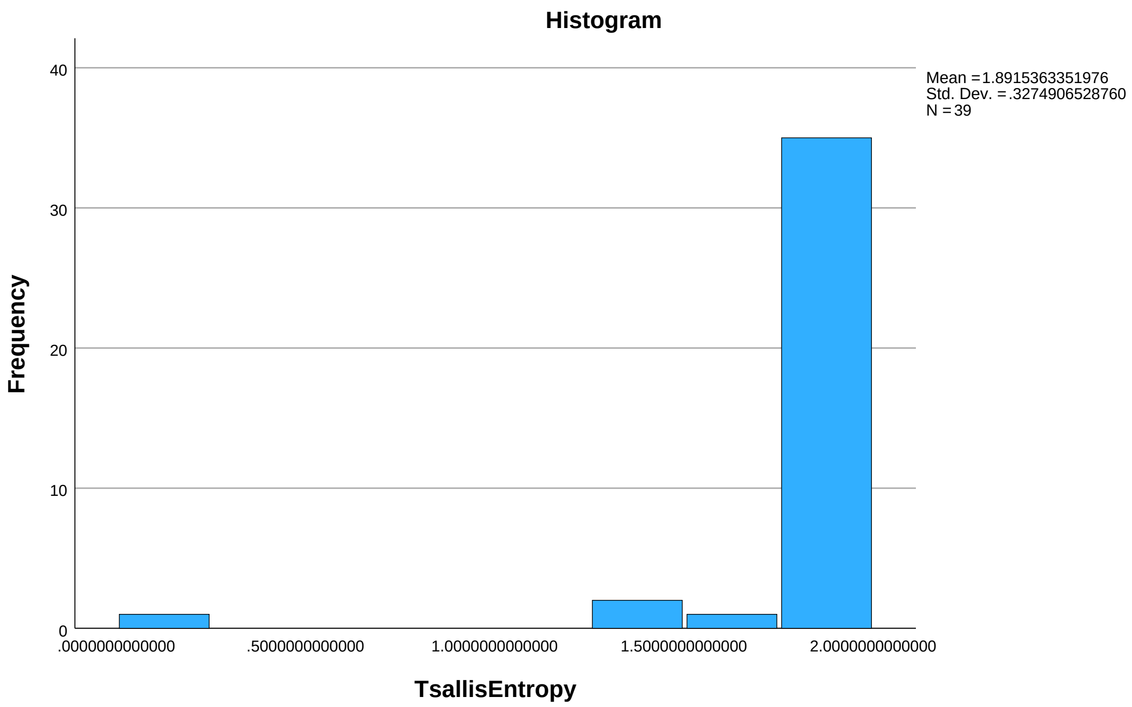

TsallisEntropy Stem-and-Leaf Plot

Frequency   Stem & Leaf

```
    8.00 Extremes    (=<1.9814)
    1.00    1984 .  8
    3.00    1985 .  123
    5.00    1985 .  56789
    7.00    1986 .  0112334
    7.00    1986 .  5677899
    5.00    1987 .  01234
     .00    1987 .
    1.00    1988 .  4
    2.00    1988 .  59

 Stem width:   .0010000
 Each leaf:       1 case(s)
```

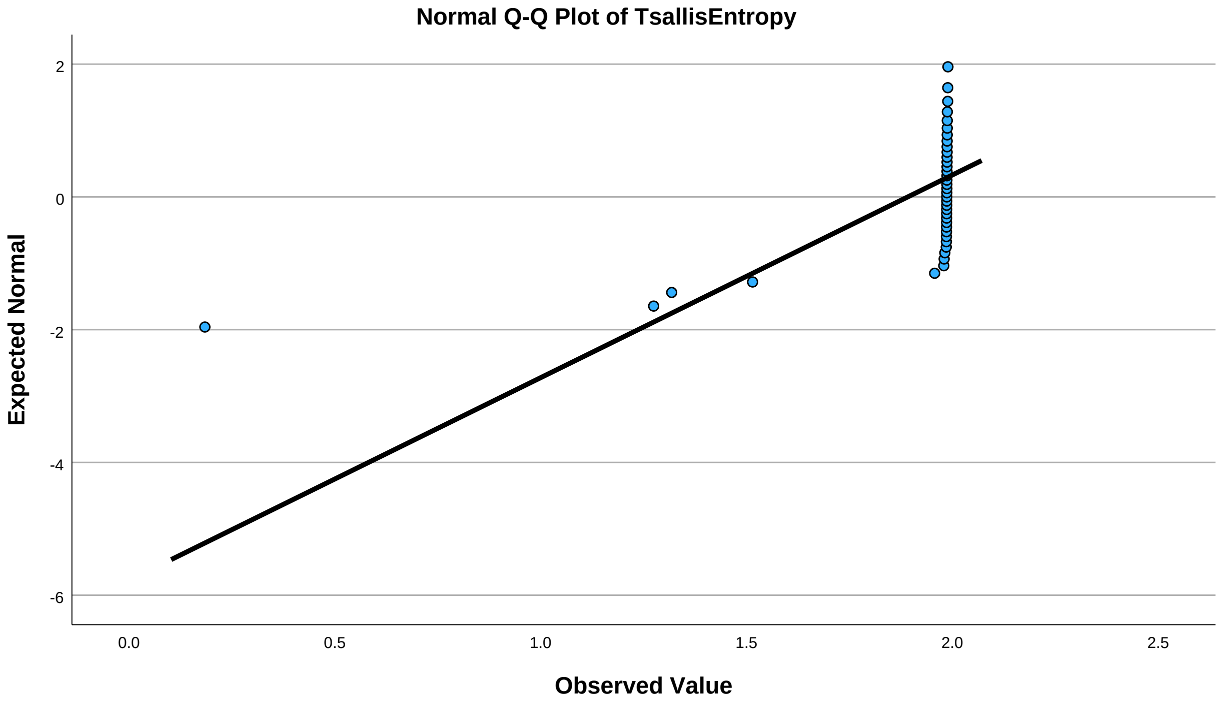

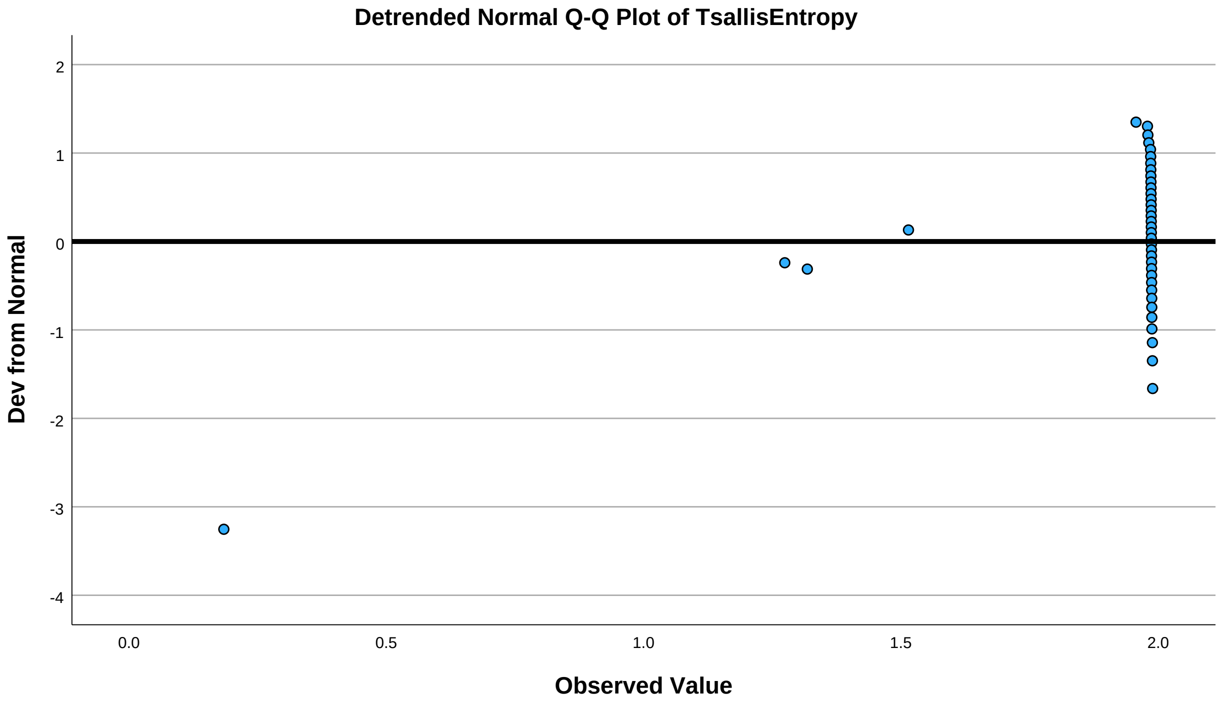
Detrended Normal Q-Q Plot of TsallisEntropy
Dev from Normal
2
1
0
-1
-2
-3
-4
0.0
0.5
1.0
1.5
2.0
Observed Value

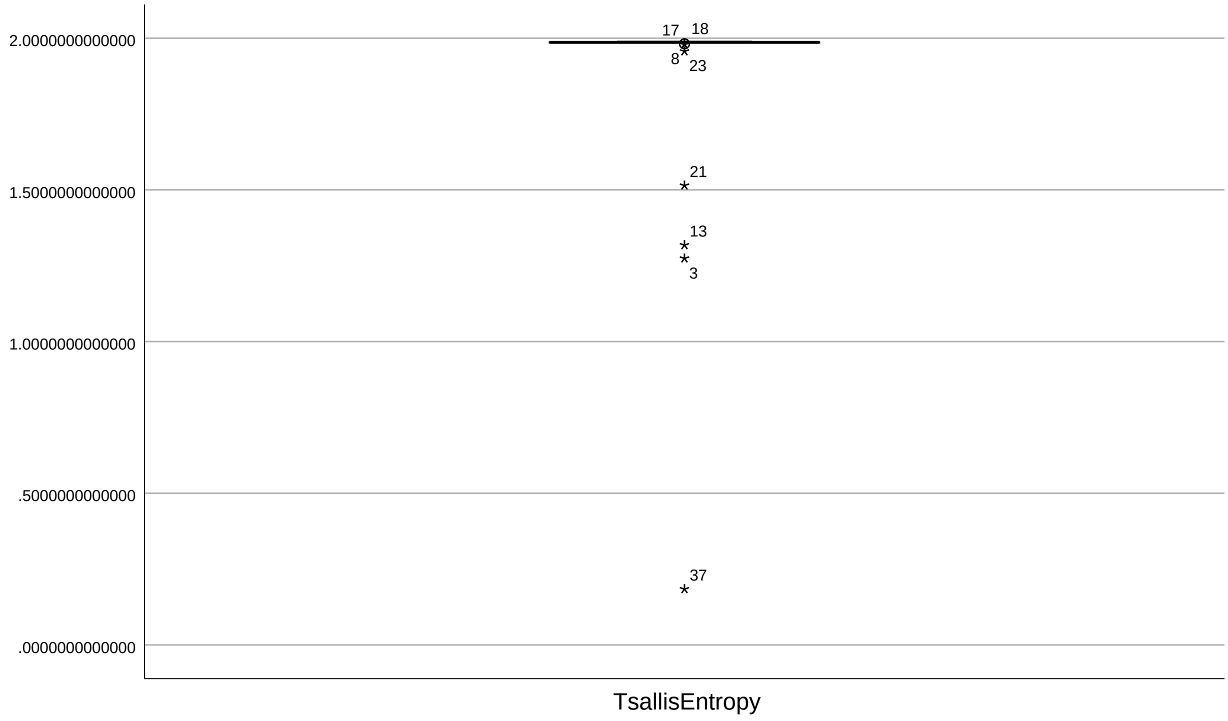
2.0000000000000
1.5000000000000
1.0000000000000
.5000000000000
.0000000000000
17
18
8
23
21
13
3
37
TsallisEntropy

PS_4_5

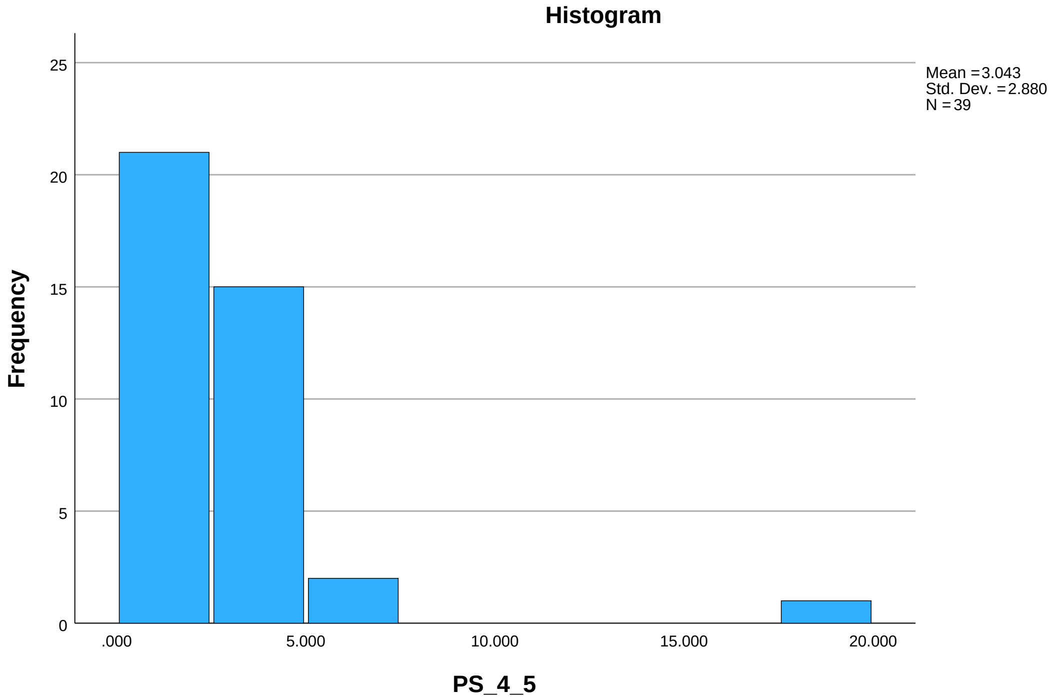


```
PS_4_5 Stem-and-Leaf Plot

 Frequency    Stem &  Leaf

     2.00        0 .  99
     9.00        1 .  003334444
     4.00        1 .  6889
     6.00        2 .  011344
     6.00        2 .  678899
     3.00        3 .  234
      .00        3 .
     2.00        4 .  14
     4.00        4 .  5899
     2.00        5 .  11
     1.00 Extremes    (>=18.7)

 Stem width:      1.000
 Each leaf:       1 case(s)
```

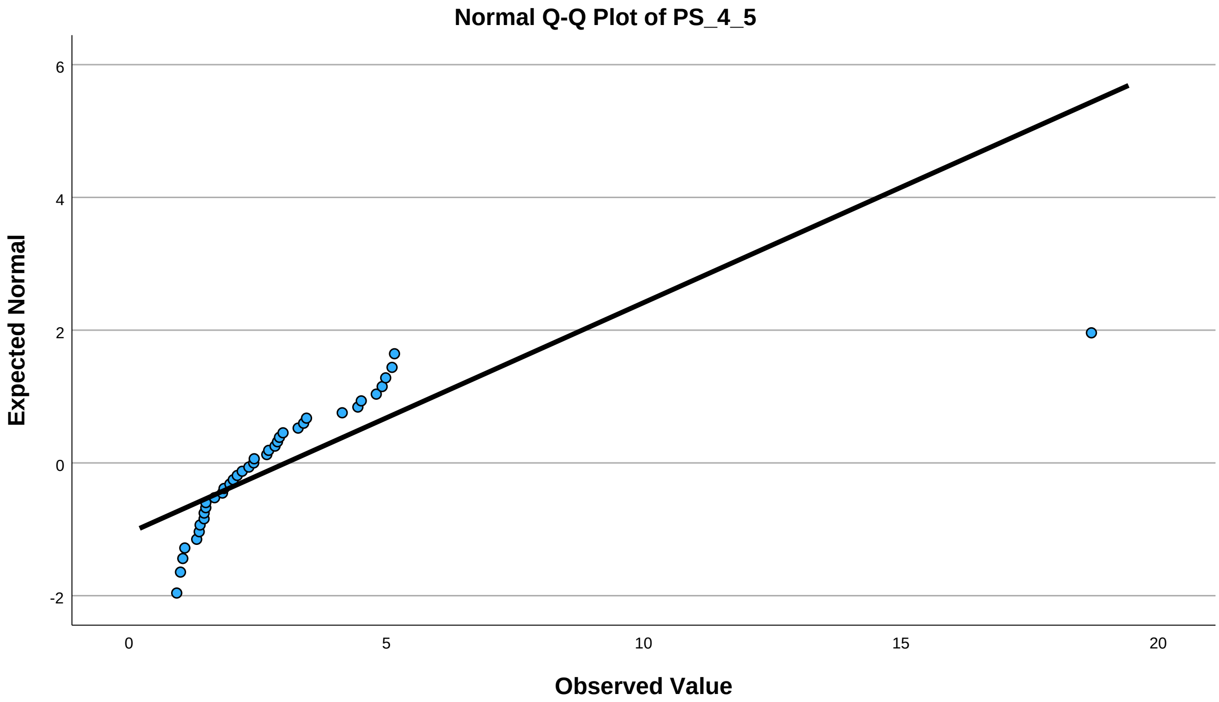

Normal Q-Q Plot of PS_4_5
Expected Normal
Observed Value
6
4
2
0
-2
0
5
10
15
20

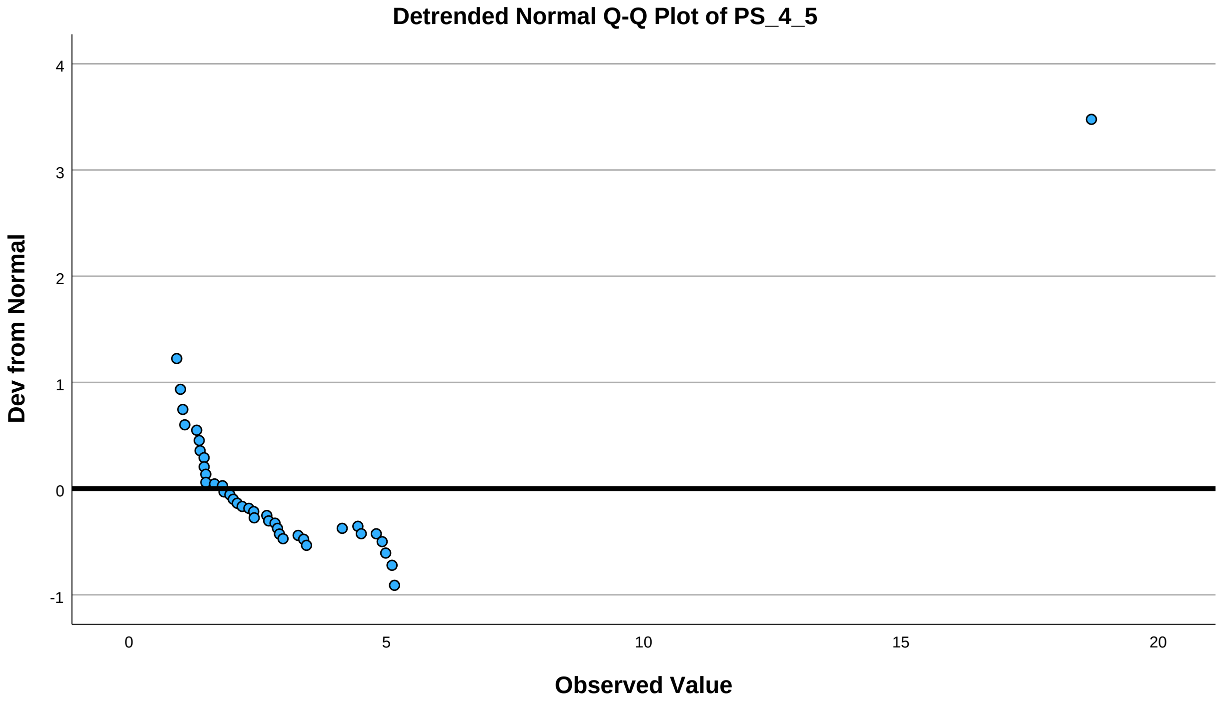

Detrended Normal Q-Q Plot of PS_4_5
Dev from Normal
Observed Value
4
3
2
1
0
-1
0
5
10
15
20

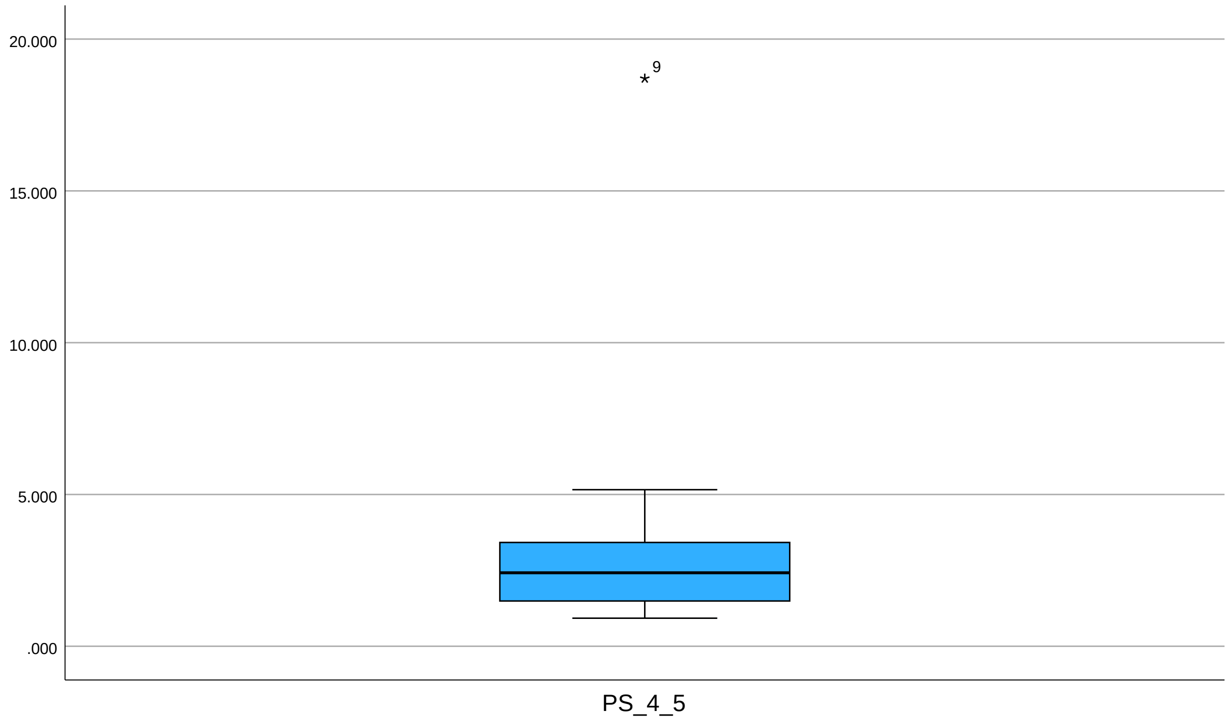

PS_5_6

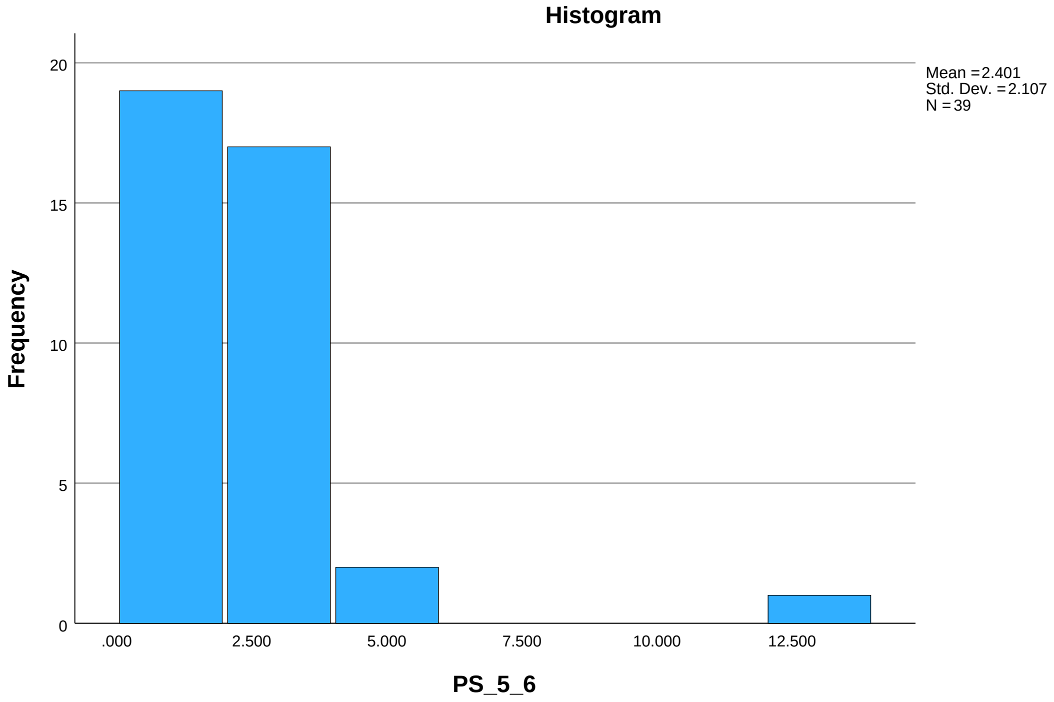

PS_5_6 Stem-and-Leaf Plot

Frequency   Stem & Leaf

```
     5.00        0 .  57999
     7.00        1 .  0123334
     7.00        1 .  5556689
     6.00        2 .  001224
     7.00        2 .  5566678
     3.00        3 .  123
     1.00        3 .  9
     1.00        4 .  0
     2.00 Extremes    (>=5.9)

 Stem width:      1.000
 Each leaf:        1 case(s)
```

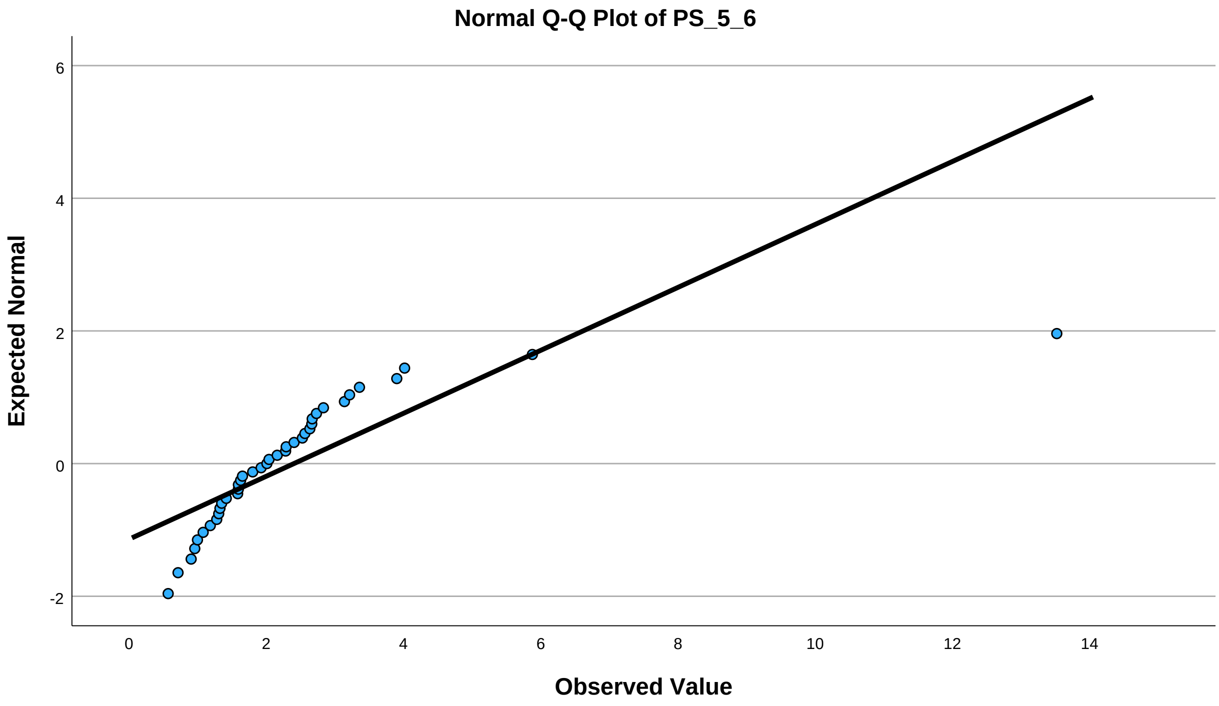

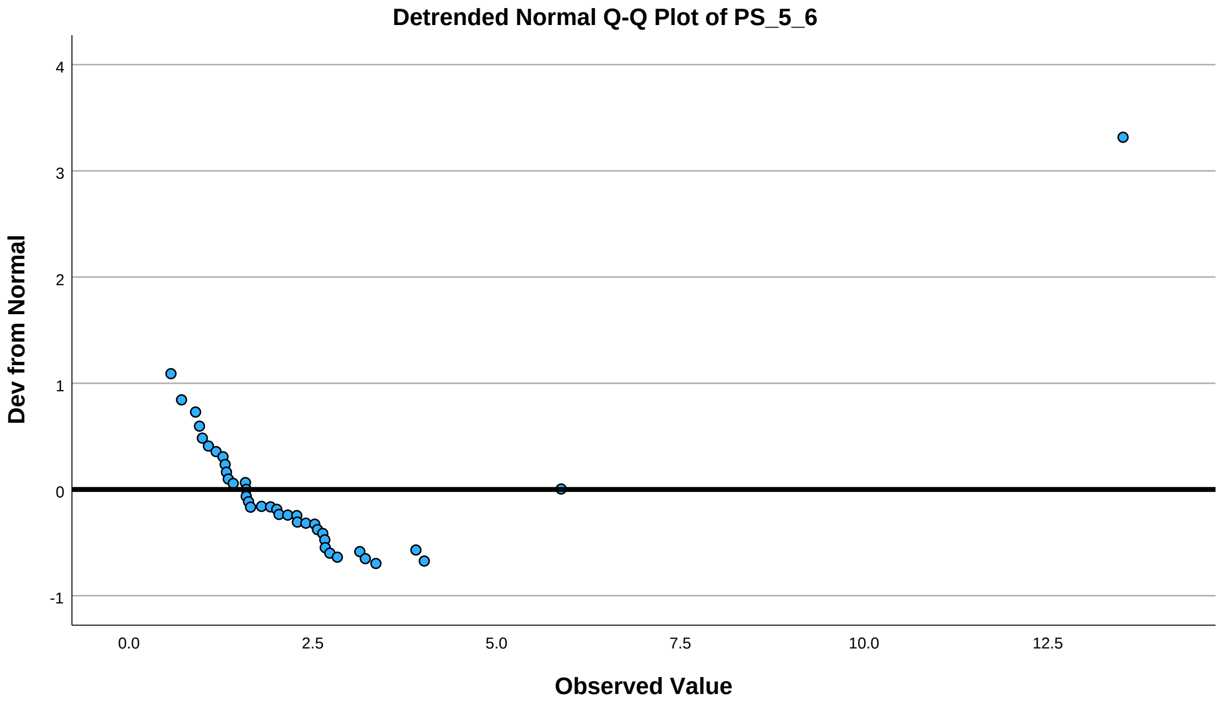
Detrended Normal Q-Q Plot of PS_5_6
Dev from Normal
4
3
2
1
0
-1
0.0
2.5
5.0
7.5
10.0
12.5
Observed Value

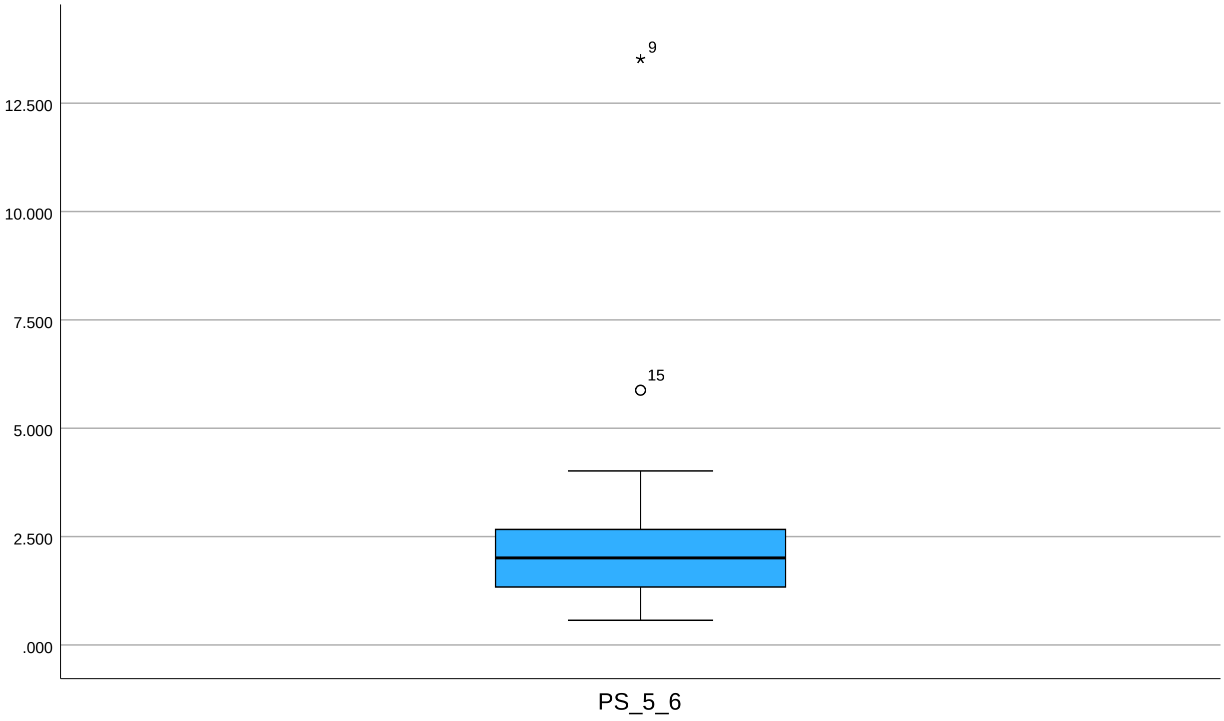
9
15
12.500
10.000
7.500
5.000
2.500
.000
PS_5_6

PS_6_7

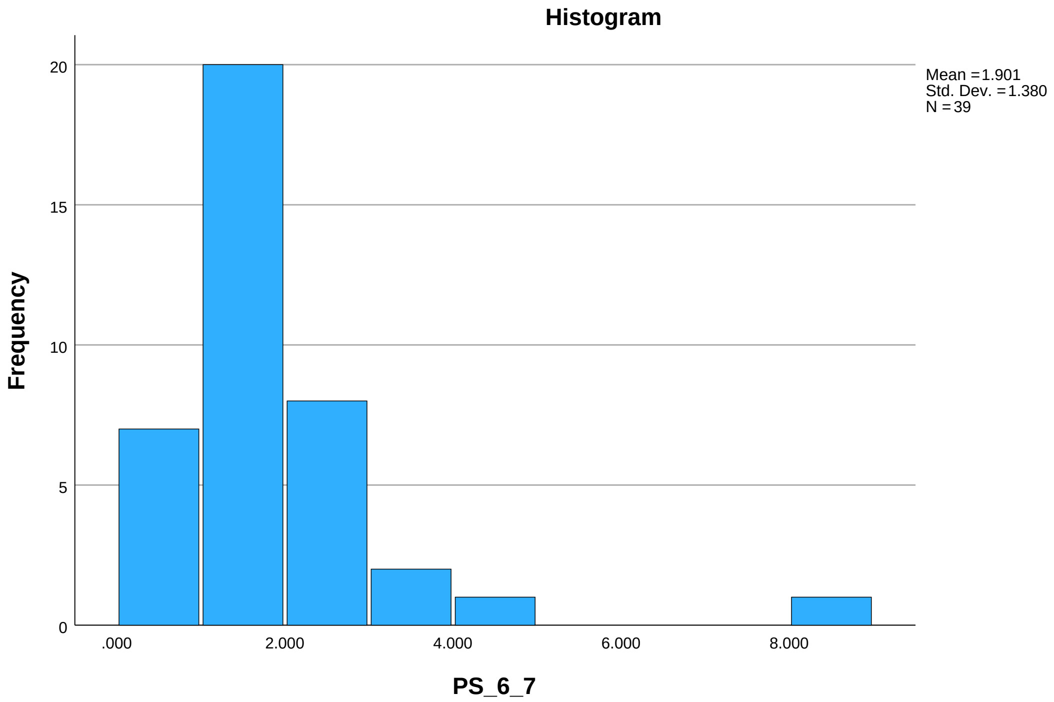


```
PS_6_7 Stem-and-Leaf Plot

 Frequency    Stem &  Leaf

     1.00        0 .  4
     6.00        0 .  578889
    11.00        1 .  00112344444
     9.00        1 .  556678888
     4.00        2 .  0112
     4.00        2 .  5567
     1.00        3 .  2
     3.00 Extremes    (>=3.9)

 Stem width:      1.000
 Each leaf:       1 case(s)
```

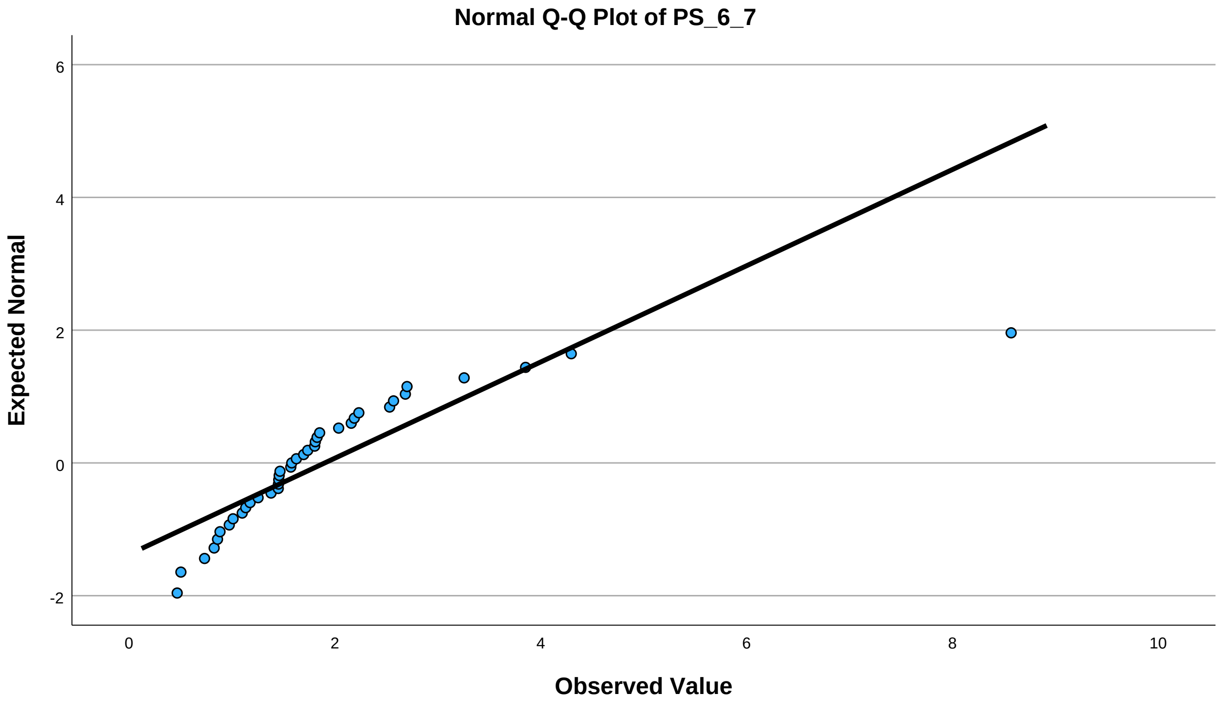
Normal Q-Q Plot of PS_6_7
Expected Normal
Observed Value
6
4
2
0
-2
0
2
4
6
8
10

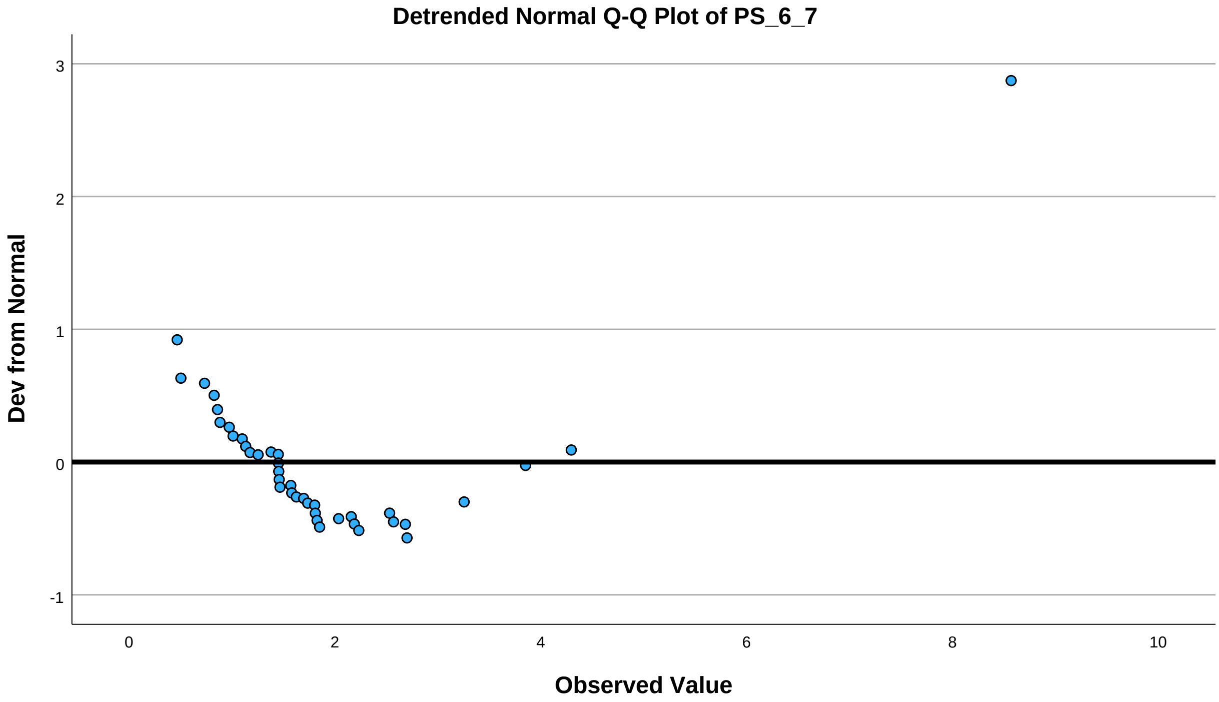
Detrended Normal Q-Q Plot of PS_6_7
Dev from Normal
Observed Value
3
2
1
0
-1
0
2
4
6
8
10

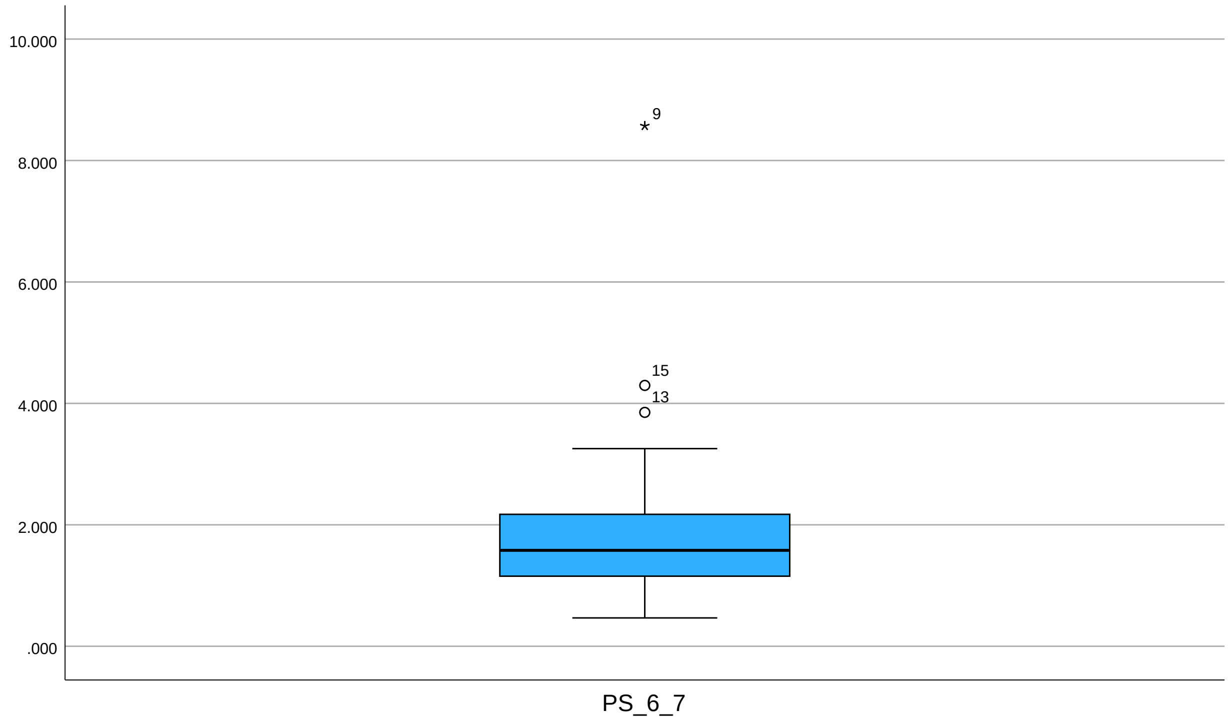

PS_7_8

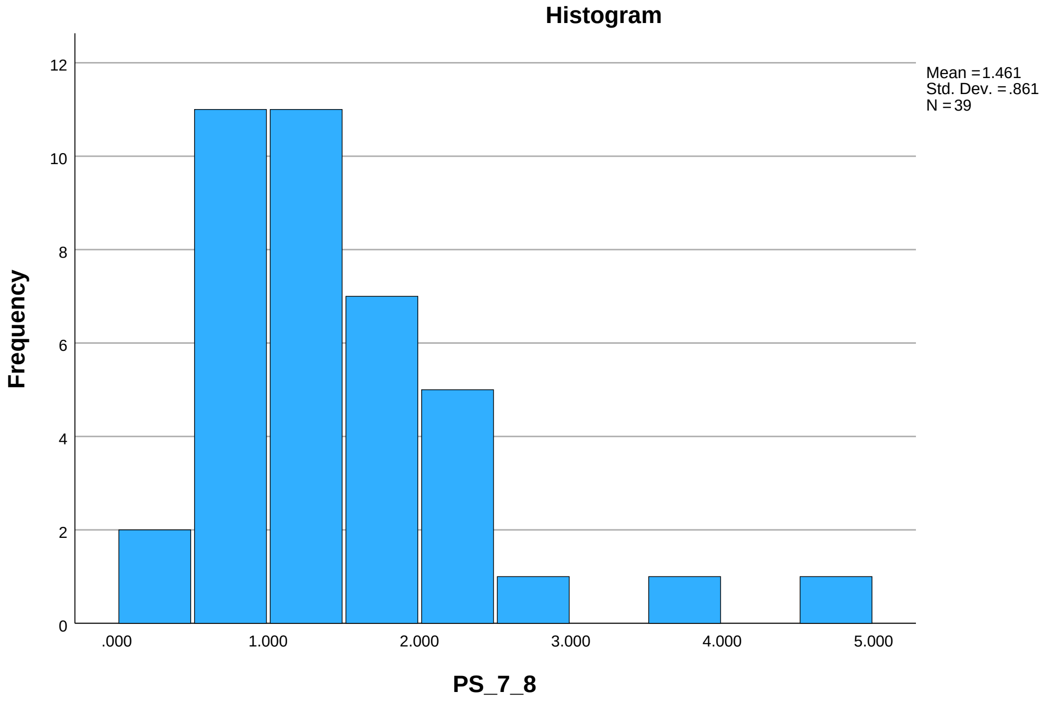


```
PS_7_8 Stem-and-Leaf Plot

 Frequency    Stem &  Leaf
```

```
  2.00      0 . 34
 11.00       0 . 66666889999
 11.00       1 . 00111122344
  7.00      1 . 5666779
  5.00      2 . 00123
  1.00     2 . 7
  2.00 Extremes    (>=3.8)

Stem width:     1.000
Each leaf:       1 case(s)
```

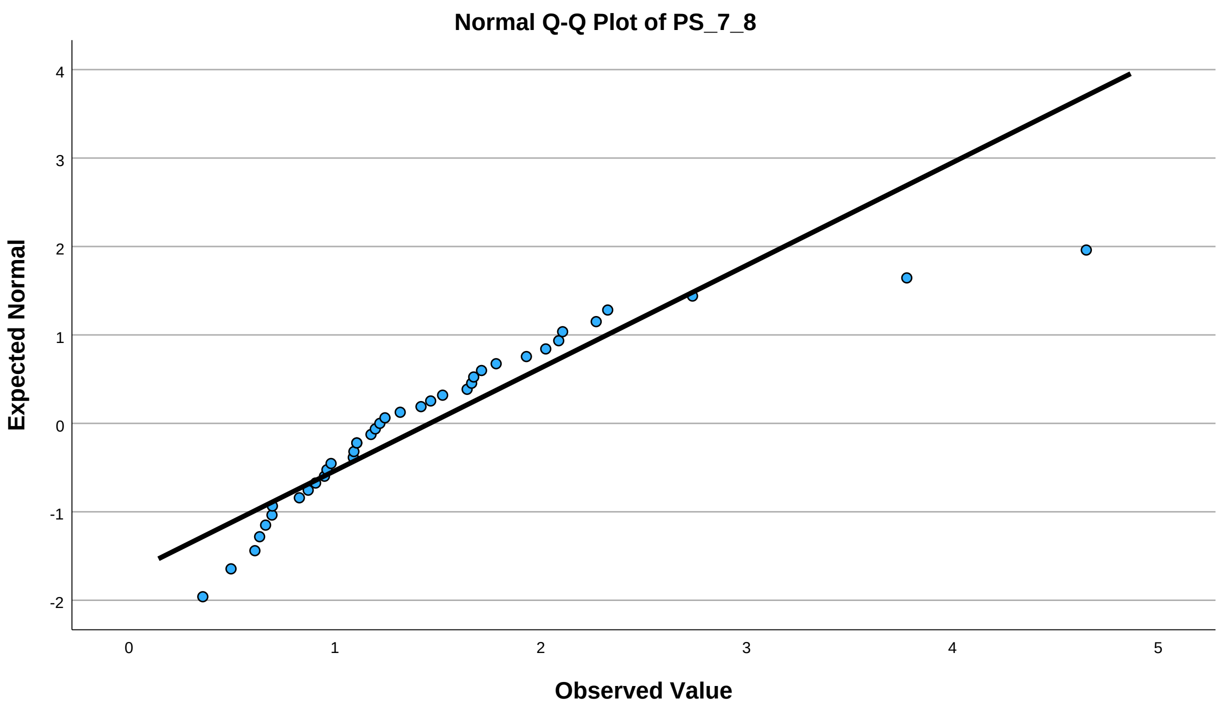

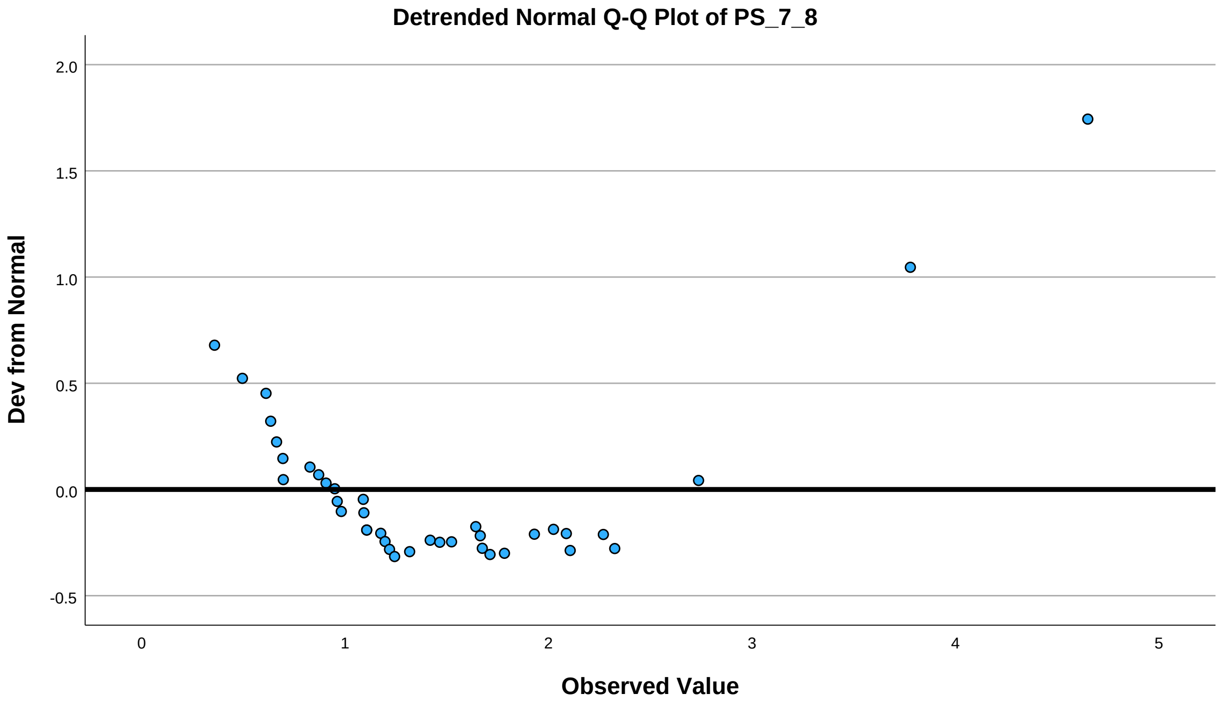

Detrended Normal Q-Q Plot of PS_7_8
Dev from Normal
2.0
1.5
1.0
0.5
0.0
-0.5
0
1
2
3
4
5
Observed Value

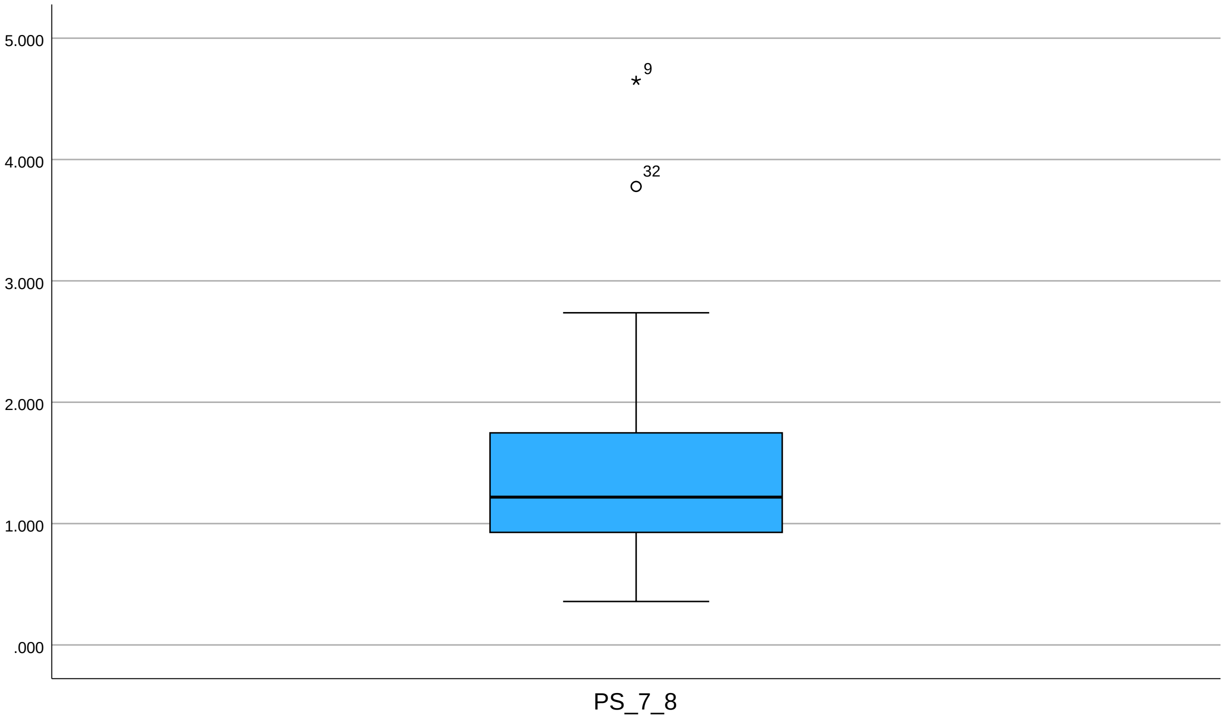

5.000
4.000
3.000
2.000
1.000
.000
9
32
PS_7_8

PS_8_9

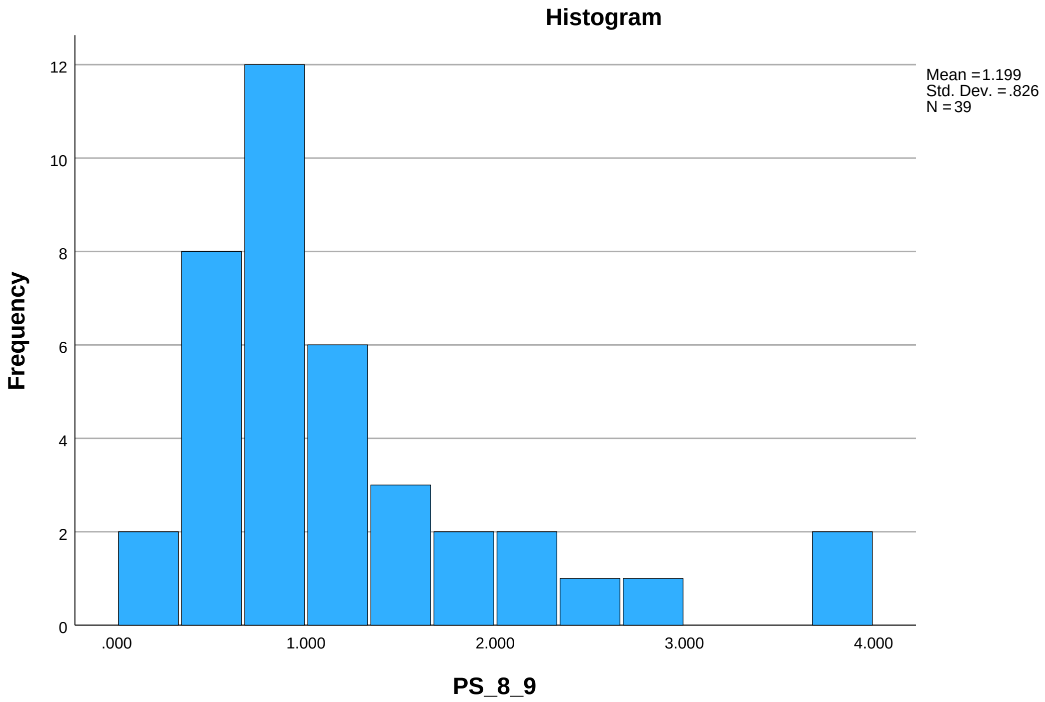


```
PS_8_9 Stem-and-Leaf Plot

 Frequency    Stem &  Leaf

     4.00        0 .  2344
    18.00        0 .  555666777778888999
     8.00        1 .  00011144
     3.00        1 .  669
     3.00        2 .  024
     3.00 Extremes    (>=2.7)

 Stem width:      1.000
 Each leaf:       1 case(s)
```

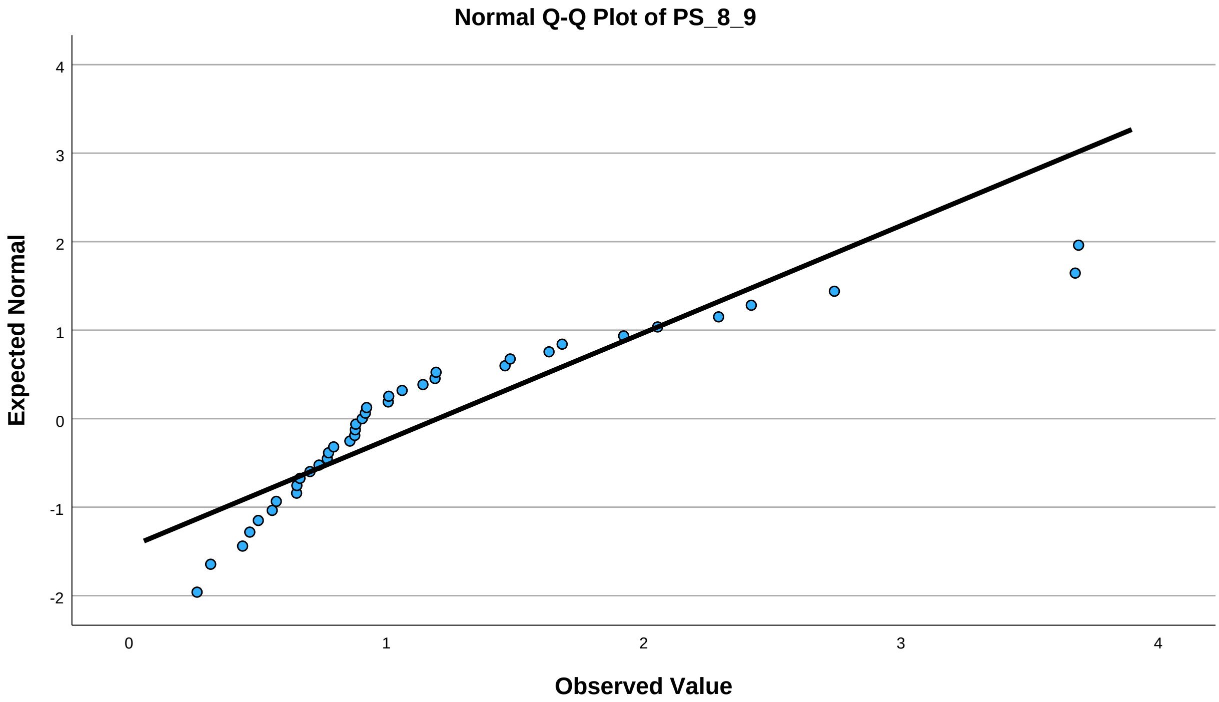
Normal Q-Q Plot of PS_8_9
Expected Normal
Observed Value
4
3
2
1
0
-1
-2
0
1
2
3
4

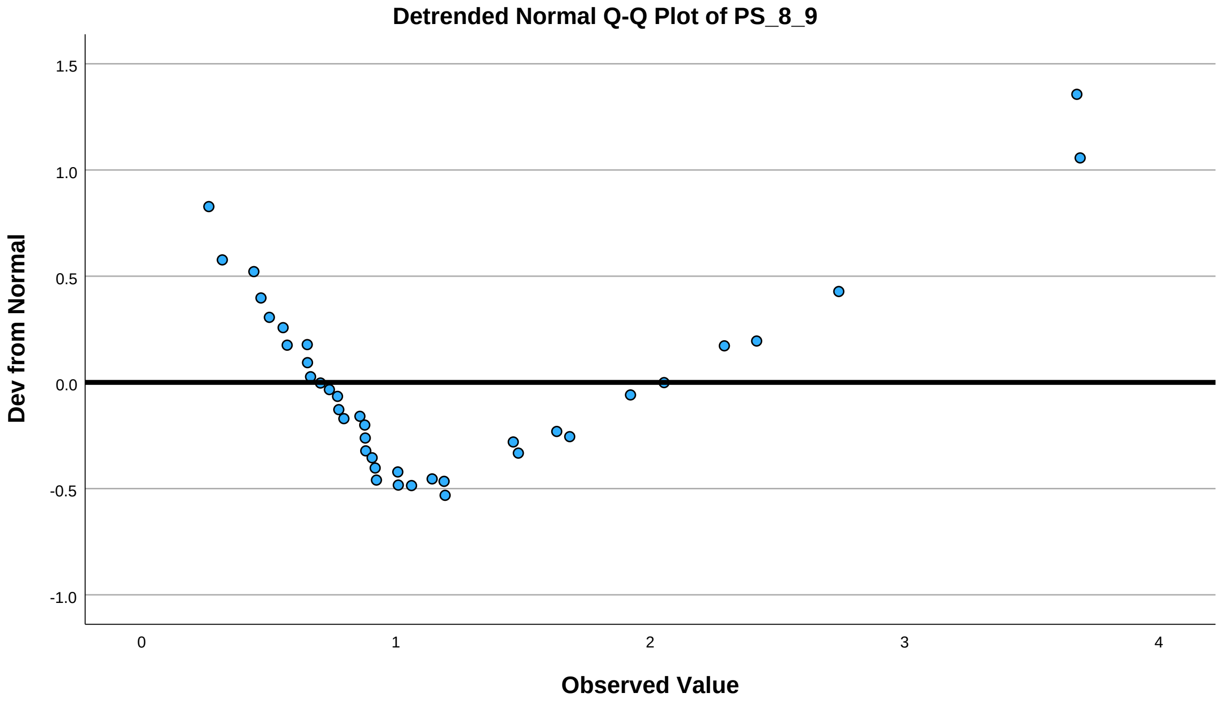
Detrended Normal Q-Q Plot of PS_8_9
Dev from Normal
Observed Value
1.5
1.0
0.5
0.0
-0.5
-1.0
0
1
2
3
4

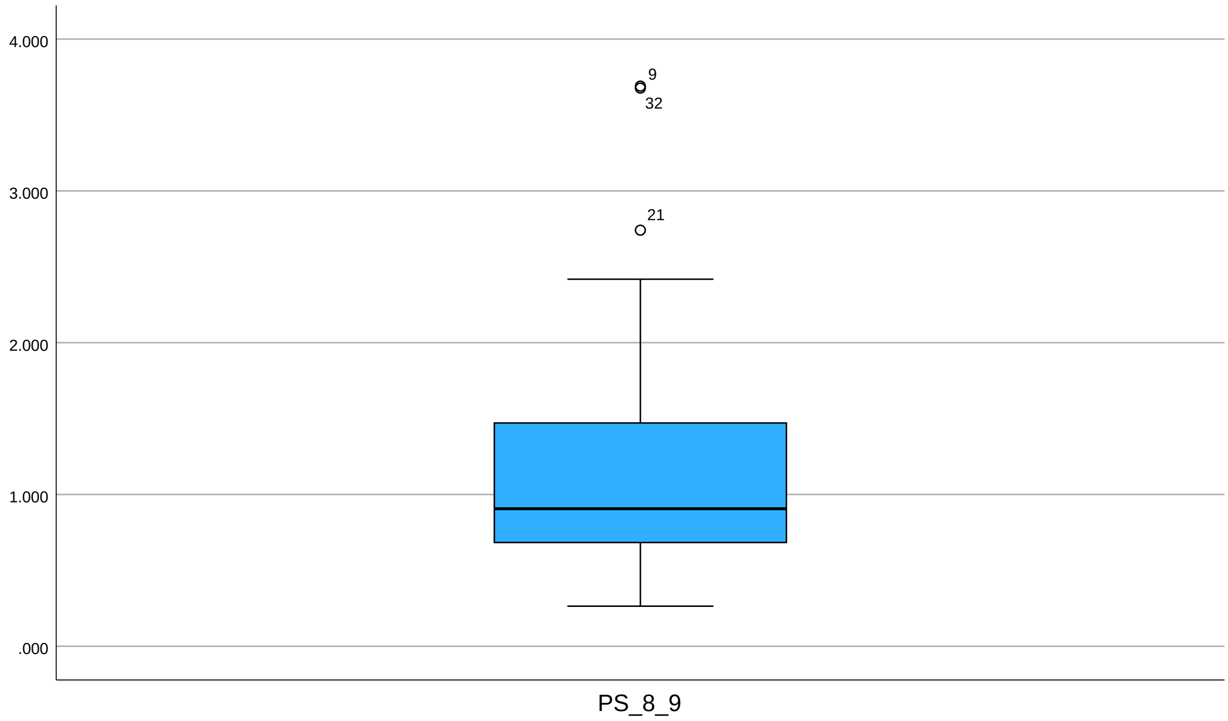

PS_9_10

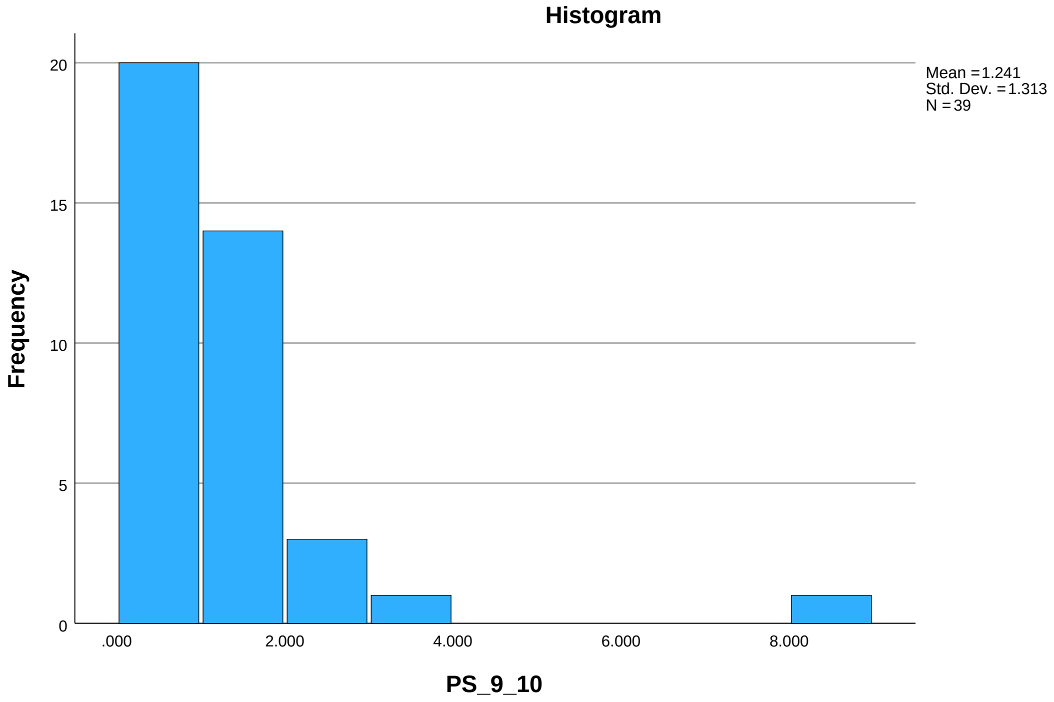

PS_9_10 Stem-and-Leaf Plot

Frequency   Stem & Leaf

```
    9.00        0 .  223334444
   11.00        0 .  55567888999
   11.00        1 .  00001111234
    3.00        1 .  789
    3.00        2 .  034
    2.00 Extremes    (>=3.3)

 Stem width:      1.000
 Each leaf:       1 case(s)
```

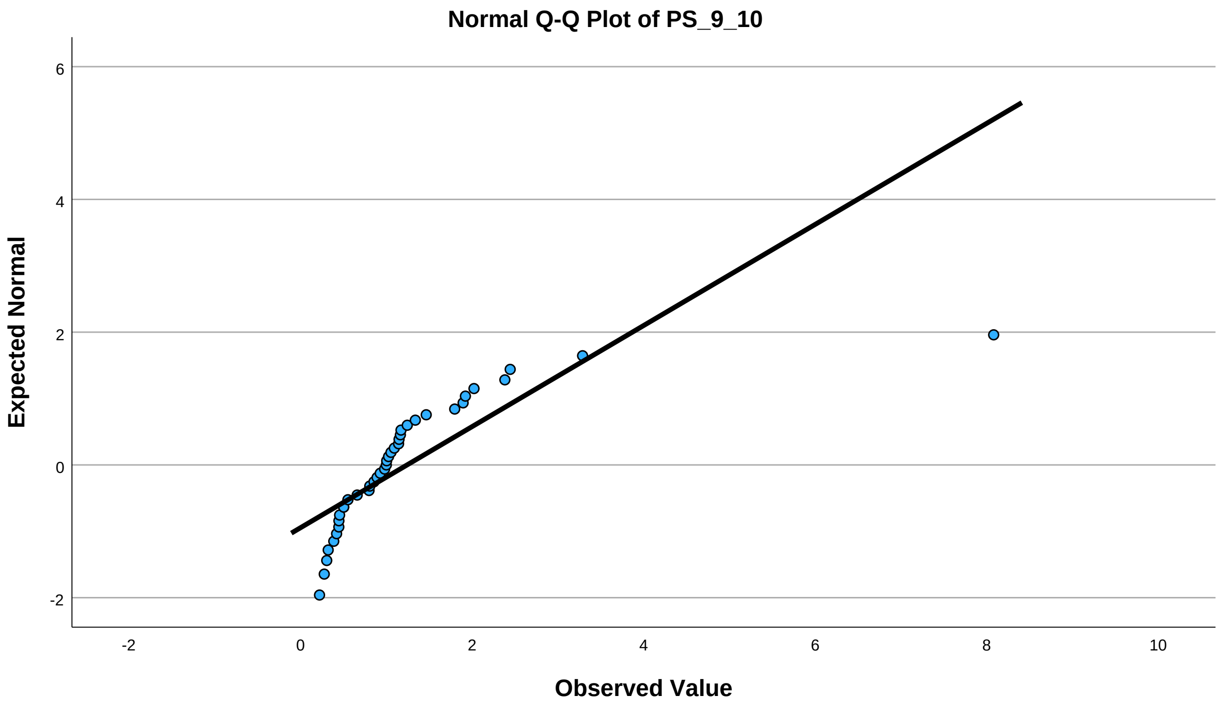

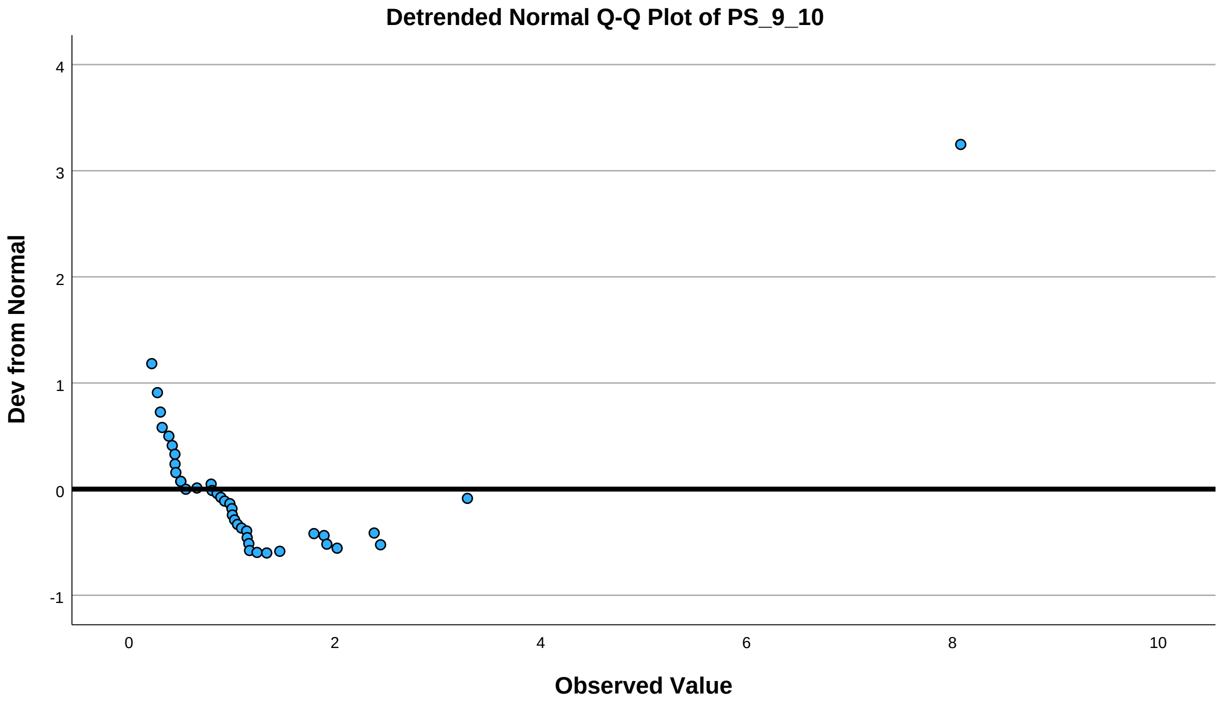

Detrended Normal Q-Q Plot of PS_9_10
Dev from Normal
Observed Value
4
3
2
1
0
-1
0
2
4
6
8
10

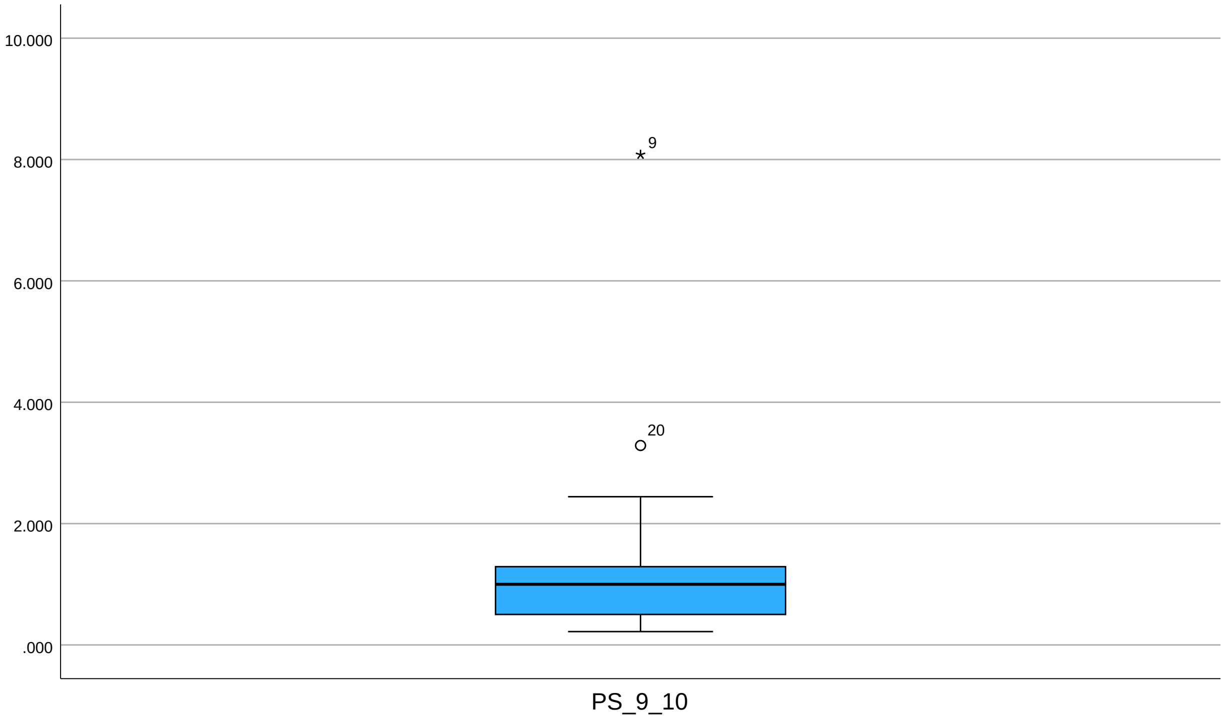

10.000
8.000
6.000
4.000
2.000
.000
9
20
PS_9_10

PS_10_11

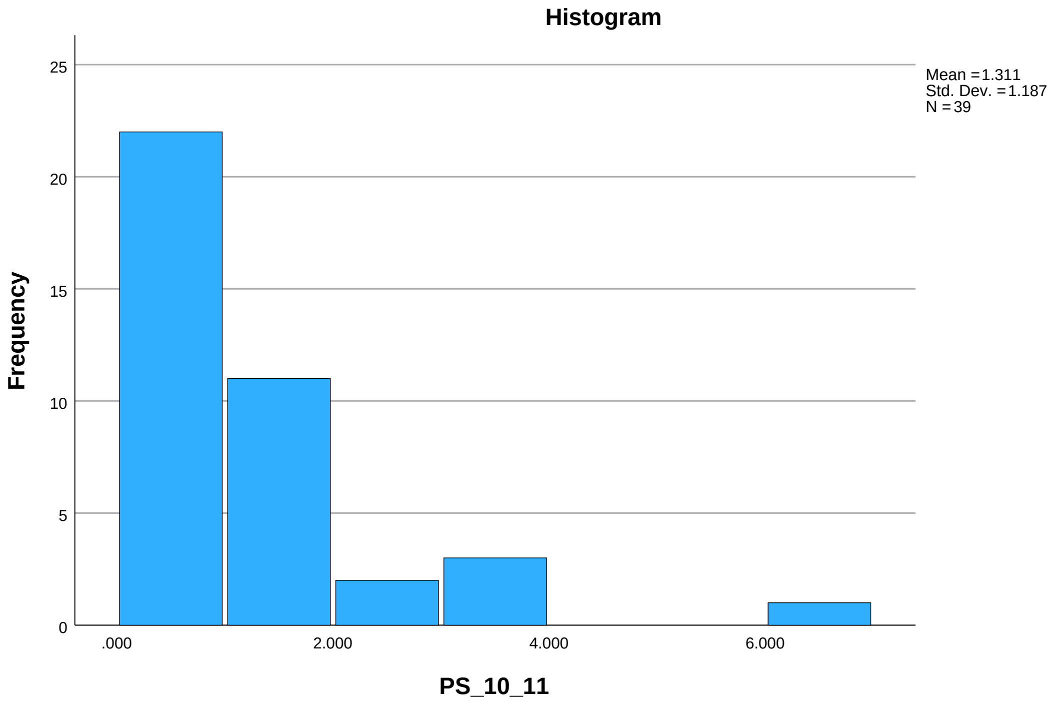


```
PS_10_11 Stem-and-Leaf Plot

 Frequency    Stem &  Leaf

     8.00        0 .  22333444
    14.00        0 .  55667777888899
     6.00        1 .  012234
     5.00        1 .  56677
      .00        2 .
     2.00        2 .  58
     1.00        3 .  0
     3.00 Extremes    (>=3.1)

 Stem width:      1.000
 Each leaf:        1 case(s)
```

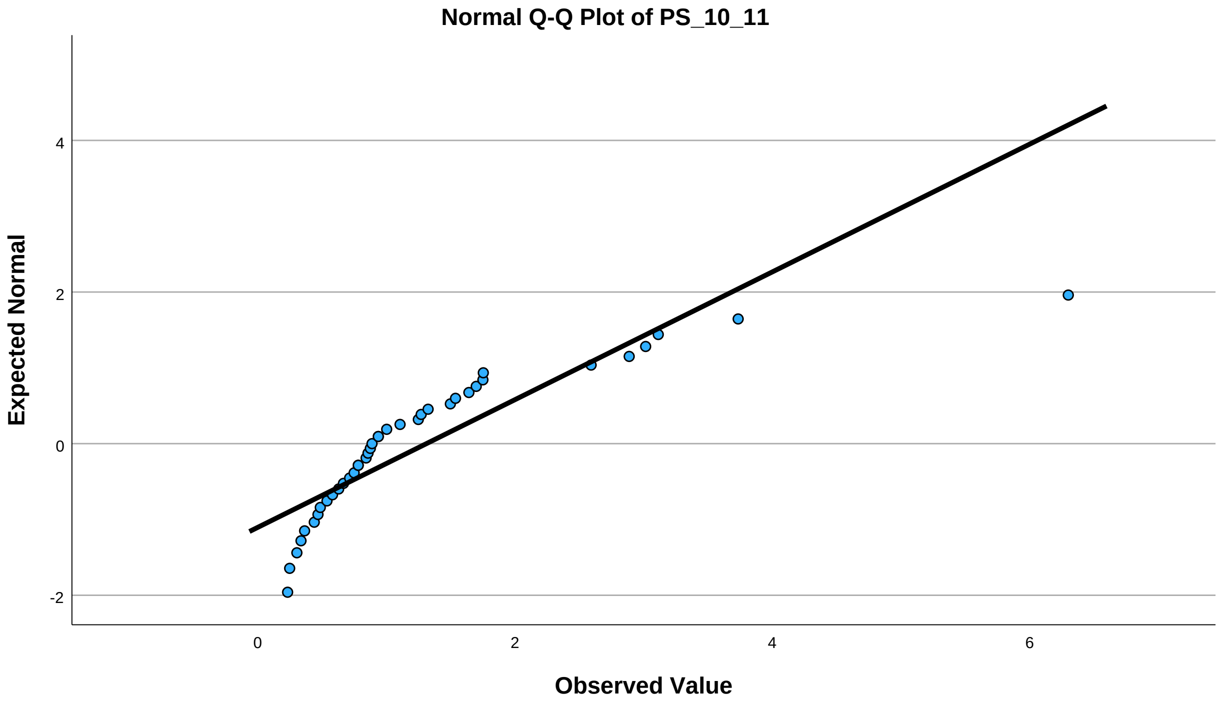
Normal Q-Q Plot of PS_10_11
Expected Normal
Observed Value
4
2
0
-2
0
2
4
6

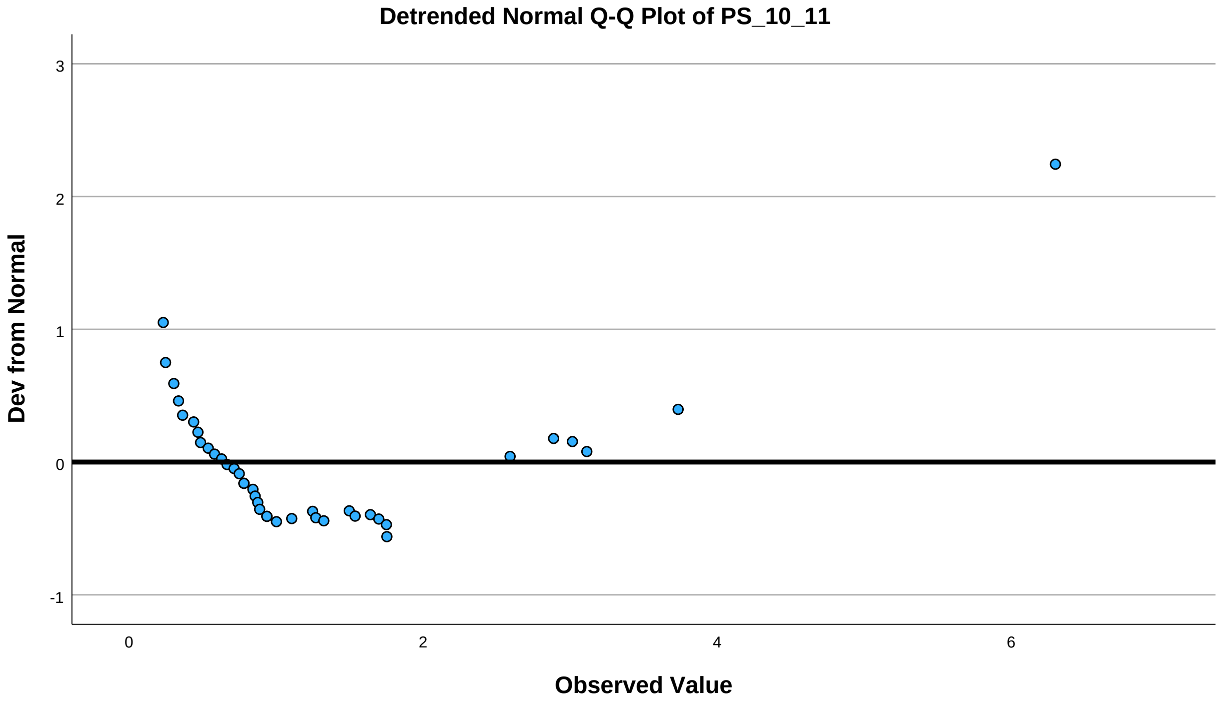
Detrended Normal Q-Q Plot of PS_10_11
Dev from Normal
Observed Value
3
2
1
0
-1
0
2
4
6

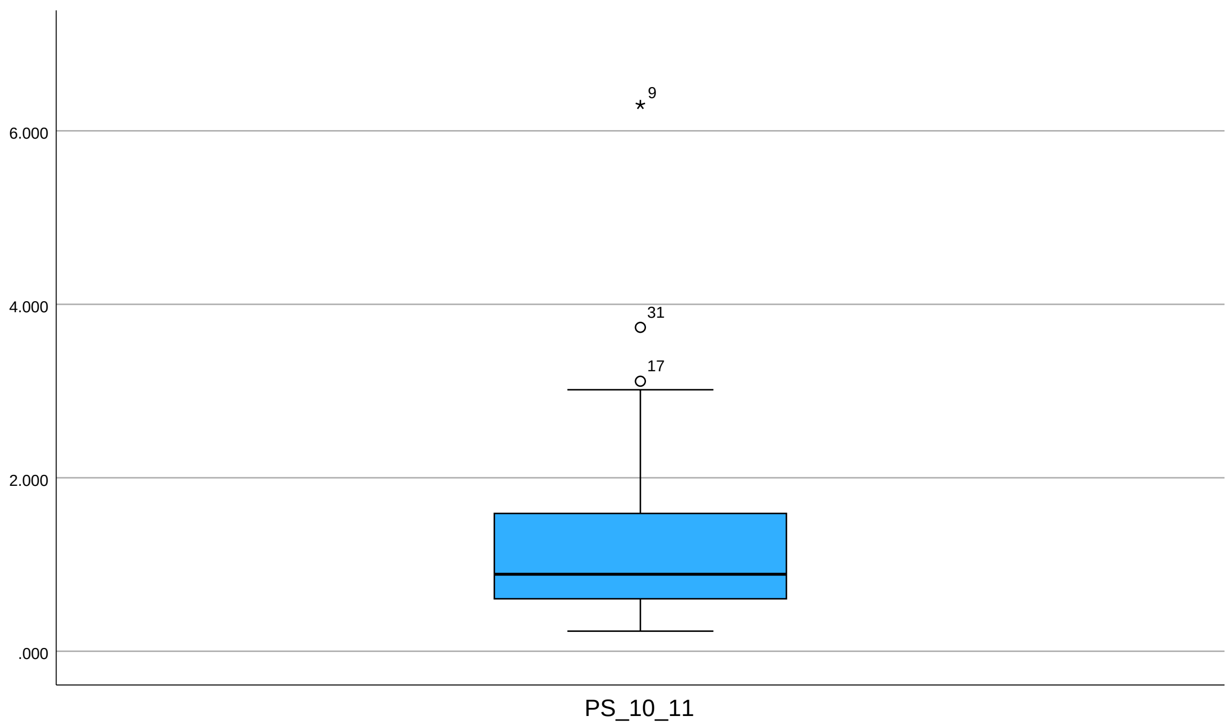

PS_11_12

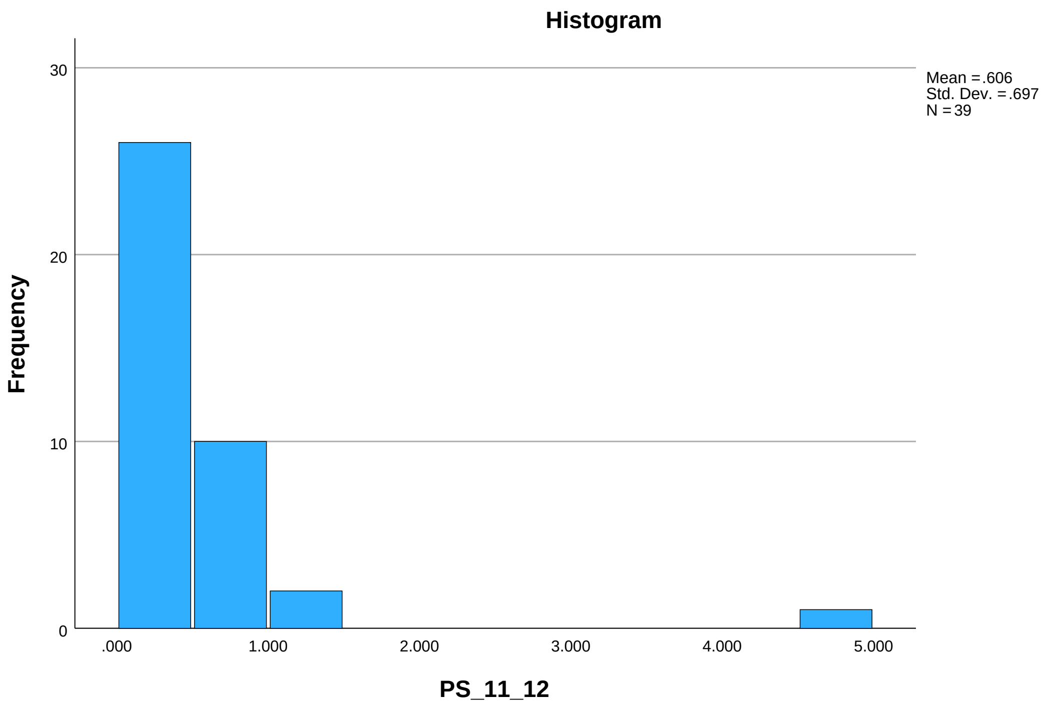

PS_11_12 Stem-and-Leaf Plot

Frequency   Stem & Leaf

```
    2.00        1 .  56
     7.00        2 .  0045699
     6.00        3 .  333679
    11.00         4 .  00112345679
     3.00        5 .  588
    1.00       6 .  2
    1.00       7 .  1
     3.00        8 .  599
    2.00        9 .  39
     3.00 Extremes    (>=1.22)

Stem width:      .100
Each leaf:       1 case(s)
```

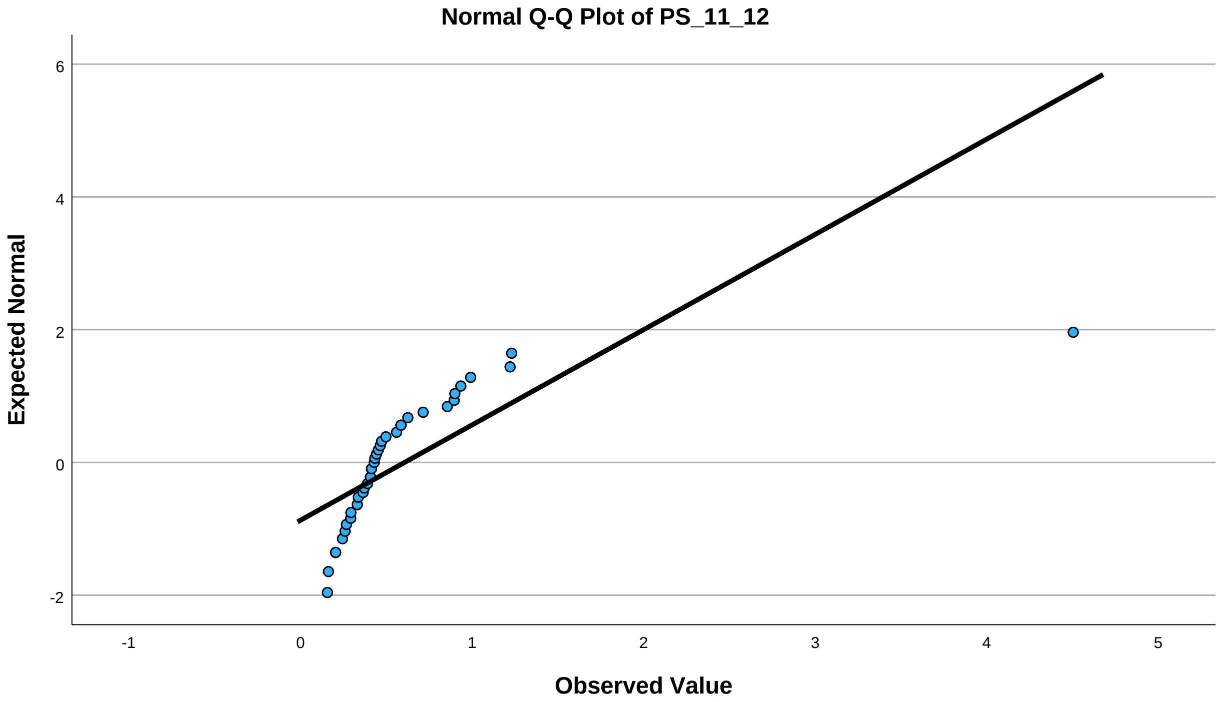

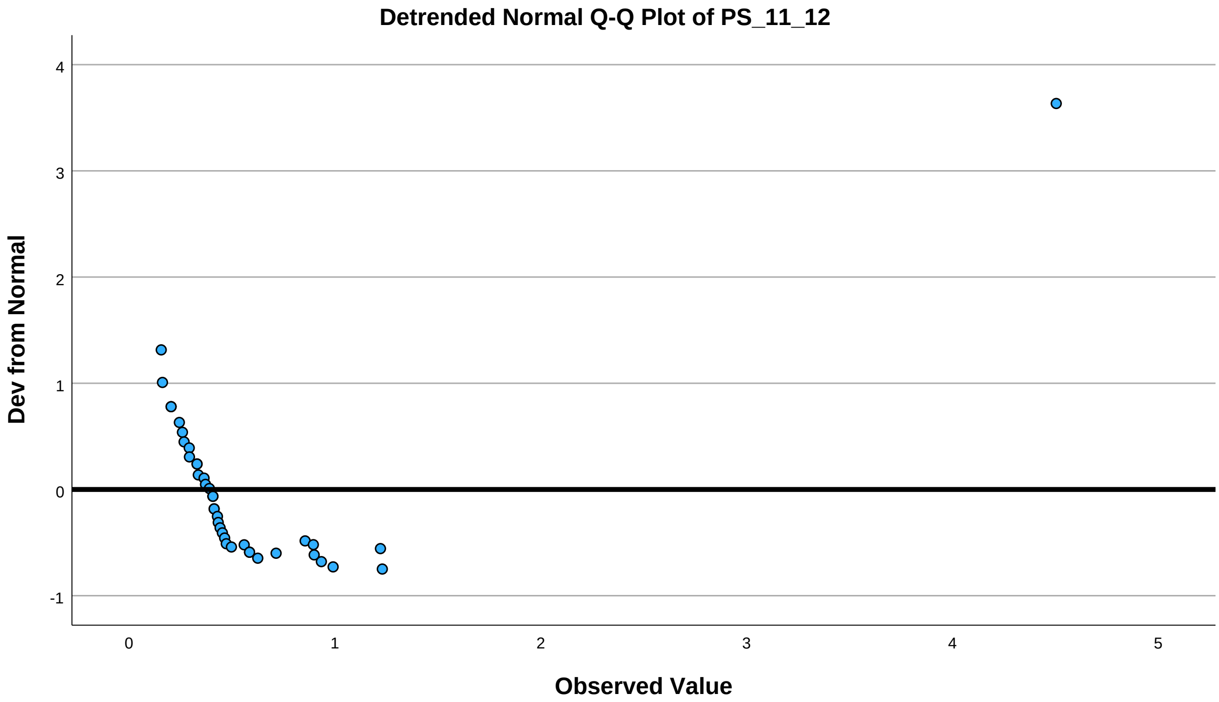
Detrended Normal Q-Q Plot of PS_11_12
Dev from Normal
4
3
2
1
0
-1
0
1
2
3
4
5
Observed Value

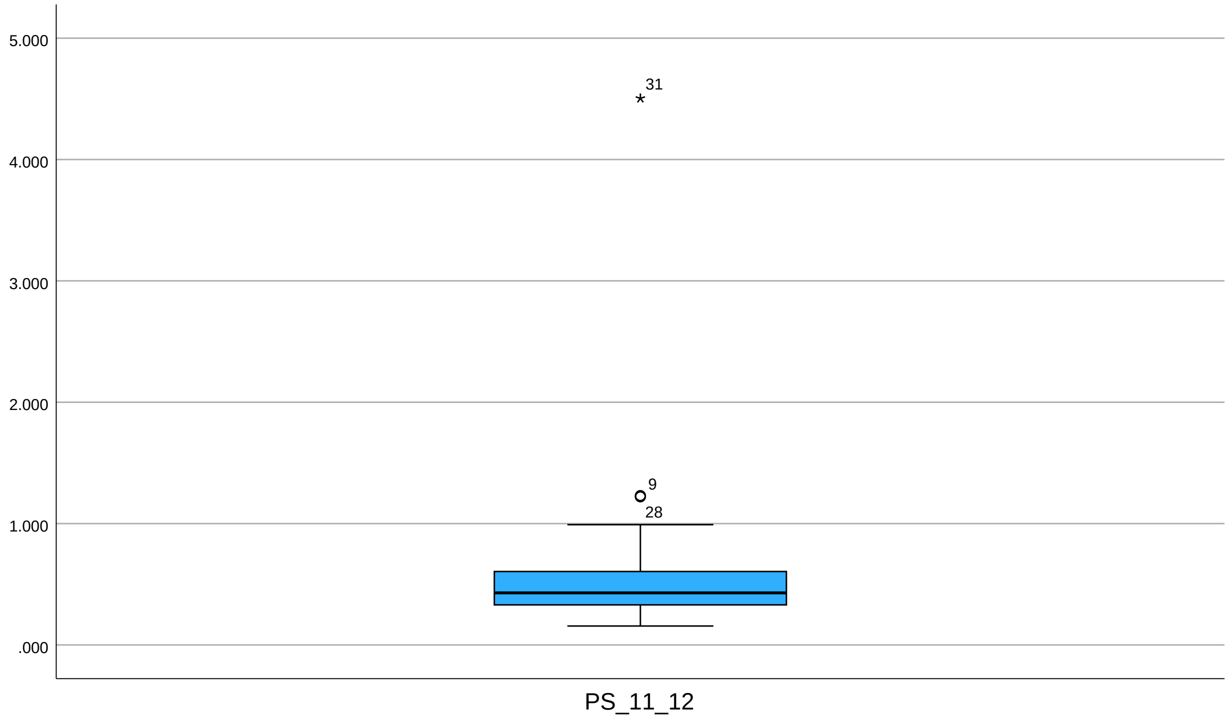
5.000
4.000
3.000
2.000
1.000
.000
31
9
28
PS_11_12

PS_12_13

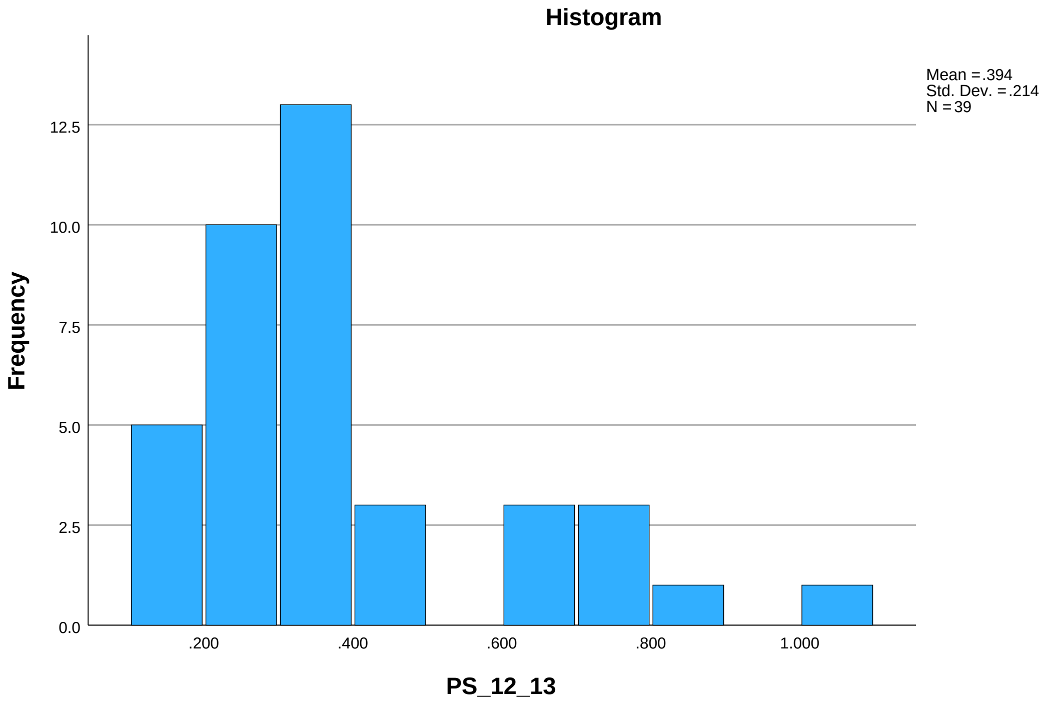


```
PS_12_13 Stem-and-Leaf Plot

 Frequency    Stem &  Leaf

     2.00        1 .  14
     3.00        1 .  599
     3.00        2 .  144
     7.00        2 .  5777899
     7.00        3 .  0001113
     6.00        3 .  567888
     1.00        4 .  2
     2.00        4 .  69
      .00        5 .
      .00        5 .
     2.00        6 .  14
     1.00        6 .  8
     5.00 Extremes    (>=.75)

 Stem width:      .100
 Each leaf:       1 case(s)
```

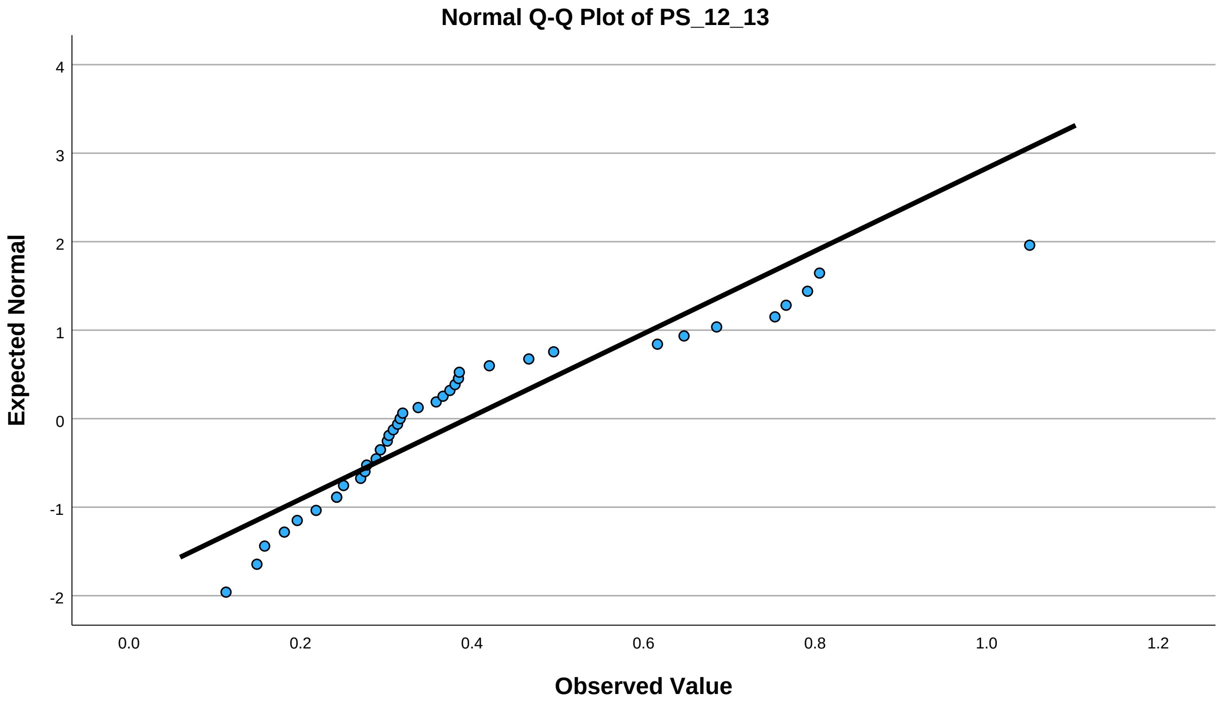

Normal Q-Q Plot of PS_12_13
Expected Normal
4
3
2
1
0
-1
-2
0.0
0.2
0.4
0.6
0.8
1.0
1.2
Observed Value

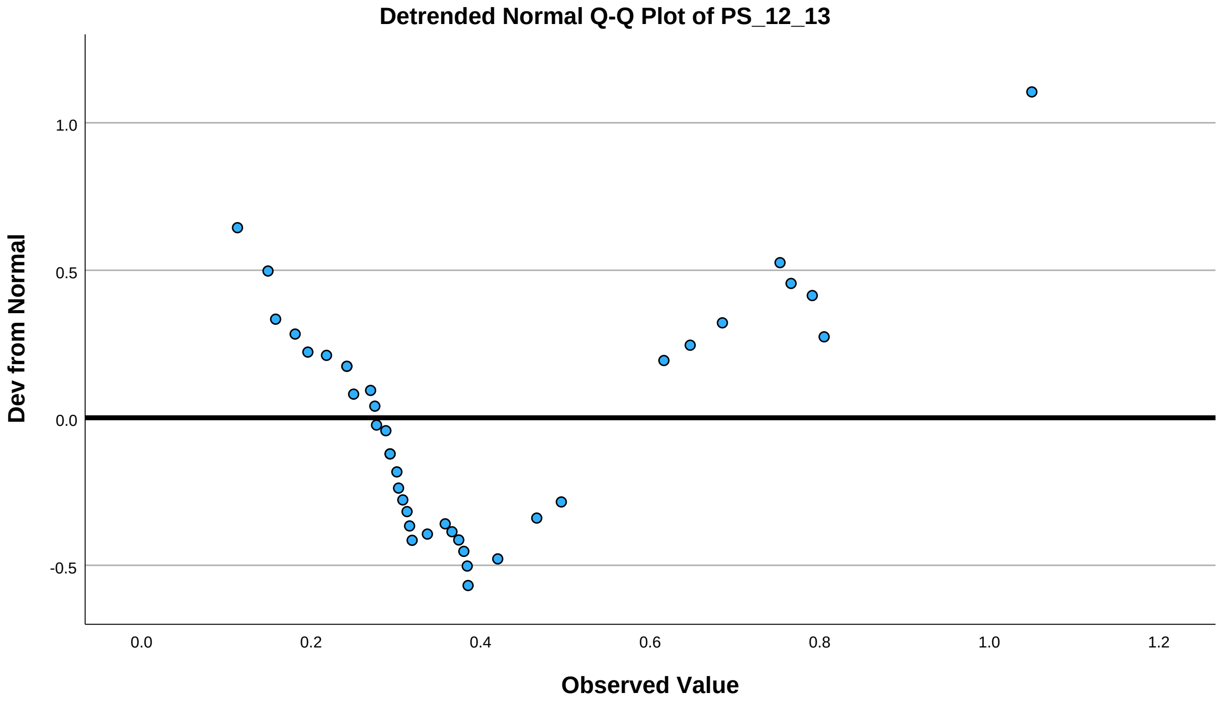

Detrended Normal Q-Q Plot of PS_12_13
Dev from Normal
1.0
0.5
0.0
-0.5
0.0
0.2
0.4
0.6
0.8
1.0
1.2
Observed Value

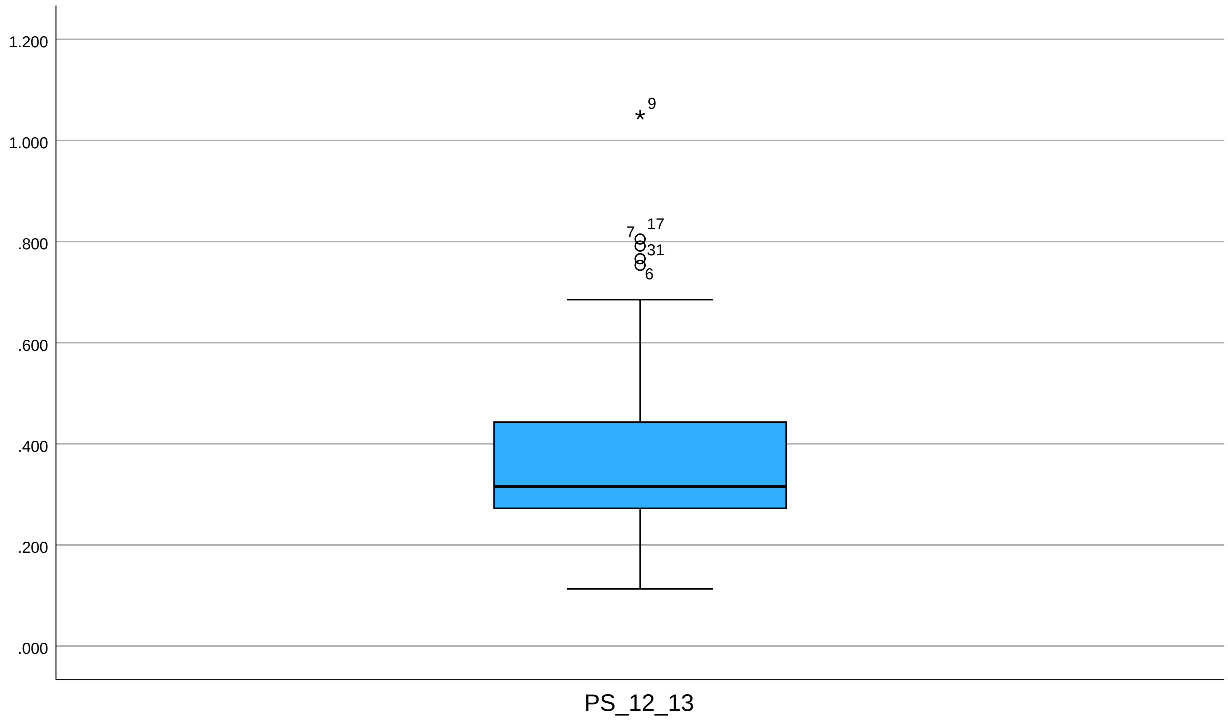

PS_13_14

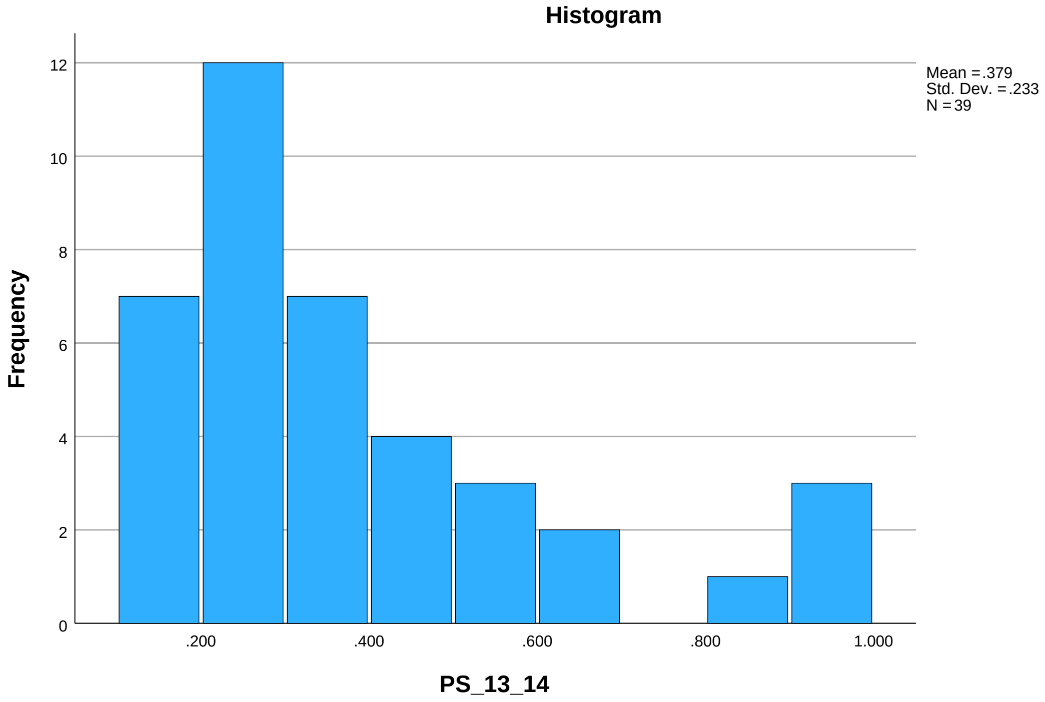

PS_13_14 Stem-and-Leaf Plot

Frequency   Stem & Leaf

```
  3.00        1 .  023
  4.00        1 .  6779
  7.00        2 .  0122333
  5.00        2 .  56688
  5.00        3 .  00123
  2.00        3 .  69
  2.00        4 .  22
  2.00        4 .  69
  1.00        5 .  2
  2.00        5 .  56
  1.00        6 .  0
  1.00        6 .  5
  4.00 Extremes    (>=.87)

Stem width:      .100
Each leaf:       1 case(s)
```

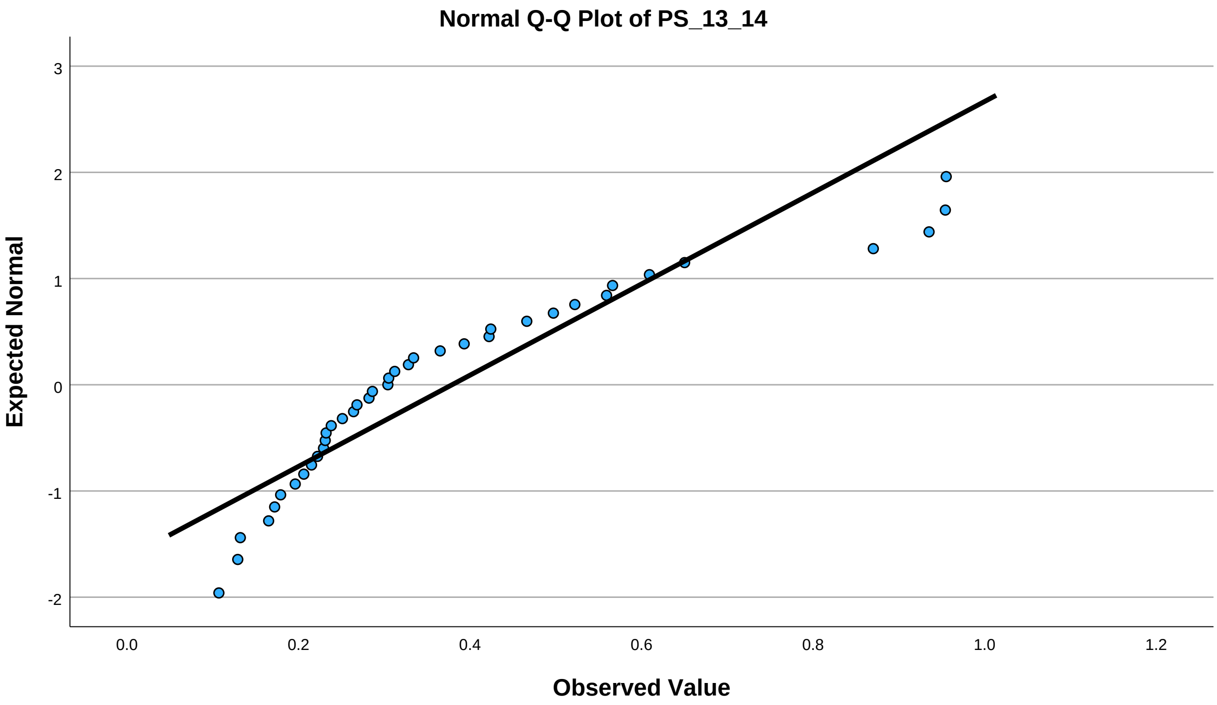

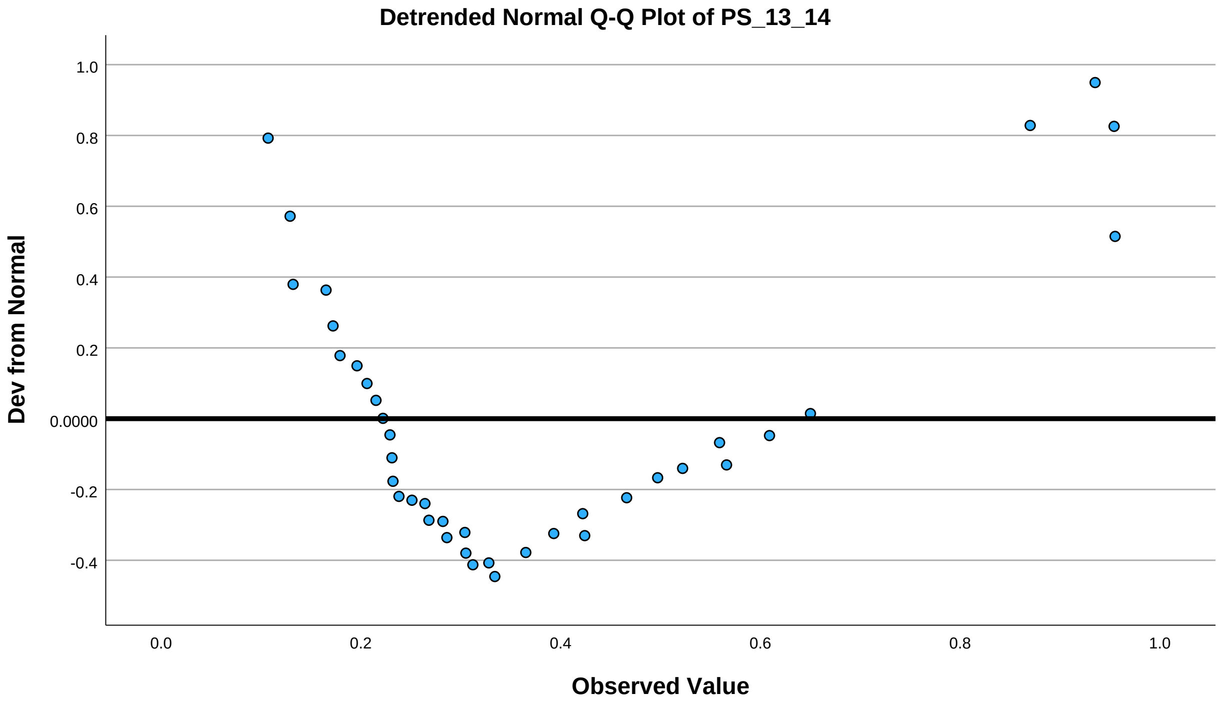
Detrended Normal Q-Q Plot of PS_13_14
Dev from Normal
Observed Value
1.0
0.8
0.6
0.4
0.2
0.0000
-0.2
-0.4
0.0
0.2
0.4
0.6
0.8
1.0

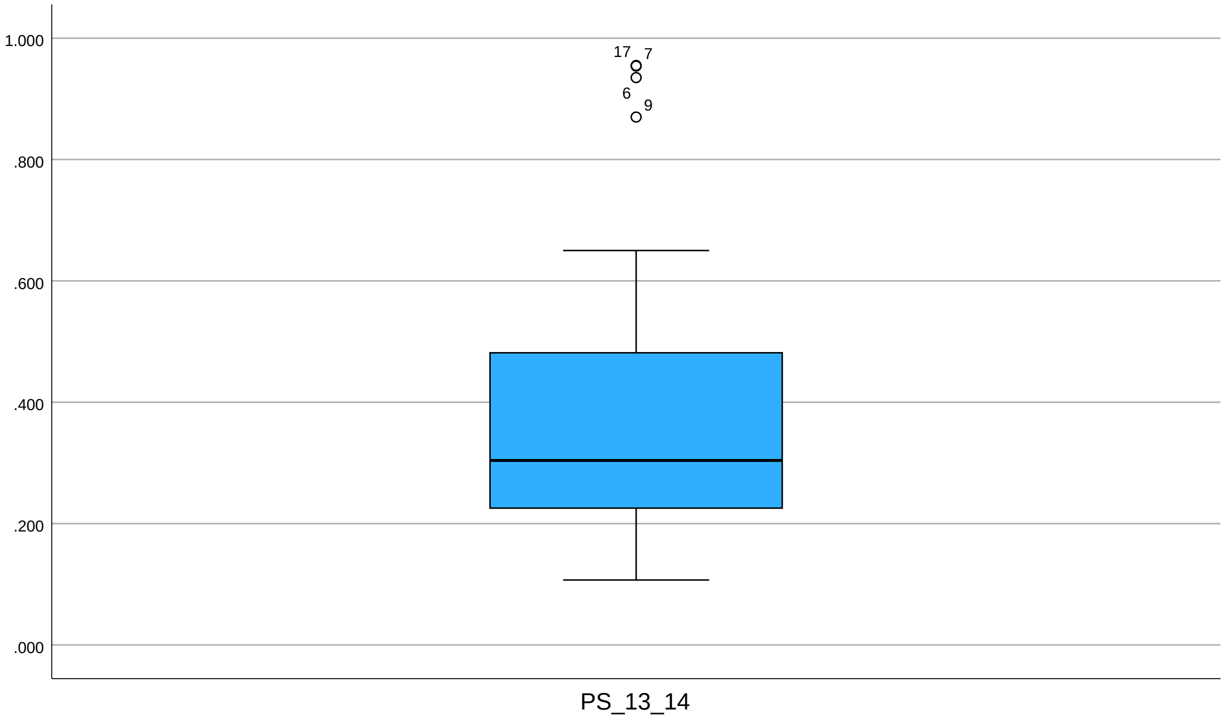
1.000
.800
.600
.400
.200
.000
17
7
6
9
PS_13_14

PS_14_15

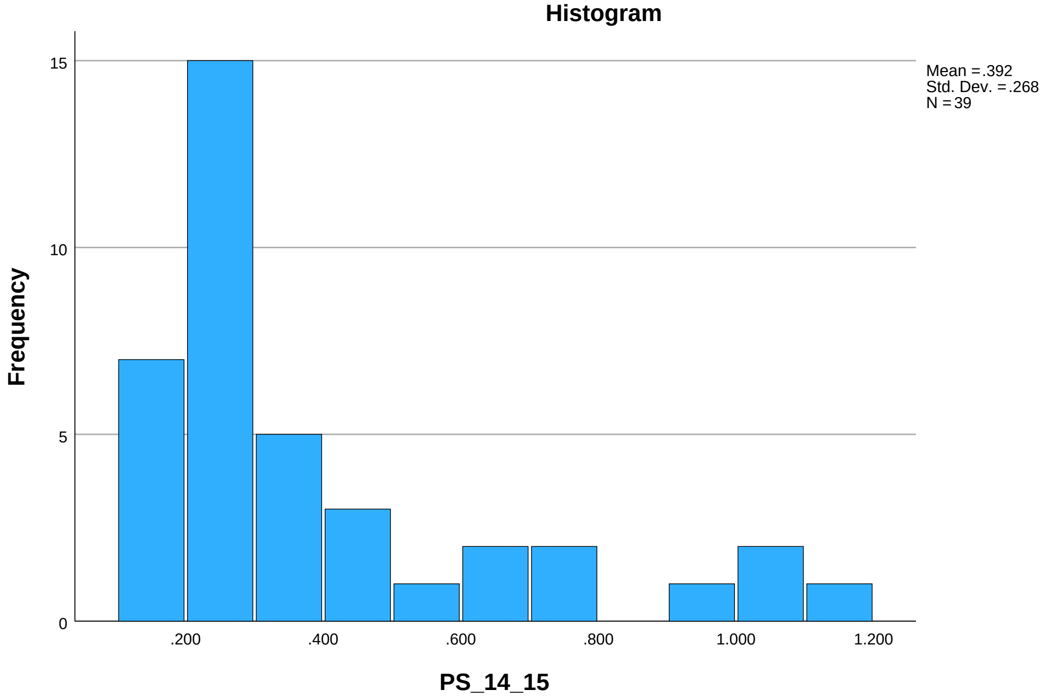


```
PS_14_15 Stem-and-Leaf Plot

 Frequency    Stem &  Leaf

     7.00        1 .  1445559
    15.00        2 .  002244556677789
     5.00        3 .  04999
     3.00        4 .  025
     1.00        5 .  1
     2.00        6 .  36
     2.00        7 .  02
     4.00 Extremes    (>=.95)

 Stem width:      .100
 Each leaf:       1 case(s)
```

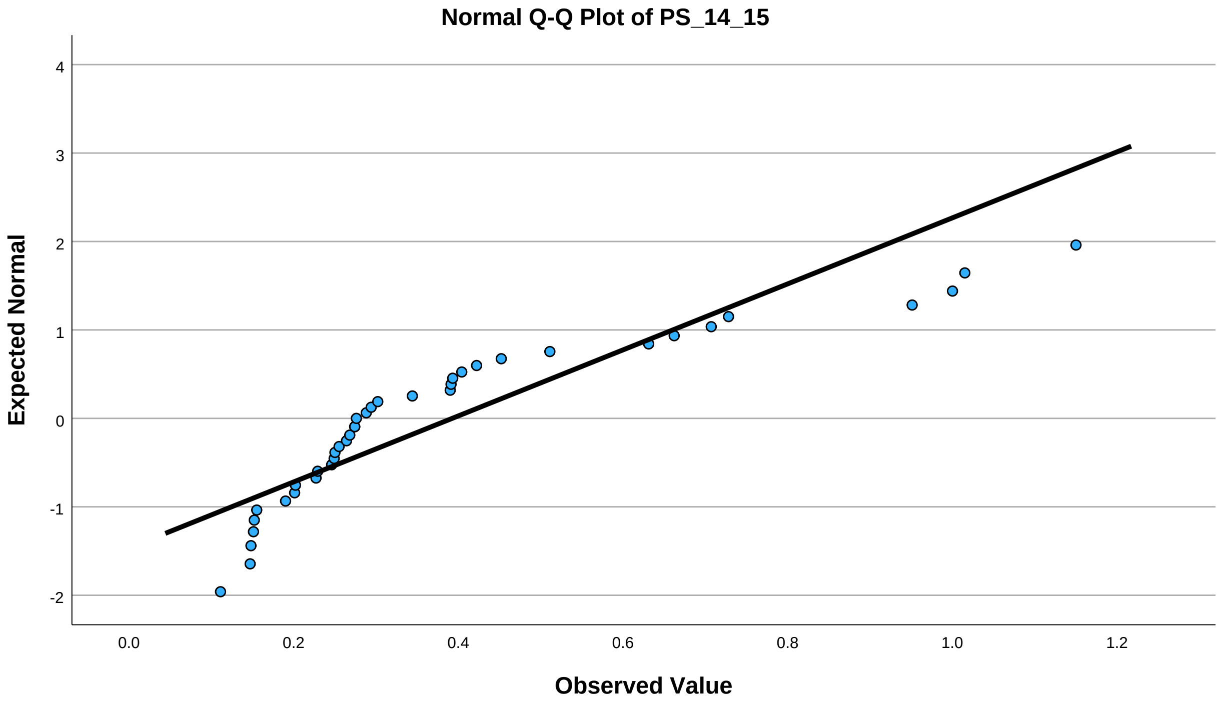
Normal Q-Q Plot of PS_14_15
Expected Normal
4
3
2
1
0
-1
-2
0.0
0.2
0.4
0.6
0.8
1.0
1.2
Observed Value

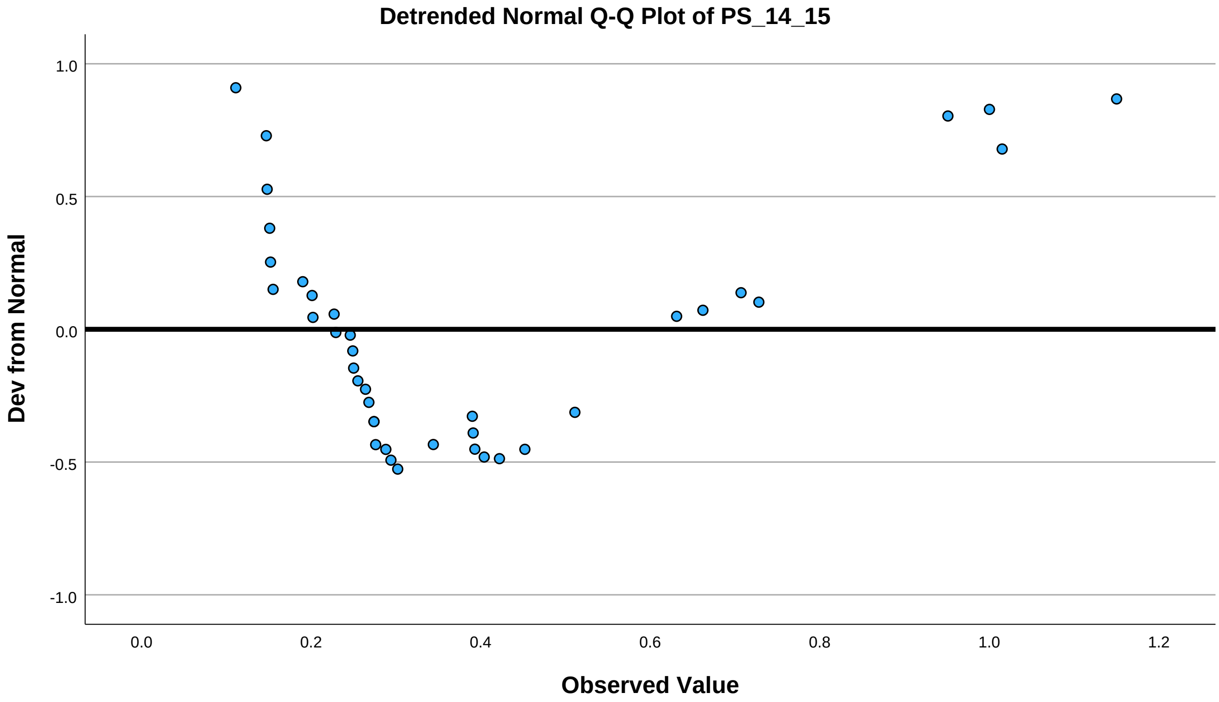
Detrended Normal Q-Q Plot of PS_14_15
Dev from Normal
1.0
0.5
0.0
-0.5
-1.0
0.0
0.2
0.4
0.6
0.8
1.0
1.2
Observed Value

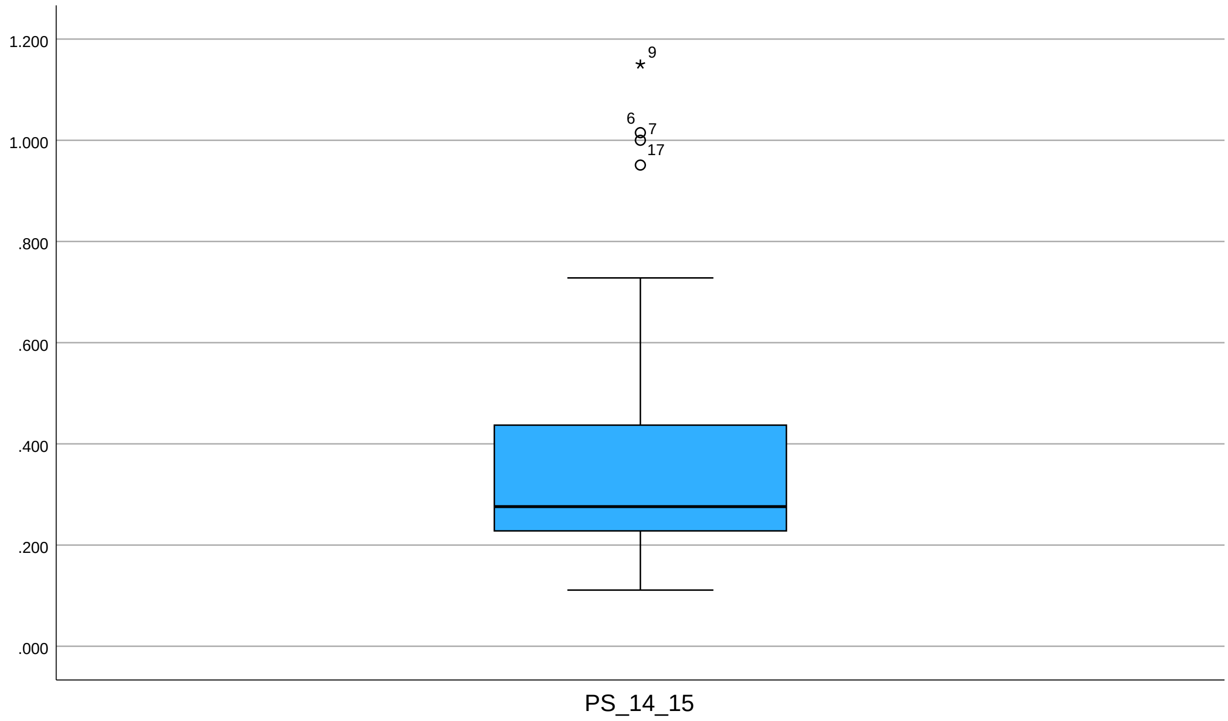

PS_15_16

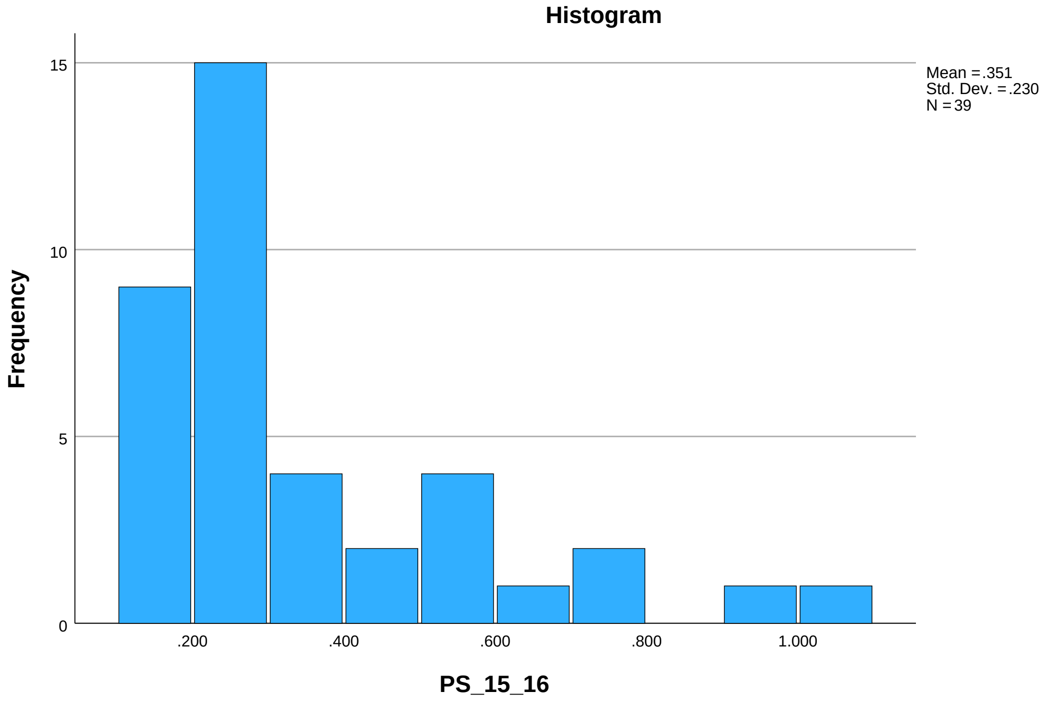

PS_15_16 Stem-and-Leaf Plot

Frequency   Stem & Leaf

```
     9.00        1 .  022356788
    15.00        2 .  011122333567999
     4.00        3 .  3359
     2.00        4 .  46
     4.00        5 .  1467
     1.00        6 .  4
     2.00        7 .  23
     2.00 Extremes    (>=1.00)

 Stem width:       .100
 Each leaf:        1 case(s)
```

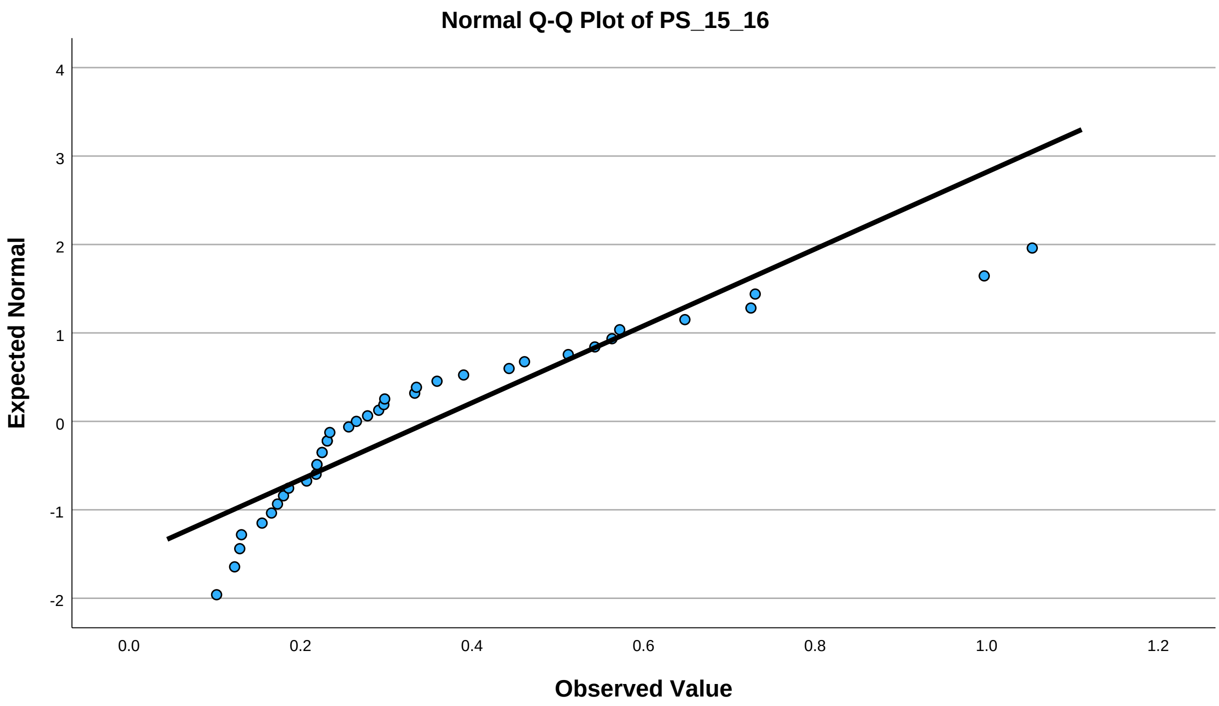

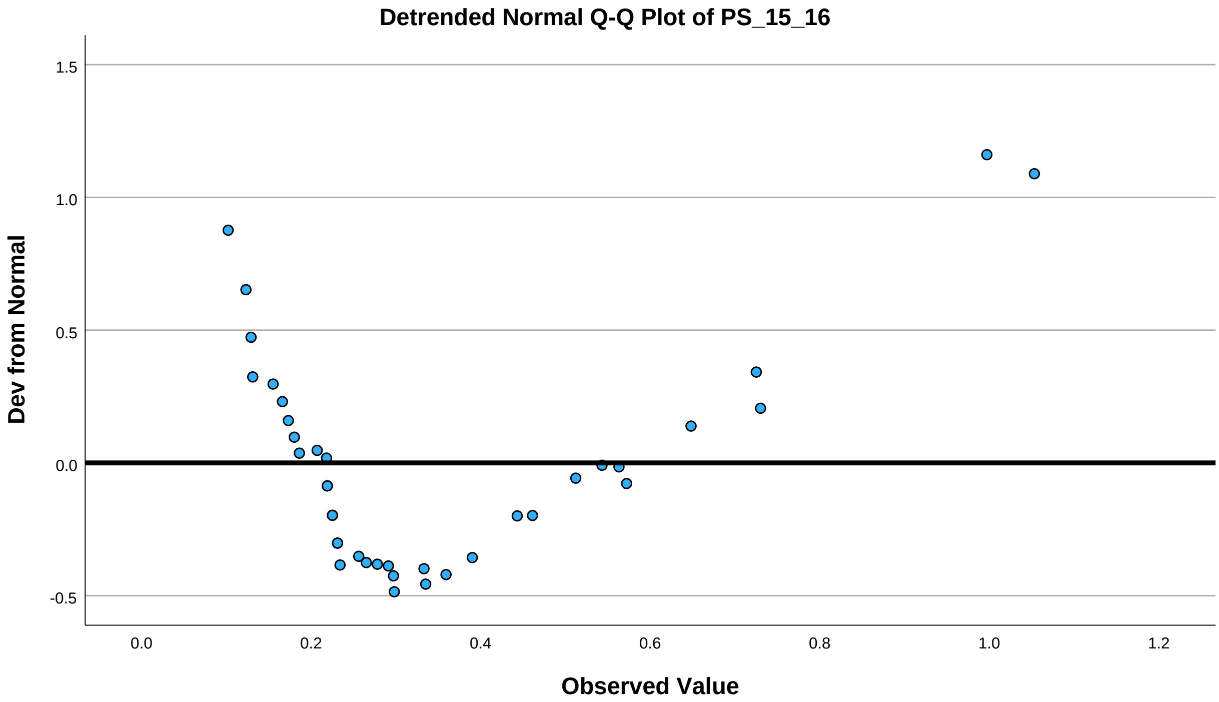
Detrended Normal Q-Q Plot of PS_15_16
Dev from Normal
1.5
1.0
0.5
0.0
-0.5
0.0
0.2
0.4
0.6
0.8
1.0
1.2
Observed Value

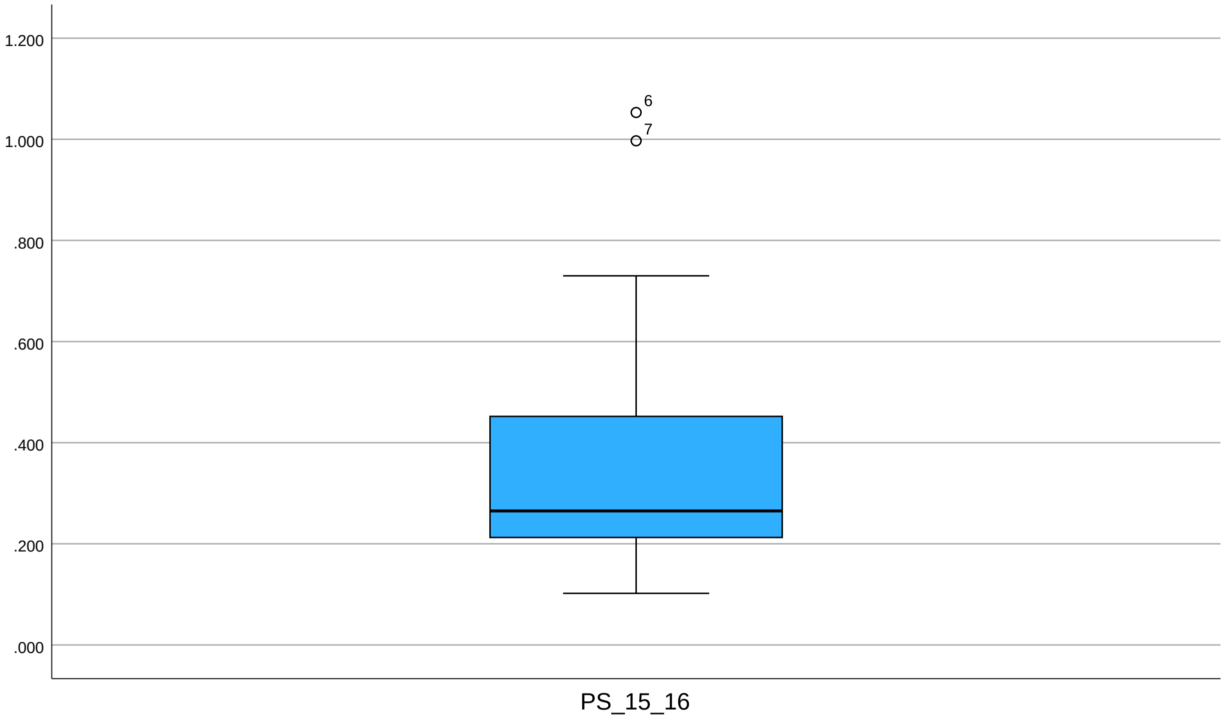
1.200
1.000
.800
.600
.400
.200
.000
6
7
PS_15_16

PS_16_17

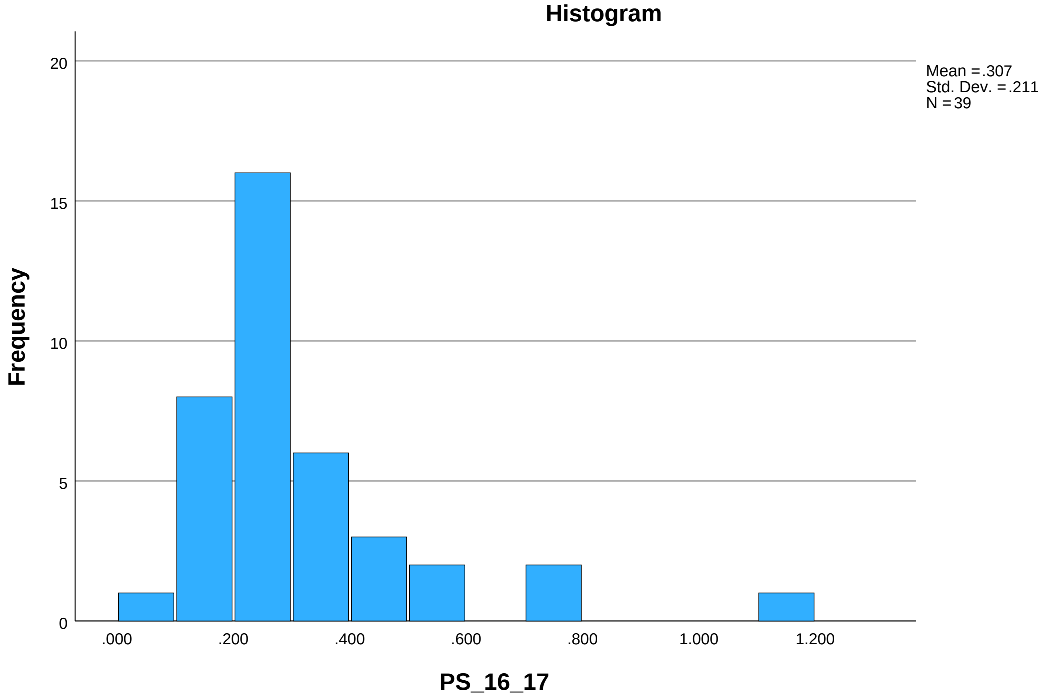

PS_16_17 Stem-and-Leaf Plot

```
 Frequency    Stem &  Leaf

     1.00        0 .  9
     5.00        1 .  12233
     3.00        1 .  679
    13.00        2 .  0001111223333
     3.00        2 .  558
     5.00        3 .  11144
     1.00        3 .  6
     2.00        4 .  02
     1.00        4 .  6
     1.00        5 .  4
     4.00 Extremes    (>=.57)

 Stem width:      .100
 Each leaf:       1 case(s)
```

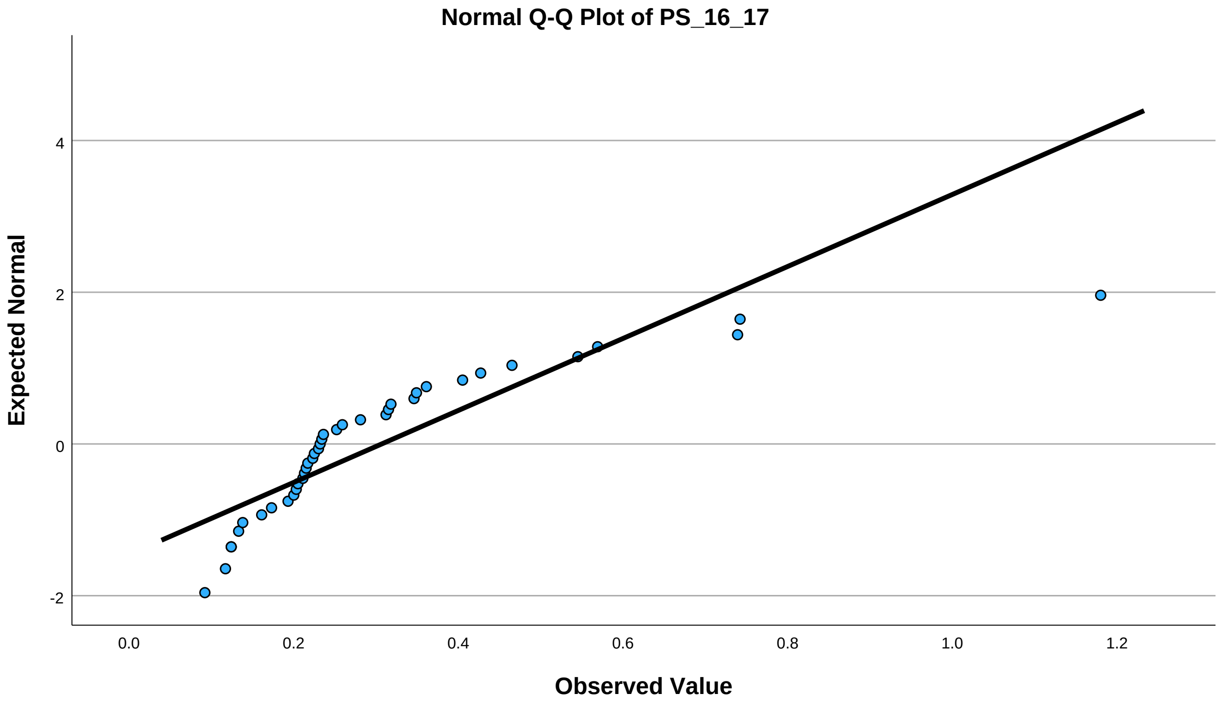
Normal Q-Q Plot of PS_16_17
Expected Normal
Observed Value
4
2
0
-2
0.0
0.2
0.4
0.6
0.8
1.0
1.2

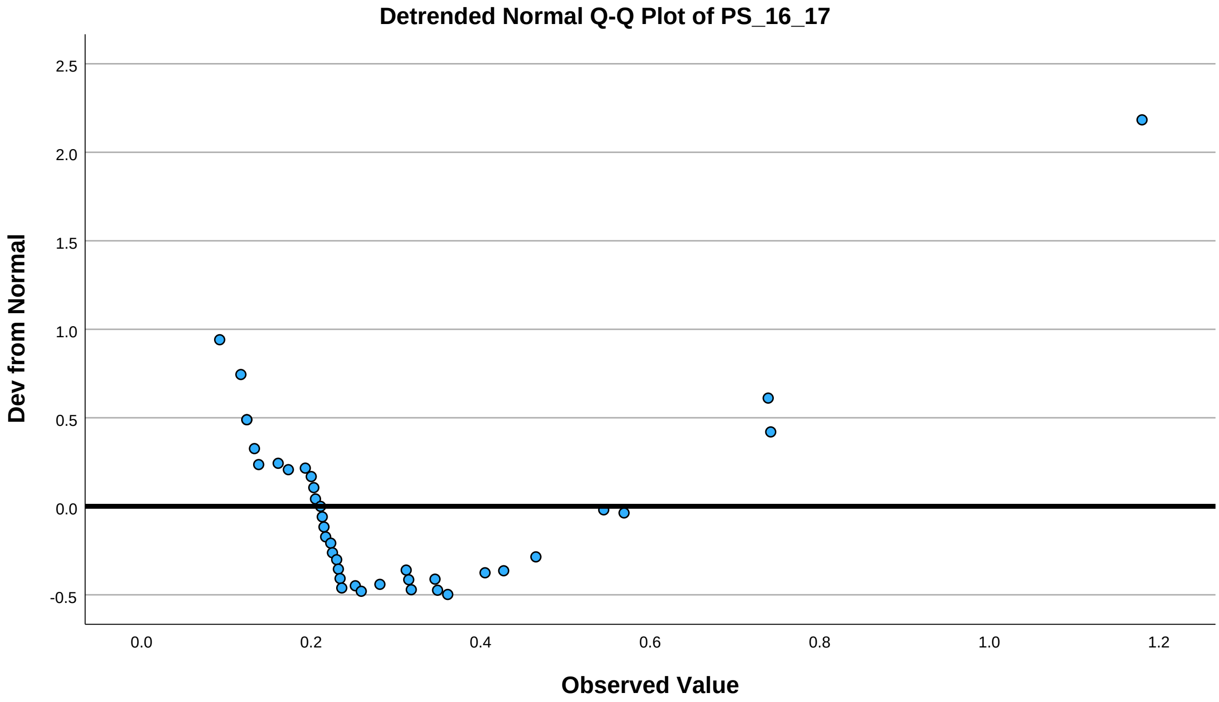
Detrended Normal Q-Q Plot of PS_16_17
Dev from Normal
Observed Value
2.5
2.0
1.5
1.0
0.5
0.0
-0.5
0.0
0.2
0.4
0.6
0.8
1.0
1.2

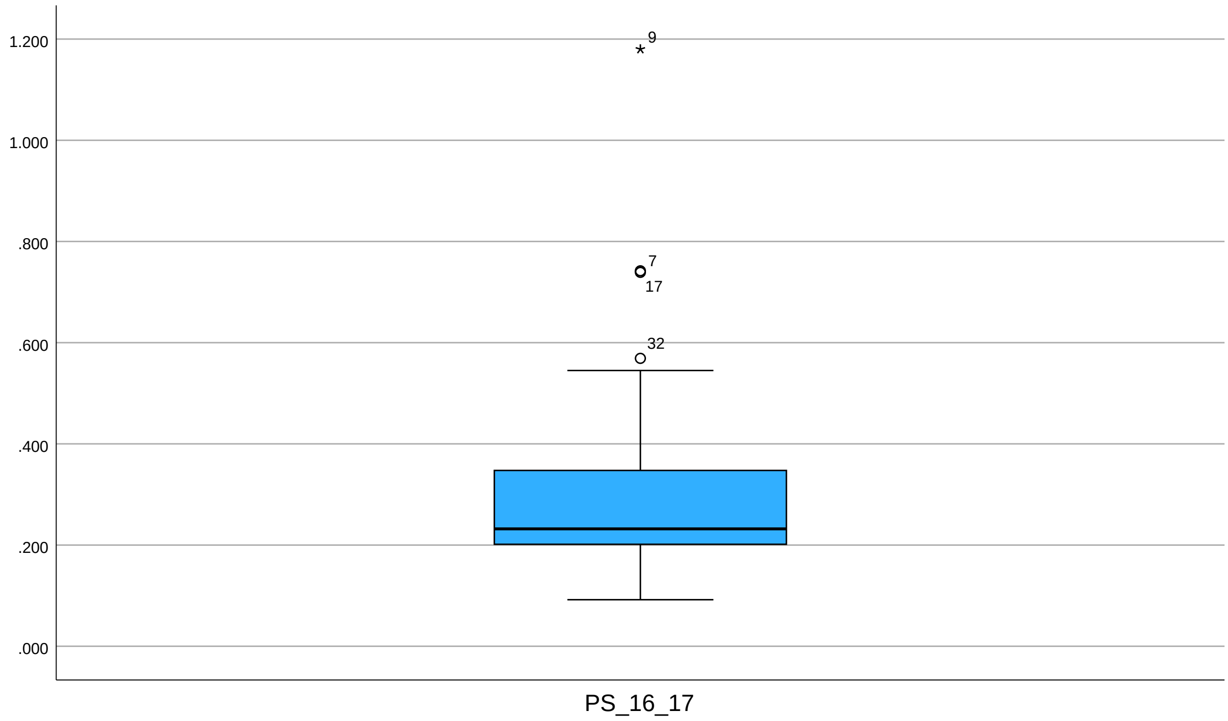

PS_17_18

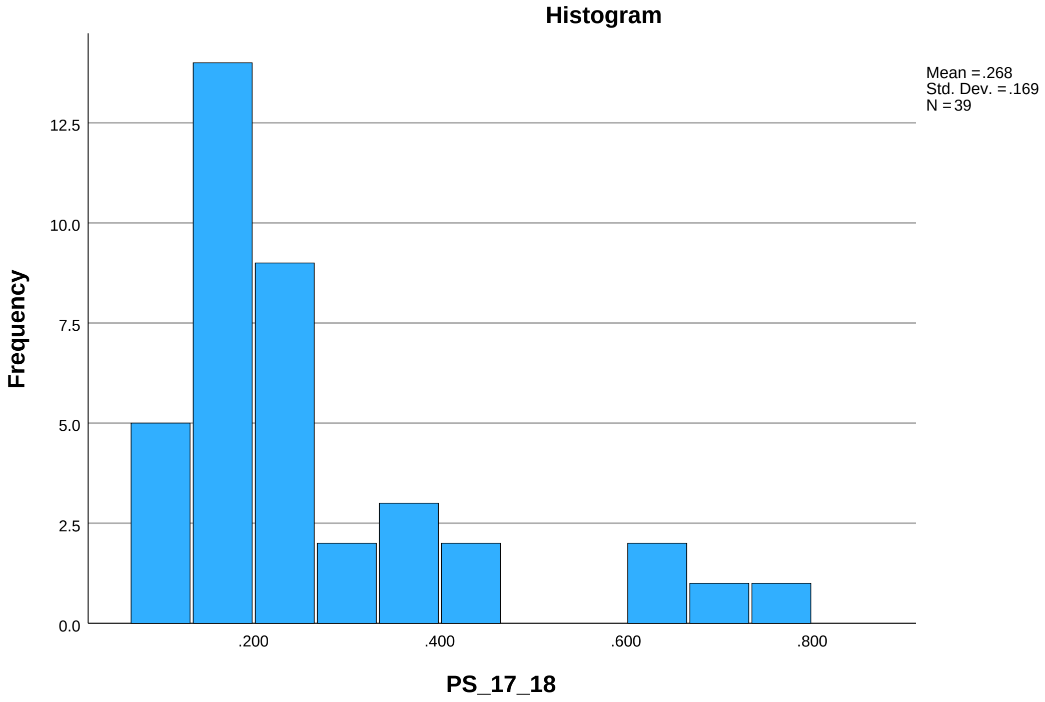

PS_17_18 Stem-and-Leaf Plot

Frequency Stem & Leaf

```
     1.00        0 .  9
      6.00         1 .  112233
     11.00          1 .  67788888999
      8.00         2 .  00002334
     3.00        2 .  666
     2.00        3 .  14
     2.00        3 .  59
     2.00        4 .  02
      4.00 Extremes     (>=.66)

 Stem width:       .100
 Each leaf:        1 case(s)
```

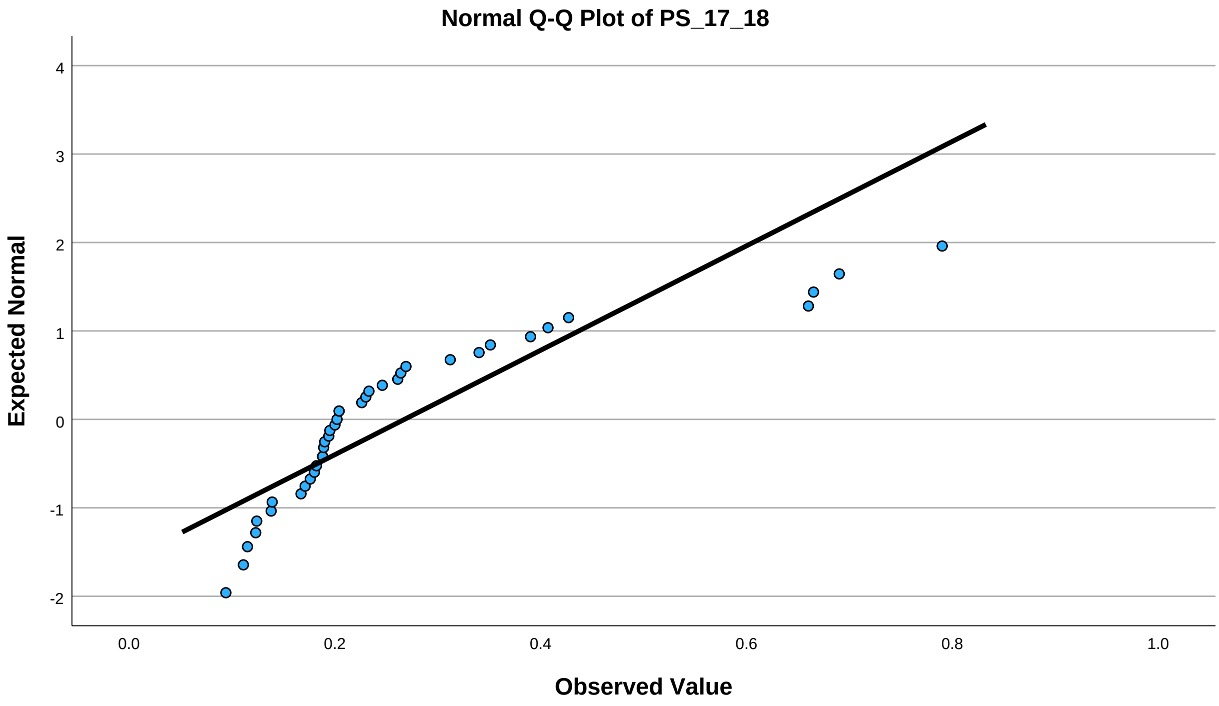

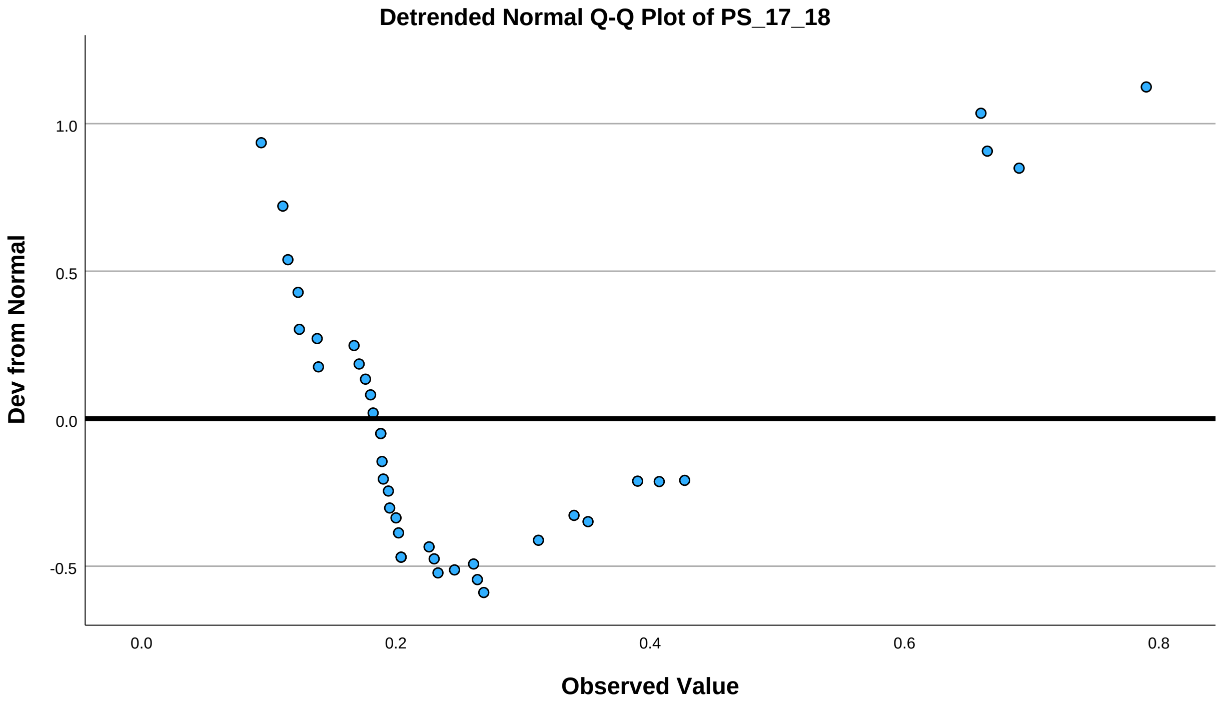
Detrended Normal Q-Q Plot of PS_17_18
Dev from Normal
1.0
0.5
0.0
-0.5
0.0
0.2
0.4
0.6
0.8
Observed Value

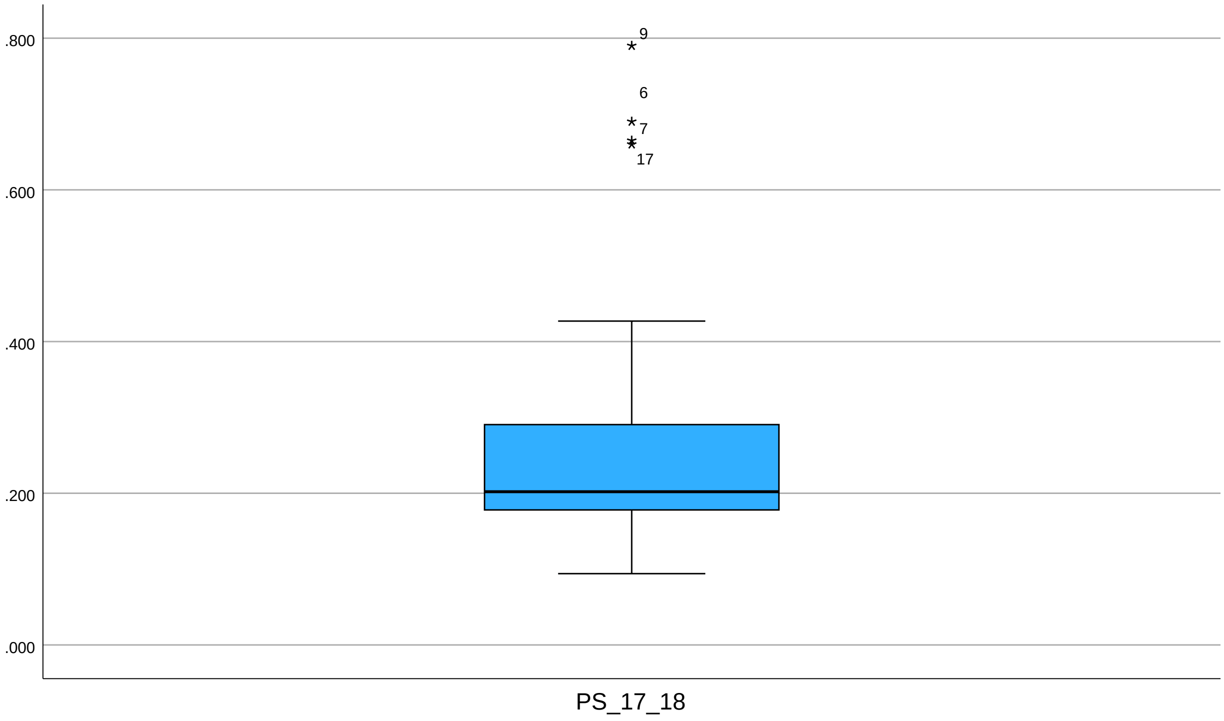
.800
.600
.400
.200
.000
9
6
7
17
PS_17_18

PS_18_19

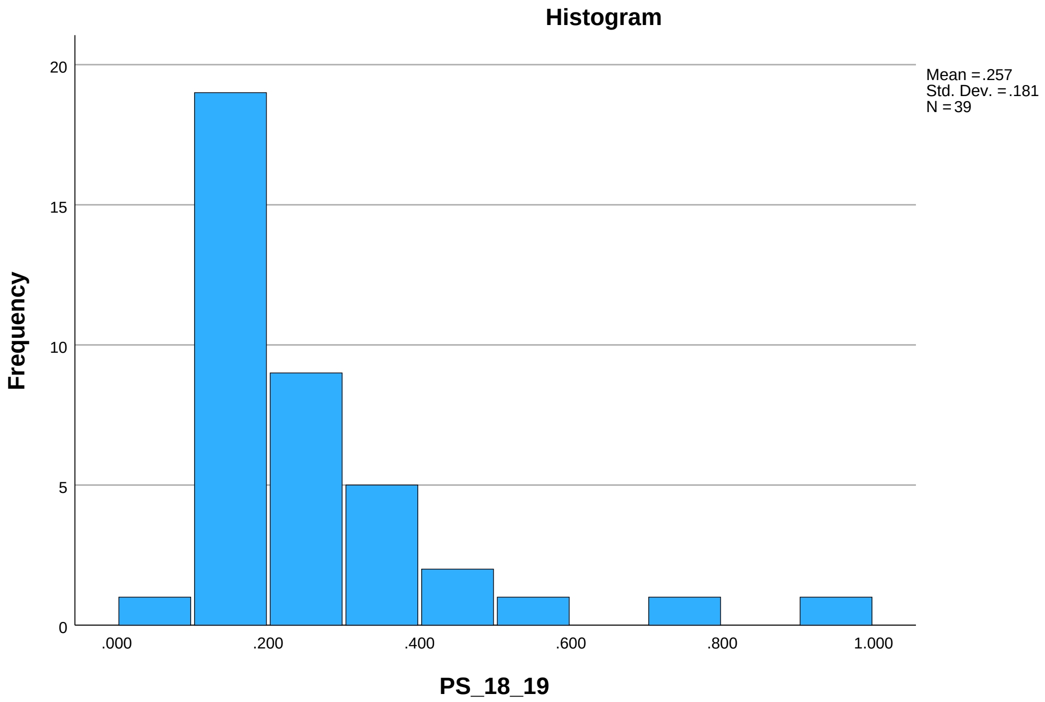


```
PS_18_19 Stem-and-Leaf Plot

 Frequency    Stem &  Leaf

     1.00        0 .  8
     8.00        1 .  00122344
    11.00        1 .  56666777799
     7.00        2 .  0111133
     2.00        2 .  59
     1.00        3 .  3
     4.00        3 .  6668
     1.00        4 .  2
     1.00        4 .  6
     3.00 Extremes    (>=.55)

 Stem width:      .100
 Each leaf:       1 case(s)
```

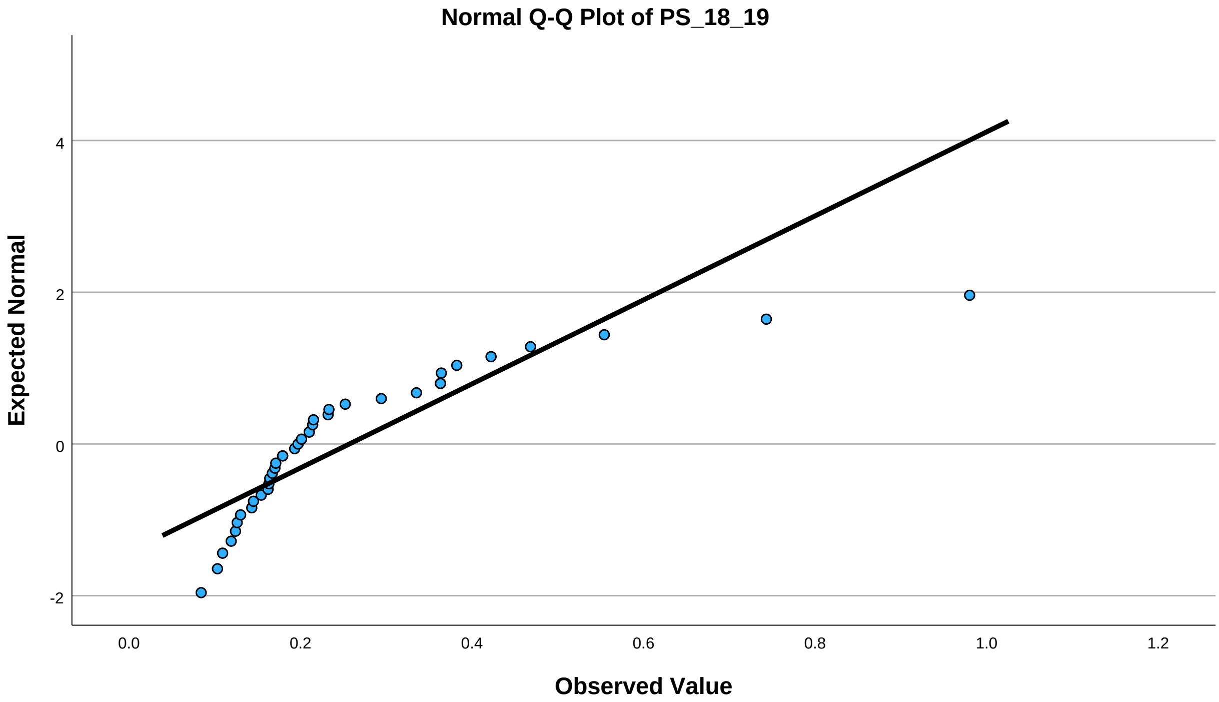
Normal Q-Q Plot of PS_18_19
Expected Normal
4
2
0
-2
0.0
0.2
0.4
0.6
0.8
1.0
1.2
Observed Value

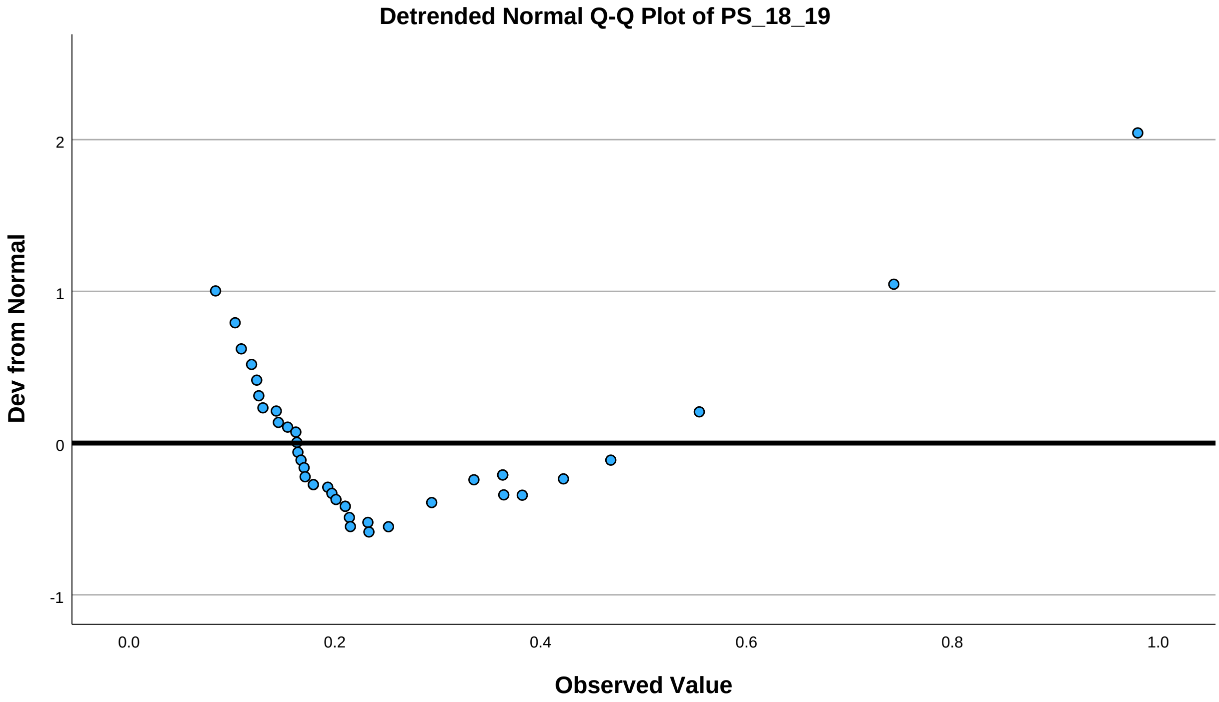
Detrended Normal Q-Q Plot of PS_18_19
Dev from Normal
2
1
0
-1
0.0
0.2
0.4
0.6
0.8
1.0
Observed Value

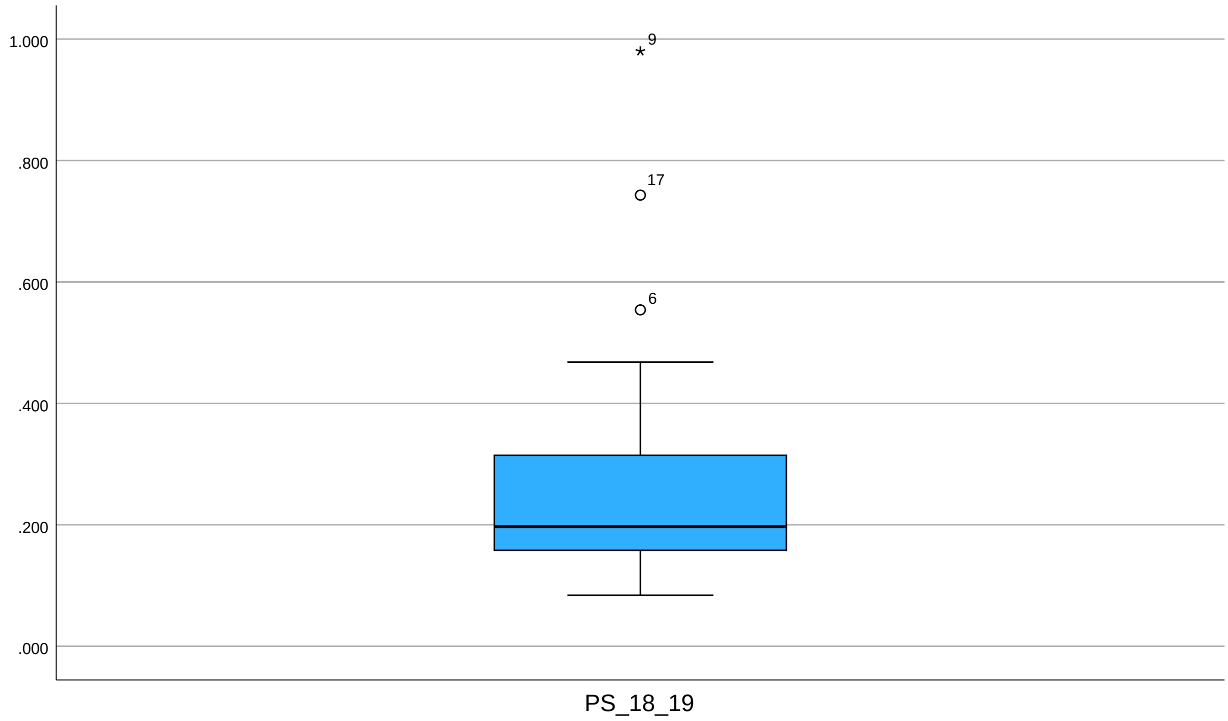

PS_19_20

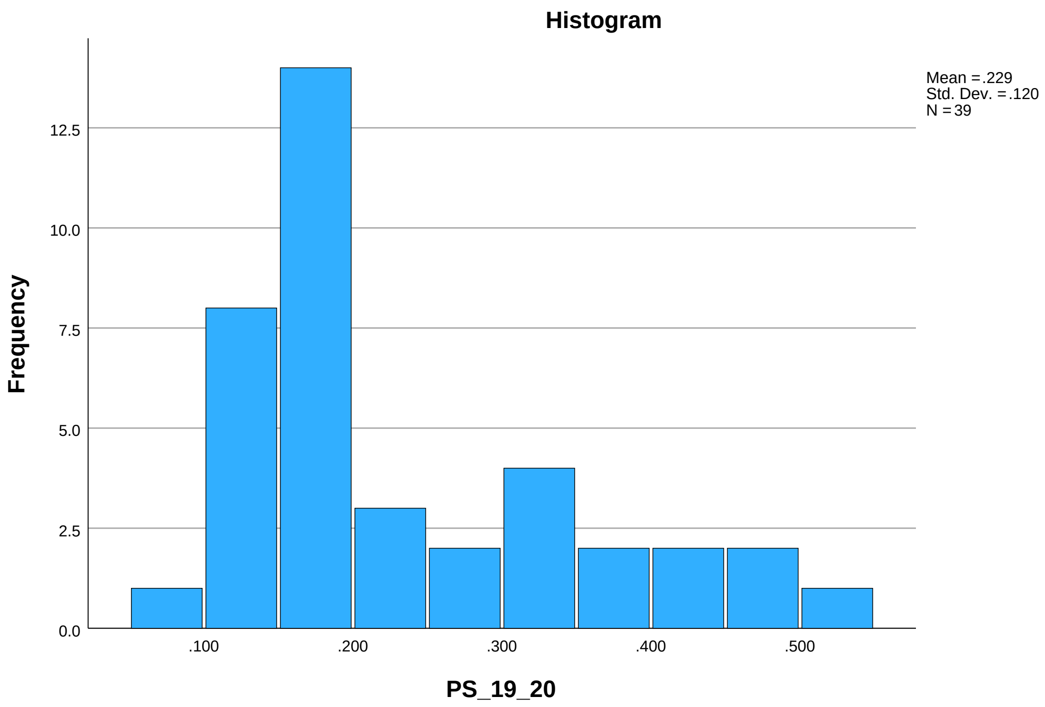

PS_19_20 Stem-and-Leaf Plot

Frequency   Stem & Leaf

```
  1.00     0 . 8
  8.00      1 . 00001113
 14.00       1 . 55566677778999
  3.00     2 . 011
  2.00     2 . 56
  4.00      3 . 0034
  2.00     3 . 78
  2.00     4 . 34
  2.00     4 . 55
  1.00 Extremes    (>=.55)

Stem width:      .100
Each leaf:       1 case(s)
```

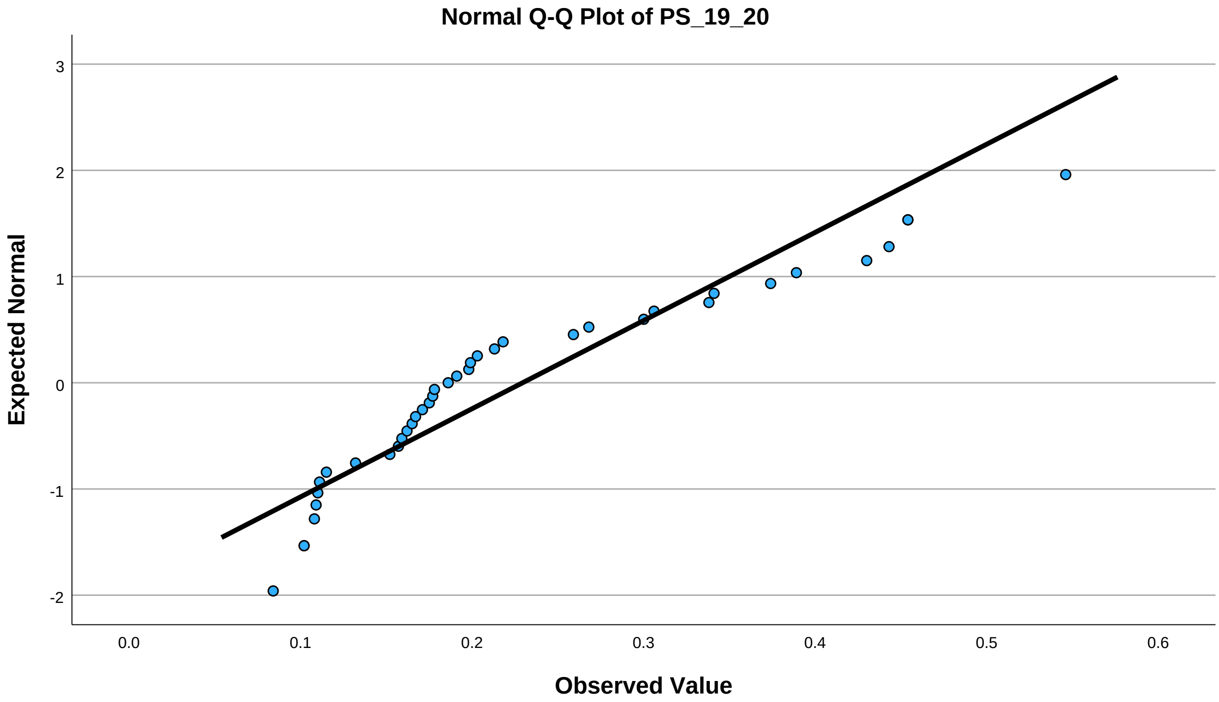

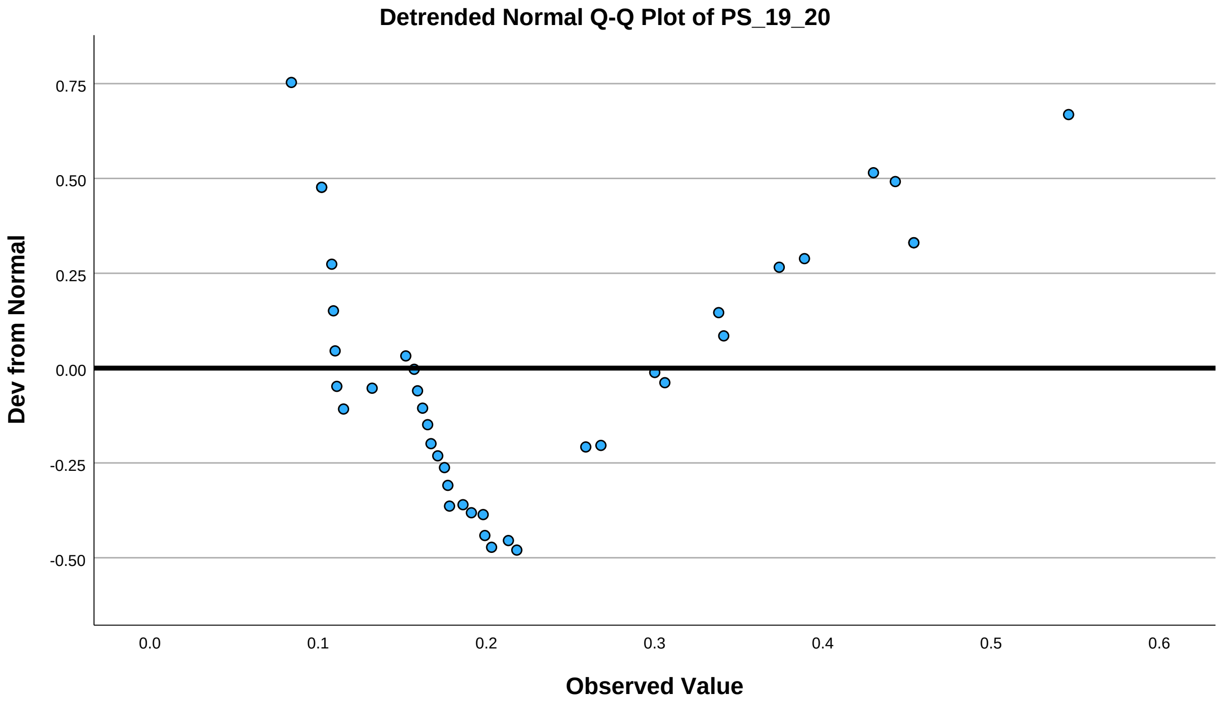

Detrended Normal Q-Q Plot of PS_19_20
Dev from Normal
0.75
0.50
0.25
0.00
-0.25
-0.50
0.0
0.1
0.2
0.3
0.4
0.5
0.6
Observed Value

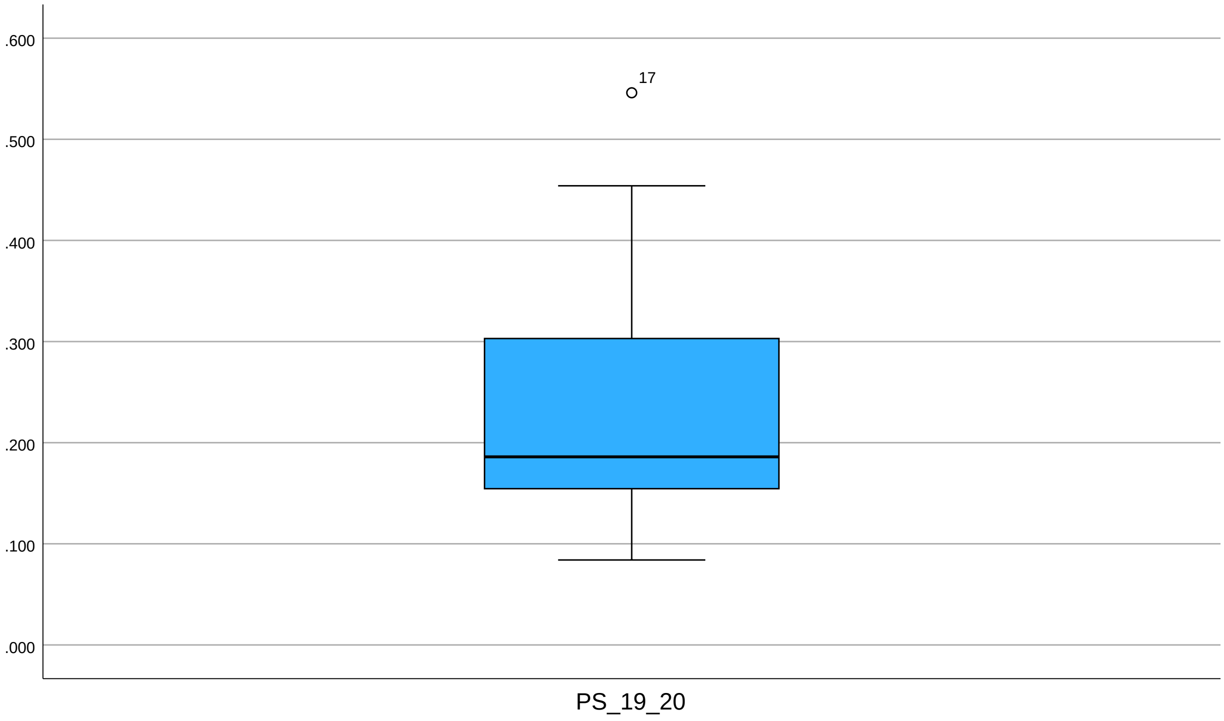

.600
.500
.400
.300
.200
.100
.000
17
PS_19_20

Explore

Case Processing Summary

	Cases					
	Valid		Missing		Total	
	N	Percent	N	Percent	N	Percent
BrainRate	39	100.0%	0	0.0%	39	100.0%
PermutationEntropy	39	100.0%	0	0.0%	39	100.0%
PermutationEntropy_01	39	100.0%	0	0.0%	39	100.0%
SampleEntropy	39	100.0%	0	0.0%	39	100.0%
SpectralEntropy_bits	39	100.0%	0	0.0%	39	100.0%
SpectralEntropy_01	39	100.0%	0	0.0%	39	100.0%
HiguchiFractalDimension	39	100.0%	0	0.0%	39	100.0%
LempelZivComplexity	39	100.0%	0	0.0%	39	100.0%
RenyiEntropy	39	100.0%	0	0.0%	39	100.0%
TsallisEntropy	39	100.0%	0	0.0%	39	100.0%
PS_4_5	39	100.0%	0	0.0%	39	100.0%
PS_5_6	39	100.0%	0	0.0%	39	100.0%
PS_6_7	39	100.0%	0	0.0%	39	100.0%
PS_7_8	39	100.0%	0	0.0%	39	100.0%
PS_8_9	39	100.0%	0	0.0%	39	100.0%
PS_9_10	39	100.0%	0	0.0%	39	100.0%
PS_10_11	39	100.0%	0	0.0%	39	100.0%
PS_11_12	39	100.0%	0	0.0%	39	100.0%
PS_12_13	39	100.0%	0	0.0%	39	100.0%
PS_13_14	39	100.0%	0	0.0%	39	100.0%
PS_14_15	39	100.0%	0	0.0%	39	100.0%
PS_15_16	39	100.0%	0	0.0%	39	100.0%
PS_16_17	39	100.0%	0	0.0%	39	100.0%
PS_17_18	39	100.0%	0	0.0%	39	100.0%
PS_18_19	39	100.0%	0	0.0%	39	100.0%
PS_19_20	39	100.0%	0	0.0%	39	100.0%

Descriptives

			Statistic	Std. Error
BrainRate	Mean		9.5785	.71501
	95% Confidence Interval for Mean	Lower Bound	8.1310	
		Upper Bound	11.0259	
	5% Trimmed Mean		9.2466	
	Median		7.7700	
	Variance		19.938	
	Std. Deviation		4.46523	
	Minimum		4.45	
	Maximum		21.68	
	Range		17.23	
	Interquartile Range		5.00	
	Skewness		1.166	.378
	Kurtosis		.617	.741
PermutationEntropy	Mean		1.4887819210	.00945692390
	95% Confidence Interval for Mean	Lower Bound	1.4696373794	
		Upper Bound	1.5079264625	
	5% Trimmed Mean		1.4898684000	
	Median		1.4914984561	
	Variance		.003	
	Std. Deviation		.05905847085	
	Minimum		1.3381091333	
	Maximum		1.6128454843	
	Range		.27473635096	
	Interquartile Range		.07351605858	
	Skewness		-.315	.378
	Kurtosis		.395	.741
PermutationEntropy_01	Mean		.83090501071	.00527800973
	95% Confidence Interval for Mean	Lower Bound	.82022023863	
		Upper Bound	.84158978280	
	5% Trimmed Mean		.83151138620	
	Median		.83242113781	
	Variance		.001	
	Std. Deviation		.03296116017	
	Minimum		.74681292679	
	Maximum		.90014620376	
	Range		.15333327697	
	Interquartile Range		.04103009352	
	Skewness		-.315	.378
	Kurtosis		.395	.741

Descriptives

			Statistic	Std. Error
SampleEntropy	Mean		.76194181826	.03588743594
	95% Confidence Interval for Mean	Lower Bound	.68929150238	
		Upper Bound	.83459213415	
	5% Trimmed Mean		.75775815435	
	Median		.73061400099	
	Variance		.050	
	Std. Deviation		.22411696564	
	Minimum		.39030440941	
	Maximum		1.2614692804	
	Range		.87116487099	
	Interquartile Range		.35504196552	
	Skewness		.182	.378
	Kurtosis		-.652	.741
SpectralEntropy_bits	Mean		4.3361976063	.09866011310
	95% Confidence Interval for Mean	Lower Bound	4.1364706491	
		Upper Bound	4.5359245635	
	5% Trimmed Mean		4.3225347264	
	Median		4.1610674020	
	Variance		.380	
	Std. Deviation		.61613220883	
	Minimum		3.3326843653	
	Maximum		5.5911084212	
	Range		2.2584240559	
	Interquartile Range		.84494945589	
	Skewness		.398	.378
	Kurtosis		-.726	.741
SpectralEntropy_01	Mean		.61846484907	.01407173231
	95% Confidence Interval for Mean	Lower Bound	.58997811631	
		Upper Bound	.64695158183	
	5% Trimmed Mean		.61651613461	
	Median		.59348631138	
	Variance		.008	
	Std. Deviation		.08787794011	
	Minimum		.47533537909	
	Maximum		.79745074828	
	Range		.32211536919	
	Interquartile Range		.12051377385	
	Skewness		.398	.378
	Kurtosis		-.726	.741

Descriptives

			Statistic	Std. Error
HiguchiFractalDimension	Mean		.48777285067	.01937625902
	95% Confidence Interval for Mean	Lower Bound	.44854766500	
		Upper Bound	.52699803634	
	5% Trimmed Mean		.48944012232	
	Median		.50561999619	
	Variance		.015	
	Std. Deviation		.12100469877	
	Minimum		.24135668218	
	Maximum		.73686118347	
	Range		.49550450129	
	Interquartile Range		.17341392152	
	Skewness		-.275	.378
	Kurtosis		-.585	.741
LempelZivComplexity	Mean		.28618195779	.00752920384
	95% Confidence Interval for Mean	Lower Bound	.27093988149	
		Upper Bound	.30142403409	
	5% Trimmed Mean		.28551728560	
	Median		.28037634935	
	Variance		.002	
	Std. Deviation		.04701986288	
	Minimum		.19472070688	
	Maximum		.39381949982	
	Range		.19909879293	
	Interquartile Range		.05745815249	
	Skewness		.416	.378
	Kurtosis		-.114	.741
RenyiEntropy	Mean		7.5691108191	.14140078716
	95% Confidence Interval for Mean	Lower Bound	7.2828598908	
		Upper Bound	7.8553617474	
	5% Trimmed Mean		7.6062225524	
	Median		7.9283095748	
	Variance		.780	
	Std. Deviation		.88304763280	
	Minimum		5.7420806091	
	Maximum		8.6850068802	
	Range		2.9429262712	
	Interquartile Range		.94019481568	
	Skewness		-.937	.378
	Kurtosis		-.340	.741

Descriptives

			Statistic	Std. Error
TsallisEntropy	Mean		1.7215912518	.06932655296
	95% Confidence Interval for Mean	Lower Bound	1.5812469826	
		Upper Bound	1.8619355210	
	5% Trimmed Mean		1.7744368155	
	Median		1.9453410232	
	Variance		.187	
	Std. Deviation		.43294418450	
	Minimum		.44450929221	
	Maximum		1.9866859281	
	Range		1.5421766359	
	Interquartile Range		.32139006919	
	Skewness		-1.898	.378
	Kurtosis		2.634	.741
PS_4_5	Mean		2.39313	.373704
	95% Confidence Interval for Mean	Lower Bound	1.63660	
		Upper Bound	3.14965	
	5% Trimmed Mean		2.07626	
	Median		1.85700	
	Variance		5.447	
	Std. Deviation		2.333783	
	Minimum		.587	
	Maximum		15.010	
	Range		14.423	
	Interquartile Range		1.795	
	Skewness		4.362	.378
	Kurtosis		23.177	.741
PS_5_6	Mean		1.70169	.183818
	95% Confidence Interval for Mean	Lower Bound	1.32957	
		Upper Bound	2.07381	
	5% Trimmed Mean		1.56367	
	Median		1.43200	
	Variance		1.318	
	Std. Deviation		1.147945	
	Minimum		.392	
	Maximum		7.120	
	Range		6.728	
	Interquartile Range		1.101	
	Skewness		2.969	.378
	Kurtosis		12.573	.741

Descriptives

			Statistic	Std. Error
PS_6_7	Mean		1.49918	.202025
	95% Confidence Interval for Mean	Lower Bound	1.09020	
		Upper Bound	1.90816	
	5% Trimmed Mean		1.33781	
	Median		1.20100	
	Variance		1.592	
	Std. Deviation		1.261647	
	Minimum		.266	
	Maximum		8.280	
	Range		8.014	
	Interquartile Range		.929	
	Skewness		4.259	.378
	Kurtosis		22.606	.741
PS_7_8	Mean		1.22359	.120186
	95% Confidence Interval for Mean	Lower Bound	.98029	
		Upper Bound	1.46689	
	5% Trimmed Mean		1.14284	
	Median		1.01700	
	Variance		.563	
	Std. Deviation		.750559	
	Minimum		.223	
	Maximum		4.120	
	Range		3.897	
	Interquartile Range		.621	
	Skewness		2.035	.378
	Kurtosis		5.277	.741
PS_8_9	Mean		1.15267	.139909
	95% Confidence Interval for Mean	Lower Bound	.86944	
		Upper Bound	1.43590	
	5% Trimmed Mean		1.04355	
	Median		.97900	
	Variance		.763	
	Std. Deviation		.873729	
	Minimum		.179	
	Maximum		4.960	
	Range		4.781	
	Interquartile Range		.543	
	Skewness		2.671	.378
	Kurtosis		9.104	.741

Descriptives

			Statistic	Std. Error
PS_9_10	Mean		1.21213	.213881
	95% Confidence Interval for Mean	Lower Bound	.77915	
		Upper Bound	1.64511	
	5% Trimmed Mean		1.00172	
	Median		.98000	
	Variance		1.784	
	Std. Deviation		1.335686	
	Minimum		.160	
	Maximum		8.430	
	Range		8.270	
	Interquartile Range		.683	
	Skewness		4.446	.378
	Kurtosis		23.322	.741
PS_10_11	Mean		1.39051	.219236
	95% Confidence Interval for Mean	Lower Bound	.94669	
		Upper Bound	1.83433	
	5% Trimmed Mean		1.17773	
	Median		1.03300	
	Variance		1.875	
	Std. Deviation		1.369130	
	Minimum		.155	
	Maximum		7.220	
	Range		7.065	
	Interquartile Range		.827	
	Skewness		2.930	.378
	Kurtosis		9.754	.741
PS_11_12	Mean		.66403	.094975
	95% Confidence Interval for Mean	Lower Bound	.47176	
		Upper Bound	.85629	
	5% Trimmed Mean		.57472	
	Median		.49800	
	Variance		.352	
	Std. Deviation		.593119	
	Minimum		.112	
	Maximum		3.548	
	Range		3.436	
	Interquartile Range		.368	
	Skewness		3.457	.378
	Kurtosis		14.880	.741

Descriptives

			Statistic	Std. Error
PS_12_13	Mean		.49782	.051129
	95% Confidence Interval for Mean	Lower Bound	.39432	
		Upper Bound	.60133	
	5% Trimmed Mean		.47041	
	Median		.43500	
	Variance		.102	
	Std. Deviation		.319300	
	Minimum		.082	
	Maximum		1.500	
	Range		1.418	
	Interquartile Range		.270	
	Skewness		1.416	.378
	Kurtosis		1.782	.741
PS_13_14	Mean		.49172	.053696
	95% Confidence Interval for Mean	Lower Bound	.38302	
		Upper Bound	.60042	
	5% Trimmed Mean		.46578	
	Median		.42000	
	Variance		.112	
	Std. Deviation		.335328	
	Minimum		.073	
	Maximum		1.430	
	Range		1.357	
	Interquartile Range		.351	
	Skewness		1.325	.378
	Kurtosis		1.148	.741
PS_14_15	Mean		.51356	.060501
	95% Confidence Interval for Mean	Lower Bound	.39109	
		Upper Bound	.63604	
	5% Trimmed Mean		.48506	
	Median		.38500	
	Variance		.143	
	Std. Deviation		.377831	
	Minimum		.083	
	Maximum		1.430	
	Range		1.347	
	Interquartile Range		.364	
	Skewness		1.303	.378
	Kurtosis		.847	.741

Descriptives

			Statistic	Std. Error
PS_15_16	Mean		.50869	.058487
	95% Confidence Interval for Mean	Lower Bound	.39029	
		Upper Bound	.62709	
	5% Trimmed Mean		.48079	
	Median		.38800	
	Variance		.133	
	Std. Deviation		.365249	
	Minimum		.087	
	Maximum		1.570	
	Range		1.483	
	Interquartile Range		.398	
	Skewness		1.238	.378
	Kurtosis		.796	.741
PS_16_17	Mean		.49946	.060642
	95% Confidence Interval for Mean	Lower Bound	.37670	
		Upper Bound	.62223	
	5% Trimmed Mean		.46267	
	Median		.41500	
	Variance		.143	
	Std. Deviation		.378711	
	Minimum		.089	
	Maximum		1.731	
	Range		1.642	
	Interquartile Range		.376	
	Skewness		1.545	.378
	Kurtosis		2.191	.741
PS_17_18	Mean		.47474	.059177
	95% Confidence Interval for Mean	Lower Bound	.35495	
		Upper Bound	.59454	
	5% Trimmed Mean		.43699	
	Median		.35800	
	Variance		.137	
	Std. Deviation		.369563	
	Minimum		.083	
	Maximum		1.641	
	Range		1.558	
	Interquartile Range		.456	
	Skewness		1.481	.378
	Kurtosis		1.871	.741

Descriptives

			Statistic	Std. Error
PS_18_19	Mean		.47772	.064070
	95% Confidence Interval for Mean	Lower Bound	.34802	
		Upper Bound	.60742	
	5% Trimmed Mean		.43698	
	Median		.33600	
	Variance		.160	
	Std. Deviation		.400116	
	Minimum		.072	
	Maximum		1.644	
	Range		1.572	
	Interquartile Range		.450	
	Skewness		1.582	.378
	Kurtosis		1.870	.741
PS_19_20	Mean		.47503	.064974
	95% Confidence Interval for Mean	Lower Bound	.34349	
		Upper Bound	.60656	
	5% Trimmed Mean		.43302	
	Median		.32600	
	Variance		.165	
	Std. Deviation		.405764	
	Minimum		.067	
	Maximum		1.680	
	Range		1.613	
	Interquartile Range		.452	
	Skewness		1.498	.378
	Kurtosis		1.642	.741

Tests of Normality

	Kolmogorov-Smirnov[a]			Shapiro-Wilk		
	Statistic	df	Sig.	Statistic	df	Sig.
BrainRate	.185	39	.002	.872	39	<.001
PermutationEntropy	.108	39	.200*	.984	39	.836
PermutationEntropy_01	.108	39	.200*	.984	39	.836
SampleEntropy	.086	39	.200*	.978	39	.623
SpectralEntropy_bits	.139	39	.056	.957	39	.146
SpectralEntropy_01	.139	39	.056	.957	39	.146
HiguchiFractalDimension	.104	39	.200*	.972	39	.434
LempelZivComplexity	.084	39	.200*	.976	39	.564
RenyiEntropy	.208	39	<.001	.859	39	<.001
TsallisEntropy	.270	39	<.001	.662	39	<.001
PS_4_5	.237	39	<.001	.547	39	<.001
PS_5_6	.164	39	.009	.736	39	<.001
PS_6_7	.241	39	<.001	.582	39	<.001
PS_7_8	.177	39	.003	.814	39	<.001
PS_8_9	.251	39	<.001	.729	39	<.001
PS_9_10	.262	39	<.001	.531	39	<.001
PS_10_11	.265	39	<.001	.662	39	<.001
PS_11_12	.231	39	<.001	.641	39	<.001
PS_12_13	.214	39	<.001	.864	39	<.001
PS_13_14	.169	39	.006	.861	39	<.001
PS_14_15	.176	39	.004	.837	39	<.001
PS_15_16	.182	39	.002	.861	39	<.001
PS_16_17	.199	39	<.001	.838	39	<.001
PS_17_18	.190	39	.001	.834	39	<.001
PS_18_19	.206	39	<.001	.804	39	<.001
PS_19_20	.225	39	<.001	.811	39	<.001

*. This is a lower bound of the true significance.

a. Lilliefors Significance Correction

BrainRate

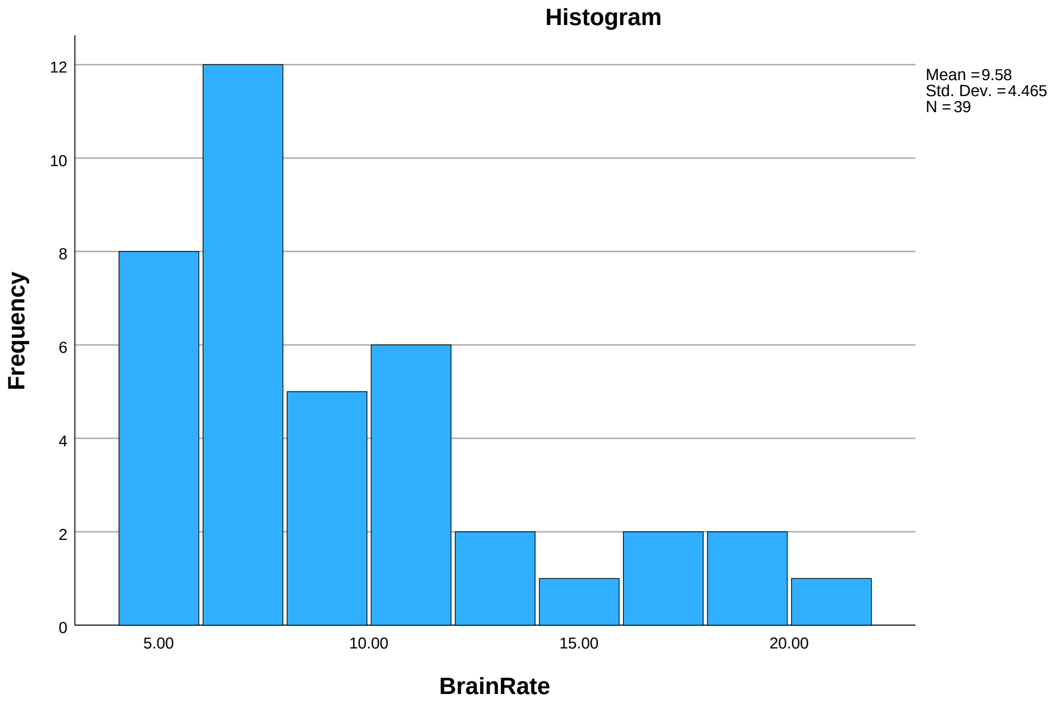

BrainRate Stem-and-Leaf Plot

```
 Frequency    Stem &  Leaf

     8.00        0 .  44555555
    12.00        0 .  666666677777
     5.00        0 .  89999
     6.00        1 .  000111
     2.00        1 .  33
     1.00        1 .  4
     2.00        1 .  67
     3.00 Extremes    (>=19)

 Stem width:      10.00
 Each leaf:        1 case(s)
```

Normal Q-Q Plot of BrainRate

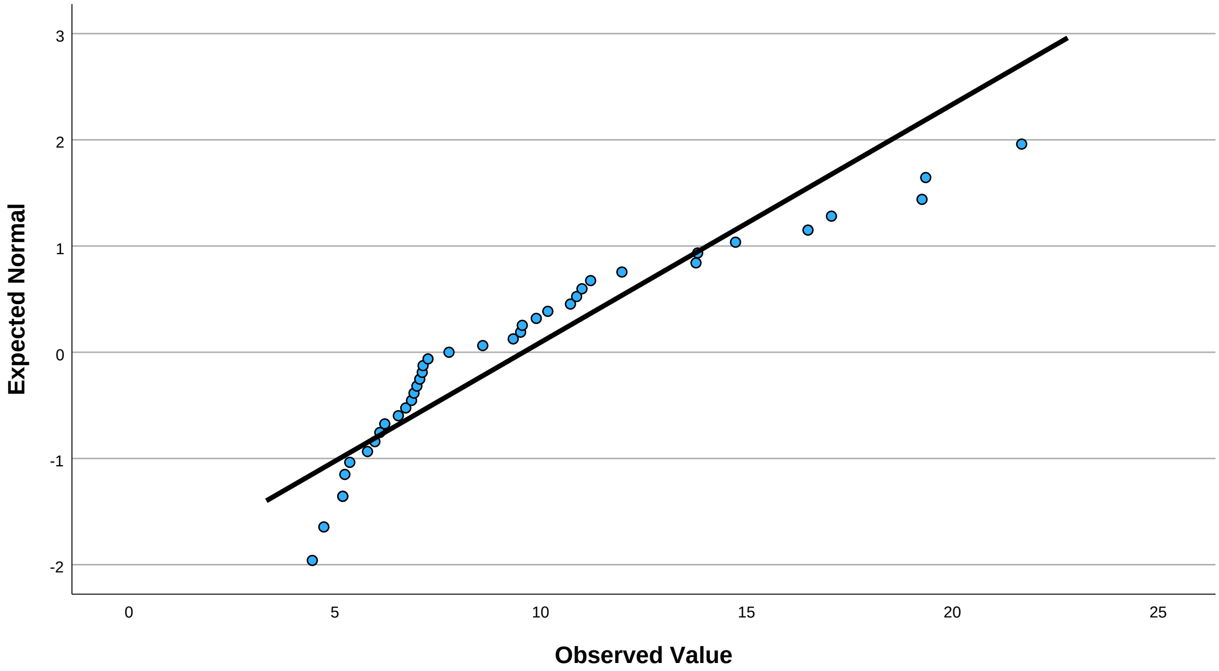

Detrended Normal Q-Q Plot of BrainRate

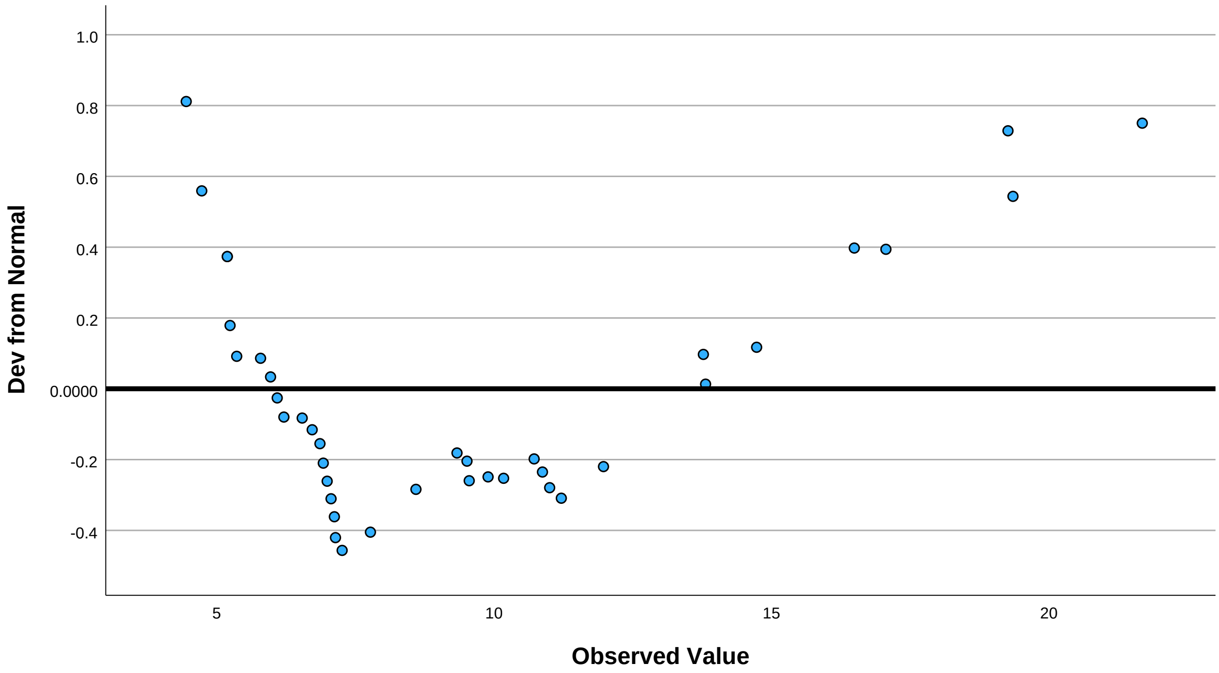

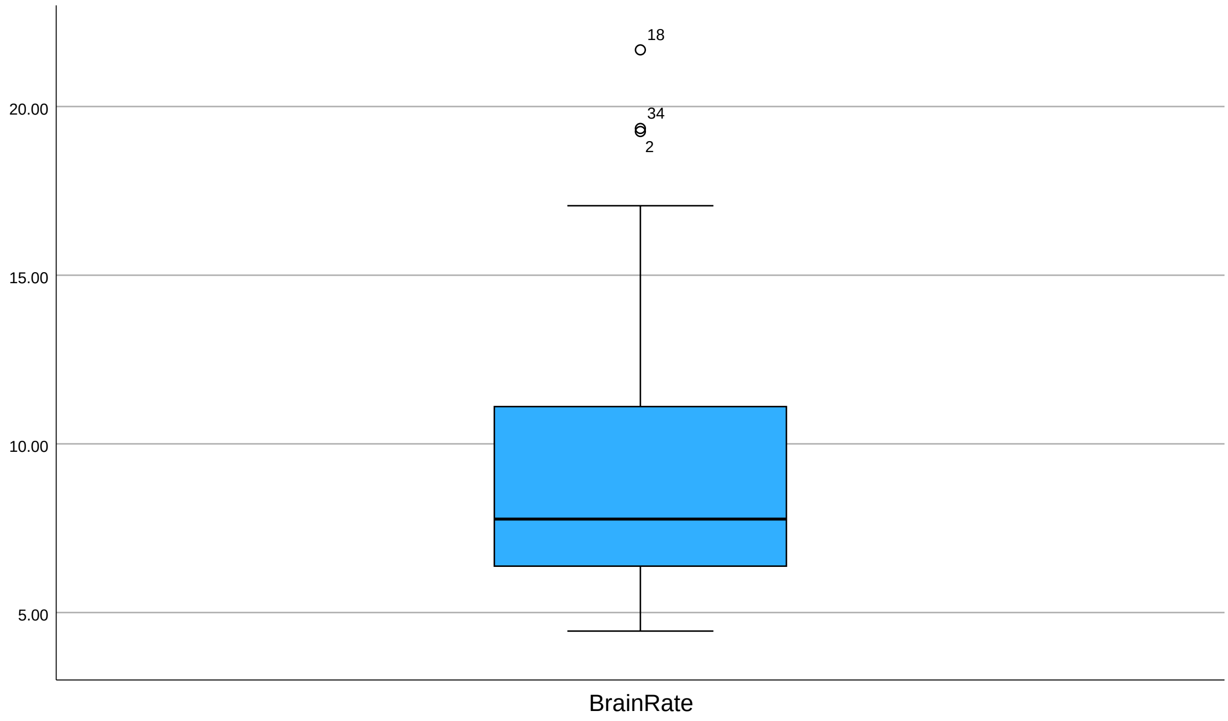

PermutationEntropy

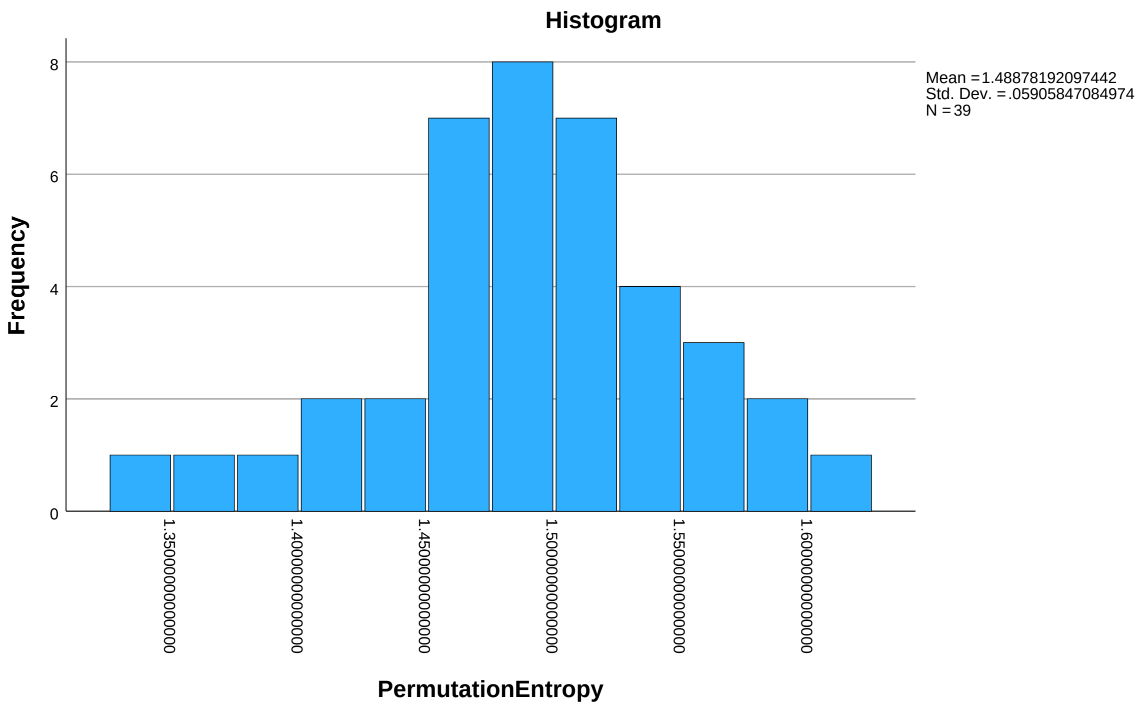

PermutationEntropy Stem-and-Leaf Plot

Frequency   Stem & Leaf

```
   1.00 Extremes    (=<1.34)
  2.00     13 .  78
  4.00      14 .  0134
 15.00       14 .  555556688889999
 11.00       15 .  00000122333
  5.00      15 .  55679
  1.00     16 .  1

Stem width:  .1000000
Each leaf:       1 case(s)
```

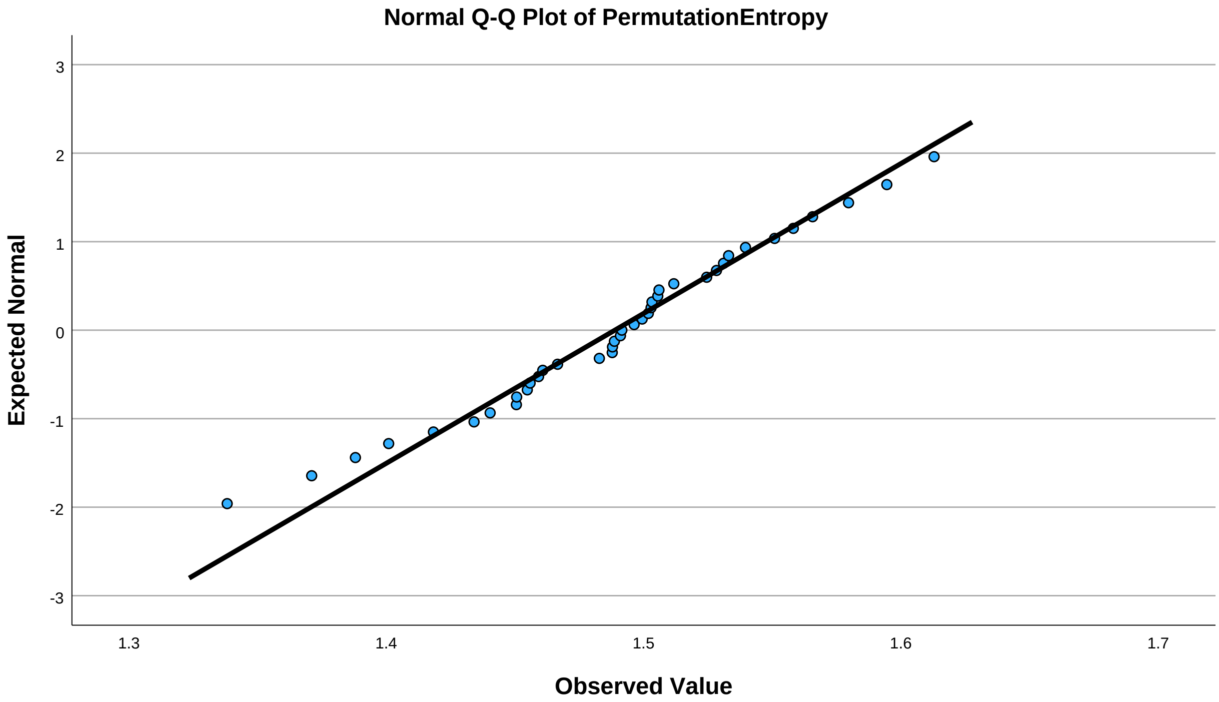

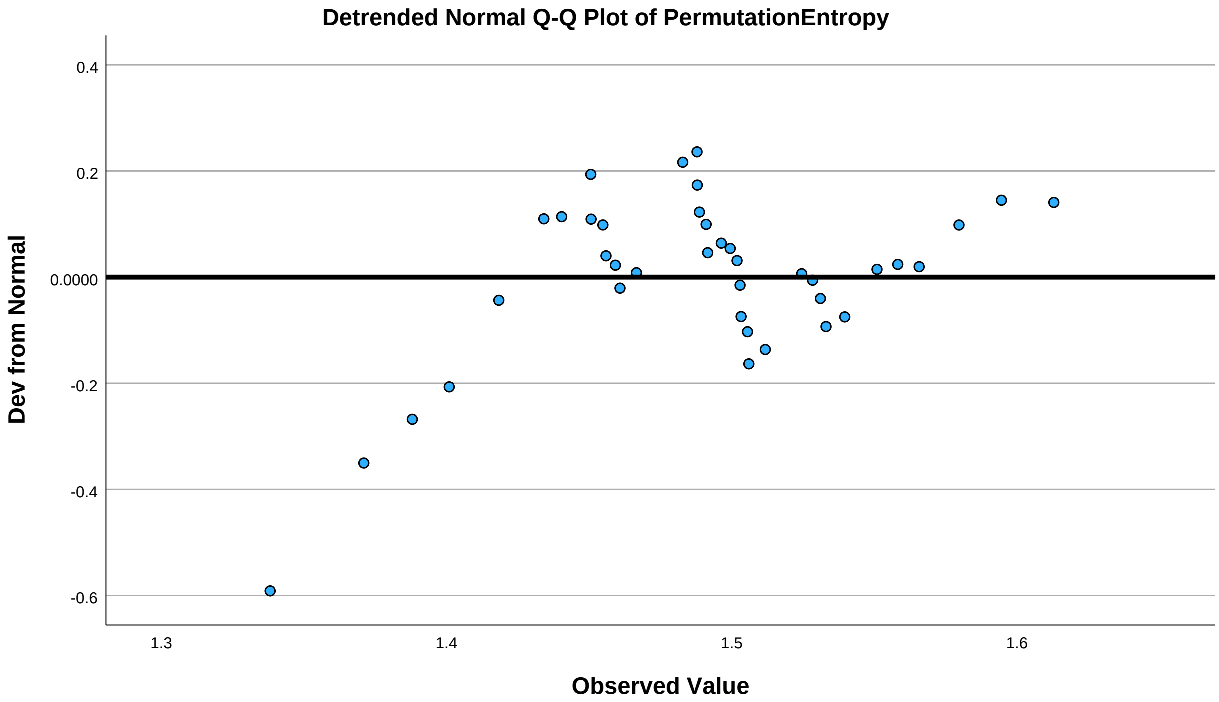

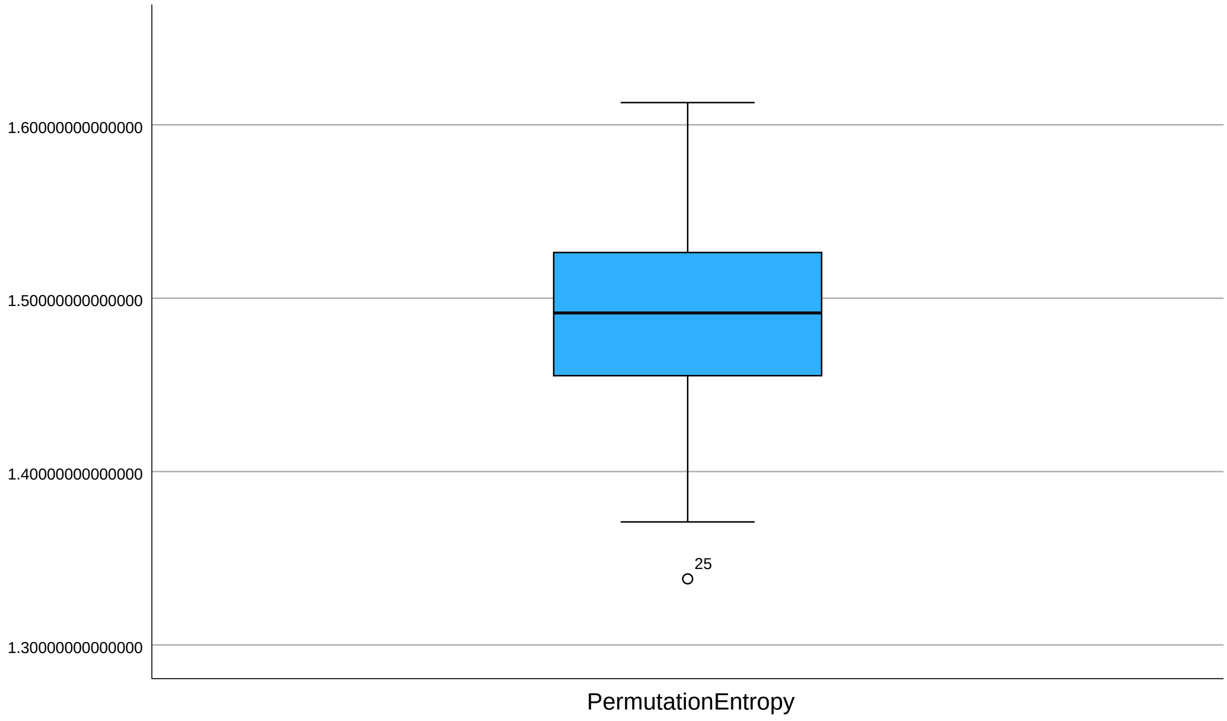

PermutationEntropy_01

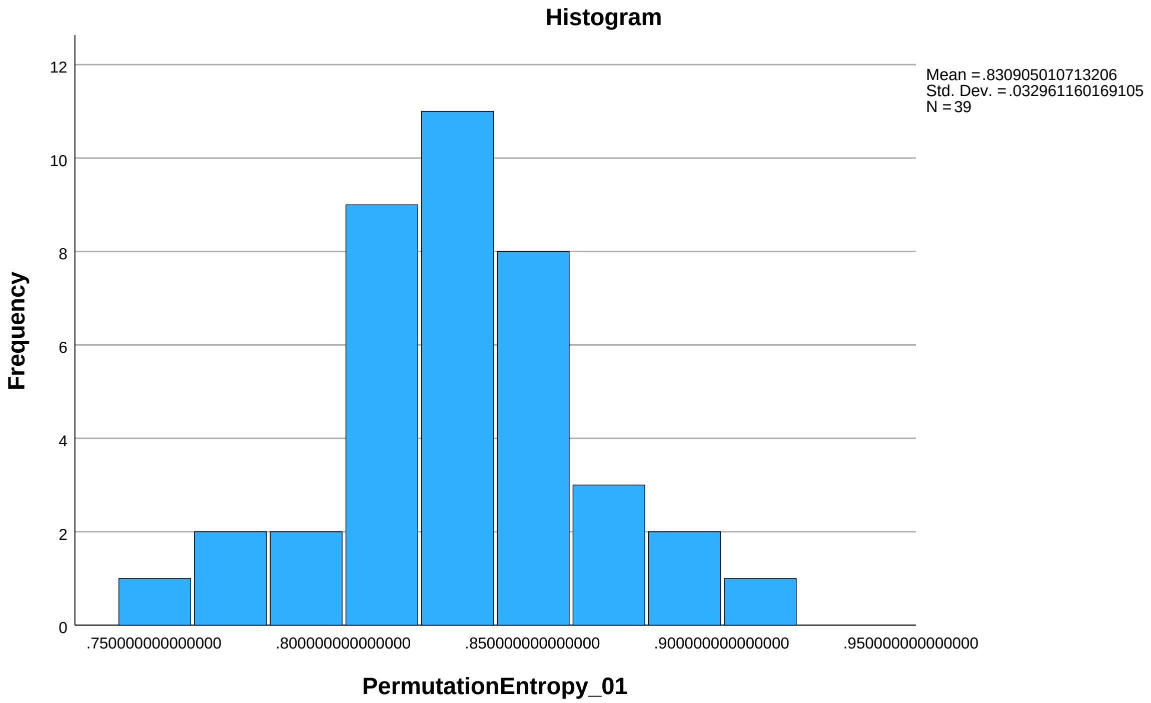


```
PermutationEntropy_01 Stem-and-Leaf Plot

 Frequency    Stem &  Leaf

     1.00 Extremes    (=<.75)
     4.00        7 .  6789
    23.00        8 .  00001111123333333333444
    10.00        8 .  5555566788
     1.00        9 .  0

 Stem width:   .1000000
 Each leaf:       1 case(s)
```

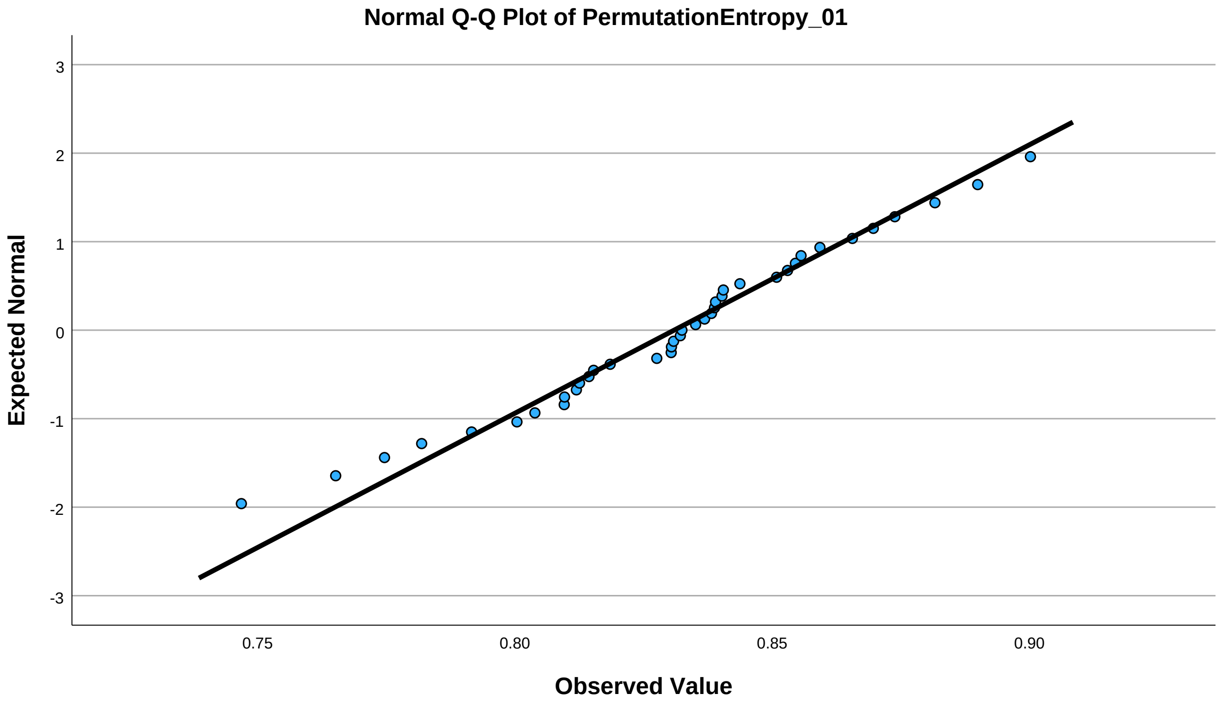
Normal Q-Q Plot of PermutationEntropy_01
Expected Normal
Observed Value
3
2
1
0
-1
-2
-3
0.75
0.80
0.85
0.90

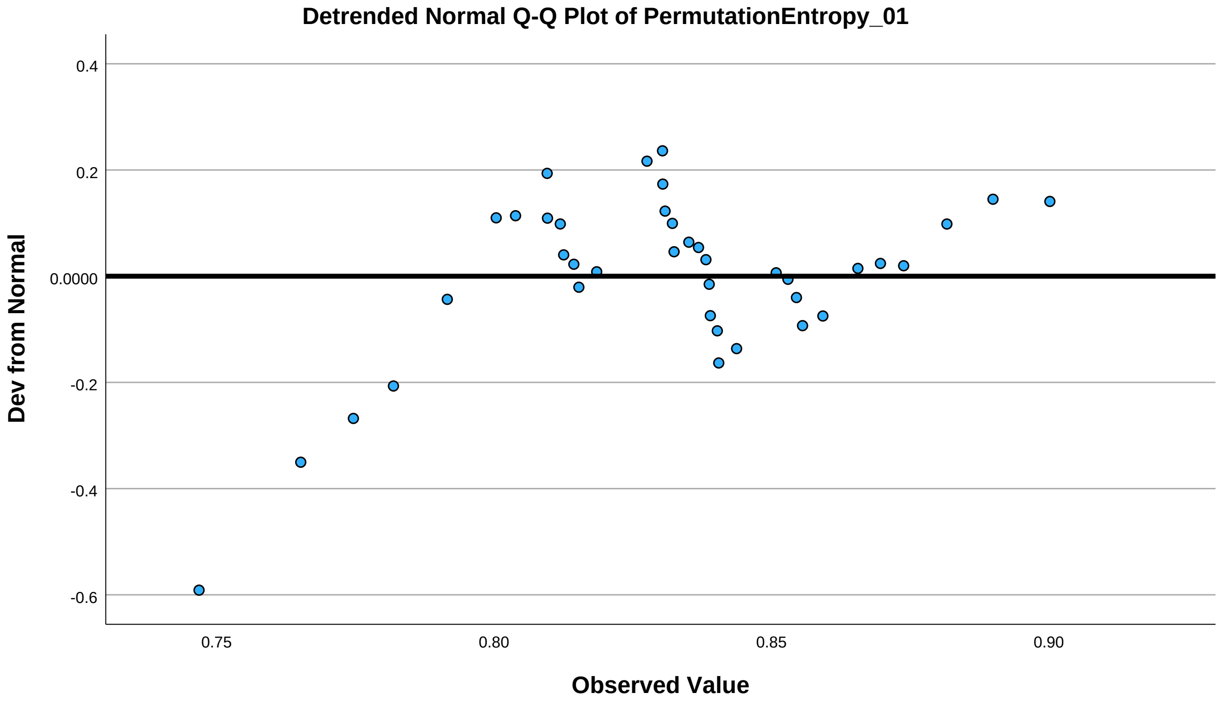
Detrended Normal Q-Q Plot of PermutationEntropy_01
Dev from Normal
Observed Value
0.4
0.2
0.0000
-0.2
-0.4
-0.6
0.75
0.80
0.85
0.90

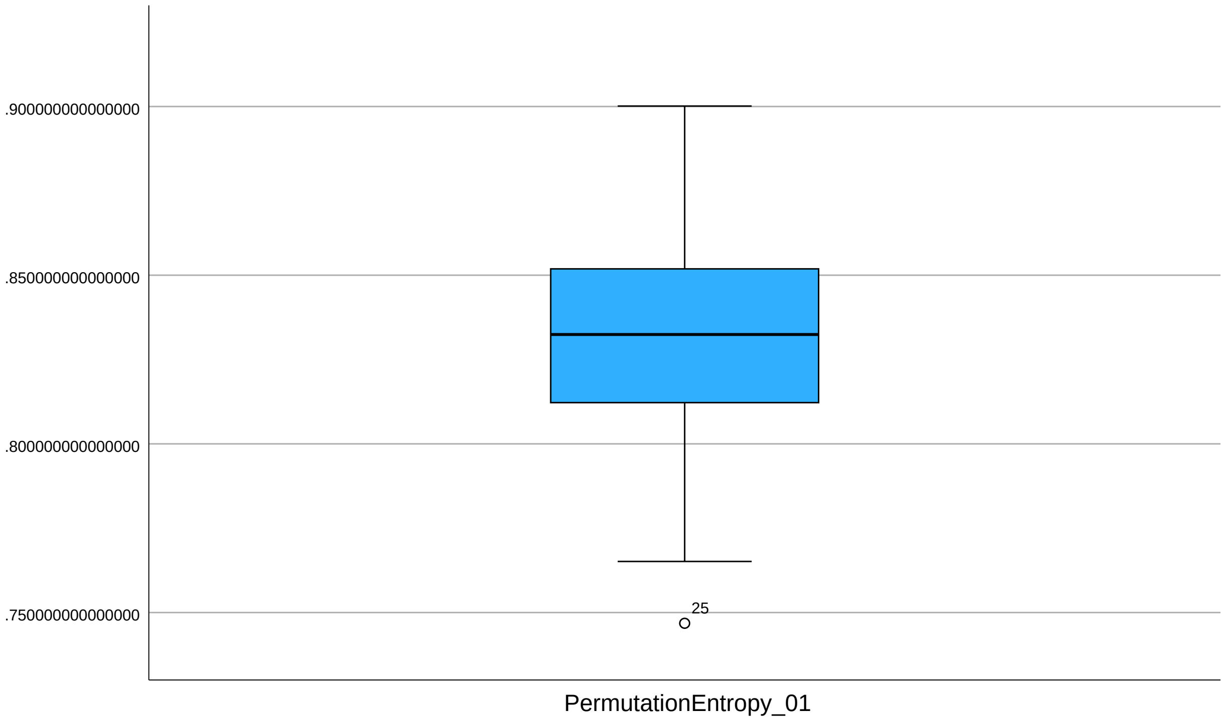

SampleEntropy

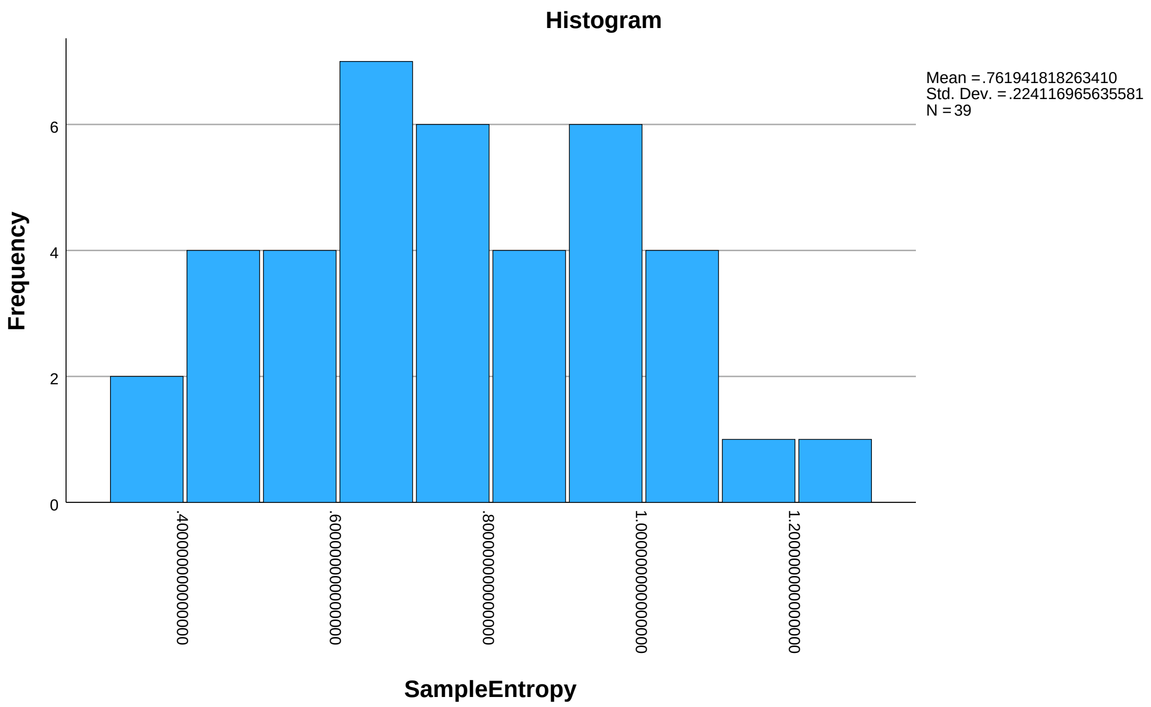


```
SampleEntropy Stem-and-Leaf Plot

 Frequency    Stem &  Leaf
```

```
 2.00      3 .  99
 4.00      4 .  0469
 4.00      5 .  1679
 7.00      6 .  3358899
 6.00      7 .  113379
 4.00      8 .  2348
 6.00      9 .  115578
 4.00     10 .  2688
 1.00     11 .  4
 1.00     12 .  6

Stem width:   .1000000
Each leaf:       1 case(s)
```

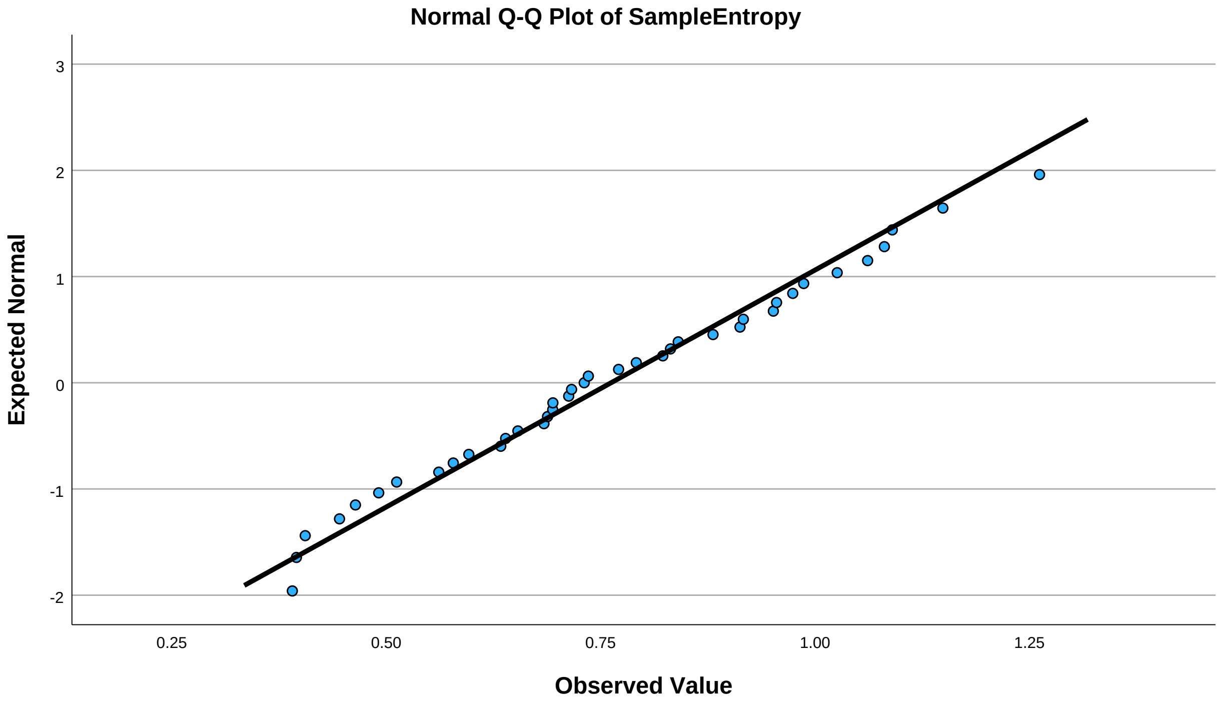

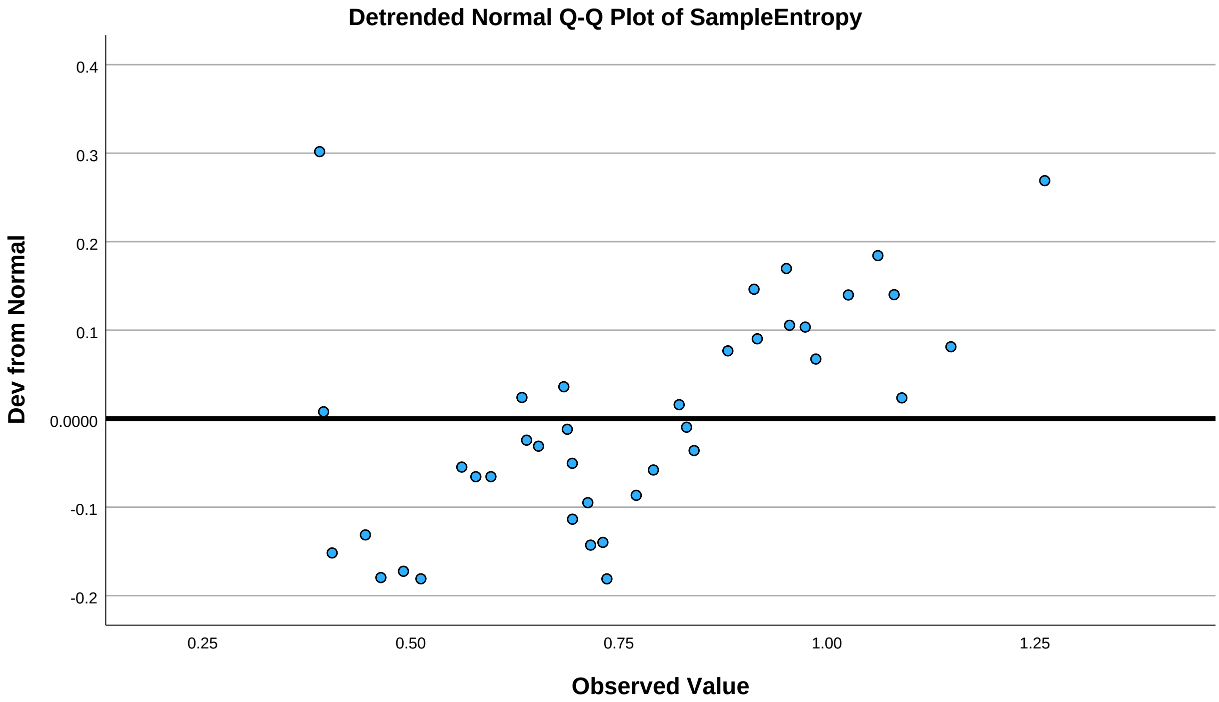

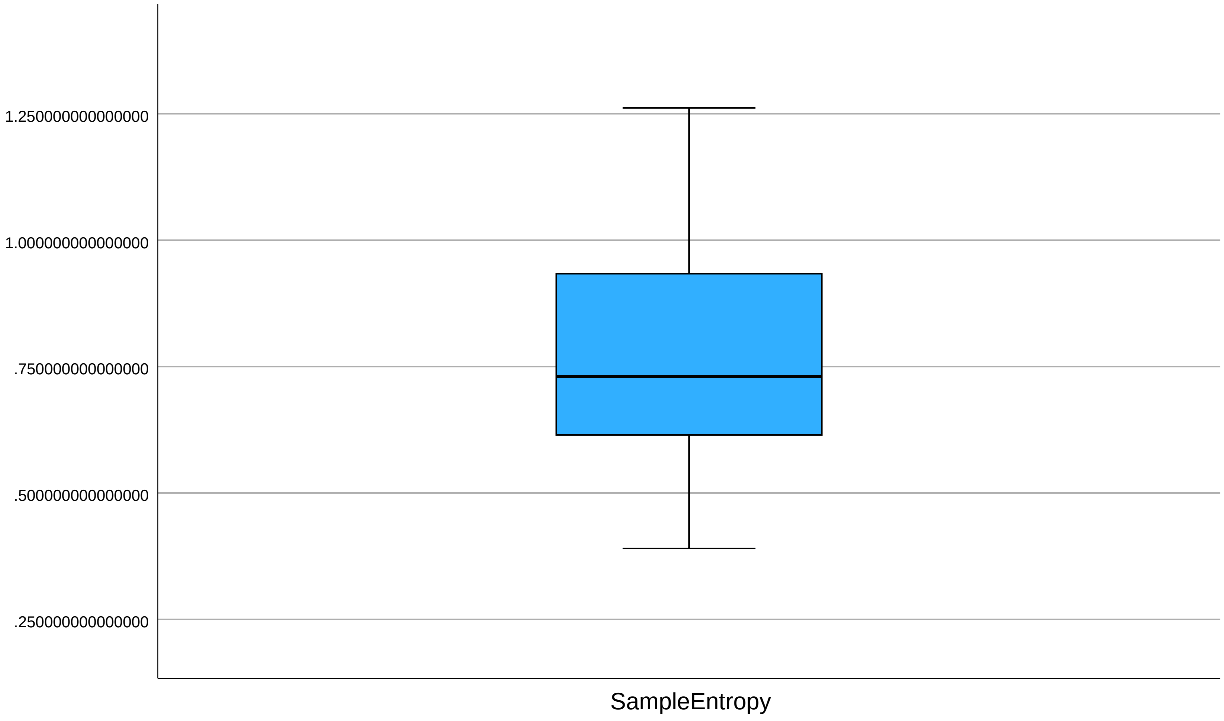

SpectralEntropy_bits

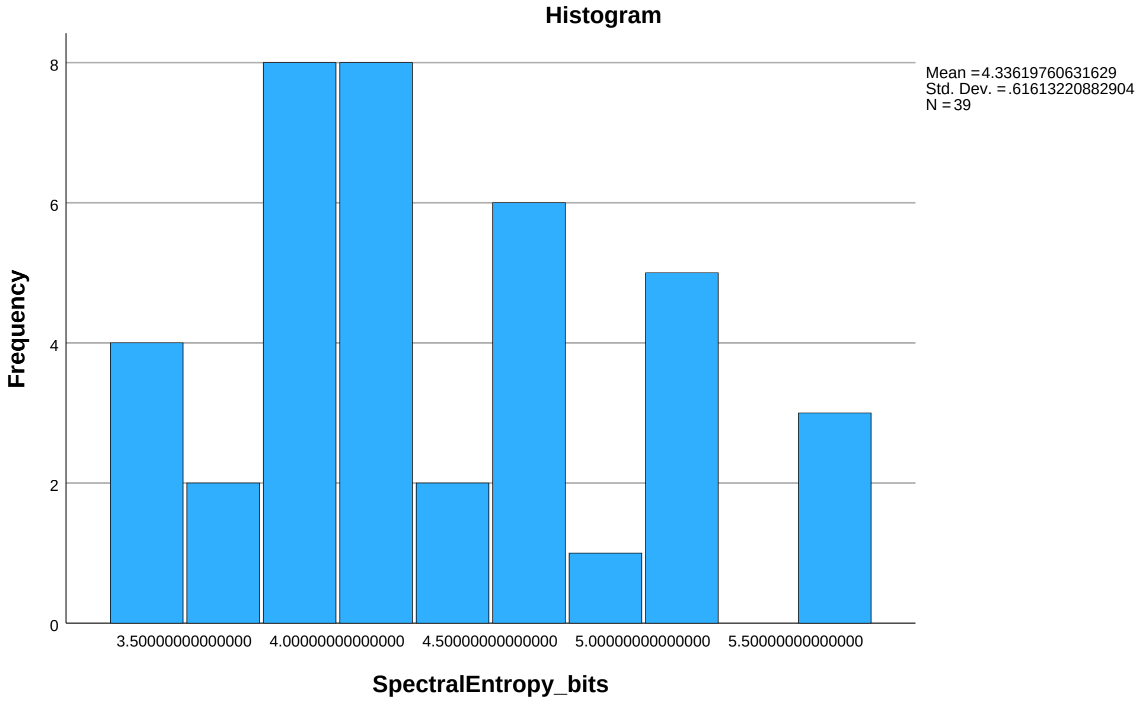


```
SpectralEntropy_bits Stem-and-Leaf Plot

 Frequency    Stem &  Leaf

     4.00        3 .  3444
    10.00        3 .  6678889999
    10.00        4 .  0001111244
     7.00        4 .  5566779
     5.00        5 .  00011
     3.00        5 .  555

 Stem width:   1.000000
 Each leaf:       1 case(s)
```

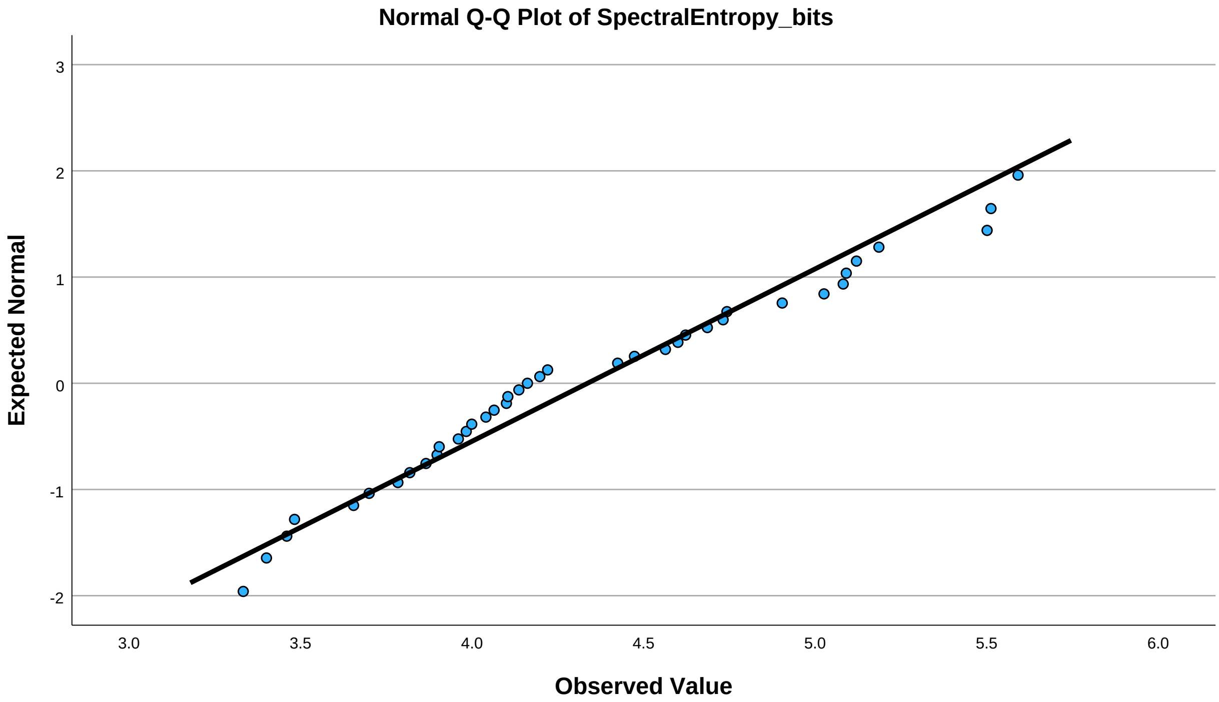
Normal Q-Q Plot of SpectralEntropy_bits
Expected Normal
3
2
1
0
-1
-2
3.0
3.5
4.0
4.5
5.0
5.5
6.0
Observed Value

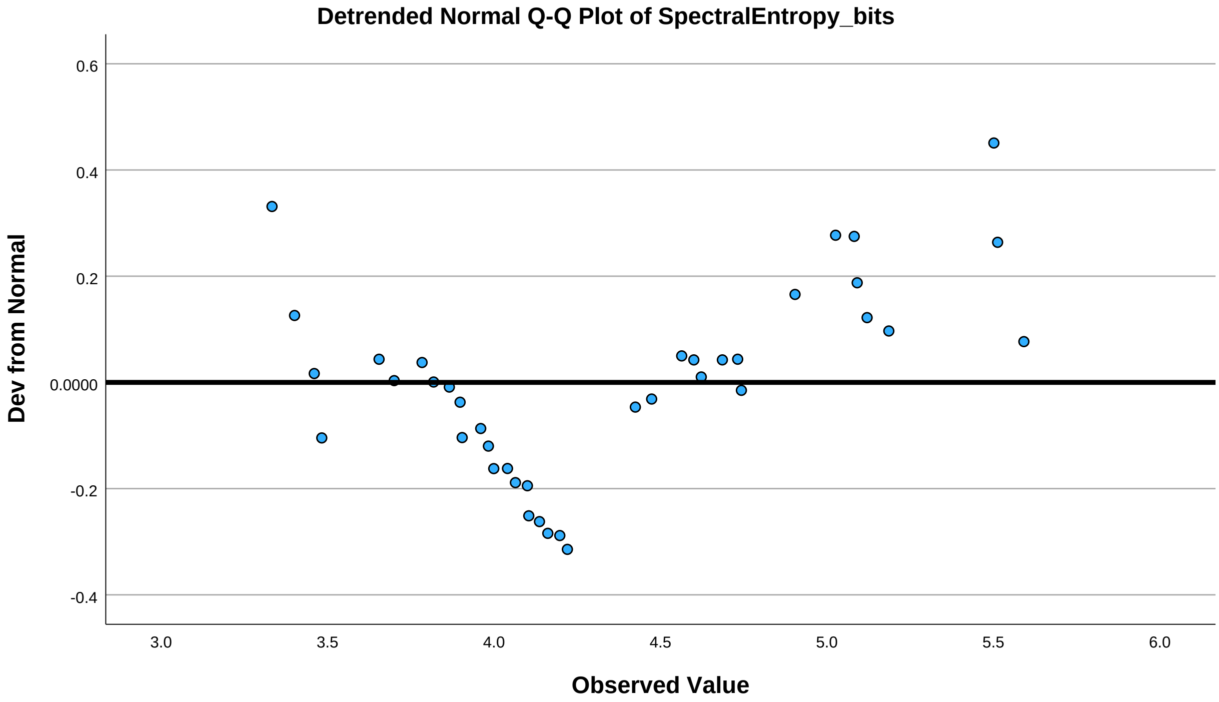
Detrended Normal Q-Q Plot of SpectralEntropy_bits
Dev from Normal
0.6
0.4
0.2
0.0000
-0.2
-0.4
3.0
3.5
4.0
4.5
5.0
5.5
6.0
Observed Value

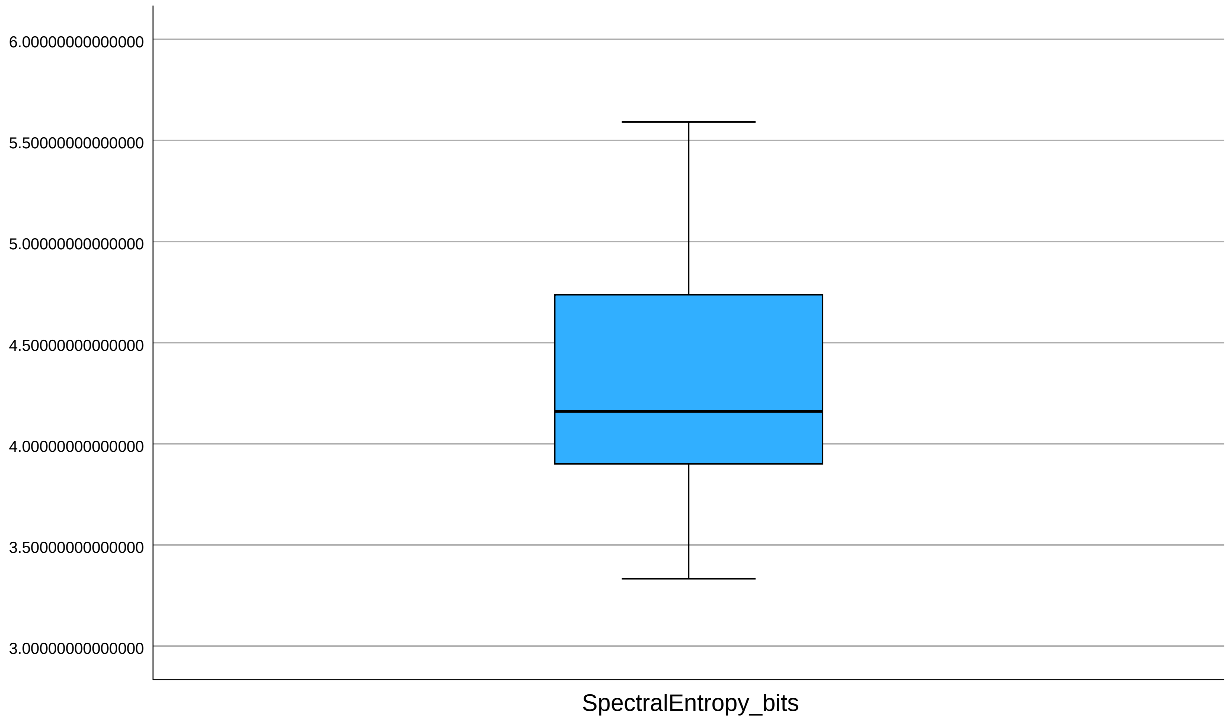

SpectralEntropy_01

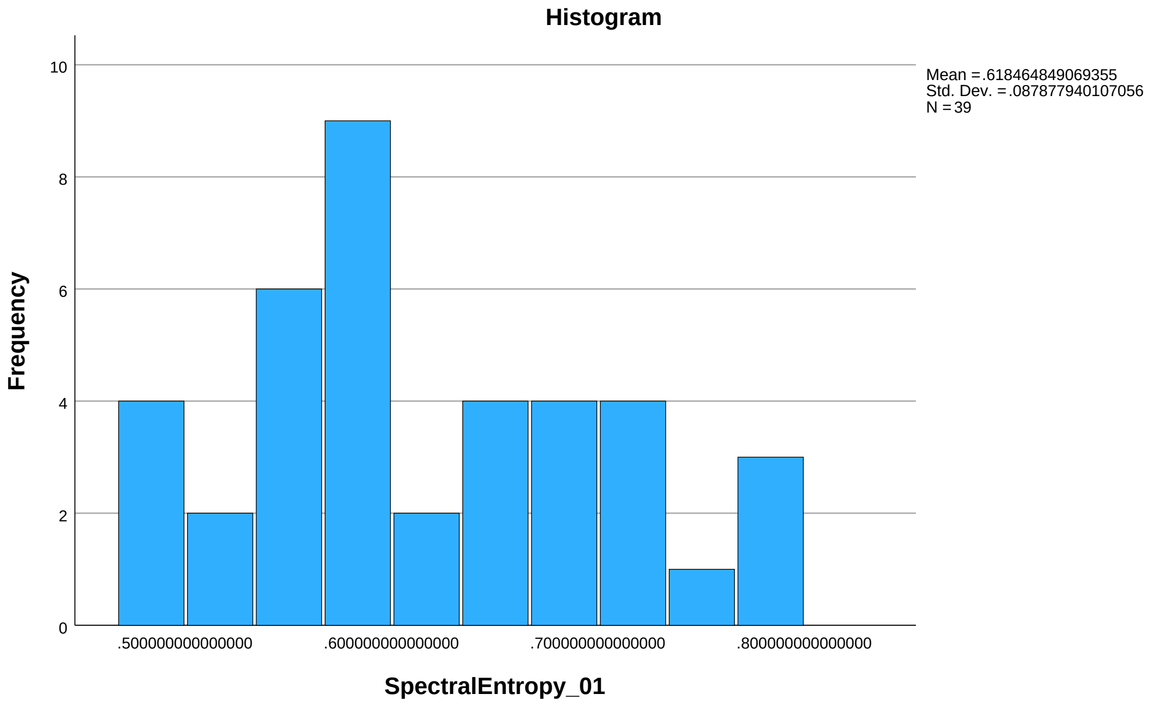


```
SpectralEntropy_01 Stem-and-Leaf Plot

 Frequency    Stem &  Leaf
```

```
   4.00      4 .  7899
   4.00      5 .  2234
  13.00      5 .  5556677788899
   3.00      6 .  033
   7.00      6 .  5556779
   5.00      7 .  12233
   3.00      7 .  889

 Stem width:   .1000000
 Each leaf:       1 case(s)
```

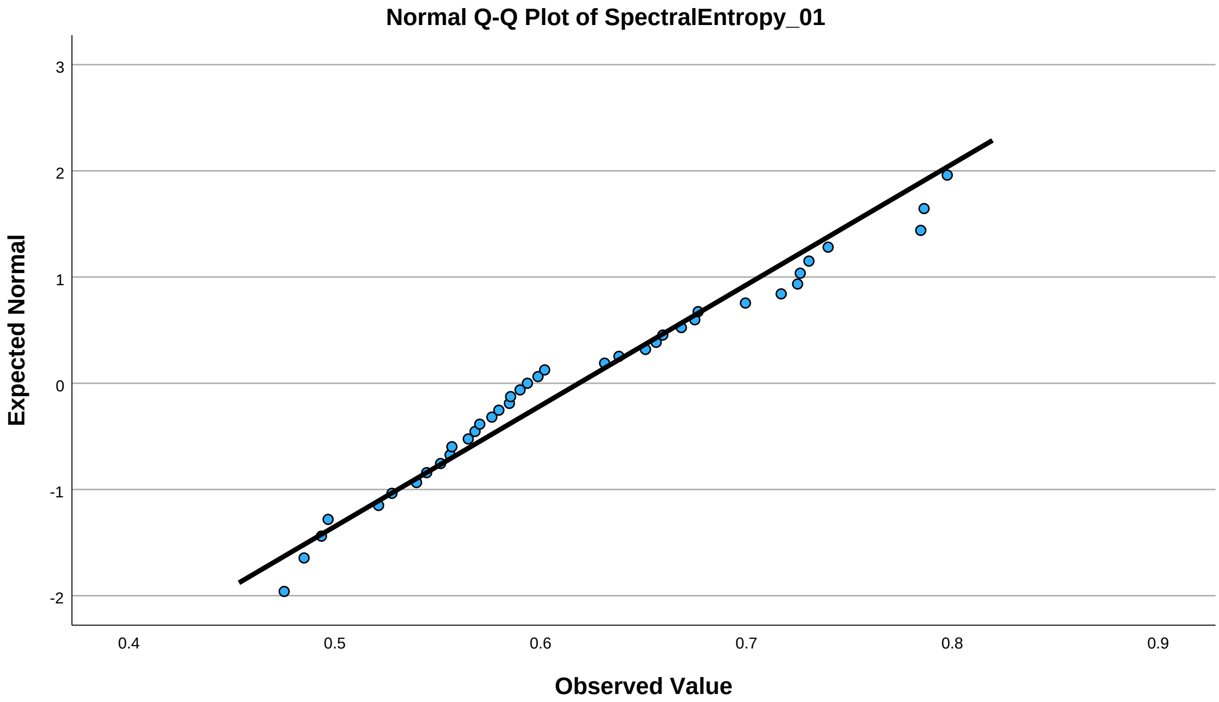

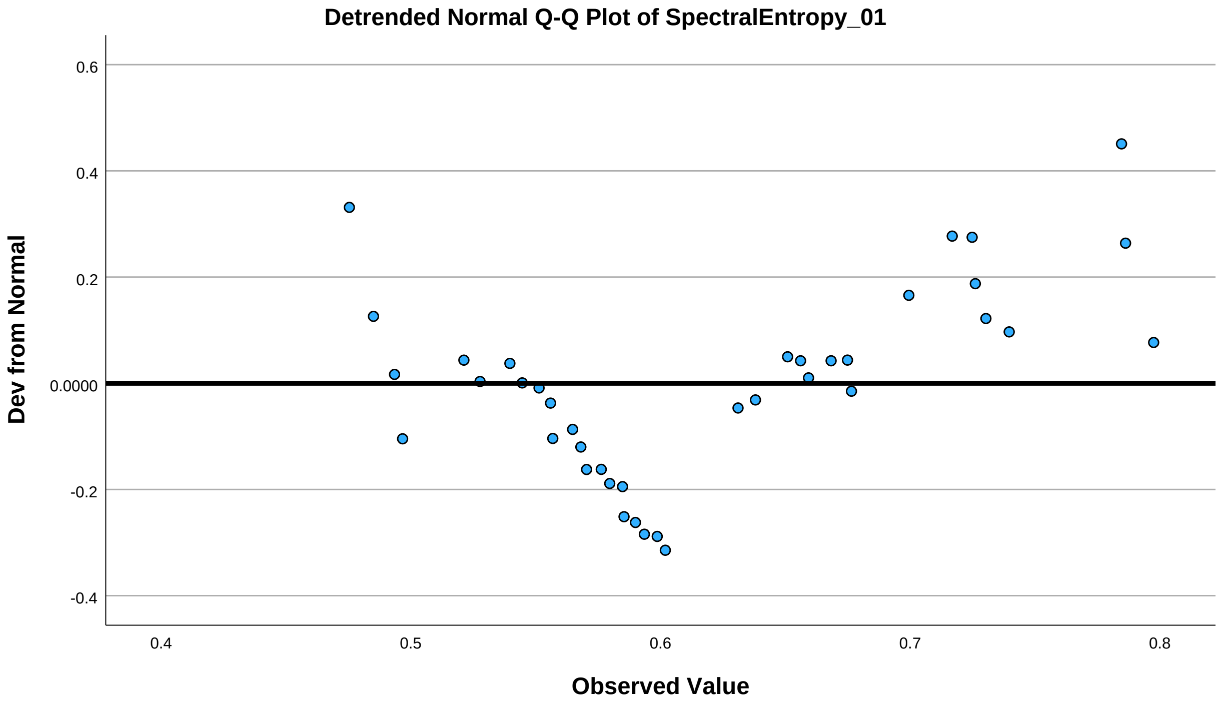

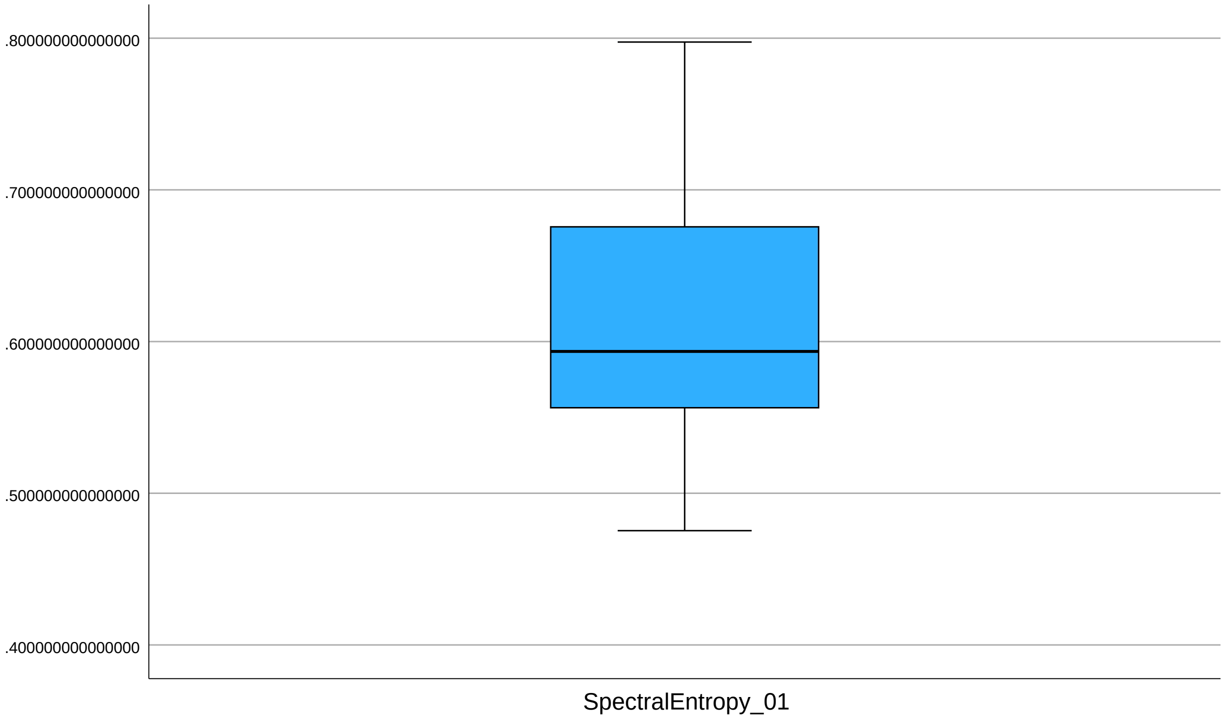

HiguchiFractalDimension

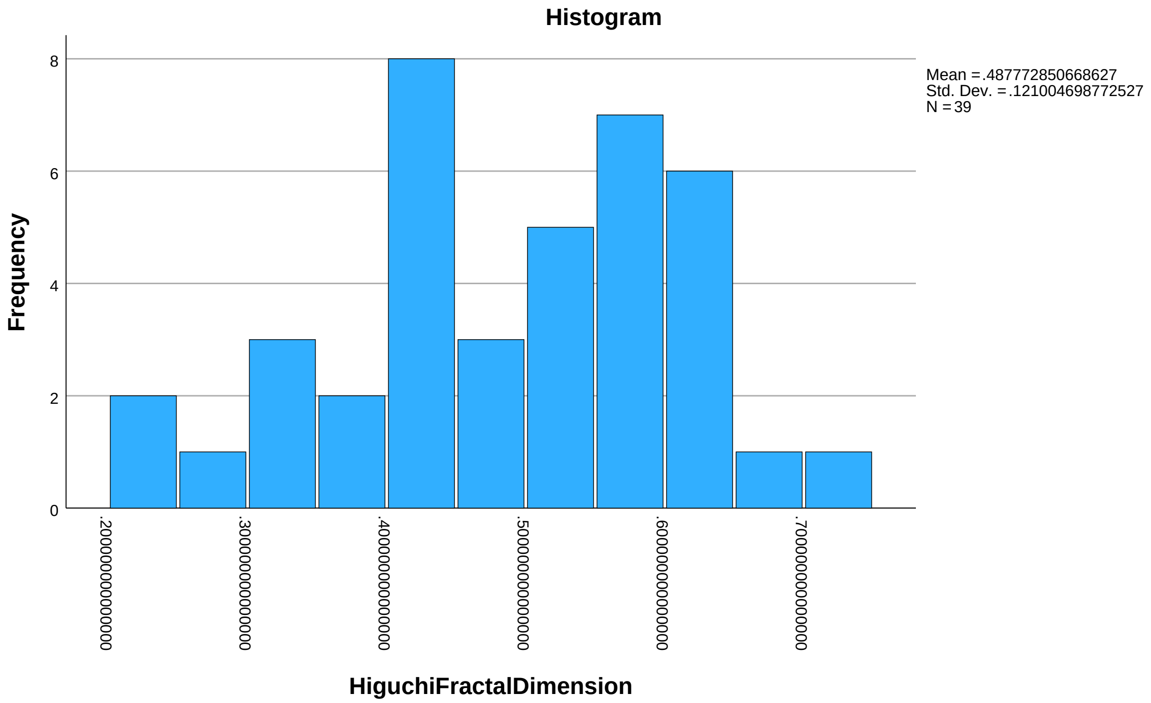


```
HiguchiFractalDimension Stem-and-Leaf Plot

 Frequency    Stem &  Leaf

     2.00        2 .  44
     1.00        2 .  9
     3.00        3 .  012
     2.00        3 .  78
     8.00        4 .  00222233
     3.00        4 .  588
     5.00        5 .  01334
     7.00        5 .  5566788
     6.00        6 .  001223
     1.00        6 .  6
     1.00        7 .  3

 Stem width:   .1000000
 Each leaf:        1 case(s)
```

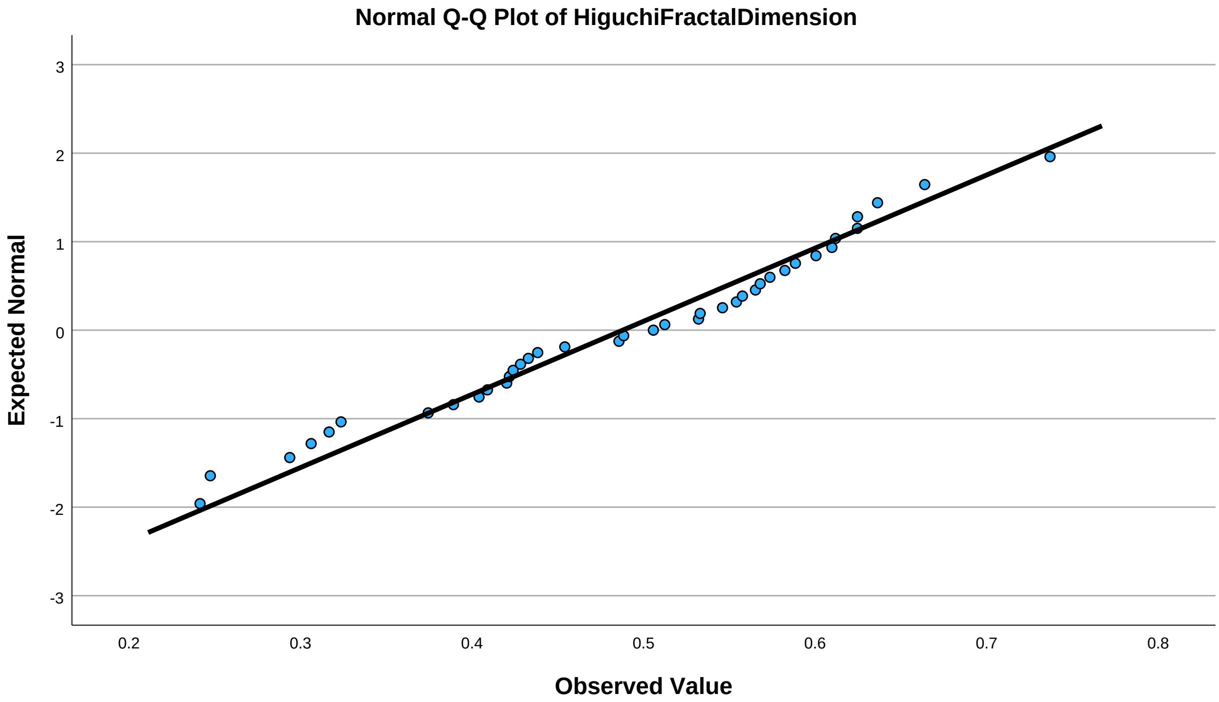
Normal Q-Q Plot of HiguchiFractalDimension
Expected Normal
3
2
1
0
-1
-2
-3
0.2
0.3
0.4
0.5
0.6
0.7
0.8
Observed Value

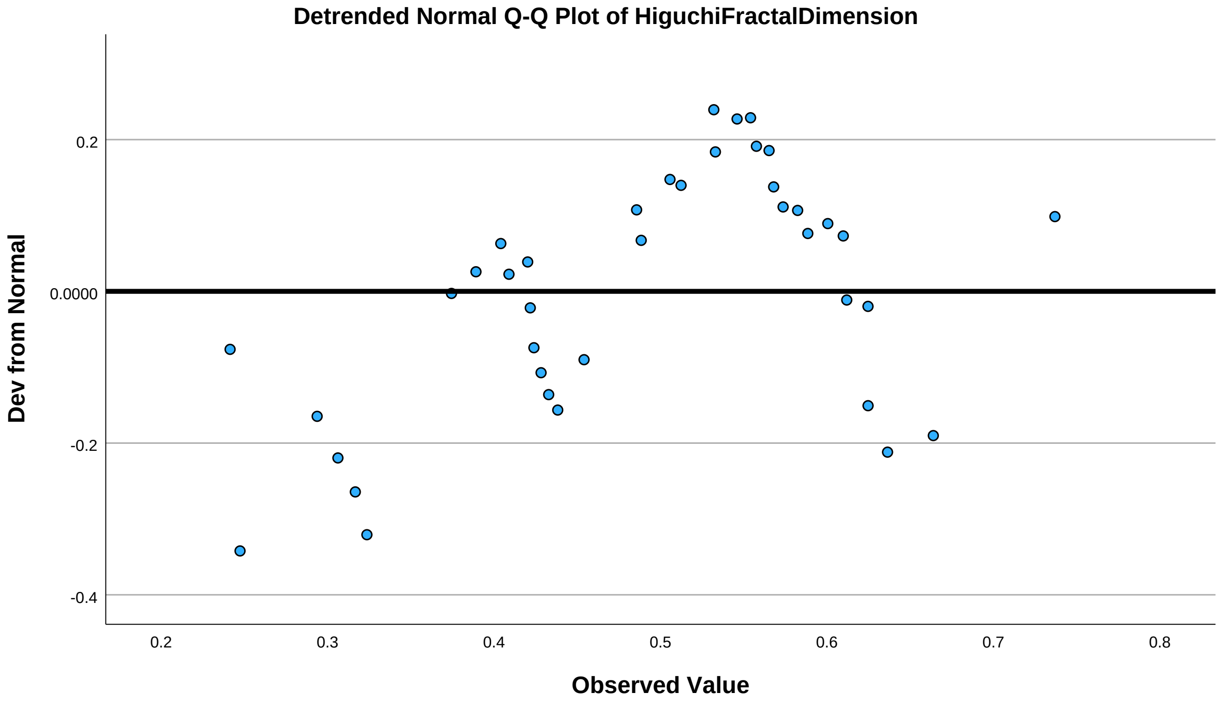
Detrended Normal Q-Q Plot of HiguchiFractalDimension
Dev from Normal
0.2
0.0000
-0.2
-0.4
0.2
0.3
0.4
0.5
0.6
0.7
0.8
Observed Value

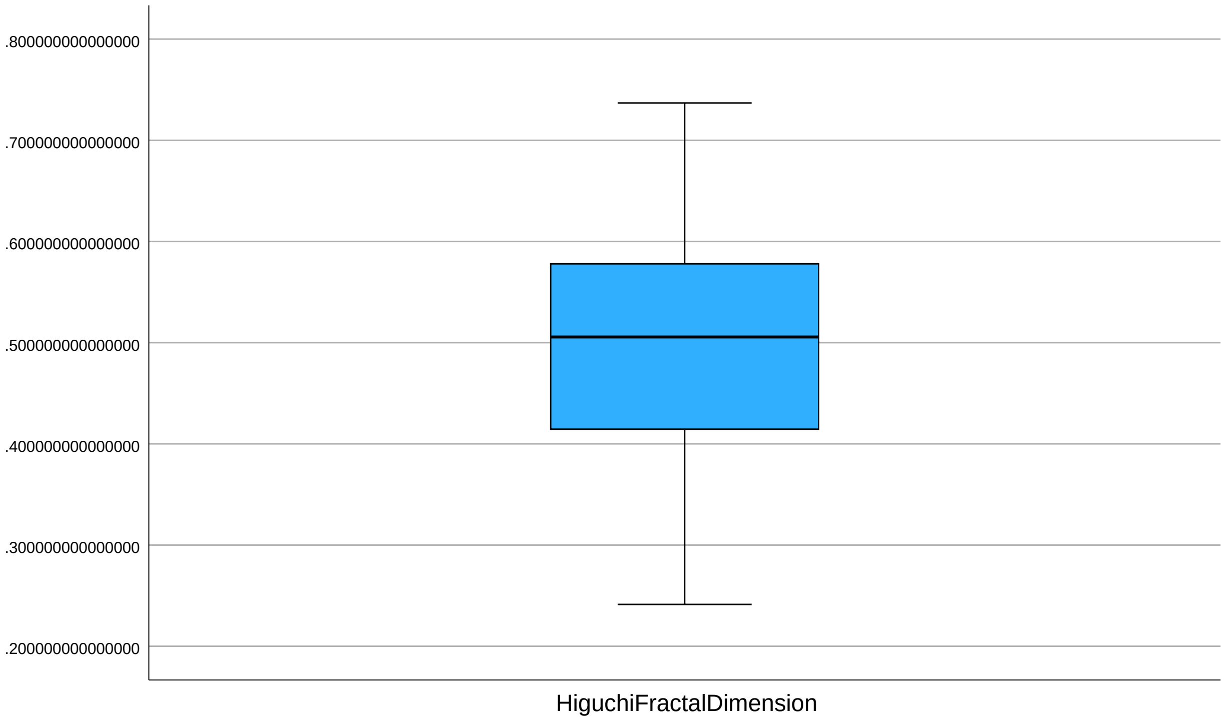

LempelZivComplexity

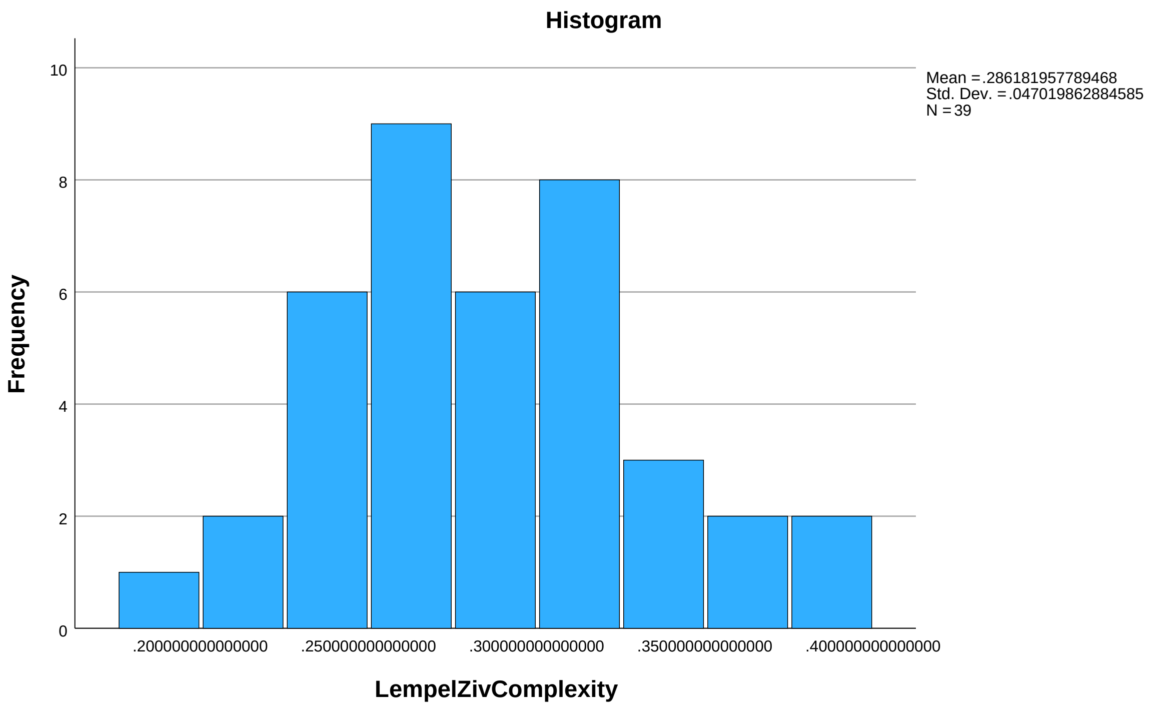


```
LempelZivComplexity Stem-and-Leaf Plot

 Frequency    Stem &  Leaf
```

```
 1.00      1 .  9
 8.00      2 .  02334444
15.00      2 .  555566777788888
11.00      3 .  00011111344
 4.00      3 .  6779

Stem width:   .1000000
Each leaf:       1 case(s)
```

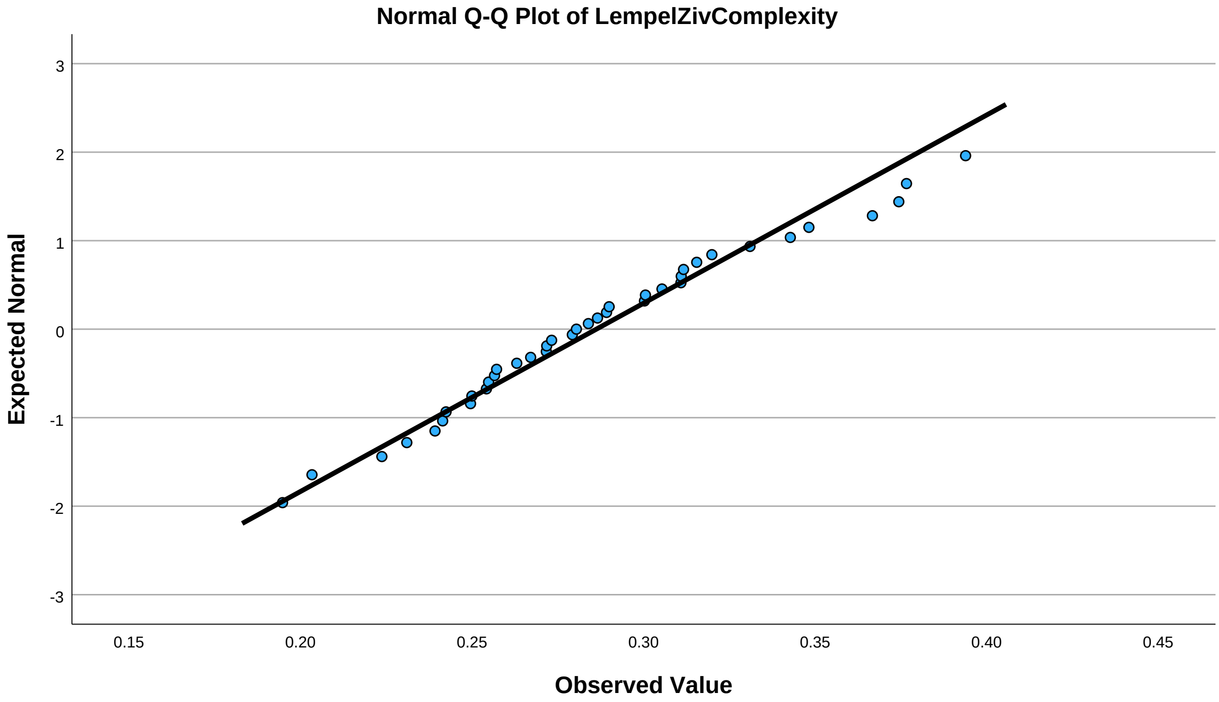

Detrended Normal Q-Q Plot of LempelZivComplexity

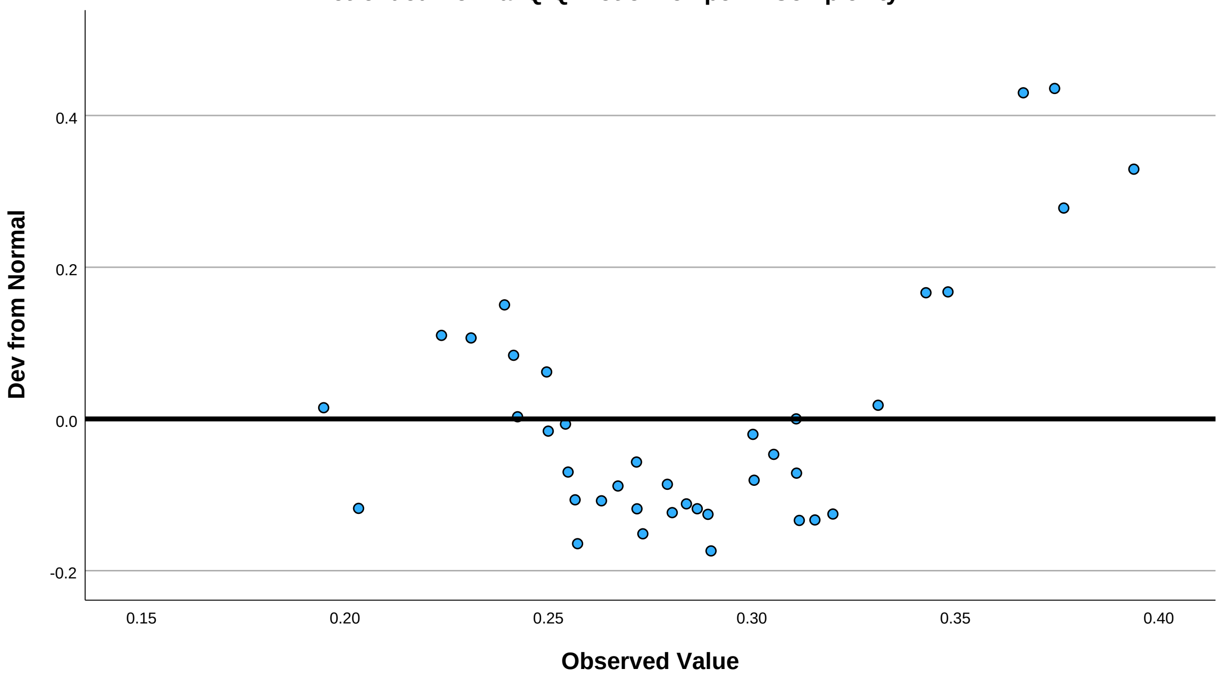

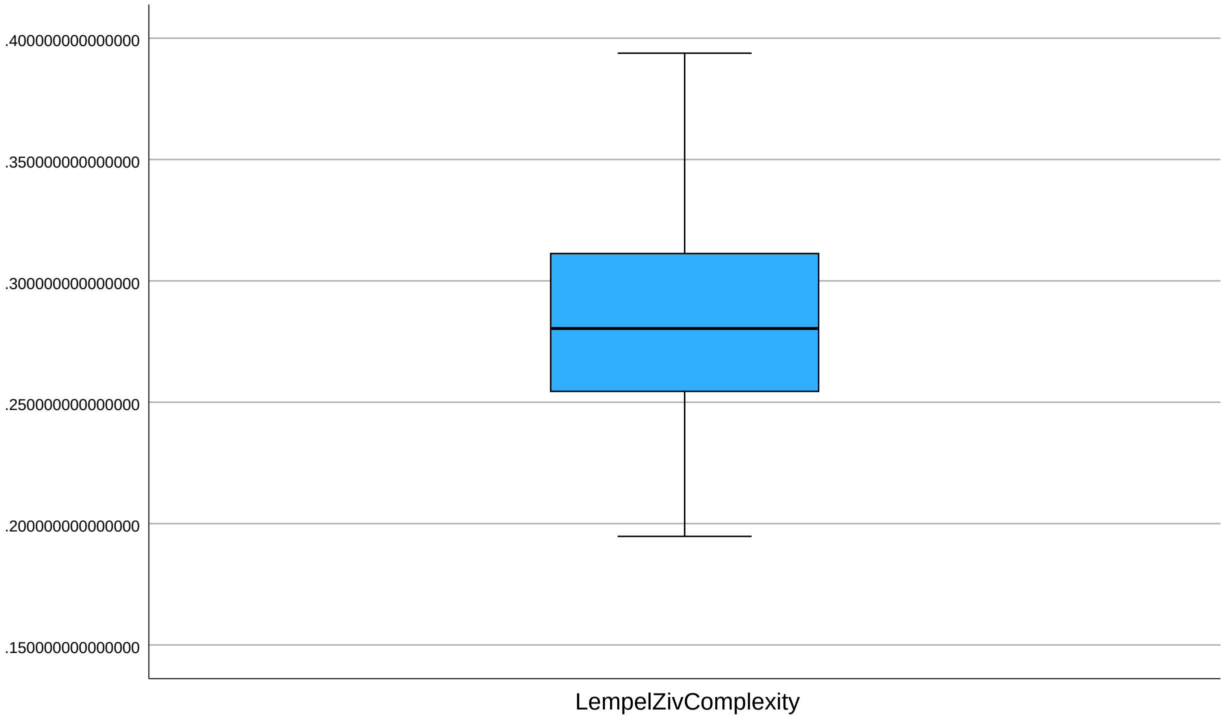

RenyiEntropy

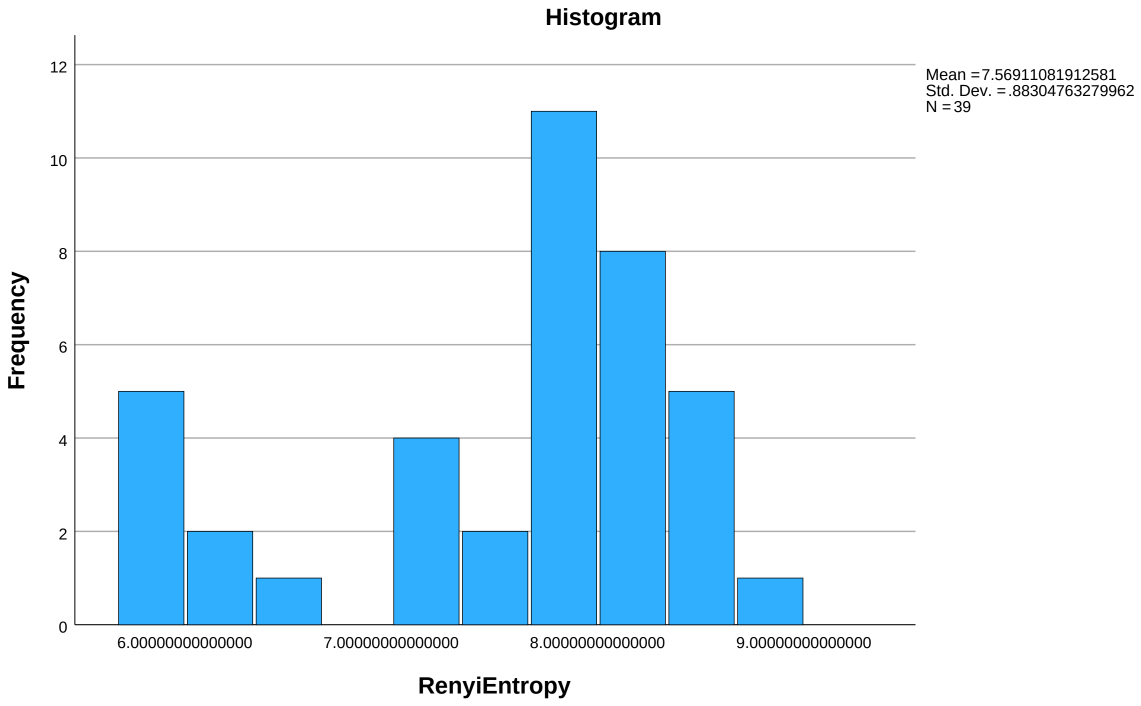


```
RenyiEntropy Stem-and-Leaf Plot

 Frequency    Stem &  Leaf

     1.00 Extremes    (=<5.7)
     4.00        5 .  8899
     2.00        6 .  11
     1.00        6 .  5
     6.00        7 .  012344
    11.00        7 .  67889999999
    10.00        8 .  0000112244
     4.00        8 .  5566

 Stem width:  1.000000
 Each leaf:       1 case(s)
```

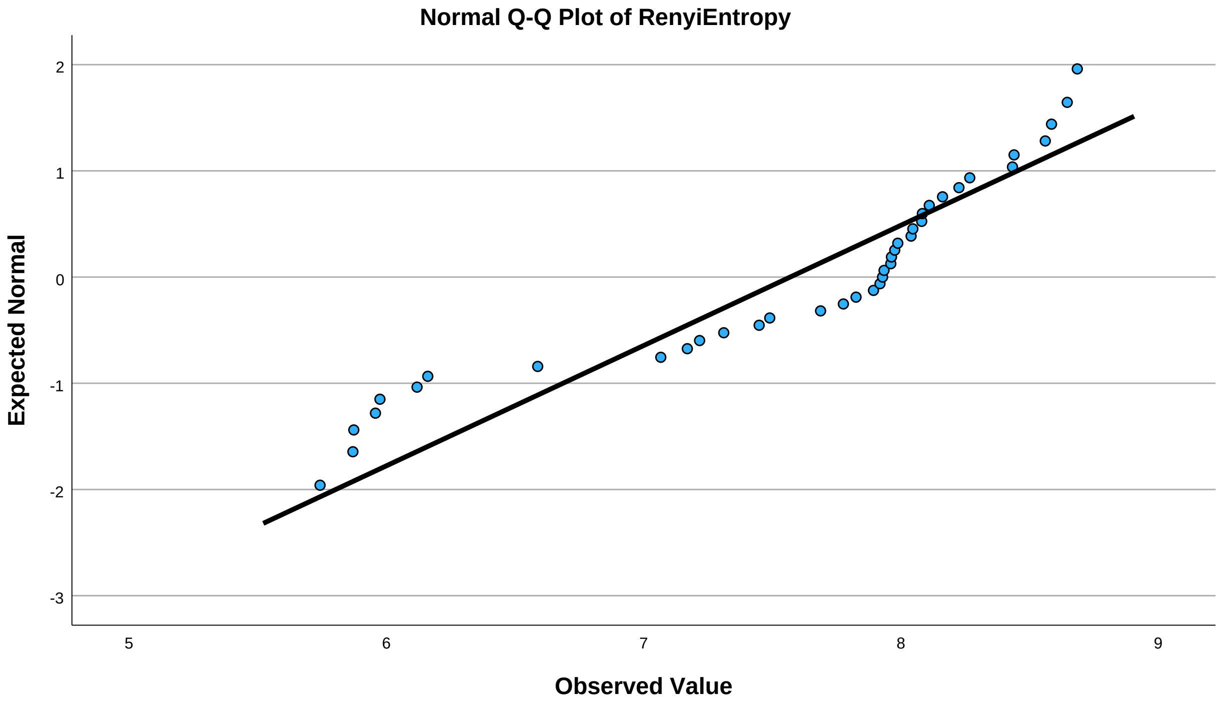
Normal Q-Q Plot of RenyiEntropy
Expected Normal
Observed Value
2
1
0
-1
-2
-3
5
6
7
8
9

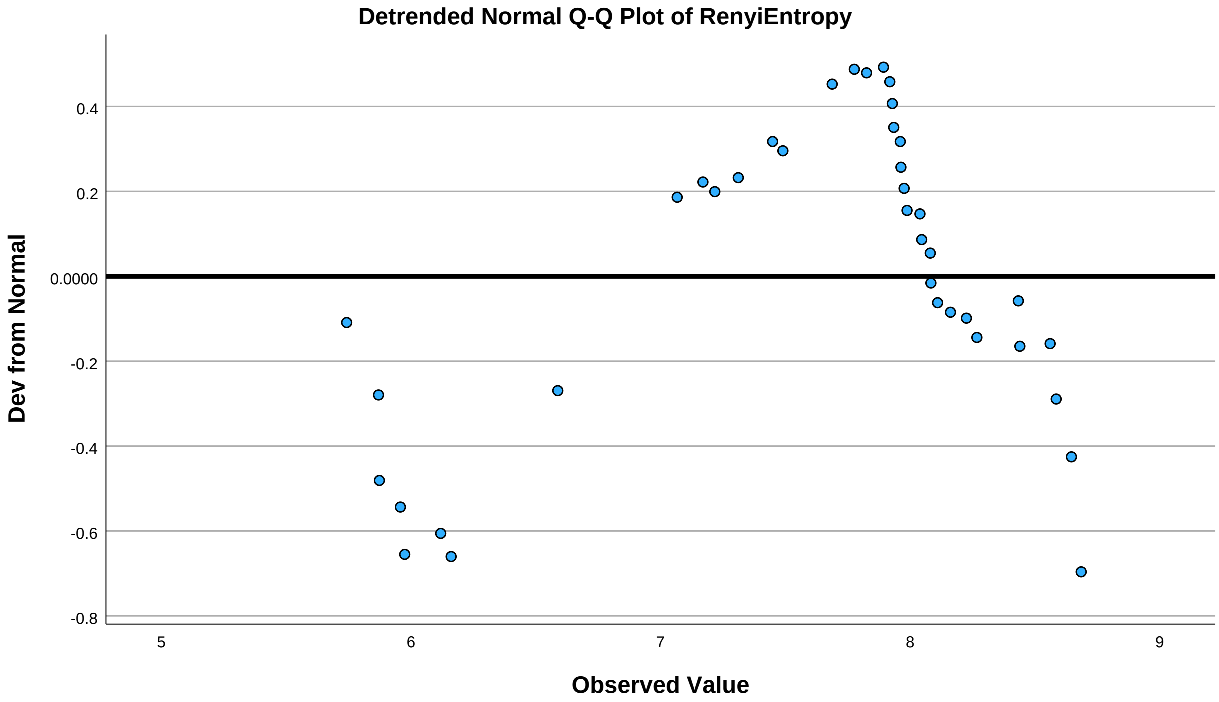
Detrended Normal Q-Q Plot of RenyiEntropy
Dev from Normal
Observed Value
0.4
0.2
0.0000
-0.2
-0.4
-0.6
-0.8
5
6
7
8
9

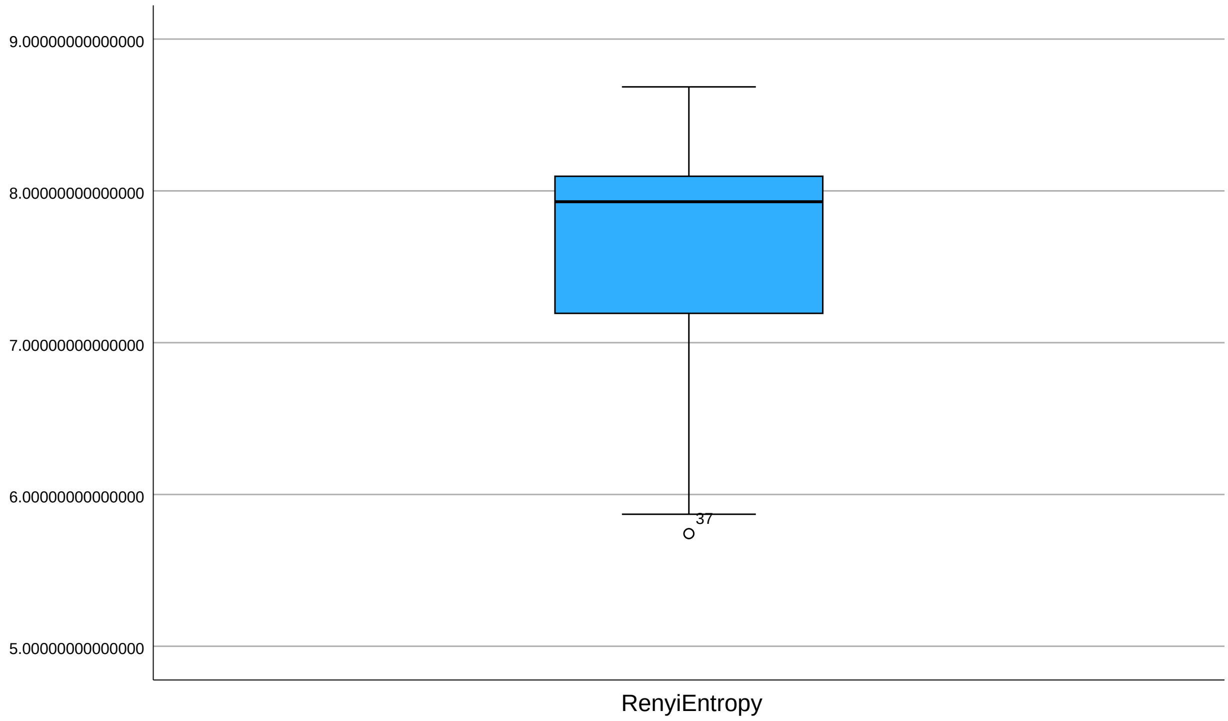

TsallisEntropy

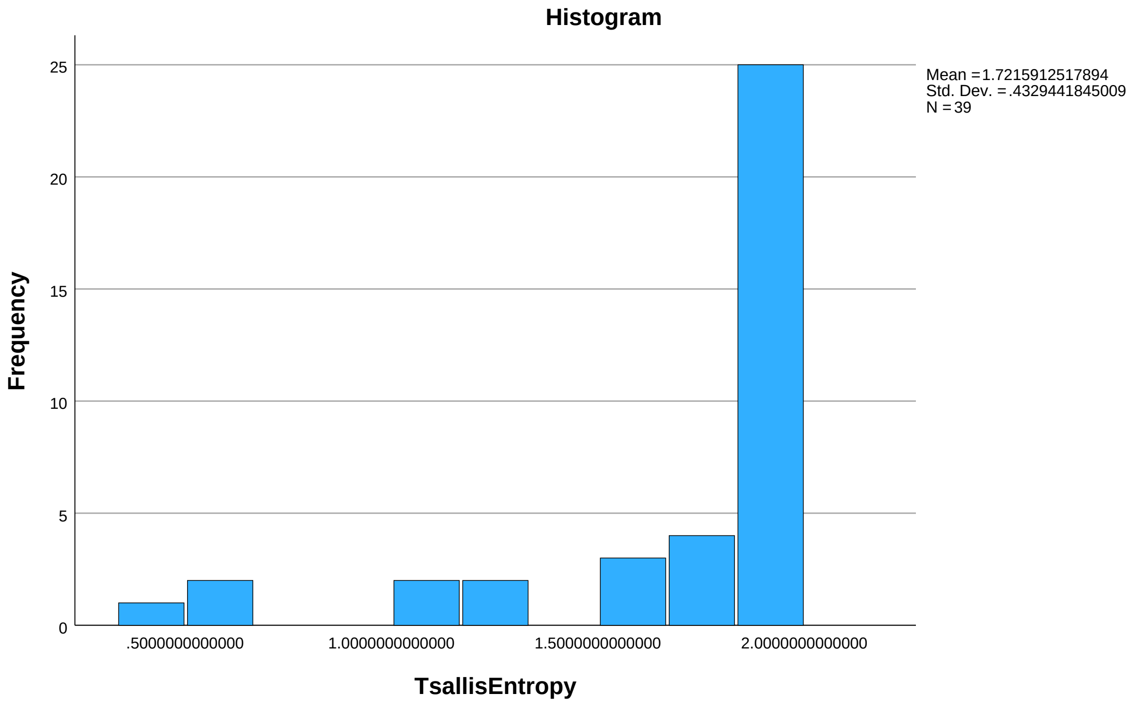

TsallisEntropy Stem-and-Leaf Plot

Frequency   Stem & Leaf

```
 7.00 Extremes    (=<1.27)
 2.00      15 .  68
  .00     16 .
 1.00      16 .  6
 1.00      17 .  4
 1.00      17 .  7
 2.00      18 .  03
 3.00      18 .  557
 3.00      19 .  024
 19.00       19 .  5667778888888888888

Stem width:   .1000000
Each leaf:       1 case(s)
```

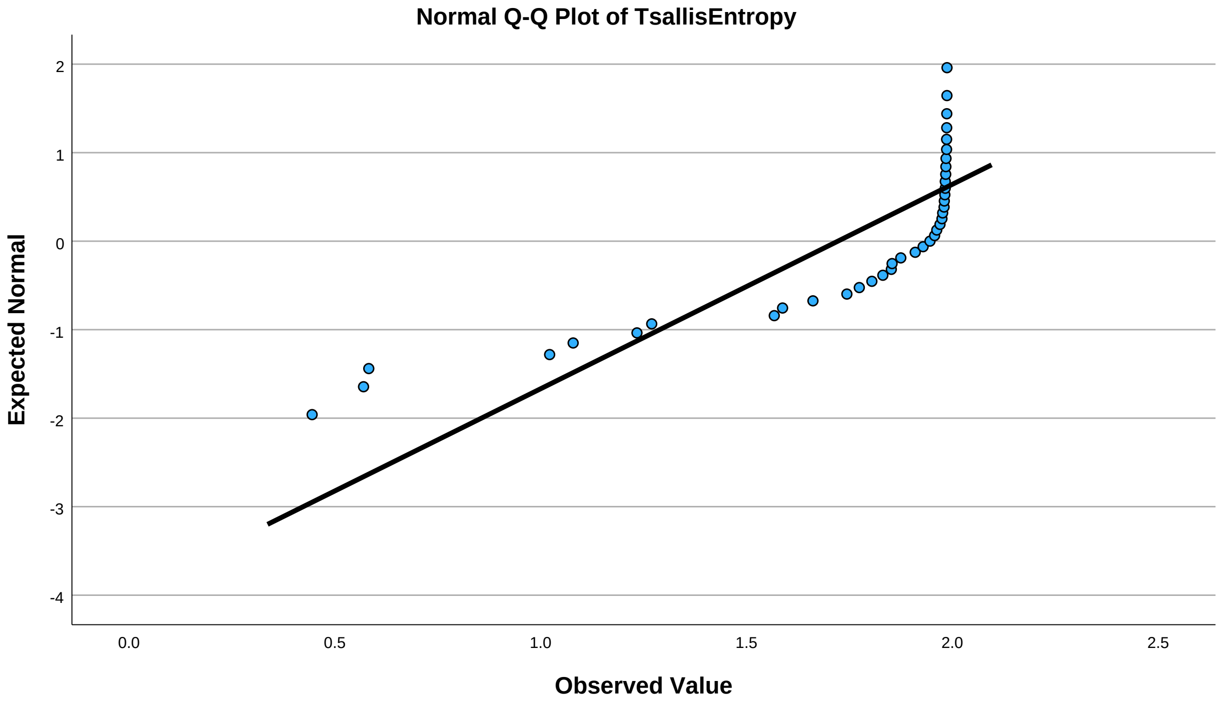

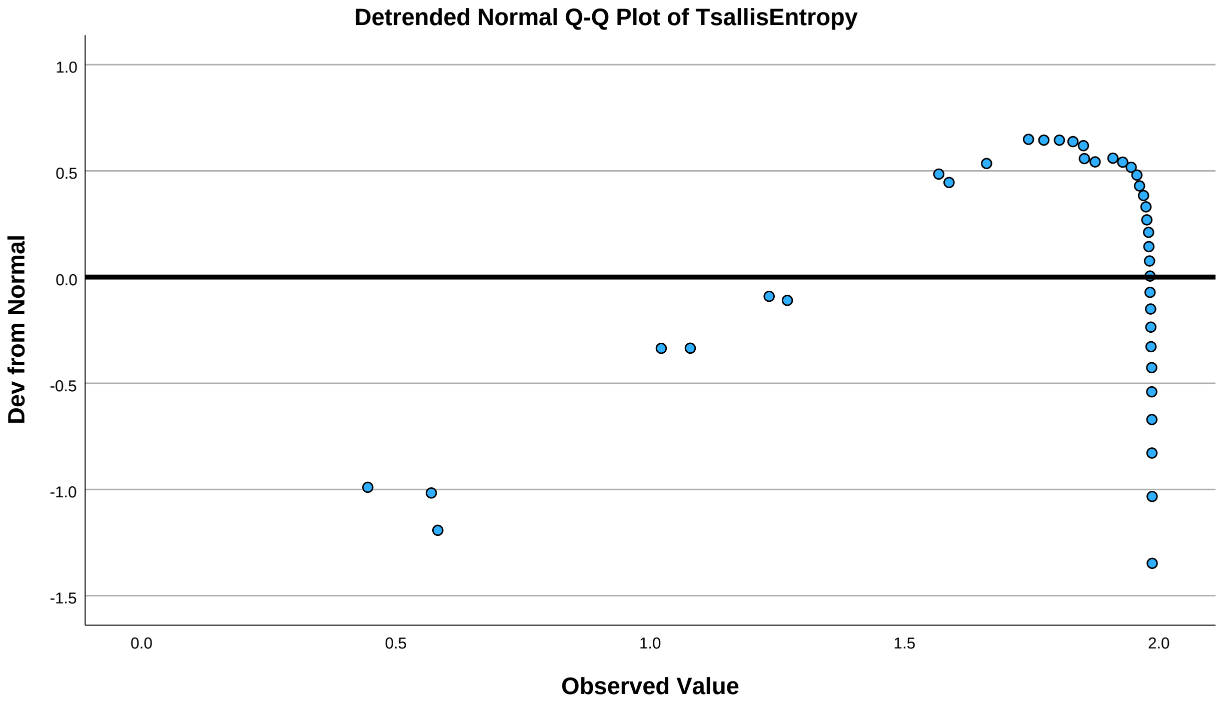

Detrended Normal Q-Q Plot of TsallisEntropy
Dev from Normal
1.0
0.5
0.0
-0.5
-1.0
-1.5
0.0
0.5
1.0
1.5
2.0
Observed Value

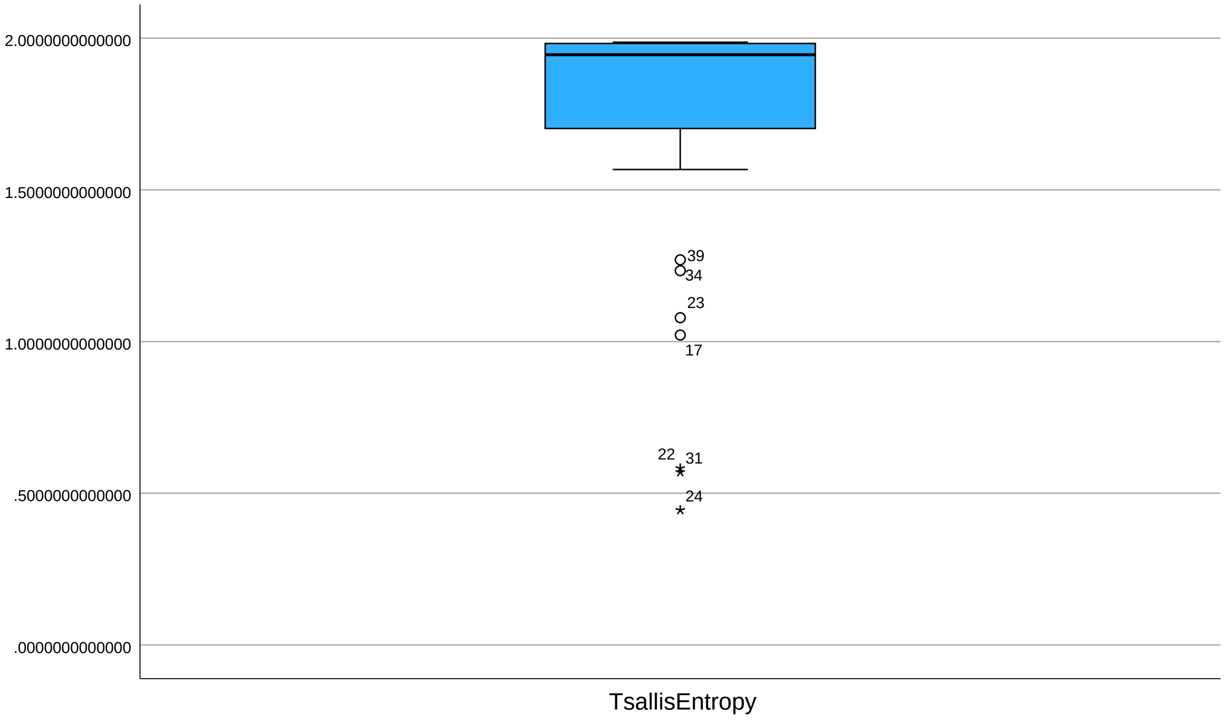

2.0000000000000
1.5000000000000
1.0000000000000
.5000000000000
.0000000000000
39
34
23
17
22
31
24
TsallisEntropy

PS_4_5

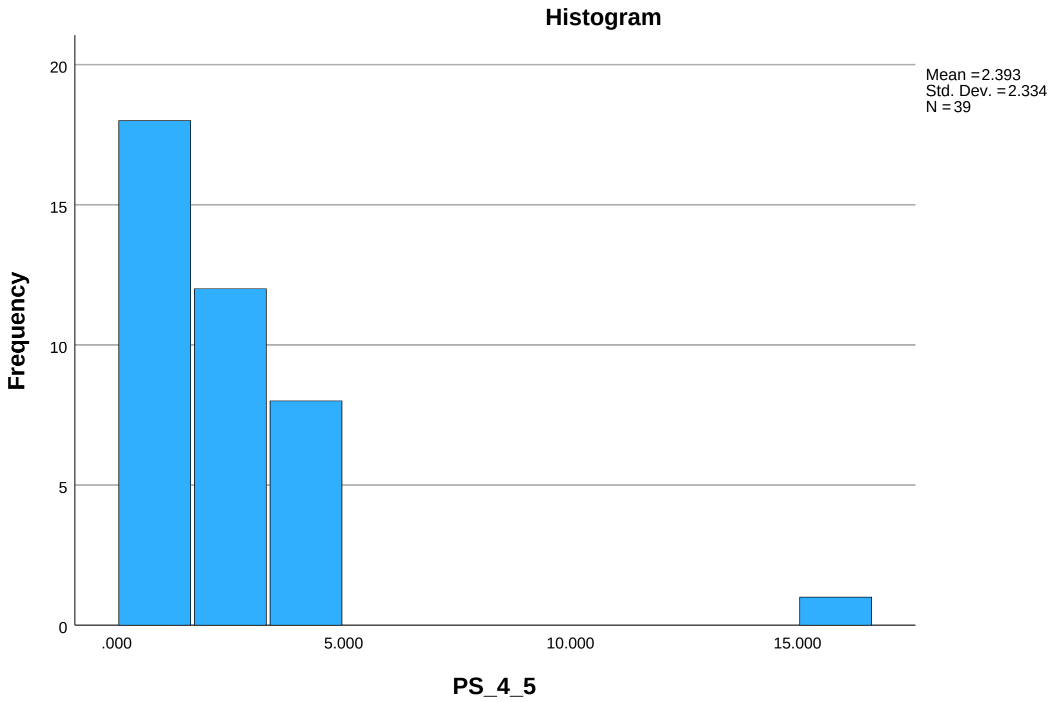


```
PS_4_5 Stem-and-Leaf Plot

 Frequency    Stem &  Leaf

     6.00        0 .  588999
     8.00        1 .  11111224
     7.00        1 .  5556789
     8.00        2 .  00111244
     1.00        2 .  9
     1.00        3 .  3
     4.00        3 .  7789
     3.00        4 .  012
     1.00 Extremes    (>=15.0)

 Stem width:      1.000
 Each leaf:       1 case(s)
```

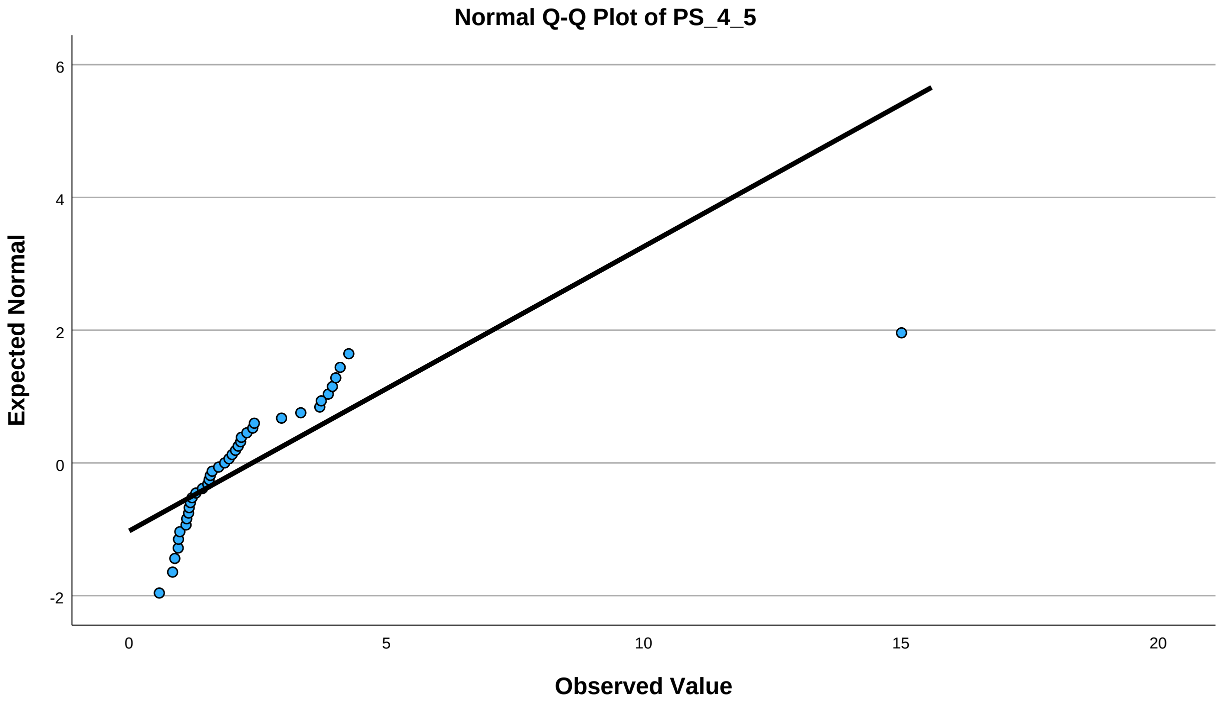
Normal Q-Q Plot of PS_4_5
Expected Normal
6
4
2
0
-2
0
5
10
15
20
Observed Value

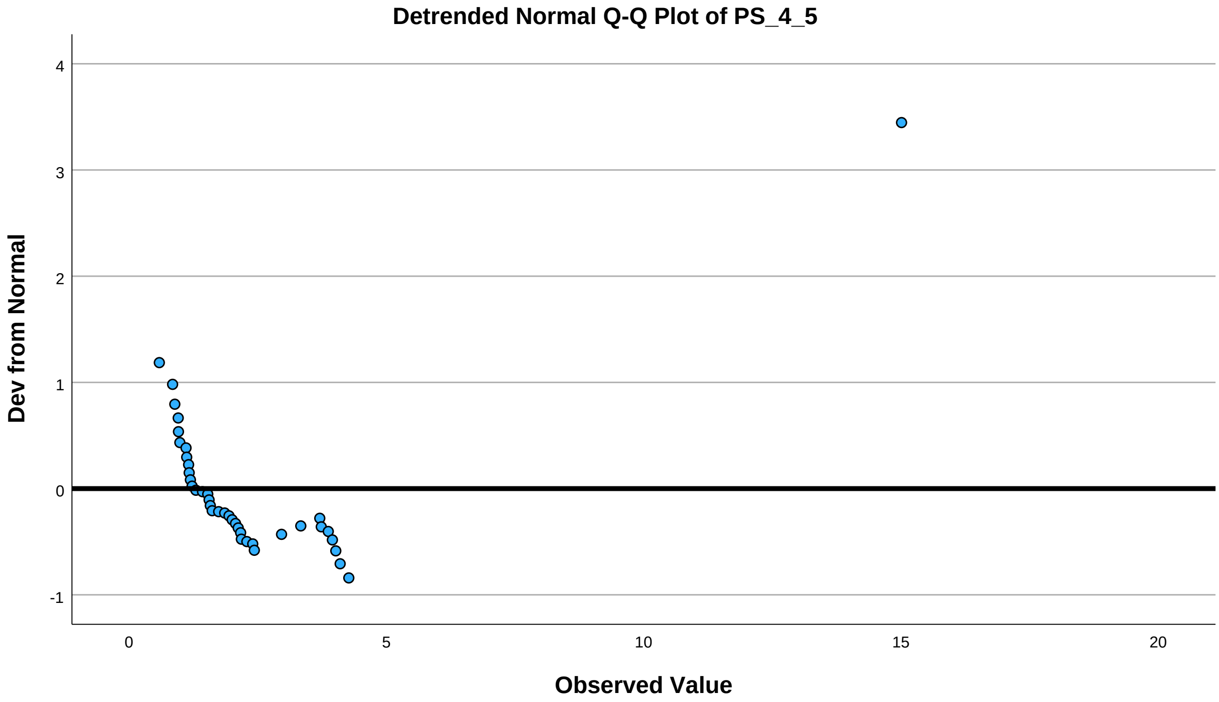
Detrended Normal Q-Q Plot of PS_4_5
Dev from Normal
4
3
2
1
0
-1
0
5
10
15
20
Observed Value

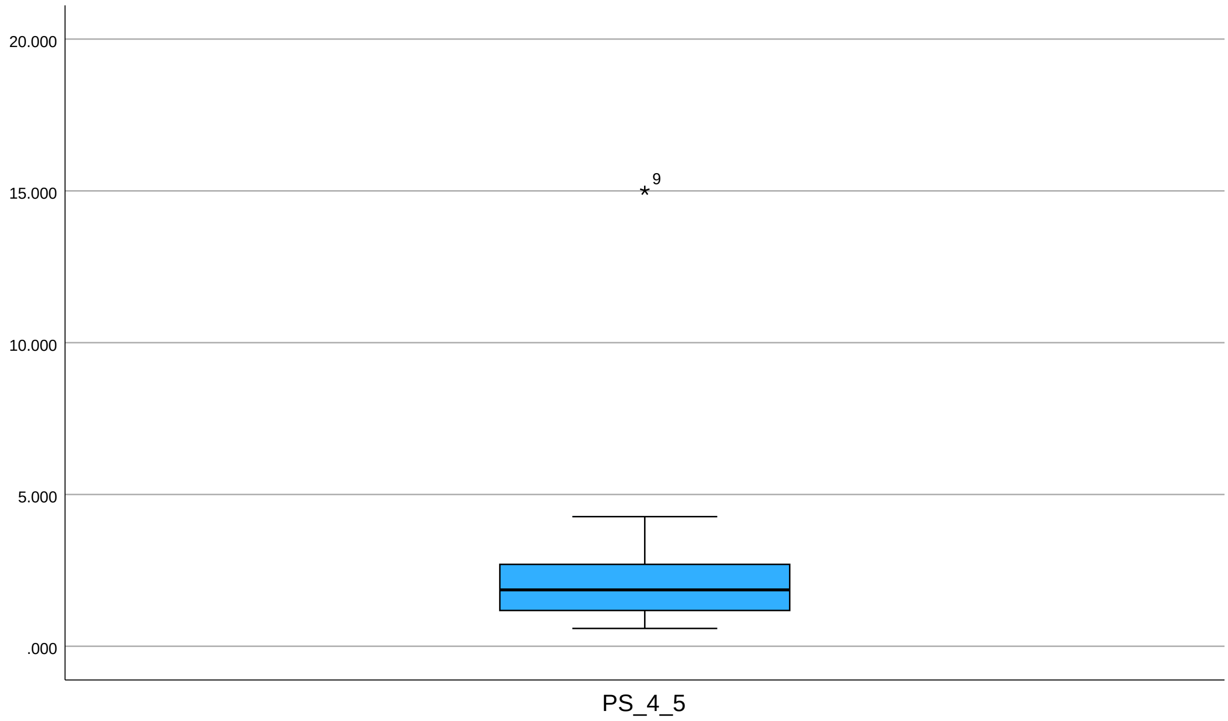

PS_5_6

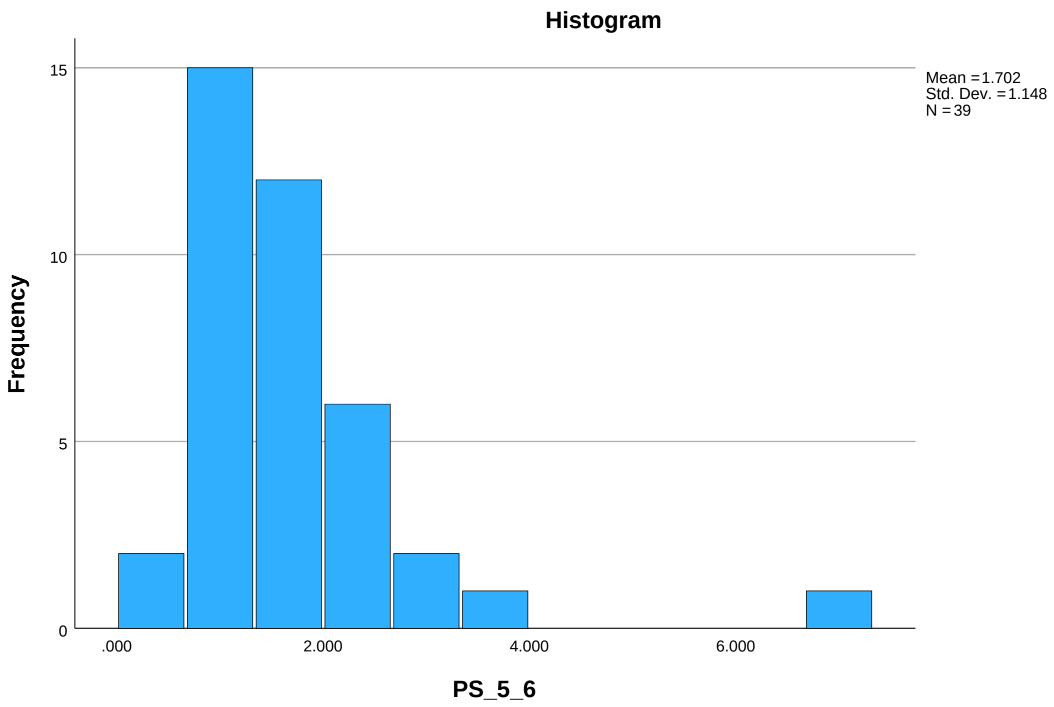


```
PS_5_6 Stem-and-Leaf Plot

 Frequency    Stem &  Leaf
```

```
 1.00      0 . 3
 7.00      0 . 5778888
14.00      1 . 00011122244444
 7.00      1 . 5567899
 4.00      2 . 1234
 4.00      2 . 6668
 2.00 Extremes    (>=3.7)

Stem width:     1.000
Each leaf:       1 case(s)
```

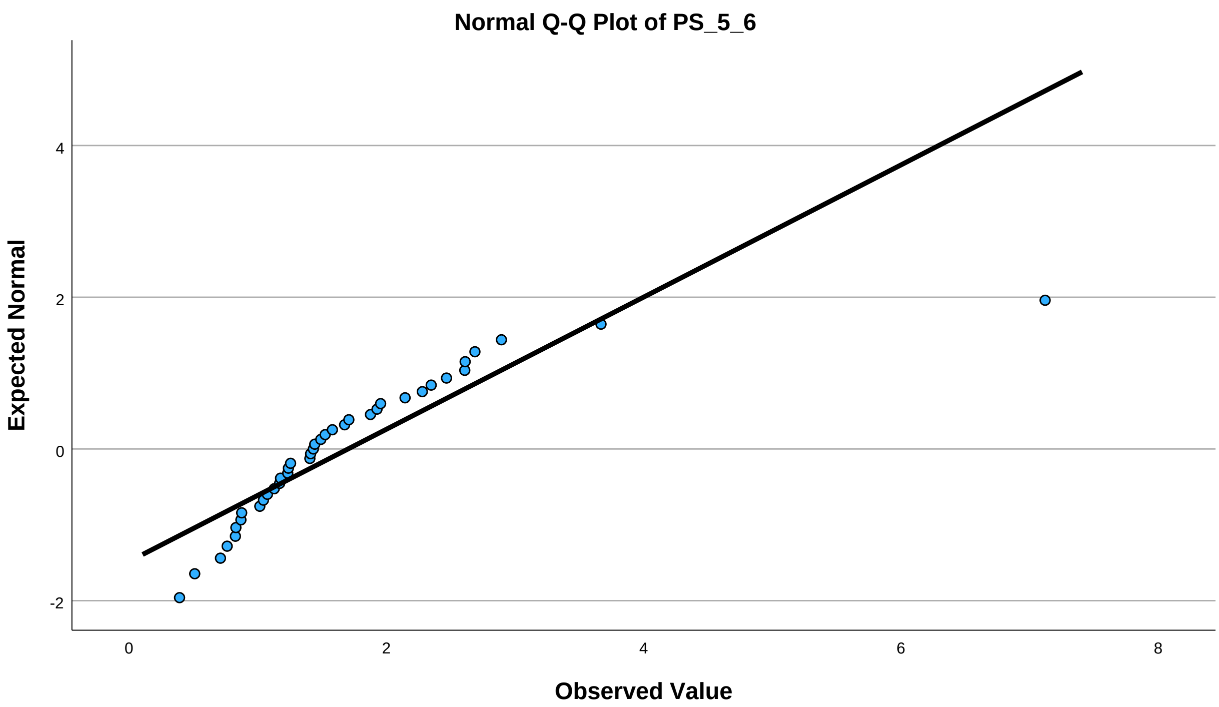

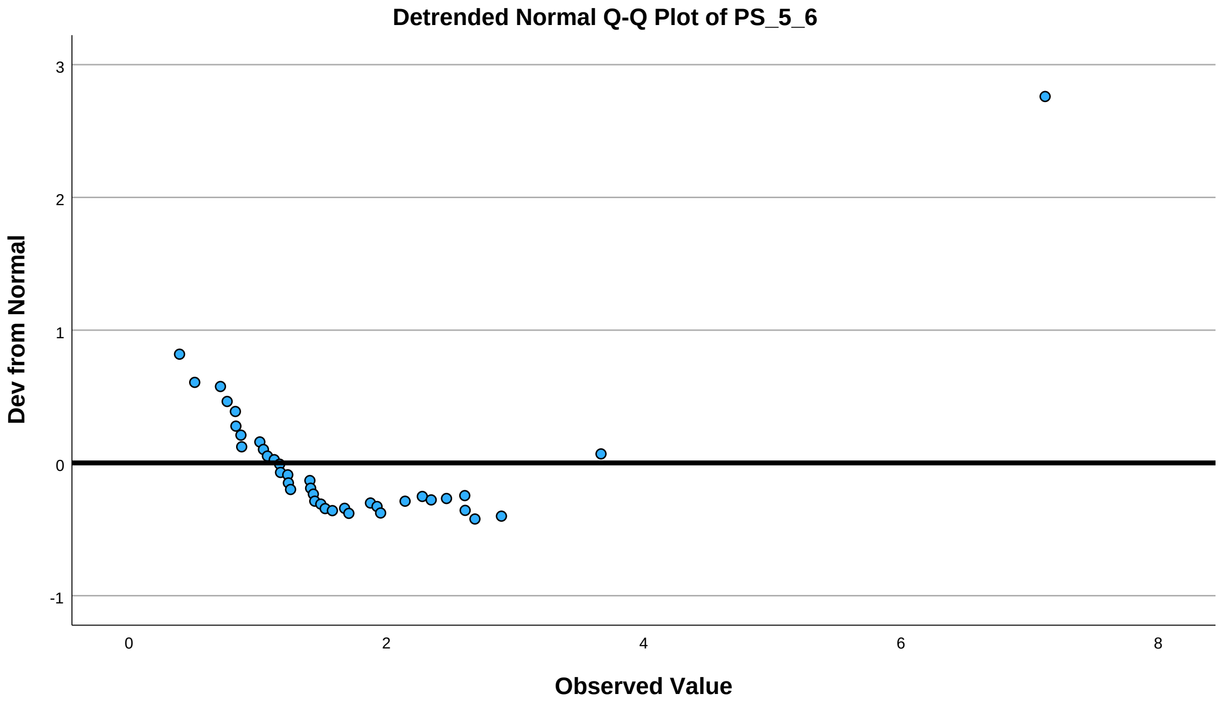
Detrended Normal Q-Q Plot of PS_5_6
Dev from Normal
3
2
1
0
-1
0
2
4
6
8
Observed Value

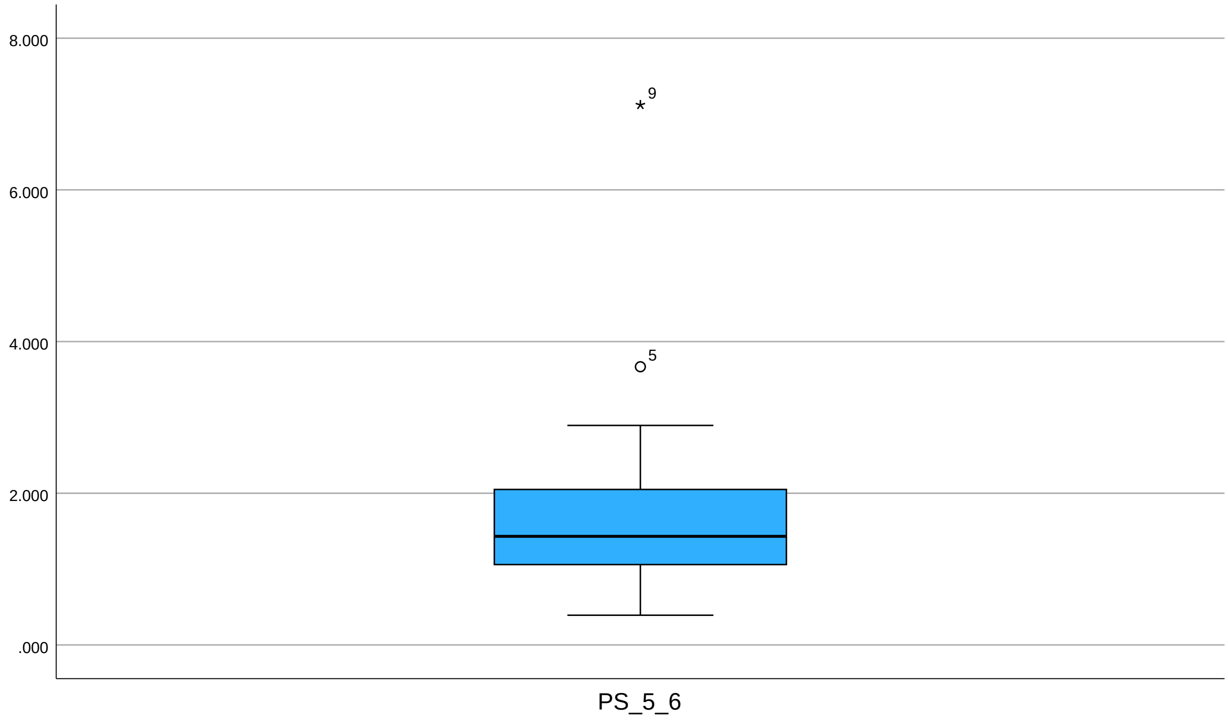
8.000
6.000
4.000
2.000
.000
9
5
PS_5_6

PS_6_7

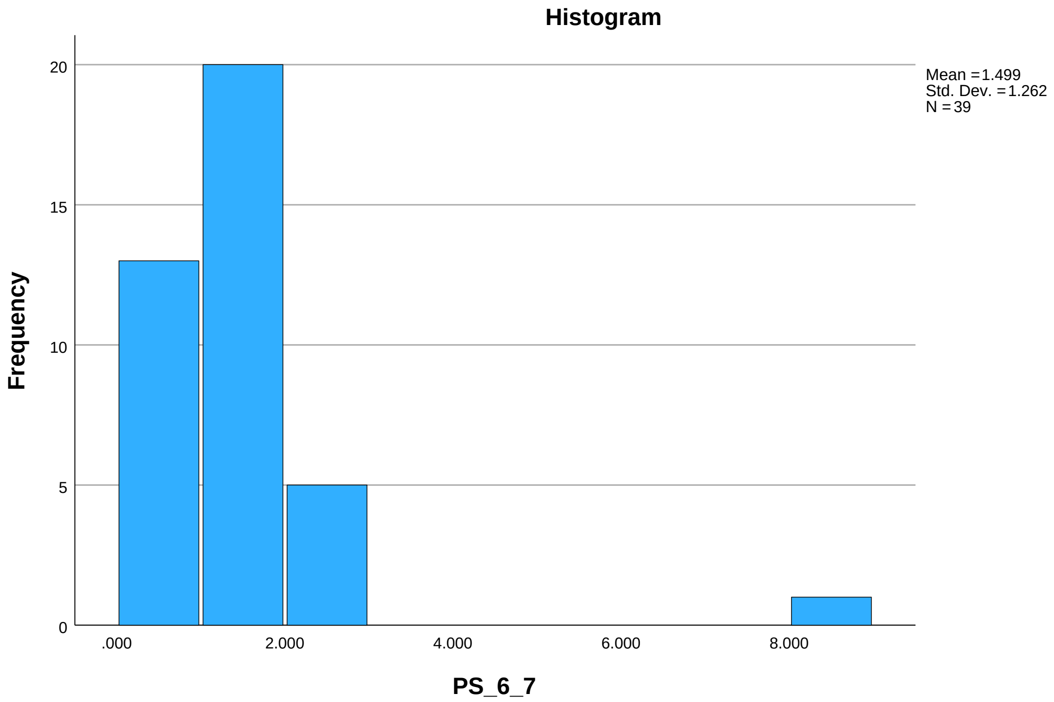


```
PS_6_7 Stem-and-Leaf Plot

 Frequency    Stem &  Leaf

     2.00        0 .  24
    11.00        0 .  56677788899
    11.00        1 .  00011122344
     9.00        1 .  566677888
     3.00        2 .  033
     2.00        2 .  56
     1.00 Extremes    (>=8.3)

 Stem width:      1.000
 Each leaf:       1 case(s)
```

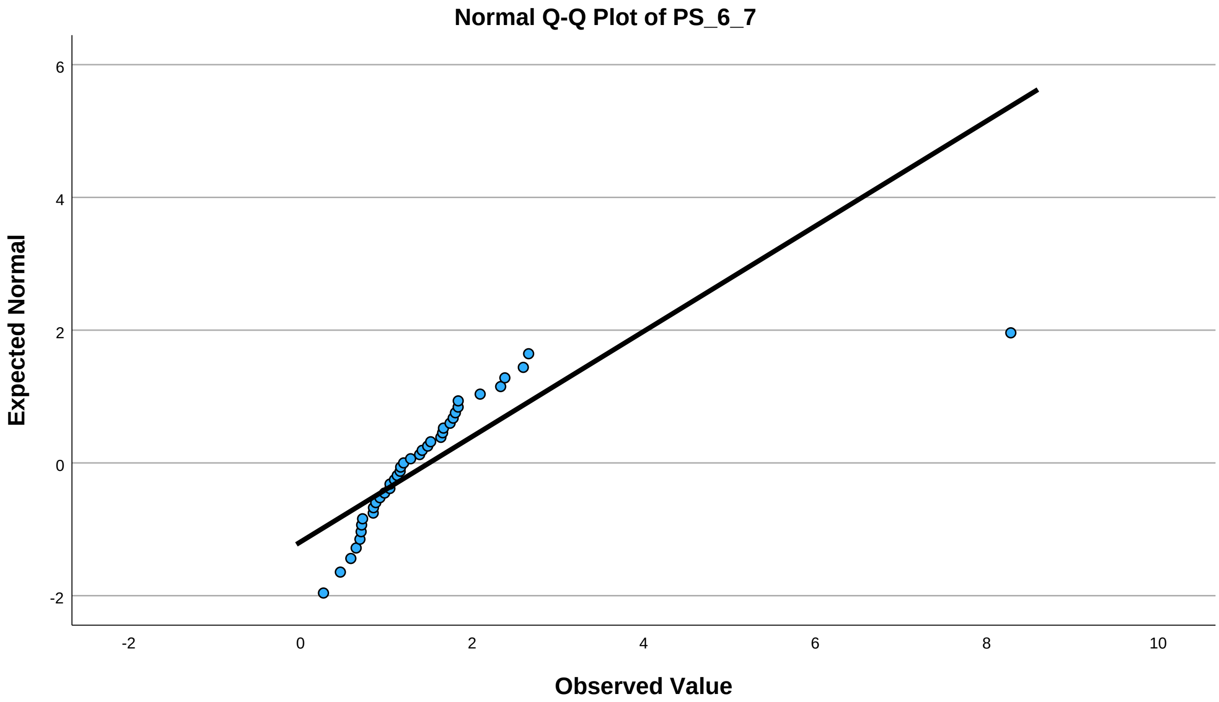
Normal Q-Q Plot of PS_6_7
Expected Normal
Observed Value
6
4
2
0
-2
-2
0
2
4
6
8
10

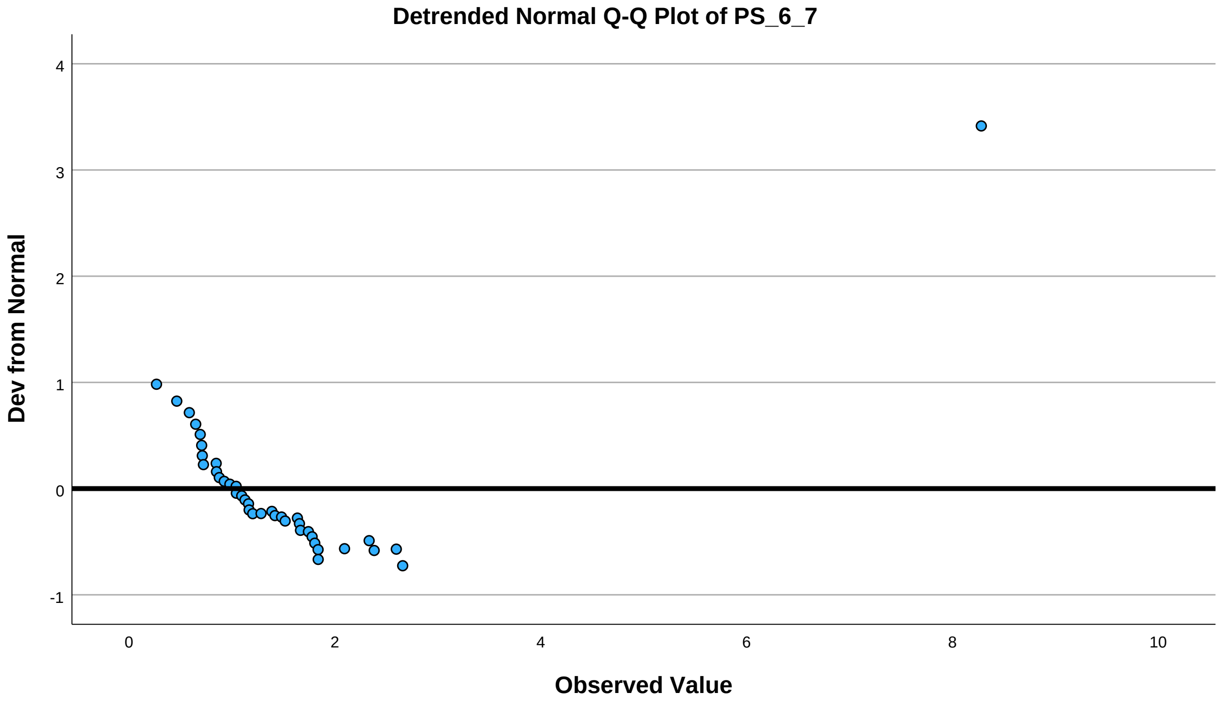
Detrended Normal Q-Q Plot of PS_6_7
Dev from Normal
Observed Value
4
3
2
1
0
-1
0
2
4
6
8
10

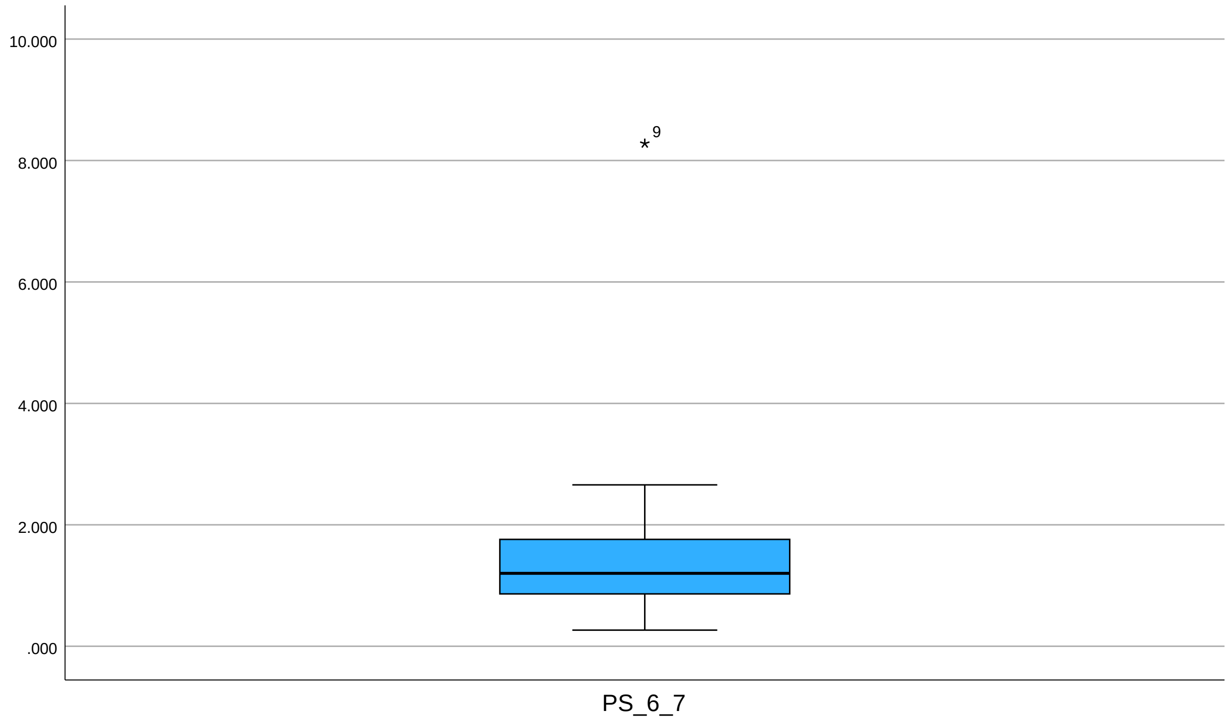

PS_7_8

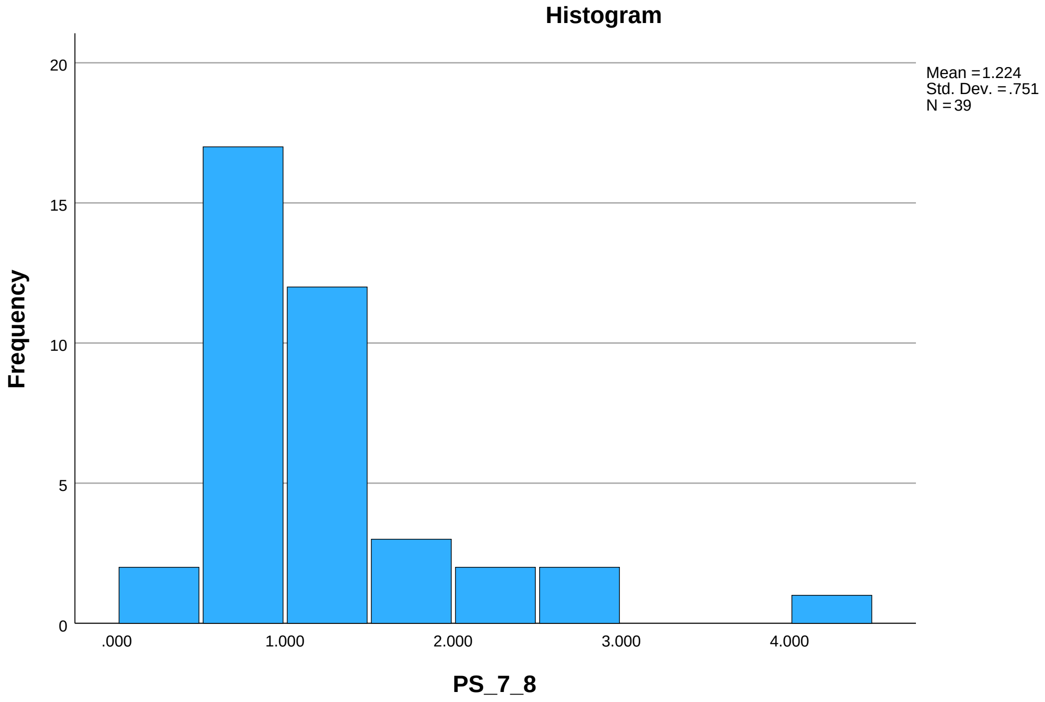

PS_7_8 Stem-and-Leaf Plot

Frequency   Stem & Leaf

```
  2.00      0 .  24
  17.00       0 .  55557778888888999
  12.00       1 .  000012233444
  3.00      1 .  566
  2.00      2 .  13
   3.00 Extremes    (>=2.6)

Stem width:    1.000
Each leaf:       1 case(s)
```

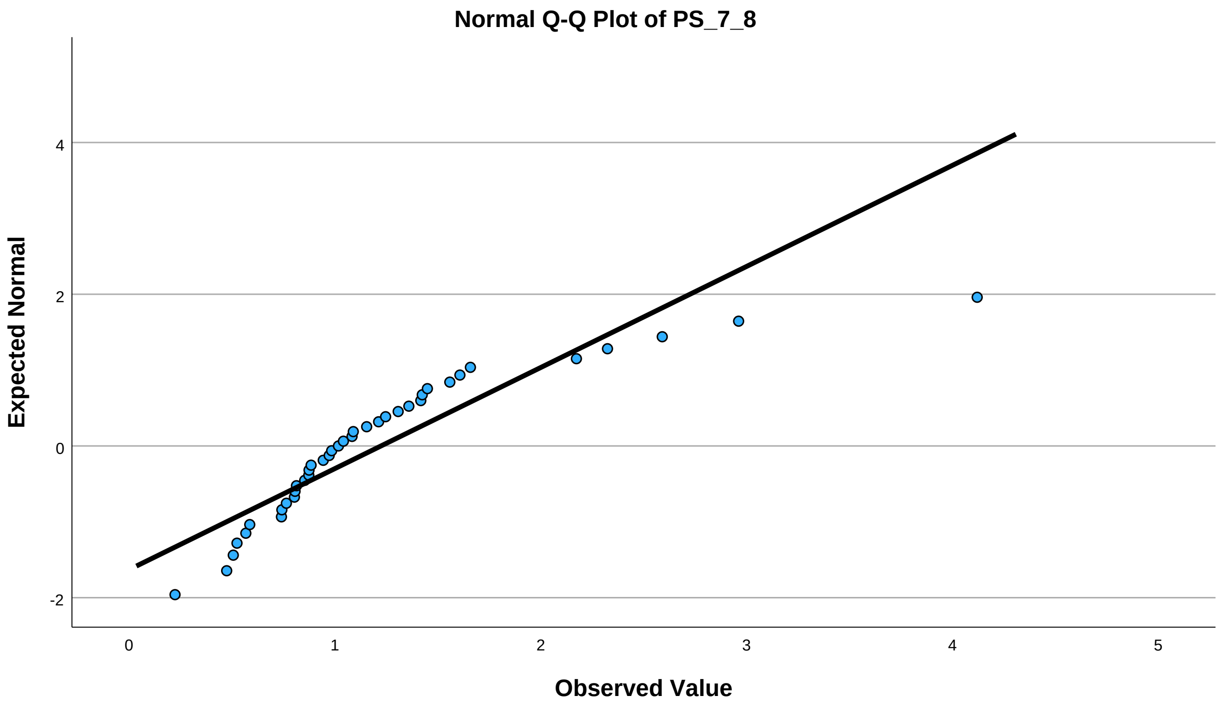

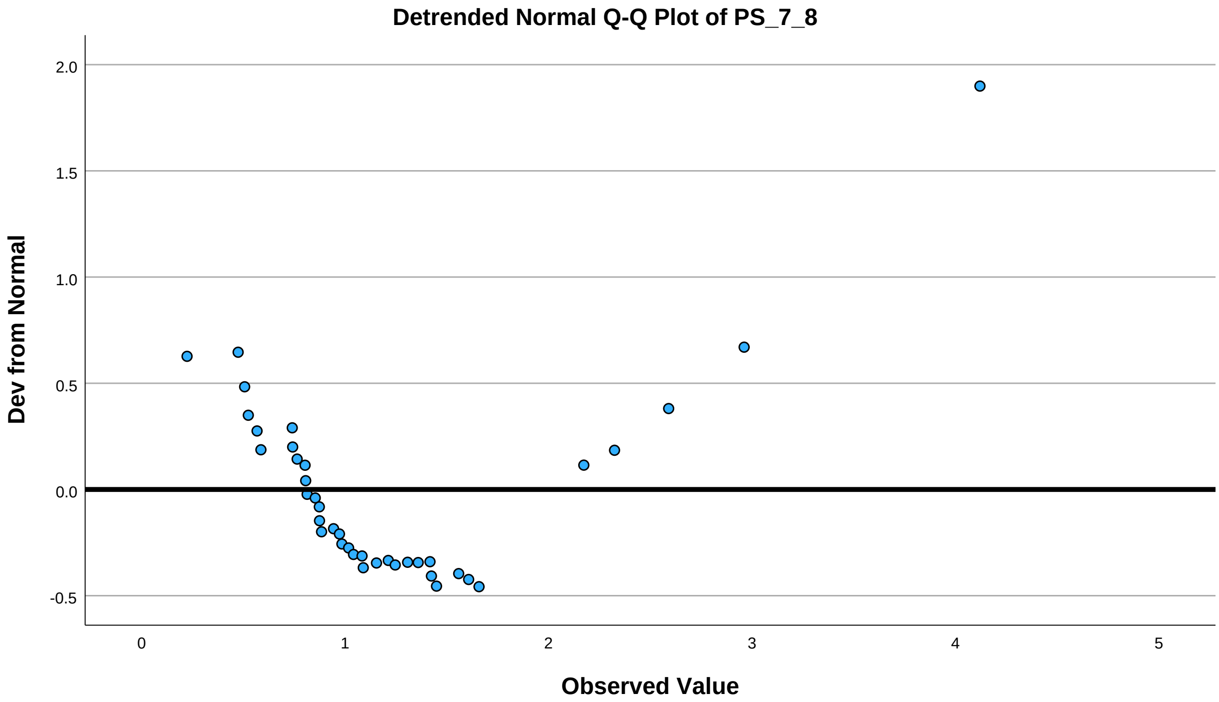

Detrended Normal Q-Q Plot of PS_7_8
Dev from Normal
2.0
1.5
1.0
0.5
0.0
-0.5
0
1
2
3
4
5
Observed Value

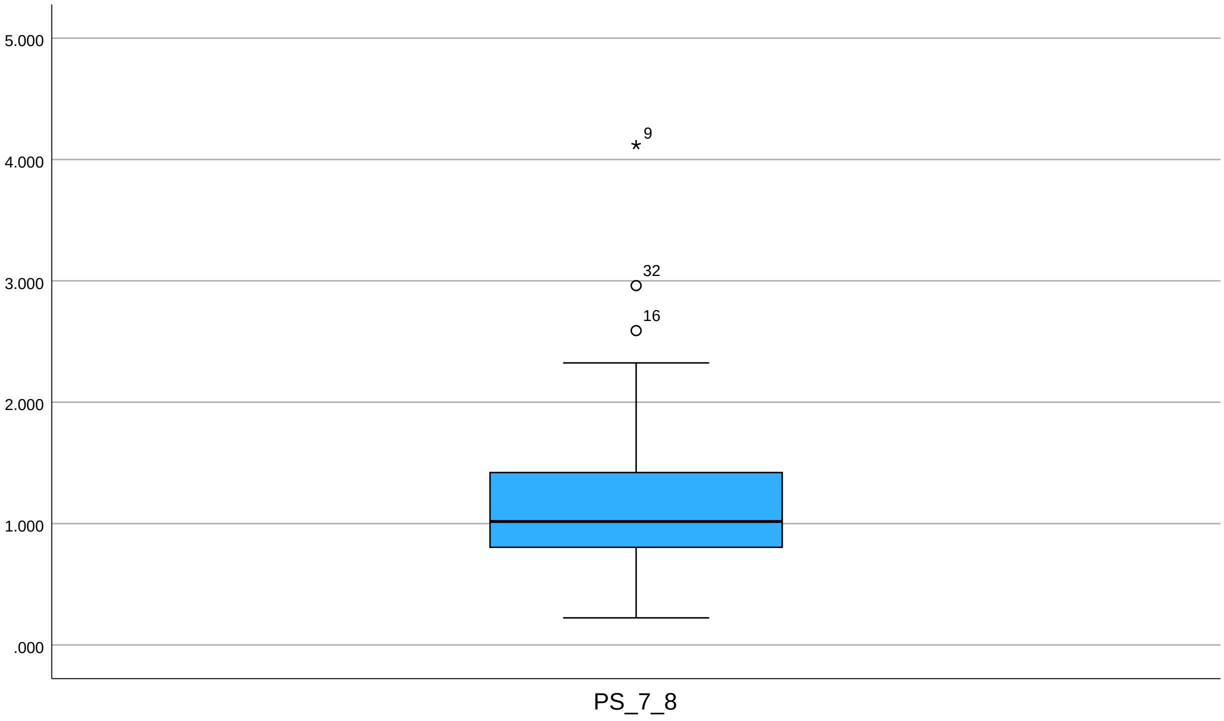

5.000
4.000
3.000
2.000
1.000
.000
9
32
16
PS_7_8

PS_8_9

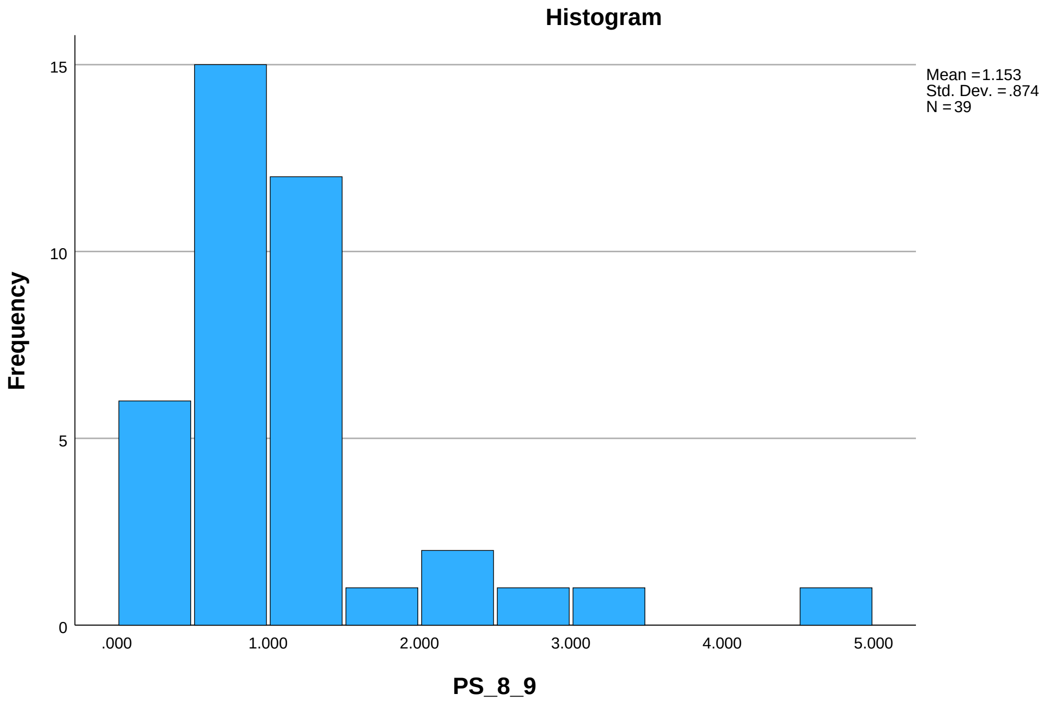


```
PS_8_9 Stem-and-Leaf Plot

 Frequency    Stem &  Leaf

     1.00        0 .  1
     2.00        0 .  33
     4.00        0 .  4445
     6.00        0 .  666677
     8.00        0 .  88888999
     8.00        1 .  00000111
     3.00        1 .  223
     1.00        1 .  4
     1.00        1 .  6
     5.00 Extremes    (>=2.1)

 Stem width:      1.000
 Each leaf:       1 case(s)
```

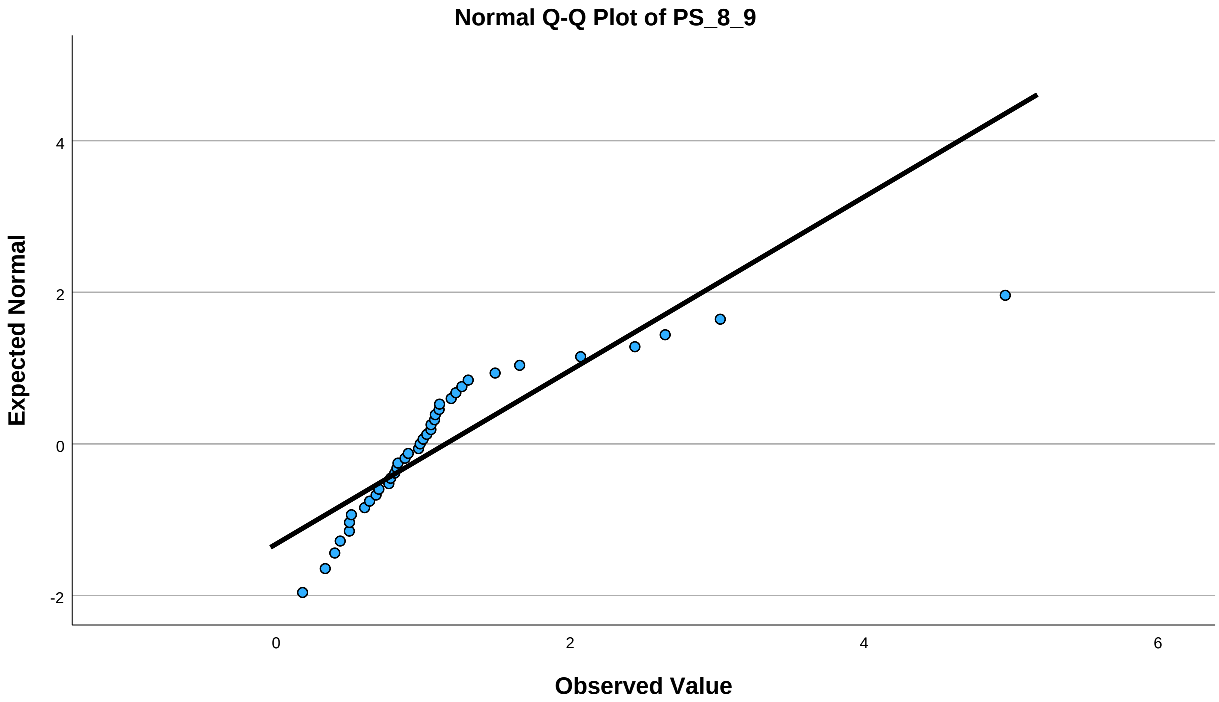

Normal Q-Q Plot of PS_8_9
Expected Normal
Observed Value
4
2
0
-2
0
2
4
6

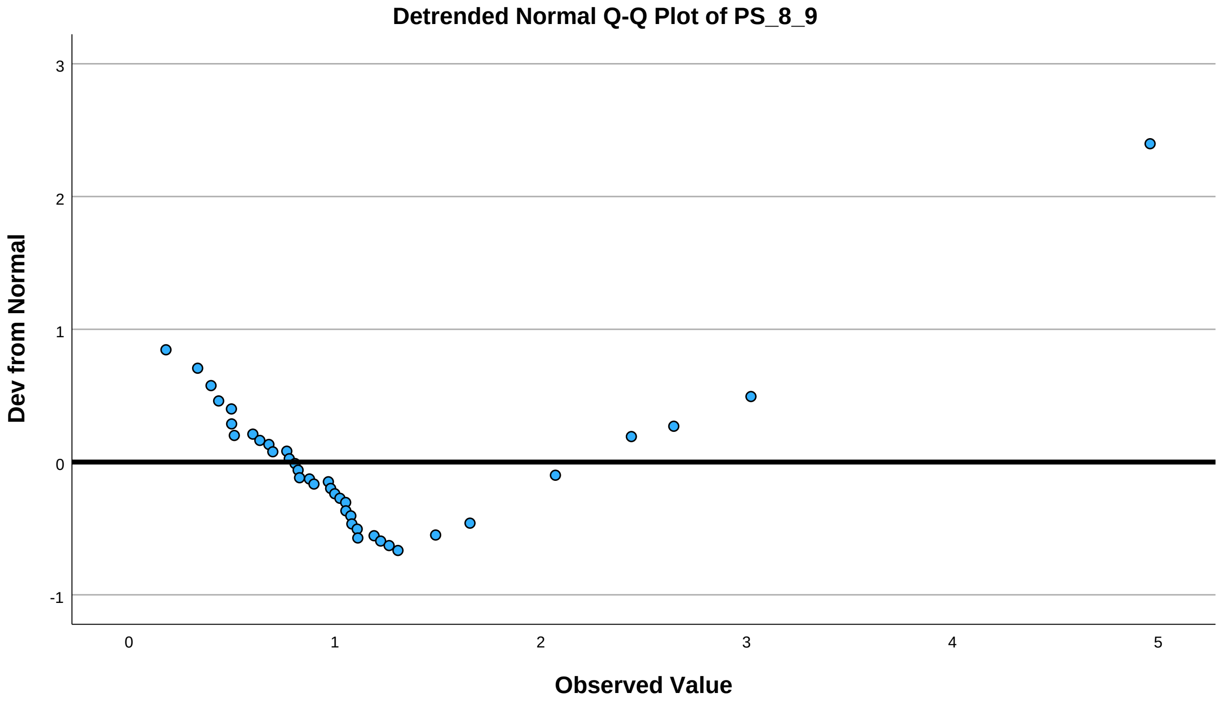

Detrended Normal Q-Q Plot of PS_8_9
Dev from Normal
Observed Value
3
2
1
0
-1
0
1
2
3
4
5

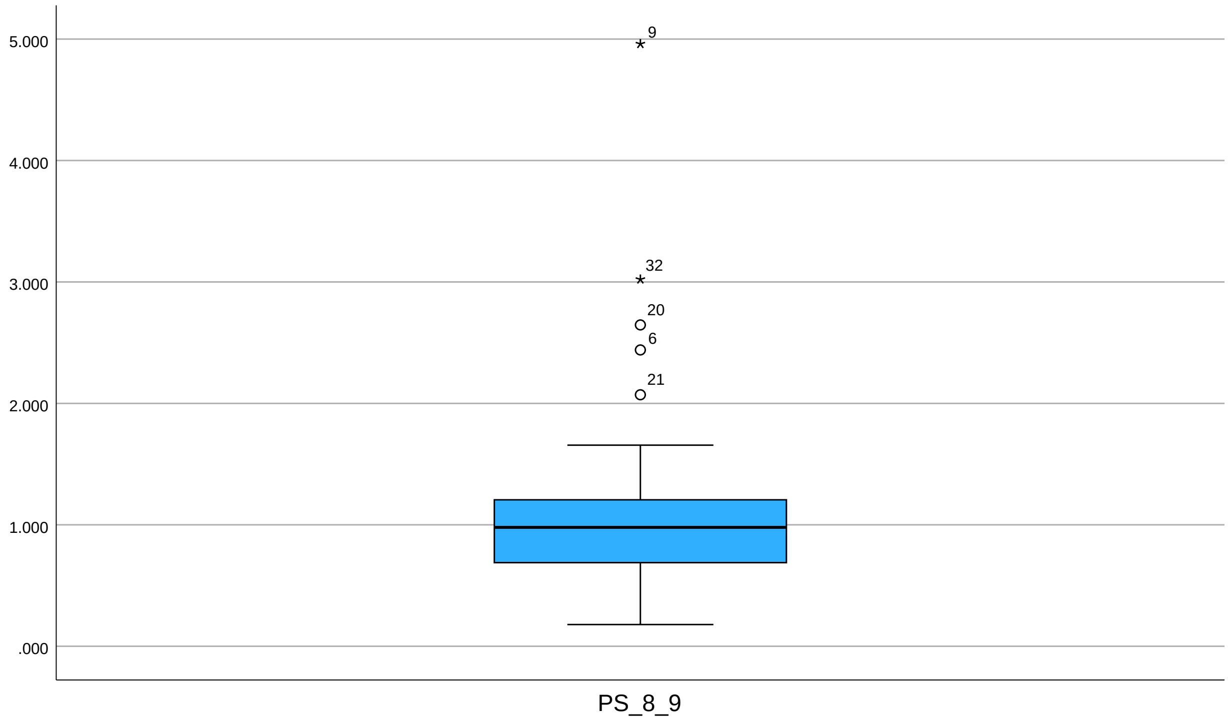

PS_9_10

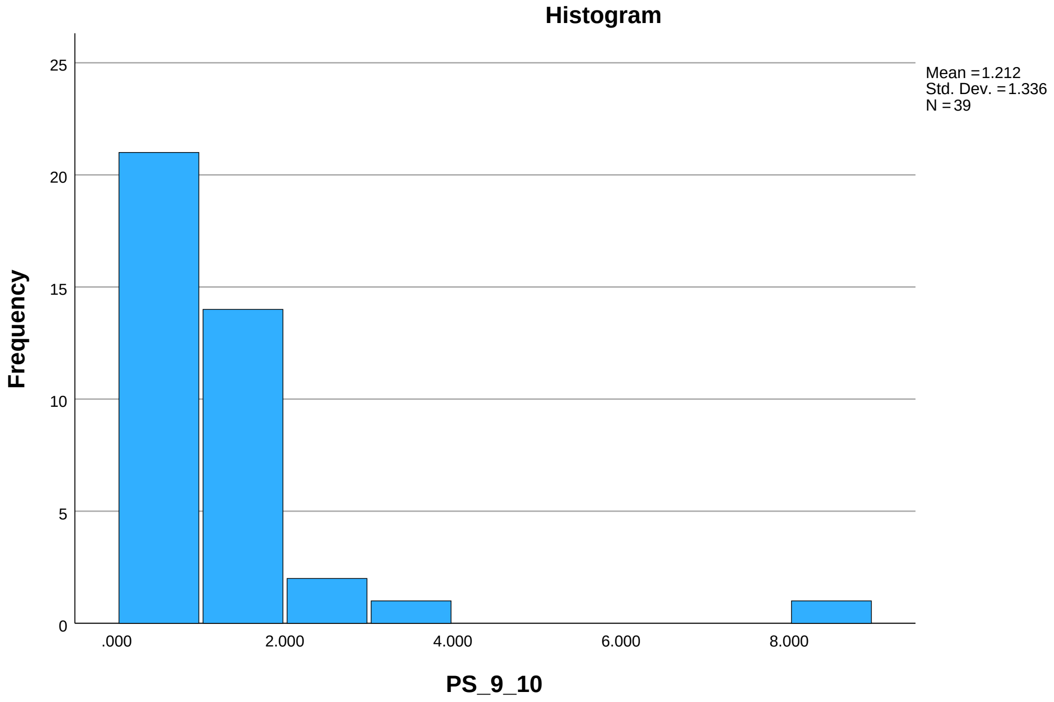

PS_9_10 Stem-and-Leaf Plot

Frequency   Stem & Leaf

```
  7.00       0 .  1223344
 14.00       0 .  55666677789999
 11.00       1 .  00000222334
  3.00       1 .  668
  2.00       2 .  00
  2.00 Extremes    (>=3.4)

Stem width:      1.000
Each leaf:       1 case(s)
```

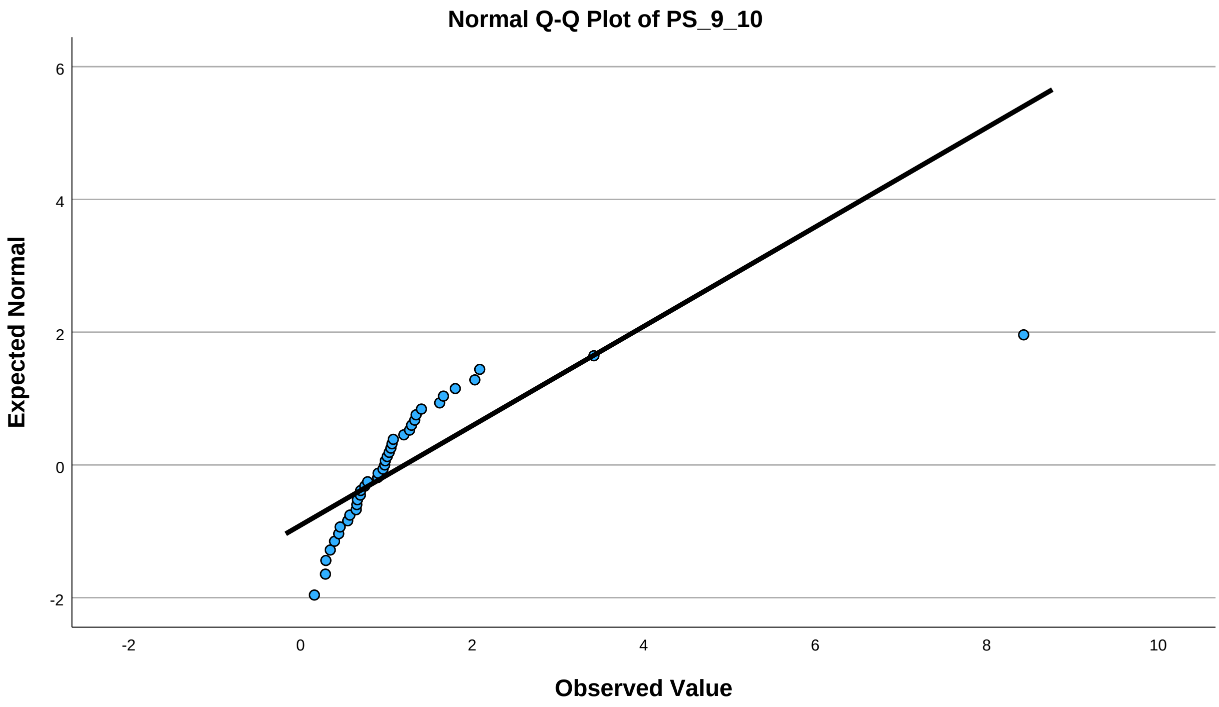

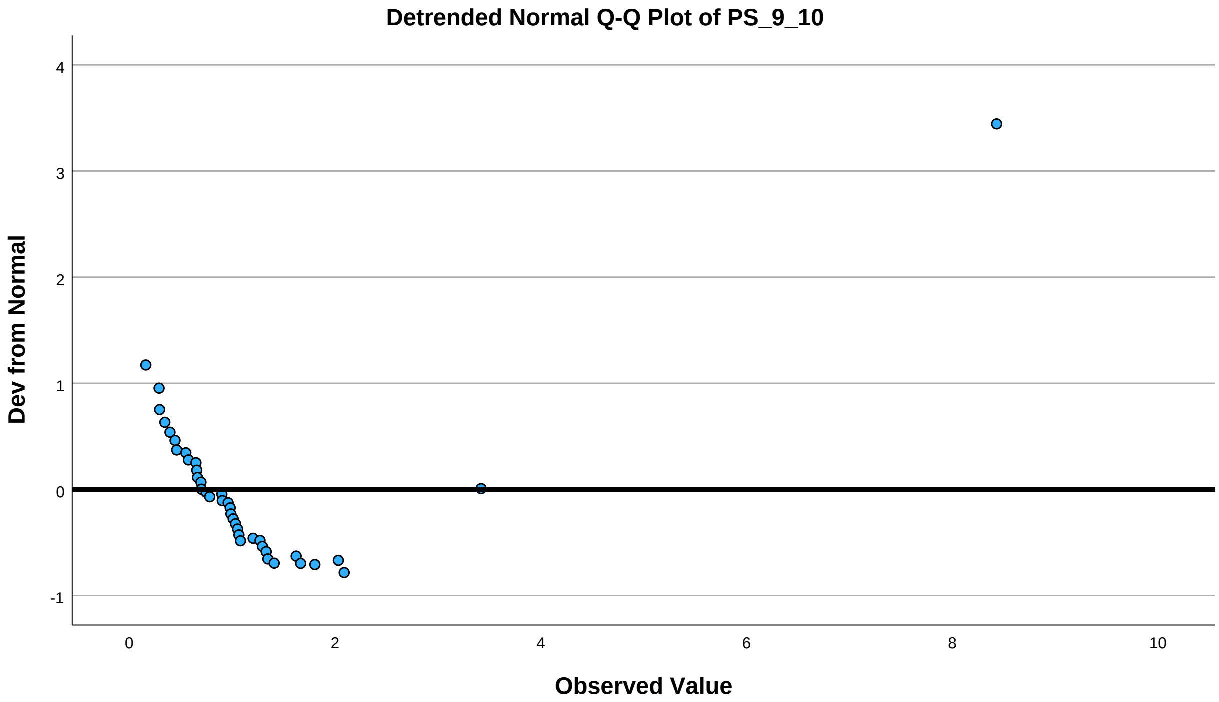
Detrended Normal Q-Q Plot of PS_9_10
Dev from Normal
4
3
2
1
0
-1
0
2
4
6
8
10
Observed Value

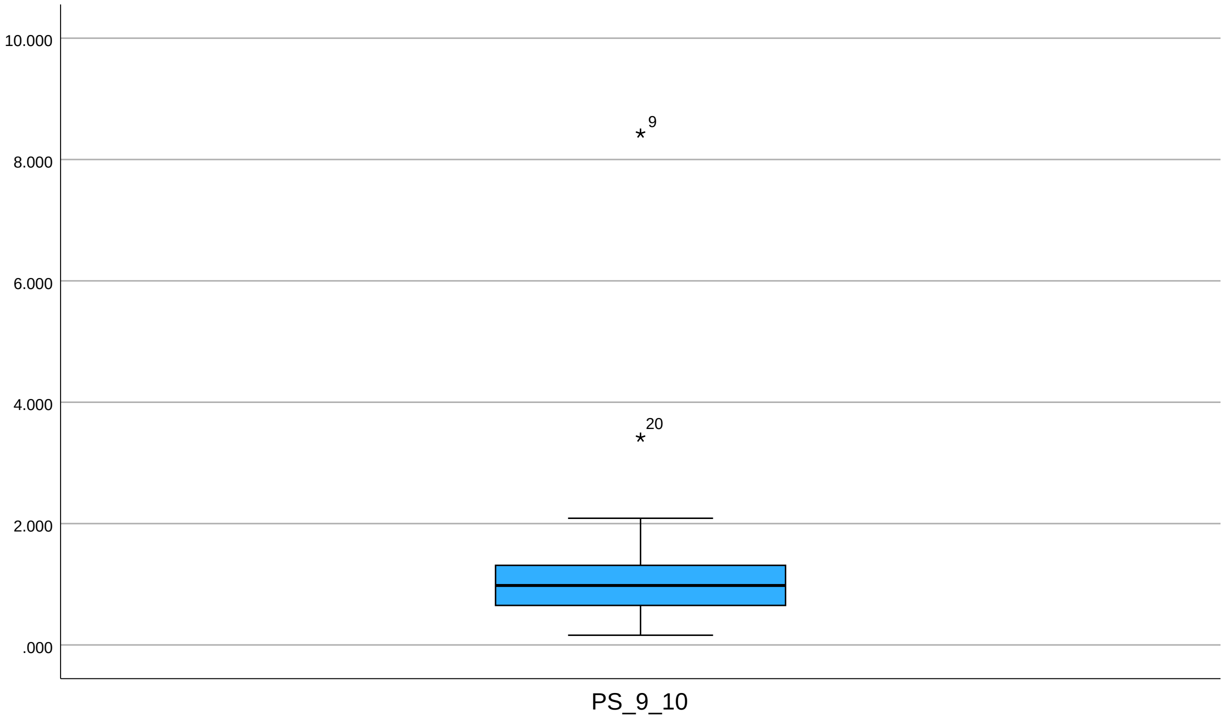
10.000
8.000
6.000
4.000
2.000
.000
9
20
PS_9_10

PS_10_11

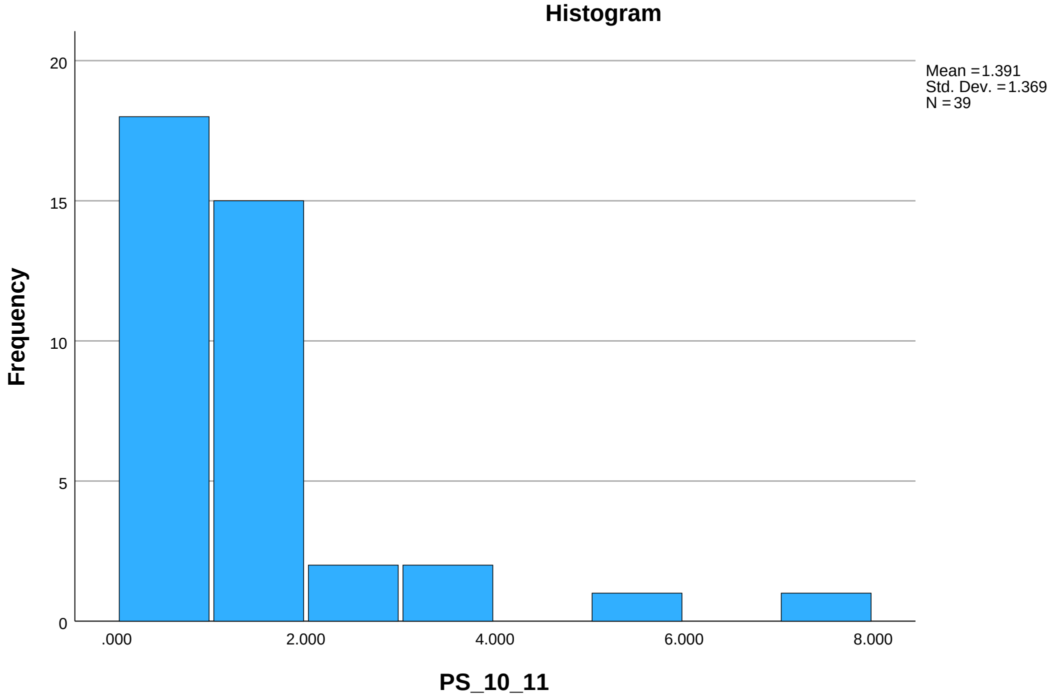

PS_10_11 Stem-and-Leaf Plot

```
 Frequency    Stem &  Leaf

     7.00        0 .  1223444
    11.00        0 .  66777888899
    10.00        1 .  0001112224
     5.00        1 .  55566
     2.00        2 .  11
     4.00 Extremes    (>=3.2)

 Stem width:      1.000
 Each leaf:       1 case(s)
```

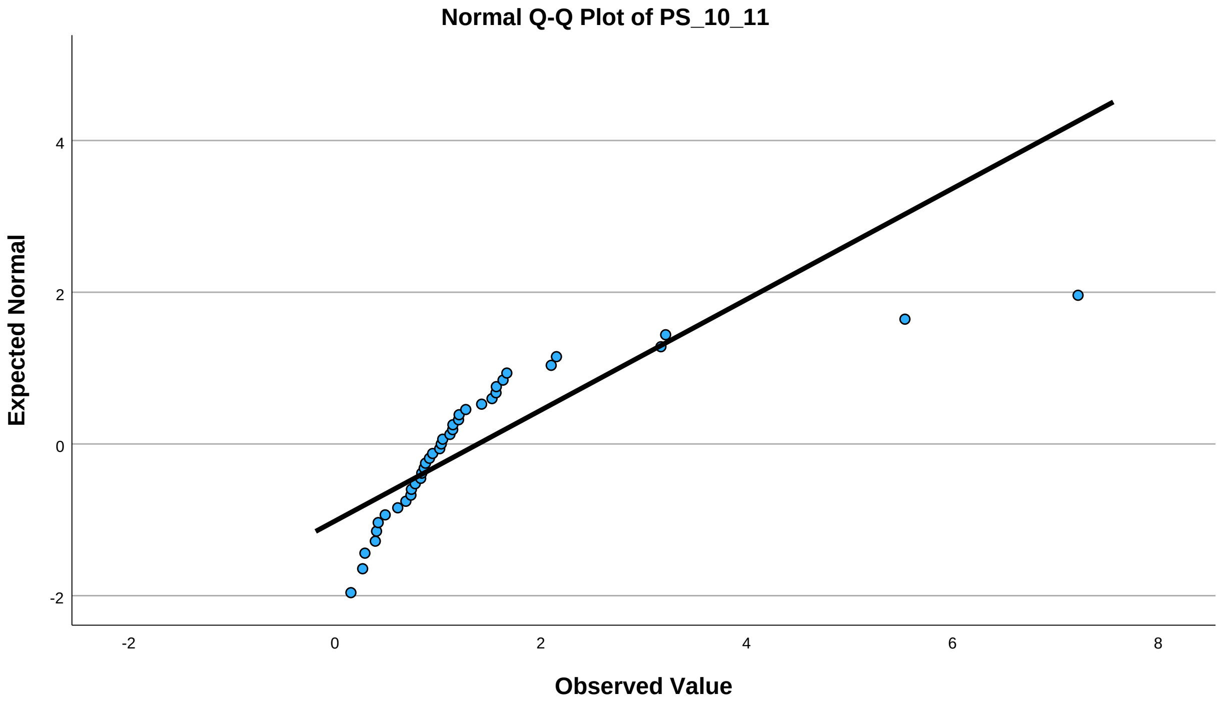
Normal Q-Q Plot of PS_10_11
Expected Normal
4
2
0
-2
-2
0
2
4
6
8
Observed Value

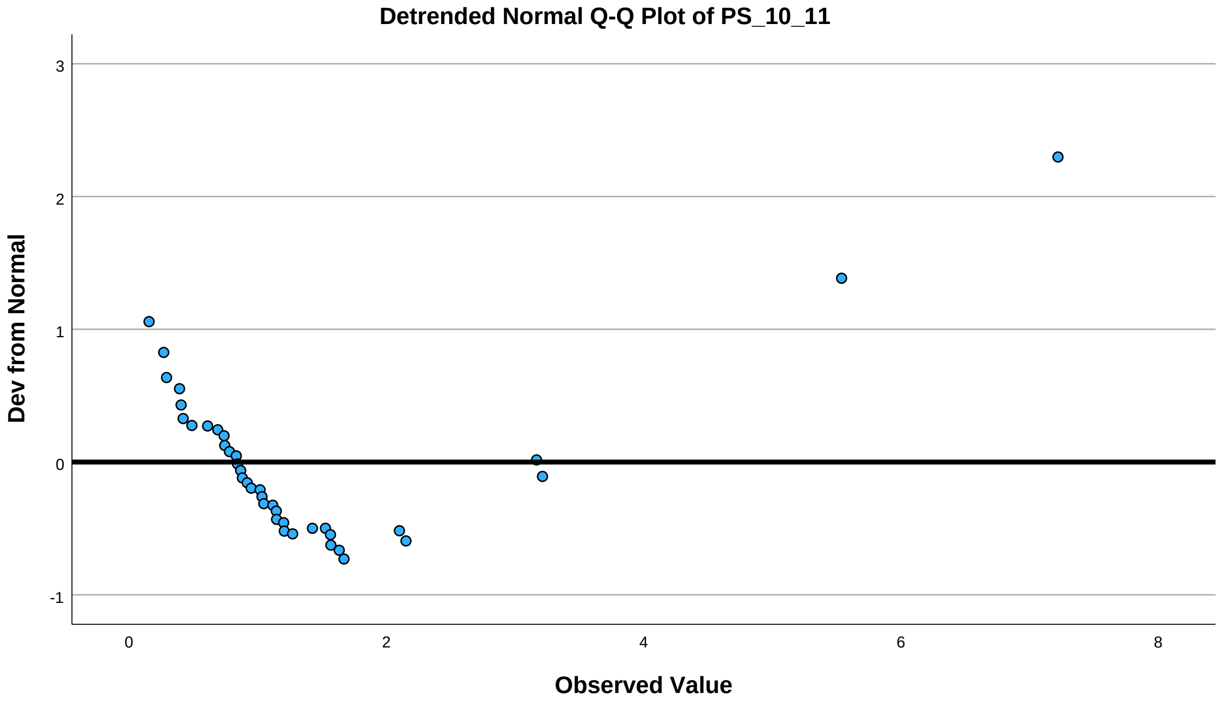
Detrended Normal Q-Q Plot of PS_10_11
Dev from Normal
3
2
1
0
-1
0
2
4
6
8
Observed Value

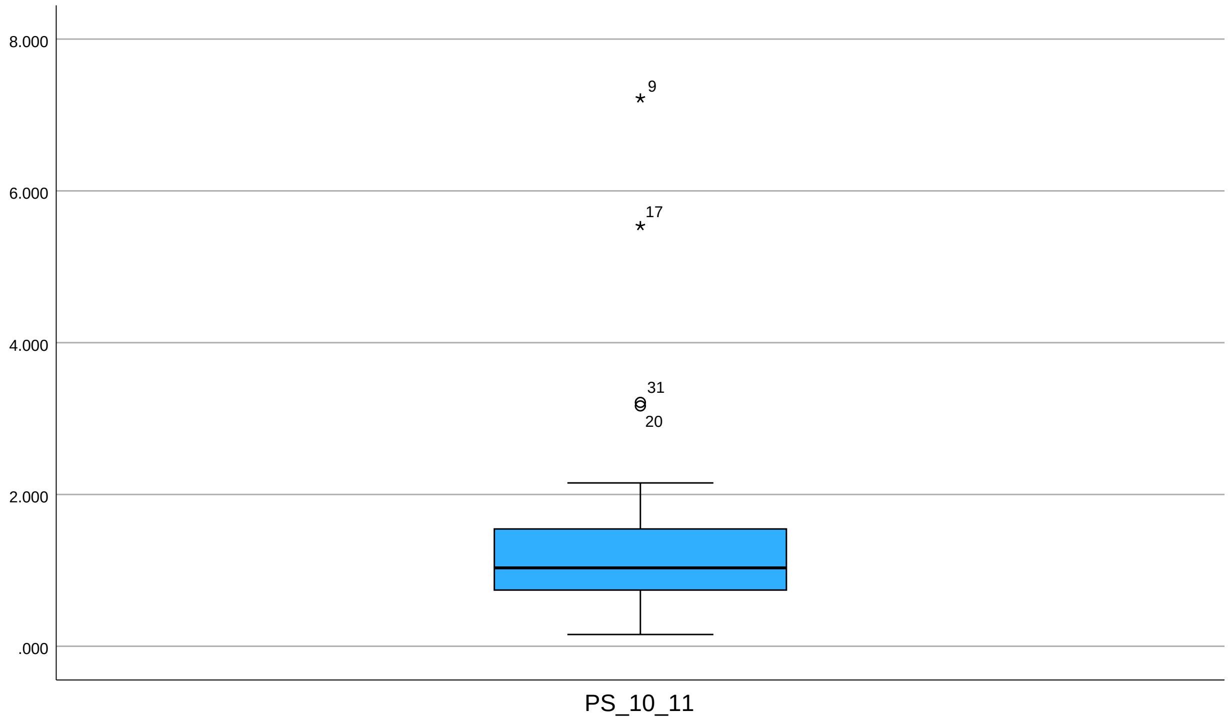

PS_11_12

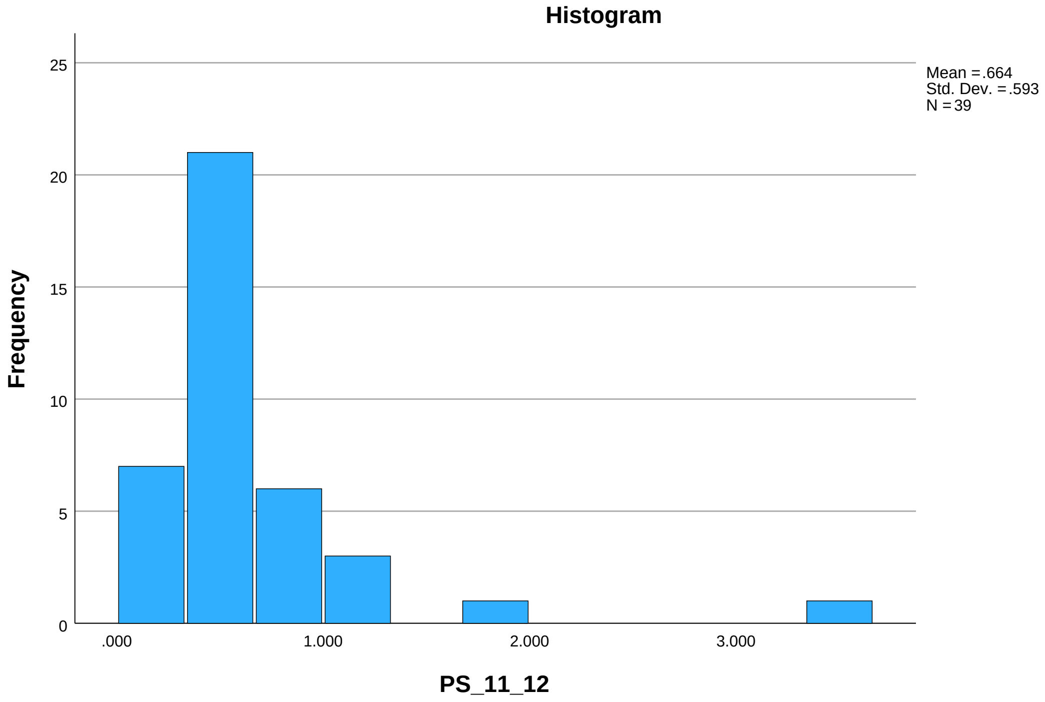


```
PS_11_12 Stem-and-Leaf Plot

 Frequency    Stem &  Leaf
```

```
    2.00        1 .  19
    4.00        2 .  2289
    7.00        3 .  0457889
    8.00        4 .  12458899
    3.00        5 .  334
    5.00        6 .  01568
    1.00        7 .  4
    2.00        8 .  47
    2.00        9 .  28
     .00       10 .
    2.00       11 .  02
    3.00 Extremes    (>=1.33)

 Stem width:       .100
 Each leaf:        1 case(s)
```

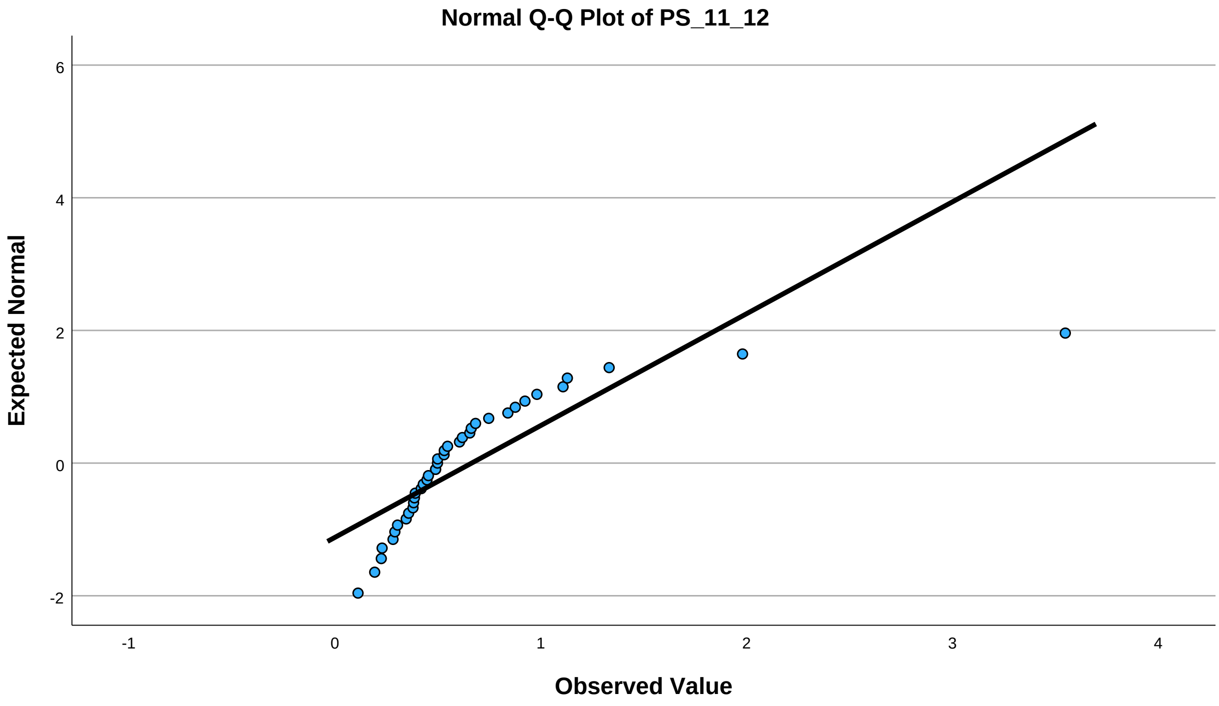

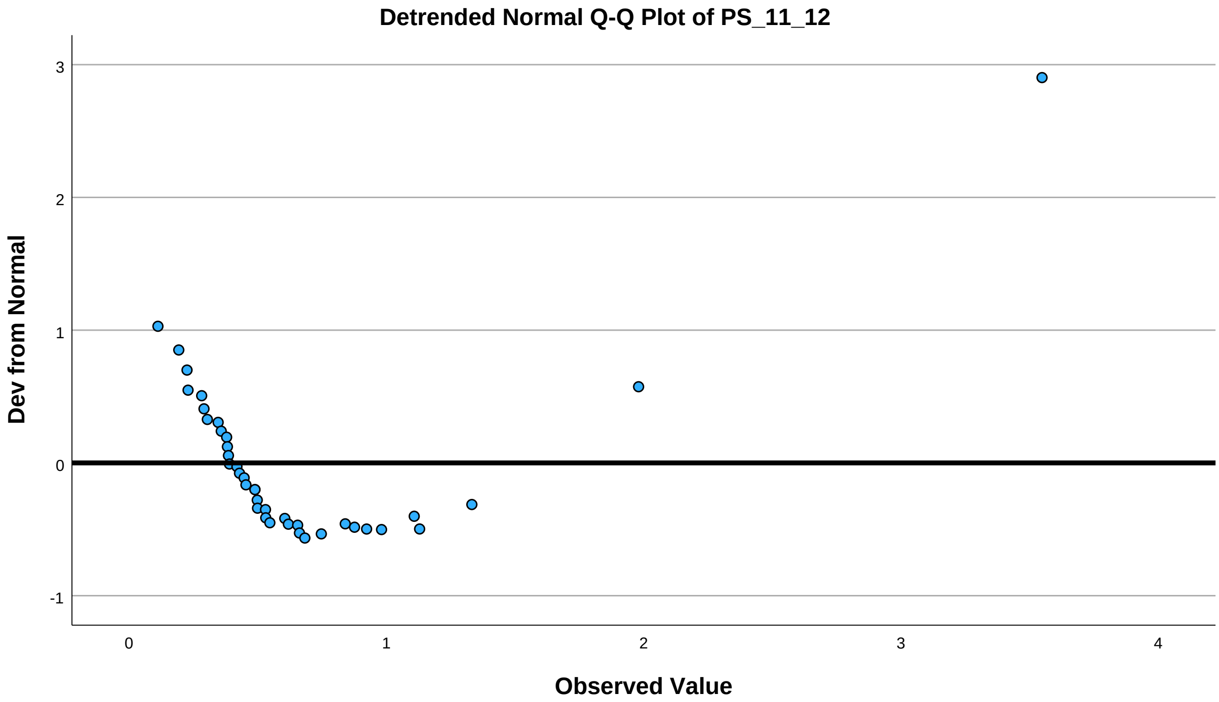

Detrended Normal Q-Q Plot of PS_11_12
Dev from Normal
3
2
1
0
-1
0
1
2
3
4
Observed Value

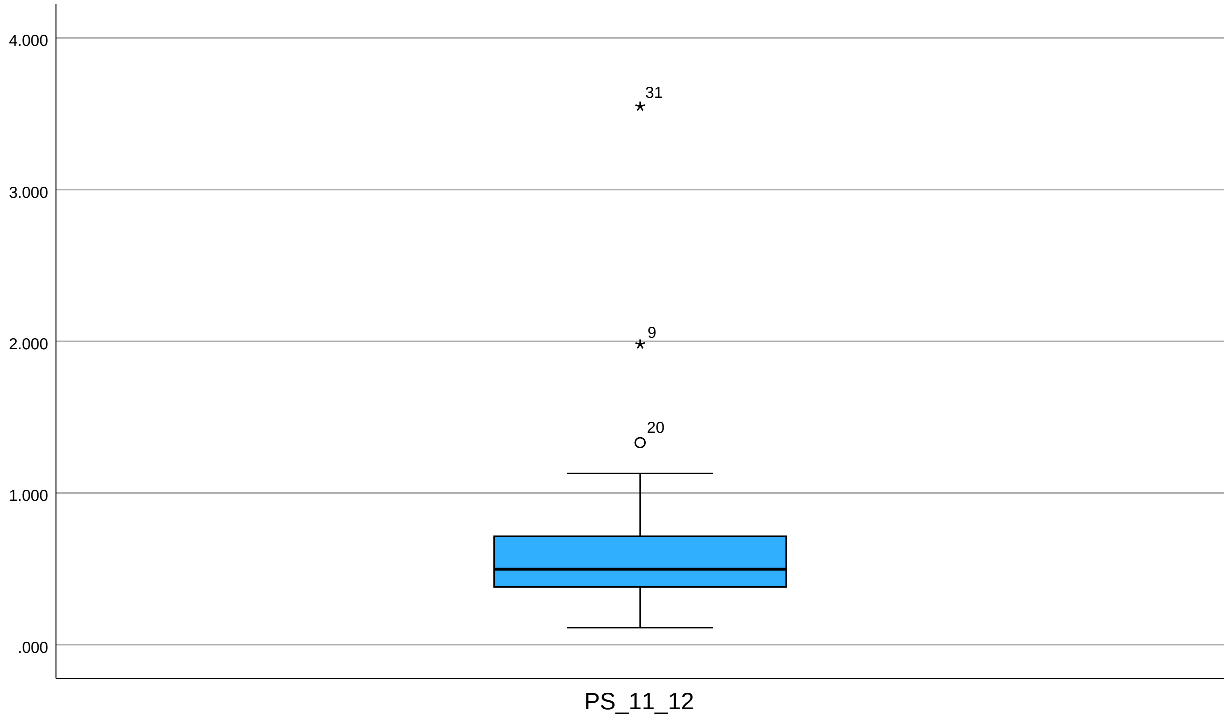

4.000
3.000
2.000
1.000
.000
31
9
20
PS_11_12

PS_12_13

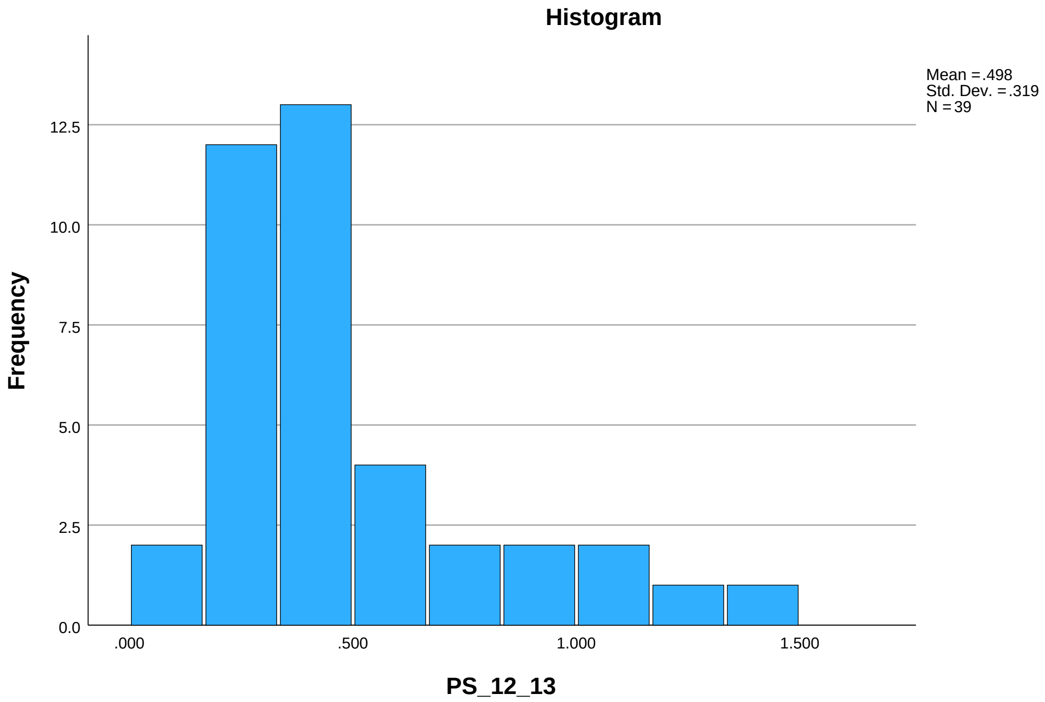


```
PS_12_13 Stem-and-Leaf Plot

 Frequency    Stem &  Leaf

     1.00        0 .  8
     3.00        1 .  579
     9.00        2 .  112378899
     5.00        3 .  26778
     9.00        4 .  035588999
     3.00        5 .  045
     1.00        6 .  1
     2.00        7 .  58
      .00        8 .
     1.00        9 .  1
     5.00 Extremes    (>=.95)

 Stem width:      .100
 Each leaf:       1 case(s)
```

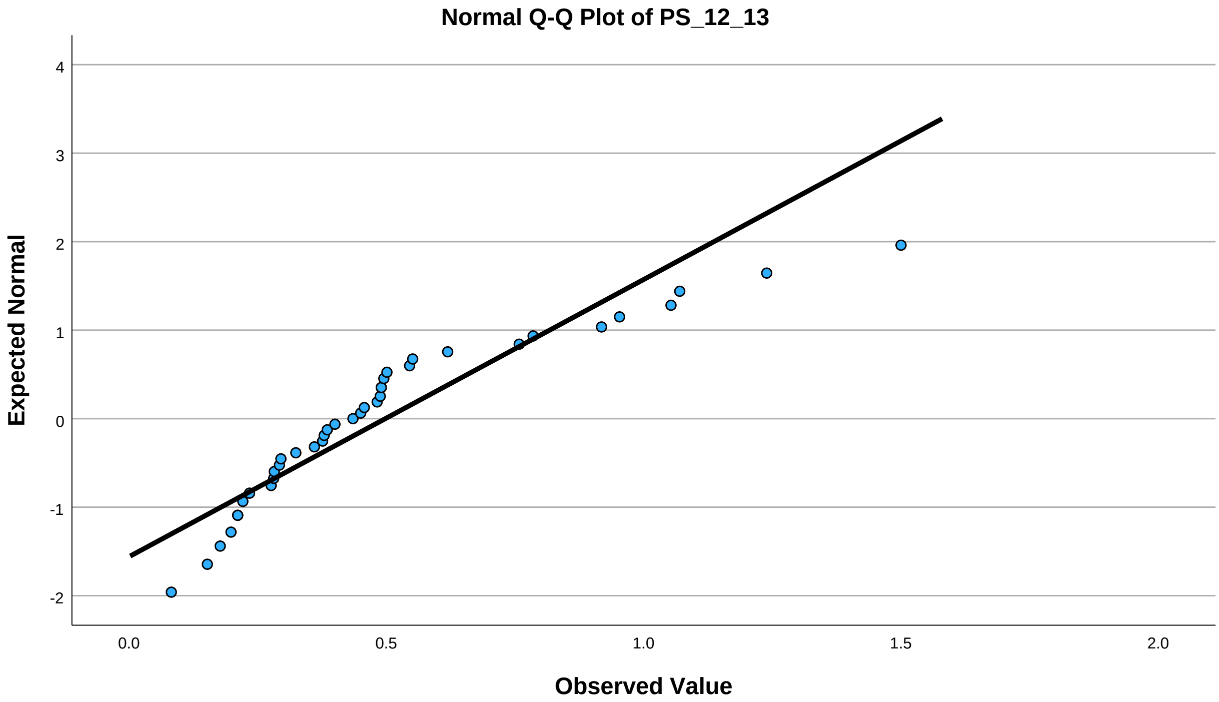
Normal Q-Q Plot of PS_12_13
Expected Normal
Observed Value
4
3
2
1
0
-1
-2
0.0
0.5
1.0
1.5
2.0

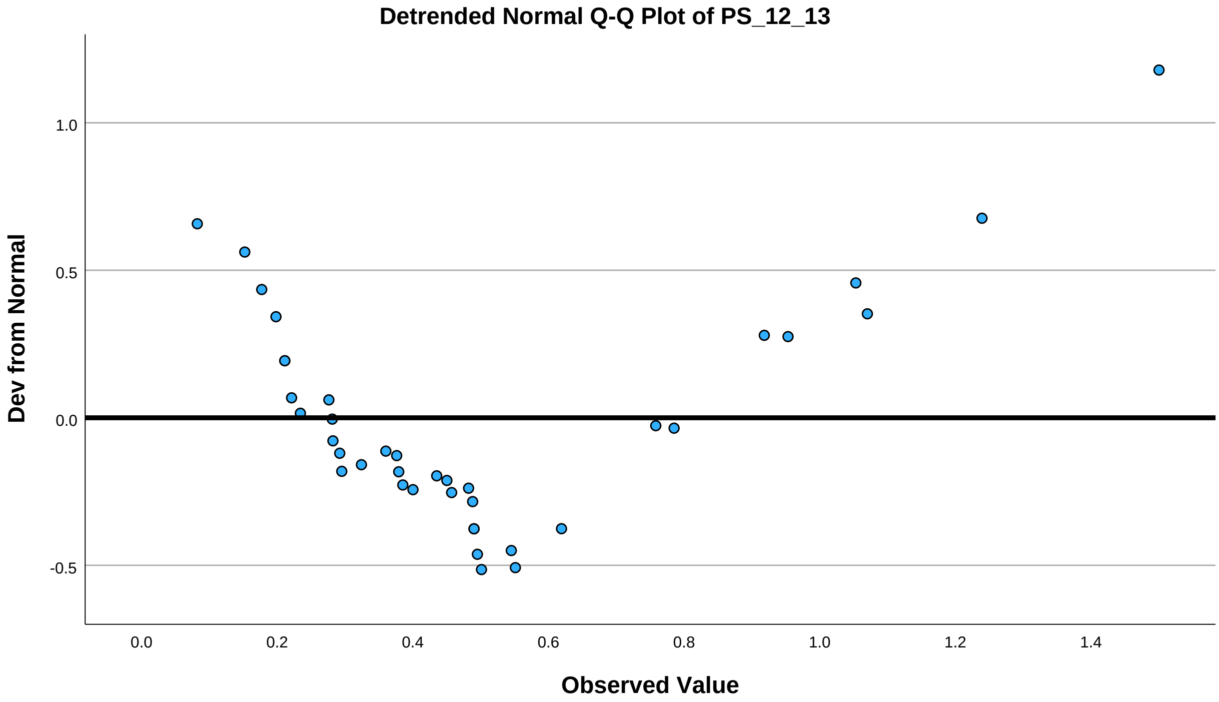
Detrended Normal Q-Q Plot of PS_12_13
Dev from Normal
Observed Value
1.0
0.5
0.0
-0.5
0.0
0.2
0.4
0.6
0.8
1.0
1.2
1.4

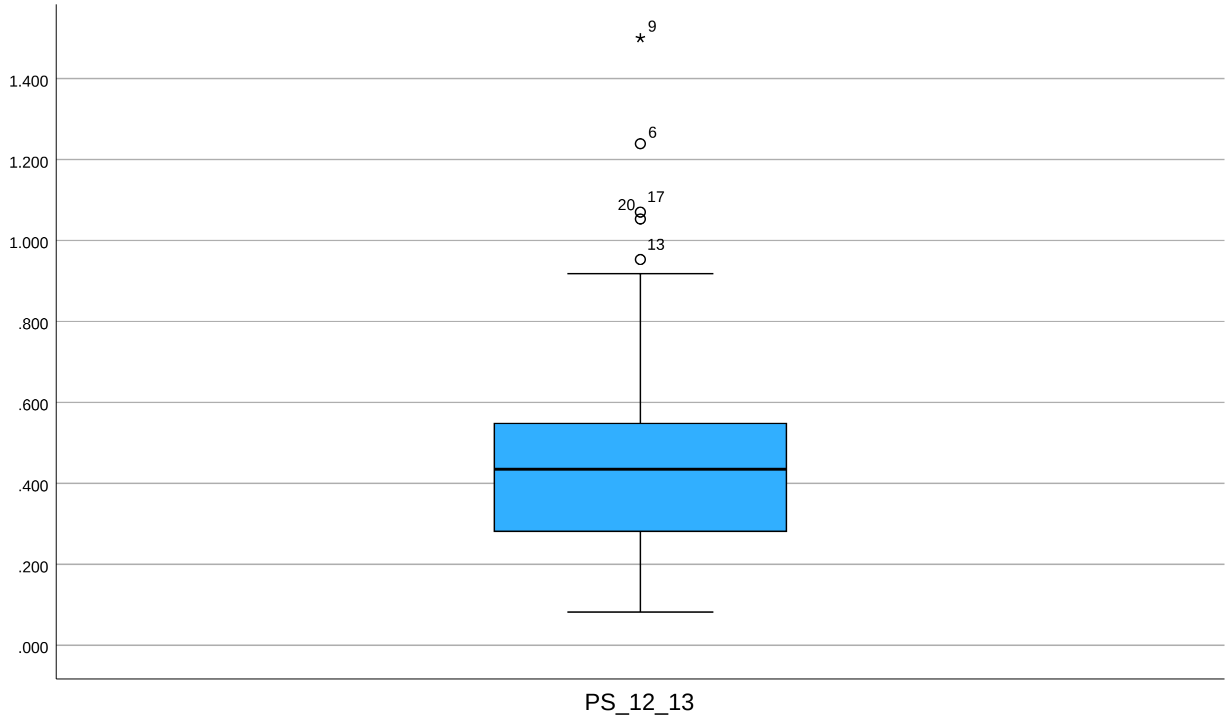

PS_13_14

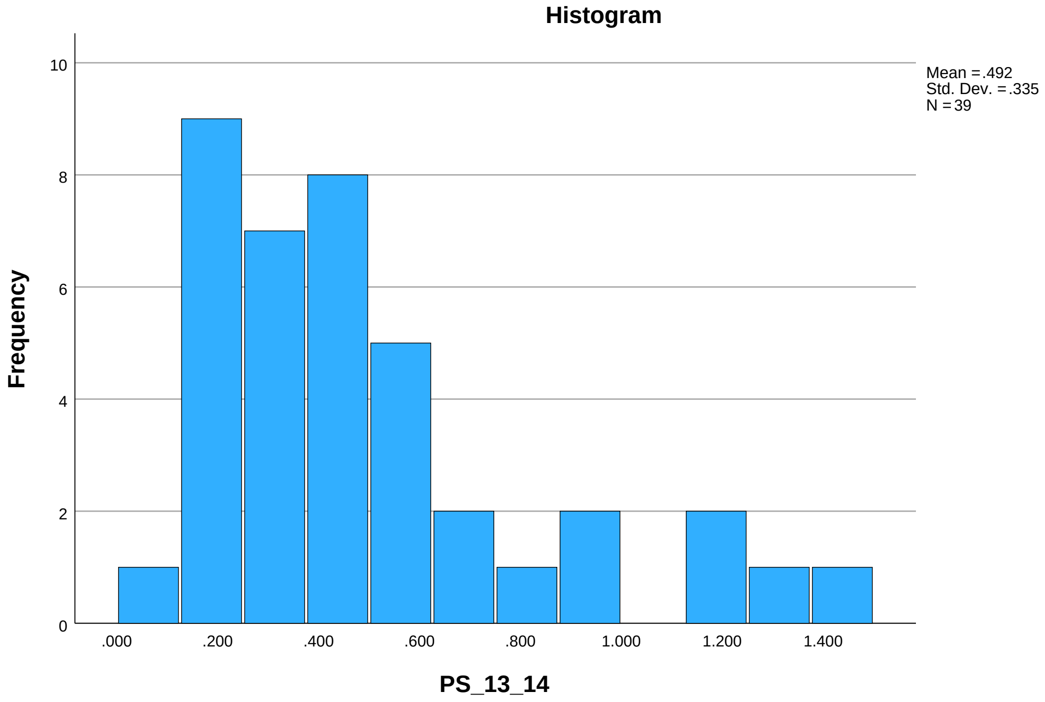

PS_13_14 Stem-and-Leaf Plot

Frequency   Stem & Leaf

```
 1.00      0 . 7
 5.00      1 . 44789
 8.00      2 . 11336788
 5.00      3 . 45579
 6.00      4 . 223569
 5.00      5 . 01469
 2.00      6 . 55
 1.00      7 . 5
  .00      8 .
 2.00      9 . 37
 4.00 Extremes    (>=1.18)

Stem width:      .100
Each leaf:       1 case(s)
```

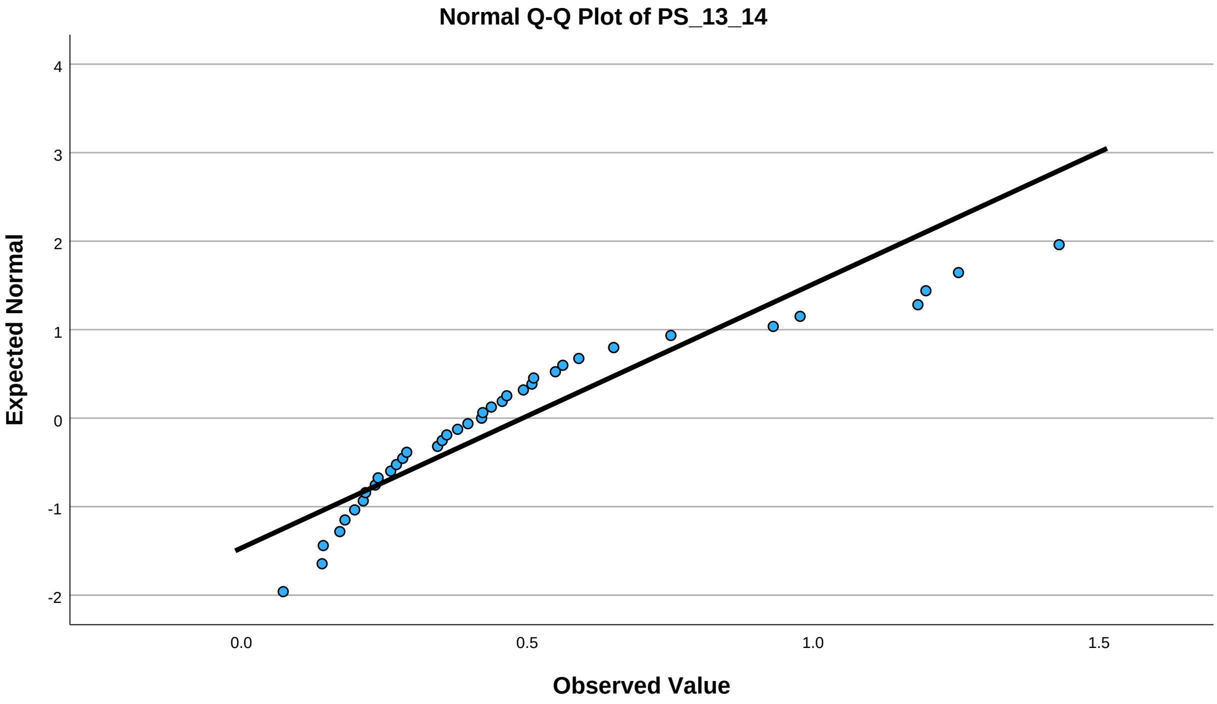

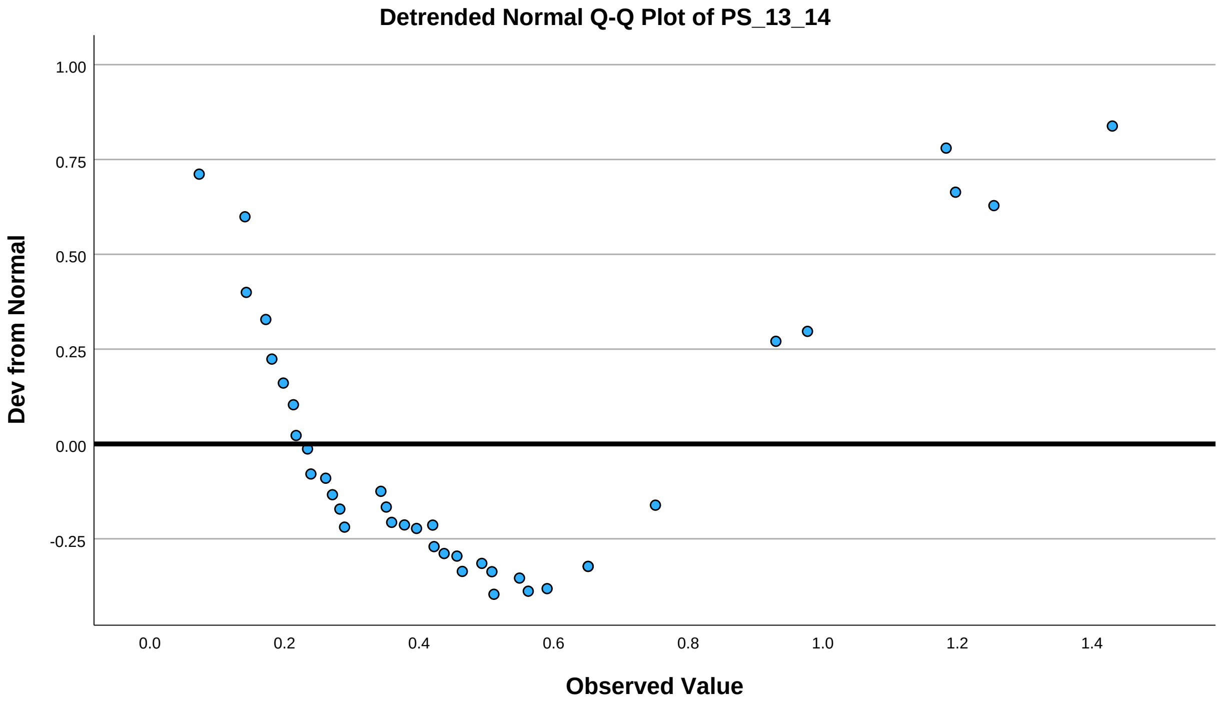
Detrended Normal Q-Q Plot of PS_13_14
Dev from Normal
1.00
0.75
0.50
0.25
0.00
-0.25
0.0
0.2
0.4
0.6
0.8
1.0
1.2
1.4
Observed Value

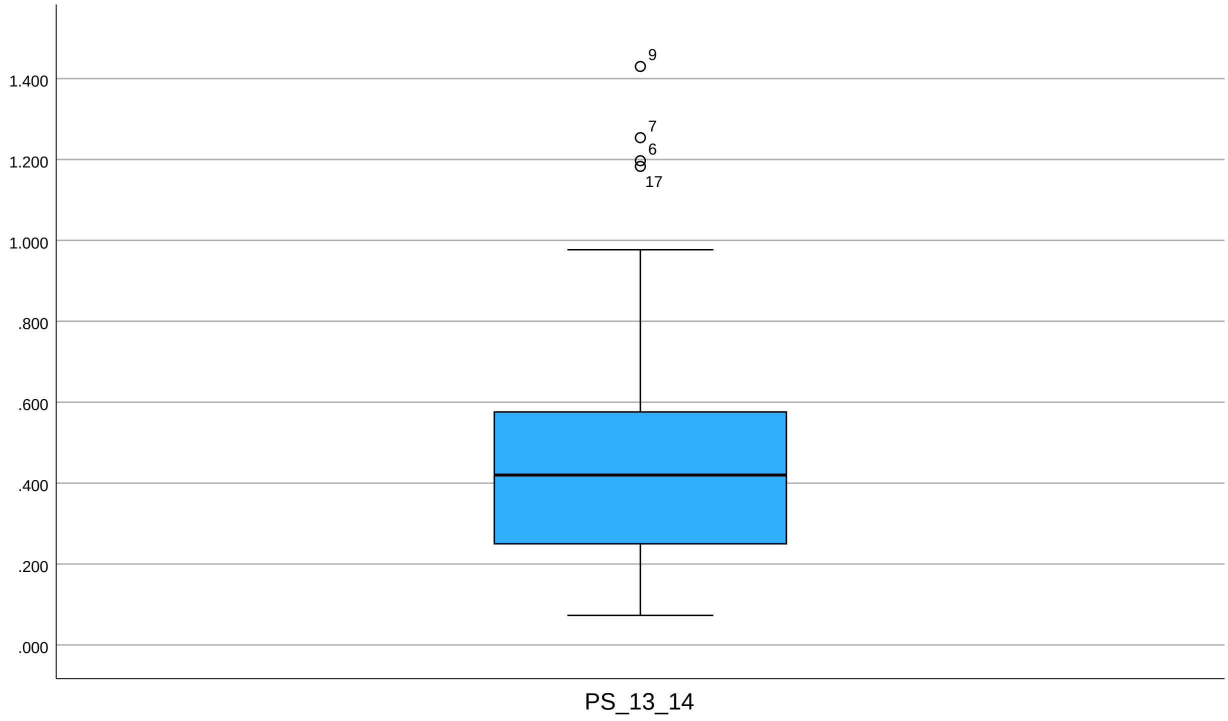
1.400
1.200
1.000
.800
.600
.400
.200
.000
9
7
6
17
PS_13_14

PS_14_15

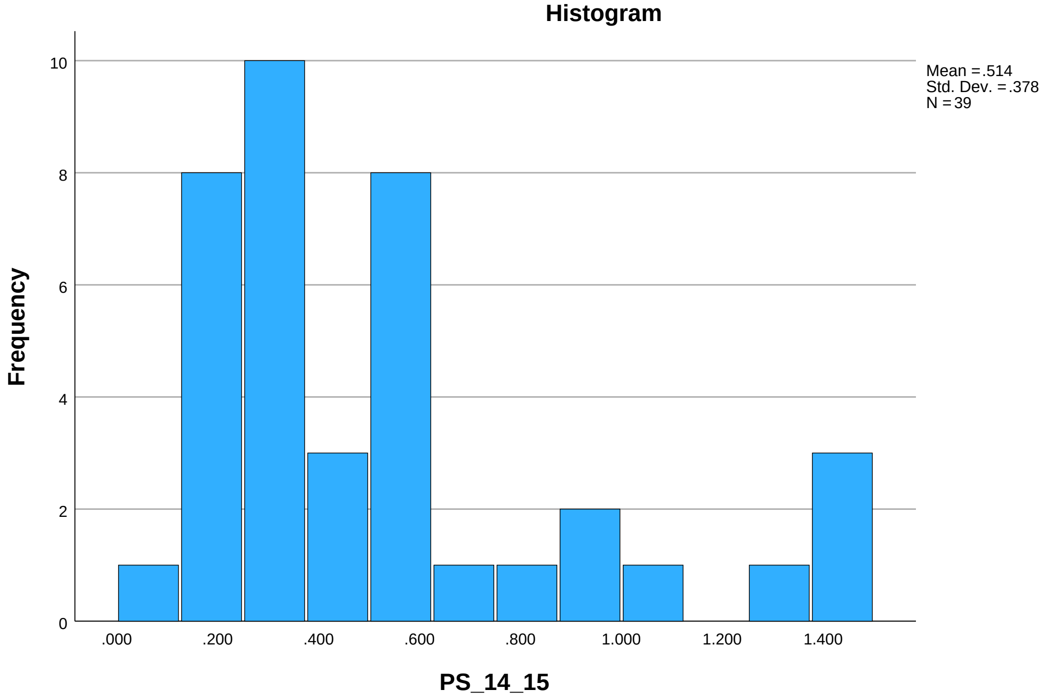


```
PS_14_15 Stem-and-Leaf Plot

 Frequency    Stem &  Leaf

     1.00        0 .  8
     7.00        1 .  4577889
     6.00        2 .  057788
     7.00        3 .  0134588
     1.00        4 .  3
     7.00        5 .  0123567
     2.00        6 .  19
     1.00        7 .  7
     1.00        8 .  9
     1.00        9 .  8
     1.00       10 .  3
     4.00 Extremes    (>=1.28)

 Stem width:      .100
 Each leaf:       1 case(s)
```

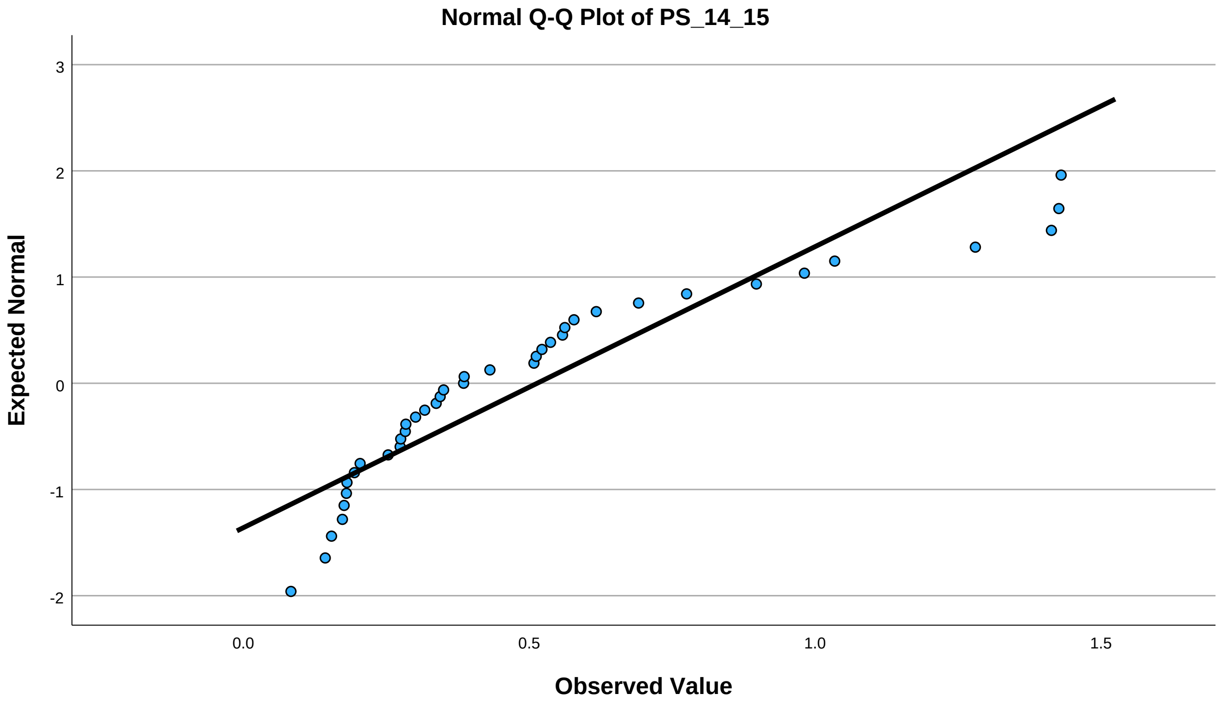

Normal Q-Q Plot of PS_14_15
Expected Normal
Observed Value
3
2
1
0
-1
-2
0.0
0.5
1.0
1.5

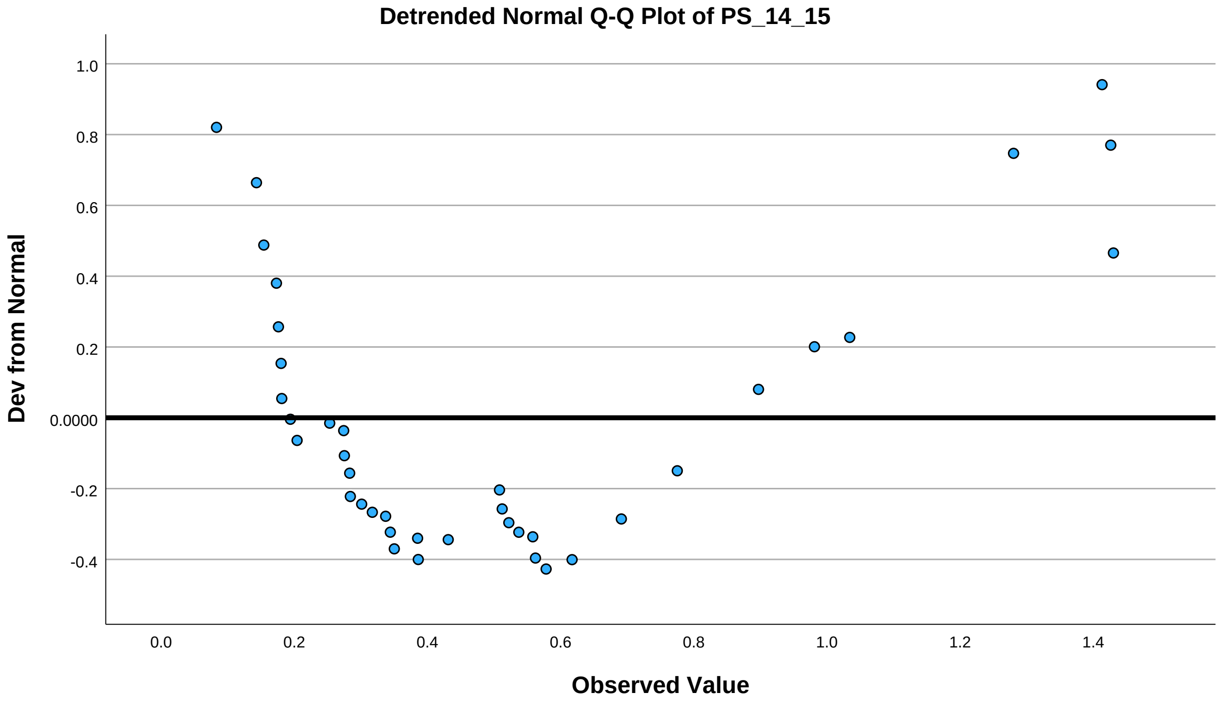

Detrended Normal Q-Q Plot of PS_14_15
Dev from Normal
Observed Value
1.0
0.8
0.6
0.4
0.2
0.0000
-0.2
-0.4
0.0
0.2
0.4
0.6
0.8
1.0
1.2
1.4

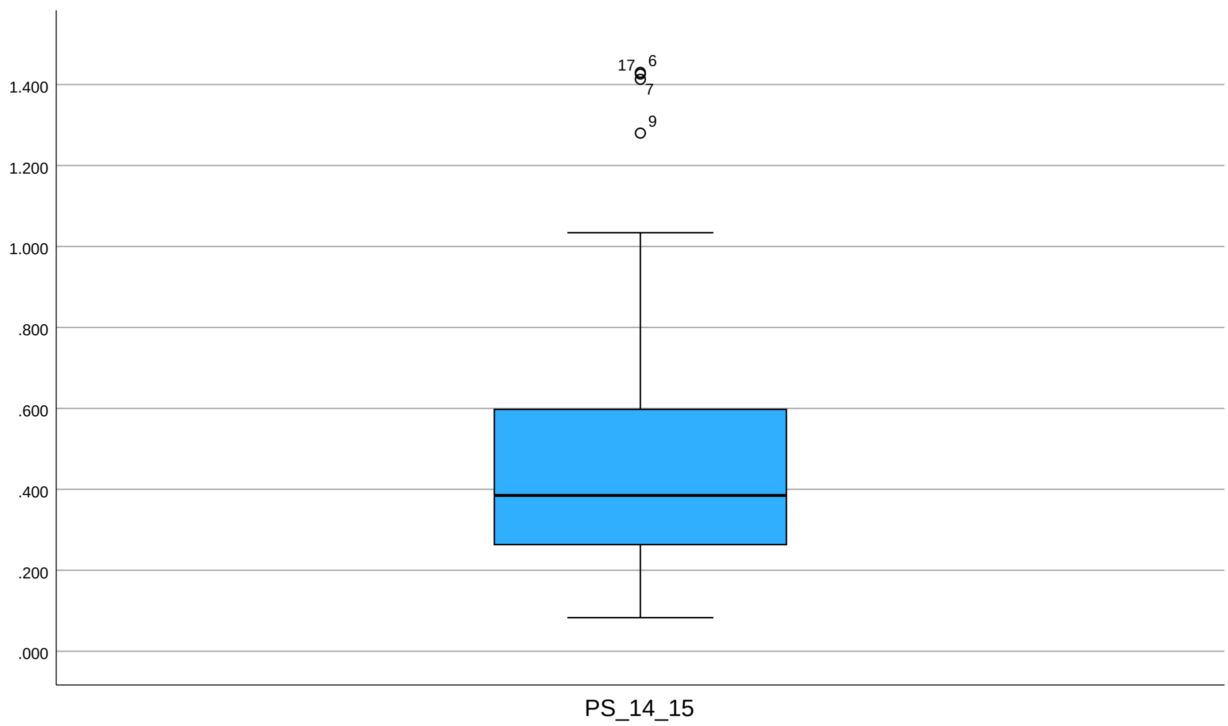

PS_15_16

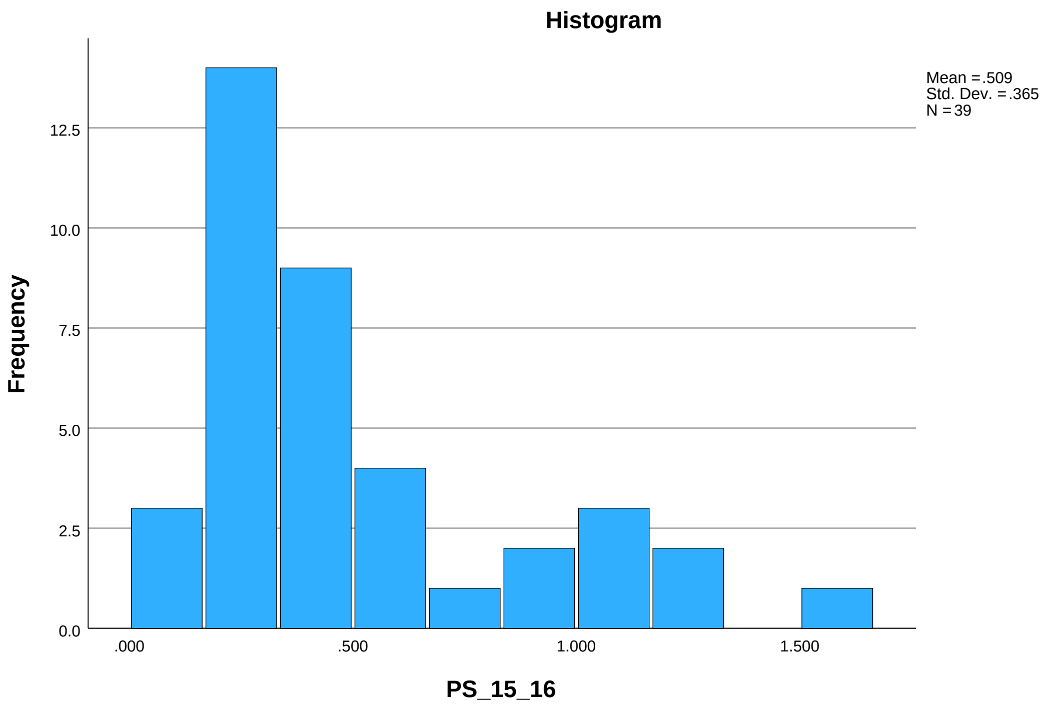

PS_15_16 Stem-and-Leaf Plot

Frequency   Stem & Leaf

```
 1.00     0 . 8
 6.00      1 . 357889
 7.00      2 . 0345568
 6.00      3 . 012578
 6.00      4 . 024889
 2.00     5 . 67
 2.00     6 . 13
 1.00     7 . 7
 1.00     8 . 5
 1.00     9 . 1
 2.00    10 . 35
 1.00    11 . 4
  3.00 Extremes    (>=1.22)

Stem width:      .100
Each leaf:       1 case(s)
```

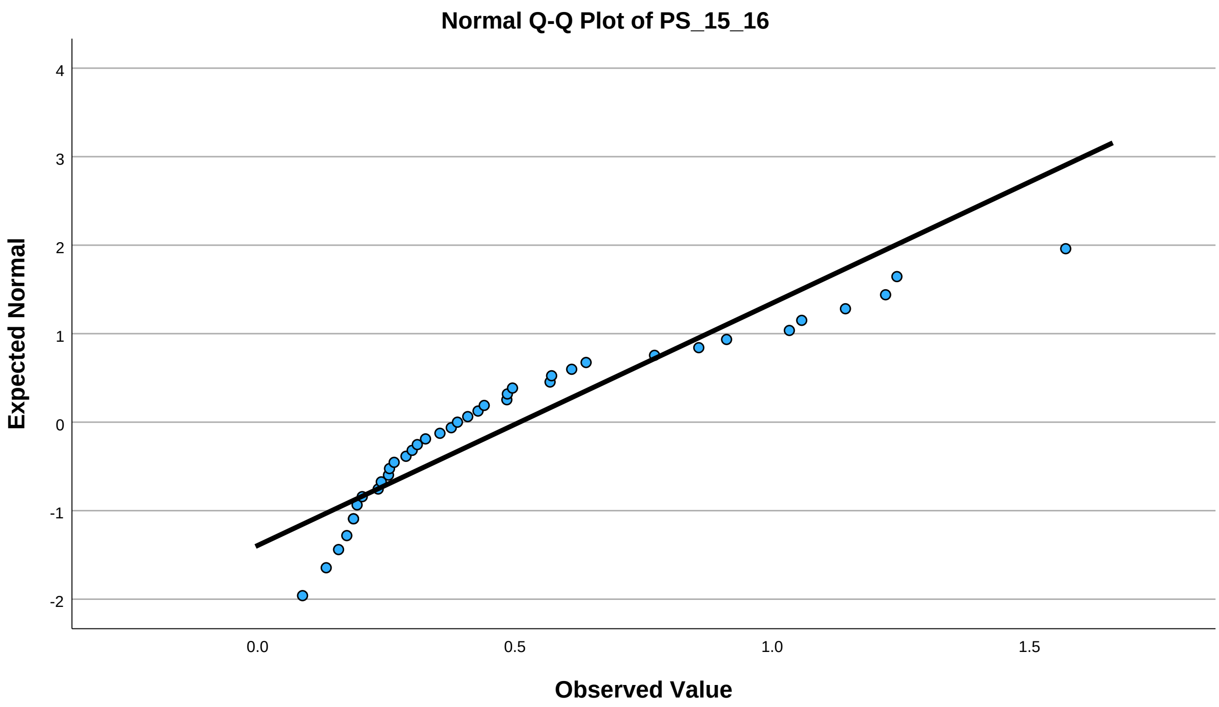

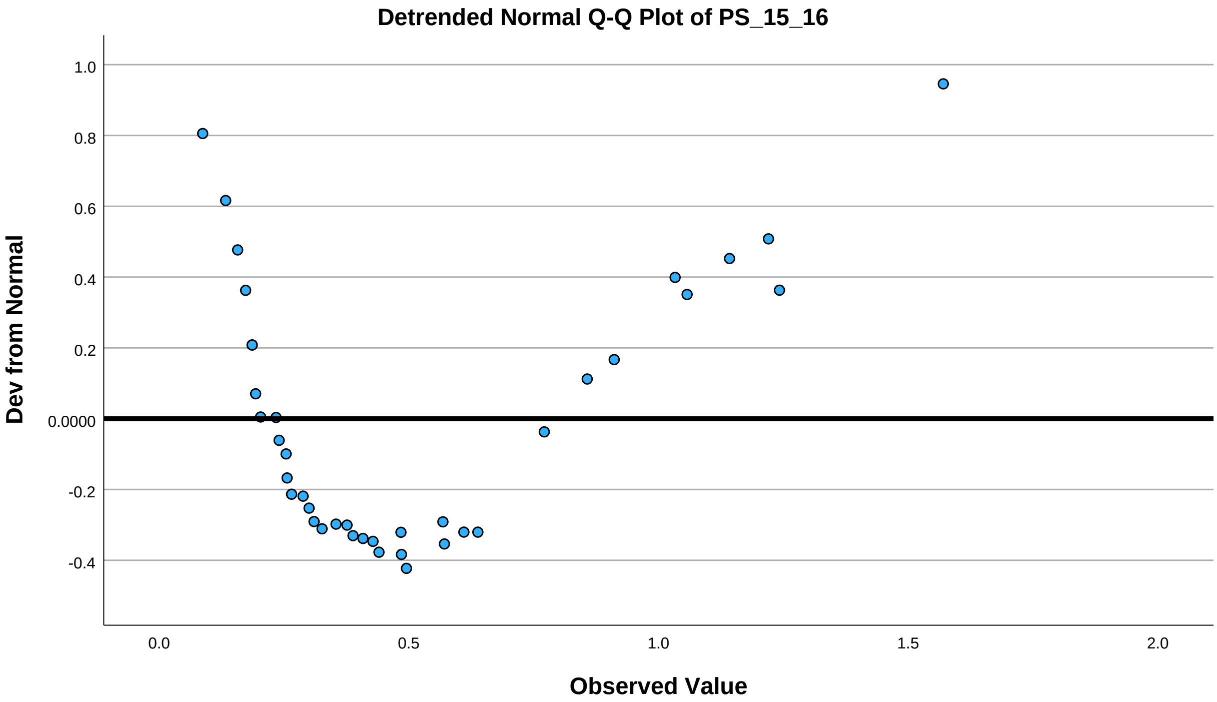

Detrended Normal Q-Q Plot of PS_15_16
Dev from Normal
1.0
0.8
0.6
0.4
0.2
0.0000
-0.2
-0.4
0.0
0.5
1.0
1.5
2.0
Observed Value

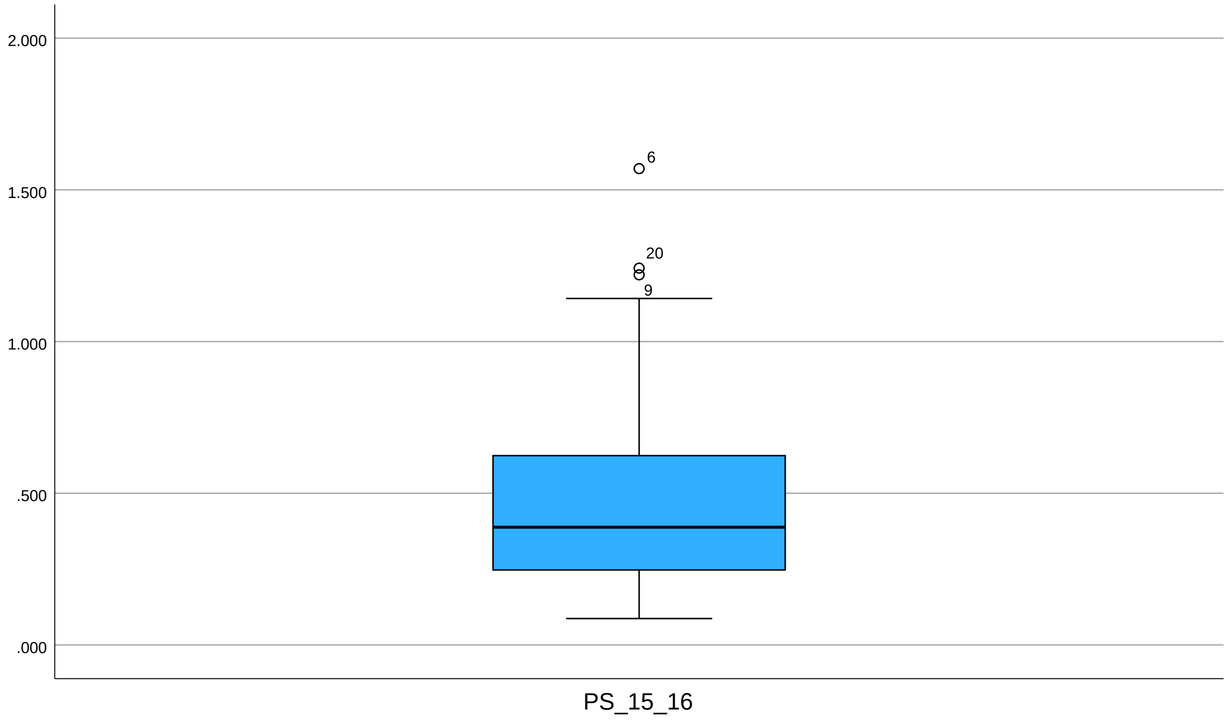

2.000
1.500
1.000
.500
.000
6
20
9
PS_15_16

PS_16_17

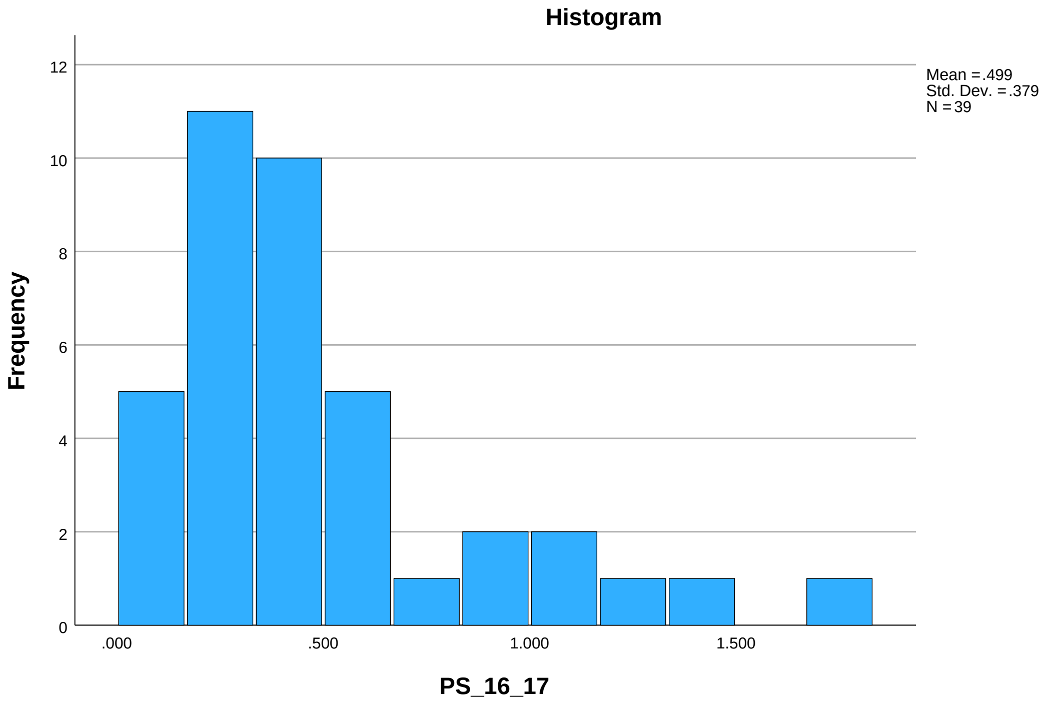

PS_16_17 Stem-and-Leaf Plot

```
 Frequency    Stem &  Leaf

     1.00        0 .  8
     8.00        1 .  35556799
     6.00        2 .  345569
     3.00        3 .  266
     8.00        4 .  01123448
     3.00        5 .  277
     2.00        6 .  13
     1.00        7 .  6
     1.00        8 .  7
     1.00        9 .  7
     2.00       10 .  24
     3.00 Extremes    (>=1.29)

 Stem width:      .100
 Each leaf:       1 case(s)
```

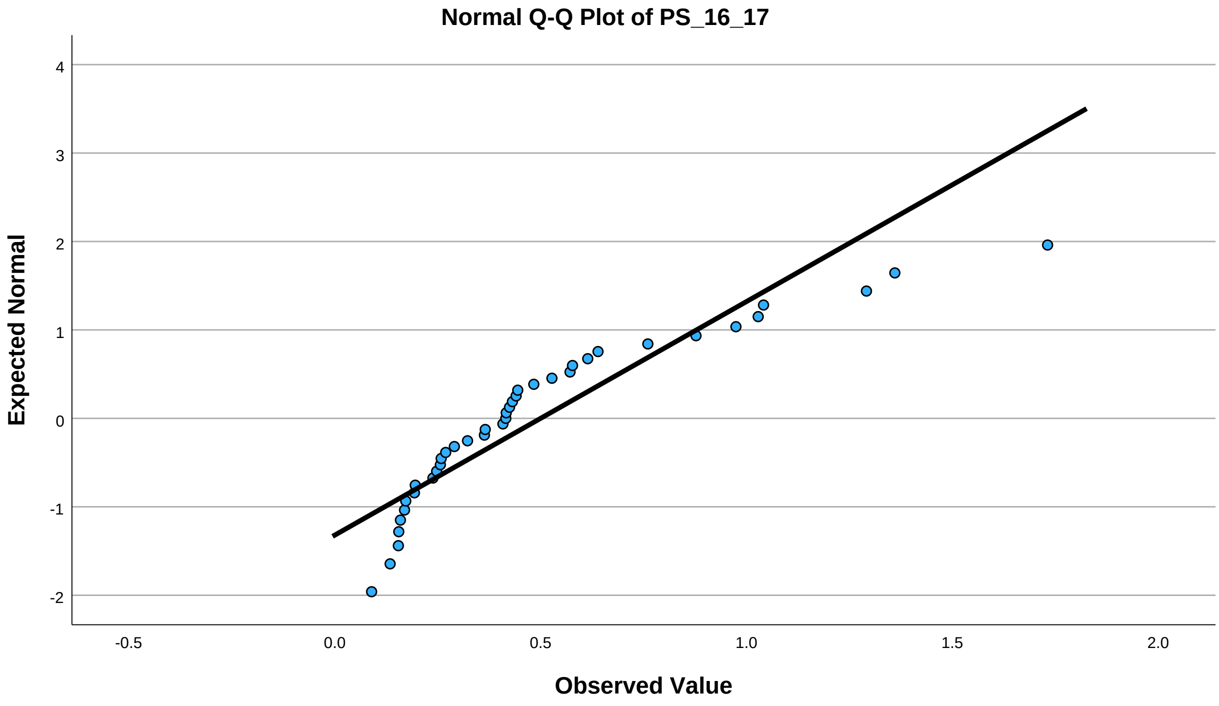

Normal Q-Q Plot of PS_16_17
Expected Normal
4
3
2
1
0
-1
-2
-0.5
0.0
0.5
1.0
1.5
2.0
Observed Value

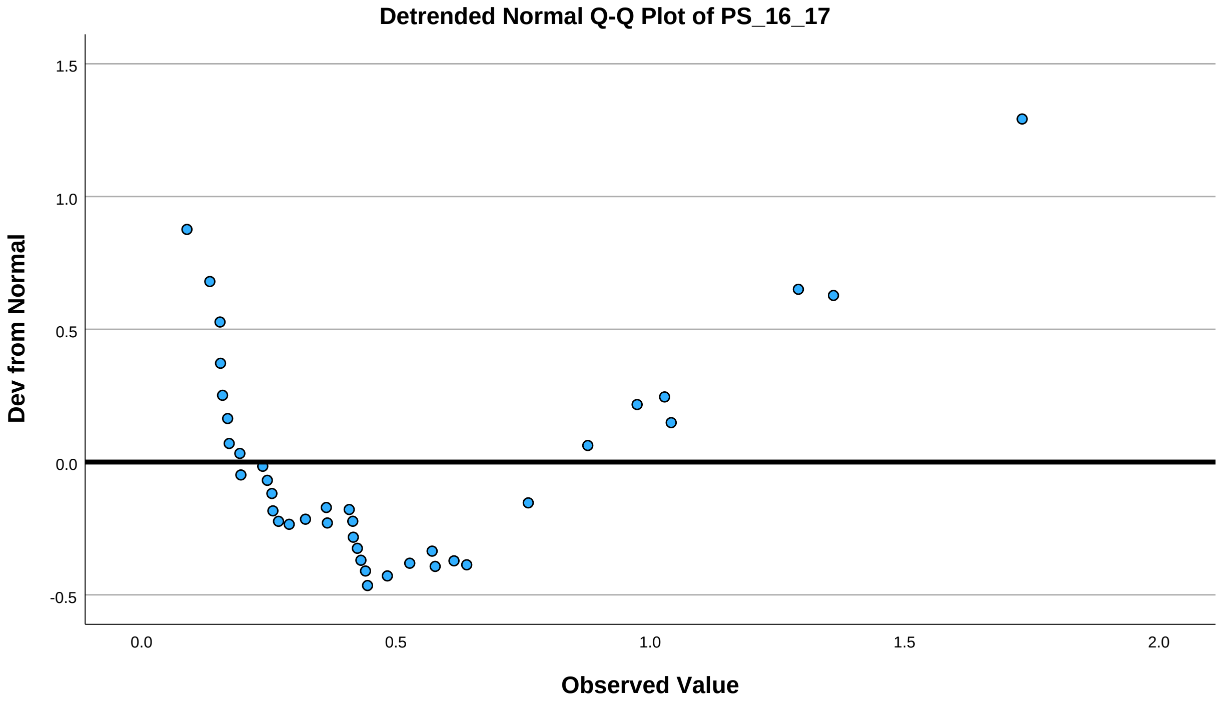

Detrended Normal Q-Q Plot of PS_16_17
Dev from Normal
1.5
1.0
0.5
0.0
-0.5
0.0
0.5
1.0
1.5
2.0
Observed Value

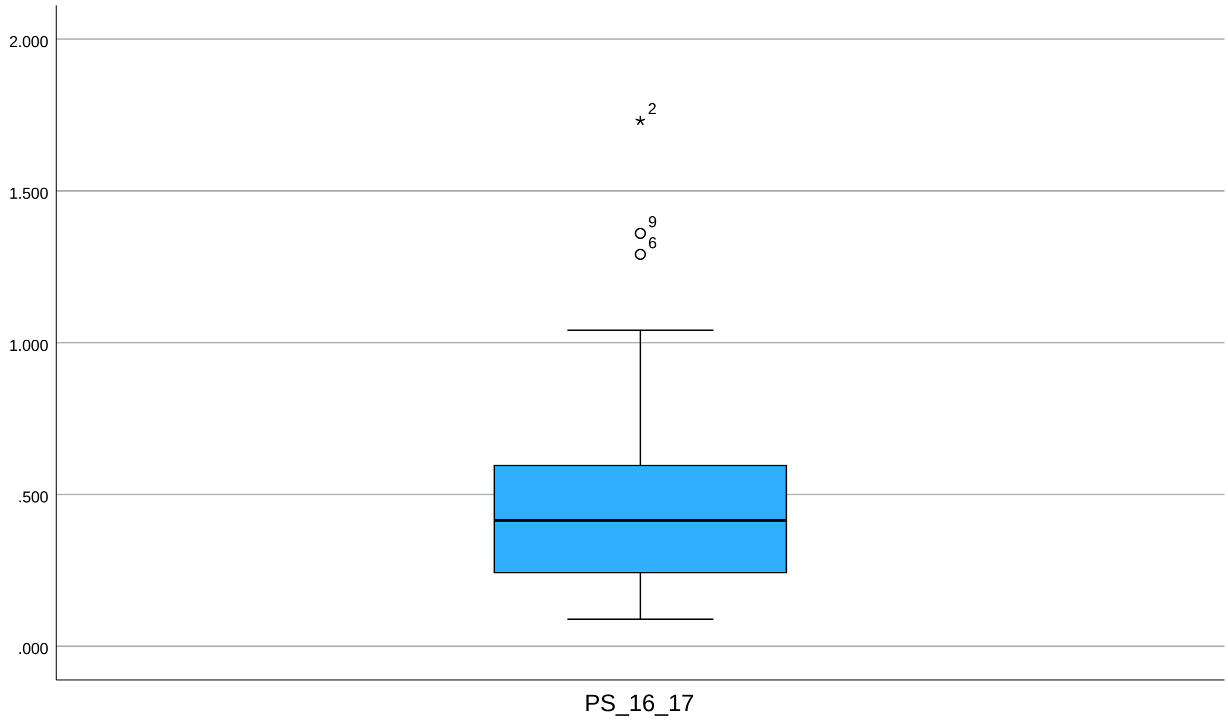

PS_17_18

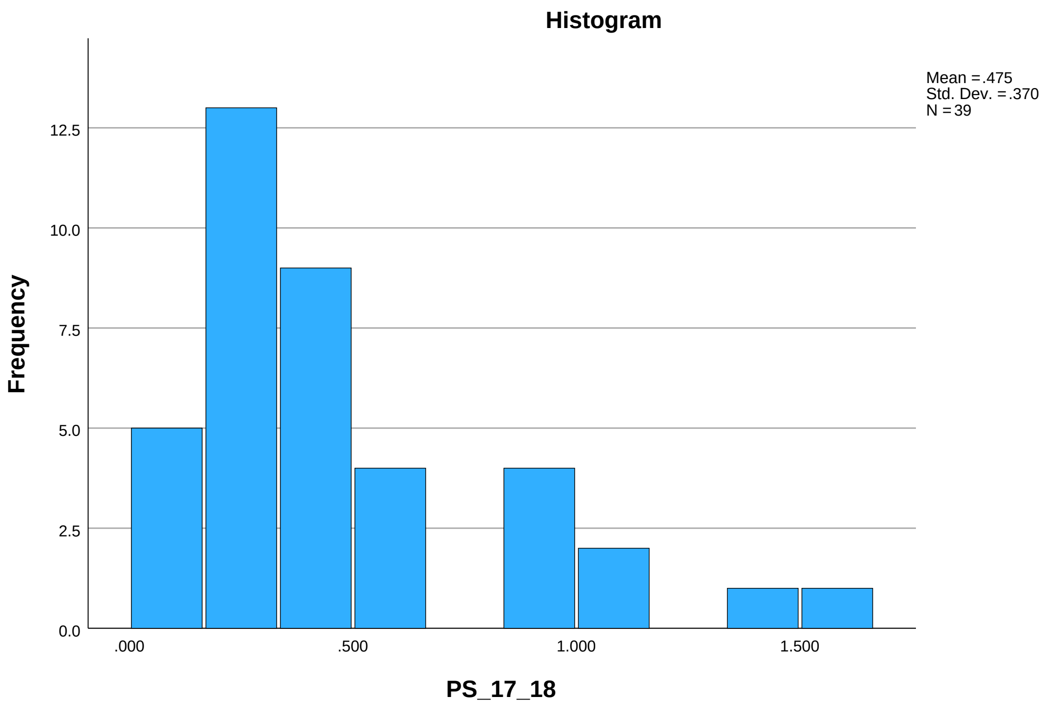

PS_17_18 Stem-and-Leaf Plot

Frequency   Stem & Leaf

```
 1.00     0 . 8
 8.00      1 . 24667779
 8.00      2 . 00112589
 6.00      3 . 255789
 4.00      4 . 1467
 1.00     5 . 0
 3.00      6 . 366
  .00     7 .
 2.00      8 . 35
 2.00      9 . 66
 2.00     10 . 07
 2.00 Extremes    (>=1.41)

Stem width:      .100
Each leaf:       1 case(s)
```

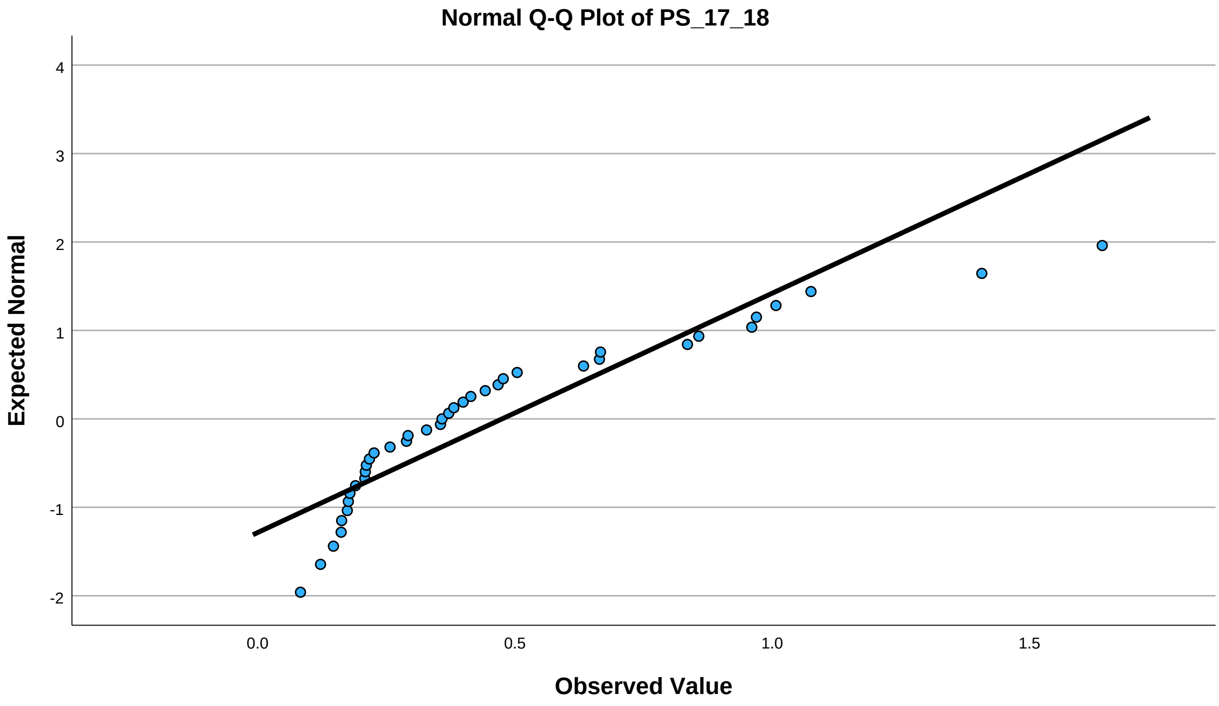

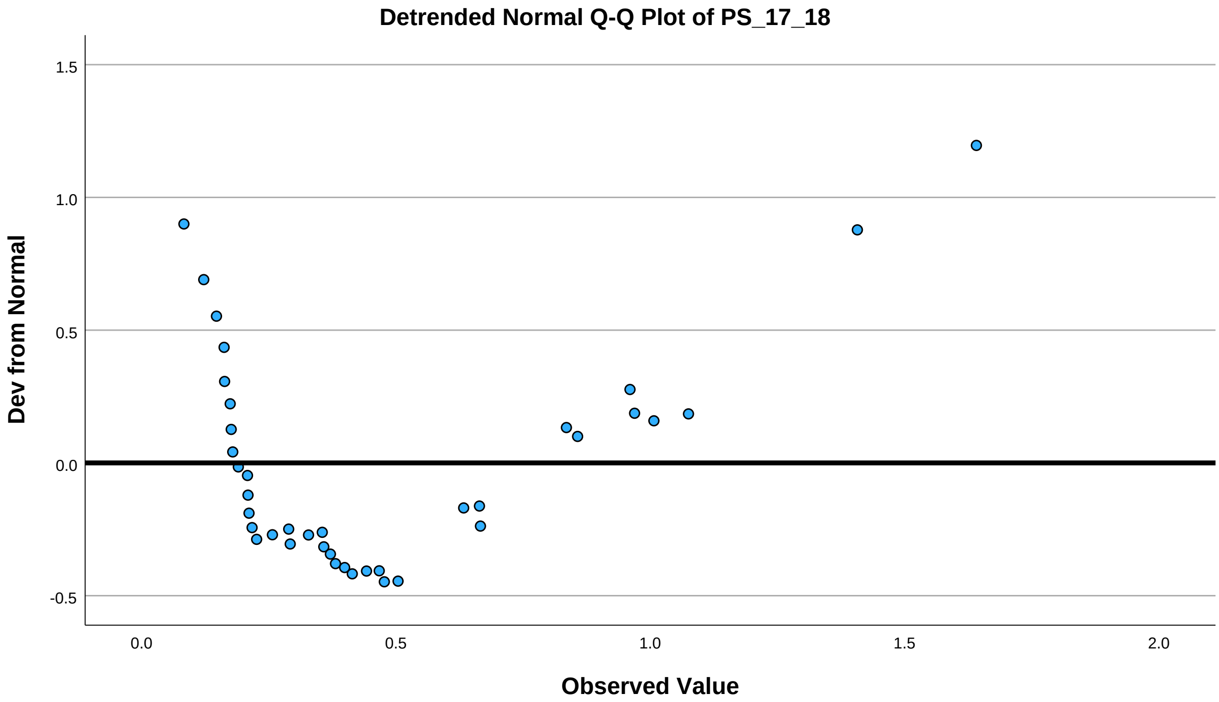
Detrended Normal Q-Q Plot of PS_17_18
Dev from Normal
1.5
1.0
0.5
0.0
-0.5
0.0
0.5
1.0
1.5
2.0
Observed Value

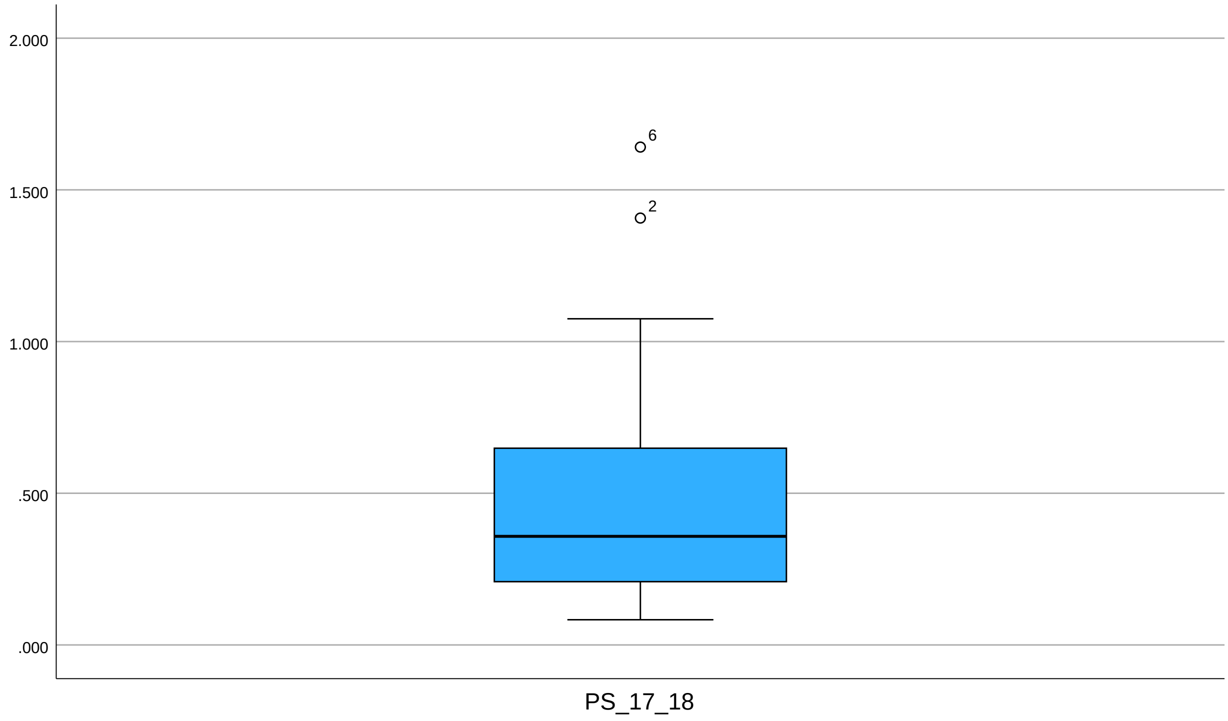
2.000
1.500
1.000
.500
.000
6
2
PS_17_18

PS_18_19

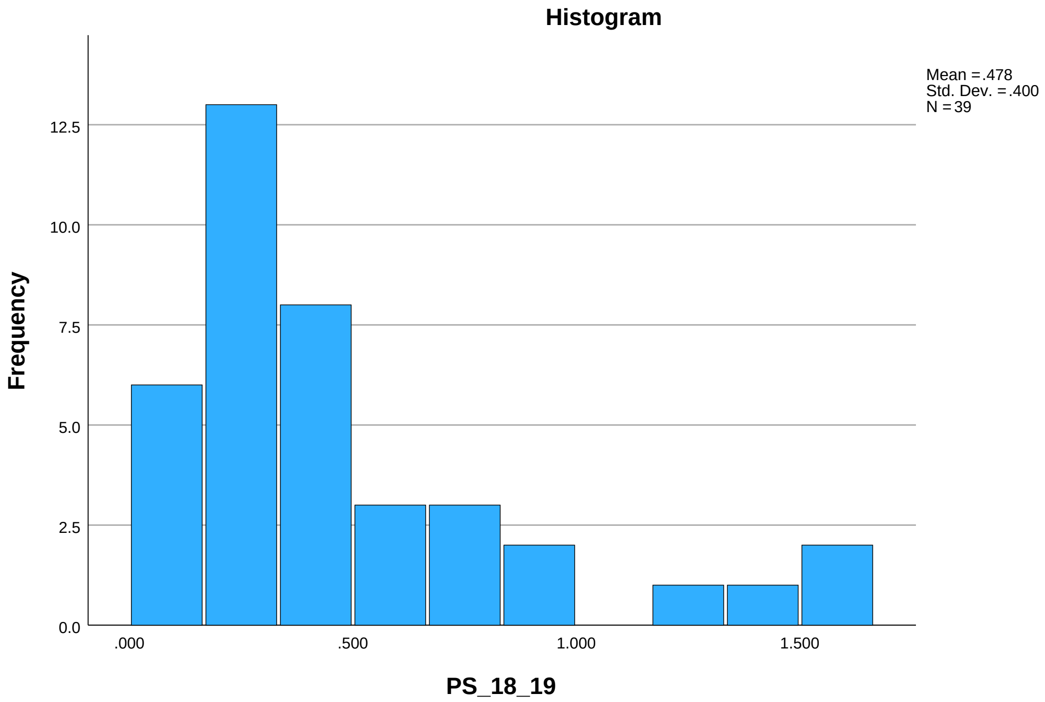


```
PS_18_19 Stem-and-Leaf Plot

 Frequency    Stem &  Leaf

     1.00        0 .  7
     8.00        1 .  23456689
     9.00        2 .  001137789
     6.00        3 .  034679
     3.00        4 .  346
     1.00        5 .  0
     3.00        6 .  057
     1.00        7 .  5
     1.00        8 .  2
     2.00        9 .  07
      .00       10 .
     1.00       11 .  8
     3.00 Extremes    (>=1.37)

 Stem width:      .100
 Each leaf:       1 case(s)
```

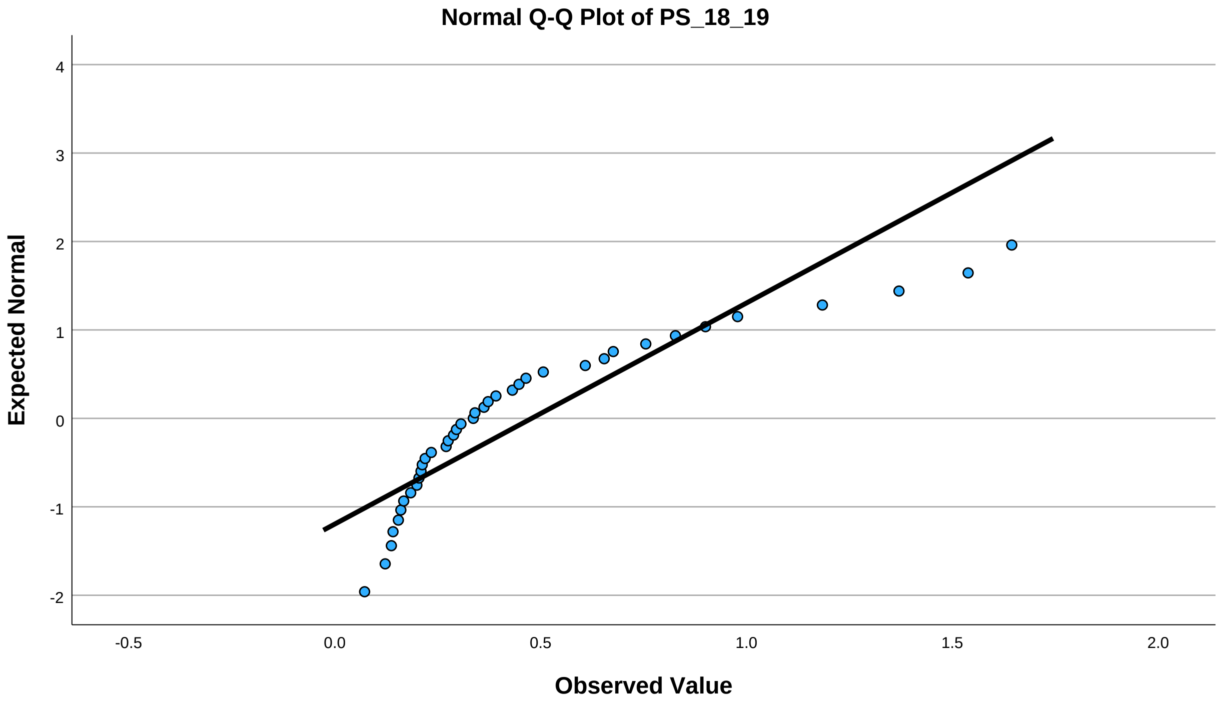

Normal Q-Q Plot of PS_18_19
Expected Normal
Observed Value
4
3
2
1
0
-1
-2
-0.5
0.0
0.5
1.0
1.5
2.0

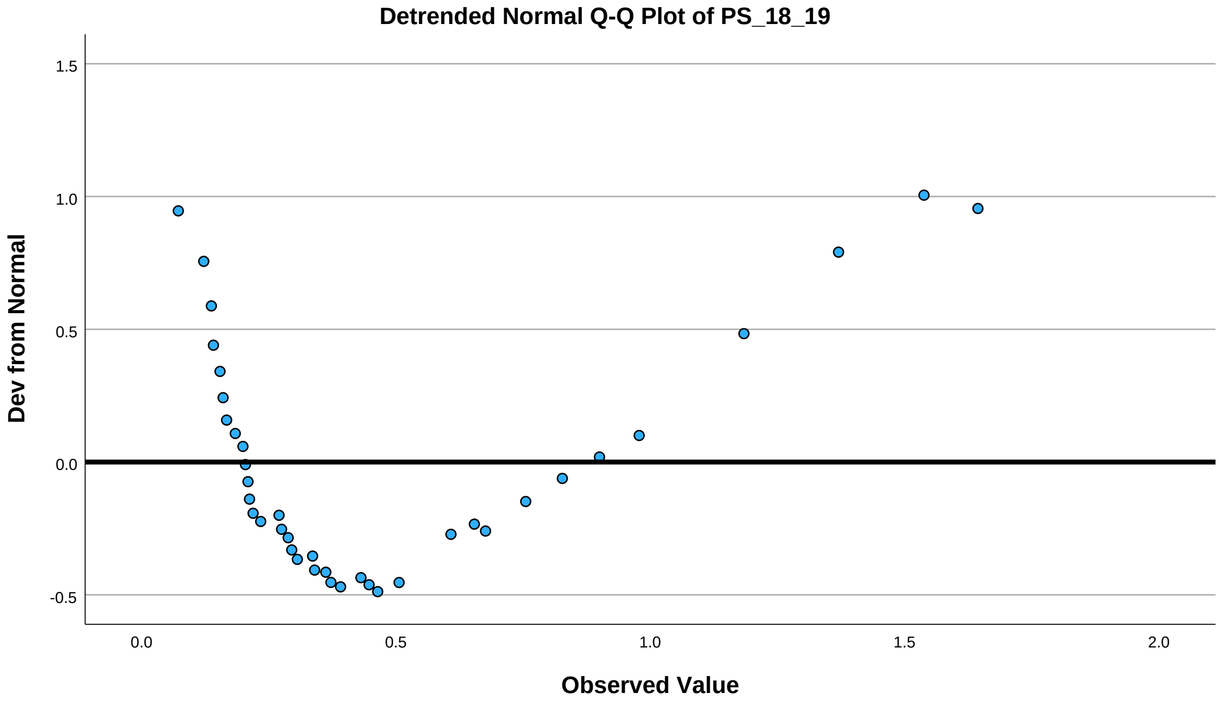

Detrended Normal Q-Q Plot of PS_18_19
Dev from Normal
Observed Value
1.5
1.0
0.5
0.0
-0.5
0.0
0.5
1.0
1.5
2.0

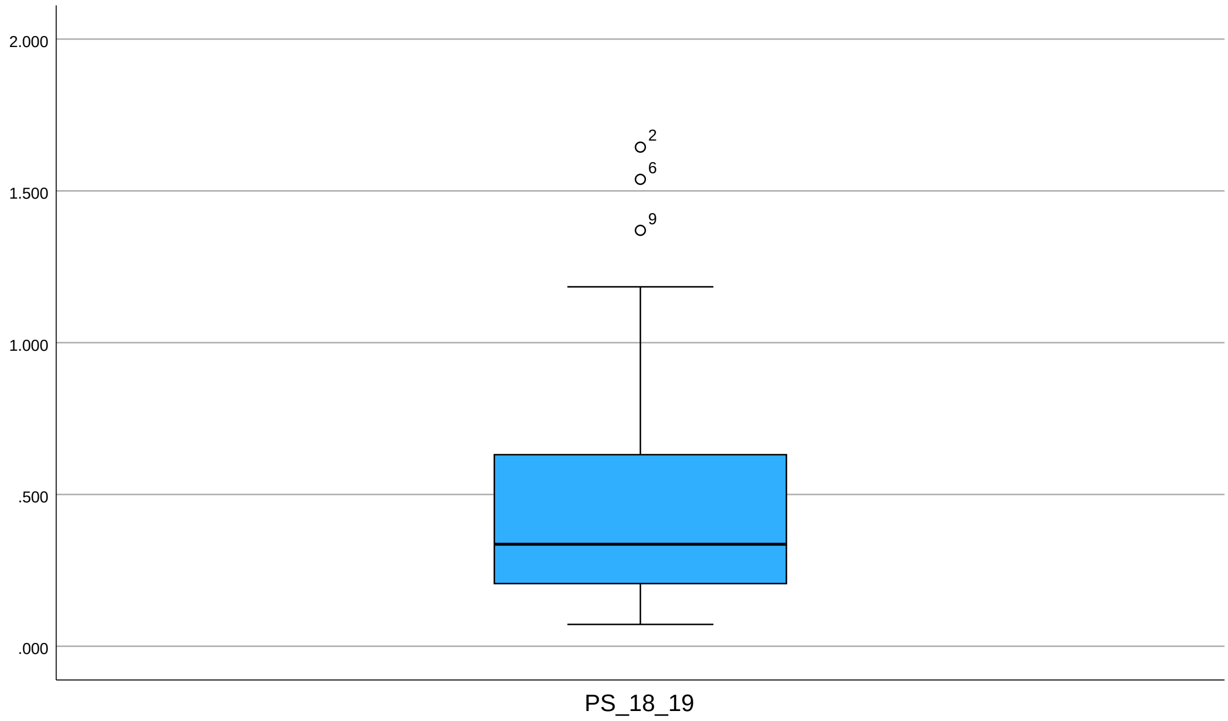

PS_19_20

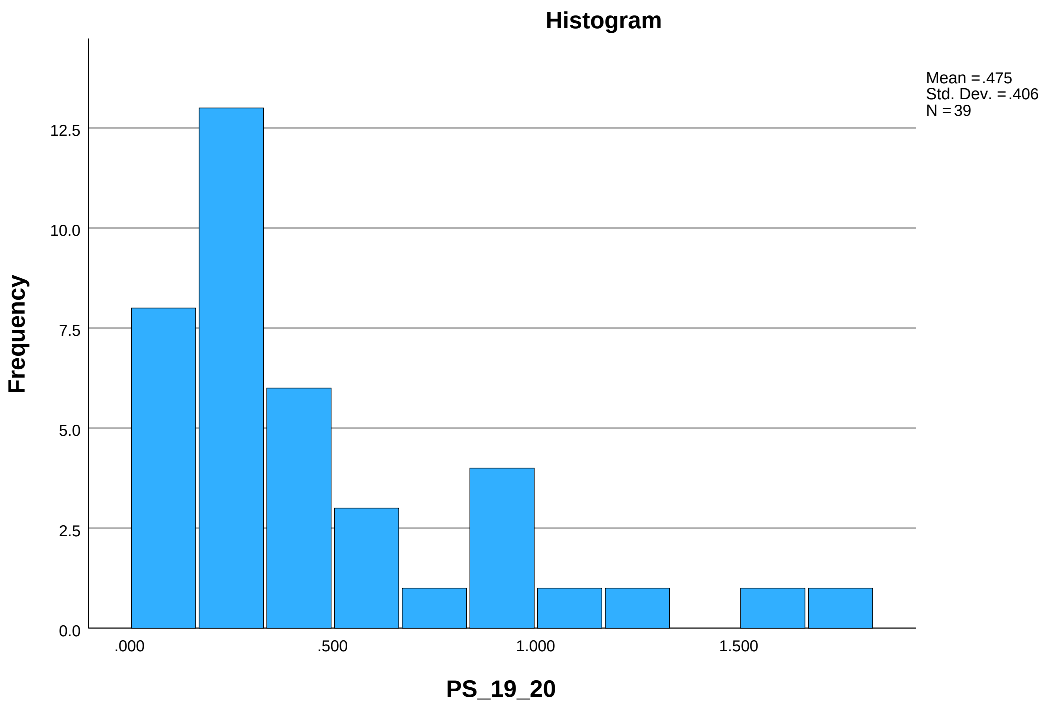

PS_19_20 Stem-and-Leaf Plot

Frequency   Stem & Leaf

```
     1.00        0 .  6
     9.00        1 .  233444678
     8.00        2 .  11223578
     7.00        3 .  0223488
     2.00        4 .  57
     1.00        5 .  5
     2.00        6 .  13
      .00        7 .
     2.00        8 .  04
     3.00        9 .  348
     1.00       10 .  3
     3.00 Extremes    (>=1.30)

 Stem width:      .100
 Each leaf:       1 case(s)
```

Normal Q-Q Plot of PS_19_20

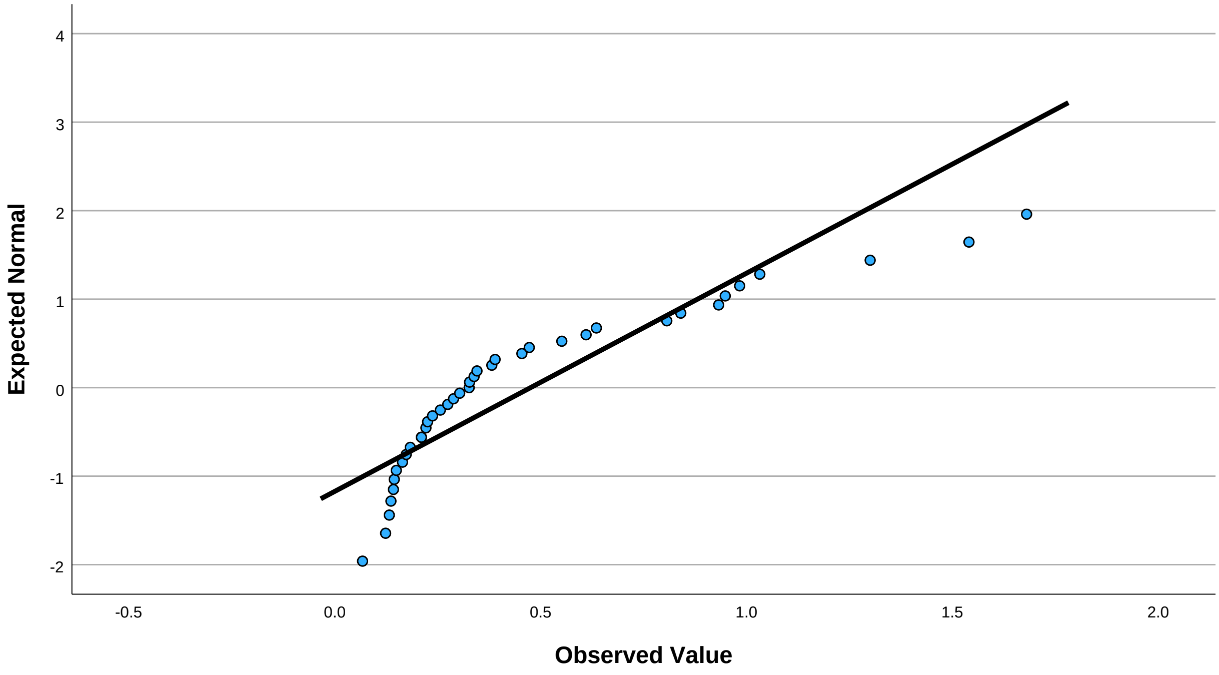

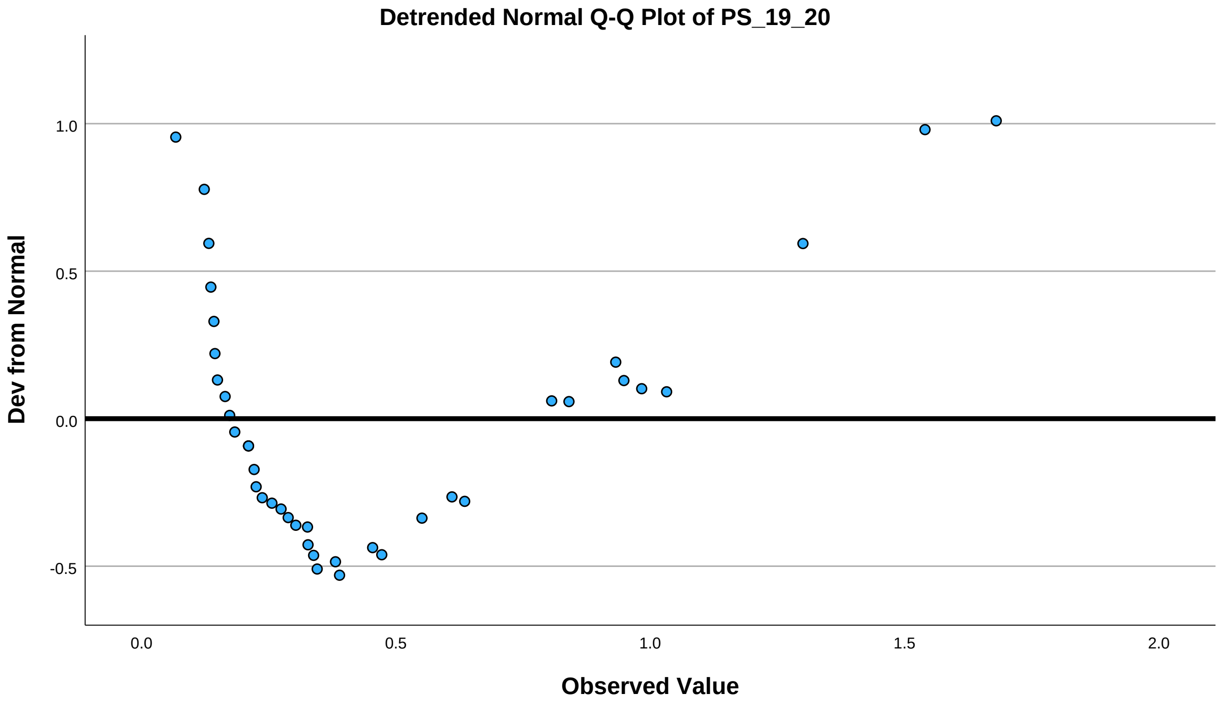
Detrended Normal Q-Q Plot of PS_19_20
Dev from Normal
1.0
0.5
0.0
-0.5
0.0
0.5
1.0
1.5
2.0
Observed Value

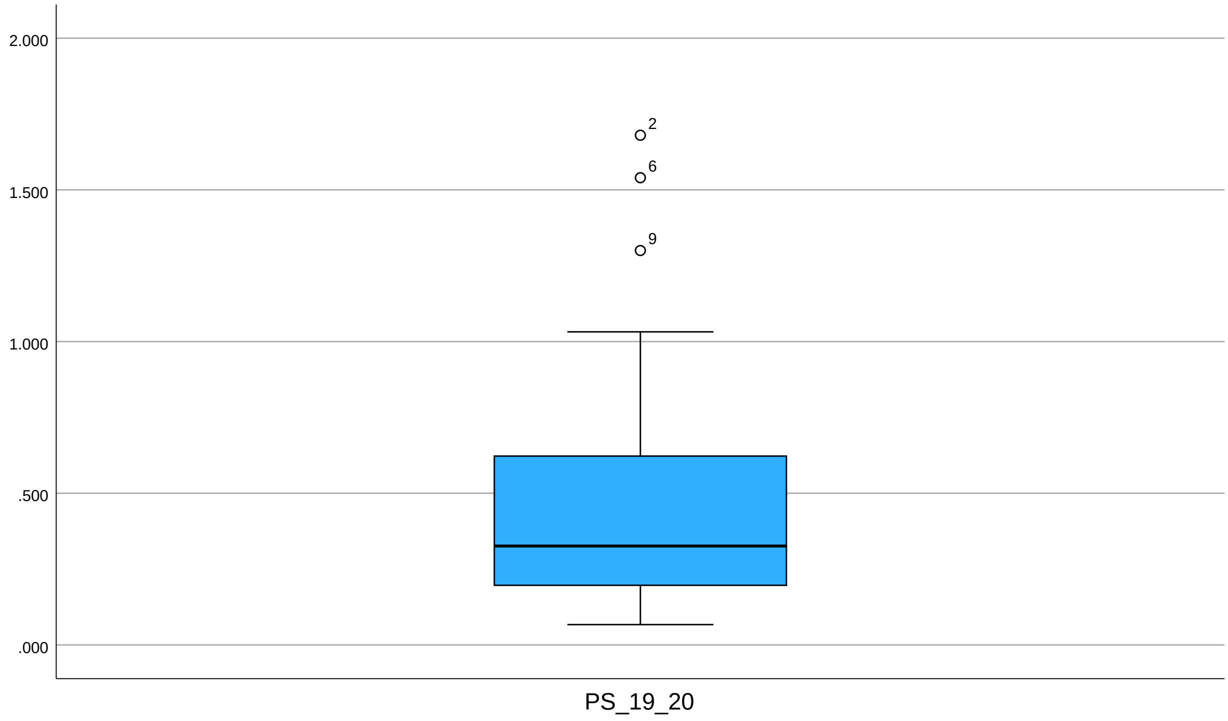
2.000
1.500
1.000
.500
.000
2
6
9
PS_19_20

Explore

Case Processing Summary

	Cases					
	Valid		Missing		Total	
	N	Percent	N	Percent	N	Percent
BrainRate	39	100.0%	0	0.0%	39	100.0%
PermutationEntropy	39	100.0%	0	0.0%	39	100.0%
PermutationEntropy_01	39	100.0%	0	0.0%	39	100.0%
SampleEntropy	39	100.0%	0	0.0%	39	100.0%
SpectralEntropy_bits	39	100.0%	0	0.0%	39	100.0%
SpectralEntropy_01	39	100.0%	0	0.0%	39	100.0%
HiguchiFractalDimension	39	100.0%	0	0.0%	39	100.0%
LempelZivComplexity	39	100.0%	0	0.0%	39	100.0%
RenyiEntropy	39	100.0%	0	0.0%	39	100.0%
TsallisEntropy	39	100.0%	0	0.0%	39	100.0%
PS_4_5	39	100.0%	0	0.0%	39	100.0%
PS_5_6	39	100.0%	0	0.0%	39	100.0%
PS_6_7	39	100.0%	0	0.0%	39	100.0%
PS_7_8	39	100.0%	0	0.0%	39	100.0%
PS_8_9	39	100.0%	0	0.0%	39	100.0%
PS_9_10	39	100.0%	0	0.0%	39	100.0%
PS_10_11	39	100.0%	0	0.0%	39	100.0%
PS_11_12	39	100.0%	0	0.0%	39	100.0%
PS_12_13	39	100.0%	0	0.0%	39	100.0%
PS_13_14	39	100.0%	0	0.0%	39	100.0%
PS_14_15	39	100.0%	0	0.0%	39	100.0%
PS_15_16	39	100.0%	0	0.0%	39	100.0%
PS_16_17	39	100.0%	0	0.0%	39	100.0%
PS_17_18	39	100.0%	0	0.0%	39	100.0%
PS_18_19	39	100.0%	0	0.0%	39	100.0%
PS_19_20	39	100.0%	0	0.0%	39	100.0%

Descriptives

			Statistic	Std. Error
BrainRate	Mean		8.0256	.74496
	95% Confidence Interval for Mean	Lower Bound	6.5176	
		Upper Bound	9.5337	
	5% Trimmed Mean		7.4706	
	Median		6.9300	
	Variance		21.643	
	Std. Deviation		4.65224	
	Minimum		2.73	
	Maximum		28.51	
	Range		25.78	
	Interquartile Range		4.33	
	Skewness		2.585	.378
	Kurtosis		9.294	.741
PermutationEntropy	Mean		1.5183060339	.00754482424
	95% Confidence Interval for Mean	Lower Bound	1.5030323357	
		Upper Bound	1.5335797320	
	5% Trimmed Mean		1.5191852561	
	Median		1.5213472239	
	Variance		.002	
	Std. Deviation		.04711741230	
	Minimum		1.3683067968	
	Maximum		1.6601015366	
	Range		.29179473971	
	Interquartile Range		.04689964561	
	Skewness		-.264	.378
	Kurtosis		3.285	.741
PermutationEntropy_01	Mean		.84738273186	.00421084659
	95% Confidence Interval for Mean	Lower Bound	.83885831861	
		Upper Bound	.85590714511	
	5% Trimmed Mean		.84787343512	
	Median		.84908005233	
	Variance		.001	
	Std. Deviation		.02629672850	
	Minimum		.76366656370	
	Maximum		.92652030871	
	Range		.16285374500	
	Interquartile Range		.02617519059	
	Skewness		-.264	.378
	Kurtosis		3.285	.741

Descriptives

			Statistic	Std. Error
SampleEntropy	Mean		.68399958978	.04349015653
	95% Confidence Interval for Mean	Lower Bound	.59595837072	
		Upper Bound	.77204080884	
	5% Trimmed Mean		.67987301939	
	Median		.63795023822	
	Variance		.074	
	Std. Deviation		.27159594046	
	Minimum		.14826237826	
	Maximum		1.3383743964	
	Range		1.1901120181	
	Interquartile Range		.39696245854	
	Skewness		.148	.378
	Kurtosis		-.263	.741
SpectralEntropy_bits	Mean		3.8846896716	.12680906274
	95% Confidence Interval for Mean	Lower Bound	3.6279781450	
		Upper Bound	4.1414011981	
	5% Trimmed Mean		3.8833004664	
	Median		3.9711662951	
	Variance		.627	
	Std. Deviation		.79192234301	
	Minimum		2.2933545510	
	Maximum		5.8951158468	
	Range		3.6017612959	
	Interquartile Range		1.0804968467	
	Skewness		.017	.378
	Kurtosis		.072	.741
SpectralEntropy_01	Mean		.55406700283	.01808657146
	95% Confidence Interval for Mean	Lower Bound	.51745265313	
		Upper Bound	.59068135253	
	5% Trimmed Mean		.55386886275	
	Median		.56640102373	
	Variance		.013	
	Std. Deviation		.11295060254	
	Minimum		.32709744919	
	Maximum		.84081083555	
	Range		.51371338636	
	Interquartile Range		.15410951711	
	Skewness		.017	.378
	Kurtosis		.072	.741

Descriptives

			Statistic	Std. Error
HiguchiFractalDimension	Mean		.46969932718	.01596291280
	95% Confidence Interval for Mean	Lower Bound	.43738409966	
		Upper Bound	.50201455470	
	5% Trimmed Mean		.47305877051	
	Median		.49679256505	
	Variance		.010	
	Std. Deviation		.09968835851	
	Minimum		.16883478234	
	Maximum		.71605925310	
	Range		.54722447076	
	Interquartile Range		.12644030614	
	Skewness		-.640	.378
	Kurtosis		1.496	.741
LempelZivComplexity	Mean		.26959924747	.00838855767
	95% Confidence Interval for Mean	Lower Bound	.25261750028	
		Upper Bound	.28658099466	
	5% Trimmed Mean		.26943210853	
	Median		.27348663139	
	Variance		.003	
	Std. Deviation		.05238652585	
	Minimum		.14846172093	
	Maximum		.41699174623	
	Range		.26853002530	
	Interquartile Range		.05086126927	
	Skewness		.274	.378
	Kurtosis		1.305	.741
RenyiEntropy	Mean		7.5064183977	.14188205954
	95% Confidence Interval for Mean	Lower Bound	7.2191931844	
		Upper Bound	7.7936436110	
	5% Trimmed Mean		7.5608182355	
	Median		7.8211085244	
	Variance		.785	
	Std. Deviation		.88605317784	
	Minimum		5.0399041724	
	Maximum		8.7048019163	
	Range		3.6648977439	
	Interquartile Range		.95425149960	
	Skewness		-1.121	.378
	Kurtosis		.493	.741

Descriptives

			Statistic	Std. Error
TsallisEntropy	Mean		1.7780119838	.06604147432
	95% Confidence Interval for Mean	Lower Bound	1.6443180086	
		Upper Bound	1.9117059590	
	5% Trimmed Mean		1.8366336665	
	Median		1.9720317333	
	Variance		.170	
	Std. Deviation		.41242887492	
	Minimum		.44703821924	
	Maximum		1.9870708781	
	Range		1.5400326588	
	Interquartile Range		.12000292895	
	Skewness		-2.225	.378
	Kurtosis		3.927	.741
PS_4_5	Mean		2.20015	.381174
	95% Confidence Interval for Mean	Lower Bound	1.42851	
		Upper Bound	2.97180	
	5% Trimmed Mean		1.85165	
	Median		1.67800	
	Variance		5.666	
	Std. Deviation		2.380428	
	Minimum		.397	
	Maximum		15.400	
	Range		15.003	
	Interquartile Range		1.311	
	Skewness		4.719	.378
	Kurtosis		25.984	.741
PS_5_6	Mean		1.58272	.276595
	95% Confidence Interval for Mean	Lower Bound	1.02278	
		Upper Bound	2.14265	
	5% Trimmed Mean		1.34746	
	Median		1.29400	
	Variance		2.984	
	Std. Deviation		1.727334	
	Minimum		.275	
	Maximum		11.330	
	Range		11.055	
	Interquartile Range		1.087	
	Skewness		4.932	.378
	Kurtosis		28.056	.741

Descriptives

			Statistic	Std. Error
PS_6_7	Mean		1.19359	.171439
	95% Confidence Interval for Mean	Lower Bound	.84653	
		Upper Bound	1.54065	
	5% Trimmed Mean		1.04986	
	Median		1.06000	
	Variance		1.146	
	Std. Deviation		1.070636	
	Minimum		.249	
	Maximum		7.020	
	Range		6.771	
	Interquartile Range		.797	
	Skewness		4.411	.378
	Kurtosis		23.909	.741
PS_7_8	Mean		1.17603	.206626
	95% Confidence Interval for Mean	Lower Bound	.75773	
		Upper Bound	1.59432	
	5% Trimmed Mean		.96416	
	Median		.97100	
	Variance		1.665	
	Std. Deviation		1.290379	
	Minimum		.225	
	Maximum		8.090	
	Range		7.865	
	Interquartile Range		.695	
	Skewness		4.413	.378
	Kurtosis		22.639	.741
PS_8_9	Mean		.96928	.143207
	95% Confidence Interval for Mean	Lower Bound	.67938	
		Upper Bound	1.25919	
	5% Trimmed Mean		.82467	
	Median		.76000	
	Variance		.800	
	Std. Deviation		.894325	
	Minimum		.190	
	Maximum		5.170	
	Range		4.980	
	Interquartile Range		.628	
	Skewness		3.391	.378
	Kurtosis		13.717	.741

Descriptives

			Statistic	Std. Error
PS_9_10	Mean		.91913	.117803
	95% Confidence Interval for Mean	Lower Bound	.68065	
		Upper Bound	1.15761	
	5% Trimmed Mean		.83247	
	Median		.83000	
	Variance		.541	
	Std. Deviation		.735677	
	Minimum		.150	
	Maximum		4.300	
	Range		4.150	
	Interquartile Range		.533	
	Skewness		2.810	.378
	Kurtosis		11.115	.741
PS_10_11	Mean		1.05331	.142790
	95% Confidence Interval for Mean	Lower Bound	.76424	
		Upper Bound	1.34237	
	5% Trimmed Mean		.93130	
	Median		.87000	
	Variance		.795	
	Std. Deviation		.891726	
	Minimum		.170	
	Maximum		5.280	
	Range		5.110	
	Interquartile Range		.673	
	Skewness		3.133	.378
	Kurtosis		13.133	.741
PS_11_12	Mean		.56608	.083743
	95% Confidence Interval for Mean	Lower Bound	.39655	
		Upper Bound	.73561	
	5% Trimmed Mean		.49105	
	Median		.43100	
	Variance		.274	
	Std. Deviation		.522974	
	Minimum		.118	
	Maximum		2.920	
	Range		2.802	
	Interquartile Range		.320	
	Skewness		3.005	.378
	Kurtosis		10.813	.741

Descriptives

			Statistic	Std. Error
PS_12_13	Mean		.43631	.066726
	95% Confidence Interval for Mean	Lower Bound	.30123	
		Upper Bound	.57139	
	5% Trimmed Mean		.37904	
	Median		.36100	
	Variance		.174	
	Std. Deviation		.416705	
	Minimum		.083	
	Maximum		2.690	
	Range		2.607	
	Interquartile Range		.264	
	Skewness		4.382	.378
	Kurtosis		23.263	.741
PS_13_14	Mean		.41218	.063213
	95% Confidence Interval for Mean	Lower Bound	.28421	
		Upper Bound	.54015	
	5% Trimmed Mean		.35591	
	Median		.33400	
	Variance		.156	
	Std. Deviation		.394766	
	Minimum		.064	
	Maximum		2.410	
	Range		2.346	
	Interquartile Range		.203	
	Skewness		3.766	.378
	Kurtosis		17.458	.741
PS_14_15	Mean		.38210	.051490
	95% Confidence Interval for Mean	Lower Bound	.27787	
		Upper Bound	.48634	
	5% Trimmed Mean		.34140	
	Median		.30700	
	Variance		.103	
	Std. Deviation		.321553	
	Minimum		.069	
	Maximum		1.670	
	Range		1.601	
	Interquartile Range		.237	
	Skewness		2.399	.378
	Kurtosis		6.613	.741

Descriptives

			Statistic	Std. Error
PS_15_16	Mean		.36364	.045134
	95% Confidence Interval for Mean	Lower Bound	.27227	
		Upper Bound	.45501	
	5% Trimmed Mean		.32932	
	Median		.27100	
	Variance		.079	
	Std. Deviation		.281859	
	Minimum		.059	
	Maximum		1.380	
	Range		1.321	
	Interquartile Range		.207	
	Skewness		2.095	.378
	Kurtosis		4.790	.741
PS_16_17	Mean		.33456	.038922
	95% Confidence Interval for Mean	Lower Bound	.25577	
		Upper Bound	.41336	
	5% Trimmed Mean		.30920	
	Median		.27500	
	Variance		.059	
	Std. Deviation		.243067	
	Minimum		.058	
	Maximum		1.152	
	Range		1.094	
	Interquartile Range		.229	
	Skewness		1.808	.378
	Kurtosis		3.481	.741
PS_17_18	Mean		.34144	.045093
	95% Confidence Interval for Mean	Lower Bound	.25015	
		Upper Bound	.43272	
	5% Trimmed Mean		.29979	
	Median		.28500	
	Variance		.079	
	Std. Deviation		.281605	
	Minimum		.066	
	Maximum		1.460	
	Range		1.394	
	Interquartile Range		.240	
	Skewness		2.624	.378
	Kurtosis		8.029	.741

Descriptives

			Statistic	Std. Error
PS_18_19	Mean		.32869	.049762
	95% Confidence Interval for Mean	Lower Bound	.22795	
		Upper Bound	.42943	
	5% Trimmed Mean		.28355	
	Median		.27300	
	Variance		.097	
	Std. Deviation		.310765	
	Minimum		.051	
	Maximum		1.777	
	Range		1.726	
	Interquartile Range		.234	
	Skewness		3.132	.378
	Kurtosis		12.329	.741
PS_19_20	Mean		.31508	.046412
	95% Confidence Interval for Mean	Lower Bound	.22112	
		Upper Bound	.40903	
	5% Trimmed Mean		.27396	
	Median		.22400	
	Variance		.084	
	Std. Deviation		.289841	
	Minimum		.050	
	Maximum		1.612	
	Range		1.562	
	Interquartile Range		.222	
	Skewness		2.859	.378
	Kurtosis		10.312	.741

Tests of Normality

	Kolmogorov-Smirnov[a]			Shapiro-Wilk		
	Statistic	df	Sig.	Statistic	df	Sig.
BrainRate	.174	39	.004	.769	39	<.001
PermutationEntropy	.113	39	.200*	.938	39	.033
PermutationEntropy_01	.113	39	.200*	.938	39	.033
SampleEntropy	.098	39	.200*	.982	39	.760
SpectralEntropy_bits	.067	39	.200*	.987	39	.922
SpectralEntropy_01	.067	39	.200*	.987	39	.922
HiguchiFractalDimension	.124	39	.134	.948	39	.072
LempelZivComplexity	.099	39	.200*	.966	39	.280
RenyiEntropy	.204	39	<.001	.880	39	<.001
TsallisEntropy	.353	39	<.001	.567	39	<.001
PS_4_5	.264	39	<.001	.505	39	<.001
PS_5_6	.255	39	<.001	.482	39	<.001
PS_6_7	.234	39	<.001	.562	39	<.001
PS_7_8	.319	39	<.001	.516	39	<.001
PS_8_9	.254	39	<.001	.631	39	<.001
PS_9_10	.257	39	<.001	.739	39	<.001
PS_10_11	.217	39	<.001	.701	39	<.001
PS_11_12	.259	39	<.001	.662	39	<.001
PS_12_13	.249	39	<.001	.555	39	<.001
PS_13_14	.289	39	<.001	.595	39	<.001
PS_14_15	.260	39	<.001	.731	39	<.001
PS_15_16	.229	39	<.001	.779	39	<.001
PS_16_17	.182	39	.002	.818	39	<.001
PS_17_18	.203	39	<.001	.717	39	<.001
PS_18_19	.241	39	<.001	.674	39	<.001
PS_19_20	.212	39	<.001	.702	39	<.001

*. This is a lower bound of the true significance.

a. Lilliefors Significance Correction

BrainRate

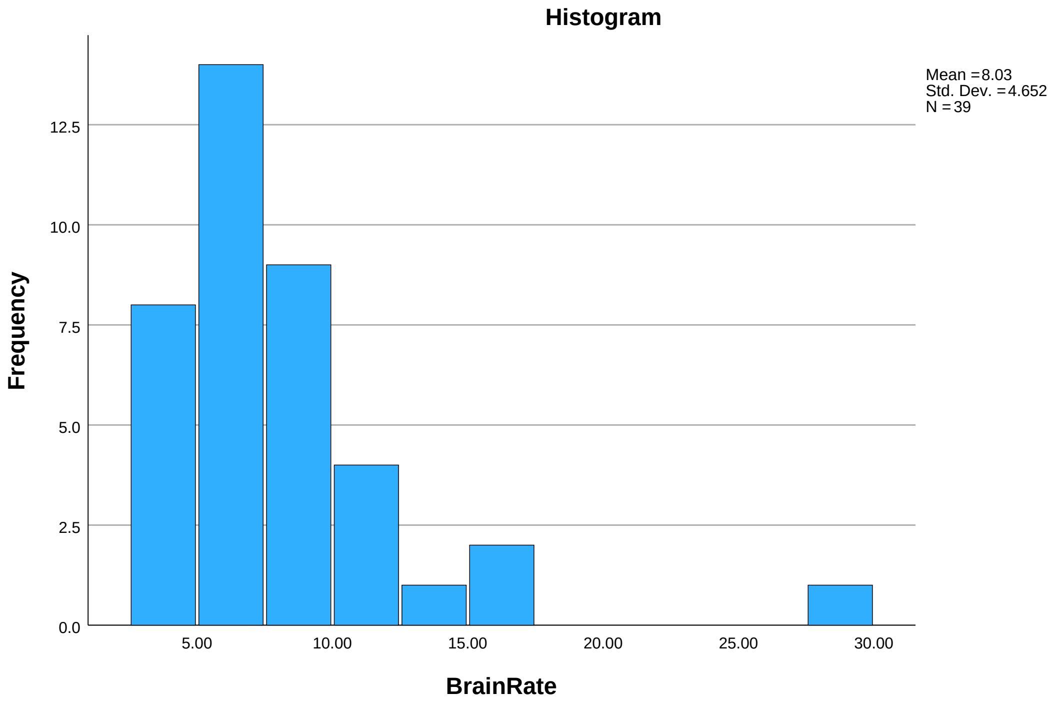


```
BrainRate Stem-and-Leaf Plot

 Frequency    Stem &  Leaf

     4.00        0 .  2333
    11.00        0 .  44445555555
     9.00        0 .  666667777
     7.00        0 .  8888999
     3.00        1 .  000
     2.00        1 .  23
     1.00        1 .  5
     2.00 Extremes    (>=17)

 Stem width:      10.00
 Each leaf:        1 case(s)
```

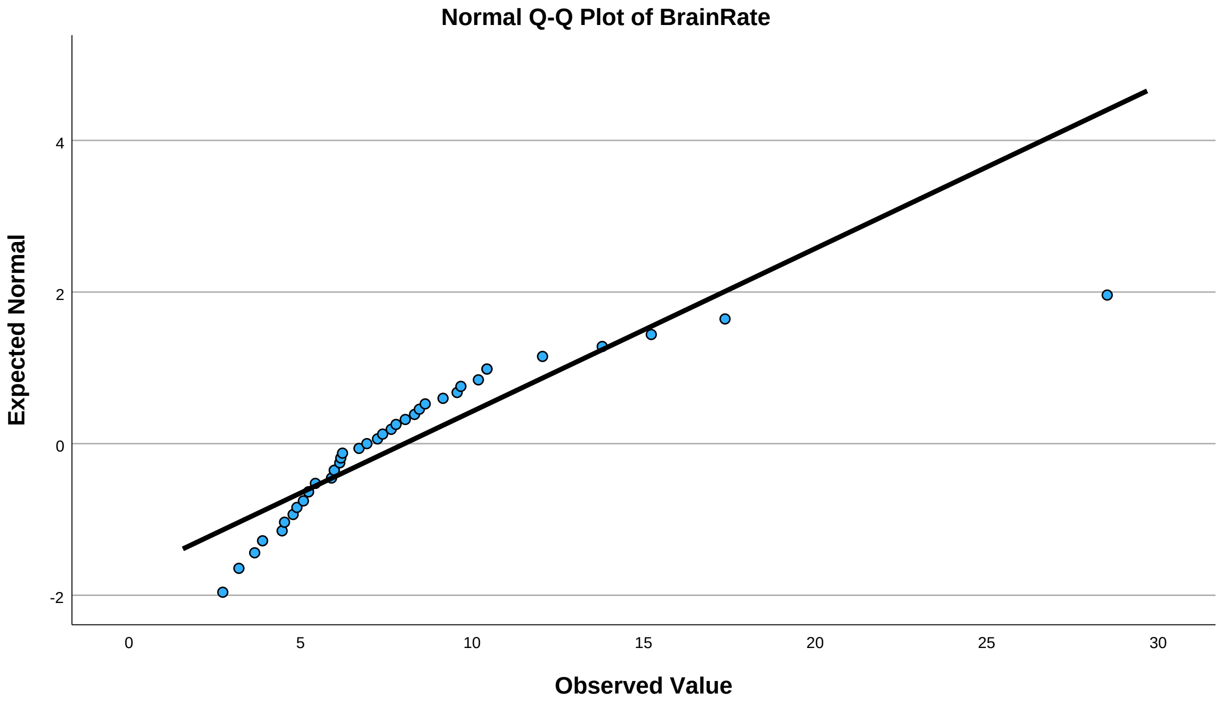
Normal Q-Q Plot of BrainRate
Expected Normal
4
2
0
-2
0
5
10
15
20
25
30
Observed Value

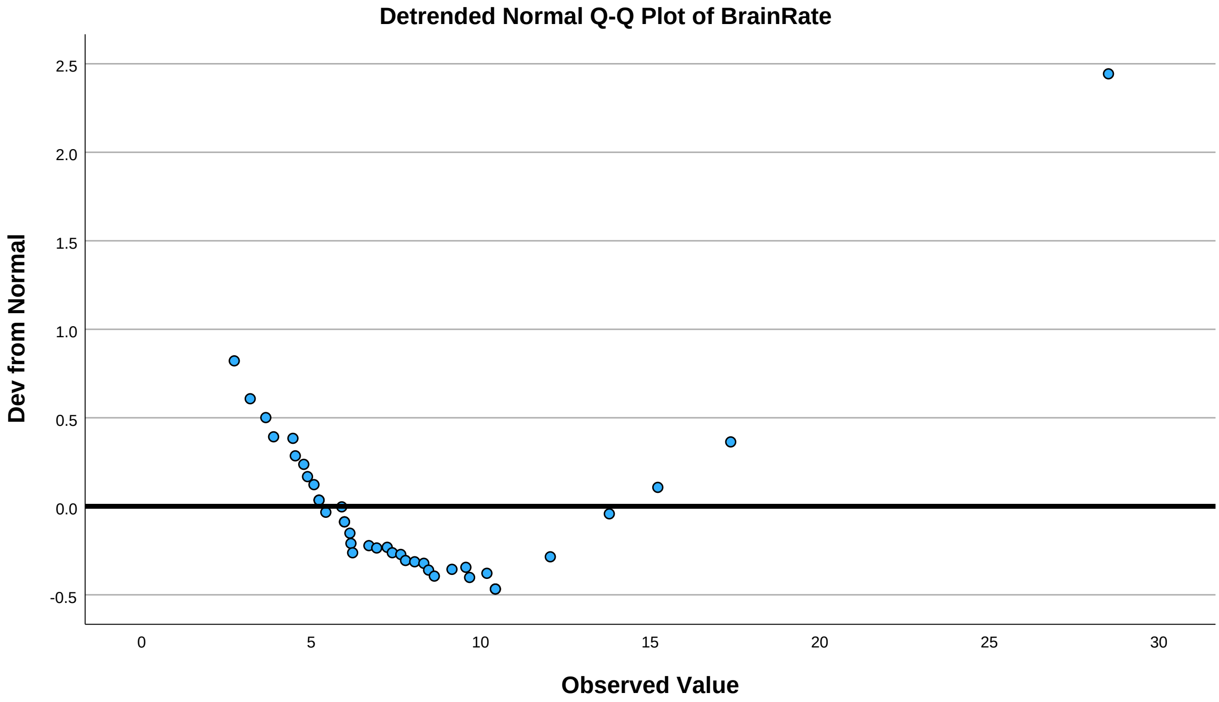
Detrended Normal Q-Q Plot of BrainRate
Dev from Normal
2.5
2.0
1.5
1.0
0.5
0.0
-0.5
0
5
10
15
20
25
30
Observed Value

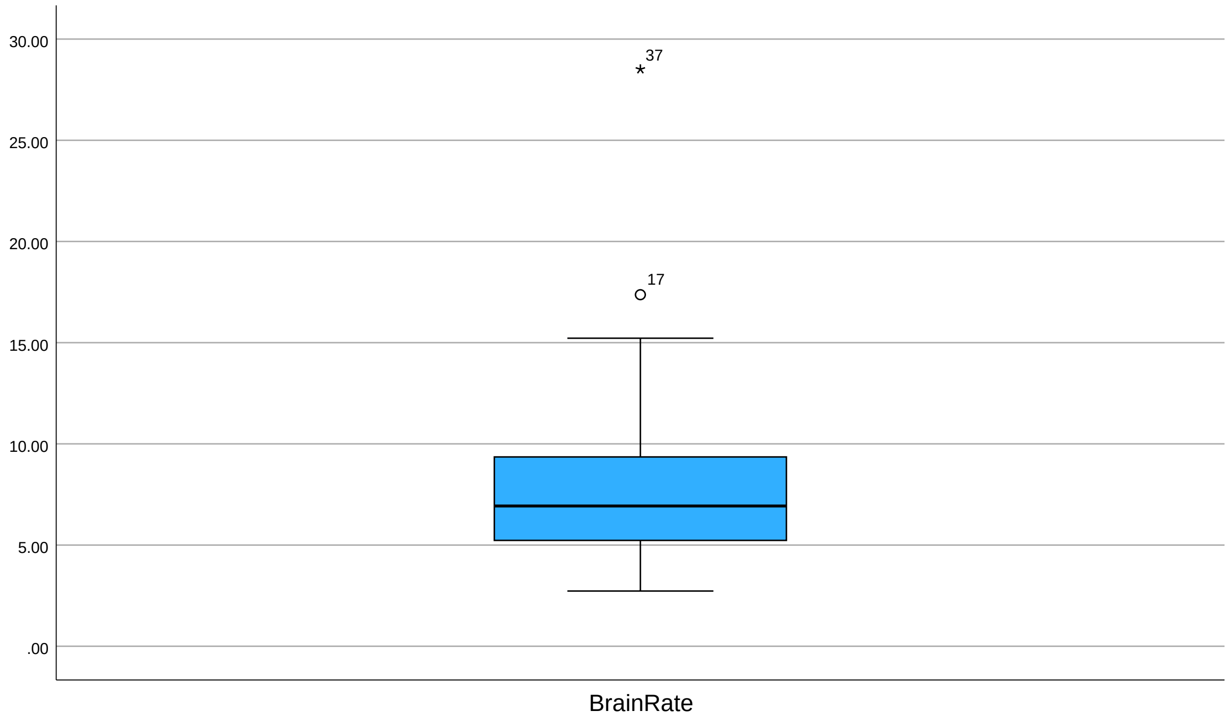

PermutationEntropy

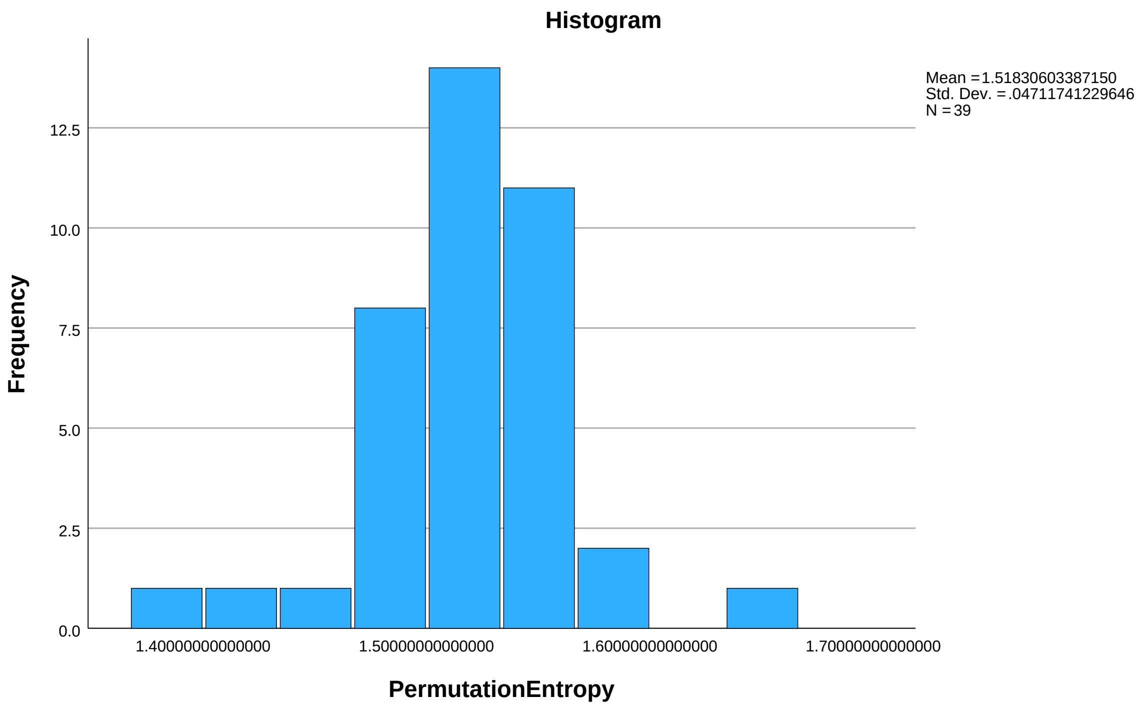


```
PermutationEntropy Stem-and-Leaf Plot

 Frequency    Stem &  Leaf
```

```
 1.00 Extremes    (=<1.37)
 1.00     14 .  3
  .00     14 .
 6.00     14 .  667777
 3.00     14 .  999
 7.00     15 .  0011111
 9.00     15 .  222222333
 7.00     15 .  4444445
 2.00     15 .  66
 2.00     15 .  88
 1.00 Extremes    (>=1.66)

Stem width:   .1000000
Each leaf:        1 case(s)
```

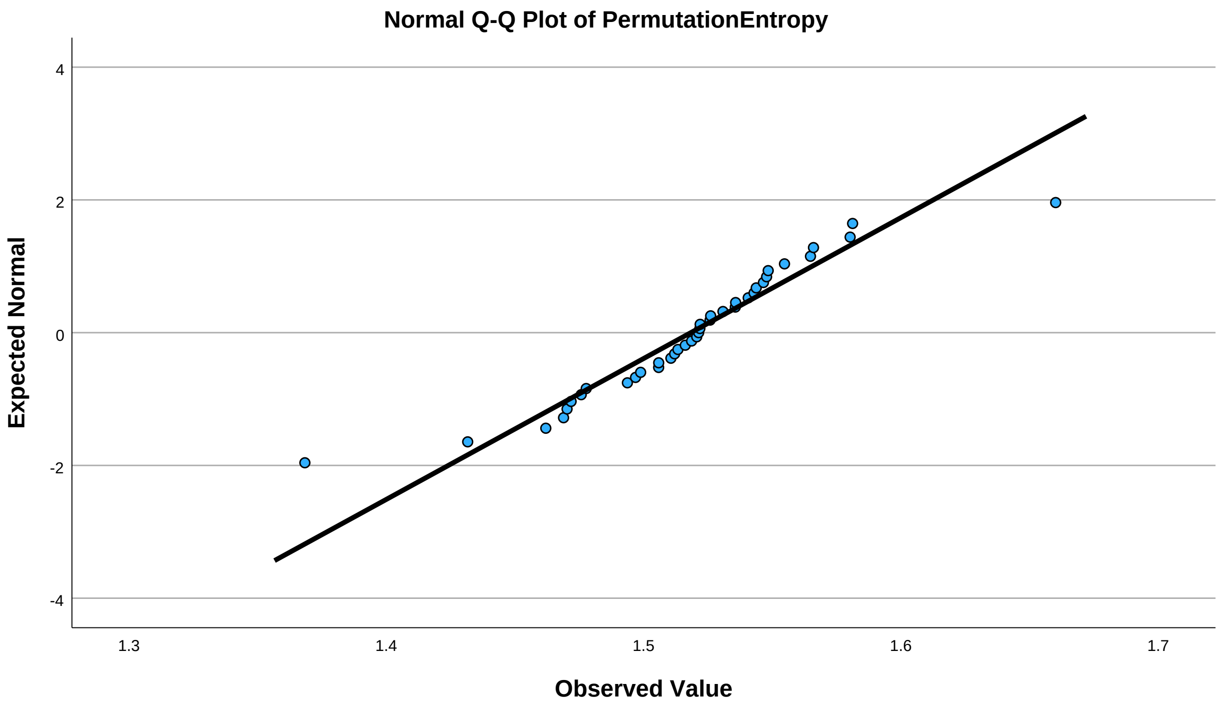

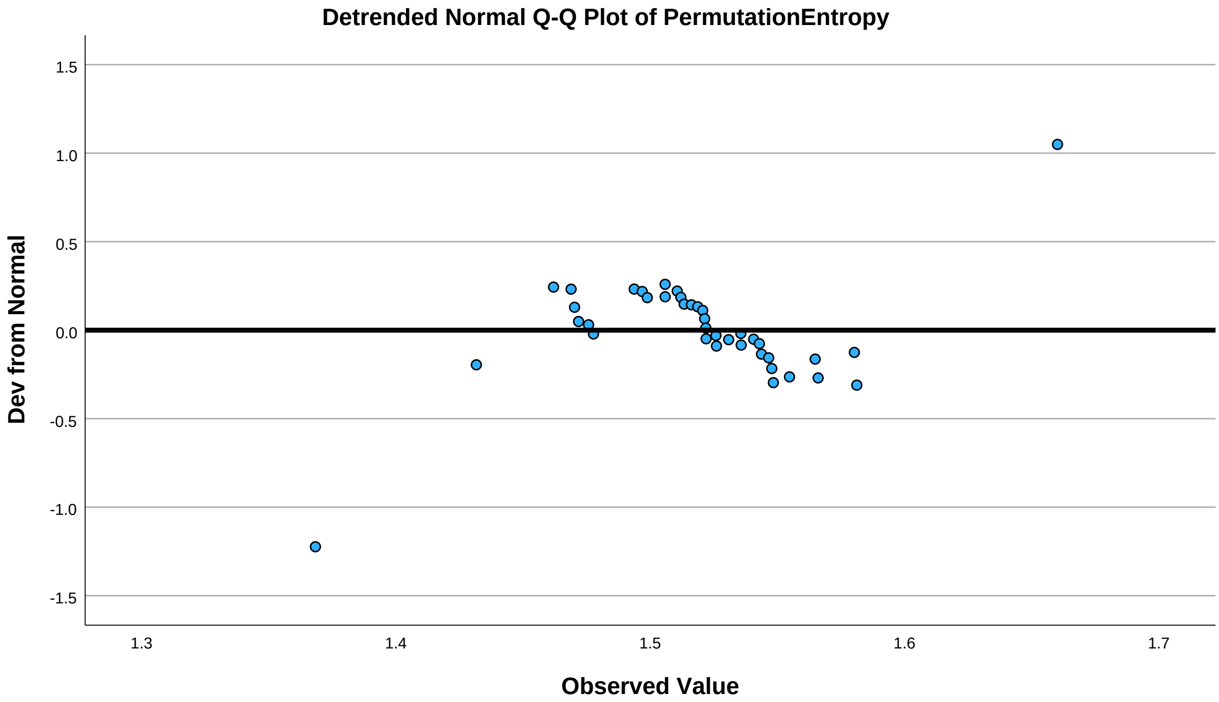

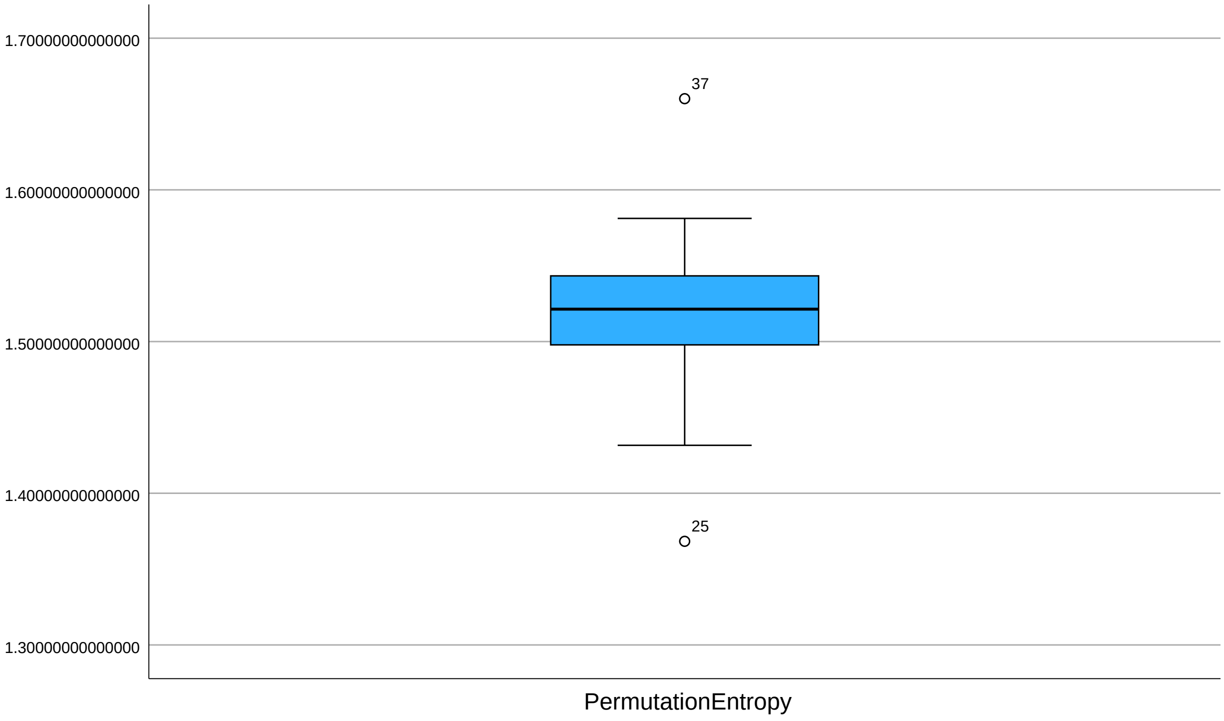

PermutationEntropy_01

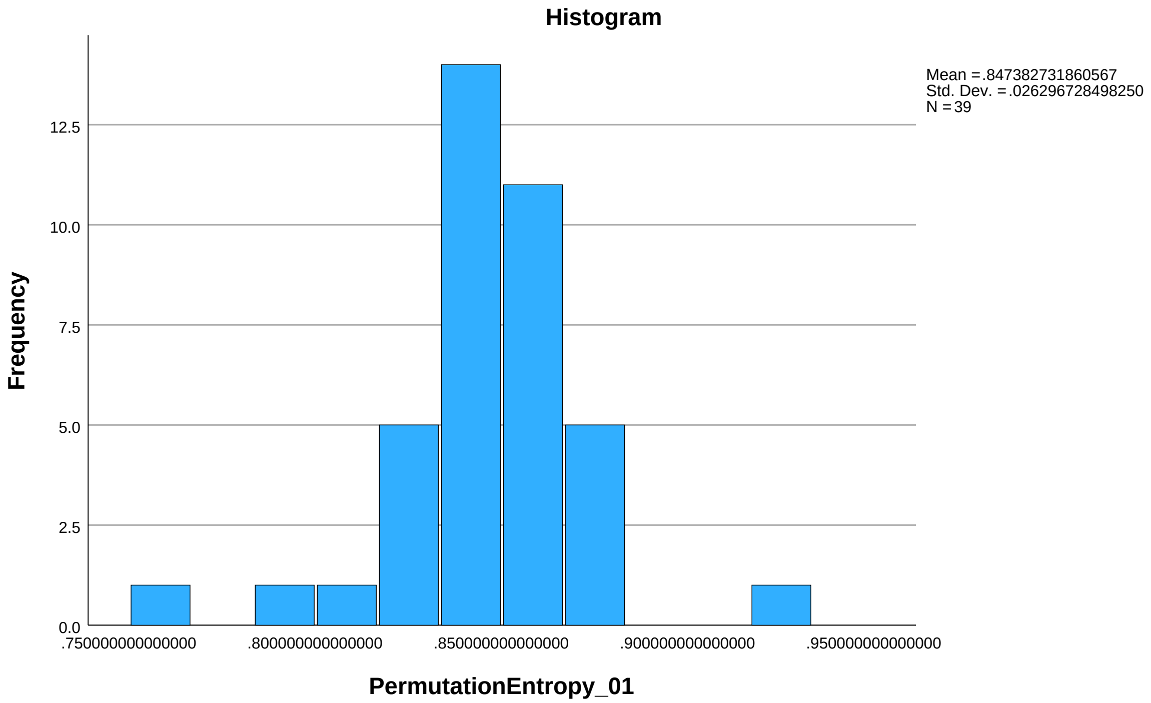


```
PermutationEntropy_01 Stem-and-Leaf Plot

 Frequency    Stem &  Leaf

     1.00 Extremes    (=<.764)
     1.00       79 .  8
      .00       80 .
     2.00       81 .  59
     4.00       82 .  0134
     3.00       83 .  356
    11.00       84 .  00334678999
     6.00       85 .  114779
     6.00       86 .  113347
     2.00       87 .  33
     2.00       88 .  12
     1.00 Extremes    (>=.927)

 Stem width:   .0100000
 Each leaf:        1 case(s)
```

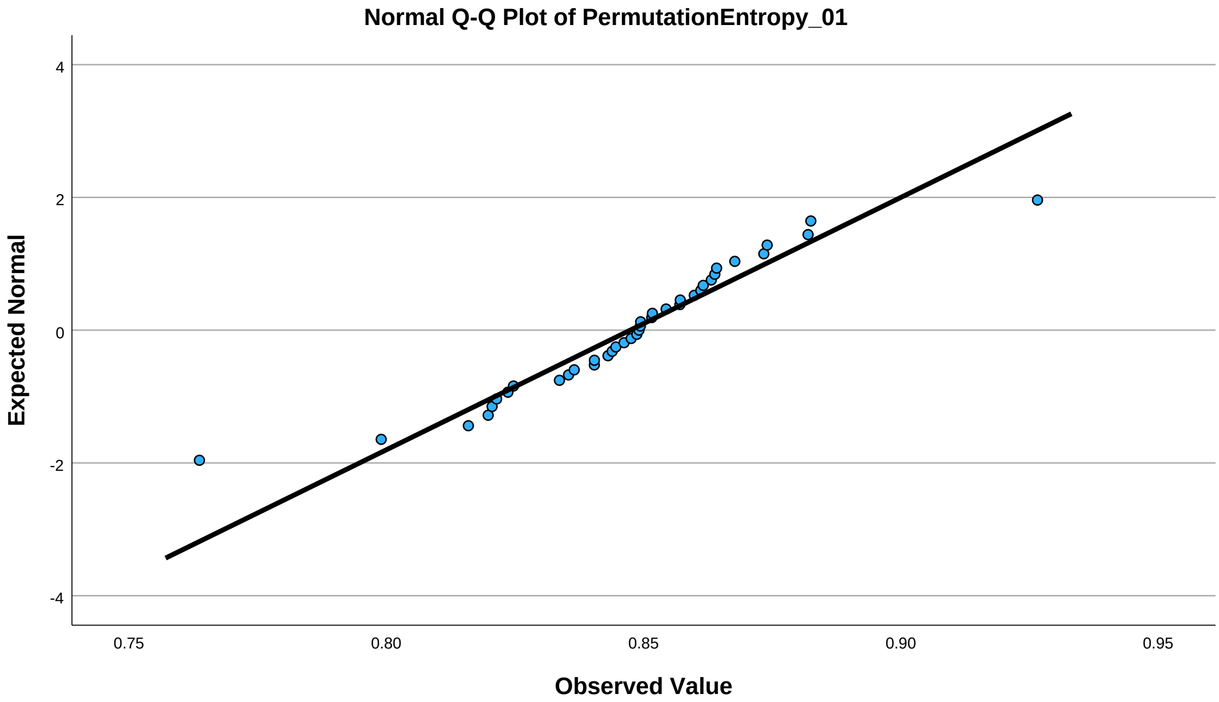
Normal Q-Q Plot of PermutationEntropy_01
Expected Normal
4
2
0
-2
-4
0.75
0.80
0.85
0.90
0.95
Observed Value

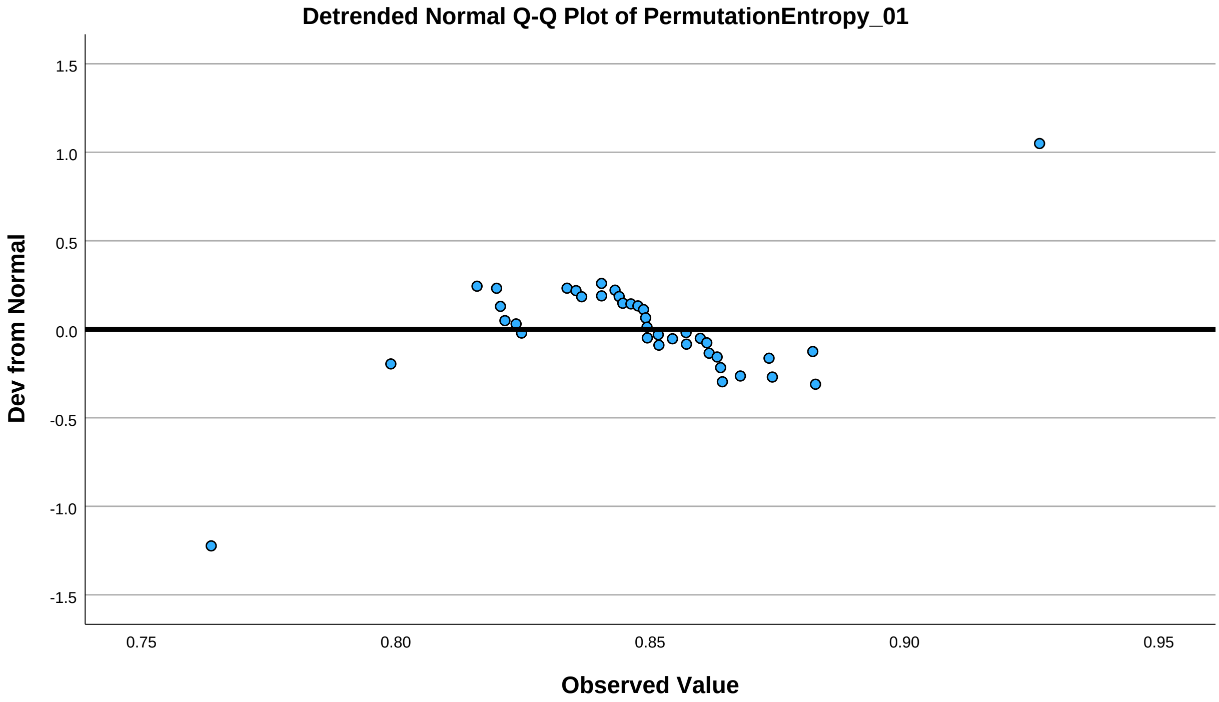
Detrended Normal Q-Q Plot of PermutationEntropy_01
Dev from Normal
1.5
1.0
0.5
0.0
-0.5
-1.0
-1.5
0.75
0.80
0.85
0.90
0.95
Observed Value

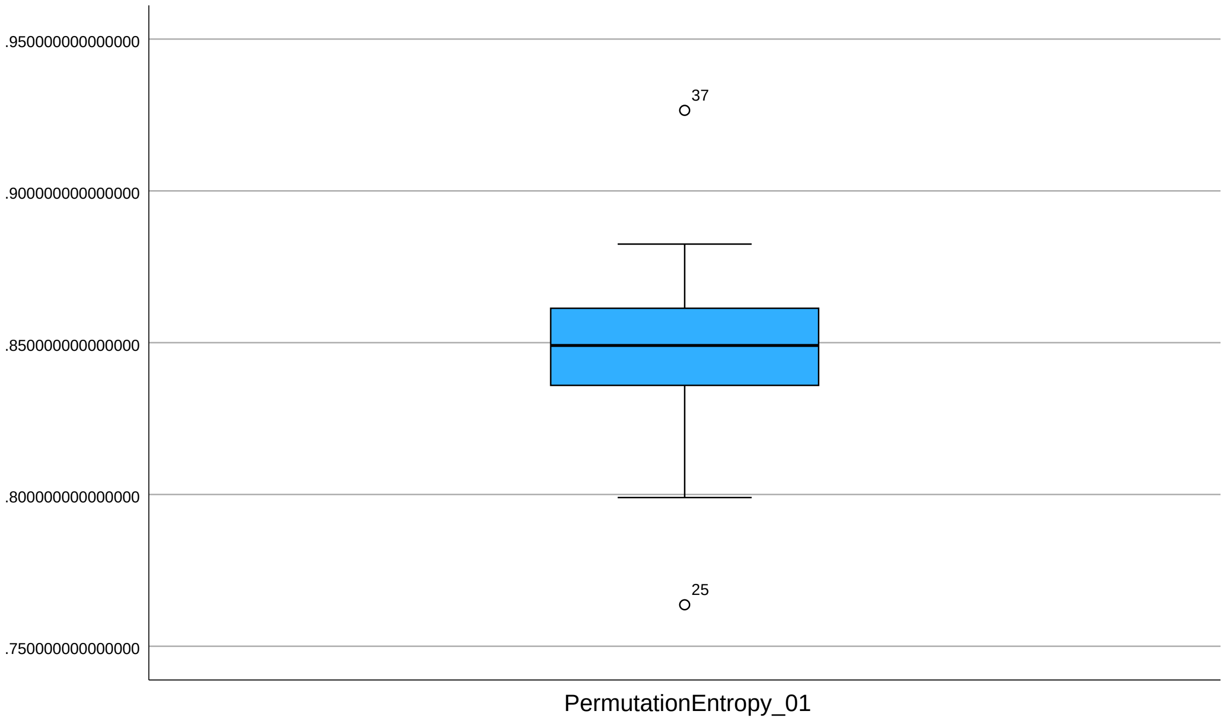

SampleEntropy

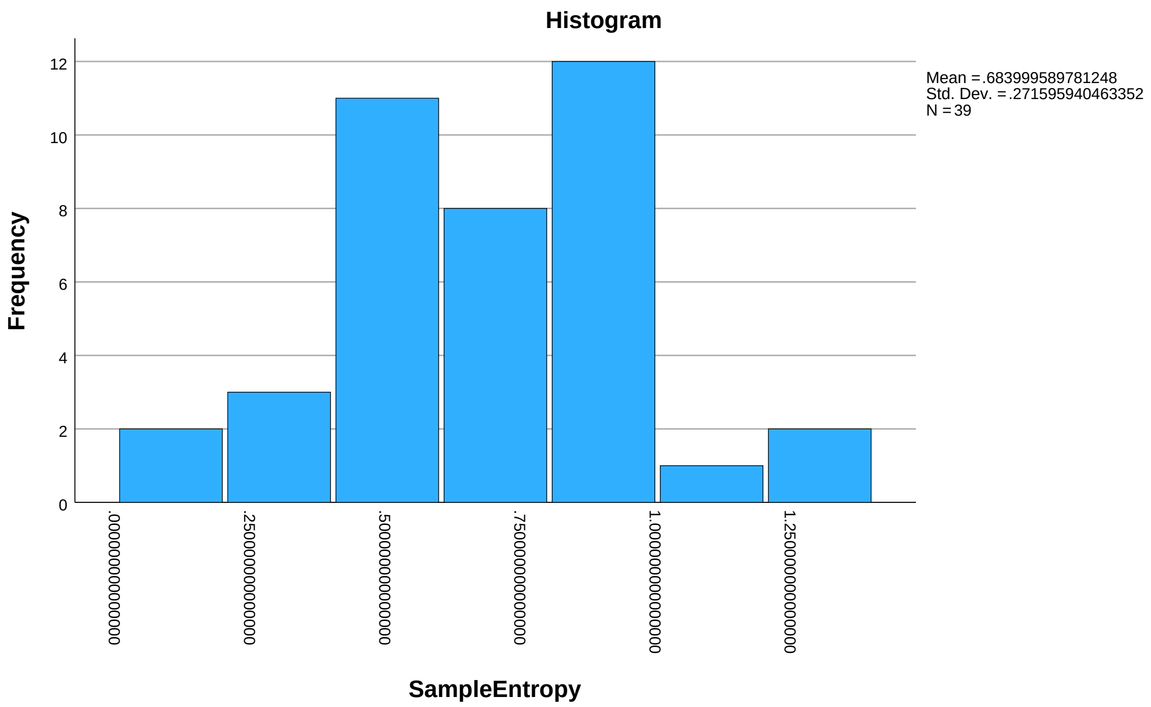


```
SampleEntropy Stem-and-Leaf Plot

 Frequency    Stem &  Leaf
```

```
 2.00     0 . 11
 3.00     0 . 233
11.00     0 . 44444445555
 8.00     0 . 66666777
12.00     0 . 888888999999
 1.00     1 . 0
 2.00     1 . 23

Stem width:  1.000000
Each leaf:       1 case(s)
```

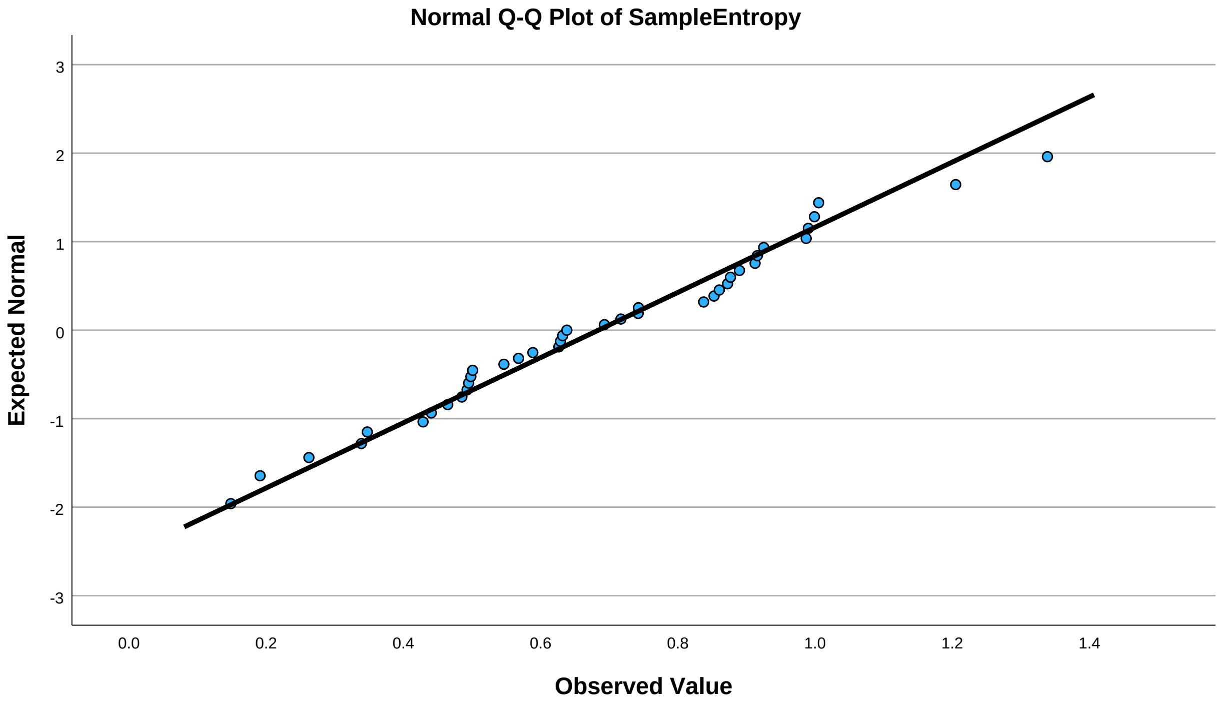

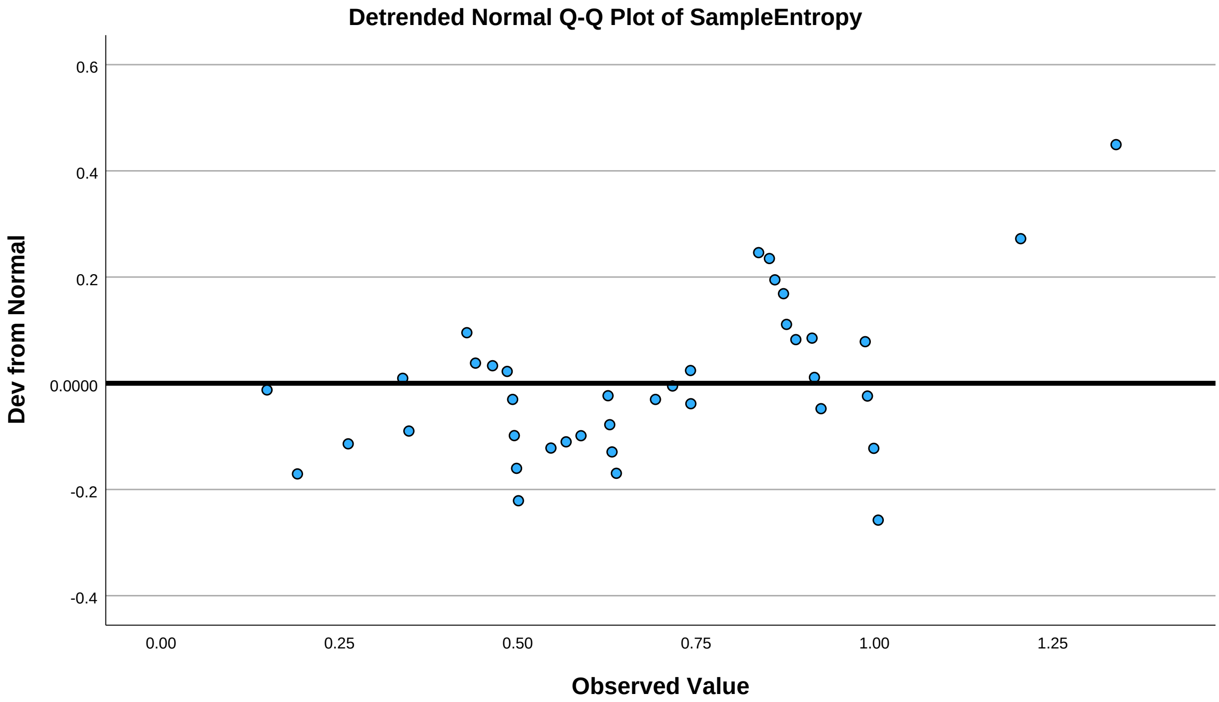

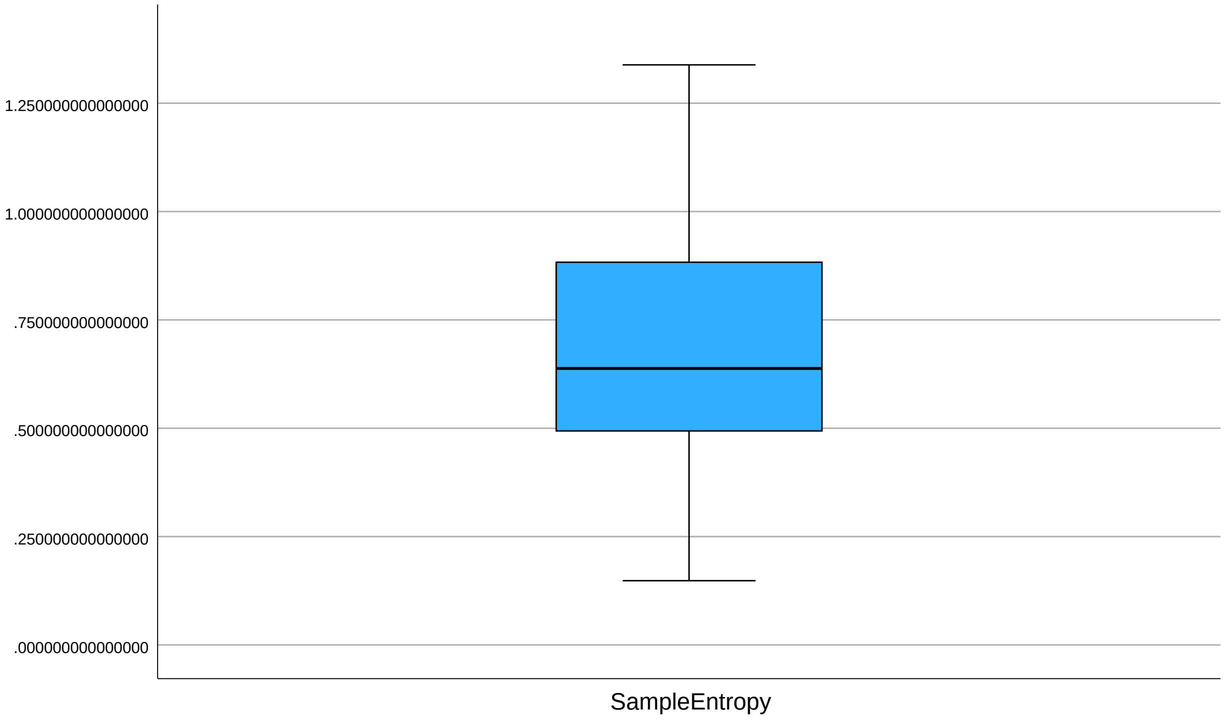

SpectralEntropy_bits

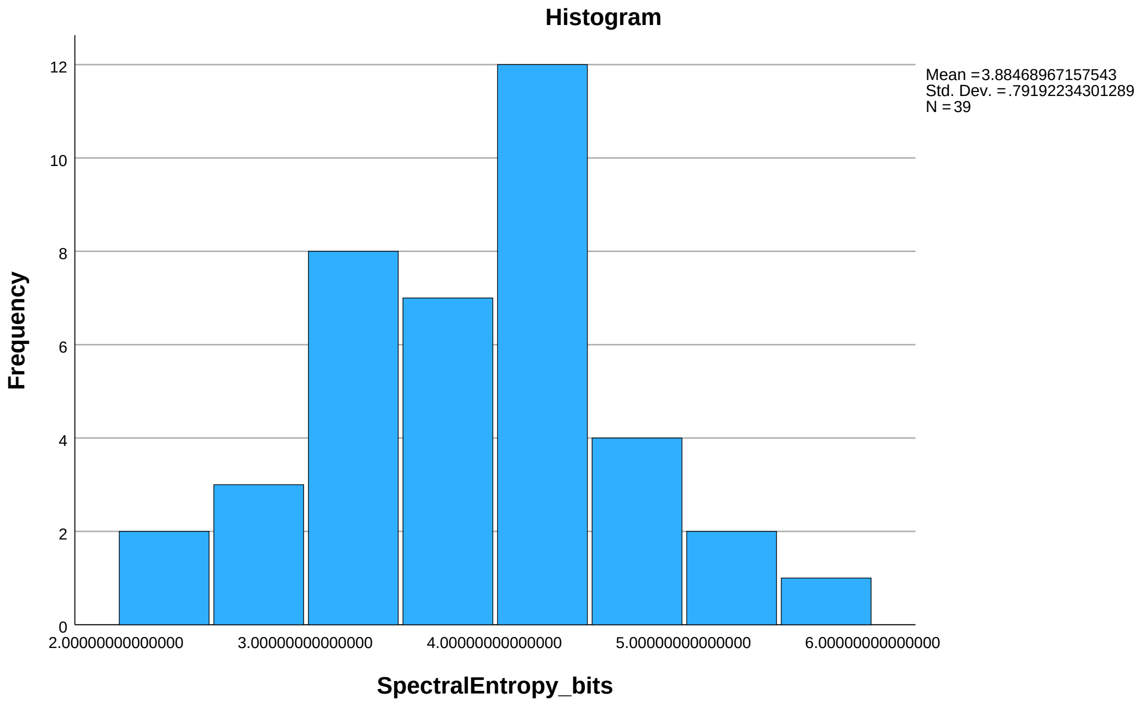


```
SpectralEntropy_bits Stem-and-Leaf Plot

 Frequency    Stem &  Leaf

     2.00        2 .  23
     3.00        2 .  578
     8.00        3 .  02233344
     7.00        3 .  5568889
    12.00        4 .  000012333444
     4.00        4 .  5679
     2.00        5 .  00
     1.00        5 .  8

 Stem width:   1.000000
 Each leaf:       1 case(s)
```

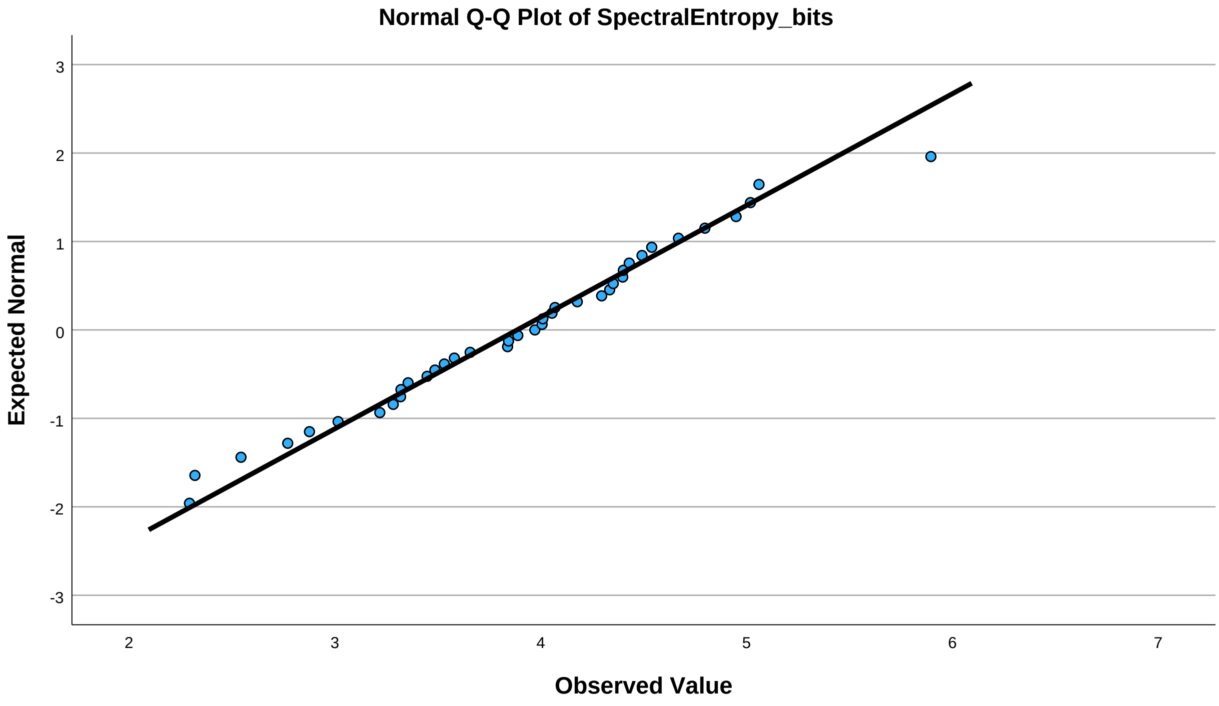

Normal Q-Q Plot of SpectralEntropy_bits
Expected Normal
Observed Value
3
2
1
0
-1
-2
-3
2
3
4
5
6
7

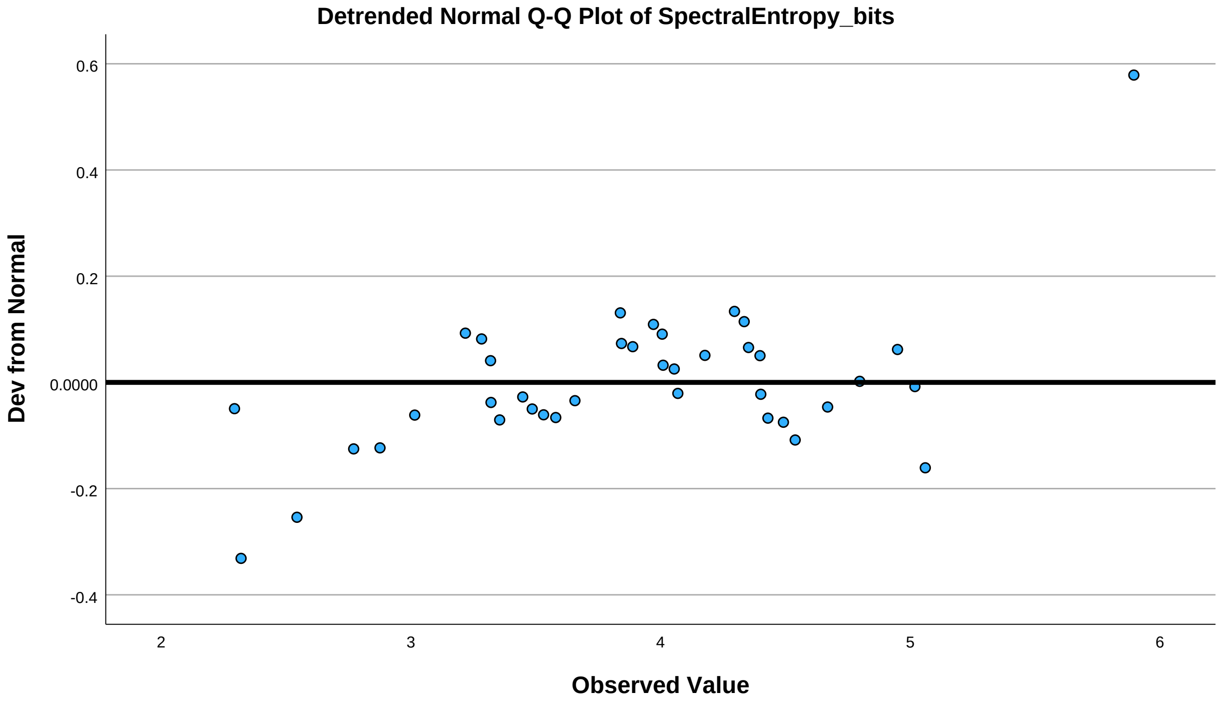

Detrended Normal Q-Q Plot of SpectralEntropy_bits
Dev from Normal
Observed Value
0.6
0.4
0.2
0.0000
-0.2
-0.4
2
3
4
5
6

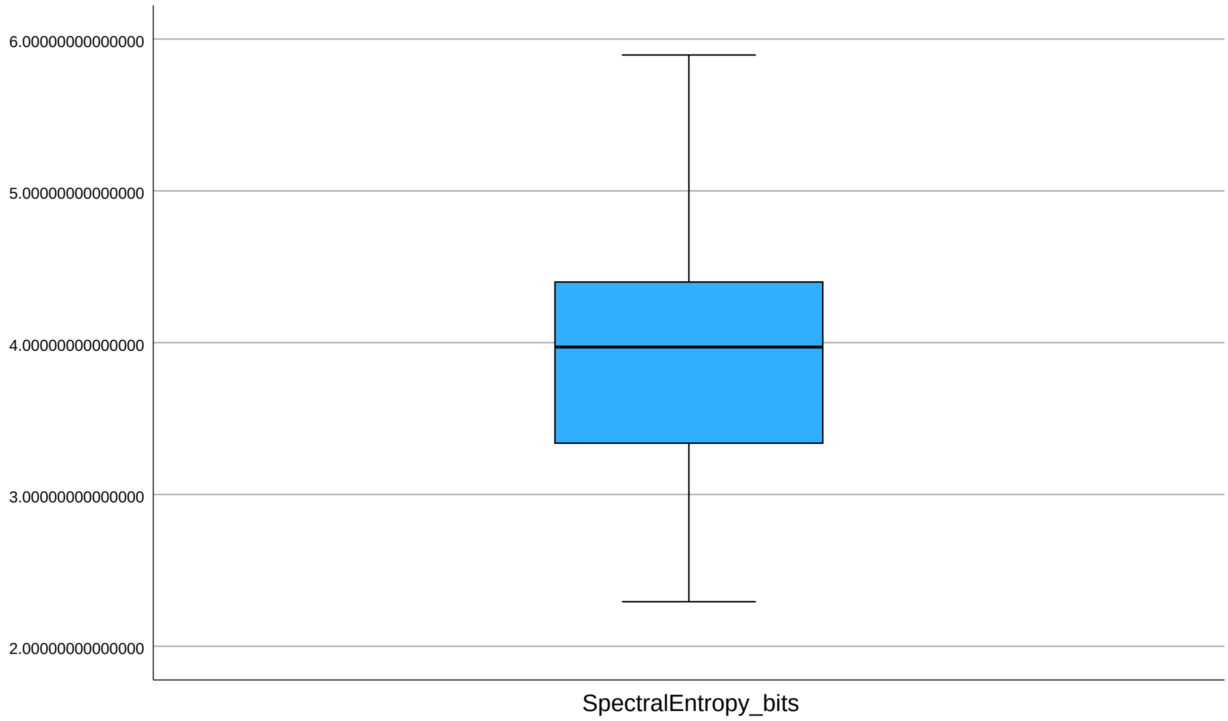

SpectralEntropy_01

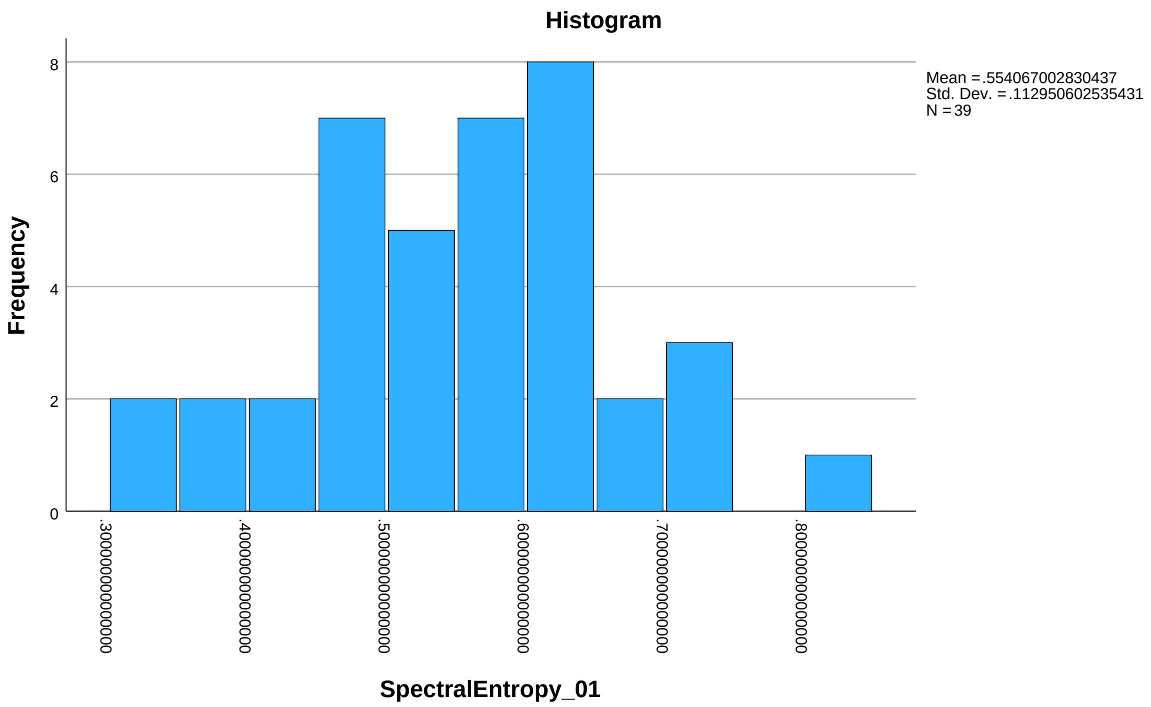

SpectralEntropy_01 Stem-and-Leaf Plot

Frequency   Stem & Leaf

```
2.00      3 .  23
2.00      3 .  69
2.00      4 .  13
7.00      4 .  5677799
5.00      5 .  01244
7.00      5 .  5677789
8.00      6 .  11222344
2.00      6 .  68
3.00      7 .  012
 .00      7 .
1.00      8 .  4

Stem width:   .1000000
Each leaf:       1 case(s)
```

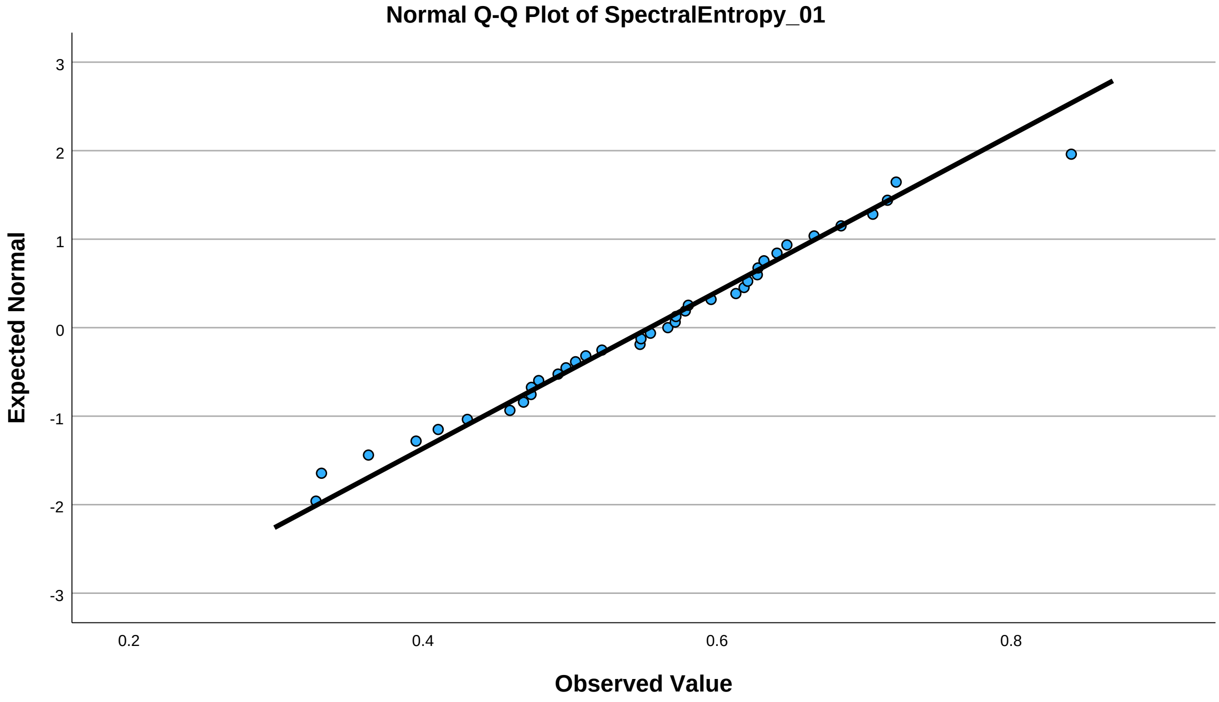

Detrended Normal Q-Q Plot of SpectralEntropy_01

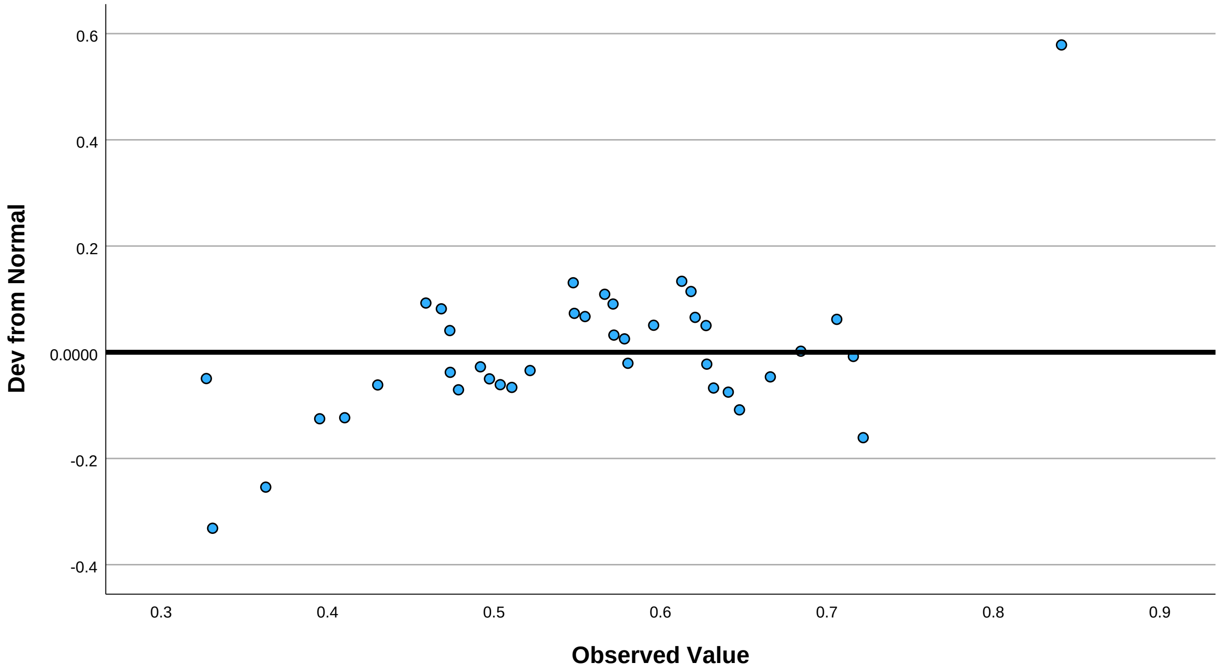

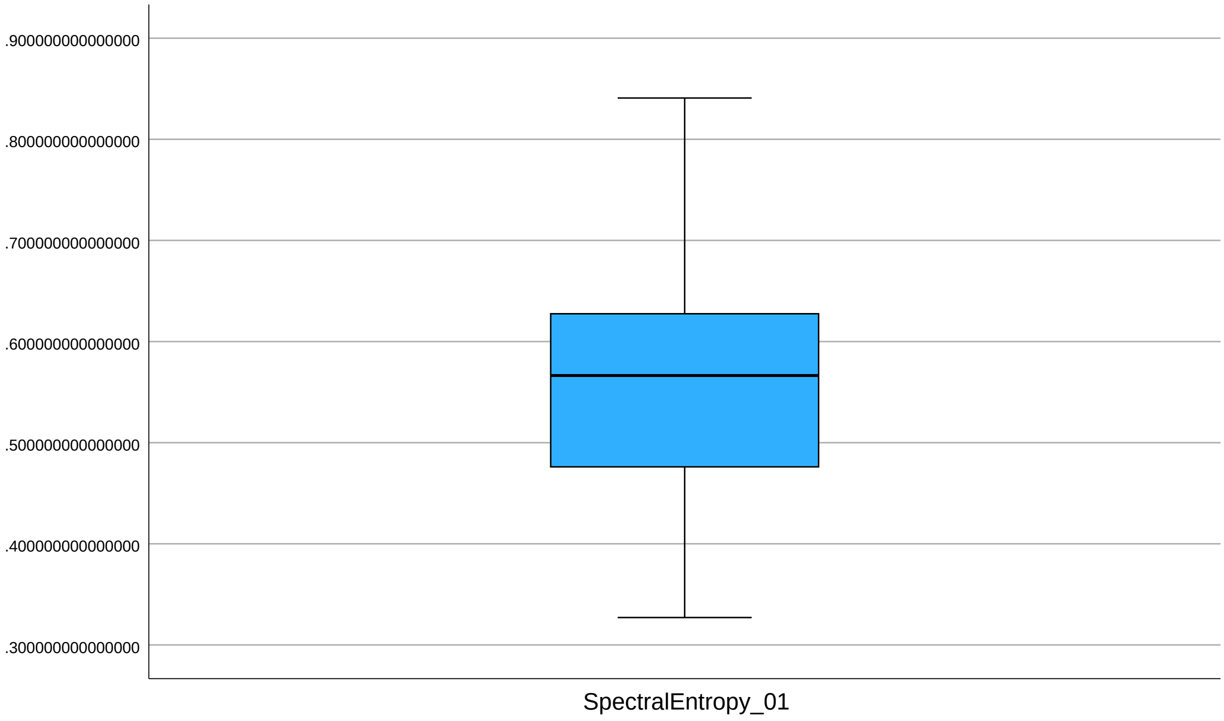

HiguchiFractalDimension

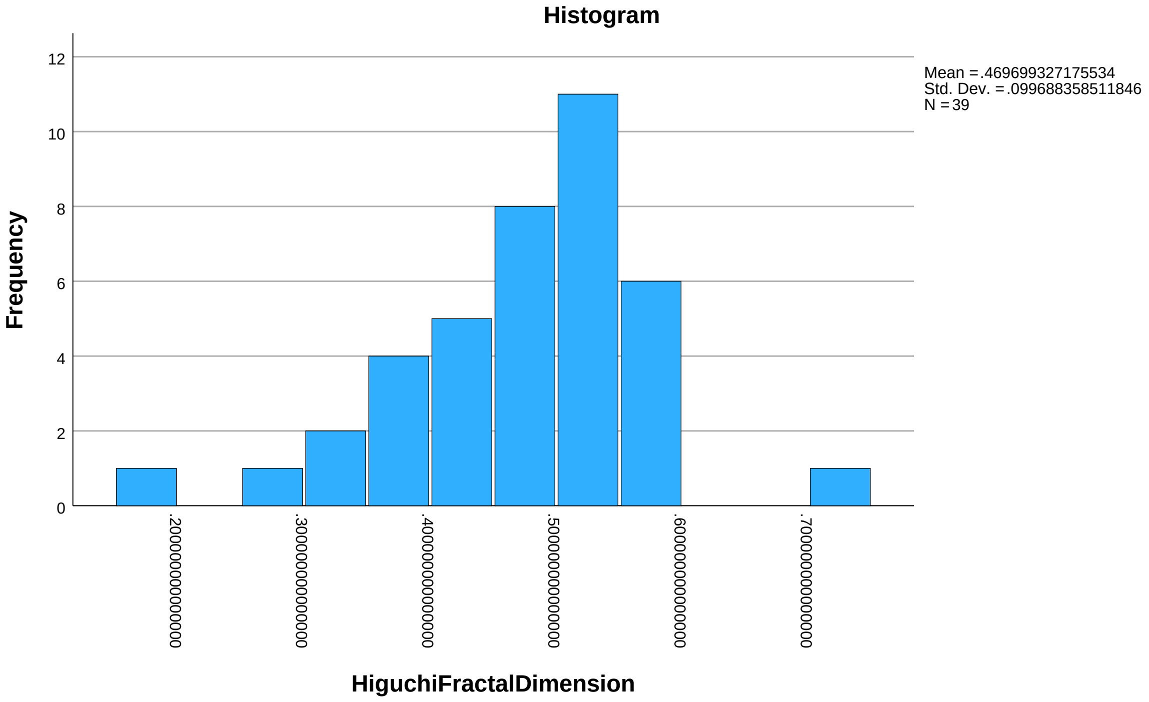


```
HiguchiFractalDimension Stem-and-Leaf Plot

 Frequency    Stem &  Leaf

     1.00 Extremes    (=<.17)
     1.00        2 .  9
     2.00        3 .  11
     4.00        3 .  5669
     5.00        4 .  00122
     8.00        4 .  56677999
    11.00        5 .  00011223344
     6.00        5 .  566777
     1.00 Extremes    (>=.72)

 Stem width:   .1000000
 Each leaf:       1 case(s)
```

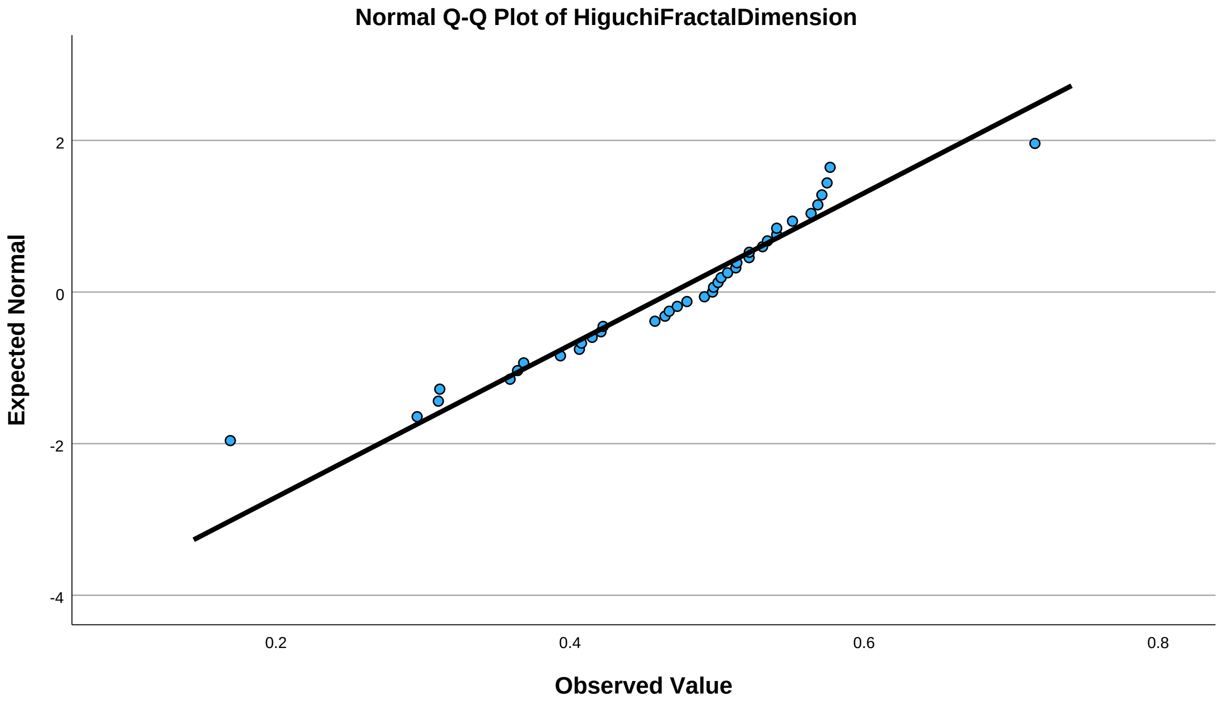

Normal Q-Q Plot of HiguchiFractalDimension
Expected Normal
2
0
-2
-4
0.2
0.4
0.6
0.8
Observed Value

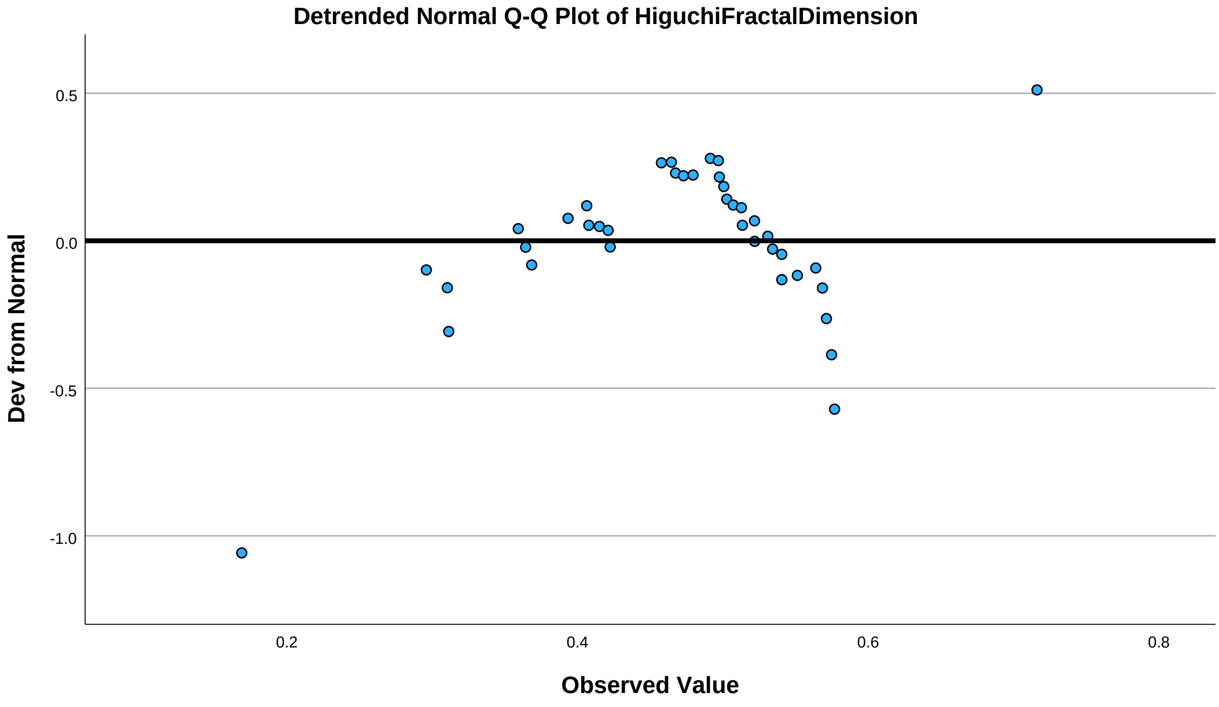

Detrended Normal Q-Q Plot of HiguchiFractalDimension
Dev from Normal
0.5
0.0
-0.5
-1.0
0.2
0.4
0.6
0.8
Observed Value

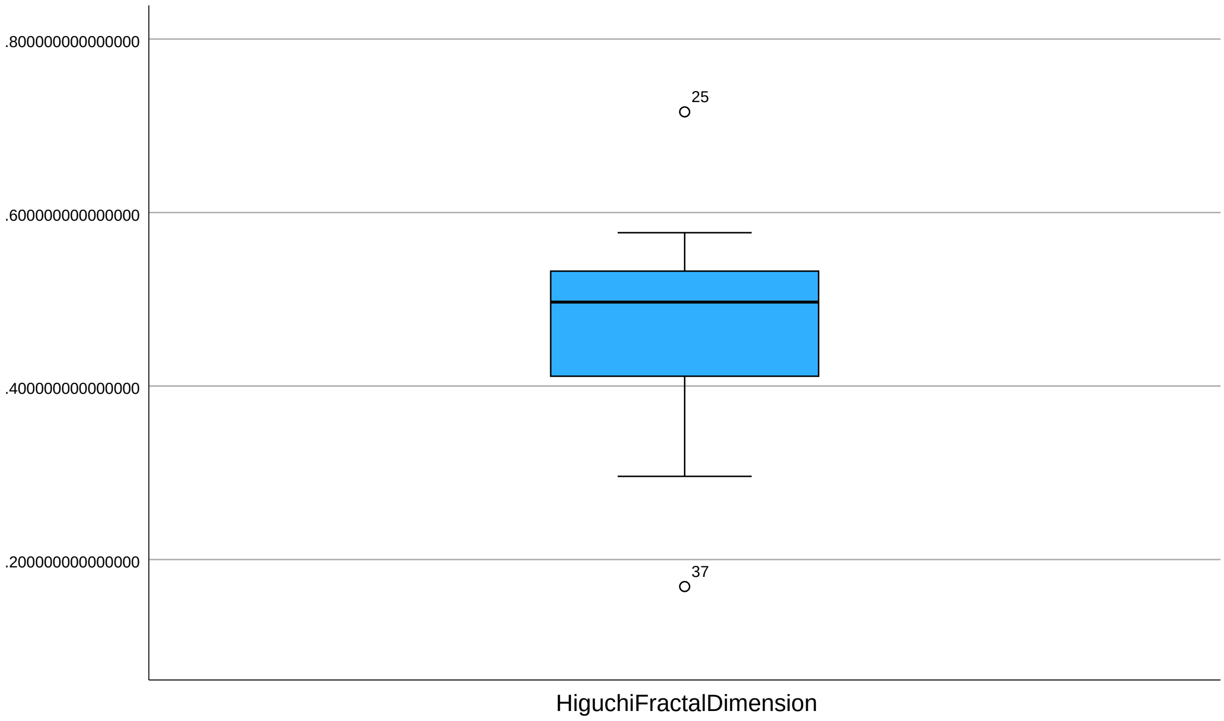

LempelZivComplexity

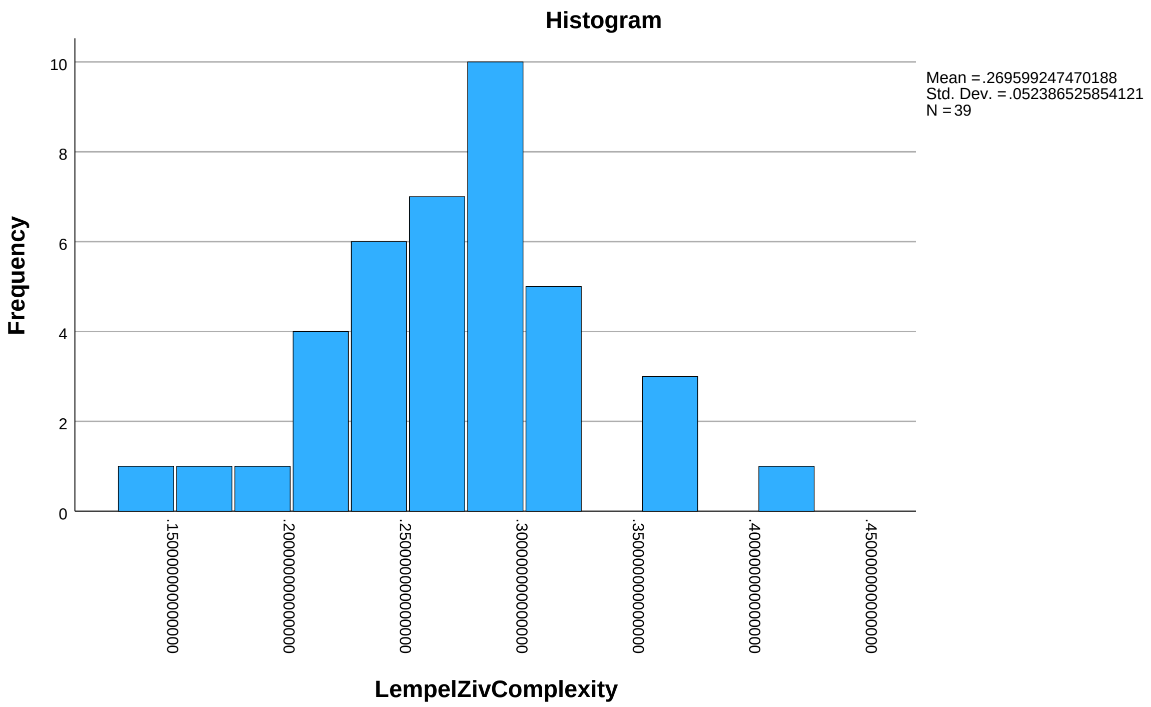


```
LempelZivComplexity Stem-and-Leaf Plot

 Frequency    Stem &  Leaf
```

```
 2.00 Extremes    (=<.16)
1.00     1 . 9
10.00       2 . 1112344444
17.00       2 .  55555677777888899
5.00      3 .  00011
3.00      3 .  556
 1.00 Extremes    (>=.42)

Stem width:   .1000000
Each leaf:       1 case(s)
```

Normal Q-Q Plot of LempelZivComplexity

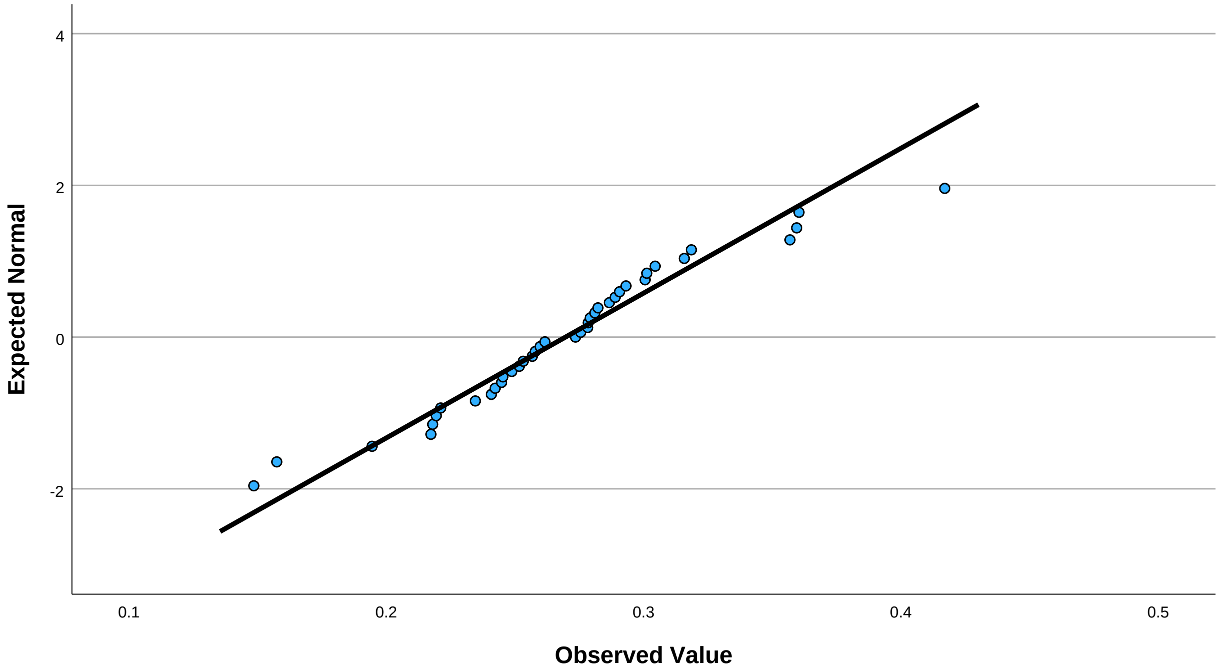

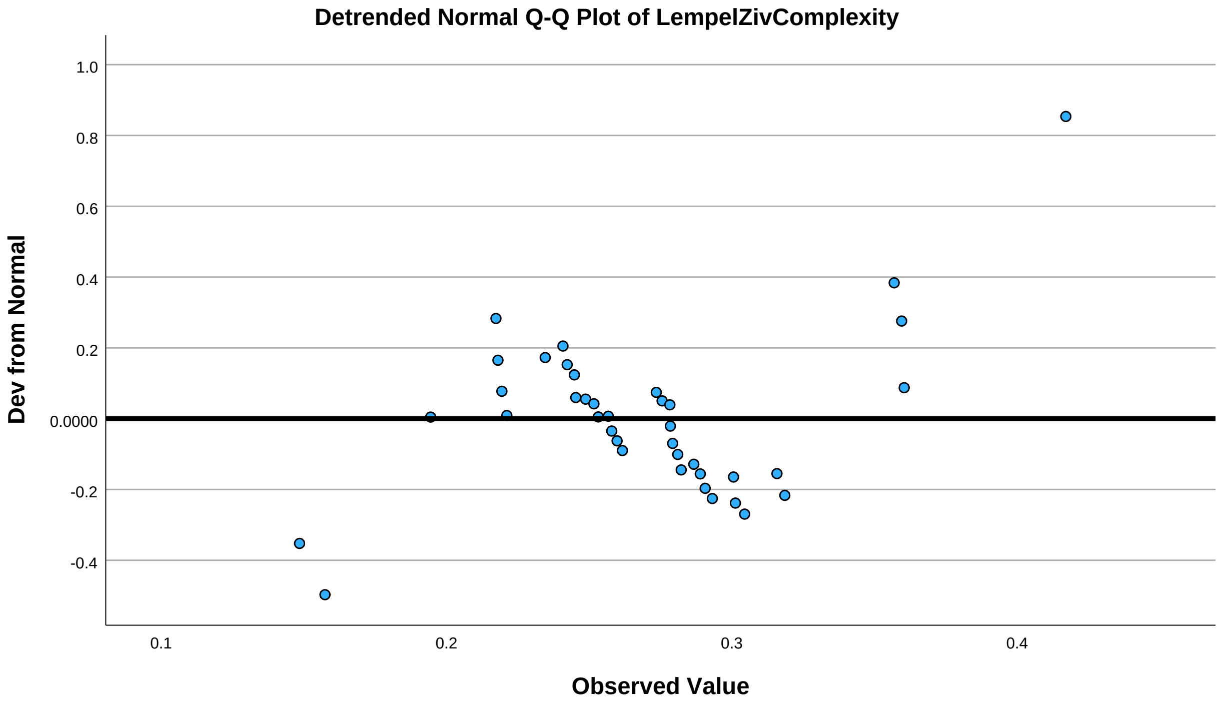

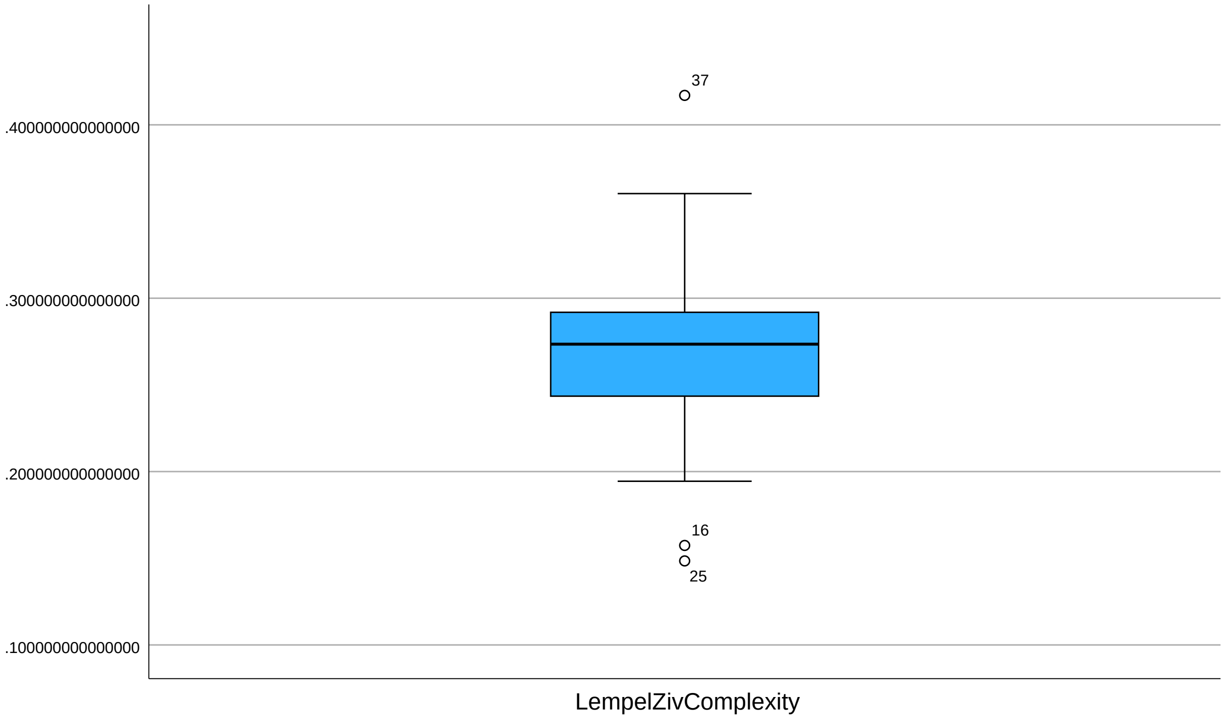

RenyiEntropy

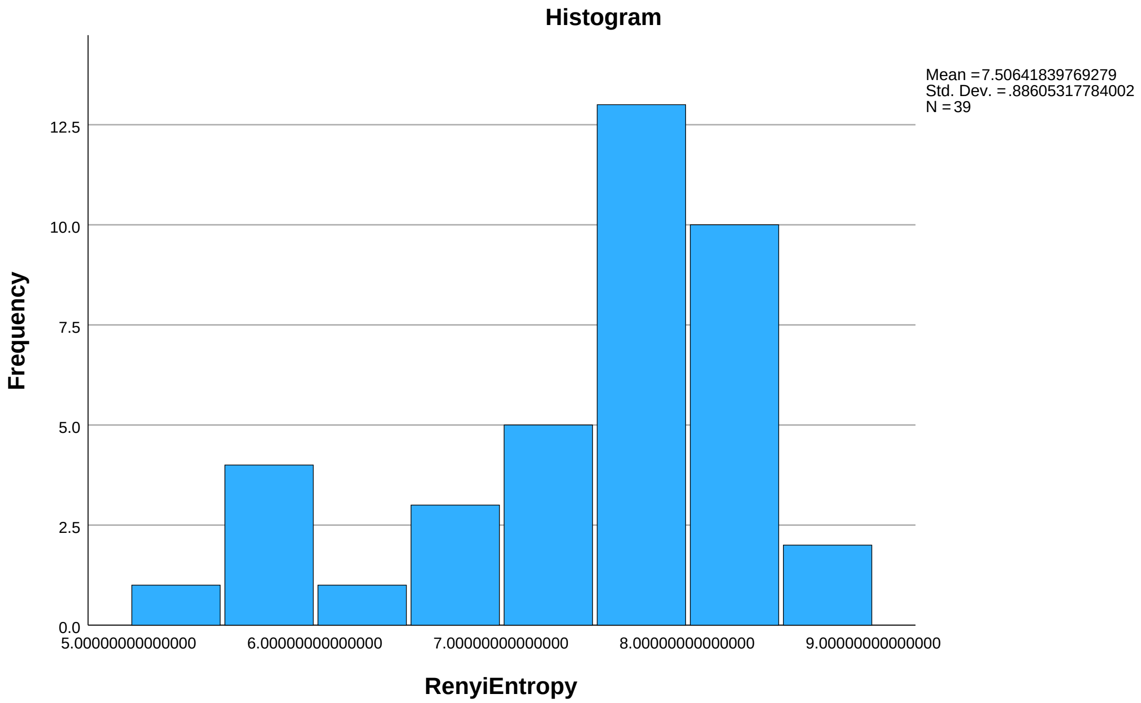


```
RenyiEntropy Stem-and-Leaf Plot

 Frequency    Stem &  Leaf

     4.00 Extremes    (=<6.0)
     1.00        5 .  9
     1.00        6 .  2
     3.00        6 .  558
     5.00        7 .  13334
    13.00        7 .  6777788889999
    10.00        8 .  0001122344
     2.00        8 .  57

 Stem width:   1.000000
 Each leaf:       1 case(s)
```

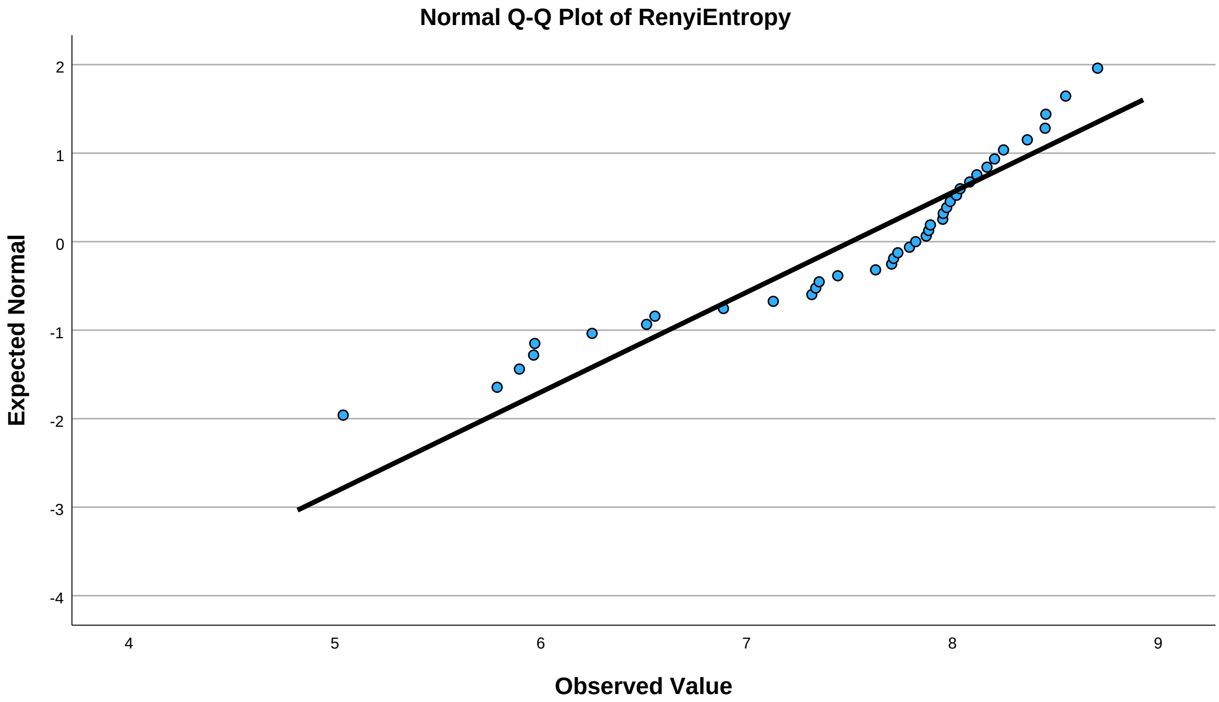

Normal Q-Q Plot of RenyiEntropy
Expected Normal
2
1
0
-1
-2
-3
-4
4
5
6
7
8
9
Observed Value

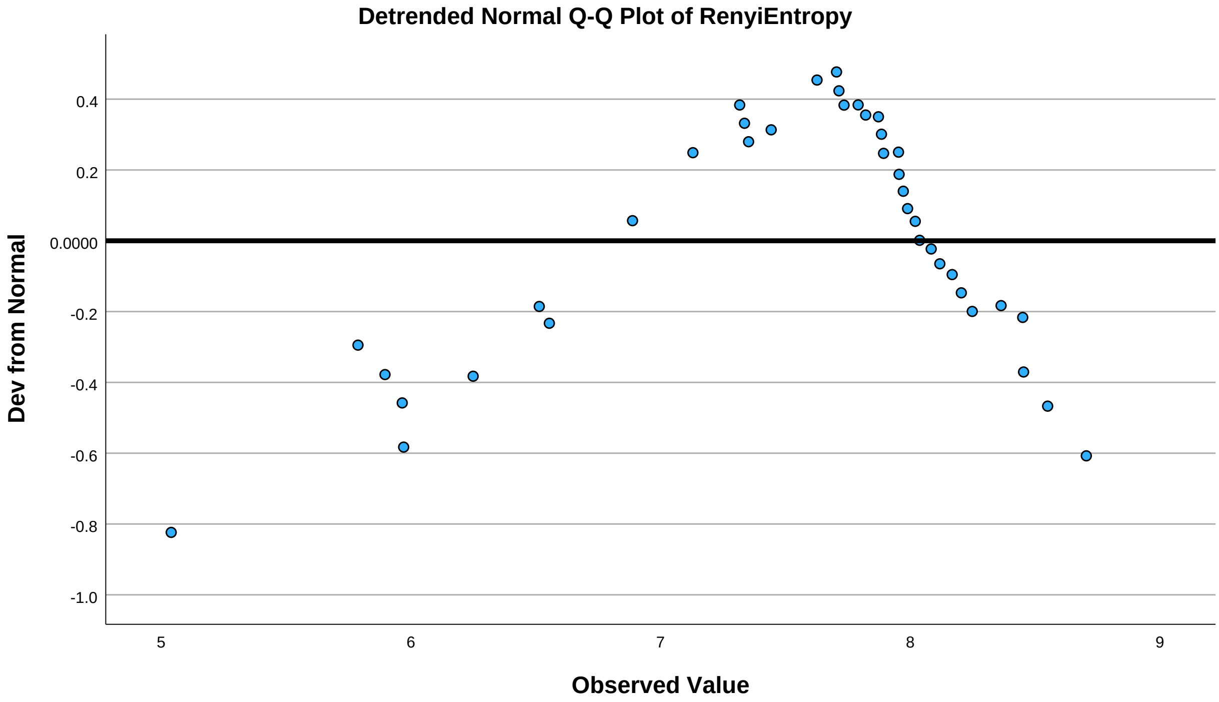

Detrended Normal Q-Q Plot of RenyiEntropy
Dev from Normal
0.4
0.2
0.0000
-0.2
-0.4
-0.6
-0.8
-1.0
5
6
7
8
9
Observed Value

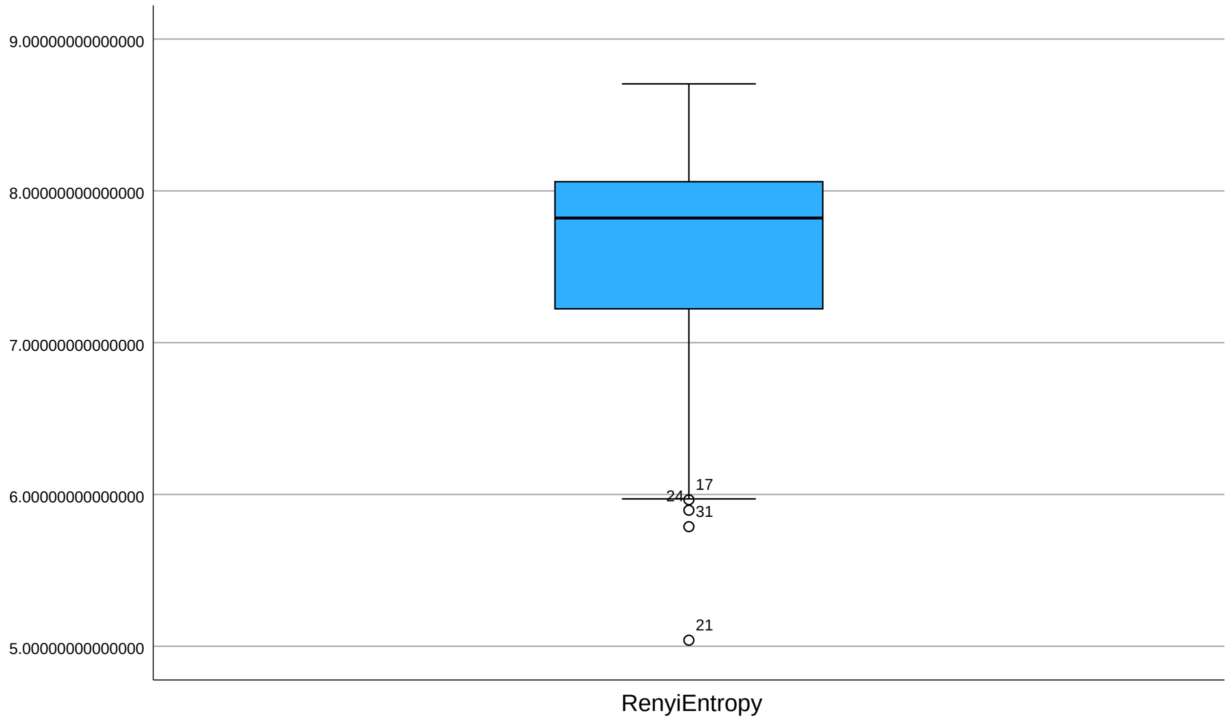

TsallisEntropy

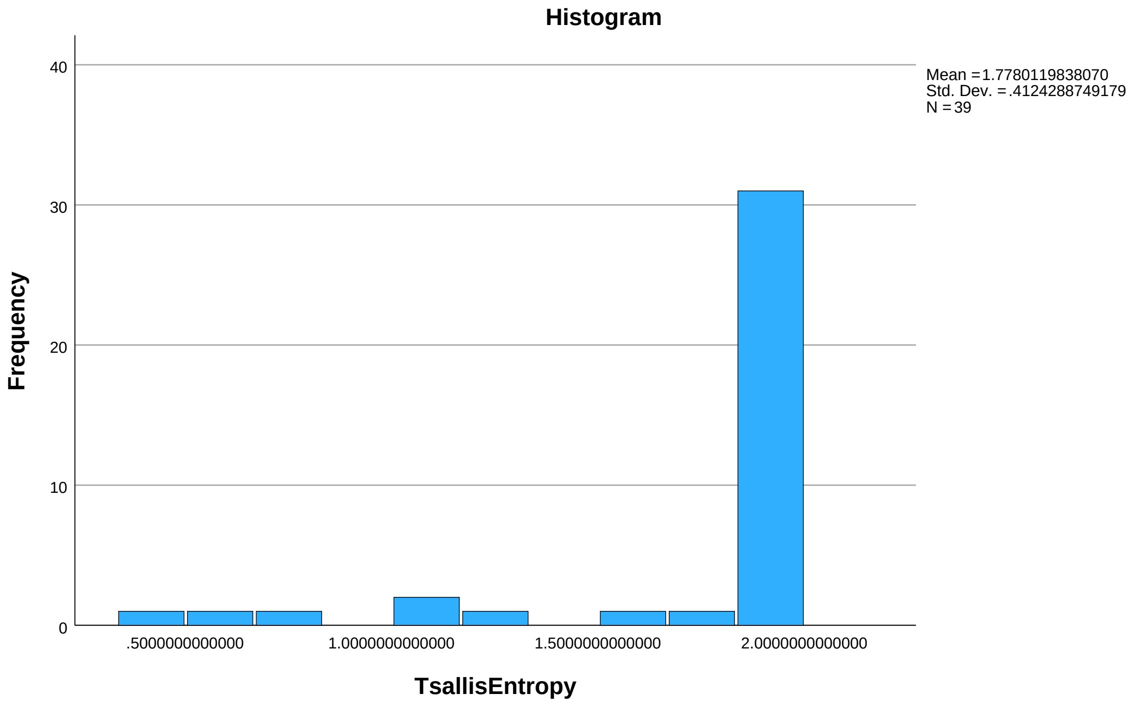


```
TsallisEntropy Stem-and-Leaf Plot

 Frequency    Stem &  Leaf
```

```
 7.00 Extremes    (=<1.56)
 1.00     18 . 0
 1.00     18 . 3
  .00     18 .
 4.00     18 . 6777
  .00     18 .
  .00     19 .
 2.00     19 . 33
 4.00     19 . 4555
 5.00     19 . 77777
15.00     19 . 888888888888888

Stem width:   .1000000
Each leaf:       1 case(s)
```

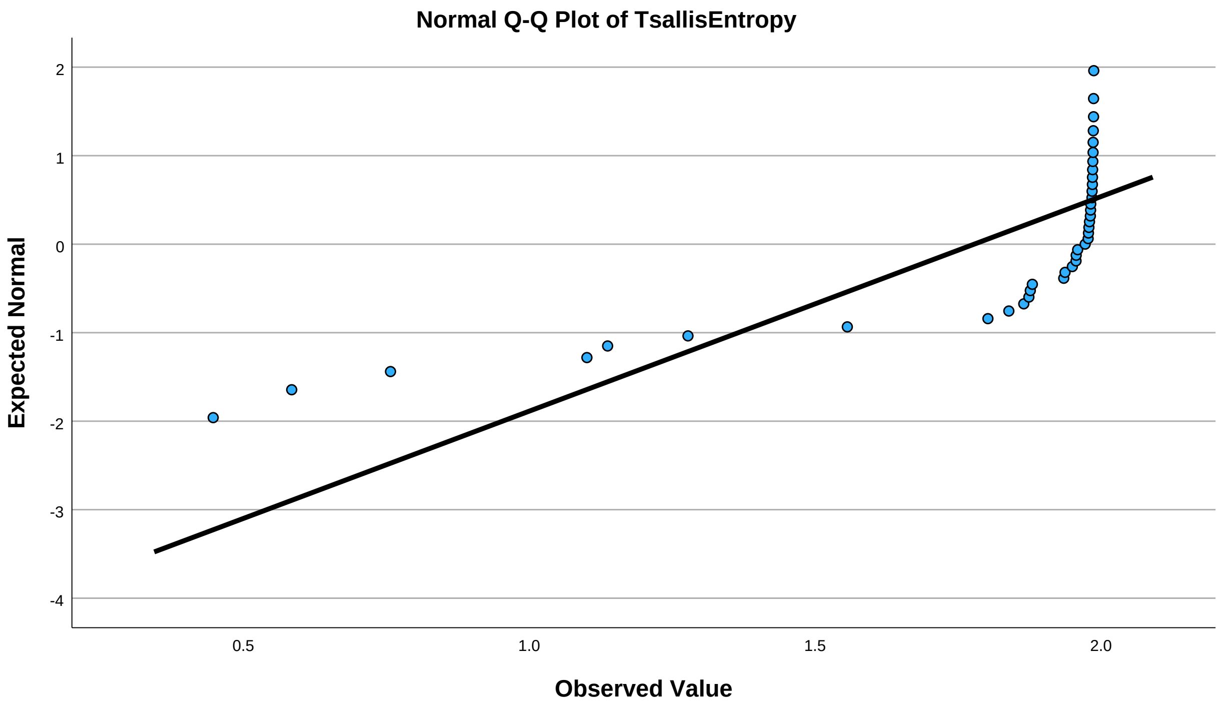

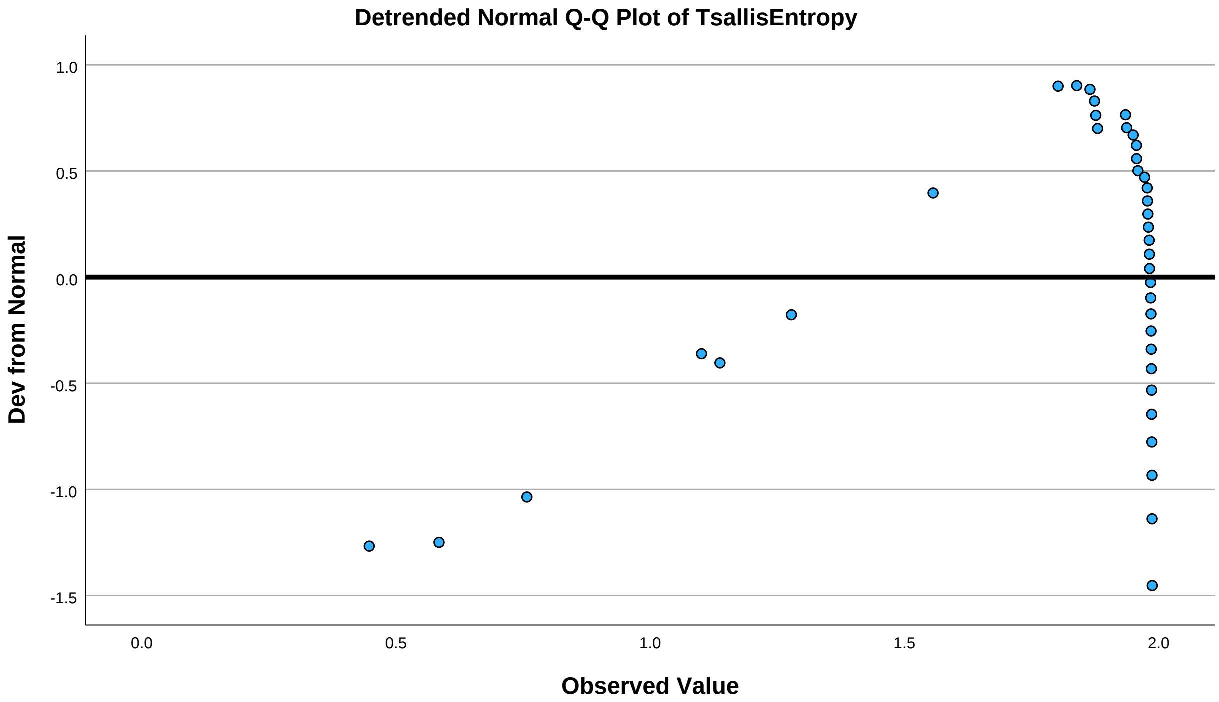

Detrended Normal Q-Q Plot of TsallisEntropy
Dev from Normal
1.0
0.5
0.0
-0.5
-1.0
-1.5
0.0
0.5
1.0
1.5
2.0
Observed Value

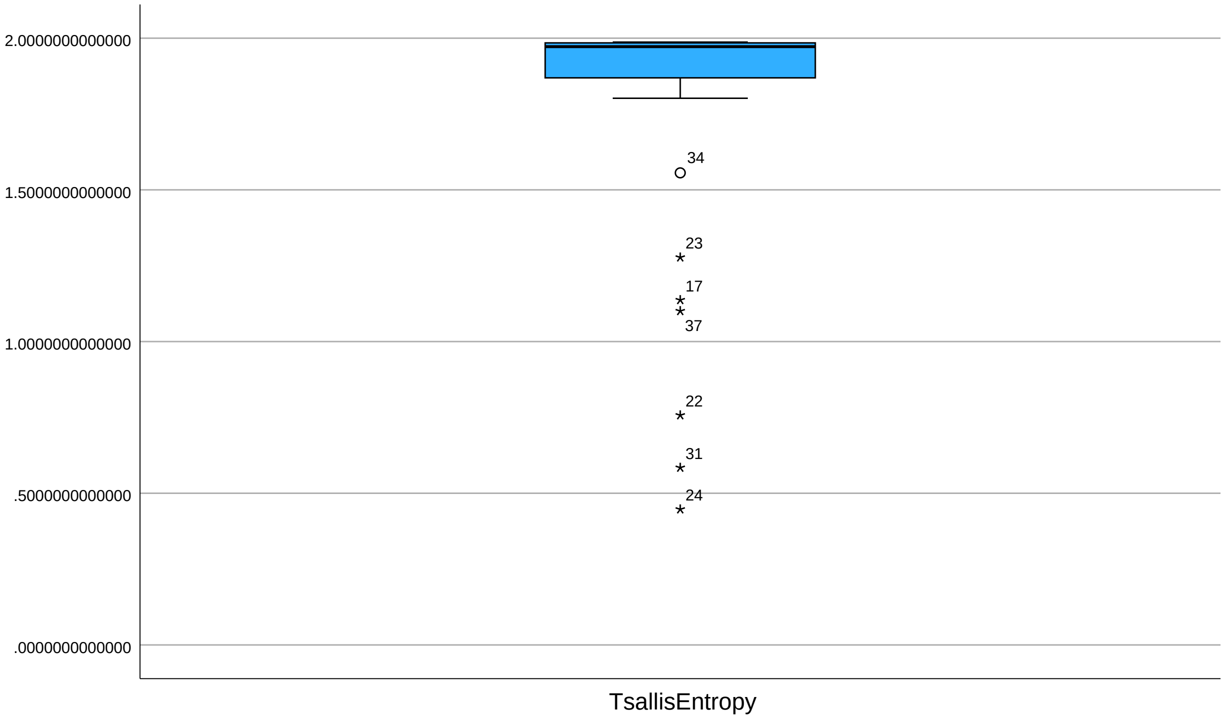

2.0000000000000
1.5000000000000
1.0000000000000
.5000000000000
.0000000000000
34
23
17
37
22
31
24
TsallisEntropy

PS_4_5

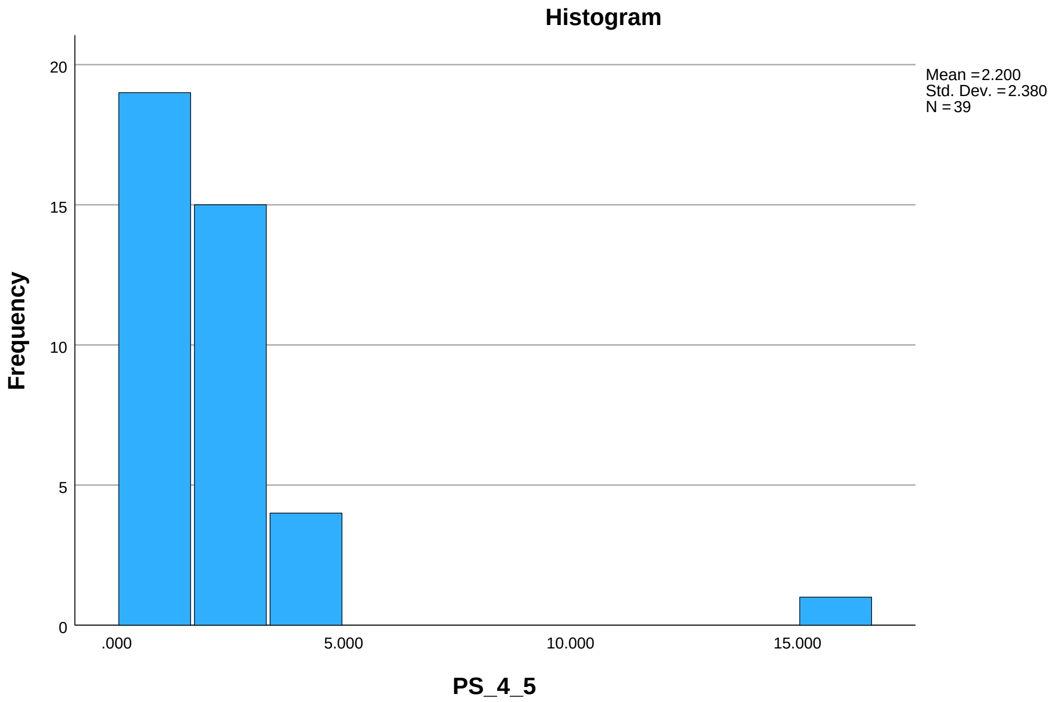


```
PS_4_5 Stem-and-Leaf Plot

 Frequency    Stem &  Leaf

     1.00        0 .  3
     7.00        0 .  6888889
    10.00        1 .  0122333333
     7.00        1 .  5667899
     5.00        2 .  33444
     3.00        2 .  557
     2.00        3 .  24
     1.00        3 .  6
     1.00        4 .  0
     2.00 Extremes    (>=4.7)

 Stem width:      1.000
 Each leaf:       1 case(s)
```

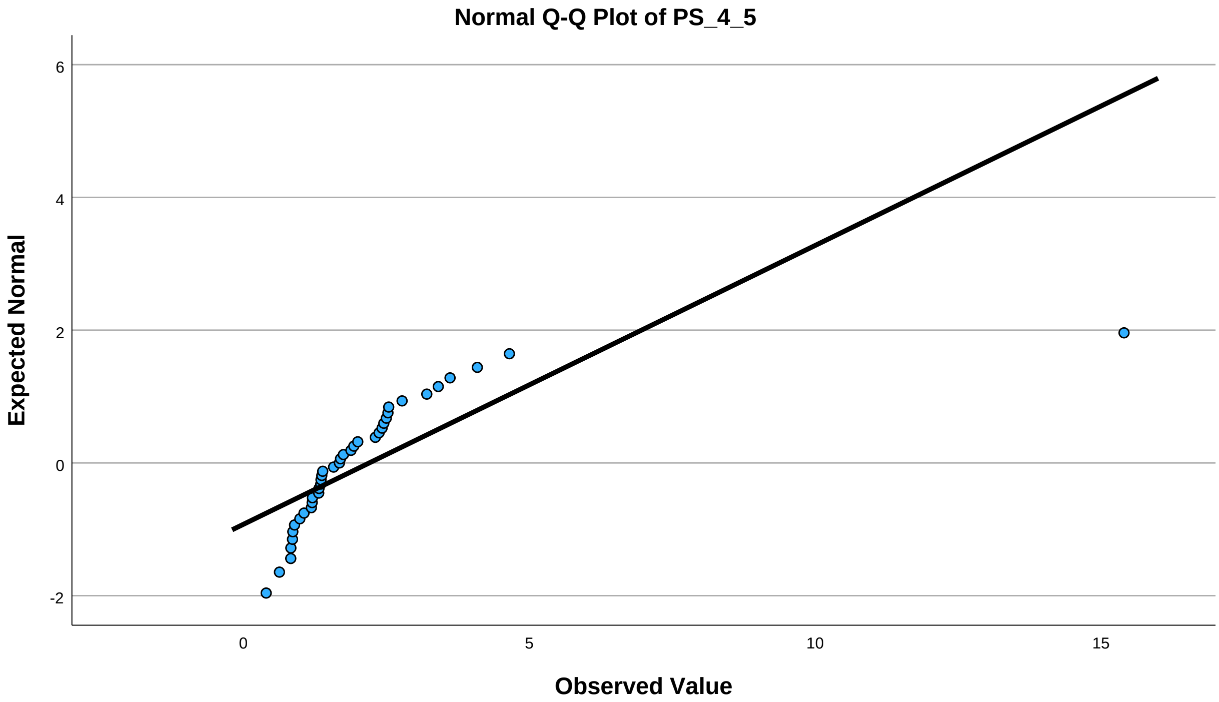
Normal Q-Q Plot of PS_4_5
Expected Normal
Observed Value
6
4
2
0
-2
0
5
10
15

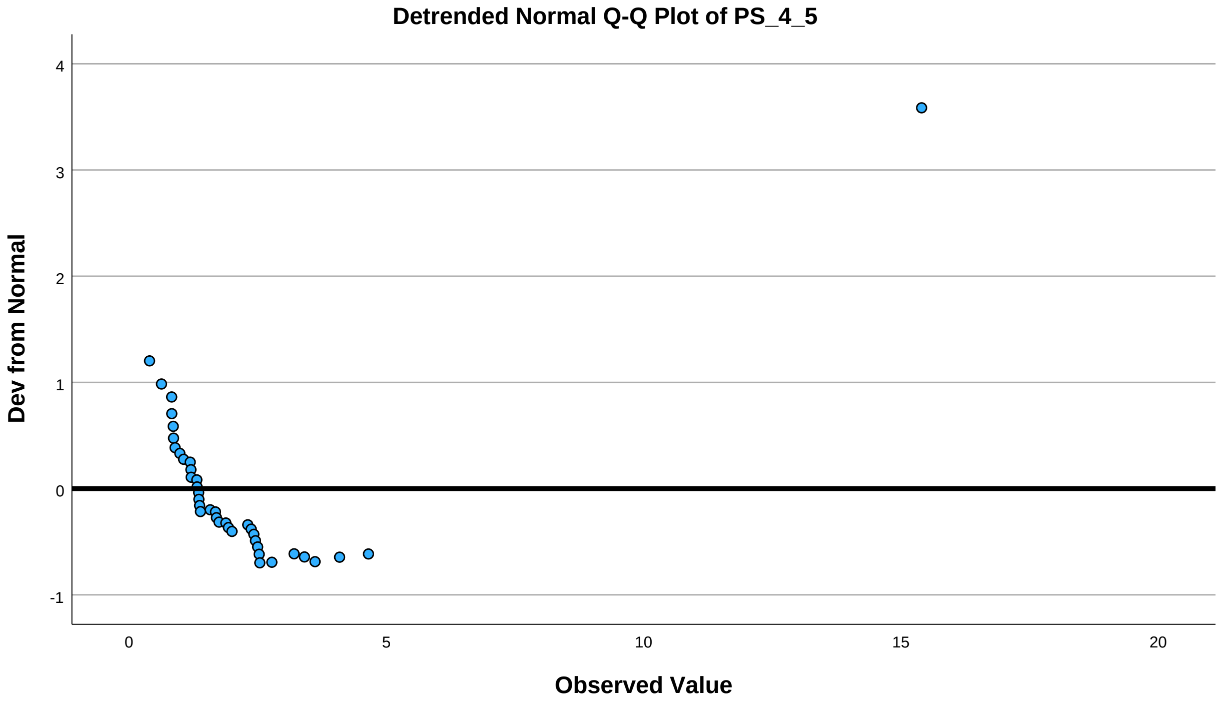
Detrended Normal Q-Q Plot of PS_4_5
Dev from Normal
Observed Value
4
3
2
1
0
-1
0
5
10
15
20

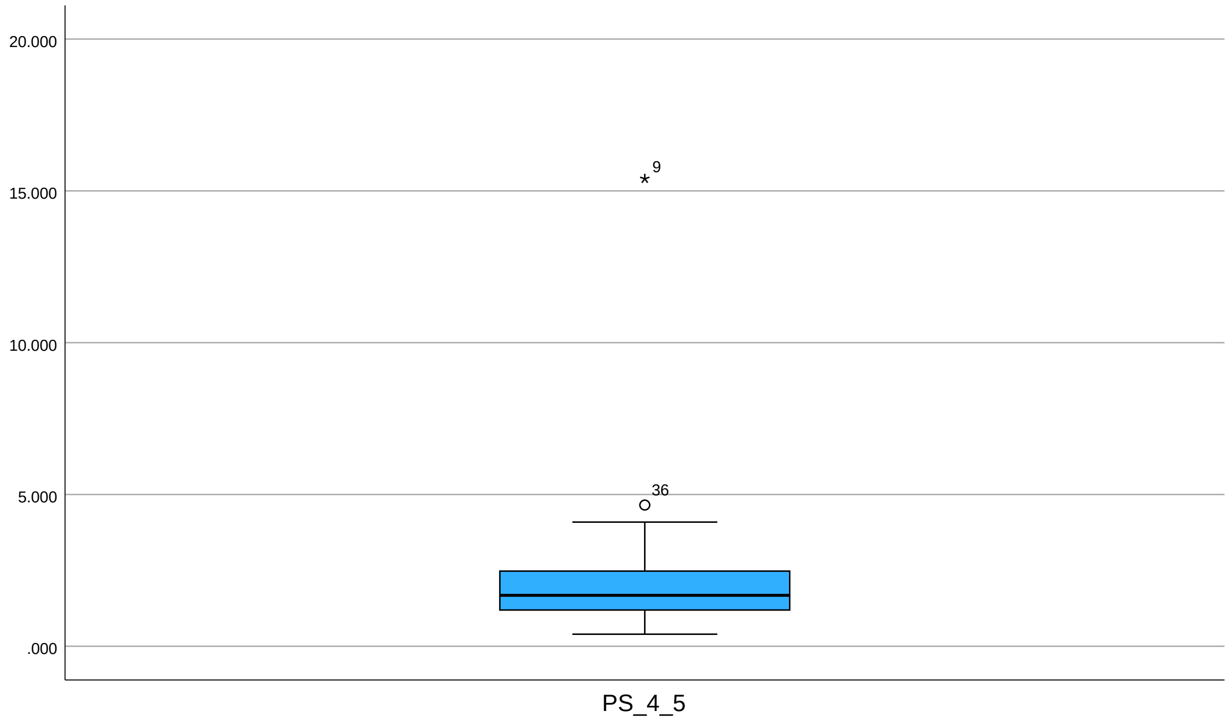

PS_5_6

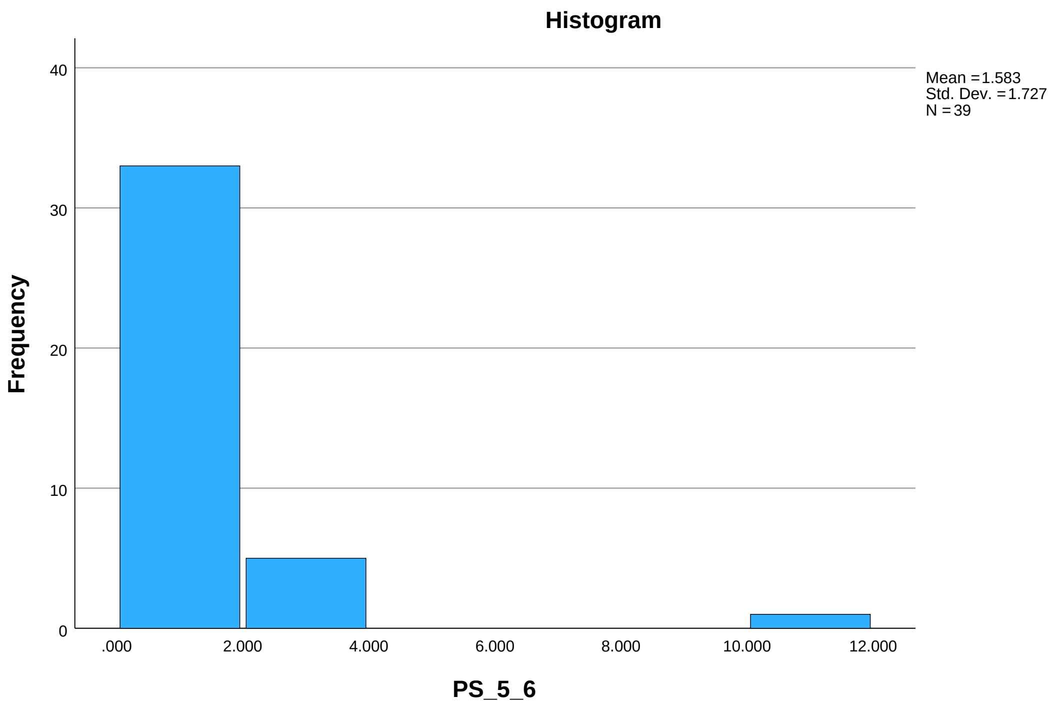

PS_5_6 Stem-and-Leaf Plot

Frequency   Stem & Leaf

```
    2.00        0 .  23
   13.00        0 .  5556667789999
    7.00        1 .  0002244
   11.00        1 .  66666778899
    3.00        2 .  134
    2.00        2 .  56
    1.00 Extremes    (>=11.3)

Stem width:      1.000
Each leaf:        1 case(s)
```

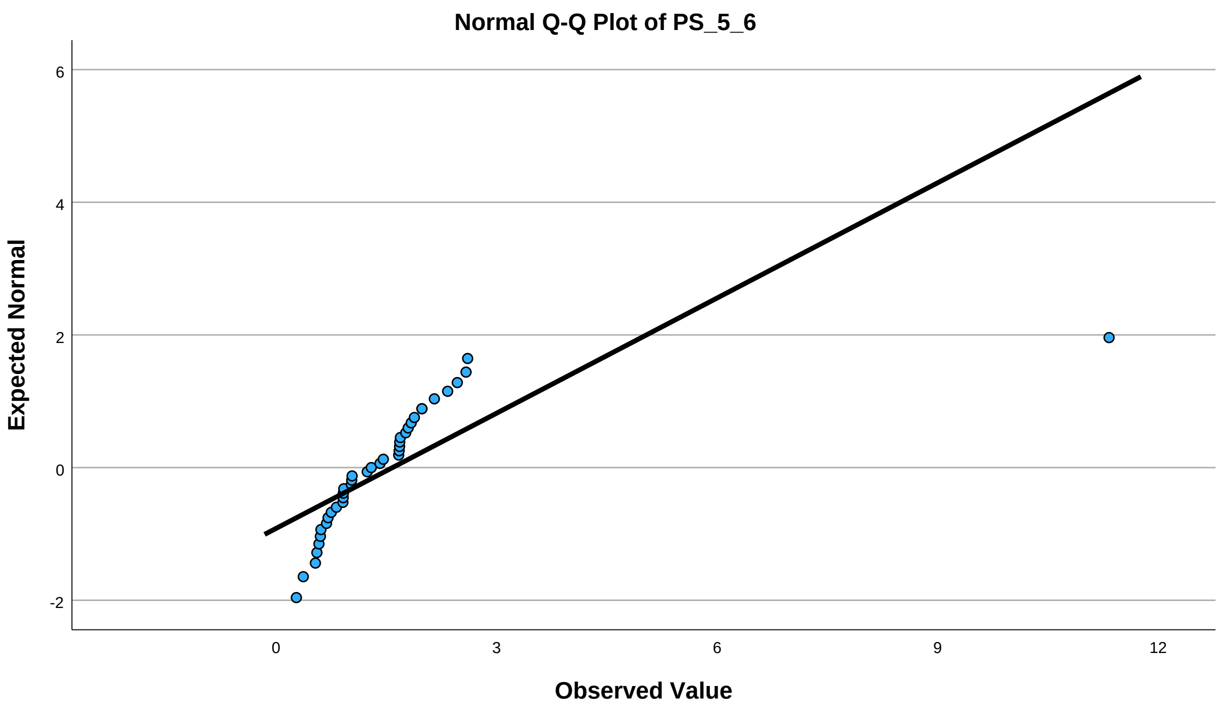

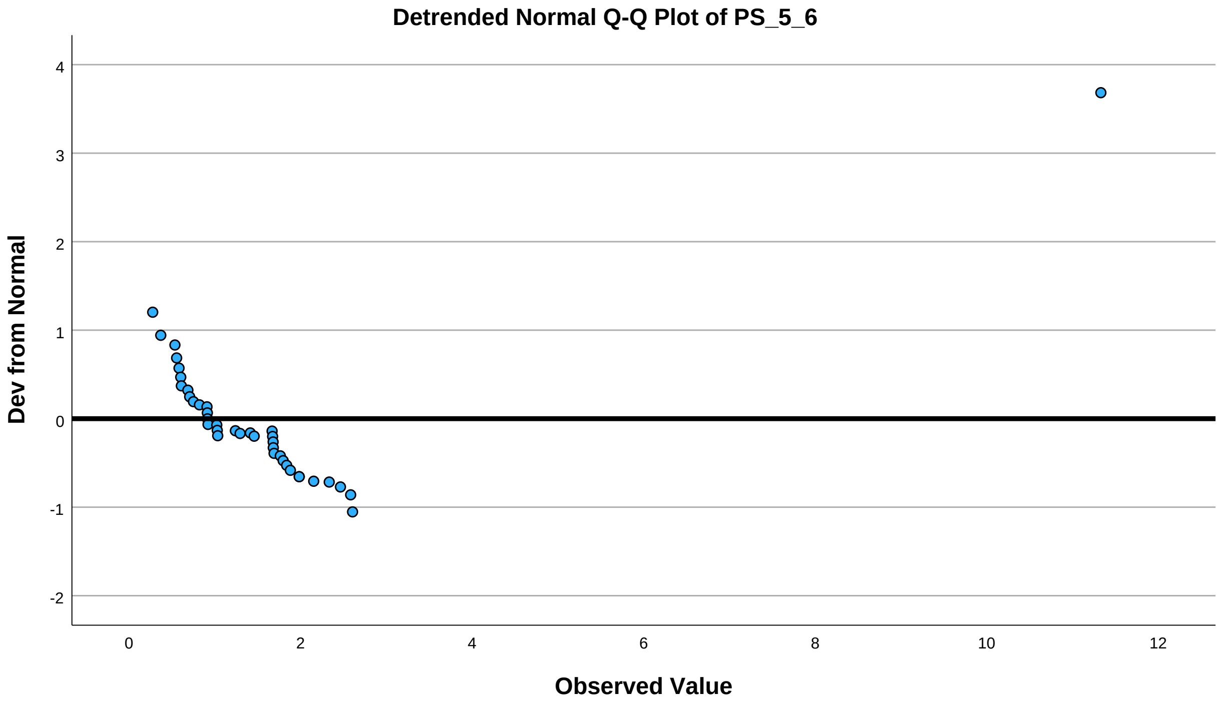
Detrended Normal Q-Q Plot of PS_5_6
Dev from Normal
4
3
2
1
0
-1
-2
0
2
4
6
8
10
12
Observed Value

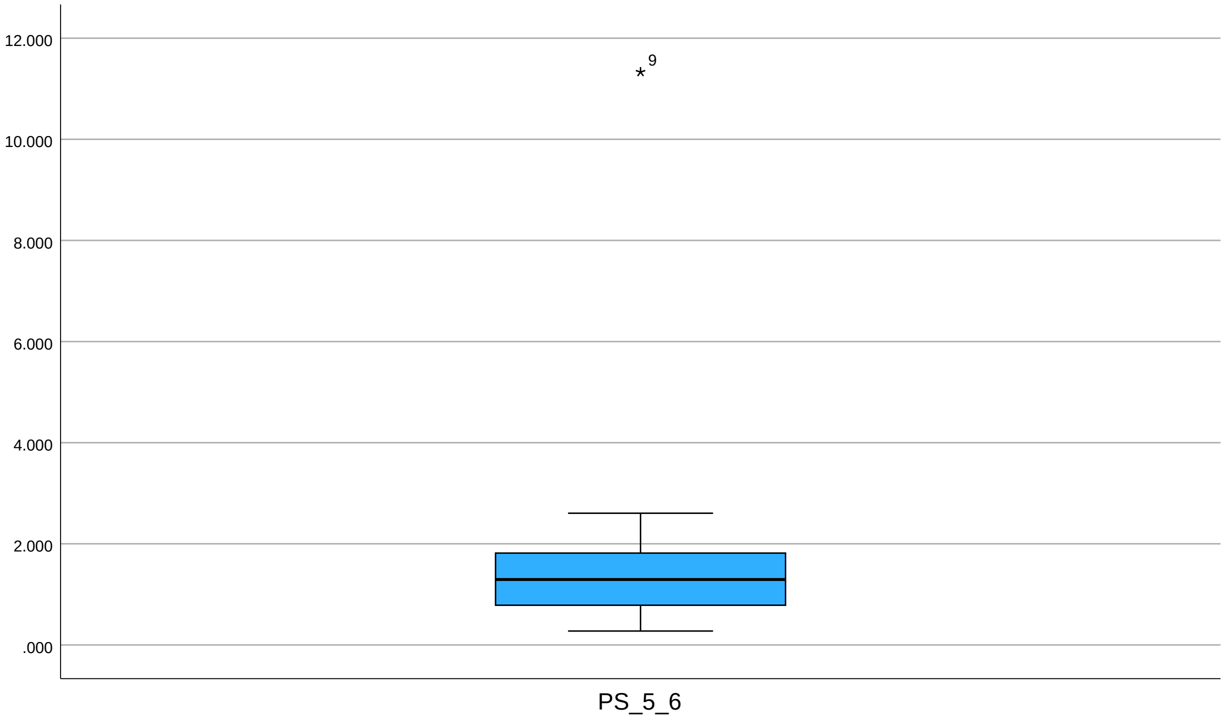
12.000
10.000
8.000
6.000
4.000
2.000
.000
9
PS_5_6

PS_6_7

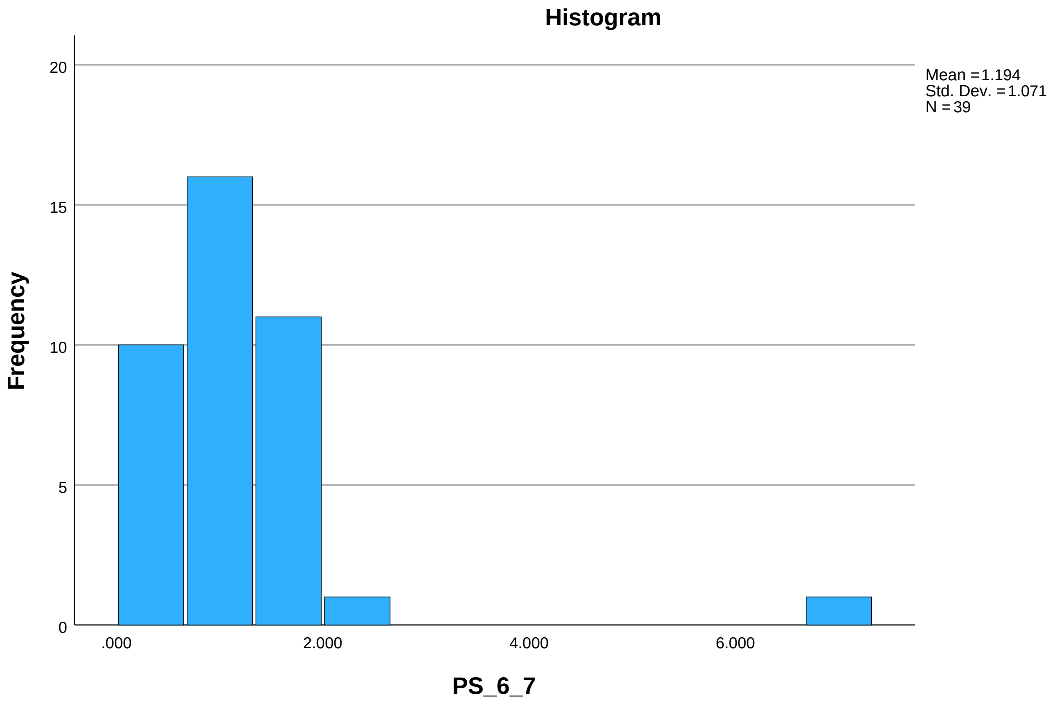


```
PS_6_7 Stem-and-Leaf Plot

 Frequency    Stem &  Leaf

     5.00        0 .  22444
    12.00        0 .  555566777889
    15.00        1 .  000011123334444
     5.00        1 .  56678
     1.00        2 .  3
     1.00 Extremes    (>=7.0)

 Stem width:      1.000
 Each leaf:       1 case(s)
```

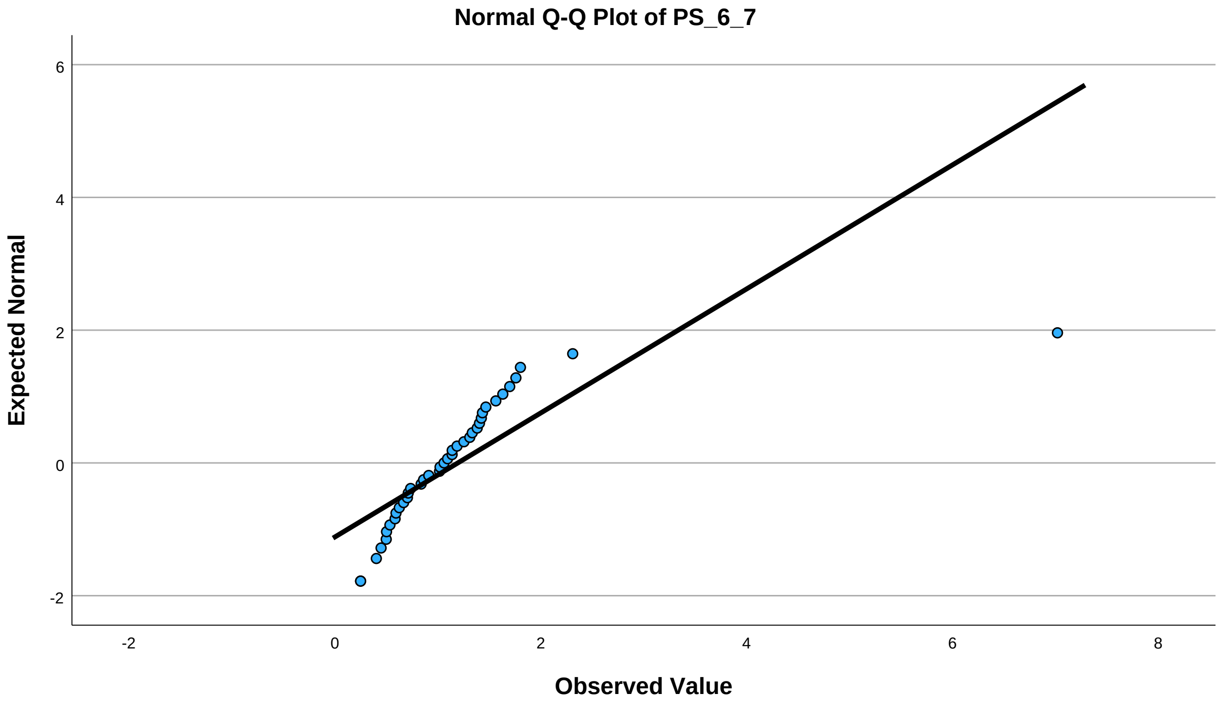

Normal Q-Q Plot of PS_6_7
Expected Normal
Observed Value
6
4
2
0
-2
-2
0
2
4
6
8

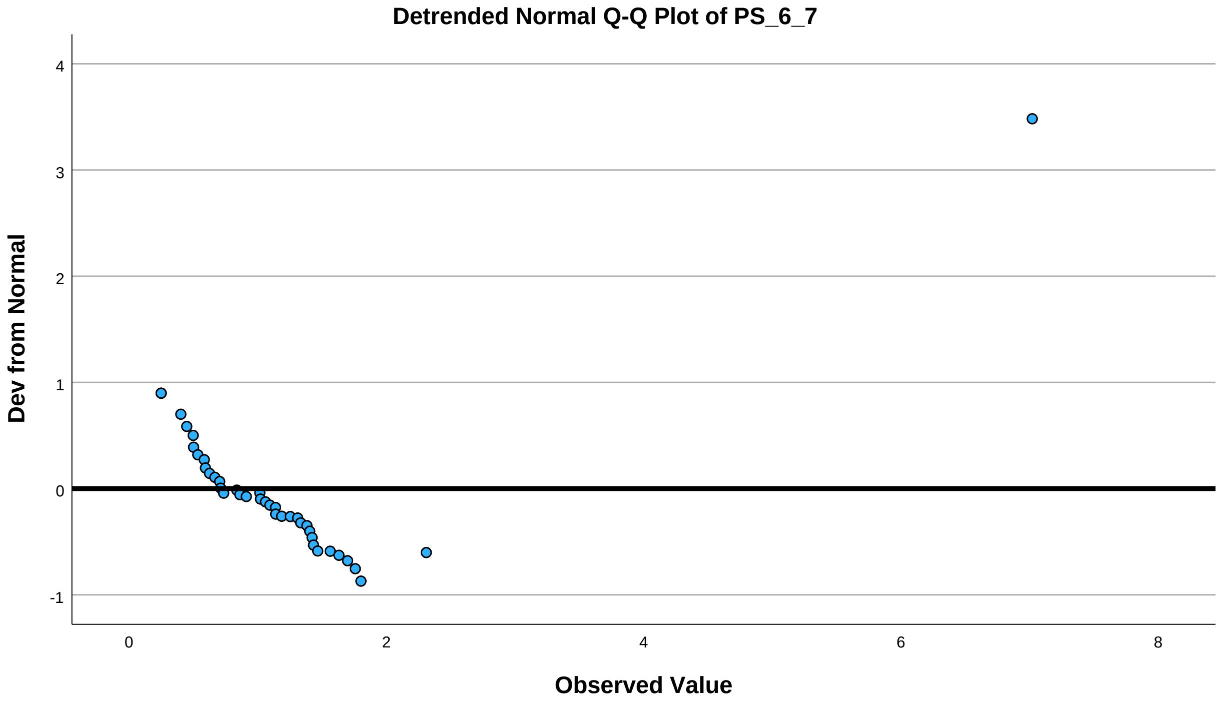

Detrended Normal Q-Q Plot of PS_6_7
Dev from Normal
Observed Value
4
3
2
1
0
-1
0
2
4
6
8

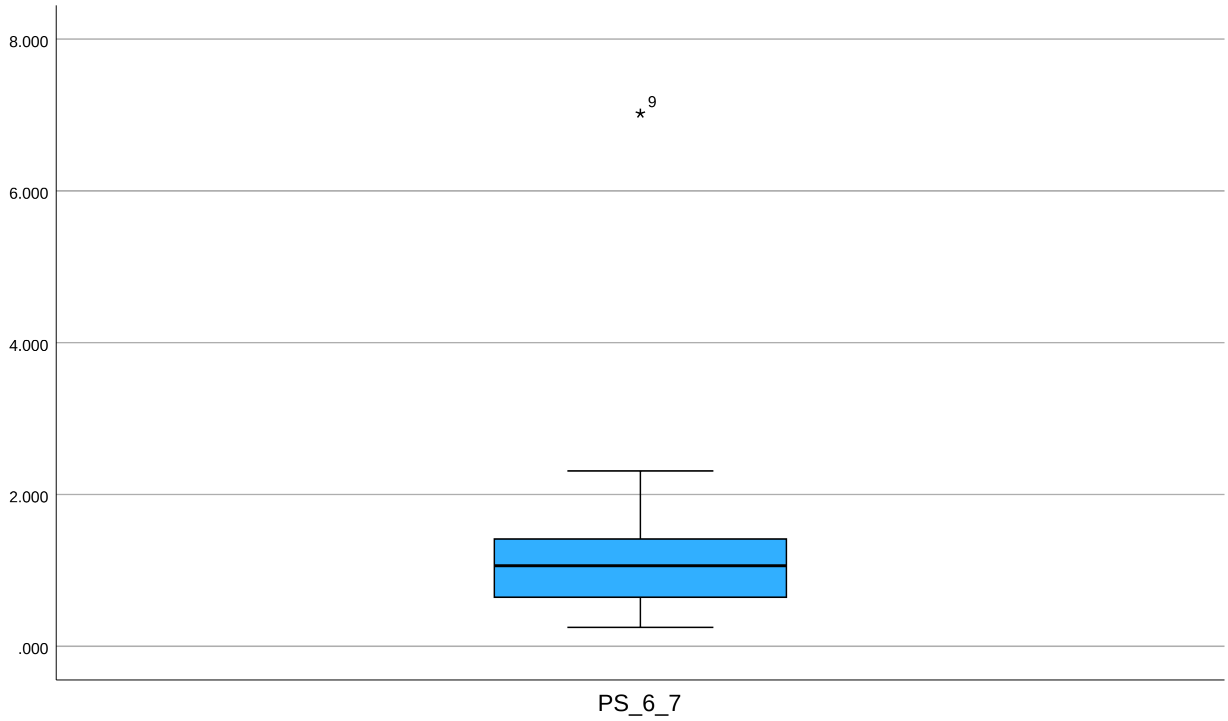

PS_7_8

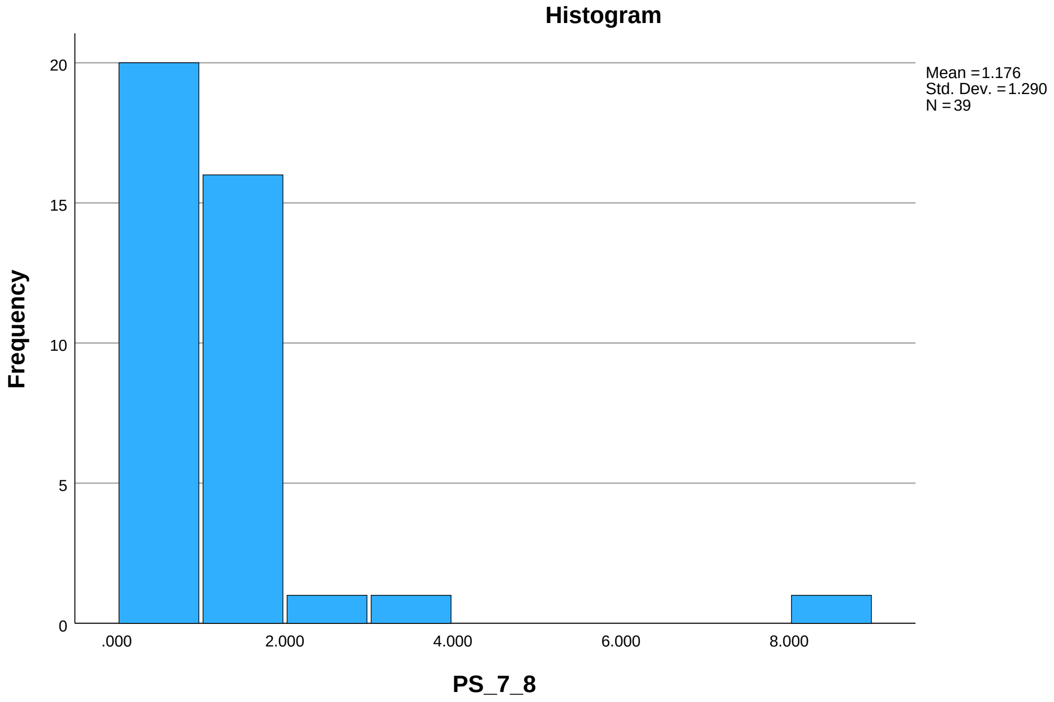

PS_7_8 Stem-and-Leaf Plot

Frequency   Stem & Leaf

```
   8.00        0 .  22334444
  12.00        0 .  566667788899
  14.00        1 .  00000012233344
   2.00        1 .  55
   1.00        2 .  2
   2.00 Extremes    (>=3.7)

 Stem width:      1.000
 Each leaf:        1 case(s)
```

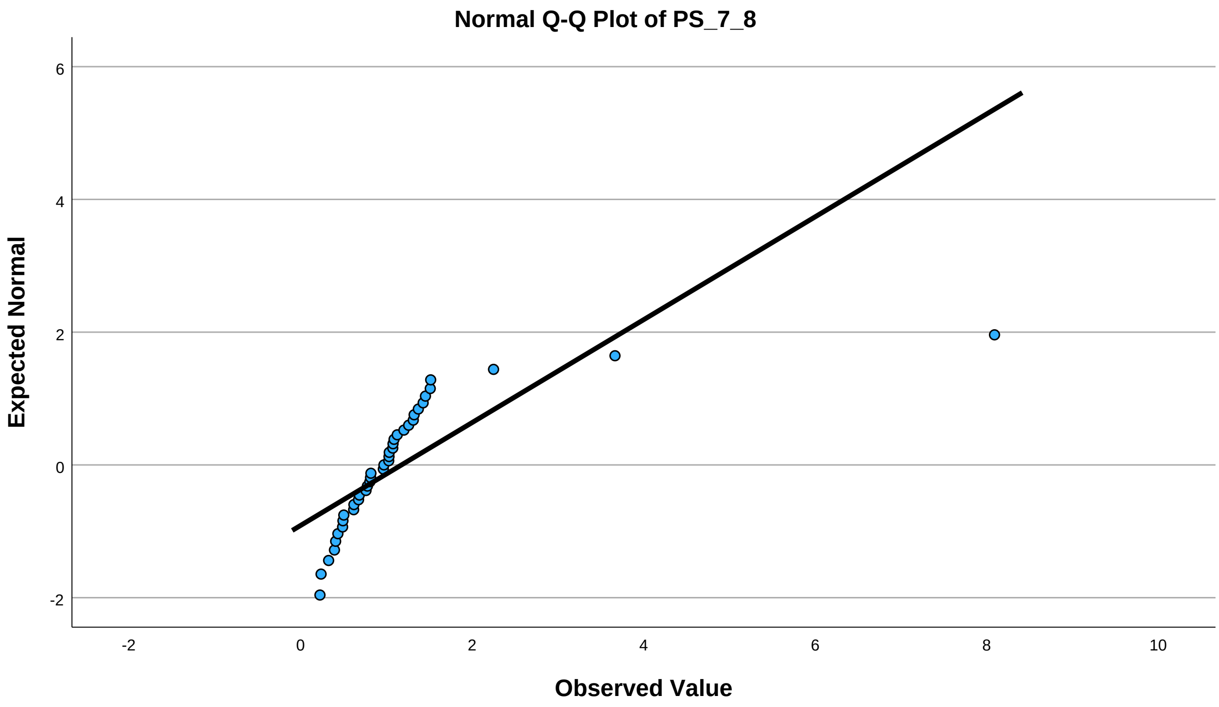

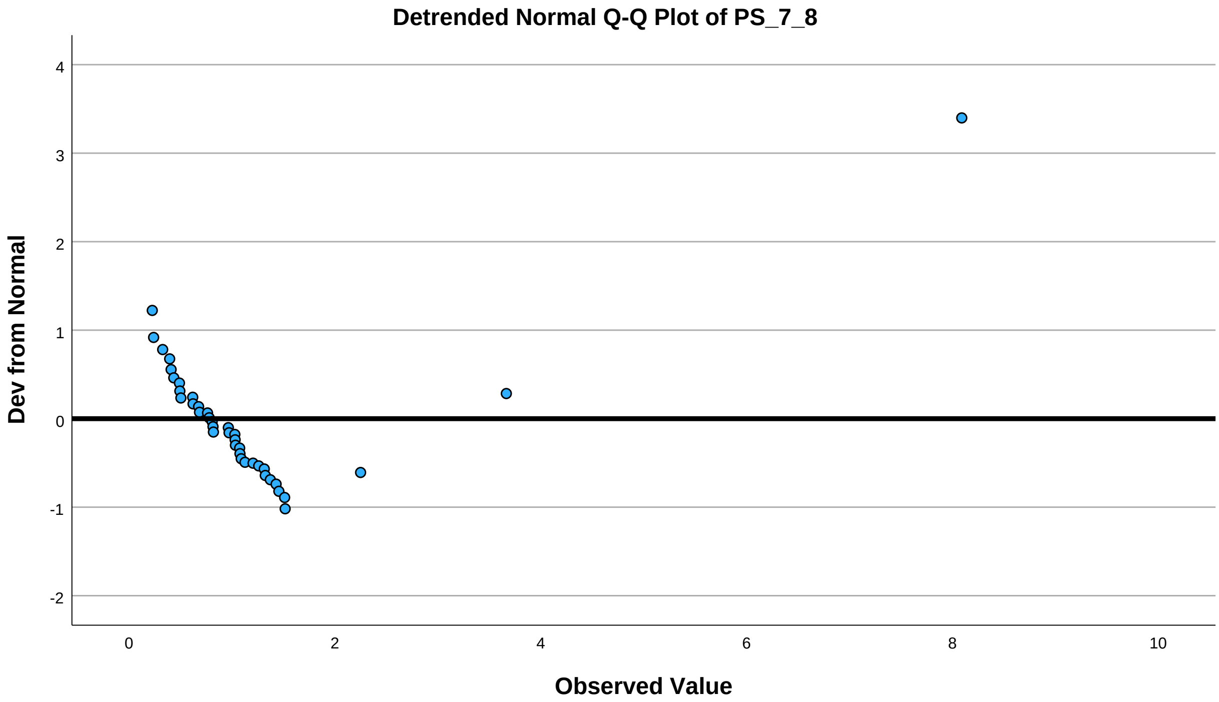

Detrended Normal Q-Q Plot of PS_7_8
Dev from Normal
4
3
2
1
0
-1
-2
0
2
4
6
8
10
Observed Value

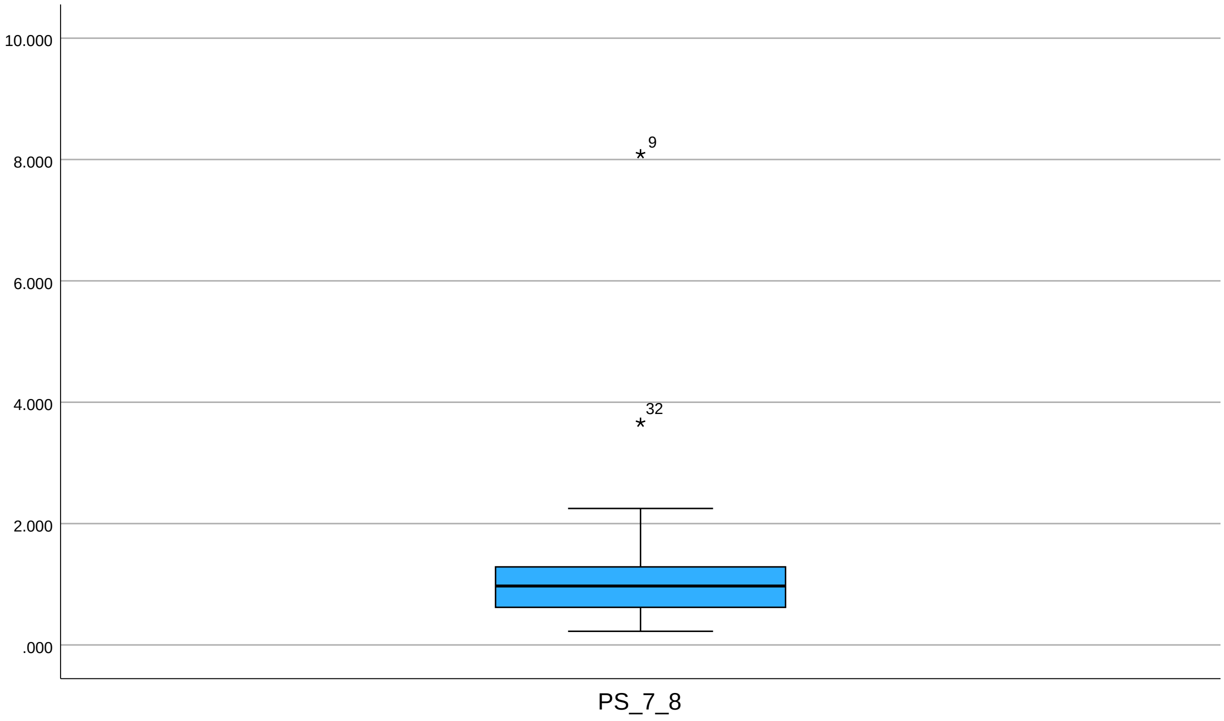

10.000
8.000
6.000
4.000
2.000
.000
9
32
PS_7_8

PS_8_9

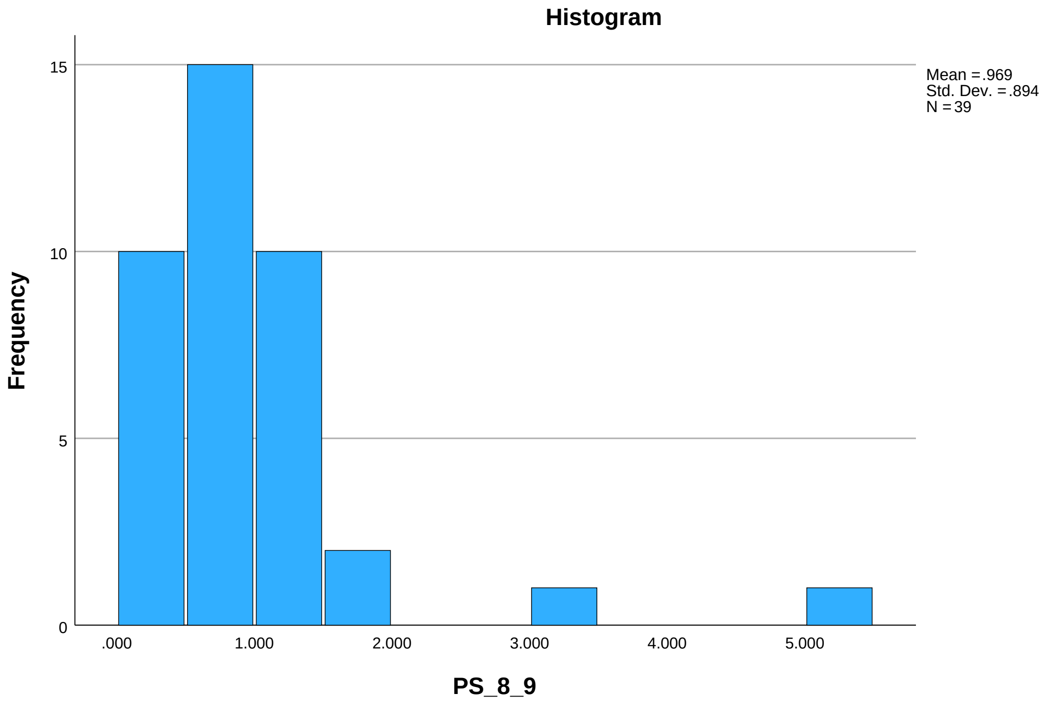


```
PS_8_9 Stem-and-Leaf Plot

 Frequency    Stem &  Leaf

     1.00        0 .  1
     5.00        0 .  22233
     6.00        0 .  444455
     9.00        0 .  666666777
     4.00        0 .  8889
     7.00        1 .  0001111
     2.00        1 .  22
     2.00        1 .  45
     1.00        1 .  7
     2.00 Extremes    (>=3.5)

 Stem width:      1.000
 Each leaf:       1 case(s)
```

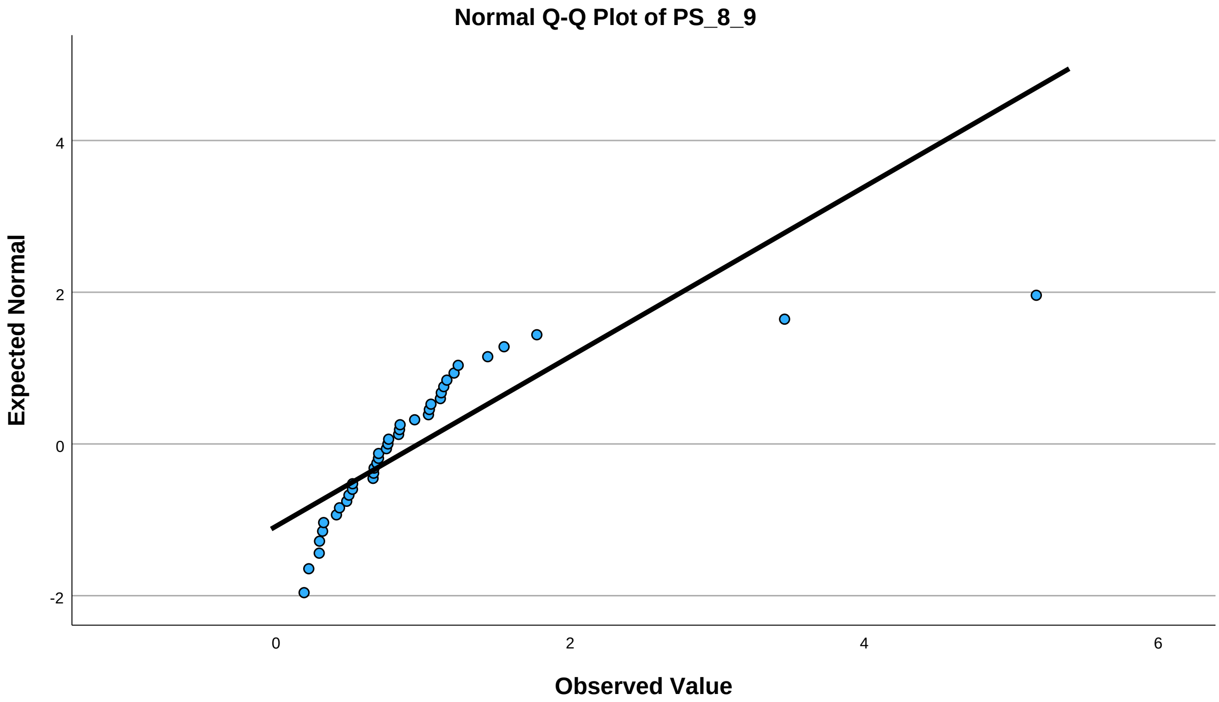
Normal Q-Q Plot of PS_8_9
Expected Normal
Observed Value
4
2
0
-2
0
2
4
6

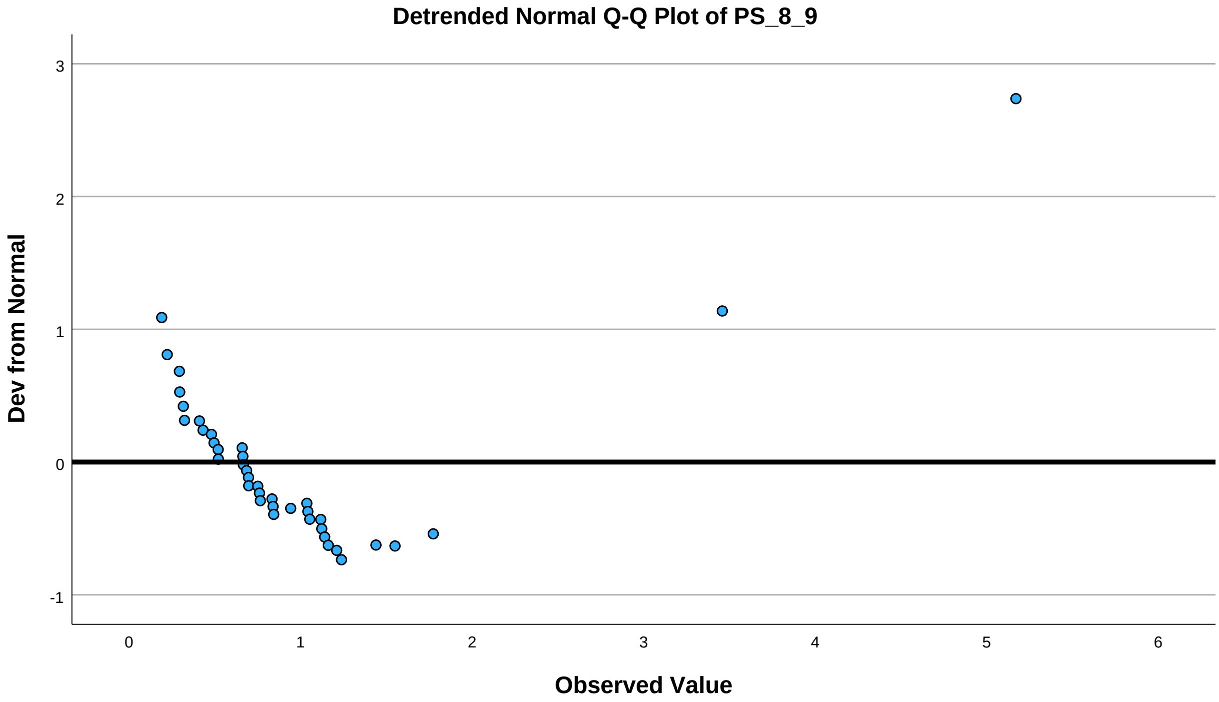
Detrended Normal Q-Q Plot of PS_8_9
Dev from Normal
Observed Value
3
2
1
0
-1
0
1
2
3
4
5
6

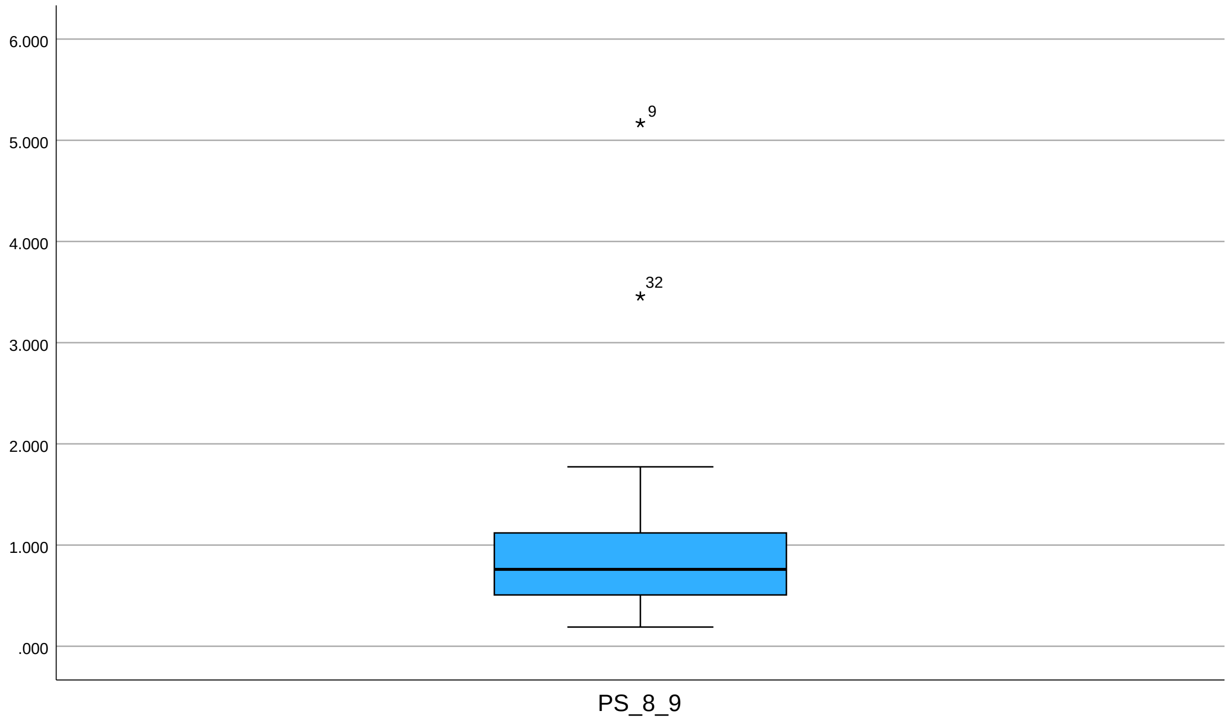

PS_9_10

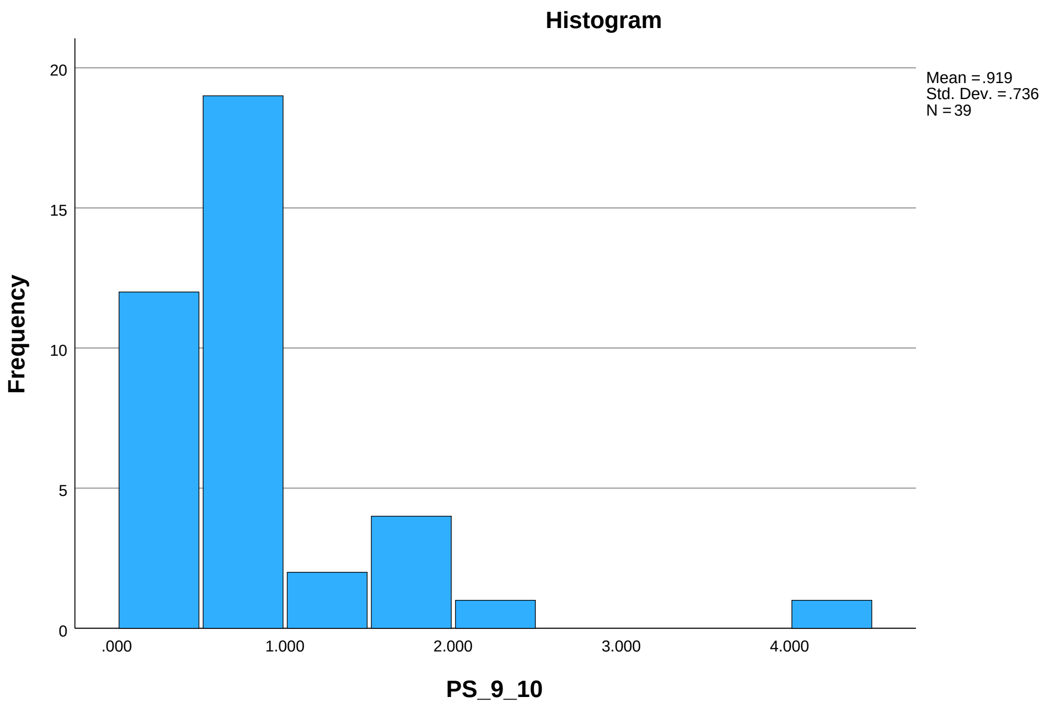


```
PS_9_10 Stem-and-Leaf Plot

 Frequency    Stem &  Leaf
```

```
  2.00      0 . 11
  3.00      0 . 223
  9.00      0 . 444444455
  5.00      0 . 66667
 12.00      0 . 888999999999
  1.00      1 . 1
  1.00      1 . 2
   .00      1 .
  2.00      1 . 67
  4.00 Extremes    (>=1.8)

Stem width:     1.000
Each leaf:       1 case(s)
```

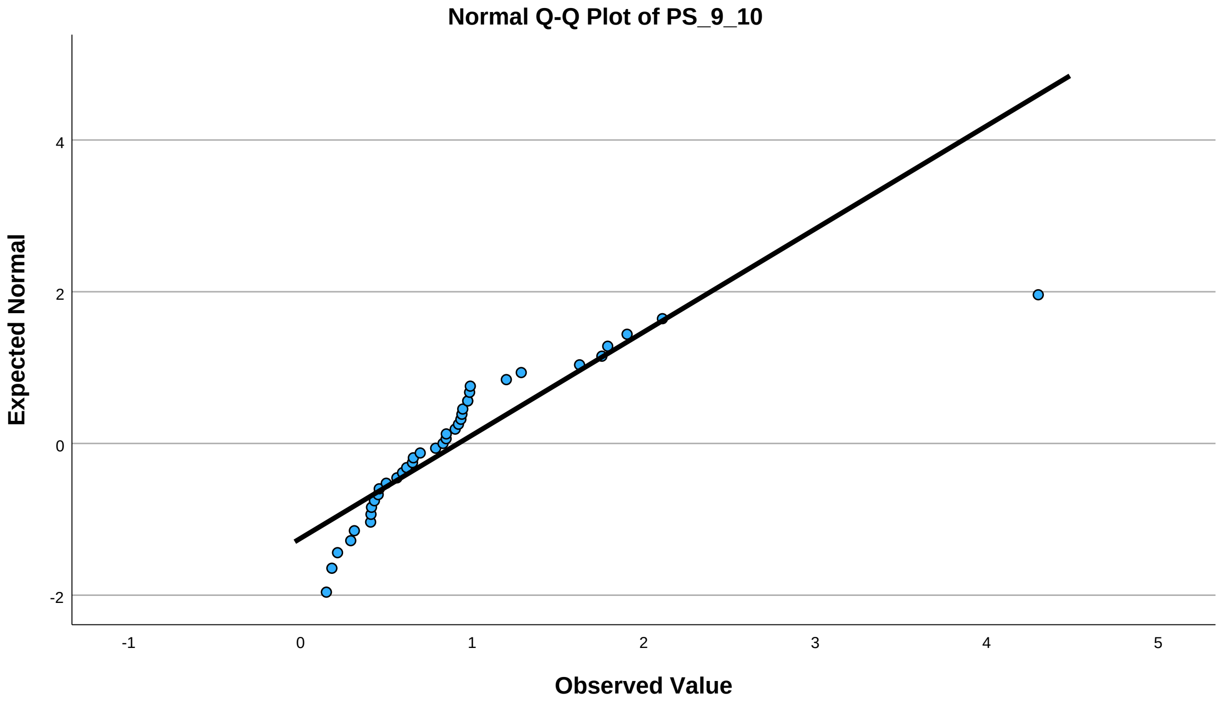

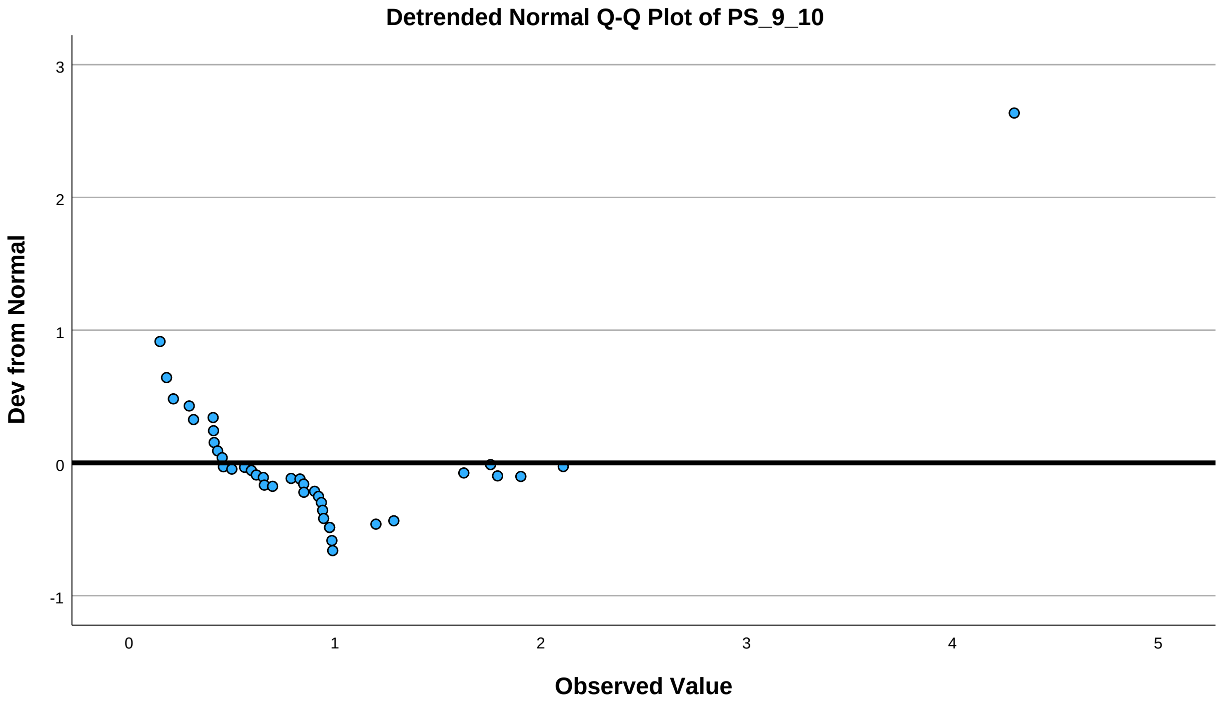
Detrended Normal Q-Q Plot of PS_9_10
Dev from Normal
3
2
1
0
-1
0
1
2
3
4
5
Observed Value

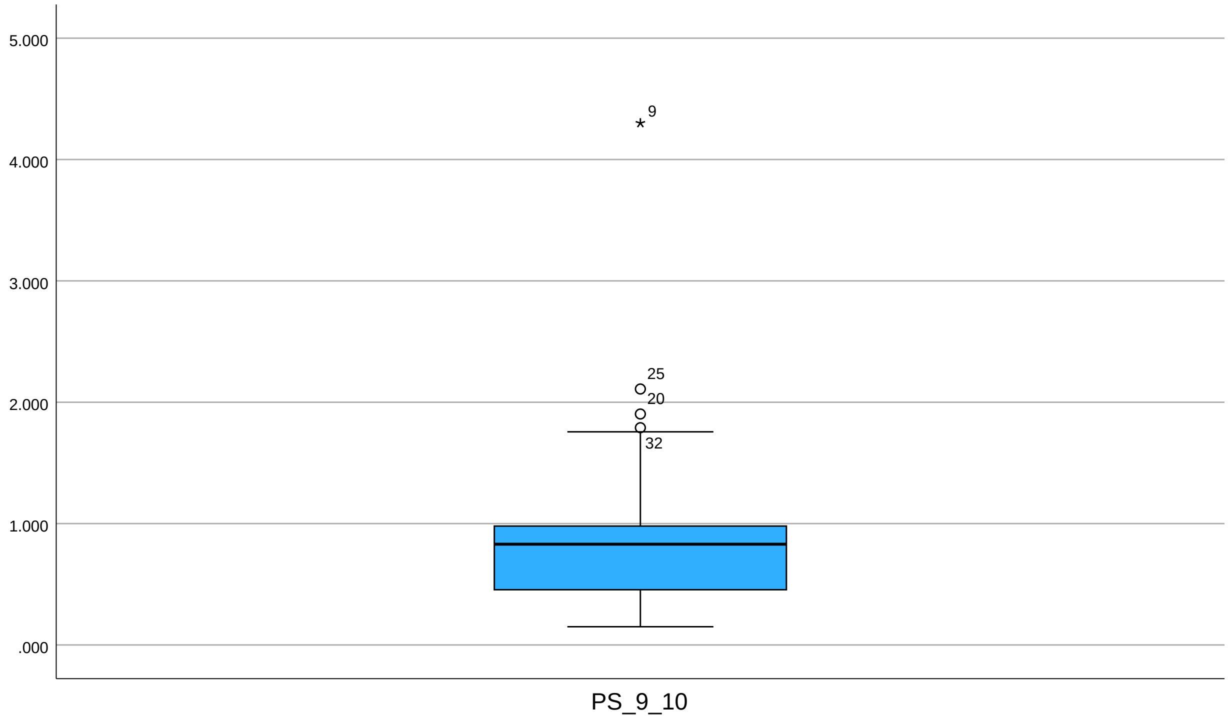
5.000
4.000
3.000
2.000
1.000
.000
9
25
20
32
PS_9_10

PS_10_11

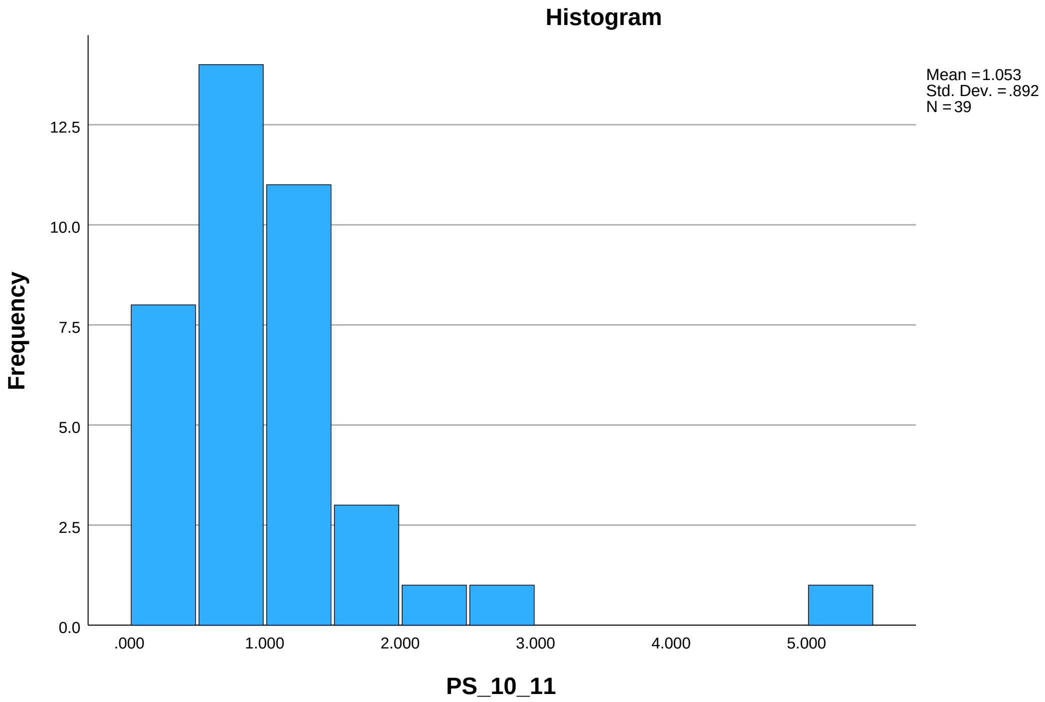


```
PS_10_11 Stem-and-Leaf Plot

 Frequency    Stem &  Leaf

     8.00        0 .  11223344
    14.00        0 .  55556778888889
    11.00        1 .  00111111233
     3.00        1 .  559
     1.00        2 .  1
     2.00 Extremes    (>=2.9)

 Stem width:      1.000
 Each leaf:       1 case(s)
```

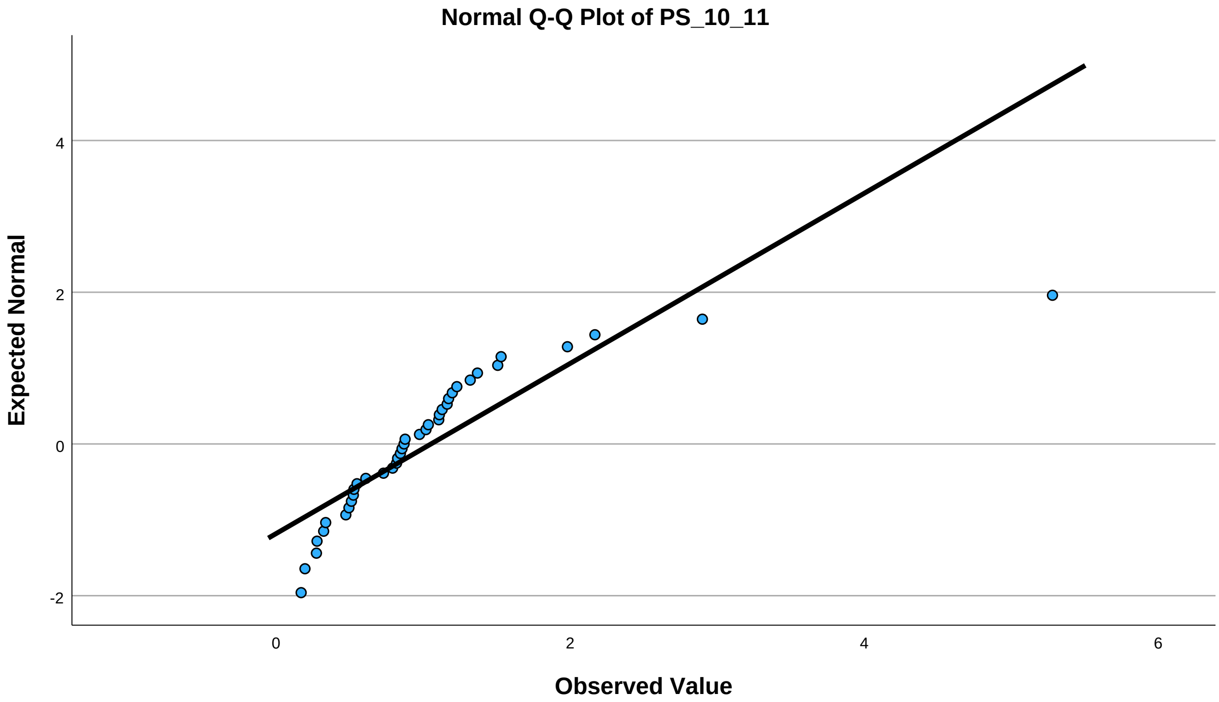

Normal Q-Q Plot of PS_10_11
Expected Normal
Observed Value
4
2
0
-2
0
2
4
6

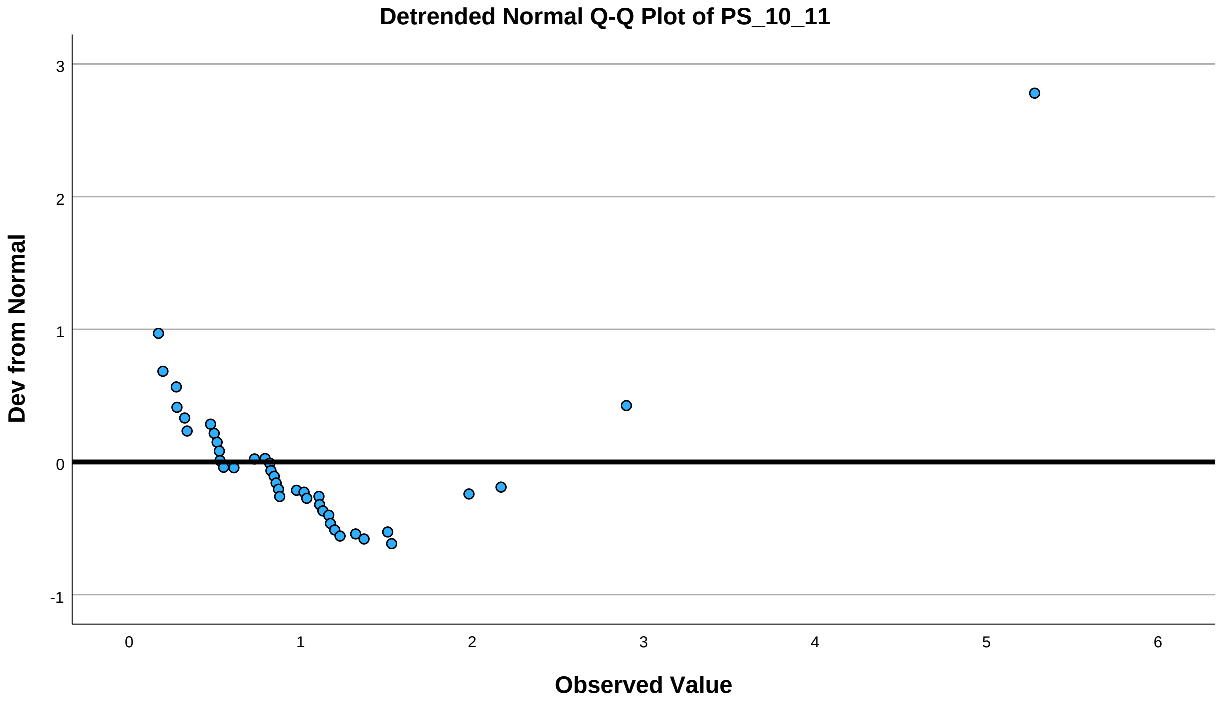

Detrended Normal Q-Q Plot of PS_10_11
Dev from Normal
Observed Value
3
2
1
0
-1
0
1
2
3
4
5
6

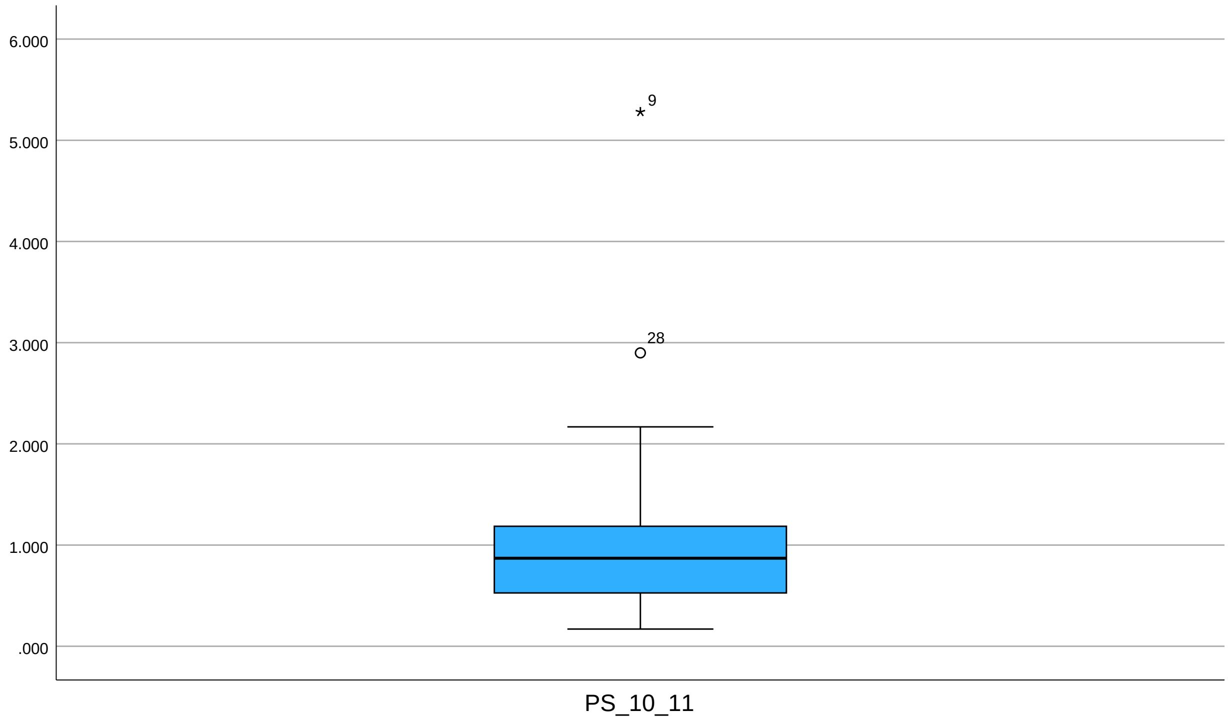

PS_11_12

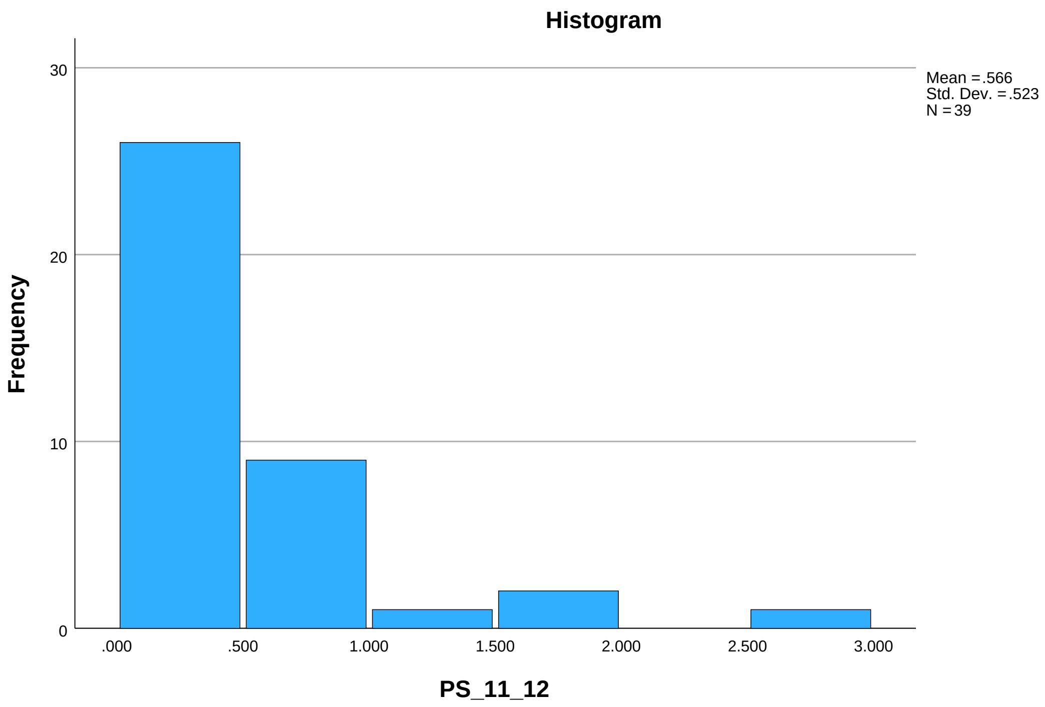

PS_11_12 Stem-and-Leaf Plot

Frequency Stem & Leaf

```
 4.00      1 . 1559
 8.00      2 . 01245889
 5.00      3 . 67899
 9.00      4 . 023556677
 3.00      5 . 266
 3.00      6 . 016
 2.00      7 . 59
  .00      8 .
 1.00      9 . 6
 4.00 Extremes    (>=1.17)

Stem width:      .100
Each leaf:       1 case(s)
```

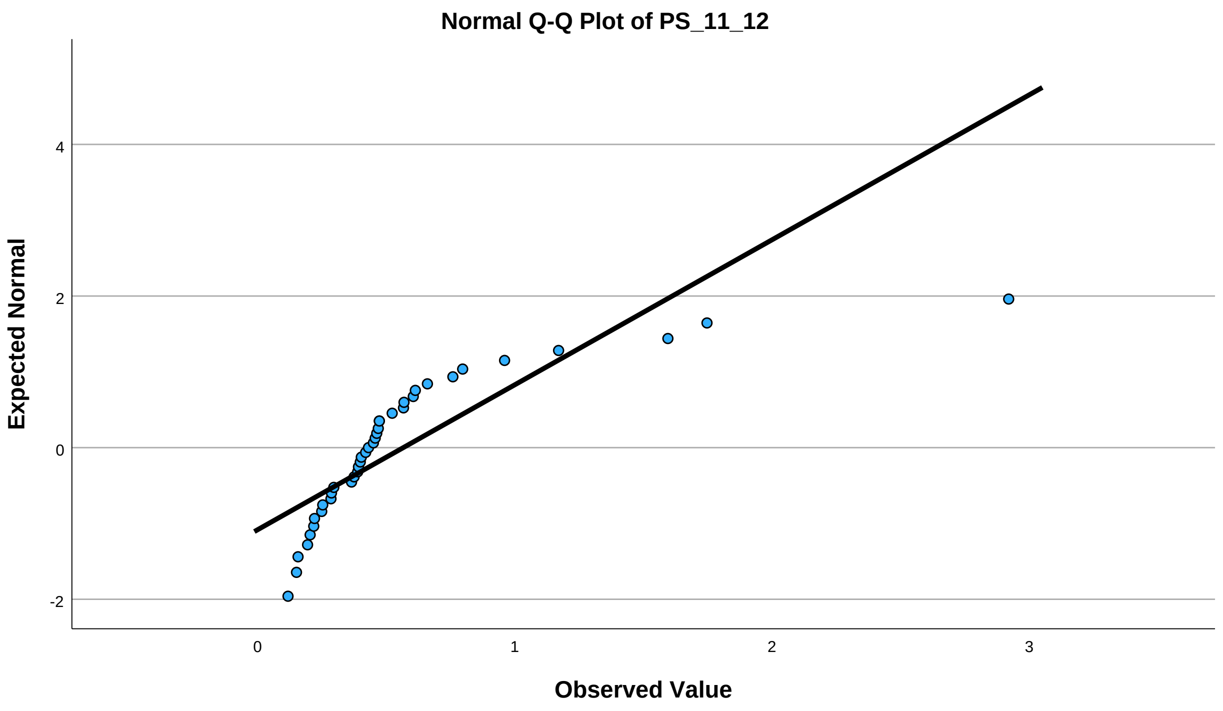

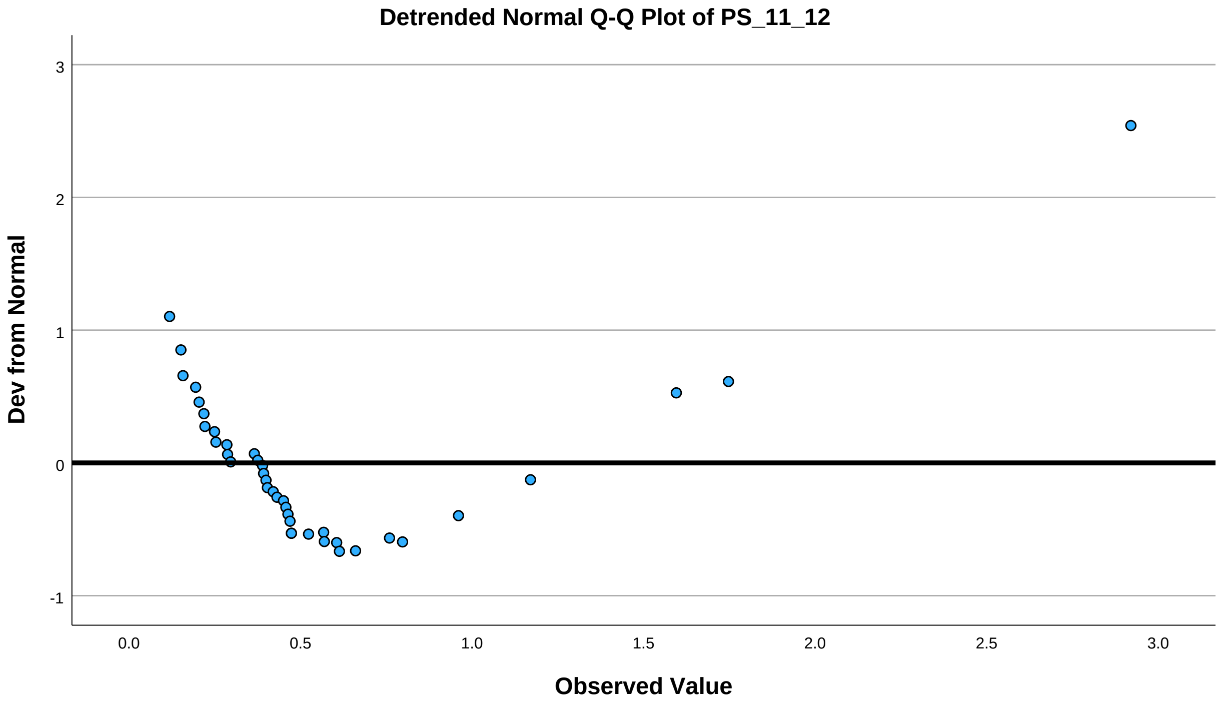
Detrended Normal Q-Q Plot of PS_11_12
Dev from Normal
3
2
1
0
-1
0.0
0.5
1.0
1.5
2.0
2.5
3.0
Observed Value

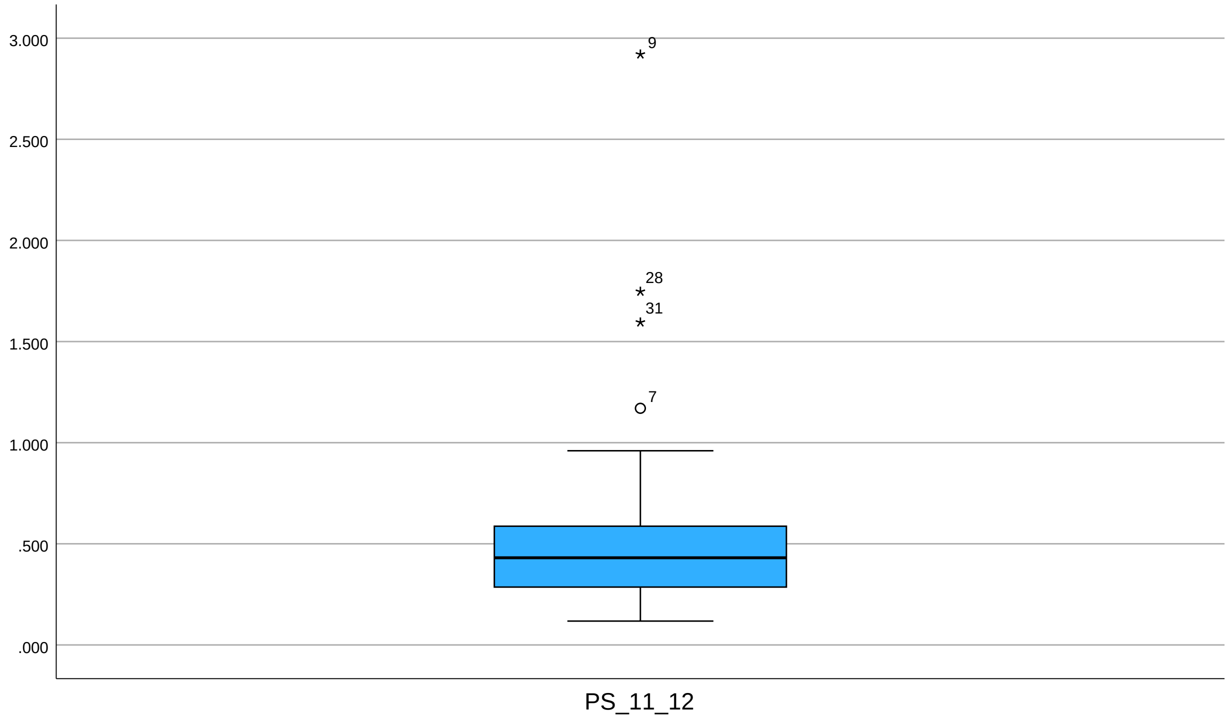
3.000
2.500
2.000
1.500
1.000
.500
.000
9
28
31
7
PS_11_12

PS_12_13

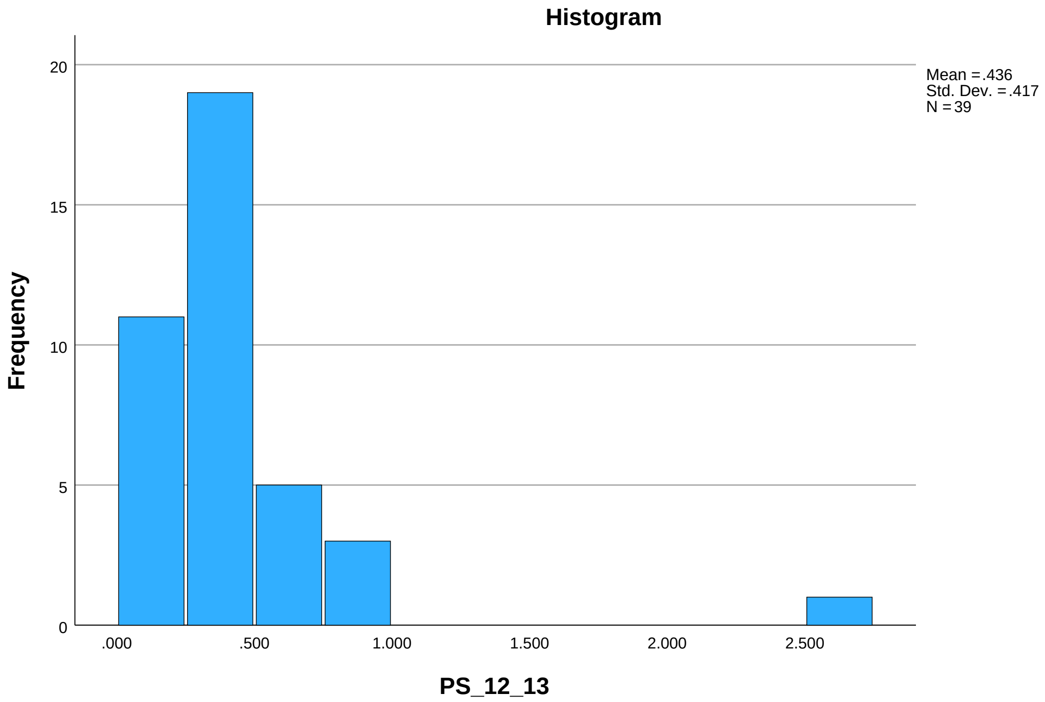


```
PS_12_13 Stem-and-Leaf Plot

 Frequency    Stem &  Leaf

    1.00        0 .  8
    5.00        1 .  27779
   11.00        2 .  00134566899
    6.00        3 .  136689
    7.00        4 .  0035789
    3.00        5 .  018
    2.00        6 .  04
     .00        7 .
    2.00        8 .  04
    2.00 Extremes    (>=.87)

 Stem width:      .100
 Each leaf:       1 case(s)
```

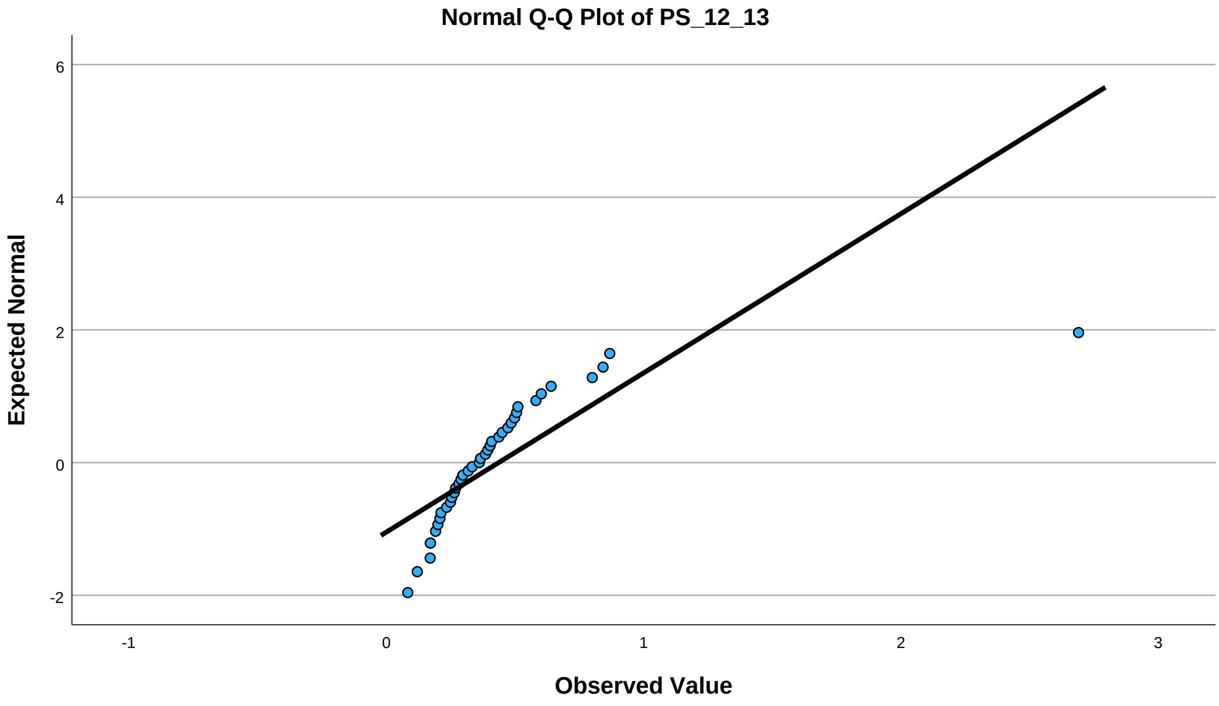
Normal Q-Q Plot of PS_12_13
Expected Normal
Observed Value
6
4
2
0
-2
-1
0
1
2
3

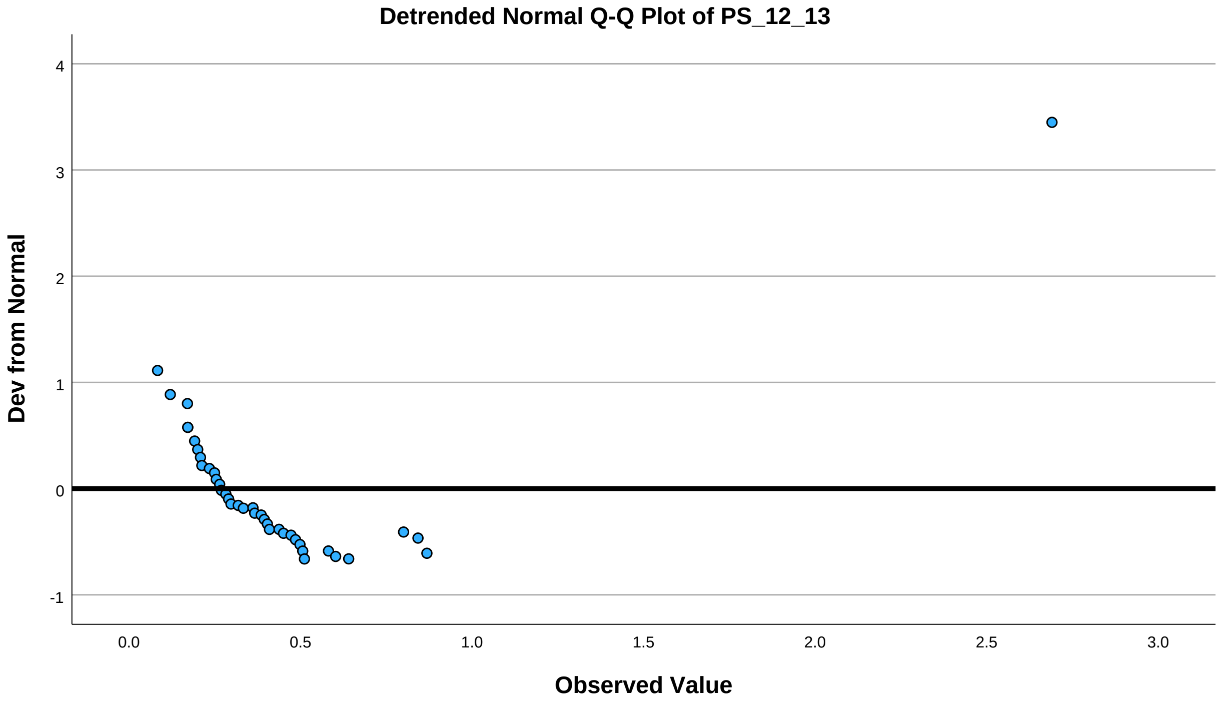
Detrended Normal Q-Q Plot of PS_12_13
Dev from Normal
Observed Value
4
3
2
1
0
-1
0.0
0.5
1.0
1.5
2.0
2.5
3.0

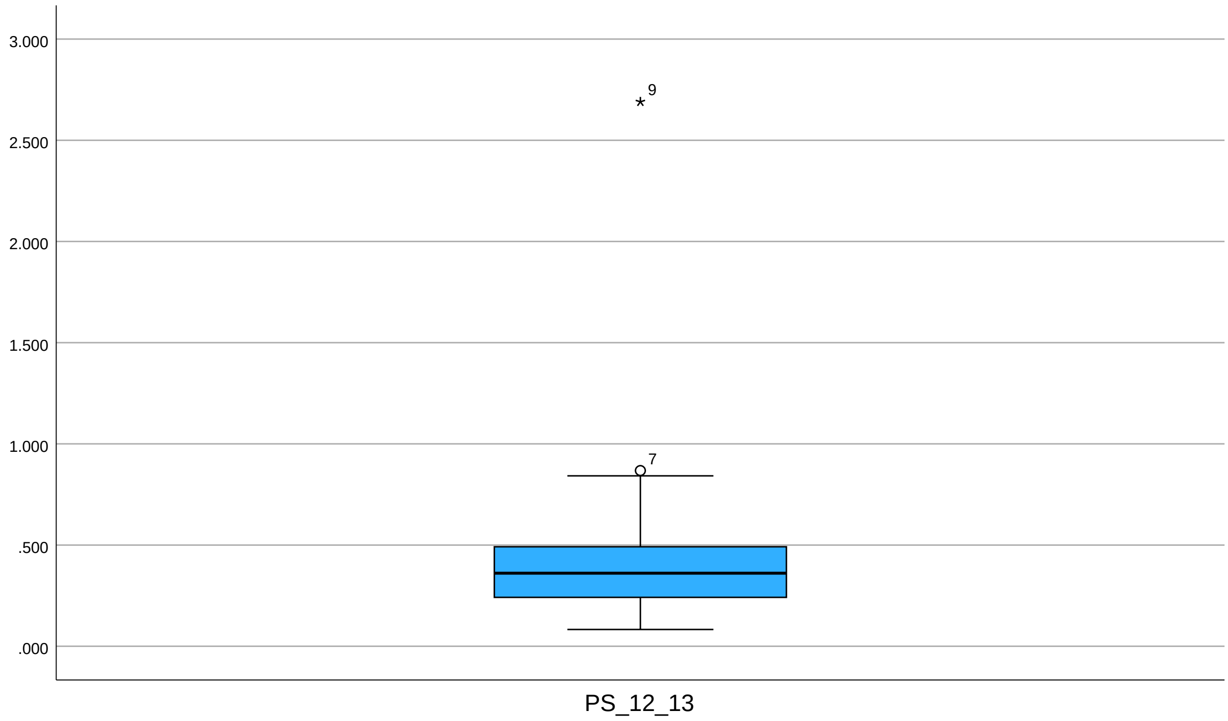

PS_13_14

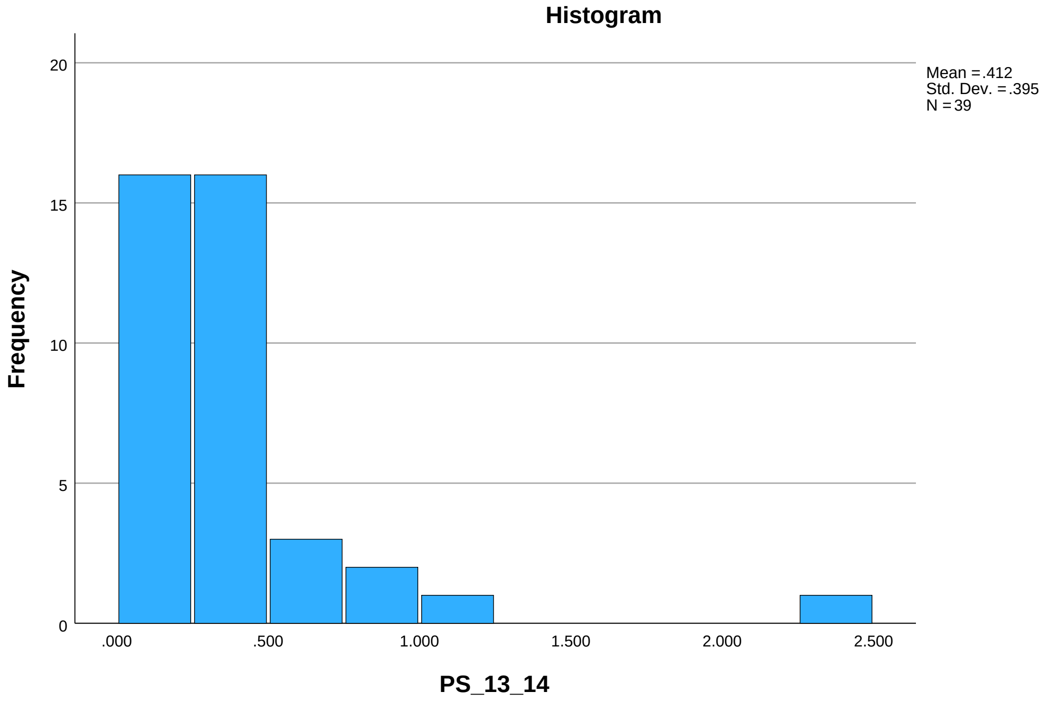


```
PS_13_14 Stem-and-Leaf Plot

 Frequency    Stem &  Leaf
```

```
 1.00      0 . 6
 1.00      1 . 1
 6.00      1 . 666779
 8.00      2 . 01233344
 1.00      2 . 9
 3.00      3 . 023
 6.00      3 . 556889
 6.00      4 . 000124
  .00      4 .
 1.00      5 . 1
 2.00      5 . 77
 4.00 Extremes    (>=.95)

Stem width:      .100
Each leaf:       1 case(s)
```

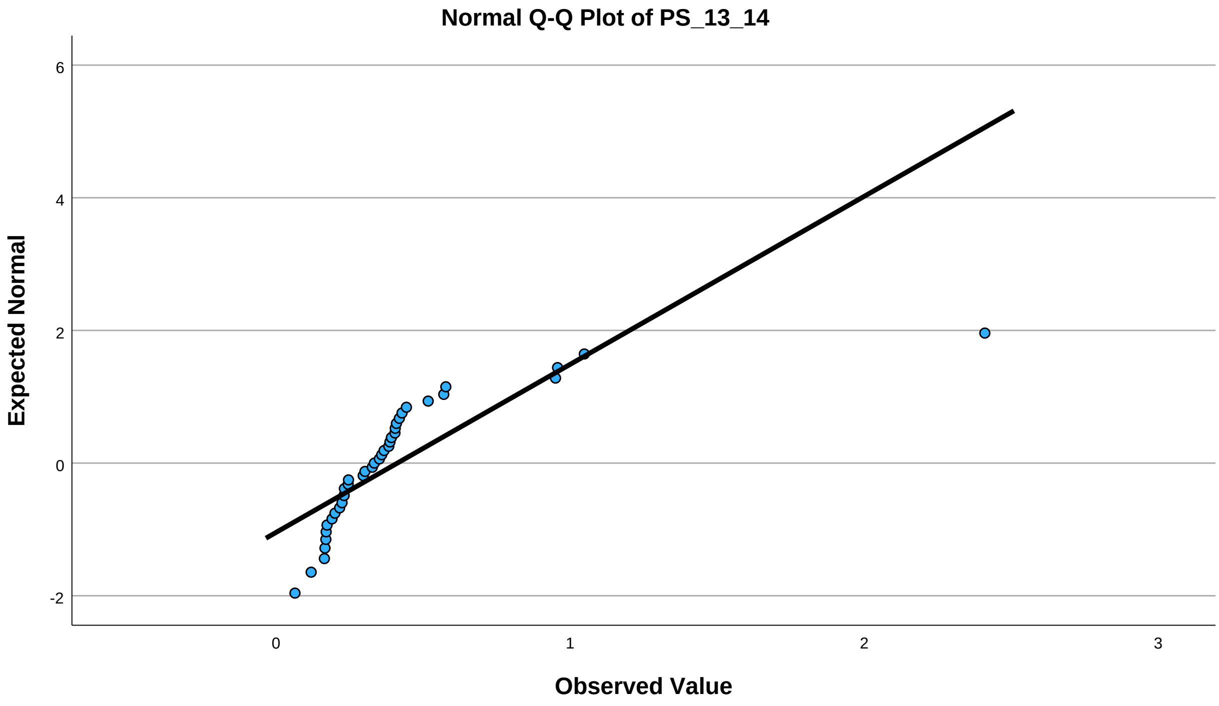

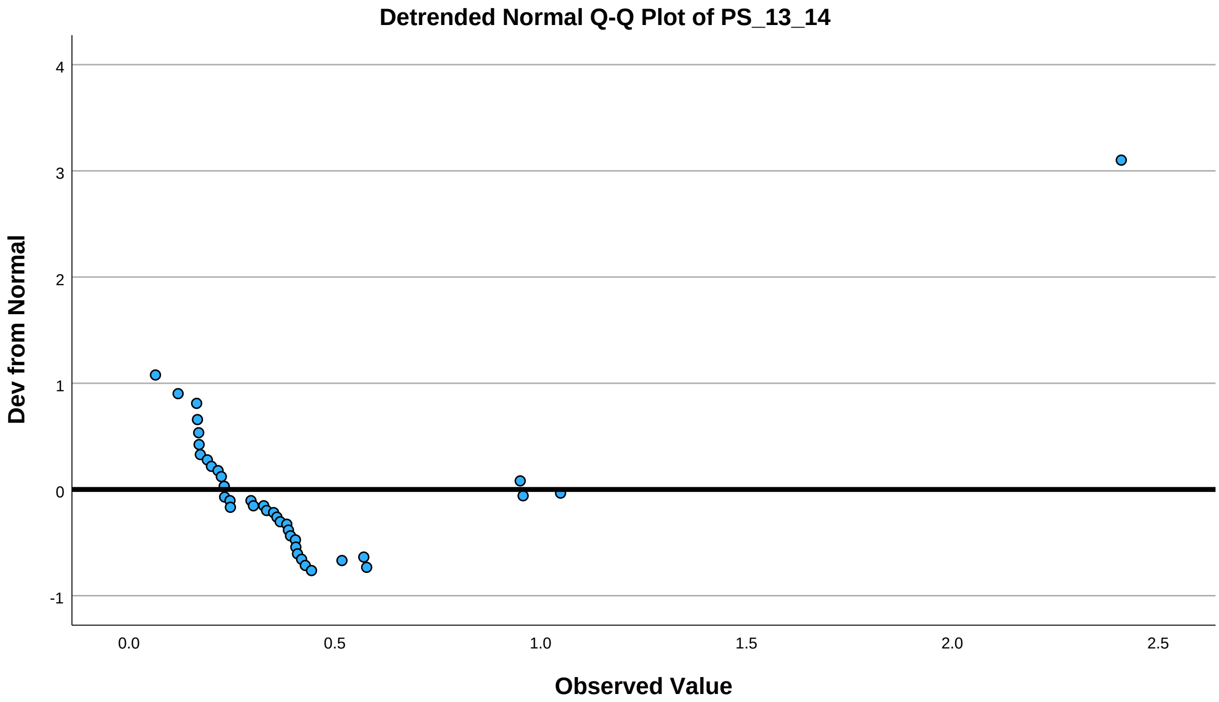
Detrended Normal Q-Q Plot of PS_13_14
Dev from Normal
Observed Value
4
3
2
1
0
-1
0.0
0.5
1.0
1.5
2.0
2.5

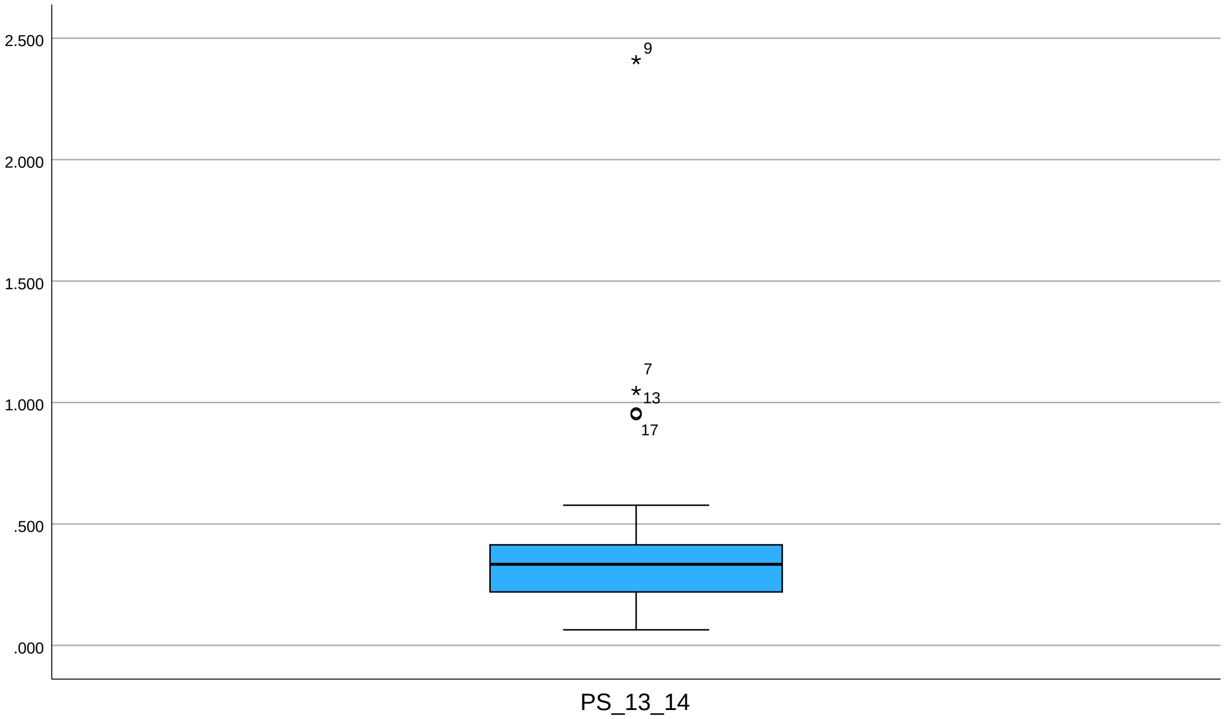
2.500
2.000
1.500
1.000
.500
.000
9
7
13
17
PS_13_14

PS_14_15

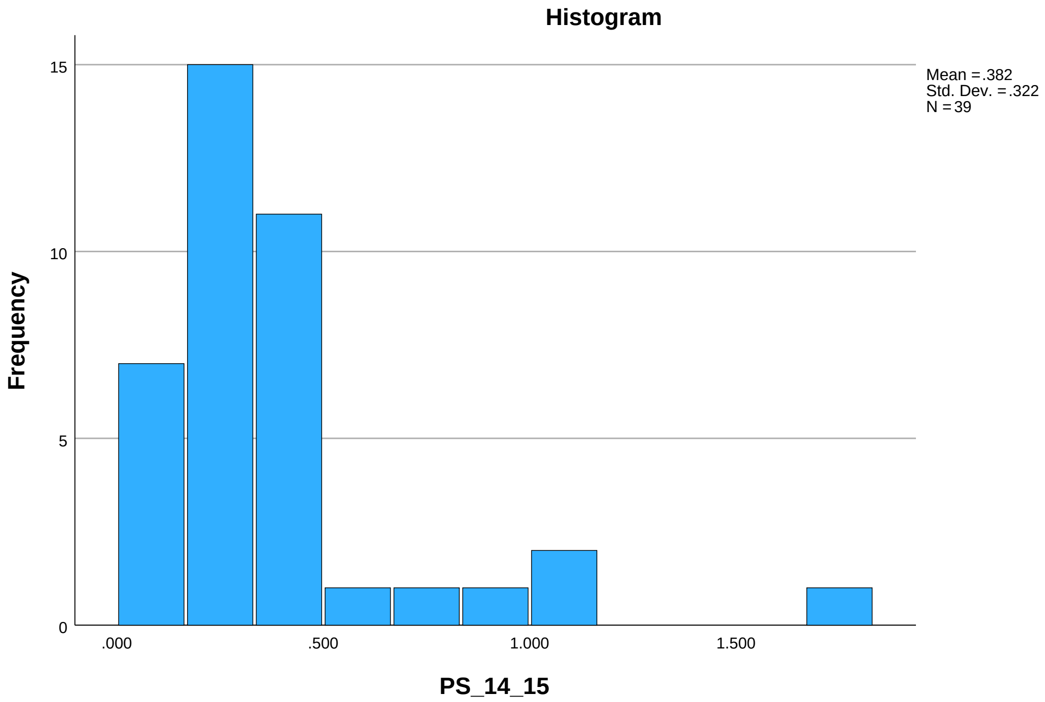


```
PS_14_15 Stem-and-Leaf Plot

 Frequency    Stem &  Leaf

     2.00        0 .  68
    10.00        1 .  3556677788
     7.00        2 .  0112478
     8.00        3 .  00156699
     6.00        4 .  001445
      .00        5 .
     2.00        6 .  37
     4.00 Extremes    (>=.87)

 Stem width:      .100
 Each leaf:       1 case(s)
```

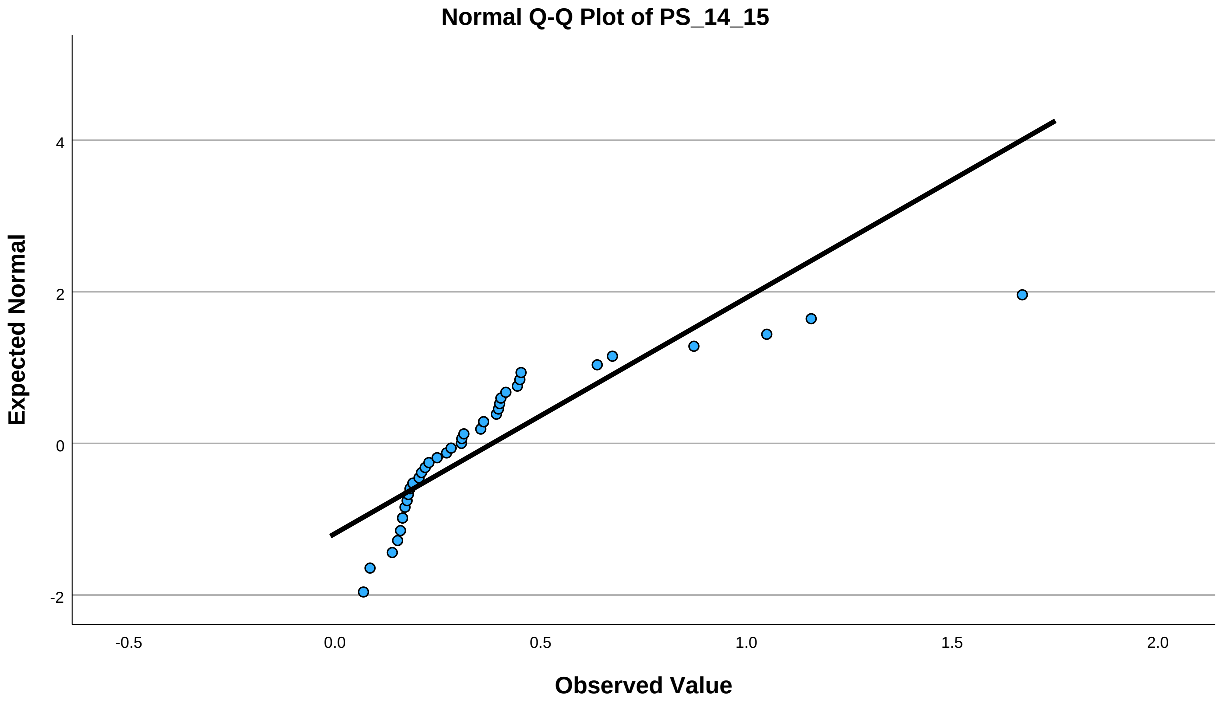
Normal Q-Q Plot of PS_14_15
Expected Normal
4
2
0
-2
-0.5
0.0
0.5
1.0
1.5
2.0
Observed Value

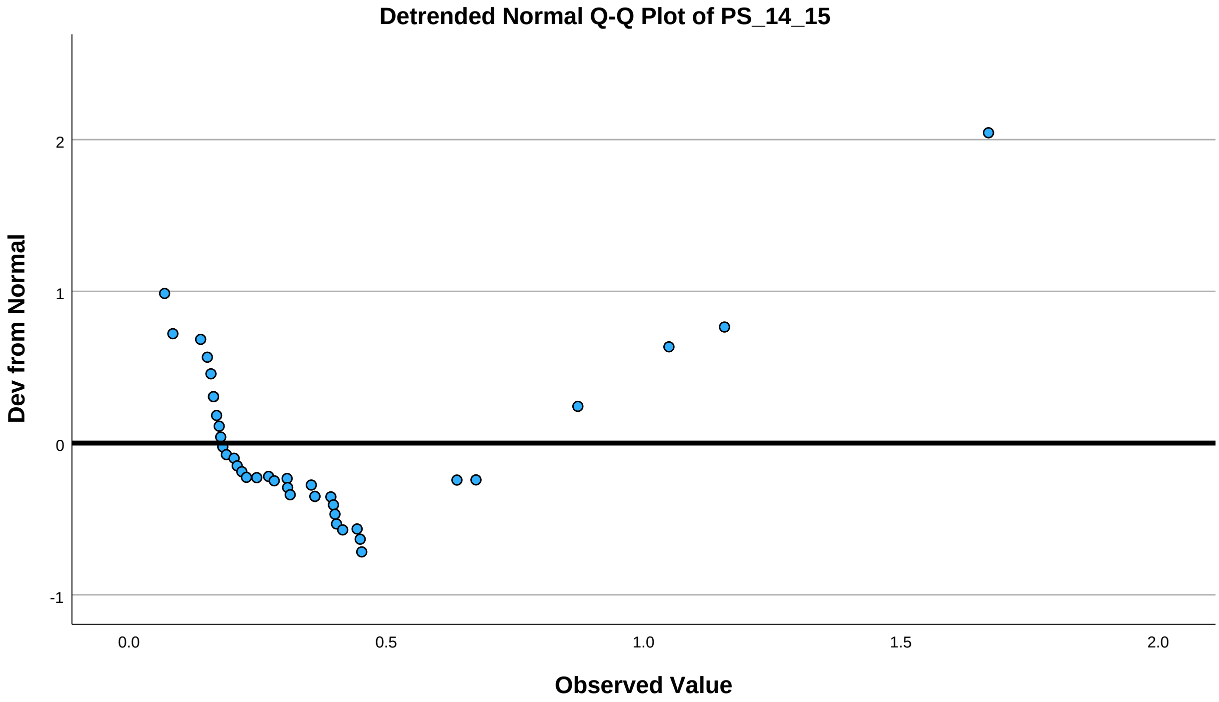
Detrended Normal Q-Q Plot of PS_14_15
Dev from Normal
2
1
0
-1
0.0
0.5
1.0
1.5
2.0
Observed Value

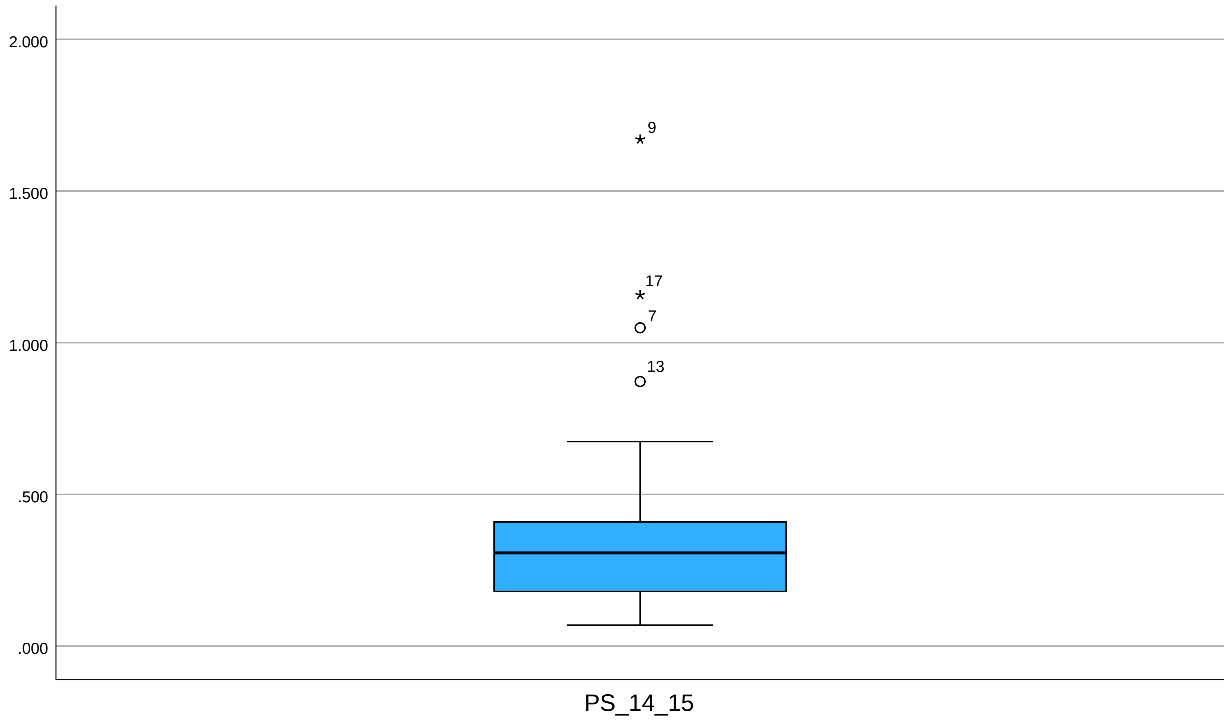

PS_15_16

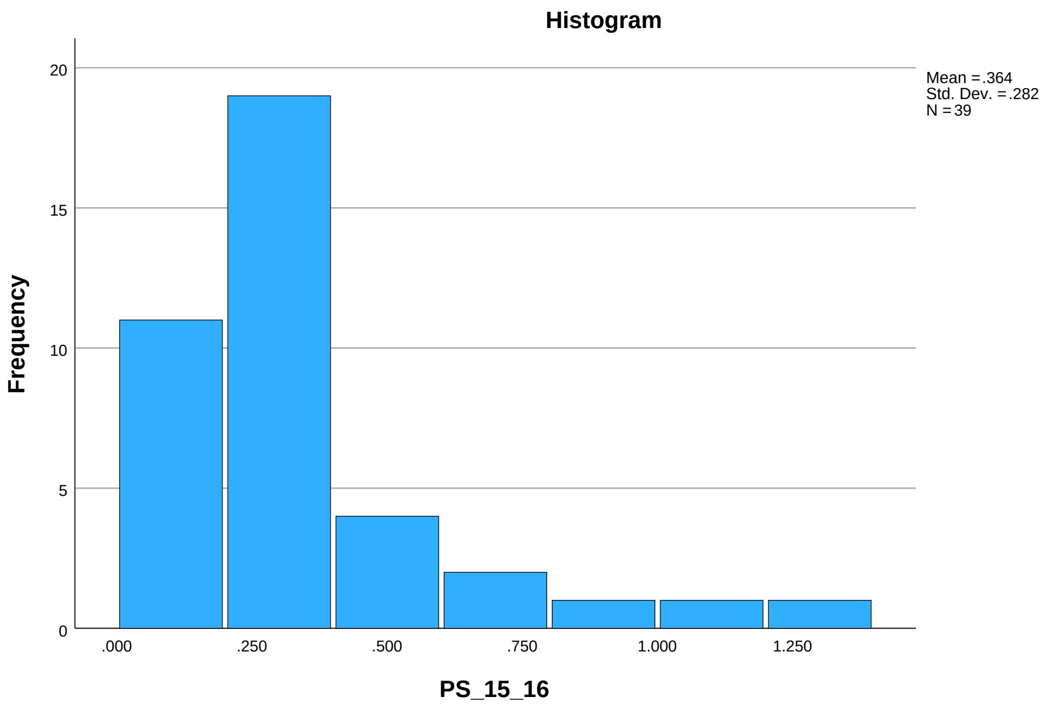

PS_15_16 Stem-and-Leaf Plot

Frequency   Stem & Leaf

```
    2.00      0 . 59
    9.00      1 . 233346789
    9.00      2 . 022346667
   10.00      3 . 0144556889
    2.00      4 . 68
    2.00      5 . 06
    1.00      6 . 7
    4.00 Extremes    (>=.70)

Stem width:     .100
Each leaf:      1 case(s)
```

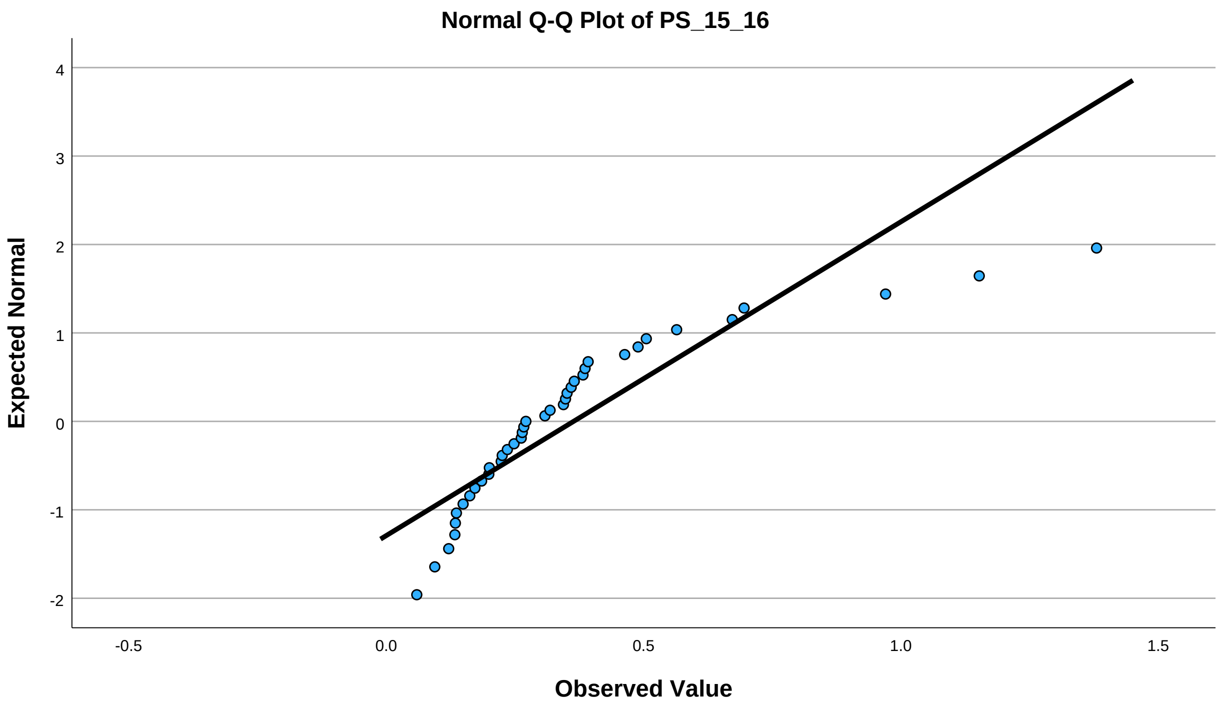

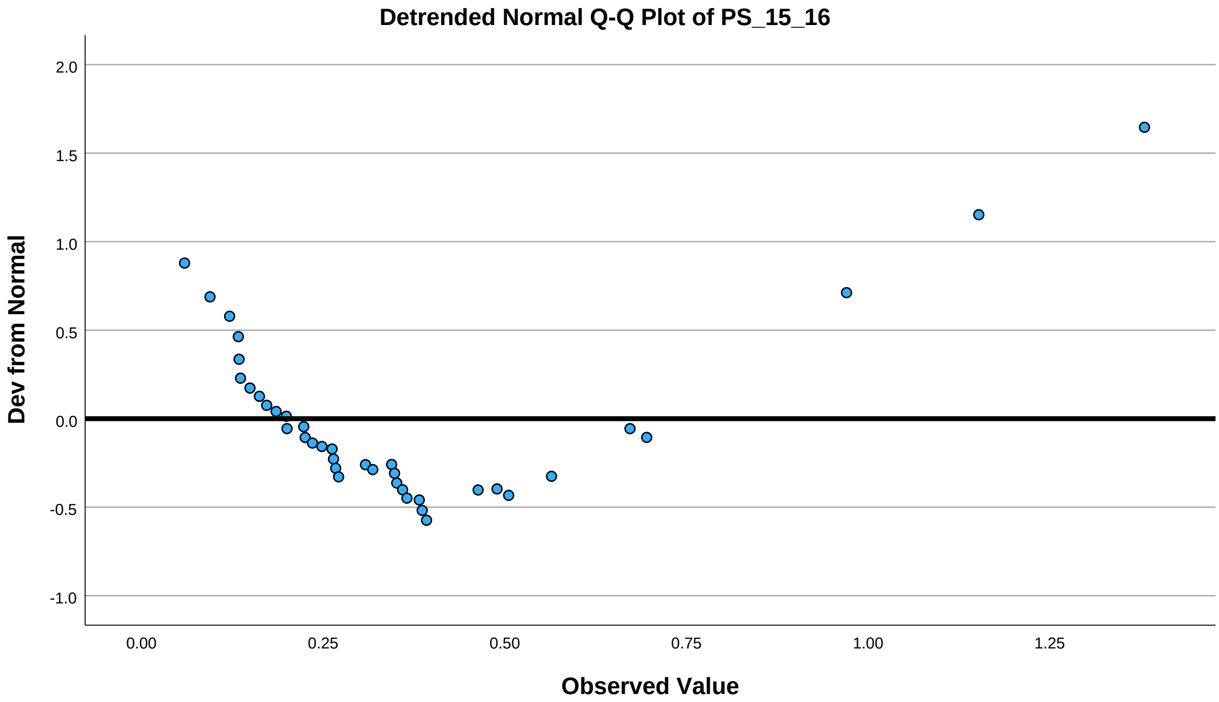
Detrended Normal Q-Q Plot of PS_15_16
Dev from Normal
2.0
1.5
1.0
0.5
0.0
-0.5
-1.0
0.00
0.25
0.50
0.75
1.00
1.25
Observed Value

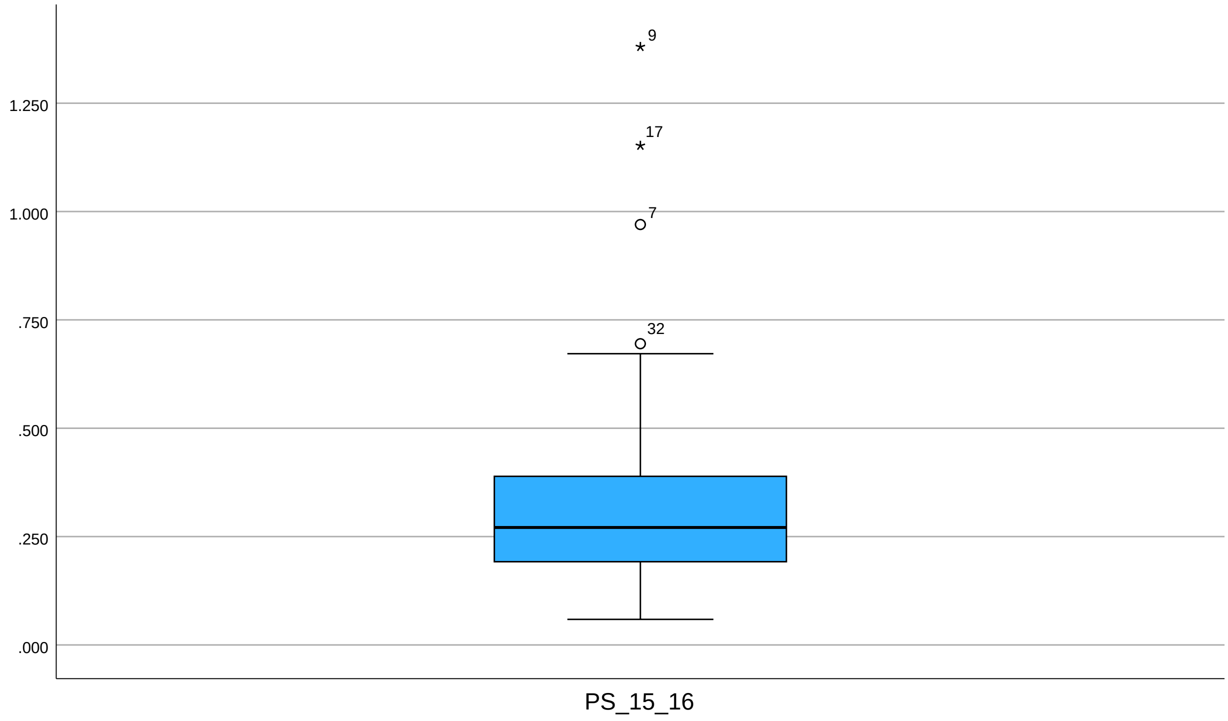
1.250
1.000
.750
.500
.250
.000
9
17
7
32
PS_15_16

PS_16_17

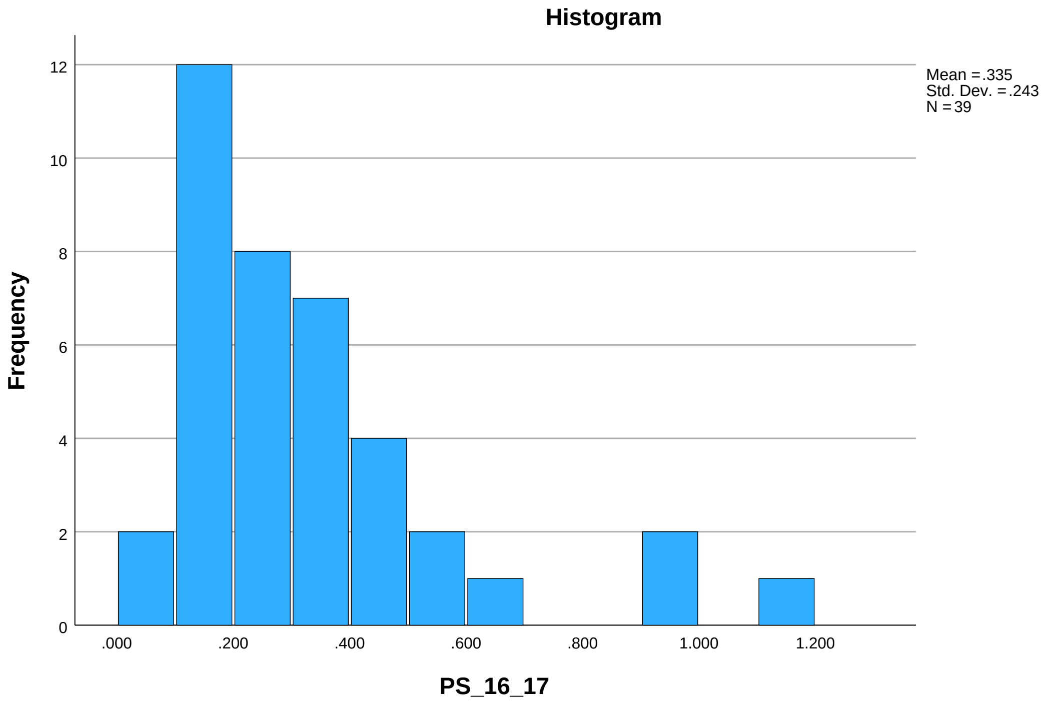


```
PS_16_17 Stem-and-Leaf Plot

 Frequency    Stem &  Leaf

     2.00        0 .  57
    12.00        1 .  023355577788
     8.00        2 .  02477799
     7.00        3 .  1155788
     4.00        4 .  0056
     2.00        5 .  08
     1.00        6 .  4
     3.00 Extremes    (>=.93)

 Stem width:      .100
 Each leaf:       1 case(s)
```

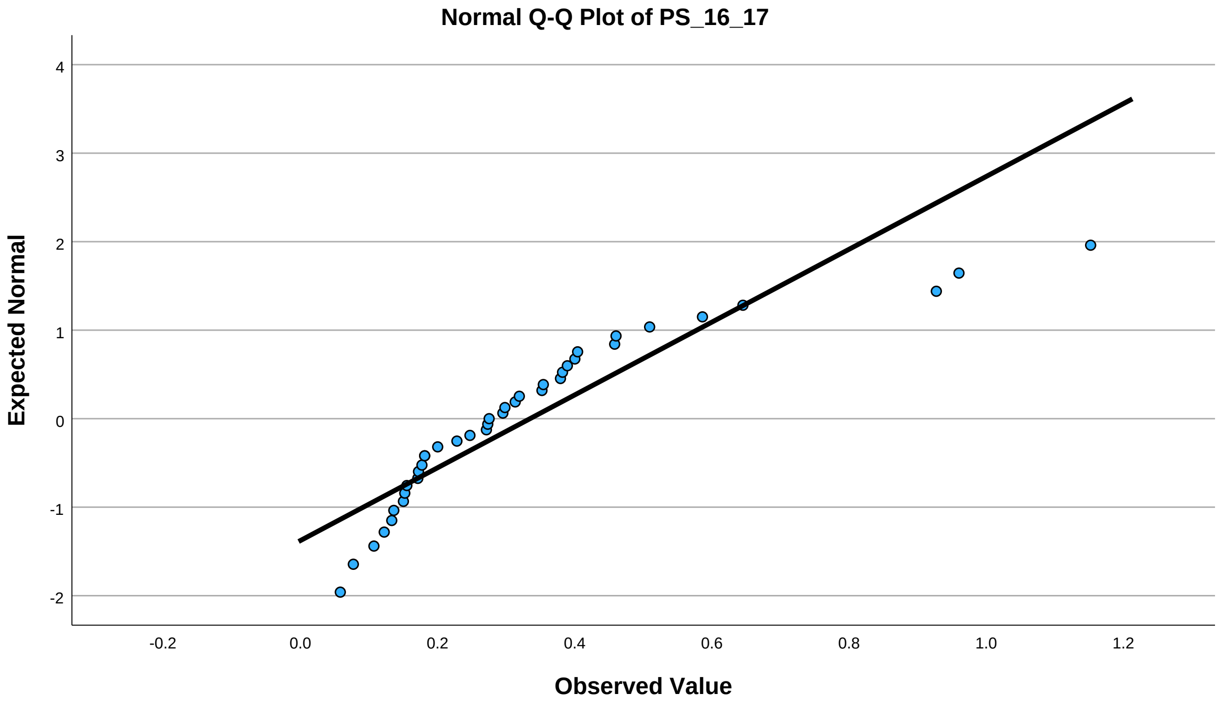

Normal Q-Q Plot of PS_16_17
Expected Normal
Observed Value
4
3
2
1
0
-1
-2
-0.2
0.0
0.2
0.4
0.6
0.8
1.0
1.2

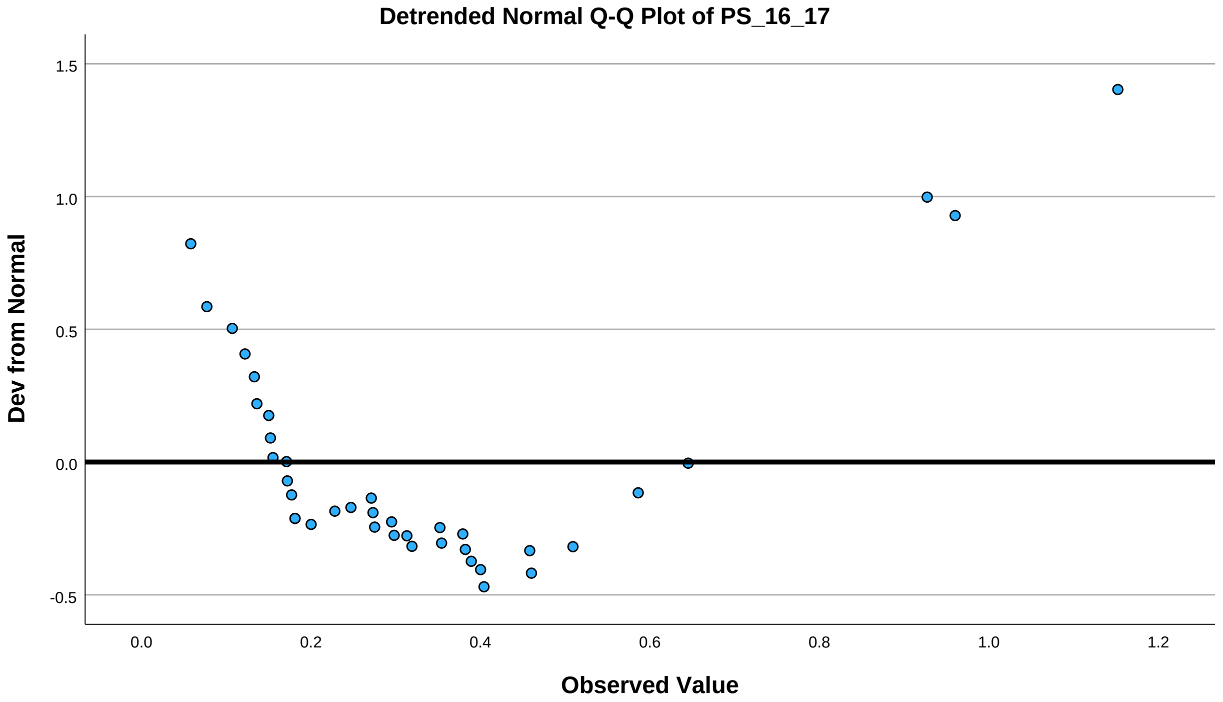

Detrended Normal Q-Q Plot of PS_16_17
Dev from Normal
Observed Value
1.5
1.0
0.5
0.0
-0.5
0.0
0.2
0.4
0.6
0.8
1.0
1.2

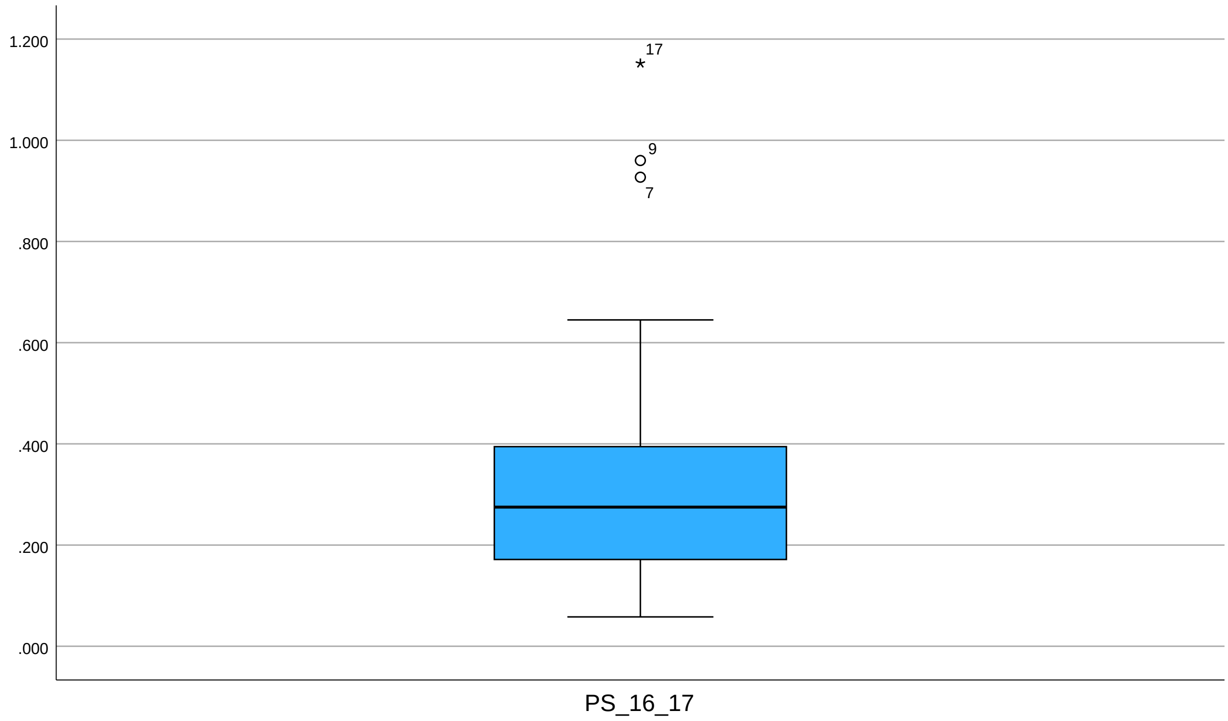

PS_17_18

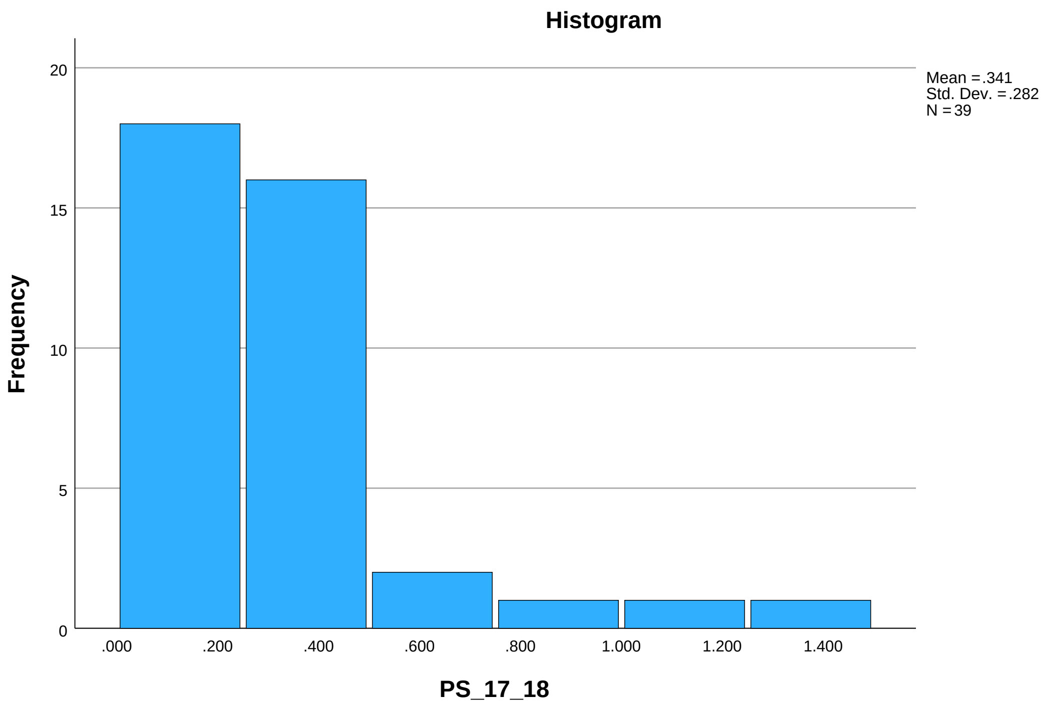

PS_17_18 Stem-and-Leaf Plot

Frequency   Stem & Leaf

```
 2.00     0 . 68
 4.00     1 . 1124
 7.00     1 . 5566777
 5.00     2 . 01334
 4.00     2 . 8889
 3.00     3 . 134
 4.00     3 . 5589
 3.00     4 . 003
 2.00     4 . 68
 1.00     5 . 2
 1.00     5 . 6
 3.00 Extremes    (>=.76)

Stem width:      .100
Each leaf:       1 case(s)
```

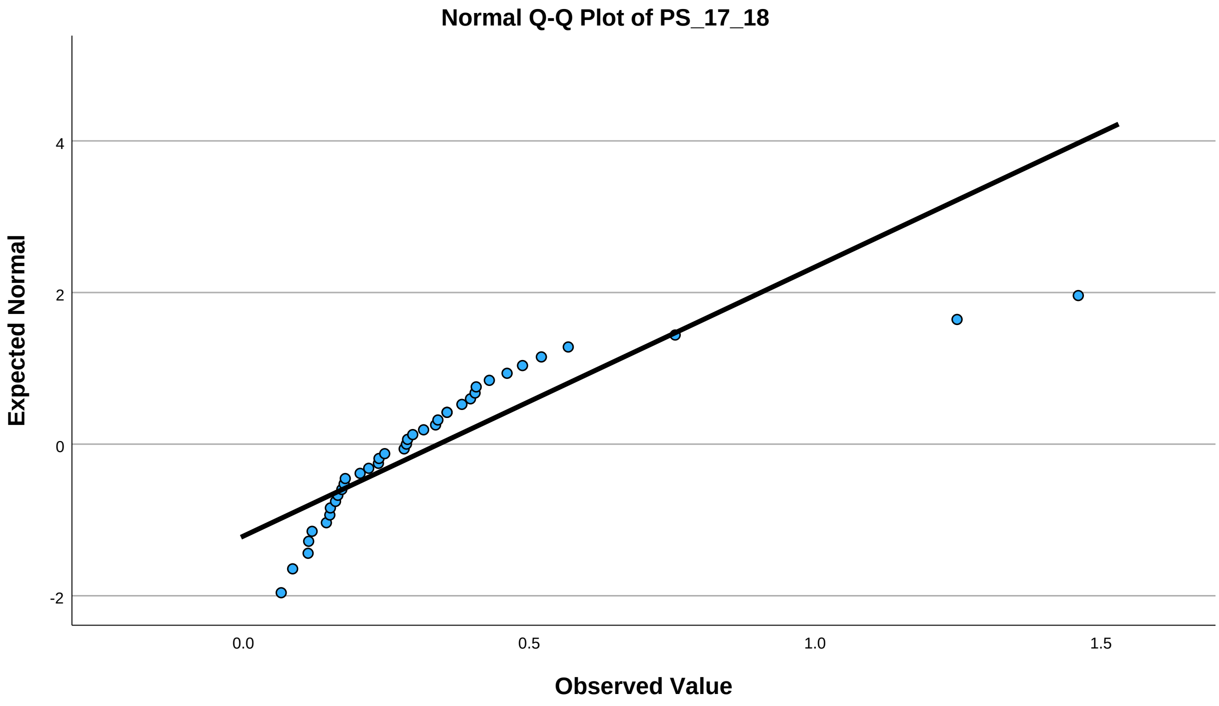

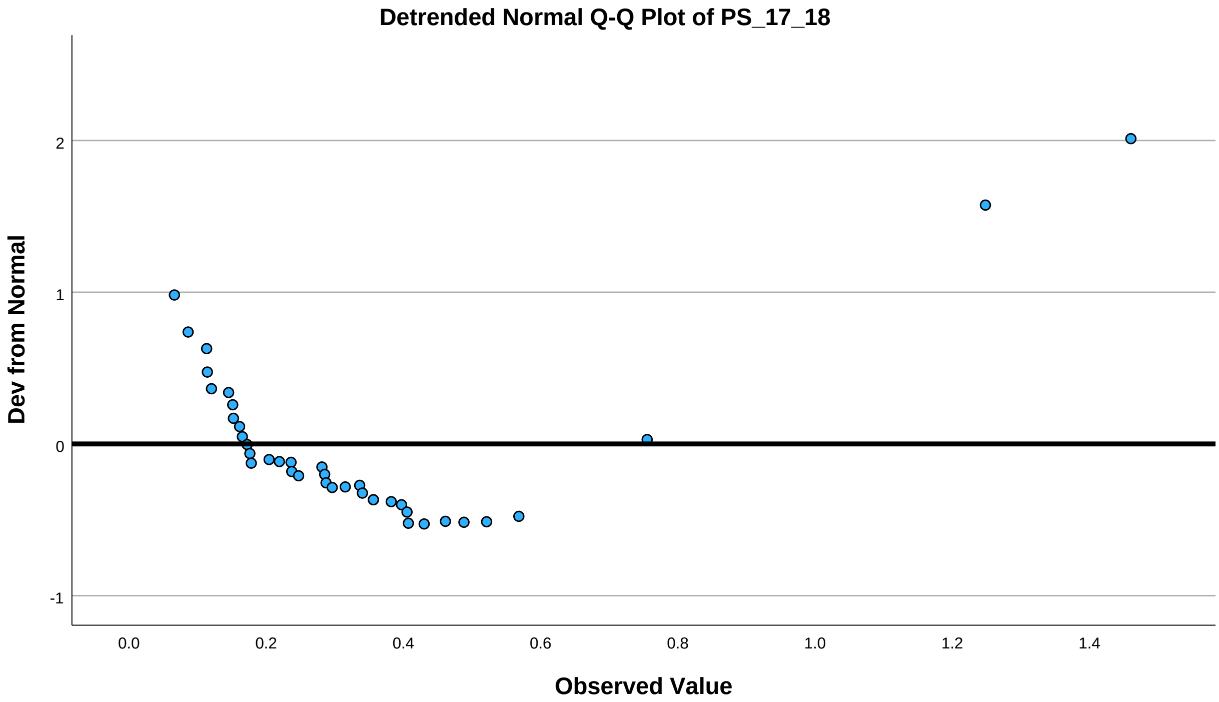
Detrended Normal Q-Q Plot of PS_17_18
Dev from Normal
2
1
0
-1
0.0
0.2
0.4
0.6
0.8
1.0
1.2
1.4
Observed Value

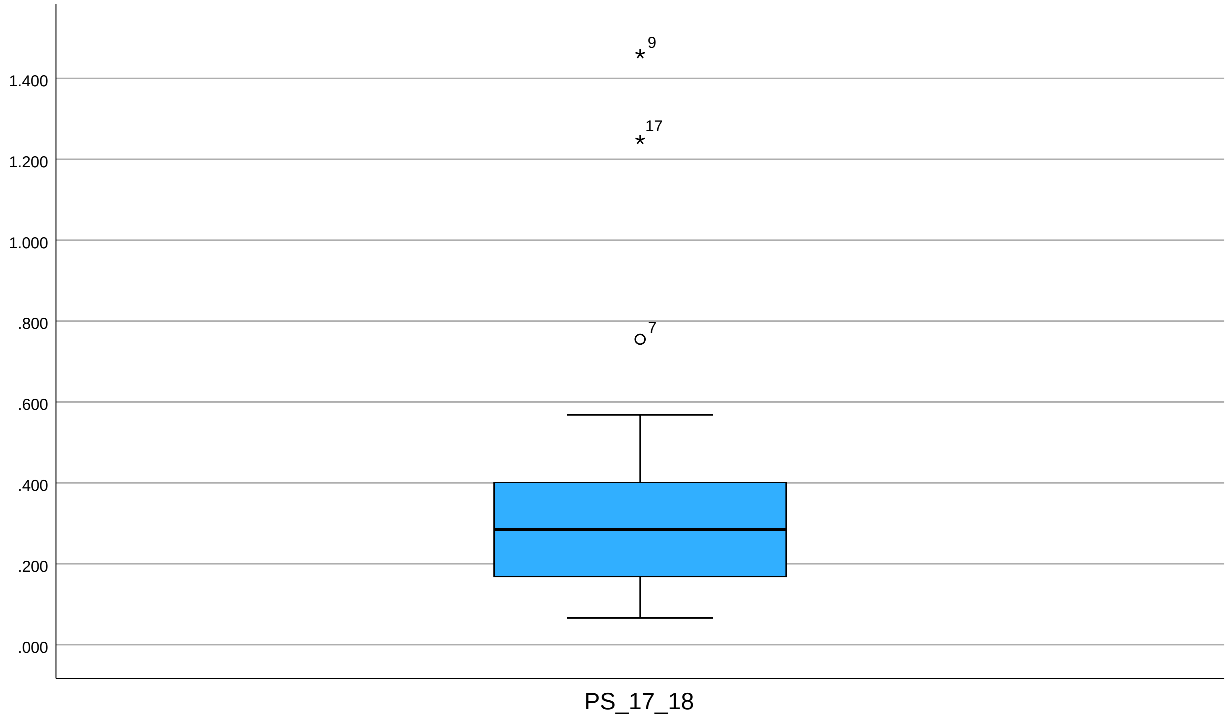
1.400
1.200
1.000
.800
.600
.400
.200
.000
9
17
7
PS_17_18

PS_18_19

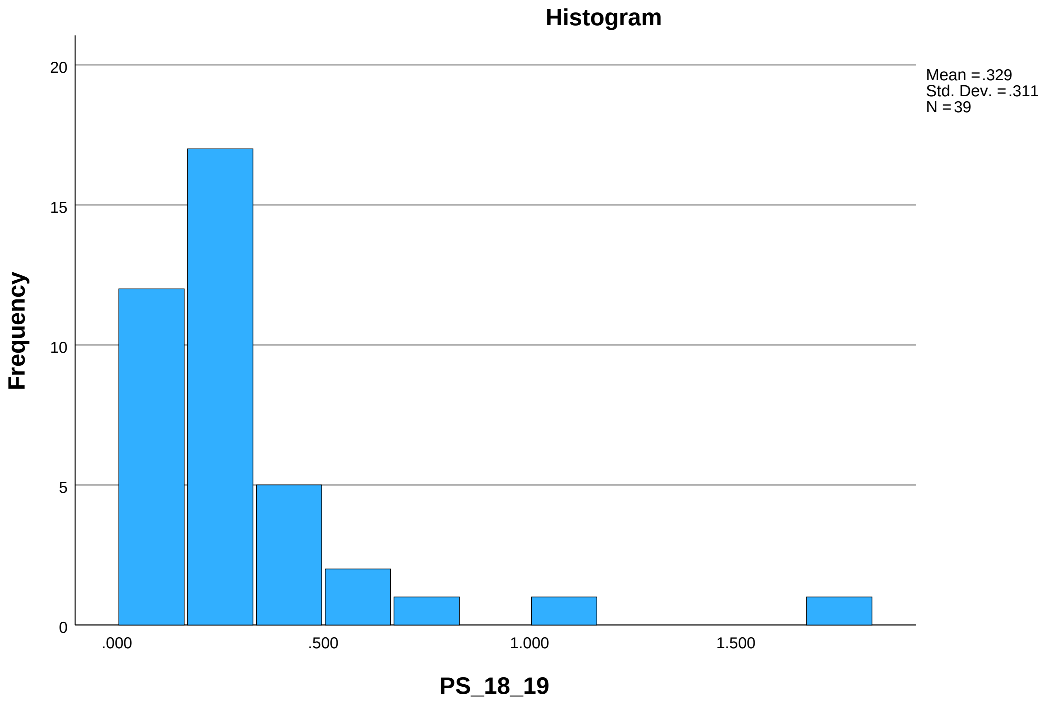

PS_18_19 Stem-and-Leaf Plot

```
 Frequency    Stem &  Leaf

     3.00        0 .  588
    13.00        1 .  0023344568999
     8.00        2 .  02577899
     7.00        3 .  0022389
     3.00        4 .  239
     1.00        5 .  7
     1.00        6 .  2
     3.00 Extremes    (>=.82)

 Stem width:      .100
 Each leaf:       1 case(s)
```

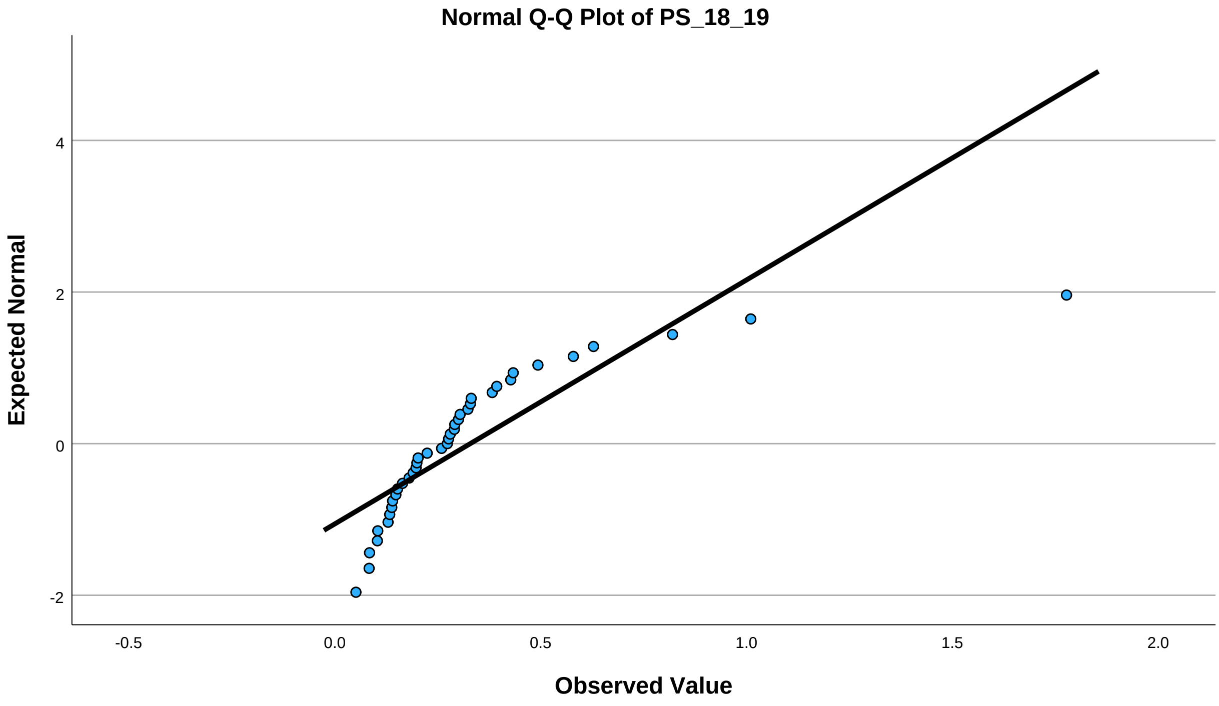
Normal Q-Q Plot of PS_18_19
Expected Normal
4
2
0
-2
-0.5
0.0
0.5
1.0
1.5
2.0
Observed Value

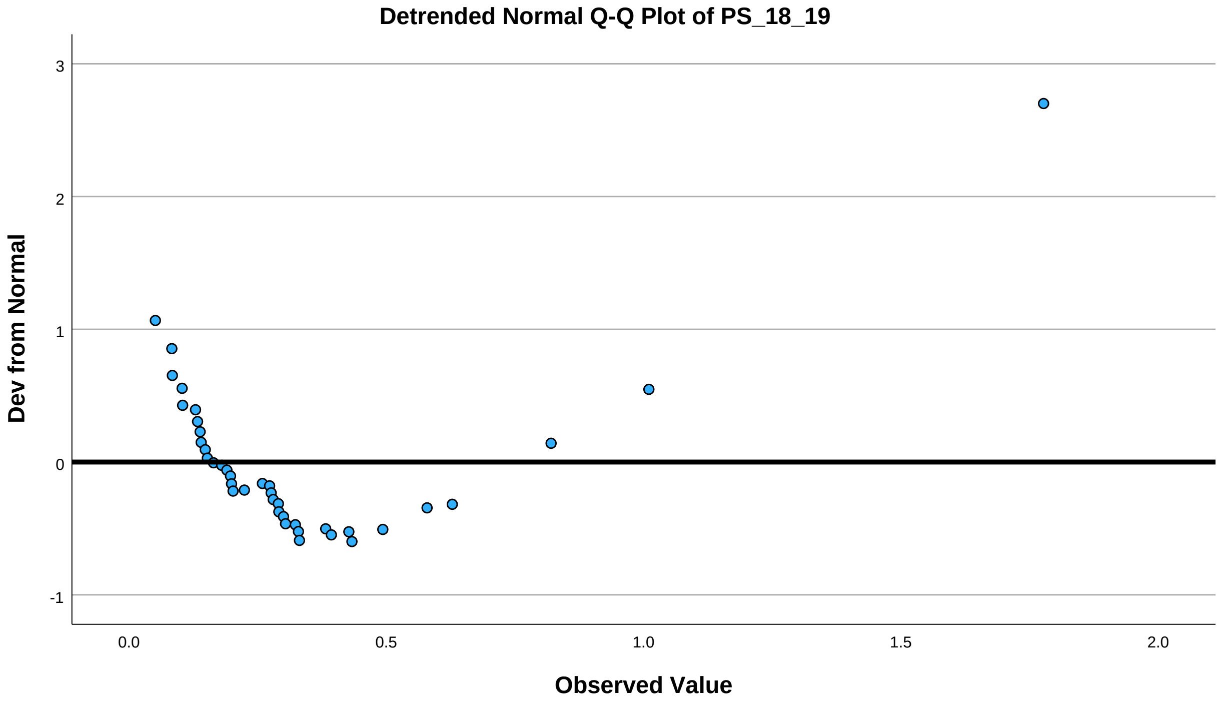
Detrended Normal Q-Q Plot of PS_18_19
Dev from Normal
3
2
1
0
-1
0.0
0.5
1.0
1.5
2.0
Observed Value

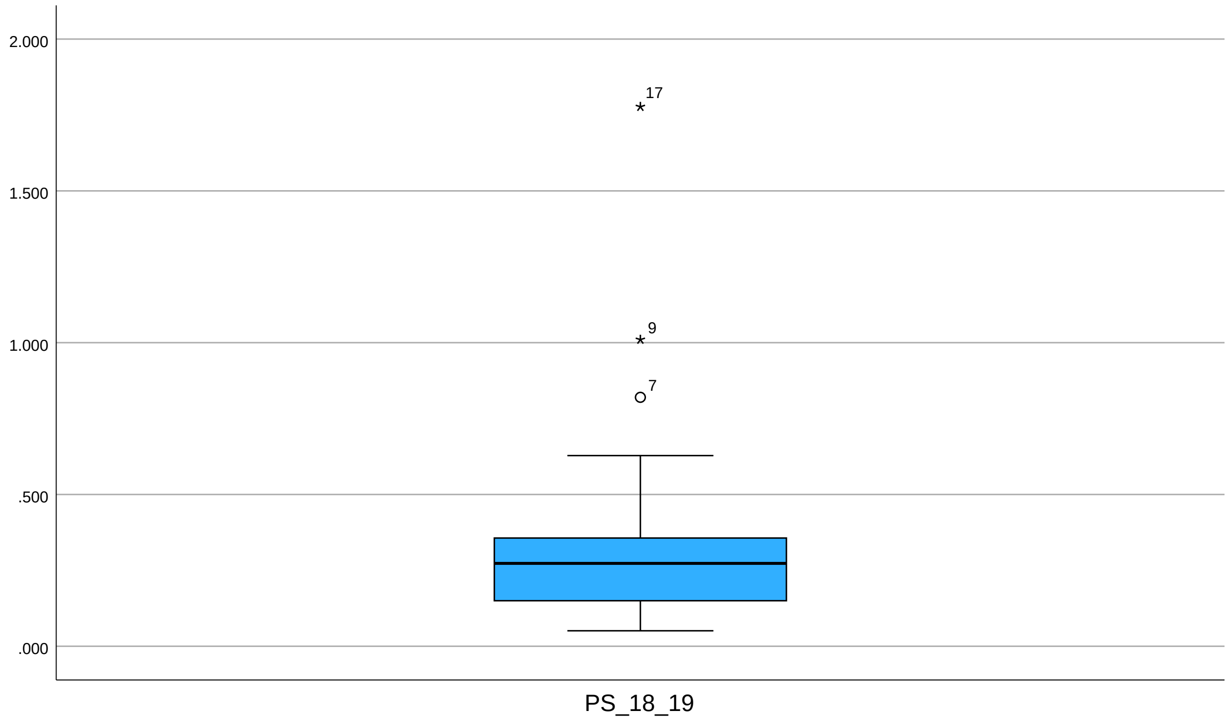

PS_19_20

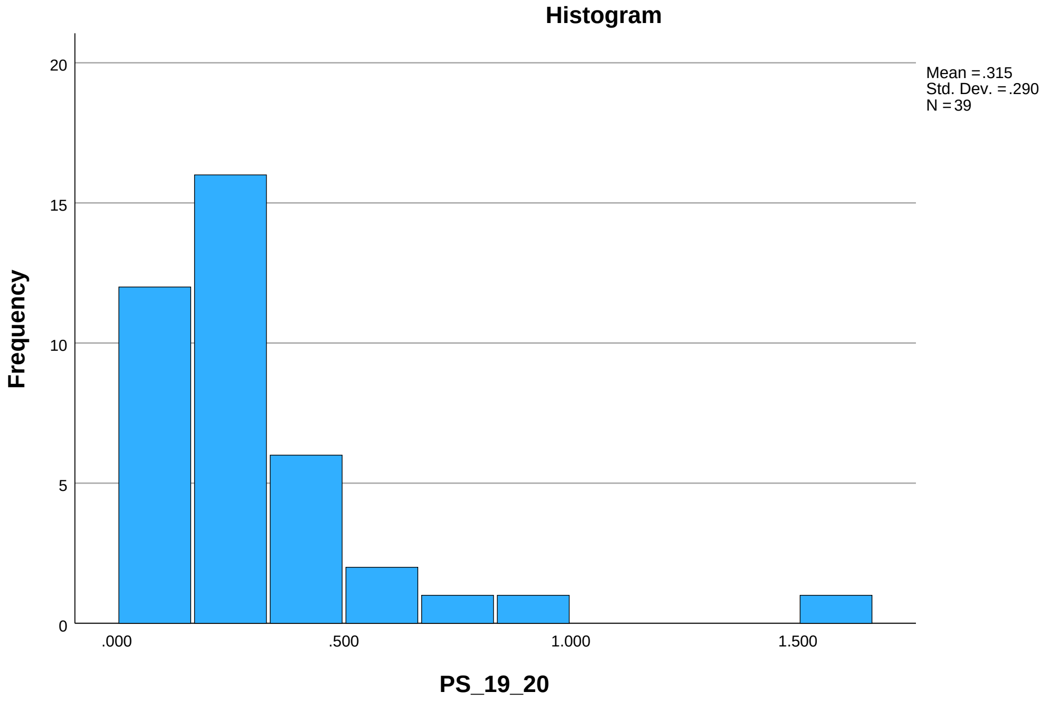


```
PS_19_20 Stem-and-Leaf Plot

 Frequency    Stem &  Leaf
```

```
 4.00      0 . 5789
12.00      1 . 033334566889
10.00      2 . 0222344889
 4.00      3 . 0246
 4.00      4 . 0479
  .00      5 .
 1.00      6 . 1
 4.00 Extremes    (>=.66)

Stem width:      .100
Each leaf:       1 case(s)
```

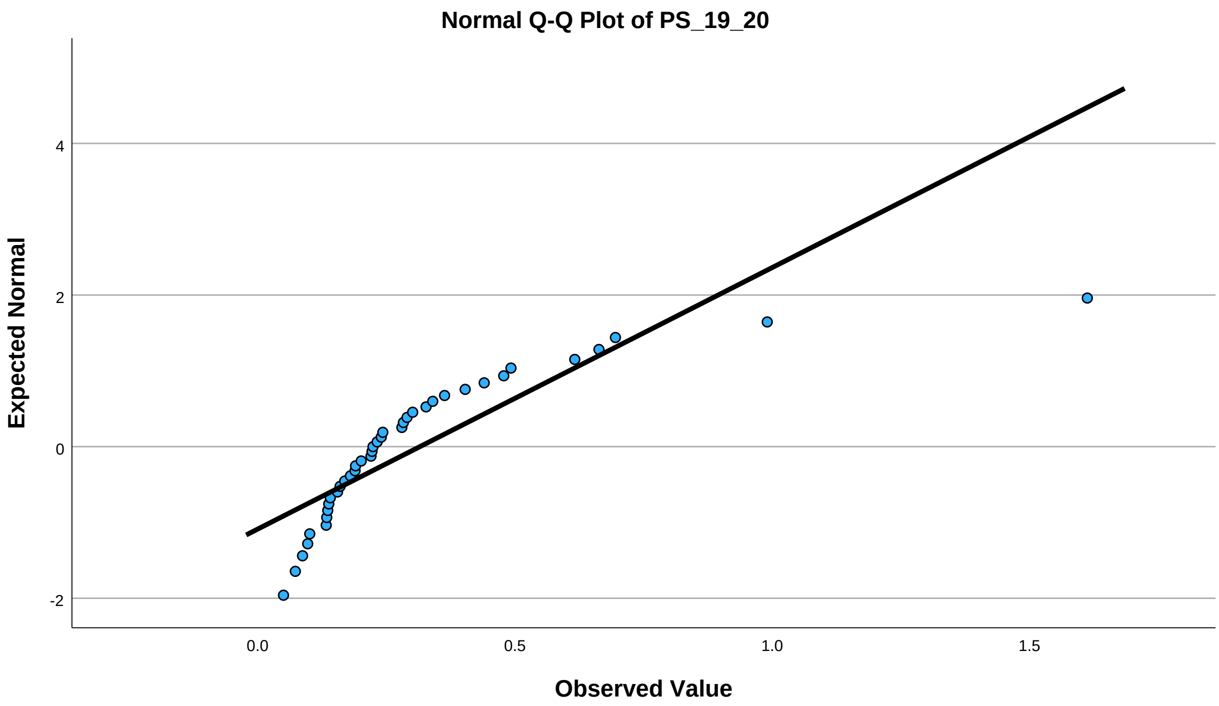

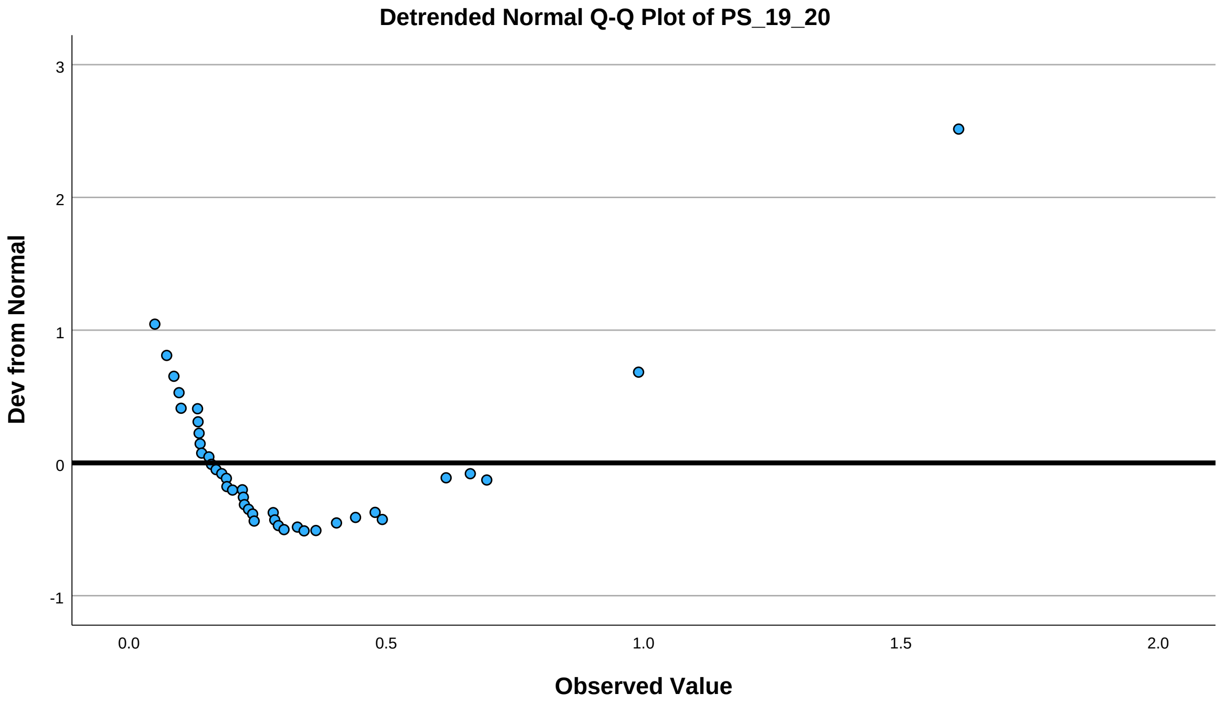
Detrended Normal Q-Q Plot of PS_19_20
Dev from Normal
3
2
1
0
-1
0.0
0.5
1.0
1.5
2.0
Observed Value

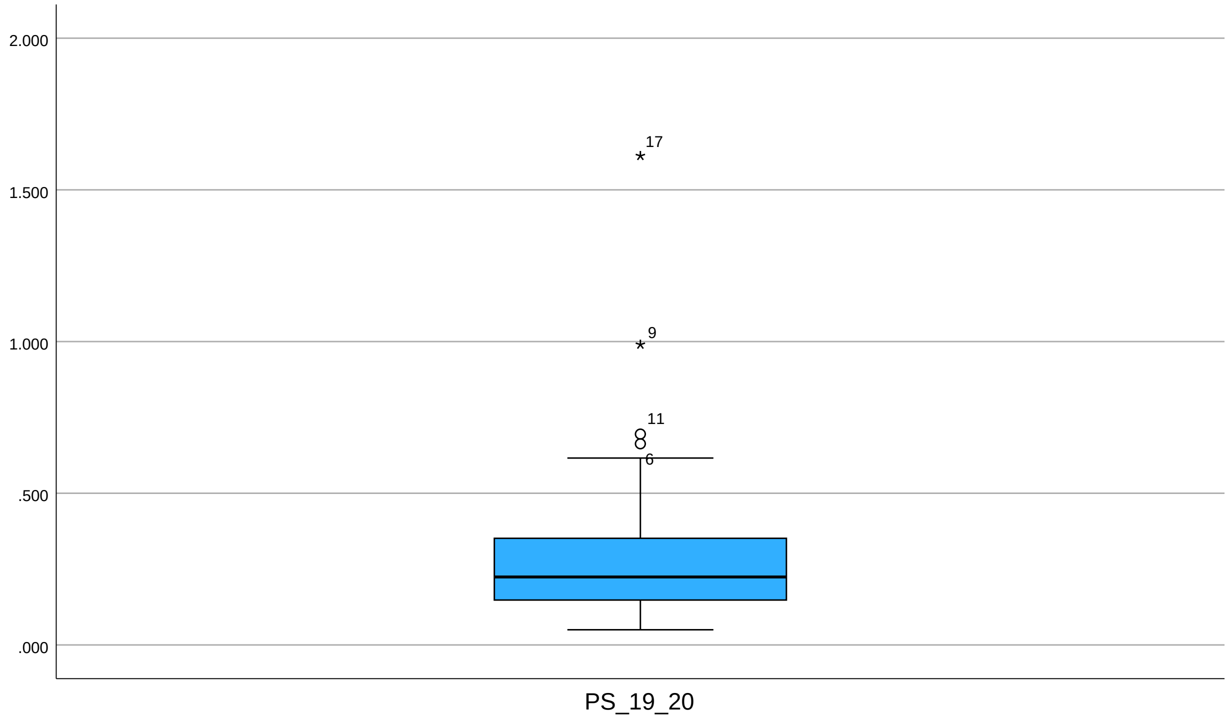
2.000
1.500
1.000
.500
.000
17
9
11
6
PS_19_20

Explore

Case Processing Summary

	Cases					
	Valid		Missing		Total	
	N	Percent	N	Percent	N	Percent
BrainRate	39	100.0%	0	0.0%	39	100.0%
PermutationEntropy	39	100.0%	0	0.0%	39	100.0%
PermutationEntropy_01	39	100.0%	0	0.0%	39	100.0%
SampleEntropy	39	100.0%	0	0.0%	39	100.0%
SpectralEntropy_bits	39	100.0%	0	0.0%	39	100.0%
SpectralEntropy_01	39	100.0%	0	0.0%	39	100.0%
HiguchiFractalDimension	39	100.0%	0	0.0%	39	100.0%
LempelZivComplexity	39	100.0%	0	0.0%	39	100.0%
RenyiEntropy	39	100.0%	0	0.0%	39	100.0%
TsallisEntropy	39	100.0%	0	0.0%	39	100.0%
PS_4_5	39	100.0%	0	0.0%	39	100.0%
PS_5_6	39	100.0%	0	0.0%	39	100.0%
PS_6_7	39	100.0%	0	0.0%	39	100.0%
PS_7_8	39	100.0%	0	0.0%	39	100.0%
PS_8_9	39	100.0%	0	0.0%	39	100.0%
PS_9_10	39	100.0%	0	0.0%	39	100.0%
PS_10_11	39	100.0%	0	0.0%	39	100.0%
PS_11_12	39	100.0%	0	0.0%	39	100.0%
PS_12_13	39	100.0%	0	0.0%	39	100.0%
PS_13_14	39	100.0%	0	0.0%	39	100.0%
PS_14_15	39	100.0%	0	0.0%	39	100.0%
PS_15_16	39	100.0%	0	0.0%	39	100.0%
PS_16_17	39	100.0%	0	0.0%	39	100.0%
PS_17_18	39	100.0%	0	0.0%	39	100.0%
PS_18_19	39	100.0%	0	0.0%	39	100.0%
PS_19_20	39	100.0%	0	0.0%	39	100.0%

Descriptives

			Statistic	Std. Error
BrainRate	Mean		13.4633	.99423
	95% Confidence Interval for Mean	Lower Bound	11.4506	
		Upper Bound	15.4760	
	5% Trimmed Mean		13.0604	
	Median		12.6000	
	Variance		38.551	
	Std. Deviation		6.20896	
	Minimum		6.04	
	Maximum		29.04	
	Range		23.00	
	Interquartile Range		8.32	
	Skewness		.969	.378
	Kurtosis		.213	.741
PermutationEntropy	Mean		1.5422212281	.01078971508
	95% Confidence Interval for Mean	Lower Bound	1.5203785918	
		Upper Bound	1.5640638643	
	5% Trimmed Mean		1.5443589177	
	Median		1.5466218408	
	Variance		.005	
	Std. Deviation		.06738174907	
	Minimum		1.3697619195	
	Maximum		1.6471706031	
	Range		.27740868365	
	Interquartile Range		.10628582168	
	Skewness		-.404	.378
	Kurtosis		-.352	.741
PermutationEntropy_01	Mean		.86073005587	.00602185464
	95% Confidence Interval for Mean	Lower Bound	.84853944848	
		Upper Bound	.87292066327	
	5% Trimmed Mean		.86192312319	
	Median		.86318608462	
	Variance		.001	
	Std. Deviation		.03760647019	
	Minimum		.76447868311	
	Maximum		.91930341735	
	Range		.15482473425	
	Interquartile Range		.05931924653	
	Skewness		-.404	.378
	Kurtosis		-.352	.741

Descriptives

			Statistic	Std. Error
SampleEntropy	Mean		.92730241390	.04371225828
	95% Confidence Interval for Mean	Lower Bound	.83881157334	
		Upper Bound	1.0157932545	
	5% Trimmed Mean		.91715423403	
	Median		.92344600580	
	Variance		.075	
	Std. Deviation		.27298296547	
	Minimum		.48915868726	
	Maximum		1.5602642699	
	Range		1.0711055826	
	Interquartile Range		.41632382896	
	Skewness		.415	.378
	Kurtosis		-.337	.741
SpectralEntropy_bits	Mean		4.7884022127	.09571710751
	95% Confidence Interval for Mean	Lower Bound	4.5946330588	
		Upper Bound	4.9821713665	
	5% Trimmed Mean		4.7771840308	
	Median		4.7261316724	
	Variance		.357	
	Std. Deviation		.59775314479	
	Minimum		3.8226342046	
	Maximum		5.9621074033	
	Range		2.1394731987	
	Interquartile Range		.82067772561	
	Skewness		.242	.378
	Kurtosis		-.634	.741
SpectralEntropy_01	Mean		.68296206045	.01365197618
	95% Confidence Interval for Mean	Lower Bound	.65532507954	
		Upper Bound	.71059904136	
	5% Trimmed Mean		.68136202933	
	Median		.67408051404	
	Variance		.007	
	Std. Deviation		.08525656394	
	Minimum		.54521613197	
	Maximum		.85036573285	
	Range		.30514960088	
	Interquartile Range		.11705193623	
	Skewness		.242	.378
	Kurtosis		-.634	.741

Descriptives

			Statistic	Std. Error
HiguchiFractalDimension	Mean		.39895167956	.02307364860
	95% Confidence Interval for Mean	Lower Bound	.35224152000	
		Upper Bound	.44566183912	
	5% Trimmed Mean		.39762384129	
	Median		.39701908828	
	Variance		.021	
	Std. Deviation		.14409488929	
	Minimum		.15818600032	
	Maximum		.68416925277	
	Range		.52598325245	
	Interquartile Range		.25099806067	
	Skewness		.124	.378
	Kurtosis		-.869	.741
LempelZivComplexity	Mean		.32514500586	.00889590161
	95% Confidence Interval for Mean	Lower Bound	.30713619455	
		Upper Bound	.34315381716	
	5% Trimmed Mean		.32405643692	
	Median		.31882230061	
	Variance		.003	
	Std. Deviation		.05555488775	
	Minimum		.23331917097	
	Maximum		.43527669182	
	Range		.20195752086	
	Interquartile Range		.07607082505	
	Skewness		.354	.378
	Kurtosis		-.713	.741
RenyiEntropy	Mean		7.3932408929	.12101655841
	95% Confidence Interval for Mean	Lower Bound	7.1482556783	
		Upper Bound	7.6382261075	
	5% Trimmed Mean		7.3935428274	
	Median		7.4287036602	
	Variance		.571	
	Std. Deviation		.75574816505	
	Minimum		6.0849811619	
	Maximum		8.6751099165	
	Range		2.5901287546	
	Interquartile Range		1.4098038748	
	Skewness		-.147	.378
	Kurtosis		-1.115	.741

Descriptives

			Statistic	Std. Error
TsallisEntropy	Mean		1.7834740746	.07162877887
	95% Confidence Interval for Mean	Lower Bound	1.6384691927	
		Upper Bound	1.9284789565	
	5% Trimmed Mean		1.8516648540	
	Median		1.9835743254	
	Variance		.200	
	Std. Deviation		.44732158068	
	Minimum		.33782484855	
	Maximum		1.9879736743	
	Range		1.6501488257	
	Interquartile Range		.14120772798	
	Skewness		-2.519	.378
	Kurtosis		5.280	.741
PS_4_5	Mean		2.59603	.349671
	95% Confidence Interval for Mean	Lower Bound	1.88815	
		Upper Bound	3.30390	
	5% Trimmed Mean		2.31163	
	Median		2.16300	
	Variance		4.769	
	Std. Deviation		2.183696	
	Minimum		.479	
	Maximum		13.770	
	Range		13.291	
	Interquartile Range		1.371	
	Skewness		3.741	.378
	Kurtosis		18.170	.741
PS_5_6	Mean		1.84887	.210940
	95% Confidence Interval for Mean	Lower Bound	1.42185	
		Upper Bound	2.27590	
	5% Trimmed Mean		1.68995	
	Median		1.52600	
	Variance		1.735	
	Std. Deviation		1.317317	
	Minimum		.304	
	Maximum		7.900	
	Range		7.596	
	Interquartile Range		1.027	
	Skewness		2.802	.378
	Kurtosis		11.166	.741

Descriptives

			Statistic	Std. Error
PS_6_7	Mean		1.46882	.224207
	95% Confidence Interval for Mean	Lower Bound	1.01494	
		Upper Bound	1.92270	
	5% Trimmed Mean		1.27790	
	Median		1.16700	
	Variance		1.960	
	Std. Deviation		1.400172	
	Minimum		.276	
	Maximum		9.030	
	Range		8.754	
	Interquartile Range		.988	
	Skewness		4.338	.378
	Kurtosis		23.089	.741
PS_7_8	Mean		1.25923	.172370
	95% Confidence Interval for Mean	Lower Bound	.91029	
		Upper Bound	1.60818	
	5% Trimmed Mean		1.10109	
	Median		1.00500	
	Variance		1.159	
	Std. Deviation		1.076453	
	Minimum		.236	
	Maximum		6.380	
	Range		6.144	
	Interquartile Range		.837	
	Skewness		3.257	.378
	Kurtosis		13.551	.741
PS_8_9	Mean		1.21241	.155348
	95% Confidence Interval for Mean	Lower Bound	.89793	
		Upper Bound	1.52690	
	5% Trimmed Mean		1.08301	
	Median		.94500	
	Variance		.941	
	Std. Deviation		.970147	
	Minimum		.231	
	Maximum		5.510	
	Range		5.279	
	Interquartile Range		.734	
	Skewness		2.726	.378
	Kurtosis		9.844	.741

Descriptives

			Statistic	Std. Error
PS_9_10	Mean		1.26241	.177705
	95% Confidence Interval for Mean	Lower Bound	.90266	
		Upper Bound	1.62216	
	5% Trimmed Mean		1.10308	
	Median		1.01700	
	Variance		1.232	
	Std. Deviation		1.109769	
	Minimum		.241	
	Maximum		6.360	
	Range		6.119	
	Interquartile Range		.918	
	Skewness		2.984	.378
	Kurtosis		11.598	.741
PS_10_11	Mean		1.58238	.221907
	95% Confidence Interval for Mean	Lower Bound	1.13316	
		Upper Bound	2.03161	
	5% Trimmed Mean		1.43809	
	Median		1.13400	
	Variance		1.920	
	Std. Deviation		1.385806	
	Minimum		.227	
	Maximum		5.920	
	Range		5.693	
	Interquartile Range		1.420	
	Skewness		1.700	.378
	Kurtosis		2.441	.741
PS_11_12	Mean		.83236	.107172
	95% Confidence Interval for Mean	Lower Bound	.61540	
		Upper Bound	1.04932	
	5% Trimmed Mean		.74724	
	Median		.64300	
	Variance		.448	
	Std. Deviation		.669290	
	Minimum		.223	
	Maximum		3.180	
	Range		2.957	
	Interquartile Range		.670	
	Skewness		1.951	.378
	Kurtosis		4.140	.741

Descriptives

			Statistic	Std. Error
PS_12_13	Mean		.67023	.079279
	95% Confidence Interval for Mean	Lower Bound	.50974	
		Upper Bound	.83072	
	5% Trimmed Mean		.61130	
	Median		.56600	
	Variance		.245	
	Std. Deviation		.495100	
	Minimum		.158	
	Maximum		2.460	
	Range		2.302	
	Interquartile Range		.426	
	Skewness		1.928	.378
	Kurtosis		4.300	.741
PS_13_14	Mean		.59826	.072670
	95% Confidence Interval for Mean	Lower Bound	.45114	
		Upper Bound	.74537	
	5% Trimmed Mean		.55915	
	Median		.45800	
	Variance		.206	
	Std. Deviation		.453824	
	Minimum		.135	
	Maximum		1.802	
	Range		1.667	
	Interquartile Range		.463	
	Skewness		1.505	.378
	Kurtosis		1.544	.741
PS_14_15	Mean		.59508	.070458
	95% Confidence Interval for Mean	Lower Bound	.45244	
		Upper Bound	.73771	
	5% Trimmed Mean		.55422	
	Median		.42700	
	Variance		.194	
	Std. Deviation		.440009	
	Minimum		.137	
	Maximum		1.808	
	Range		1.671	
	Interquartile Range		.578	
	Skewness		1.227	.378
	Kurtosis		.908	.741

Descriptives

			Statistic	Std. Error
PS_15_16	Mean		.60556	.078538
	95% Confidence Interval for Mean	Lower Bound	.44657	
		Upper Bound	.76456	
	5% Trimmed Mean		.55740	
	Median		.42500	
	Variance		.241	
	Std. Deviation		.490472	
	Minimum		.117	
	Maximum		2.285	
	Range		2.168	
	Interquartile Range		.517	
	Skewness		1.567	.378
	Kurtosis		2.569	.741
PS_16_17	Mean		.63821	.094306
	95% Confidence Interval for Mean	Lower Bound	.44729	
		Upper Bound	.82912	
	5% Trimmed Mean		.57134	
	Median		.37100	
	Variance		.347	
	Std. Deviation		.588940	
	Minimum		.128	
	Maximum		2.466	
	Range		2.338	
	Interquartile Range		.580	
	Skewness		1.805	.378
	Kurtosis		2.944	.741
PS_17_18	Mean		.62328	.090257
	95% Confidence Interval for Mean	Lower Bound	.44057	
		Upper Bound	.80600	
	5% Trimmed Mean		.56377	
	Median		.38700	
	Variance		.318	
	Std. Deviation		.563657	
	Minimum		.114	
	Maximum		2.307	
	Range		2.193	
	Interquartile Range		.609	
	Skewness		1.636	.378
	Kurtosis		2.294	.741

Descriptives

			Statistic	Std. Error
PS_18_19	Mean		.64749	.103562
	95% Confidence Interval for Mean	Lower Bound	.43784	
		Upper Bound	.85714	
	5% Trimmed Mean		.56384	
	Median		.39500	
	Variance		.418	
	Std. Deviation		.646744	
	Minimum		.098	
	Maximum		2.932	
	Range		2.834	
	Interquartile Range		.643	
	Skewness		2.145	.378
	Kurtosis		4.720	.741
PS_19_20	Mean		.64887	.102769
	95% Confidence Interval for Mean	Lower Bound	.44083	
		Upper Bound	.85692	
	5% Trimmed Mean		.57492	
	Median		.42200	
	Variance		.412	
	Std. Deviation		.641790	
	Minimum		.086	
	Maximum		2.669	
	Range		2.583	
	Interquartile Range		.575	
	Skewness		1.835	.378
	Kurtosis		2.937	.741

Tests of Normality

	Kolmogorov-Smirnov[a]			Shapiro-Wilk		
	Statistic	df	Sig.	Statistic	df	Sig.
BrainRate	.165	39	.009	.902	39	.003
PermutationEntropy	.079	39	.200*	.969	39	.344
PermutationEntropy_01	.079	39	.200*	.969	39	.344
SampleEntropy	.078	39	.200*	.971	39	.412
SpectralEntropy_bits	.074	39	.200*	.964	39	.243
SpectralEntropy_01	.074	39	.200*	.964	39	.243
HiguchiFractalDimension	.087	39	.200*	.967	39	.313
LempelZivComplexity	.093	39	.200*	.962	39	.214
RenyiEntropy	.128	39	.109	.950	39	.083
TsallisEntropy	.343	39	<.001	.515	39	<.001
PS_4_5	.234	39	<.001	.638	39	<.001
PS_5_6	.190	39	.001	.751	39	<.001
PS_6_7	.197	39	<.001	.567	39	<.001
PS_7_8	.227	39	<.001	.674	39	<.001
PS_8_9	.213	39	<.001	.740	39	<.001
PS_9_10	.220	39	<.001	.705	39	<.001
PS_10_11	.187	39	.001	.796	39	<.001
PS_11_12	.193	39	<.001	.789	39	<.001
PS_12_13	.228	39	<.001	.806	39	<.001
PS_13_14	.180	39	.003	.807	39	<.001
PS_14_15	.174	39	.005	.858	39	<.001
PS_15_16	.189	39	.001	.835	39	<.001
PS_16_17	.214	39	<.001	.769	39	<.001
PS_17_18	.186	39	.002	.795	39	<.001
PS_18_19	.216	39	<.001	.739	39	<.001
PS_19_20	.245	39	<.001	.762	39	<.001

*. This is a lower bound of the true significance.

a. Lilliefors Significance Correction

BrainRate

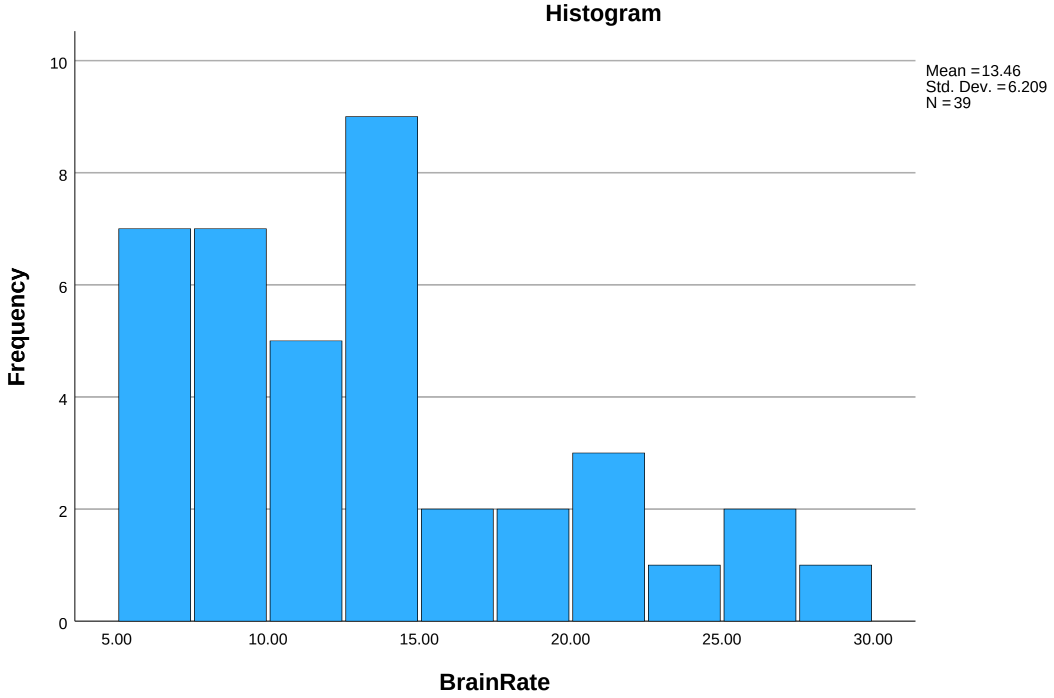


```
BrainRate Stem-and-Leaf Plot

 Frequency    Stem &  Leaf

    14.00        0 .  66666777899999
    14.00        1 .  01111223333334
     4.00        1 .  5789
     4.00        2 .  0013
     2.00        2 .  67
     1.00 Extremes    (>=29)

 Stem width:      10.00
 Each leaf:        1 case(s)
```

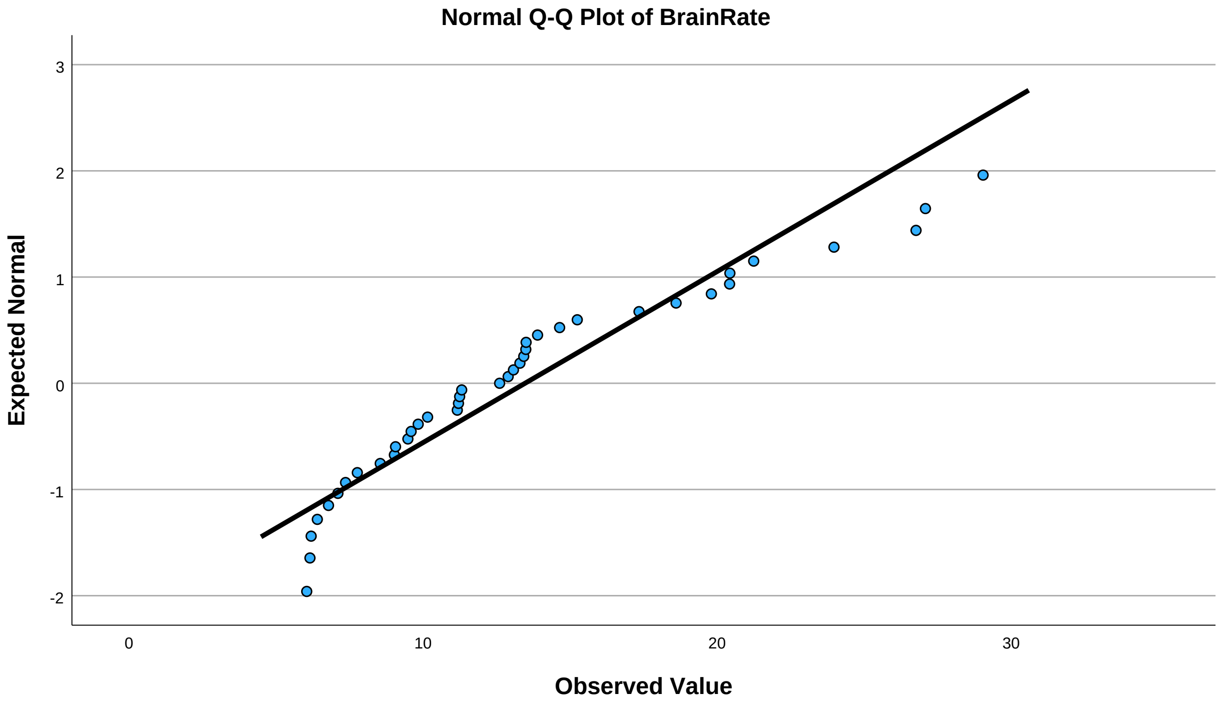
Normal Q-Q Plot of BrainRate
Expected Normal
3
2
1
0
-1
-2
0
10
20
30
Observed Value

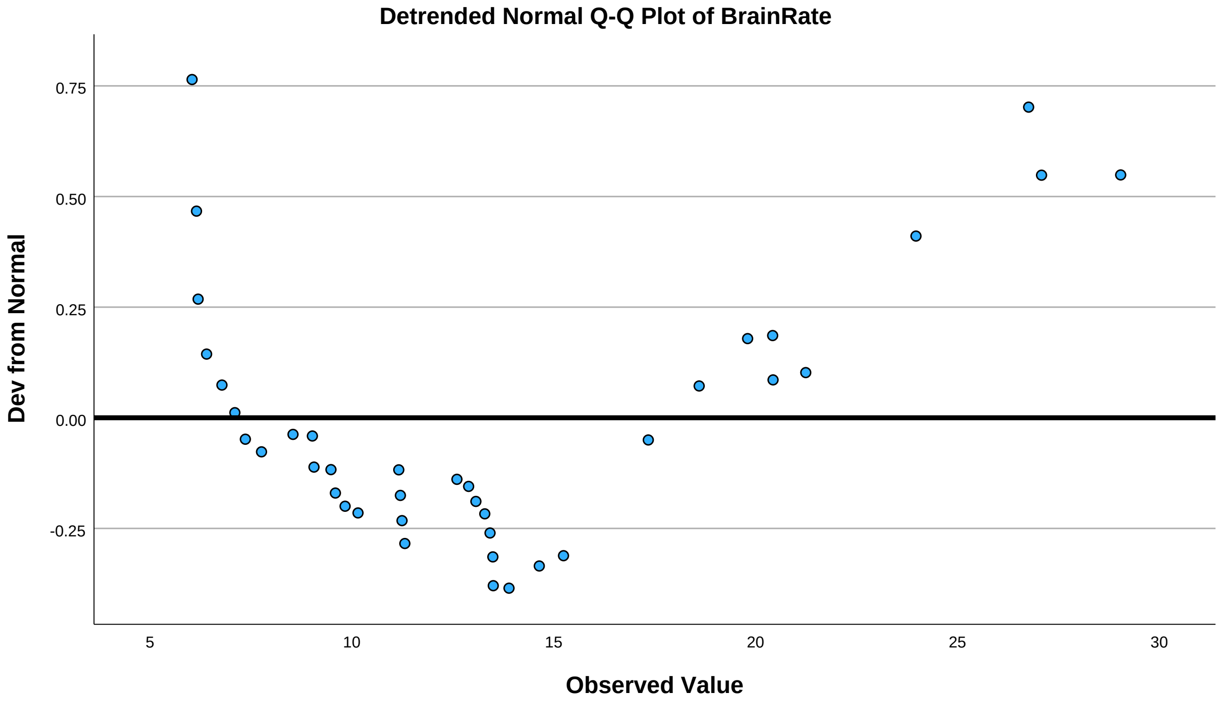
Detrended Normal Q-Q Plot of BrainRate
Dev from Normal
0.75
0.50
0.25
0.00
-0.25
5
10
15
20
25
30
Observed Value

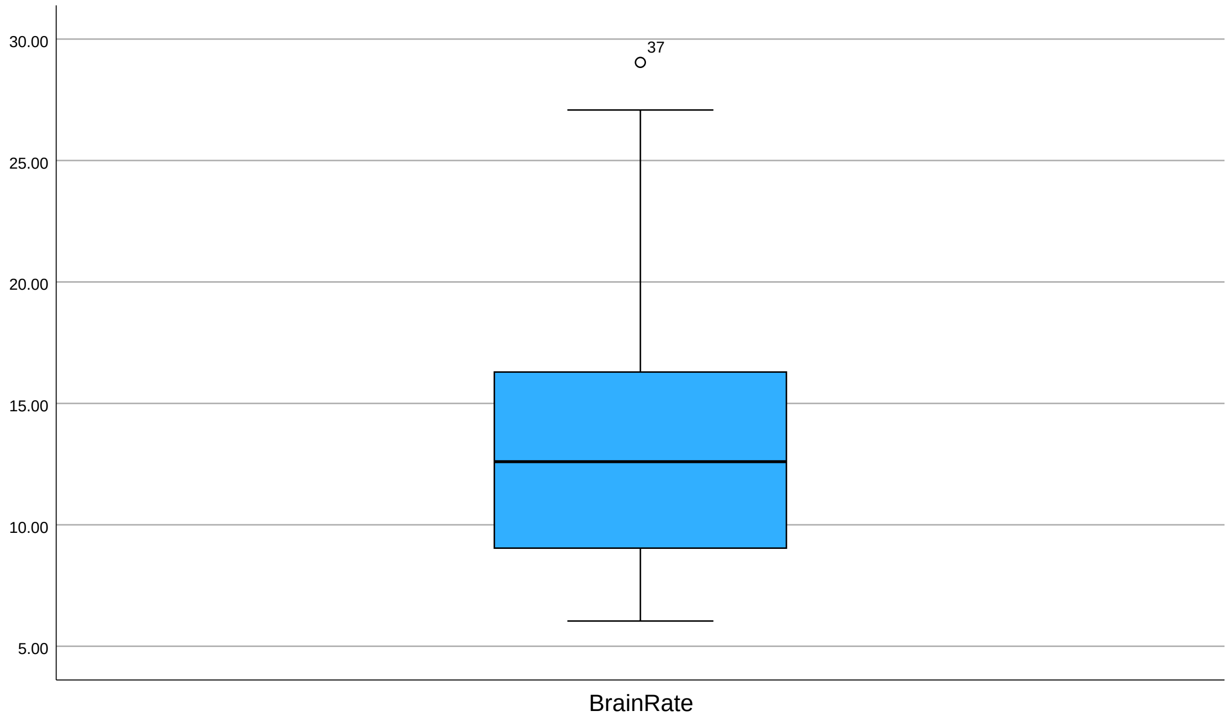

PermutationEntropy

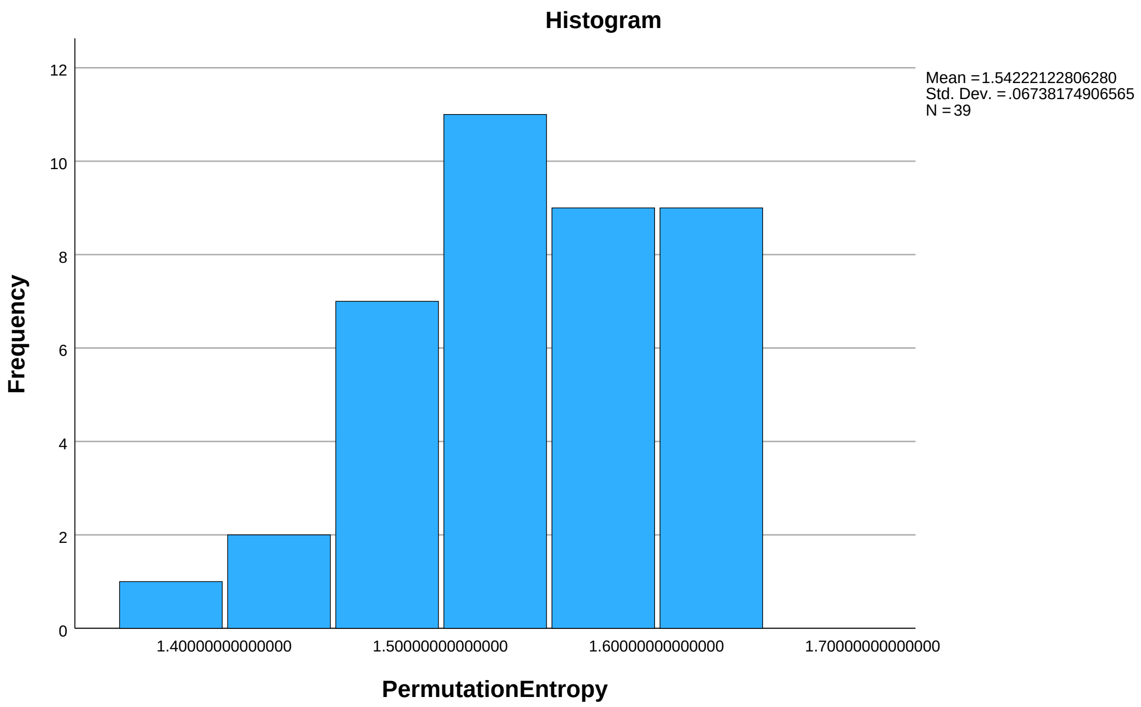

PermutationEntropy Stem-and-Leaf Plot

Frequency Stem & Leaf

```
  1.00      13 .  6
  2.00      14 .  34
  7.00      14 .  5556688
 11.00      15 .  11112334444
  9.00      15 .  555678999
  9.00      16 .  011123444

Stem width:   .1000000
Each leaf:        1 case(s)
```

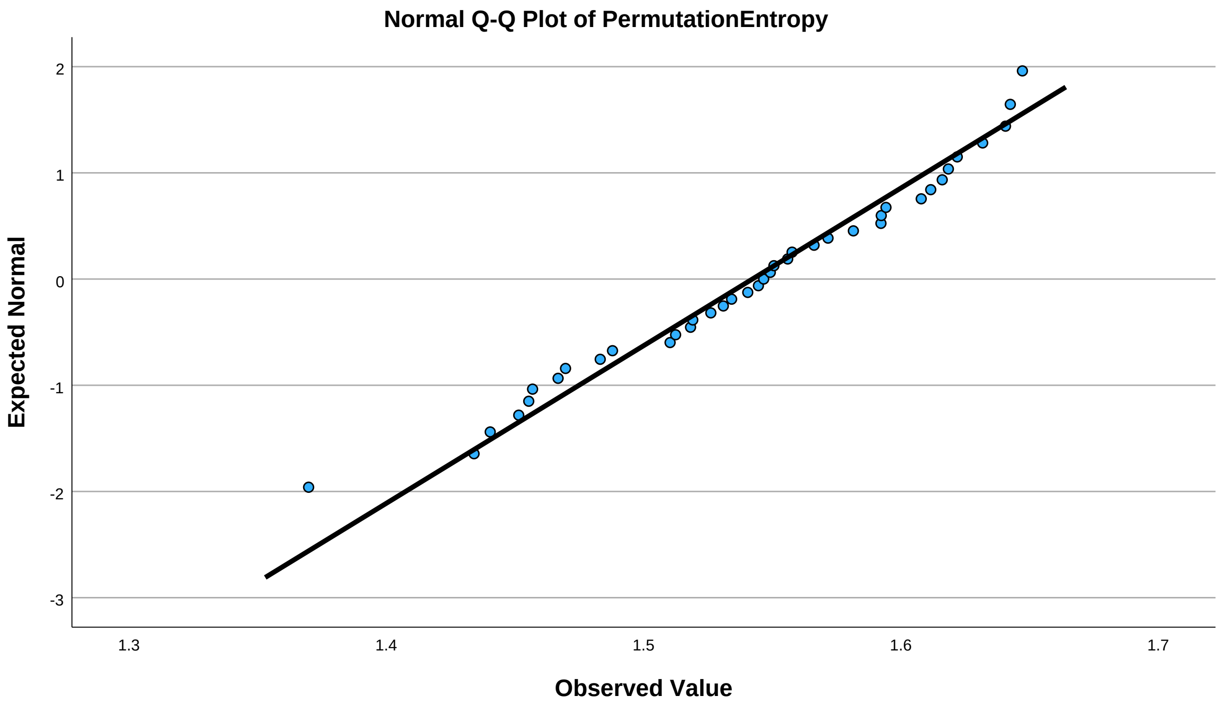

Detrended Normal Q-Q Plot of PermutationEntropy

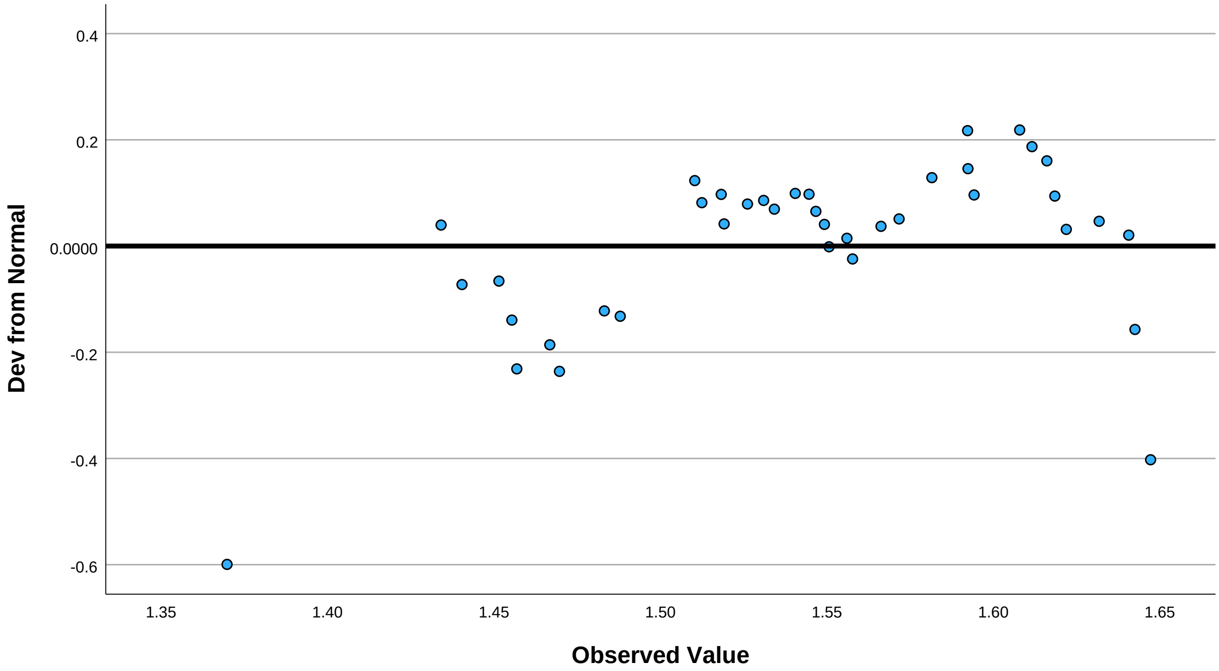

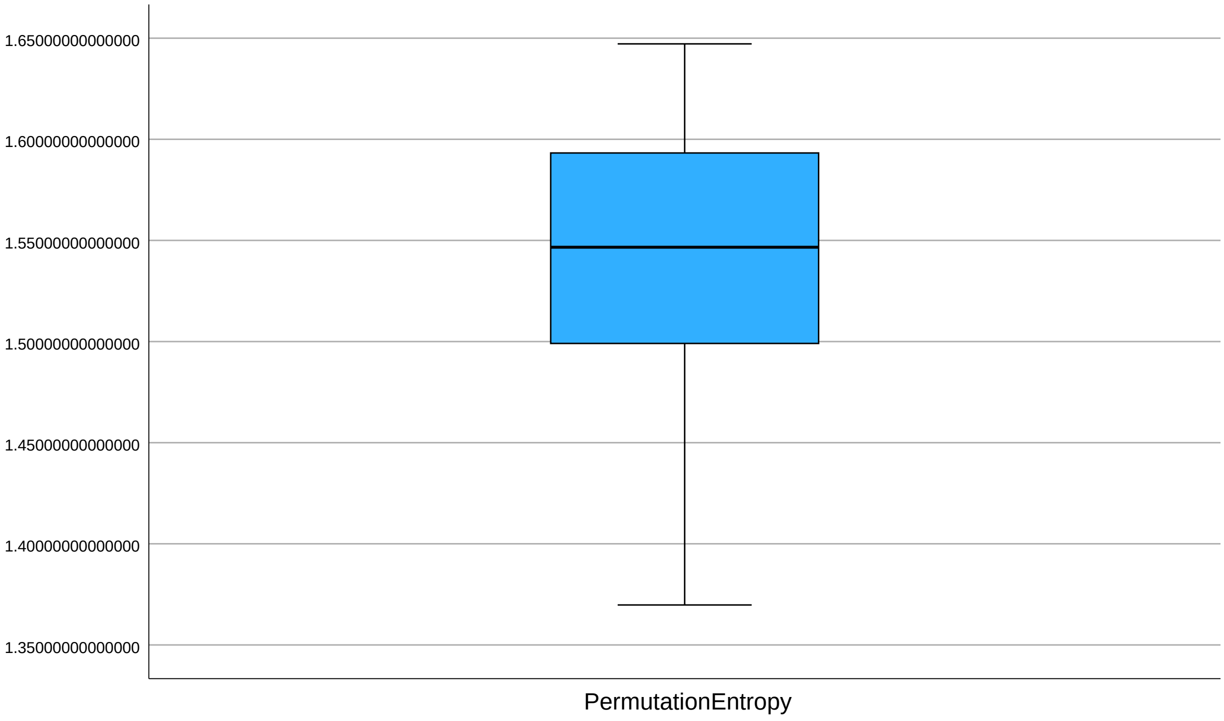

PermutationEntropy_01

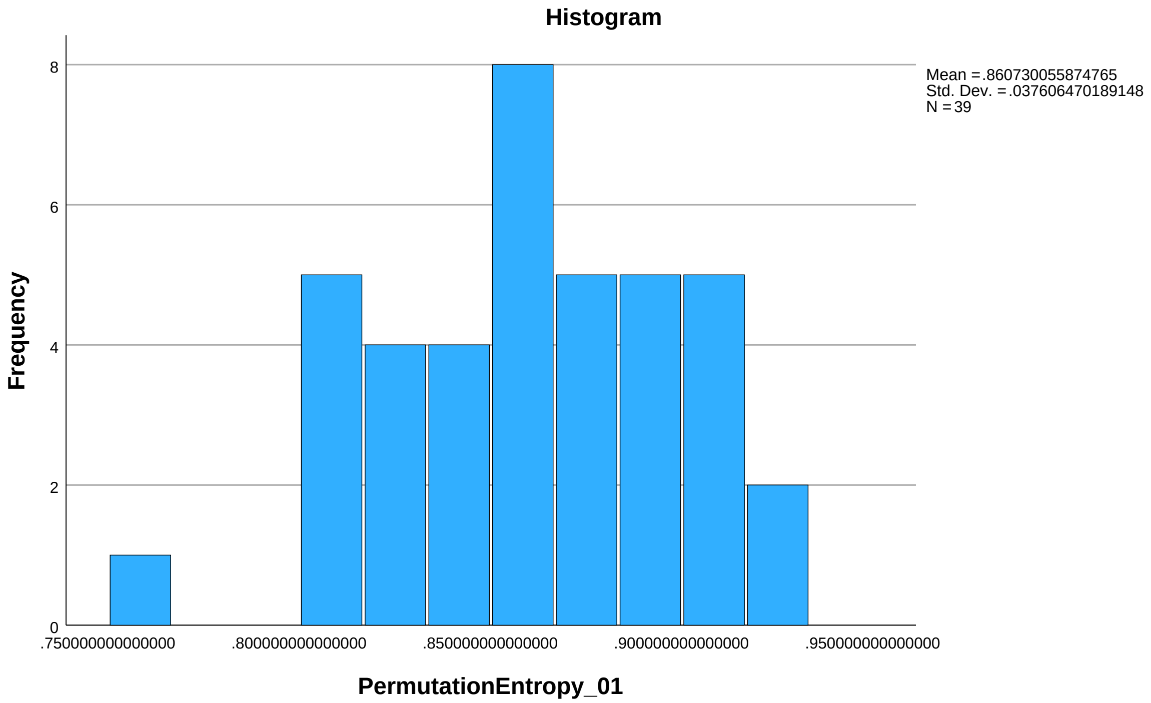


```
PermutationEntropy_01 Stem-and-Leaf Plot

 Frequency    Stem &  Leaf

     1.00        7 .  6
    13.00        8 .  0011112234444
    18.00        8 .  555566666677888899
     7.00        9 .  0001111

 Stem width:   .1000000
 Each leaf:        1 case(s)
```

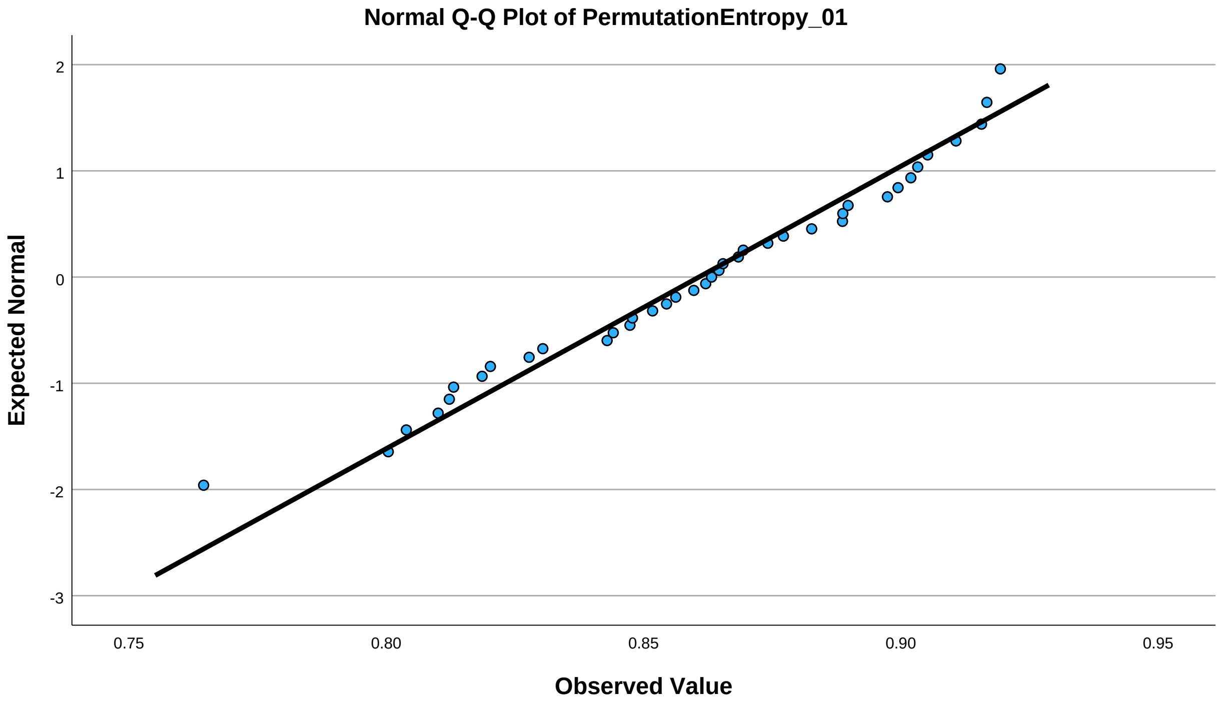
Normal Q-Q Plot of PermutationEntropy_01
Expected Normal
2
1
0
-1
-2
-3
0.75
0.80
0.85
0.90
0.95
Observed Value

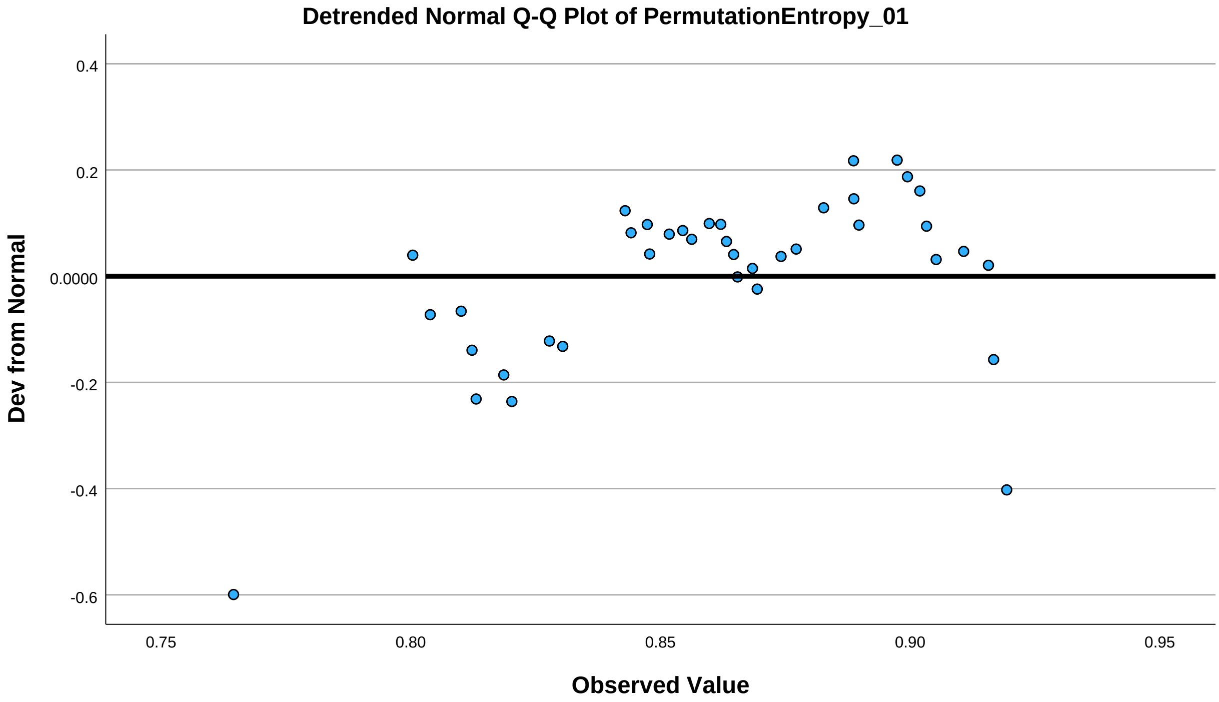
Detrended Normal Q-Q Plot of PermutationEntropy_01
Dev from Normal
0.4
0.2
0.0000
-0.2
-0.4
-0.6
0.75
0.80
0.85
0.90
0.95
Observed Value

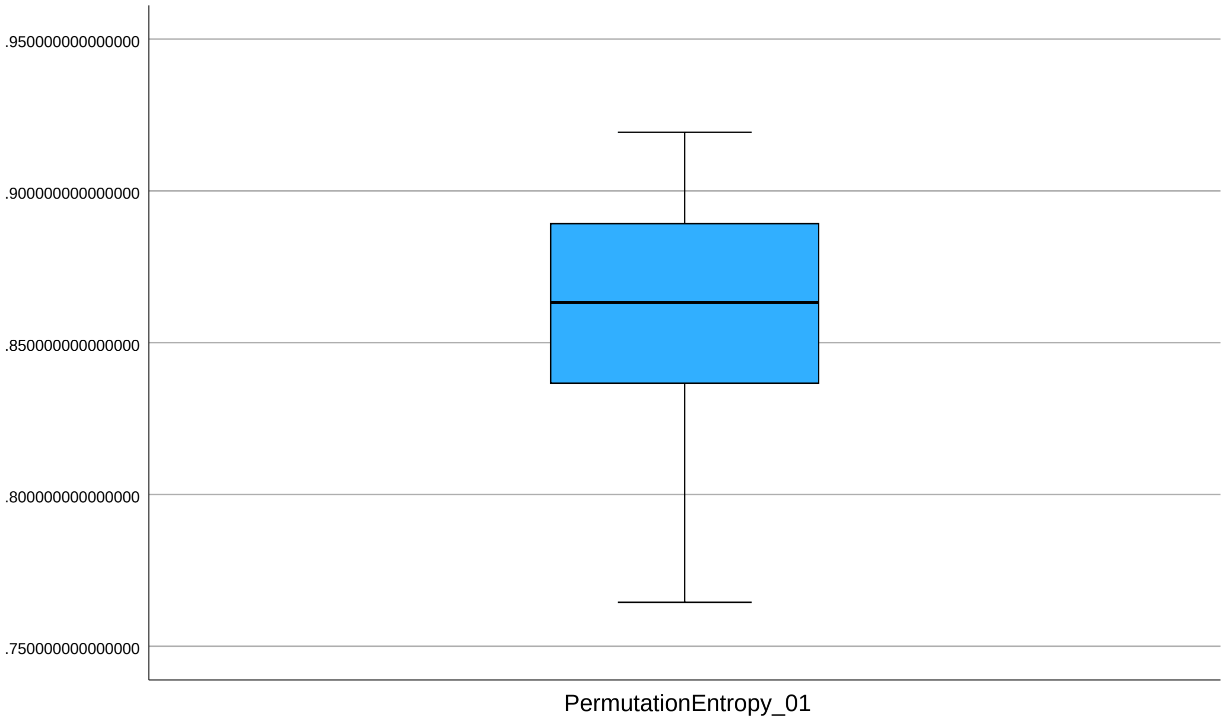

SampleEntropy

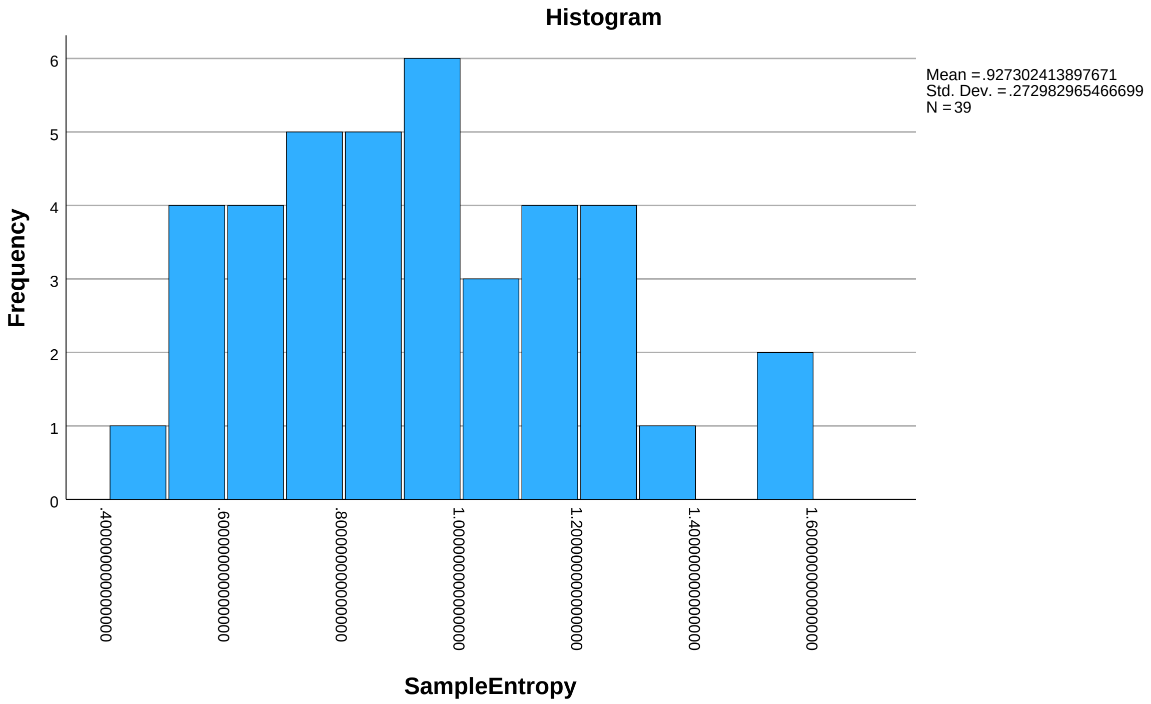

SampleEntropy Stem-and-Leaf Plot

Frequency   Stem & Leaf

```
 1.00      4 . 8
 4.00      5 . 1557
 4.00      6 . 2357
 5.00      7 . 14579
 5.00      8 . 35689
 6.00      9 . 233367
 3.00     10 . 244
 4.00     11 . 3357
 4.00     12 . 0236
 1.00     13 . 9
  .00     14 .
 2.00     15 . 16

Stem width:   .1000000
Each leaf:        1 case(s)
```

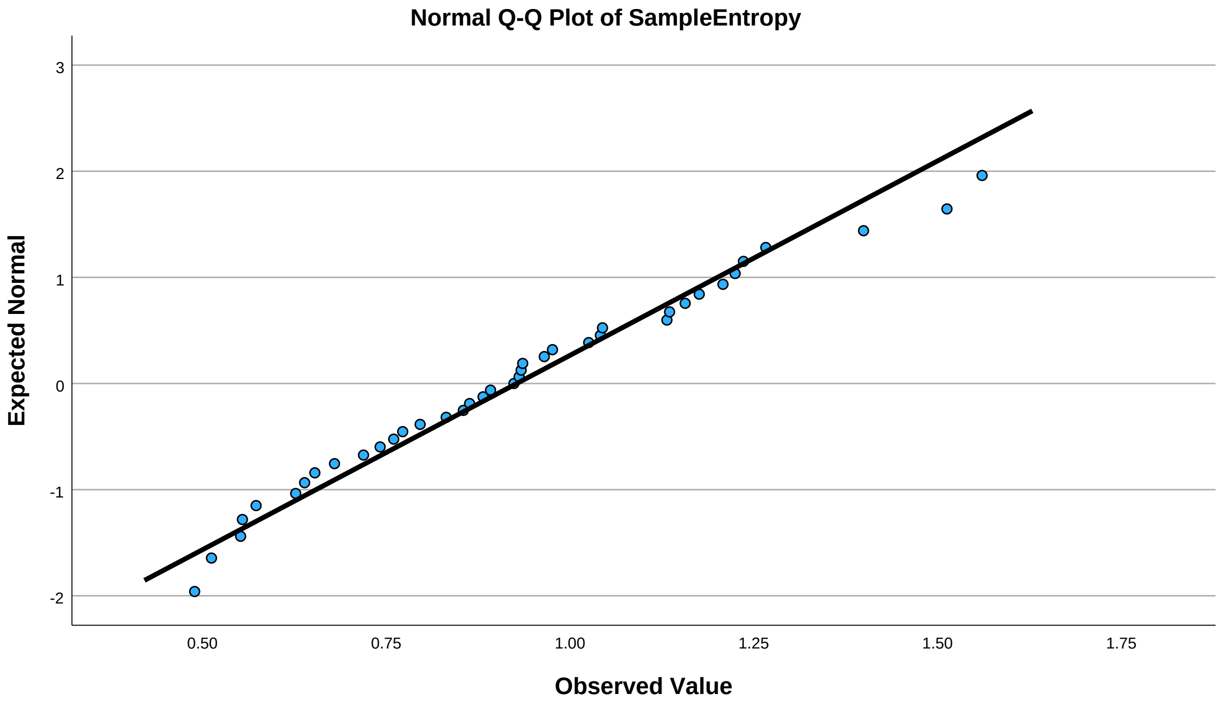

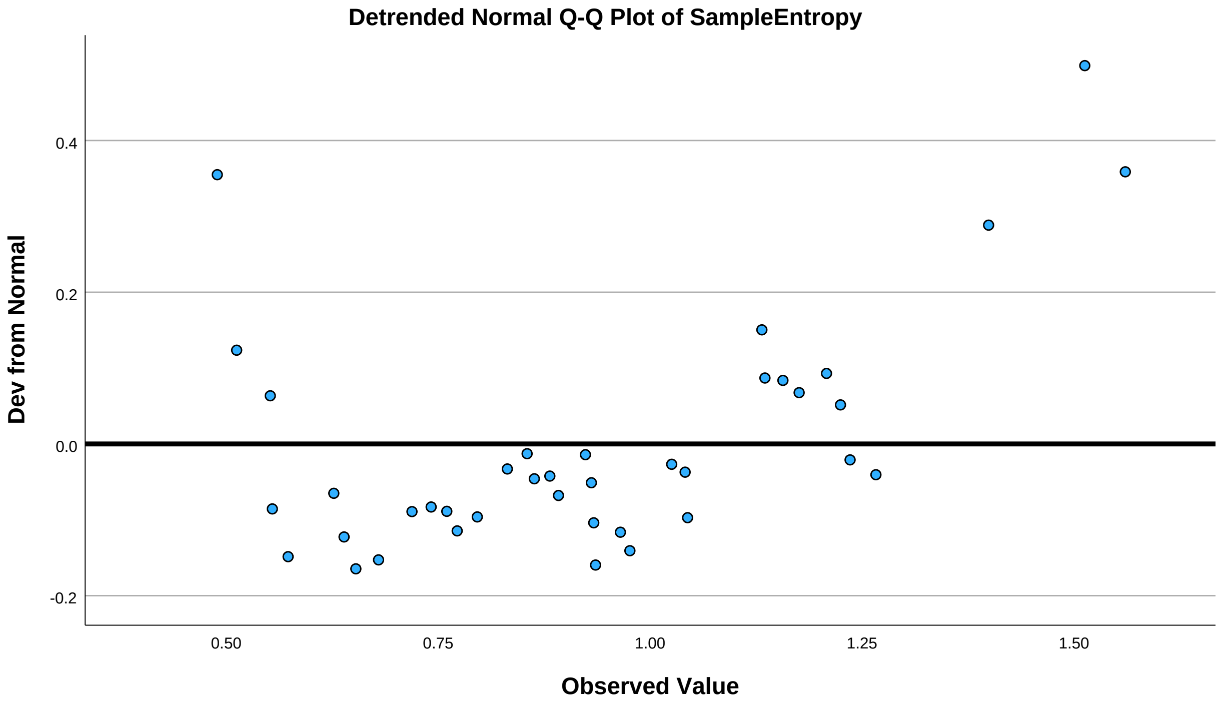

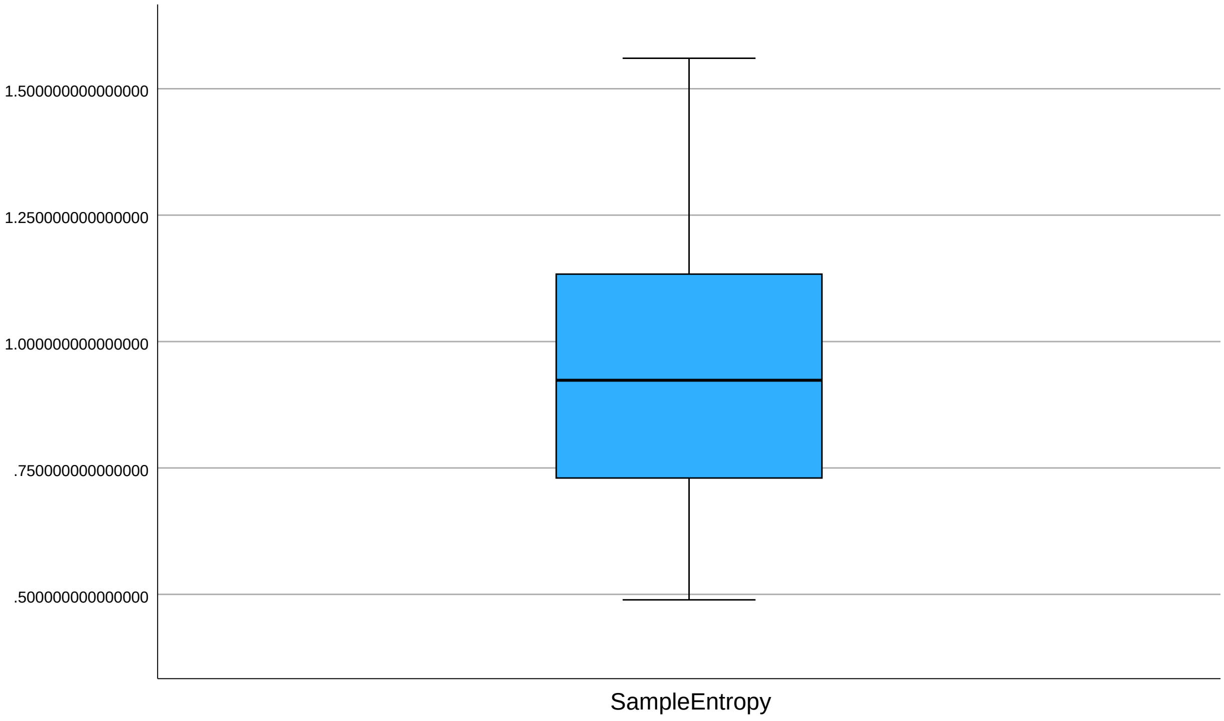

SpectralEntropy_bits

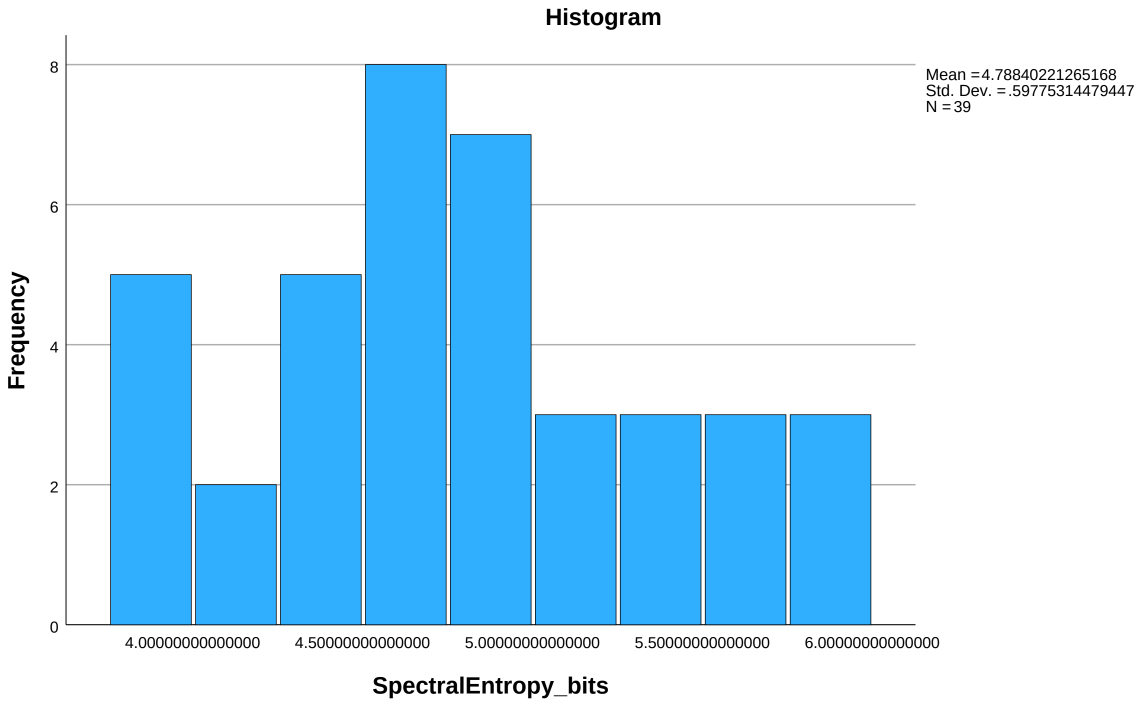


```
SpectralEntropy_bits Stem-and-Leaf Plot

 Frequency    Stem &  Leaf

     5.00        3 .  88999
     7.00        4 .  0023344
    15.00        4 .  555567777899999
     6.00        5 .  011222
     6.00        5 .  567889

 Stem width:  1.000000
 Each leaf:       1 case(s)
```

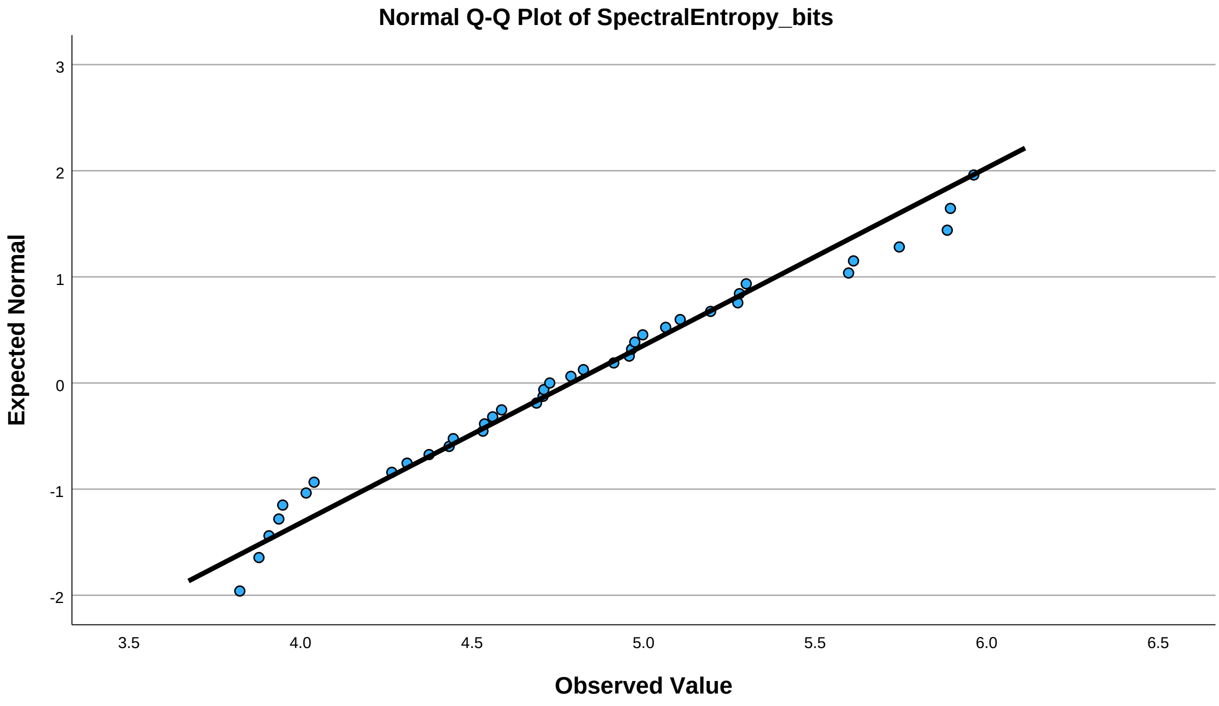

Normal Q-Q Plot of SpectralEntropy_bits
Expected Normal
Observed Value
3
2
1
0
-1
-2
3.5
4.0
4.5
5.0
5.5
6.0
6.5

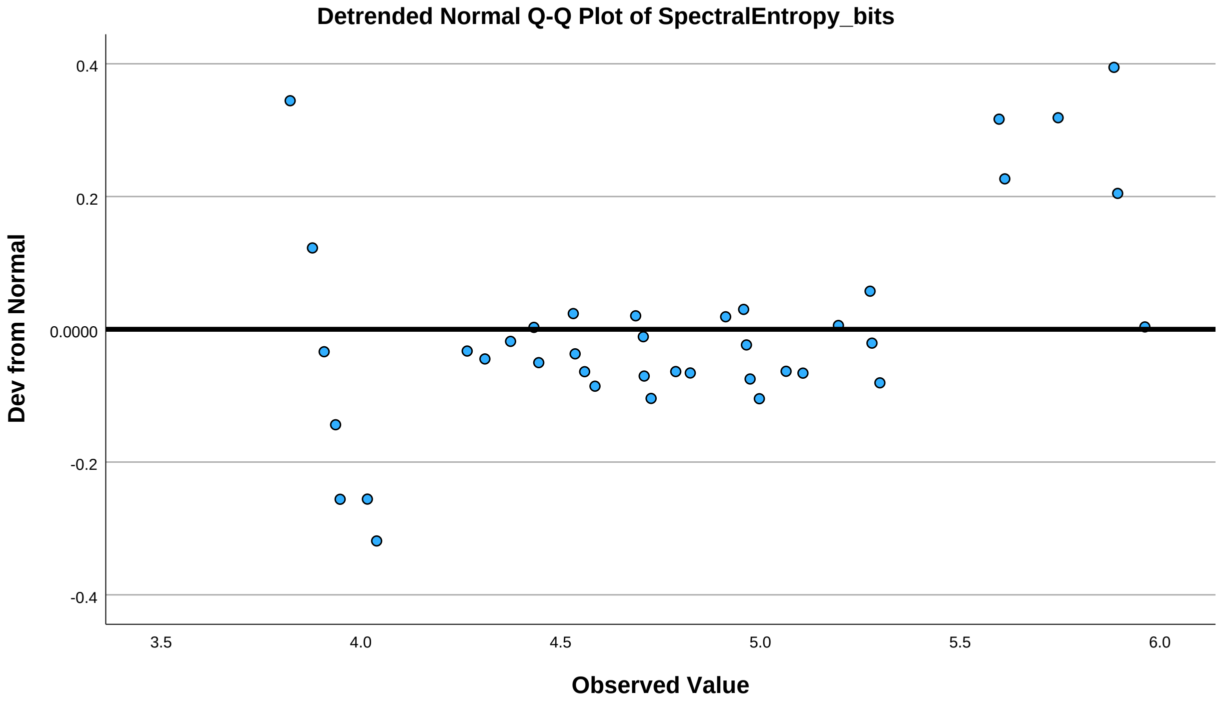

Detrended Normal Q-Q Plot of SpectralEntropy_bits
Dev from Normal
Observed Value
0.4
0.2
0.0000
-0.2
-0.4
3.5
4.0
4.5
5.0
5.5
6.0

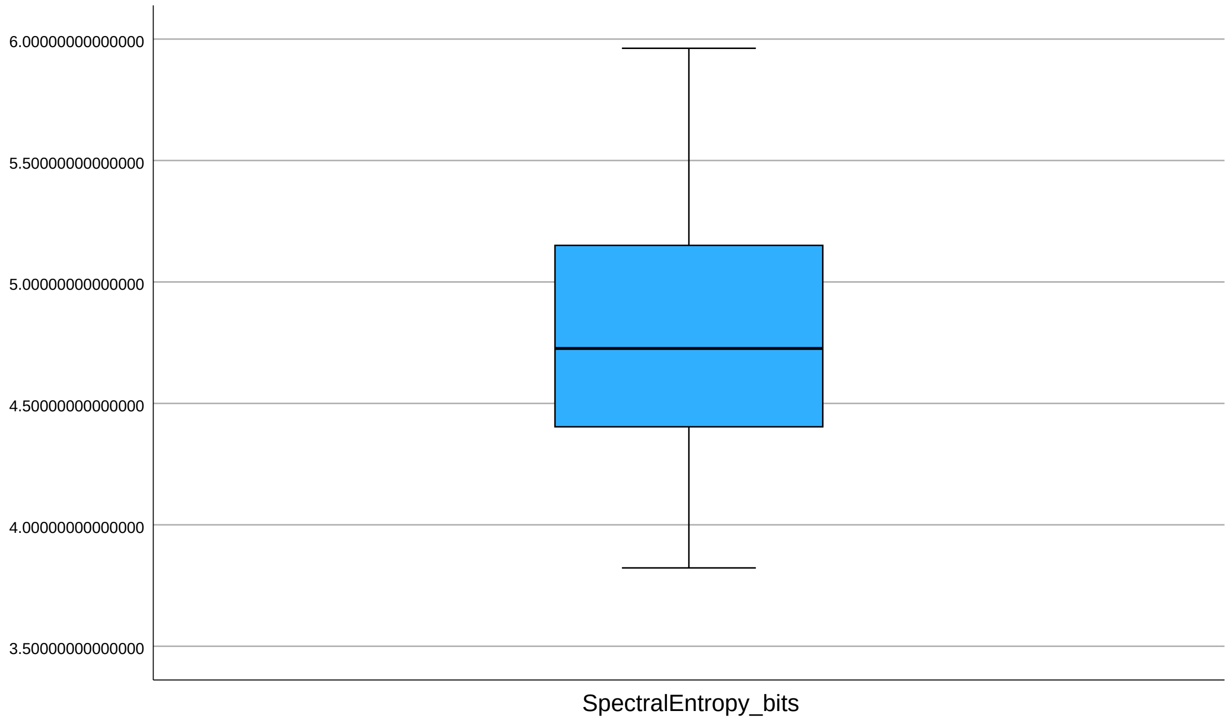

SpectralEntropy_01

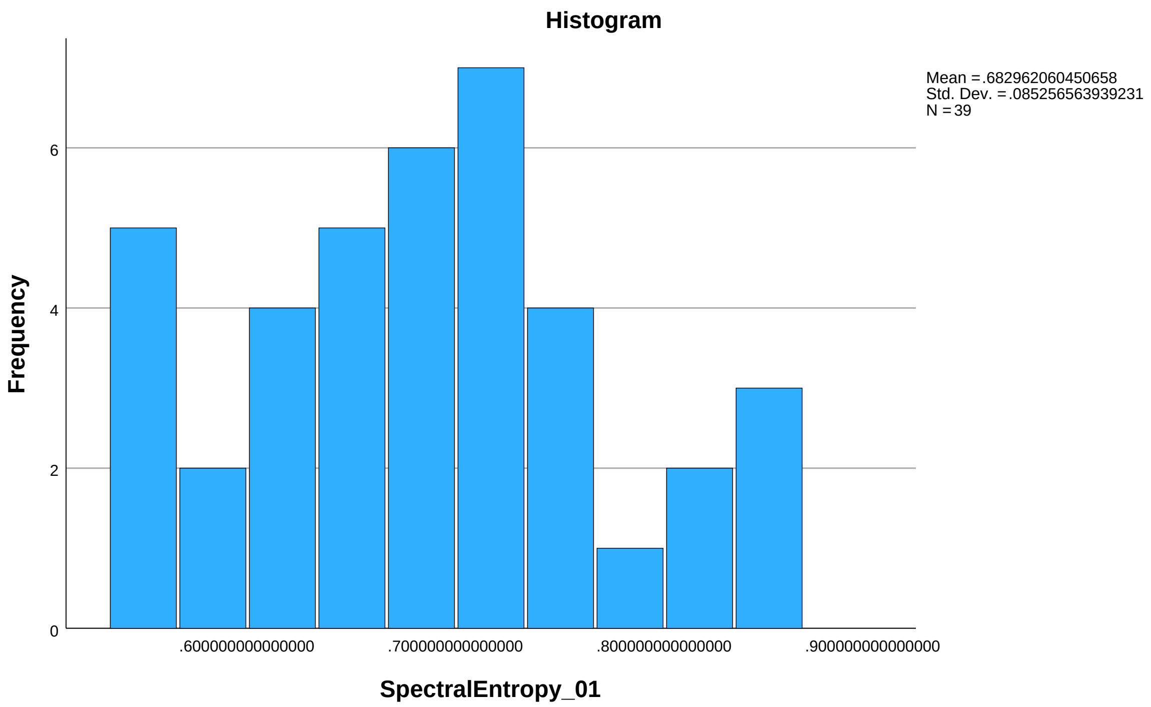

SpectralEntropy_01 Stem-and-Leaf Plot

Frequency   Stem & Leaf

```
 1.00      5 . 4
 6.00      5 . 556677
 7.00      6 . 0123344
 8.00      6 . 55677788
 8.00      7 . 00001224
 4.00      7 . 5559
 4.00      8 . 0134
 1.00      8 . 5

Stem width:   .1000000
Each leaf:       1 case(s)
```

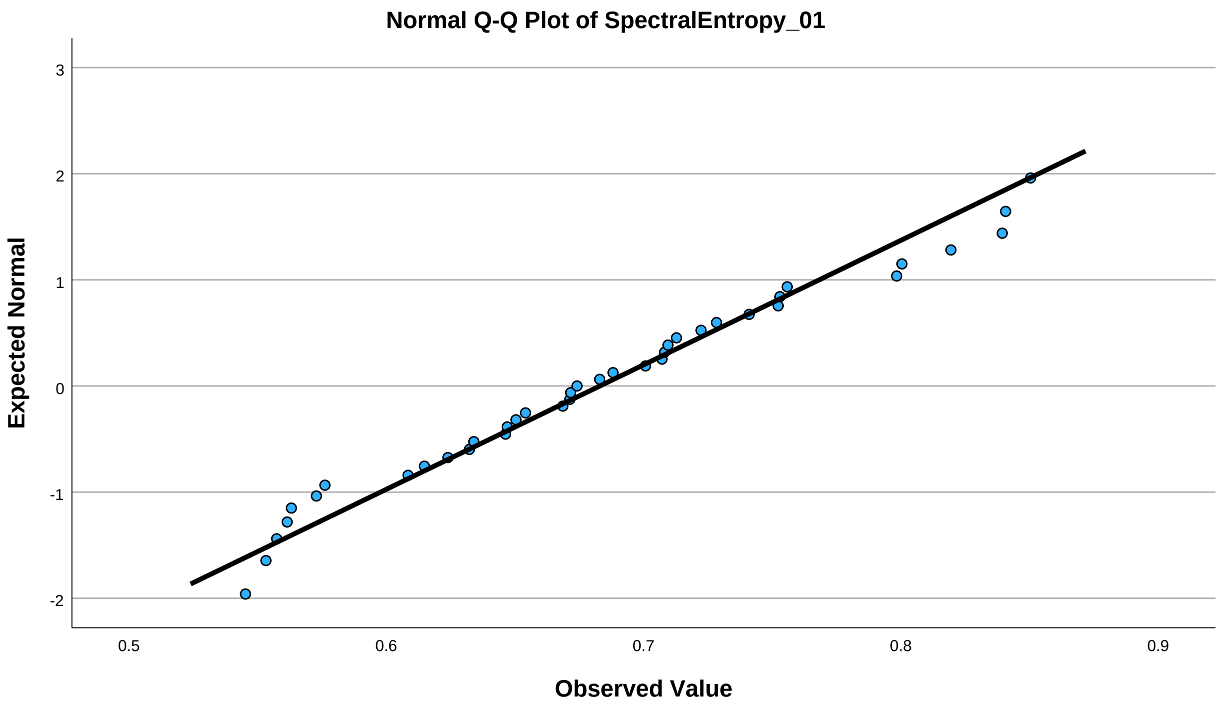

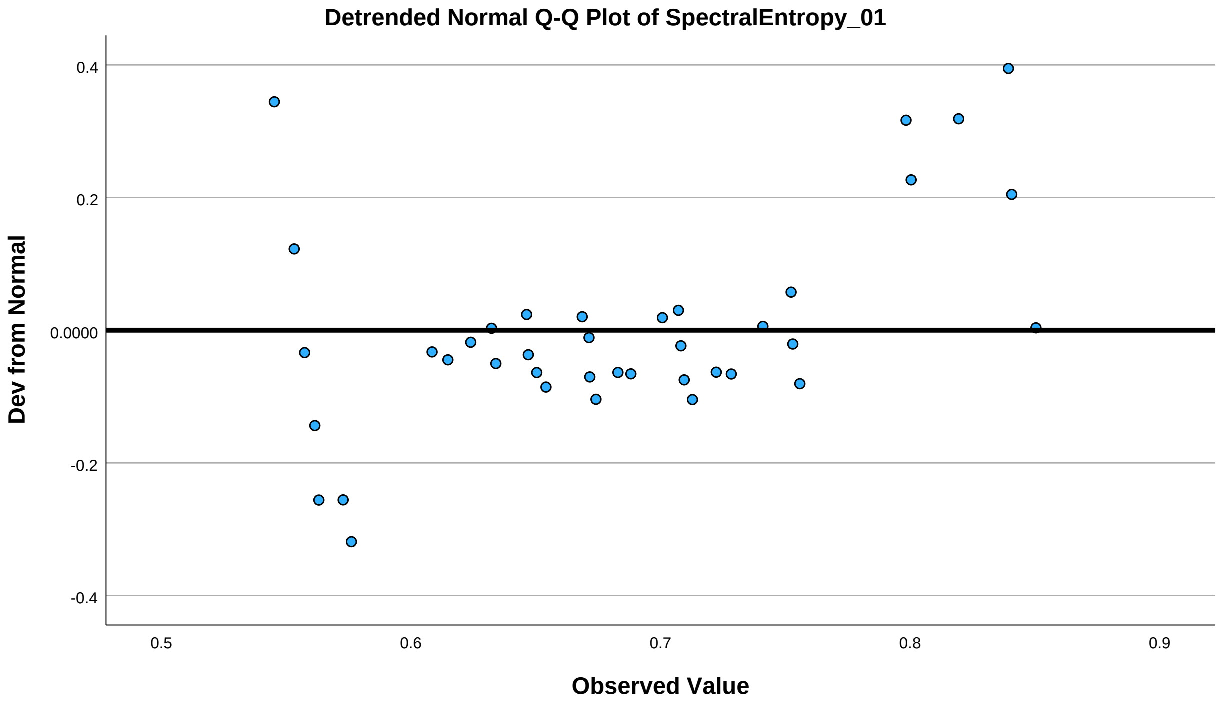

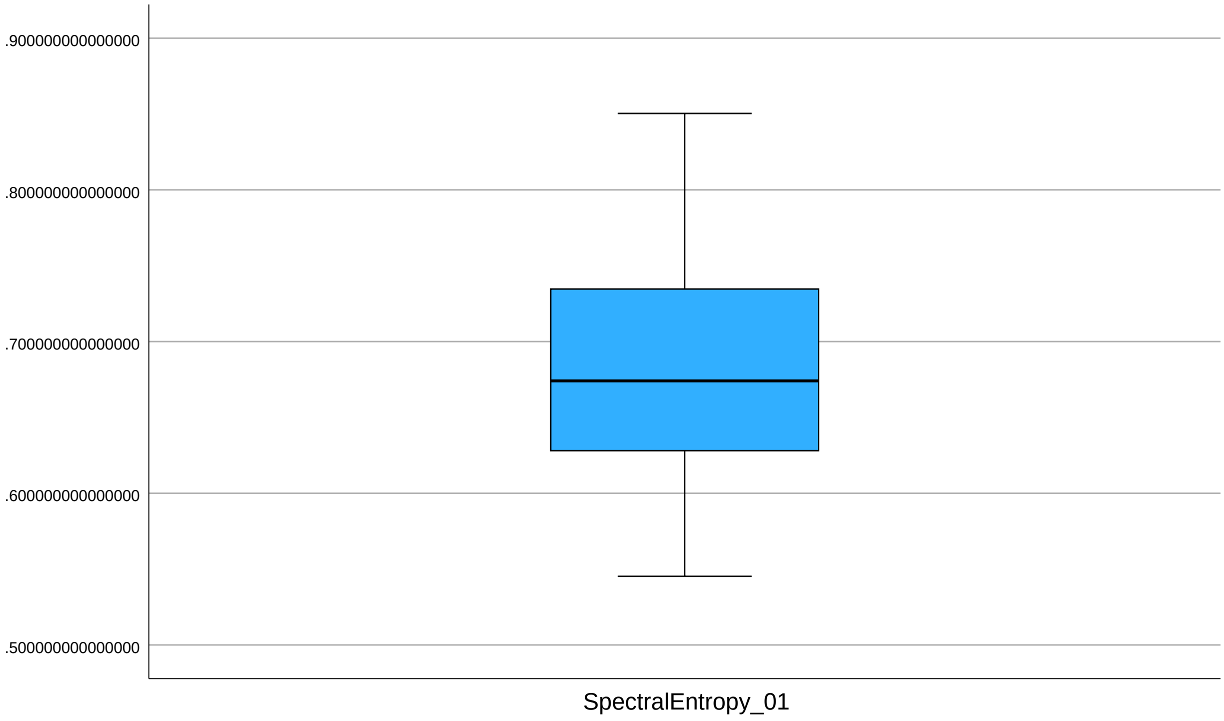

HiguchiFractalDimension

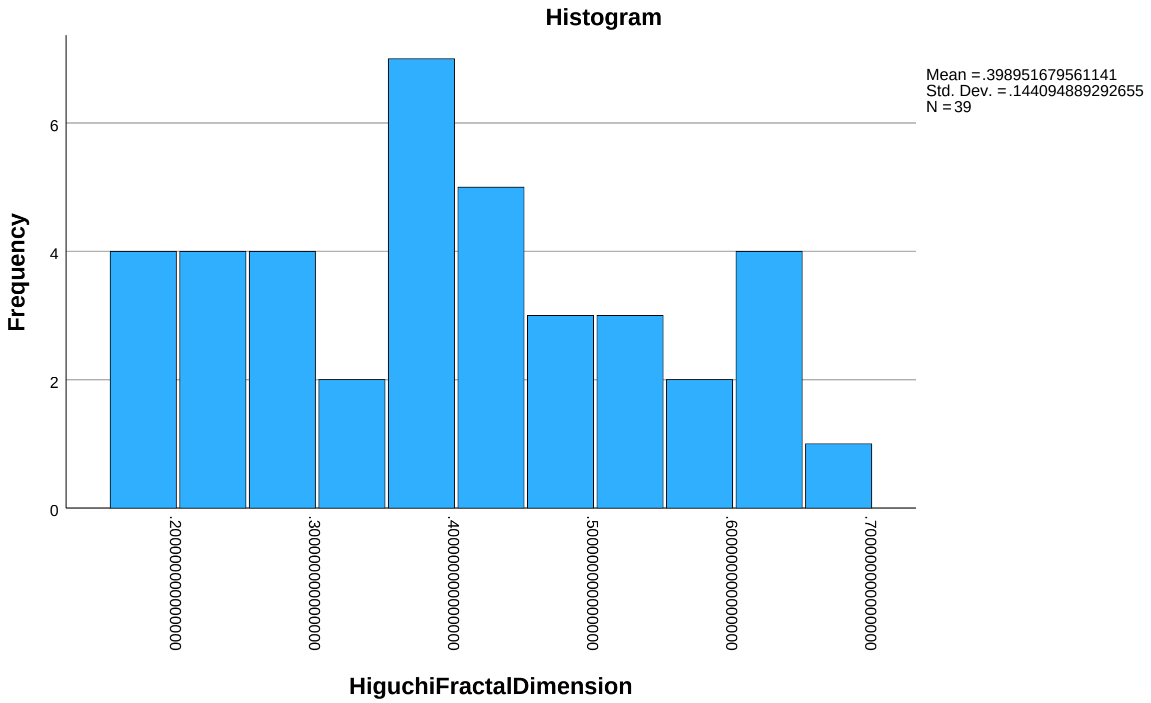


```
HiguchiFractalDimension Stem-and-Leaf Plot

 Frequency    Stem &  Leaf

     4.00        1 .  5699
     4.00        2 .  0244
     4.00        2 .  5689
     2.00        3 .  34
     7.00        3 .  7788999
     5.00        4 .  01244
     3.00        4 .  578
     3.00        5 .  123
     2.00        5 .  69
     4.00        6 .  0013
     1.00        6 .  8

 Stem width:   .1000000
 Each leaf:        1 case(s)
```

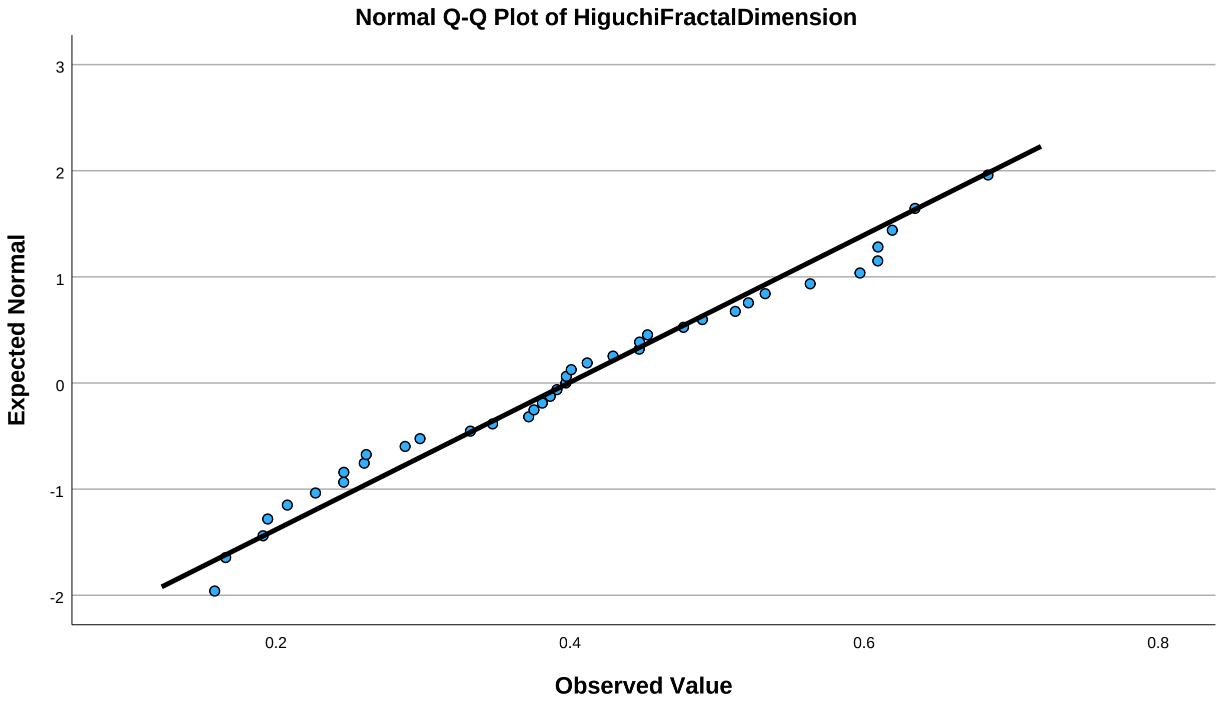
Normal Q-Q Plot of HiguchiFractalDimension
Expected Normal
3
2
1
0
-1
-2
0.2
0.4
0.6
0.8
Observed Value

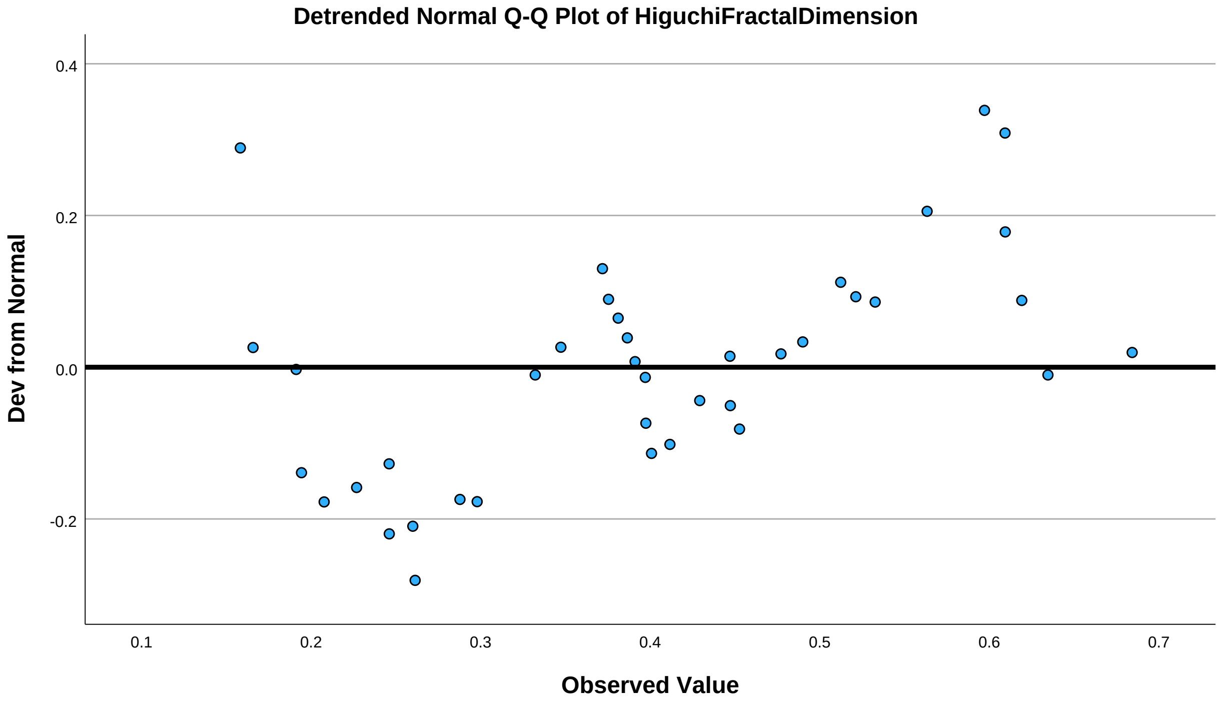
Detrended Normal Q-Q Plot of HiguchiFractalDimension
Dev from Normal
0.4
0.2
0.0
-0.2
0.1
0.2
0.3
0.4
0.5
0.6
0.7
Observed Value

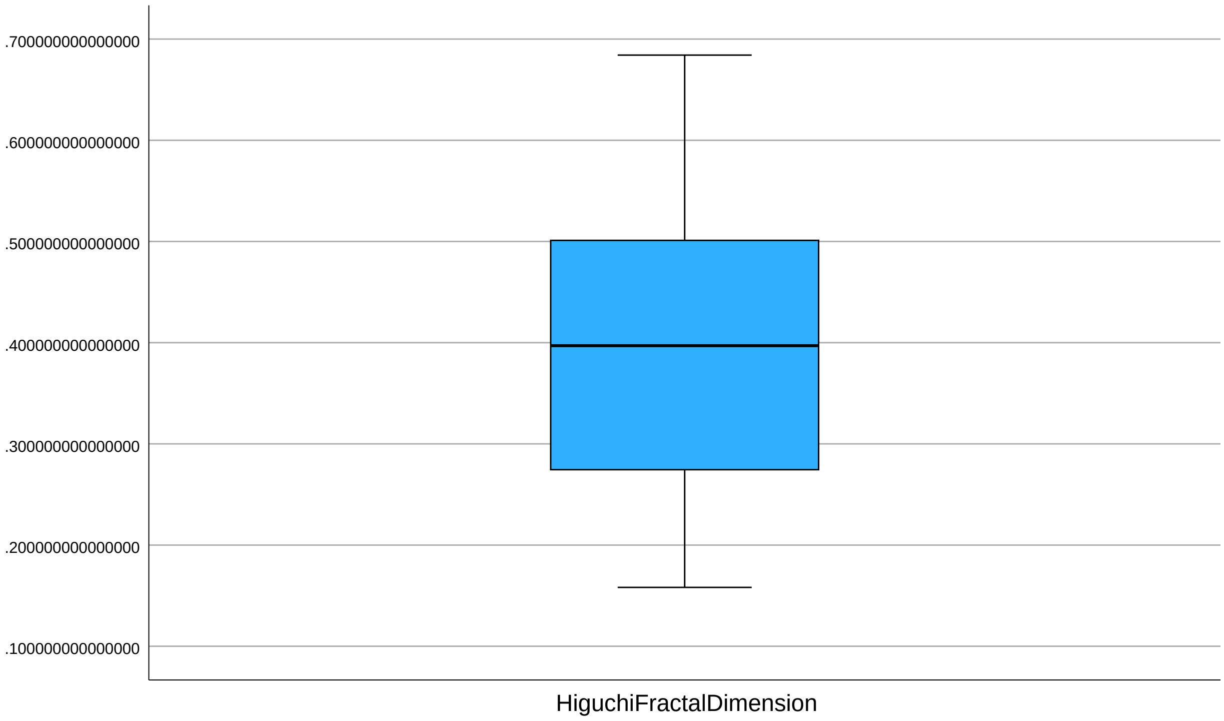

LempelZivComplexity

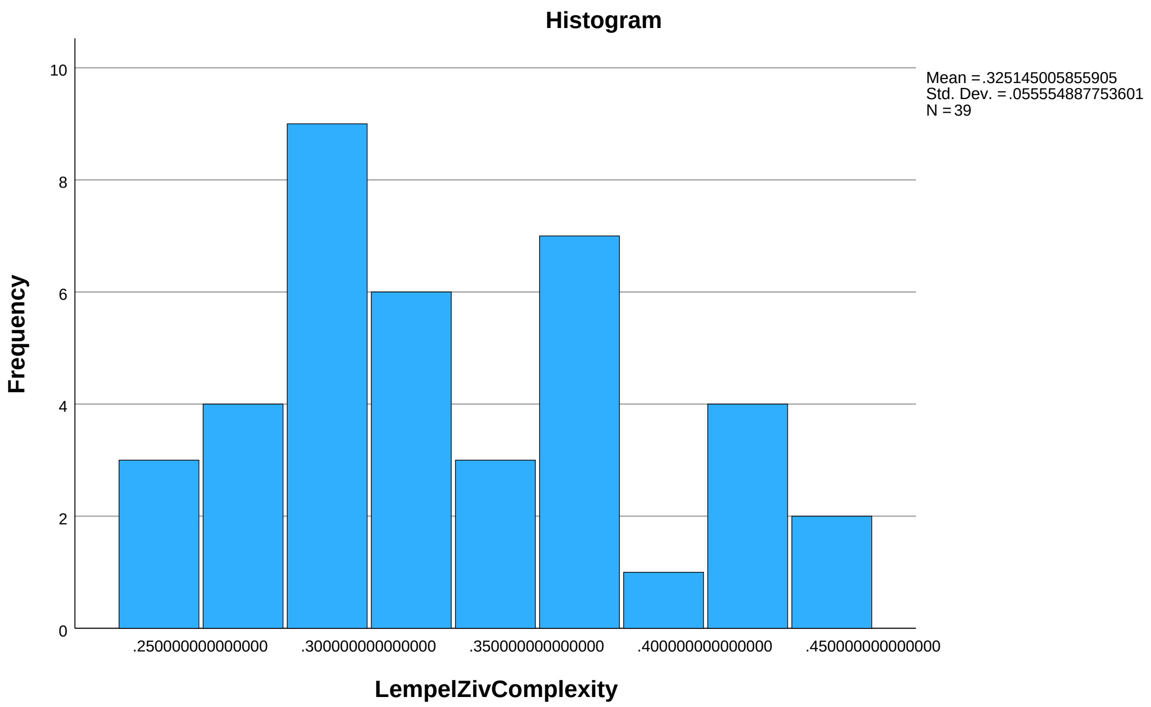


```
LempelZivComplexity Stem-and-Leaf Plot

 Frequency    Stem &  Leaf
```

```
    3.00        2 .  333
   13.00        2 .  5666888889999
    9.00        3 .  001122334
    8.00        3 .  55555778
    6.00        4 .  011133

 Stem width:   .1000000
 Each leaf:        1 case(s)
```

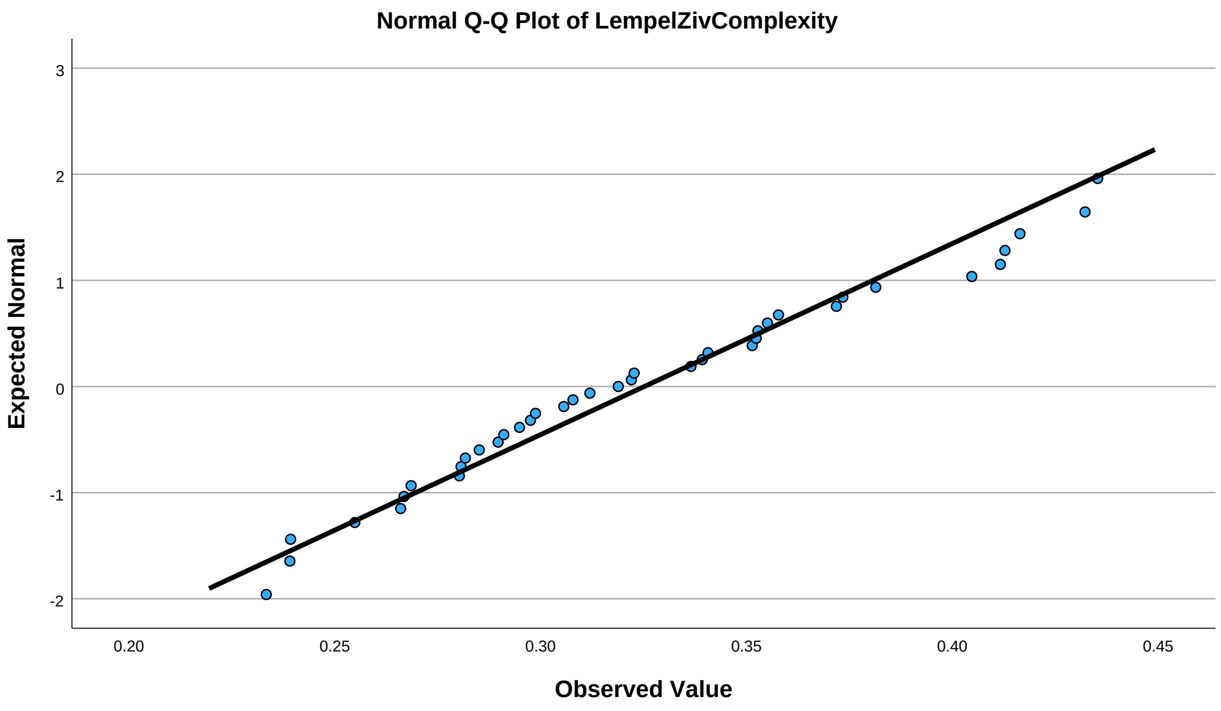

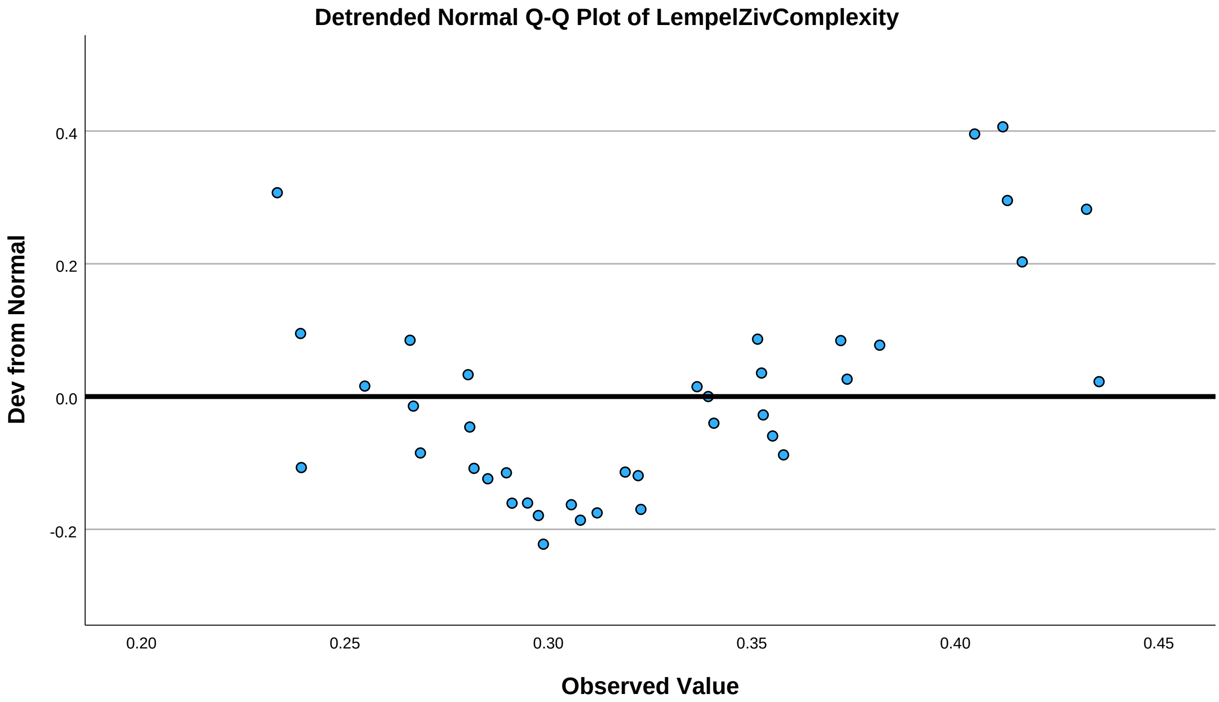

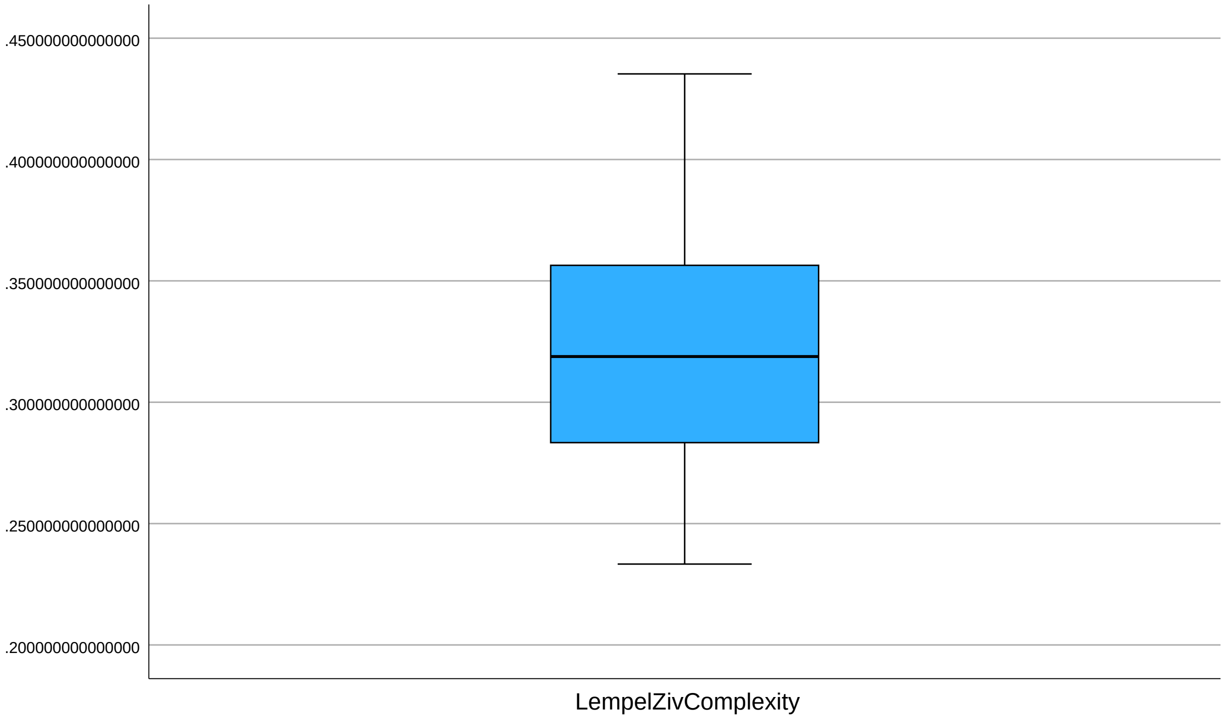

RenyiEntropy

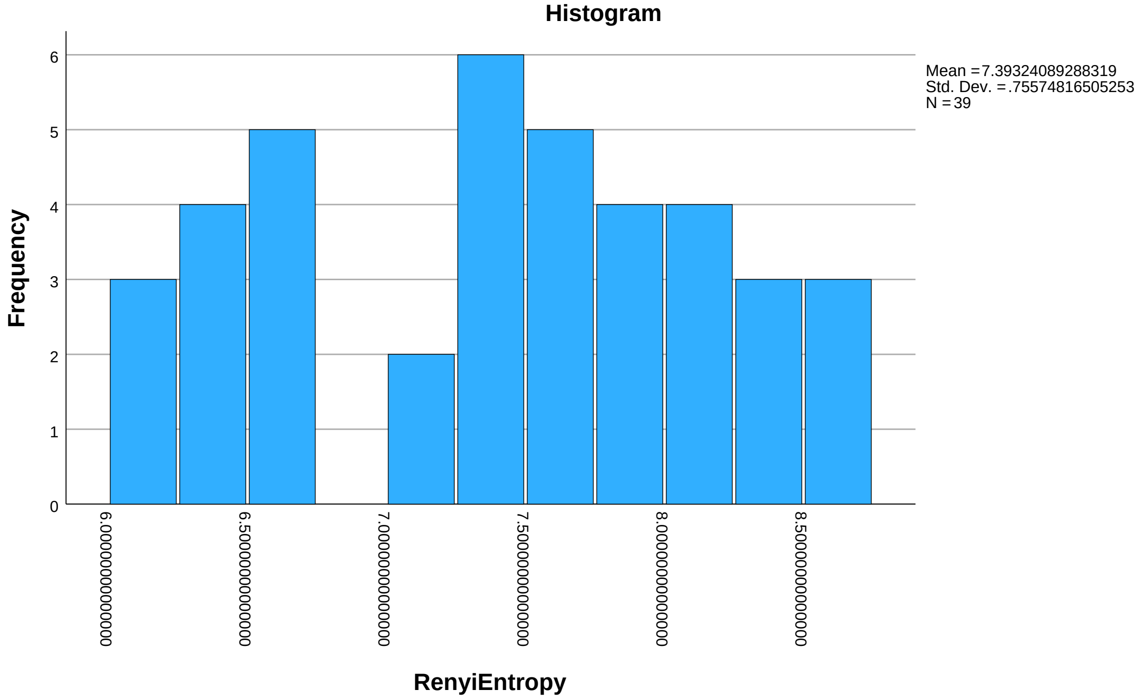


```
RenyiEntropy Stem-and-Leaf Plot

 Frequency    Stem &  Leaf

     7.00        6 .  0124444
     5.00        6 .  55667
     8.00        7 .  01233344
     9.00        7 .  556777889
     7.00        8 .  0001223
     3.00        8 .  566

 Stem width:   1.000000
 Each leaf:       1 case(s)
```

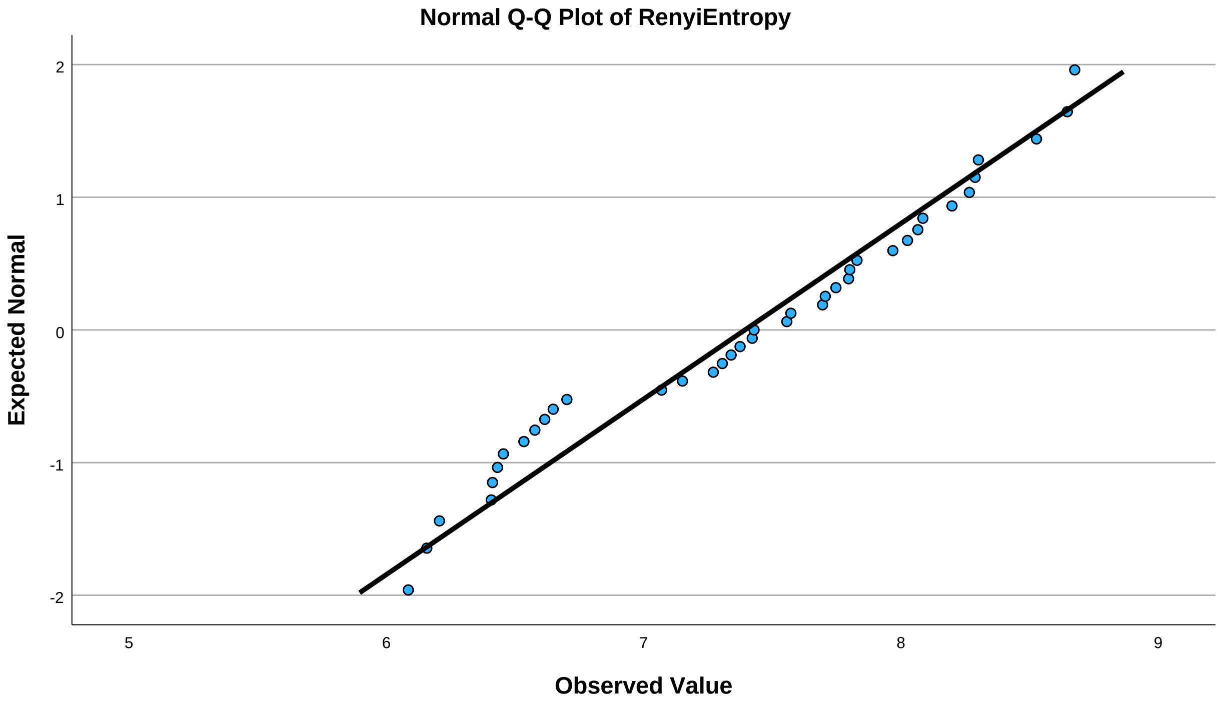
Normal Q-Q Plot of RenyiEntropy
Expected Normal
2
1
0
-1
-2
5
6
7
8
9
Observed Value

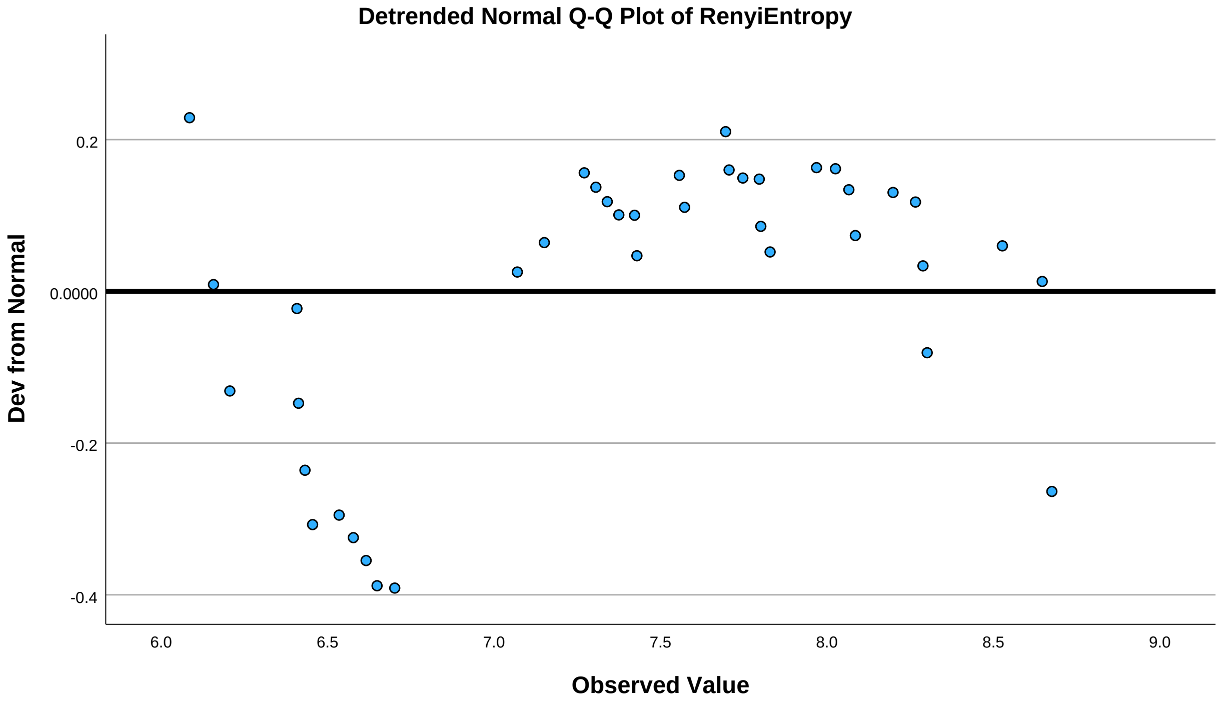
Detrended Normal Q-Q Plot of RenyiEntropy
Dev from Normal
0.2
0.0000
-0.2
-0.4
6.0
6.5
7.0
7.5
8.0
8.5
9.0
Observed Value

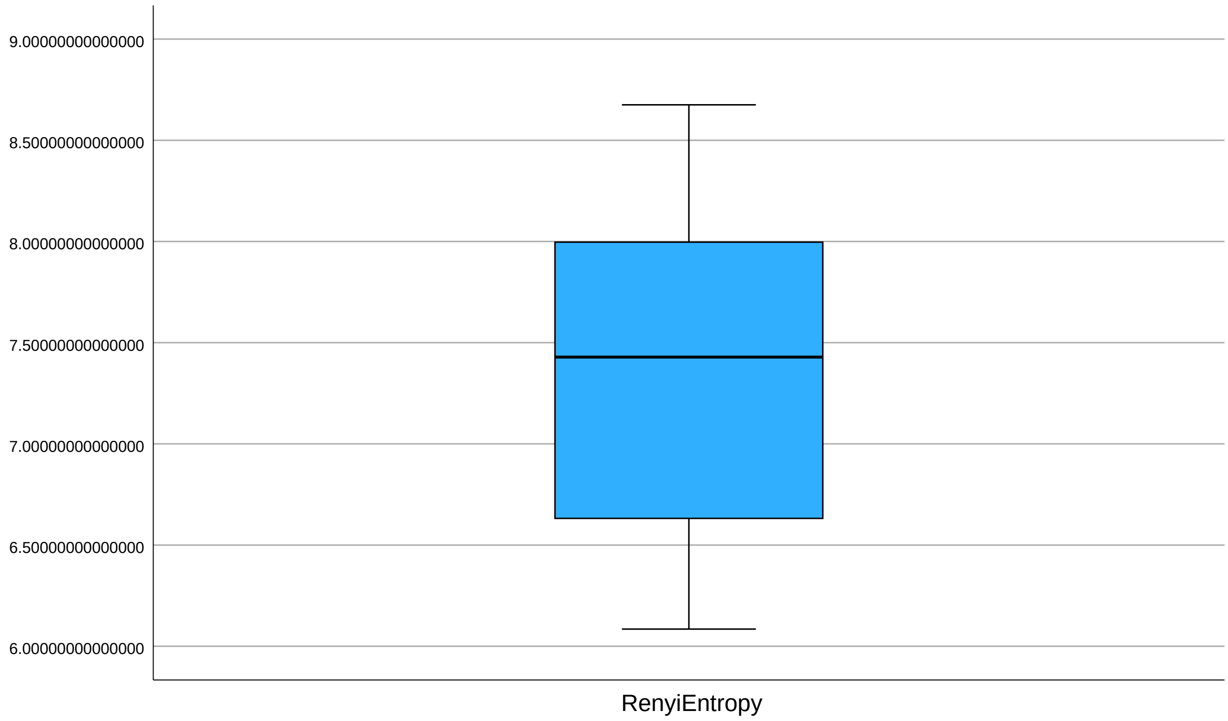

TsallisEntropy

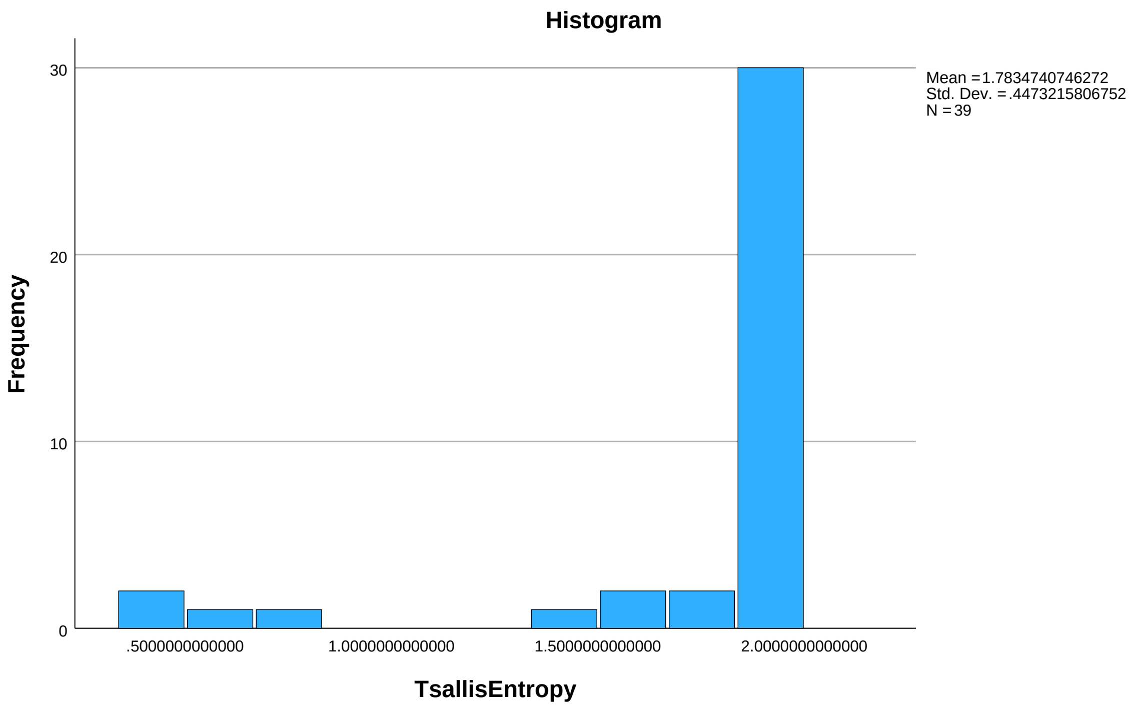

TsallisEntropy Stem-and-Leaf Plot

Frequency Stem & Leaf

```
   7.00 Extremes    (=<1.67)
   2.00     17 .  56
   1.00     18 .  4
   1.00     18 .  8
   2.00     19 .  24
  26.00       19 .  55677888888888888888888888

Stem width:   .1000000
Each leaf:       1 case(s)
```

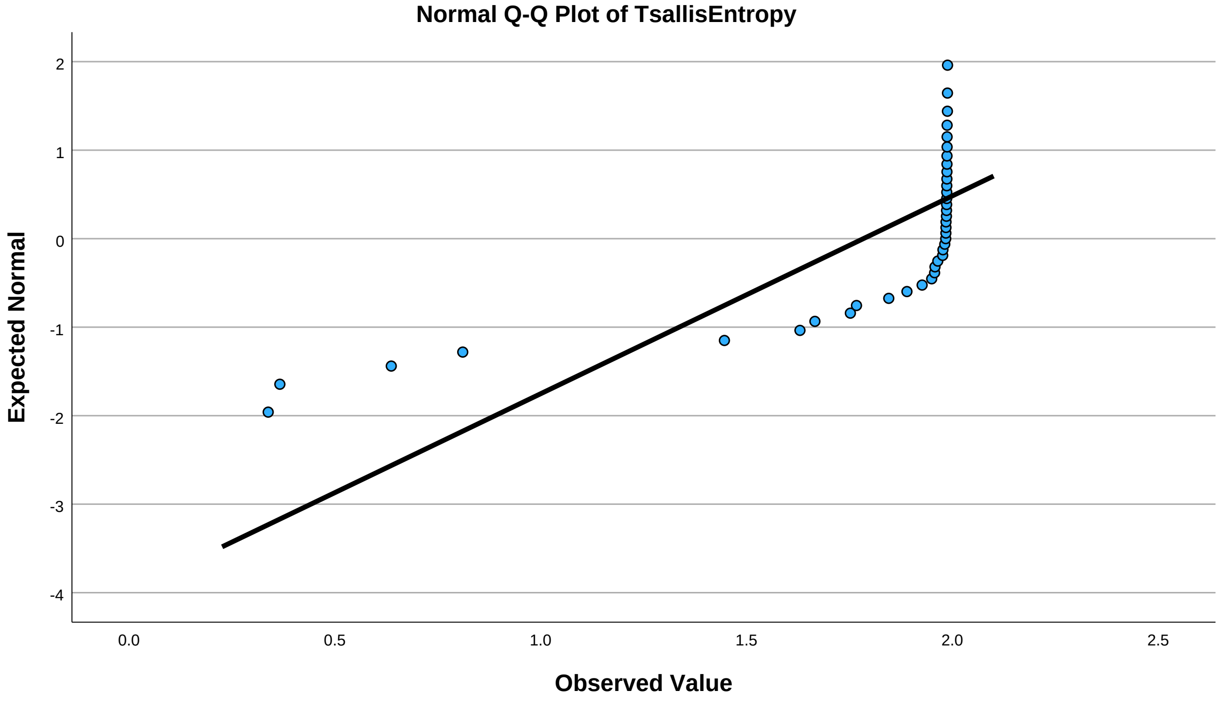

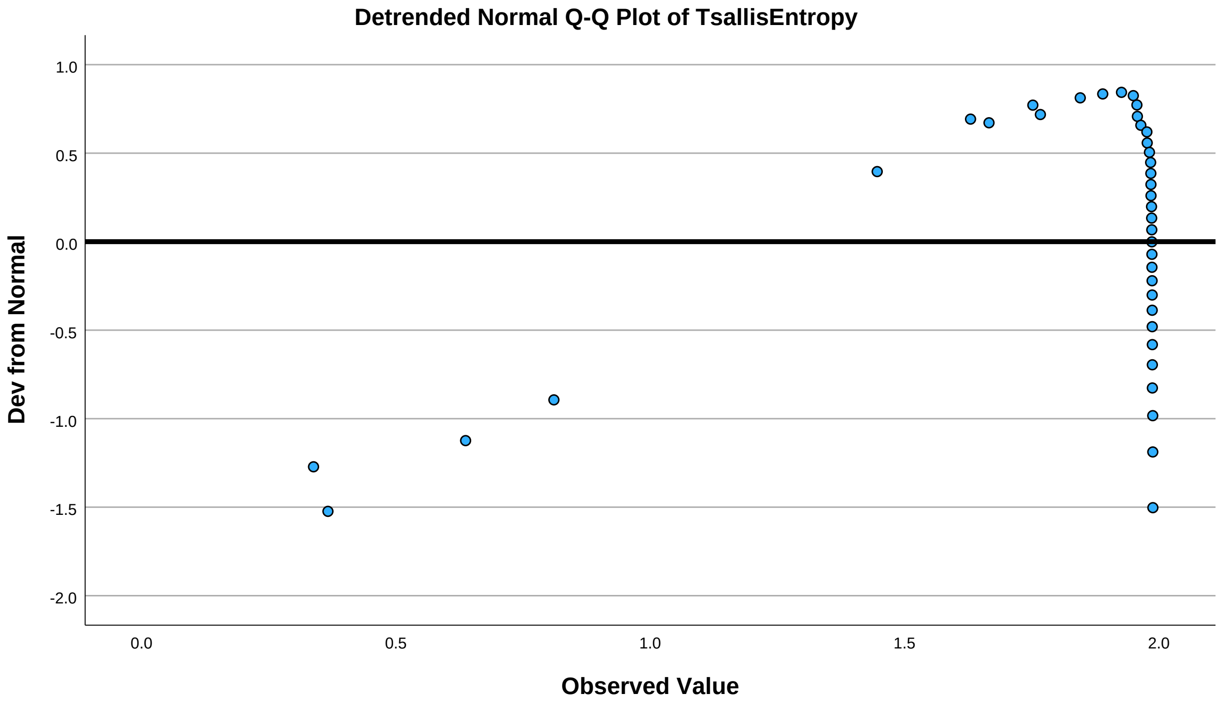

Detrended Normal Q-Q Plot of TsallisEntropy
Dev from Normal
1.0
0.5
0.0
-0.5
-1.0
-1.5
-2.0
0.0
0.5
1.0
1.5
2.0
Observed Value

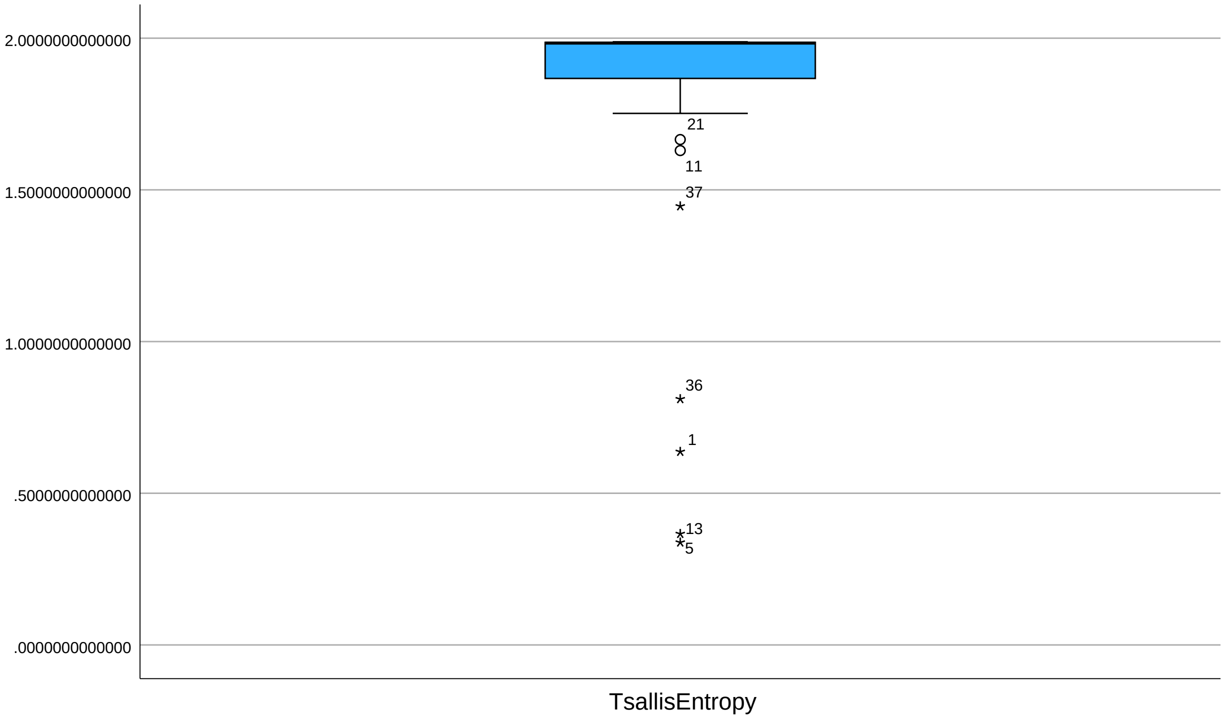

2.0000000000000
1.5000000000000
1.0000000000000
.5000000000000
.0000000000000
21
11
37
36
1
13
5
TsallisEntropy

PS_4_5

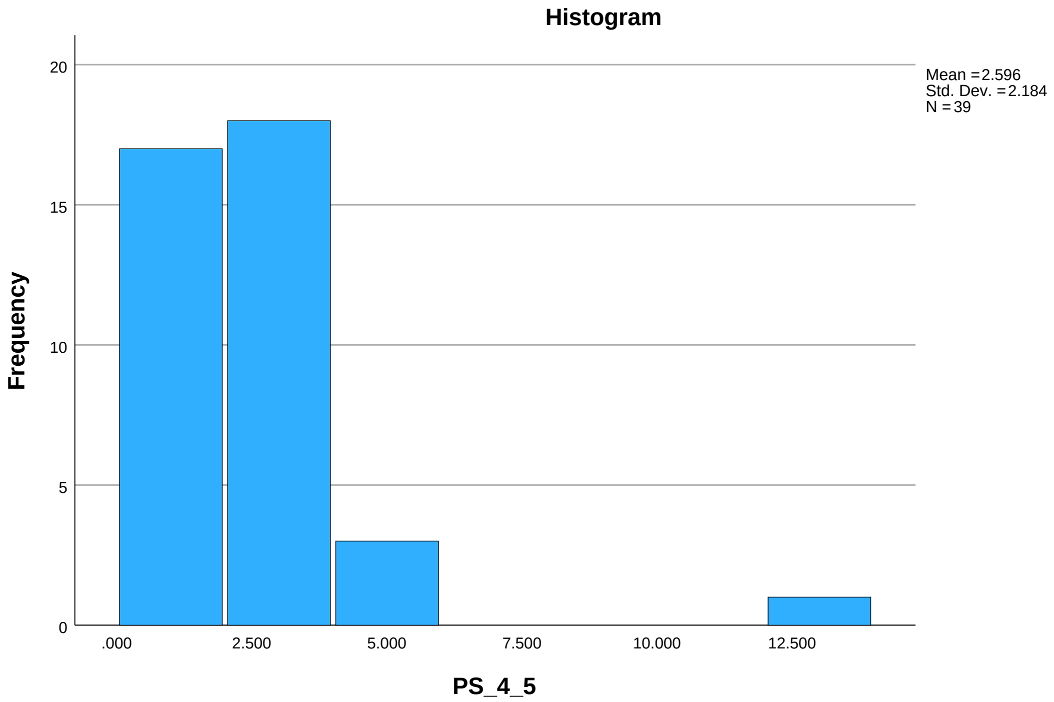


```
PS_4_5 Stem-and-Leaf Plot

 Frequency    Stem &  Leaf

     1.00        0 .  4
     1.00        0 .  5
     9.00        1 .  000111344
     6.00        1 .  557799
     9.00        2 .  001233444
     4.00        2 .  5667
     1.00        3 .  4
     4.00        3 .  5889
     1.00        4 .  3
     3.00 Extremes    (>=5.1)

 Stem width:      1.000
 Each leaf:       1 case(s)
```

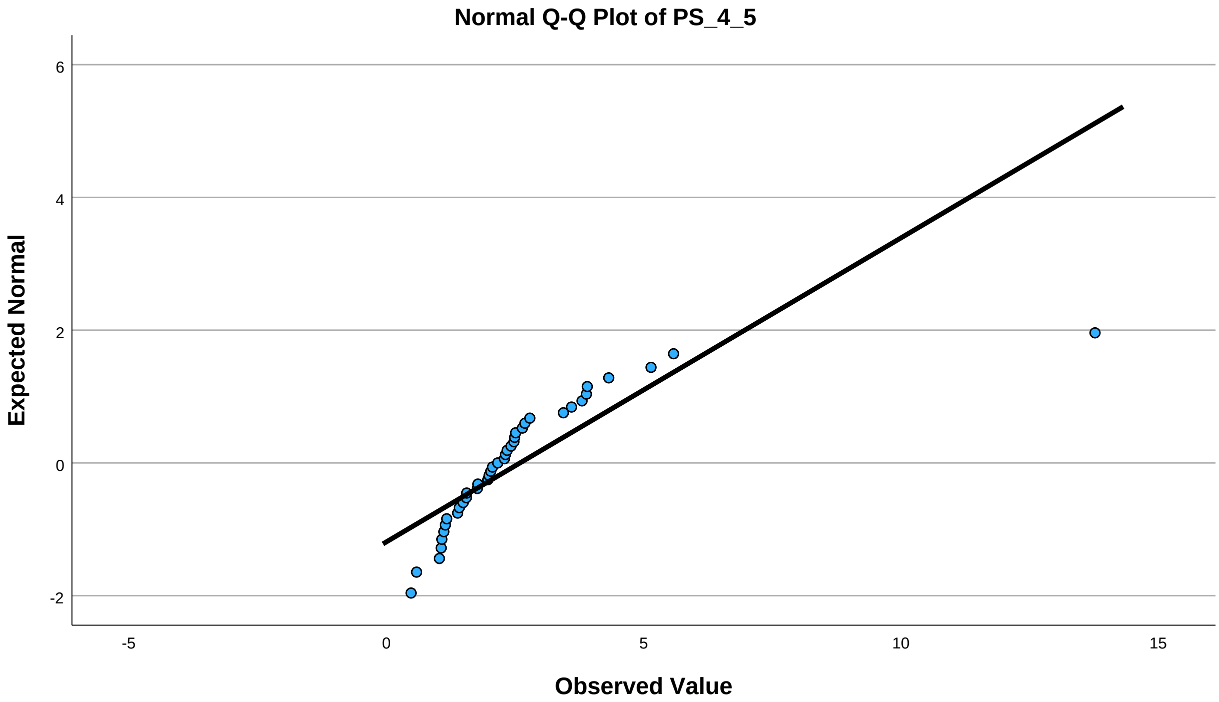
Normal Q-Q Plot of PS_4_5
Expected Normal
6
4
2
0
-2
-5
0
5
10
15
Observed Value

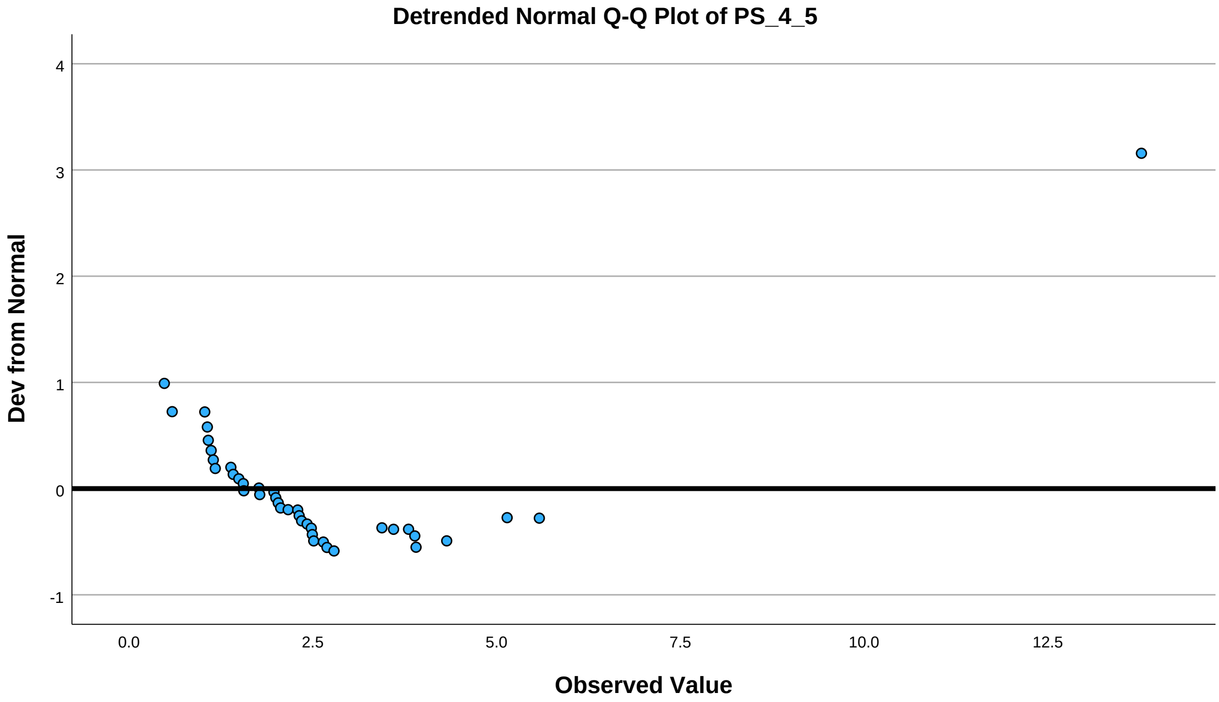
Detrended Normal Q-Q Plot of PS_4_5
Dev from Normal
4
3
2
1
0
-1
0.0
2.5
5.0
7.5
10.0
12.5
Observed Value

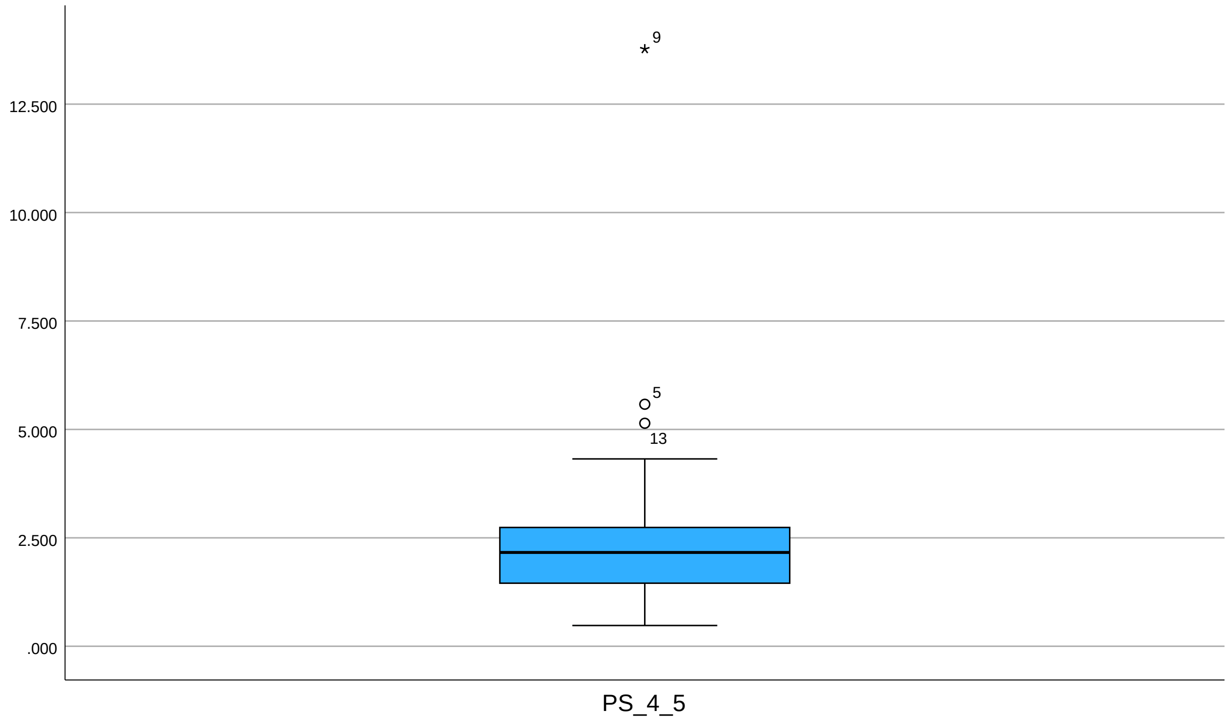

PS_5_6

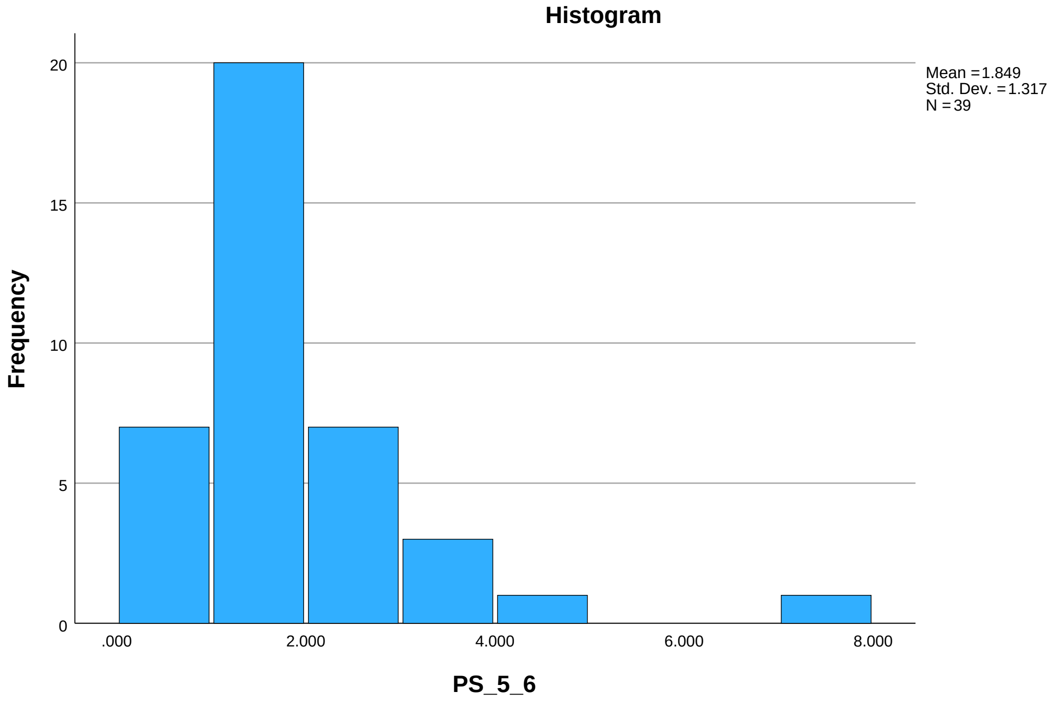


```
PS_5_6 Stem-and-Leaf Plot

 Frequency    Stem &  Leaf
```

```
 1.00      0 .  3
 6.00      0 .  566678
10.00      1 .  0012233344
10.00      1 .  5555557889
 5.00      2 .  01122
 2.00      2 .  68
 2.00      3 .  02
 1.00      3 .  5
 2.00 Extremes    (>=4.2)

Stem width:      1.000
Each leaf:       1 case(s)
```

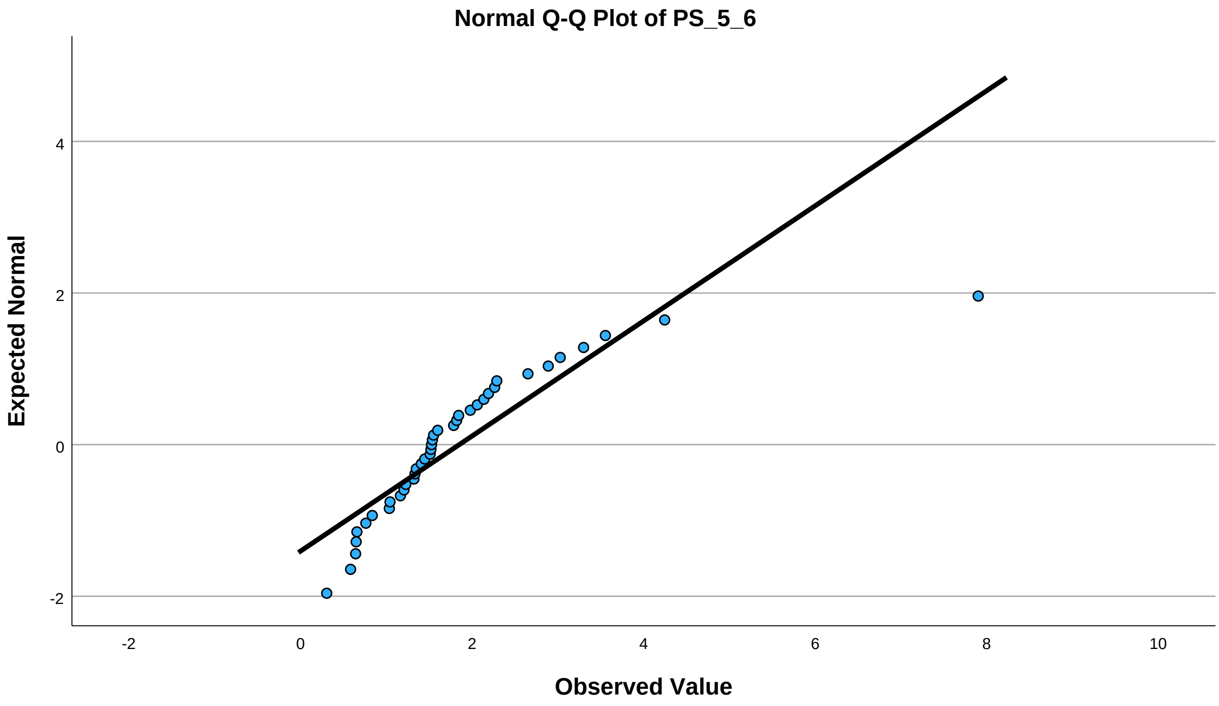

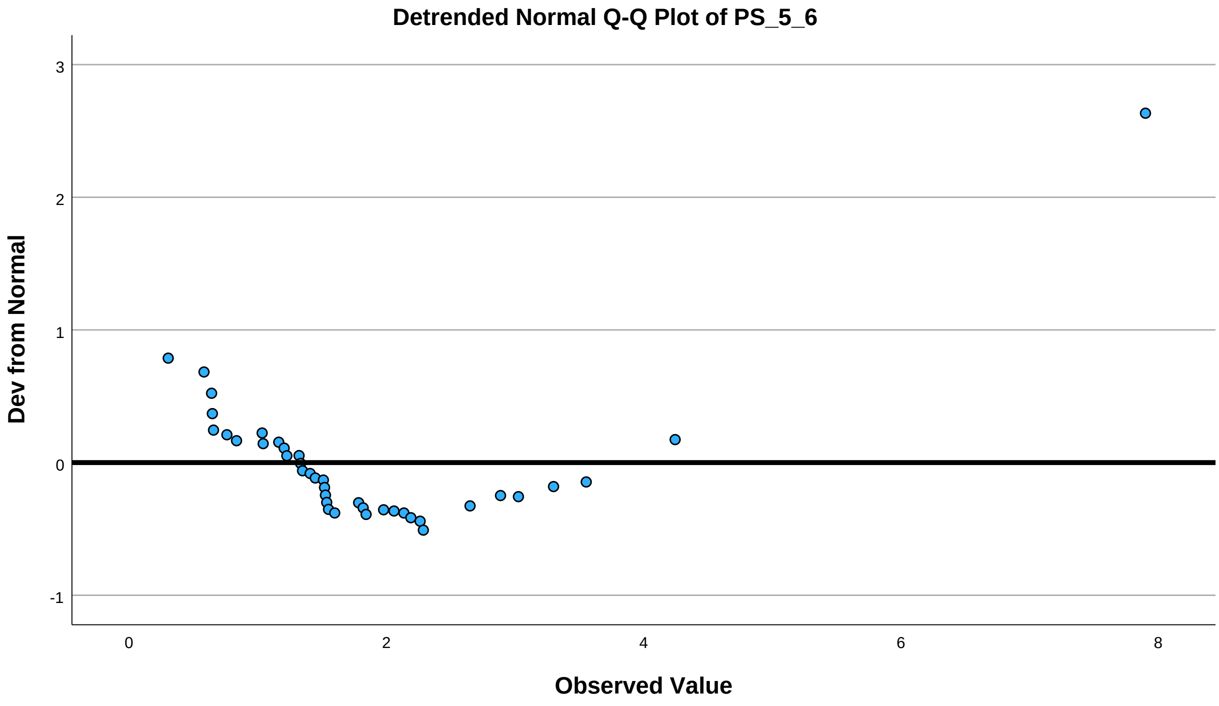
Detrended Normal Q-Q Plot of PS_5_6
Dev from Normal
3
2
1
0
-1
0
2
4
6
8
Observed Value

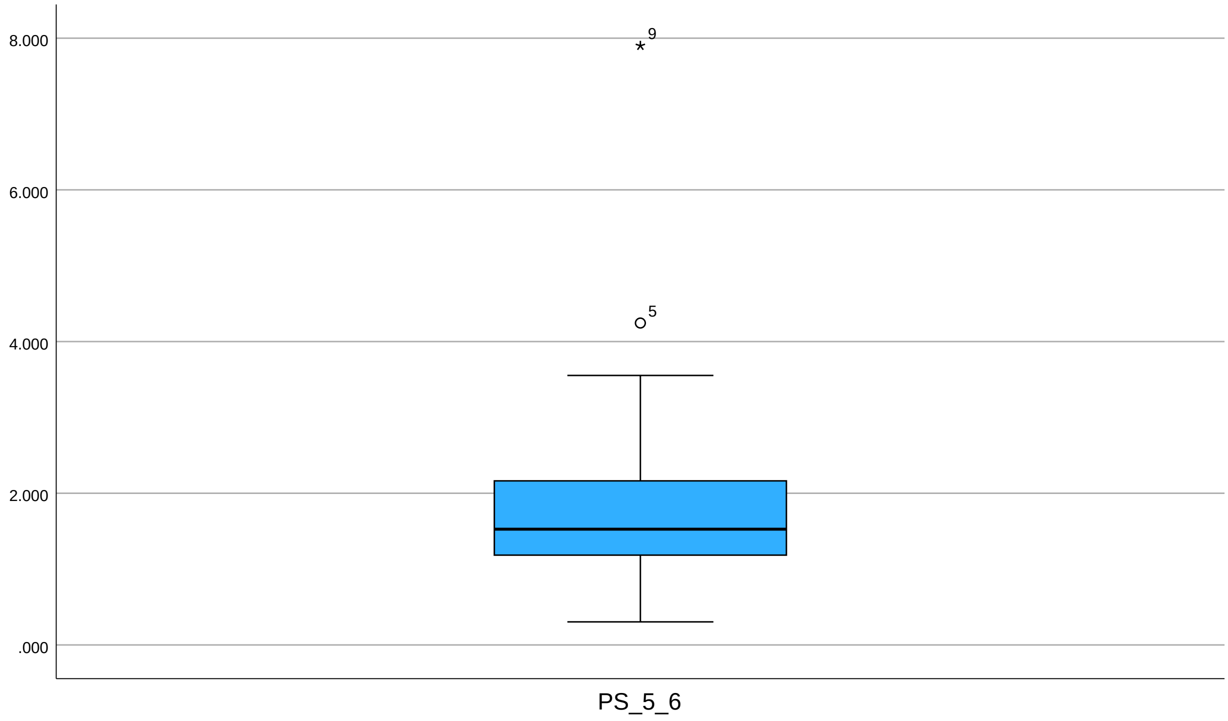
8.000
6.000
4.000
2.000
.000
9
5
PS_5_6

PS_6_7

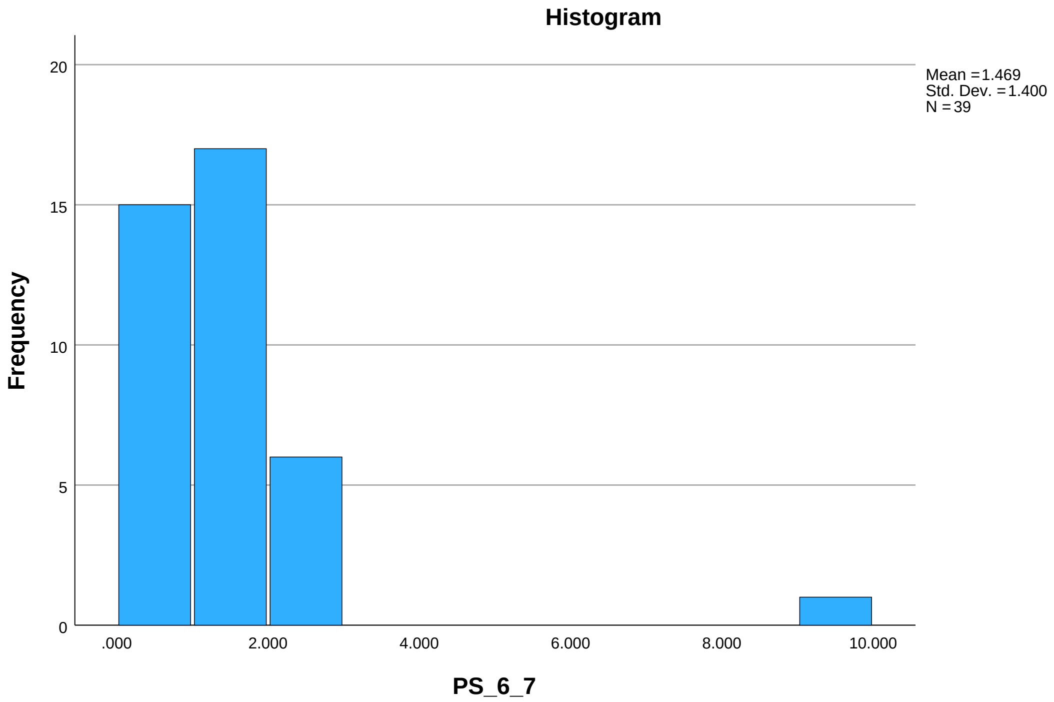


```
PS_6_7 Stem-and-Leaf Plot

 Frequency    Stem &  Leaf

     4.00        0 .  2344
    11.00        0 .  55667899999
    12.00        1 .  000111223344
     5.00        1 .  66899
     4.00        2 .  0124
     2.00        2 .  58
     1.00 Extremes    (>=9.0)

 Stem width:      1.000
 Each leaf:       1 case(s)
```

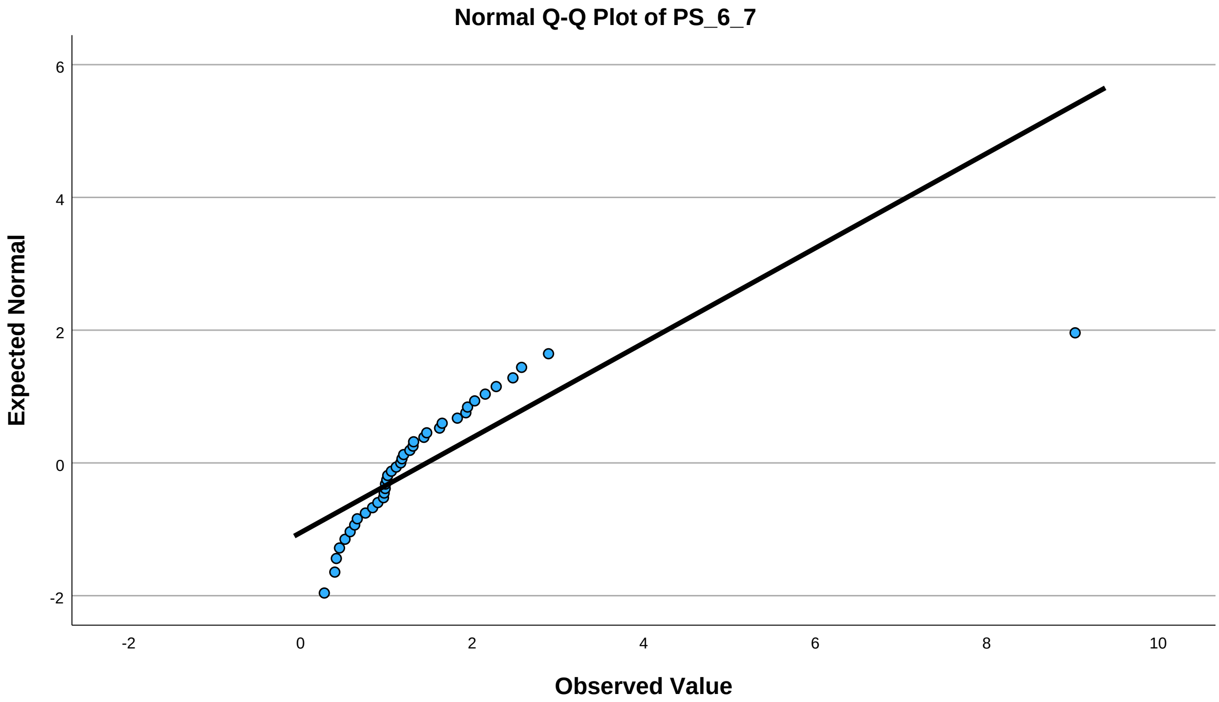

Normal Q-Q Plot of PS_6_7
Expected Normal
Observed Value
6
4
2
0
-2
-2
0
2
4
6
8
10

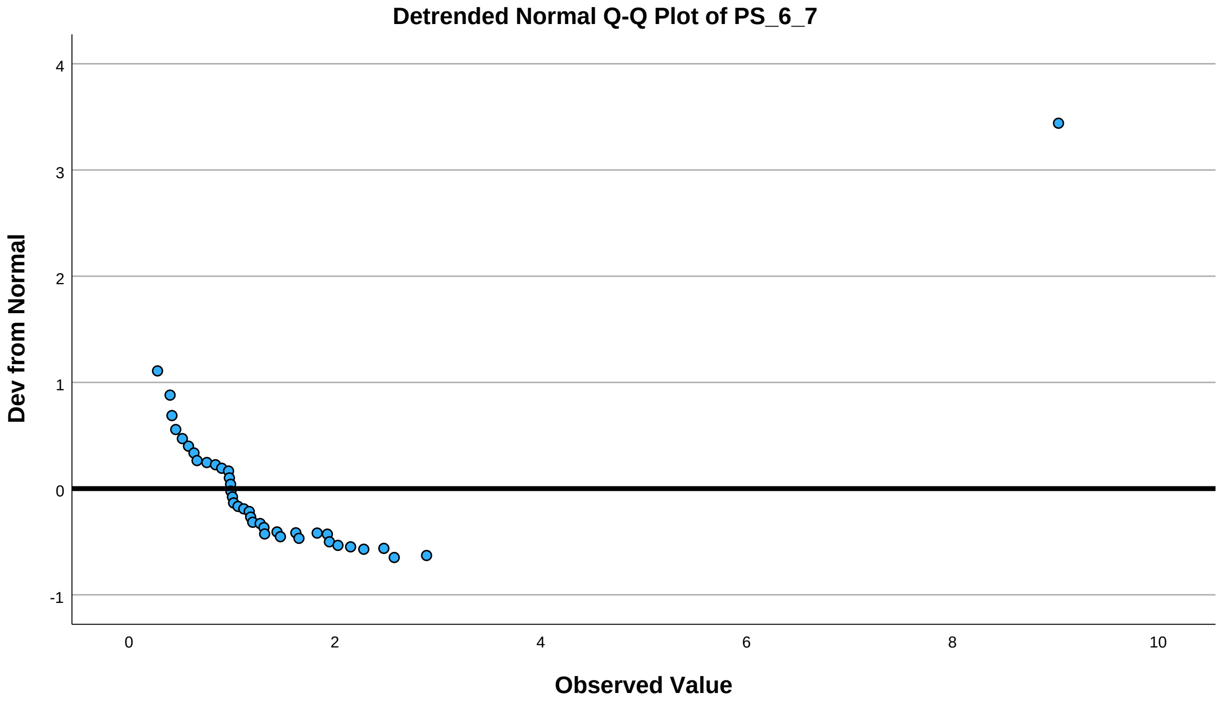

Detrended Normal Q-Q Plot of PS_6_7
Dev from Normal
Observed Value
4
3
2
1
0
-1
0
2
4
6
8
10

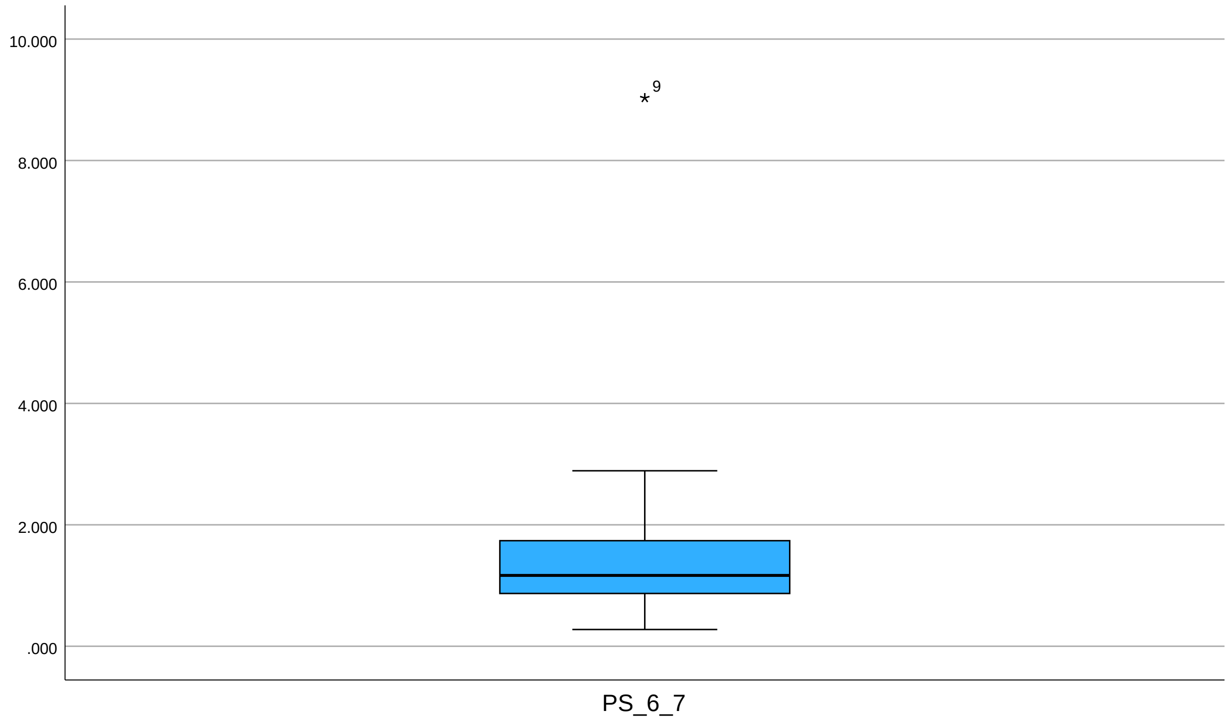

PS_7_8

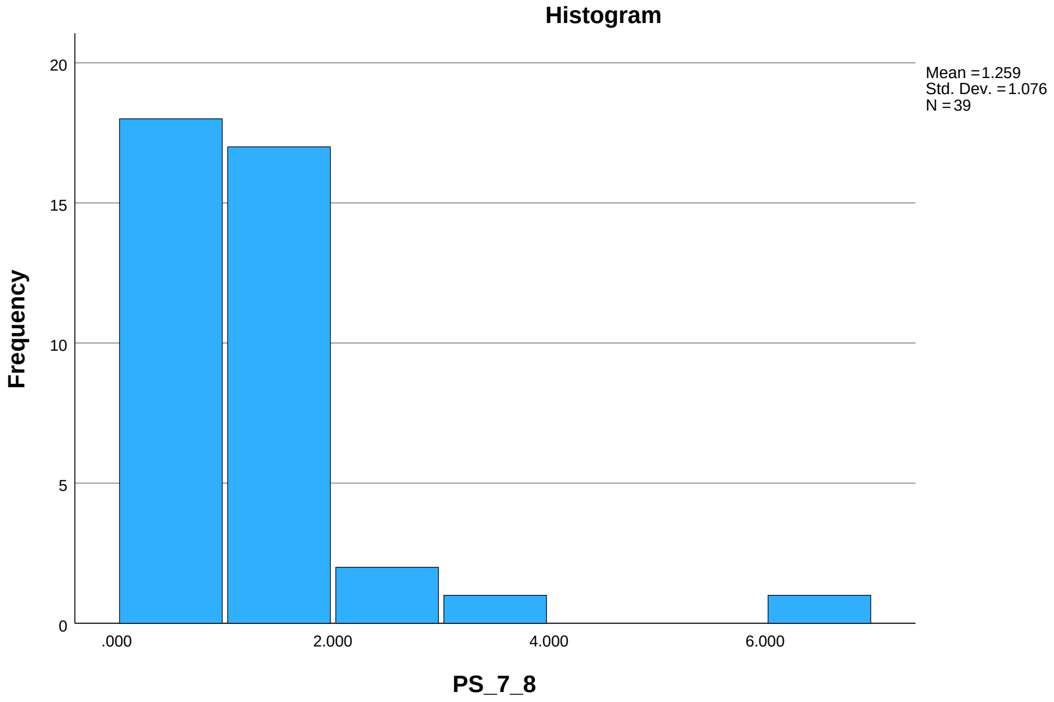

PS_7_8 Stem-and-Leaf Plot

Frequency   Stem & Leaf

```
 5.00      0 . 23334
13.00       0 . 5566677788889
12.00       1 . 000001122334
 5.00      1 . 56999
1.00     2 . 0
 3.00 Extremes    (>=2.5)

Stem width:     1.000
Each leaf:       1 case(s)
```

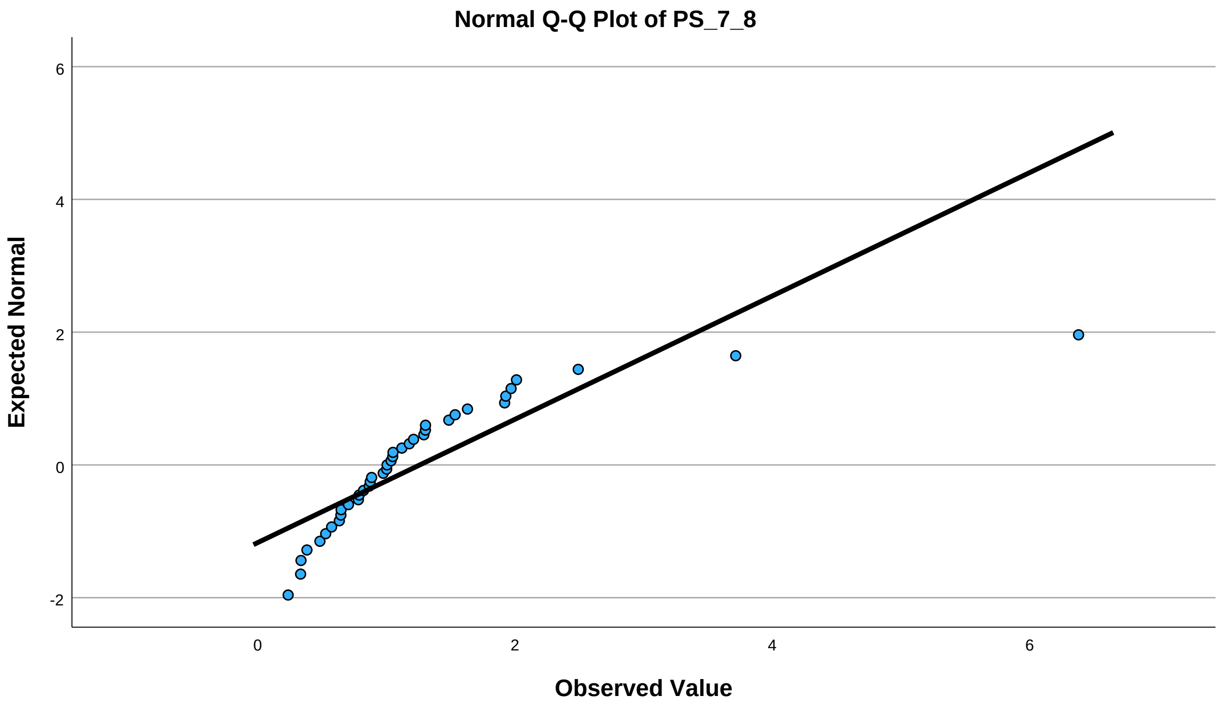

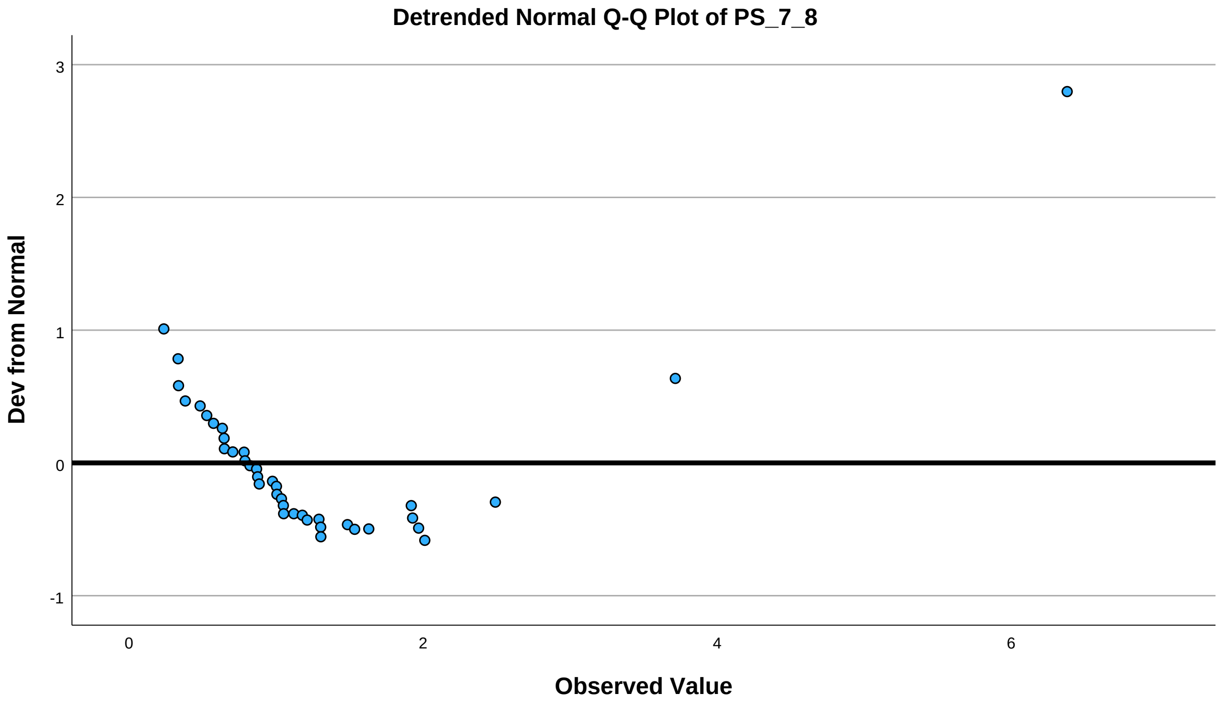

Detrended Normal Q-Q Plot of PS_7_8
Dev from Normal
3
2
1
0
-1
0
2
4
6
Observed Value

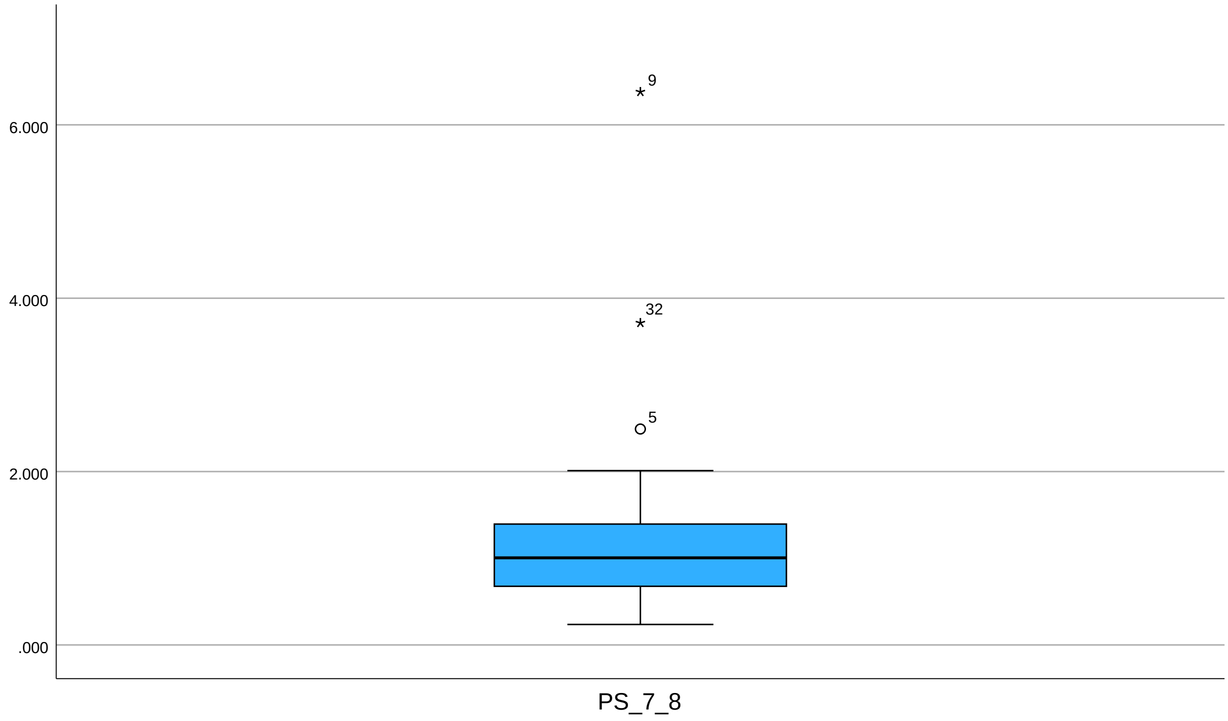

9
32
5
6.000
4.000
2.000
.000
PS_7_8

PS_8_9

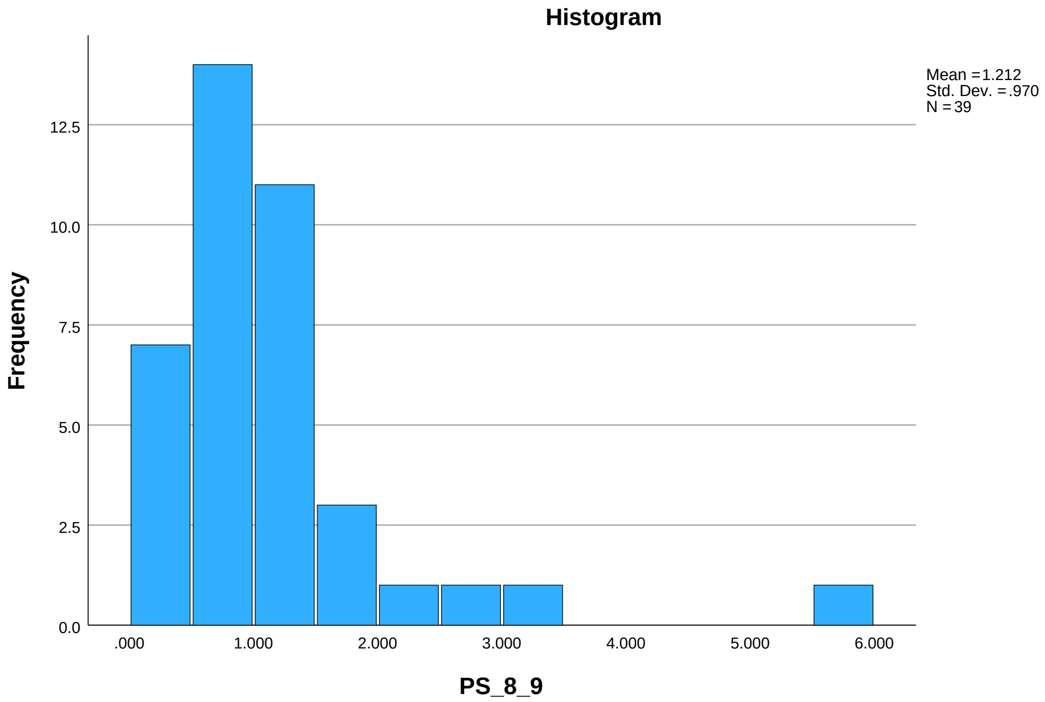


```
PS_8_9 Stem-and-Leaf Plot

 Frequency    Stem &  Leaf

     7.00        0 .  2233344
    14.00        0 .  55667788889999
    11.00        1 .  11223333444
     3.00        1 .  689
     1.00        2 .  2
     3.00 Extremes    (>=2.7)

 Stem width:      1.000
 Each leaf:       1 case(s)
```

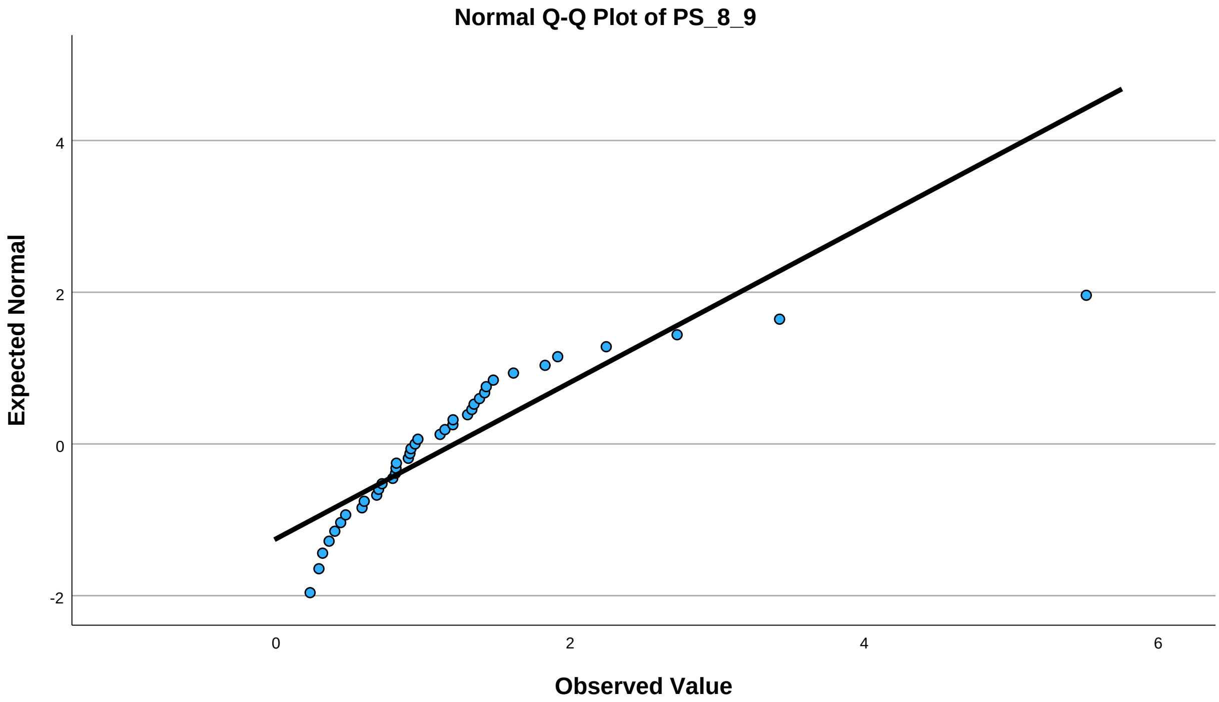
Normal Q-Q Plot of PS_8_9
Expected Normal
Observed Value
4
2
0
-2
0
2
4
6

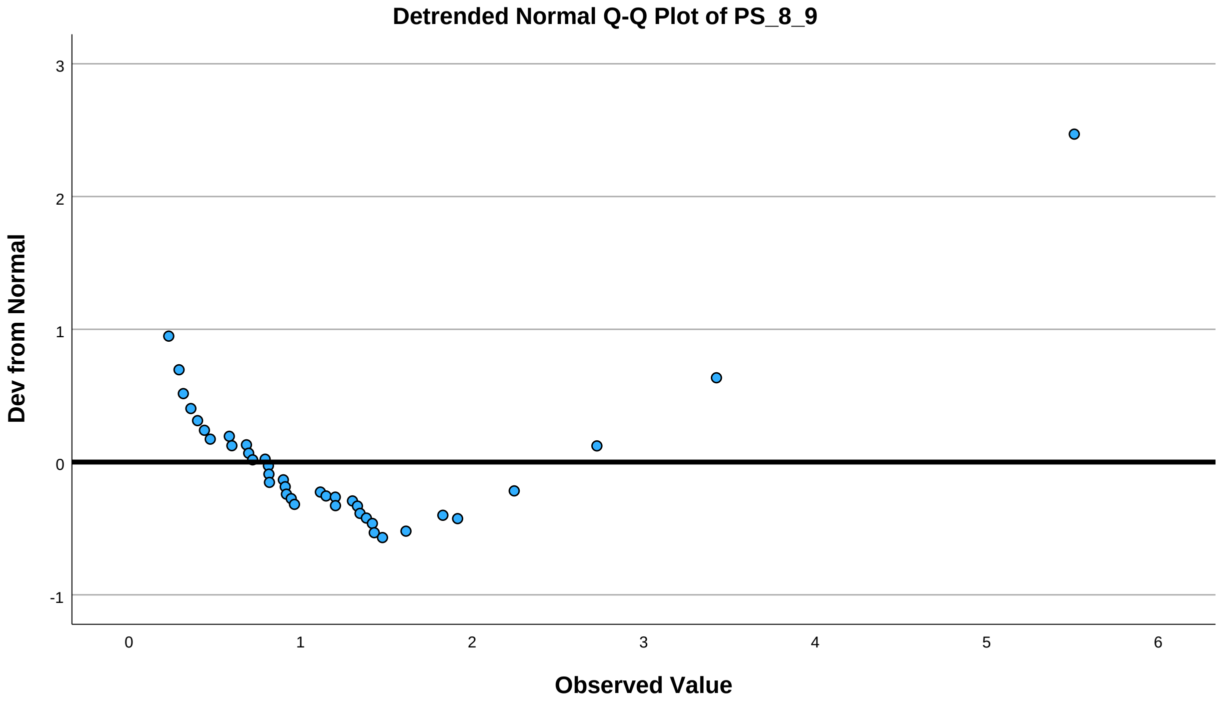
Detrended Normal Q-Q Plot of PS_8_9
Dev from Normal
Observed Value
3
2
1
0
-1
0
1
2
3
4
5
6

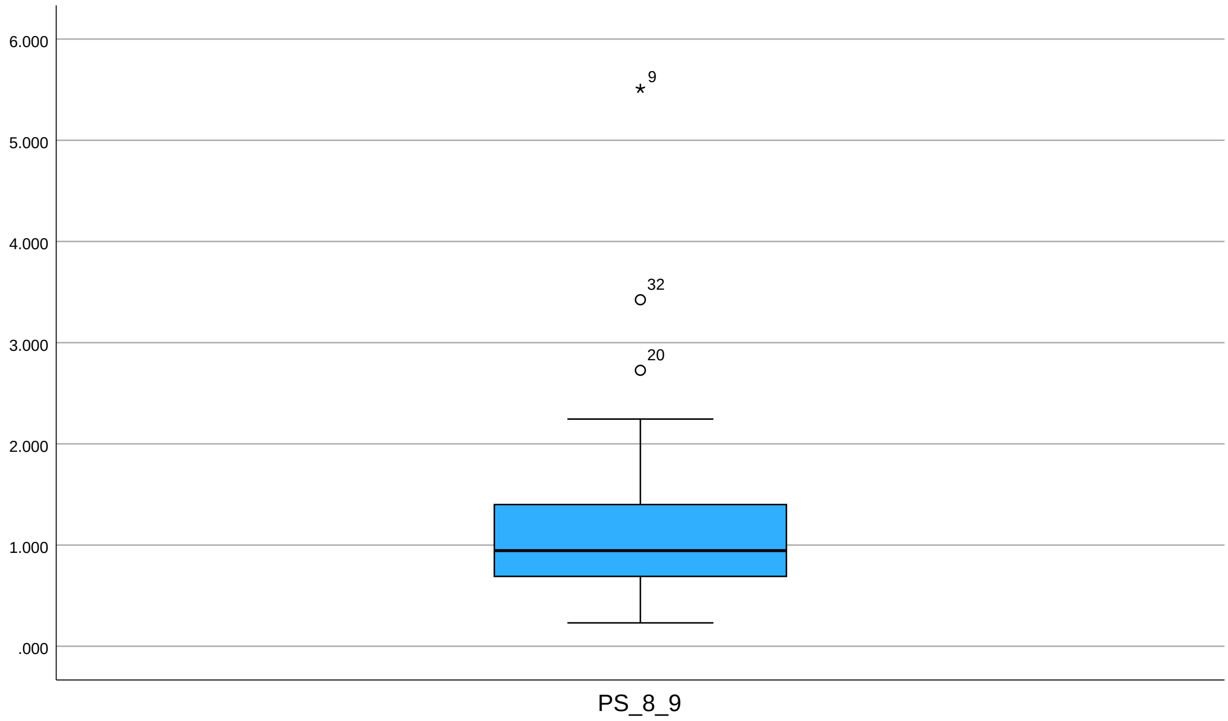

PS_9_10

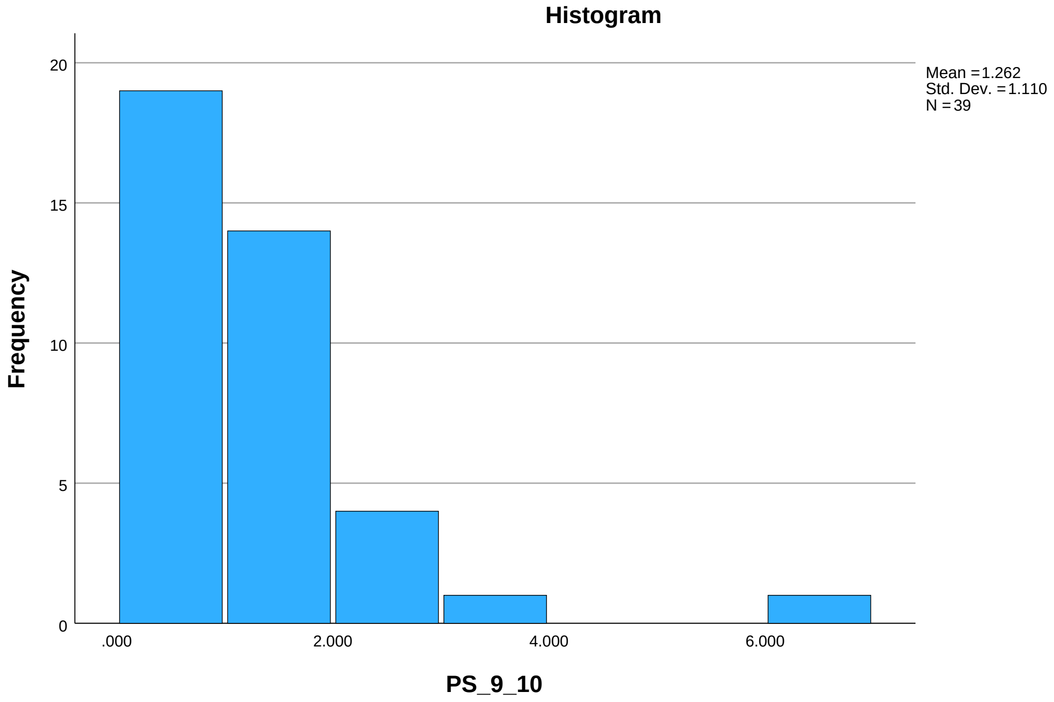

PS_9_10 Stem-and-Leaf Plot

Frequency Stem & Leaf

```
    7.00        0 .  2233444
   12.00        0 .  555566788999
   11.00        1 .  00002233334
    3.00        1 .  559
    4.00        2 .  0134
    2.00 Extremes    (>=3.9)

 Stem width:     1.000
 Each leaf:       1 case(s)
```

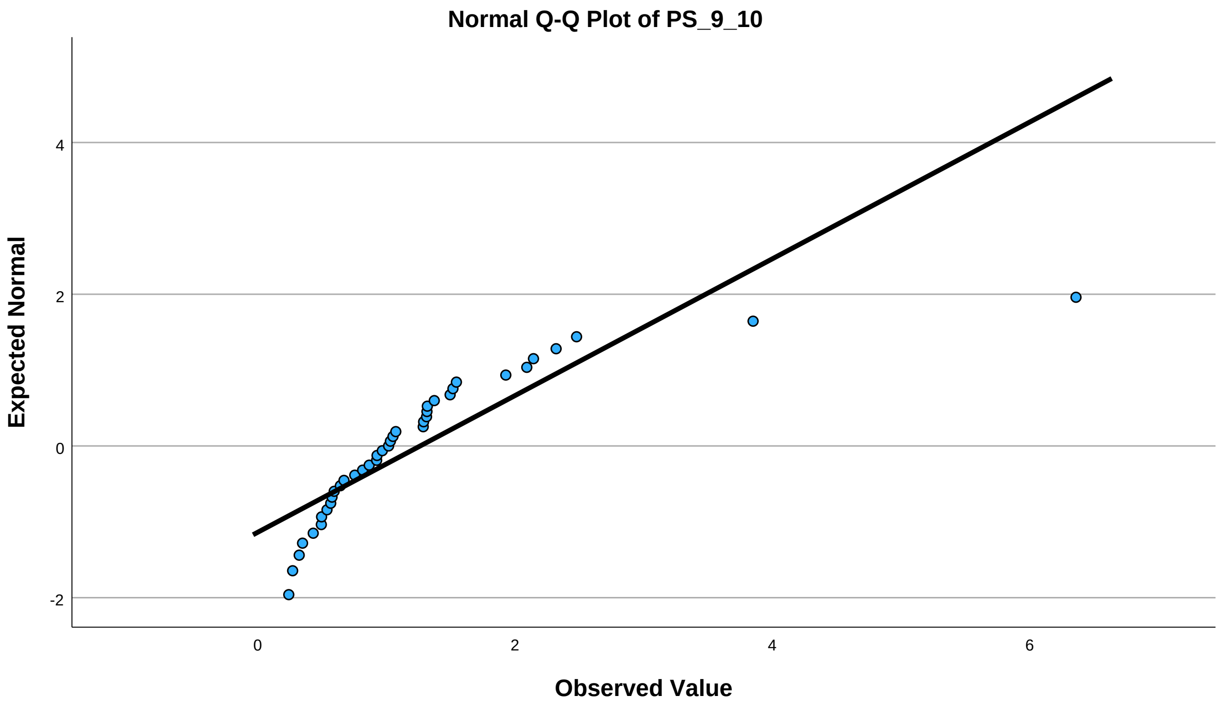

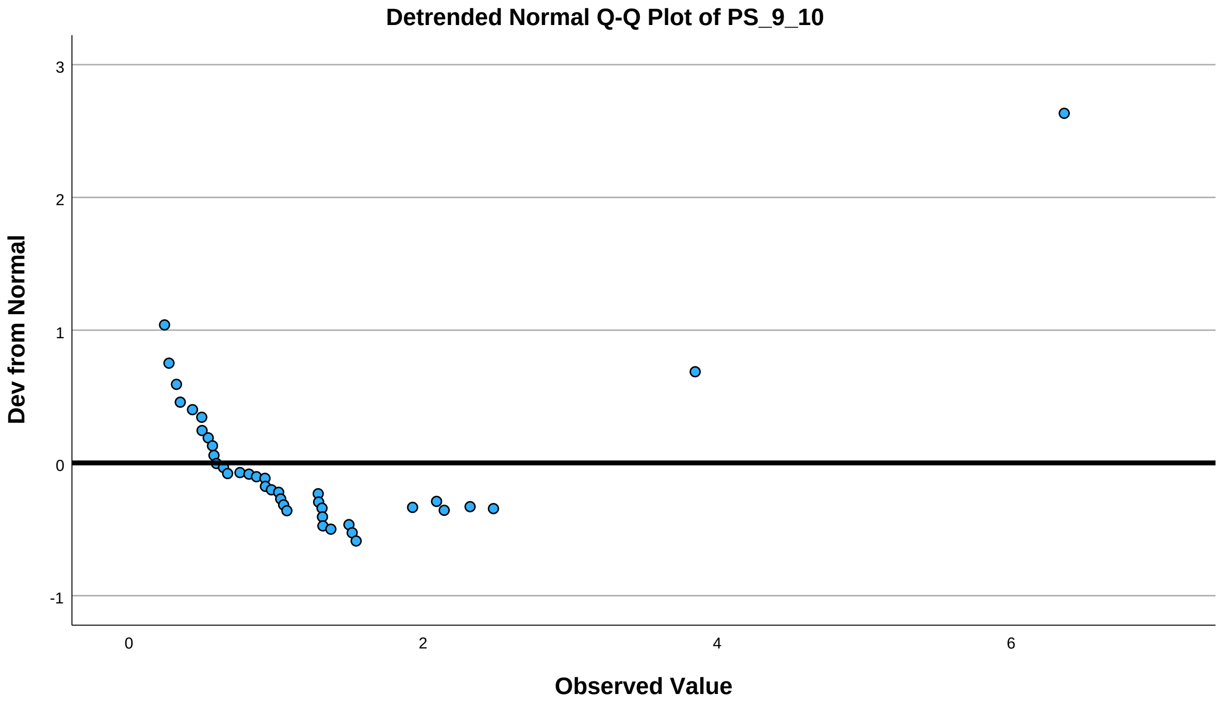

Detrended Normal Q-Q Plot of PS_9_10
Dev from Normal
3
2
1
0
-1
0
2
4
6
Observed Value

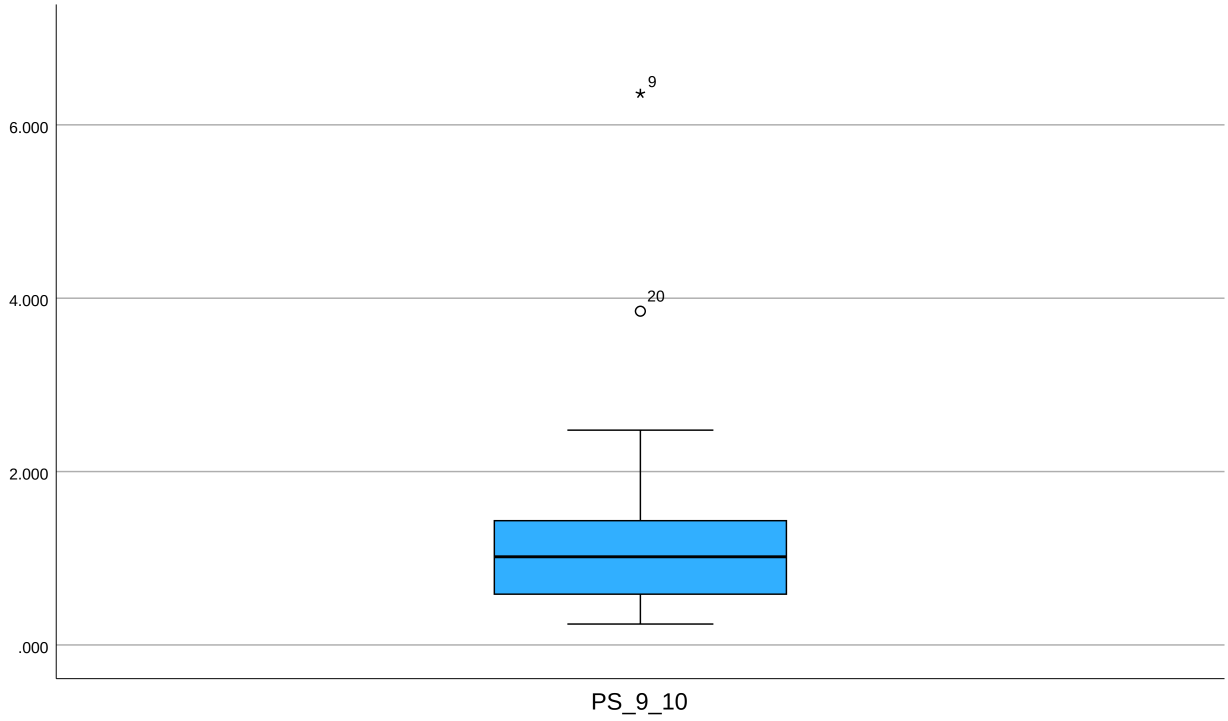

9
20
6.000
4.000
2.000
.000
PS_9_10

PS_10_11

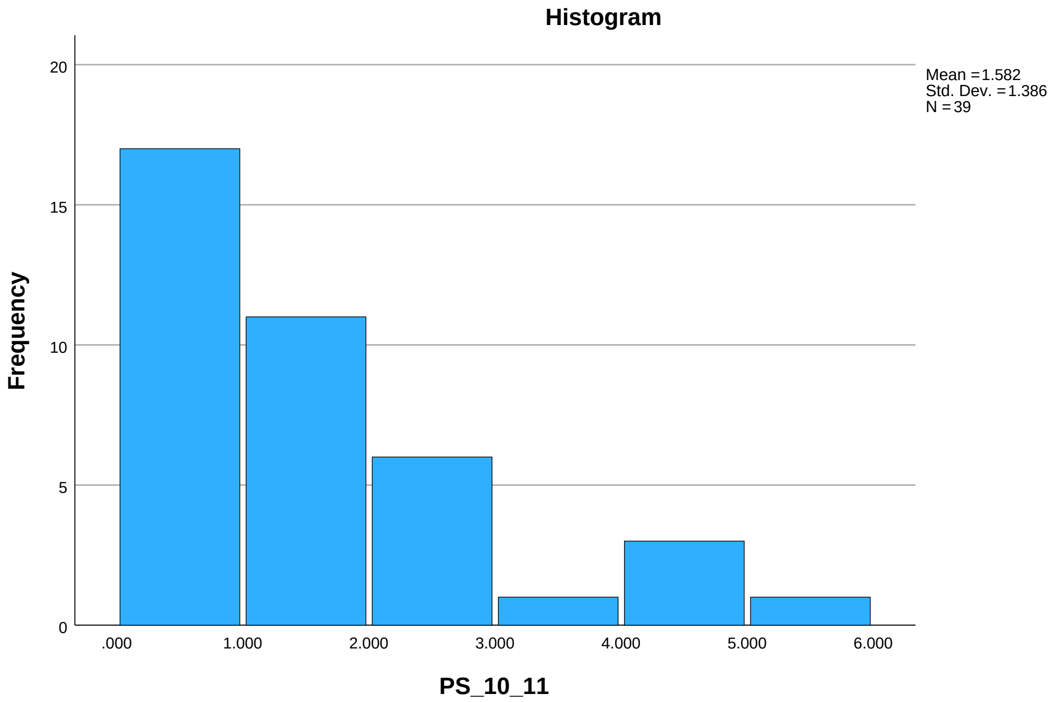


```
PS_10_11 Stem-and-Leaf Plot

 Frequency    Stem &  Leaf

     9.00        0 .  233334444
     8.00        0 .  67888999
     8.00        1 .  00111234
     3.00        1 .  569
     5.00        2 .  00012
     1.00        2 .  7
     1.00        3 .  3
     4.00 Extremes    (>=4.5)

 Stem width:      1.000
 Each leaf:        1 case(s)
```

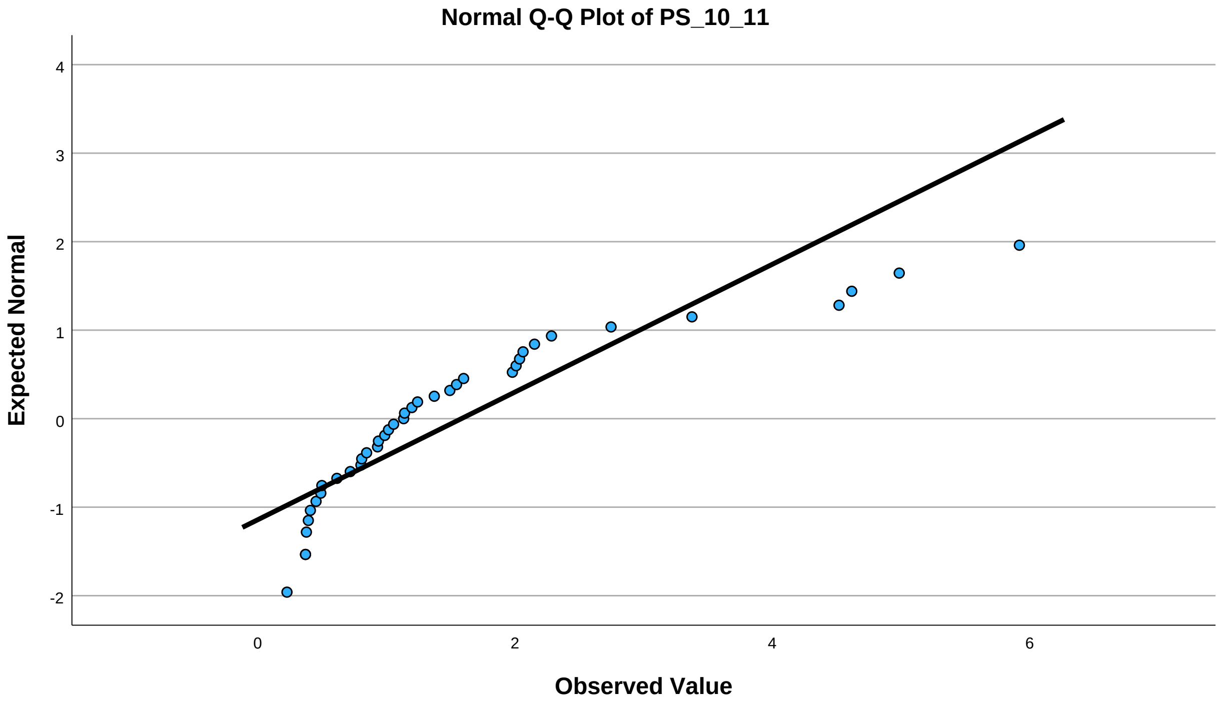

Normal Q-Q Plot of PS_10_11
Expected Normal
Observed Value
4
3
2
1
0
-1
-2
0
2
4
6

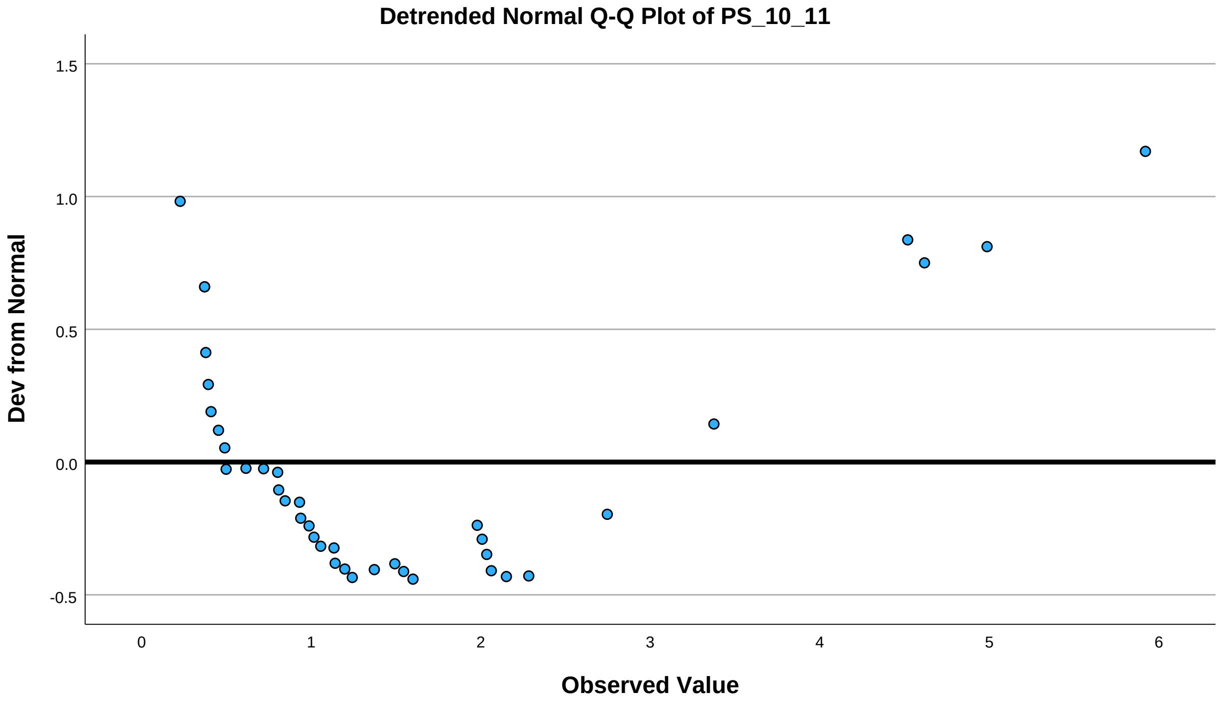

Detrended Normal Q-Q Plot of PS_10_11
Dev from Normal
Observed Value
1.5
1.0
0.5
0.0
-0.5
0
1
2
3
4
5
6

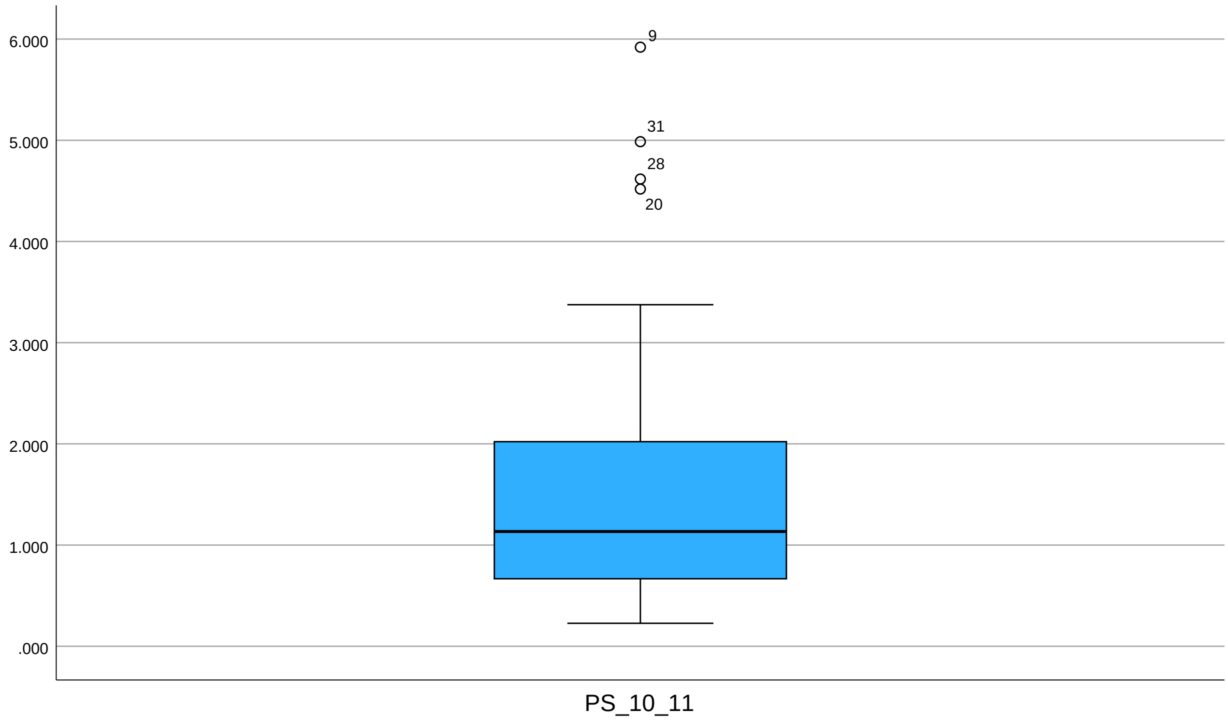

PS_11_12

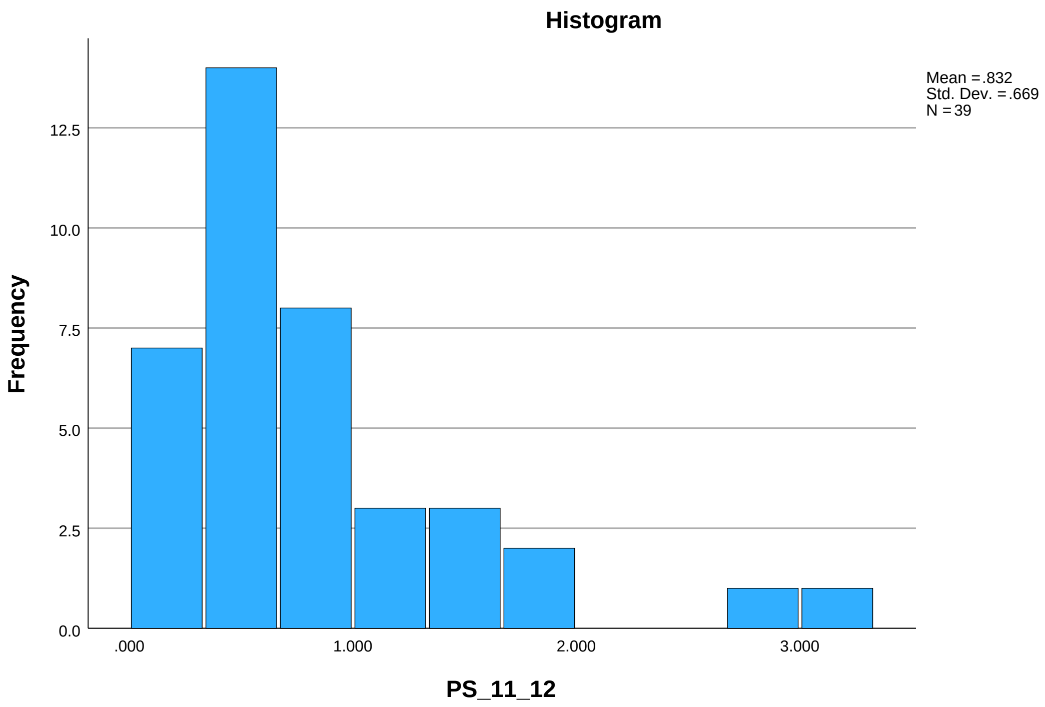

PS_11_12 Stem-and-Leaf Plot

Frequency   Stem & Leaf

```
11.00       0 .  22222223333
 7.00      0 .  4444555
 5.00      0 .  66667
 6.00      0 .  888889
 2.00     1 .  00
 3.00     1 .  233
 1.00     1 .  4
  .00     1 .
 1.00     1 .  8
  3.00 Extremes    (>=2.0)

Stem width:     1.000
Each leaf:       1 case(s)
```

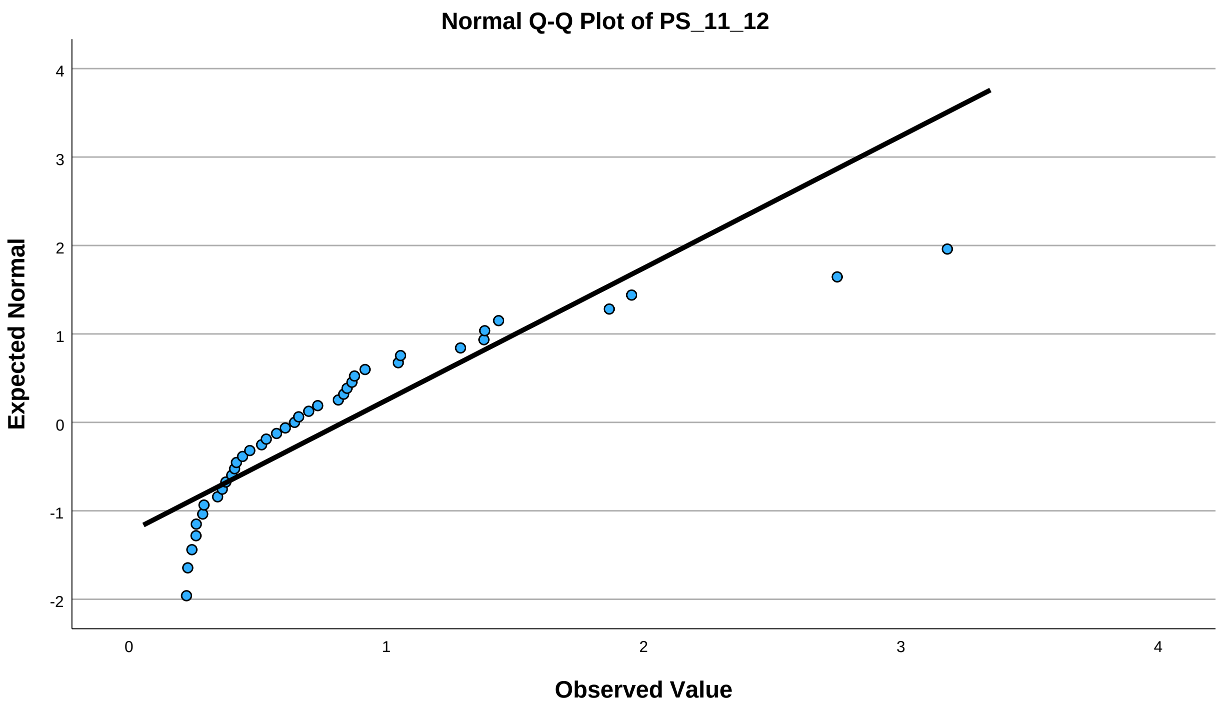

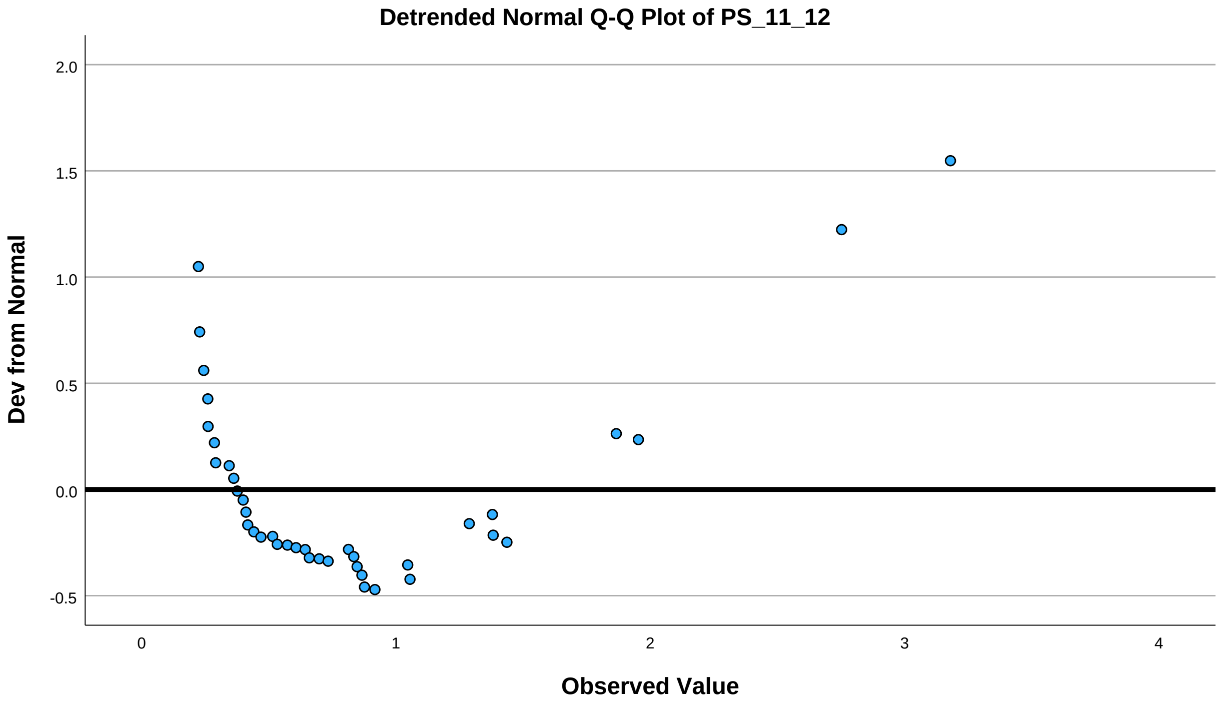
Detrended Normal Q-Q Plot of PS_11_12
Dev from Normal
2.0
1.5
1.0
0.5
0.0
-0.5
0
1
2
3
4
Observed Value

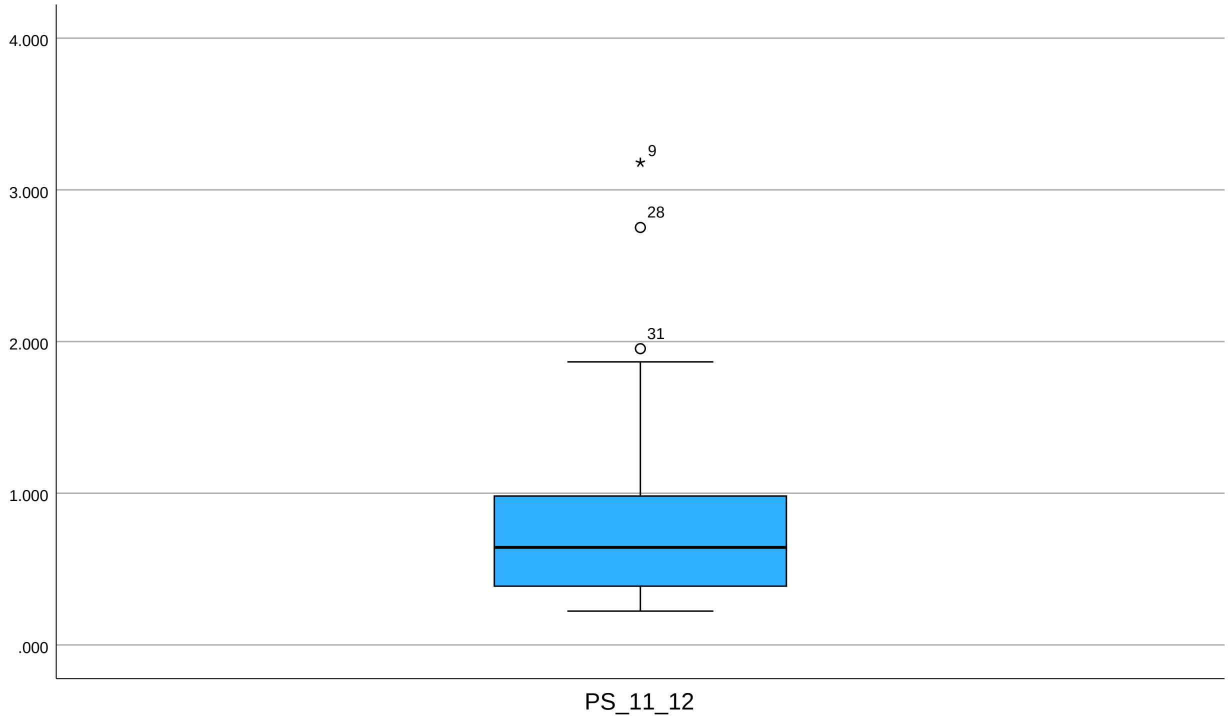
4.000
3.000
2.000
1.000
.000
9
28
31
PS_11_12

PS_12_13

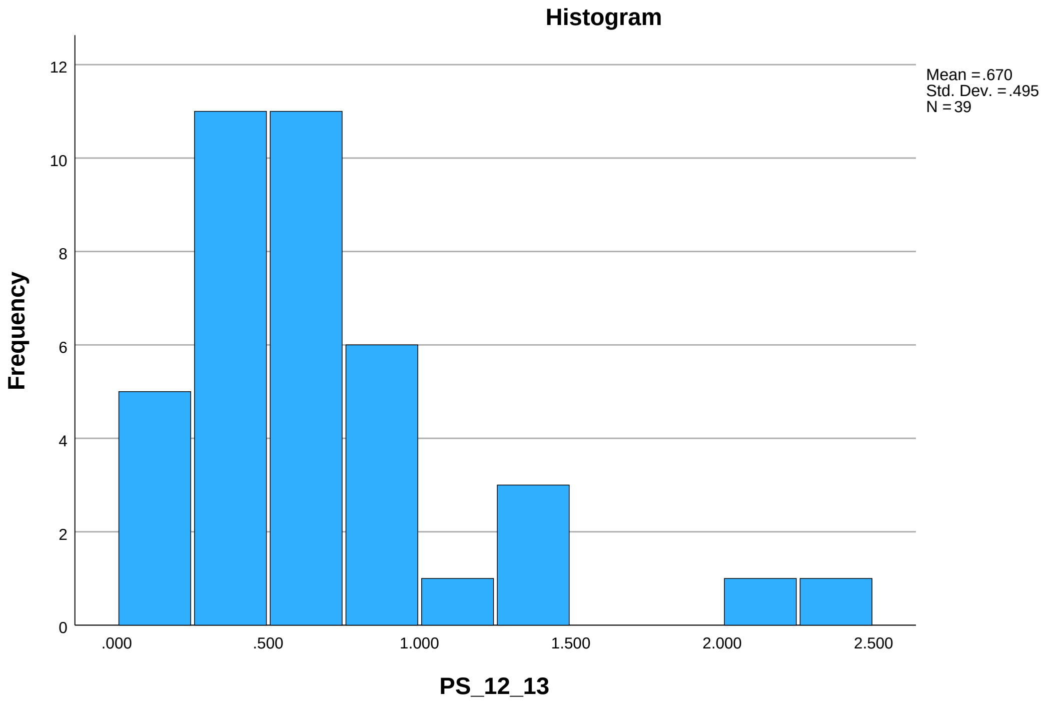


```
PS_12_13 Stem-and-Leaf Plot

 Frequency    Stem &  Leaf

     3.00        0 .  111
    10.00        0 .  2222233333
     7.00        0 .  4445555
    12.00        0 .  666666777777
     1.00        0 .  9
     1.00        1 .  1
     2.00        1 .  33
     3.00 Extremes    (>=1.5)

 Stem width:      1.000
 Each leaf:       1 case(s)
```

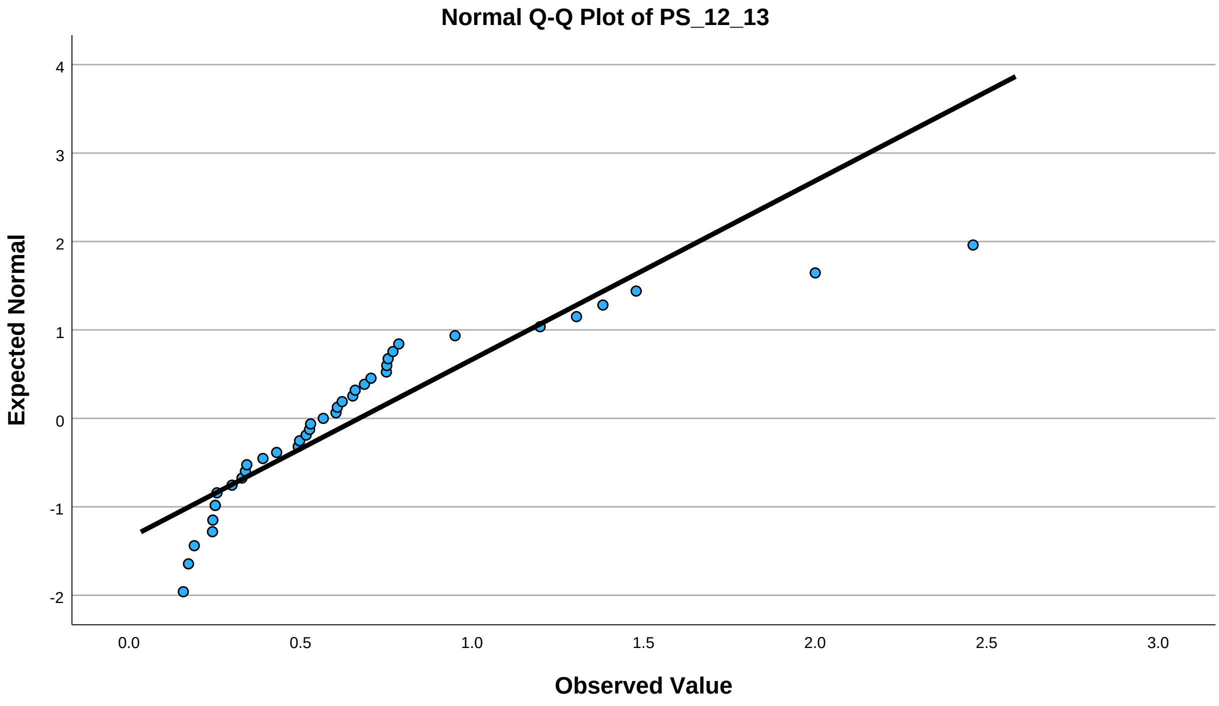
Normal Q-Q Plot of PS_12_13
Expected Normal
Observed Value
4
3
2
1
0
-1
-2
0.0
0.5
1.0
1.5
2.0
2.5
3.0

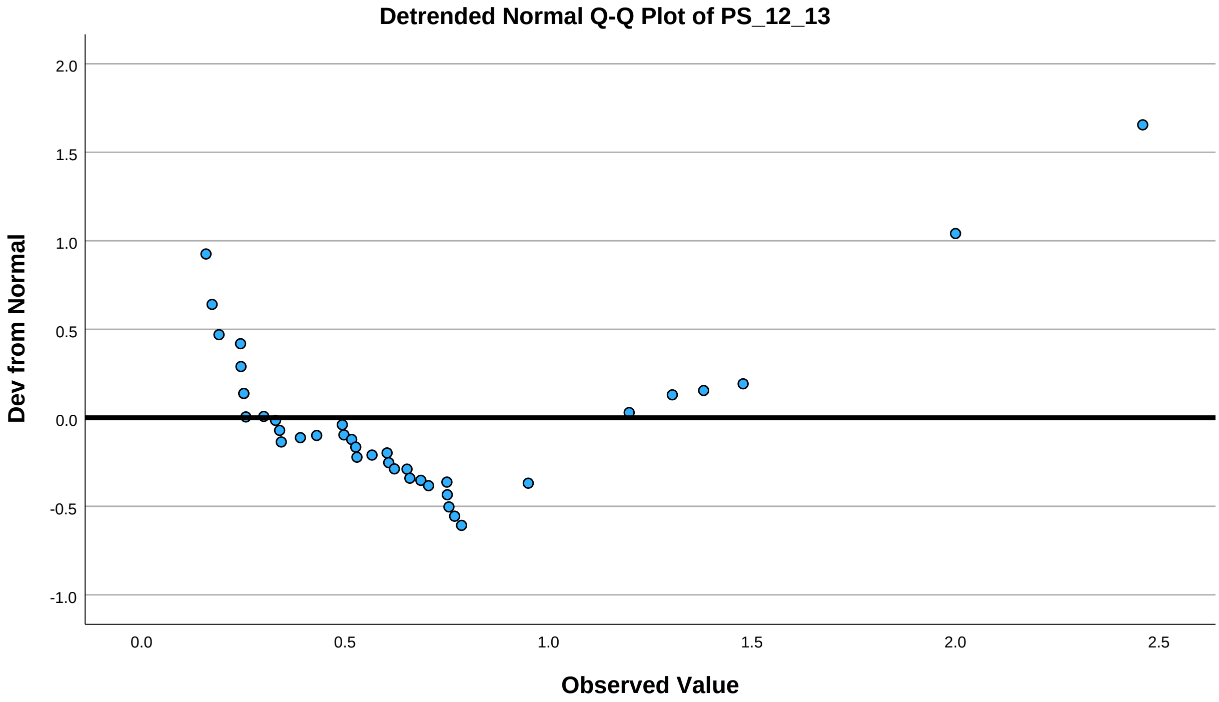
Detrended Normal Q-Q Plot of PS_12_13
Dev from Normal
Observed Value
2.0
1.5
1.0
0.5
0.0
-0.5
-1.0
0.0
0.5
1.0
1.5
2.0
2.5

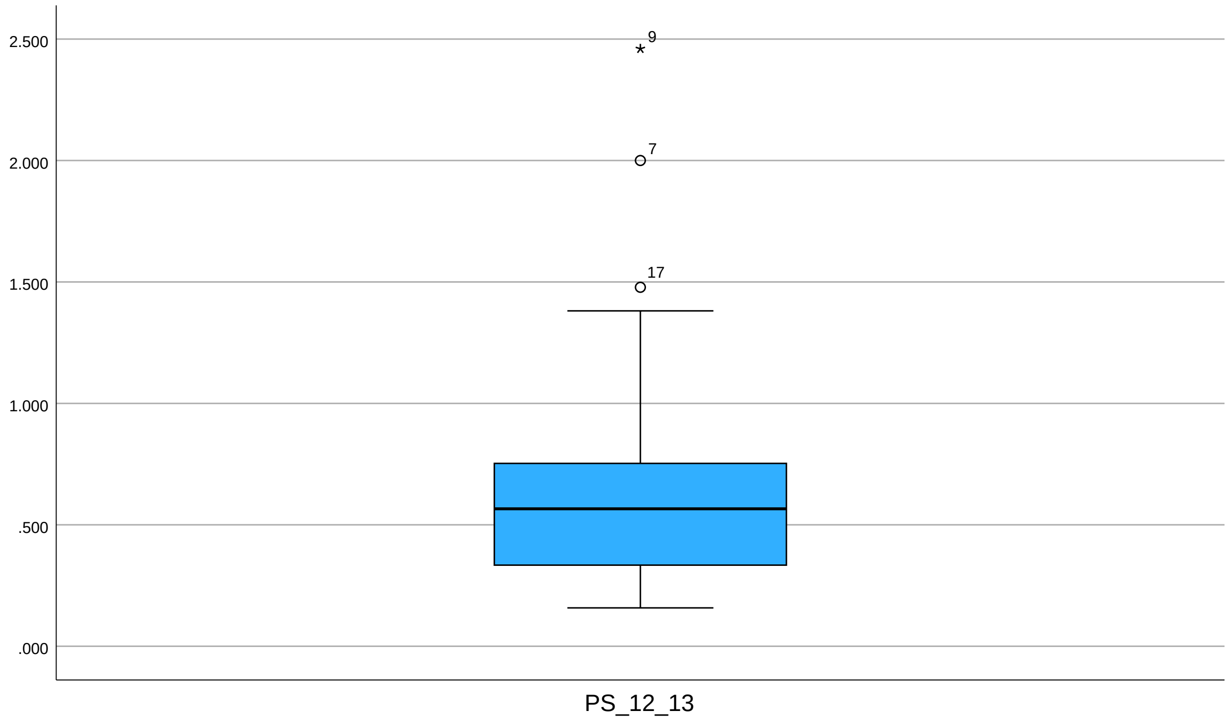

PS_13_14

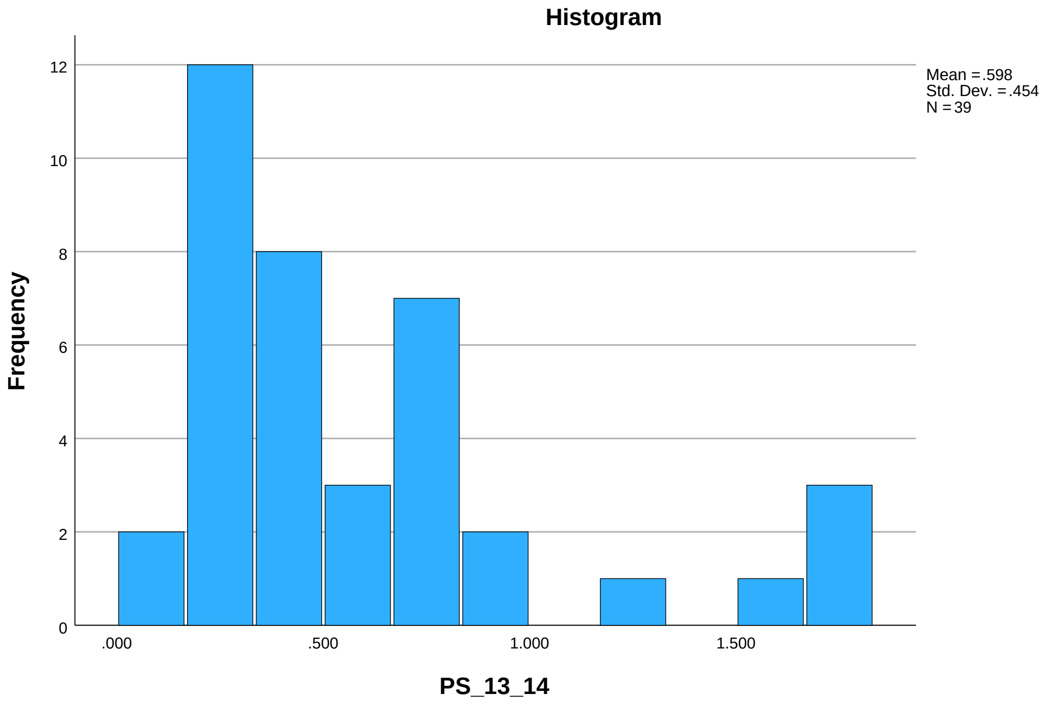

PS_13_14 Stem-and-Leaf Plot

Frequency   Stem & Leaf

```
     3.00        1 .  357
    11.00        2 .  12334566789
     1.00        3 .  3
     7.00        4 .  0012566
     3.00        5 .  469
     2.00        6 .  68
     4.00        7 .  1225
     2.00        8 .  23
     1.00        9 .  8
      .00       10 .
      .00       11 .
     1.00       12 .  4
     4.00 Extremes    (>=1.65)

 Stem width:       .100
 Each leaf:        1 case(s)
```

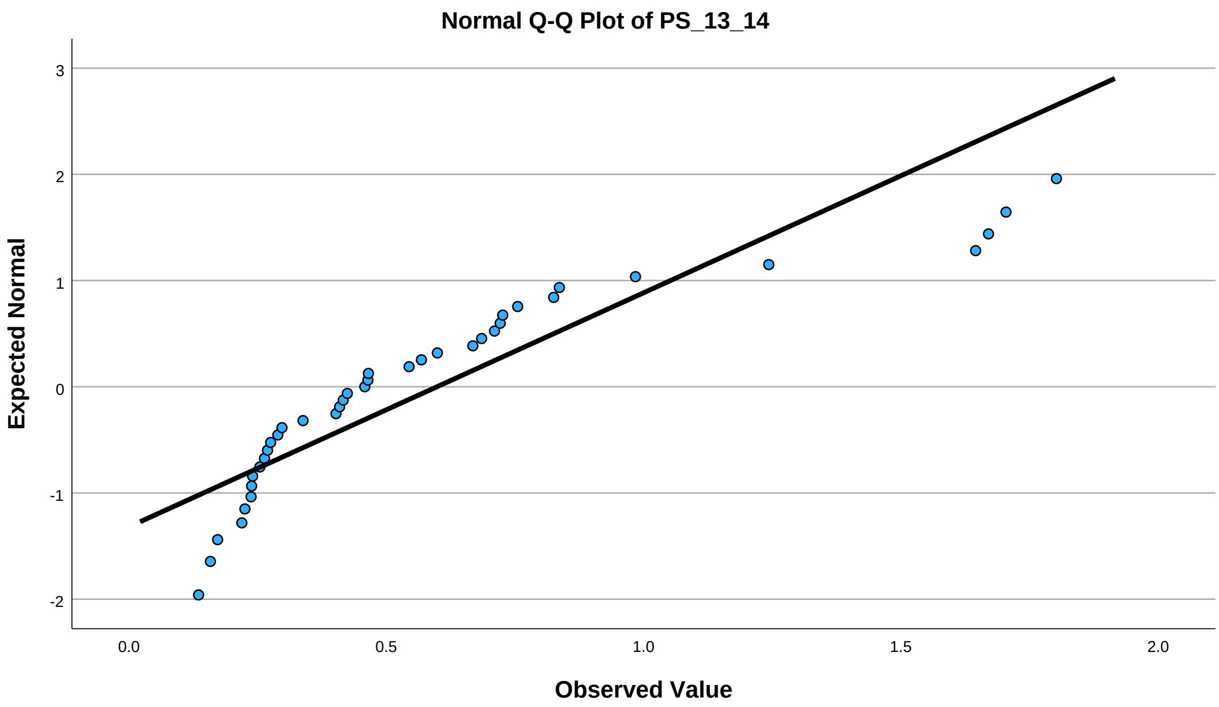

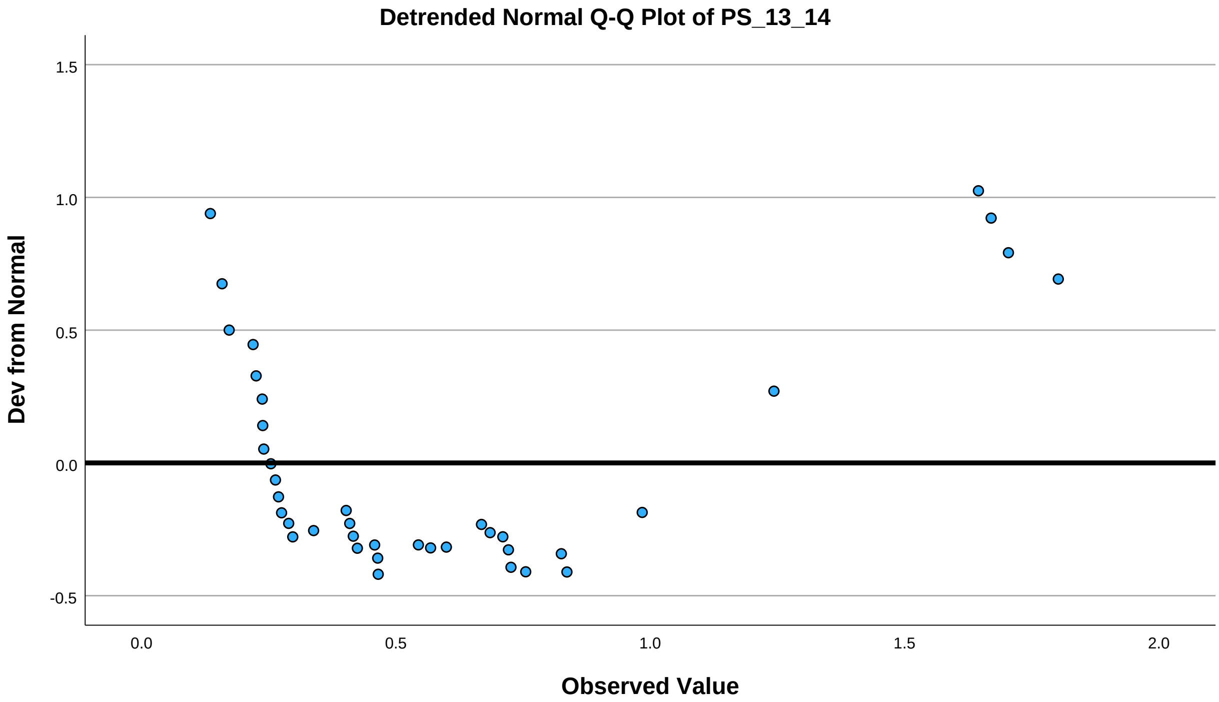
Detrended Normal Q-Q Plot of PS_13_14
Dev from Normal
1.5
1.0
0.5
0.0
-0.5
0.0
0.5
1.0
1.5
2.0
Observed Value

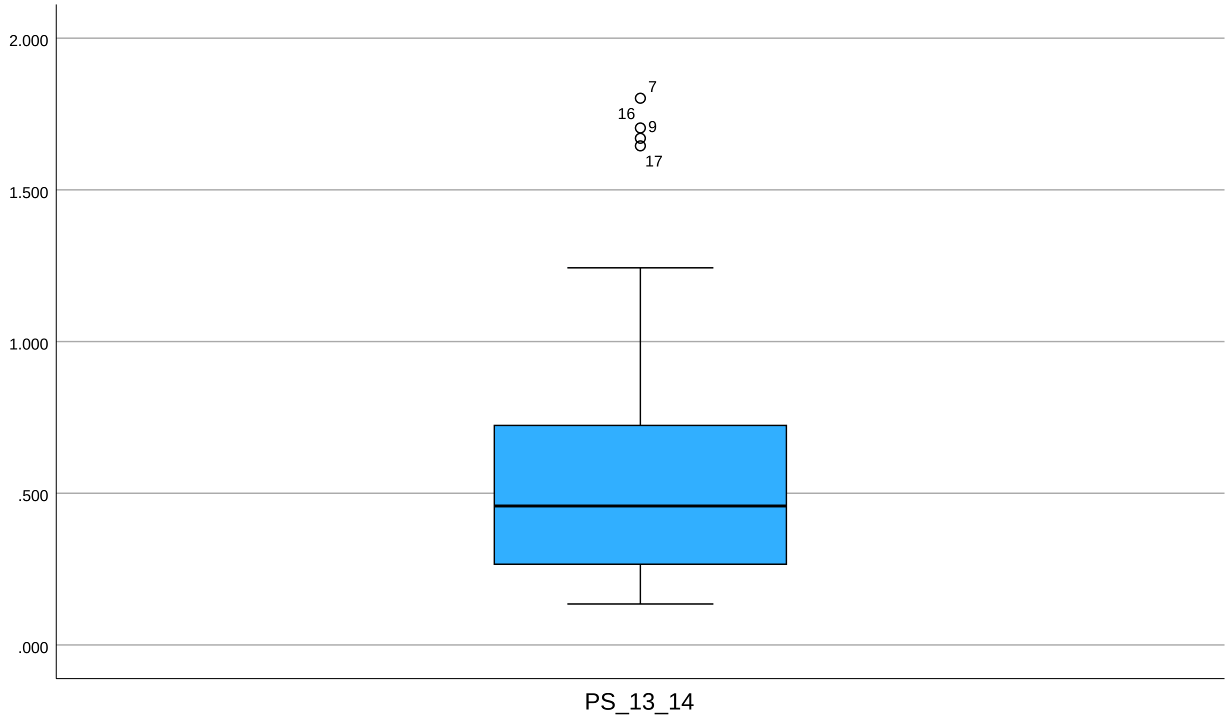
2.000
1.500
1.000
.500
.000
7
16
9
17
PS_13_14

PS_14_15

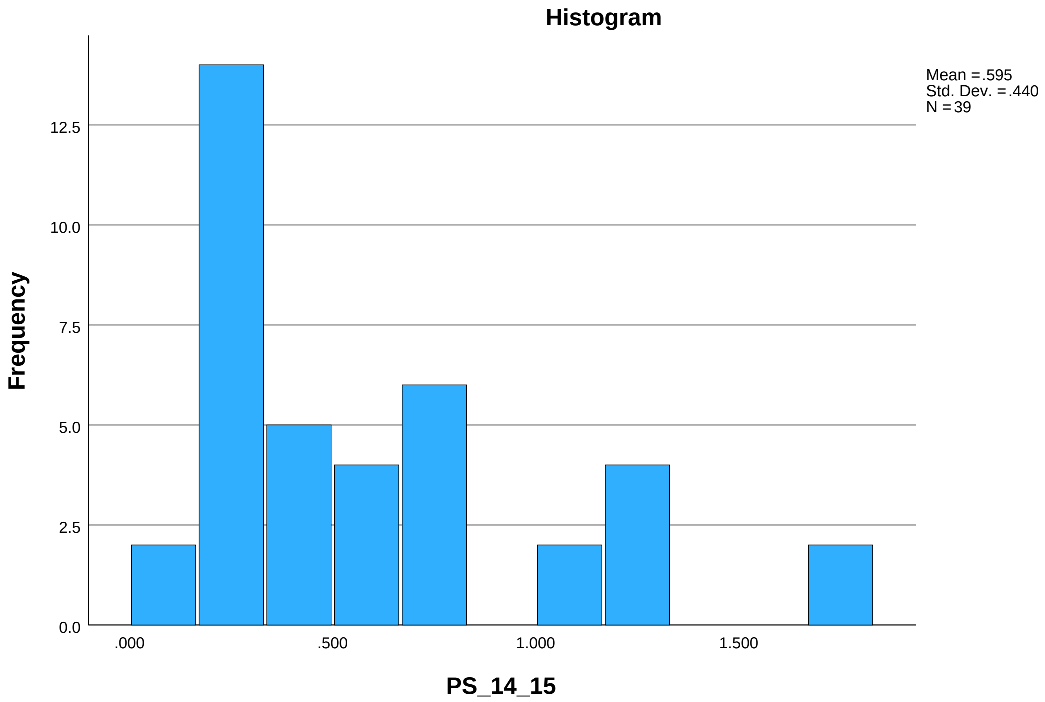


```
PS_14_15 Stem-and-Leaf Plot

 Frequency    Stem &  Leaf

     5.00        1 .  35788
     7.00        2 .  1222378
     6.00        3 .  001157
     3.00        4 .  224
     2.00        5 .  36
     3.00        6 .  137
     3.00        7 .  017
     2.00        8 .  03
      .00        9 .
     2.00       10 .  34
     1.00       11 .  6
     3.00       12 .  089
     2.00 Extremes    (>=1.75)

 Stem width:      .100
 Each leaf:       1 case(s)
```

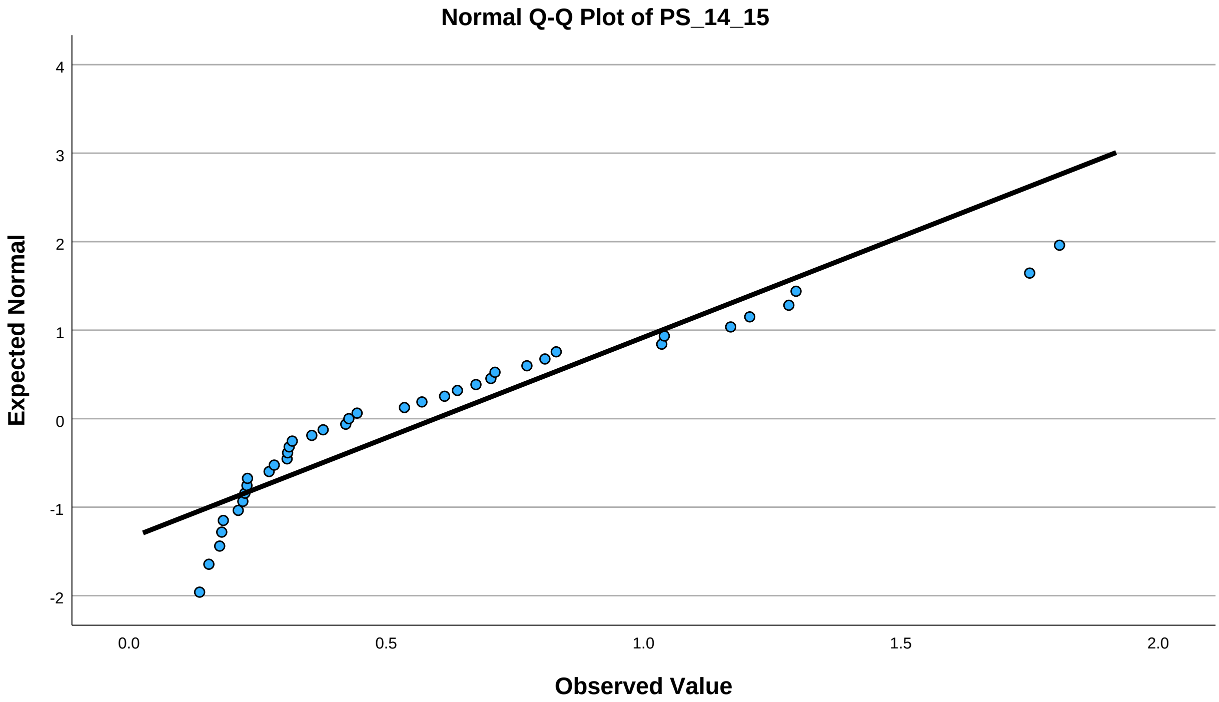
Normal Q-Q Plot of PS_14_15
Expected Normal
4
3
2
1
0
-1
-2
0.0
0.5
1.0
1.5
2.0
Observed Value

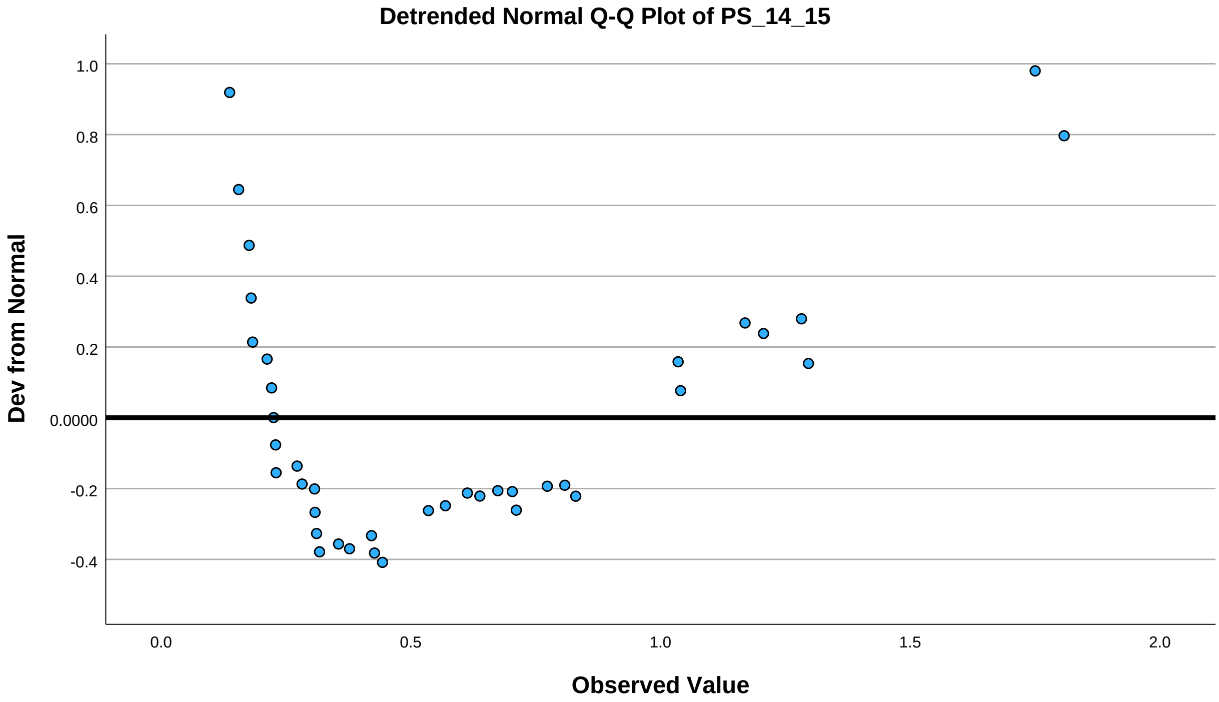
Detrended Normal Q-Q Plot of PS_14_15
Dev from Normal
1.0
0.8
0.6
0.4
0.2
0.0000
-0.2
-0.4
0.0
0.5
1.0
1.5
2.0
Observed Value

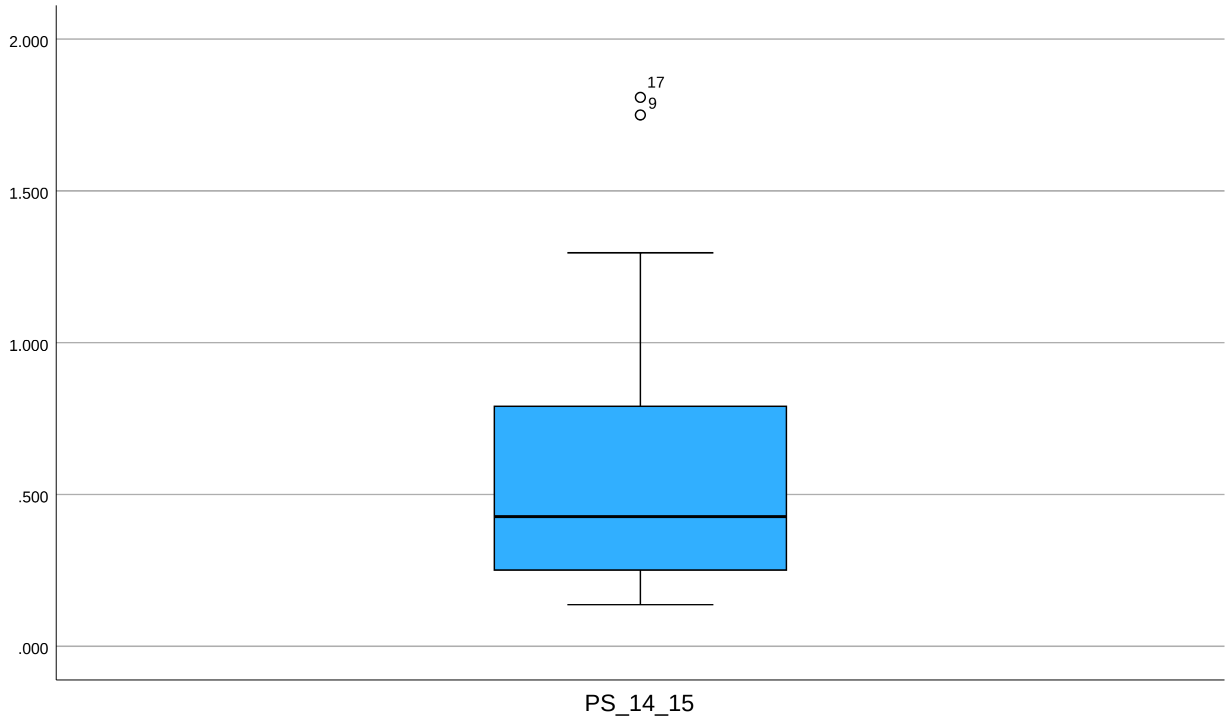

PS_15_16

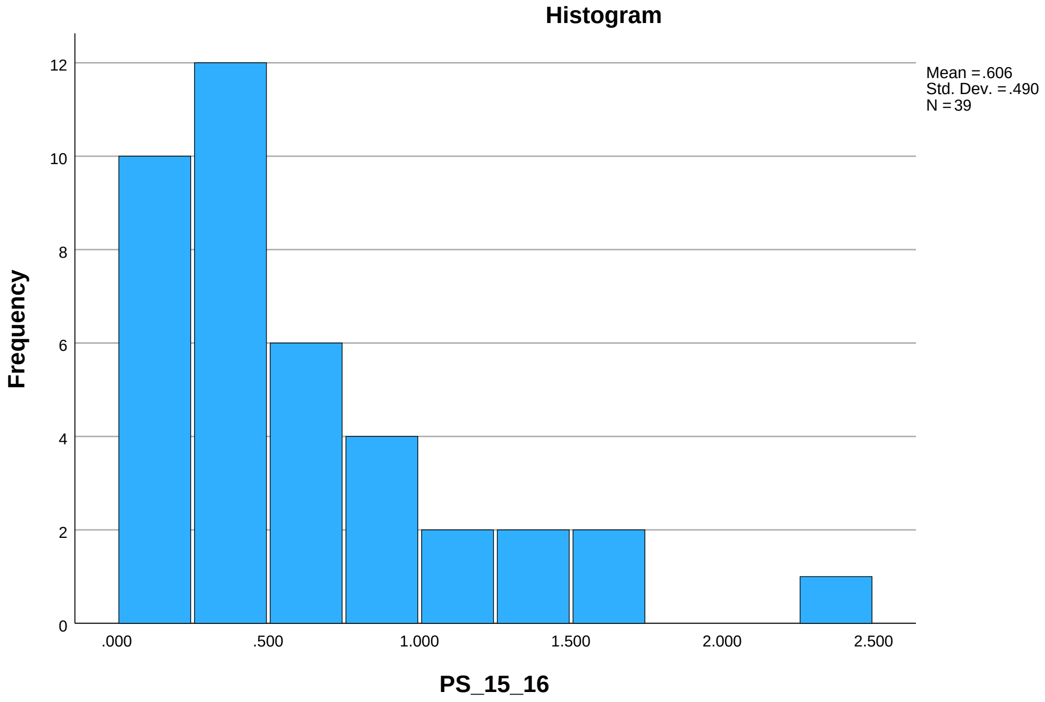

PS_15_16 Stem-and-Leaf Plot

Frequency   Stem & Leaf

```
     7.00        0 .  1111111
    10.00        0 .  2222223333
     6.00        0 .  444445
     8.00        0 .  66677777
     1.00        0 .  8
     1.00        1 .  0
     3.00        1 .  223
     3.00 Extremes    (>=1.6)

 Stem width:      1.000
 Each leaf:        1 case(s)
```

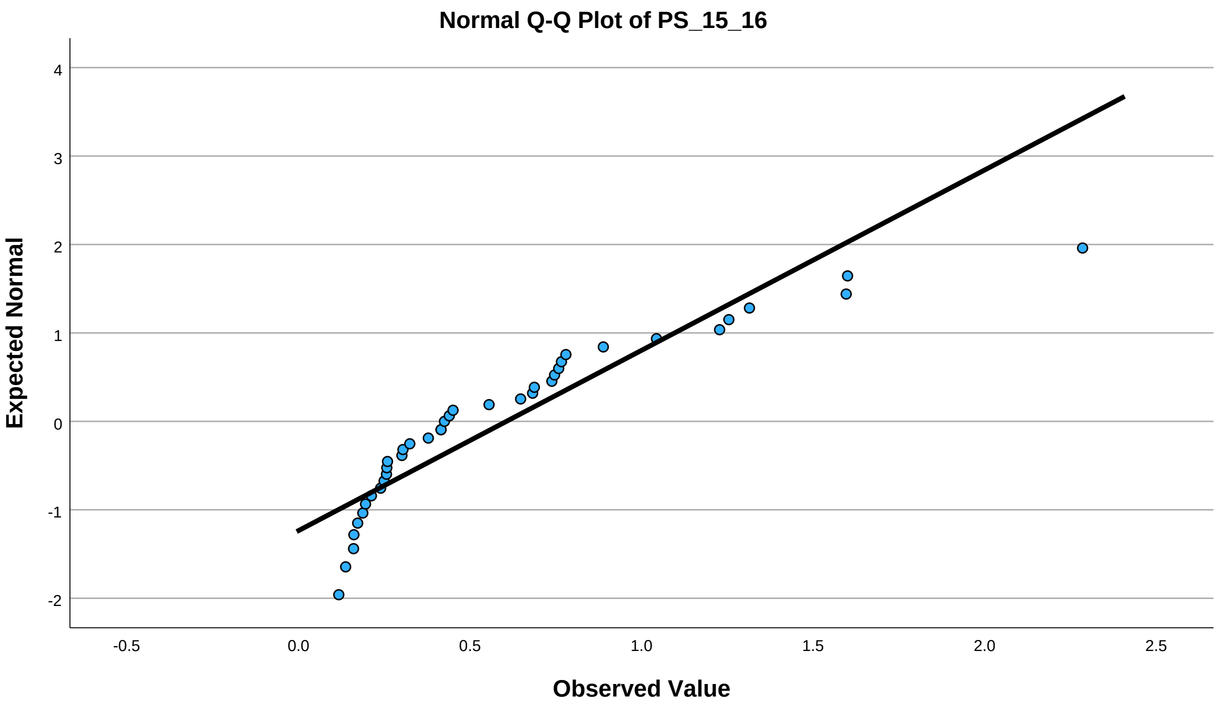

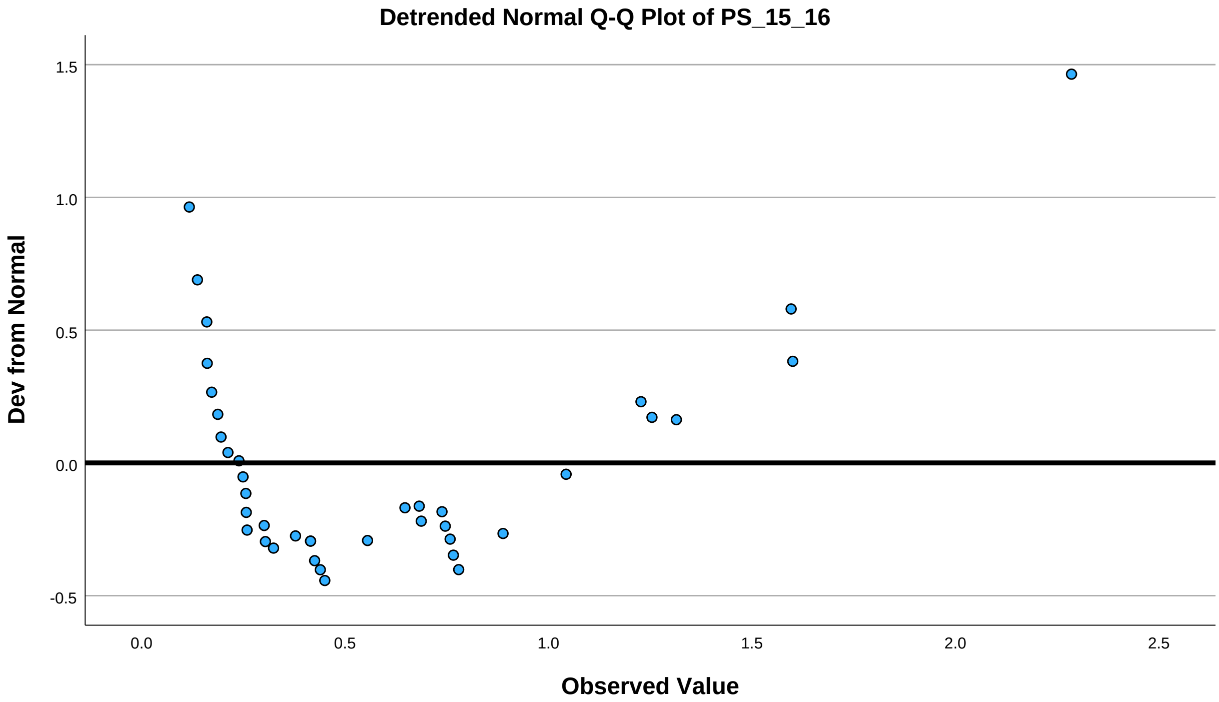

Detrended Normal Q-Q Plot of PS_15_16
Dev from Normal
1.5
1.0
0.5
0.0
-0.5
0.0
0.5
1.0
1.5
2.0
2.5
Observed Value

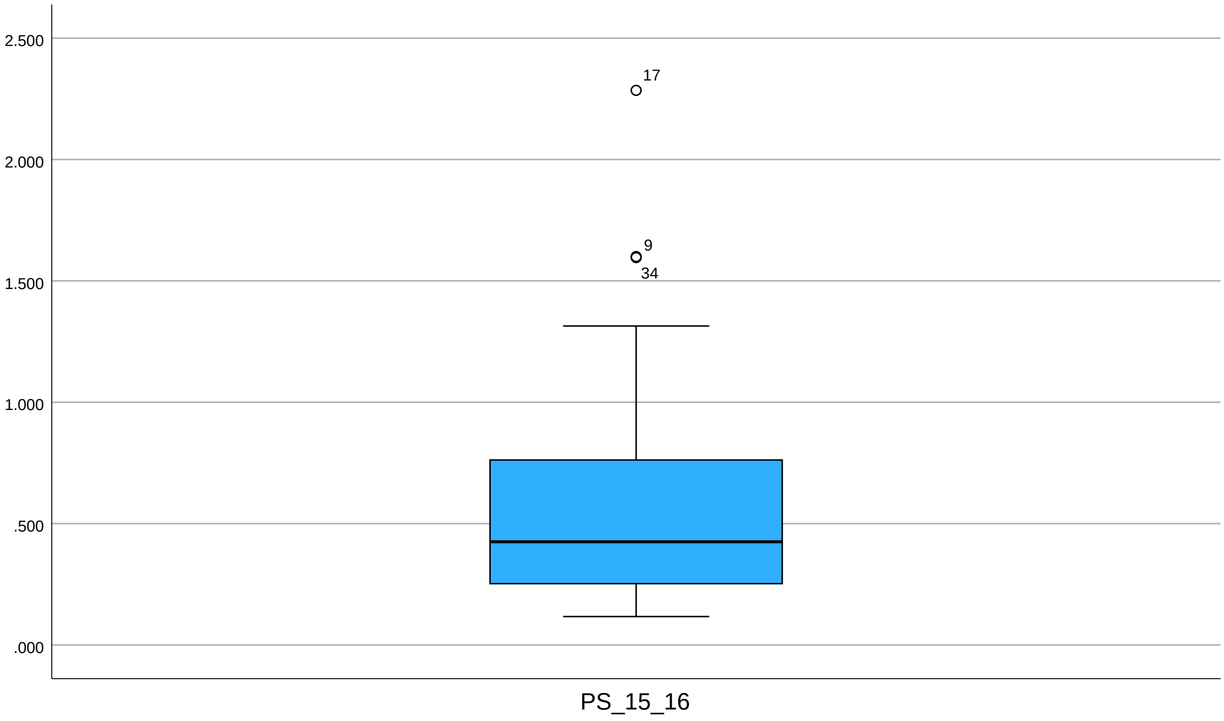

2.500
2.000
1.500
1.000
.500
.000
17
9
34
PS_15_16

PS_16_17

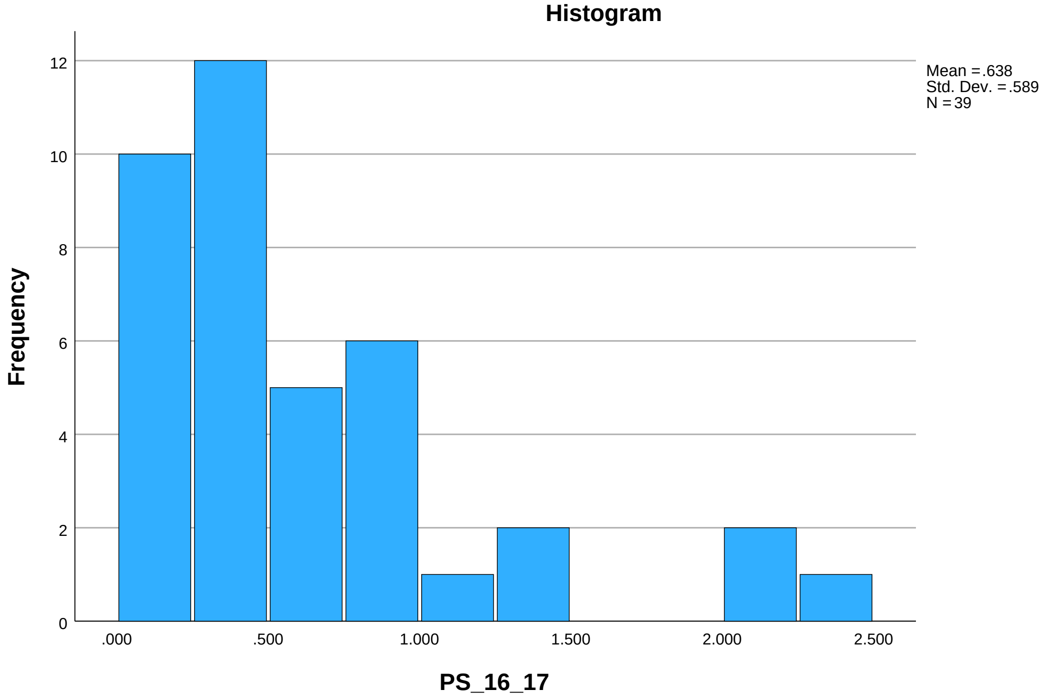

PS_16_17 Stem-and-Leaf Plot

```
 Frequency    Stem &  Leaf

     6.00        0 .  111111
    15.00        0 .  222222222233333
     2.00        0 .  45
     6.00        0 .  667777
     4.00        0 .  8899
     1.00        1 .  1
     1.00        1 .  2
     1.00        1 .  4
     3.00 Extremes    (>=2.2)

 Stem width:      1.000
 Each leaf:       1 case(s)
```

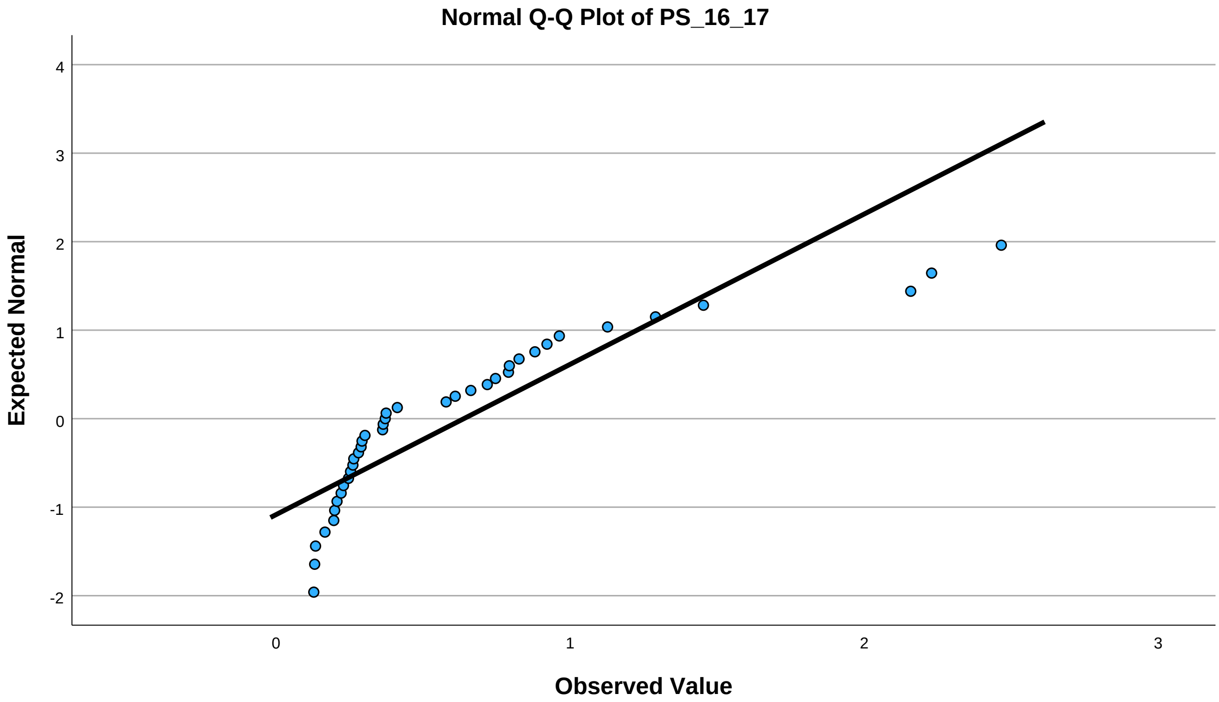
Normal Q-Q Plot of PS_16_17
Expected Normal
4
3
2
1
0
-1
-2
0
1
2
3
Observed Value

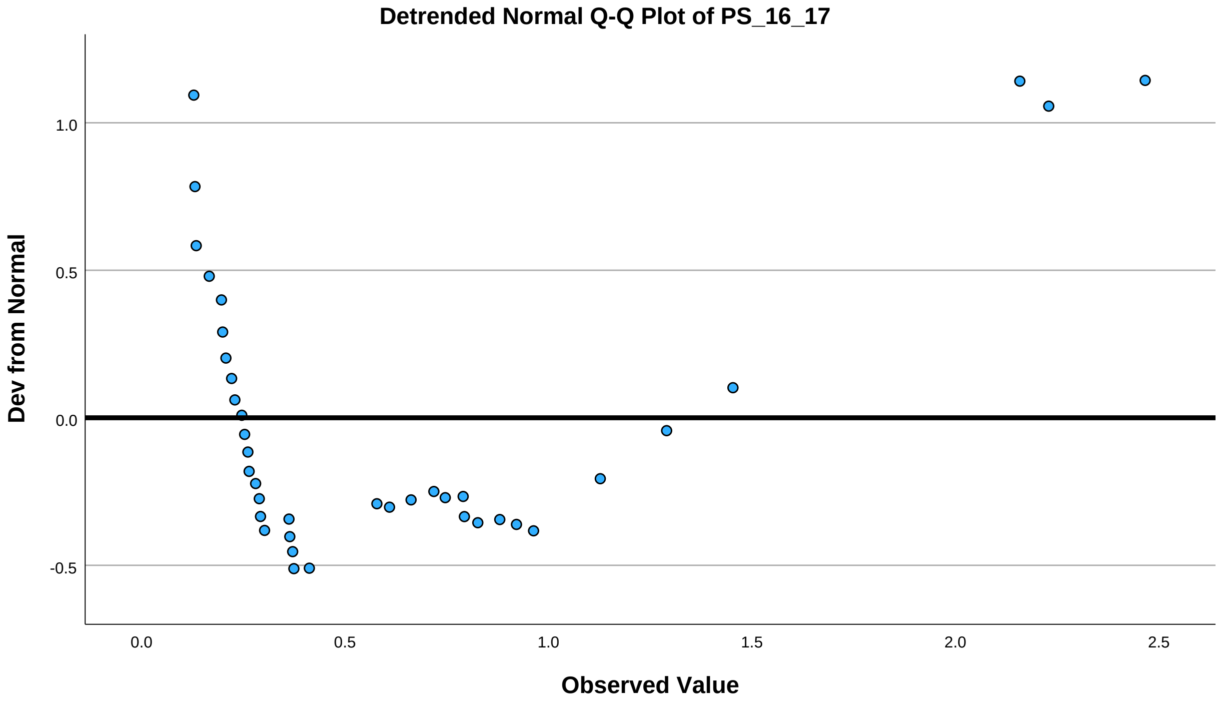
Detrended Normal Q-Q Plot of PS_16_17
Dev from Normal
1.0
0.5
0.0
-0.5
0.0
0.5
1.0
1.5
2.0
2.5
Observed Value

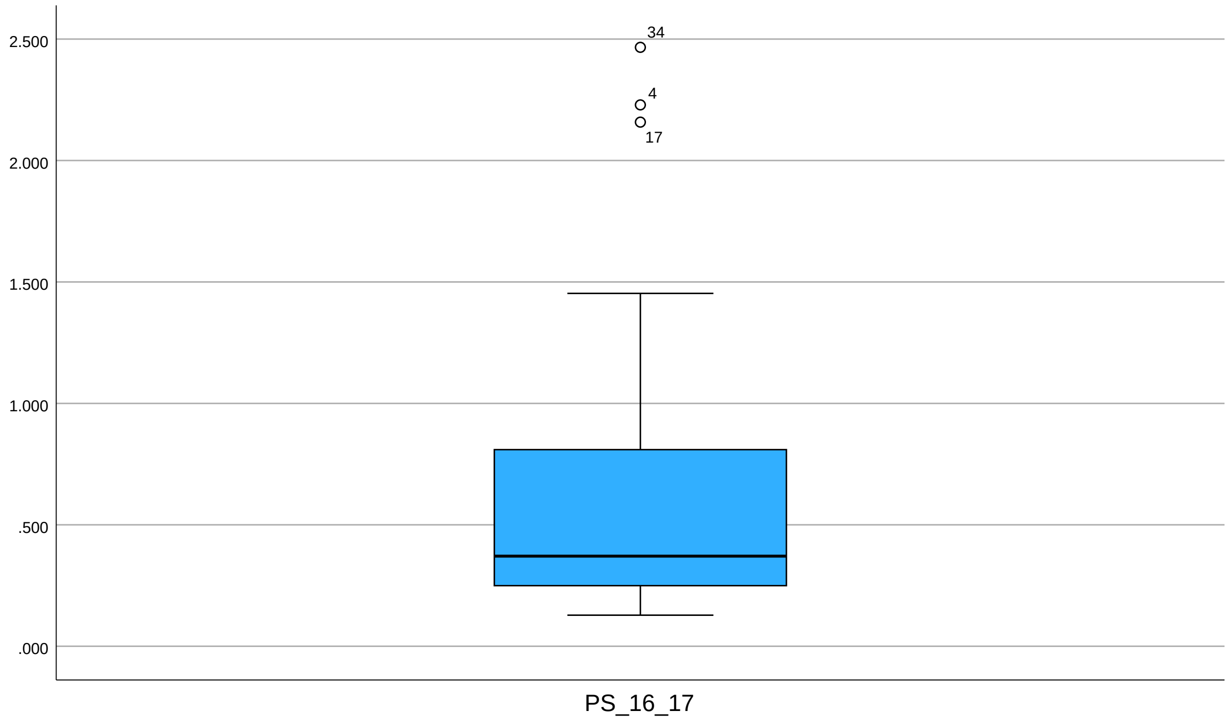

PS_17_18

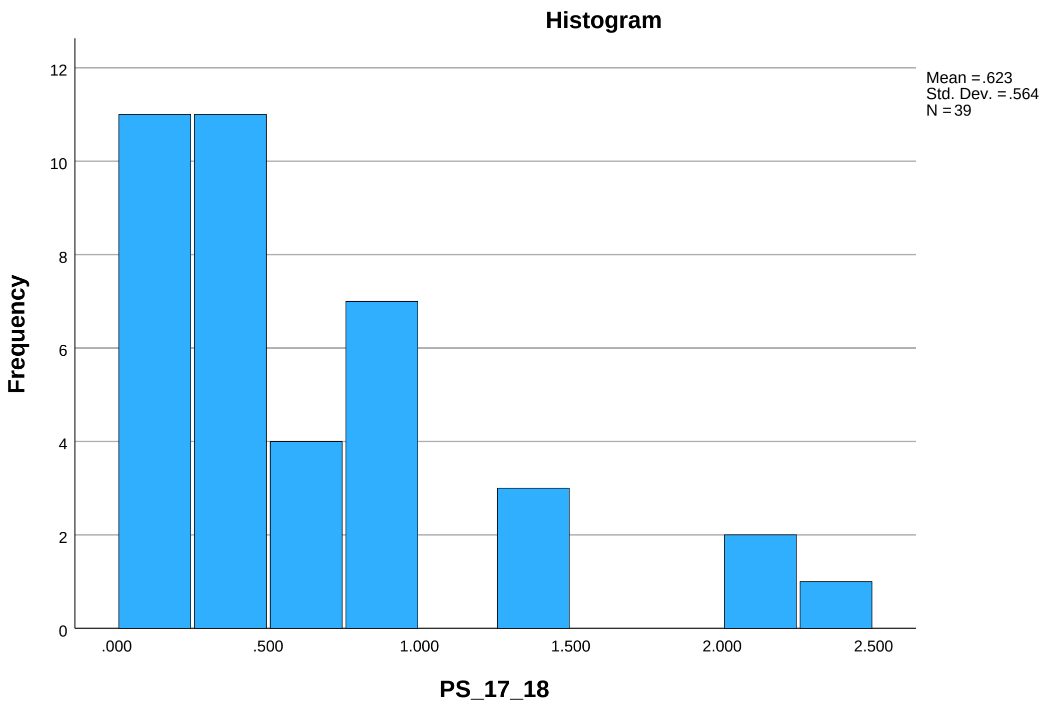

PS_17_18 Stem-and-Leaf Plot

Frequency   Stem & Leaf

```
  8.00       0 .  11111111
 12.00        0 .  222222223333
  4.00       0 .  4455
  4.00       0 .  7777
  5.00       0 .  88889
   .00     1 .
  3.00       1 .  233
  3.00 Extremes    (>=2.1)

Stem width:     1.000
Each leaf:       1 case(s)
```

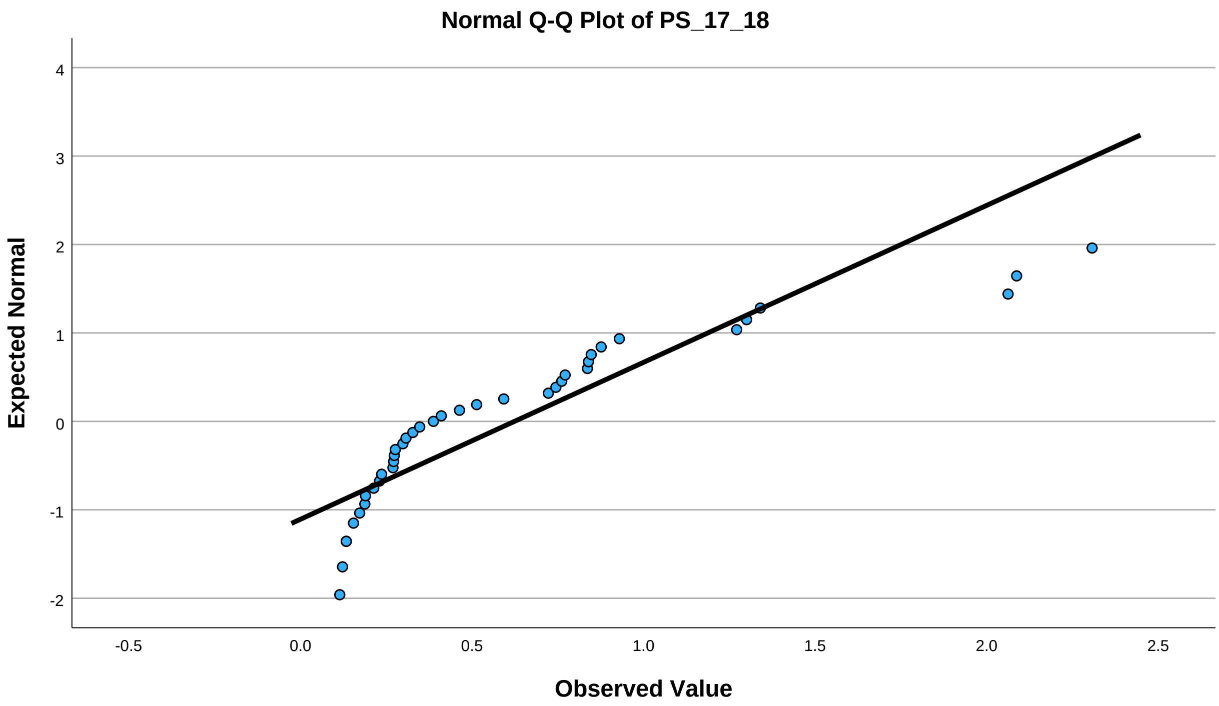

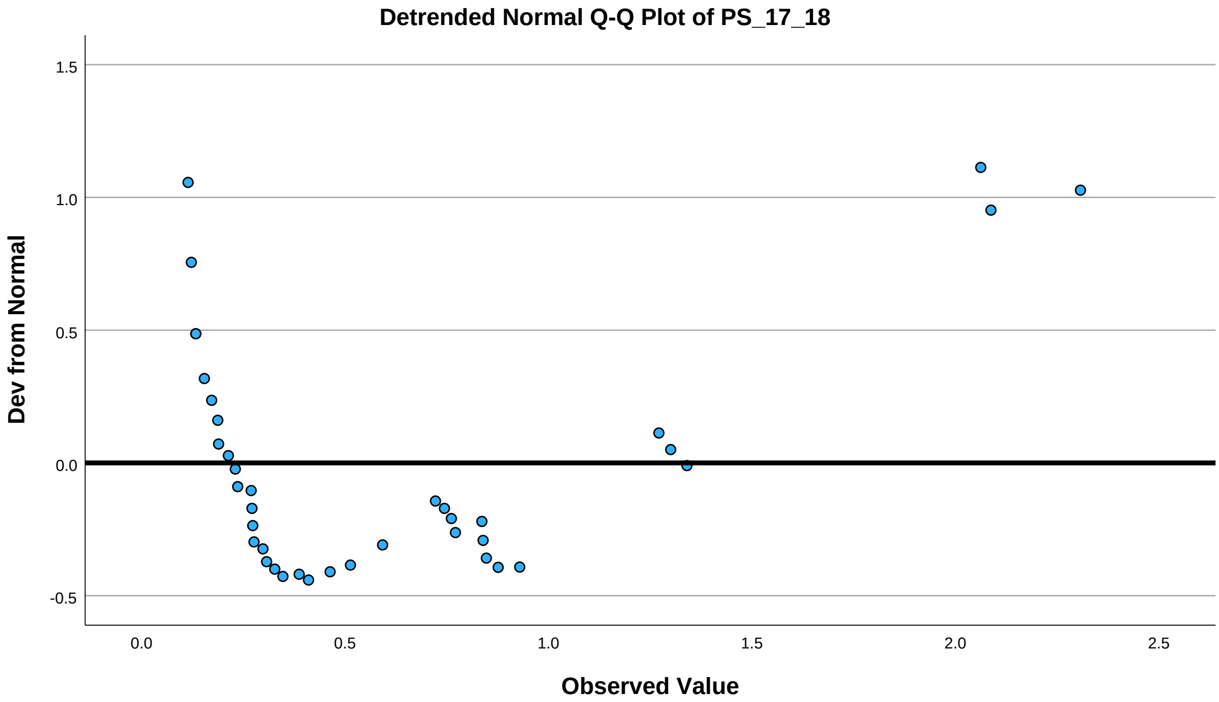
Detrended Normal Q-Q Plot of PS_17_18
Dev from Normal
Observed Value
1.5
1.0
0.5
0.0
-0.5
0.0
0.5
1.0
1.5
2.0
2.5

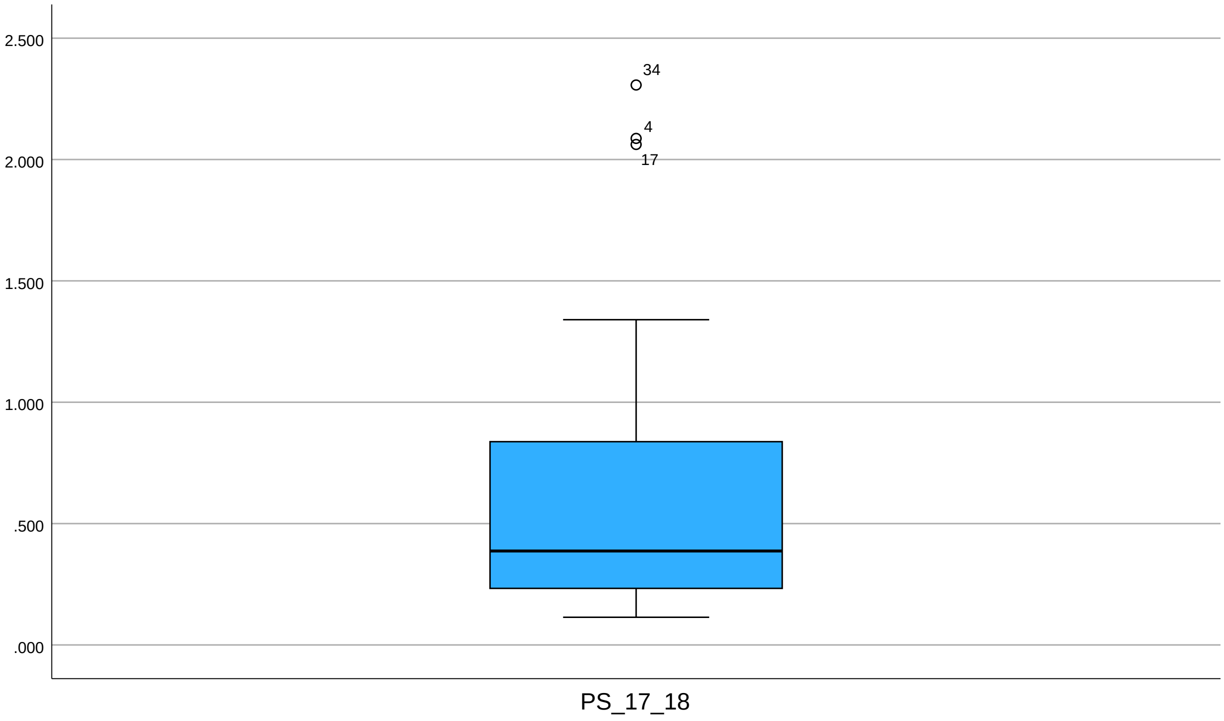
2.500
2.000
1.500
1.000
.500
.000
34
4
17
PS_17_18

PS_18_19

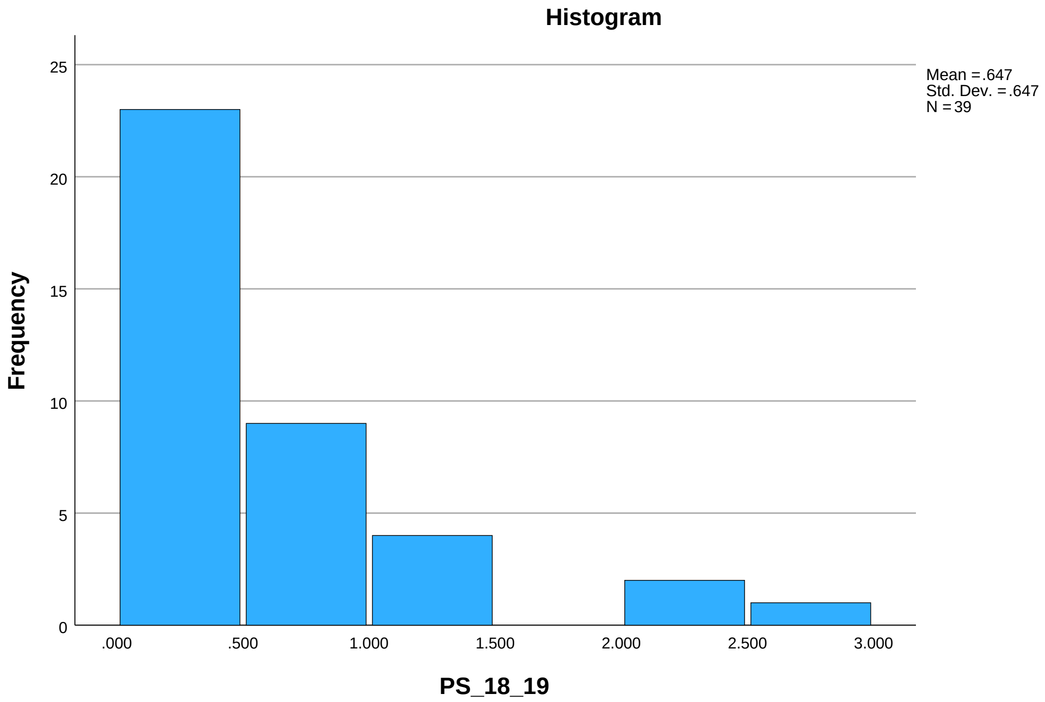


```
PS_18_19 Stem-and-Leaf Plot

 Frequency    Stem &  Leaf

     7.00        0 .  0111111
    13.00        0 .  2222223333333
     4.00        0 .  4445
     5.00        0 .  67777
     3.00        0 .  889
     2.00        1 .  01
     2.00        1 .  22
     3.00 Extremes    (>=2.3)

 Stem width:      1.000
 Each leaf:       1 case(s)
```

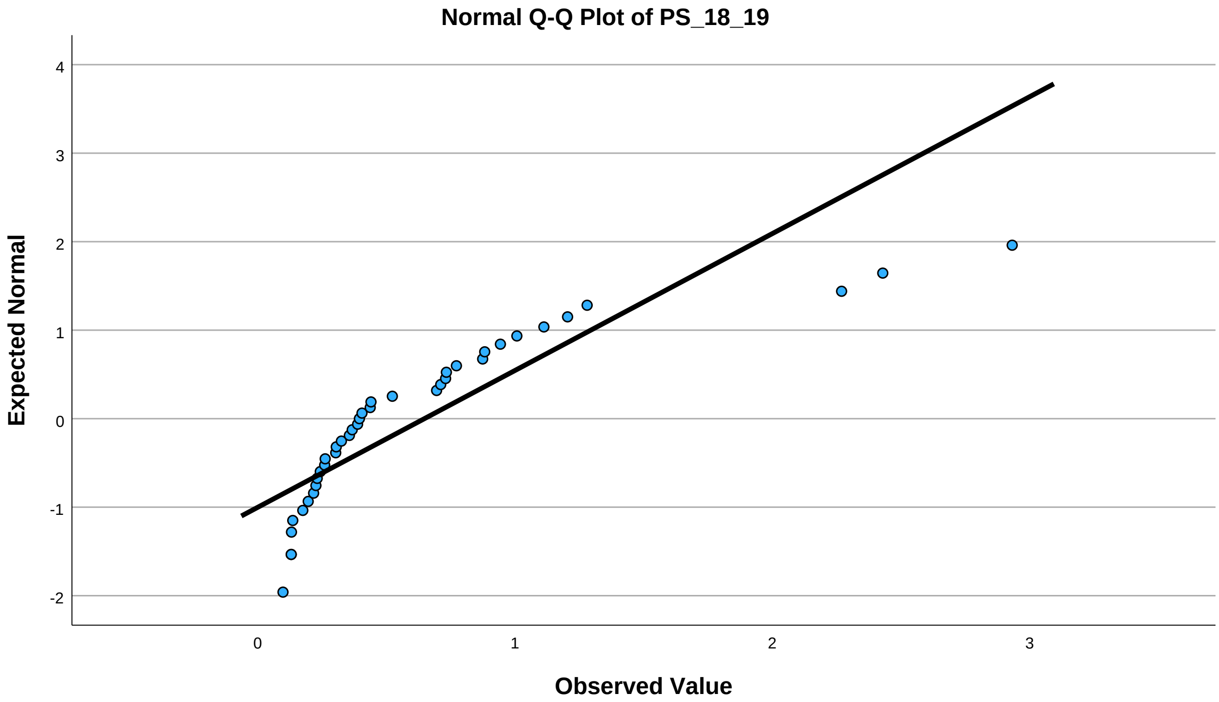
Normal Q-Q Plot of PS_18_19
Expected Normal
Observed Value
4
3
2
1
0
-1
-2
0
1
2
3

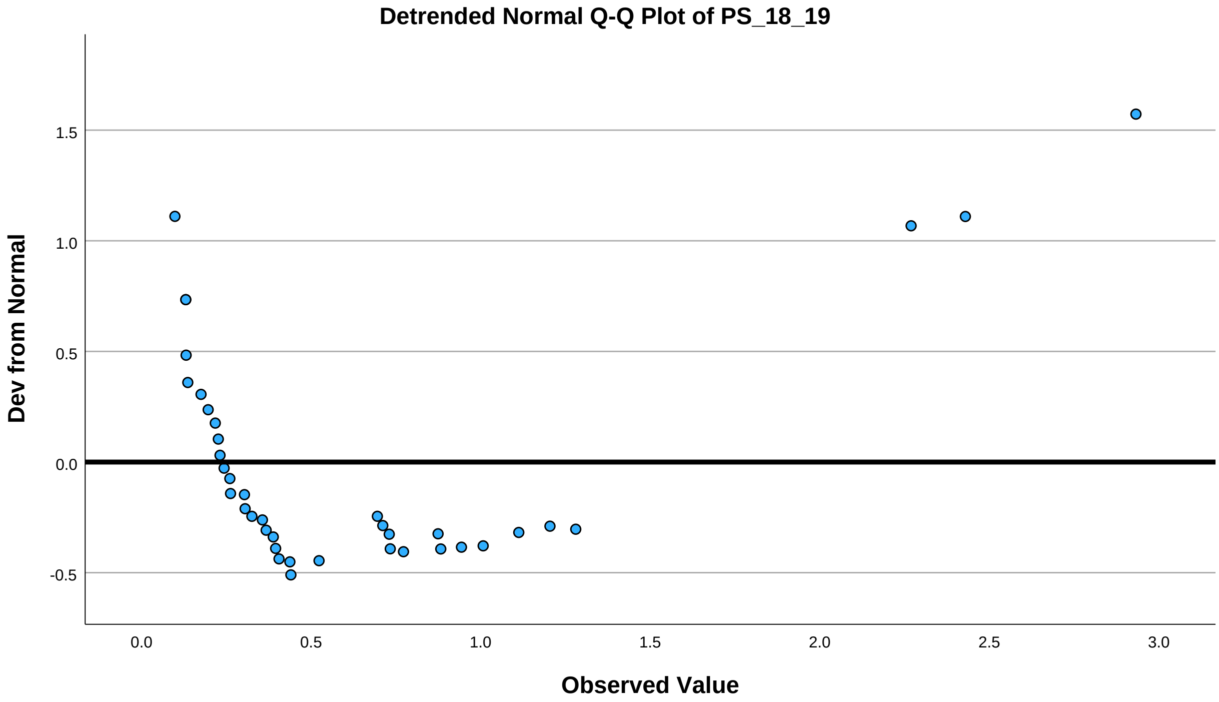
Detrended Normal Q-Q Plot of PS_18_19
Dev from Normal
Observed Value
1.5
1.0
0.5
0.0
-0.5
0.0
0.5
1.0
1.5
2.0
2.5
3.0

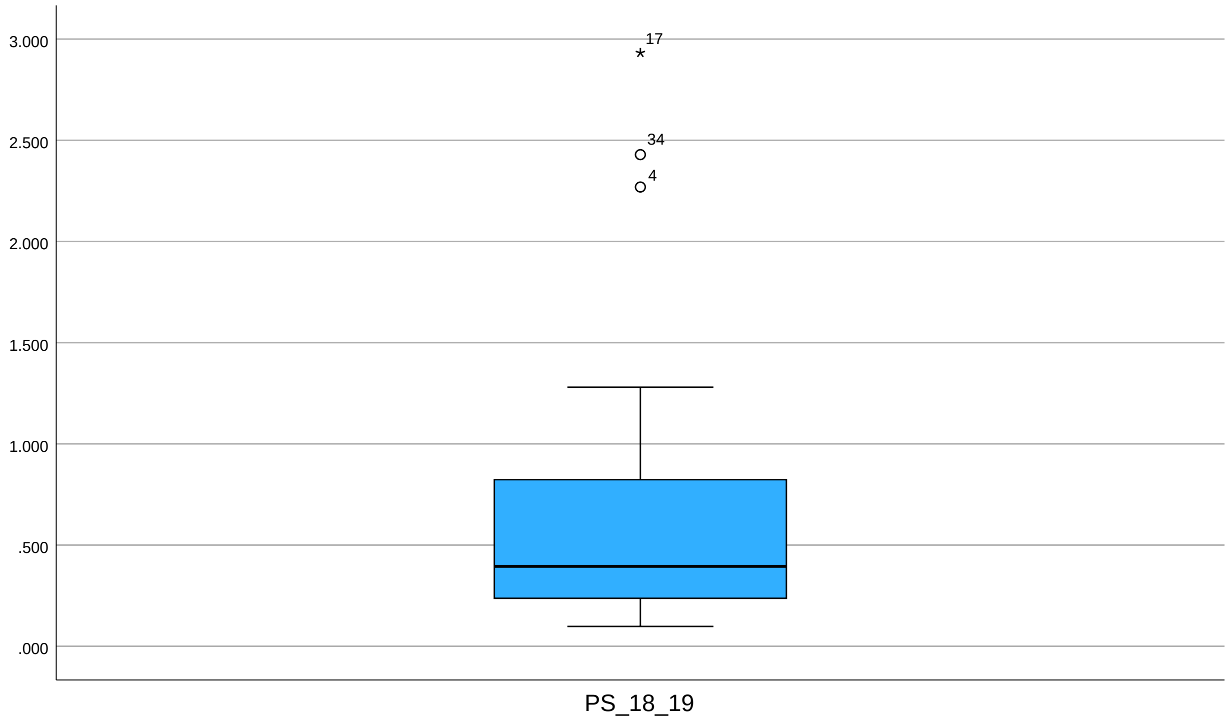

PS_19_20

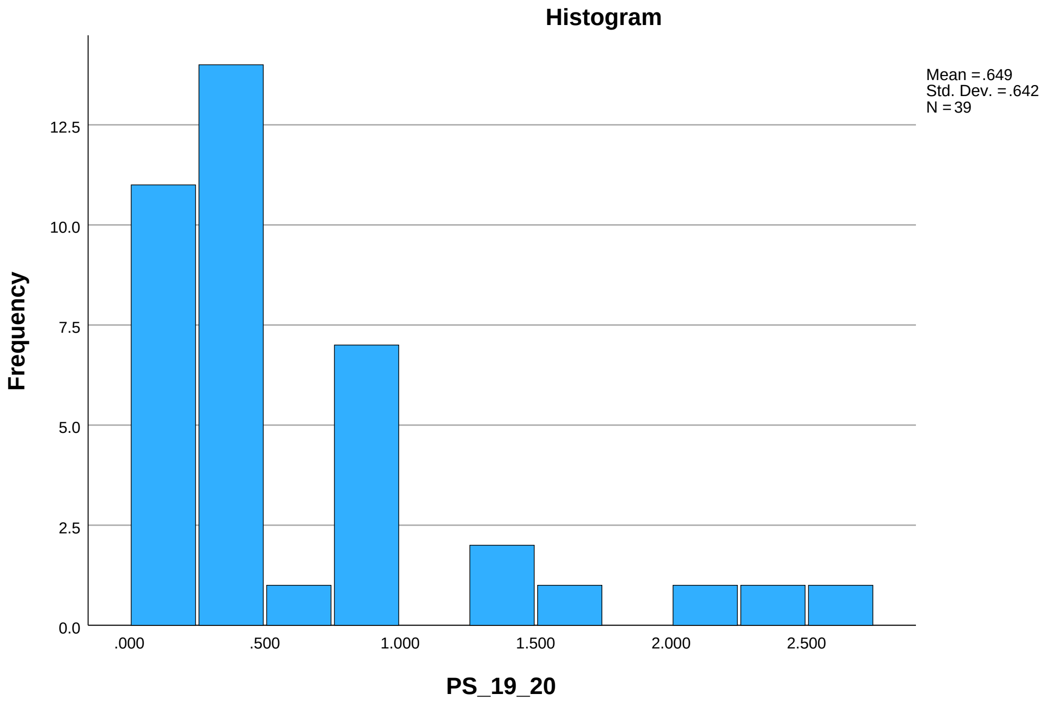

PS_19_20 Stem-and-Leaf Plot

Frequency   Stem & Leaf

```
 8.00        0 .  01111111
11.00        0 .  22222223333
 6.00        0 .  444444
 4.00        0 .  6777
 4.00        0 .  8899
  .00        1 .
 1.00        1 .  3
 2.00        1 .  45
 3.00 Extremes    (>=2.2)

Stem width:     1.000
Each leaf:      1 case(s)
```

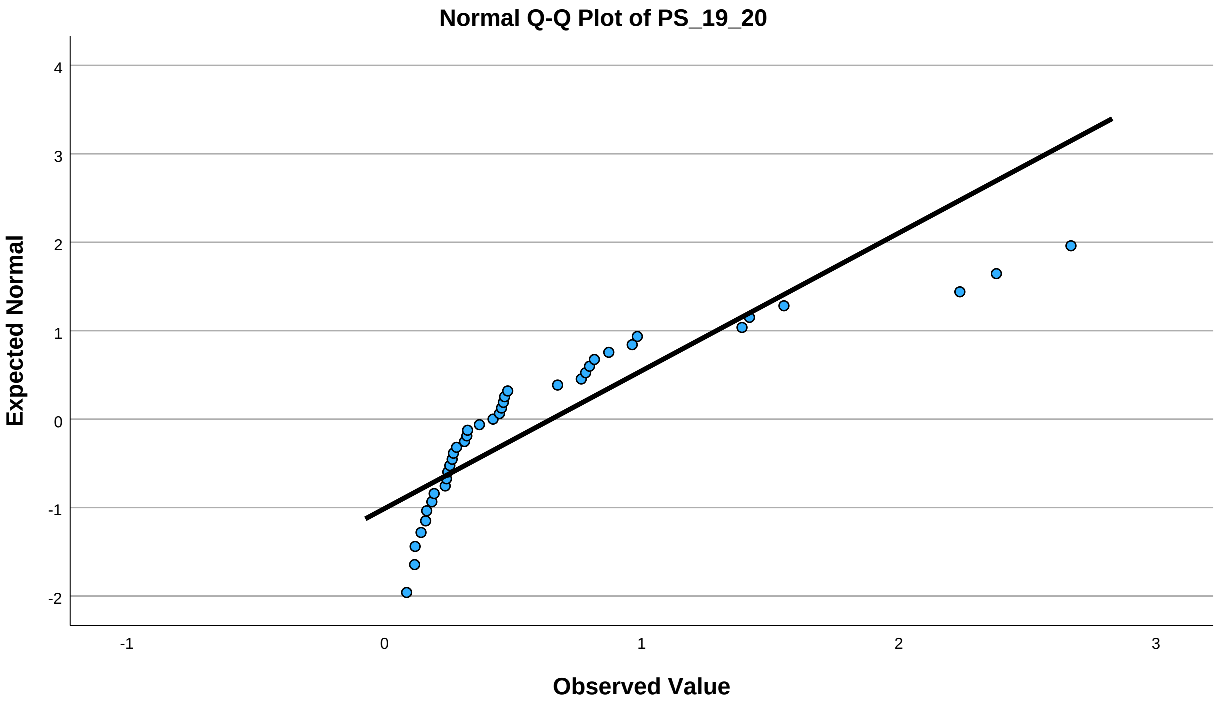

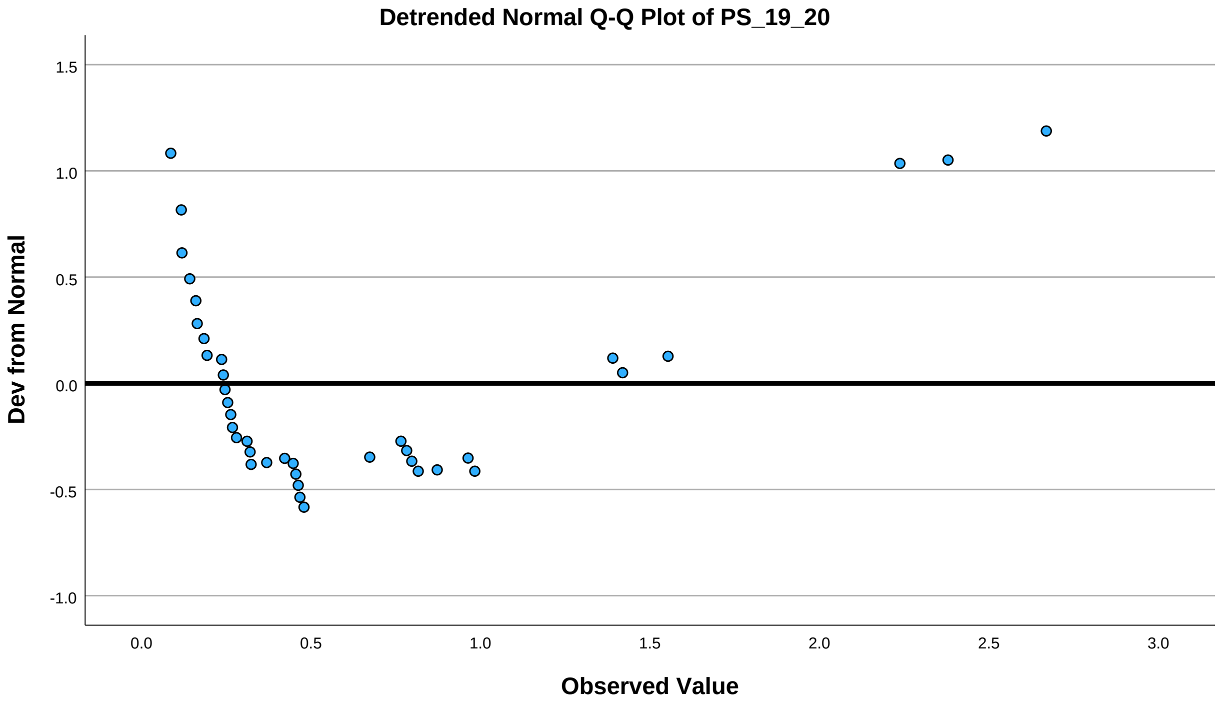

Detrended Normal Q-Q Plot of PS_19_20
Dev from Normal
1.5
1.0
0.5
0.0
-0.5
-1.0
0.0
0.5
1.0
1.5
2.0
2.5
3.0
Observed Value

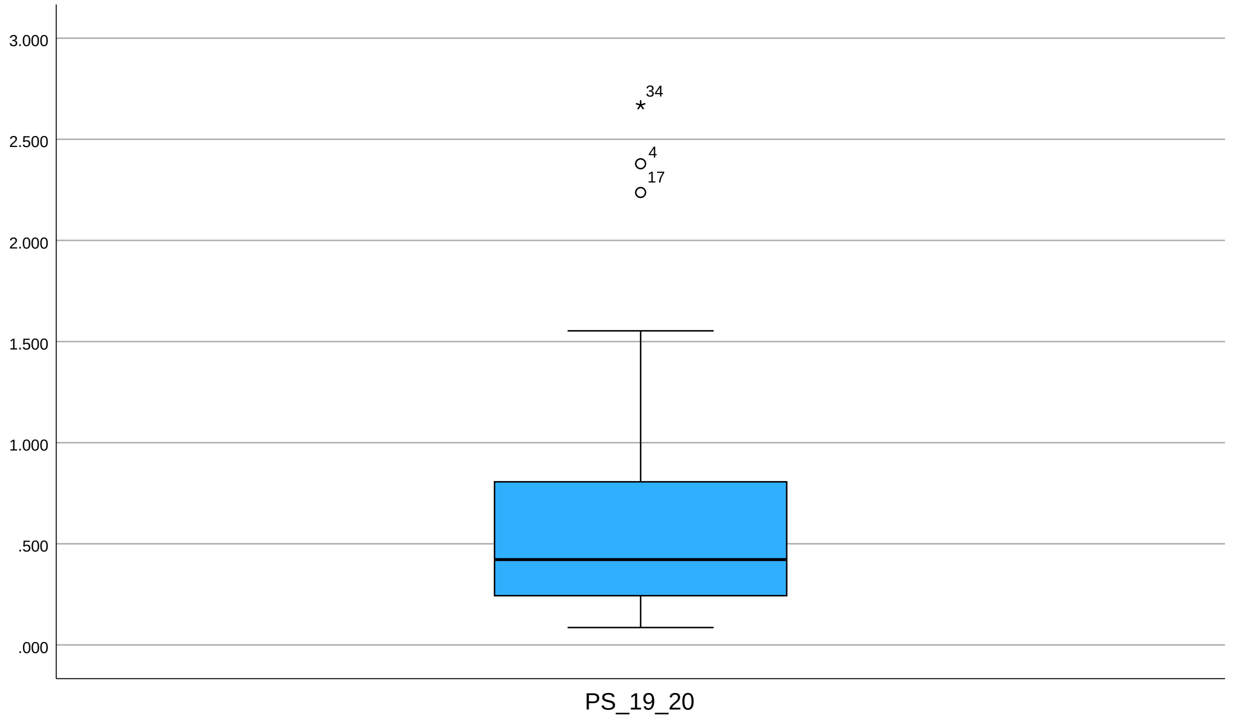

3.000
2.500
2.000
1.500
1.000
.500
.000
34
4
17
PS_19_20

Explore

Case Processing Summary

	Cases					
	Valid		Missing		Total	
	N	Percent	N	Percent	N	Percent
BrainRate	39	100.0%	0	0.0%	39	100.0%
PermutationEntropy	39	100.0%	0	0.0%	39	100.0%
PermutationEntropy_01	39	100.0%	0	0.0%	39	100.0%
SampleEntropy	39	100.0%	0	0.0%	39	100.0%
SpectralEntropy_bits	39	100.0%	0	0.0%	39	100.0%
SpectralEntropy_01	39	100.0%	0	0.0%	39	100.0%
HiguchiFractalDimension	39	100.0%	0	0.0%	39	100.0%
LempelZivComplexity	39	100.0%	0	0.0%	39	100.0%
RenyiEntropy	39	100.0%	0	0.0%	39	100.0%
TsallisEntropy	39	100.0%	0	0.0%	39	100.0%
PS_4_5	39	100.0%	0	0.0%	39	100.0%
PS_5_6	39	100.0%	0	0.0%	39	100.0%
PS_6_7	39	100.0%	0	0.0%	39	100.0%
PS_7_8	39	100.0%	0	0.0%	39	100.0%
PS_8_9	39	100.0%	0	0.0%	39	100.0%
PS_9_10	39	100.0%	0	0.0%	39	100.0%
PS_10_11	39	100.0%	0	0.0%	39	100.0%
PS_11_12	39	100.0%	0	0.0%	39	100.0%
PS_12_13	39	100.0%	0	0.0%	39	100.0%
PS_13_14	39	100.0%	0	0.0%	39	100.0%
PS_14_15	39	100.0%	0	0.0%	39	100.0%
PS_15_16	39	100.0%	0	0.0%	39	100.0%
PS_16_17	39	100.0%	0	0.0%	39	100.0%
PS_17_18	39	100.0%	0	0.0%	39	100.0%
PS_18_19	39	100.0%	0	0.0%	39	100.0%
PS_19_20	39	100.0%	0	0.0%	39	100.0%

Descriptives

			Statistic	Std. Error
BrainRate	Mean		8.3451	.63639
	95% Confidence Interval for Mean	Lower Bound	7.0568	
		Upper Bound	9.6334	
	5% Trimmed Mean		7.7820	
	Median		7.1100	
	Variance		15.794	
	Std. Deviation		3.97423	
	Minimum		4.53	
	Maximum		26.17	
	Range		21.64	
	Interquartile Range		3.27	
	Skewness		2.903	.378
	Kurtosis		10.624	.741
PermutationEntropy	Mean		1.4473493562	.01200002309
	95% Confidence Interval for Mean	Lower Bound	1.4230565795	
		Upper Bound	1.4716421330	
	5% Trimmed Mean		1.4462197353	
	Median		1.4469045723	
	Variance		.006	
	Std. Deviation		.07494012020	
	Minimum		1.2855664986	
	Maximum		1.6485852114	
	Range		.36301871283	
	Interquartile Range		.10910193542	
	Skewness		.144	.378
	Kurtosis		.358	.741
PermutationEntropy_01	Mean		.80778105605	.00669734041
	95% Confidence Interval for Mean	Lower Bound	.79422299922	
		Upper Bound	.82133911288	
	5% Trimmed Mean		.80715060260	
	Median		.80753281740	
	Variance		.002	
	Std. Deviation		.04182487744	
	Minimum		.71748832401	
	Maximum		.92009292527	
	Range		.20260460127	
	Interquartile Range		.06089094953	
	Skewness		.144	.378
	Kurtosis		.358	.741

Descriptives

			Statistic	Std. Error
SampleEntropy	Mean		.67516127378	.02874889103
	95% Confidence Interval for Mean	Lower Bound	.61696218656	
		Upper Bound	.73336036100	
	5% Trimmed Mean		.67307534172	
	Median		.66775737473	
	Variance		.032	
	Std. Deviation		.17953676694	
	Minimum		.30357504025	
	Maximum		1.2023377053	
	Range		.89876266507	
	Interquartile Range		.15664848662	
	Skewness		.087	.378
	Kurtosis		1.402	.741
SpectralEntropy_bits	Mean		4.2033962994	.08201726338
	95% Confidence Interval for Mean	Lower Bound	4.0373610301	
		Upper Bound	4.3694315687	
	5% Trimmed Mean		4.1742662381	
	Median		4.1149715622	
	Variance		.262	
	Std. Deviation		.51219764563	
	Minimum		3.3170165249	
	Maximum		5.7787192252	
	Range		2.4617027003	
	Interquartile Range		.57901222983	
	Skewness		.857	.378
	Kurtosis		1.693	.741
SpectralEntropy_01	Mean		.59952361352	.01169798958
	95% Confidence Interval for Mean	Lower Bound	.57584227169	
		Upper Bound	.62320495536	
	5% Trimmed Mean		.59536884001	
	Median		.58691173632	
	Variance		.005	
	Std. Deviation		.07305392151	
	Minimum		.47310070036	
	Maximum		.82420937372	
	Range		.35110867336	
	Interquartile Range		.08258357756	
	Skewness		.857	.378
	Kurtosis		1.693	.741

Descriptives

			Statistic	Std. Error
HiguchiFractalDimension	Mean		.59564549322	.01886085244
	95% Confidence Interval for Mean	Lower Bound	.55746369361	
		Upper Bound	.63382729283	
	5% Trimmed Mean		.60428011433	
	Median		.61558351775	
	Variance		.014	
	Std. Deviation		.11778598575	
	Minimum		.17785354966	
	Maximum		.78304752433	
	Range		.60519397467	
	Interquartile Range		.14303406623	
	Skewness		-1.328	.378
	Kurtosis		2.968	.741
LempelZivComplexity	Mean		.27237698214	.00609151402
	95% Confidence Interval for Mean	Lower Bound	.26004535671	
		Upper Bound	.28470860758	
	5% Trimmed Mean		.26818698499	
	Median		.26268775401	
	Variance		.001	
	Std. Deviation		.03804149287	
	Minimum		.22161016670	
	Maximum		.42554055196	
	Range		.20393038526	
	Interquartile Range		.03202181785	
	Skewness		2.148	.378
	Kurtosis		6.432	.741
RenyiEntropy	Mean		7.6233478408	.15889252885
	95% Confidence Interval for Mean	Lower Bound	7.3016867327	
		Upper Bound	7.9450089489	
	5% Trimmed Mean		7.6651308239	
	Median		7.9205361341	
	Variance		.985	
	Std. Deviation		.99228352461	
	Minimum		5.6973524916	
	Maximum		8.7921446422	
	Range		3.0947921507	
	Interquartile Range		1.8584700250	
	Skewness		-.816	.378
	Kurtosis		-.765	.741

Descriptives

			Statistic	Std. Error
TsallisEntropy	Mean		1.9010554801	.04642252976
	95% Confidence Interval for Mean	Lower Bound	1.8070779818	
		Upper Bound	1.9950329785	
	5% Trimmed Mean		1.9588303845	
	Median		1.9859101582	
	Variance		.084	
	Std. Deviation		.28990860546	
	Minimum		.75627520939	
	Maximum		1.9886862121	
	Range		1.2324110027	
	Interquartile Range		.00510367553	
	Skewness		-3.541	.378
	Kurtosis		11.595	.741
PS_4_5	Mean		2.10482	.310633
	95% Confidence Interval for Mean	Lower Bound	1.47598	
		Upper Bound	2.73366	
	5% Trimmed Mean		1.85143	
	Median		1.67000	
	Variance		3.763	
	Std. Deviation		1.939904	
	Minimum		.388	
	Maximum		12.490	
	Range		12.102	
	Interquartile Range		1.180	
	Skewness		4.234	.378
	Kurtosis		22.215	.741
PS_5_6	Mean		1.54418	.180374
	95% Confidence Interval for Mean	Lower Bound	1.17903	
		Upper Bound	1.90933	
	5% Trimmed Mean		1.41626	
	Median		1.27500	
	Variance		1.269	
	Std. Deviation		1.126437	
	Minimum		.284	
	Maximum		7.400	
	Range		7.116	
	Interquartile Range		.891	
	Skewness		3.787	.378
	Kurtosis		19.471	.741

Descriptives

			Statistic	Std. Error
PS_6_7	Mean		1.25033	.159207
	95% Confidence Interval for Mean	Lower Bound	.92804	
		Upper Bound	1.57263	
	5% Trimmed Mean		1.13005	
	Median		1.07300	
	Variance		.989	
	Std. Deviation		.994246	
	Minimum		.205	
	Maximum		6.580	
	Range		6.375	
	Interquartile Range		.648	
	Skewness		4.190	.378
	Kurtosis		22.349	.741
PS_7_8	Mean		1.08962	.182635
	95% Confidence Interval for Mean	Lower Bound	.71989	
		Upper Bound	1.45934	
	5% Trimmed Mean		.91464	
	Median		.80600	
	Variance		1.301	
	Std. Deviation		1.140557	
	Minimum		.186	
	Maximum		7.360	
	Range		7.174	
	Interquartile Range		.515	
	Skewness		4.636	.378
	Kurtosis		25.081	.741
PS_8_9	Mean		1.07931	.189856
	95% Confidence Interval for Mean	Lower Bound	.69497	
		Upper Bound	1.46365	
	5% Trimmed Mean		.90004	
	Median		.76200	
	Variance		1.406	
	Std. Deviation		1.185647	
	Minimum		.164	
	Maximum		7.310	
	Range		7.146	
	Interquartile Range		.588	
	Skewness		4.129	.378
	Kurtosis		20.593	.741

Descriptives

			Statistic	Std. Error
PS_9_10	Mean		1.71172	.497609
	95% Confidence Interval for Mean	Lower Bound	.70436	
		Upper Bound	2.71907	
	5% Trimmed Mean		1.22101	
	Median		1.00300	
	Variance		9.657	
	Std. Deviation		3.107566	
	Minimum		.151	
	Maximum		19.790	
	Range		19.639	
	Interquartile Range		1.133	
	Skewness		5.450	.378
	Kurtosis		32.076	.741
PS_10_11	Mean		2.50321	.425681
	95% Confidence Interval for Mean	Lower Bound	1.64146	
		Upper Bound	3.36495	
	5% Trimmed Mean		2.20052	
	Median		1.48700	
	Variance		7.067	
	Std. Deviation		2.658379	
	Minimum		.223	
	Maximum		11.059	
	Range		10.836	
	Interquartile Range		2.955	
	Skewness		1.833	.378
	Kurtosis		3.034	.741
PS_11_12	Mean		.97444	.194738
	95% Confidence Interval for Mean	Lower Bound	.58021	
		Upper Bound	1.36866	
	5% Trimmed Mean		.78956	
	Median		.53400	
	Variance		1.479	
	Std. Deviation		1.216141	
	Minimum		.180	
	Maximum		5.765	
	Range		5.585	
	Interquartile Range		.549	
	Skewness		2.665	.378
	Kurtosis		7.068	.741

Descriptives

			Statistic	Std. Error
PS_12_13	Mean		.42382	.049107
	95% Confidence Interval for Mean	Lower Bound	.32441	
		Upper Bound	.52323	
	5% Trimmed Mean		.39057	
	Median		.34100	
	Variance		.094	
	Std. Deviation		.306670	
	Minimum		.113	
	Maximum		1.320	
	Range		1.207	
	Interquartile Range		.285	
	Skewness		1.949	.378
	Kurtosis		3.578	.741
PS_13_14	Mean		.33003	.038575
	95% Confidence Interval for Mean	Lower Bound	.25193	
		Upper Bound	.40812	
	5% Trimmed Mean		.29648	
	Median		.25000	
	Variance		.058	
	Std. Deviation		.240903	
	Minimum		.120	
	Maximum		1.157	
	Range		1.037	
	Interquartile Range		.204	
	Skewness		2.286	.378
	Kurtosis		5.340	.741
PS_14_15	Mean		.30033	.038547
	95% Confidence Interval for Mean	Lower Bound	.22230	
		Upper Bound	.37837	
	5% Trimmed Mean		.26908	
	Median		.22700	
	Variance		.058	
	Std. Deviation		.240723	
	Minimum		.112	
	Maximum		1.080	
	Range		.968	
	Interquartile Range		.162	
	Skewness		2.125	.378
	Kurtosis		3.980	.741

Descriptives

			Statistic	Std. Error
PS_15_16	Mean		.29338	.042426
	95% Confidence Interval for Mean	Lower Bound	.20750	
		Upper Bound	.37927	
	5% Trimmed Mean		.25913	
	Median		.18500	
	Variance		.070	
	Std. Deviation		.264953	
	Minimum		.091	
	Maximum		1.334	
	Range		1.243	
	Interquartile Range		.170	
	Skewness		2.390	.378
	Kurtosis		5.953	.741
PS_16_17	Mean		.25754	.036407
	95% Confidence Interval for Mean	Lower Bound	.18384	
		Upper Bound	.33124	
	5% Trimmed Mean		.22535	
	Median		.17400	
	Variance		.052	
	Std. Deviation		.227362	
	Minimum		.081	
	Maximum		1.223	
	Range		1.142	
	Interquartile Range		.130	
	Skewness		2.730	.378
	Kurtosis		8.396	.741
PS_17_18	Mean		.24821	.037075
	95% Confidence Interval for Mean	Lower Bound	.17315	
		Upper Bound	.32326	
	5% Trimmed Mean		.21309	
	Median		.16000	
	Variance		.054	
	Std. Deviation		.231534	
	Minimum		.082	
	Maximum		1.138	
	Range		1.056	
	Interquartile Range		.098	
	Skewness		2.626	.378
	Kurtosis		6.896	.741

Descriptives

			Statistic	Std. Error
PS_18_19	Mean		.24441	.037211
	95% Confidence Interval for Mean	Lower Bound	.16908	
		Upper Bound	.31974	
	5% Trimmed Mean		.20854	
	Median		.17000	
	Variance		.054	
	Std. Deviation		.232383	
	Minimum		.074	
	Maximum		1.210	
	Range		1.136	
	Interquartile Range		.134	
	Skewness		2.841	.378
	Kurtosis		8.619	.741
PS_19_20	Mean		.22538	.026622
	95% Confidence Interval for Mean	Lower Bound	.17149	
		Upper Bound	.27928	
	5% Trimmed Mean		.20618	
	Median		.18600	
	Variance		.028	
	Std. Deviation		.166254	
	Minimum		.072	
	Maximum		.781	
	Range		.709	
	Interquartile Range		.128	
	Skewness		1.902	.378
	Kurtosis		3.348	.741

Tests of Normality

	Kolmogorov-Smirnov[a]			Shapiro-Wilk		
	Statistic	df	Sig.	Statistic	df	Sig.
BrainRate	.223	39	<.001	.704	39	<.001
PermutationEntropy	.066	39	.200*	.986	39	.894
PermutationEntropy_01	.066	39	.200*	.986	39	.894
SampleEntropy	.166	39	.008	.938	39	.034
SpectralEntropy_bits	.091	39	.200*	.949	39	.079
SpectralEntropy_01	.091	39	.200*	.949	39	.079
HiguchiFractalDimension	.140	39	.051	.917	39	.007
LempelZivComplexity	.183	39	.002	.815	39	<.001
RenyiEntropy	.178	39	.003	.858	39	<.001
TsallisEntropy	.458	39	<.001	.326	39	<.001
PS_4_5	.217	39	<.001	.580	39	<.001
PS_5_6	.191	39	.001	.646	39	<.001
PS_6_7	.207	39	<.001	.595	39	<.001
PS_7_8	.257	39	<.001	.511	39	<.001
PS_8_9	.263	39	<.001	.565	39	<.001
PS_9_10	.319	39	<.001	.380	39	<.001
PS_10_11	.225	39	<.001	.765	39	<.001
PS_11_12	.336	39	<.001	.610	39	<.001
PS_12_13	.187	39	.001	.762	39	<.001
PS_13_14	.243	39	<.001	.720	39	<.001
PS_14_15	.248	39	<.001	.703	39	<.001
PS_15_16	.253	39	<.001	.673	39	<.001
PS_16_17	.265	39	<.001	.654	39	<.001
PS_17_18	.297	39	<.001	.635	39	<.001
PS_18_19	.285	39	<.001	.631	39	<.001
PS_19_20	.231	39	<.001	.771	39	<.001

*. This is a lower bound of the true significance.

a. Lilliefors Significance Correction

BrainRate

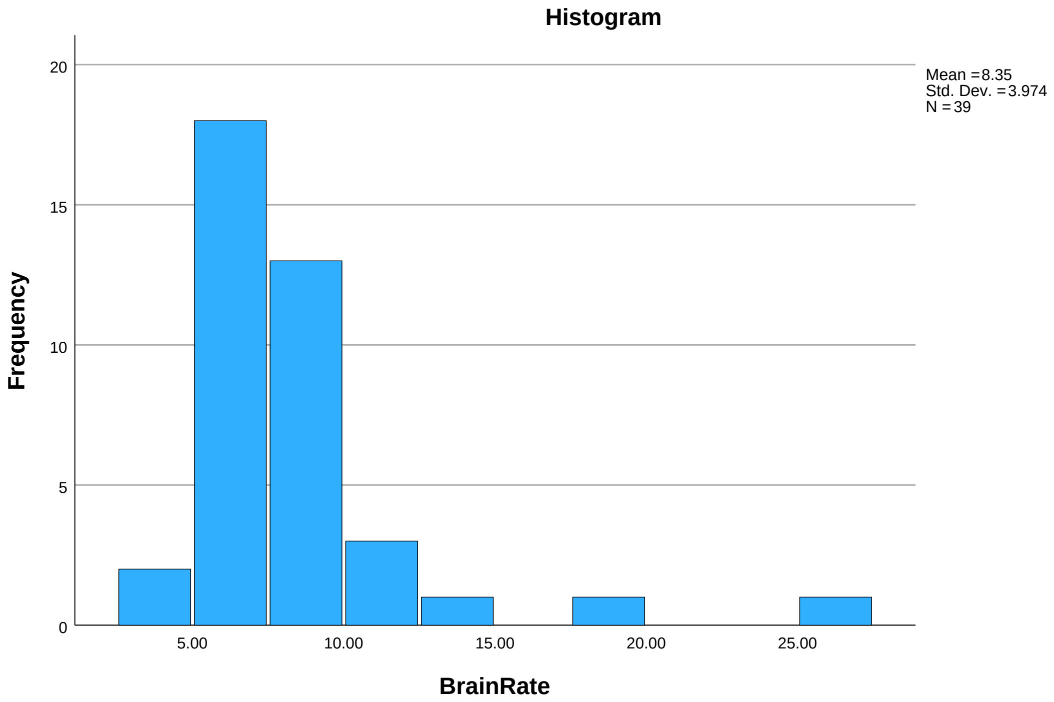


```
BrainRate Stem-and-Leaf Plot

 Frequency    Stem &  Leaf

     2.00        4 .  57
     5.00        5 .  02268
    11.00        6 .  00123345889
     5.00        7 .  01556
     5.00        8 .  01289
     5.00        9 .  34455
     1.00       10 .  1
     1.00       11 .  2
     1.00       12 .  0
     3.00 Extremes    (>=14.7)

 Stem width:      1.00
 Each leaf:       1 case(s)
```

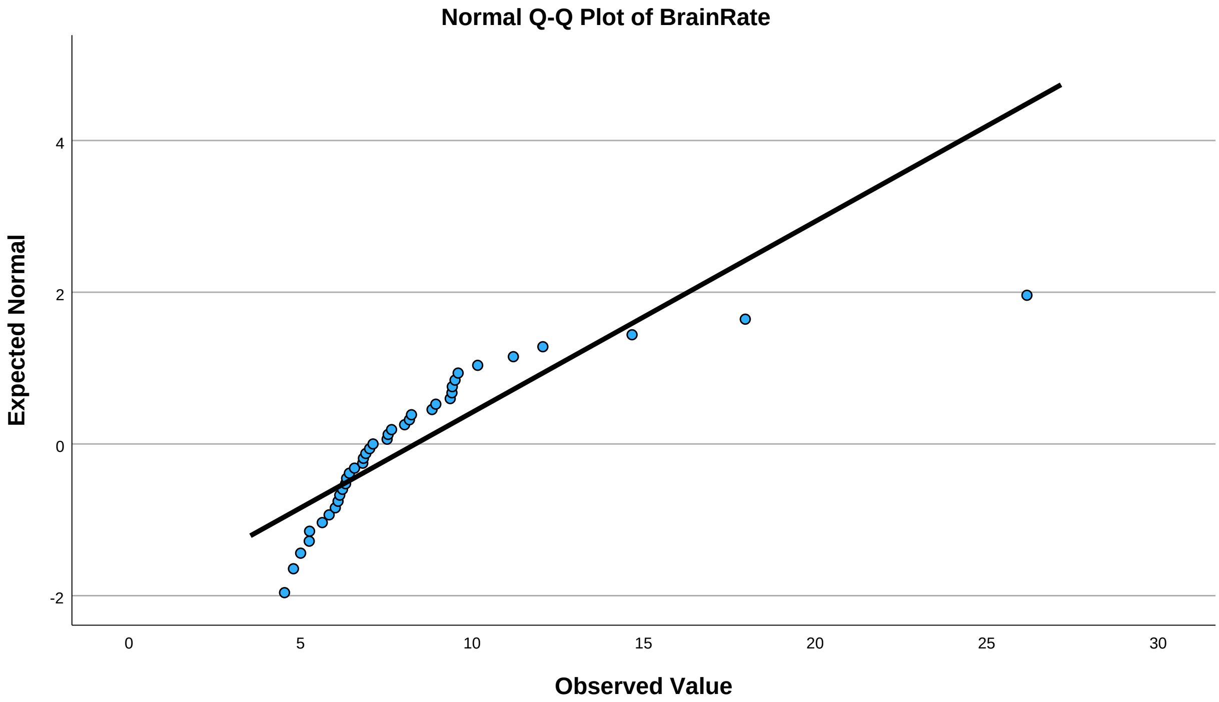
Normal Q-Q Plot of BrainRate
Expected Normal
Observed Value
4
2
0
-2
0
5
10
15
20
25
30

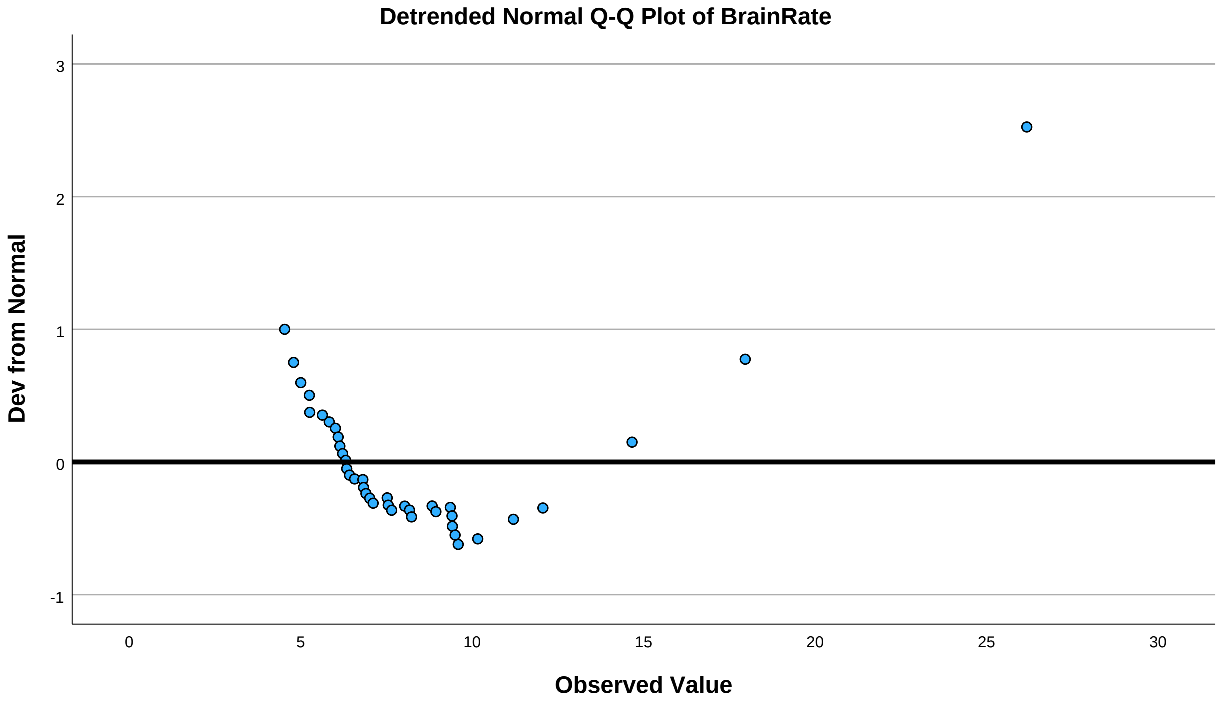
Detrended Normal Q-Q Plot of BrainRate
Dev from Normal
Observed Value
3
2
1
0
-1
0
5
10
15
20
25
30

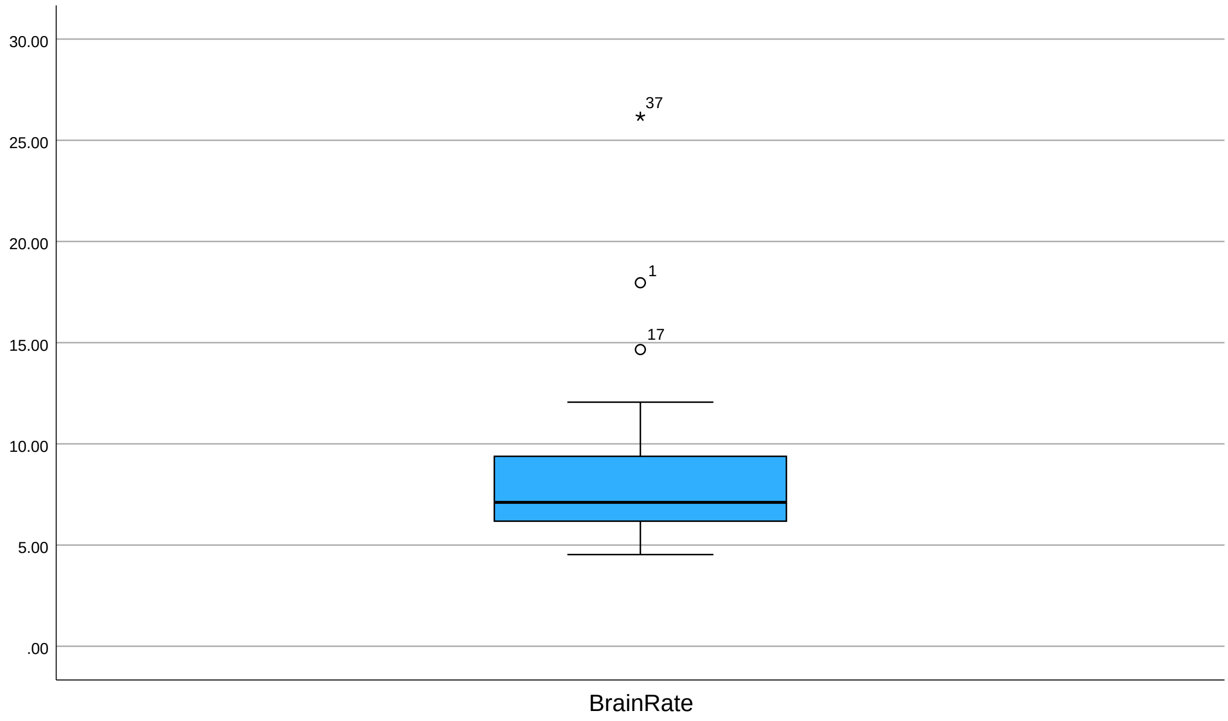

PermutationEntropy

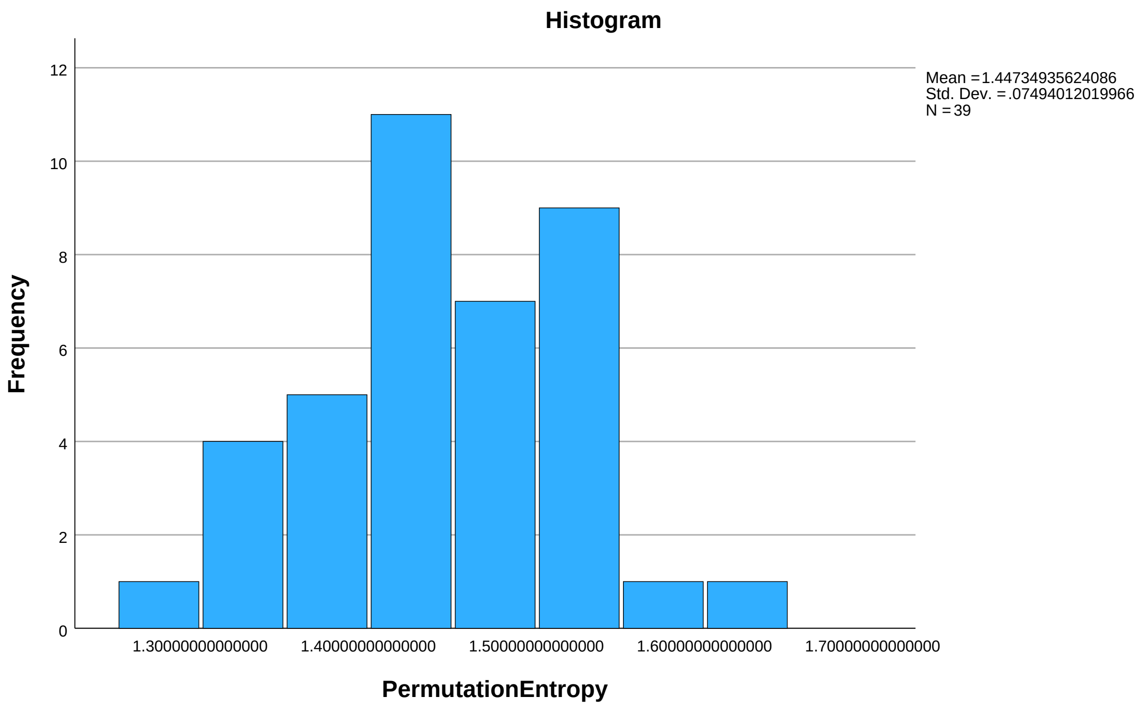

PermutationEntropy Stem-and-Leaf Plot

Frequency   Stem & Leaf

```
 1.00      12 .  8
 4.00      13 .  2334
 5.00      13 .  78899
11.00      14 .  00122233444
 7.00      14 .  5566777
 9.00      15 .  001122233
 1.00      15 .  7
 1.00      16 .  4

Stem width:   .1000000
Each leaf:        1 case(s)
```

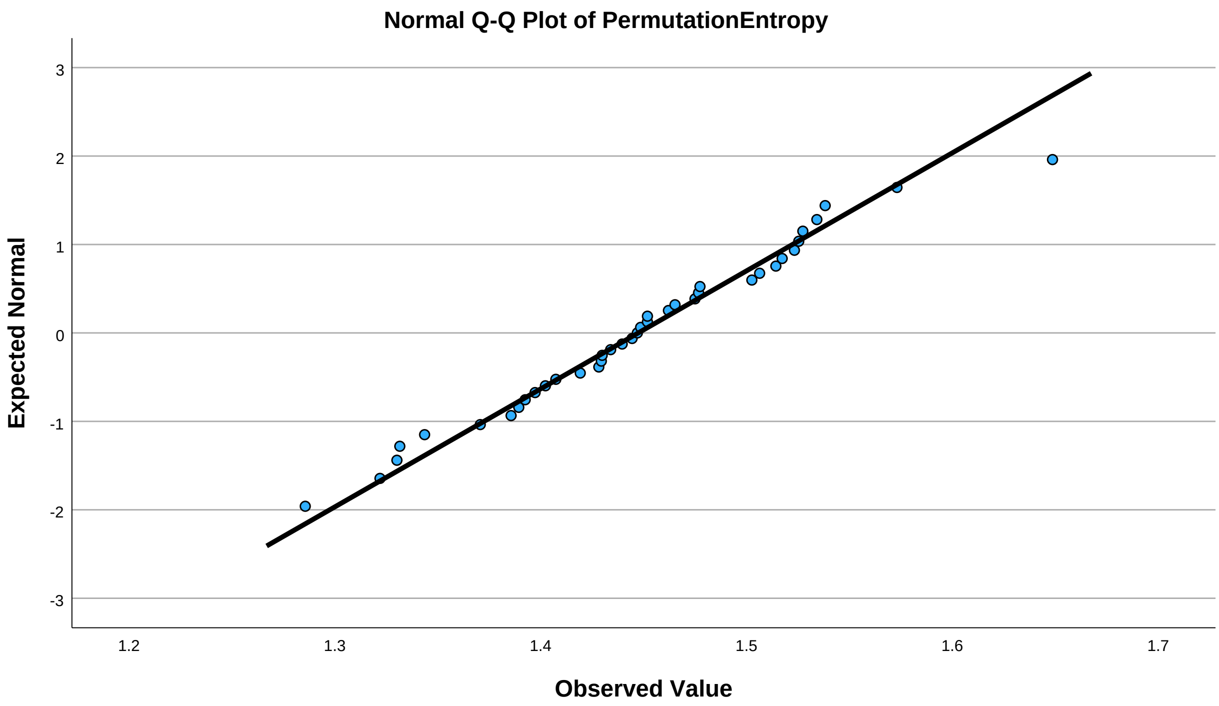

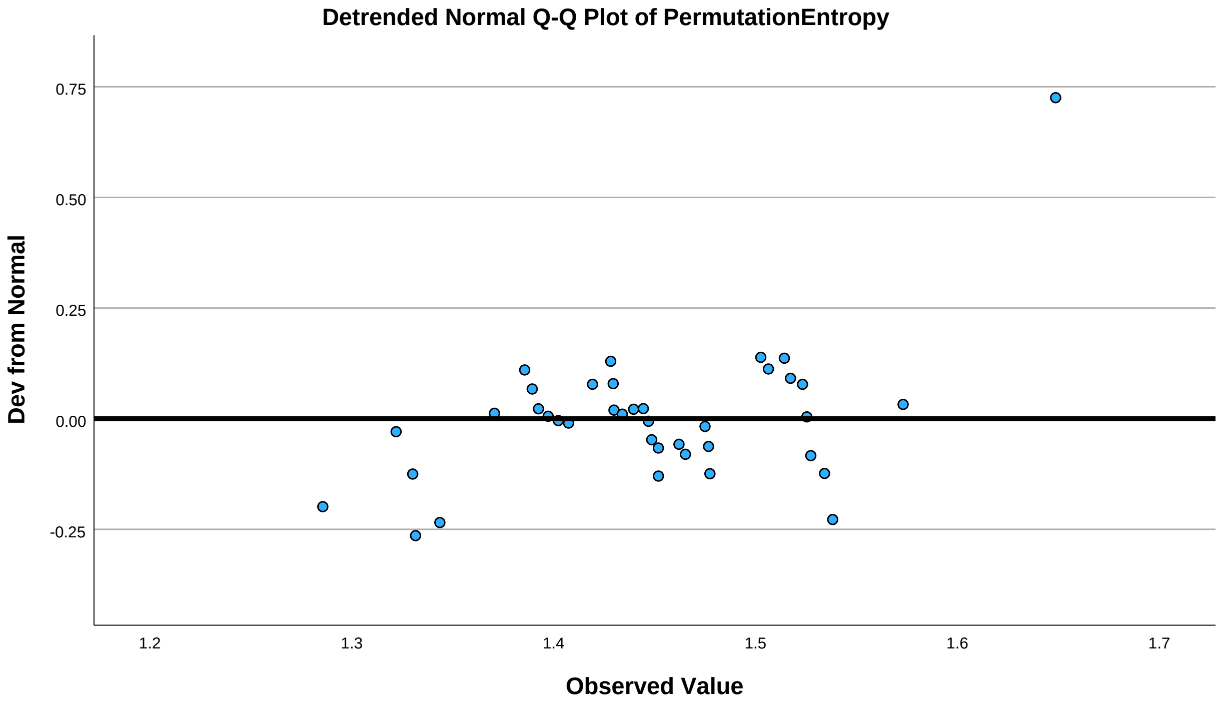

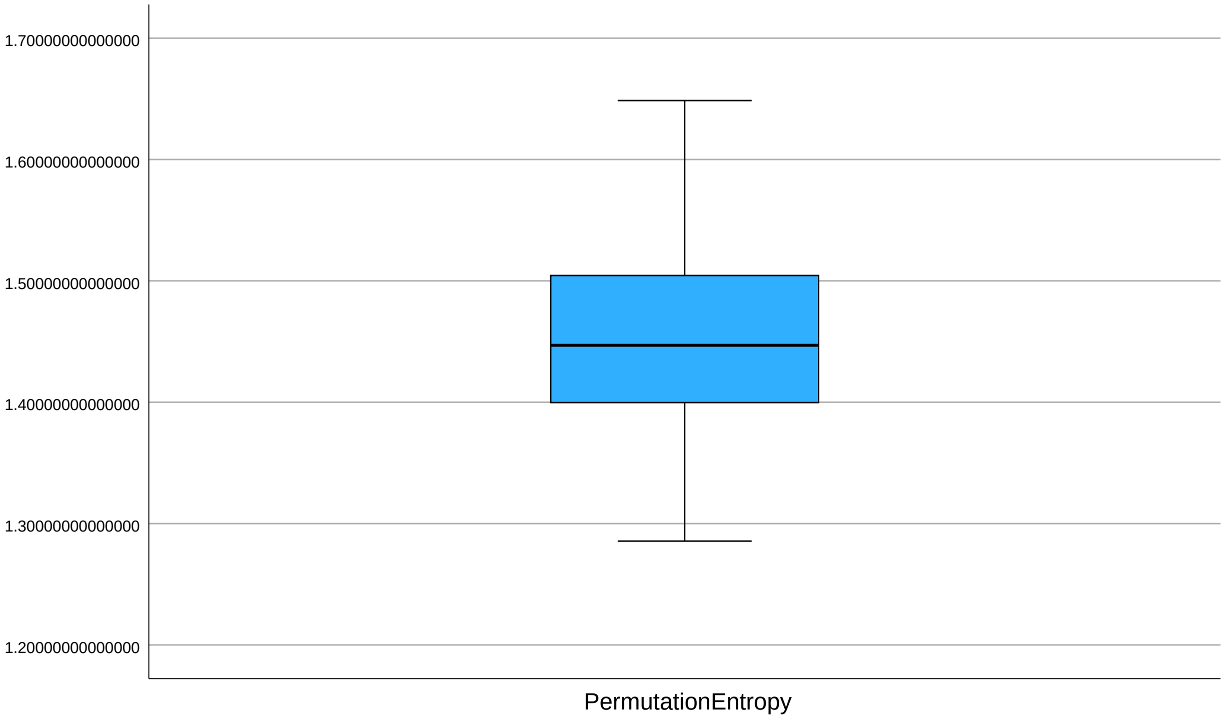

PermutationEntropy_01

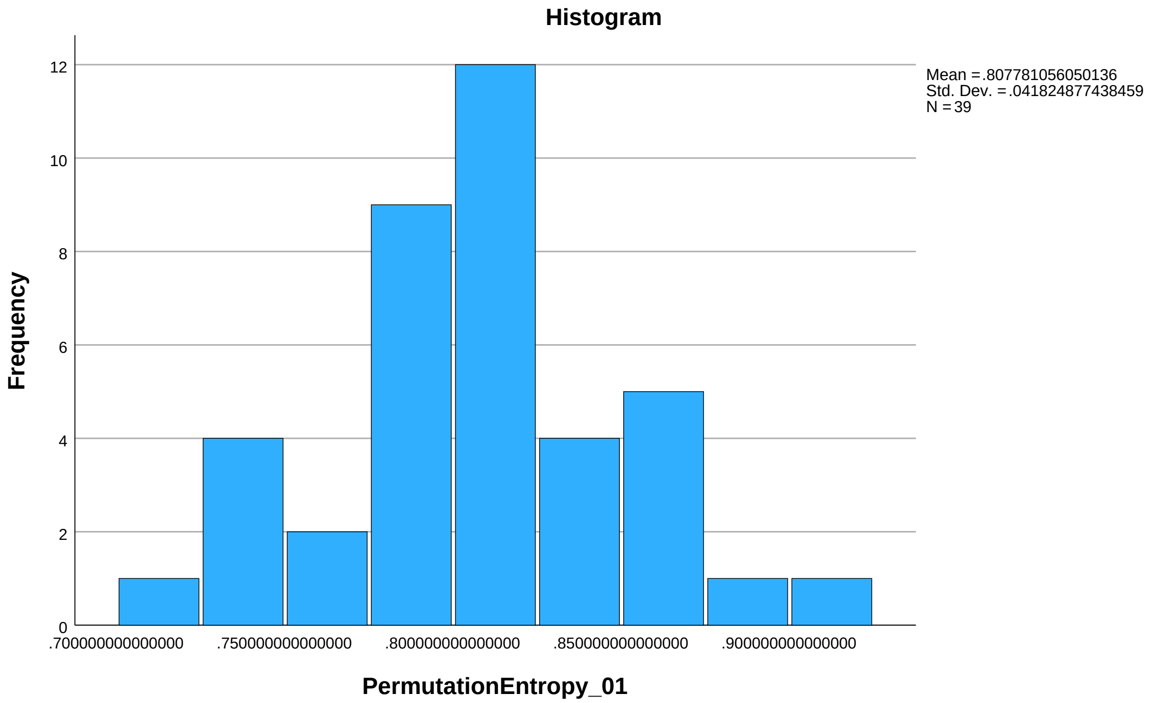


```
PermutationEntropy_01 Stem-and-Leaf Plot

 Frequency    Stem &  Leaf

     5.00        7 .  13444
    11.00        7 .  67777889999
    16.00        8 .  0000011112223444
     6.00        8 .  555557
     1.00        9 .  2

 Stem width:   .1000000
 Each leaf:       1 case(s)
```

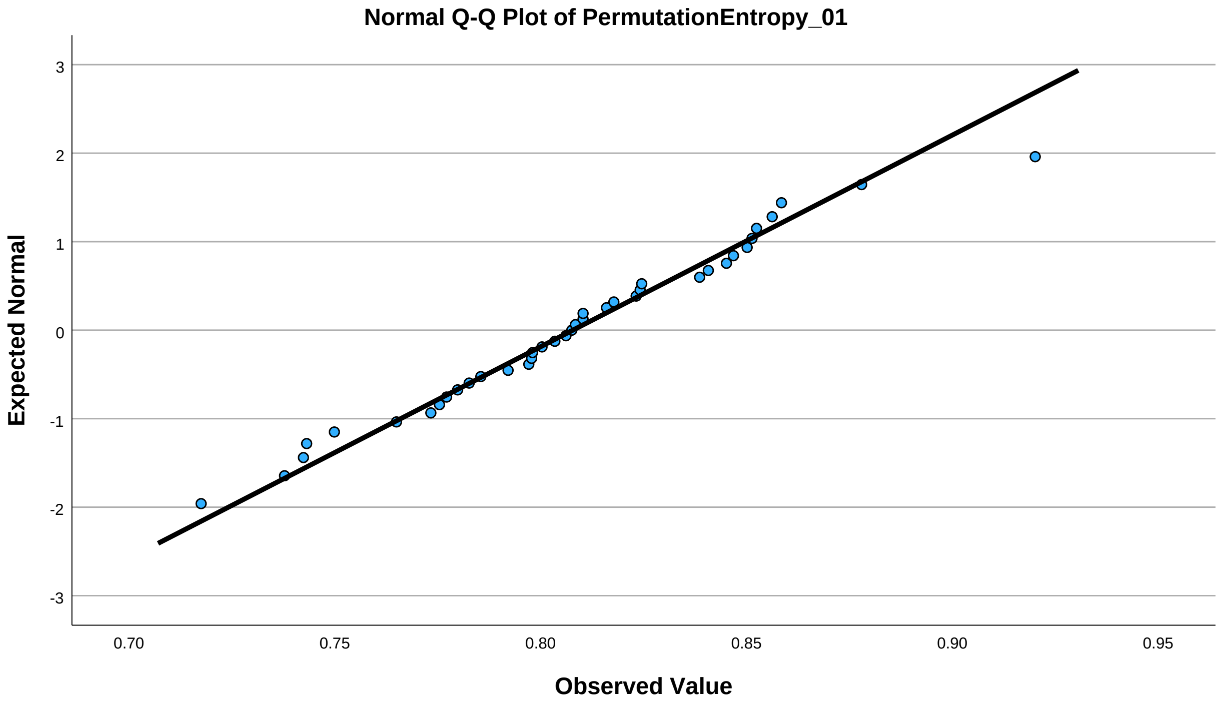
Normal Q-Q Plot of PermutationEntropy_01
Expected Normal
3
2
1
0
-1
-2
-3
0.70
0.75
0.80
0.85
0.90
0.95
Observed Value

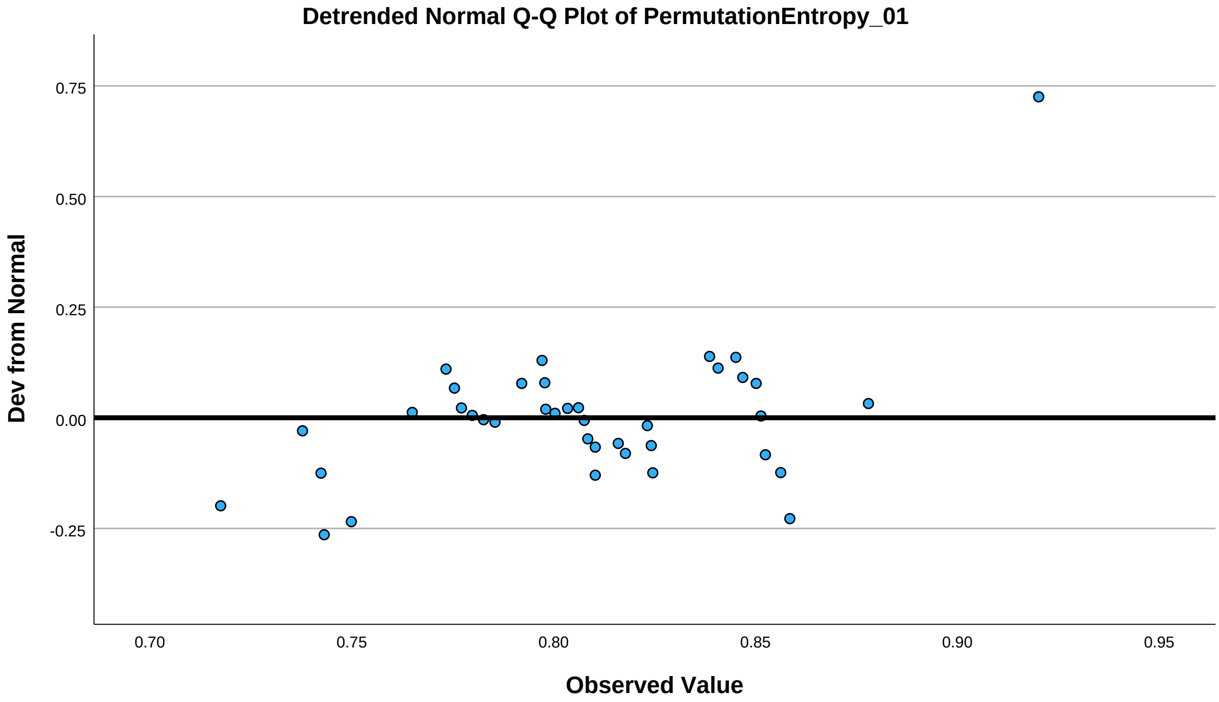
Detrended Normal Q-Q Plot of PermutationEntropy_01
Dev from Normal
0.75
0.50
0.25
0.00
-0.25
0.70
0.75
0.80
0.85
0.90
0.95
Observed Value

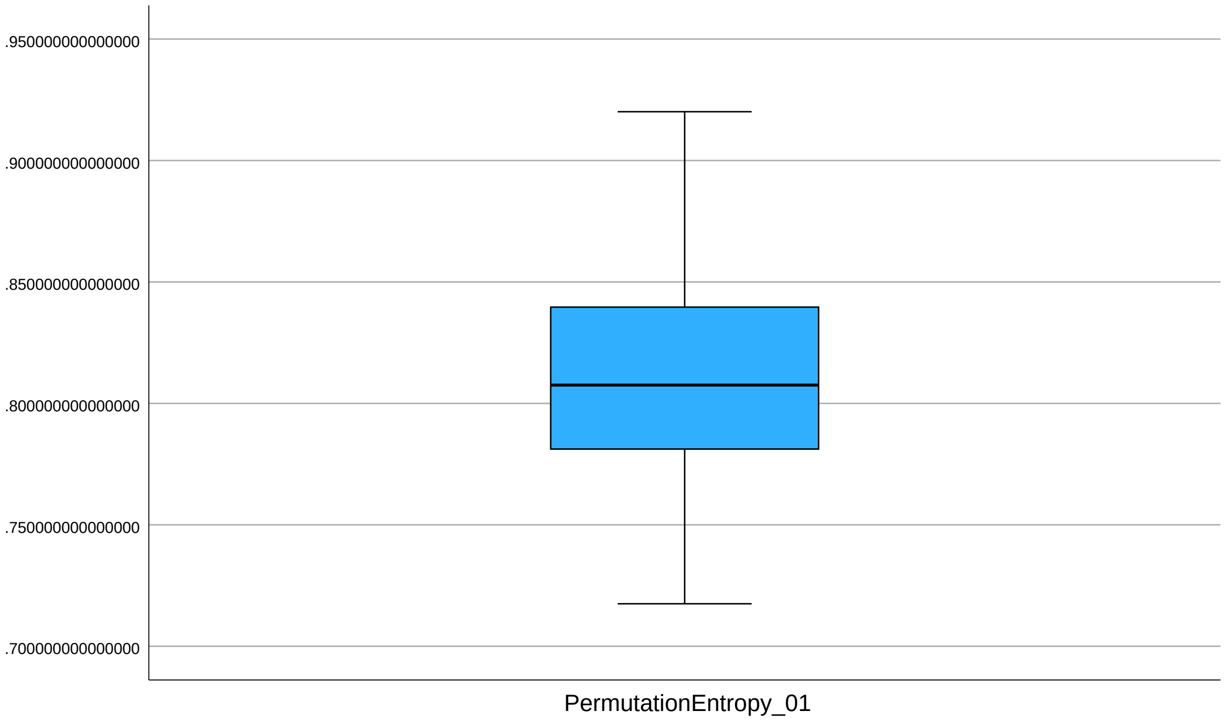

SampleEntropy

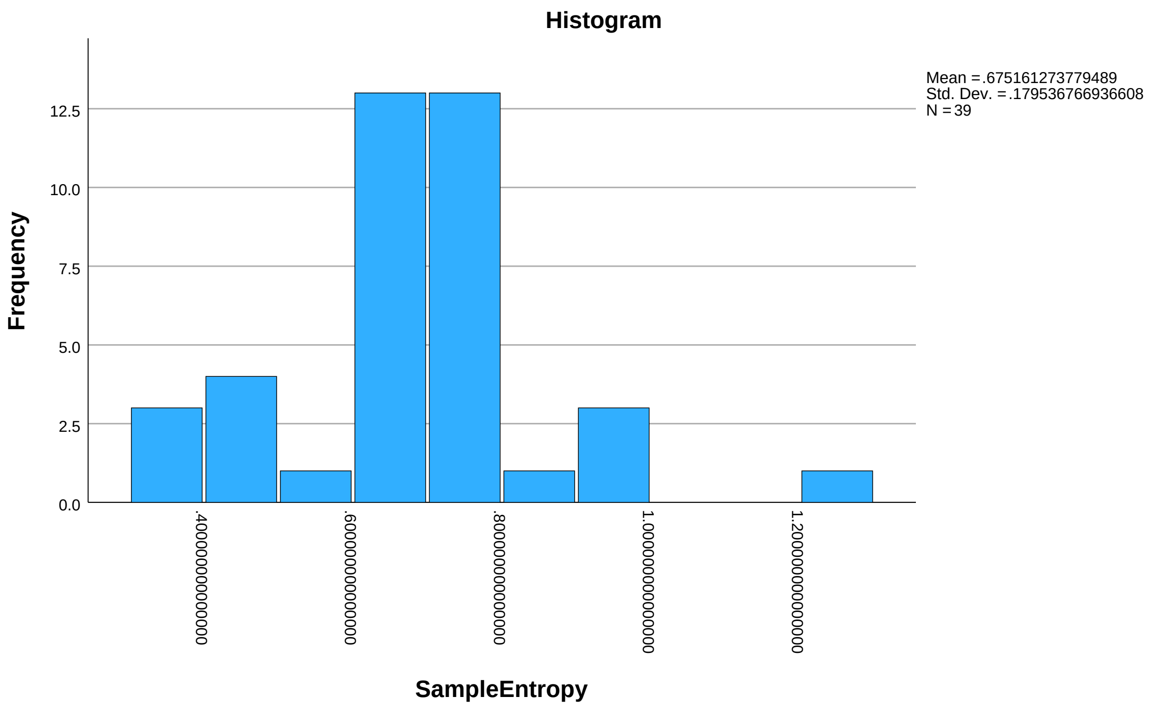


```
SampleEntropy Stem-and-Leaf Plot

 Frequency    Stem &  Leaf
```

```
  3.00 Extremes    (=<.33)
 3.00      4 . 124
 1.00     4 . 8
  .00     5 .
 1.00     5 . 9
  8.00       6 .  11224444
  5.00      6 .  66669
  7.00       7 .  0112234
  6.00       7 .  777888
 1.00     8 . 1
  .00     8 .
 2.00     9 . 23
 1.00     9 . 5
  1.00 Extremes    (>=1.20)

Stem width:   .1000000
Each leaf:       1 case(s)
```

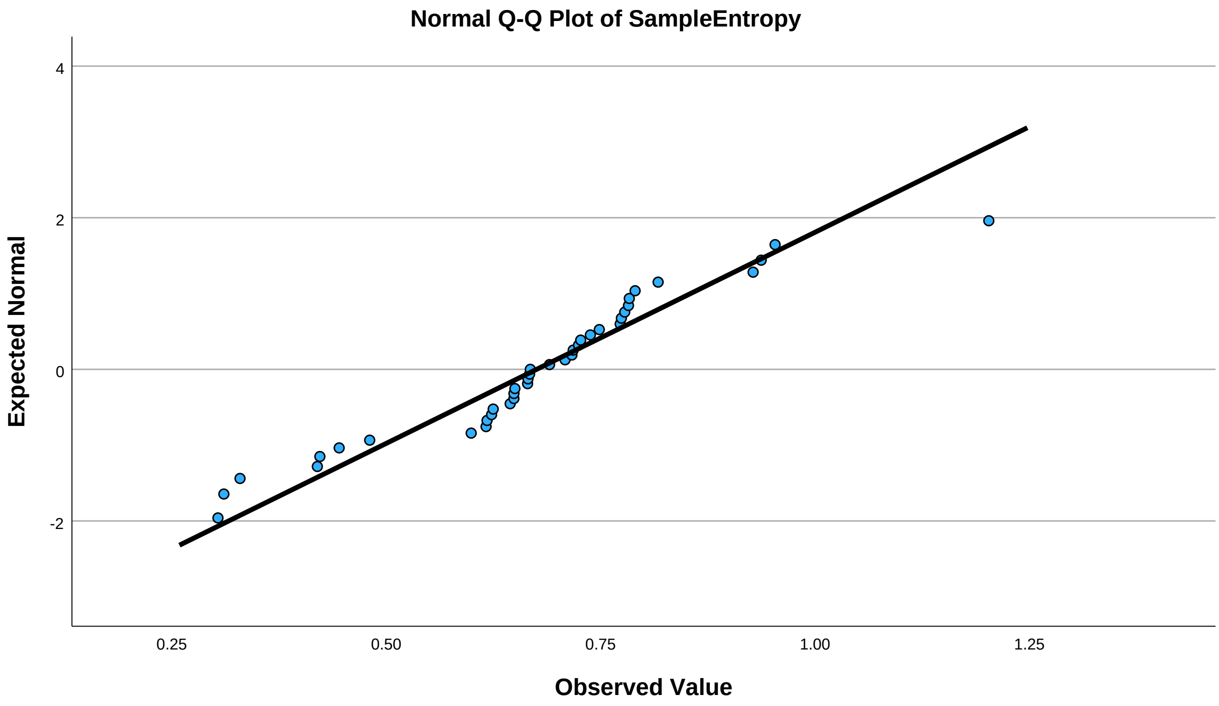

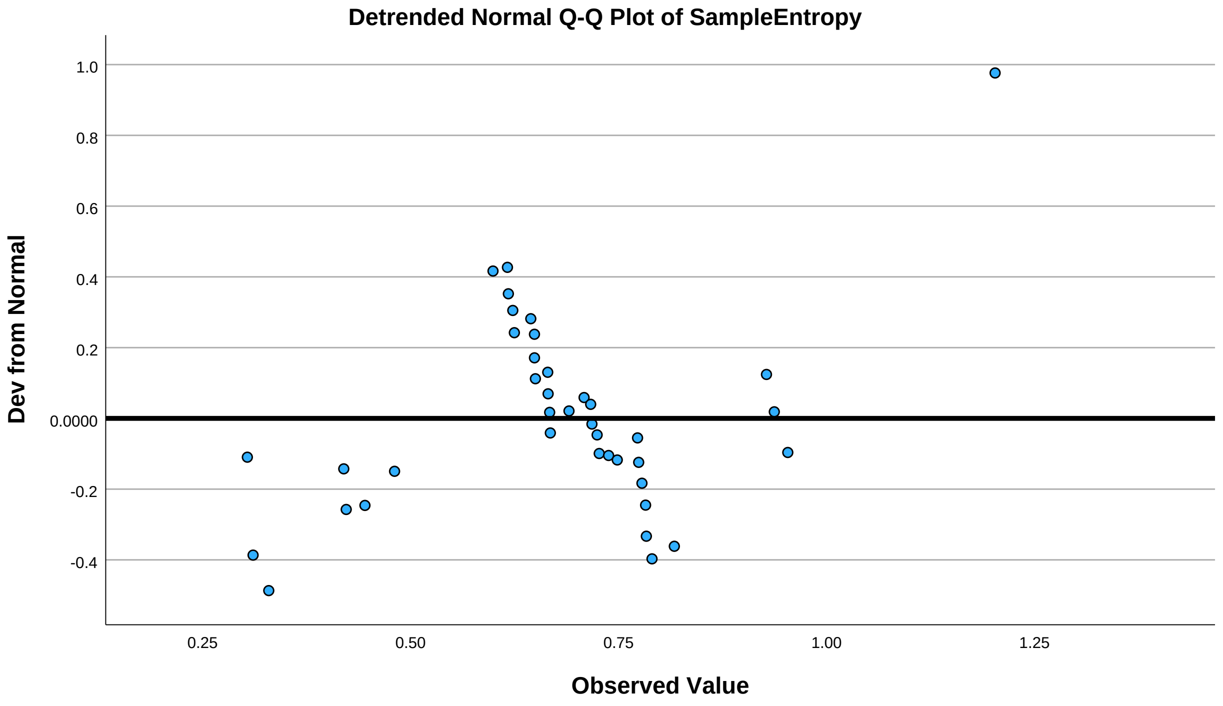

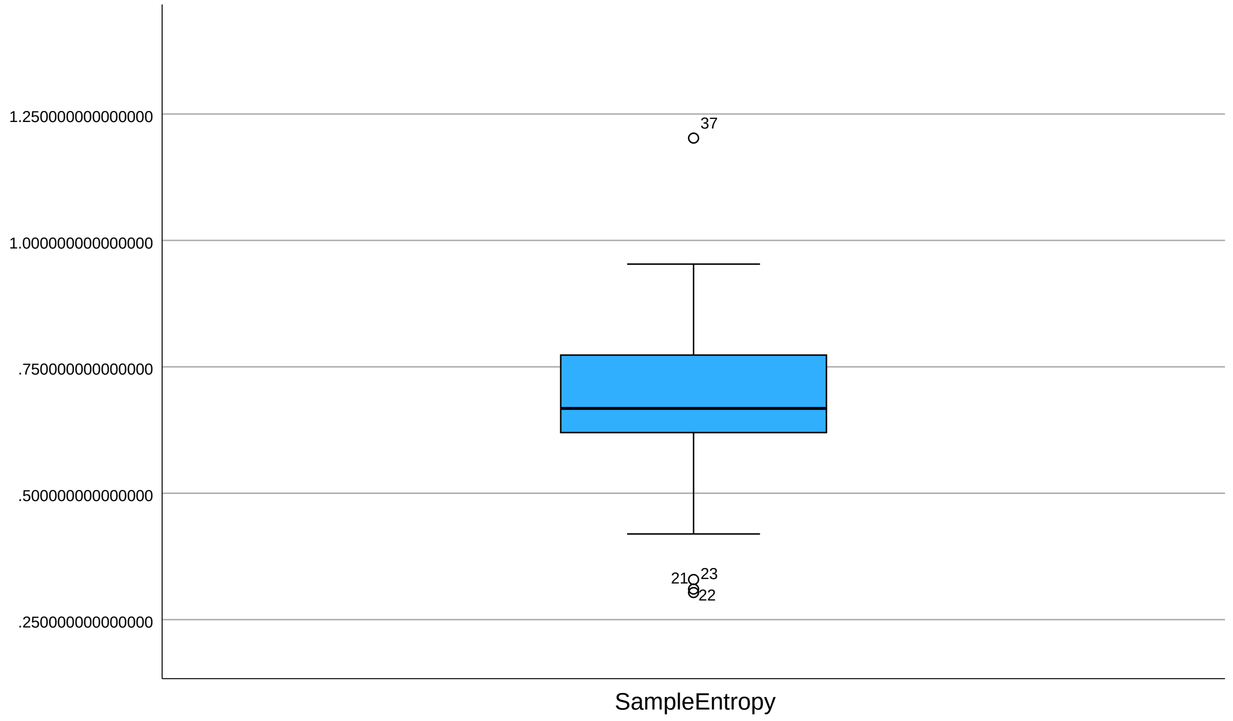

SpectralEntropy_bits

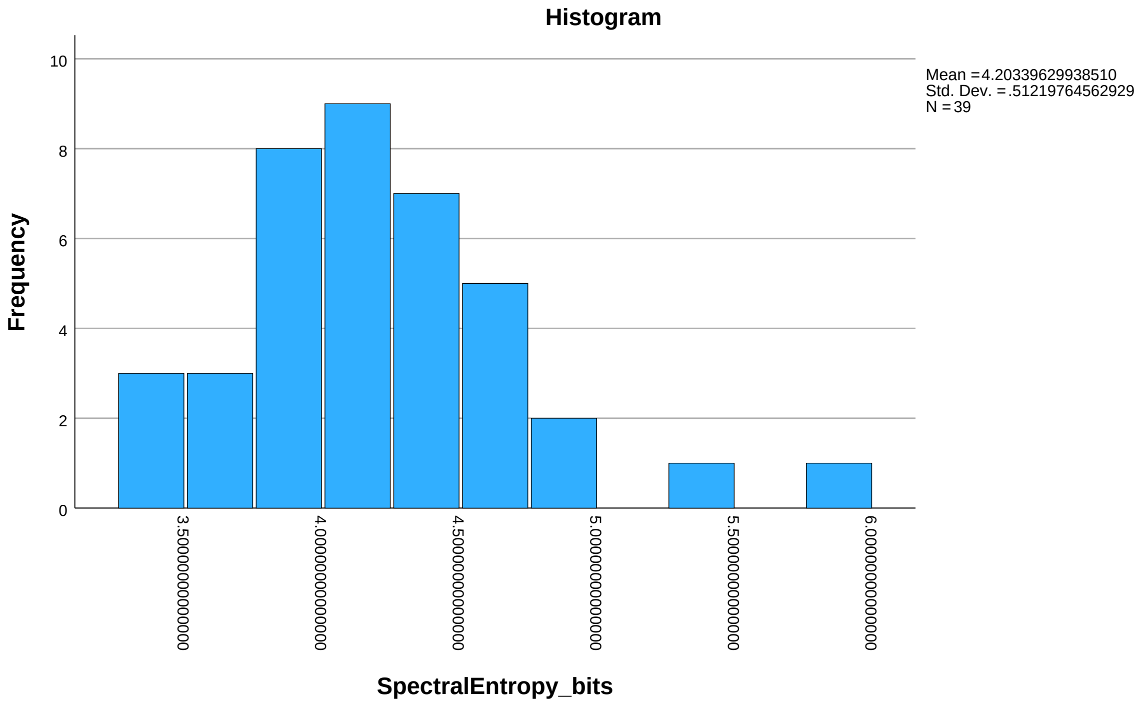

SpectralEntropy_bits Stem-and-Leaf Plot

```
 Frequency    Stem &  Leaf

     3.00        3 .  333
      .00      3 .
     5.00        3 .  67777
     6.00        3 .  889999
     7.00        4 .  0001111
     6.00        4 .  222333
     6.00        4 .  444555
     2.00      4 .  67
     2.00      4 .  89
     2.00 Extremes    (>=5.4)

 Stem width:   1.000000
 Each leaf:       1 case(s)
```

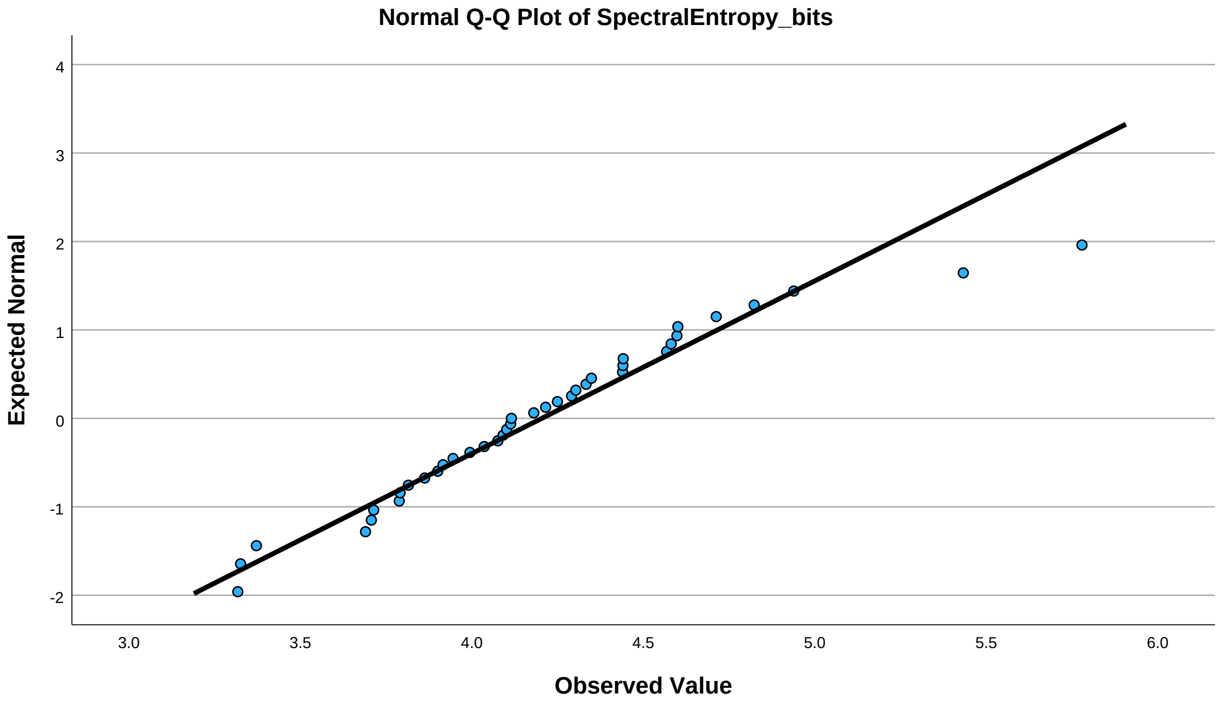

Normal Q-Q Plot of SpectralEntropy_bits
Expected Normal
Observed Value
4
3
2
1
0
-1
-2
3.0
3.5
4.0
4.5
5.0
5.5
6.0

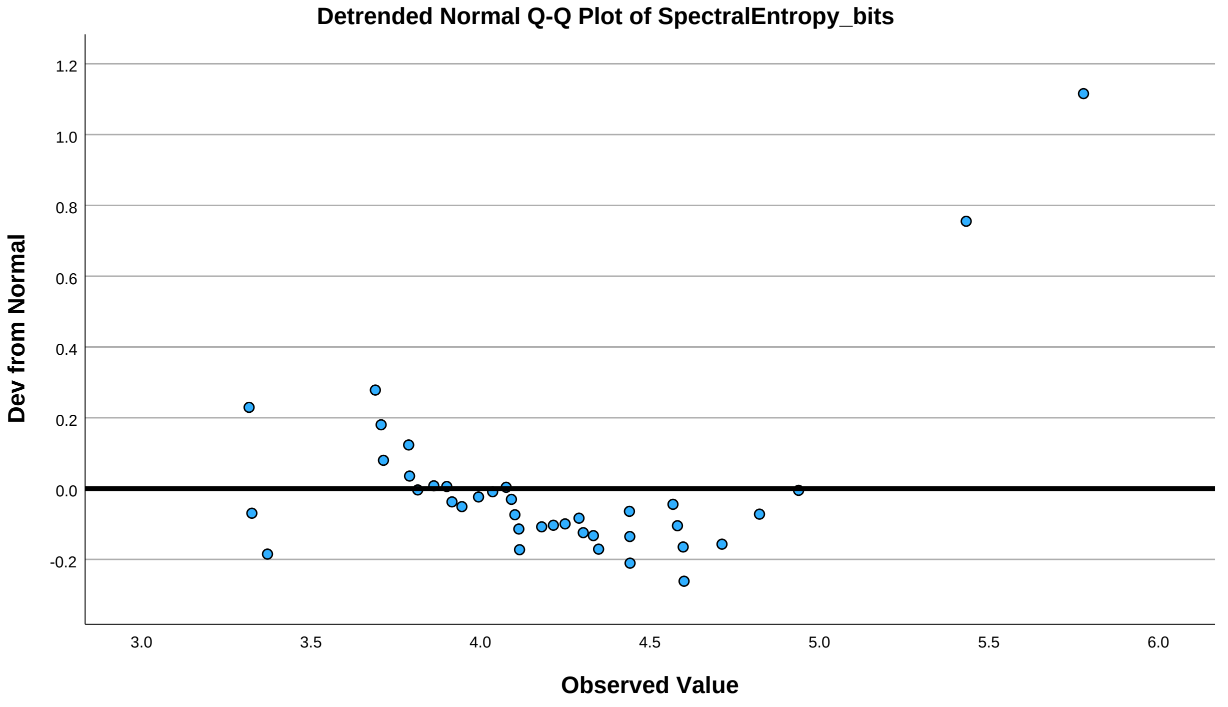

Detrended Normal Q-Q Plot of SpectralEntropy_bits
Dev from Normal
Observed Value
1.2
1.0
0.8
0.6
0.4
0.2
0.0
-0.2
3.0
3.5
4.0
4.5
5.0
5.5
6.0

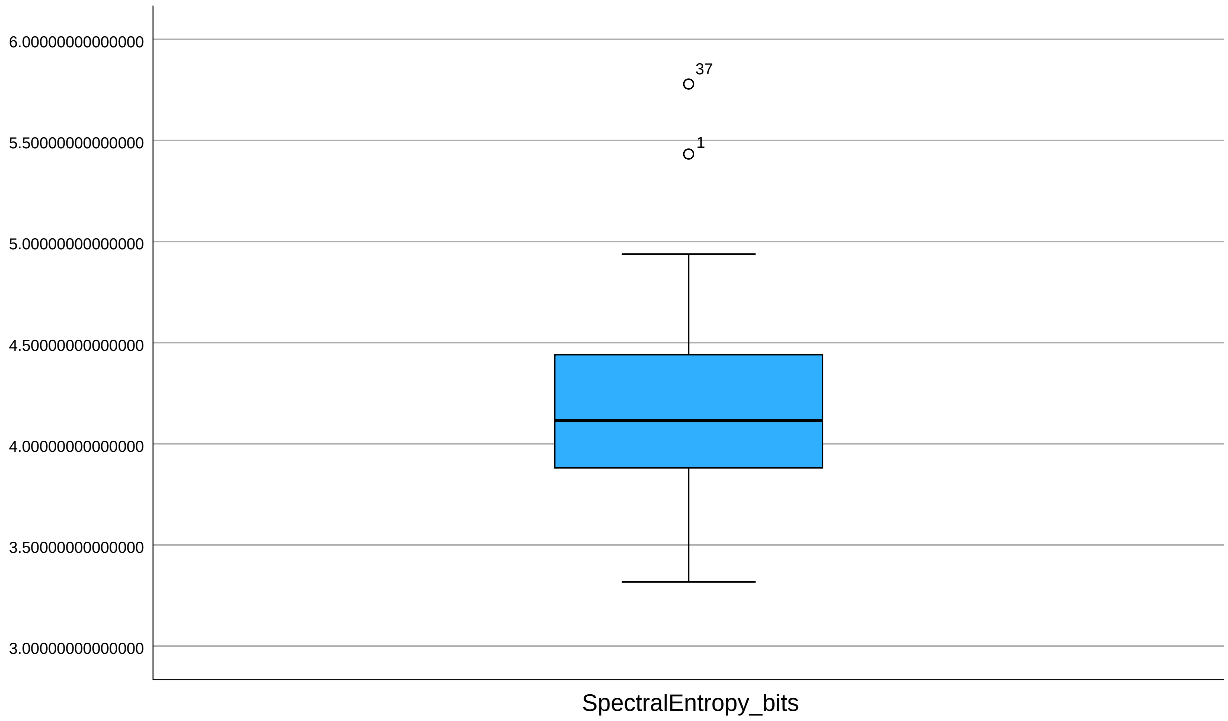

SpectralEntropy_01

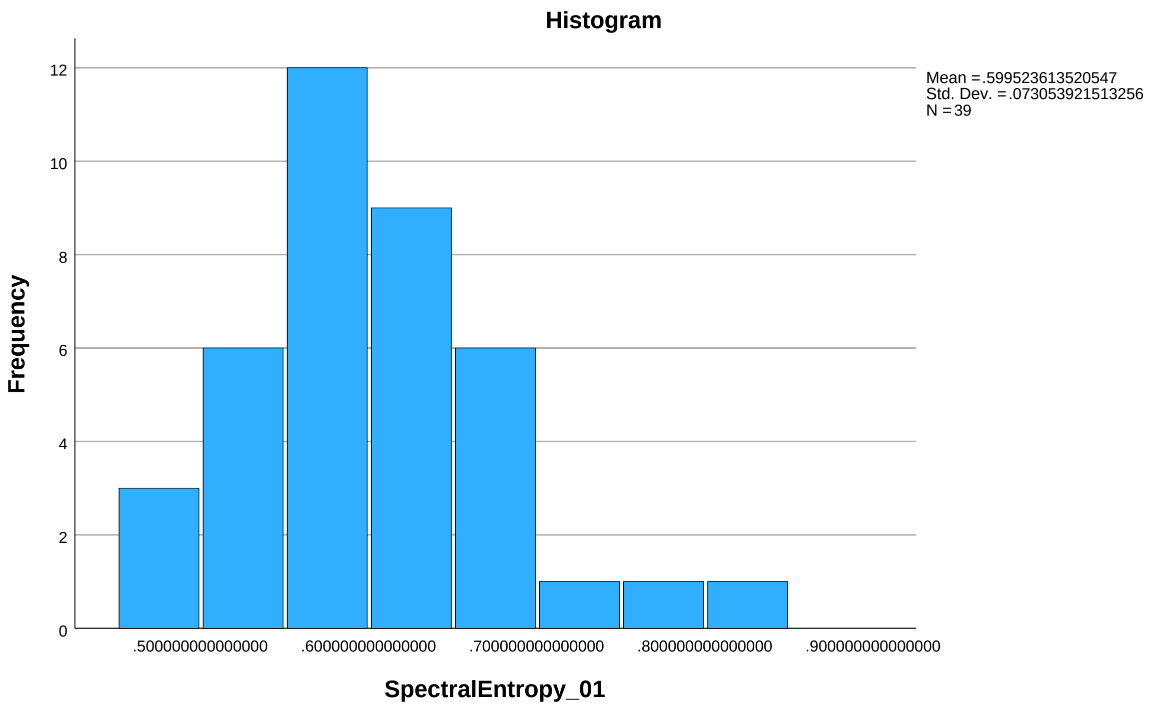


```
SpectralEntropy_01 Stem-and-Leaf Plot

 Frequency    Stem &  Leaf
```

```
     3.00        4 .  778
     6.00        5 .  222444
    12.00        5 .  555667888889
     9.00        6 .  001112333
     6.00        6 .  555578
     1.00        7 .  0
     2.00 Extremes    (>=.77)

 Stem width:   .1000000
 Each leaf:       1 case(s)
```

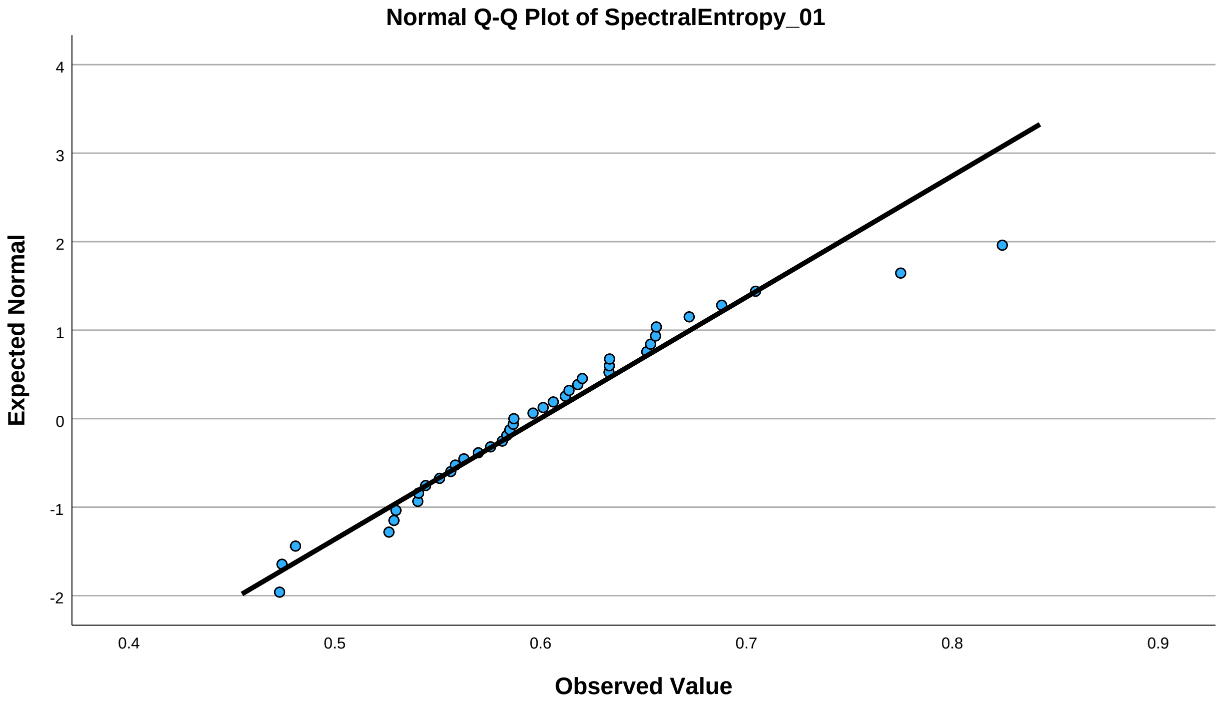

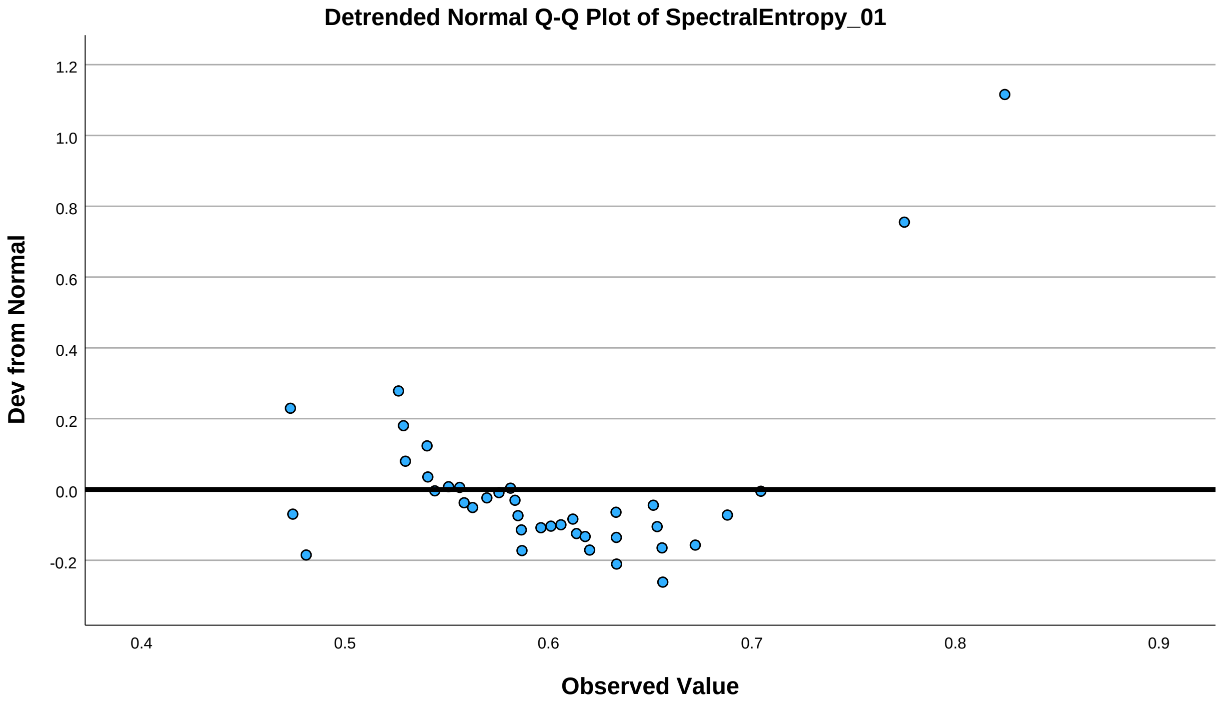

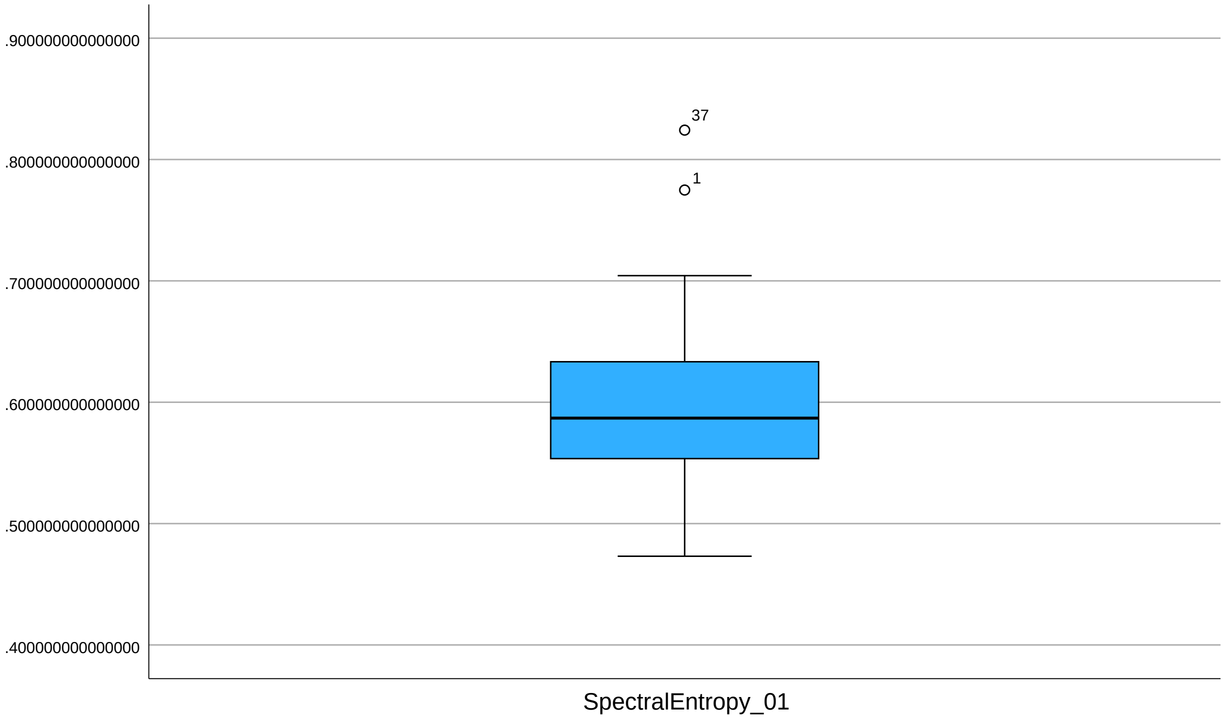

HiguchiFractalDimension

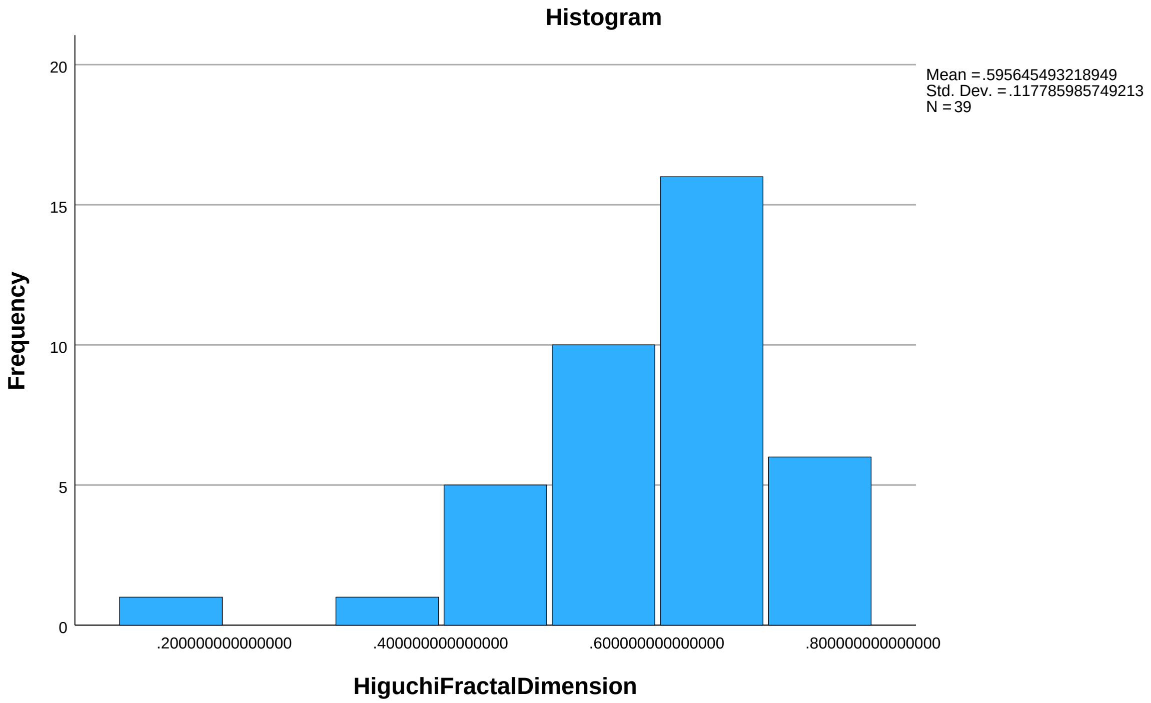


```
HiguchiFractalDimension Stem-and-Leaf Plot

 Frequency    Stem &  Leaf

     1.00 Extremes    (=<.18)
     1.00        3 .  6
     2.00        4 .  24
     3.00        4 .  578
     3.00        5 .  023
     7.00        5 .  6788899
     8.00        6 .  00112224
     8.00        6 .  55677899
     4.00        7 .  0223
     2.00        7 .  58

 Stem width:   .1000000
 Each leaf:        1 case(s)
```

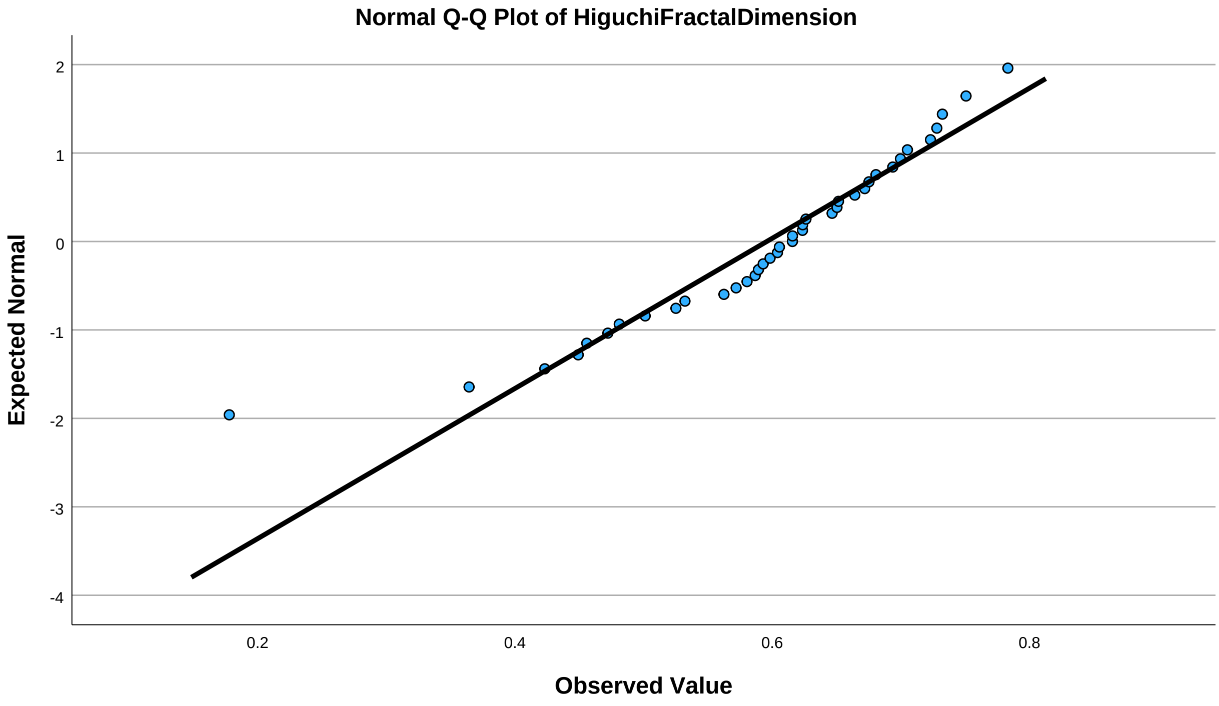

Normal Q-Q Plot of HiguchiFractalDimension
Expected Normal
2
1
0
-1
-2
-3
-4
0.2
0.4
0.6
0.8
Observed Value

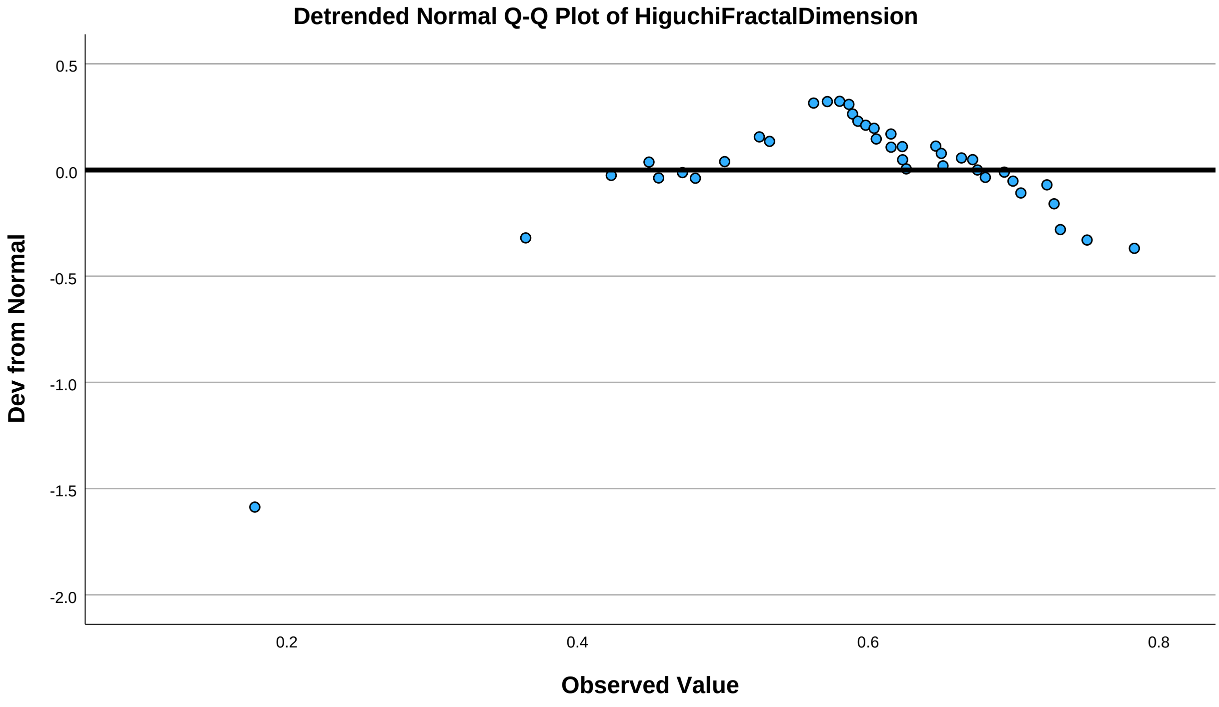

Detrended Normal Q-Q Plot of HiguchiFractalDimension
Dev from Normal
0.5
0.0
-0.5
-1.0
-1.5
-2.0
0.2
0.4
0.6
0.8
Observed Value

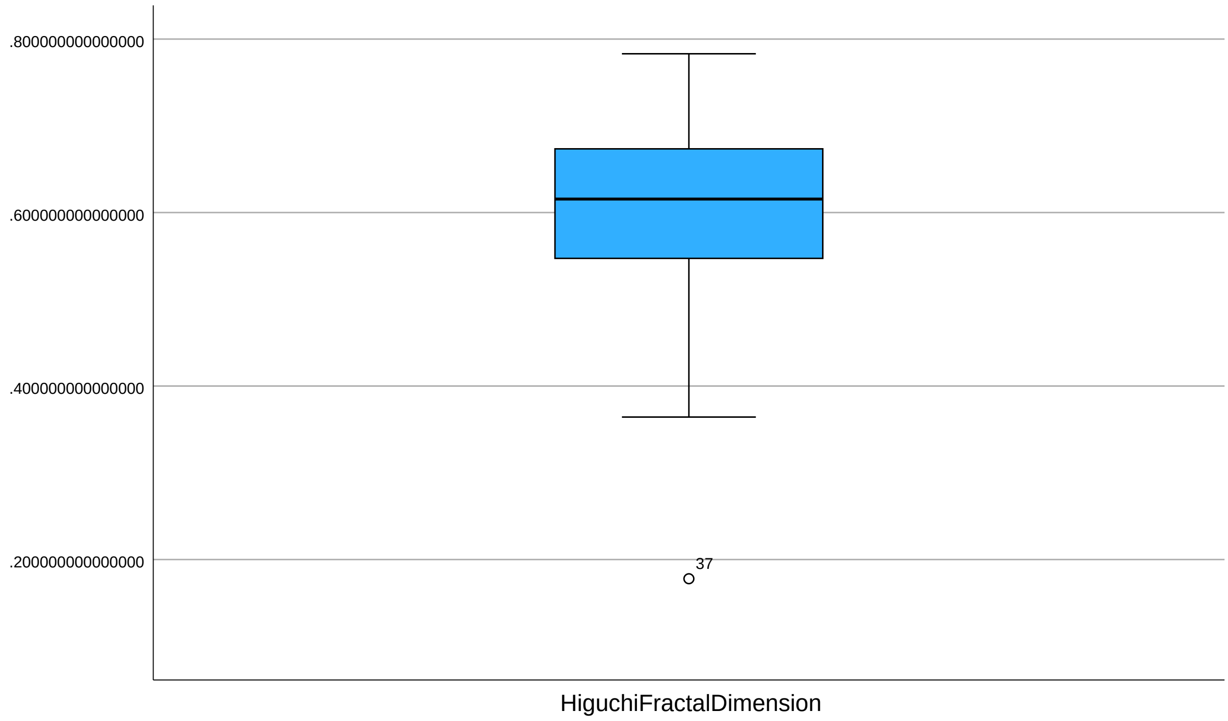

LempelZivComplexity

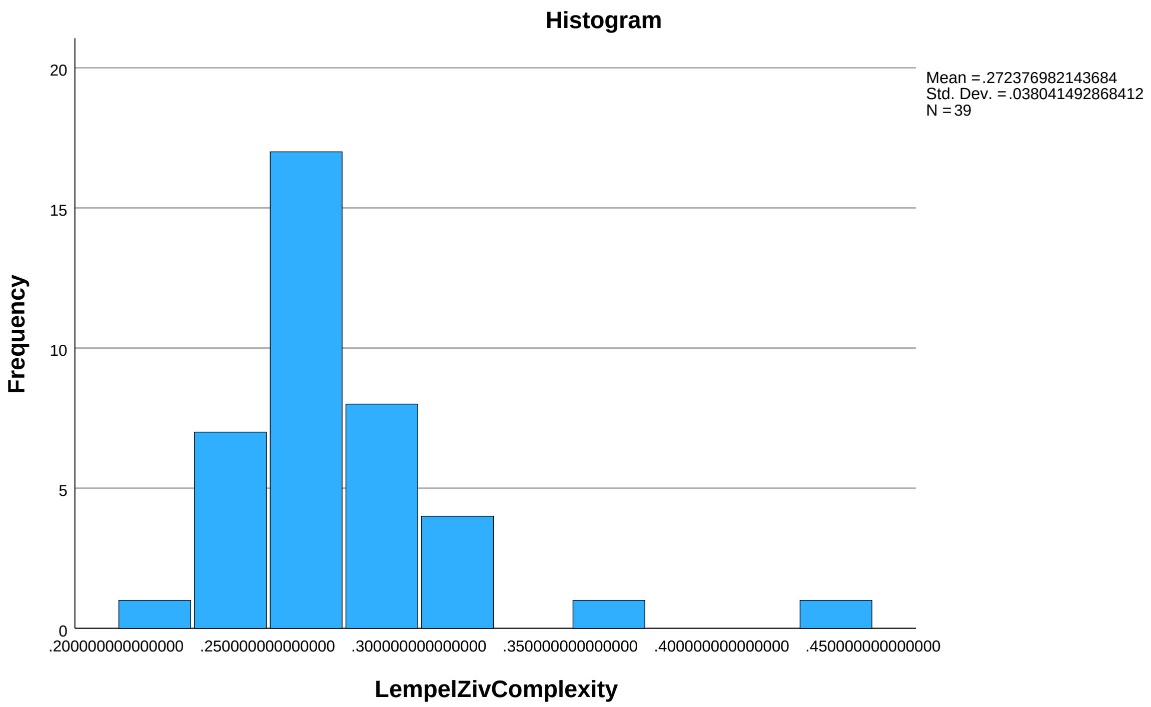


```
LempelZivComplexity Stem-and-Leaf Plot

 Frequency    Stem &  Leaf
```

```
     2.00       22 .  16
     2.00       23 .  58
     4.00       24 .  0236
    10.00       25 .  0234455568
     6.00       26 .  223557
     5.00       27 .  07778
     2.00       28 .  46
     2.00       29 .  47
     1.00       30 .  5
     2.00       31 .  18
     1.00       32 .  0
     2.00 Extremes    (>=.365)

 Stem width:   .0100000
 Each leaf:        1 case(s)
```

Normal Q-Q Plot of LempelZivComplexity

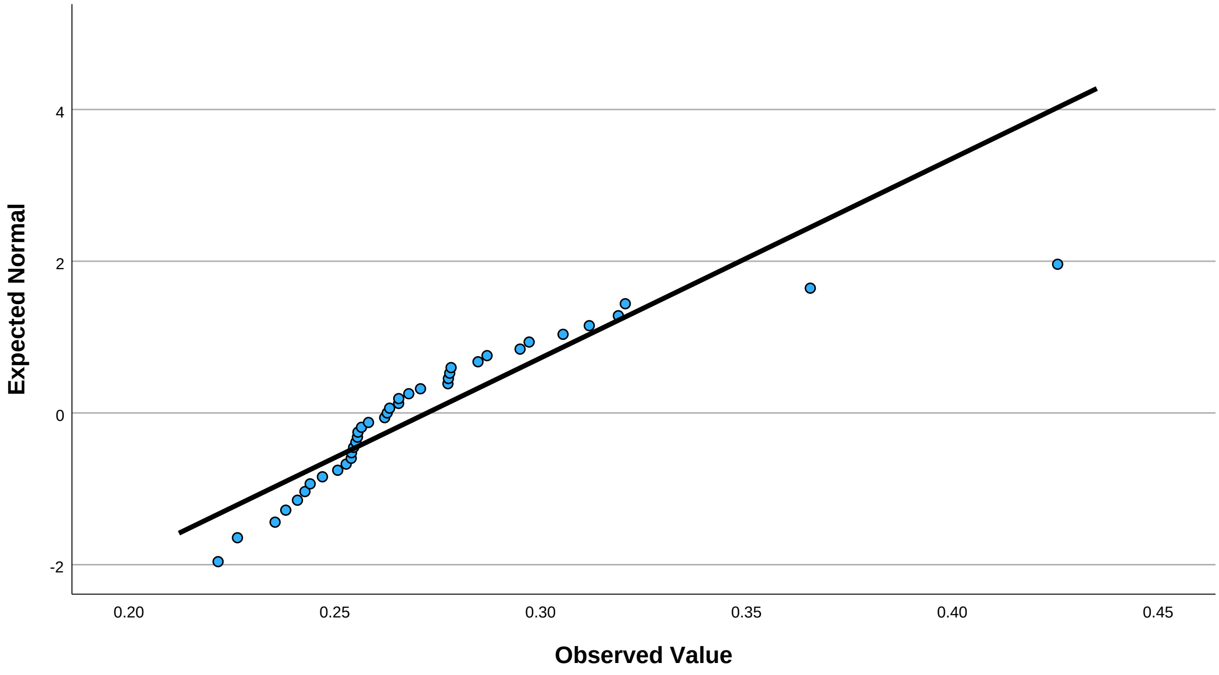

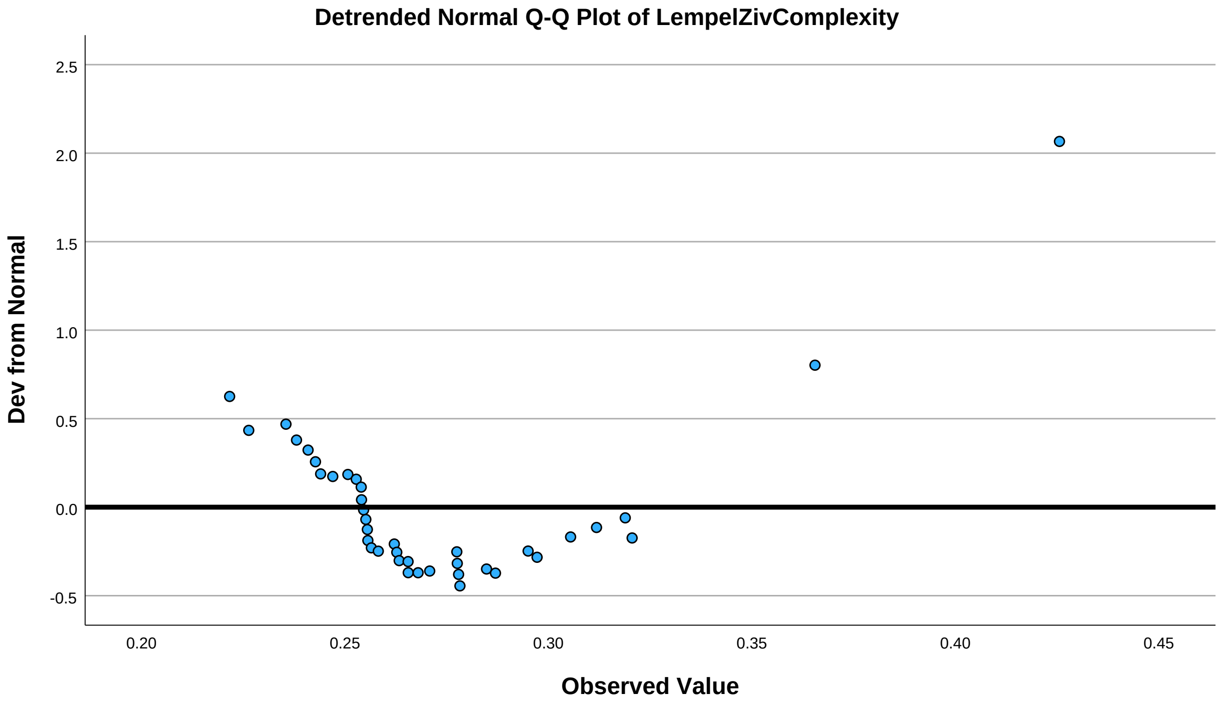

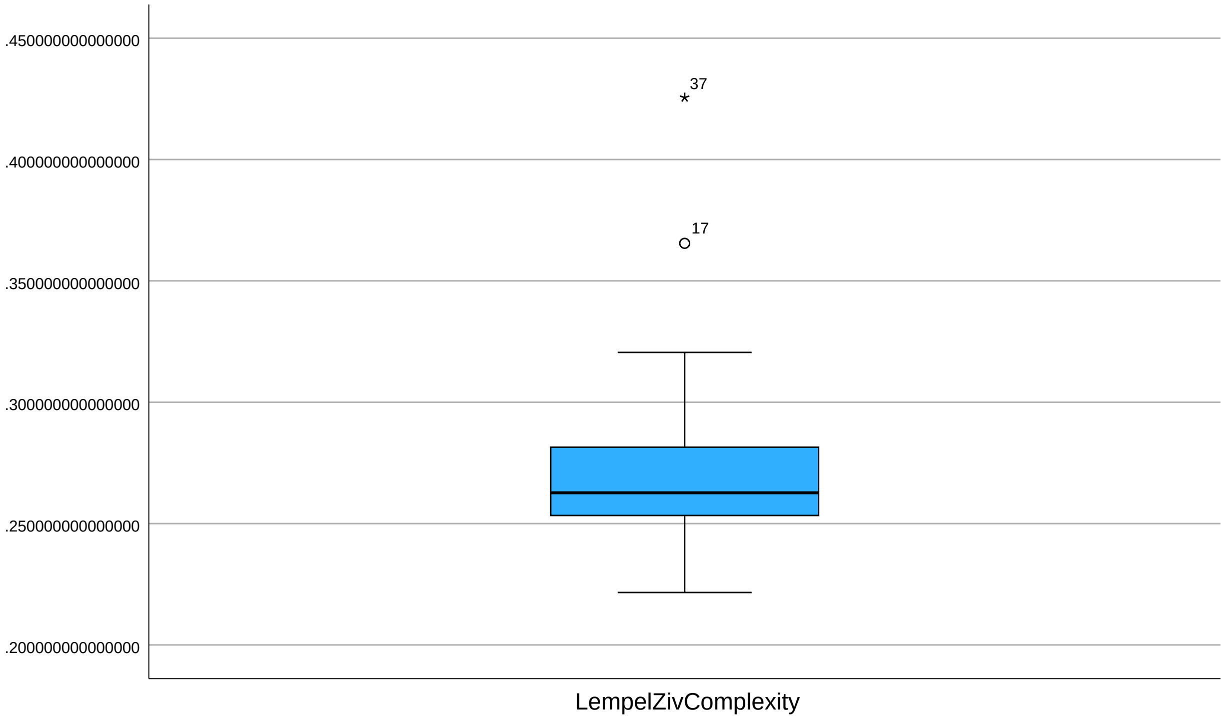

RenyiEntropy

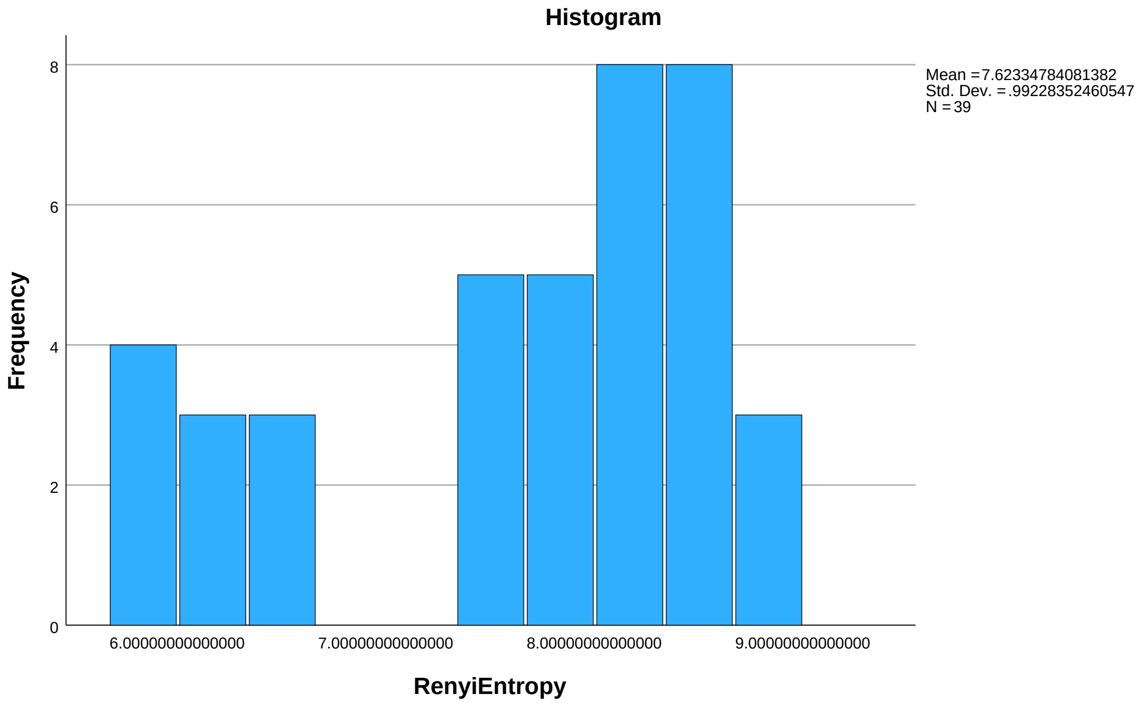


```
RenyiEntropy Stem-and-Leaf Plot

 Frequency    Stem &  Leaf

     4.00        5 .  6789
     5.00        6 .  00134
     1.00        6 .  5
     2.00        7 .  34
     8.00        7 .  56668889
    12.00        8 .  000122224444
     7.00        8 .  5556777

 Stem width:   1.000000
 Each leaf:       1 case(s)
```

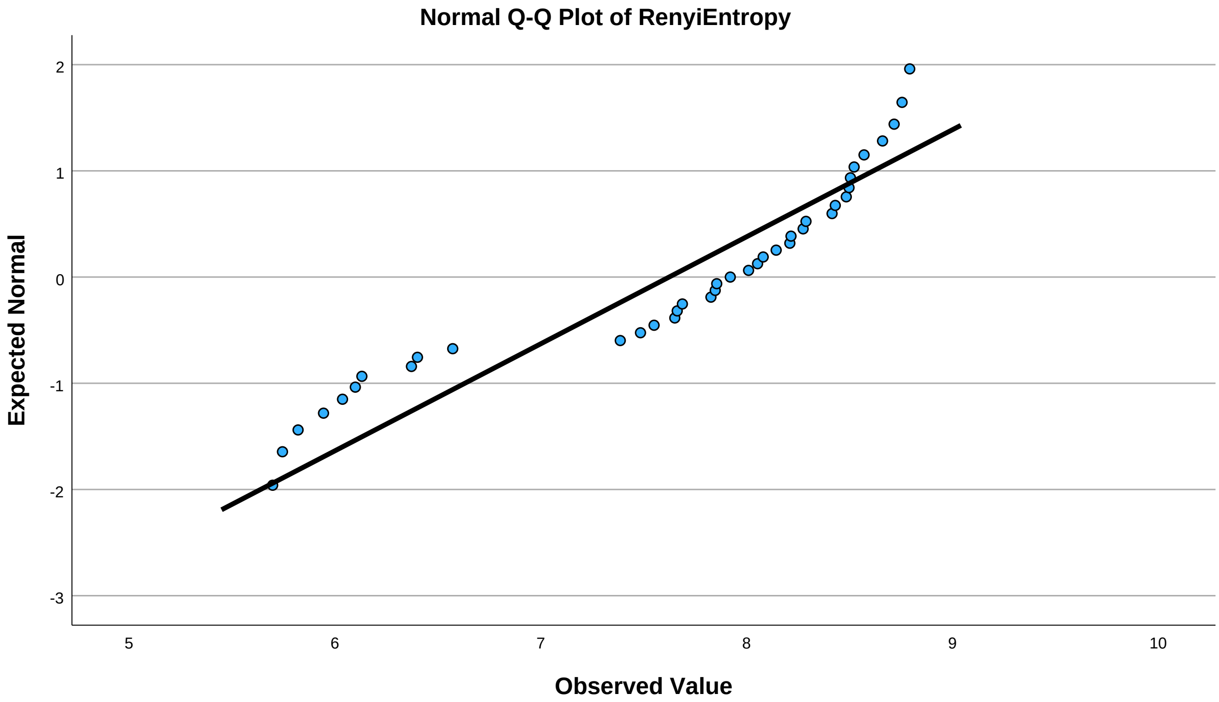
Normal Q-Q Plot of RenyiEntropy
Expected Normal
2
1
0
-1
-2
-3
5
6
7
8
9
10
Observed Value

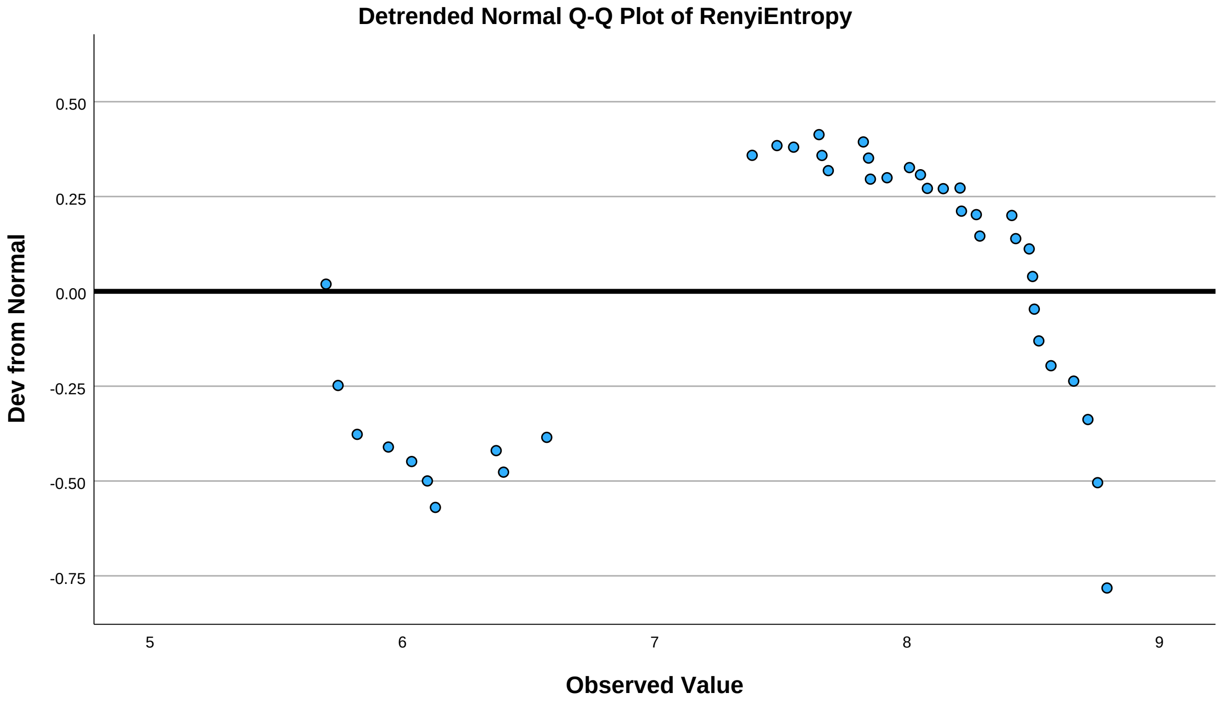
Detrended Normal Q-Q Plot of RenyiEntropy
Dev from Normal
0.50
0.25
0.00
-0.25
-0.50
-0.75
5
6
7
8
9
Observed Value

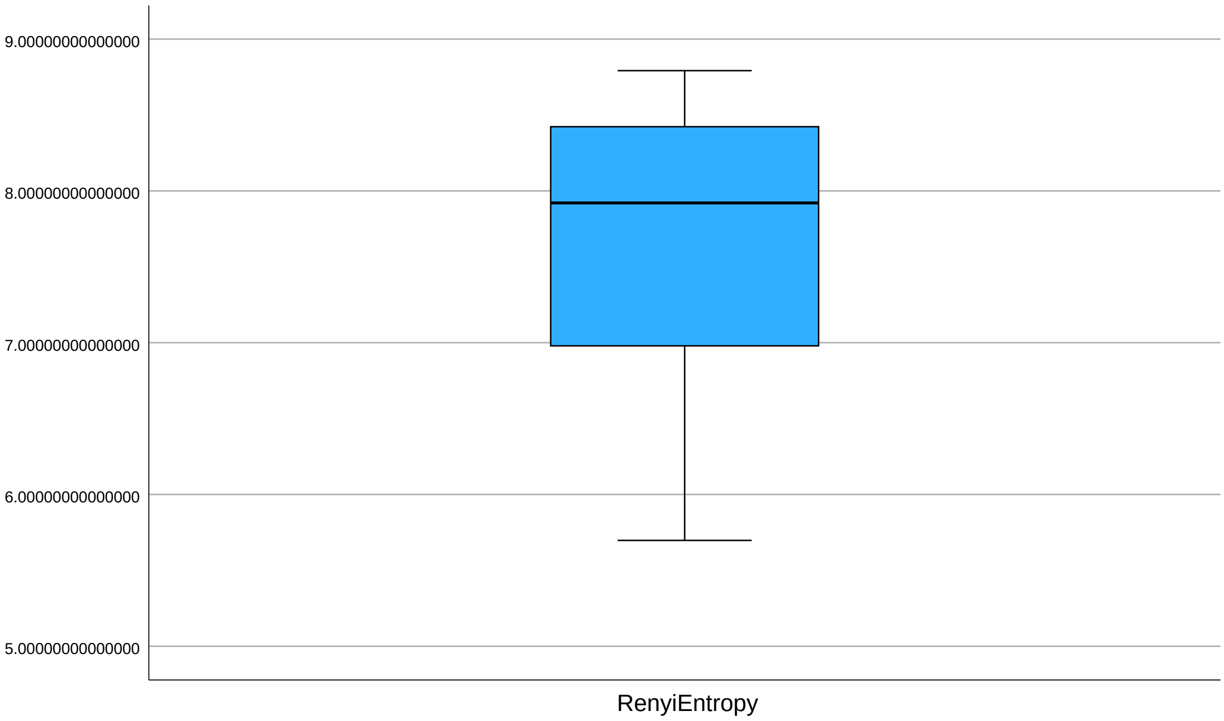

TsallisEntropy

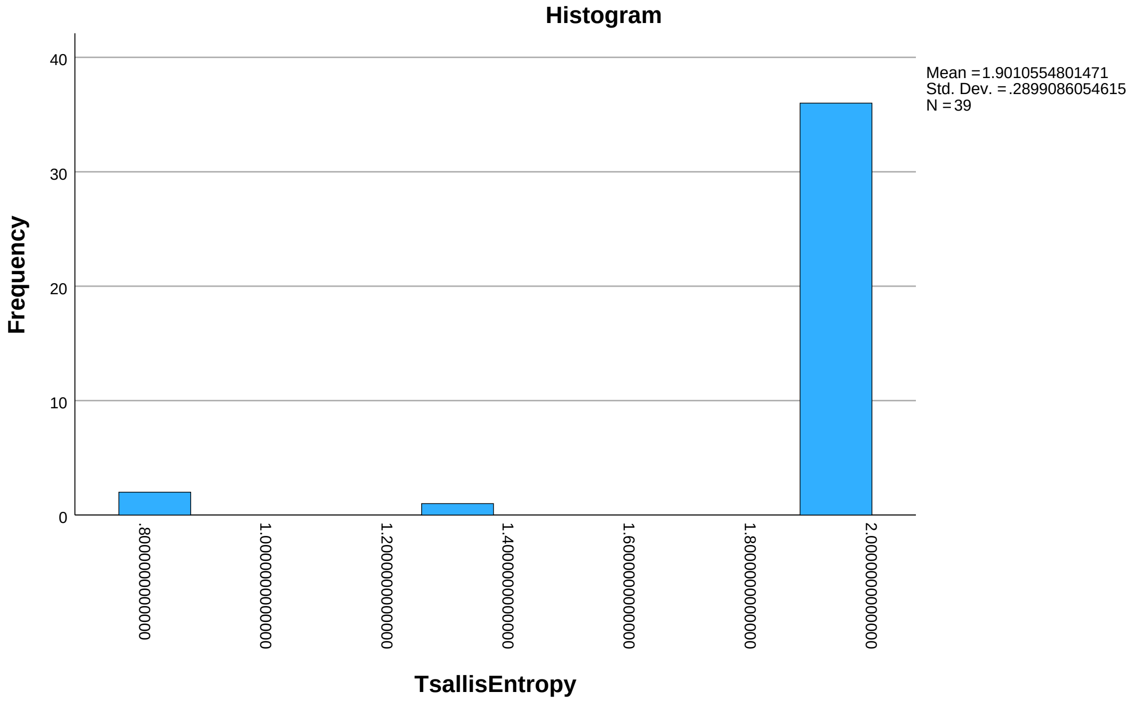

TsallisEntropy Stem-and-Leaf Plot

Frequency   Stem & Leaf

```
  7.00 Extremes    (=<1.9687)
  1.00    1979 .  8
   .00    1980 .
  2.00    1981 .  07
   .00    1982 .
  1.00    1983 .  4
  2.00    1984 .  58
  7.00    1985 .  2445789
 13.00    1986 .  1234447778999
  4.00    1987 .  0116
  2.00    1988 .  26

Stem width:  .0010000
Each leaf:       1 case(s)
```

Normal Q-Q Plot of TsallisEntropy

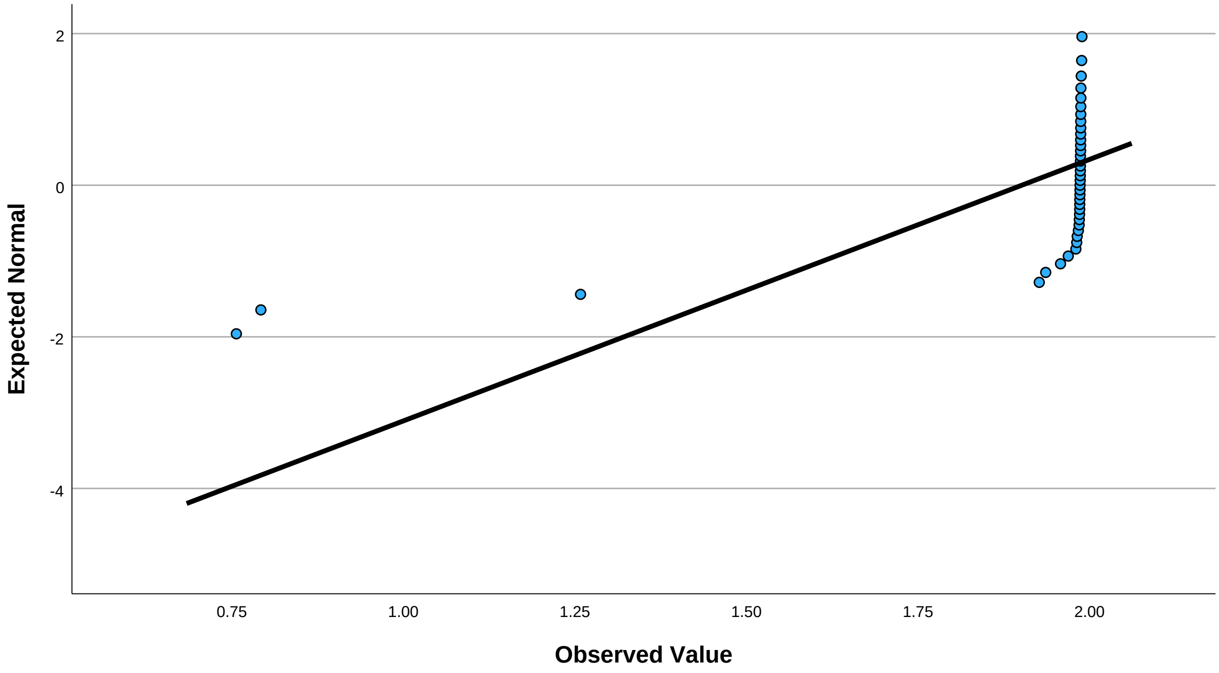

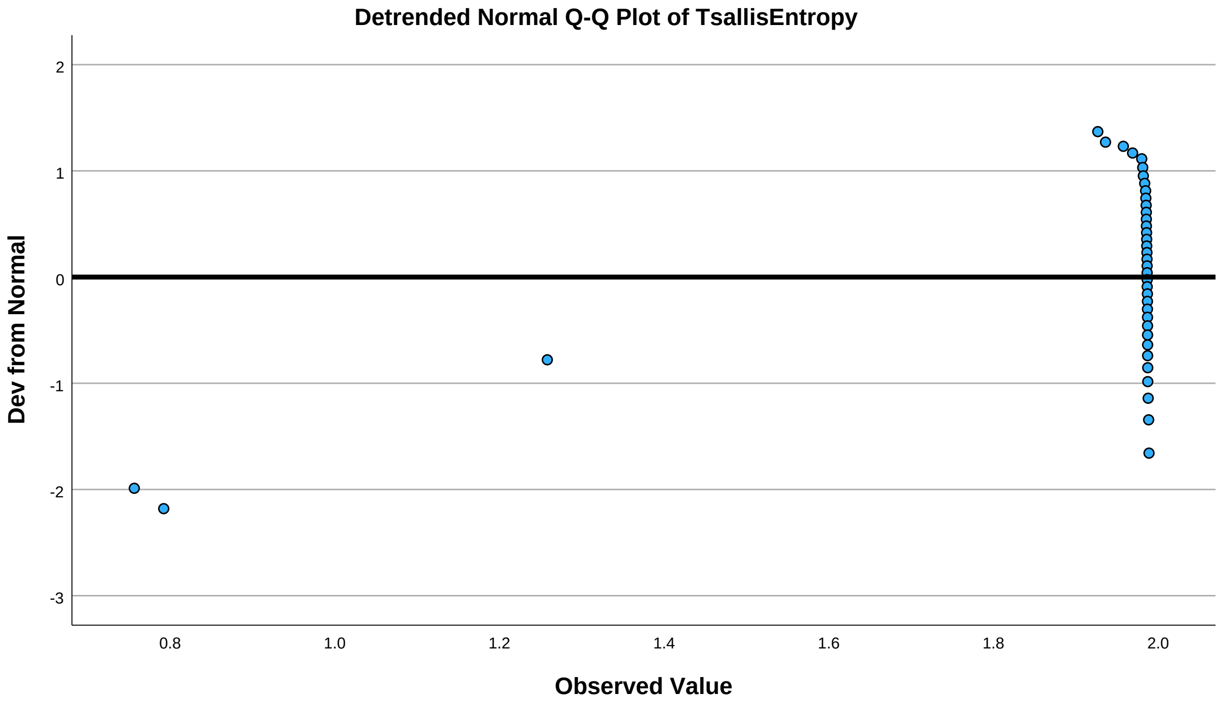

Detrended Normal Q-Q Plot of TsallisEntropy
Dev from Normal
Observed Value
2
1
0
-1
-2
-3
0.8
1.0
1.2
1.4
1.6
1.8
2.0

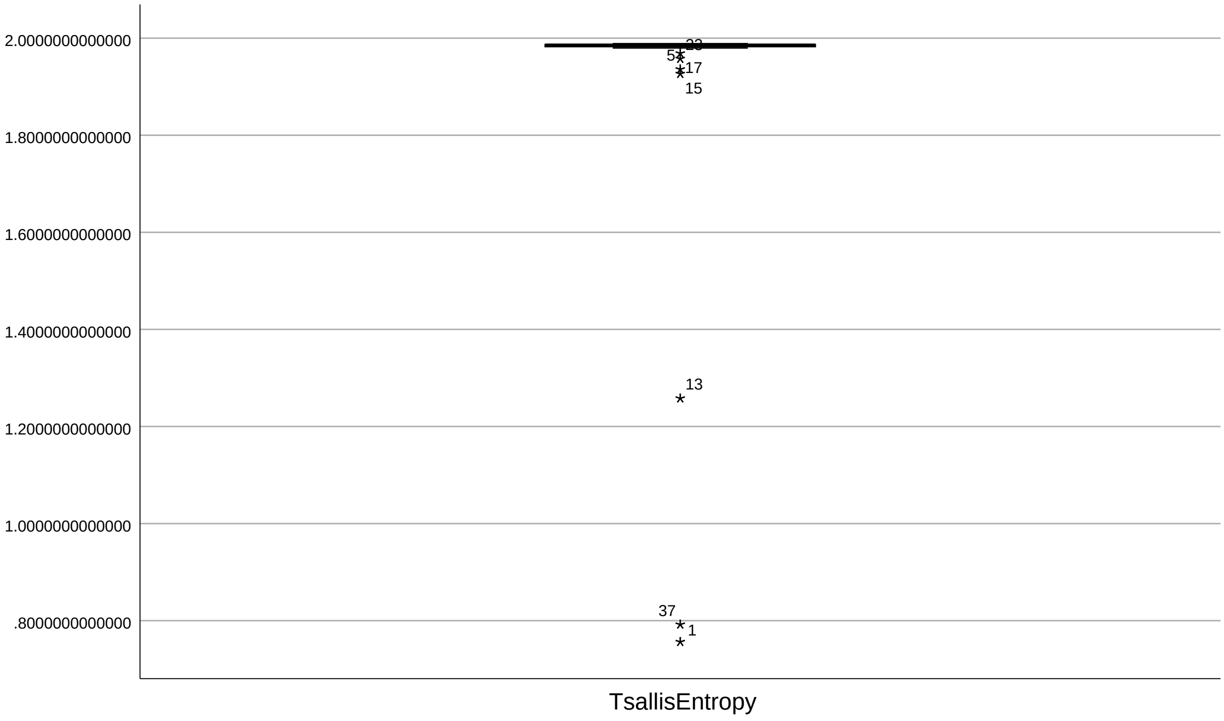

2.0000000000000
1.8000000000000
1.6000000000000
1.4000000000000
1.2000000000000
1.0000000000000
.8000000000000
17
15
13
37
1
TsallisEntropy

PS_4_5

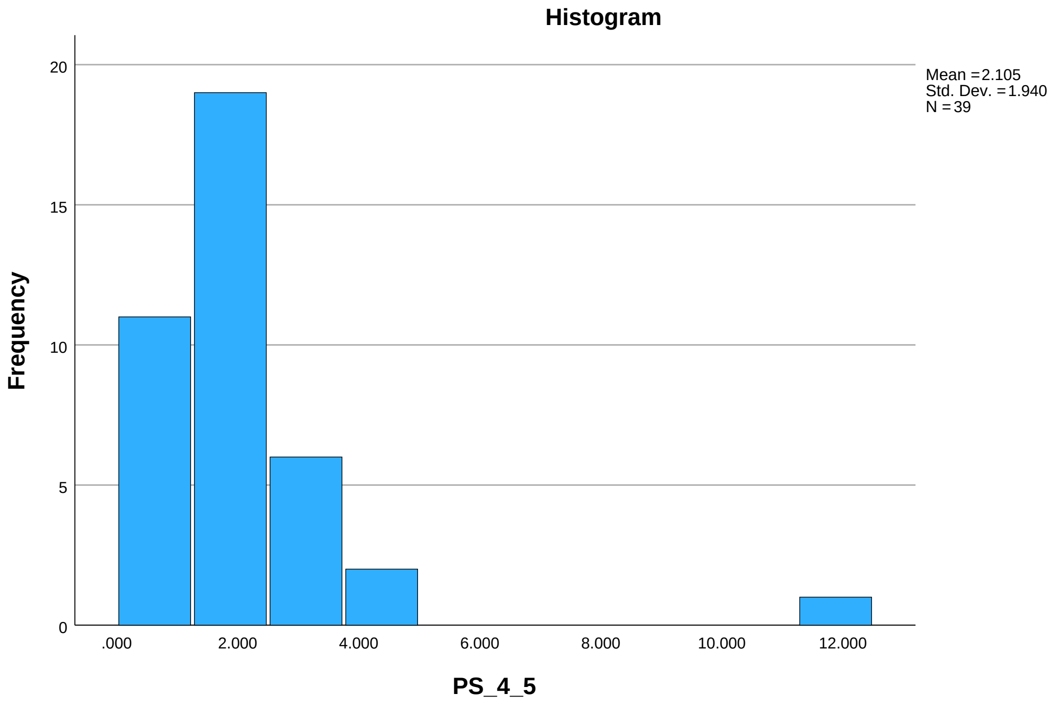


```
PS_4_5 Stem-and-Leaf Plot

 Frequency    Stem &  Leaf

     2.00        0 .  34
     5.00        0 .  67889
     9.00        1 .  111223444
     9.00        1 .  666667899
     5.00        2 .  02223
     4.00        2 .  5899
     1.00        3 .  3
     3.00        3 .  799
     1.00 Extremes    (>=12.5)

 Stem width:      1.000
 Each leaf:       1 case(s)
```

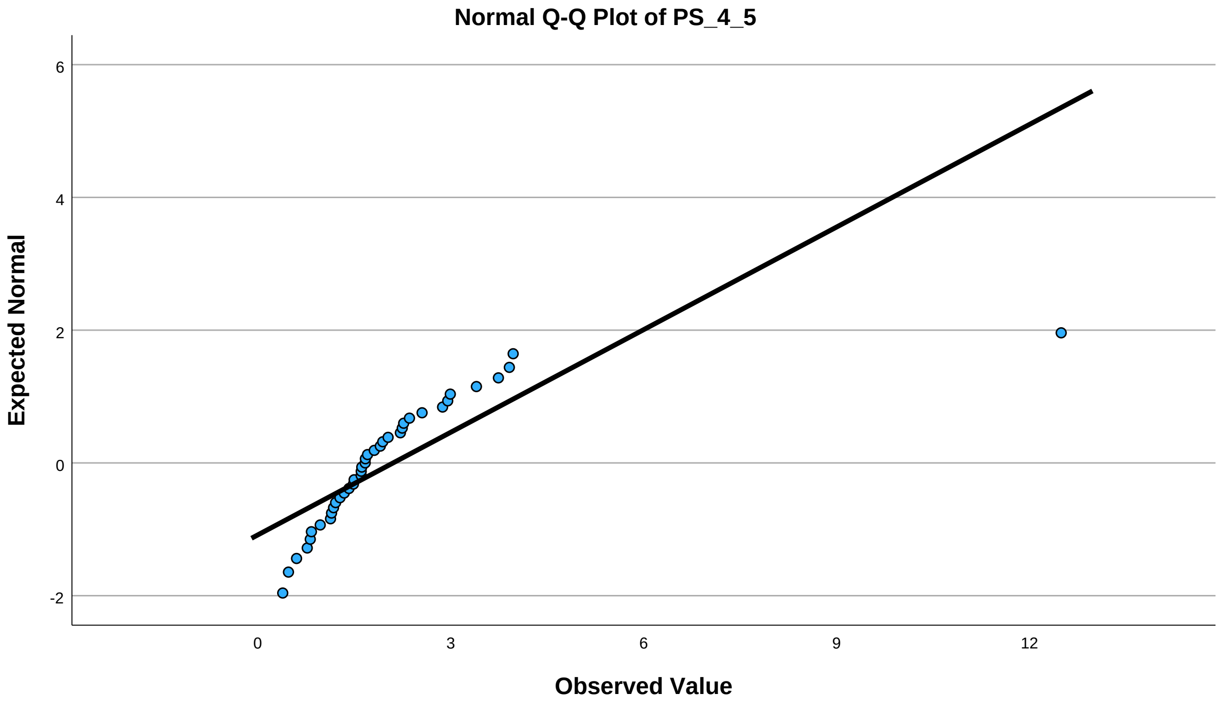

Normal Q-Q Plot of PS_4_5
Expected Normal
Observed Value
6
4
2
0
-2
0
3
6
9
12

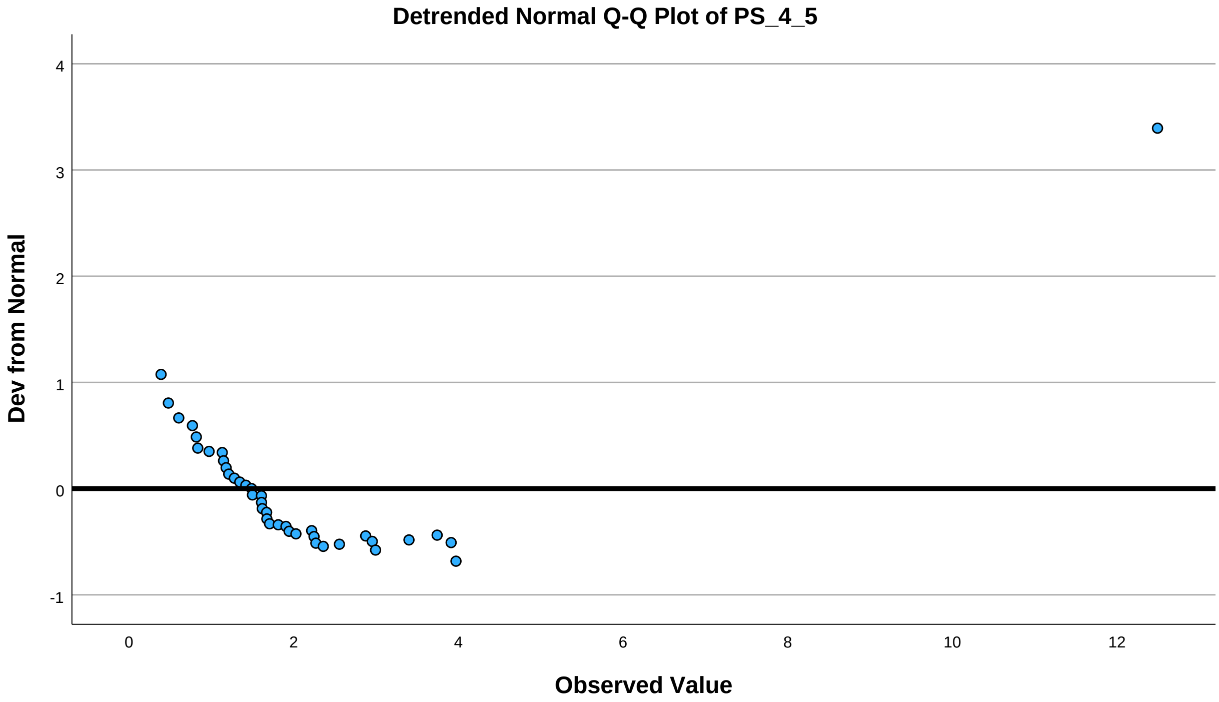

Detrended Normal Q-Q Plot of PS_4_5
Dev from Normal
Observed Value
4
3
2
1
0
-1
0
2
4
6
8
10
12

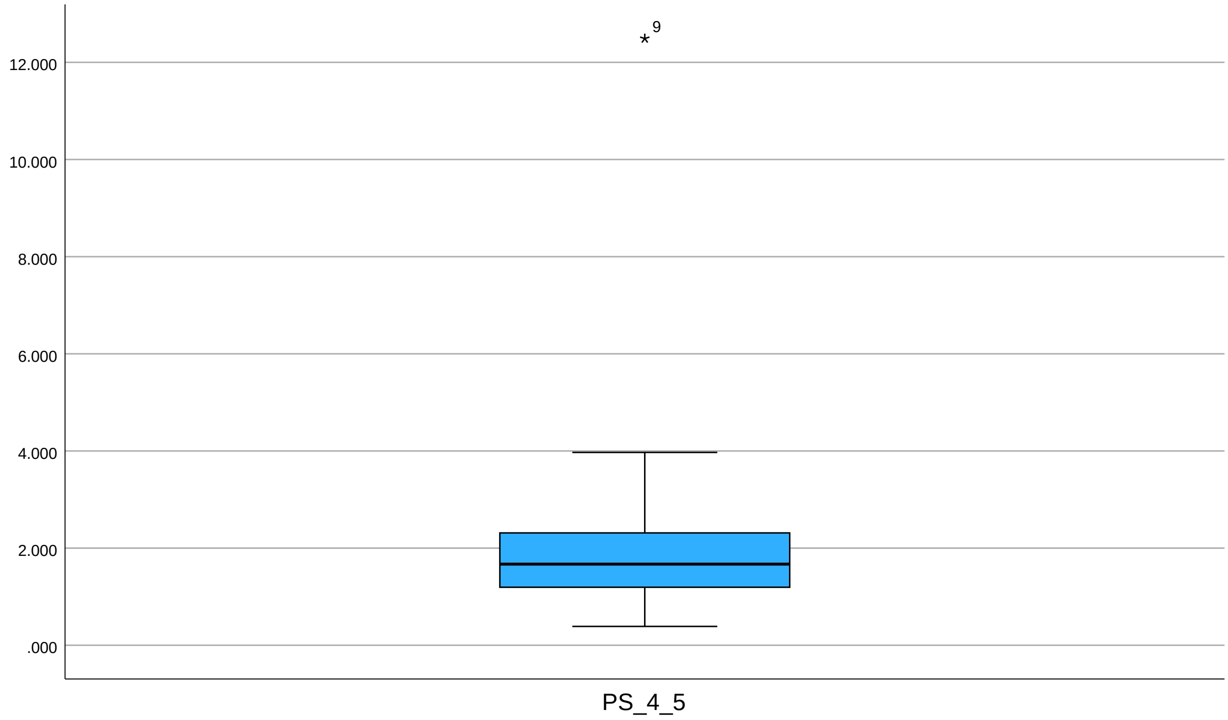

PS_5_6

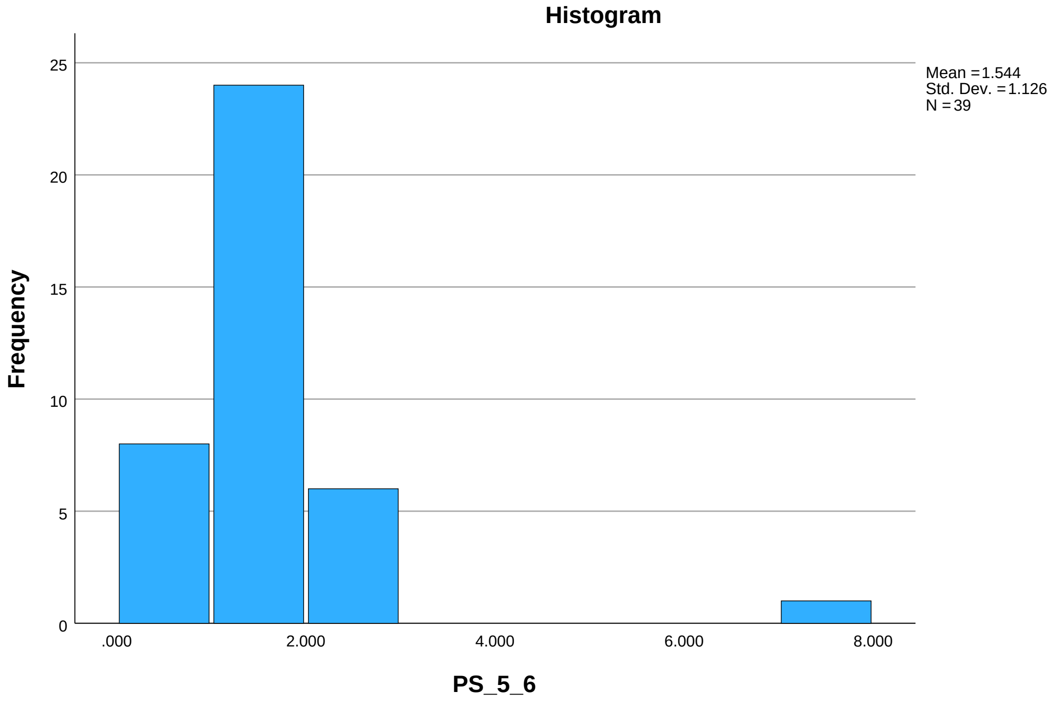


```
PS_5_6 Stem-and-Leaf Plot

 Frequency    Stem &  Leaf
```

```
  3.00        0 .  244
  5.00        0 .  56779
 13.00        1 .  0000011122223
 11.00        1 .  55666788999
  5.00        2 .  11223
  1.00        2 .  5
  1.00 Extremes    (>=7.4)

Stem width:      1.000
Each leaf:       1 case(s)
```

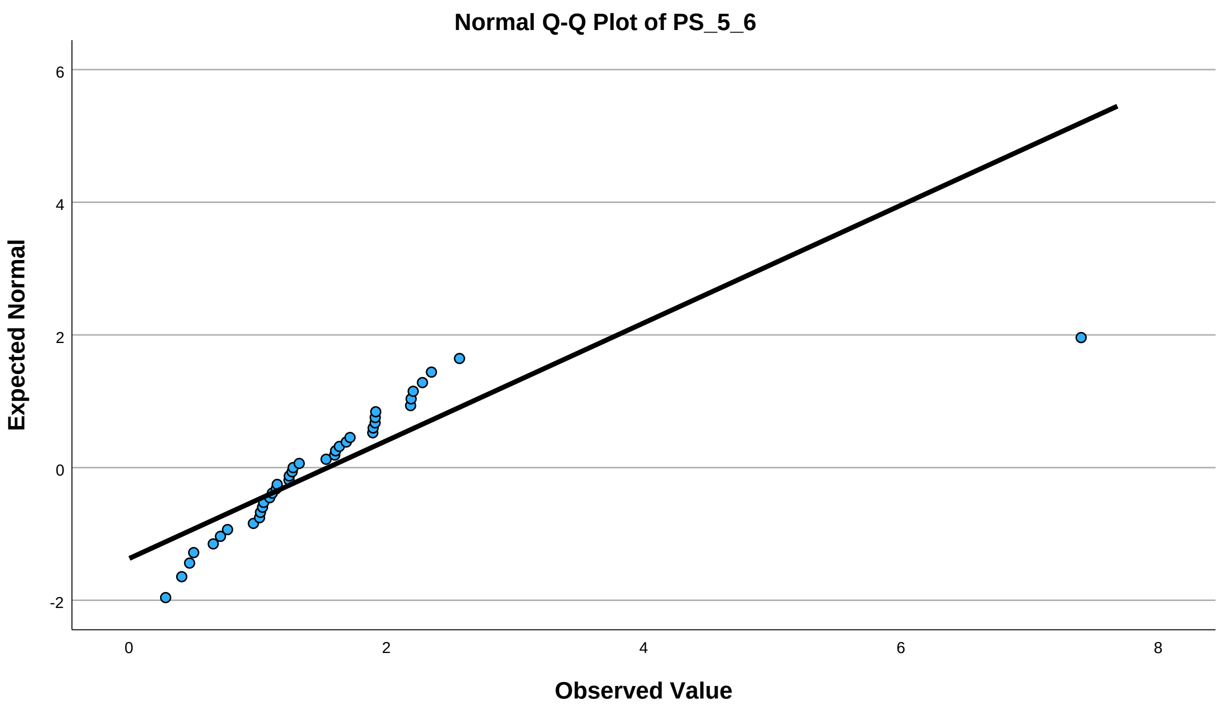

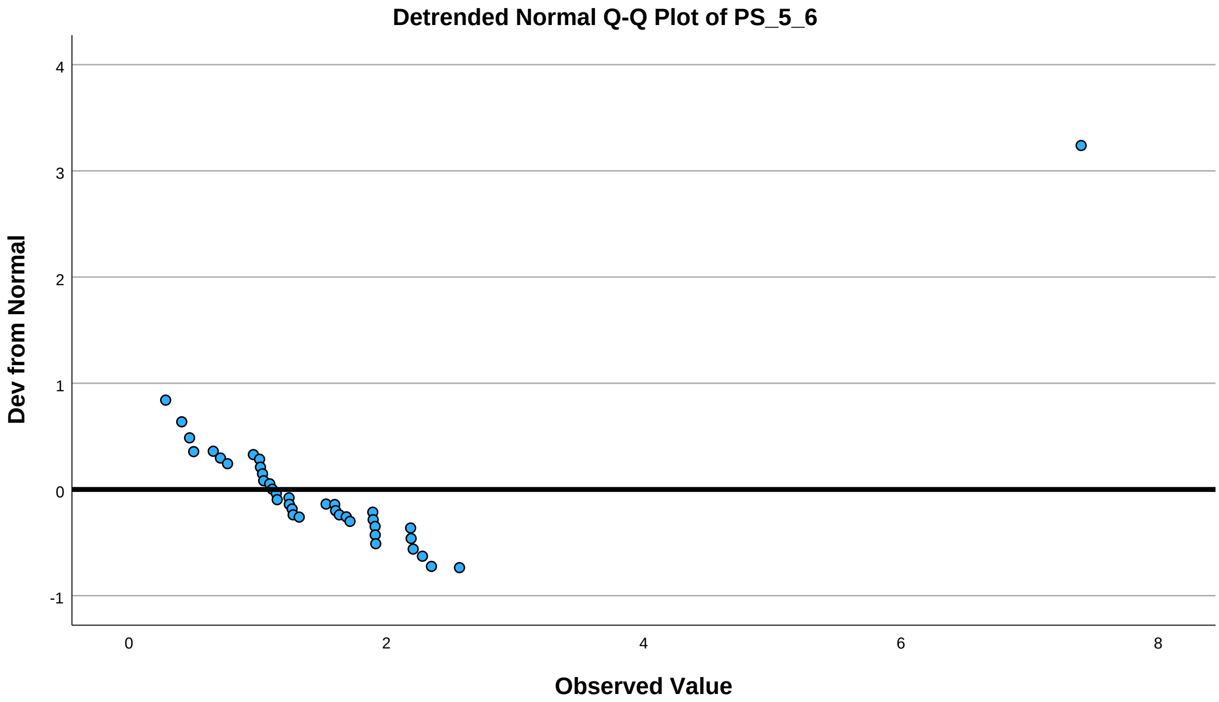

Detrended Normal Q-Q Plot of PS_5_6
Dev from Normal
4
3
2
1
0
-1
0
2
4
6
8
Observed Value

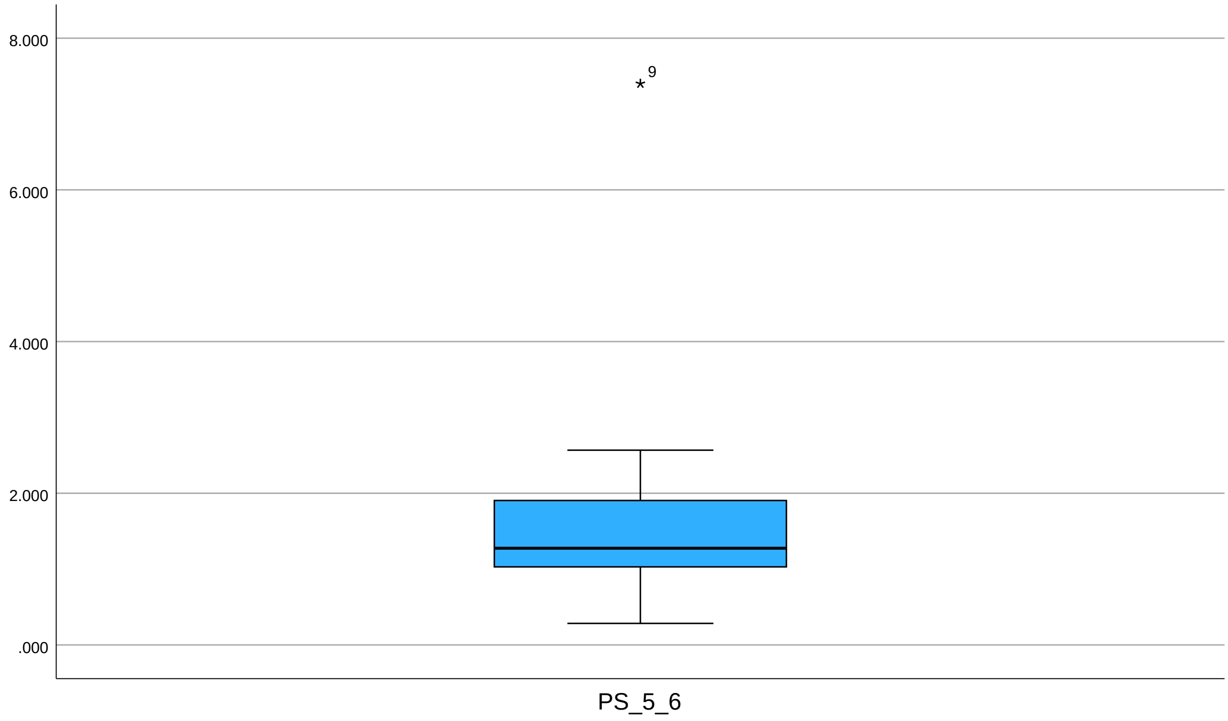

8.000
6.000
4.000
2.000
.000
9
PS_5_6

PS_6_7

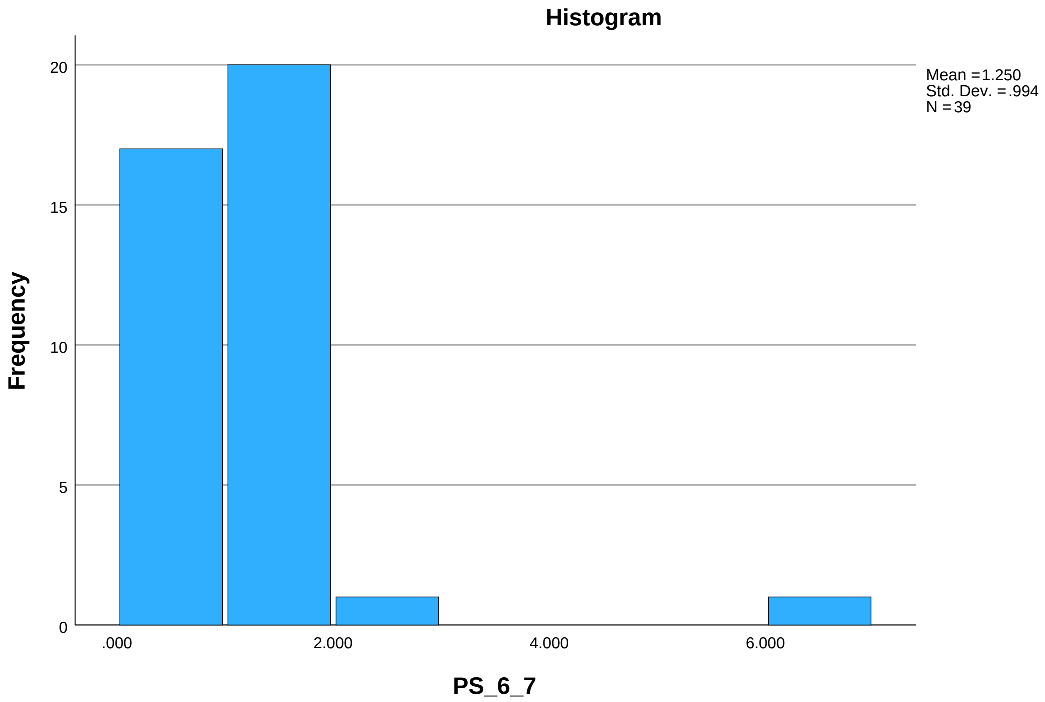


```
PS_6_7 Stem-and-Leaf Plot

 Frequency    Stem &  Leaf

     4.00        0 .  2344
    13.00        0 .  5566788899999
    13.00        1 .  0000112223344
     7.00        1 .  6678888
     1.00        2 .  0
     1.00 Extremes    (>=6.6)

 Stem width:      1.000
 Each leaf:       1 case(s)
```

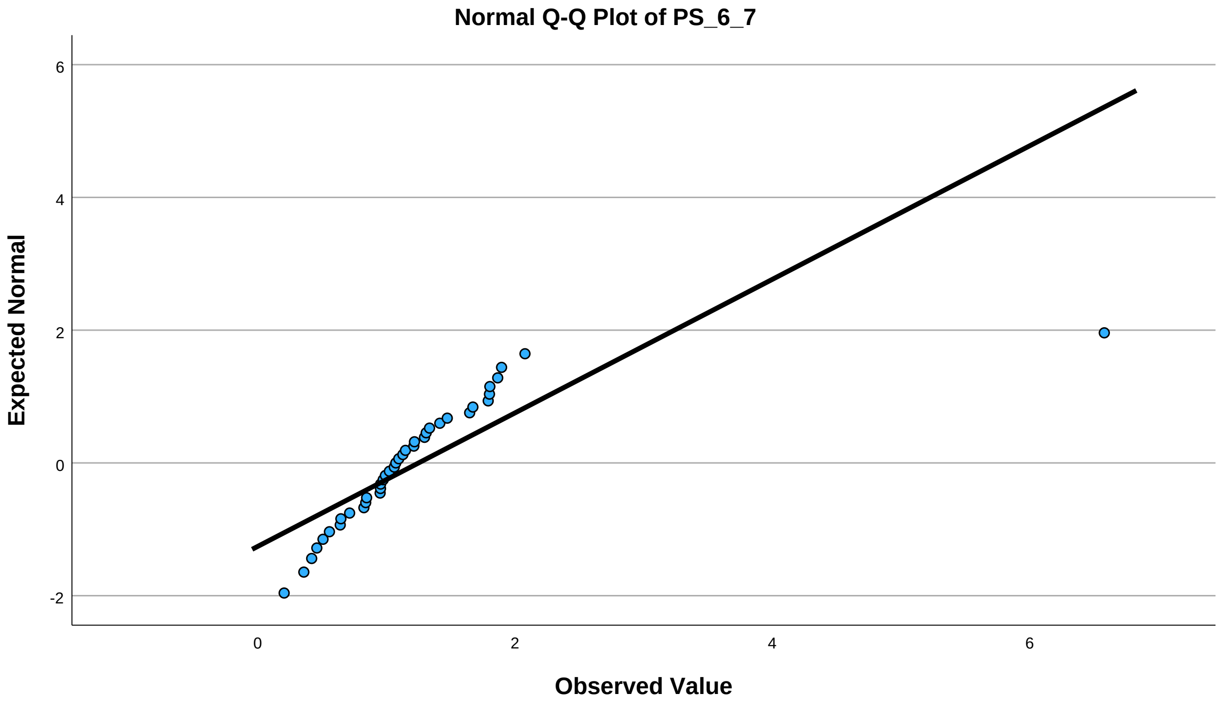
Normal Q-Q Plot of PS_6_7
Expected Normal
6
4
2
0
-2
0
2
4
6
Observed Value

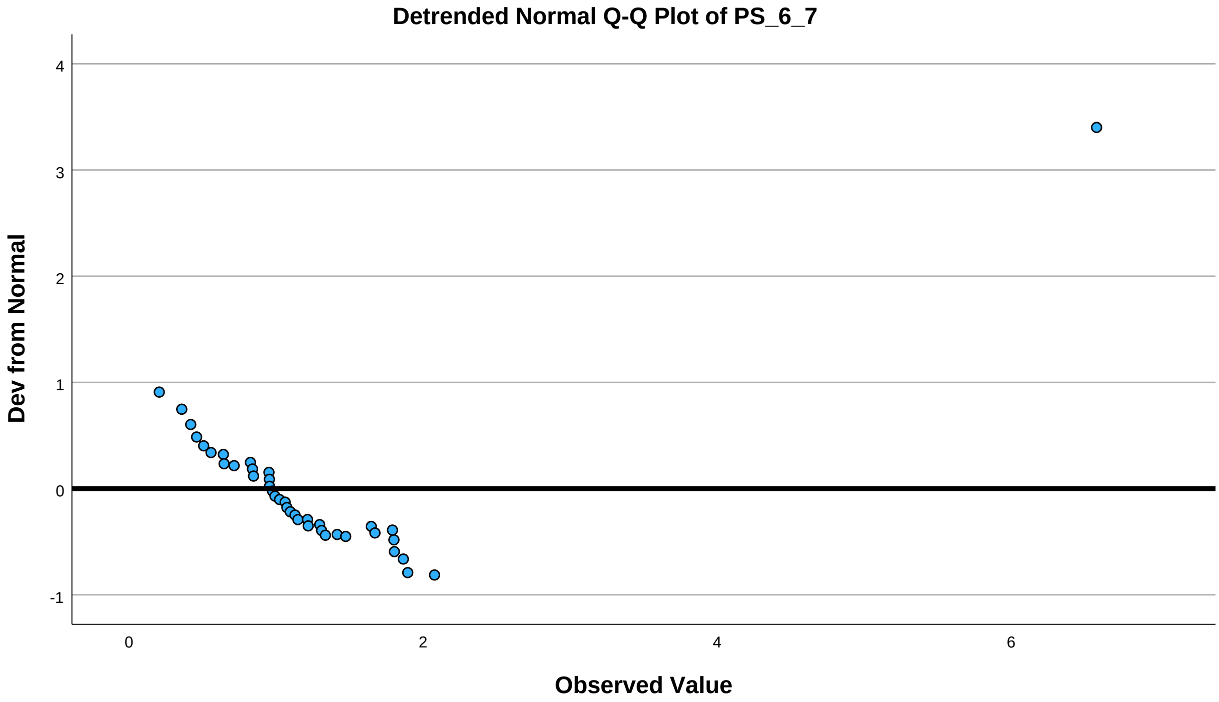
Detrended Normal Q-Q Plot of PS_6_7
Dev from Normal
4
3
2
1
0
-1
0
2
4
6
Observed Value

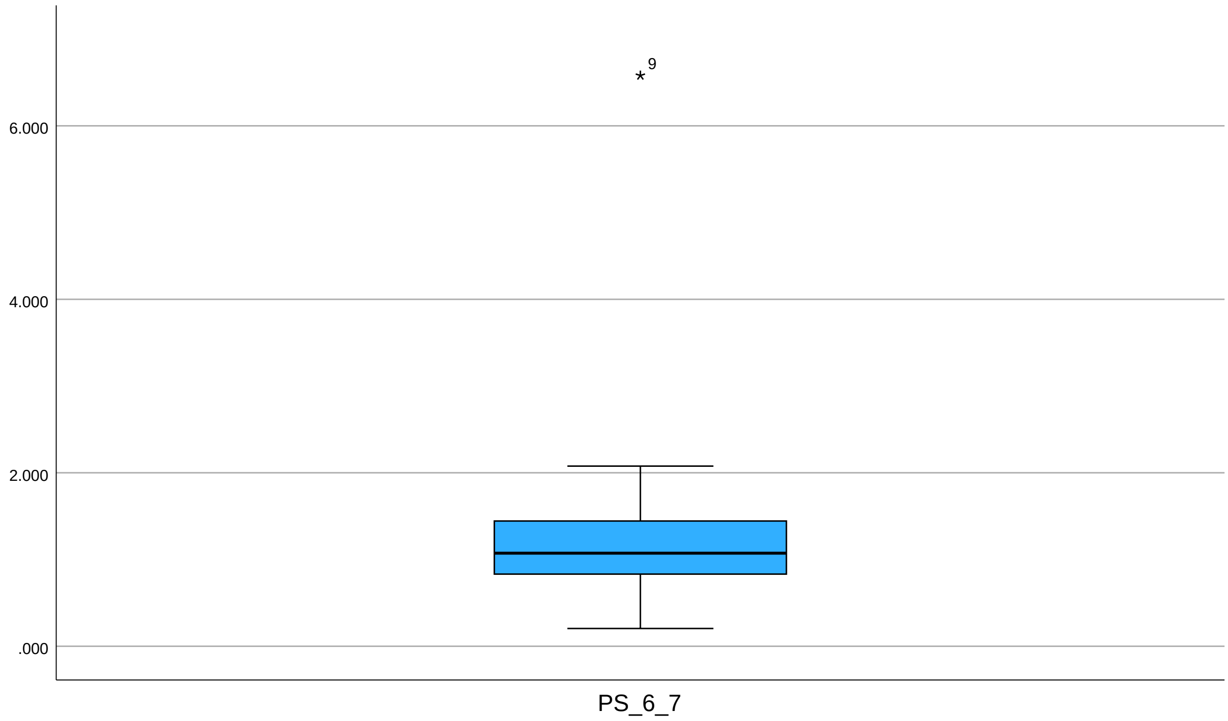

PS_7_8

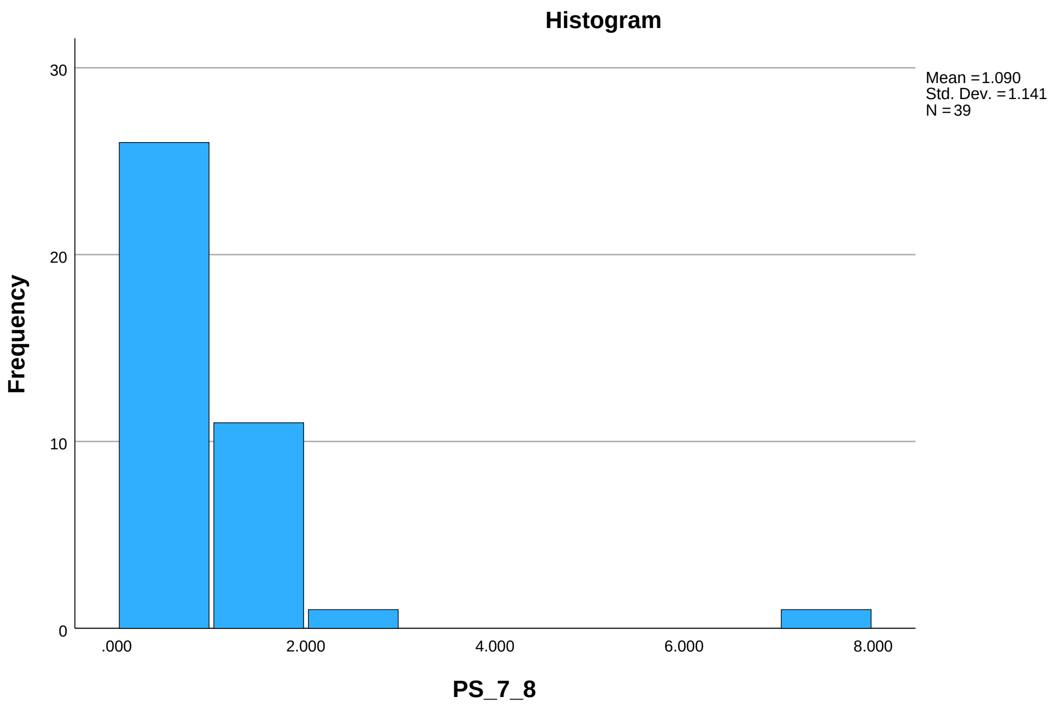

PS_7_8 Stem-and-Leaf Plot

Frequency   Stem & Leaf

```
  1.00      0 . 1
  3.00      0 . 233
  4.00      0 . 4455
 11.00      0 . 66667777777
  7.00      0 . 8889999
  5.00      1 . 00111
  1.00      1 . 3
  3.00      1 . 455
  1.00      1 . 7
  1.00      1 . 8
  2.00 Extremes    (>=2.7)

Stem width:     1.000
Each leaf:        1 case(s)
```

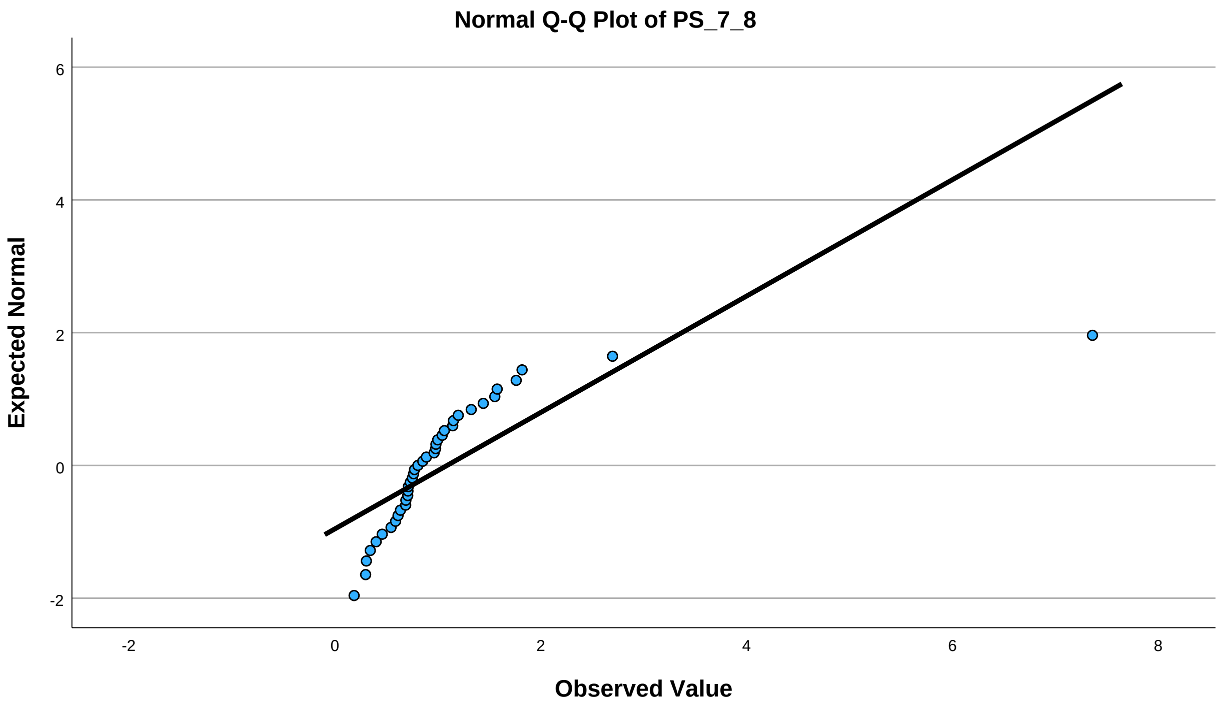

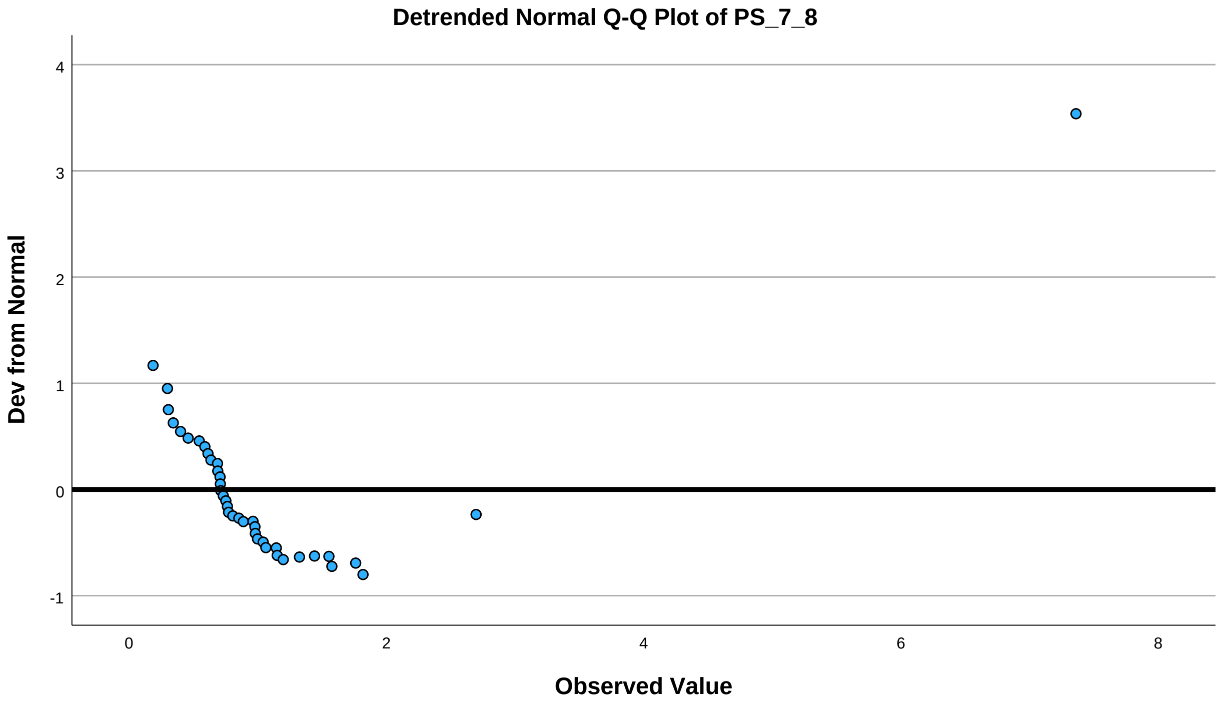
Detrended Normal Q-Q Plot of PS_7_8
Dev from Normal
Observed Value
4
3
2
1
0
-1
0
2
4
6
8

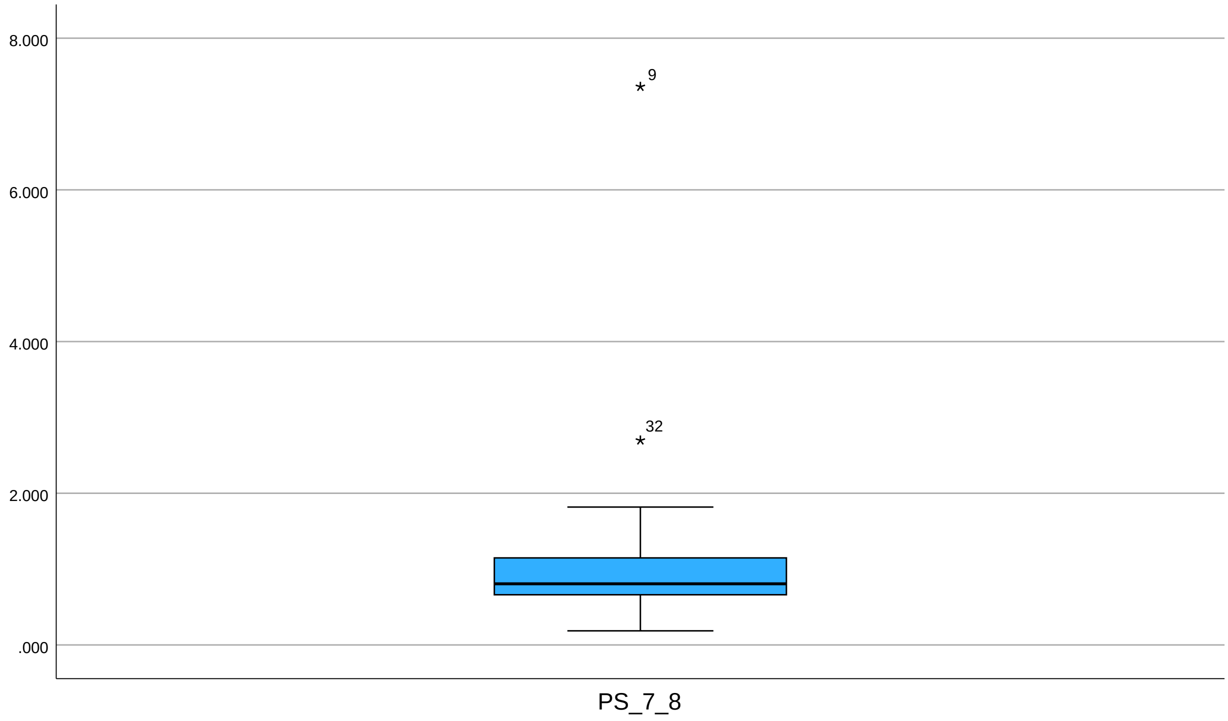
8.000
6.000
4.000
2.000
.000
9
32
PS_7_8

PS_8_9

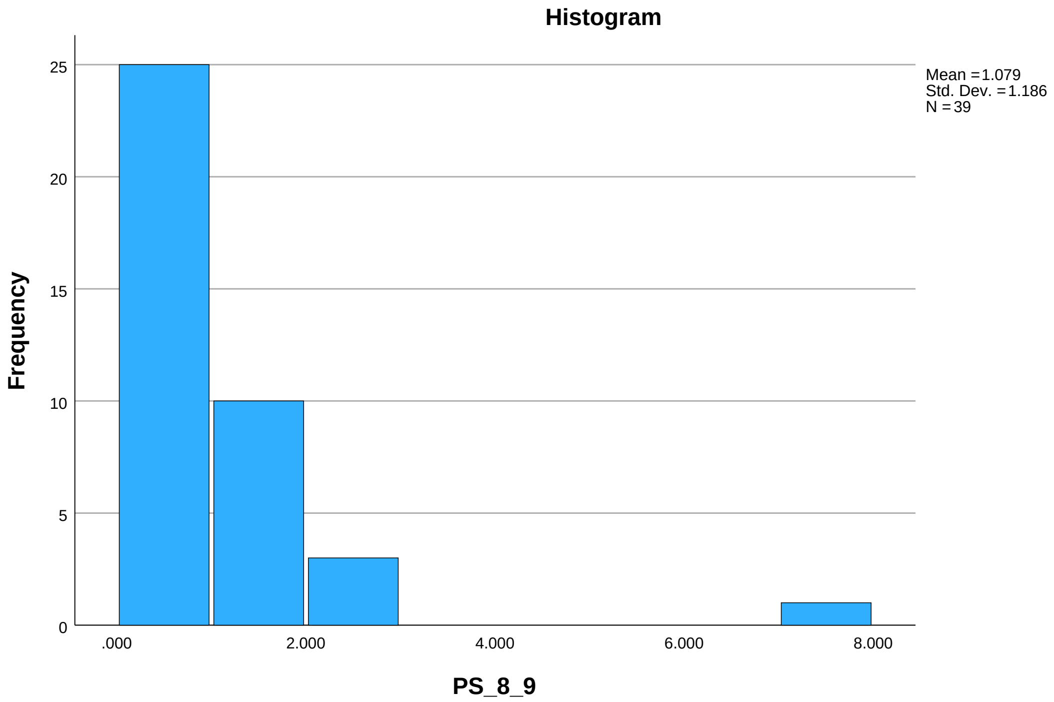


```
PS_8_9 Stem-and-Leaf Plot

 Frequency    Stem &  Leaf

     1.00        0 .  1
     6.00        0 .  222333
     5.00        0 .  45555
     8.00        0 .  66666777
     5.00        0 .  88999
     5.00        1 .  00001
     3.00        1 .  233
     1.00        1 .  4
     1.00        1 .  7
     4.00 Extremes    (>=2.0)

 Stem width:      1.000
 Each leaf:        1 case(s)
```

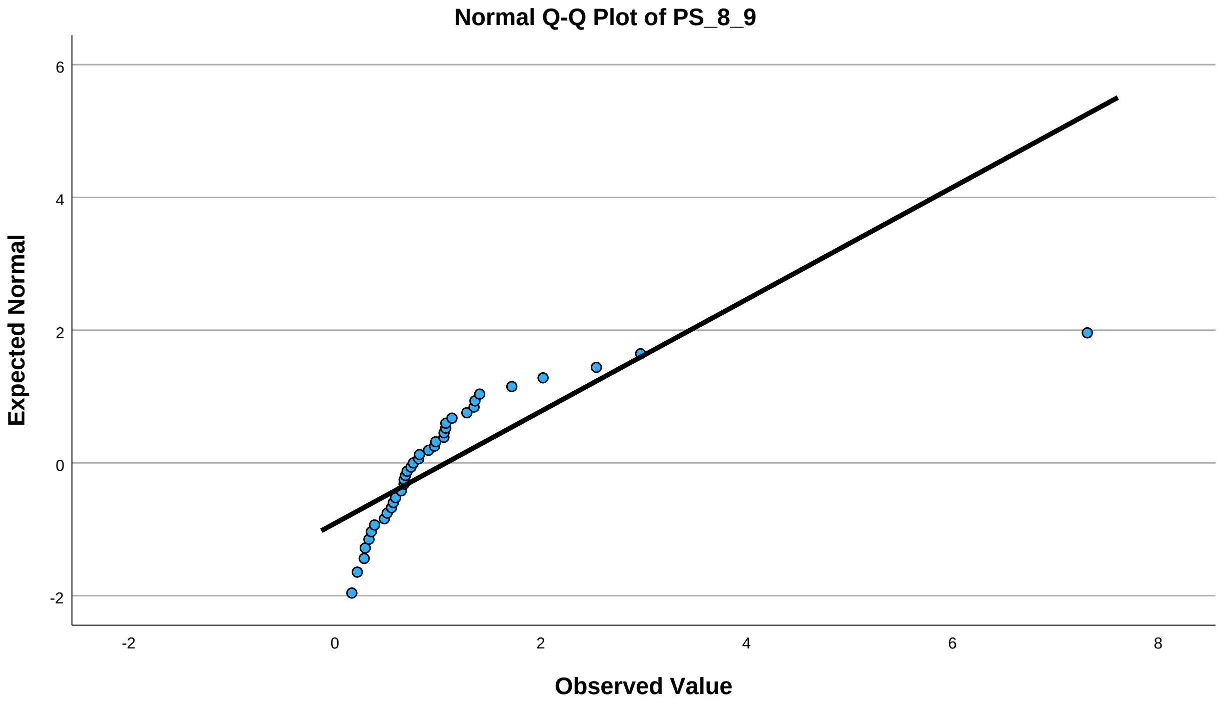
Normal Q-Q Plot of PS_8_9
Expected Normal
Observed Value
6
4
2
0
-2
-2
0
2
4
6
8

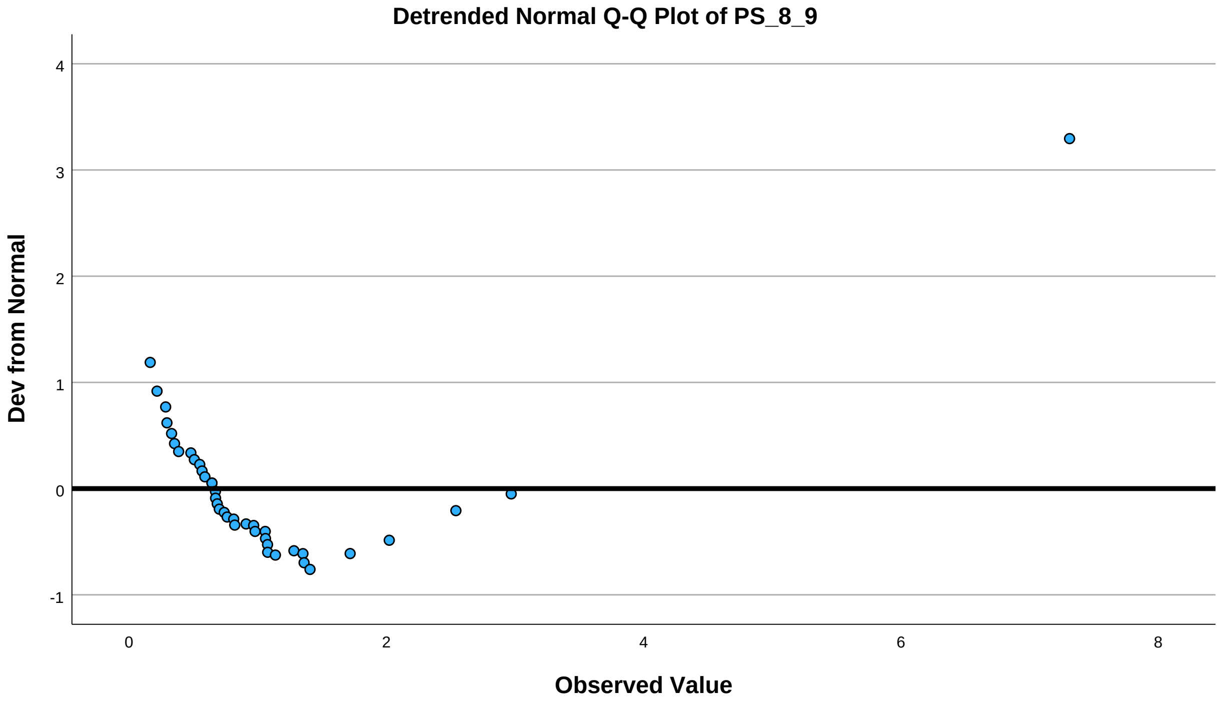
Detrended Normal Q-Q Plot of PS_8_9
Dev from Normal
Observed Value
4
3
2
1
0
-1
0
2
4
6
8

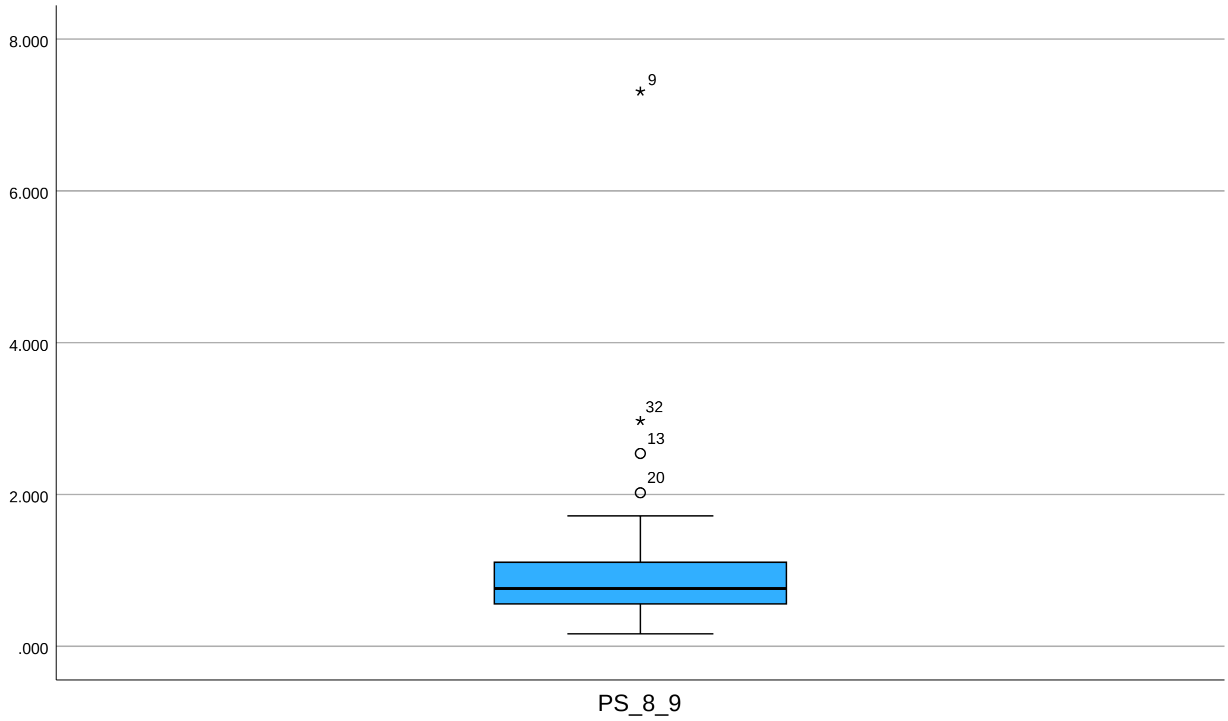

PS_9_10

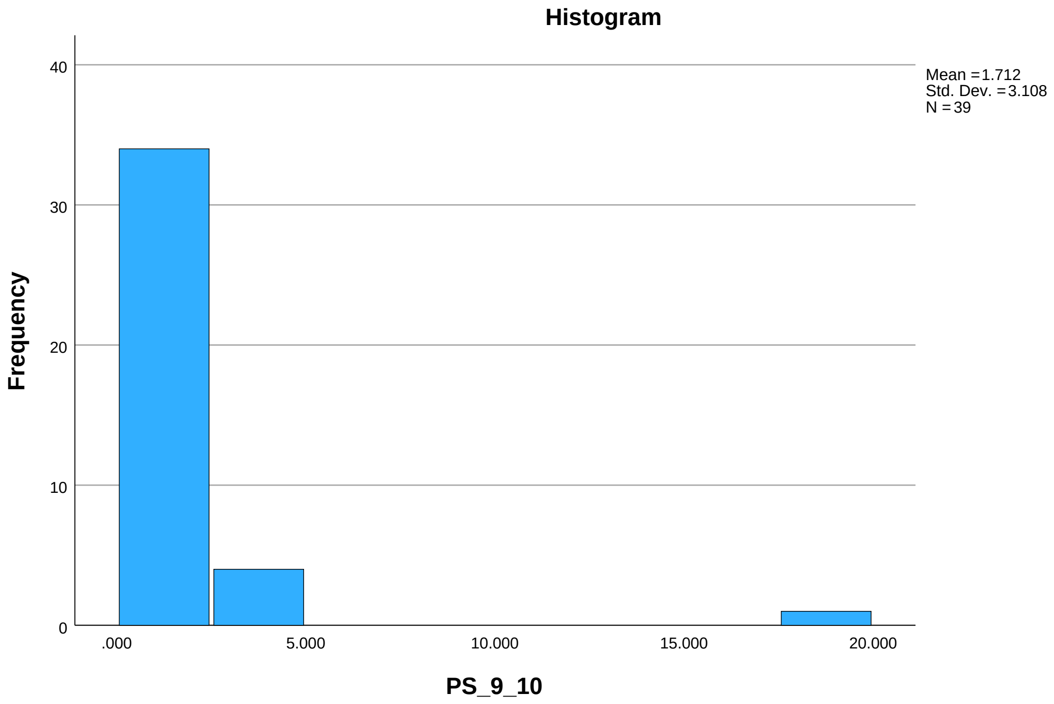


```
PS_9_10 Stem-and-Leaf Plot

 Frequency    Stem &  Leaf
```

```
    6.00        0 .  112233
   13.00        0 .  5566666677888
    7.00        1 .  0022234
    7.00        1 .  5667779
    1.00        2 .  2
     .00        2 .
    2.00        3 .  01
    3.00 Extremes    (>=3.3)

 Stem width:      1.000
 Each leaf:        1 case(s)
```

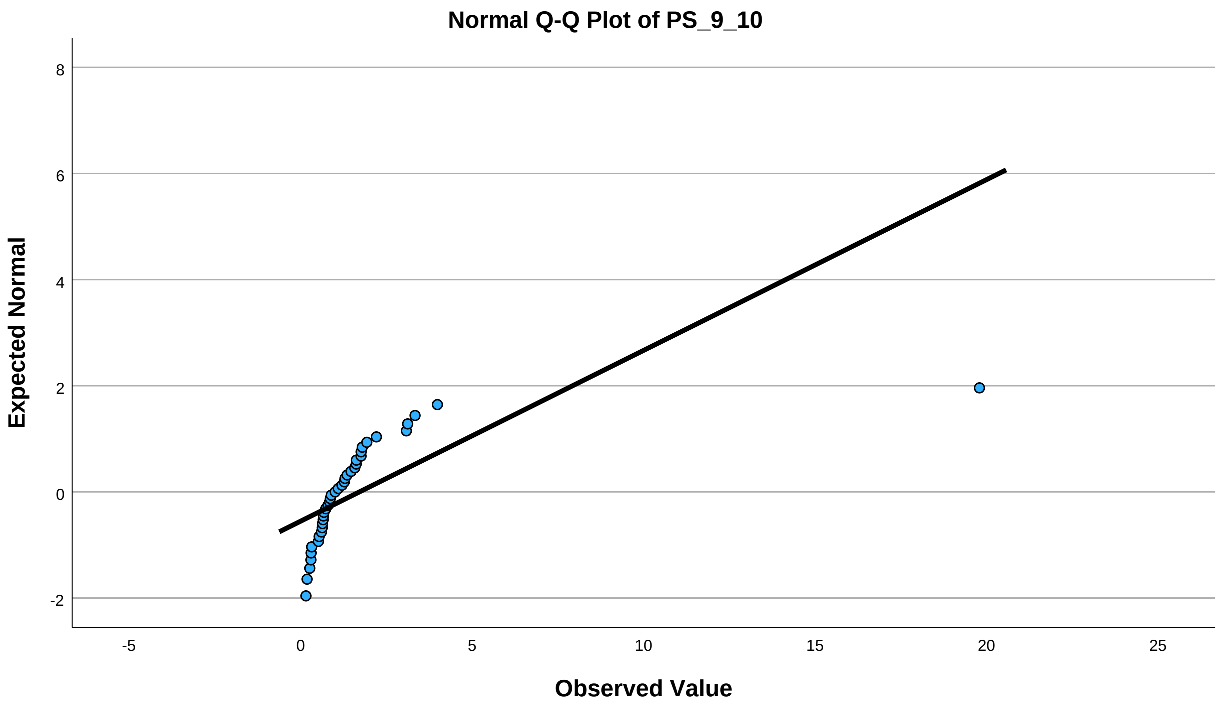

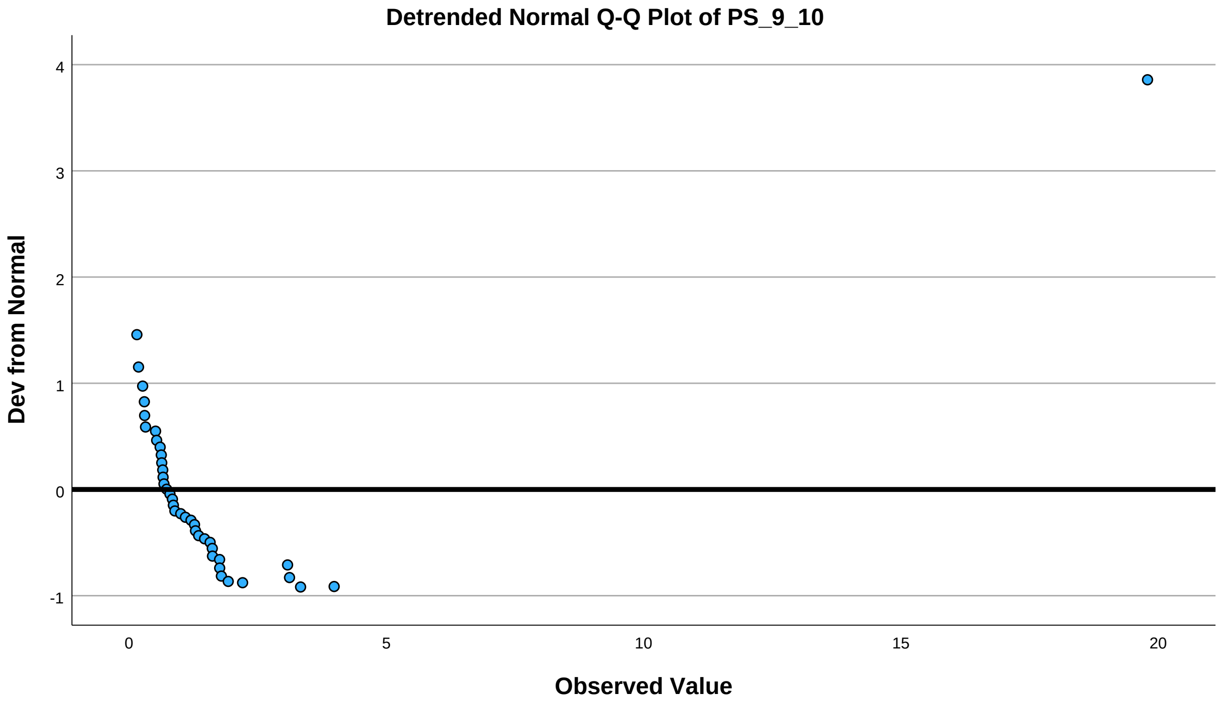
Detrended Normal Q-Q Plot of PS_9_10
Dev from Normal
4
3
2
1
0
-1
0
5
10
15
20
Observed Value

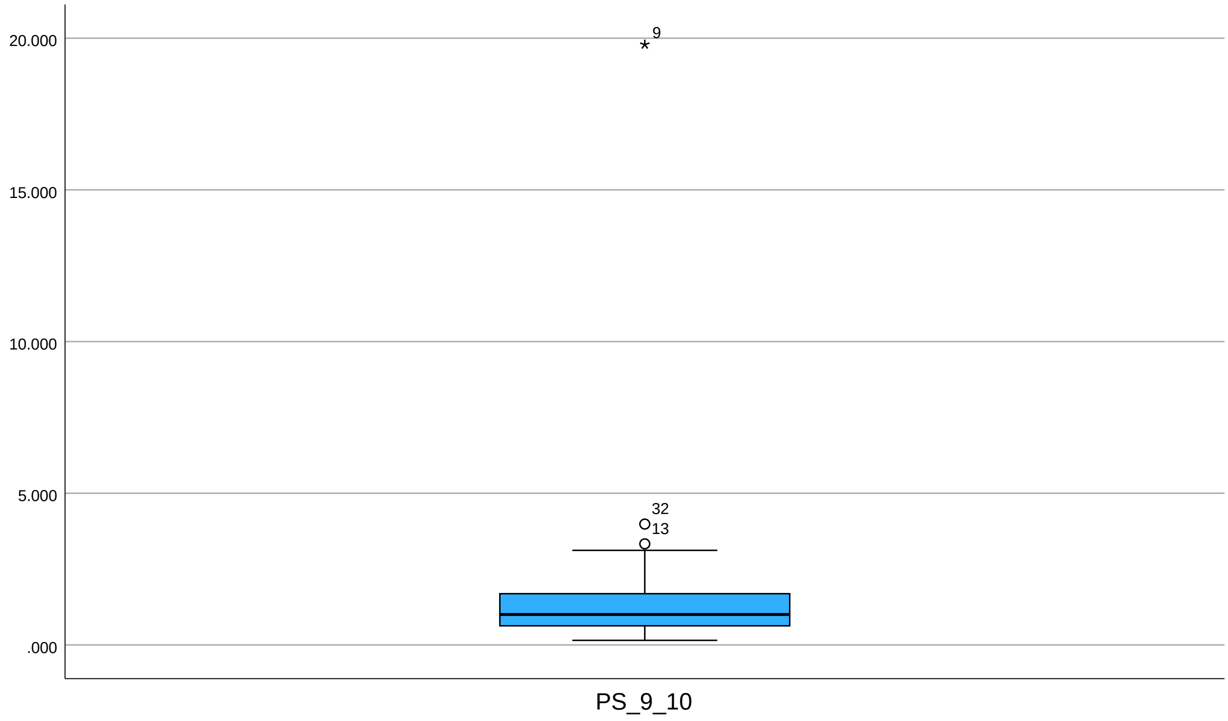
20.000
15.000
10.000
5.000
.000
9
32
13
PS_9_10

PS_10_11

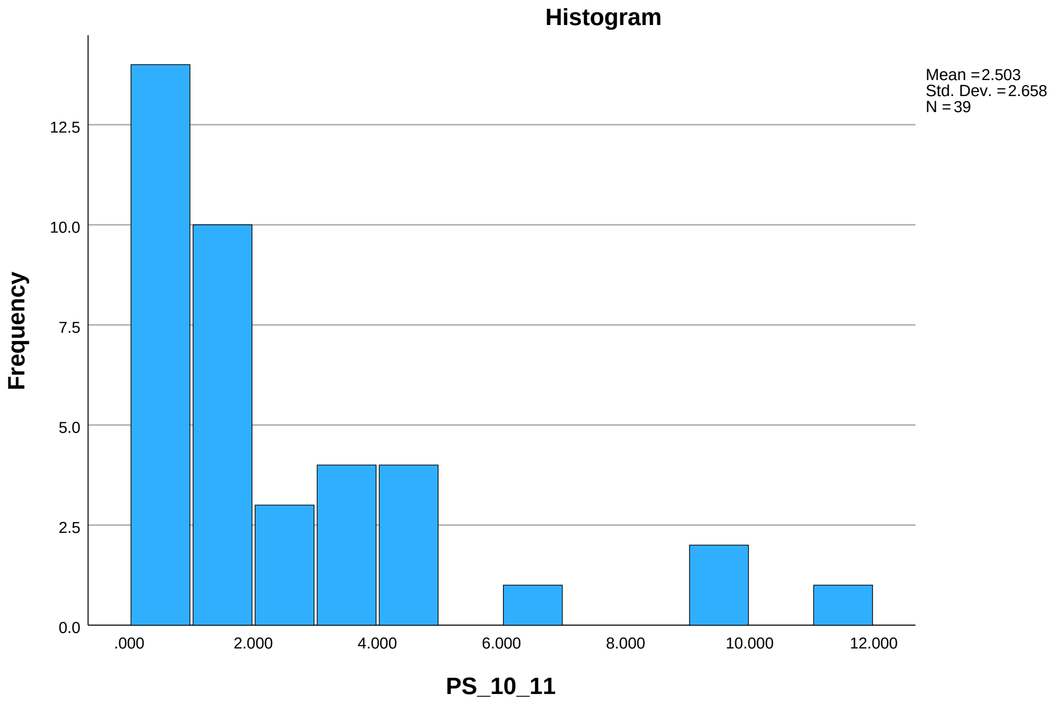


```
PS_10_11 Stem-and-Leaf Plot

 Frequency    Stem &  Leaf

    14.00        0 .  22334466677788
    10.00        1 .  0002446778
     3.00        2 .  122
     4.00        3 .  3569
     4.00        4 .  1255
      .00        5 .
     1.00        6 .  8
     3.00 Extremes    (>=9.2)

 Stem width:     1.000
 Each leaf:        1 case(s)
```

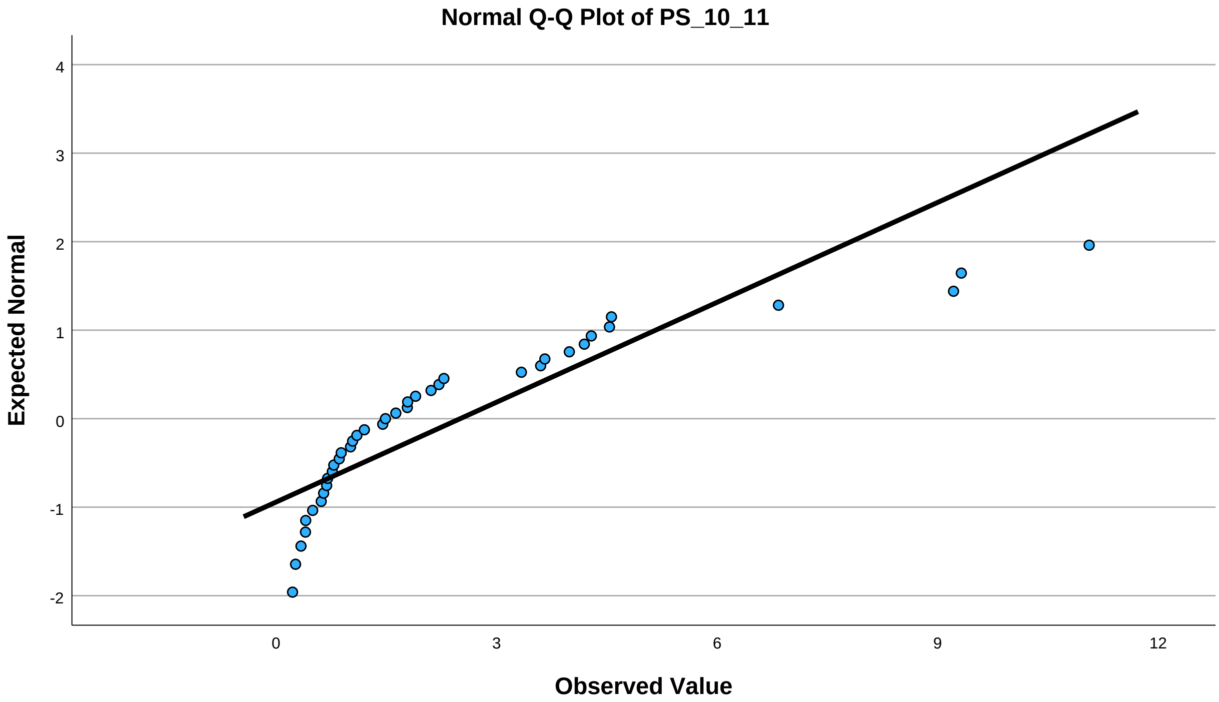
Normal Q-Q Plot of PS_10_11
Expected Normal
Observed Value
4
3
2
1
0
-1
-2
0
3
6
9
12

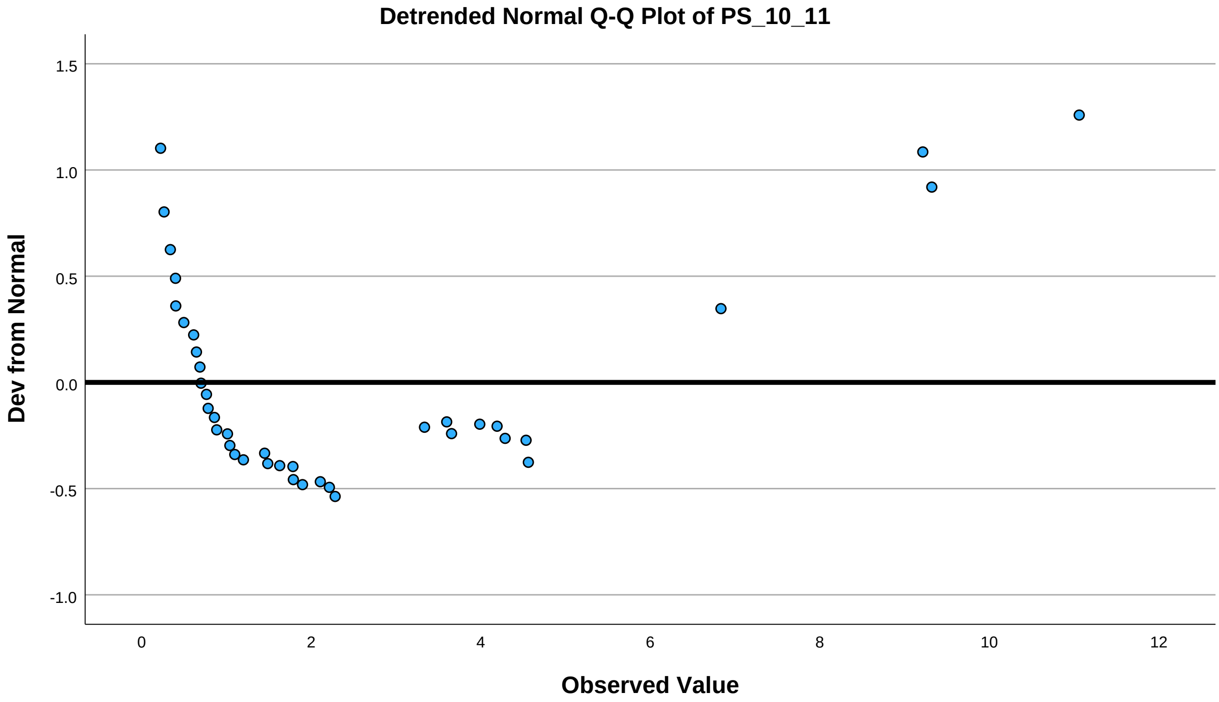
Detrended Normal Q-Q Plot of PS_10_11
Dev from Normal
Observed Value
1.5
1.0
0.5
0.0
-0.5
-1.0
0
2
4
6
8
10
12

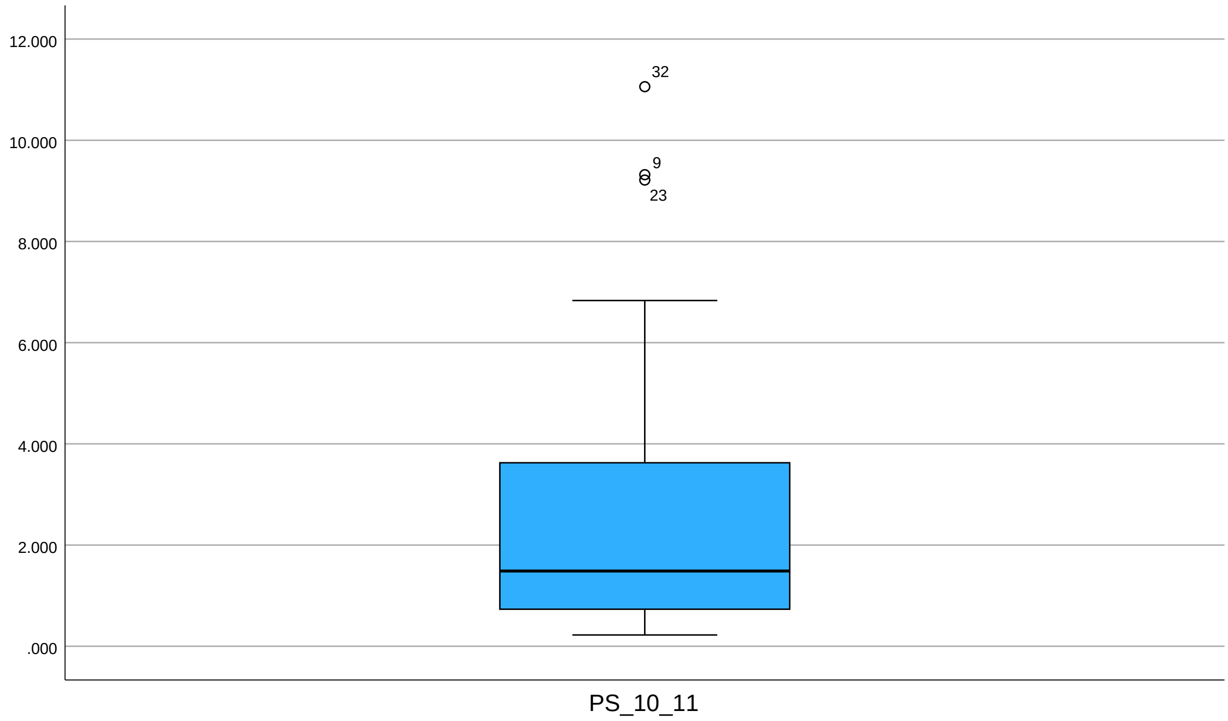

PS_11_12

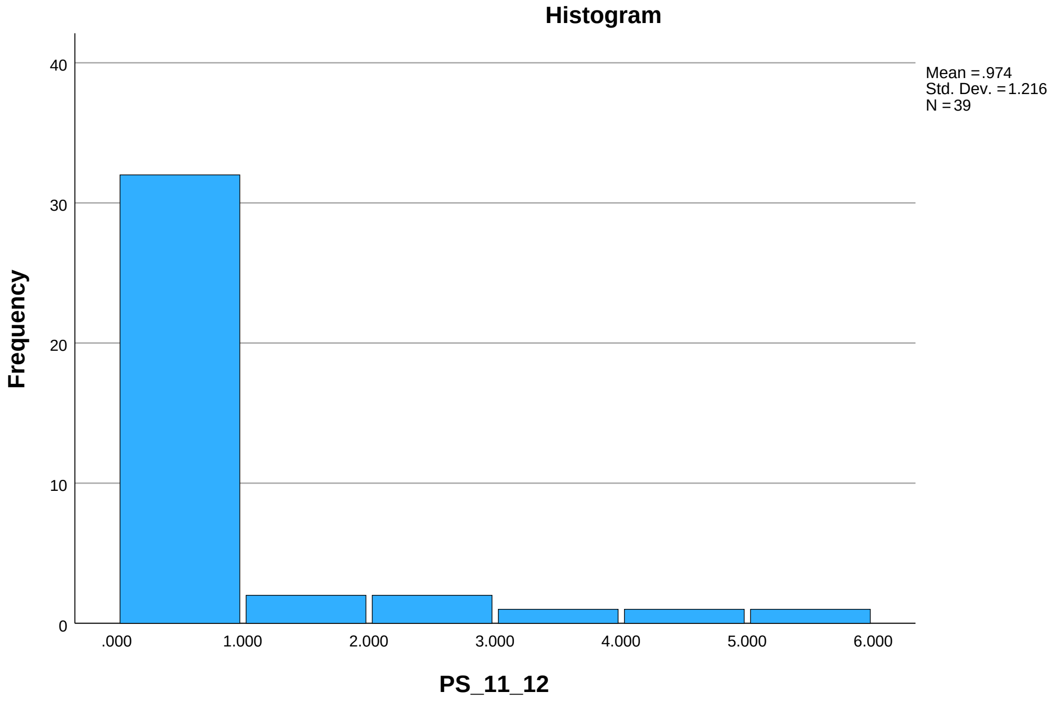

PS_11_12 Stem-and-Leaf Plot

Frequency   Stem & Leaf

```
  3.00      0 . 111
 11.00      0 . 22222333333
  8.00      0 . 44455555
  3.00      0 . 677
  7.00      0 . 8888999
  1.00      1 . 0
   .00      1 .
  1.00      1 . 4
  5.00 Extremes    (>=2.5)

Stem width:    1.000
Each leaf:       1 case(s)
```

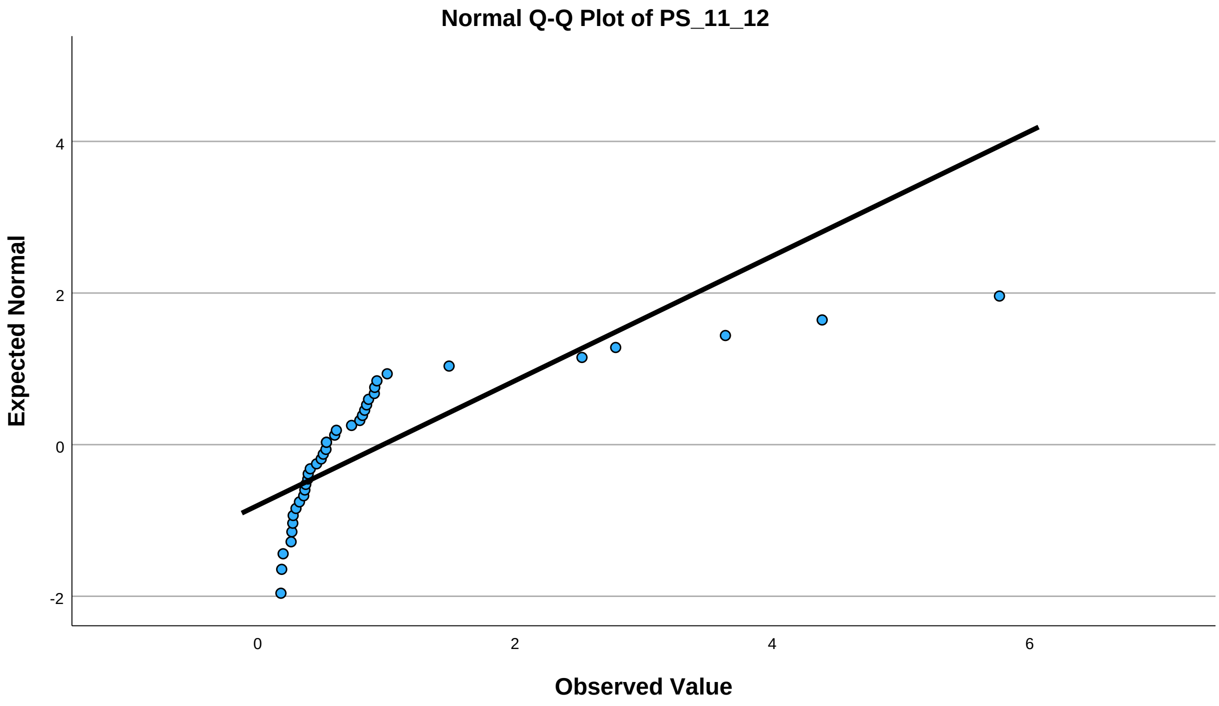

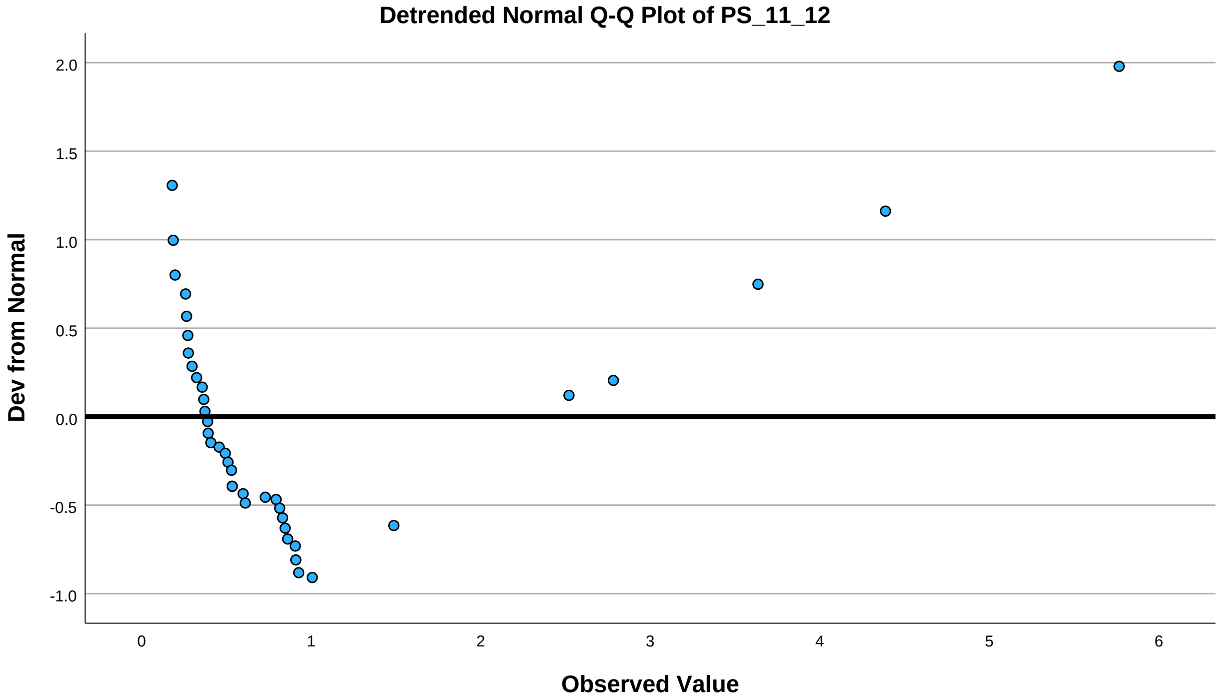
Detrended Normal Q-Q Plot of PS_11_12
Dev from Normal
2.0
1.5
1.0
0.5
0.0
-0.5
-1.0
0
1
2
3
4
5
6
Observed Value

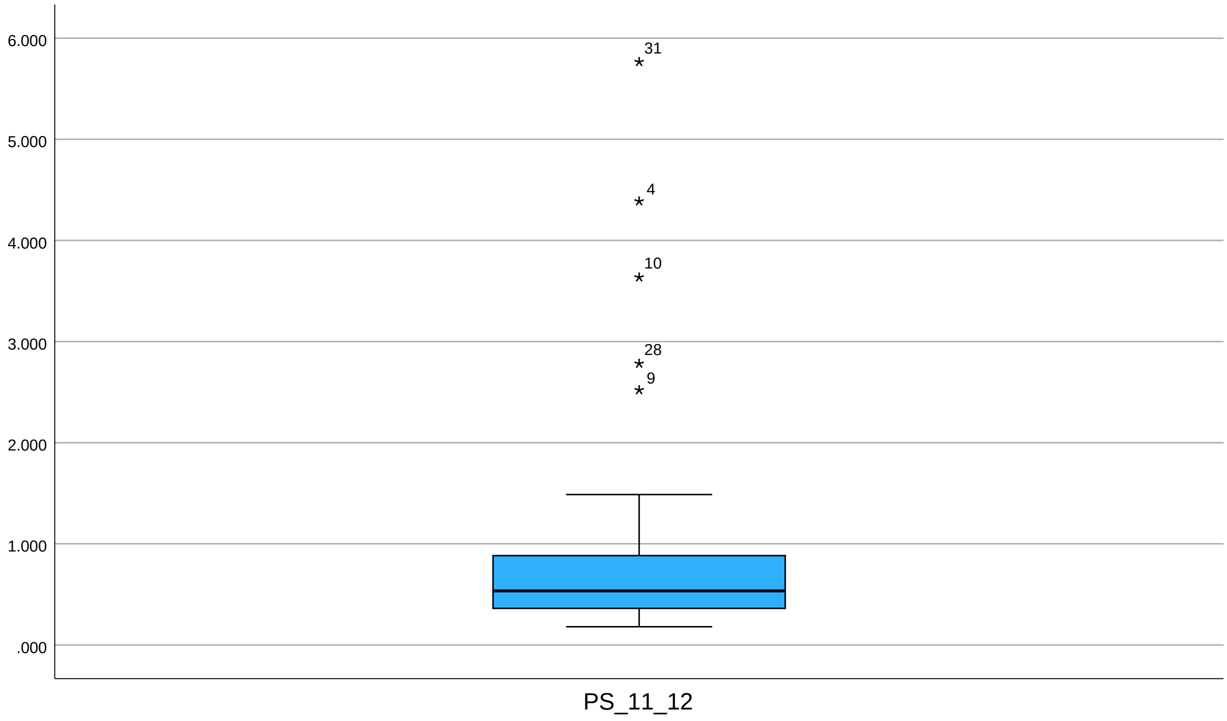
6.000
5.000
4.000
3.000
2.000
1.000
.000
31
4
10
28
9
PS_11_12

PS_12_13

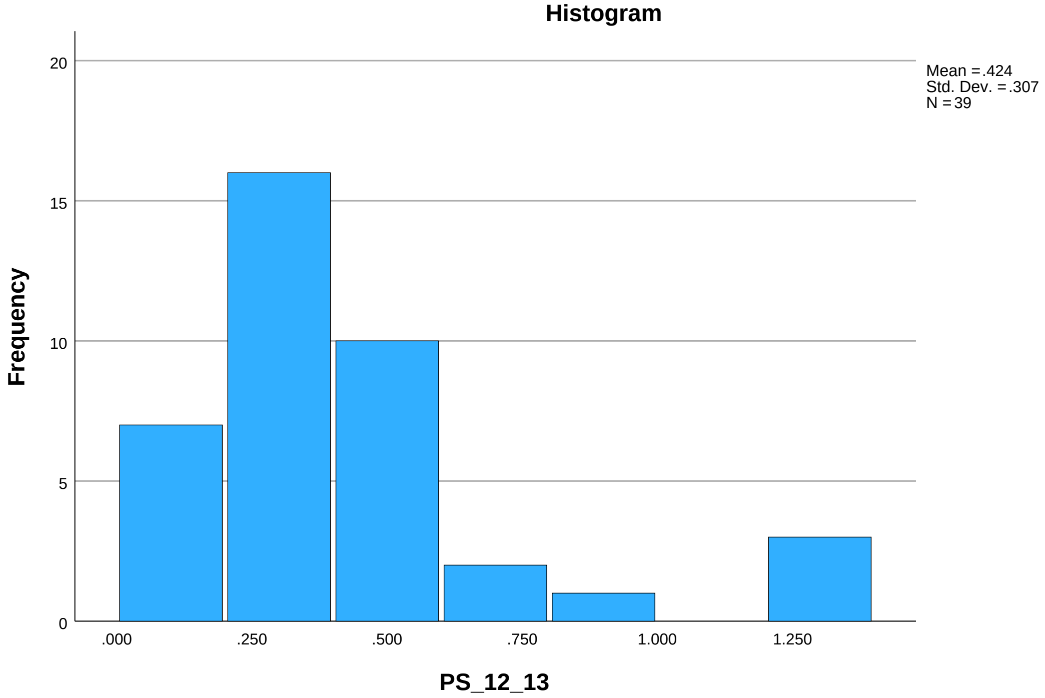


```
PS_12_13 Stem-and-Leaf Plot

 Frequency    Stem &  Leaf

     7.00        1 .  1456789
    10.00        2 .  0015566679
     6.00        3 .  114456
     6.00        4 .  011458
     4.00        5 .  0057
     1.00        6 .  1
     1.00        7 .  5
     1.00        8 .  1
     3.00 Extremes    (>=1.30)

 Stem width:      .100
 Each leaf:       1 case(s)
```

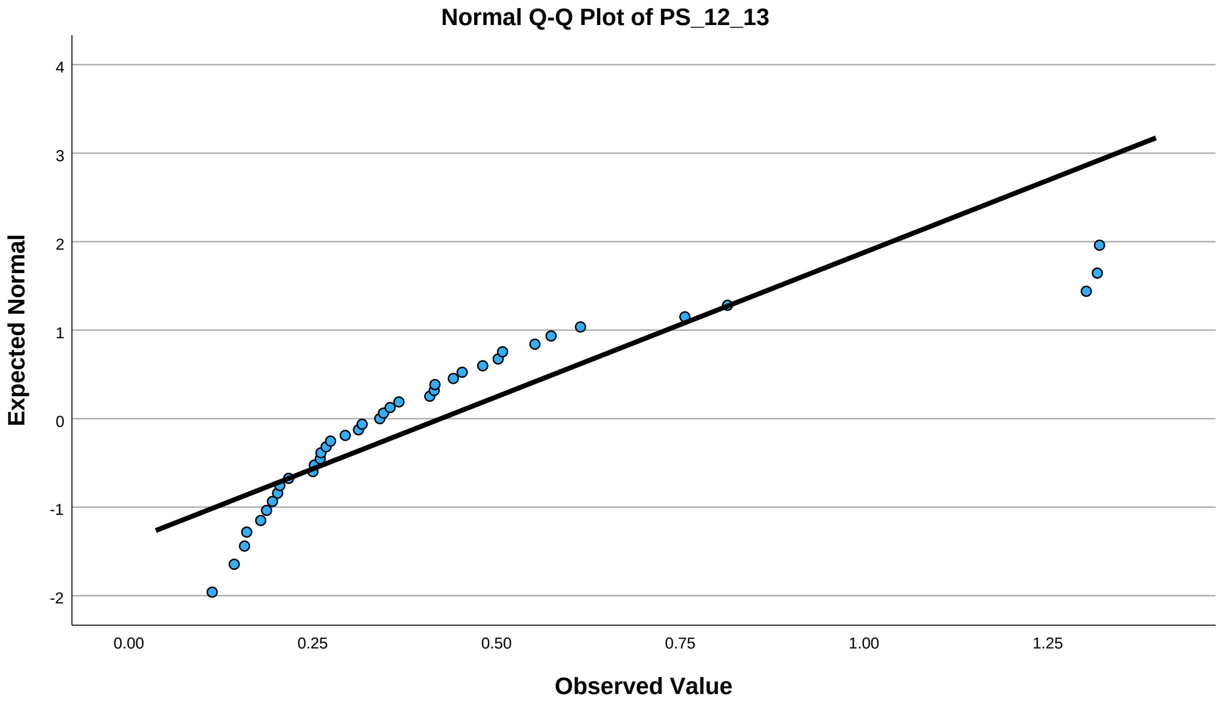
Normal Q-Q Plot of PS_12_13
Expected Normal
4
3
2
1
0
-1
-2
0.00
0.25
0.50
0.75
1.00
1.25
Observed Value

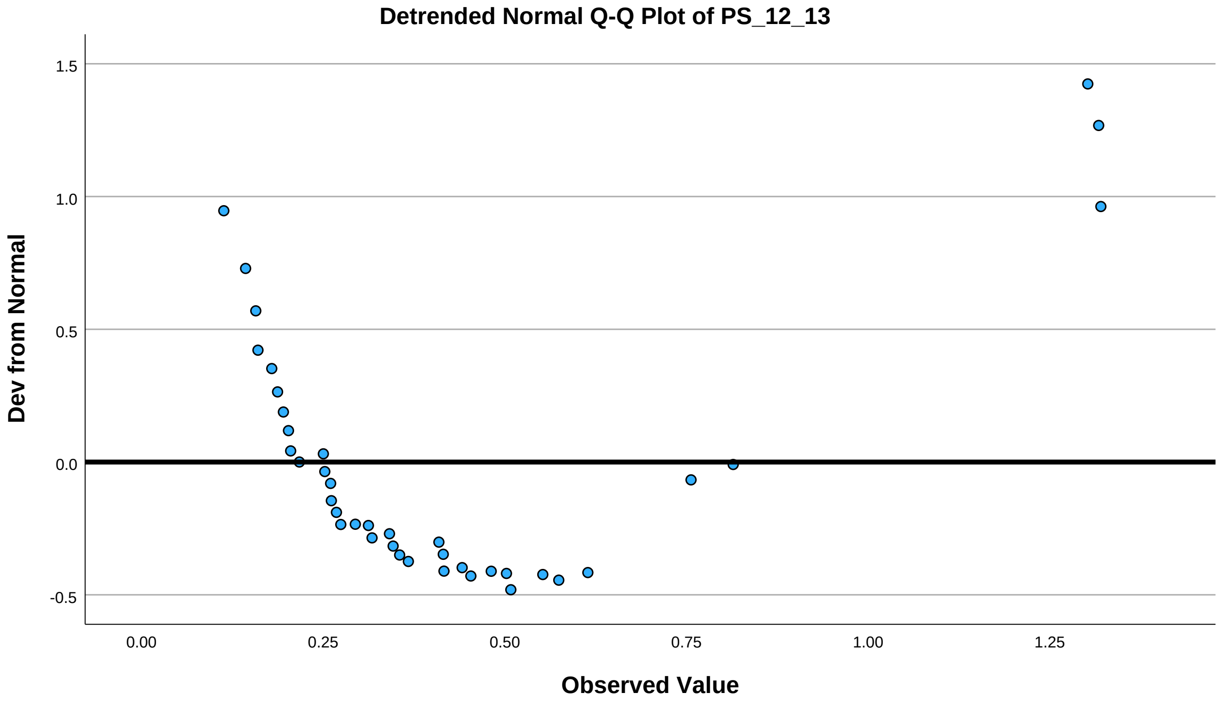
Detrended Normal Q-Q Plot of PS_12_13
Dev from Normal
1.5
1.0
0.5
0.0
-0.5
0.00
0.25
0.50
0.75
1.00
1.25
Observed Value

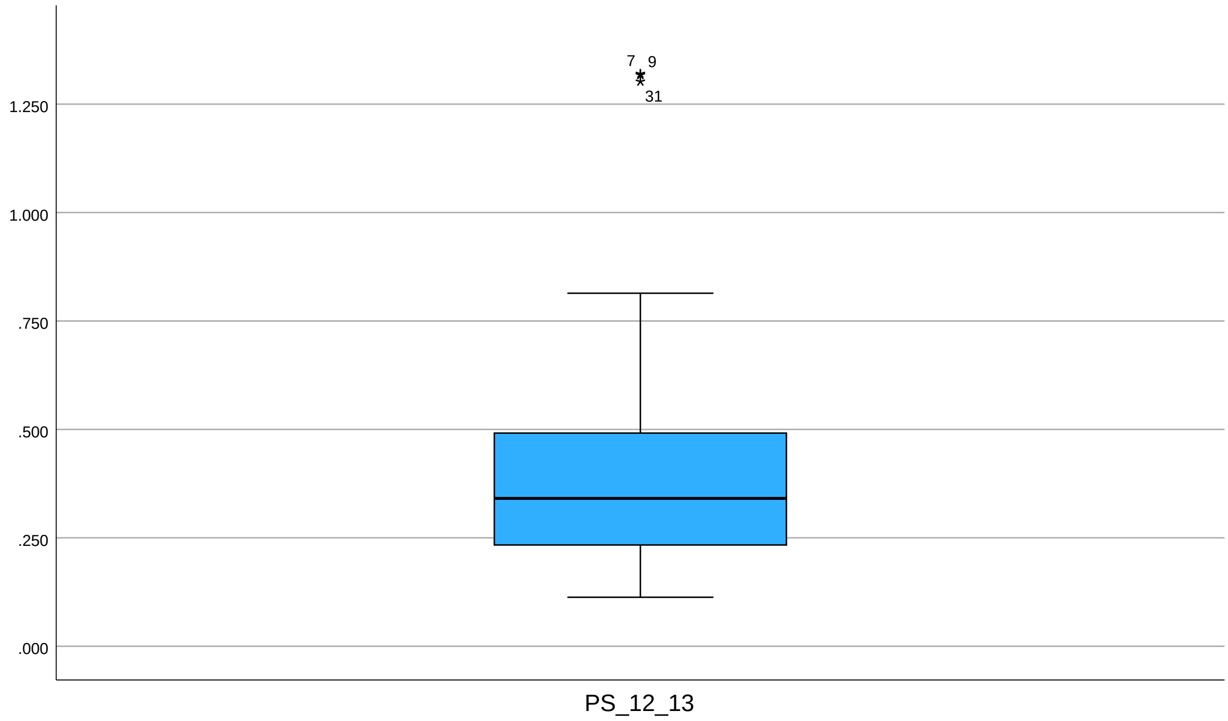

PS_13_14

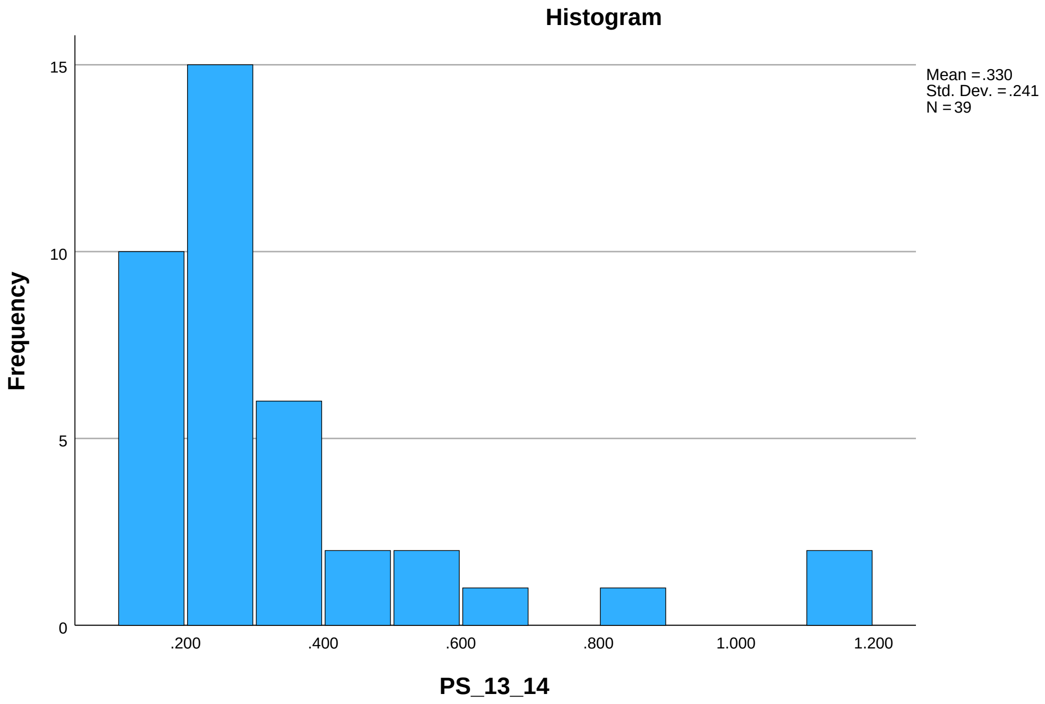


```
PS_13_14 Stem-and-Leaf Plot

 Frequency    Stem &  Leaf
```

```
     5.00        1 .  23444
     5.00        1 .  56777
     9.00        2 .  001112234
     6.00        2 .  577789
     3.00        3 .  001
     3.00        3 .  589
     1.00        4 .  0
     1.00        4 .  5
     1.00        5 .  1
     1.00        5 .  7
     4.00 Extremes    (>=.63)

 Stem width:      .100
 Each leaf:       1 case(s)
```

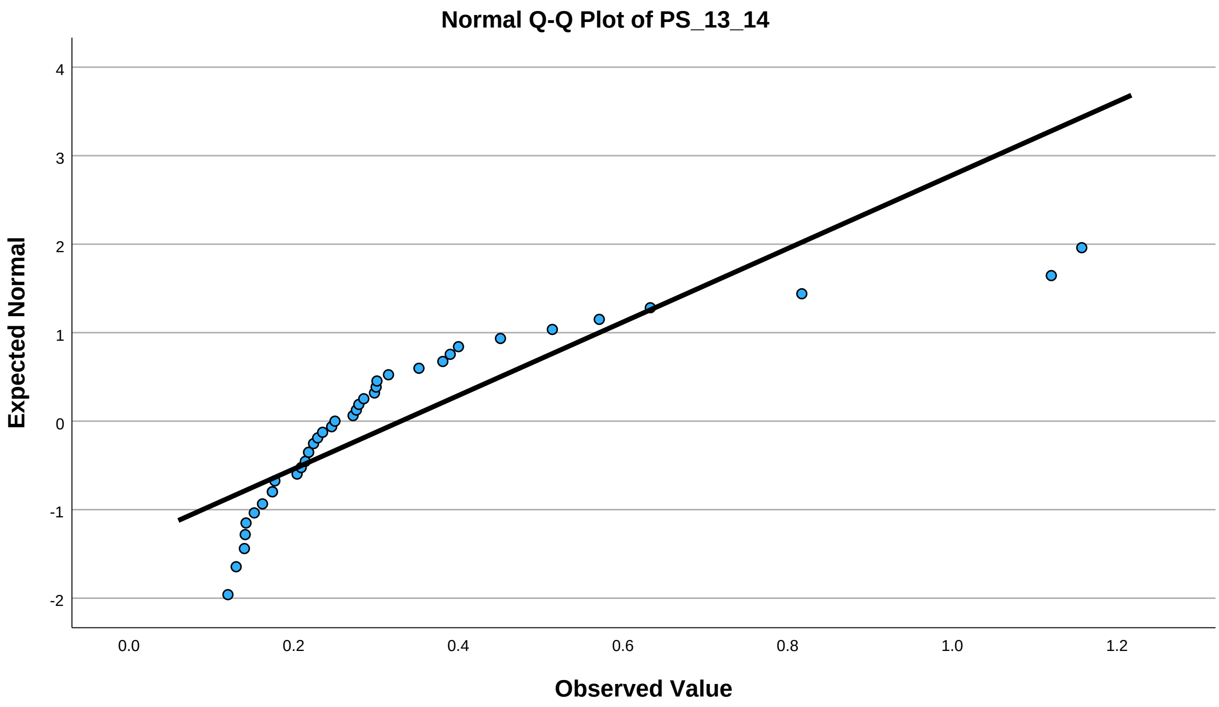

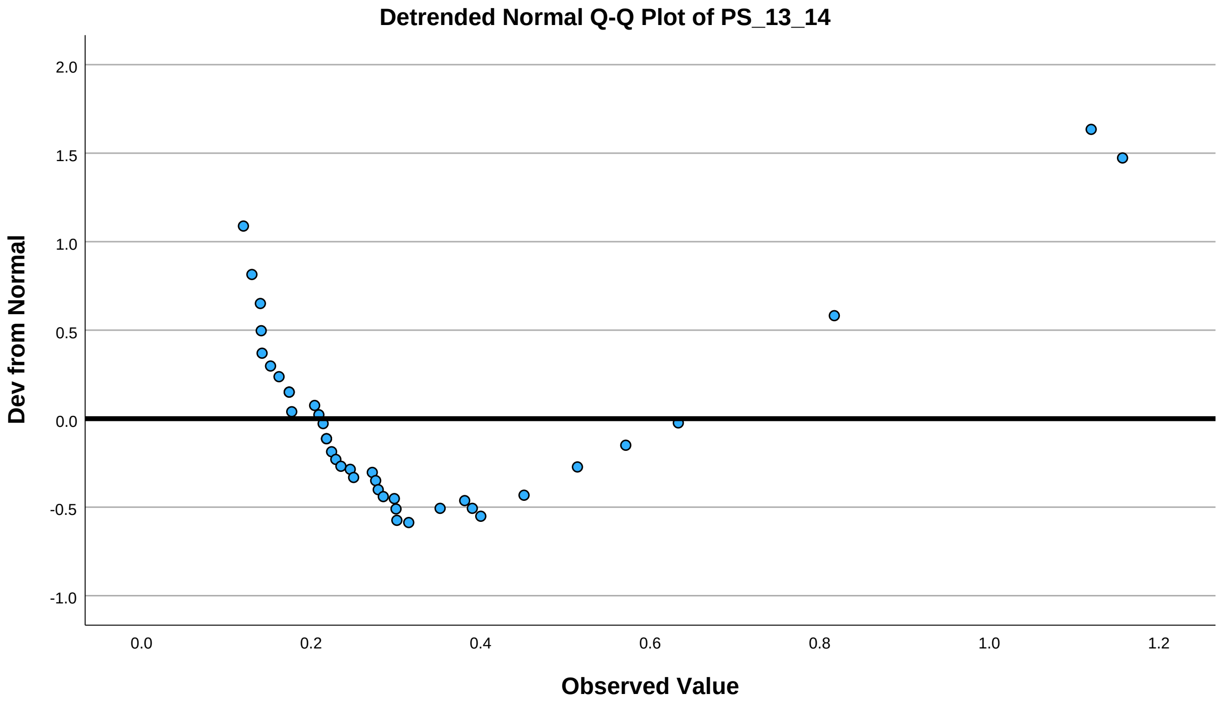
Detrended Normal Q-Q Plot of PS_13_14
Dev from Normal
Observed Value
2.0
1.5
1.0
0.5
0.0
-0.5
-1.0
0.0
0.2
0.4
0.6
0.8
1.0
1.2

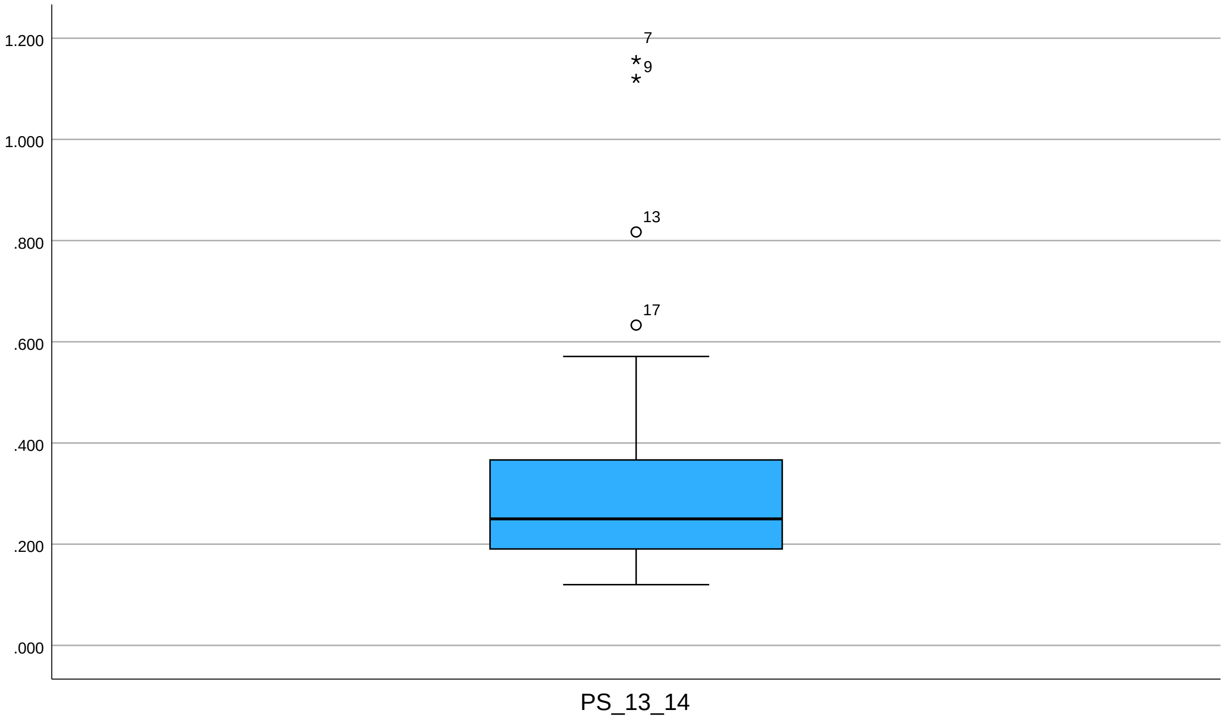
1.200
1.000
.800
.600
.400
.200
.000
7
9
13
17
PS_13_14

PS_14_15

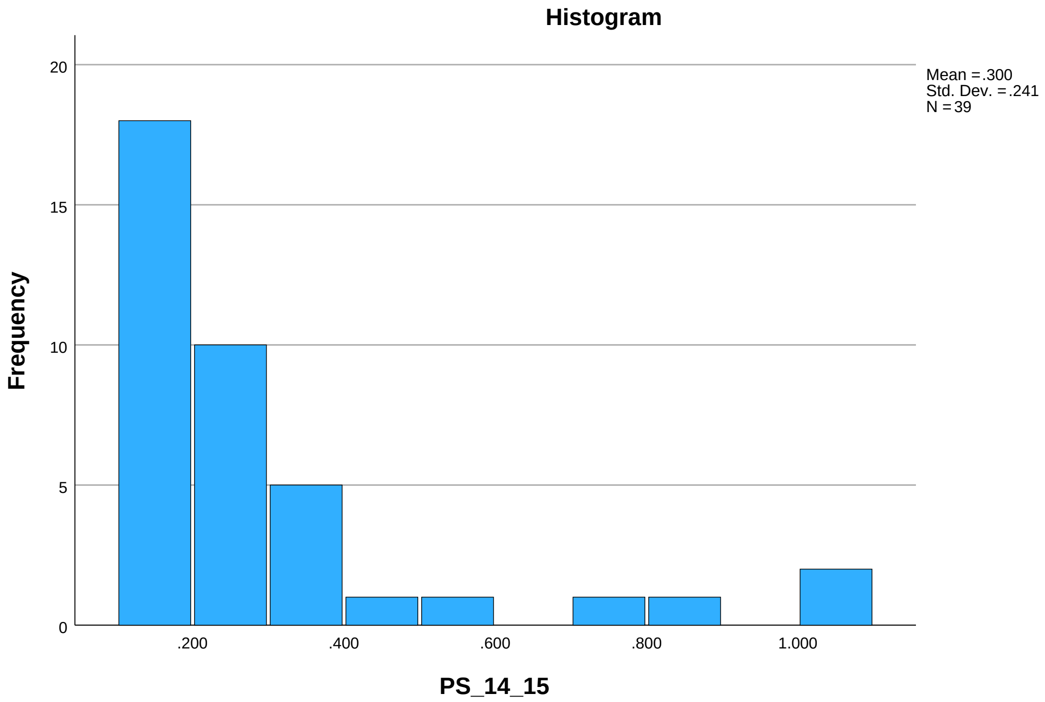


```
PS_14_15 Stem-and-Leaf Plot

 Frequency    Stem &  Leaf

    8.00        1 .  11122344
   10.00        1 .  5666688889
    6.00        2 .  023444
    4.00        2 .  5588
    3.00        3 .  123
    2.00        3 .  89
    1.00        4 .  4
    5.00 Extremes    (>=.59)

 Stem width:      .100
 Each leaf:       1 case(s)
```

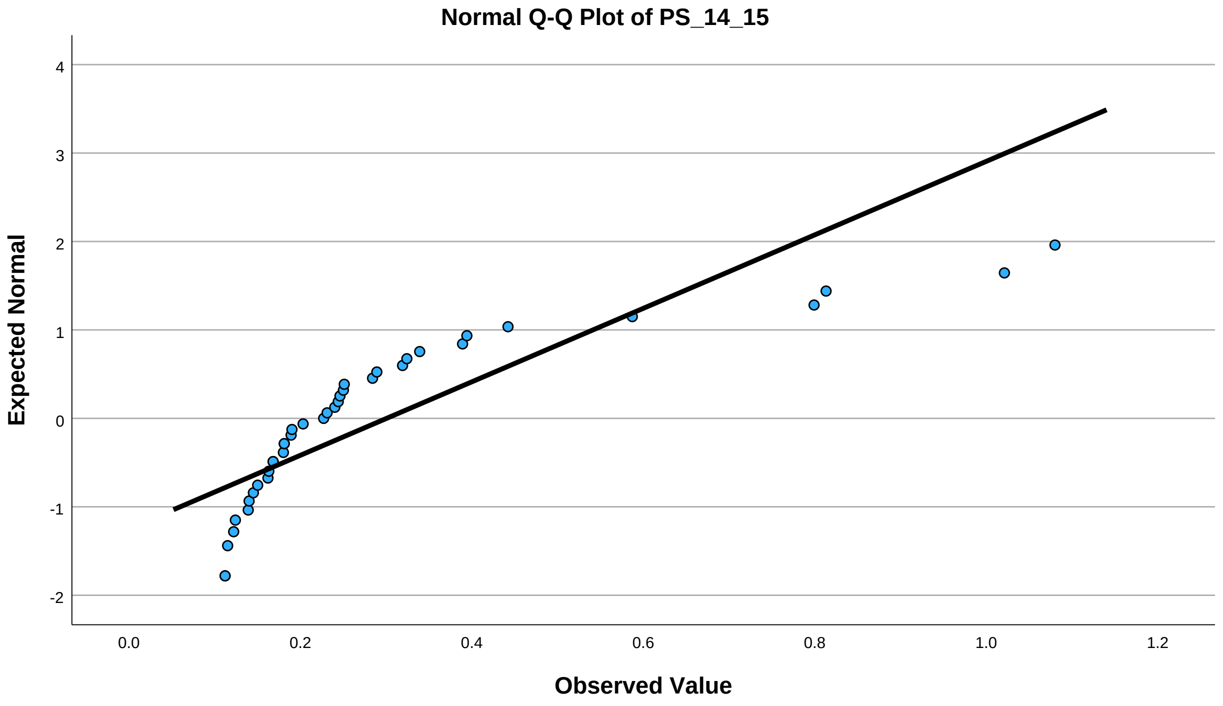
Normal Q-Q Plot of PS_14_15
Expected Normal
4
3
2
1
0
-1
-2
0.0
0.2
0.4
0.6
0.8
1.0
1.2
Observed Value

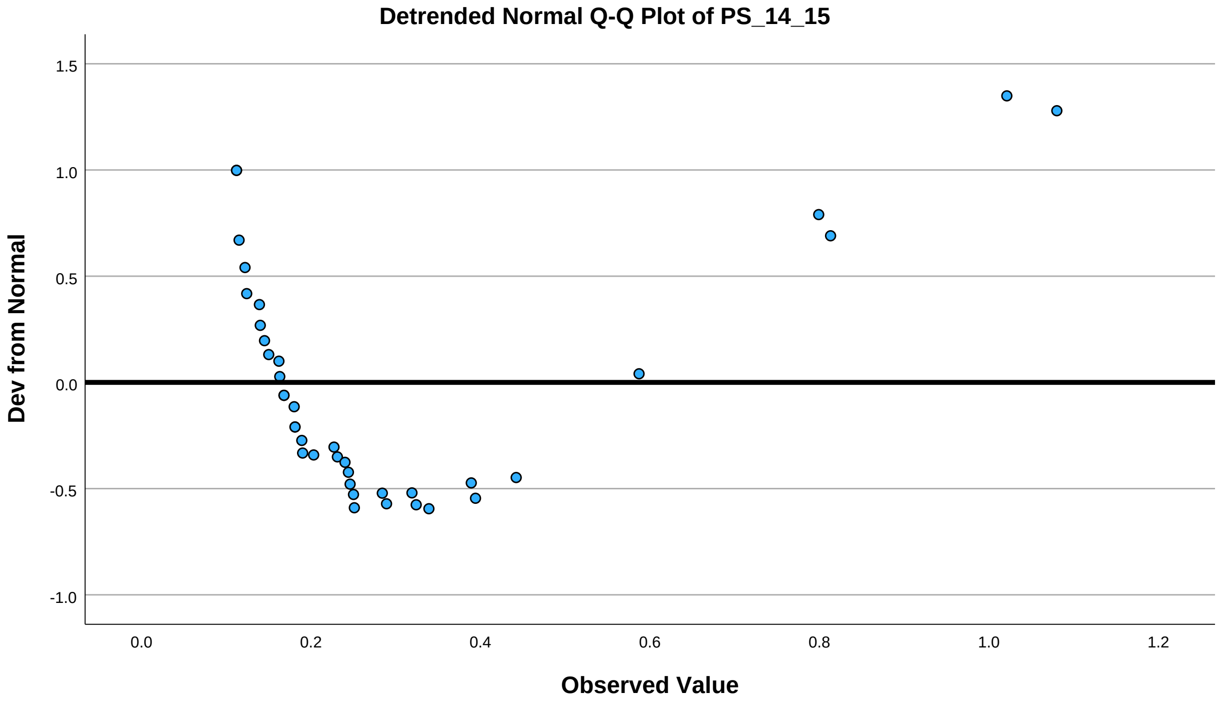
Detrended Normal Q-Q Plot of PS_14_15
Dev from Normal
1.5
1.0
0.5
0.0
-0.5
-1.0
0.0
0.2
0.4
0.6
0.8
1.0
1.2
Observed Value

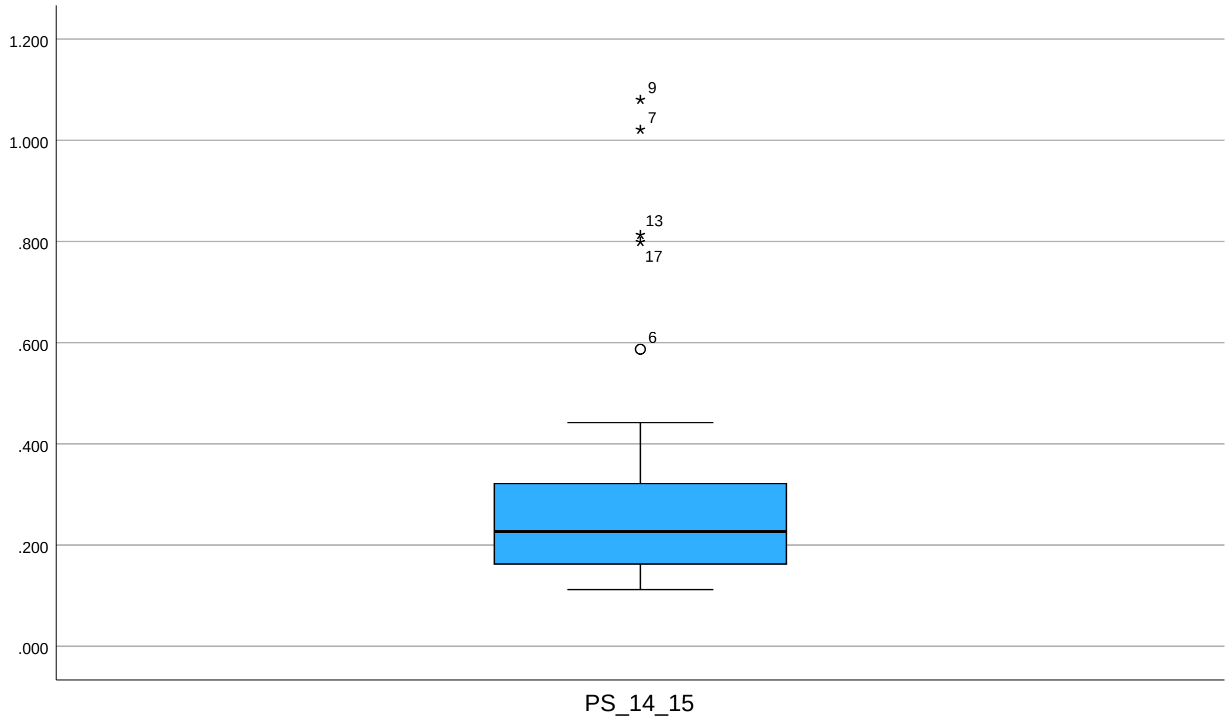

PS_15_16

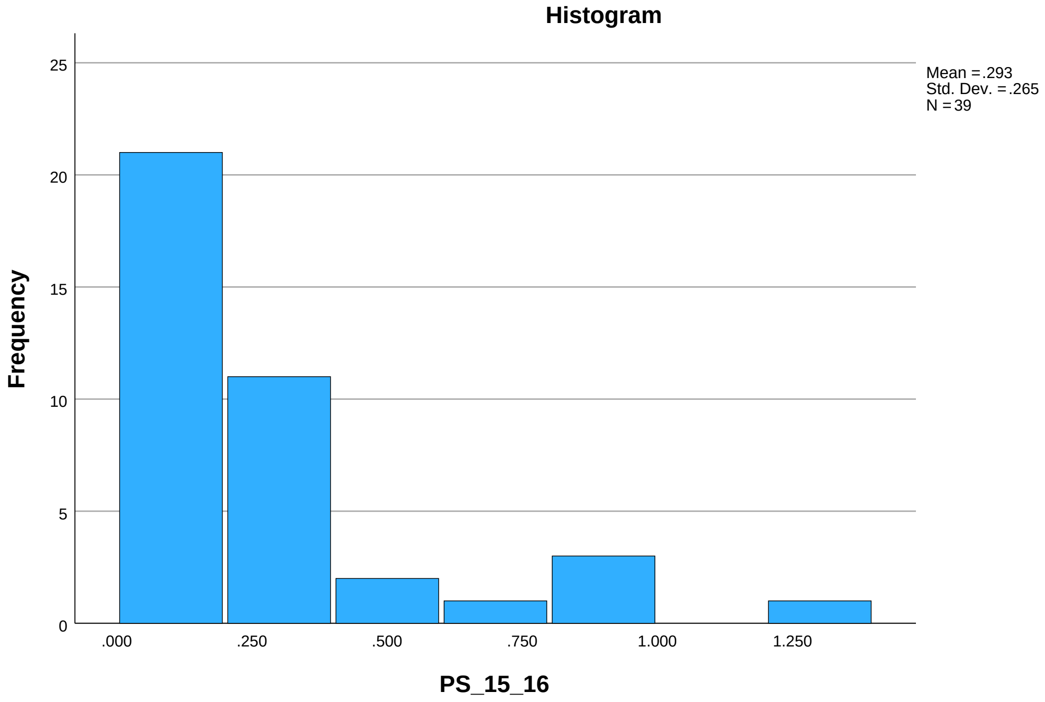


```
PS_15_16 Stem-and-Leaf Plot

 Frequency    Stem &  Leaf
```

```
 1.00     0 . 9
10.00       1 . 0122223344
10.00       1 . 5556677789
 5.00      2 . 11134
 3.00      2 . 689
 3.00      3 . 124
  .00     3 .
 1.00     4 . 0
 1.00     4 . 7
  5.00 Extremes    (>=.70)

Stem width:      .100
Each leaf:       1 case(s)
```

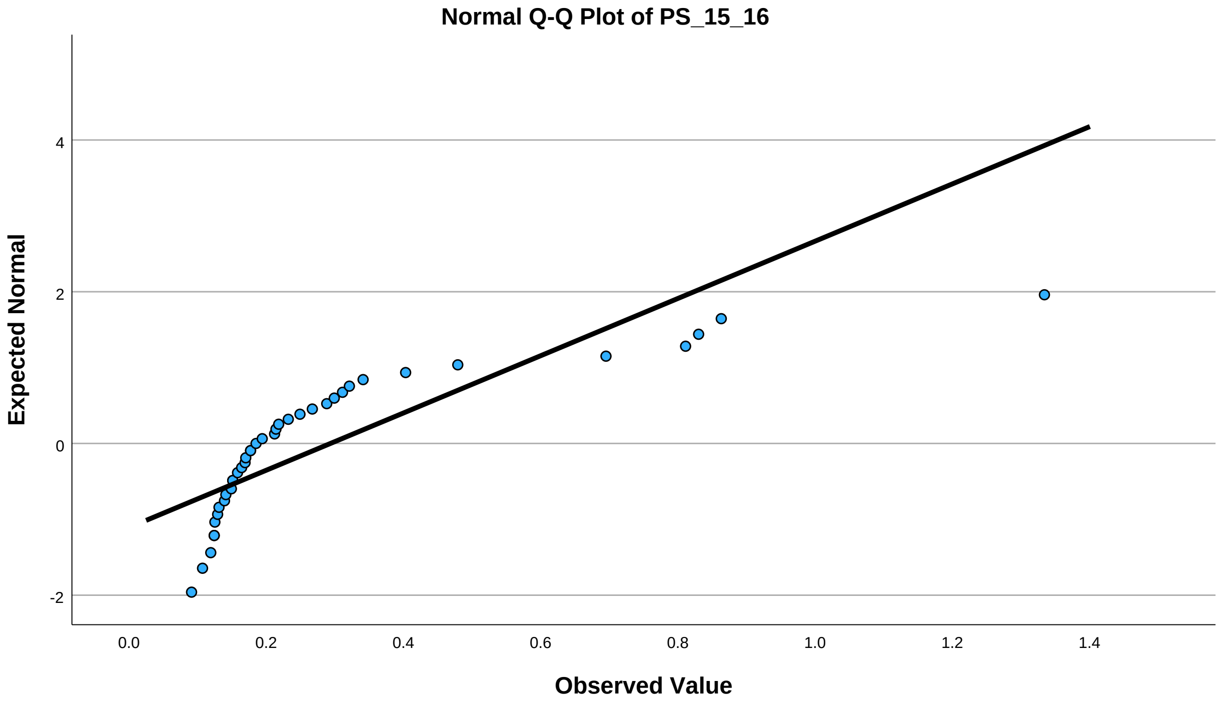

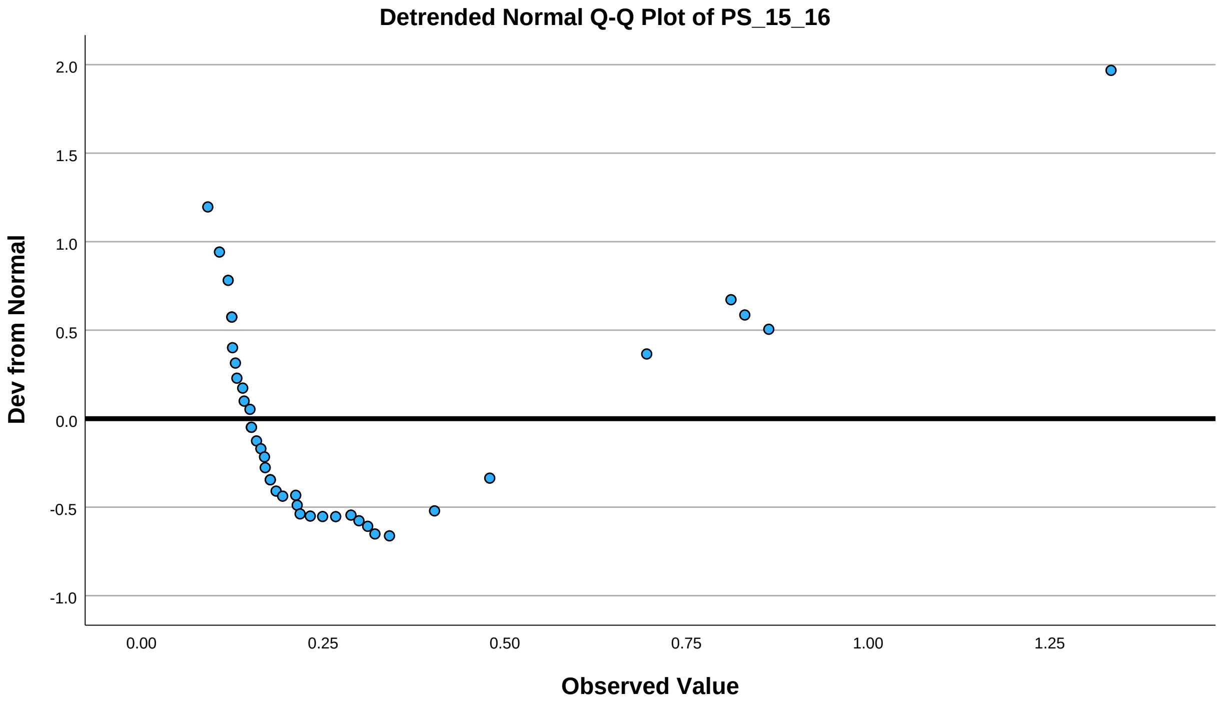
Detrended Normal Q-Q Plot of PS_15_16
Dev from Normal
2.0
1.5
1.0
0.5
0.0
-0.5
-1.0
0.00
0.25
0.50
0.75
1.00
1.25
Observed Value

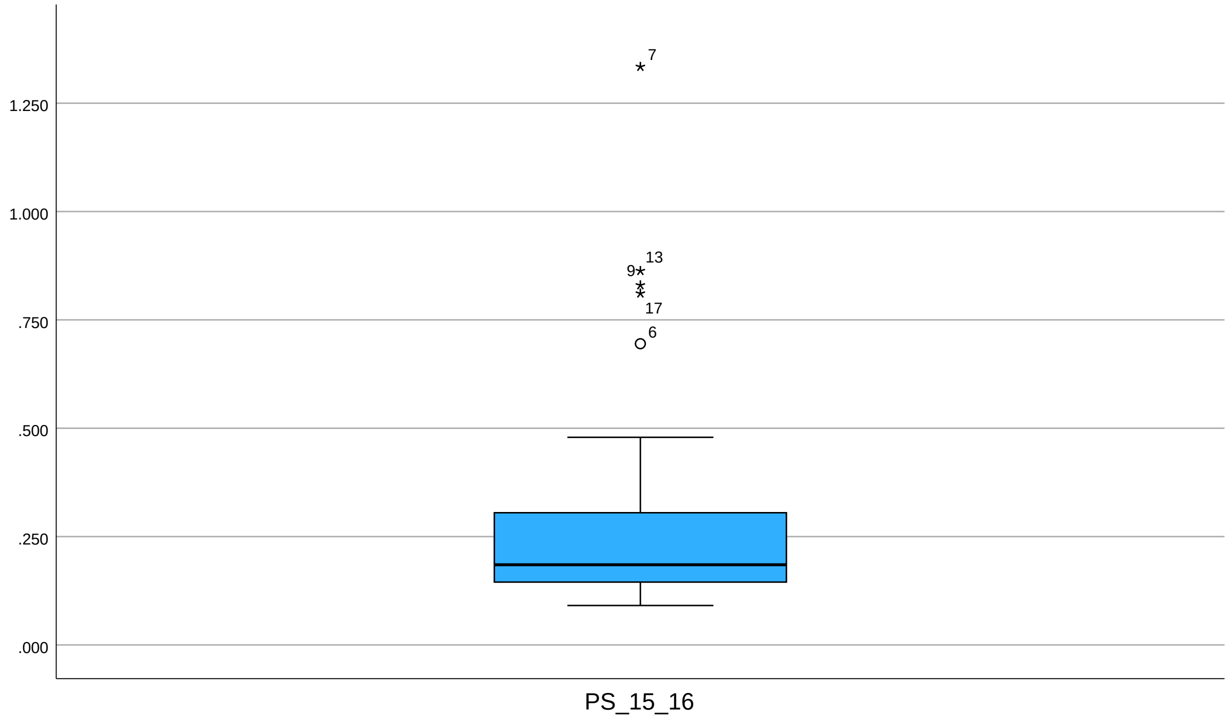
1.250
1.000
.750
.500
.250
.000
7
13
9
17
6
PS_15_16

PS_16_17

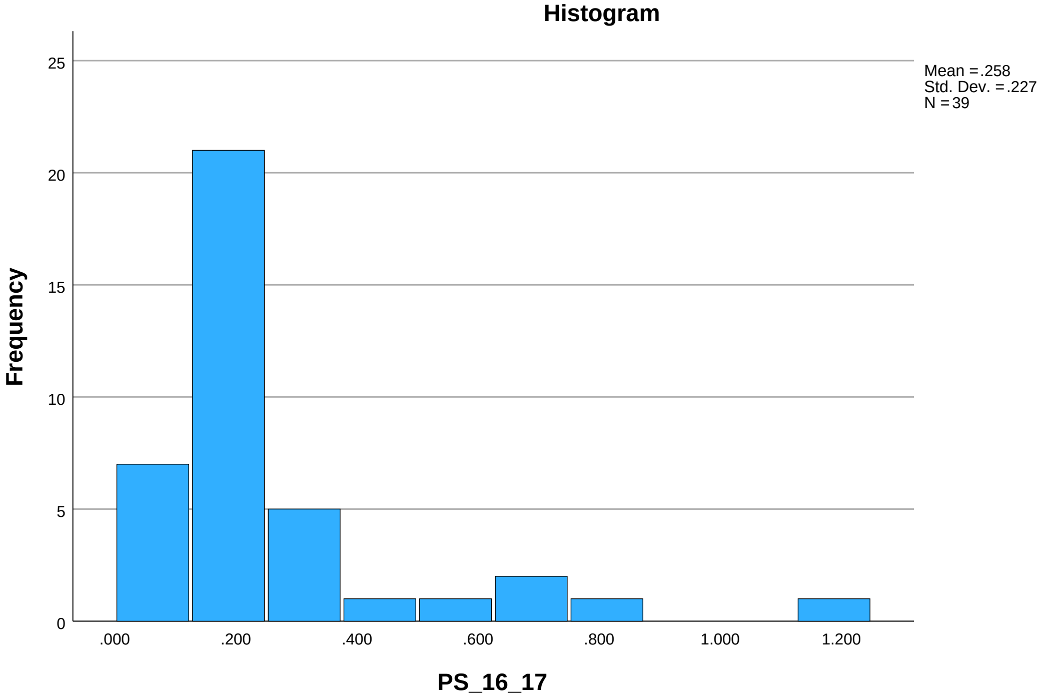


```
PS_16_17 Stem-and-Leaf Plot

 Frequency    Stem &  Leaf

     1.00        0 .  8
    13.00        1 .  0001222233444
     9.00        1 .  566677889
     5.00        2 .  00234
     3.00        2 .  568
     2.00        3 .  03
     1.00        3 .  8
     5.00 Extremes    (>=.50)

 Stem width:      .100
 Each leaf:       1 case(s)
```

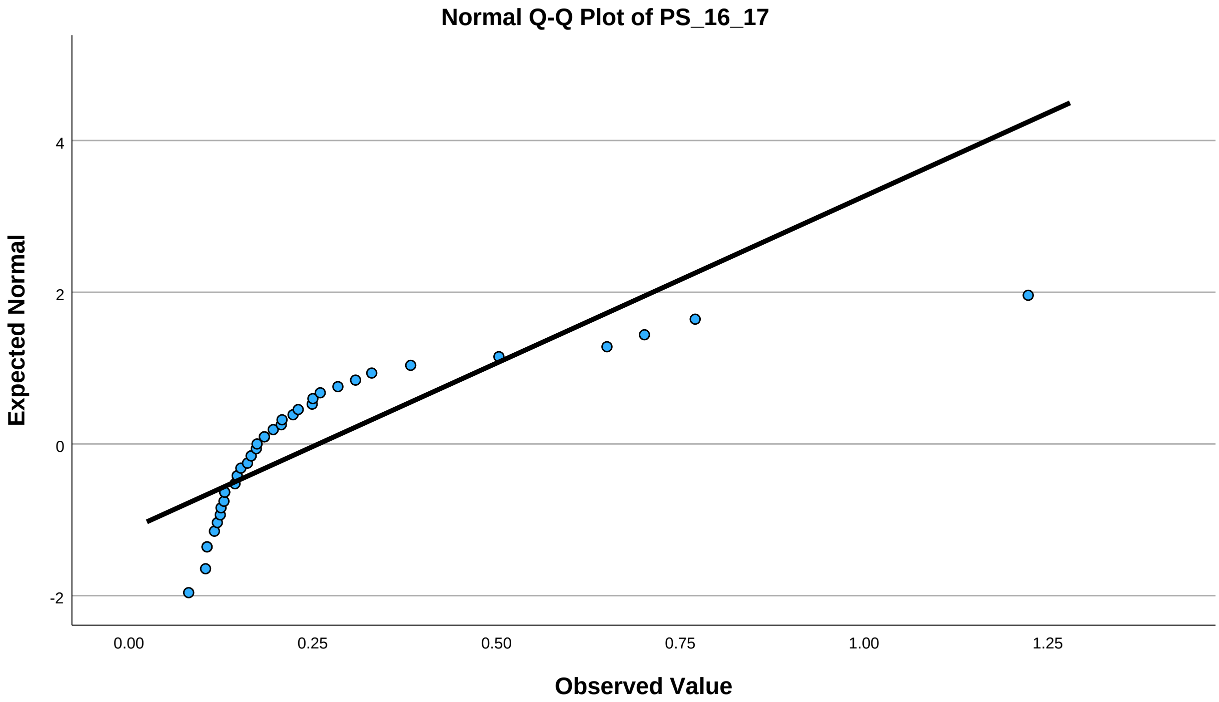
Normal Q-Q Plot of PS_16_17
Expected Normal
4
2
0
-2
0.00
0.25
0.50
0.75
1.00
1.25
Observed Value

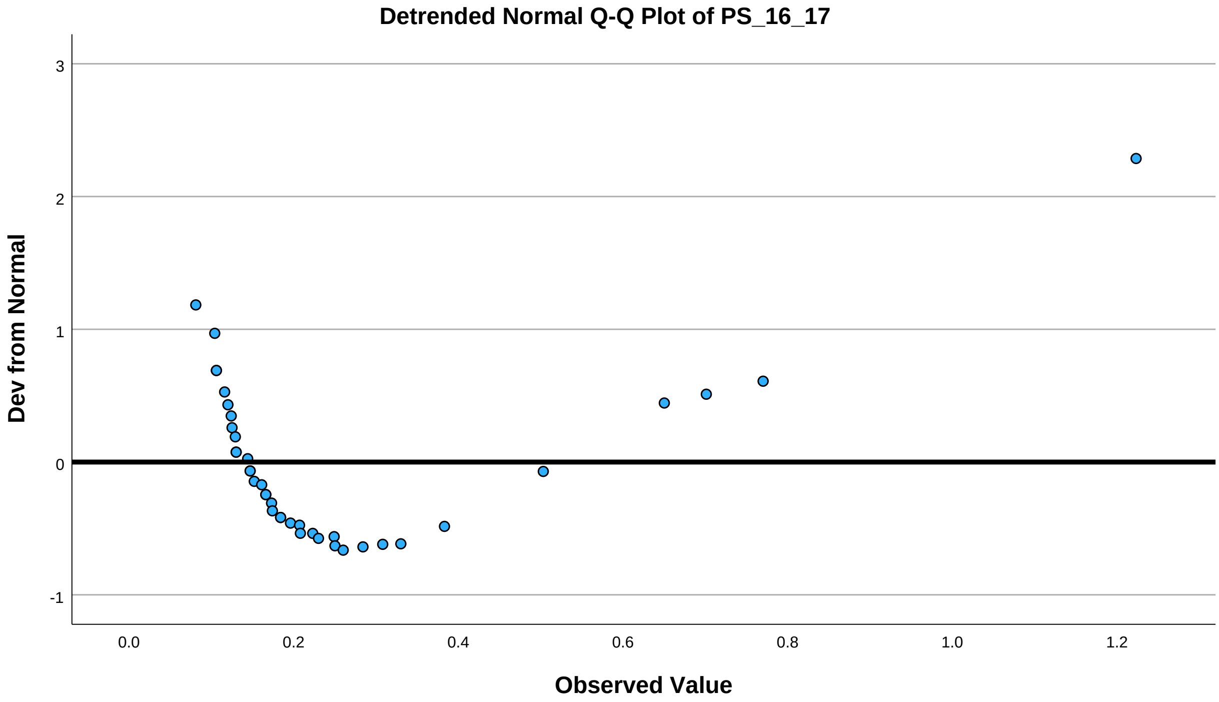
Detrended Normal Q-Q Plot of PS_16_17
Dev from Normal
3
2
1
0
-1
0.0
0.2
0.4
0.6
0.8
1.0
1.2
Observed Value

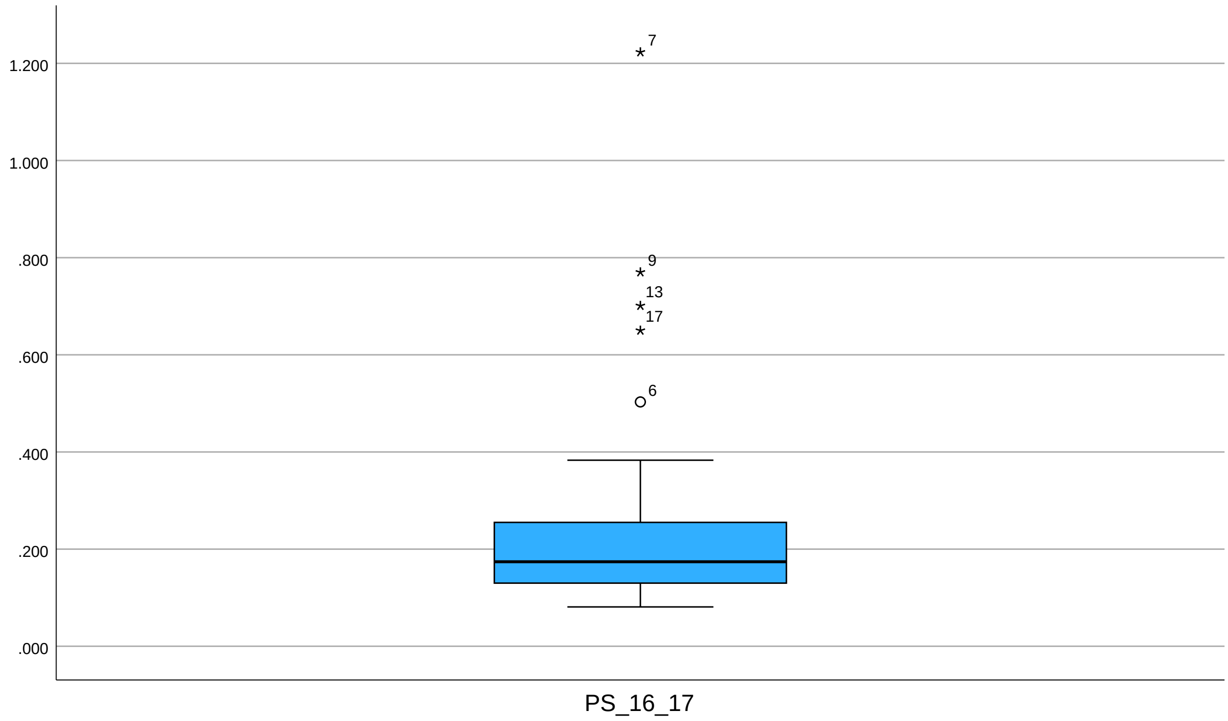

PS_17_18

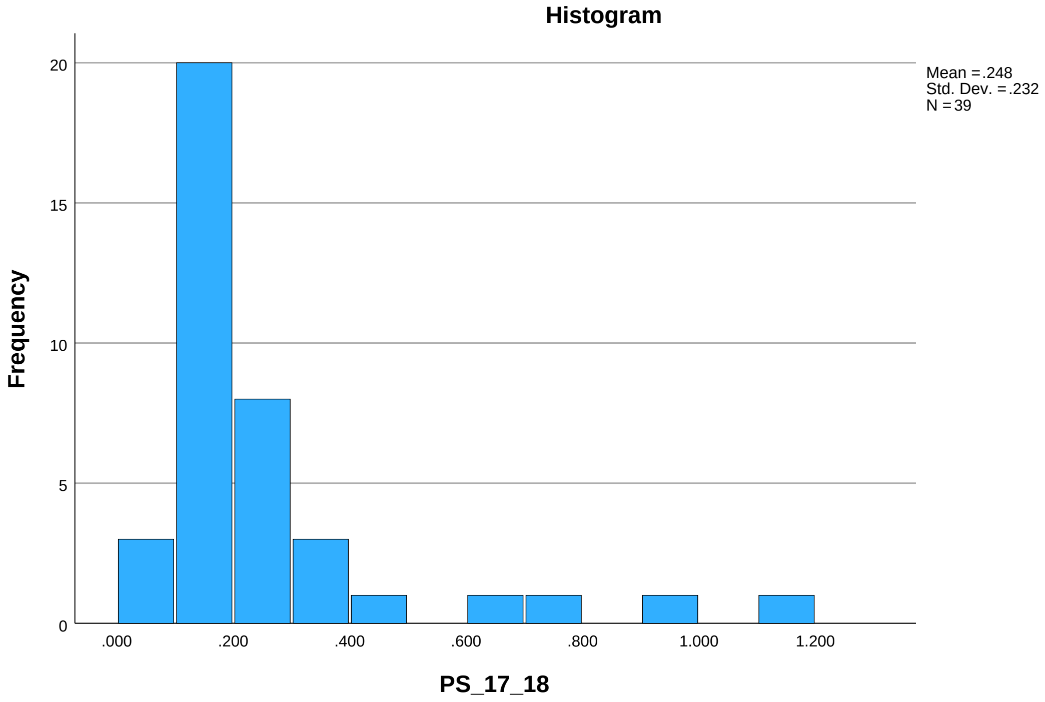

PS_17_18 Stem-and-Leaf Plot

Frequency Stem & Leaf

```
    3.00        0 .  888
   14.00        1 .  00012333333444
    6.00        1 .  556788
    8.00        2 .  00112234
     .00        2 .
    2.00        3 .  02
    6.00 Extremes    (>=.39)

 Stem width:       .100
 Each leaf:        1 case(s)
```

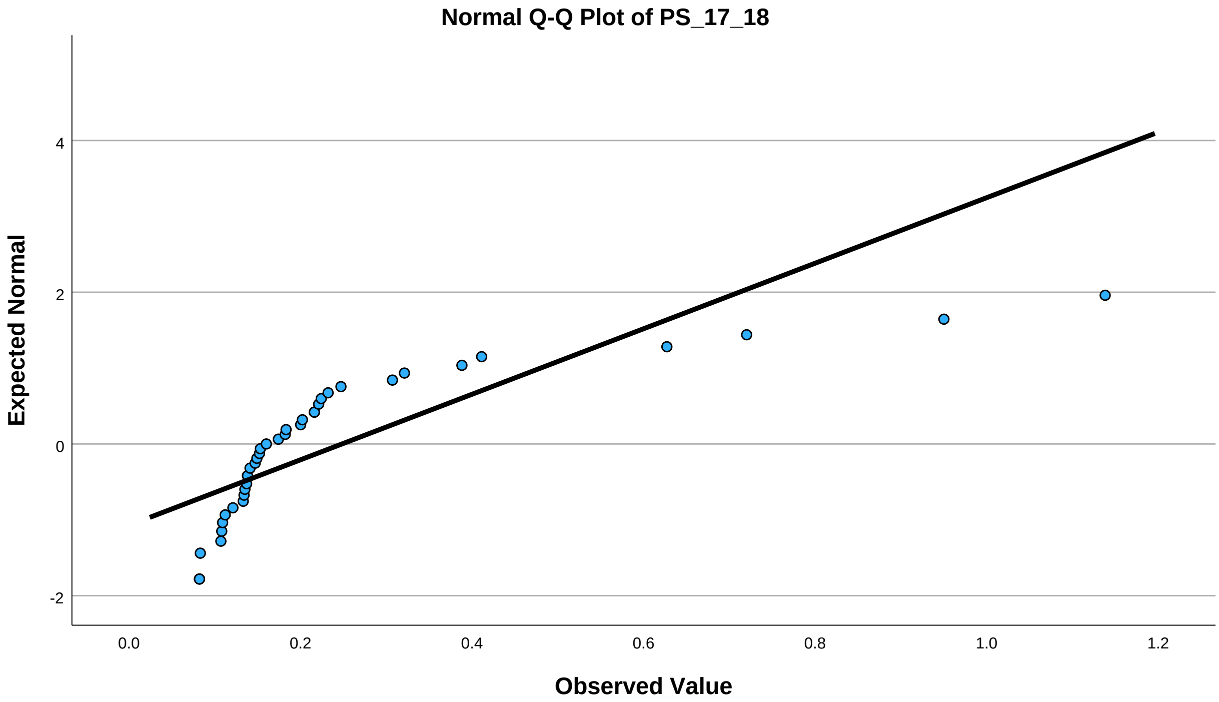

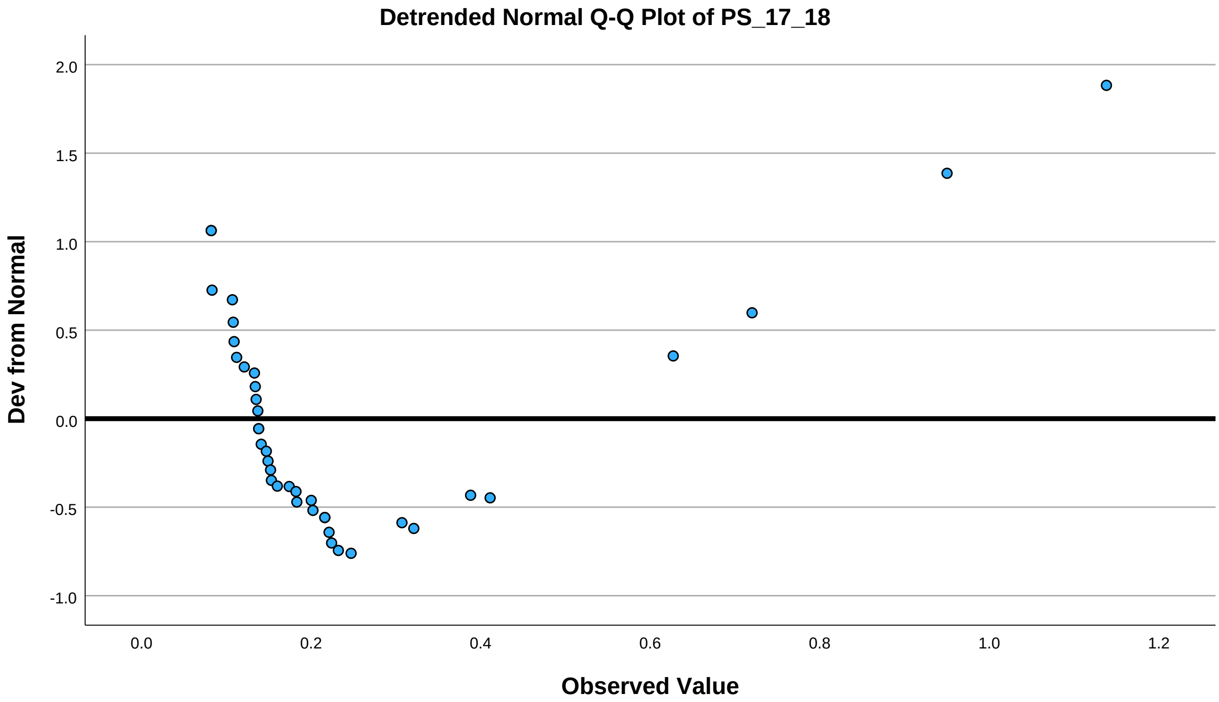
Detrended Normal Q-Q Plot of PS_17_18
Dev from Normal
2.0
1.5
1.0
0.5
0.0
-0.5
-1.0
0.0
0.2
0.4
0.6
0.8
1.0
1.2
Observed Value

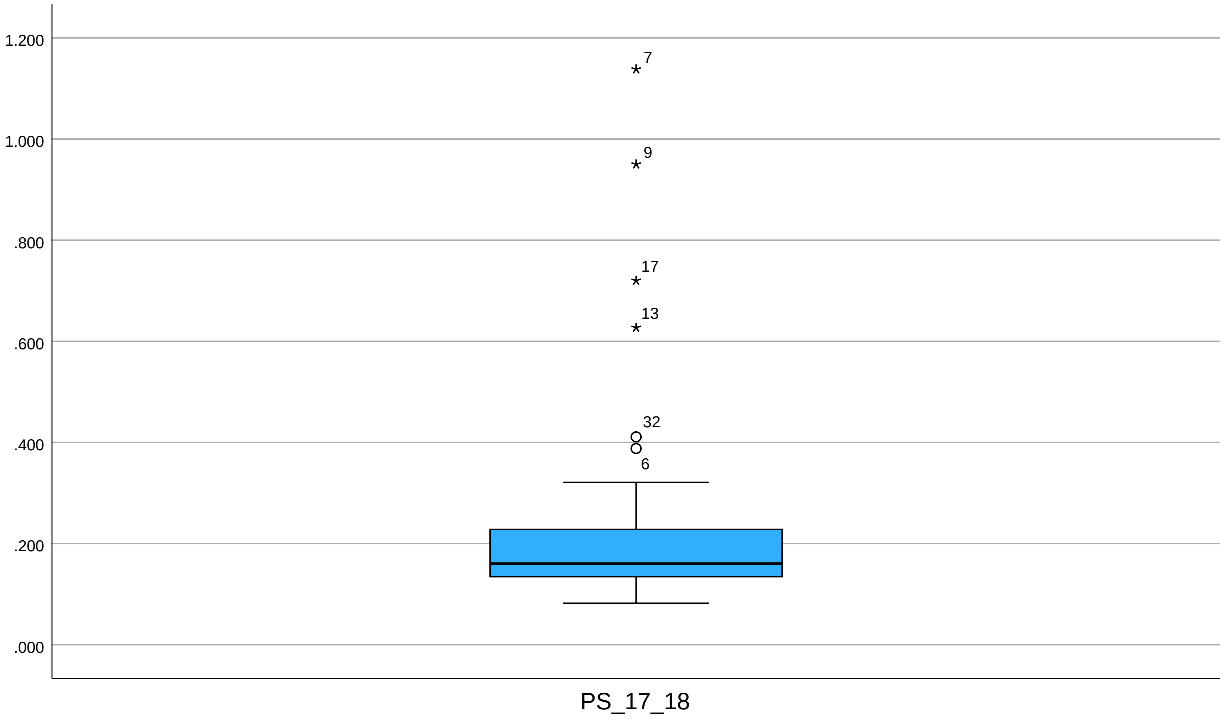
1.200
1.000
.800
.600
.400
.200
.000
7
9
17
13
32
6
PS_17_18

PS_18_19

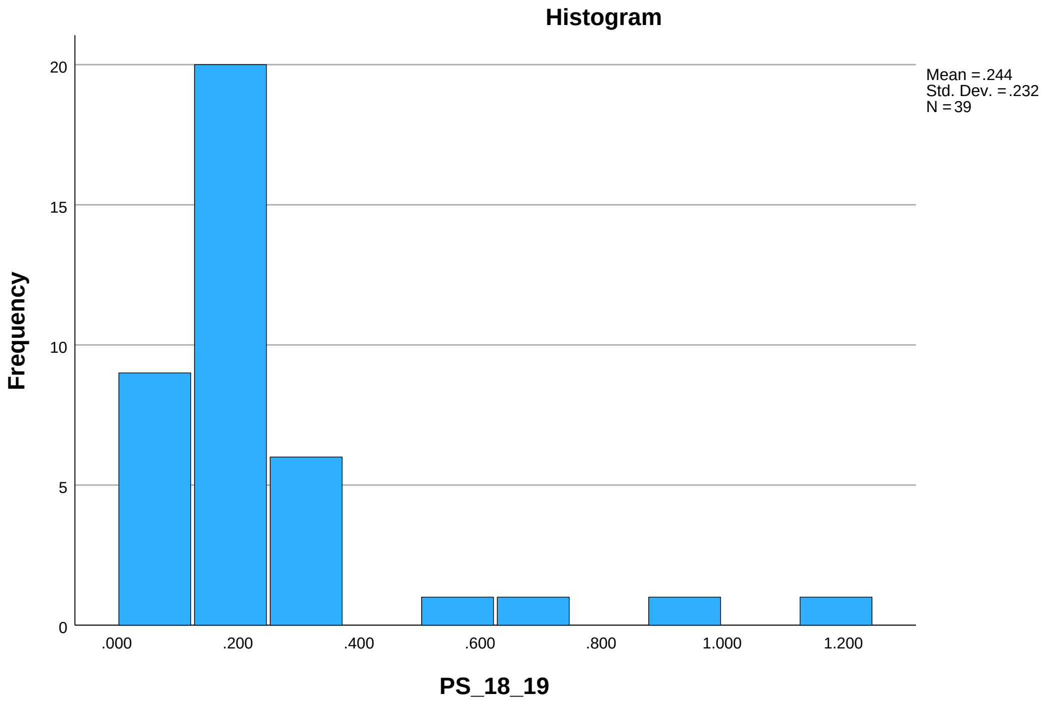


```
PS_18_19 Stem-and-Leaf Plot

 Frequency    Stem &  Leaf

     6.00        0 .  789999
    10.00        1 .  0222223444
     8.00        1 .  56677778
     5.00        2 .  01244
     4.00        2 .  6669
      .00        3 .
     2.00        3 .  66
     4.00 Extremes    (>=.55)

 Stem width:      .100
 Each leaf:       1 case(s)
```

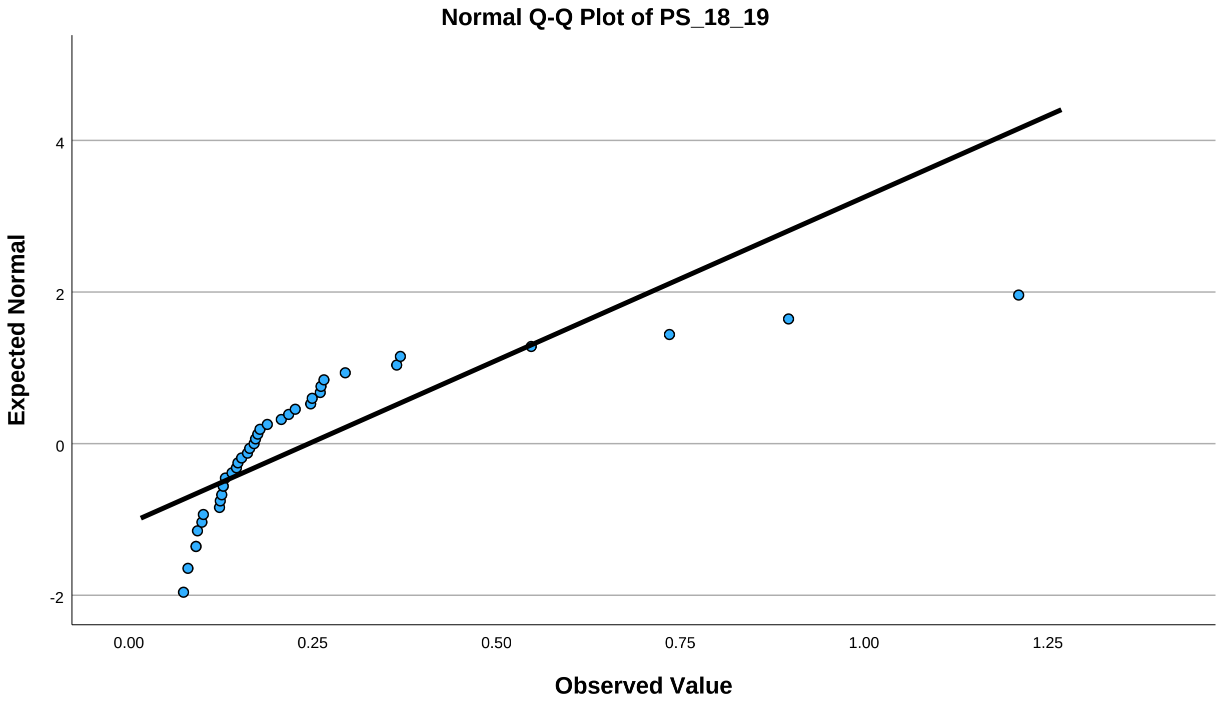
Normal Q-Q Plot of PS_18_19
Expected Normal
4
2
0
-2
0.00
0.25
0.50
0.75
1.00
1.25
Observed Value

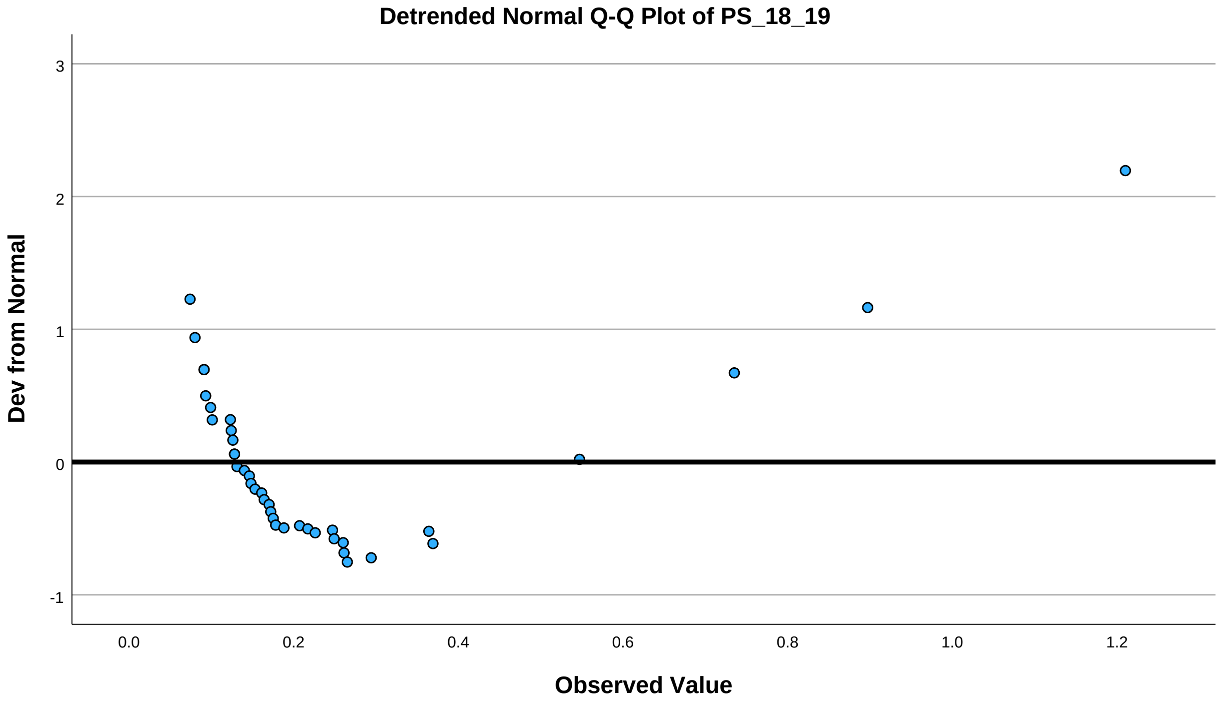
Detrended Normal Q-Q Plot of PS_18_19
Dev from Normal
3
2
1
0
-1
0.0
0.2
0.4
0.6
0.8
1.0
1.2
Observed Value

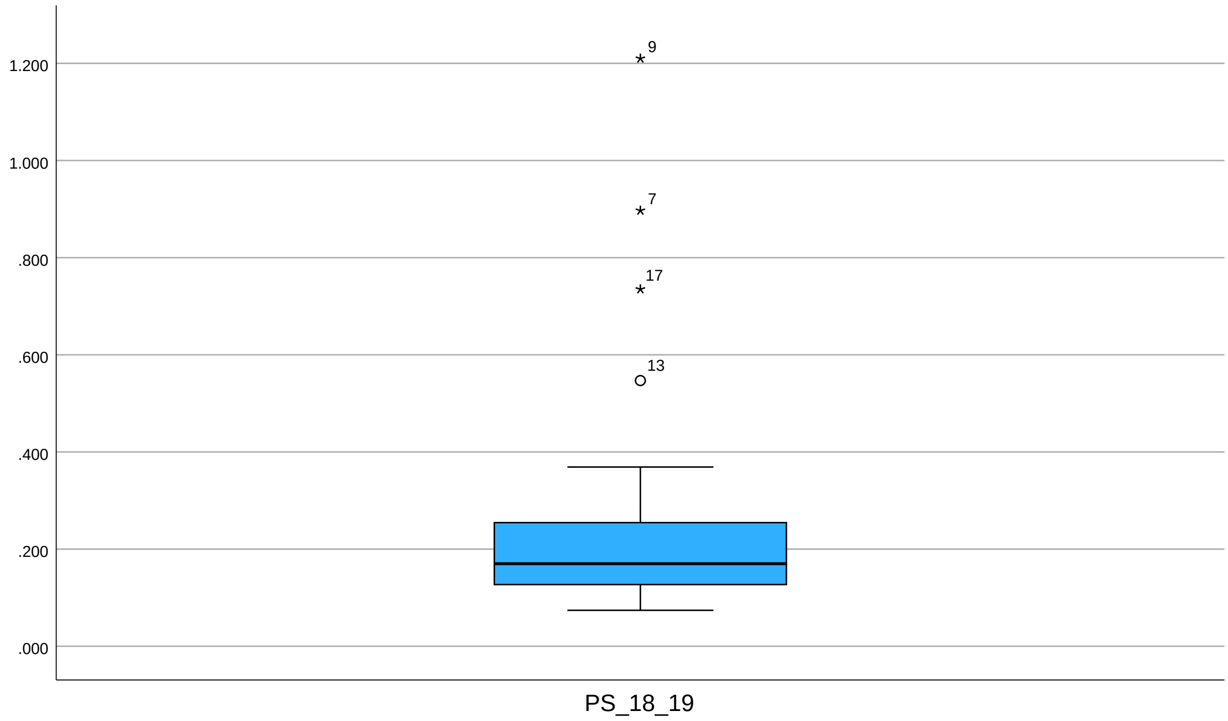

PS_19_20

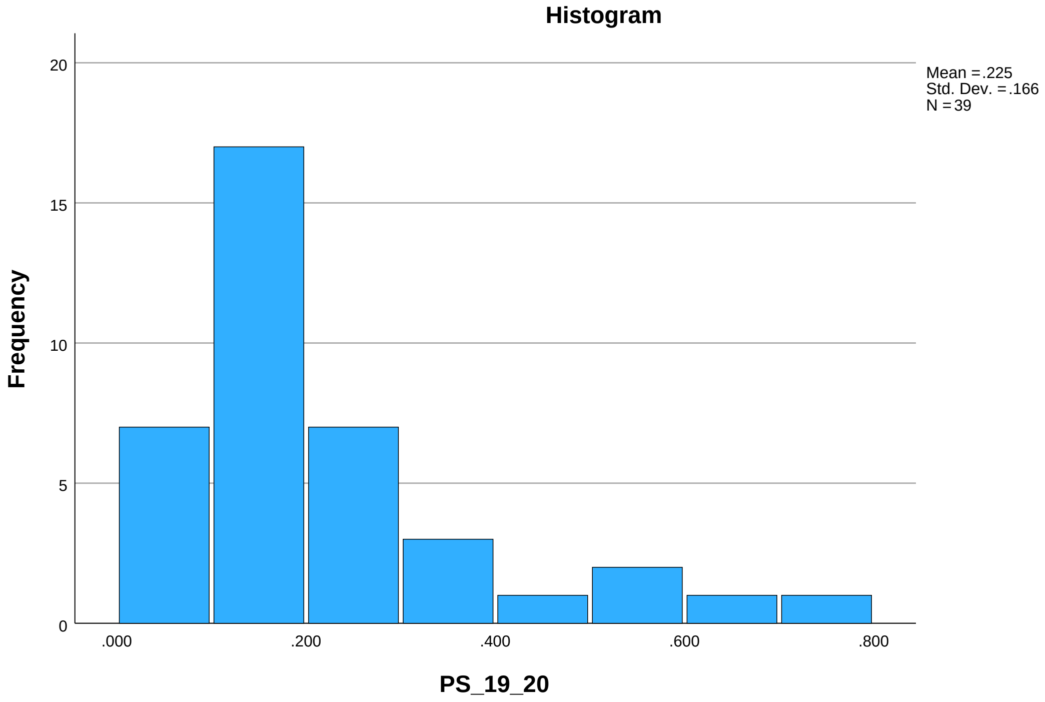

PS_19_20 Stem-and-Leaf Plot

Frequency   Stem & Leaf

```
  7.00        0 .  7788899
  9.00        1 .  002233444
  8.00        1 .  56888899
  6.00        2 .  001244
  1.00        2 .  5
  2.00        3 .  11
  1.00        3 .  5
  5.00 Extremes    (>=.46)

Stem width:      .100
Each leaf:       1 case(s)
```

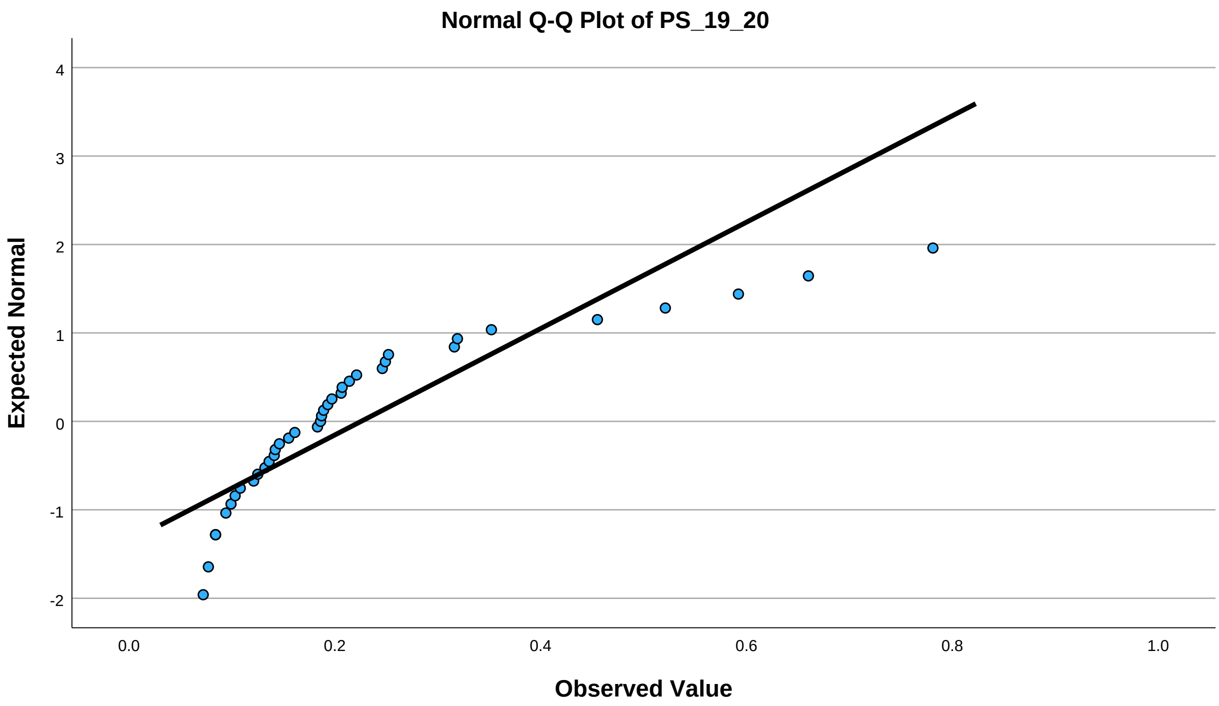

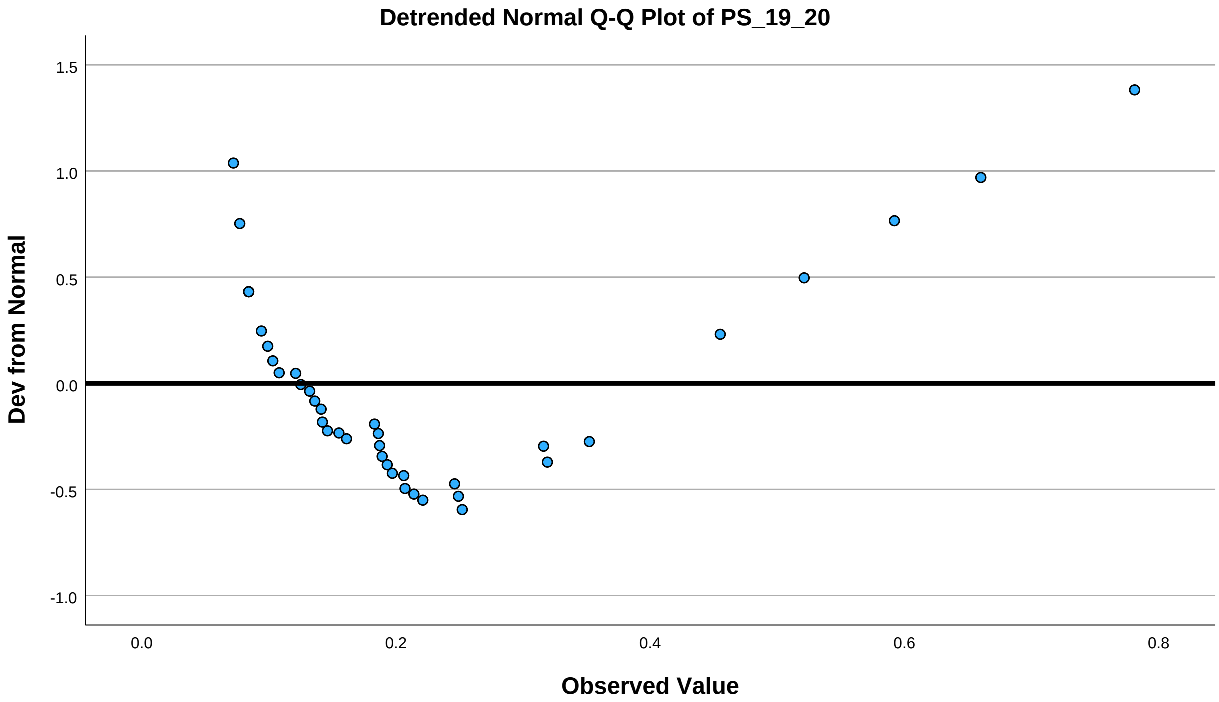
Detrended Normal Q-Q Plot of PS_19_20
Dev from Normal
1.5
1.0
0.5
0.0
-0.5
-1.0
0.0
0.2
0.4
0.6
0.8
Observed Value

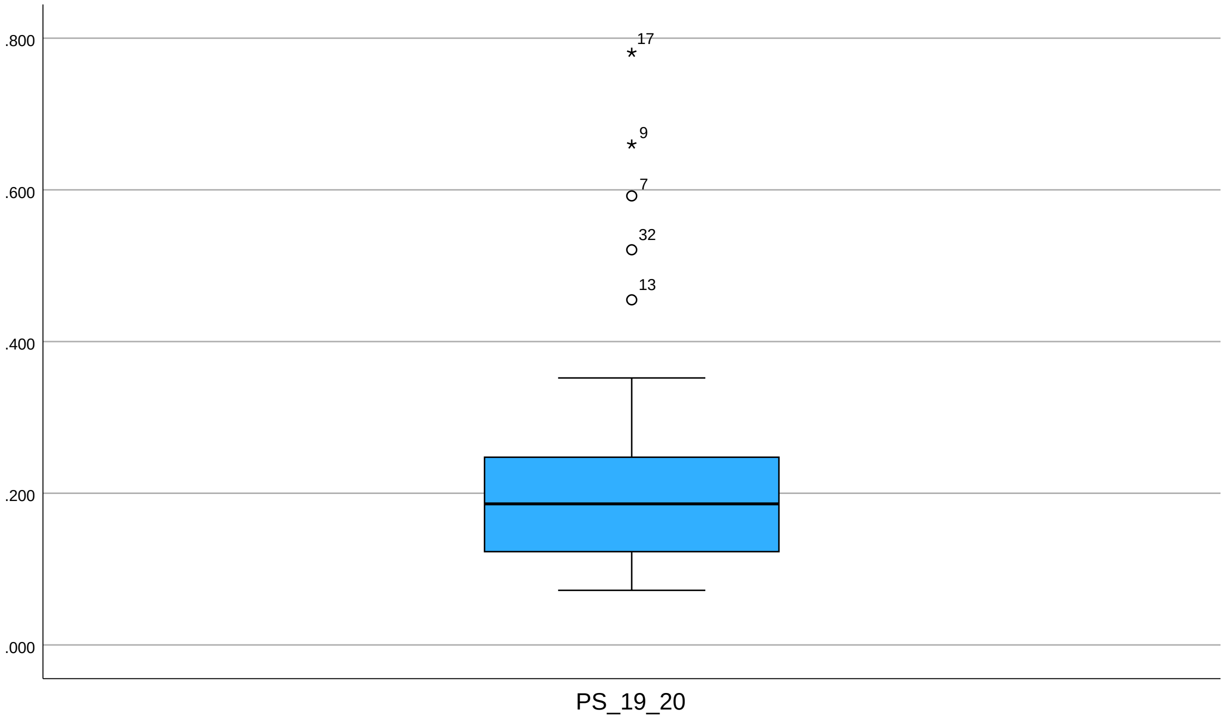
.800
.600
.400
.200
.000
17
9
7
32
13
PS_19_20

Explore

Case Processing Summary

	Cases					
	Valid		Missing		Total	
	N	Percent	N	Percent	N	Percent
BrainRate	39	100.0%	0	0.0%	39	100.0%
PermutationEntropy	39	100.0%	0	0.0%	39	100.0%
PermutationEntropy_01	39	100.0%	0	0.0%	39	100.0%
SampleEntropy	39	100.0%	0	0.0%	39	100.0%
SpectralEntropy_bits	39	100.0%	0	0.0%	39	100.0%
SpectralEntropy_01	39	100.0%	0	0.0%	39	100.0%
HiguchiFractalDimension	39	100.0%	0	0.0%	39	100.0%
LempelZivComplexity	39	100.0%	0	0.0%	39	100.0%
RenyiEntropy	39	100.0%	0	0.0%	39	100.0%
TsallisEntropy	39	100.0%	0	0.0%	39	100.0%
PS_4_5	39	100.0%	0	0.0%	39	100.0%
PS_5_6	39	100.0%	0	0.0%	39	100.0%
PS_6_7	39	100.0%	0	0.0%	39	100.0%
PS_7_8	39	100.0%	0	0.0%	39	100.0%
PS_8_9	39	100.0%	0	0.0%	39	100.0%
PS_9_10	39	100.0%	0	0.0%	39	100.0%
PS_10_11	39	100.0%	0	0.0%	39	100.0%
PS_11_12	39	100.0%	0	0.0%	39	100.0%
PS_12_13	39	100.0%	0	0.0%	39	100.0%
PS_13_14	39	100.0%	0	0.0%	39	100.0%
PS_14_15	39	100.0%	0	0.0%	39	100.0%
PS_15_16	39	100.0%	0	0.0%	39	100.0%
PS_16_17	39	100.0%	0	0.0%	39	100.0%
PS_17_18	39	100.0%	0	0.0%	39	100.0%
PS_18_19	39	100.0%	0	0.0%	39	100.0%
PS_19_20	39	100.0%	0	0.0%	39	100.0%

Descriptives

			Statistic	Std. Error
BrainRate	Mean		5.3513	.13164
	95% Confidence Interval for Mean	Lower Bound	5.0848	
		Upper Bound	5.6178	
	5% Trimmed Mean		5.3731	
	Median		5.4300	
	Variance		.676	
	Std. Deviation		.82211	
	Minimum		3.20	
	Maximum		6.93	
	Range		3.73	
	Interquartile Range		1.11	
	Skewness		-.391	.378
	Kurtosis		.257	.741
PermutationEntropy	Mean		1.4306114762	.00794694685
	95% Confidence Interval for Mean	Lower Bound	1.4145237233	
		Upper Bound	1.4466992290	
	5% Trimmed Mean		1.4295788822	
	Median		1.4379029151	
	Variance		.002	
	Std. Deviation		.04962866715	
	Minimum		1.3286004361	
	Maximum		1.5780427998	
	Range		.24944236371	
	Interquartile Range		.06463724200	
	Skewness		.284	.378
	Kurtosis		.813	.741
PermutationEntropy_01	Mean		.79843946731	.00443527548
	95% Confidence Interval for Mean	Lower Bound	.78946072151	
		Upper Bound	.80741821312	
	5% Trimmed Mean		.79786316568	
	Median		.80250889687	
	Variance		.001	
	Std. Deviation		.02769828652	
	Minimum		.74150602183	
	Maximum		.88072245572	
	Range		.13921643390	
	Interquartile Range		.03607473163	
	Skewness		.284	.378
	Kurtosis		.813	.741

Descriptives

			Statistic	Std. Error
SampleEntropy	Mean		.54928132975	.02127940508
	95% Confidence Interval for Mean	Lower Bound	.50620342629	
		Upper Bound	.59235923320	
	5% Trimmed Mean		.55874141310	
	Median		.58465105335	
	Variance		.018	
	Std. Deviation		.13288984213	
	Minimum		.17283672213	
	Maximum		.73570001153	
	Range		.56286328940	
	Interquartile Range		.10179981190	
	Skewness		-1.130	.378
	Kurtosis		.916	.741
SpectralEntropy_bits	Mean		3.7032188258	.05275179625
	95% Confidence Interval for Mean	Lower Bound	3.5964283973	
		Upper Bound	3.8100092543	
	5% Trimmed Mean		3.7341105702	
	Median		3.7868954344	
	Variance		.109	
	Std. Deviation		.32943486202	
	Minimum		2.5082352059	
	Maximum		4.1942497041	
	Range		1.6860144982	
	Interquartile Range		.34577120916	
	Skewness		-1.630	.378
	Kurtosis		4.284	.741
SpectralEntropy_01	Mean		.52818410970	.00752390335
	95% Confidence Interval for Mean	Lower Bound	.51295276366	
		Upper Bound	.54341545574	
	5% Trimmed Mean		.53259014923	
	Median		.54011876900	
	Variance		.002	
	Std. Deviation		.04698676138	
	Minimum		.35774552936	
	Maximum		.59821904944	
	Range		.24047352008	
	Interquartile Range		.04931678814	
	Skewness		-1.630	.378
	Kurtosis		4.284	.741

Descriptives

			Statistic	Std. Error
HiguchiFractalDimension	Mean		.66327731039	.00917668369
	95% Confidence Interval for Mean	Lower Bound	.64470008549	
		Upper Bound	.68185453529	
	5% Trimmed Mean		.66729799999	
	Median		.66402006857	
	Variance		.003	
	Std. Deviation		.05730837126	
	Minimum		.44704287961	
	Maximum		.75514817477	
	Range		.30810529516	
	Interquartile Range		.07992333269	
	Skewness		-1.358	.378
	Kurtosis		3.948	.741
LempelZivComplexity	Mean		.23709776172	.00273100591
	95% Confidence Interval for Mean	Lower Bound	.23156912929	
		Upper Bound	.24262639414	
	5% Trimmed Mean		.23719720725	
	Median		.23693640130	
	Variance		.000	
	Std. Deviation		.01705512644	
	Minimum		.20178488700	
	Maximum		.26845624665	
	Range		.06667135964	
	Interquartile Range		.02455735368	
	Skewness		-.018	.378
	Kurtosis		-.526	.741
RenyiEntropy	Mean		7.5856777114	.18121388515
	95% Confidence Interval for Mean	Lower Bound	7.2188293799	
		Upper Bound	7.9525260429	
	5% Trimmed Mean		7.6518361557	
	Median		7.7593320774	
	Variance		1.281	
	Std. Deviation		1.1316803500	
	Minimum		5.1226922559	
	Maximum		8.8176257835	
	Range		3.6949335276	
	Interquartile Range		1.3233326764	
	Skewness		-.873	.378
	Kurtosis		-.419	.741

Descriptives

			Statistic	Std. Error
TsallisEntropy	Mean		1.5778705181	.08369092593
	95% Confidence Interval for Mean	Lower Bound	1.4084470961	
		Upper Bound	1.7472939401	
	5% Trimmed Mean		1.6181161786	
	Median		1.7895695467	
	Variance		.273	
	Std. Deviation		.52264966494	
	Minimum		.42826267388	
	Maximum		1.9869651945	
	Range		1.5587025207	
	Interquartile Range		.78627584855	
	Skewness		-1.118	.378
	Kurtosis		-.085	.741
PS_4_5	Mean		3.12726	.361726
	95% Confidence Interval for Mean	Lower Bound	2.39498	
		Upper Bound	3.85953	
	5% Trimmed Mean		2.84934	
	Median		2.56900	
	Variance		5.103	
	Std. Deviation		2.258977	
	Minimum		.782	
	Maximum		14.310	
	Range		13.528	
	Interquartile Range		1.365	
	Skewness		3.412	.378
	Kurtosis		15.645	.741
PS_5_6	Mean		2.49949	.235859
	95% Confidence Interval for Mean	Lower Bound	2.02201	
		Upper Bound	2.97696	
	5% Trimmed Mean		2.34513	
	Median		2.08100	
	Variance		2.170	
	Std. Deviation		1.472942	
	Minimum		.578	
	Maximum		8.830	
	Range		8.252	
	Interquartile Range		1.321	
	Skewness		2.323	.378
	Kurtosis		8.152	.741

Descriptives

			Statistic	Std. Error
PS_6_7	Mean		2.09656	.235378
	95% Confidence Interval for Mean	Lower Bound	1.62007	
		Upper Bound	2.57306	
	5% Trimmed Mean		1.93651	
	Median		1.87100	
	Variance		2.161	
	Std. Deviation		1.469934	
	Minimum		.313	
	Maximum		9.560	
	Range		9.247	
	Interquartile Range		1.100	
	Skewness		3.541	.378
	Kurtosis		17.520	.741
PS_7_8	Mean		1.62885	.207341
	95% Confidence Interval for Mean	Lower Bound	1.20911	
		Upper Bound	2.04859	
	5% Trimmed Mean		1.45750	
	Median		1.34900	
	Variance		1.677	
	Std. Deviation		1.294845	
	Minimum		.243	
	Maximum		8.150	
	Range		7.907	
	Interquartile Range		.768	
	Skewness		3.616	.378
	Kurtosis		17.011	.741
PS_8_9	Mean		1.36023	.158021
	95% Confidence Interval for Mean	Lower Bound	1.04033	
		Upper Bound	1.68013	
	5% Trimmed Mean		1.24748	
	Median		1.14800	
	Variance		.974	
	Std. Deviation		.986838	
	Minimum		.211	
	Maximum		5.700	
	Range		5.489	
	Interquartile Range		.778	
	Skewness		2.565	.378
	Kurtosis		9.202	.741

Descriptives

			Statistic	Std. Error
PS_9_10	Mean		1.45315	.264632
	95% Confidence Interval for Mean	Lower Bound	.91743	
		Upper Bound	1.98887	
	5% Trimmed Mean		1.16900	
	Median		1.14500	
	Variance		2.731	
	Std. Deviation		1.652629	
	Minimum		.182	
	Maximum		9.190	
	Range		9.008	
	Interquartile Range		.845	
	Skewness		3.519	.378
	Kurtosis		13.981	.741
PS_10_11	Mean		1.73231	.385087
	95% Confidence Interval for Mean	Lower Bound	.95274	
		Upper Bound	2.51188	
	5% Trimmed Mean		1.30815	
	Median		1.04400	
	Variance		5.783	
	Std. Deviation		2.404869	
	Minimum		.191	
	Maximum		13.740	
	Range		13.549	
	Interquartile Range		1.074	
	Skewness		3.888	.378
	Kurtosis		17.255	.741
PS_11_12	Mean		.56759	.078734
	95% Confidence Interval for Mean	Lower Bound	.40820	
		Upper Bound	.72698	
	5% Trimmed Mean		.50234	
	Median		.41800	
	Variance		.242	
	Std. Deviation		.491693	
	Minimum		.125	
	Maximum		2.970	
	Range		2.845	
	Interquartile Range		.431	
	Skewness		3.370	.378
	Kurtosis		14.759	.741

Descriptives

			Statistic	Std. Error
PS_12_13	Mean		.35703	.042276
	95% Confidence Interval for Mean	Lower Bound	.27144	
		Upper Bound	.44261	
	5% Trimmed Mean		.32133	
	Median		.29700	
	Variance		.070	
	Std. Deviation		.264012	
	Minimum		.102	
	Maximum		1.710	
	Range		1.608	
	Interquartile Range		.171	
	Skewness		3.795	.378
	Kurtosis		18.362	.741
PS_13_14	Mean		.30433	.033243
	95% Confidence Interval for Mean	Lower Bound	.23704	
		Upper Bound	.37163	
	5% Trimmed Mean		.27751	
	Median		.23700	
	Variance		.043	
	Std. Deviation		.207604	
	Minimum		.094	
	Maximum		1.150	
	Range		1.056	
	Interquartile Range		.140	
	Skewness		2.508	.378
	Kurtosis		7.345	.741
PS_14_15	Mean		.28082	.031428
	95% Confidence Interval for Mean	Lower Bound	.21720	
		Upper Bound	.34444	
	5% Trimmed Mean		.25642	
	Median		.20400	
	Variance		.039	
	Std. Deviation		.196267	
	Minimum		.079	
	Maximum		.990	
	Range		.911	
	Interquartile Range		.142	
	Skewness		2.149	.378
	Kurtosis		4.893	.741

Descriptives

			Statistic	Std. Error
PS_15_16	Mean		.27621	.034493
	95% Confidence Interval for Mean	Lower Bound	.20638	
		Upper Bound	.34603	
	5% Trimmed Mean		.24937	
	Median		.19700	
	Variance		.046	
	Std. Deviation		.215412	
	Minimum		.071	
	Maximum		.960	
	Range		.889	
	Interquartile Range		.151	
	Skewness		2.039	.378
	Kurtosis		3.892	.741
PS_16_17	Mean		.21464	.023132
	95% Confidence Interval for Mean	Lower Bound	.16781	
		Upper Bound	.26147	
	5% Trimmed Mean		.19544	
	Median		.16300	
	Variance		.021	
	Std. Deviation		.144462	
	Minimum		.061	
	Maximum		.710	
	Range		.649	
	Interquartile Range		.147	
	Skewness		2.210	.378
	Kurtosis		5.547	.741
PS_17_18	Mean		.19990	.020983
	95% Confidence Interval for Mean	Lower Bound	.15742	
		Upper Bound	.24237	
	5% Trimmed Mean		.18301	
	Median		.15200	
	Variance		.017	
	Std. Deviation		.131036	
	Minimum		.057	
	Maximum		.660	
	Range		.603	
	Interquartile Range		.131	
	Skewness		2.062	.378
	Kurtosis		4.907	.741

Descriptives

			Statistic	Std. Error
PS_18_19	Mean		.17923	.019347
	95% Confidence Interval for Mean	Lower Bound	.14006	
		Upper Bound	.21840	
	5% Trimmed Mean		.16344	
	Median		.15300	
	Variance		.015	
	Std. Deviation		.120825	
	Minimum		.055	
	Maximum		.750	
	Range		.695	
	Interquartile Range		.098	
	Skewness		3.077	.378
	Kurtosis		12.772	.741
PS_19_20	Mean		.18303	.021323
	95% Confidence Interval for Mean	Lower Bound	.13986	
		Upper Bound	.22619	
	5% Trimmed Mean		.16450	
	Median		.14300	
	Variance		.018	
	Std. Deviation		.133160	
	Minimum		.060	
	Maximum		.810	
	Range		.750	
	Interquartile Range		.089	
	Skewness		3.110	.378
	Kurtosis		12.650	.741

Tests of Normality

	Kolmogorov-Smirnov[a]			Shapiro-Wilk		
	Statistic	df	Sig.	Statistic	df	Sig.
BrainRate	.120	39	.171	.981	39	.743
PermutationEntropy	.085	39	.200*	.974	39	.496
PermutationEntropy_01	.085	39	.200*	.974	39	.496
SampleEntropy	.238	39	<.001	.895	39	.002
SpectralEntropy_bits	.149	39	.029	.873	39	<.001
SpectralEntropy_01	.149	39	.029	.873	39	<.001
HiguchiFractalDimension	.111	39	.200*	.904	39	.003
LempelZivComplexity	.061	39	.200*	.983	39	.815
RenyiEntropy	.181	39	.002	.863	39	<.001
TsallisEntropy	.229	39	<.001	.772	39	<.001
PS_4_5	.228	39	<.001	.678	39	<.001
PS_5_6	.157	39	.017	.810	39	<.001
PS_6_7	.176	39	.004	.682	39	<.001
PS_7_8	.236	39	<.001	.650	39	<.001
PS_8_9	.192	39	<.001	.770	39	<.001
PS_9_10	.305	39	<.001	.580	39	<.001
PS_10_11	.290	39	<.001	.534	39	<.001
PS_11_12	.232	39	<.001	.654	39	<.001
PS_12_13	.215	39	<.001	.625	39	<.001
PS_13_14	.210	39	<.001	.728	39	<.001
PS_14_15	.200	39	<.001	.759	39	<.001
PS_15_16	.244	39	<.001	.737	39	<.001
PS_16_17	.198	39	<.001	.756	39	<.001
PS_17_18	.195	39	<.001	.786	39	<.001
PS_18_19	.202	39	<.001	.710	39	<.001
PS_19_20	.232	39	<.001	.691	39	<.001

*. This is a lower bound of the true significance.

a. Lilliefors Significance Correction

BrainRate

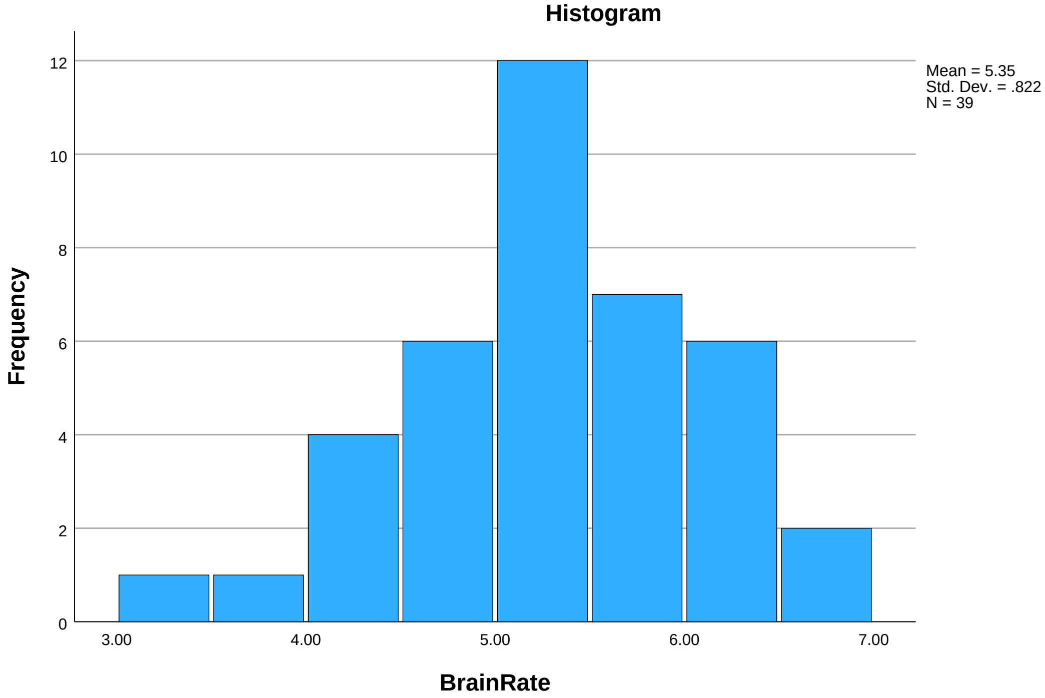


```
BrainRate Stem-and-Leaf Plot

 Frequency    Stem &  Leaf

     1.00 Extremes    (=<3.2)
     1.00        3 .  6
     4.00        4 .  1333
     6.00        4 .  577789
    12.00        5 .  002344444444
     7.00        5 .  5678889
     6.00        6 .  022244
     2.00        6 .  89

 Stem width:      1.00
 Each leaf:       1 case(s)
```

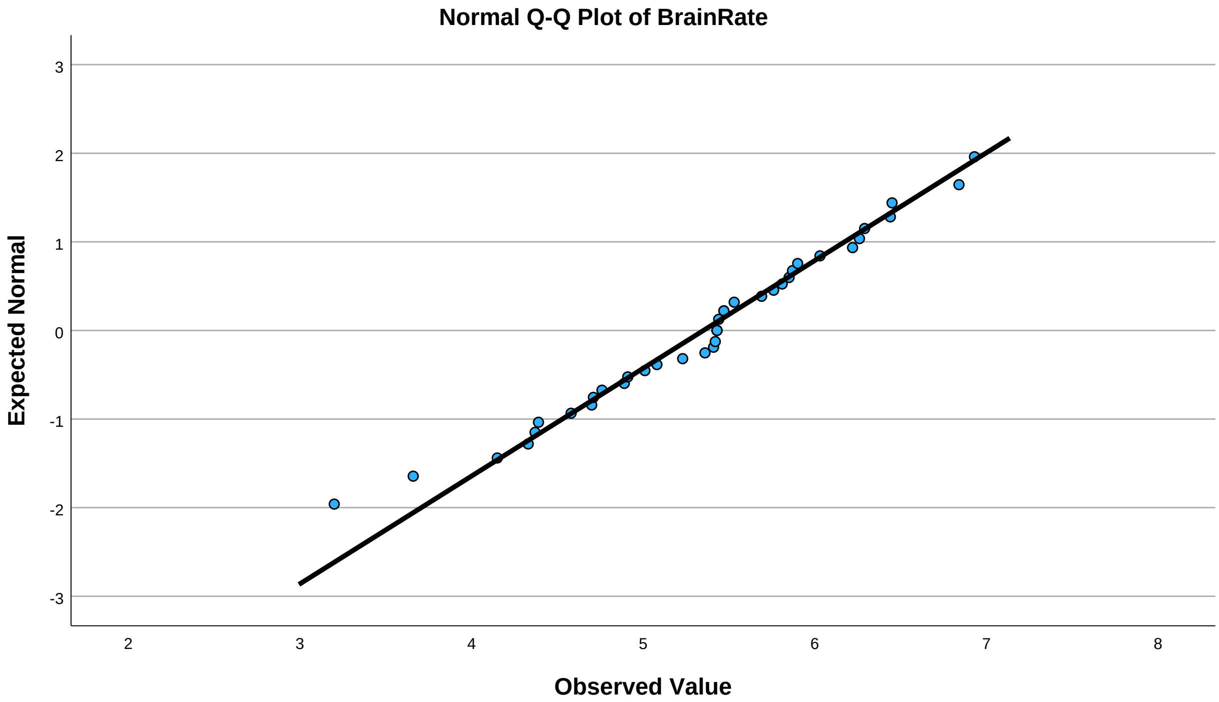
Normal Q-Q Plot of BrainRate
Expected Normal
Observed Value
3
2
1
0
-1
-2
-3
2
3
4
5
6
7
8

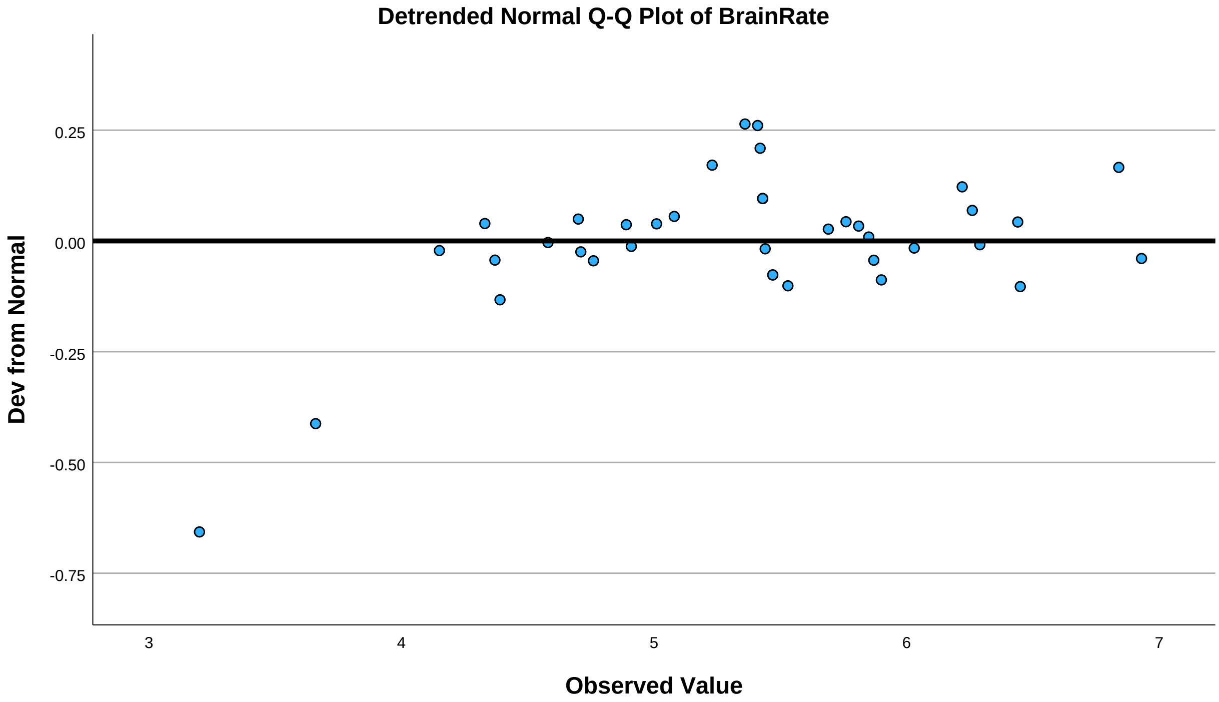
Detrended Normal Q-Q Plot of BrainRate
Dev from Normal
Observed Value
0.25
0.00
-0.25
-0.50
-0.75
3
4
5
6
7

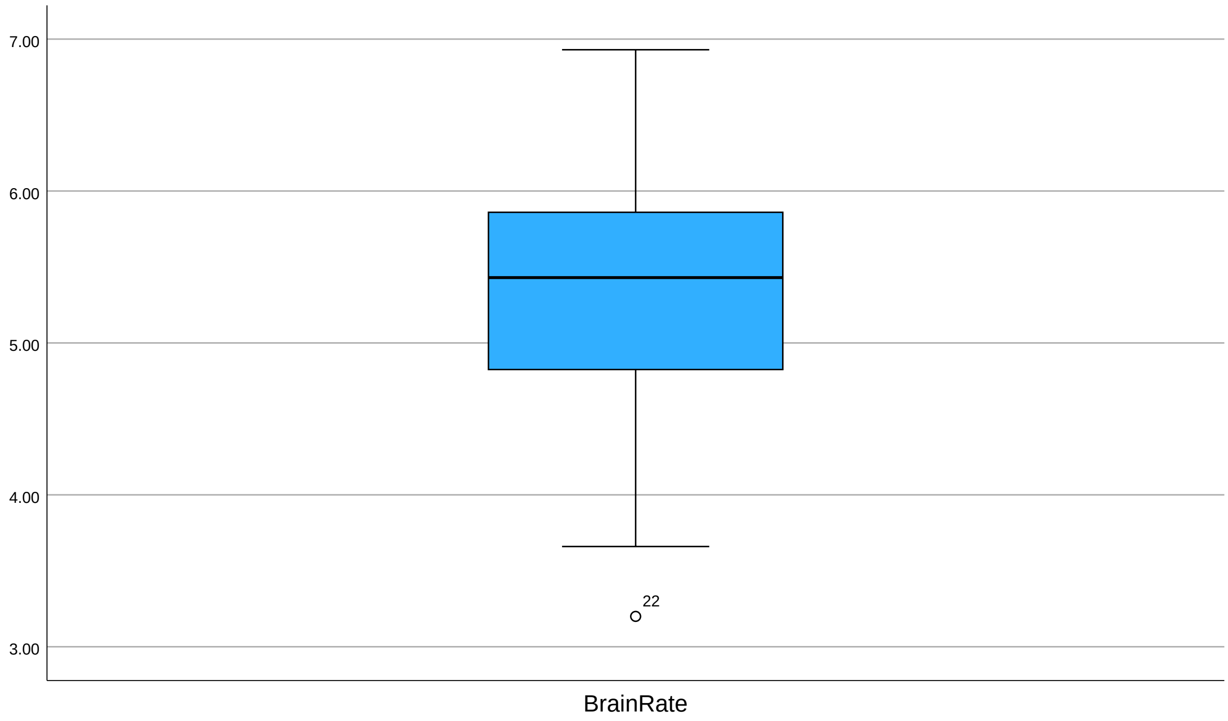

PermutationEntropy

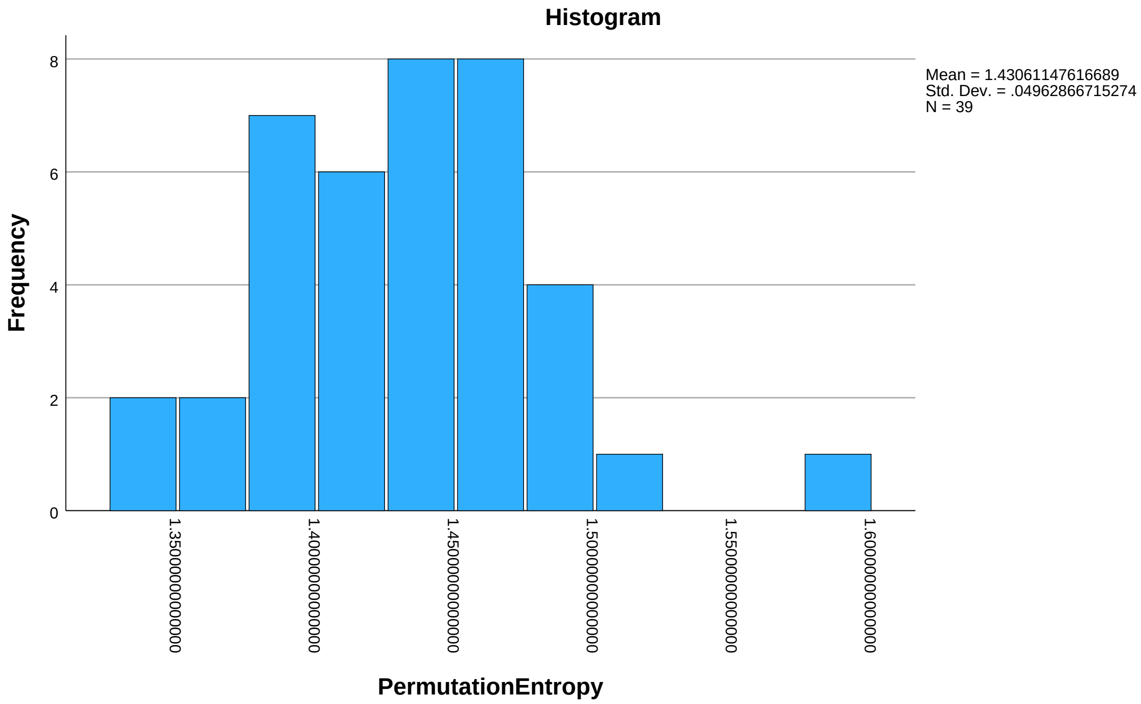


```
PermutationEntropy Stem-and-Leaf Plot

 Frequency    Stem &  Leaf
```

```
     2.00       13 .  24
      9.00       13 .  567778899
     14.00       14 .  00111133334444
     12.00       14 .  555566677889
     1.00      15 .  0
      1.00 Extremes    (>=1.58)

 Stem width:   .1000000
 Each leaf:       1 case(s)
```

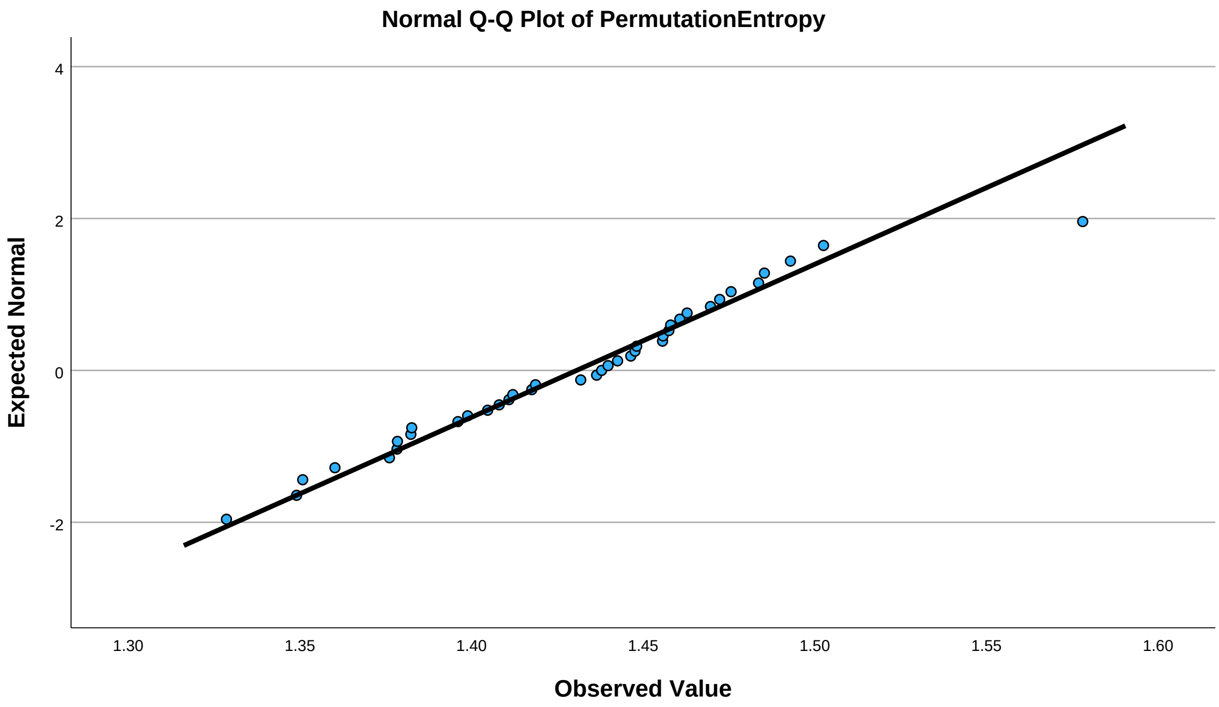

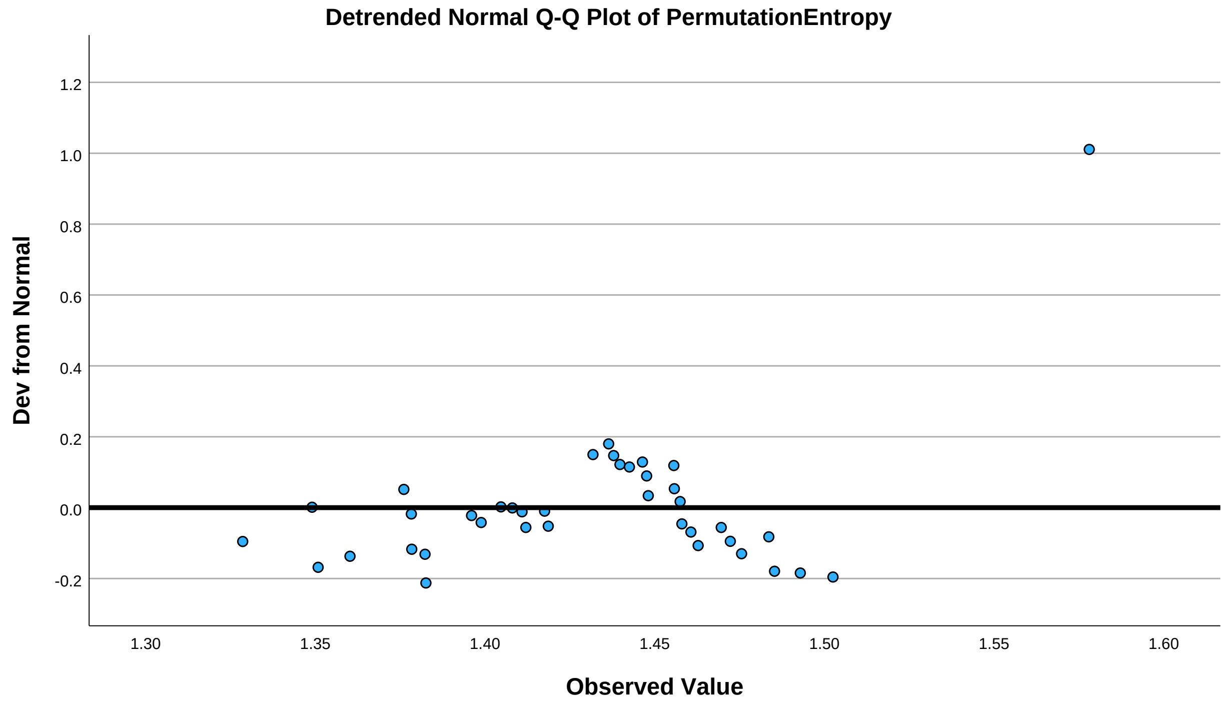

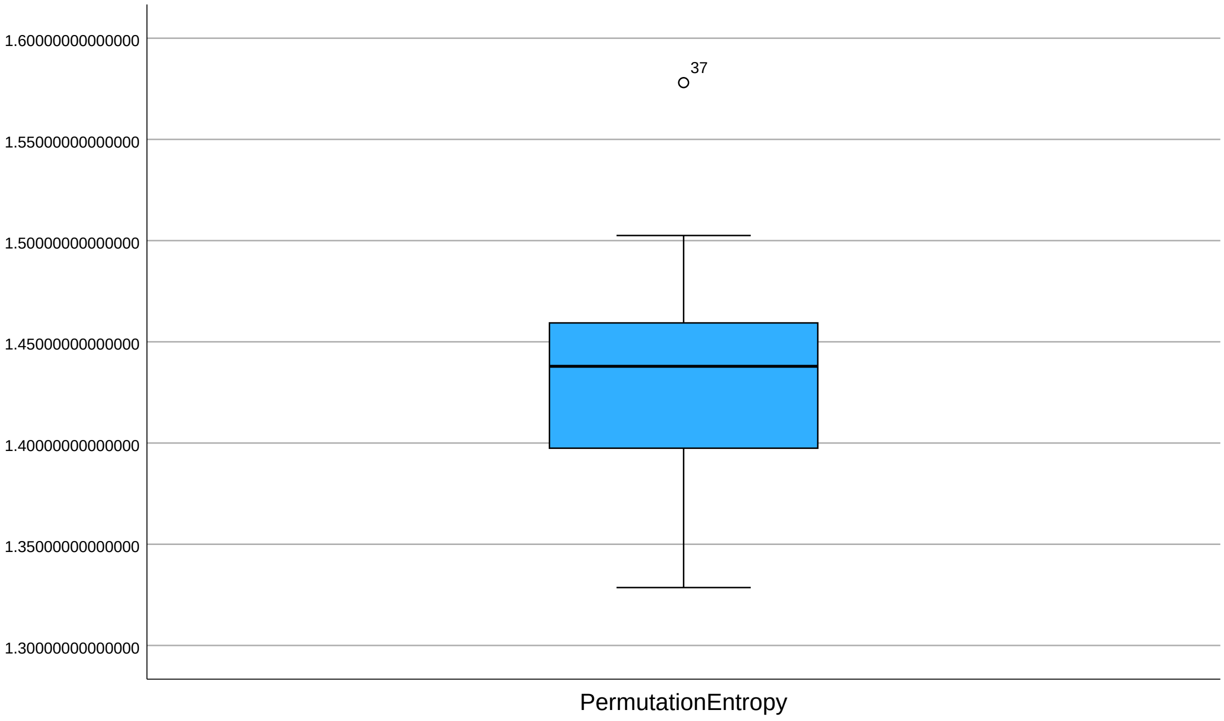

PermutationEntropy_01

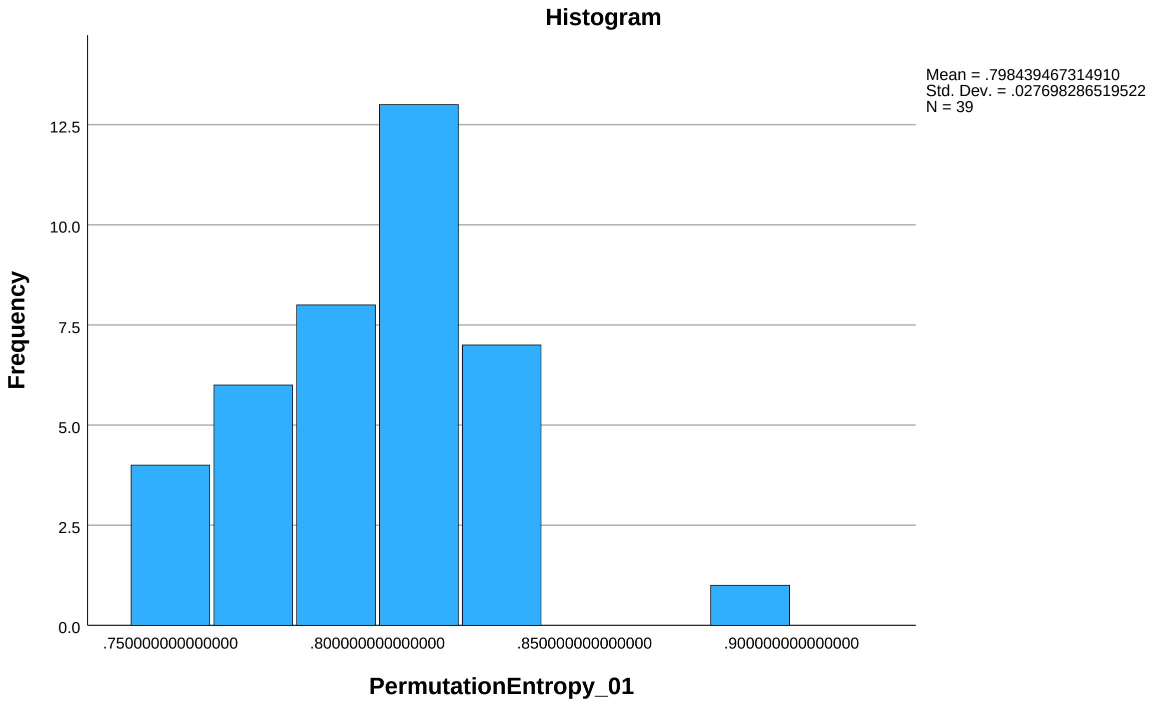


```
PermutationEntropy_01 Stem-and-Leaf Plot

 Frequency    Stem &  Leaf

     1.00      74 .  1
     3.00      75 .  239
     3.00      76 .  899
     3.00      77 .  119
     5.00      78 .  03578
     3.00      79 .  119
     7.00      80 .  1235778
     6.00      81 .  223356
     5.00      82 .  01388
     2.00      83 .  38
     1.00 Extremes    (>=.881)

 Stem width:   .0100000
 Each leaf:        1 case(s)
```

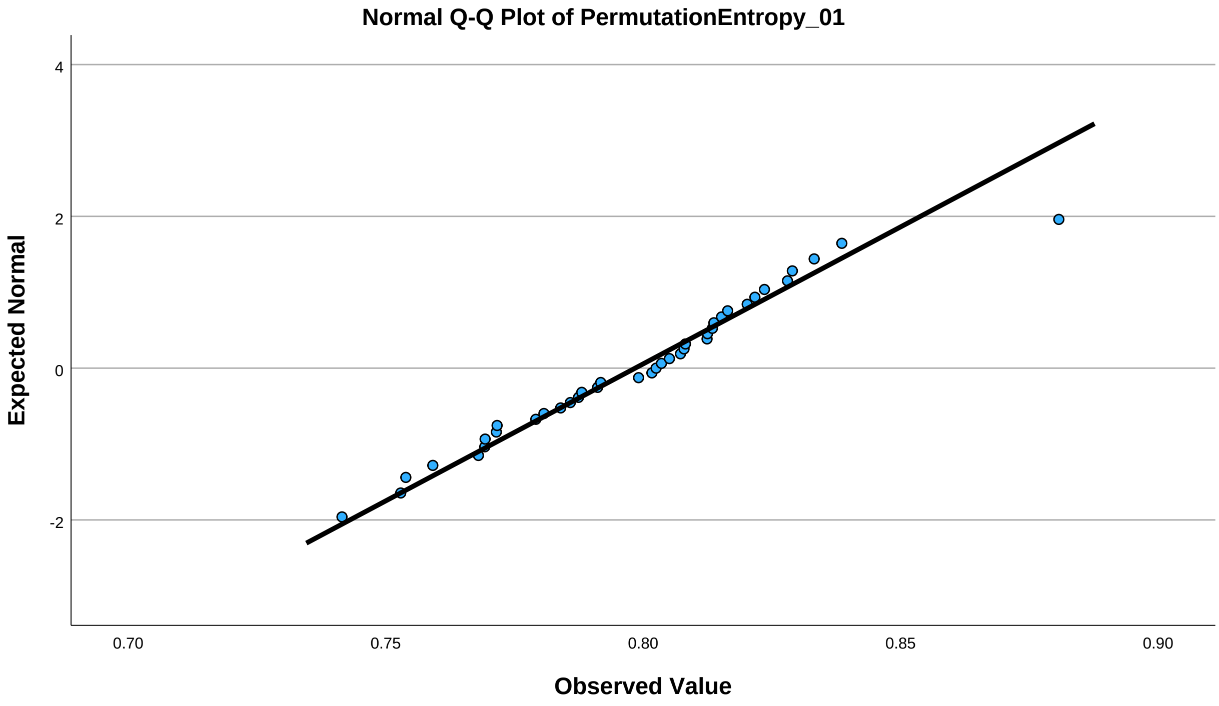
Normal Q-Q Plot of PermutationEntropy_01
Expected Normal
4
2
0
-2
0.70
0.75
0.80
0.85
0.90
Observed Value

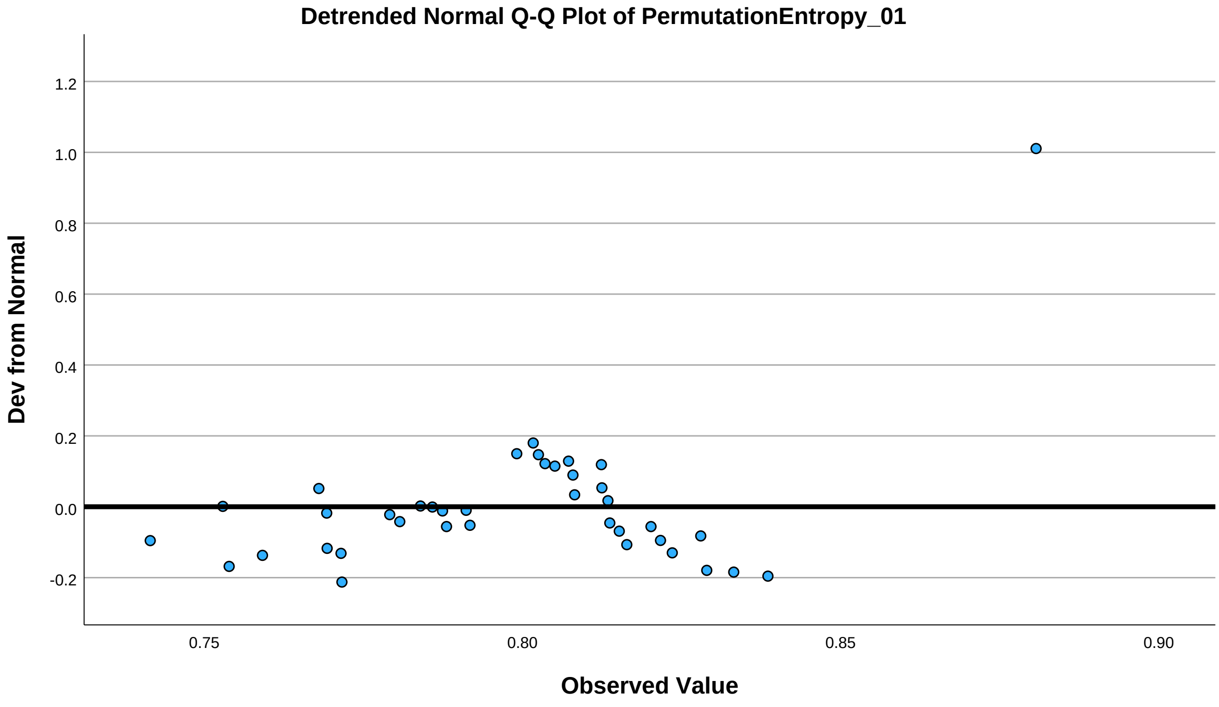
Detrended Normal Q-Q Plot of PermutationEntropy_01
Dev from Normal
1.2
1.0
0.8
0.6
0.4
0.2
0.0
-0.2
0.75
0.80
0.85
0.90
Observed Value

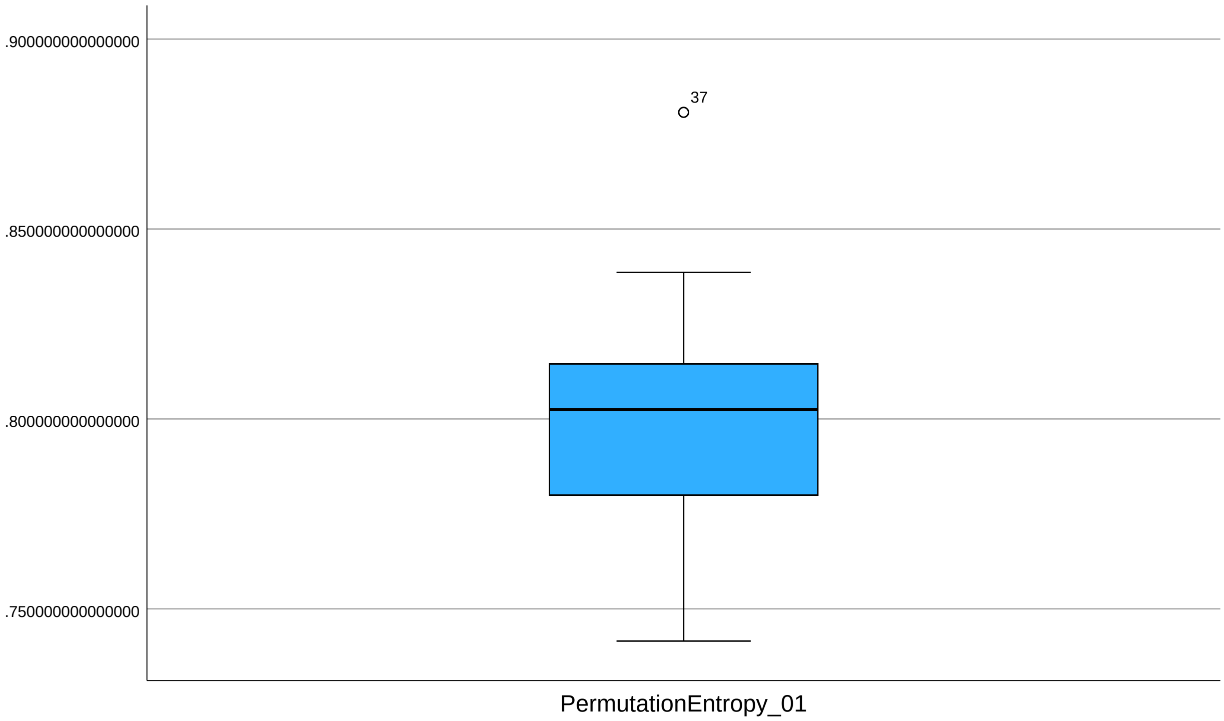

SampleEntropy

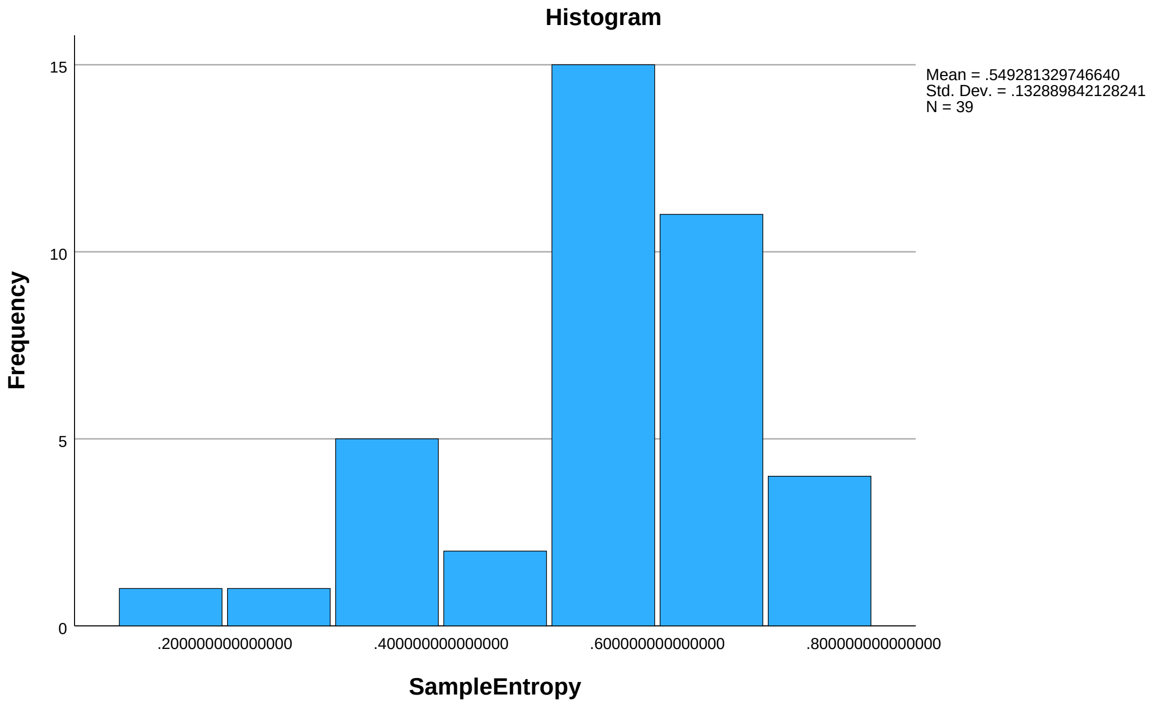


```
SampleEntropy Stem-and-Leaf Plot

 Frequency    Stem &  Leaf
```

```
 7.00 Extremes    (=<.39)
 2.00      4 . 12
  .00     4 .
 4.00      5 . 2444
11.00       5 . 55556788899
 8.00      6 . 01122234
 3.00      6 . 569
 4.00      7 . 0113

Stem width:  .1000000
Each leaf:       1 case(s)
```

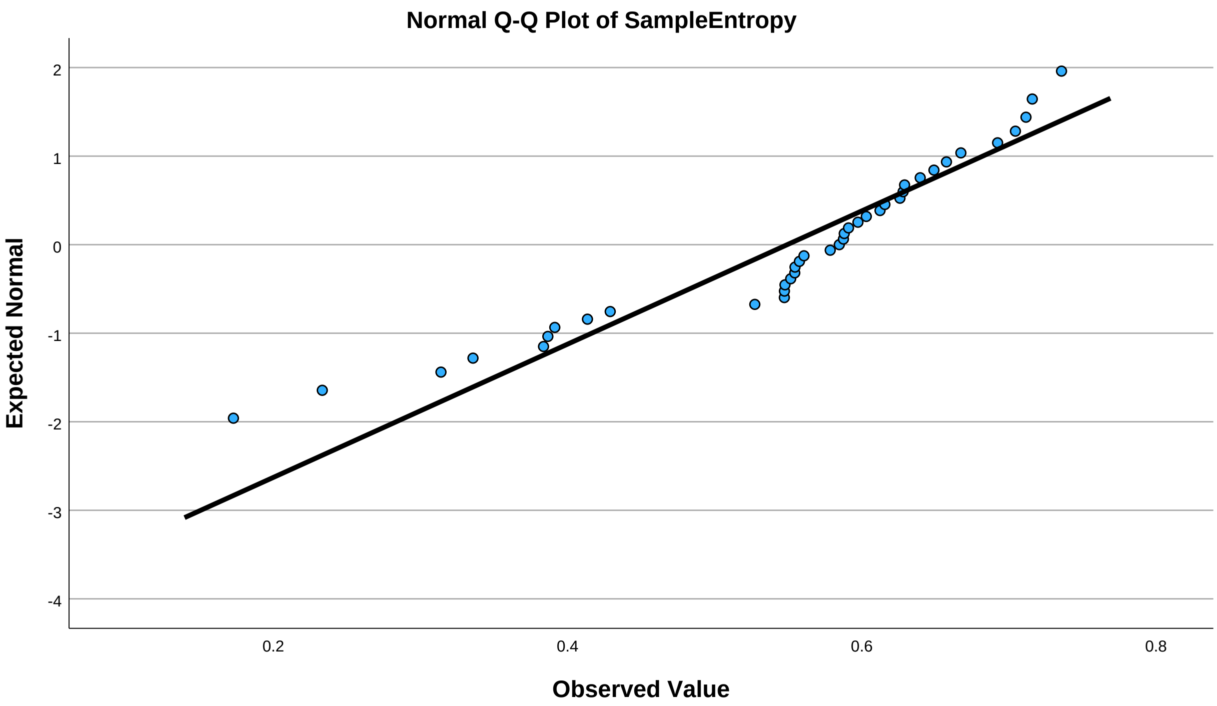

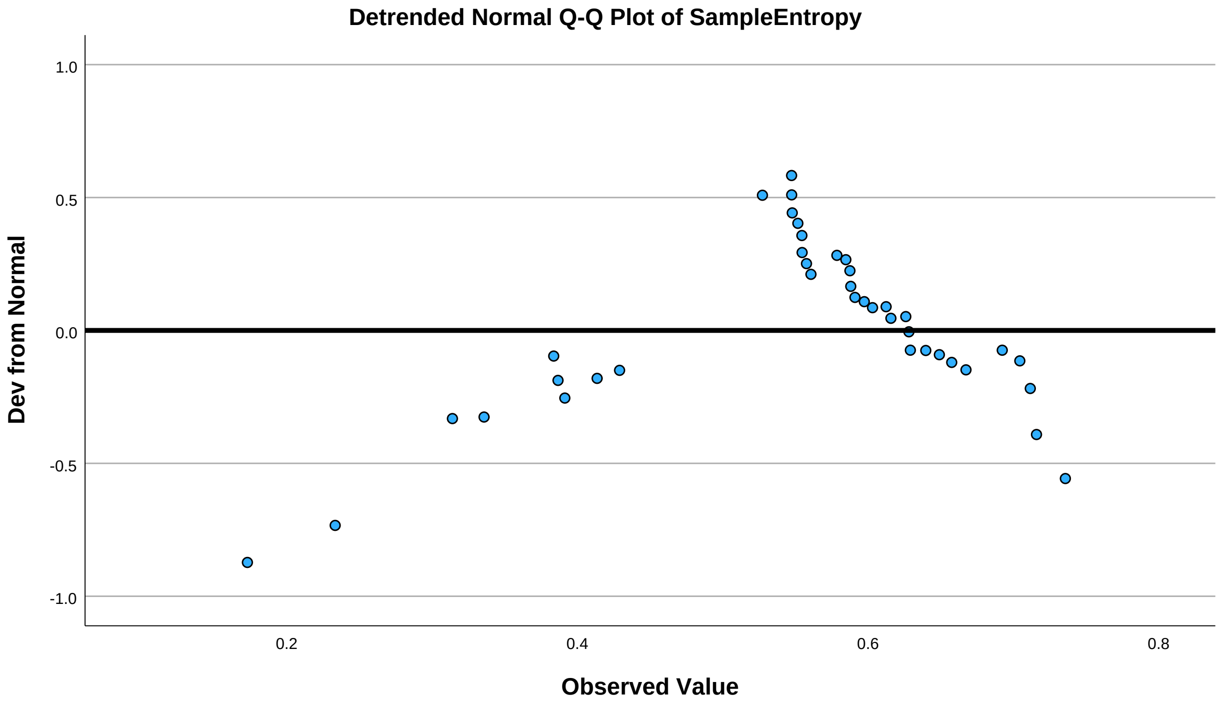

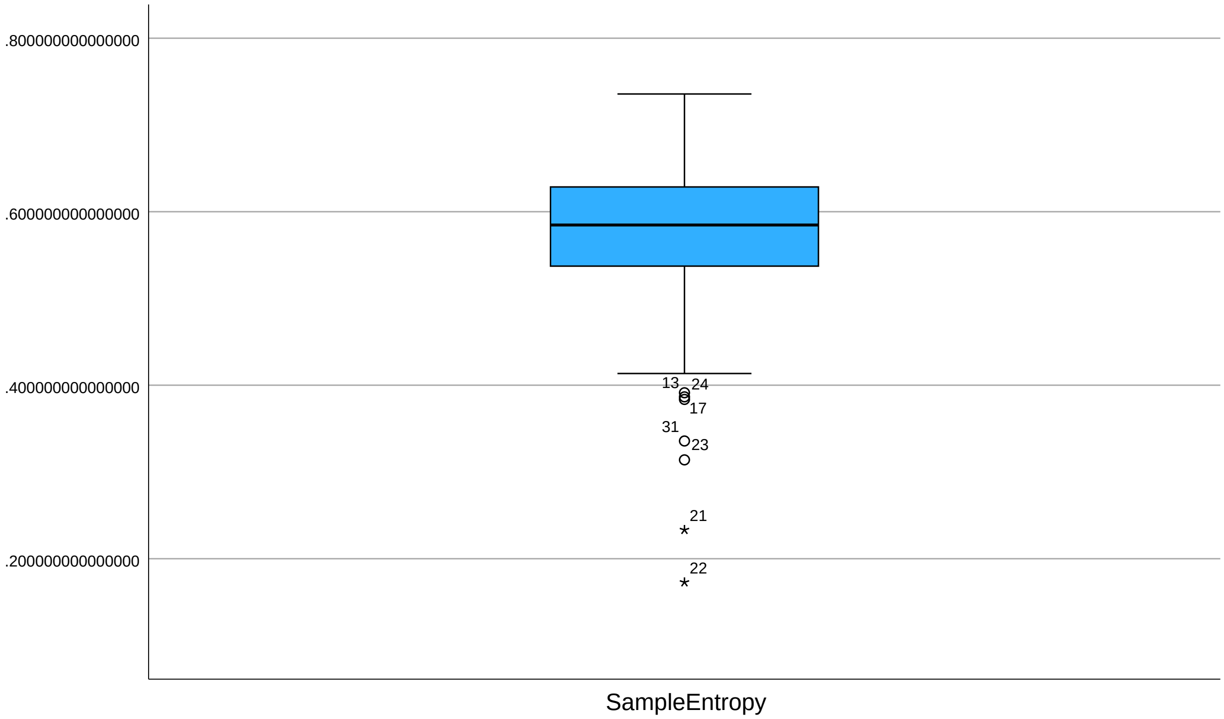

SpectralEntropy_bits

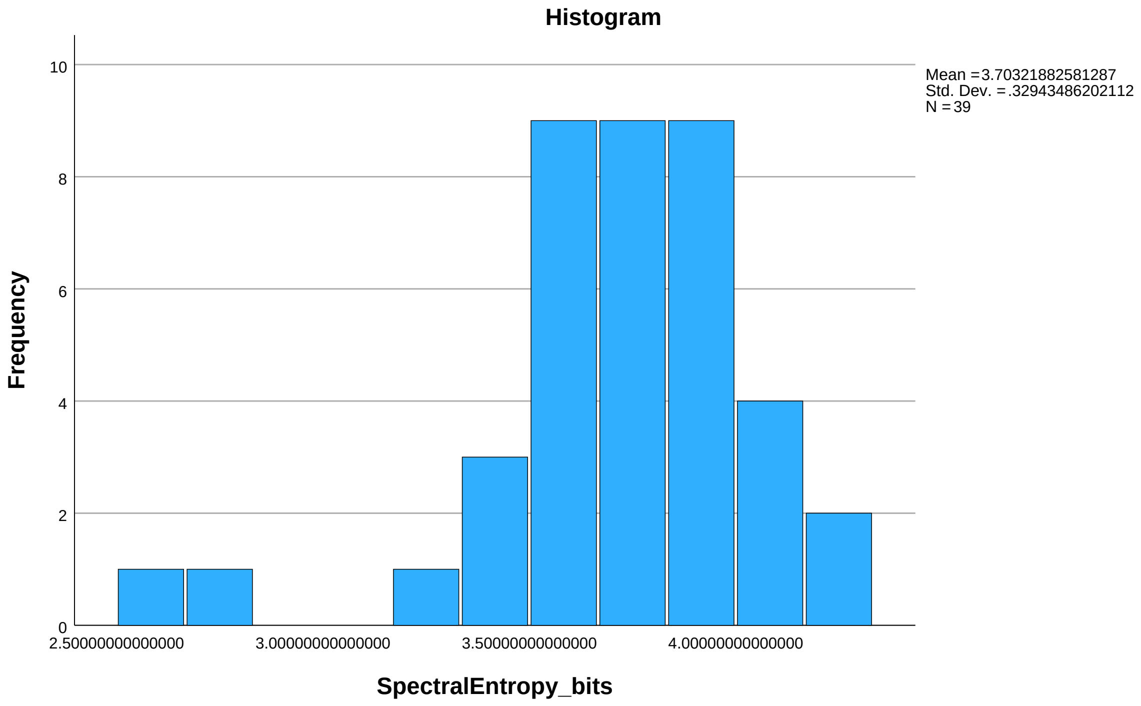


```
SpectralEntropy_bits Stem-and-Leaf Plot

 Frequency    Stem &  Leaf

     2.00 Extremes    (=<2.82)
     1.00       32 .  7
     1.00       33 .  9
     2.00       34 .  48
     9.00       35 .  112467799
     1.00       36 .  6
     5.00       37 .  17889
     9.00       38 .  013344789
     3.00       39 .  025
     4.00       40 .  0357
     2.00       41 .  89

 Stem width:   .1000000
 Each leaf:        1 case(s)
```

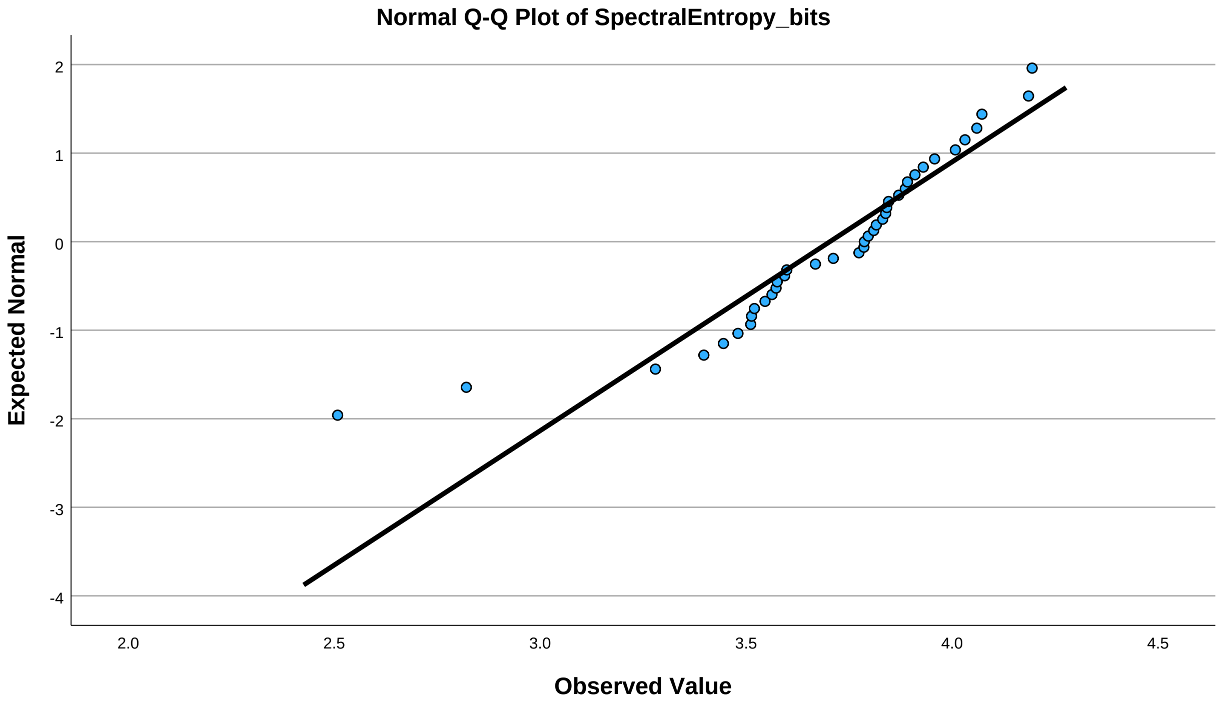
Normal Q-Q Plot of SpectralEntropy_bits
Expected Normal
Observed Value
2
1
0
-1
-2
-3
-4
2.0
2.5
3.0
3.5
4.0
4.5

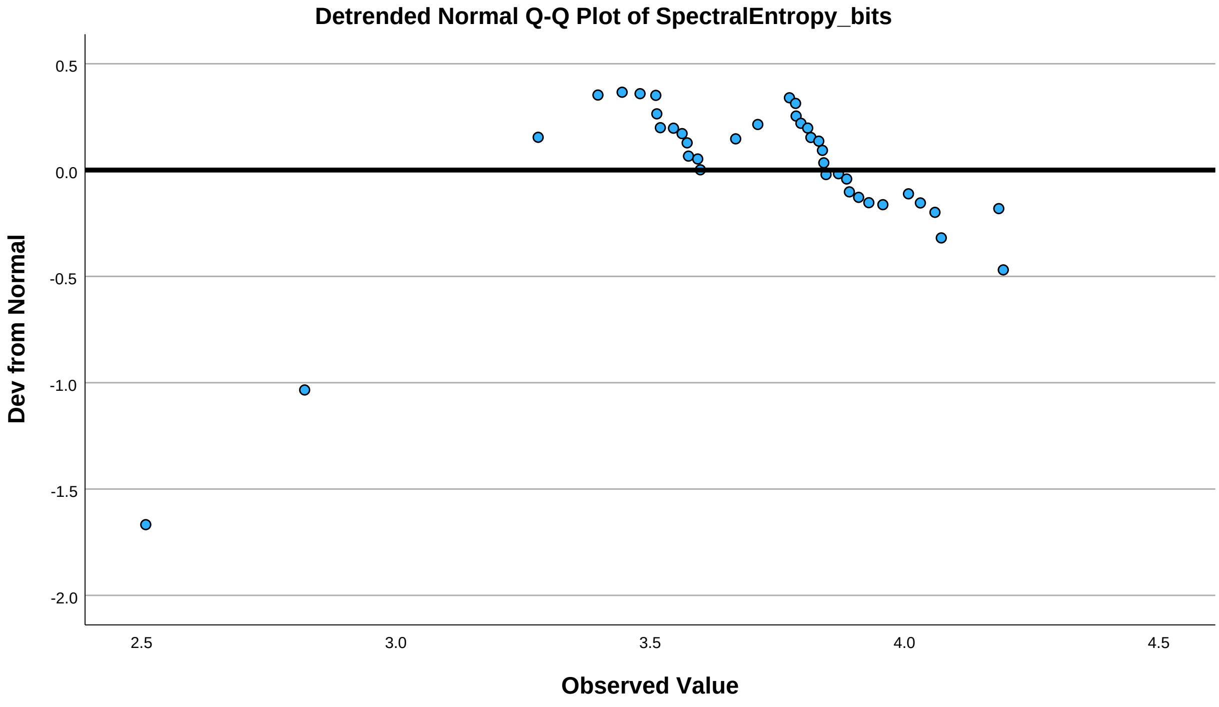
Detrended Normal Q-Q Plot of SpectralEntropy_bits
Dev from Normal
Observed Value
0.5
0.0
-0.5
-1.0
-1.5
-2.0
2.5
3.0
3.5
4.0
4.5

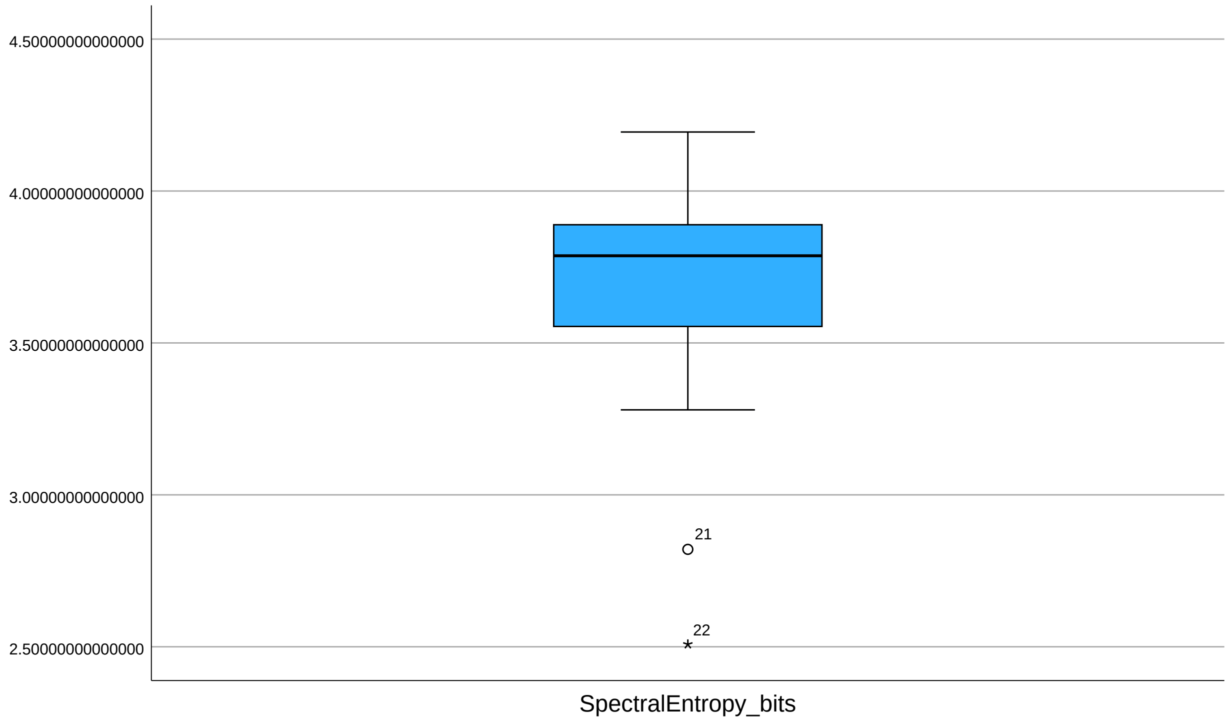

SpectralEntropy_01

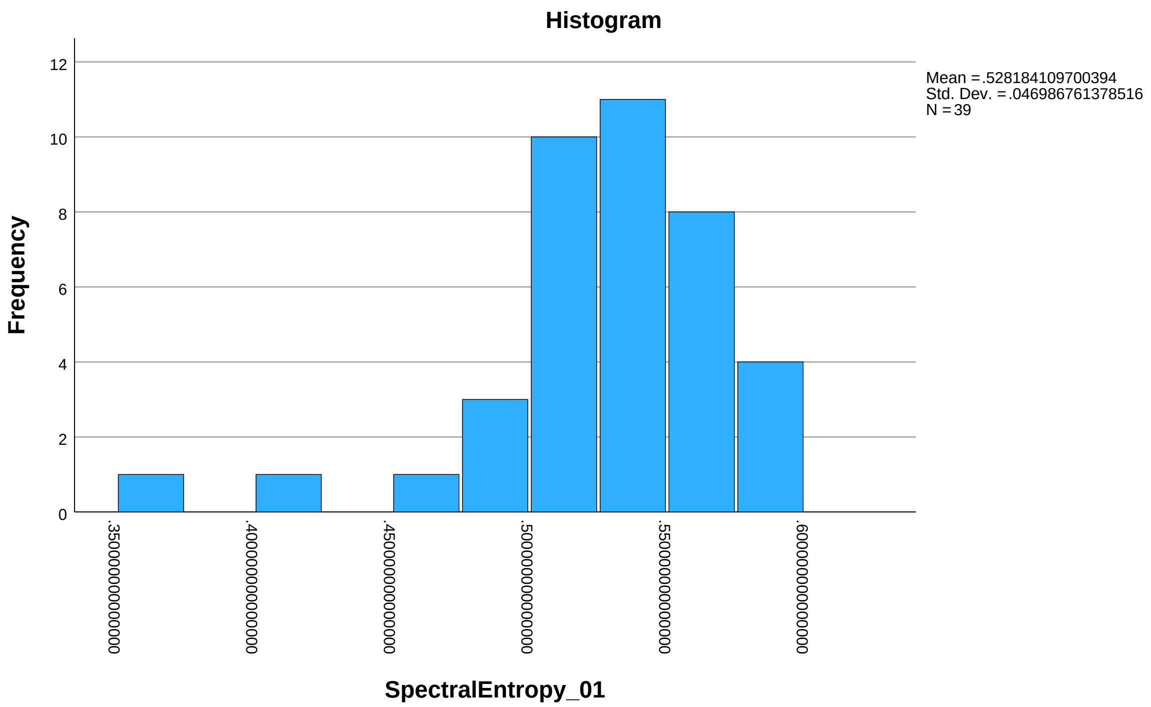


```
SpectralEntropy_01 Stem-and-Leaf Plot

 Frequency    Stem &  Leaf
```

```
    2.00 Extremes    (=<.40)
   1.00      4 . 6
   3.00      4 . 899
   9.00      5 . 000000011
   4.00      5 . 2233
  12.00      5 . 444444445555
   5.00      5 . 66777
   3.00      5 . 899

Stem width:   .1000000
Each leaf:       1 case(s)
```

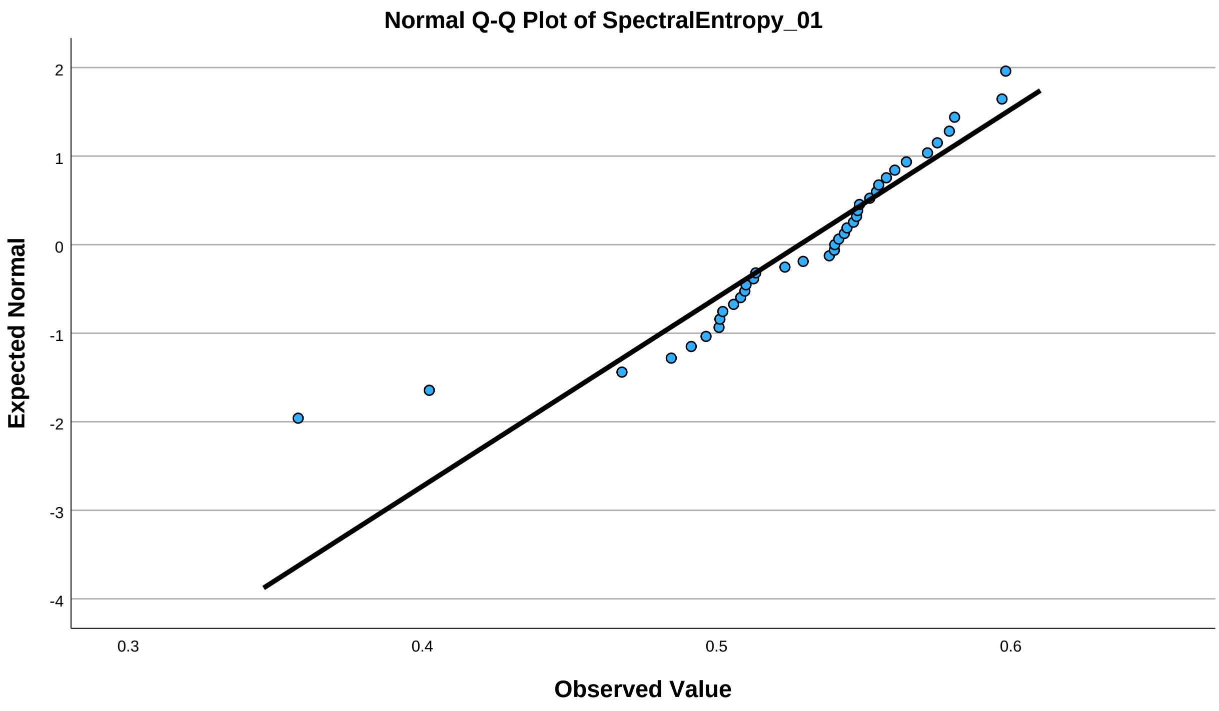

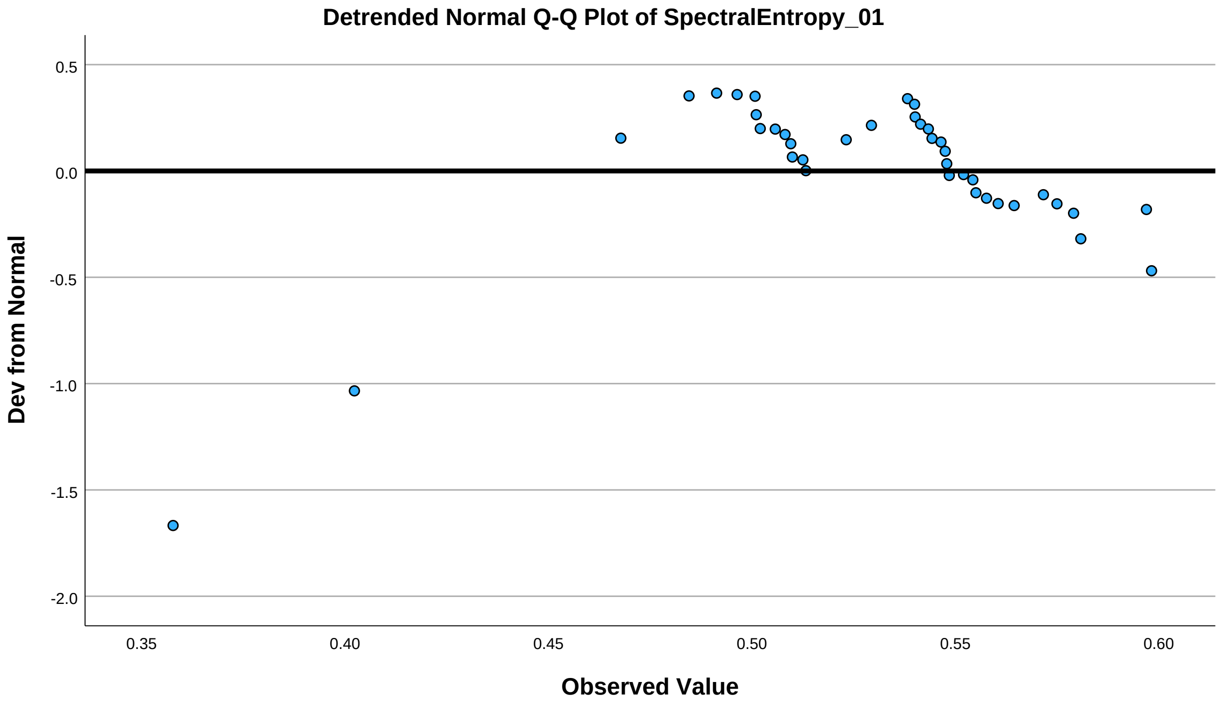

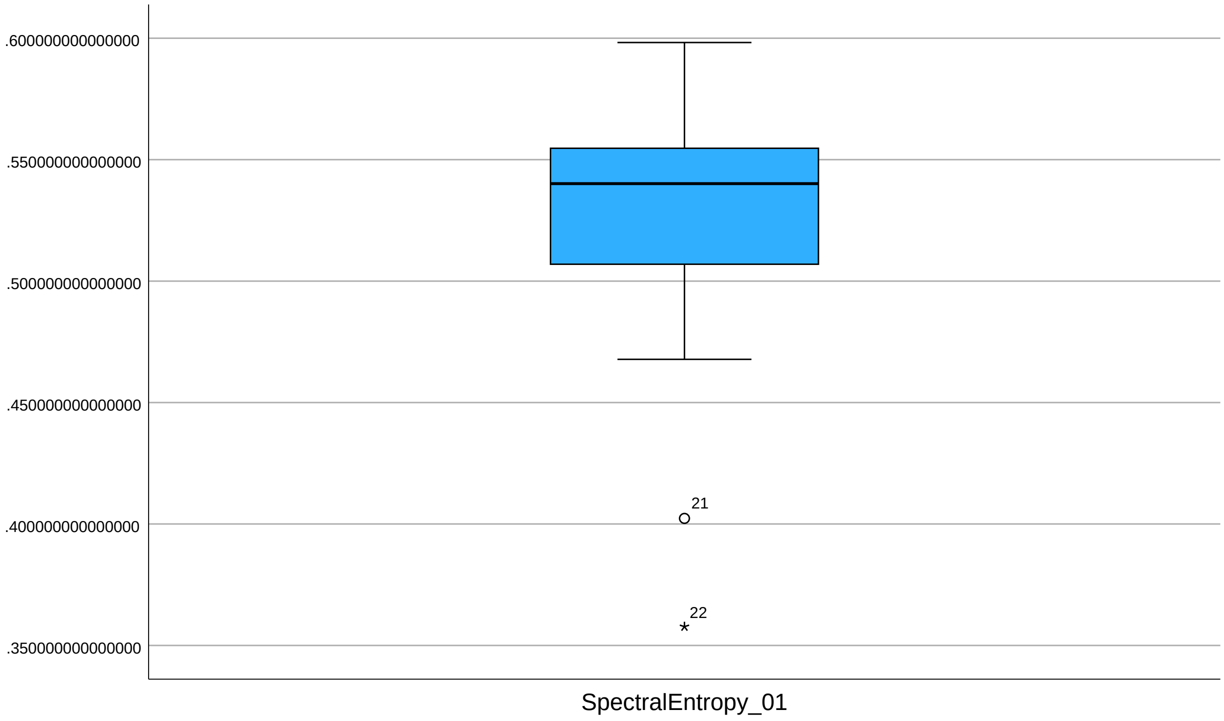

HiguchiFractalDimension

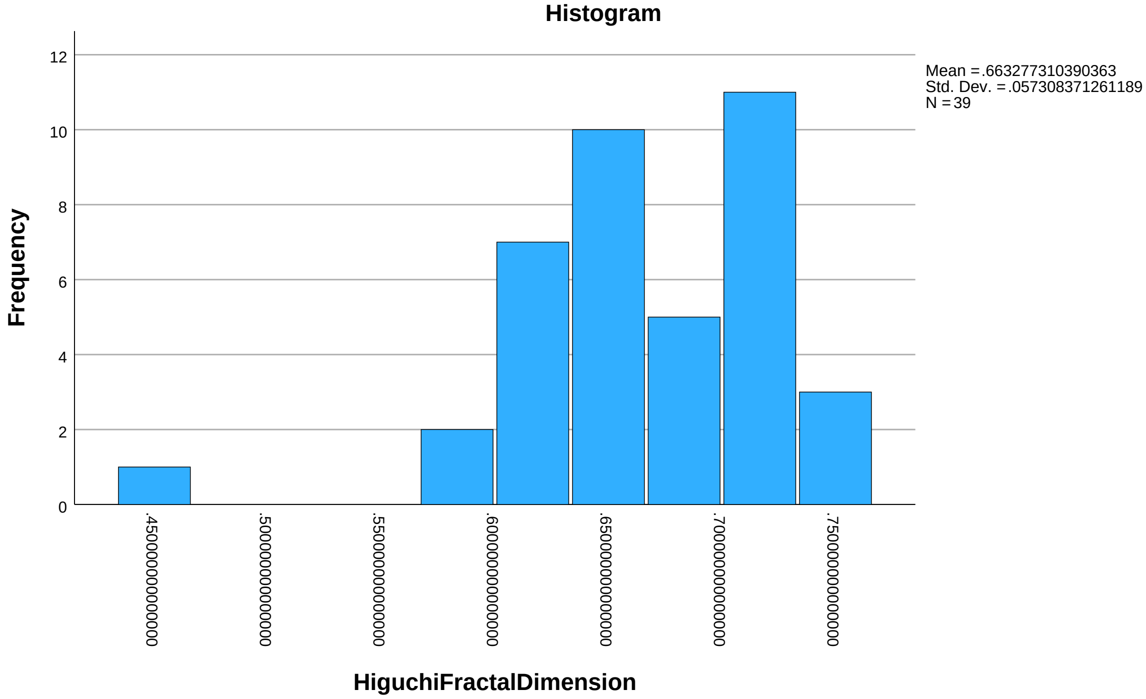


```
HiguchiFractalDimension Stem-and-Leaf Plot

 Frequency    Stem &  Leaf

     1.00 Extremes    (=<.45)
     2.00        5 .  69
    15.00        6 .  011122233444444
     7.00        6 .  6678888
    13.00        7 .  0000011112234
     1.00        7 .  5

 Stem width:   .1000000
 Each leaf:        1 case(s)
```

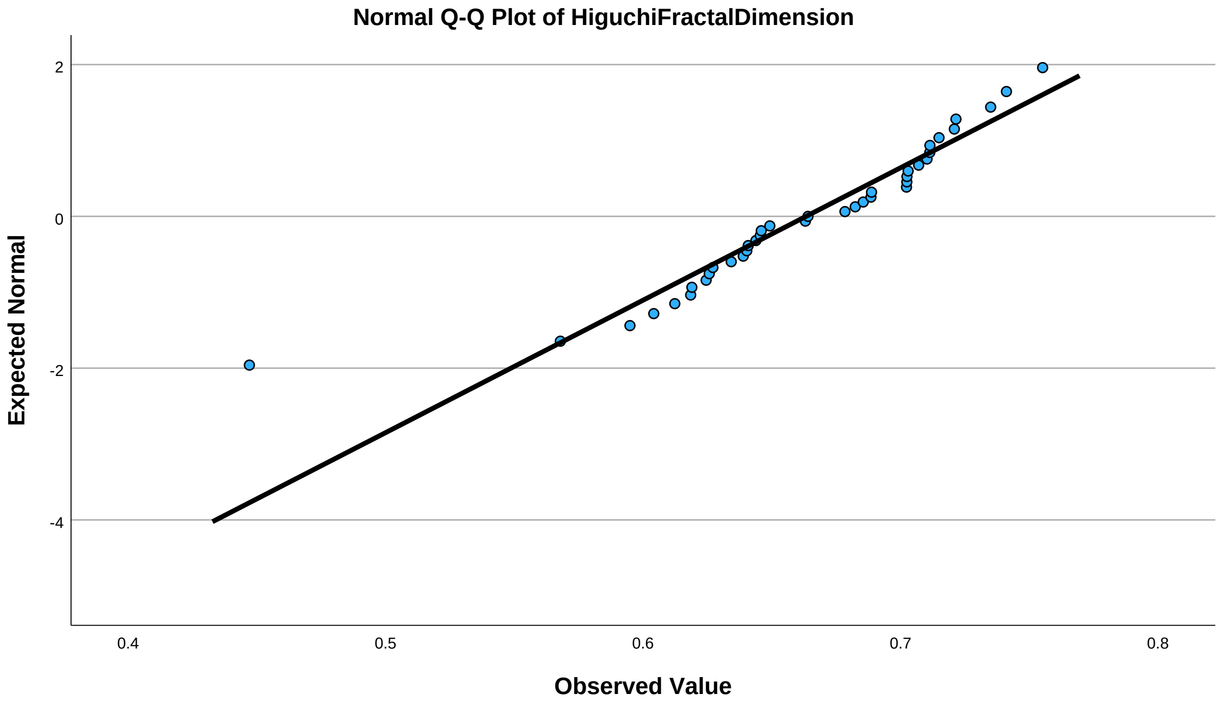
Normal Q-Q Plot of HiguchiFractalDimension
Expected Normal
Observed Value
2
0
-2
-4
0.4
0.5
0.6
0.7
0.8

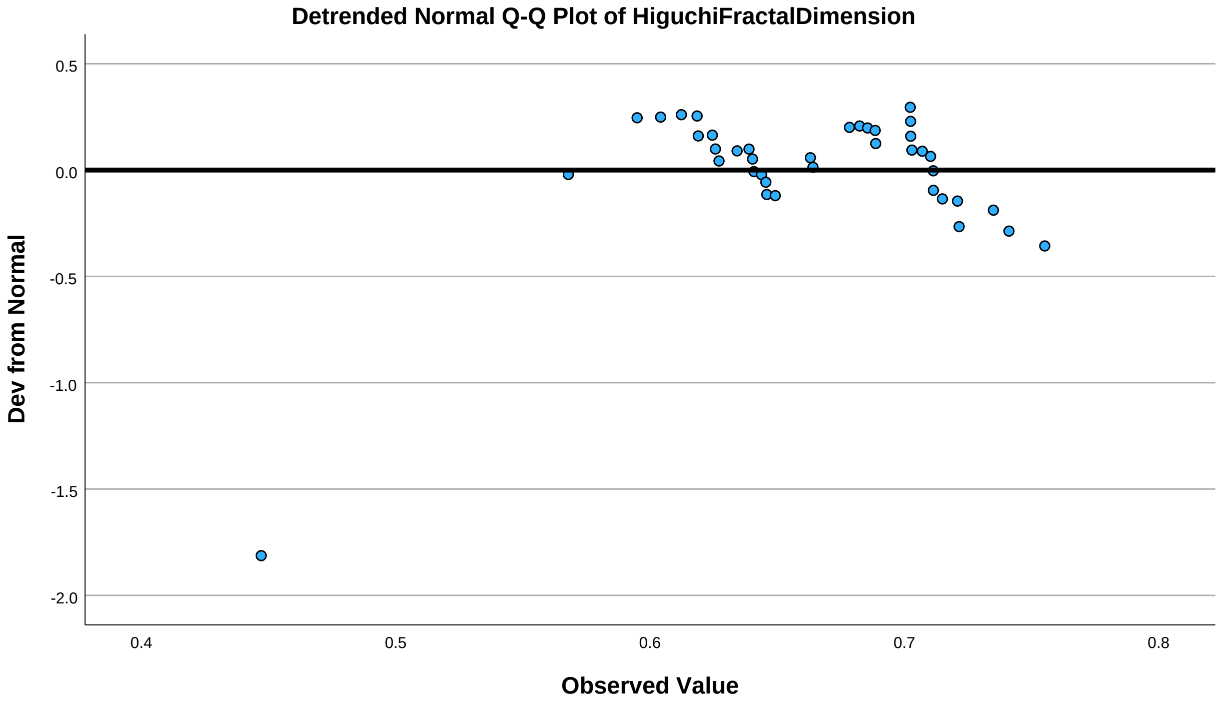
Detrended Normal Q-Q Plot of HiguchiFractalDimension
Dev from Normal
Observed Value
0.5
0.0
-0.5
-1.0
-1.5
-2.0
0.4
0.5
0.6
0.7
0.8

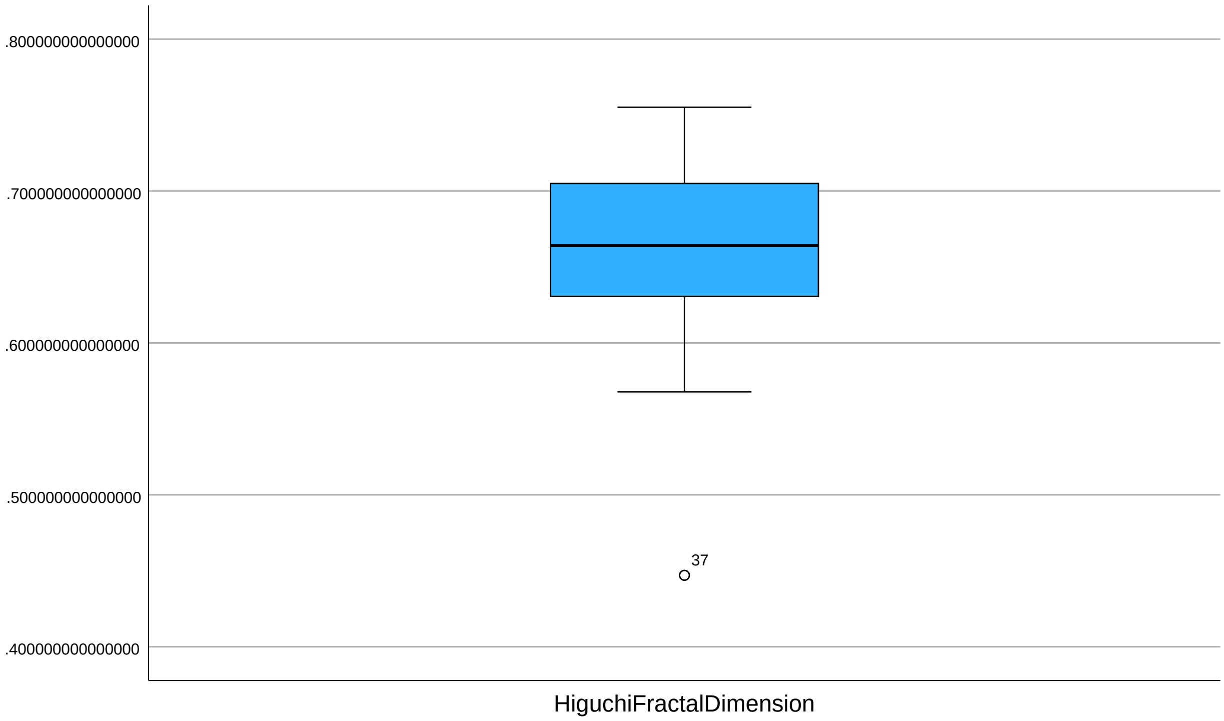

LempelZivComplexity

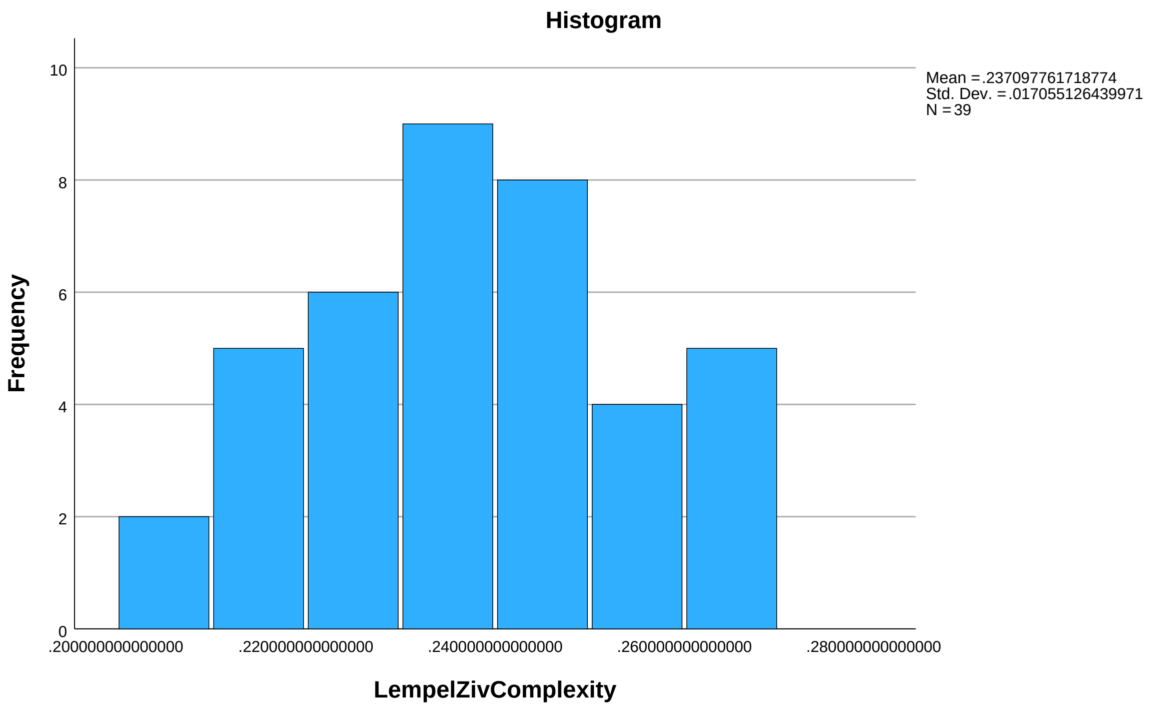


```
LempelZivComplexity Stem-and-Leaf Plot

 Frequency    Stem &  Leaf
```

```
  2.00      20 .  16
  5.00      21 .  02789
  6.00      22 .  223479
  9.00      23 .  114555689
  8.00      24 .  01136677
  4.00      25 .  0126
  5.00      26 .  22488

Stem width:   .0100000
Each leaf:        1 case(s)
```

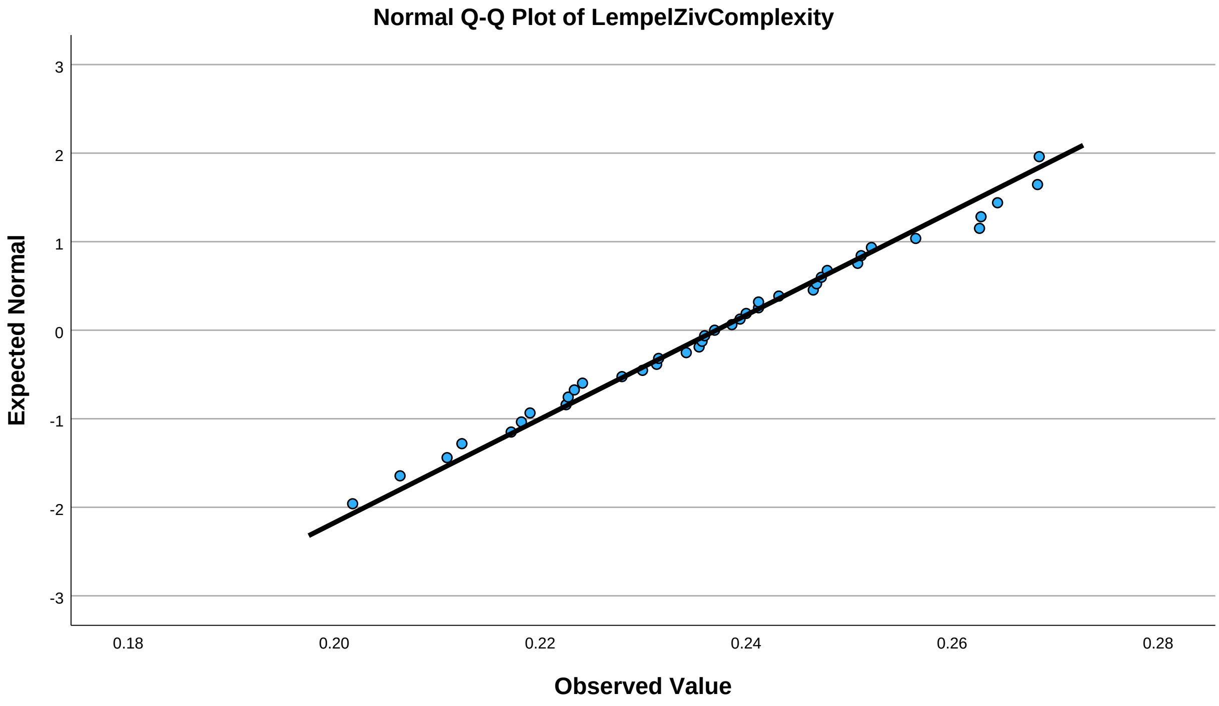

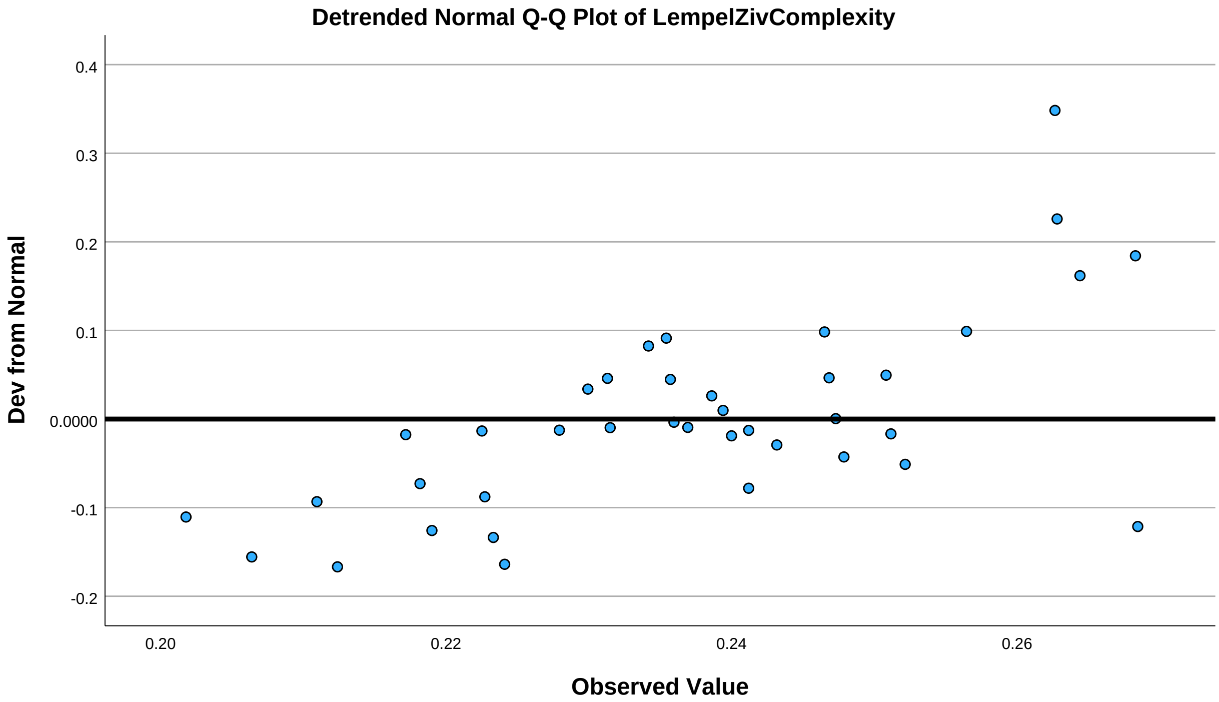

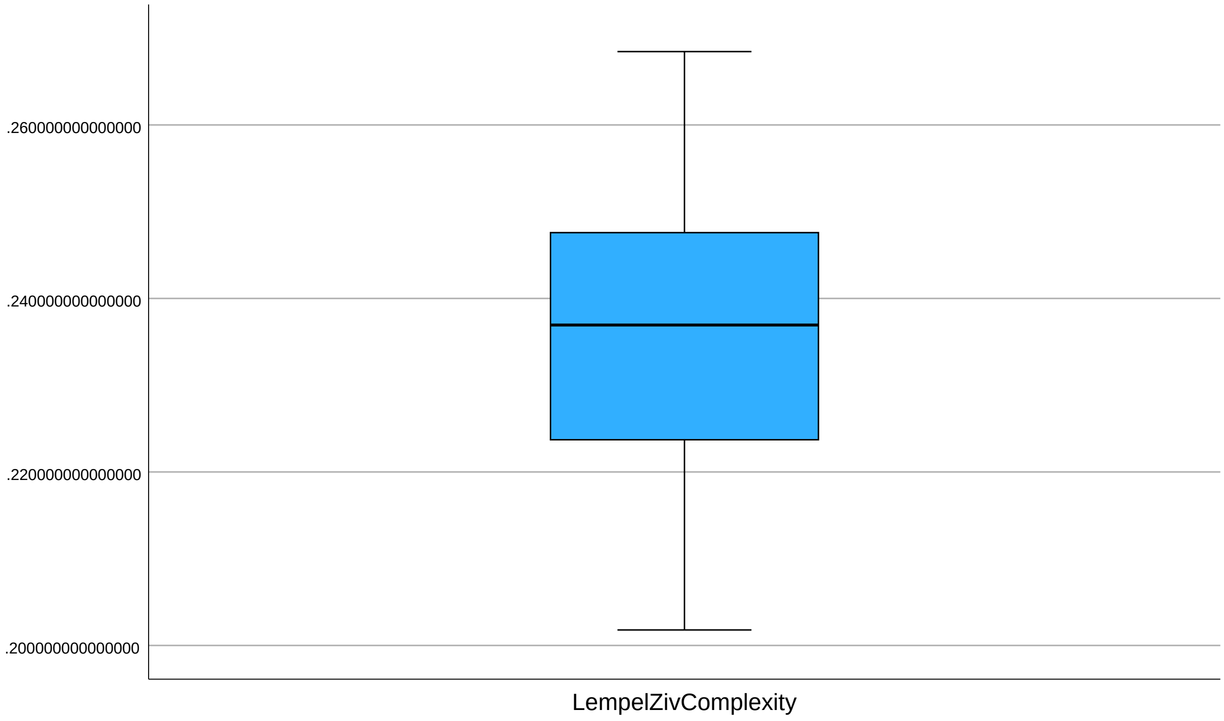

RenyiEntropy

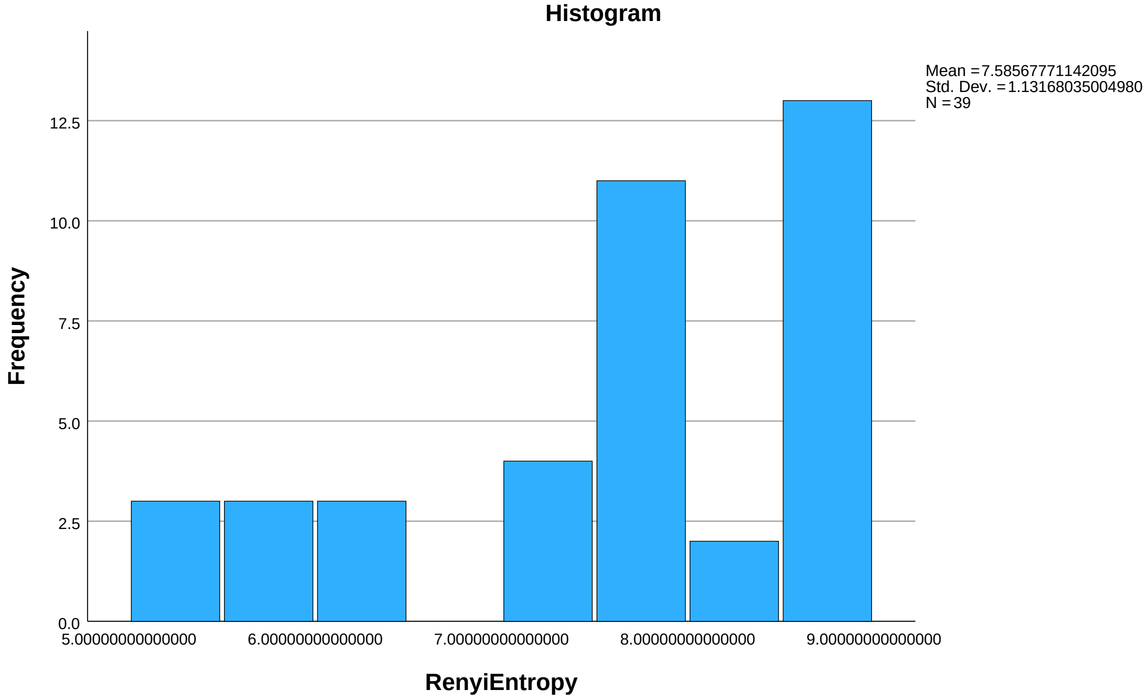


```
RenyiEntropy Stem-and-Leaf Plot

 Frequency    Stem &  Leaf

     3.00 Extremes    (=<5.4)
     3.00        5 .  779
     3.00        6 .  013
      .00        6 .
     4.00        7 .  2444
    11.00        7 .  56666678899
     2.00        8 .  24
    13.00        8 .  5555666667788

 Stem width:   1.000000
 Each leaf:        1 case(s)
```

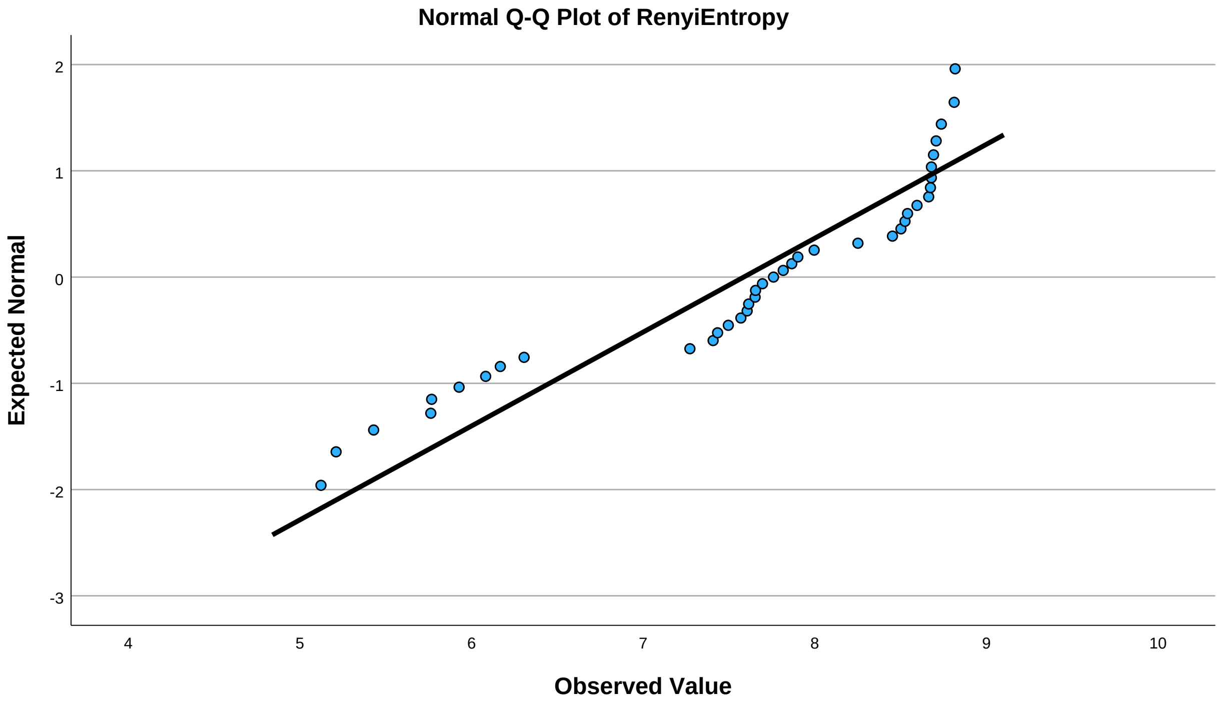

Normal Q-Q Plot of RenyiEntropy
Expected Normal
2
1
0
-1
-2
-3
4
5
6
7
8
9
10
Observed Value

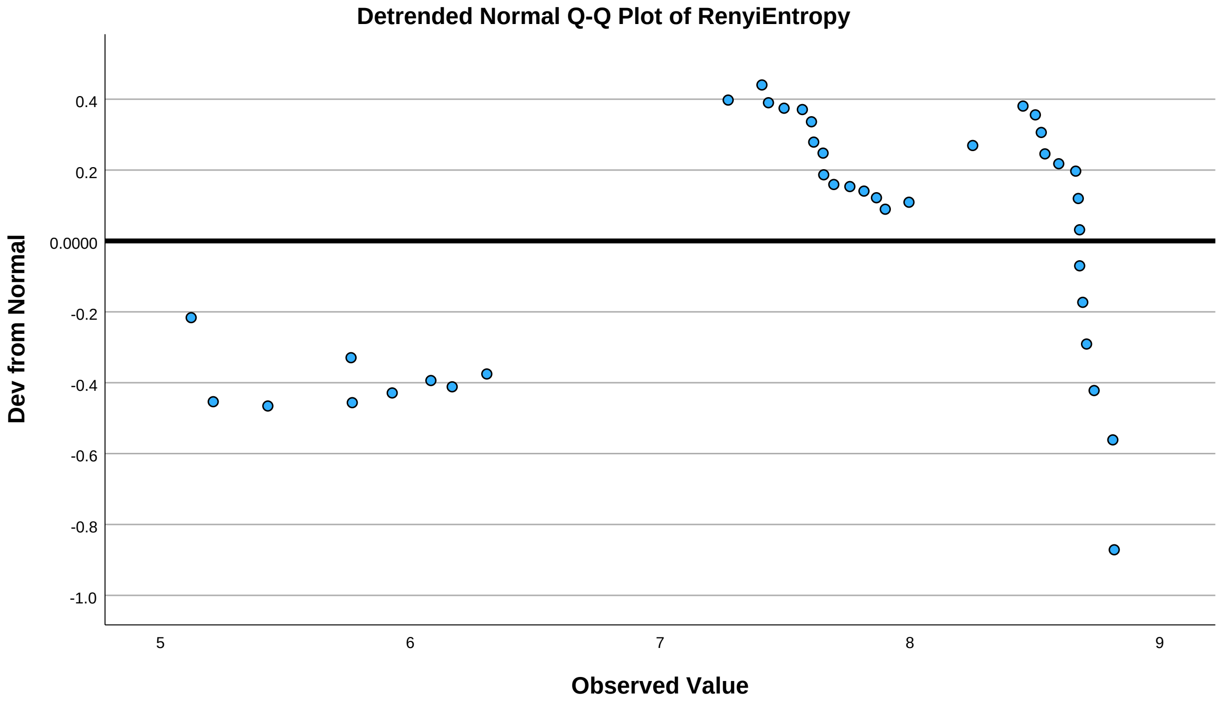

Detrended Normal Q-Q Plot of RenyiEntropy
Dev from Normal
0.4
0.2
0.0000
-0.2
-0.4
-0.6
-0.8
-1.0
5
6
7
8
9
Observed Value

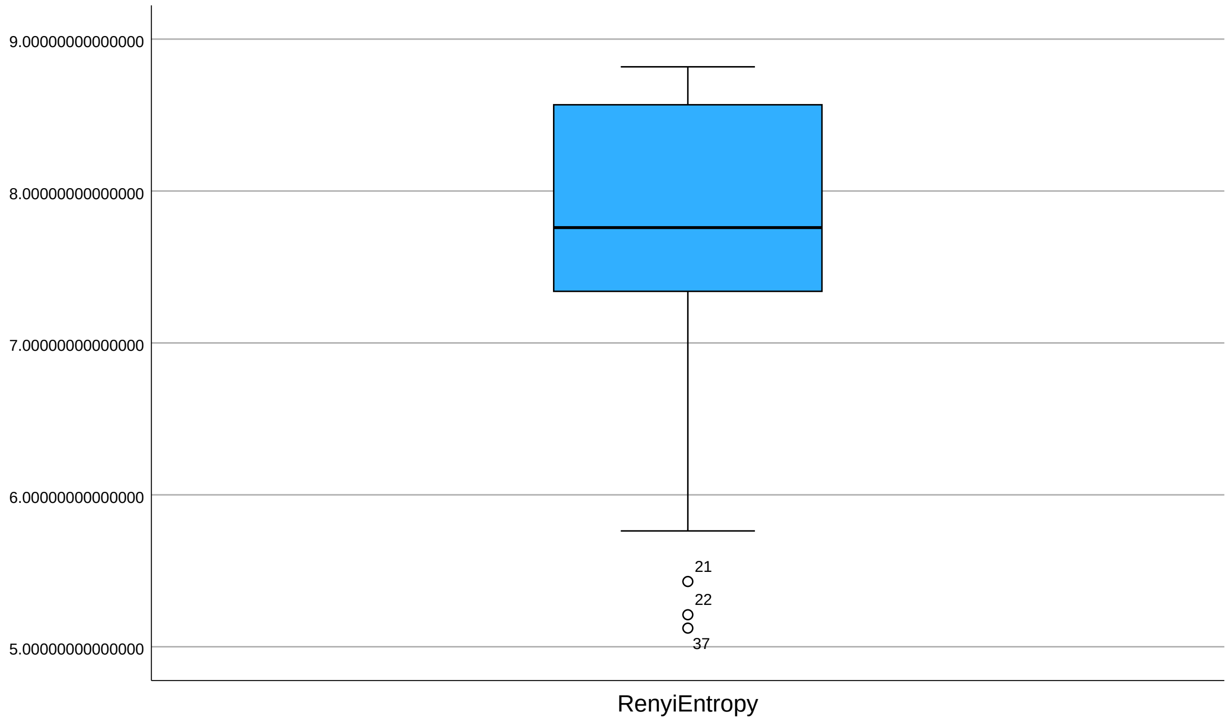

TsallisEntropy

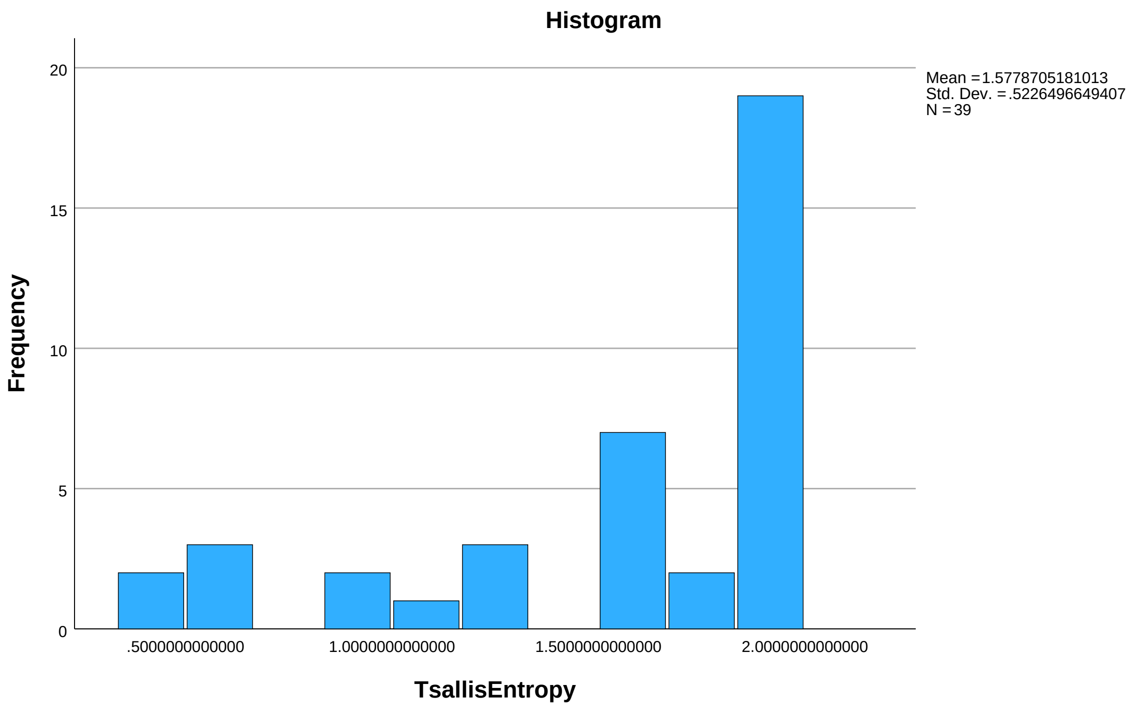

TsallisEntropy Stem-and-Leaf Plot

Frequency Stem & Leaf

```
 5.00      0 . 44555
  .00     0 .
 2.00      0 . 99
 3.00      1 . 011
 1.00      1 . 2
 3.00      1 . 555
 6.00      1 . 666677
19.00       1 . 8999999999999999999

Stem width:  1.000000
Each leaf:       1 case(s)
```

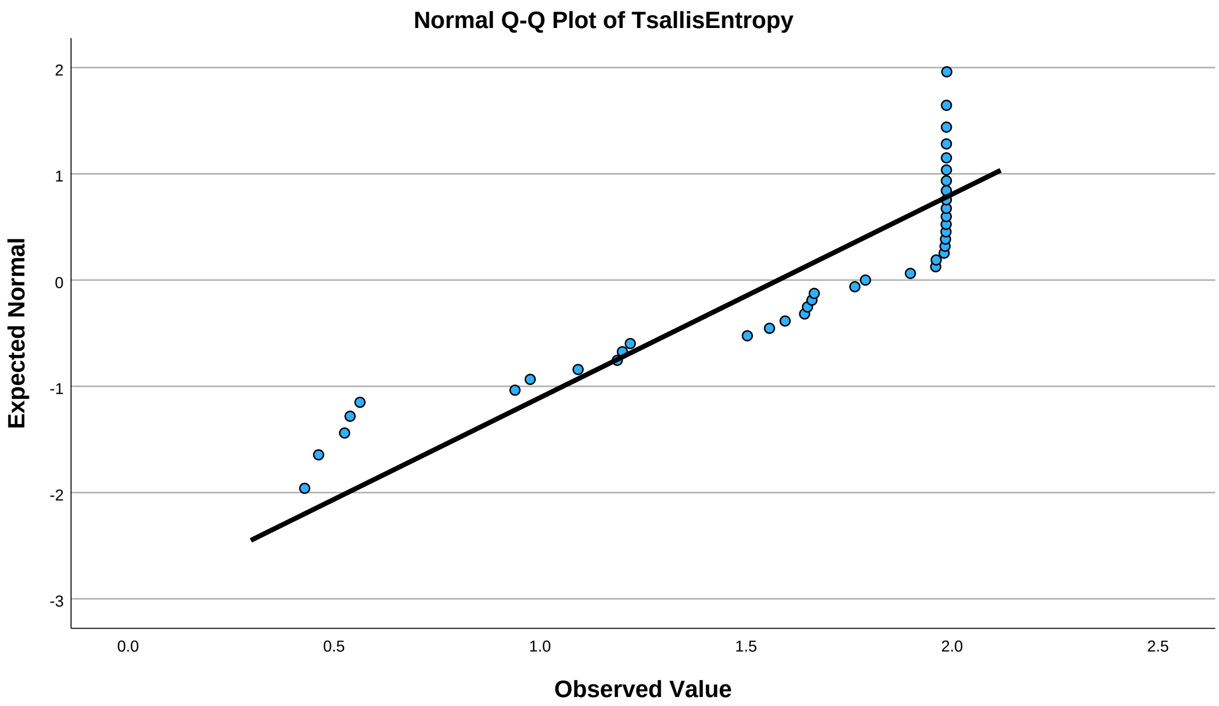

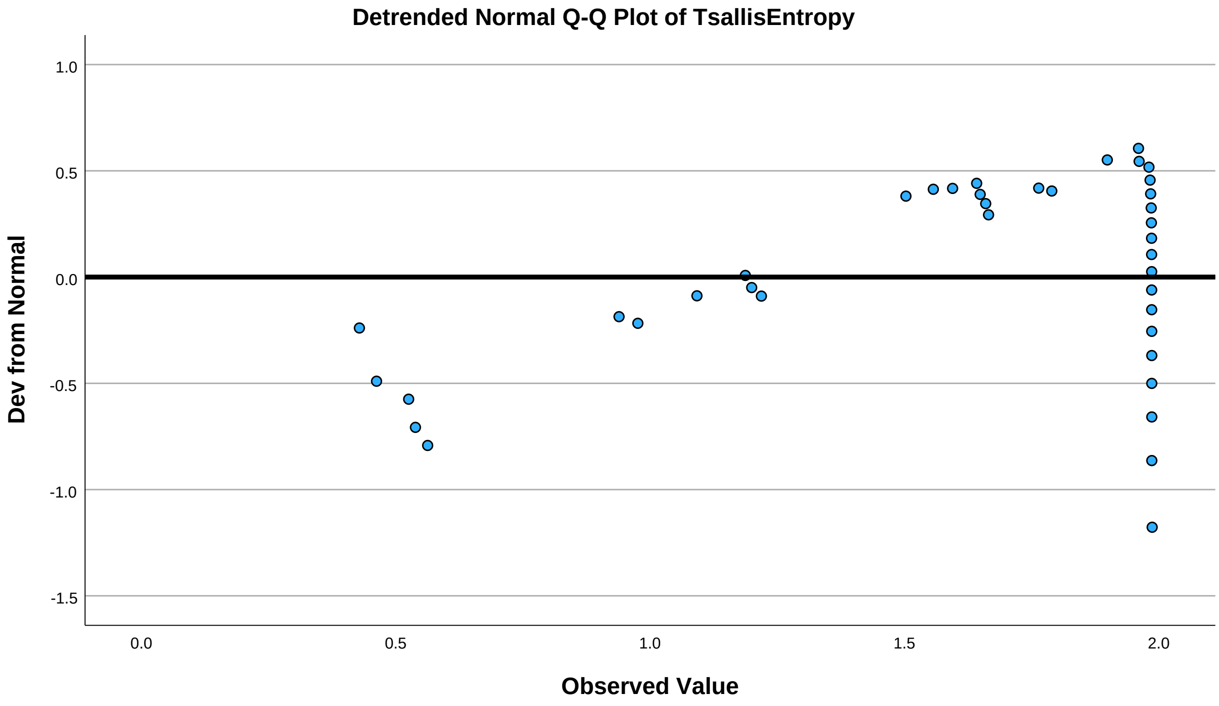
Detrended Normal Q-Q Plot of TsallisEntropy
1.0
0.5
0.0
-0.5
-1.0
-1.5
Dev from Normal
0.0
0.5
1.0
1.5
2.0
Observed Value

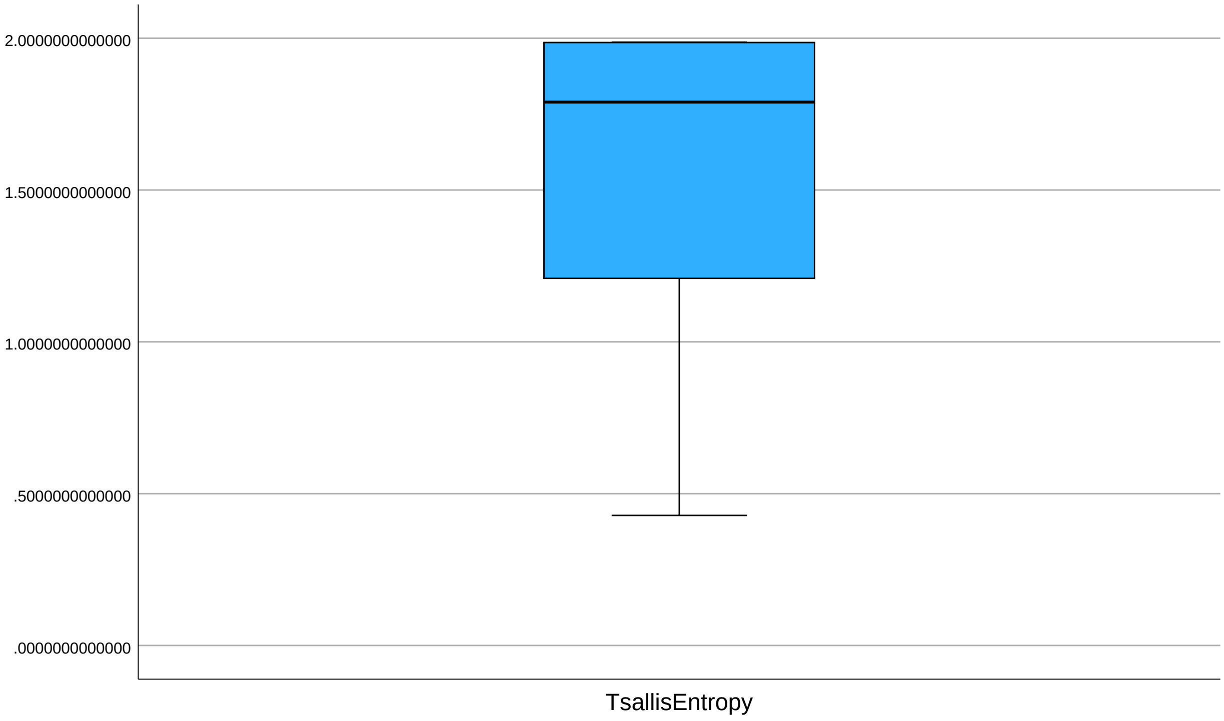
2.0000000000000
1.5000000000000
1.0000000000000
.5000000000000
.0000000000000
TsallisEntropy

PS_4_5

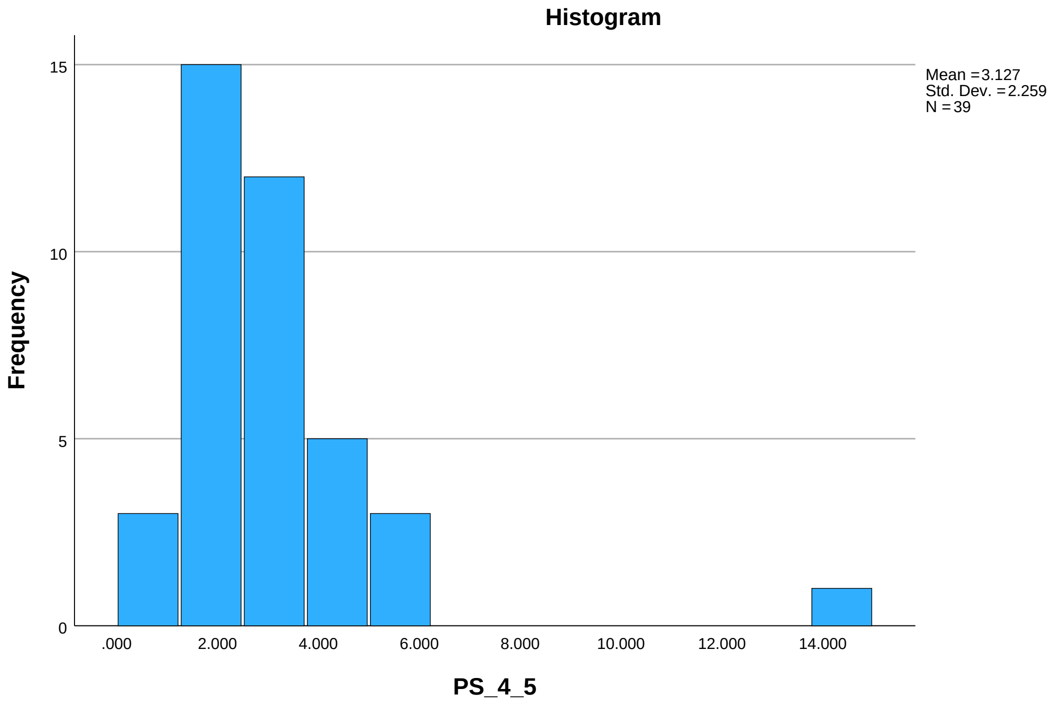


```
PS_4_5 Stem-and-Leaf Plot

 Frequency    Stem &  Leaf

     1.00        0 .  7
     4.00        1 .  0134
     5.00        1 .  56779
     8.00        2 .  11123444
     7.00        2 .  5577889
     5.00        3 .  00133
      .00        3 .
     3.00        4 .  223
     2.00        4 .  55
     4.00 Extremes    (>=5.5)

 Stem width:      1.000
 Each leaf:       1 case(s)
```

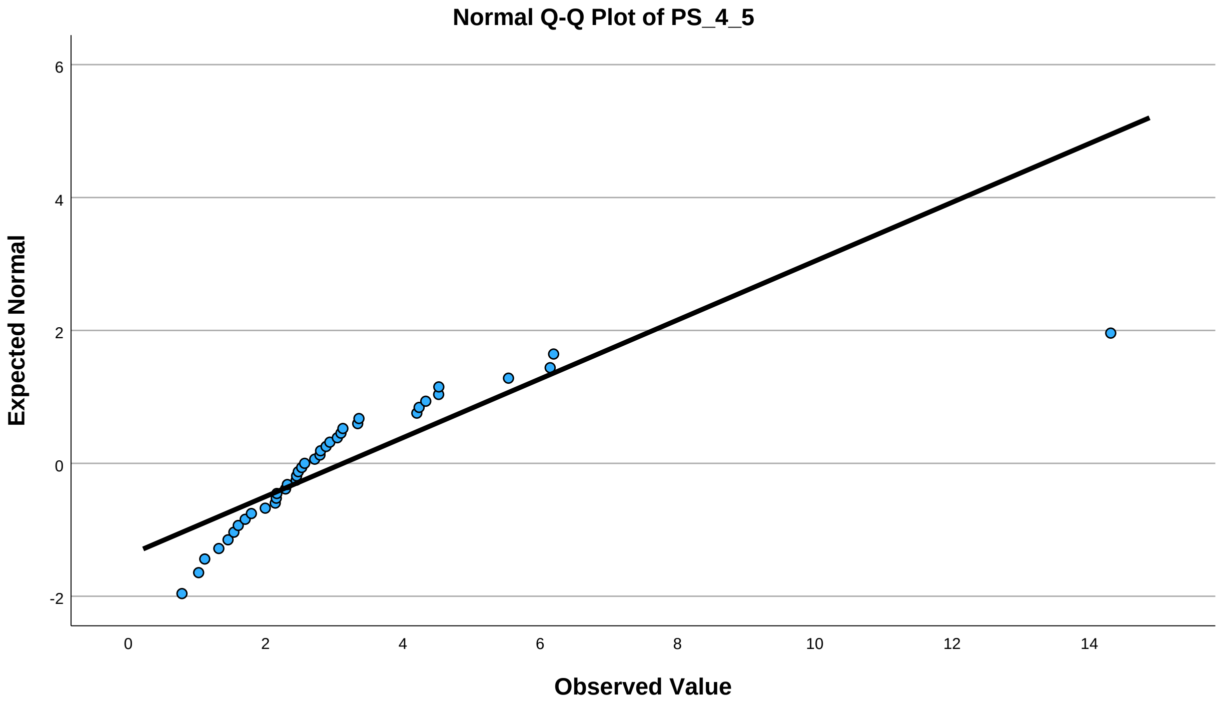
Normal Q-Q Plot of PS_4_5
Expected Normal
Observed Value
6
4
2
0
-2
0
2
4
6
8
10
12
14

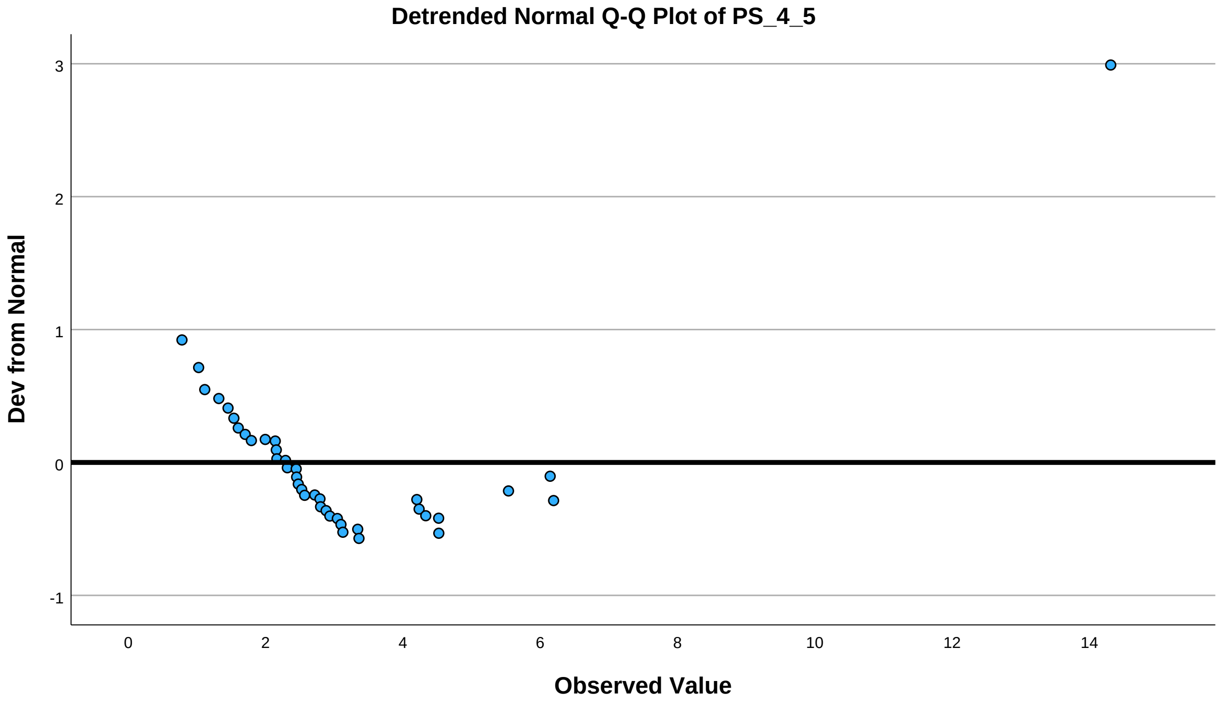
Detrended Normal Q-Q Plot of PS_4_5
Dev from Normal
Observed Value
3
2
1
0
-1
0
2
4
6
8
10
12
14

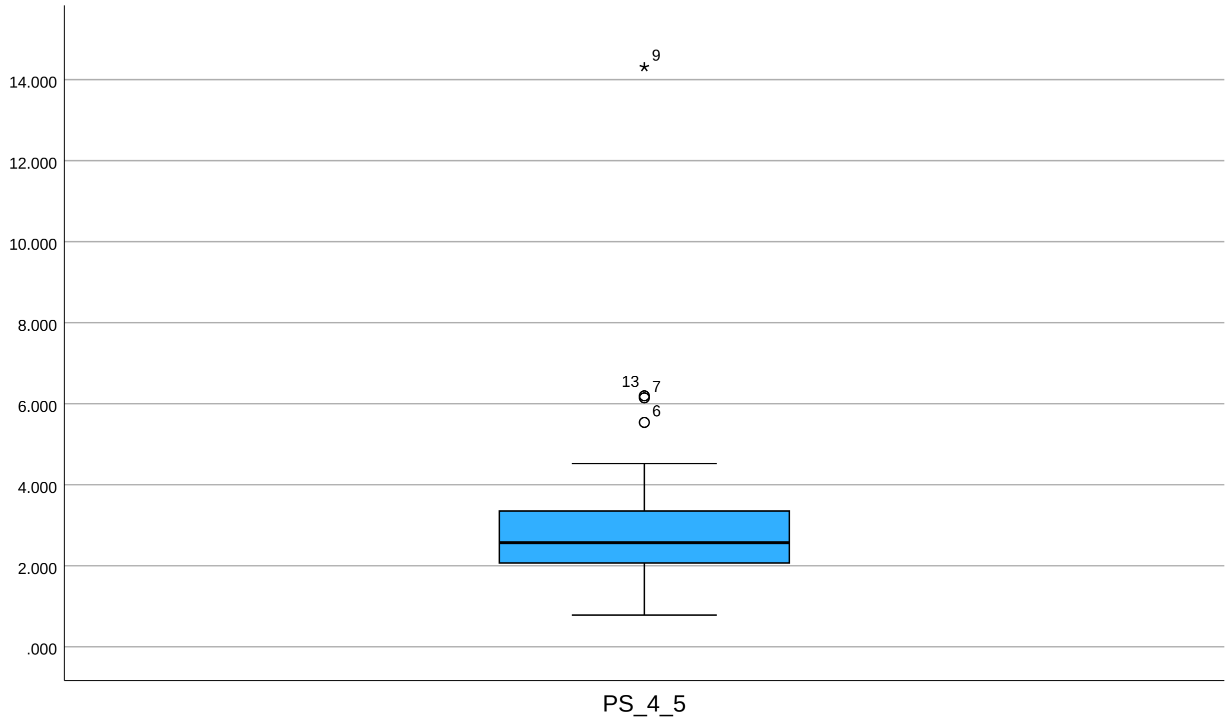

PS_5_6

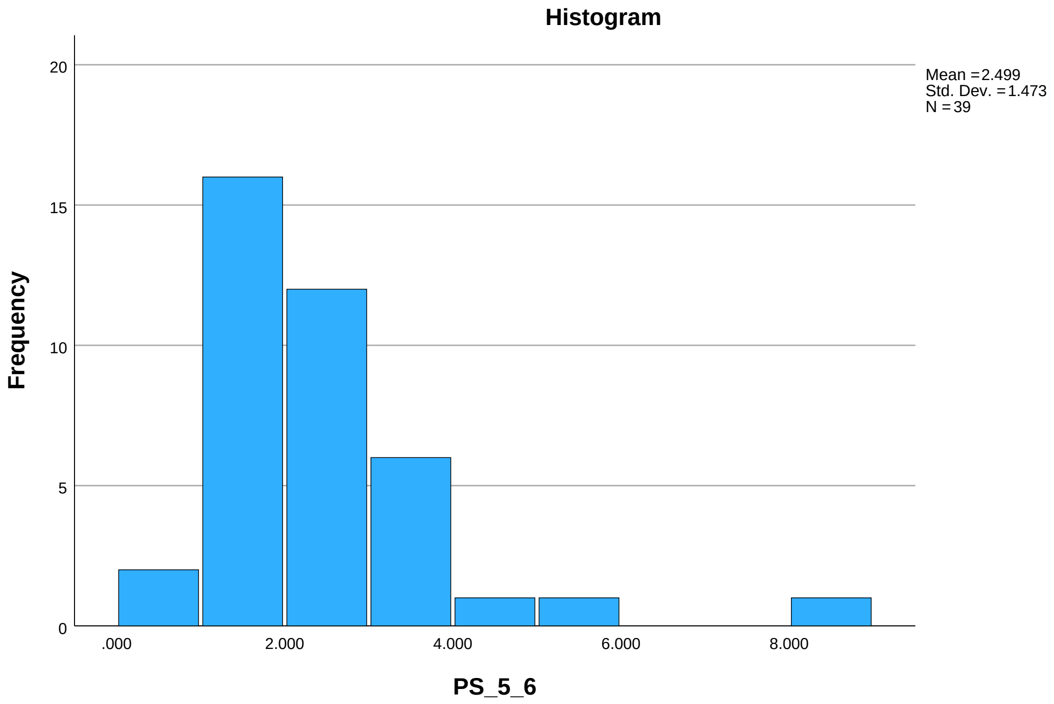


```
PS_5_6 Stem-and-Leaf Plot

 Frequency    Stem &  Leaf
```

```
  2.00        0 .  57
  5.00        1 .  01134
 11.00        1 .  55667888899
  6.00        2 .  000344
  6.00        2 .  567789
  3.00        3 .  344
  3.00        3 .  699
   .00        4 .
  1.00        4 .  6
  2.00 Extremes    (>=5.3)

Stem width:      1.000
Each leaf:       1 case(s)
```

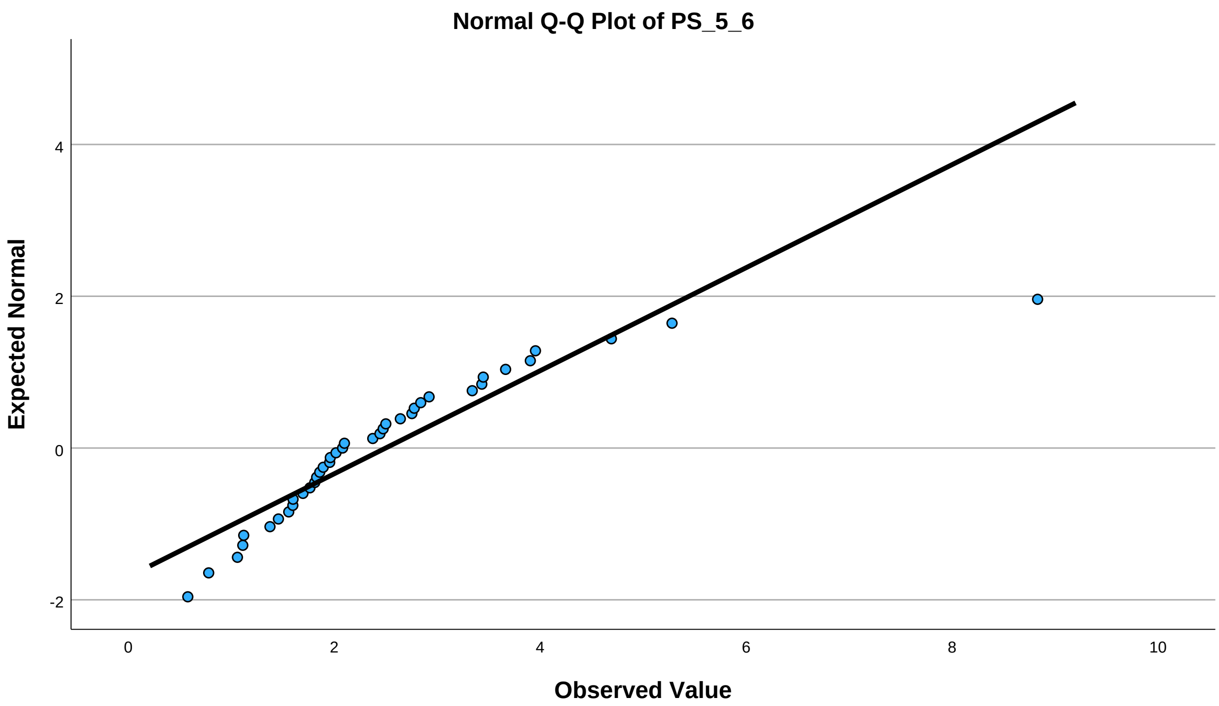

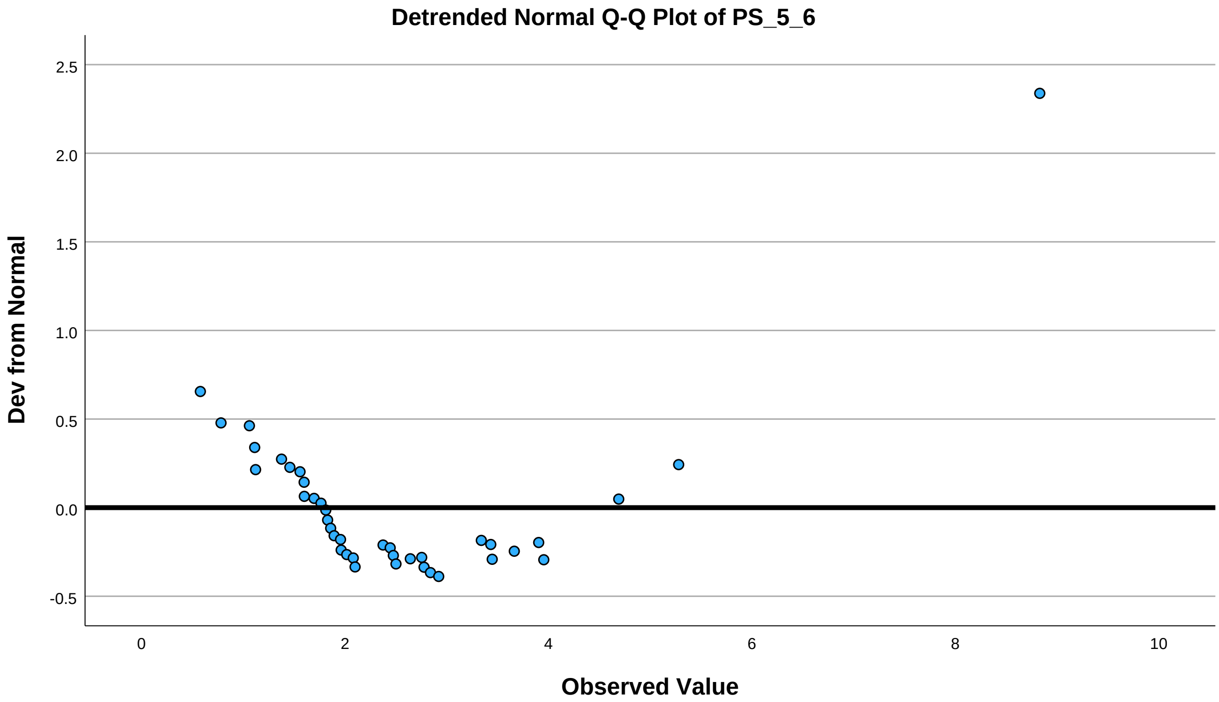
Detrended Normal Q-Q Plot of PS_5_6
Dev from Normal
2.5
2.0
1.5
1.0
0.5
0.0
-0.5
0
2
4
6
8
10
Observed Value

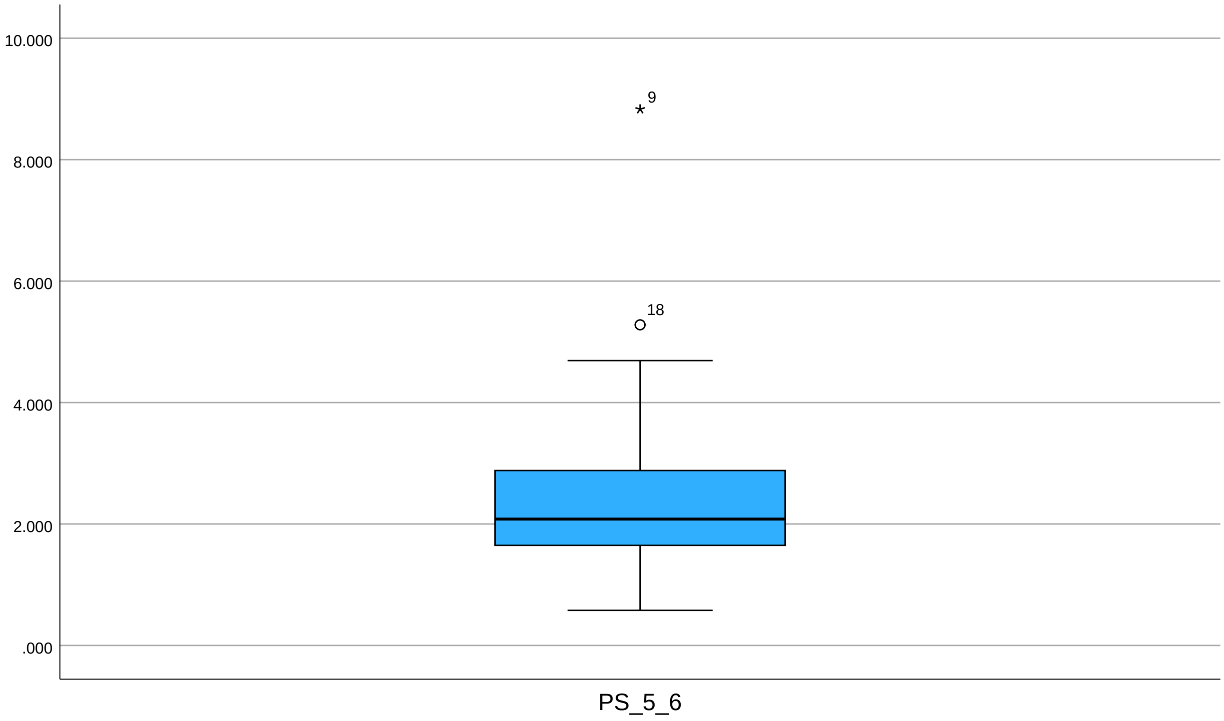
10.000
8.000
6.000
4.000
2.000
.000
9
18
PS_5_6

PS_6_7

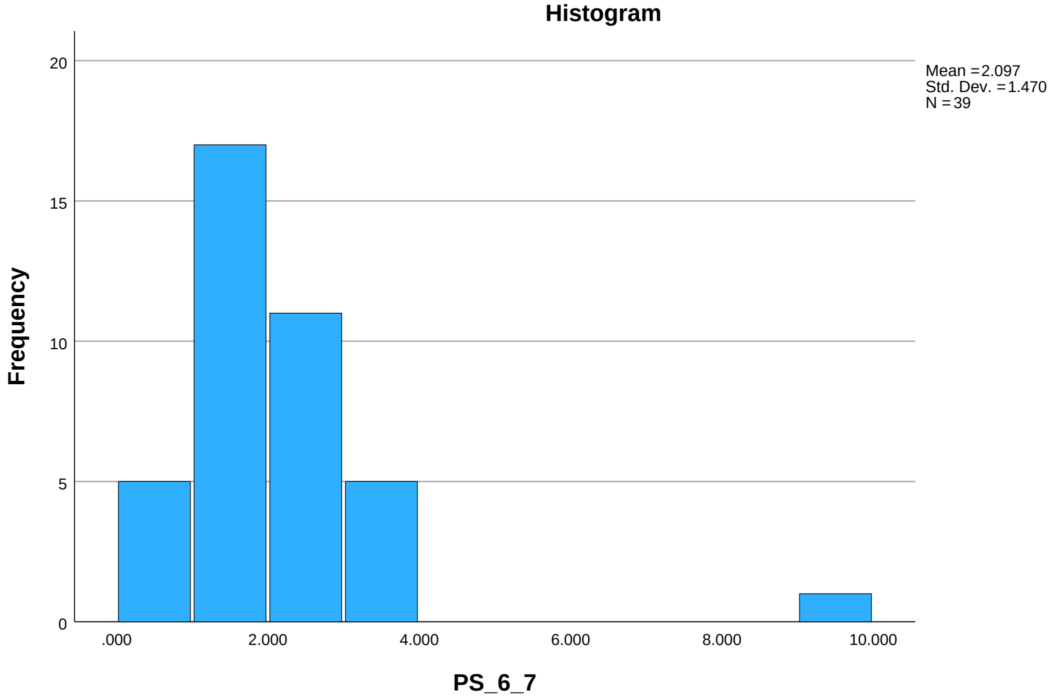


```
PS_6_7 Stem-and-Leaf Plot

 Frequency    Stem &  Leaf

     2.00        0 .  34
     3.00        0 .  778
     7.00        1 .  0122344
    10.00        1 .  5566777889
     8.00        2 .  00122344
     3.00        2 .  577
     4.00        3 .  0134
     1.00        3 .  6
     1.00 Extremes    (>=9.6)

 Stem width:      1.000
 Each leaf:       1 case(s)
```

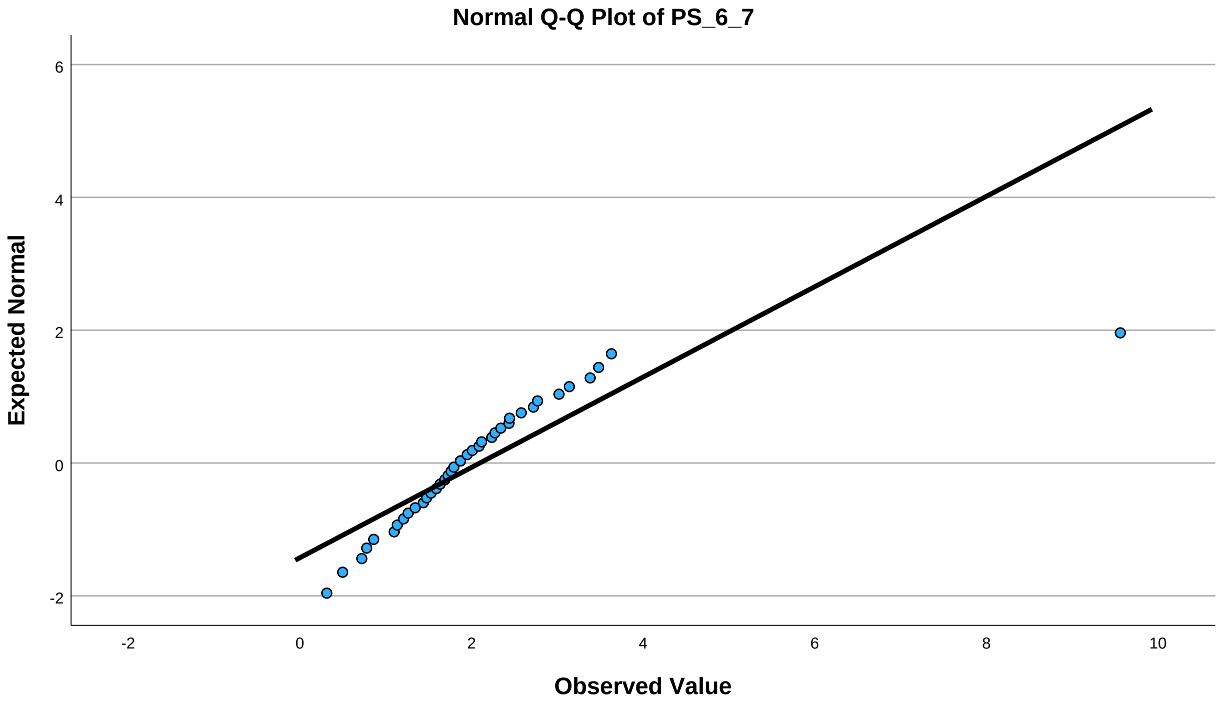
Normal Q-Q Plot of PS_6_7
Expected Normal
Observed Value
6
4
2
0
-2
-2
0
2
4
6
8
10

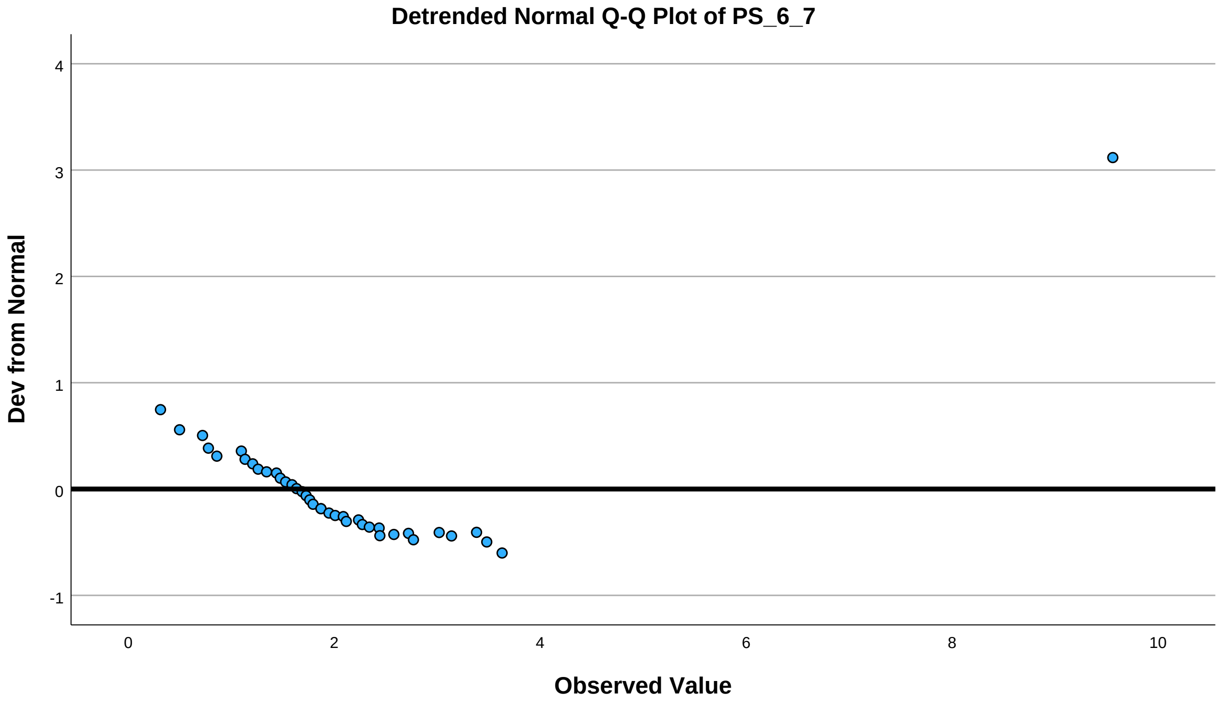
Detrended Normal Q-Q Plot of PS_6_7
Dev from Normal
Observed Value
4
3
2
1
0
-1
0
2
4
6
8
10

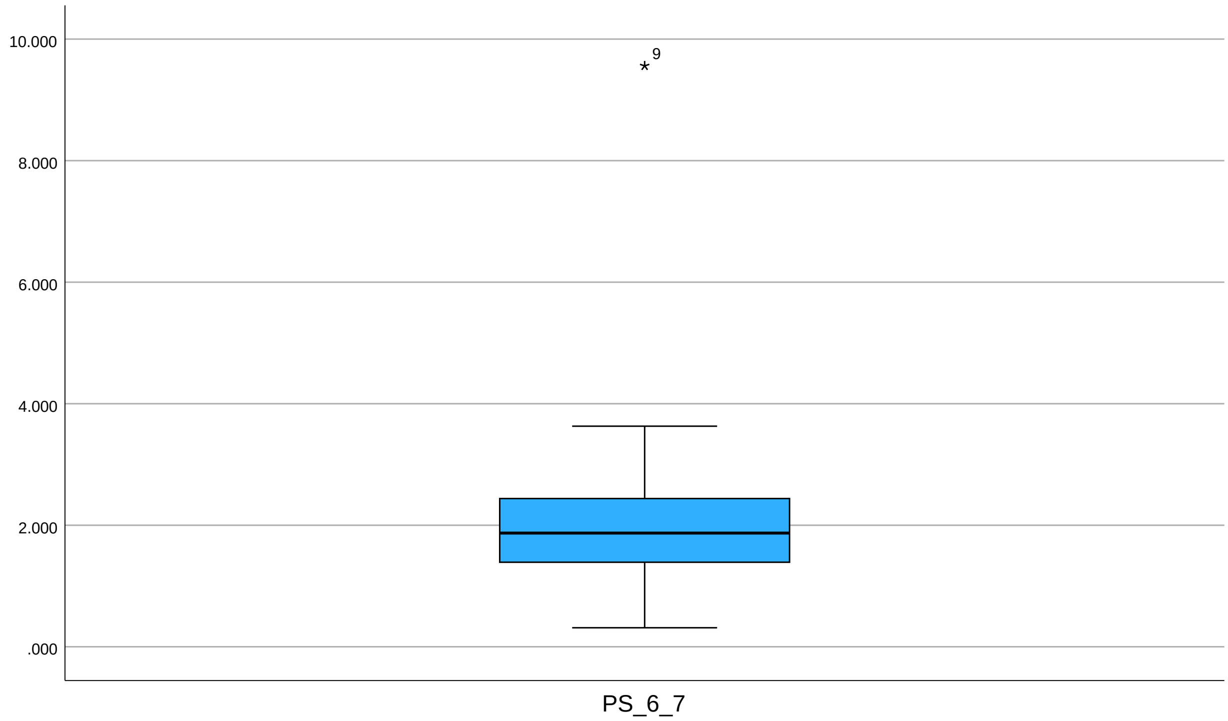

PS_7_8

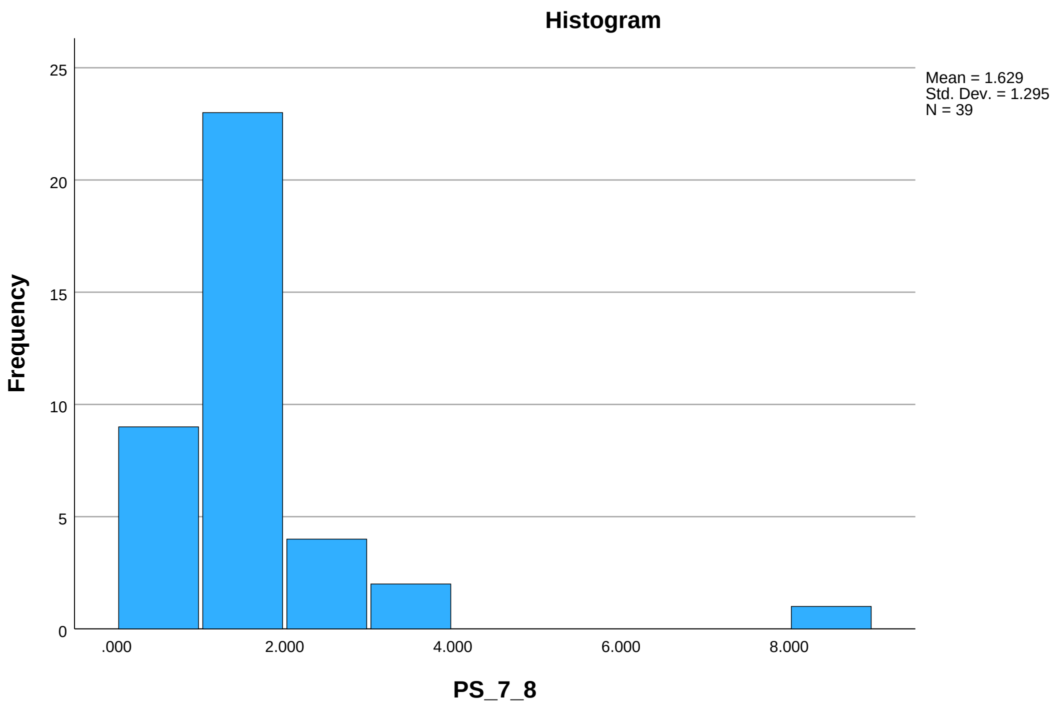


```
PS_7_8 Stem-and-Leaf Plot

 Frequency    Stem &  Leaf
```

```
   2.00        0 .  24
   7.00        0 .  5568999
  15.00        1 .  001112222334444
   8.00        1 .  55678889
   2.00        2 .  01
   2.00        2 .  78
   3.00 Extremes    (>=3.2)

 Stem width:      1.000
 Each leaf:       1 case(s)
```

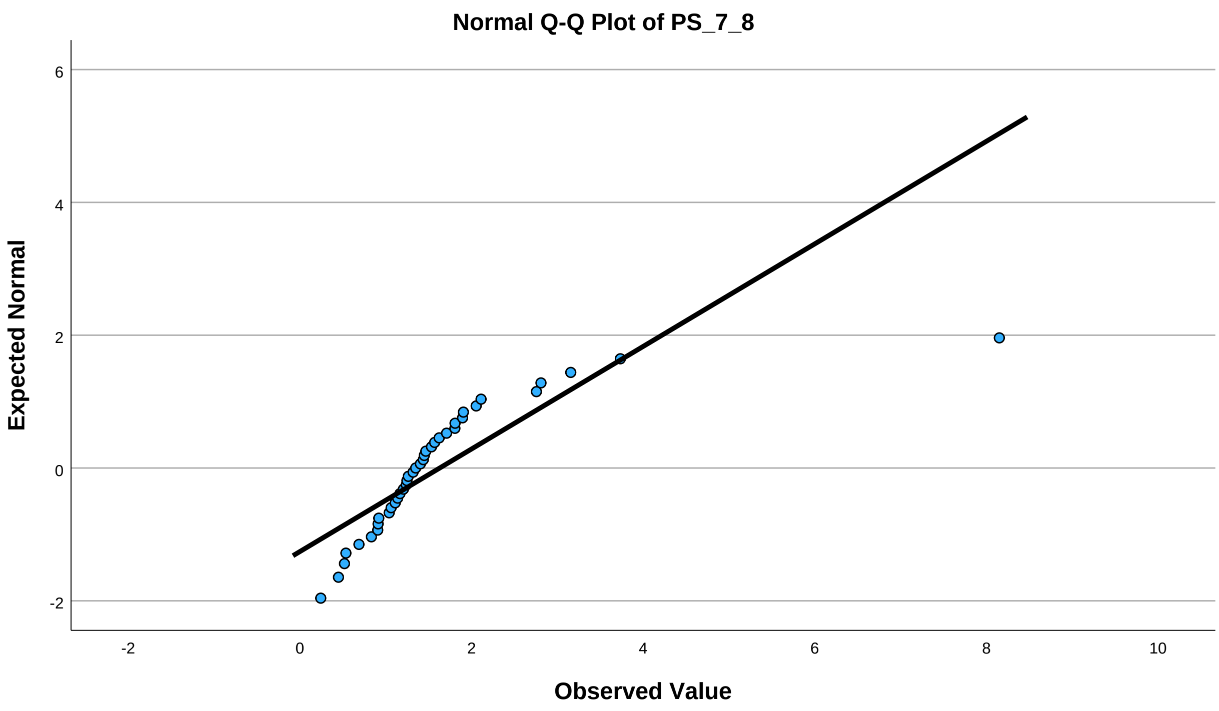

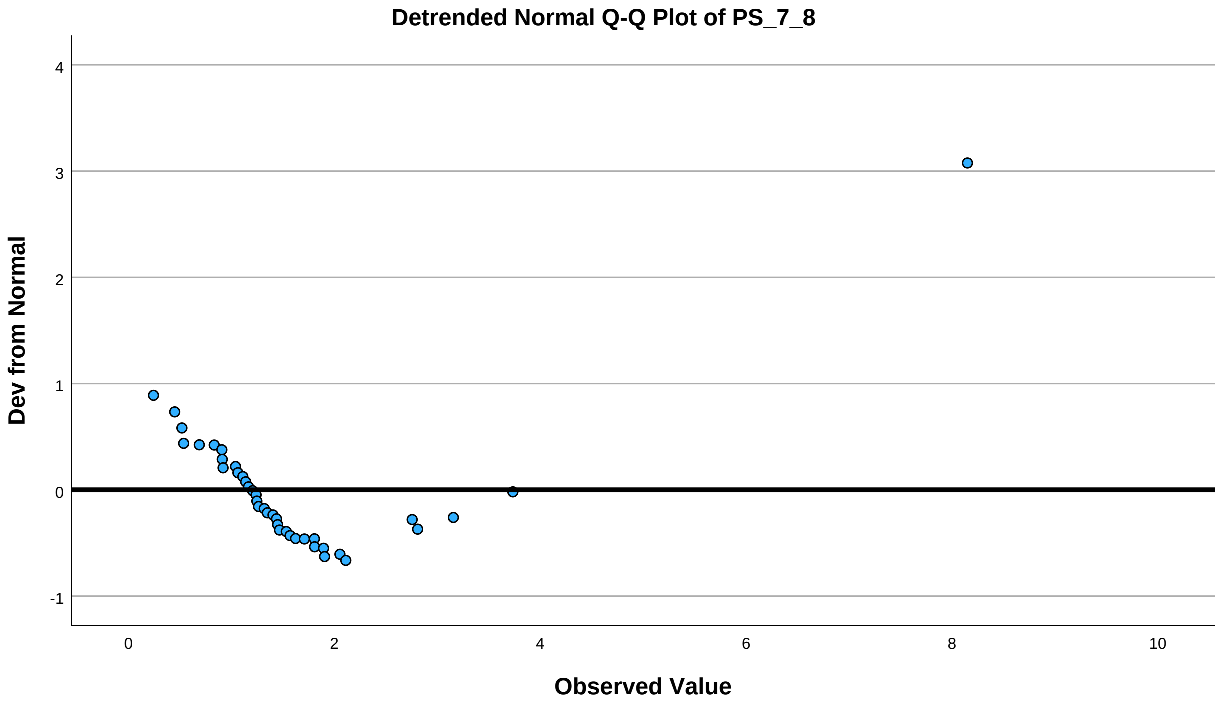
Detrended Normal Q-Q Plot of PS_7_8
Dev from Normal
4
3
2
1
0
-1
0
2
4
6
8
10
Observed Value

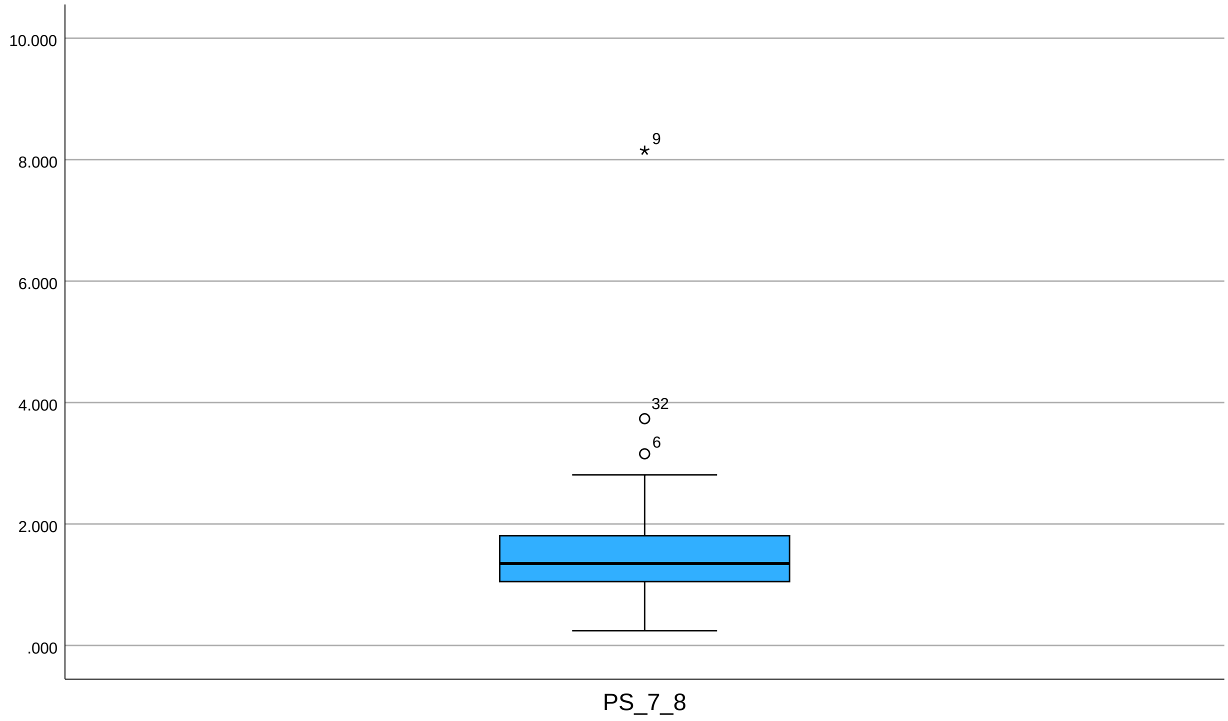
10.000
8.000
6.000
4.000
2.000
.000
9
32
6
PS_7_8

PS_8_9

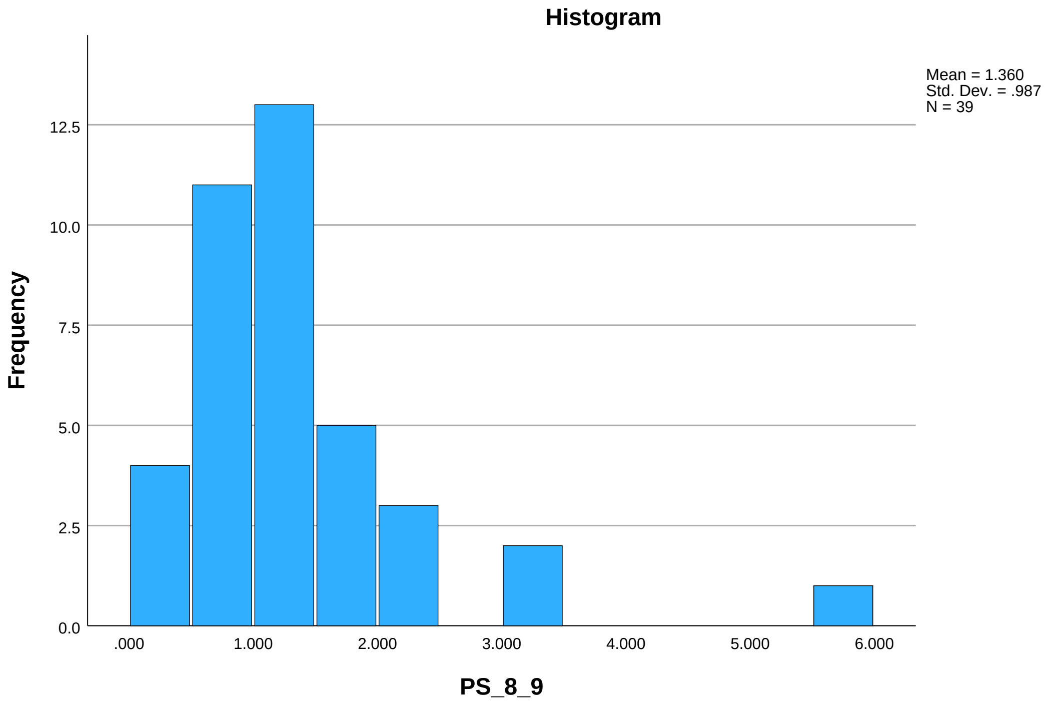


```
PS_8_9 Stem-and-Leaf Plot

 Frequency    Stem &  Leaf

     4.00        0 .  2344
    11.00        0 .  55567888899
    13.00        1 .  0001111123444
     5.00        1 .  56678
     3.00        2 .  234
     3.00 Extremes    (>=3.1)

 Stem width:      1.000
 Each leaf:       1 case(s)
```

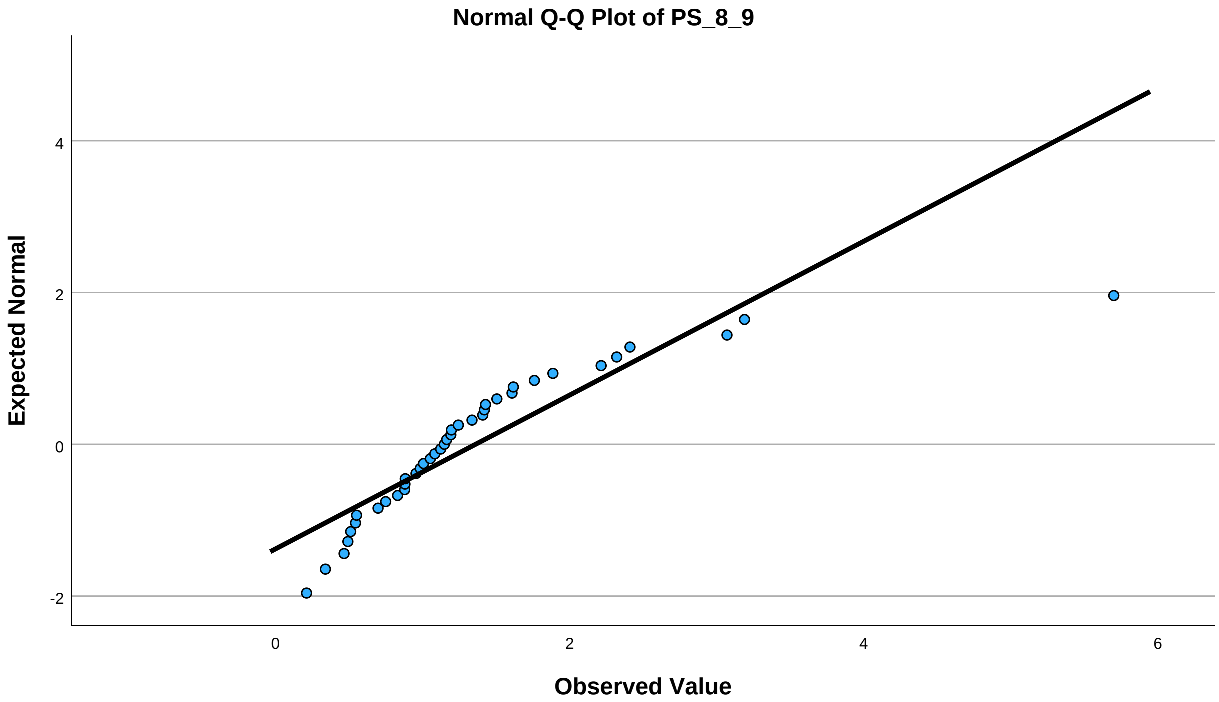
Normal Q-Q Plot of PS_8_9
Expected Normal
4
2
0
-2
0
2
4
6
Observed Value

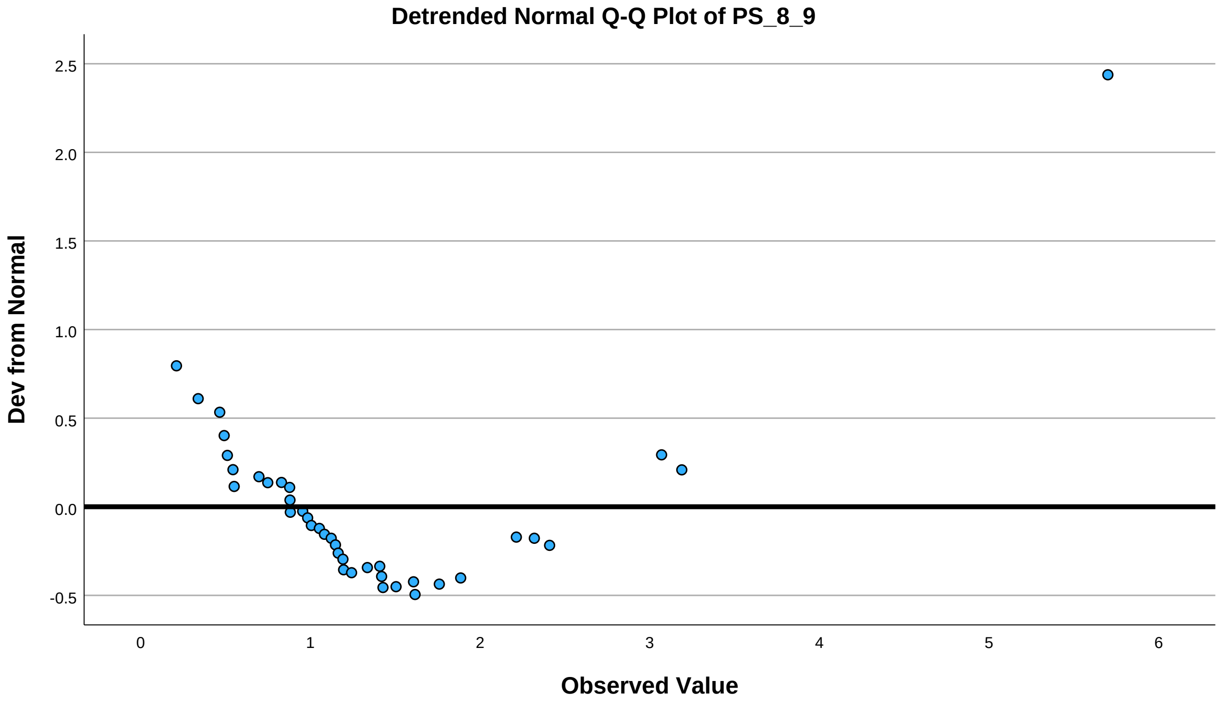
Detrended Normal Q-Q Plot of PS_8_9
Dev from Normal
2.5
2.0
1.5
1.0
0.5
0.0
-0.5
0
1
2
3
4
5
6
Observed Value

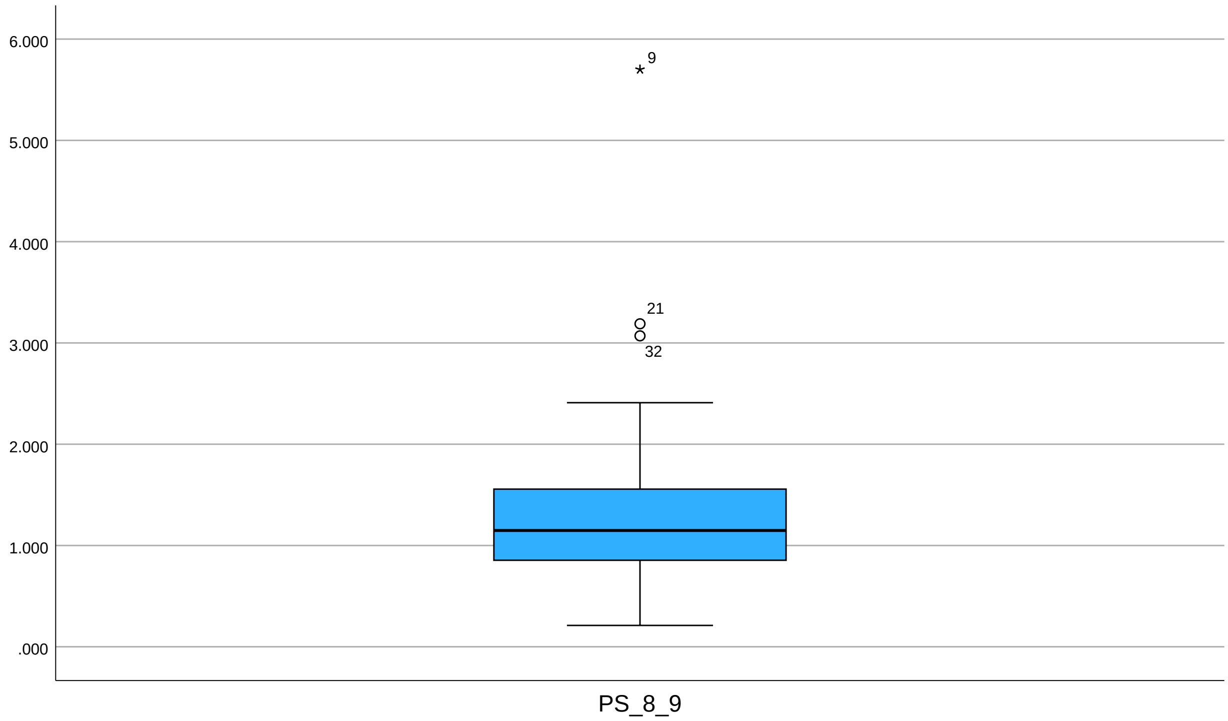

PS_9_10

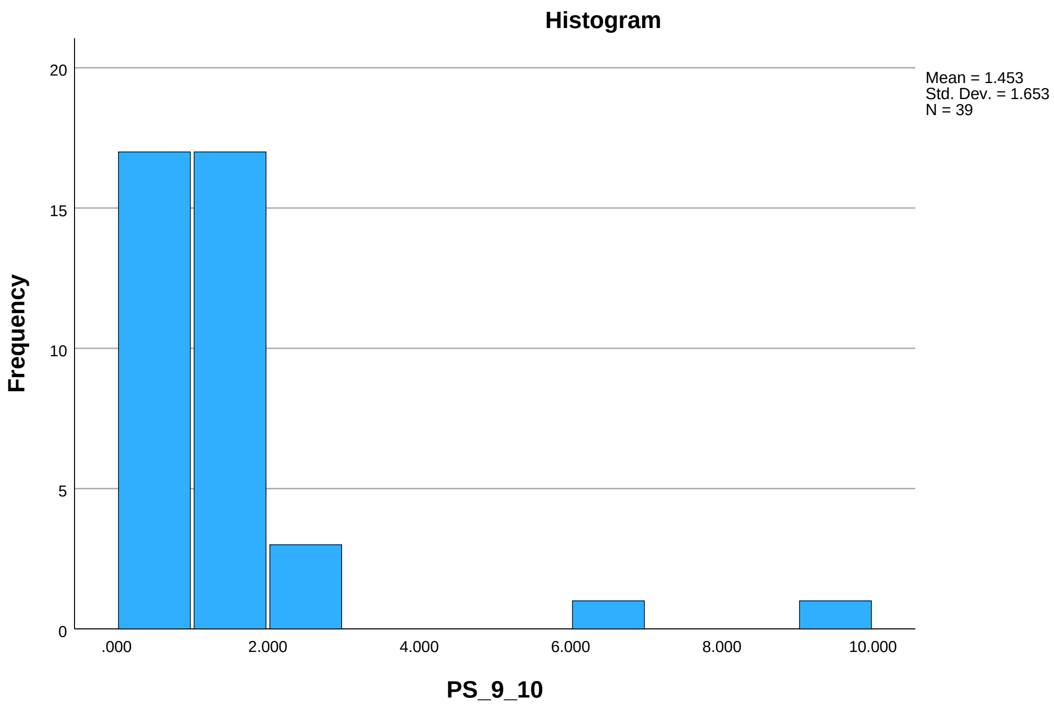

PS_9_10 Stem-and-Leaf Plot

Frequency   Stem & Leaf

```
   5.00        0 .  12234
  12.00        0 .  555566667899
  14.00        1 .  00112223333444
   3.00        1 .  567
   1.00        2 .  4
   4.00 Extremes    (>=2.9)

 Stem width:      1.000
 Each leaf:        1 case(s)
```

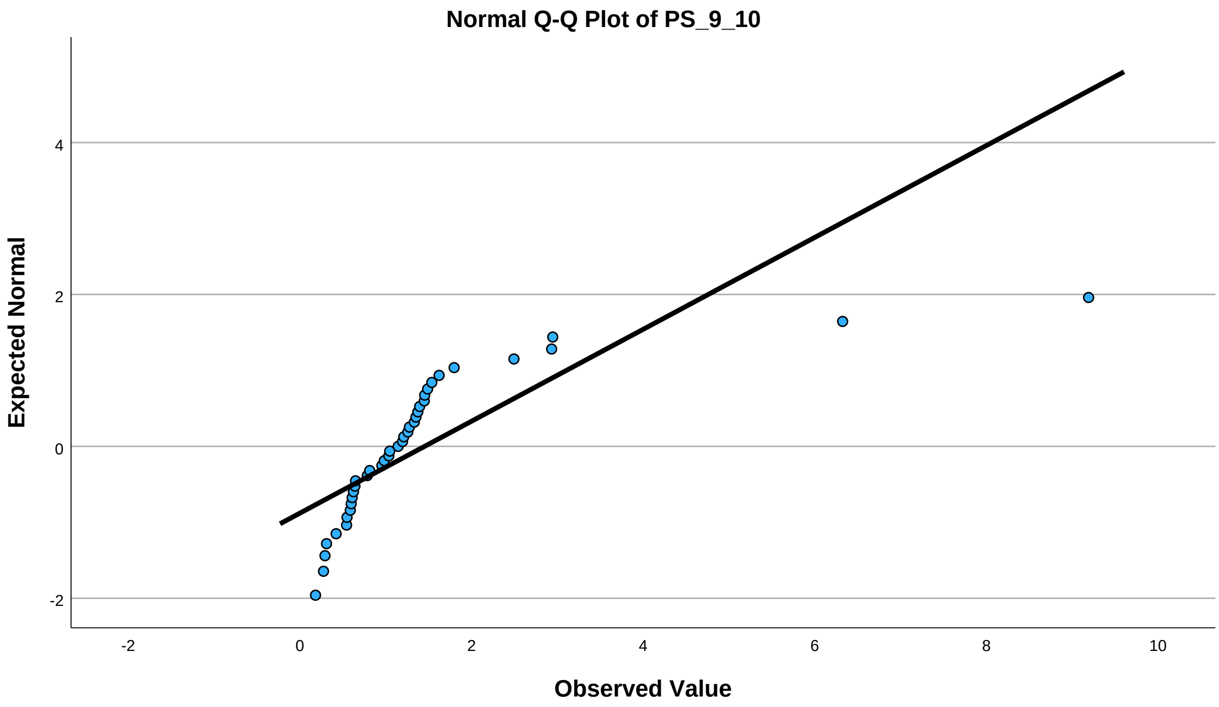

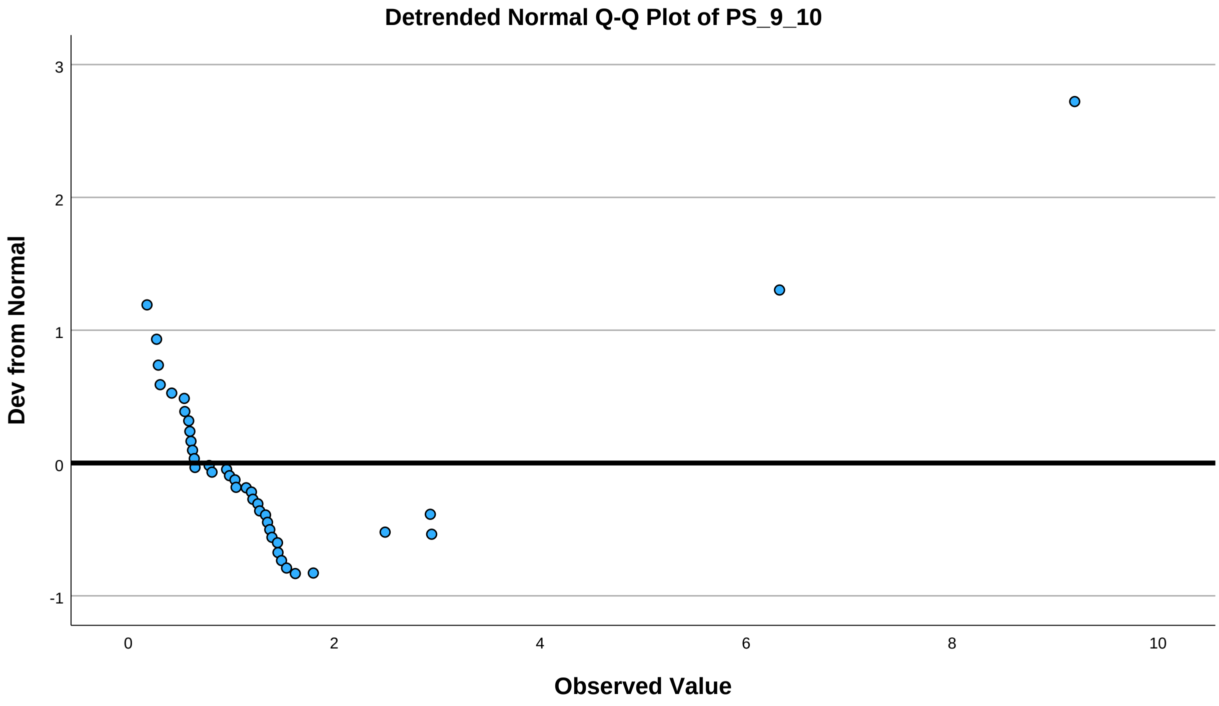
Detrended Normal Q-Q Plot of PS_9_10
Dev from Normal
3
2
1
0
-1
0
2
4
6
8
10
Observed Value

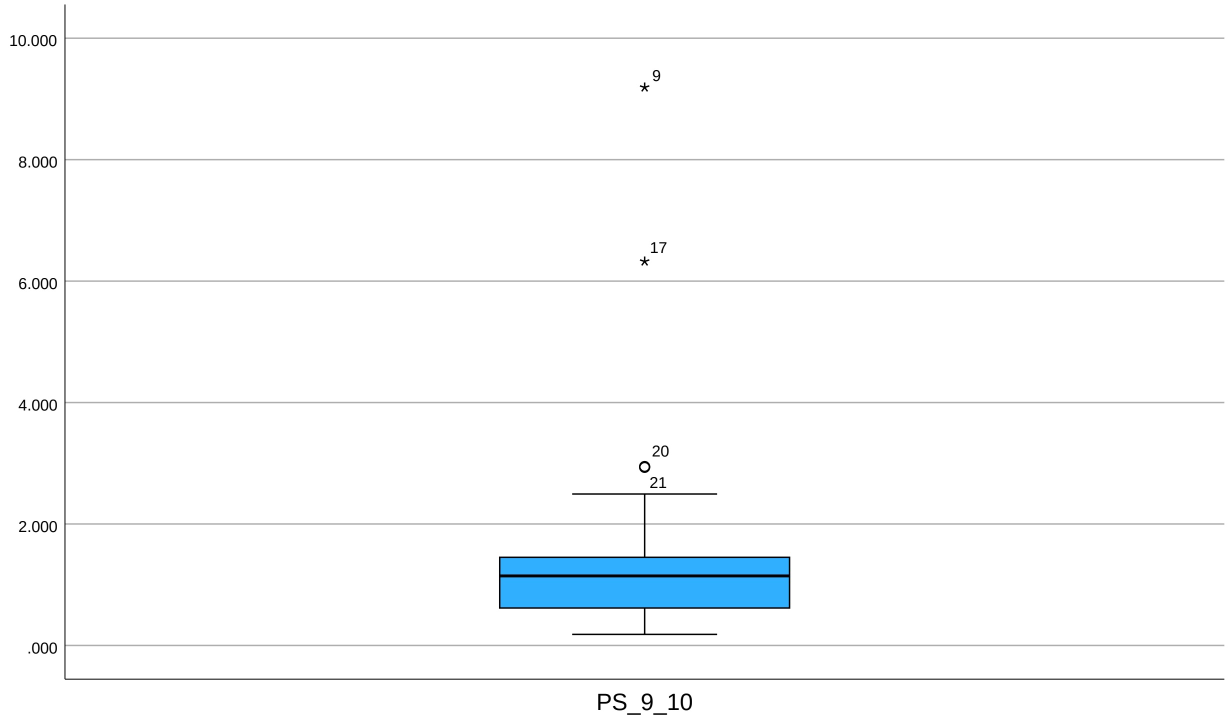
10.000
8.000
6.000
4.000
2.000
.000
9
17
20
21
PS_9_10

PS_10_11

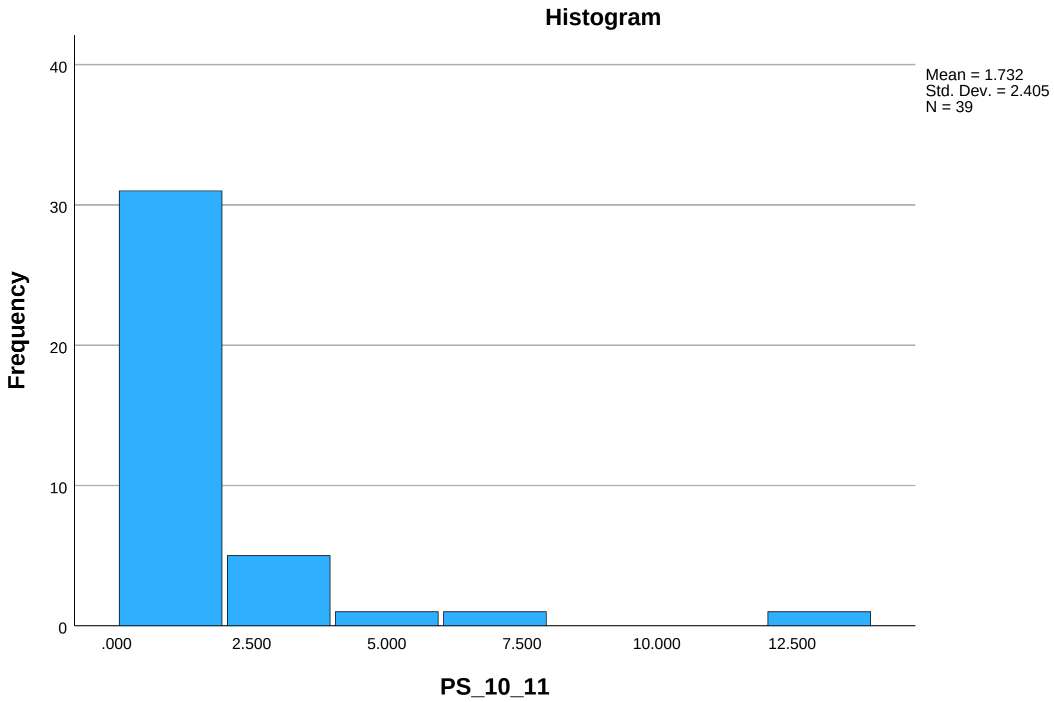


```
PS_10_11 Stem-and-Leaf Plot

 Frequency    Stem &  Leaf

     6.00        0 .  123444
    13.00        0 .  5556777778889
     7.00        1 .  0222334
     5.00        1 .  55678
     3.00        2 .  004
      .00        2 .
     1.00        3 .  1
     4.00 Extremes    (>=3.6)

 Stem width:      1.000
 Each leaf:       1 case(s)
```

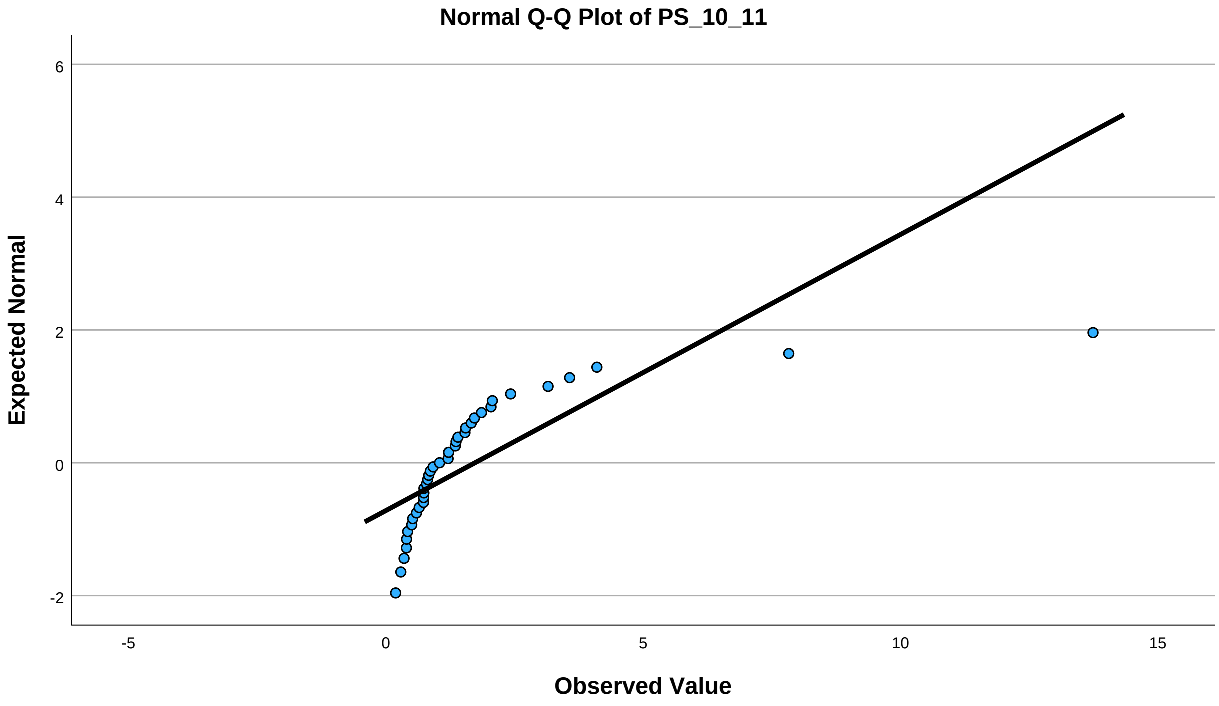
Normal Q-Q Plot of PS_10_11
Expected Normal
Observed Value
6
4
2
0
-2
-5
0
5
10
15

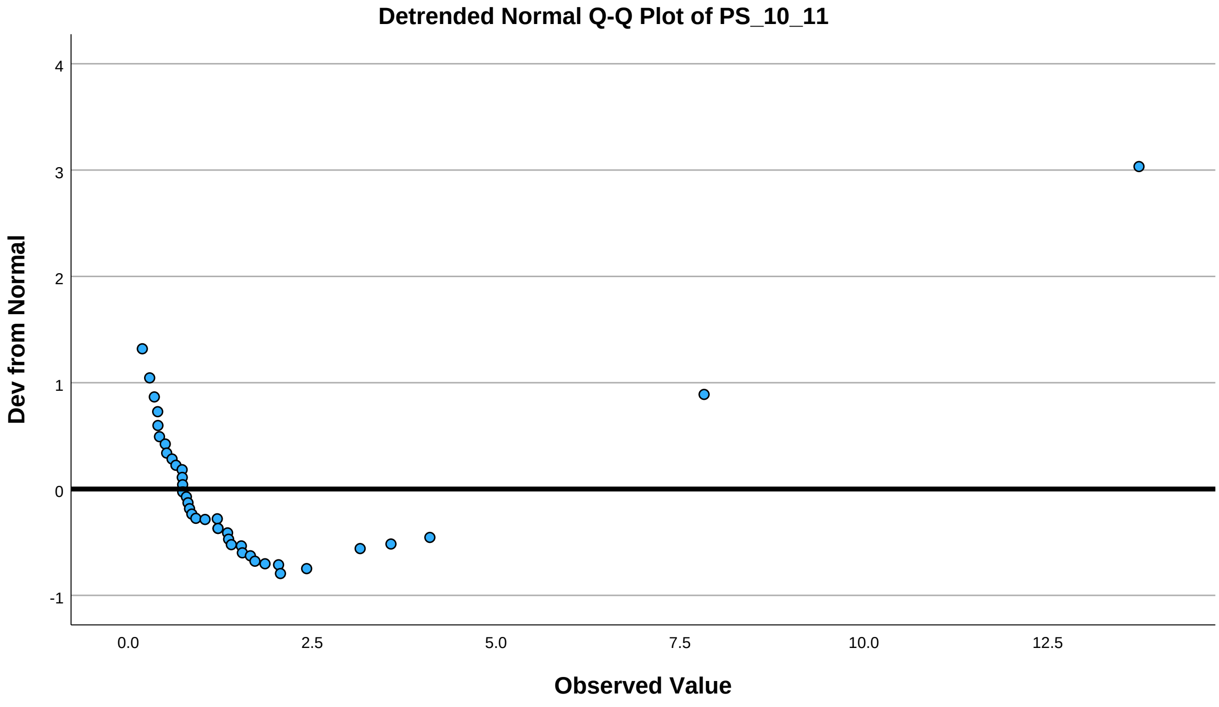
Detrended Normal Q-Q Plot of PS_10_11
Dev from Normal
Observed Value
4
3
2
1
0
-1
0.0
2.5
5.0
7.5
10.0
12.5

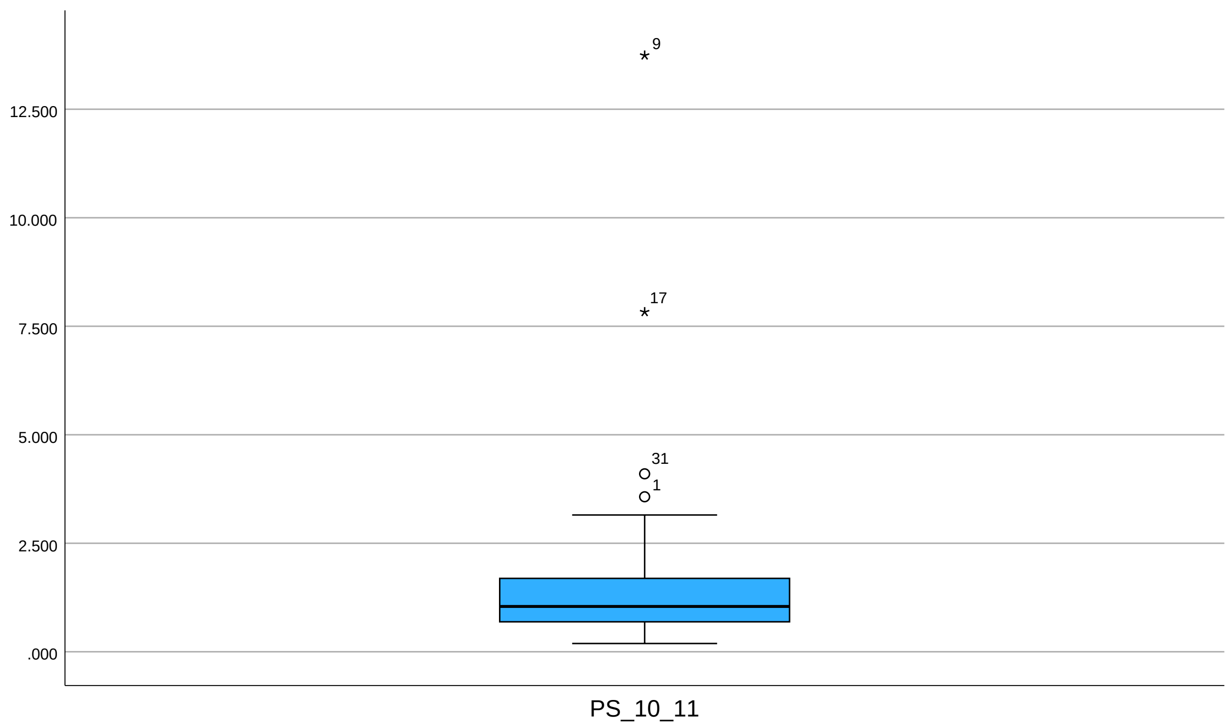

PS_11_12

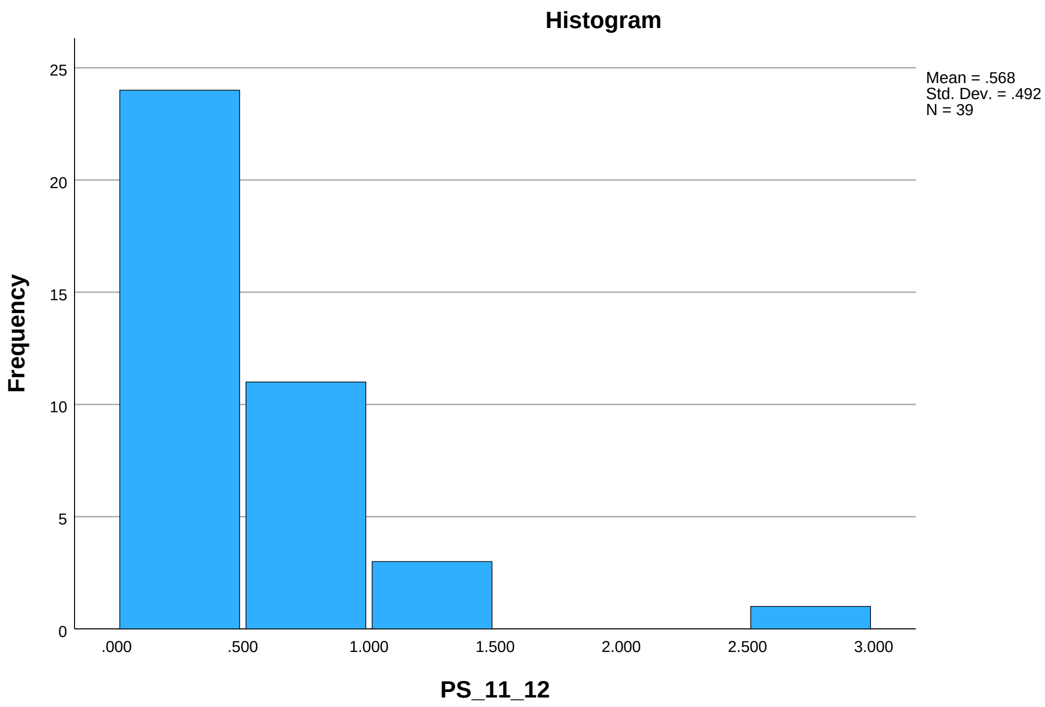


```
PS_11_12 Stem-and-Leaf Plot

 Frequency    Stem &  Leaf
```

```
   2.00      1 . 29
   7.00      2 . 0125899
  10.00      3 . 0222355568
   5.00      4 . 13449
   5.00      5 . 23368
    .00      6 .
   2.00      7 . 37
   3.00      8 . 034
   1.00      9 . 2
   4.00 Extremes    (>=1.21)

Stem width:      .100
Each leaf:       1 case(s)
```

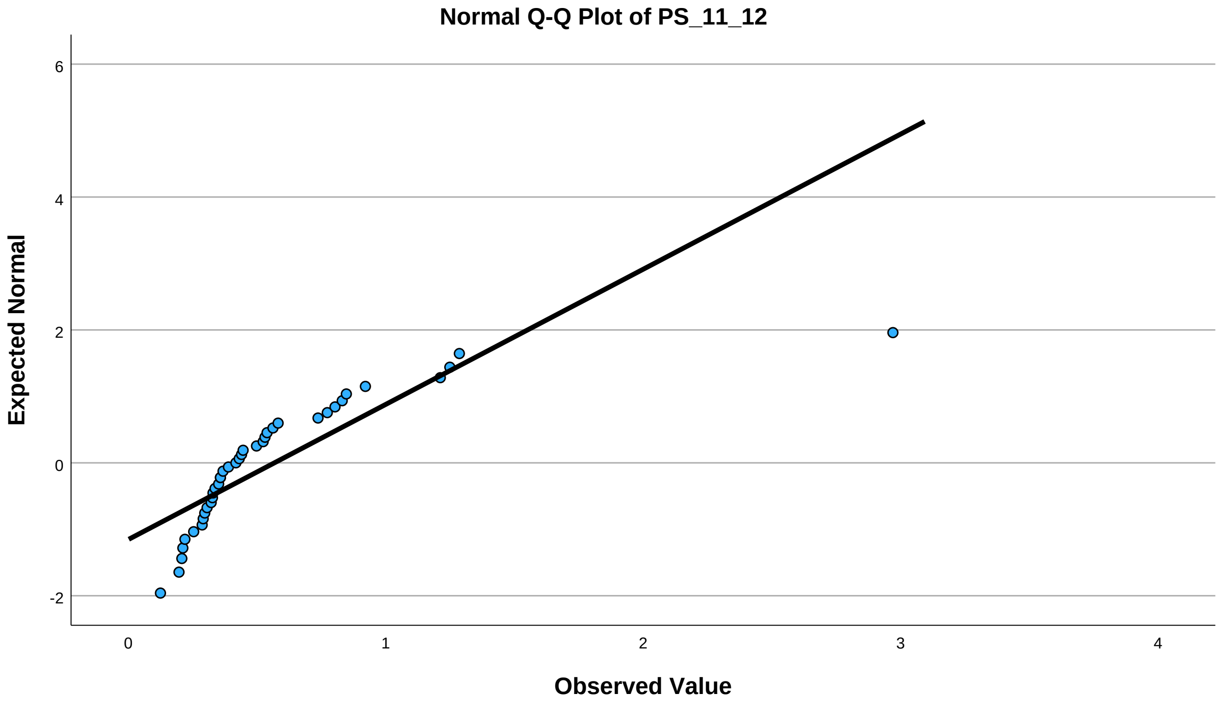

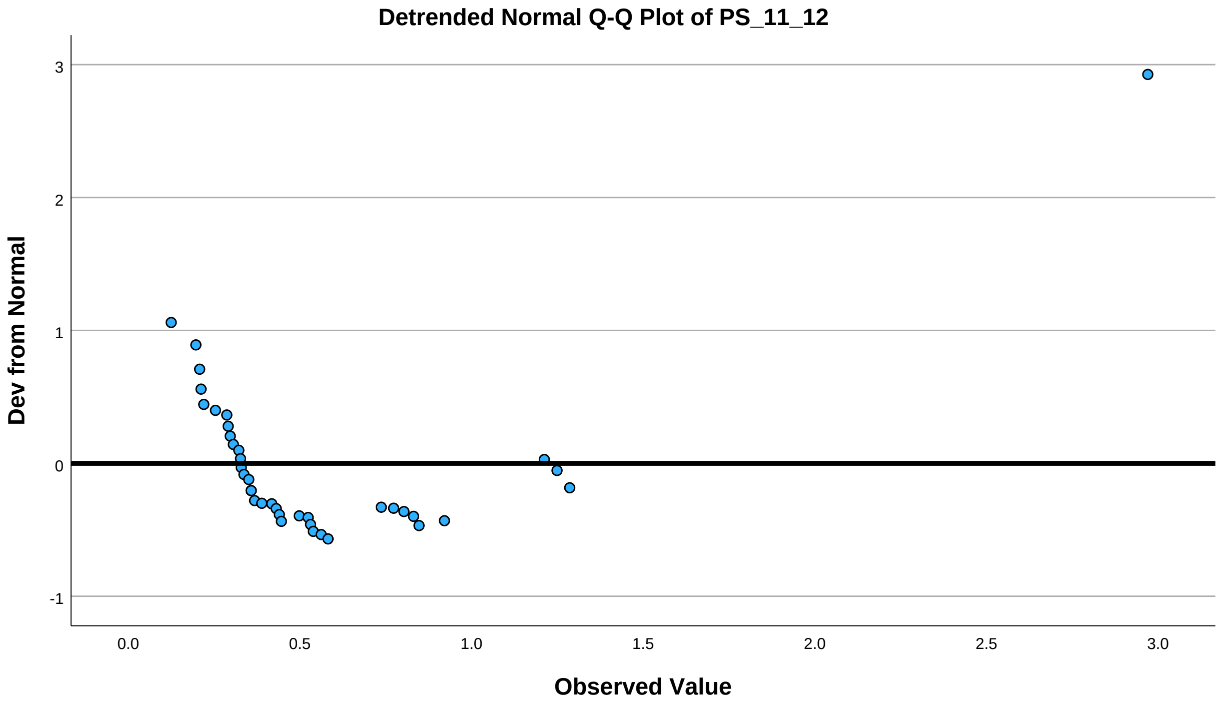
Detrended Normal Q-Q Plot of PS_11_12
Dev from Normal
3
2
1
0
-1
0.0
0.5
1.0
1.5
2.0
2.5
3.0
Observed Value

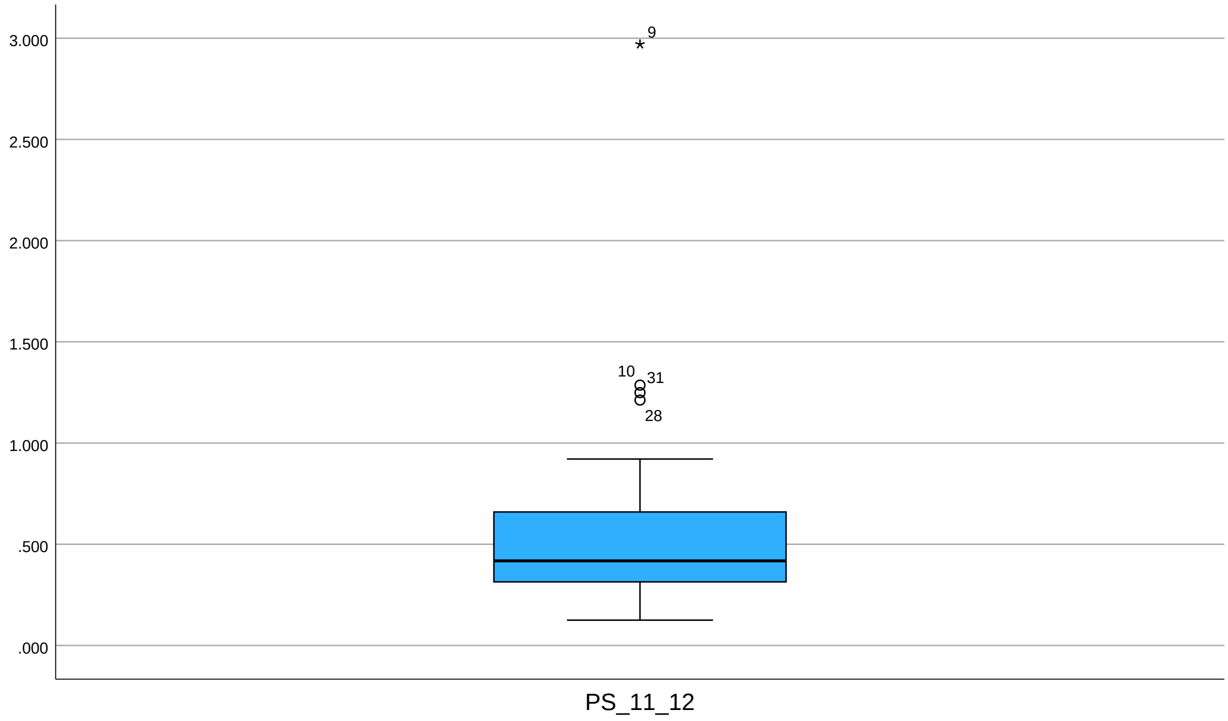
3.000
2.500
2.000
1.500
1.000
.500
.000
9
10
31
28
PS_11_12

PS_12_13

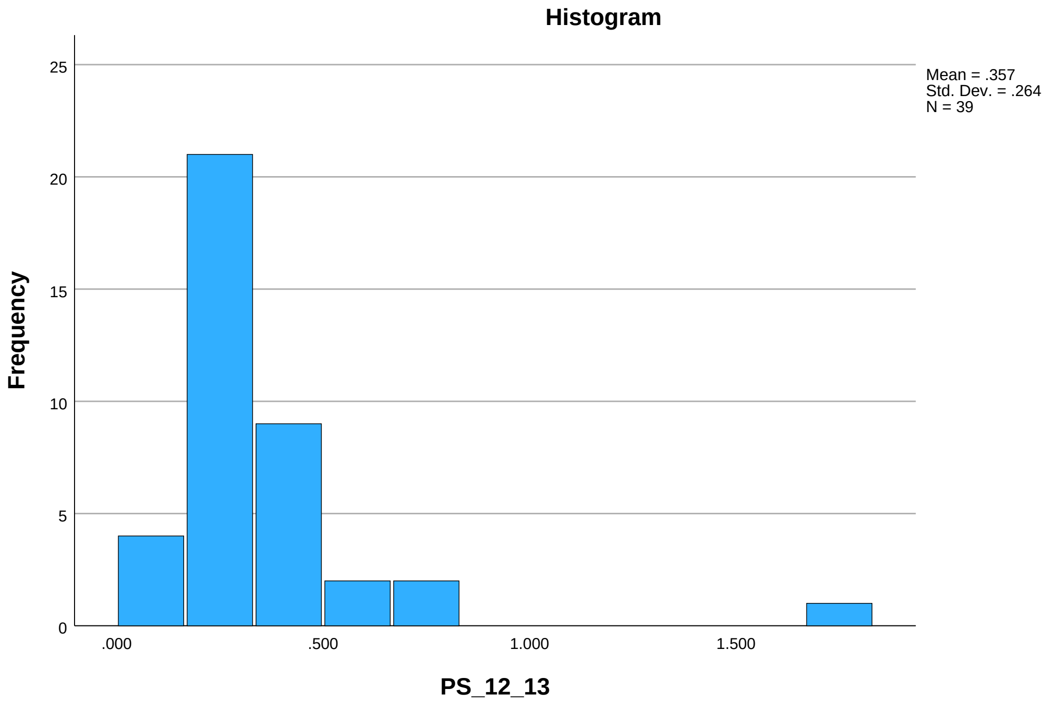


```
PS_12_13 Stem-and-Leaf Plot

 Frequency    Stem &  Leaf

     3.00        1 .  023
     2.00        1 .  58
     9.00        2 .  012233444
     6.00        2 .  567799
     5.00        3 .  00011
     3.00        3 .  589
     4.00        4 .  0012
     2.00        4 .  79
     2.00        5 .  13
     3.00 Extremes    (>=.70)

 Stem width:      .100
 Each leaf:       1 case(s)
```

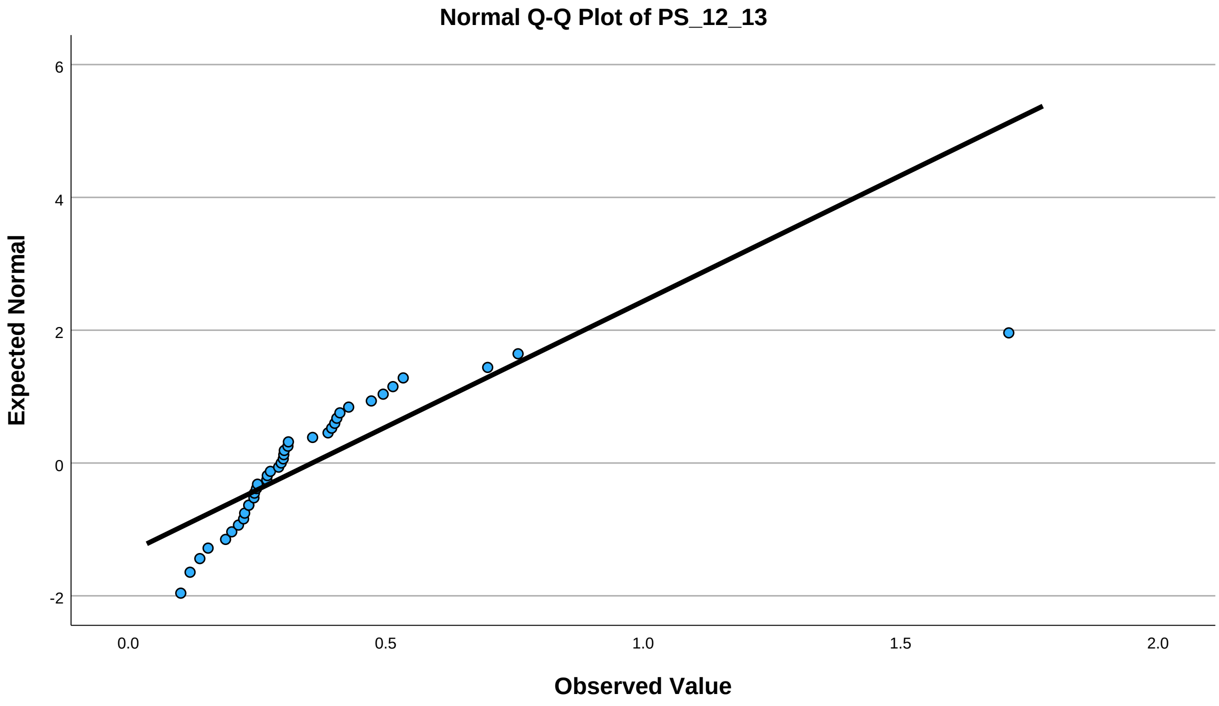

Normal Q-Q Plot of PS_12_13
Expected Normal
Observed Value
6
4
2
0
-2
0.0
0.5
1.0
1.5
2.0

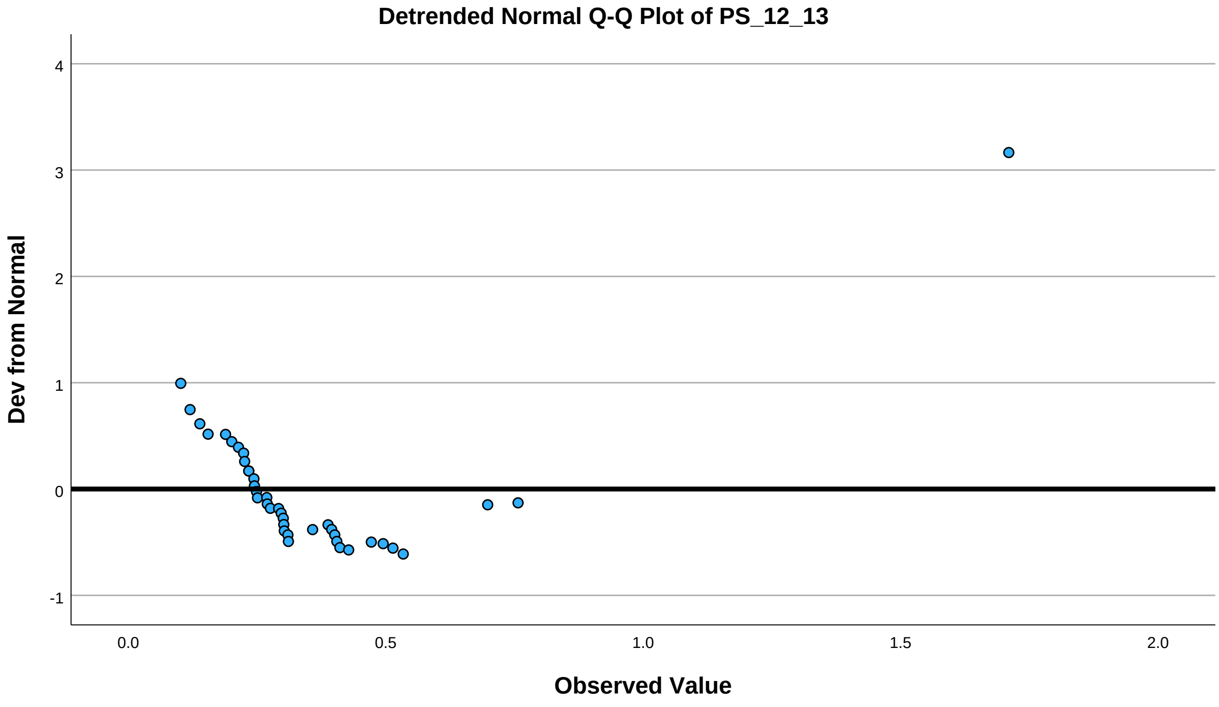

Detrended Normal Q-Q Plot of PS_12_13
Dev from Normal
Observed Value
4
3
2
1
0
-1
0.0
0.5
1.0
1.5
2.0

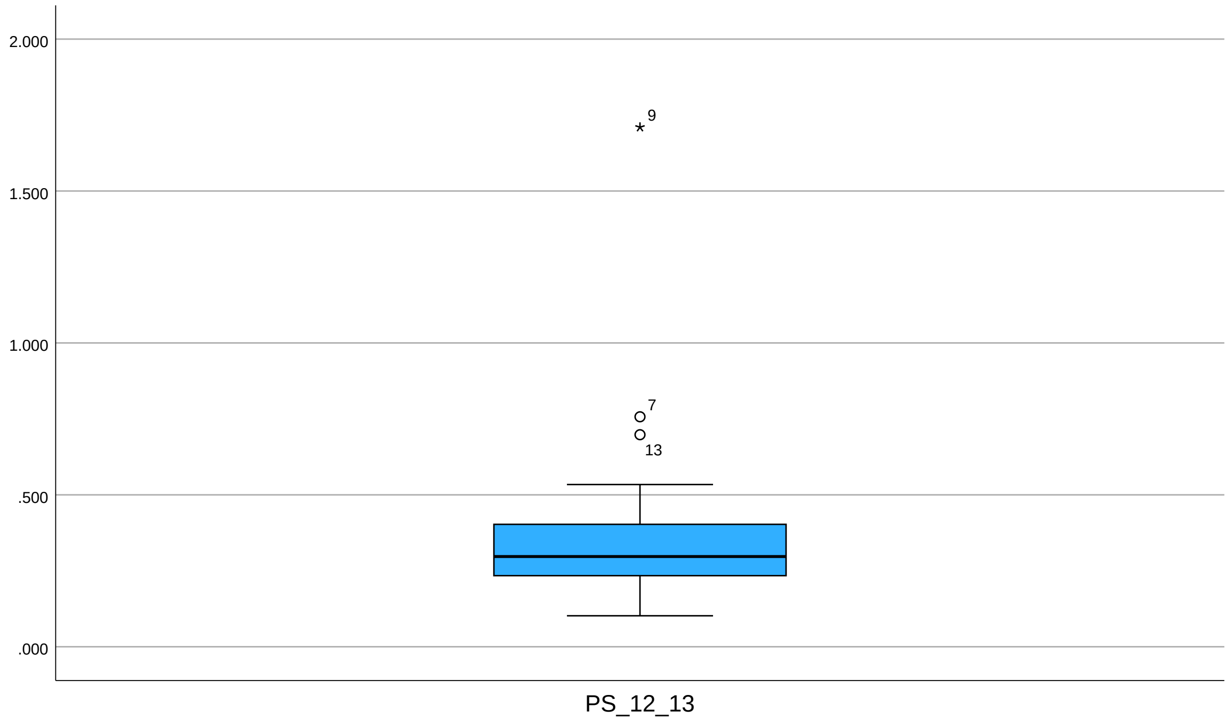

PS_13_14

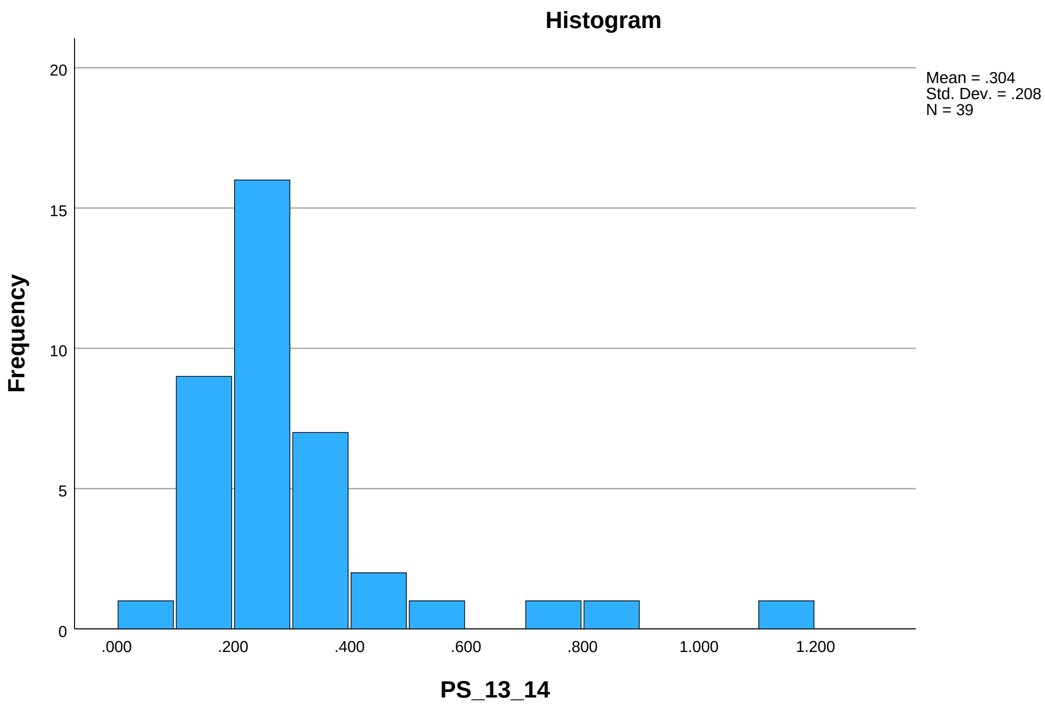

PS_13_14 Stem-and-Leaf Plot

Frequency   Stem & Leaf

```
  1.00      0 . 9
  3.00      1 . 044
  6.00      1 . 556699
 11.00      2 . 00011111134
  5.00      2 . 56899
  4.00      3 . 1123
  3.00      3 . 668
   .00      4 .
  2.00      4 . 58
  1.00      5 . 0
  3.00 Extremes    (>=.76)

Stem width:      .100
Each leaf:       1 case(s)
```

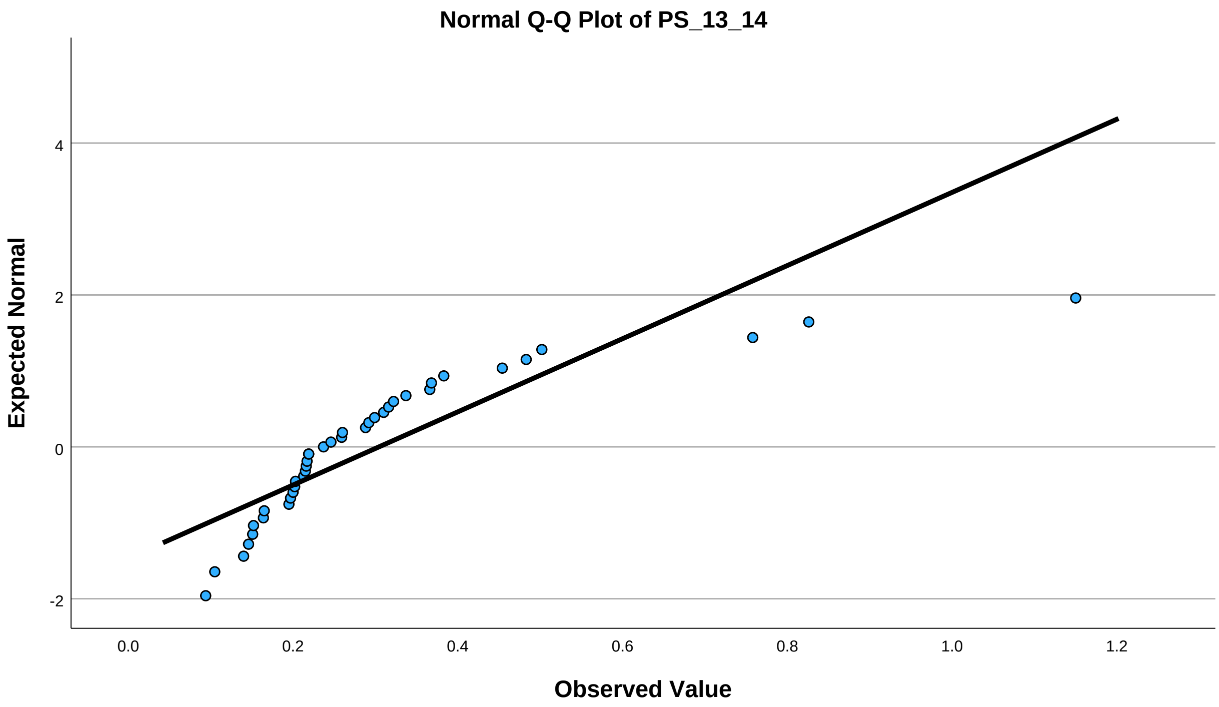

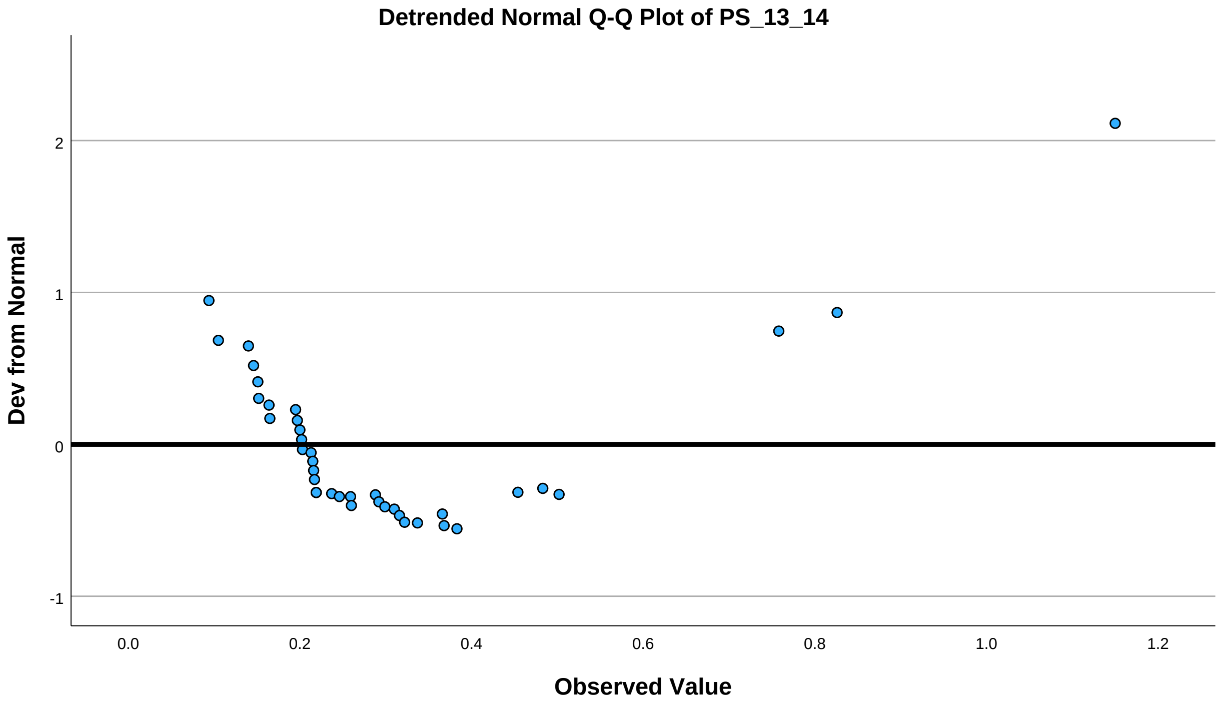
Detrended Normal Q-Q Plot of PS_13_14
Dev from Normal
2
1
0
-1
0.0
0.2
0.4
0.6
0.8
1.0
1.2
Observed Value

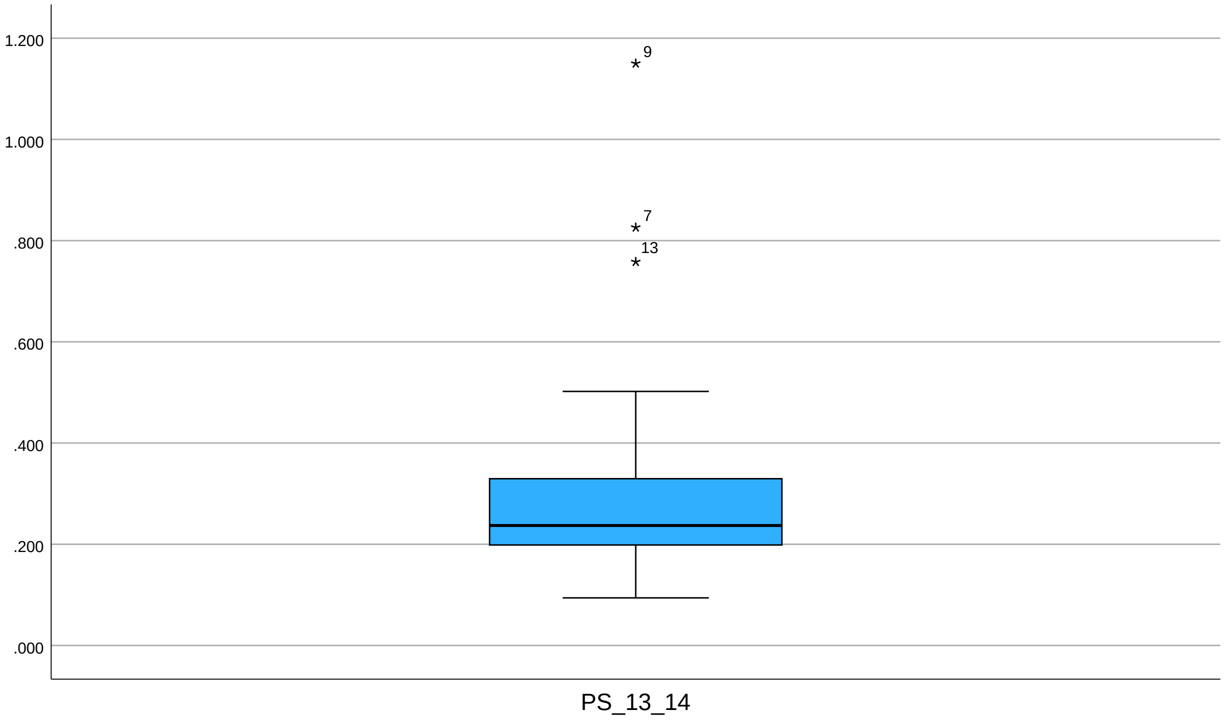
1.200
1.000
.800
.600
.400
.200
.000
9
7
13
PS_13_14

PS_14_15

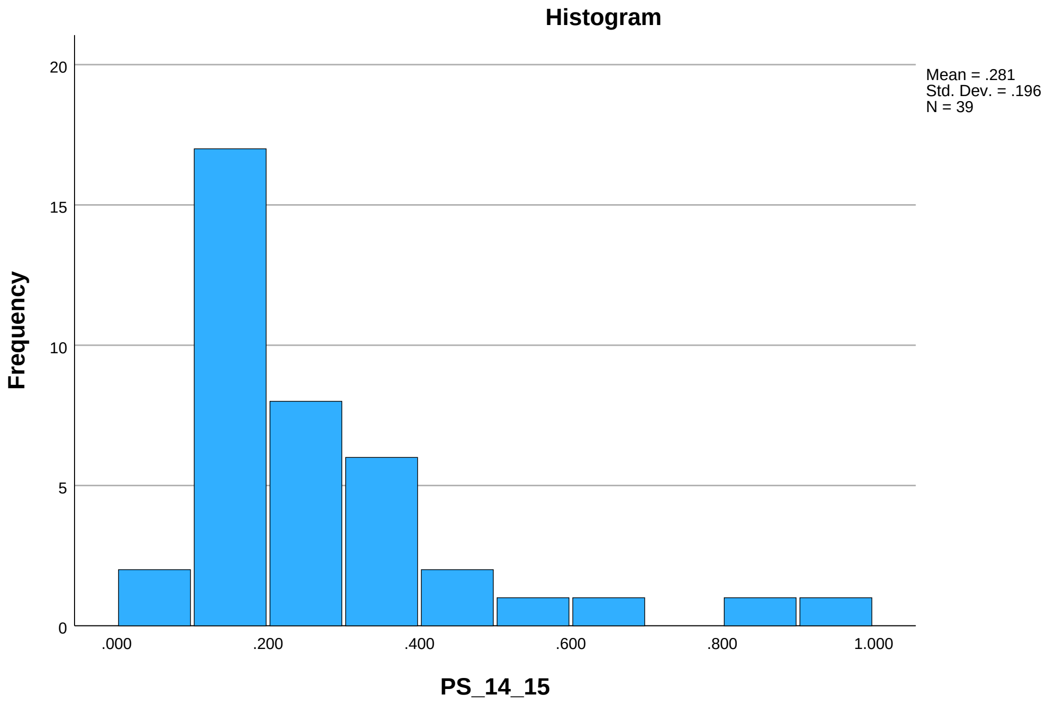


```
PS_14_15 Stem-and-Leaf Plot

 Frequency    Stem &  Leaf

     2.00        0 .  78
     5.00        1 .  02344
    12.00        1 .  677788889999
     5.00        2 .  00234
     3.00        2 .  679
     3.00        3 .  011
     3.00        3 .  567
     1.00        4 .  0
     1.00        4 .  9
     4.00 Extremes    (>=.56)

 Stem width:      .100
 Each leaf:       1 case(s)
```

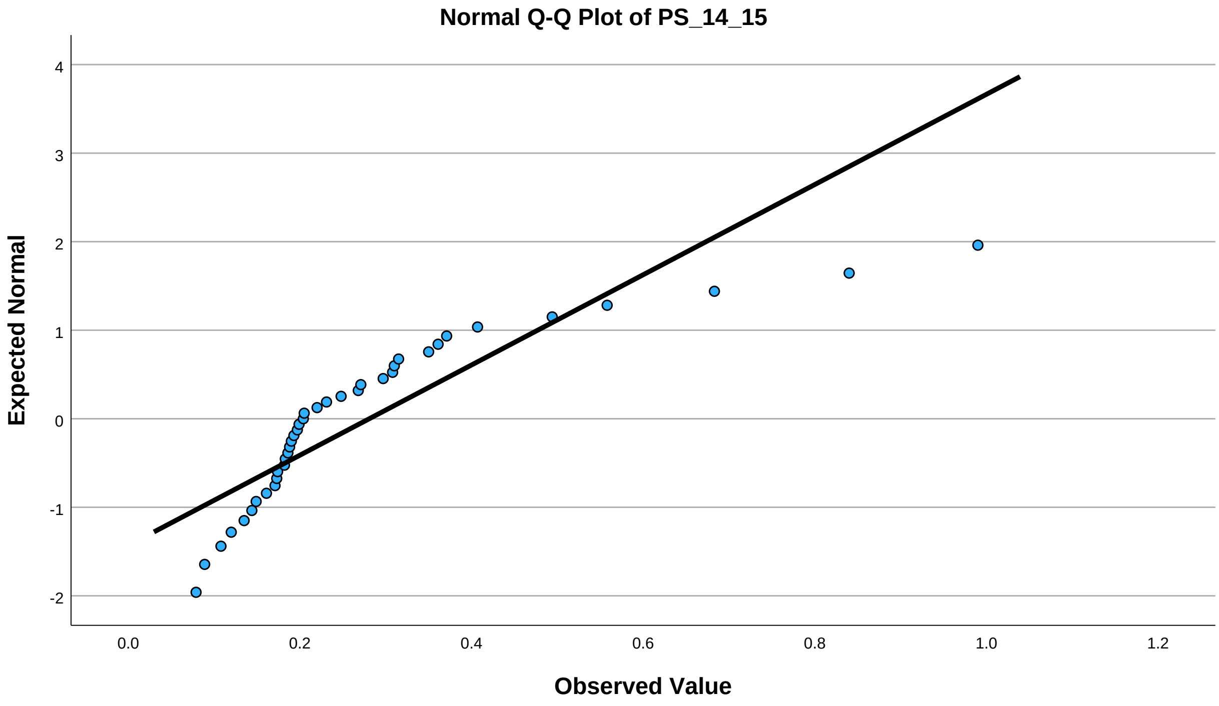

Normal Q-Q Plot of PS_14_15
Expected Normal
Observed Value
4
3
2
1
0
-1
-2
0.0
0.2
0.4
0.6
0.8
1.0
1.2

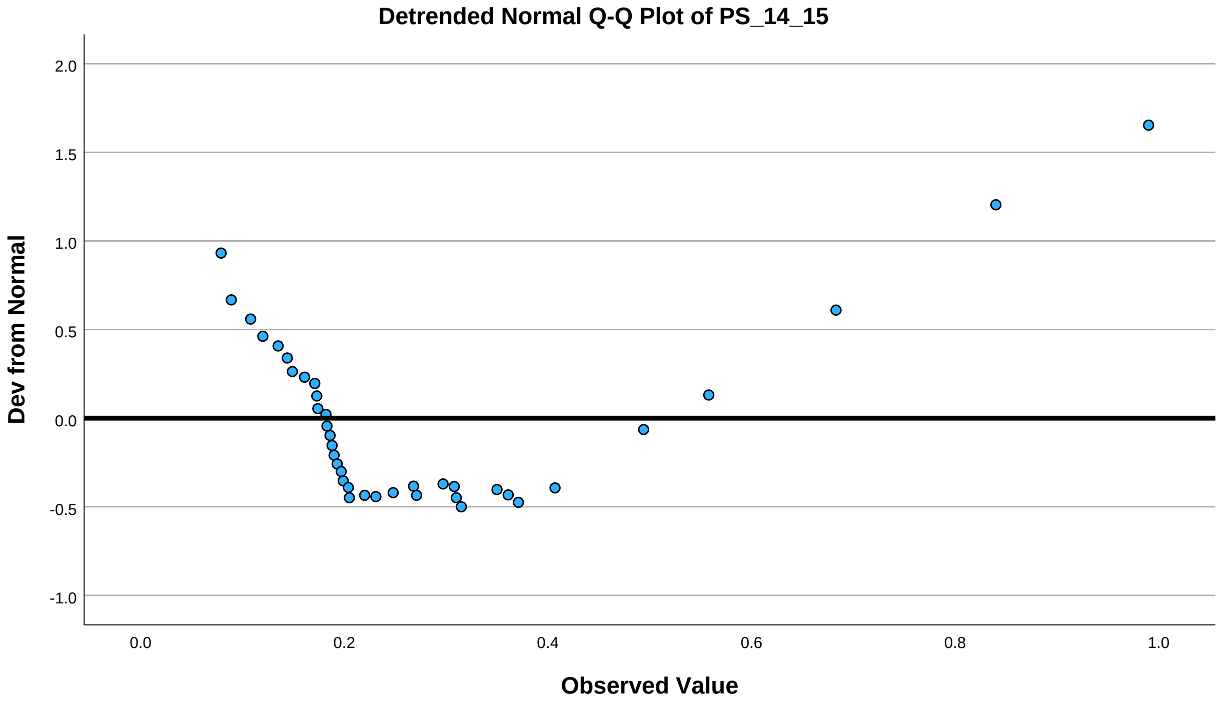

Detrended Normal Q-Q Plot of PS_14_15
Dev from Normal
Observed Value
2.0
1.5
1.0
0.5
0.0
-0.5
-1.0
0.0
0.2
0.4
0.6
0.8
1.0

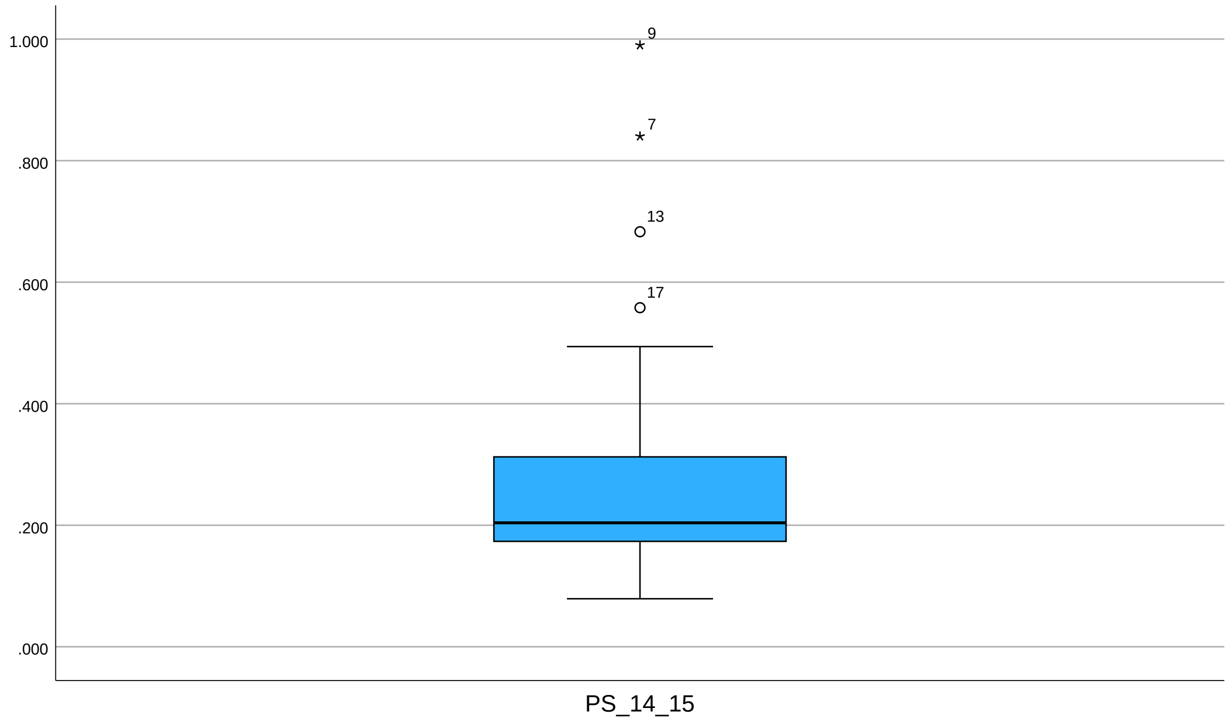

PS_15_16

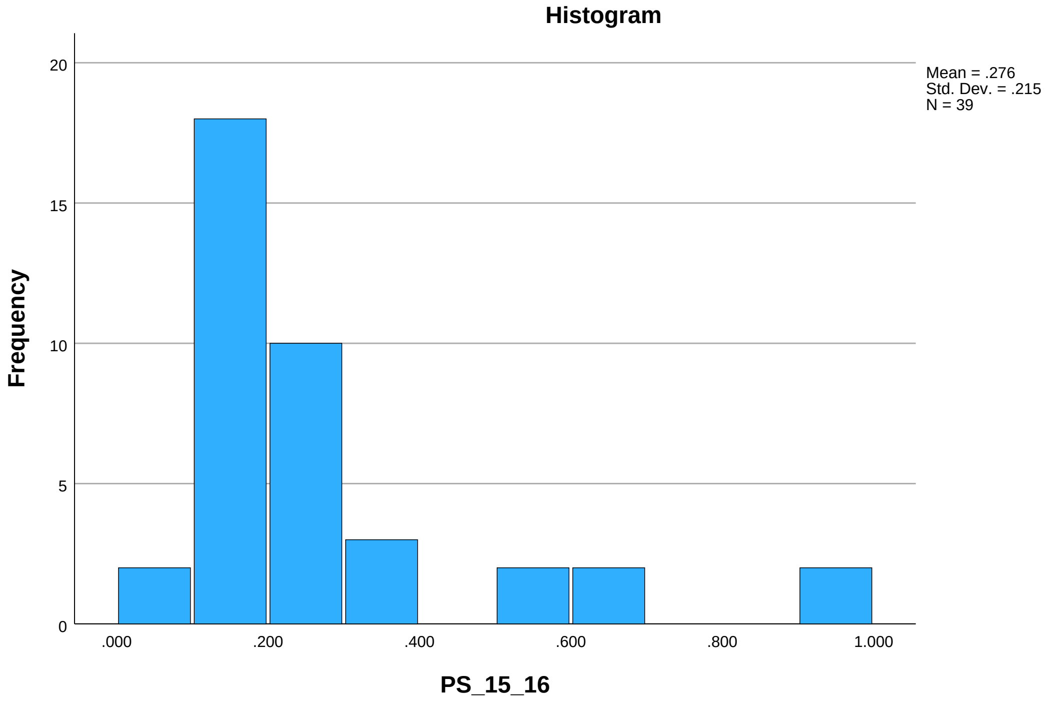


```
PS_15_16 Stem-and-Leaf Plot

 Frequency    Stem &  Leaf
```

```
  2.00      0 . 78
  8.00       1 . 01233334
 10.00       1 . 5566677889
  5.00      2 . 01233
  5.00      2 . 58899
  2.00     3 . 04
  1.00     3 . 8
   .00     4 .
   .00     4 .
  1.00     5 . 1
  5.00 Extremes    (>=.58)

Stem width:      .100
Each leaf:       1 case(s)
```

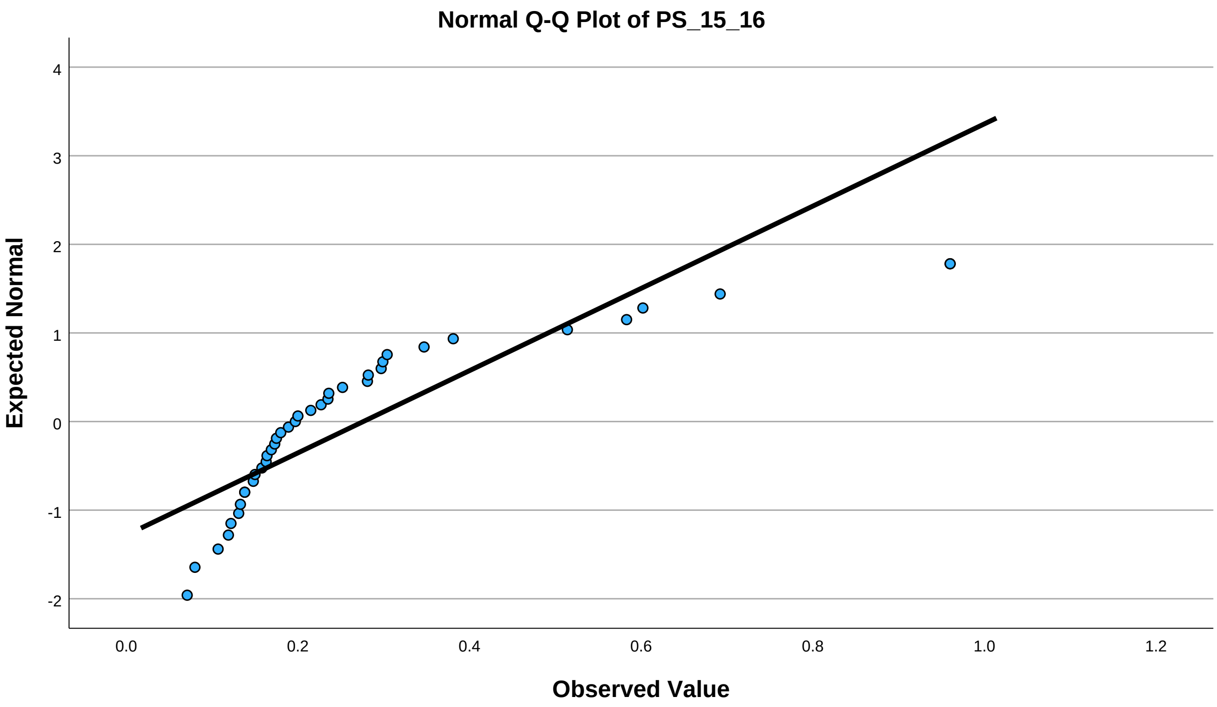

Detrended Normal Q-Q Plot of PS_15_16

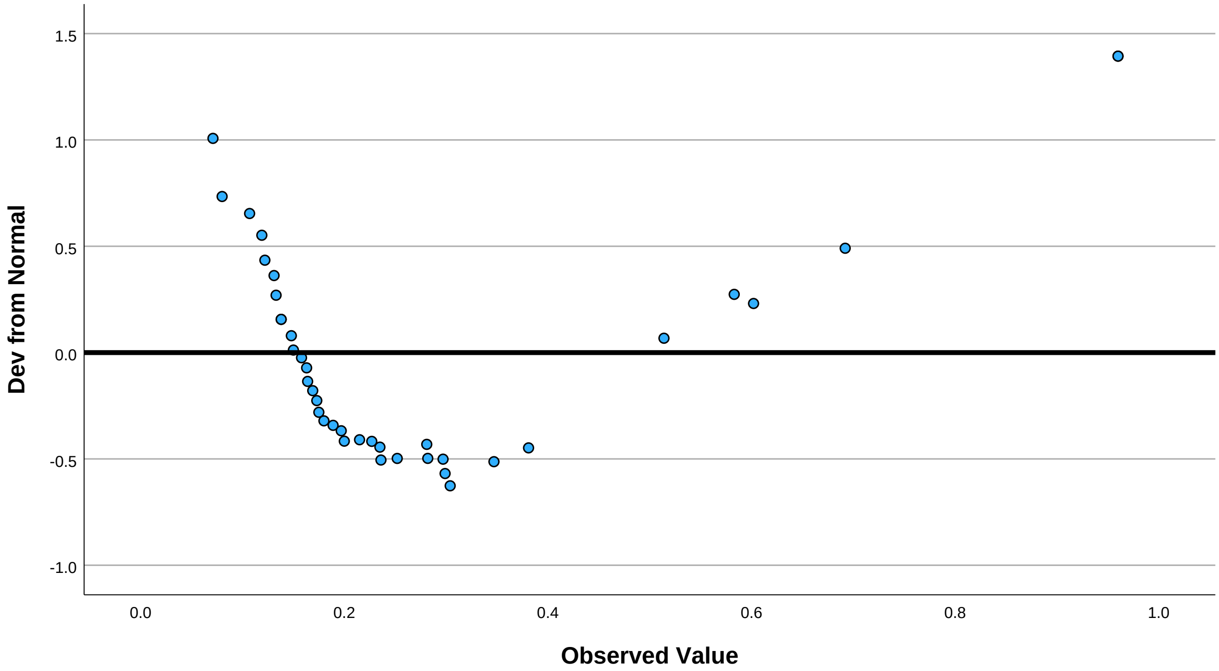

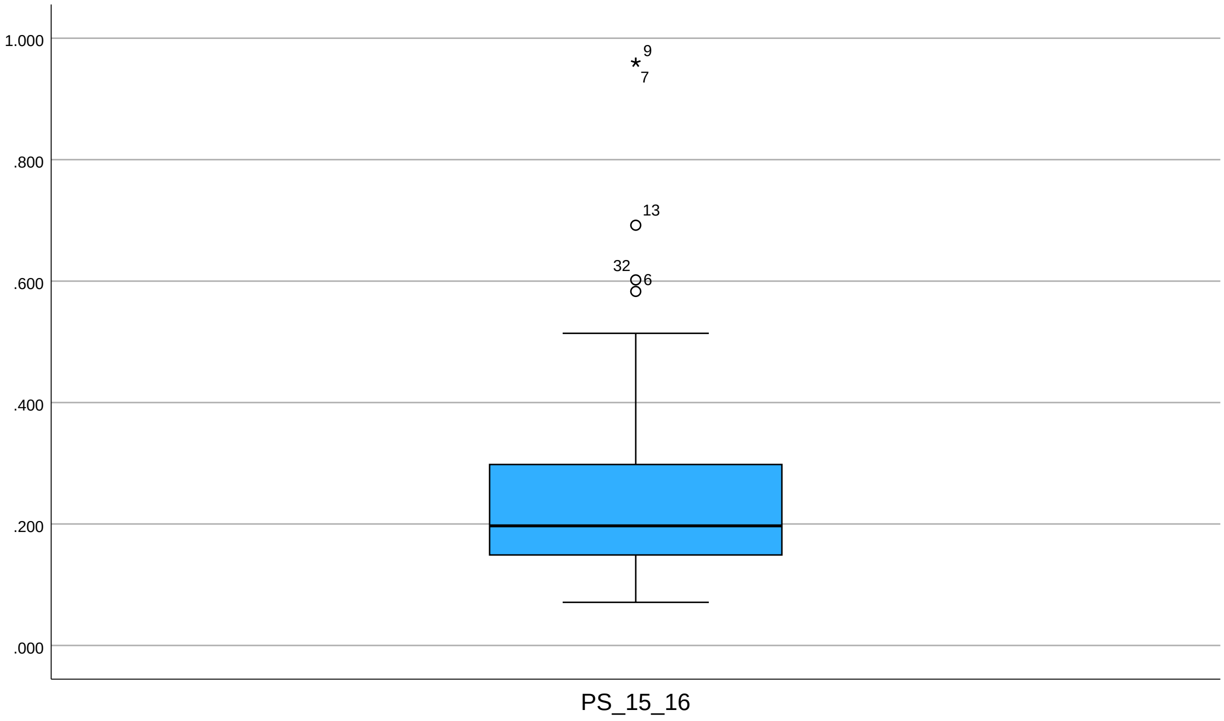

PS_16_17

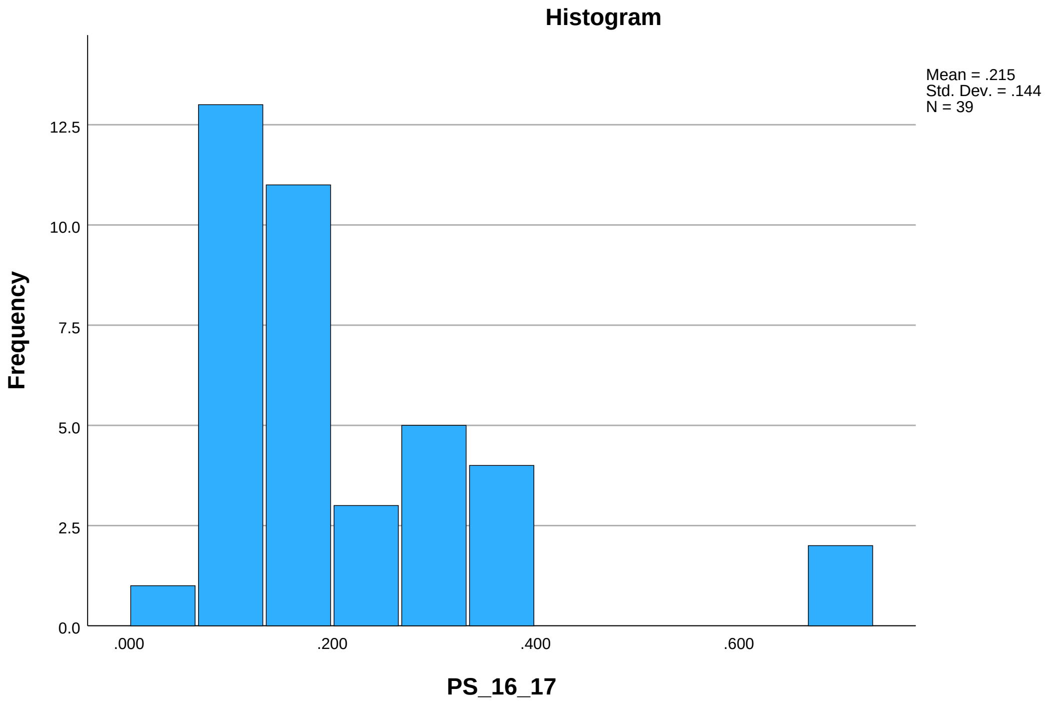


```
PS_16_17 Stem-and-Leaf Plot

 Frequency    Stem &  Leaf

     4.00        0 .  6799
    12.00        1 .  012222333334
     9.00        1 .  556677899
     3.00        2 .  112
     4.00        2 .  6778
     3.00        3 .  144
     1.00        3 .  8
     1.00        4 .  0
     2.00 Extremes    (>=.71)

 Stem width:      .100
 Each leaf:       1 case(s)
```

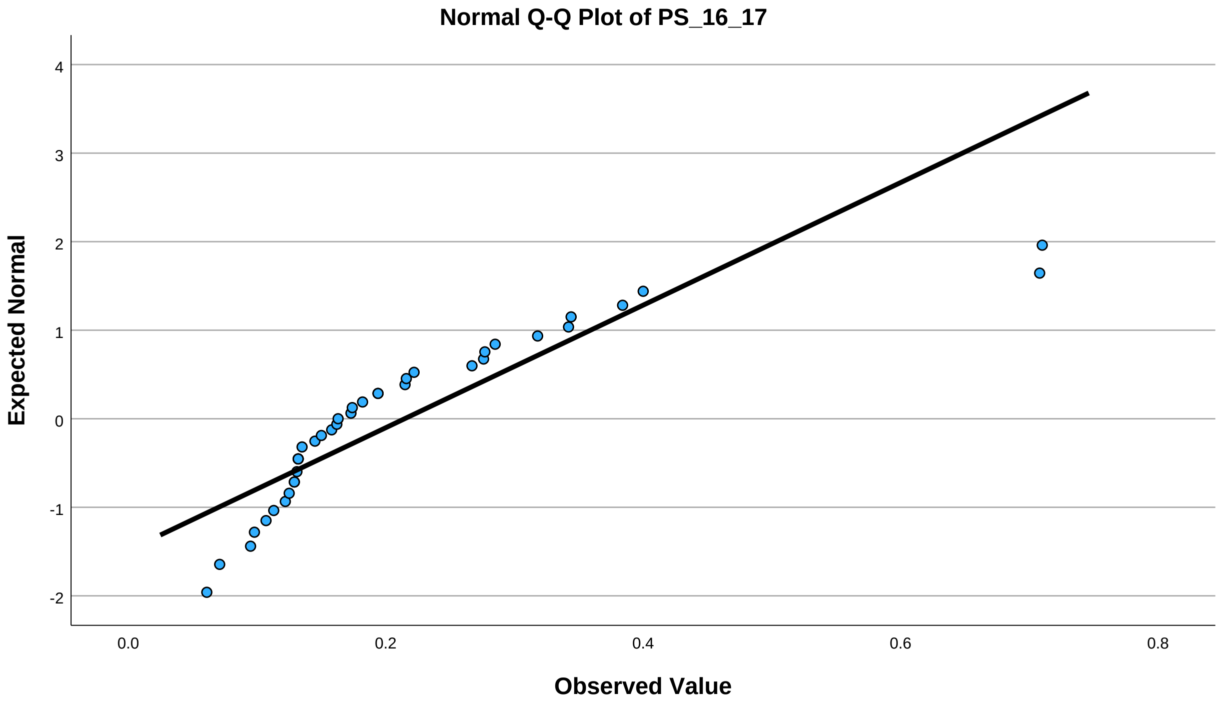
Normal Q-Q Plot of PS_16_17
Expected Normal
4
3
2
1
0
-1
-2
0.0
0.2
0.4
0.6
0.8
Observed Value

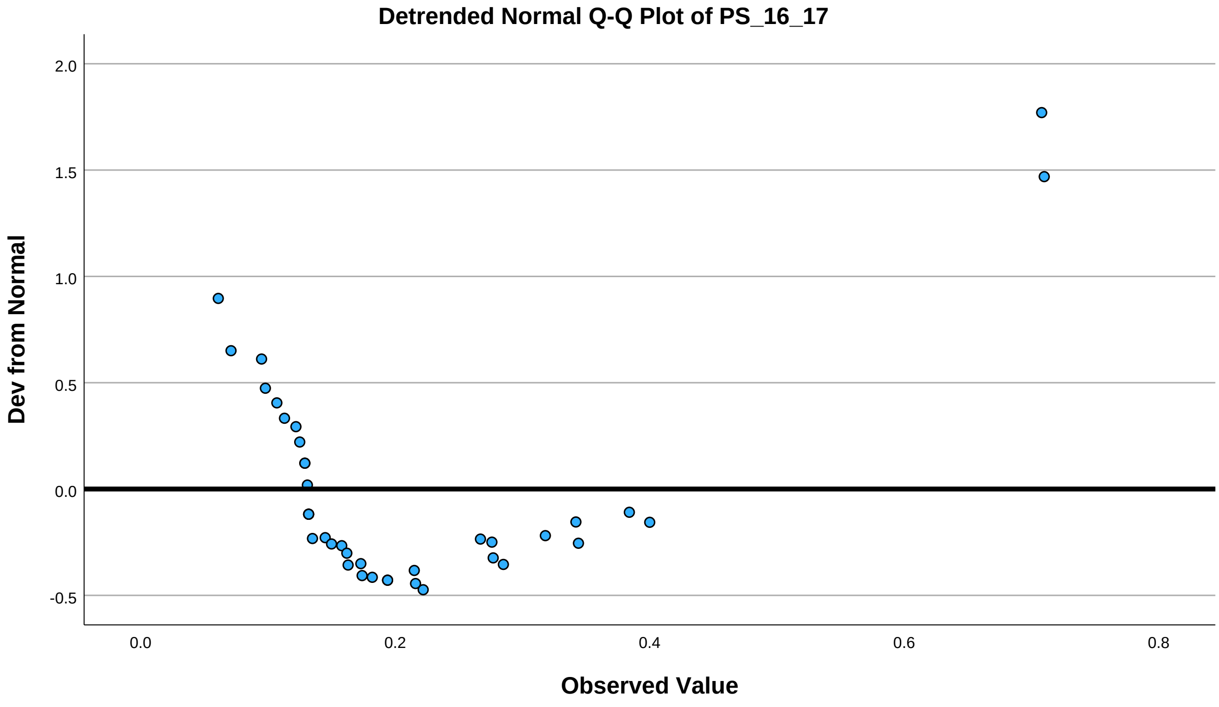
Detrended Normal Q-Q Plot of PS_16_17
Dev from Normal
2.0
1.5
1.0
0.5
0.0
-0.5
0.0
0.2
0.4
0.6
0.8
Observed Value

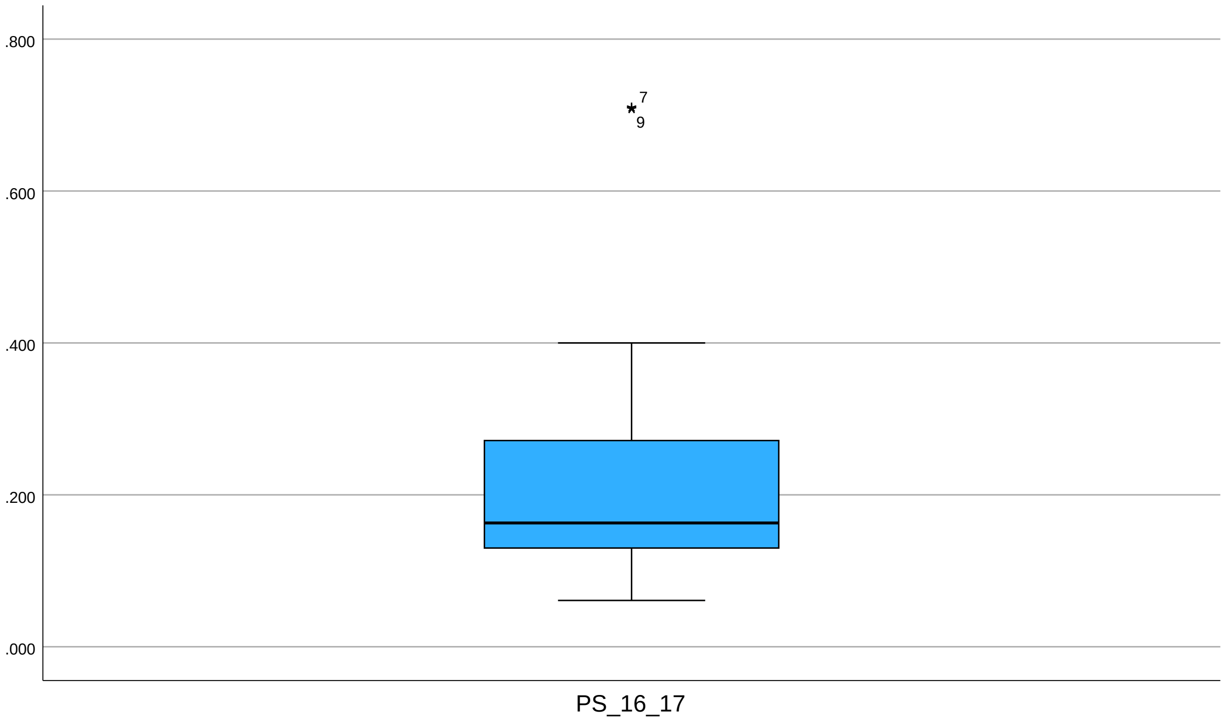

PS_17_18

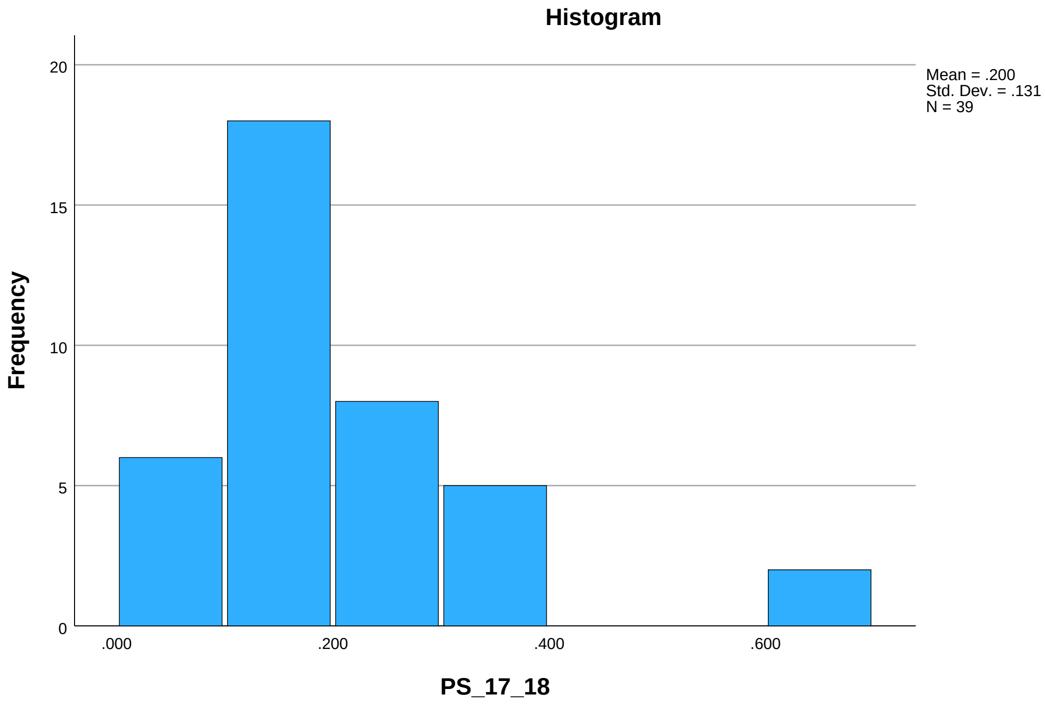

PS_17_18 Stem-and-Leaf Plot

Frequency Stem & Leaf

```
 6.00      0 . 578899
10.00      1 . 0011123344
 8.00      1 . 55555569
 6.00      2 . 000144
 2.00      2 . 56
 3.00      3 . 013
 2.00      3 . 66
 2.00 Extremes    (>=.61)

Stem width:      .100
Each leaf:       1 case(s)
```

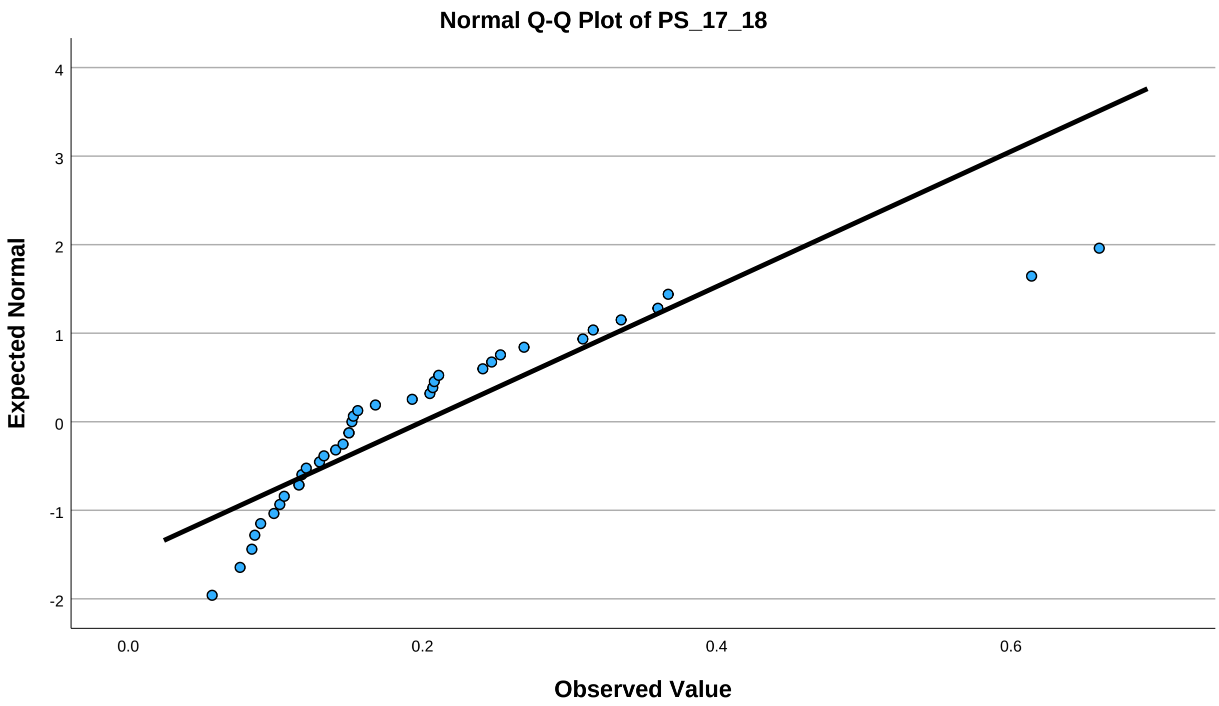

Detrended Normal Q-Q Plot of PS_17_18

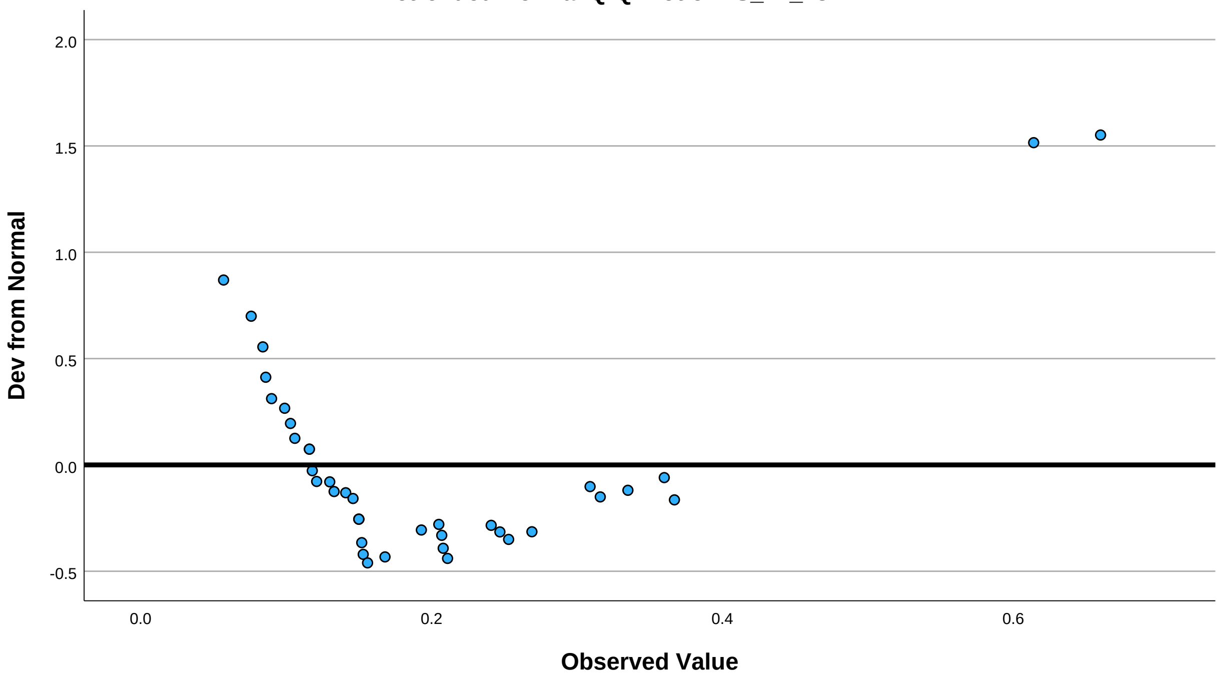

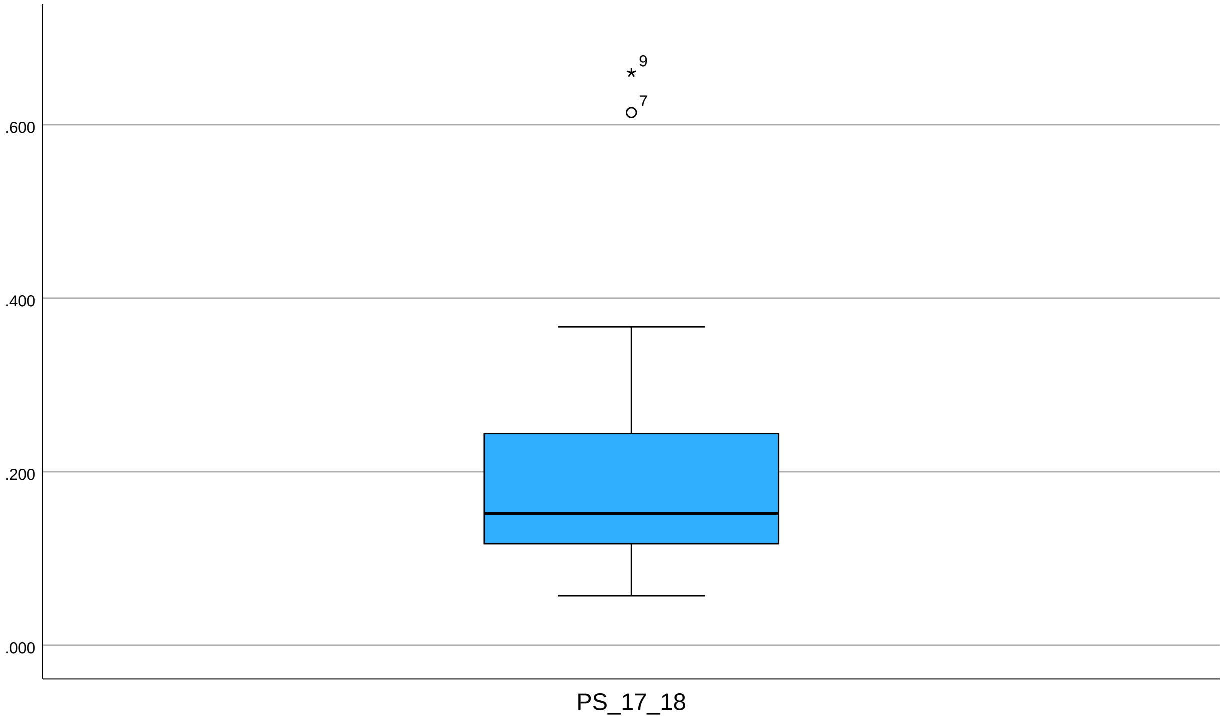

PS_18_19

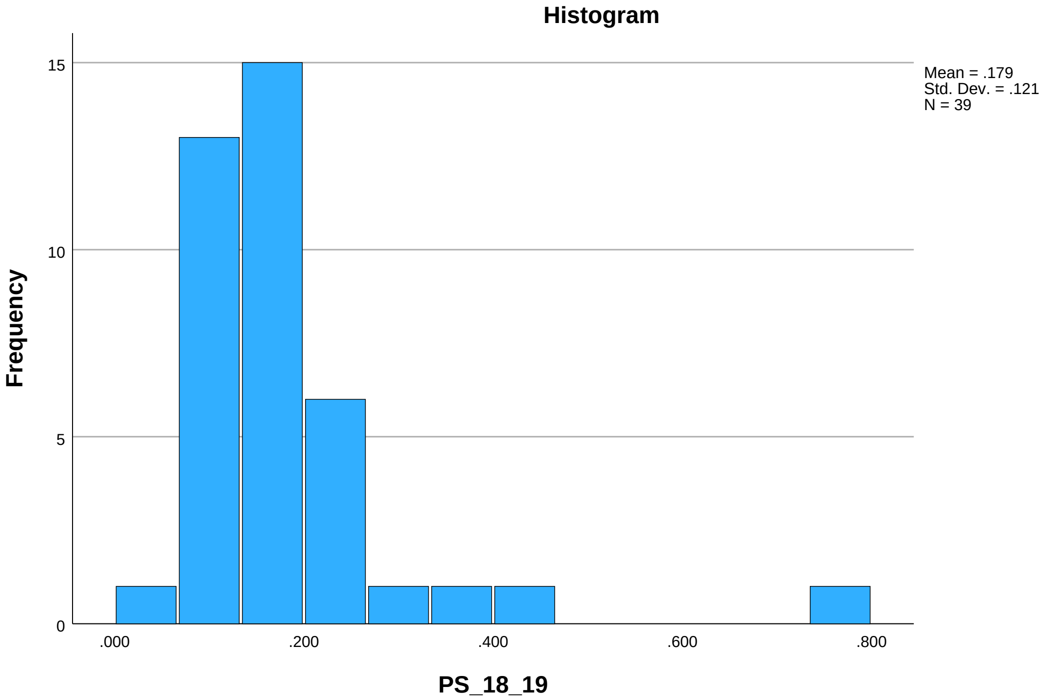

PS_18_19 Stem-and-Leaf Plot

```
 Frequency    Stem &  Leaf

     7.00        0 .  5678888
    12.00        1 .  011112333344
    10.00        1 .  5666667889
     4.00        2 .  1124
     2.00        2 .  66
     1.00        3 .  0
     3.00 Extremes    (>=.35)

 Stem width:      .100
 Each leaf:       1 case(s)
```

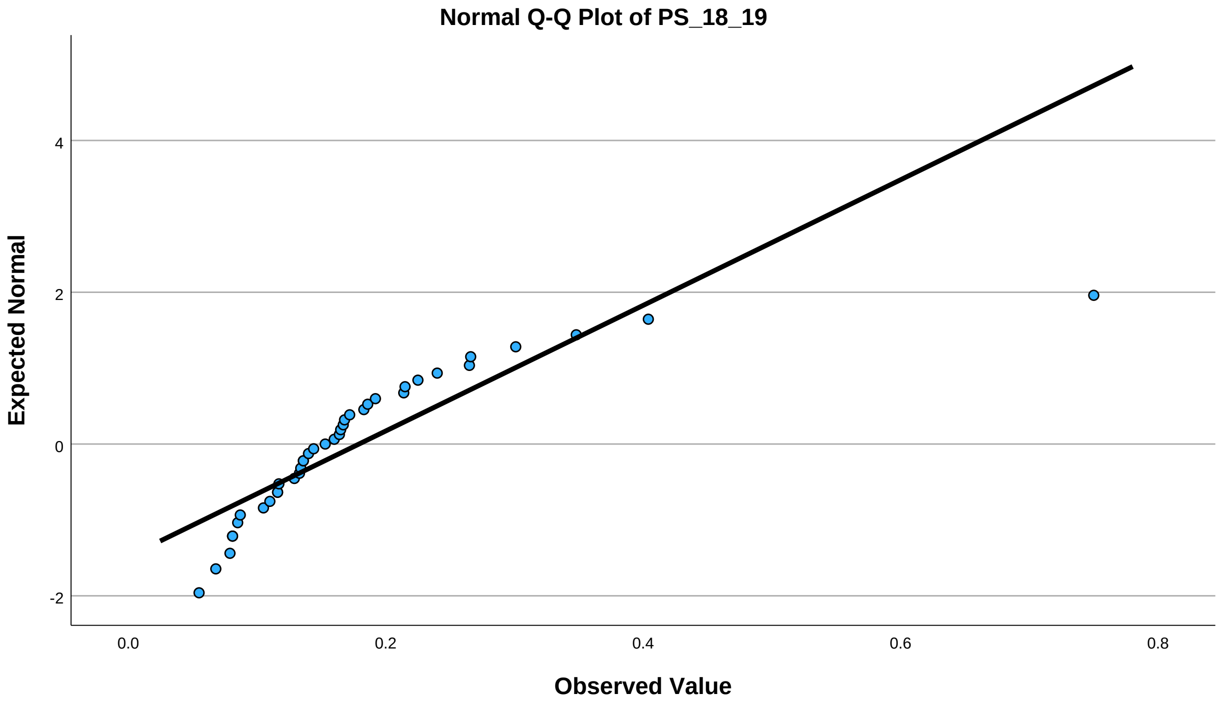
Normal Q-Q Plot of PS_18_19
Expected Normal
4
2
0
-2
0.0
0.2
0.4
0.6
0.8
Observed Value

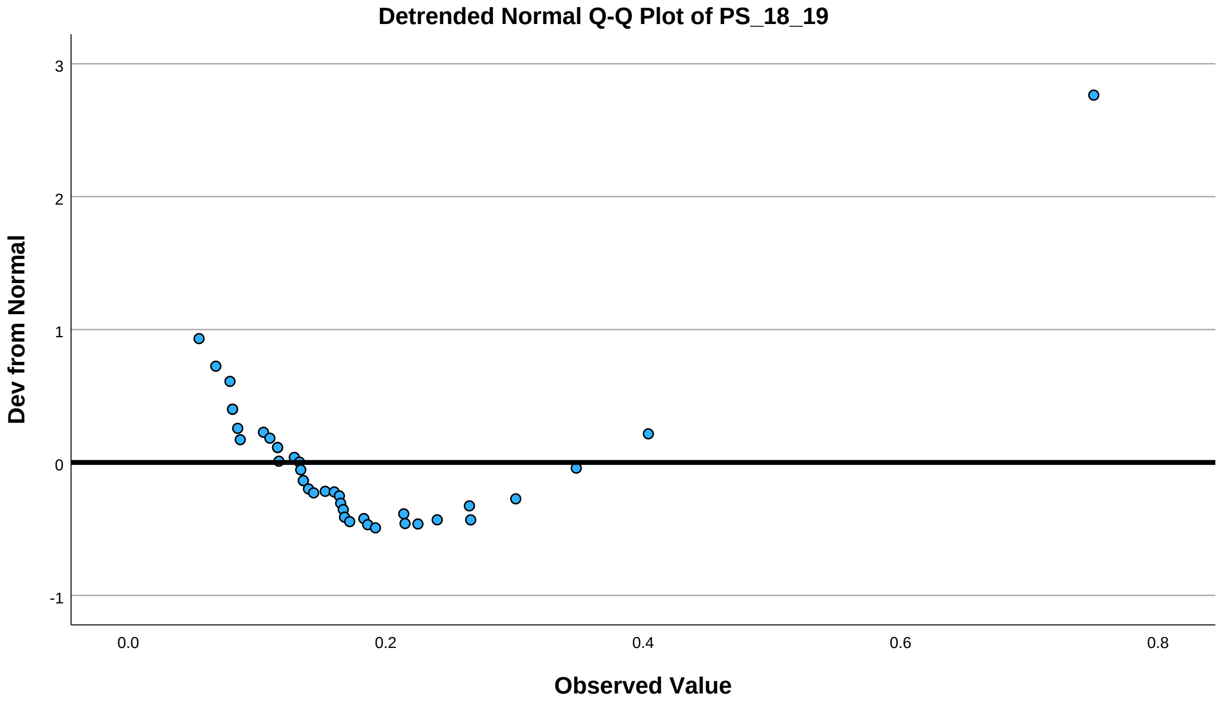
Detrended Normal Q-Q Plot of PS_18_19
Dev from Normal
3
2
1
0
-1
0.0
0.2
0.4
0.6
0.8
Observed Value

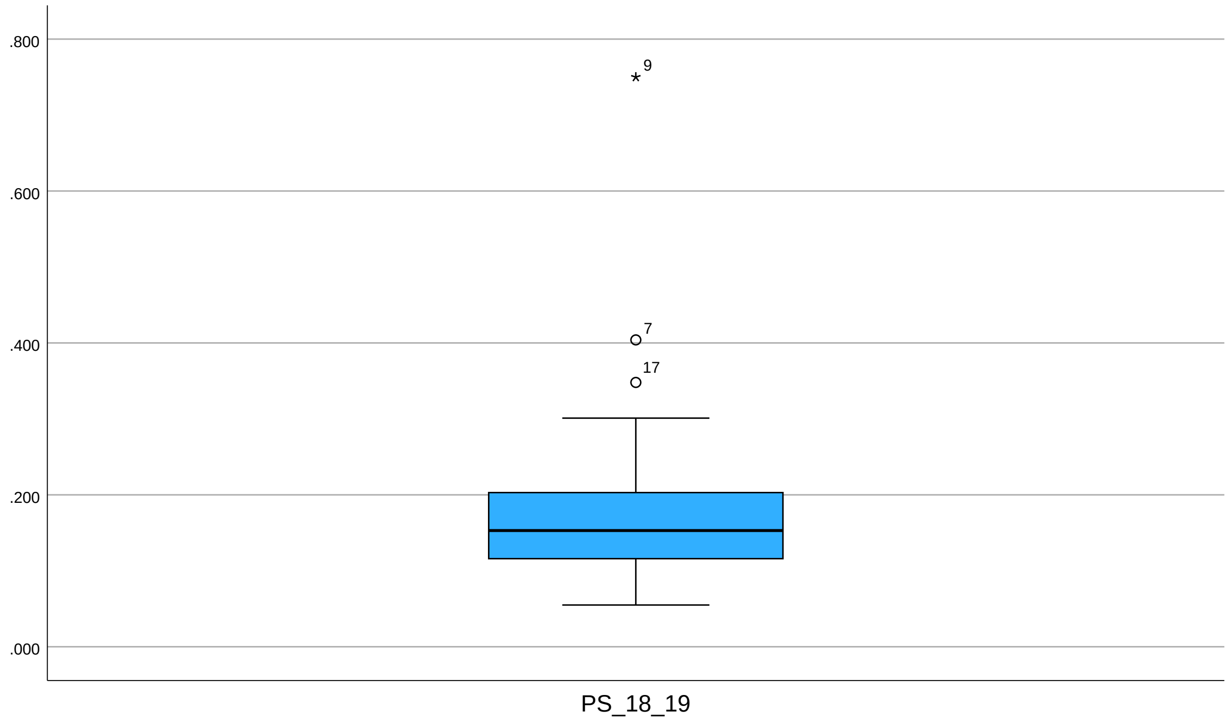

PS_19_20

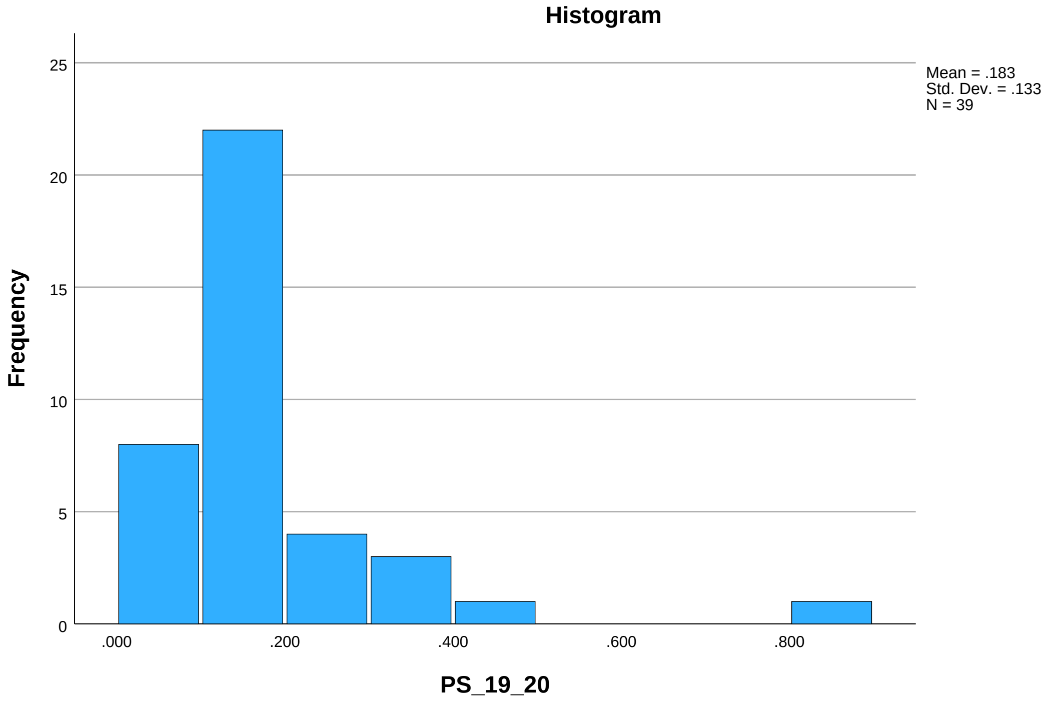


```
PS_19_20 Stem-and-Leaf Plot

 Frequency    Stem &  Leaf
```

```
  8.00       0 .  67778899
 13.00        1 .  0011113344444
  9.00       1 .  55789999
 3.00      2 .  024
 1.00      2 .  5
 1.00      3 .  2
  4.00 Extremes    (>=.33)

Stem width:      .100
Each leaf:       1 case(s)
```

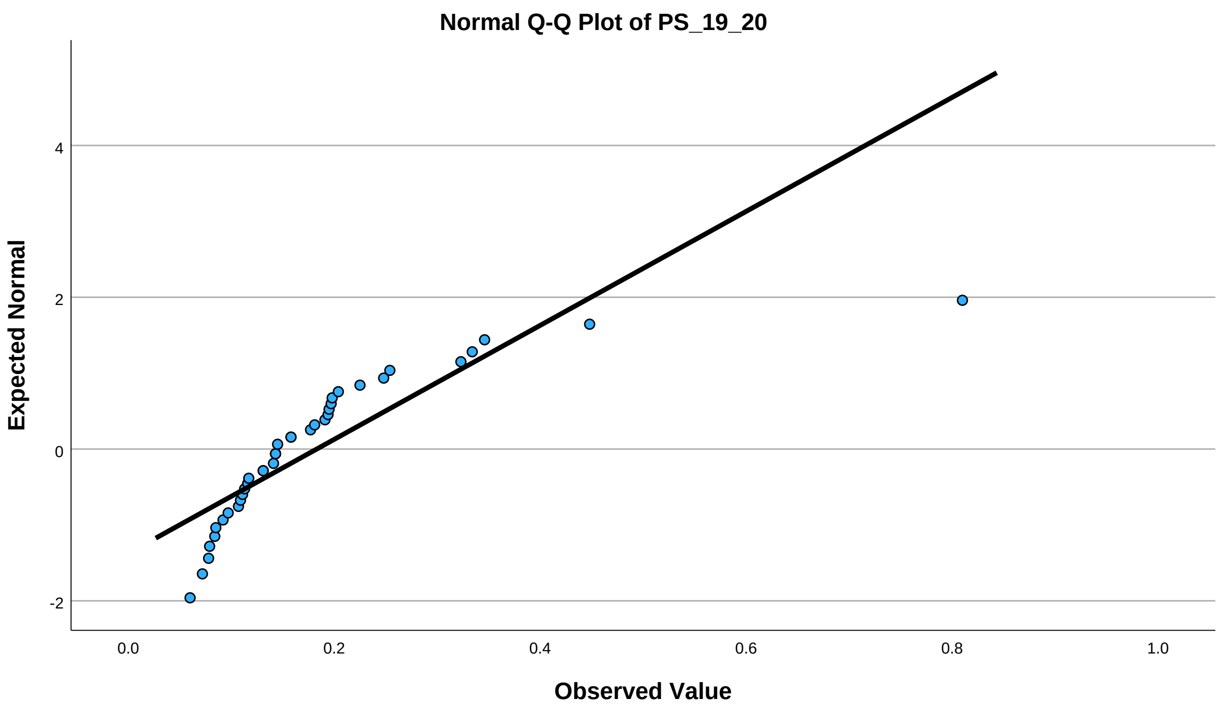

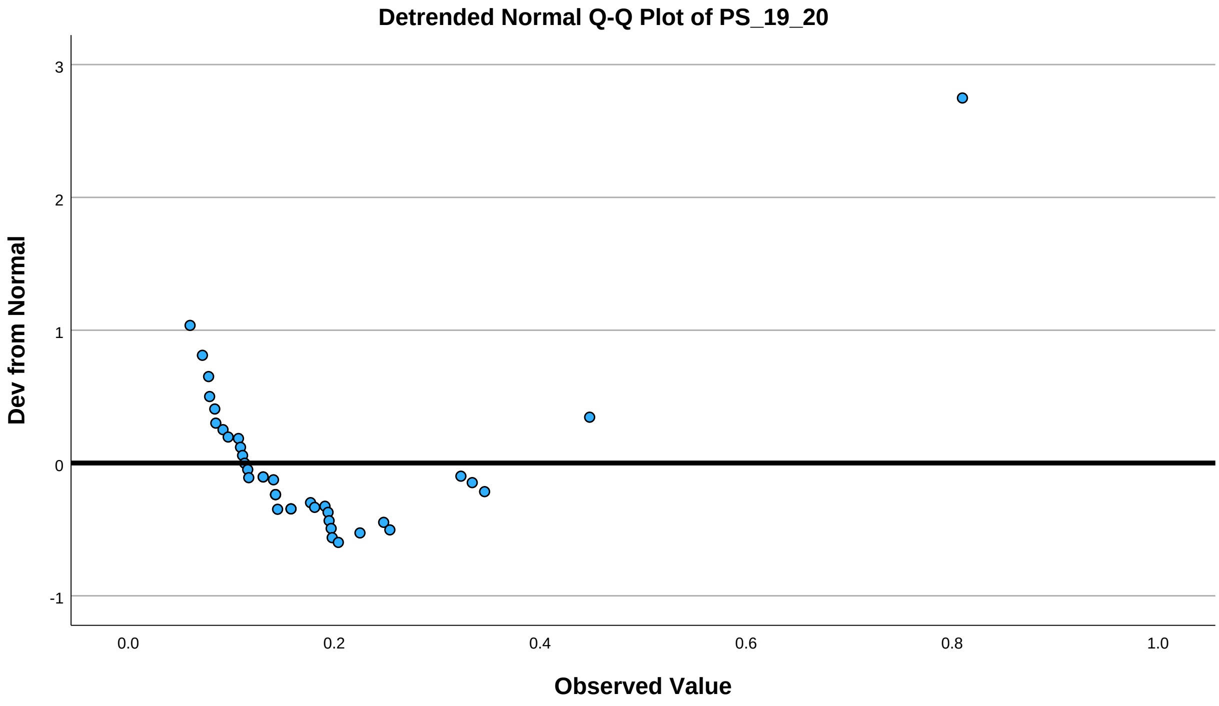
Detrended Normal Q-Q Plot of PS_19_20
Dev from Normal
3
2
1
0
-1
0.0
0.2
0.4
0.6
0.8
1.0
Observed Value

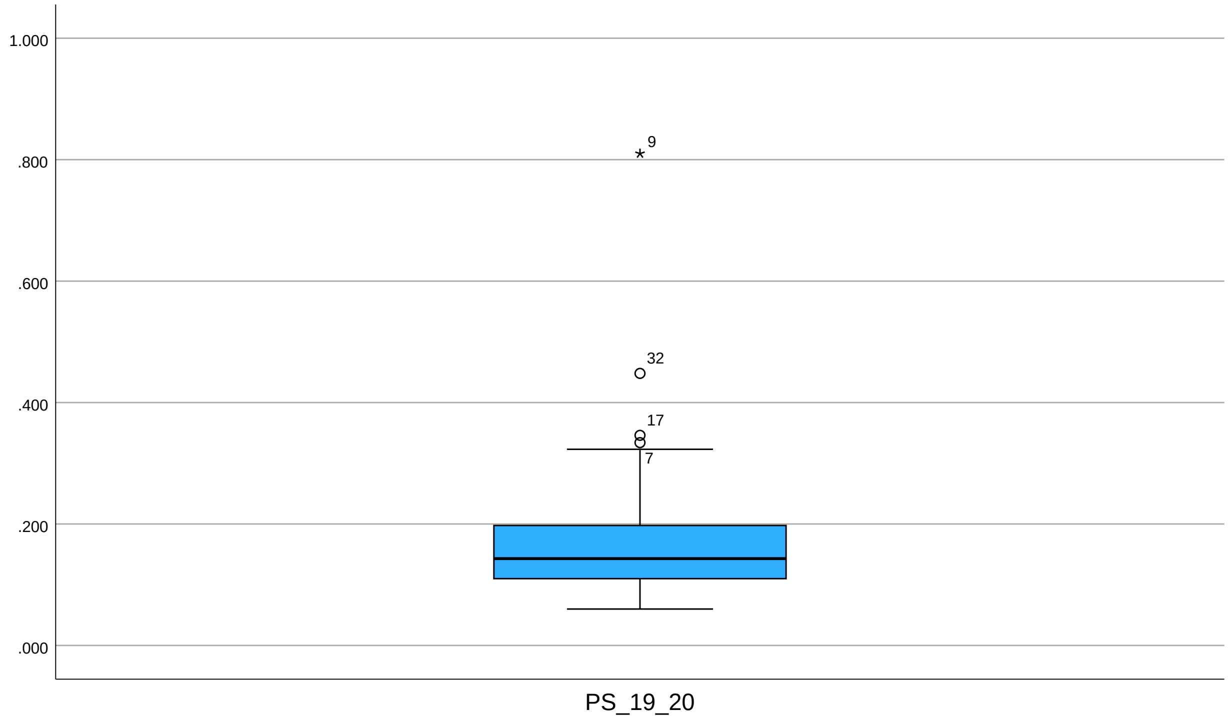
1.000
.800
.600
.400
.200
.000
9
32
17
7
PS_19_20

Explore

[DataSet14] C:\Users\aleks\OneDrive\Desktop\Results\SPSS Files\Channel11.sav

Case Processing Summary

	Cases					
	Valid		Missing		Total	
	N	Percent	N	Percent	N	Percent
BrainRate	39	100.0%	0	0.0%	39	100.0%
PermutationEntropy	39	100.0%	0	0.0%	39	100.0%
PermutationEntropy_01	39	100.0%	0	0.0%	39	100.0%
SampleEntropy	39	100.0%	0	0.0%	39	100.0%
SpectralEntropy_bits	39	100.0%	0	0.0%	39	100.0%
SpectralEntropy_01	39	100.0%	0	0.0%	39	100.0%
HiguchiFractalDimension	39	100.0%	0	0.0%	39	100.0%
LempelZivComplexity	39	100.0%	0	0.0%	39	100.0%
RenyiEntropy	39	100.0%	0	0.0%	39	100.0%
TsallisEntropy	39	100.0%	0	0.0%	39	100.0%
PS_4_5	39	100.0%	0	0.0%	39	100.0%
PS_5_6	39	100.0%	0	0.0%	39	100.0%
PS_6_7	39	100.0%	0	0.0%	39	100.0%
PS_7_8	39	100.0%	0	0.0%	39	100.0%
PS_8_9	39	100.0%	0	0.0%	39	100.0%
PS_9_10	39	100.0%	0	0.0%	39	100.0%
PS_10_11	39	100.0%	0	0.0%	39	100.0%
PS_11_12	39	100.0%	0	0.0%	39	100.0%
PS_12_13	39	100.0%	0	0.0%	39	100.0%
PS_13_14	39	100.0%	0	0.0%	39	100.0%
PS_14_15	39	100.0%	0	0.0%	39	100.0%
PS_15_16	39	100.0%	0	0.0%	39	100.0%
PS_16_17	39	100.0%	0	0.0%	39	100.0%
PS_17_18	39	100.0%	0	0.0%	39	100.0%
PS_18_19	39	100.0%	0	0.0%	39	100.0%
PS_19_20	39	100.0%	0	0.0%	39	100.0%

Descriptives

			Statistic	Std. Error
BrainRate	Mean		7.5572	.46379
	95% Confidence Interval for Mean	Lower Bound	6.6183	
		Upper Bound	8.4961	
	5% Trimmed Mean		7.2564	
	Median		7.1800	
	Variance		8.389	
	Std. Deviation		2.89636	
	Minimum		3.33	
	Maximum		21.53	
	Range		18.20	
	Interquartile Range		2.34	
	Skewness		3.048	.378
	Kurtosis		13.971	.741
PermutationEntropy	Mean		1.4380112555	.01008346275
	95% Confidence Interval for Mean	Lower Bound	1.4175983523	
		Upper Bound	1.4584241586	
	5% Trimmed Mean		1.4363357938	
	Median		1.4449575857	
	Variance		.004	
	Std. Deviation		.06297120467	
	Minimum		1.3184006460	
	Maximum		1.6354229873	
	Range		.31702234125	
	Interquartile Range		.08033278573	
	Skewness		.345	.378
	Kurtosis		1.327	.741
PermutationEntropy_01	Mean		.80256936277	.00562768771
	95% Confidence Interval for Mean	Lower Bound	.79117670461	
		Upper Bound	.81396202093	
	5% Trimmed Mean		.80163426979	
	Median		.80644618351	
	Variance		.001	
	Std. Deviation		.03514489849	
	Minimum		.73581341060	
	Maximum		.91274694811	
	Range		.17693353750	
	Interquartile Range		.04483458138	
	Skewness		.345	.378
	Kurtosis		1.327	.741

Descriptives

			Statistic	Std. Error
SampleEntropy	Mean		.62397499004	.02610580285
	95% Confidence Interval for Mean	Lower Bound	.57112655510	
		Upper Bound	.67682342498	
	5% Trimmed Mean		.62980228825	
	Median		.65002635724	
	Variance		.027	
	Std. Deviation		.16303068656	
	Minimum		.17289101621	
	Maximum		.95685259451	
	Range		.78396157829	
	Interquartile Range		.12189156728	
	Skewness		-.740	.378
	Kurtosis		.821	.741
SpectralEntropy_bits	Mean		4.0695212633	.08241406119
	95% Confidence Interval for Mean	Lower Bound	3.9026827188	
		Upper Bound	4.2363598078	
	5% Trimmed Mean		4.0885585126	
	Median		4.1753789075	
	Variance		.265	
	Std. Deviation		.51467564714	
	Minimum		2.3347400893	
	Maximum		5.5092499238	
	Range		3.1745098344	
	Interquartile Range		.45986209201	
	Skewness		-.694	.378
	Kurtosis		3.826	.741
SpectralEntropy_01	Mean		.58042923372	.01175458421
	95% Confidence Interval for Mean	Lower Bound	.55663332205	
		Upper Bound	.60422514539	
	5% Trimmed Mean		.58314448579	
	Median		.59552753824	
	Variance		.005	
	Std. Deviation		.07340735486	
	Minimum		.33300020157	
	Maximum		.78577540323	
	Range		.45277520166	
	Interquartile Range		.06558938617	
	Skewness		-.694	.378
	Kurtosis		3.826	.741

Descriptives

			Statistic	Std. Error
HiguchiFractalDimension	Mean		.60342696283	.01516341688
	95% Confidence Interval for Mean	Lower Bound	.57273023019	
		Upper Bound	.63412369547	
	5% Trimmed Mean		.61267660955	
	Median		.61585030692	
	Variance		.009	
	Std. Deviation		.09469550808	
	Minimum		.23291228262	
	Maximum		.73074333177	
	Range		.49783104916	
	Interquartile Range		.10564125939	
	Skewness		-1.784	.378
	Kurtosis		5.107	.741
LempelZivComplexity	Mean		.26617659662	.00492762629
	95% Confidence Interval for Mean	Lower Bound	.25620113871	
		Upper Bound	.27615205452	
	5% Trimmed Mean		.26407954833	
	Median		.26304292157	
	Variance		.001	
	Std. Deviation		.03077301632	
	Minimum		.21301505990	
	Maximum		.40167513596	
	Range		.18866007606	
	Interquartile Range		.02808306170	
	Skewness		2.155	.378
	Kurtosis		9.187	.741
RenyiEntropy	Mean		7.3194826085	.17578899758
	95% Confidence Interval for Mean	Lower Bound	6.9636163877	
		Upper Bound	7.6753488292	
	5% Trimmed Mean		7.3610532685	
	Median		7.5620927605	
	Variance		1.205	
	Std. Deviation		1.0978019380	
	Minimum		5.0022082218	
	Maximum		8.7516068842	
	Range		3.7493986624	
	Interquartile Range		2.2865395748	
	Skewness		-.645	.378
	Kurtosis		-.763	.741

Descriptives

			Statistic	Std. Error
TsallisEntropy	Mean		1.5192436960	.08638248654
	95% Confidence Interval for Mean	Lower Bound	1.3443714944	
		Upper Bound	1.6941158977	
	5% Trimmed Mean		1.5545684865	
	Median		1.7027392803	
	Variance		.291	
	Std. Deviation		.53945845556	
	Minimum		.41094292253	
	Maximum		1.9868108448	
	Range		1.5758679223	
	Interquartile Range		.80197281126	
	Skewness		-.923	.378
	Kurtosis		-.466	.741
PS_4_5	Mean		1.83308	.211773
	95% Confidence Interval for Mean	Lower Bound	1.40437	
		Upper Bound	2.26179	
	5% Trimmed Mean		1.67728	
	Median		1.49800	
	Variance		1.749	
	Std. Deviation		1.322521	
	Minimum		.475	
	Maximum		7.820	
	Range		7.345	
	Interquartile Range		.850	
	Skewness		2.766	.378
	Kurtosis		10.391	.741
PS_5_6	Mean		1.51956	.194052
	95% Confidence Interval for Mean	Lower Bound	1.12673	
		Upper Bound	1.91240	
	5% Trimmed Mean		1.36805	
	Median		1.25900	
	Variance		1.469	
	Std. Deviation		1.211855	
	Minimum		.321	
	Maximum		7.910	
	Range		7.589	
	Interquartile Range		.822	
	Skewness		4.019	.378
	Kurtosis		20.767	.741

Descriptives

			Statistic	Std. Error
PS_6_7	Mean		1.20305	.158930
	95% Confidence Interval for Mean	Lower Bound	.88131	
		Upper Bound	1.52479	
	5% Trimmed Mean		1.07977	
	Median		1.06300	
	Variance		.985	
	Std. Deviation		.992519	
	Minimum		.210	
	Maximum		6.590	
	Range		6.380	
	Interquartile Range		.611	
	Skewness		4.354	.378
	Kurtosis		23.632	.741
PS_7_8	Mean		.96746	.103257
	95% Confidence Interval for Mean	Lower Bound	.75843	
		Upper Bound	1.17649	
	5% Trimmed Mean		.88583	
	Median		.81800	
	Variance		.416	
	Std. Deviation		.644841	
	Minimum		.198	
	Maximum		3.600	
	Range		3.402	
	Interquartile Range		.376	
	Skewness		2.403	.378
	Kurtosis		7.488	.741
PS_8_9	Mean		.98390	.140085
	95% Confidence Interval for Mean	Lower Bound	.70031	
		Upper Bound	1.26748	
	5% Trimmed Mean		.86218	
	Median		.74300	
	Variance		.765	
	Std. Deviation		.874828	
	Minimum		.160	
	Maximum		5.240	
	Range		5.080	
	Interquartile Range		.607	
	Skewness		3.360	.378
	Kurtosis		14.631	.741

Descriptives

			Statistic	Std. Error
PS_9_10	Mean		1.71272	.515180
	95% Confidence Interval for Mean	Lower Bound	.66979	
		Upper Bound	2.75565	
	5% Trimmed Mean		1.18713	
	Median		.92300	
	Variance		10.351	
	Std. Deviation		3.217301	
	Minimum		.187	
	Maximum		20.340	
	Range		20.153	
	Interquartile Range		1.234	
	Skewness		5.384	.378
	Kurtosis		31.420	.741
PS_10_11	Mean		2.81423	.587587
	95% Confidence Interval for Mean	Lower Bound	1.62472	
		Upper Bound	4.00374	
	5% Trimmed Mean		2.29789	
	Median		1.31900	
	Variance		13.465	
	Std. Deviation		3.669477	
	Minimum		.282	
	Maximum		21.050	
	Range		20.768	
	Interquartile Range		3.305	
	Skewness		3.494	.378
	Kurtosis		15.865	.741
PS_11_12	Mean		.94046	.180084
	95% Confidence Interval for Mean	Lower Bound	.57590	
		Upper Bound	1.30502	
	5% Trimmed Mean		.78255	
	Median		.58800	
	Variance		1.265	
	Std. Deviation		1.124622	
	Minimum		.168	
	Maximum		4.627	
	Range		4.459	
	Interquartile Range		.531	
	Skewness		2.480	.378
	Kurtosis		5.355	.741

Descriptives

			Statistic	Std. Error
PS_12_13	Mean		.40033	.042830
	95% Confidence Interval for Mean	Lower Bound	.31363	
		Upper Bound	.48704	
	5% Trimmed Mean		.36530	
	Median		.32400	
	Variance		.072	
	Std. Deviation		.267473	
	Minimum		.136	
	Maximum		1.460	
	Range		1.324	
	Interquartile Range		.229	
	Skewness		2.319	.378
	Kurtosis		6.290	.741
PS_13_14	Mean		.33036	.042218
	95% Confidence Interval for Mean	Lower Bound	.24489	
		Upper Bound	.41582	
	5% Trimmed Mean		.29202	
	Median		.24400	
	Variance		.070	
	Std. Deviation		.263652	
	Minimum		.126	
	Maximum		1.390	
	Range		1.264	
	Interquartile Range		.184	
	Skewness		2.629	.378
	Kurtosis		7.476	.741
PS_14_15	Mean		.30362	.039487
	95% Confidence Interval for Mean	Lower Bound	.22368	
		Upper Bound	.38355	
	5% Trimmed Mean		.27179	
	Median		.20000	
	Variance		.061	
	Std. Deviation		.246595	
	Minimum		.101	
	Maximum		1.159	
	Range		1.058	
	Interquartile Range		.167	
	Skewness		2.223	.378
	Kurtosis		4.632	.741

Descriptives

			Statistic	Std. Error
PS_15_16	Mean		.30938	.047922
	95% Confidence Interval for Mean	Lower Bound	.21237	
		Upper Bound	.40640	
	5% Trimmed Mean		.26731	
	Median		.19400	
	Variance		.090	
	Std. Deviation		.299276	
	Minimum		.082	
	Maximum		1.714	
	Range		1.632	
	Interquartile Range		.172	
	Skewness		3.185	.378
	Kurtosis		12.496	.741
PS_16_17	Mean		.28656	.046080
	95% Confidence Interval for Mean	Lower Bound	.19328	
		Upper Bound	.37985	
	5% Trimmed Mean		.24204	
	Median		.19800	
	Variance		.083	
	Std. Deviation		.287768	
	Minimum		.079	
	Maximum		1.717	
	Range		1.638	
	Interquartile Range		.146	
	Skewness		3.648	.378
	Kurtosis		16.185	.741
PS_17_18	Mean		.25187	.037600
	95% Confidence Interval for Mean	Lower Bound	.17575	
		Upper Bound	.32799	
	5% Trimmed Mean		.21736	
	Median		.17900	
	Variance		.055	
	Std. Deviation		.234811	
	Minimum		.070	
	Maximum		1.432	
	Range		1.362	
	Interquartile Range		.156	
	Skewness		3.673	.378
	Kurtosis		16.862	.741

Descriptives

			Statistic	Std. Error
PS_18_19	Mean		.23259	.030116
	95% Confidence Interval for Mean	Lower Bound	.17162	
		Upper Bound	.29356	
	5% Trimmed Mean		.20779	
	Median		.16400	
	Variance		.035	
	Std. Deviation		.188075	
	Minimum		.083	
	Maximum		.830	
	Range		.747	
	Interquartile Range		.166	
	Skewness		2.313	.378
	Kurtosis		5.118	.741
PS_19_20	Mean		.23162	.032782
	95% Confidence Interval for Mean	Lower Bound	.16525	
		Upper Bound	.29798	
	5% Trimmed Mean		.20176	
	Median		.18200	
	Variance		.042	
	Std. Deviation		.204726	
	Minimum		.072	
	Maximum		1.240	
	Range		1.168	
	Interquartile Range		.121	
	Skewness		3.519	.378
	Kurtosis		15.516	.741

Tests of Normality

	Kolmogorov-Smirnov[a]			Shapiro-Wilk		
	Statistic	df	Sig.	Statistic	df	Sig.
BrainRate	.193	39	<.001	.738	39	<.001
PermutationEntropy	.117	39	.193	.950	39	.083
PermutationEntropy_01	.117	39	.193	.950	39	.083
SampleEntropy	.206	39	<.001	.944	39	.050
SpectralEntropy_bits	.133	39	.079	.912	39	.005
SpectralEntropy_01	.133	39	.079	.912	39	.005
HiguchiFractalDimension	.131	39	.092	.868	39	<.001
LempelZivComplexity	.155	39	.020	.830	39	<.001
RenyiEntropy	.152	39	.023	.904	39	.003
TsallisEntropy	.222	39	<.001	.813	39	<.001
PS_4_5	.232	39	<.001	.731	39	<.001
PS_5_6	.215	39	<.001	.615	39	<.001
PS_6_7	.201	39	<.001	.573	39	<.001
PS_7_8	.224	39	<.001	.765	39	<.001
PS_8_9	.228	39	<.001	.664	39	<.001
PS_9_10	.318	39	<.001	.383	39	<.001
PS_10_11	.245	39	<.001	.628	39	<.001
PS_11_12	.327	39	<.001	.601	39	<.001
PS_12_13	.241	39	<.001	.752	39	<.001
PS_13_14	.228	39	<.001	.676	39	<.001
PS_14_15	.240	39	<.001	.702	39	<.001
PS_15_16	.248	39	<.001	.630	39	<.001
PS_16_17	.269	39	<.001	.588	39	<.001
PS_17_18	.239	39	<.001	.611	39	<.001
PS_18_19	.222	39	<.001	.689	39	<.001
PS_19_20	.253	39	<.001	.630	39	<.001

a. Lilliefors Significance Correction

BrainRate

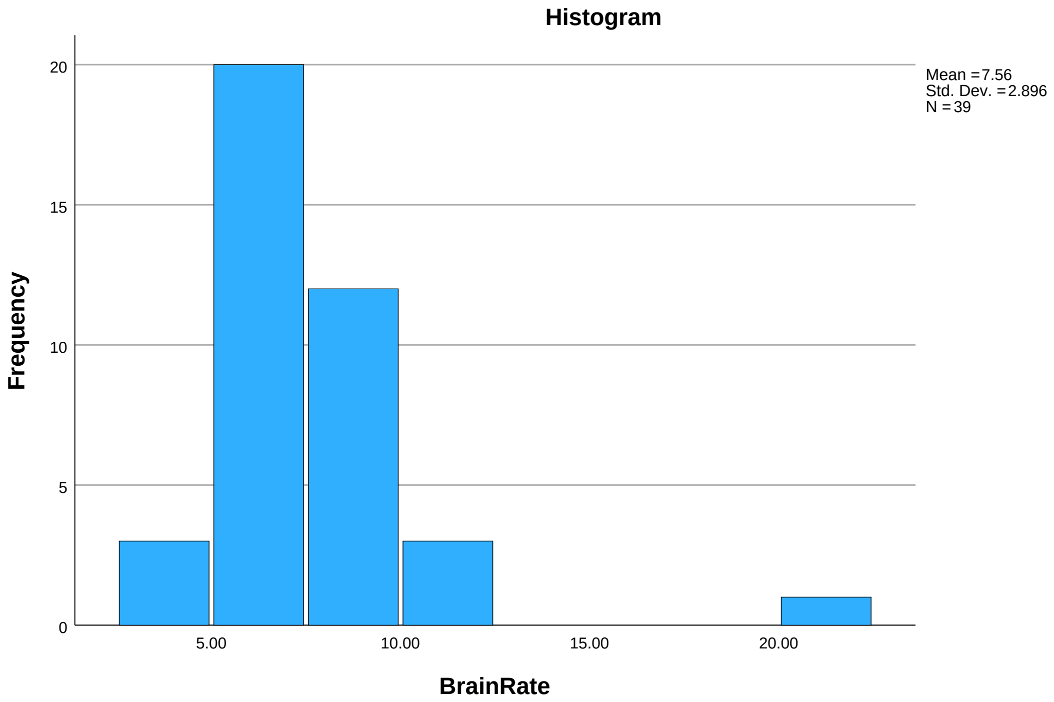


```
BrainRate Stem-and-Leaf Plot

 Frequency    Stem &  Leaf

     2.00        3 .  39
     1.00        4 .  9
     6.00        5 .  134488
    10.00        6 .  0122677788
     7.00        7 .  1223578
     6.00        8 .  123345
     3.00        9 .  009
     2.00       10 .  13
     2.00 Extremes    (>=12.0)

 Stem width:      1.00
 Each leaf:       1 case(s)
```

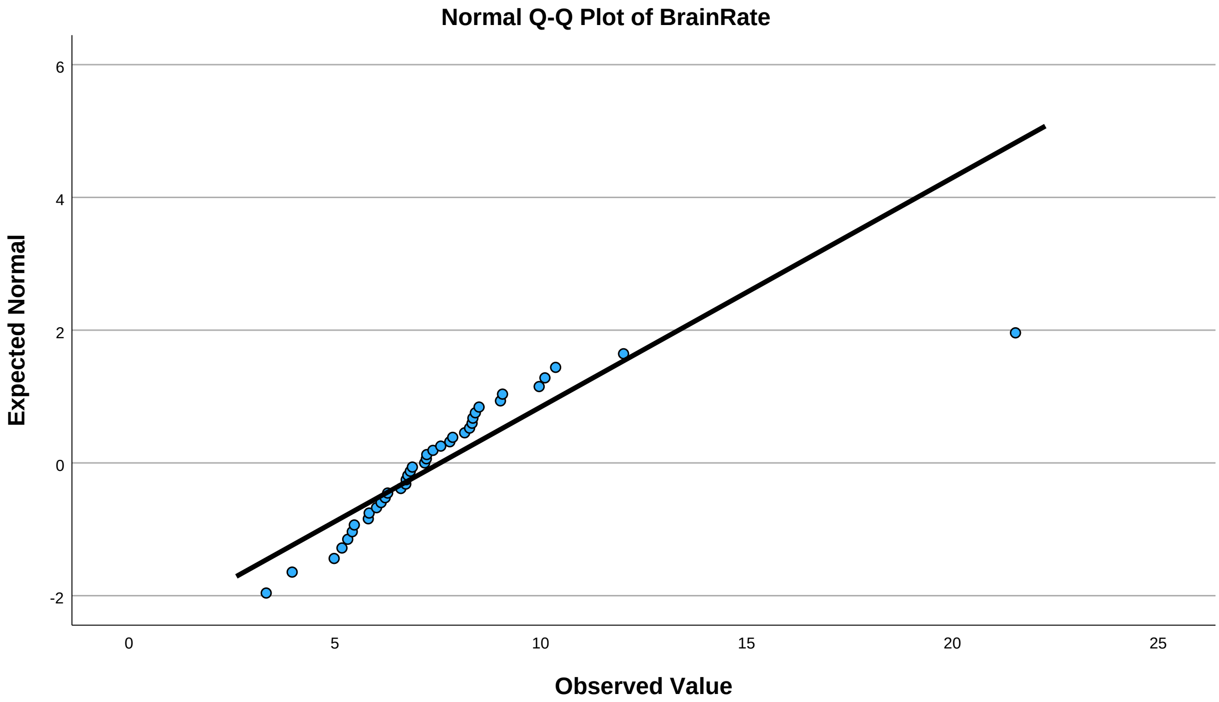

Normal Q-Q Plot of BrainRate
Expected Normal
6
4
2
0
-2
0
5
10
15
20
25
Observed Value

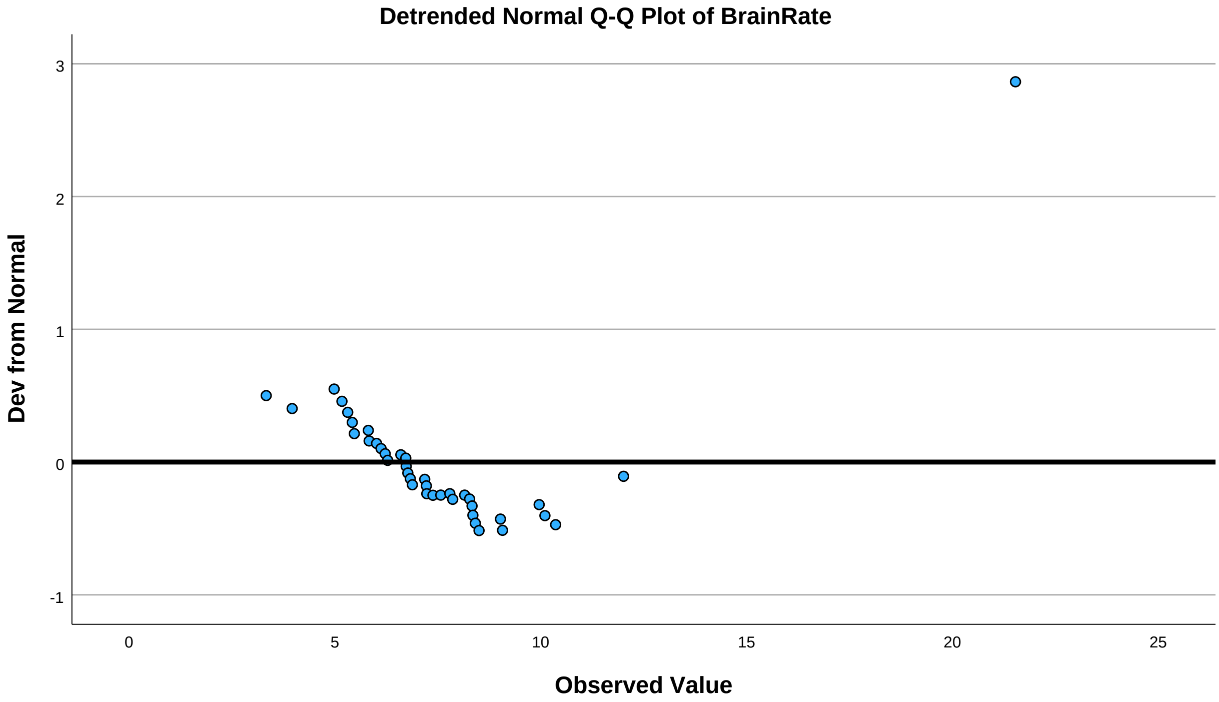

Detrended Normal Q-Q Plot of BrainRate
Dev from Normal
3
2
1
0
-1
0
5
10
15
20
25
Observed Value

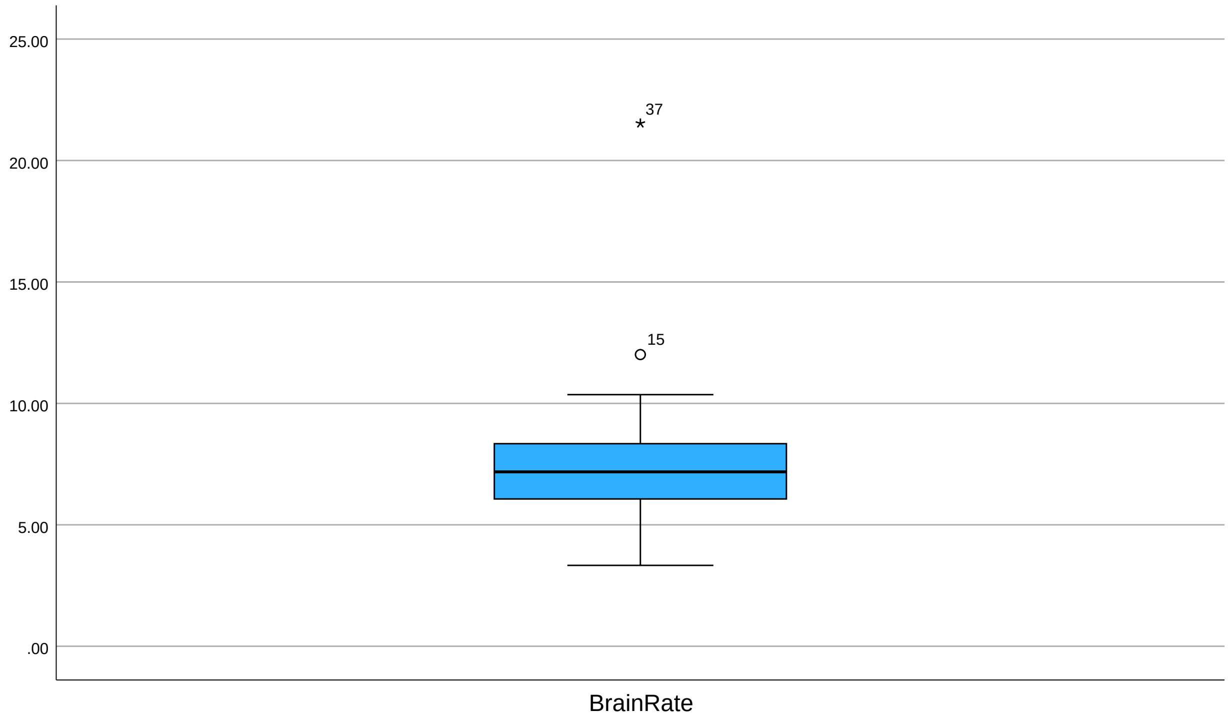

PermutationEntropy

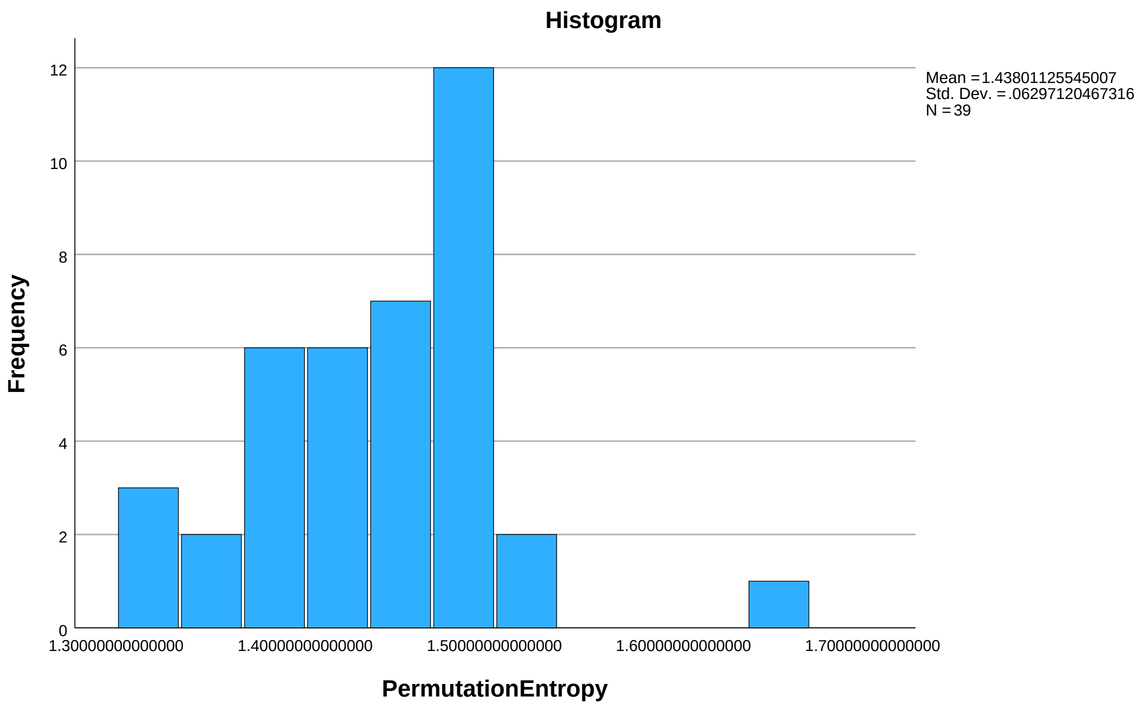

PermutationEntropy Stem-and-Leaf Plot

Frequency   Stem & Leaf

```
   4.00      13 .  1223
   7.00      13 .  6788899
  10.00      14 .  0001233444
  15.00      14 .  555777777888999
   2.00      15 .  02
   1.00 Extremes    (>=1.64)

 Stem width:   .1000000
 Each leaf:       1 case(s)
```

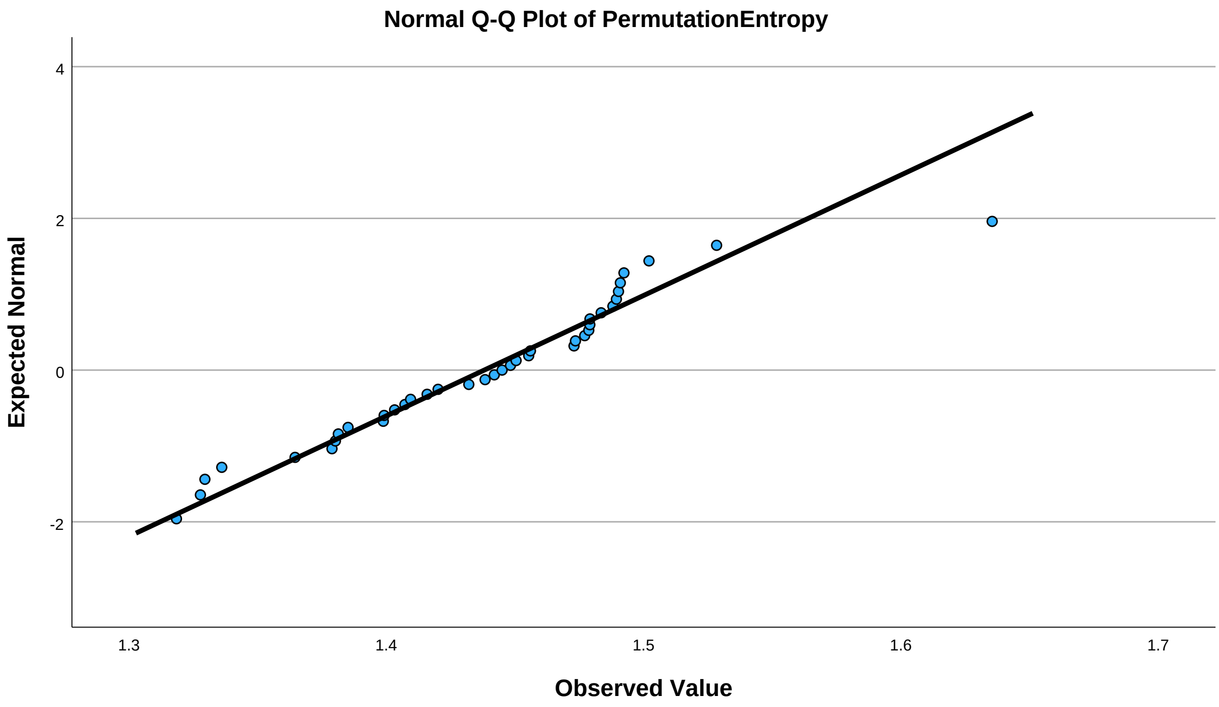

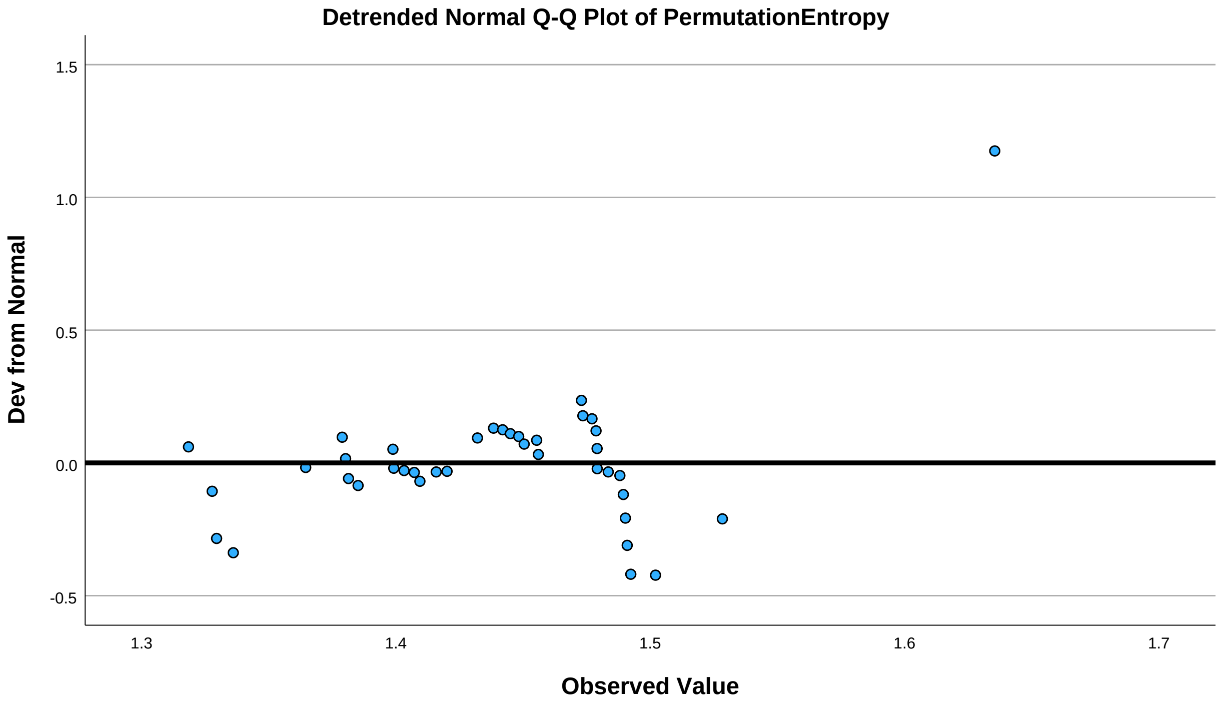

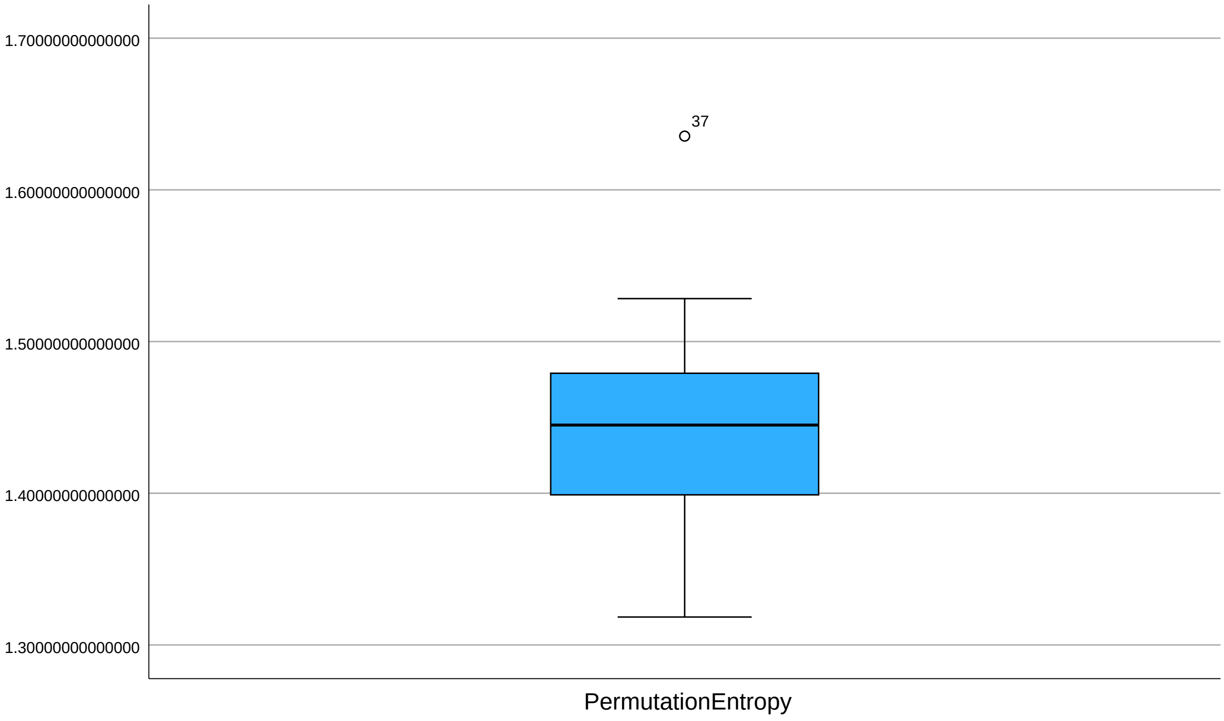

PermutationEntropy_01

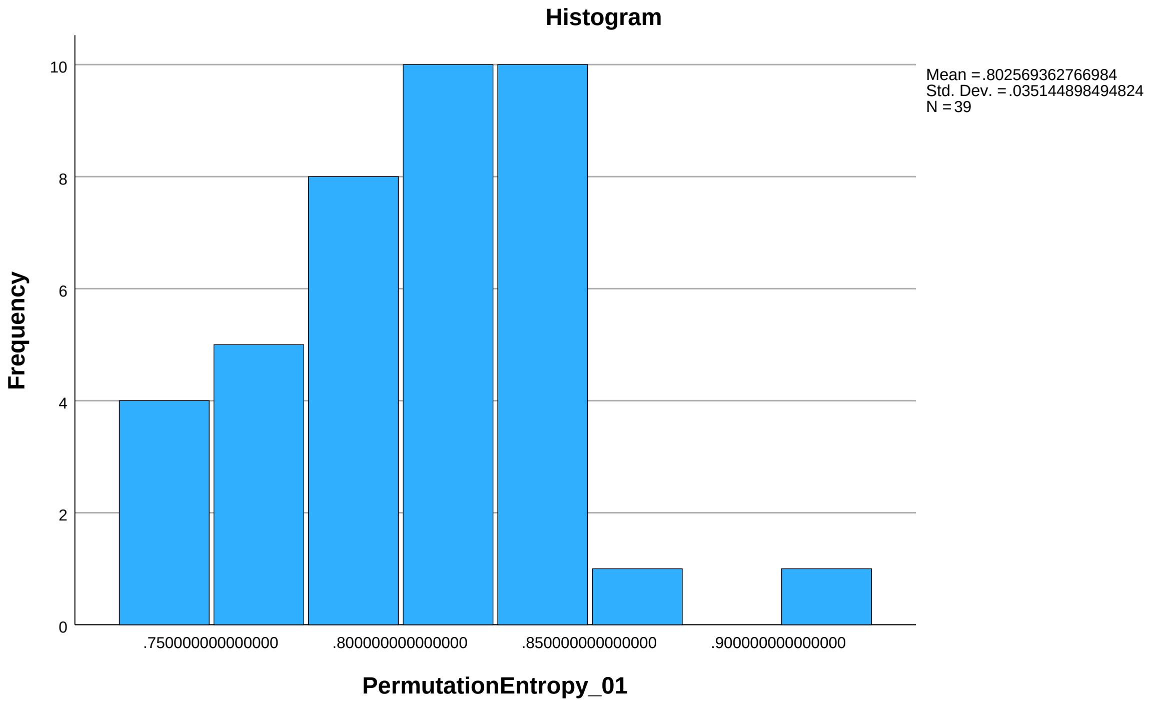


```
PermutationEntropy_01 Stem-and-Leaf Plot

 Frequency    Stem &  Leaf

     1.00       73 .  5
     3.00       74 .  115
      .00       75 .
     2.00       76 .  19
     3.00       77 .  003
     5.00       78 .  00356
     3.00       79 .  029
     5.00       80 .  24689
     2.00       81 .  22
     7.00       82 .  2245557
     6.00       83 .  011228
      .00       84 .
     1.00       85 .  2
     1.00 Extremes    (>=.913)

 Stem width:   .0100000
 Each leaf:        1 case(s)
```

Normal Q-Q Plot of PermutationEntropy_01

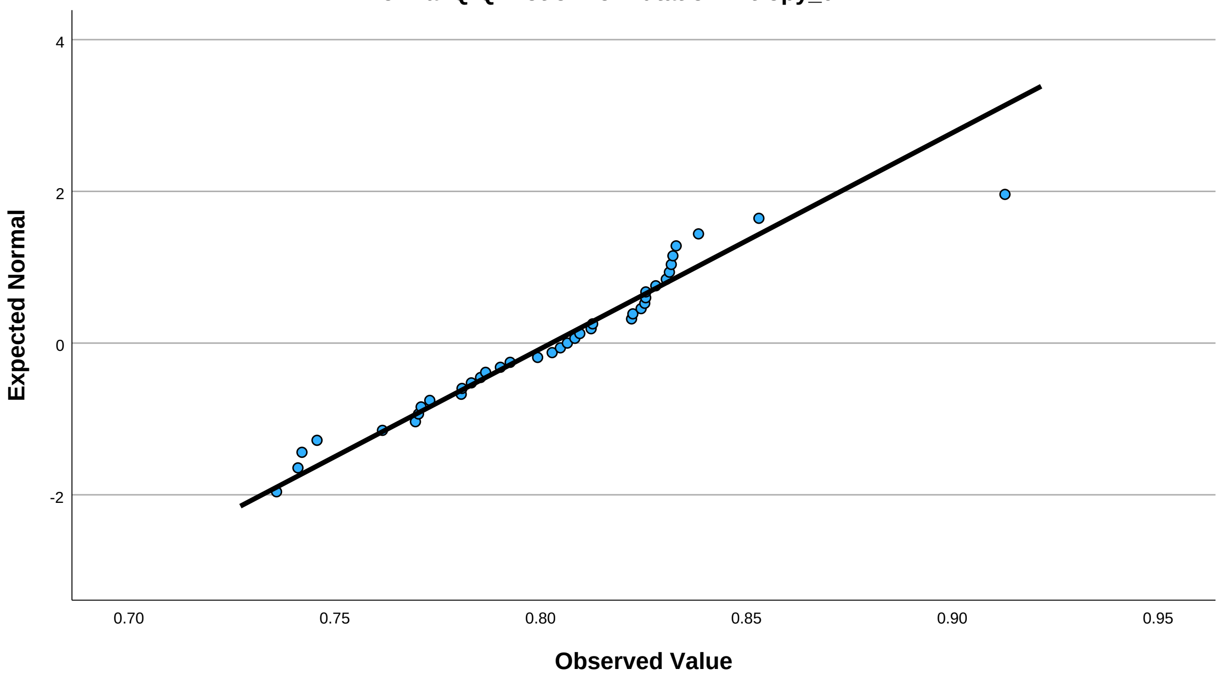

Detrended Normal Q-Q Plot of PermutationEntropy_01

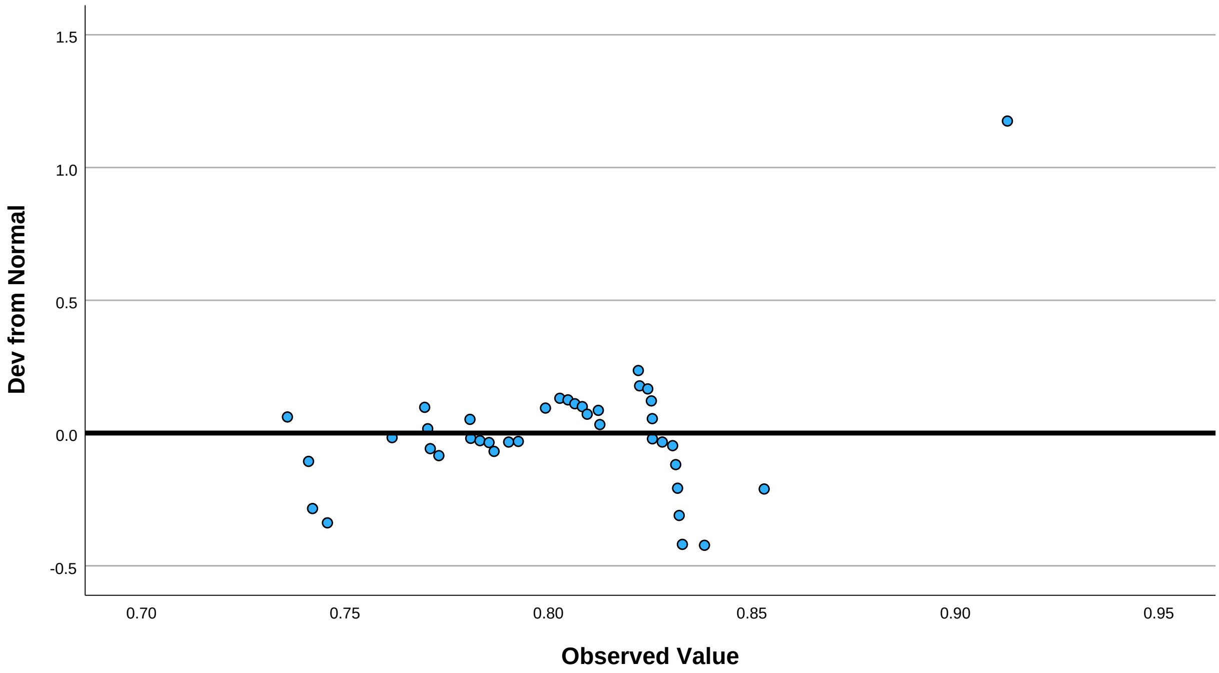

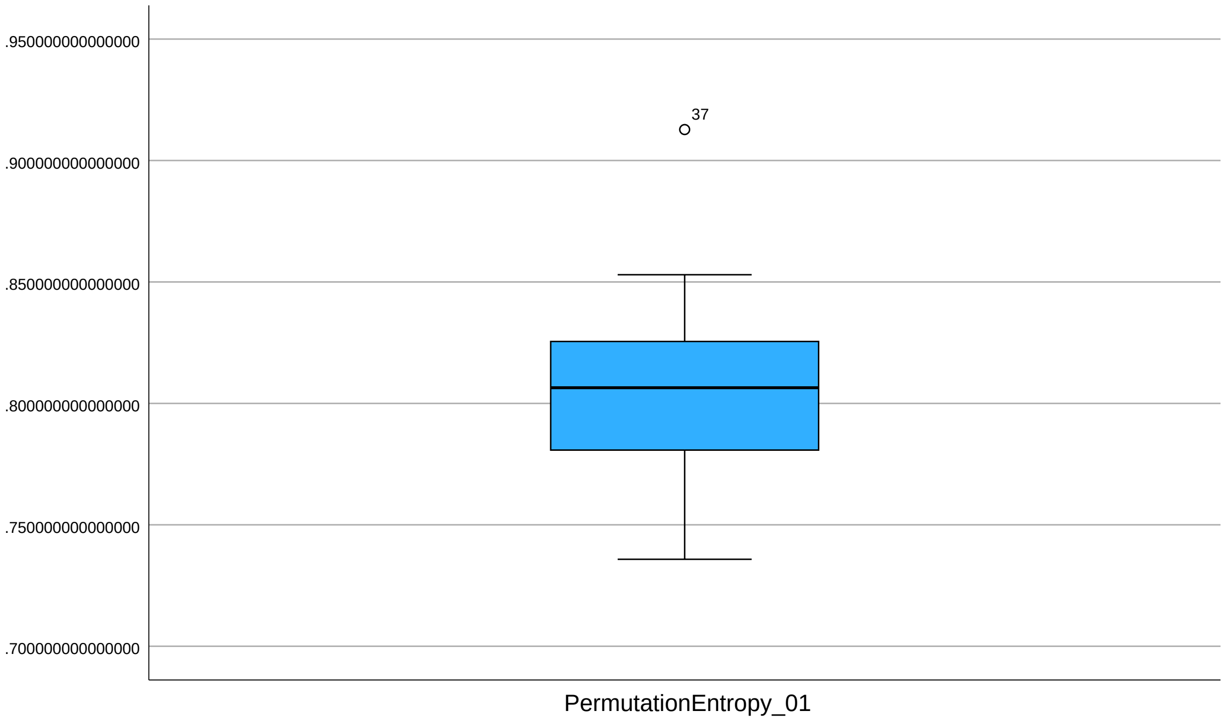

SampleEntropy

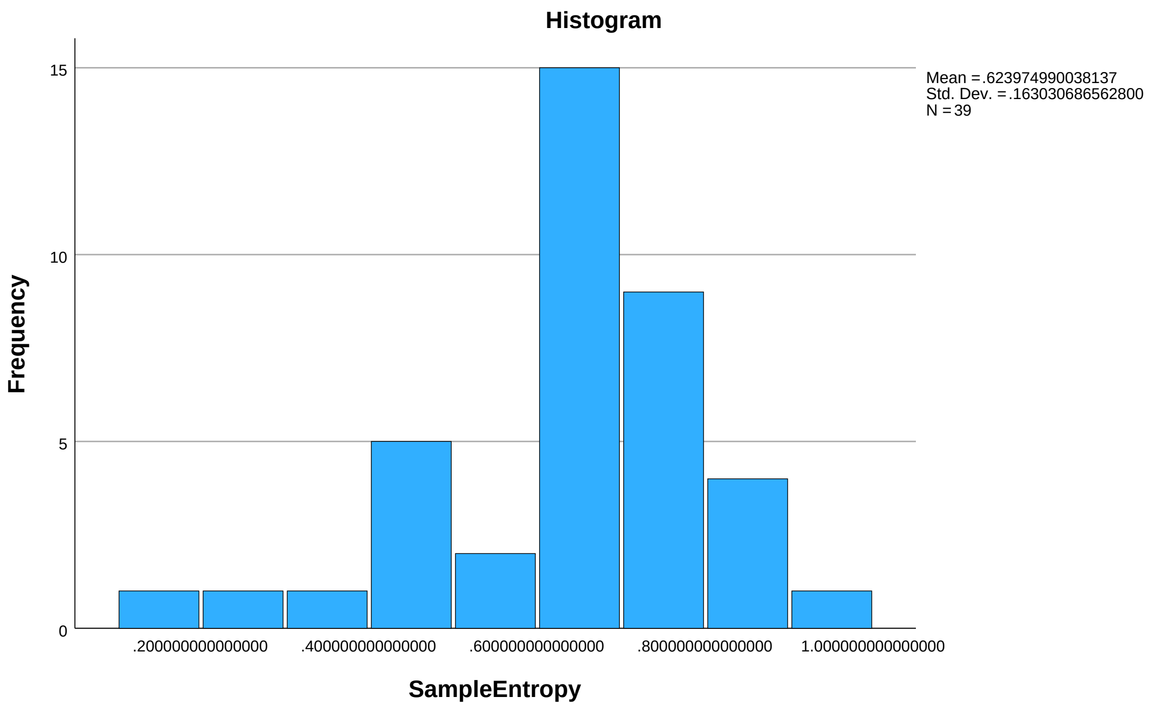

SampleEntropy Stem-and-Leaf Plot

Frequency   Stem & Leaf

```
  7.00 Extremes    (=<.43)
 1.00     4 . 8
 1.00     5 . 2
 1.00     5 . 9
 9.00     6 . 000001234
 6.00     6 . 556668
 8.00     7 . 00002223
 1.00     7 . 5
 3.00     8 . 114
 1.00     8 . 6
  1.00 Extremes    (>=.96)

Stem width:  .1000000
Each leaf:       1 case(s)
```

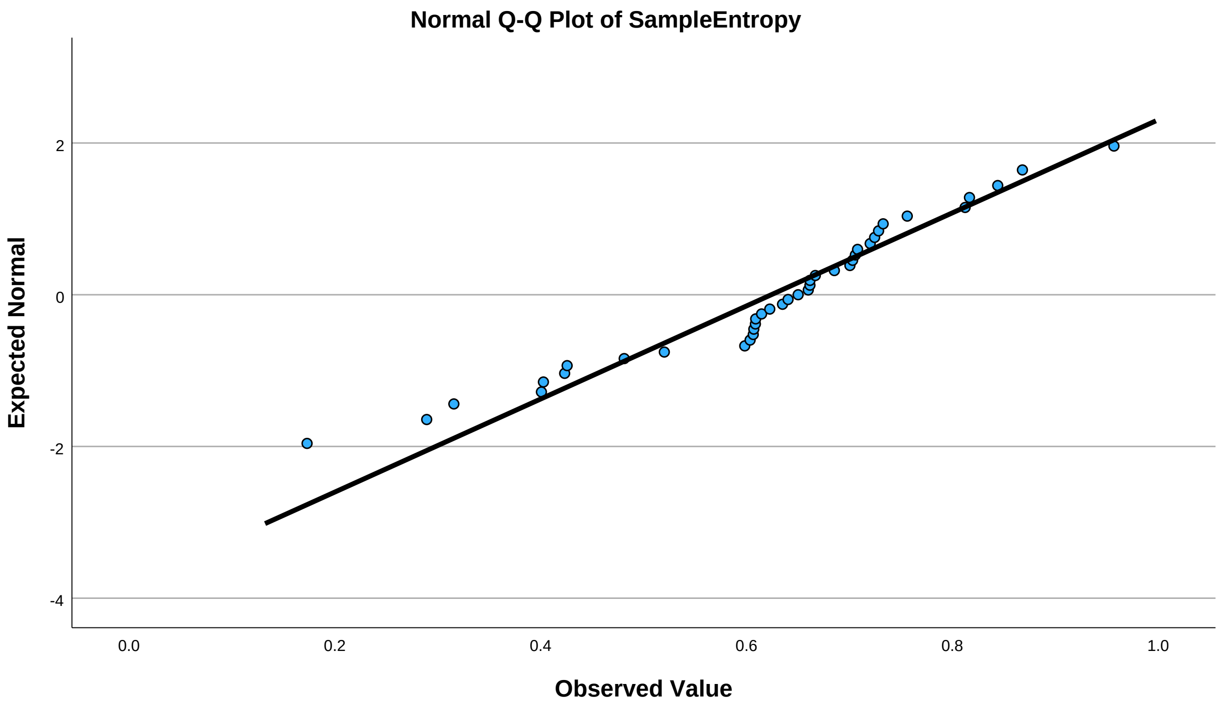

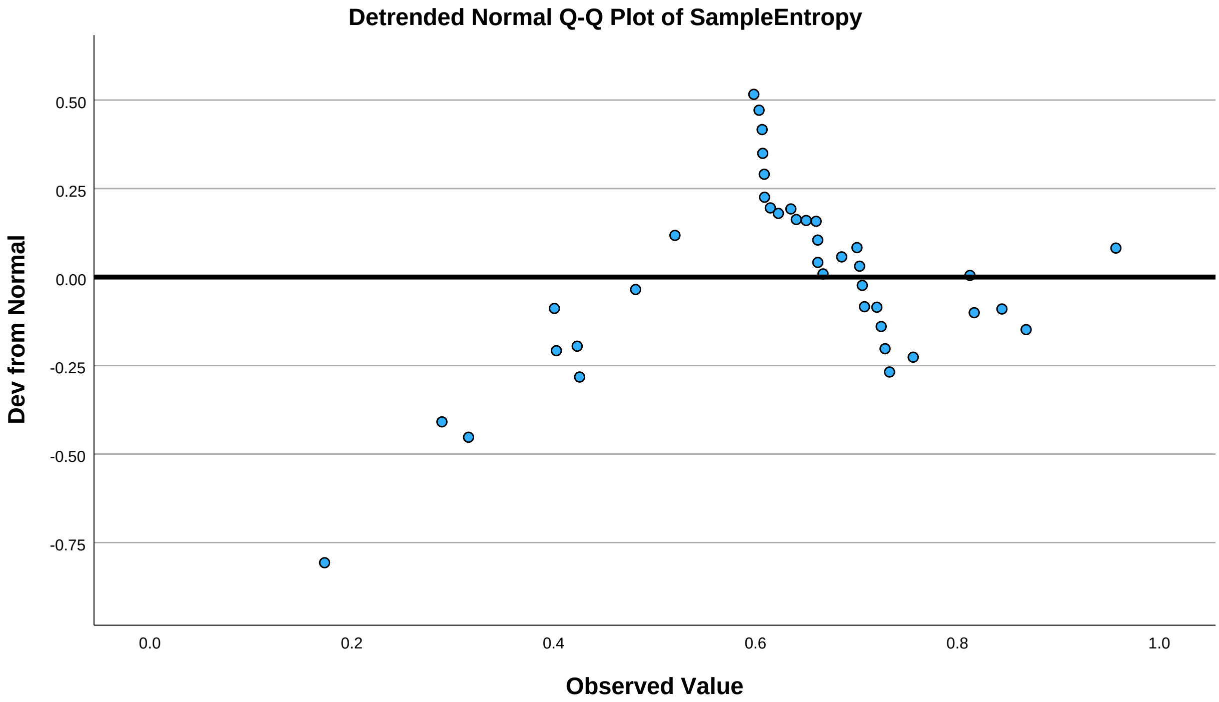

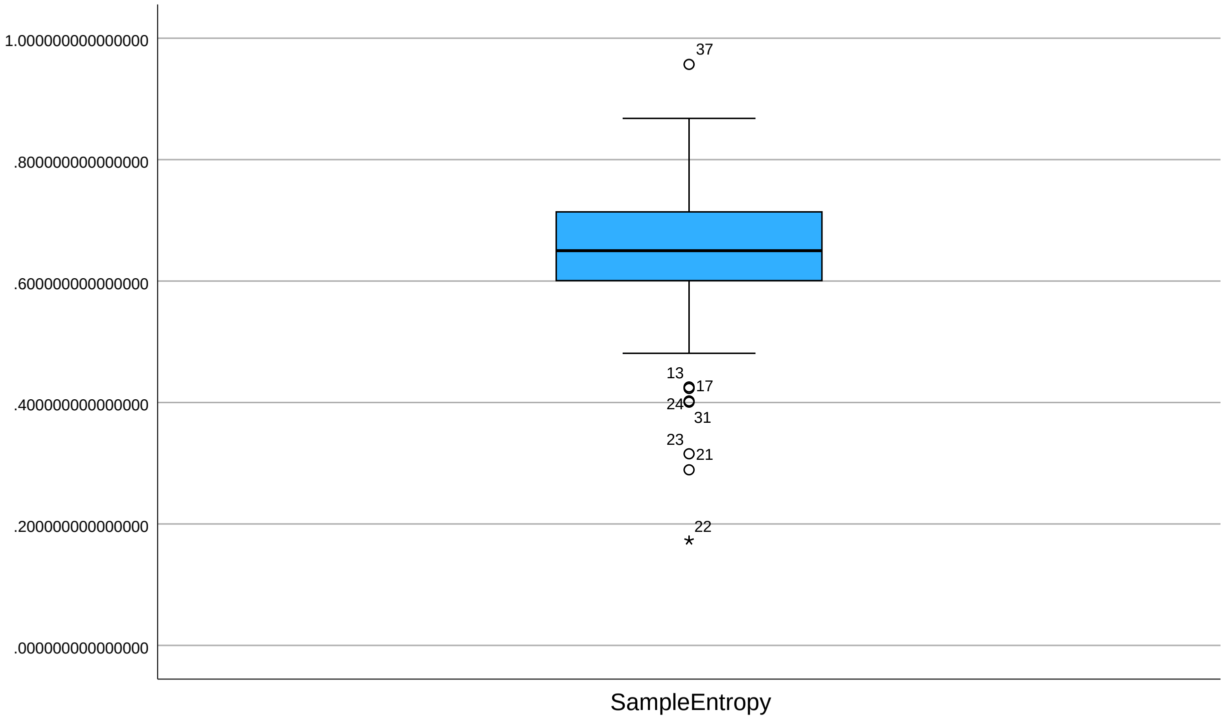

SpectralEntropy_bits

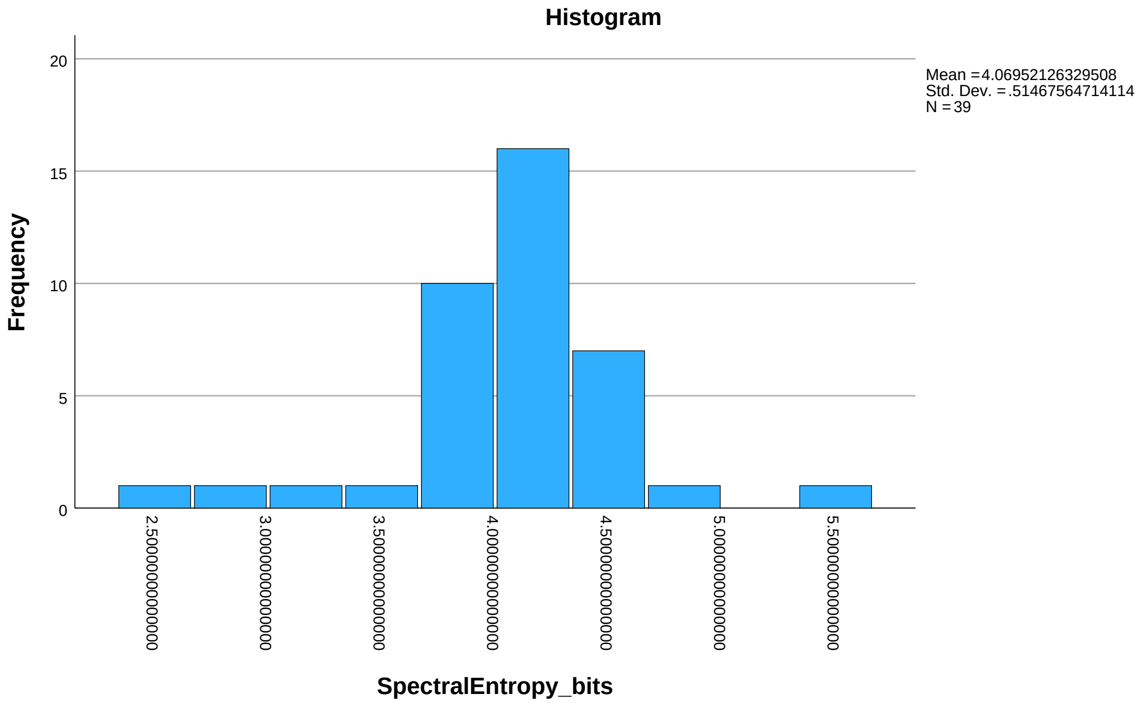


```
SpectralEntropy_bits Stem-and-Leaf Plot

 Frequency    Stem &  Leaf

     2.00 Extremes    (=<2.9)
     1.00        3 .  3
      .00        3 .
     6.00        3 .  667777
     5.00        3 .  88889
     9.00        4 .  000011111
    10.00        4 .  2222222333
     1.00        4 .  4
     3.00        4 .  666
     1.00        4 .  8
     1.00 Extremes    (>=5.5)

 Stem width:  1.000000
 Each leaf:       1 case(s)
```

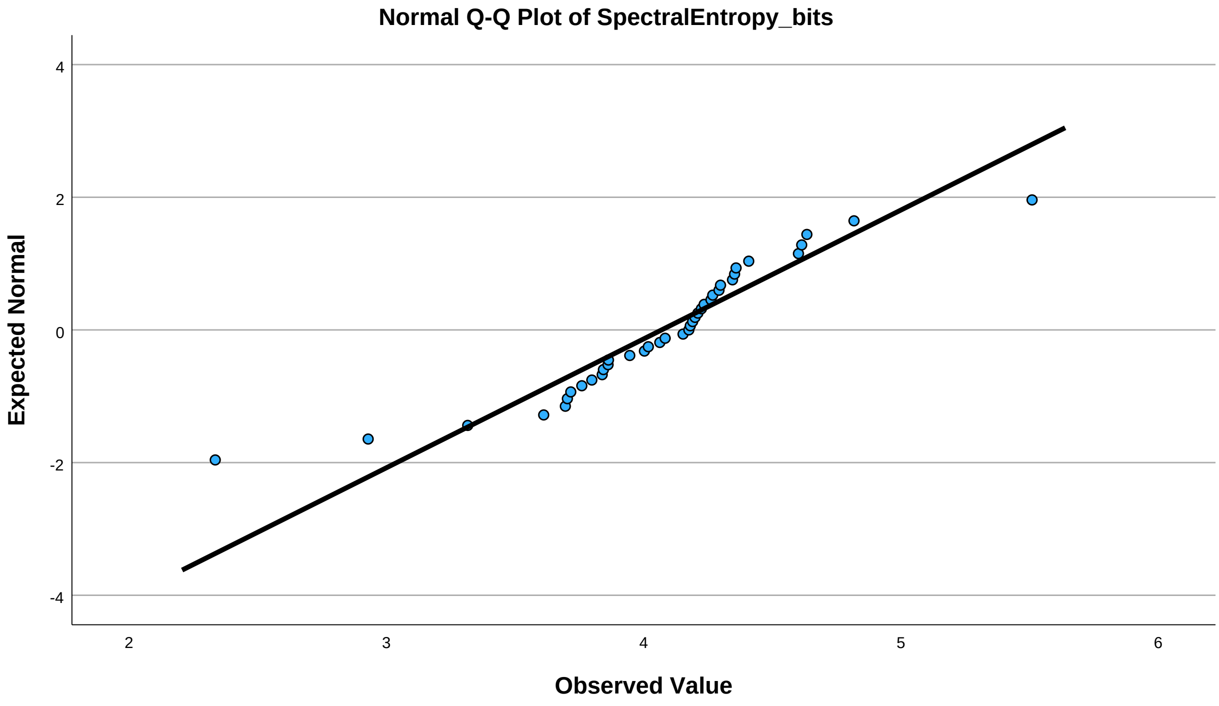

Normal Q-Q Plot of SpectralEntropy_bits
Expected Normal
Observed Value
4
2
0
-2
-4
2
3
4
5
6

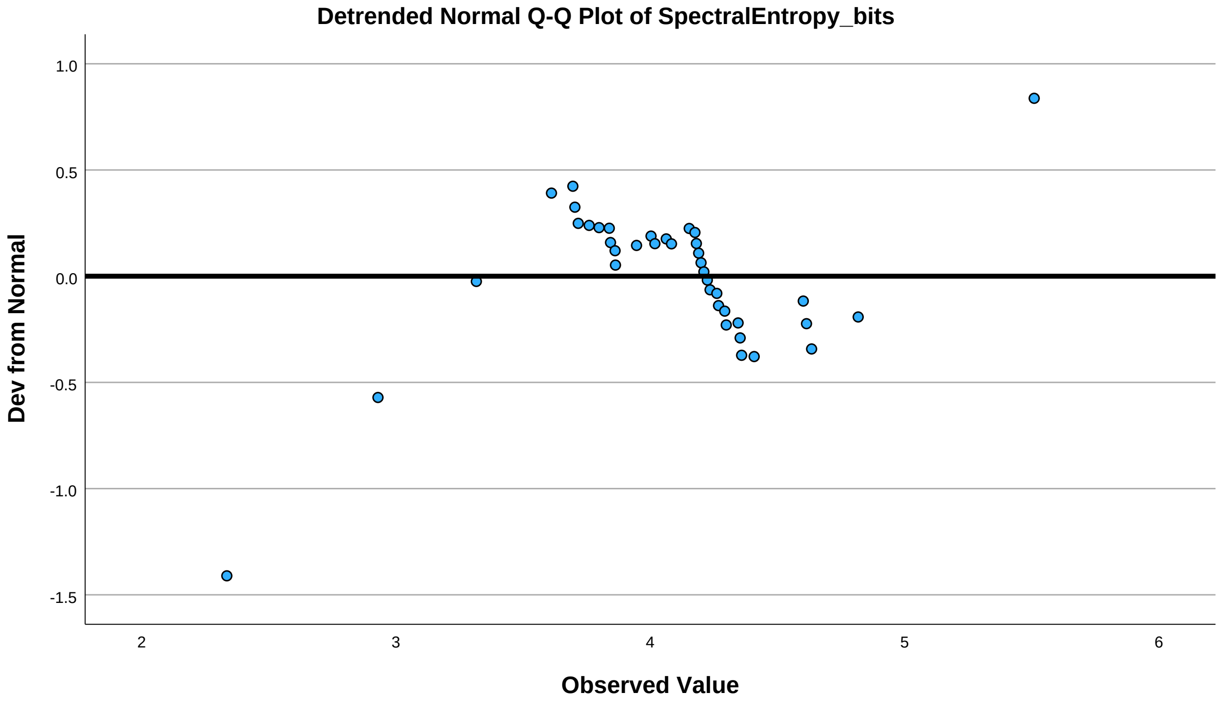

Detrended Normal Q-Q Plot of SpectralEntropy_bits
Dev from Normal
Observed Value
1.0
0.5
0.0
-0.5
-1.0
-1.5
2
3
4
5
6

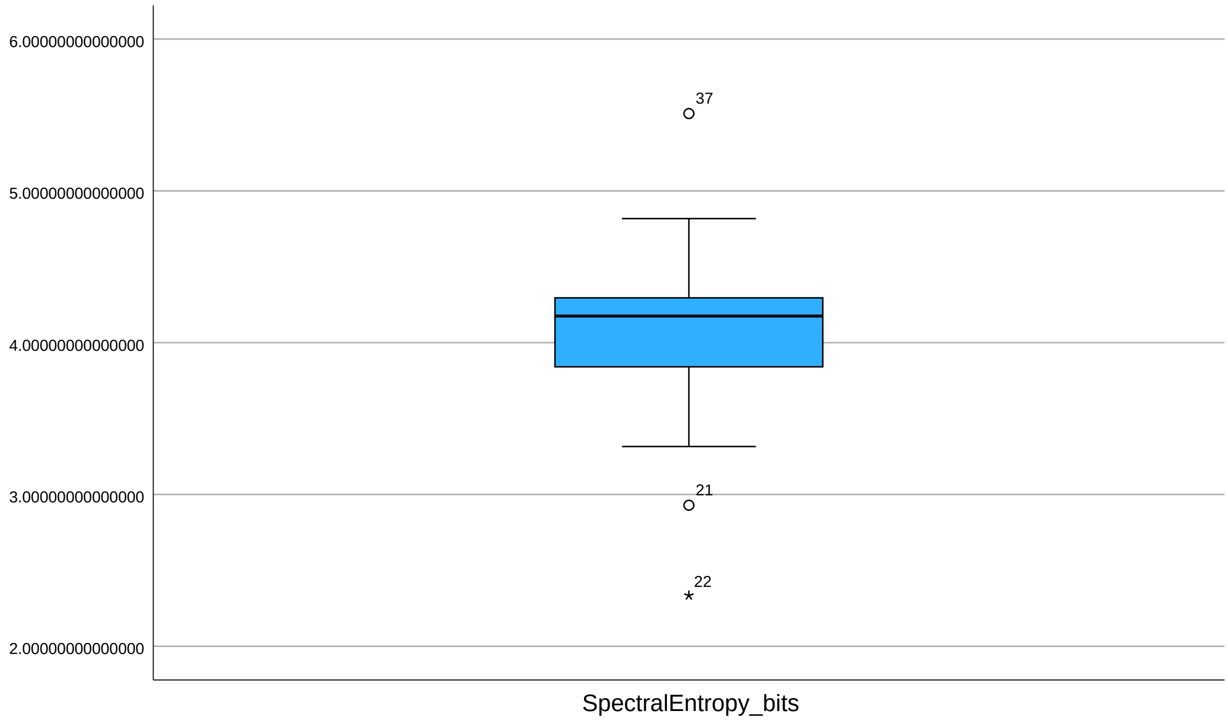

SpectralEntropy_01

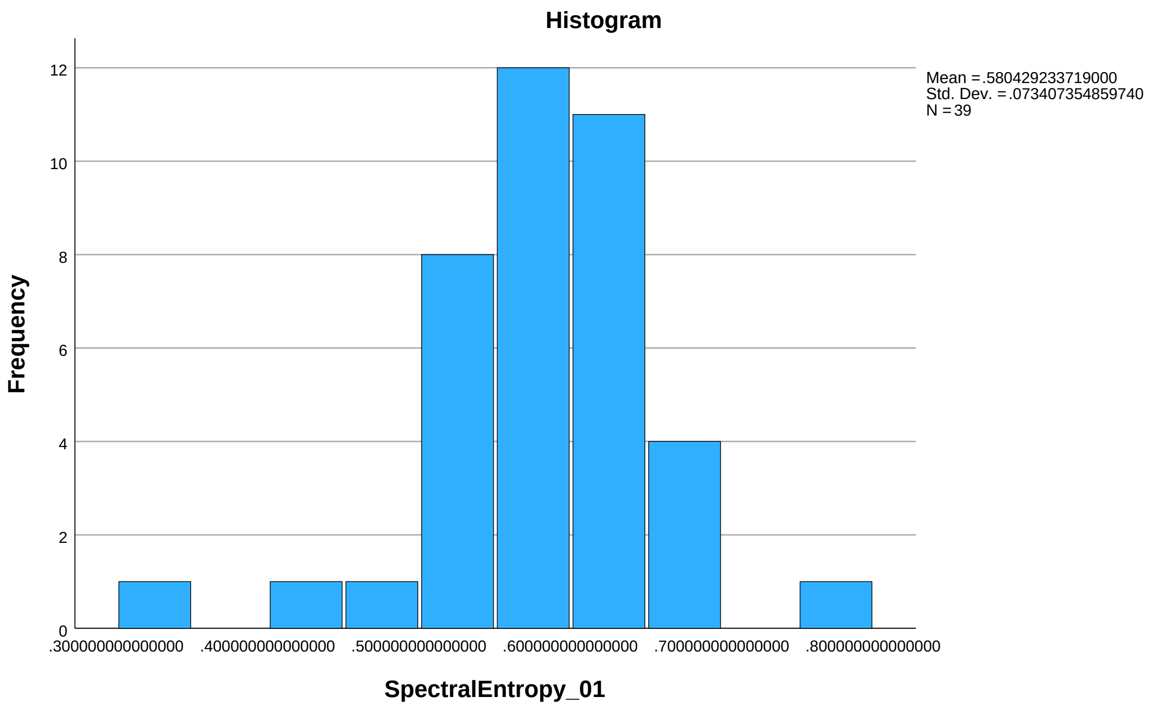


```
SpectralEntropy_01 Stem-and-Leaf Plot

 Frequency    Stem &  Leaf
```

```
  2.00 Extremes    (=<.42)
 1.00      4 . 7
  8.00       5 .  12233444
 12.00        5 .  556777899999
 11.00        6 .  00000111222
  4.00       6 .  5568
  1.00 Extremes    (>=.79)

Stem width:   .1000000
Each leaf:       1 case(s)
```

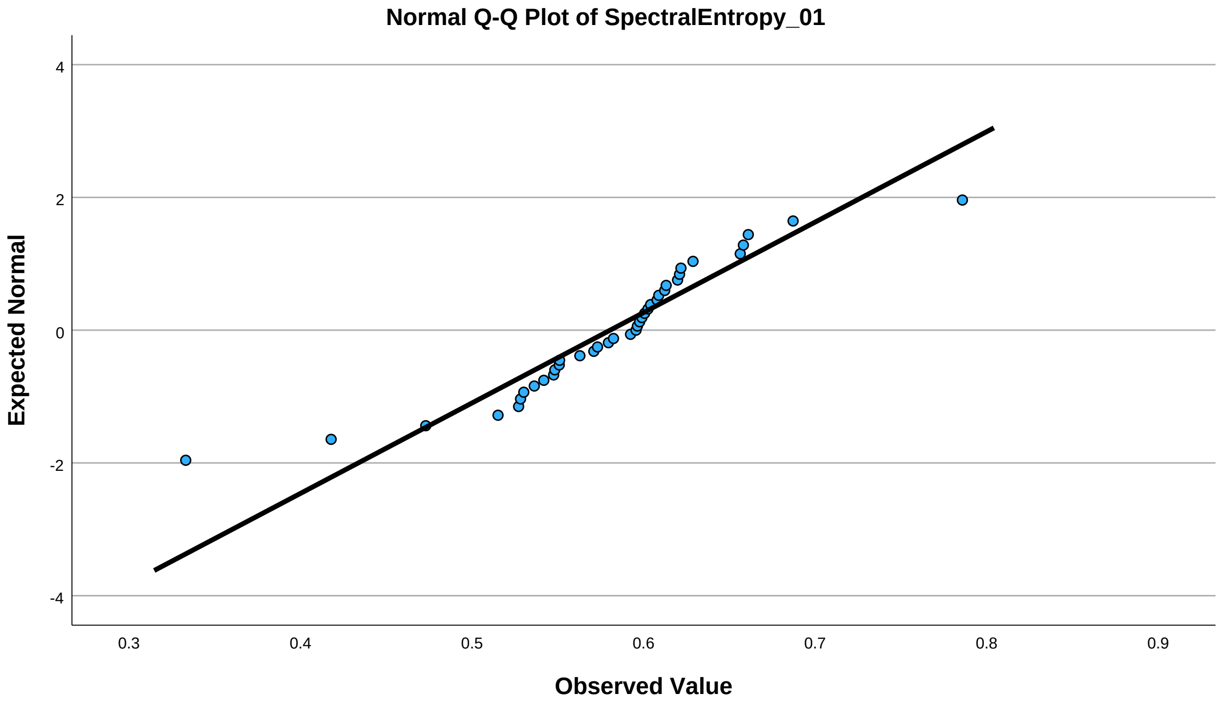

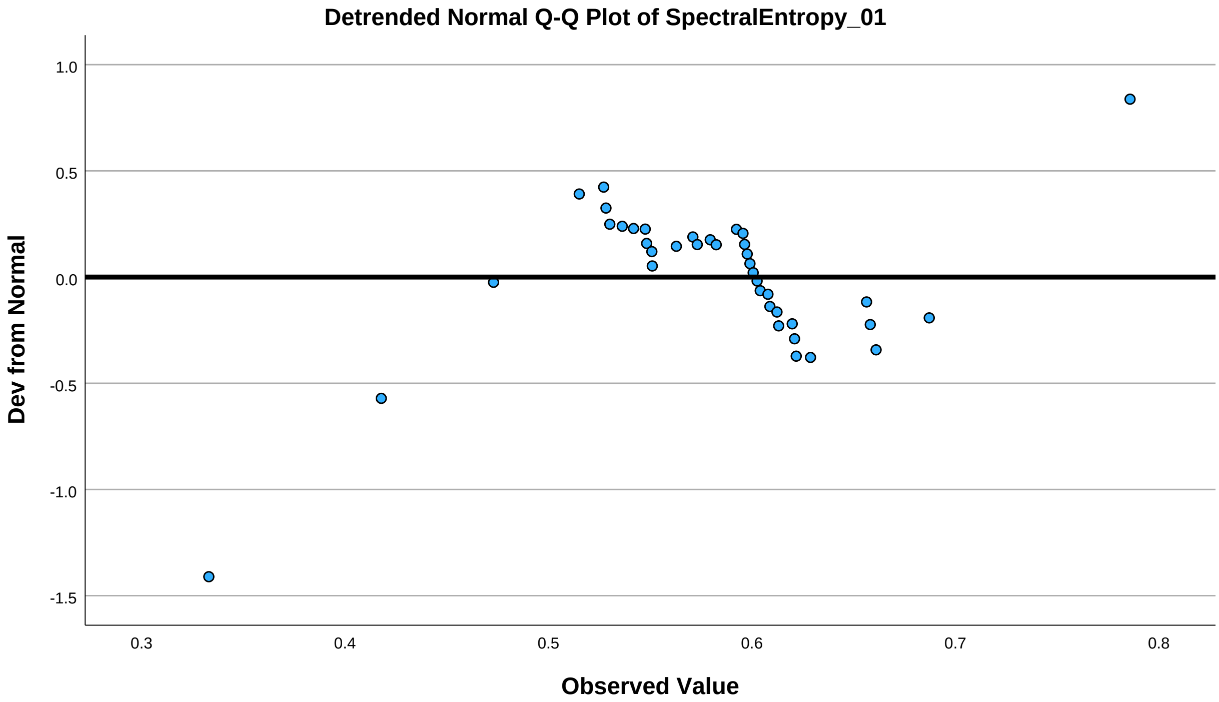

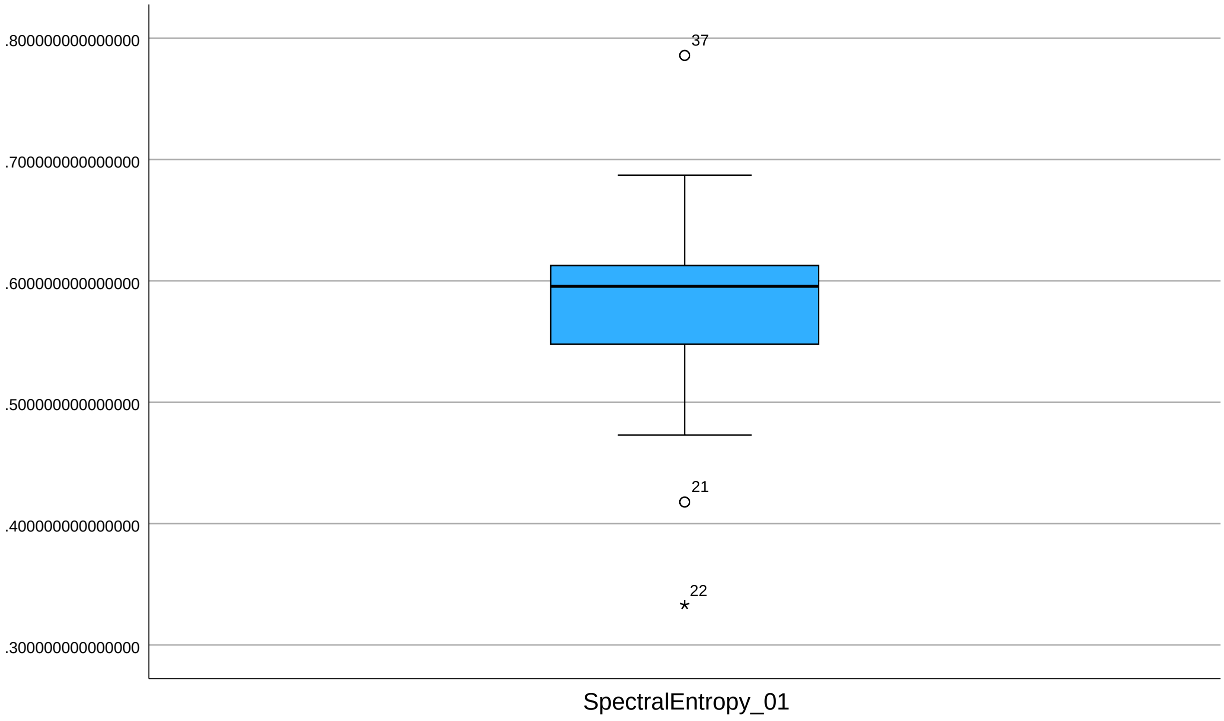

HiguchiFractalDimension

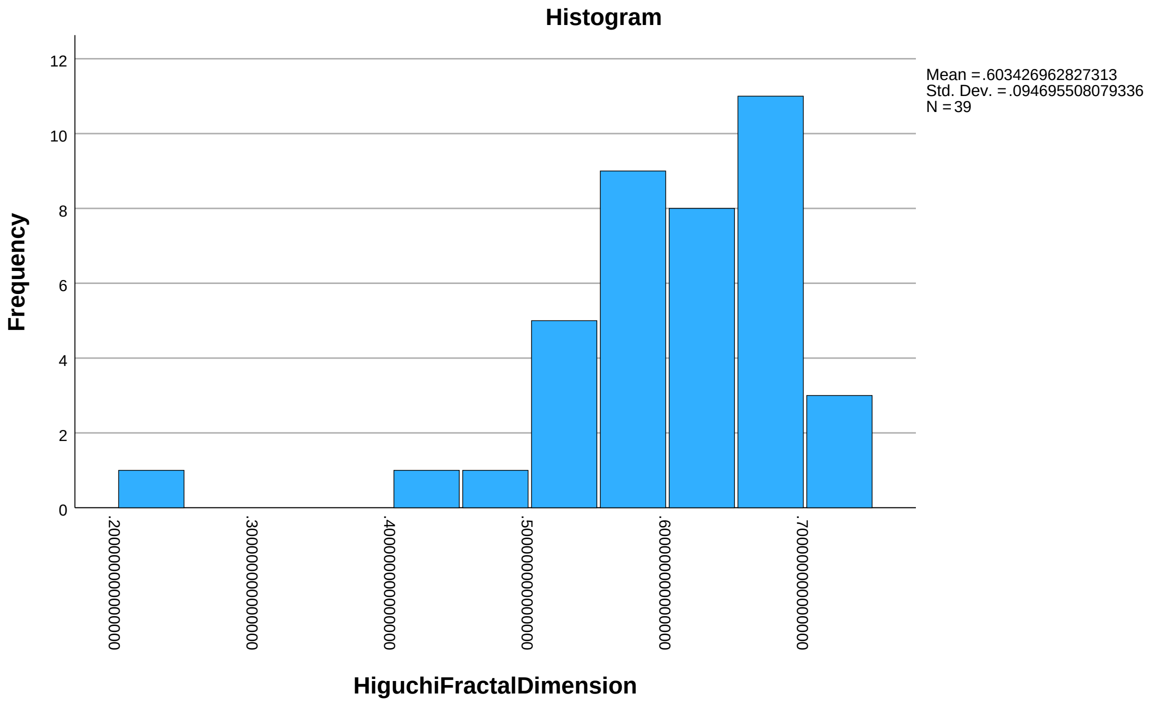


```
HiguchiFractalDimension Stem-and-Leaf Plot

 Frequency    Stem &  Leaf

     2.00 Extremes    (=<.41)
     1.00        4 .  5
     5.00        5 .  00234
     9.00        5 .  667778899
     8.00        6 .  00112344
    11.00        6 .  55567789999
     3.00        7 .  003

 Stem width:   .1000000
 Each leaf:        1 case(s)
```

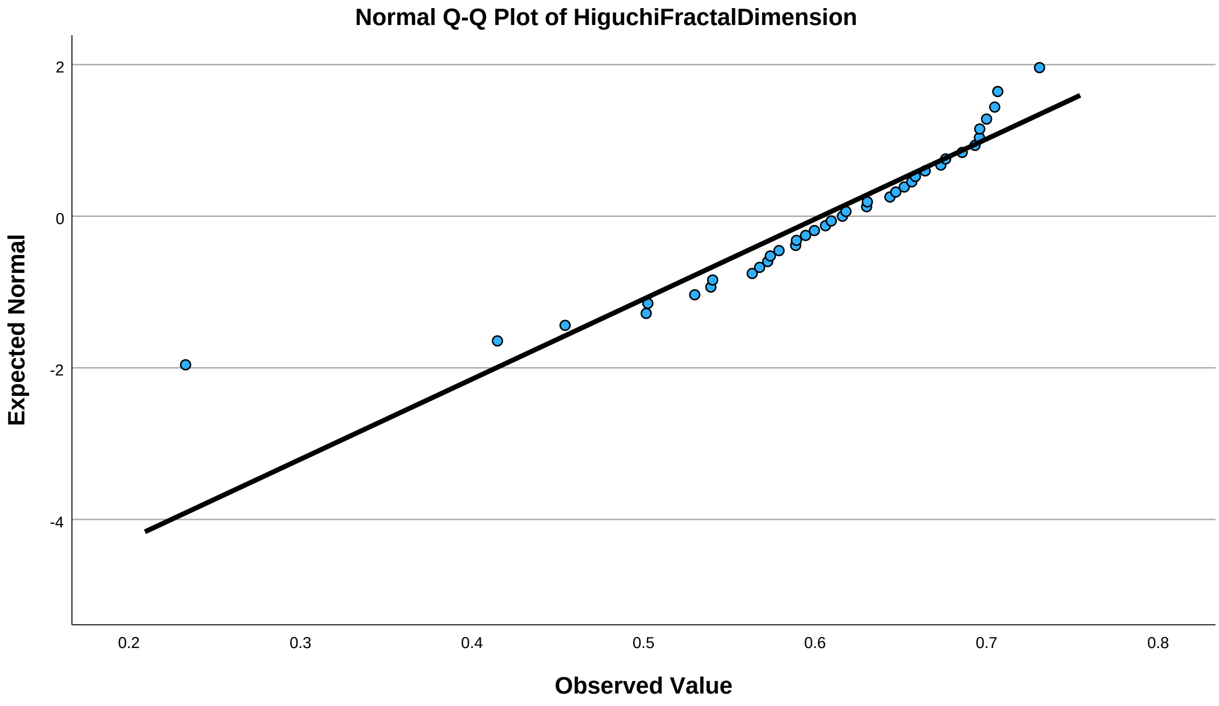
Normal Q-Q Plot of HiguchiFractalDimension
Expected Normal
Observed Value
2
0
-2
-4
0.2
0.3
0.4
0.5
0.6
0.7
0.8

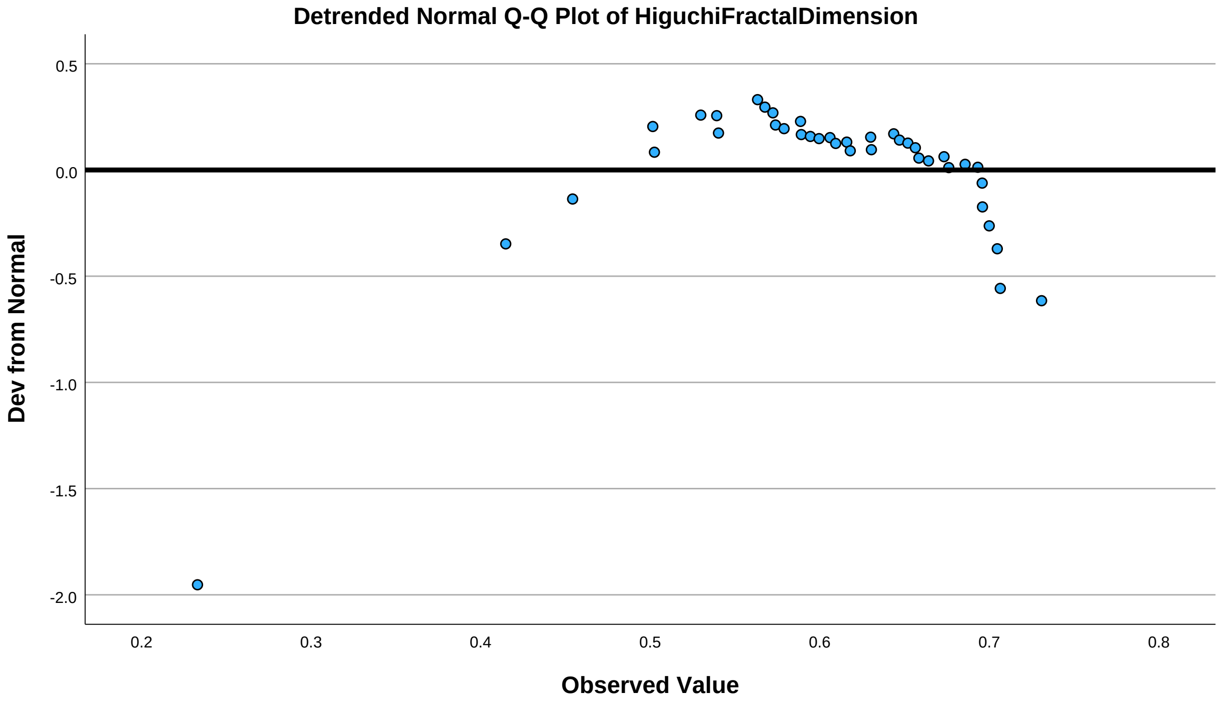
Detrended Normal Q-Q Plot of HiguchiFractalDimension
Dev from Normal
Observed Value
0.5
0.0
-0.5
-1.0
-1.5
-2.0
0.2
0.3
0.4
0.5
0.6
0.7
0.8

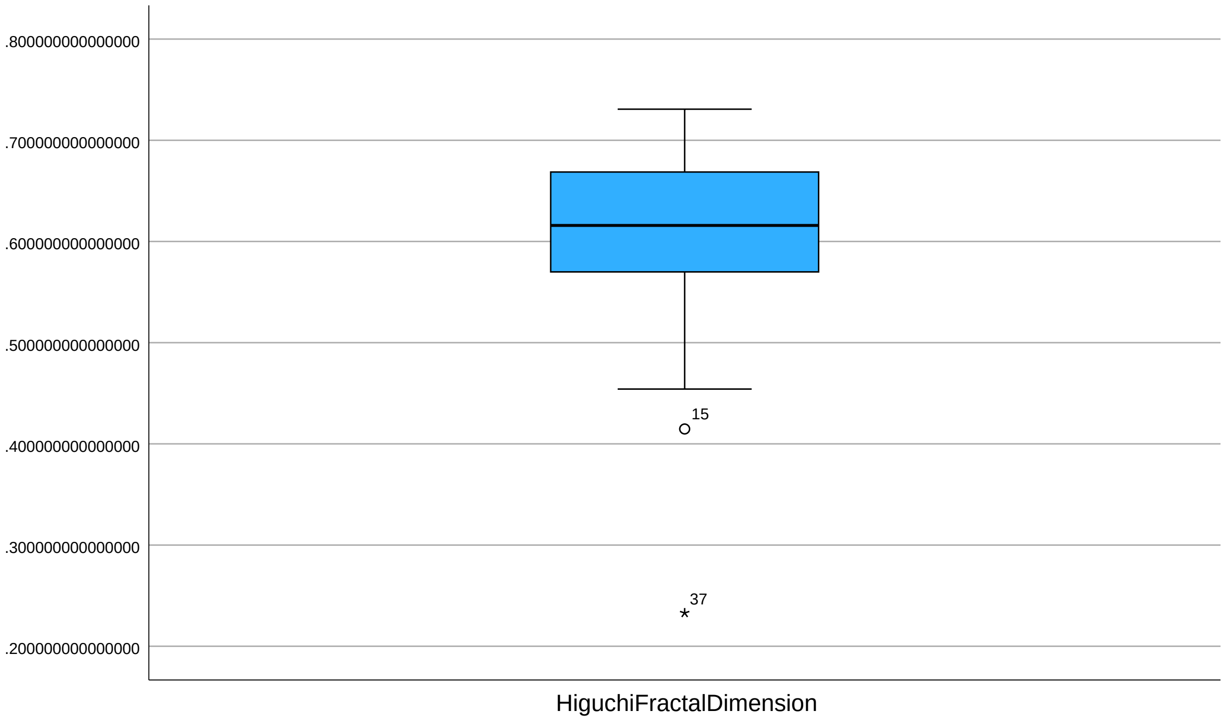

LempelZivComplexity

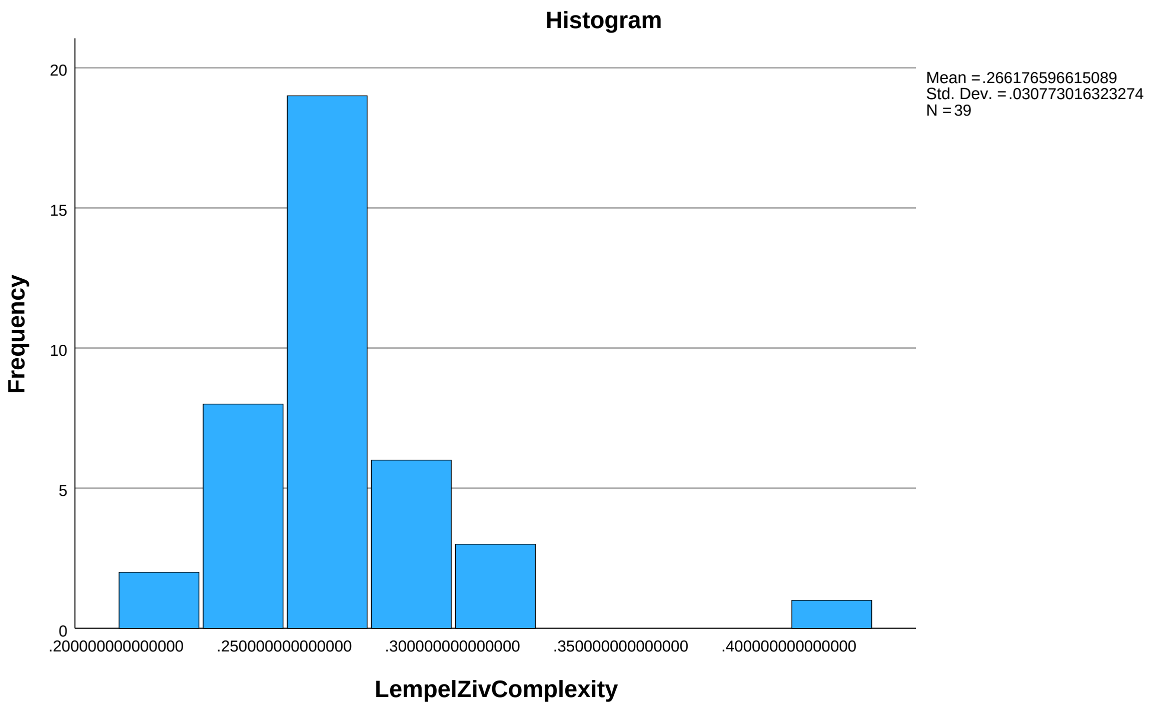


```
LempelZivComplexity Stem-and-Leaf Plot

 Frequency    Stem &  Leaf
```

```
    2.00      21 .  37
    1.00      22 .  7
    2.00      23 .  89
    5.00      24 .  15778
    6.00      25 .  014589
    8.00      26 .  12333366
    7.00      27 .  0001468
    2.00      28 .  45
    2.00      29 .  03
    3.00      30 .  015
    1.00 Extremes    (>=.402)

 Stem width:   .0100000
 Each leaf:        1 case(s)
```

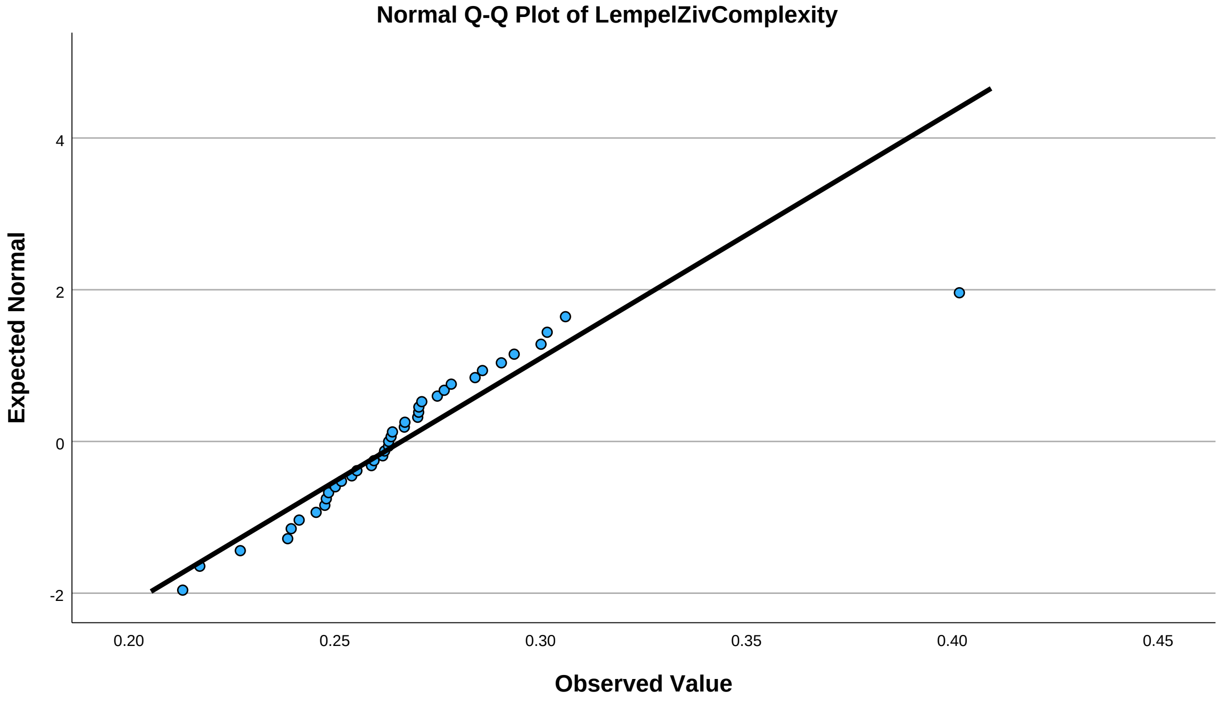

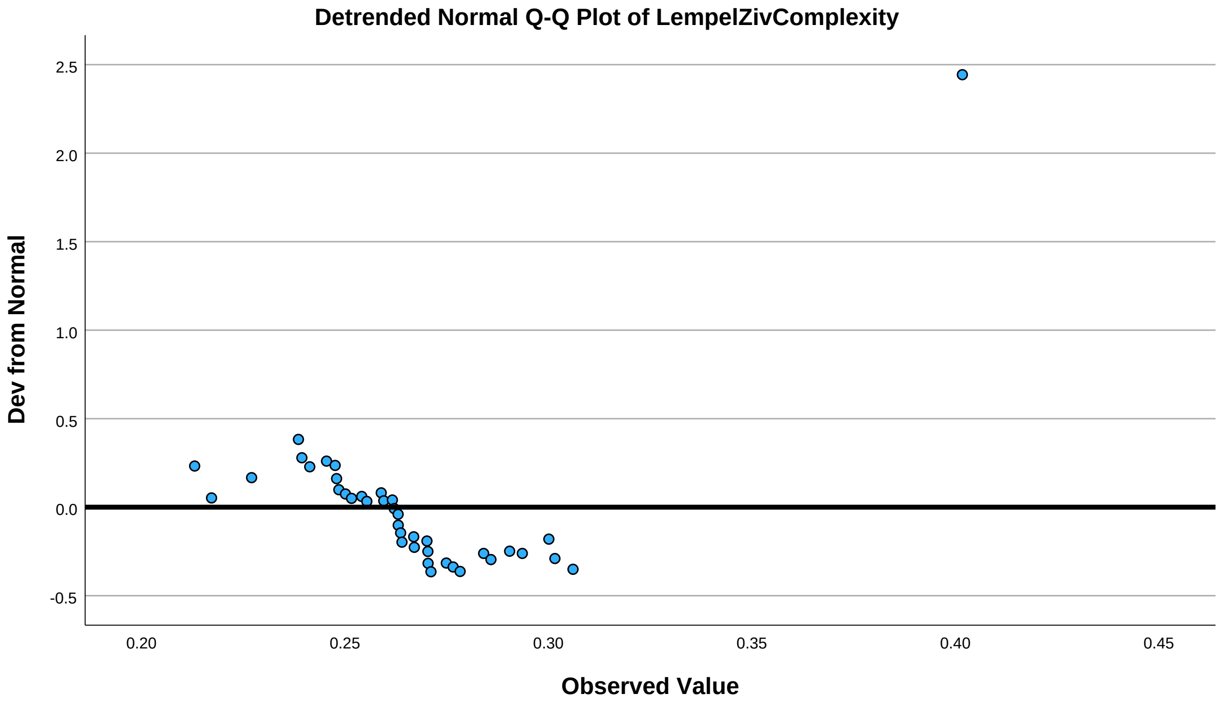

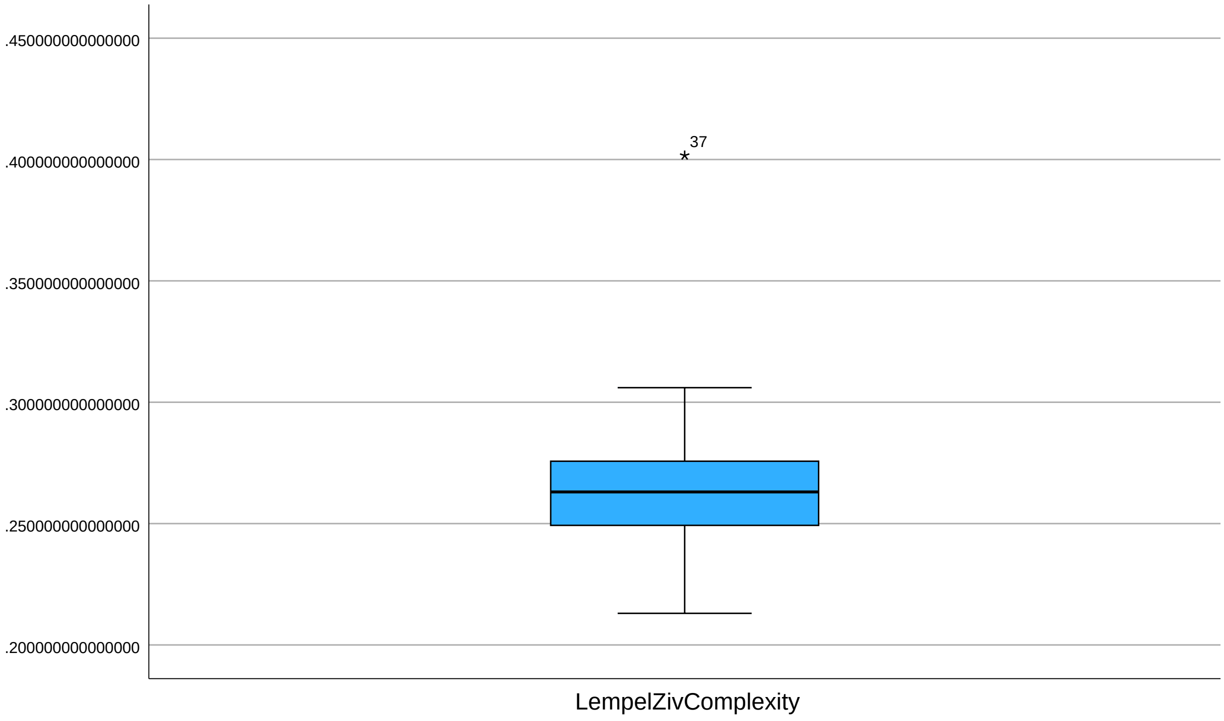

RenyiEntropy

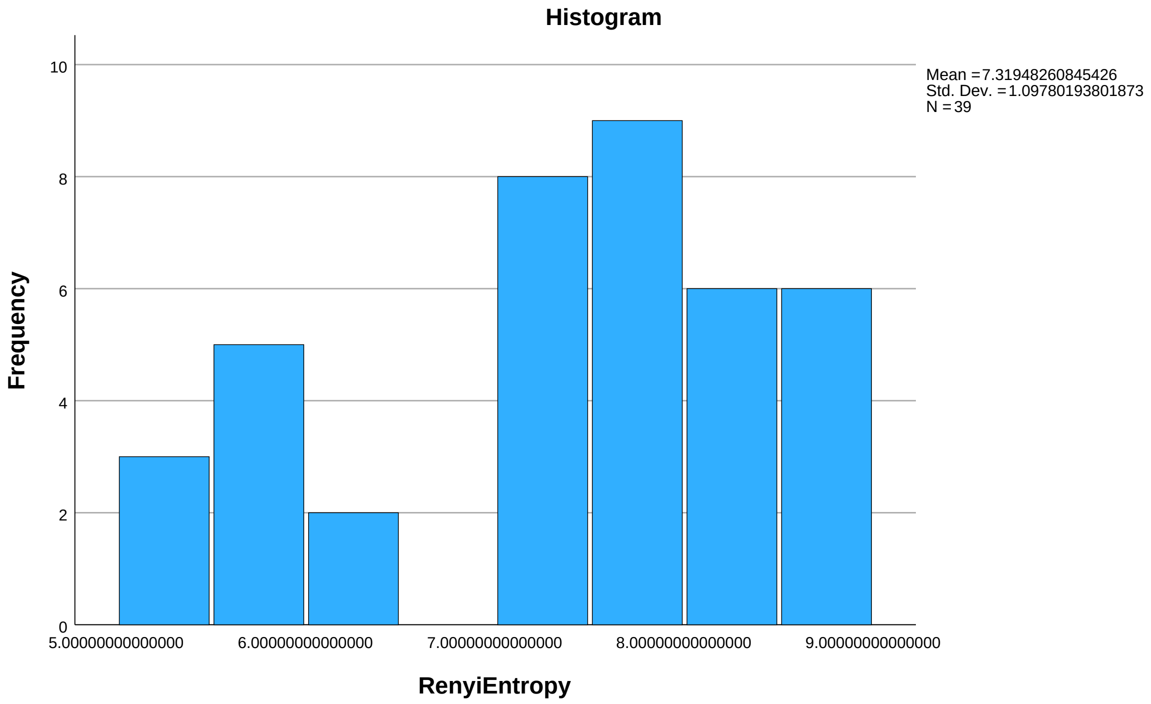


```
RenyiEntropy Stem-and-Leaf Plot

 Frequency    Stem &  Leaf

     3.00        5 .  034
     5.00        5 .  57789
     2.00        6 .  00
      .00        6 .
     8.00        7 .  01123444
     9.00        7 .  556667778
     6.00        8 .  013334
     6.00        8 .  555677

 Stem width:  1.000000
 Each leaf:       1 case(s)
```

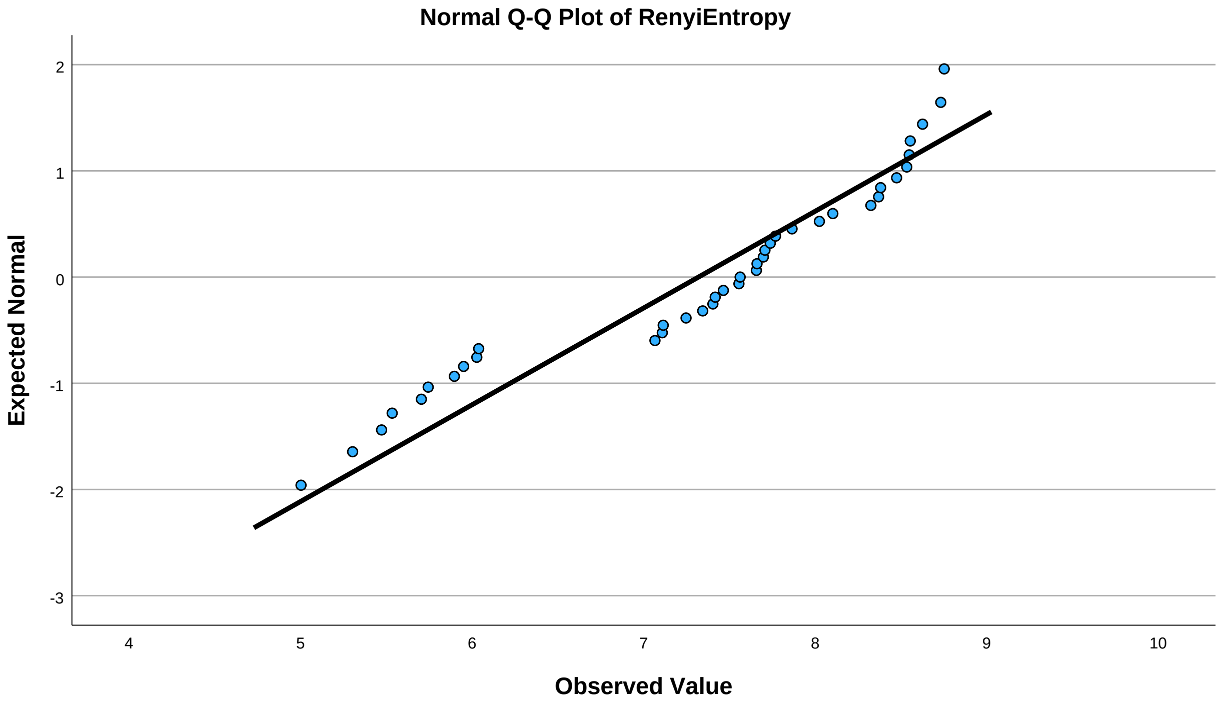
Normal Q-Q Plot of RenyiEntropy
Expected Normal
2
1
0
-1
-2
-3
4
5
6
7
8
9
10
Observed Value

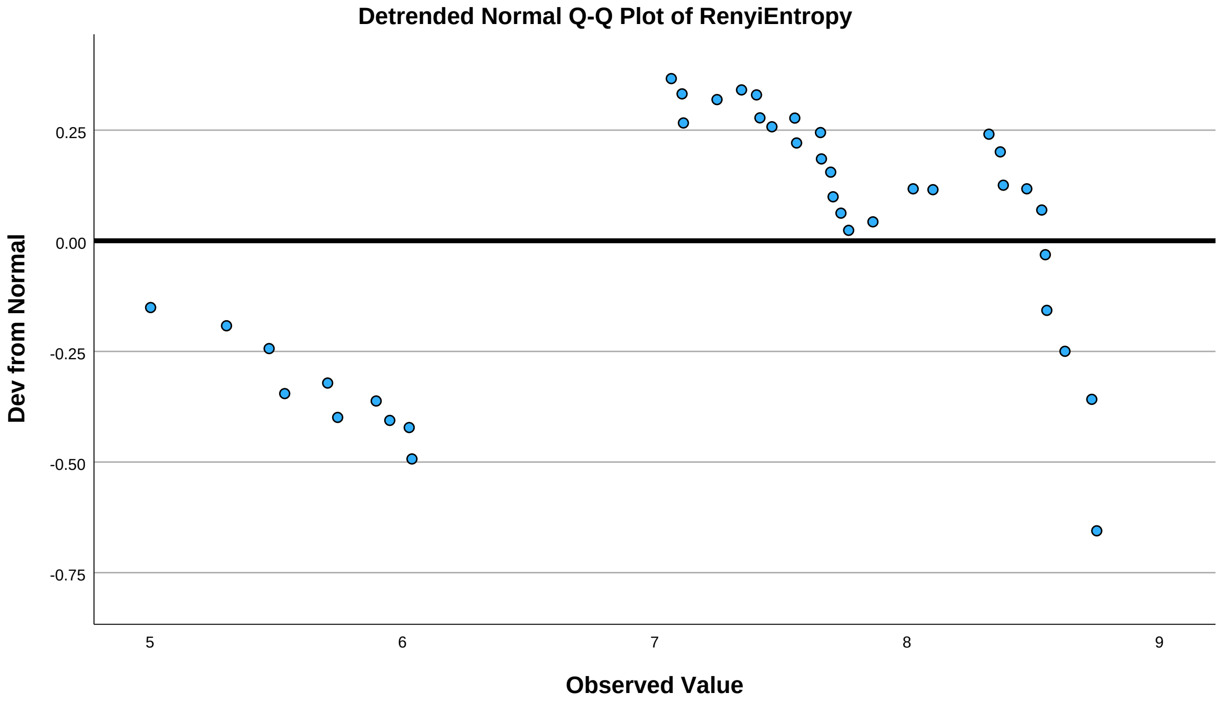
Detrended Normal Q-Q Plot of RenyiEntropy
Dev from Normal
0.25
0.00
-0.25
-0.50
-0.75
5
6
7
8
9
Observed Value

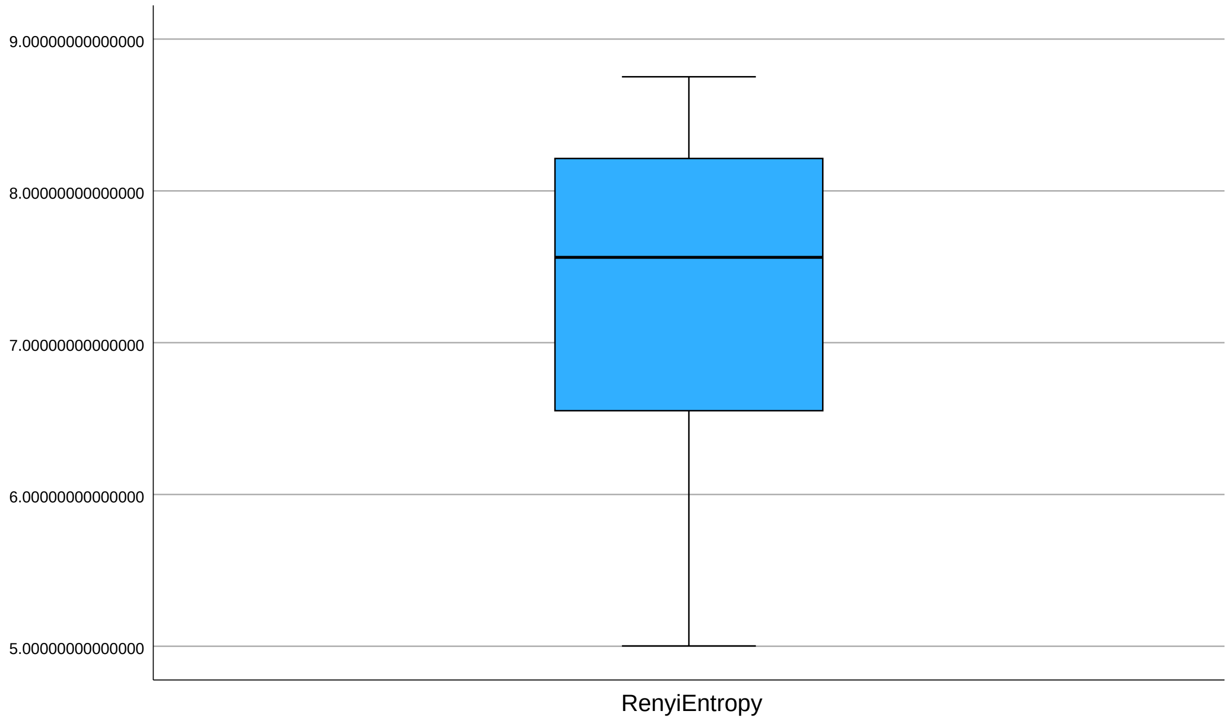

TsallisEntropy

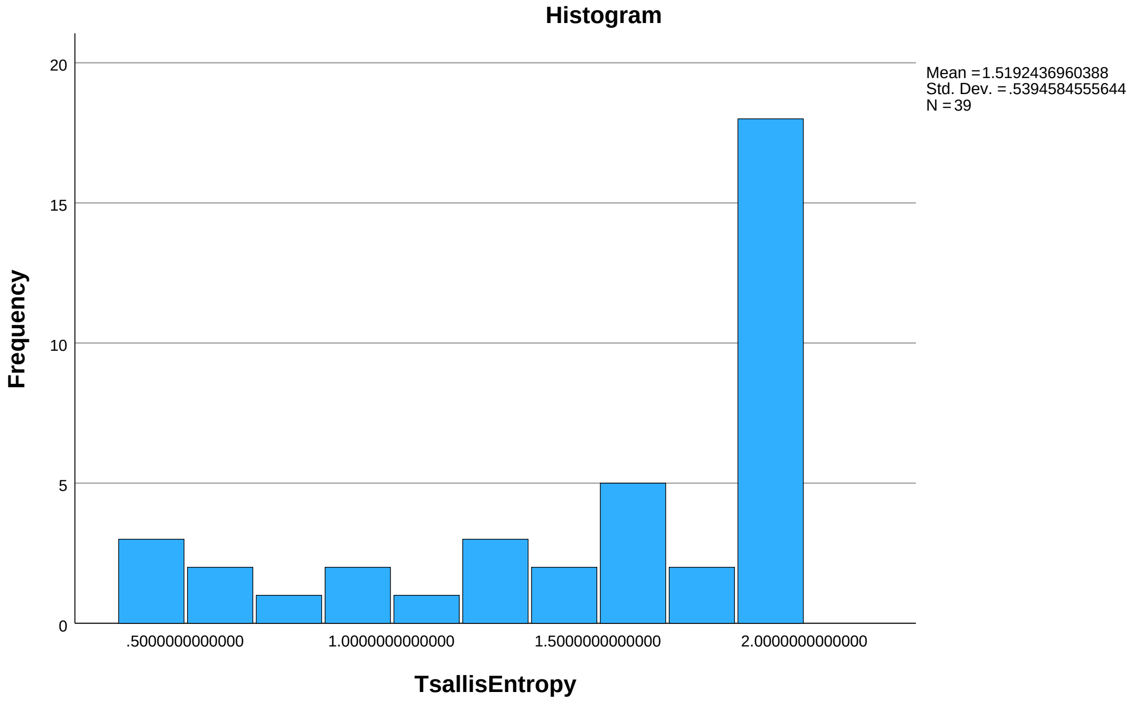

TsallisEntropy Stem-and-Leaf Plot

Frequency Stem & Leaf

```
 5.00      0 . 44455
 1.00     0 . 7
 2.00     0 . 99
 2.00     1 . 01
 2.00     1 . 22
 7.00      1 . 4455555
 2.00     1 . 77
18.00        1 .  999999999999999999

Stem width:  1.000000
Each leaf:       1 case(s)
```

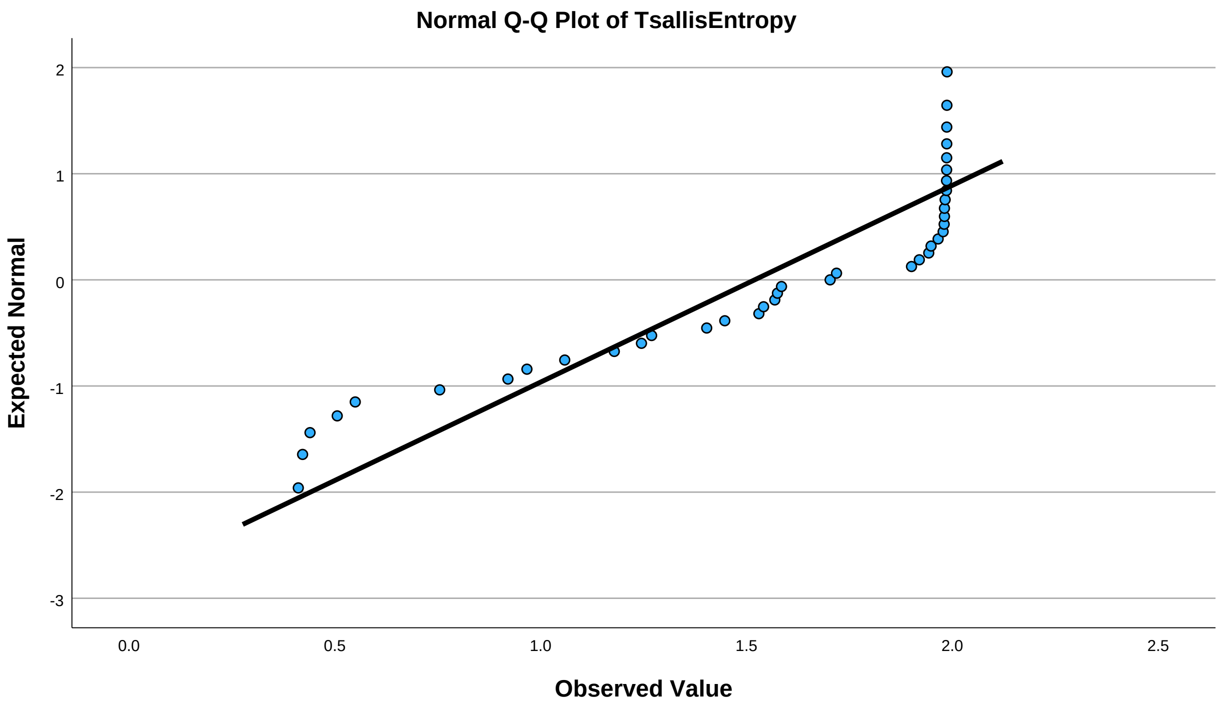

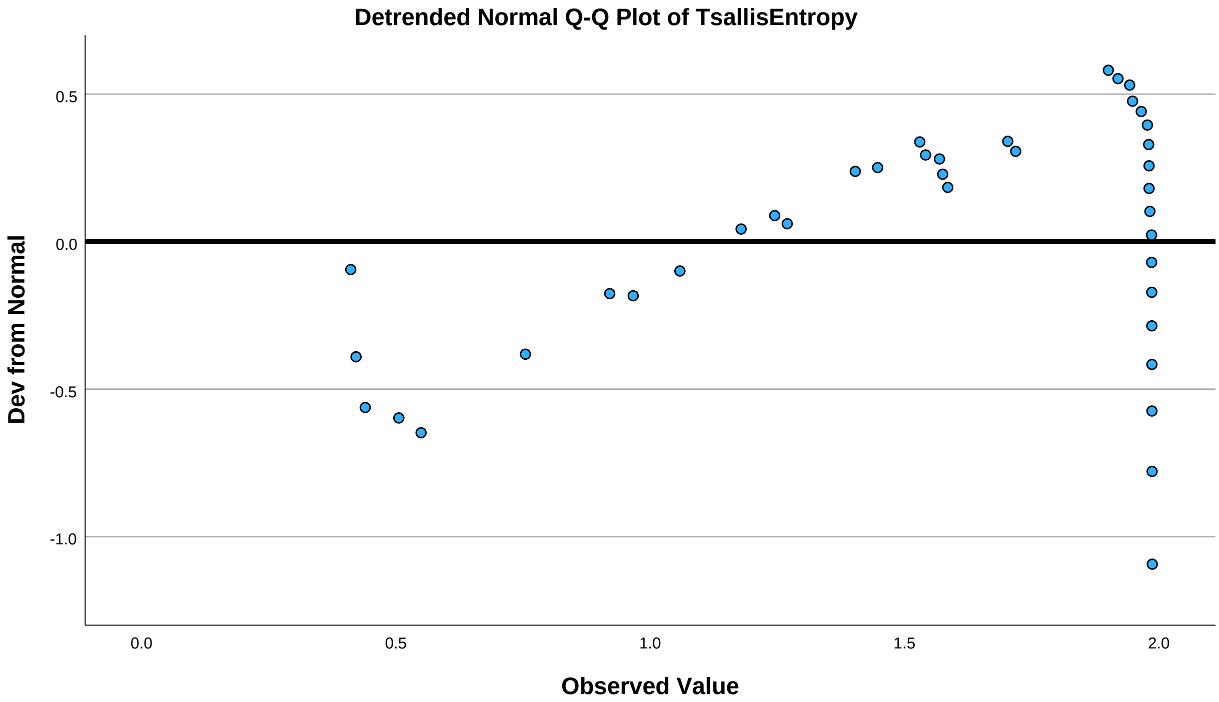
Detrended Normal Q-Q Plot of TsallisEntropy
Dev from Normal
0.5
0.0
-0.5
-1.0
0.0
0.5
1.0
1.5
2.0
Observed Value

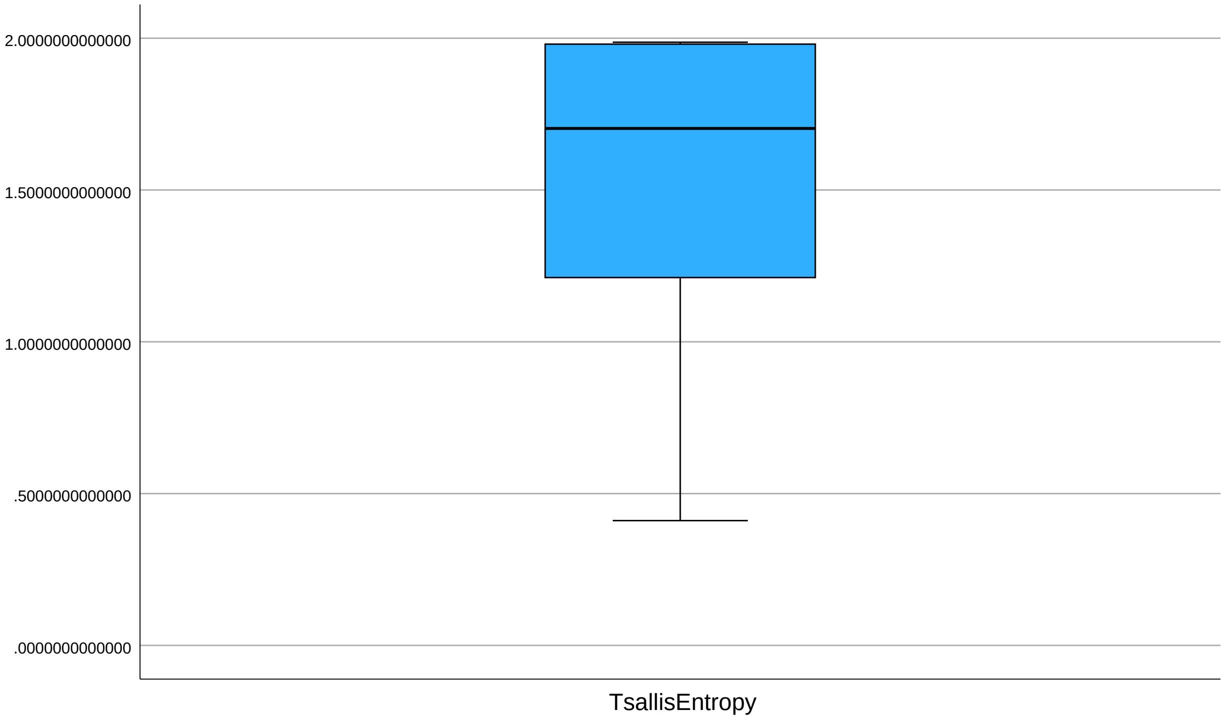
2.0000000000000
1.5000000000000
1.0000000000000
.5000000000000
.0000000000000
TsallisEntropy

PS_4_5

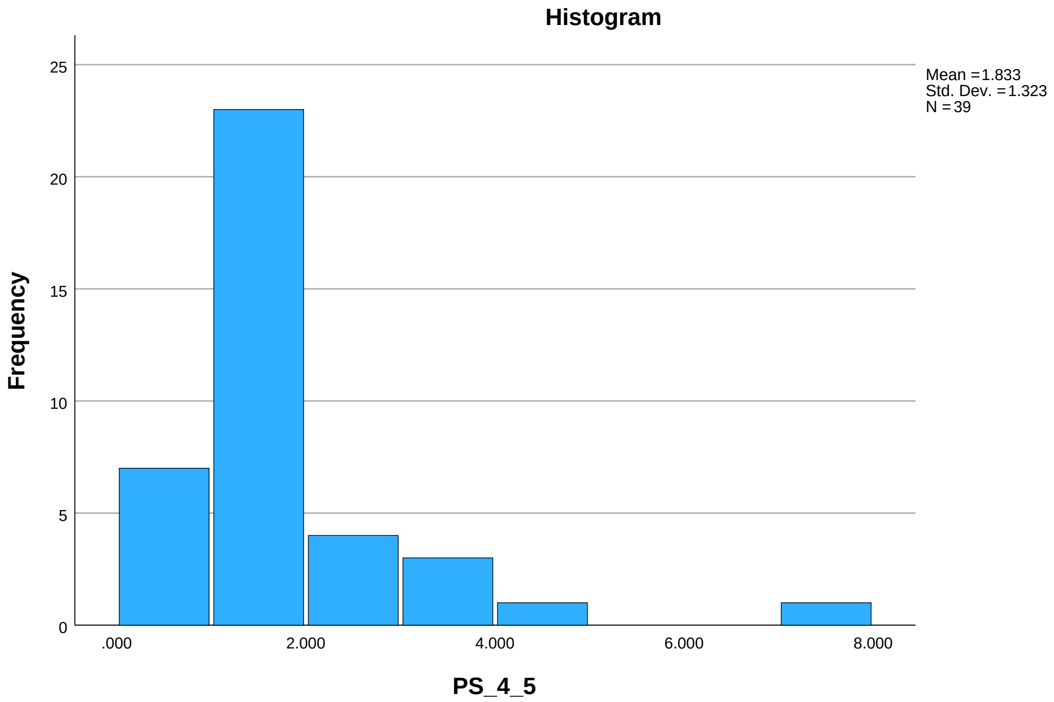


```
PS_4_5 Stem-and-Leaf Plot

 Frequency    Stem &  Leaf

     1.00        0 .  4
     6.00        0 .  558889
    13.00        1 .  0011112333444
    10.00        1 .  5555667899
     1.00        2 .  3
     3.00        2 .  788
     1.00        3 .  0
     4.00 Extremes    (>=3.6)

 Stem width:      1.000
 Each leaf:       1 case(s)
```

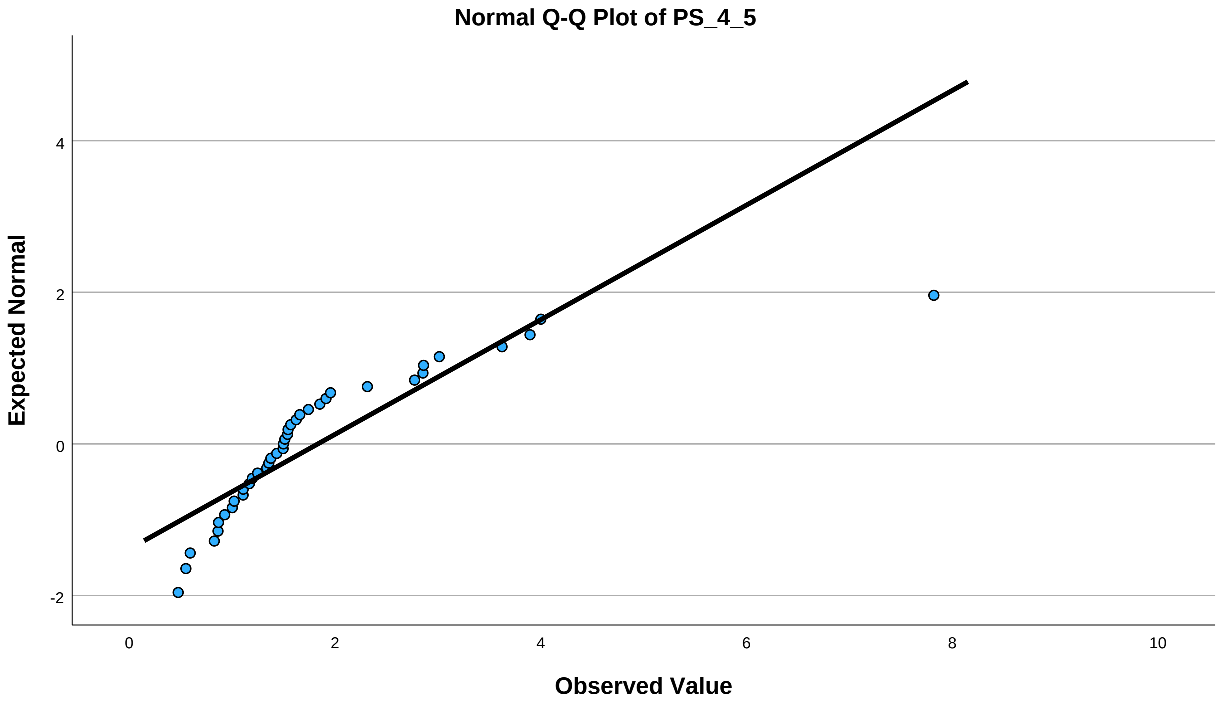
Normal Q-Q Plot of PS_4_5
Expected Normal
Observed Value
4
2
0
-2
0
2
4
6
8
10

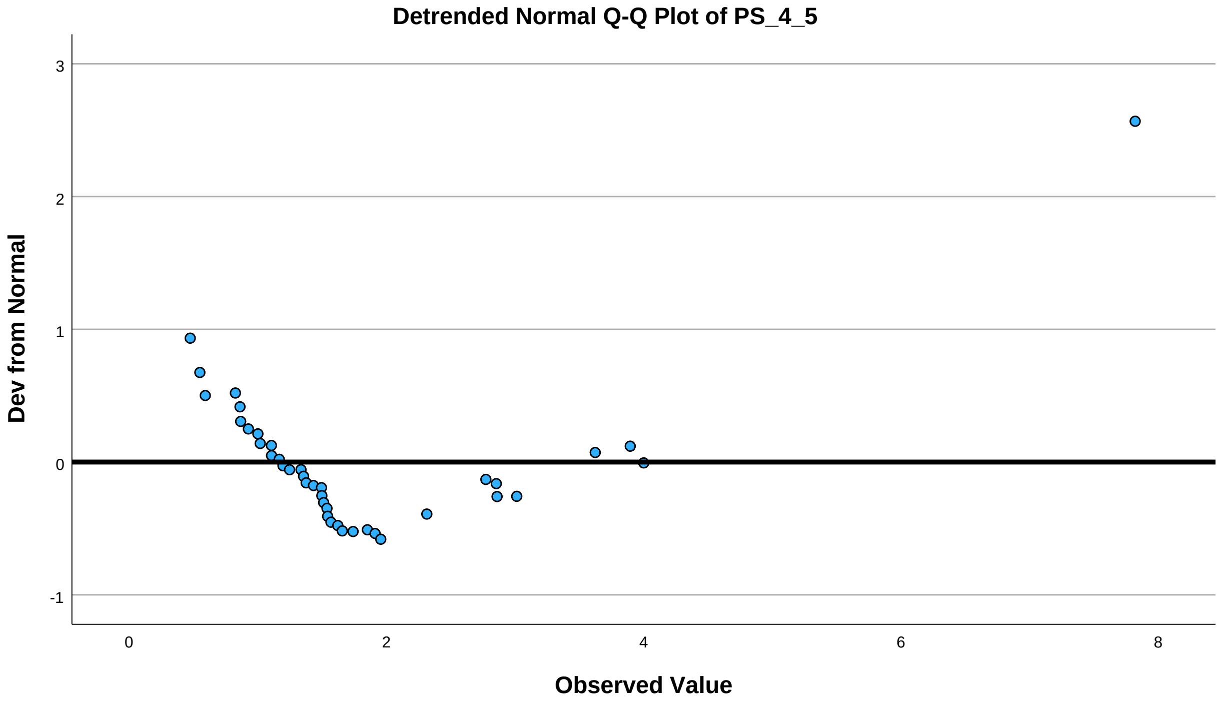
Detrended Normal Q-Q Plot of PS_4_5
Dev from Normal
Observed Value
3
2
1
0
-1
0
2
4
6
8

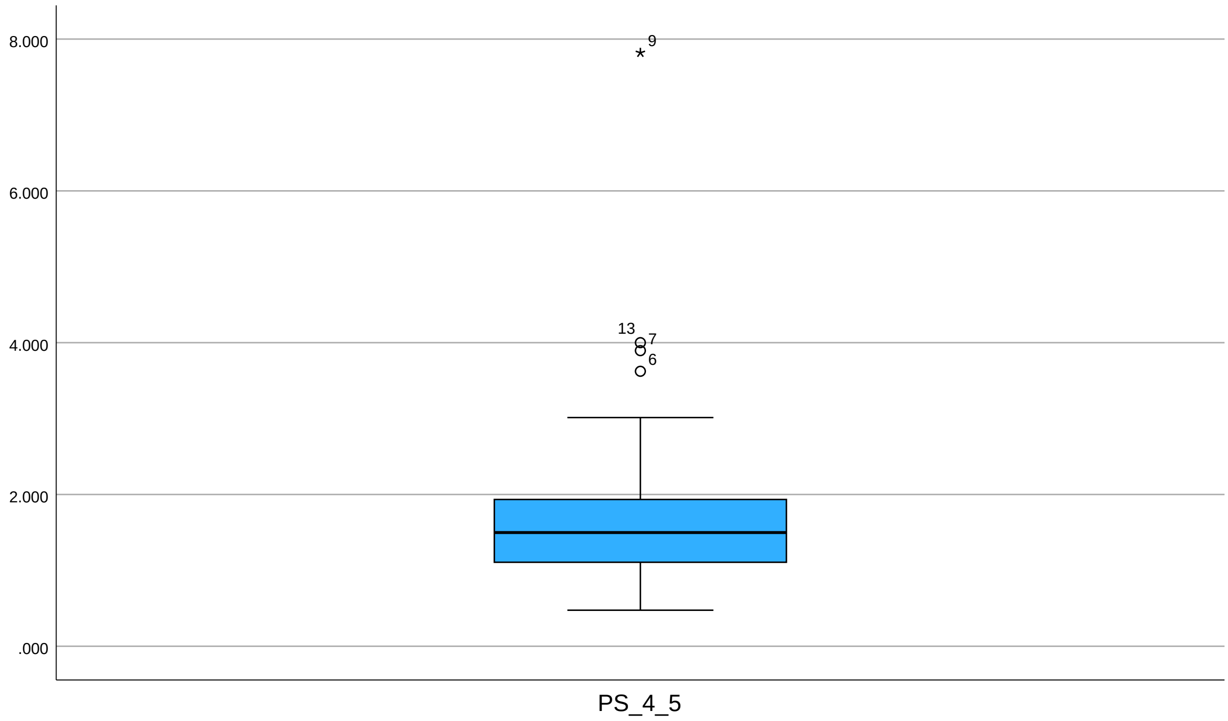

PS_5_6

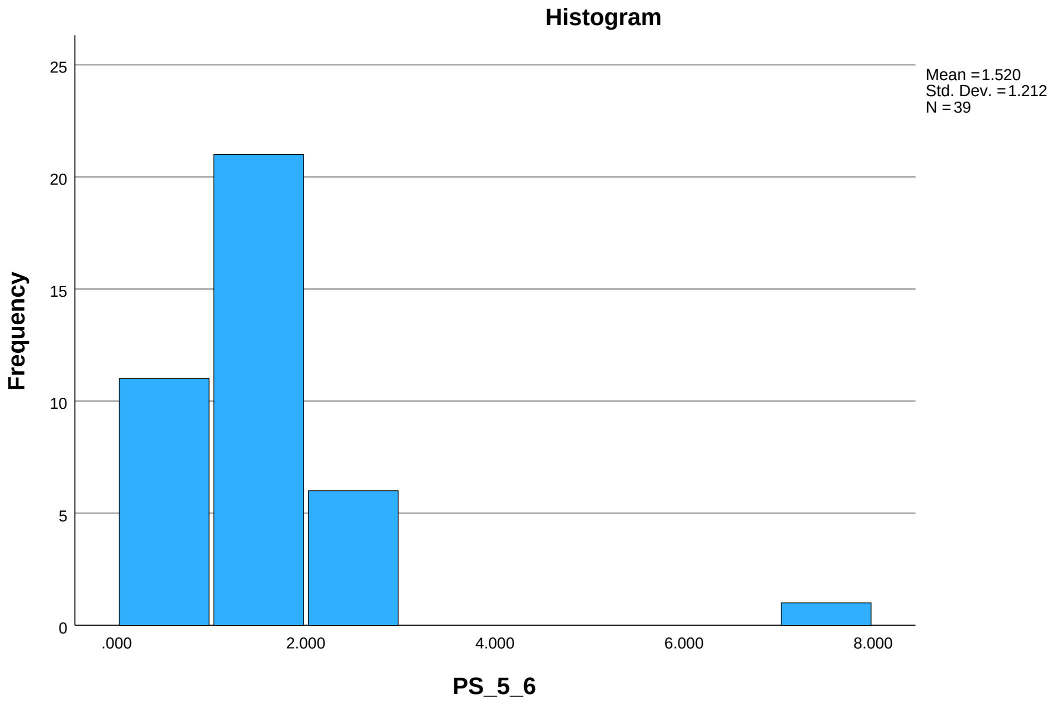


```
PS_5_6 Stem-and-Leaf Plot

 Frequency    Stem &  Leaf
```

```
    3.00        0 .  344
    8.00        0 .  56668999
   12.00        1 .  000111112234
    9.00        1 .  555668888
    4.00        2 .  0224
    2.00        2 .  67
    1.00 Extremes    (>=7.9)

Stem width:      1.000
Each leaf:        1 case(s)
```

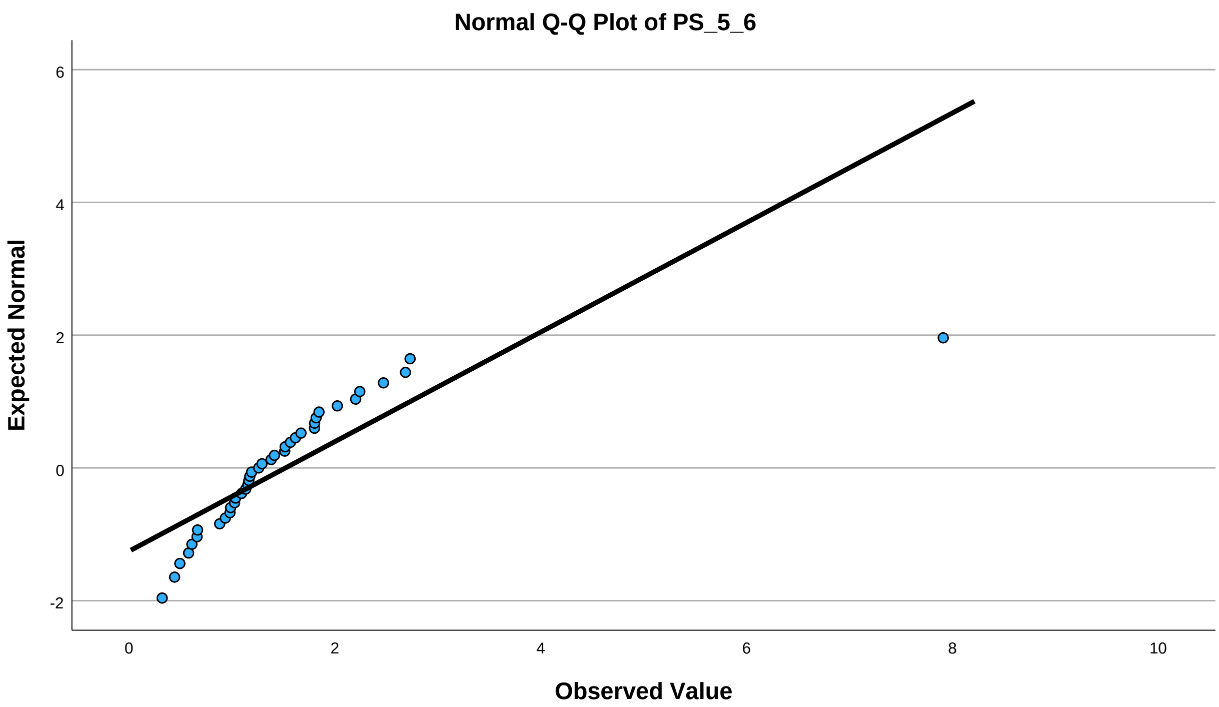

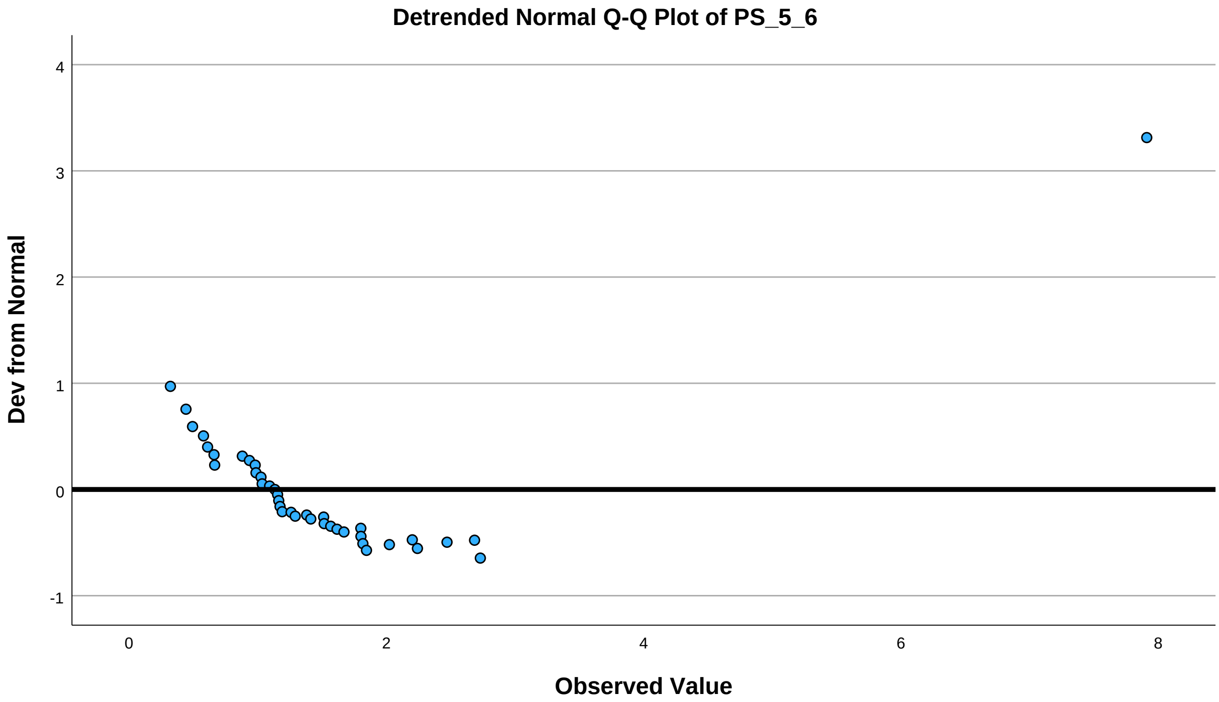

Detrended Normal Q-Q Plot of PS_5_6
Dev from Normal
4
3
2
1
0
-1
0
2
4
6
8
Observed Value

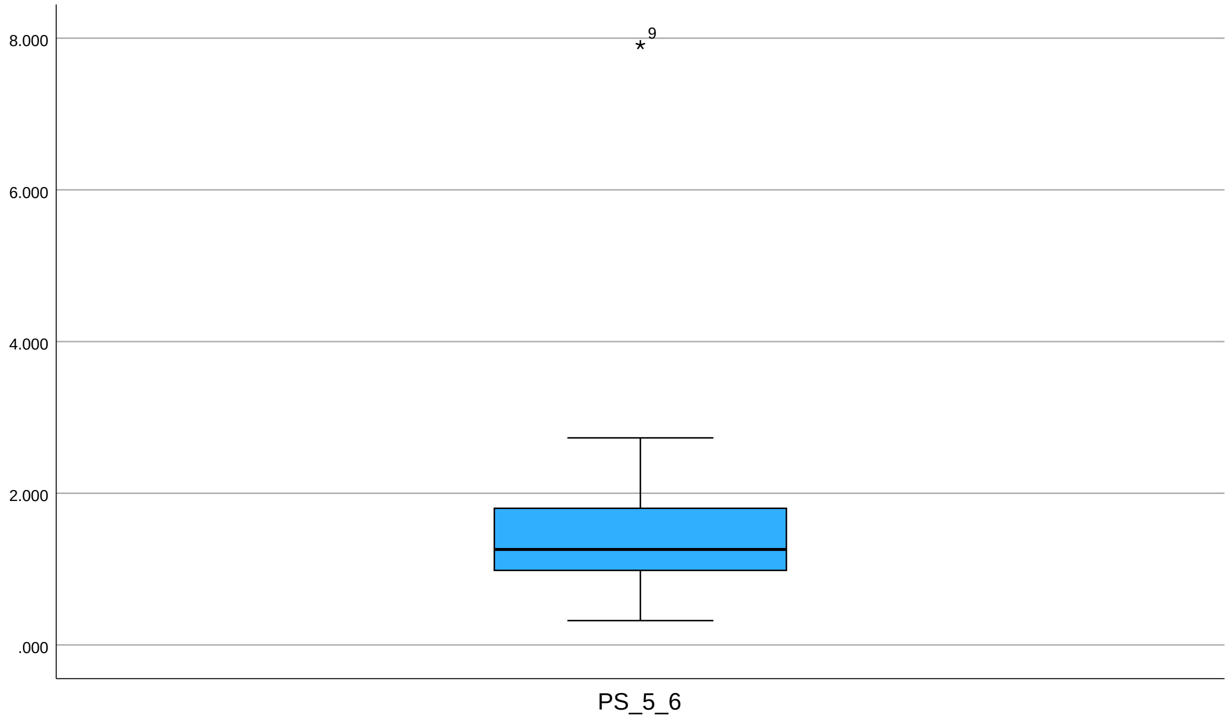

8.000
6.000
4.000
2.000
.000
9
PS_5_6

PS_6_7

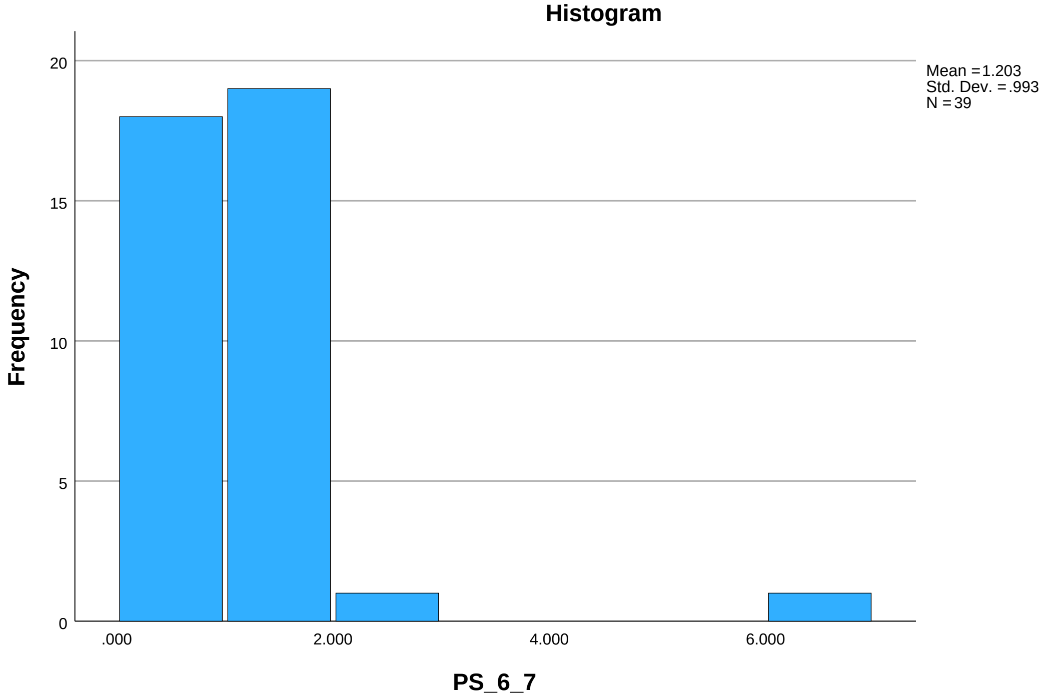


```
PS_6_7 Stem-and-Leaf Plot

 Frequency    Stem &  Leaf

     5.00        0 .  23344
    13.00        0 .  5567788999999
    13.00        1 .  0011111222334
     6.00        1 .  566788
     1.00        2 .  0
     1.00 Extremes    (>=6.6)

 Stem width:      1.000
 Each leaf:       1 case(s)
```

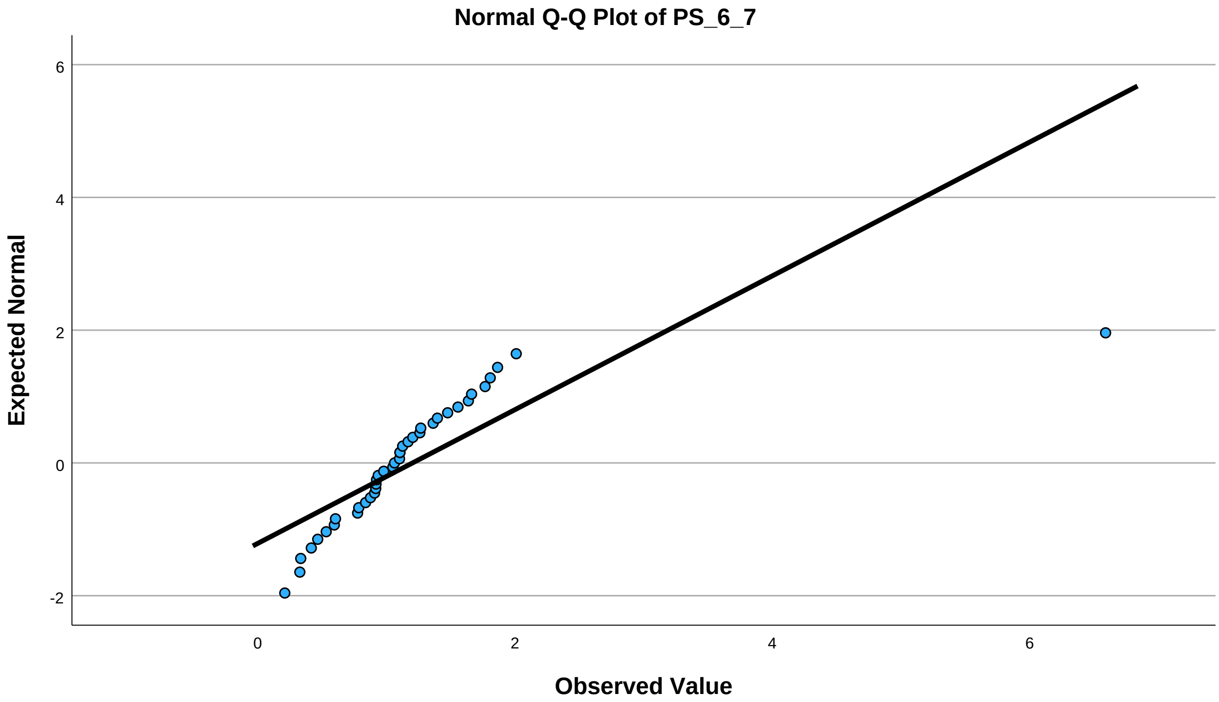
Normal Q-Q Plot of PS_6_7
Expected Normal
Observed Value
6
4
2
0
-2
0
2
4
6

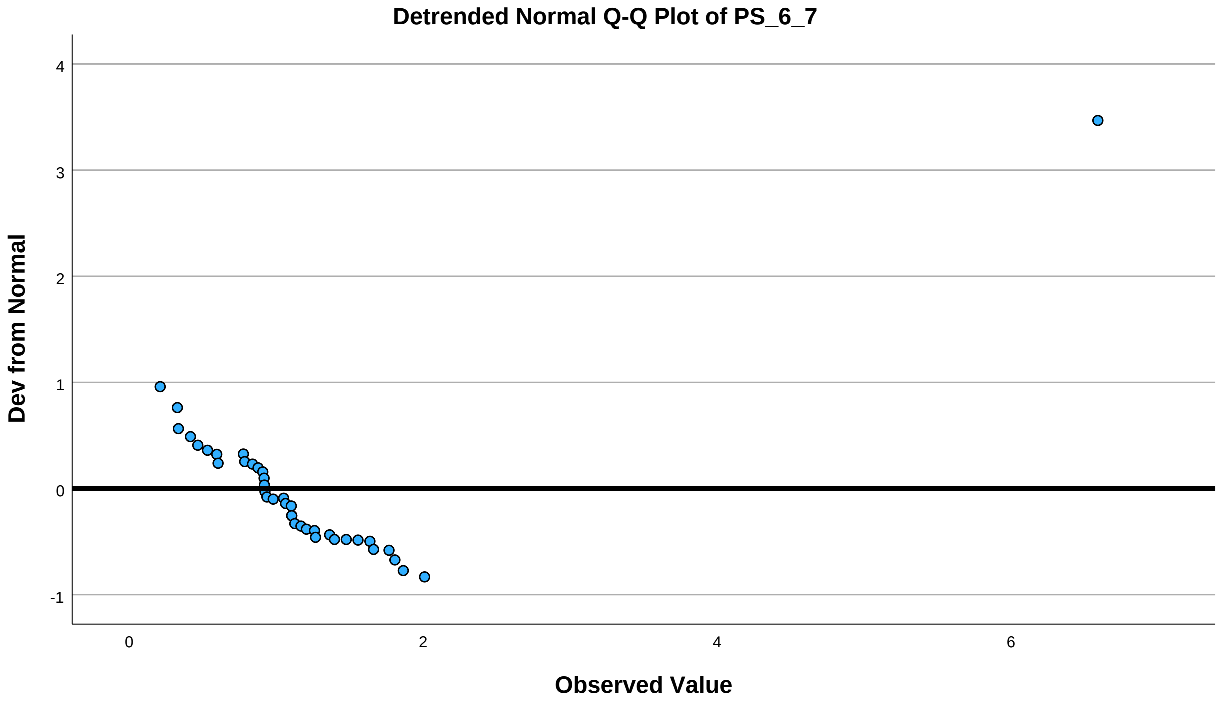
Detrended Normal Q-Q Plot of PS_6_7
Dev from Normal
Observed Value
4
3
2
1
0
-1
0
2
4
6

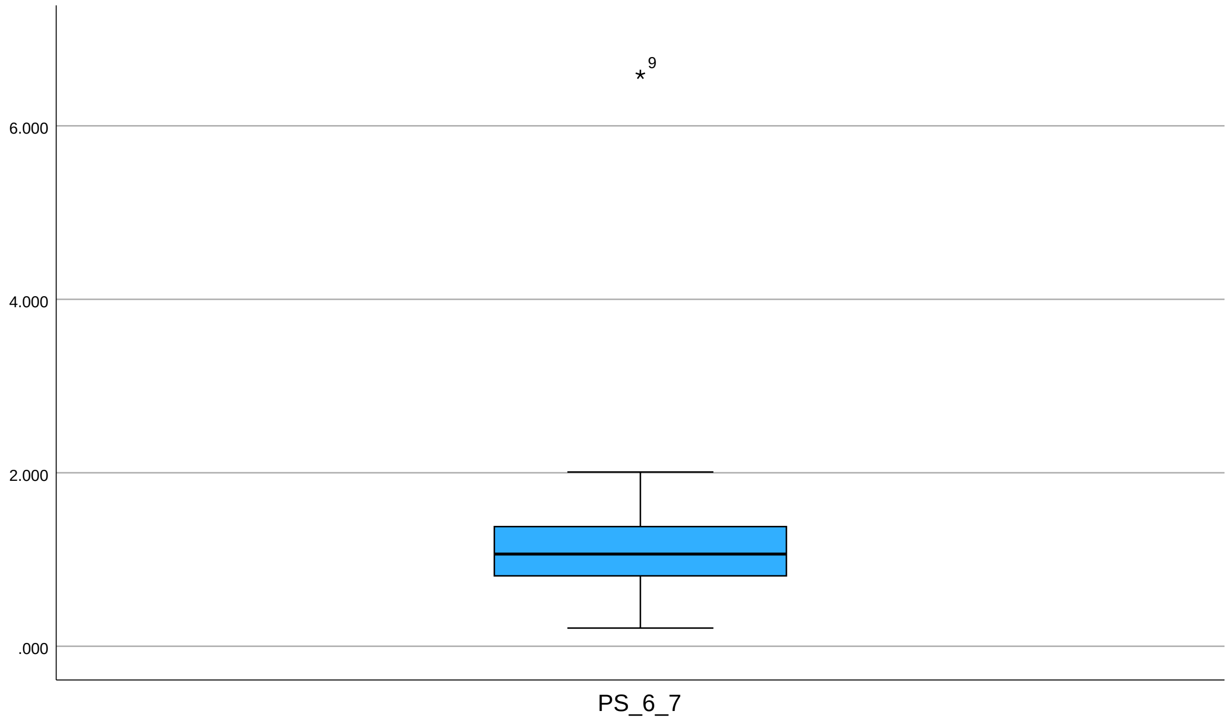

PS_7_8

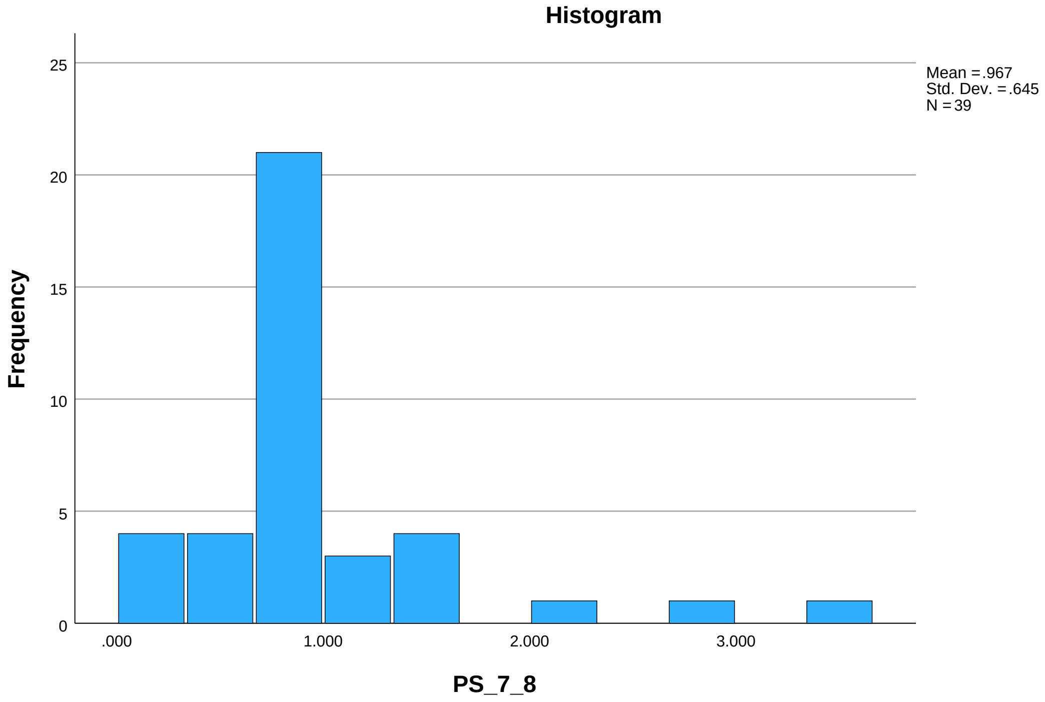

PS_7_8 Stem-and-Leaf Plot

Frequency   Stem & Leaf

```
 1.00      0 . 1
 4.00      0 . 2333
 2.00      0 . 44
11.00      0 . 66667777777
11.00      0 . 88888999999
 1.00      1 . 0
 5.00      1 . 22333
 1.00      1 . 4
 3.00 Extremes    (>=2.0)

Stem width:     1.000
Each leaf:       1 case(s)
```

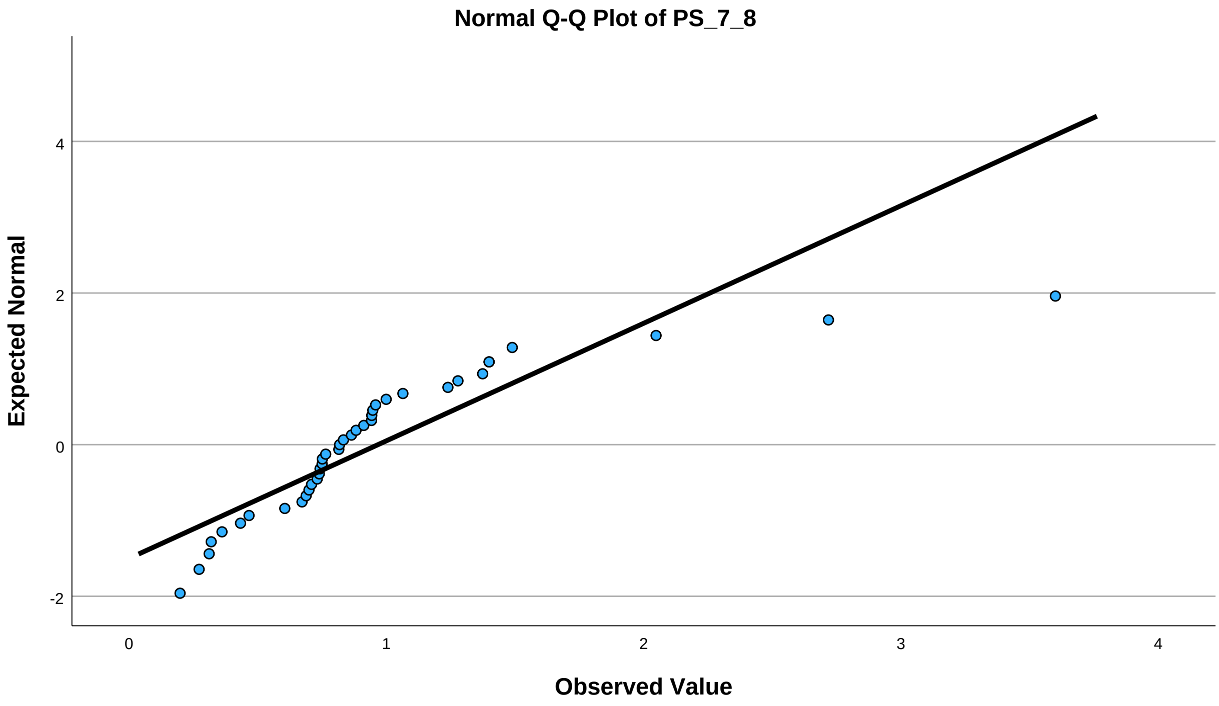

Detrended Normal Q-Q Plot of PS_7_8

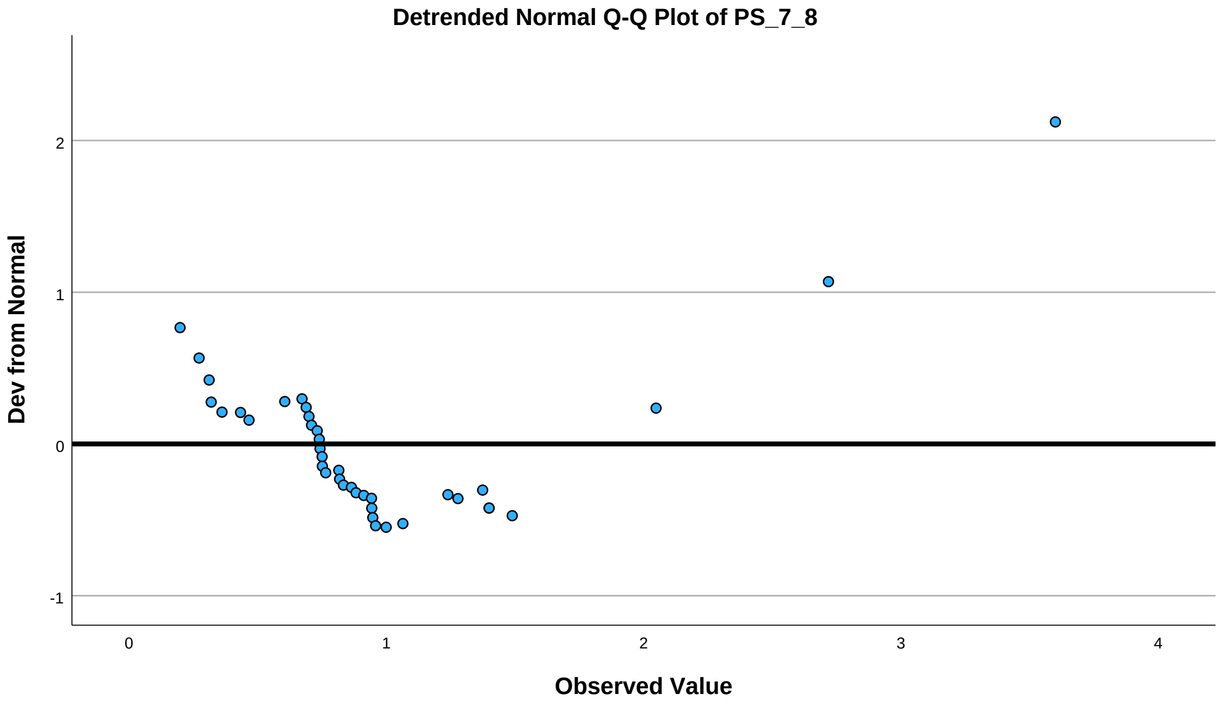

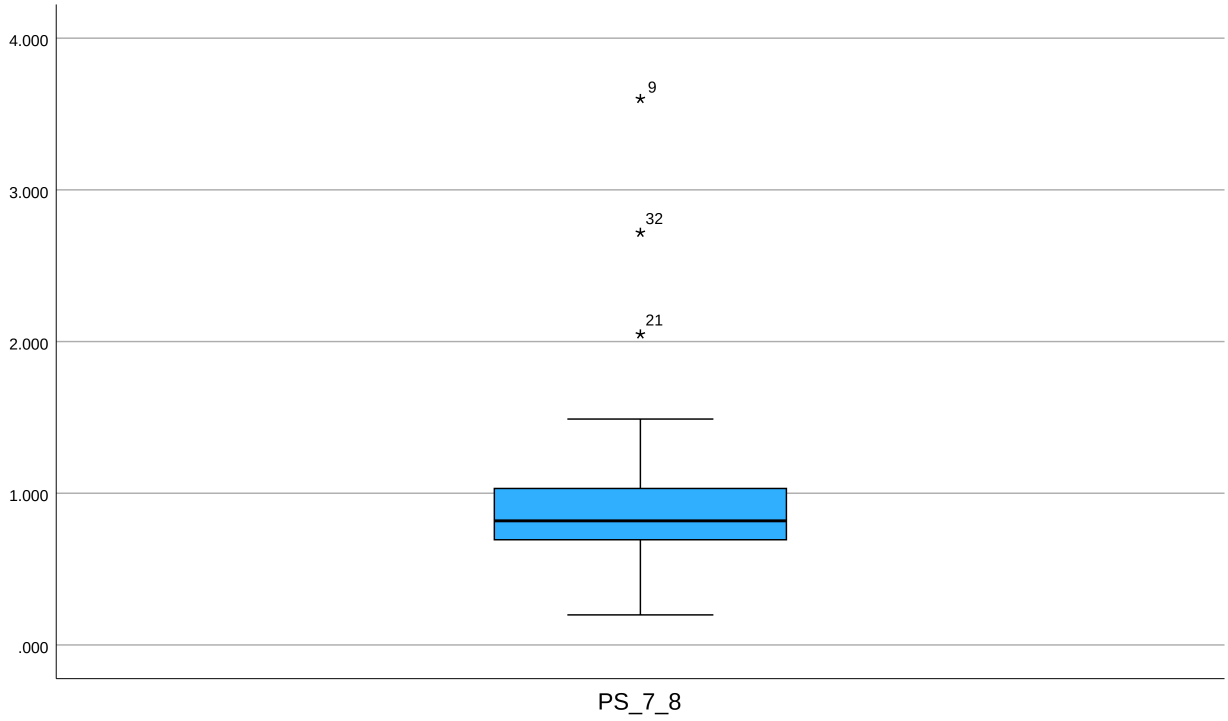

PS_8_9

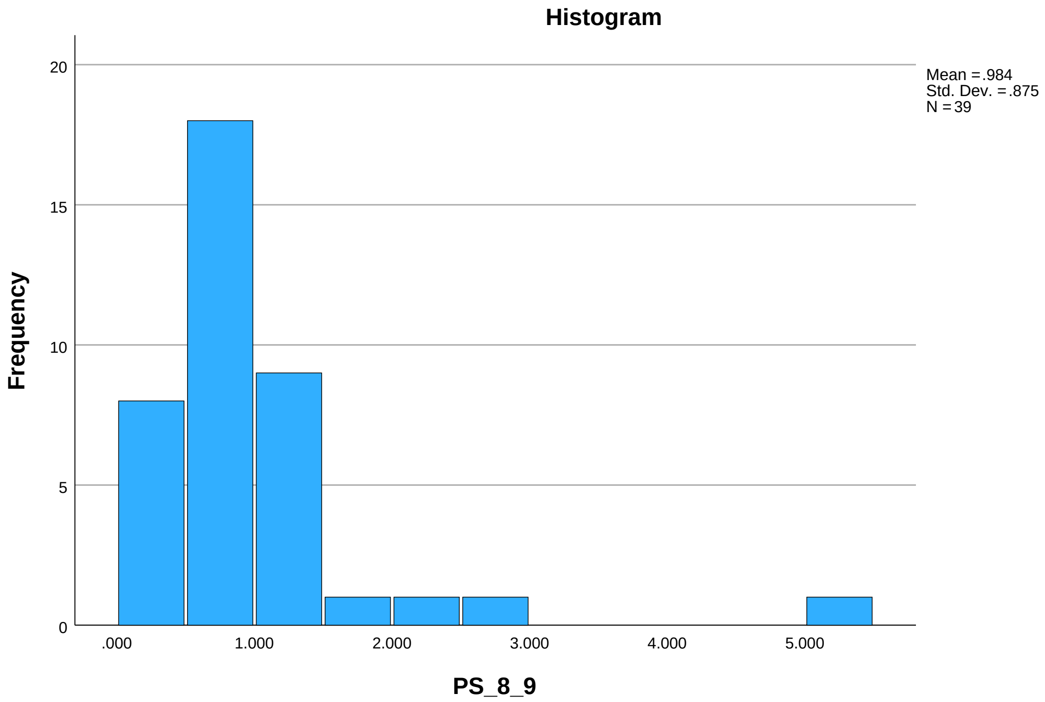


```
PS_8_9 Stem-and-Leaf Plot

 Frequency    Stem &  Leaf

     1.00        0 .  1
     6.00        0 .  223333
     2.00        0 .  45
    16.00        0 .  6666666677777777
     1.00        0 .  9
     3.00        1 .  000
     3.00        1 .  223
     3.00        1 .  444
      .00        1 .
     1.00        1 .  8
     3.00 Extremes    (>=2.1)

 Stem width:      1.000
 Each leaf:       1 case(s)
```

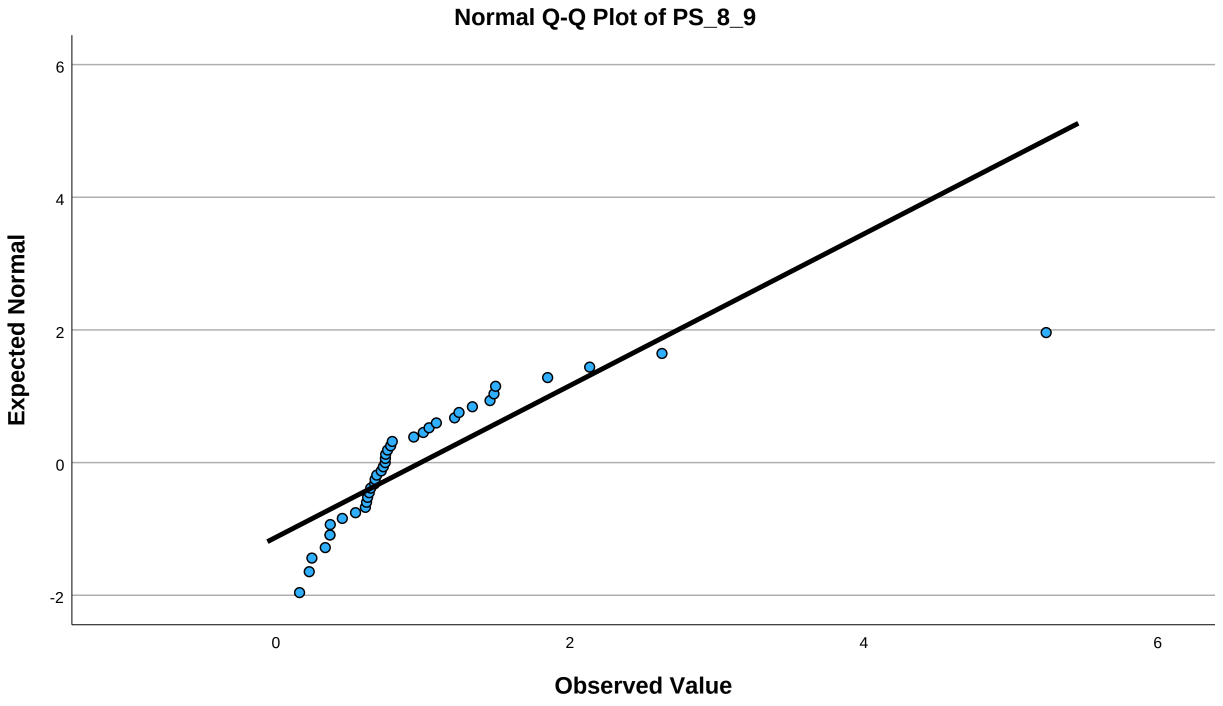

Normal Q-Q Plot of PS_8_9
Expected Normal
Observed Value
6
4
2
0
-2
0
2
4
6

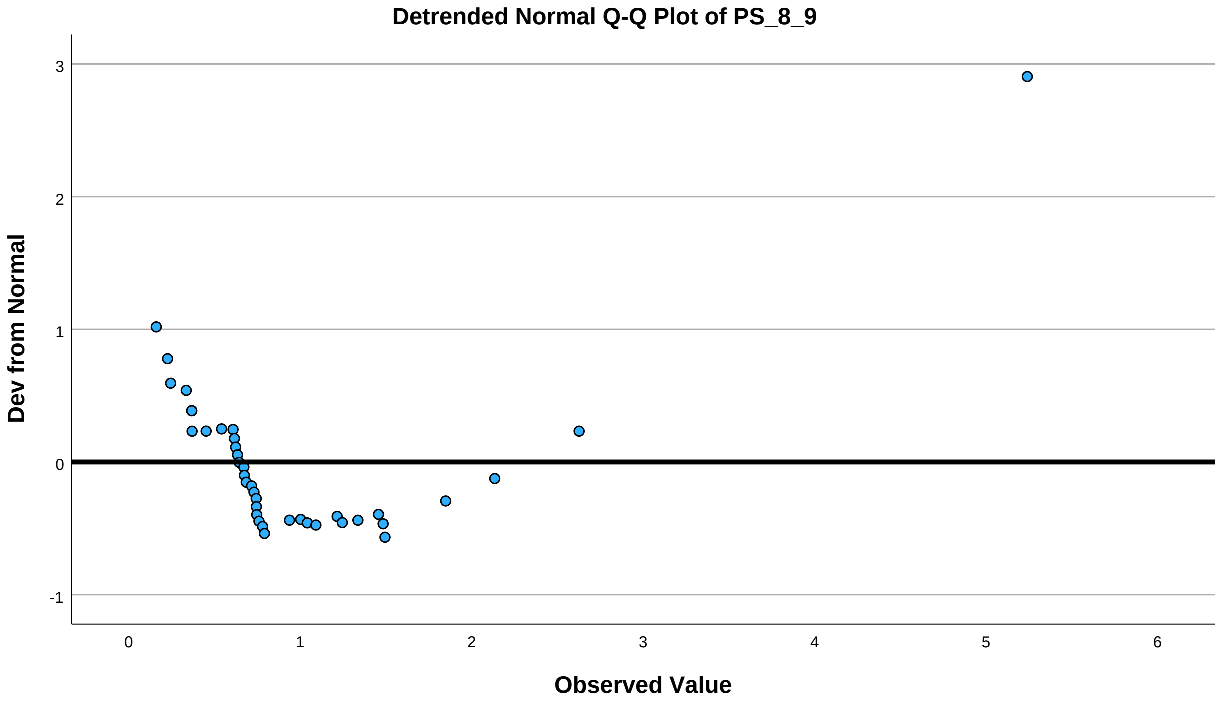

Detrended Normal Q-Q Plot of PS_8_9
Dev from Normal
Observed Value
3
2
1
0
-1
0
1
2
3
4
5
6

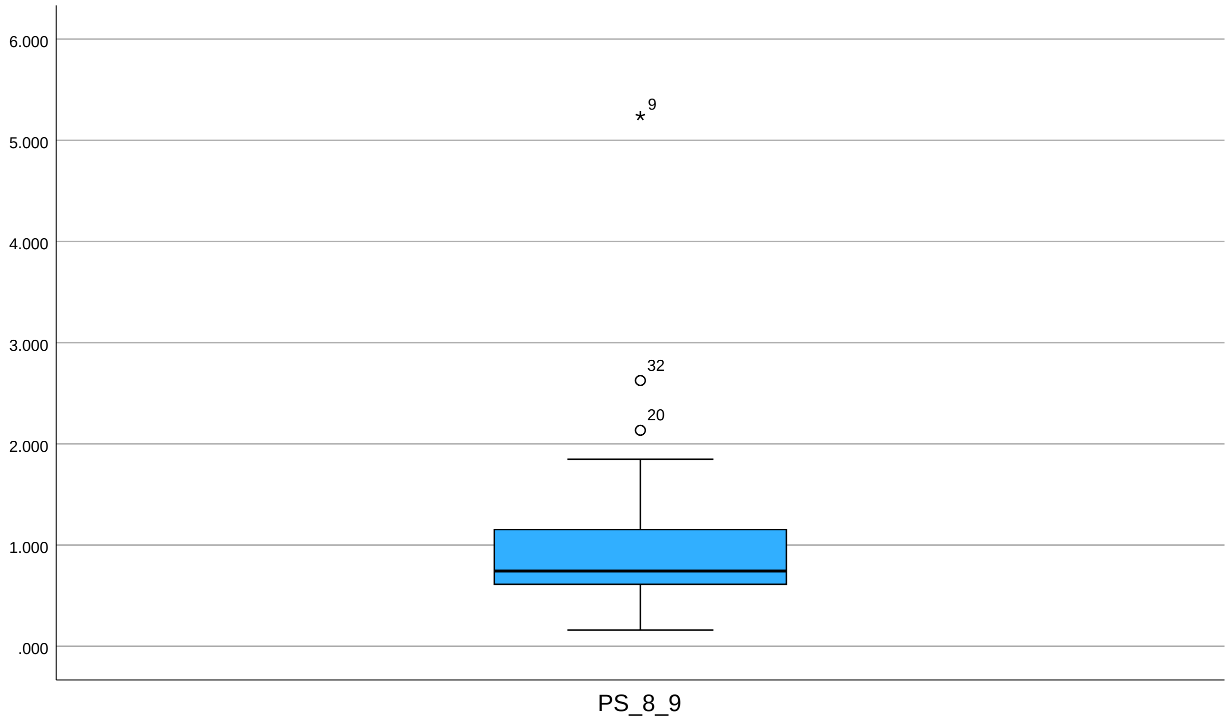

PS_9_10

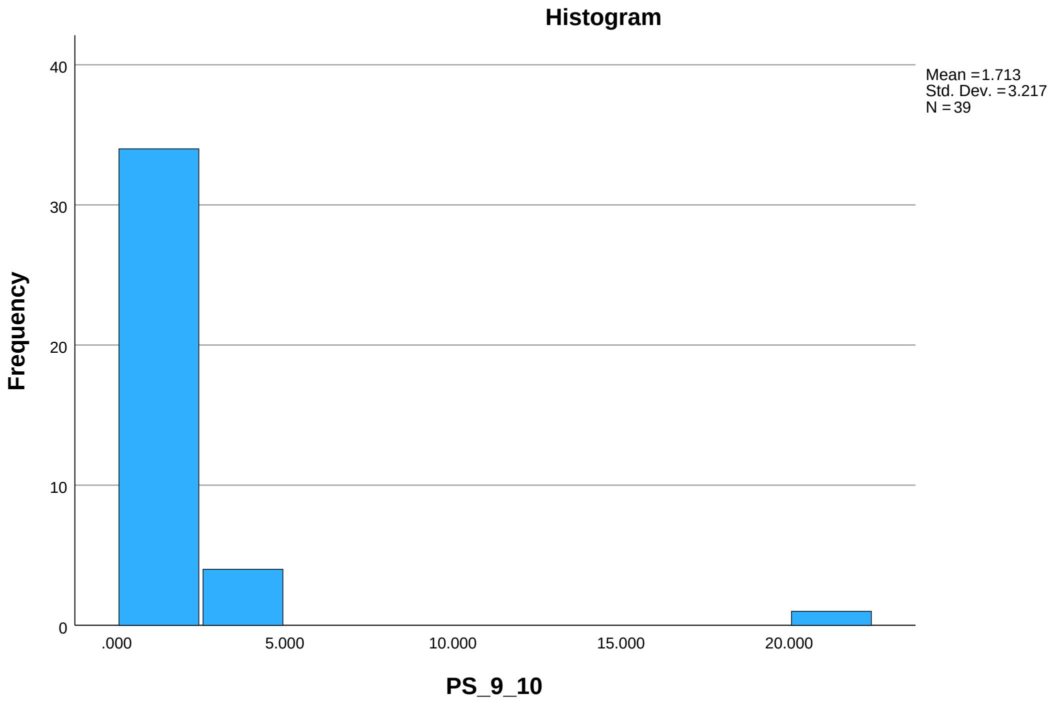

PS_9_10 Stem-and-Leaf Plot

```
 Frequency    Stem &  Leaf
```

```
     7.00        0 .  1222334
    15.00        0 .  555555567889999
     6.00        1 .  122444
     3.00        1 .  777
     3.00        2 .  004
     2.00        2 .  67
     3.00 Extremes    (>=3.9)

 Stem width:      1.000
 Each leaf:        1 case(s)
```

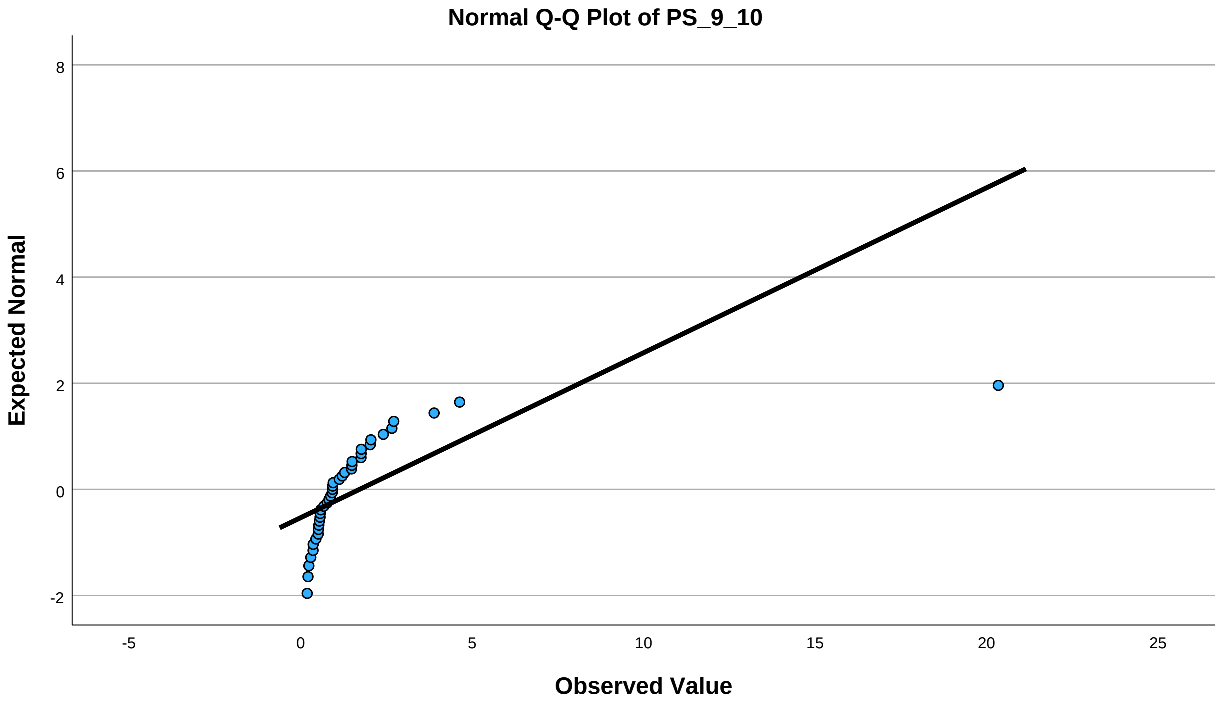

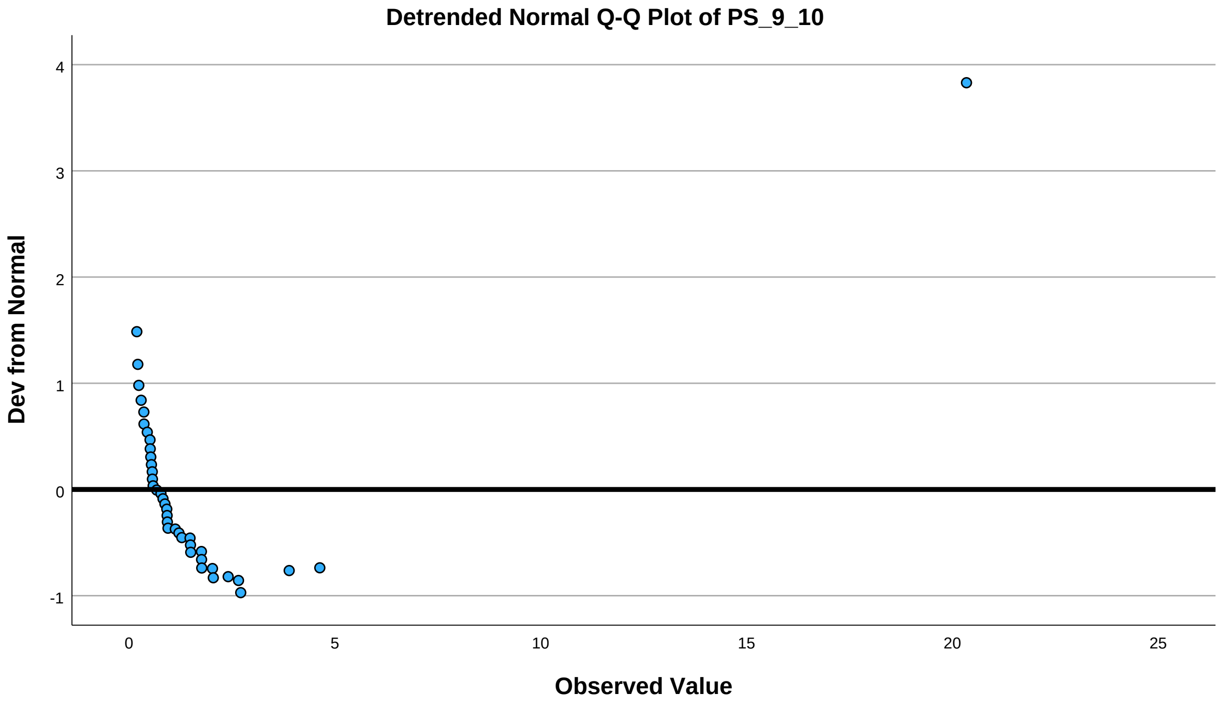

Detrended Normal Q-Q Plot of PS_9_10
Dev from Normal
4
3
2
1
0
-1
0
5
10
15
20
25
Observed Value

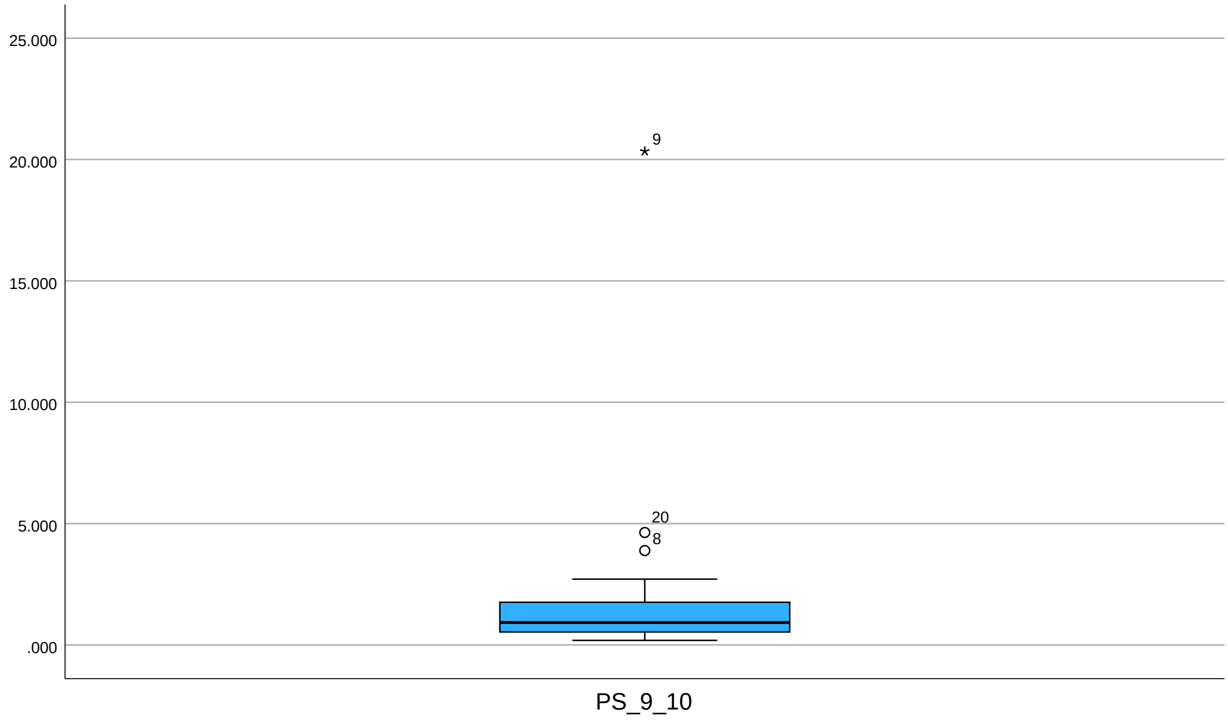

25.000
20.000
15.000
10.000
5.000
.000
9
20
8
PS_9_10

PS_10_11

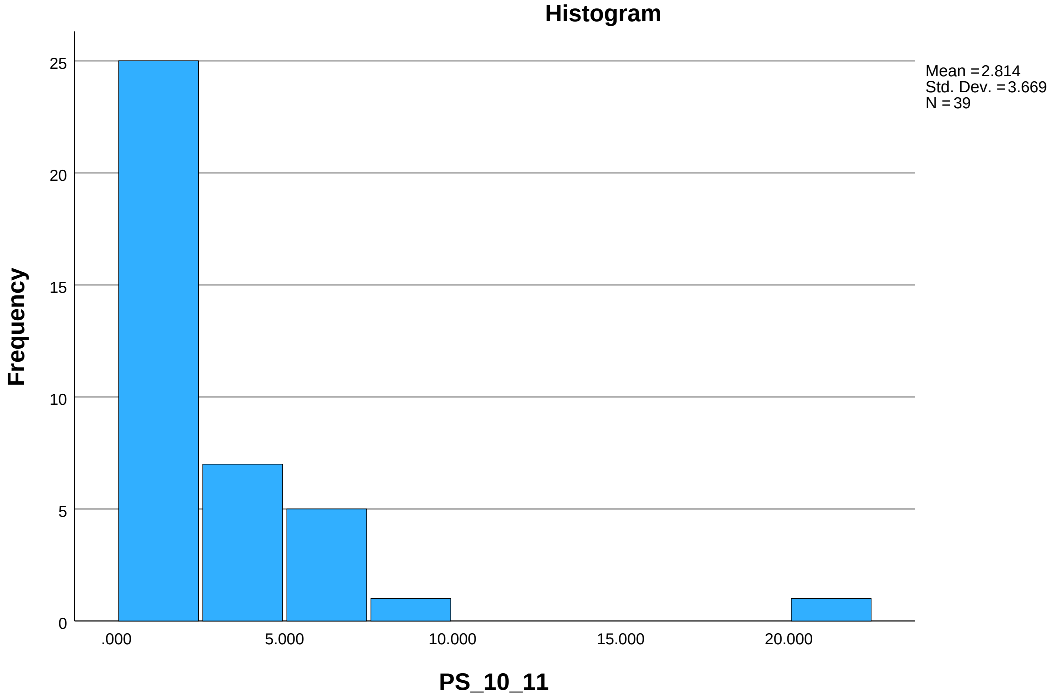


```
PS_10_11 Stem-and-Leaf Plot

 Frequency    Stem &  Leaf

    15.00        0 .  233444556777899
     7.00        1 .  1123378
     4.00        2 .  0347
     3.00        3 .  222
     3.00        4 .  001
     2.00        5 .  13
     3.00        6 .  589
     1.00        7 .  8
     1.00 Extremes    (>=21.1)

 Stem width:      1.000
 Each leaf:       1 case(s)
```

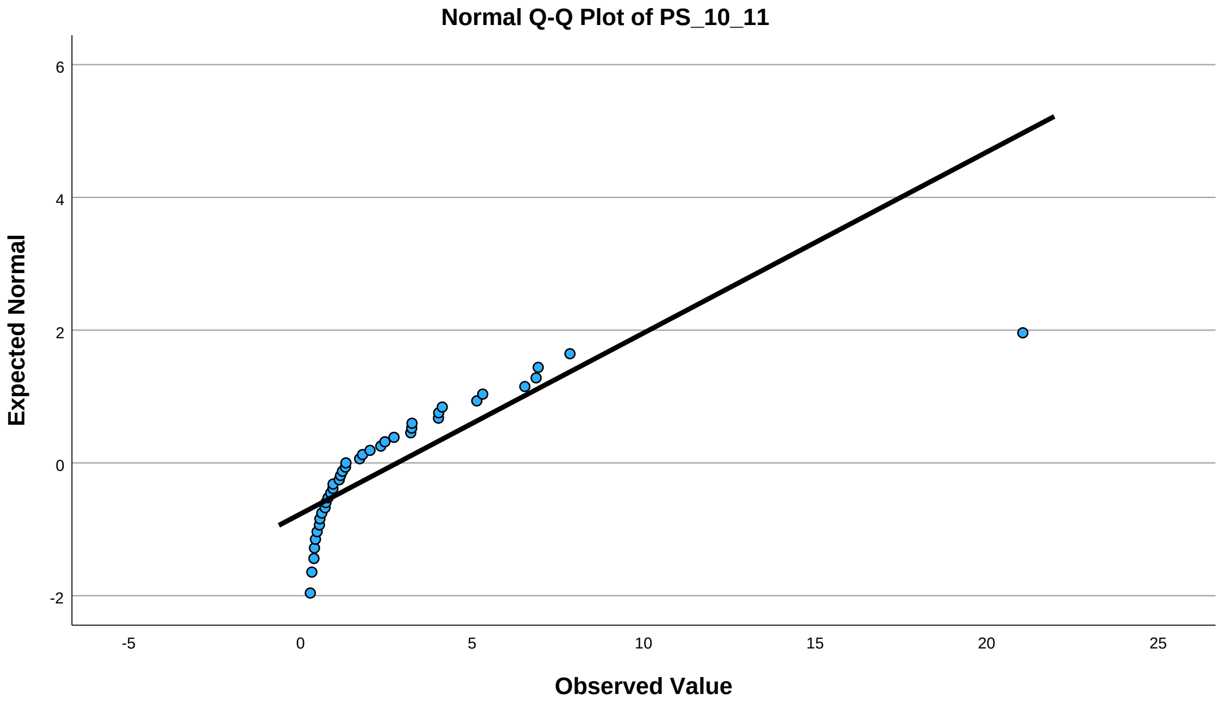
Normal Q-Q Plot of PS_10_11
Expected Normal
Observed Value
6
4
2
0
-2
-5
0
5
10
15
20
25

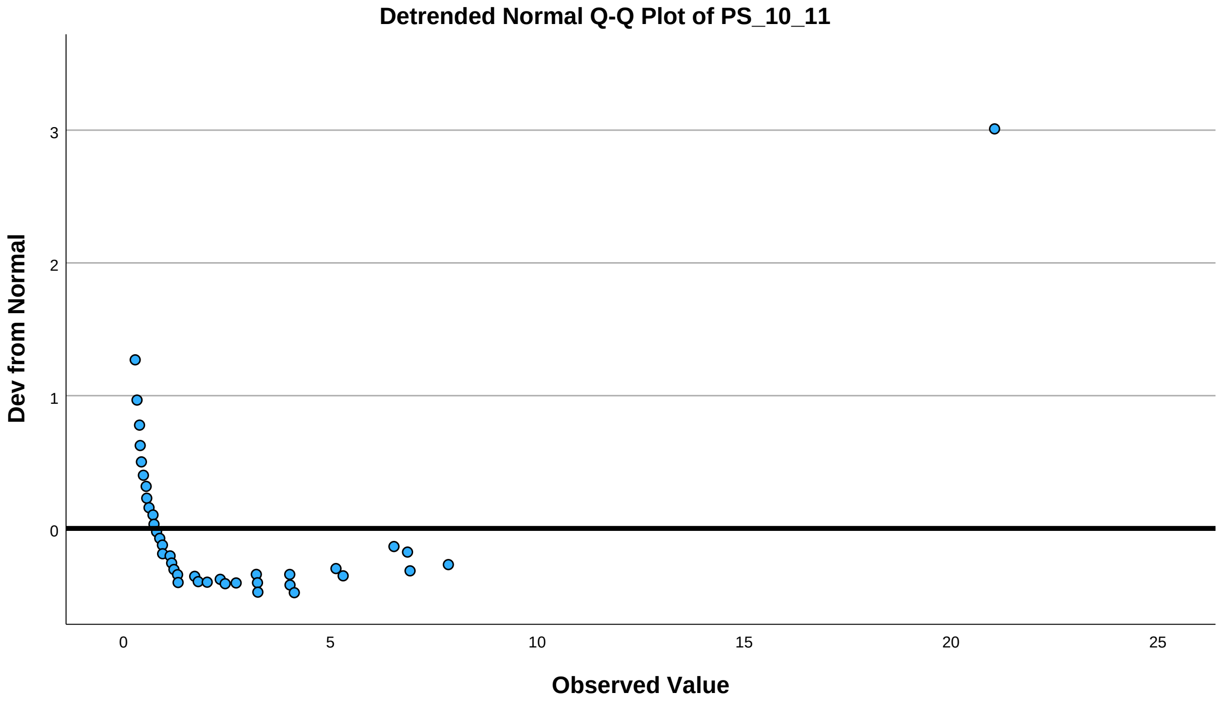
Detrended Normal Q-Q Plot of PS_10_11
Dev from Normal
Observed Value
3
2
1
0
0
5
10
15
20
25

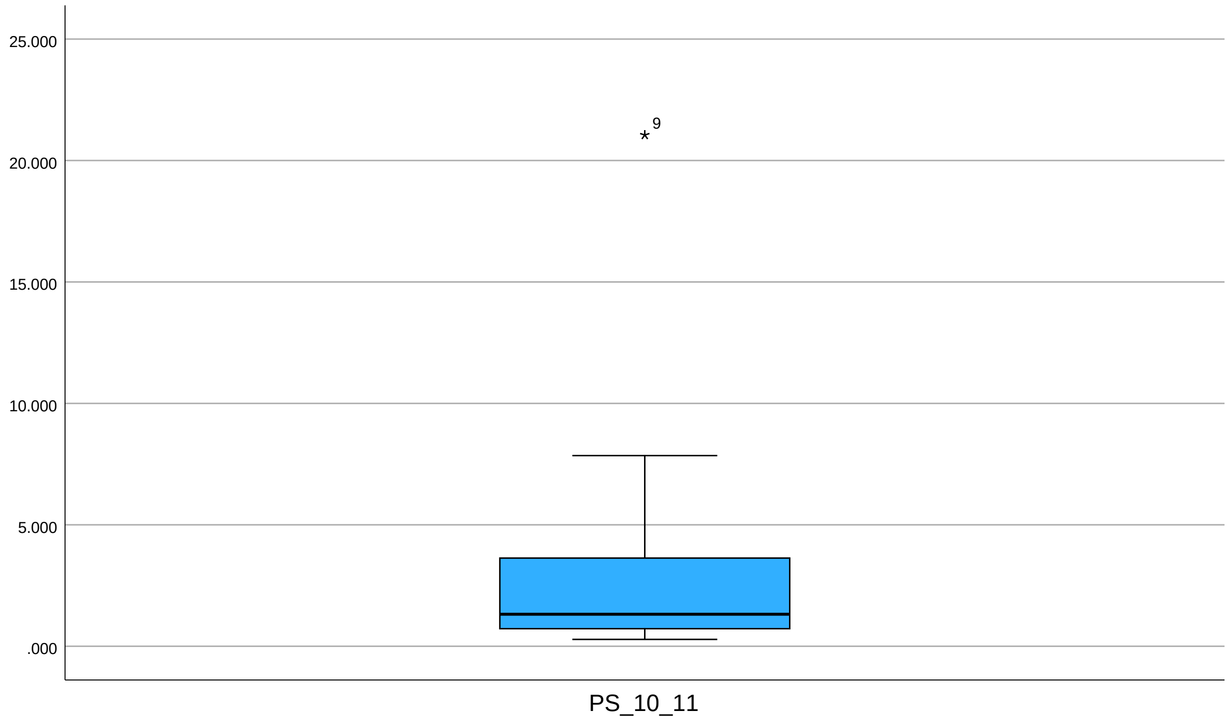

PS_11_12

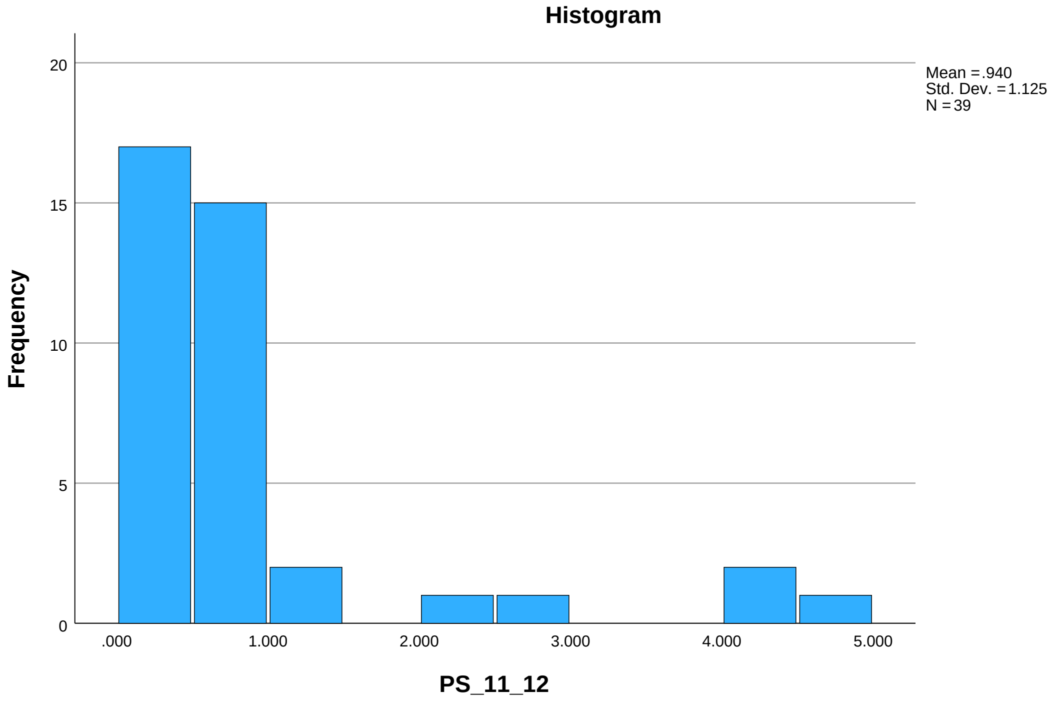

PS_11_12 Stem-and-Leaf Plot

Frequency Stem & Leaf

```
  2.00      1 . 69
  2.00      2 . 18
 10.00      3 . 0133455899
  3.00      4 . 045
  3.00      5 . 348
  7.00      6 . 0012889
  1.00      7 . 0
  2.00      8 . 88
  2.00      9 . 12
   .00     10 .
   .00     11 .
  2.00     12 . 34
  5.00 Extremes    (>=2.09)

 Stem width:      .100
 Each leaf:       1 case(s)
```

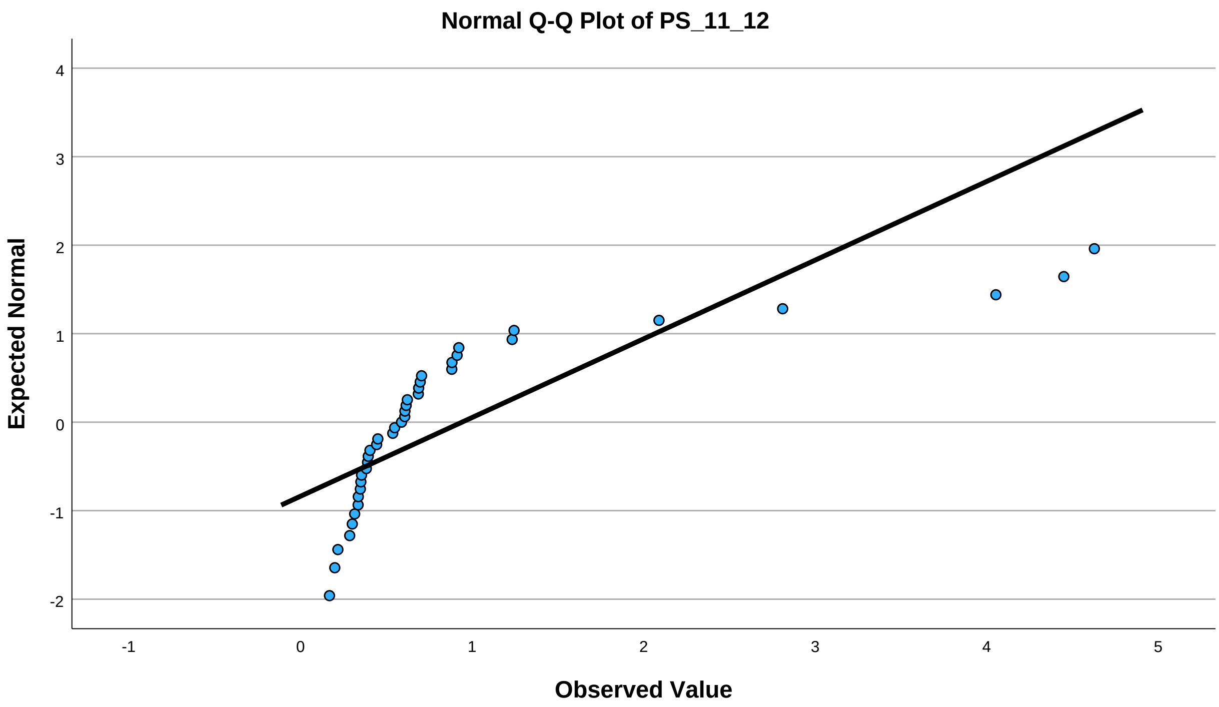

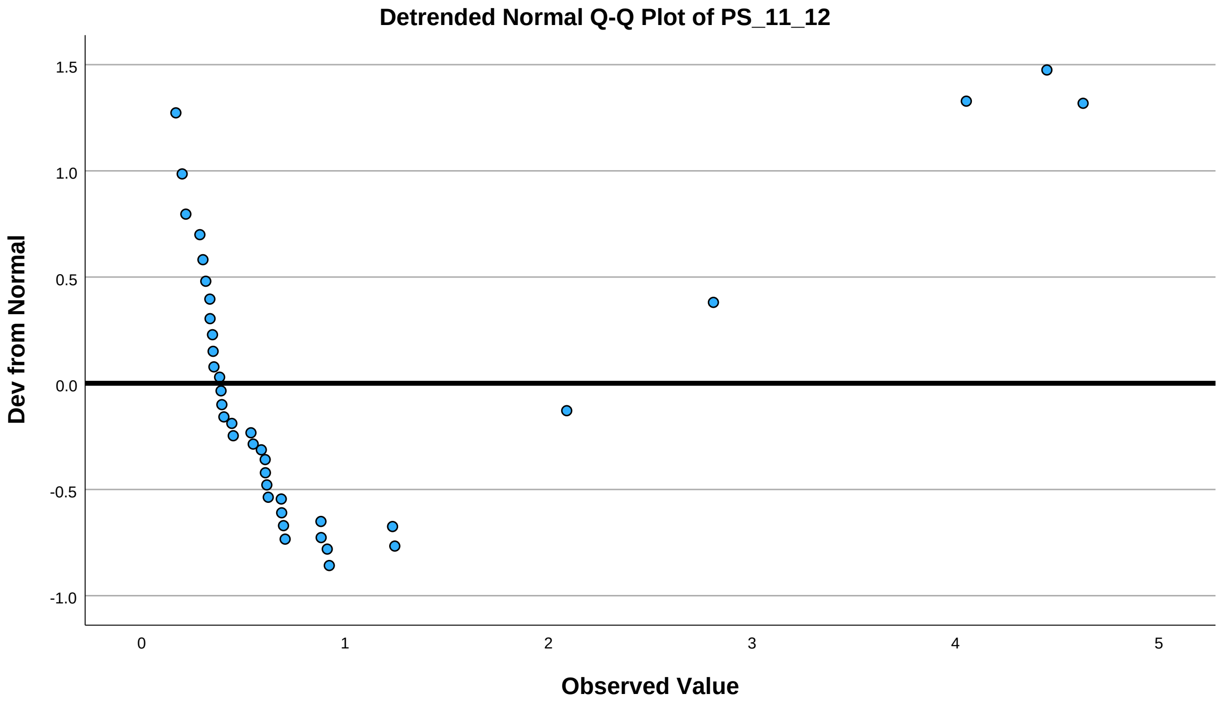

Detrended Normal Q-Q Plot of PS_11_12
Dev from Normal
1.5
1.0
0.5
0.0
-0.5
-1.0
0
1
2
3
4
5
Observed Value

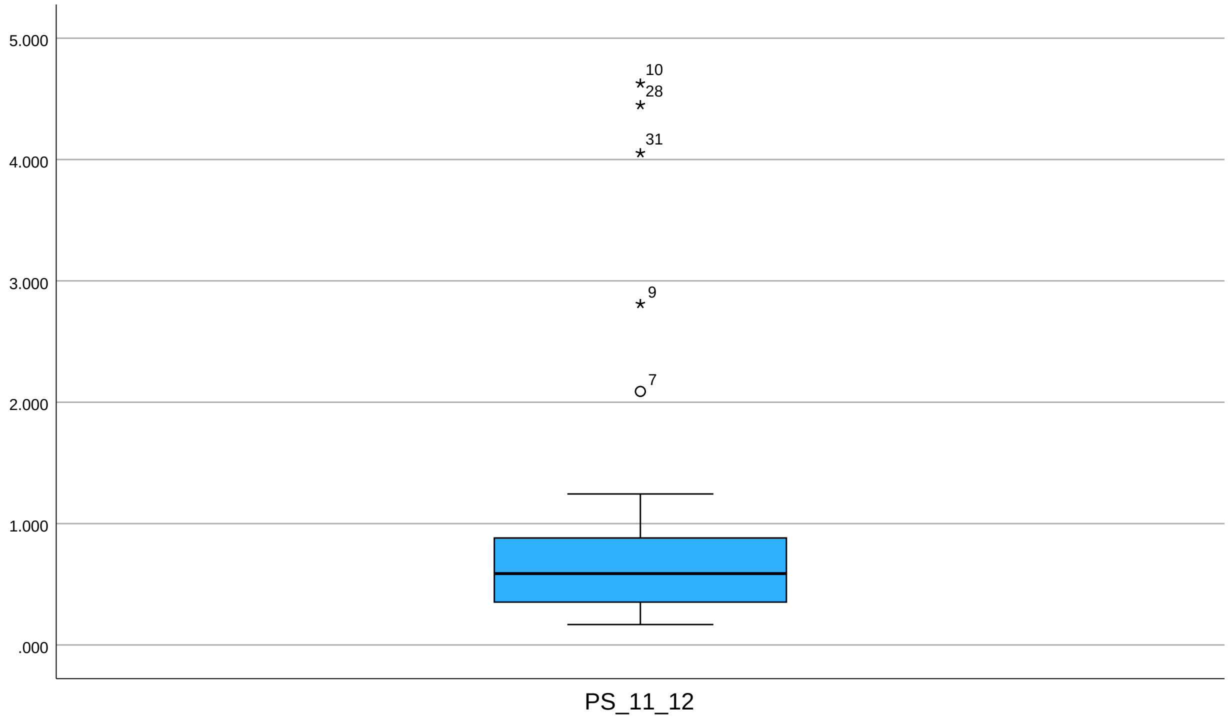

5.000
4.000
3.000
2.000
1.000
.000
10
28
31
9
7
PS_11_12

PS_12_13

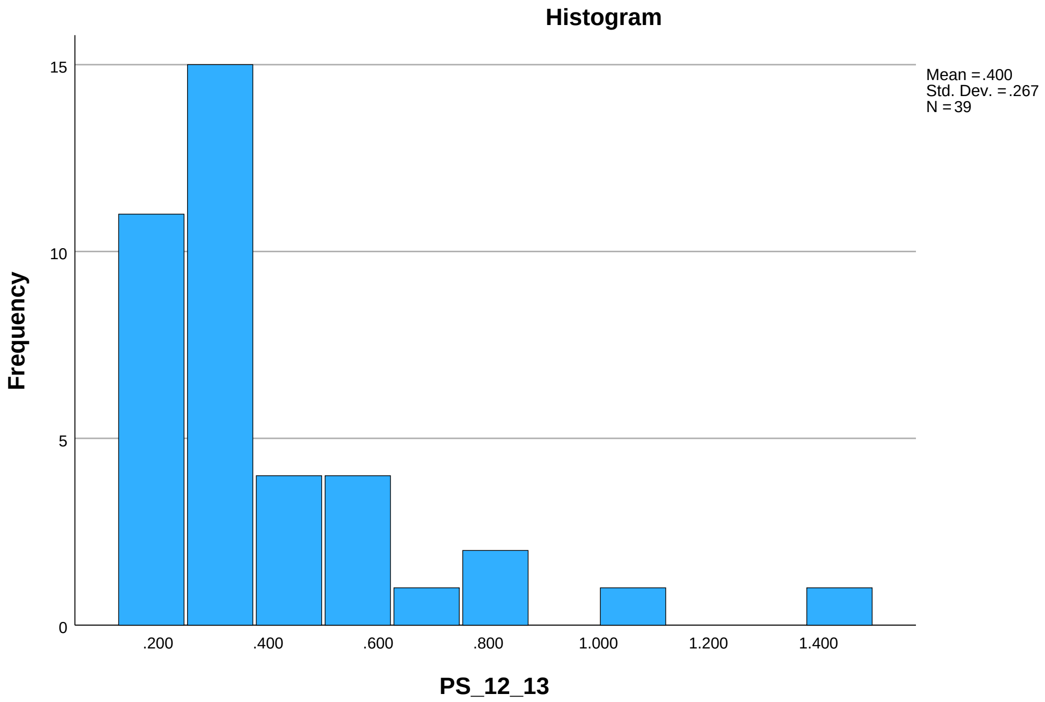


```
PS_12_13 Stem-and-Leaf Plot

 Frequency    Stem &  Leaf

     1.00        1 .  3
     4.00        1 .  7778
     6.00        2 .  113333
     7.00        2 .  5677789
     4.00        3 .  2233
     6.00        3 .  666689
     1.00        4 .  0
     1.00        4 .  6
     2.00        5 .  04
     2.00        5 .  56
      .00        6 .
     1.00        6 .  7
     4.00 Extremes    (>=.82)

 Stem width:      .100
 Each leaf:       1 case(s)
```

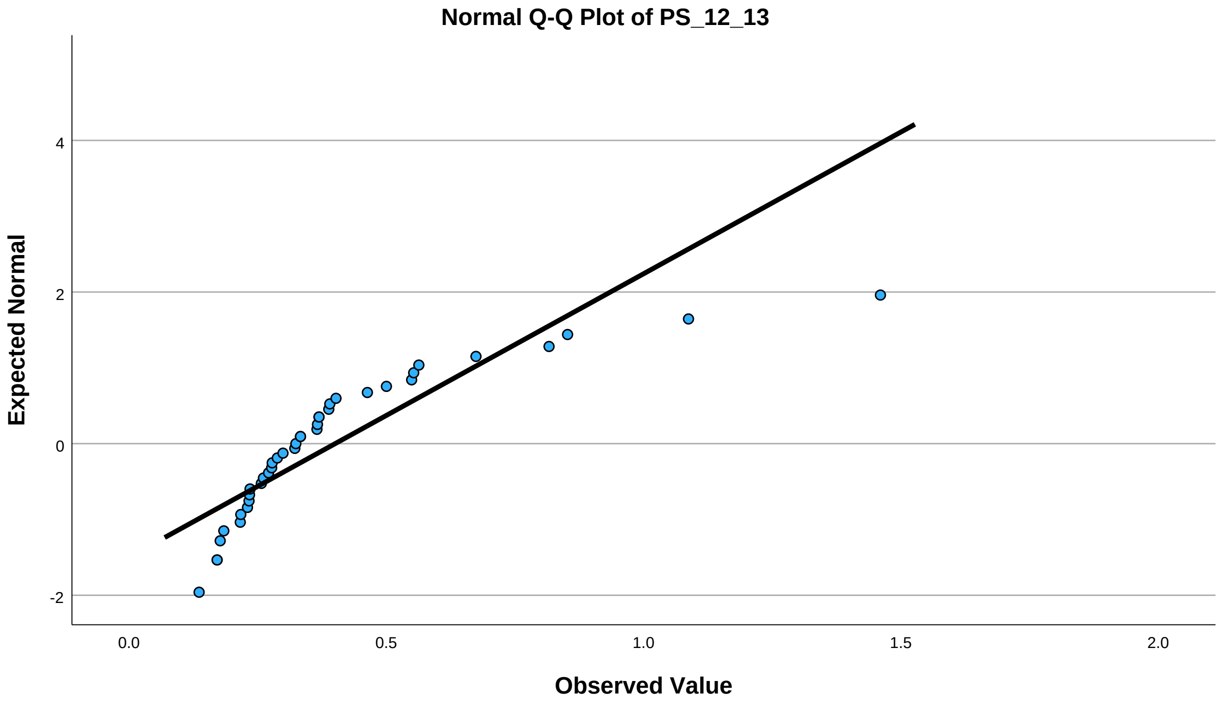
Normal Q-Q Plot of PS_12_13
Expected Normal
Observed Value
4
2
0
-2
0.0
0.5
1.0
1.5
2.0

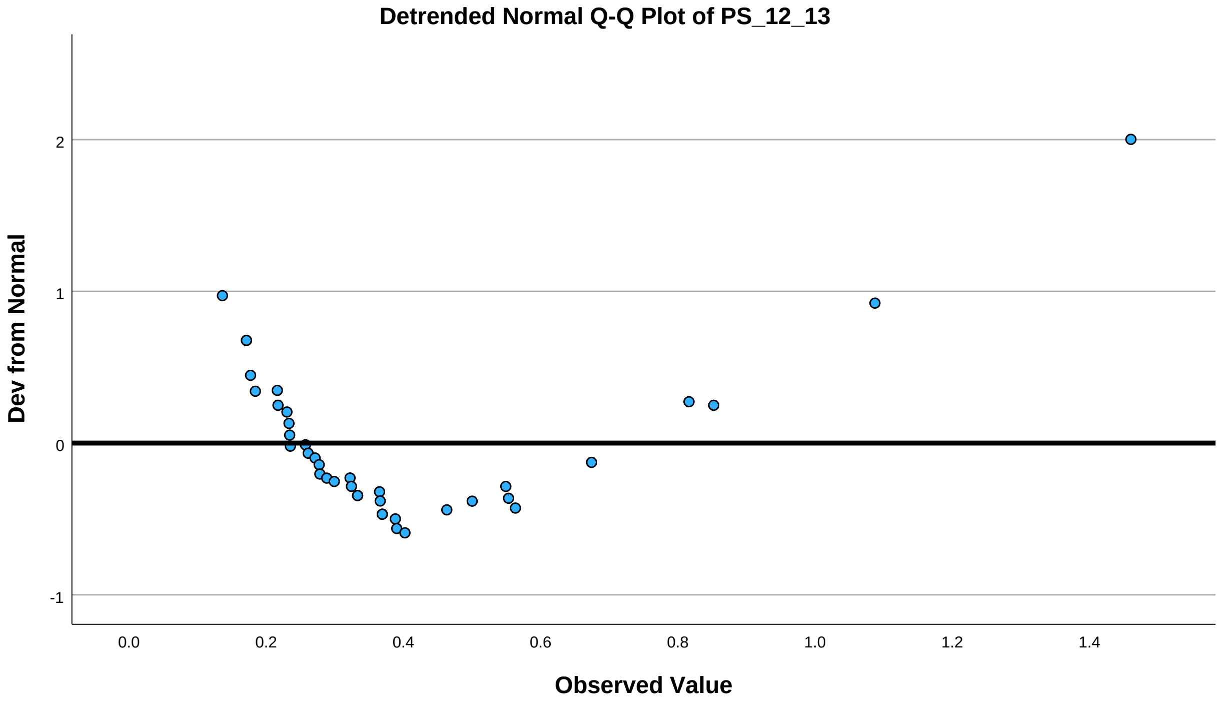
Detrended Normal Q-Q Plot of PS_12_13
Dev from Normal
Observed Value
2
1
0
-1
0.0
0.2
0.4
0.6
0.8
1.0
1.2
1.4

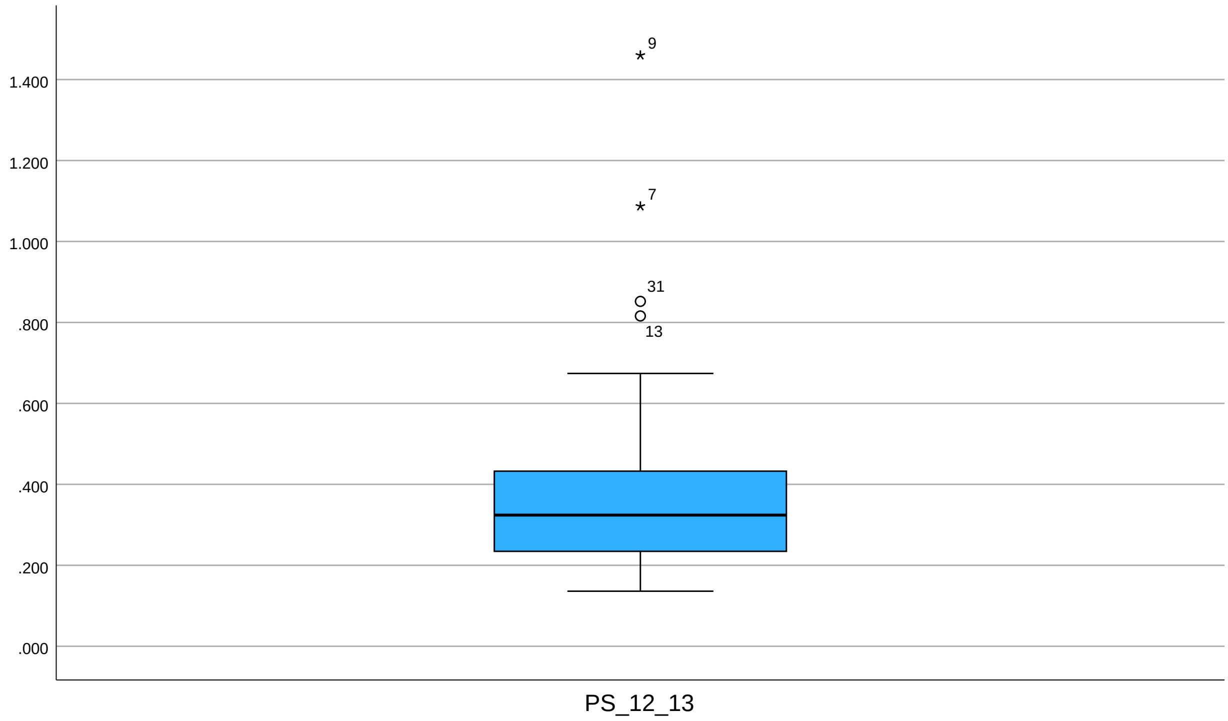

PS_13_14

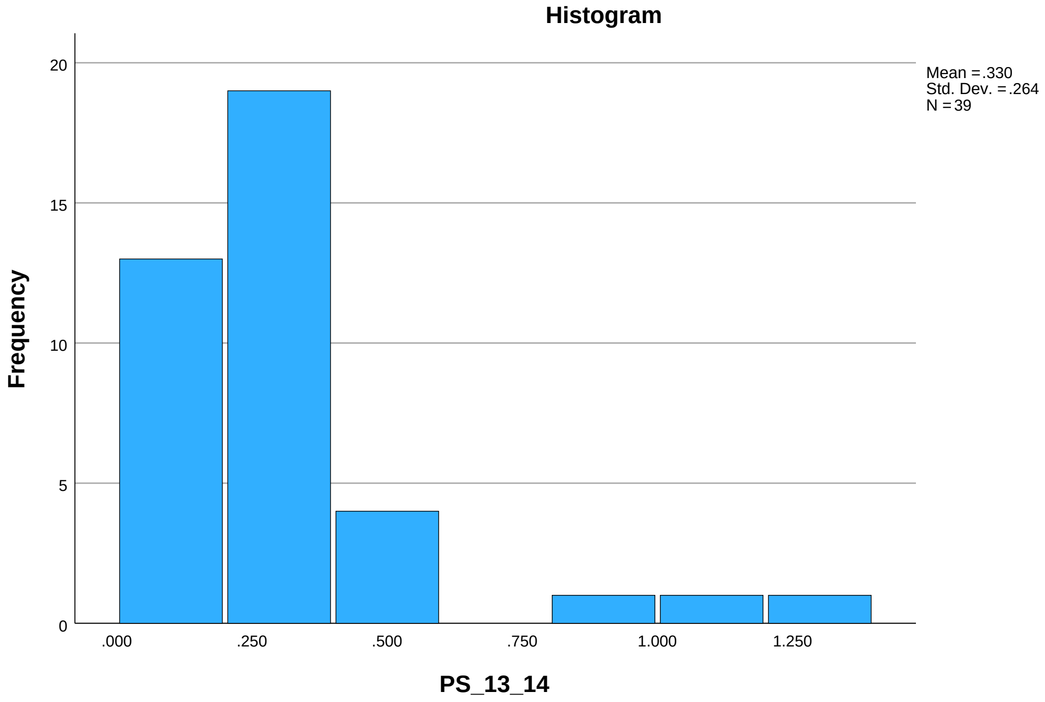

PS_13_14 Stem-and-Leaf Plot

Frequency   Stem & Leaf

```
 5.00     1 . 23344
 8.00     1 . 56777888
 9.00     2 . 001223444
 3.00     2 . 569
 3.00     3 . 234
 4.00     3 . 5689
 1.00     4 . 4
 1.00     4 . 9
 1.00     5 . 1
 1.00     5 . 9
 3.00 Extremes    (>=.94)

Stem width:      .100
Each leaf:       1 case(s)
```

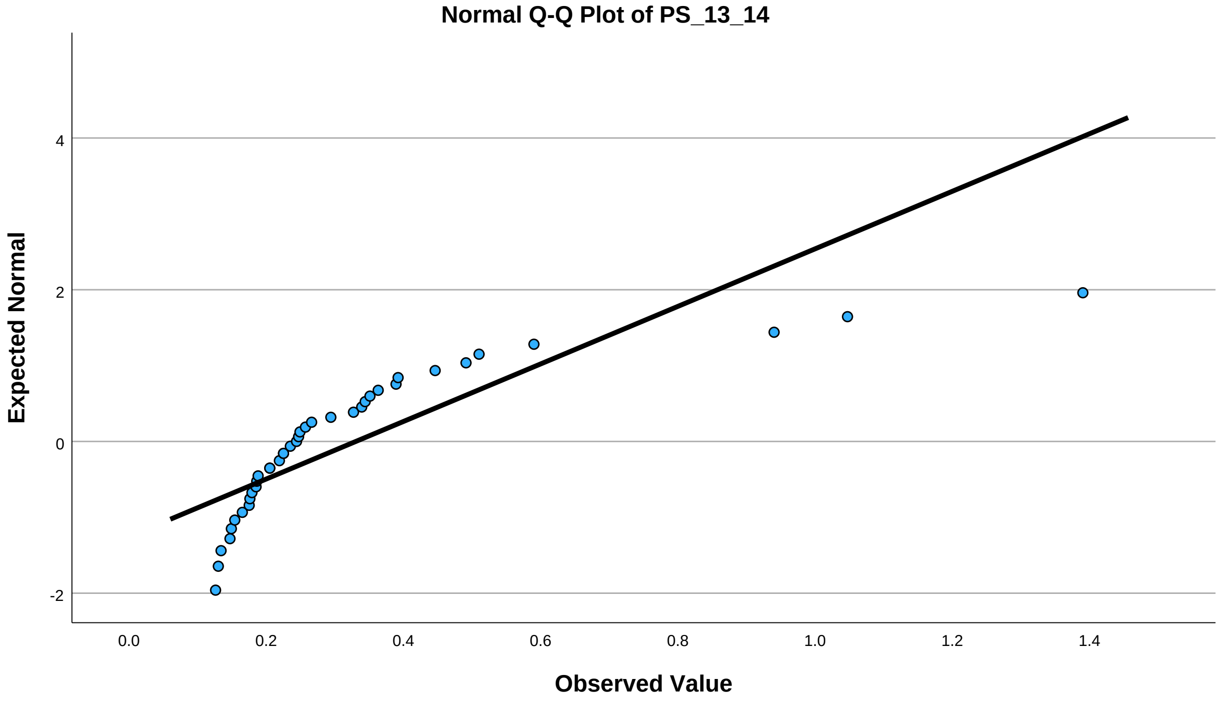

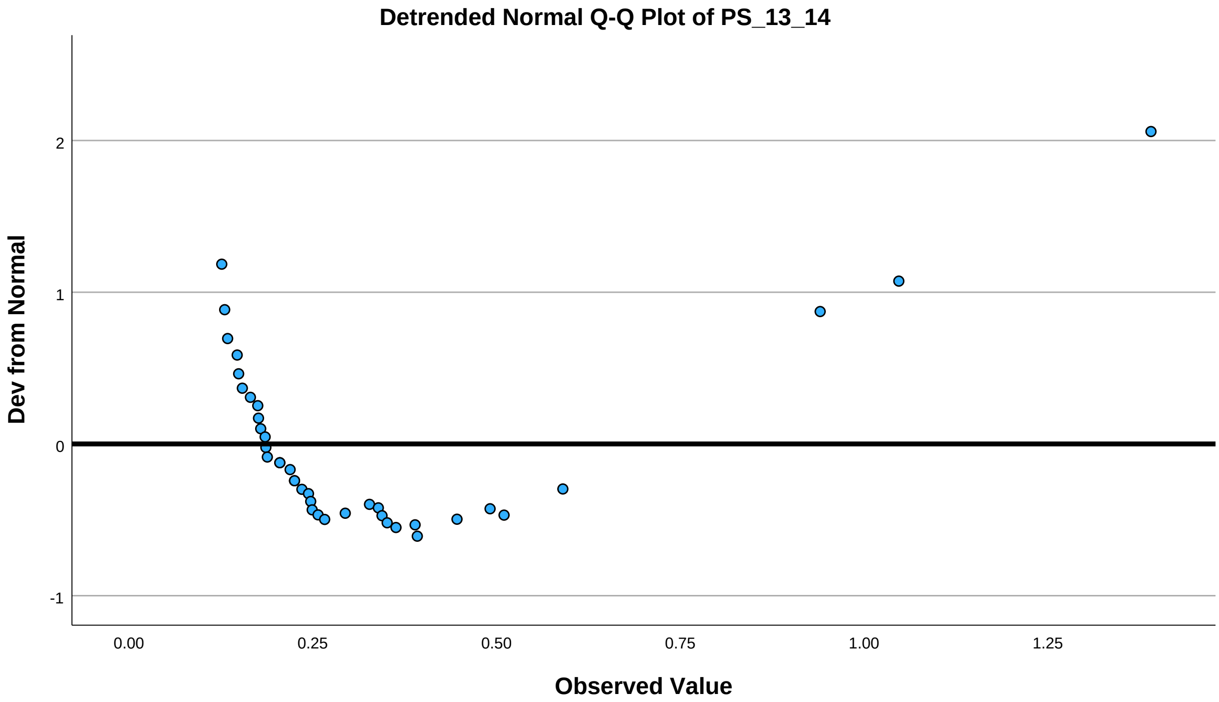

Detrended Normal Q-Q Plot of PS_13_14
Dev from Normal
2
1
0
-1
0.00
0.25
0.50
0.75
1.00
1.25
Observed Value

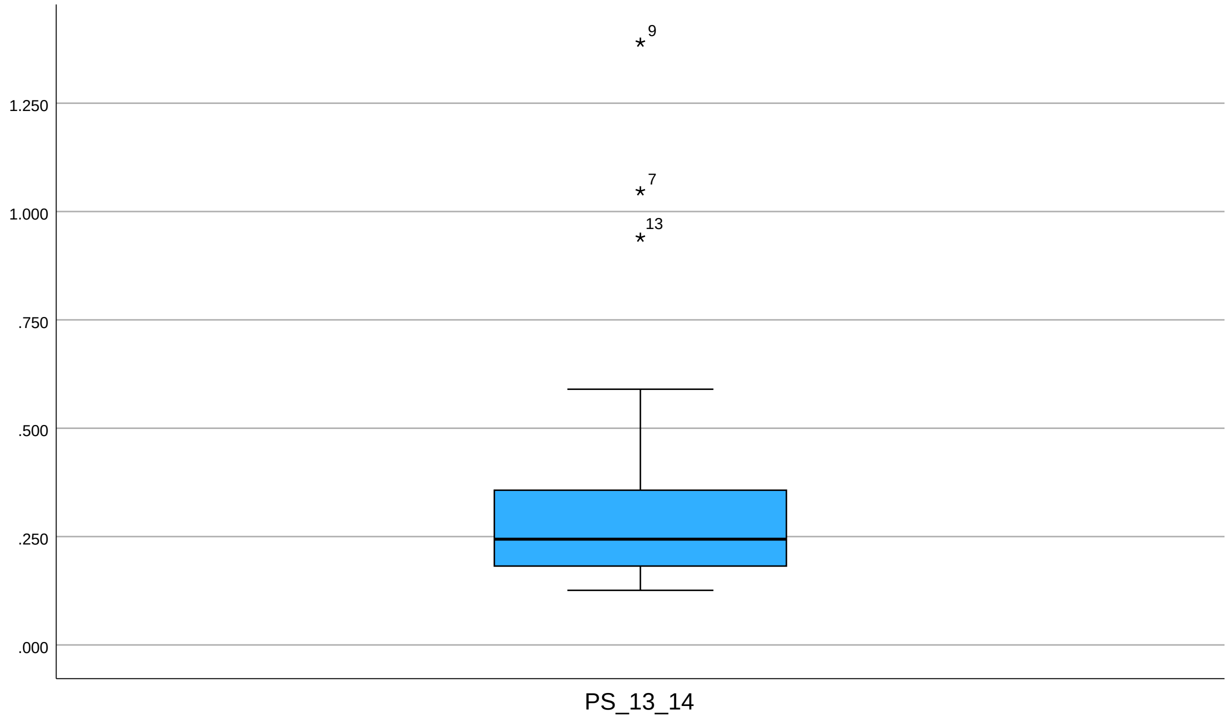

9
7
13
1.250
1.000
.750
.500
.250
.000
PS_13_14

PS_14_15

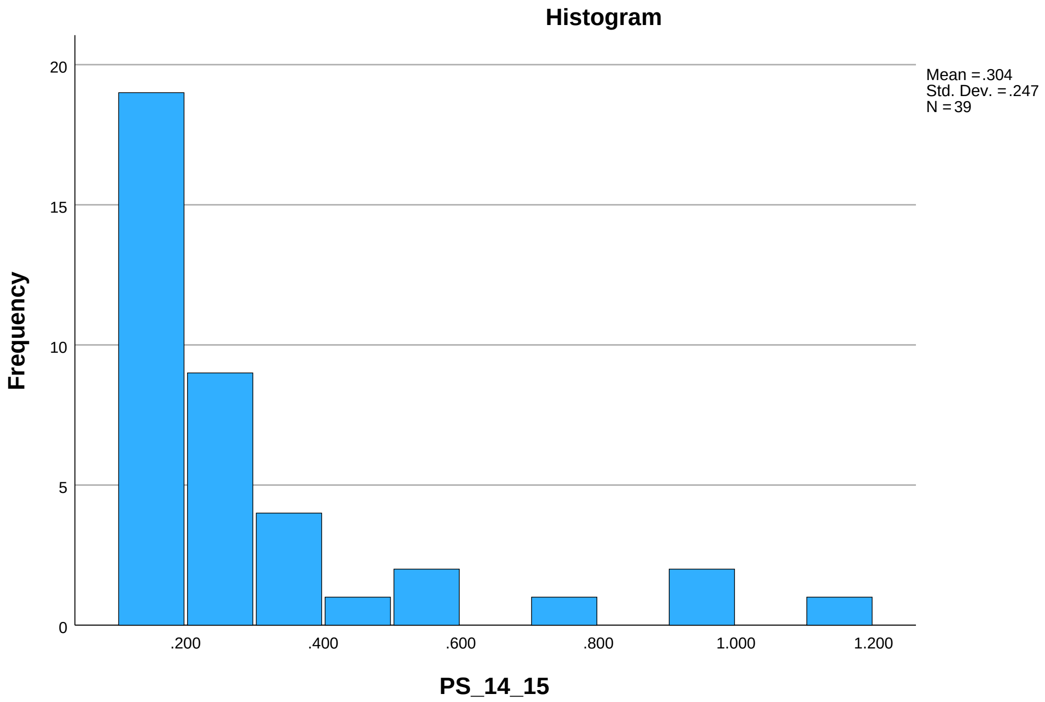


```
PS_14_15 Stem-and-Leaf Plot

 Frequency    Stem &  Leaf

     9.00        1 .  012333334
    10.00        1 .  5668899999
     4.00        2 .  0123
     5.00        2 .  57899
     2.00        3 .  12
     2.00        3 .  57
     1.00        4 .  1
      .00        4 .
     2.00        5 .  00
     4.00 Extremes    (>=.71)

 Stem width:      .100
 Each leaf:       1 case(s)
```

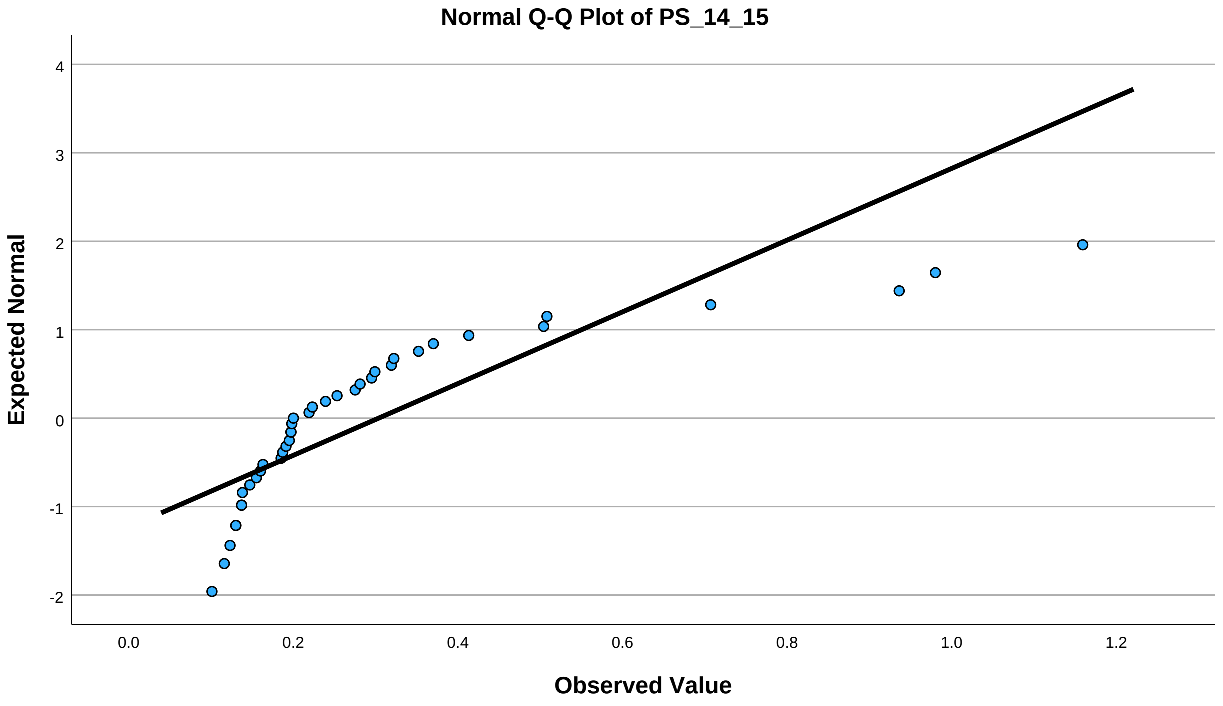

Normal Q-Q Plot of PS_14_15
Expected Normal
4
3
2
1
0
-1
-2
0.0
0.2
0.4
0.6
0.8
1.0
1.2
Observed Value

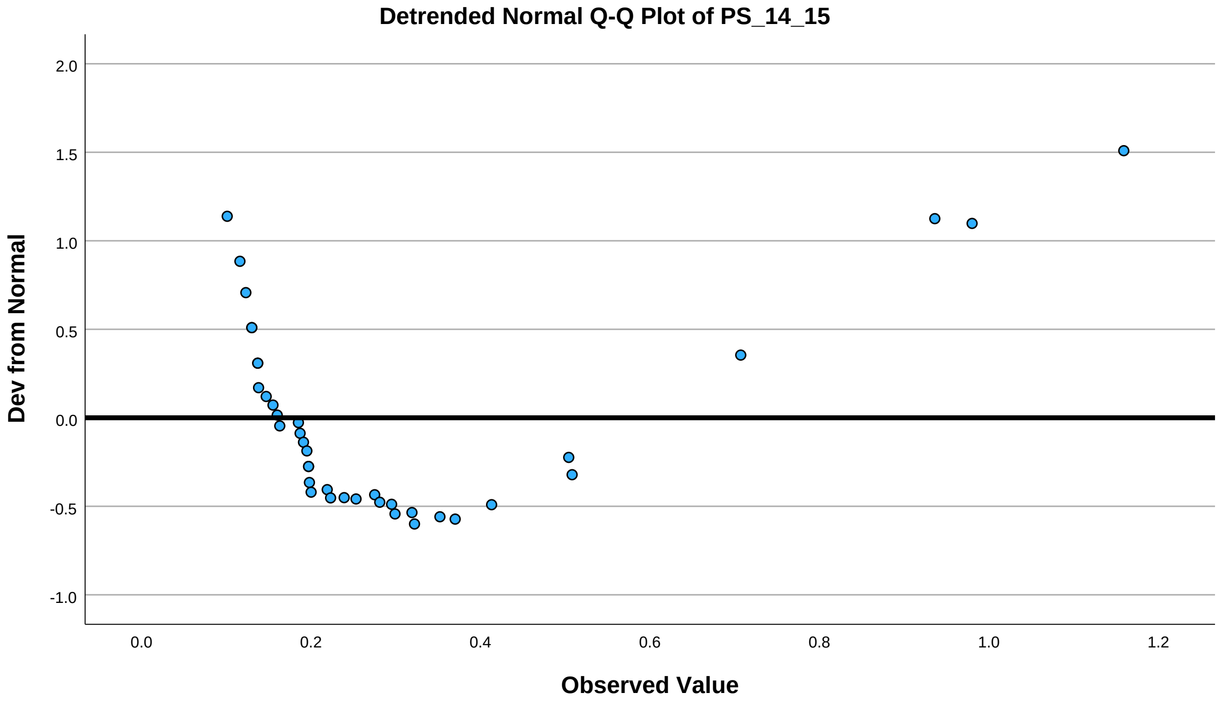

Detrended Normal Q-Q Plot of PS_14_15
Dev from Normal
2.0
1.5
1.0
0.5
0.0
-0.5
-1.0
0.0
0.2
0.4
0.6
0.8
1.0
1.2
Observed Value

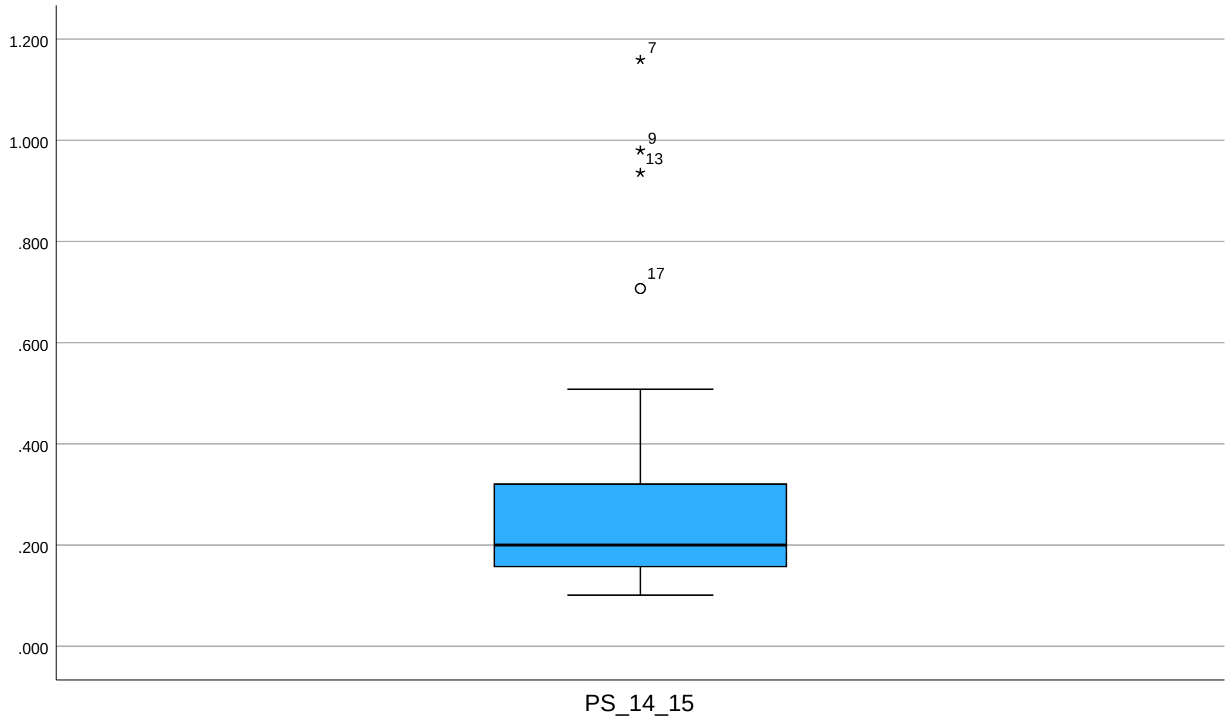

PS_15_16

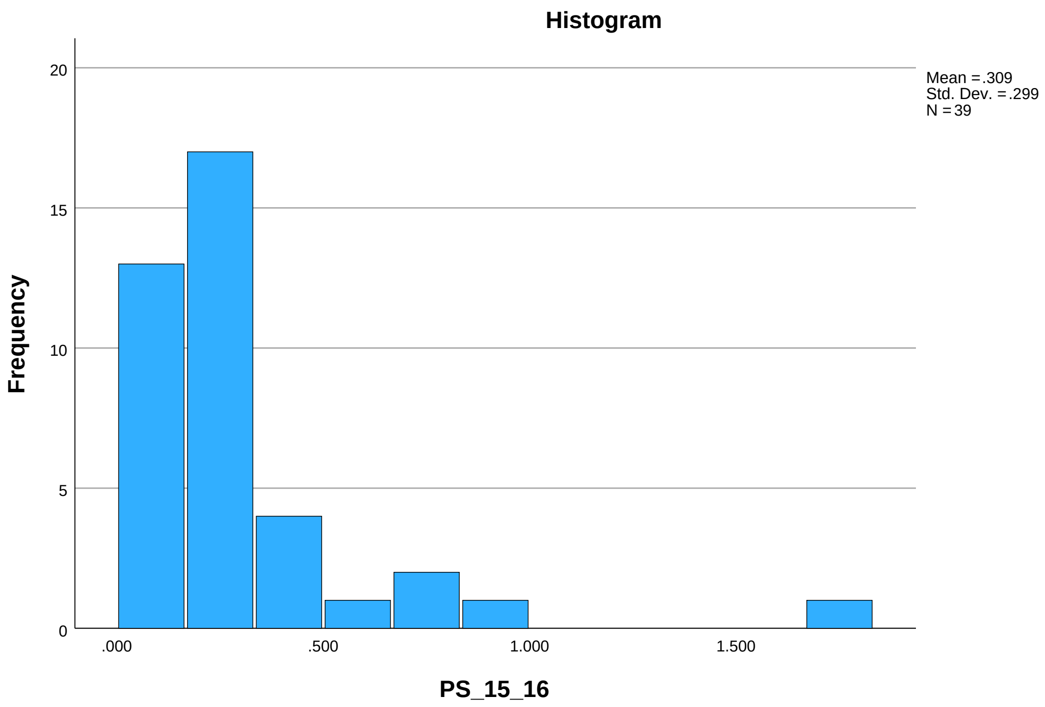


```
PS_15_16 Stem-and-Leaf Plot

 Frequency    Stem &  Leaf
```

```
  2.00      0 . 89
  6.00      1 . 123334
 12.00      1 . 556667788889
  3.00      2 . 000
  5.00      2 . 56788
  2.00      3 . 03
  1.00      3 . 5
  1.00      4 . 0
  2.00      4 . 66
  5.00 Extremes    (>=.60)

Stem width:     .100
Each leaf:      1 case(s)
```

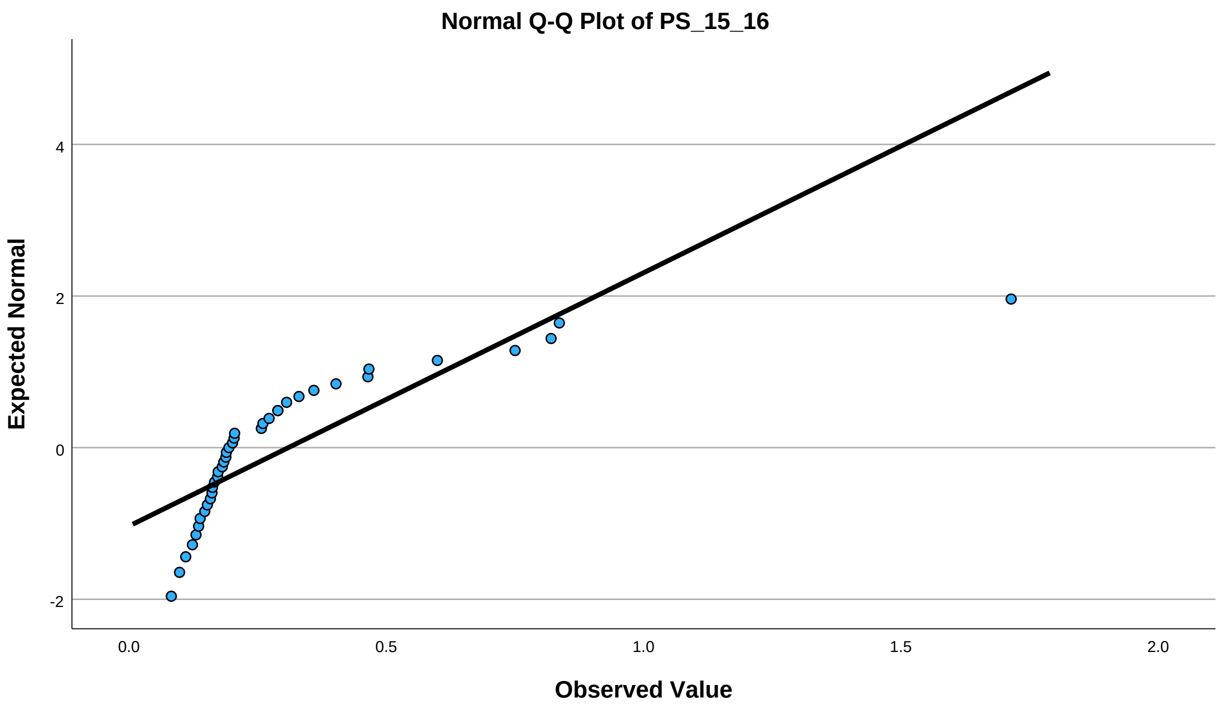

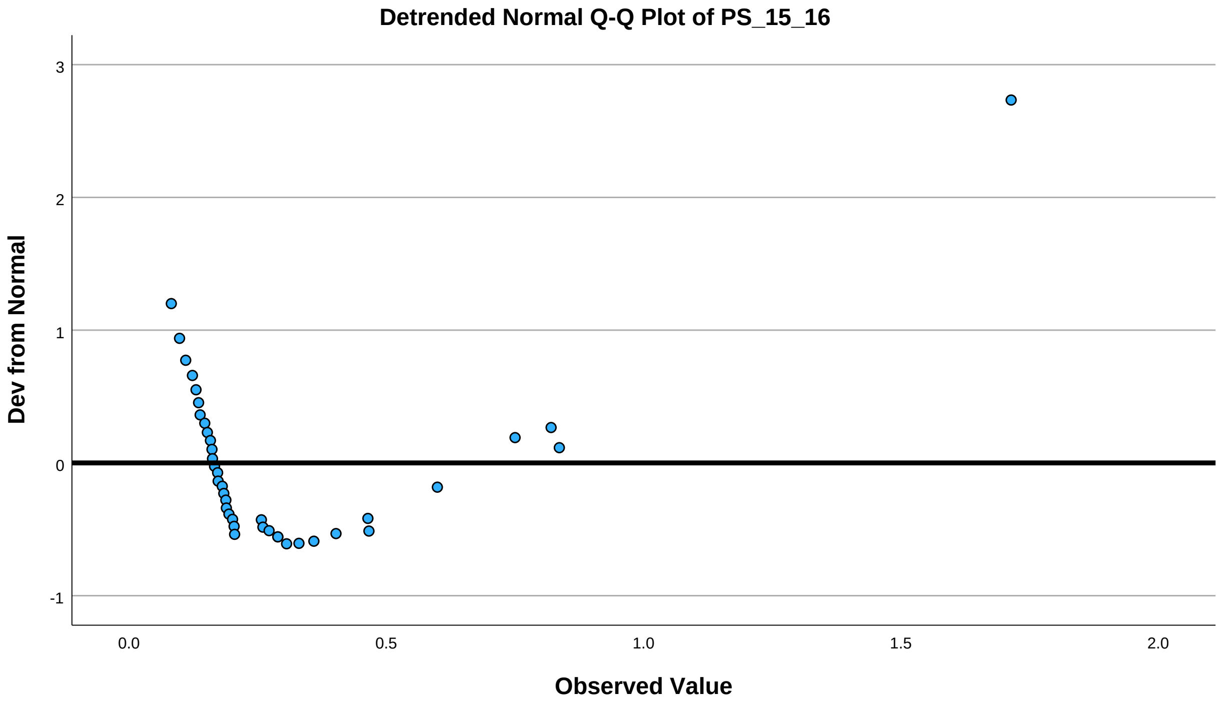

Detrended Normal Q-Q Plot of PS_15_16
Dev from Normal
3
2
1
0
-1
0.0
0.5
1.0
1.5
2.0
Observed Value

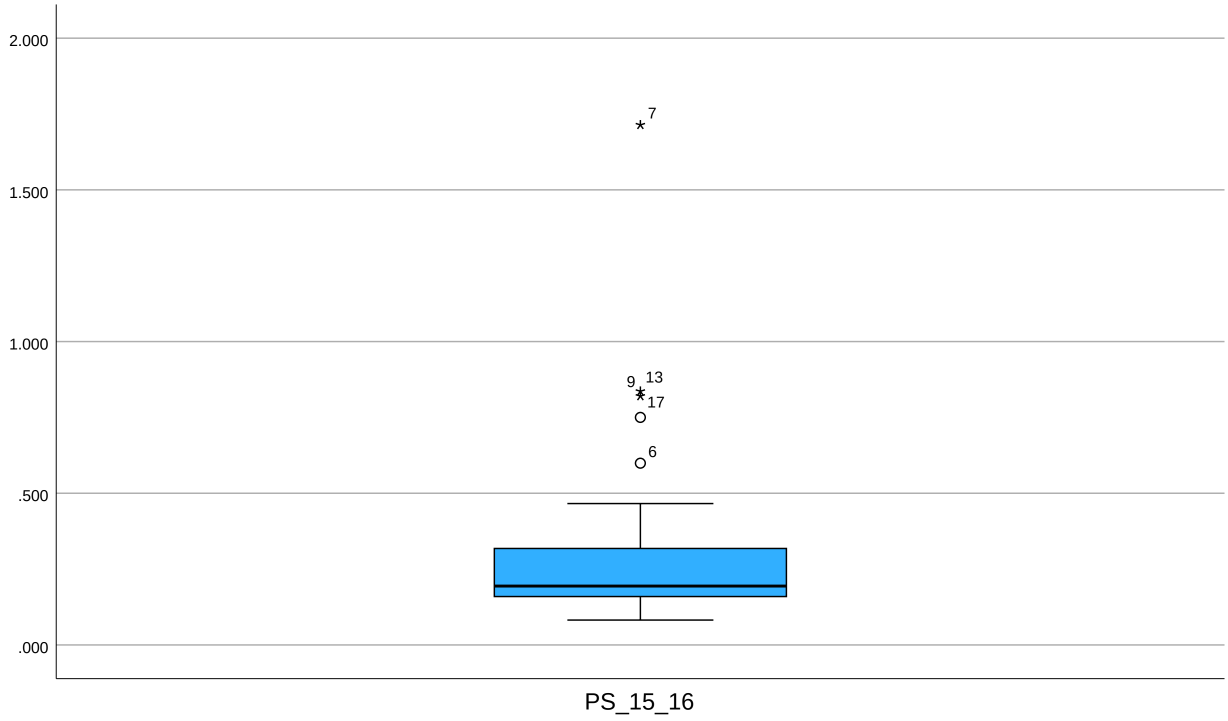

2.000
1.500
1.000
.500
.000
7
9
13
17
6
PS_15_16

PS_16_17

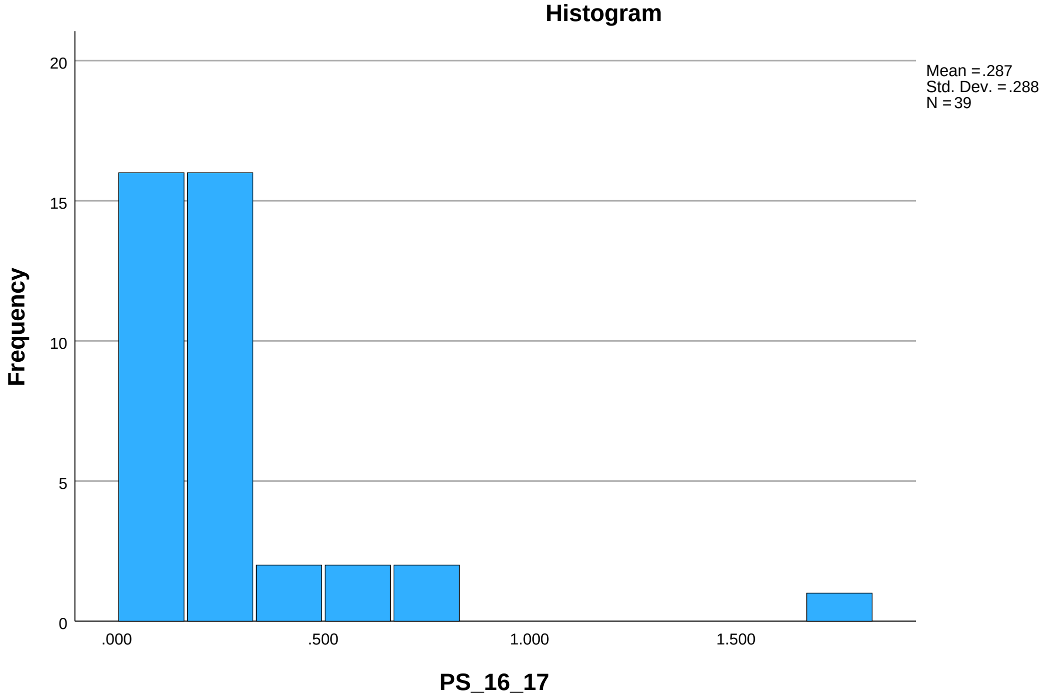

PS_16_17 Stem-and-Leaf Plot

```
 Frequency    Stem &  Leaf

     1.00        0 .  7
    12.00        1 .  002223334444
     8.00        1 .  55678899
     3.00        2 .  034
     6.00        2 .  566788
     2.00        3 .  13
     1.00        3 .  6
     1.00        4 .  1
     5.00 Extremes    (>=.55)

 Stem width:      .100
 Each leaf:       1 case(s)
```

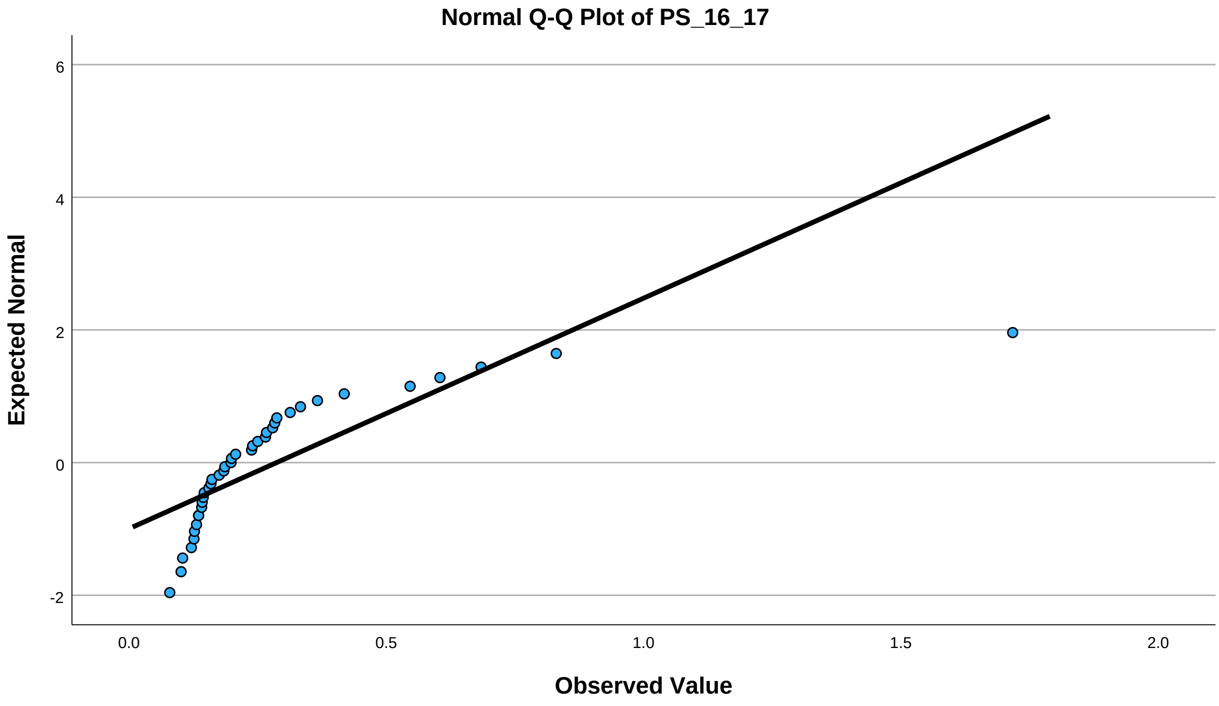
Normal Q-Q Plot of PS_16_17
Expected Normal
6
4
2
0
-2
0.0
0.5
1.0
1.5
2.0
Observed Value

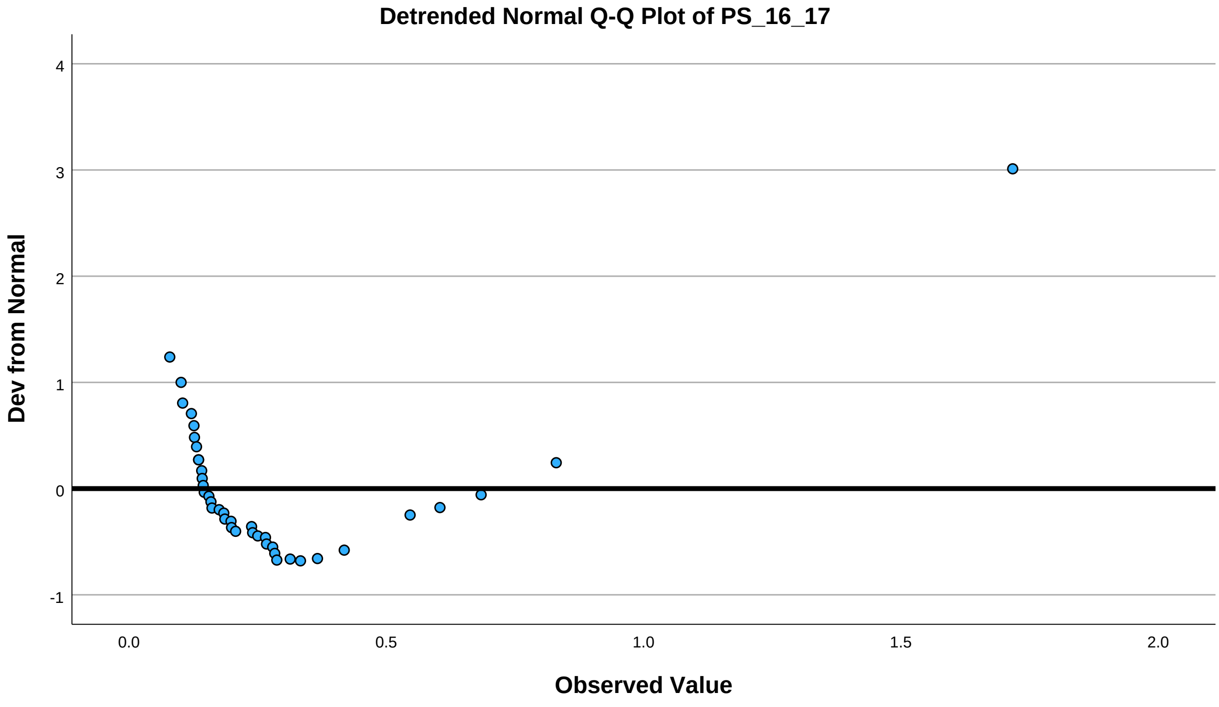
Detrended Normal Q-Q Plot of PS_16_17
Dev from Normal
4
3
2
1
0
-1
0.0
0.5
1.0
1.5
2.0
Observed Value

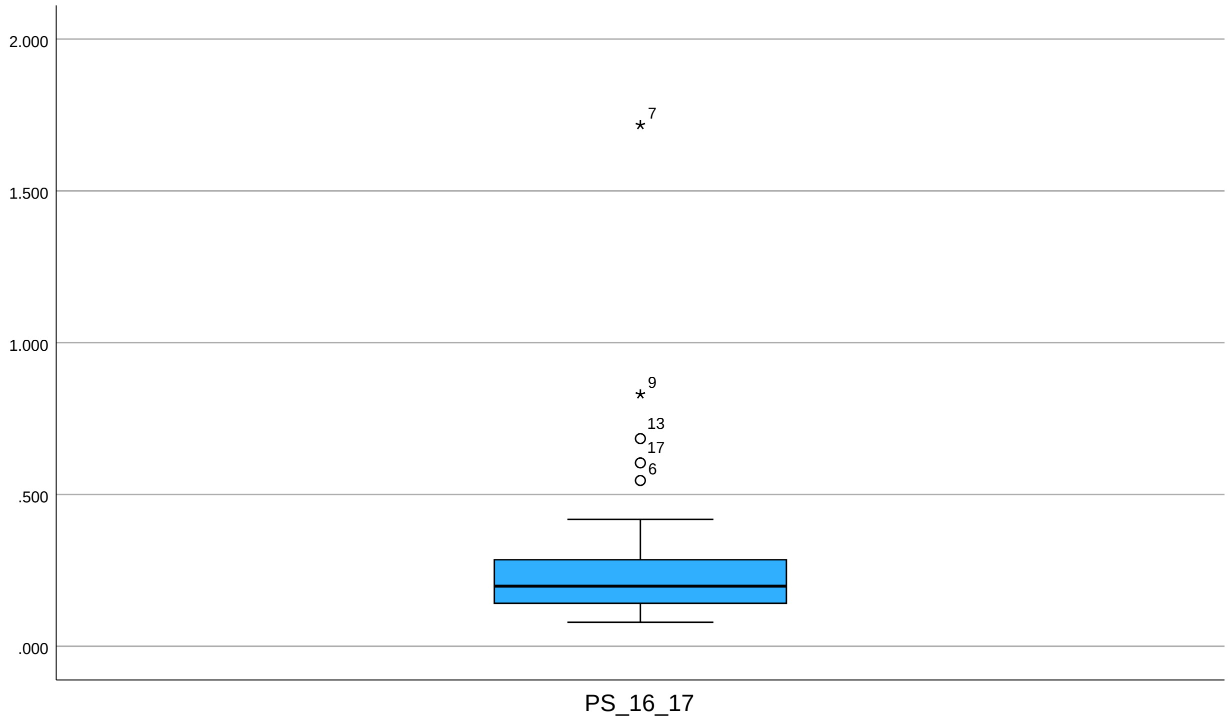

PS_17_18

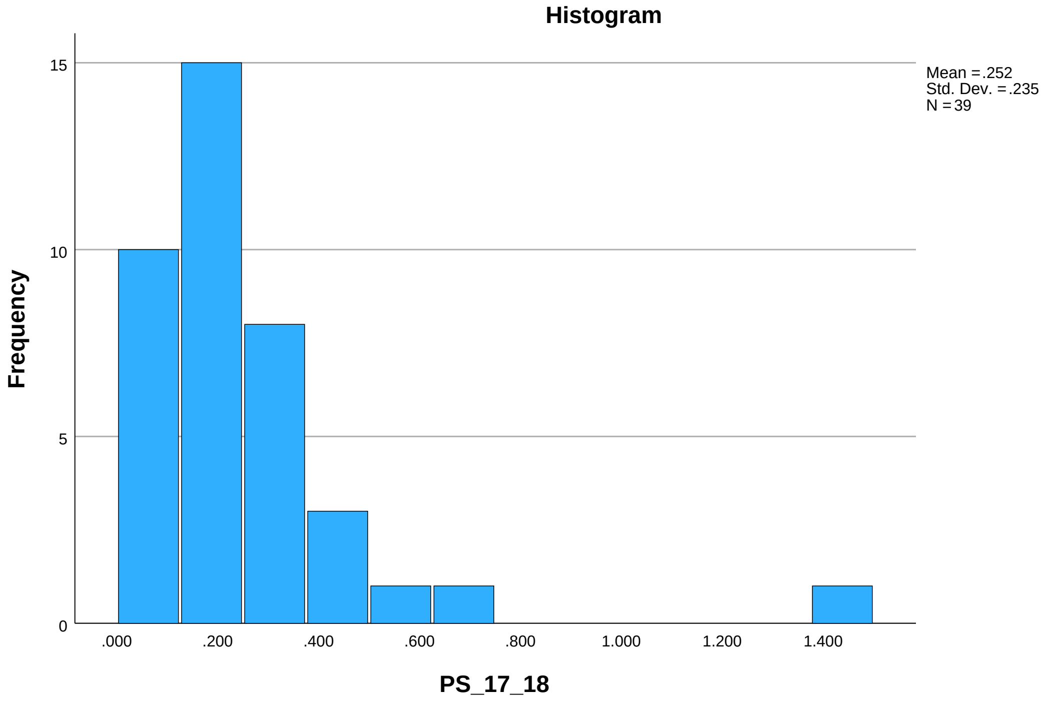

PS_17_18 Stem-and-Leaf Plot

Frequency Stem & Leaf

```
    3.00        0 .  789
   10.00         1 .  0111122224
    9.00        1 .  555567788
    3.00        2 .  023
    6.00        2 .  555789
    2.00        3 .  02
    1.00        3 .  9
    1.00        4 .  2
    1.00        4 .  8
    3.00 Extremes    (>=.58)

Stem width:      .100
Each leaf:       1 case(s)
```

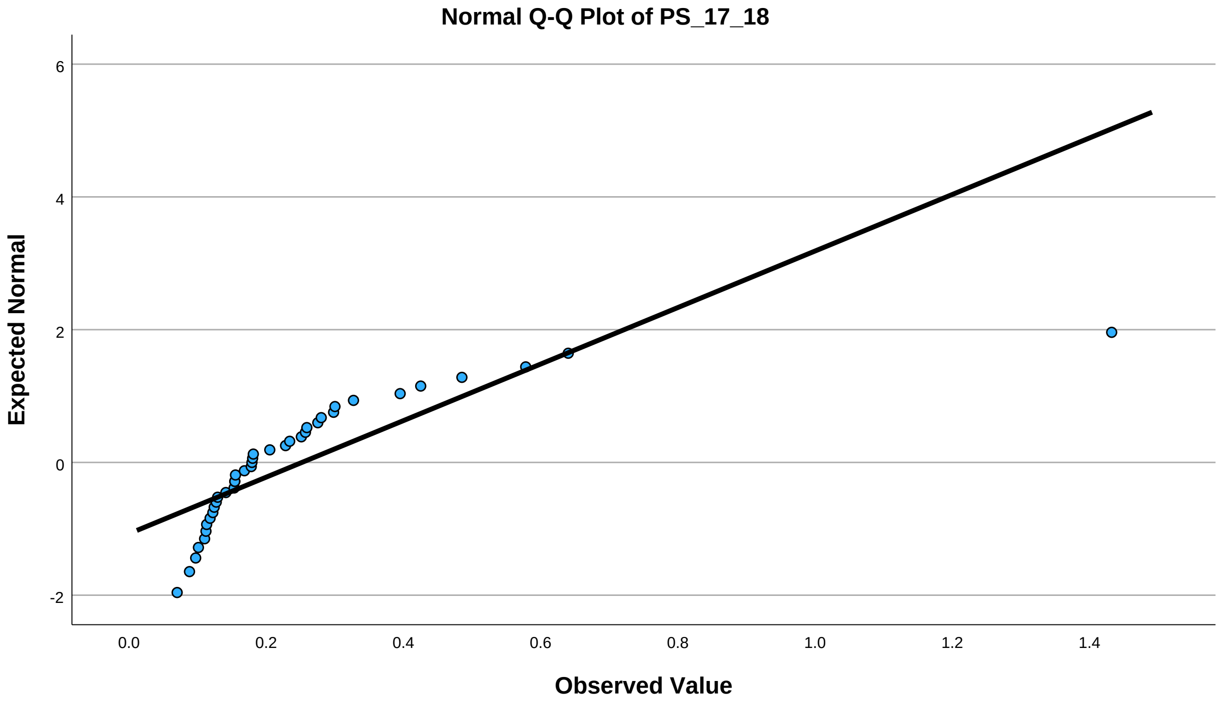

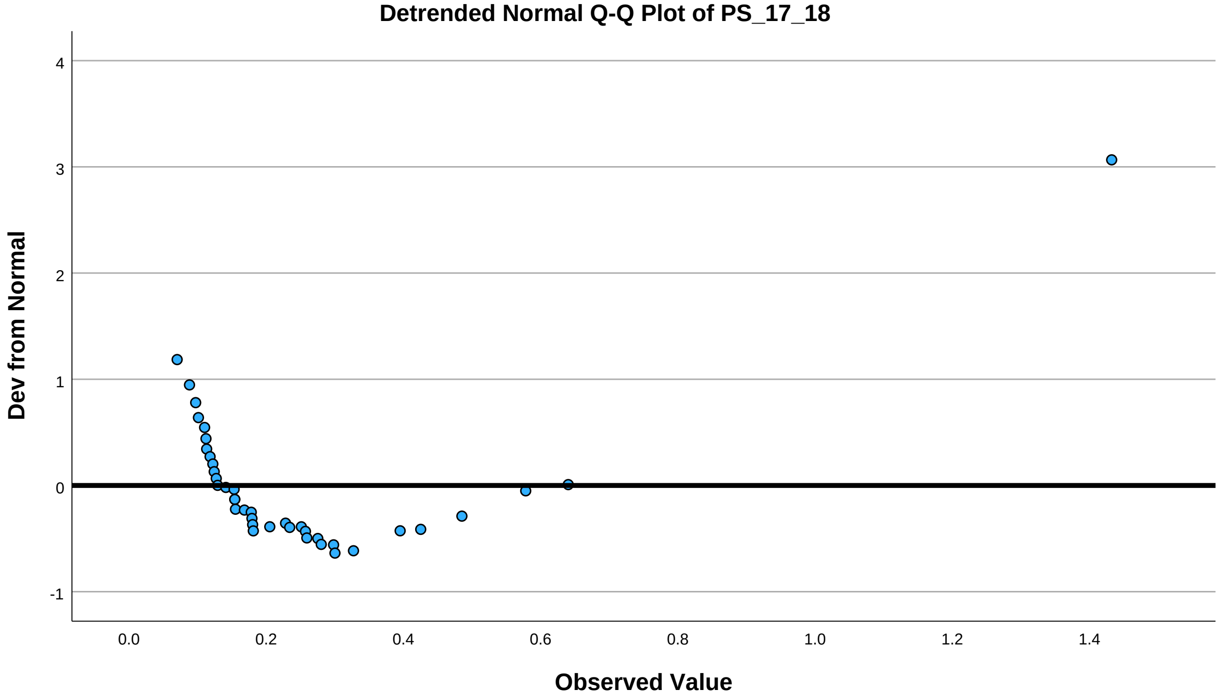

Detrended Normal Q-Q Plot of PS_17_18
Dev from Normal
4
3
2
1
0
-1
0.0
0.2
0.4
0.6
0.8
1.0
1.2
1.4
Observed Value

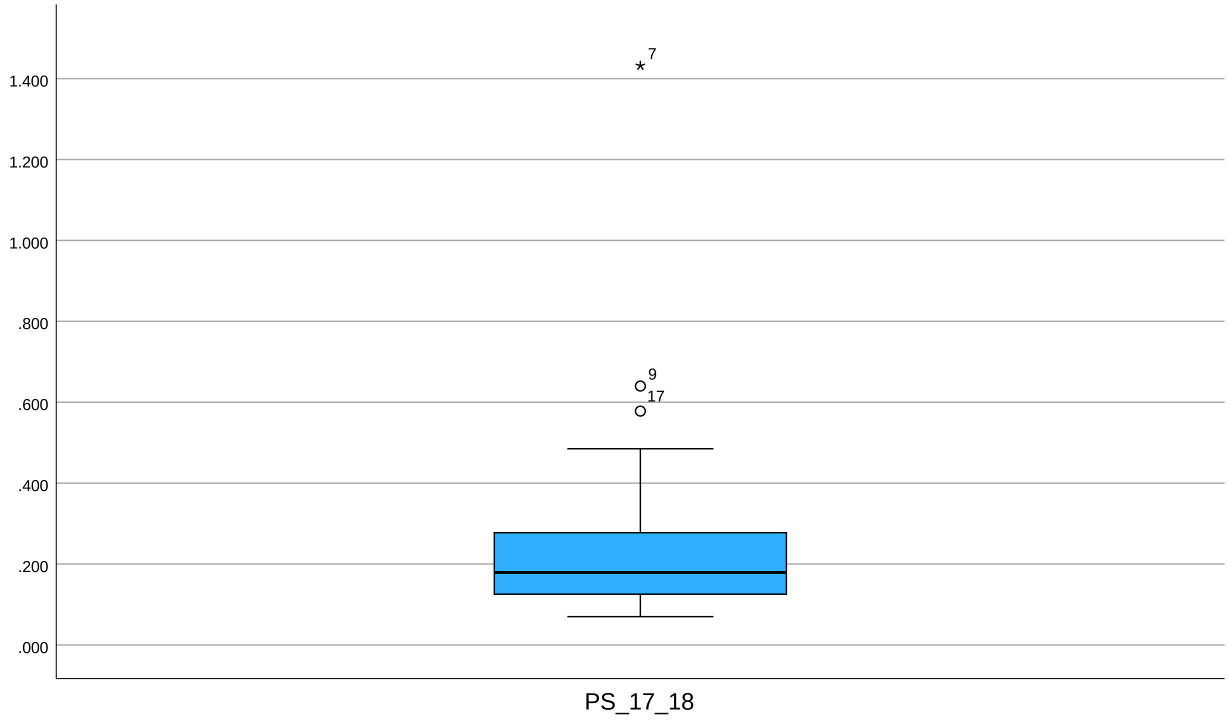

7
1.400
1.200
1.000
.800
9
.600
17
.400
.200
.000
PS_17_18

PS_18_19

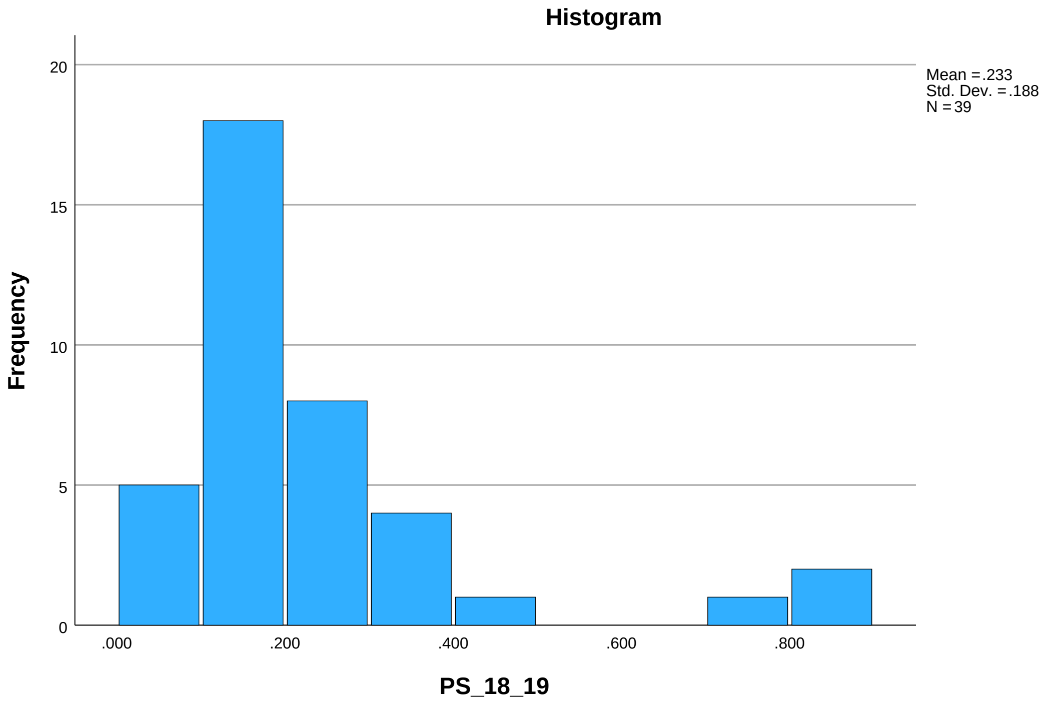


```
PS_18_19 Stem-and-Leaf Plot

 Frequency    Stem &  Leaf

     5.00        0 .  88999
    12.00        1 .  001111144444
     6.00        1 .  556799
     6.00        2 .  001244
     2.00        2 .  78
     3.00        3 .  224
     1.00        3 .  5
     1.00        4 .  0
     3.00 Extremes    (>=.77)

 Stem width:      .100
 Each leaf:       1 case(s)
```

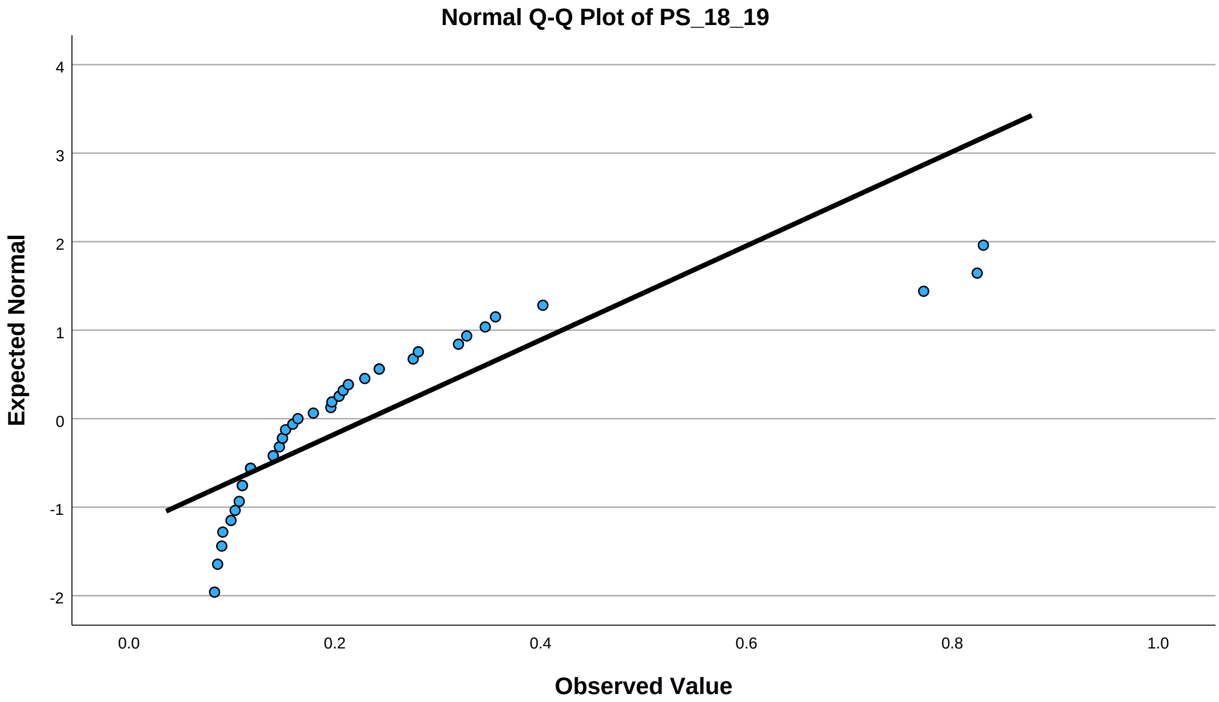
Normal Q-Q Plot of PS_18_19
Expected Normal
Observed Value
4
3
2
1
0
-1
-2
0.0
0.2
0.4
0.6
0.8
1.0

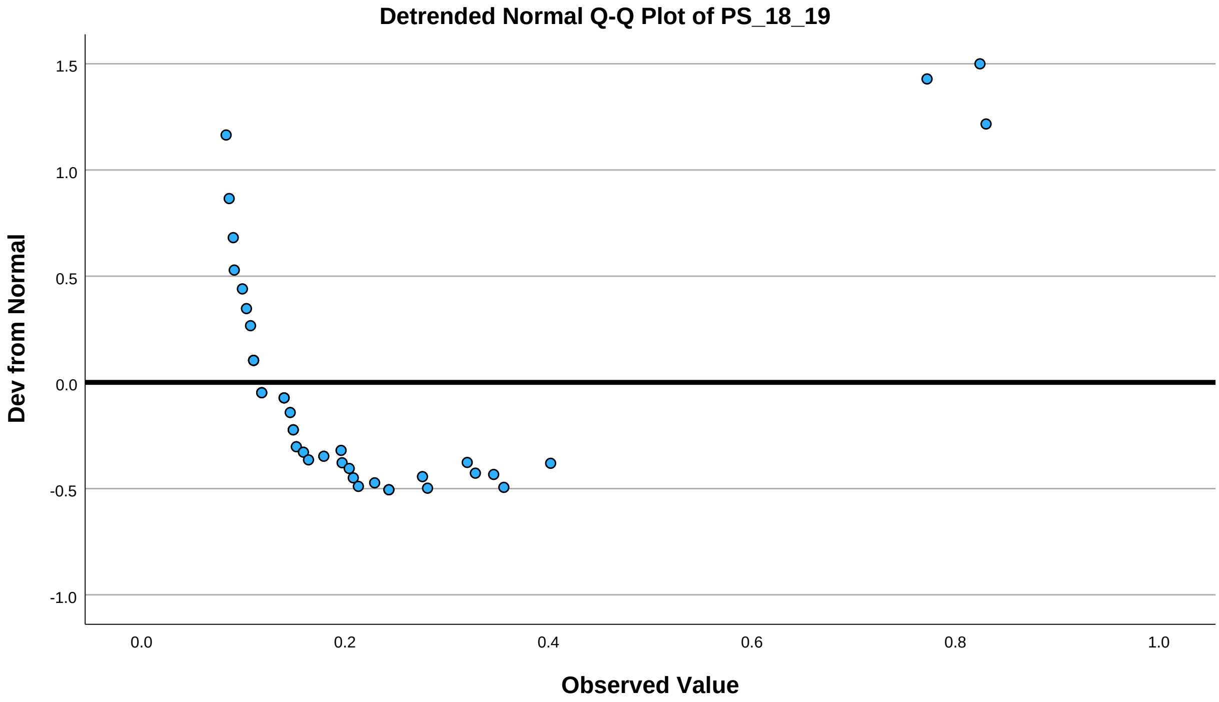
Detrended Normal Q-Q Plot of PS_18_19
Dev from Normal
Observed Value
1.5
1.0
0.5
0.0
-0.5
-1.0
0.0
0.2
0.4
0.6
0.8
1.0

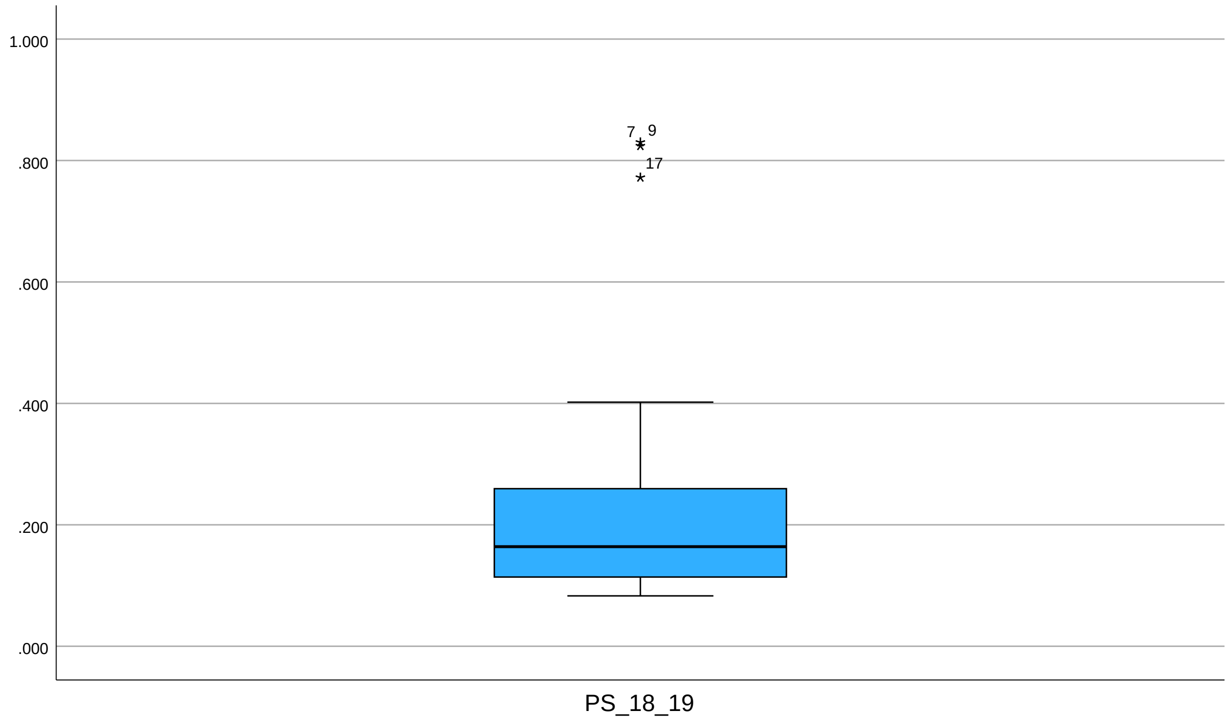

PS_19_20

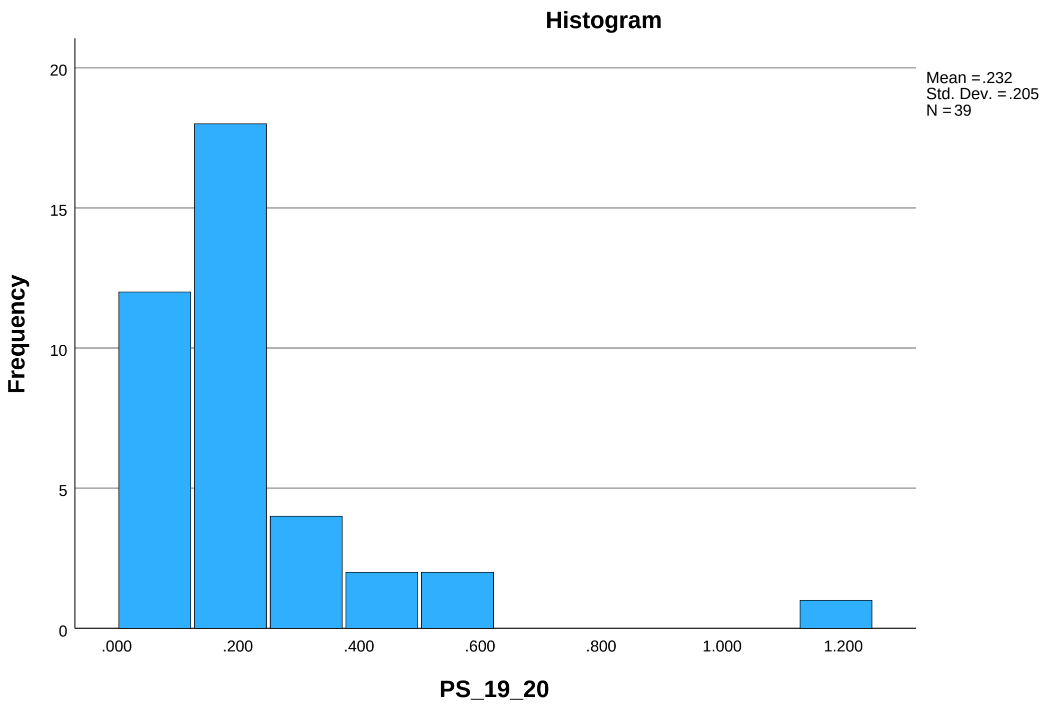


```
PS_19_20 Stem-and-Leaf Plot

 Frequency    Stem &  Leaf
```

```
   6.00        0 .  777899
   8.00        1 .  01112244
   8.00        1 .  55668889
   8.00        2 .  01112234
   2.00        2 .  79
   2.00        3 .  13
   2.00        3 .  89
   3.00 Extremes    (>=.57)

 Stem width:      .100
 Each leaf:       1 case(s)
```

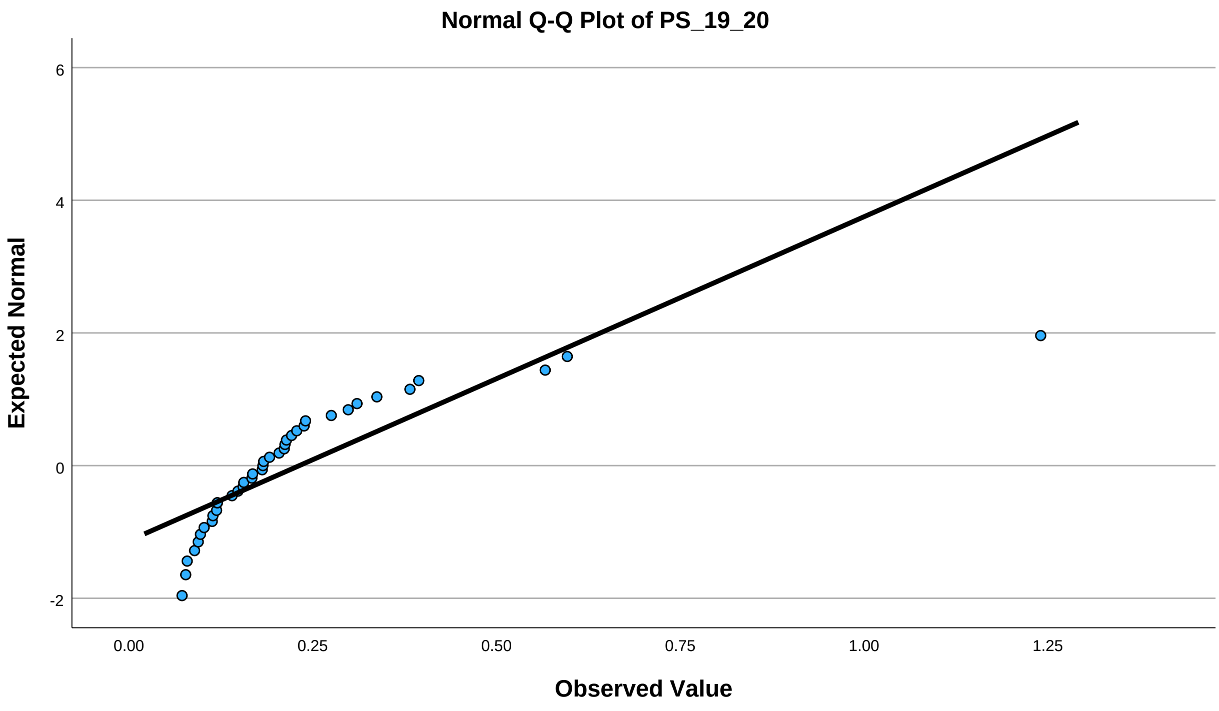

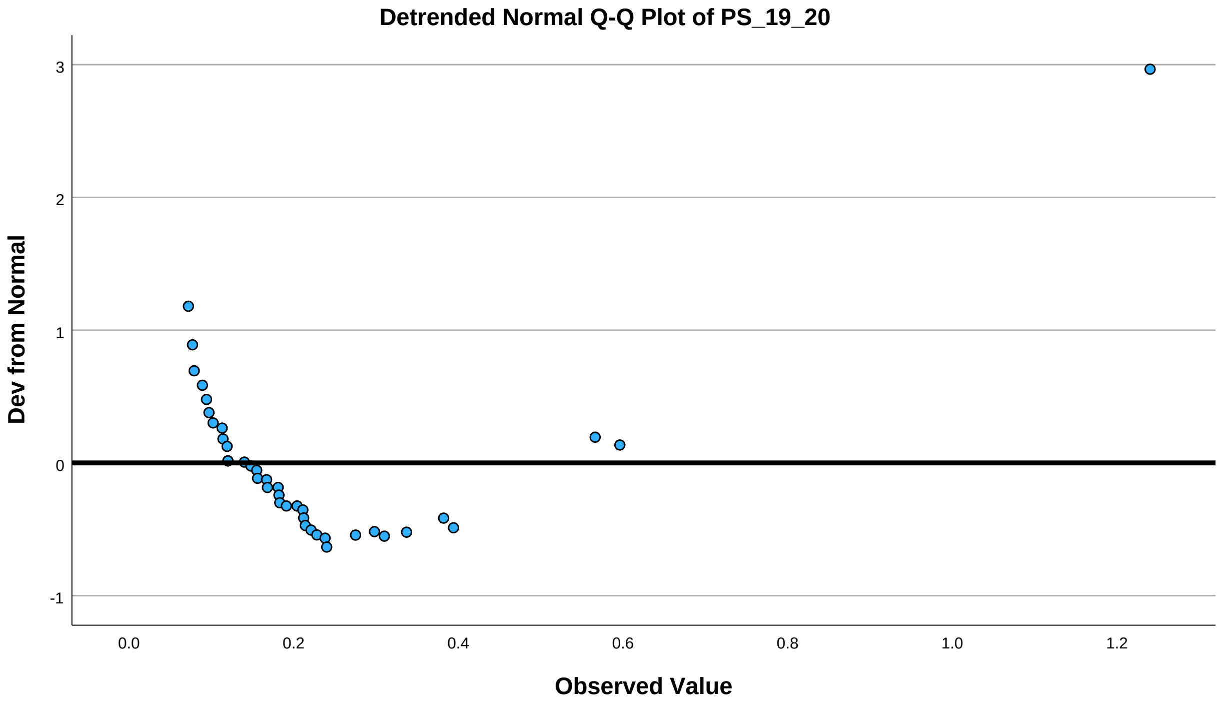

Detrended Normal Q-Q Plot of PS_19_20
Dev from Normal
3
2
1
0
-1
0.0
0.2
0.4
0.6
0.8
1.0
1.2
Observed Value

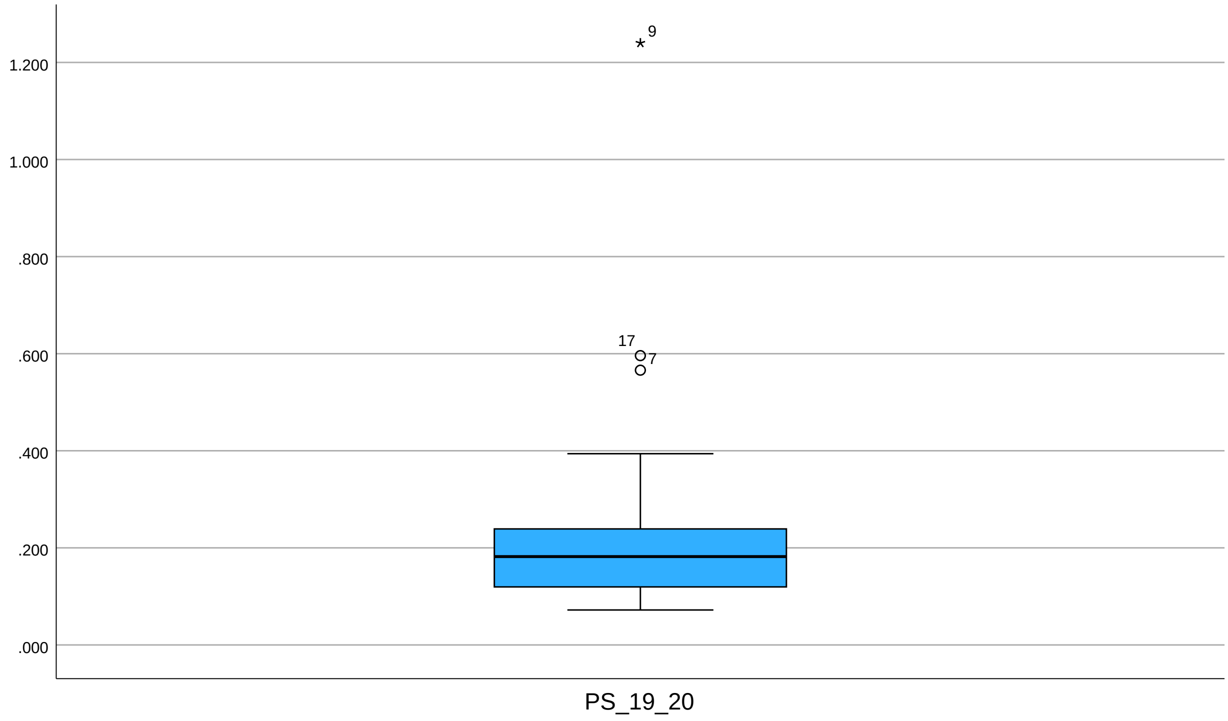

9
17
7
1.200
1.000
.800
.600
.400
.200
.000
PS_19_20

Explore

Case Processing Summary

	Cases					
	Valid		Missing		Total	
	N	Percent	N	Percent	N	Percent
BrainRate	39	100.0%	0	0.0%	39	100.0%
PermutationEntropy	39	100.0%	0	0.0%	39	100.0%
PermutationEntropy_01	39	100.0%	0	0.0%	39	100.0%
SampleEntropy	39	100.0%	0	0.0%	39	100.0%
SpectralEntropy_bits	39	100.0%	0	0.0%	39	100.0%
SpectralEntropy_01	39	100.0%	0	0.0%	39	100.0%
HiguchiFractalDimension	39	100.0%	0	0.0%	39	100.0%
LempelZivComplexity	39	100.0%	0	0.0%	39	100.0%
RenyiEntropy	39	100.0%	0	0.0%	39	100.0%
TsallisEntropy	39	100.0%	0	0.0%	39	100.0%
PS_4_5	39	100.0%	0	0.0%	39	100.0%
PS_5_6	39	100.0%	0	0.0%	39	100.0%
PS_6_7	39	100.0%	0	0.0%	39	100.0%
PS_7_8	39	100.0%	0	0.0%	39	100.0%
PS_8_9	39	100.0%	0	0.0%	39	100.0%
PS_9_10	39	100.0%	0	0.0%	39	100.0%
PS_10_11	39	100.0%	0	0.0%	39	100.0%
PS_11_12	39	100.0%	0	0.0%	39	100.0%
PS_12_13	39	100.0%	0	0.0%	39	100.0%
PS_13_14	39	100.0%	0	0.0%	39	100.0%
PS_14_15	39	100.0%	0	0.0%	39	100.0%
PS_15_16	39	100.0%	0	0.0%	39	100.0%
PS_16_17	39	100.0%	0	0.0%	39	100.0%
PS_17_18	39	100.0%	0	0.0%	39	100.0%
PS_18_19	39	100.0%	0	0.0%	39	100.0%
PS_19_20	39	100.0%	0	0.0%	39	100.0%

Descriptives

			Statistic	Std. Error
BrainRate	Mean		12.5500	1.03972
	95% Confidence Interval for Mean	Lower Bound	10.4452	
		Upper Bound	14.6548	
	5% Trimmed Mean		12.3093	
	Median		10.2700	
	Variance		42.160	
	Std. Deviation		6.49306	
	Minimum		4.31	
	Maximum		25.40	
	Range		21.09	
	Interquartile Range		9.64	
	Skewness		.734	.378
	Kurtosis		-.785	.741
PermutationEntropy	Mean		1.5302845291	.00984192244
	95% Confidence Interval for Mean	Lower Bound	1.5103605987	
		Upper Bound	1.5502084595	
	5% Trimmed Mean		1.5330254494	
	Median		1.5457071372	
	Variance		.004	
	Std. Deviation		.06146278596	
	Minimum		1.3803061205	
	Maximum		1.6337832108	
	Range		.25347709025	
	Interquartile Range		.08608341435	
	Skewness		-.568	.378
	Kurtosis		-.247	.741
PermutationEntropy_01	Mean		.85406805734	.00549288150
	95% Confidence Interval for Mean	Lower Bound	.84294830008	
		Upper Bound	.86518781459	
	5% Trimmed Mean		.85559779409	
	Median		.86267557879	
	Variance		.001	
	Std. Deviation		.03430303398	
	Minimum		.77036351375	
	Maximum		.91183177141	
	Range		.14146825765	
	Interquartile Range		.04804406832	
	Skewness		-.568	.378
	Kurtosis		-.247	.741

Descriptives

			Statistic	Std. Error
SampleEntropy	Mean		.85480162680	.04899284333
	95% Confidence Interval for Mean	Lower Bound	.75562080069	
		Upper Bound	.95398245291	
	5% Trimmed Mean		.85404615491	
	Median		.78229159210	
	Variance		.094	
	Std. Deviation		.30596020853	
	Minimum		.21830571034	
	Maximum		1.4987008666	
	Range		1.2803951562	
	Interquartile Range		.44732858881	
	Skewness		.150	.378
	Kurtosis		-.633	.741
SpectralEntropy_bits	Mean		4.5947667188	.12264031672
	95% Confidence Interval for Mean	Lower Bound	4.3464943773	
		Upper Bound	4.8430390602	
	5% Trimmed Mean		4.6183479277	
	Median		4.5872713704	
	Variance		.587	
	Std. Deviation		.76588853245	
	Minimum		2.8291338808	
	Maximum		5.7465868520	
	Range		2.9174529712	
	Interquartile Range		1.0714510062	
	Skewness		-.142	.378
	Kurtosis		-.660	.741
SpectralEntropy_01	Mean		.65534414324	.01749198995
	95% Confidence Interval for Mean	Lower Bound	.61993346086	
		Upper Bound	.69075482562	
	5% Trimmed Mean		.65870749292	
	Median		.65427509383	
	Variance		.012	
	Std. Deviation		.10923744225	
	Minimum		.40351478817	
	Maximum		.81962638532	
	Range		.41611159715	
	Interquartile Range		.15281932351	
	Skewness		-.142	.378
	Kurtosis		-.660	.741

Descriptives

			Statistic	Std. Error
HiguchiFractalDimension	Mean		.41414938590	.02295866203
	95% Confidence Interval for Mean	Lower Bound	.36767200448	
		Upper Bound	.46062676733	
	5% Trimmed Mean		.41287744901	
	Median		.40868391601	
	Variance		.021	
	Std. Deviation		.14337679843	
	Minimum		.19116957696	
	Maximum		.66318678153	
	Range		.47201720458	
	Interquartile Range		.24701760008	
	Skewness		.073	.378
	Kurtosis		-1.274	.741
LempelZivComplexity	Mean		.31776492638	.00913929429
	95% Confidence Interval for Mean	Lower Bound	.29926339236	
		Upper Bound	.33626646040	
	5% Trimmed Mean		.31694650820	
	Median		.30644450408	
	Variance		.003	
	Std. Deviation		.05707487455	
	Minimum		.23108334575	
	Maximum		.42145587338	
	Range		.19037252763	
	Interquartile Range		.10595103773	
	Skewness		.295	.378
	Kurtosis		-1.301	.741
RenyiEntropy	Mean		7.2665511171	.14146373850
	95% Confidence Interval for Mean	Lower Bound	6.9801727505	
		Upper Bound	7.5529294837	
	5% Trimmed Mean		7.2895394678	
	Median		7.3807739409	
	Variance		.780	
	Std. Deviation		.88344076376	
	Minimum		5.1186499191	
	Maximum		8.8438764754	
	Range		3.7252265562	
	Interquartile Range		1.0342192290	
	Skewness		-.521	.378
	Kurtosis		-.125	.741

Descriptives

			Statistic	Std. Error
TsallisEntropy	Mean		1.6154900568	.07755955993
	95% Confidence Interval for Mean	Lower Bound	1.4584789363	
		Upper Bound	1.7725011773	
	5% Trimmed Mean		1.6523574350	
	Median		1.8582849101	
	Variance		.235	
	Std. Deviation		.48435929653	
	Minimum		.56027902904	
	Maximum		1.9864636945	
	Range		1.4261846654	
	Interquartile Range		.86500223450	
	Skewness		-1.099	.378
	Kurtosis		-.435	.741
PS_4_5	Mean		2.90310	.499722
	95% Confidence Interval for Mean	Lower Bound	1.89147	
		Upper Bound	3.91474	
	5% Trimmed Mean		2.46868	
	Median		2.00400	
	Variance		9.739	
	Std. Deviation		3.120764	
	Minimum		.431	
	Maximum		19.970	
	Range		19.539	
	Interquartile Range		2.335	
	Skewness		4.516	.378
	Kurtosis		24.410	.741
PS_5_6	Mean		1.85326	.224374
	95% Confidence Interval for Mean	Lower Bound	1.39903	
		Upper Bound	2.30748	
	5% Trimmed Mean		1.68990	
	Median		1.44900	
	Variance		1.963	
	Std. Deviation		1.401217	
	Minimum		.349	
	Maximum		8.460	
	Range		8.111	
	Interquartile Range		1.449	
	Skewness		2.959	.378
	Kurtosis		12.437	.741

Descriptives

			Statistic	Std. Error
PS_6_7	Mean		1.50364	.207269
	95% Confidence Interval for Mean	Lower Bound	1.08405	
		Upper Bound	1.92323	
	5% Trimmed Mean		1.32136	
	Median		1.15300	
	Variance		1.675	
	Std. Deviation		1.294393	
	Minimum		.273	
	Maximum		7.890	
	Range		7.617	
	Interquartile Range		1.173	
	Skewness		3.427	.378
	Kurtosis		15.533	.741
PS_7_8	Mean		1.28646	.150960
	95% Confidence Interval for Mean	Lower Bound	.98086	
		Upper Bound	1.59206	
	5% Trimmed Mean		1.16671	
	Median		1.07700	
	Variance		.889	
	Std. Deviation		.942745	
	Minimum		.221	
	Maximum		4.790	
	Range		4.569	
	Interquartile Range		.919	
	Skewness		2.107	.378
	Kurtosis		5.547	.741
PS_8_9	Mean		1.18377	.140499
	95% Confidence Interval for Mean	Lower Bound	.89934	
		Upper Bound	1.46819	
	5% Trimmed Mean		1.08788	
	Median		.91700	
	Variance		.770	
	Std. Deviation		.877413	
	Minimum		.200	
	Maximum		4.500	
	Range		4.300	
	Interquartile Range		.808	
	Skewness		1.985	.378
	Kurtosis		4.860	.741

Descriptives

			Statistic	Std. Error
PS_9_10	Mean		1.18418	.156470
	95% Confidence Interval for Mean	Lower Bound	.86742	
		Upper Bound	1.50094	
	5% Trimmed Mean		1.05617	
	Median		1.02900	
	Variance		.955	
	Std. Deviation		.977152	
	Minimum		.186	
	Maximum		5.400	
	Range		5.214	
	Interquartile Range		.820	
	Skewness		2.632	.378
	Kurtosis		8.935	.741
PS_10_11	Mean		1.47236	.198258
	95% Confidence Interval for Mean	Lower Bound	1.07101	
		Upper Bound	1.87371	
	5% Trimmed Mean		1.30924	
	Median		1.23400	
	Variance		1.533	
	Std. Deviation		1.238121	
	Minimum		.194	
	Maximum		6.004	
	Range		5.810	
	Interquartile Range		1.289	
	Skewness		2.178	.378
	Kurtosis		5.612	.741
PS_11_12	Mean		.87792	.123331
	95% Confidence Interval for Mean	Lower Bound	.62825	
		Upper Bound	1.12759	
	5% Trimmed Mean		.76786	
	Median		.75300	
	Variance		.593	
	Std. Deviation		.770203	
	Minimum		.179	
	Maximum		4.300	
	Range		4.121	
	Interquartile Range		.668	
	Skewness		2.807	.378
	Kurtosis		10.157	.741

Descriptives

			Statistic	Std. Error
PS_12_13	Mean		.69497	.084489
	95% Confidence Interval for Mean	Lower Bound	.52393	
		Upper Bound	.86601	
	5% Trimmed Mean		.63613	
	Median		.52500	
	Variance		.278	
	Std. Deviation		.527636	
	Minimum		.138	
	Maximum		2.660	
	Range		2.522	
	Interquartile Range		.525	
	Skewness		1.944	.378
	Kurtosis		4.567	.741
PS_13_14	Mean		.66036	.079284
	95% Confidence Interval for Mean	Lower Bound	.49986	
		Upper Bound	.82086	
	5% Trimmed Mean		.61986	
	Median		.51200	
	Variance		.245	
	Std. Deviation		.495127	
	Minimum		.124	
	Maximum		1.950	
	Range		1.826	
	Interquartile Range		.588	
	Skewness		1.282	.378
	Kurtosis		.783	.741
PS_14_15	Mean		.65523	.076404
	95% Confidence Interval for Mean	Lower Bound	.50056	
		Upper Bound	.80990	
	5% Trimmed Mean		.63051	
	Median		.47800	
	Variance		.228	
	Std. Deviation		.477145	
	Minimum		.115	
	Maximum		1.753	
	Range		1.638	
	Interquartile Range		.864	
	Skewness		.859	.378
	Kurtosis		-.648	.741

Descriptives

			Statistic	Std. Error
PS_15_16	Mean		.65815	.080070
	95% Confidence Interval for Mean	Lower Bound	.49606	
		Upper Bound	.82025	
	5% Trimmed Mean		.61783	
	Median		.44900	
	Variance		.250	
	Std. Deviation		.500035	
	Minimum		.104	
	Maximum		2.021	
	Range		1.917	
	Interquartile Range		.730	
	Skewness		1.127	.378
	Kurtosis		.542	.741
PS_16_17	Mean		.68705	.093567
	95% Confidence Interval for Mean	Lower Bound	.49764	
		Upper Bound	.87647	
	5% Trimmed Mean		.63613	
	Median		.44200	
	Variance		.341	
	Std. Deviation		.584323	
	Minimum		.090	
	Maximum		2.360	
	Range		2.270	
	Interquartile Range		.808	
	Skewness		1.284	.378
	Kurtosis		.909	.741
PS_17_18	Mean		.71841	.119341
	95% Confidence Interval for Mean	Lower Bound	.47682	
		Upper Bound	.96000	
	5% Trimmed Mean		.63813	
	Median		.43000	
	Variance		.555	
	Std. Deviation		.745286	
	Minimum		.088	
	Maximum		3.035	
	Range		2.947	
	Interquartile Range		.703	
	Skewness		1.828	.378
	Kurtosis		2.610	.741

Descriptives

			Statistic	Std. Error
PS_18_19	Mean		.80531	.143079
	95% Confidence Interval for Mean	Lower Bound	.51566	
		Upper Bound	1.09496	
	5% Trimmed Mean		.71154	
	Median		.40900	
	Variance		.798	
	Std. Deviation		.893526	
	Minimum		.088	
	Maximum		3.438	
	Range		3.350	
	Interquartile Range		.800	
	Skewness		1.762	.378
	Kurtosis		2.093	.741
PS_19_20	Mean		.85638	.176944
	95% Confidence Interval for Mean	Lower Bound	.49818	
		Upper Bound	1.21459	
	5% Trimmed Mean		.69091	
	Median		.38400	
	Variance		1.221	
	Std. Deviation		1.105017	
	Minimum		.085	
	Maximum		4.865	
	Range		4.780	
	Interquartile Range		.843	
	Skewness		2.356	.378
	Kurtosis		5.600	.741

Tests of Normality

	Kolmogorov-Smirnov[a]			Shapiro-Wilk		
	Statistic	df	Sig.	Statistic	df	Sig.
BrainRate	.186	39	.002	.875	39	<.001
PermutationEntropy	.180	39	.003	.950	39	.083
PermutationEntropy_01	.180	39	.003	.950	39	.083
SampleEntropy	.106	39	.200*	.973	39	.449
SpectralEntropy_bits	.084	39	.200*	.963	39	.232
SpectralEntropy_01	.084	39	.200*	.963	39	.232
HiguchiFractalDimension	.110	39	.200*	.946	39	.061
LempelZivComplexity	.188	39	.001	.922	39	.010
RenyiEntropy	.116	39	.200*	.965	39	.263
TsallisEntropy	.303	39	<.001	.740	39	<.001
PS_4_5	.226	39	<.001	.531	39	<.001
PS_5_6	.186	39	.002	.727	39	<.001
PS_6_7	.177	39	.003	.669	39	<.001
PS_7_8	.192	39	<.001	.801	39	<.001
PS_8_9	.198	39	<.001	.814	39	<.001
PS_9_10	.230	39	<.001	.738	39	<.001
PS_10_11	.185	39	.002	.776	39	<.001
PS_11_12	.201	39	<.001	.719	39	<.001
PS_12_13	.186	39	.002	.812	39	<.001
PS_13_14	.203	39	<.001	.845	39	<.001
PS_14_15	.200	39	<.001	.859	39	<.001
PS_15_16	.188	39	.001	.870	39	<.001
PS_16_17	.212	39	<.001	.840	39	<.001
PS_17_18	.235	39	<.001	.742	39	<.001
PS_18_19	.269	39	<.001	.725	39	<.001
PS_19_20	.250	39	<.001	.674	39	<.001

*. This is a lower bound of the true significance.

a. Lilliefors Significance Correction

BrainRate

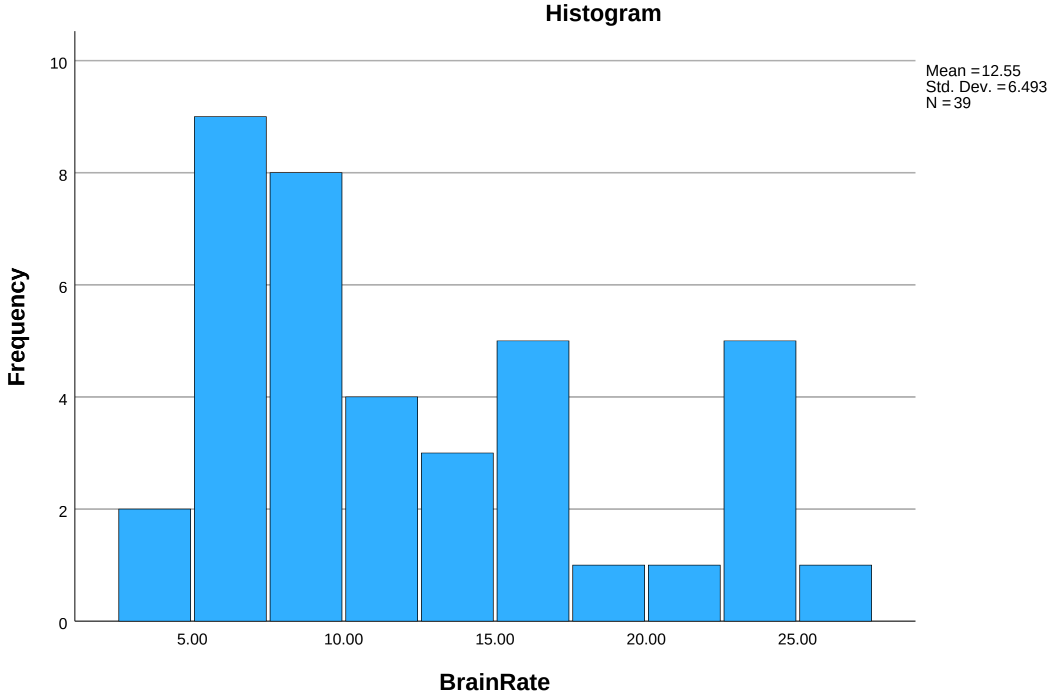


```
BrainRate Stem-and-Leaf Plot

 Frequency    Stem &  Leaf

     2.00        0 .  44
    17.00        0 .  56677777777788889
     7.00        1 .  0001334
     6.00        1 .  566777
     6.00        2 .  133444
     1.00        2 .  5

 Stem width:      10.00
 Each leaf:        1 case(s)
```

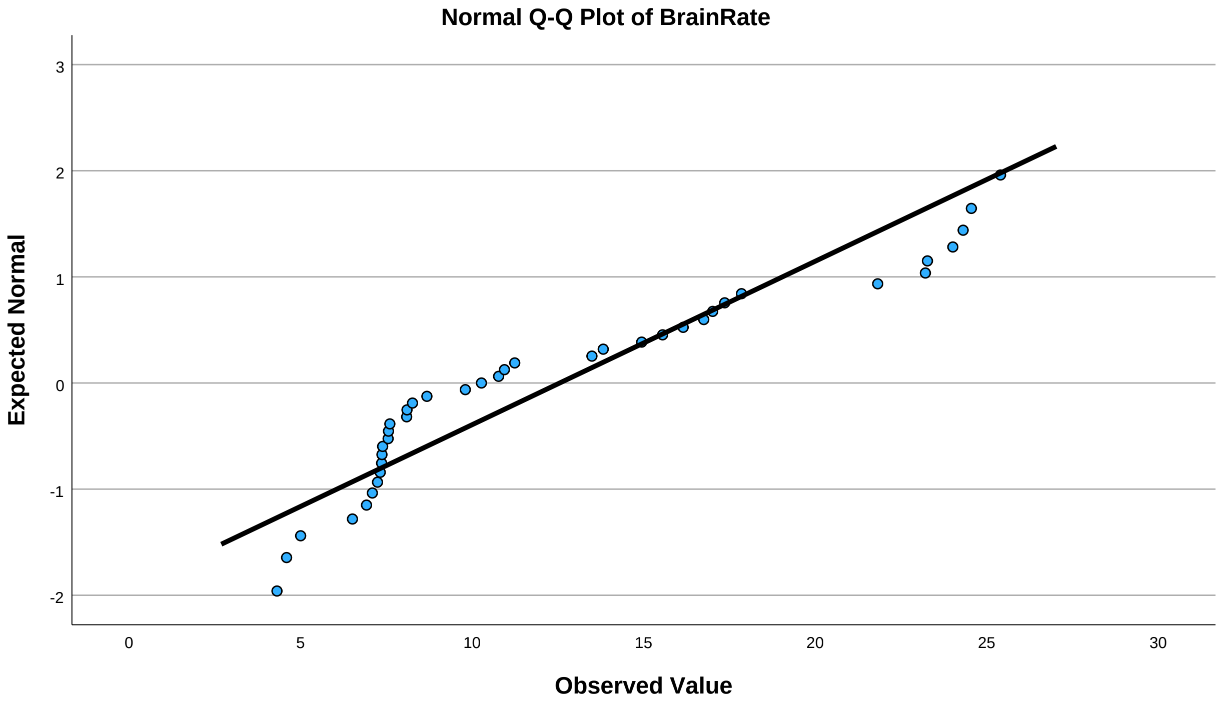
Normal Q-Q Plot of BrainRate
Expected Normal
Observed Value
3
2
1
0
-1
-2
0
5
10
15
20
25
30

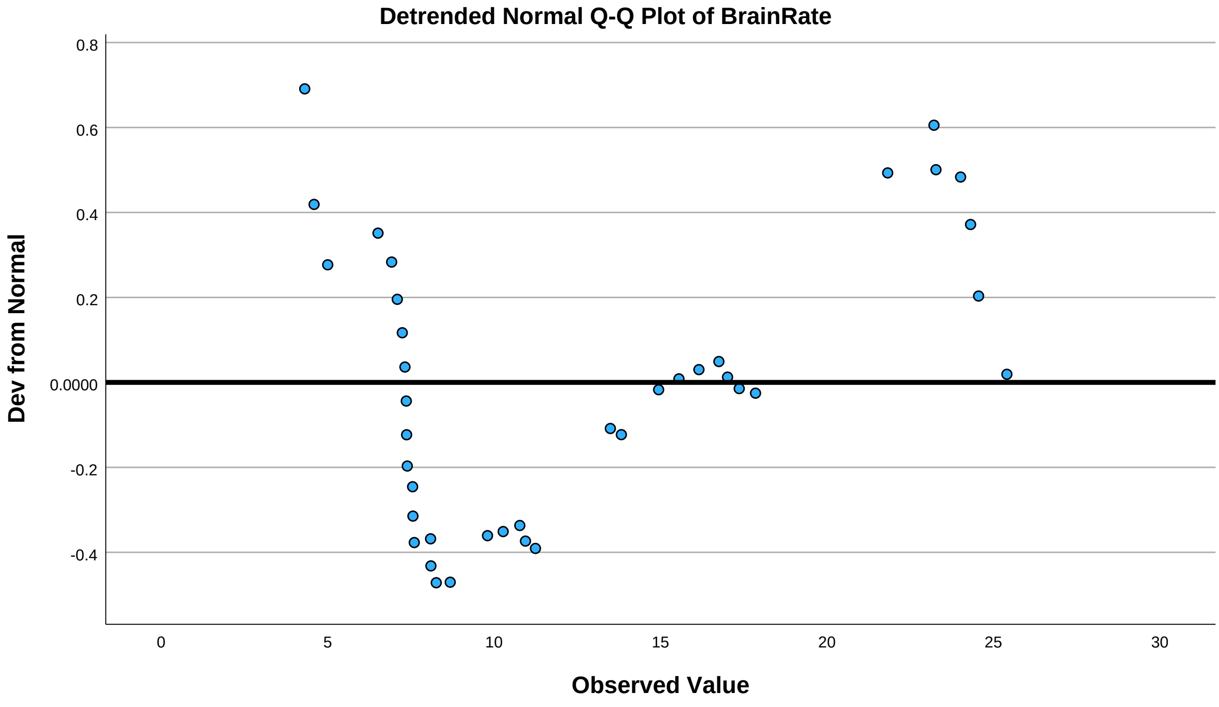
Detrended Normal Q-Q Plot of BrainRate
Dev from Normal
Observed Value
0.8
0.6
0.4
0.2
0.0000
-0.2
-0.4
0
5
10
15
20
25
30

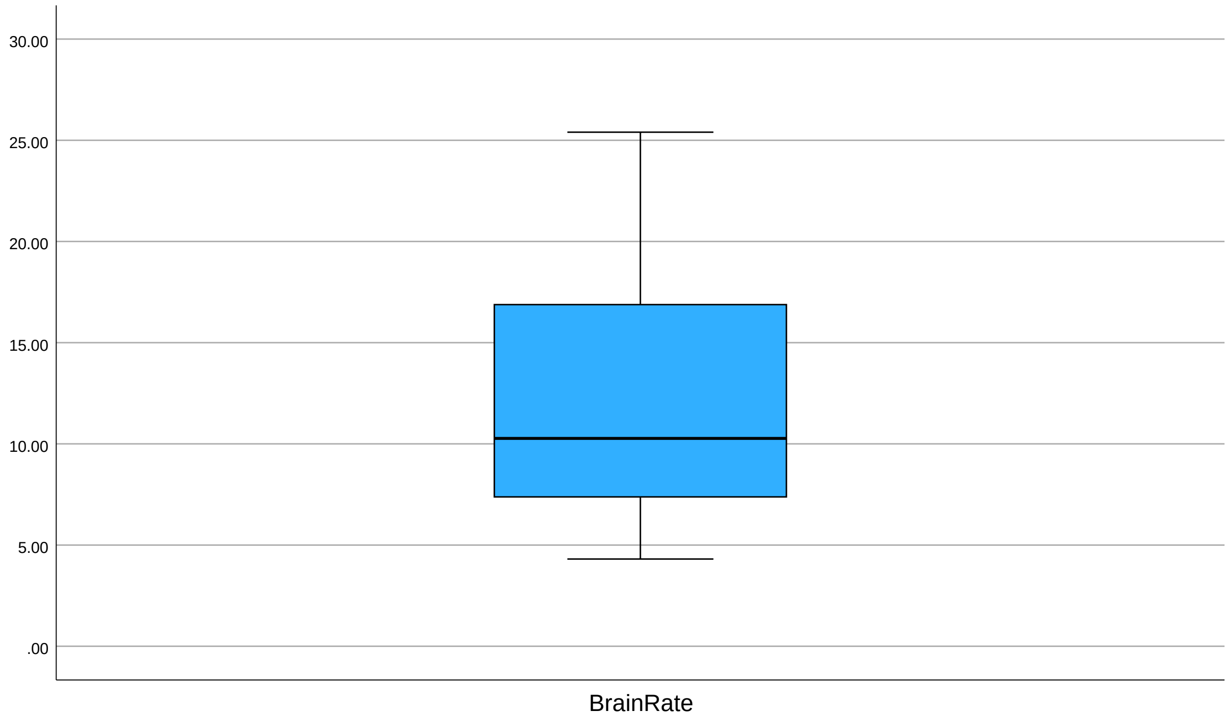

PermutationEntropy

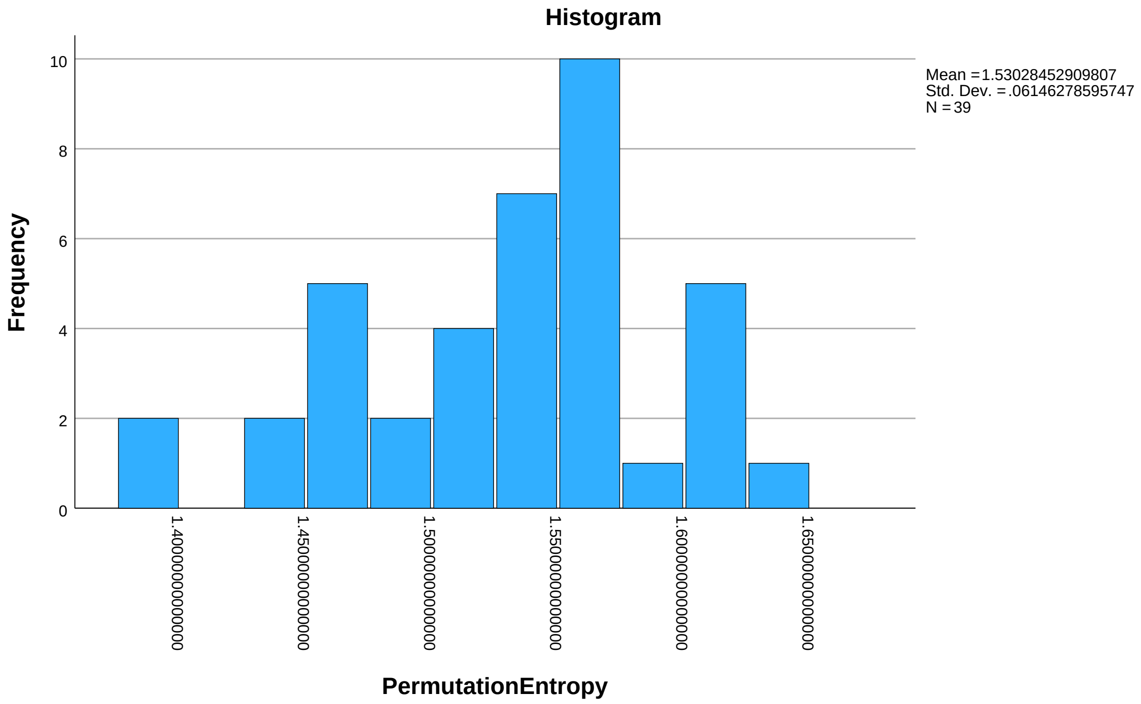

PermutationEntropy Stem-and-Leaf Plot

```
 Frequency    Stem &  Leaf
```

```
  2.00     13 .  89
  2.00     14 .  44
  7.00      14 .  5666688
 11.00       15 .  00004444444
 11.00       15 .  56666666679
  6.00      16 .  000013

Stem width:   .1000000
Each leaf:        1 case(s)
```

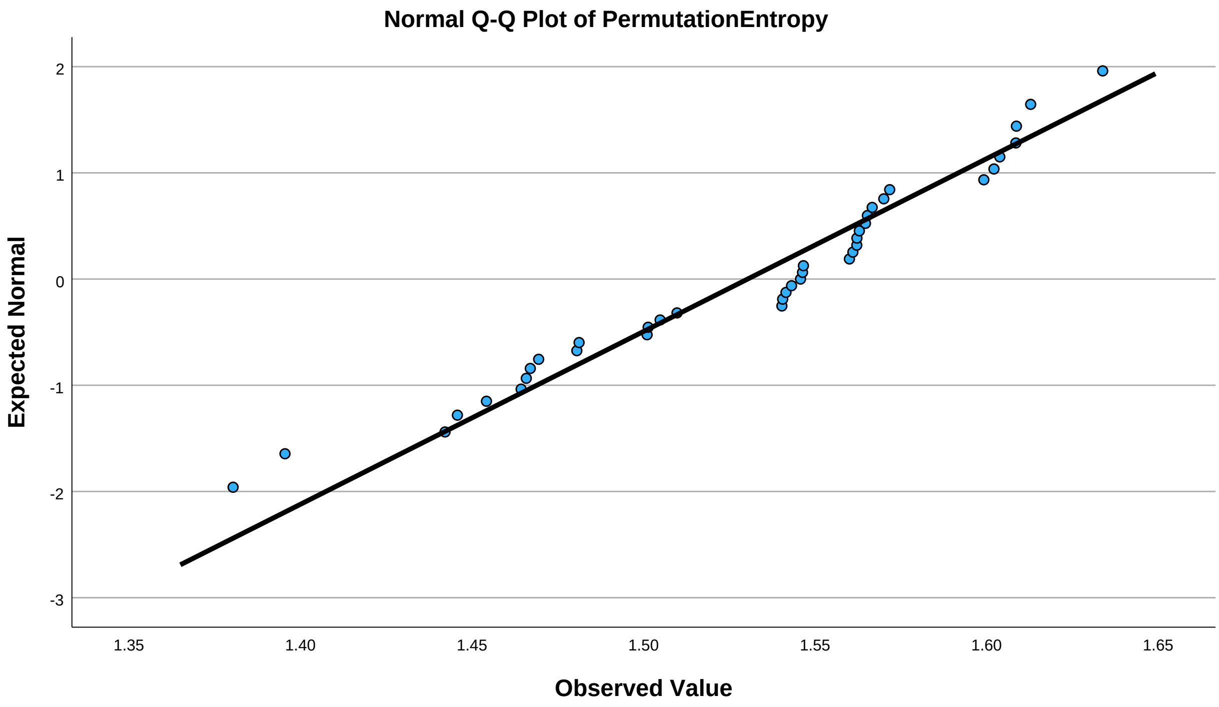

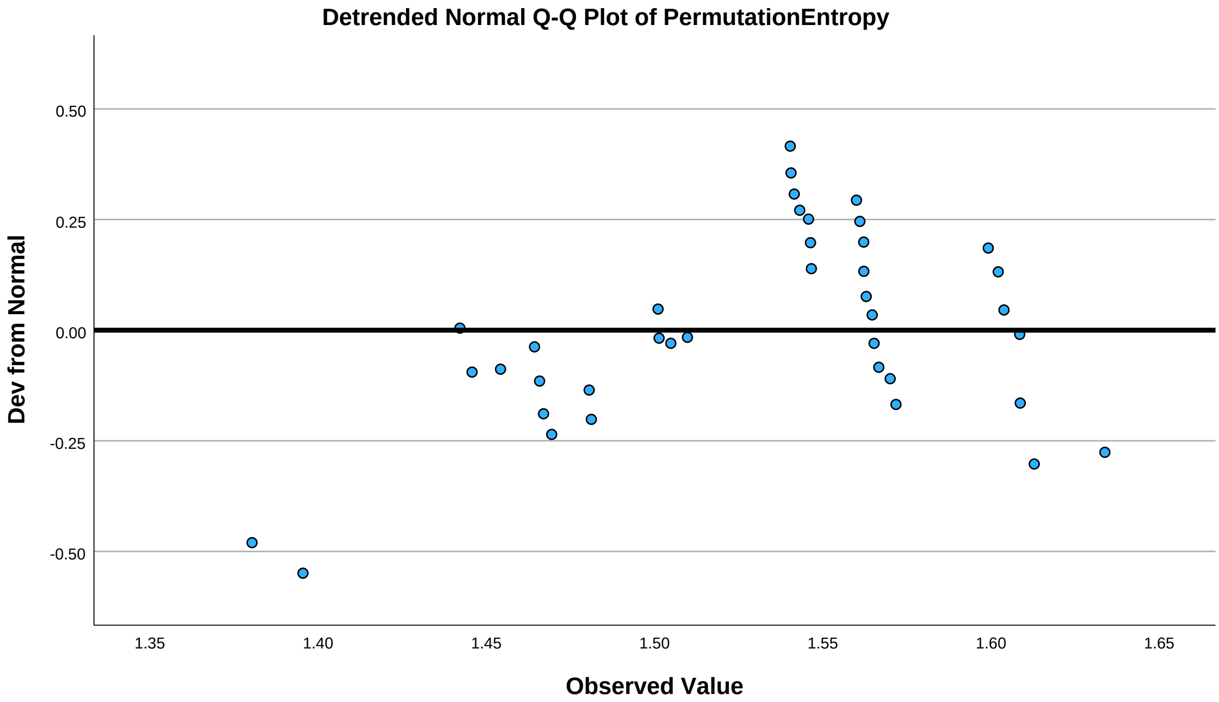

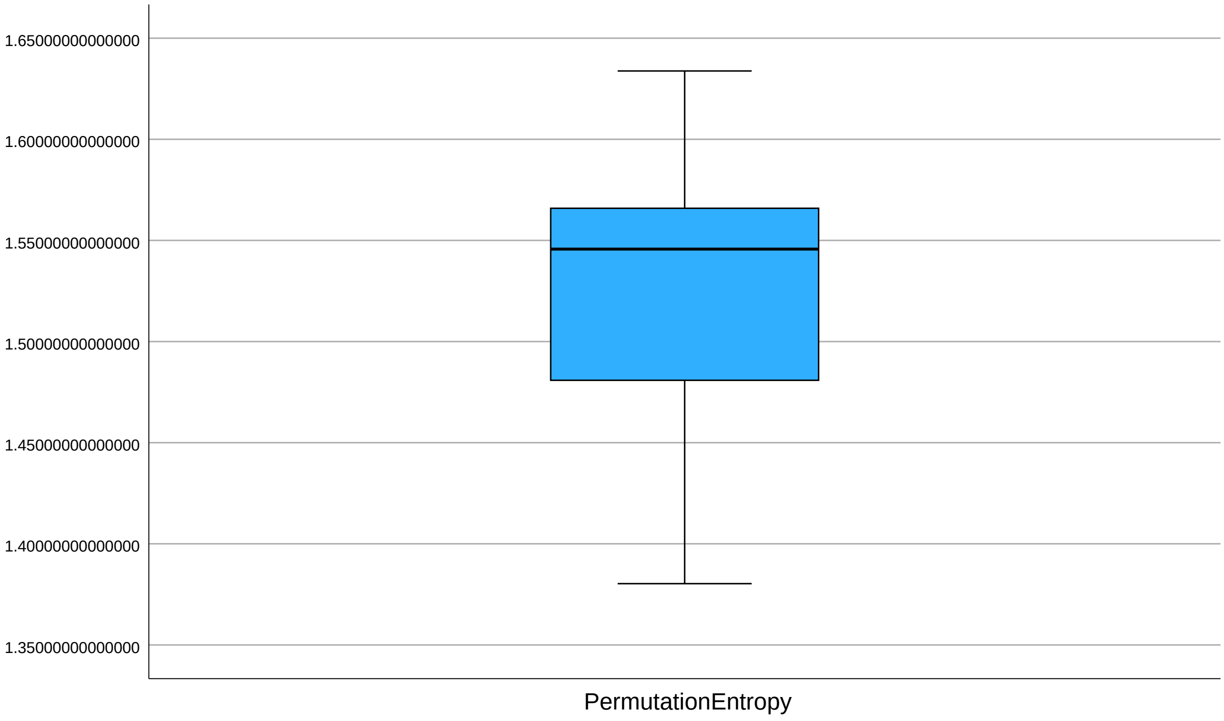

PermutationEntropy_01

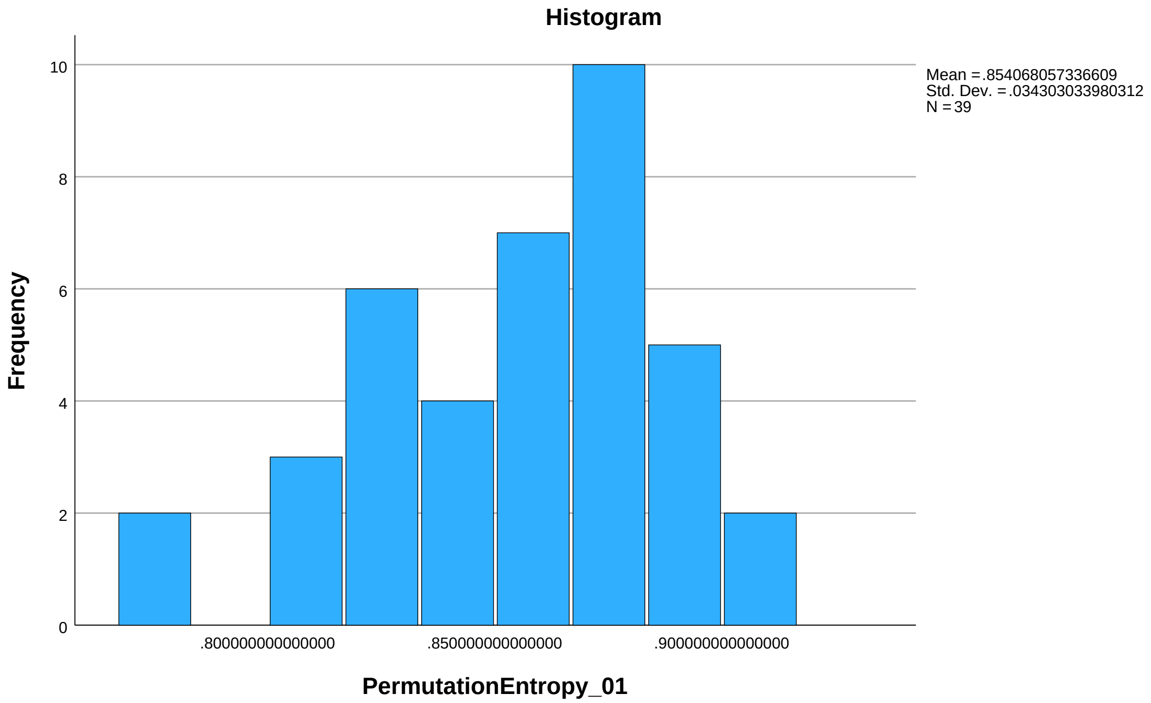


```
PermutationEntropy_01 Stem-and-Leaf Plot

 Frequency    Stem &  Leaf

    2.00      7 .  77
   13.00      8 .  0011112223334
   22.00      8 .  5566666777777777799999
    2.00      9 .  01

 Stem width:   .1000000
 Each leaf:      1 case(s)
```

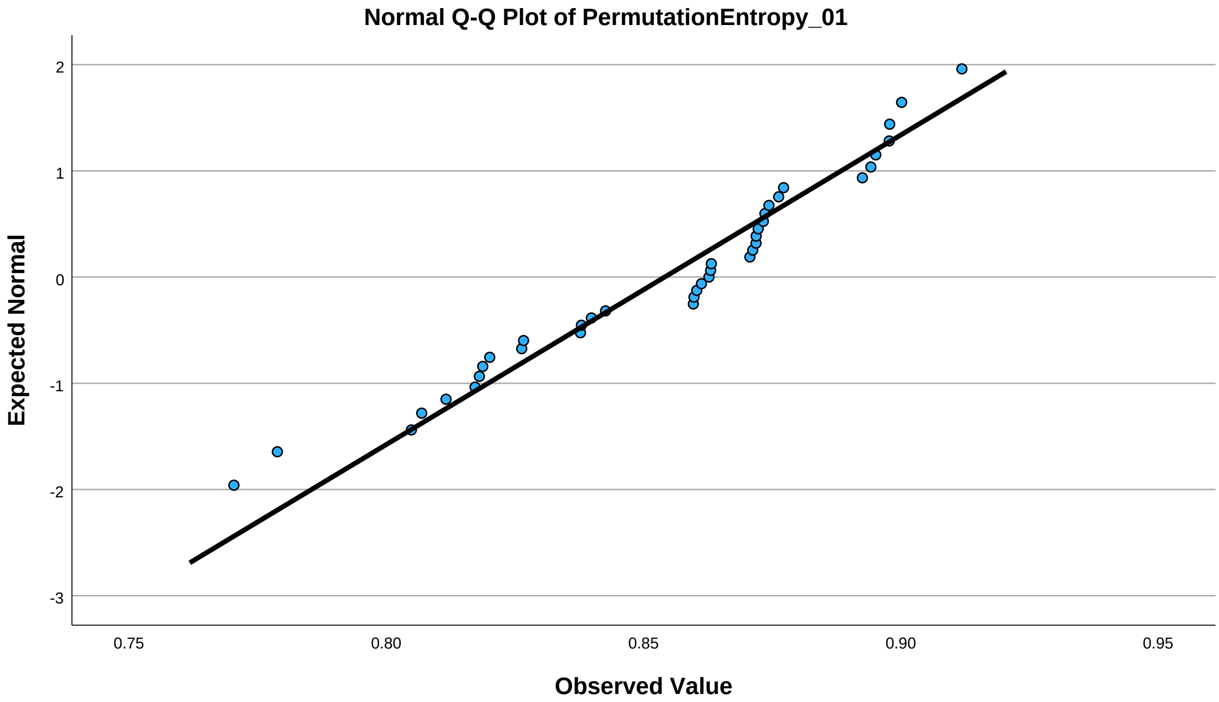

Normal Q-Q Plot of PermutationEntropy_01
Expected Normal
2
1
0
-1
-2
-3
0.75
0.80
0.85
0.90
0.95
Observed Value

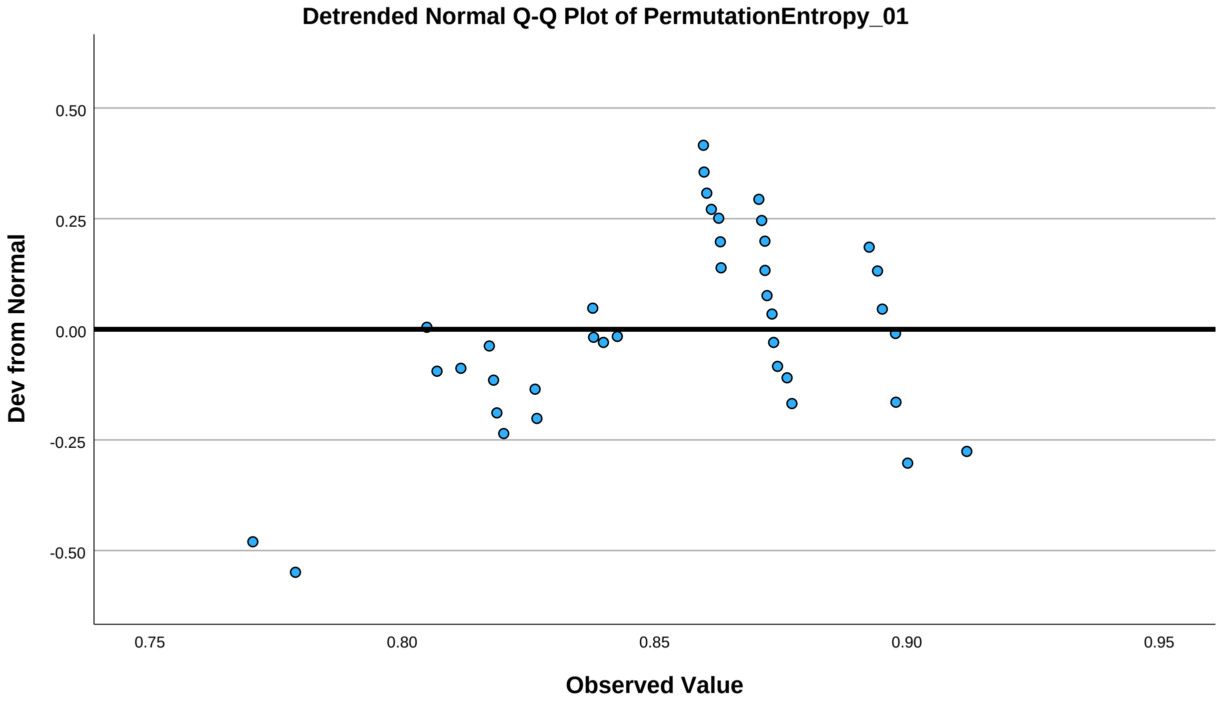

Detrended Normal Q-Q Plot of PermutationEntropy_01
Dev from Normal
0.50
0.25
0.00
-0.25
-0.50
0.75
0.80
0.85
0.90
0.95
Observed Value

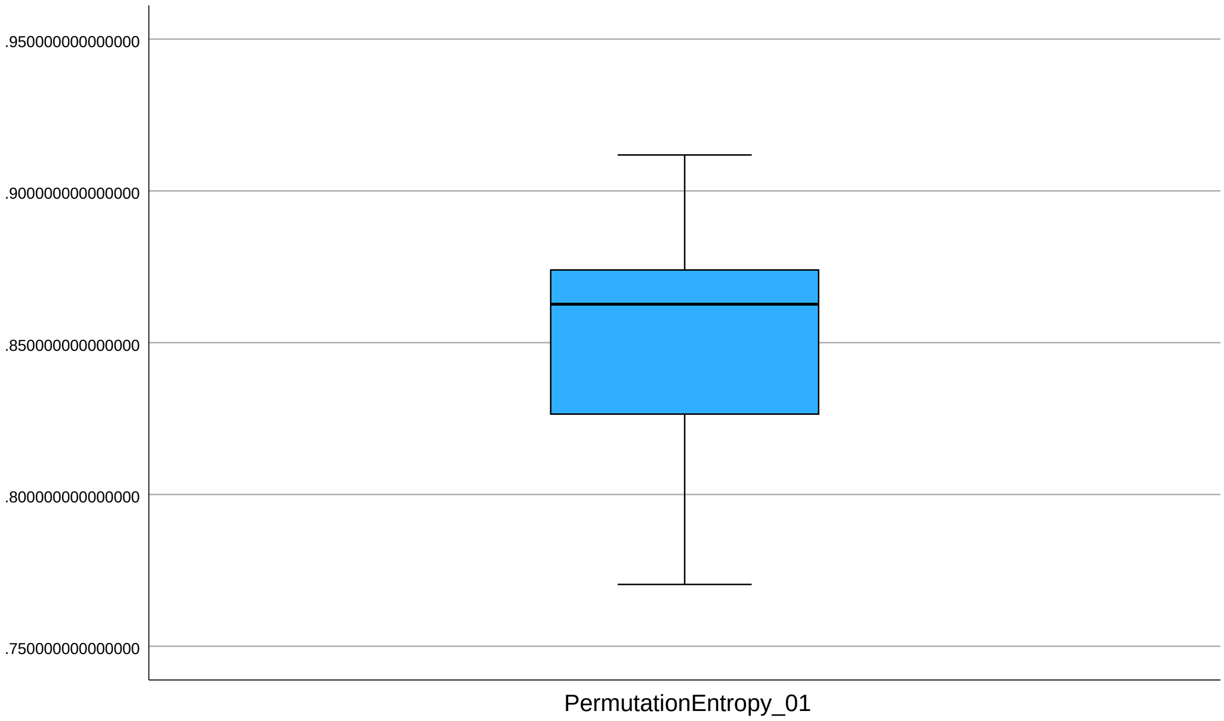

SampleEntropy

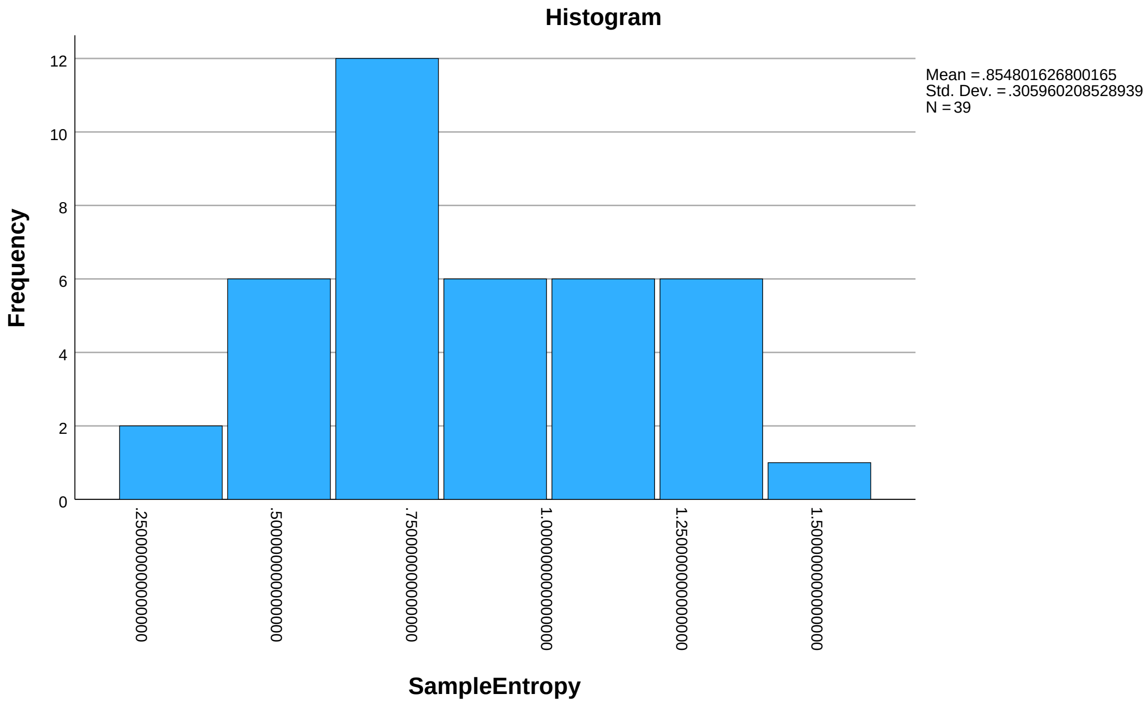


```
SampleEntropy Stem-and-Leaf Plot

 Frequency    Stem &  Leaf
```

```
 2.00      0 . 23
 6.00      0 . 445555
12.00      0 . 666677777777
 6.00      0 . 888889
 6.00      1 . 001111
 6.00      1 . 222223
 1.00      1 . 4

Stem width:  1.000000
Each leaf:       1 case(s)
```

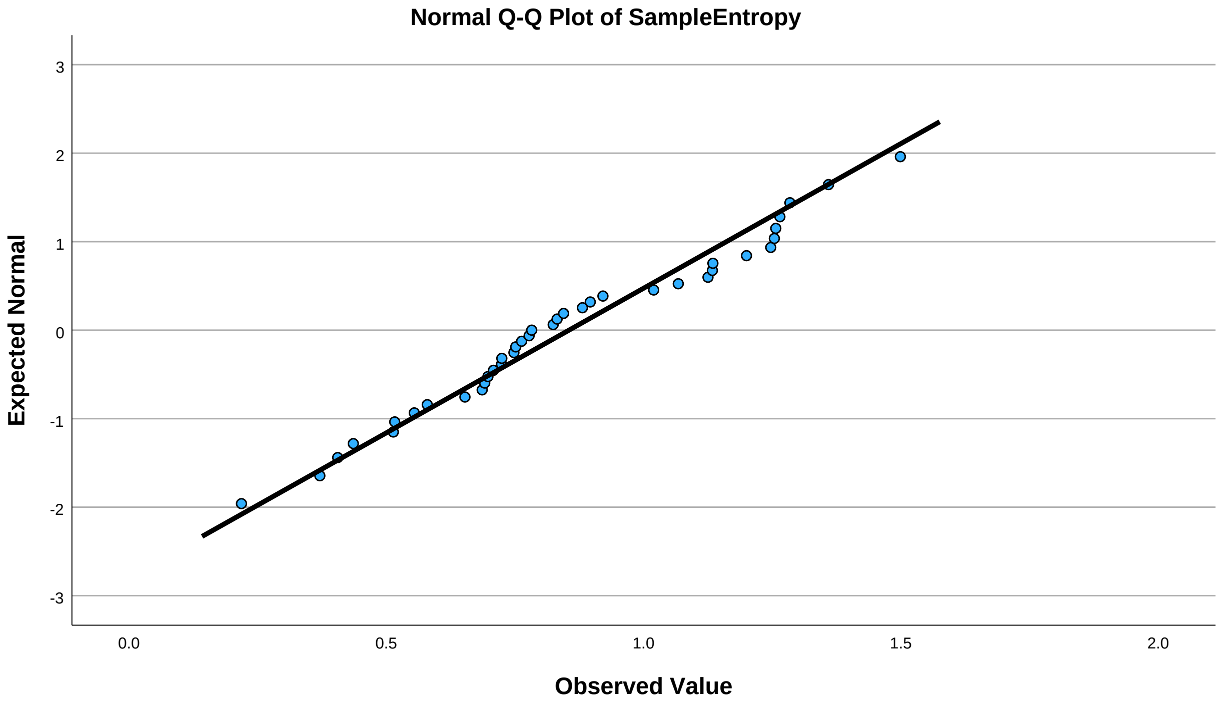

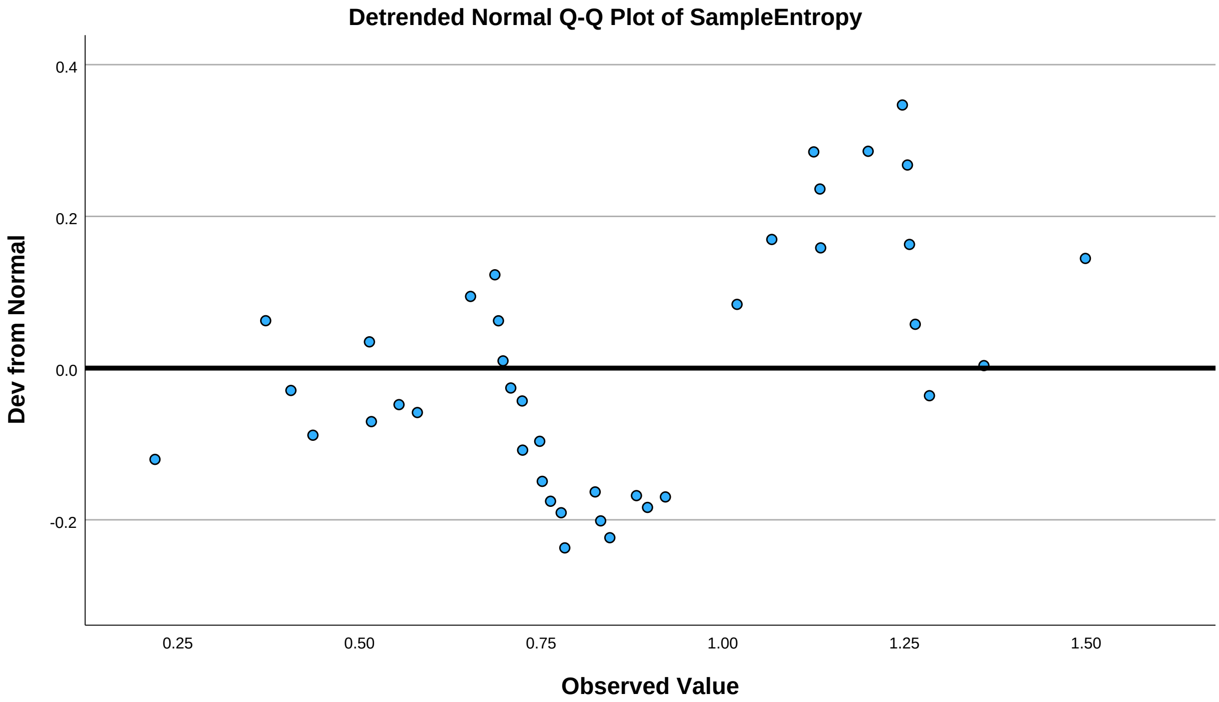

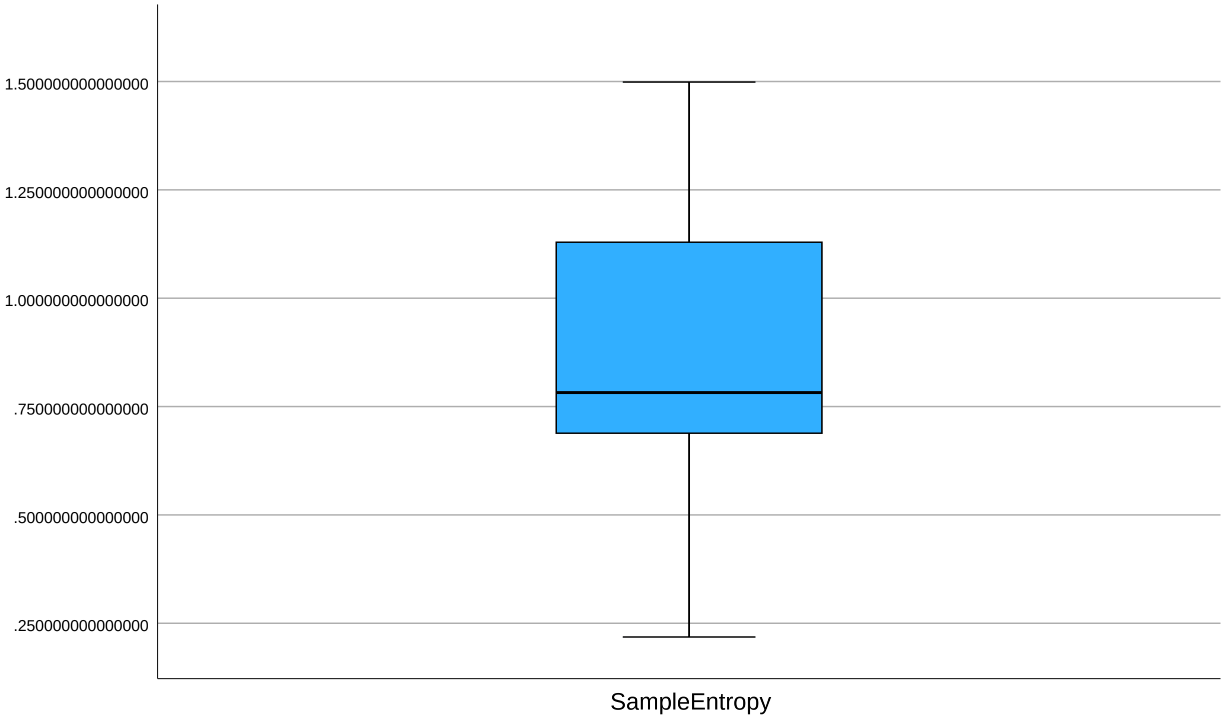

SpectralEntropy_bits

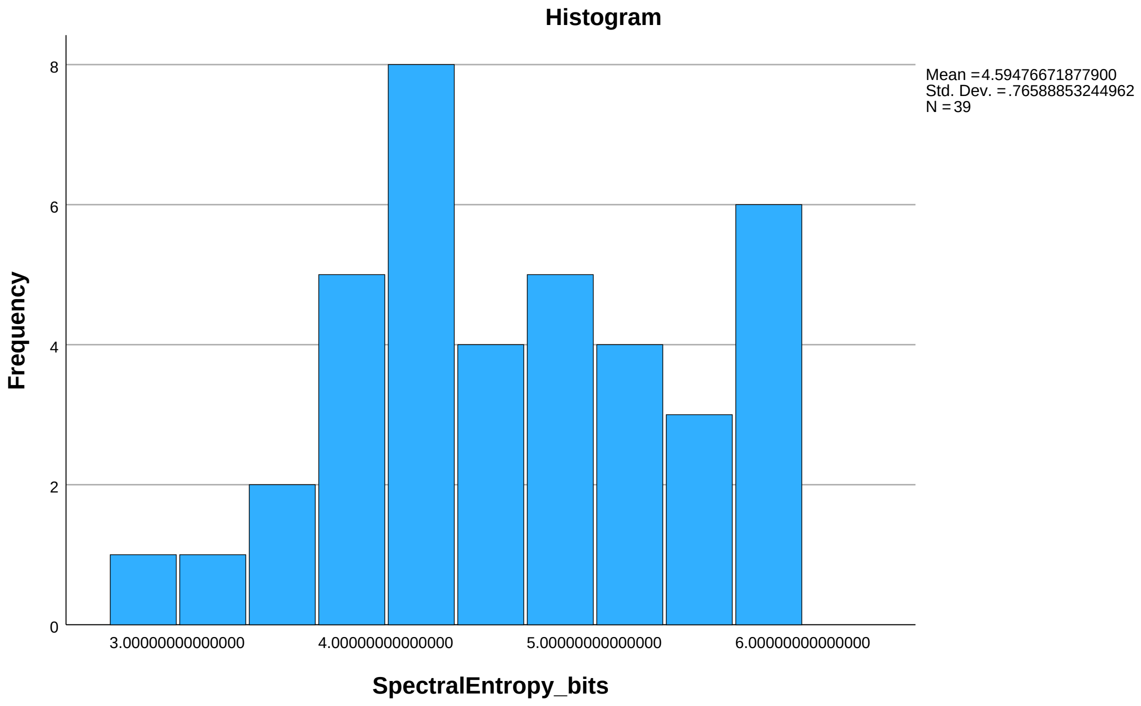


```
SpectralEntropy_bits Stem-and-Leaf Plot

 Frequency    Stem &  Leaf

     1.00        2 .  8
     2.00        3 .  24
     6.00        3 .  577799
    10.00        4 .  1112222344
     7.00        4 .  5666789
     7.00        5 .  0012344
     6.00        5 .  667777

 Stem width:  1.000000
 Each leaf:       1 case(s)
```

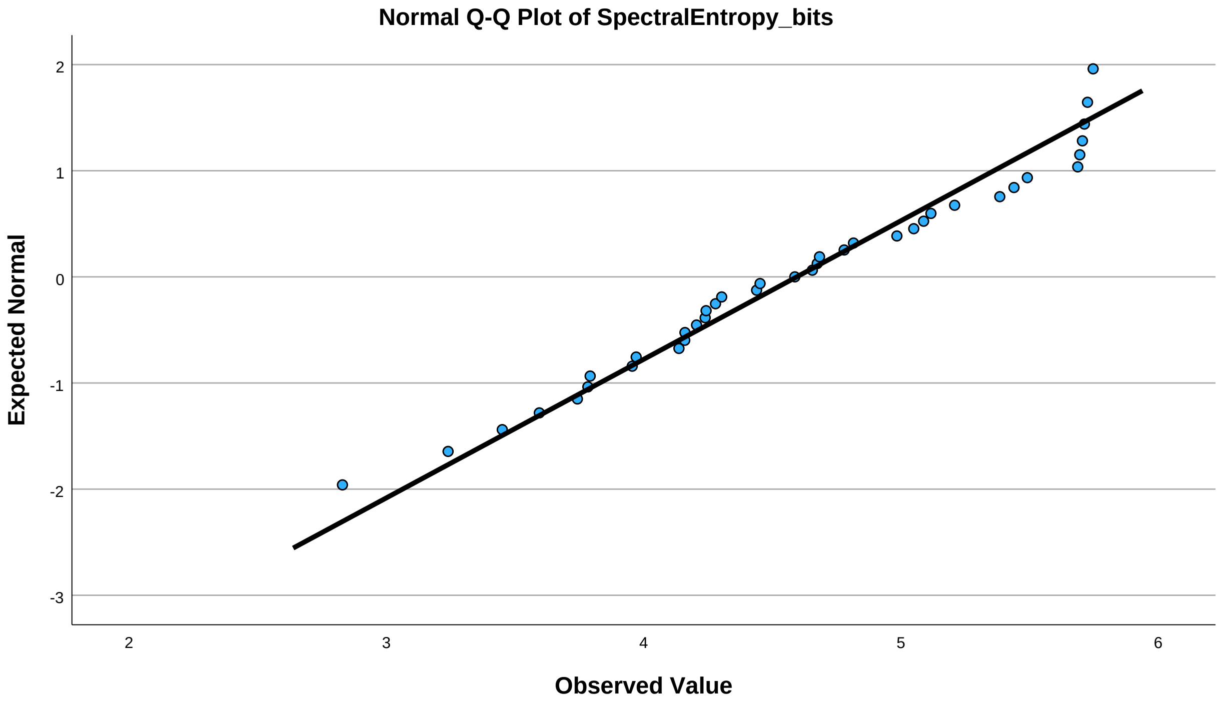
Normal Q-Q Plot of SpectralEntropy_bits
Expected Normal
Observed Value
2
1
0
-1
-2
-3
2
3
4
5
6

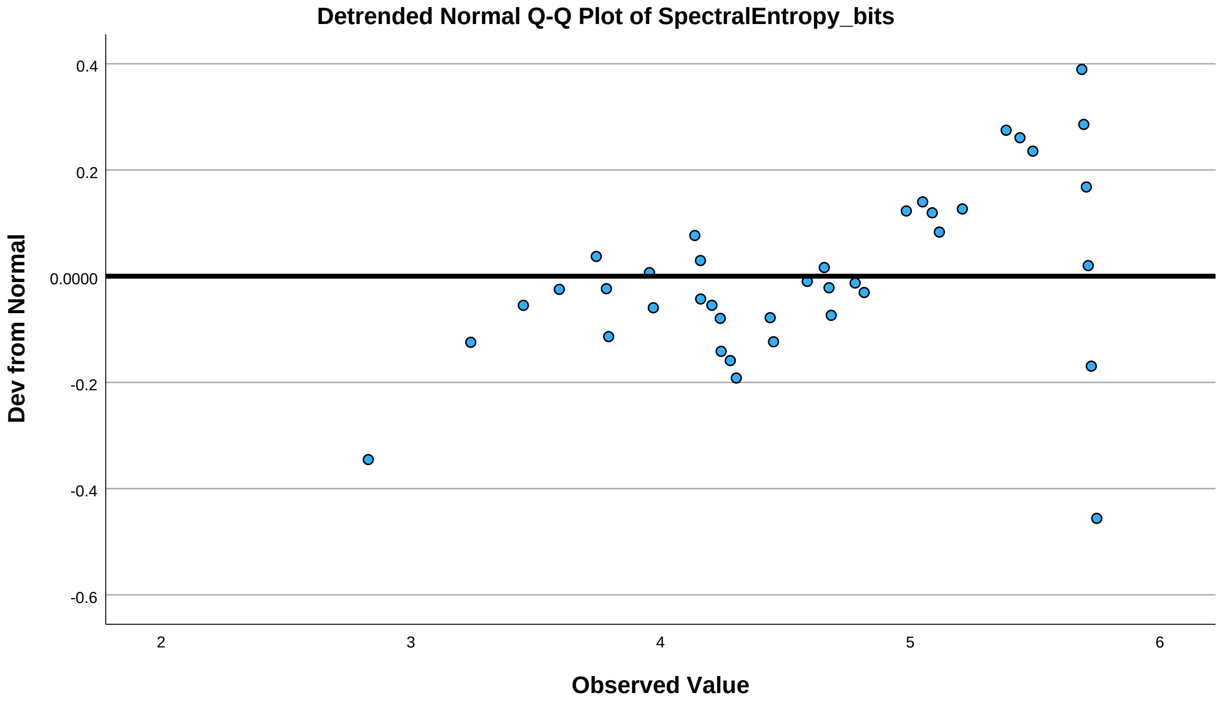
Detrended Normal Q-Q Plot of SpectralEntropy_bits
Dev from Normal
Observed Value
0.4
0.2
0.0000
-0.2
-0.4
-0.6
2
3
4
5
6

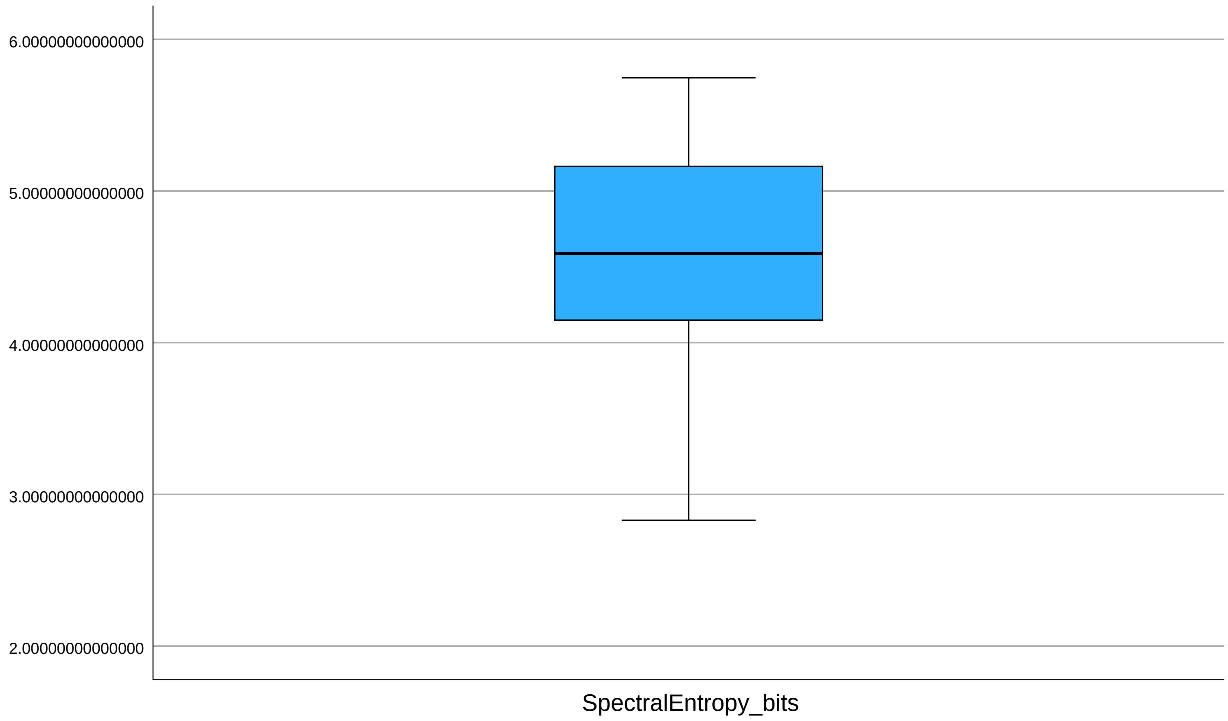

SpectralEntropy_01

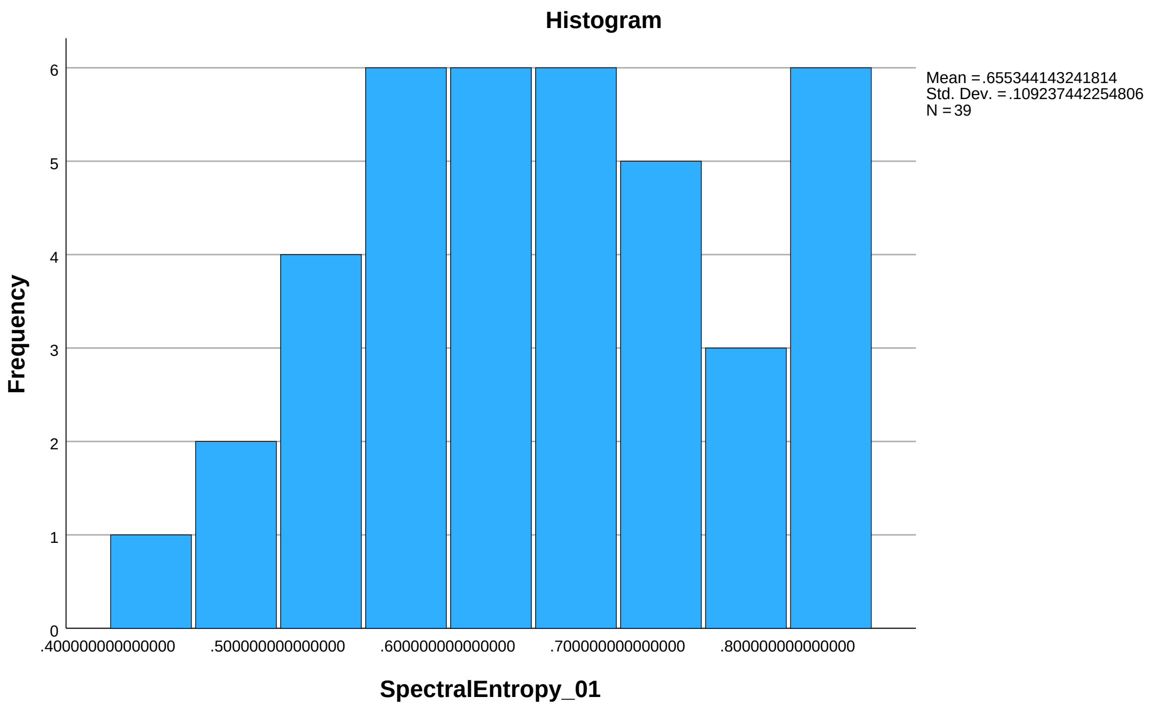


```
SpectralEntropy_01 Stem-and-Leaf Plot

 Frequency    Stem &  Leaf
```

```
 1.00     4 . 0
 2.00     4 . 69
 4.00     5 . 1334
 6.00     5 . 669999
 6.00     6 . 001133
 6.00     6 . 566688
 5.00     7 . 12224
 3.00     7 . 678
 6.00     8 . 111111

Stem width:  .1000000
Each leaf:        1 case(s)
```

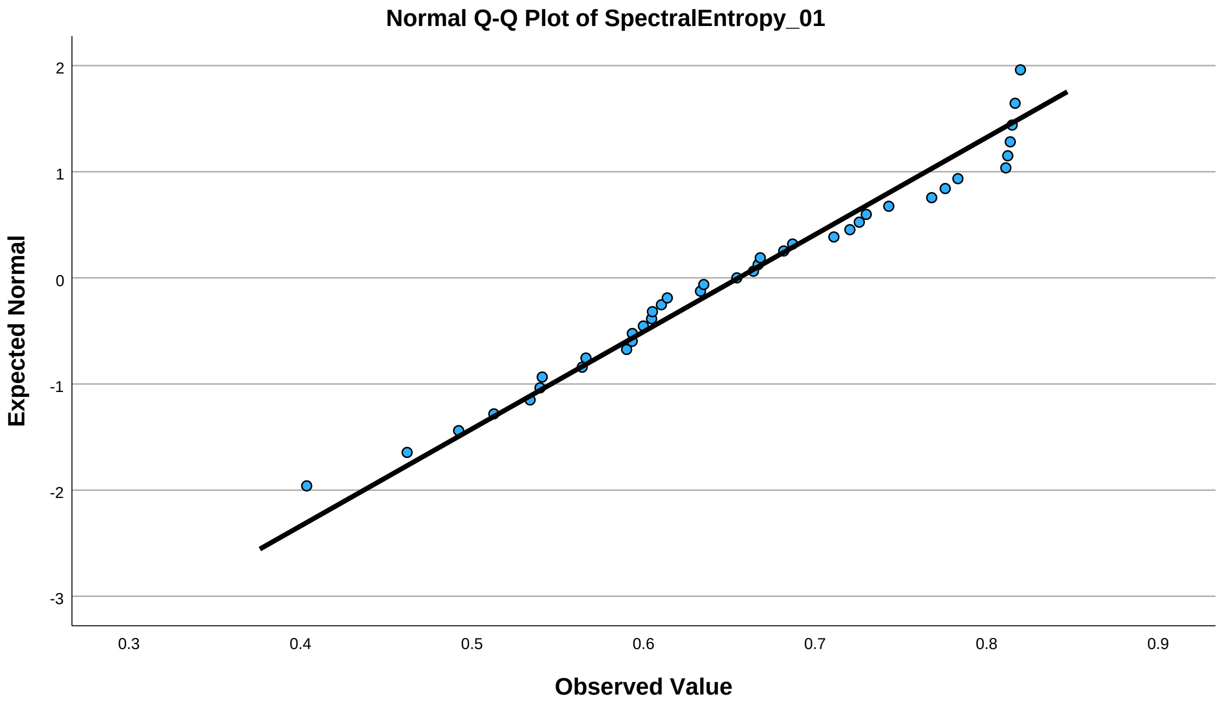

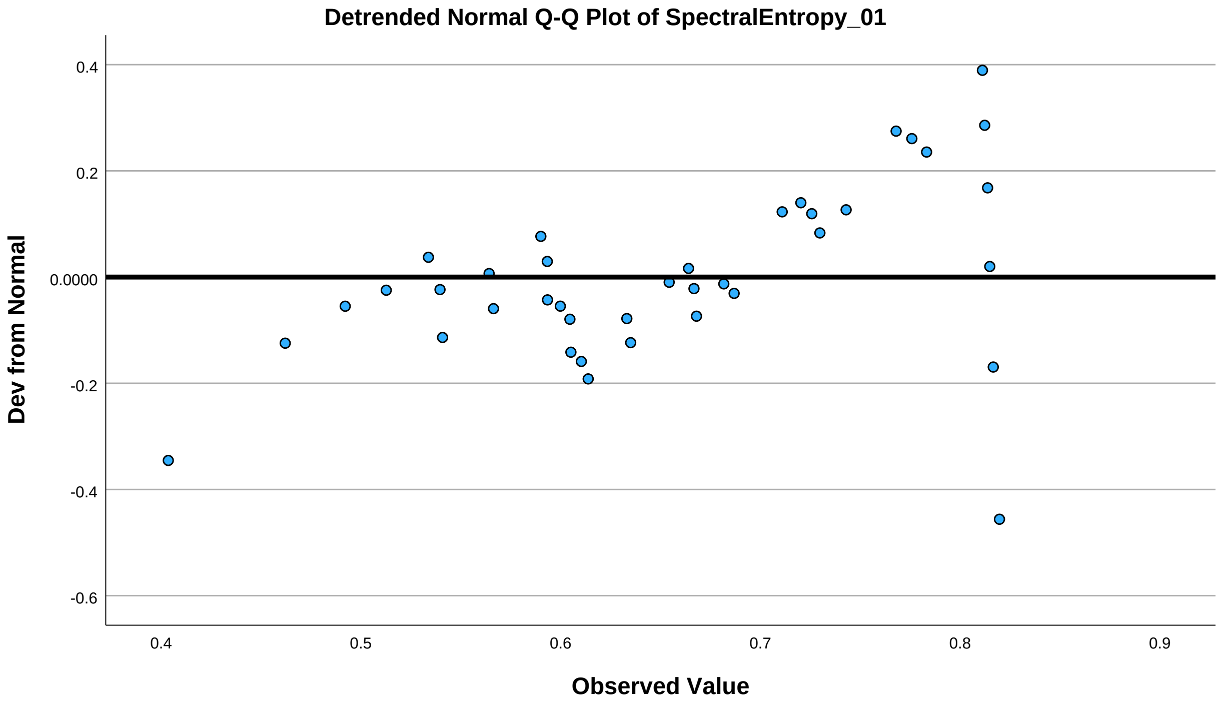

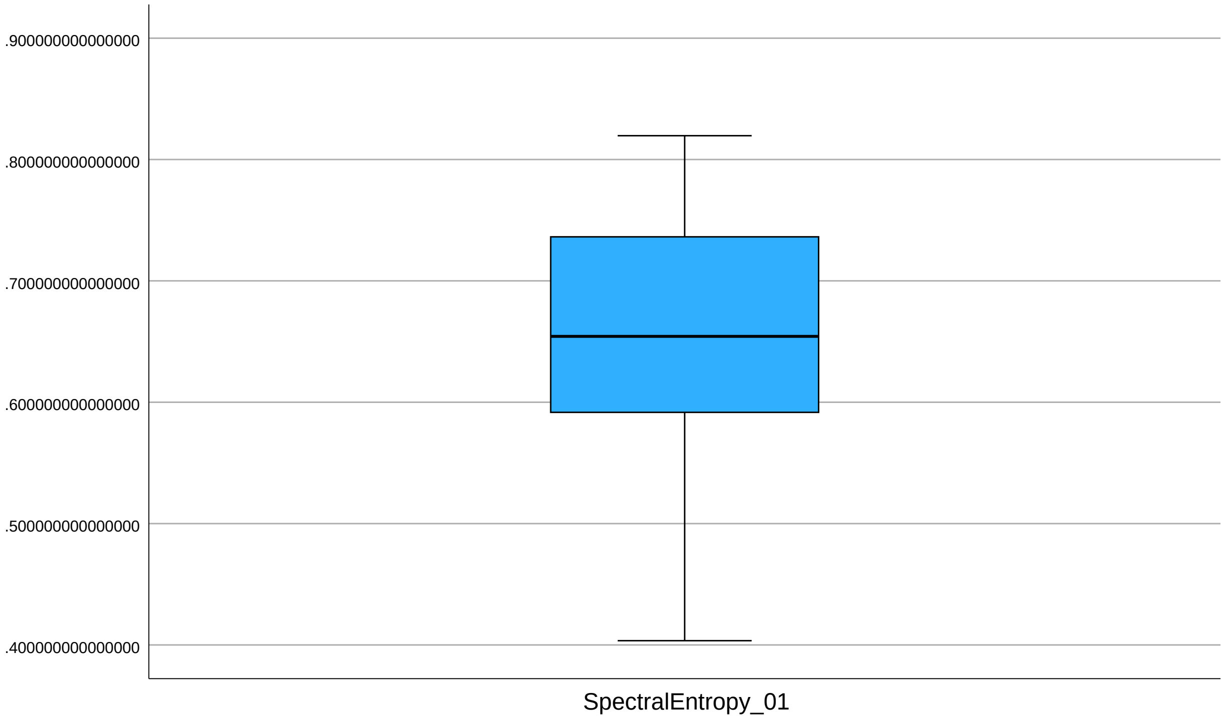

HiguchiFractalDimension

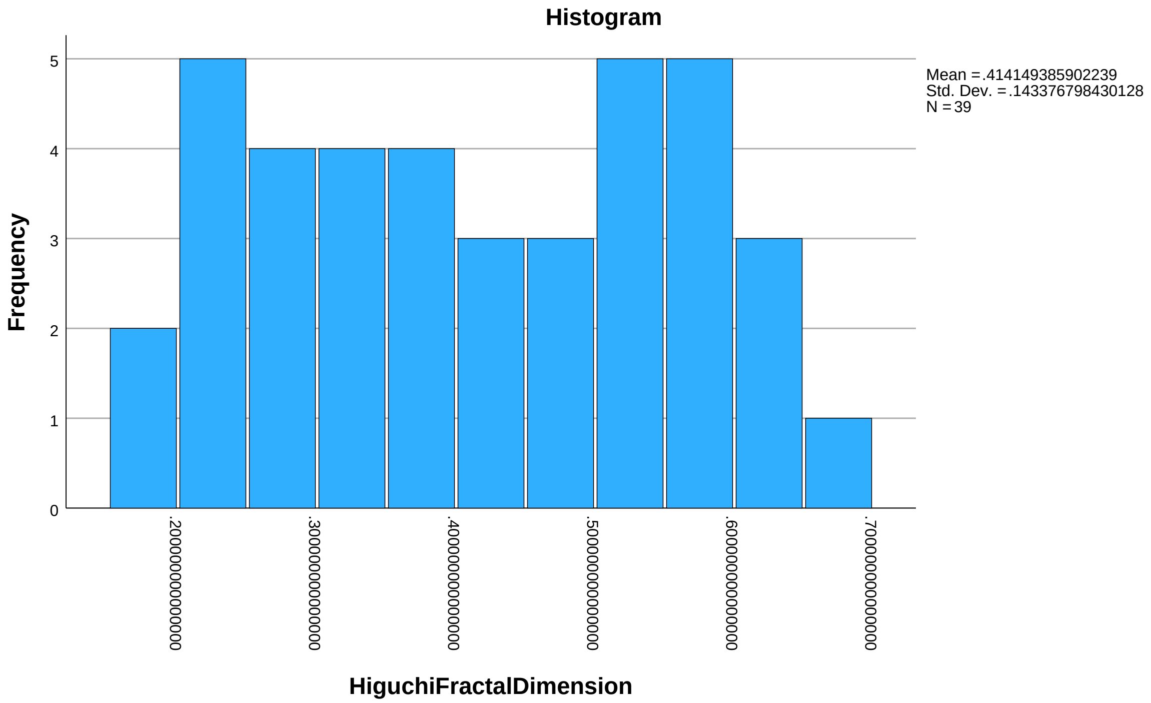


```
HiguchiFractalDimension Stem-and-Leaf Plot

 Frequency    Stem &  Leaf

     2.00        1 .  99
     5.00        2 .  12344
     4.00        2 .  5778
     4.00        3 .  1233
     4.00        3 .  5689
     3.00        4 .  014
     3.00        4 .  557
     5.00        5 .  11112
     5.00        5 .  57899
     3.00        6 .  024
     1.00        6 .  6

 Stem width:   .1000000
 Each leaf:        1 case(s)
```

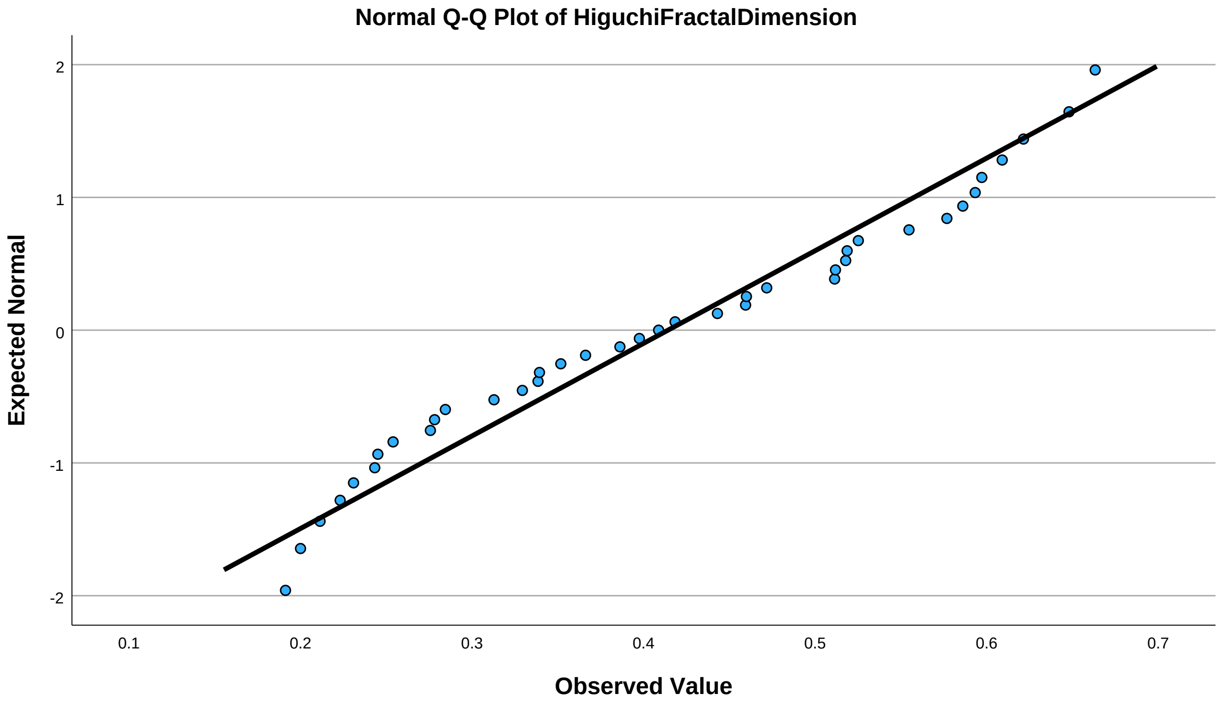
Normal Q-Q Plot of HiguchiFractalDimension
Expected Normal
2
1
0
-1
-2
0.1
0.2
0.3
0.4
0.5
0.6
0.7
Observed Value

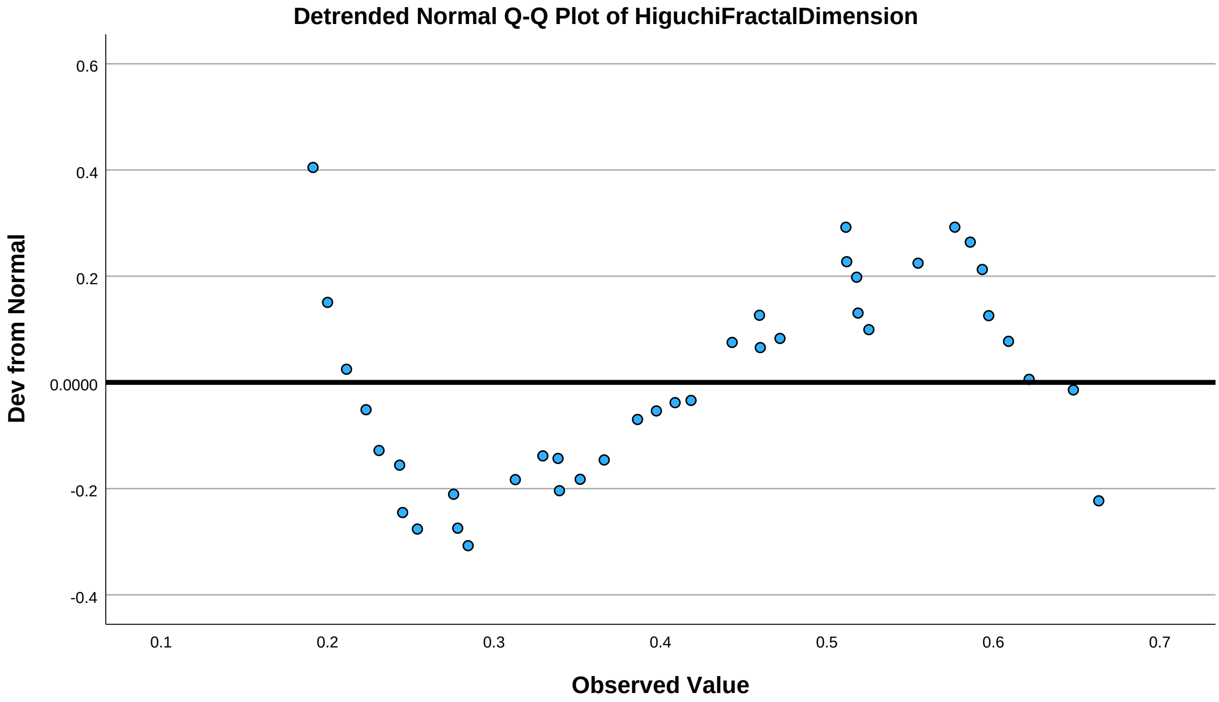
Detrended Normal Q-Q Plot of HiguchiFractalDimension
Dev from Normal
0.6
0.4
0.2
0.0000
-0.2
-0.4
0.1
0.2
0.3
0.4
0.5
0.6
0.7
Observed Value

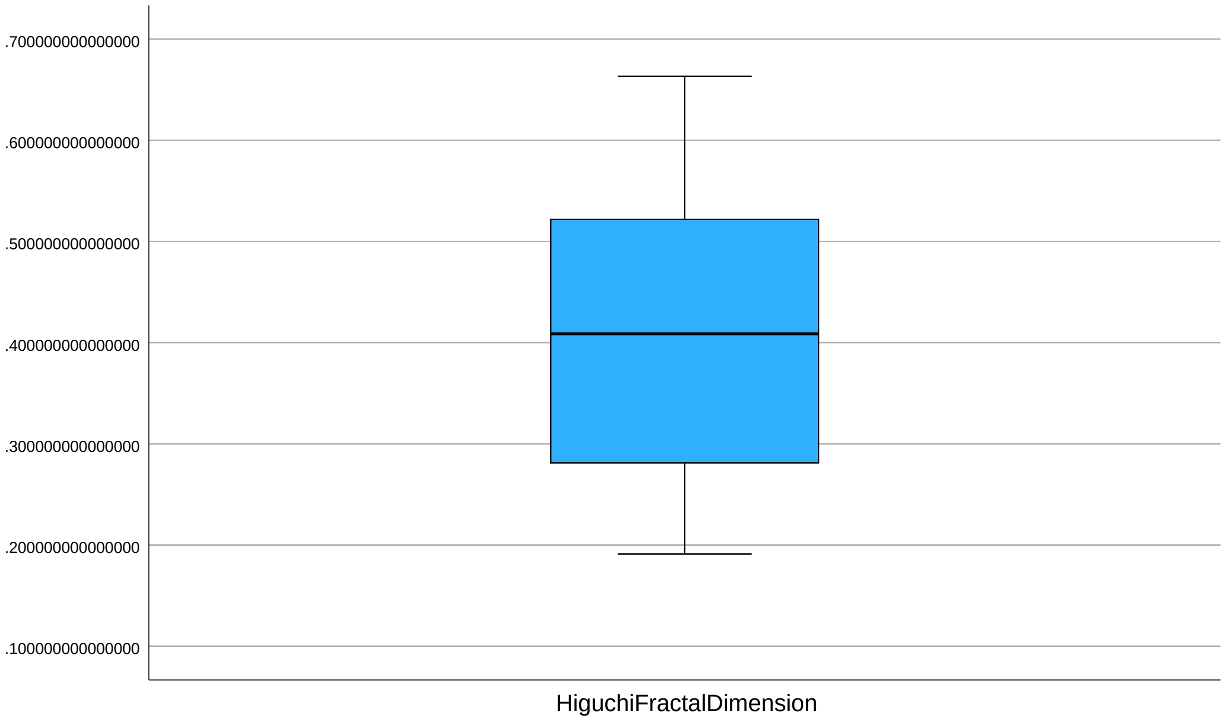

LempelZivComplexity

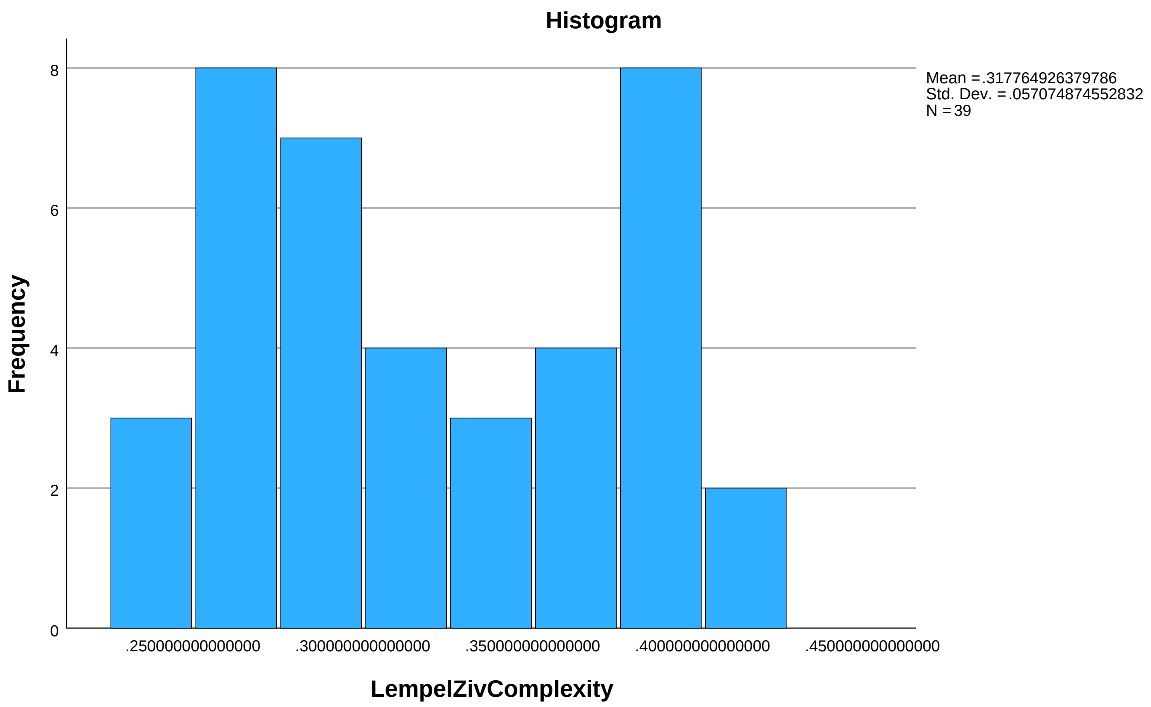


```
LempelZivComplexity Stem-and-Leaf Plot

 Frequency    Stem &  Leaf
```

```
   3.00       2 .  334
  15.00       2 .  555666777778888
   7.00       3 .  0001223
  12.00       3 .  557777889999
   2.00       4 .  12

Stem width:   .1000000
Each leaf:       1 case(s)
```

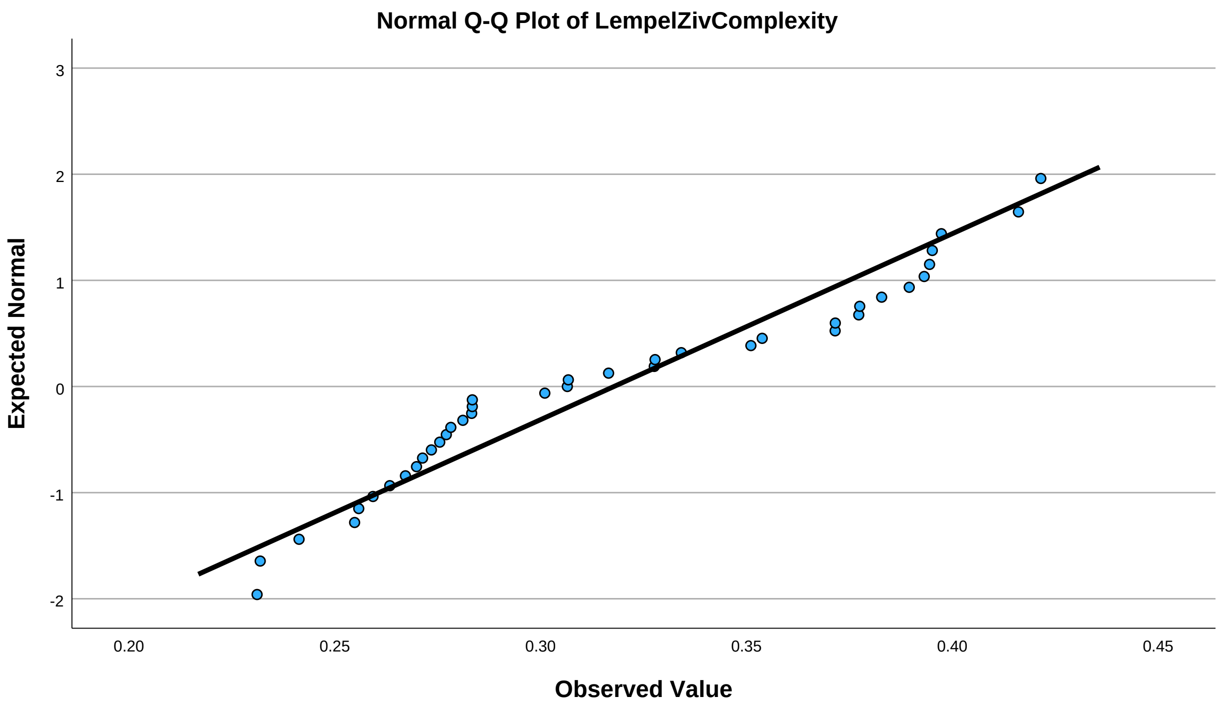

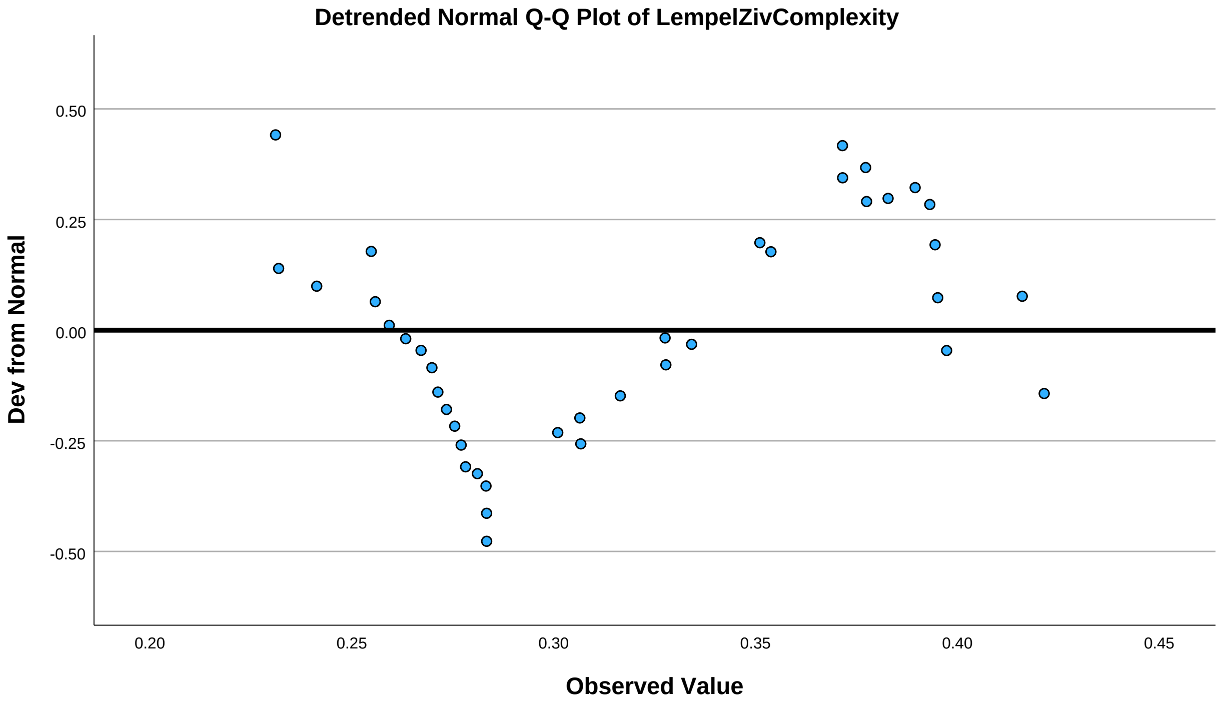

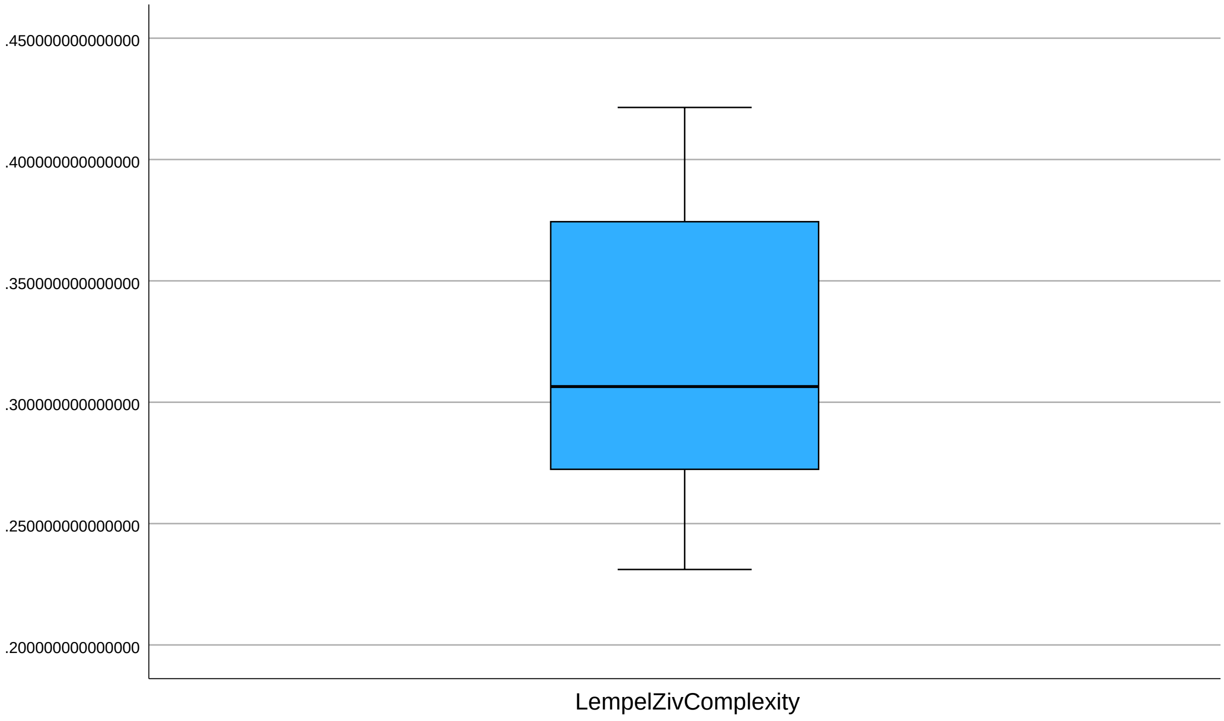

RenyiEntropy

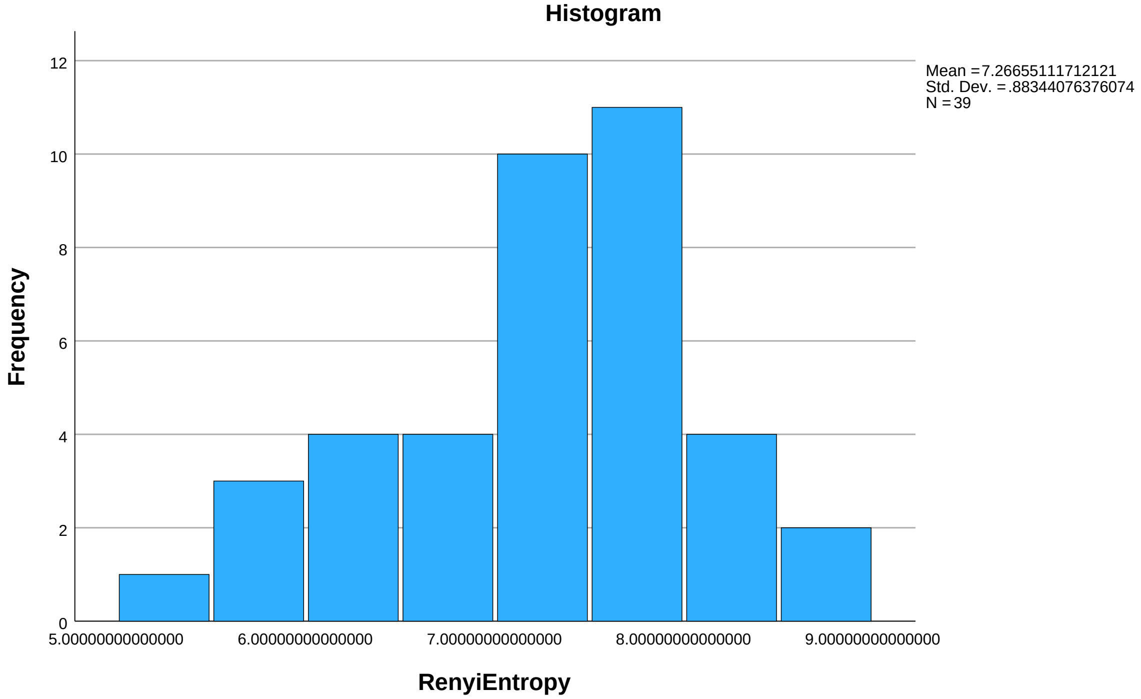


```
RenyiEntropy Stem-and-Leaf Plot

 Frequency    Stem &  Leaf

     1.00 Extremes    (=<5.1)
     3.00        5 .  589
     4.00        6 .  0012
     4.00        6 .  5799
    10.00        7 .  0111233344
    11.00        7 .  56677778899
     4.00        8 .  1334
     2.00        8 .  78

 Stem width:   1.000000
 Each leaf:       1 case(s)
```

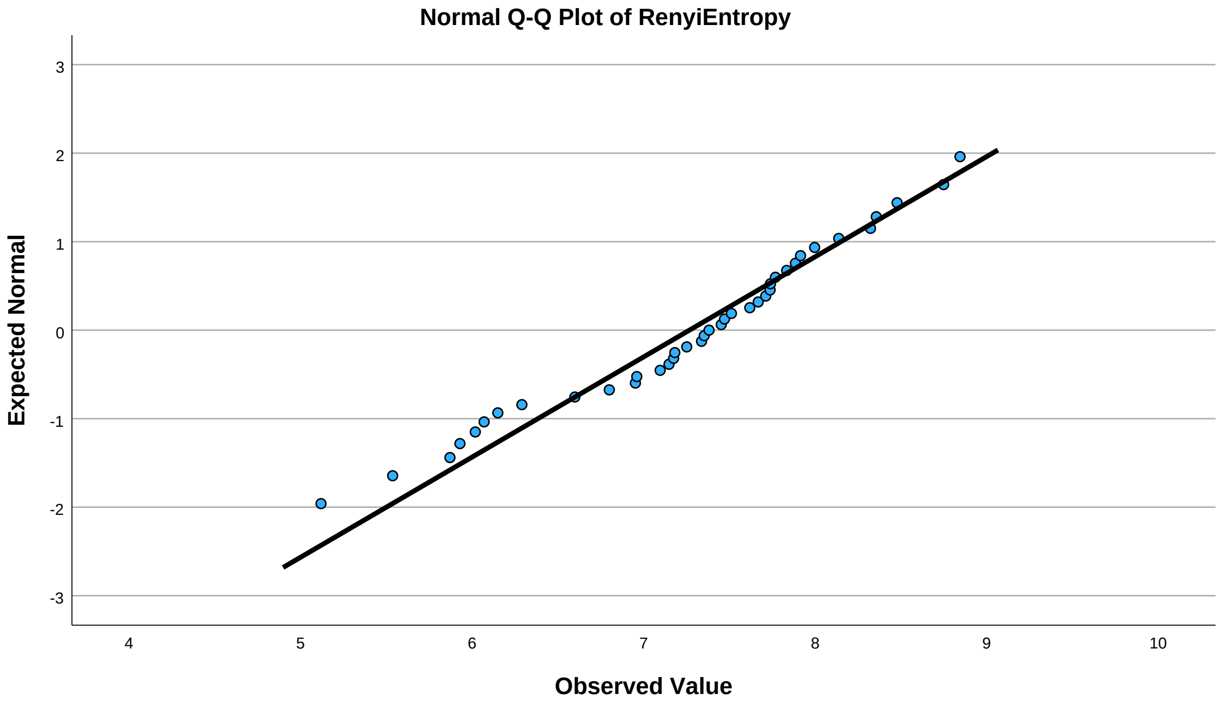
Normal Q-Q Plot of RenyiEntropy
Expected Normal
3
2
1
0
-1
-2
-3
4
5
6
7
8
9
10
Observed Value

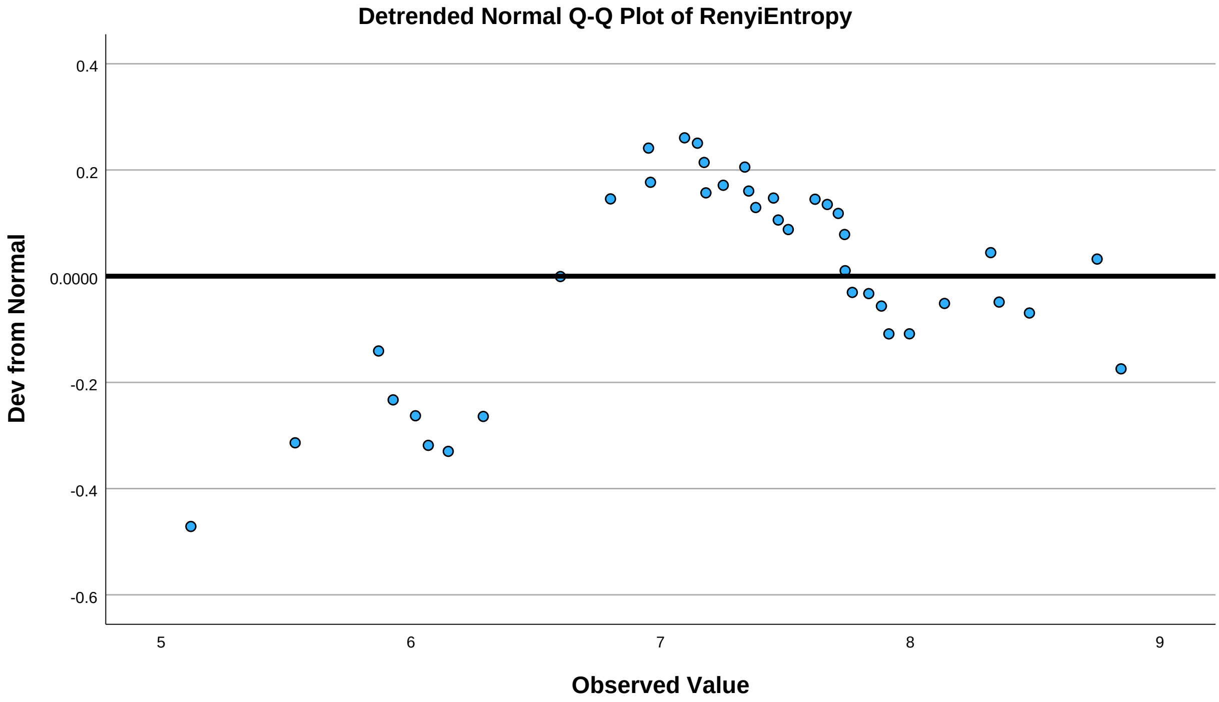
Detrended Normal Q-Q Plot of RenyiEntropy
Dev from Normal
0.4
0.2
0.0000
-0.2
-0.4
-0.6
5
6
7
8
9
Observed Value

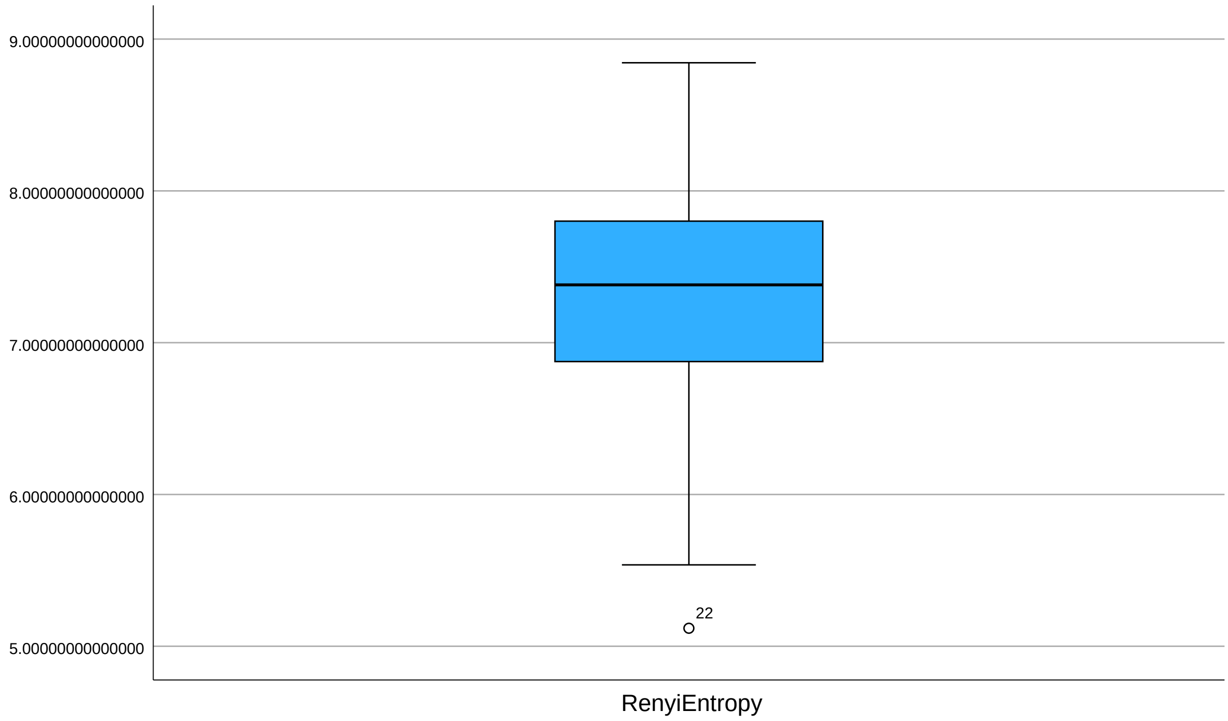

TsallisEntropy

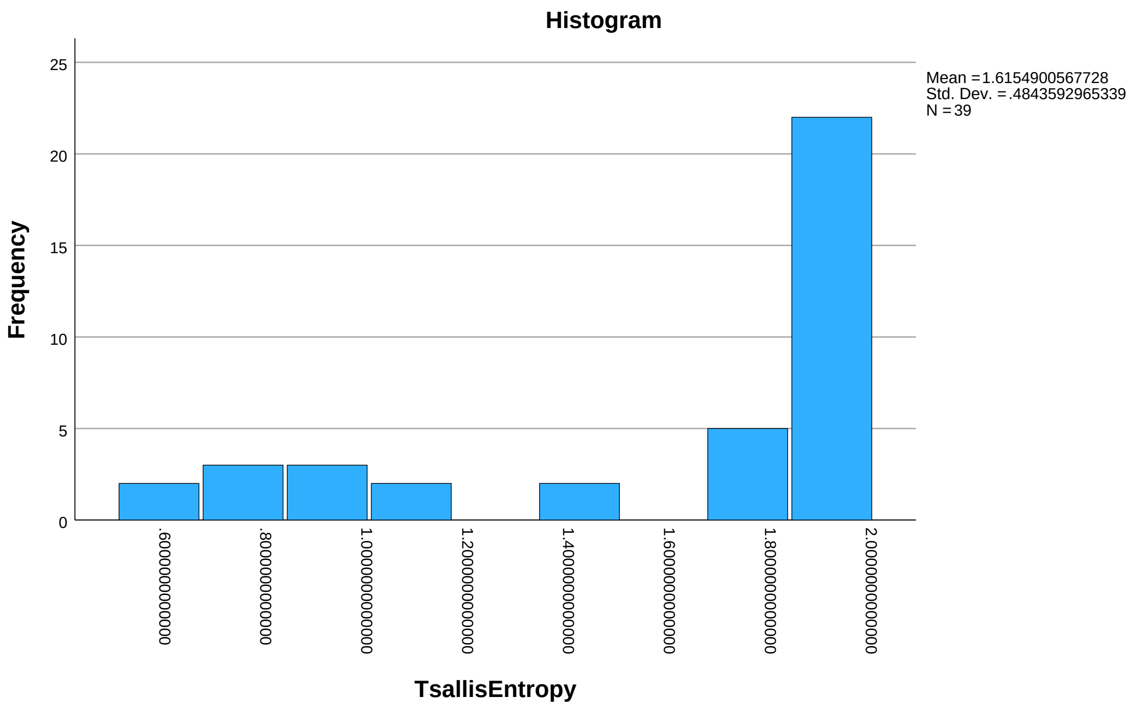

TsallisEntropy Stem-and-Leaf Plot

Frequency   Stem & Leaf

```
 1.00      0 .  5
 4.00      0 .  6677
 3.00      0 .  999
 2.00      1 .  01
 1.00      1 .  3
 1.00      1 .  4
 2.00      1 .  77
25.00      1 .  8888888899999999999999999

Stem width:    1.000000
Each leaf:        1 case(s)
```

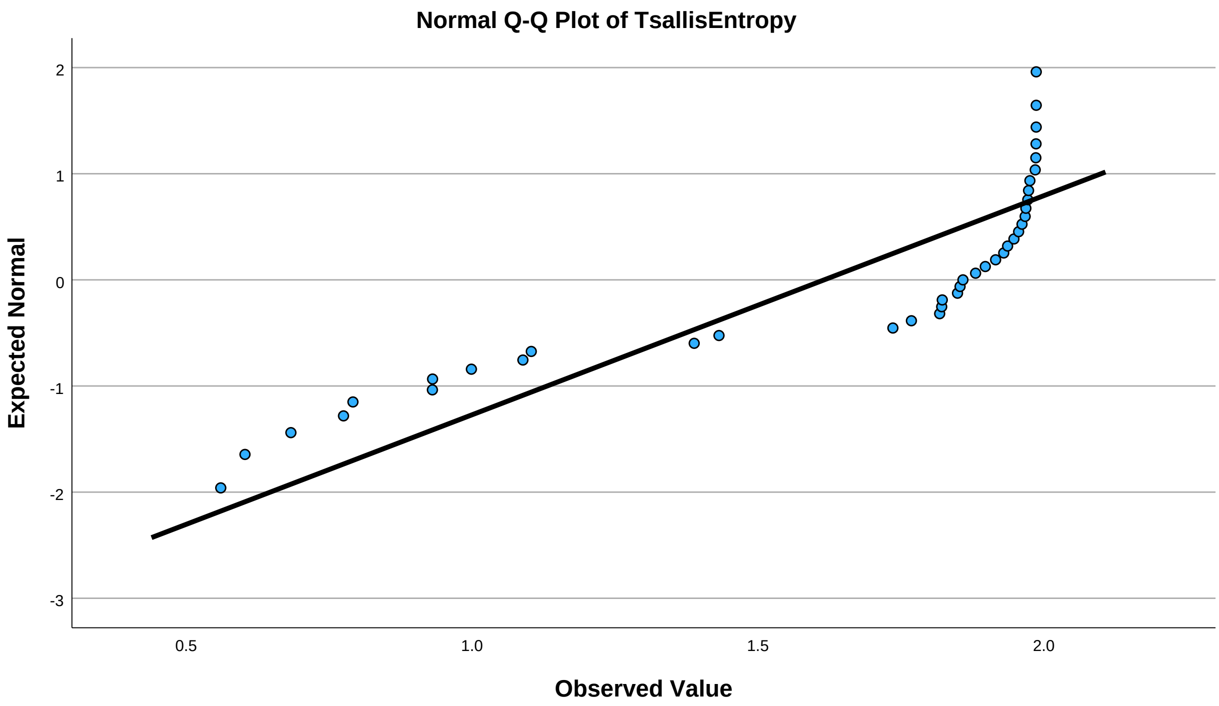

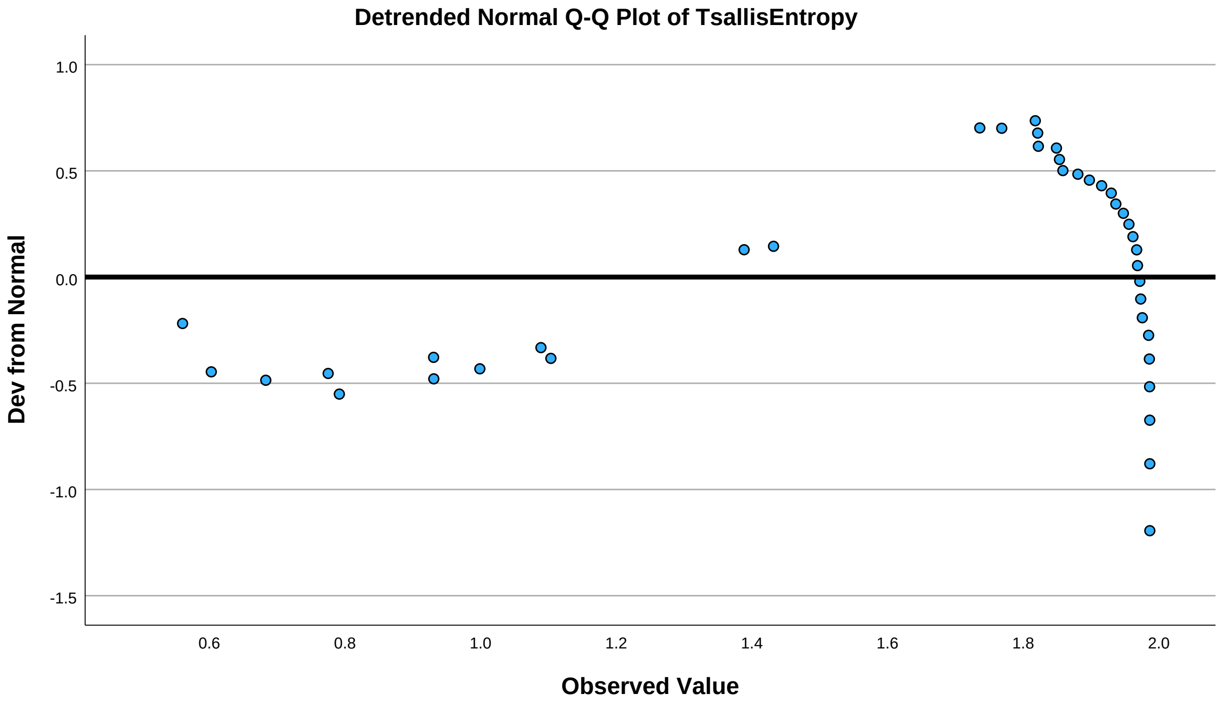
Detrended Normal Q-Q Plot of TsallisEntropy
Dev from Normal
1.0
0.5
0.0
-0.5
-1.0
-1.5
0.6
0.8
1.0
1.2
1.4
1.6
1.8
2.0
Observed Value

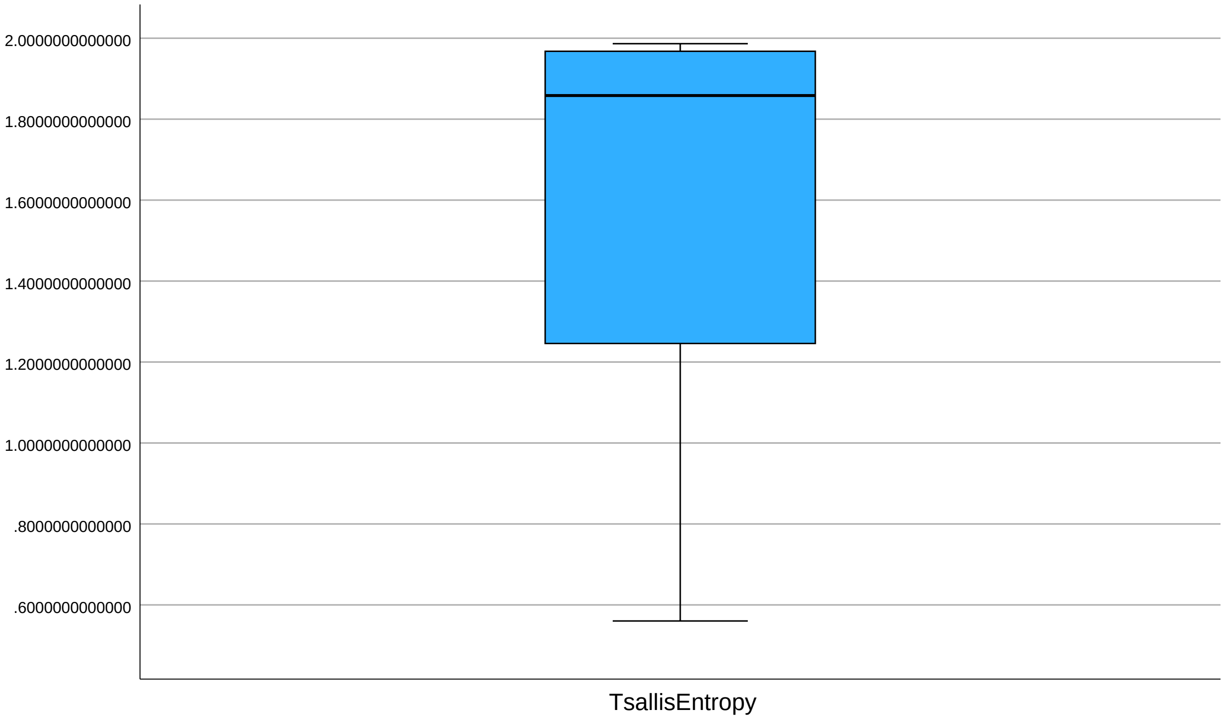
2.0000000000000
1.8000000000000
1.6000000000000
1.4000000000000
1.2000000000000
1.0000000000000
.8000000000000
.6000000000000
TsallisEntropy

PS_4_5

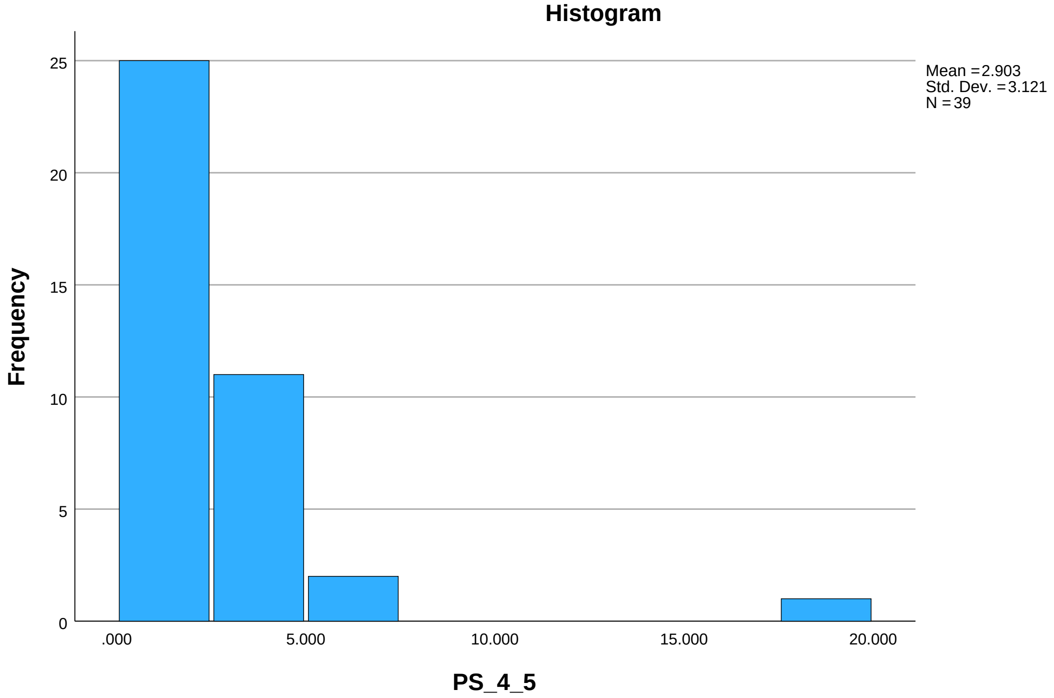


```
PS_4_5 Stem-and-Leaf Plot

 Frequency    Stem &  Leaf

     1.00        0 .  4
     2.00        0 .  89
     7.00        1 .  0012244
     9.00        1 .  556777789
     6.00        2 .  000223
     1.00        2 .  9
     2.00        3 .  12
     3.00        3 .  578
     2.00        4 .  11
     3.00        4 .  567
      .00        5 .
     2.00        5 .  56
     1.00 Extremes    (>=20.0)

 Stem width:      1.000
 Each leaf:       1 case(s)
```

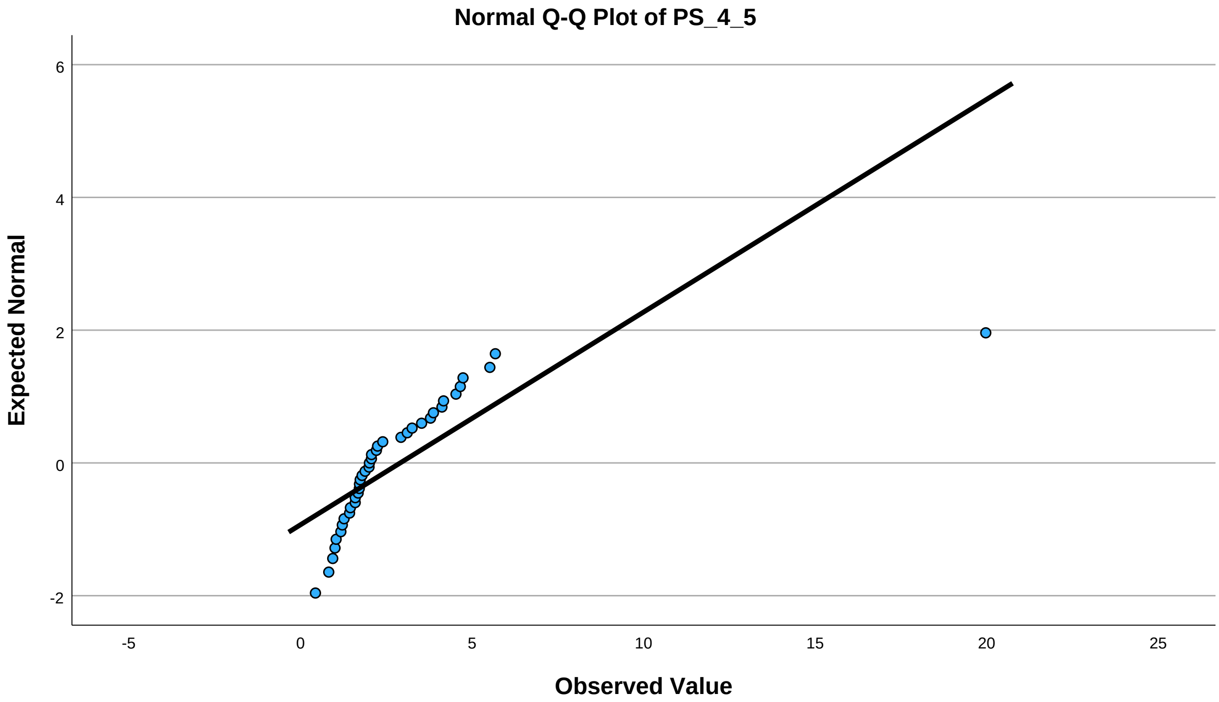
Normal Q-Q Plot of PS_4_5
Expected Normal
Observed Value
6
4
2
0
-2
-5
0
5
10
15
20
25

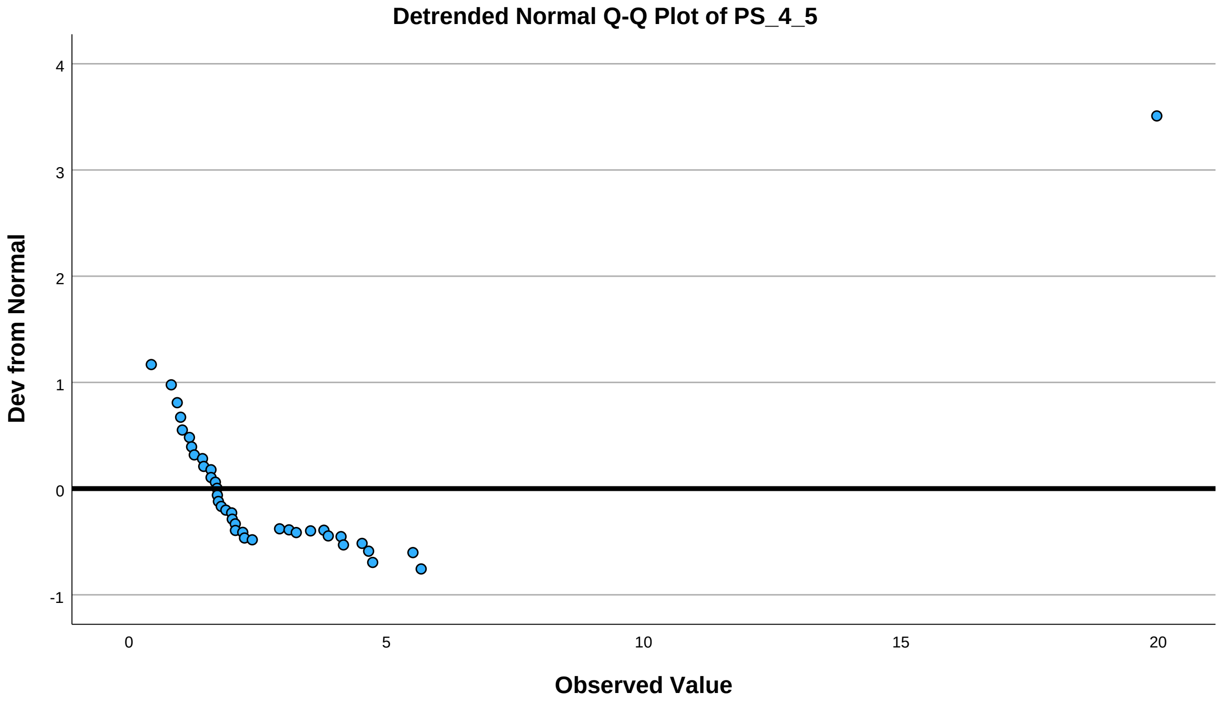
Detrended Normal Q-Q Plot of PS_4_5
Dev from Normal
Observed Value
4
3
2
1
0
-1
0
5
10
15
20

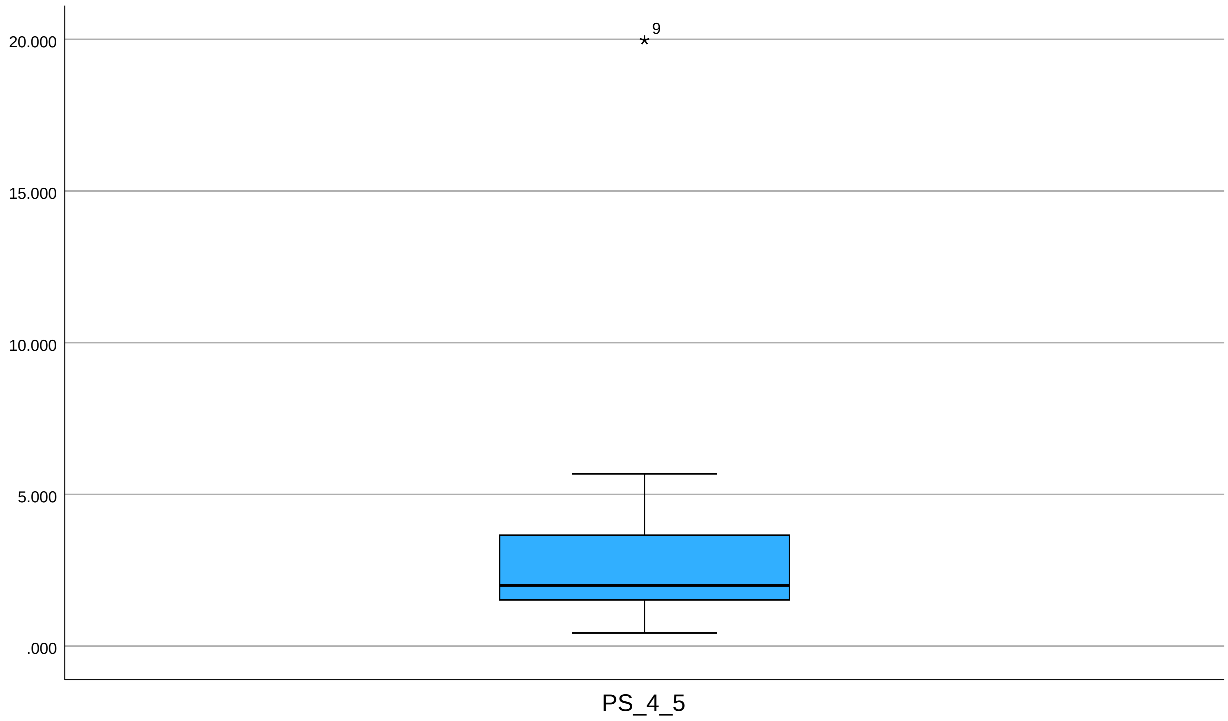

PS_5_6

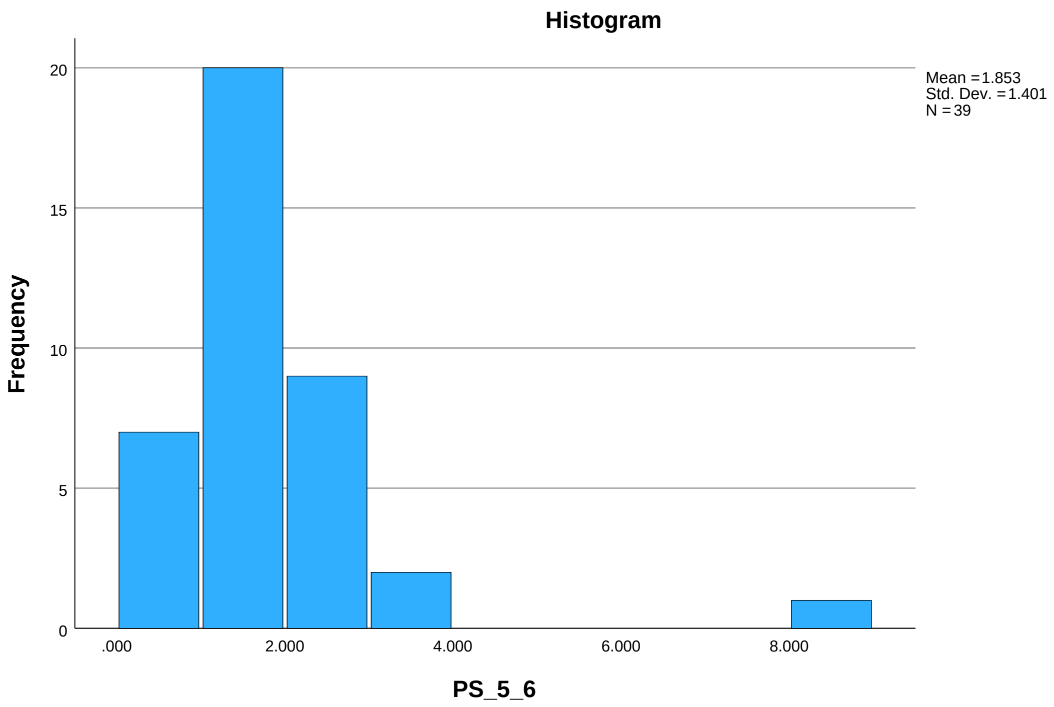

PS_5_6 Stem-and-Leaf Plot

Frequency   Stem & Leaf

```
  1.00      0 . 3
  6.00      0 . 556688
 15.00      1 . 000112222334444
  5.00      1 . 56779
  2.00      2 . 03
  7.00      2 . 5678999
   .00      3 .
  2.00      3 . 88
  1.00 Extremes    (>=8.5)

Stem width:     1.000
Each leaf:       1 case(s)
```

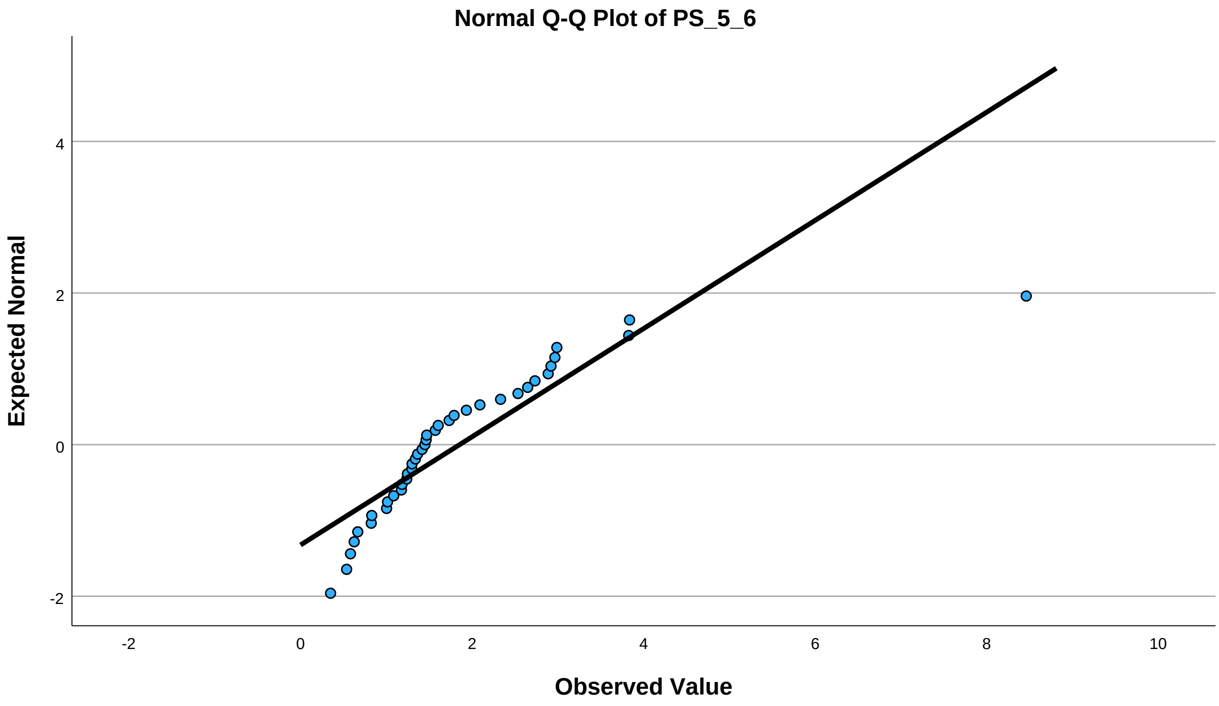

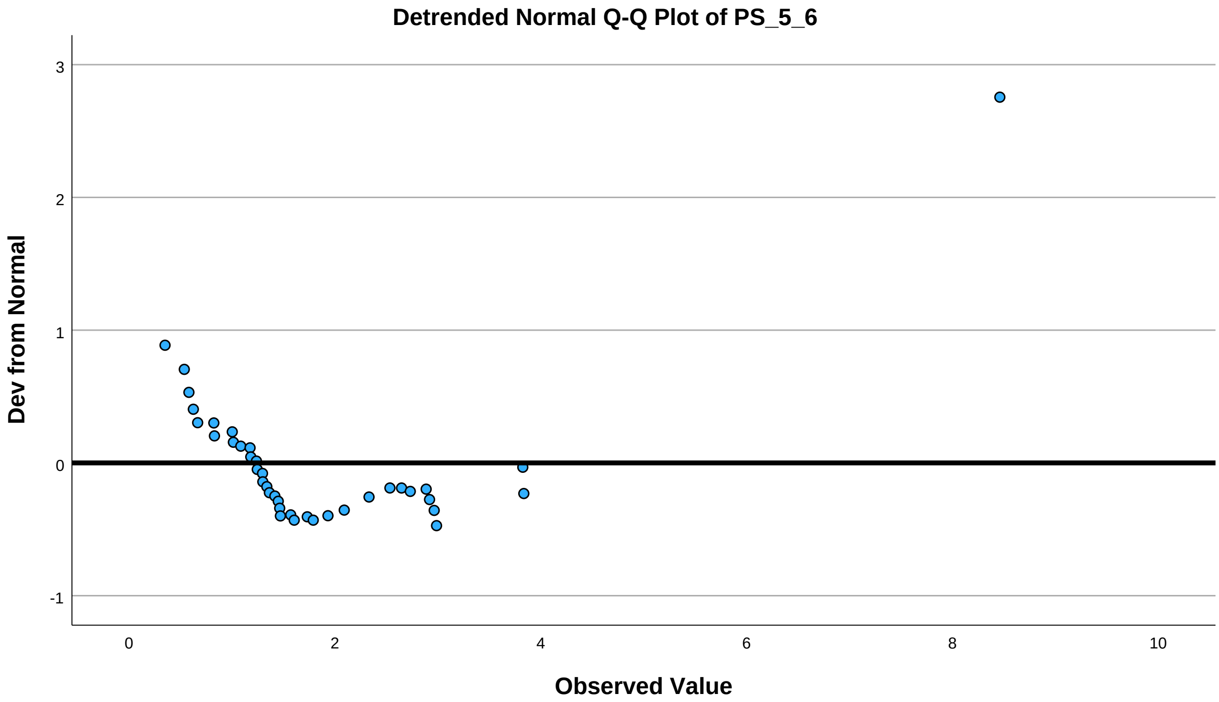

Detrended Normal Q-Q Plot of PS_5_6
Dev from Normal
3
2
1
0
-1
0
2
4
6
8
10
Observed Value

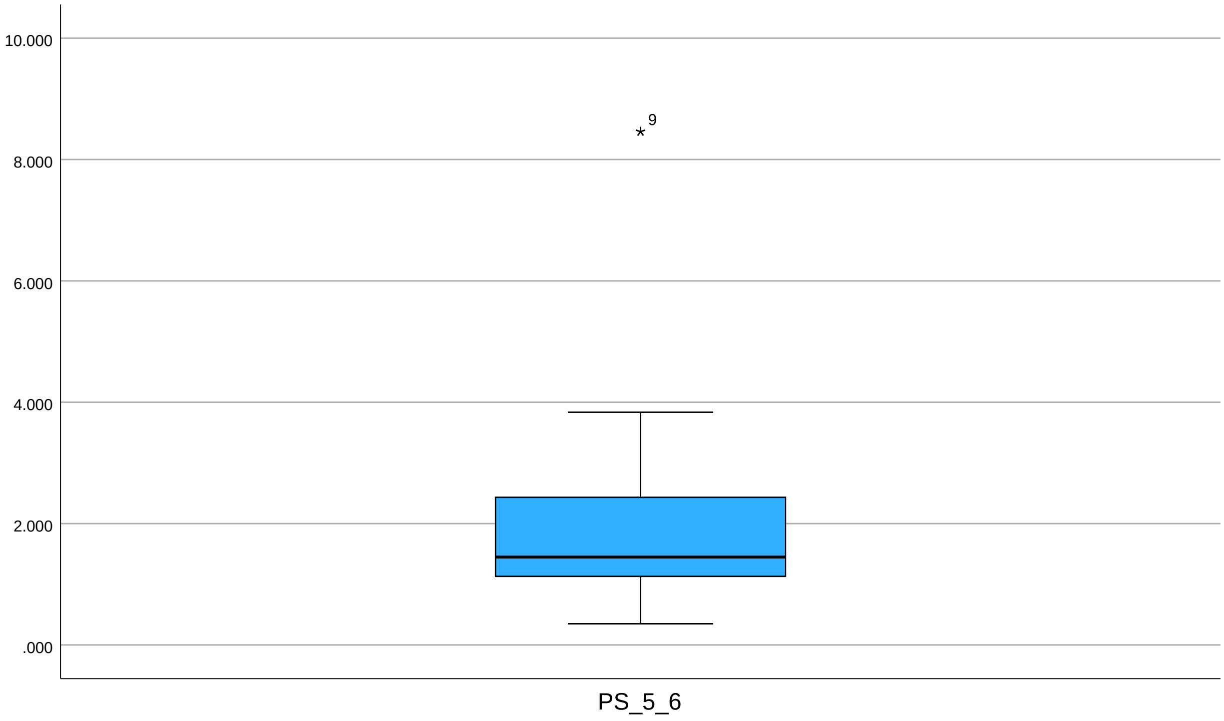

10.000
8.000
6.000
4.000
2.000
.000
9
PS_5_6

PS_6_7

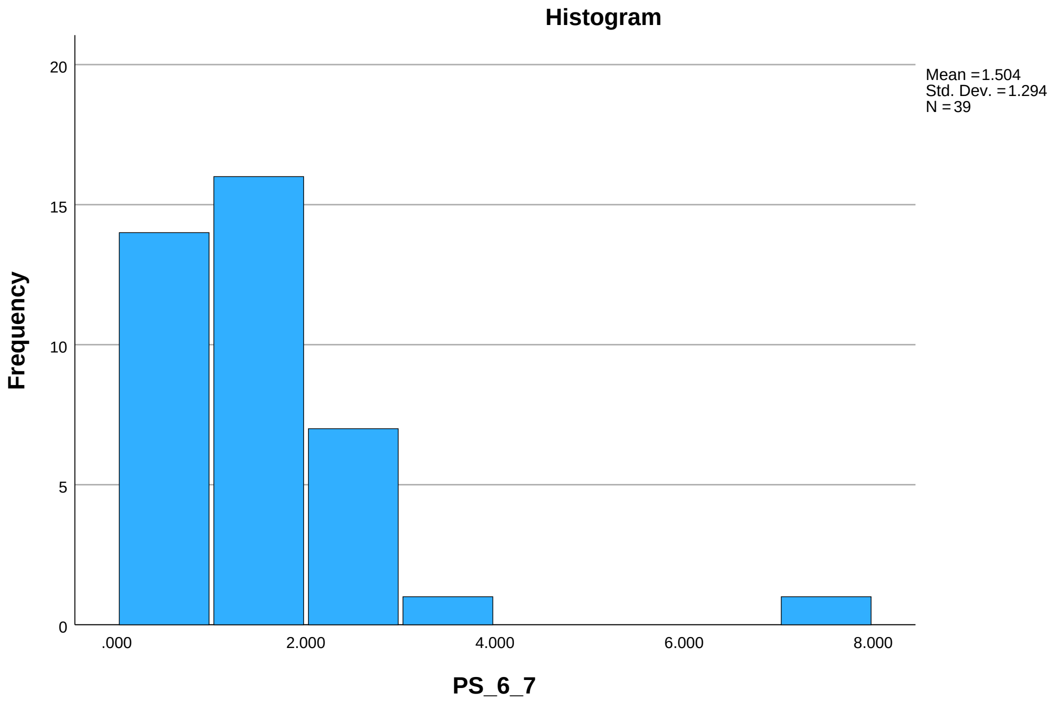


```
PS_6_7 Stem-and-Leaf Plot

 Frequency    Stem &  Leaf

     5.00        0 .  24444
     9.00        0 .  666778899
    12.00        1 .  000111112344
     4.00        1 .  6679
     6.00        2 .  122233
     1.00        2 .  6
     2.00 Extremes    (>=3.9)

 Stem width:      1.000
 Each leaf:       1 case(s)
```

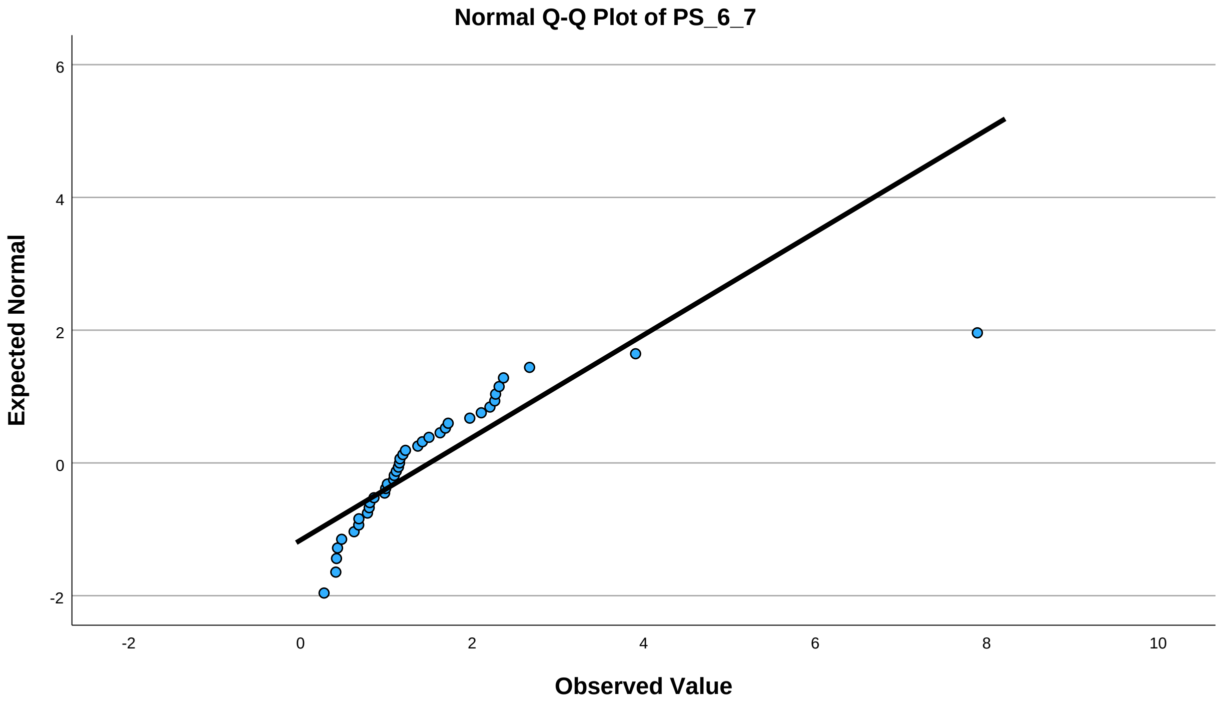
Normal Q-Q Plot of PS_6_7
Expected Normal
Observed Value
6
4
2
0
-2
-2
0
2
4
6
8
10

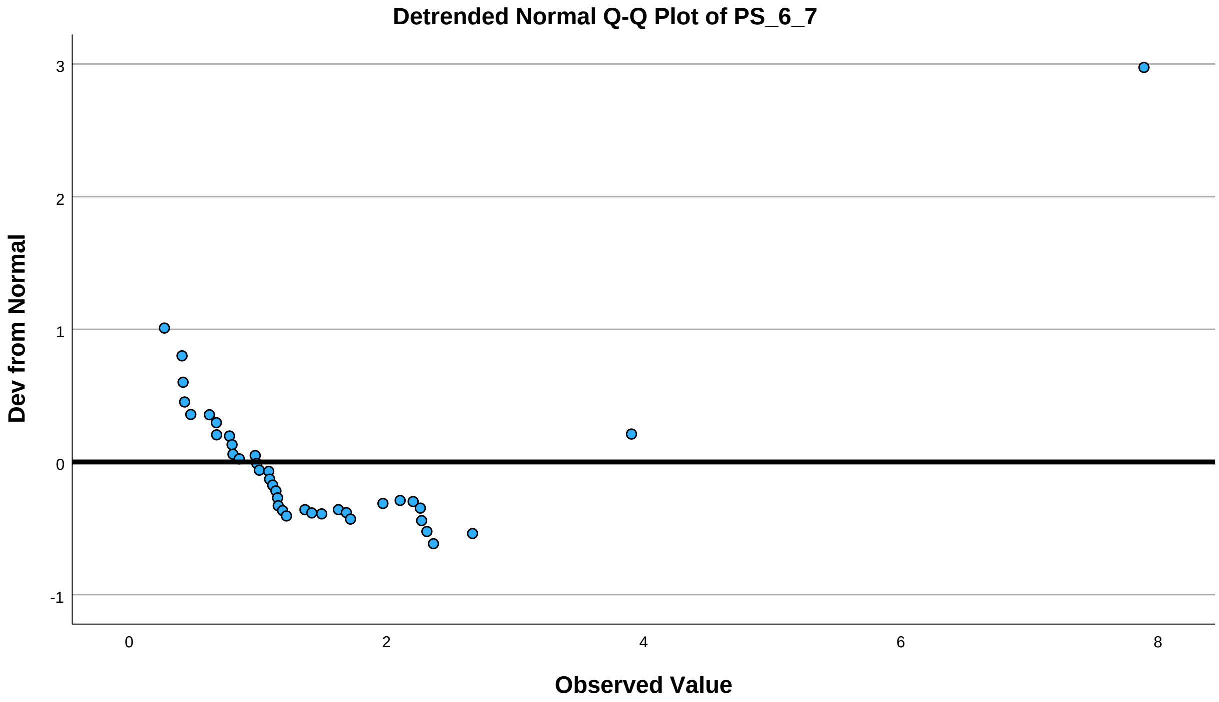
Detrended Normal Q-Q Plot of PS_6_7
Dev from Normal
Observed Value
3
2
1
0
-1
0
2
4
6
8

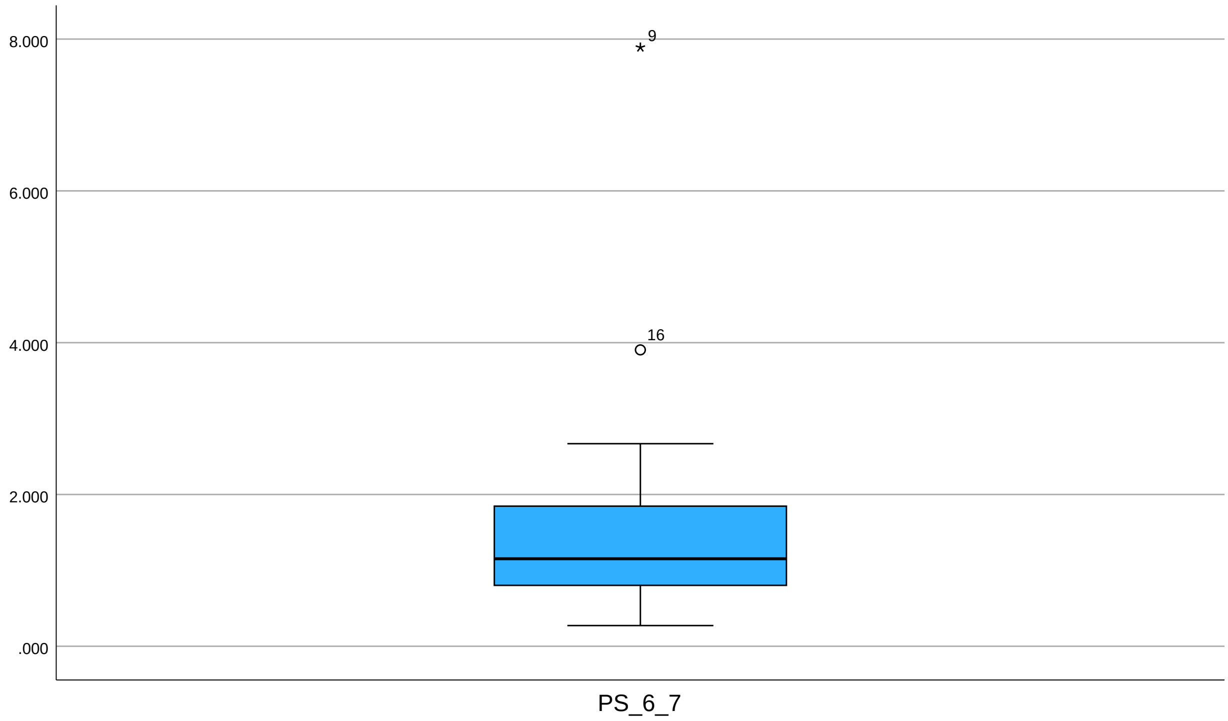

PS_7_8

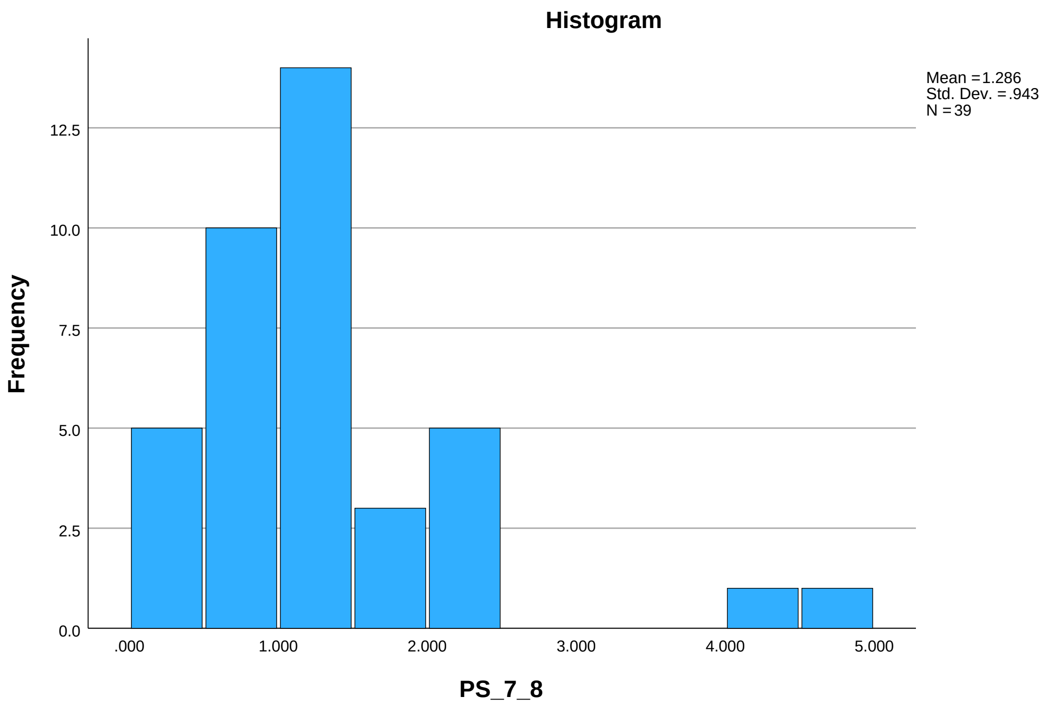

PS_7_8 Stem-and-Leaf Plot

Frequency Stem & Leaf

```
  5.00      0 . 23334
 10.00       0 . 5556677789
 14.00       1 .  00000111113344
  3.00      1 . 678
  5.00      2 . 00134
  2.00 Extremes    (>=4.1)

Stem width:     1.000
Each leaf:       1 case(s)
```

Normal Q-Q Plot of PS_7_8

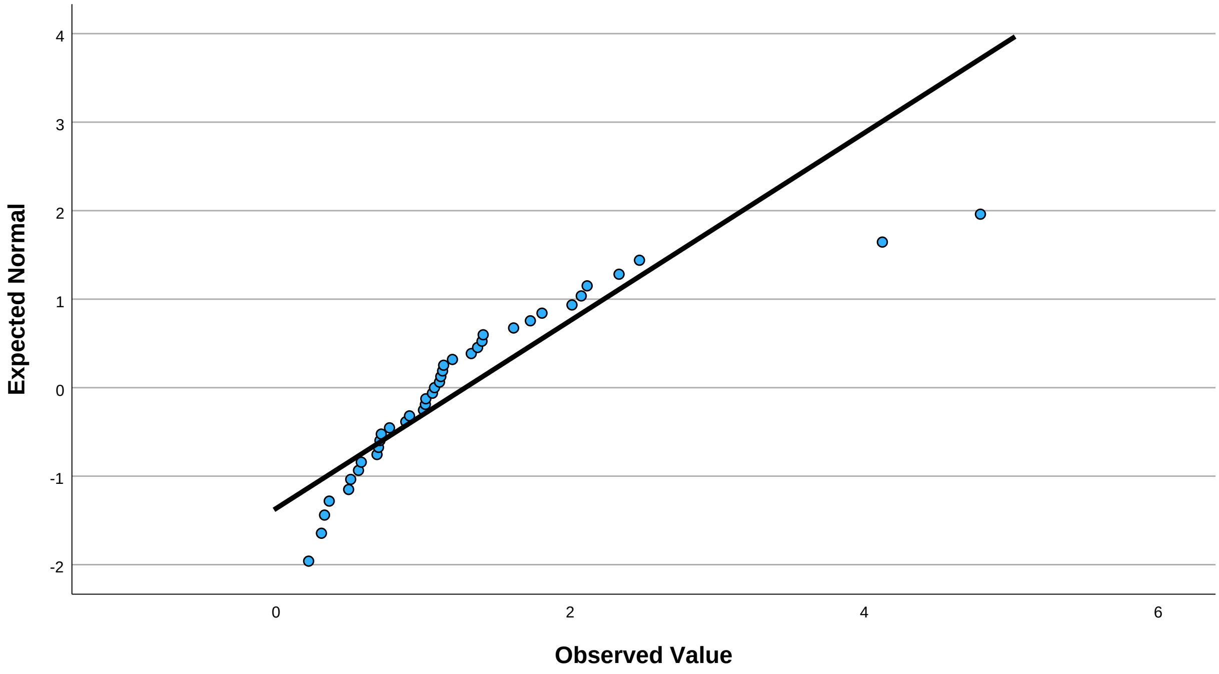

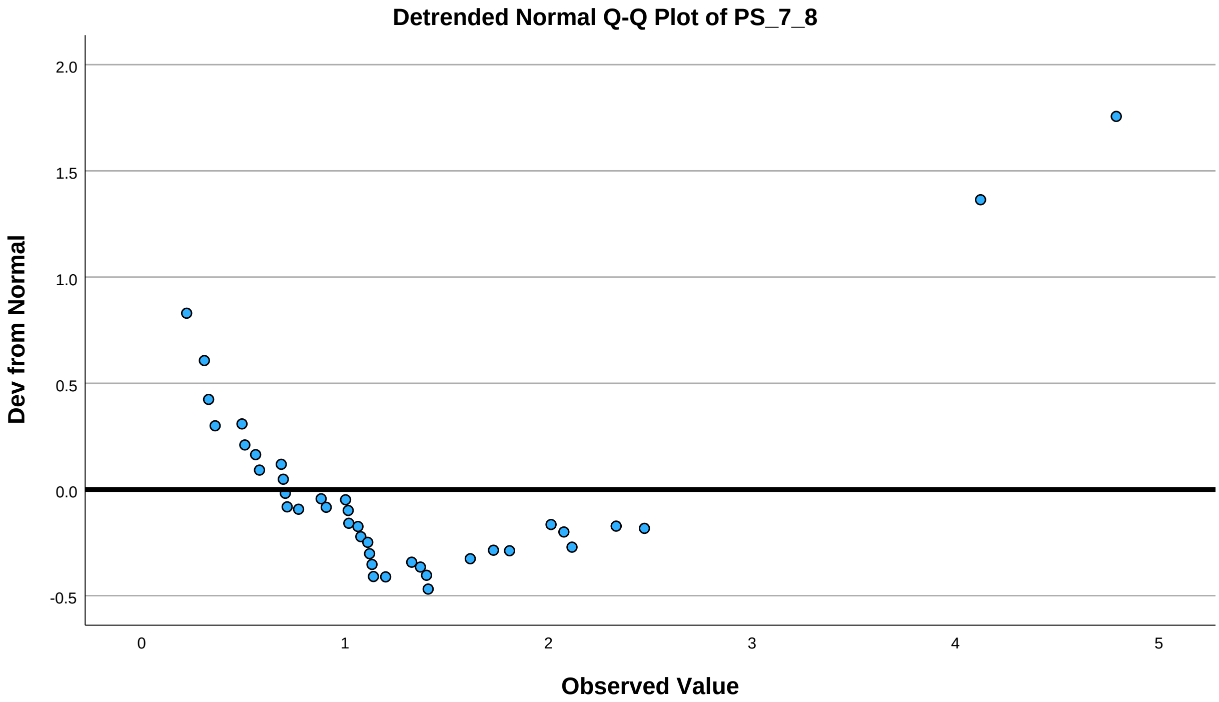
Detrended Normal Q-Q Plot of PS_7_8
Dev from Normal
2.0
1.5
1.0
0.5
0.0
-0.5
0
1
2
3
4
5
Observed Value

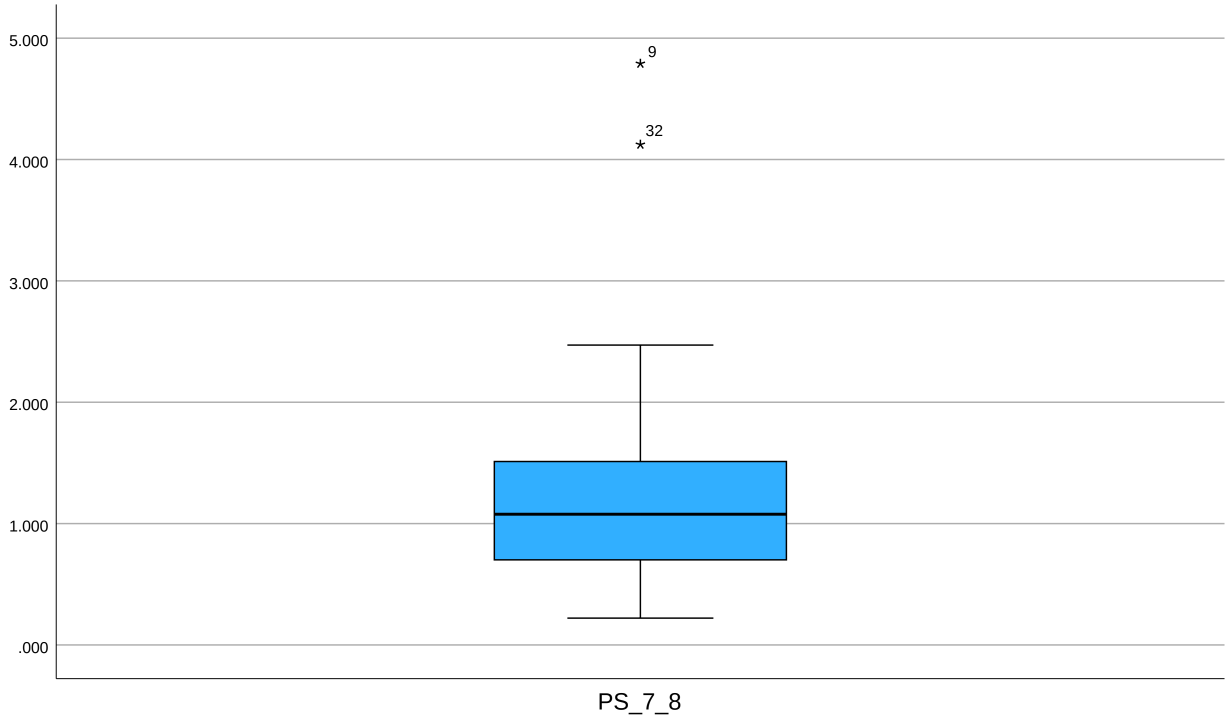
5.000
4.000
3.000
2.000
1.000
.000
9
32
PS_7_8

PS_8_9

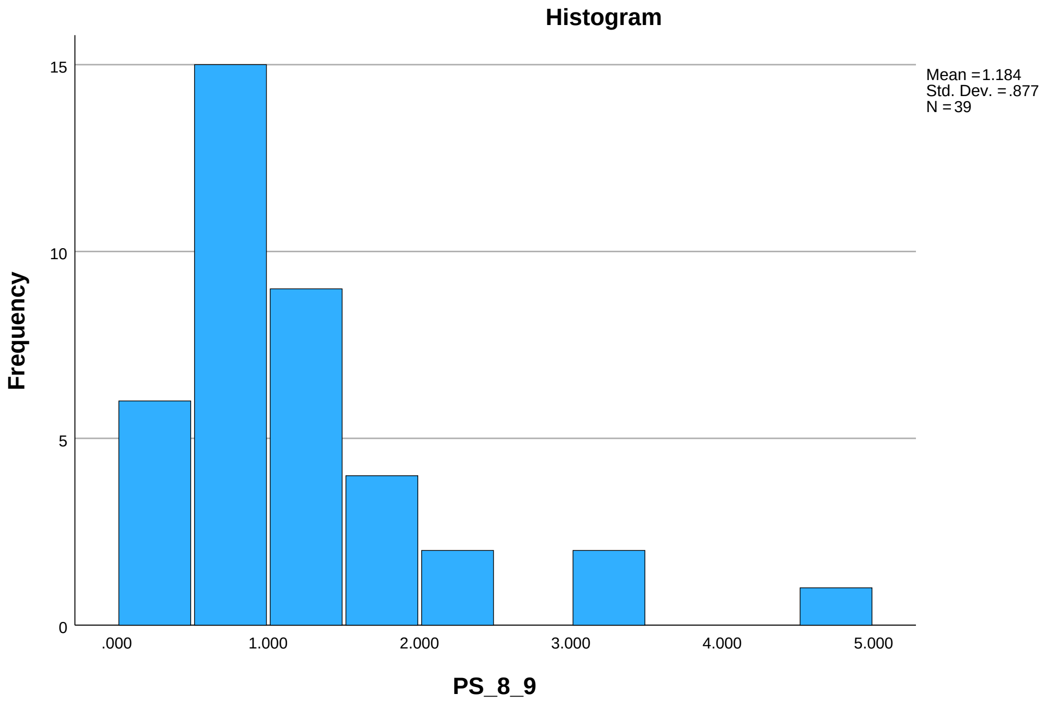


```
PS_8_9 Stem-and-Leaf Plot

 Frequency    Stem &  Leaf

     6.00        0 .  222334
    15.00        0 .  555667777788899
     9.00        1 .  000111334
     4.00        1 .  5889
     2.00        2 .  01
     3.00 Extremes    (>=3.1)

 Stem width:      1.000
 Each leaf:       1 case(s)
```

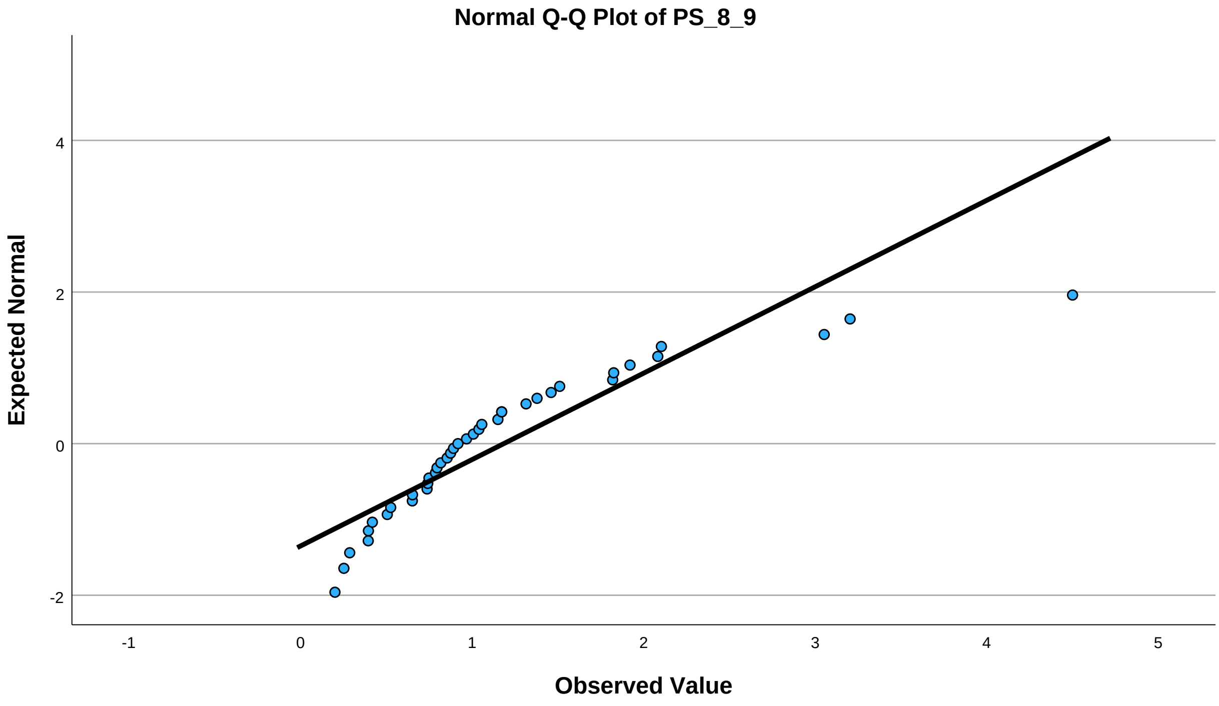
Normal Q-Q Plot of PS_8_9
Expected Normal
4
2
0
-2
-1
0
1
2
3
4
5
Observed Value

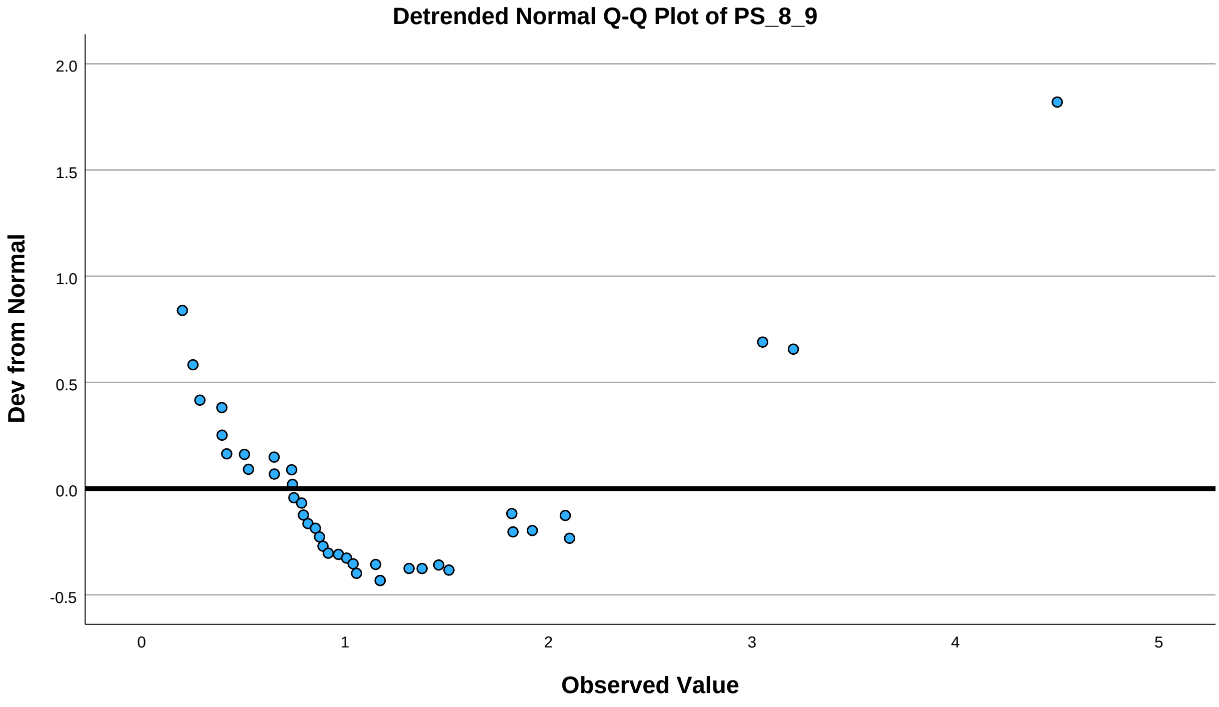
Detrended Normal Q-Q Plot of PS_8_9
Dev from Normal
2.0
1.5
1.0
0.5
0.0
-0.5
0
1
2
3
4
5
Observed Value

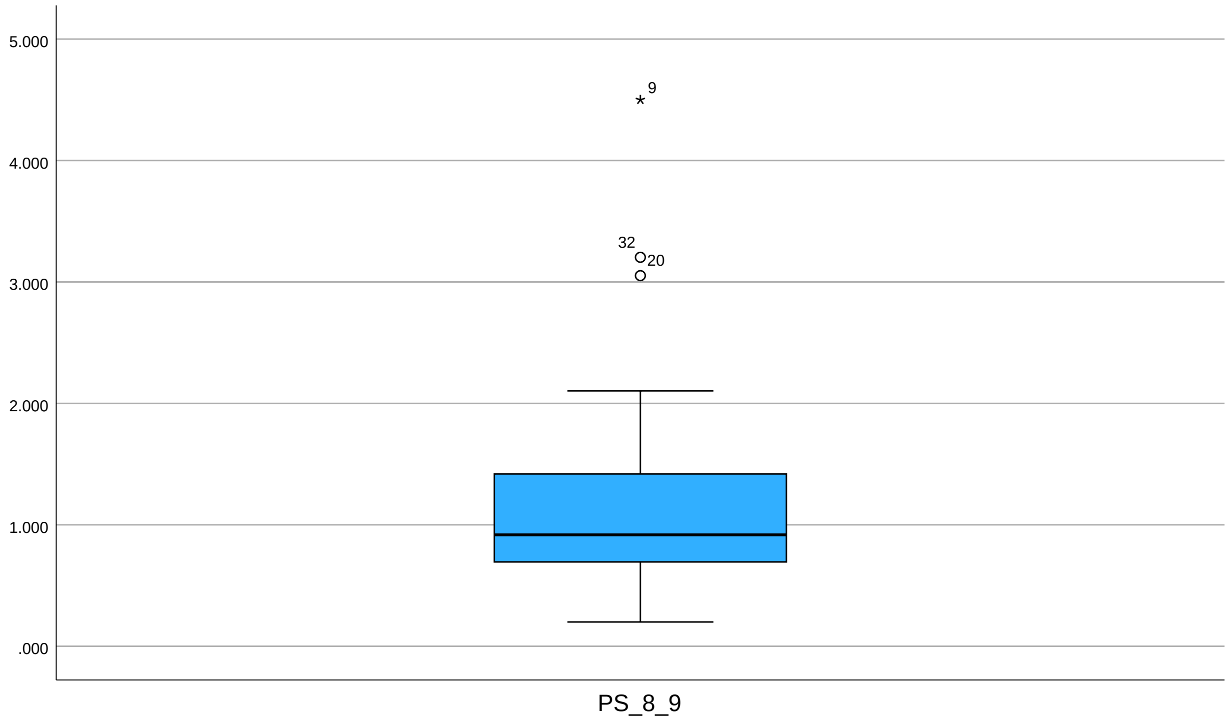

PS_9_10

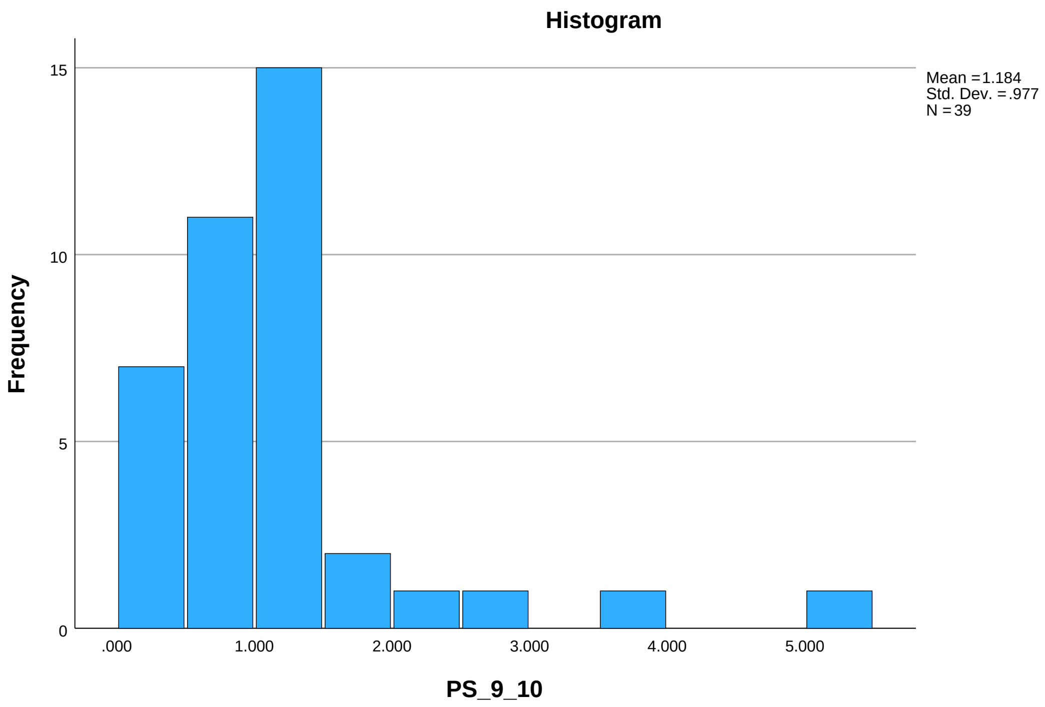

PS_9_10 Stem-and-Leaf Plot

Frequency   Stem & Leaf

```
    1.00        0 .  1
    3.00        0 .  222
    6.00        0 .  444555
    5.00        0 .  66677
    3.00        0 .  999
    7.00        1 .  0000111
    5.00        1 .  22233
    3.00        1 .  444
    2.00        1 .  66
    4.00 Extremes    (>=2.5)

Stem width:      1.000
Each leaf:       1 case(s)
```

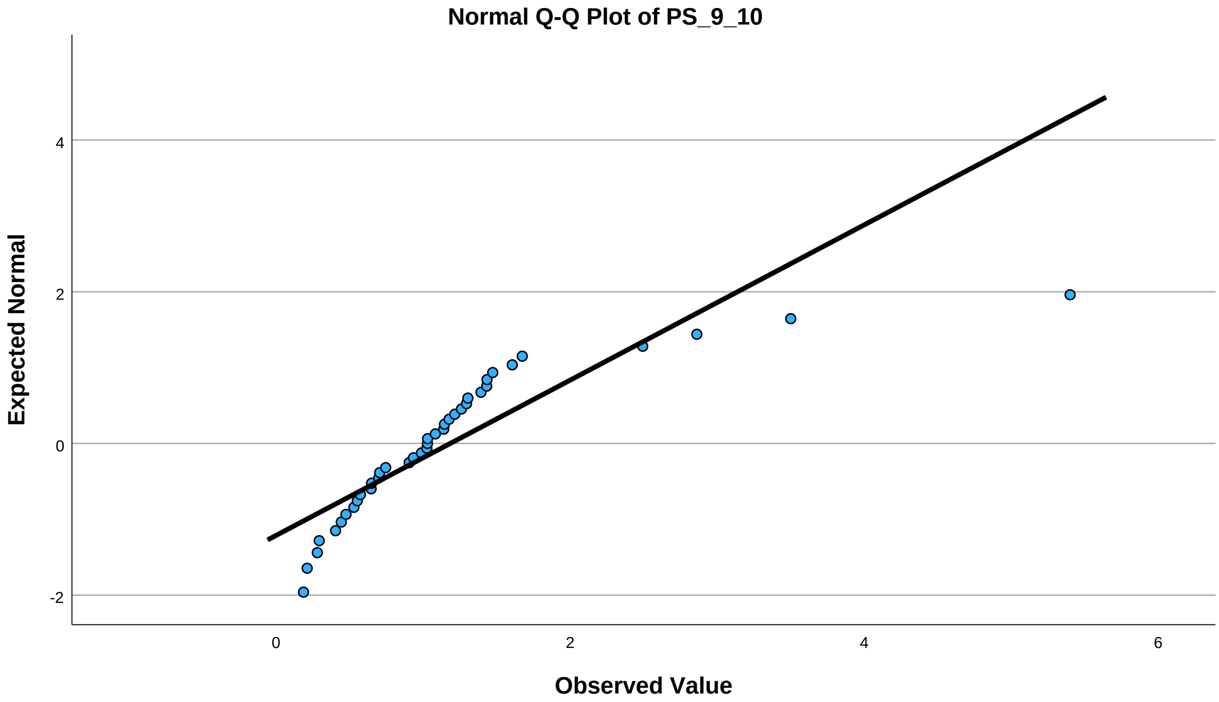

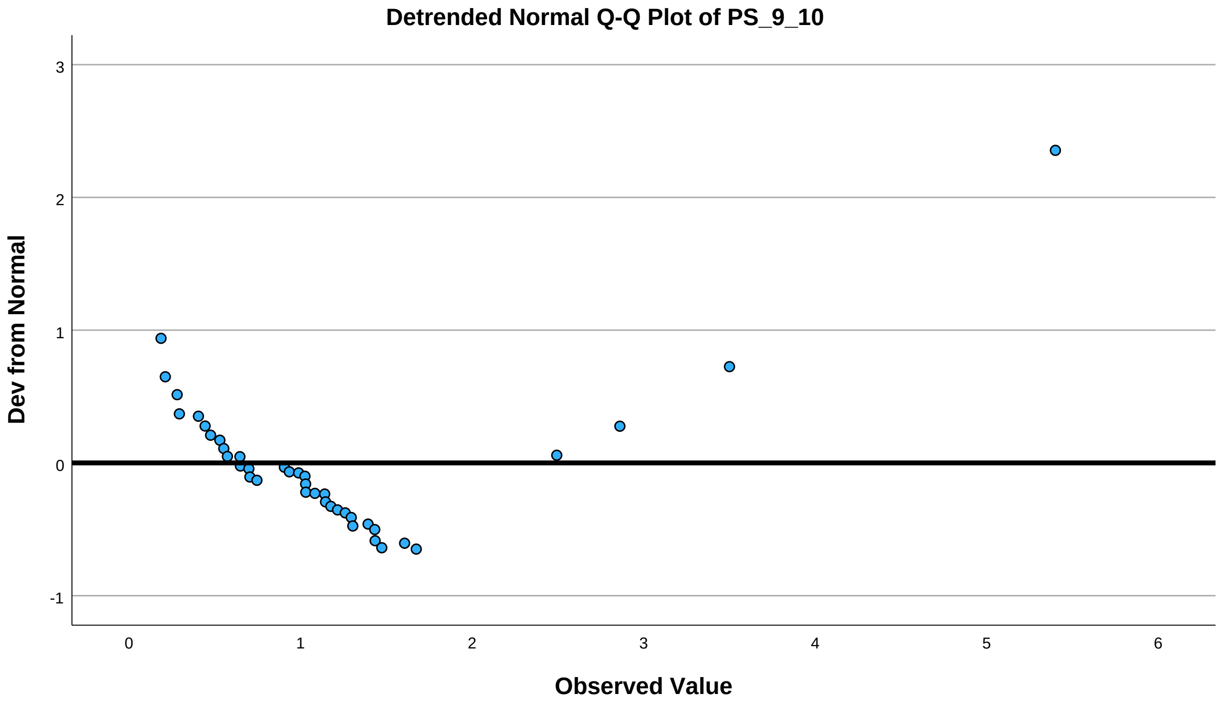
Detrended Normal Q-Q Plot of PS_9_10
Dev from Normal
3
2
1
0
-1
0
1
2
3
4
5
6
Observed Value

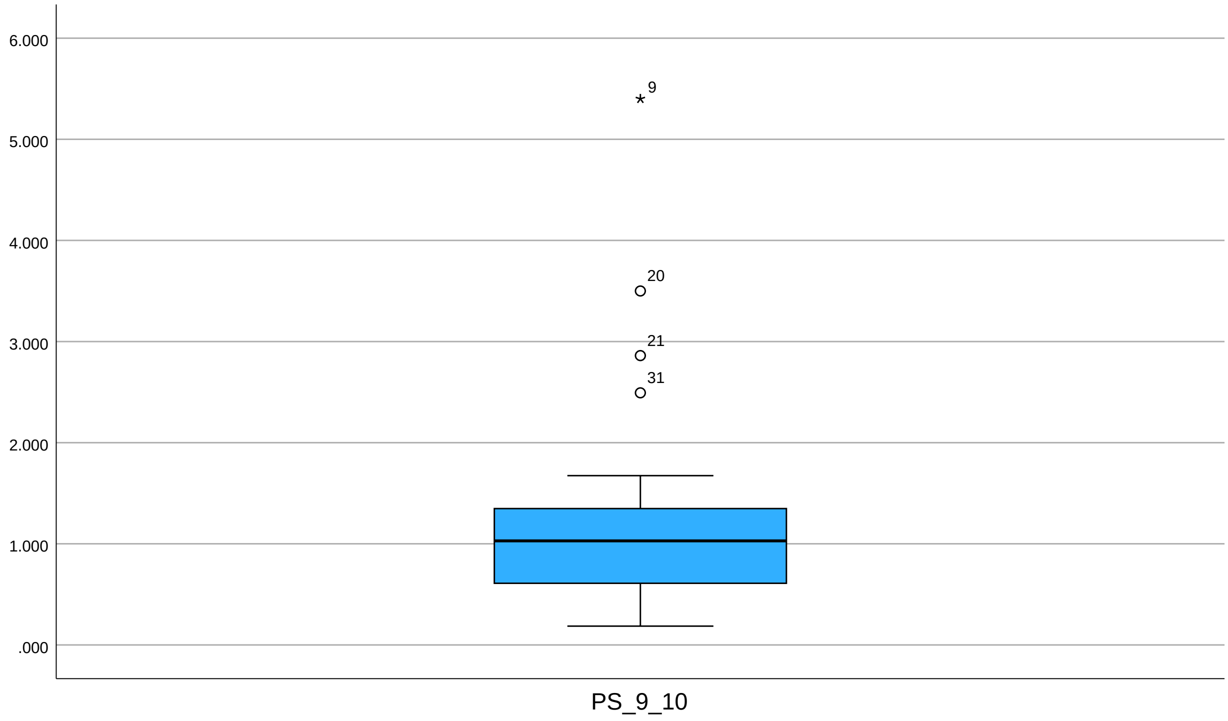
6.000
5.000
4.000
3.000
2.000
1.000
.000
9
20
21
31
PS_9_10

PS_10_11

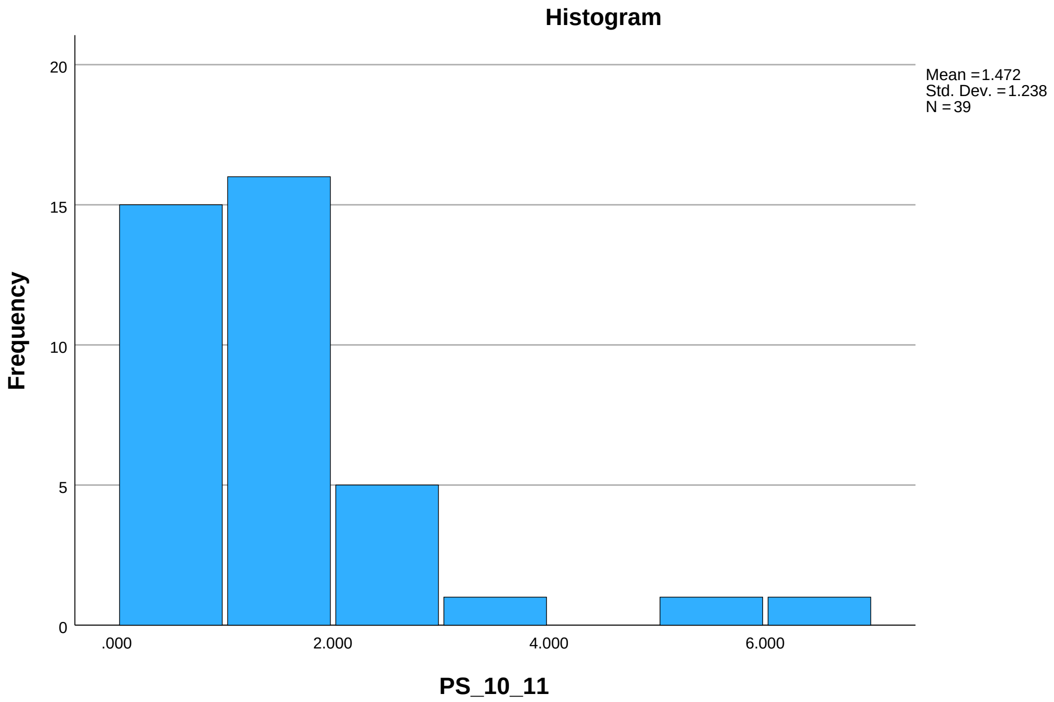

PS_10_11 Stem-and-Leaf Plot

```
 Frequency    Stem &  Leaf

     8.00        0 .  12333344
     7.00        0 .  5678889
    12.00        1 .  001122334444
     4.00        1 .  6899
     5.00        2 .  11124
     3.00 Extremes    (>=3.8)

 Stem width:      1.000
 Each leaf:       1 case(s)
```

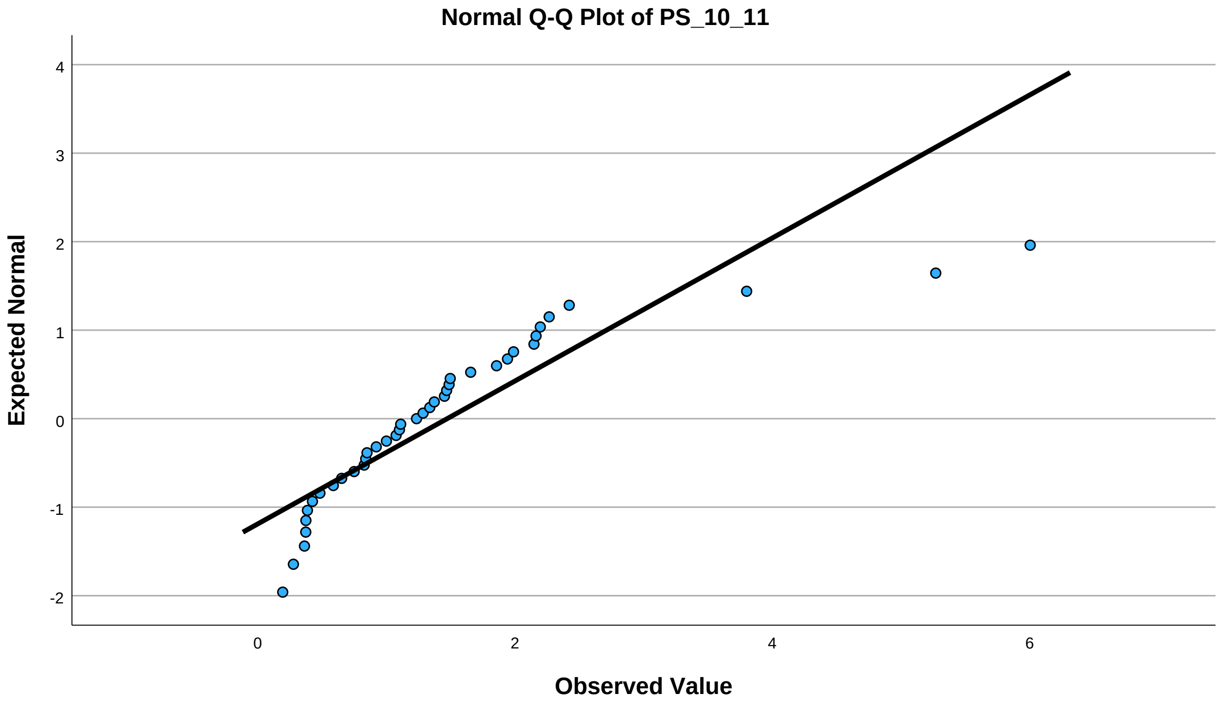
Normal Q-Q Plot of PS_10_11
Expected Normal
Observed Value
4
3
2
1
0
-1
-2
0
2
4
6

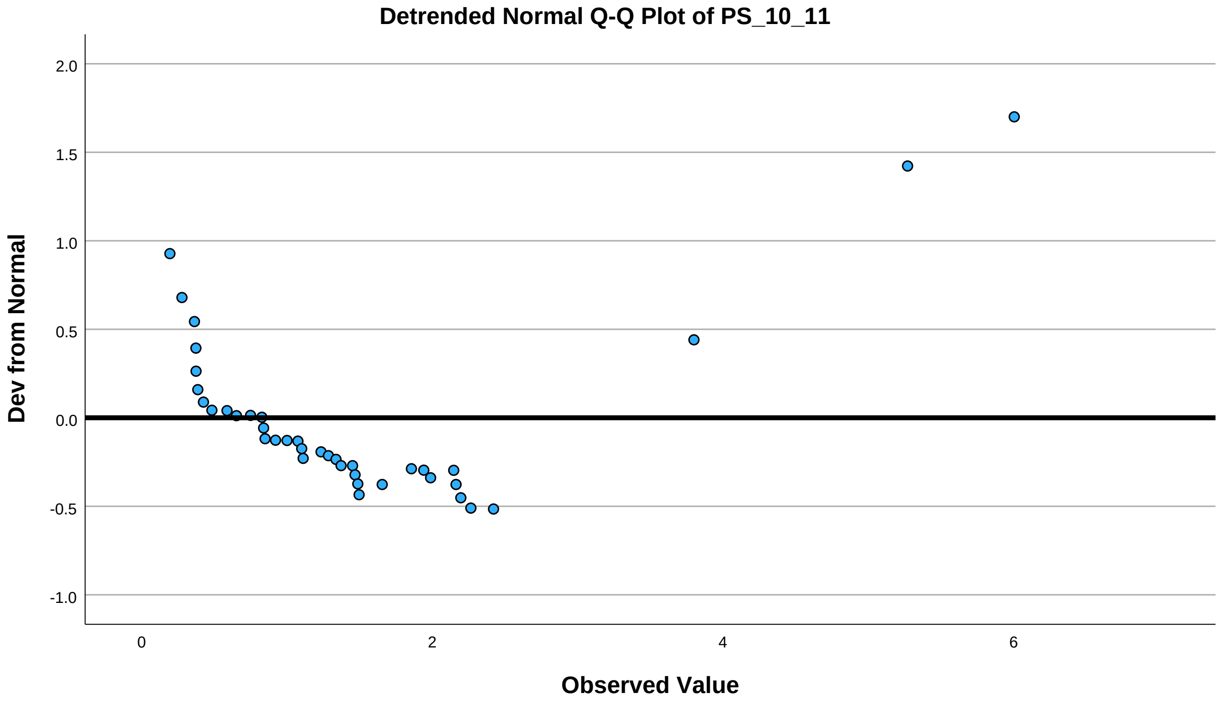
Detrended Normal Q-Q Plot of PS_10_11
Dev from Normal
Observed Value
2.0
1.5
1.0
0.5
0.0
-0.5
-1.0
0
2
4
6

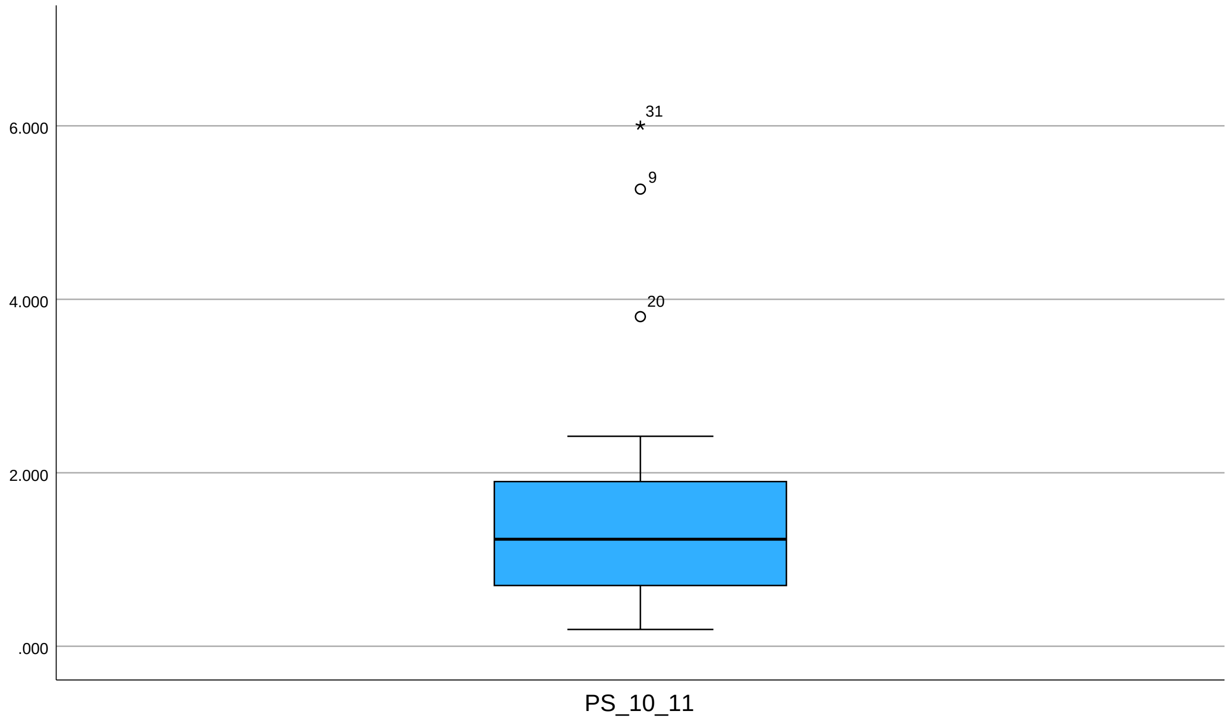

PS_11_12

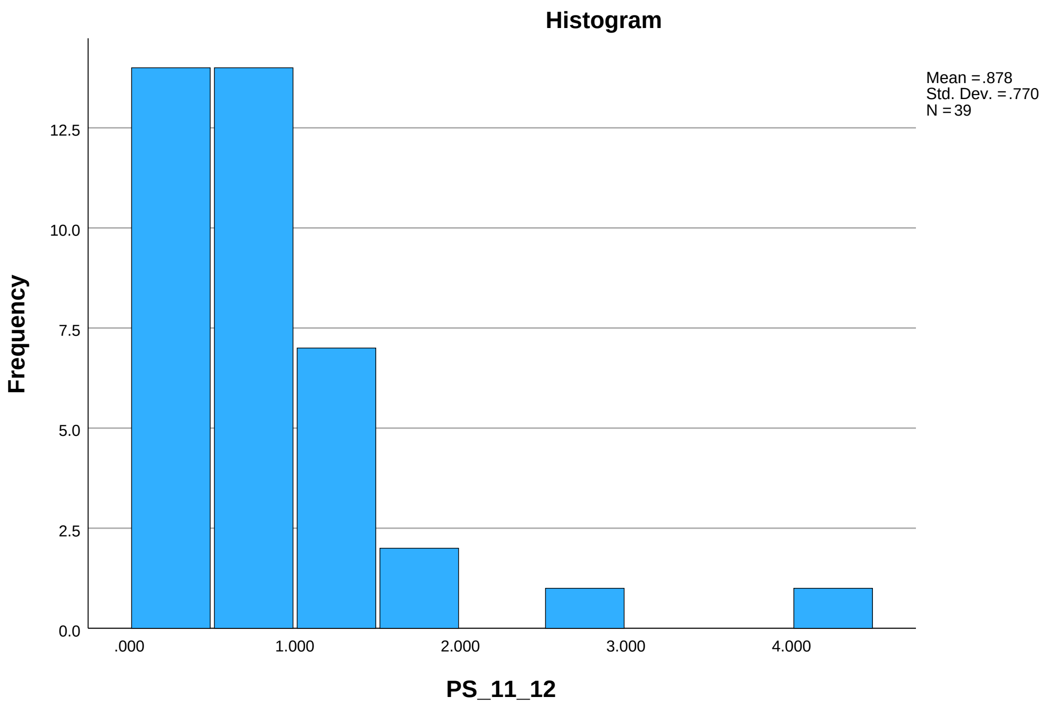

PS_11_12 Stem-and-Leaf Plot

Frequency   Stem & Leaf

```
   1.00      0 . 1
  10.00      0 . 2222333333
   4.00      0 . 4445
   6.00      0 . 666777
   7.00      0 . 8888899
   4.00      1 . 0001
   2.00      1 . 22
   1.00      1 . 4
   1.00      1 . 6
   1.00      1 . 9
   2.00 Extremes    (>=2.8)

 Stem width:      1.000
 Each leaf:       1 case(s)
```

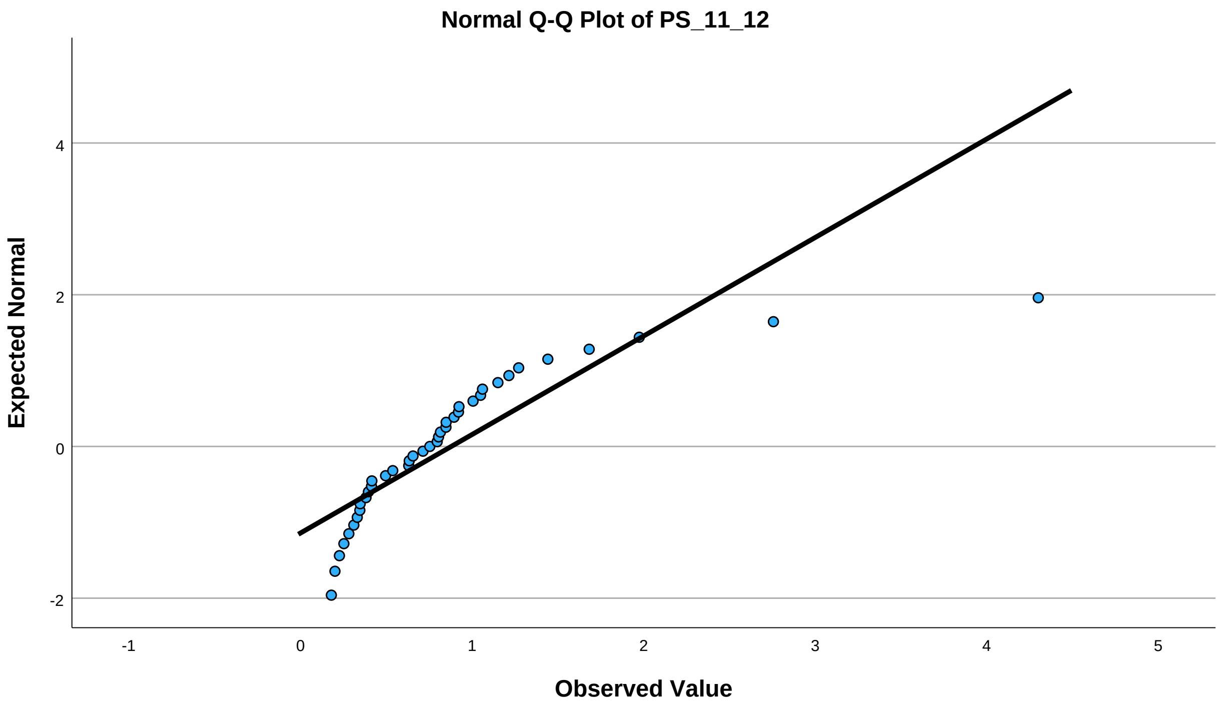

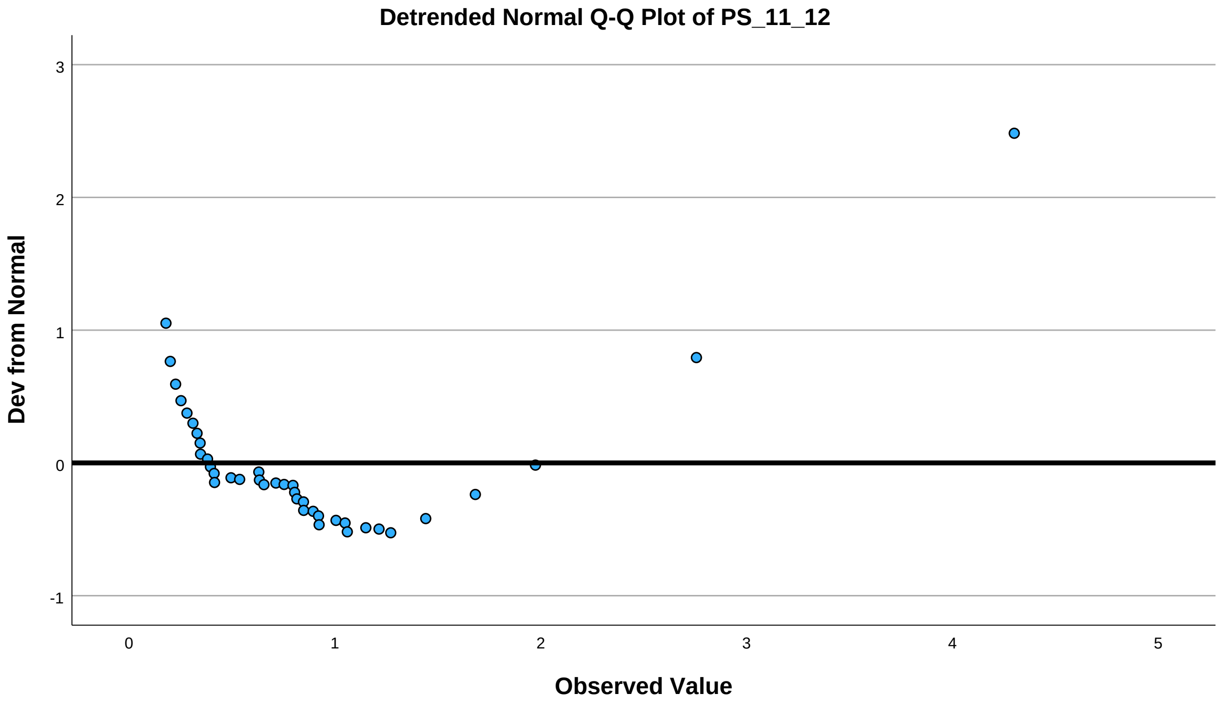
Detrended Normal Q-Q Plot of PS_11_12
Dev from Normal
3
2
1
0
-1
0
1
2
3
4
5
Observed Value

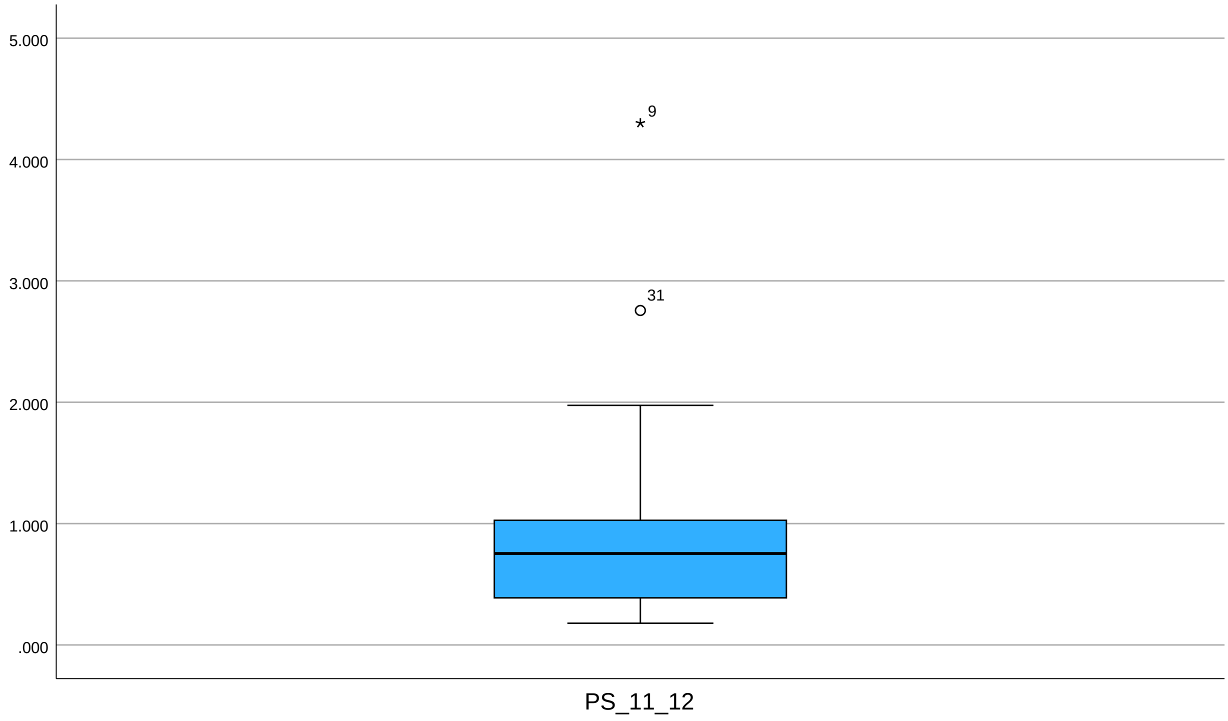
5.000
4.000
3.000
2.000
1.000
.000
9
31
PS_11_12

PS_12_13

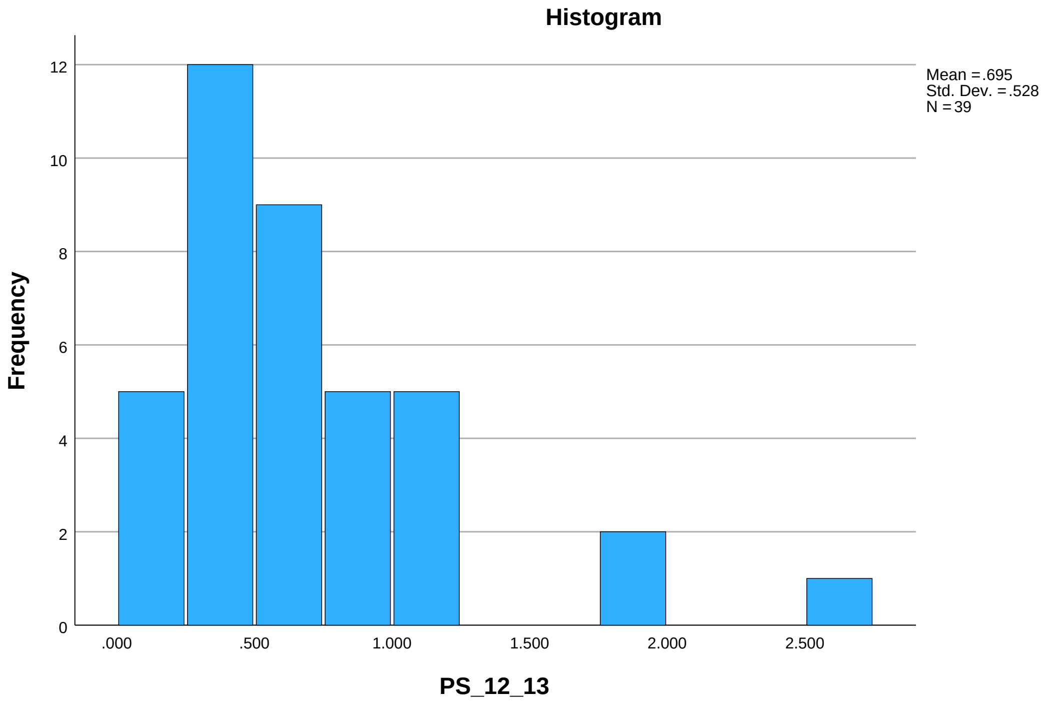


```
PS_12_13 Stem-and-Leaf Plot

 Frequency    Stem &  Leaf

     3.00        1 .  359
     5.00        2 .  24558
     4.00        3 .  1589
     5.00        4 .  02677
     6.00        5 .  002369
     2.00        6 .  05
     2.00        7 .  49
     3.00        8 .  348
     1.00        9 .  8
     2.00       10 .  69
     2.00       11 .  18
     1.00       12 .  0
     3.00 Extremes    (>=1.84)

 Stem width:      .100
 Each leaf:       1 case(s)
```

Normal Q-Q Plot of PS_12_13

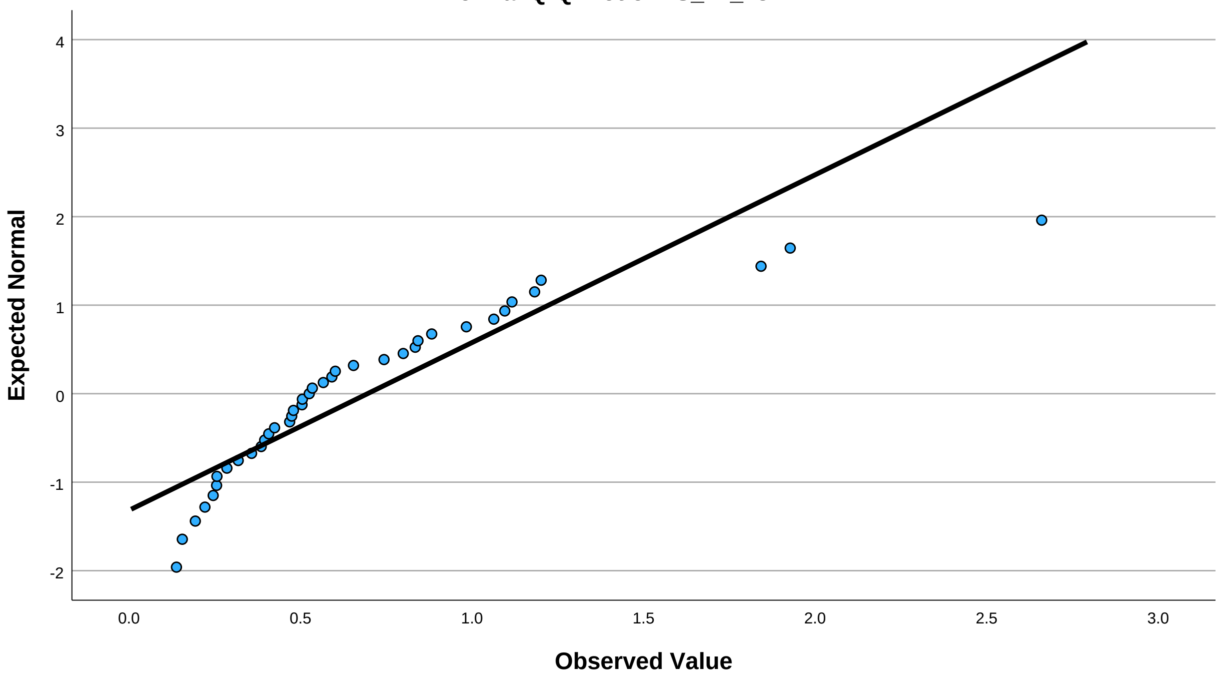

Detrended Normal Q-Q Plot of PS_12_13

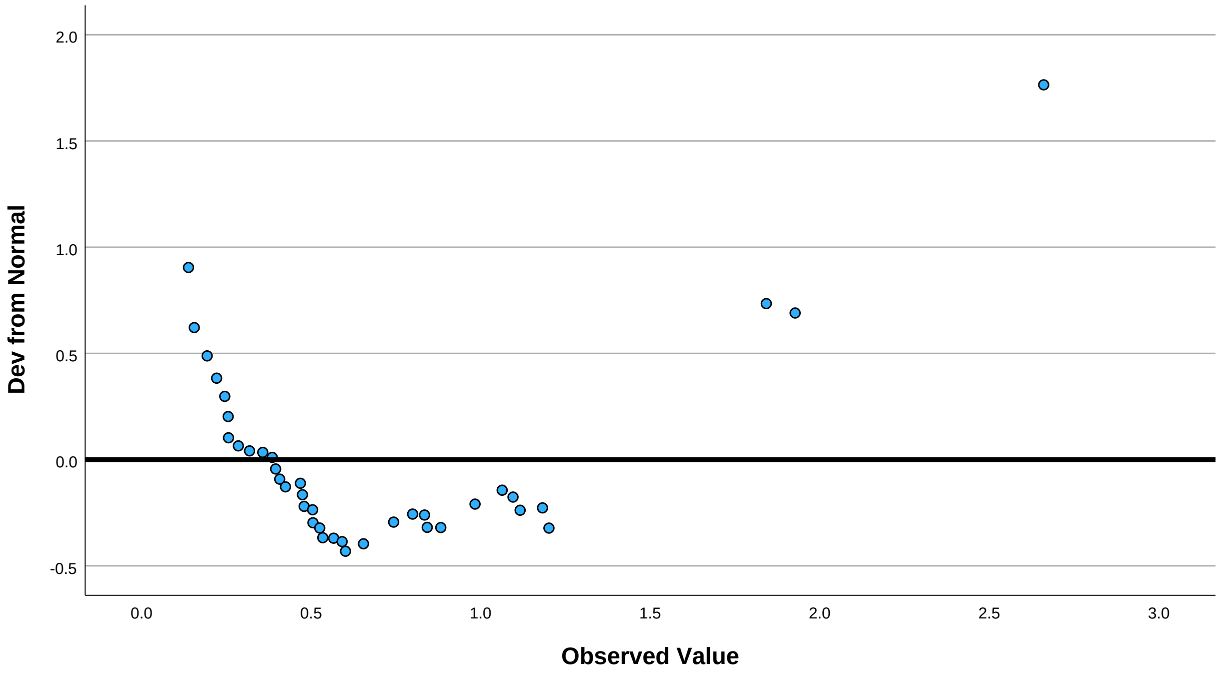

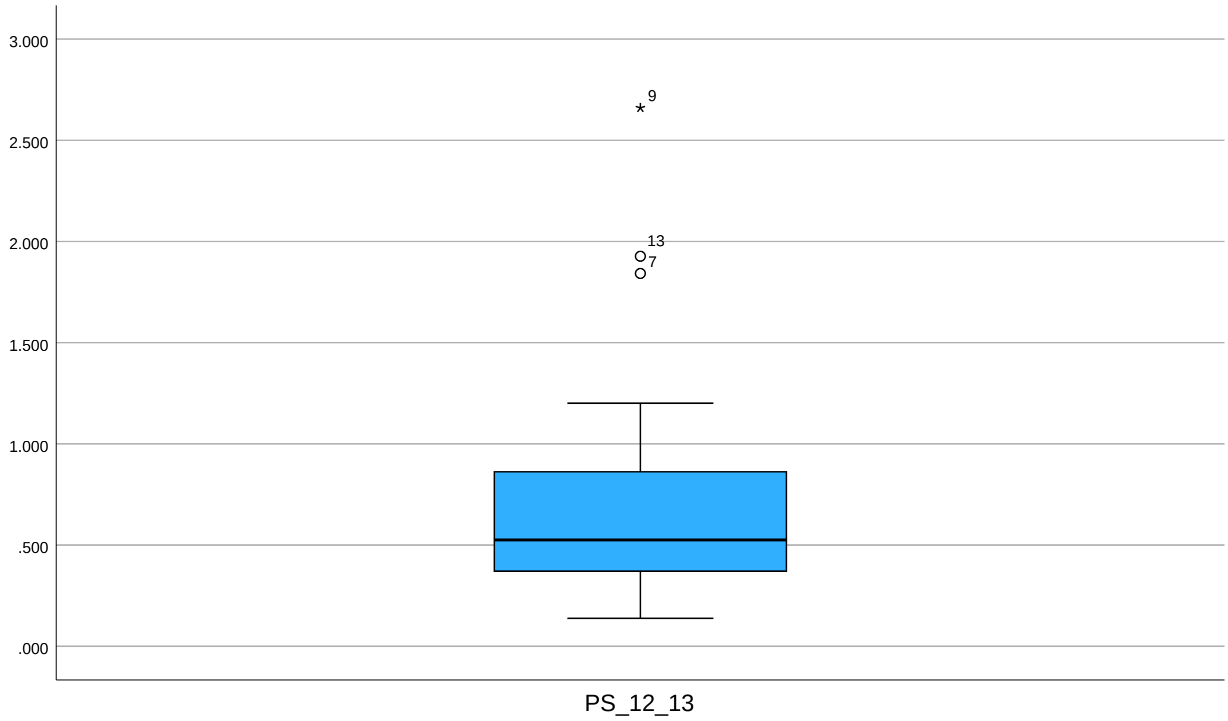

PS_13_14

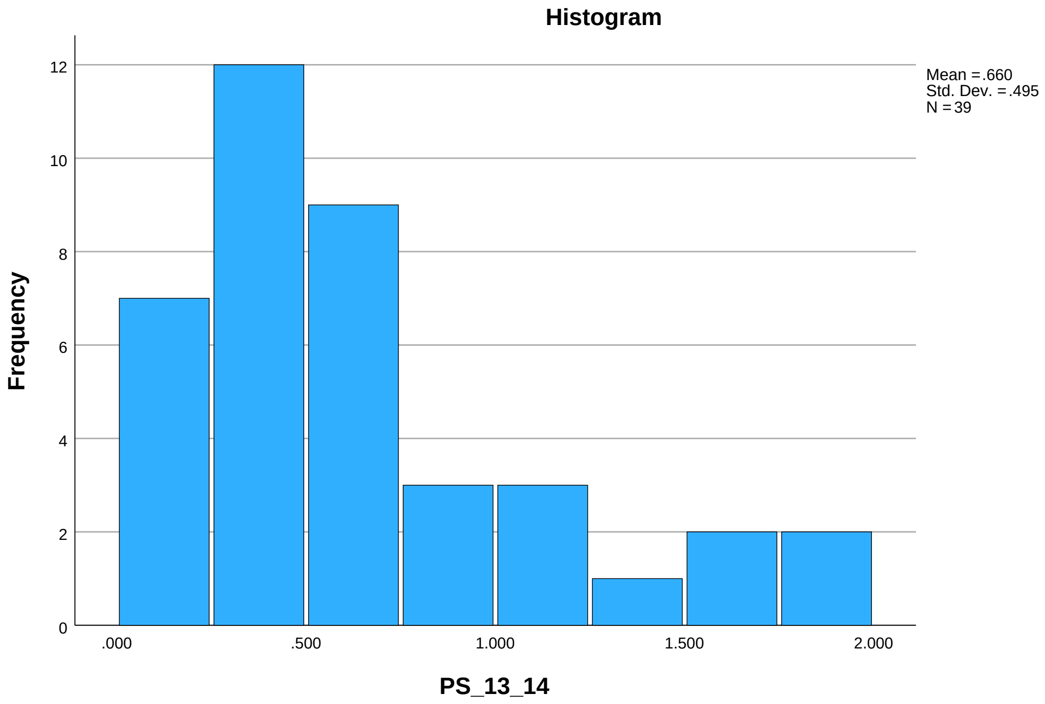

PS_13_14 Stem-and-Leaf Plot

Frequency Stem & Leaf

```
  2.00     0 . 11
  14.00      0 .  22222222333333
   9.00      0 .  444555555
  3.00     0 . 667
  3.00     0 . 889
  1.00     1 . 0
  2.00     1 . 22
  2.00     1 . 45
  1.00     1 . 6
   2.00 Extremes    (>=1.9)

Stem width:     1.000
Each leaf:       1 case(s)
```

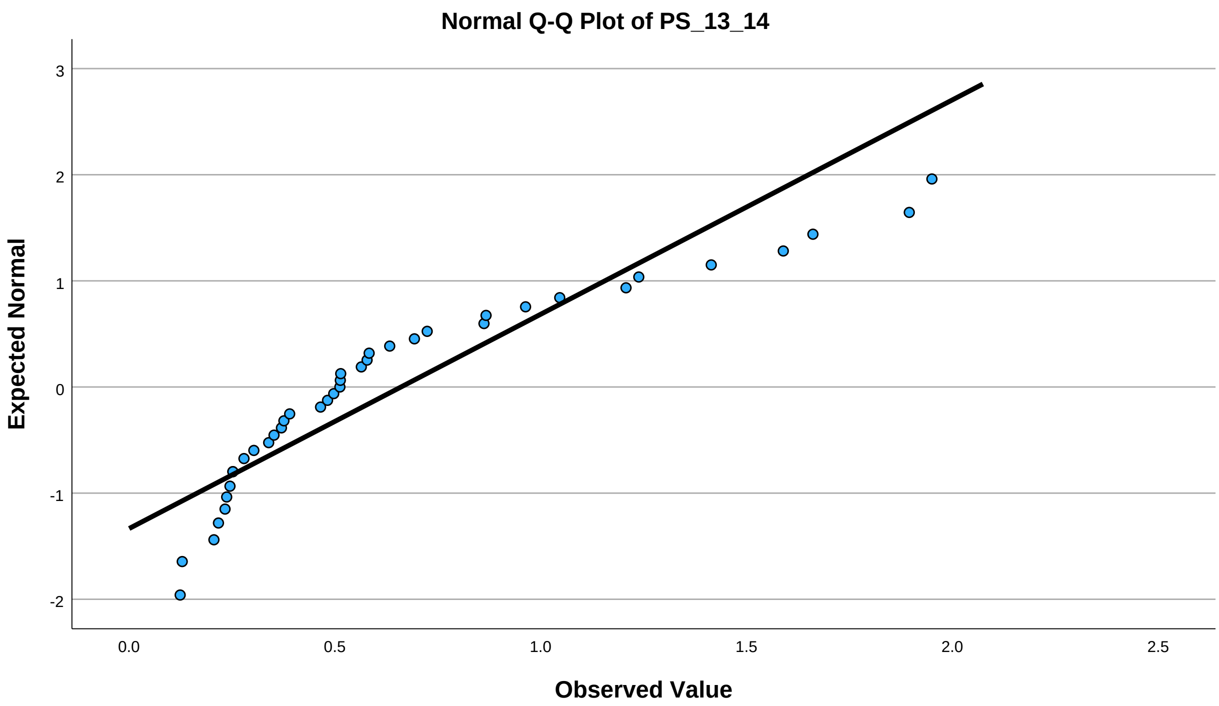

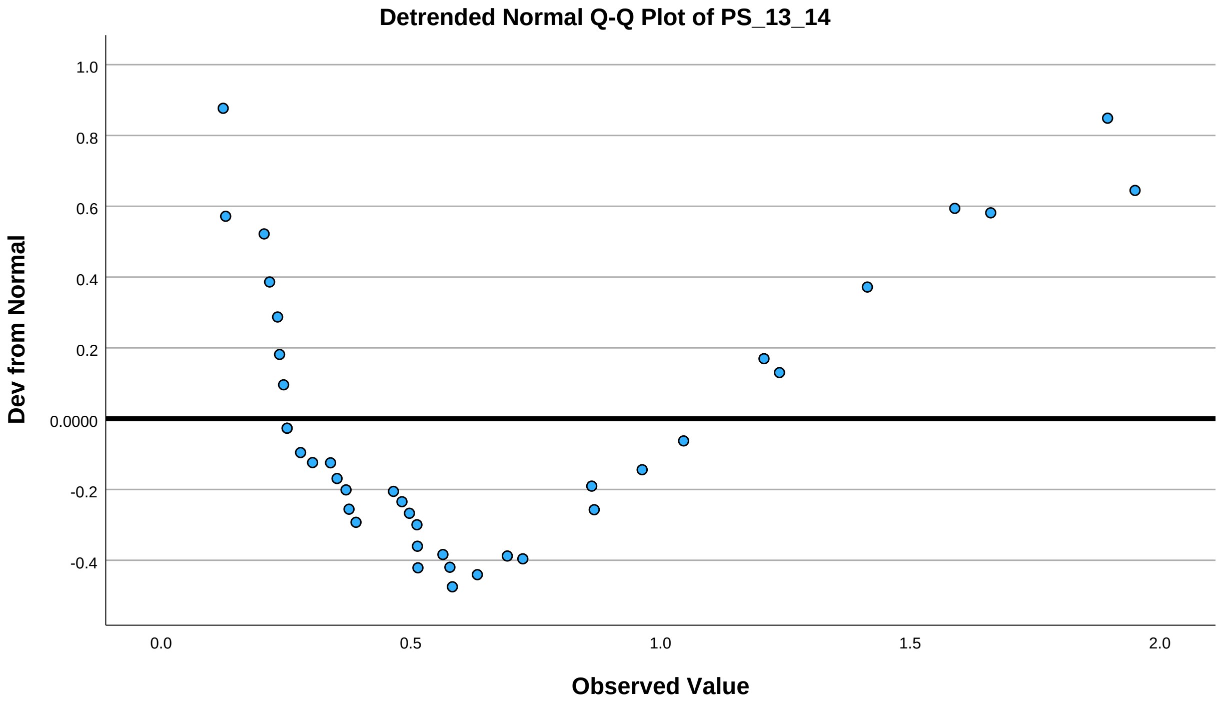
Detrended Normal Q-Q Plot of PS_13_14
Dev from Normal
1.0
0.8
0.6
0.4
0.2
0.0000
-0.2
-0.4
0.0
0.5
1.0
1.5
2.0
Observed Value

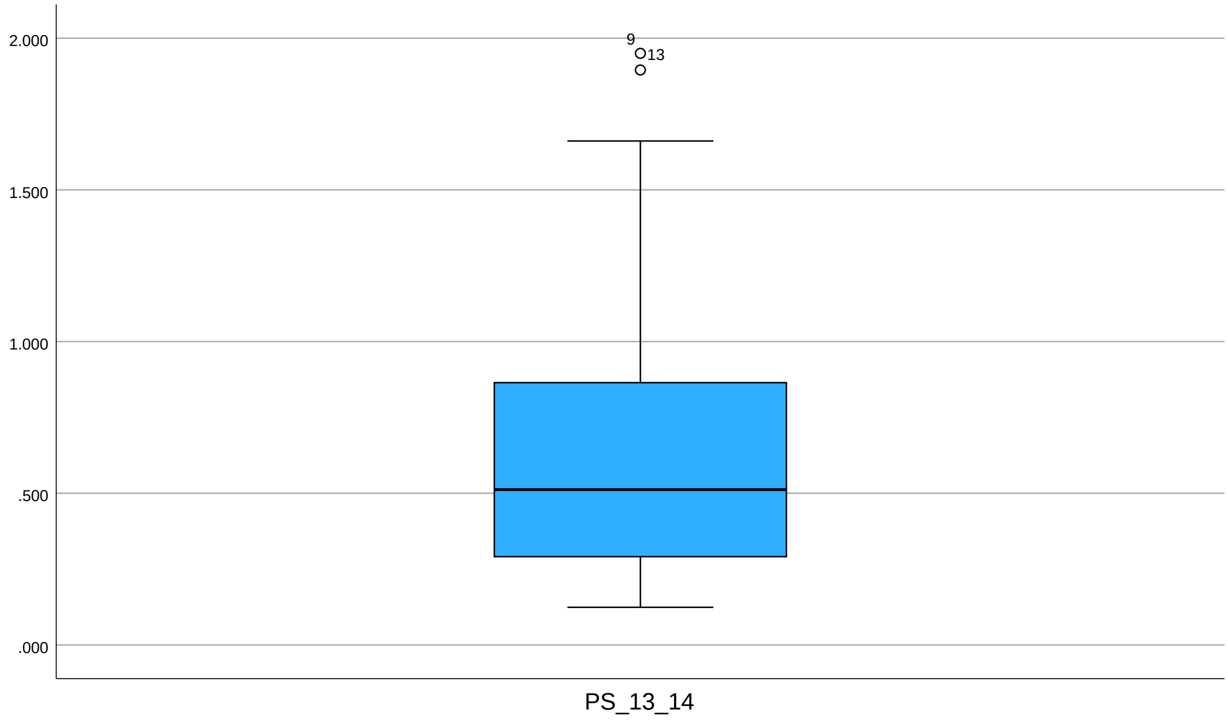
2.000
1.500
1.000
.500
.000
9
13
PS_13_14

PS_14_15

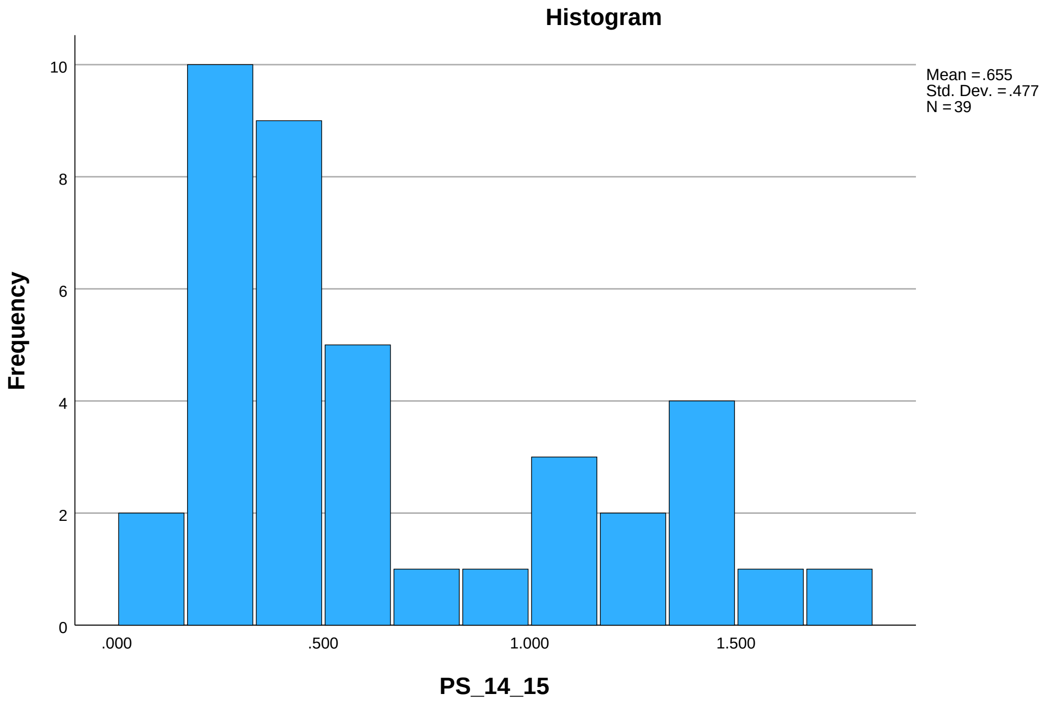


```
PS_14_15 Stem-and-Leaf Plot

 Frequency    Stem &  Leaf

     4.00        0 .  1111
    12.00        0 .  222222223333
     9.00        0 .  444445555
     2.00        0 .  66
     1.00        0 .  8
     3.00        1 .  011
     3.00        1 .  223
     4.00        1 .  4445
     1.00        1 .  7

 Stem width:      1.000
 Each leaf:       1 case(s)
```

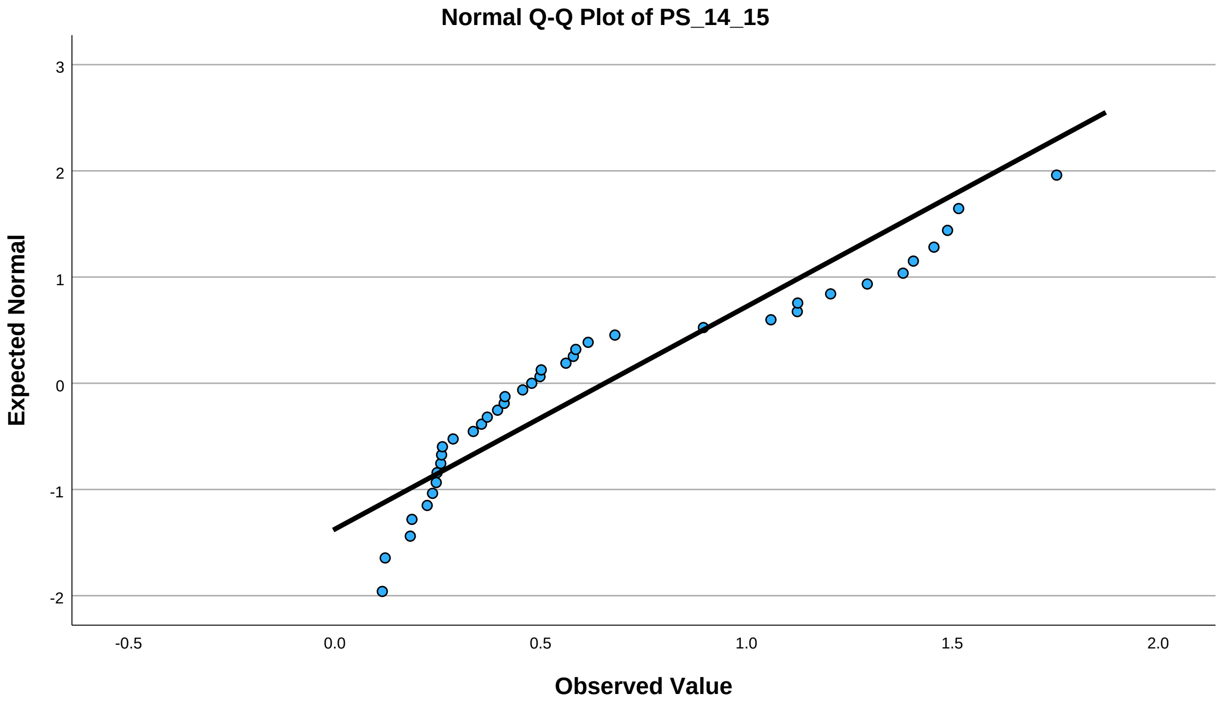
Normal Q-Q Plot of PS_14_15
Expected Normal
Observed Value
3
2
1
0
-1
-2
-0.5
0.0
0.5
1.0
1.5
2.0

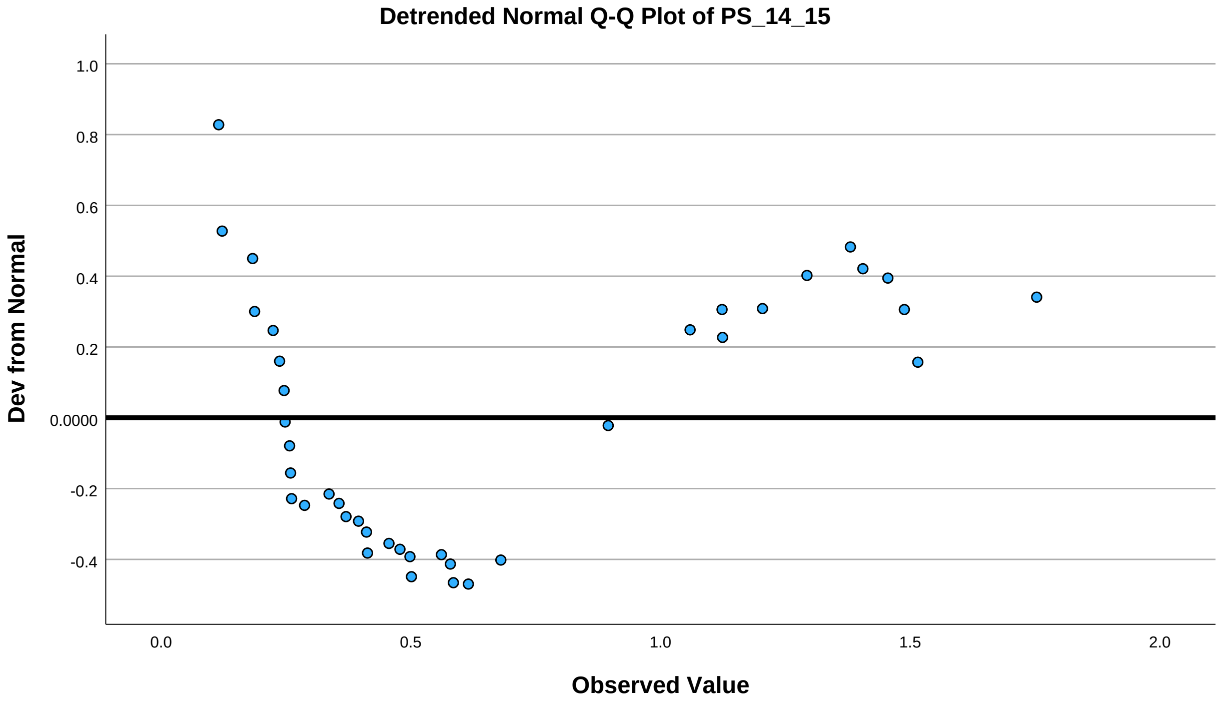
Detrended Normal Q-Q Plot of PS_14_15
Dev from Normal
Observed Value
1.0
0.8
0.6
0.4
0.2
0.0000
-0.2
-0.4
0.0
0.5
1.0
1.5
2.0

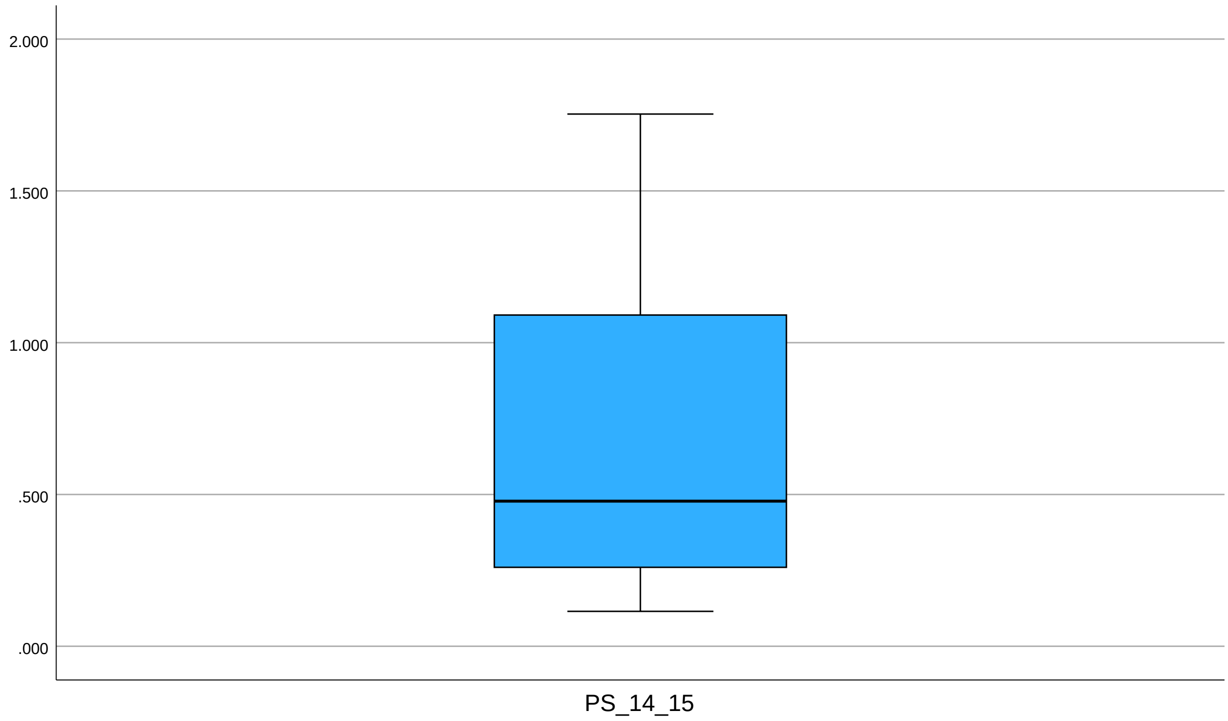

PS_15_16

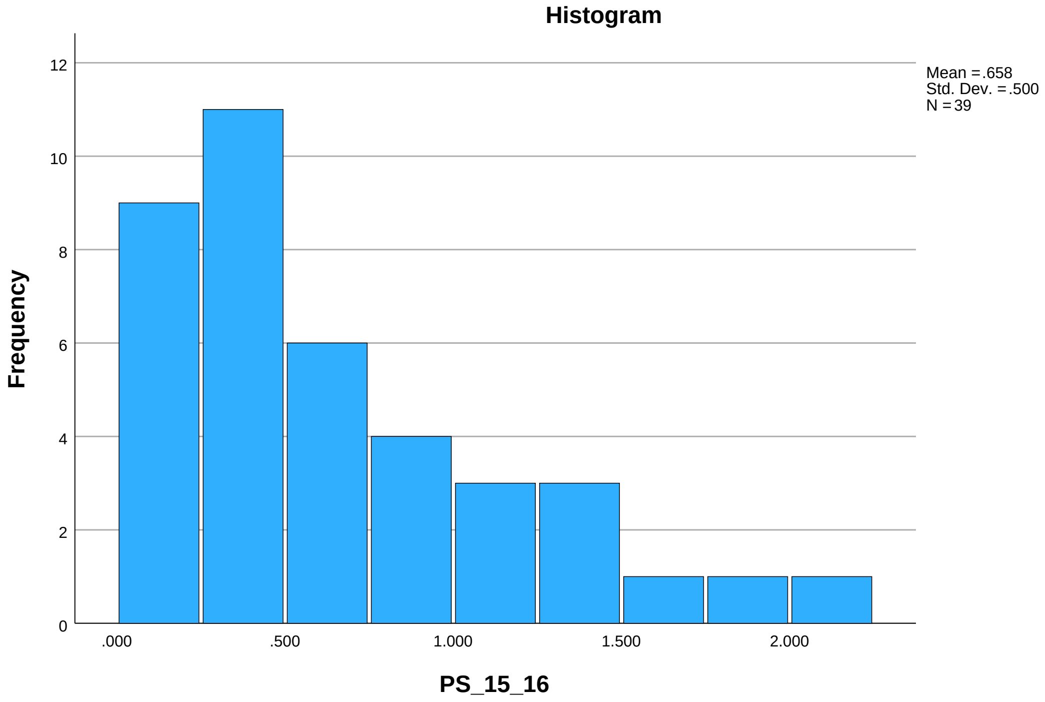


```
PS_15_16 Stem-and-Leaf Plot

 Frequency    Stem &  Leaf
```

```
    6.00        0 .  111111
   10.00        0 .  2222233333
    8.00        0 .  44445555
    2.00        0 .  66
    4.00        0 .  8999
    2.00        1 .  11
    4.00        1 .  2222
     .00        1 .
    1.00        1 .  7
    1.00        1 .  8
    1.00 Extremes    (>=2.0)

 Stem width:     1.000
 Each leaf:        1 case(s)
```

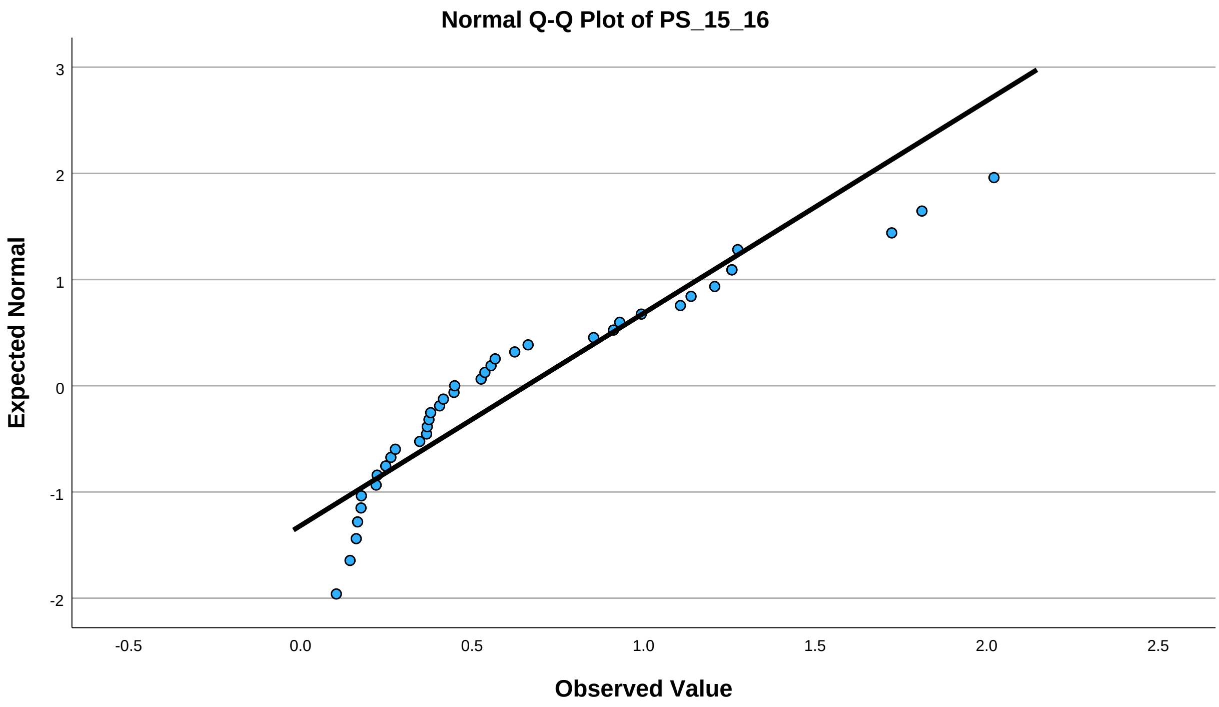

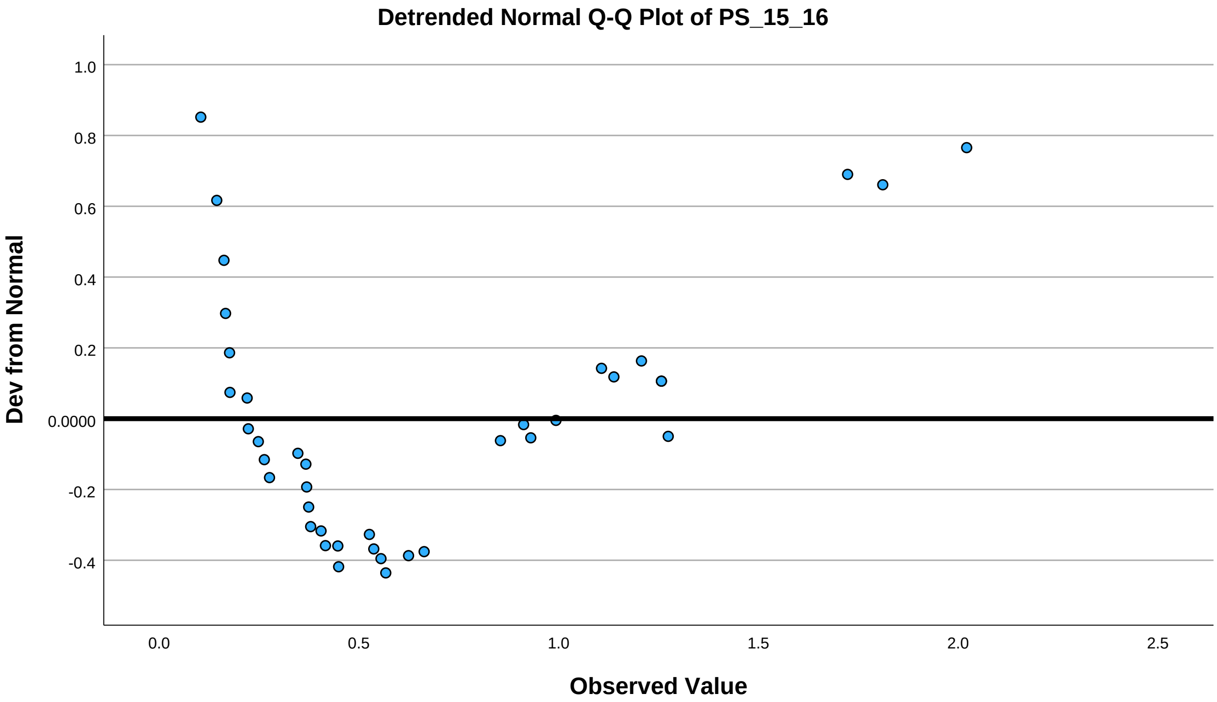
Detrended Normal Q-Q Plot of PS_15_16
Dev from Normal
1.0
0.8
0.6
0.4
0.2
0.0000
-0.2
-0.4
0.0
0.5
1.0
1.5
2.0
2.5
Observed Value

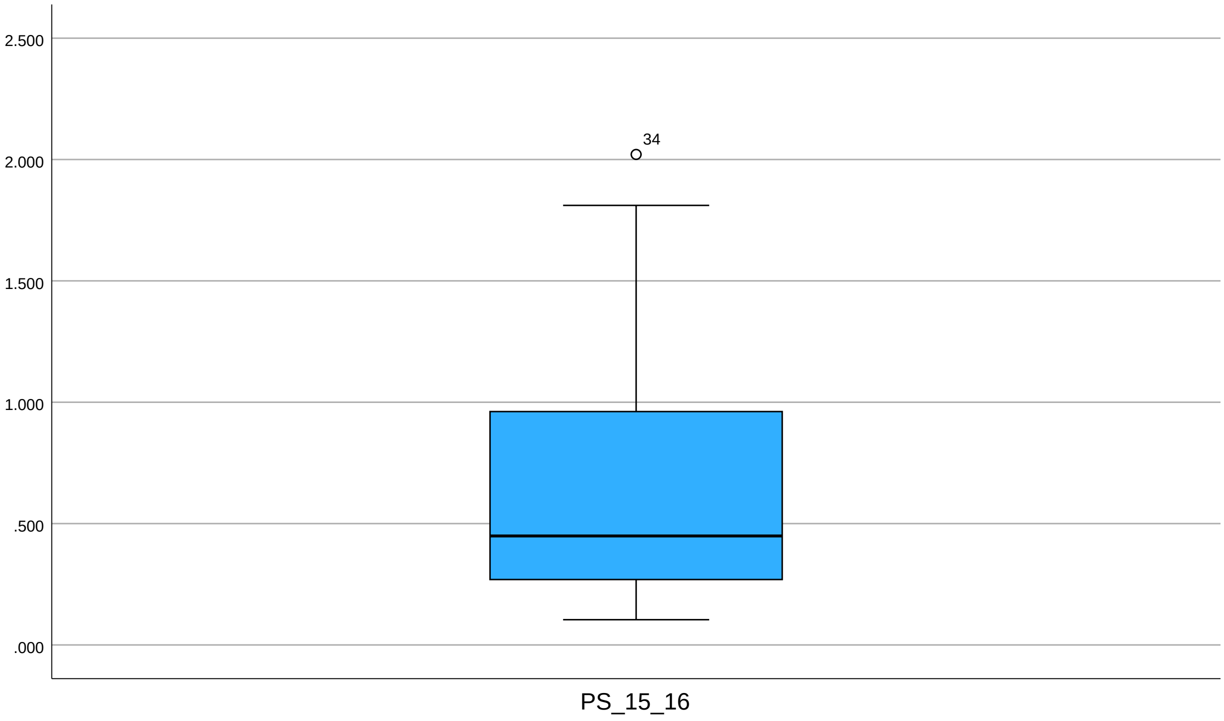
2.500
2.000
1.500
1.000
.500
.000
34
PS_15_16

PS_16_17

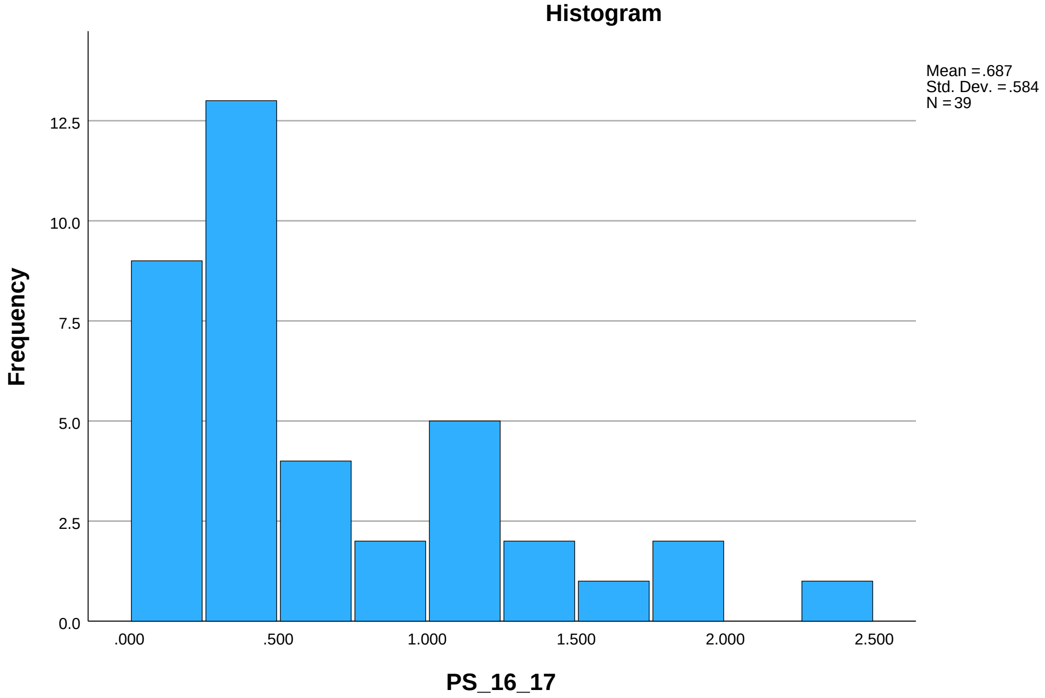


```
PS_16_17 Stem-and-Leaf Plot

 Frequency    Stem &  Leaf

     6.00        0 .  011111
    12.00        0 .  222222233333
     6.00        0 .  444455
     3.00        0 .  667
     1.00        0 .  9
     4.00        1 .  0011
     1.00        1 .  2
     3.00        1 .  445
      .00        1 .
     2.00        1 .  99
     1.00 Extremes    (>=2.4)

 Stem width:      1.000
 Each leaf:       1 case(s)
```

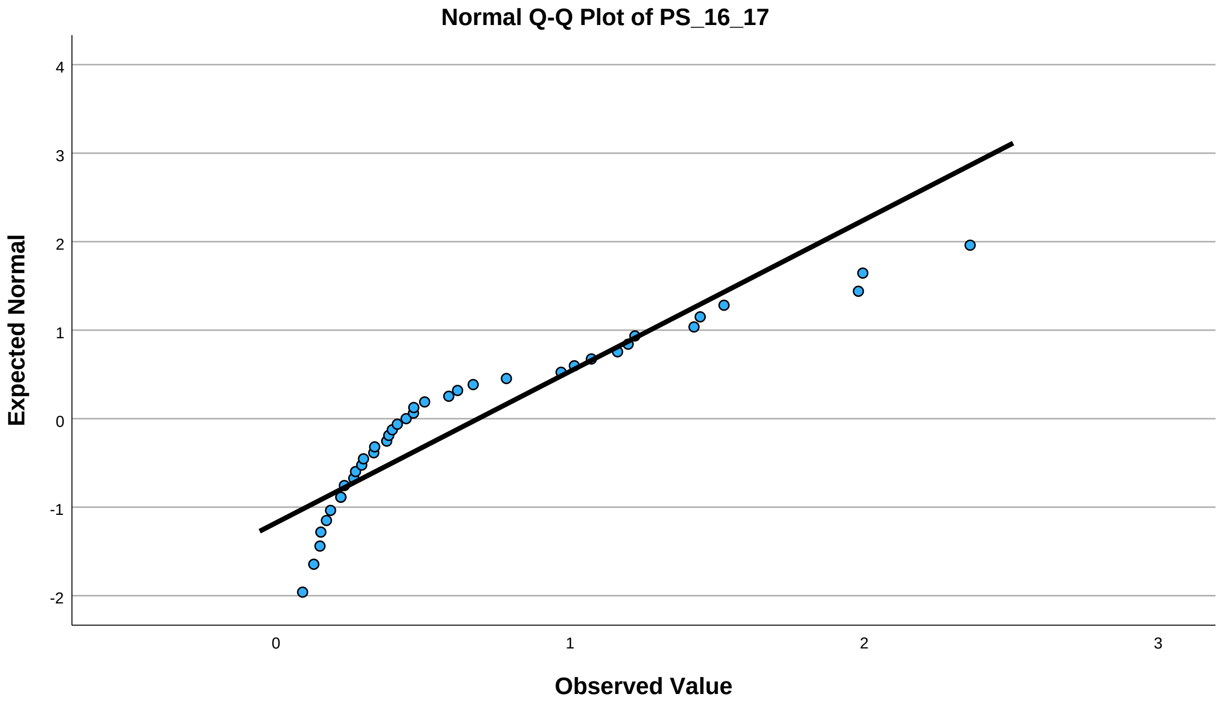
Normal Q-Q Plot of PS_16_17
Expected Normal
4
3
2
1
0
-1
-2
0
1
2
3
Observed Value

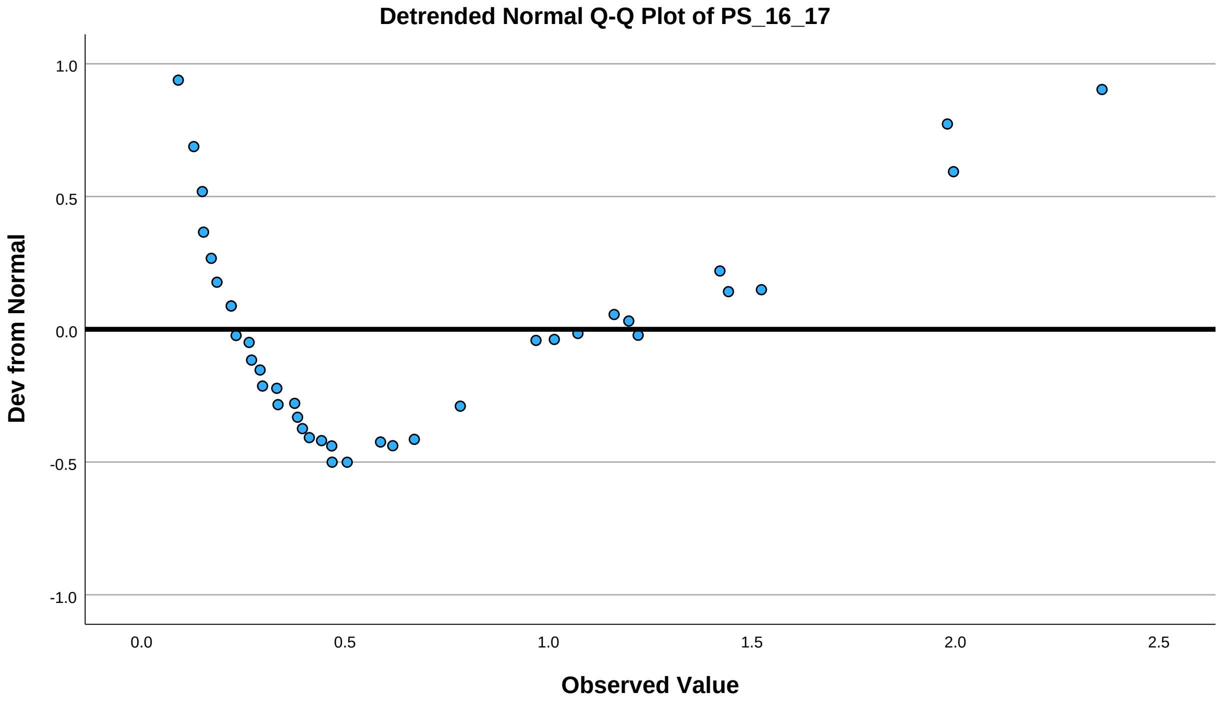
Detrended Normal Q-Q Plot of PS_16_17
Dev from Normal
1.0
0.5
0.0
-0.5
-1.0
0.0
0.5
1.0
1.5
2.0
2.5
Observed Value

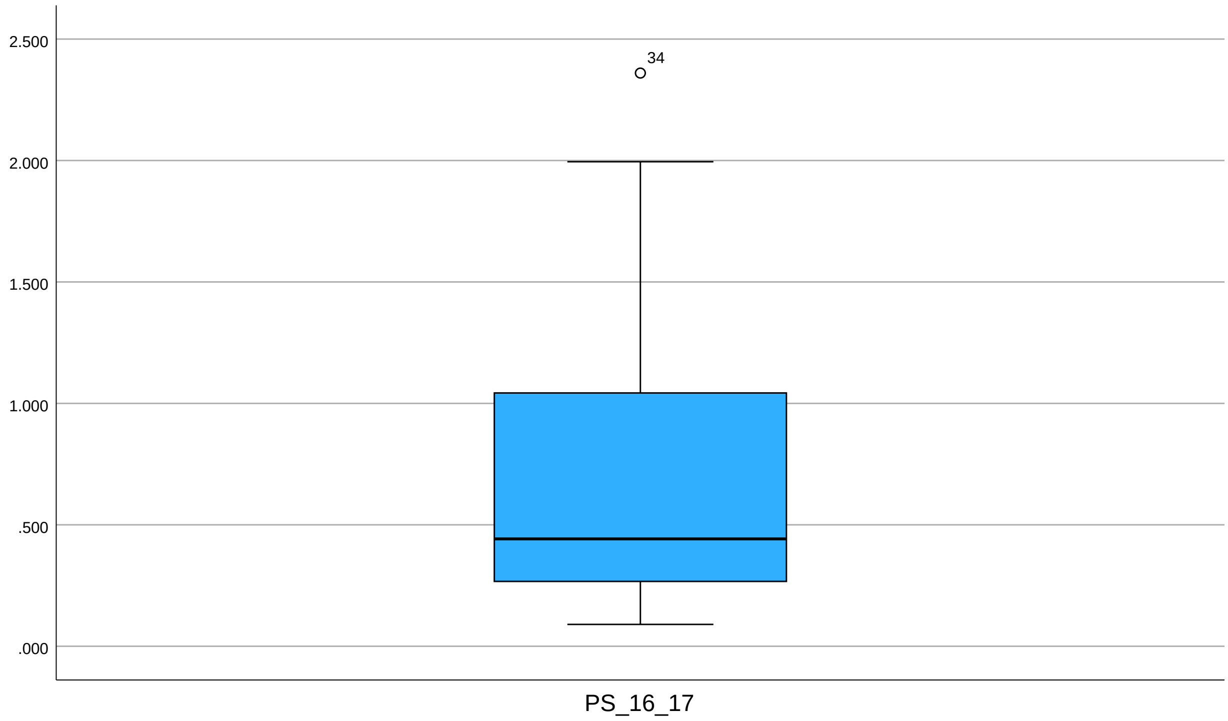

PS_17_18

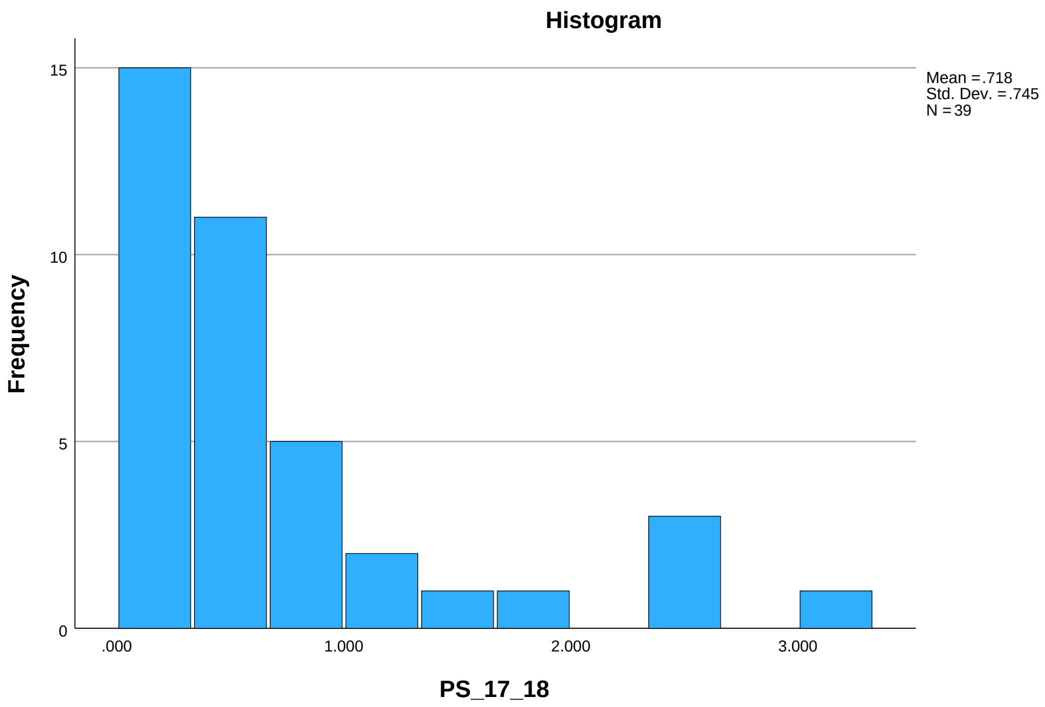

PS_17_18 Stem-and-Leaf Plot

Frequency   Stem & Leaf

```
    8.00        0 .  01111111
   11.00        0 .  22223333333
    5.00        0 .  44445
    5.00        0 .  66667
    2.00        0 .  99
    1.00        1 .  0
    1.00        1 .  2
    1.00        1 .  4
    1.00        1 .  6
    4.00 Extremes    (>=2.4)

Stem width:       1.000
Each leaf:        1 case(s)
```

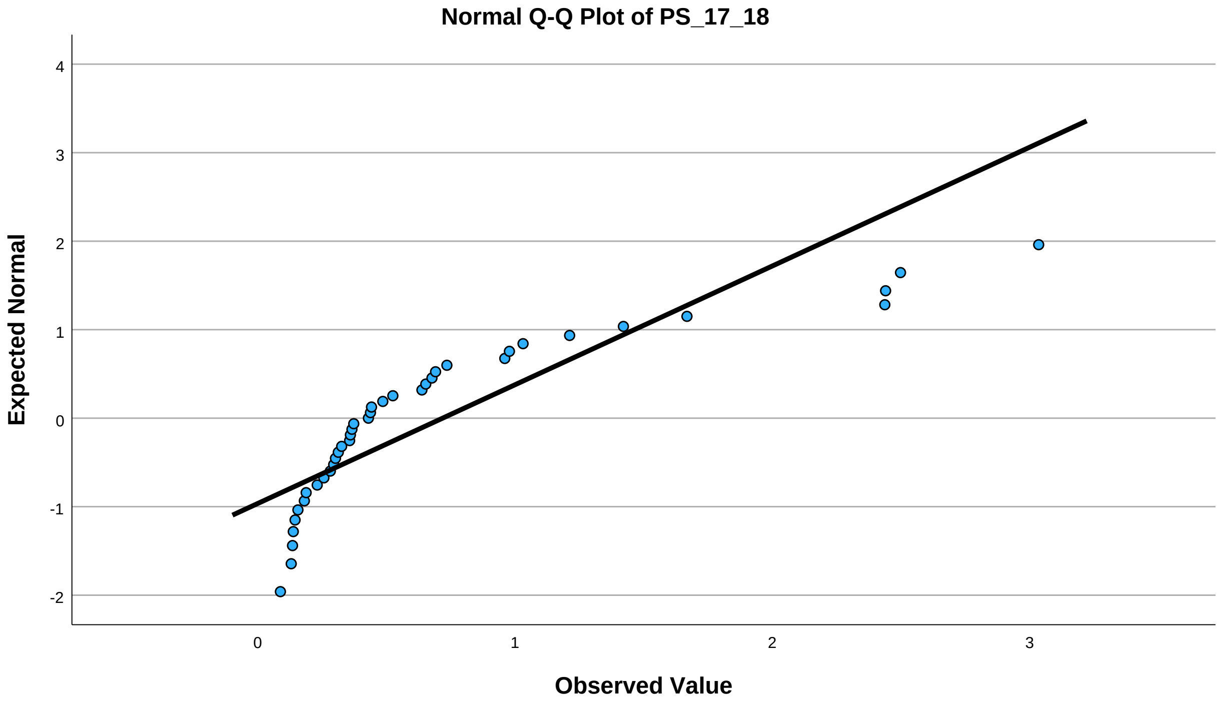

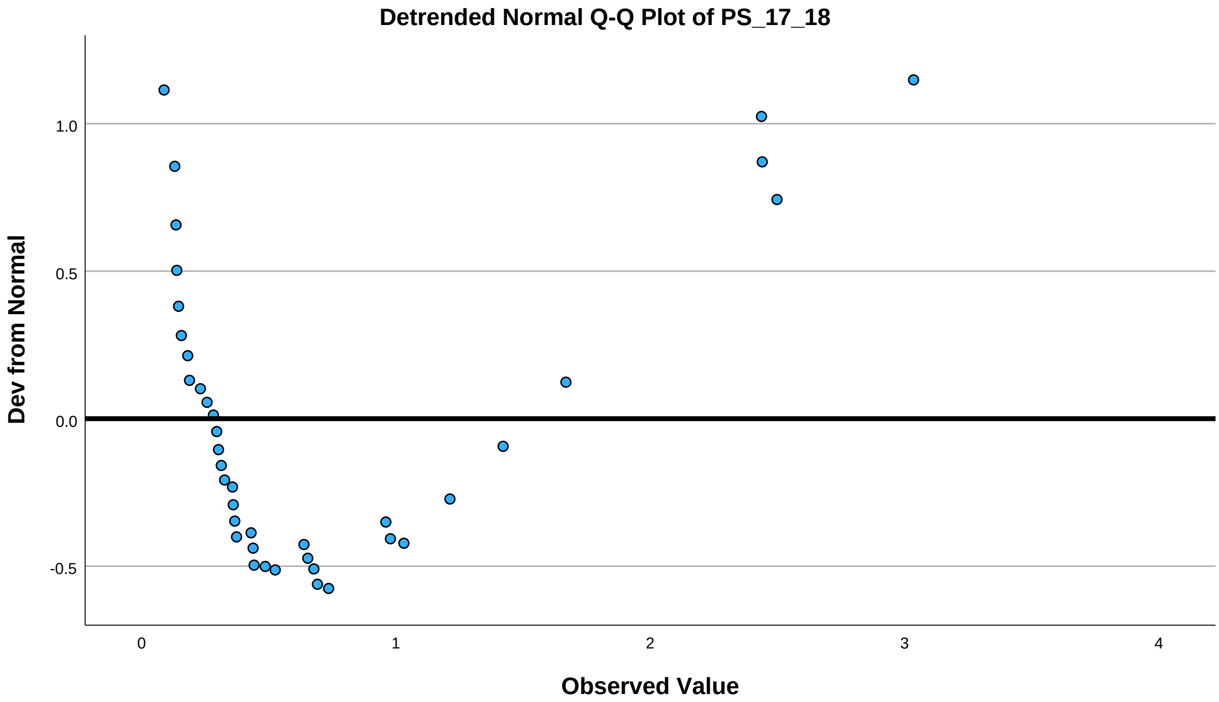
Detrended Normal Q-Q Plot of PS_17_18
Dev from Normal
1.0
0.5
0.0
-0.5
0
1
2
3
4
Observed Value

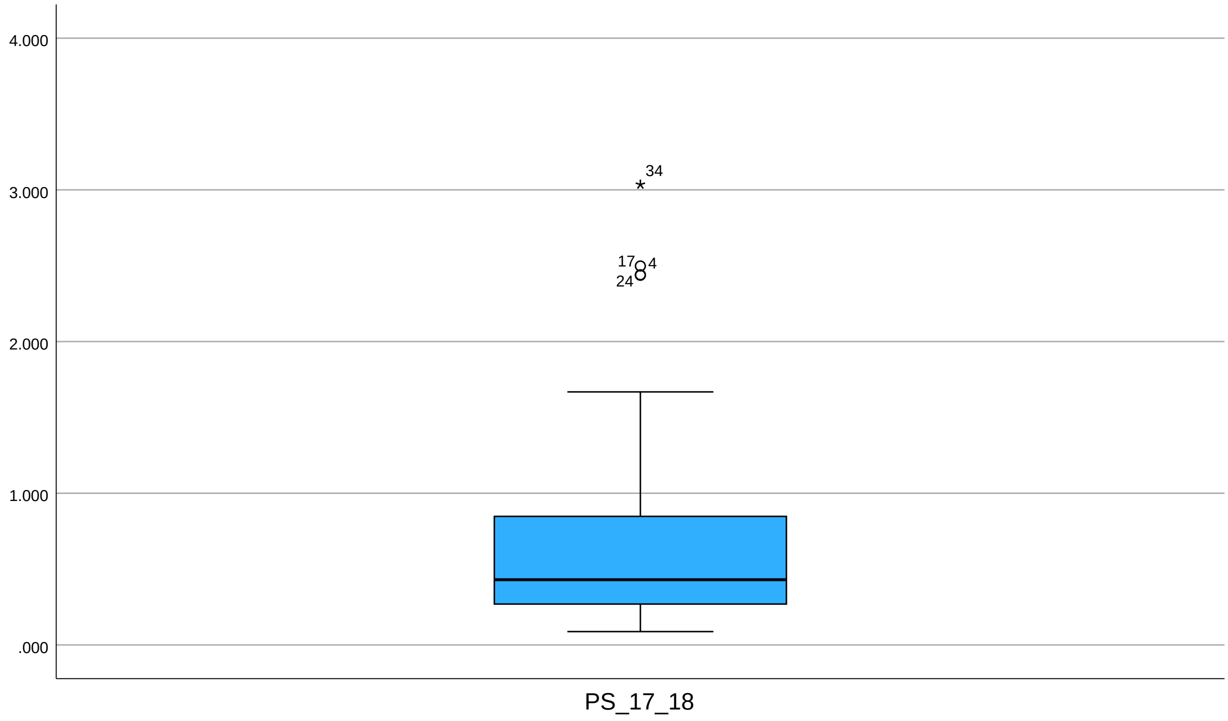
4.000
3.000
2.000
1.000
.000
34
17
4
24
PS_17_18

PS_18_19

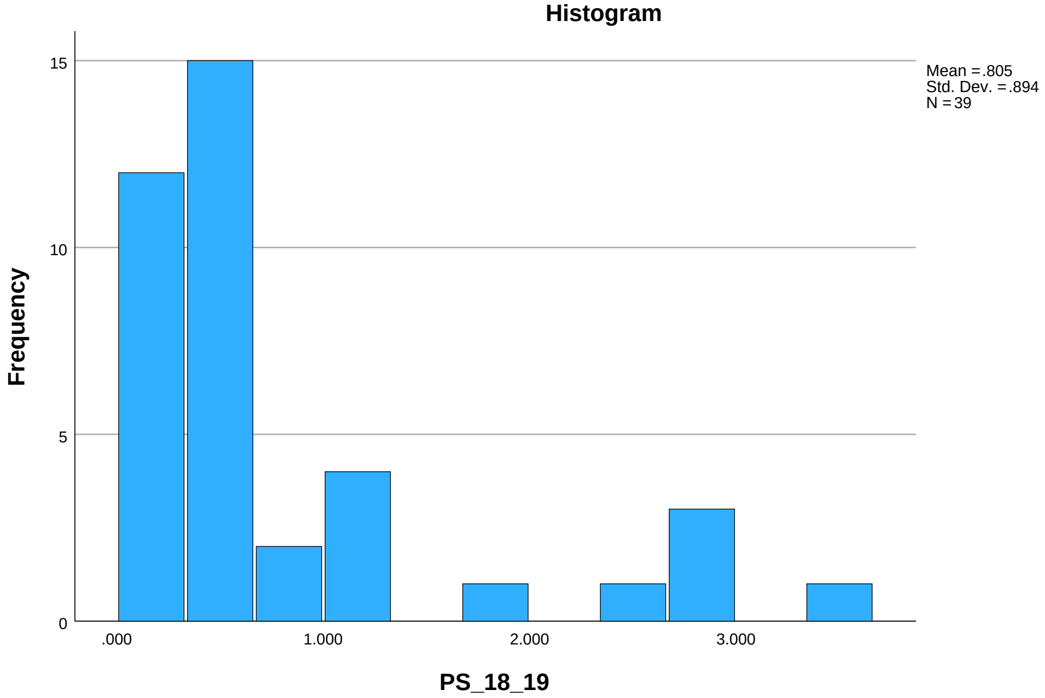


```
PS_18_19 Stem-and-Leaf Plot

 Frequency    Stem &  Leaf

     7.00        0 .  0111111
    12.00        0 .  222233333333
     6.00        0 .  444445
     3.00        0 .  667
     1.00        0 .  8
     2.00        1 .  01
     2.00        1 .  23
      .00        1 .
     1.00        1 .  7
     5.00 Extremes    (>=2.4)

 Stem width:      1.000
 Each leaf:        1 case(s)
```

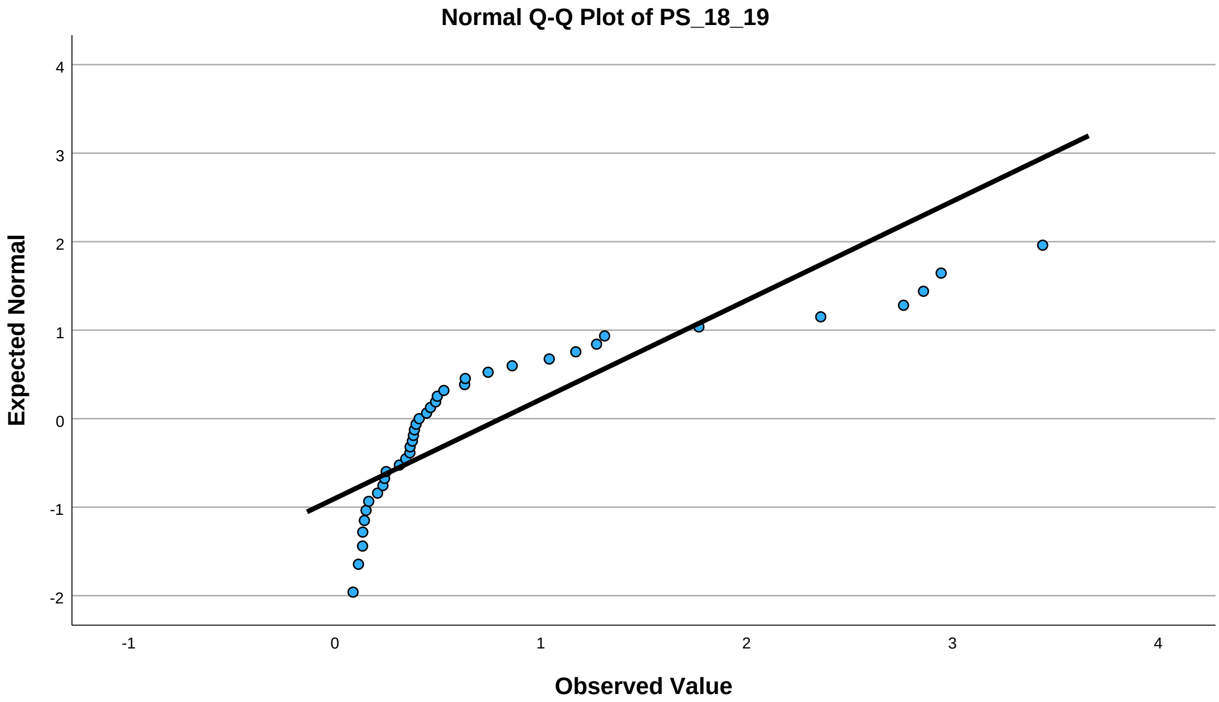
Normal Q-Q Plot of PS_18_19
Expected Normal
Observed Value
4
3
2
1
0
-1
-2
-1
0
1
2
3
4

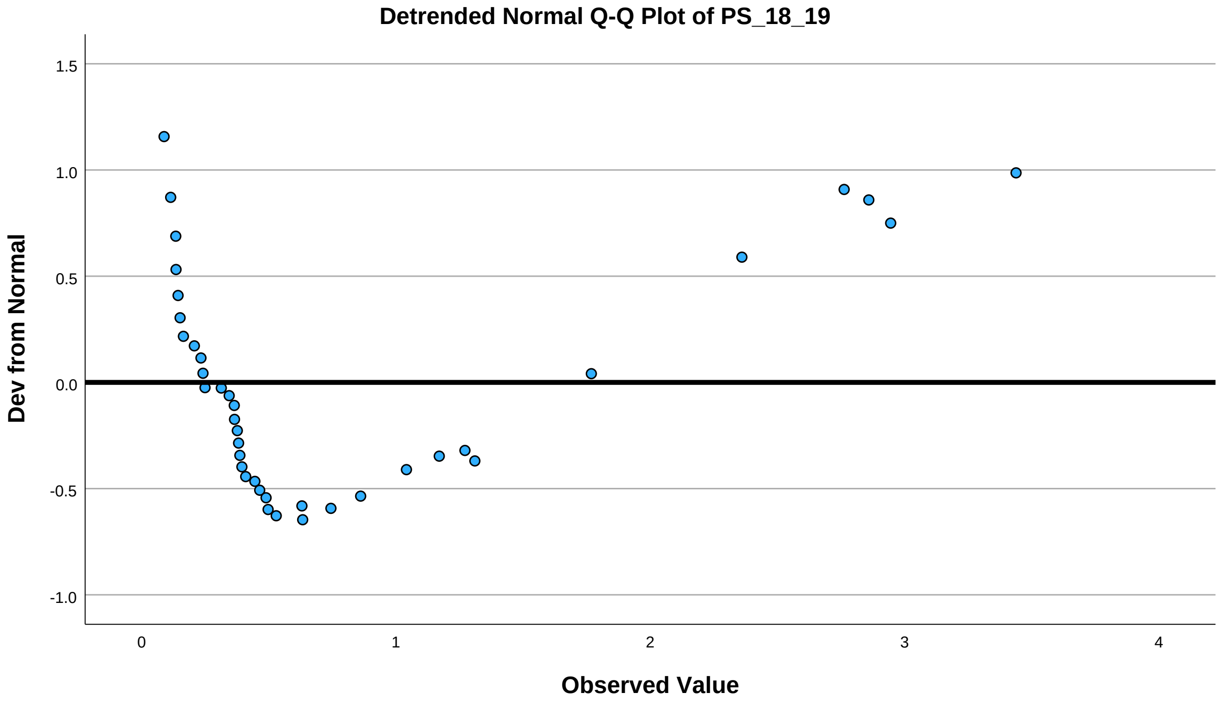
Detrended Normal Q-Q Plot of PS_18_19
Dev from Normal
Observed Value
1.5
1.0
0.5
0.0
-0.5
-1.0
0
1
2
3
4

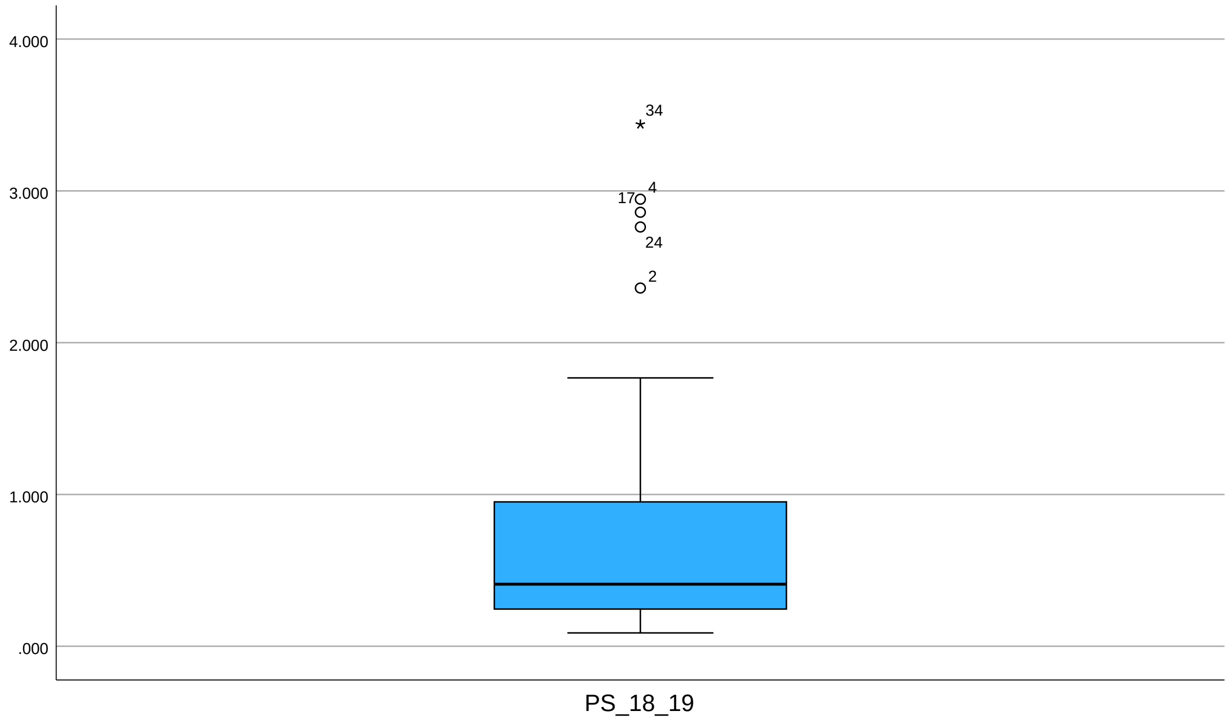

PS_19_20

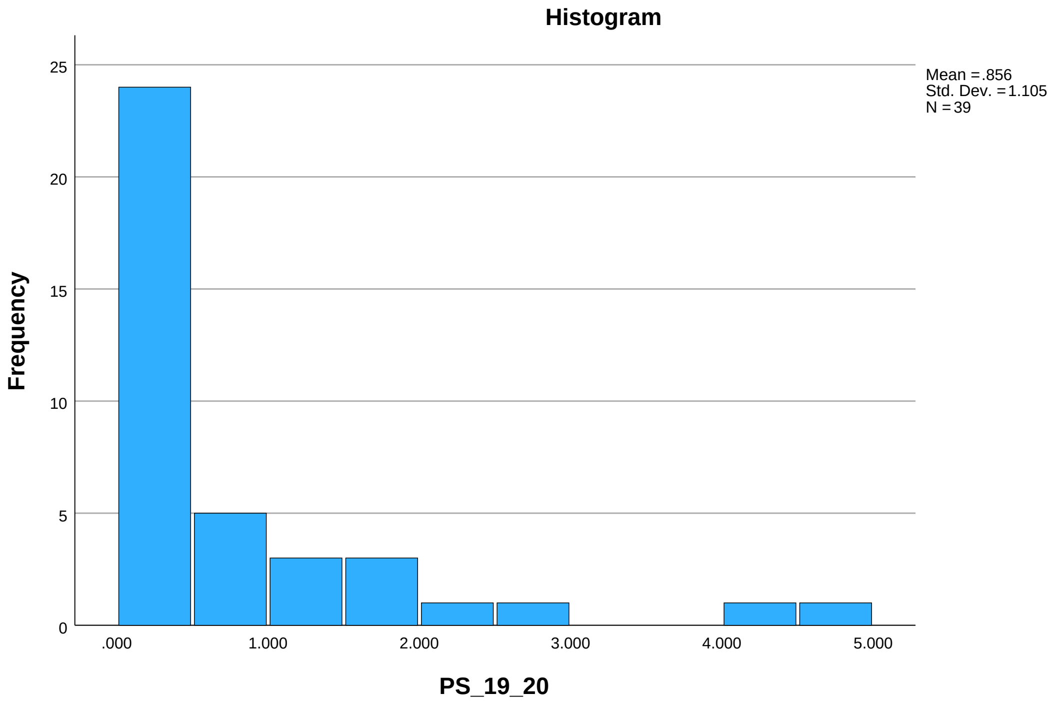

PS_19_20 Stem-and-Leaf Plot

Frequency   Stem & Leaf

```
10.00      0 . 0111111111
11.00      0 . 22333333333
 4.00      0 . 4445
 3.00      0 . 677
 1.00      0 . 8
 3.00      1 . 011
  .00      1 .
  .00      1 .
 1.00      1 . 6
 2.00      1 . 99
 4.00 Extremes    (>=2.5)

Stem width:    1.000
Each leaf:       1 case(s)
```

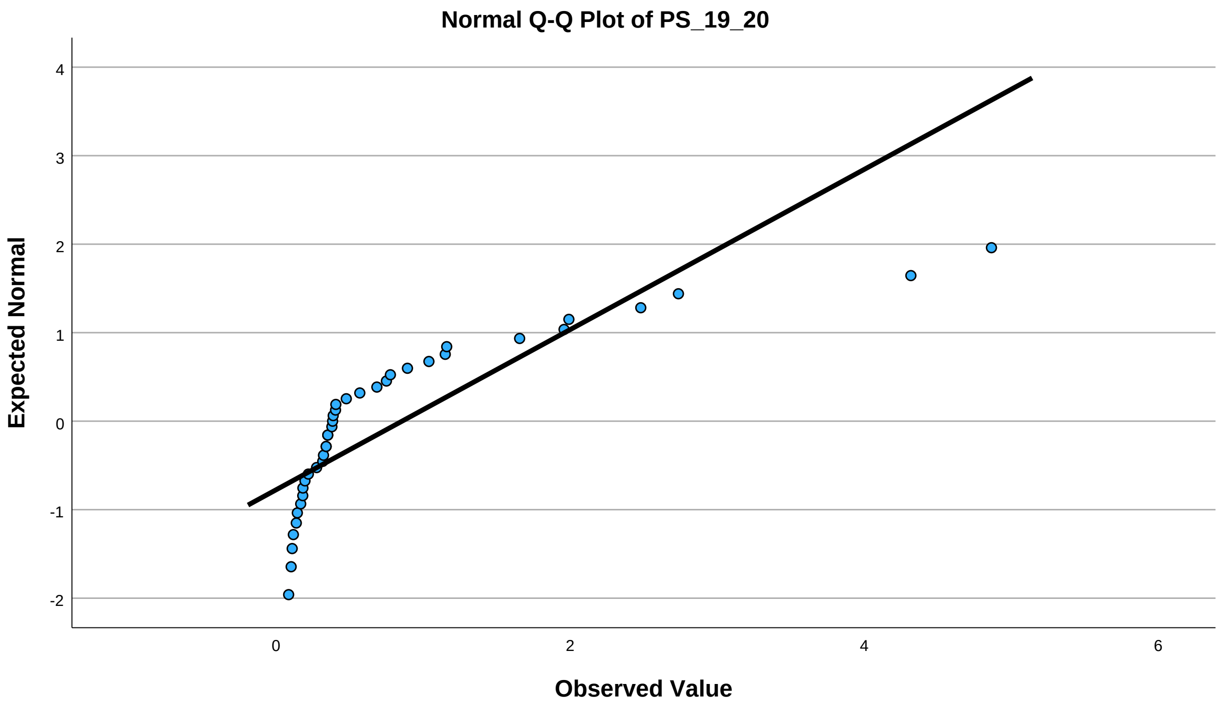

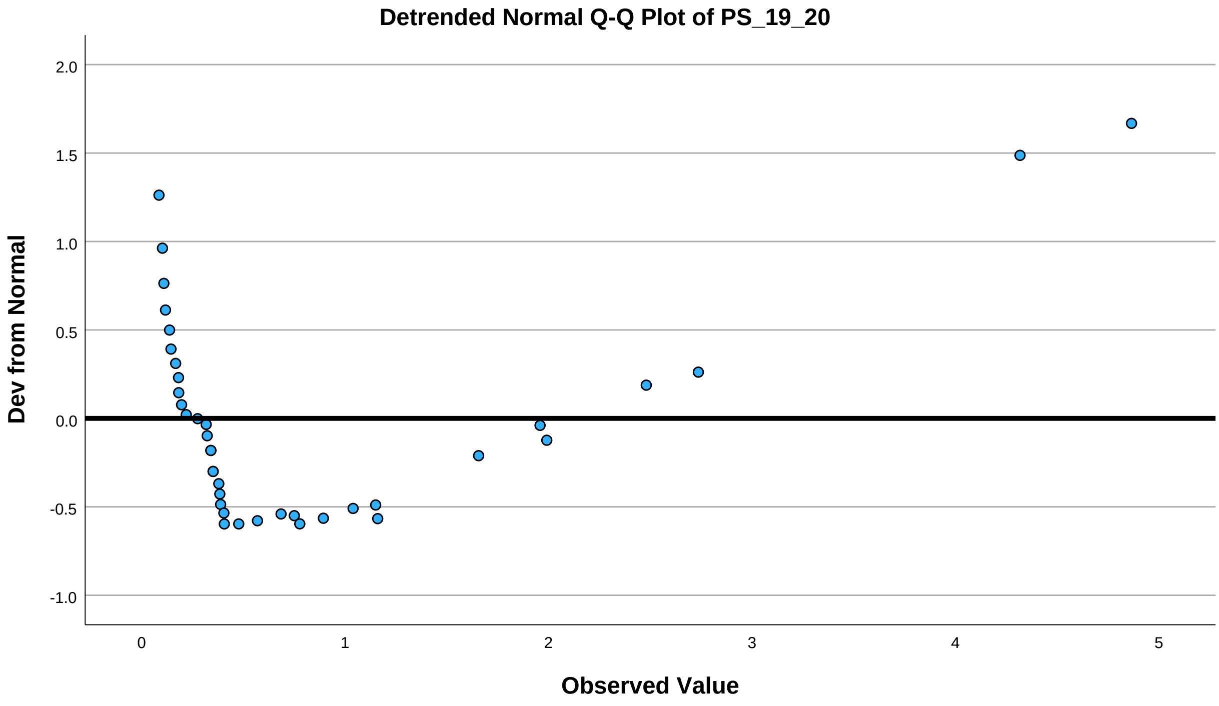

Detrended Normal Q-Q Plot of PS_19_20
Dev from Normal
2.0
1.5
1.0
0.5
0.0
-0.5
-1.0
0
1
2
3
4
5
Observed Value

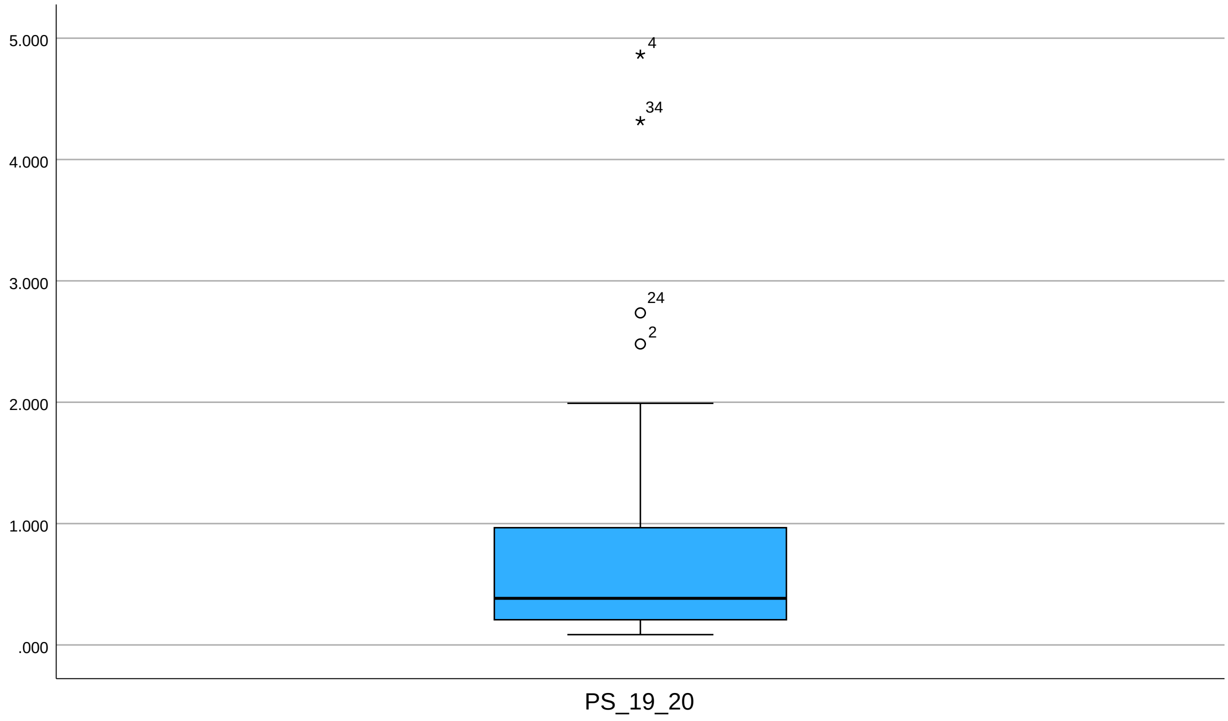

5.000
4.000
3.000
2.000
1.000
.000
4
34
24
2
PS_19_20

Explore

Case Processing Summary

	Cases					
	Valid		Missing		Total	
	N	Percent	N	Percent	N	Percent
BrainRate	39	100.0%	0	0.0%	39	100.0%
PermutationEntropy	39	100.0%	0	0.0%	39	100.0%
PermutationEntropy_01	39	100.0%	0	0.0%	39	100.0%
SampleEntropy	39	100.0%	0	0.0%	39	100.0%
SpectralEntropy_bits	39	100.0%	0	0.0%	39	100.0%
SpectralEntropy_01	39	100.0%	0	0.0%	39	100.0%
HiguchiFractalDimension	39	100.0%	0	0.0%	39	100.0%
LempelZivComplexity	39	100.0%	0	0.0%	39	100.0%
RenyiEntropy	39	100.0%	0	0.0%	39	100.0%
TsallisEntropy	39	100.0%	0	0.0%	39	100.0%
PS_4_5	39	100.0%	0	0.0%	39	100.0%
PS_5_6	39	100.0%	0	0.0%	39	100.0%
PS_6_7	39	100.0%	0	0.0%	39	100.0%
PS_7_8	39	100.0%	0	0.0%	39	100.0%
PS_8_9	39	100.0%	0	0.0%	39	100.0%
PS_9_10	39	100.0%	0	0.0%	39	100.0%
PS_10_11	39	100.0%	0	0.0%	39	100.0%
PS_11_12	39	100.0%	0	0.0%	39	100.0%
PS_12_13	39	100.0%	0	0.0%	39	100.0%
PS_13_14	39	100.0%	0	0.0%	39	100.0%
PS_14_15	39	100.0%	0	0.0%	39	100.0%
PS_15_16	39	100.0%	0	0.0%	39	100.0%
PS_16_17	39	100.0%	0	0.0%	39	100.0%
PS_17_18	39	100.0%	0	0.0%	39	100.0%
PS_18_19	39	100.0%	0	0.0%	39	100.0%
PS_19_20	39	100.0%	0	0.0%	39	100.0%

Descriptives

			Statistic	Std. Error
BrainRate	Mean		7.5567	.27958
	95% Confidence Interval for Mean	Lower Bound	6.9907	
		Upper Bound	8.1226	
	5% Trimmed Mean		7.5174	
	Median		7.3300	
	Variance		3.048	
	Std. Deviation		1.74595	
	Minimum		4.37	
	Maximum		11.29	
	Range		6.92	
	Interquartile Range		2.90	
	Skewness		.568	.378
	Kurtosis		-.616	.741
PermutationEntropy	Mean		1.4628855330	.00992659886
	95% Confidence Interval for Mean	Lower Bound	1.4427901842	
		Upper Bound	1.4829808818	
	5% Trimmed Mean		1.4644563272	
	Median		1.4786085057	
	Variance		.004	
	Std. Deviation		.06199159004	
	Minimum		1.3148325351	
	Maximum		1.5824809610	
	Range		.26764842594	
	Interquartile Range		.09476918740	
	Skewness		-.384	.378
	Kurtosis		-.458	.741
PermutationEntropy_01	Mean		.81645196138	.00554014031
	95% Confidence Interval for Mean	Lower Bound	.80523653367	
		Upper Bound	.82766738910	
	5% Trimmed Mean		.81732863832	
	Median		.82522711956	
	Variance		.001	
	Std. Deviation		.03459816516	
	Minimum		.73382200997	
	Maximum		.88319944067	
	Range		.14937743070	
	Interquartile Range		.05289169056	
	Skewness		-.384	.378
	Kurtosis		-.458	.741

Descriptives

			Statistic	Std. Error
SampleEntropy	Mean		.70873620463	.02192131761
	95% Confidence Interval for Mean	Lower Bound	.66435881721	
		Upper Bound	.75311359206	
	5% Trimmed Mean		.70710358424	
	Median		.71260935312	
	Variance		.019	
	Std. Deviation		.13689858457	
	Minimum		.34698205245	
	Maximum		1.0623721184	
	Range		.71539006595	
	Interquartile Range		.15210326214	
	Skewness		.210	.378
	Kurtosis		1.128	.741
SpectralEntropy_bits	Mean		4.1667509401	.05330366930
	95% Confidence Interval for Mean	Lower Bound	4.0588433031	
		Upper Bound	4.2746585772	
	5% Trimmed Mean		4.1788350987	
	Median		4.0745104598	
	Variance		.111	
	Std. Deviation		.33288130806	
	Minimum		3.0730905388	
	Maximum		4.7687386255	
	Range		1.6956480867	
	Interquartile Range		.46065928589	
	Skewness		-.580	.378
	Kurtosis		1.702	.741
SpectralEntropy_01	Mean		.59429694522	.00760261611
	95% Confidence Interval for Mean	Lower Bound	.57890625353	
		Upper Bound	.60968763690	
	5% Trimmed Mean		.59602048921	
	Median		.58114083474	
	Variance		.002	
	Std. Deviation		.04747832240	
	Minimum		.43830993161	
	Maximum		.68015747482	
	Range		.24184754322	
	Interquartile Range		.06570308865	
	Skewness		-.580	.378
	Kurtosis		1.702	.741

Descriptives

			Statistic	Std. Error
HiguchiFractalDimension	Mean		.58026215172	.01459019185
	95% Confidence Interval for Mean	Lower Bound	.55072585248	
		Upper Bound	.60979845096	
	5% Trimmed Mean		.58161664119	
	Median		.59232797240	
	Variance		.008	
	Std. Deviation		.09111571893	
	Minimum		.37150267158	
	Maximum		.74347562711	
	Range		.37197295553	
	Interquartile Range		.13052903746	
	Skewness		-.190	.378
	Kurtosis		-.596	.741
LempelZivComplexity	Mean		.26841162865	.00401409863
	95% Confidence Interval for Mean	Lower Bound	.26028551080	
		Upper Bound	.27653774650	
	5% Trimmed Mean		.26762707398	
	Median		.27042472619	
	Variance		.001	
	Std. Deviation		.02506803793	
	Minimum		.22714482140	
	Maximum		.31981038681	
	Range		.09266556541	
	Interquartile Range		.03437069852	
	Skewness		.548	.378
	Kurtosis		-.387	.741
RenyiEntropy	Mean		7.8008309572	.13659363903
	95% Confidence Interval for Mean	Lower Bound	7.5243115915	
		Upper Bound	8.0773503228	
	5% Trimmed Mean		7.8562431960	
	Median		8.0026022490	
	Variance		.728	
	Std. Deviation		.85302700235	
	Minimum		5.6097972681	
	Maximum		8.8109867393	
	Range		3.2011894712	
	Interquartile Range		.92206383571	
	Skewness		-1.086	.378
	Kurtosis		.232	.741

Descriptives

			Statistic	Std. Error
TsallisEntropy	Mean		1.8933044331	.04969635382
	95% Confidence Interval for Mean	Lower Bound	1.7926994245	
		Upper Bound	1.9939094417	
	5% Trimmed Mean		1.9547313872	
	Median		1.9860286520	
	Variance		.096	
	Std. Deviation		.31035363011	
	Minimum		.38361289086	
	Maximum		1.9887258051	
	Range		1.6051129143	
	Interquartile Range		.00649499670	
	Skewness		-3.970	.378
	Kurtosis		16.333	.741
PS_4_5	Mean		3.22779	.428074
	95% Confidence Interval for Mean	Lower Bound	2.36120	
		Upper Bound	4.09438	
	5% Trimmed Mean		2.87183	
	Median		2.32000	
	Variance		7.147	
	Std. Deviation		2.673320	
	Minimum		.670	
	Maximum		12.780	
	Range		12.110	
	Interquartile Range		1.114	
	Skewness		2.213	.378
	Kurtosis		5.028	.741
PS_5_6	Mean		2.69079	.388600
	95% Confidence Interval for Mean	Lower Bound	1.90412	
		Upper Bound	3.47747	
	5% Trimmed Mean		2.35110	
	Median		1.98700	
	Variance		5.889	
	Std. Deviation		2.426805	
	Minimum		.492	
	Maximum		13.690	
	Range		13.198	
	Interquartile Range		1.015	
	Skewness		3.036	.378
	Kurtosis		11.038	.741

Descriptives

			Statistic	Std. Error
PS_6_7	Mean		1.94749	.241455
	95% Confidence Interval for Mean	Lower Bound	1.45869	
		Upper Bound	2.43629	
	5% Trimmed Mean		1.76738	
	Median		1.36000	
	Variance		2.274	
	Std. Deviation		1.507885	
	Minimum		.373	
	Maximum		8.660	
	Range		8.287	
	Interquartile Range		1.230	
	Skewness		2.675	.378
	Kurtosis		9.627	.741
PS_7_8	Mean		1.65400	.200725
	95% Confidence Interval for Mean	Lower Bound	1.24765	
		Upper Bound	2.06035	
	5% Trimmed Mean		1.50626	
	Median		1.25100	
	Variance		1.571	
	Std. Deviation		1.253527	
	Minimum		.384	
	Maximum		5.995	
	Range		5.611	
	Interquartile Range		.660	
	Skewness		2.004	.378
	Kurtosis		3.946	.741
PS_8_9	Mean		1.51779	.182290
	95% Confidence Interval for Mean	Lower Bound	1.14877	
		Upper Bound	1.88682	
	5% Trimmed Mean		1.41196	
	Median		1.06700	
	Variance		1.296	
	Std. Deviation		1.138400	
	Minimum		.284	
	Maximum		4.968	
	Range		4.684	
	Interquartile Range		.902	
	Skewness		1.714	.378
	Kurtosis		2.260	.741

Descriptives

			Statistic	Std. Error
PS_9_10	Mean		1.79808	.273270
	95% Confidence Interval for Mean	Lower Bound	1.24487	
		Upper Bound	2.35128	
	5% Trimmed Mean		1.59342	
	Median		1.24900	
	Variance		2.912	
	Std. Deviation		1.706570	
	Minimum		.265	
	Maximum		7.127	
	Range		6.862	
	Interquartile Range		1.137	
	Skewness		2.173	.378
	Kurtosis		4.126	.741
PS_10_11	Mean		2.11867	.290336
	95% Confidence Interval for Mean	Lower Bound	1.53091	
		Upper Bound	2.70642	
	5% Trimmed Mean		1.90648	
	Median		1.57500	
	Variance		3.288	
	Std. Deviation		1.813150	
	Minimum		.303	
	Maximum		9.470	
	Range		9.167	
	Interquartile Range		1.639	
	Skewness		2.298	.378
	Kurtosis		6.511	.741
PS_11_12	Mean		1.05695	.127481
	95% Confidence Interval for Mean	Lower Bound	.79888	
		Upper Bound	1.31502	
	5% Trimmed Mean		.96251	
	Median		.77900	
	Variance		.634	
	Std. Deviation		.796118	
	Minimum		.256	
	Maximum		3.800	
	Range		3.544	
	Interquartile Range		.608	
	Skewness		1.960	.378
	Kurtosis		3.869	.741

Descriptives

			Statistic	Std. Error
PS_12_13	Mean		.84962	.126311
	95% Confidence Interval for Mean	Lower Bound	.59391	
		Upper Bound	1.10532	
	5% Trimmed Mean		.72872	
	Median		.57000	
	Variance		.622	
	Std. Deviation		.788812	
	Minimum		.175	
	Maximum		4.324	
	Range		4.149	
	Interquartile Range		.508	
	Skewness		2.999	.378
	Kurtosis		10.424	.741
PS_13_14	Mean		.78403	.138361
	95% Confidence Interval for Mean	Lower Bound	.50393	
		Upper Bound	1.06412	
	5% Trimmed Mean		.64906	
	Median		.48400	
	Variance		.747	
	Std. Deviation		.864062	
	Minimum		.156	
	Maximum		5.093	
	Range		4.937	
	Interquartile Range		.586	
	Skewness		3.706	.378
	Kurtosis		16.510	.741
PS_14_15	Mean		.69567	.110039
	95% Confidence Interval for Mean	Lower Bound	.47290	
		Upper Bound	.91843	
	5% Trimmed Mean		.59851	
	Median		.46200	
	Variance		.472	
	Std. Deviation		.687196	
	Minimum		.163	
	Maximum		4.105	
	Range		3.942	
	Interquartile Range		.524	
	Skewness		3.552	.378
	Kurtosis		15.878	.741

Descriptives

			Statistic	Std. Error
PS_15_16	Mean		.57644	.090970
	95% Confidence Interval for Mean	Lower Bound	.39228	
		Upper Bound	.76060	
	5% Trimmed Mean		.49600	
	Median		.41700	
	Variance		.323	
	Std. Deviation		.568109	
	Minimum		.129	
	Maximum		3.528	
	Range		3.399	
	Interquartile Range		.413	
	Skewness		3.959	.378
	Kurtosis		19.462	.741
PS_16_17	Mean		.47805	.059413
	95% Confidence Interval for Mean	Lower Bound	.35778	
		Upper Bound	.59833	
	5% Trimmed Mean		.42603	
	Median		.34400	
	Variance		.138	
	Std. Deviation		.371036	
	Minimum		.102	
	Maximum		2.035	
	Range		1.933	
	Interquartile Range		.350	
	Skewness		2.618	.378
	Kurtosis		8.362	.741
PS_17_18	Mean		.40285	.048086
	95% Confidence Interval for Mean	Lower Bound	.30550	
		Upper Bound	.50019	
	5% Trimmed Mean		.36526	
	Median		.32800	
	Variance		.090	
	Std. Deviation		.300298	
	Minimum		.107	
	Maximum		1.462	
	Range		1.355	
	Interquartile Range		.226	
	Skewness		2.113	.378
	Kurtosis		4.560	.741

Descriptives

			Statistic	Std. Error
PS_18_19	Mean		.38336	.058764
	95% Confidence Interval for Mean	Lower Bound	.26440	
		Upper Bound	.50232	
	5% Trimmed Mean		.32805	
	Median		.27000	
	Variance		.135	
	Std. Deviation		.366983	
	Minimum		.077	
	Maximum		2.170	
	Range		2.093	
	Interquartile Range		.246	
	Skewness		3.474	.378
	Kurtosis		14.847	.741
PS_19_20	Mean		.35331	.047955
	95% Confidence Interval for Mean	Lower Bound	.25623	
		Upper Bound	.45039	
	5% Trimmed Mean		.30768	
	Median		.26000	
	Variance		.090	
	Std. Deviation		.299482	
	Minimum		.078	
	Maximum		1.450	
	Range		1.372	
	Interquartile Range		.160	
	Skewness		2.670	.378
	Kurtosis		7.695	.741

Tests of Normality

	Kolmogorov-Smirnov[a]			Shapiro-Wilk		
	Statistic	df	Sig.	Statistic	df	Sig.
BrainRate	.158	39	.016	.935	39	.026
PermutationEntropy	.113	39	.200*	.973	39	.466
PermutationEntropy_01	.113	39	.200*	.973	39	.466
SampleEntropy	.141	39	.050	.962	39	.210
SpectralEntropy_bits	.122	39	.149	.940	39	.037
SpectralEntropy_01	.122	39	.149	.940	39	.037
HiguchiFractalDimension	.075	39	.200*	.979	39	.675
LempelZivComplexity	.118	39	.189	.935	39	.026
RenyiEntropy	.181	39	.003	.874	39	<.001
TsallisEntropy	.438	39	<.001	.346	39	<.001
PS_4_5	.310	39	<.001	.718	39	<.001
PS_5_6	.303	39	<.001	.651	39	<.001
PS_6_7	.218	39	<.001	.736	39	<.001
PS_7_8	.284	39	<.001	.760	39	<.001
PS_8_9	.215	39	<.001	.781	39	<.001
PS_9_10	.247	39	<.001	.699	39	<.001
PS_10_11	.204	39	<.001	.765	39	<.001
PS_11_12	.205	39	<.001	.779	39	<.001
PS_12_13	.232	39	<.001	.655	39	<.001
PS_13_14	.257	39	<.001	.574	39	<.001
PS_14_15	.227	39	<.001	.618	39	<.001
PS_15_16	.216	39	<.001	.587	39	<.001
PS_16_17	.205	39	<.001	.723	39	<.001
PS_17_18	.251	39	<.001	.754	39	<.001
PS_18_19	.255	39	<.001	.620	39	<.001
PS_19_20	.256	39	<.001	.674	39	<.001

*. This is a lower bound of the true significance.

a. Lilliefors Significance Correction

BrainRate

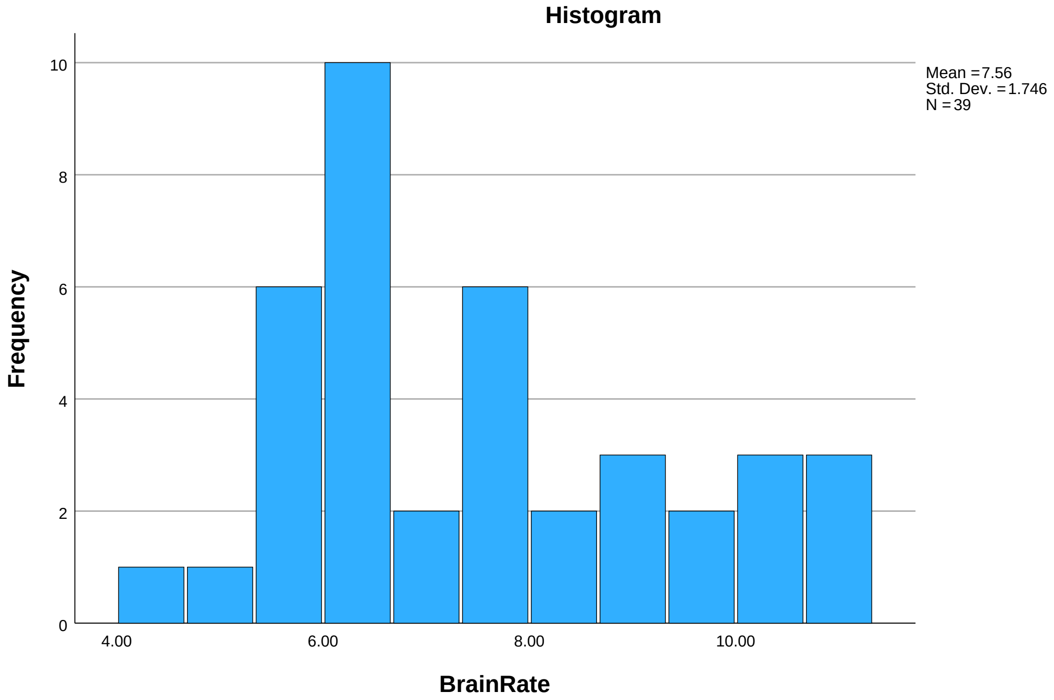

BrainRate Stem-and-Leaf Plot

```
 Frequency    Stem &  Leaf

     1.00        4 .  3
     7.00        5 .  2888999
    11.00        6 .  00245555669
     7.00        7 .  3355667
     4.00        8 .  1269
     3.00        9 .  039
     5.00       10 .  01377
     1.00       11 .  2

 Stem width:      1.00
 Each leaf:       1 case(s)
```

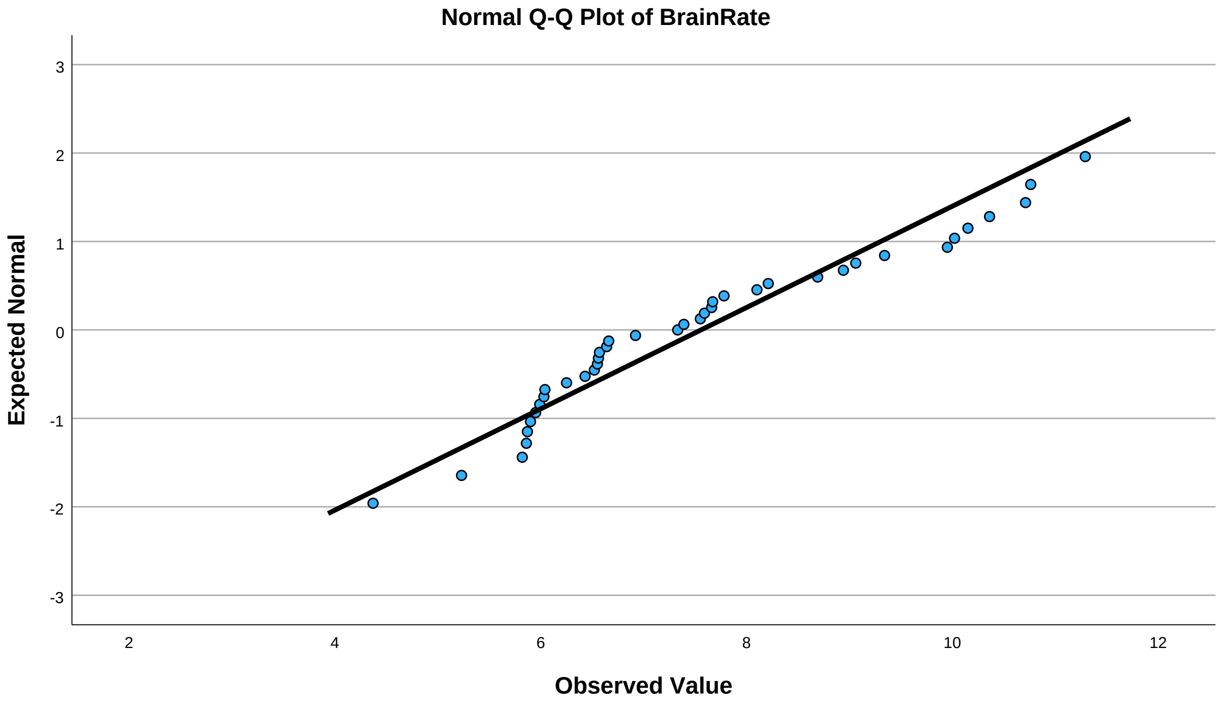
Normal Q-Q Plot of BrainRate
Expected Normal
3
2
1
0
-1
-2
-3
2
4
6
8
10
12
Observed Value

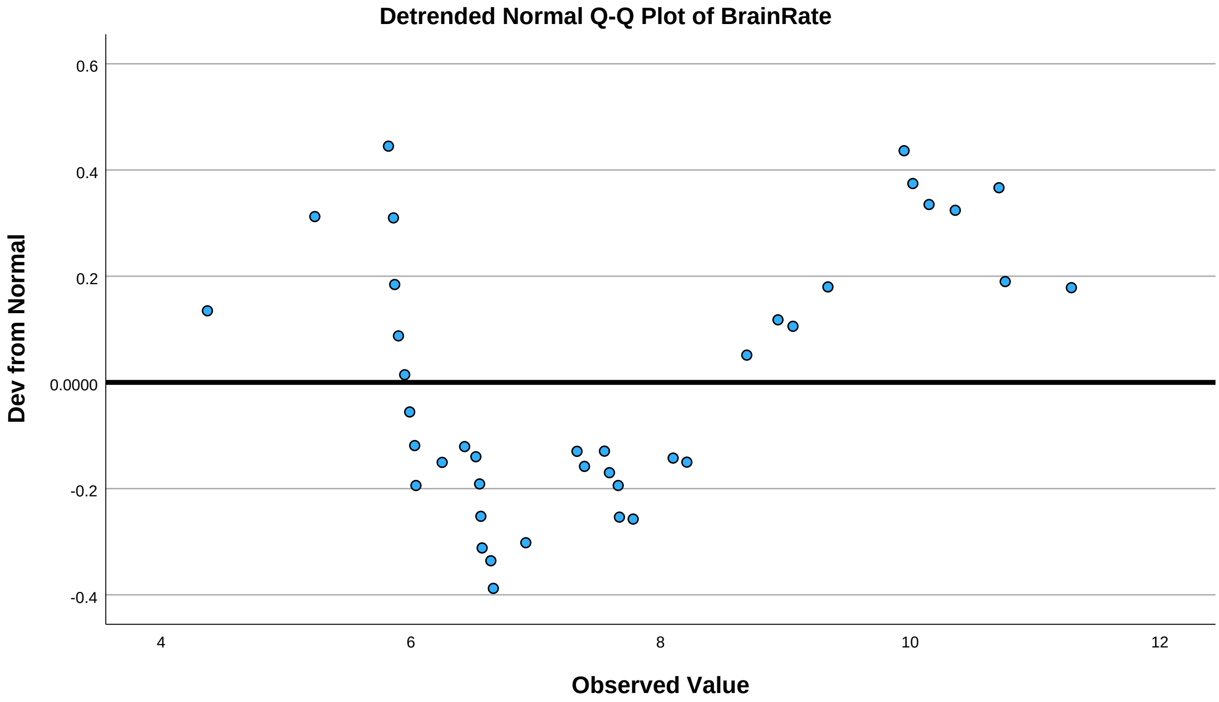
Detrended Normal Q-Q Plot of BrainRate
Dev from Normal
0.6
0.4
0.2
0.0000
-0.2
-0.4
4
6
8
10
12
Observed Value

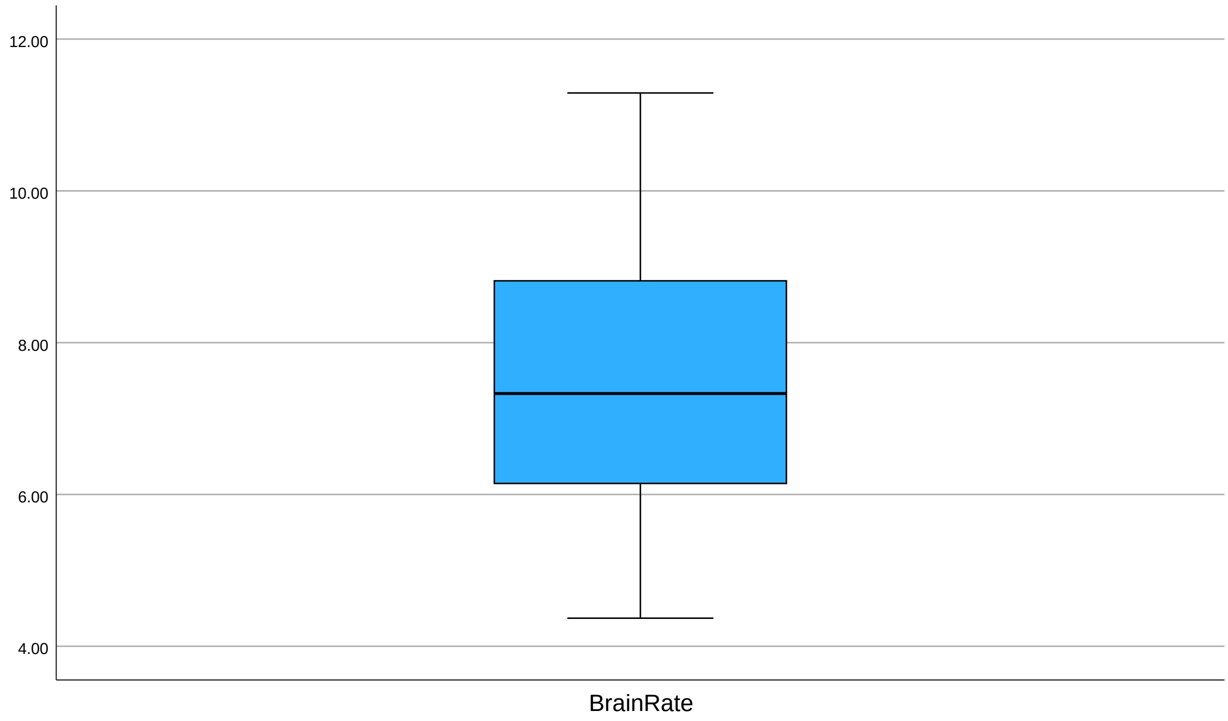

PermutationEntropy

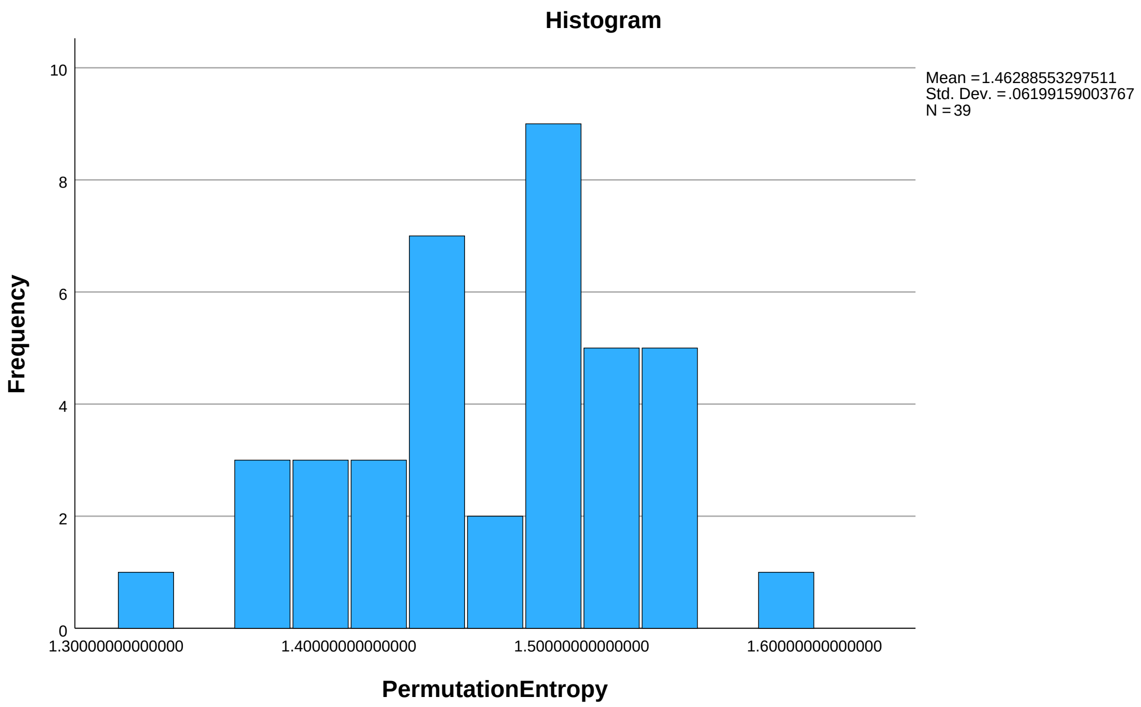

PermutationEntropy Stem-and-Leaf Plot

Frequency   Stem & Leaf

```
    1.00      13 .  1
    6.00      13 .  557889
   10.00      14 .  1112233334
   11.00      14 .  67777899999
   10.00      15 .  1112223344
    1.00      15 .  8

 Stem width:   .1000000
 Each leaf:        1 case(s)
```

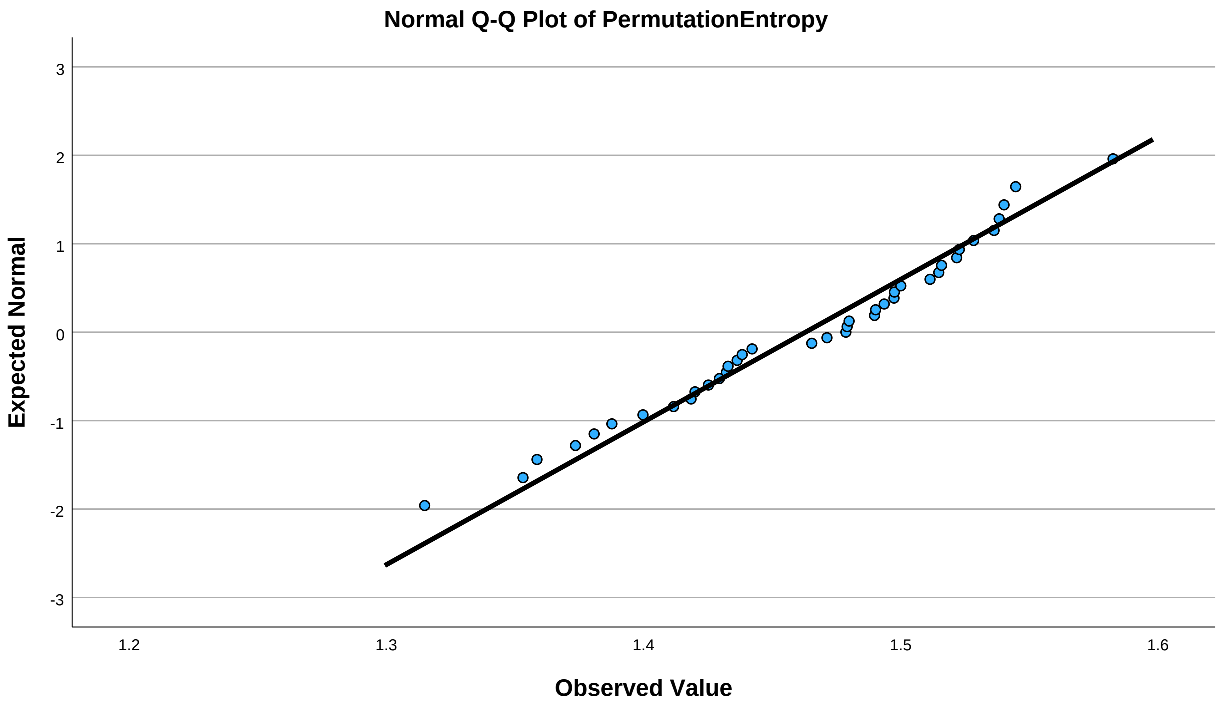

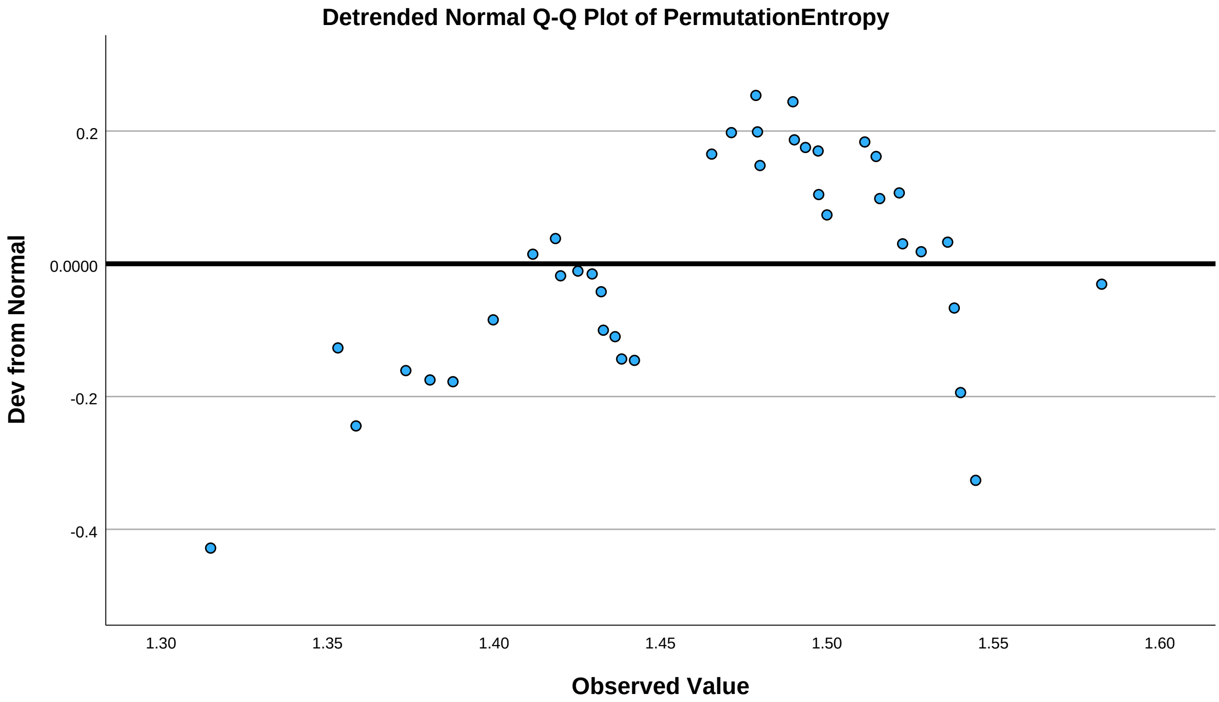

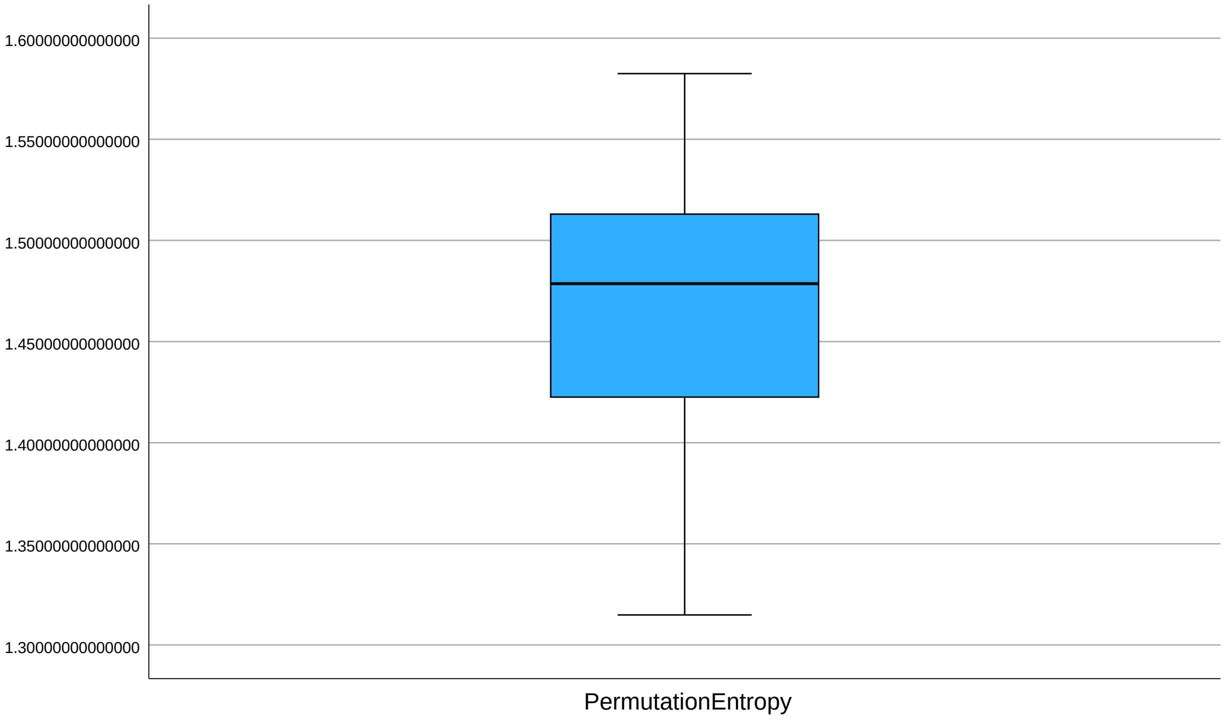

PermutationEntropy_01

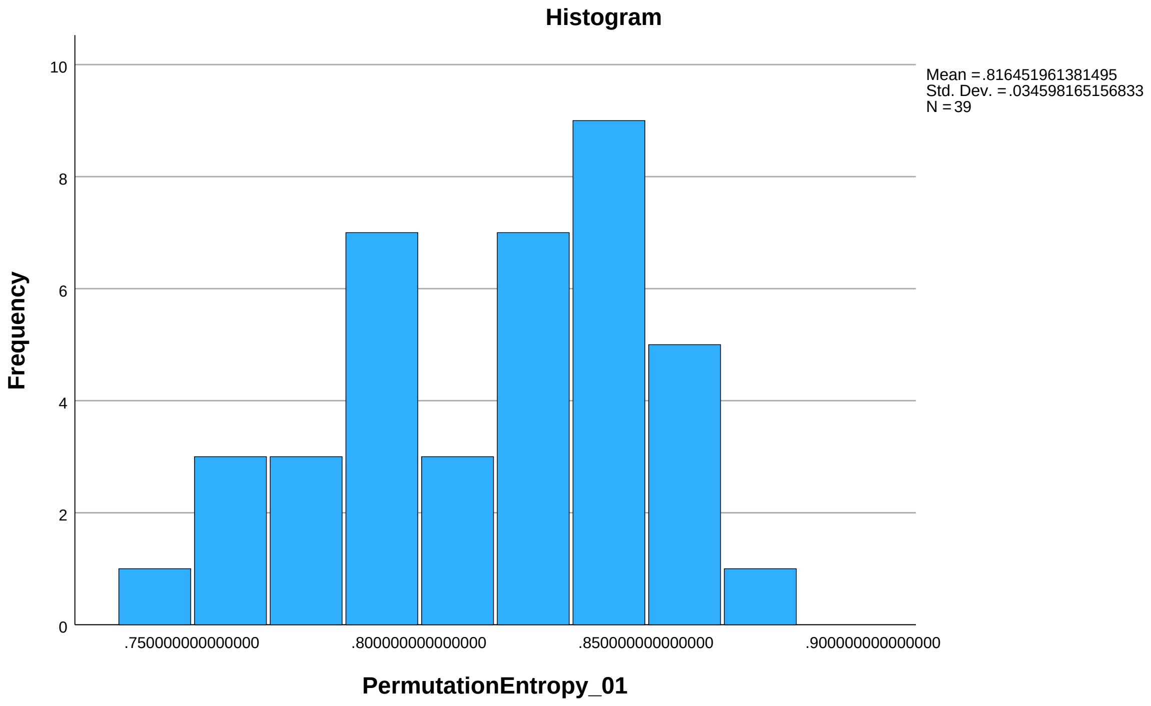


```
PermutationEntropy_01 Stem-and-Leaf Plot

 Frequency    Stem &  Leaf

     1.00        7 .  3
     2.00        7 .  55
     3.00        7 .  677
     8.00        7 .  88999999
     4.00        8 .  0001
    10.00        8 .  2222333333
     9.00        8 .  444445555
     1.00        8 .  6
     1.00        8 .  8

 Stem width:   .1000000
 Each leaf:       1 case(s)
```

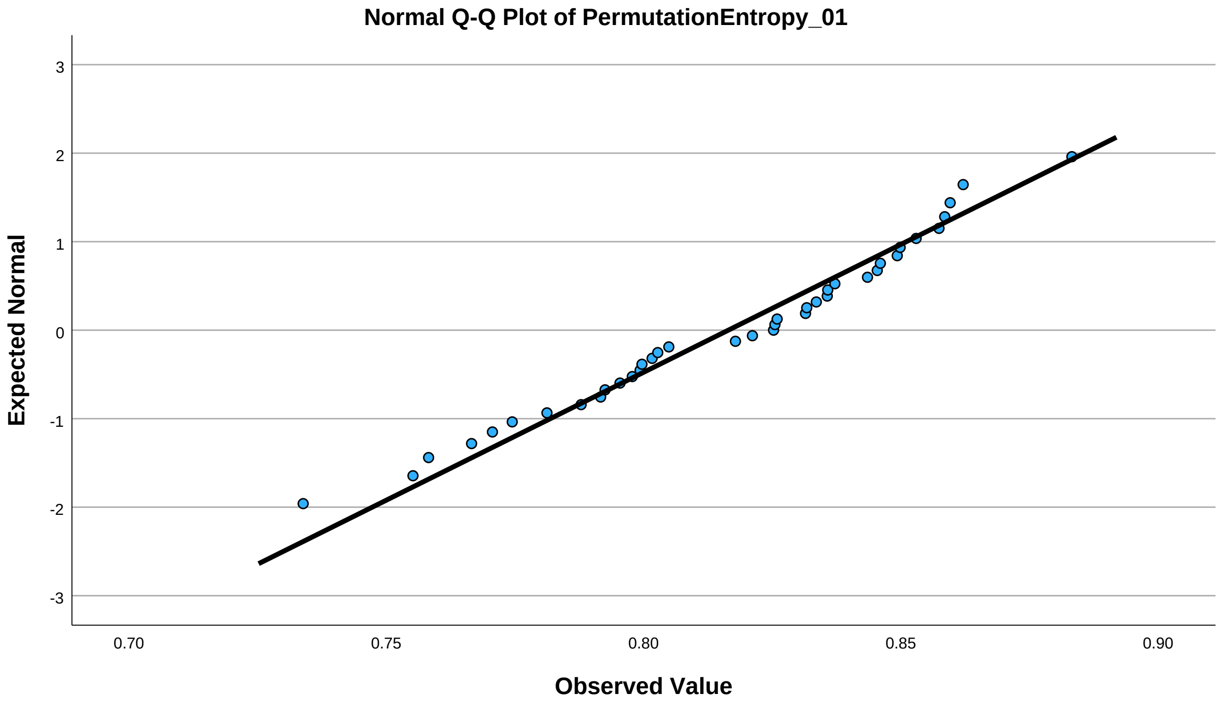
Normal Q-Q Plot of PermutationEntropy_01
Expected Normal
3
2
1
0
-1
-2
-3
0.70
0.75
0.80
0.85
0.90
Observed Value

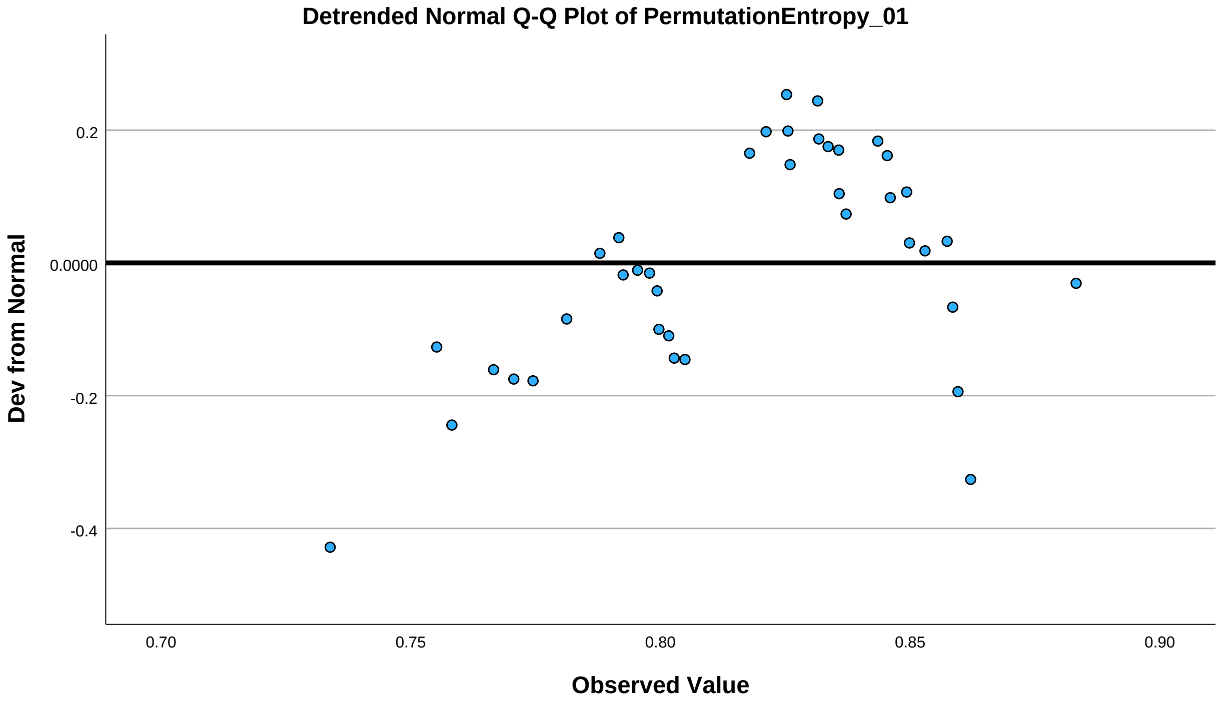
Detrended Normal Q-Q Plot of PermutationEntropy_01
Dev from Normal
0.2
0.0000
-0.2
-0.4
0.70
0.75
0.80
0.85
0.90
Observed Value

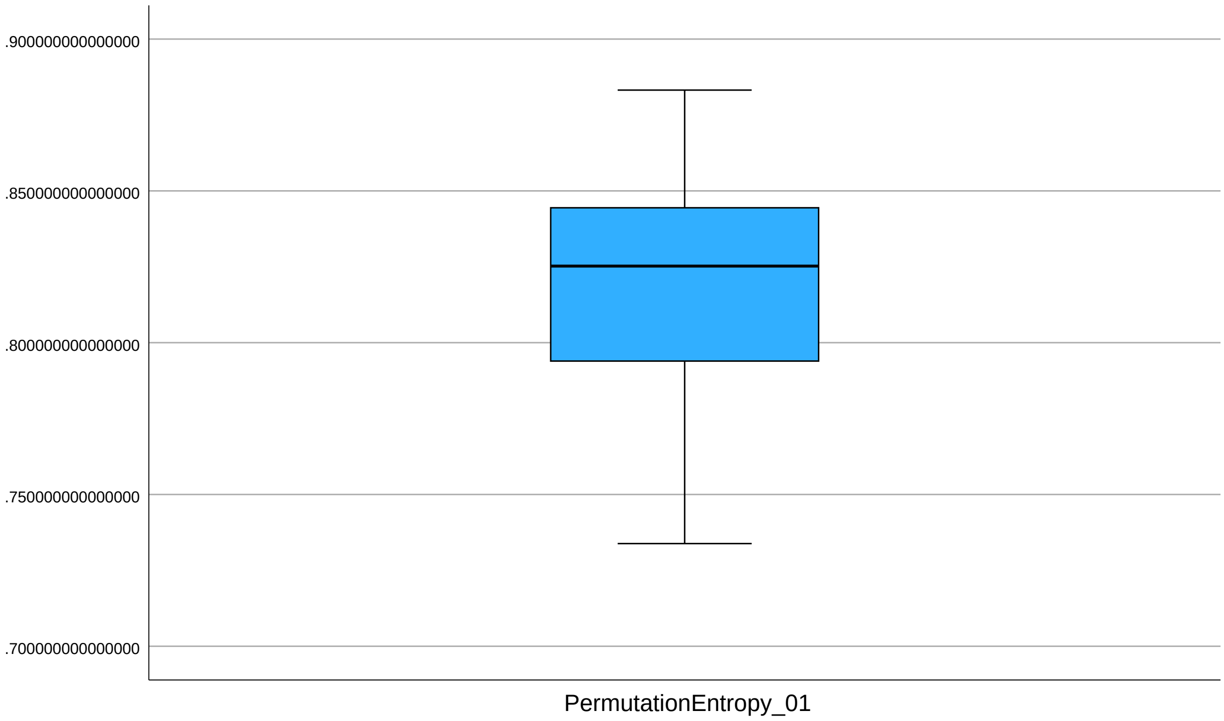

SampleEntropy

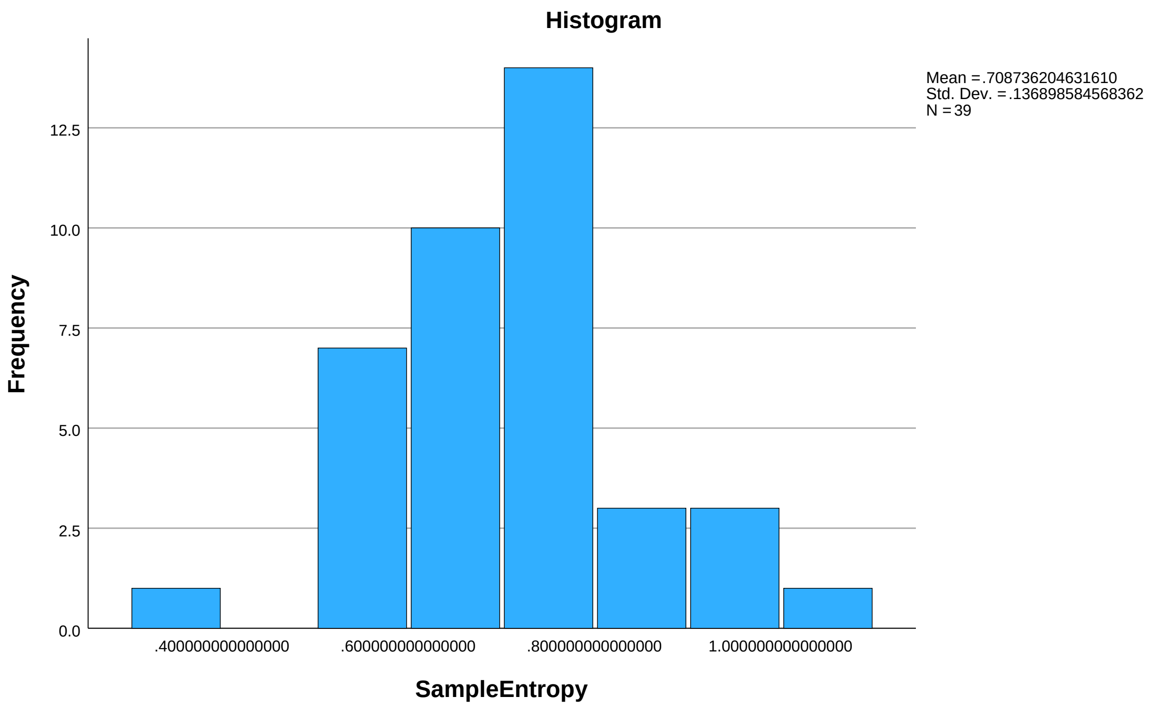


```
SampleEntropy Stem-and-Leaf Plot

 Frequency    Stem &  Leaf
```

```
 1.00 Extremes    (=<.35)
 2.00      5 .  12
 5.00      5 .  55799
 4.00      6 .  0111
 6.00      6 .  566789
 4.00      7 .  1124
10.00      7 .  5556666677
 3.00      8 .  002
  .00      8 .
 1.00      9 .  3
 2.00      9 .  67
 1.00 Extremes    (>=1.06)

Stem width:   .1000000
Each leaf:       1 case(s)
```

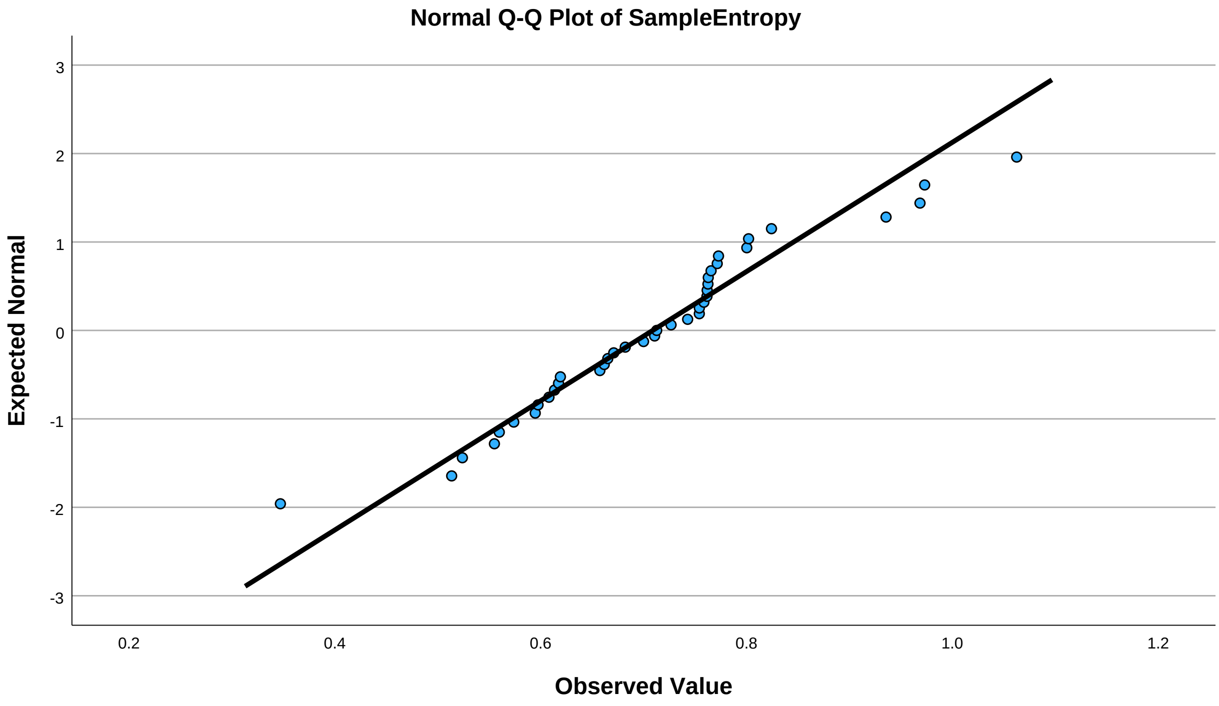

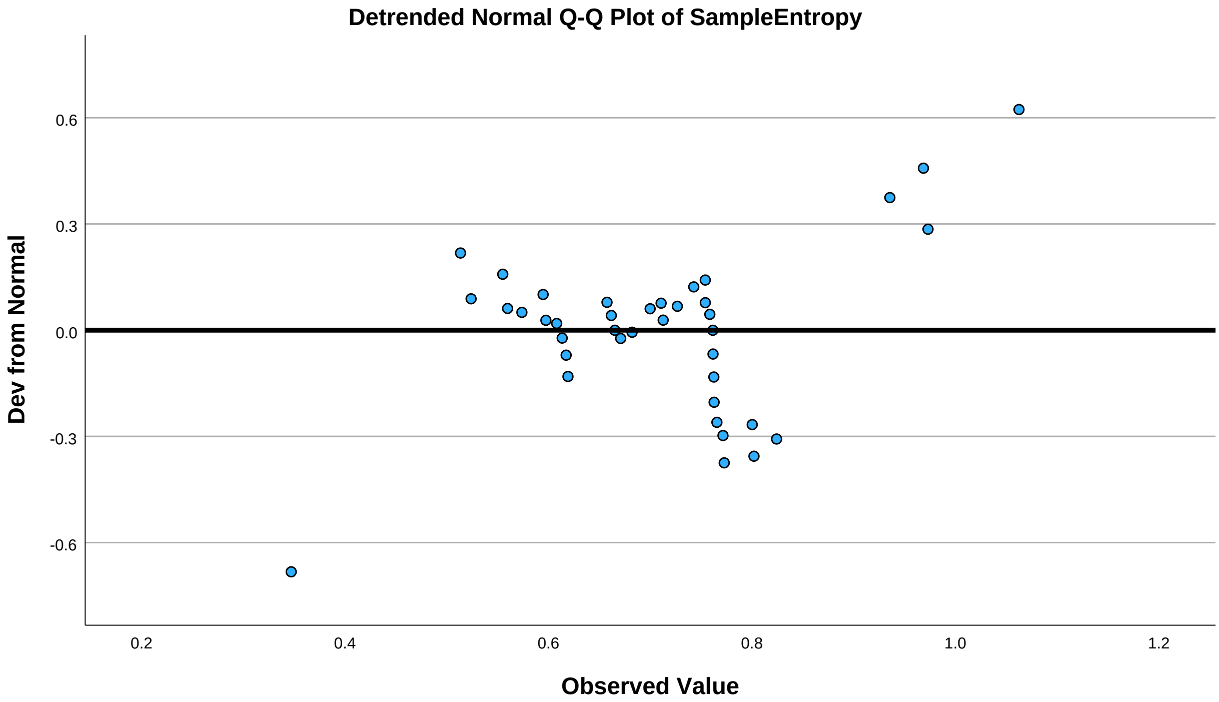

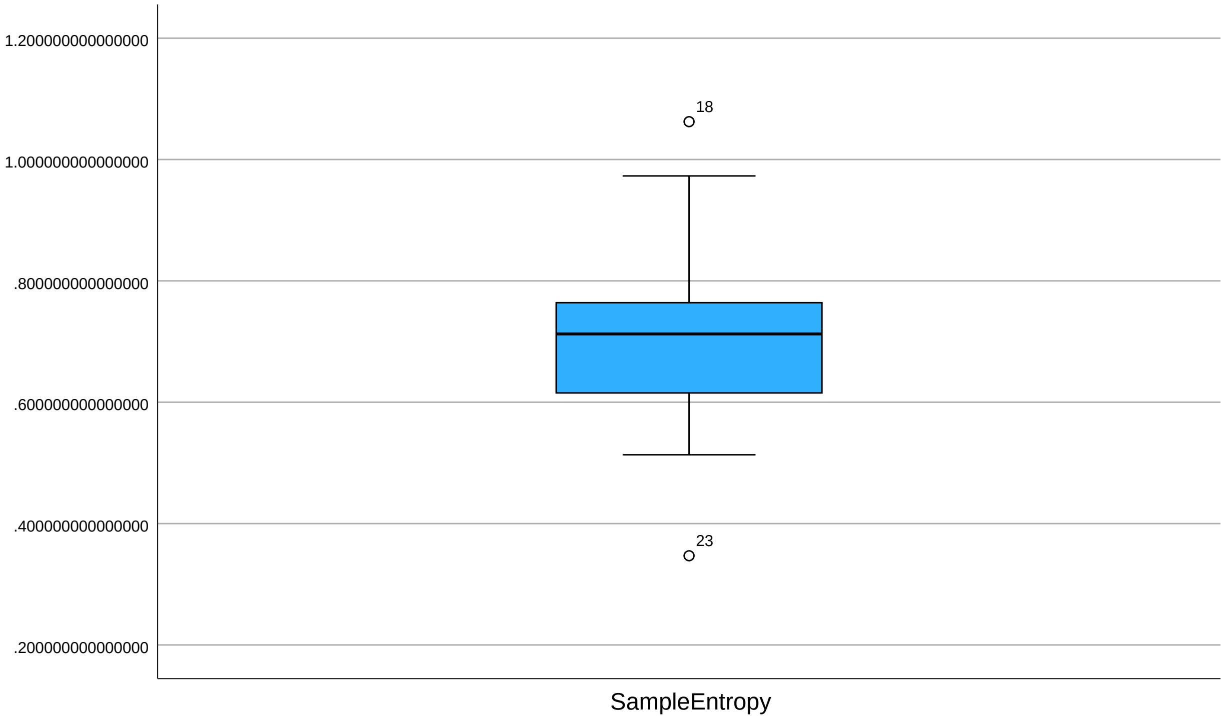

SpectralEntropy_bits

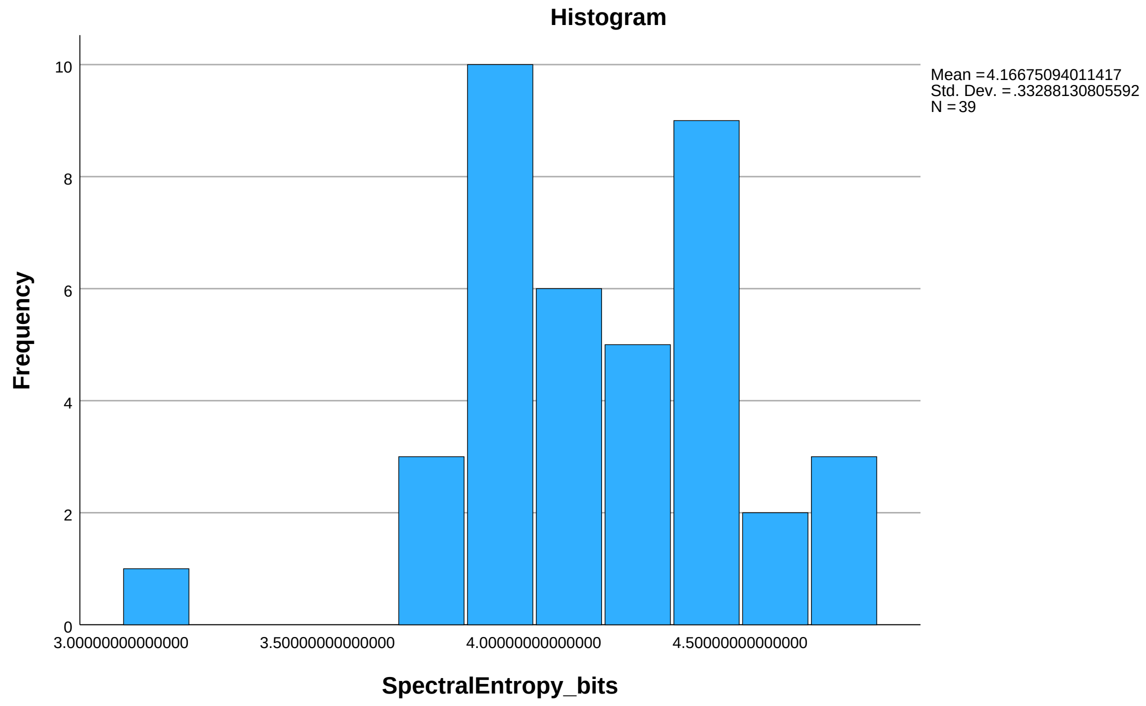


```
SpectralEntropy_bits Stem-and-Leaf Plot

 Frequency    Stem &  Leaf

     1.00 Extremes    (=<3.07)
     1.00       36 .  7
      .00       37 .
     3.00       38 .  127
     9.00       39 .  224467789
     6.00       40 .  003457
     1.00       41 .  7
     2.00       42 .  49
     6.00       43 .  024455
     5.00       44 .  22268
     1.00       45 .  7
     2.00       46 .  58
     2.00       47 .  36

 Stem width:   .1000000
 Each leaf:        1 case(s)
```

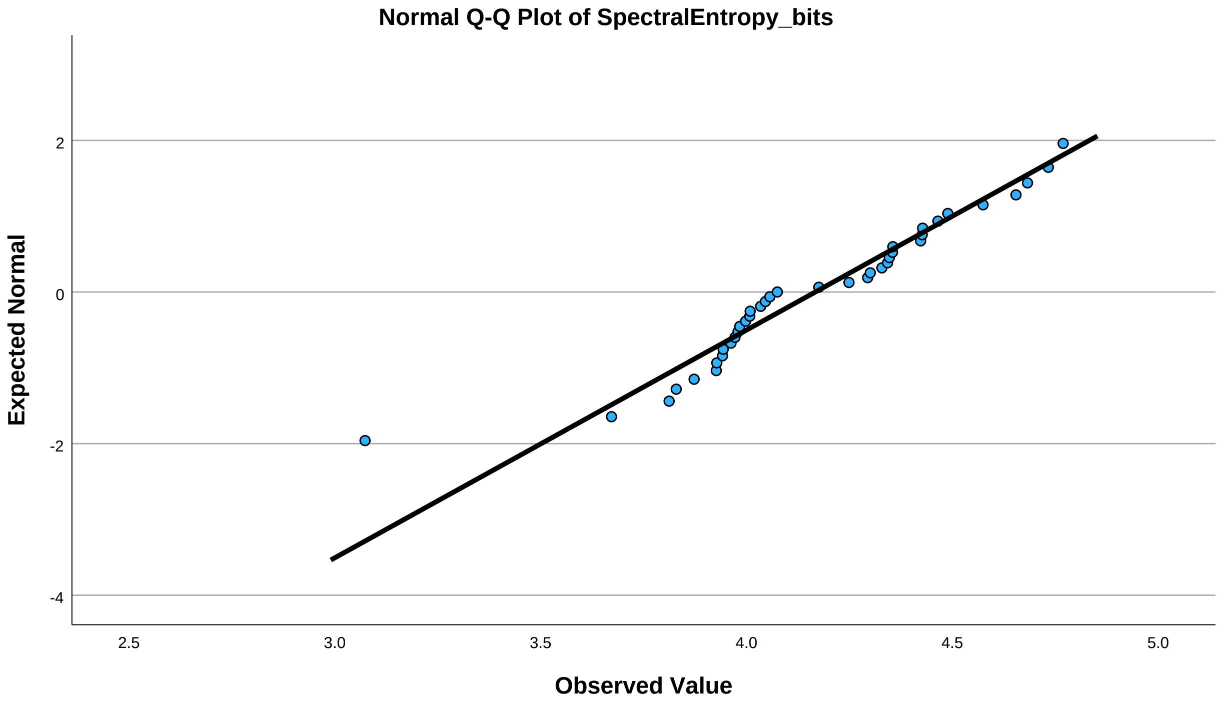
Normal Q-Q Plot of SpectralEntropy_bits
Expected Normal
2
0
-2
-4
2.5
3.0
3.5
4.0
4.5
5.0
Observed Value

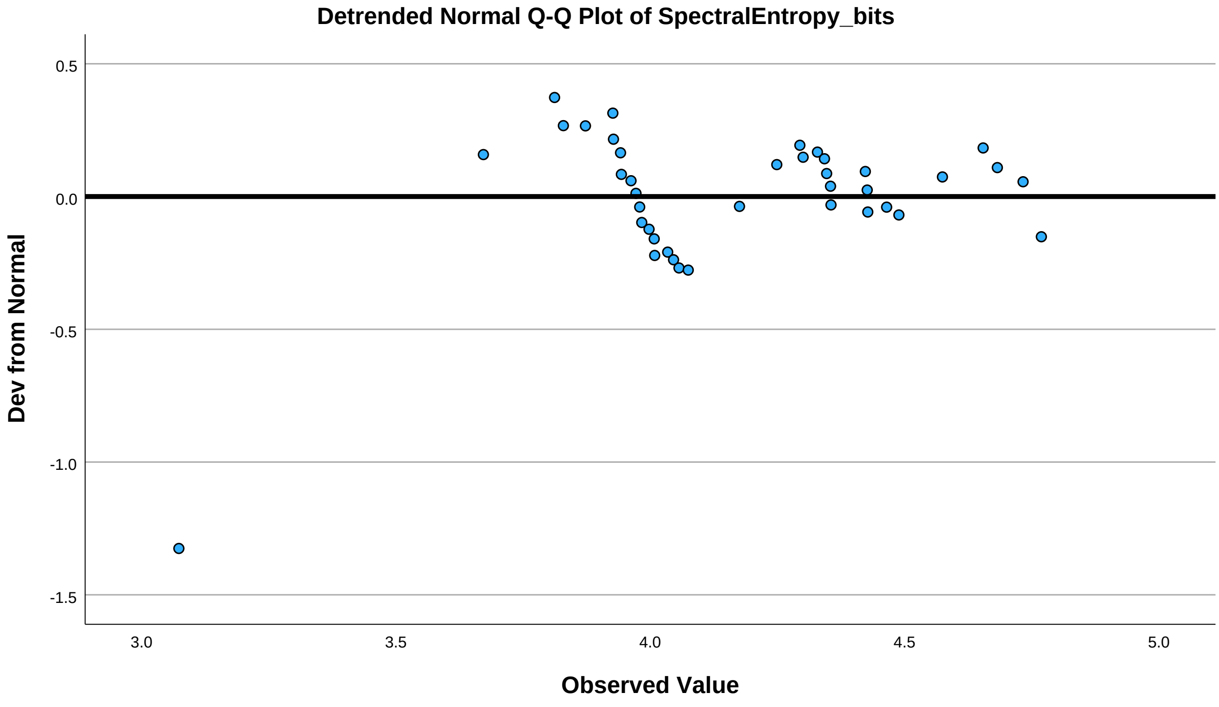
Detrended Normal Q-Q Plot of SpectralEntropy_bits
Dev from Normal
0.5
0.0
-0.5
-1.0
-1.5
3.0
3.5
4.0
4.5
5.0
Observed Value

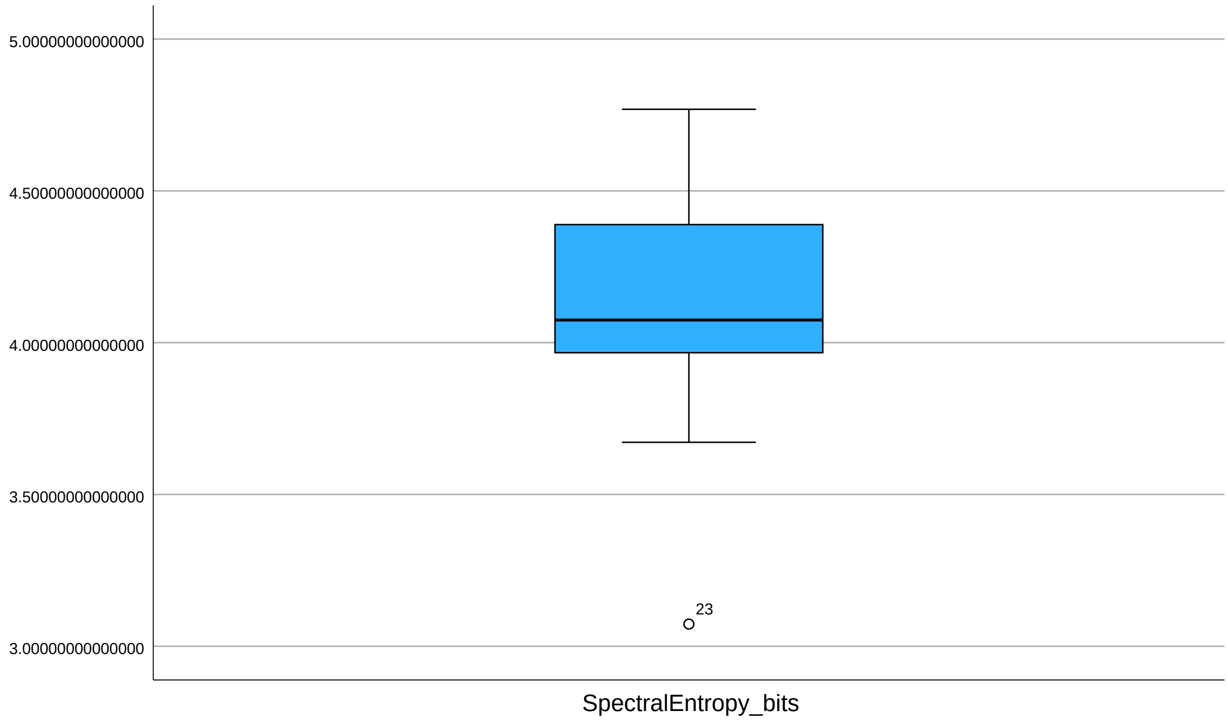

SpectralEntropy_01

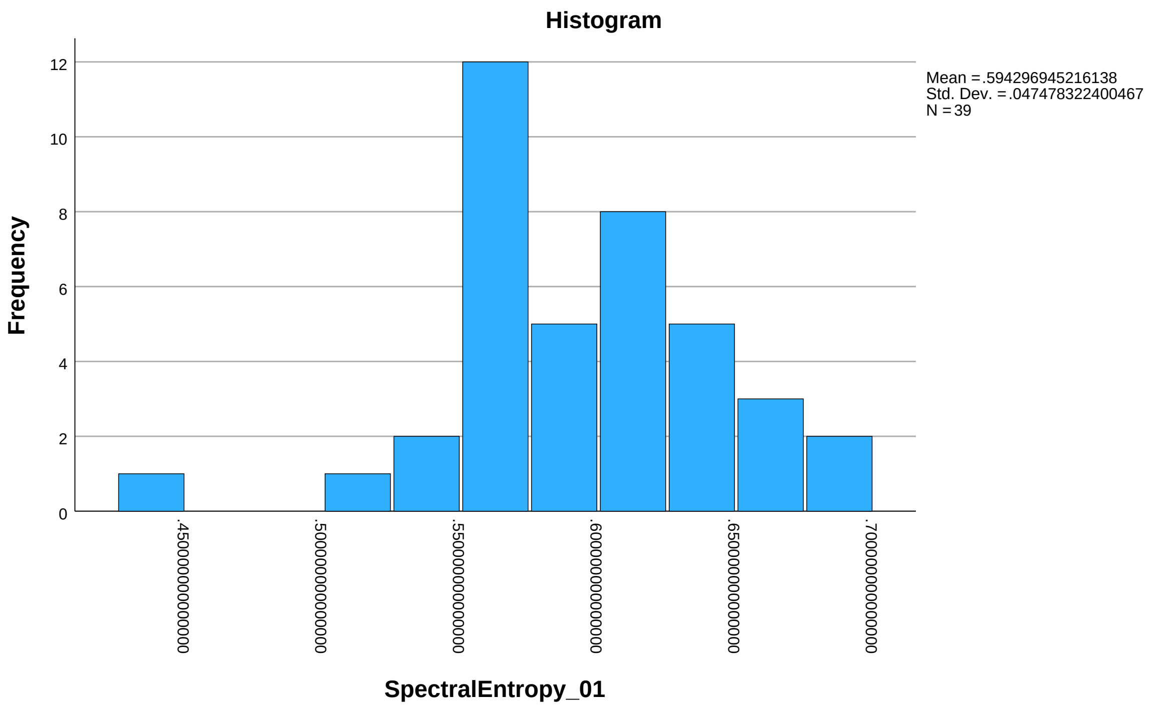

SpectralEntropy_01 Stem-and-Leaf Plot

Frequency Stem & Leaf

```
 1.00 Extremes    (=<.44)
 1.00      5 . 2
 4.00      5 . 4455
13.00      5 . 6666666777777
 2.00      5 . 89
 6.00      6 . 011111
 6.00      6 . 223333
 2.00      6 . 45
 3.00      6 . 667
 1.00      6 . 8

Stem width:   .1000000
Each leaf:       1 case(s)
```

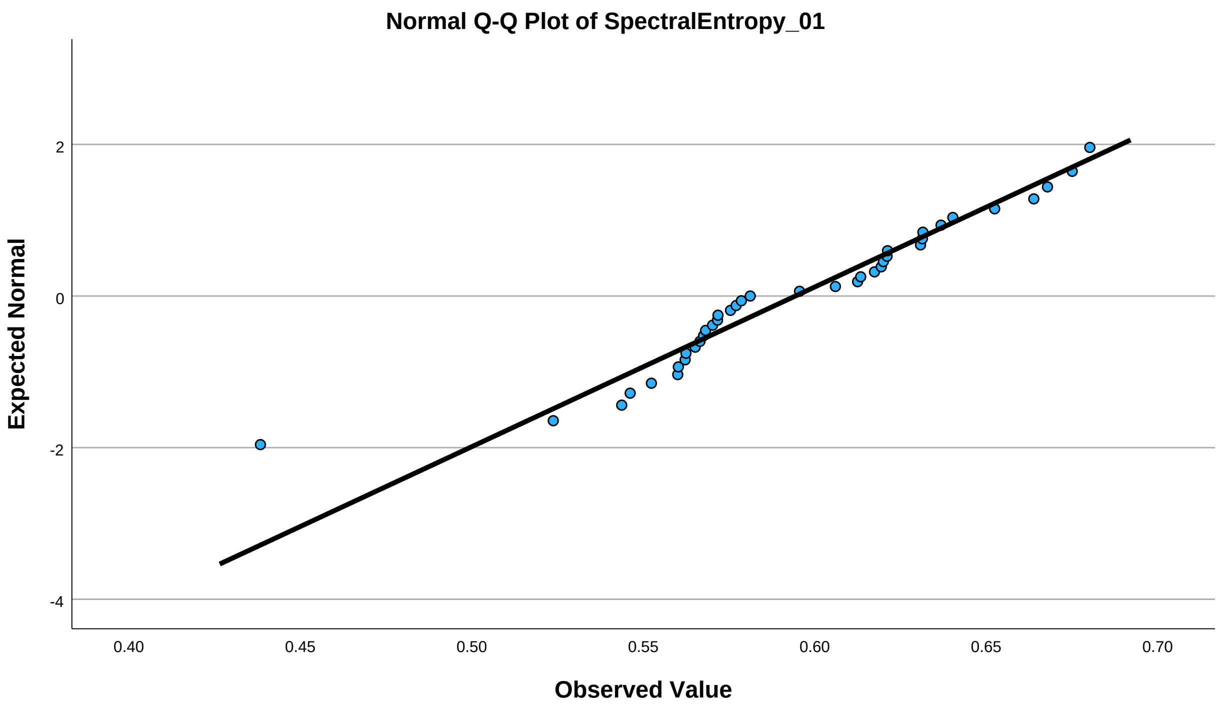

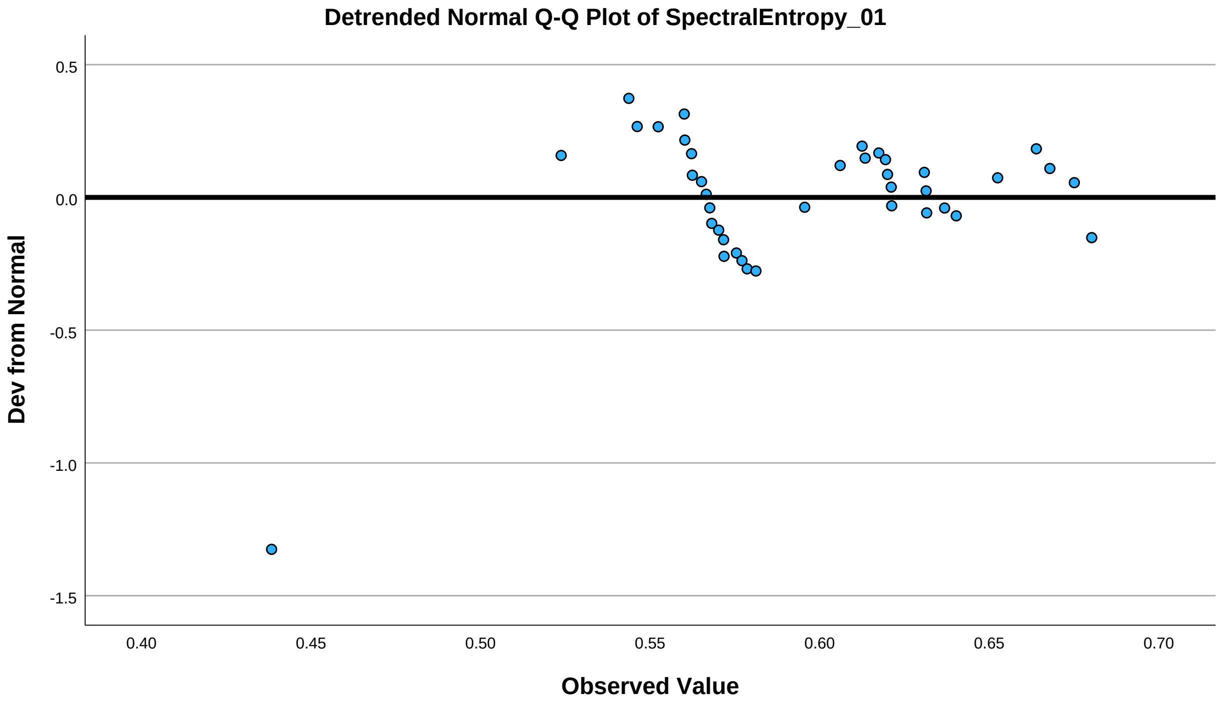

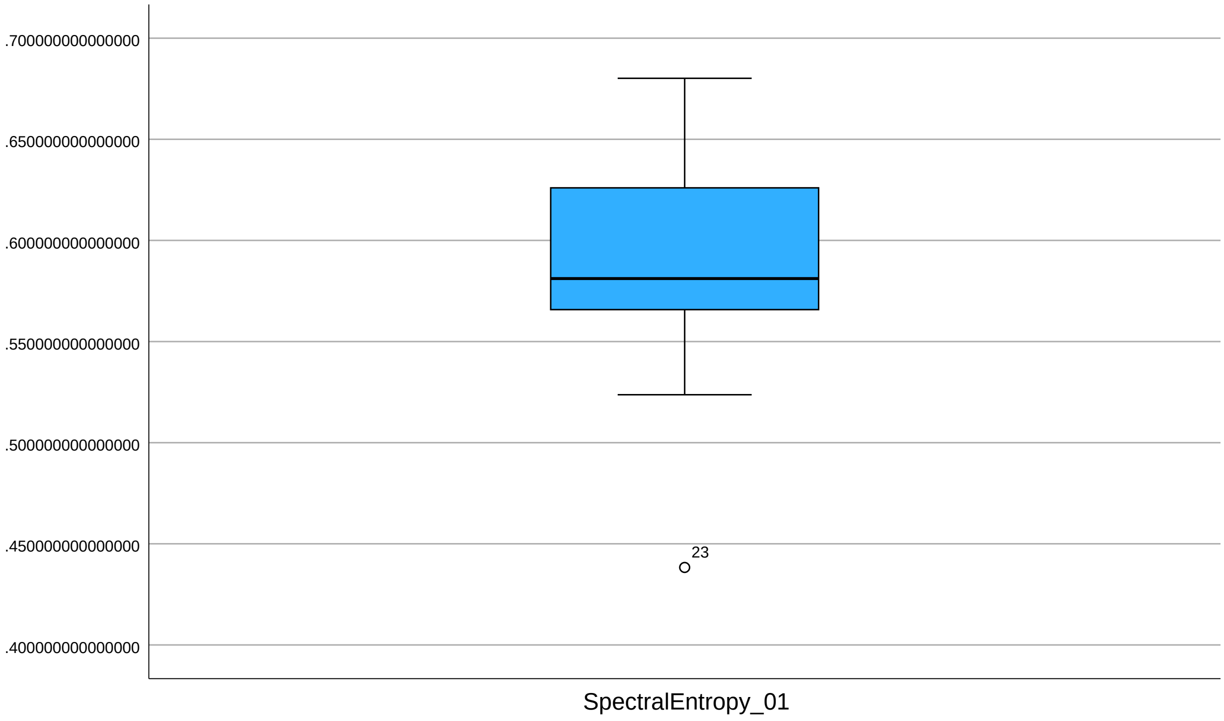

HiguchiFractalDimension

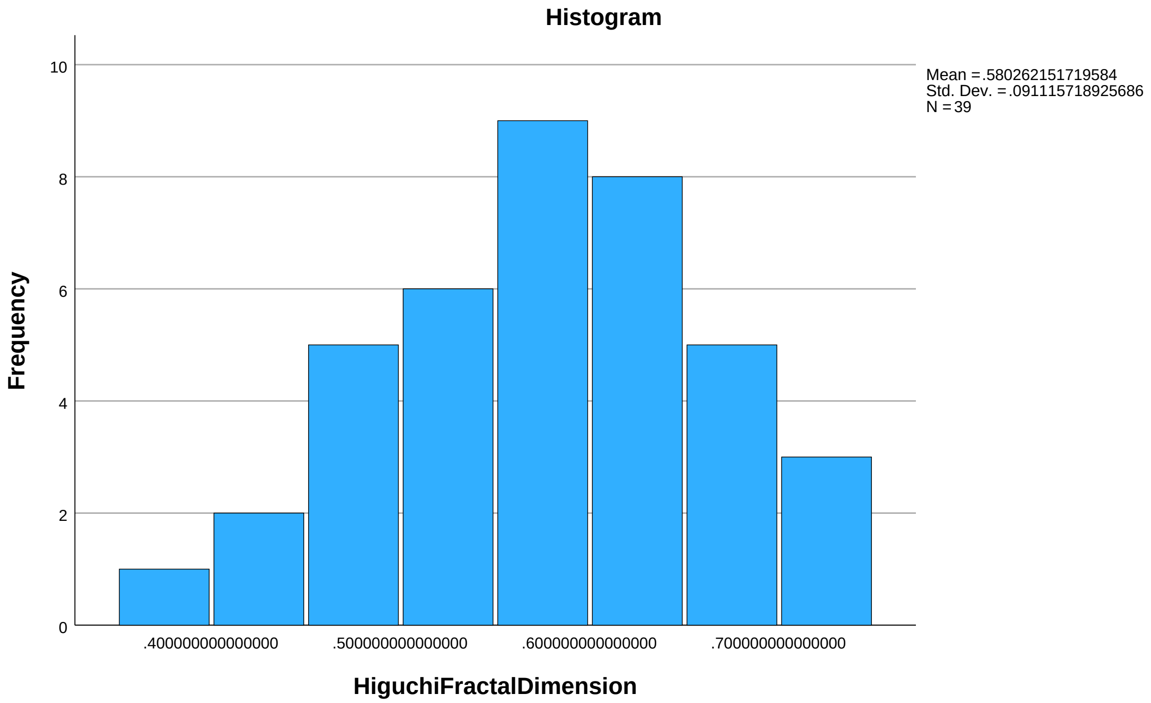


```
HiguchiFractalDimension Stem-and-Leaf Plot

 Frequency    Stem &  Leaf

    1.00       3 .  7
    2.00       4 .  24
    5.00       4 .  66779
    6.00       5 .  011123
    9.00       5 .  677789999
    8.00       6 .  01233344
    5.00       6 .  59999
    3.00       7 .  134

 Stem width:   .1000000
 Each leaf:        1 case(s)
```

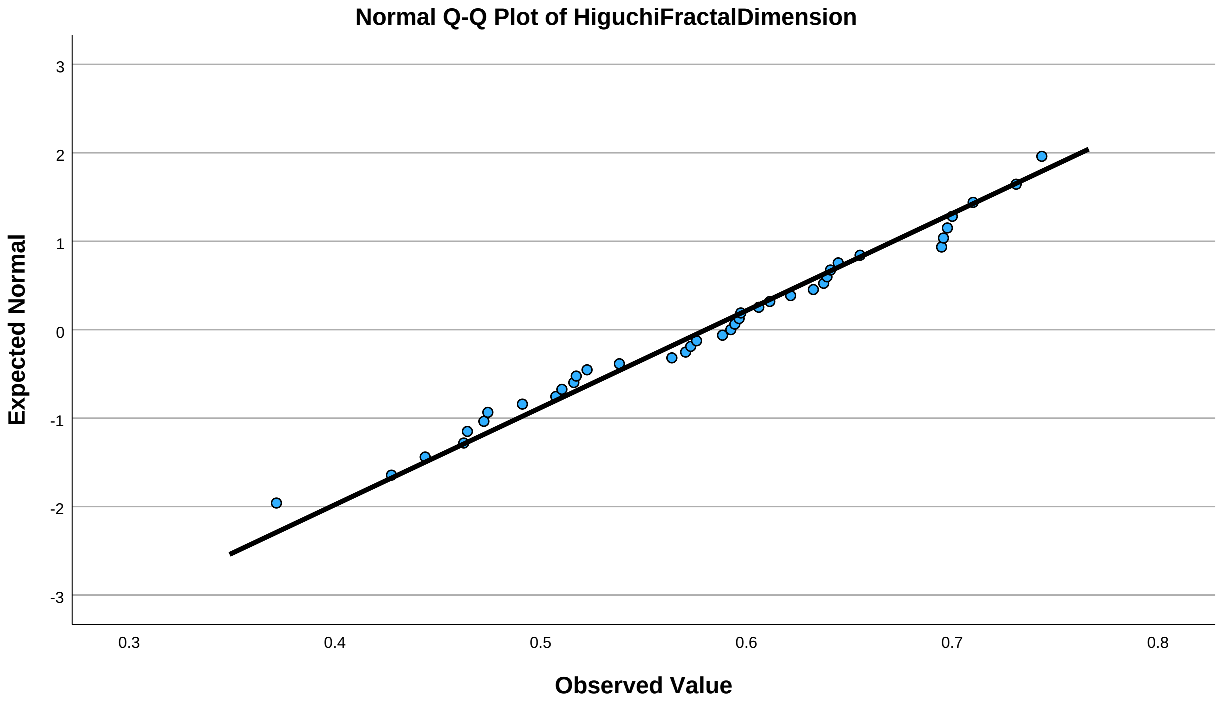

Normal Q-Q Plot of HiguchiFractalDimension
Expected Normal
3
2
1
0
-1
-2
-3
0.3
0.4
0.5
0.6
0.7
0.8
Observed Value

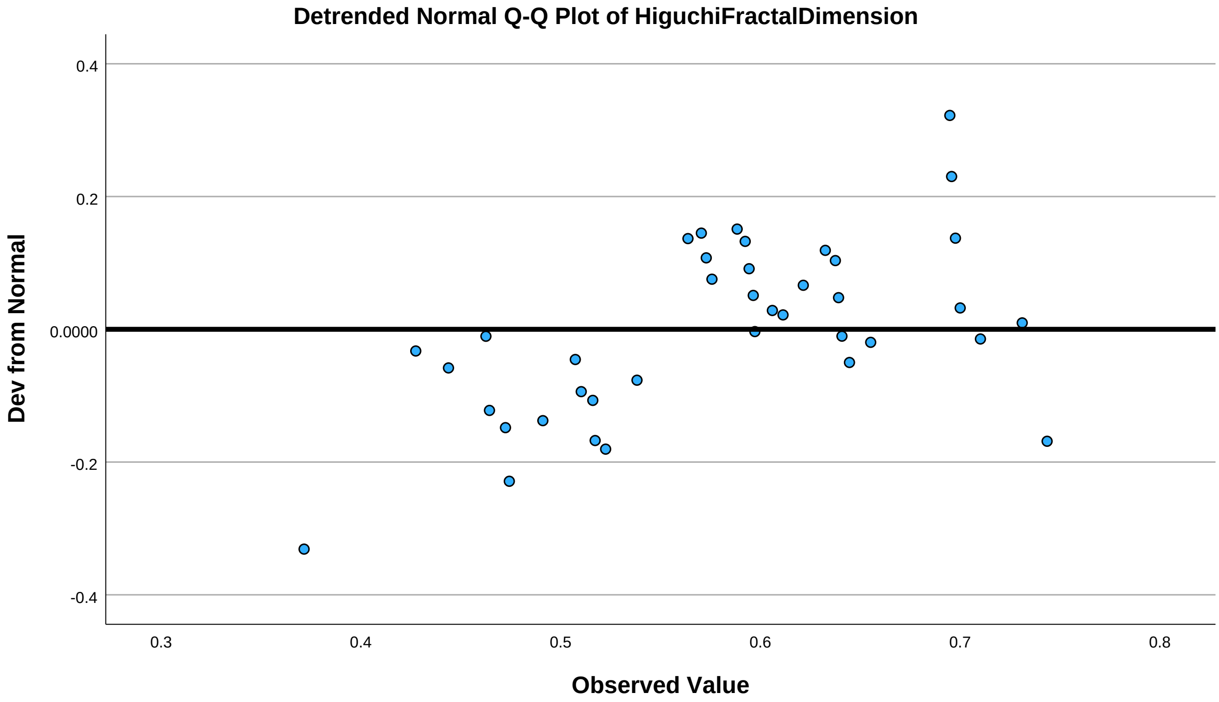

Detrended Normal Q-Q Plot of HiguchiFractalDimension
Dev from Normal
0.4
0.2
0.0000
-0.2
-0.4
0.3
0.4
0.5
0.6
0.7
0.8
Observed Value

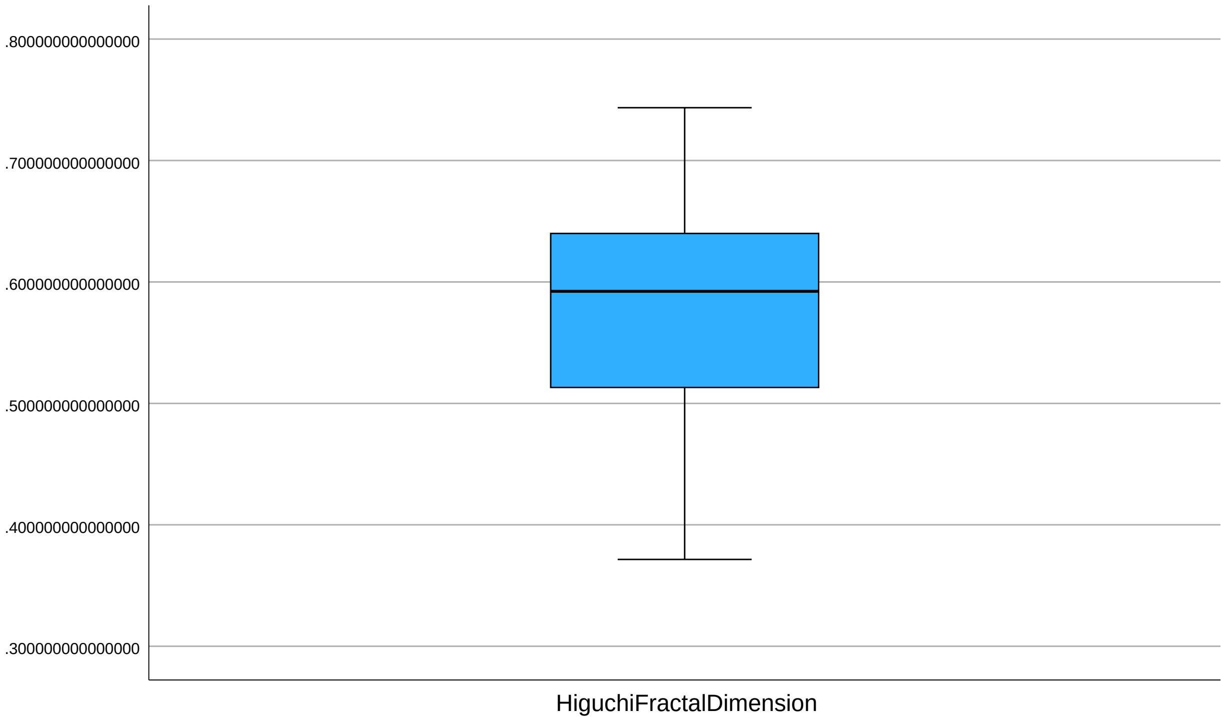

LempelZivComplexity

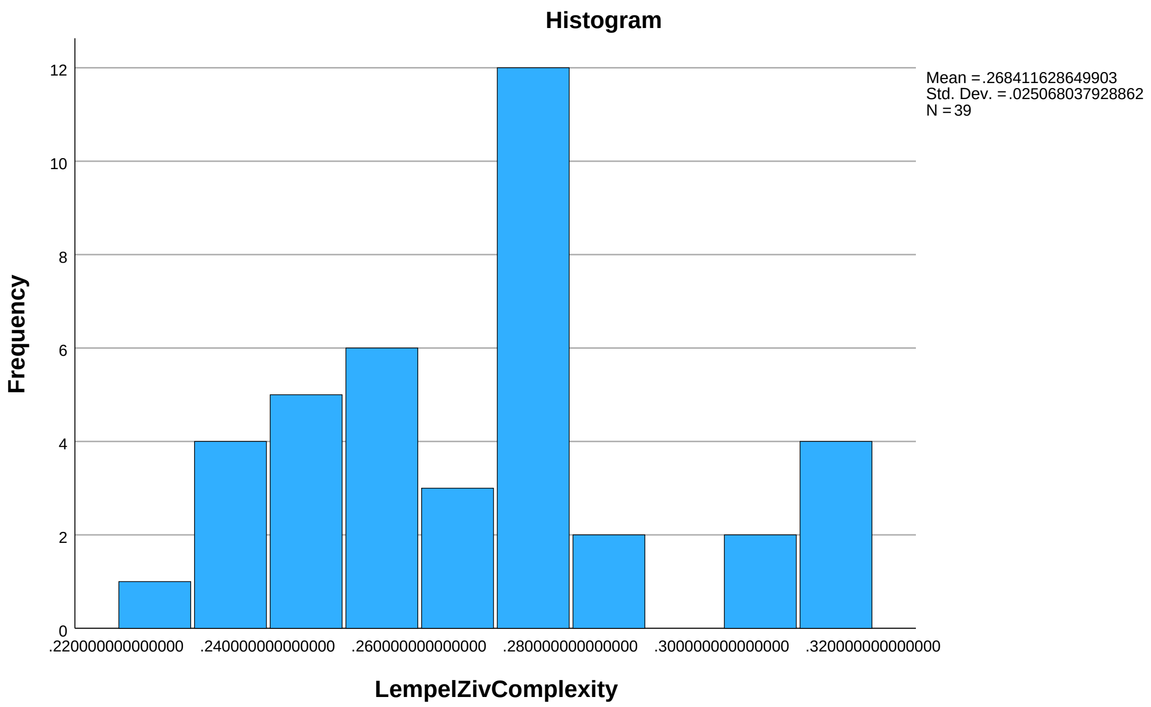


```
LempelZivComplexity Stem-and-Leaf Plot

 Frequency    Stem &  Leaf
```

```
 1.00     22 .  7
 4.00     23 .  5699
 5.00     24 .  13455
 6.00     25 .  001136
 3.00     26 .  268
12.00     27 .  002455677799
 2.00     28 .  35
  .00     29 .
 2.00     30 .  38
 4.00     31 .  1899

Stem width:   .0100000
Each leaf:       1 case(s)
```

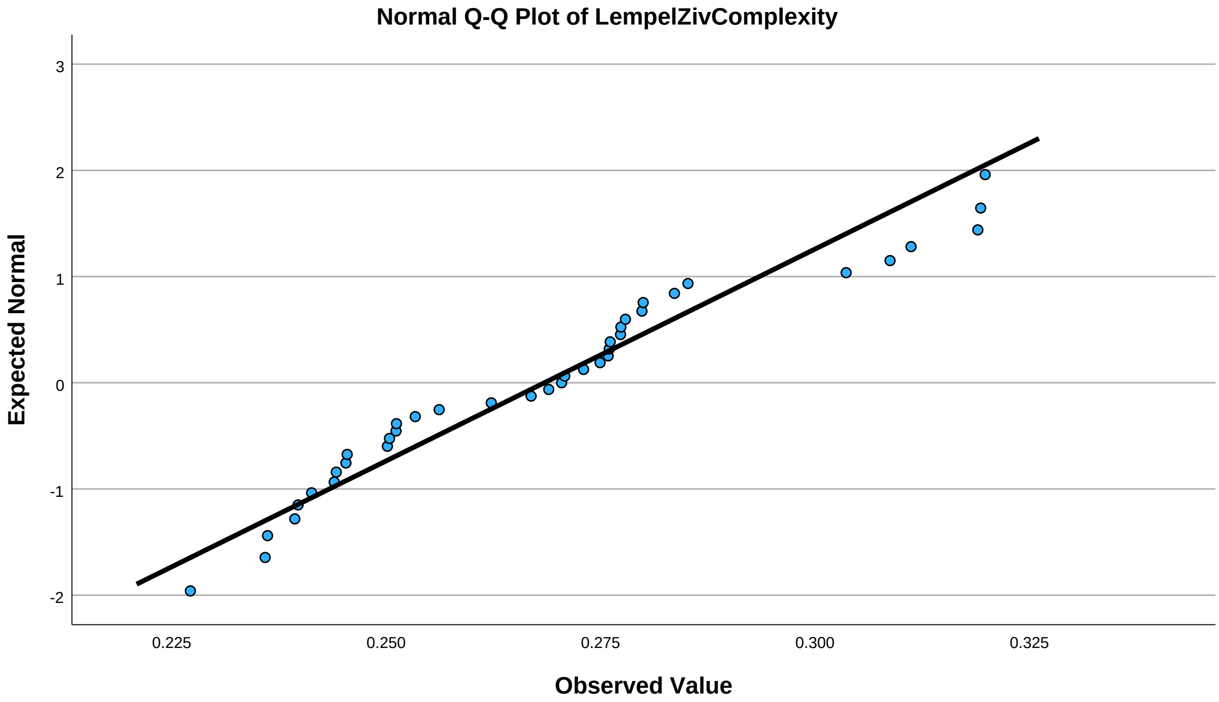

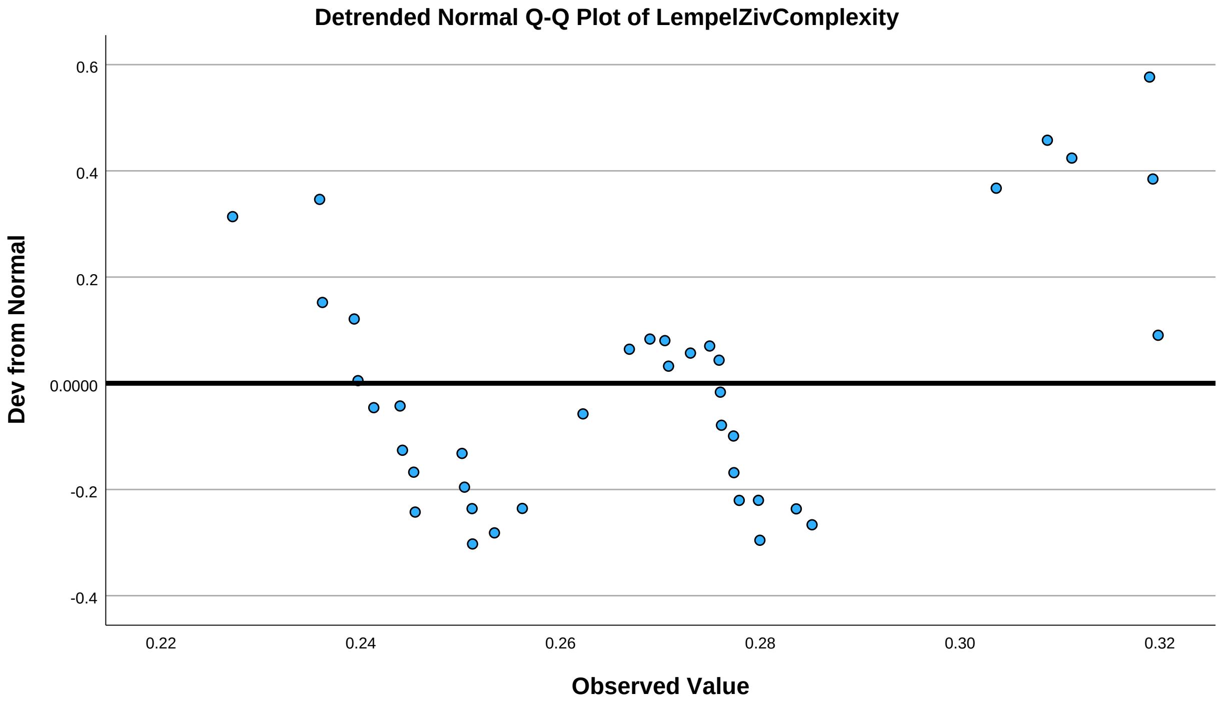

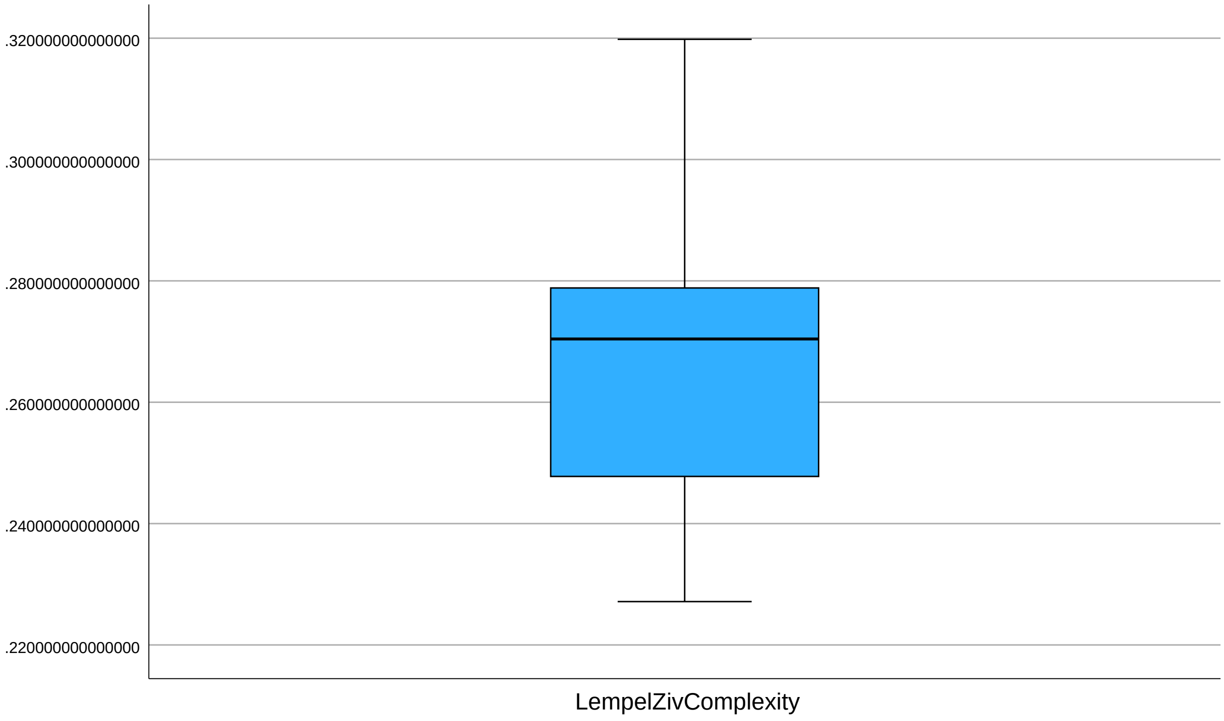

RenyiEntropy

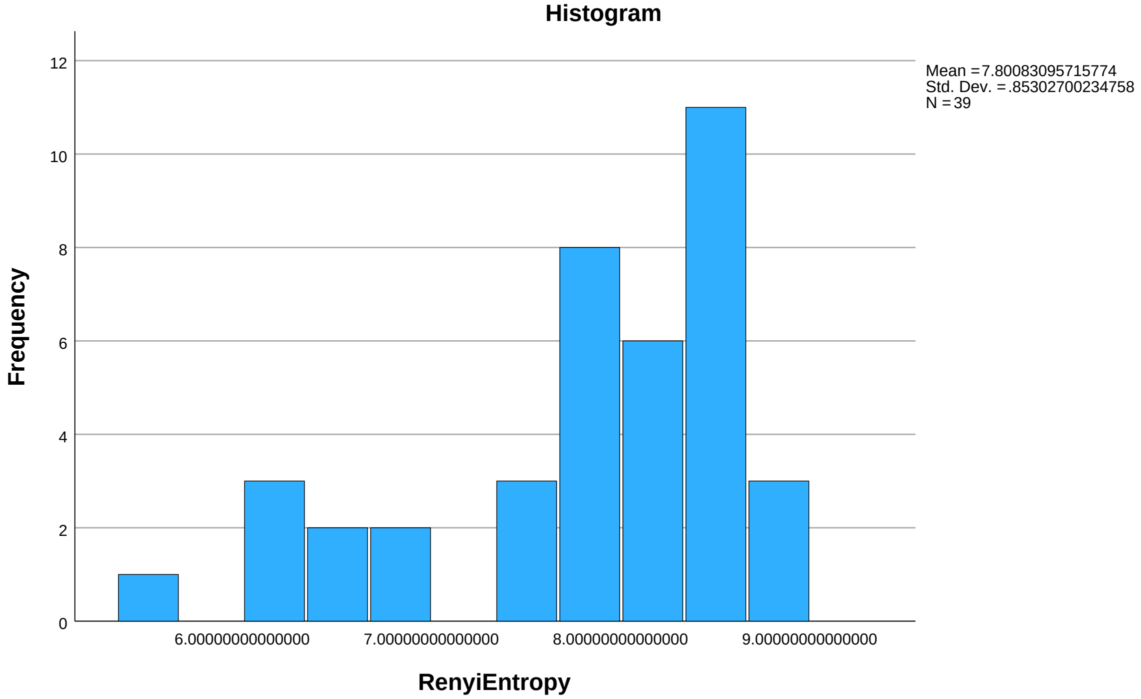


```
RenyiEntropy Stem-and-Leaf Plot

 Frequency    Stem &  Leaf

     4.00 Extremes    (=<6.2)
     4.00        6 .  5678
     2.00        7 .  34
     9.00        7 .  577778999
    13.00        8 .  0012223333444
     7.00        8 .  5566778

 Stem width:   1.000000
 Each leaf:        1 case(s)
```

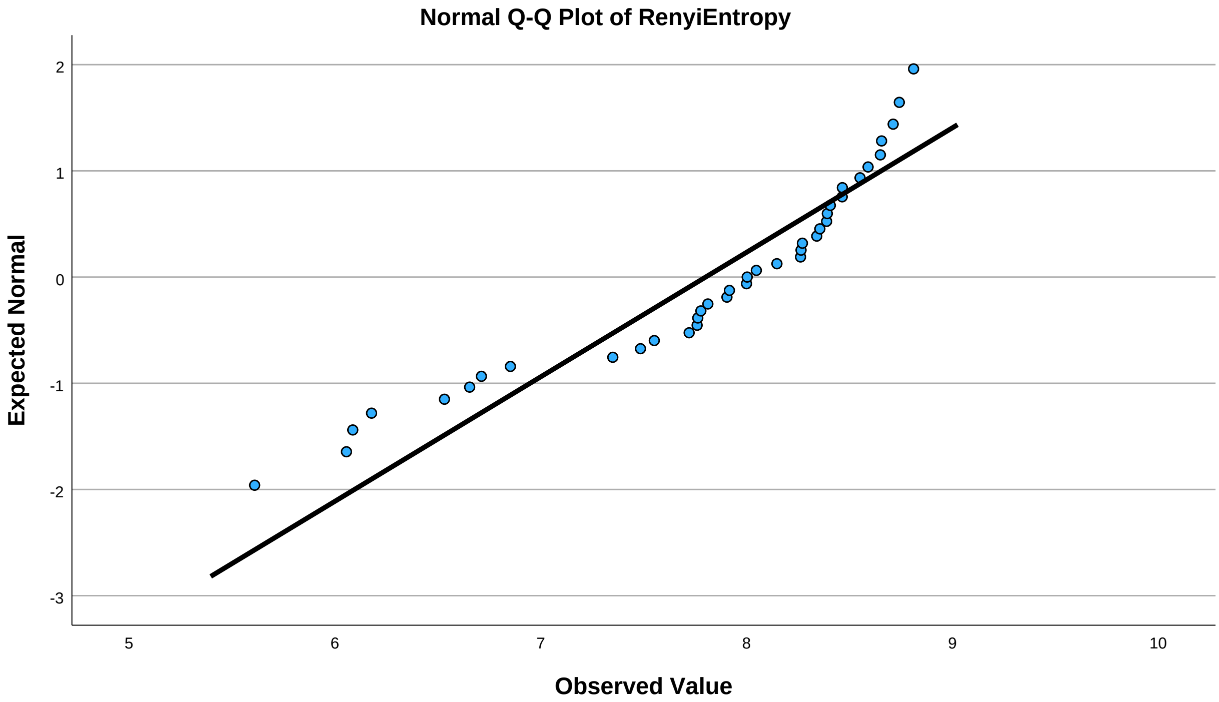

Normal Q-Q Plot of RenyiEntropy
Expected Normal
2
1
0
-1
-2
-3
5
6
7
8
9
10
Observed Value

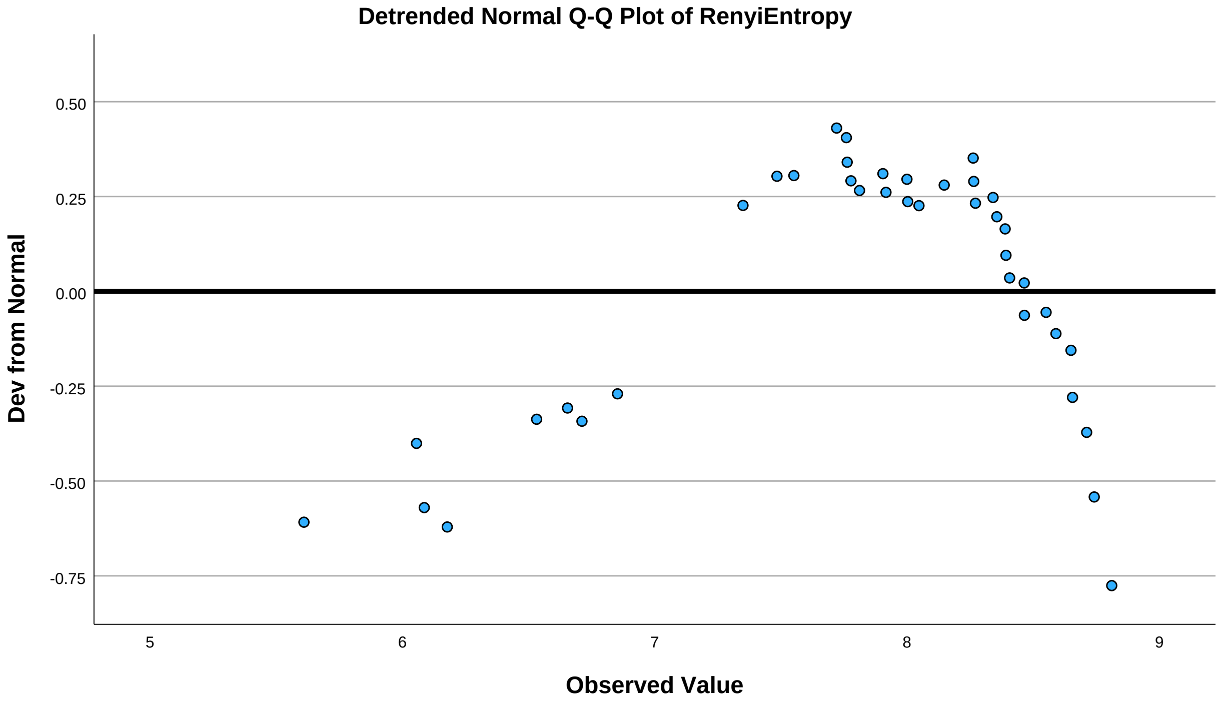

Detrended Normal Q-Q Plot of RenyiEntropy
Dev from Normal
0.50
0.25
0.00
-0.25
-0.50
-0.75
5
6
7
8
9
Observed Value

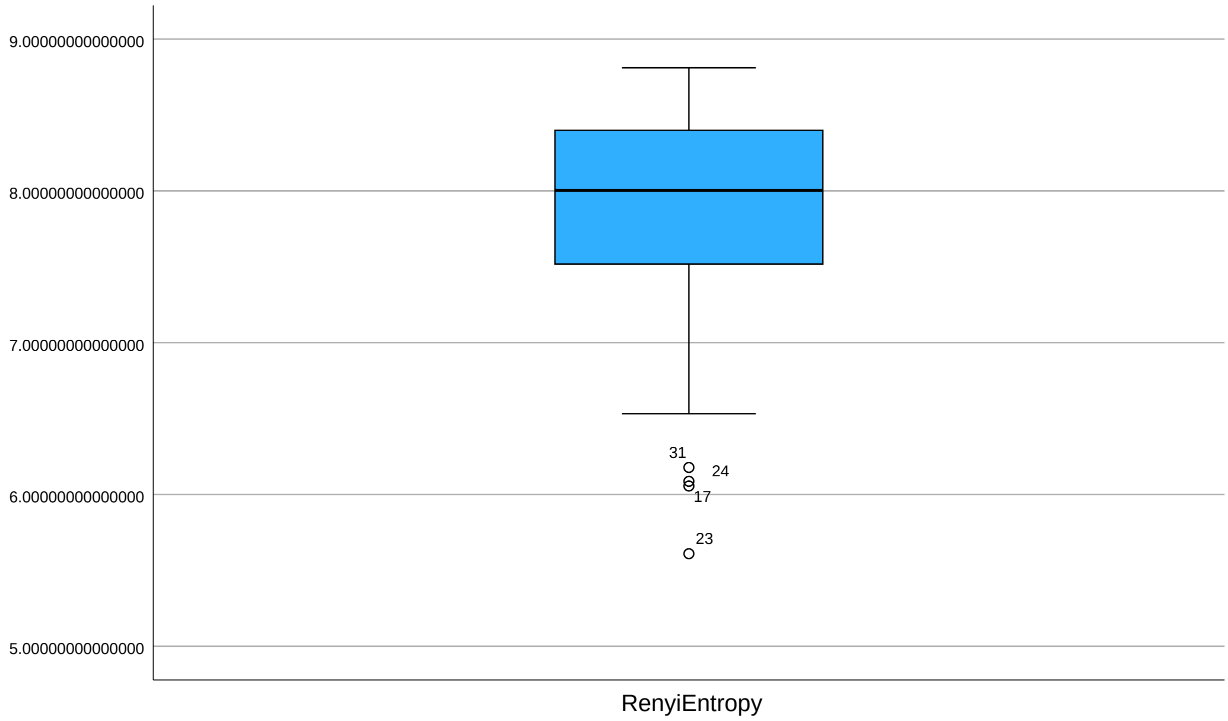

TsallisEntropy

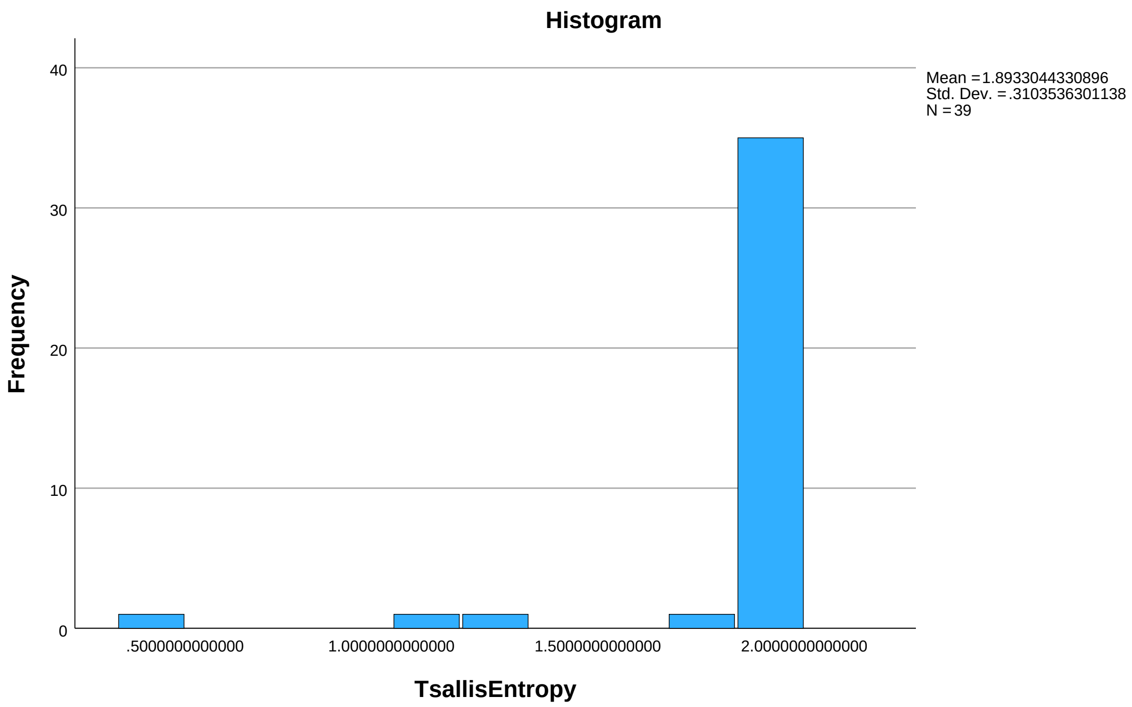

TsallisEntropy Stem-and-Leaf Plot

Frequency Stem & Leaf

```
  8.00 Extremes    (=<1.9672)
  1.00    1977 .  3
   .00    1978 .
   .00    1979 .
  1.00    1980 .  4
   .00    1981 .
   .00    1982 .
   .00    1983 .
  2.00    1984 .  45
  6.00    1985 .  034669
 12.00    1986 .  000112236789
  7.00    1987 .  0022339
  2.00    1988 .  27

Stem width:   .0010000
Each leaf:       1 case(s)
```

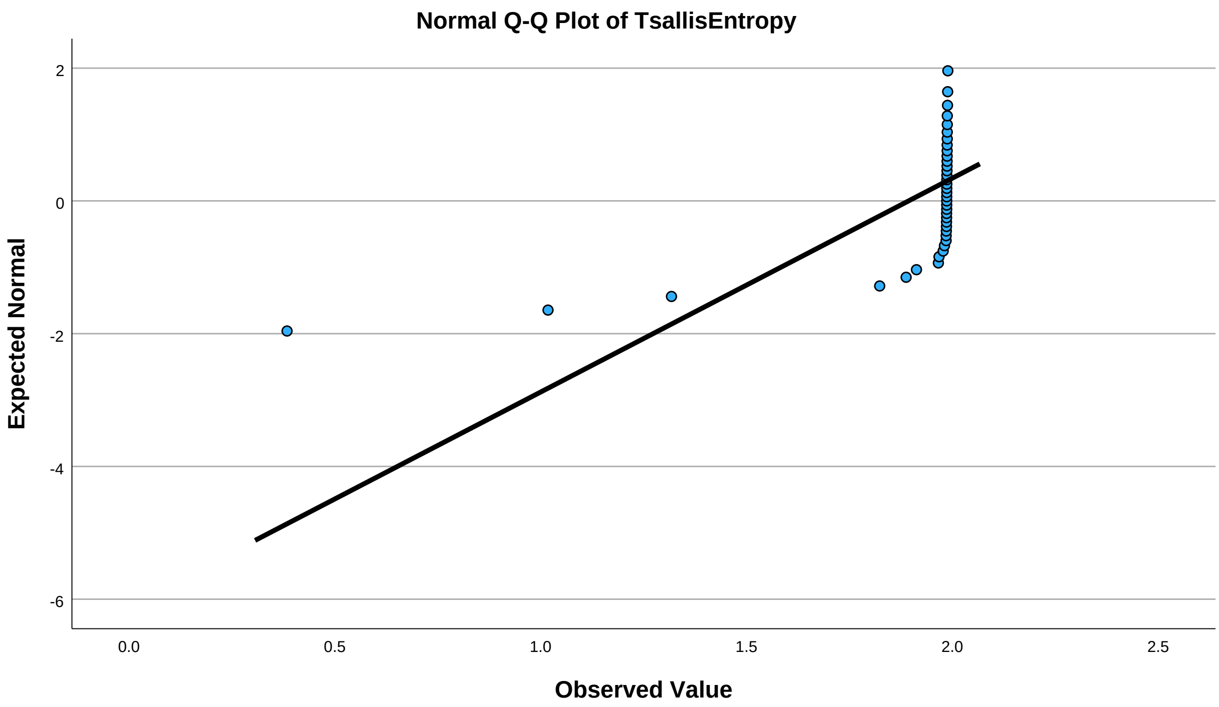

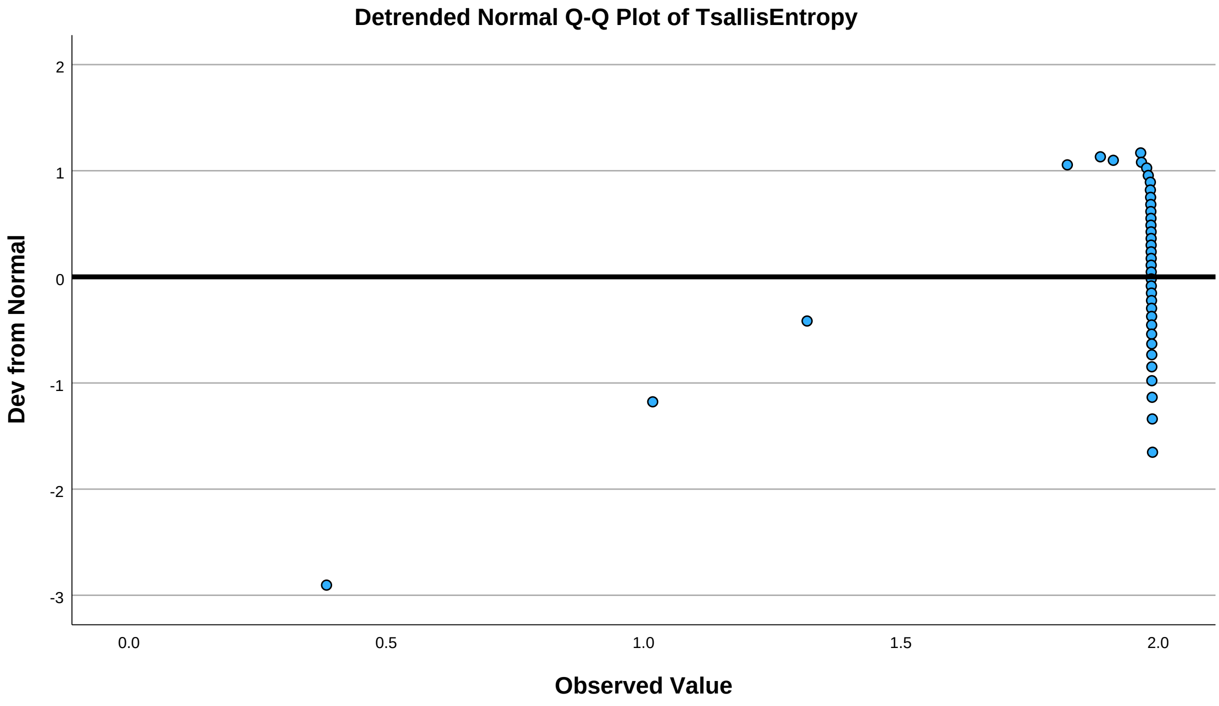

Detrended Normal Q-Q Plot of TsallisEntropy
2
1
0
-1
-2
-3
Dev from Normal
0.0
0.5
1.0
1.5
2.0
Observed Value

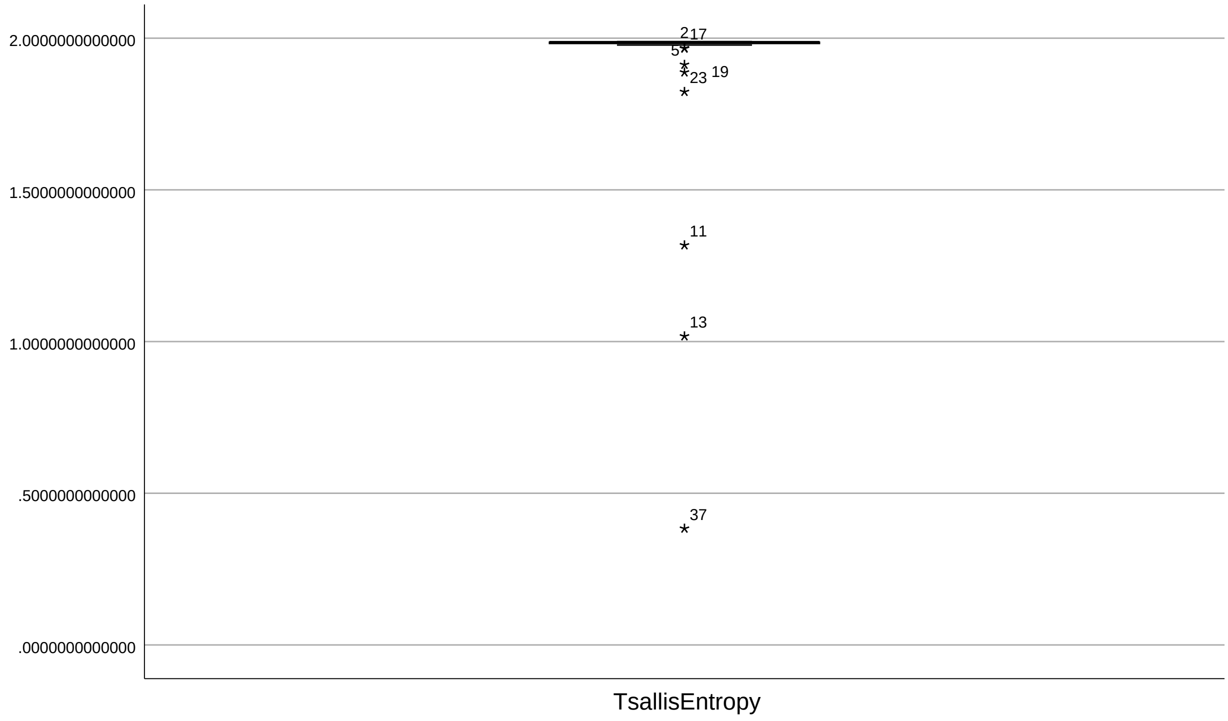

2.0000000000000
1.5000000000000
1.0000000000000
.5000000000000
.0000000000000
2
17
5
23
19
11
13
37
TsallisEntropy

PS_4_5

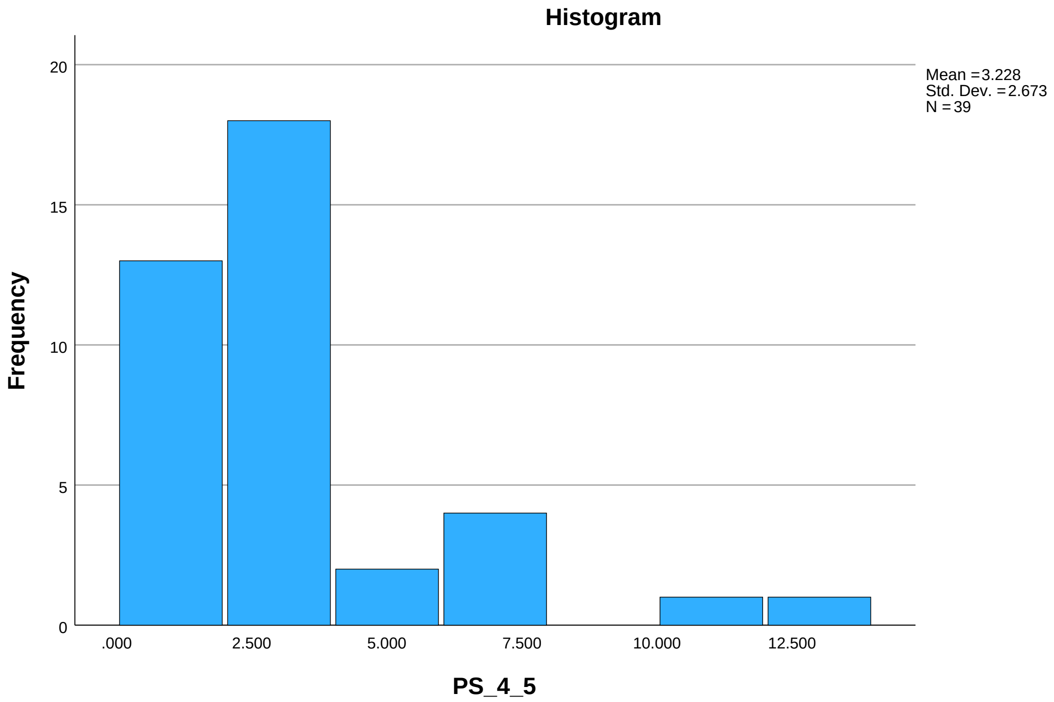


```
PS_4_5 Stem-and-Leaf Plot

 Frequency    Stem &  Leaf

     2.00        0 .  68
     5.00        1 .  01233
     6.00        1 .  668889
     9.00        2 .  122223344
     8.00        2 .  55677899
     1.00        3 .  3
      .00        3 .
     1.00        4 .  2
     7.00 Extremes    (>=5.9)

 Stem width:      1.000
 Each leaf:        1 case(s)
```

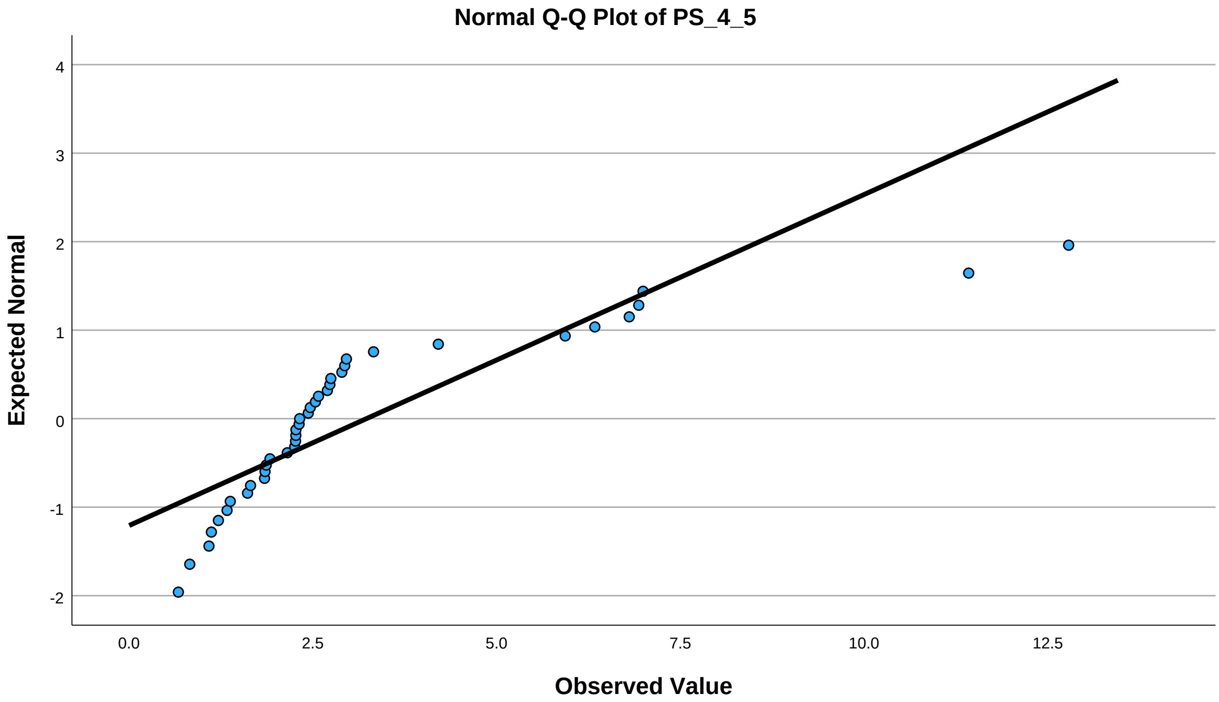
Normal Q-Q Plot of PS_4_5
Expected Normal
4
3
2
1
0
-1
-2
0.0
2.5
5.0
7.5
10.0
12.5
Observed Value

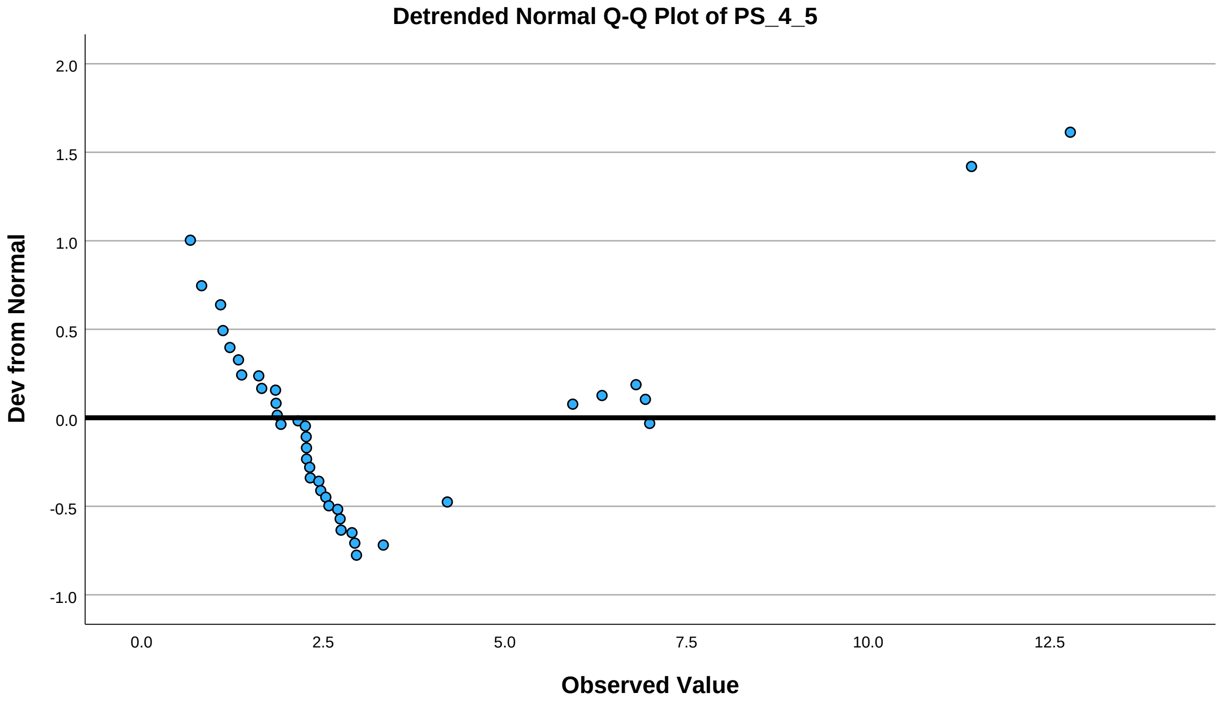
Detrended Normal Q-Q Plot of PS_4_5
Dev from Normal
2.0
1.5
1.0
0.5
0.0
-0.5
-1.0
0.0
2.5
5.0
7.5
10.0
12.5
Observed Value

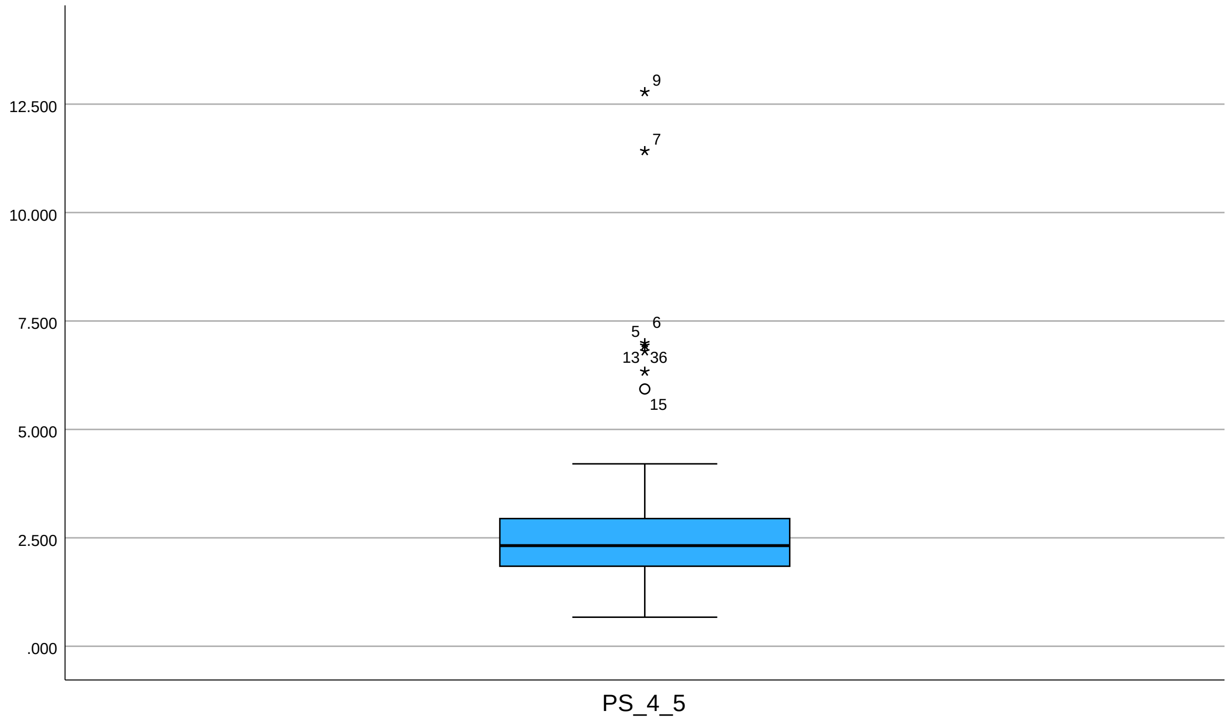

PS_5_6

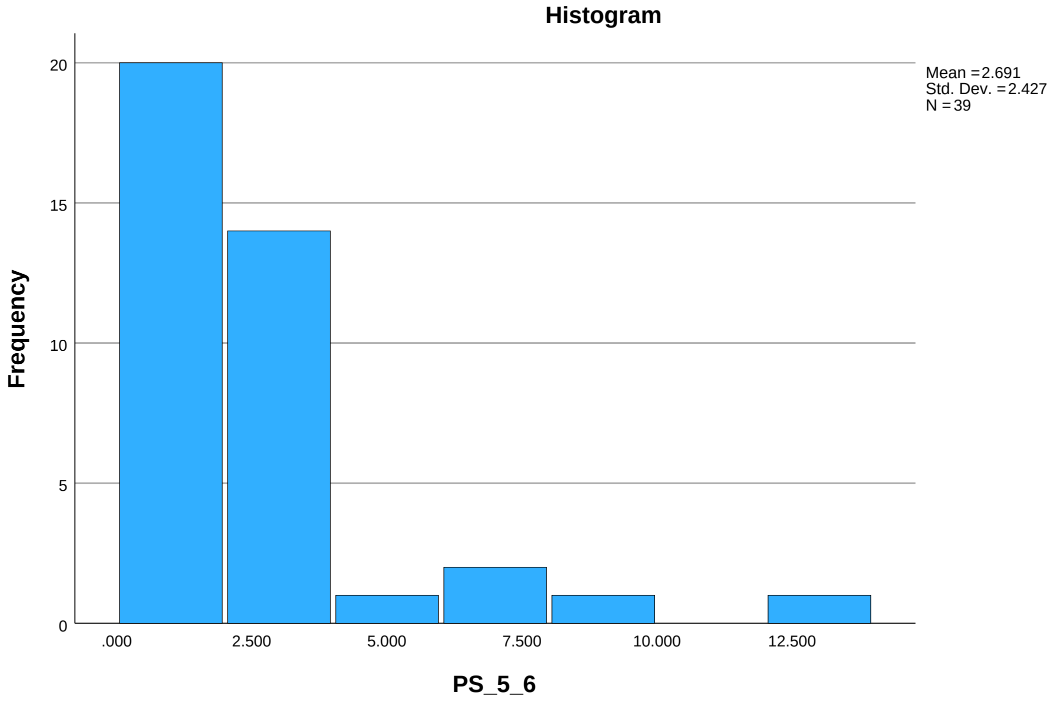

PS_5_6 Stem-and-Leaf Plot

Frequency   Stem & Leaf

```
 1.00      0 .  4
 3.00      0 .  579
 5.00      1 .  12224
11.00      1 .  66777789999
 7.00      2 .  0122223
 5.00      2 .  56677
  .00      3 .
 2.00      3 .  58
 5.00 Extremes    (>=4.6)

Stem width:     1.000
Each leaf:      1 case(s)
```

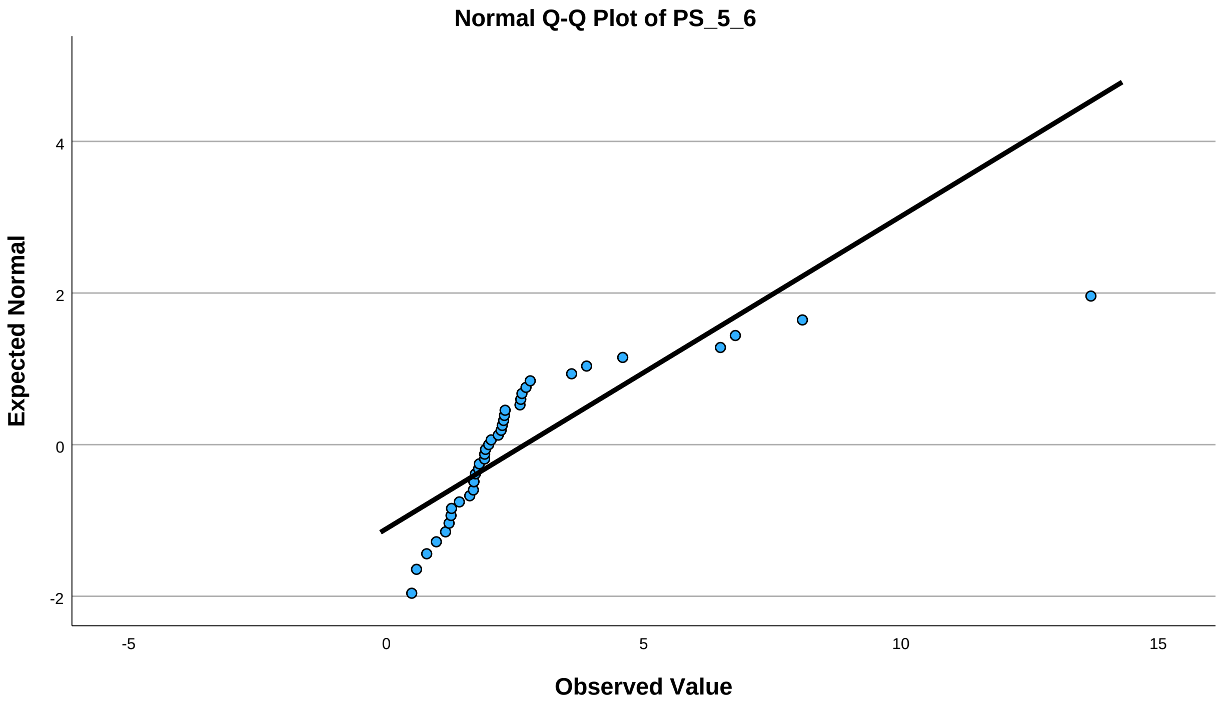

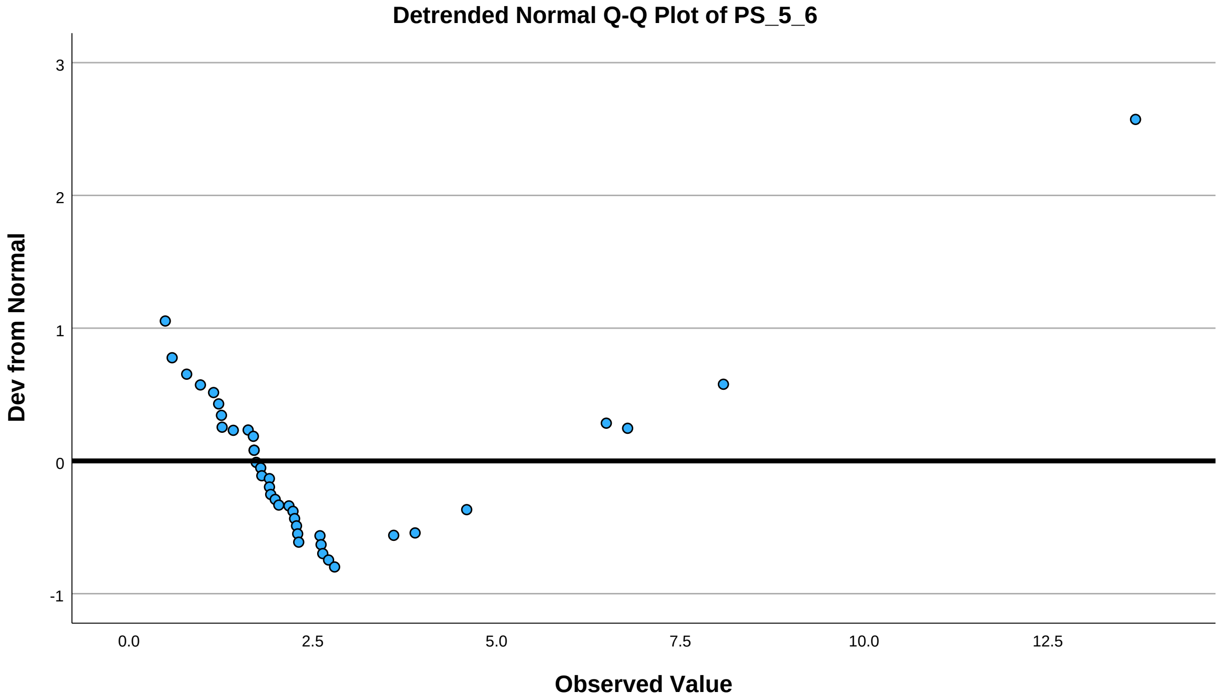
Detrended Normal Q-Q Plot of PS_5_6
Dev from Normal
3
2
1
0
-1
0.0
2.5
5.0
7.5
10.0
12.5
Observed Value

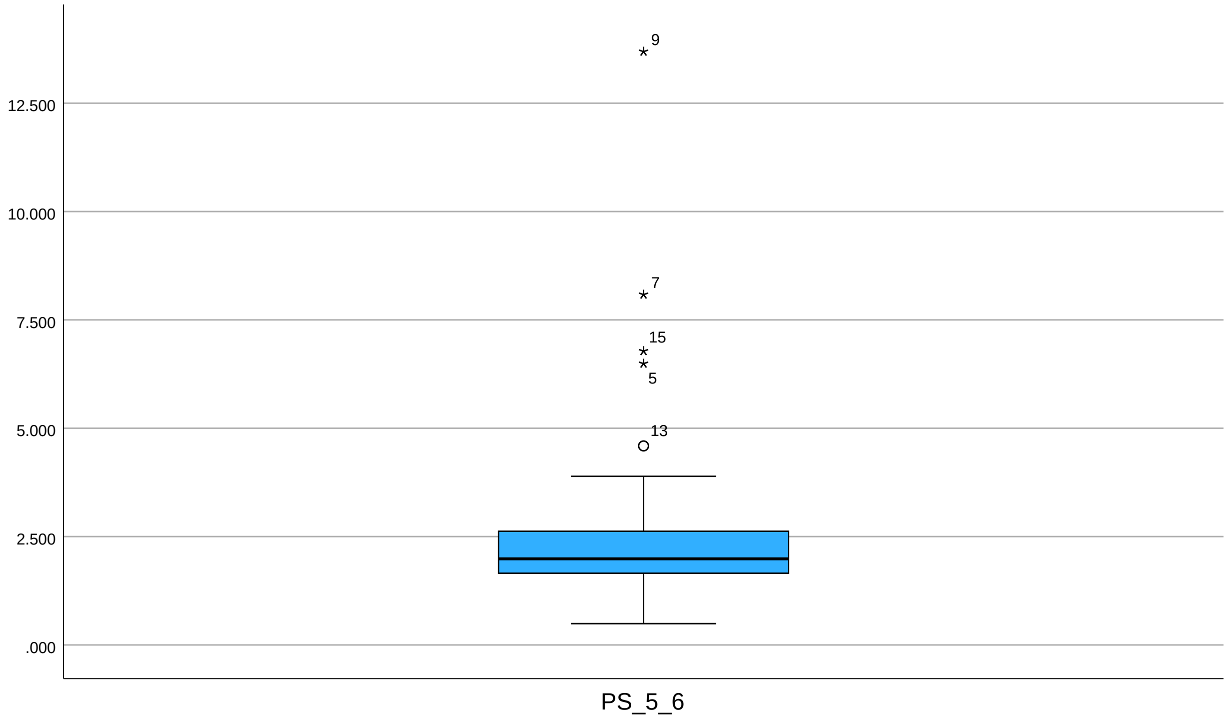
9
12.500
10.000
7
7.500
15
5
5.000
13
2.500
.000
PS_5_6

PS_6_7

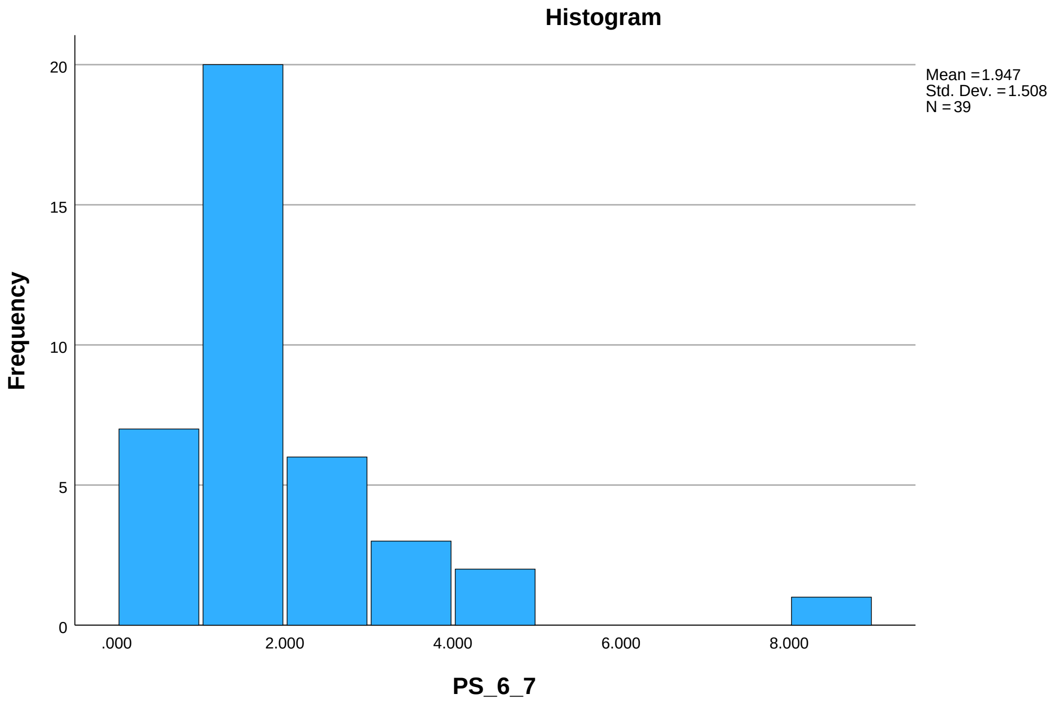


```
PS_6_7 Stem-and-Leaf Plot

 Frequency    Stem &  Leaf

     2.00        0 .  34
     5.00        0 .  67899
    14.00        1 .  00112223333334
     6.00        1 .  556689
     3.00        2 .  003
     3.00        2 .  777
      .00        3 .
     3.00        3 .  567
     3.00 Extremes    (>=4.1)

 Stem width:      1.000
 Each leaf:       1 case(s)
```

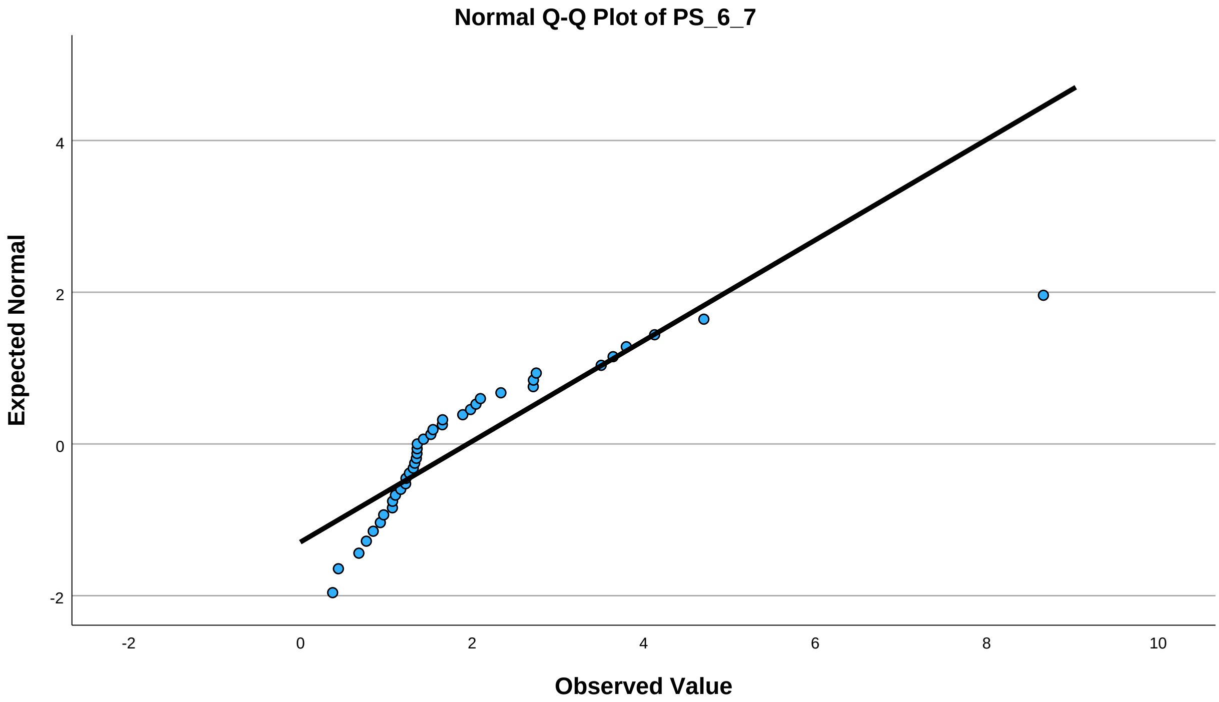
Normal Q-Q Plot of PS_6_7
Expected Normal
4
2
0
-2
-2
0
2
4
6
8
10
Observed Value

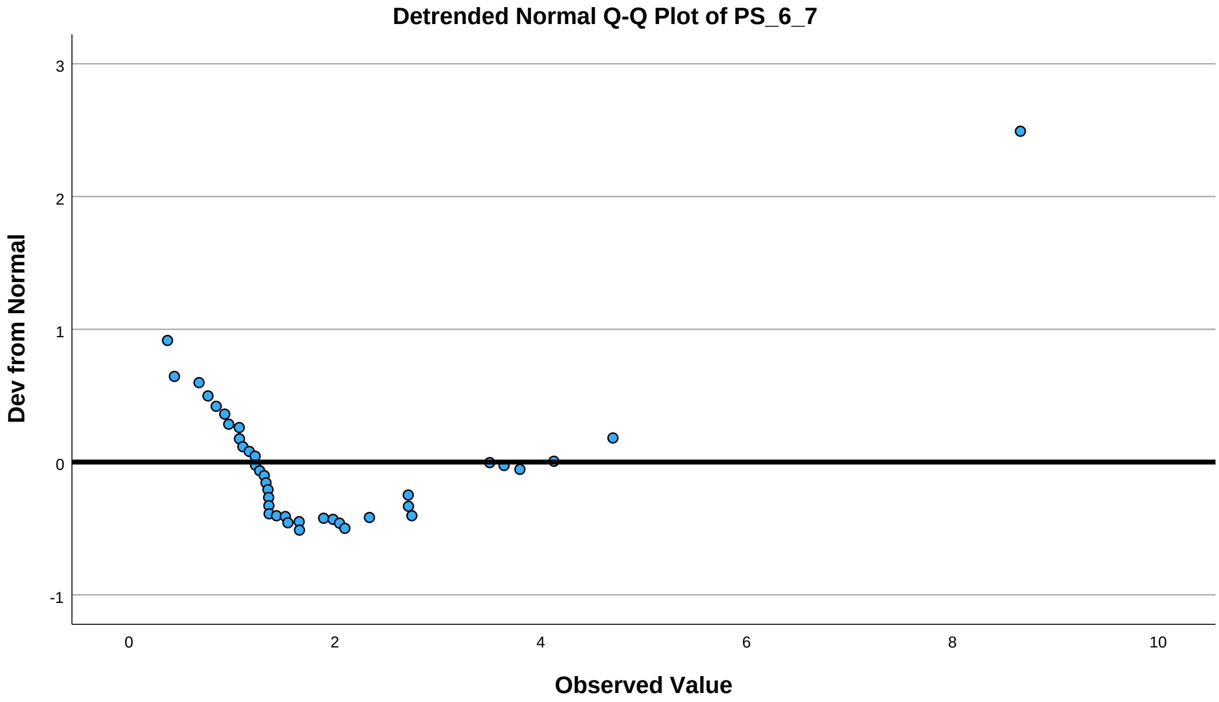
Detrended Normal Q-Q Plot of PS_6_7
Dev from Normal
3
2
1
0
-1
0
2
4
6
8
10
Observed Value

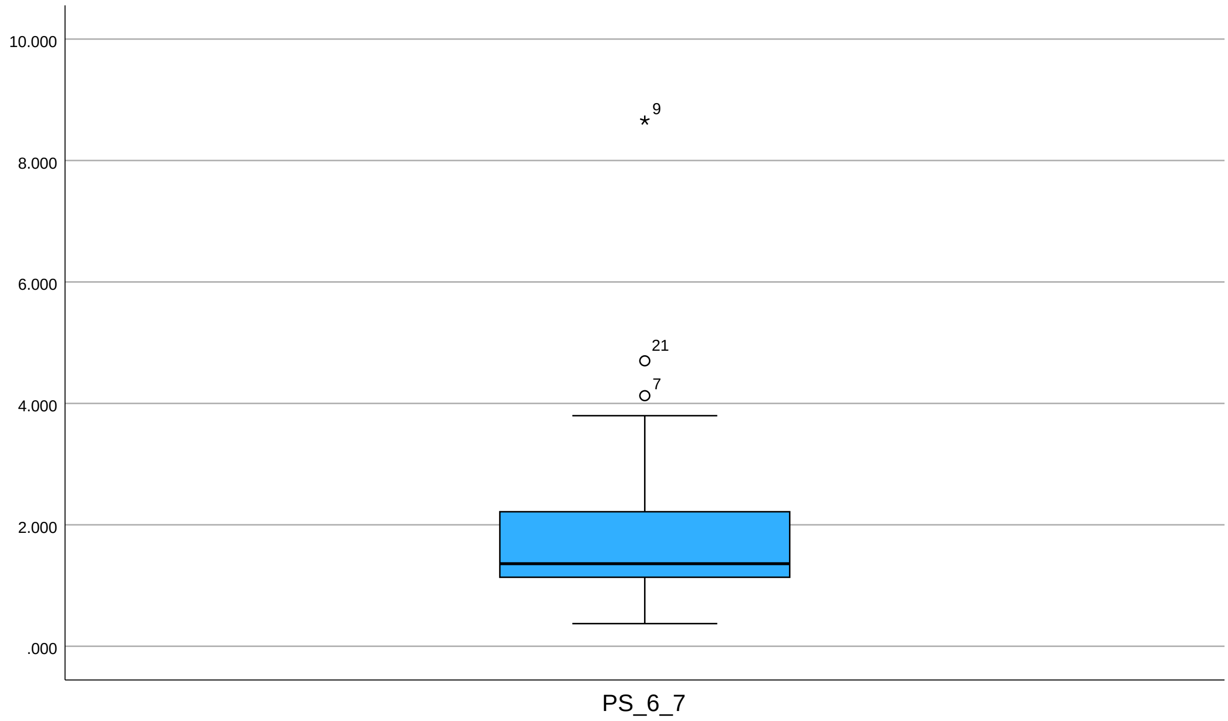

PS_7_8

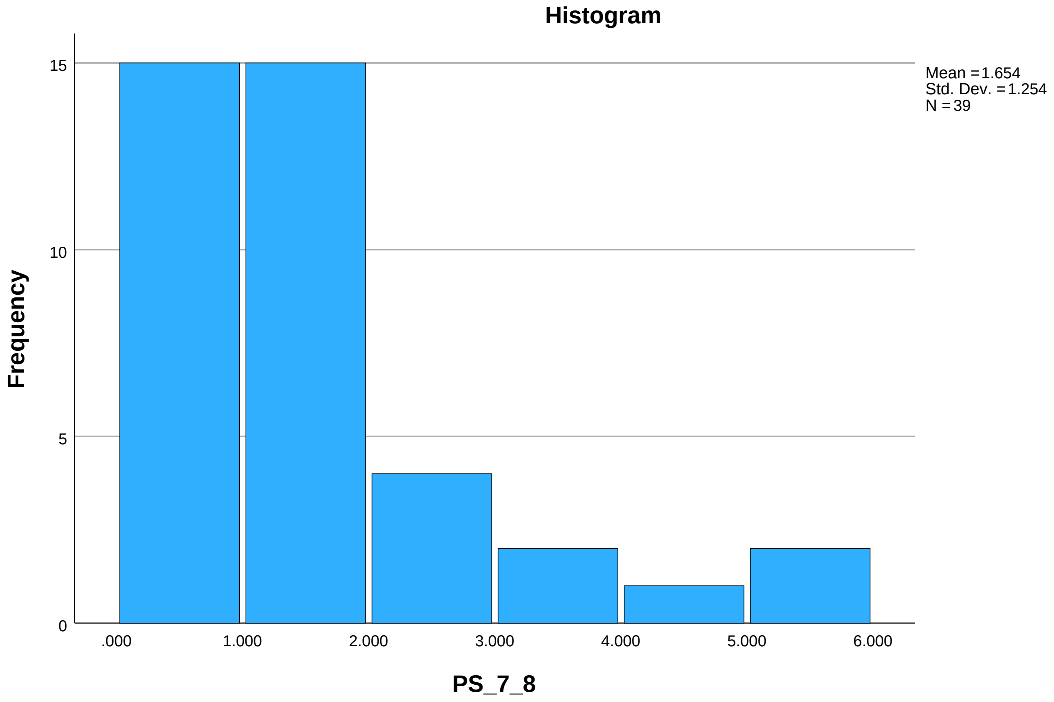

PS_7_8 Stem-and-Leaf Plot

Frequency   Stem & Leaf

```
  2.00     0 . 34
 13.00     0 . 5578899999999
 12.00     1 . 012222224444
  3.00     1 . 556
  1.00     2 . 1
  1.00     2 . 5
  7.00 Extremes    (>=2.6)

Stem width:     1.000
Each leaf:       1 case(s)
```

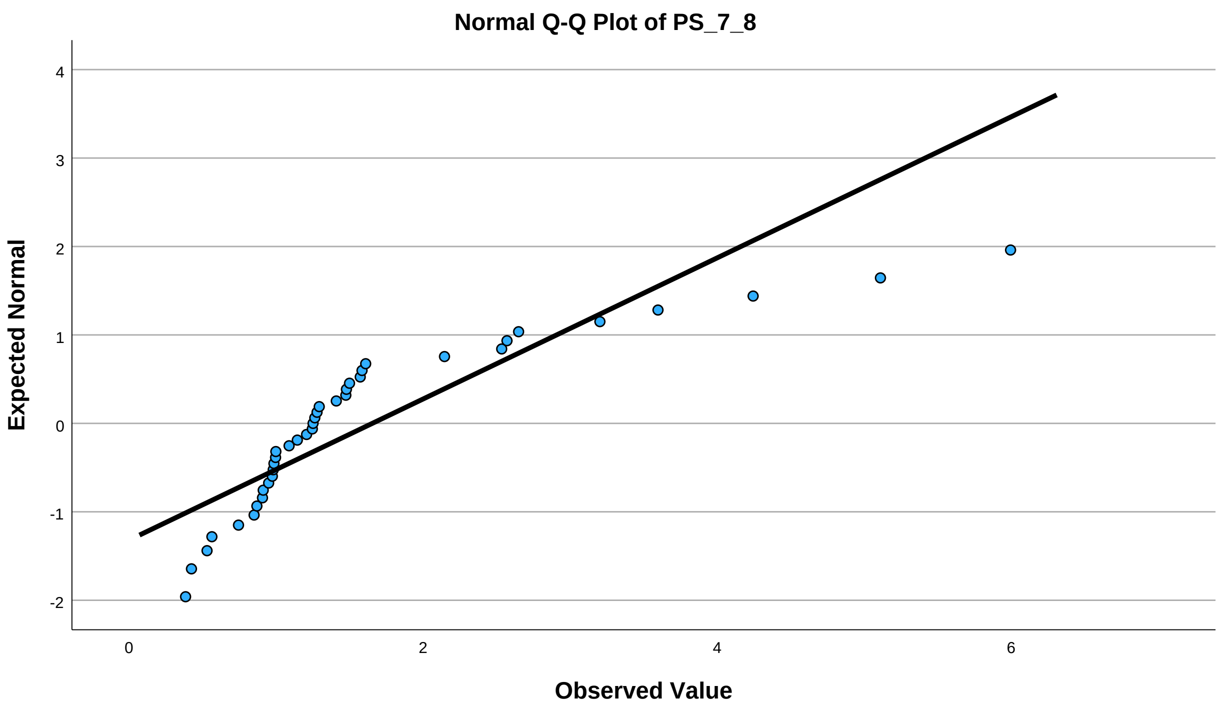

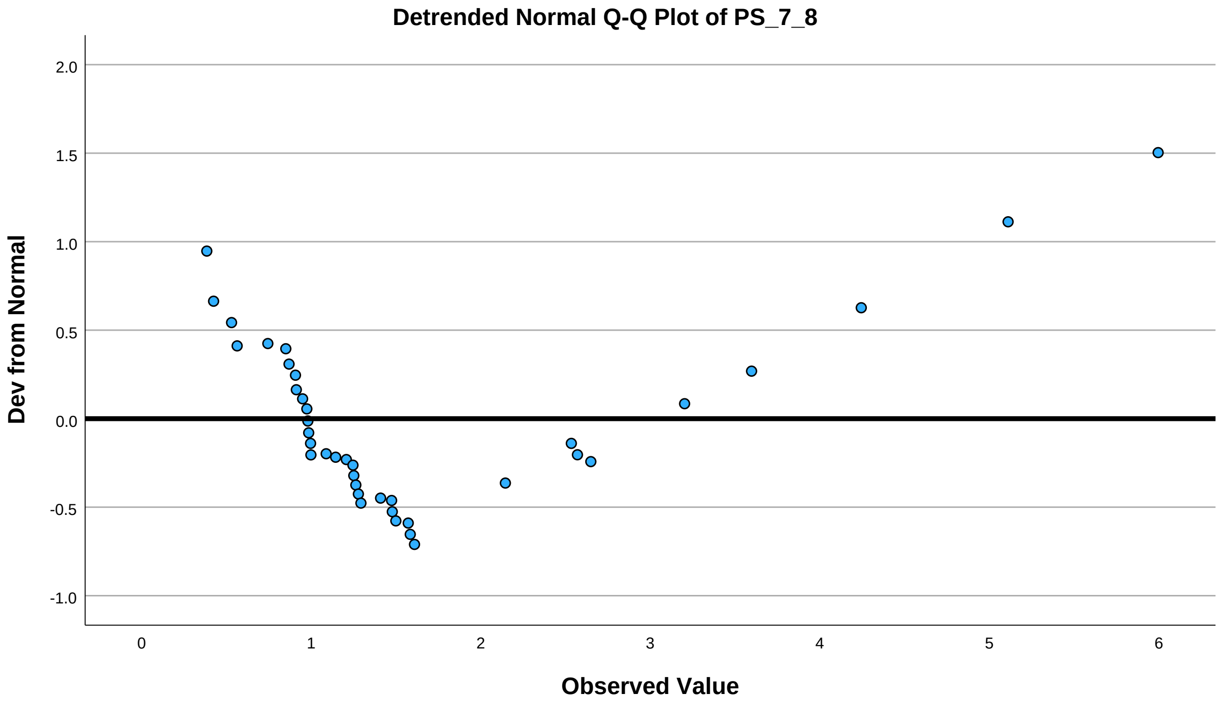
Detrended Normal Q-Q Plot of PS_7_8
Dev from Normal
2.0
1.5
1.0
0.5
0.0
-0.5
-1.0
0
1
2
3
4
5
6
Observed Value

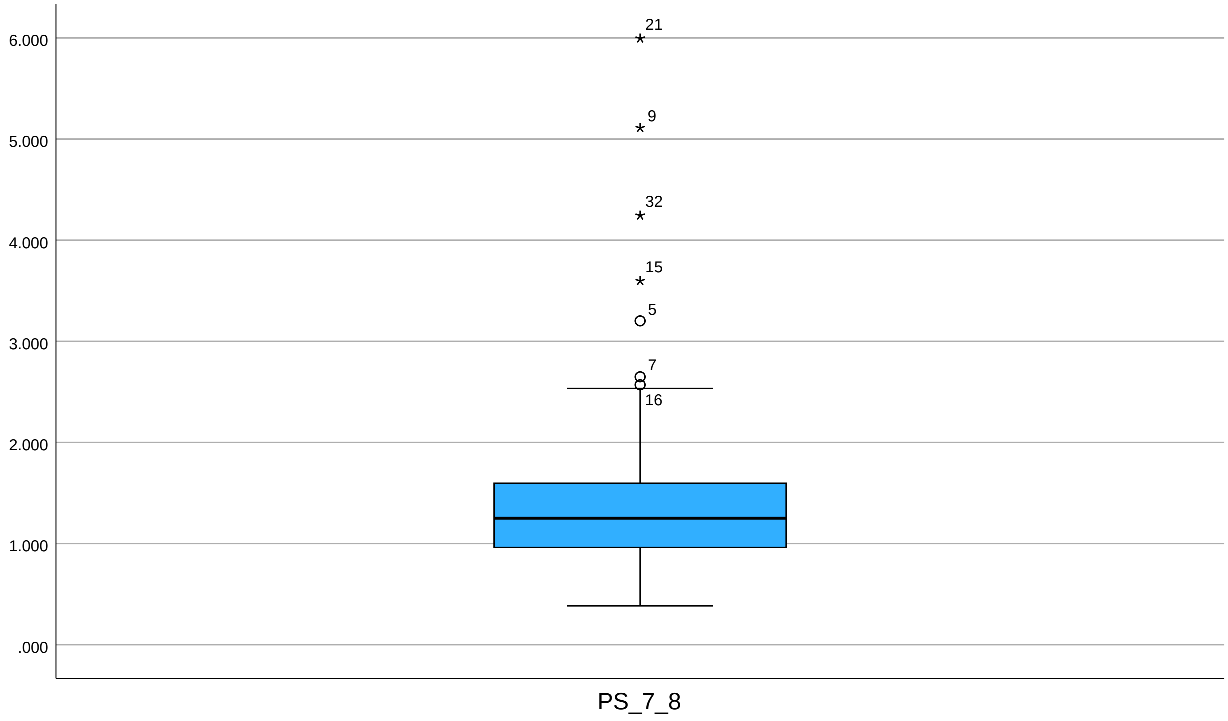
6.000
5.000
4.000
3.000
2.000
1.000
.000
21
9
32
15
5
7
16
PS_7_8

PS_8_9

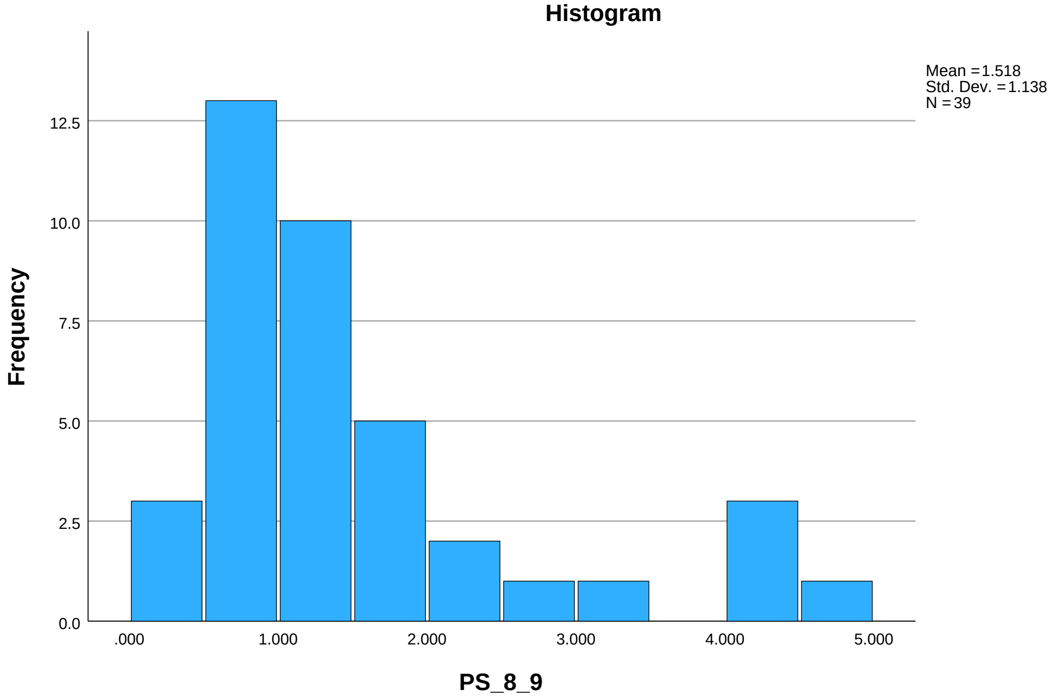


```
PS_8_9 Stem-and-Leaf Plot

 Frequency    Stem &  Leaf

     3.00        0 .  234
    13.00        0 .  5677888888999
    10.00        1 .  0000122334
     5.00        1 .  55678
     2.00        2 .  01
     1.00        2 .  5
     5.00 Extremes    (>=3.4)

 Stem width:      1.000
 Each leaf:       1 case(s)
```

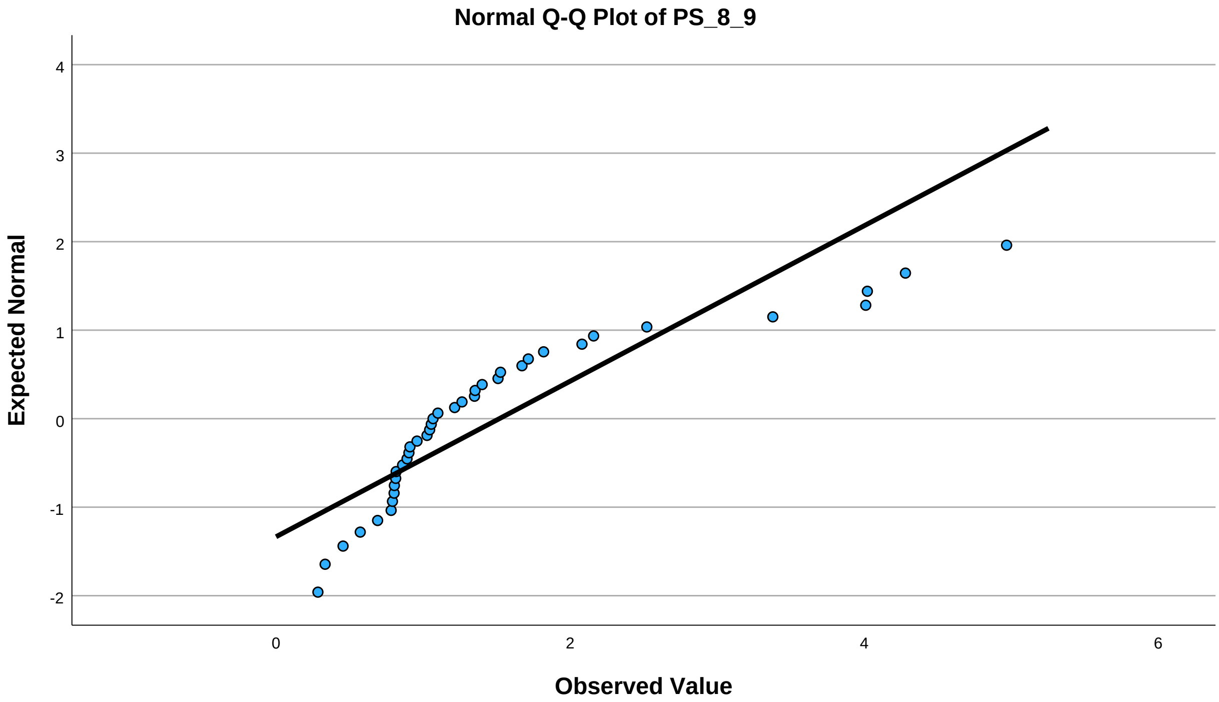
Normal Q-Q Plot of PS_8_9
Expected Normal
Observed Value
4
3
2
1
0
-1
-2
0
2
4
6

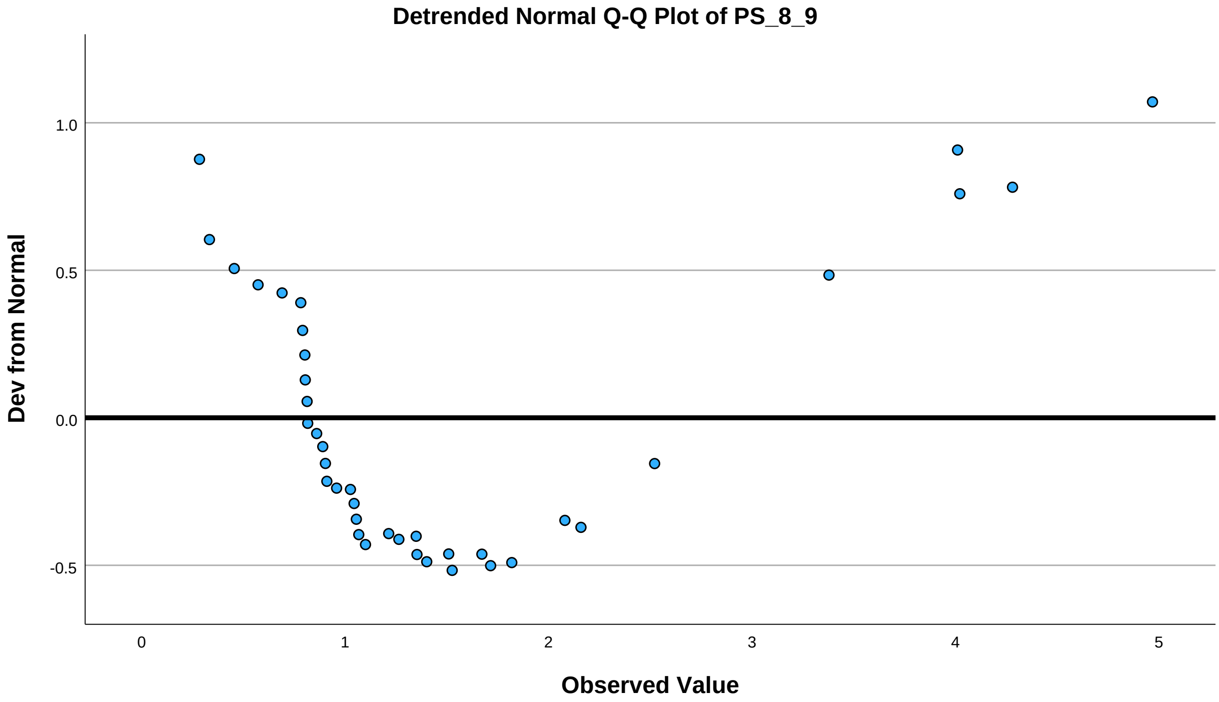
Detrended Normal Q-Q Plot of PS_8_9
Dev from Normal
Observed Value
1.0
0.5
0.0
-0.5
0
1
2
3
4
5

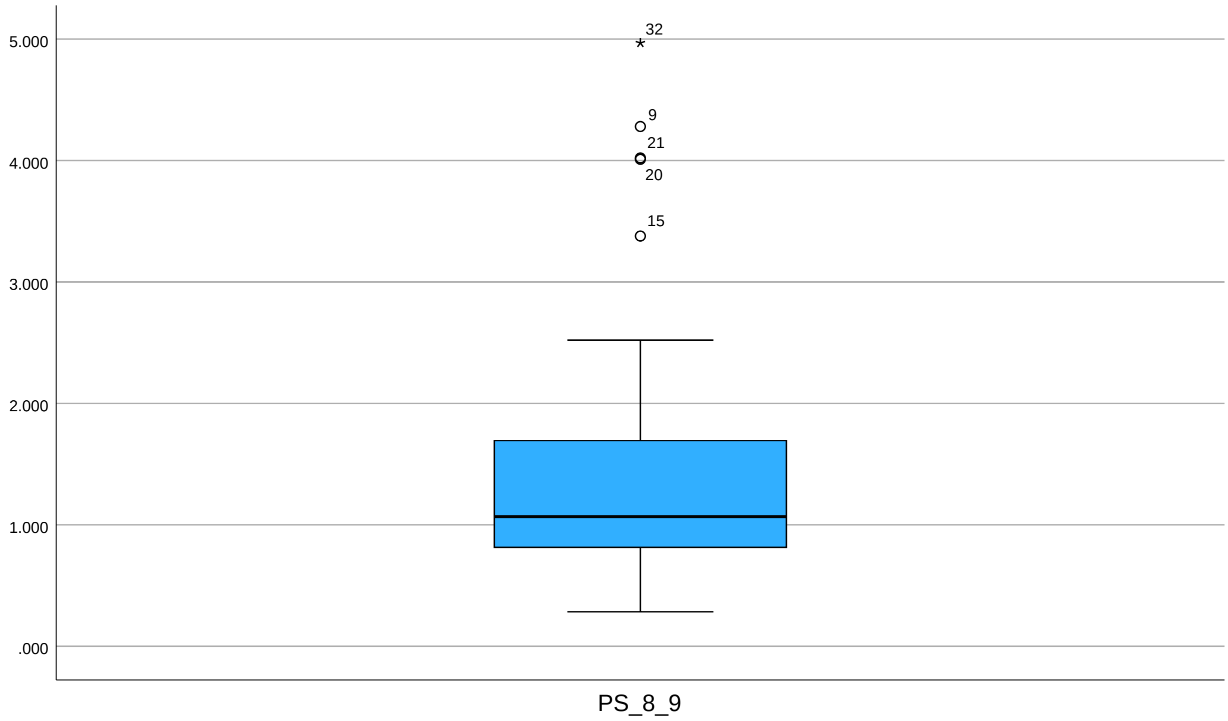

PS_9_10

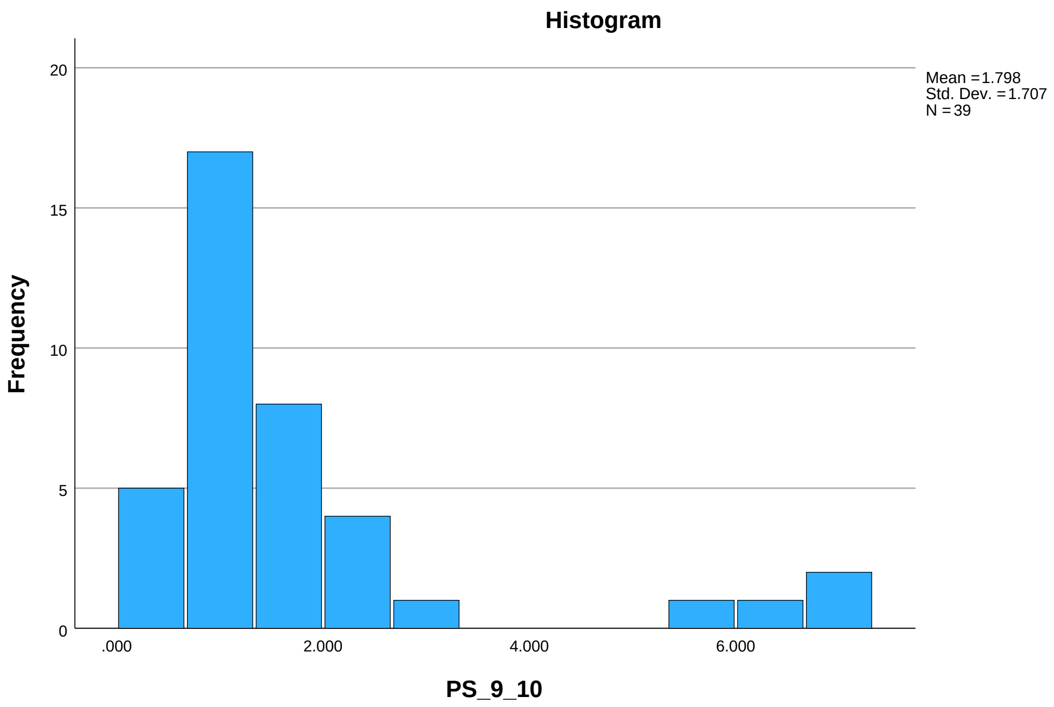

PS_9_10 Stem-and-Leaf Plot

Frequency   Stem & Leaf

```
 3.00       0 .  223
11.00       0 .  55677888899
10.00       1 .  0011122333
 6.00       1 .  67789
 3.00       2 .  024
 2.00       2 .  68
 4.00 Extremes    (>=5.5)

Stem width:      1.000
Each leaf:       1 case(s)
```

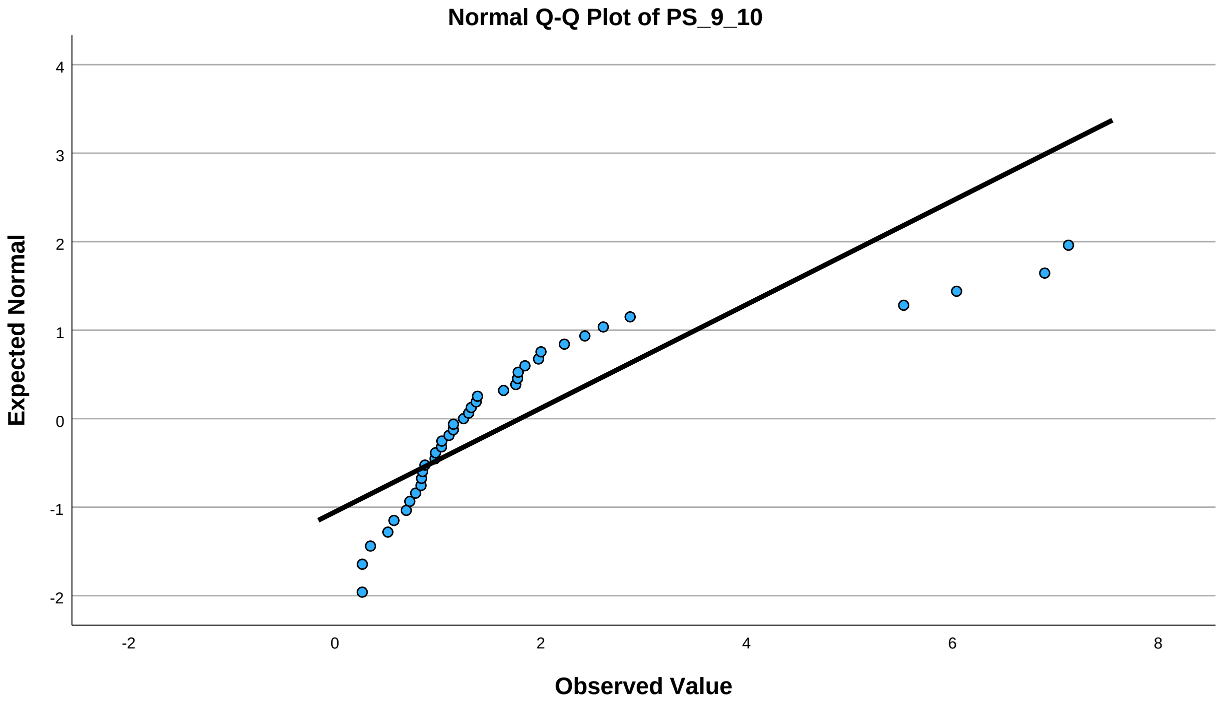

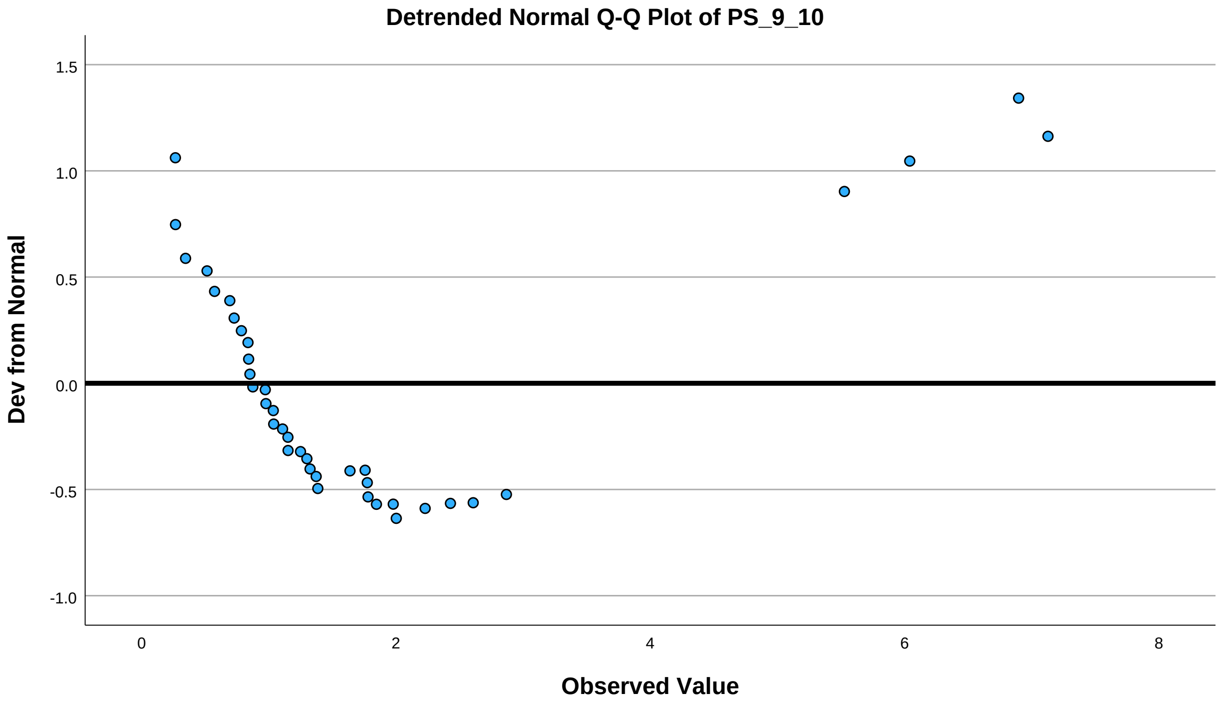

Detrended Normal Q-Q Plot of PS_9_10
Dev from Normal
1.5
1.0
0.5
0.0
-0.5
-1.0
0
2
4
6
8
Observed Value

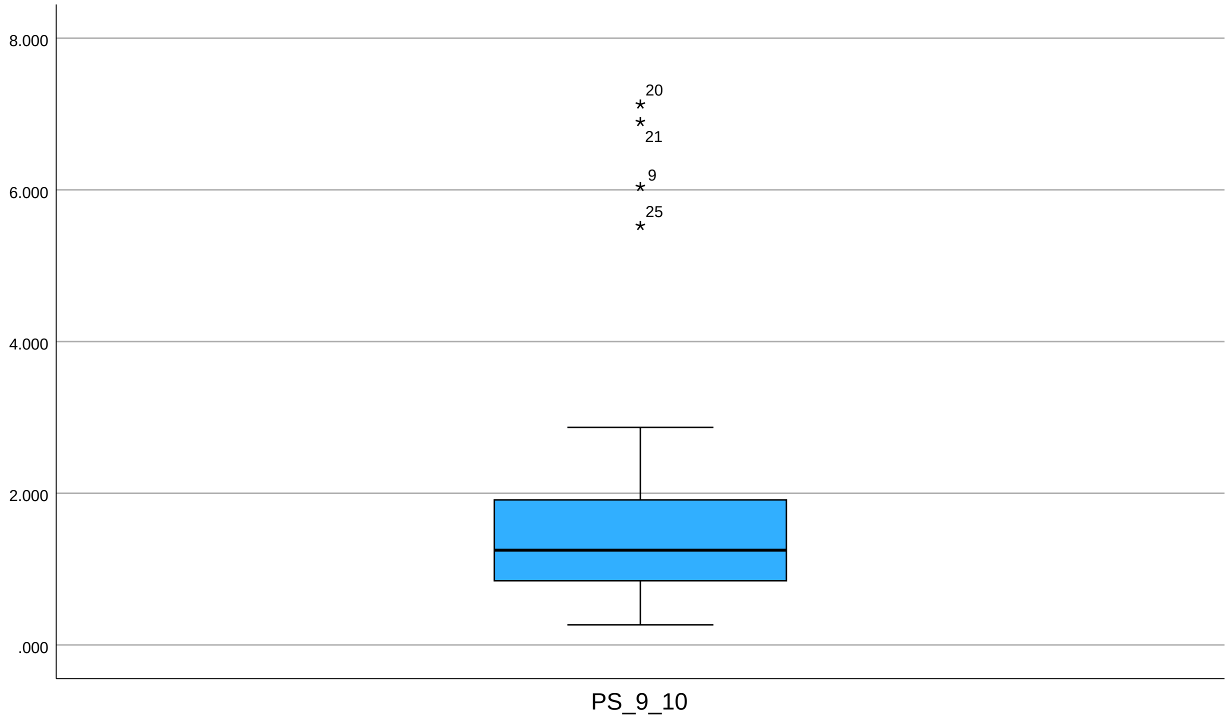

8.000
6.000
4.000
2.000
.000
20
21
9
25
PS_9_10

PS_10_11

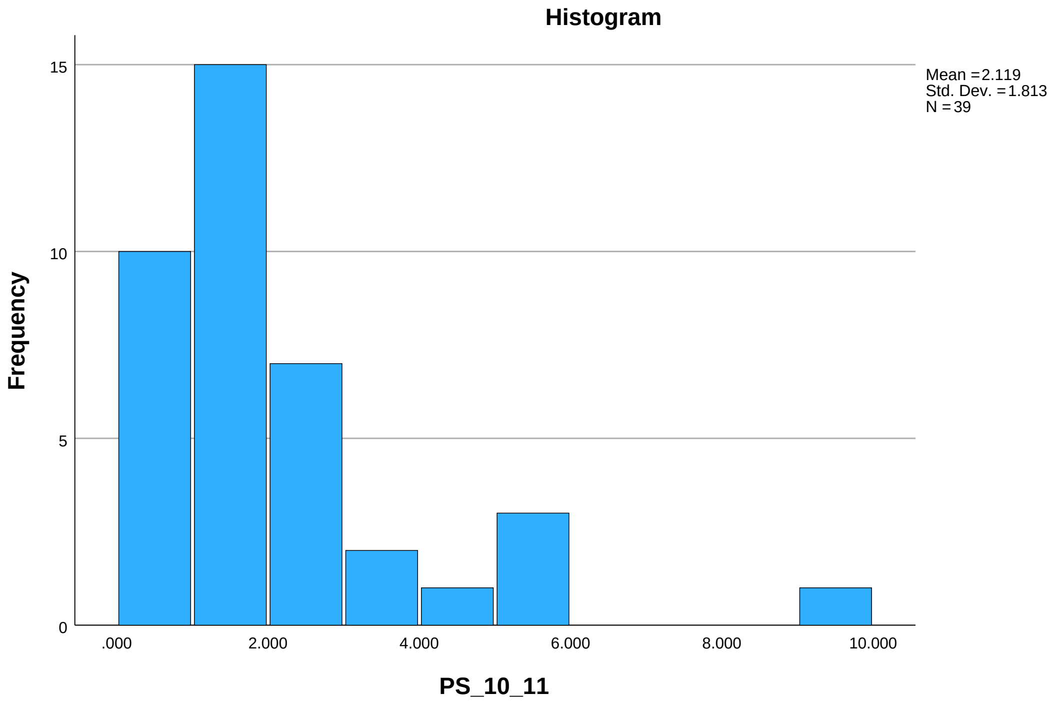


```
PS_10_11 Stem-and-Leaf Plot

 Frequency    Stem &  Leaf

     3.00        0 .  334
     7.00        0 .  6777889
     8.00        1 .  22223344
     7.00        1 .  5566788
     4.00        2 .  0123
     3.00        2 .  579
     2.00        3 .  01
      .00        3 .
     1.00        4 .  1
     4.00 Extremes    (>=5.3)

 Stem width:      1.000
 Each leaf:       1 case(s)
```

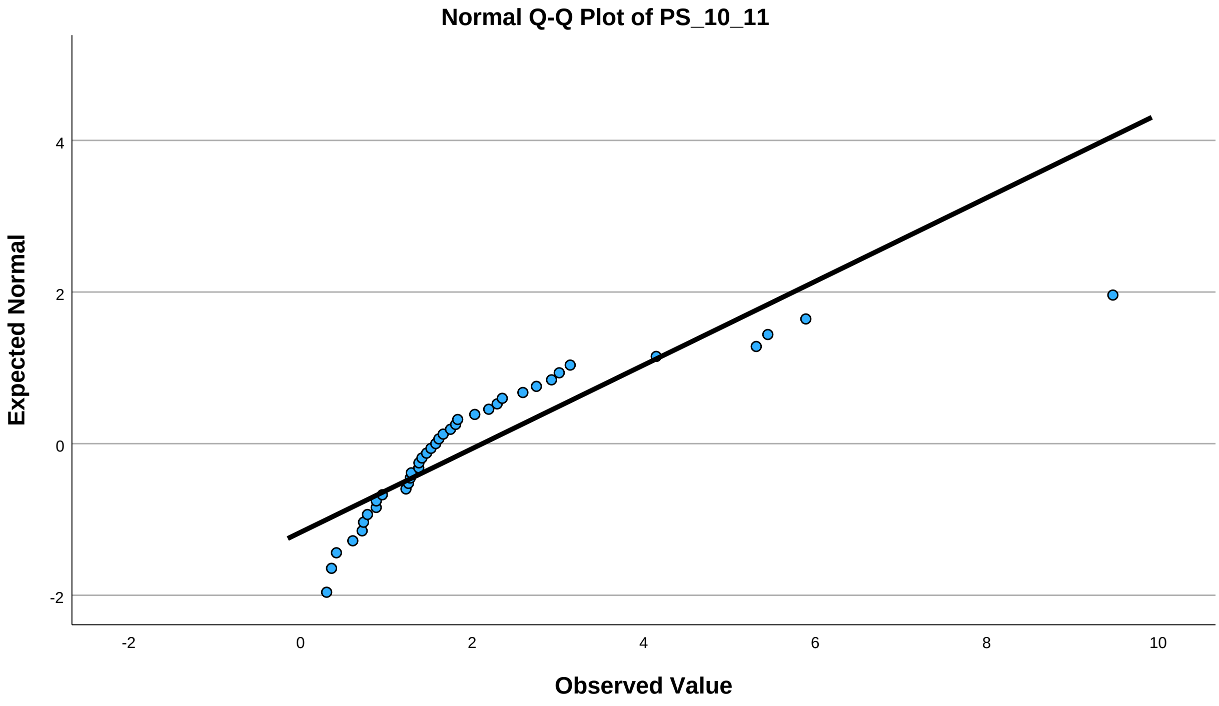
Normal Q-Q Plot of PS_10_11
Expected Normal
Observed Value
4
2
0
-2
-2
0
2
4
6
8
10

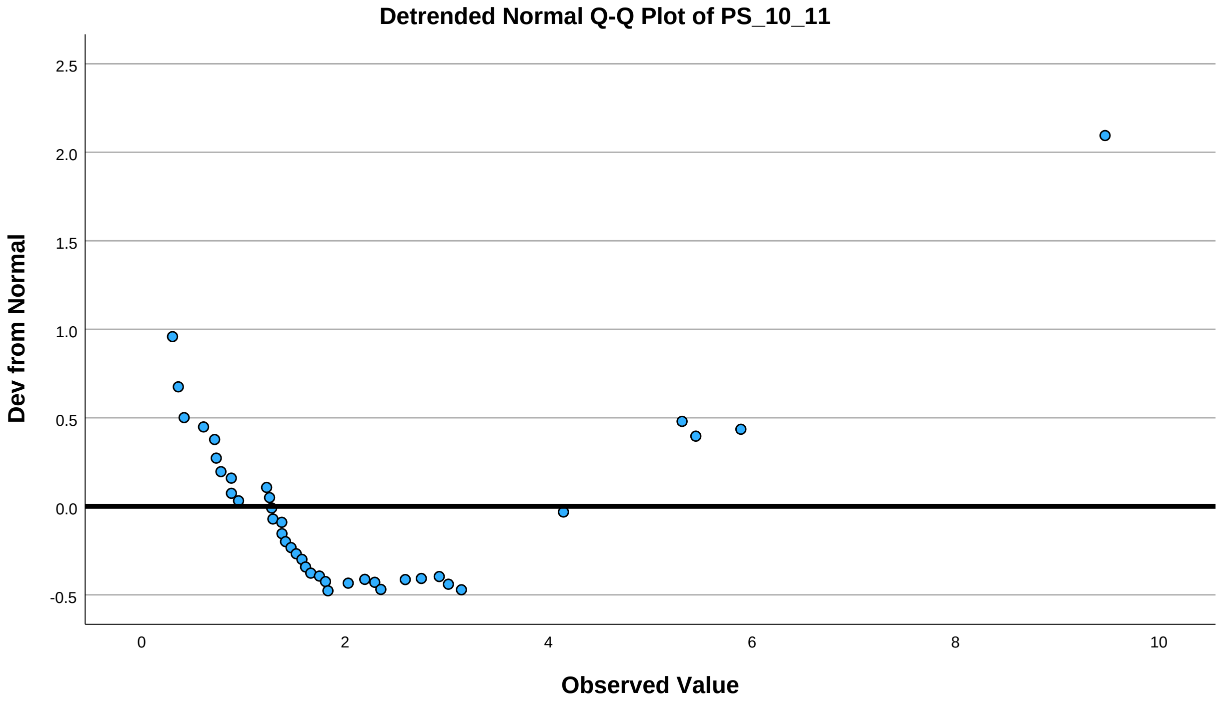
Detrended Normal Q-Q Plot of PS_10_11
Dev from Normal
Observed Value
2.5
2.0
1.5
1.0
0.5
0.0
-0.5
0
2
4
6
8
10

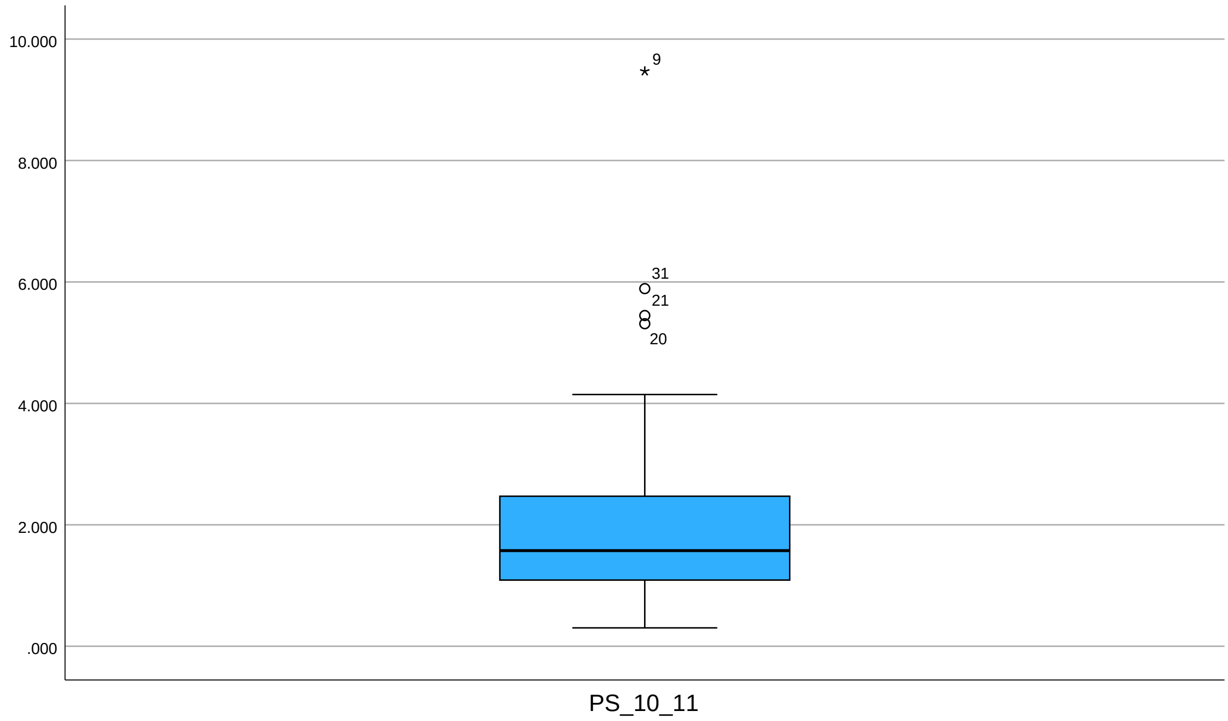

PS_11_12

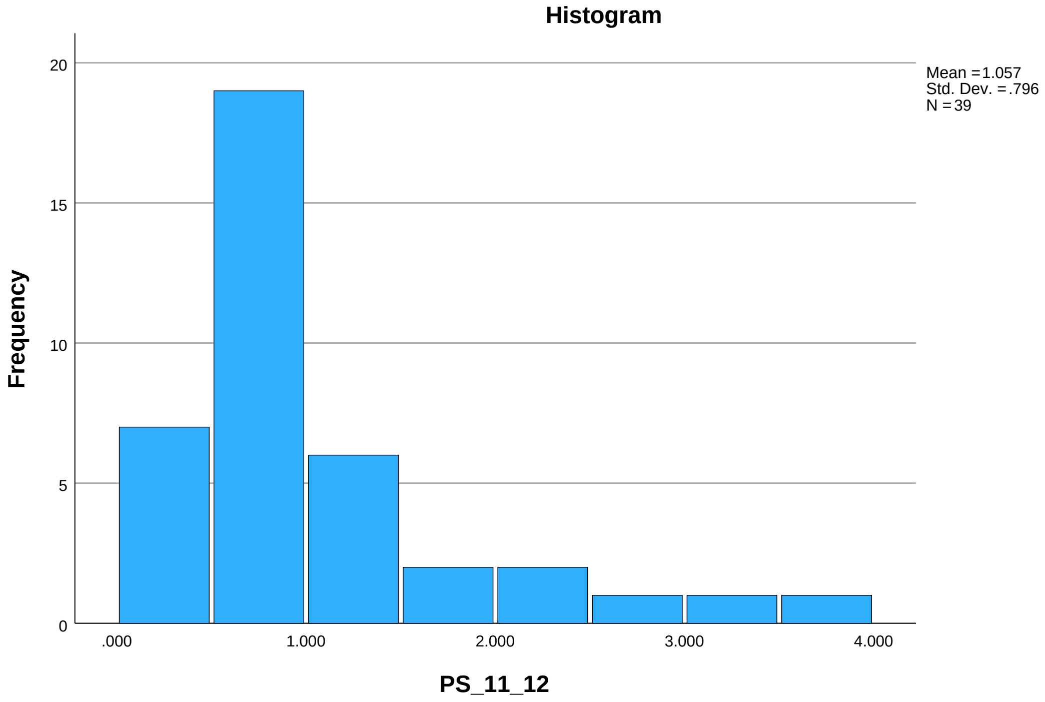

PS_11_12 Stem-and-Leaf Plot

Frequency   Stem & Leaf

```
  7.00      0 .  2233444
 19.00       0 .  5555666667777889999
  6.00      1 .  011123
  2.00     1 .  78
  2.00     2 .  00
  3.00 Extremes    (>=2.7)

Stem width:     1.000
Each leaf:       1 case(s)
```

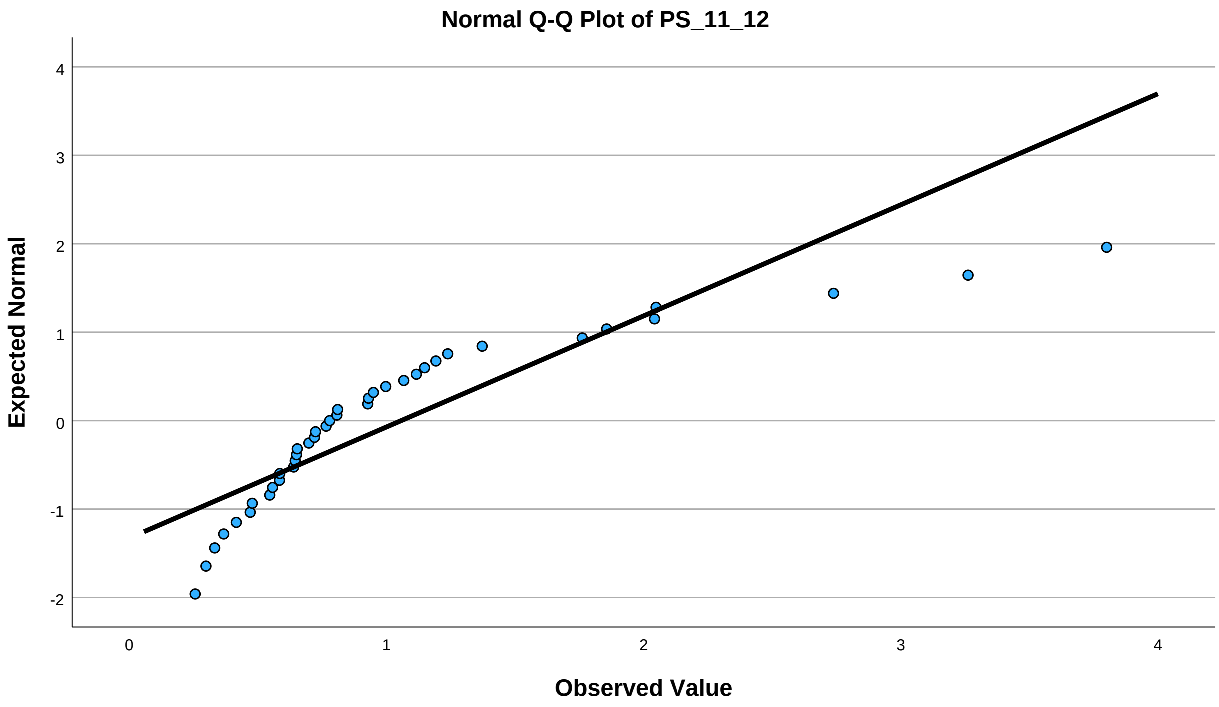

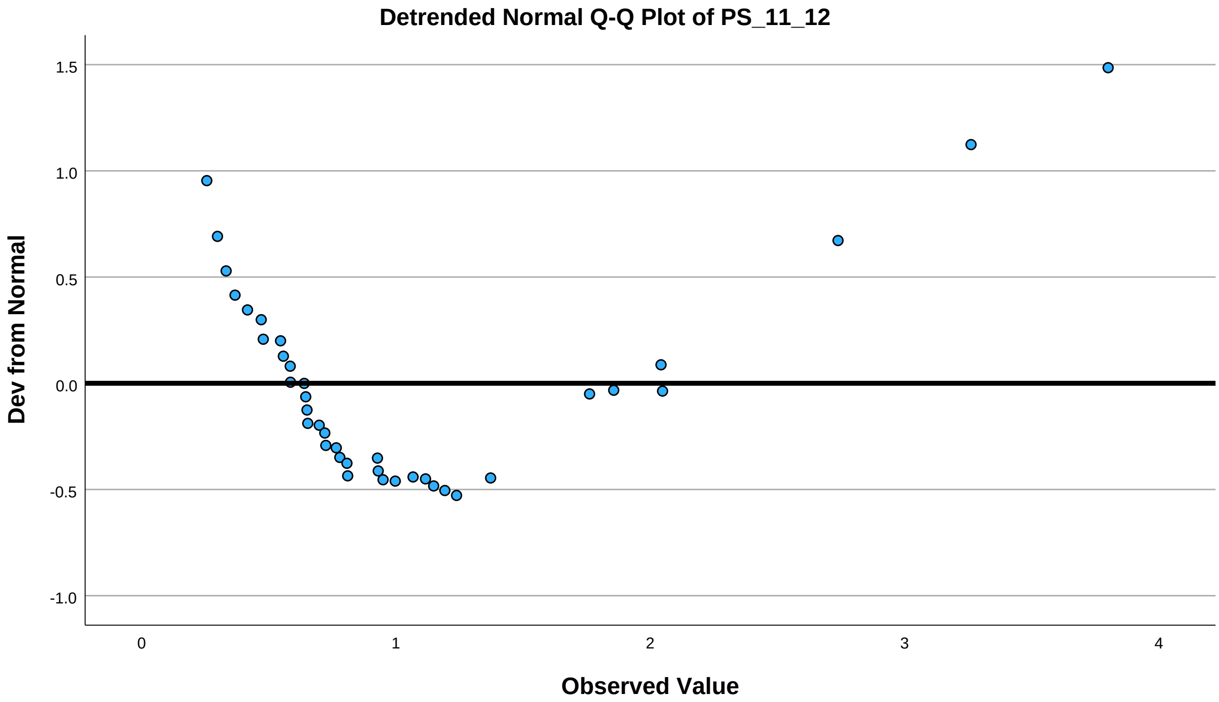
Detrended Normal Q-Q Plot of PS_11_12
Dev from Normal
Observed Value
1.5
1.0
0.5
0.0
-0.5
-1.0
0
1
2
3
4

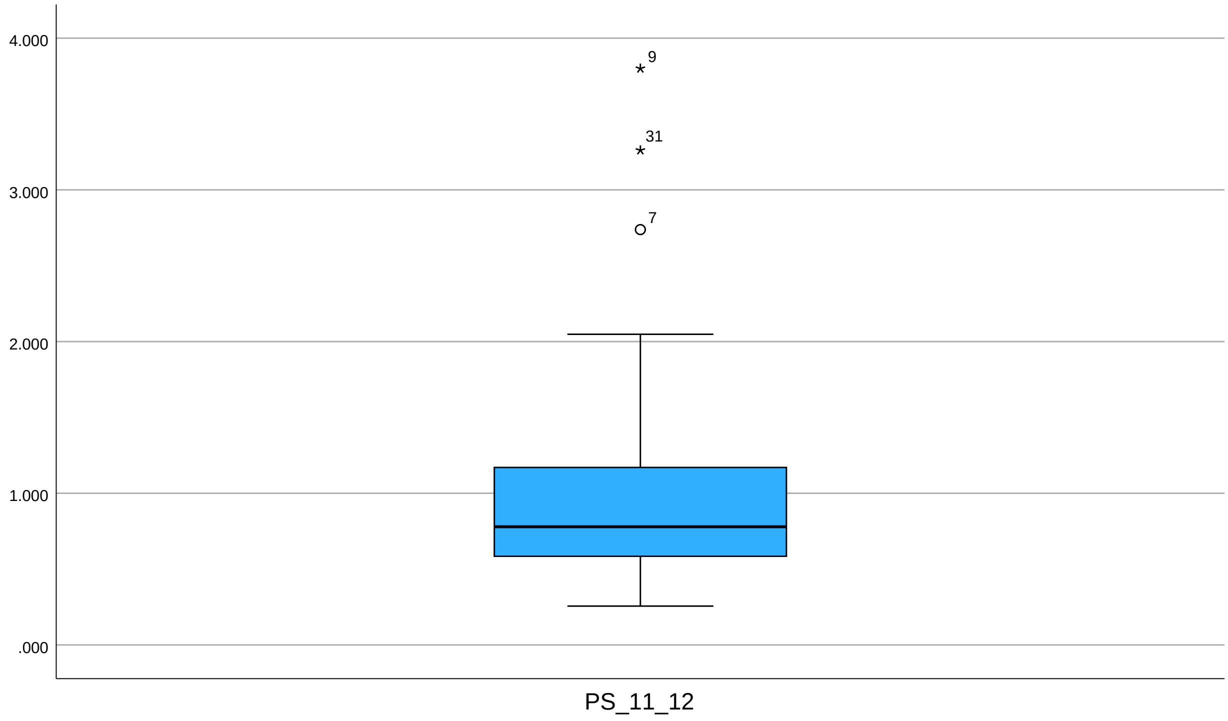
4.000
3.000
2.000
1.000
.000
9
31
7
PS_11_12

PS_12_13

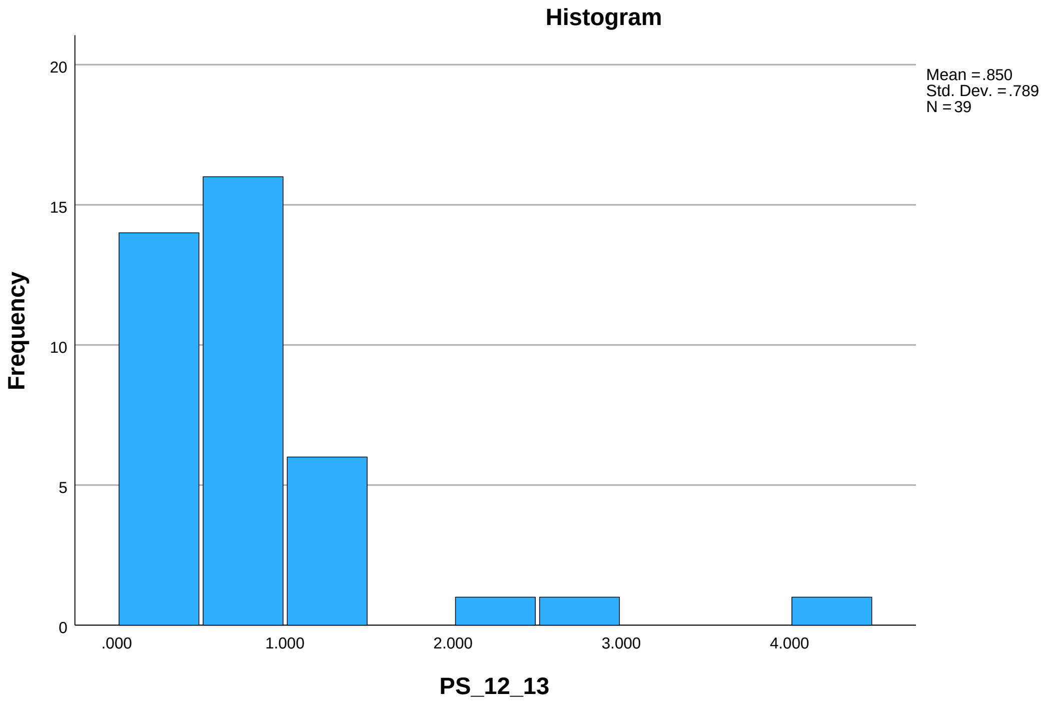


```
PS_12_13 Stem-and-Leaf Plot

 Frequency    Stem &  Leaf

     1.00        0 .  1
     5.00        0 .  22233
    15.00        0 .  444444445555555
     4.00        0 .  6667
     5.00        0 .  88889
     2.00        1 .  00
     4.00        1 .  2333
     3.00 Extremes    (>=2.3)

 Stem width:      1.000
 Each leaf:        1 case(s)
```

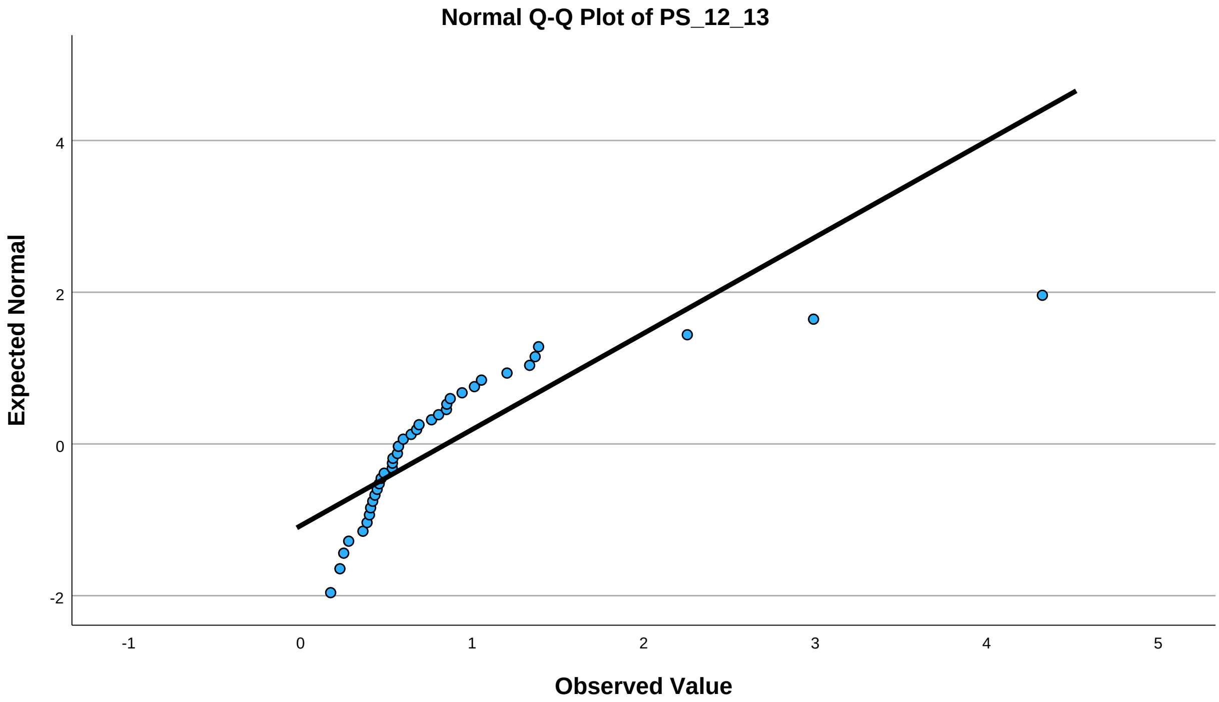
Normal Q-Q Plot of PS_12_13
Expected Normal
Observed Value
4
2
0
-2
-1
0
1
2
3
4
5

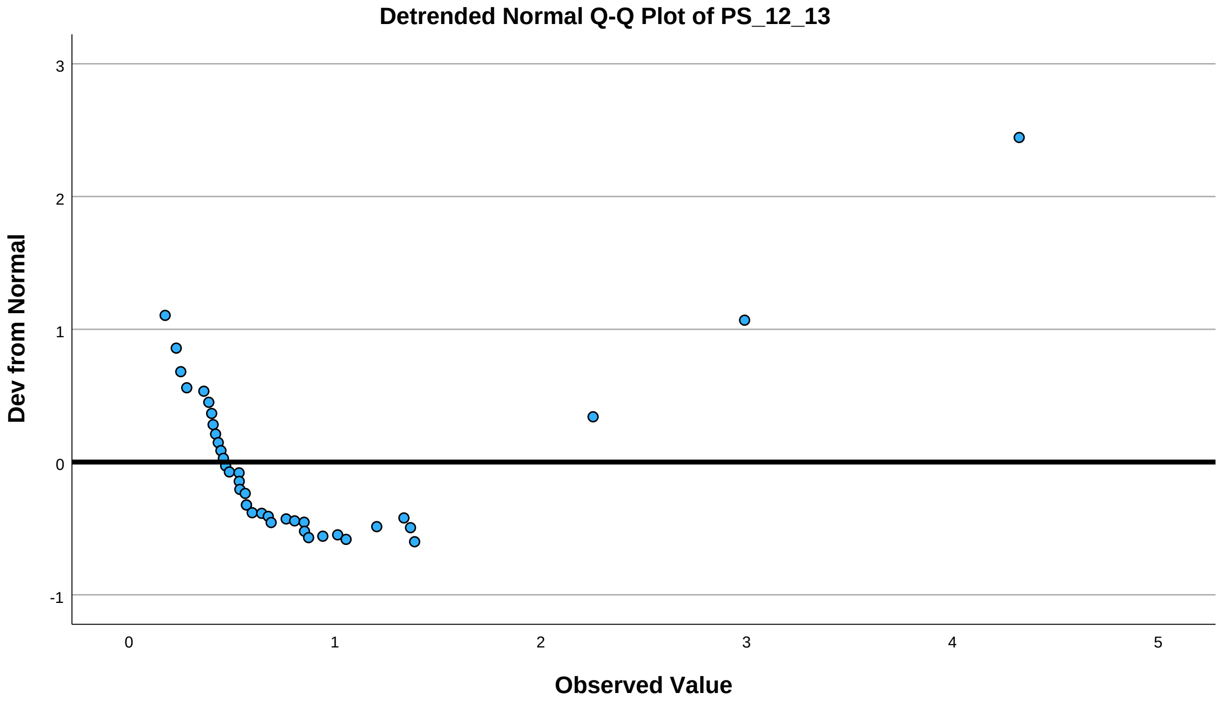
Detrended Normal Q-Q Plot of PS_12_13
Dev from Normal
Observed Value
3
2
1
0
-1
0
1
2
3
4
5

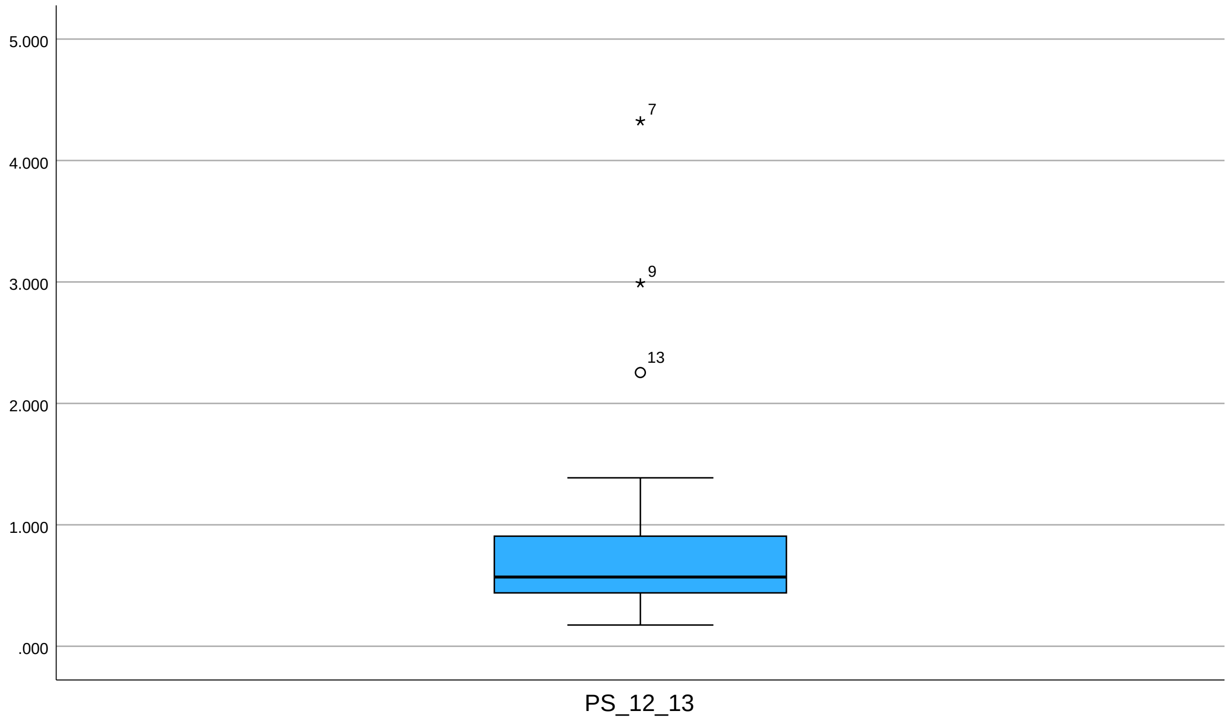

PS_13_14

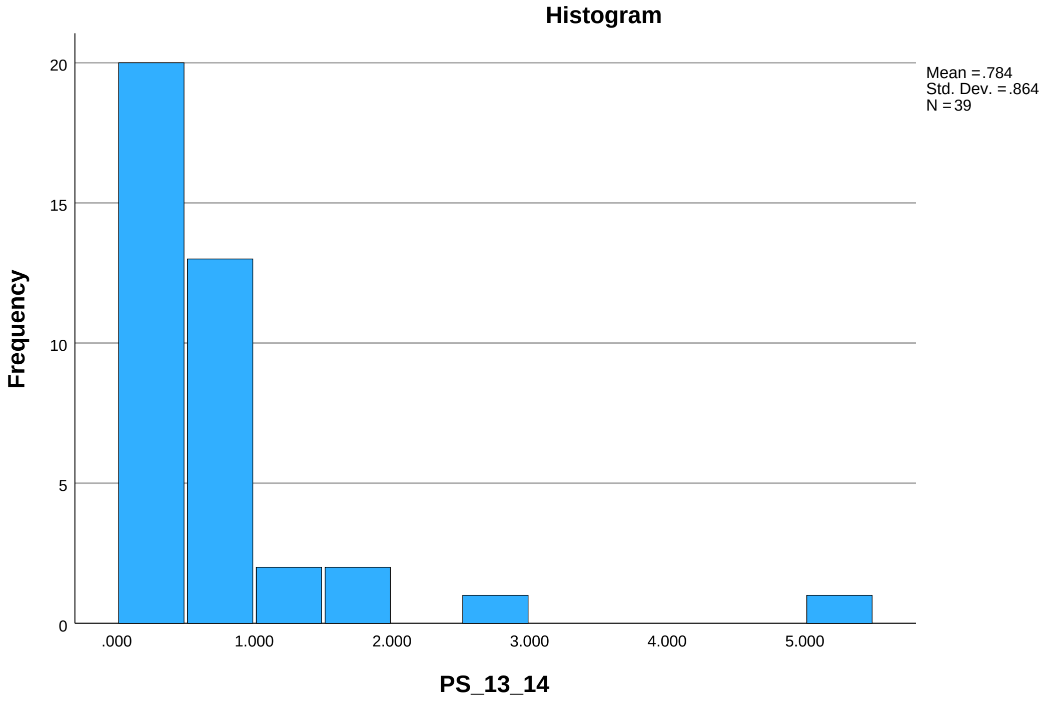

PS_13_14 Stem-and-Leaf Plot

Frequency   Stem & Leaf

```
 2.00        1 .  58
 2.00        2 .  34
 7.00        3 .  2467789
 9.00        4 .  001126668
 5.00        5 .  22467
 2.00        6 .  04
  .00        7 .
 2.00        8 .  77
 4.00        9 .  6779
 1.00       10 .  8
 1.00       11 .  1
 4.00 Extremes    (>=1.86)

Stem width:       .100
Each leaf:        1 case(s)
```

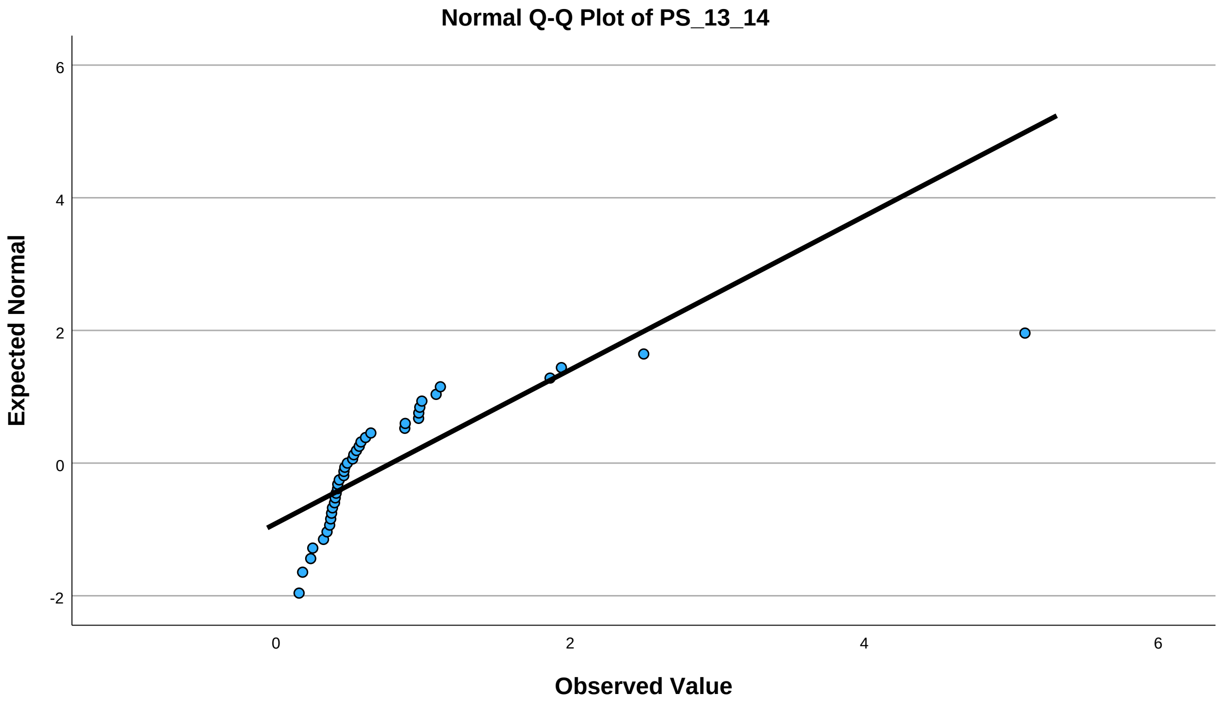

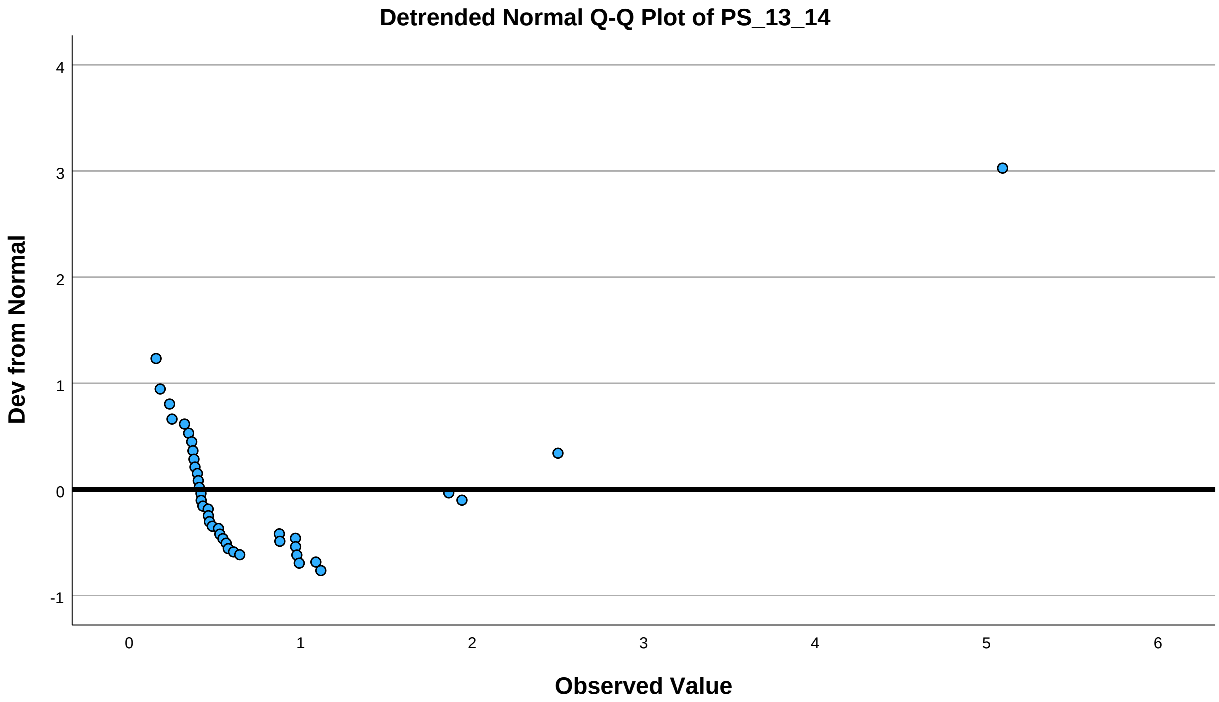
Detrended Normal Q-Q Plot of PS_13_14
Dev from Normal
4
3
2
1
0
-1
0
1
2
3
4
5
6
Observed Value

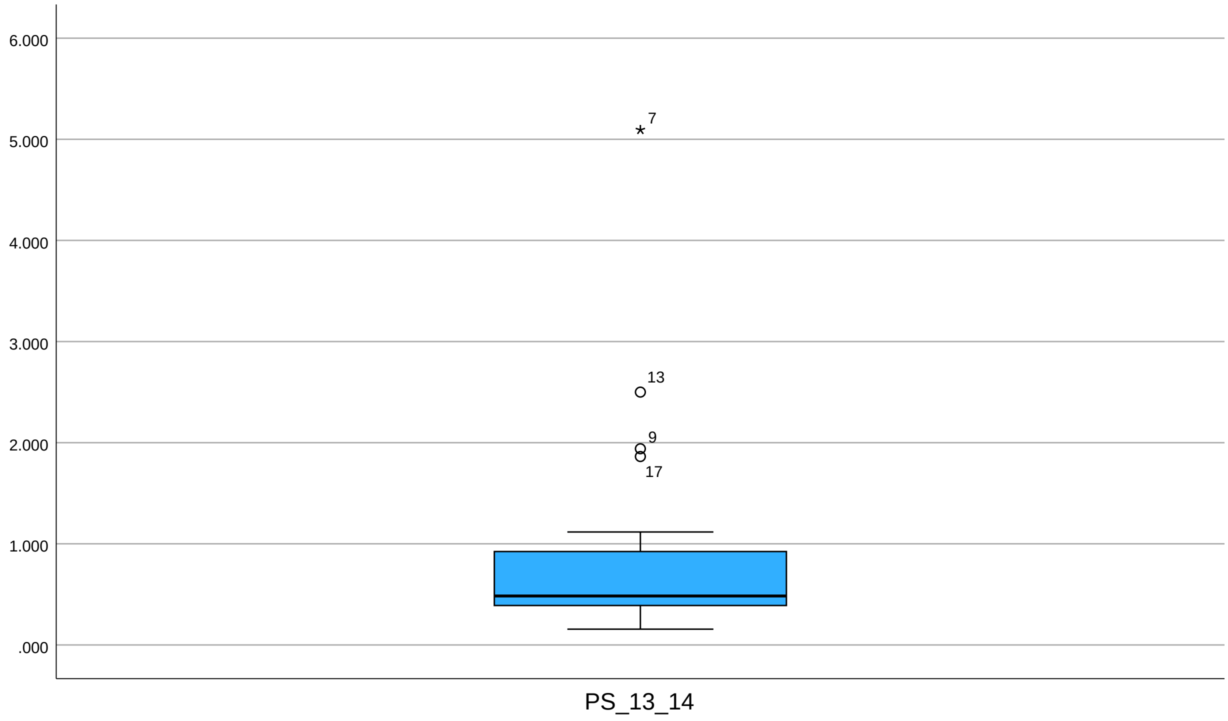
6.000
5.000
4.000
3.000
2.000
1.000
.000
7
13
9
17
PS_13_14

PS_14_15

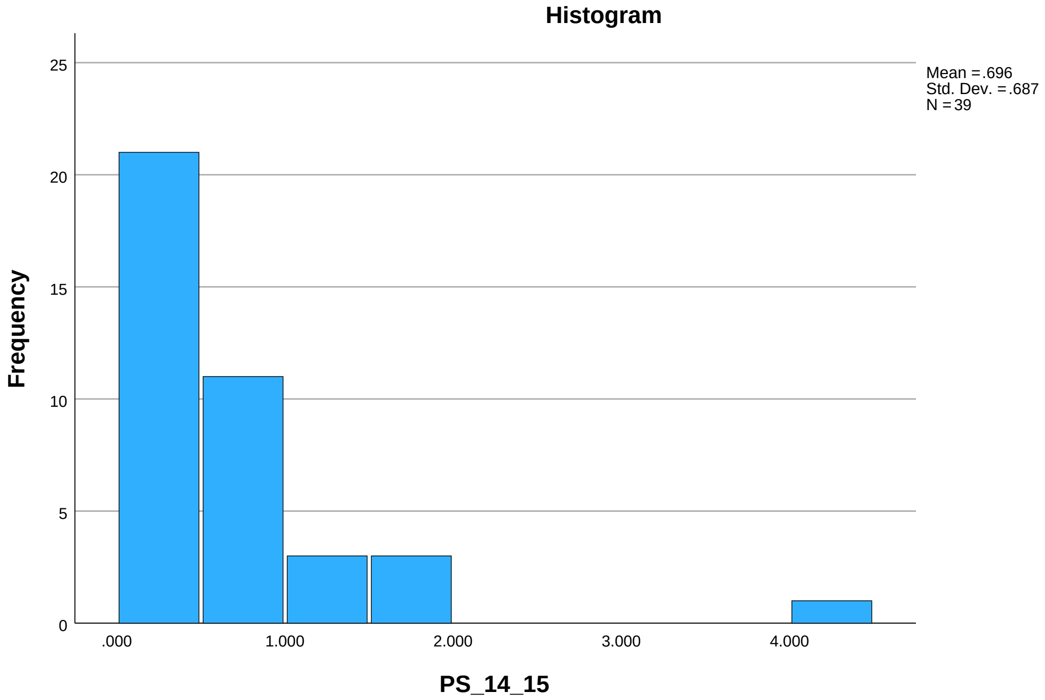


```
PS_14_15 Stem-and-Leaf Plot

 Frequency    Stem &  Leaf

     2.00        1 .  69
     2.00        2 .  45
    12.00        3 .  000013445789
     5.00        4 .  11669
     4.00        5 .  1389
     1.00        6 .  0
     3.00        7 .  012
     3.00        8 .  678
      .00        9 .
     2.00       10 .  04
      .00       11 .
     1.00       12 .  3
     4.00 Extremes    (>=1.56)

 Stem width:      .100
 Each leaf:       1 case(s)
```

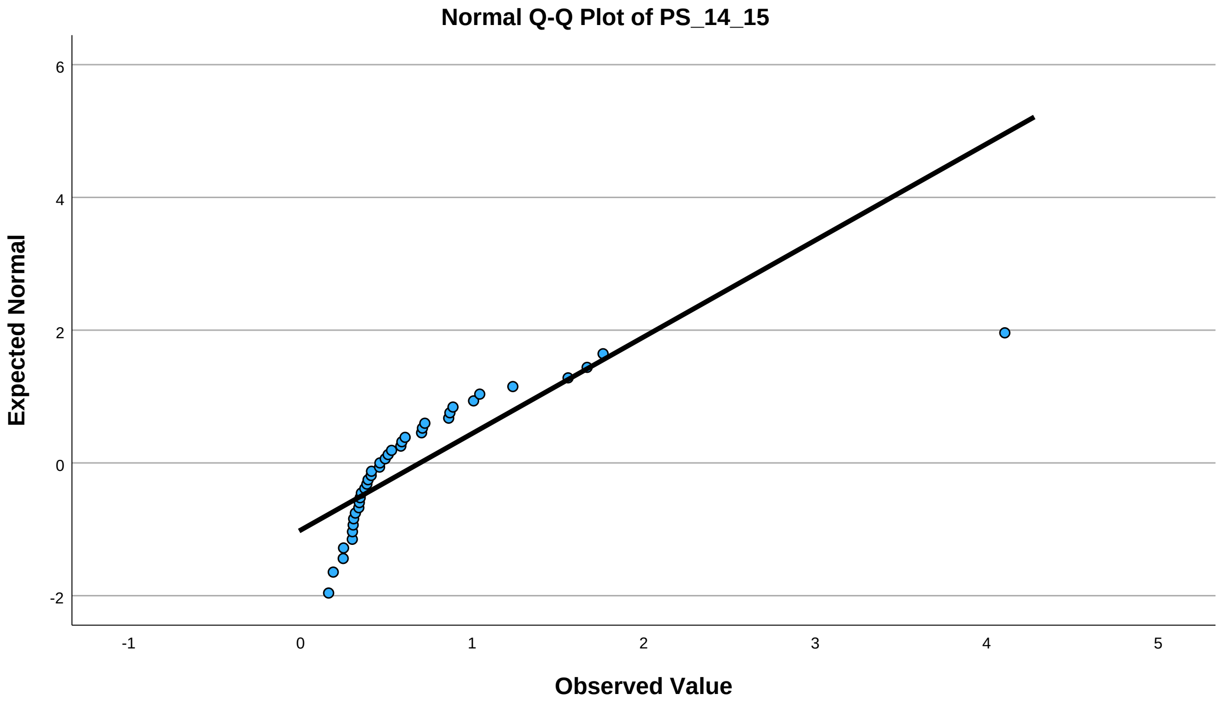
Normal Q-Q Plot of PS_14_15
Expected Normal
6
4
2
0
-2
-1
0
1
2
3
4
5
Observed Value

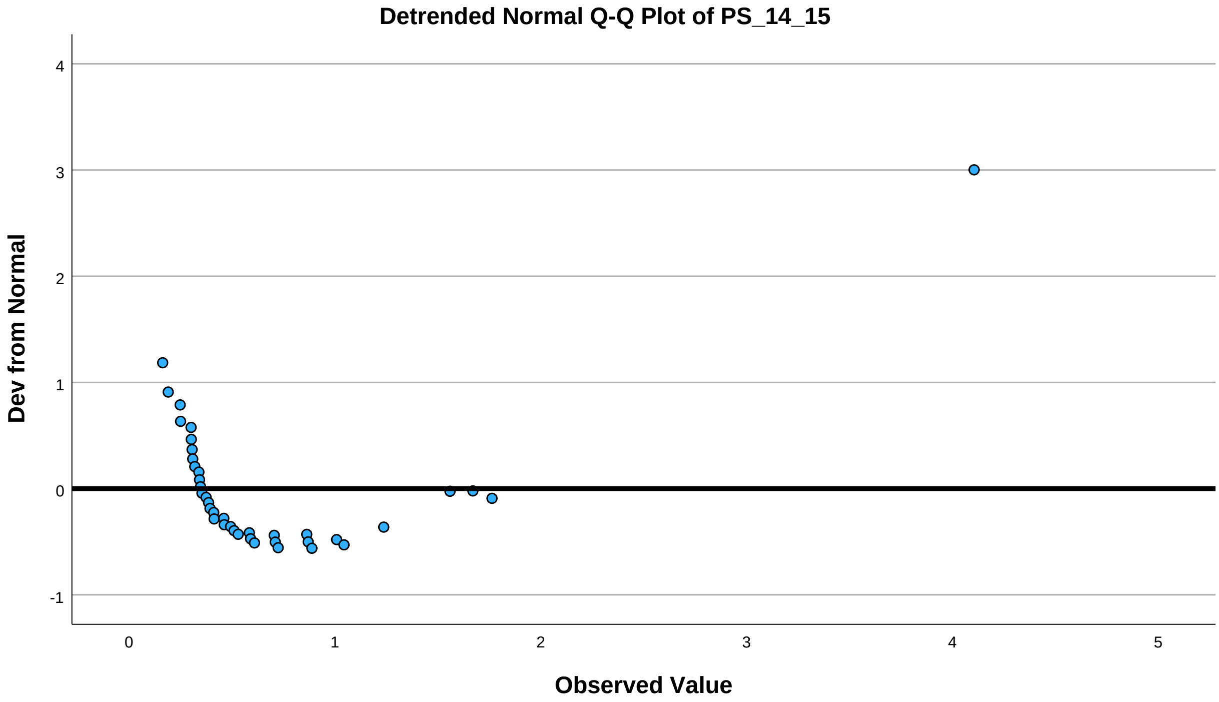
Detrended Normal Q-Q Plot of PS_14_15
Dev from Normal
4
3
2
1
0
-1
0
1
2
3
4
5
Observed Value

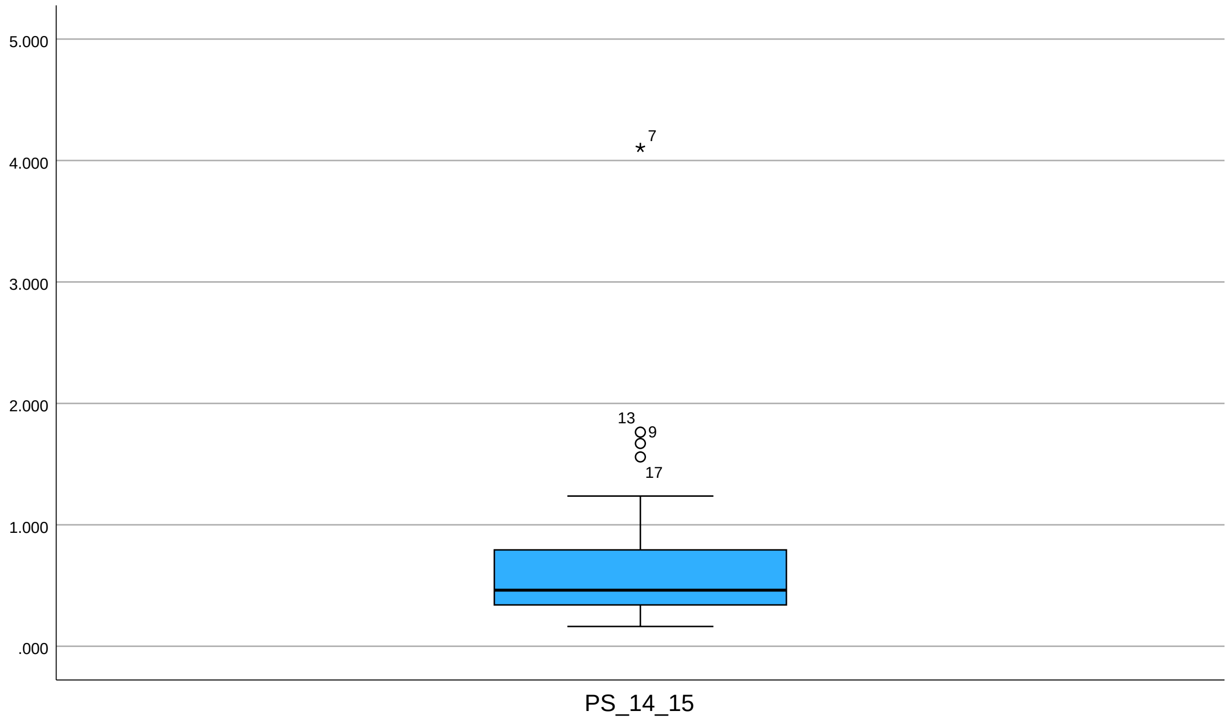

PS_15_16

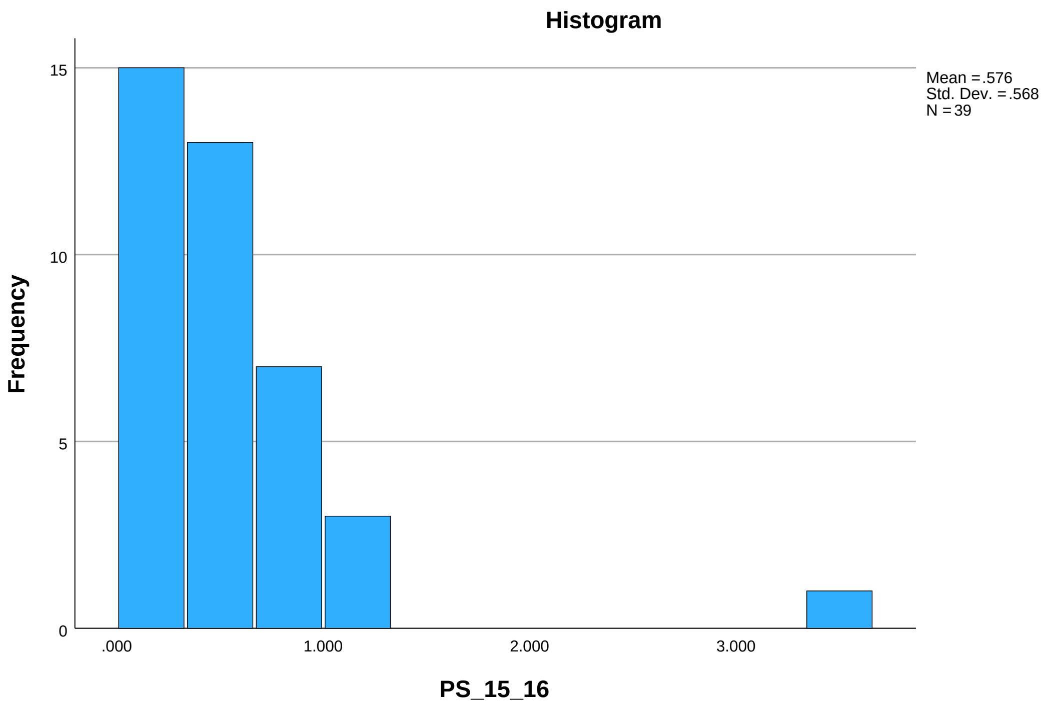

PS_15_16 Stem-and-Leaf Plot

Frequency Stem & Leaf

```
  2.00      1 . 25
  8.00      2 . 13445788
  8.00      3 . 01122466
  7.00      4 . 0113578
  2.00      5 . 06
  3.00      6 . 189
  3.00      7 . 456
   .00      8 .
  2.00      9 . 11
   .00     10 .
  1.00     11 . 3
  1.00     12 . 7
  2.00 Extremes    (>=1.33)

Stem width:      .100
Each leaf:       1 case(s)
```

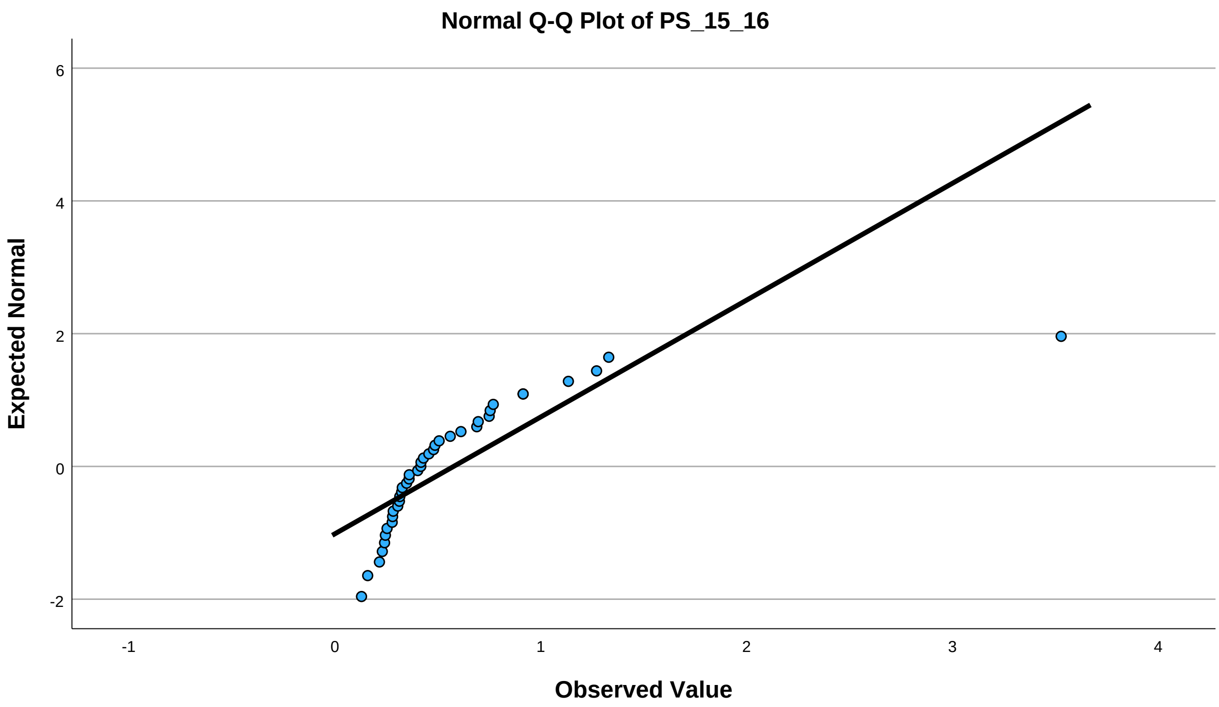

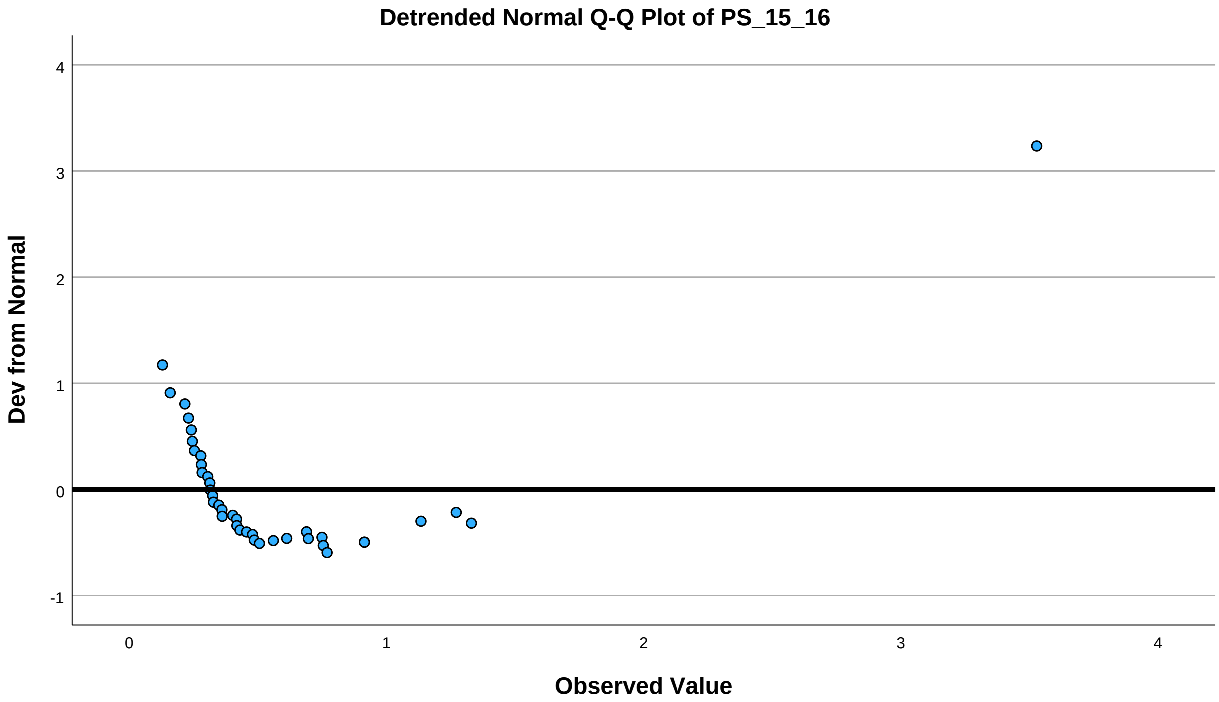
Detrended Normal Q-Q Plot of PS_15_16
Dev from Normal
4
3
2
1
0
-1
0
1
2
3
4
Observed Value

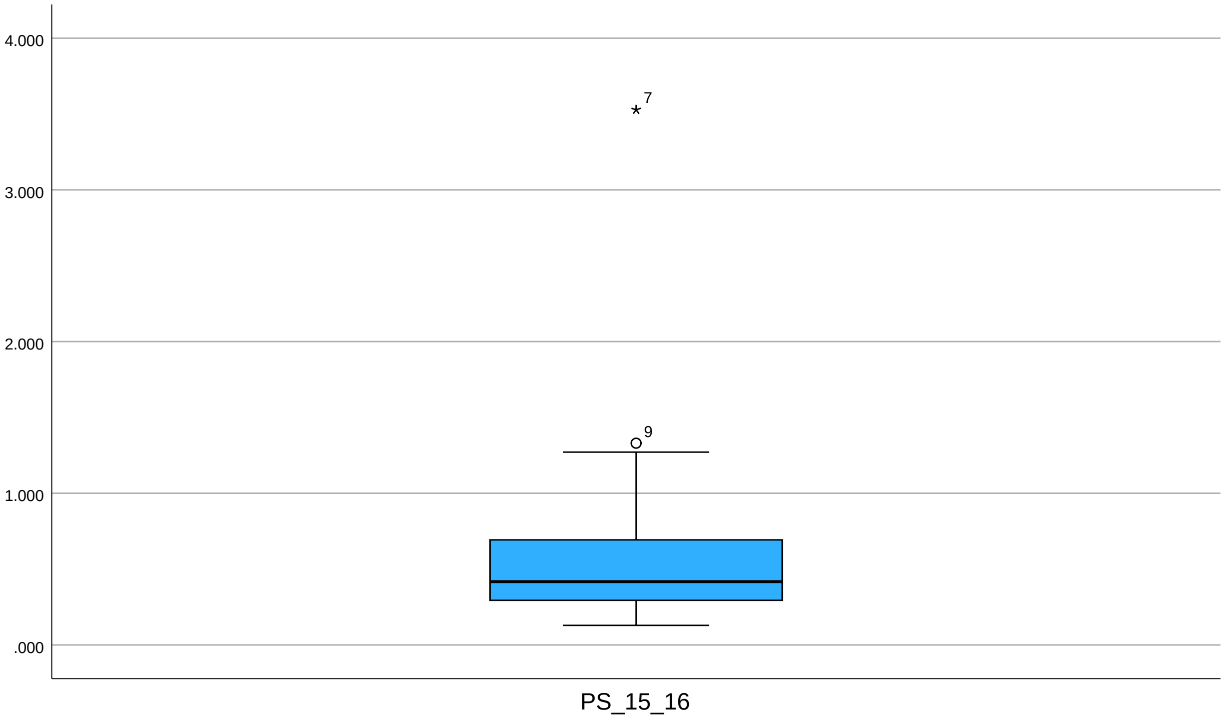
4.000
3.000
2.000
1.000
.000
7
9
PS_15_16

PS_16_17

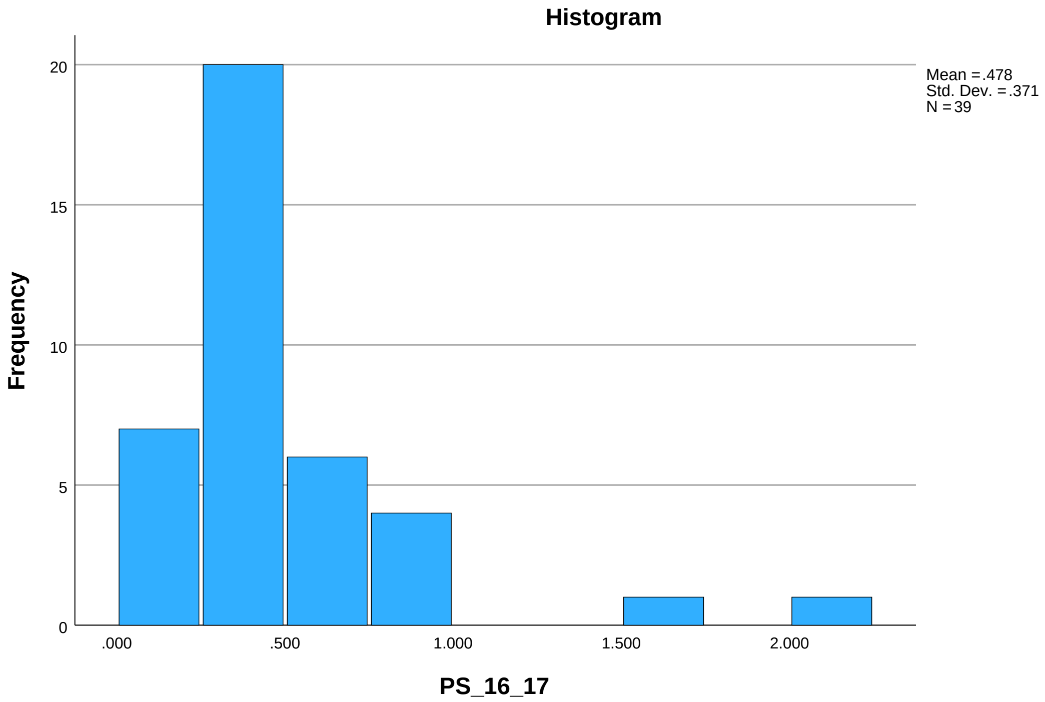


```
PS_16_17 Stem-and-Leaf Plot

 Frequency    Stem &  Leaf

     3.00        1 .  039
    11.00        2 .  11245558899
     9.00        3 .  002234567
     4.00        4 .  1446
     1.00        5 .  1
     4.00        6 .  0014
     3.00        7 .  068
     1.00        8 .  1
     1.00        9 .  9
     2.00 Extremes    (>=1.50)

 Stem width:       .100
 Each leaf:        1 case(s)
```

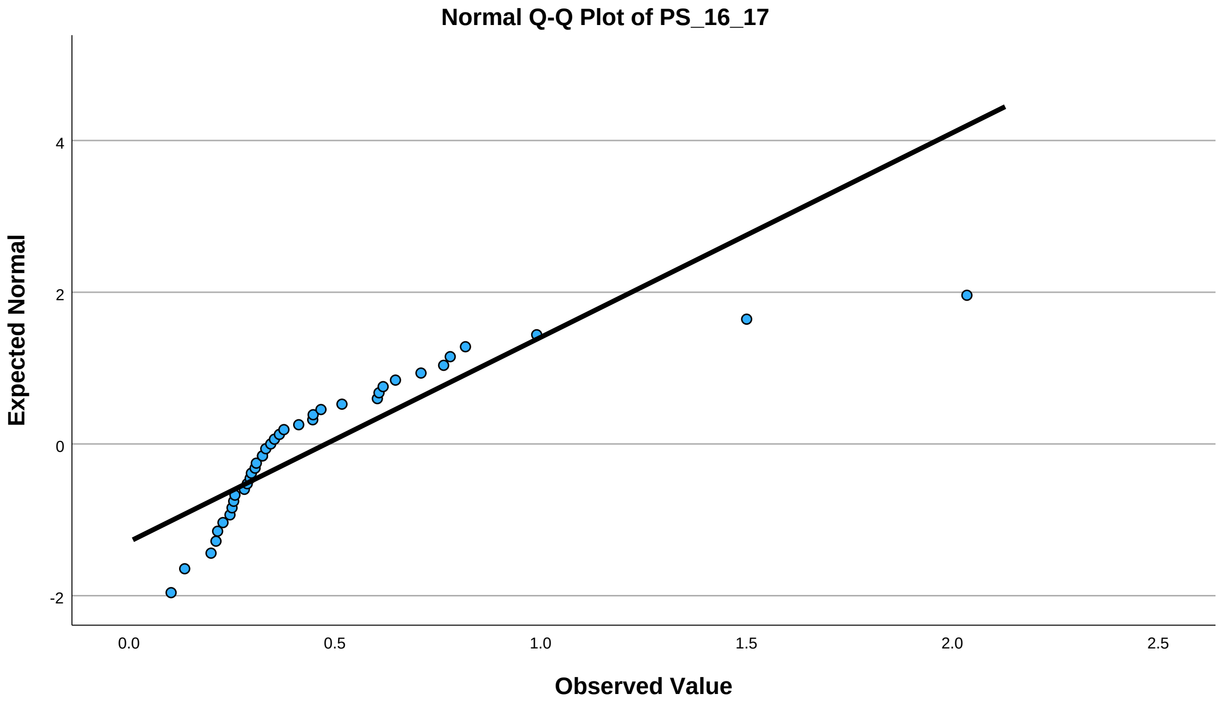
Normal Q-Q Plot of PS_16_17
Expected Normal
Observed Value
4
2
0
-2
0.0
0.5
1.0
1.5
2.0
2.5

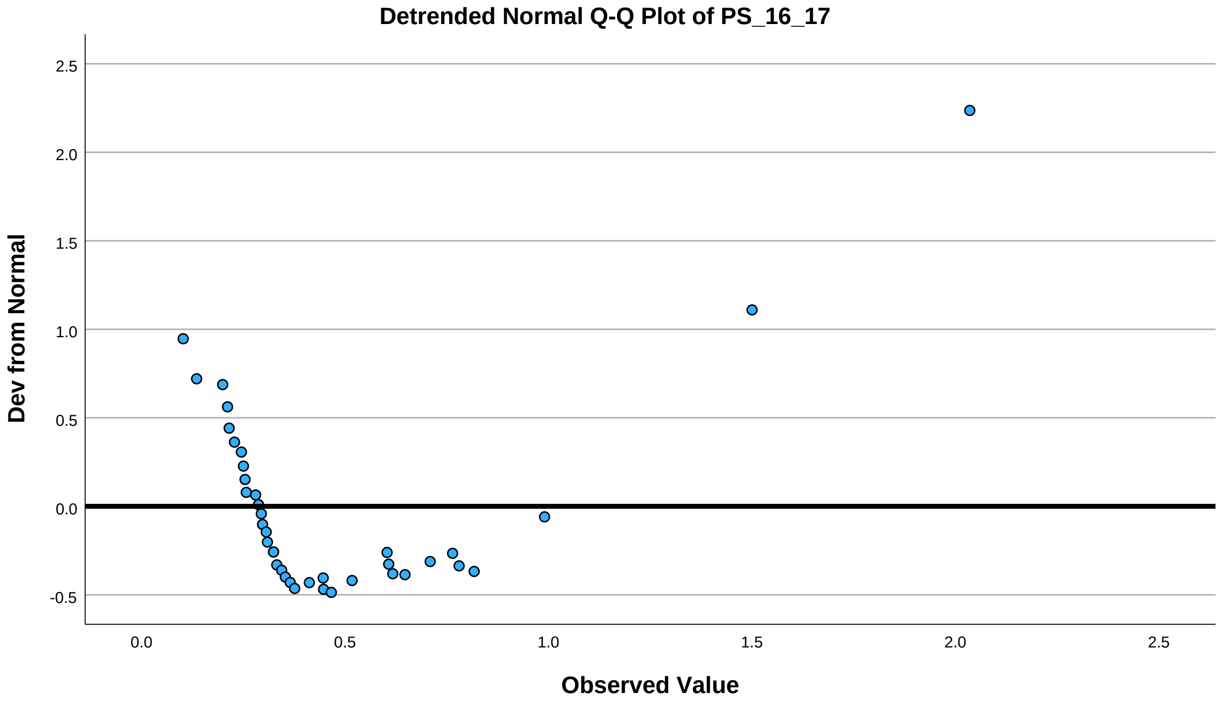
Detrended Normal Q-Q Plot of PS_16_17
Dev from Normal
Observed Value
2.5
2.0
1.5
1.0
0.5
0.0
-0.5
0.0
0.5
1.0
1.5
2.0
2.5

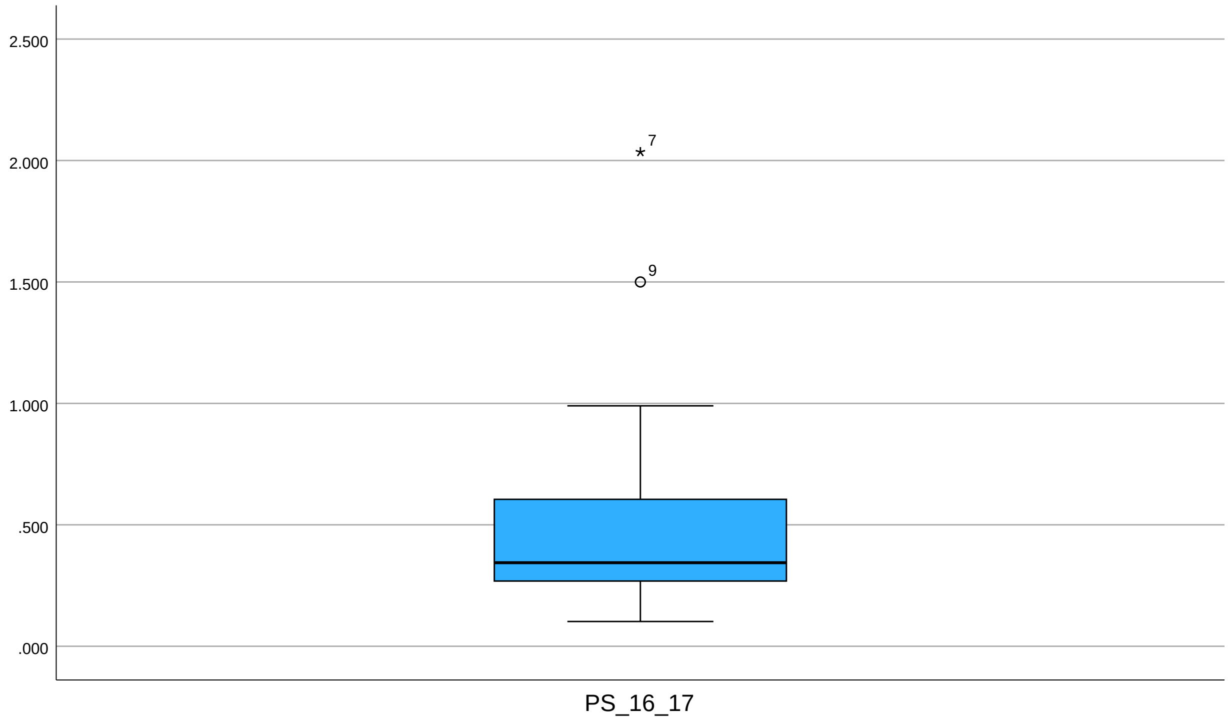

PS_17_18

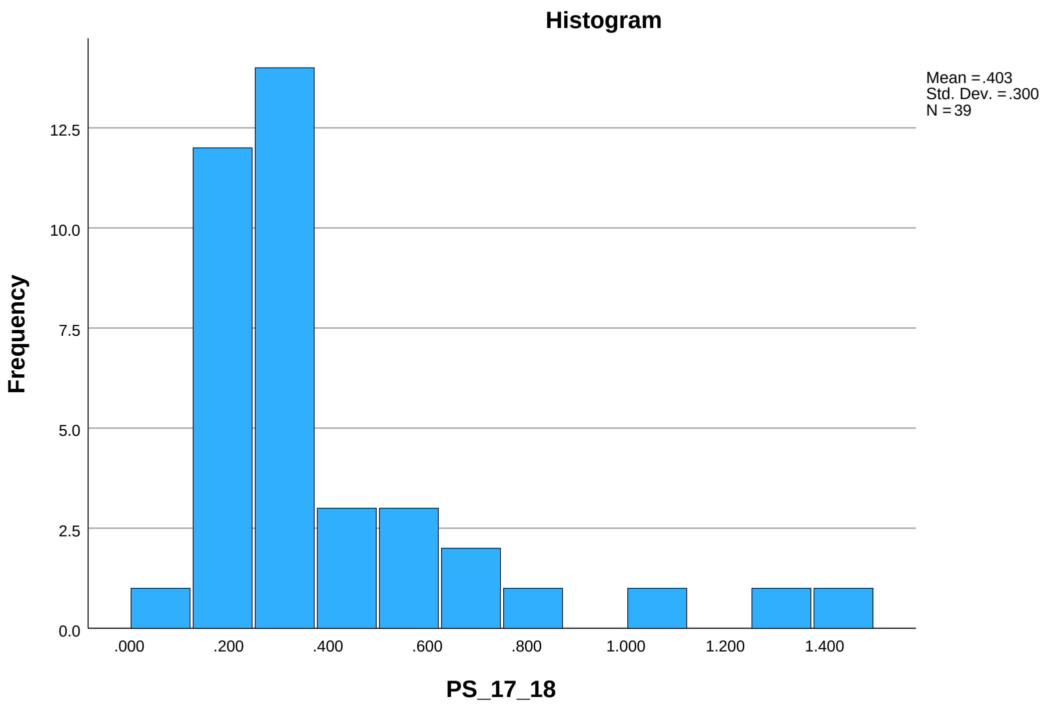


```
PS_17_18 Stem-and-Leaf Plot

 Frequency    Stem &  Leaf
```

```
 2.00      1 . 03
 5.00      1 . 55788
 6.00      2 . 012333
 5.00      2 . 56668
 7.00      3 . 1234444
 3.00      3 . 567
 1.00      4 . 1
 1.00      4 . 5
 1.00      5 . 4
 1.00      5 . 5
 1.00      6 . 1
 2.00      6 . 57
 4.00 Extremes    (>=.78)

Stem width:      .100
Each leaf:       1 case(s)
```

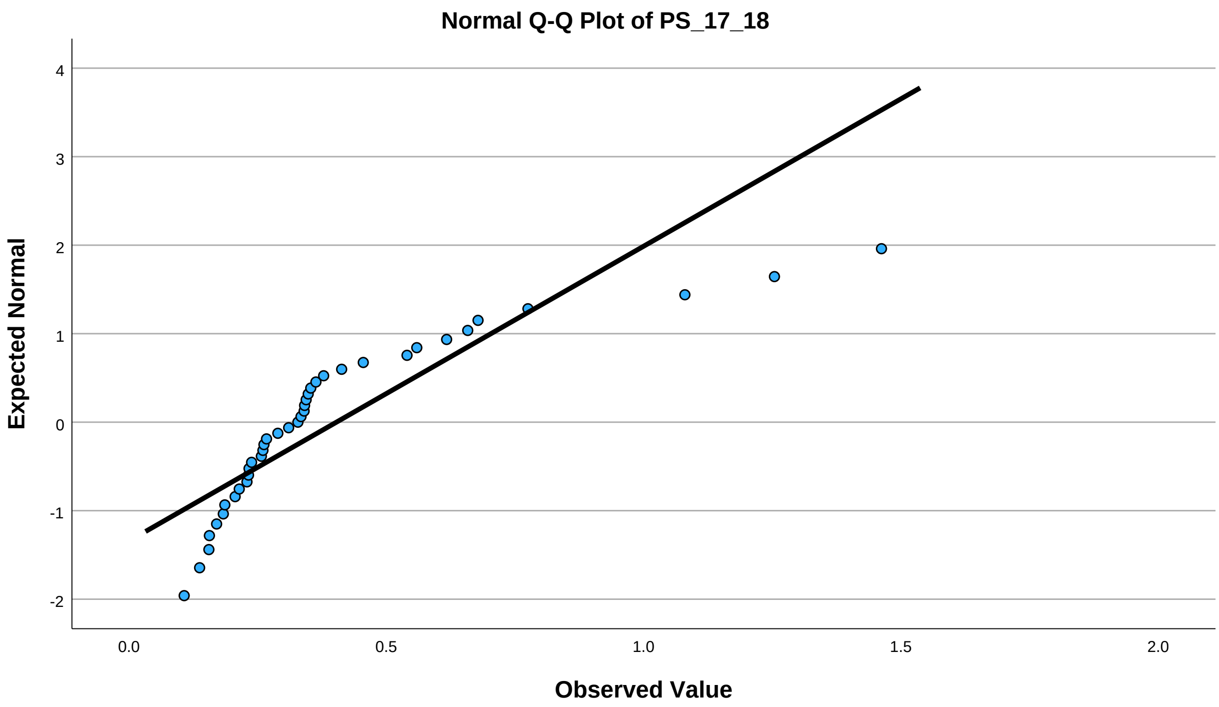

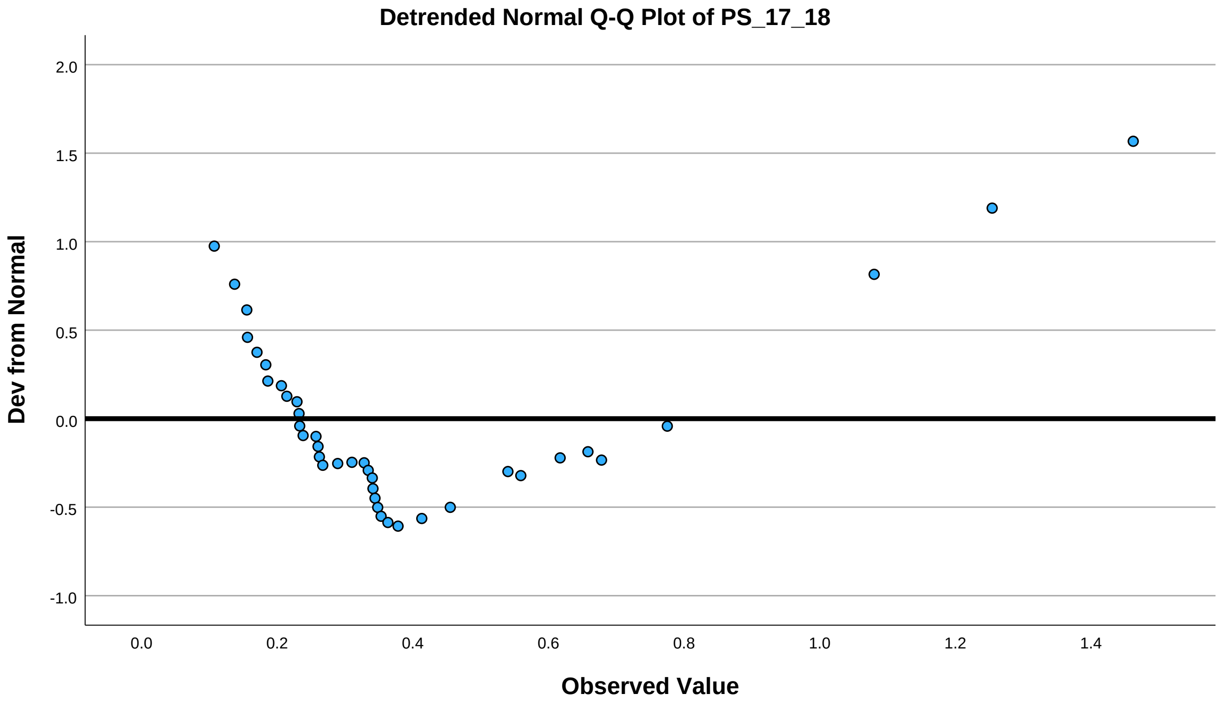

Detrended Normal Q-Q Plot of PS_17_18
Dev from Normal
2.0
1.5
1.0
0.5
0.0
-0.5
-1.0
0.0
0.2
0.4
0.6
0.8
1.0
1.2
1.4
Observed Value

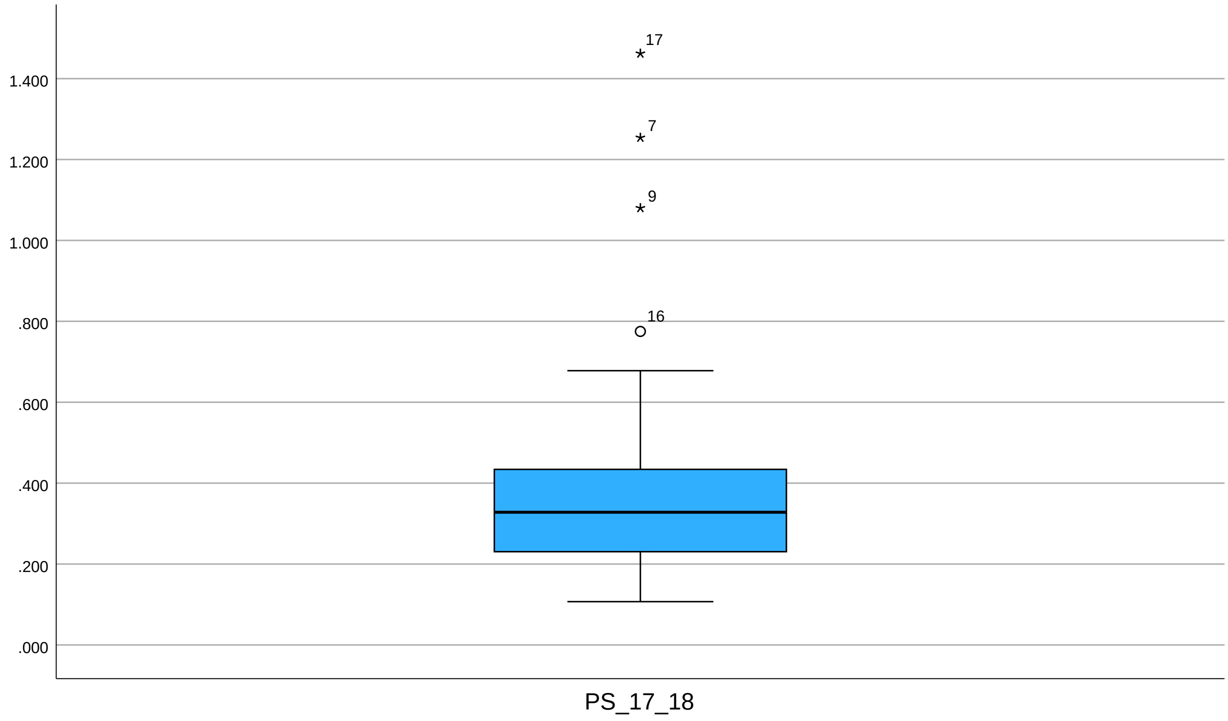

17
7
9
16
1.400
1.200
1.000
.800
.600
.400
.200
.000
PS_17_18

PS_18_19

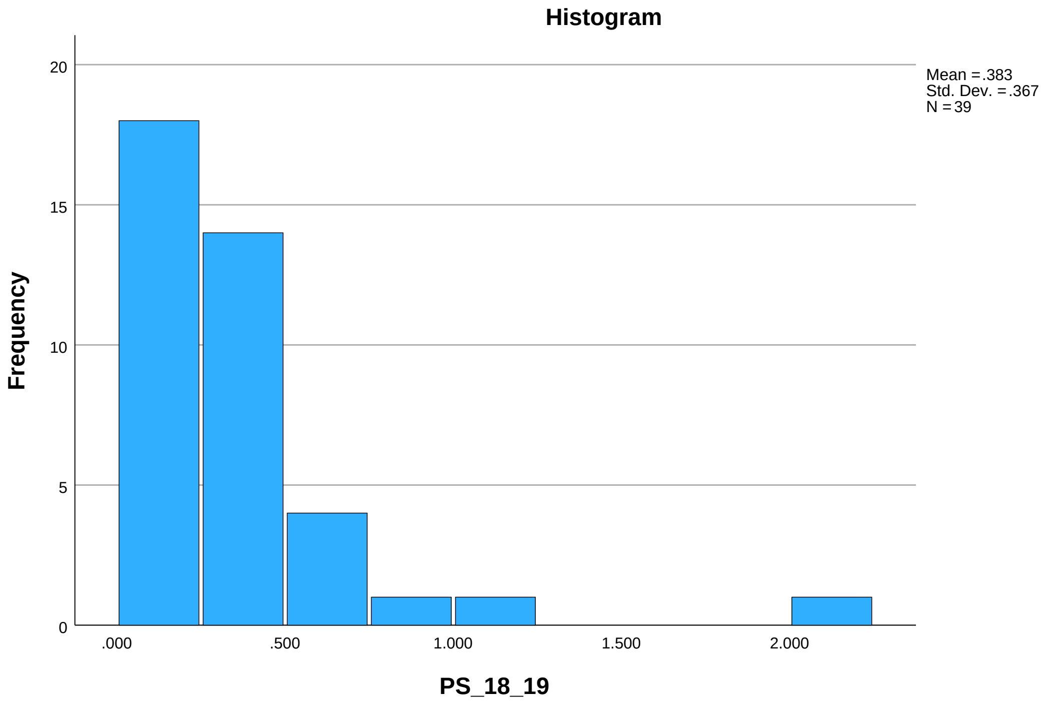


```
PS_18_19 Stem-and-Leaf Plot

 Frequency    Stem &  Leaf

     1.00        0 .  7
     7.00        1 .  2455666
    16.00        2 .  0111122233678999
     3.00        3 .  112
     5.00        4 .  24556
     1.00        5 .  2
     2.00        6 .  01
     1.00        7 .  0
     3.00 Extremes    (>=.94)

 Stem width:      .100
 Each leaf:       1 case(s)
```

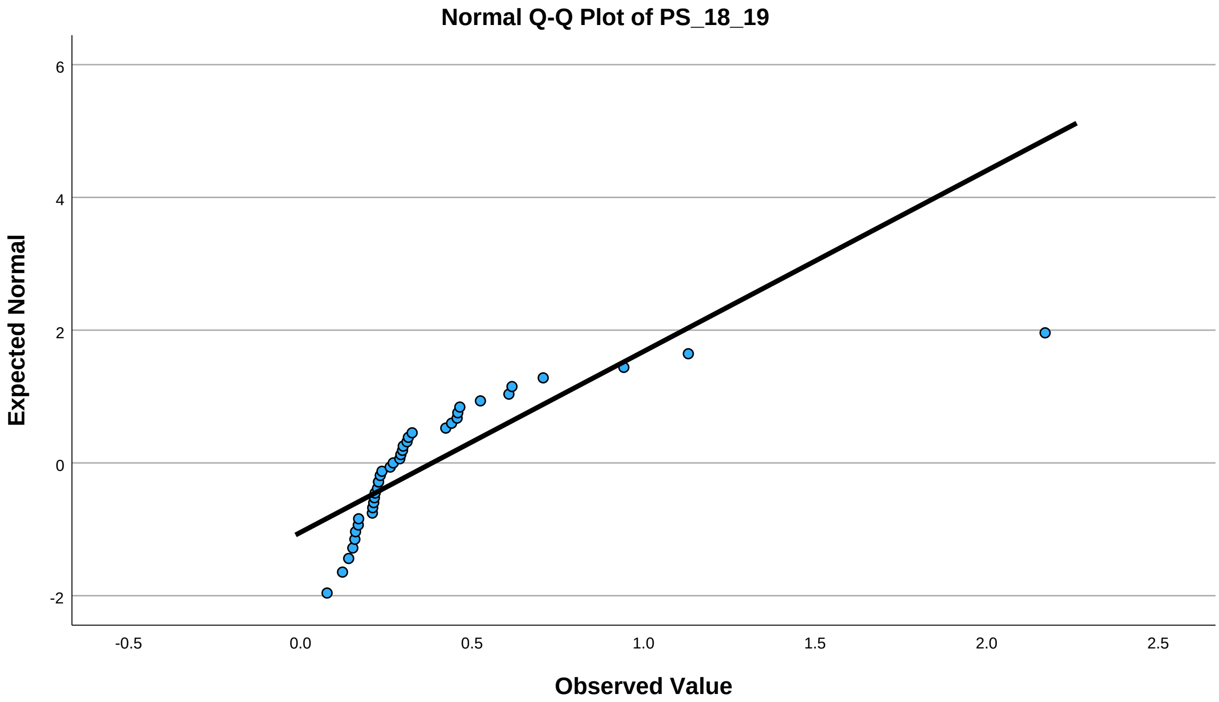
Normal Q-Q Plot of PS_18_19
Expected Normal
Observed Value
6
4
2
0
-2
-0.5
0.0
0.5
1.0
1.5
2.0
2.5

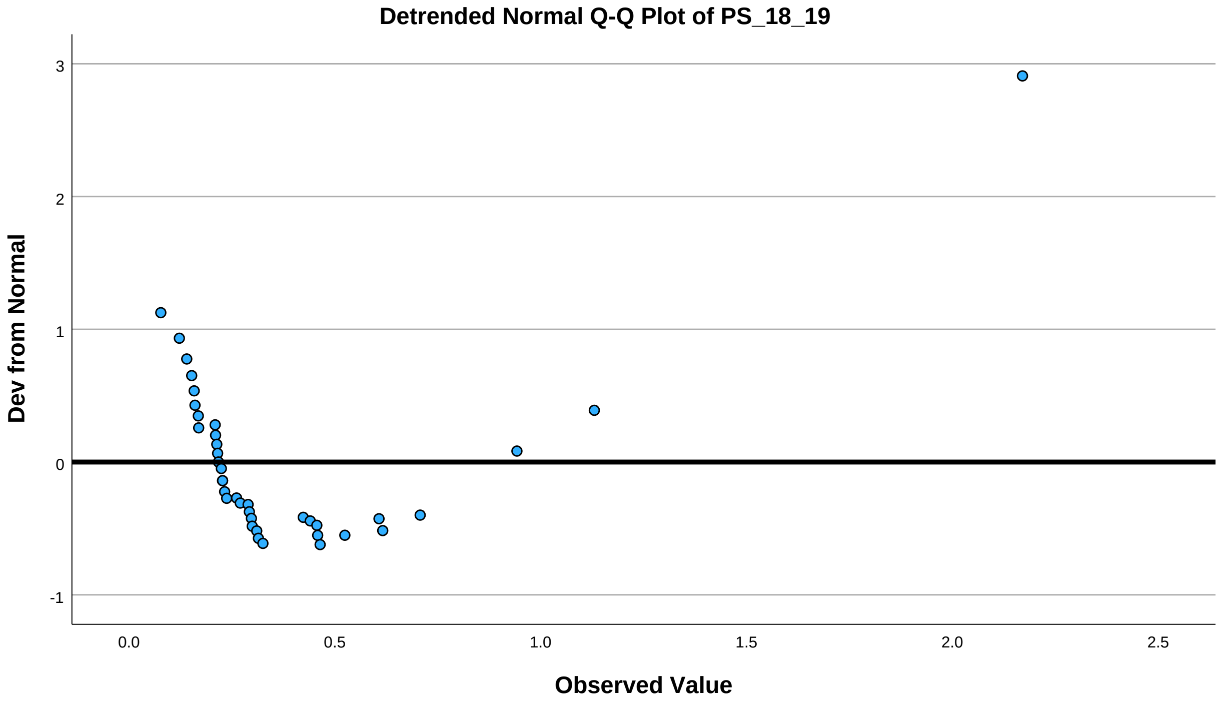
Detrended Normal Q-Q Plot of PS_18_19
Dev from Normal
Observed Value
3
2
1
0
-1
0.0
0.5
1.0
1.5
2.0
2.5

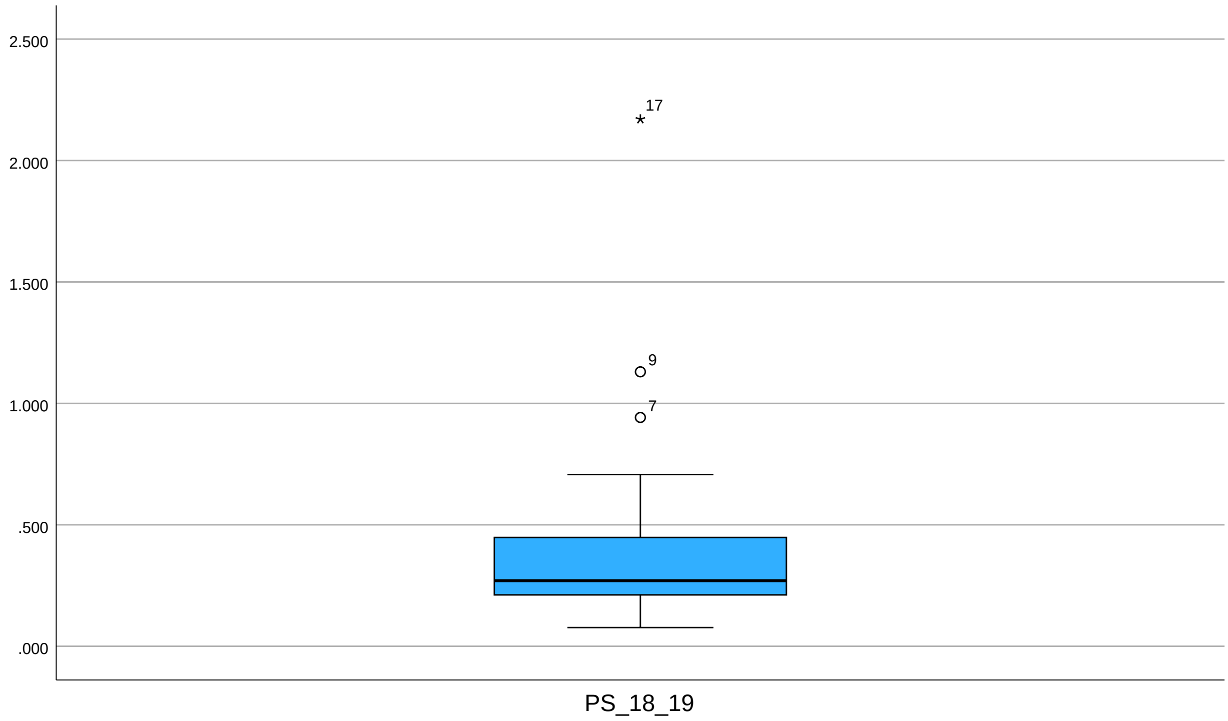

PS_19_20

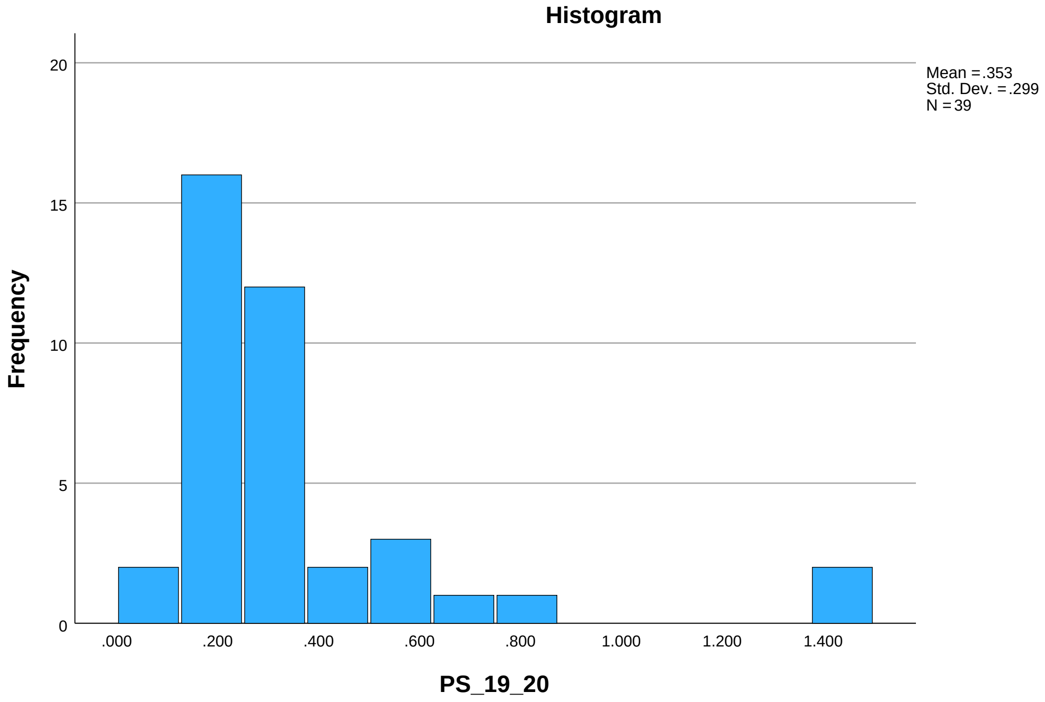


```
PS_19_20 Stem-and-Leaf Plot

 Frequency    Stem &  Leaf
```

```
     1.00        0 .  7
     5.00        1 .  02344
     2.00        1 .  55
    10.00        2 .  0012223344
     6.00        2 .  566667
     4.00        3 .  1234
     3.00        3 .  568
      .00        4 .
     1.00        4 .  8
     1.00        5 .  1
     2.00        5 .  68
     4.00 Extremes    (>=.63)

 Stem width:      .100
 Each leaf:       1 case(s)
```

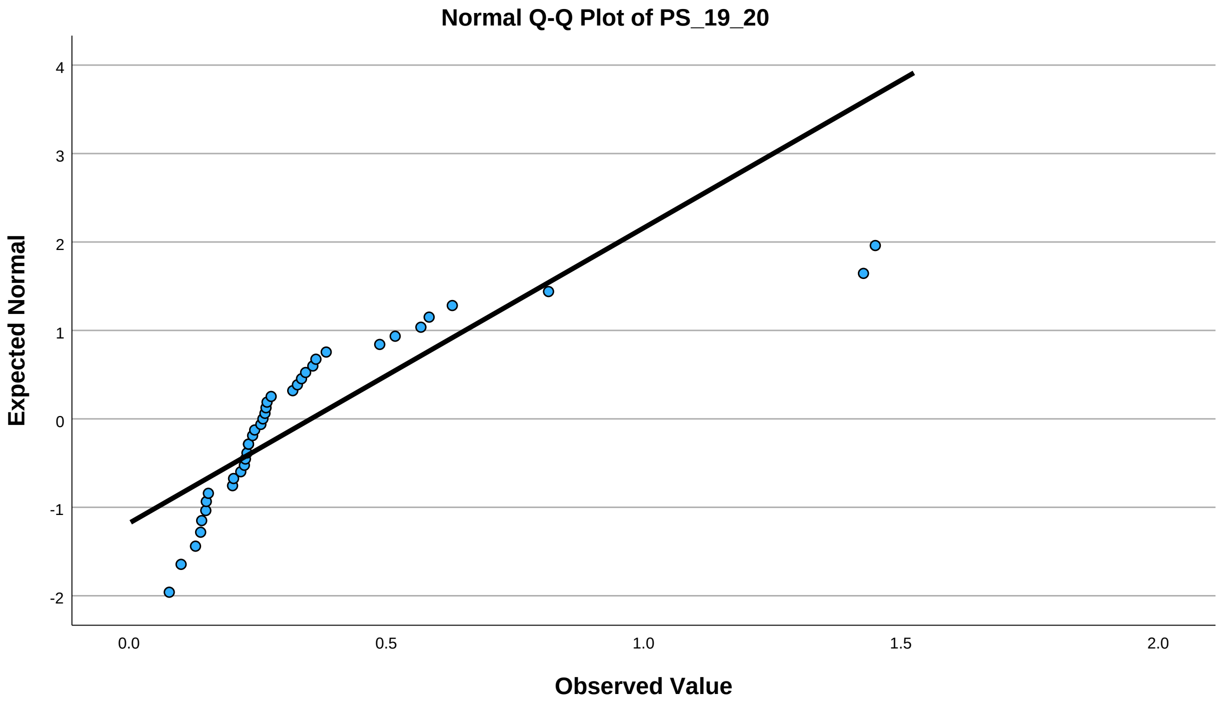

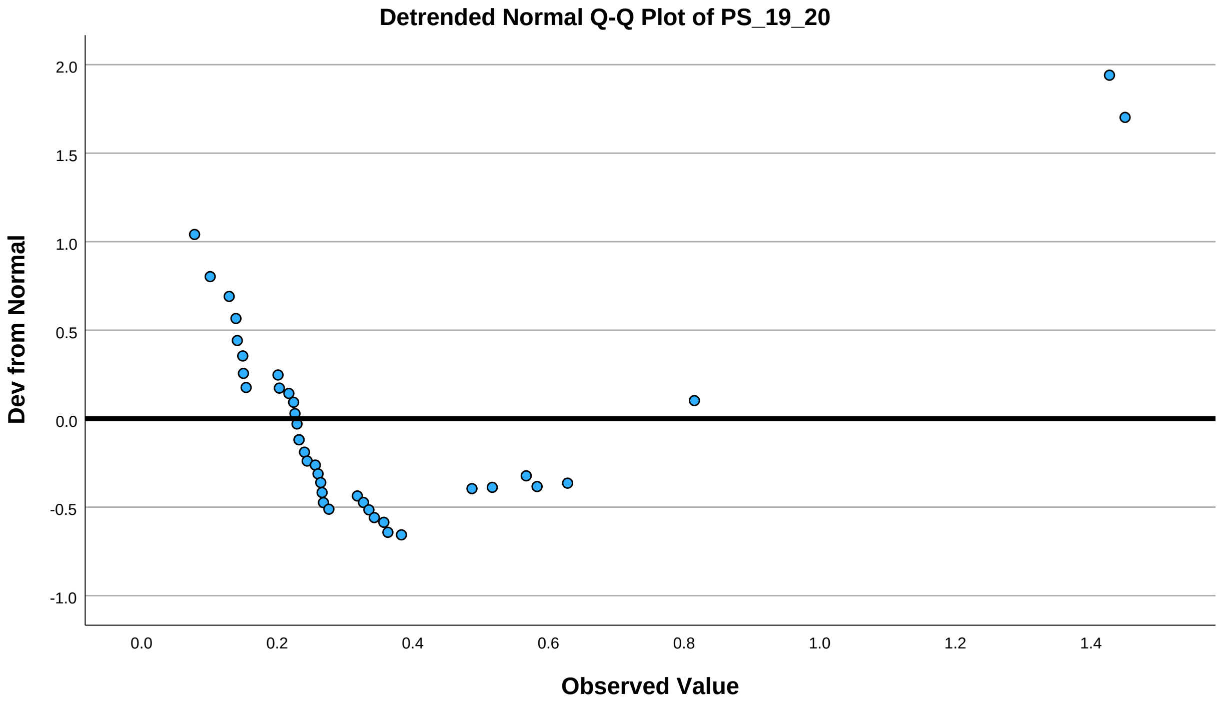
Detrended Normal Q-Q Plot of PS_19_20
Dev from Normal
2.0
1.5
1.0
0.5
0.0
-0.5
-1.0
0.0
0.2
0.4
0.6
0.8
1.0
1.2
1.4
Observed Value

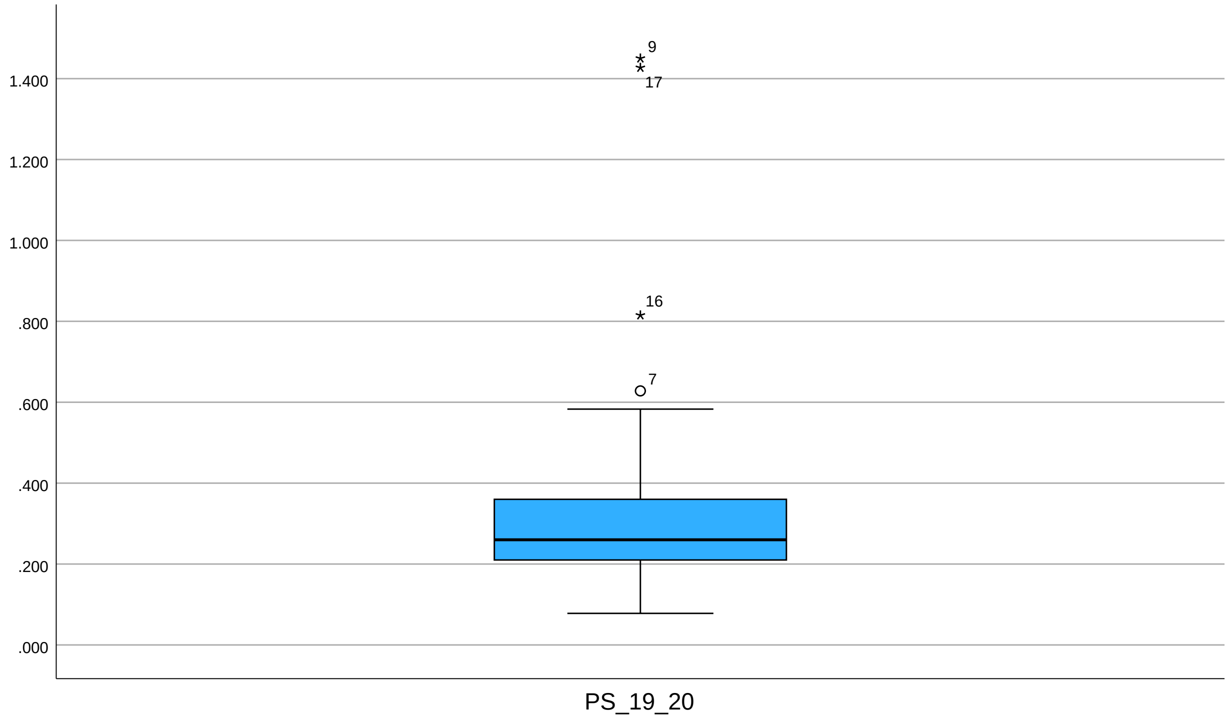
1.400
1.200
1.000
.800
.600
.400
.200
.000
9
17
16
7
PS_19_20

Explore

Case Processing Summary

	Cases					
	Valid		Missing		Total	
	N	Percent	N	Percent	N	Percent
BrainRate	39	100.0%	0	0.0%	39	100.0%
PermutationEntropy	39	100.0%	0	0.0%	39	100.0%
PermutationEntropy_01	39	100.0%	0	0.0%	39	100.0%
SampleEntropy	39	100.0%	0	0.0%	39	100.0%
SpectralEntropy_bits	39	100.0%	0	0.0%	39	100.0%
SpectralEntropy_01	39	100.0%	0	0.0%	39	100.0%
HiguchiFractalDimension	39	100.0%	0	0.0%	39	100.0%
LempelZivComplexity	39	100.0%	0	0.0%	39	100.0%
RenyiEntropy	39	100.0%	0	0.0%	39	100.0%
TsallisEntropy	39	100.0%	0	0.0%	39	100.0%
PS_4_5	39	100.0%	0	0.0%	39	100.0%
PS_5_6	39	100.0%	0	0.0%	39	100.0%
PS_6_7	39	100.0%	0	0.0%	39	100.0%
PS_7_8	39	100.0%	0	0.0%	39	100.0%
PS_8_9	39	100.0%	0	0.0%	39	100.0%
PS_9_10	39	100.0%	0	0.0%	39	100.0%
PS_10_11	39	100.0%	0	0.0%	39	100.0%
PS_11_12	39	100.0%	0	0.0%	39	100.0%
PS_12_13	39	100.0%	0	0.0%	39	100.0%
PS_13_14	39	100.0%	0	0.0%	39	100.0%
PS_14_15	39	100.0%	0	0.0%	39	100.0%
PS_15_16	39	100.0%	0	0.0%	39	100.0%
PS_16_17	39	100.0%	0	0.0%	39	100.0%
PS_17_18	39	100.0%	0	0.0%	39	100.0%
PS_18_19	39	100.0%	0	0.0%	39	100.0%
PS_19_20	39	100.0%	0	0.0%	39	100.0%

Descriptives

			Statistic	Std. Error
BrainRate	Mean		6.5321	.21134
	95% Confidence Interval for Mean	Lower Bound	6.1042	
		Upper Bound	6.9599	
	5% Trimmed Mean		6.4465	
	Median		6.3000	
	Variance		1.742	
	Std. Deviation		1.31982	
	Minimum		4.61	
	Maximum		10.89	
	Range		6.28	
	Interquartile Range		1.64	
	Skewness		1.009	.378
	Kurtosis		1.693	.741
PermutationEntropy	Mean		1.4412011355	.00973711340
	95% Confidence Interval for Mean	Lower Bound	1.4214893799	
		Upper Bound	1.4609128910	
	5% Trimmed Mean		1.4405602295	
	Median		1.4463861295	
	Variance		.004	
	Std. Deviation		.06080825369	
	Minimum		1.3232604066	
	Maximum		1.6122198420	
	Range		.28895943542	
	Interquartile Range		.09527307336	
	Skewness		.250	.378
	Kurtosis		.358	.741
PermutationEntropy_01	Mean		.80434966870	.00543438646
	95% Confidence Interval for Mean	Lower Bound	.79334832847	
		Upper Bound	.81535100894	
	5% Trimmed Mean		.80399197226	
	Median		.80724346895	
	Variance		.001	
	Std. Deviation		.03393773257	
	Minimum		.73852569462	
	Maximum		.89979702617	
	Range		.16127133155	
	Interquartile Range		.05317291467	
	Skewness		.250	.378
	Kurtosis		.358	.741

Descriptives

			Statistic	Std. Error
SampleEntropy	Mean		.63085059907	.02125850244
	95% Confidence Interval for Mean	Lower Bound	.58781501080	
		Upper Bound	.67388618734	
	5% Trimmed Mean		.63813629418	
	Median		.64895986209	
	Variance		.018	
	Std. Deviation		.13275930519	
	Minimum		.28616699916	
	Maximum		.86842120186	
	Range		.58225420270	
	Interquartile Range		.16847675399	
	Skewness		-.842	.378
	Kurtosis		.517	.741
SpectralEntropy_bits	Mean		3.9512687905	.04638941012
	95% Confidence Interval for Mean	Lower Bound	3.8573583393	
		Upper Bound	4.0451792416	
	5% Trimmed Mean		3.9594806377	
	Median		3.9471966195	
	Variance		.084	
	Std. Deviation		.28970177333	
	Minimum		3.2227364096	
	Maximum		4.4899728166	
	Range		1.2672364070	
	Interquartile Range		.35625065793	
	Skewness		-.347	.378
	Kurtosis		-.029	.741
SpectralEntropy_01	Mean		.56356307484	.00661644651
	95% Confidence Interval for Mean	Lower Bound	.55016877913	
		Upper Bound	.57695737054	
	5% Trimmed Mean		.56473431732	
	Median		.56298226769	
	Variance		.002	
	Std. Deviation		.04131969522	
	Minimum		.45965368005	
	Maximum		.64039755852	
	Range		.18074387847	
	Interquartile Range		.05081145496	
	Skewness		-.347	.378
	Kurtosis		-.029	.741

Descriptives

			Statistic	Std. Error
HiguchiFractalDimension	Mean		.62871706079	.01234713362
	95% Confidence Interval for Mean	Lower Bound	.60372159555	
		Upper Bound	.65371252603	
	5% Trimmed Mean		.63349327877	
	Median		.63908057147	
	Variance		.006	
	Std. Deviation		.07710782474	
	Minimum		.37611865042	
	Maximum		.76842000158	
	Range		.39230135116	
	Interquartile Range		.10288975310	
	Skewness		-.976	.378
	Kurtosis		1.687	.741
LempelZivComplexity	Mean		.25400583431	.00343808150
	95% Confidence Interval for Mean	Lower Bound	.24704580218	
		Upper Bound	.26096586644	
	5% Trimmed Mean		.25329331797	
	Median		.25104324399	
	Variance		.000	
	Std. Deviation		.02147081210	
	Minimum		.21487979002	
	Maximum		.31155647554	
	Range		.09667668553	
	Interquartile Range		.03054446745	
	Skewness		.582	.378
	Kurtosis		.329	.741
RenyiEntropy	Mean		7.7358752138	.15630254717
	95% Confidence Interval for Mean	Lower Bound	7.4194572495	
		Upper Bound	8.0522931781	
	5% Trimmed Mean		7.7823936675	
	Median		8.0208421455	
	Variance		.953	
	Std. Deviation		.97610909424	
	Minimum		5.6805012209	
	Maximum		8.8668131757	
	Range		3.1863119548	
	Interquartile Range		1.5072460329	
	Skewness		-.859	.378
	Kurtosis		-.622	.741

Descriptives

			Statistic	Std. Error
TsallisEntropy	Mean		1.9160670940	.04690910341
	95% Confidence Interval for Mean	Lower Bound	1.8211045789	
		Upper Bound	2.0110296092	
	5% Trimmed Mean		1.9804218988	
	Median		1.9858933231	
	Variance		.086	
	Std. Deviation		.29294725688	
	Minimum		.47484153597	
	Maximum		1.9882689232	
	Range		1.5134273872	
	Interquartile Range		.00323845935	
	Skewness		-4.423	.378
	Kurtosis		19.141	.741
PS_4_5	Mean		2.56874	.412990
	95% Confidence Interval for Mean	Lower Bound	1.73269	
		Upper Bound	3.40480	
	5% Trimmed Mean		2.21156	
	Median		1.95500	
	Variance		6.652	
	Std. Deviation		2.579120	
	Minimum		.509	
	Maximum		16.200	
	Range		15.691	
	Interquartile Range		1.219	
	Skewness		4.138	.378
	Kurtosis		21.003	.741
PS_5_6	Mean		2.01221	.332605
	95% Confidence Interval for Mean	Lower Bound	1.33888	
		Upper Bound	2.68553	
	5% Trimmed Mean		1.72593	
	Median		1.59100	
	Variance		4.314	
	Std. Deviation		2.077121	
	Minimum		.378	
	Maximum		13.470	
	Range		13.092	
	Interquartile Range		1.397	
	Skewness		4.621	.378
	Kurtosis		25.378	.741

Descriptives

			Statistic	Std. Error
PS_6_7	Mean		1.46841	.186181
	95% Confidence Interval for Mean	Lower Bound	1.09151	
		Upper Bound	1.84531	
	5% Trimmed Mean		1.32985	
	Median		1.14500	
	Variance		1.352	
	Std. Deviation		1.162702	
	Minimum		.321	
	Maximum		7.290	
	Range		6.969	
	Interquartile Range		1.250	
	Skewness		3.456	.378
	Kurtosis		16.373	.741
PS_7_8	Mean		1.28936	.171503
	95% Confidence Interval for Mean	Lower Bound	.94217	
		Upper Bound	1.63655	
	5% Trimmed Mean		1.13415	
	Median		.97800	
	Variance		1.147	
	Std. Deviation		1.071035	
	Minimum		.276	
	Maximum		6.081	
	Range		5.805	
	Interquartile Range		.692	
	Skewness		2.919	.378
	Kurtosis		10.658	.741
PS_8_9	Mean		1.33785	.204443
	95% Confidence Interval for Mean	Lower Bound	.92397	
		Upper Bound	1.75172	
	5% Trimmed Mean		1.17482	
	Median		.91100	
	Variance		1.630	
	Std. Deviation		1.276745	
	Minimum		.204	
	Maximum		6.213	
	Range		6.009	
	Interquartile Range		.969	
	Skewness		2.304	.378
	Kurtosis		5.638	.741

Descriptives

			Statistic	Std. Error
PS_9_10	Mean		1.80387	.305176
	95% Confidence Interval for Mean	Lower Bound	1.18608	
		Upper Bound	2.42167	
	5% Trimmed Mean		1.50412	
	Median		1.18600	
	Variance		3.632	
	Std. Deviation		1.905823	
	Minimum		.210	
	Maximum		9.770	
	Range		9.560	
	Interquartile Range		1.779	
	Skewness		2.810	.378
	Kurtosis		9.255	.741
PS_10_11	Mean		2.17938	.309763
	95% Confidence Interval for Mean	Lower Bound	1.55230	
		Upper Bound	2.80647	
	5% Trimmed Mean		1.99414	
	Median		1.69400	
	Variance		3.742	
	Std. Deviation		1.934469	
	Minimum		.240	
	Maximum		8.050	
	Range		7.810	
	Interquartile Range		2.021	
	Skewness		1.581	.378
	Kurtosis		1.928	.741
PS_11_12	Mean		.85862	.119038
	95% Confidence Interval for Mean	Lower Bound	.61764	
		Upper Bound	1.09959	
	5% Trimmed Mean		.76777	
	Median		.57000	
	Variance		.553	
	Std. Deviation		.743390	
	Minimum		.220	
	Maximum		3.318	
	Range		3.098	
	Interquartile Range		.545	
	Skewness		1.954	.378
	Kurtosis		3.408	.741

Descriptives

			Statistic	Std. Error
PS_12_13	Mean		.55441	.074377
	95% Confidence Interval for Mean	Lower Bound	.40384	
		Upper Bound	.70498	
	5% Trimmed Mean		.49135	
	Median		.40800	
	Variance		.216	
	Std. Deviation		.464486	
	Minimum		.153	
	Maximum		2.540	
	Range		2.387	
	Interquartile Range		.231	
	Skewness		2.697	.378
	Kurtosis		8.504	.741
PS_13_14	Mean		.49238	.076299
	95% Confidence Interval for Mean	Lower Bound	.33793	
		Upper Bound	.64684	
	5% Trimmed Mean		.42035	
	Median		.33800	
	Variance		.227	
	Std. Deviation		.476486	
	Minimum		.120	
	Maximum		2.231	
	Range		2.111	
	Interquartile Range		.288	
	Skewness		2.628	.378
	Kurtosis		6.837	.741
PS_14_15	Mean		.46690	.080546
	95% Confidence Interval for Mean	Lower Bound	.30384	
		Upper Bound	.62996	
	5% Trimmed Mean		.38401	
	Median		.28700	
	Variance		.253	
	Std. Deviation		.503012	
	Minimum		.118	
	Maximum		2.468	
	Range		2.350	
	Interquartile Range		.255	
	Skewness		2.855	.378
	Kurtosis		8.523	.741

Descriptives

			Statistic	Std. Error
PS_15_16	Mean		.41538	.064544
	95% Confidence Interval for Mean	Lower Bound	.28472	
		Upper Bound	.54605	
	5% Trimmed Mean		.35858	
	Median		.25700	
	Variance		.162	
	Std. Deviation		.403079	
	Minimum		.098	
	Maximum		2.164	
	Range		2.066	
	Interquartile Range		.311	
	Skewness		2.710	.378
	Kurtosis		8.880	.741
PS_16_17	Mean		.34641	.049662
	95% Confidence Interval for Mean	Lower Bound	.24587	
		Upper Bound	.44695	
	5% Trimmed Mean		.30372	
	Median		.22400	
	Variance		.096	
	Std. Deviation		.310141	
	Minimum		.091	
	Maximum		1.402	
	Range		1.311	
	Interquartile Range		.181	
	Skewness		2.231	.378
	Kurtosis		4.716	.741
PS_17_18	Mean		.28600	.037394
	95% Confidence Interval for Mean	Lower Bound	.21030	
		Upper Bound	.36170	
	5% Trimmed Mean		.26014	
	Median		.19100	
	Variance		.055	
	Std. Deviation		.233523	
	Minimum		.080	
	Maximum		.960	
	Range		.880	
	Interquartile Range		.139	
	Skewness		1.824	.378
	Kurtosis		2.395	.741

Descriptives

			Statistic	Std. Error
PS_18_19	Mean		.24954	.033486
	95% Confidence Interval for Mean	Lower Bound	.18175	
		Upper Bound	.31733	
	5% Trimmed Mean		.22312	
	Median		.18100	
	Variance		.044	
	Std. Deviation		.209119	
	Minimum		.064	
	Maximum		.915	
	Range		.851	
	Interquartile Range		.106	
	Skewness		2.182	.378
	Kurtosis		4.141	.741
PS_19_20	Mean		.22415	.027605
	95% Confidence Interval for Mean	Lower Bound	.16827	
		Upper Bound	.28004	
	5% Trimmed Mean		.20250	
	Median		.15800	
	Variance		.030	
	Std. Deviation		.172392	
	Minimum		.067	
	Maximum		.890	
	Range		.823	
	Interquartile Range		.146	
	Skewness		2.242	.378
	Kurtosis		5.518	.741

Tests of Normality

	Kolmogorov-Smirnov[a]			Shapiro-Wilk		
	Statistic	df	Sig.	Statistic	df	Sig.
BrainRate	.105	39	.200*	.940	39	.037
PermutationEntropy	.068	39	.200*	.980	39	.686
PermutationEntropy_01	.068	39	.200*	.980	39	.686
SampleEntropy	.110	39	.200*	.946	39	.060
SpectralEntropy_bits	.069	39	.200*	.987	39	.917
SpectralEntropy_01	.069	39	.200*	.987	39	.917
HiguchiFractalDimension	.107	39	.200*	.944	39	.053
LempelZivComplexity	.085	39	.200*	.970	39	.371
RenyiEntropy	.154	39	.020	.862	39	<.001
TsallisEntropy	.481	39	<.001	.257	39	<.001
PS_4_5	.303	39	<.001	.568	39	<.001
PS_5_6	.220	39	<.001	.527	39	<.001
PS_6_7	.191	39	<.001	.670	39	<.001
PS_7_8	.235	39	<.001	.695	39	<.001
PS_8_9	.247	39	<.001	.719	39	<.001
PS_9_10	.241	39	<.001	.684	39	<.001
PS_10_11	.212	39	<.001	.812	39	<.001
PS_11_12	.234	39	<.001	.744	39	<.001
PS_12_13	.270	39	<.001	.678	39	<.001
PS_13_14	.260	39	<.001	.642	39	<.001
PS_14_15	.278	39	<.001	.615	39	<.001
PS_15_16	.263	39	<.001	.675	39	<.001
PS_16_17	.272	39	<.001	.685	39	<.001
PS_17_18	.266	39	<.001	.719	39	<.001
PS_18_19	.311	39	<.001	.684	39	<.001
PS_19_20	.265	39	<.001	.735	39	<.001

*. This is a lower bound of the true significance.

a. Lilliefors Significance Correction

BrainRate

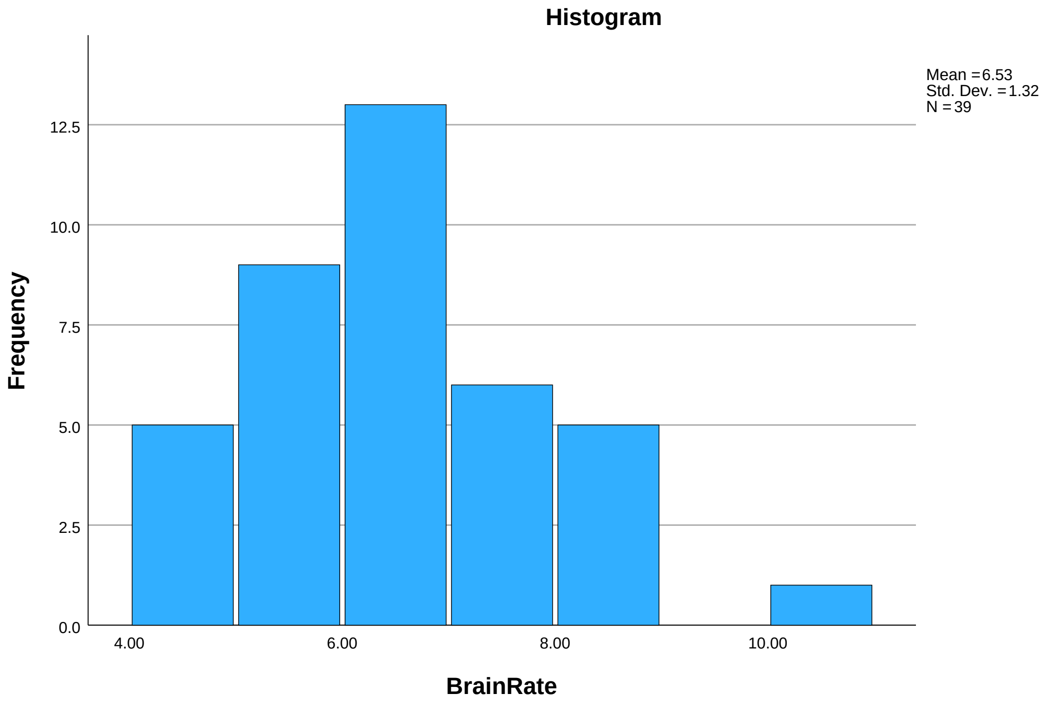


```
BrainRate Stem-and-Leaf Plot

 Frequency    Stem &  Leaf

     5.00        4 .  67899
     3.00        5 .  234
     6.00        5 .  555678
     7.00        6 .  0011233
     6.00        6 .  777899
     4.00        7 .  0114
     2.00        7 .  57
     4.00        8 .  0114
     1.00        8 .  9
     1.00 Extremes    (>=10.9)

 Stem width:      1.00
 Each leaf:       1 case(s)
```

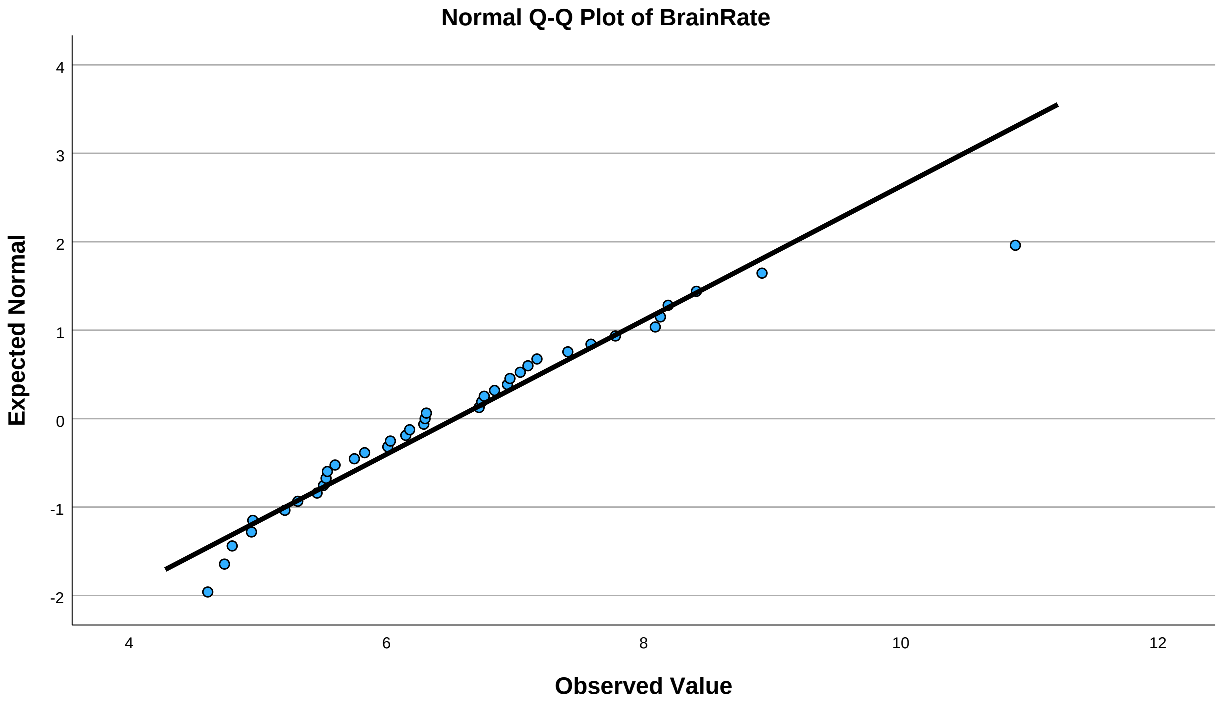
Normal Q-Q Plot of BrainRate
Expected Normal
Observed Value
4
3
2
1
0
-1
-2
4
6
8
10
12

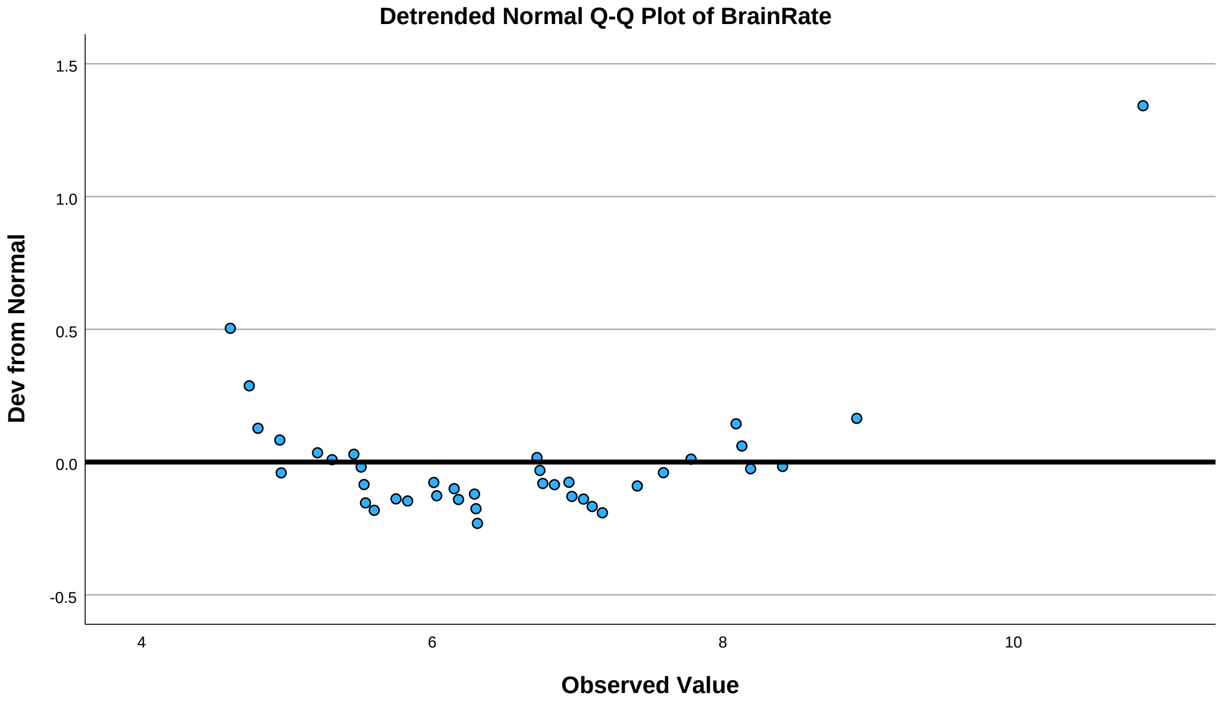
Detrended Normal Q-Q Plot of BrainRate
Dev from Normal
Observed Value
1.5
1.0
0.5
0.0
-0.5
4
6
8
10

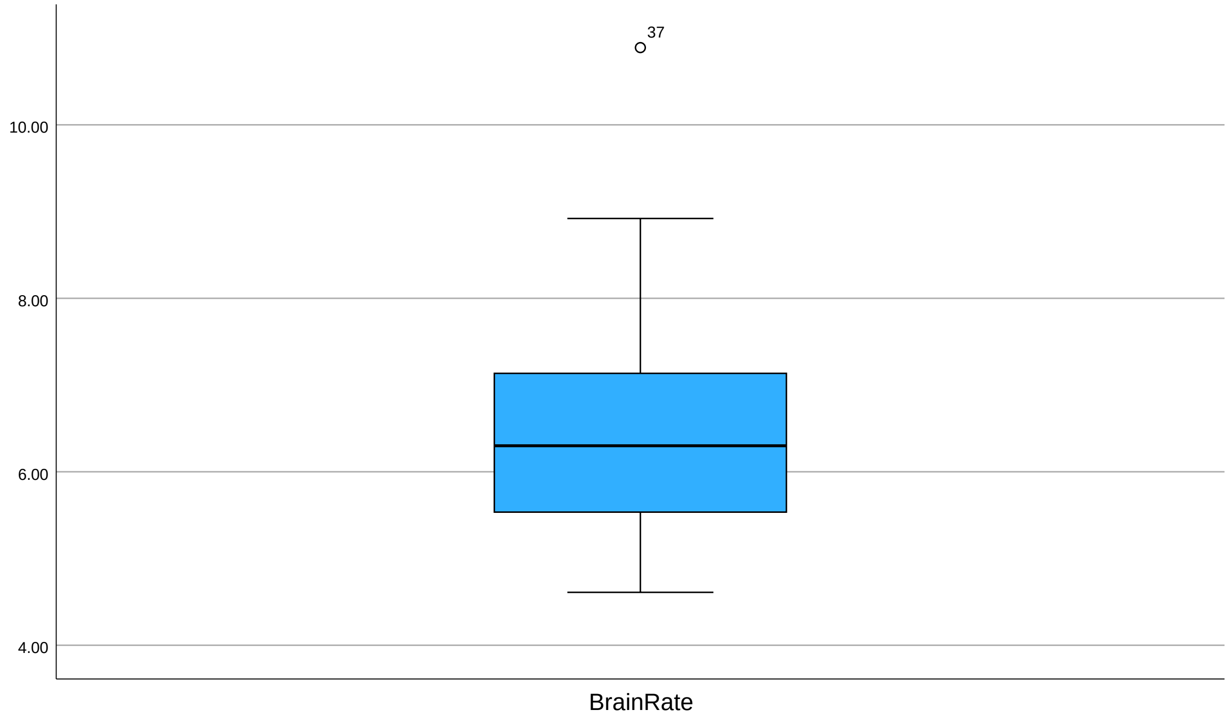

PermutationEntropy

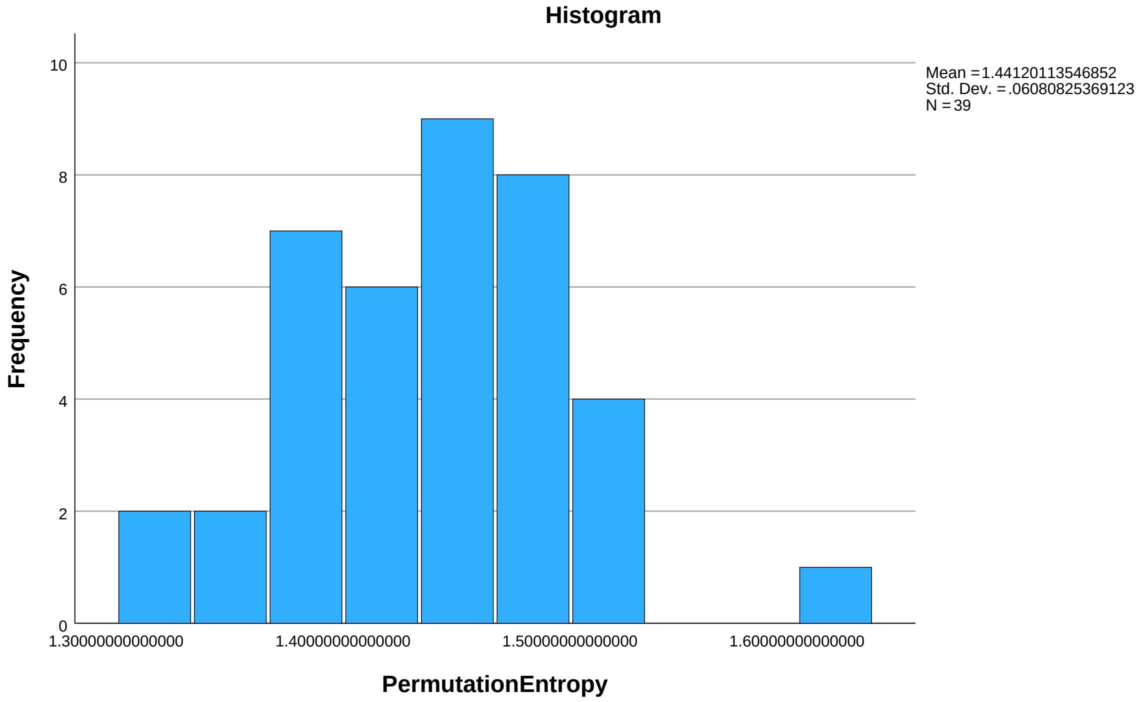


```
PermutationEntropy Stem-and-Leaf Plot

 Frequency    Stem &  Leaf
```

```
 2.00     13 .  22
  9.00      13 .  567788999
 10.00      14 .  0011123444
 13.00      14 .  5556677899999
  4.00     15 .  0022
   .00    15 .
  1.00     16 .  1

Stem width:   .1000000
Each leaf:       1 case(s)
```

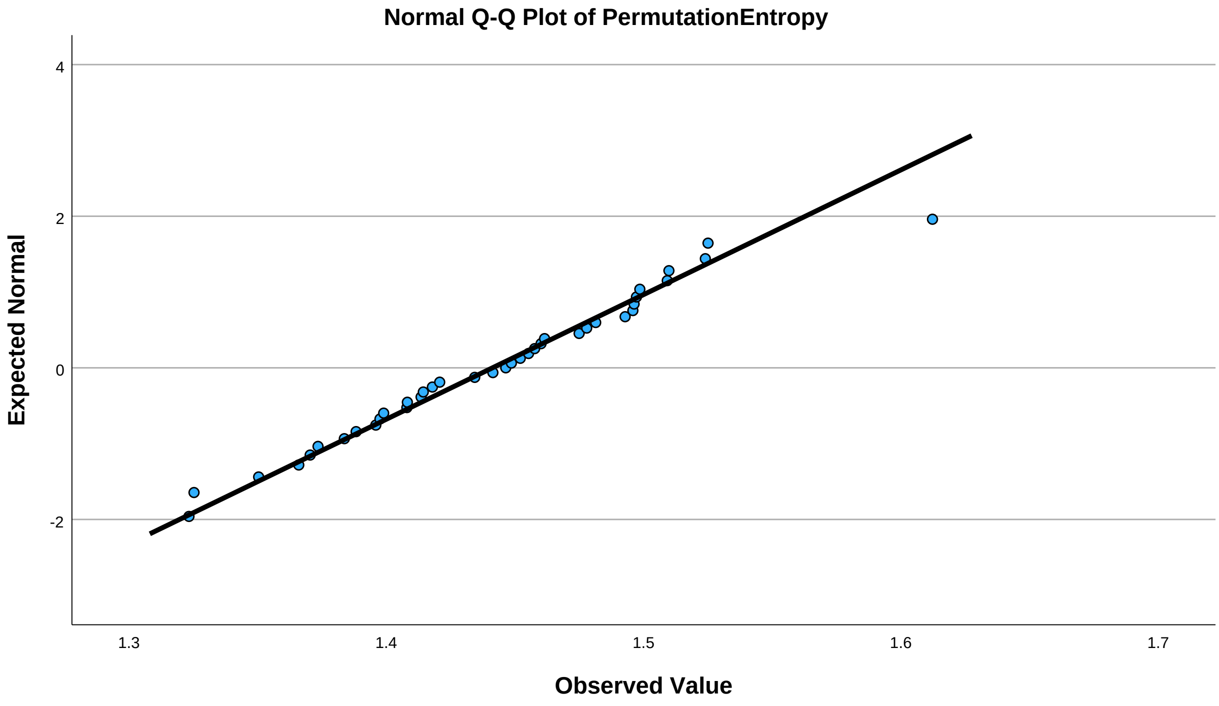

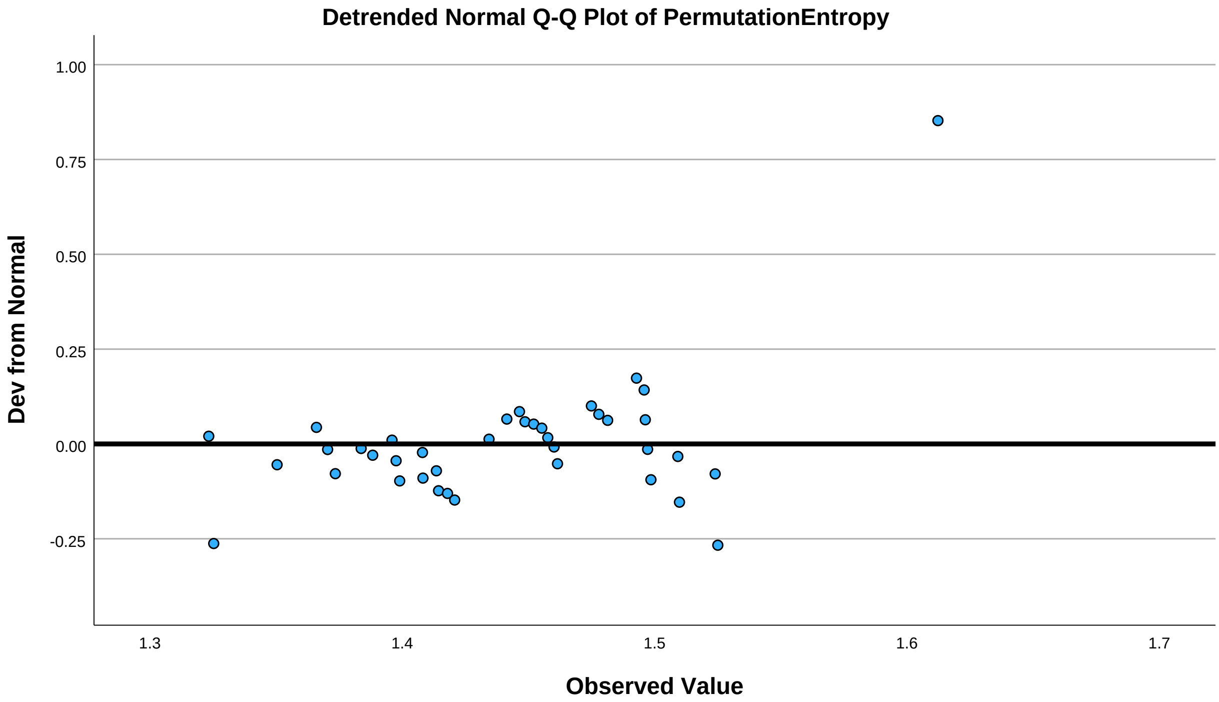

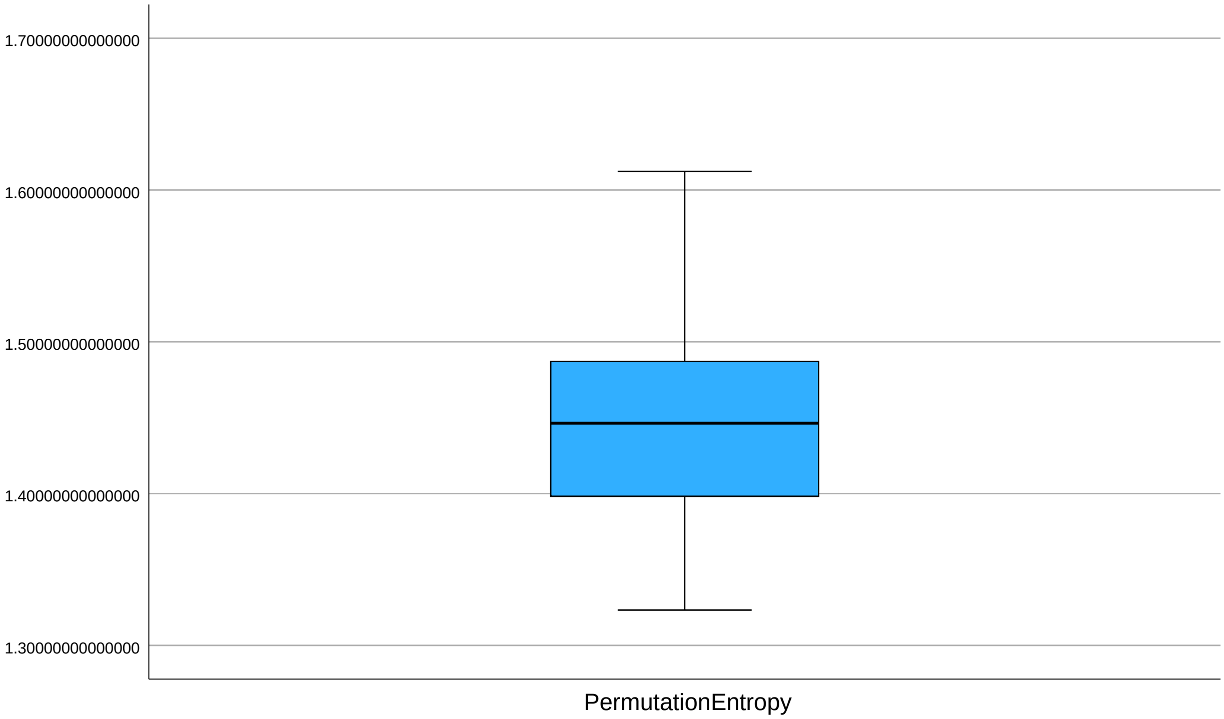

PermutationEntropy_01

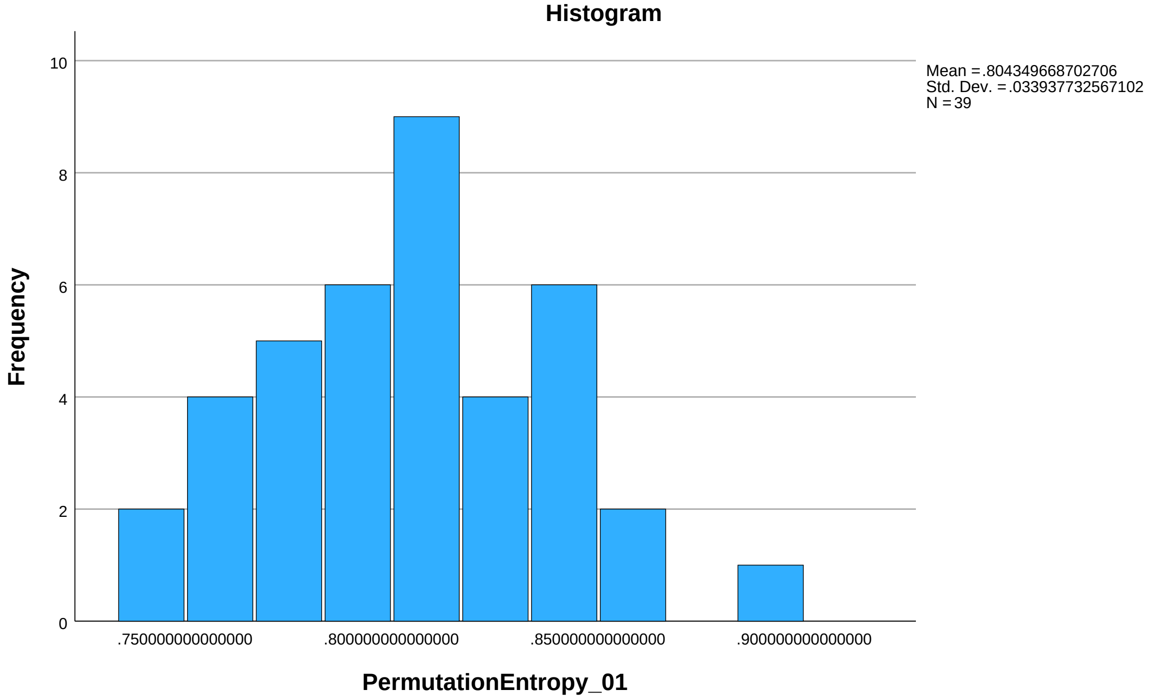


```
PermutationEntropy_01 Stem-and-Leaf Plot

 Frequency    Stem &  Leaf

     2.00        7 .  33
     1.00        7 .  5
     7.00        7 .  6667777
     7.00        7 .  8888899
     9.00        8 .  000011111
     8.00        8 .  22233333
     4.00        8 .  4455
      .00        8 .
     1.00        8 .  9

 Stem width:   .1000000
 Each leaf:       1 case(s)
```

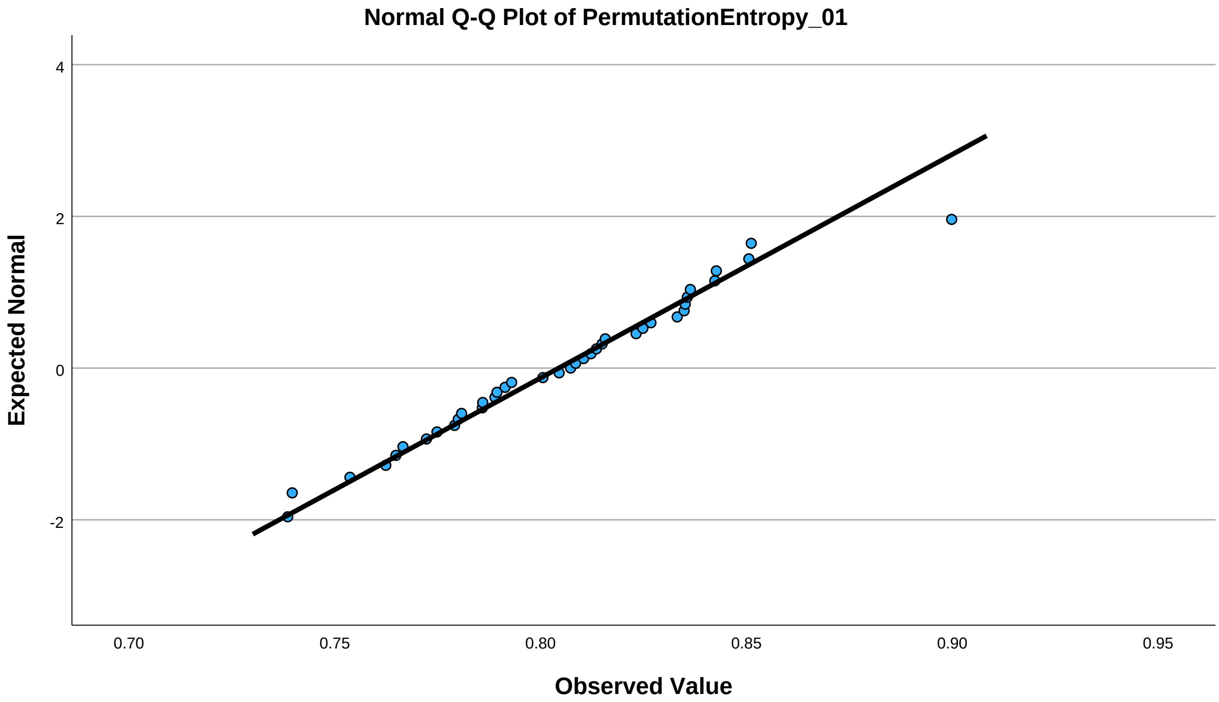
Normal Q-Q Plot of PermutationEntropy_01
Expected Normal
4
2
0
-2
0.70
0.75
0.80
0.85
0.90
0.95
Observed Value

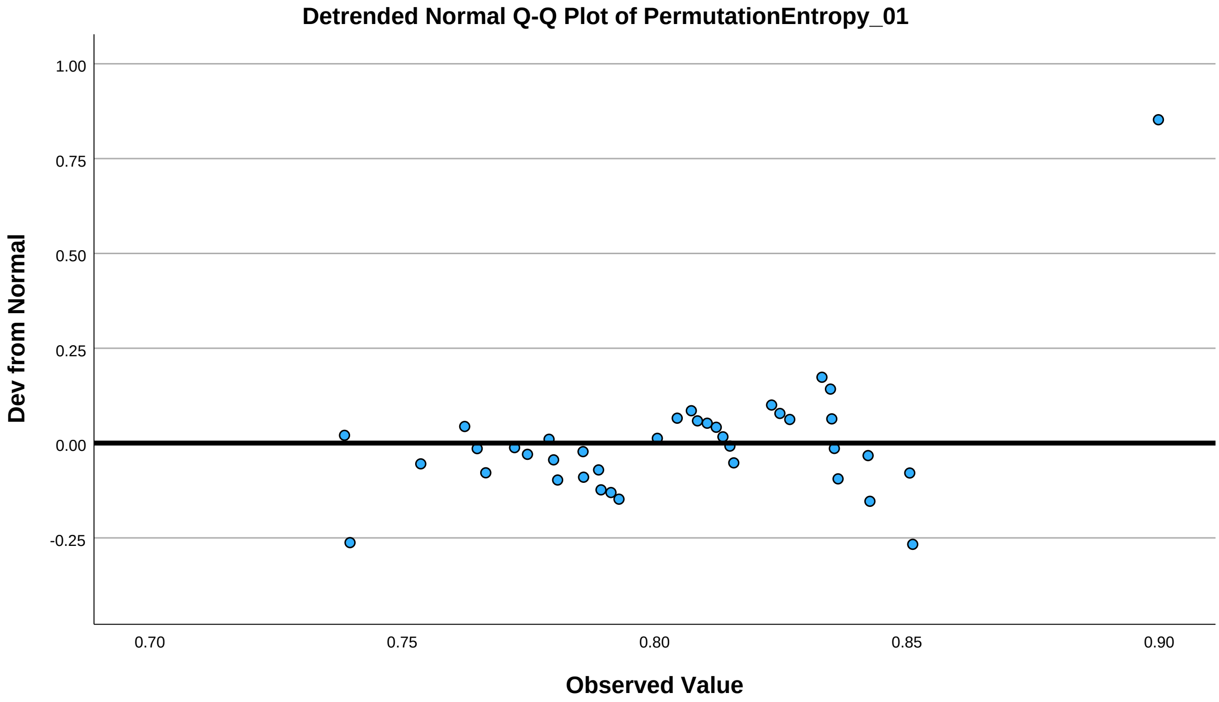
Detrended Normal Q-Q Plot of PermutationEntropy_01
Dev from Normal
1.00
0.75
0.50
0.25
0.00
-0.25
0.70
0.75
0.80
0.85
0.90
Observed Value

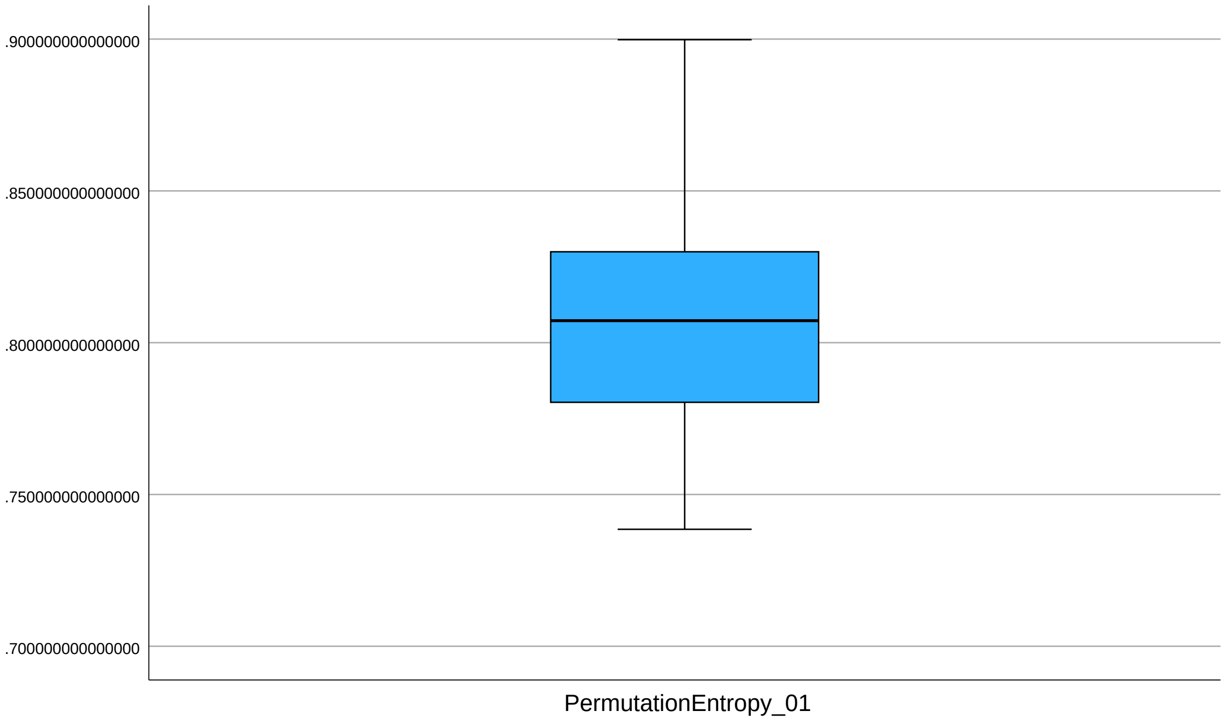

SampleEntropy

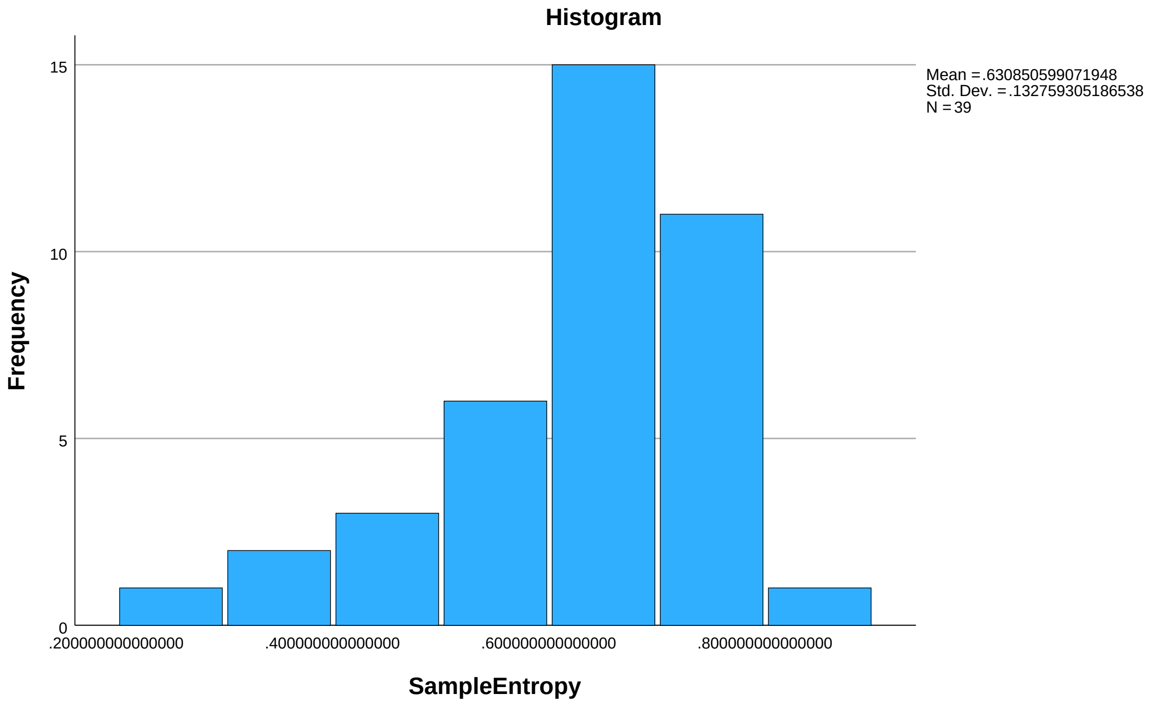


```
SampleEntropy Stem-and-Leaf Plot

 Frequency    Stem &  Leaf
```

```
  2.00 Extremes    (=<.31)
 1.00     3 . 8
 1.00     4 . 3
 2.00      4 . 59
 2.00      5 . 01
 4.00      5 . 5669
 8.00       6 . 00122344
 7.00       6 . 5668999
 4.00      7 . 1233
 7.00       7 . 5667779
  .00     8 .
 1.00     8 . 6

Stem width:  .1000000
Each leaf:       1 case(s)
```

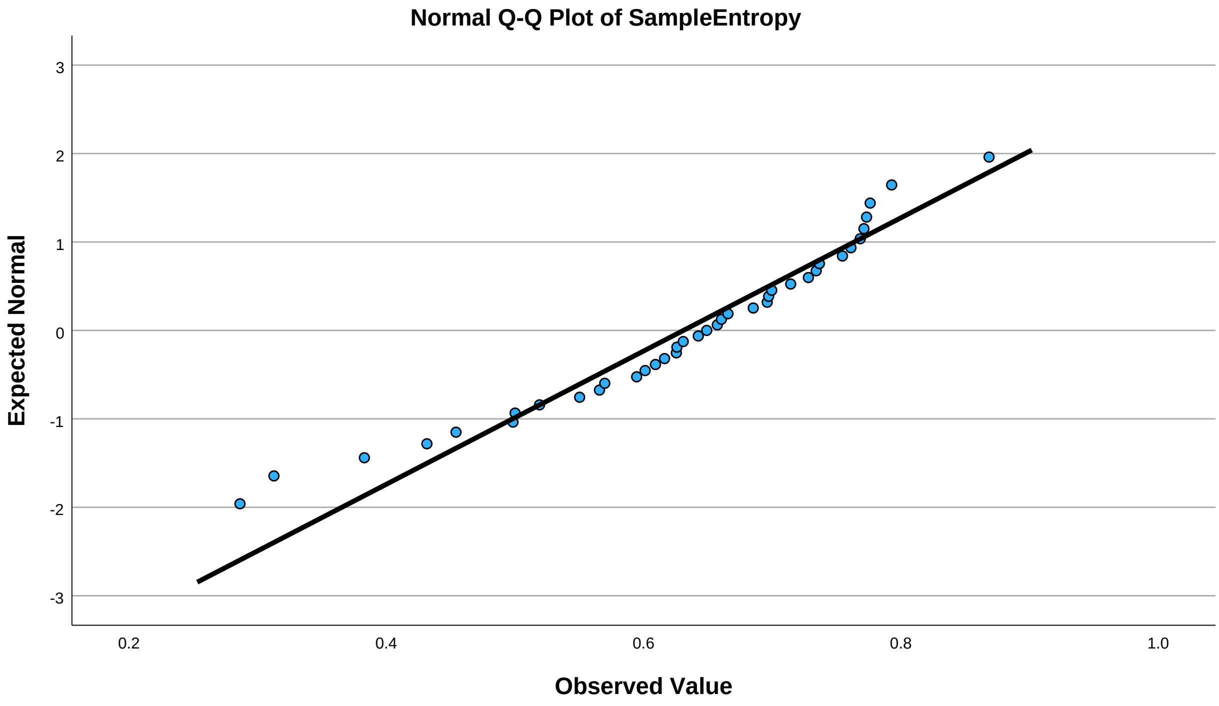

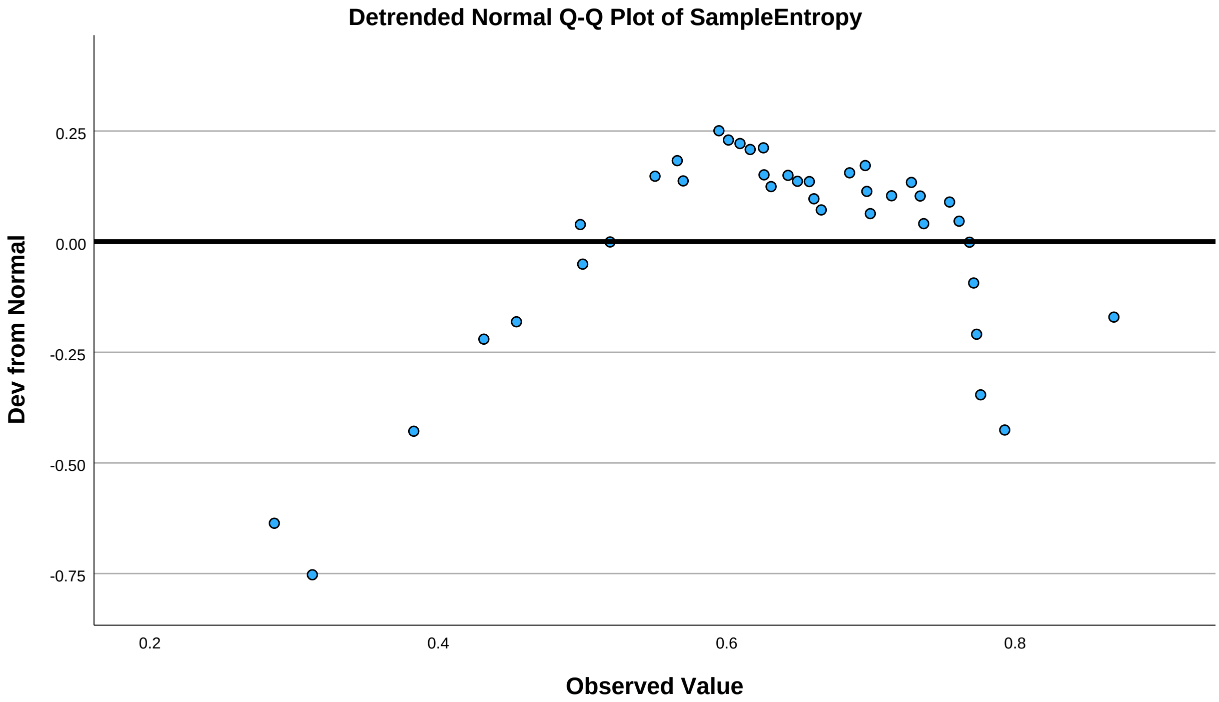

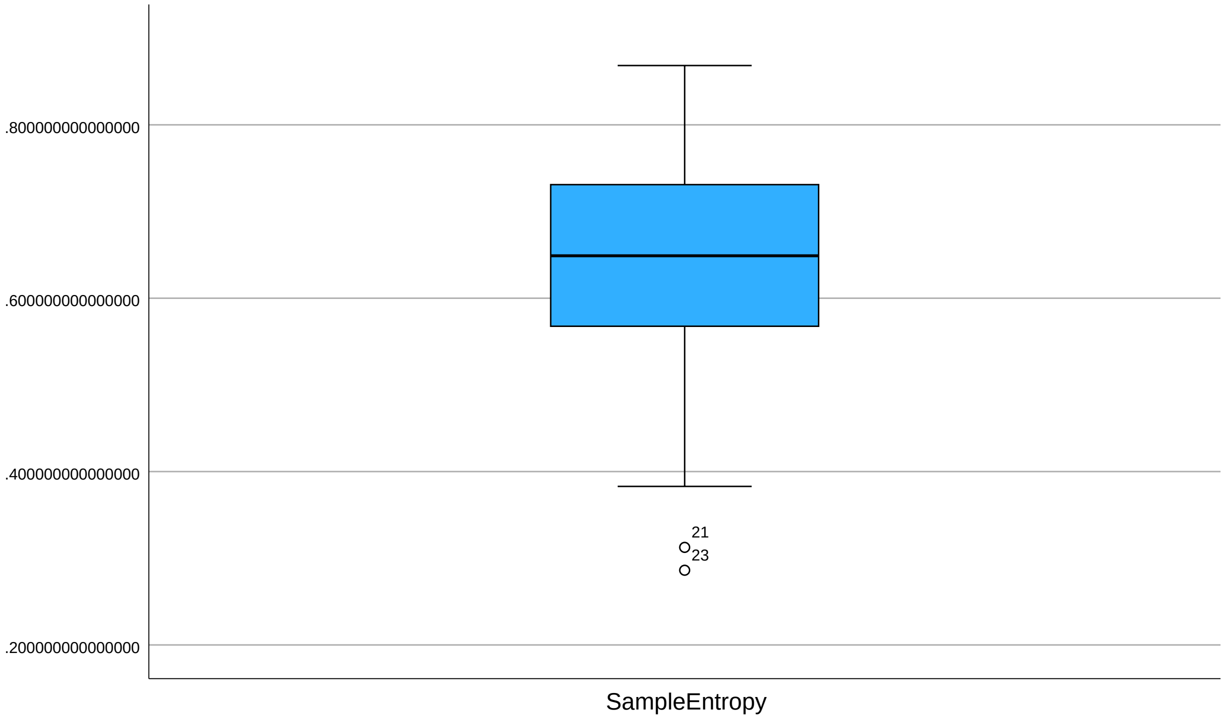

SpectralEntropy_bits

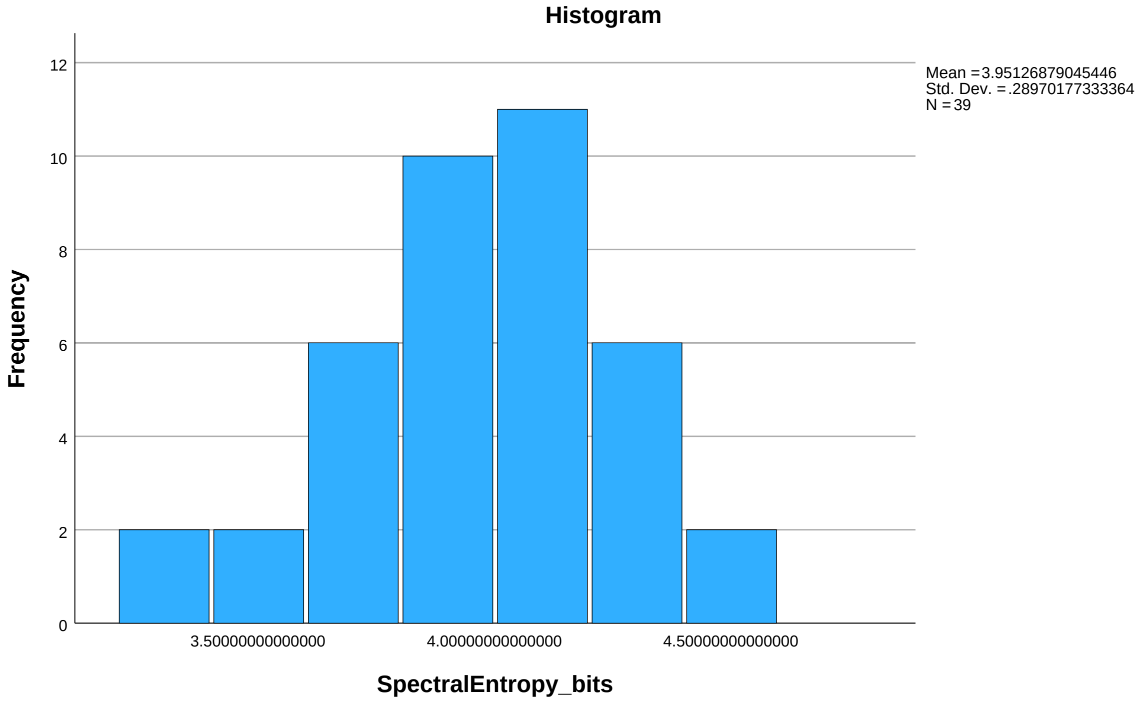


```
SpectralEntropy_bits Stem-and-Leaf Plot

 Frequency    Stem &  Leaf

     1.00 Extremes    (=<3.22)
     1.00       33 .  7
     1.00       34 .  8
     1.00       35 .  8
     3.00       36 .  015
     3.00       37 .  059
     6.00       38 .  122259
     4.00       39 .  1344
     8.00       40 .  02346699
     3.00       41 .  156
     3.00       42 .  135
     3.00       43 .  168
     2.00       44 .  28

 Stem width:   .1000000
 Each leaf:        1 case(s)
```

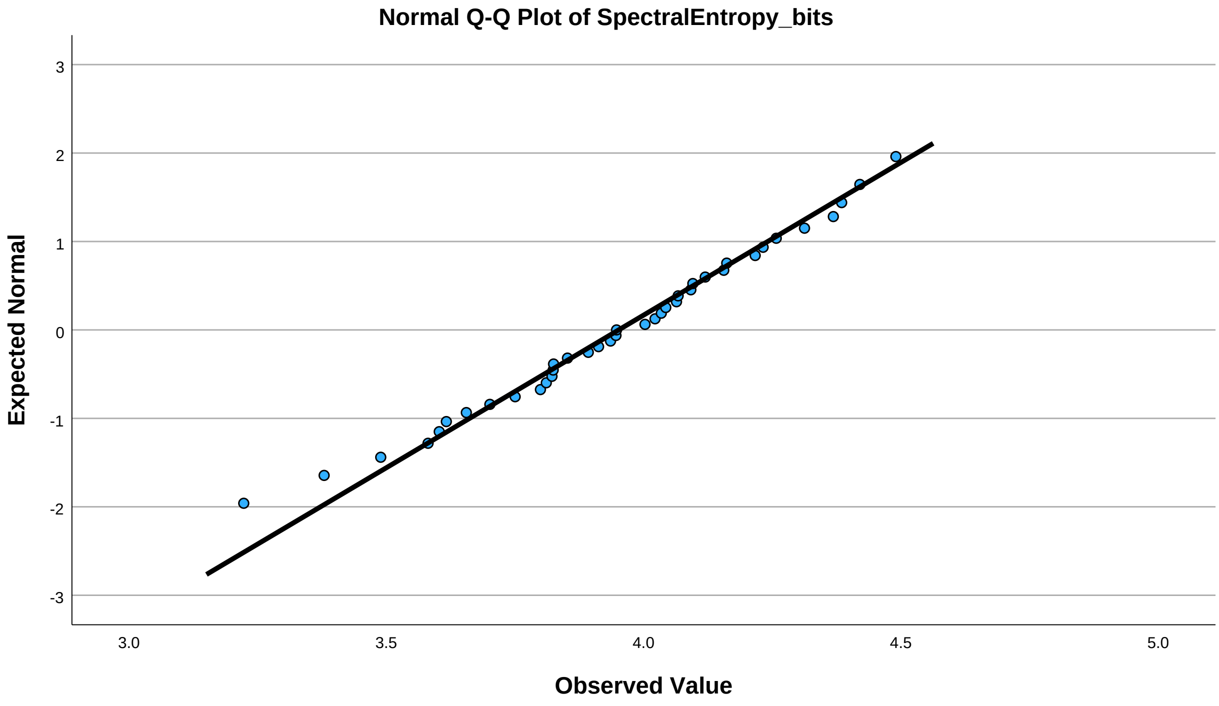

Normal Q-Q Plot of SpectralEntropy_bits
Expected Normal
3
2
1
0
-1
-2
-3
3.0
3.5
4.0
4.5
5.0
Observed Value

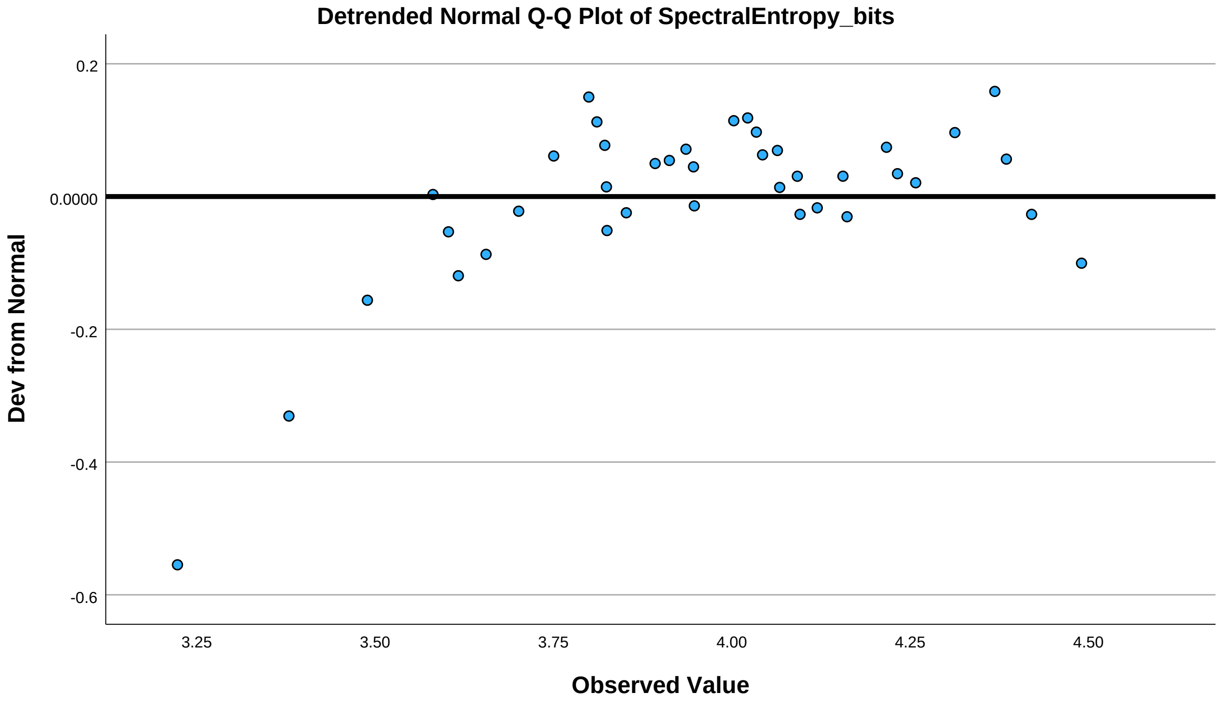

Detrended Normal Q-Q Plot of SpectralEntropy_bits
Dev from Normal
0.2
0.0000
-0.2
-0.4
-0.6
3.25
3.50
3.75
4.00
4.25
4.50
Observed Value

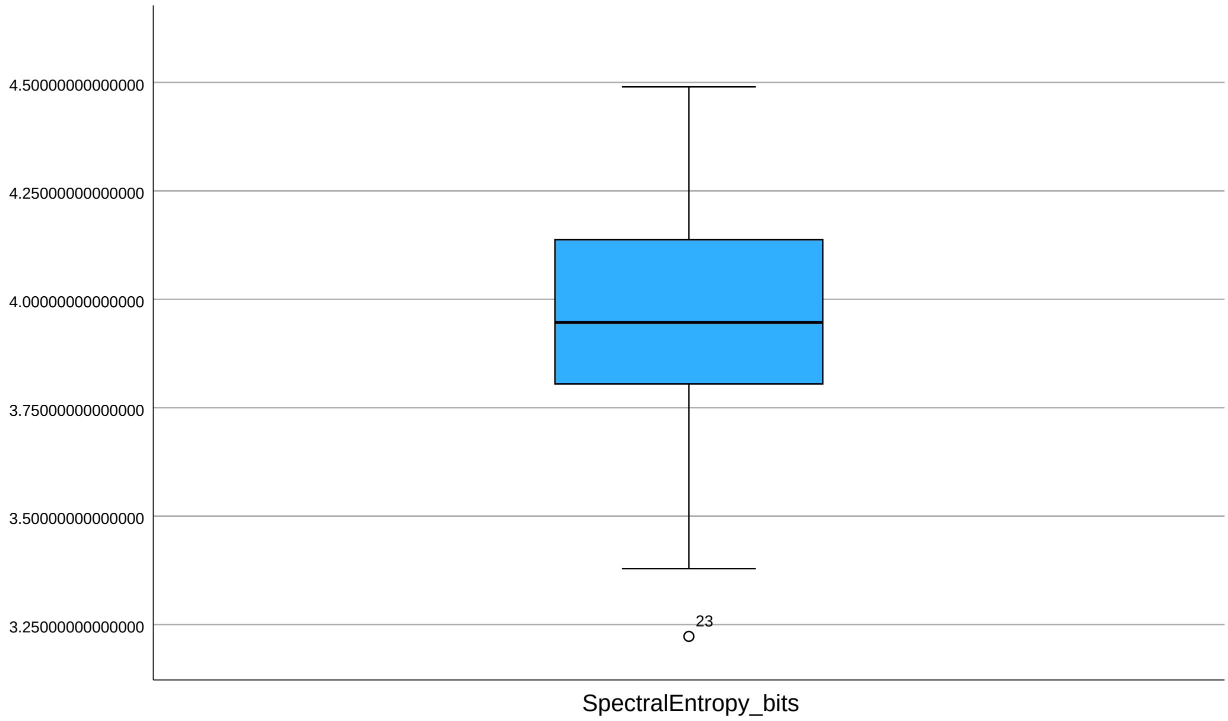

SpectralEntropy_01

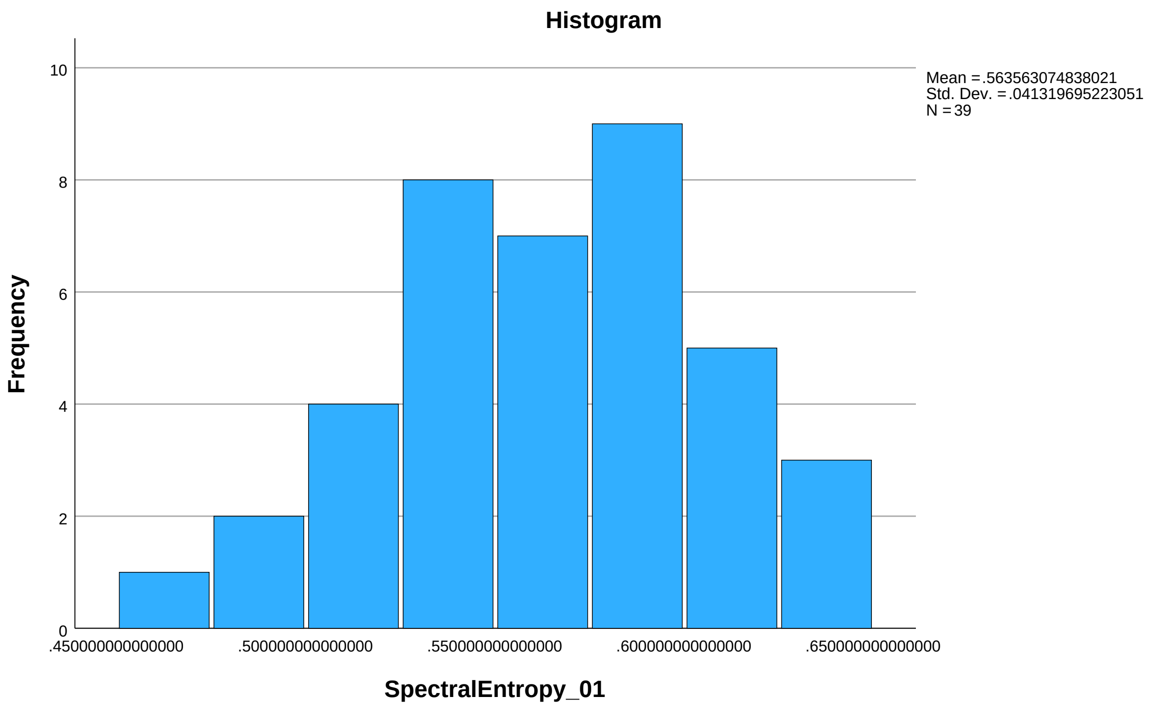


```
SpectralEntropy_01 Stem-and-Leaf Plot

 Frequency    Stem &  Leaf
```

```
 1.00 Extremes    (=<.46)
 2.00     4 . 89
12.00     5 . 111223444444
16.00     5 . 5566677777888899
 8.00     6 . 00012234

Stem width:   .1000000
Each leaf:       1 case(s)
```

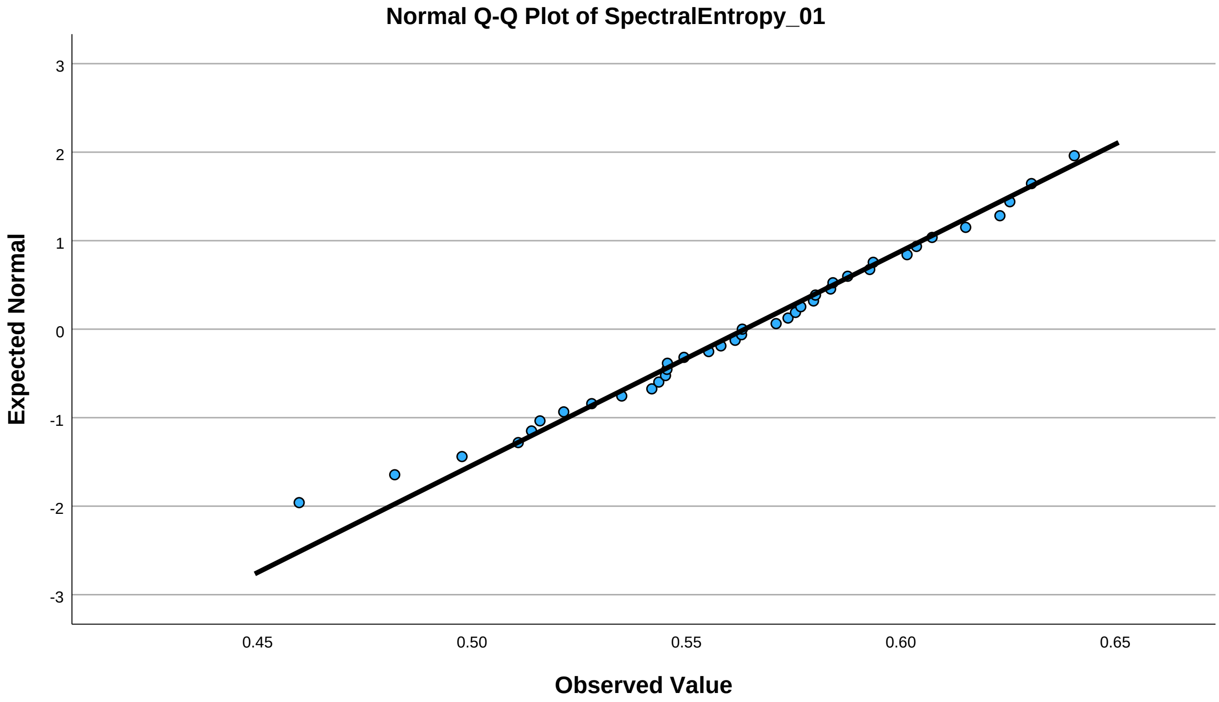

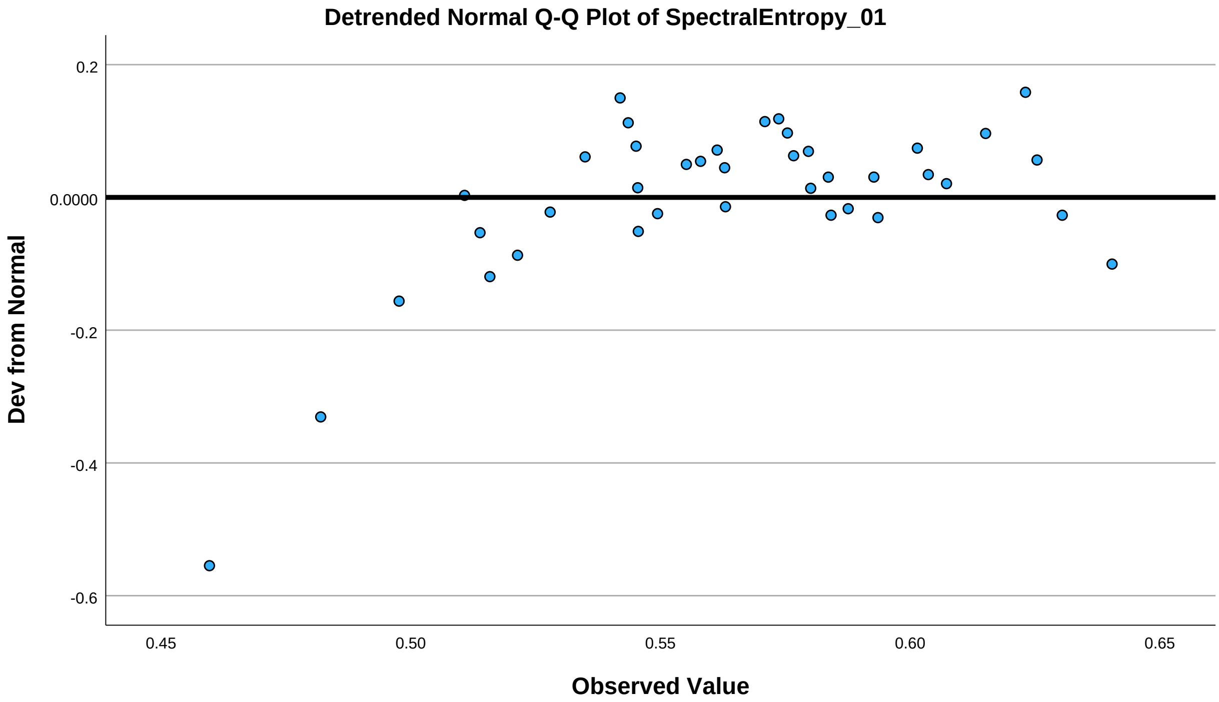

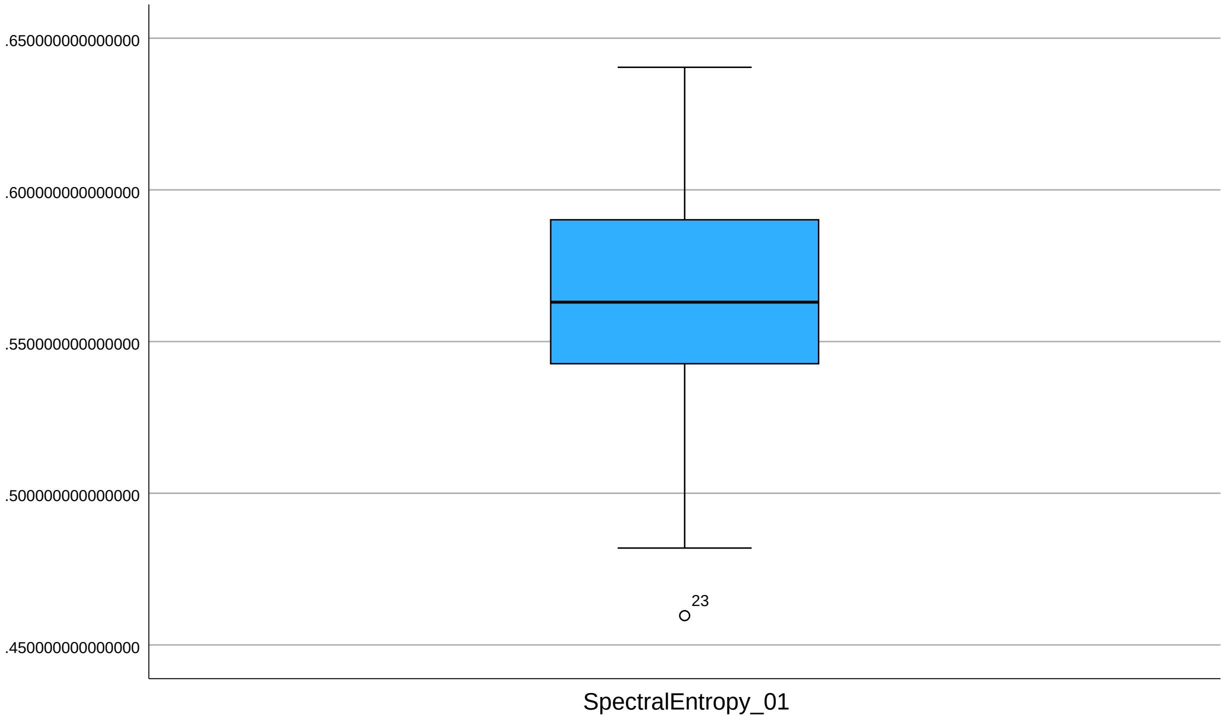

HiguchiFractalDimension

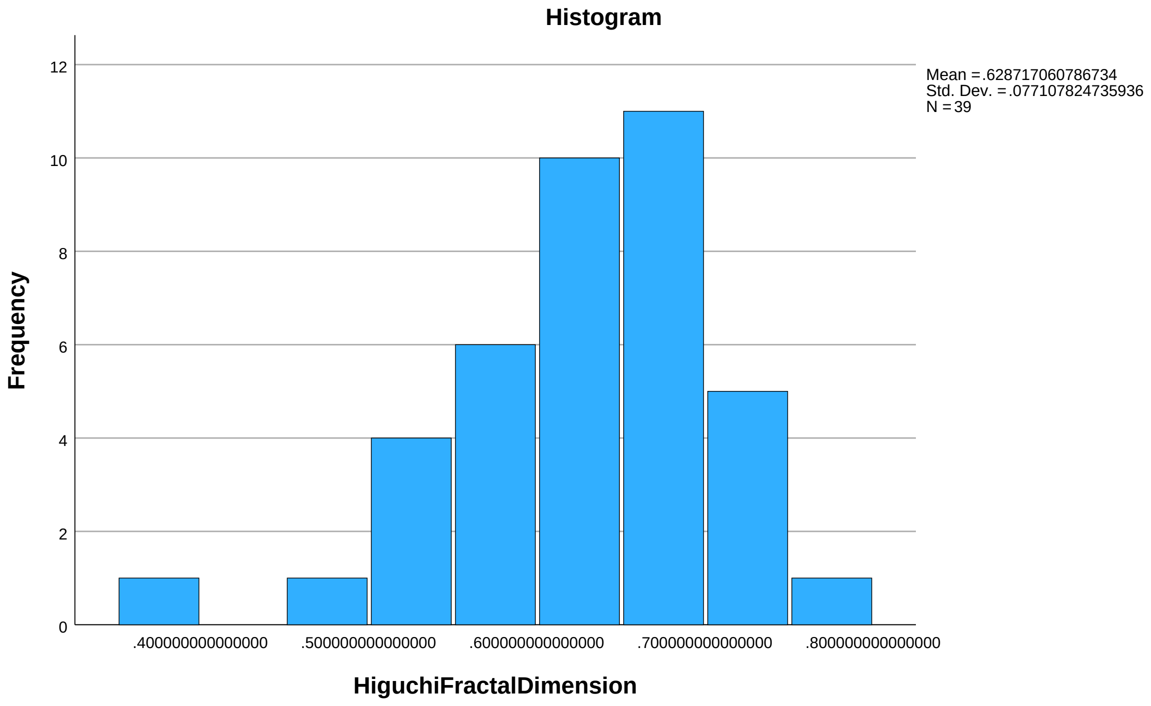


```
HiguchiFractalDimension Stem-and-Leaf Plot

 Frequency    Stem &  Leaf

     1.00 Extremes    (=<.38)
     1.00        4 .  7
     4.00        5 .  3334
     6.00        5 .  557889
    10.00        6 .  0001333344
    11.00        6 .  56667788999
     5.00        7 .  01112
     1.00        7 .  6

 Stem width:   .1000000
 Each leaf:        1 case(s)
```

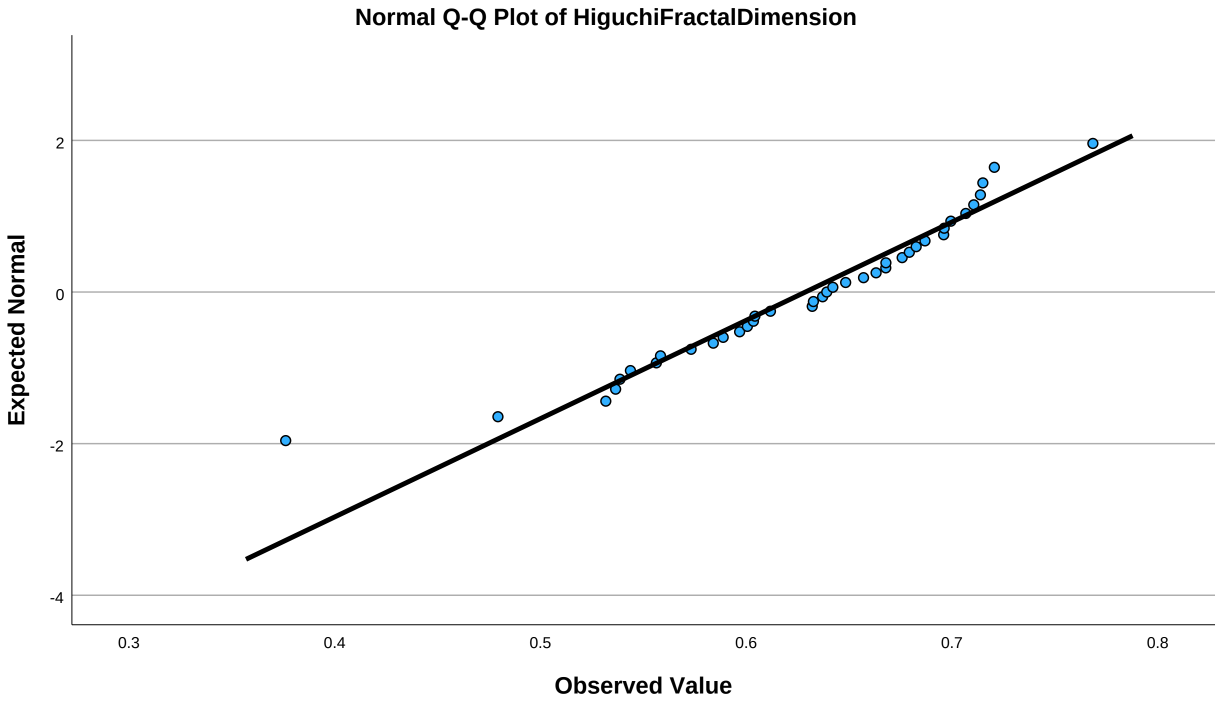
Normal Q-Q Plot of HiguchiFractalDimension
Expected Normal
2
0
-2
-4
0.3
0.4
0.5
0.6
0.7
0.8
Observed Value

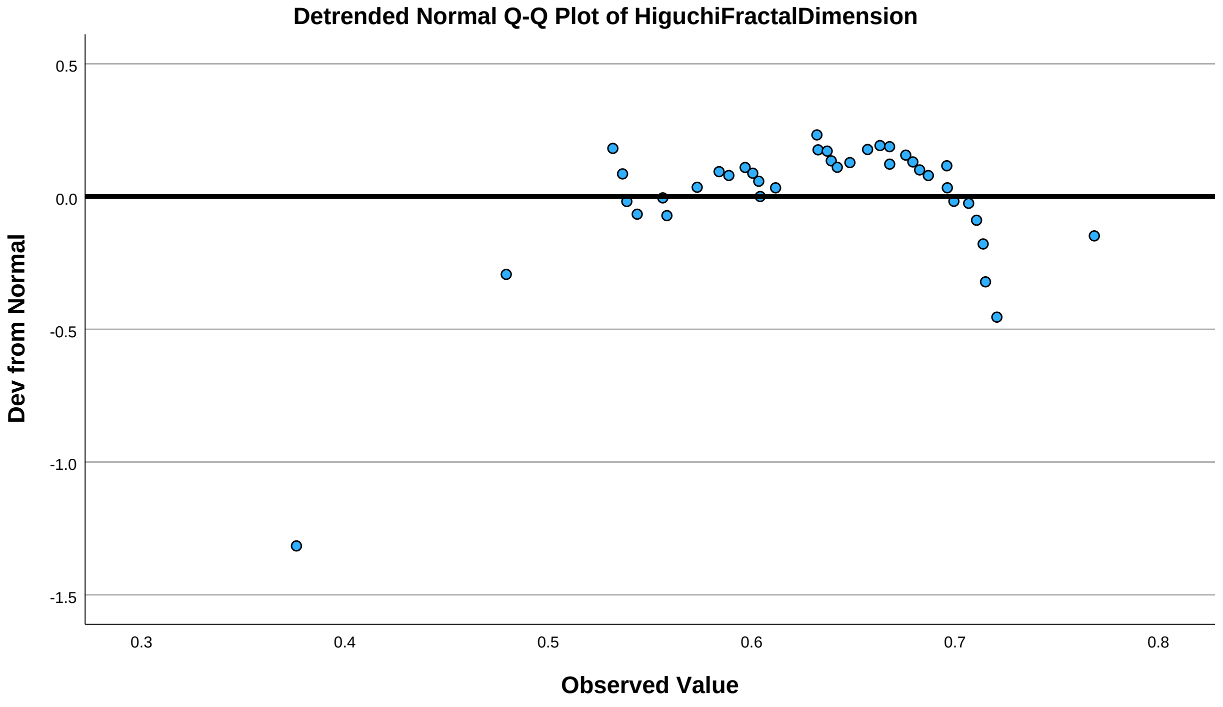
Detrended Normal Q-Q Plot of HiguchiFractalDimension
Dev from Normal
0.5
0.0
-0.5
-1.0
-1.5
0.3
0.4
0.5
0.6
0.7
0.8
Observed Value

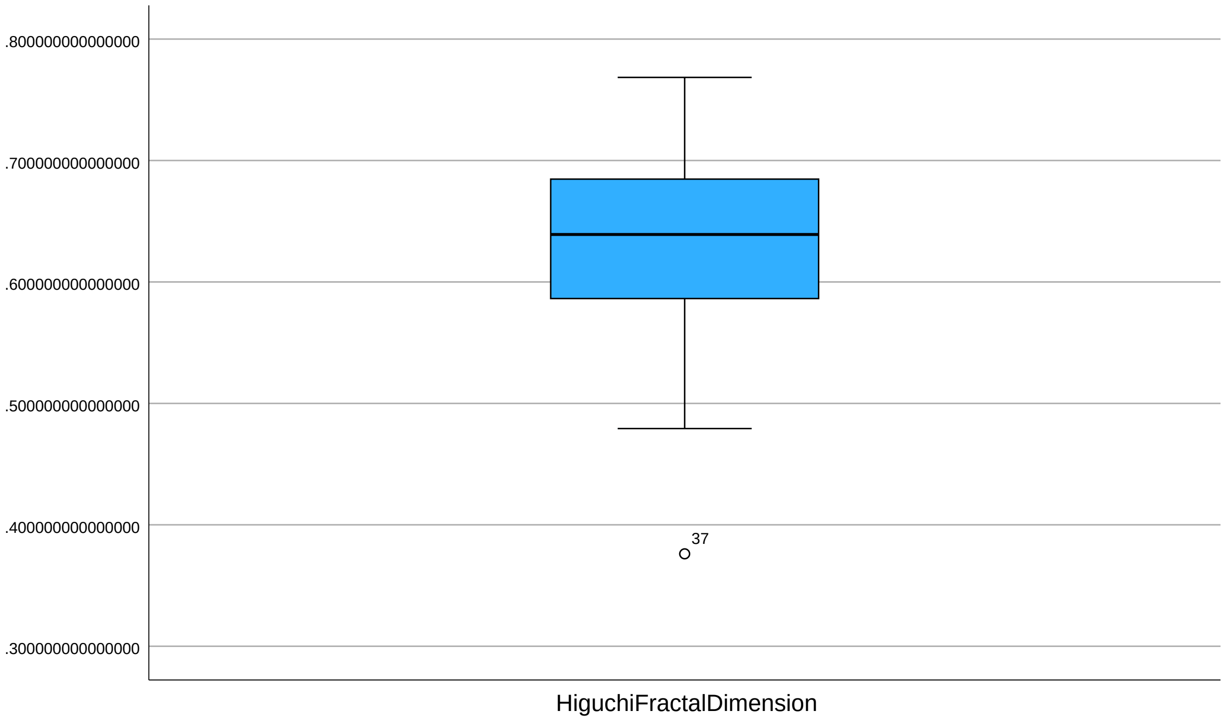

LempelZivComplexity

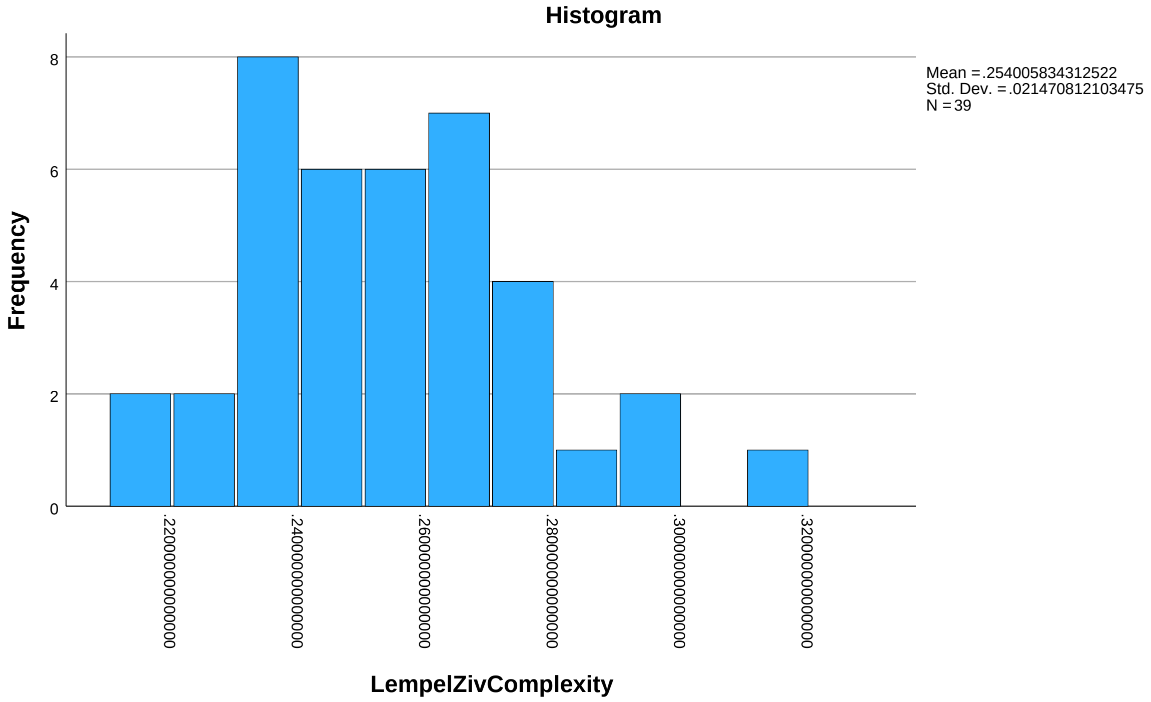

LempelZivComplexity Stem-and-Leaf Plot

Frequency   Stem & Leaf

```
 2.00      21 .  48
 2.00      22 .  59
 8.00      23 .  22567777
 6.00      24 .  134558
 6.00      25 .  012668
 7.00      26 .  0246779
 4.00      27 .  1124
 1.00      28 .  3
 2.00      29 .  56
  .00      30 .
 1.00      31 .  1

Stem width:   .0100000
Each leaf:        1 case(s)
```

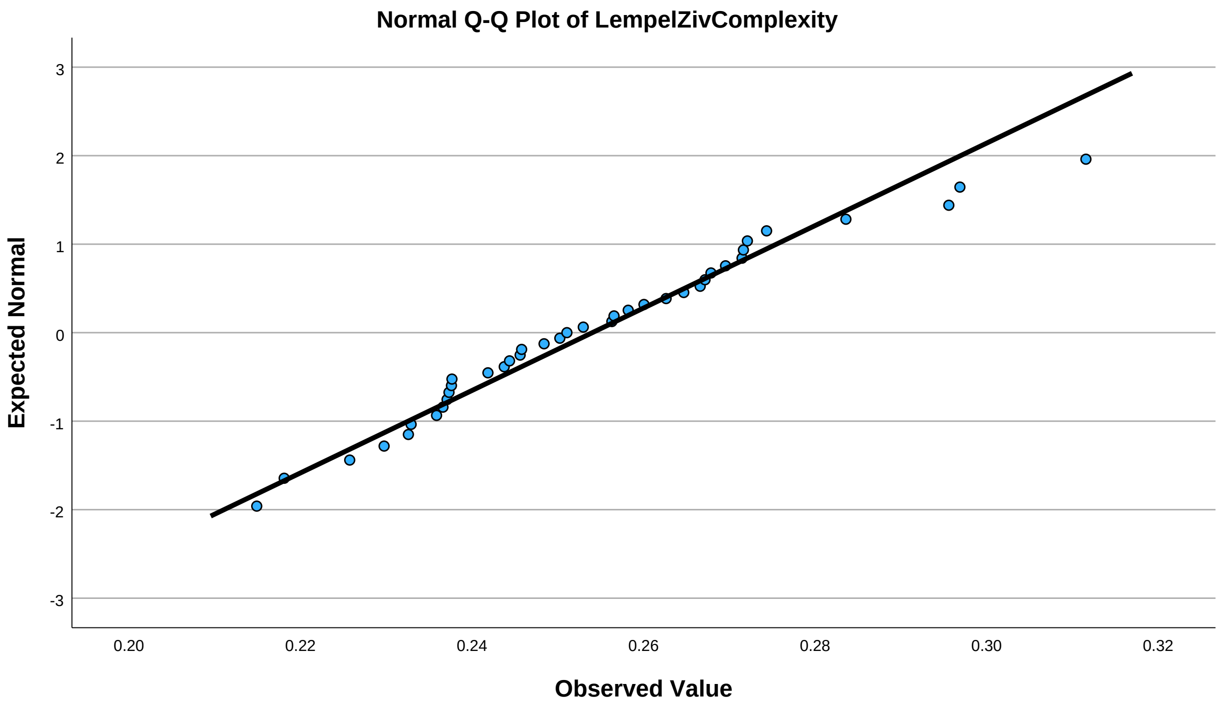

Detrended Normal Q-Q Plot of LempelZivComplexity

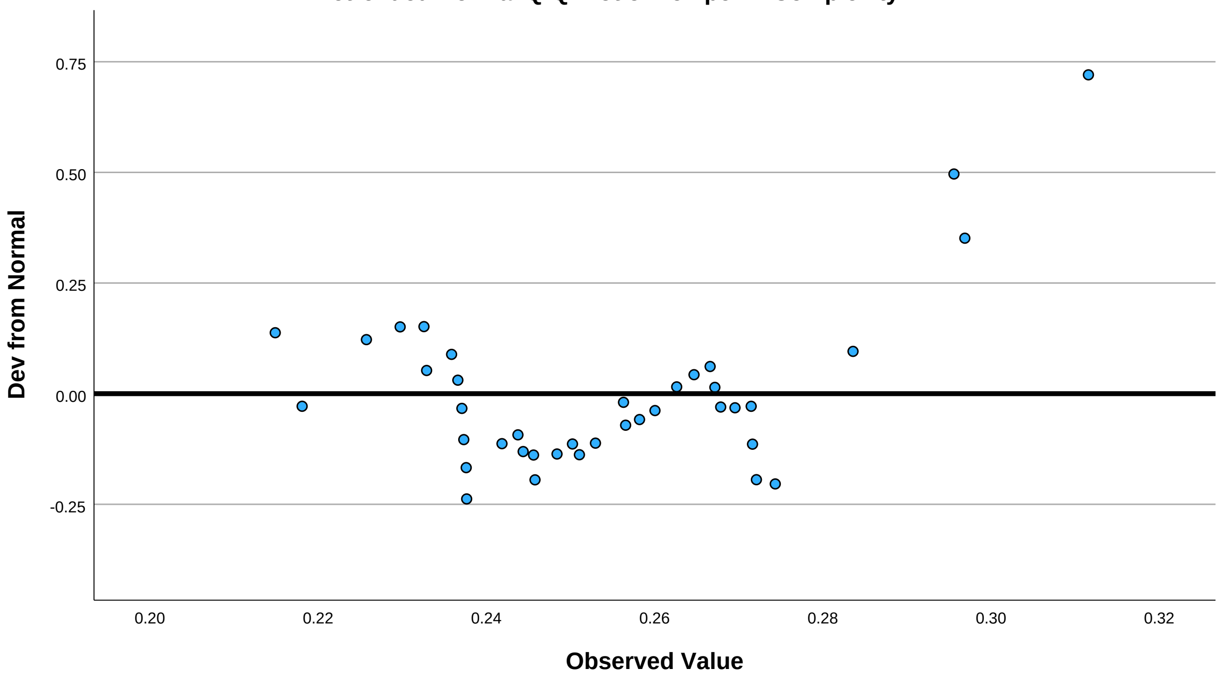

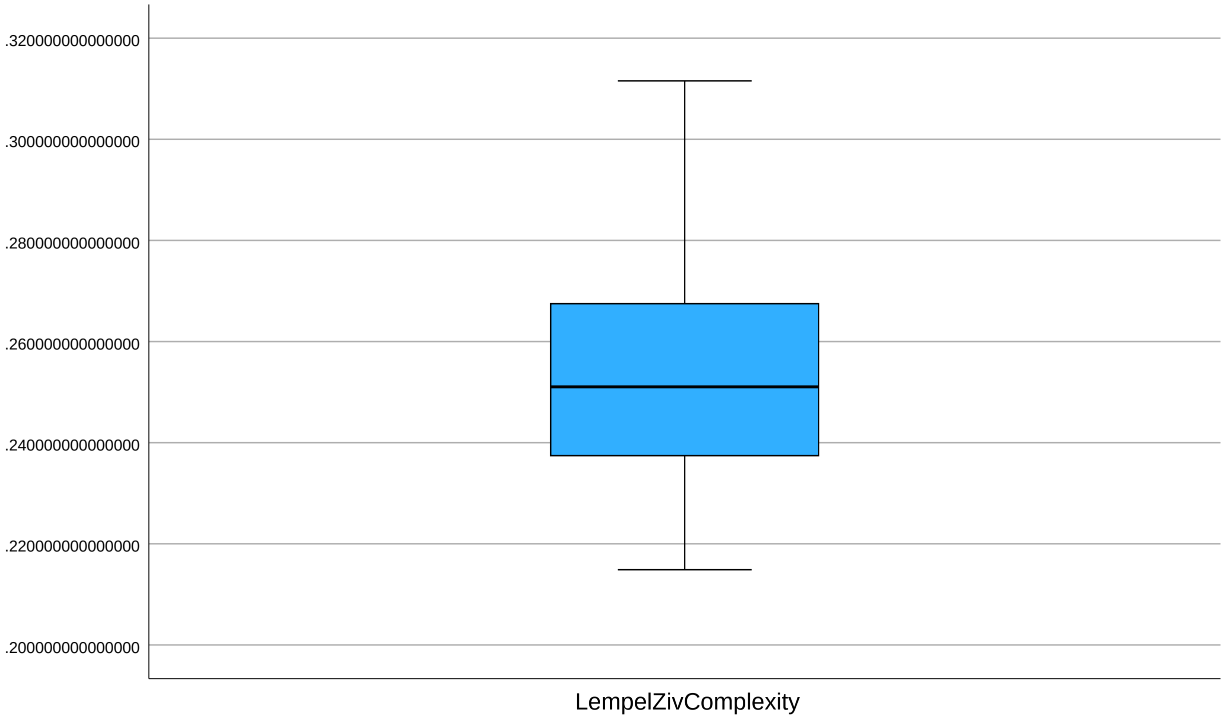

RenyiEntropy

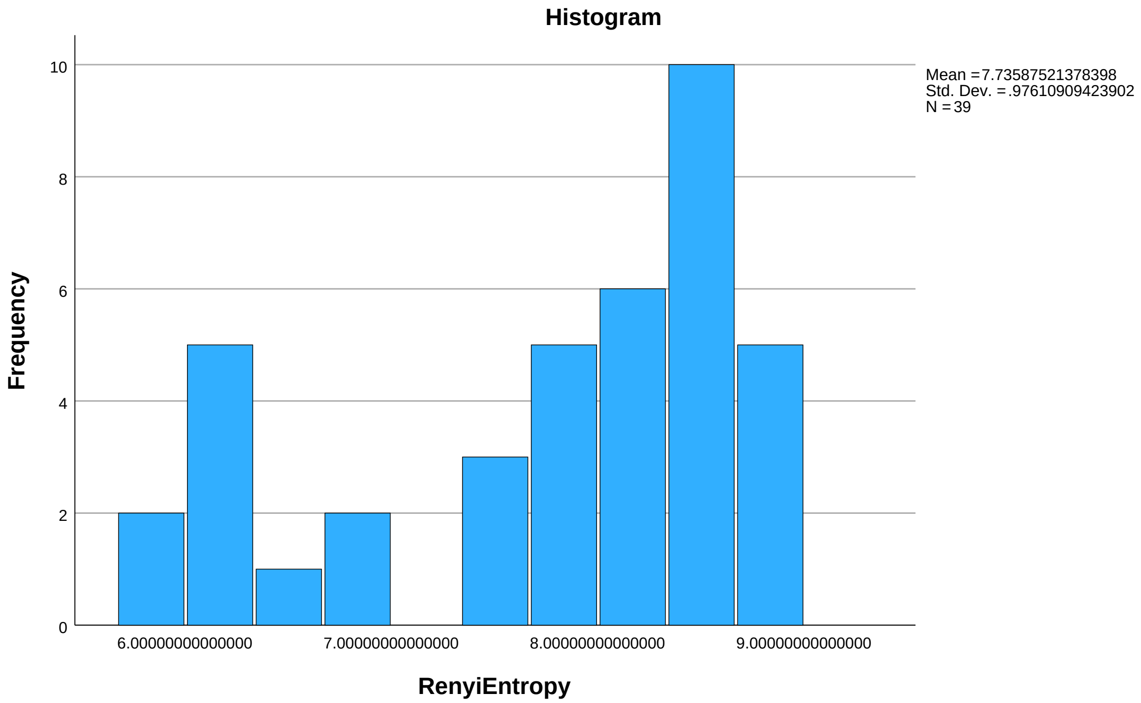


```
RenyiEntropy Stem-and-Leaf Plot

 Frequency    Stem &  Leaf

     2.00        5 .  68
     5.00        6 .  00011
     3.00        6 .  679
     1.00        7 .  4
     7.00        7 .  5667788
    12.00        8 .  000223333444
     9.00        8 .  556666788

 Stem width:   1.000000
 Each leaf:        1 case(s)
```

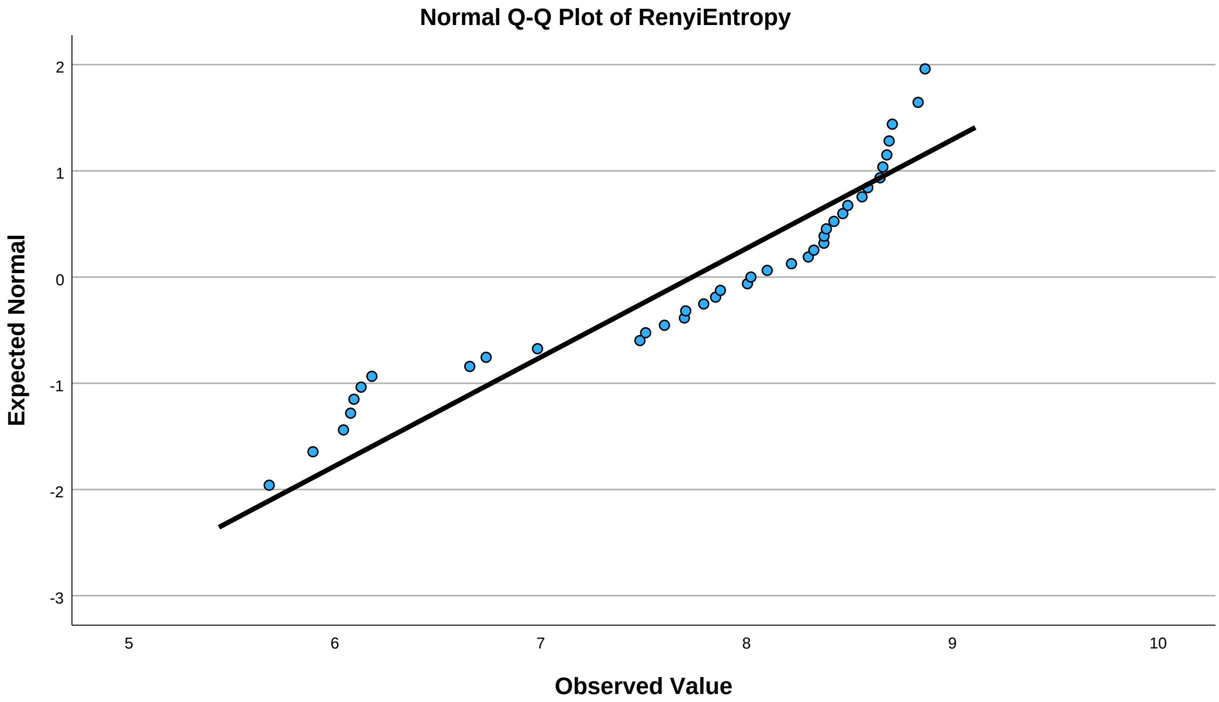
Normal Q-Q Plot of RenyiEntropy
Expected Normal
2
1
0
-1
-2
-3
5
6
7
8
9
10
Observed Value

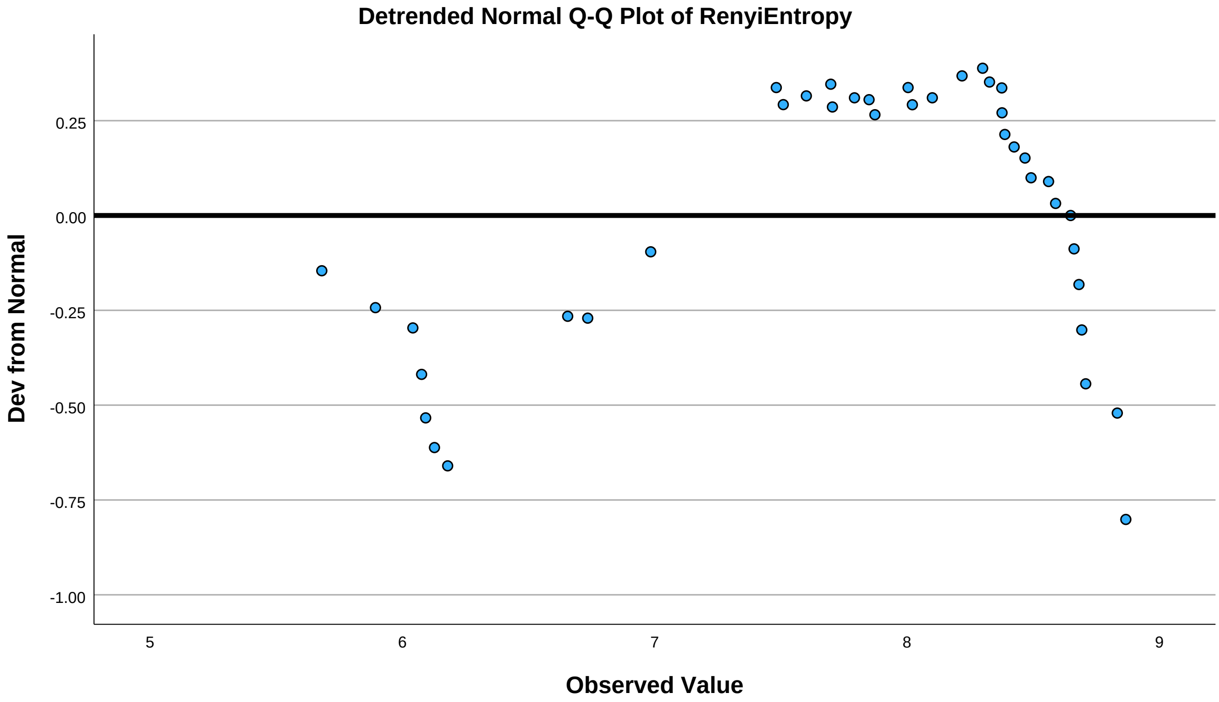
Detrended Normal Q-Q Plot of RenyiEntropy
Dev from Normal
0.25
0.00
-0.25
-0.50
-0.75
-1.00
5
6
7
8
9
Observed Value

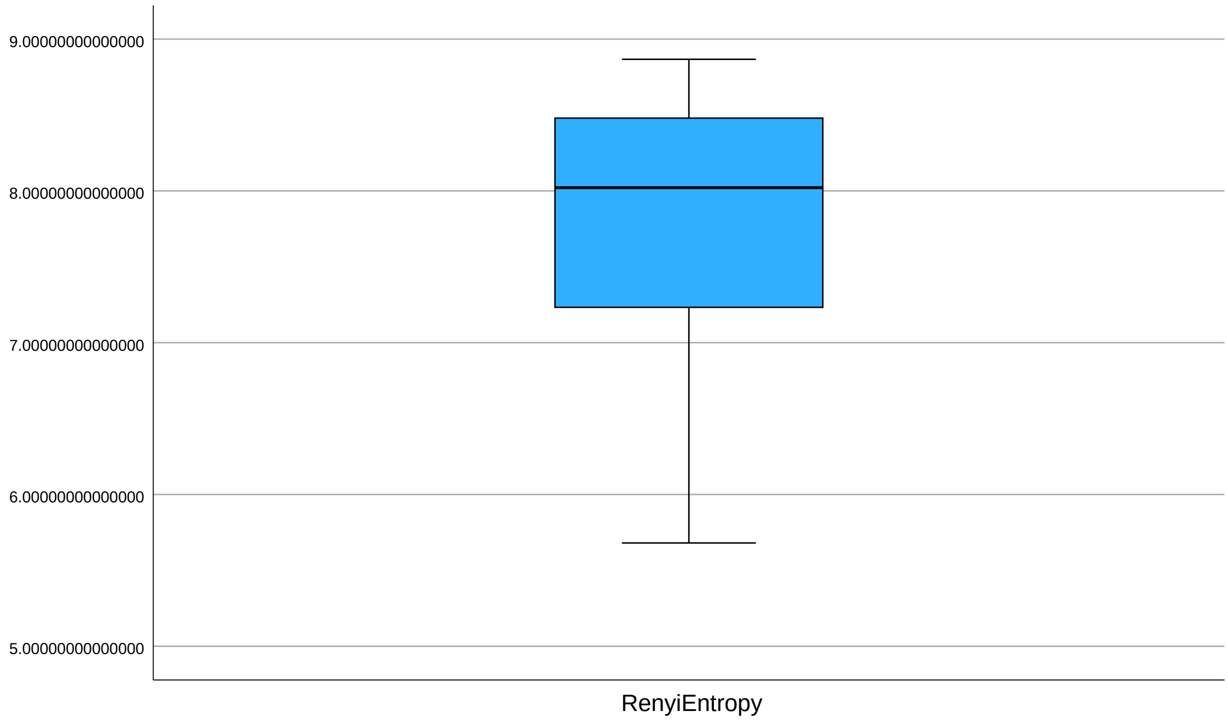

TsallisEntropy

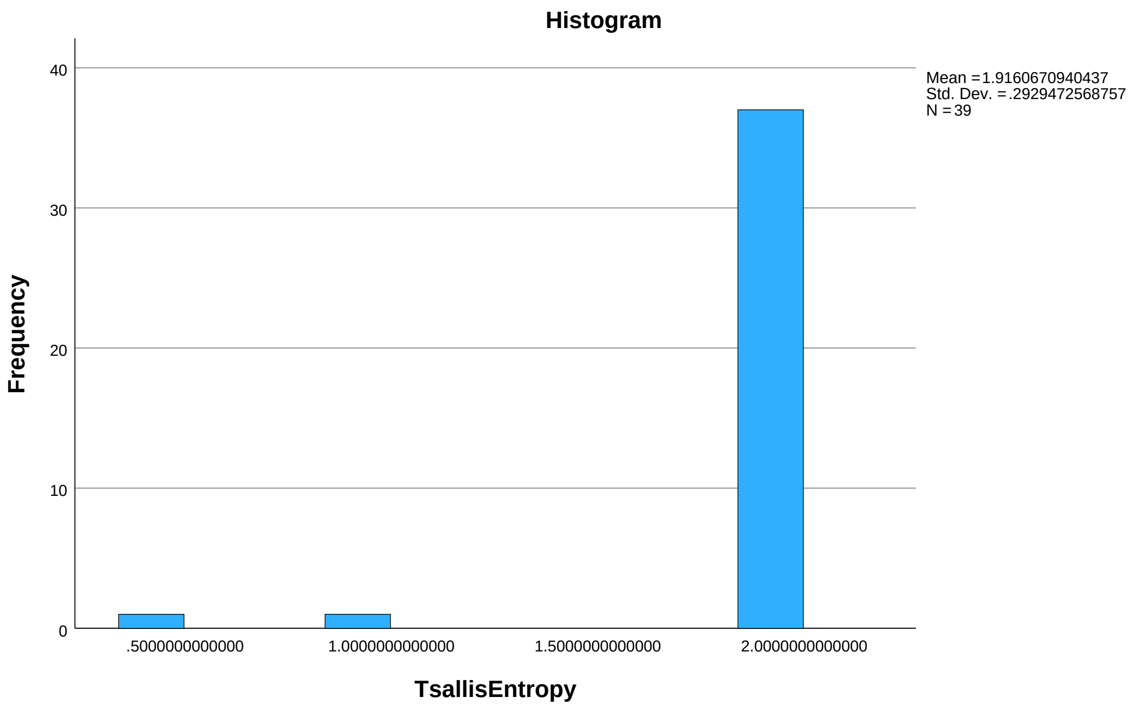


```
TsallisEntropy Stem-and-Leaf Plot

 Frequency    Stem &  Leaf
```

```
     7.00 Extremes    (=<1.9740)
    2.00     1982 .  66
    2.00     1983 .  44
    3.00     1984 .  069
    7.00     1985 .  2346889
   12.00     1986 .  011114567889
    3.00     1987 .  035
    3.00     1988 .  022

 Stem width:   .0010000
 Each leaf:       1 case(s)
```

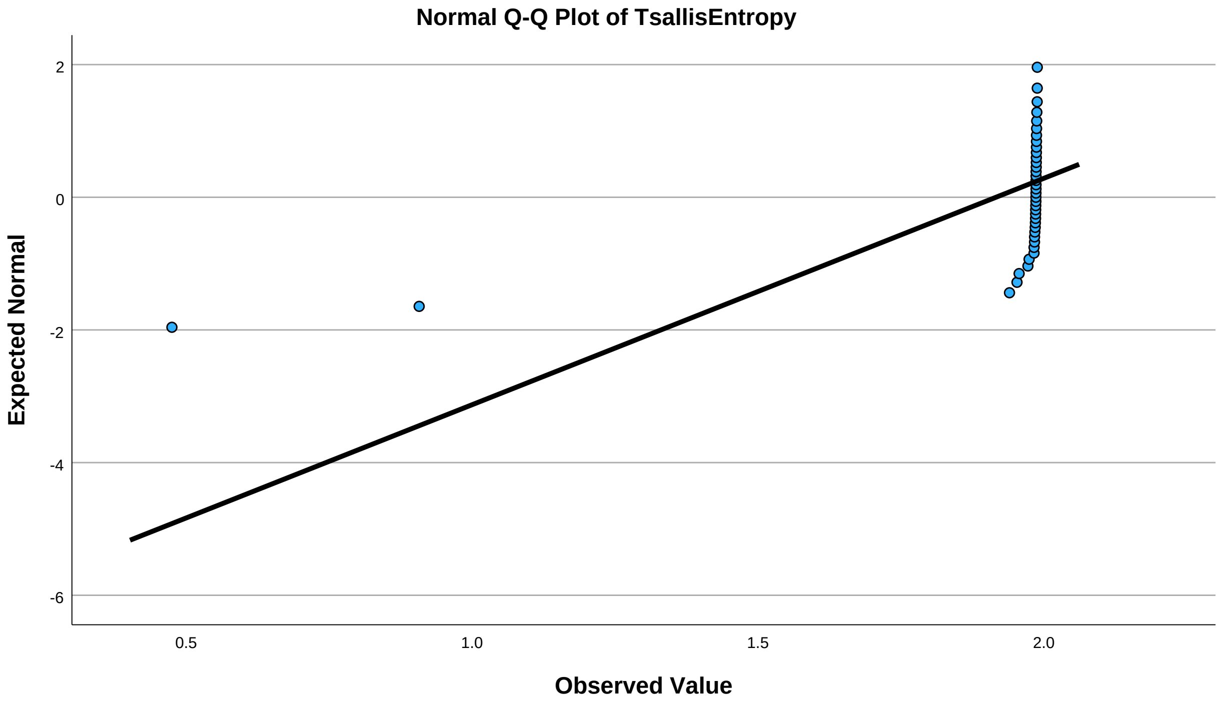

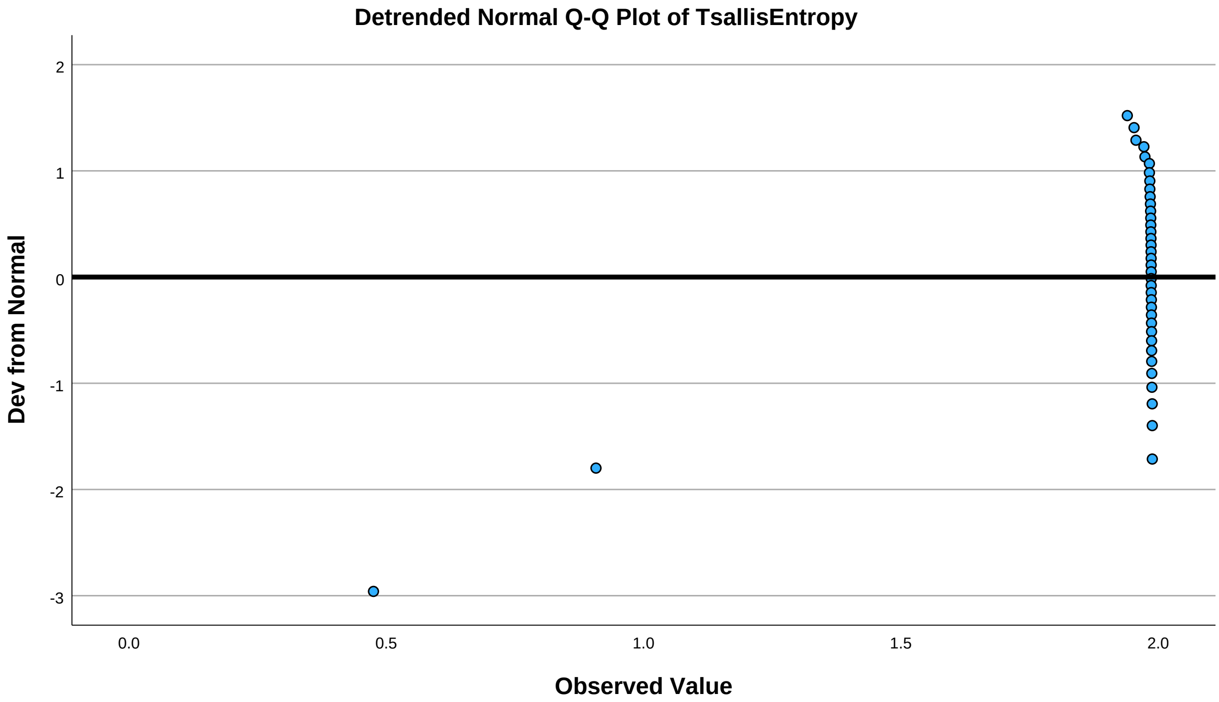
Detrended Normal Q-Q Plot of TsallisEntropy
Dev from Normal
2
1
0
-1
-2
-3
0.0
0.5
1.0
1.5
2.0
Observed Value

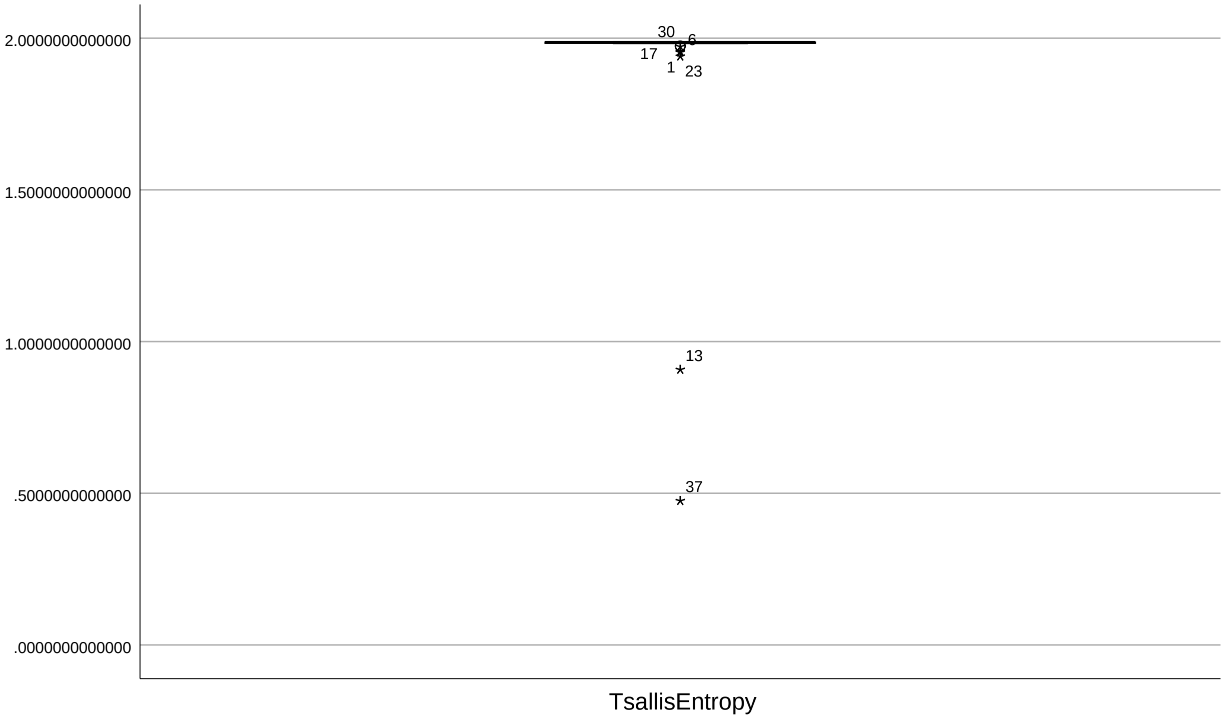
2.0000000000000
1.5000000000000
1.0000000000000
.5000000000000
.0000000000000
30
6
17
1
23
13
37
TsallisEntropy

PS_4_5

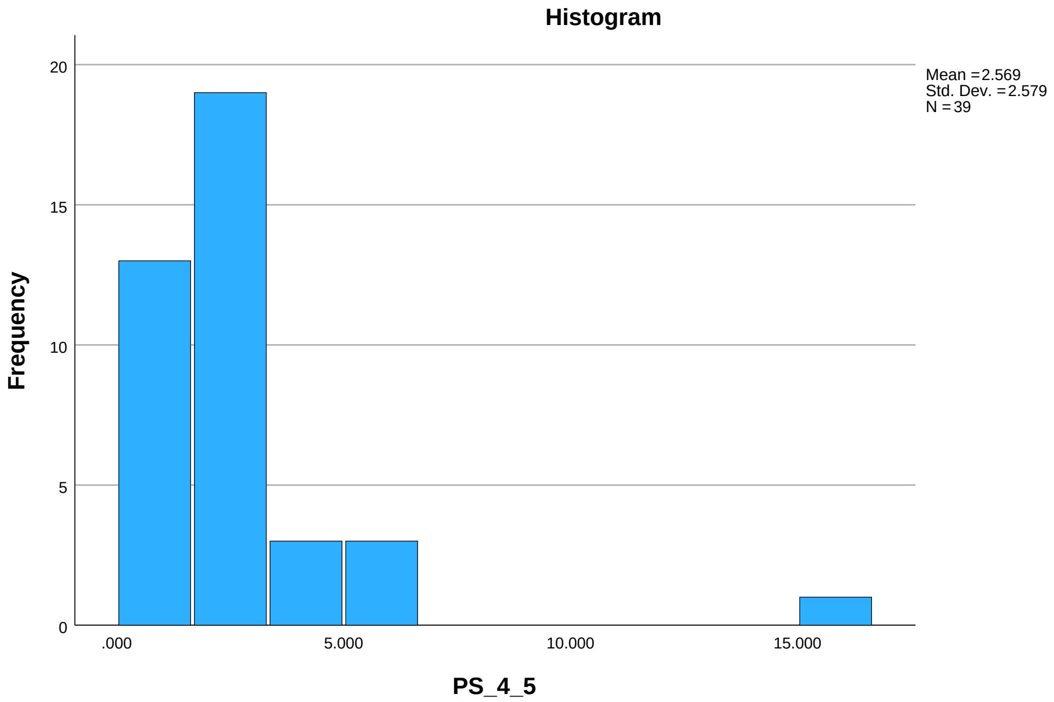


```
PS_4_5 Stem-and-Leaf Plot

 Frequency    Stem &  Leaf

     5.00        0 .  56779
     7.00        1 .  0012344
     8.00        1 .  57778999
     7.00        2 .  0011244
     5.00        2 .  55566
      .00        3 .
     1.00        3 .  9
     1.00        4 .  2
     5.00 Extremes    (>=4.5)

 Stem width:      1.000
 Each leaf:       1 case(s)
```

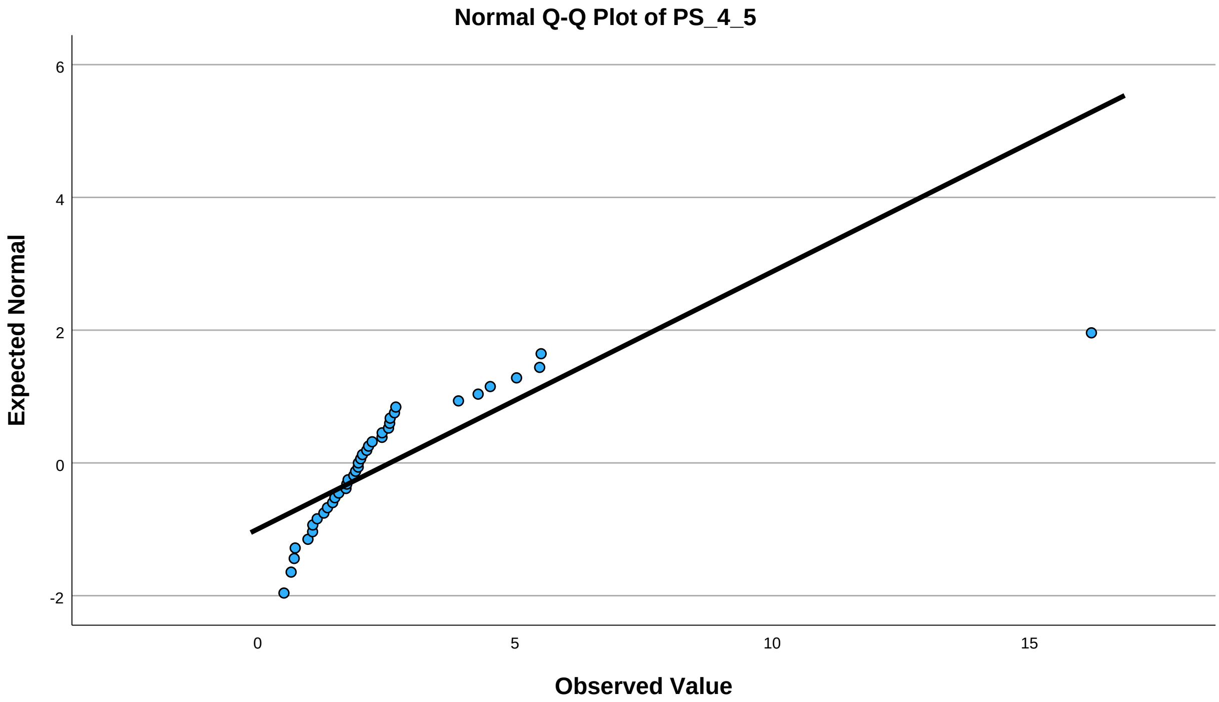
Normal Q-Q Plot of PS_4_5
Expected Normal
6
4
2
0
-2
0
5
10
15
Observed Value

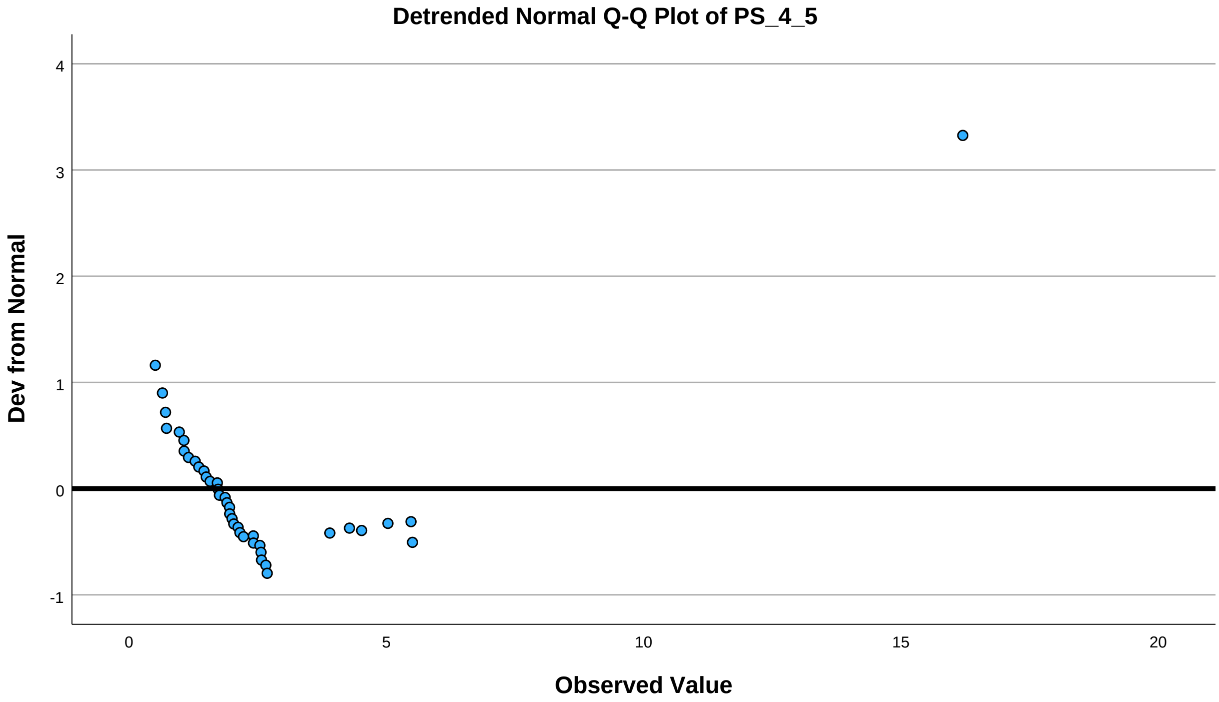
Detrended Normal Q-Q Plot of PS_4_5
Dev from Normal
4
3
2
1
0
-1
0
5
10
15
20
Observed Value

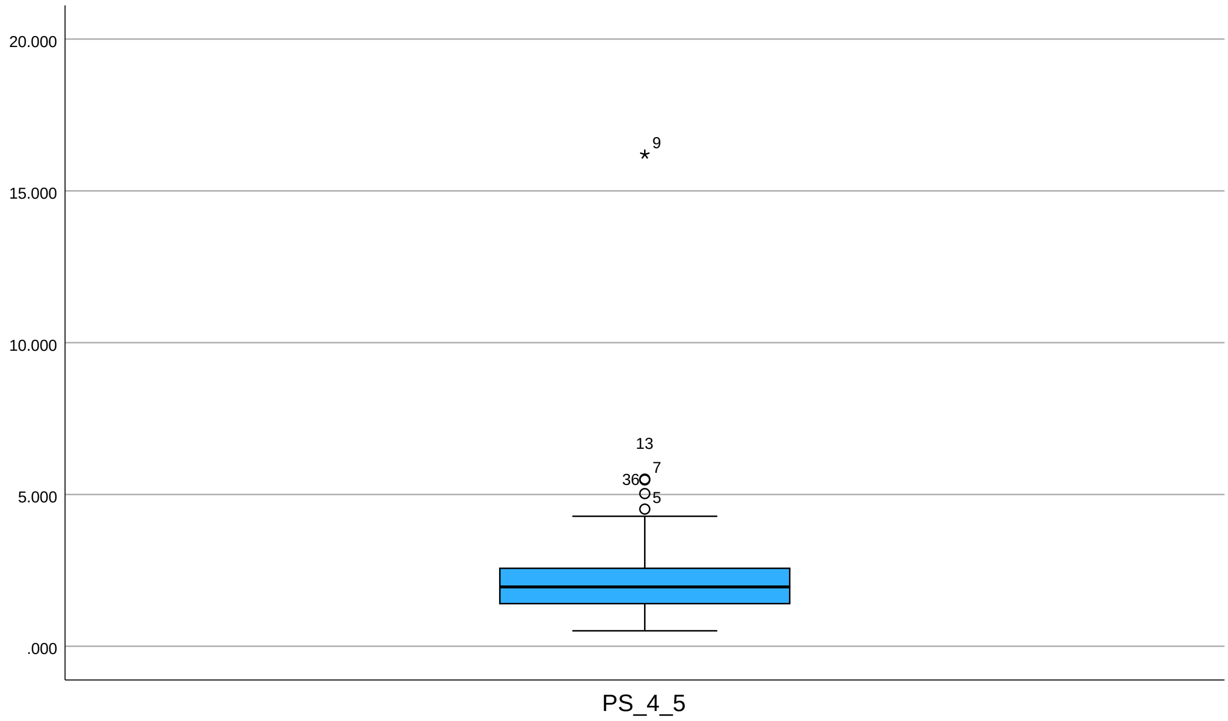

PS_5_6

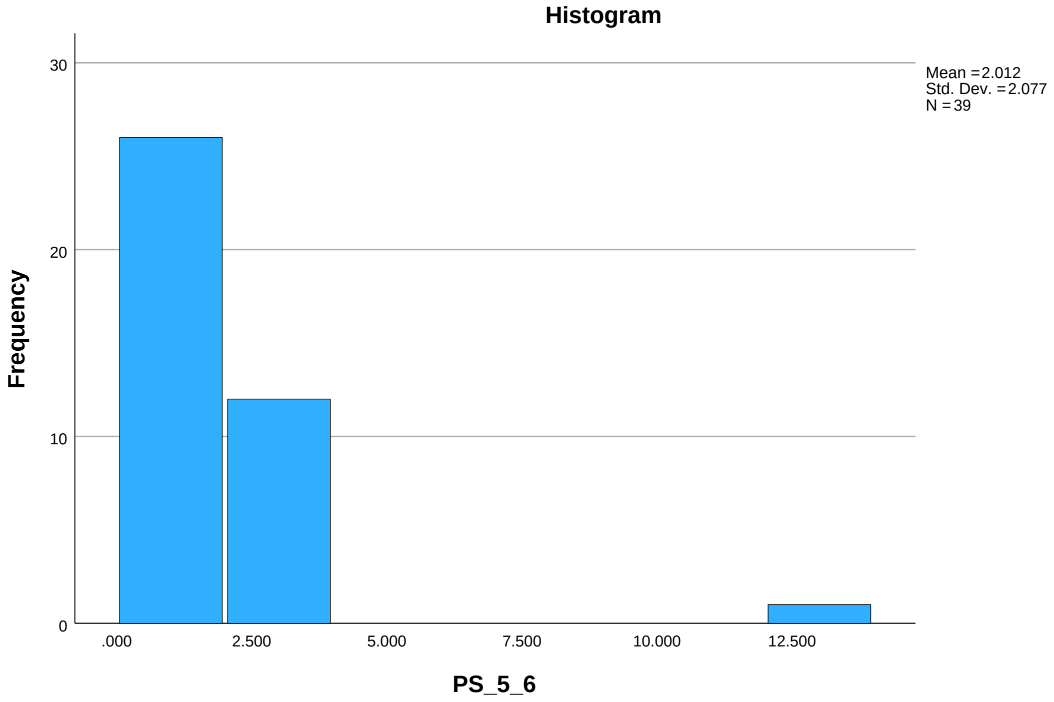

PS_5_6 Stem-and-Leaf Plot

Frequency   Stem & Leaf

```
     2.00        0 .  34
     7.00        0 .  5578889
     8.00        1 .  02222444
     9.00        1 .  555666778
     4.00        2 .  0114
     3.00        2 .  557
     4.00        3 .  1133
     1.00        3 .  8
     1.00 Extremes    (>=13.5)

 Stem width:      1.000
 Each leaf:       1 case(s)
```

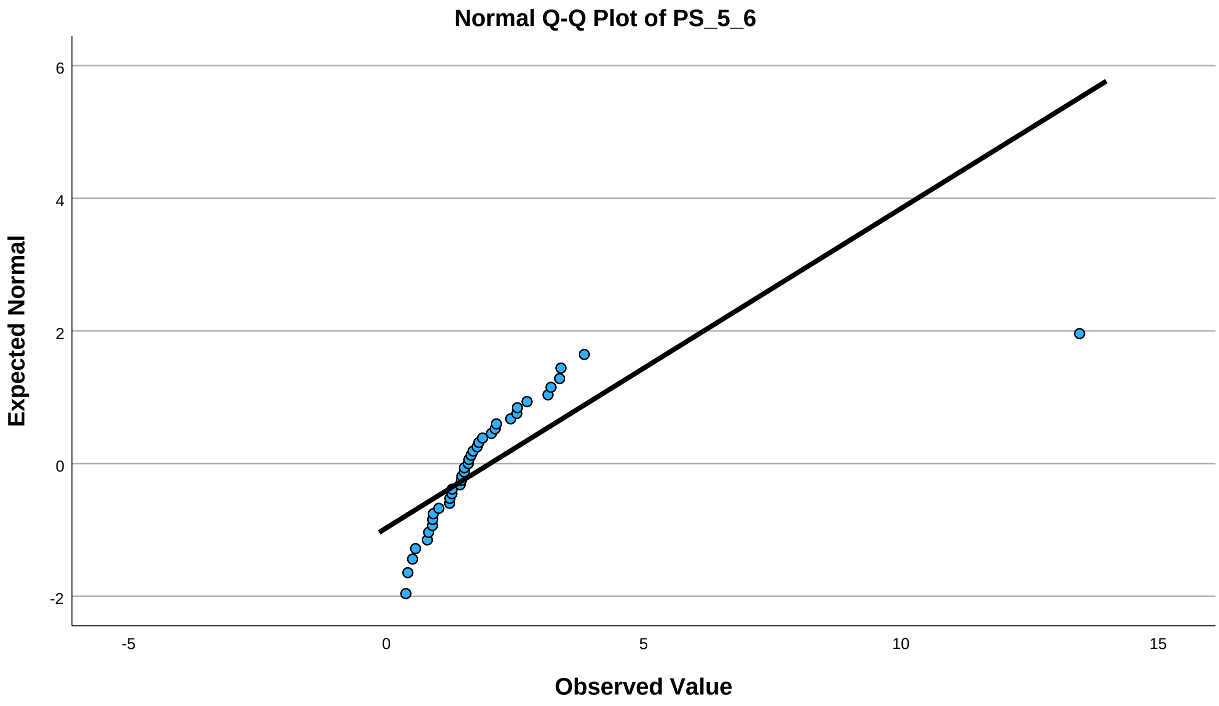

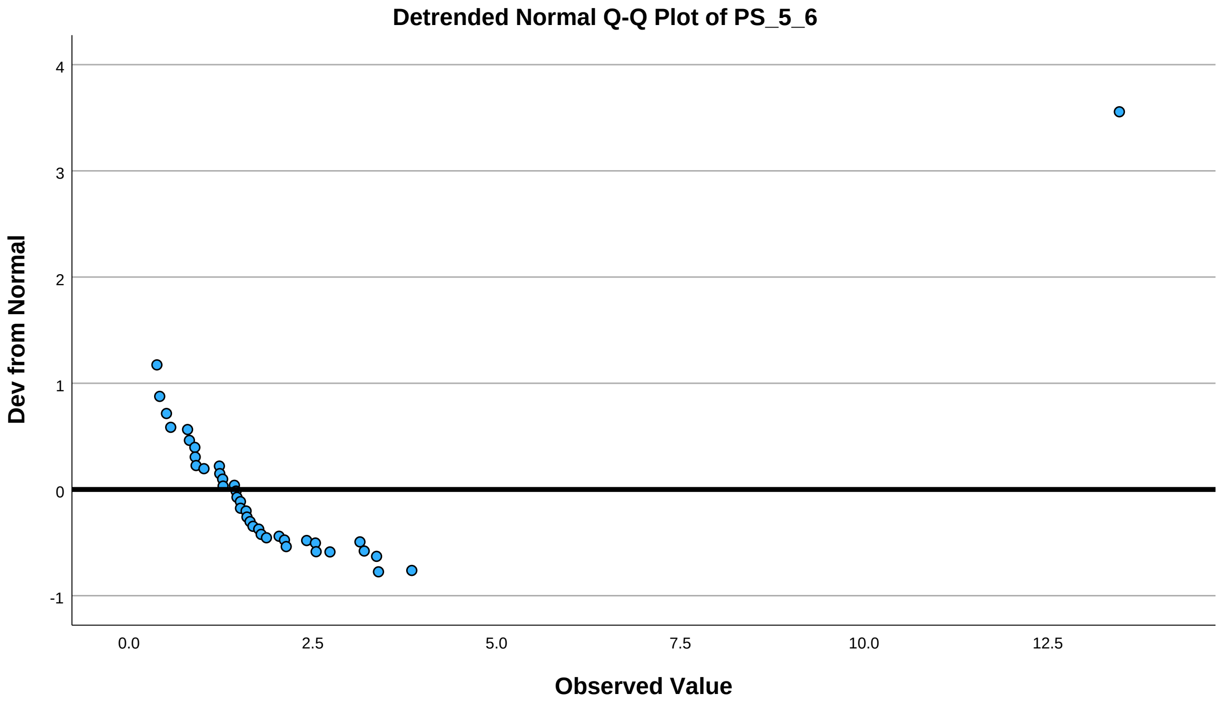
Detrended Normal Q-Q Plot of PS_5_6
Dev from Normal
4
3
2
1
0
-1
0.0
2.5
5.0
7.5
10.0
12.5
Observed Value

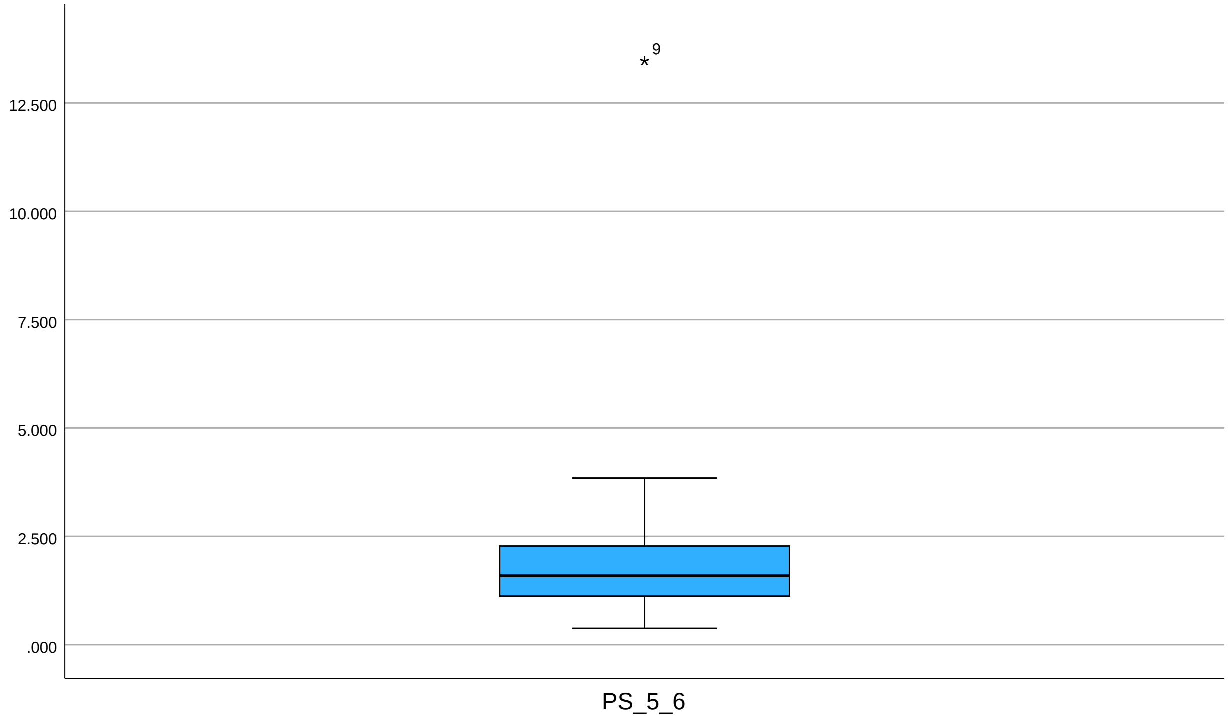
9
12.500
10.000
7.500
5.000
2.500
.000
PS_5_6

PS_6_7

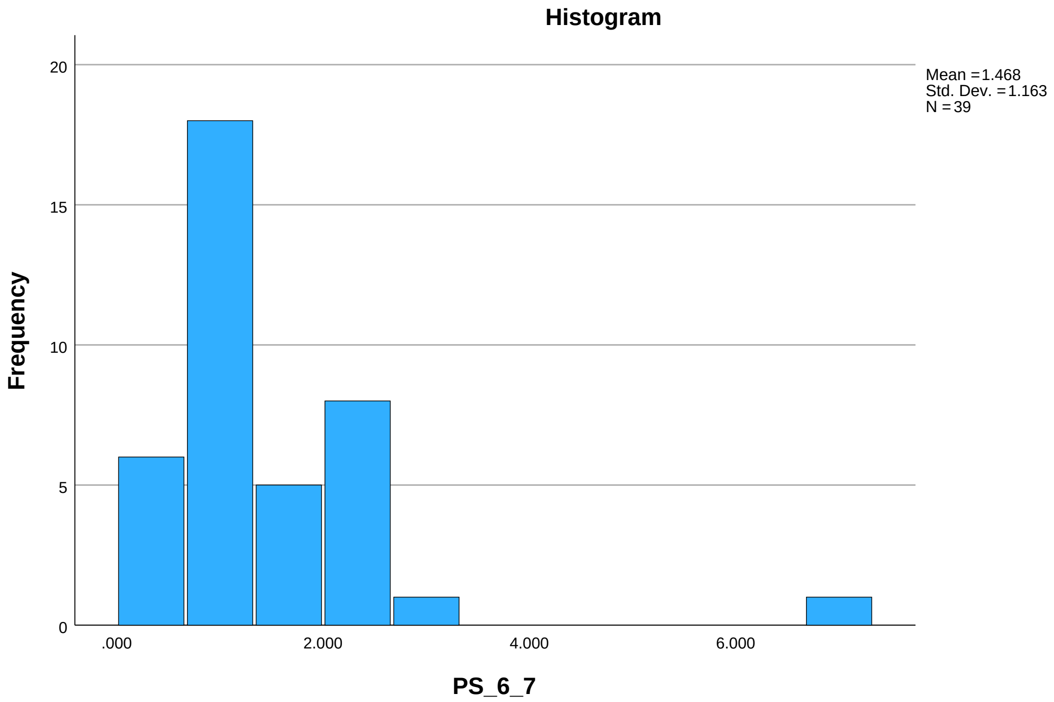

PS_6_7 Stem-and-Leaf Plot

```
 Frequency    Stem &  Leaf

     4.00        0 .  3344
    11.00        0 .  66788889999
    11.00        1 .  00111122233
     3.00        1 .  556
     8.00        2 .  11233334
     1.00        2 .  8
     1.00 Extremes    (>=7.3)

 Stem width:      1.000
 Each leaf:       1 case(s)
```

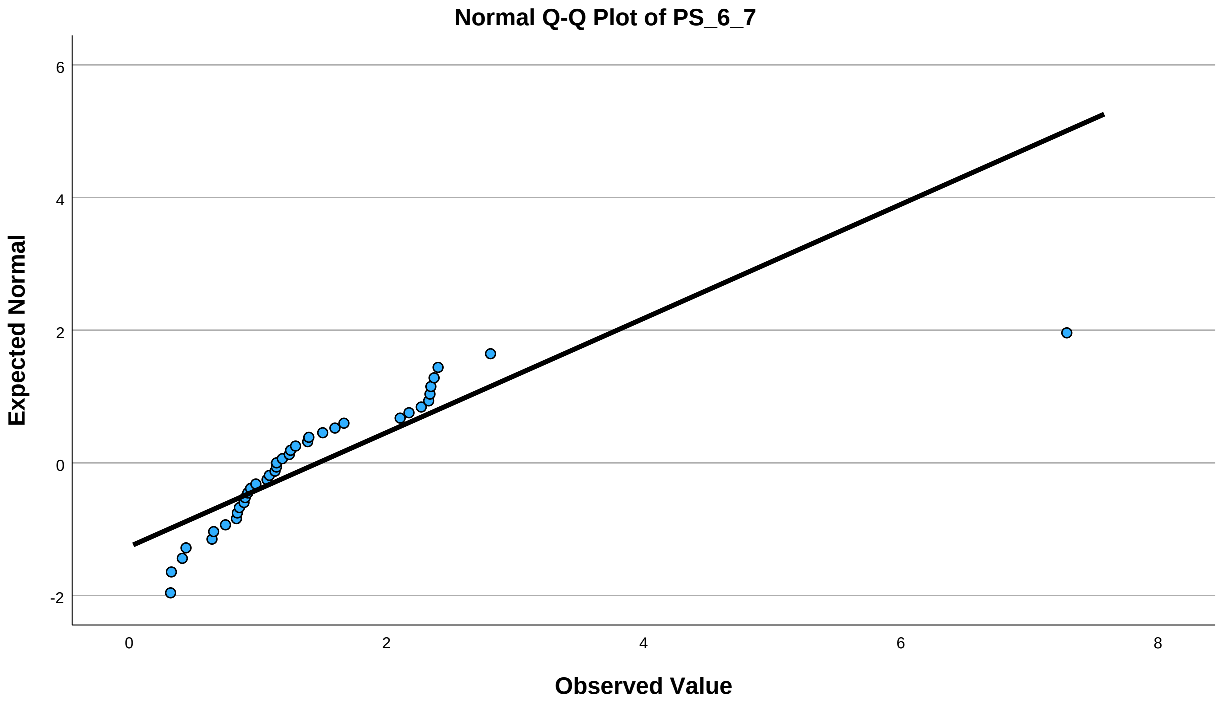
Normal Q-Q Plot of PS_6_7
Expected Normal
Observed Value
6
4
2
0
-2
0
2
4
6
8

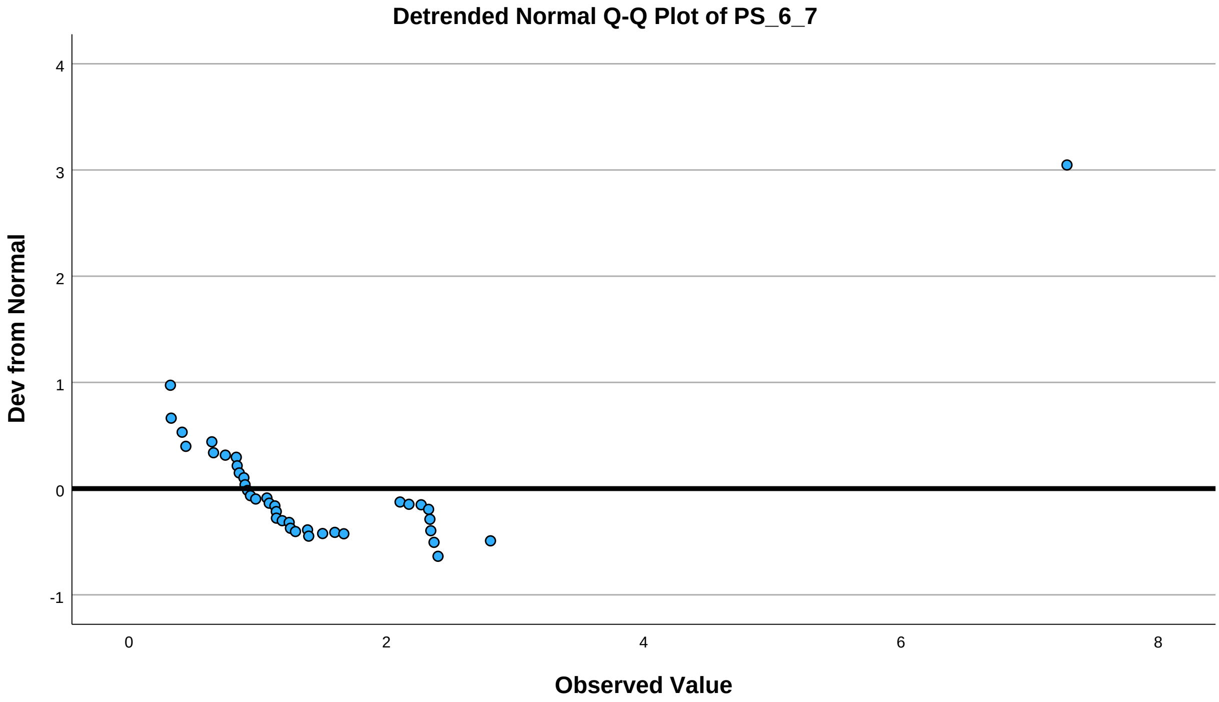
Detrended Normal Q-Q Plot of PS_6_7
Dev from Normal
Observed Value
4
3
2
1
0
-1
0
2
4
6
8

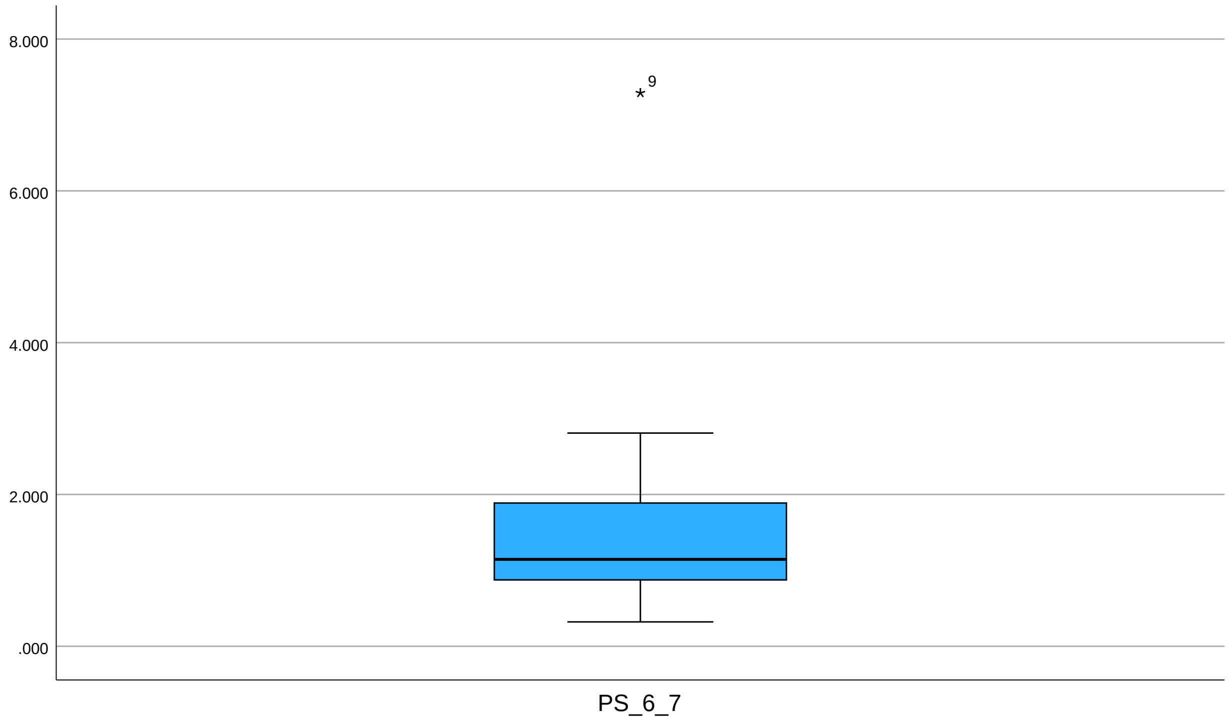

PS_7_8

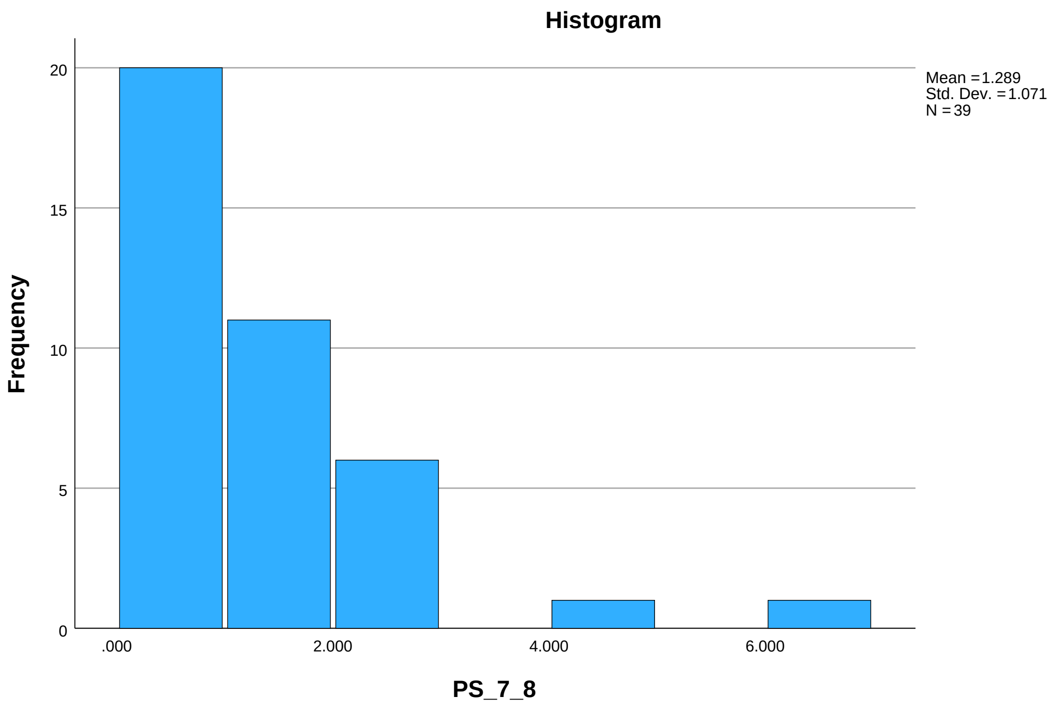


```
PS_7_8 Stem-and-Leaf Plot

 Frequency    Stem &  Leaf
```

```
  4.00      0 .  2233
 16.00       0 .  5566677788899999
 10.00       1 .  0001111333
 1.00     1 . 5
  5.00      2 .  00011
  3.00 Extremes    (>=2.6)

Stem width:     1.000
Each leaf:       1 case(s)
```

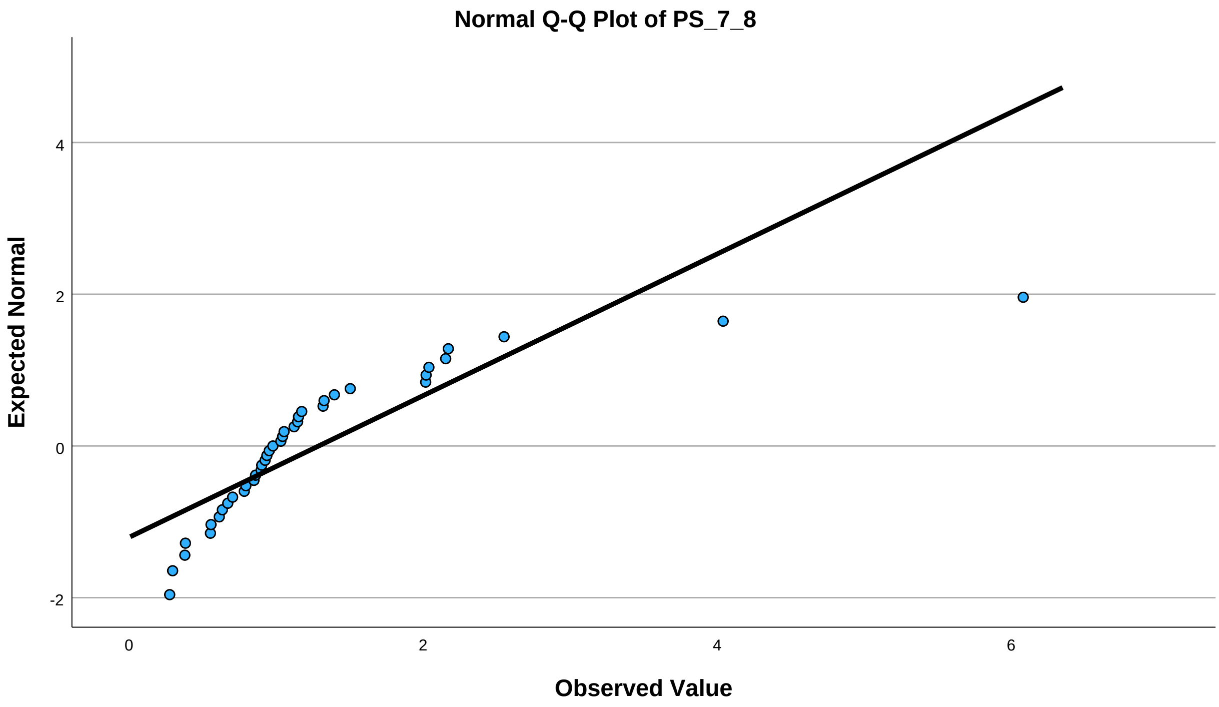

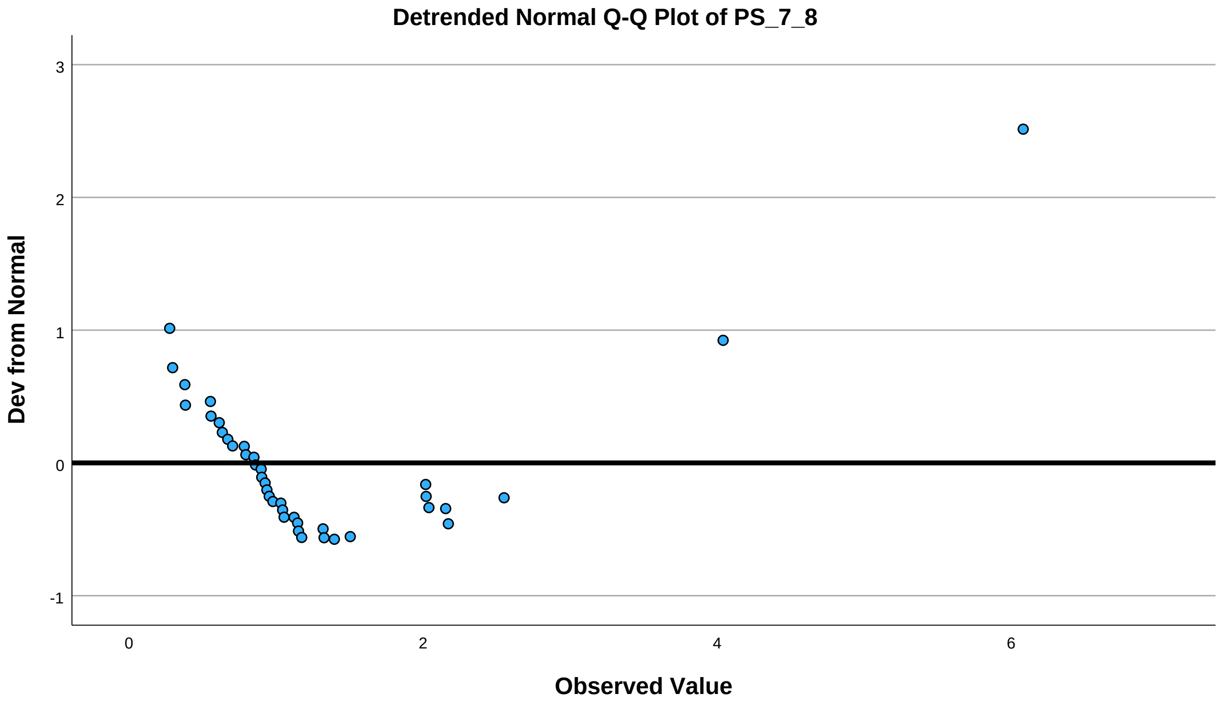

Detrended Normal Q-Q Plot of PS_7_8
Dev from Normal
3
2
1
0
-1
0
2
4
6
Observed Value

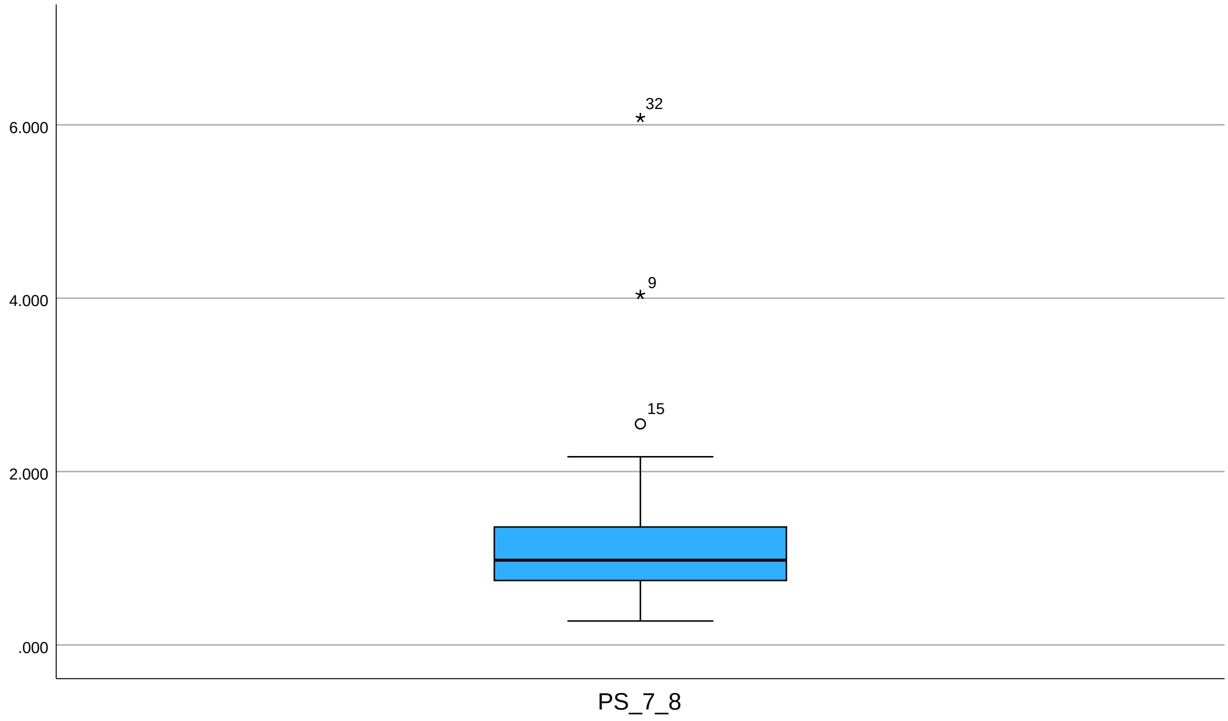

6.000
4.000
2.000
.000
32
9
15
PS_7_8

PS_8_9

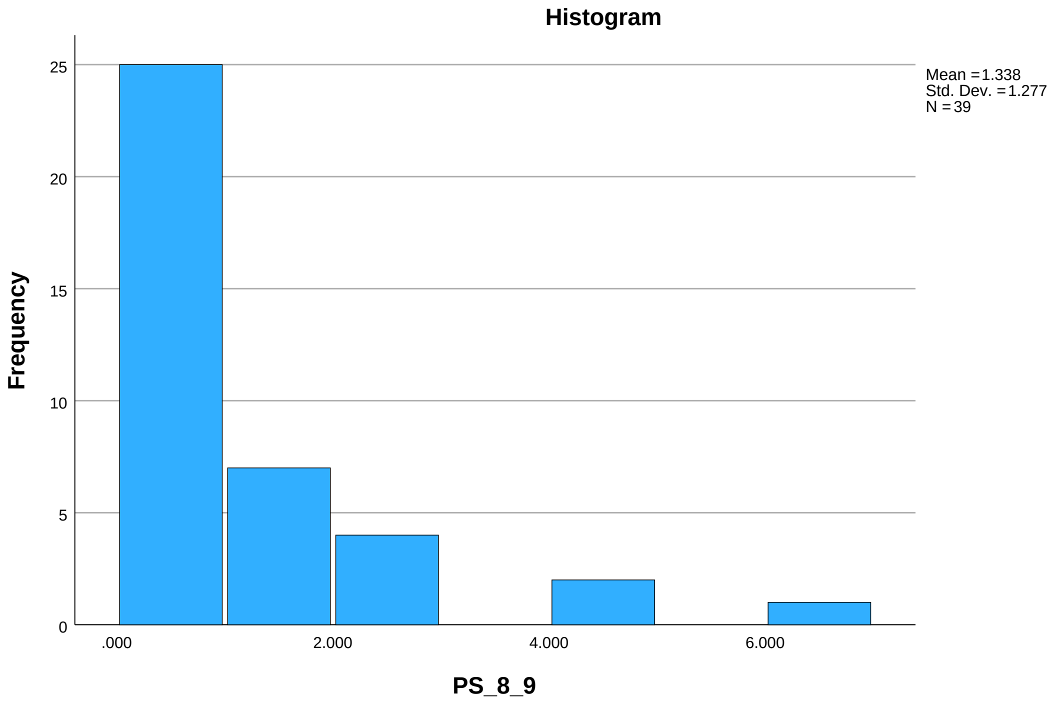

PS_8_9 Stem-and-Leaf Plot

```
 Frequency    Stem &  Leaf

     6.00        0 .  223344
    19.00        0 .  5555566677889999999
     4.00        1 .  0234
     3.00        1 .  566
     1.00        2 .  1
     3.00        2 .  677
     3.00 Extremes    (>=4.3)

 Stem width:      1.000
 Each leaf:       1 case(s)
```

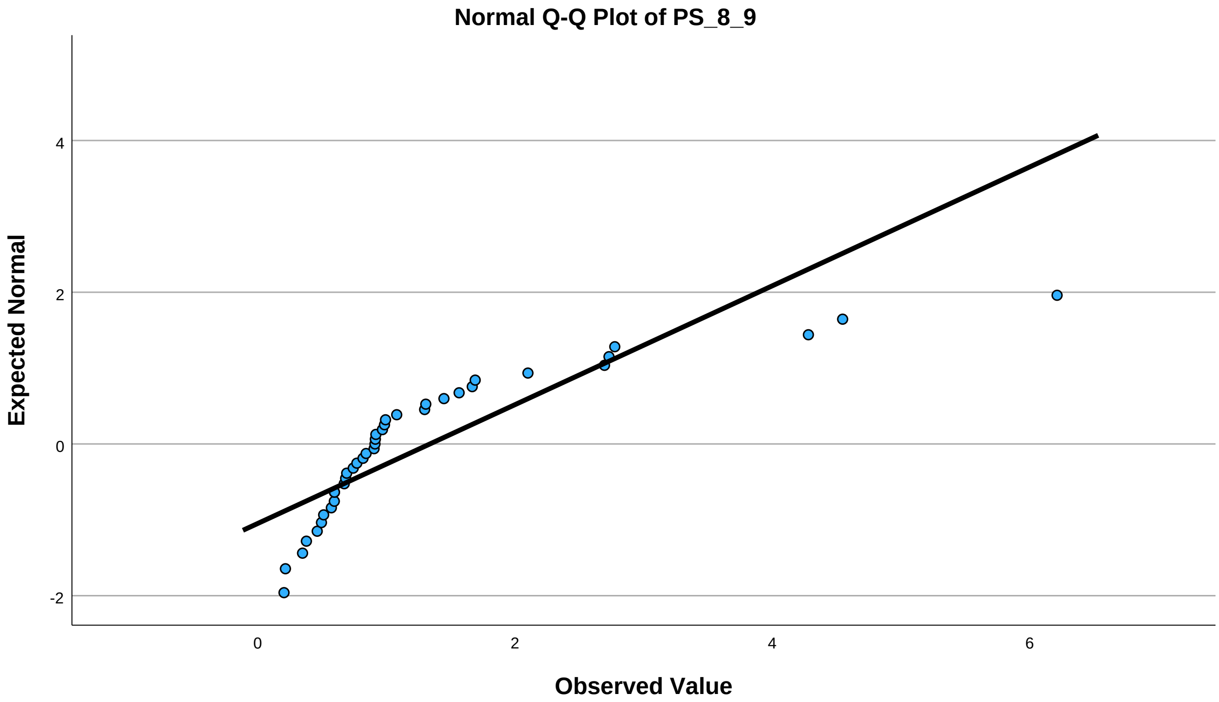
Normal Q-Q Plot of PS_8_9
Expected Normal
4
2
0
-2
0
2
4
6
Observed Value

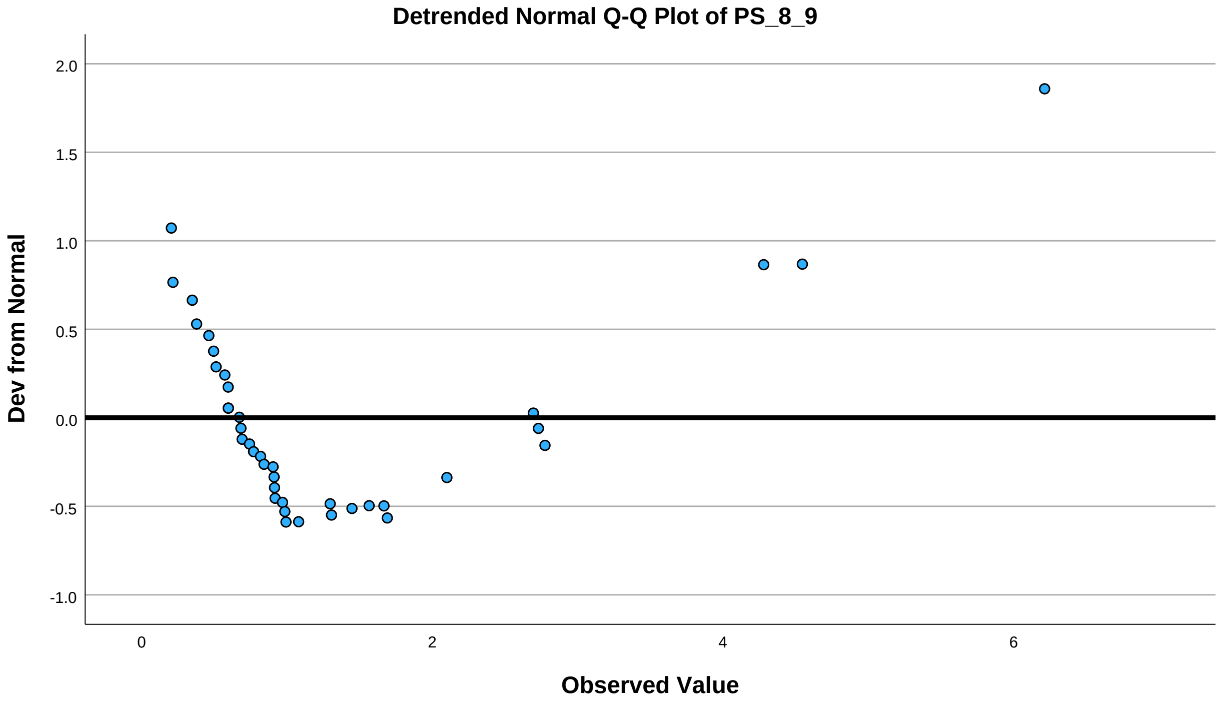
Detrended Normal Q-Q Plot of PS_8_9
Dev from Normal
2.0
1.5
1.0
0.5
0.0
-0.5
-1.0
0
2
4
6
Observed Value

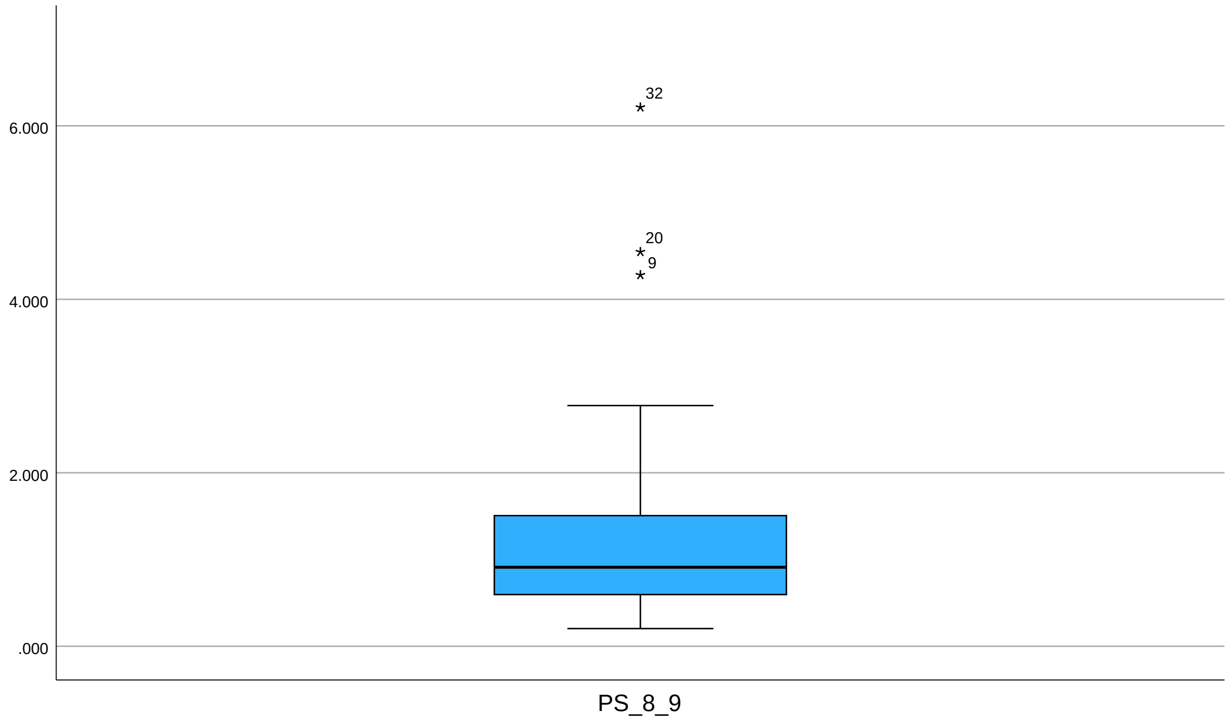

PS_9_10

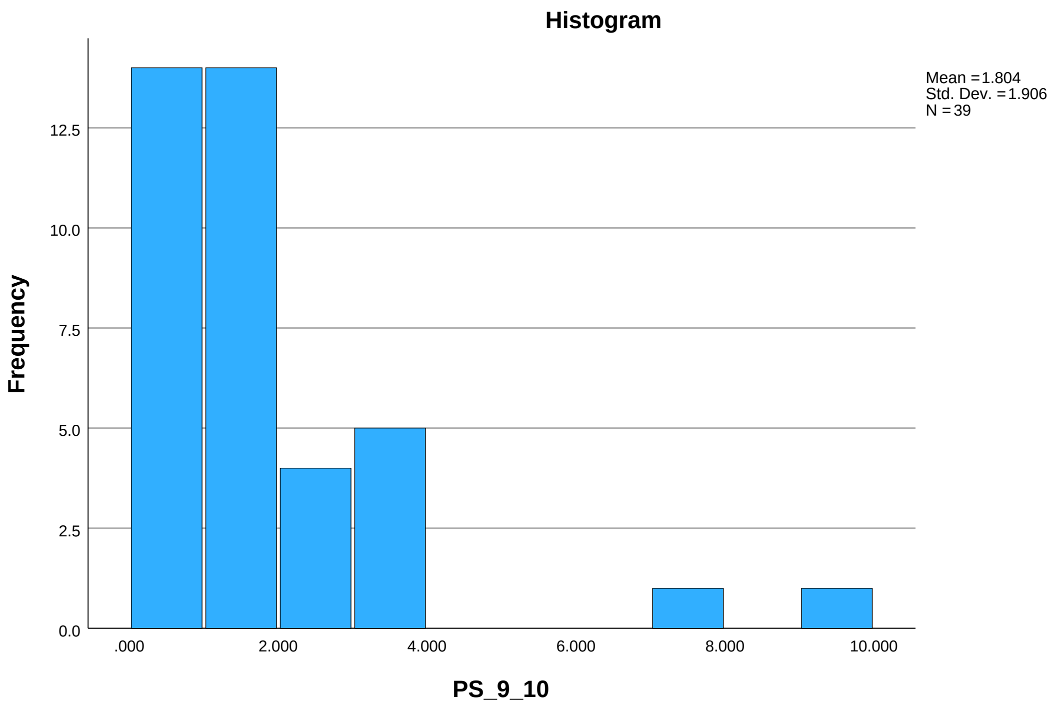


```
PS_9_10 Stem-and-Leaf Plot

 Frequency    Stem &  Leaf
```

```
     6.00        0 .  222444
     8.00        0 .  55666688
     8.00        1 .  01111133
     6.00        1 .  555666
     3.00        2 .  144
     1.00        2 .  6
     5.00        3 .  02334
     2.00 Extremes    (>=7.8)

 Stem width:      1.000
 Each leaf:        1 case(s)
```

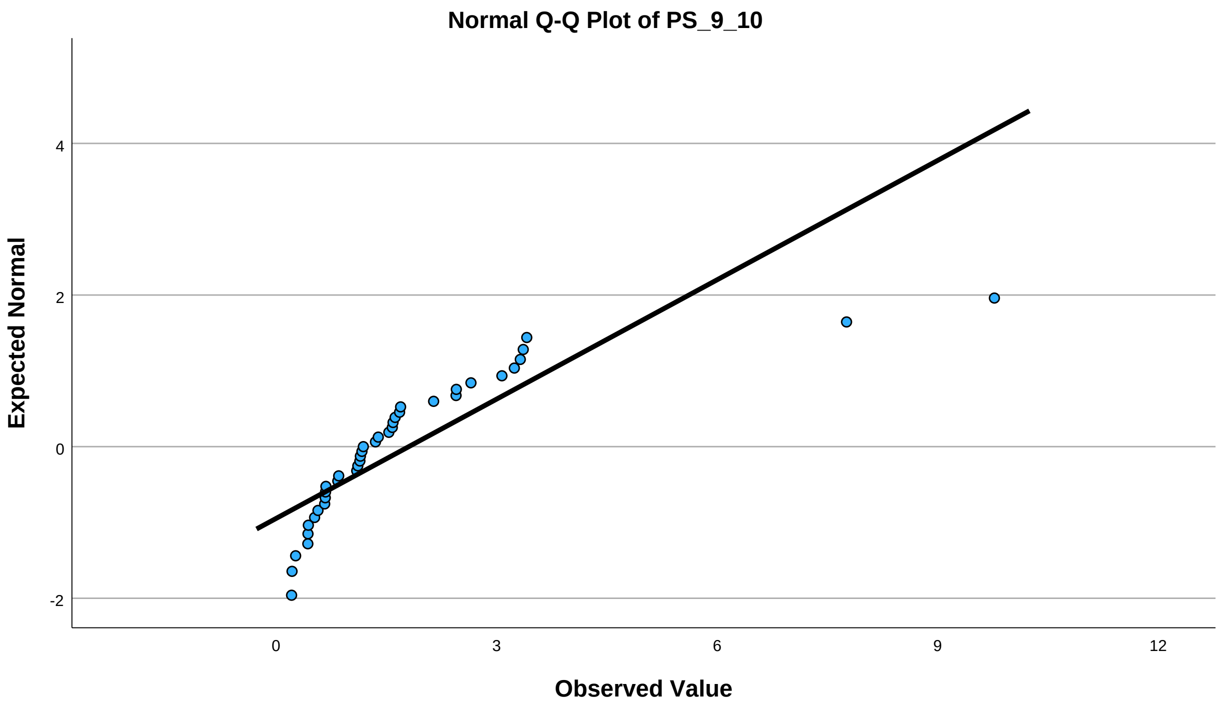

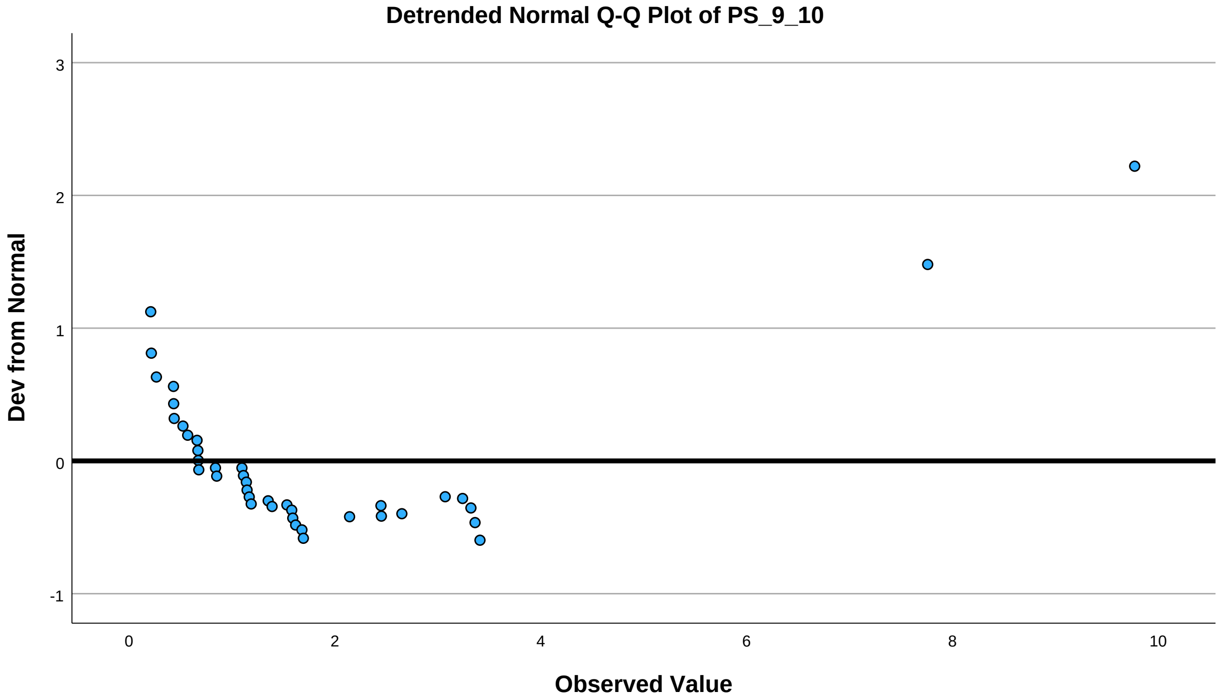
Detrended Normal Q-Q Plot of PS_9_10
Dev from Normal
3
2
1
0
-1
0
2
4
6
8
10
Observed Value

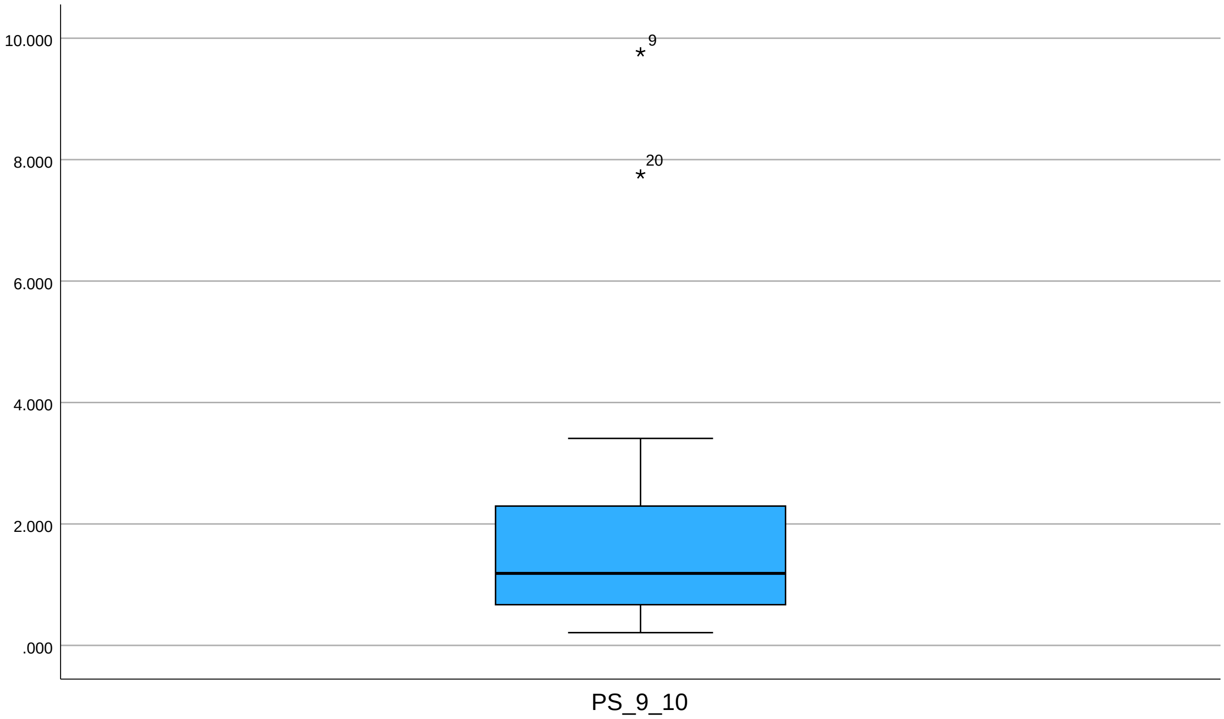
10.000
8.000
6.000
4.000
2.000
.000
9
20
PS_9_10

PS_10_11

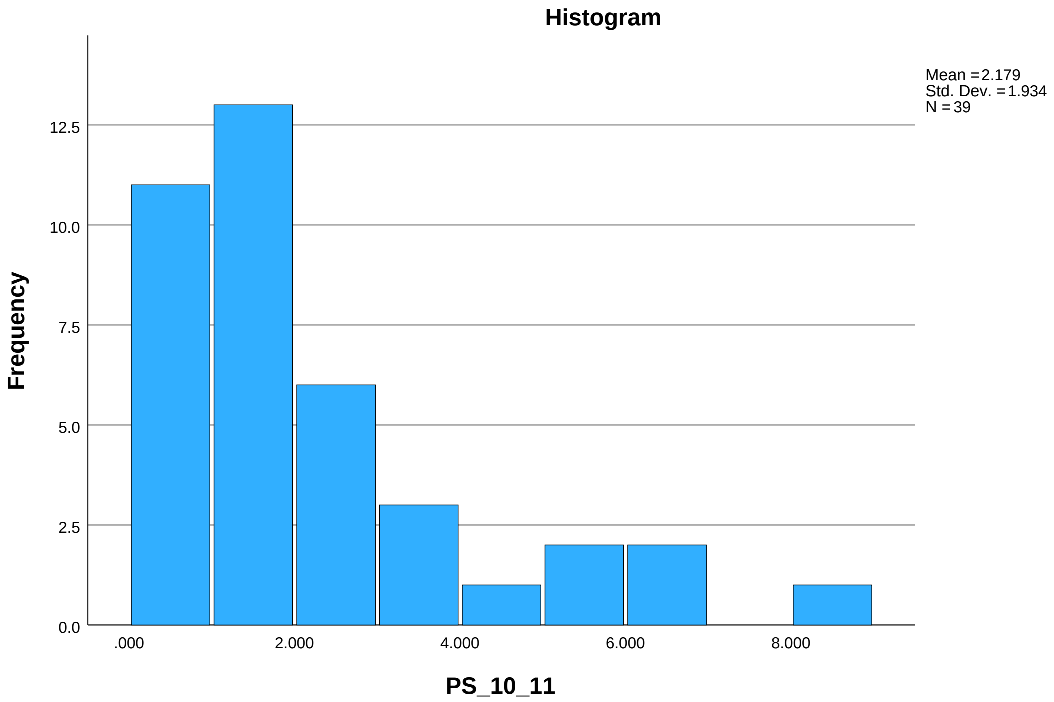


```
PS_10_11 Stem-and-Leaf Plot

 Frequency    Stem &  Leaf

     5.00        0 .  22344
     6.00        0 .  567778
     5.00        1 .  00122
     8.00        1 .  55667779
     4.00        2 .  0002
     2.00        2 .  67
     3.00        3 .  011
      .00        3 .
     1.00        4 .  1
     5.00 Extremes    (>=5.6)

 Stem width:      1.000
 Each leaf:       1 case(s)
```

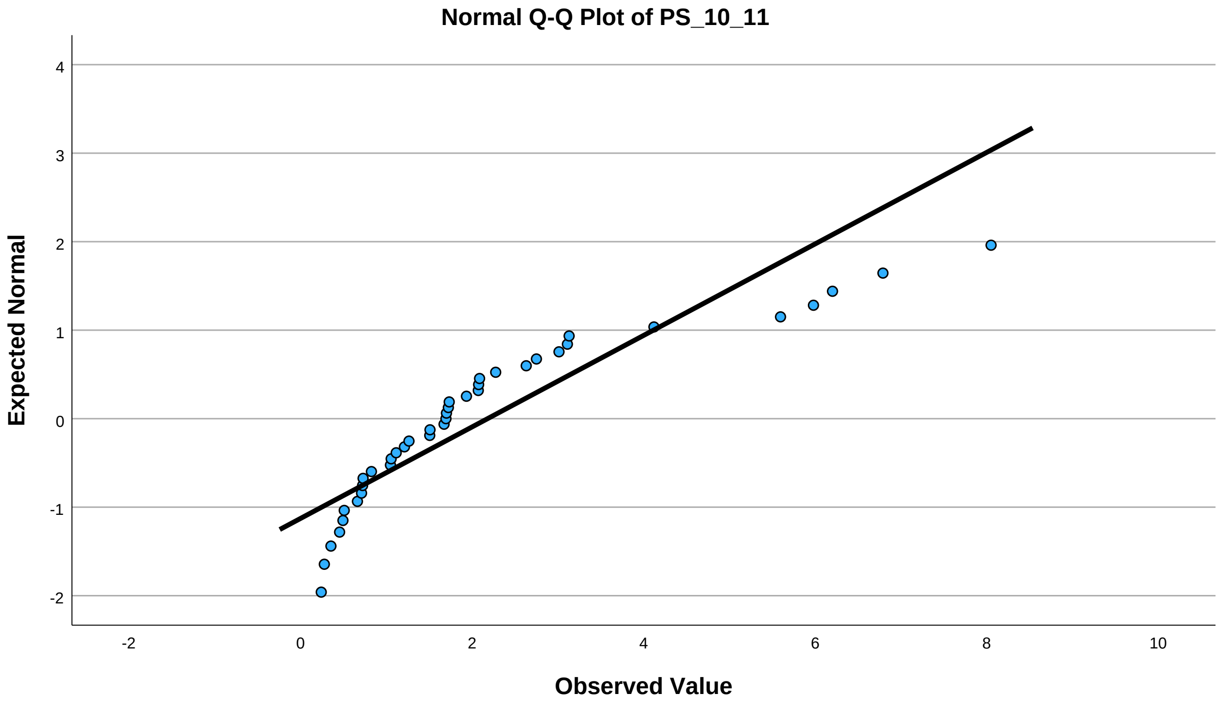

Normal Q-Q Plot of PS_10_11
Expected Normal
4
3
2
1
0
-1
-2
-2
0
2
4
6
8
10
Observed Value

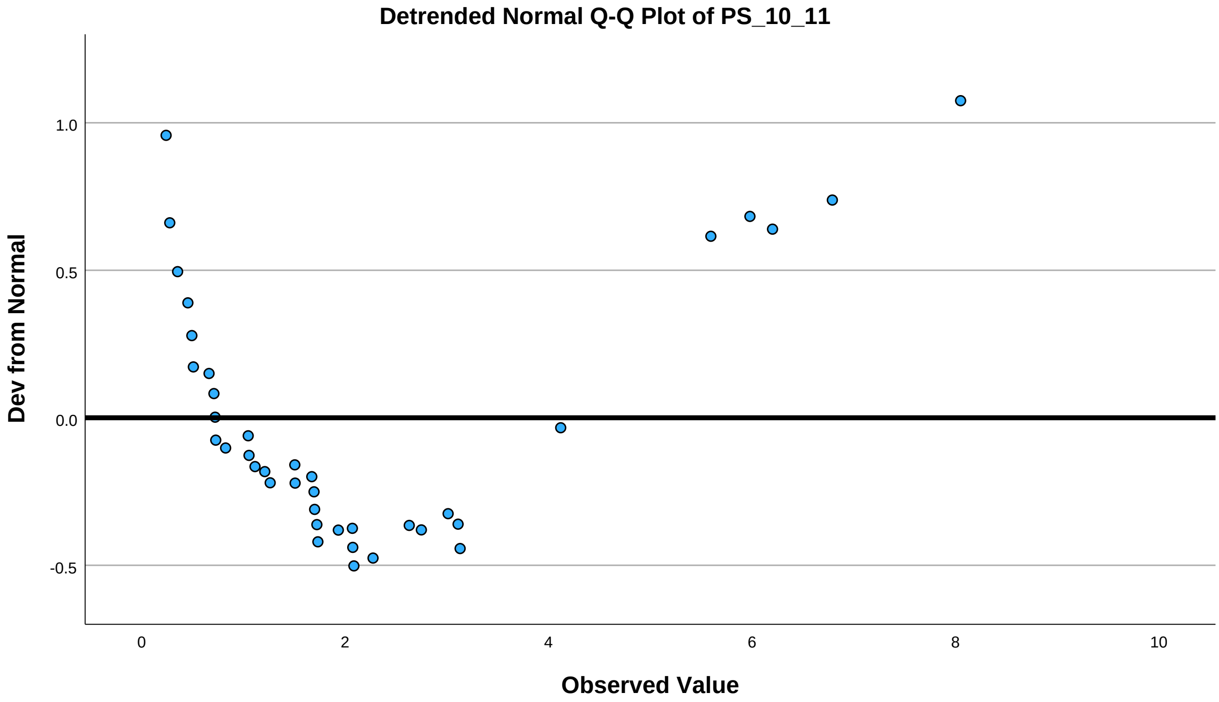

Detrended Normal Q-Q Plot of PS_10_11
Dev from Normal
1.0
0.5
0.0
-0.5
0
2
4
6
8
10
Observed Value

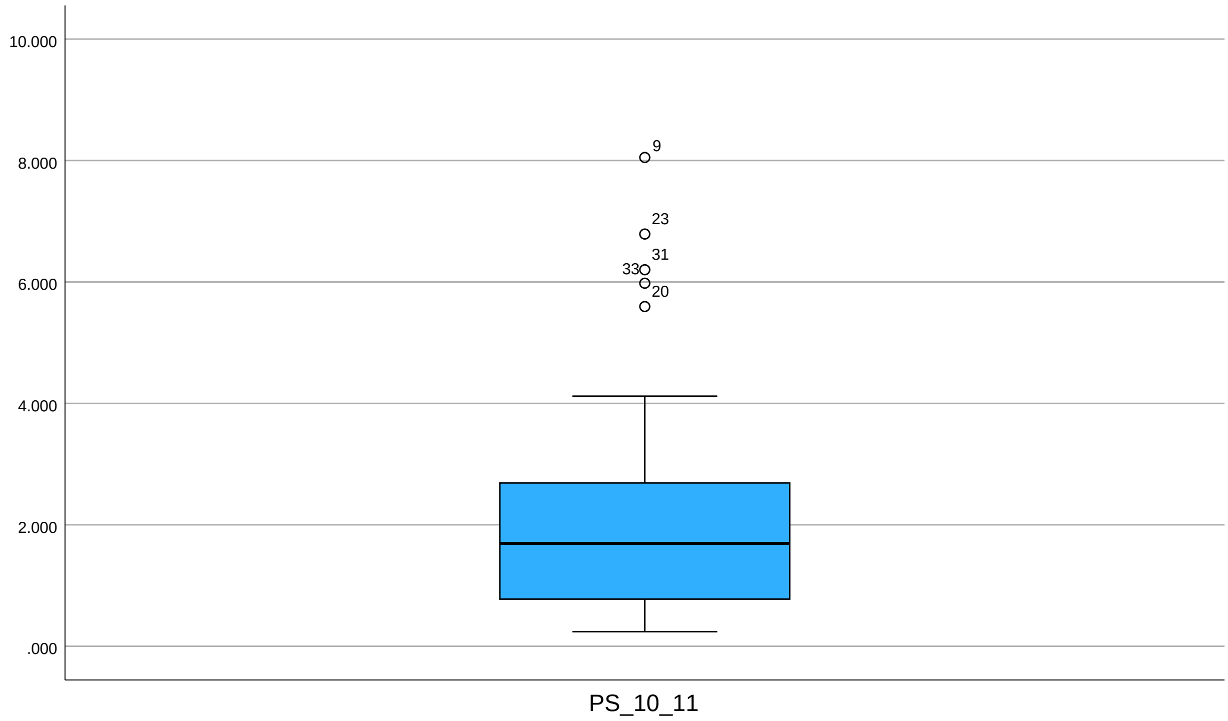

PS_11_12

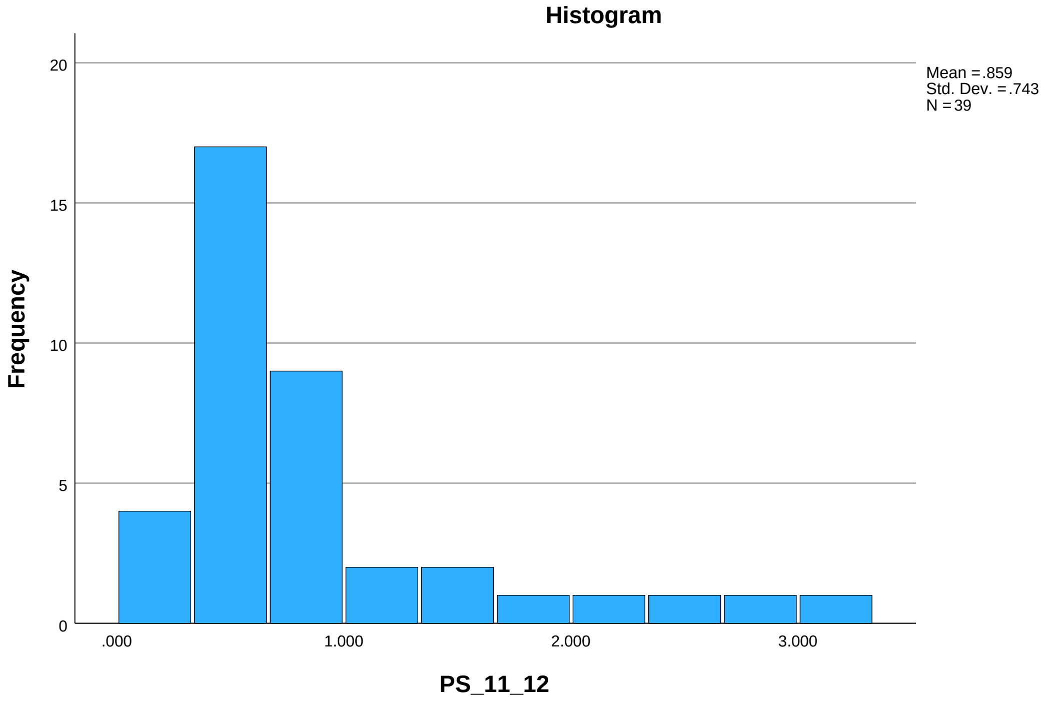

PS_11_12 Stem-and-Leaf Plot

Frequency   Stem & Leaf

```
   10.00        0 .  2222333333
   11.00        0 .  44444455555
    5.00        0 .  66677
    4.00        0 .  8889
    1.00        1 .  0
    1.00        1 .  2
    1.00        1 .  4
    1.00        1 .  6
     5.00 Extremes    (>=1.8)

 Stem width:      1.000
 Each leaf:        1 case(s)
```

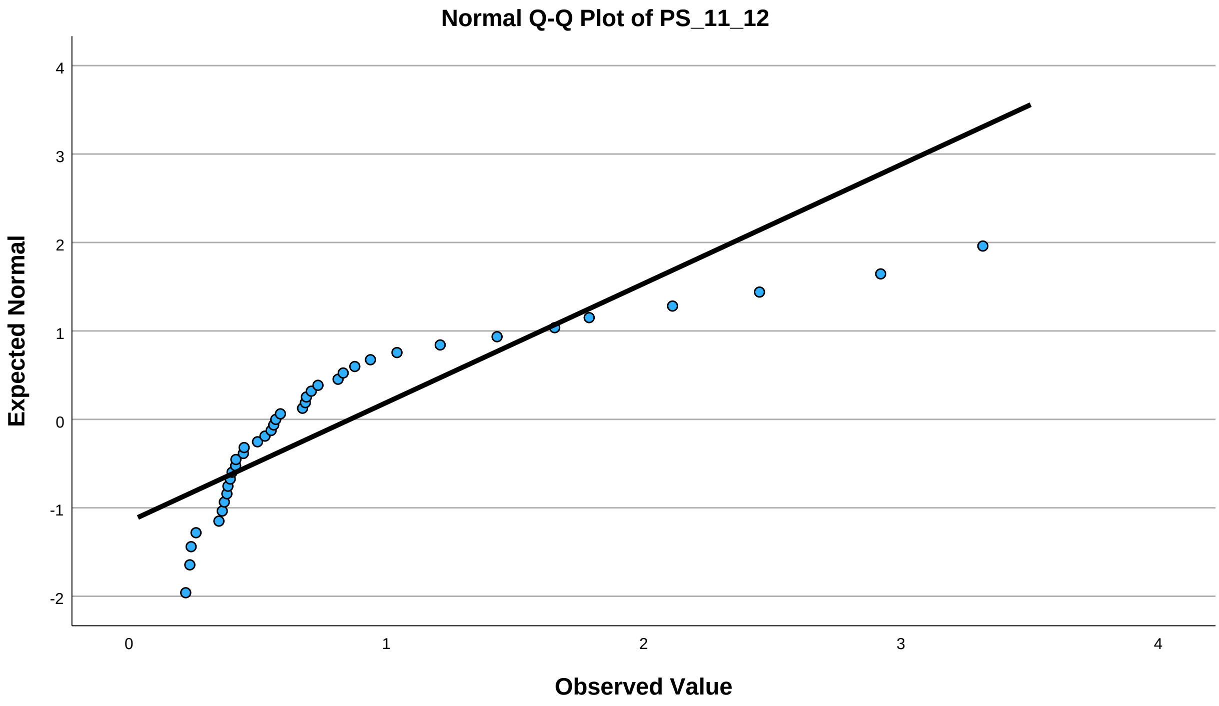

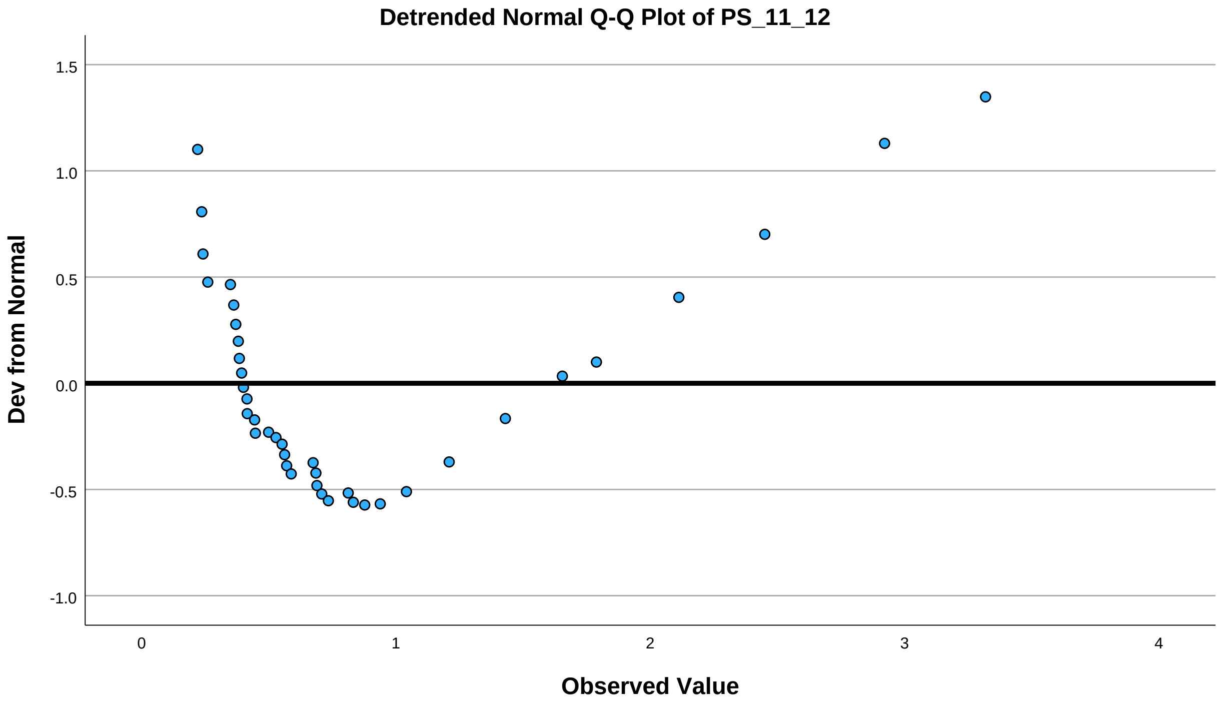
Detrended Normal Q-Q Plot of PS_11_12
Dev from Normal
1.5
1.0
0.5
0.0
-0.5
-1.0
0
1
2
3
4
Observed Value

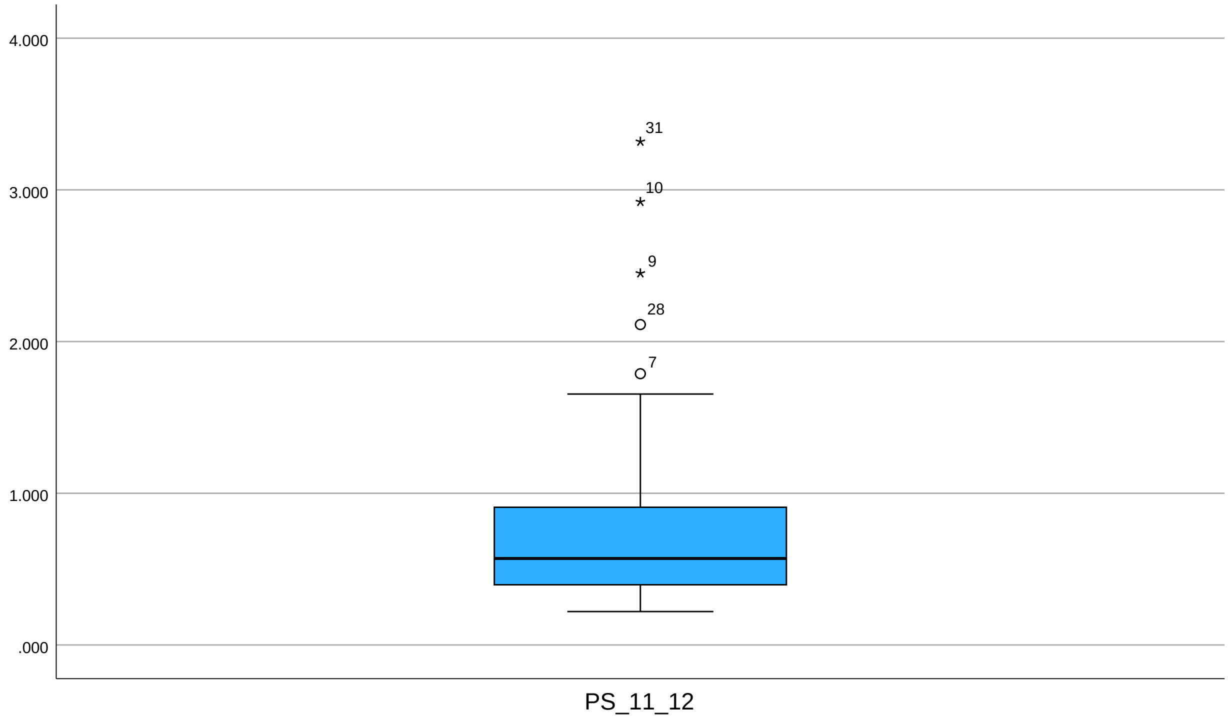
4.000
3.000
2.000
1.000
.000
31
10
9
28
7
PS_11_12

PS_12_13

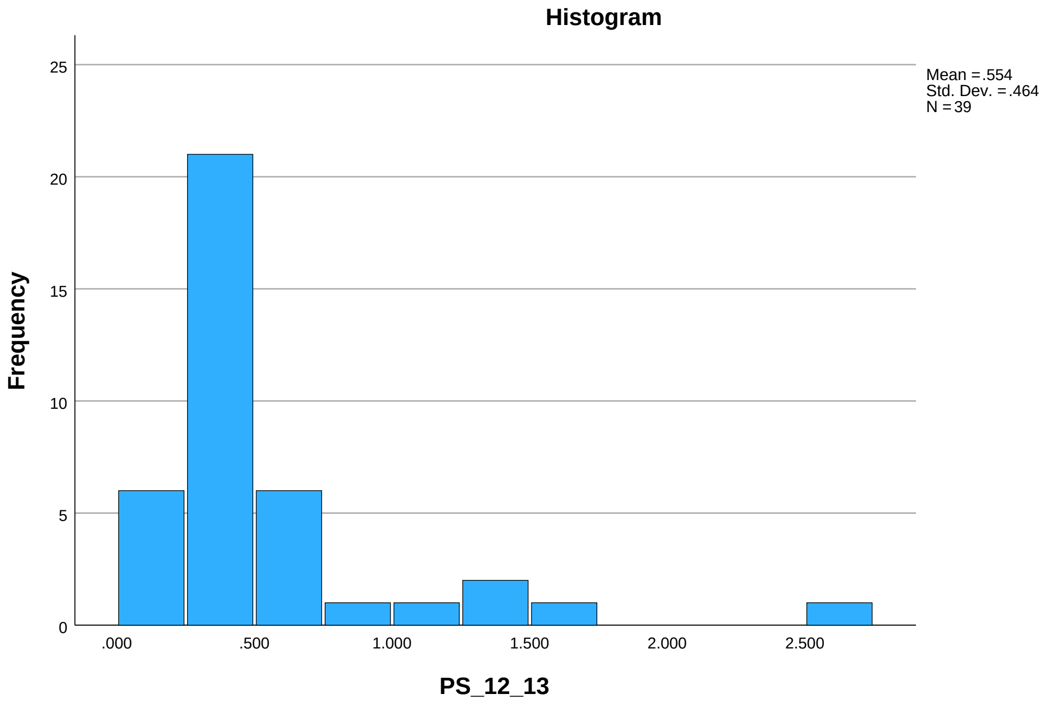


```
PS_12_13 Stem-and-Leaf Plot

 Frequency    Stem &  Leaf

     4.00        1 .  5889
     2.00        2 .  02
     1.00        2 .  8
     5.00        3 .  00344
     7.00        3 .  5588899
     7.00        4 .  0023334
     1.00        4 .  6
     2.00        5 .  23
     1.00        5 .  6
     1.00        6 .  2
     2.00        6 .  69
     6.00 Extremes    (>=.95)

 Stem width:      .100
 Each leaf:       1 case(s)
```

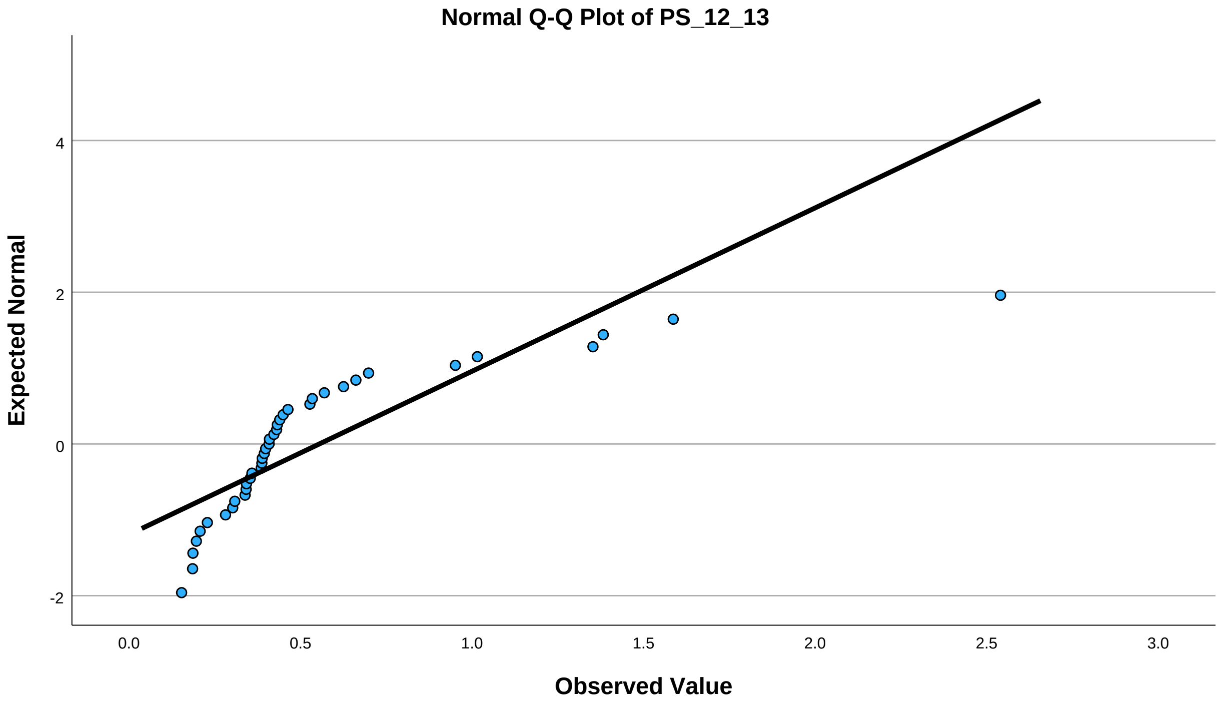
Normal Q-Q Plot of PS_12_13
Expected Normal
4
2
0
-2
0.0
0.5
1.0
1.5
2.0
2.5
3.0
Observed Value

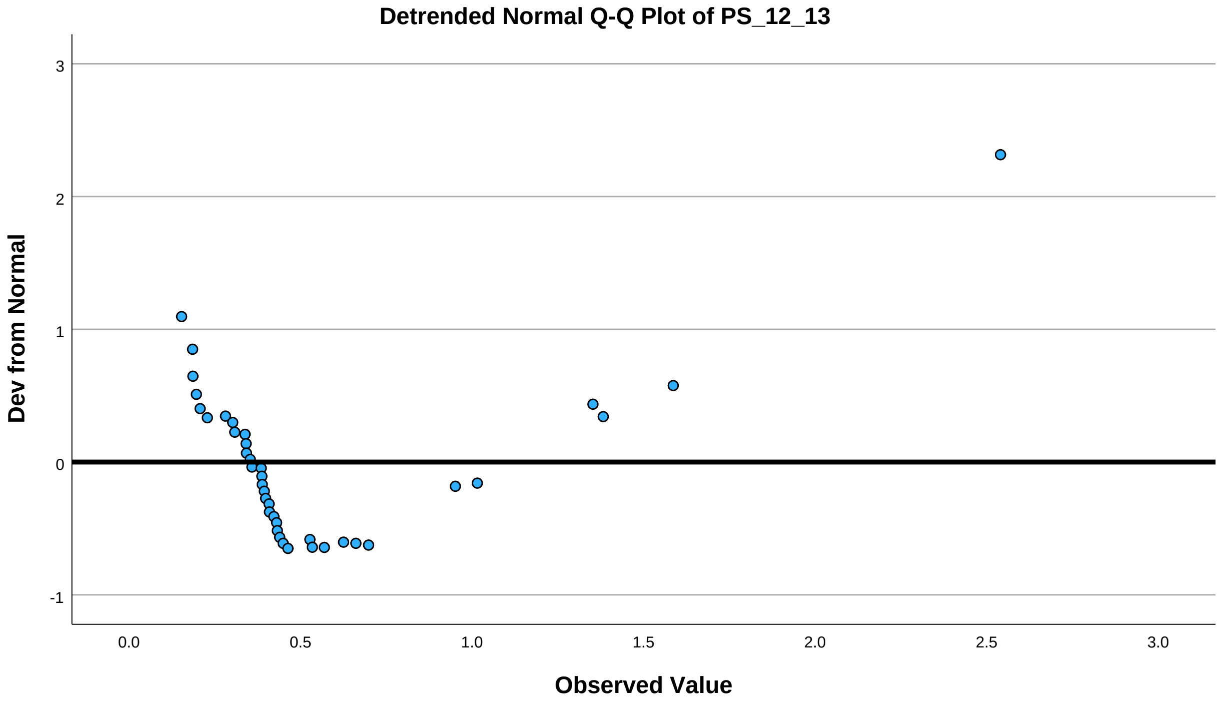
Detrended Normal Q-Q Plot of PS_12_13
Dev from Normal
3
2
1
0
-1
0.0
0.5
1.0
1.5
2.0
2.5
3.0
Observed Value

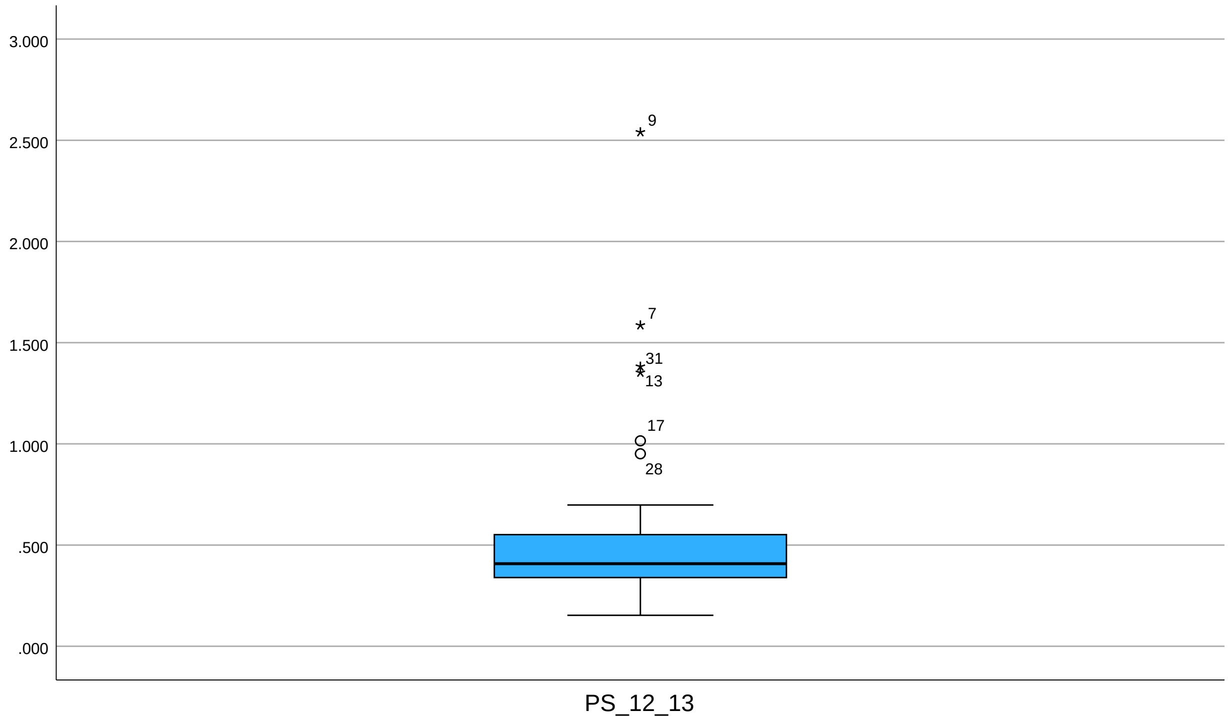

PS_13_14

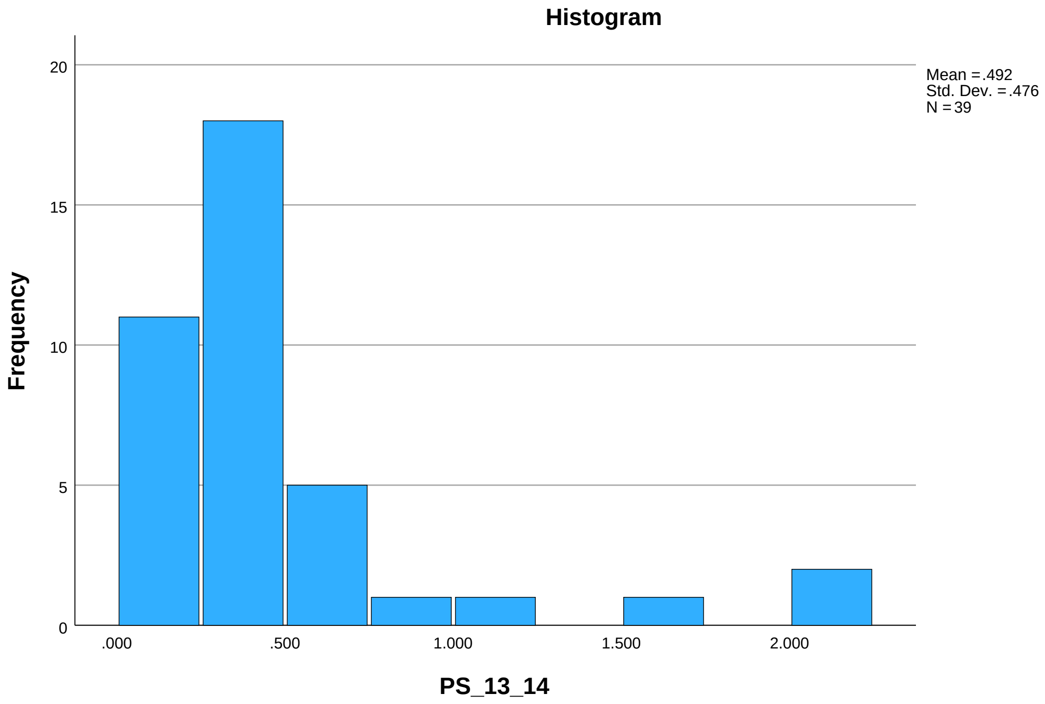

PS_13_14 Stem-and-Leaf Plot

Frequency   Stem & Leaf

```
 5.00      1 . 22479
12.00       2 . 034444557999
 9.00       3 . 233566678
 3.00      4 . 199
 3.00      5 . 347
 2.00      6 . 26
  .00     7 .
 1.00      8 . 7
 4.00 Extremes    (>=1.12)

Stem width:      .100
Each leaf:       1 case(s)
```

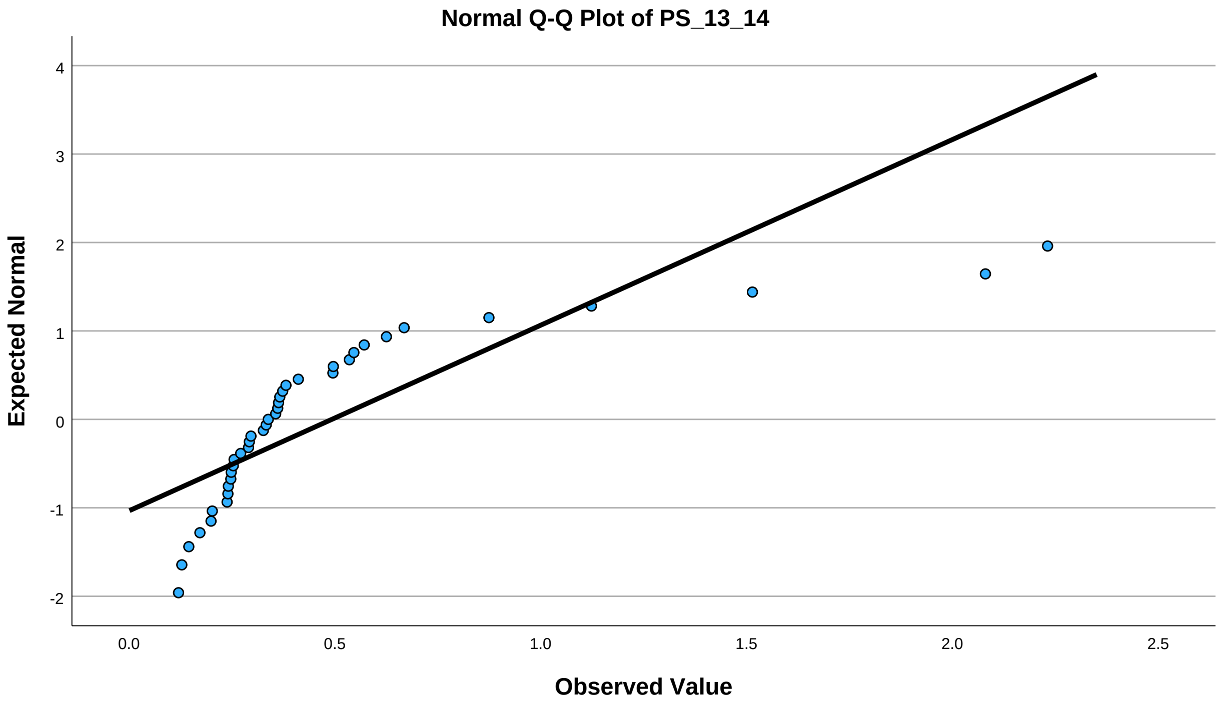

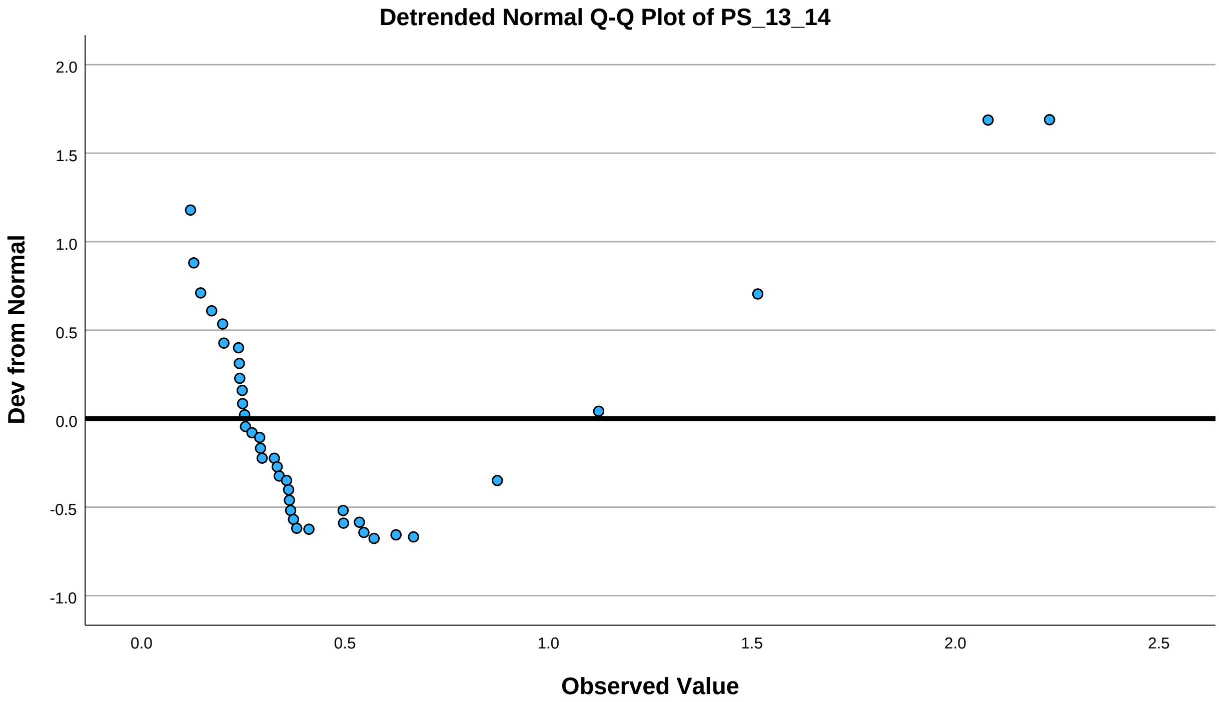
Detrended Normal Q-Q Plot of PS_13_14
Dev from Normal
2.0
1.5
1.0
0.5
0.0
-0.5
-1.0
0.0
0.5
1.0
1.5
2.0
2.5
Observed Value

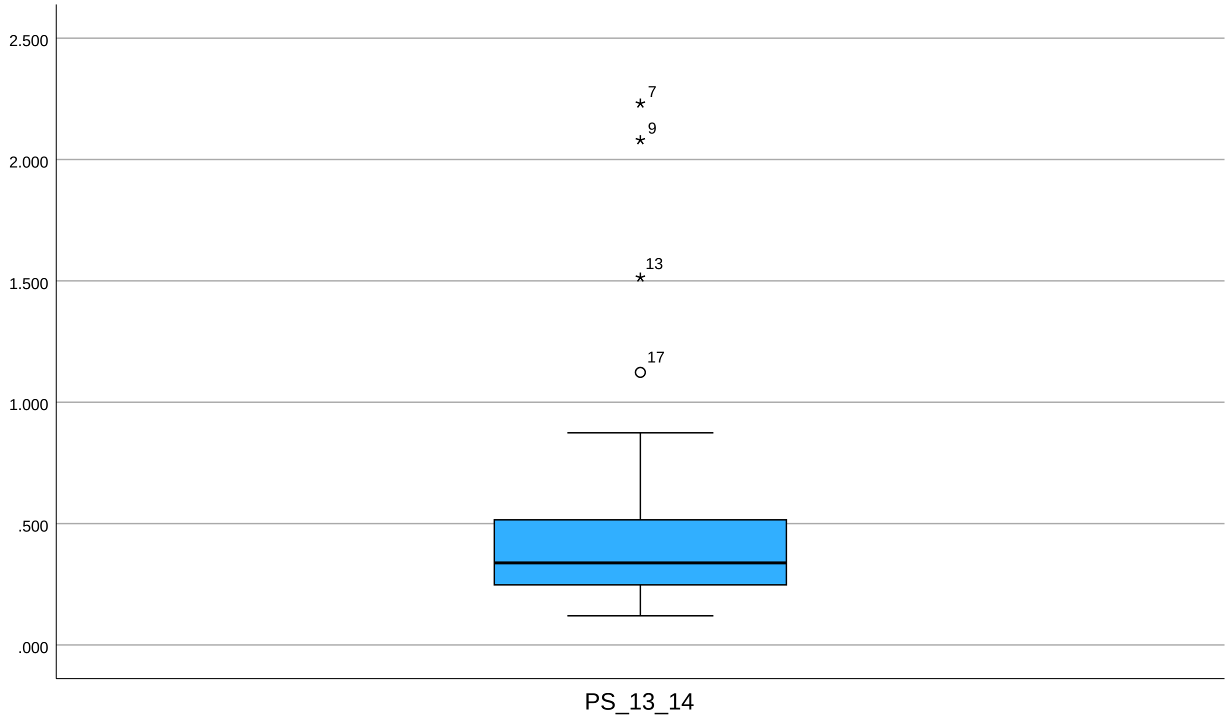
2.500
2.000
1.500
1.000
.500
.000
7
9
13
17
PS_13_14

PS_14_15

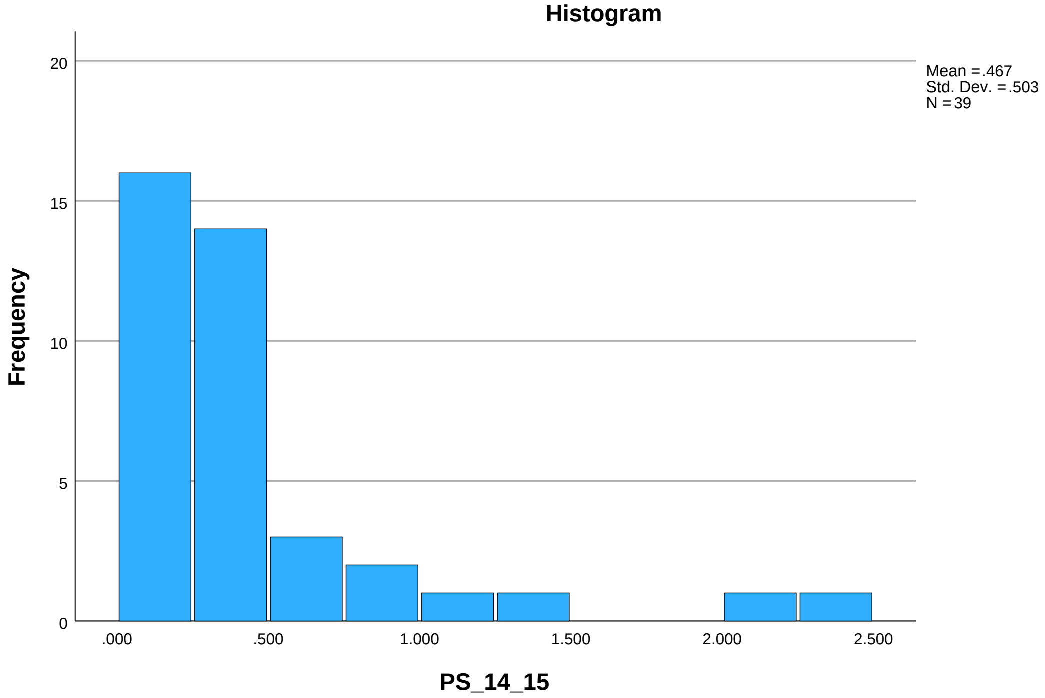

PS_14_15 Stem-and-Leaf Plot

```
 Frequency    Stem &  Leaf

     6.00        1 .  123679
    14.00        2 .  00011222335568
     8.00        3 .  01133559
     2.00        4 .  46
     1.00        5 .  6
     1.00        6 .  4
     2.00        7 .  06
     1.00        8 .  0
     4.00 Extremes    (>=1.01)

 Stem width:      .100
 Each leaf:       1 case(s)
```

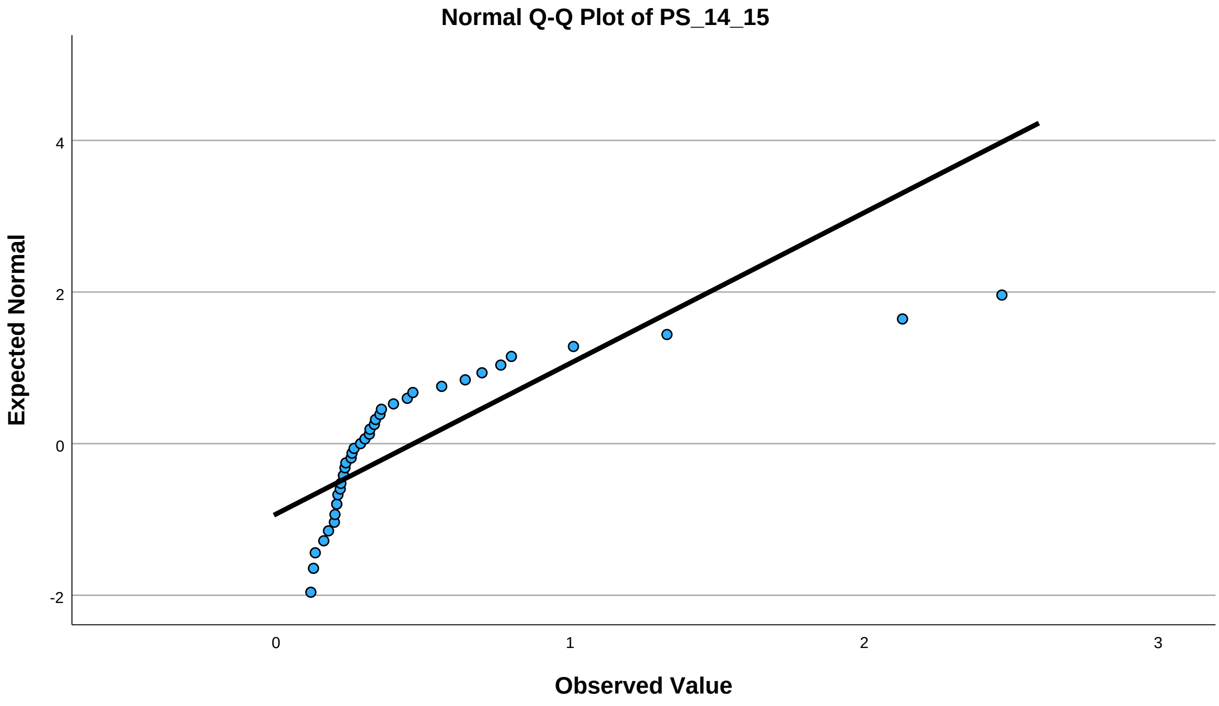
Normal Q-Q Plot of PS_14_15
Expected Normal
4
2
0
-2
0
1
2
3
Observed Value

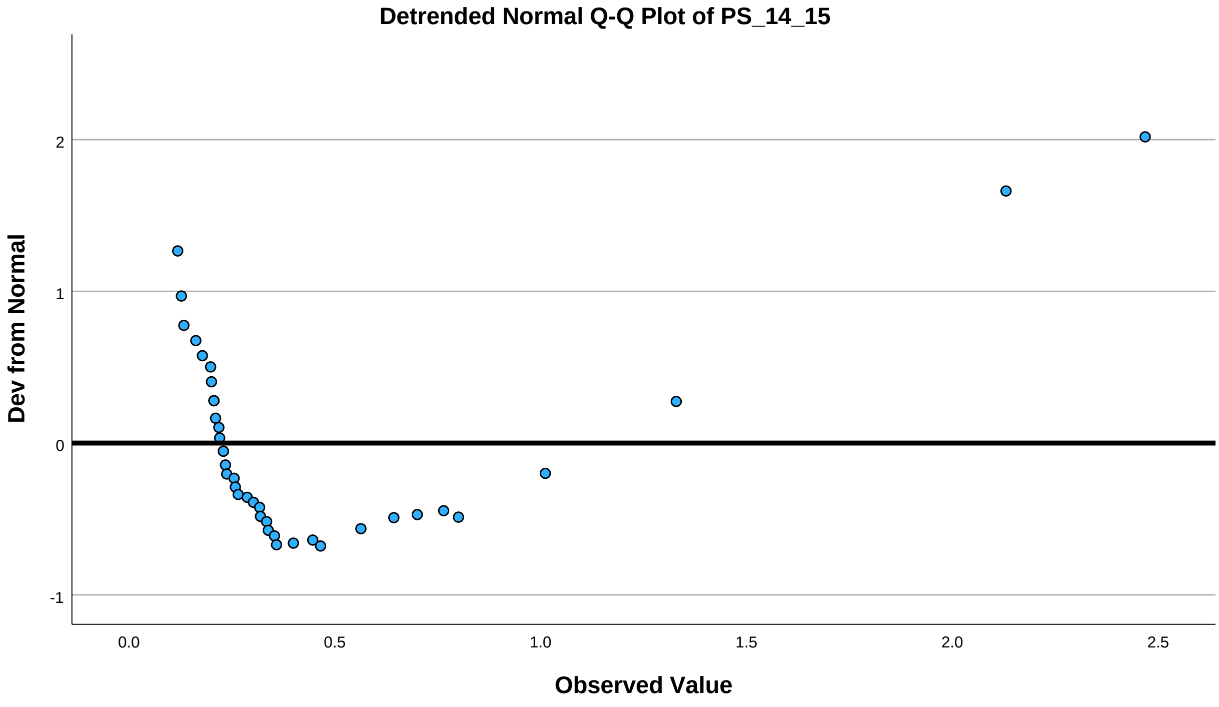
Detrended Normal Q-Q Plot of PS_14_15
Dev from Normal
2
1
0
-1
0.0
0.5
1.0
1.5
2.0
2.5
Observed Value

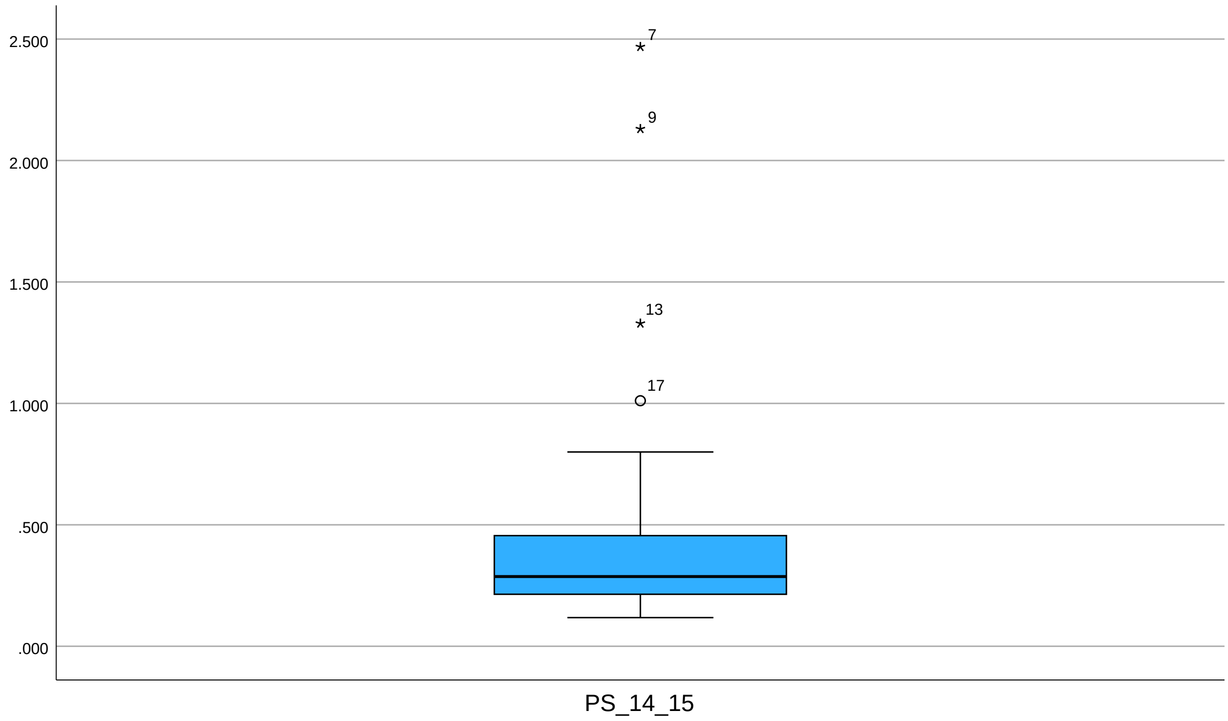

PS_15_16

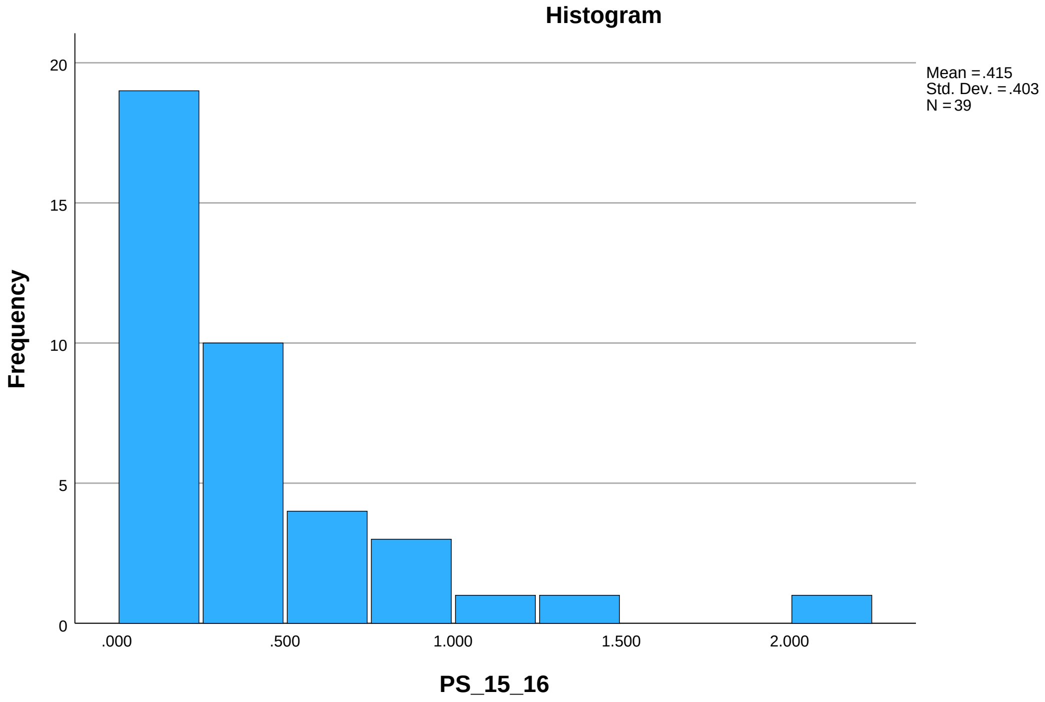

PS_15_16 Stem-and-Leaf Plot

Frequency   Stem & Leaf

```
 1.00      0 . 9
10.00      1 . 0168888899
11.00      2 . 00012234578
 6.00      3 . 000147
 1.00      4 . 7
 3.00      5 . 028
 1.00      6 . 3
  .00      7 .
 1.00      8 . 4
 2.00      9 . 00
 3.00 Extremes    (>=1.05)

Stem width:      .100
Each leaf:       1 case(s)
```

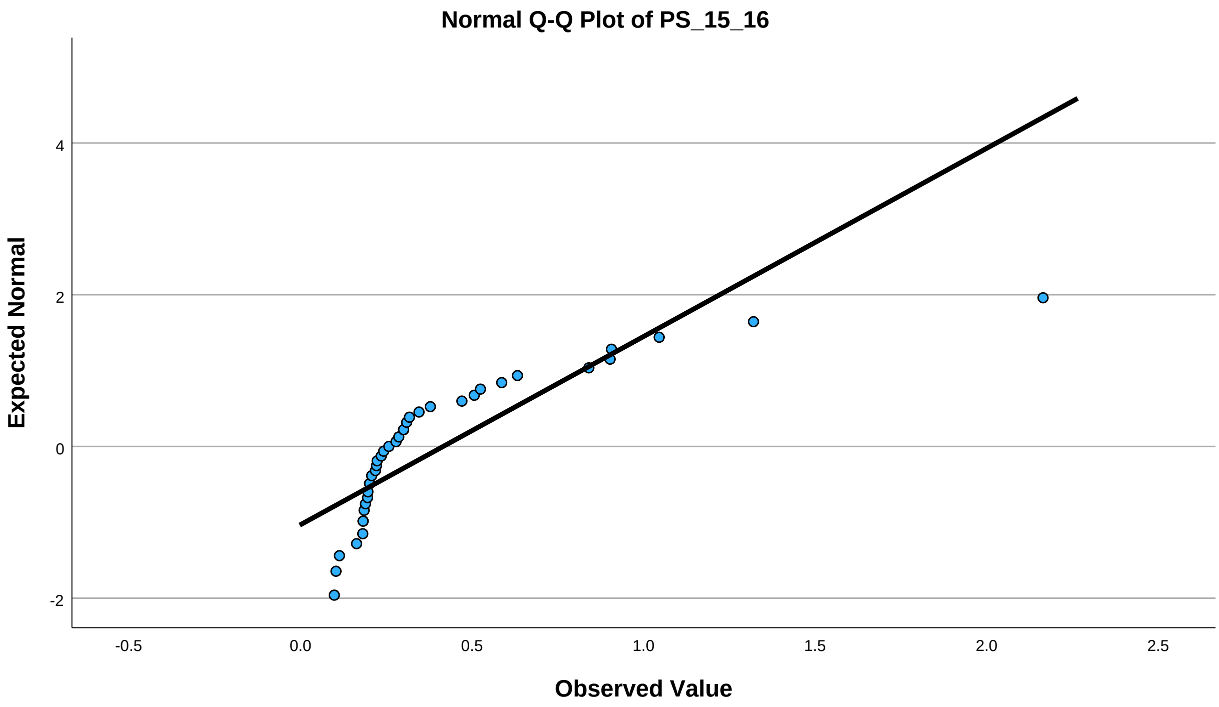

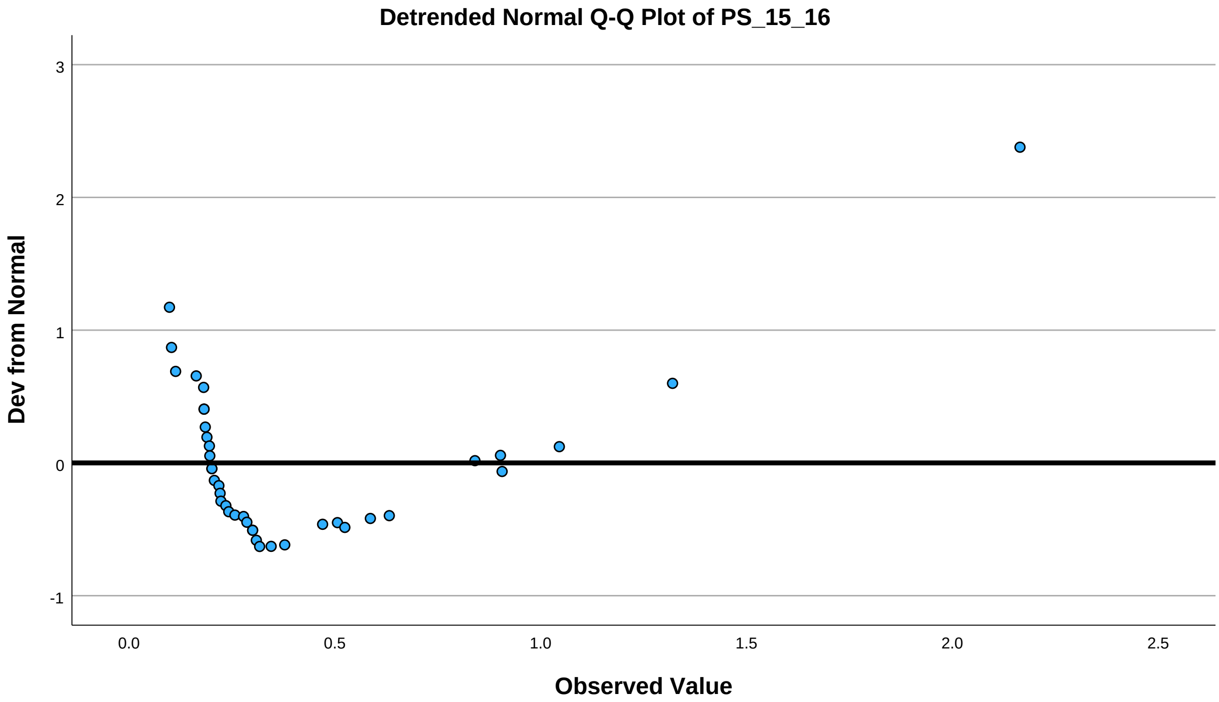
Detrended Normal Q-Q Plot of PS_15_16
Dev from Normal
3
2
1
0
-1
0.0
0.5
1.0
1.5
2.0
2.5
Observed Value

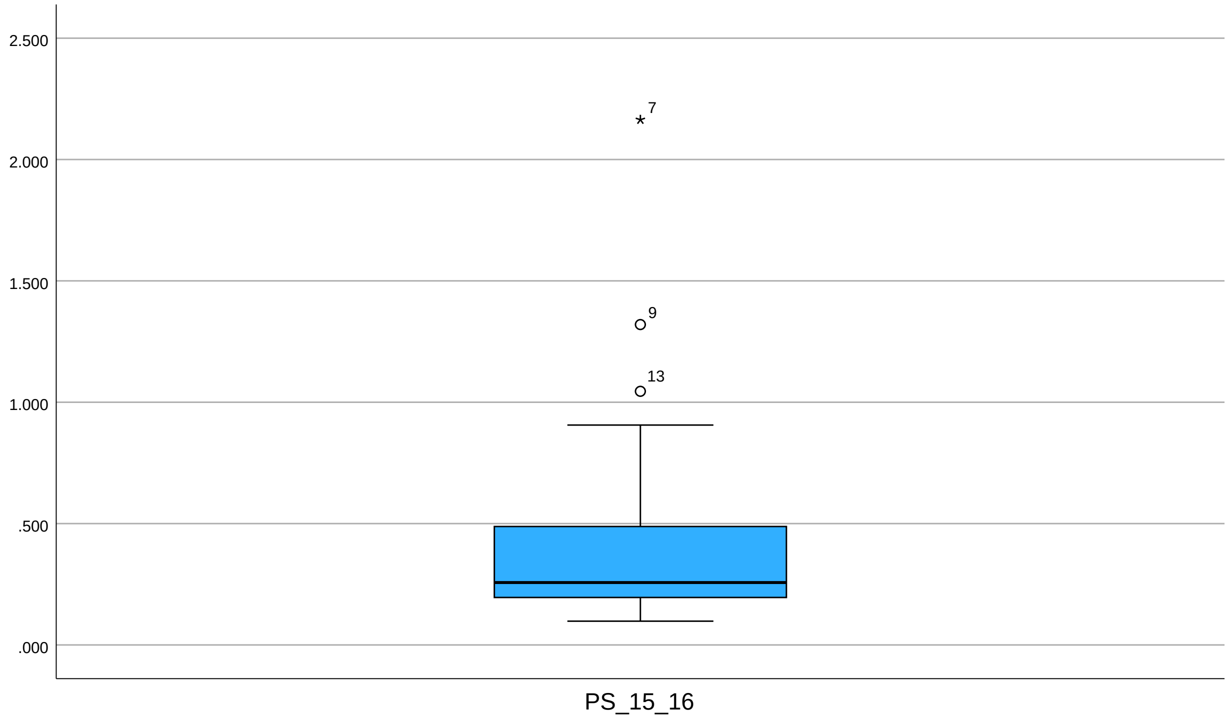
2.500
2.000
1.500
1.000
.500
.000
7
9
13
PS_15_16

PS_16_17

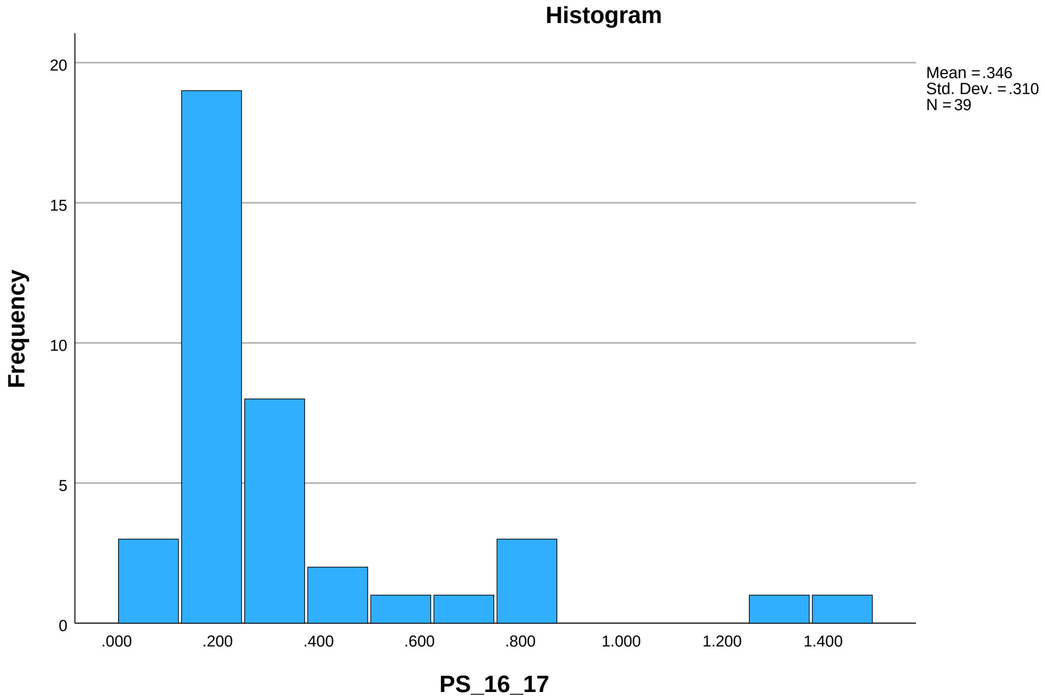


```
PS_16_17 Stem-and-Leaf Plot

 Frequency    Stem &  Leaf

     2.00        0 .  99
     2.00        1 .  14
    11.00        1 .  56667777789
     7.00        2 .  0111234
     5.00        2 .  55688
     2.00        3 .  02
     1.00        3 .  5
     2.00        4 .  14
      .00        4 .
     1.00        5 .  3
     6.00 Extremes    (>=.66)

 Stem width:      .100
 Each leaf:       1 case(s)
```

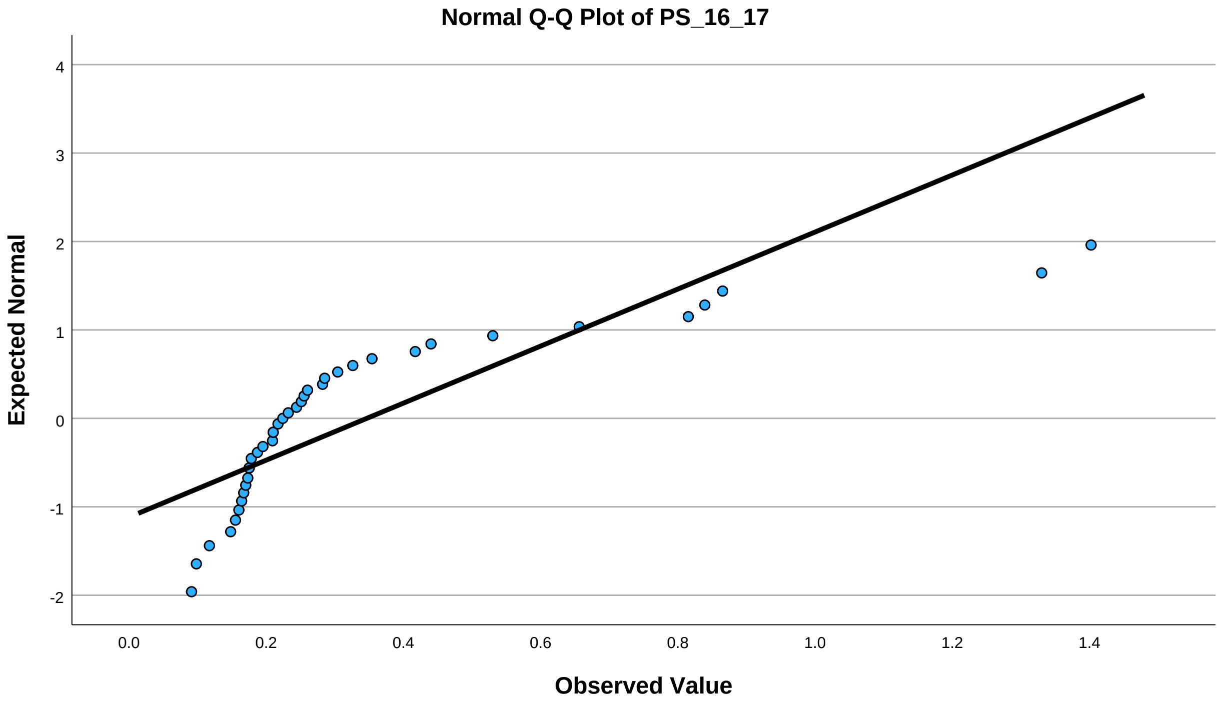

Normal Q-Q Plot of PS_16_17
Expected Normal
4
3
2
1
0
-1
-2
0.0
0.2
0.4
0.6
0.8
1.0
1.2
1.4
Observed Value

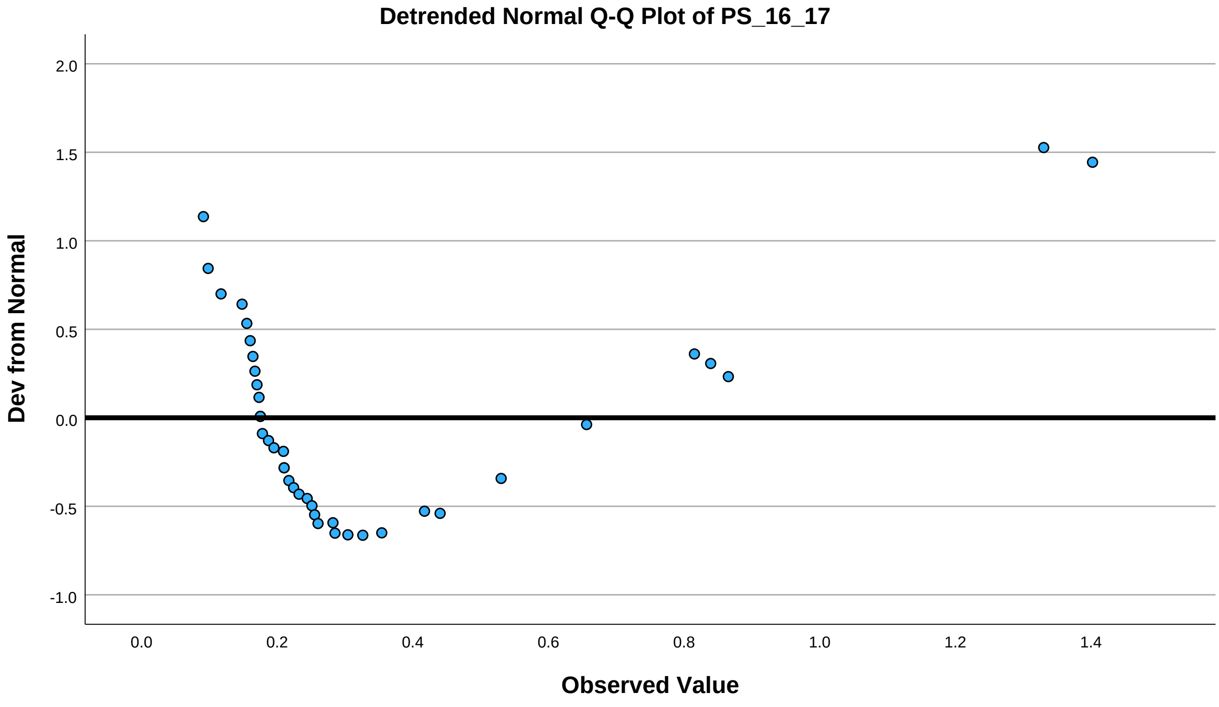

Detrended Normal Q-Q Plot of PS_16_17
Dev from Normal
2.0
1.5
1.0
0.5
0.0
-0.5
-1.0
0.0
0.2
0.4
0.6
0.8
1.0
1.2
1.4
Observed Value

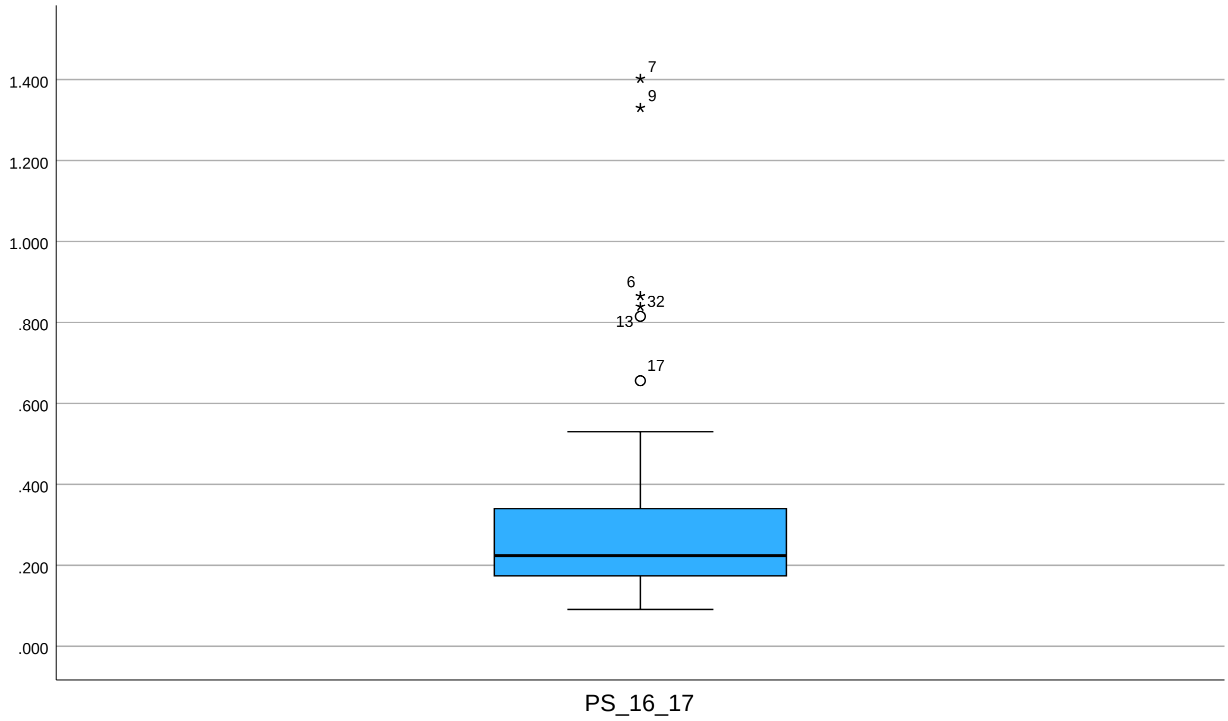

PS_17_18

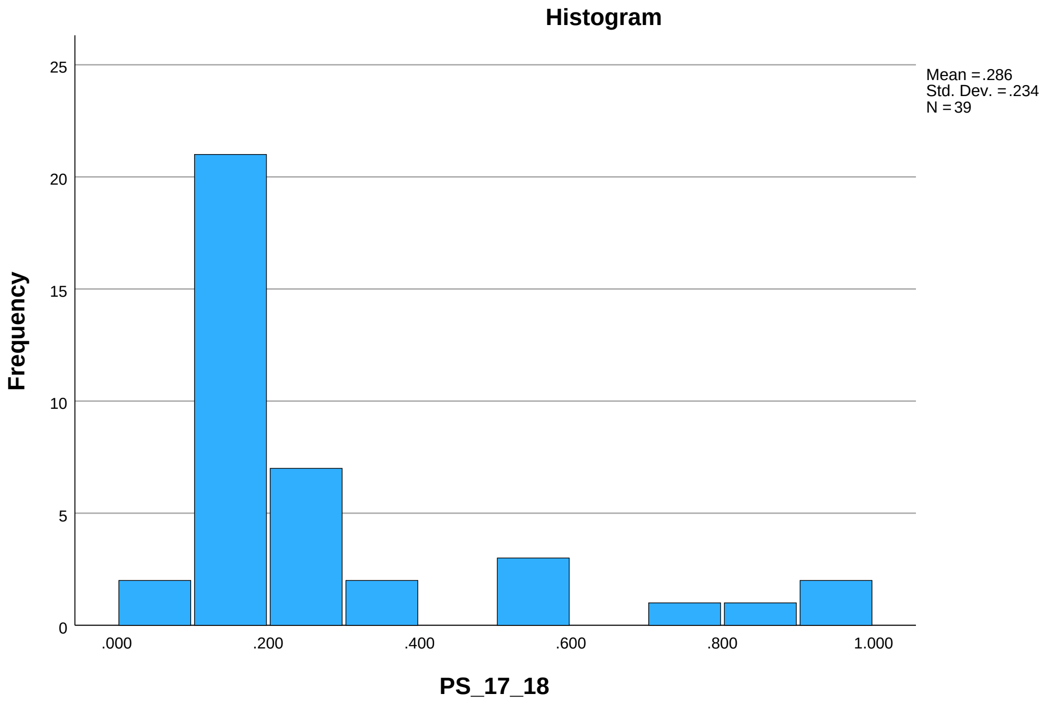


```
PS_17_18 Stem-and-Leaf Plot

 Frequency    Stem &  Leaf
```

```
  2.00      0 .  89
   7.00      1 .  0034444
  14.00       1 .  55555667779999
   4.00      2 .  1344
   3.00      2 .  678
    .00     3 .
  2.00      3 .  78
   7.00 Extremes    (>=.52)

Stem width:      .100
Each leaf:       1 case(s)
```

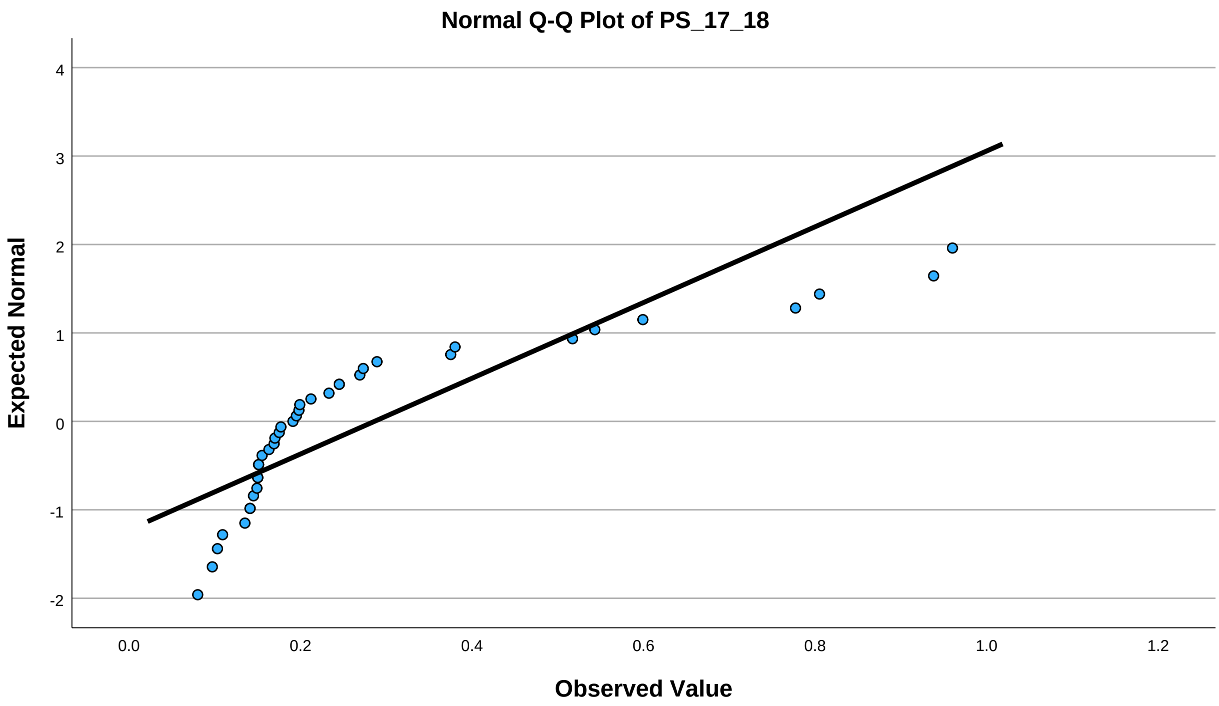

Detrended Normal Q-Q Plot of PS_17_18

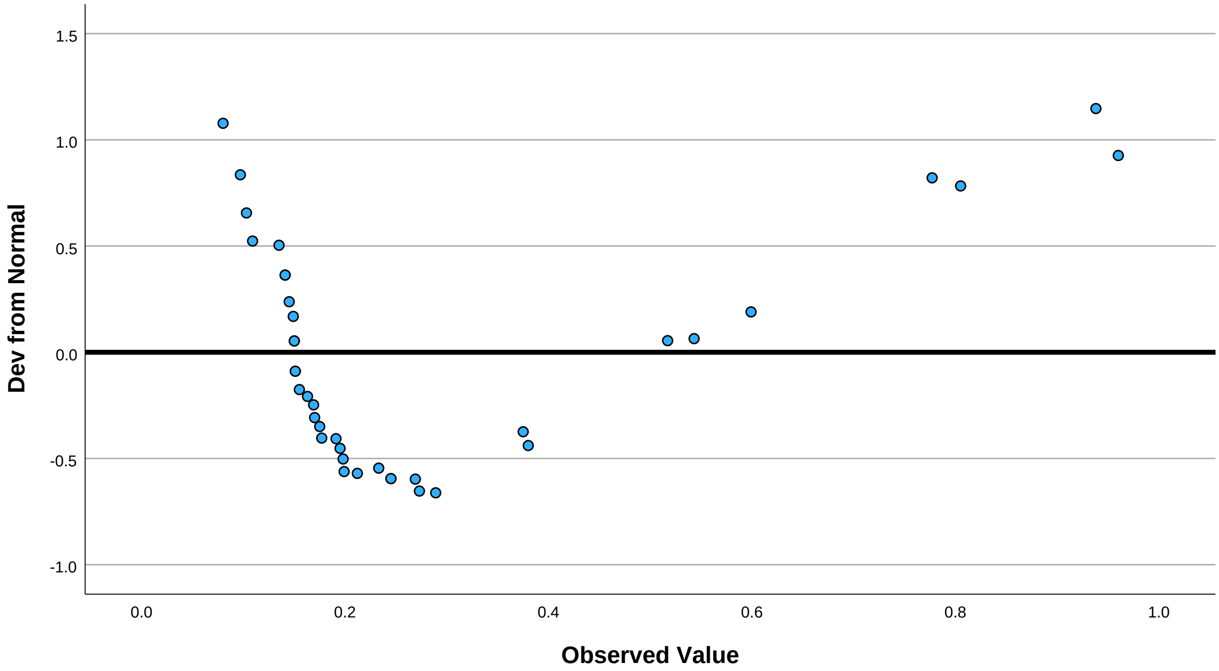

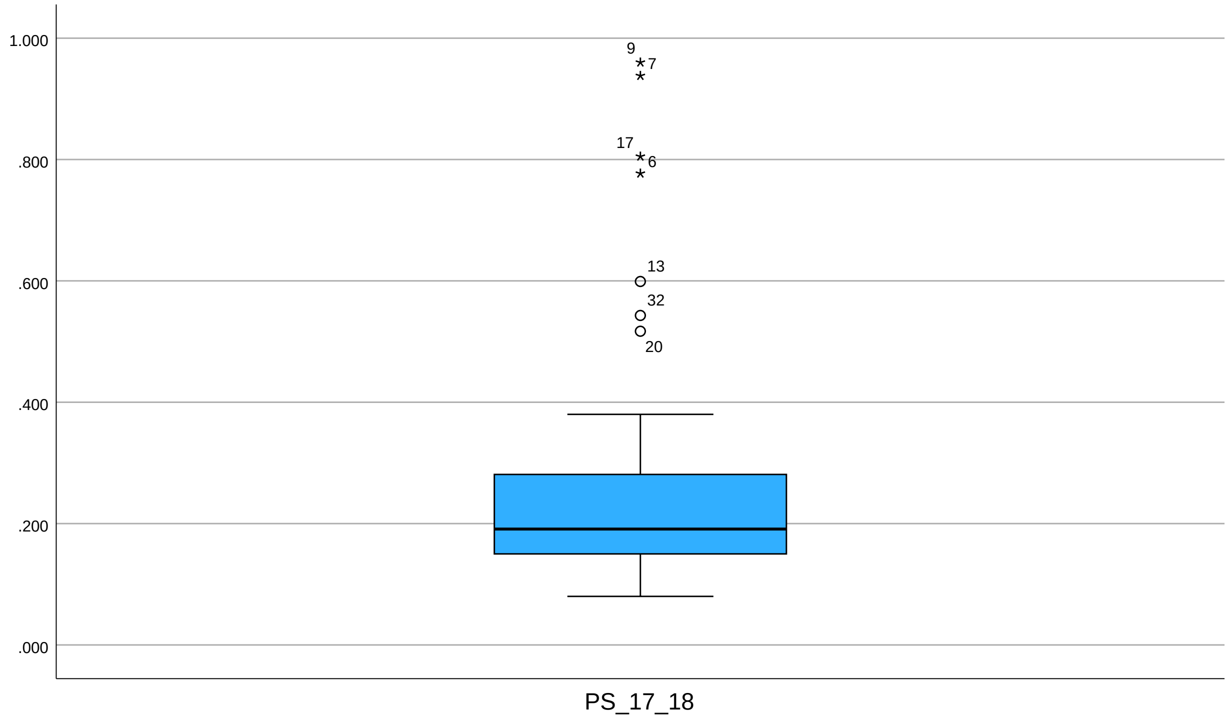

PS_18_19

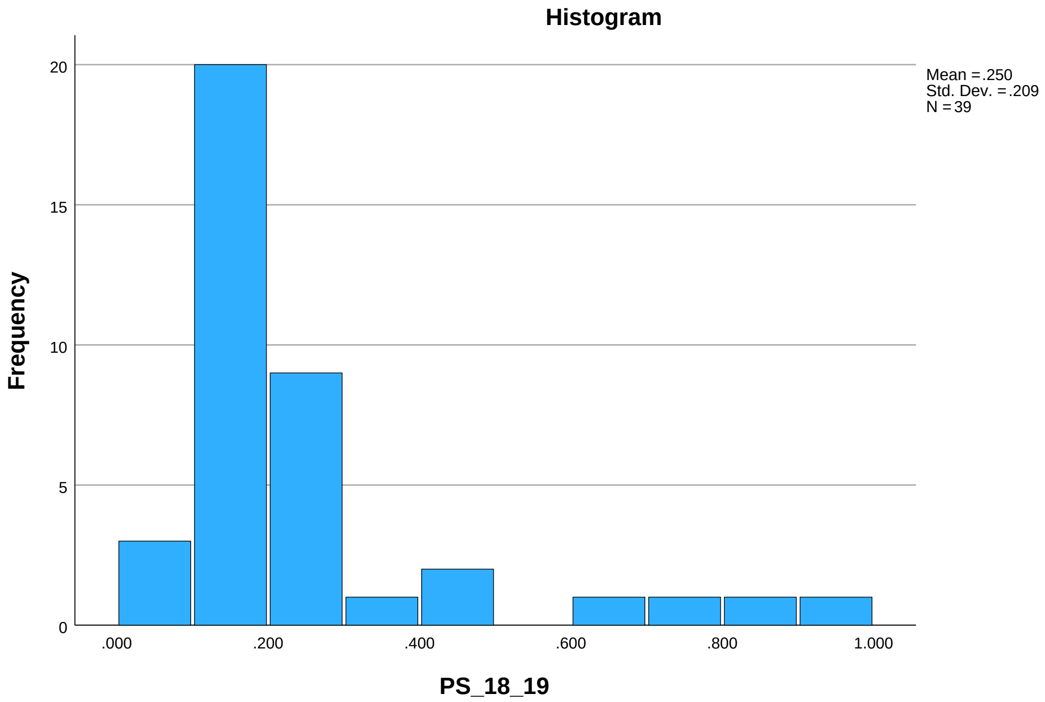

PS_18_19 Stem-and-Leaf Plot

```
 Frequency    Stem &  Leaf

     3.00        0 .  699
    11.00        1 .  01122223344
     9.00        1 .  566778889
     8.00        2 .  00112234
     1.00        2 .  9
     7.00 Extremes    (>=.39)

 Stem width:      .100
 Each leaf:       1 case(s)
```

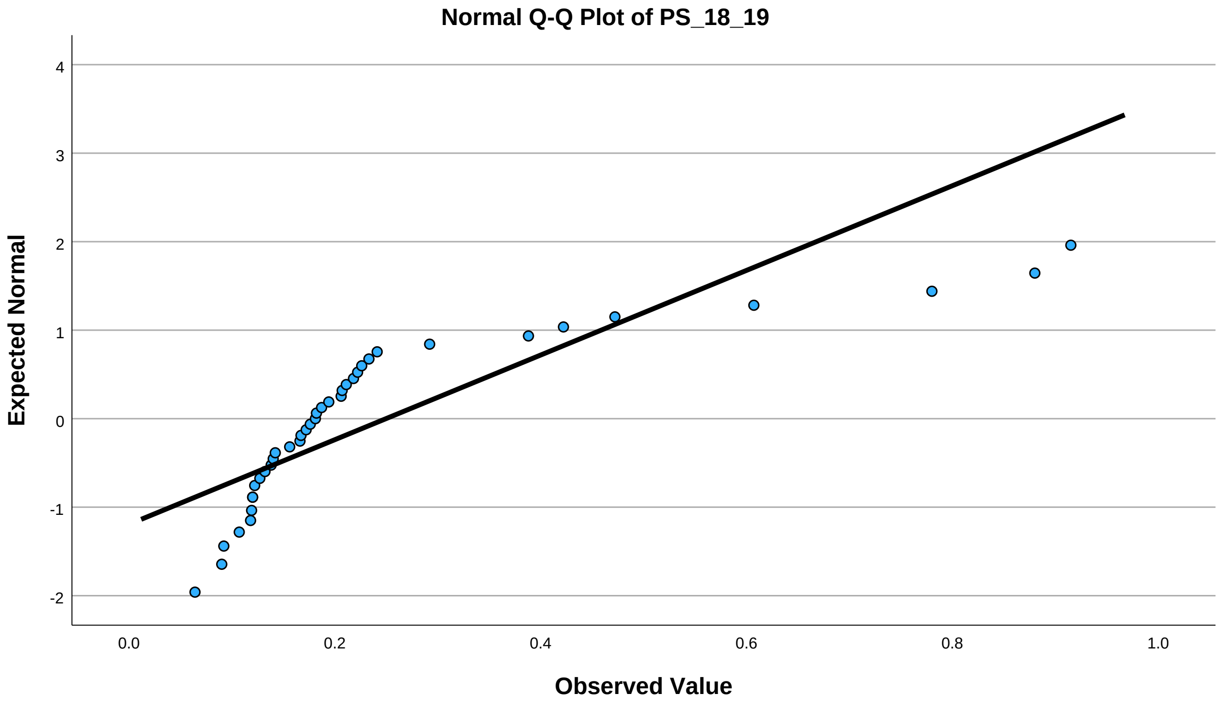

Normal Q-Q Plot of PS_18_19
Expected Normal
Observed Value
4
3
2
1
0
-1
-2
0.0
0.2
0.4
0.6
0.8
1.0

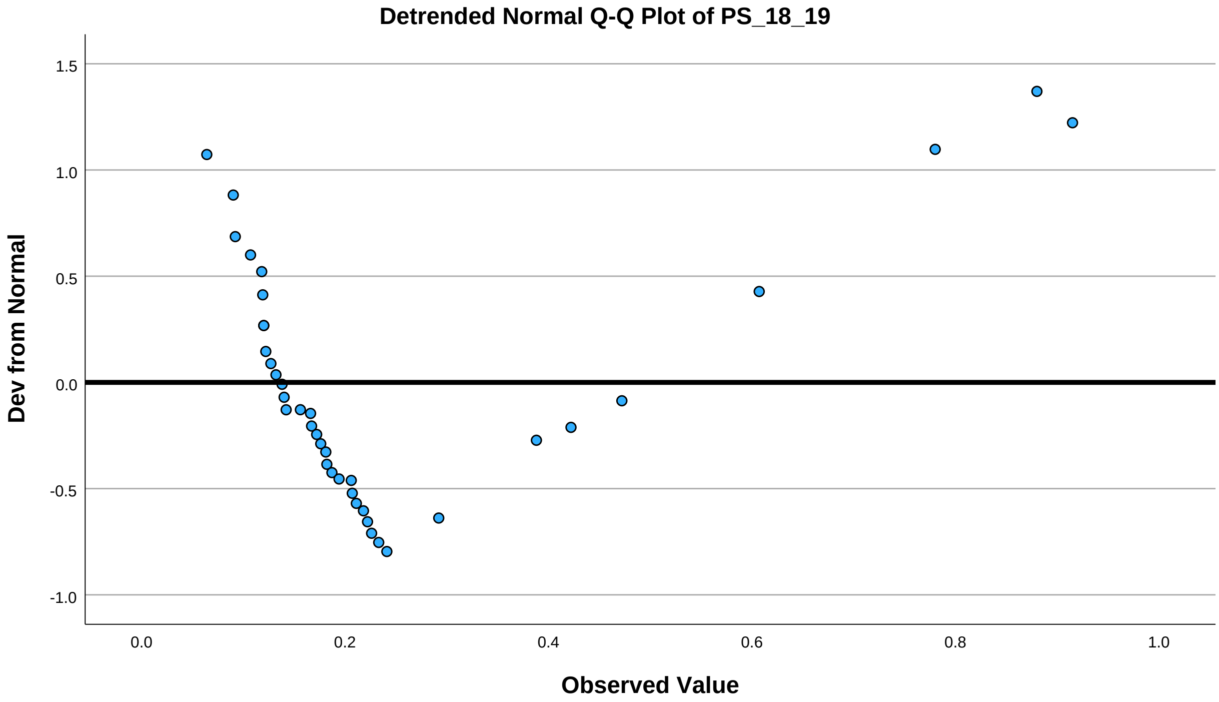

Detrended Normal Q-Q Plot of PS_18_19
Dev from Normal
Observed Value
1.5
1.0
0.5
0.0
-0.5
-1.0
0.0
0.2
0.4
0.6
0.8
1.0

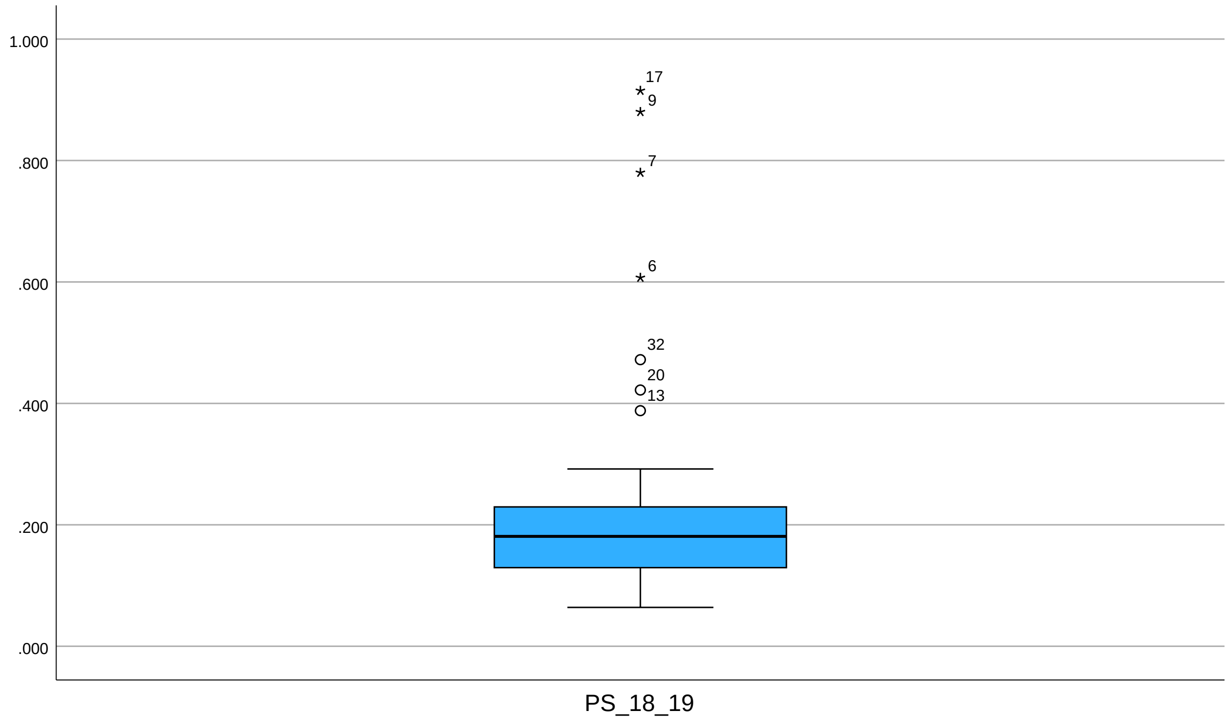

PS_19_20

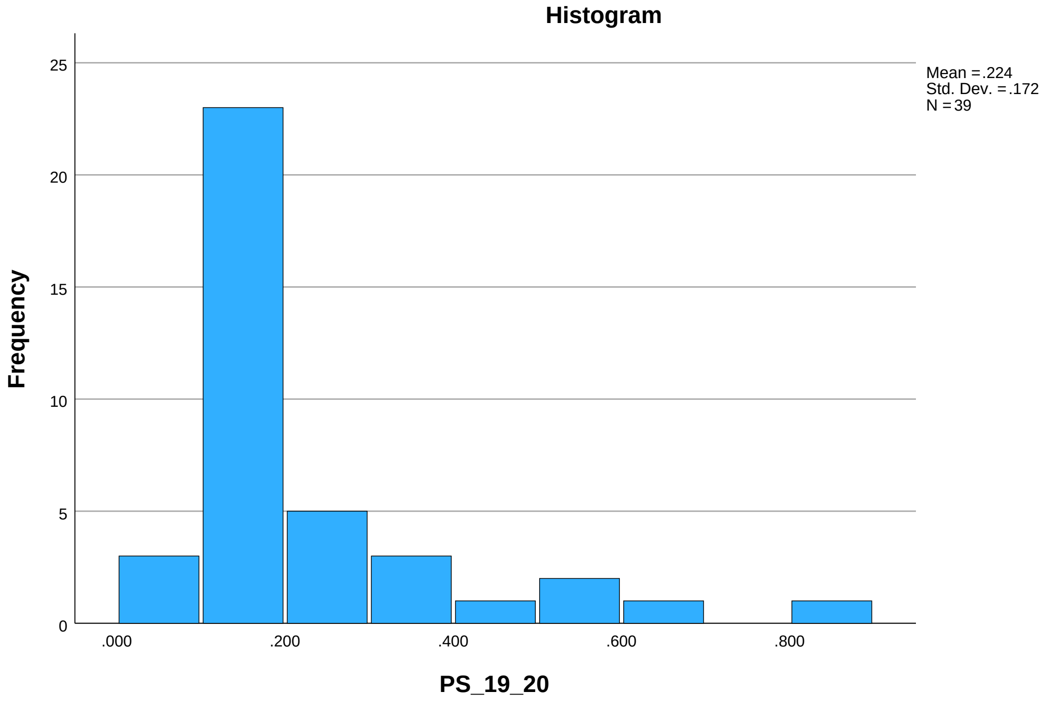


```
PS_19_20 Stem-and-Leaf Plot

 Frequency    Stem &  Leaf
```

```
 3.00      0 . 678
12.00      1 . 000012222444
11.00      1 . 55555666778
 3.00      2 . 033
 2.00      2 . 78
 3.00      3 . 223
  .00      3 .
 1.00      4 . 3
 4.00 Extremes    (>=.51)

Stem width:      .100
Each leaf:       1 case(s)
```

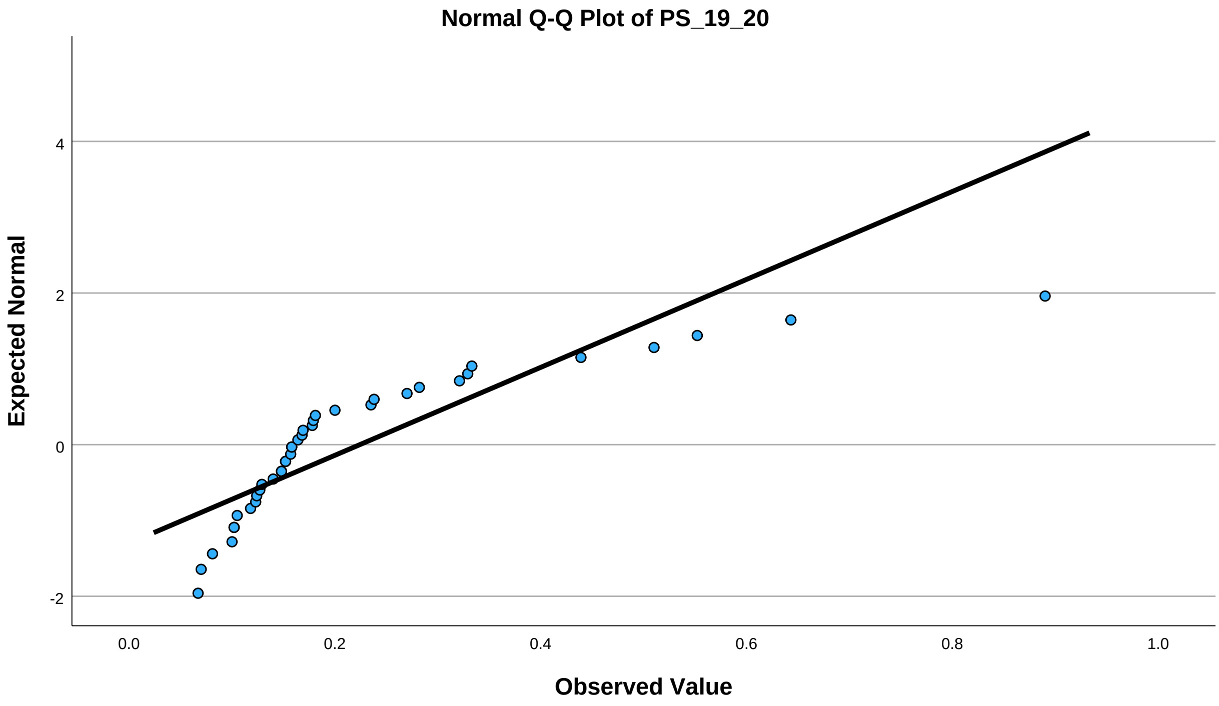

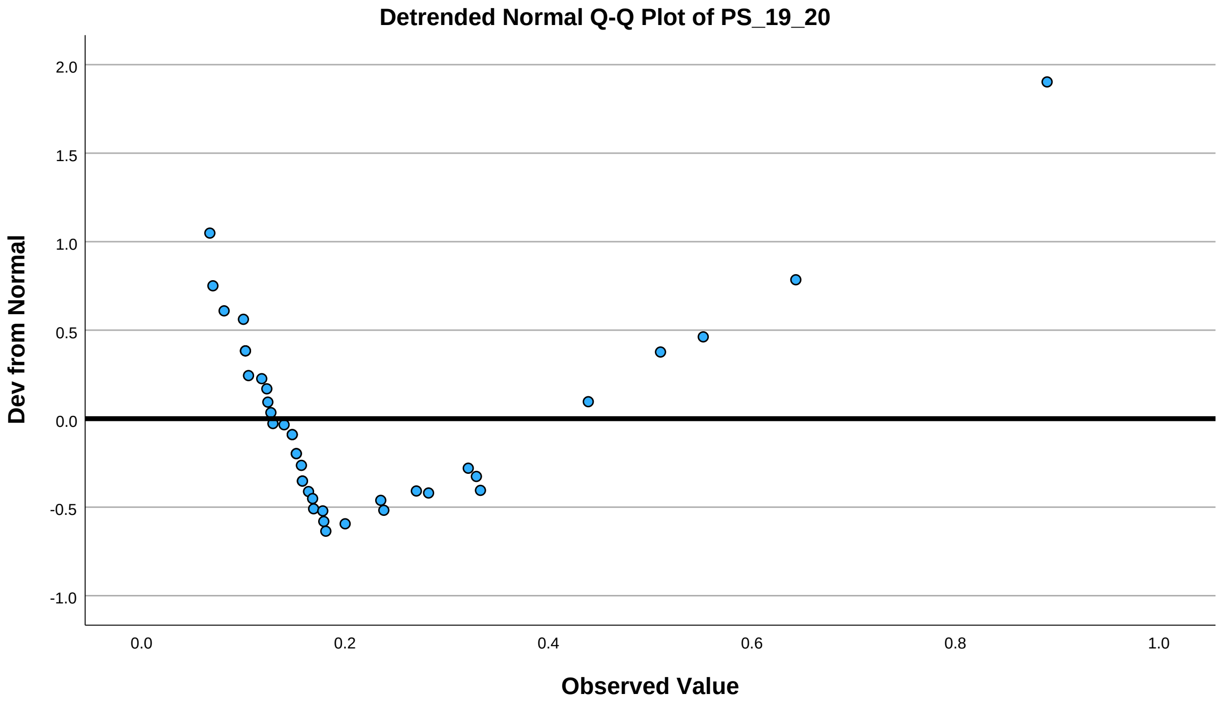
Detrended Normal Q-Q Plot of PS_19_20
Dev from Normal
Observed Value
2.0
1.5
1.0
0.5
0.0
-0.5
-1.0
0.0
0.2
0.4
0.6
0.8
1.0

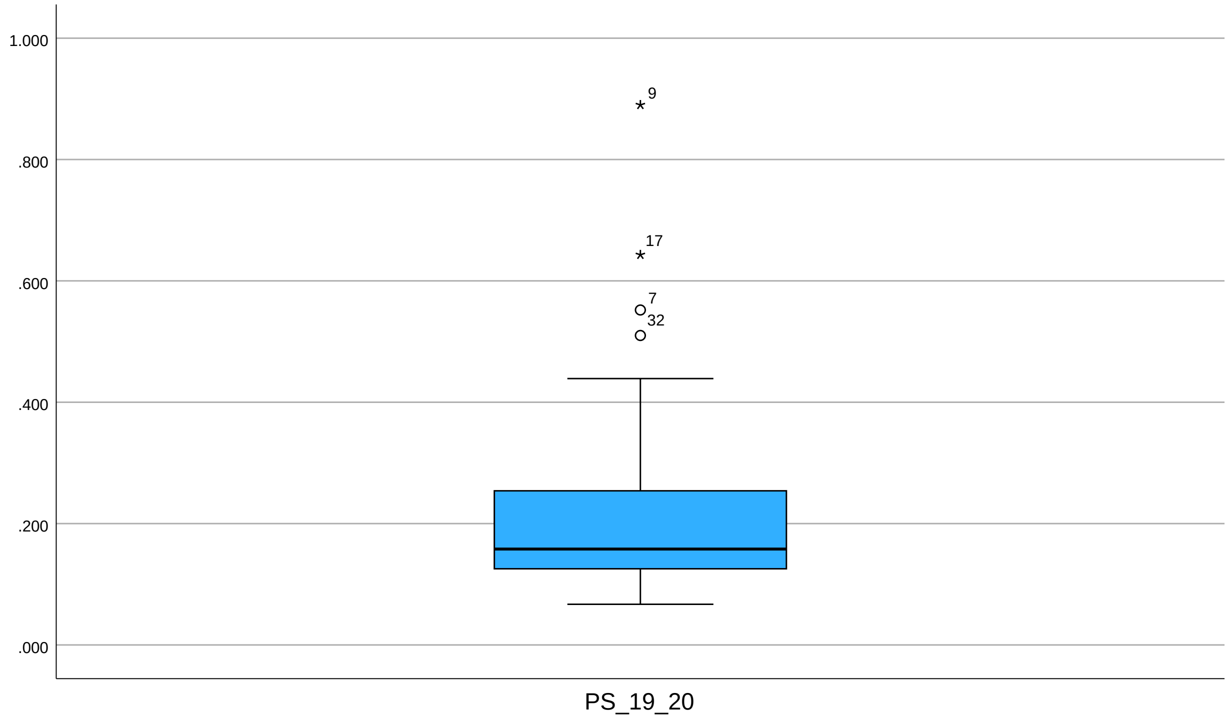
1.000
.800
.600
.400
.200
.000
9
17
7
32
PS_19_20

Explore

Case Processing Summary

	Cases					
	Valid		Missing		Total	
	N	Percent	N	Percent	N	Percent
BrainRate	39	100.0%	0	0.0%	39	100.0%
PermutationEntropy	39	100.0%	0	0.0%	39	100.0%
PermutationEntropy_01	39	100.0%	0	0.0%	39	100.0%
SampleEntropy	39	100.0%	0	0.0%	39	100.0%
SpectralEntropy_bits	39	100.0%	0	0.0%	39	100.0%
SpectralEntropy_01	39	100.0%	0	0.0%	39	100.0%
HiguchiFractalDimension	39	100.0%	0	0.0%	39	100.0%
LempelZivComplexity	39	100.0%	0	0.0%	39	100.0%
RenyiEntropy	39	100.0%	0	0.0%	39	100.0%
TsallisEntropy	39	100.0%	0	0.0%	39	100.0%
PS_4_5	39	100.0%	0	0.0%	39	100.0%
PS_5_6	39	100.0%	0	0.0%	39	100.0%
PS_6_7	39	100.0%	0	0.0%	39	100.0%
PS_7_8	39	100.0%	0	0.0%	39	100.0%
PS_8_9	39	100.0%	0	0.0%	39	100.0%
PS_9_10	39	100.0%	0	0.0%	39	100.0%
PS_10_11	39	100.0%	0	0.0%	39	100.0%
PS_11_12	39	100.0%	0	0.0%	39	100.0%
PS_12_13	39	100.0%	0	0.0%	39	100.0%
PS_13_14	39	100.0%	0	0.0%	39	100.0%
PS_14_15	39	100.0%	0	0.0%	39	100.0%
PS_15_16	39	100.0%	0	0.0%	39	100.0%
PS_16_17	39	100.0%	0	0.0%	39	100.0%
PS_17_18	39	100.0%	0	0.0%	39	100.0%
PS_18_19	39	100.0%	0	0.0%	39	100.0%
PS_19_20	39	100.0%	0	0.0%	39	100.0%

Descriptives

			Statistic	Std. Error
BrainRate	Mean		5.6331	.18162
	95% Confidence Interval for Mean	Lower Bound	5.2654	
		Upper Bound	6.0007	
	5% Trimmed Mean		5.5817	
	Median		5.6900	
	Variance		1.286	
	Std. Deviation		1.13422	
	Minimum		3.64	
	Maximum		8.95	
	Range		5.31	
	Interquartile Range		1.28	
	Skewness		.656	.378
	Kurtosis		.603	.741
PermutationEntropy	Mean		1.4367030782	.00938349041
	95% Confidence Interval for Mean	Lower Bound	1.4177071950	
		Upper Bound	1.4556989614	
	5% Trimmed Mean		1.4360290107	
	Median		1.4391867825	
	Variance		.003	
	Std. Deviation		.05859987884	
	Minimum		1.3151863090	
	Maximum		1.6153175697	
	Range		.30013126066	
	Interquartile Range		.08482375318	
	Skewness		.258	.378
	Kurtosis		1.223	.741
PermutationEntropy_01	Mean		.80183925514	.00523702571
	95% Confidence Interval for Mean	Lower Bound	.79123745085	
		Upper Bound	.81244105943	
	5% Trimmed Mean		.80146305091	
	Median		.80322543691	
	Variance		.001	
	Std. Deviation		.03270521509	
	Minimum		.73401945496	
	Maximum		.90152590089	
	Range		.16750644594	
	Interquartile Range		.04734103804	
	Skewness		.258	.378
	Kurtosis		1.223	.741

Descriptives

			Statistic	Std. Error
SampleEntropy	Mean		.58449677489	.02015793579
	95% Confidence Interval for Mean	Lower Bound	.54368916732	
		Upper Bound	.62530438247	
	5% Trimmed Mean		.58885503212	
	Median		.61503356178	
	Variance		.016	
	Std. Deviation		.12588626868	
	Minimum		.26747967831	
	Maximum		.79004859477	
	Range		.52256891646	
	Interquartile Range		.13603377534	
	Skewness		-.763	.378
	Kurtosis		-.006	.741
SpectralEntropy_bits	Mean		3.7235287646	.04752213392
	95% Confidence Interval for Mean	Lower Bound	3.6273252340	
		Upper Bound	3.8197322952	
	5% Trimmed Mean		3.7296914586	
	Median		3.7450514330	
	Variance		.088	
	Std. Deviation		.29677563118	
	Minimum		3.1112162644	
	Maximum		4.2138001999	
	Range		1.1025839355	
	Interquartile Range		.46926449934	
	Skewness		-.382	.378
	Kurtosis		-.475	.741
SpectralEntropy_01	Mean		.53108088341	.00677800507
	95% Confidence Interval for Mean	Lower Bound	.51735952949	
		Upper Bound	.54480223733	
	5% Trimmed Mean		.53195985849	
	Median		.53415062679	
	Variance		.002	
	Std. Deviation		.04232862812	
	Minimum		.44374774216	
	Maximum		.60100750502	
	Range		.15725976286	
	Interquartile Range		.06693043632	
	Skewness		-.382	.378
	Kurtosis		-.475	.741

Descriptives

			Statistic	Std. Error
HiguchiFractalDimension	Mean		.66028644179	.01071411593
	95% Confidence Interval for Mean	Lower Bound	.63859684802	
		Upper Bound	.68197603555	
	5% Trimmed Mean		.66450061993	
	Median		.66436839530	
	Variance		.004	
	Std. Deviation		.06690963255	
	Minimum		.41177057659	
	Maximum		.77490817887	
	Range		.36313760228	
	Interquartile Range		.08353564862	
	Skewness		-1.312	.378
	Kurtosis		3.766	.741
LempelZivComplexity	Mean		.23506643338	.00326435766
	95% Confidence Interval for Mean	Lower Bound	.22845808678	
		Upper Bound	.24167477998	
	5% Trimmed Mean		.23476382541	
	Median		.23183280828	
	Variance		.000	
	Std. Deviation		.02038590707	
	Minimum		.18955084963	
	Maximum		.28662245883	
	Range		.09707160919	
	Interquartile Range		.02426033216	
	Skewness		.235	.378
	Kurtosis		.391	.741
RenyiEntropy	Mean		7.9132042384	.15583652167
	95% Confidence Interval for Mean	Lower Bound	7.5977296934	
		Upper Bound	8.2286787834	
	5% Trimmed Mean		7.9776080765	
	Median		8.2678488983	
	Variance		.947	
	Std. Deviation		.97319876592	
	Minimum		5.7343114410	
	Maximum		8.8068925791	
	Range		3.0725811381	
	Interquartile Range		1.1390557823	
	Skewness		-1.065	.378
	Kurtosis		-.320	.741

Descriptives

			Statistic	Std. Error
TsallisEntropy	Mean		1.9152195490	.04757160571
	95% Confidence Interval for Mean	Lower Bound	1.8189158681	
		Upper Bound	2.0115232300	
	5% Trimmed Mean		1.9732421271	
	Median		1.9859528474	
	Variance		.088	
	Std. Deviation		.29708458243	
	Minimum		.22710177944	
	Maximum		1.9884387407	
	Range		1.7613369613	
	Interquartile Range		.00152468410	
	Skewness		-5.240	.378
	Kurtosis		29.182	.741
PS_4_5	Mean		2.87169	.415409
	95% Confidence Interval for Mean	Lower Bound	2.03074	
		Upper Bound	3.71264	
	5% Trimmed Mean		2.53443	
	Median		2.12200	
	Variance		6.730	
	Std. Deviation		2.594229	
	Minimum		.547	
	Maximum		15.430	
	Range		14.883	
	Interquartile Range		1.468	
	Skewness		3.307	.378
	Kurtosis		14.189	.741
PS_5_6	Mean		2.19254	.337148
	95% Confidence Interval for Mean	Lower Bound	1.51002	
		Upper Bound	2.87506	
	5% Trimmed Mean		1.91744	
	Median		1.44800	
	Variance		4.433	
	Std. Deviation		2.105488	
	Minimum		.357	
	Maximum		12.220	
	Range		11.863	
	Interquartile Range		1.554	
	Skewness		3.177	.378
	Kurtosis		13.150	.741

Descriptives

			Statistic	Std. Error
PS_6_7	Mean		1.69397	.239614
	95% Confidence Interval for Mean	Lower Bound	1.20890	
		Upper Bound	2.17905	
	5% Trimmed Mean		1.49265	
	Median		1.28200	
	Variance		2.239	
	Std. Deviation		1.496389	
	Minimum		.269	
	Maximum		9.170	
	Range		8.901	
	Interquartile Range		1.321	
	Skewness		3.523	.378
	Kurtosis		16.323	.741
PS_7_8	Mean		1.82323	.366342
	95% Confidence Interval for Mean	Lower Bound	1.08161	
		Upper Bound	2.56485	
	5% Trimmed Mean		1.45123	
	Median		1.18500	
	Variance		5.234	
	Std. Deviation		2.287803	
	Minimum		.240	
	Maximum		13.140	
	Range		12.900	
	Interquartile Range		.893	
	Skewness		3.748	.378
	Kurtosis		16.258	.741
PS_8_9	Mean		1.70108	.339660
	95% Confidence Interval for Mean	Lower Bound	1.01347	
		Upper Bound	2.38868	
	5% Trimmed Mean		1.35698	
	Median		1.11400	
	Variance		4.499	
	Std. Deviation		2.121176	
	Minimum		.216	
	Maximum		12.185	
	Range		11.969	
	Interquartile Range		1.137	
	Skewness		3.716	.378
	Kurtosis		16.152	.741

Descriptives

			Statistic	Std. Error
PS_9_10	Mean		2.15026	.364780
	95% Confidence Interval for Mean	Lower Bound	1.41180	
		Upper Bound	2.88871	
	5% Trimmed Mean		1.83007	
	Median		1.34200	
	Variance		5.190	
	Std. Deviation		2.278049	
	Minimum		.239	
	Maximum		10.236	
	Range		9.997	
	Interquartile Range		1.568	
	Skewness		2.376	.378
	Kurtosis		5.642	.741
PS_10_11	Mean		2.13087	.282474
	95% Confidence Interval for Mean	Lower Bound	1.55903	
		Upper Bound	2.70271	
	5% Trimmed Mean		1.95734	
	Median		1.74900	
	Variance		3.112	
	Std. Deviation		1.764052	
	Minimum		.266	
	Maximum		8.310	
	Range		8.044	
	Interquartile Range		2.241	
	Skewness		1.558	.378
	Kurtosis		2.832	.741
PS_11_12	Mean		.71813	.101564
	95% Confidence Interval for Mean	Lower Bound	.51252	
		Upper Bound	.92373	
	5% Trimmed Mean		.63880	
	Median		.48100	
	Variance		.402	
	Std. Deviation		.634267	
	Minimum		.143	
	Maximum		3.392	
	Range		3.249	
	Interquartile Range		.424	
	Skewness		2.553	.378
	Kurtosis		7.761	.741

Descriptives

			Statistic	Std. Error
PS_12_13	Mean		.44954	.047907
	95% Confidence Interval for Mean	Lower Bound	.35256	
		Upper Bound	.54652	
	5% Trimmed Mean		.41341	
	Median		.36400	
	Variance		.090	
	Std. Deviation		.299180	
	Minimum		.116	
	Maximum		1.450	
	Range		1.334	
	Interquartile Range		.173	
	Skewness		2.014	.378
	Kurtosis		4.198	.741
PS_13_14	Mean		.39744	.048598
	95% Confidence Interval for Mean	Lower Bound	.29905	
		Upper Bound	.49582	
	5% Trimmed Mean		.35982	
	Median		.27900	
	Variance		.092	
	Std. Deviation		.303496	
	Minimum		.091	
	Maximum		1.531	
	Range		1.440	
	Interquartile Range		.230	
	Skewness		2.262	.378
	Kurtosis		5.386	.741
PS_14_15	Mean		.40923	.072054
	95% Confidence Interval for Mean	Lower Bound	.26336	
		Upper Bound	.55510	
	5% Trimmed Mean		.34160	
	Median		.25700	
	Variance		.202	
	Std. Deviation		.449978	
	Minimum		.081	
	Maximum		2.611	
	Range		2.530	
	Interquartile Range		.230	
	Skewness		3.544	.378
	Kurtosis		15.135	.741

Descriptives

			Statistic	Std. Error
PS_15_16	Mean		.38838	.072373
	95% Confidence Interval for Mean	Lower Bound	.24187	
		Upper Bound	.53490	
	5% Trimmed Mean		.31182	
	Median		.23400	
	Variance		.204	
	Std. Deviation		.451971	
	Minimum		.069	
	Maximum		2.498	
	Range		2.429	
	Interquartile Range		.204	
	Skewness		3.408	.378
	Kurtosis		13.242	.741
PS_16_17	Mean		.29454	.040447
	95% Confidence Interval for Mean	Lower Bound	.21266	
		Upper Bound	.37642	
	5% Trimmed Mean		.26249	
	Median		.18400	
	Variance		.064	
	Std. Deviation		.252591	
	Minimum		.074	
	Maximum		1.204	
	Range		1.130	
	Interquartile Range		.150	
	Skewness		2.154	.378
	Kurtosis		4.447	.741
PS_17_18	Mean		.22969	.028304
	95% Confidence Interval for Mean	Lower Bound	.17239	
		Upper Bound	.28699	
	5% Trimmed Mean		.20998	
	Median		.15900	
	Variance		.031	
	Std. Deviation		.176759	
	Minimum		.065	
	Maximum		.949	
	Range		.884	
	Interquartile Range		.139	
	Skewness		2.256	.378
	Kurtosis		6.214	.741

Descriptives

			Statistic	Std. Error
PS_18_19	Mean		.19131	.019672
	95% Confidence Interval for Mean	Lower Bound	.15148	
		Upper Bound	.23113	
	5% Trimmed Mean		.17830	
	Median		.15500	
	Variance		.015	
	Std. Deviation		.122850	
	Minimum		.056	
	Maximum		.589	
	Range		.533	
	Interquartile Range		.099	
	Skewness		1.705	.378
	Kurtosis		2.461	.741
PS_19_20	Mean		.16213	.016086
	95% Confidence Interval for Mean	Lower Bound	.12956	
		Upper Bound	.19469	
	5% Trimmed Mean		.14976	
	Median		.14300	
	Variance		.010	
	Std. Deviation		.100460	
	Minimum		.055	
	Maximum		.560	
	Range		.505	
	Interquartile Range		.082	
	Skewness		2.218	.378
	Kurtosis		5.982	.741

Tests of Normality

	Kolmogorov-Smirnov[a]			Shapiro-Wilk		
	Statistic	df	Sig.	Statistic	df	Sig.
BrainRate	.085	39	.200*	.970	39	.365
PermutationEntropy	.103	39	.200*	.962	39	.206
PermutationEntropy_01	.103	39	.200*	.962	39	.206
SampleEntropy	.141	39	.049	.936	39	.028
SpectralEntropy_bits	.117	39	.193	.965	39	.252
SpectralEntropy_01	.117	39	.193	.965	39	.252
HiguchiFractalDimension	.097	39	.200*	.922	39	.010
LempelZivComplexity	.078	39	.200*	.989	39	.969
RenyiEntropy	.195	39	<.001	.802	39	<.001
TsallisEntropy	.460	39	<.001	.263	39	<.001
PS_4_5	.250	39	<.001	.663	39	<.001
PS_5_6	.205	39	<.001	.670	39	<.001
PS_6_7	.207	39	<.001	.657	39	<.001
PS_7_8	.293	39	<.001	.548	39	<.001
PS_8_9	.271	39	<.001	.562	39	<.001
PS_9_10	.239	39	<.001	.696	39	<.001
PS_10_11	.164	39	.010	.854	39	<.001
PS_11_12	.252	39	<.001	.703	39	<.001
PS_12_13	.276	39	<.001	.773	39	<.001
PS_13_14	.215	39	<.001	.735	39	<.001
PS_14_15	.283	39	<.001	.585	39	<.001
PS_15_16	.285	39	<.001	.576	39	<.001
PS_16_17	.260	39	<.001	.709	39	<.001
PS_17_18	.241	39	<.001	.732	39	<.001
PS_18_19	.239	39	<.001	.793	39	<.001
PS_19_20	.230	39	<.001	.771	39	<.001

*. This is a lower bound of the true significance.

a. Lilliefors Significance Correction

BrainRate

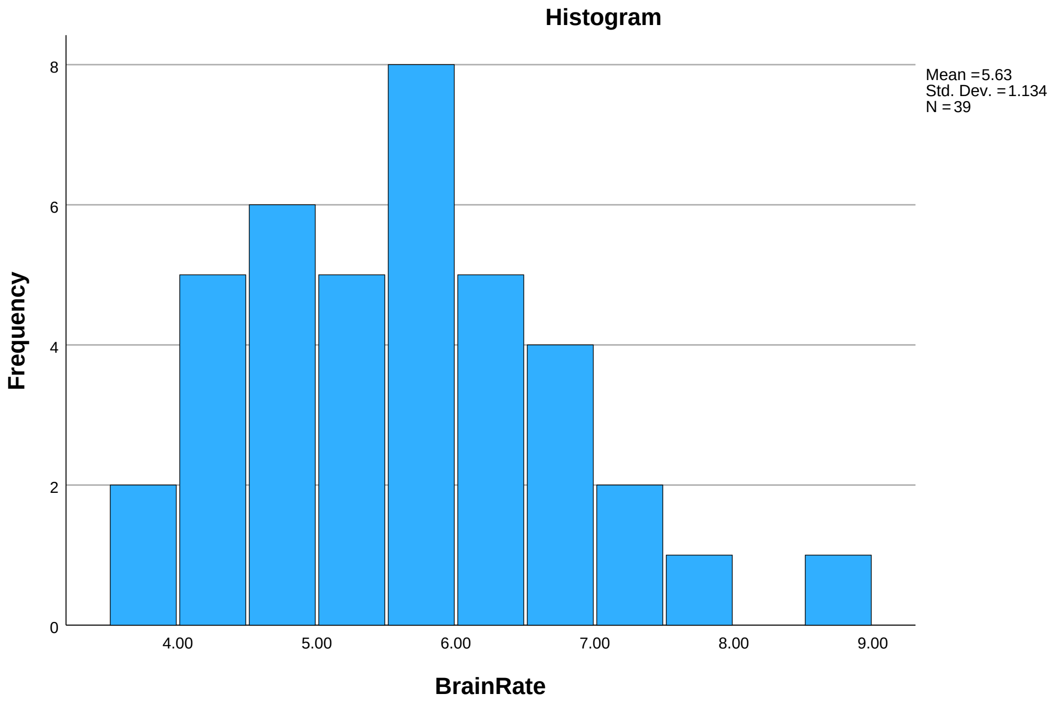


```
BrainRate Stem-and-Leaf Plot

 Frequency    Stem &  Leaf

     2.00        3 .  69
     5.00        4 .  12344
     6.00        4 .  559999
     5.00        5 .  00123
     8.00        5 .  56788889
     5.00        6 .  01114
     4.00        6 .  7789
     2.00        7 .  11
     1.00        7 .  8
     1.00 Extremes    (>=9.0)

 Stem width:      1.00
 Each leaf:       1 case(s)
```

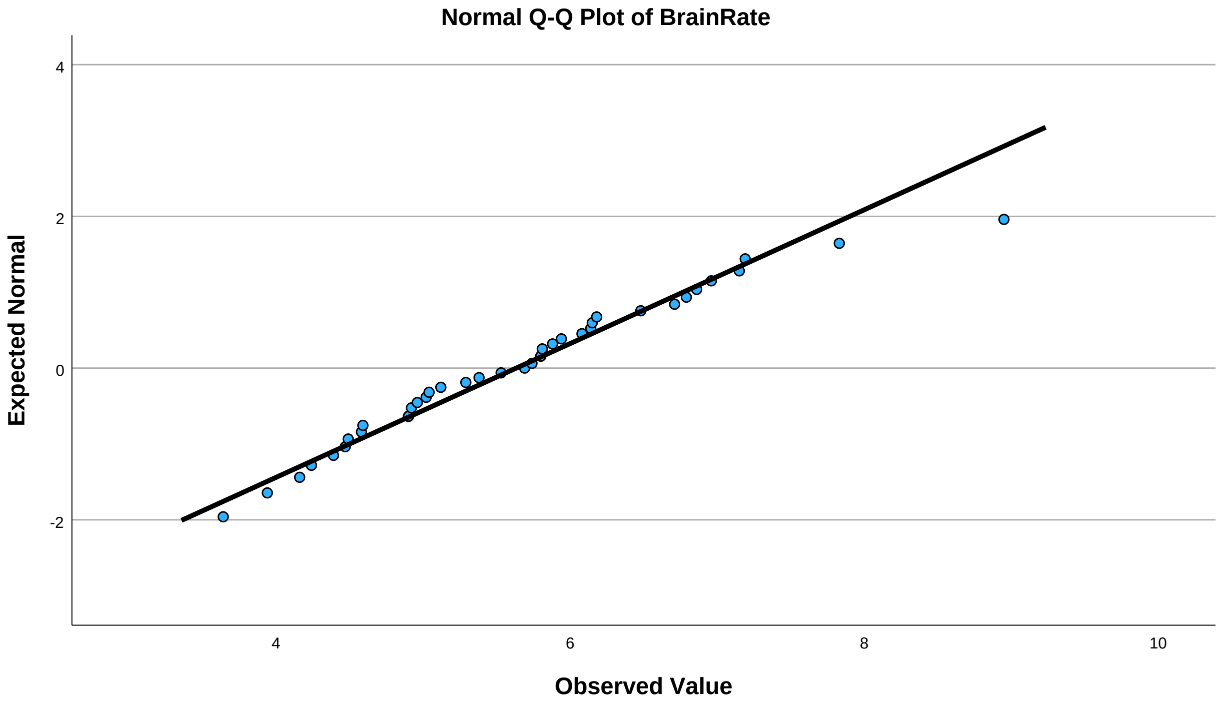

Normal Q-Q Plot of BrainRate
Expected Normal
Observed Value
4
2
0
-2
4
6
8
10

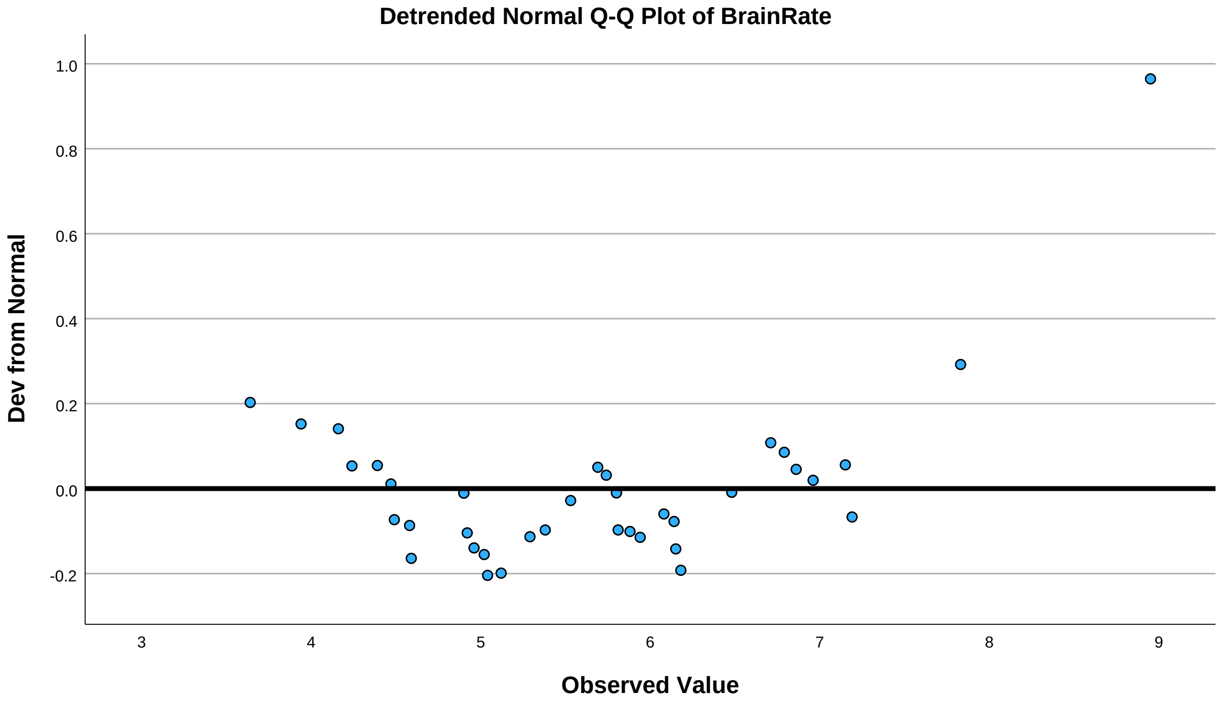

Detrended Normal Q-Q Plot of BrainRate
Dev from Normal
Observed Value
1.0
0.8
0.6
0.4
0.2
0.0
-0.2
3
4
5
6
7
8
9

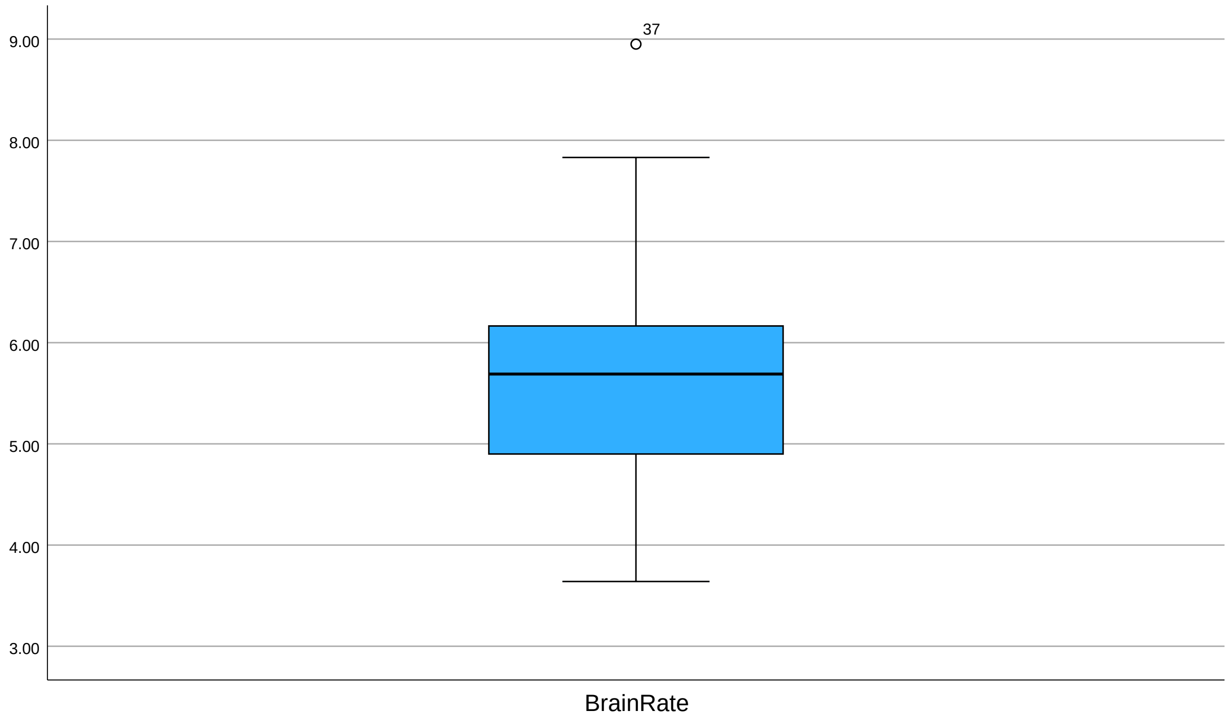

PermutationEntropy

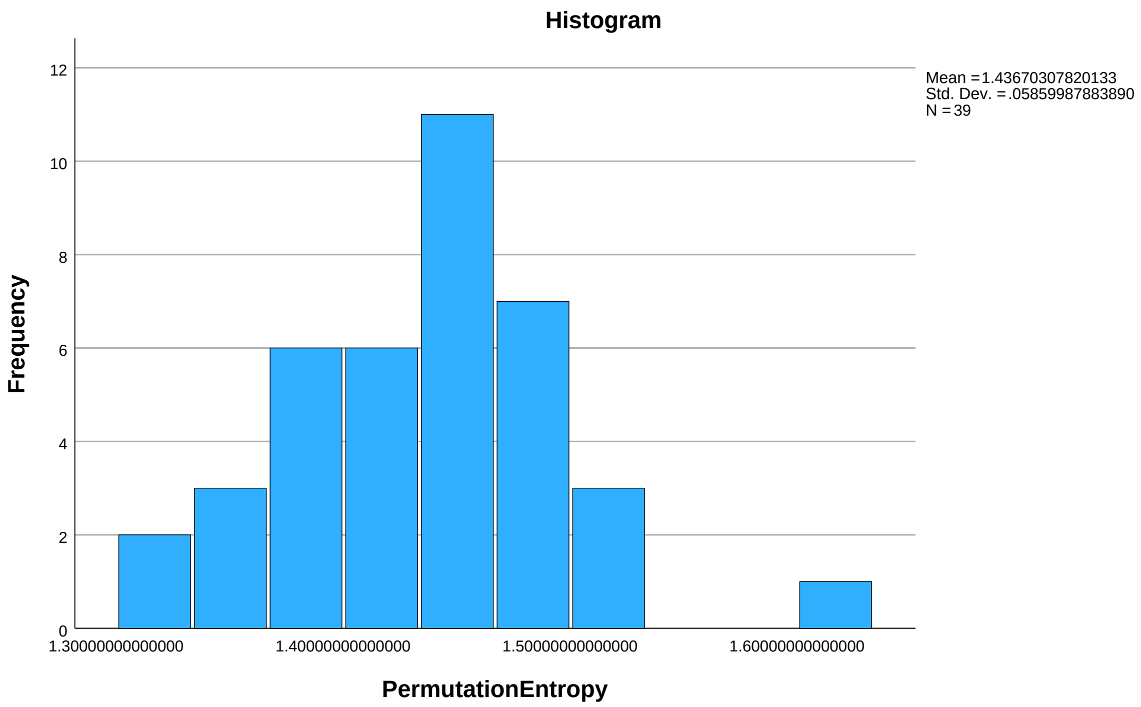


```
PermutationEntropy Stem-and-Leaf Plot

 Frequency    Stem &  Leaf
```

```
    4.00      13 .  1344
    7.00      13 .  6789999
   14.00      14 .  02223333344444
   10.00      14 .  5666889999
    3.00      15 .  000
    1.00 Extremes    (>=1.62)

 Stem width:   .1000000
 Each leaf:      1 case(s)
```

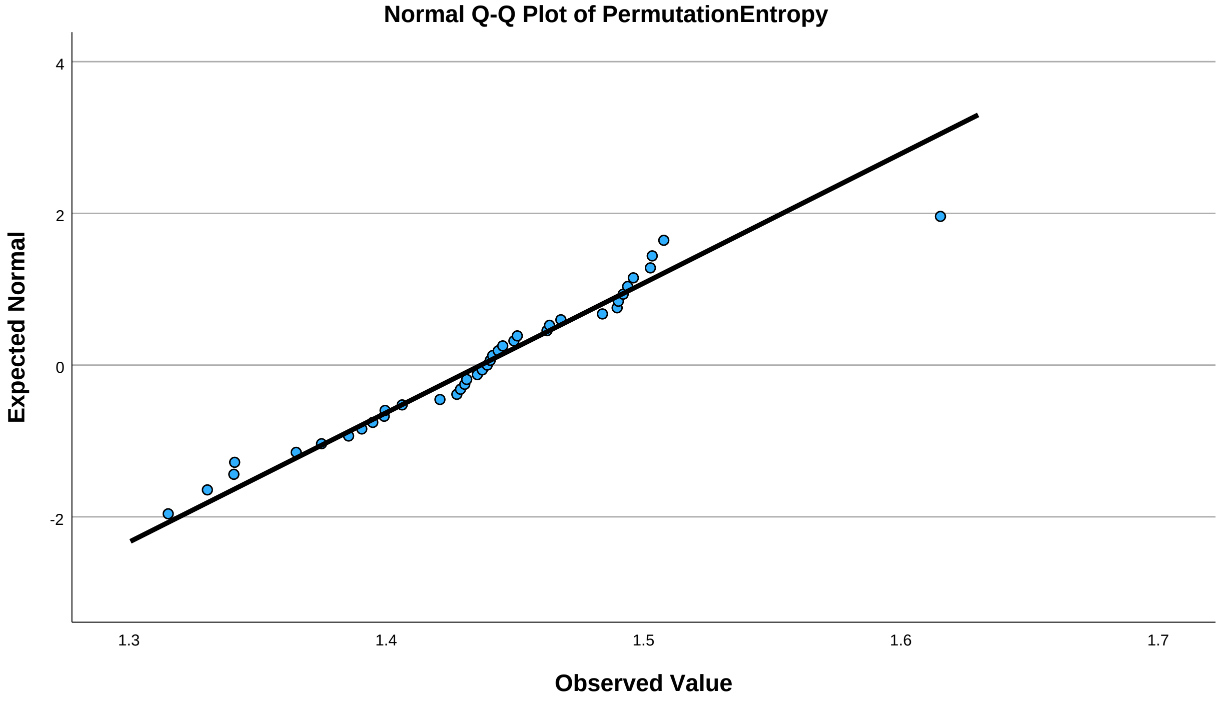

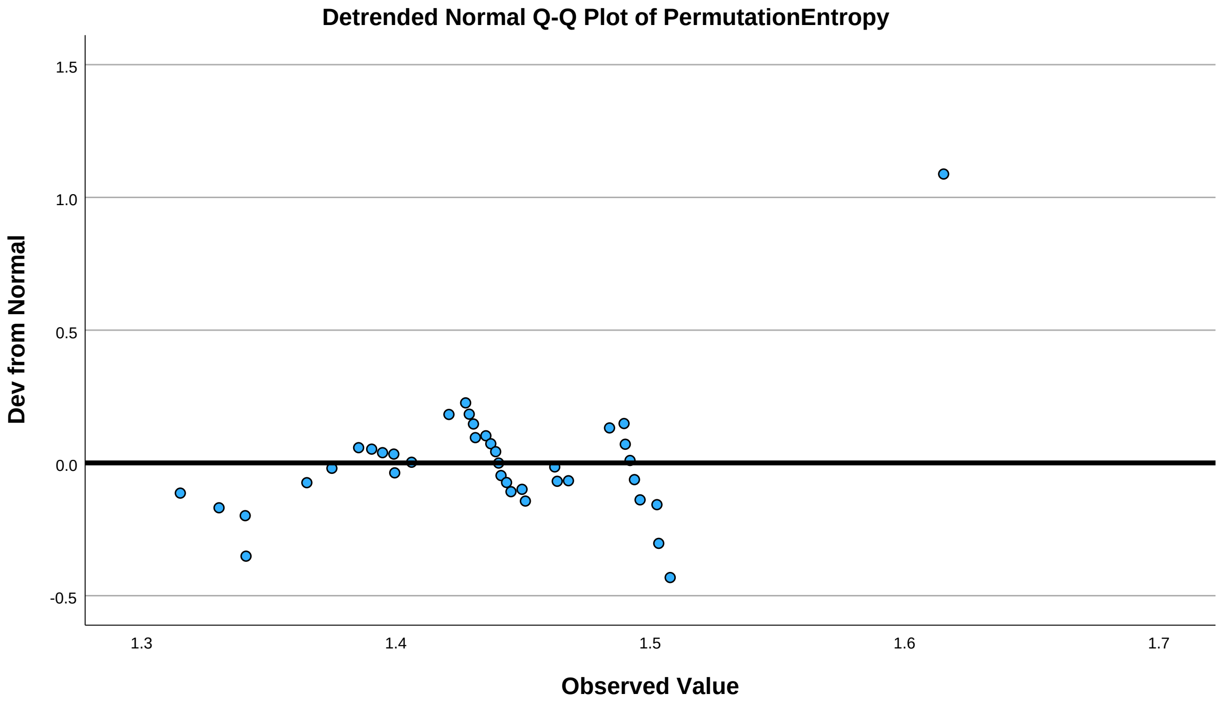

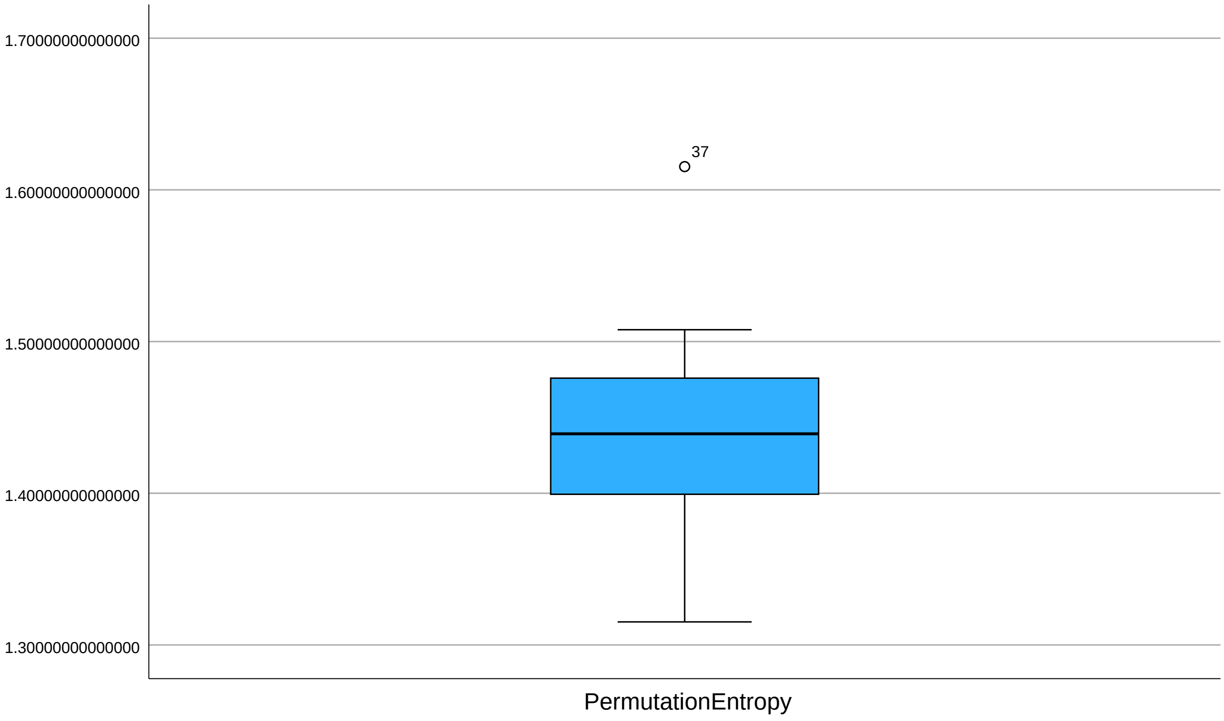

PermutationEntropy_01

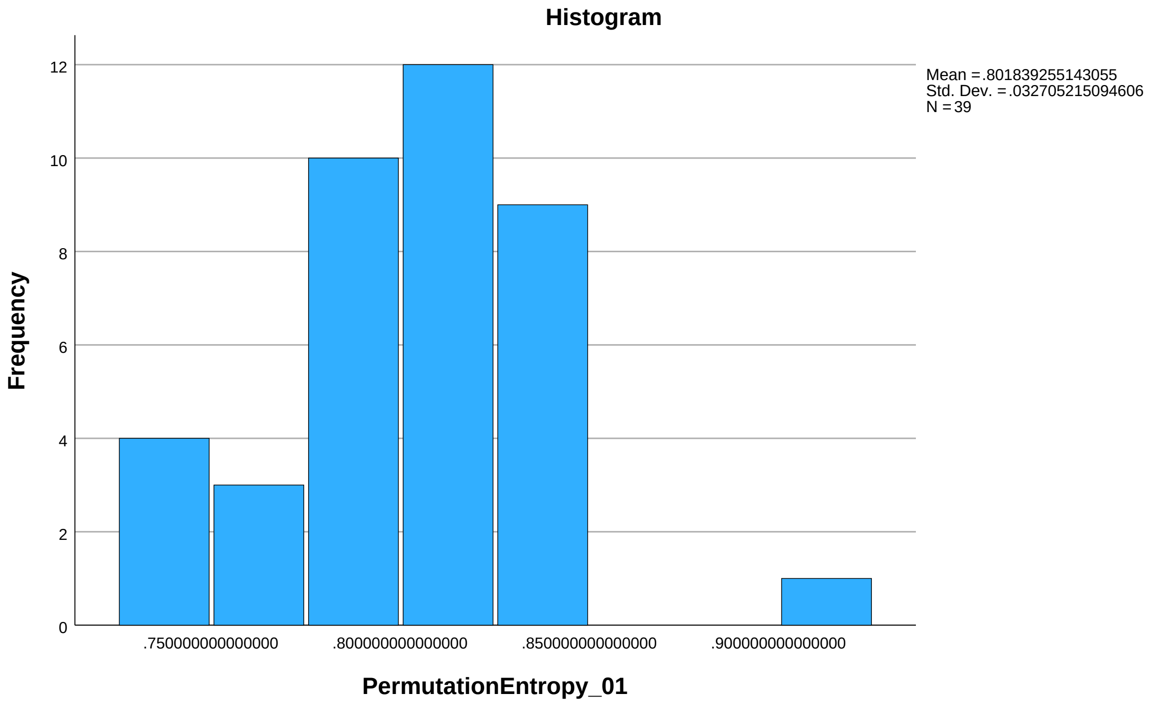


```
PermutationEntropy_01 Stem-and-Leaf Plot

 Frequency    Stem &  Leaf

     1.00       73 .  4
     3.00       74 .  288
      .00       75 .
     2.00       76 .  17
     3.00       77 .  368
     3.00       78 .  014
     5.00       79 .  26788
     9.00       80 .  123345699
     3.00       81 .  669
     1.00       82 .  8
     7.00       83 .  1123489
     1.00       84 .  1
     1.00 Extremes    (>=.902)

 Stem width:   .0100000
 Each leaf:       1 case(s)
```

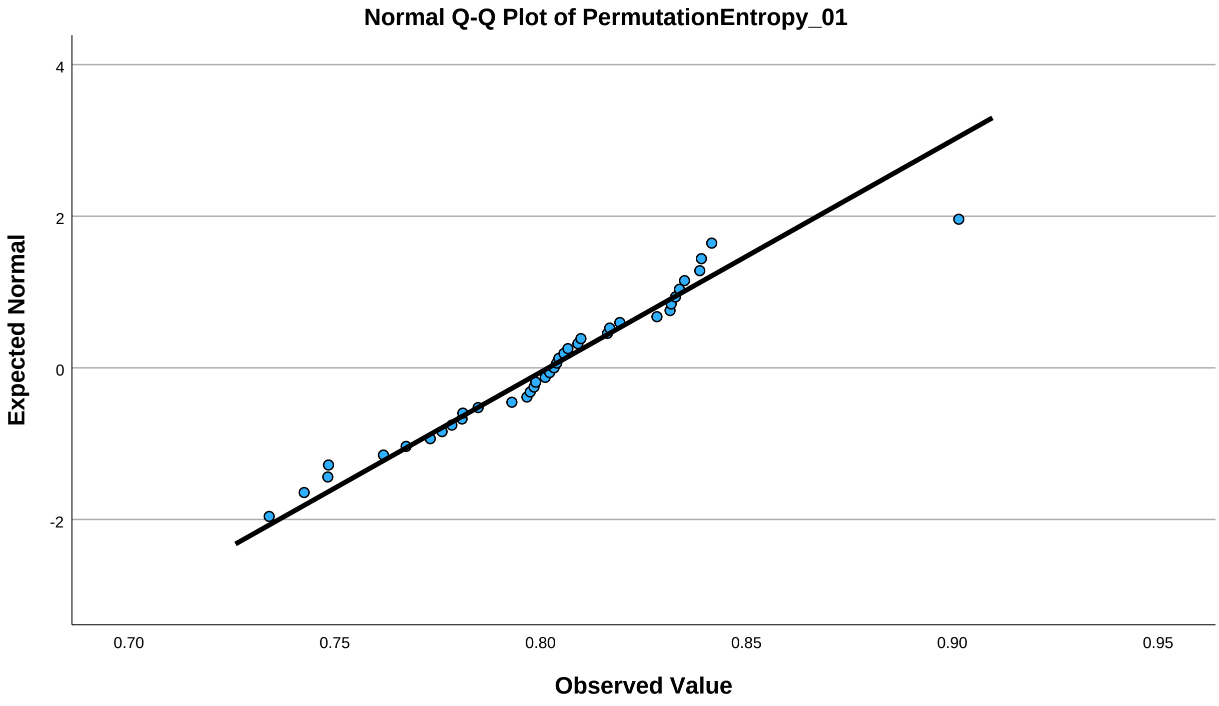
Normal Q-Q Plot of PermutationEntropy_01
Expected Normal
4
2
0
-2
0.70
0.75
0.80
0.85
0.90
0.95
Observed Value

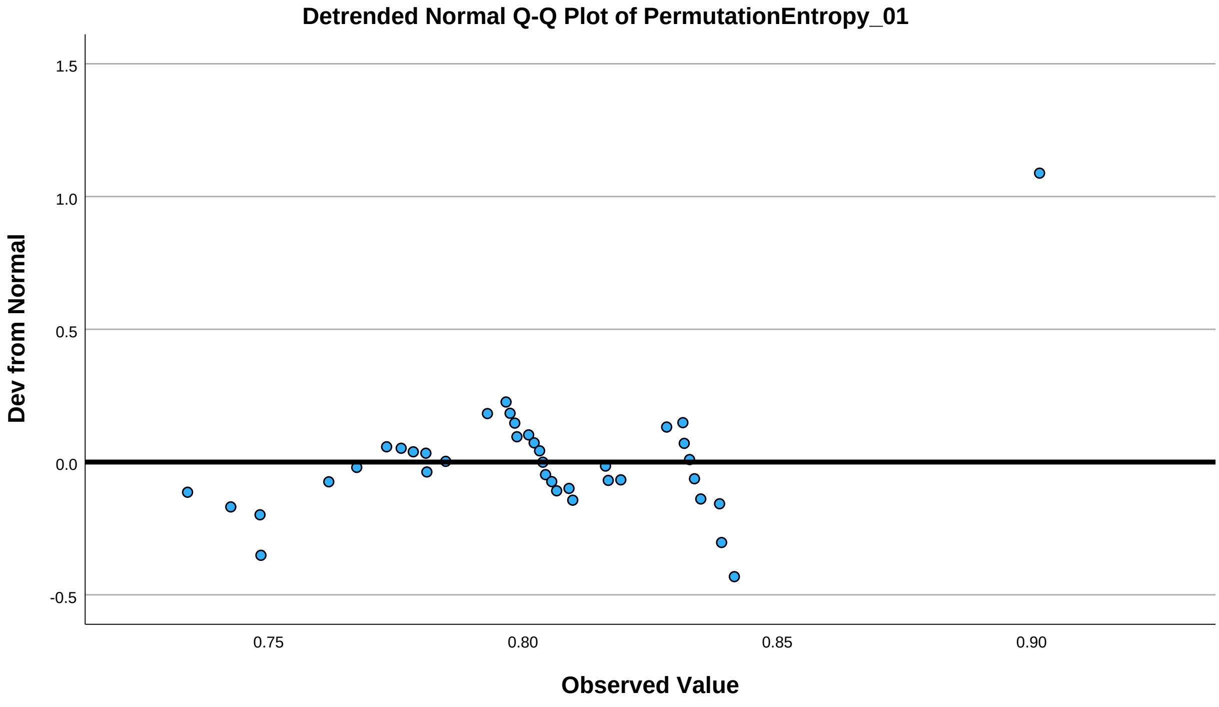
Detrended Normal Q-Q Plot of PermutationEntropy_01
Dev from Normal
1.5
1.0
0.5
0.0
-0.5
0.75
0.80
0.85
0.90
Observed Value

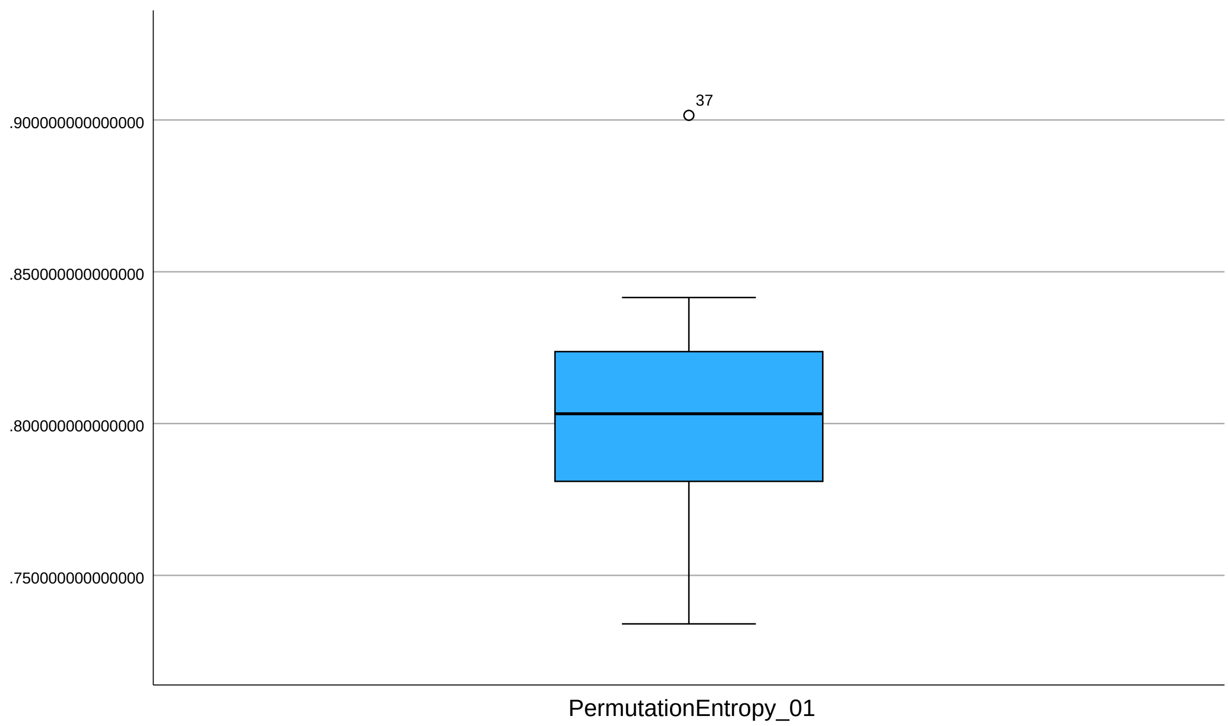

SampleEntropy

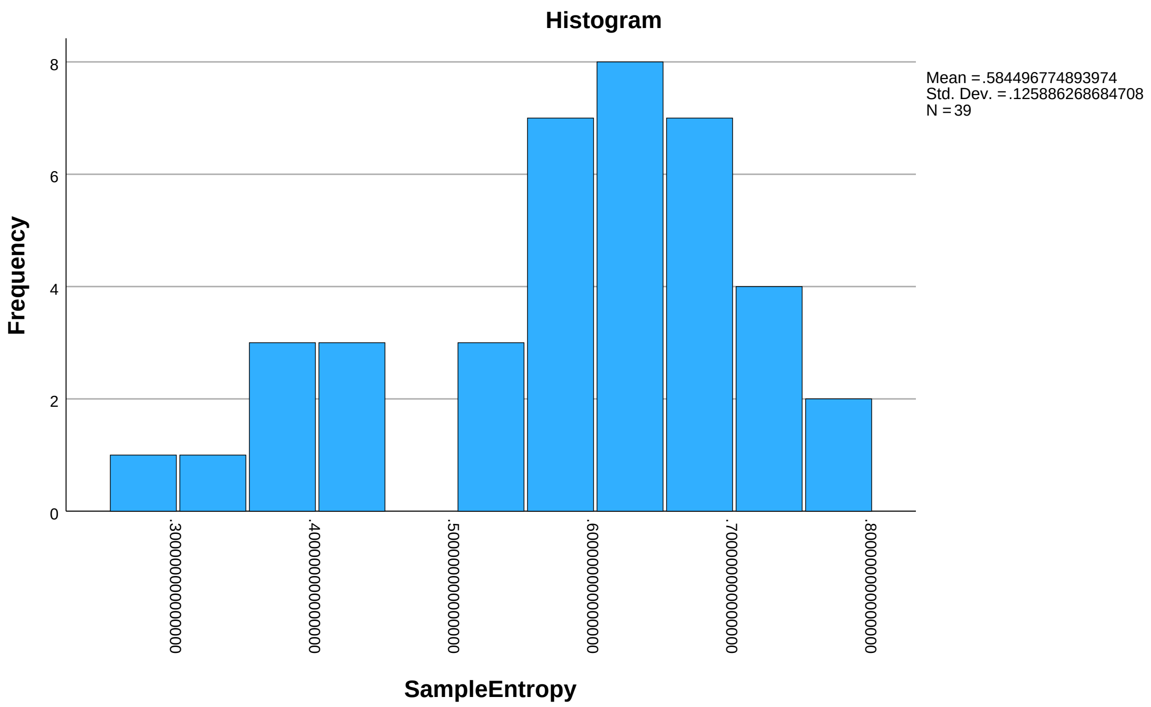


```
SampleEntropy Stem-and-Leaf Plot

 Frequency    Stem &  Leaf
```

```
 2.00 Extremes    (=<.35)
 3.00      3 . 578
 3.00      4 . 123
  .00      4 .
 3.00      5 . 034
 7.00      5 . 6668999
 8.00      6 . 11122234
 7.00      6 . 5567888
 4.00      7 . 0014
 2.00      7 . 79

Stem width:   .1000000
Each leaf:       1 case(s)
```

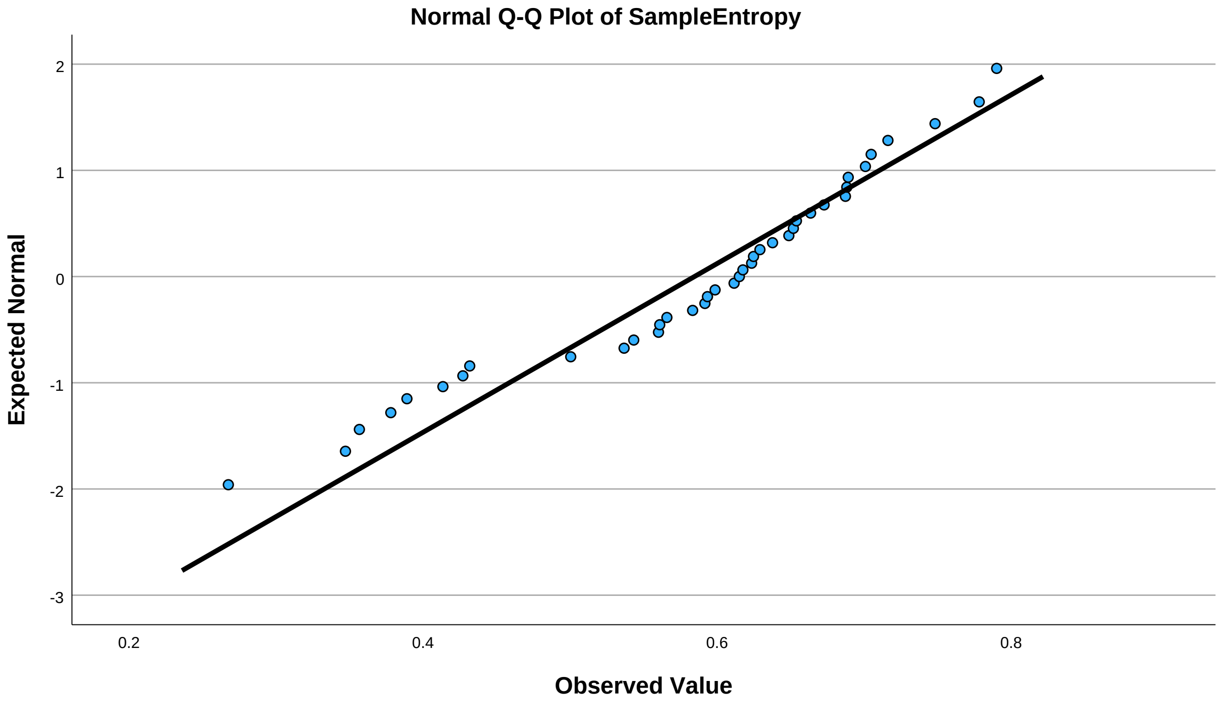

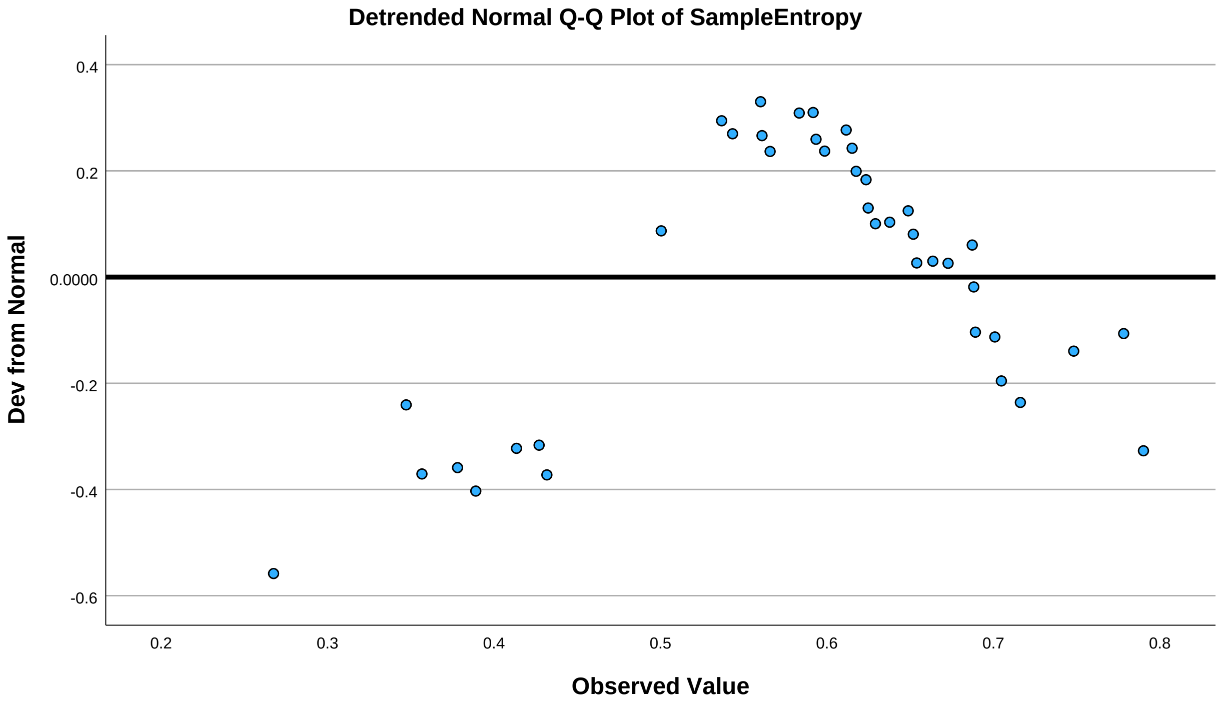

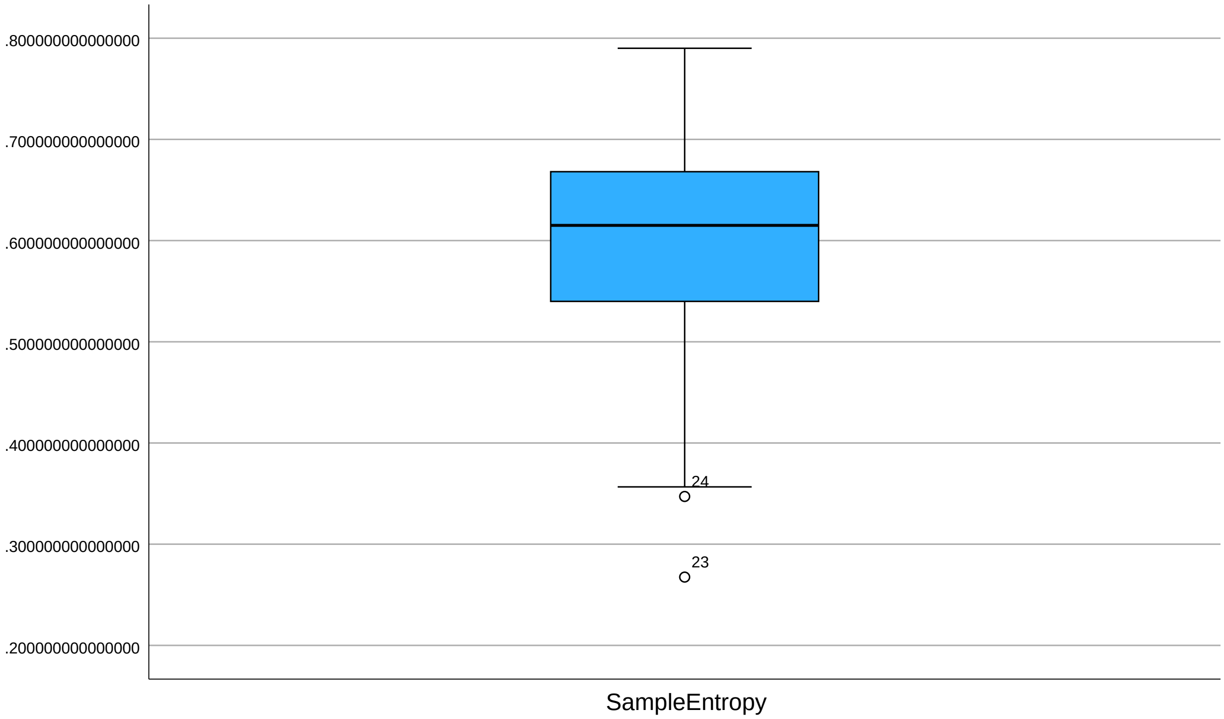

SpectralEntropy_bits

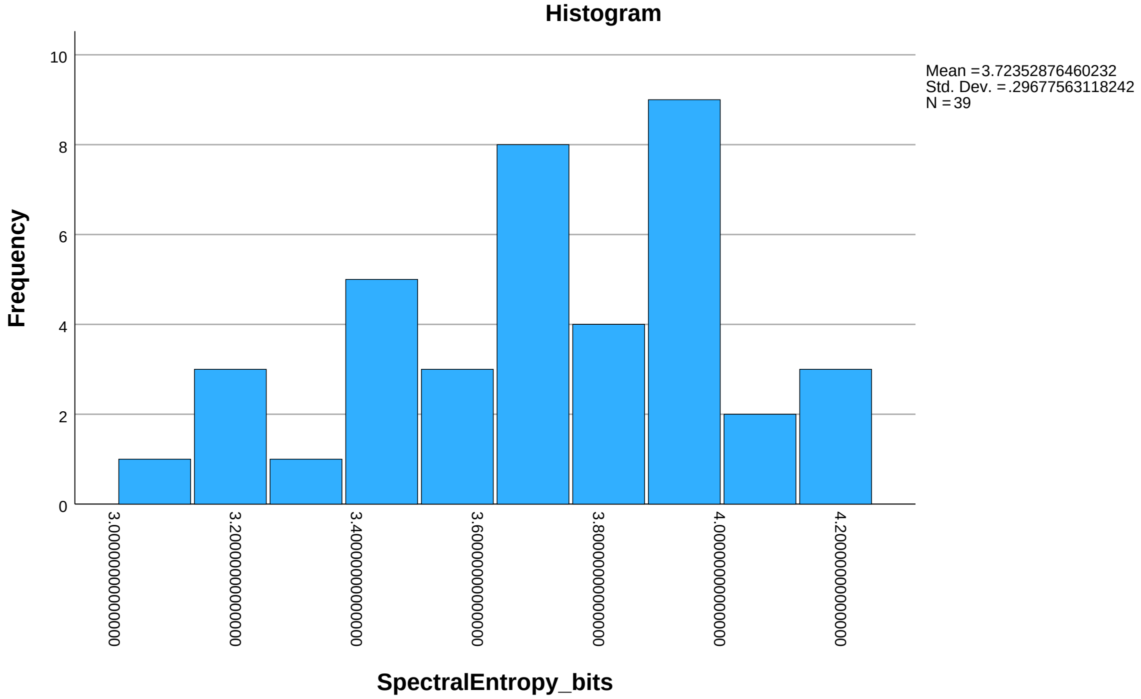


```
SpectralEntropy_bits Stem-and-Leaf Plot

 Frequency    Stem &  Leaf

     3.00       31 .  145
     1.00       32 .  1
     2.00       33 .  68
     4.00       34 .  3477
     3.00       35 .  688
     2.00       36 .  36
     7.00       37 .  2234449
     5.00       38 .  14499
     7.00       39 .  1346679
     1.00       40 .  6
     1.00       41 .  1
     3.00       42 .  001

 Stem width:   .1000000
 Each leaf:        1 case(s)
```

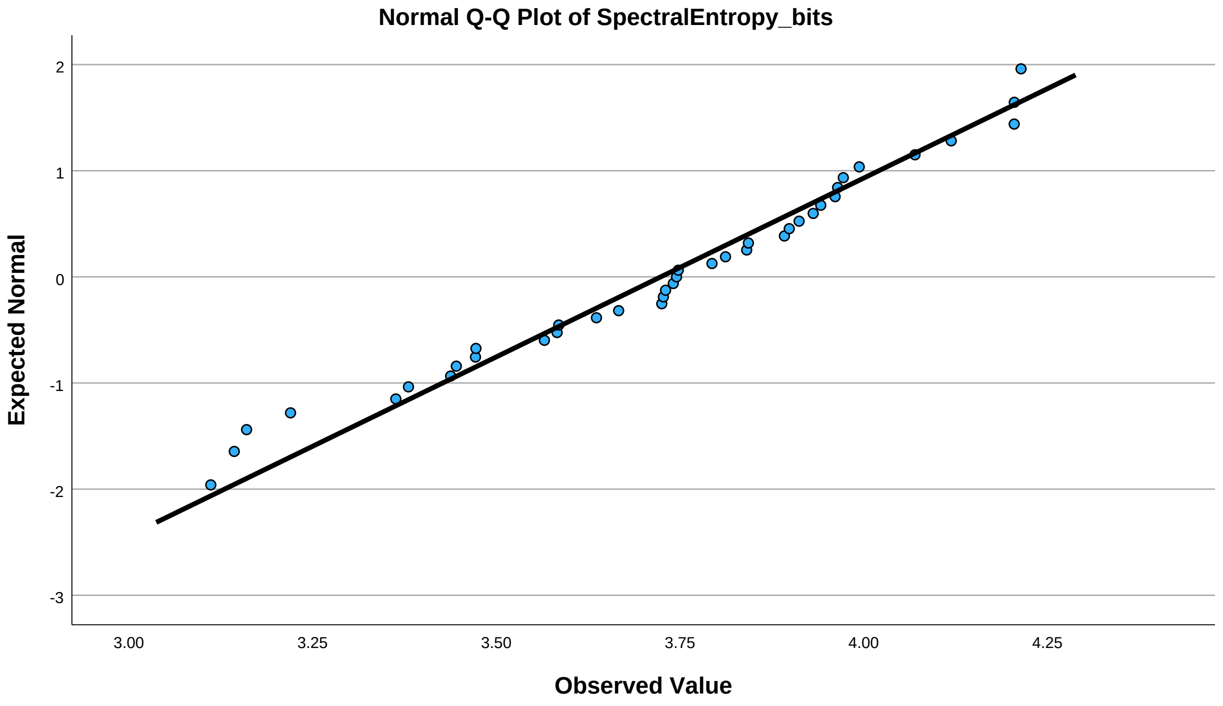

Normal Q-Q Plot of SpectralEntropy_bits
Expected Normal
2
1
0
-1
-2
-3
3.00
3.25
3.50
3.75
4.00
4.25
Observed Value

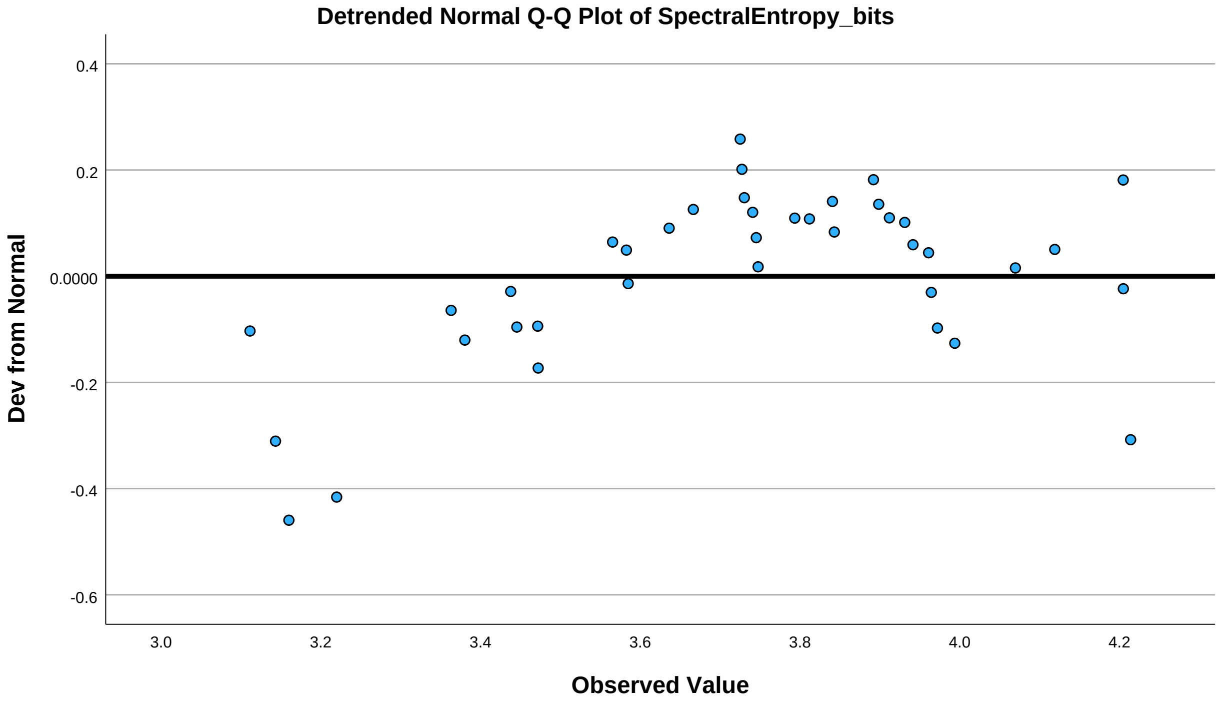

Detrended Normal Q-Q Plot of SpectralEntropy_bits
Dev from Normal
0.4
0.2
0.0000
-0.2
-0.4
-0.6
3.0
3.2
3.4
3.6
3.8
4.0
4.2
Observed Value

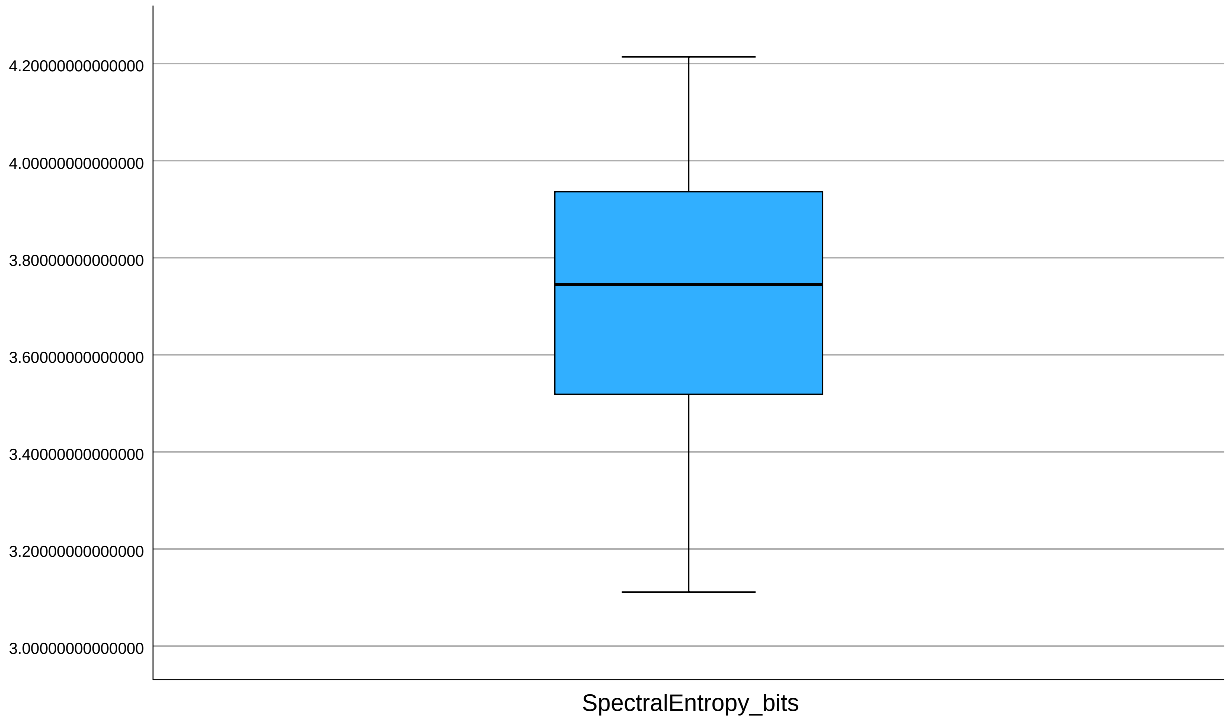

SpectralEntropy_01

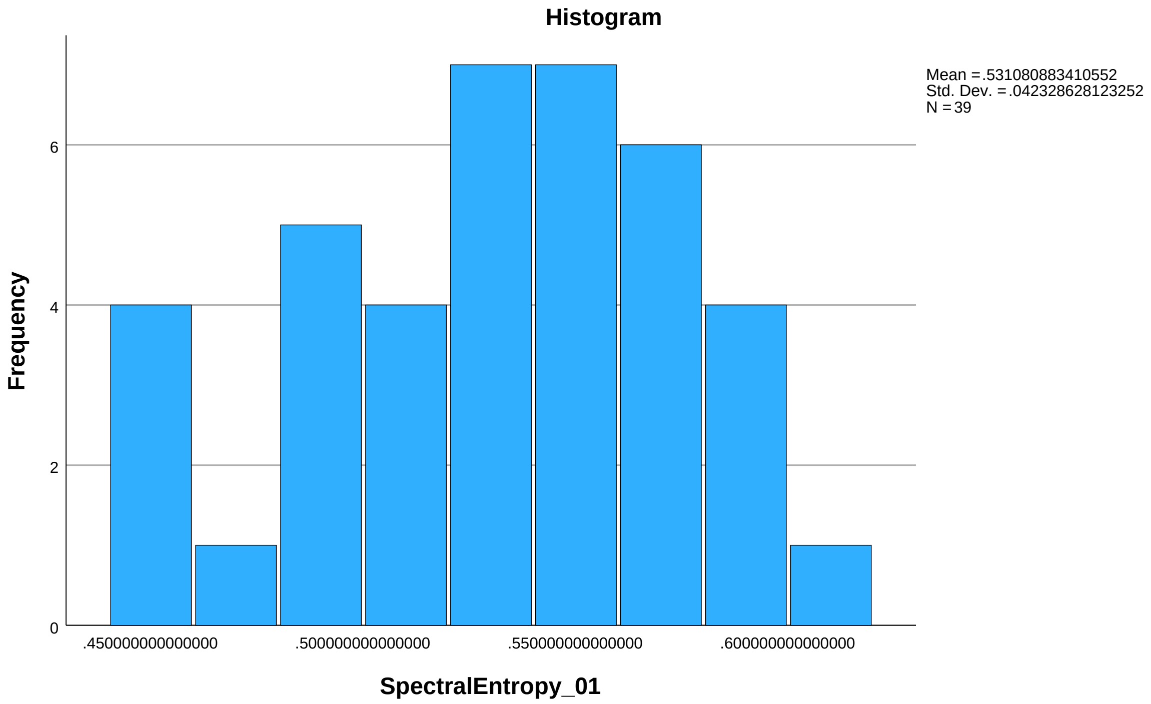


```
SpectralEntropy_01 Stem-and-Leaf Plot

 Frequency    Stem &  Leaf
```

```
 2.00       4 .  44
  8.00        4 .  5578999
 15.00        5 .  011123333334444
 13.00        5 .  5556666668899
 1.00       6 .  0

Stem width:   .1000000
Each leaf:        1 case(s)
```

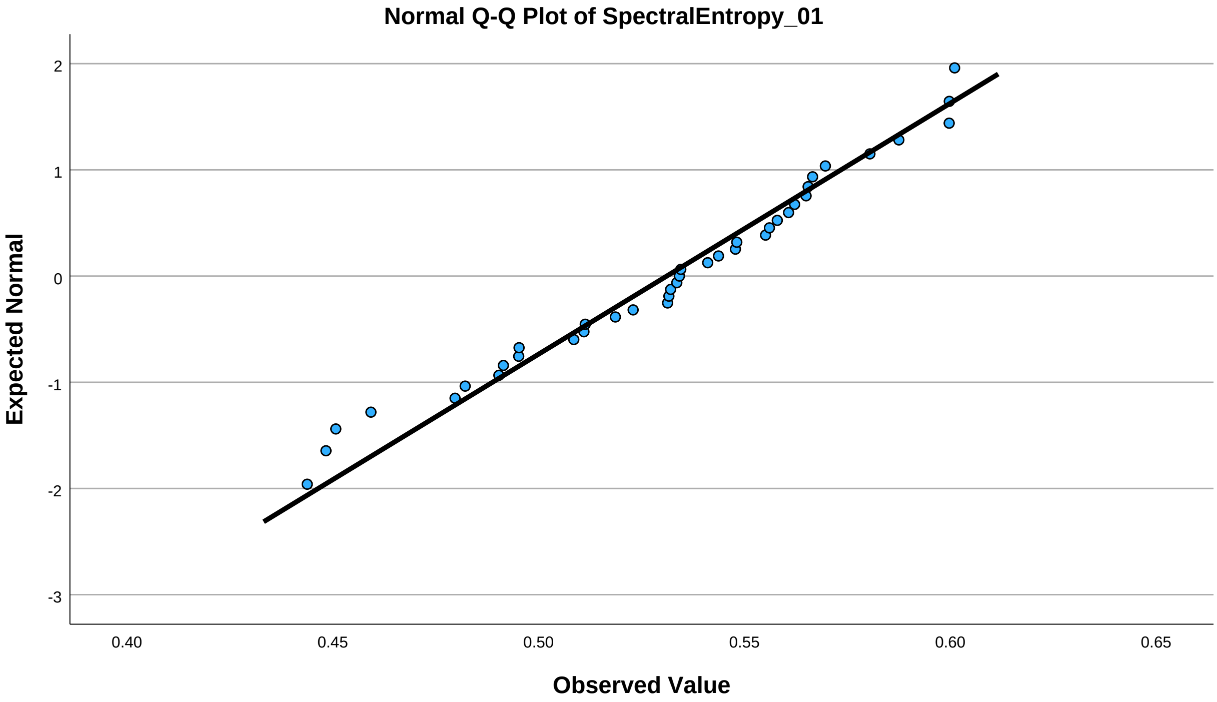

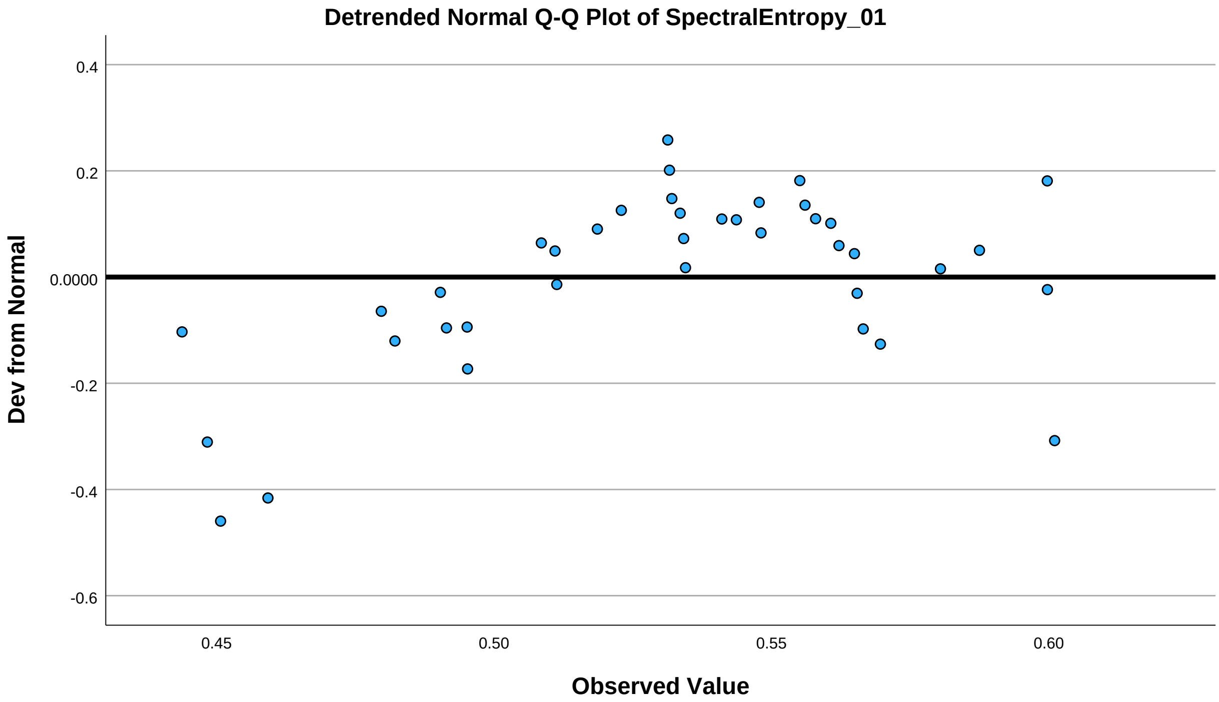

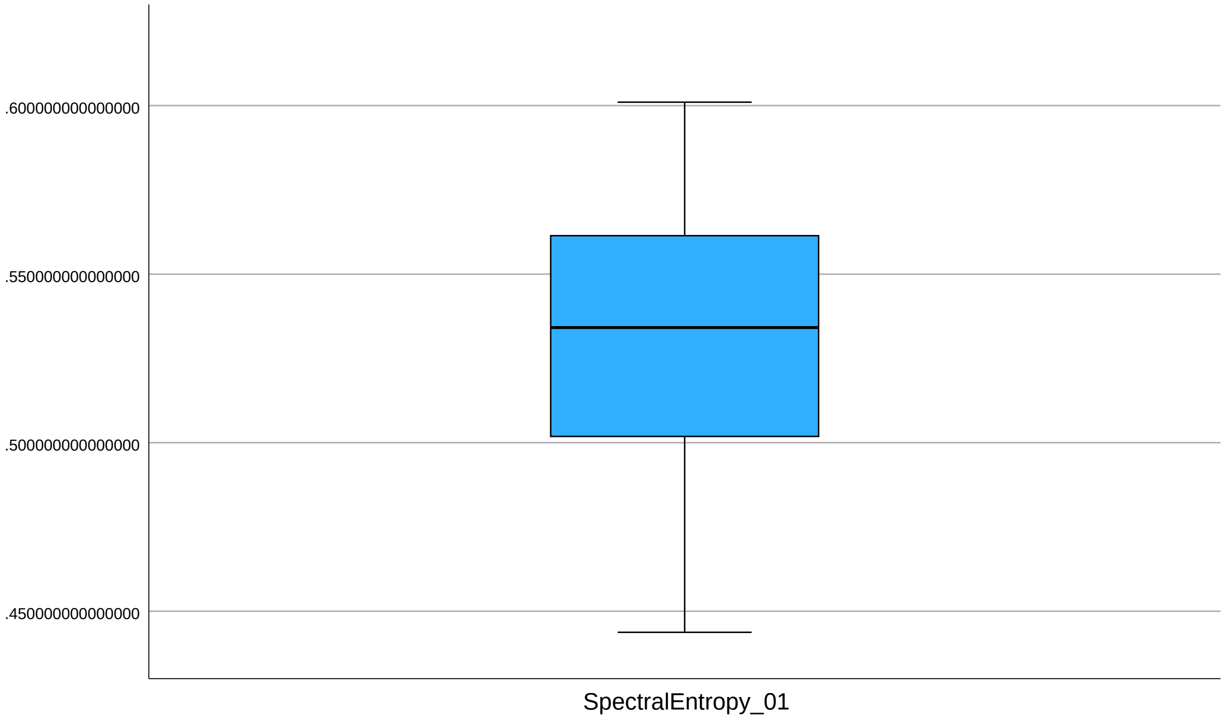

HiguchiFractalDimension

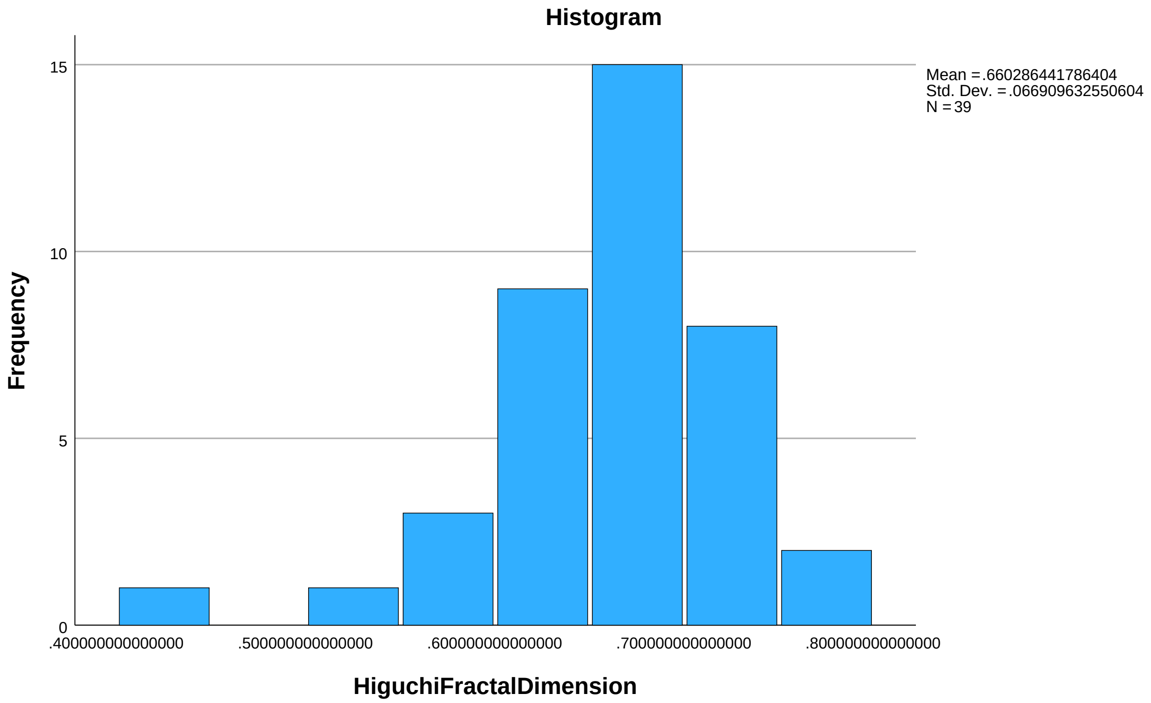


```
HiguchiFractalDimension Stem-and-Leaf Plot

 Frequency    Stem &  Leaf

     1.00 Extremes    (=<.41)
     1.00        5 .  4
     3.00        5 .  789
     9.00        6 .  000112244
    15.00        6 .  555566778899999
     8.00        7 .  00022333
     2.00        7 .  67

 Stem width:   .1000000
 Each leaf:        1 case(s)
```

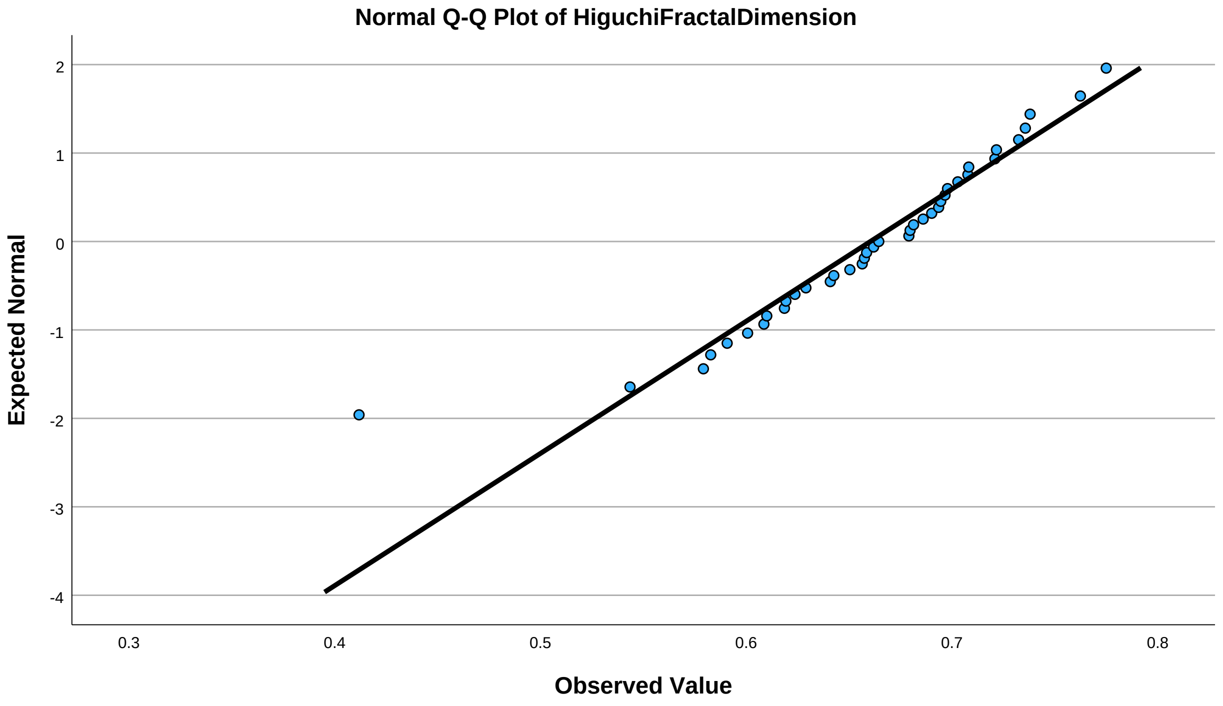

Normal Q-Q Plot of HiguchiFractalDimension
Expected Normal
2
1
0
-1
-2
-3
-4
0.3
0.4
0.5
0.6
0.7
0.8
Observed Value

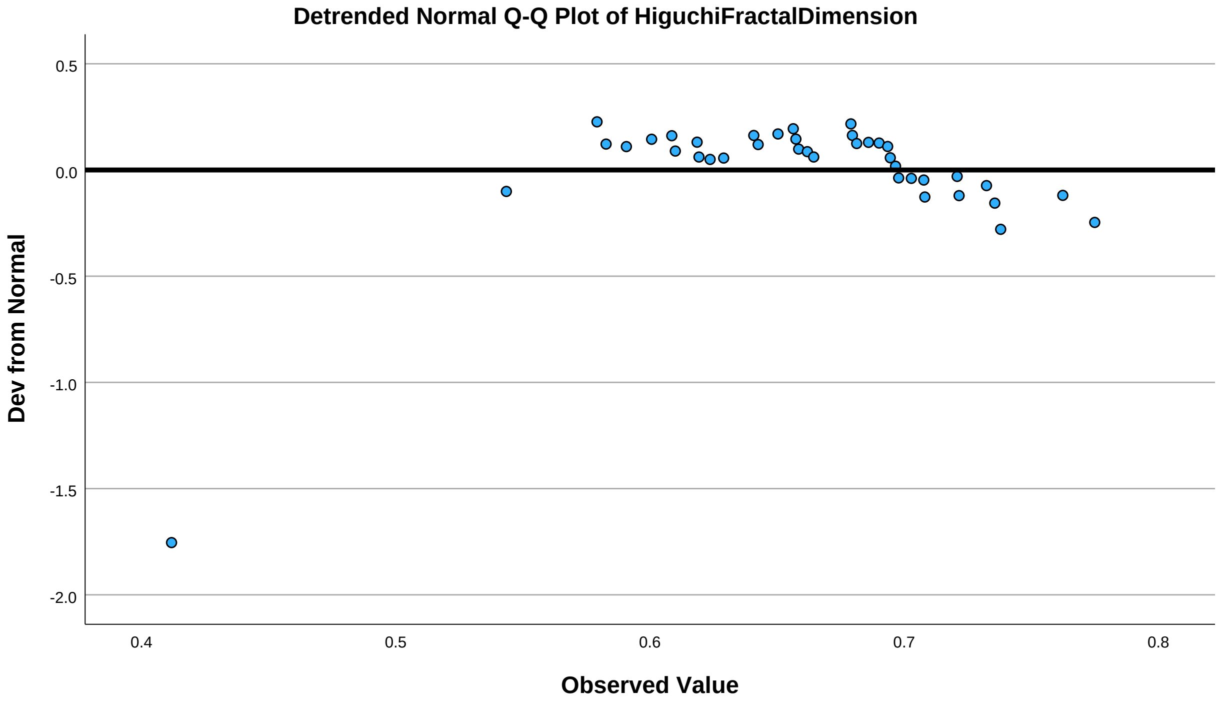

Detrended Normal Q-Q Plot of HiguchiFractalDimension
Dev from Normal
0.5
0.0
-0.5
-1.0
-1.5
-2.0
0.4
0.5
0.6
0.7
0.8
Observed Value

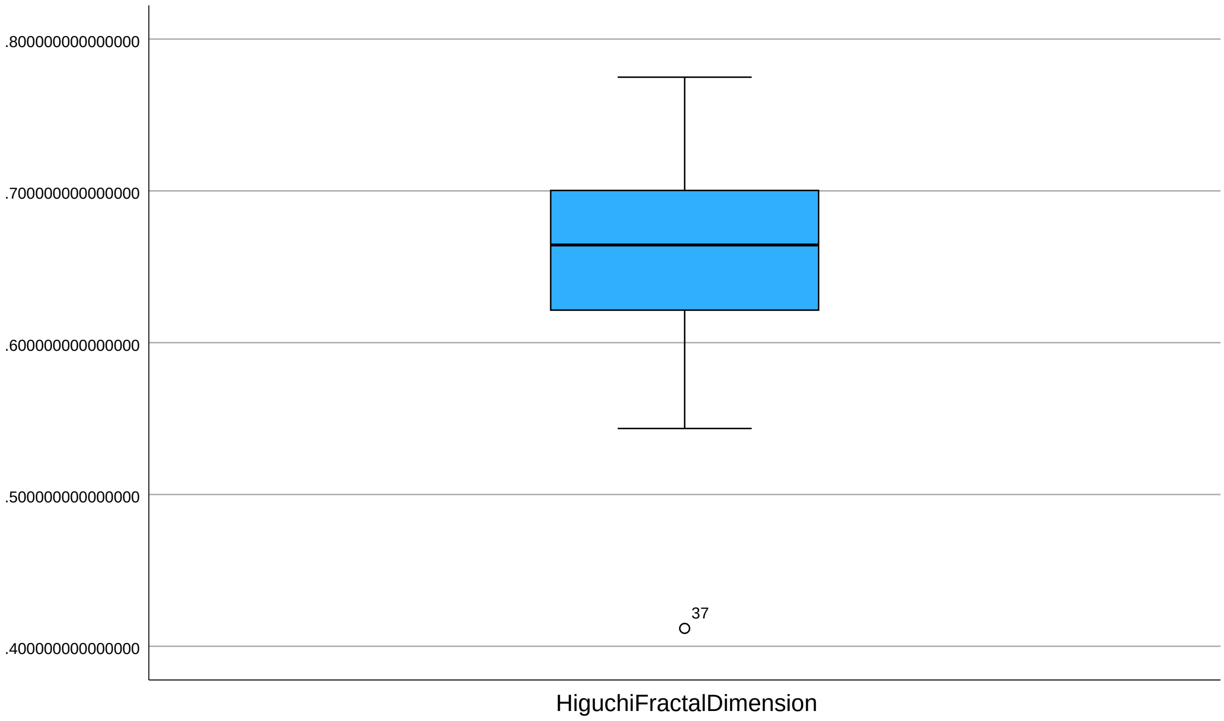

LempelZivComplexity

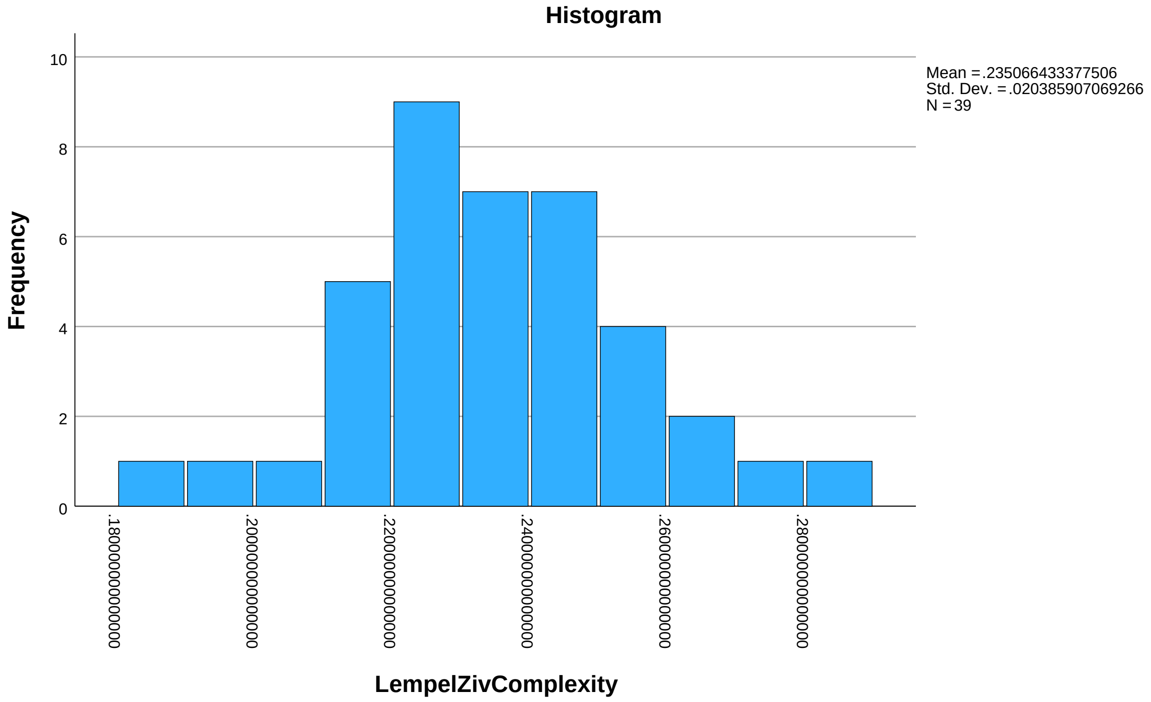

LempelZivComplexity Stem-and-Leaf Plot

Frequency   Stem & Leaf

```
 1.00     18 . 9
 1.00     19 . 7
 1.00     20 . 5
 5.00     21 . 03567
 9.00     22 . 033567899
 7.00     23 . 0113468
 7.00     24 . 1457778
 4.00     25 . 1267
 2.00     26 . 05
 1.00     27 . 7
 1.00 Extremes    (>=.287)

Stem width:   .0100000
Each leaf:        1 case(s)
```

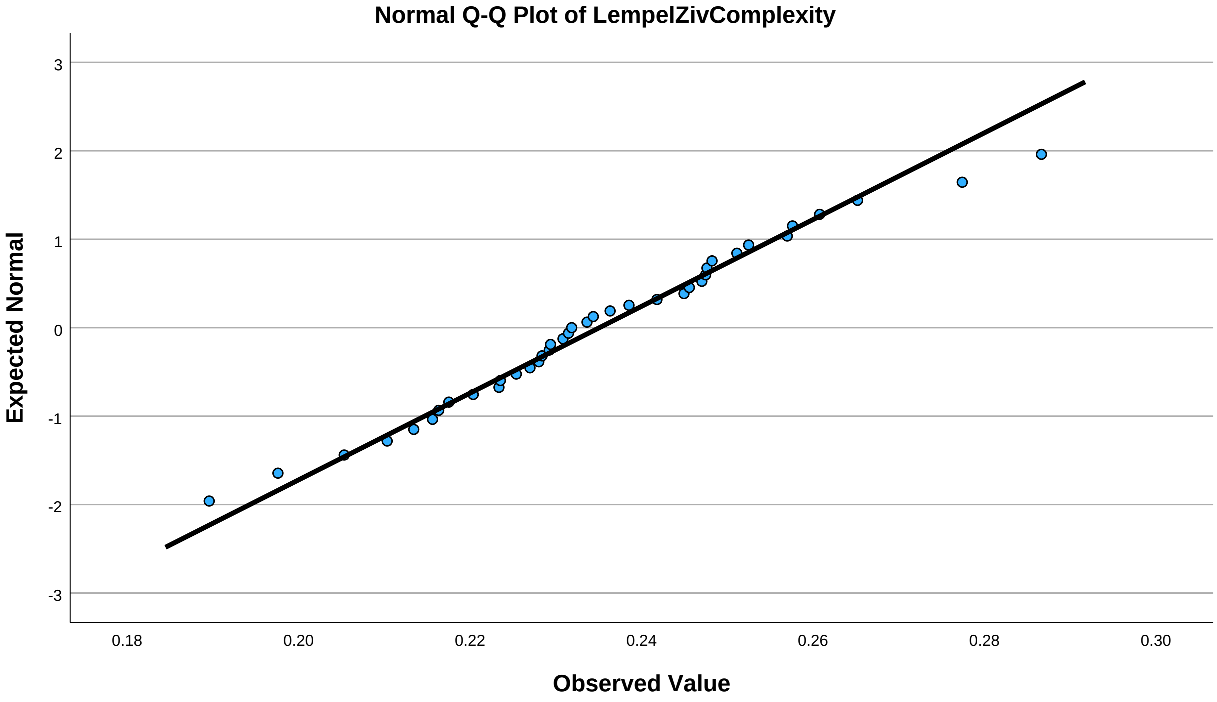

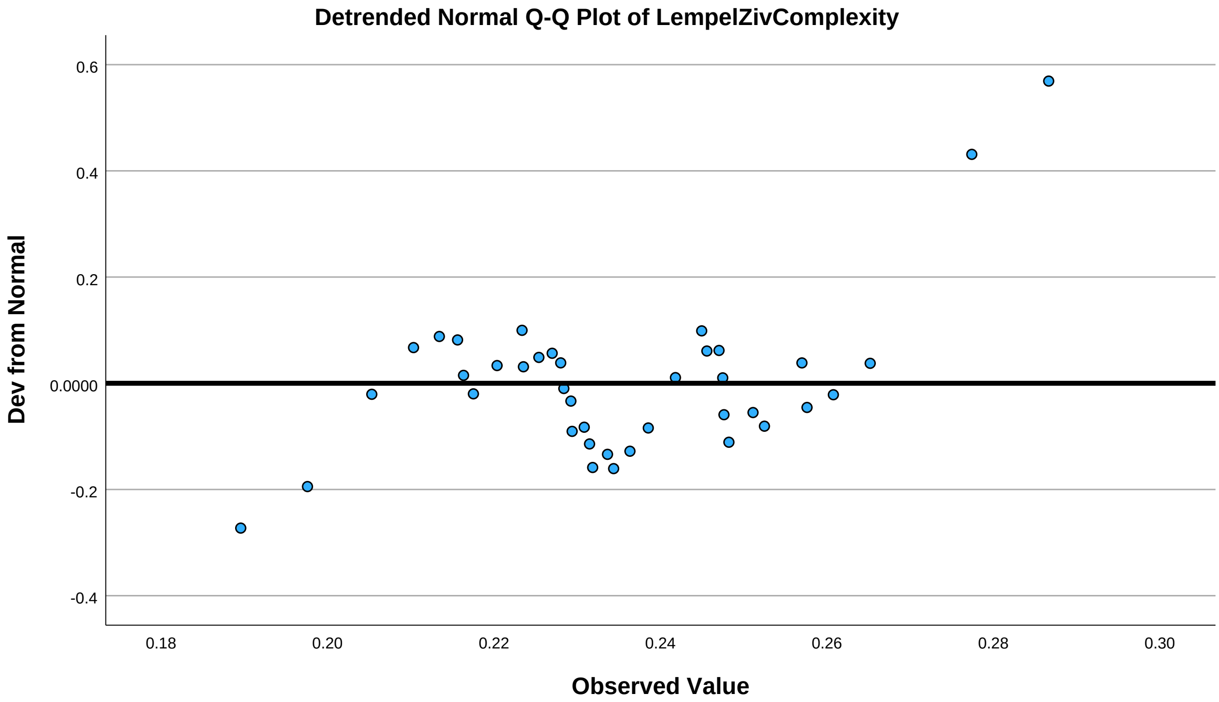

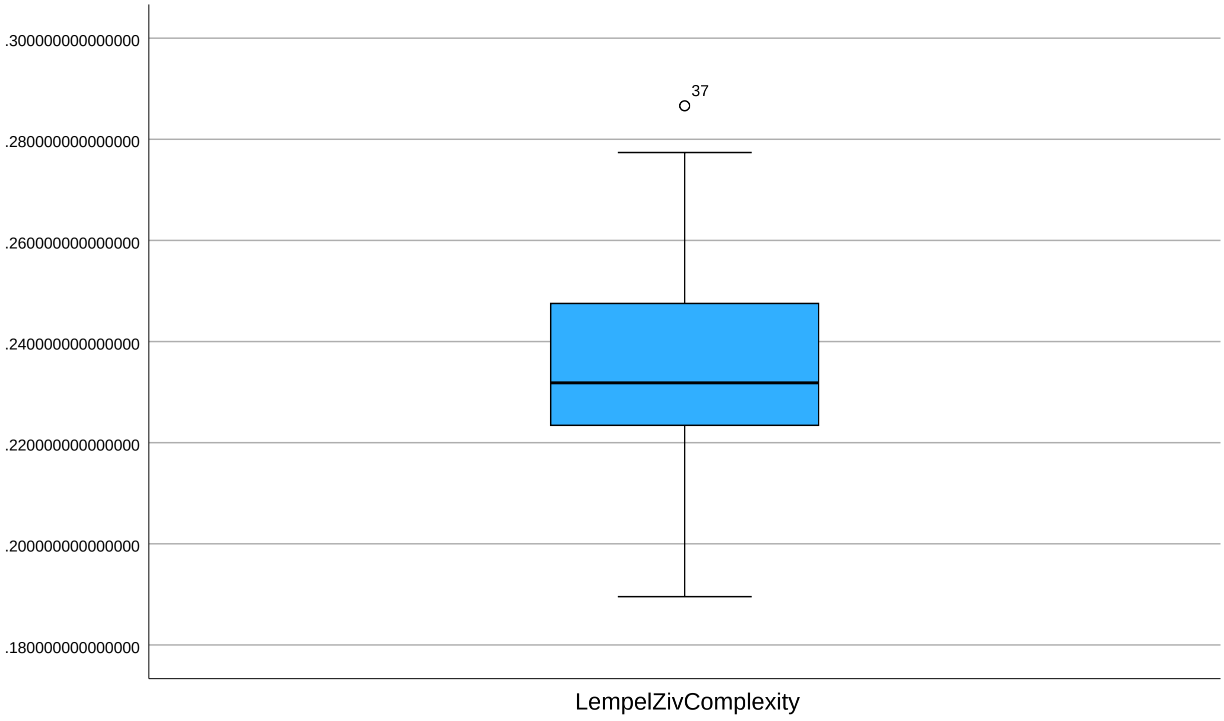

RenyiEntropy

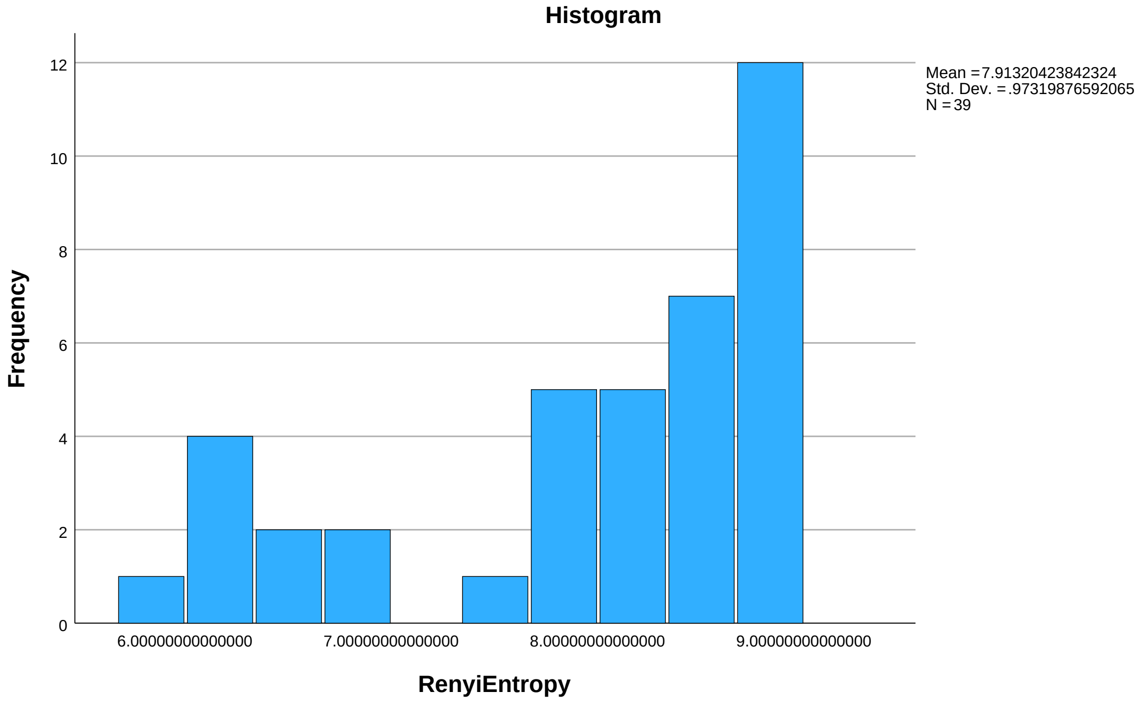


```
RenyiEntropy Stem-and-Leaf Plot

 Frequency    Stem &  Leaf

     3.00 Extremes    (=<6.1)
     3.00        6 .  113
     3.00        6 .  677
      .00        7 .
     6.00        7 .  578899
     8.00        8 .  00122334
    16.00        8 .  5666666677777778

 Stem width:   1.000000
 Each leaf:        1 case(s)
```

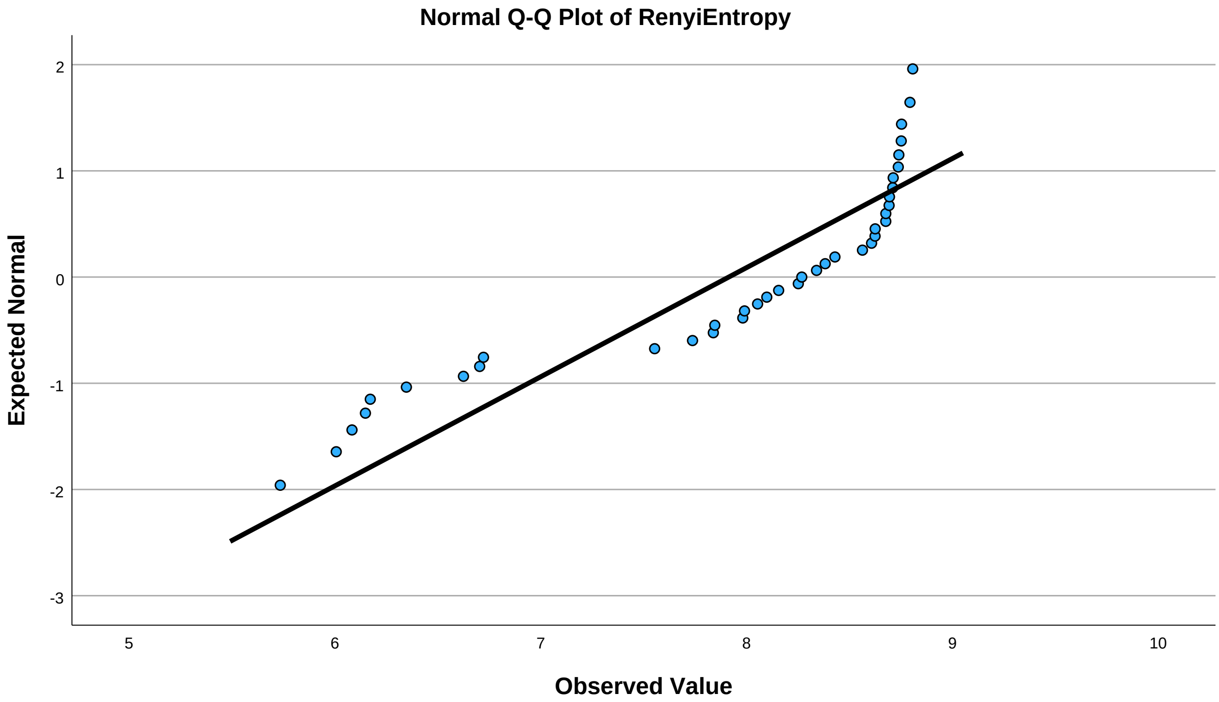
Normal Q-Q Plot of RenyiEntropy
Expected Normal
Observed Value
2
1
0
-1
-2
-3
5
6
7
8
9
10

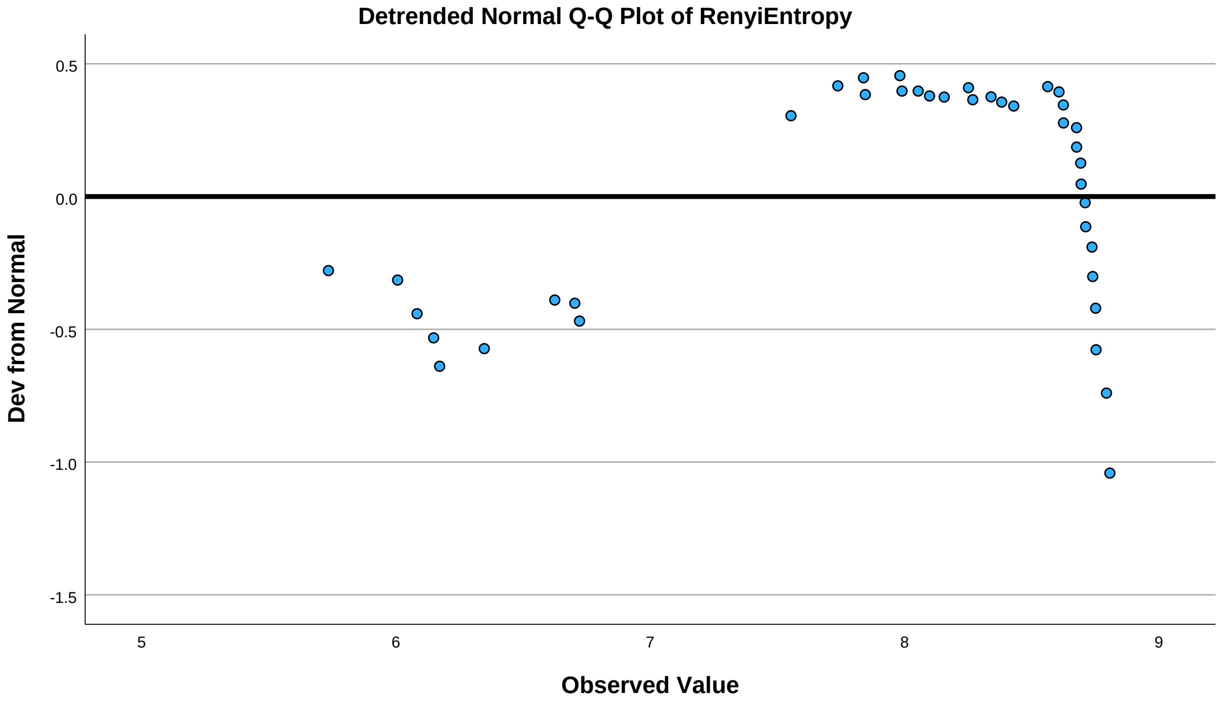
Detrended Normal Q-Q Plot of RenyiEntropy
Dev from Normal
Observed Value
0.5
0.0
-0.5
-1.0
-1.5
5
6
7
8
9

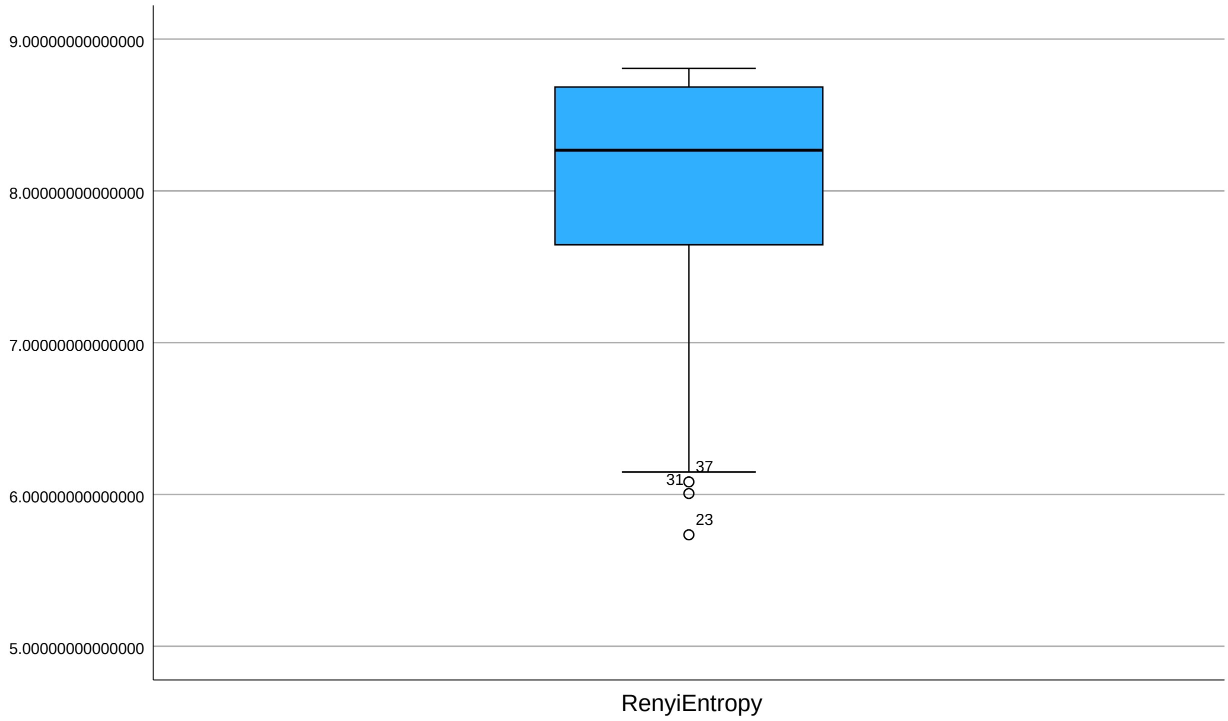

TsallisEntropy

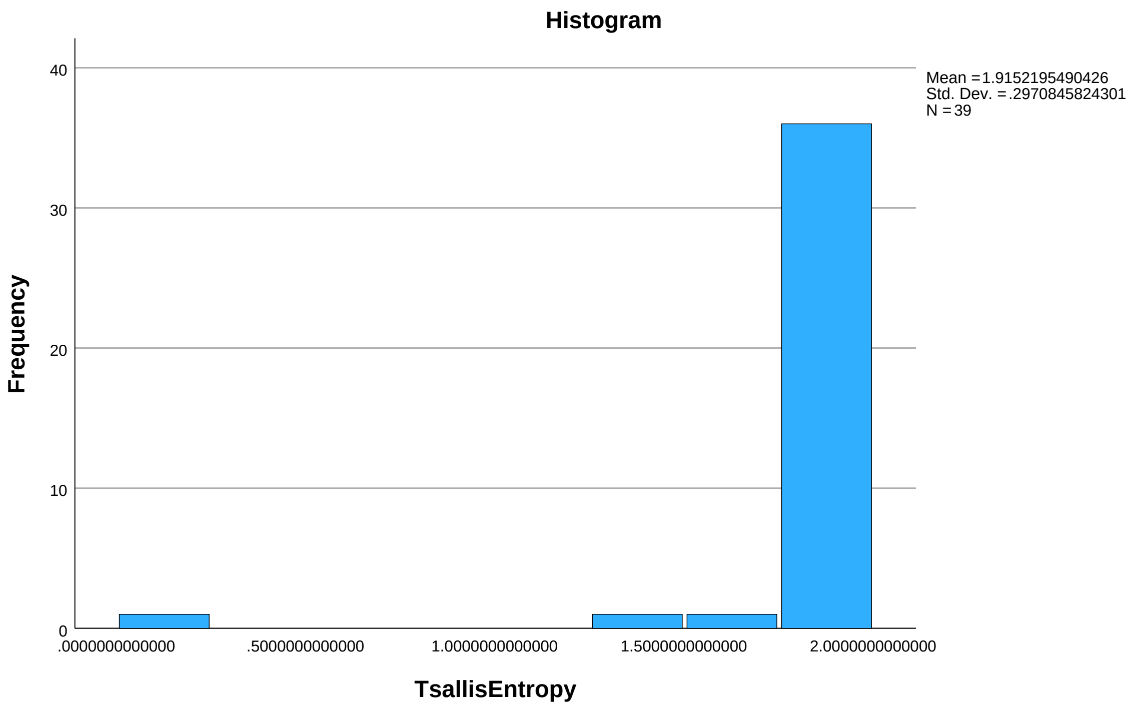


```
TsallisEntropy Stem-and-Leaf Plot

 Frequency    Stem &  Leaf
```

```
  7.00 Extremes    (=<1.9817)
  1.00    1983 .  3
   .00    1983 .
   .00    1984 .
  1.00    1984 .  7
  4.00    1985 .  0133
  8.00    1985 .  55667899
  8.00    1986 .  00122234
  5.00    1986 .  55679
  3.00    1987 .  000
   .00    1987 .
  2.00    1988 .  14

Stem width:  .0010000
Each leaf:       1 case(s)
```

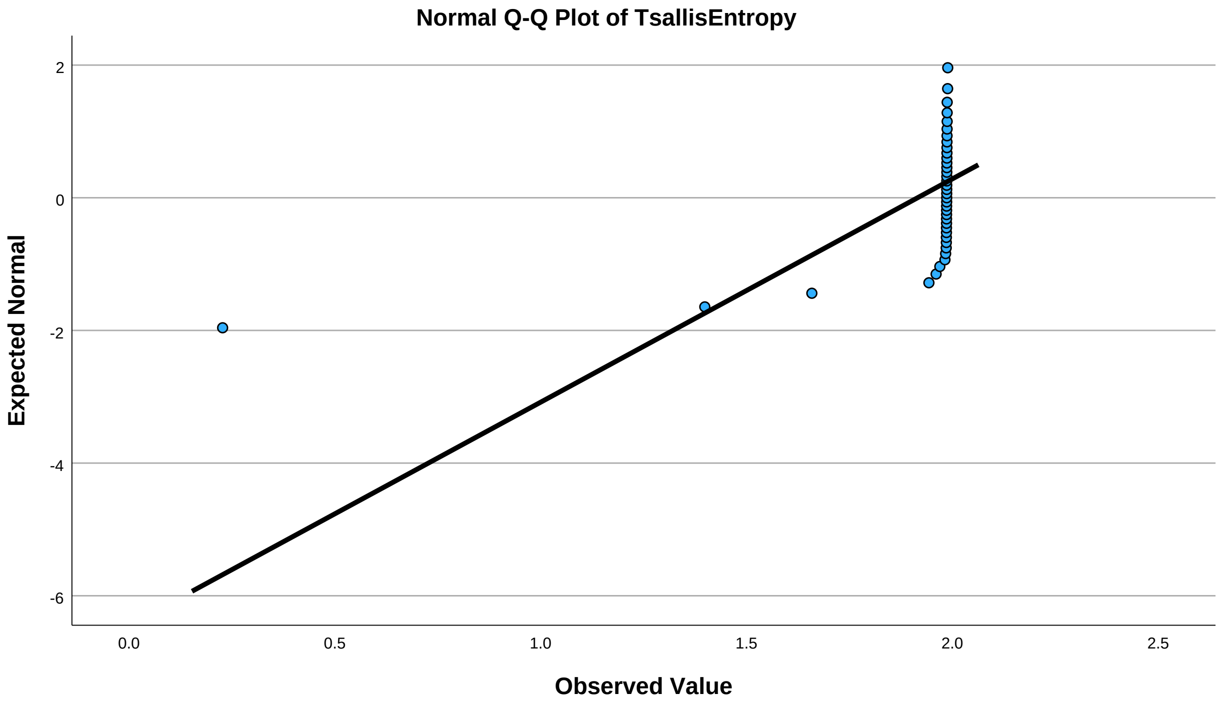

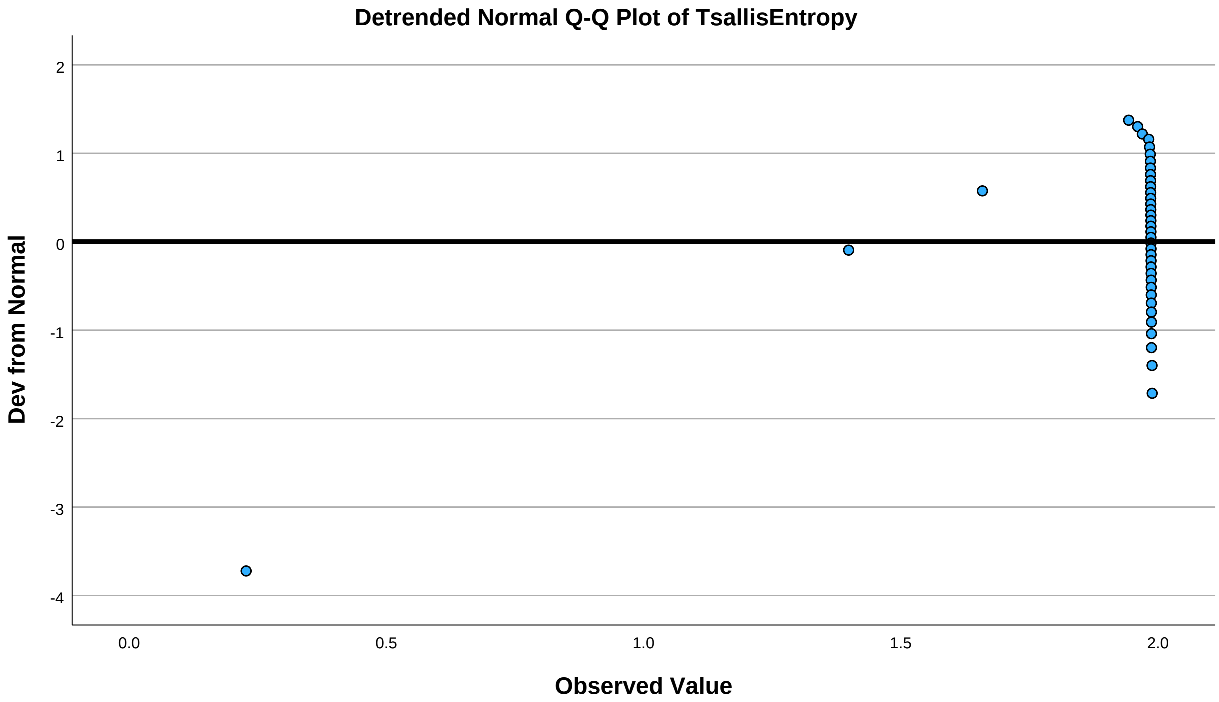
Detrended Normal Q-Q Plot of TsallisEntropy
Dev from Normal
2
1
0
-1
-2
-3
-4
0.0
0.5
1.0
1.5
2.0
Observed Value

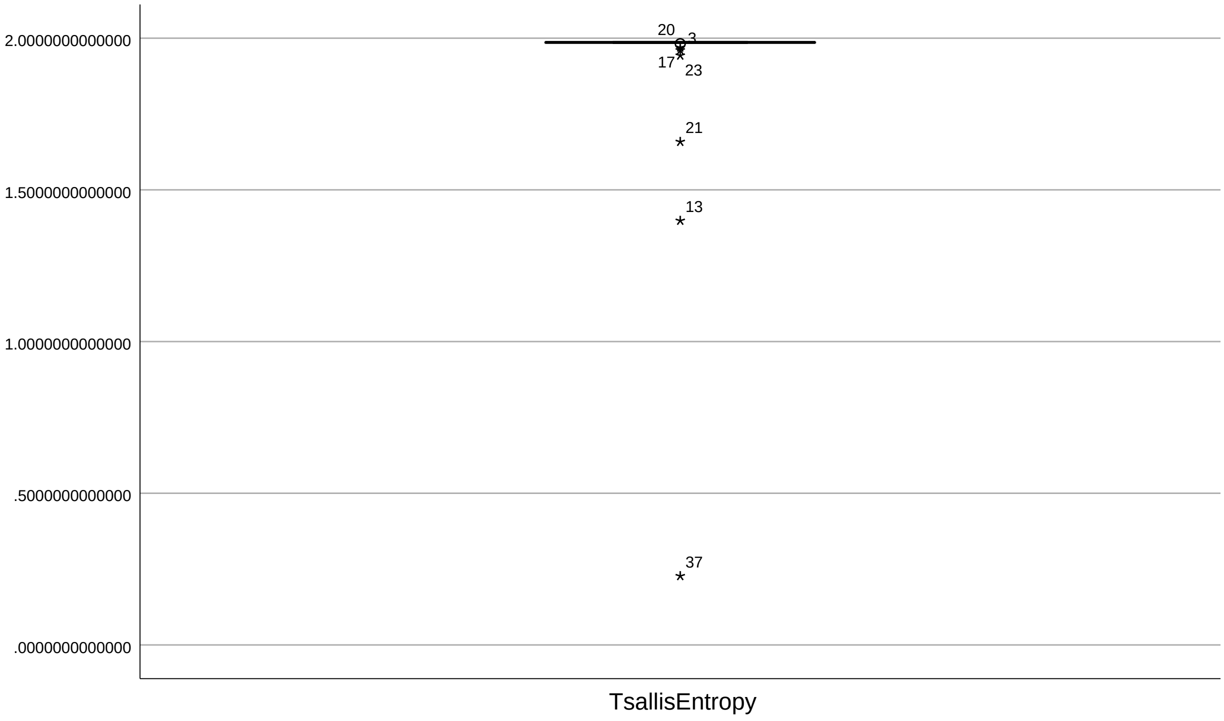
2.0000000000000
1.5000000000000
1.0000000000000
.5000000000000
.0000000000000
20
3
17
23
21
13
37
TsallisEntropy

PS_4_5

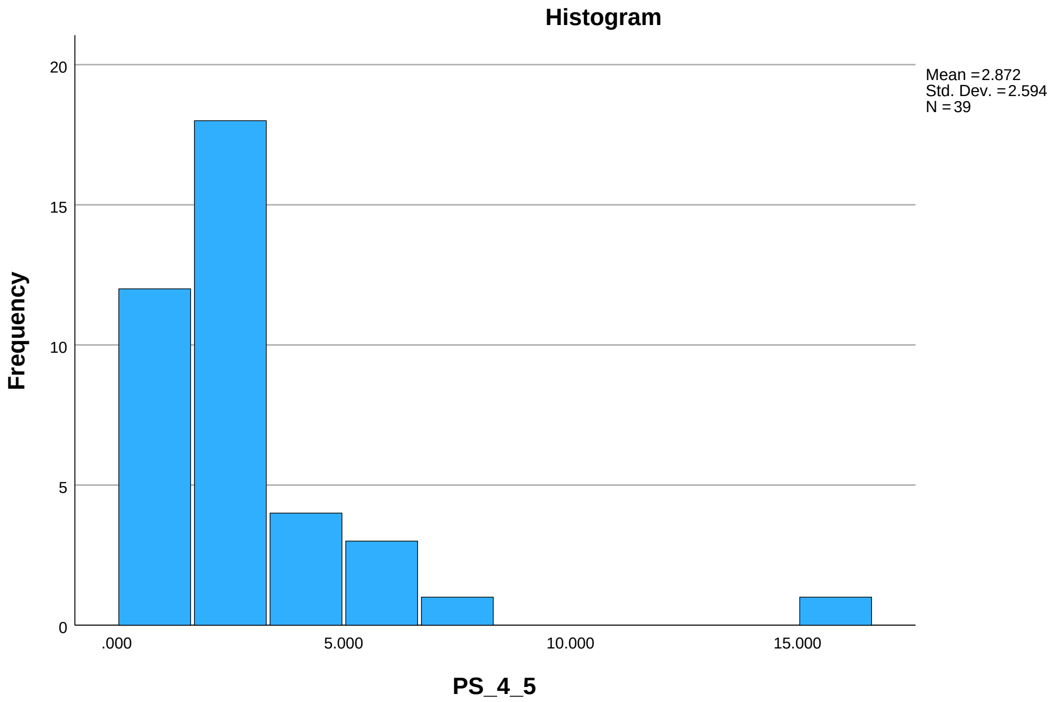

PS_4_5 Stem-and-Leaf Plot

```
 Frequency    Stem &  Leaf

     4.00        0 .  5689
     4.00        1 .  0033
     9.00        1 .  555568899
     7.00        2 .  0012223
     6.00        2 .  578899
     1.00        3 .  4
     1.00        3 .  7
     1.00        4 .  0
     1.00        4 .  8
     5.00 Extremes    (>=5.1)

 Stem width:      1.000
 Each leaf:       1 case(s)
```

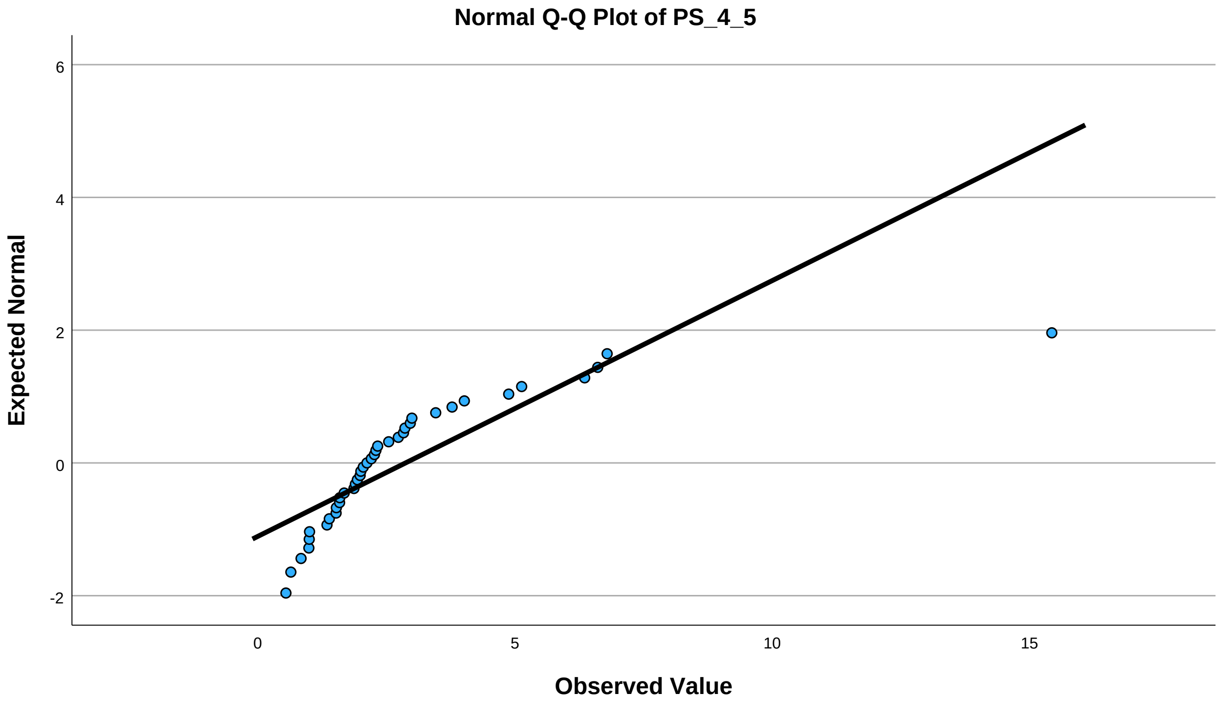
Normal Q-Q Plot of PS_4_5
Expected Normal
Observed Value
6
4
2
0
-2
0
5
10
15

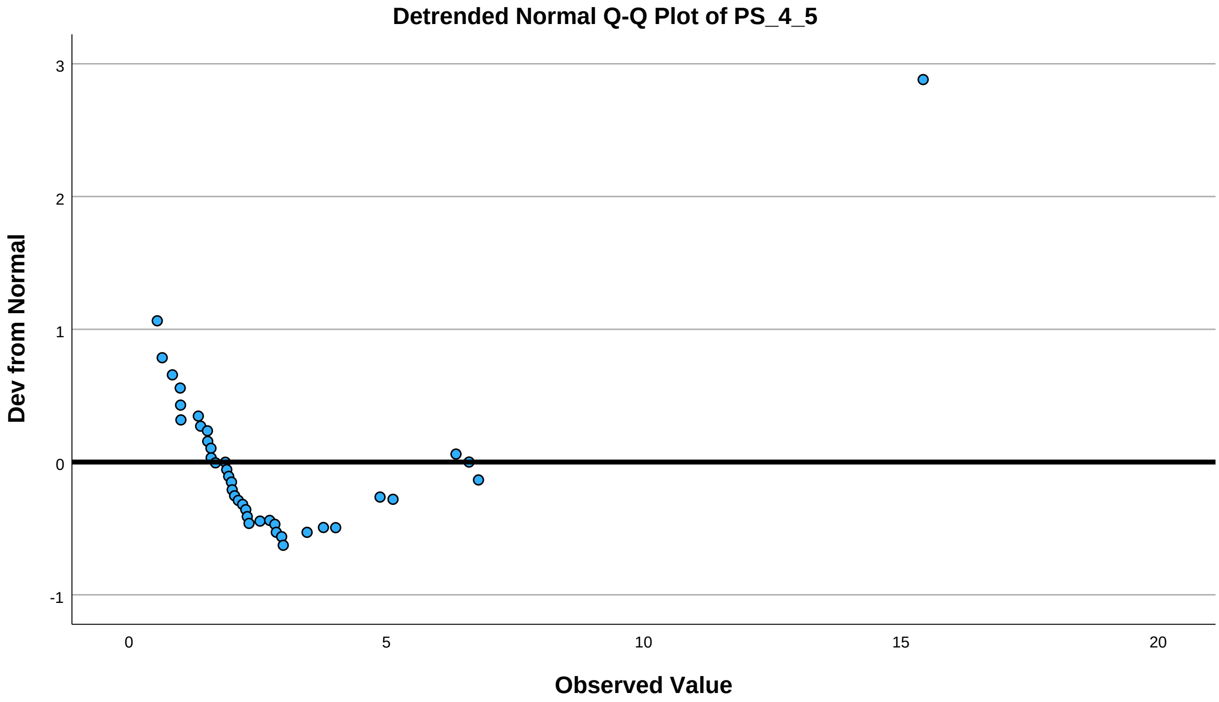
Detrended Normal Q-Q Plot of PS_4_5
Dev from Normal
Observed Value
3
2
1
0
-1
0
5
10
15
20

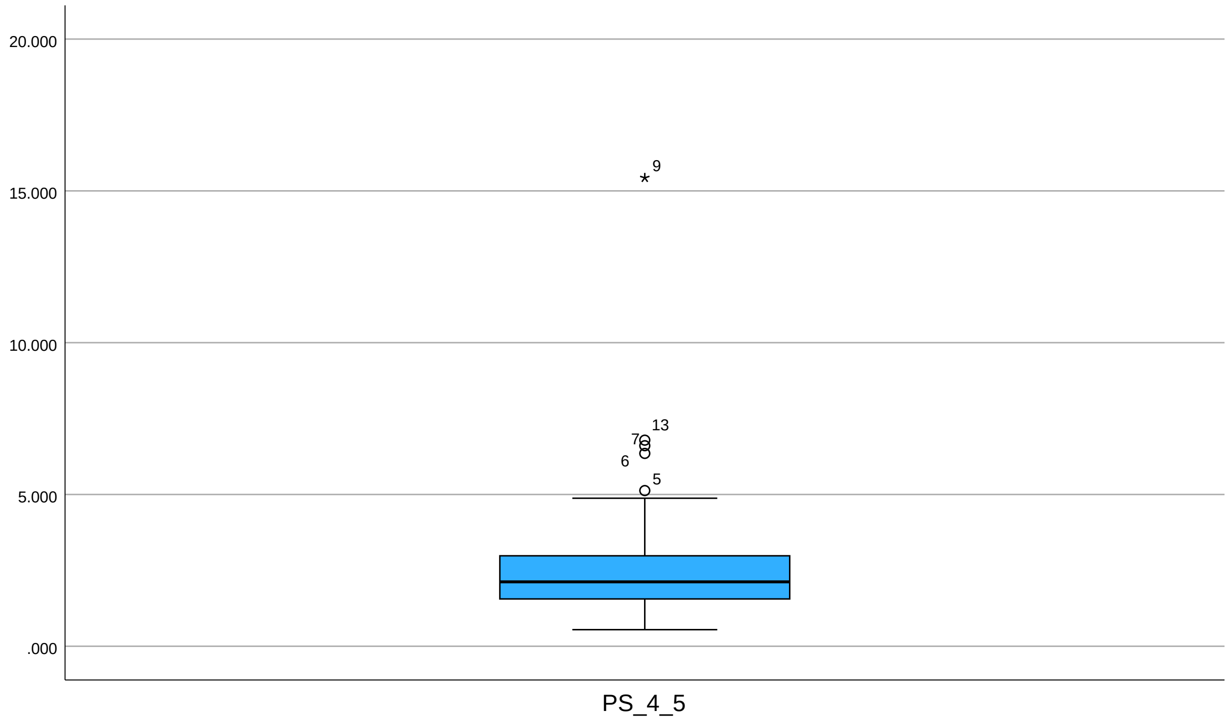

PS_5_6

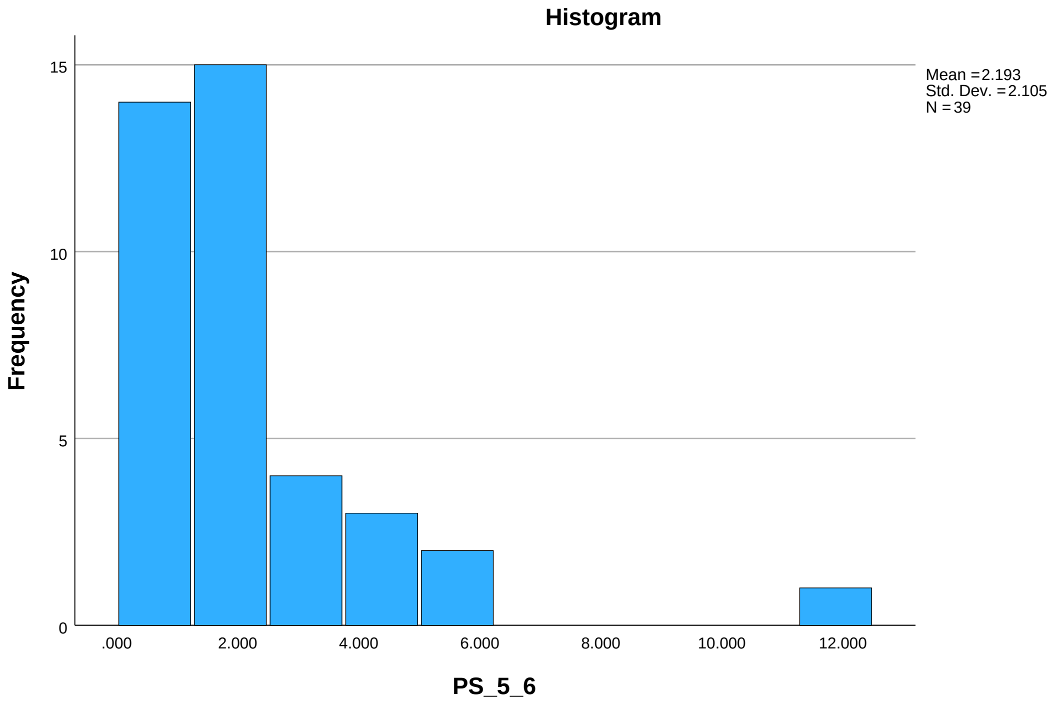


```
PS_5_6 Stem-and-Leaf Plot

 Frequency    Stem &  Leaf
```

```
  2.00      0 . 33
   8.00      0 . 66899999
  11.00      1 . 01112334444
   4.00      1 . 5678
   4.00      2 . 2234
   3.00      2 . 579
  1.00     3 . 0
   .00     3 .
  2.00      4 . 03
   4.00 Extremes    (>=4.8)

Stem width:     1.000
Each leaf:       1 case(s)
```

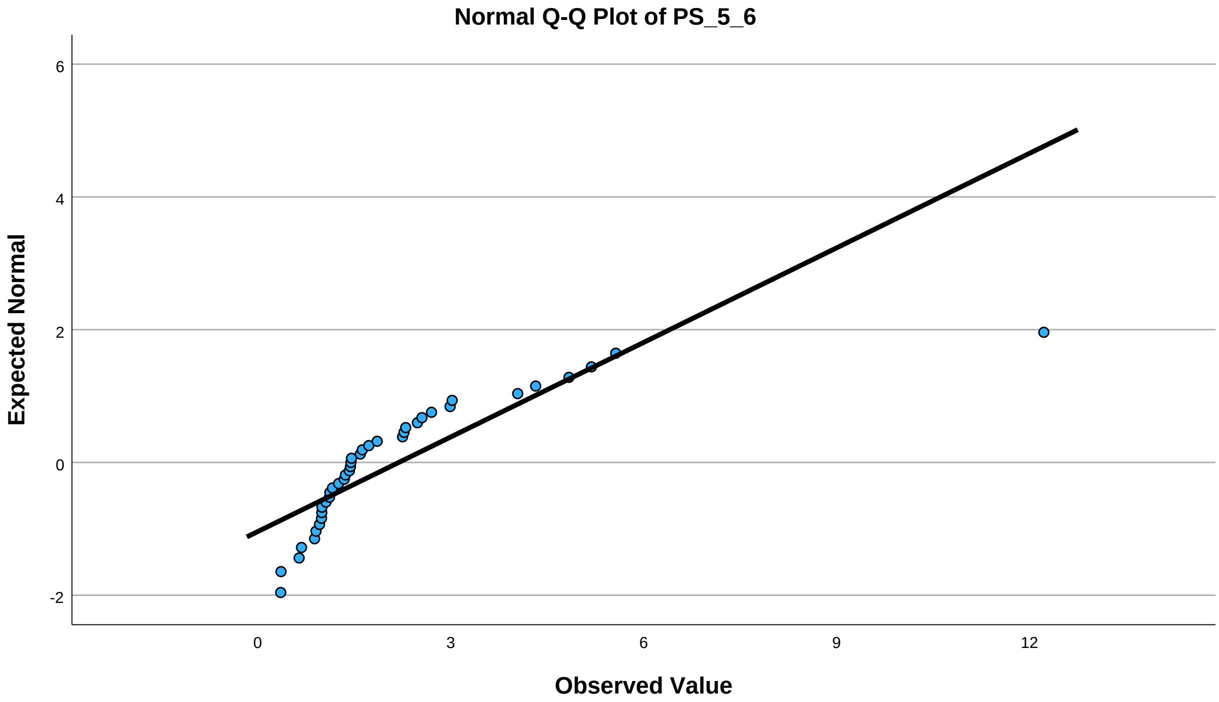

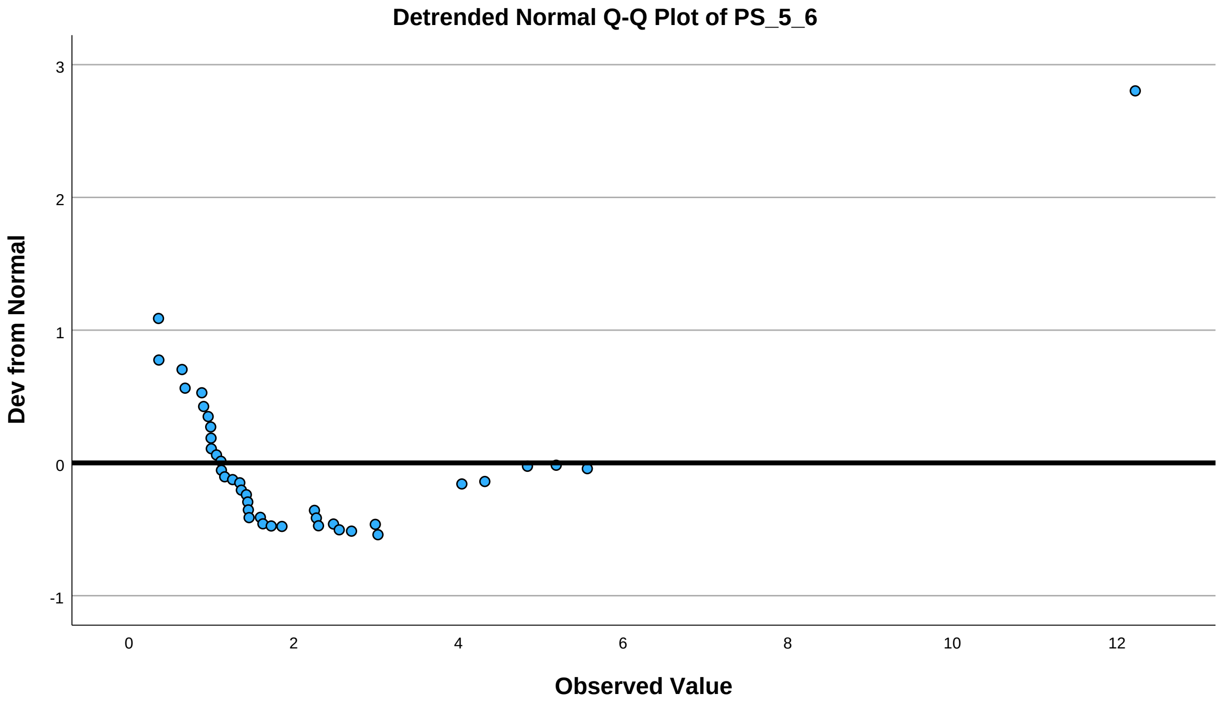
Detrended Normal Q-Q Plot of PS_5_6
Dev from Normal
Observed Value
3
2
1
0
-1
0
2
4
6
8
10
12

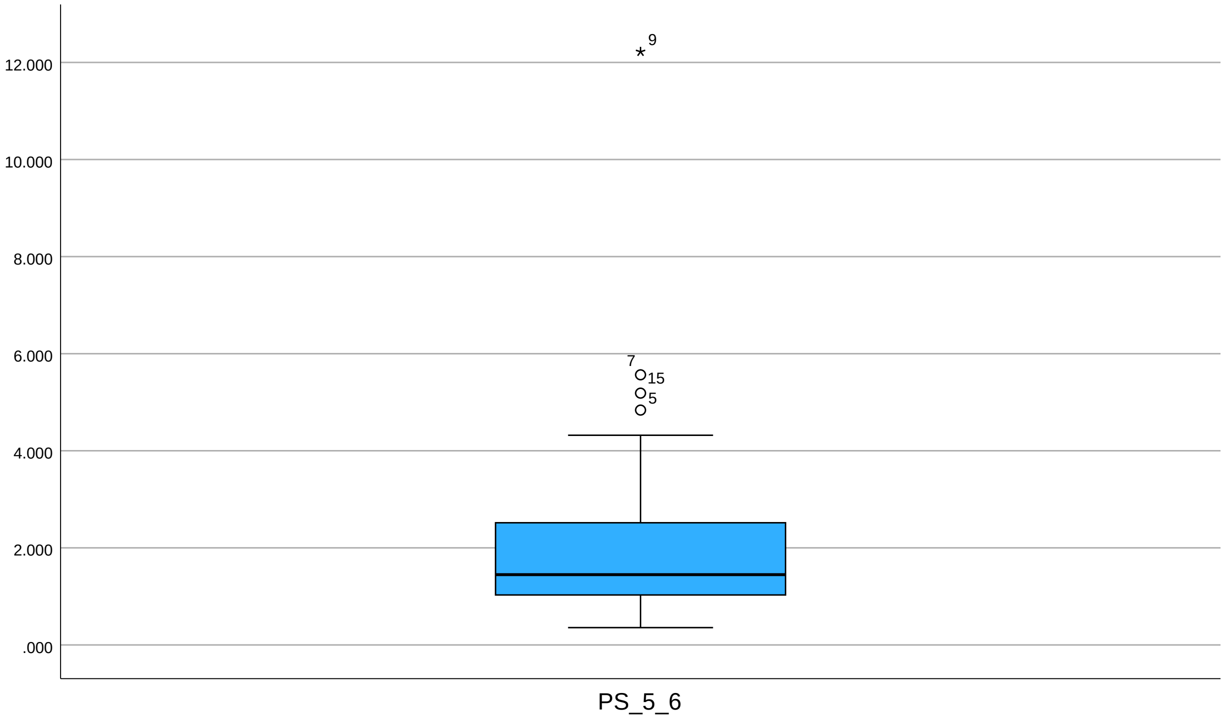
12.000
10.000
8.000
6.000
4.000
2.000
.000
9
7
15
5
PS_5_6

PS_6_7

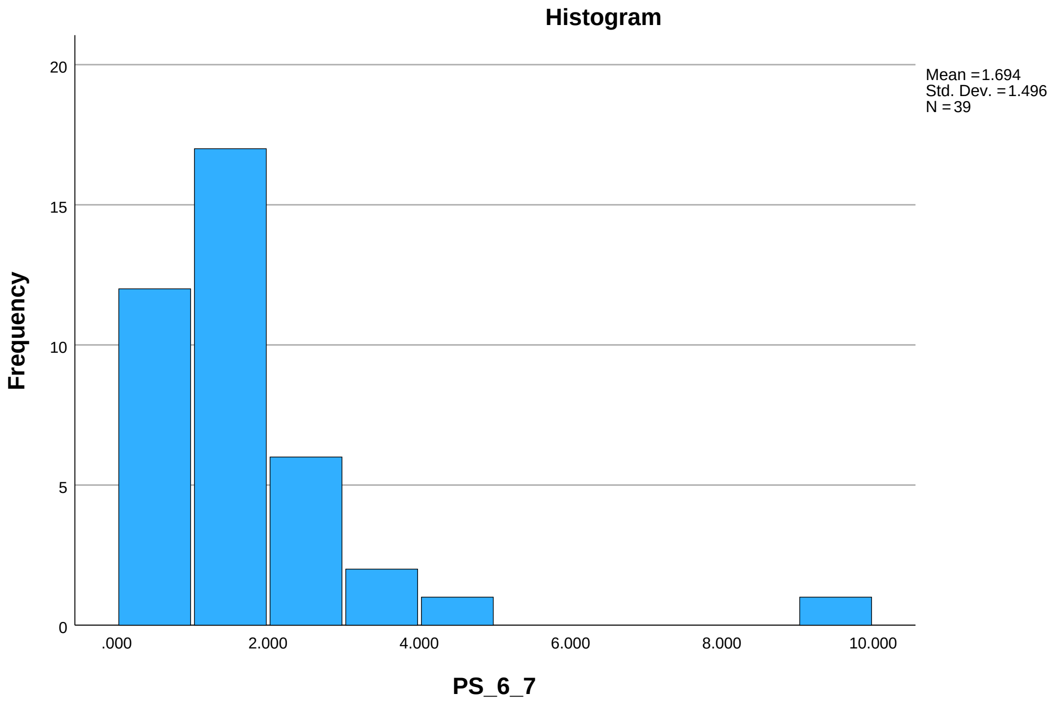


```
PS_6_7 Stem-and-Leaf Plot

 Frequency    Stem &  Leaf

     3.00        0 .  234
     9.00        0 .  577888999
    13.00        1 .  0011111222344
     4.00        1 .  6889
     3.00        2 .  224
     3.00        2 .  559
     2.00        3 .  01
     2.00 Extremes    (>=4.1)

 Stem width:      1.000
 Each leaf:       1 case(s)
```

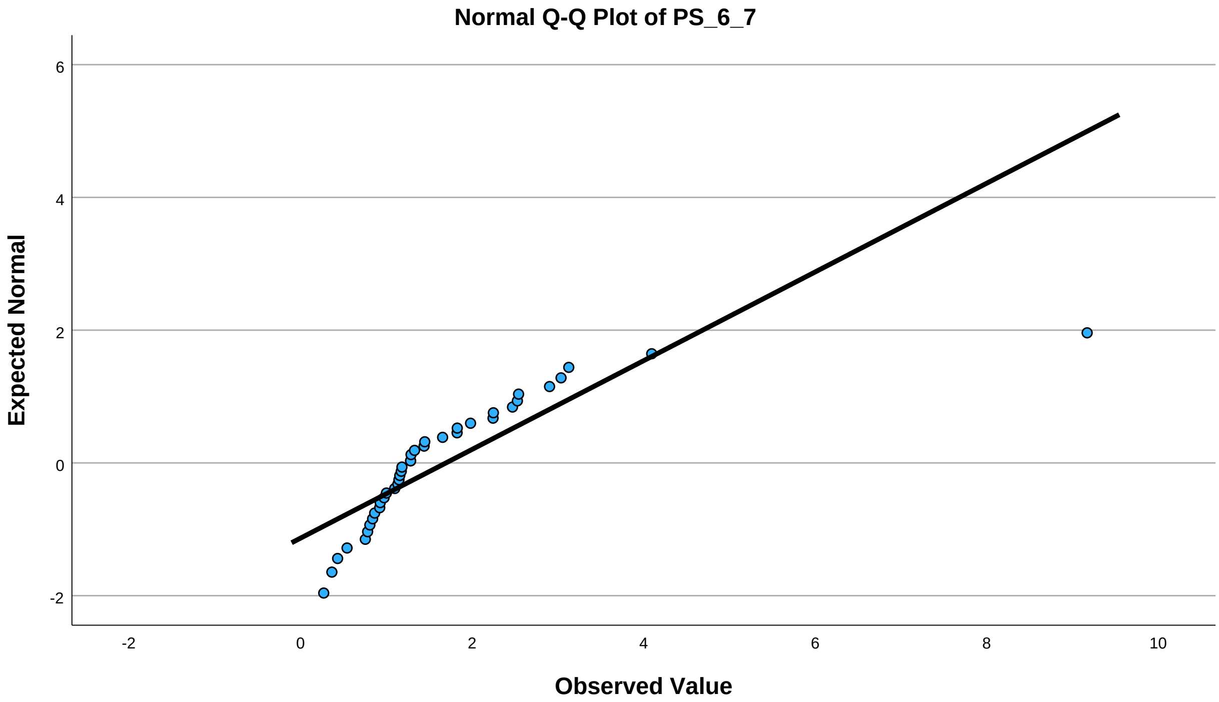
Normal Q-Q Plot of PS_6_7
Expected Normal
Observed Value
6
4
2
0
-2
-2
0
2
4
6
8
10

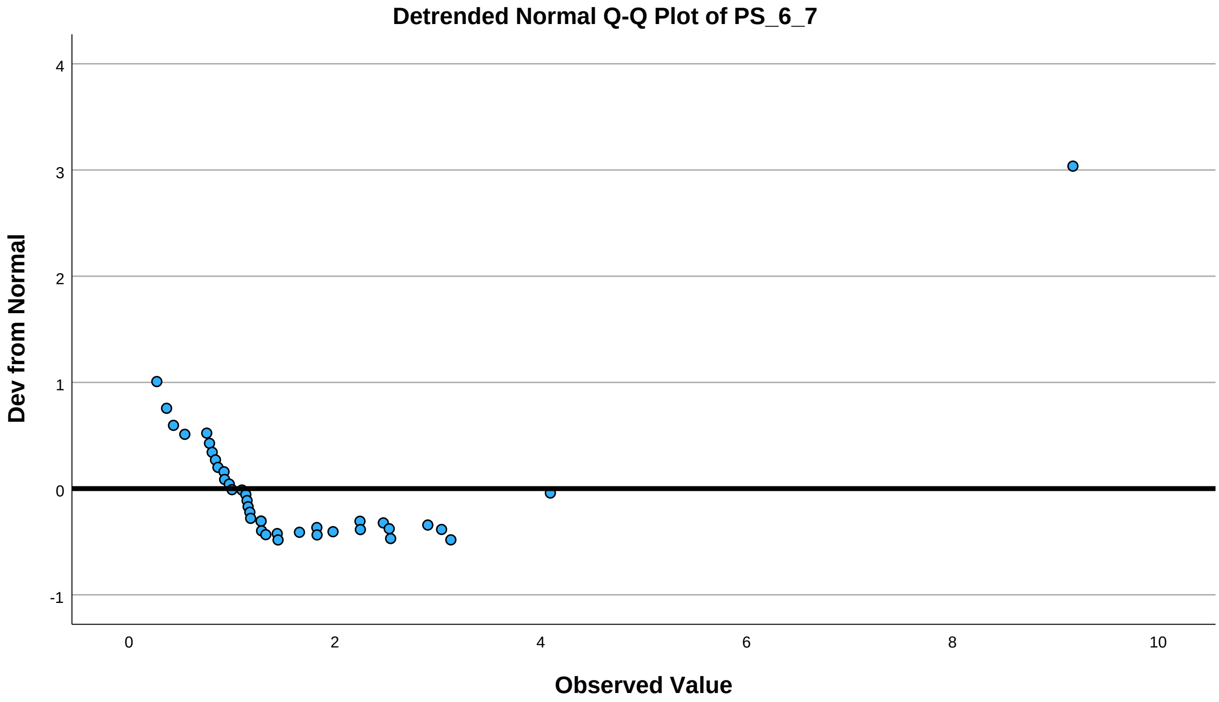
Detrended Normal Q-Q Plot of PS_6_7
Dev from Normal
Observed Value
4
3
2
1
0
-1
0
2
4
6
8
10

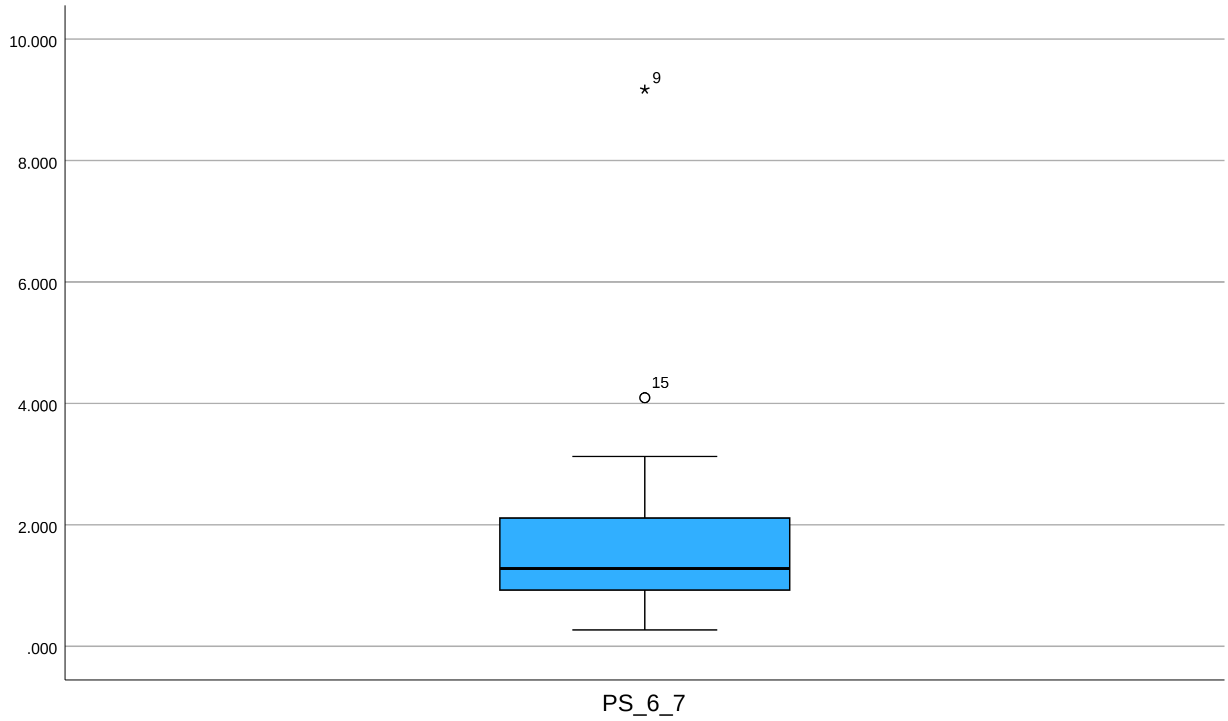

PS_7_8

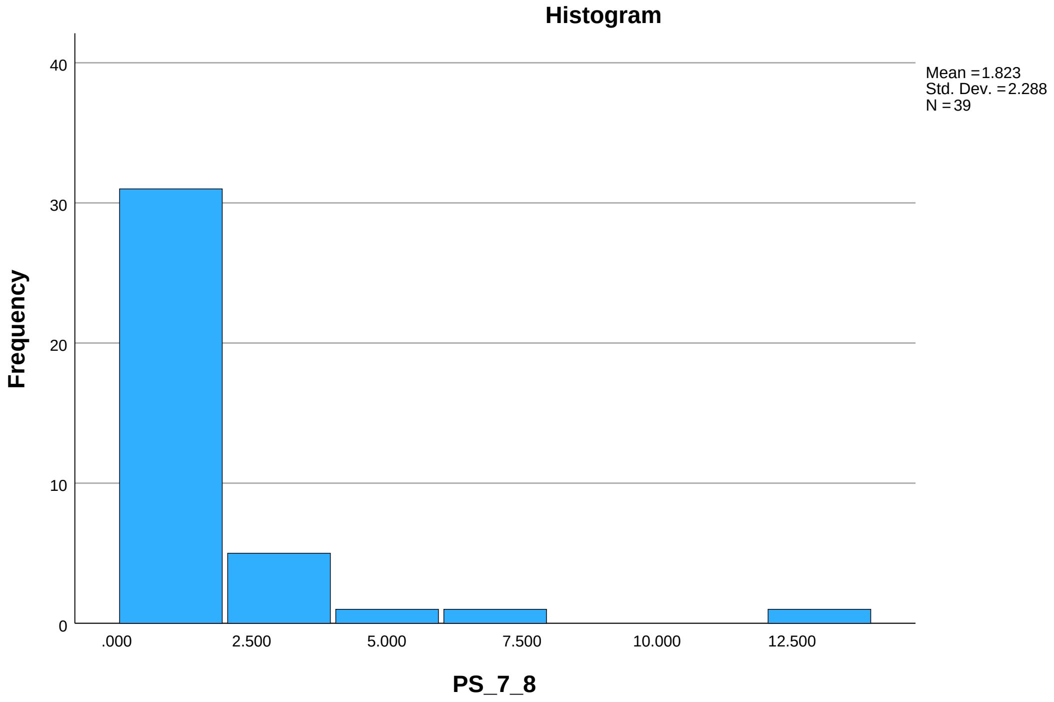


```
PS_7_8 Stem-and-Leaf Plot

 Frequency    Stem &  Leaf
```

```
     4.00        0 .  2344
    14.00        0 .  67777888888899
     8.00        1 .  11233344
     5.00        1 .  66668
     1.00        2 .  4
     3.00        2 .  558
     4.00 Extremes    (>=3.0)

 Stem width:      1.000
 Each leaf:        1 case(s)
```

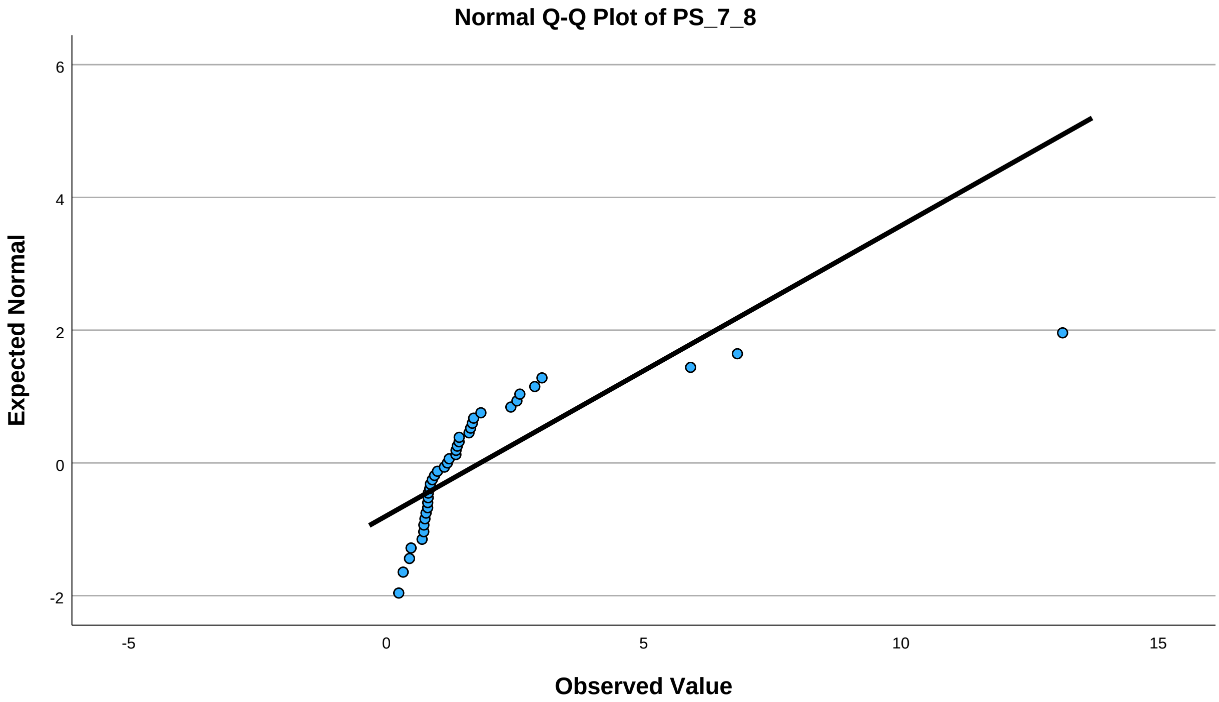

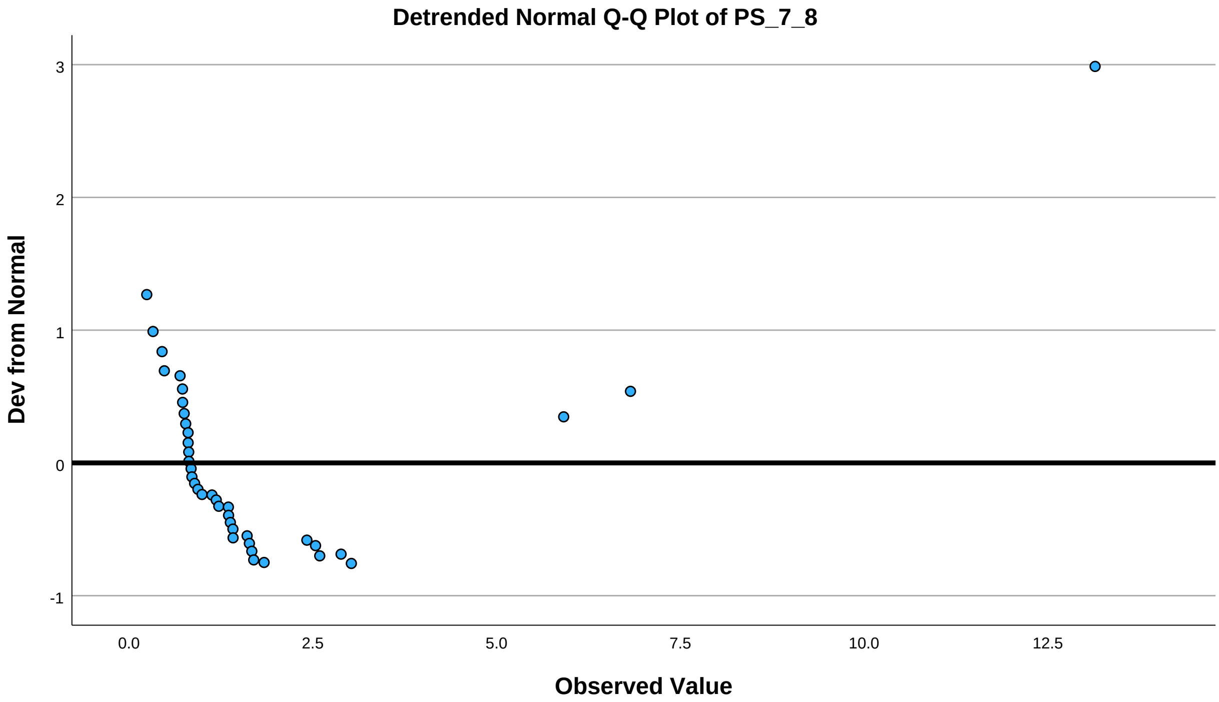
Detrended Normal Q-Q Plot of PS_7_8
Dev from Normal
3
2
1
0
-1
0.0
2.5
5.0
7.5
10.0
12.5
Observed Value

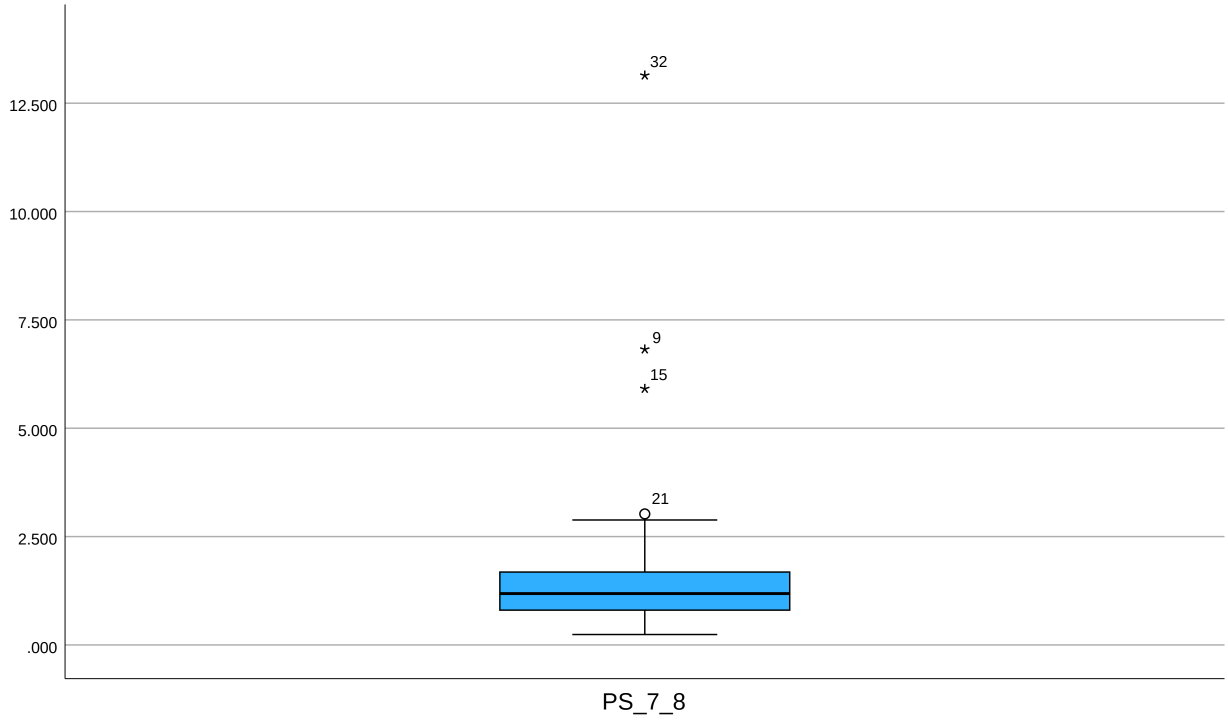
32
9
15
21
12.500
10.000
7.500
5.000
2.500
.000
PS_7_8

PS_8_9

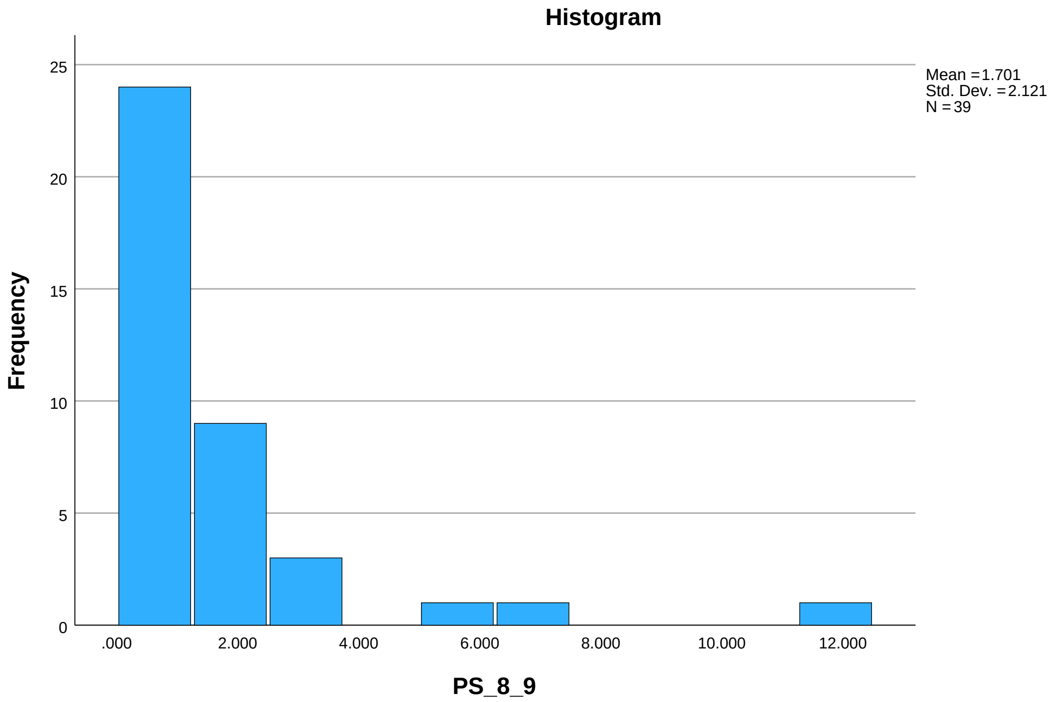


```
PS_8_9 Stem-and-Leaf Plot

 Frequency    Stem &  Leaf

     3.00        0 .  223
    14.00        0 .  55556777788899
    11.00        1 .  00111122224
     3.00        1 .  789
     2.00        2 .  13
     3.00        2 .  778
     3.00 Extremes    (>=5.2)

 Stem width:      1.000
 Each leaf:       1 case(s)
```

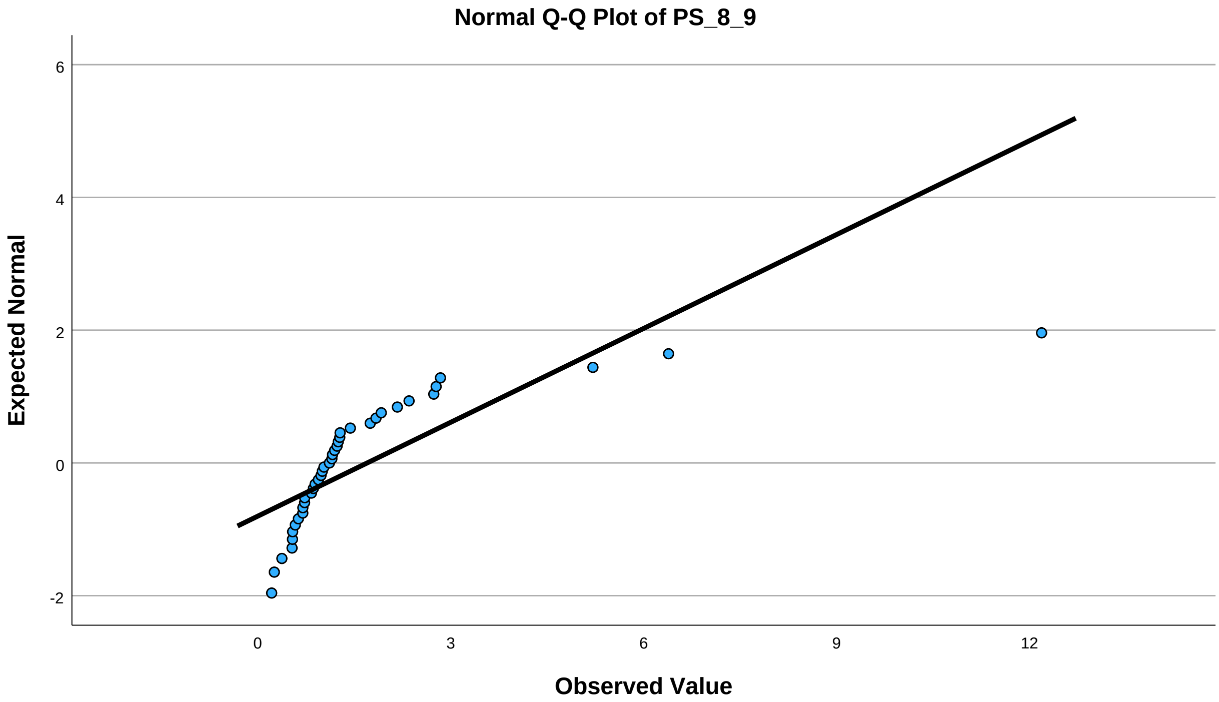
Normal Q-Q Plot of PS_8_9
Expected Normal
6
4
2
0
-2
0
3
6
9
12
Observed Value

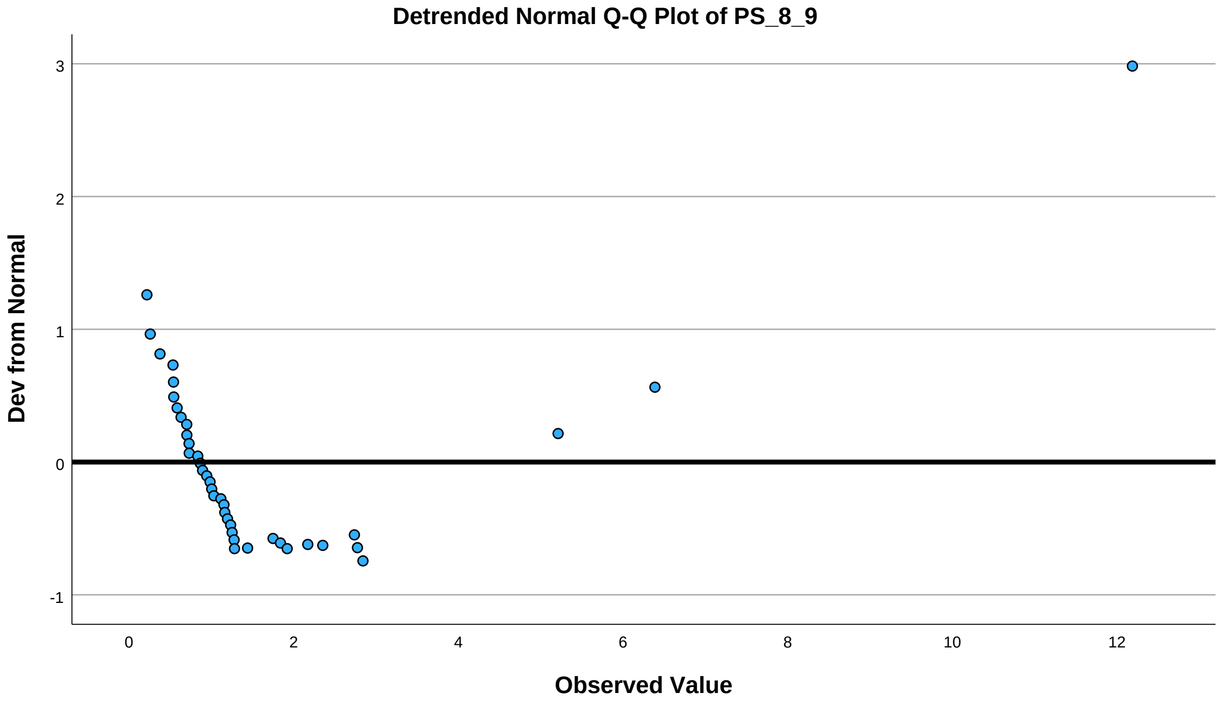
Detrended Normal Q-Q Plot of PS_8_9
Dev from Normal
3
2
1
0
-1
0
2
4
6
8
10
12
Observed Value

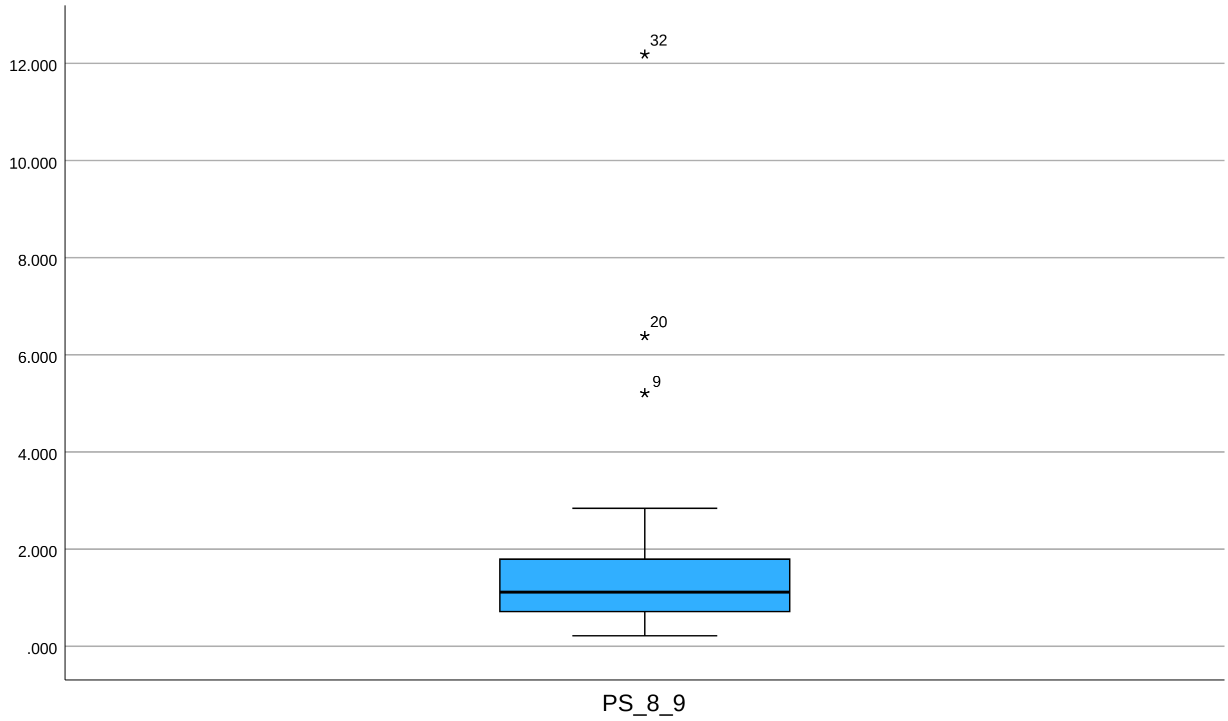

PS_9_10

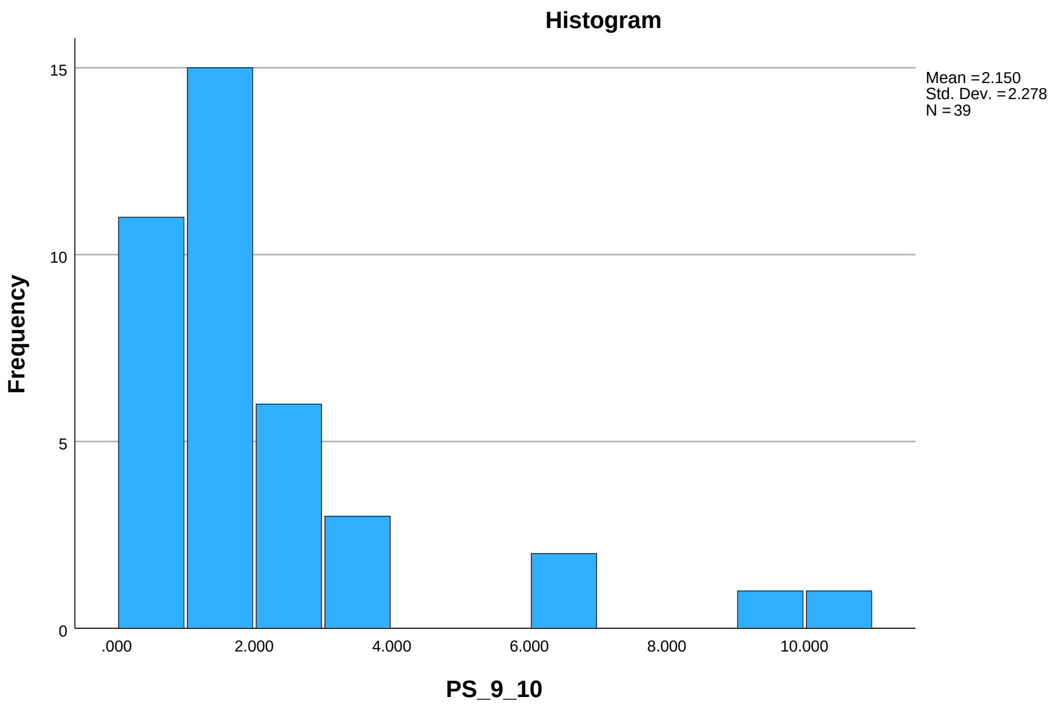

PS_9_10 Stem-and-Leaf Plot

Frequency   Stem & Leaf

```
    4.00        0 .  2224
     7.00        0 .  5556688
    10.00         1 .  1122222334
     5.00        1 .  5579
     5.00        2 .  12344
    1.00       2 .  9
    3.00       3 .  112
     4.00 Extremes      (>=6.4)

Stem width:      1.000
Each leaf:        1 case(s)
```

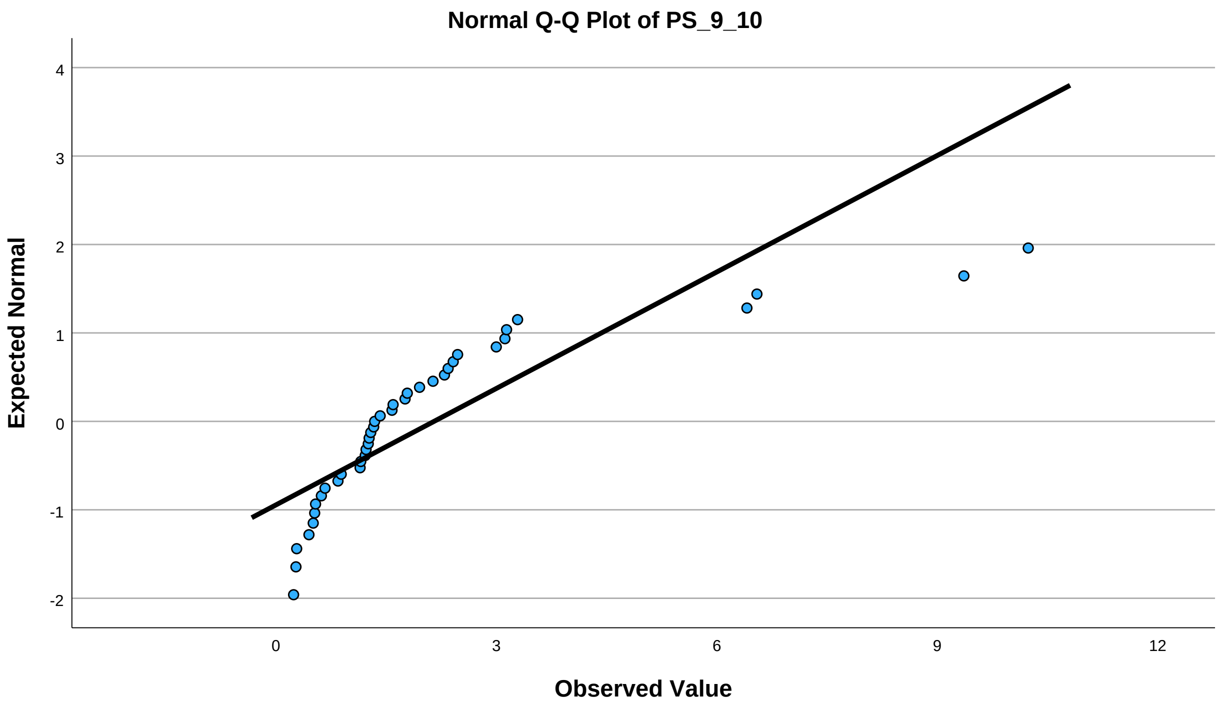

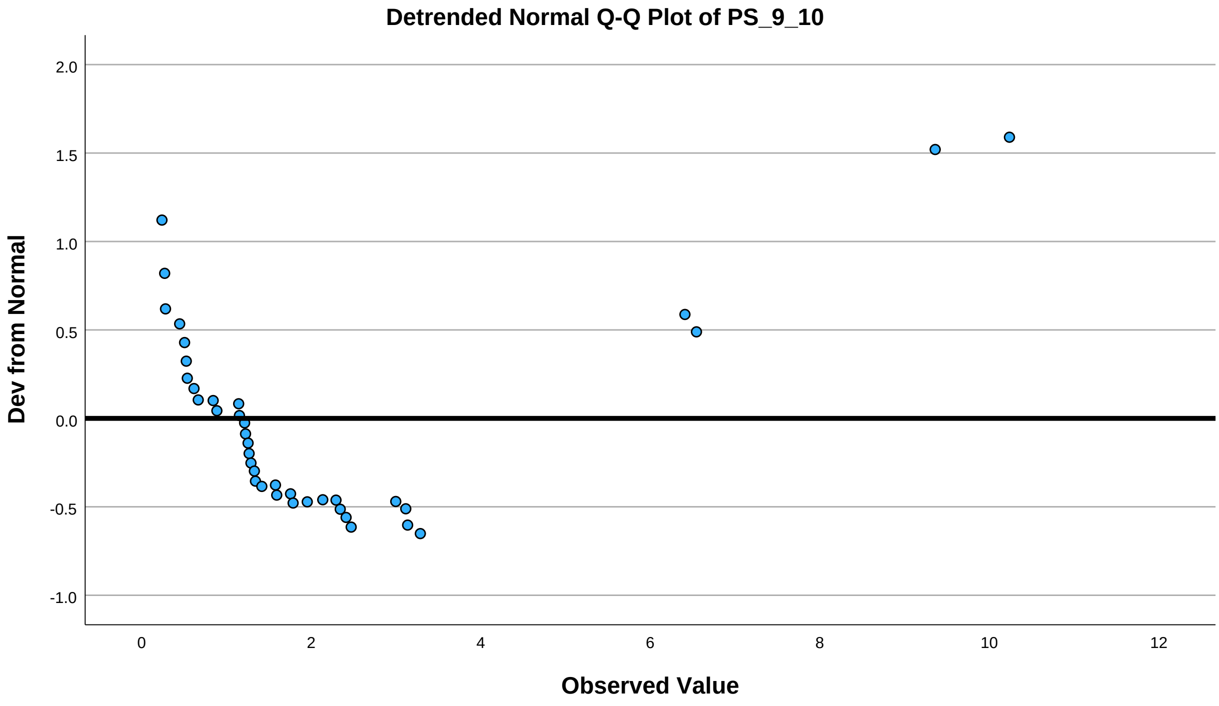
Detrended Normal Q-Q Plot of PS_9_10
Dev from Normal
2.0
1.5
1.0
0.5
0.0
-0.5
-1.0
0
2
4
6
8
10
12
Observed Value

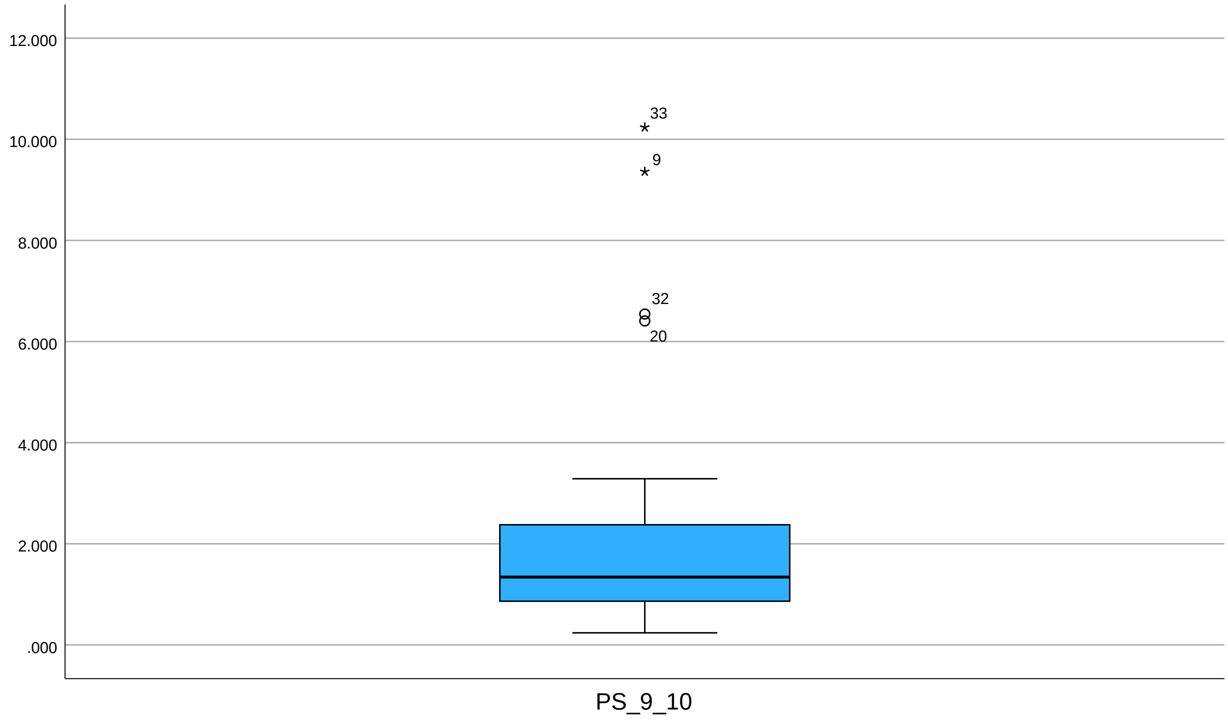
12.000
10.000
8.000
6.000
4.000
2.000
.000
33
9
32
20
PS_9_10

PS_10_11

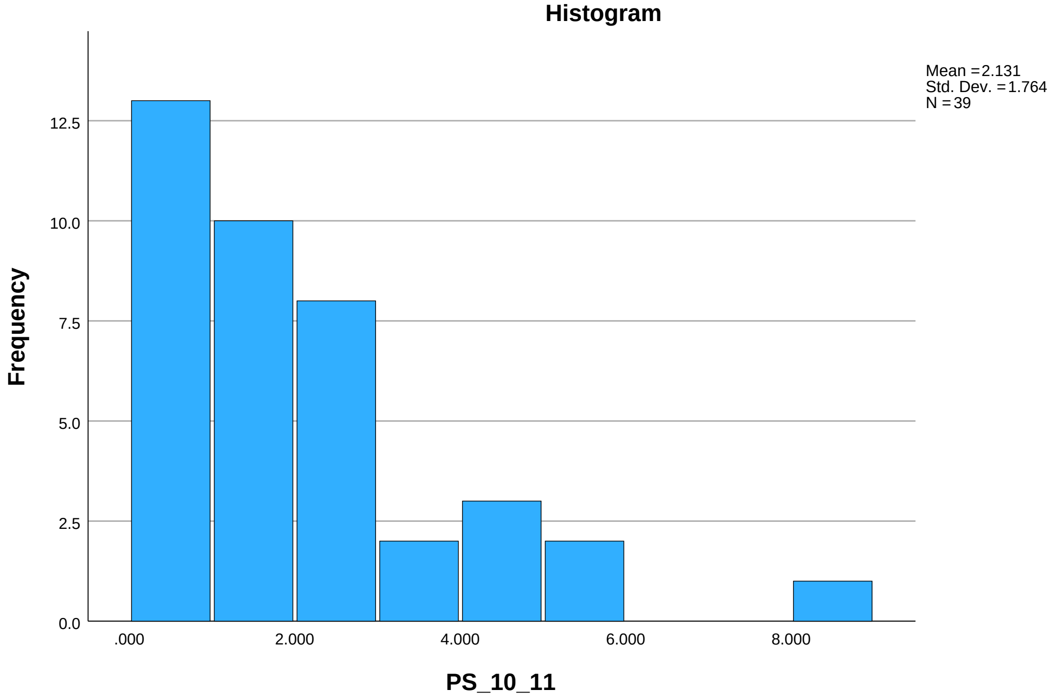

PS_10_11 Stem-and-Leaf Plot

```
 Frequency    Stem &  Leaf

     3.00        0 .  223
    10.00        0 .  5566667899
     4.00        1 .  0022
     6.00        1 .  567799
     4.00        2 .  0122
     4.00        2 .  5699
      .00        3 .
     2.00        3 .  78
     3.00        4 .  033
      .00        4 .
     1.00        5 .  2
     2.00 Extremes    (>=5.9)

 Stem width:      1.000
 Each leaf:       1 case(s)
```

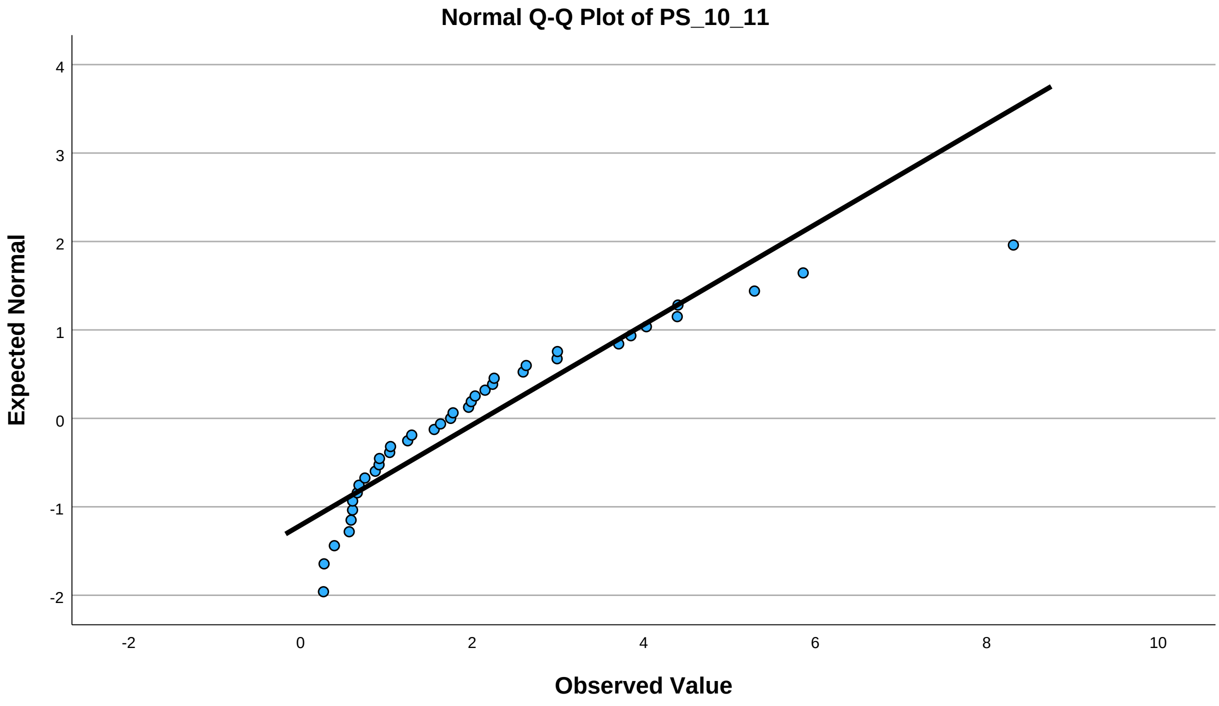

Normal Q-Q Plot of PS_10_11
Expected Normal
Observed Value
4
3
2
1
0
-1
-2
-2
0
2
4
6
8
10

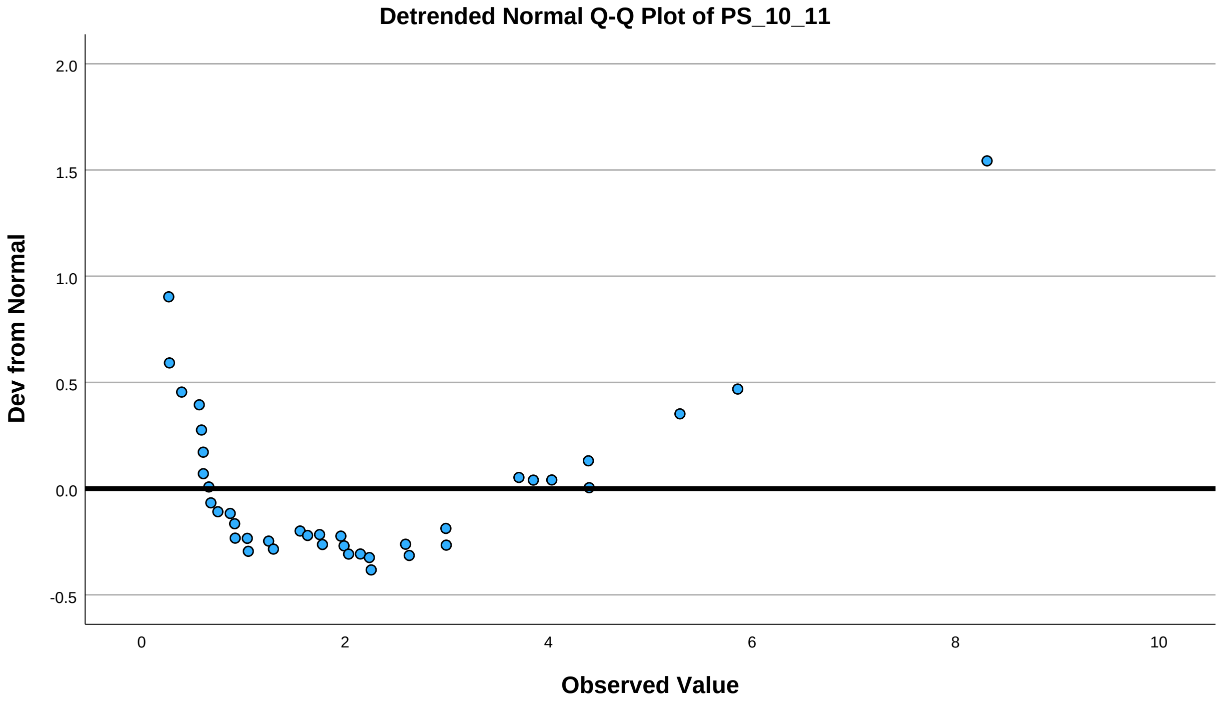

Detrended Normal Q-Q Plot of PS_10_11
Dev from Normal
Observed Value
2.0
1.5
1.0
0.5
0.0
-0.5
0
2
4
6
8
10

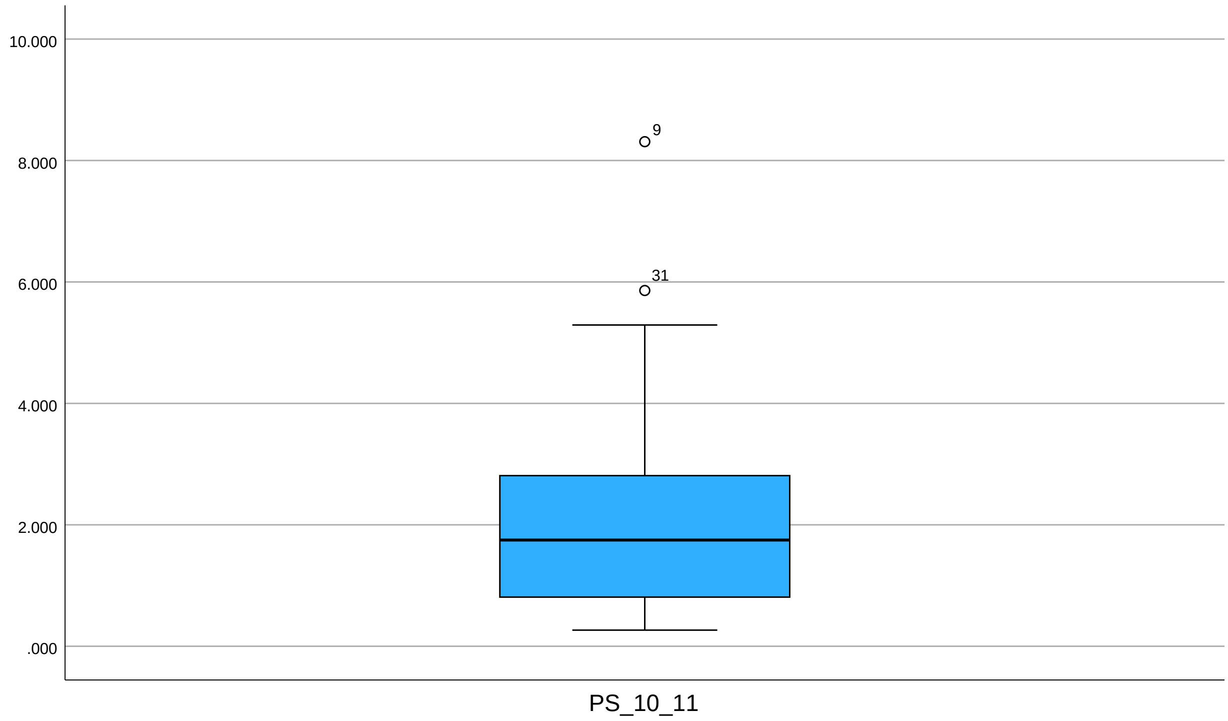

PS_11_12

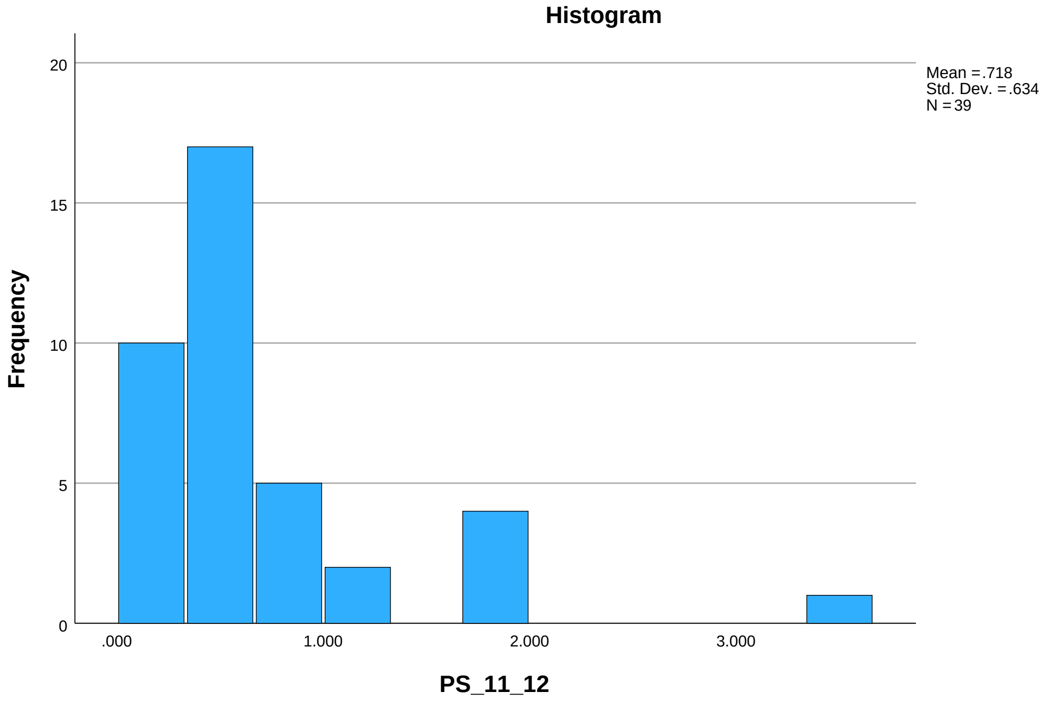

PS_11_12 Stem-and-Leaf Plot

Frequency   Stem & Leaf

```
    2.00        1 .  49
    4.00        2 .  4689
    6.00        3 .  011259
    8.00        4 .  01335578
    2.00        5 .  79
    5.00        6 .  00344
    3.00        7 .  245
    2.00        8 .  02
     .00        9 .
    1.00       10 .  8
    1.00       11 .  0
    5.00 Extremes    (>=1.72)

 Stem width:      .100
 Each leaf:       1 case(s)
```

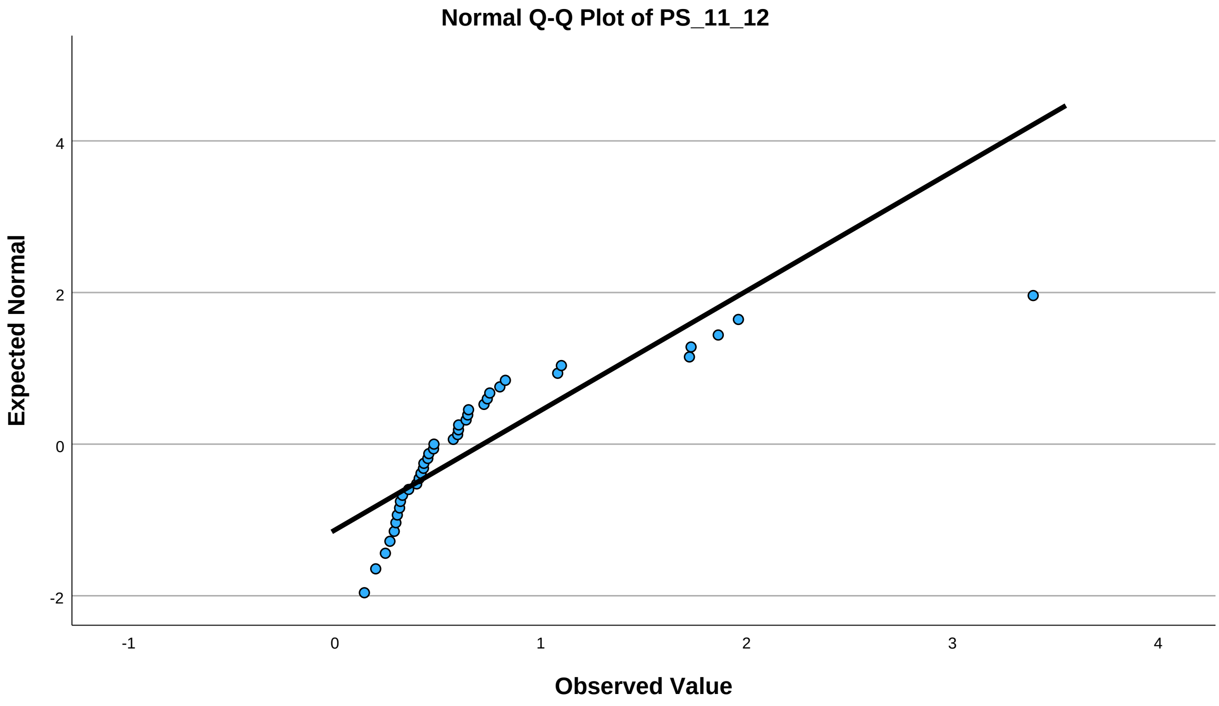

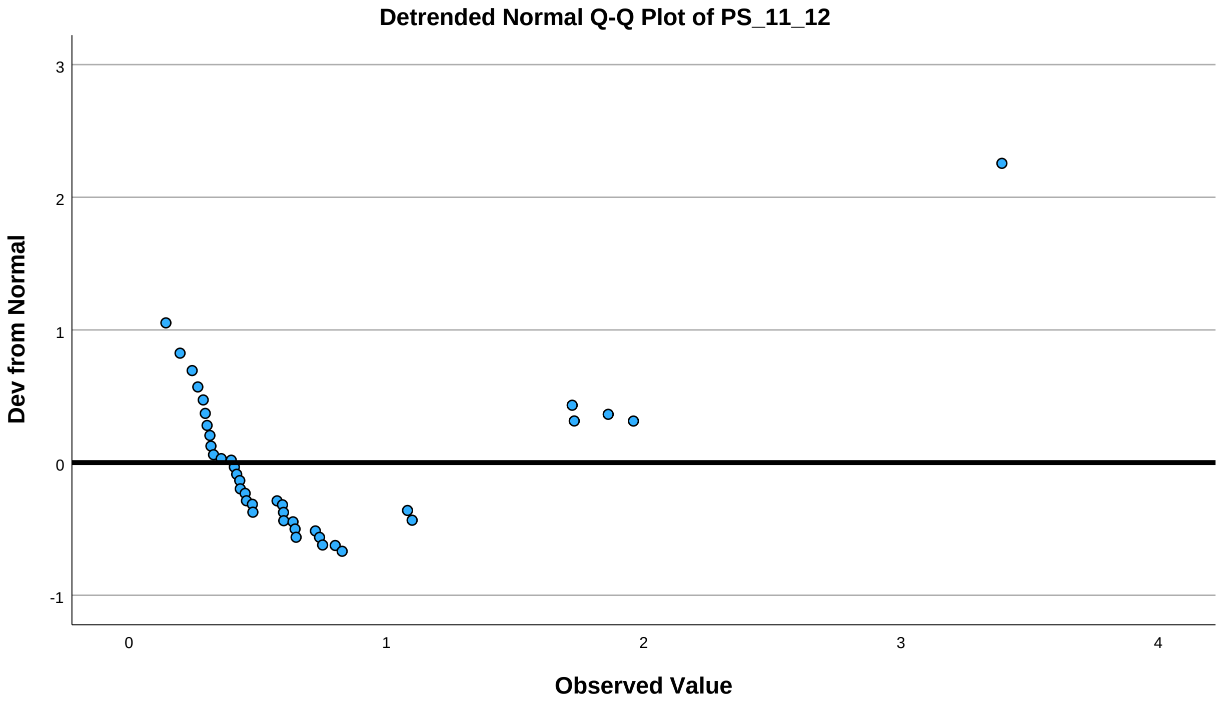
Detrended Normal Q-Q Plot of PS_11_12
Dev from Normal
Observed Value
3
2
1
0
-1
0
1
2
3
4

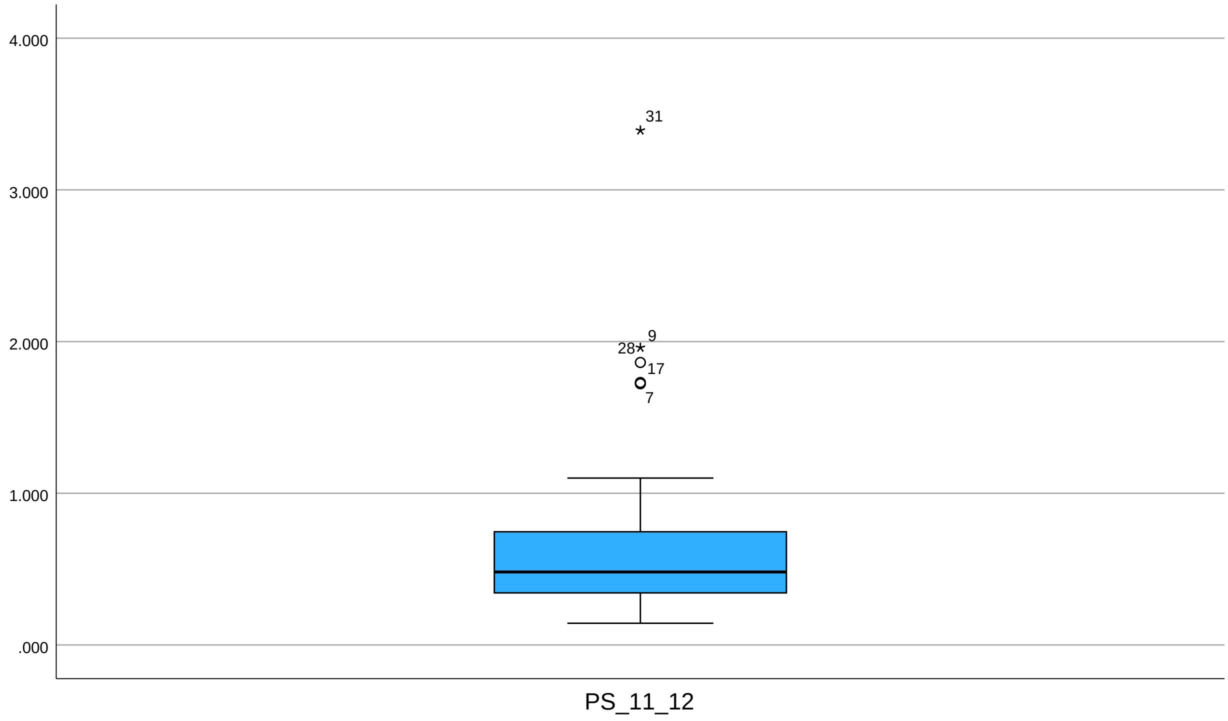
4.000
3.000
2.000
1.000
.000
31
9
28
17
7
PS_11_12

PS_12_13

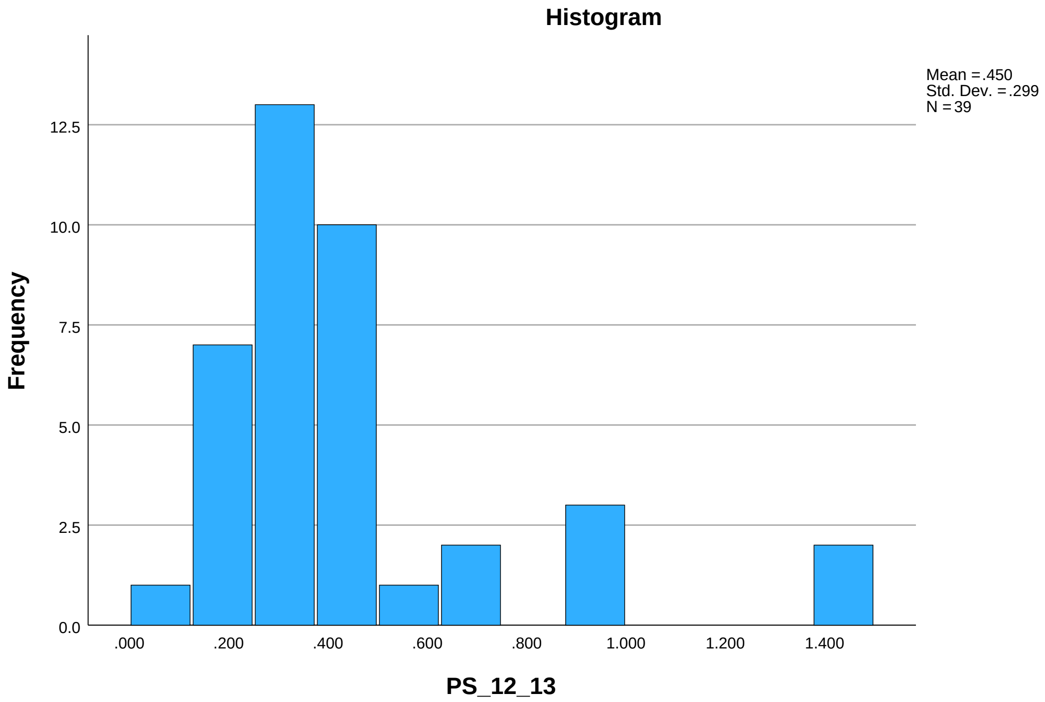


```
PS_12_13 Stem-and-Leaf Plot

 Frequency    Stem &  Leaf

     1.00        1 .  1
     3.00        1 .  588
     4.00        2 .  0124
     3.00        2 .  688
     6.00        3 .  012224
     5.00        3 .  55667
     6.00        4 .  011344
     3.00        4 .  556
      .00        5 .
      .00        5 .
     1.00        6 .  2
     2.00        6 .  56
     5.00 Extremes    (>=.88)

 Stem width:      .100
 Each leaf:       1 case(s)
```

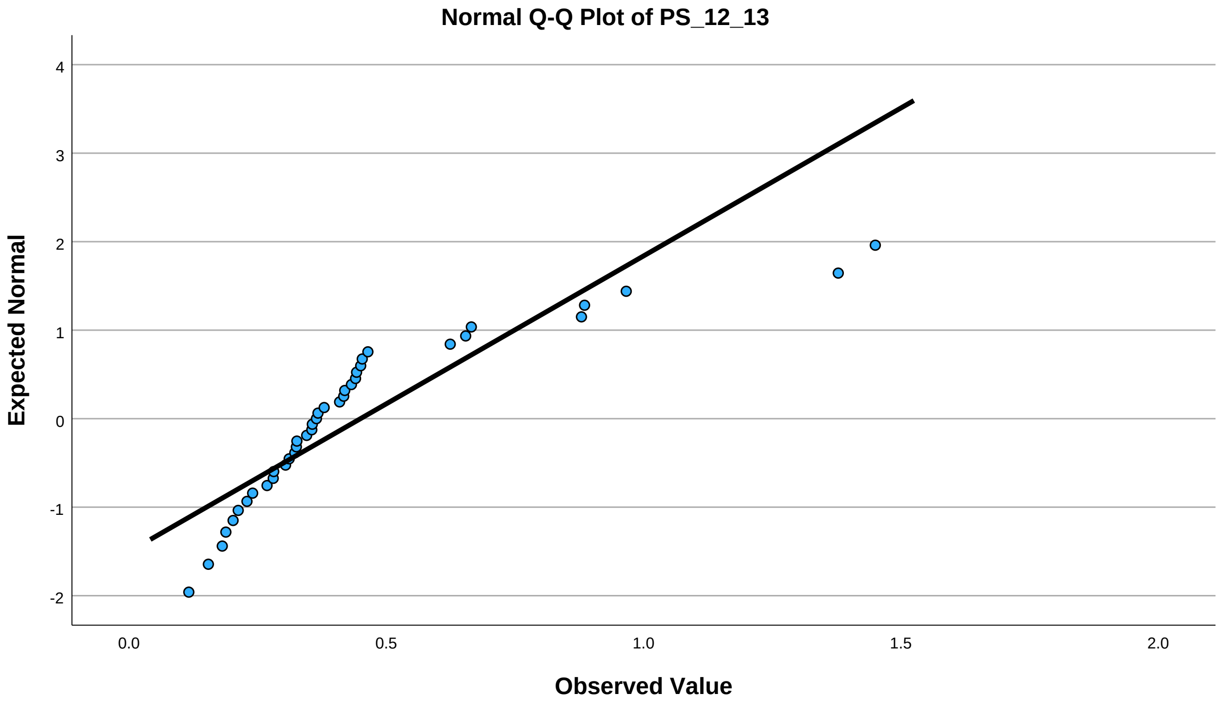
Normal Q-Q Plot of PS_12_13
Expected Normal
4
3
2
1
0
-1
-2
0.0
0.5
1.0
1.5
2.0
Observed Value

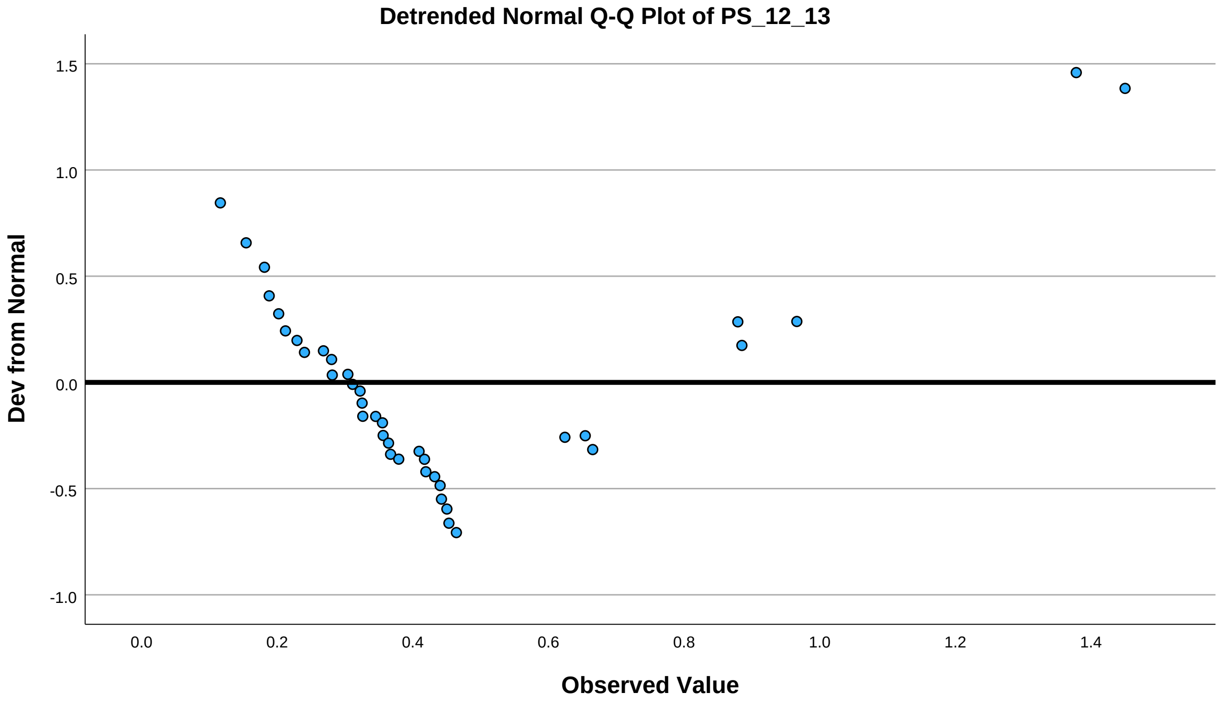
Detrended Normal Q-Q Plot of PS_12_13
Dev from Normal
1.5
1.0
0.5
0.0
-0.5
-1.0
0.0
0.2
0.4
0.6
0.8
1.0
1.2
1.4
Observed Value

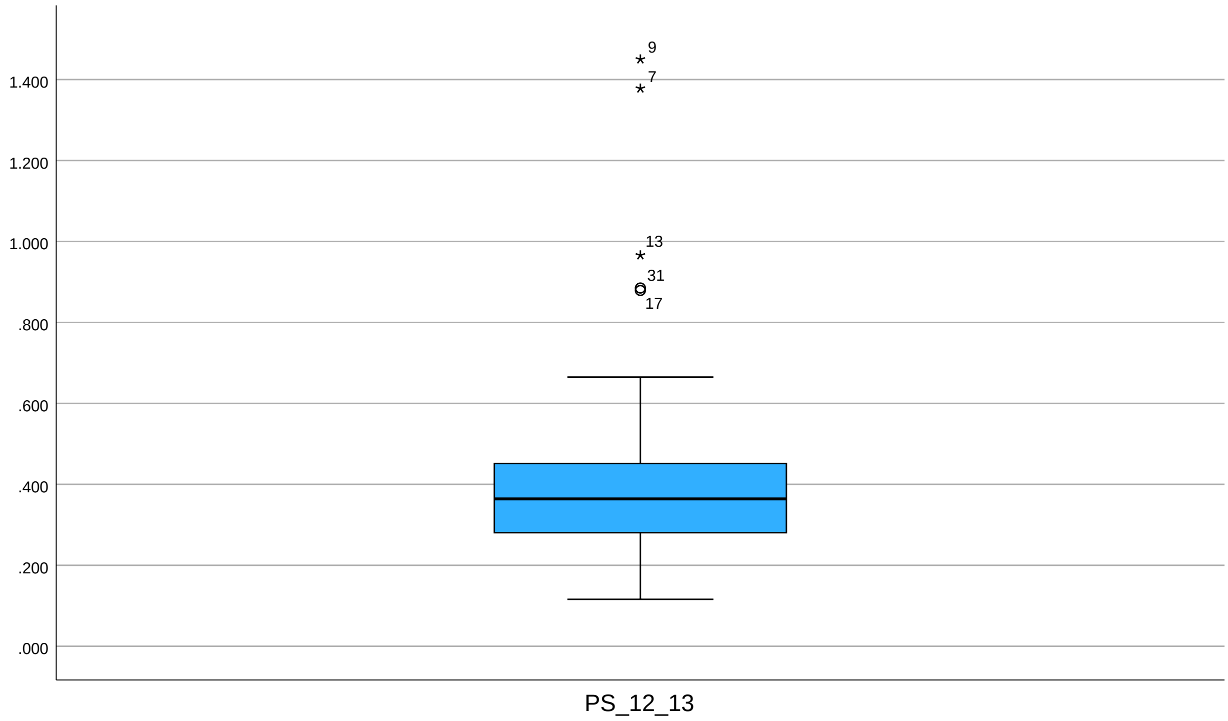

PS_13_14

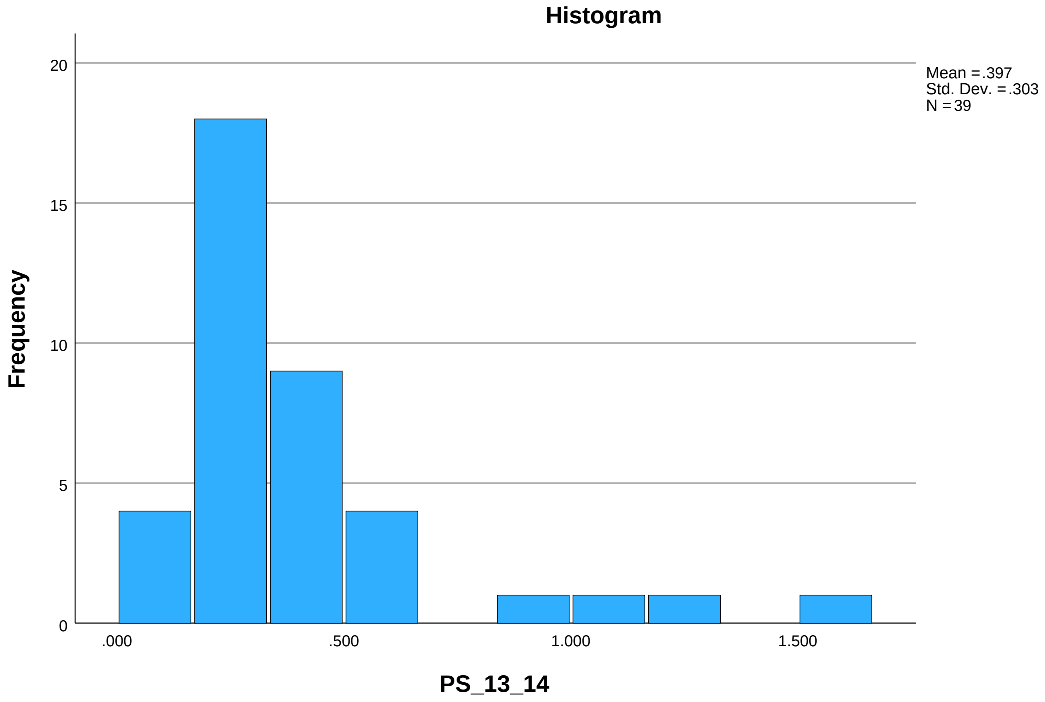

PS_13_14 Stem-and-Leaf Plot

Frequency Stem & Leaf

```
 1.00     0 . 9
 4.00     1 . 2566
15.00     2 . 001233444456777
 7.00     3 . 0133468
 4.00     4 . 0567
 2.00     5 . 34
 2.00     6 . 11
 4.00 Extremes    (>=.85)

Stem width:      .100
Each leaf:       1 case(s)
```

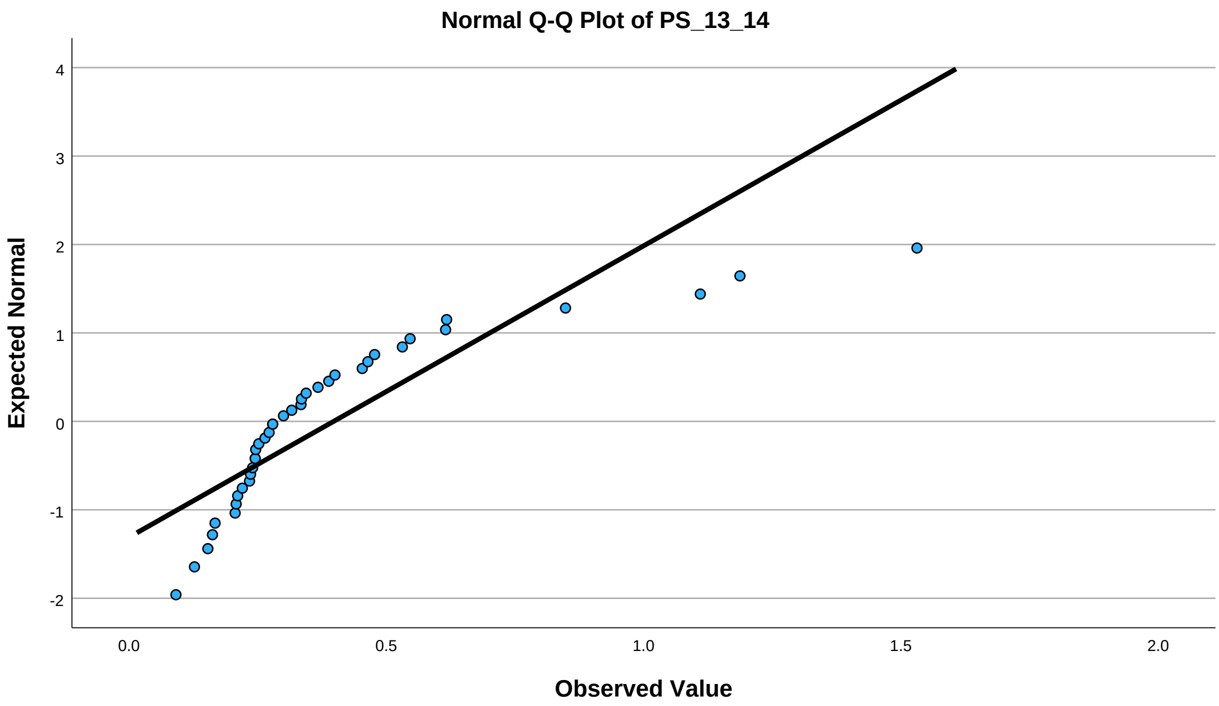

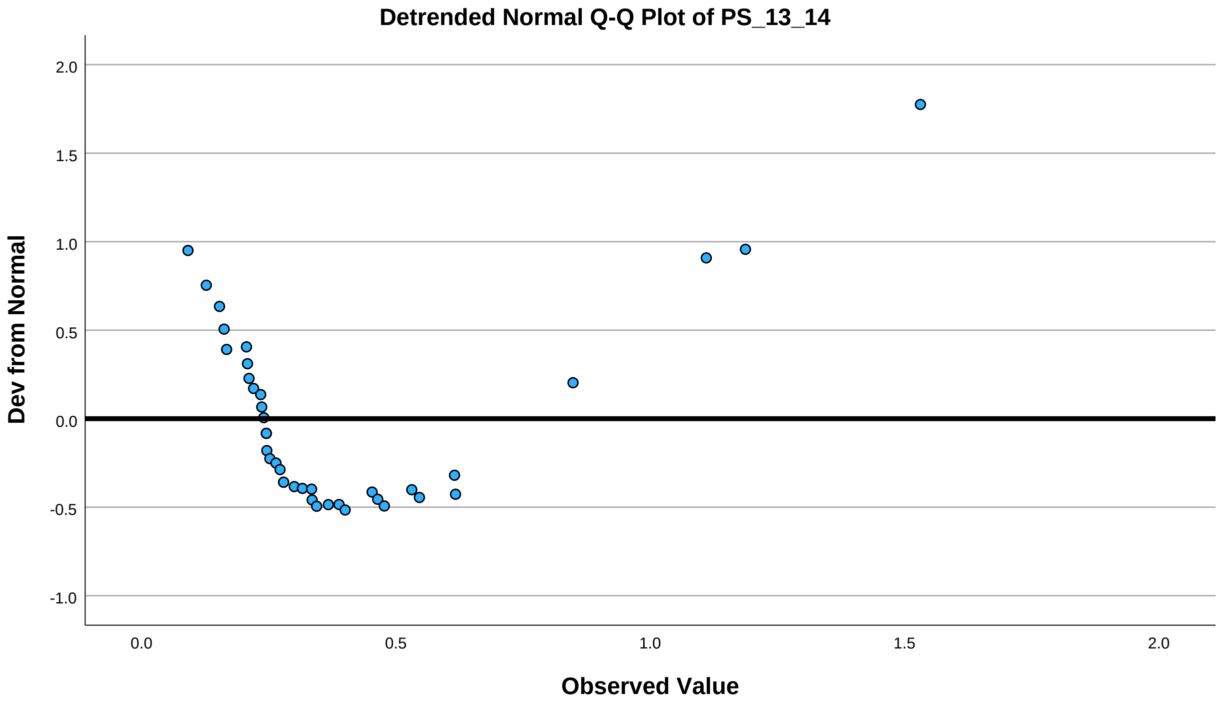
Detrended Normal Q-Q Plot of PS_13_14
Dev from Normal
2.0
1.5
1.0
0.5
0.0
-0.5
-1.0
0.0
0.5
1.0
1.5
2.0
Observed Value

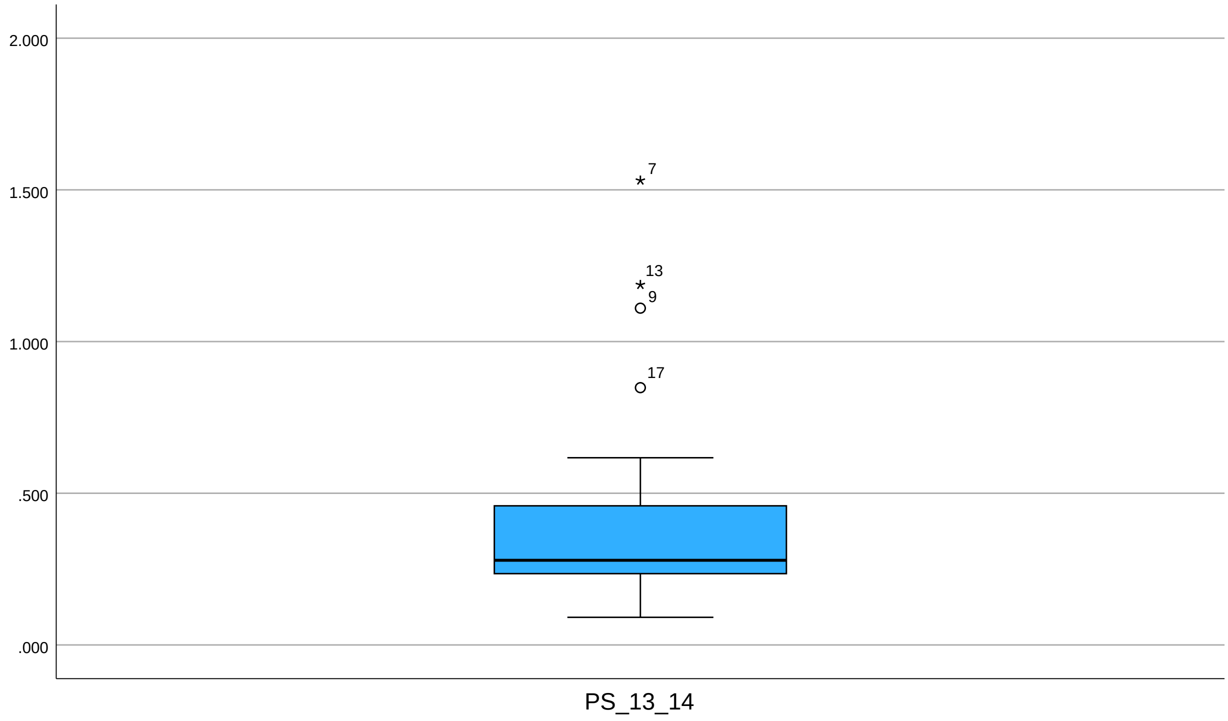
2.000
1.500
1.000
.500
.000
7
13
9
17
PS_13_14

PS_14_15

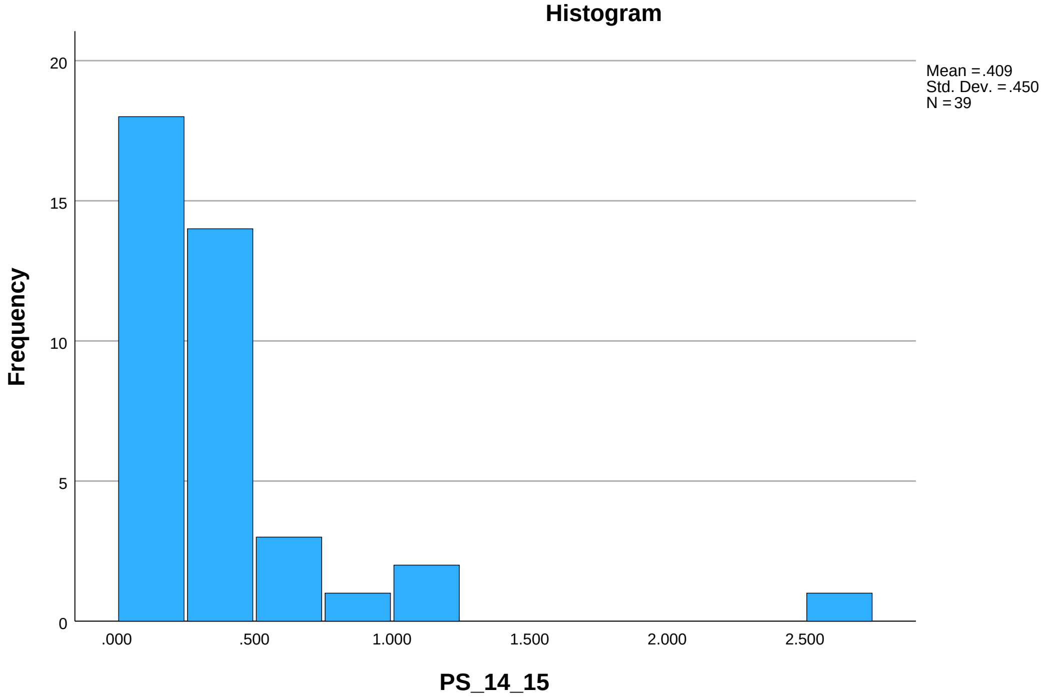


```
PS_14_15 Stem-and-Leaf Plot

 Frequency    Stem &  Leaf

     1.00        0 .  8
    11.00        1 .  22366689999
    11.00        2 .  00122455779
     6.00        3 .  245566
     3.00        4 .  255
     1.00        5 .  0
     1.00        6 .  9
     5.00 Extremes    (>=.73)

 Stem width:      .100
 Each leaf:       1 case(s)
```

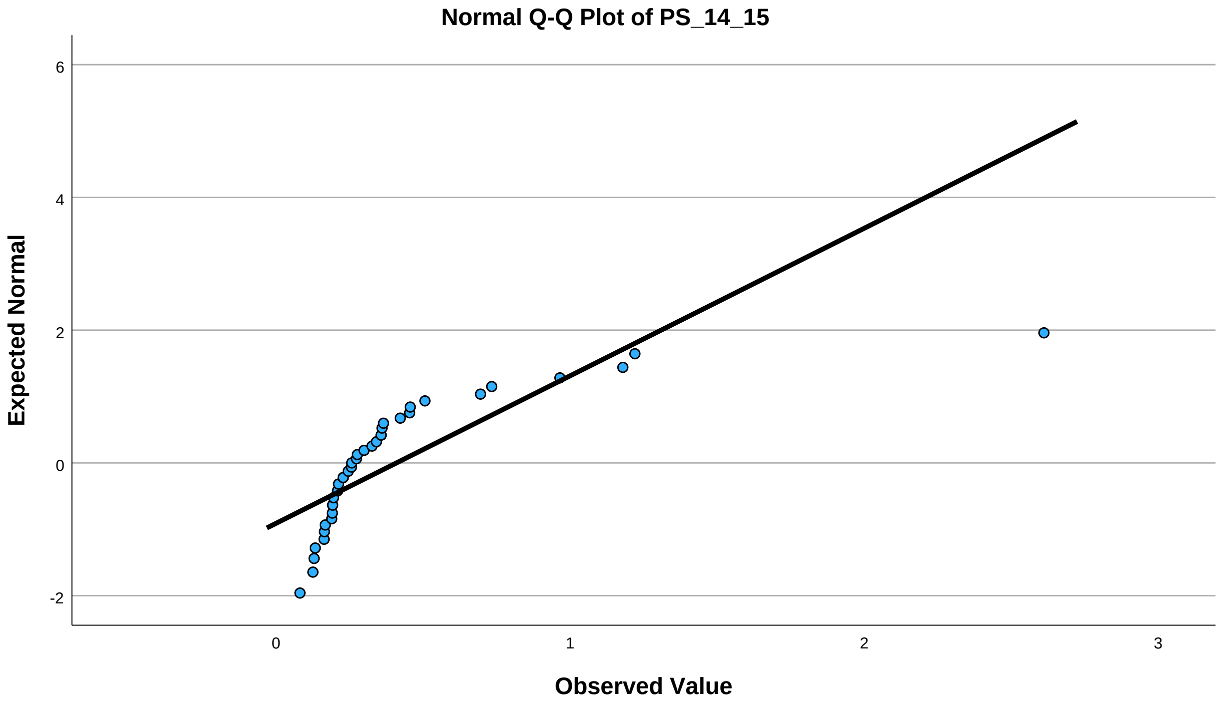
Normal Q-Q Plot of PS_14_15
Expected Normal
6
4
2
0
-2
0
1
2
3
Observed Value

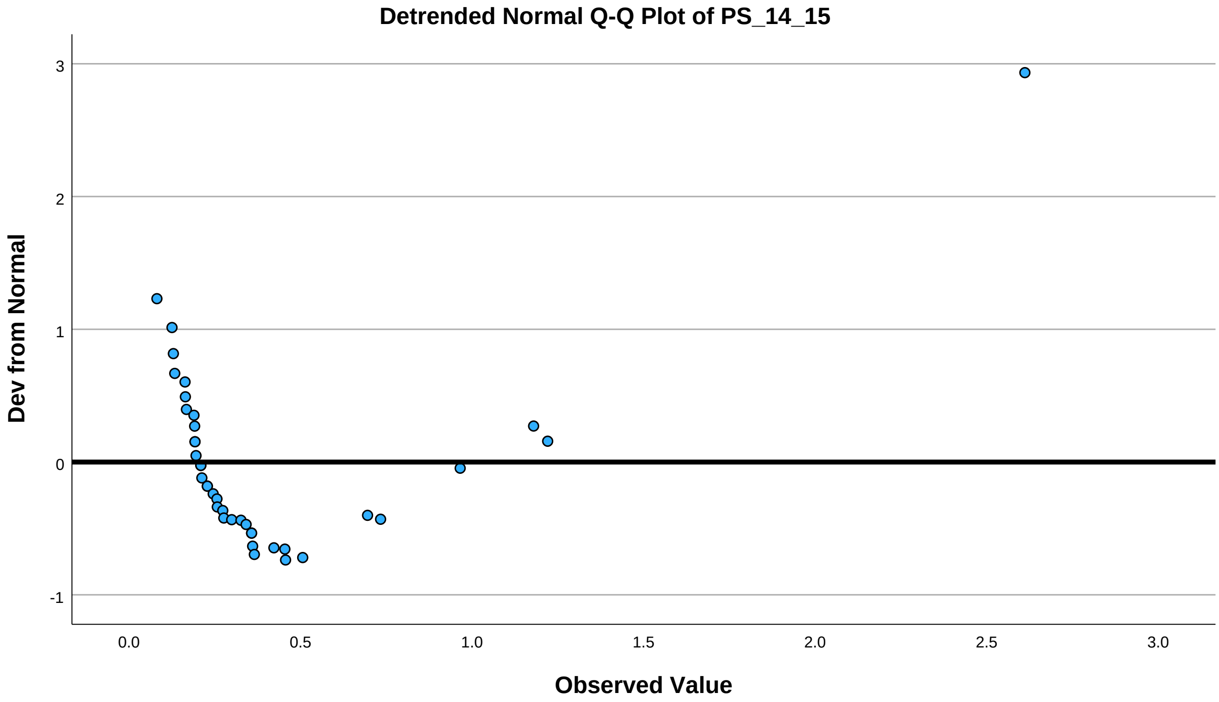
Detrended Normal Q-Q Plot of PS_14_15
Dev from Normal
3
2
1
0
-1
0.0
0.5
1.0
1.5
2.0
2.5
3.0
Observed Value

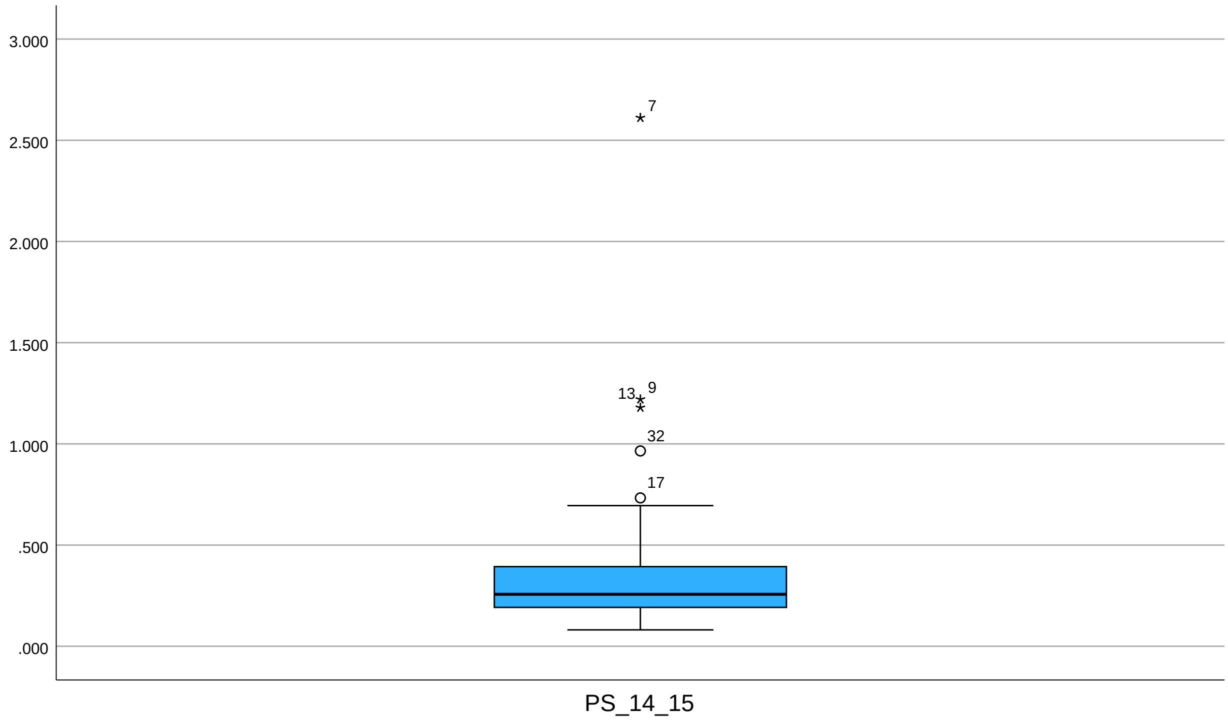

PS_15_16

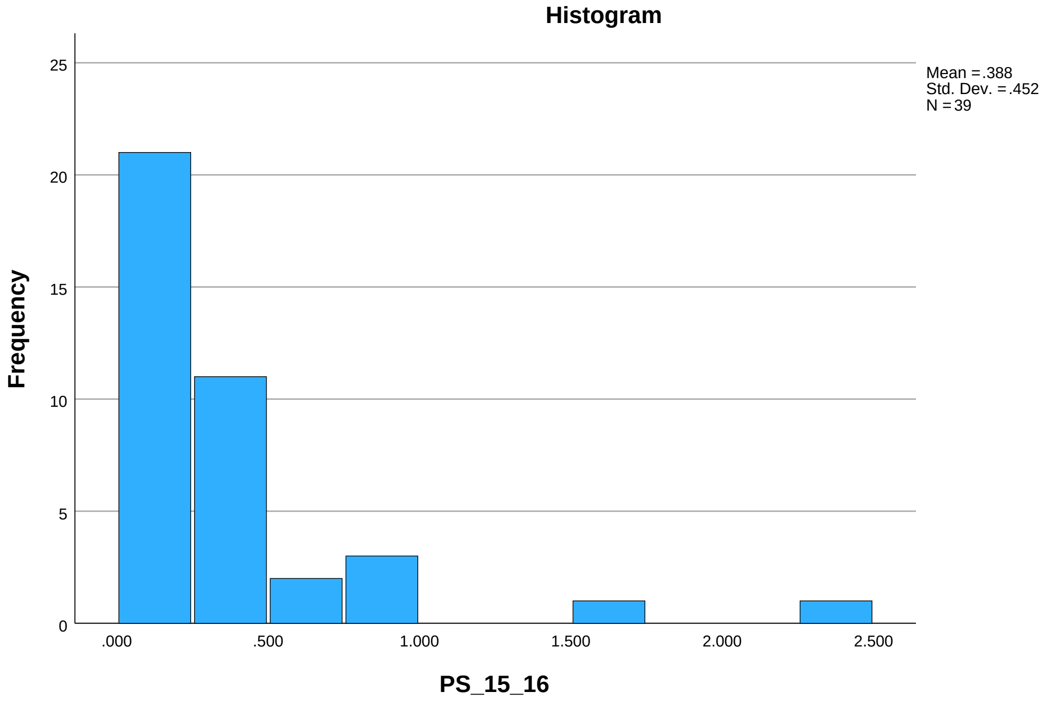


```
PS_15_16 Stem-and-Leaf Plot

 Frequency    Stem &  Leaf
```

```
 2.00      0 . 69
 3.00      1 . 234
 9.00      1 . 556668999
 7.00      2 . 0112234
 5.00      2 . 66779
 2.00      3 . 02
 2.00      3 . 57
 1.00      4 . 3
 1.00      4 . 7
 1.00      5 . 2
 6.00 Extremes    (>=.65)

Stem width:      .100
Each leaf:       1 case(s)
```

Normal Q-Q Plot of PS_15_16

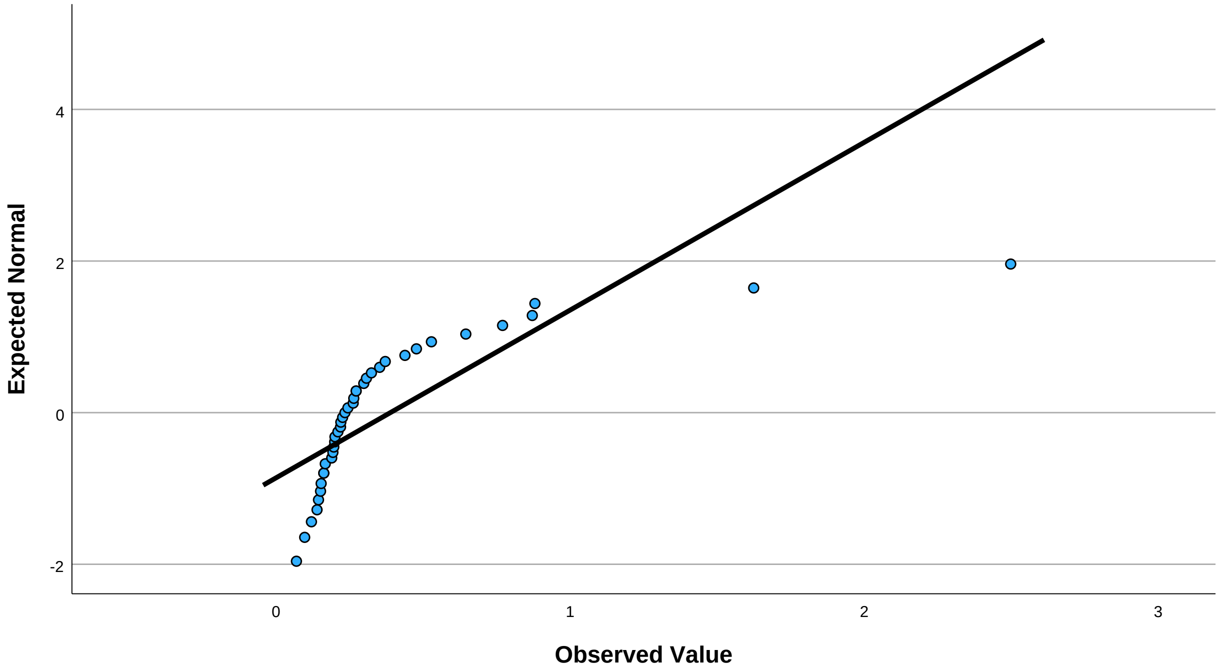

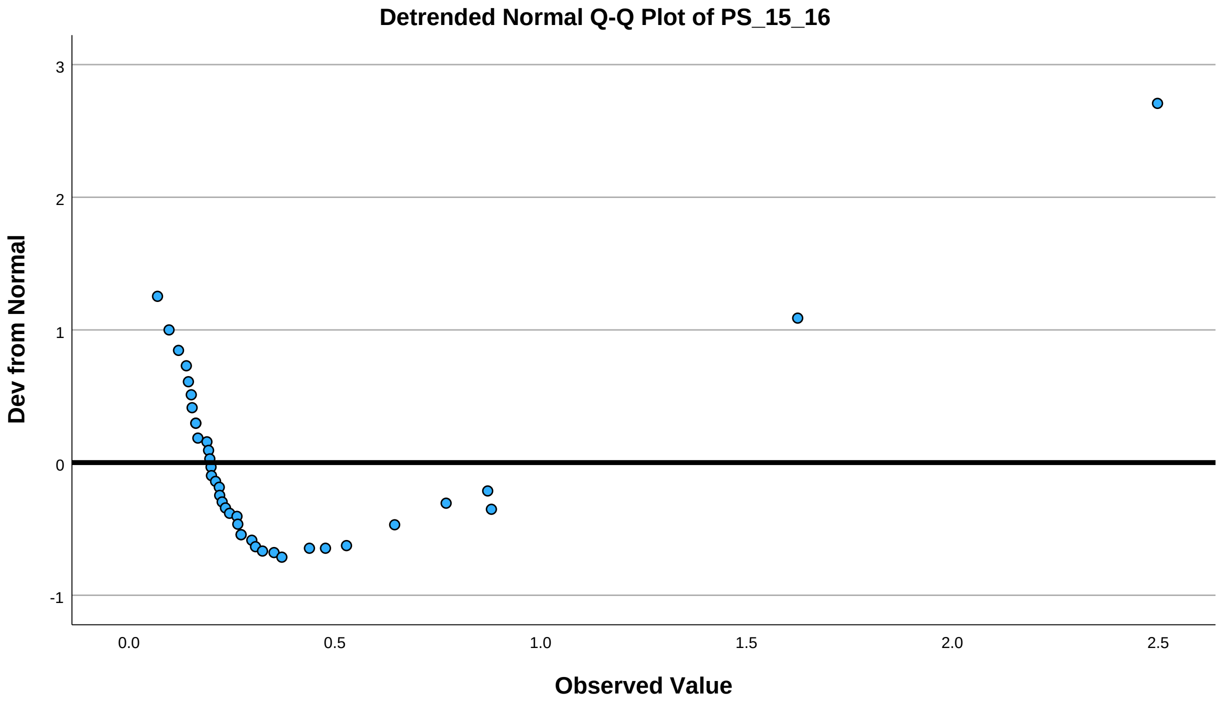
Detrended Normal Q-Q Plot of PS_15_16
Dev from Normal
3
2
1
0
-1
0.0
0.5
1.0
1.5
2.0
2.5
Observed Value

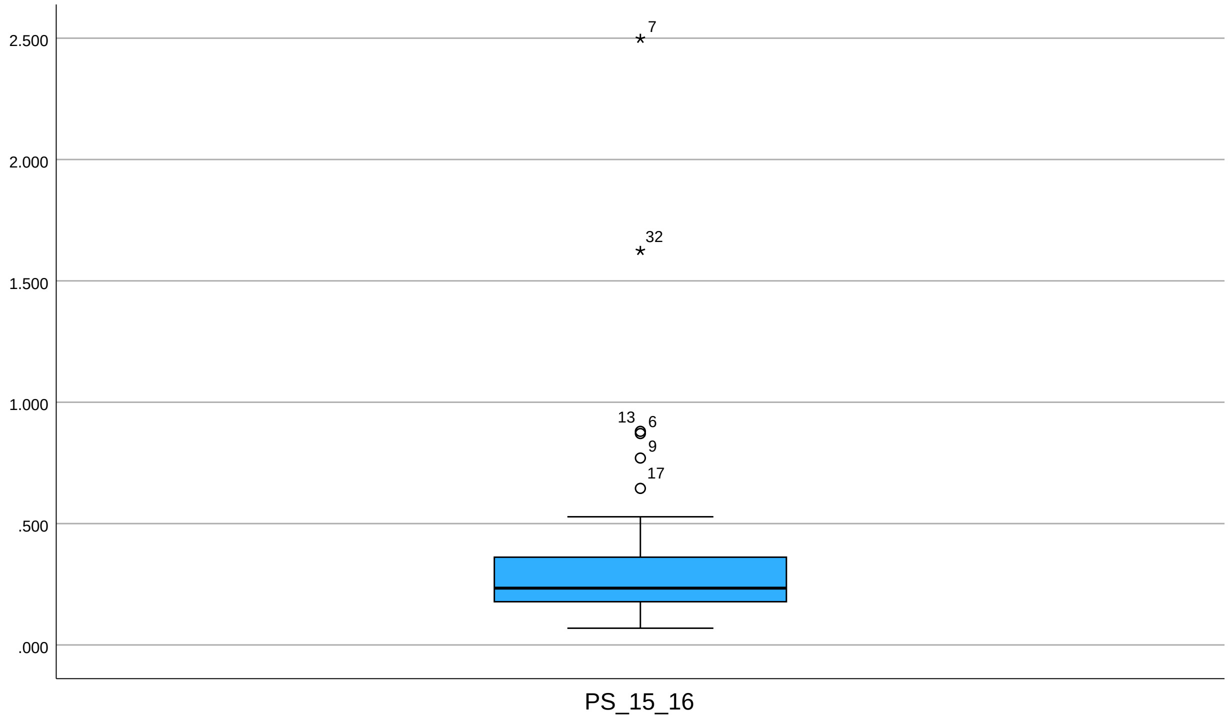
2.500
2.000
1.500
1.000
.500
.000
7
32
13
6
9
17
PS_15_16

PS_16_17

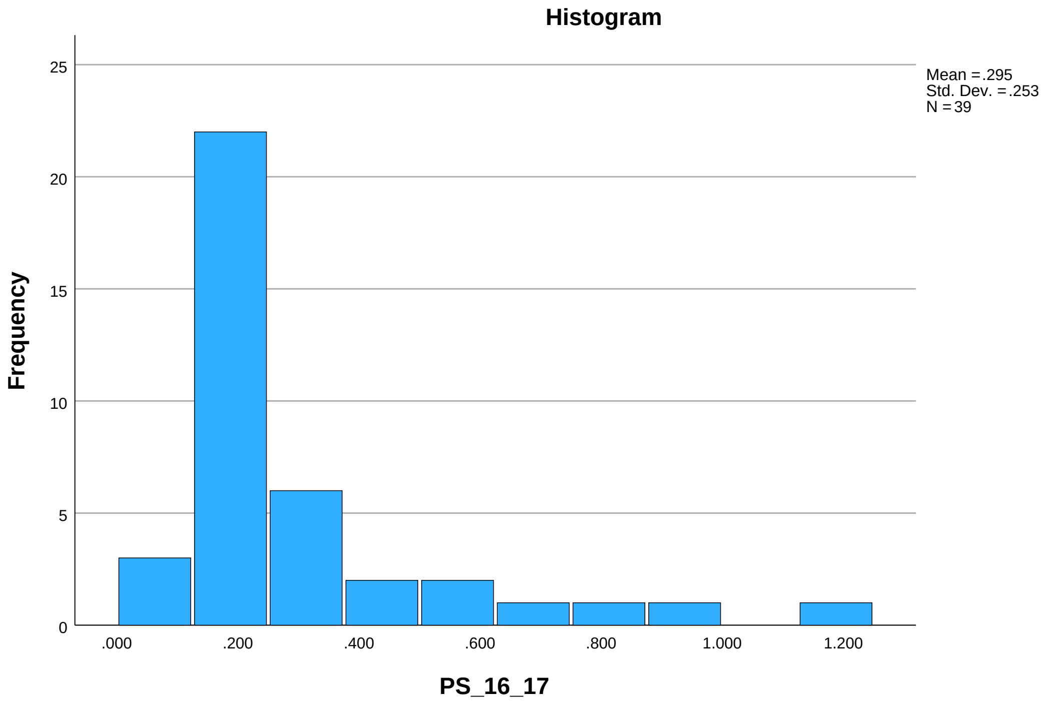

PS_16_17 Stem-and-Leaf Plot

```
 Frequency    Stem &  Leaf

     2.00        0 .  78
     5.00        1 .  13333
    13.00        1 .  5555556777888
     5.00        2 .  00223
     4.00        2 .  6778
     2.00        3 .  01
     1.00        3 .  9
      .00        4 .
     1.00        4 .  6
     6.00 Extremes    (>=.58)

 Stem width:      .100
 Each leaf:       1 case(s)
```

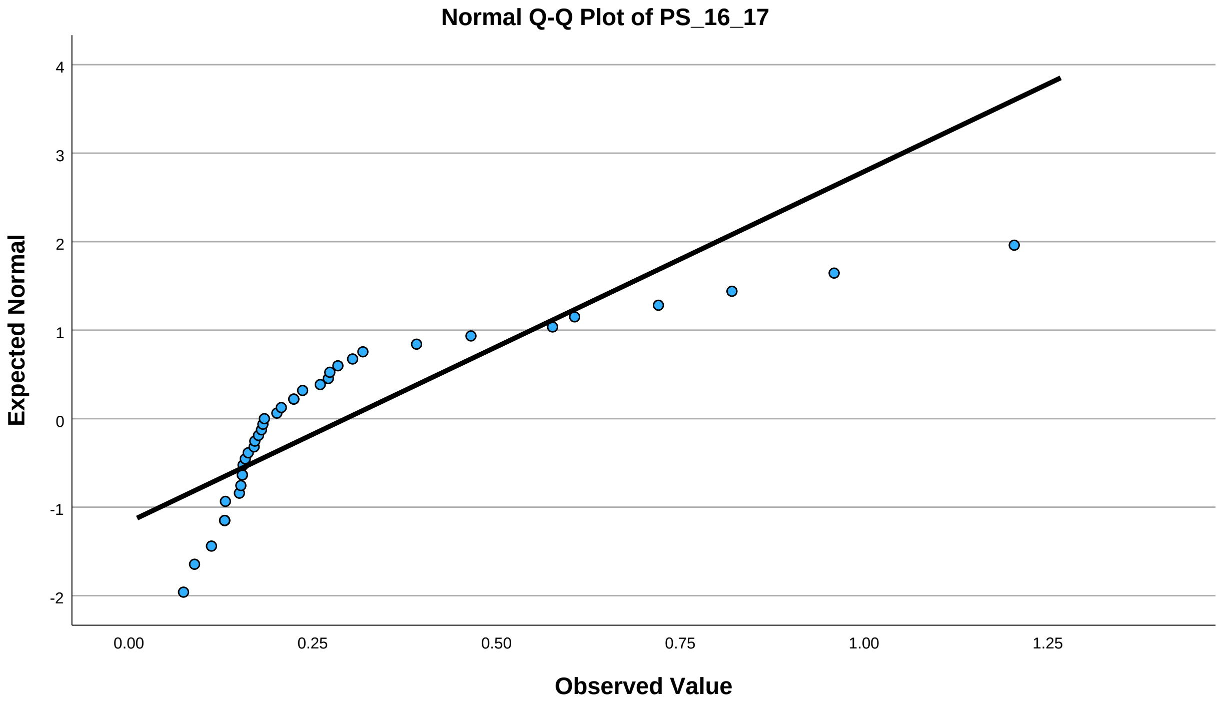
Normal Q-Q Plot of PS_16_17
Expected Normal
4
3
2
1
0
-1
-2
0.00
0.25
0.50
0.75
1.00
1.25
Observed Value

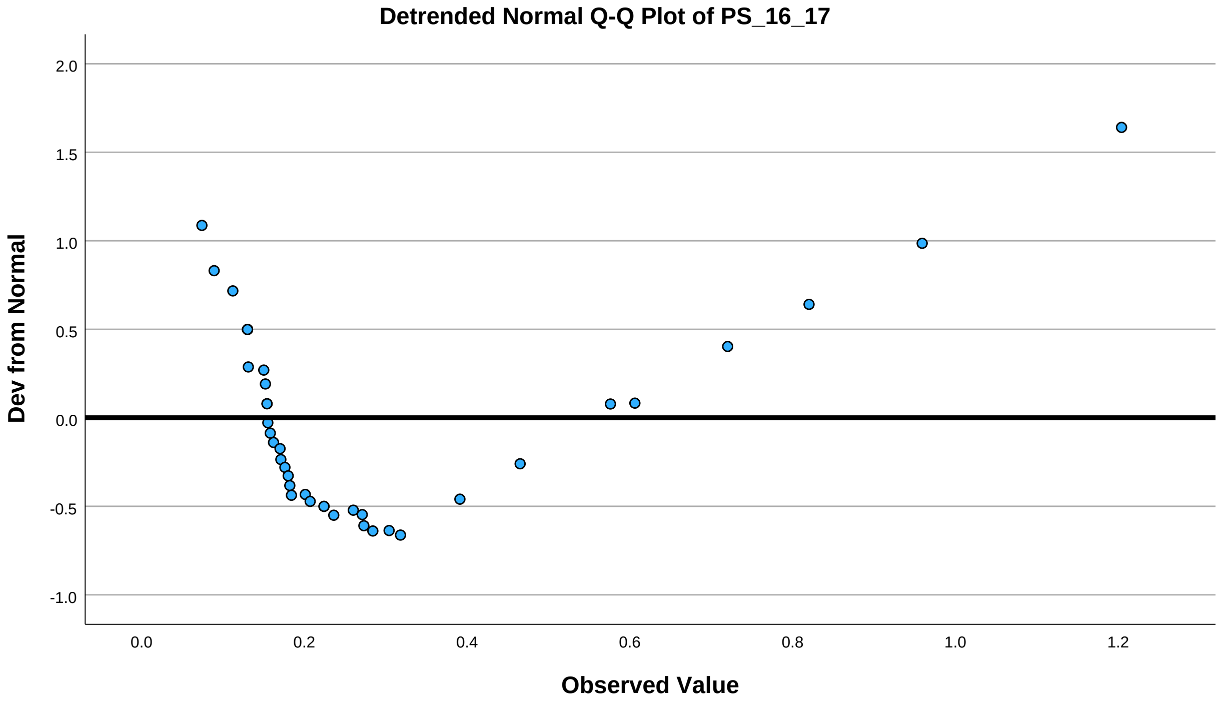
Detrended Normal Q-Q Plot of PS_16_17
Dev from Normal
2.0
1.5
1.0
0.5
0.0
-0.5
-1.0
0.0
0.2
0.4
0.6
0.8
1.0
1.2
Observed Value

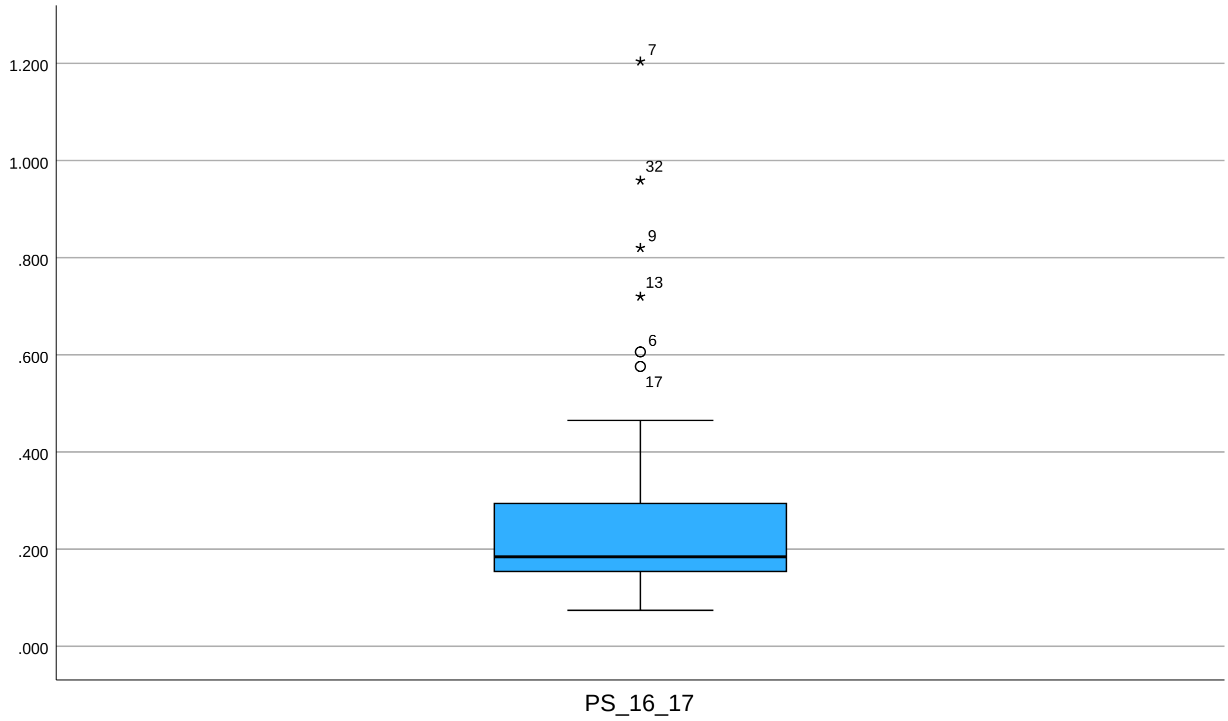

PS_17_18

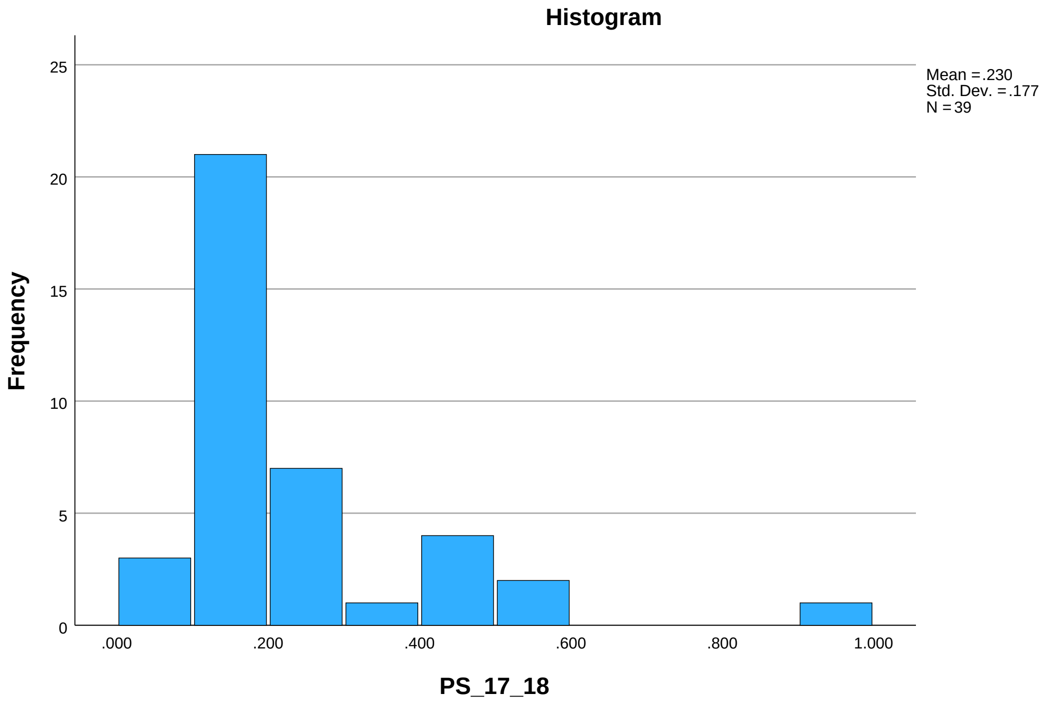

PS_17_18 Stem-and-Leaf Plot

Frequency   Stem & Leaf

```
 3.00      0 . 679
16.00       1 .  0011222333333444
 5.00      1 . 56667
 4.00      2 . 0223
 3.00      2 . 566
 1.00      3 . 1
  .00      3 .
 1.00      4 . 3
  6.00 Extremes    (>=.47)

Stem width:      .100
Each leaf:       1 case(s)
```

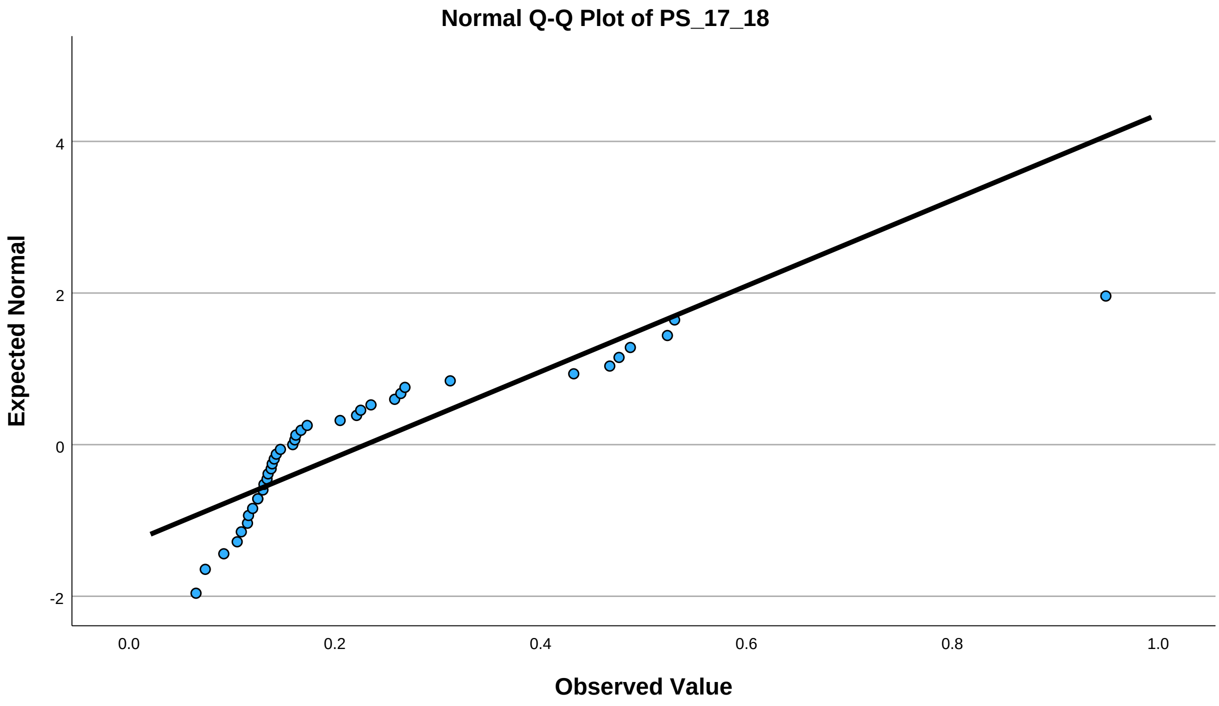

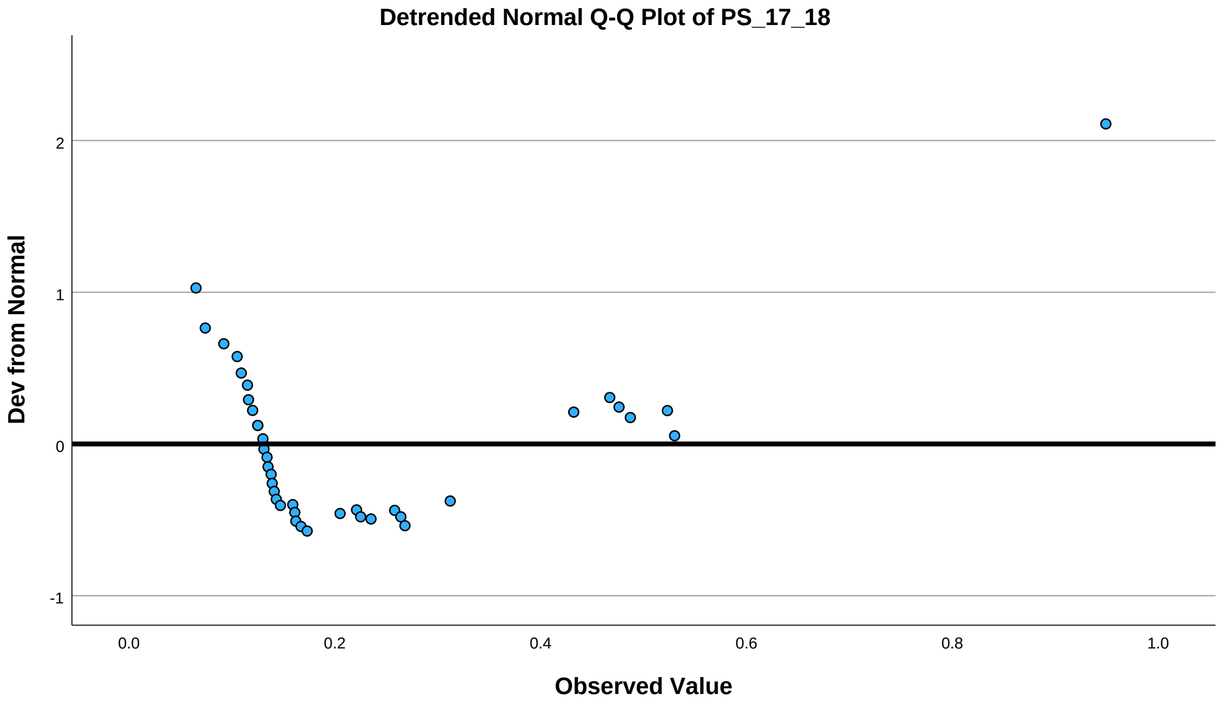
Detrended Normal Q-Q Plot of PS_17_18
Dev from Normal
Observed Value
2
1
0
-1
0.0
0.2
0.4
0.6
0.8
1.0

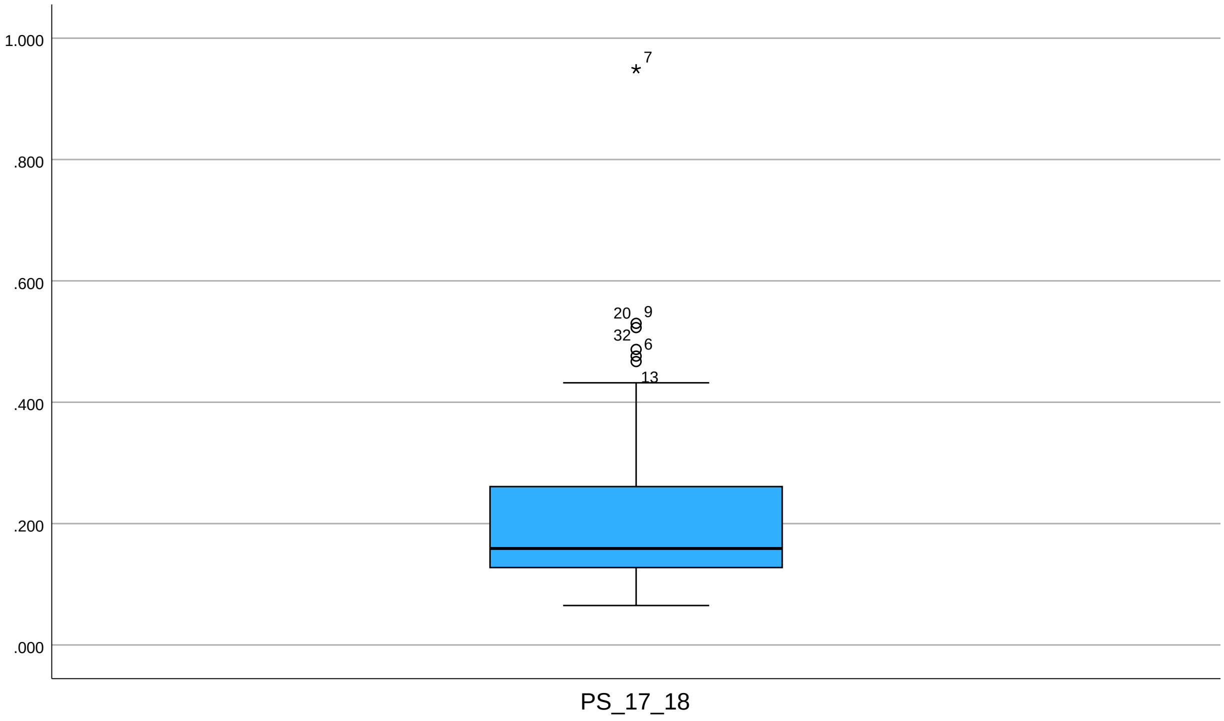
1.000
.800
.600
.400
.200
.000
7
20
9
32
6
13
PS_17_18

PS_18_19

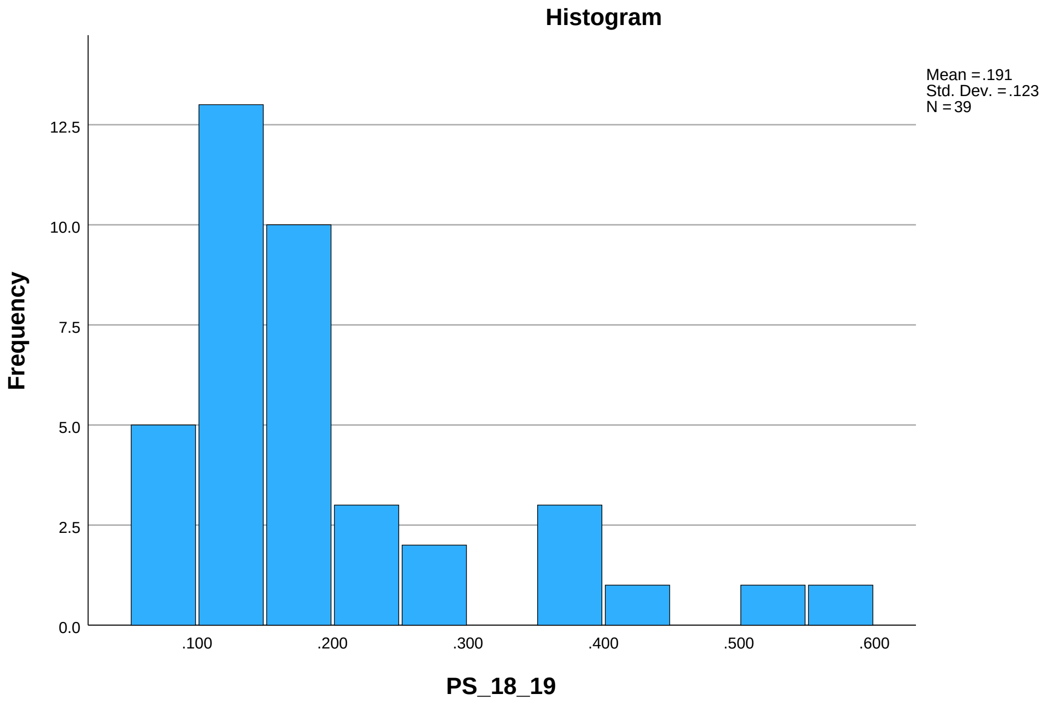


```
PS_18_19 Stem-and-Leaf Plot

 Frequency    Stem &  Leaf

     5.00        0 .  58888
    13.00        1 .  0001111122244
    10.00        1 .  5556666688
     3.00        2 .  012
     2.00        2 .  69
     6.00 Extremes    (>=.37)

 Stem width:      .100
 Each leaf:       1 case(s)
```

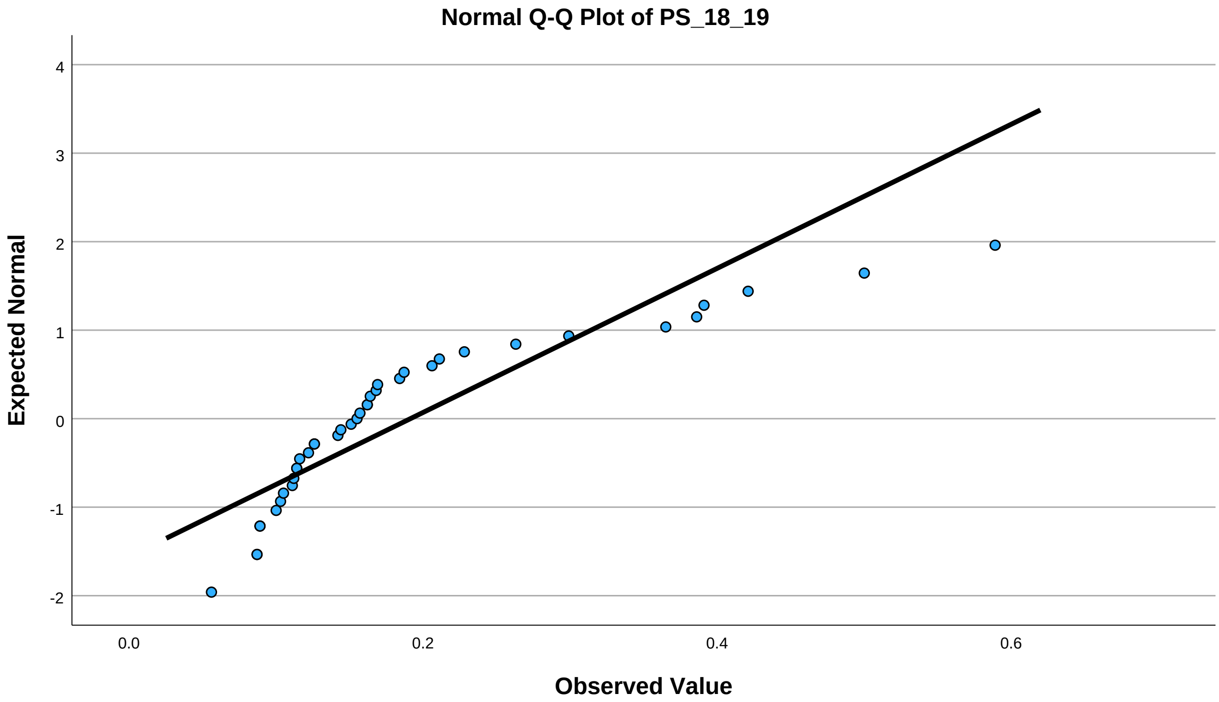
Normal Q-Q Plot of PS_18_19
Expected Normal
4
3
2
1
0
-1
-2
0.0
0.2
0.4
0.6
Observed Value

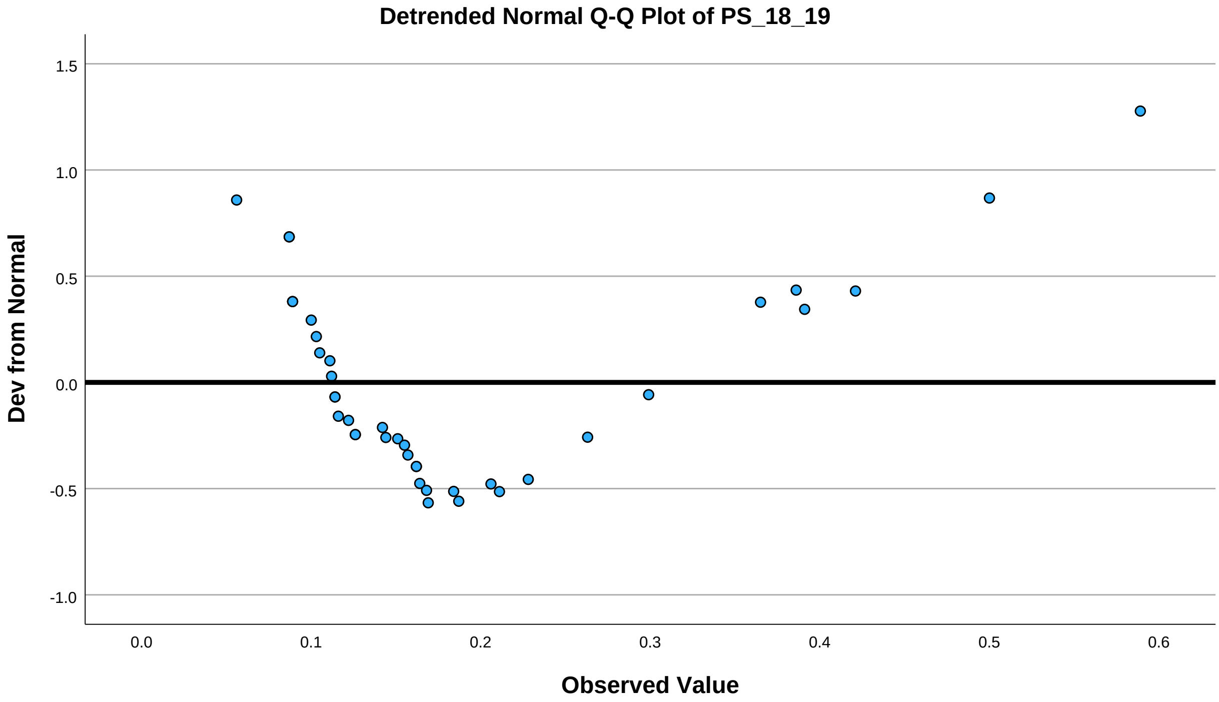
Detrended Normal Q-Q Plot of PS_18_19
Dev from Normal
1.5
1.0
0.5
0.0
-0.5
-1.0
0.0
0.1
0.2
0.3
0.4
0.5
0.6
Observed Value

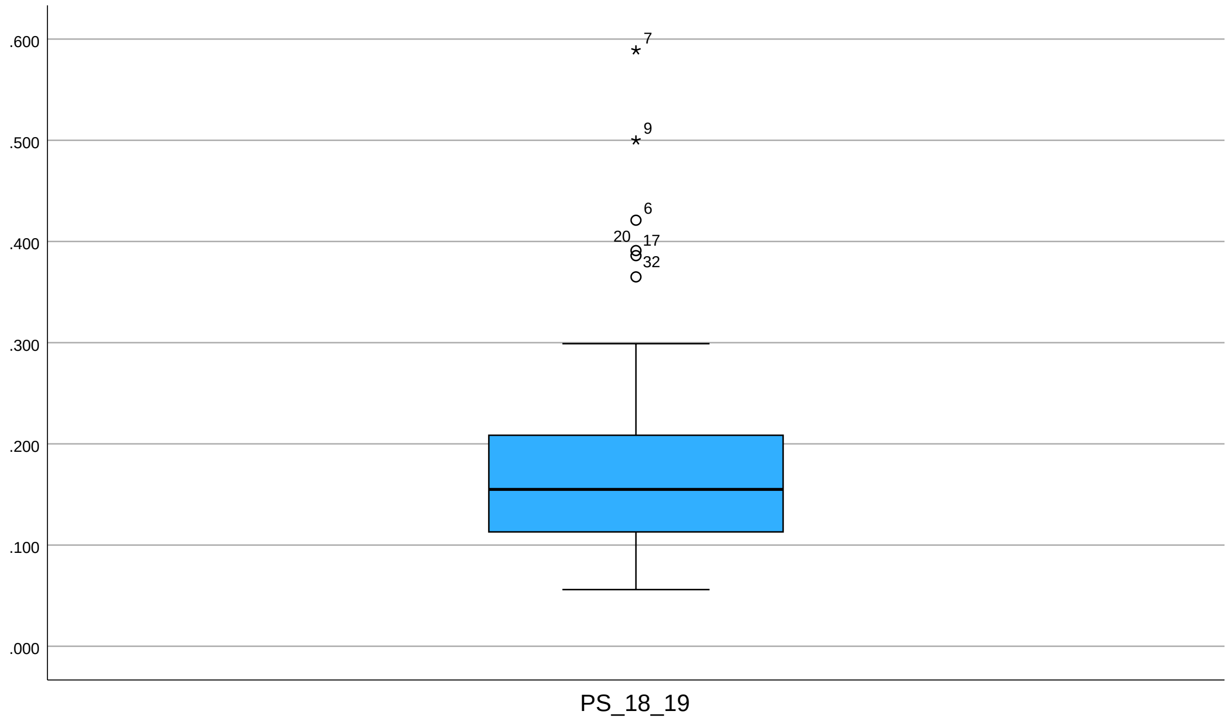

PS_19_20

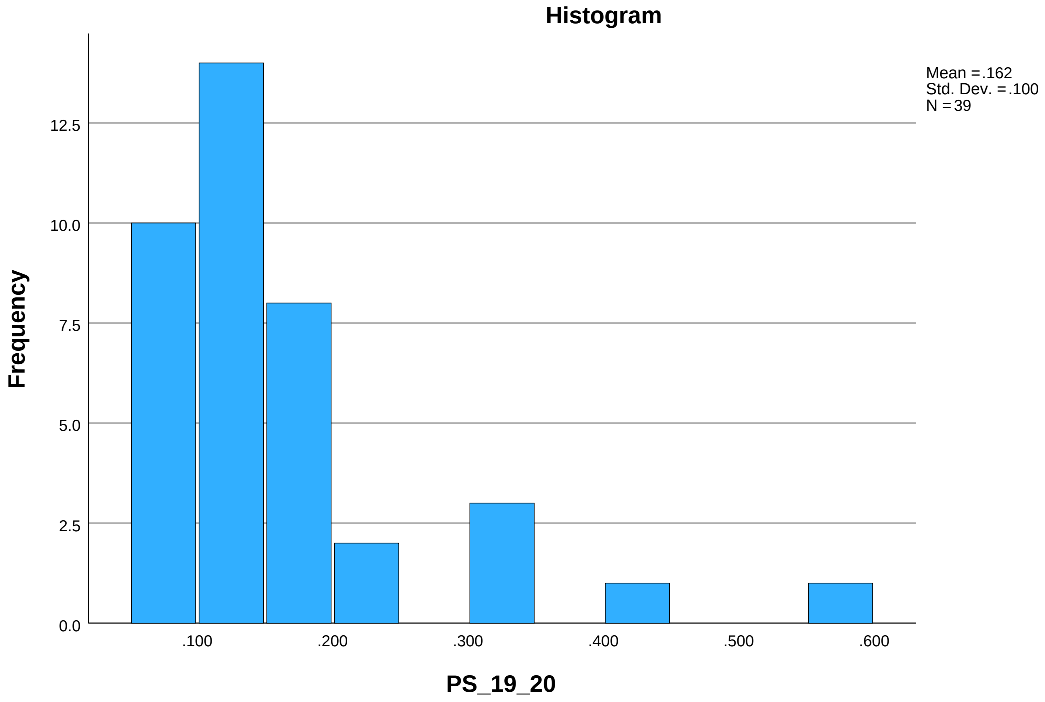


```
PS_19_20 Stem-and-Leaf Plot

 Frequency    Stem &  Leaf
```

```
  10.00        0 .  5777888889
  14.00        1 .  00012233344444
   8.00        1 .  5566778
  2.00       2 .  33
   5.00 Extremes    (>=.31)

Stem width:      .100
Each leaf:       1 case(s)
```

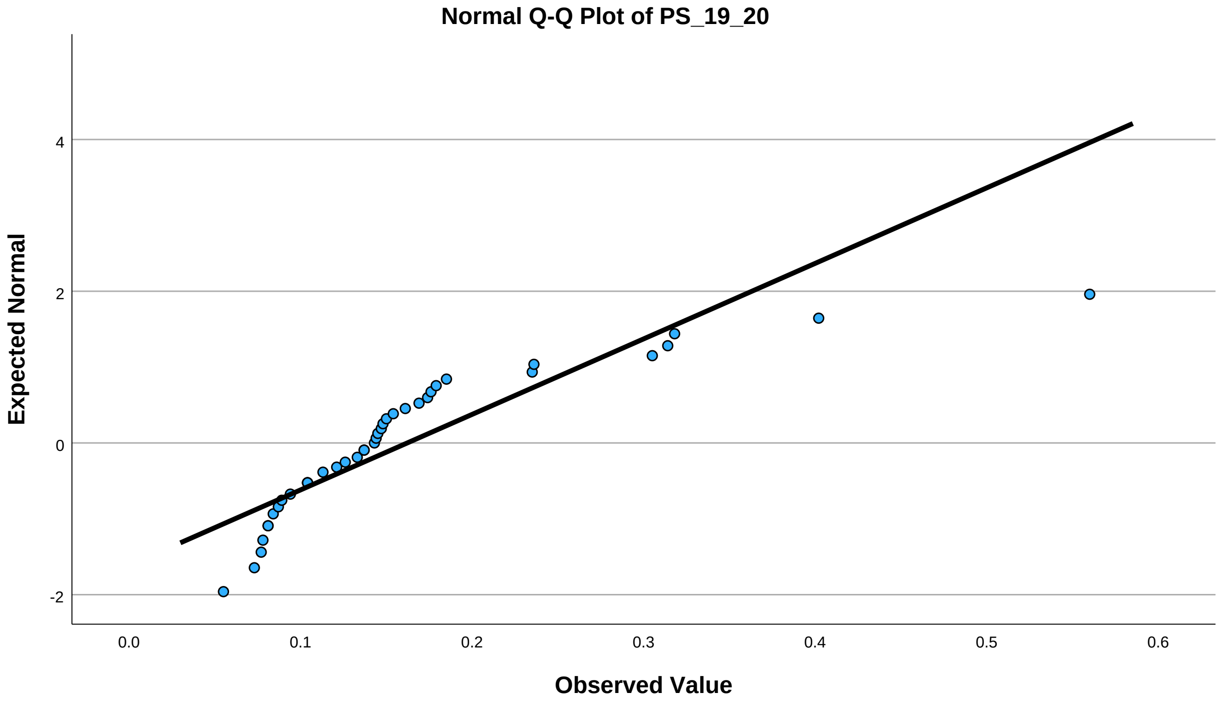

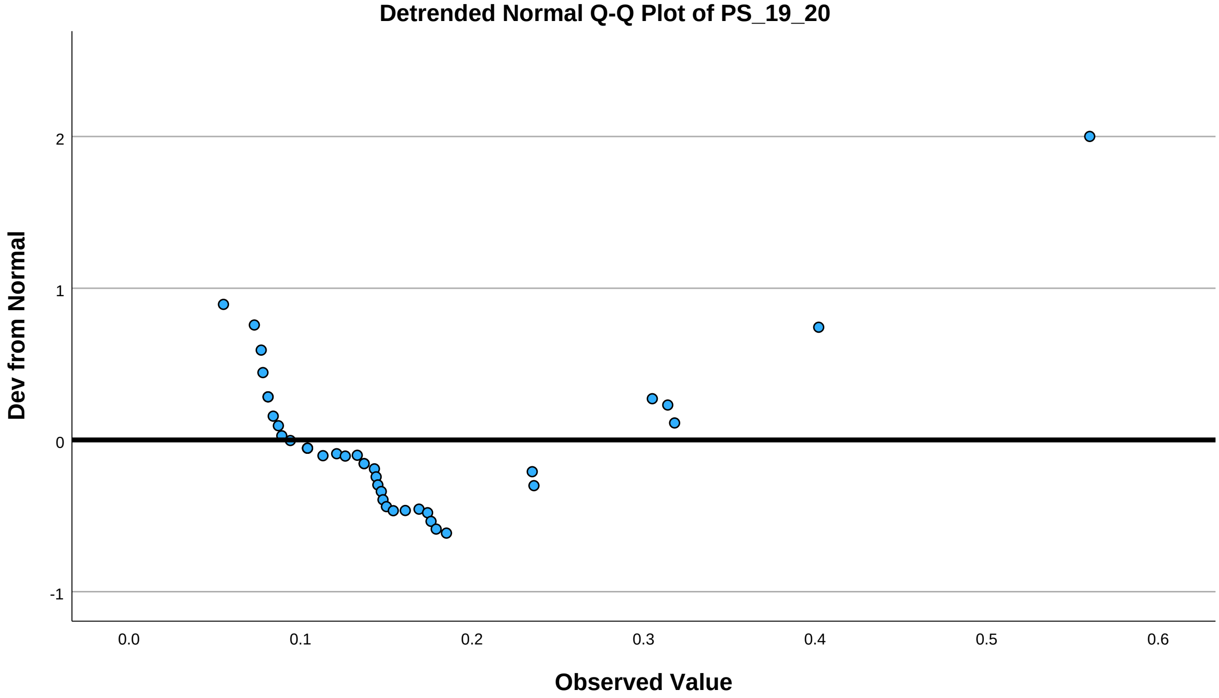
Detrended Normal Q-Q Plot of PS_19_20
Dev from Normal
2
1
0
-1
0.0
0.1
0.2
0.3
0.4
0.5
0.6
Observed Value

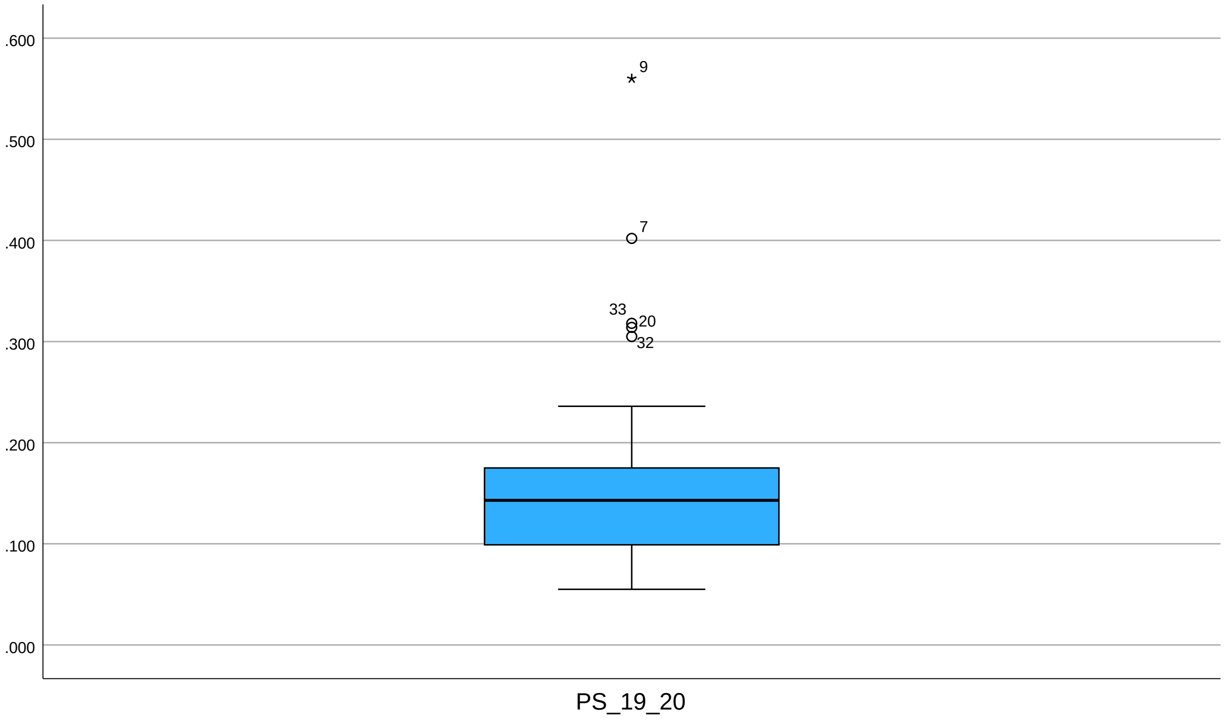
.600
.500
.400
.300
.200
.100
.000
9
7
33
20
32
PS_19_20

Explore

Case Processing Summary

	Cases					
	Valid		Missing		Total	
	N	Percent	N	Percent	N	Percent
BrainRate	39	100.0%	0	0.0%	39	100.0%
PermutationEntropy	39	100.0%	0	0.0%	39	100.0%
PermutationEntropy_01	39	100.0%	0	0.0%	39	100.0%
SampleEntropy	39	100.0%	0	0.0%	39	100.0%
SpectralEntropy_bits	39	100.0%	0	0.0%	39	100.0%
SpectralEntropy_01	39	100.0%	0	0.0%	39	100.0%
HiguchiFractalDimension	39	100.0%	0	0.0%	39	100.0%
LempelZivComplexity	39	100.0%	0	0.0%	39	100.0%
RenyiEntropy	39	100.0%	0	0.0%	39	100.0%
TsallisEntropy	39	100.0%	0	0.0%	39	100.0%
PS_4_5	39	100.0%	0	0.0%	39	100.0%
PS_5_6	39	100.0%	0	0.0%	39	100.0%
PS_6_7	39	100.0%	0	0.0%	39	100.0%
PS_7_8	39	100.0%	0	0.0%	39	100.0%
PS_8_9	39	100.0%	0	0.0%	39	100.0%
PS_9_10	39	100.0%	0	0.0%	39	100.0%
PS_10_11	39	100.0%	0	0.0%	39	100.0%
PS_11_12	39	100.0%	0	0.0%	39	100.0%
PS_12_13	39	100.0%	0	0.0%	39	100.0%
PS_13_14	39	100.0%	0	0.0%	39	100.0%
PS_14_15	39	100.0%	0	0.0%	39	100.0%
PS_15_16	39	100.0%	0	0.0%	39	100.0%
PS_16_17	39	100.0%	0	0.0%	39	100.0%
PS_17_18	39	100.0%	0	0.0%	39	100.0%
PS_18_19	39	100.0%	0	0.0%	39	100.0%
PS_19_20	39	100.0%	0	0.0%	39	100.0%

Descriptives

			Statistic	Std. Error
BrainRate	Mean		6.3667	.20400
	95% Confidence Interval for Mean	Lower Bound	5.9537	
		Upper Bound	6.7796	
	5% Trimmed Mean		6.3813	
	Median		6.4700	
	Variance		1.623	
	Std. Deviation		1.27397	
	Minimum		3.06	
	Maximum		9.24	
	Range		6.18	
	Interquartile Range		2.01	
	Skewness		-.158	.378
	Kurtosis		.300	.741
PermutationEntropy	Mean		1.4527167949	.00941623478
	95% Confidence Interval for Mean	Lower Bound	1.4336546242	
		Upper Bound	1.4717789657	
	5% Trimmed Mean		1.4531059013	
	Median		1.4576179533	
	Variance		.003	
	Std. Deviation		.05880436735	
	Minimum		1.3287874986	
	Maximum		1.6116373344	
	Range		.28284983587	
	Interquartile Range		.07610693341	
	Skewness		-.128	.378
	Kurtosis		.718	.741
PermutationEntropy_01	Mean		.81077668063	.00525530069
	95% Confidence Interval for Mean	Lower Bound	.80013788058	
		Upper Bound	.82141548068	
	5% Trimmed Mean		.81099384505	
	Median		.81351206916	
	Variance		.001	
	Std. Deviation		.03281934230	
	Minimum		.74161042337	
	Maximum		.89947192249	
	Range		.15786149911	
	Interquartile Range		.04247608829	
	Skewness		-.128	.378
	Kurtosis		.718	.741

Descriptives

			Statistic	Std. Error
SampleEntropy	Mean		.59793669299	.02677948771
	95% Confidence Interval for Mean	Lower Bound	.54372445435	
		Upper Bound	.65214893163	
	5% Trimmed Mean		.60581624547	
	Median		.63006152036	
	Variance		.028	
	Std. Deviation		.16723784716	
	Minimum		.15890268279	
	Maximum		.90948428453	
	Range		.75058160174	
	Interquartile Range		.23318467332	
	Skewness		-.766	.378
	Kurtosis		.442	.741
SpectralEntropy_bits	Mean		3.8592365900	.06560013641
	95% Confidence Interval for Mean	Lower Bound	3.7264360567	
		Upper Bound	3.9920371233	
	5% Trimmed Mean		3.8955056006	
	Median		3.9498617527	
	Variance		.168	
	Std. Deviation		.40967272055	
	Minimum		2.3892952622	
	Maximum		4.4483407711	
	Range		2.0590455089	
	Interquartile Range		.54698170259	
	Skewness		-1.485	.378
	Kurtosis		3.432	.741
SpectralEntropy_01	Mean		.55043667098	.00935644132
	95% Confidence Interval for Mean	Lower Bound	.53149554578	
		Upper Bound	.56937779619	
	5% Trimmed Mean		.55560966128	
	Median		.56336239131	
	Variance		.003	
	Std. Deviation		.05843095732	
	Minimum		.34078131761	
	Maximum		.63445964722	
	Range		.29367832961	
	Interquartile Range		.07801511528	
	Skewness		-1.485	.378
	Kurtosis		3.432	.741

Descriptives

			Statistic	Std. Error
HiguchiFractalDimension	Mean		.62165866919	.01144017422
	95% Confidence Interval for Mean	Lower Bound	.59849924726	
		Upper Bound	.64481809113	
	5% Trimmed Mean		.62498171561	
	Median		.63126850507	
	Variance		.005	
	Std. Deviation		.07144386513	
	Minimum		.38777225980	
	Maximum		.77220748161	
	Range		.38443522182	
	Interquartile Range		.09770109551	
	Skewness		-.811	.378
	Kurtosis		2.108	.741
LempelZivComplexity	Mean		.25441091559	.00326839420
	95% Confidence Interval for Mean	Lower Bound	.24779439745	
		Upper Bound	.26102743374	
	5% Trimmed Mean		.25398270695	
	Median		.25589263773	
	Variance		.000	
	Std. Deviation		.02041111524	
	Minimum		.21527107224	
	Maximum		.30466993759	
	Range		.08939886535	
	Interquartile Range		.02547122618	
	Skewness		.421	.378
	Kurtosis		-.008	.741
RenyiEntropy	Mean		7.4334057724	.18616748193
	95% Confidence Interval for Mean	Lower Bound	7.0565294085	
		Upper Bound	7.8102821363	
	5% Trimmed Mean		7.4956907230	
	Median		7.6017393256	
	Variance		1.352	
	Std. Deviation		1.1626155520	
	Minimum		4.8690472855	
	Maximum		8.7378204276	
	Range		3.8687731421	
	Interquartile Range		2.1308959253	
	Skewness		-.739	.378
	Kurtosis		-.644	.741

Descriptives

			Statistic	Std. Error
TsallisEntropy	Mean		1.5260780077	.08947497653
	95% Confidence Interval for Mean	Lower Bound	1.3449453874	
		Upper Bound	1.7072106280	
	5% Trimmed Mean		1.5655280488	
	Median		1.8107661442	
	Variance		.312	
	Std. Deviation		.55877104937	
	Minimum		.32861911077	
	Maximum		1.9864193160	
	Range		1.6578002052	
	Interquartile Range		.94892617792	
	Skewness		-.917	.378
	Kurtosis		-.613	.741
PS_4_5	Mean		2.48238	.357984
	95% Confidence Interval for Mean	Lower Bound	1.75768	
		Upper Bound	3.20708	
	5% Trimmed Mean		2.15560	
	Median		1.76000	
	Variance		4.998	
	Std. Deviation		2.235607	
	Minimum		.536	
	Maximum		13.160	
	Range		12.624	
	Interquartile Range		1.549	
	Skewness		3.246	.378
	Kurtosis		13.568	.741
PS_5_6	Mean		1.85421	.245309
	95% Confidence Interval for Mean	Lower Bound	1.35760	
		Upper Bound	2.35081	
	5% Trimmed Mean		1.65891	
	Median		1.32600	
	Variance		2.347	
	Std. Deviation		1.531955	
	Minimum		.343	
	Maximum		8.900	
	Range		8.557	
	Interquartile Range		1.144	
	Skewness		2.930	.378
	Kurtosis		11.310	.741

Descriptives

			Statistic	Std. Error
PS_6_7	Mean		1.48851	.244455
	95% Confidence Interval for Mean	Lower Bound	.99364	
		Upper Bound	1.98339	
	5% Trimmed Mean		1.27123	
	Median		1.11400	
	Variance		2.331	
	Std. Deviation		1.526622	
	Minimum		.299	
	Maximum		9.880	
	Range		9.581	
	Interquartile Range		1.070	
	Skewness		4.588	.378
	Kurtosis		24.983	.741
PS_7_8	Mean		1.34946	.216568
	95% Confidence Interval for Mean	Lower Bound	.91104	
		Upper Bound	1.78788	
	5% Trimmed Mean		1.11719	
	Median		.96500	
	Variance		1.829	
	Std. Deviation		1.352466	
	Minimum		.267	
	Maximum		6.950	
	Range		6.683	
	Interquartile Range		.639	
	Skewness		3.231	.378
	Kurtosis		10.992	.741
PS_8_9	Mean		1.39921	.231032
	95% Confidence Interval for Mean	Lower Bound	.93151	
		Upper Bound	1.86690	
	5% Trimmed Mean		1.15954	
	Median		.91700	
	Variance		2.082	
	Std. Deviation		1.442793	
	Minimum		.248	
	Maximum		7.598	
	Range		7.350	
	Interquartile Range		.611	
	Skewness		3.108	.378
	Kurtosis		10.724	.741

Descriptives

			Statistic	Std. Error
PS_9_10	Mean		1.69633	.244080
	95% Confidence Interval for Mean	Lower Bound	1.20222	
		Upper Bound	2.19045	
	5% Trimmed Mean		1.50369	
	Median		1.38600	
	Variance		2.323	
	Std. Deviation		1.524279	
	Minimum		.242	
	Maximum		8.340	
	Range		8.098	
	Interquartile Range		1.515	
	Skewness		2.513	.378
	Kurtosis		8.784	.741
PS_10_11	Mean		2.45610	.400252
	95% Confidence Interval for Mean	Lower Bound	1.64584	
		Upper Bound	3.26637	
	5% Trimmed Mean		2.13319	
	Median		1.58000	
	Variance		6.248	
	Std. Deviation		2.499571	
	Minimum		.285	
	Maximum		12.630	
	Range		12.345	
	Interquartile Range		2.764	
	Skewness		2.254	.378
	Kurtosis		6.509	.741
PS_11_12	Mean		.89926	.160696
	95% Confidence Interval for Mean	Lower Bound	.57394	
		Upper Bound	1.22457	
	5% Trimmed Mean		.74003	
	Median		.62000	
	Variance		1.007	
	Std. Deviation		1.003546	
	Minimum		.161	
	Maximum		5.801	
	Range		5.640	
	Interquartile Range		.564	
	Skewness		3.627	.378
	Kurtosis		15.390	.741

Descriptives

			Statistic	Std. Error
PS_12_13	Mean		.52692	.060962
	95% Confidence Interval for Mean	Lower Bound	.40351	
		Upper Bound	.65033	
	5% Trimmed Mean		.48485	
	Median		.37600	
	Variance		.145	
	Std. Deviation		.380707	
	Minimum		.139	
	Maximum		1.830	
	Range		1.691	
	Interquartile Range		.219	
	Skewness		1.953	.378
	Kurtosis		3.501	.741
PS_13_14	Mean		.46131	.058944
	95% Confidence Interval for Mean	Lower Bound	.34198	
		Upper Bound	.58063	
	5% Trimmed Mean		.41892	
	Median		.32700	
	Variance		.136	
	Std. Deviation		.368105	
	Minimum		.108	
	Maximum		1.580	
	Range		1.472	
	Interquartile Range		.325	
	Skewness		2.071	.378
	Kurtosis		3.946	.741
PS_14_15	Mean		.45685	.066328
	95% Confidence Interval for Mean	Lower Bound	.32257	
		Upper Bound	.59112	
	5% Trimmed Mean		.40188	
	Median		.32000	
	Variance		.172	
	Std. Deviation		.414221	
	Minimum		.088	
	Maximum		1.896	
	Range		1.808	
	Interquartile Range		.318	
	Skewness		2.368	.378
	Kurtosis		5.466	.741

Descriptives

			Statistic	Std. Error
PS_15_16	Mean		.41159	.059995
	95% Confidence Interval for Mean	Lower Bound	.29014	
		Upper Bound	.53304	
	5% Trimmed Mean		.36164	
	Median		.28000	
	Variance		.140	
	Std. Deviation		.374667	
	Minimum		.082	
	Maximum		1.938	
	Range		1.856	
	Interquartile Range		.340	
	Skewness		2.402	.378
	Kurtosis		6.792	.741
PS_16_17	Mean		.34041	.045744
	95% Confidence Interval for Mean	Lower Bound	.24781	
		Upper Bound	.43302	
	5% Trimmed Mean		.30293	
	Median		.23400	
	Variance		.082	
	Std. Deviation		.285674	
	Minimum		.094	
	Maximum		1.467	
	Range		1.373	
	Interquartile Range		.205	
	Skewness		2.290	.378
	Kurtosis		5.947	.741
PS_17_18	Mean		.28431	.037846
	95% Confidence Interval for Mean	Lower Bound	.20769	
		Upper Bound	.36092	
	5% Trimmed Mean		.25309	
	Median		.18900	
	Variance		.056	
	Std. Deviation		.236350	
	Minimum		.079	
	Maximum		1.339	
	Range		1.260	
	Interquartile Range		.160	
	Skewness		2.785	.378
	Kurtosis		9.845	.741

Descriptives

			Statistic	Std. Error
PS_18_19	Mean		.23833	.027005
	95% Confidence Interval for Mean	Lower Bound	.18366	
		Upper Bound	.29300	
	5% Trimmed Mean		.21868	
	Median		.17800	
	Variance		.028	
	Std. Deviation		.168648	
	Minimum		.073	
	Maximum		.795	
	Range		.722	
	Interquartile Range		.114	
	Skewness		1.903	.378
	Kurtosis		3.112	.741
PS_19_20	Mean		.21756	.021788
	95% Confidence Interval for Mean	Lower Bound	.17346	
		Upper Bound	.26167	
	5% Trimmed Mean		.20313	
	Median		.18300	
	Variance		.019	
	Std. Deviation		.136066	
	Minimum		.070	
	Maximum		.621	
	Range		.551	
	Interquartile Range		.117	
	Skewness		1.874	.378
	Kurtosis		3.240	.741

Tests of Normality

	Kolmogorov-Smirnov[a]			Shapiro-Wilk		
	Statistic	df	Sig.	Statistic	df	Sig.
BrainRate	.081	39	.200*	.985	39	.883
PermutationEntropy	.095	39	.200*	.965	39	.252
PermutationEntropy_01	.095	39	.200*	.965	39	.252
SampleEntropy	.175	39	.004	.948	39	.070
SpectralEntropy_bits	.110	39	.200*	.896	39	.002
SpectralEntropy_01	.110	39	.200*	.896	39	.002
HiguchiFractalDimension	.092	39	.200*	.954	39	.108
LempelZivComplexity	.102	39	.200*	.972	39	.419
RenyiEntropy	.144	39	.042	.890	39	.001
TsallisEntropy	.227	39	<.001	.795	39	<.001
PS_4_5	.216	39	<.001	.668	39	<.001
PS_5_6	.211	39	<.001	.707	39	<.001
PS_6_7	.218	39	<.001	.524	39	<.001
PS_7_8	.276	39	<.001	.587	39	<.001
PS_8_9	.287	39	<.001	.625	39	<.001
PS_9_10	.170	39	.006	.768	39	<.001
PS_10_11	.193	39	<.001	.765	39	<.001
PS_11_12	.250	39	<.001	.574	39	<.001
PS_12_13	.250	39	<.001	.757	39	<.001
PS_13_14	.224	39	<.001	.729	39	<.001
PS_14_15	.215	39	<.001	.692	39	<.001
PS_15_16	.257	39	<.001	.722	39	<.001
PS_16_17	.227	39	<.001	.732	39	<.001
PS_17_18	.237	39	<.001	.690	39	<.001
PS_18_19	.287	39	<.001	.746	39	<.001
PS_19_20	.209	39	<.001	.778	39	<.001

*. This is a lower bound of the true significance.

a. Lilliefors Significance Correction

BrainRate

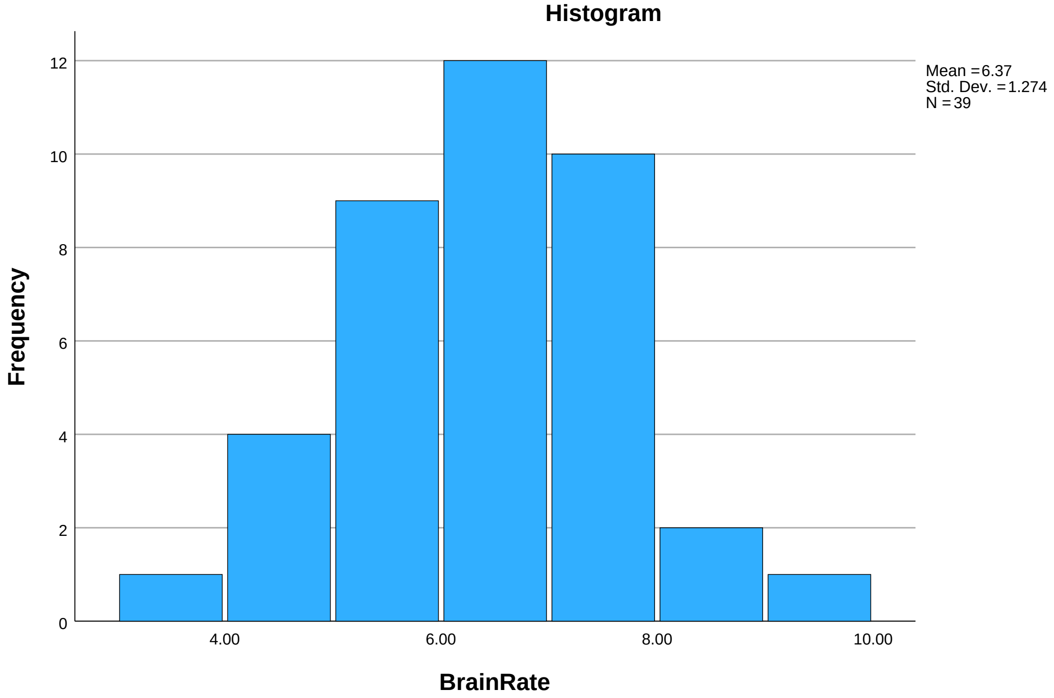


```
BrainRate Stem-and-Leaf Plot

 Frequency    Stem &  Leaf

     1.00        3 .  0
     4.00        4 .  0889
     9.00        5 .  012223579
    12.00        6 .  113344556666
    10.00        7 .  1122344577
     2.00        8 .  46
     1.00        9 .  2

 Stem width:      1.00
 Each leaf:       1 case(s)
```

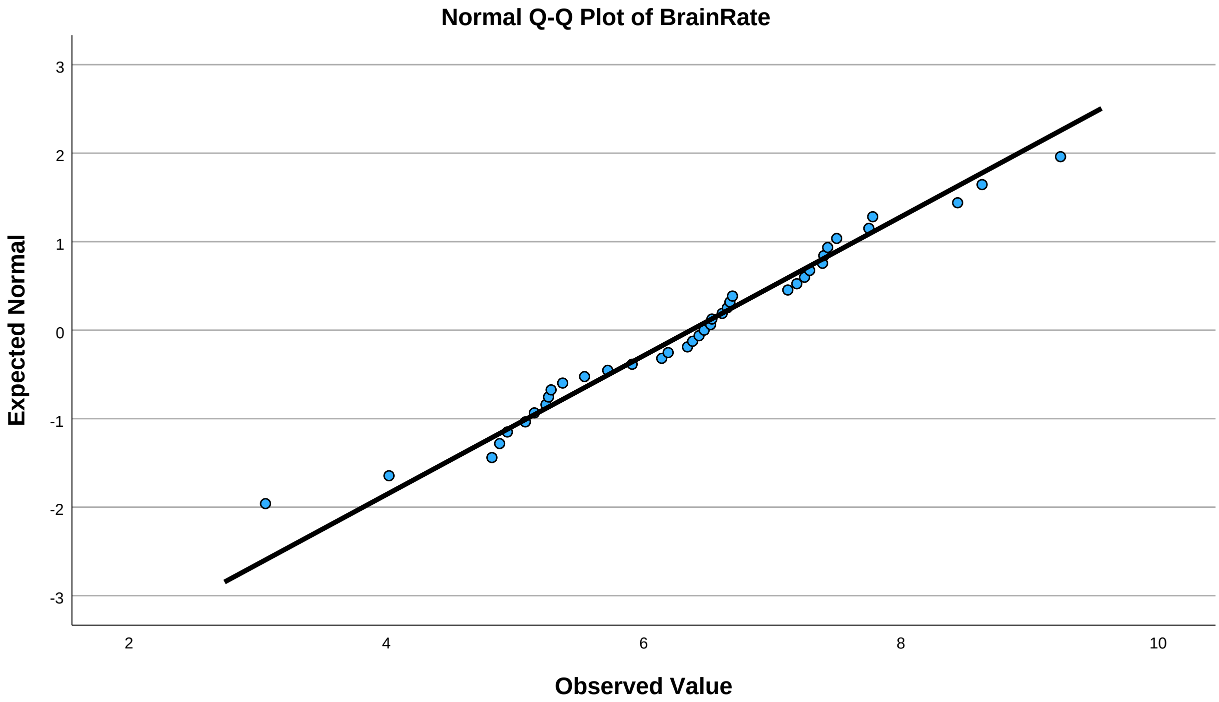
Normal Q-Q Plot of BrainRate
Expected Normal
3
2
1
0
-1
-2
-3
2
4
6
8
10
Observed Value

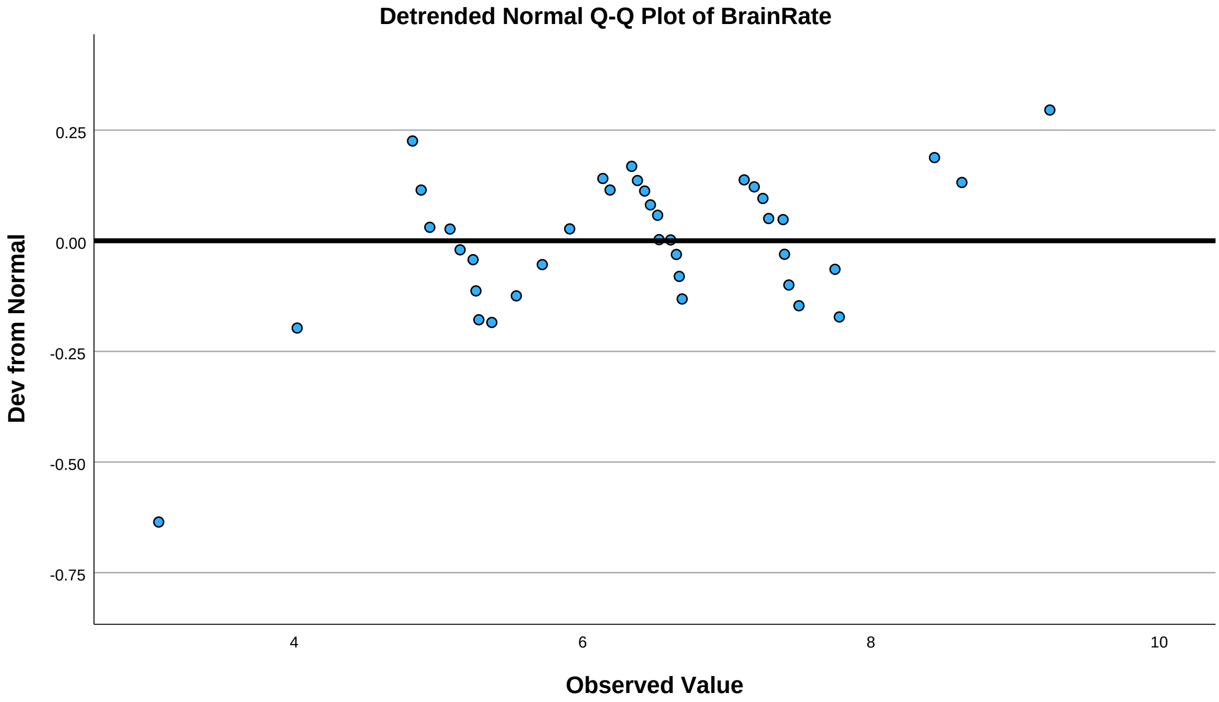
Detrended Normal Q-Q Plot of BrainRate
Dev from Normal
0.25
0.00
-0.25
-0.50
-0.75
4
6
8
10
Observed Value

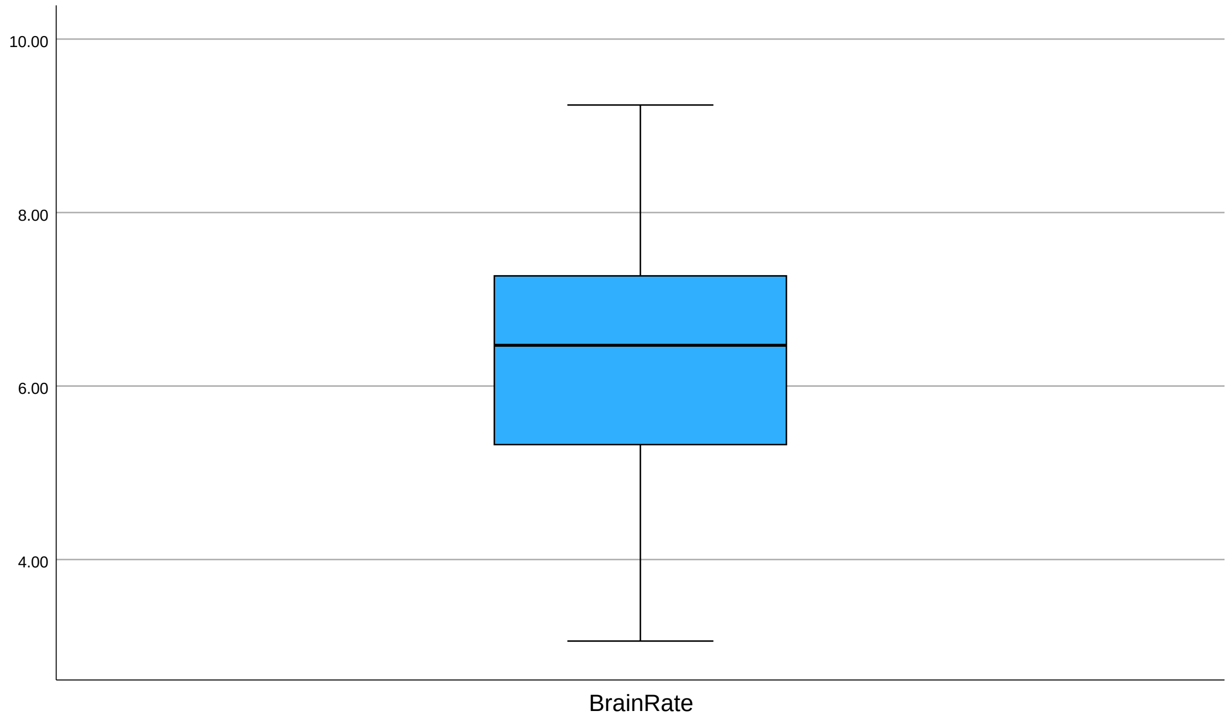

PermutationEntropy

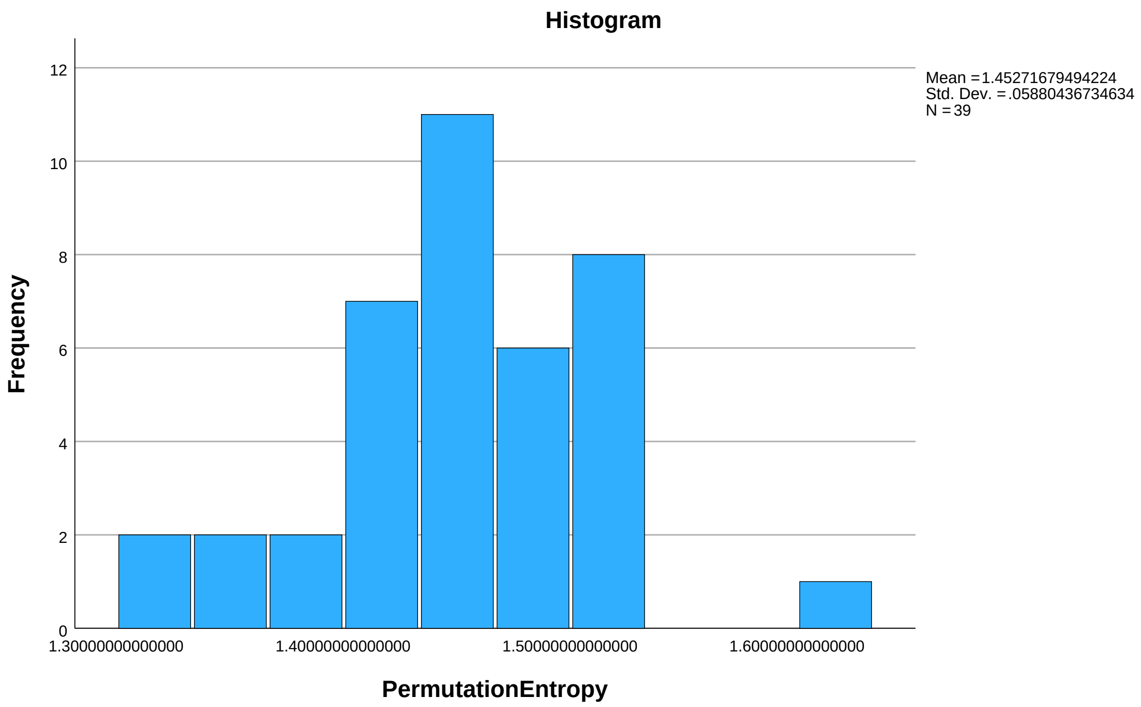

PermutationEntropy Stem-and-Leaf Plot

Frequency   Stem & Leaf

```
 1.00 Extremes    (=<1.33)
 3.00     13 .  244
 2.00     13 .  88
11.00      14 .  01123334444
13.00      14 .  5555666777789
 8.00      15 .  00011122
 1.00 Extremes    (>=1.61)

Stem width:   .1000000
Each leaf:        1 case(s)
```

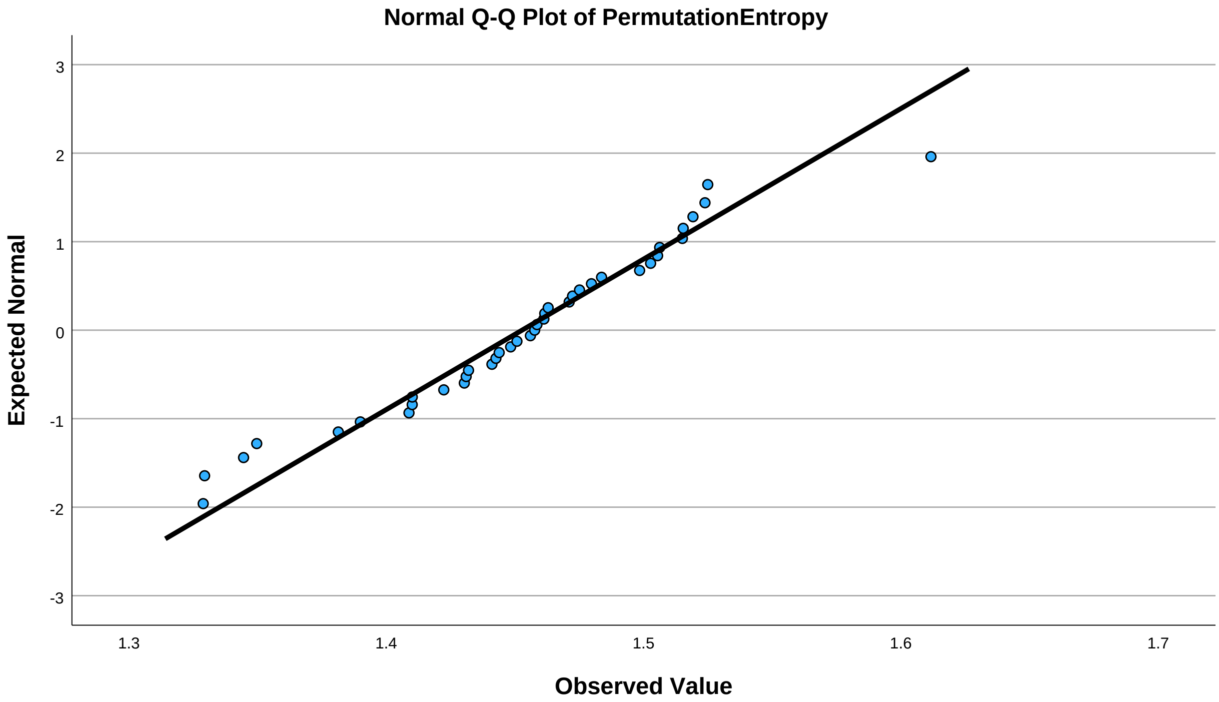

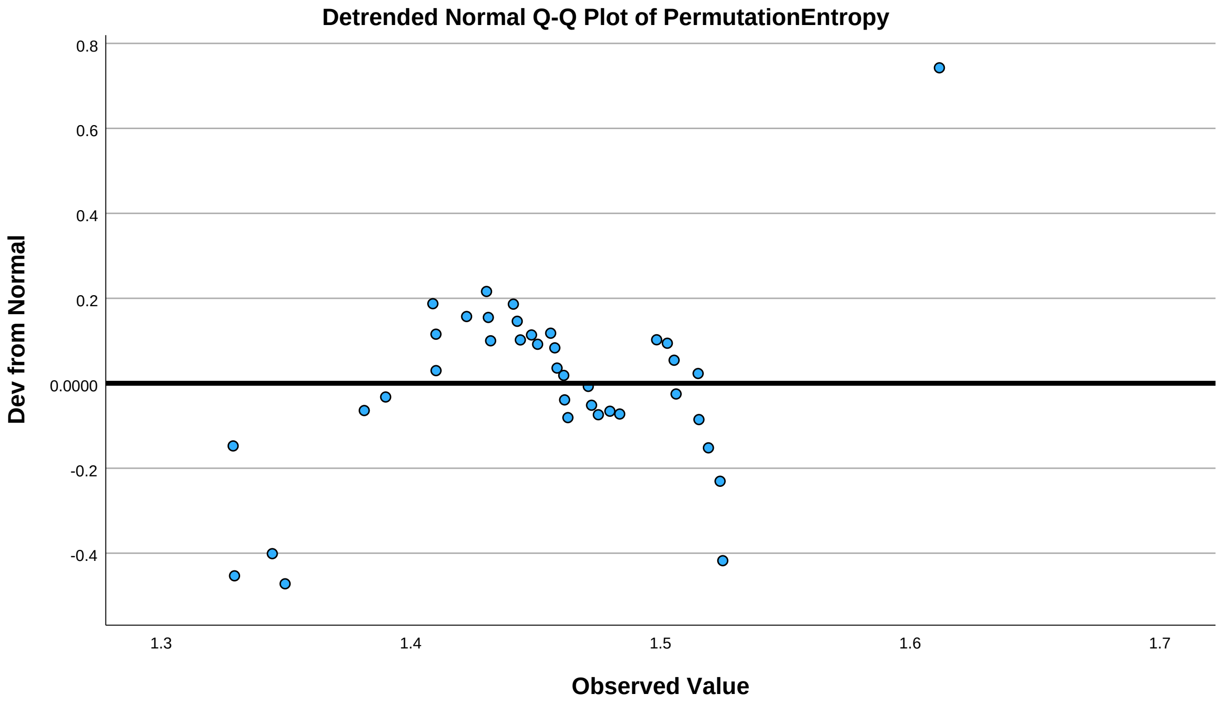

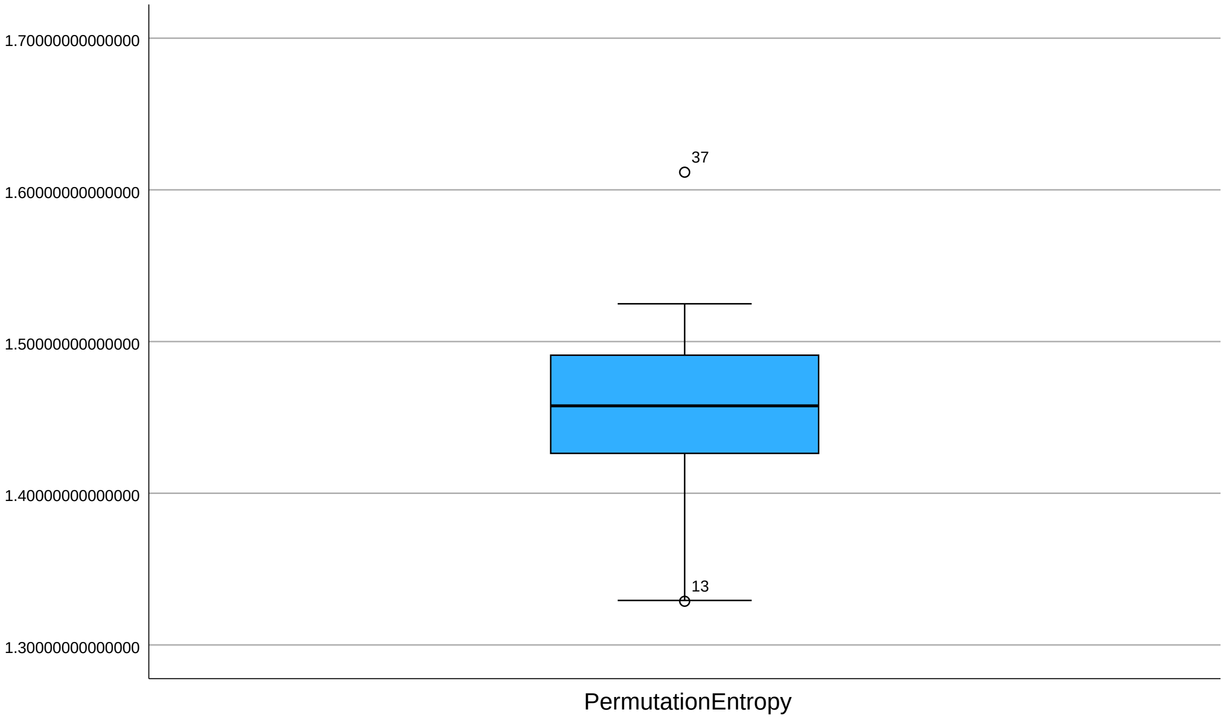

PermutationEntropy_01

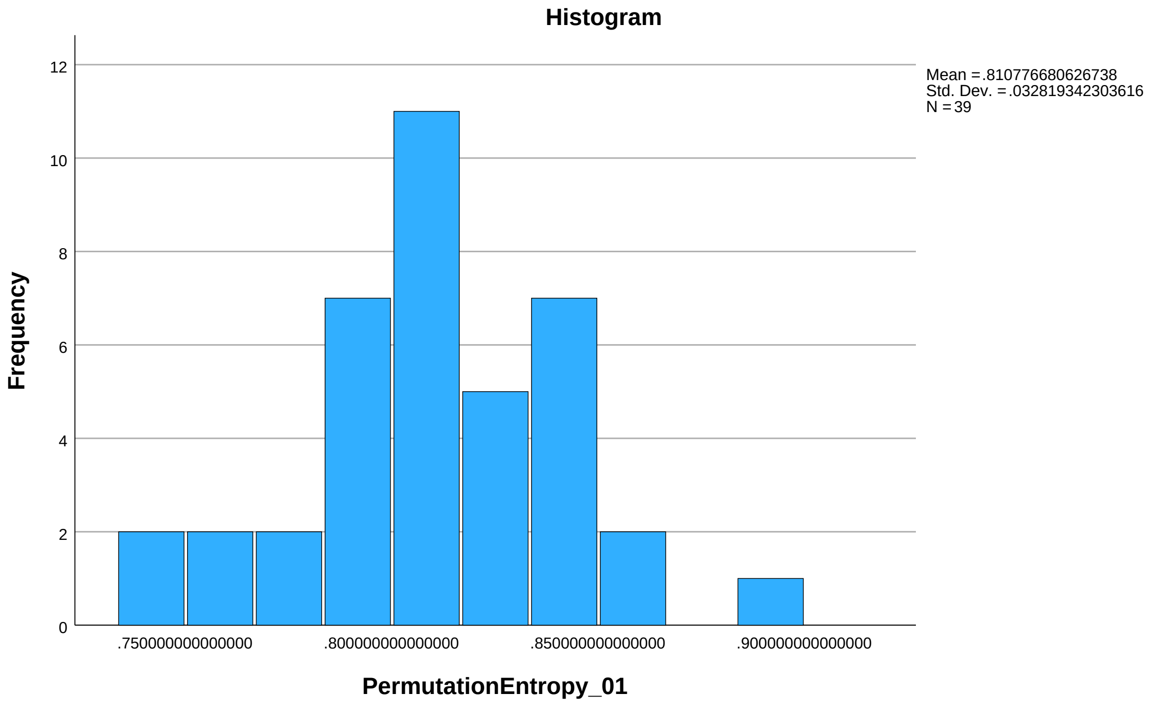


```
PermutationEntropy_01 Stem-and-Leaf Plot

 Frequency    Stem &  Leaf

     1.00 Extremes    (=<.742)
     1.00       74 .  1
     2.00       75 .  03
      .00       76 .
     2.00       77 .  05
     3.00       78 .  666
     4.00       79 .  3889
     5.00       80 .  45589
     6.00       81 .  234556
     5.00       82 .  01358
     2.00       83 .  68
     5.00       84 .  00557
     2.00       85 .  01
     1.00 Extremes    (>=.899)

 Stem width:   .0100000
 Each leaf:       1 case(s)
```

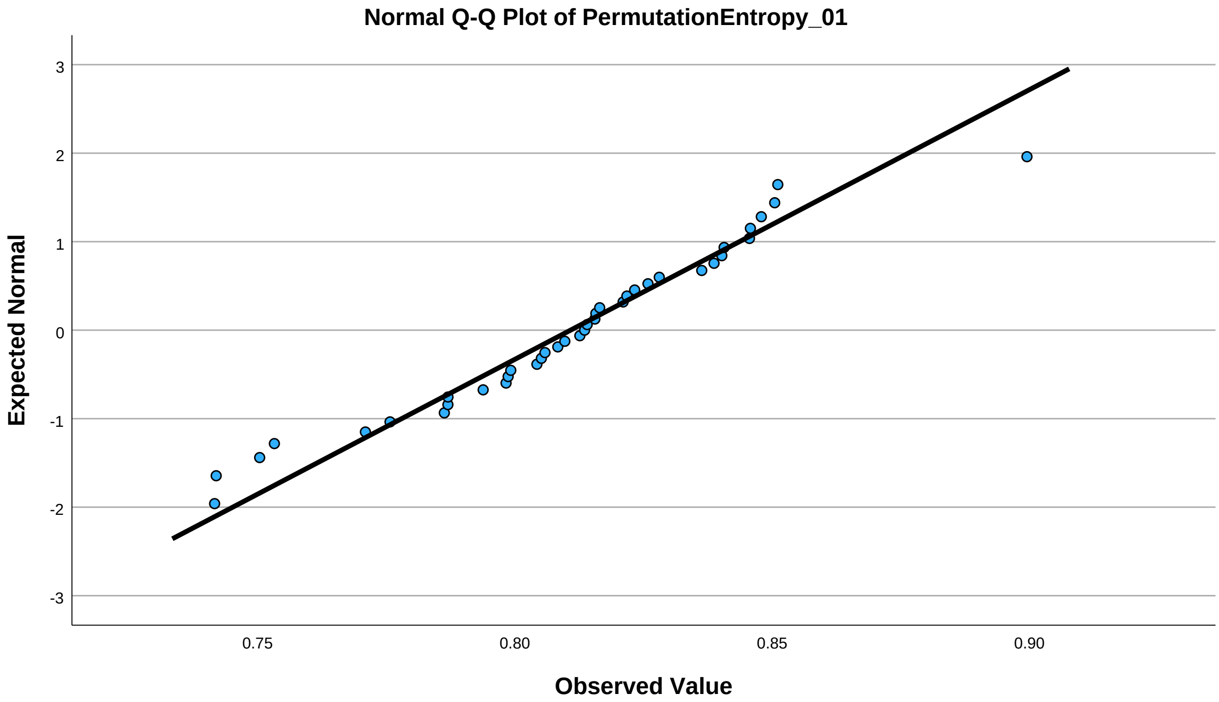
Normal Q-Q Plot of PermutationEntropy_01
Expected Normal
3
2
1
0
-1
-2
-3
0.75
0.80
0.85
0.90
Observed Value

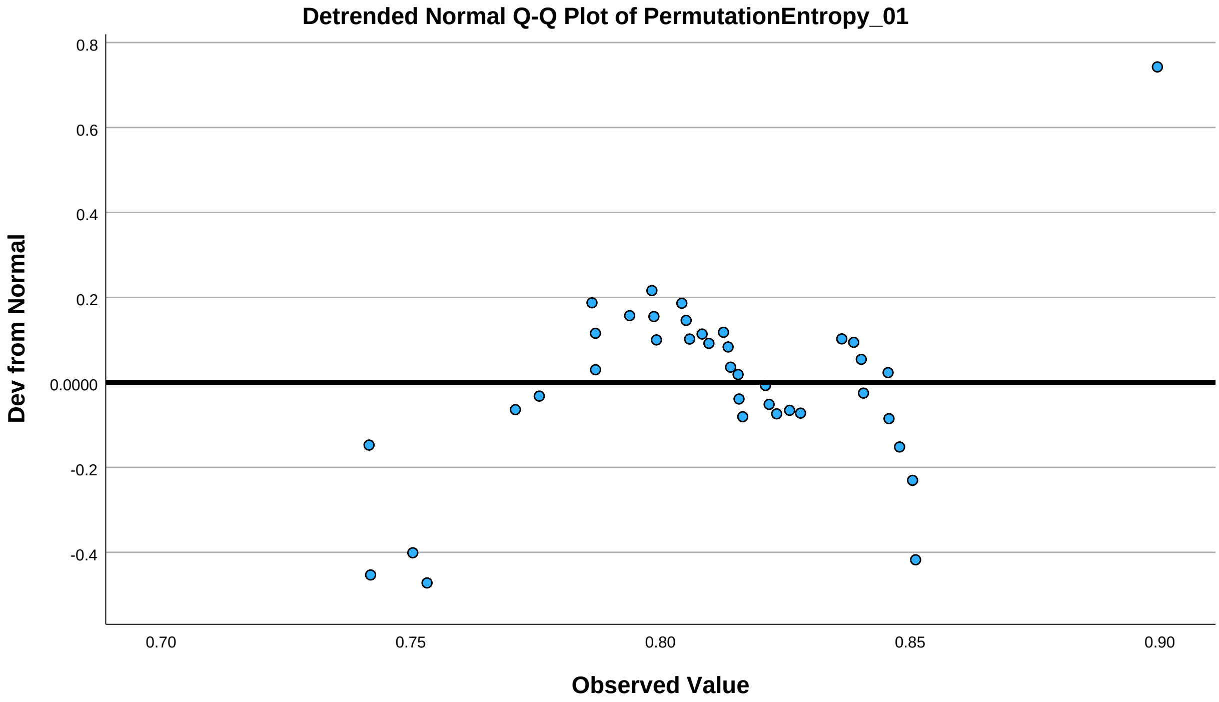
Detrended Normal Q-Q Plot of PermutationEntropy_01
Dev from Normal
0.8
0.6
0.4
0.2
0.0000
-0.2
-0.4
0.70
0.75
0.80
0.85
0.90
Observed Value

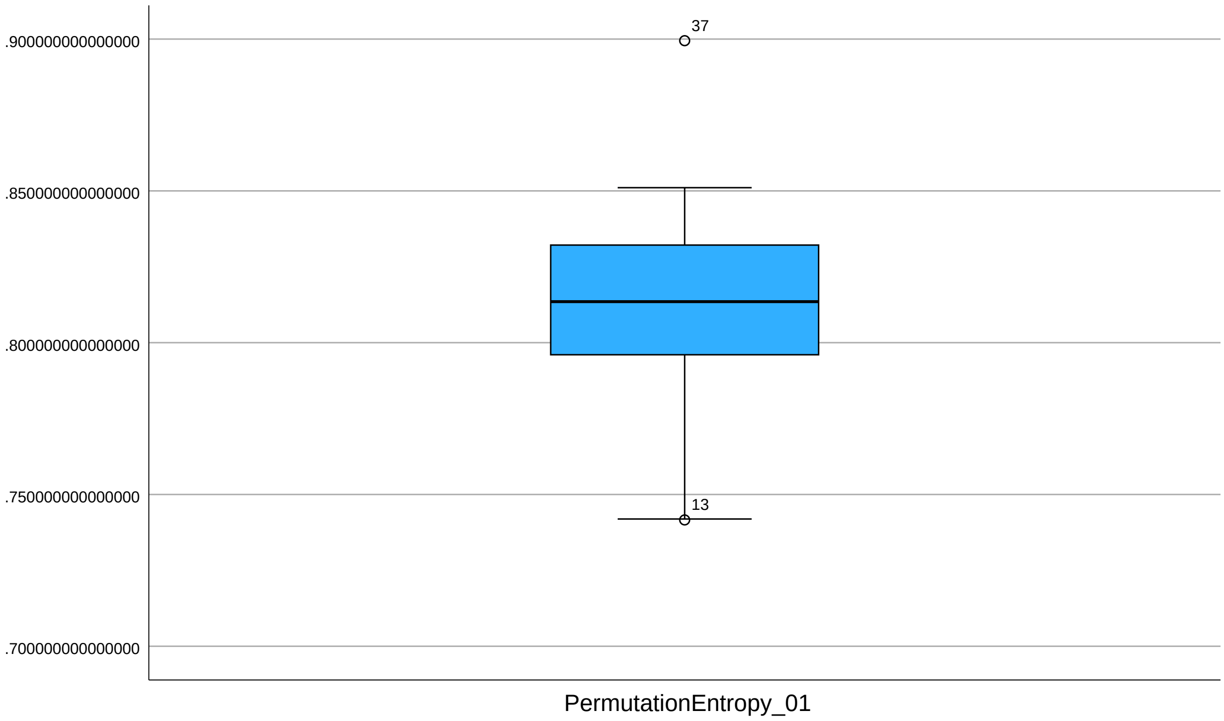

SampleEntropy

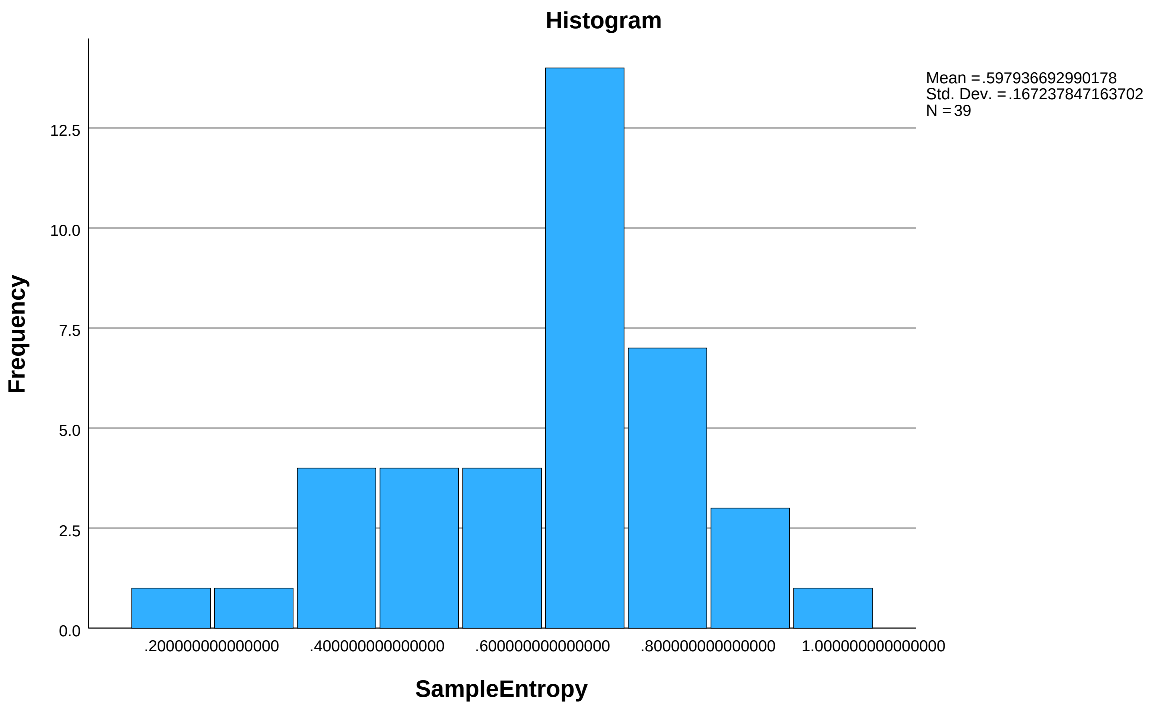


```
SampleEntropy Stem-and-Leaf Plot

 Frequency    Stem &  Leaf
```

```
 2.00 Extremes    (=<.21)
 4.00      3 .  3688
 4.00      4 .  1367
 4.00      5 .  4668
14.00      6 .  11222333355688
 7.00      7 .  001248
 3.00      8 .  013
 1.00      9 .  0

Stem width:   .1000000
Each leaf:       1 case(s)
```

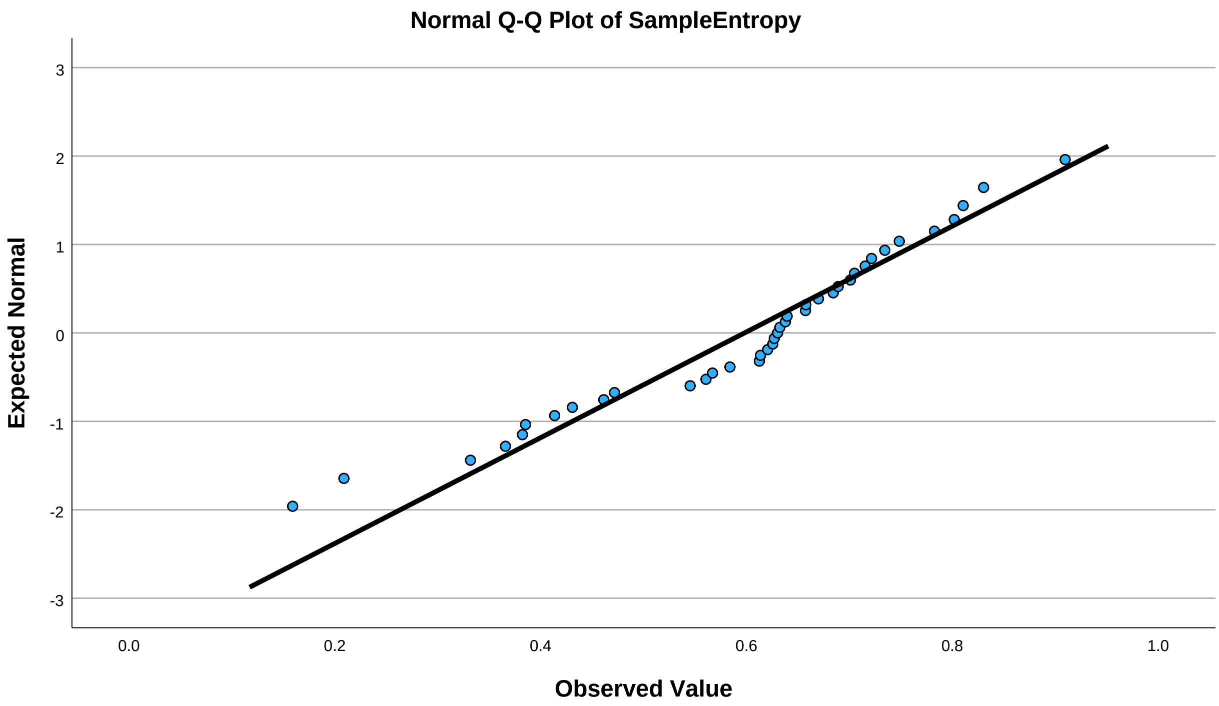

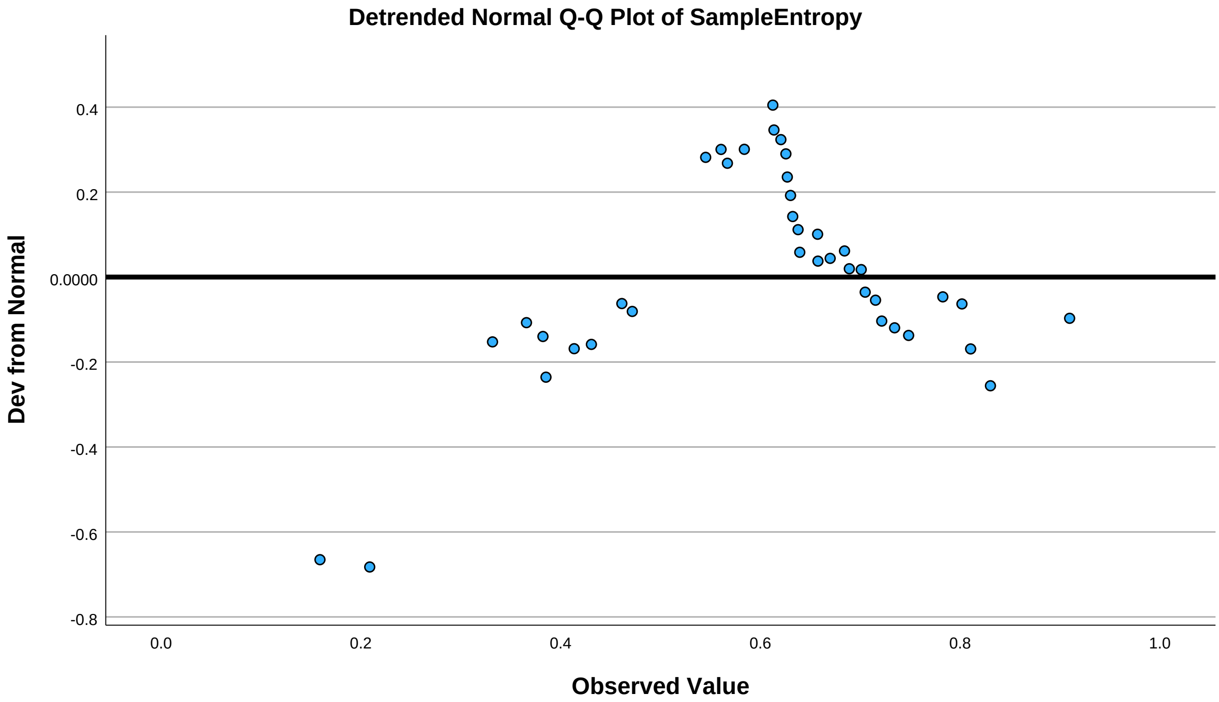

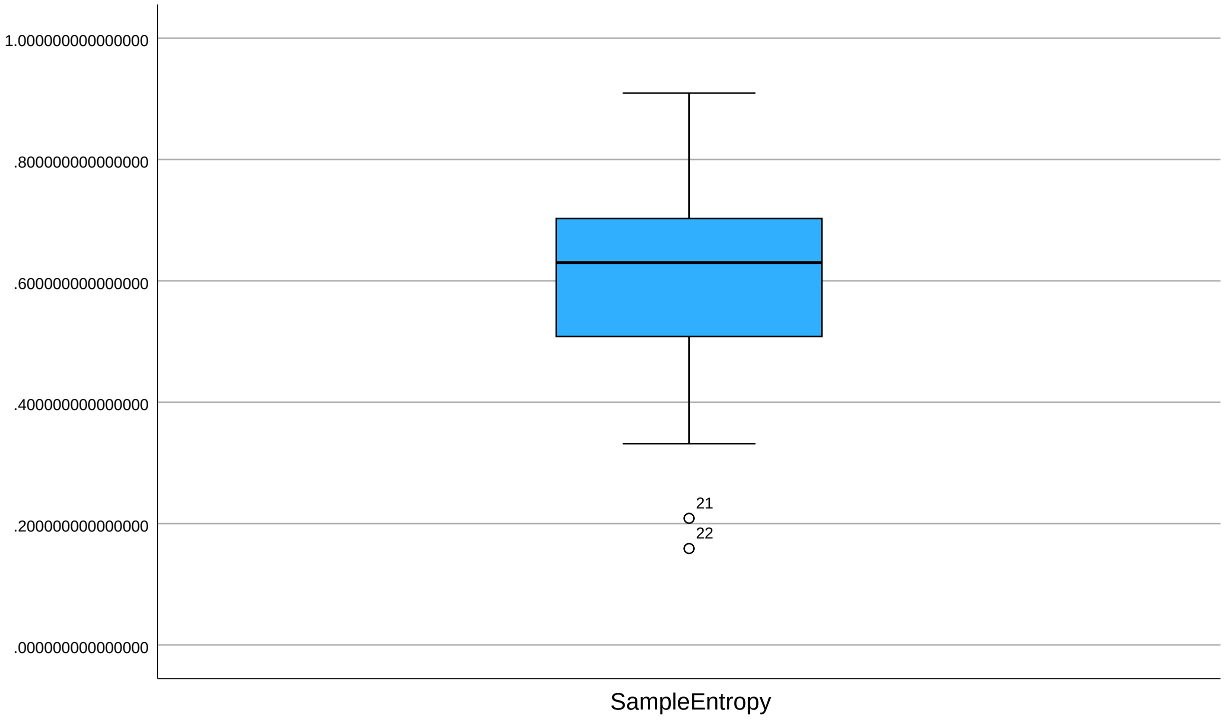

SpectralEntropy_bits

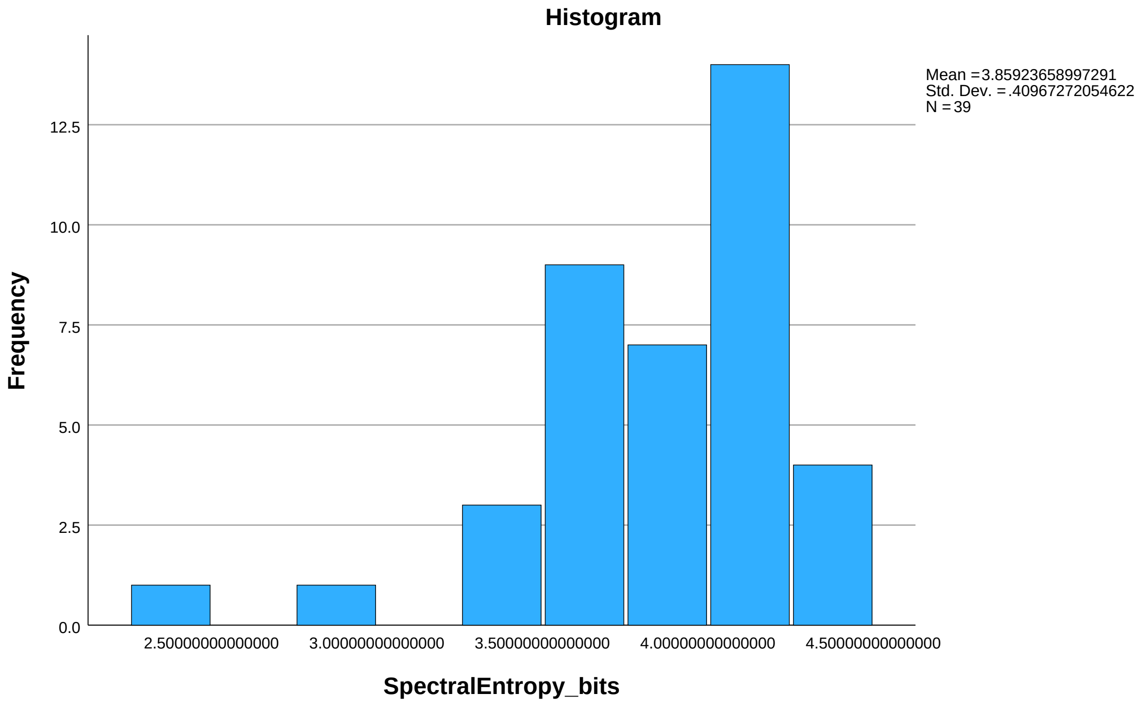


```
SpectralEntropy_bits Stem-and-Leaf Plot

 Frequency    Stem &  Leaf

     2.00 Extremes    (=<2.90)
     2.00       33 .  06
     1.00       34 .  1
     2.00       35 .  66
     4.00       36 .  0019
     5.00       37 .  22389
     1.00       38 .  1
     4.00       39 .  0346
     6.00       40 .  122349
     6.00       41 .  446779
     3.00       42 .  227
     1.00       43 .  2
     2.00       44 .  04

 Stem width:   .1000000
 Each leaf:       1 case(s)
```

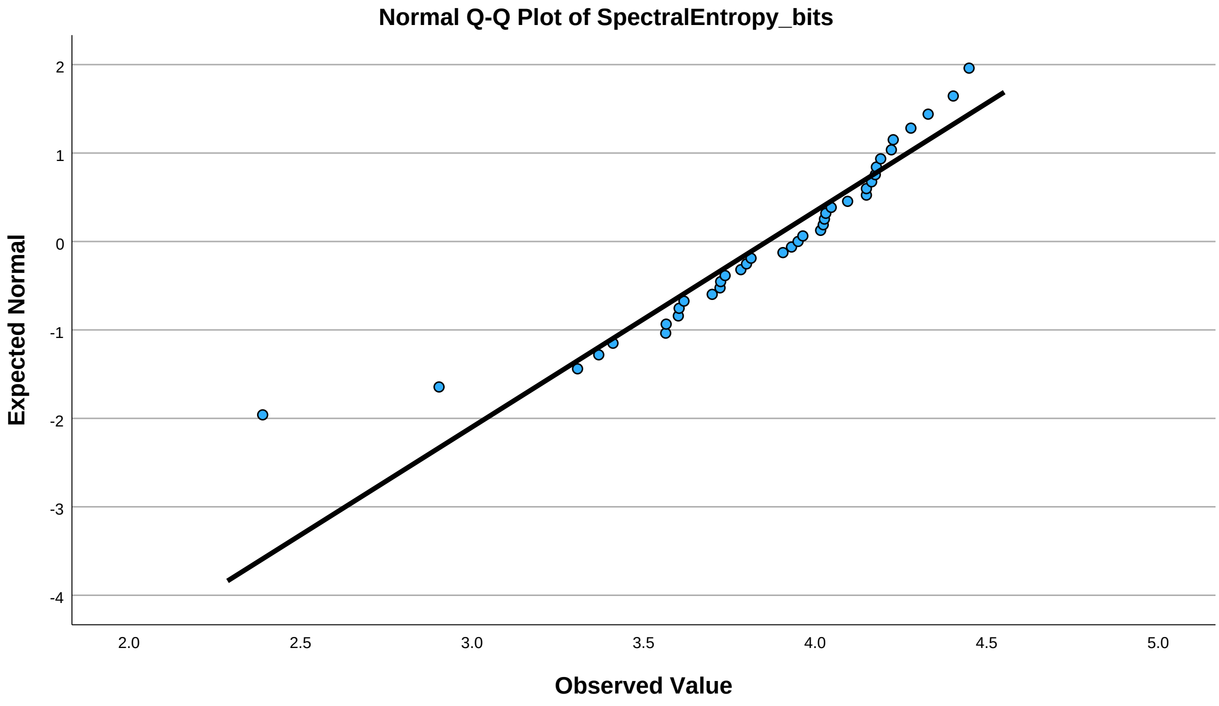
Normal Q-Q Plot of SpectralEntropy_bits
Expected Normal
Observed Value
2
1
0
-1
-2
-3
-4
2.0
2.5
3.0
3.5
4.0
4.5
5.0

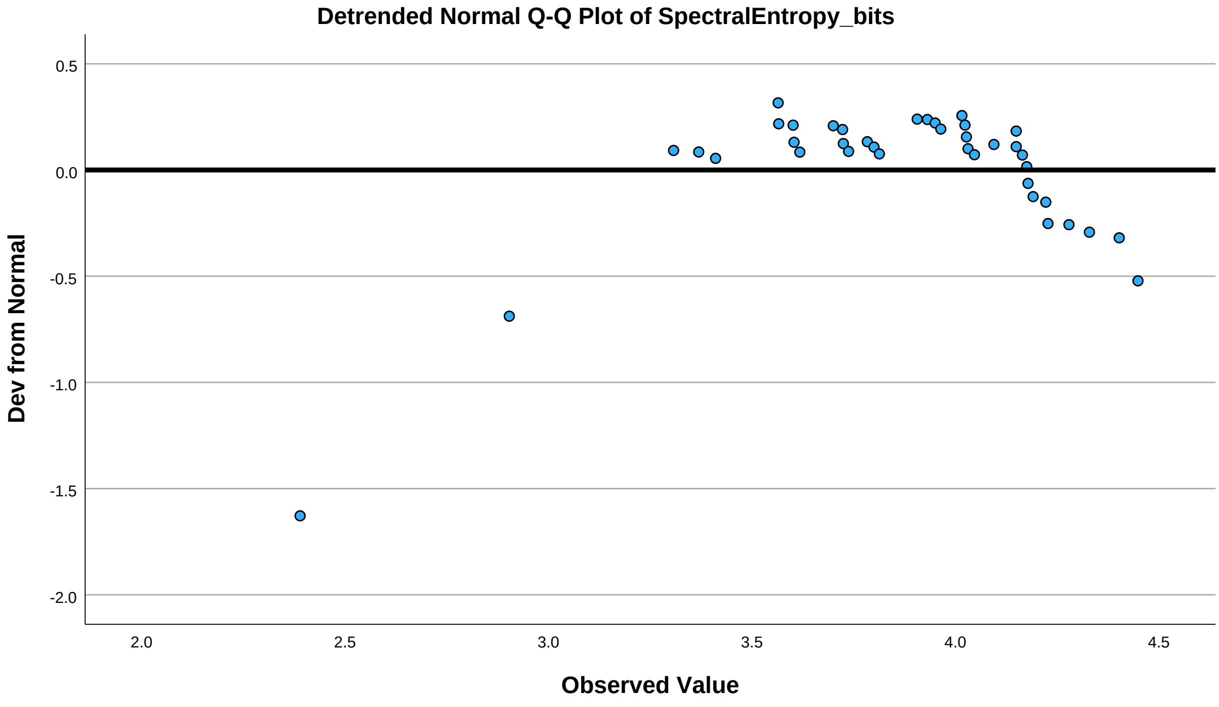
Detrended Normal Q-Q Plot of SpectralEntropy_bits
Dev from Normal
Observed Value
0.5
0.0
-0.5
-1.0
-1.5
-2.0
2.0
2.5
3.0
3.5
4.0
4.5

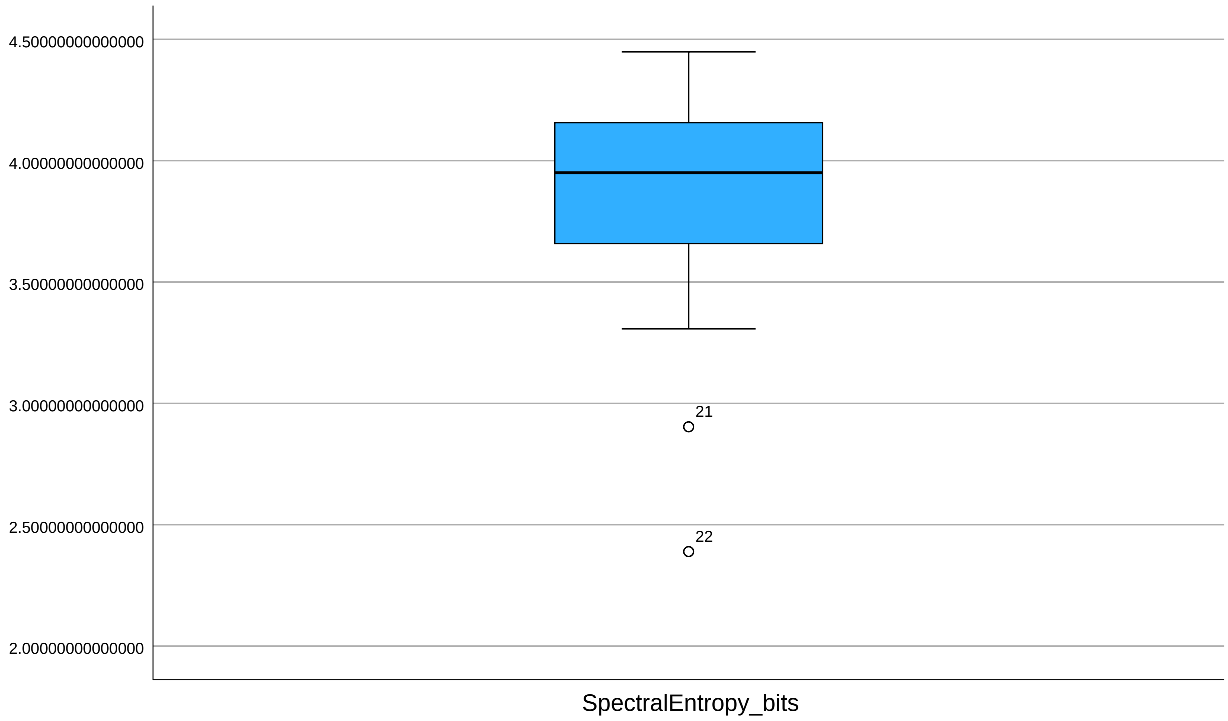

SpectralEntropy_01

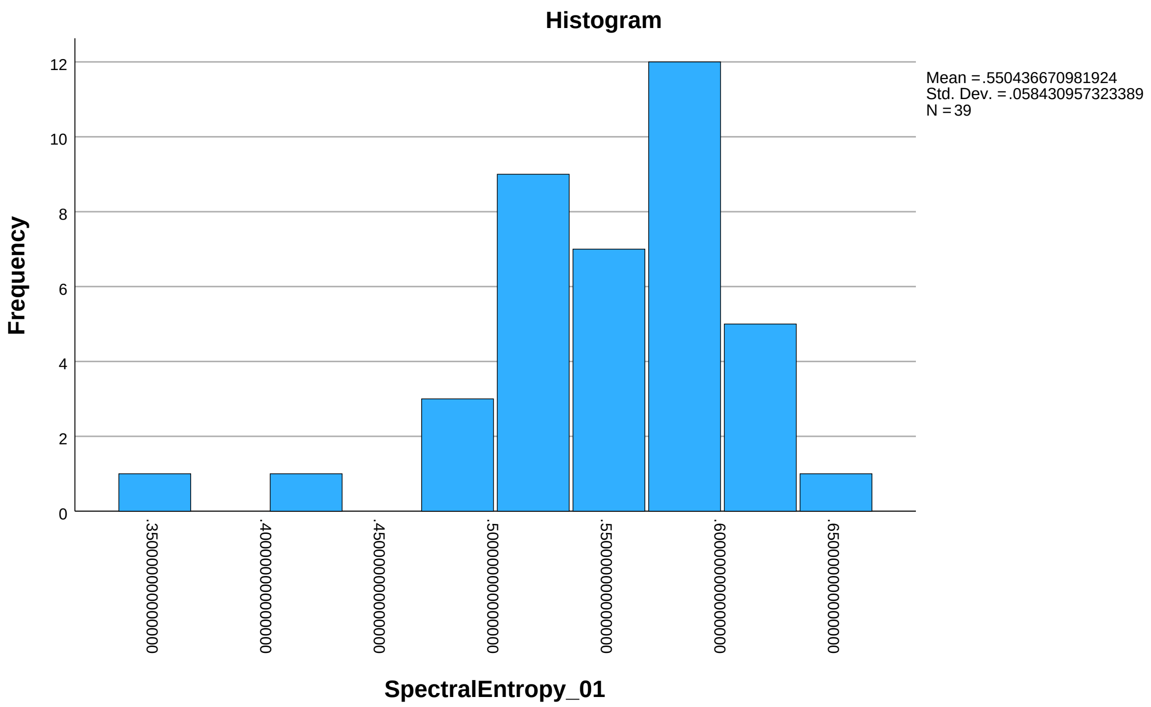

SpectralEntropy_01 Stem-and-Leaf Plot

Frequency   Stem & Leaf

```
  2.00 Extremes    (=<.41)
  3.00      4 .  788
 12.00       5 .  001112333344
 16.00       5 .  5666777778999999
  6.00      6 .  001123

Stem width:   .1000000
Each leaf:       1 case(s)
```

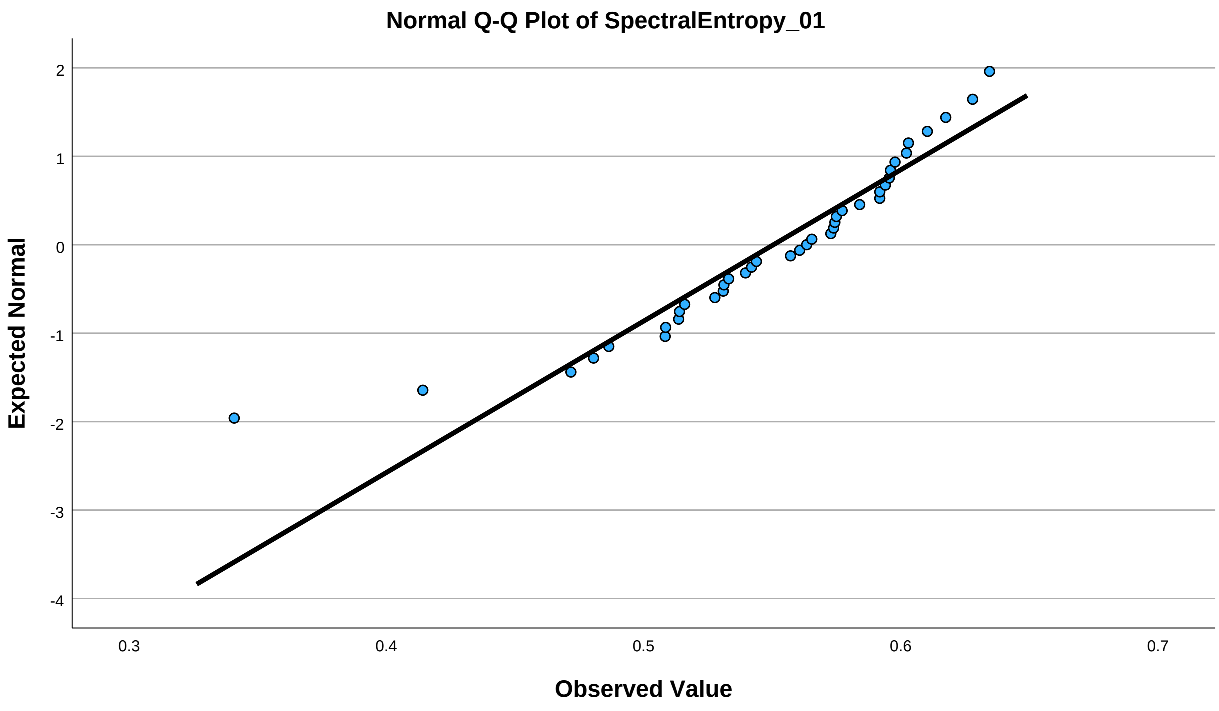

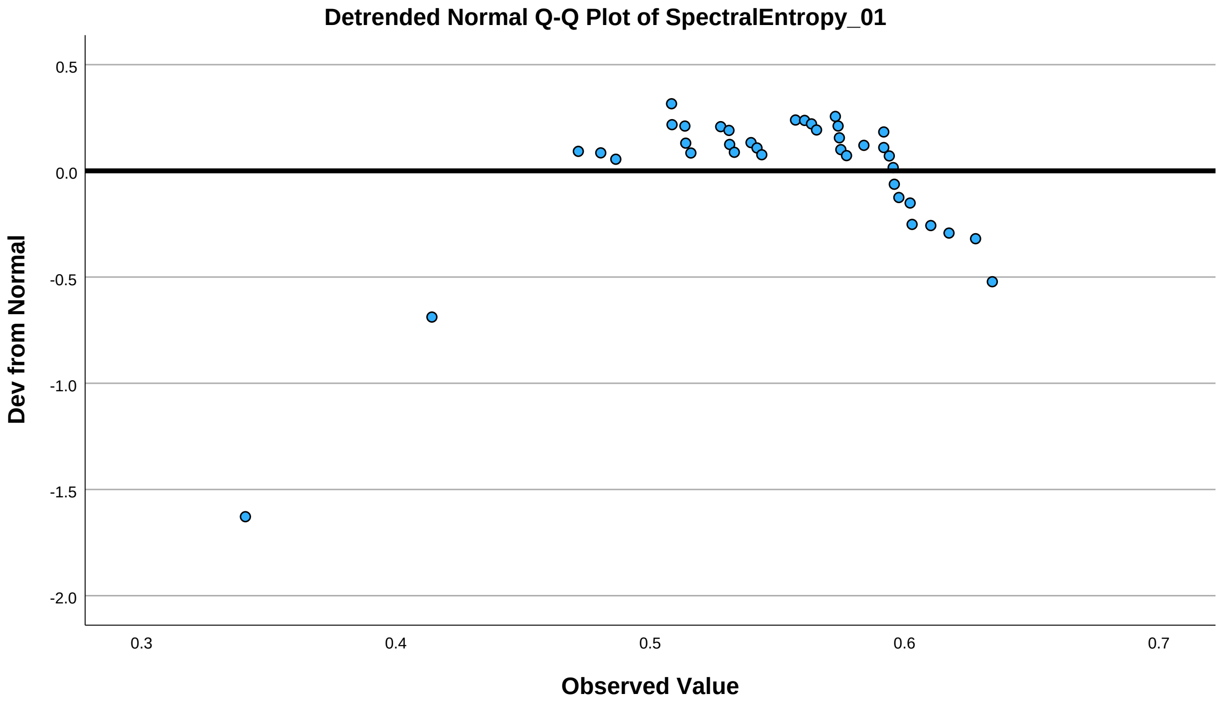

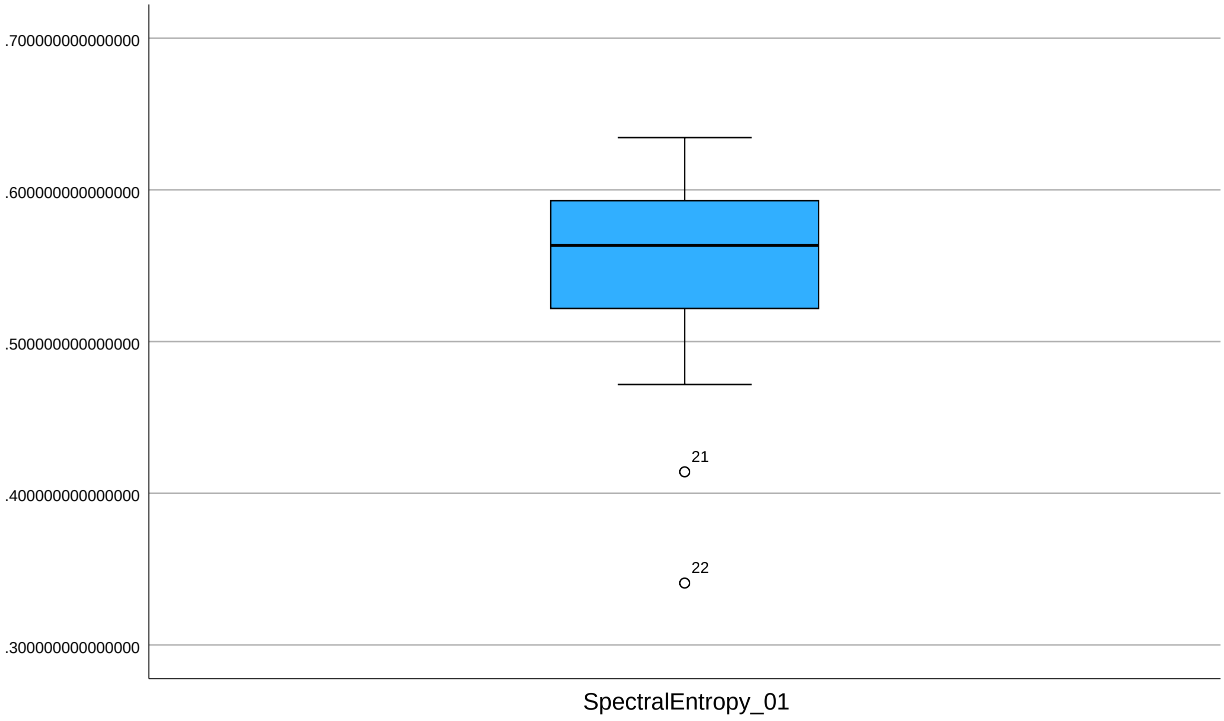

HiguchiFractalDimension

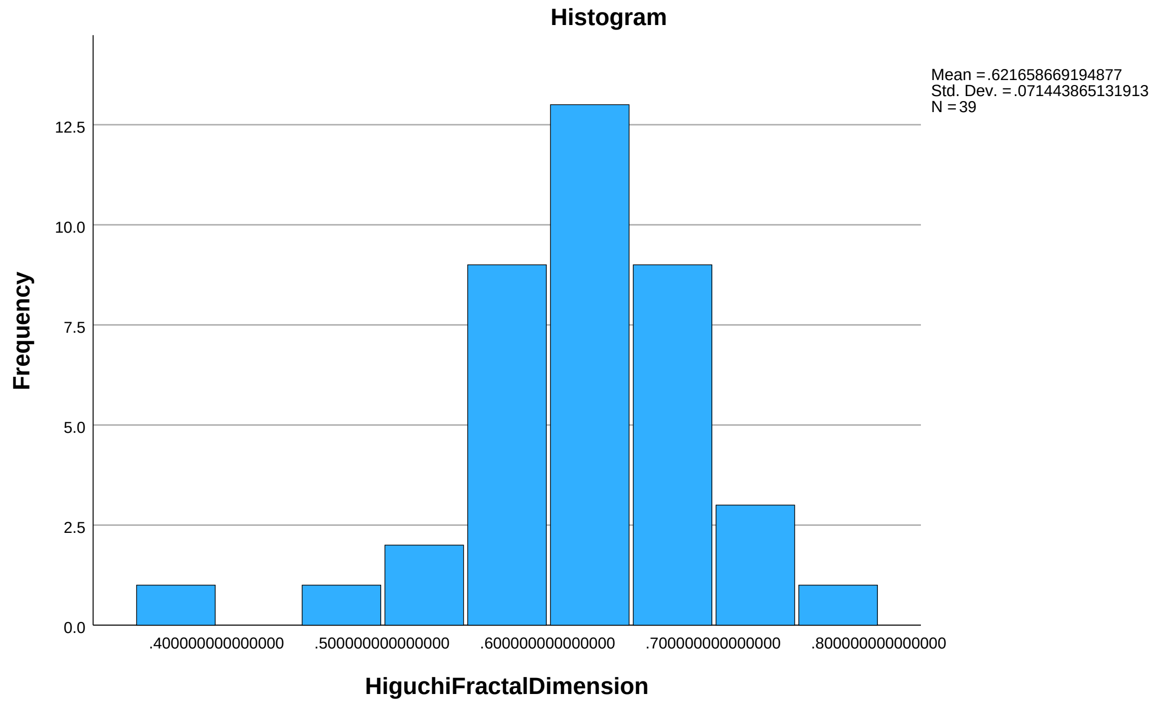


```
HiguchiFractalDimension Stem-and-Leaf Plot

 Frequency    Stem &  Leaf

     1.00 Extremes    (=<.39)
     1.00        4 .  7
     2.00        5 .  44
     9.00        5 .  555677789
    13.00        6 .  0012233334444
     9.00        6 .  567777788
     3.00        7 .  013
     1.00        7 .  7

 Stem width:   .1000000
 Each leaf:        1 case(s)
```

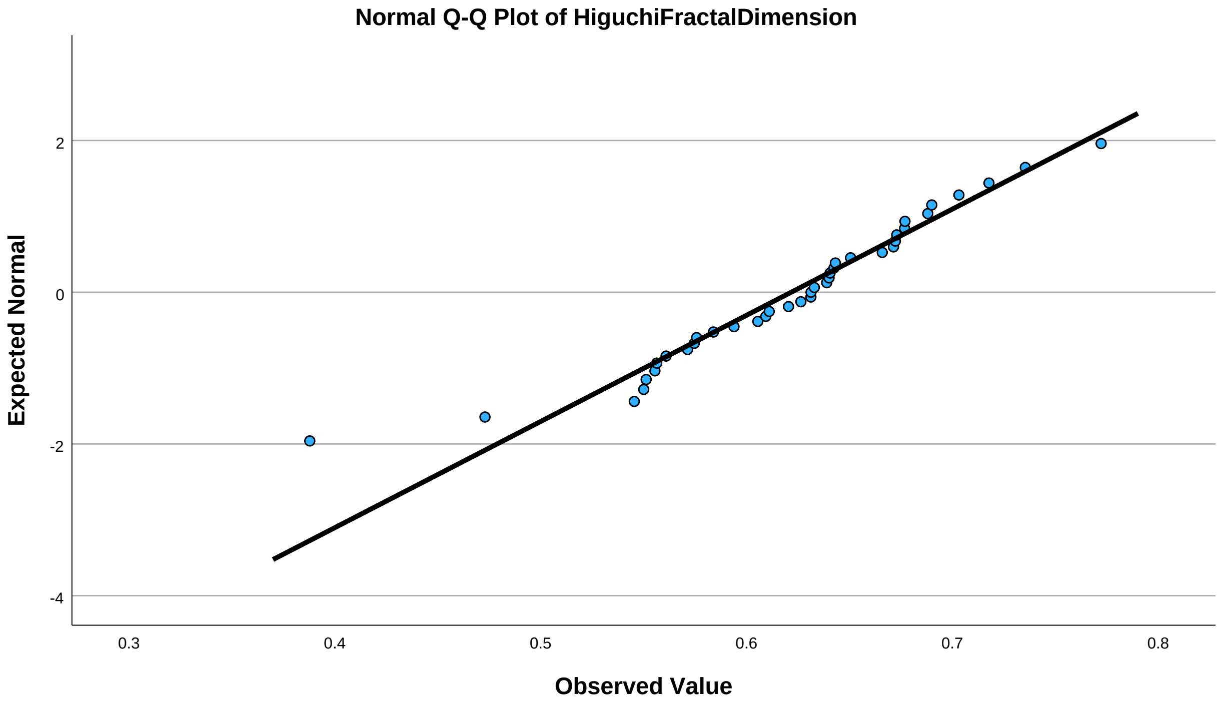
Normal Q-Q Plot of HiguchiFractalDimension
Expected Normal
2
0
-2
-4
0.3
0.4
0.5
0.6
0.7
0.8
Observed Value

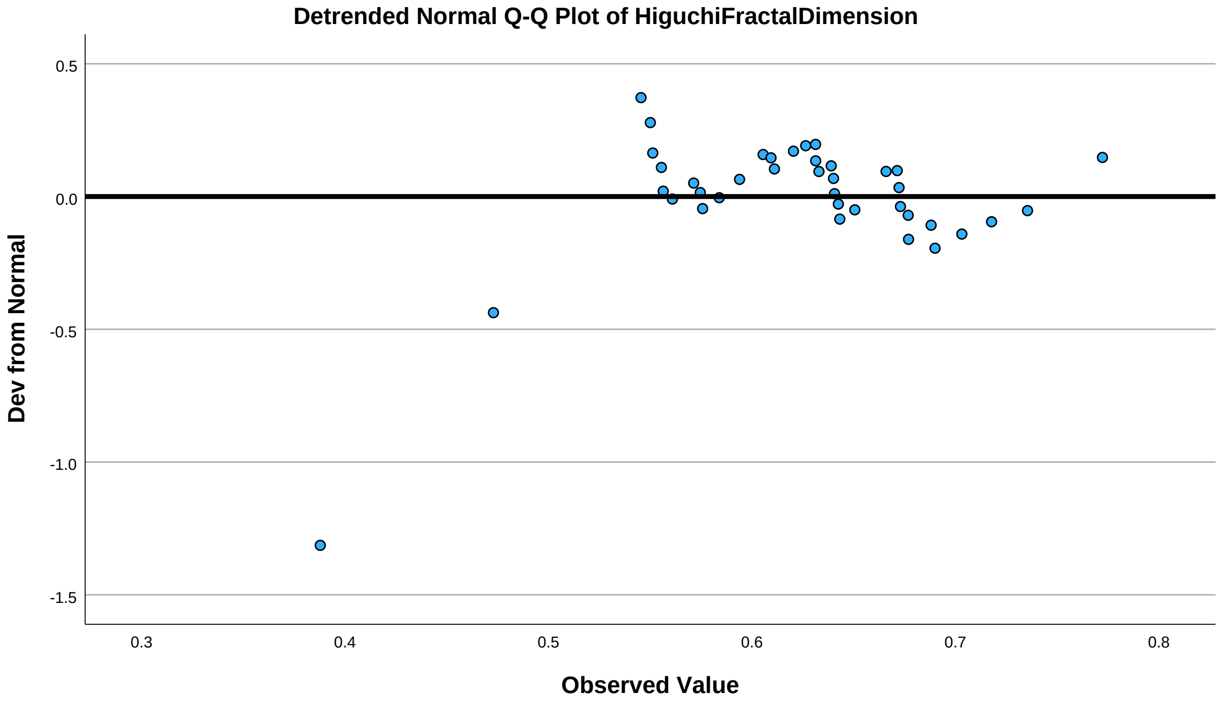
Detrended Normal Q-Q Plot of HiguchiFractalDimension
Dev from Normal
0.5
0.0
-0.5
-1.0
-1.5
0.3
0.4
0.5
0.6
0.7
0.8
Observed Value

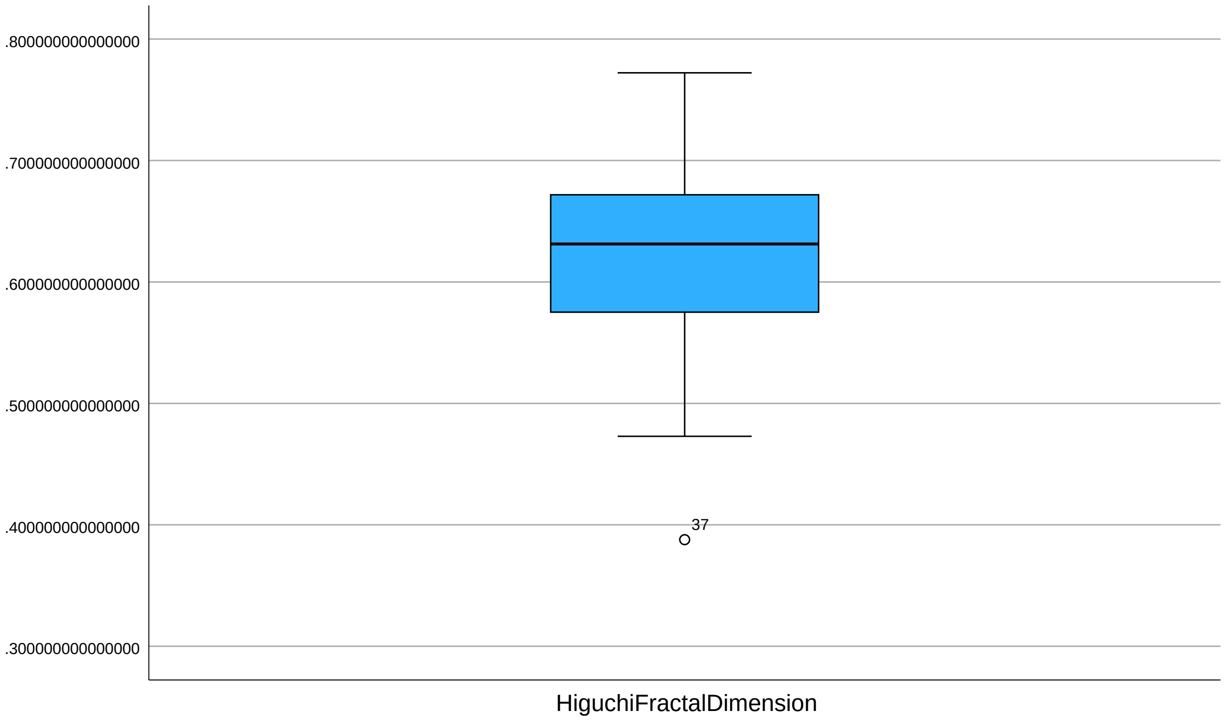

LempelZivComplexity

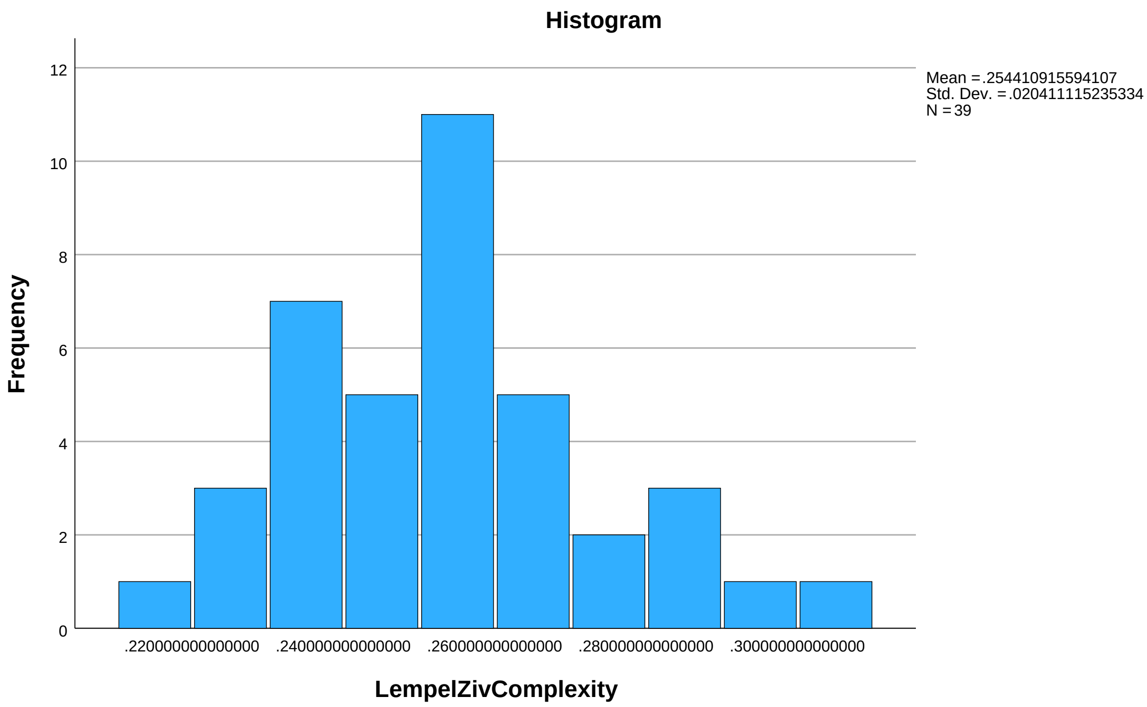


```
LempelZivComplexity Stem-and-Leaf Plot

 Frequency    Stem &  Leaf
```

```
     1.00        21 .  5
     3.00        22 .  158
     7.00        23 .  1257899
     5.00        24 .  11228
    11.00        25 .  34557778889
     5.00        26 .  04557
     2.00        27 .  35
     3.00        28 .  689
     1.00        29 .  1
     1.00 Extremes    (>=.305)

 Stem width:   .0100000
 Each leaf:       1 case(s)
```

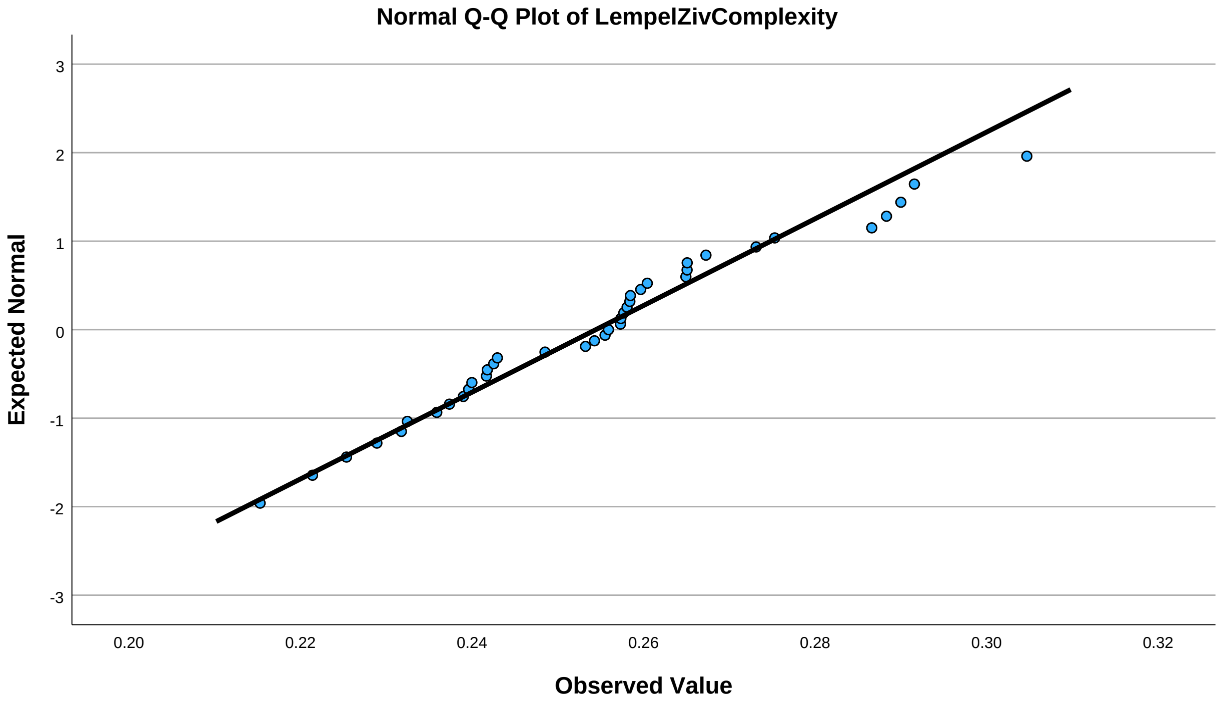

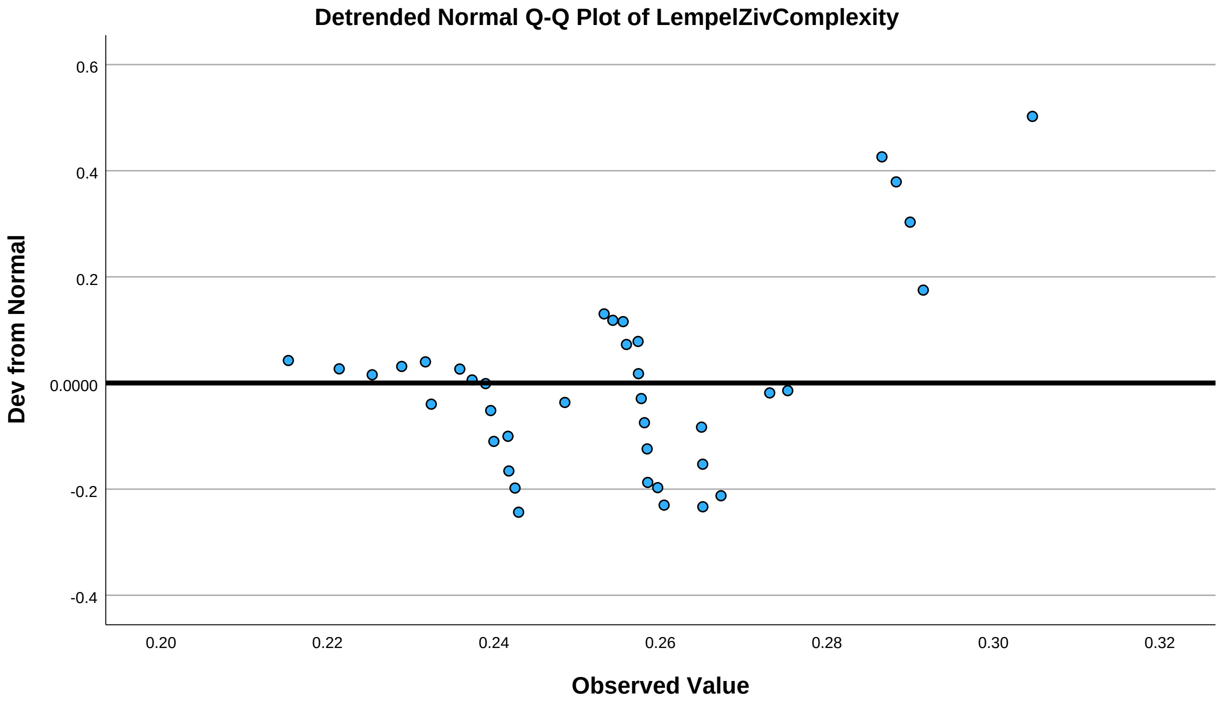

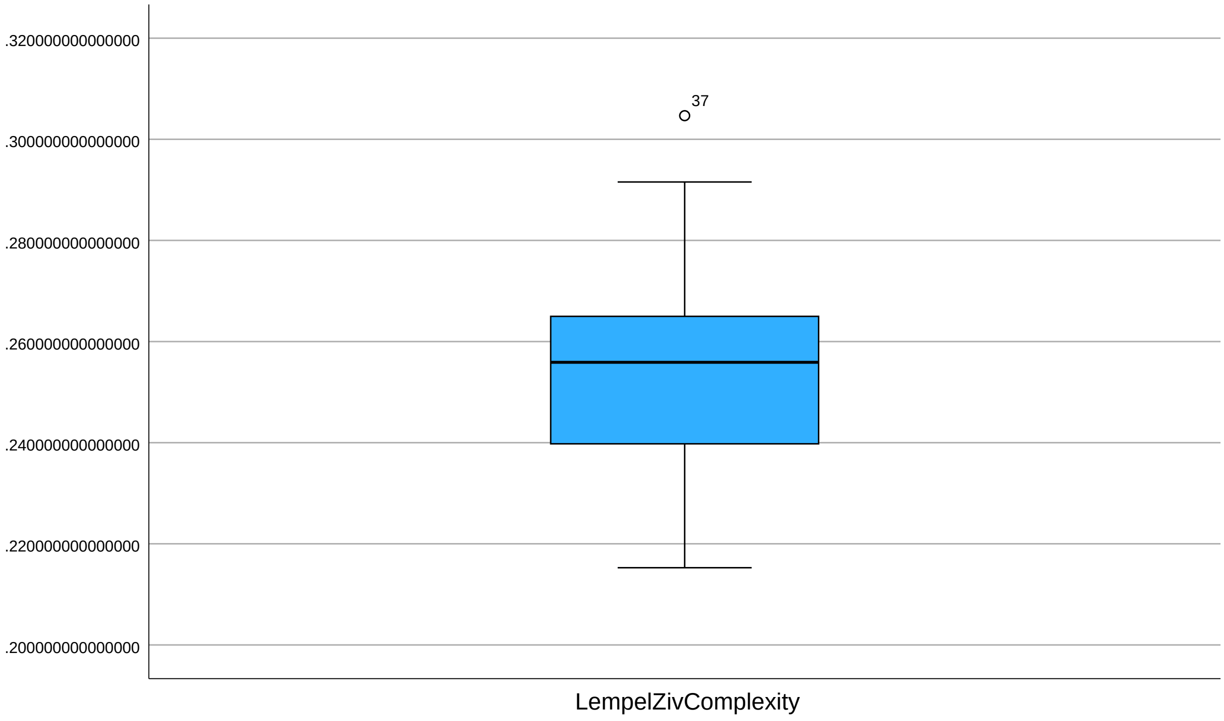

RenyiEntropy

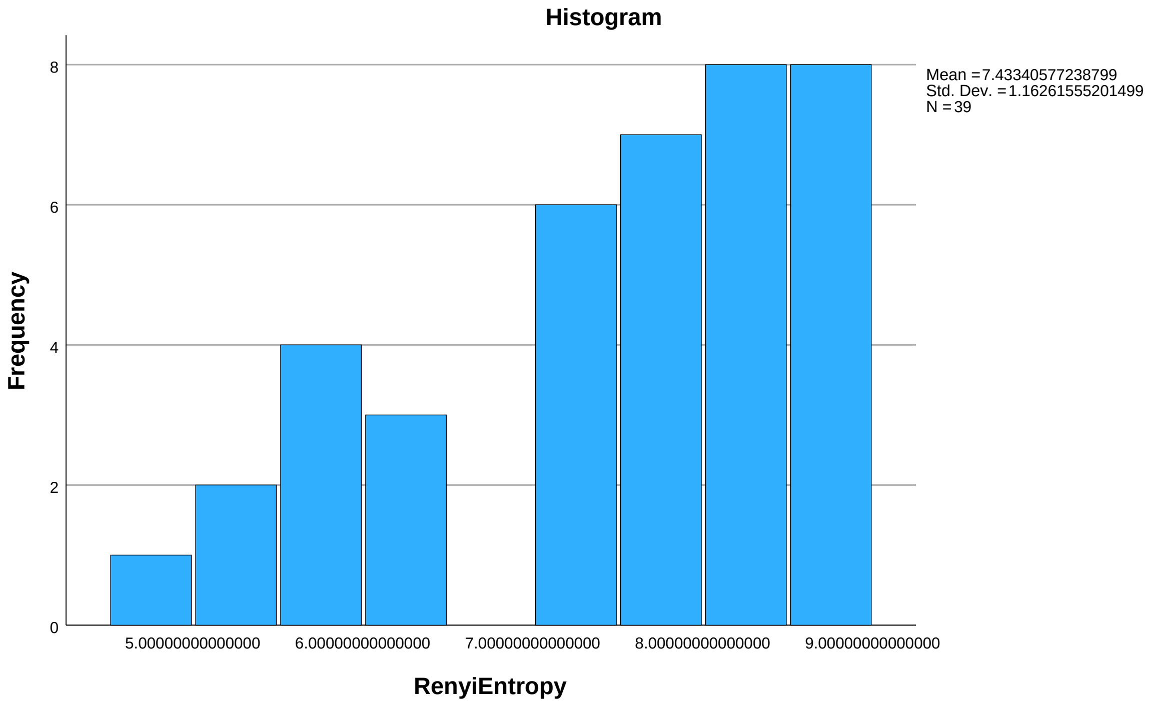


```
RenyiEntropy Stem-and-Leaf Plot

 Frequency    Stem &  Leaf

     1.00        4 .  8
     2.00        5 .  12
     4.00        5 .  6789
     3.00        6 .  013
      .00        6 .
     6.00        7 .  012334
     7.00        7 .  5556777
     8.00        8 .  00144444
     8.00        8 .  55666677

 Stem width:   1.000000
 Each leaf:        1 case(s)
```

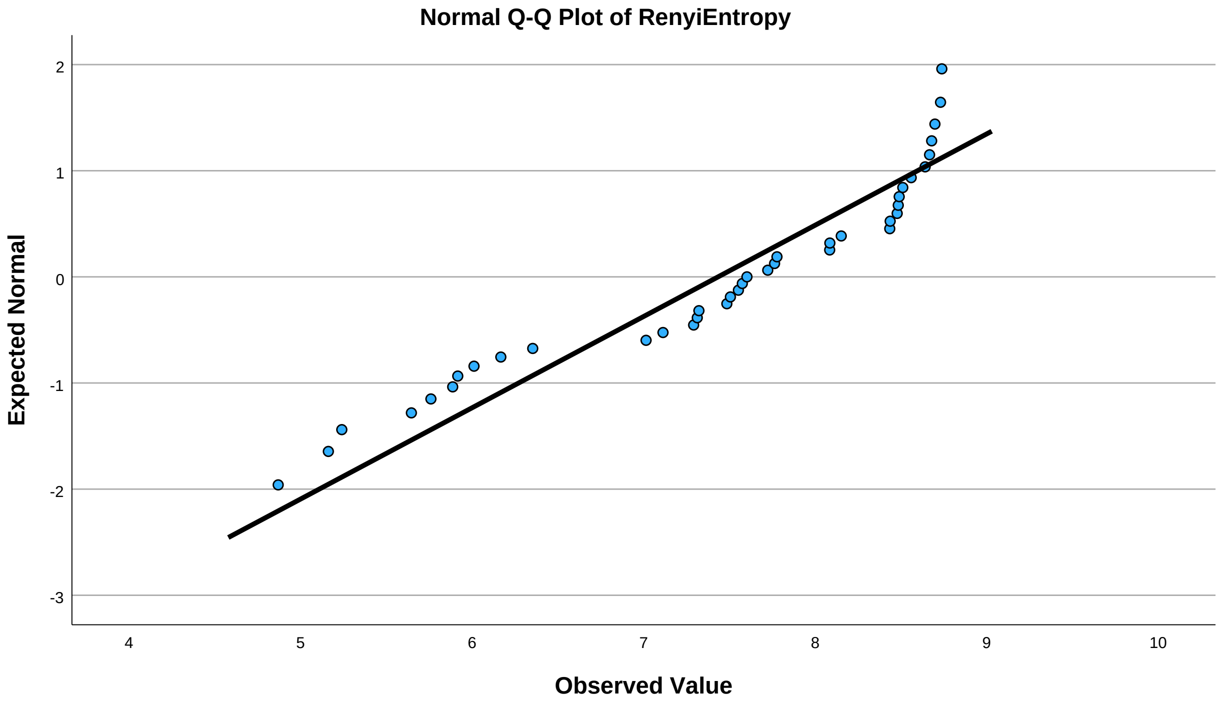
Normal Q-Q Plot of RenyiEntropy
Expected Normal
2
1
0
-1
-2
-3
4
5
6
7
8
9
10
Observed Value

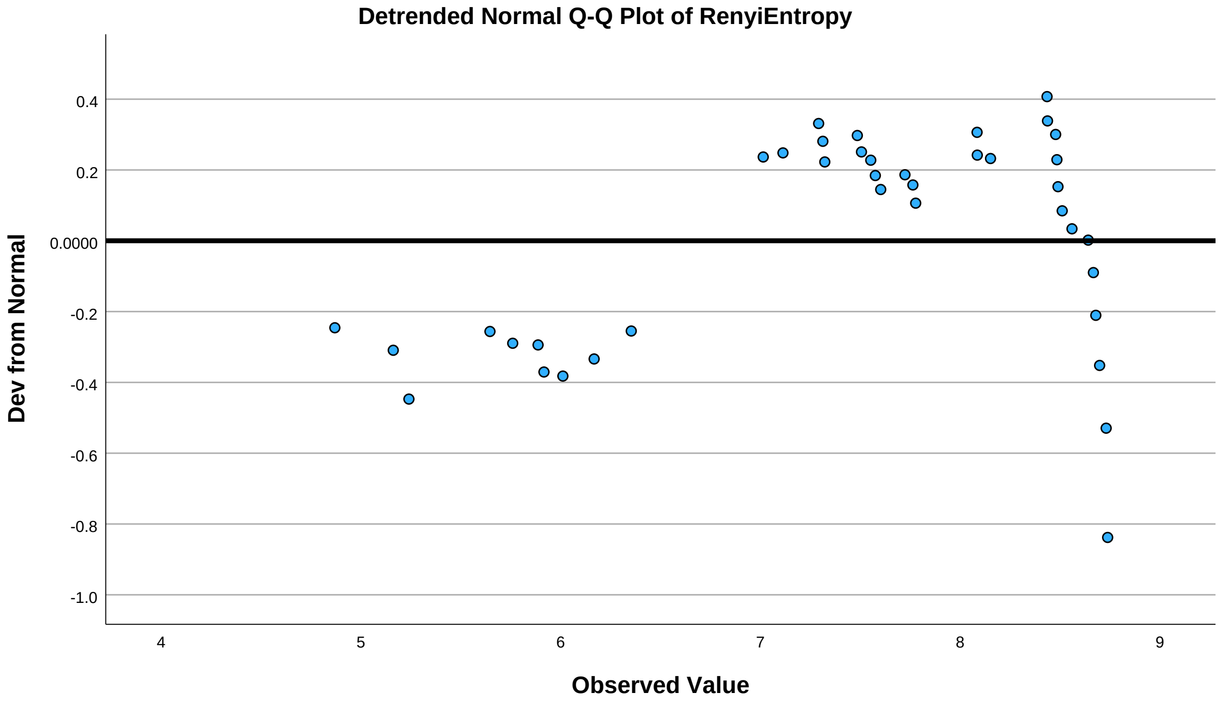
Detrended Normal Q-Q Plot of RenyiEntropy
Dev from Normal
0.4
0.2
0.0000
-0.2
-0.4
-0.6
-0.8
-1.0
4
5
6
7
8
9
Observed Value

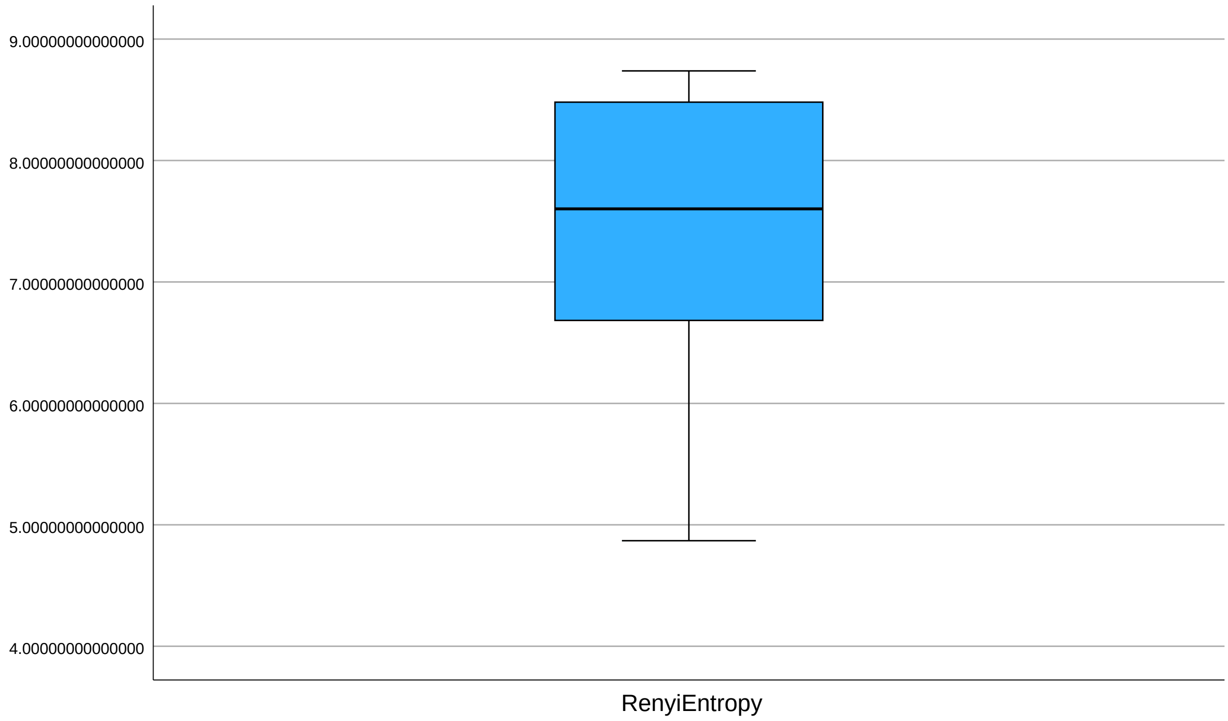

TsallisEntropy

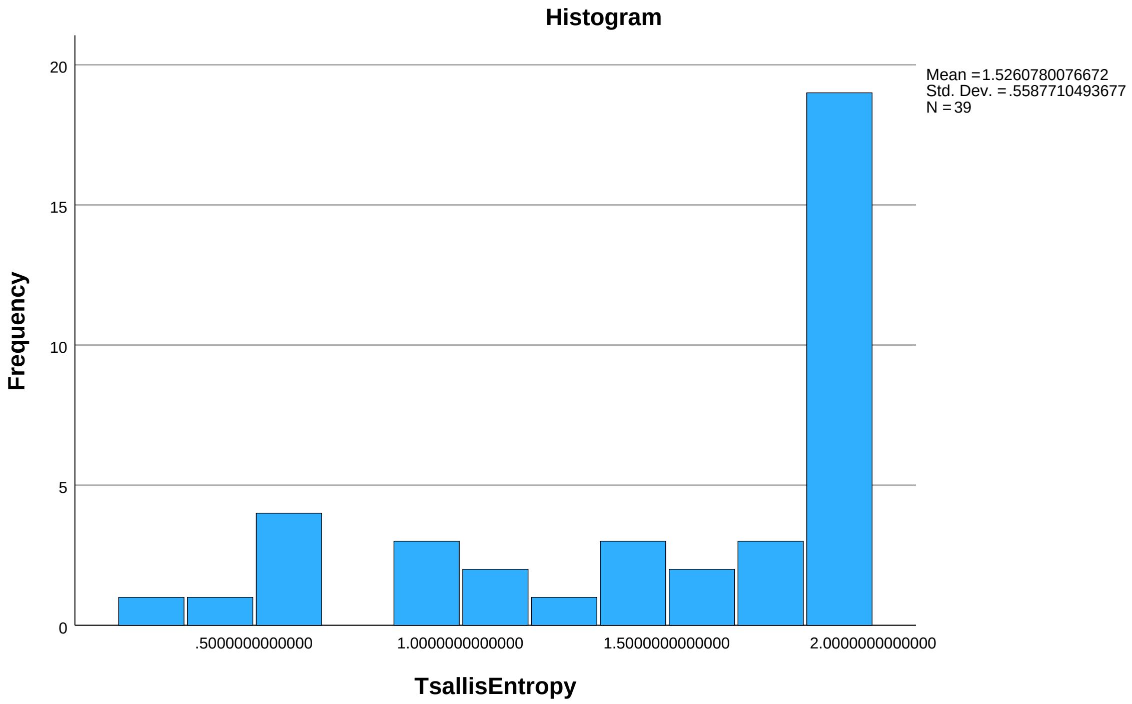

TsallisEntropy Stem-and-Leaf Plot

Frequency   Stem & Leaf

```
  2.00     0 . 33
  3.00     0 . 555
  1.00     0 . 6
  3.00     0 . 899
  2.00     1 . 00
  2.00     1 . 23
  2.00     1 . 44
  4.00     1 . 6667
 20.00     1 .  88999999999999999999

Stem width:  1.000000
Each leaf:       1 case(s)
```

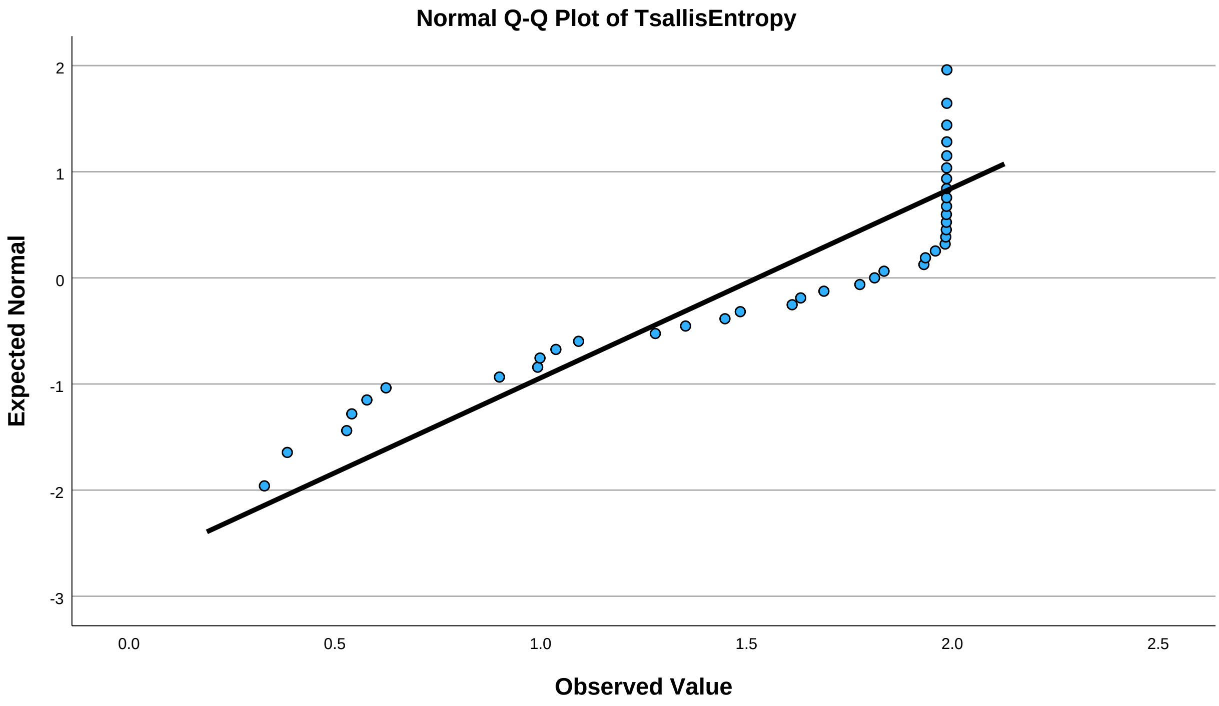

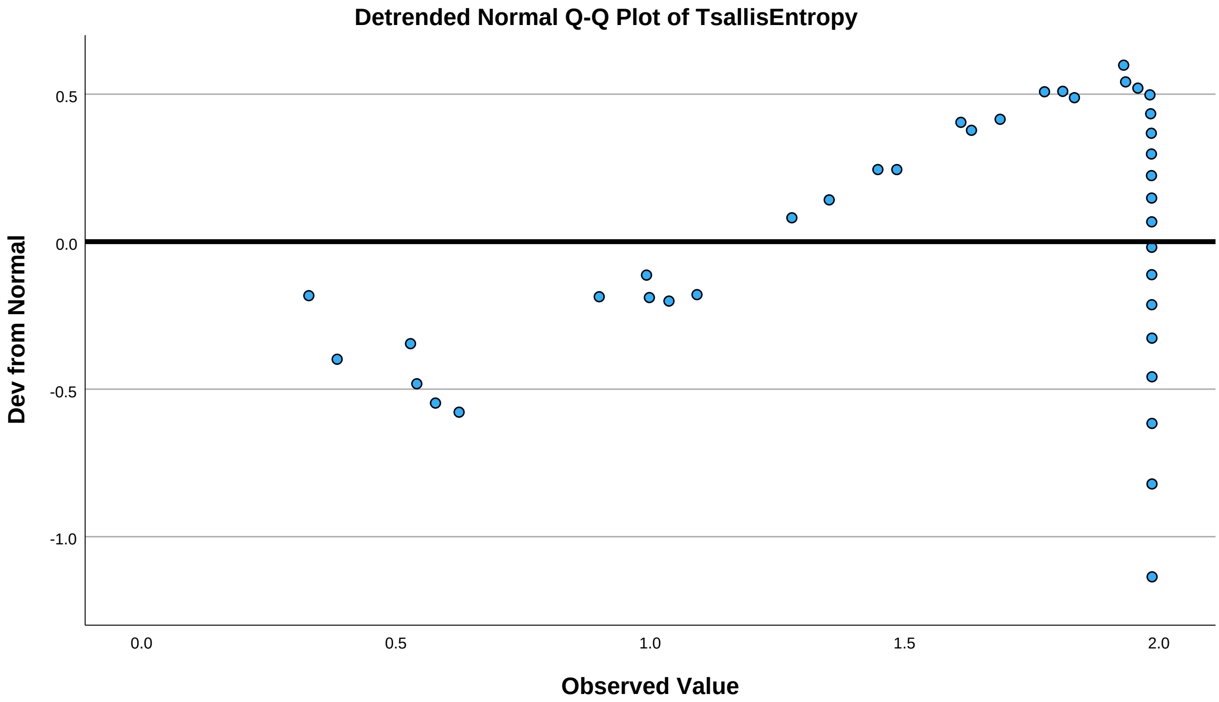
Detrended Normal Q-Q Plot of TsallisEntropy
Dev from Normal
0.5
0.0
-0.5
-1.0
0.0
0.5
1.0
1.5
2.0
Observed Value

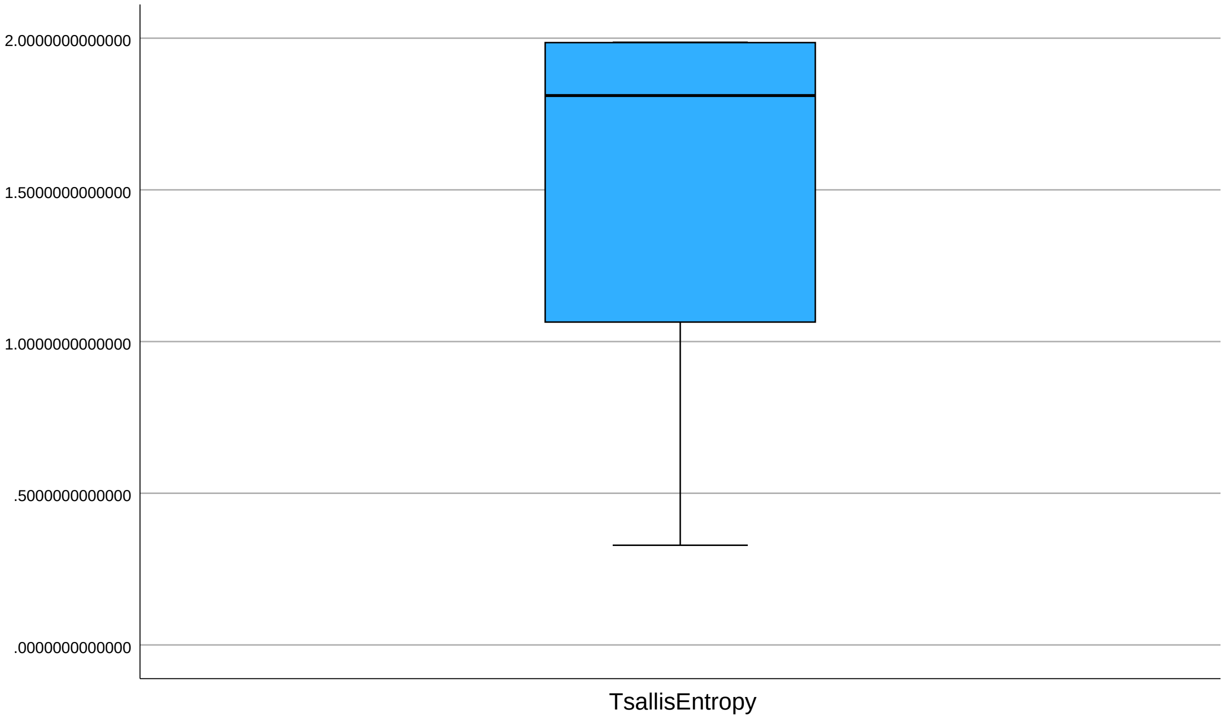
2.0000000000000
1.5000000000000
1.0000000000000
.5000000000000
.0000000000000
TsallisEntropy

PS_4_5

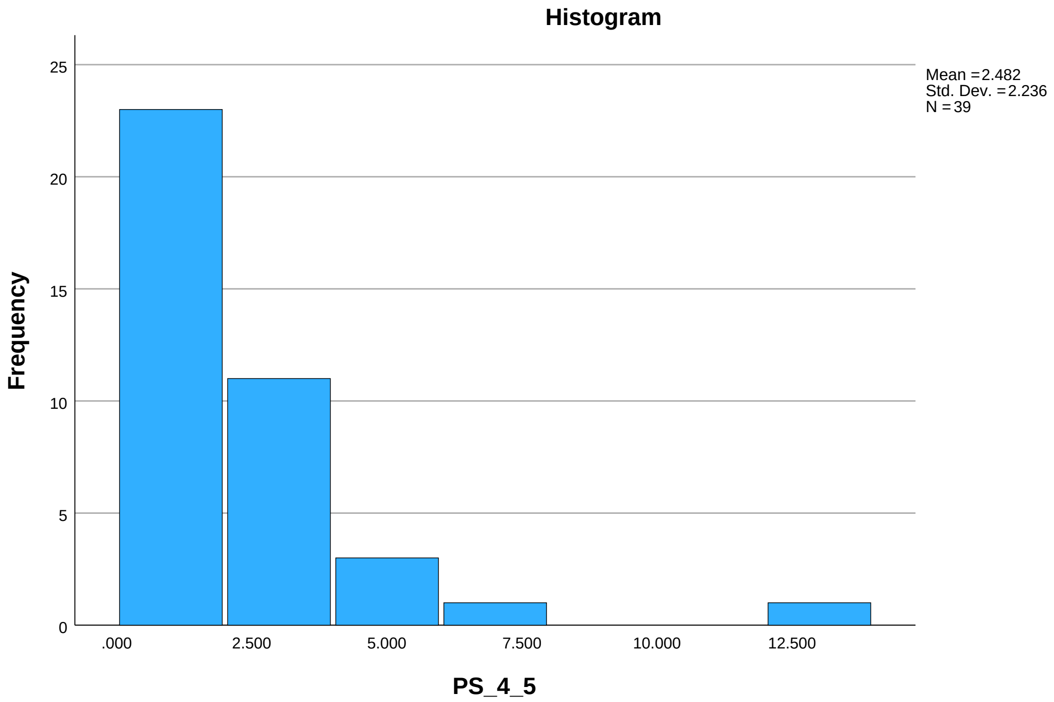


```
PS_4_5 Stem-and-Leaf Plot

 Frequency    Stem &  Leaf

     6.00        0 .  566799
     7.00        1 .  0123344
    10.00        1 .  5566677888
     4.00        2 .  1233
     3.00        2 .  568
      .00        3 .
     4.00        3 .  6779
     1.00        4 .  2
     2.00        4 .  57
     2.00 Extremes    (>=7.2)

 Stem width:      1.000
 Each leaf:        1 case(s)
```

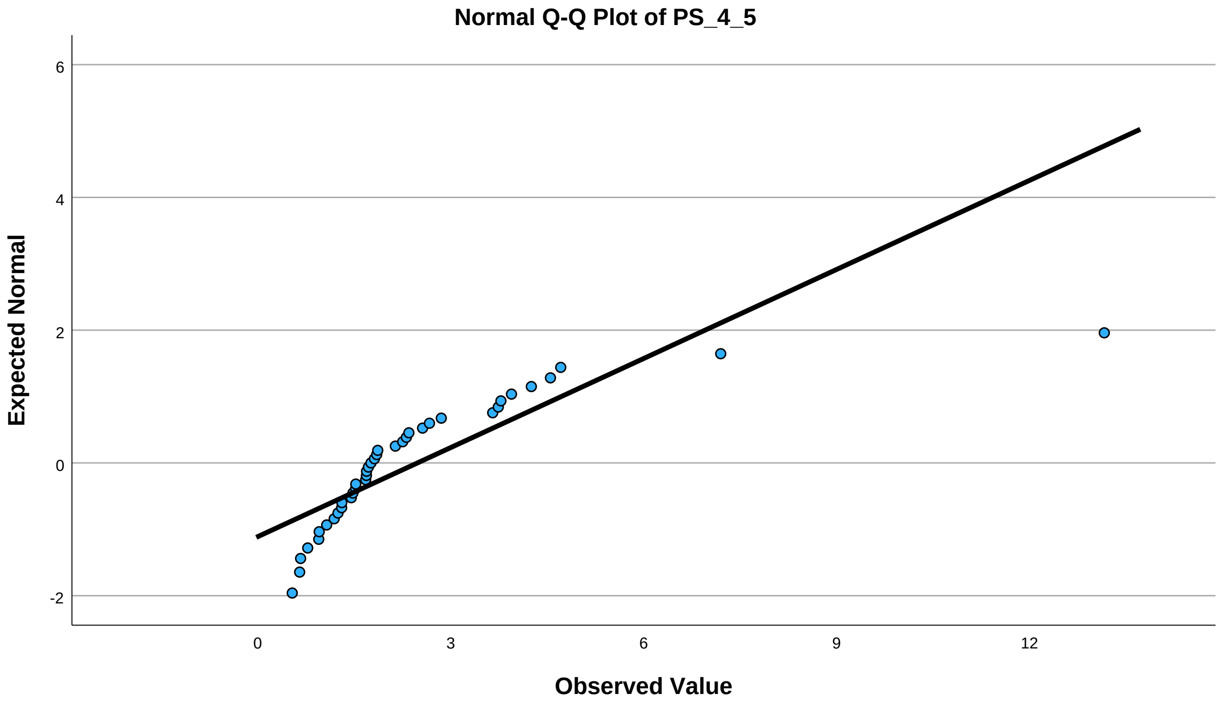
Normal Q-Q Plot of PS_4_5
Expected Normal
Observed Value
6
4
2
0
-2
0
3
6
9
12

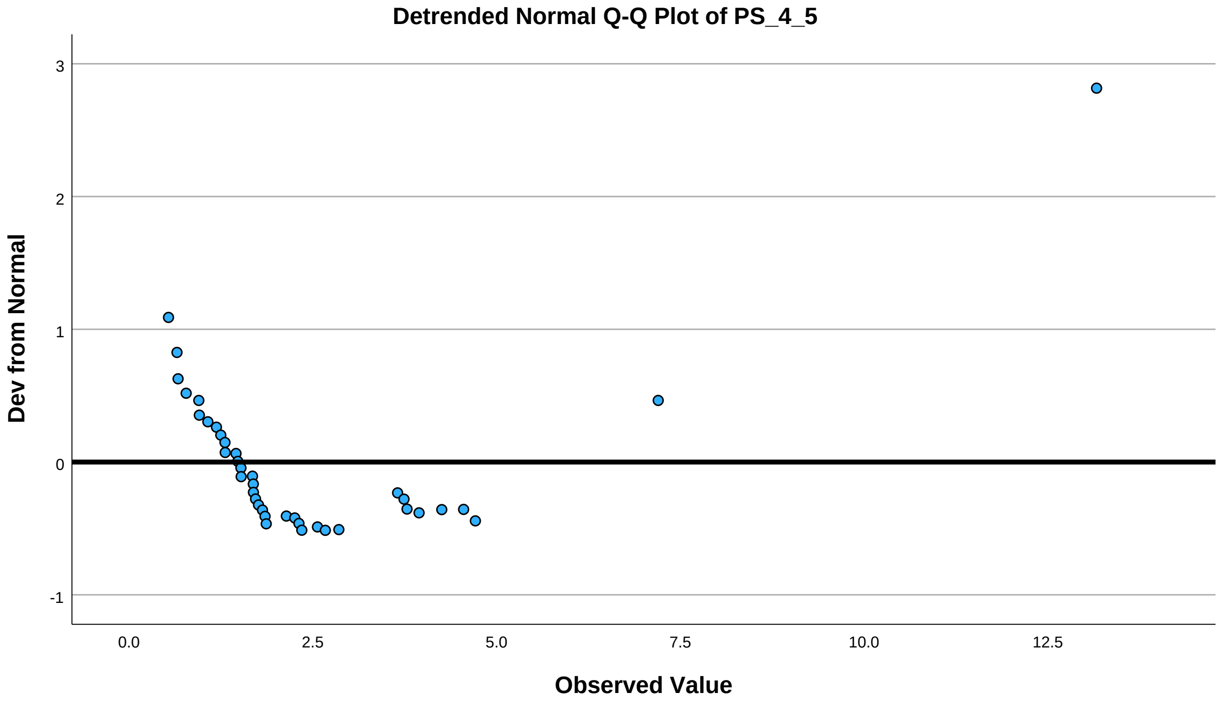
Detrended Normal Q-Q Plot of PS_4_5
Dev from Normal
Observed Value
3
2
1
0
-1
0.0
2.5
5.0
7.5
10.0
12.5

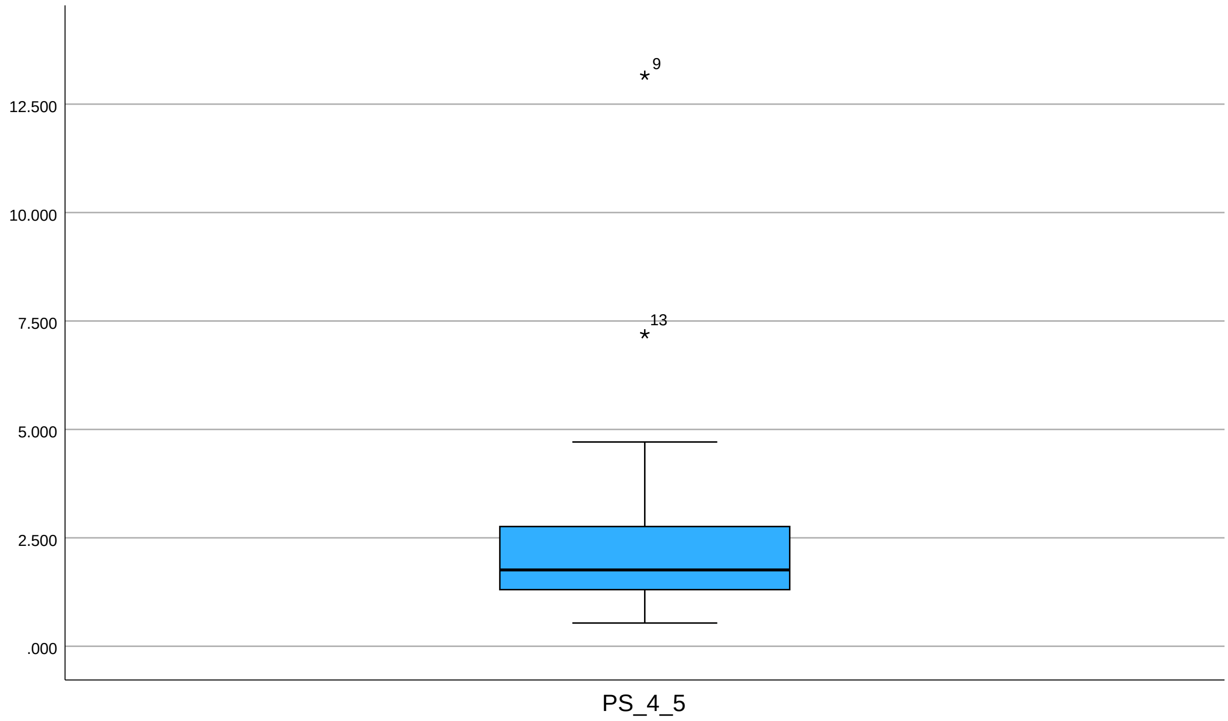

PS_5_6

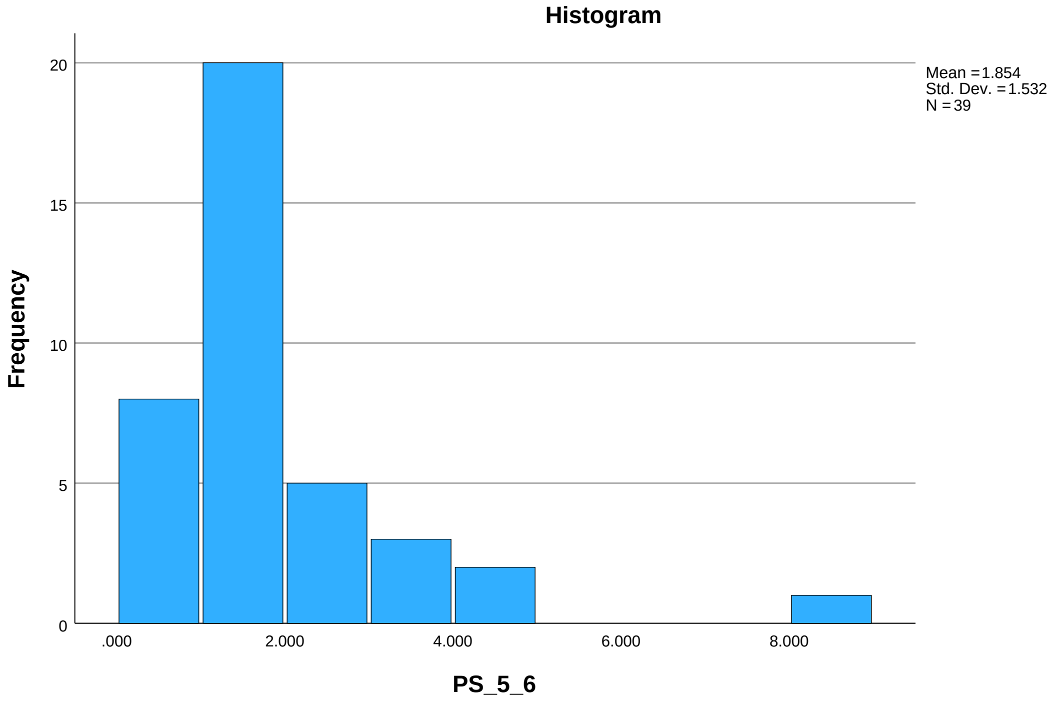


```
PS_5_6 Stem-and-Leaf Plot

 Frequency    Stem &  Leaf
```

```
  2.00      0 .  34
  6.00      0 .  567899
 15.00      1 .  011111122333344
  5.00      1 .  55789
  4.00      2 .  2224
  1.00      2 .  7
  3.00      3 .  134
  3.00 Extremes    (>=4.3)

Stem width:     1.000
Each leaf:       1 case(s)
```

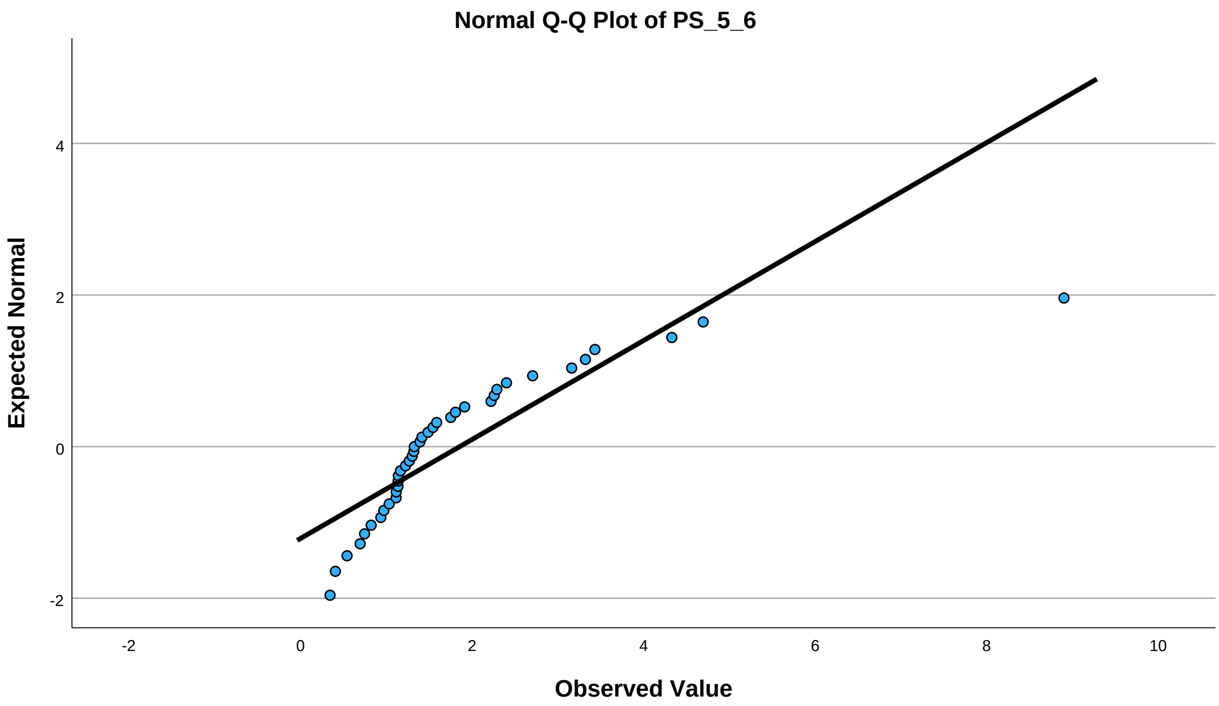

Detrended Normal Q-Q Plot of PS_5_6

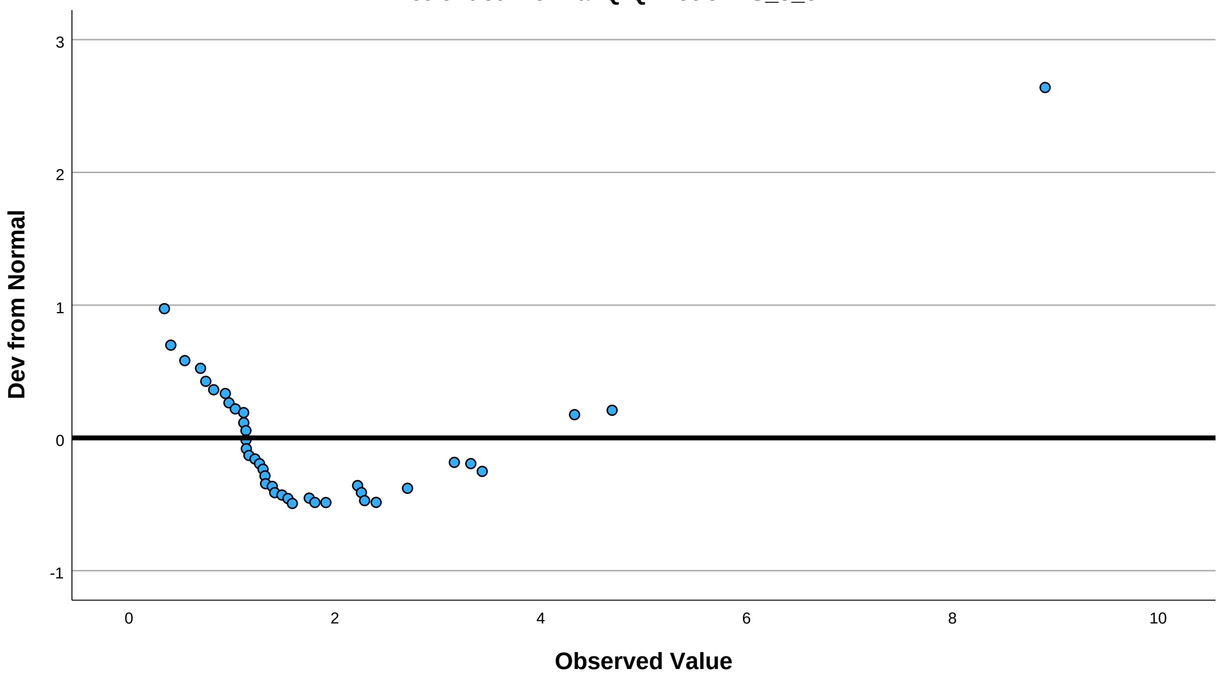

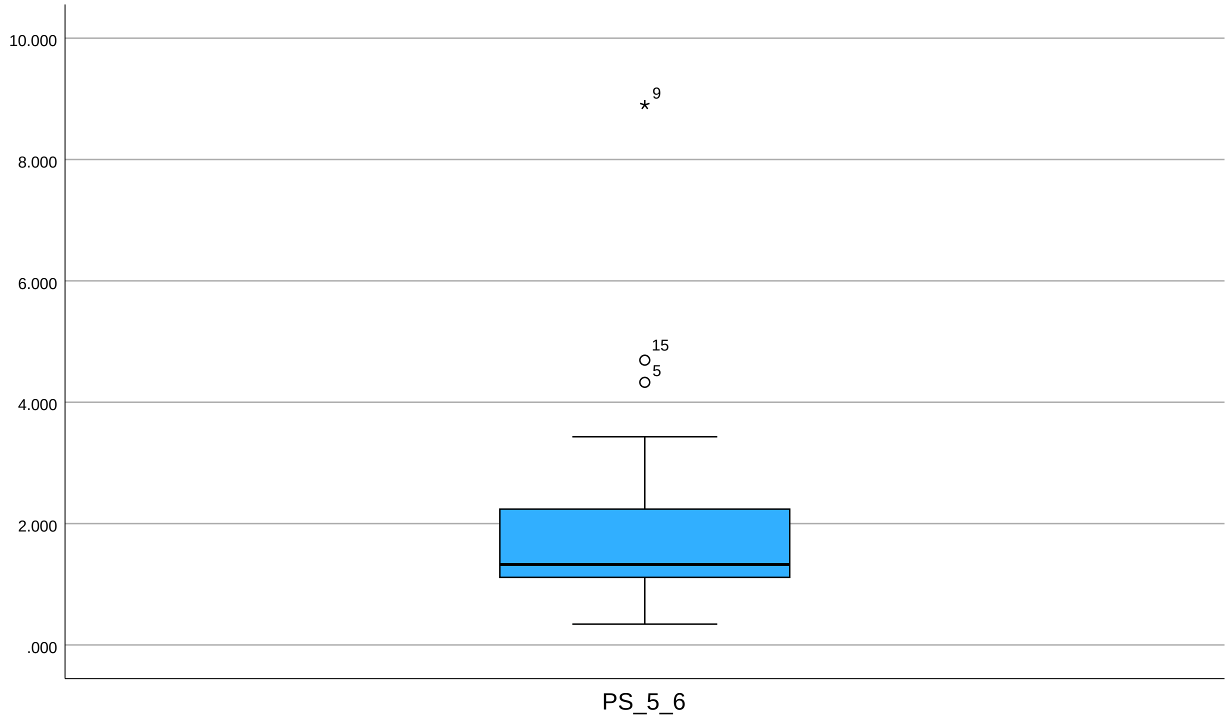

PS_6_7

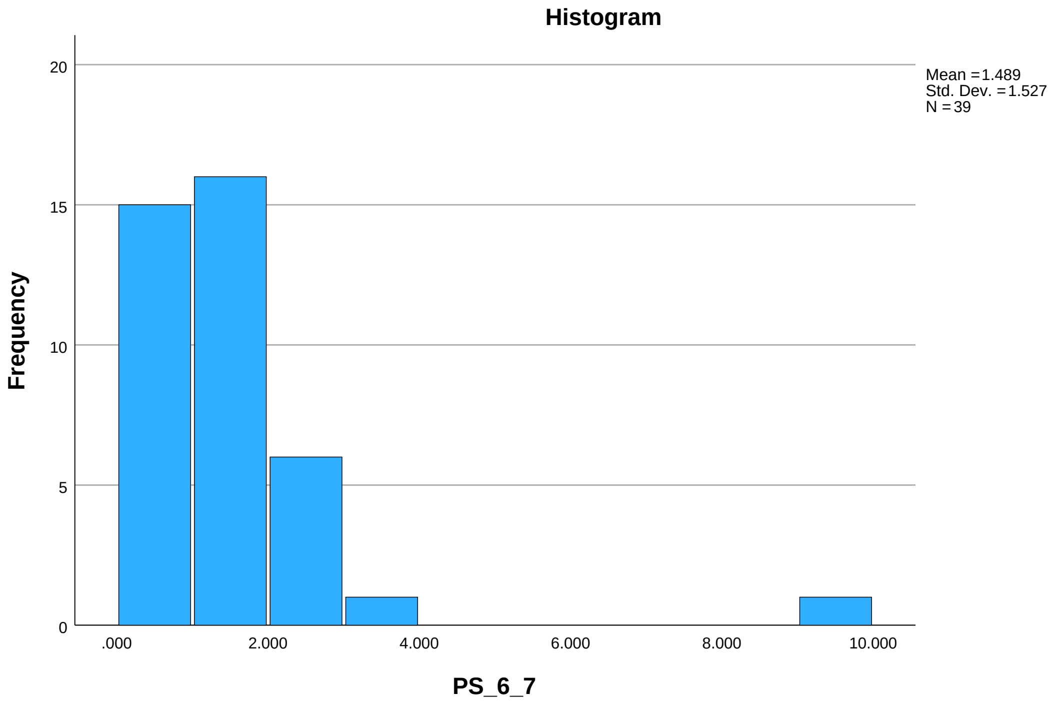


```
PS_6_7 Stem-and-Leaf Plot

 Frequency    Stem &  Leaf

     4.00        0 .  2344
    11.00        0 .  67777788889
    12.00        1 .  000111112224
     4.00        1 .  6688
     6.00        2 .  012344
      .00        2 .
     1.00        3 .  0
     1.00 Extremes    (>=9.9)

 Stem width:      1.000
 Each leaf:       1 case(s)
```

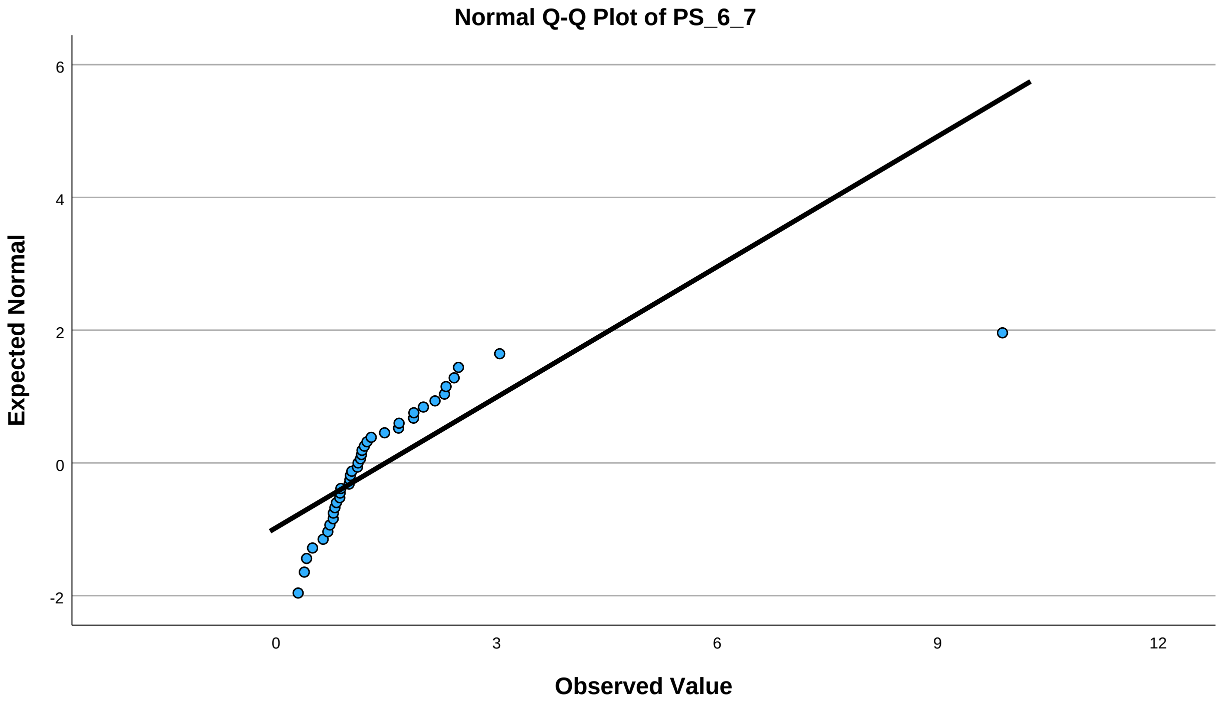
Normal Q-Q Plot of PS_6_7
Expected Normal
6
4
2
0
-2
0
3
6
9
12
Observed Value

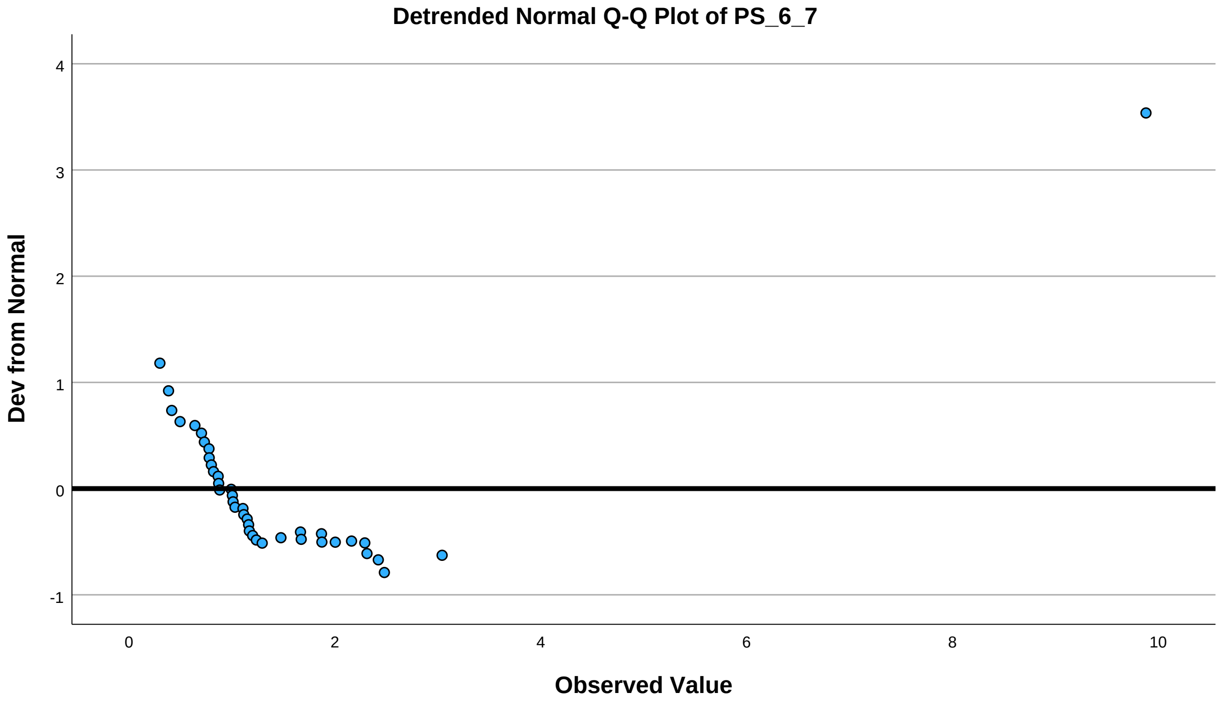
Detrended Normal Q-Q Plot of PS_6_7
Dev from Normal
4
3
2
1
0
-1
0
2
4
6
8
10
Observed Value

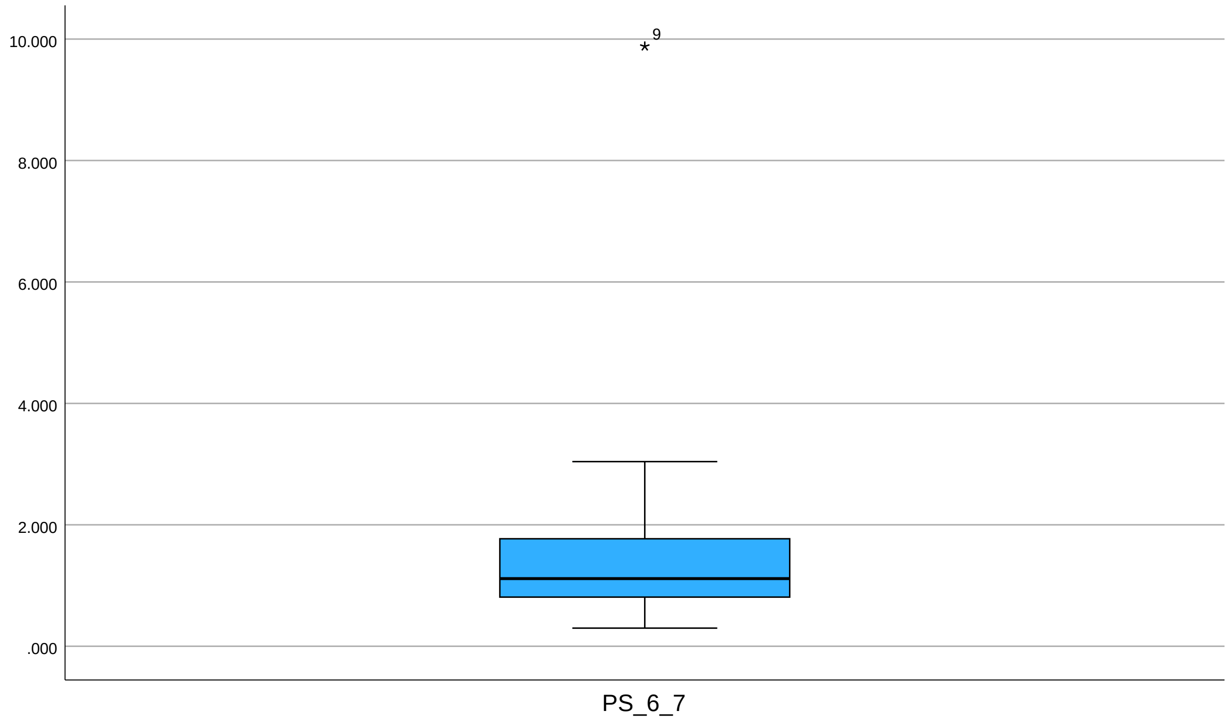

PS_7_8

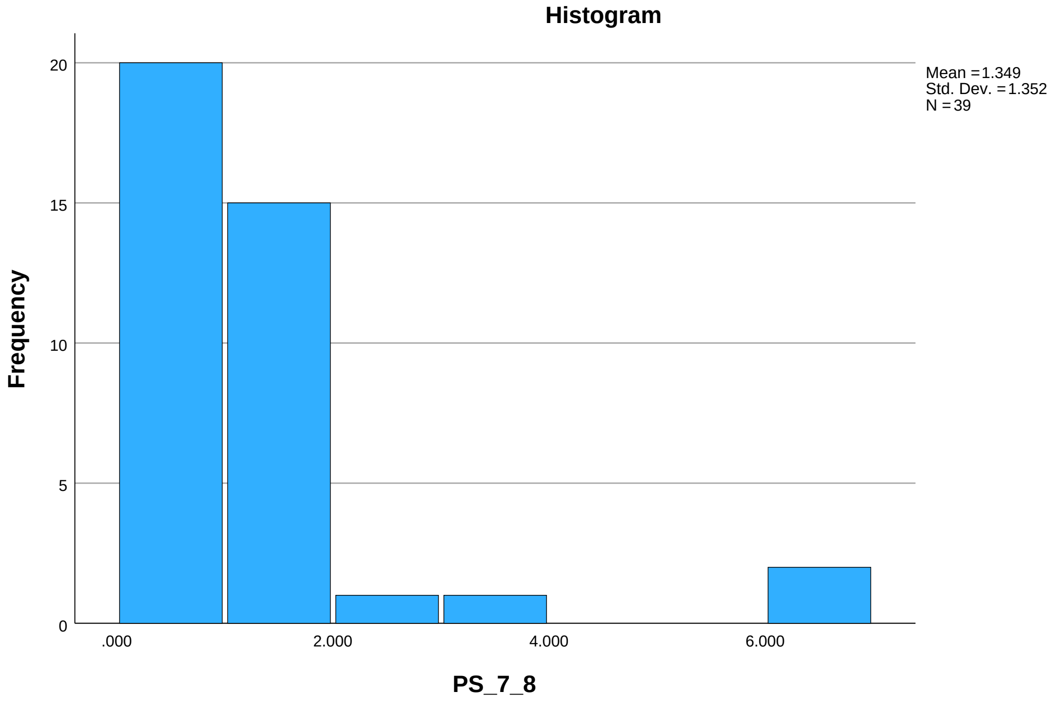

PS_7_8 Stem-and-Leaf Plot

Frequency   Stem & Leaf

```
  5.00      0 . 23344
 15.00       0 . 556777888888899
 10.00       1 . 0001112234
  5.00      1 . 55677
 1.00     2 . 2
  3.00 Extremes    (>=3.2)

Stem width:     1.000
Each leaf:       1 case(s)
```

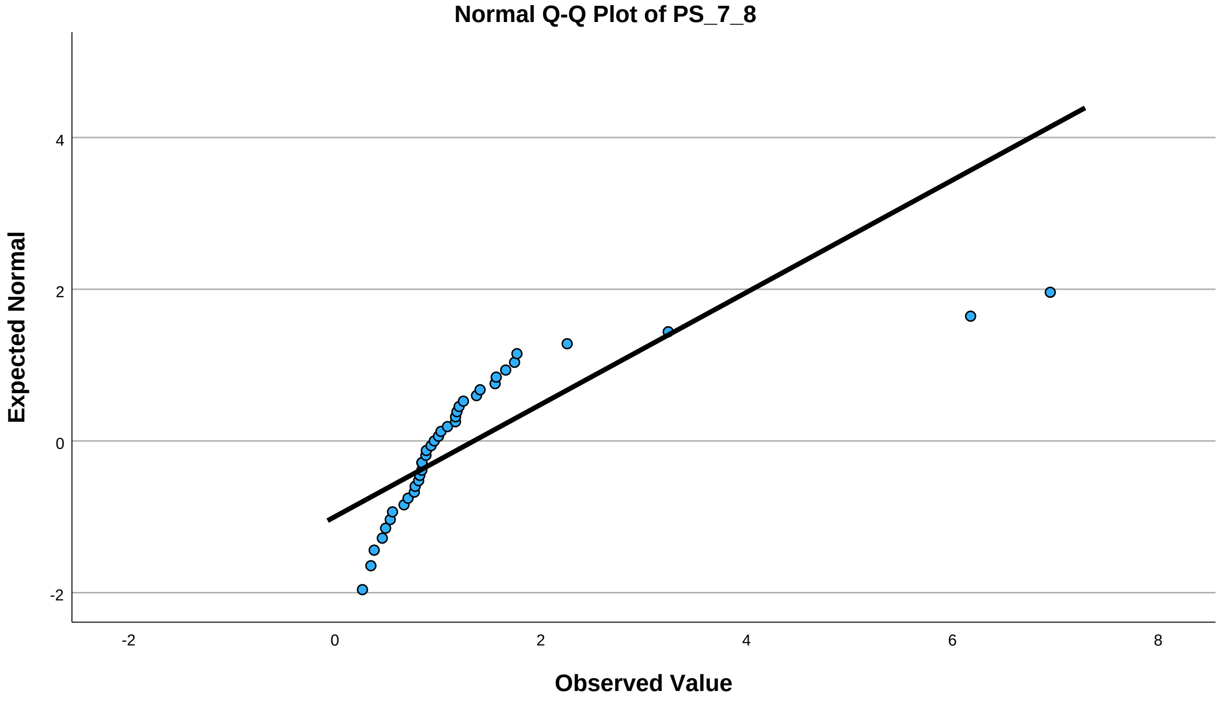

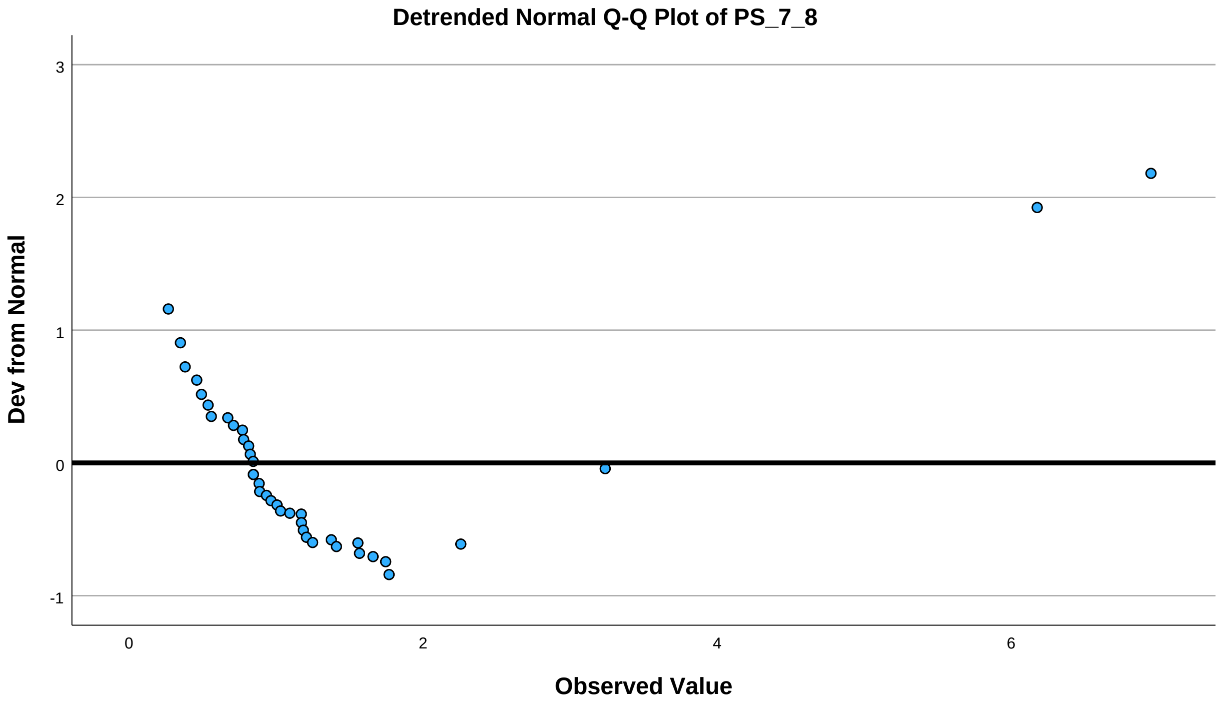
Detrended Normal Q-Q Plot of PS_7_8
Dev from Normal
3
2
1
0
-1
0
2
4
6
Observed Value

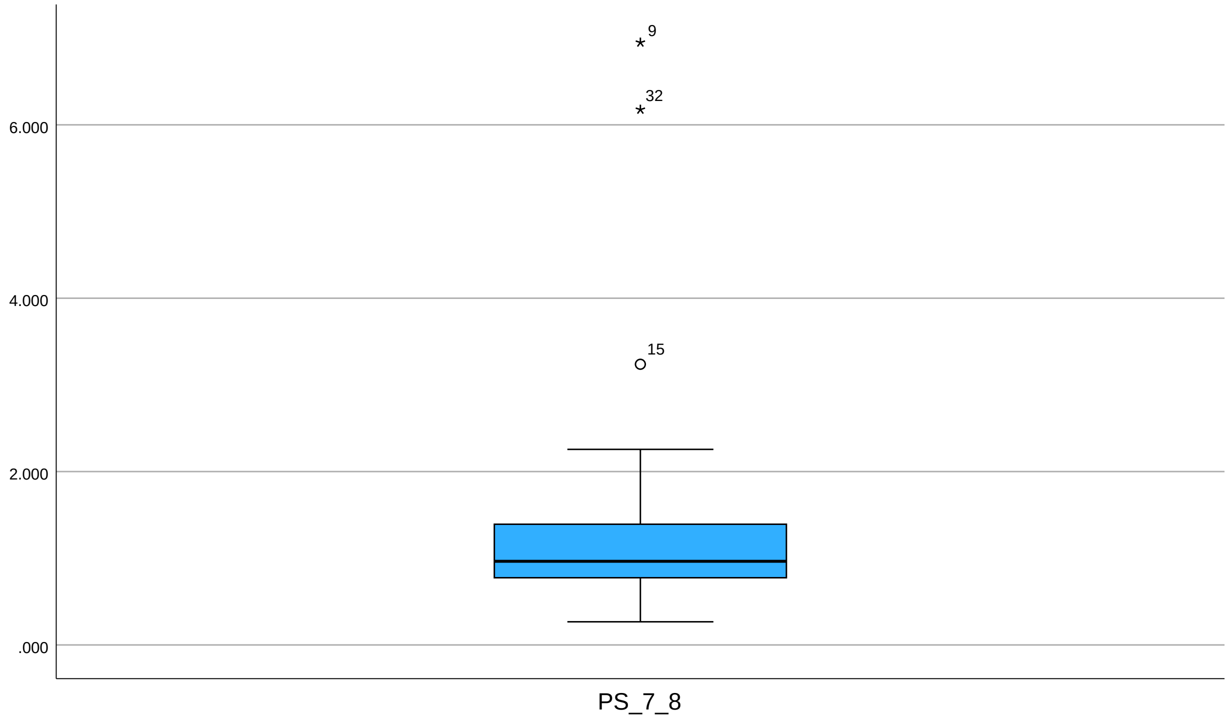
9
32
15
6.000
4.000
2.000
.000
PS_7_8

PS_8_9

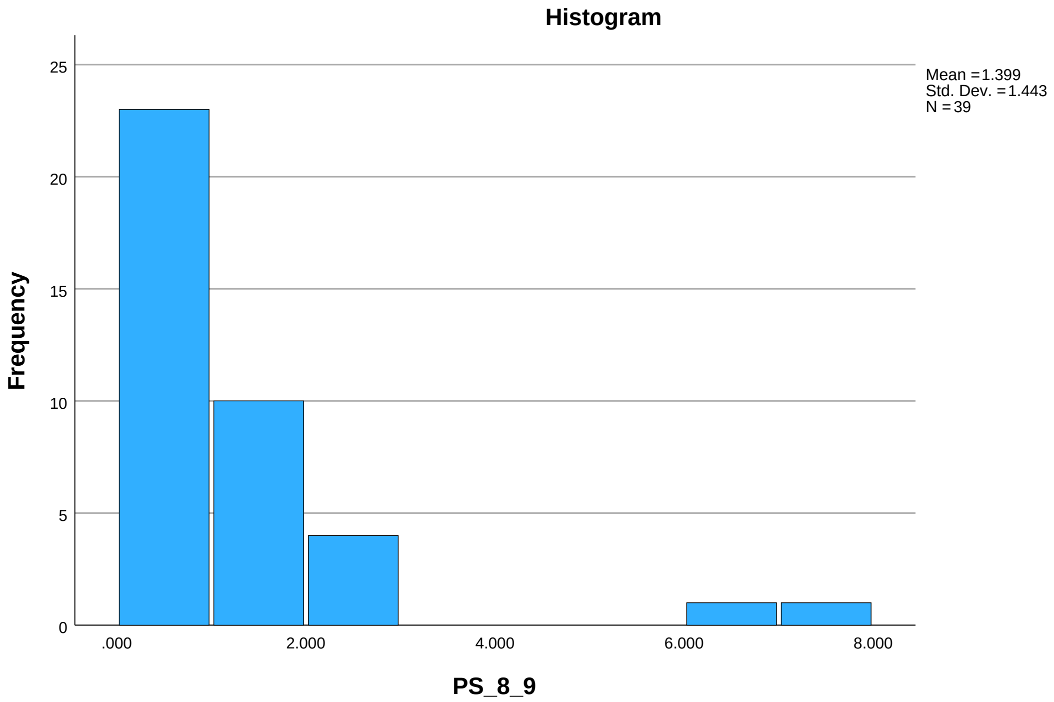


```
PS_8_9 Stem-and-Leaf Plot

 Frequency    Stem &  Leaf

     6.00        0 .  223344
    17.00        0 .  66777778888899999
     7.00        1 .  2223333
     3.00        1 .  789
     1.00        2 .  1
     5.00 Extremes    (>=2.5)

 Stem width:      1.000
 Each leaf:       1 case(s)
```

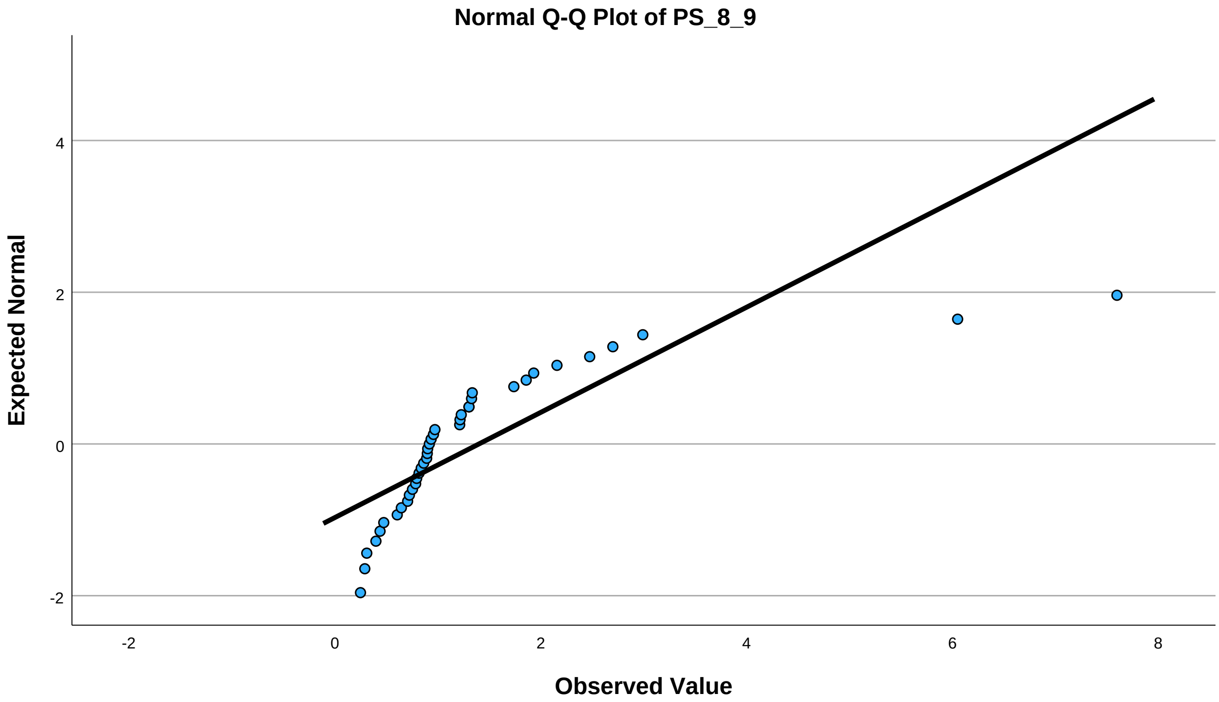
Normal Q-Q Plot of PS_8_9
Expected Normal
Observed Value
4
2
0
-2
-2
0
2
4
6
8

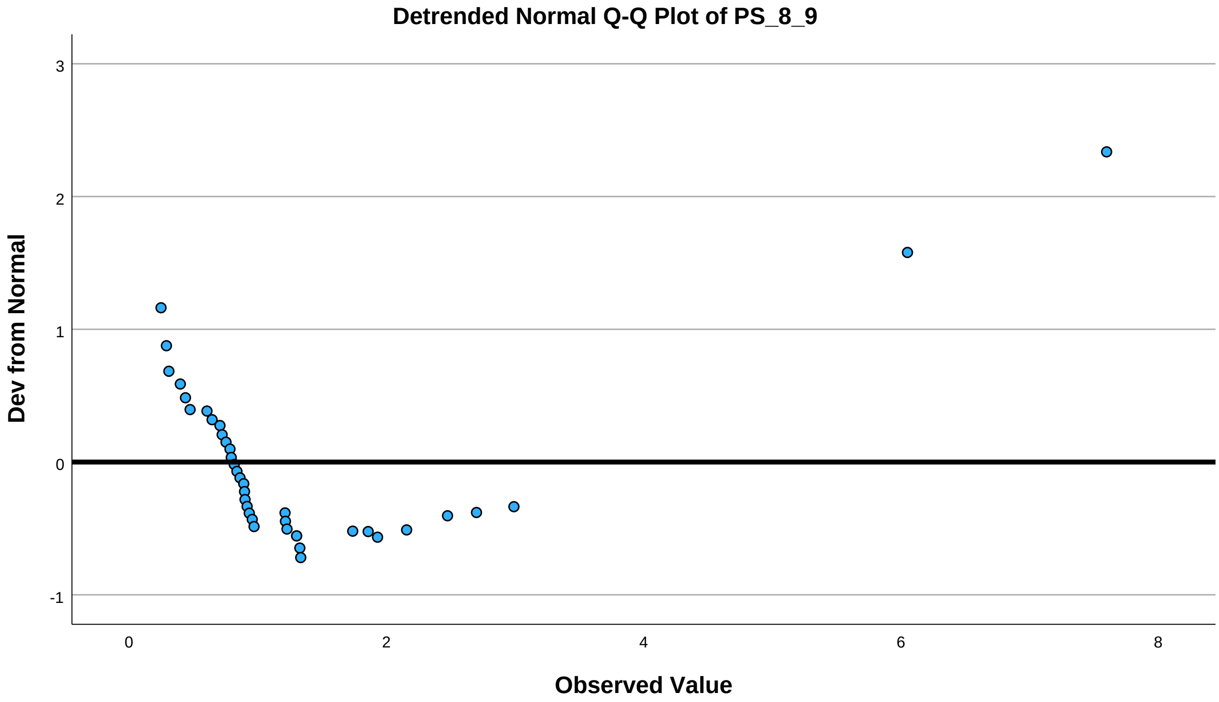
Detrended Normal Q-Q Plot of PS_8_9
Dev from Normal
Observed Value
3
2
1
0
-1
0
2
4
6
8

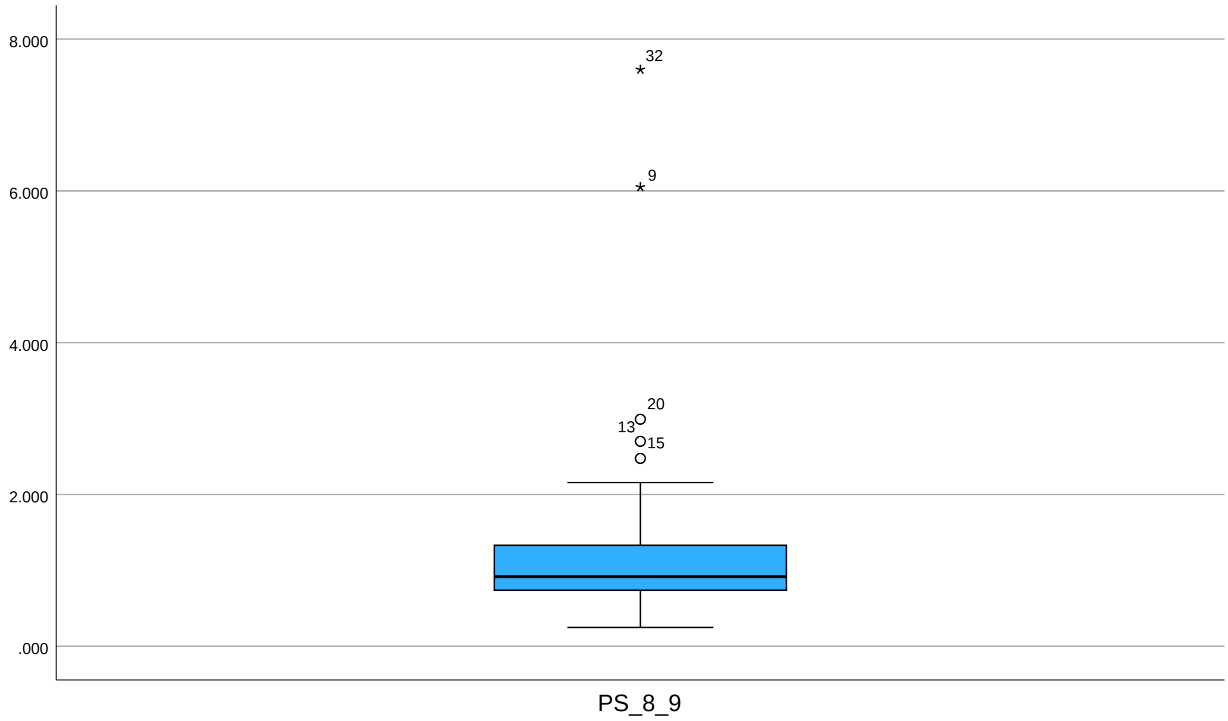

PS_9_10

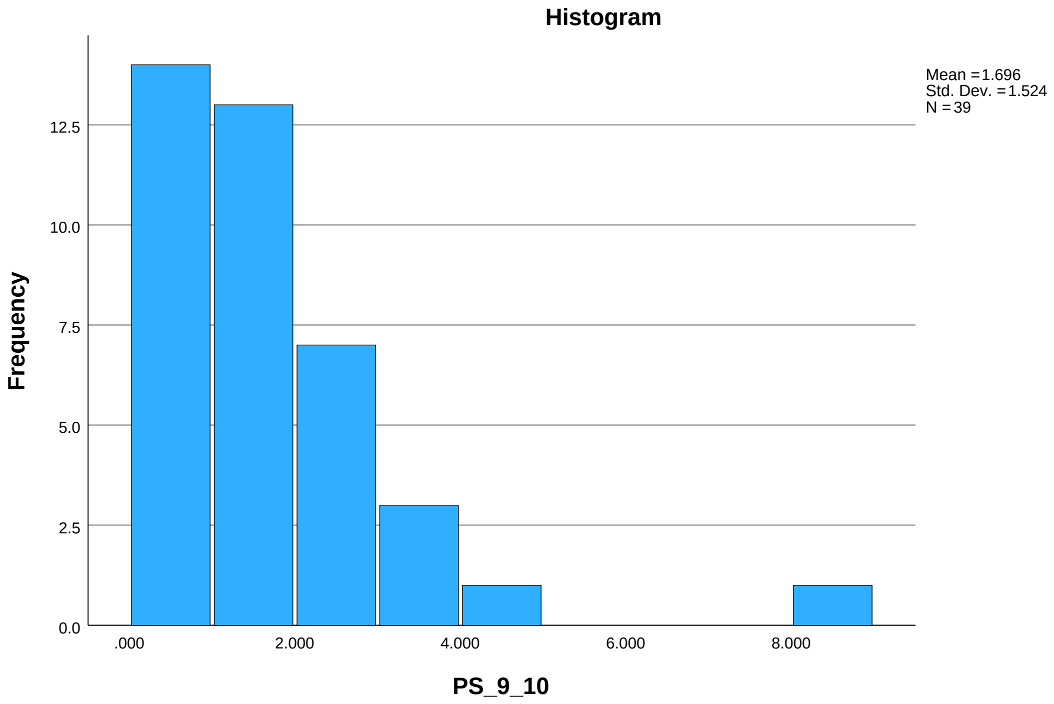


```
PS_9_10 Stem-and-Leaf Plot

 Frequency    Stem &  Leaf
```

```
5.00      0 . 22234
 9.00      0 . 555577799
10.00      1 . 0000333444
 3.00      1 . 679
 5.00      2 . 01223
 2.00      2 . 58
 2.00      3 . 34
 1.00      3 . 7
 2.00 Extremes    (>=4.8)

Stem width:     1.000
Each leaf:       1 case(s)
```

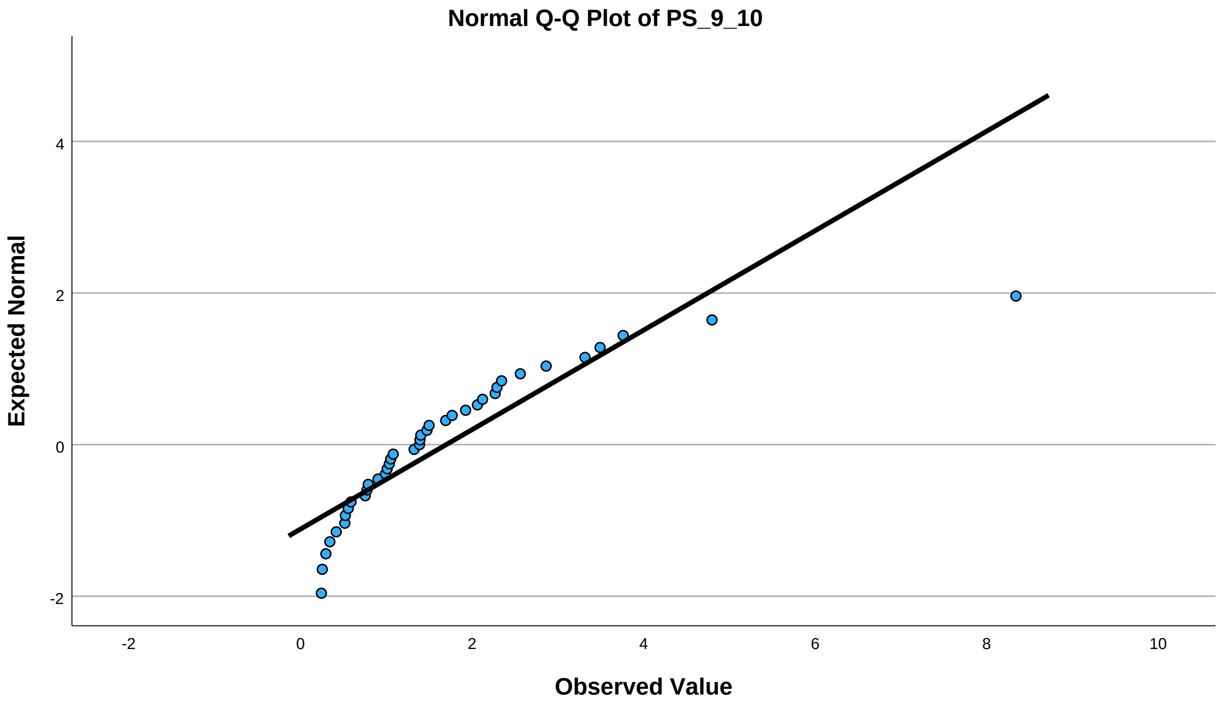

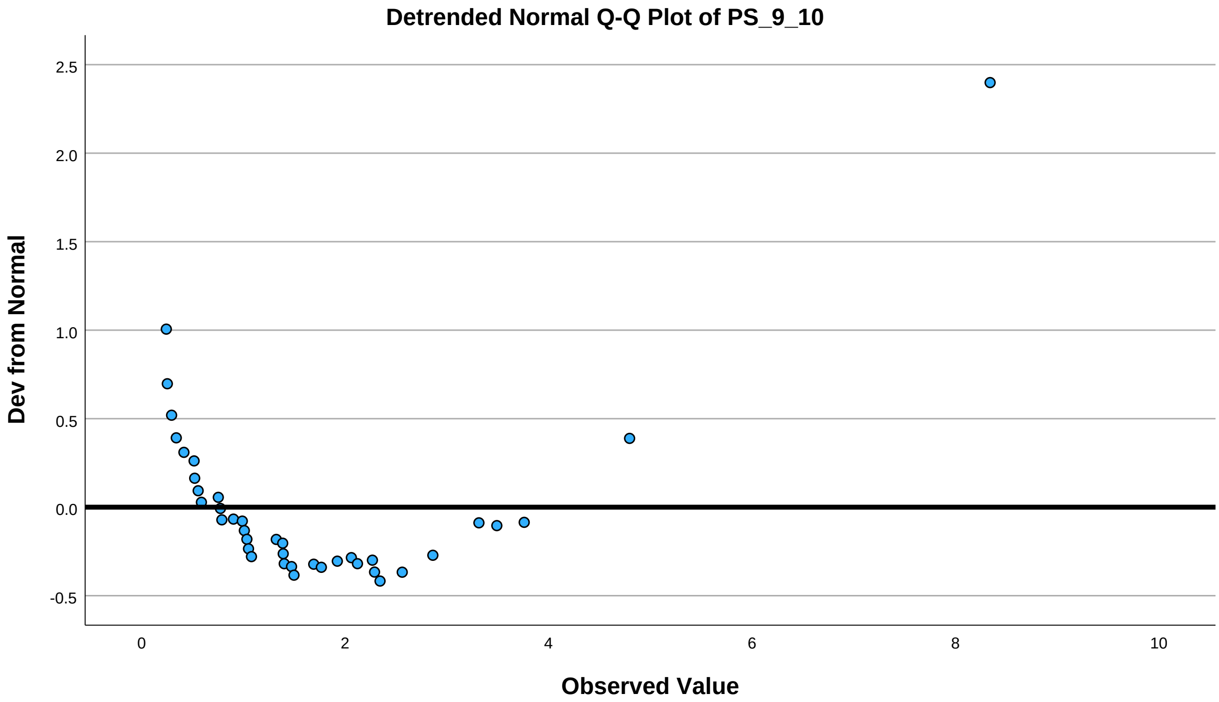
Detrended Normal Q-Q Plot of PS_9_10
Dev from Normal
Observed Value
2.5
2.0
1.5
1.0
0.5
0.0
-0.5
0
2
4
6
8
10

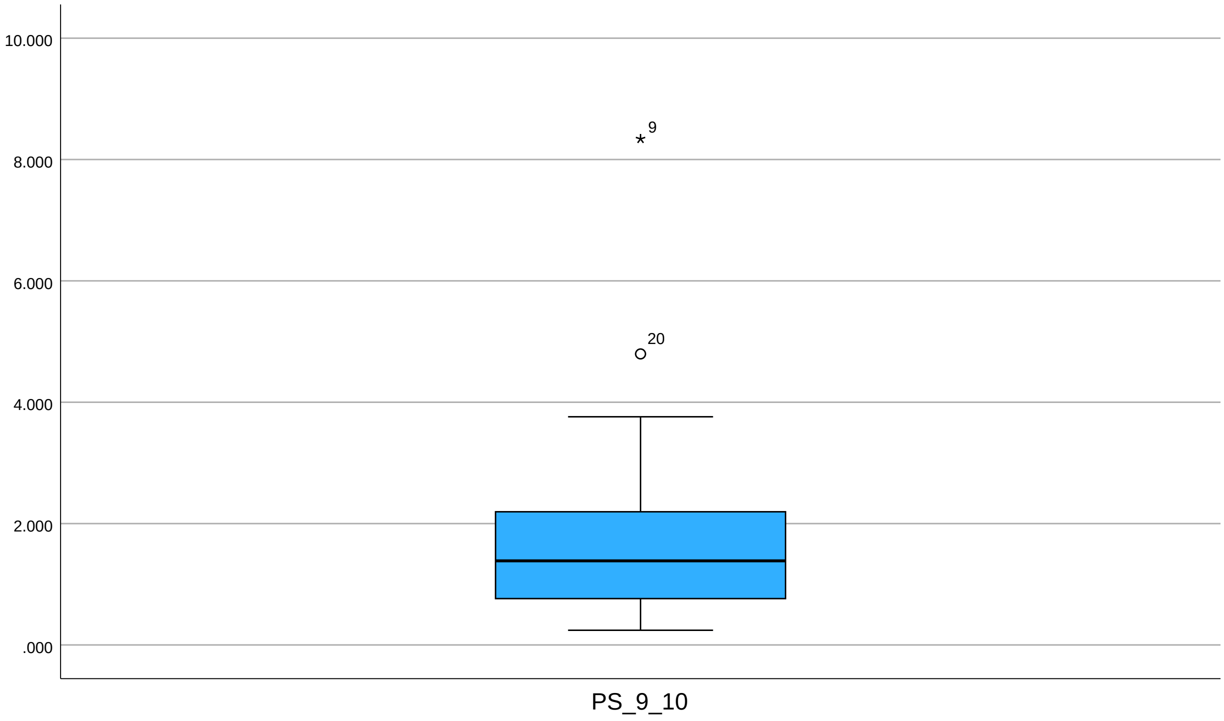
10.000
8.000
6.000
4.000
2.000
.000
9
20
PS_9_10

PS_10_11

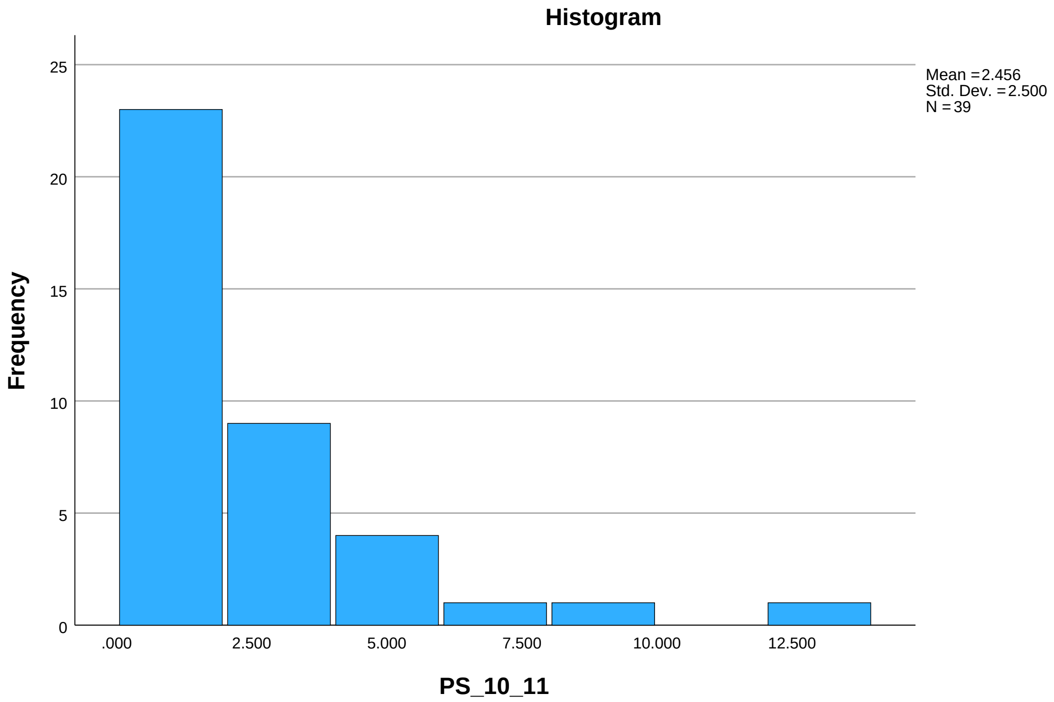


```
PS_10_11 Stem-and-Leaf Plot

 Frequency    Stem &  Leaf

    13.00        0 .  2235566677889
    10.00        1 .  0011135689
     4.00        2 .  2458
     5.00        3 .  02555
     3.00        4 .  479
     1.00        5 .  3
     1.00        6 .  5
     2.00 Extremes    (>=8.1)

 Stem width:     1.000
 Each leaf:       1 case(s)
```

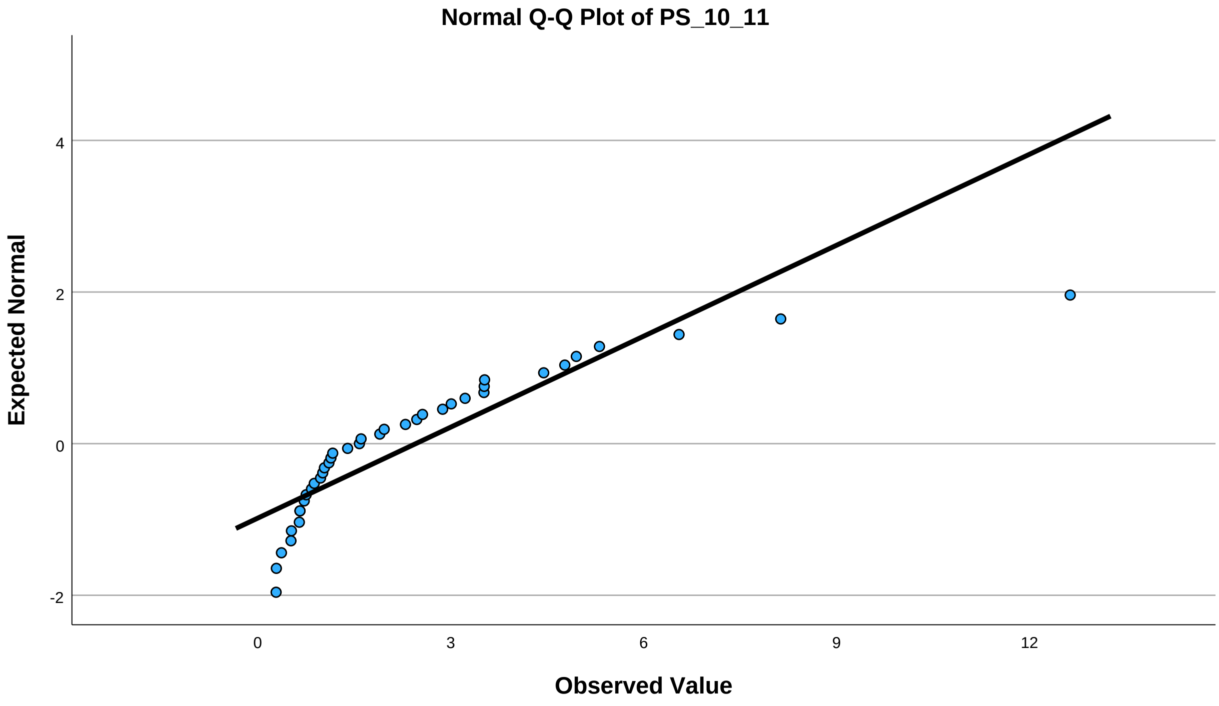
Normal Q-Q Plot of PS_10_11
Expected Normal
4
2
0
-2
0
3
6
9
12
Observed Value

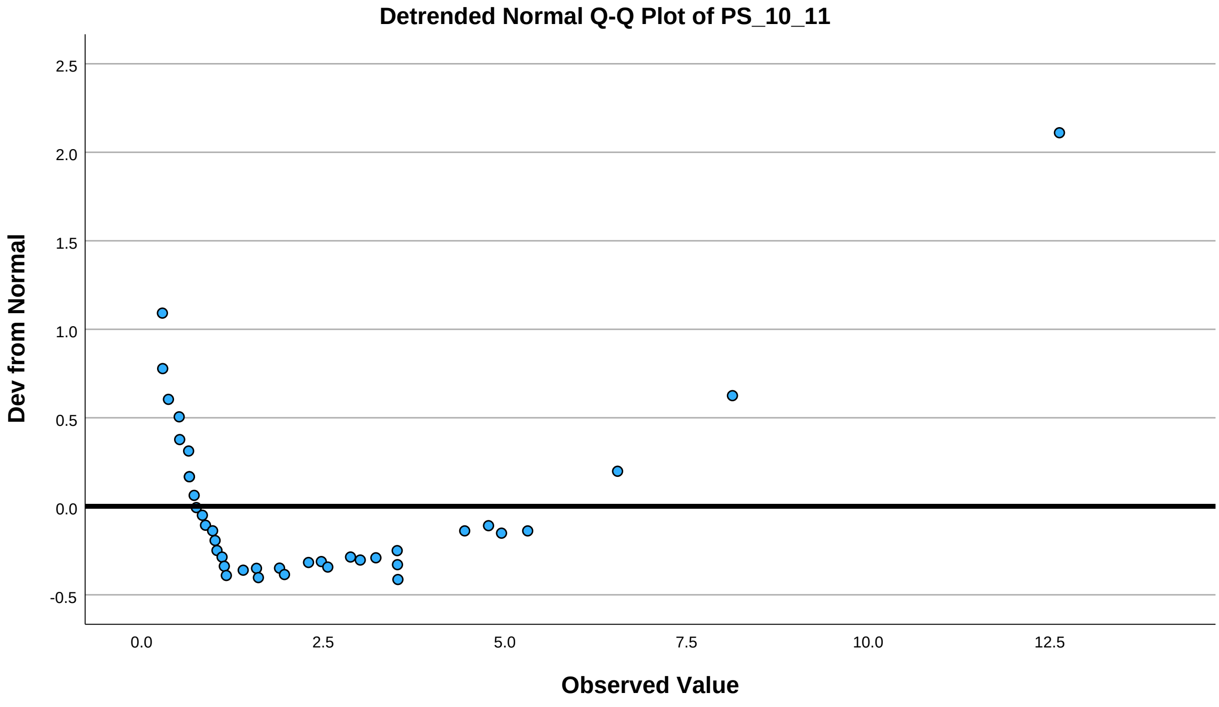
Detrended Normal Q-Q Plot of PS_10_11
Dev from Normal
2.5
2.0
1.5
1.0
0.5
0.0
-0.5
0.0
2.5
5.0
7.5
10.0
12.5
Observed Value

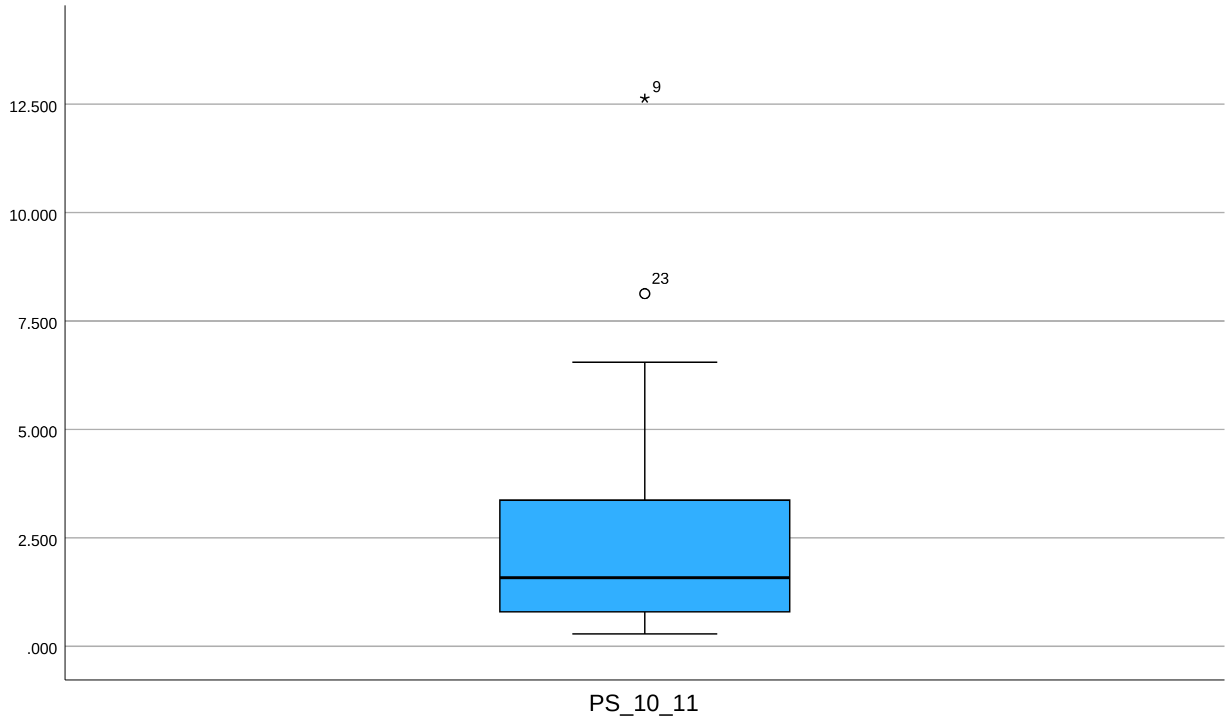

PS_11_12

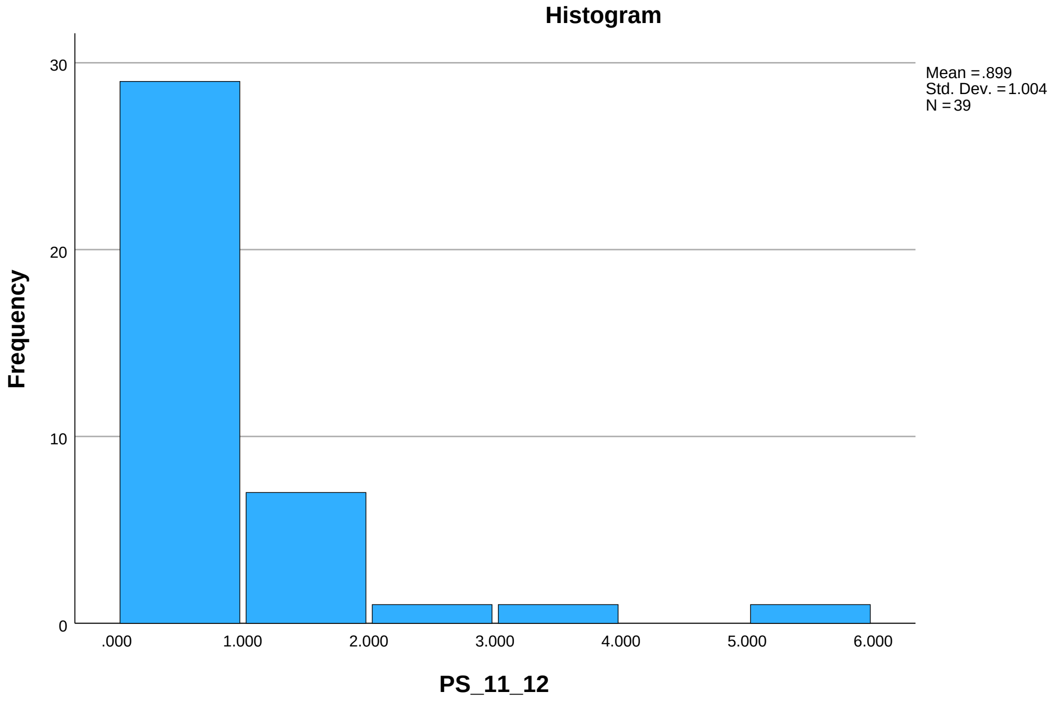

PS_11_12 Stem-and-Leaf Plot

Frequency   Stem & Leaf

```
   1.00      0 . 1
   6.00      0 . 223333
  11.00      0 . 44444444455
   8.00      0 . 66666677
   3.00      0 . 889
   4.00      1 . 0001
   1.00      1 . 2
   2.00      1 . 44
   3.00 Extremes    (>=2.8)

Stem width:     1.000
Each leaf:      1 case(s)
```

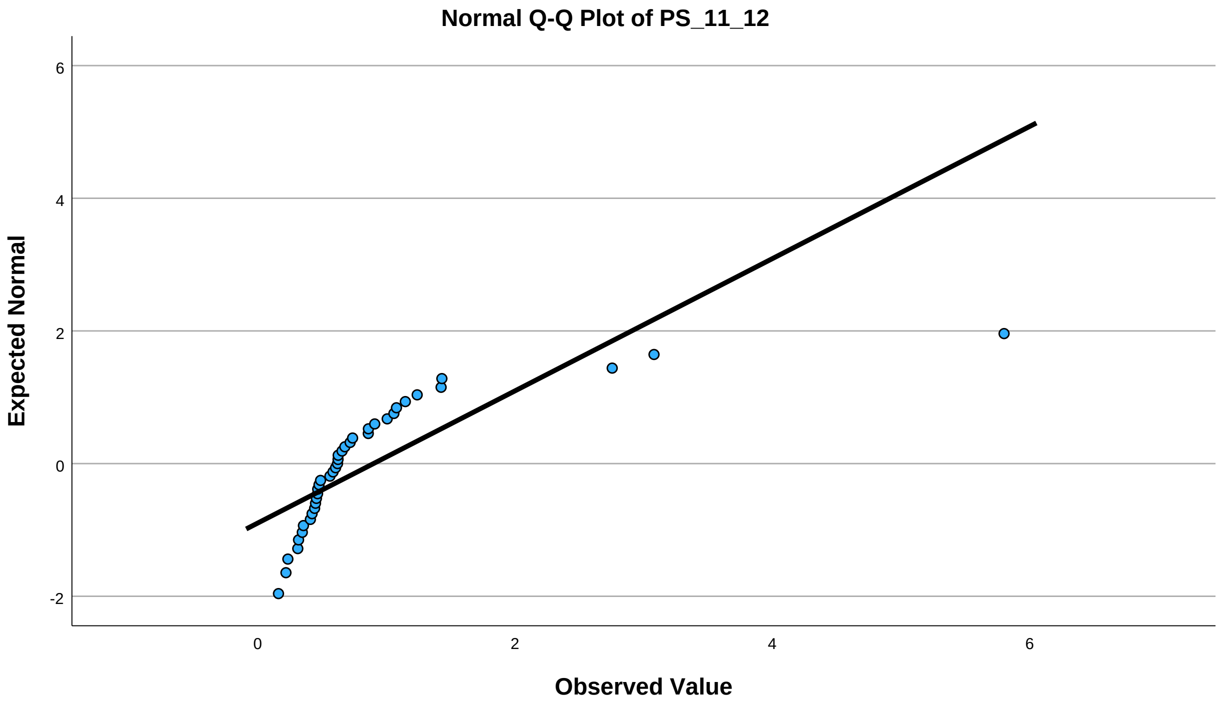

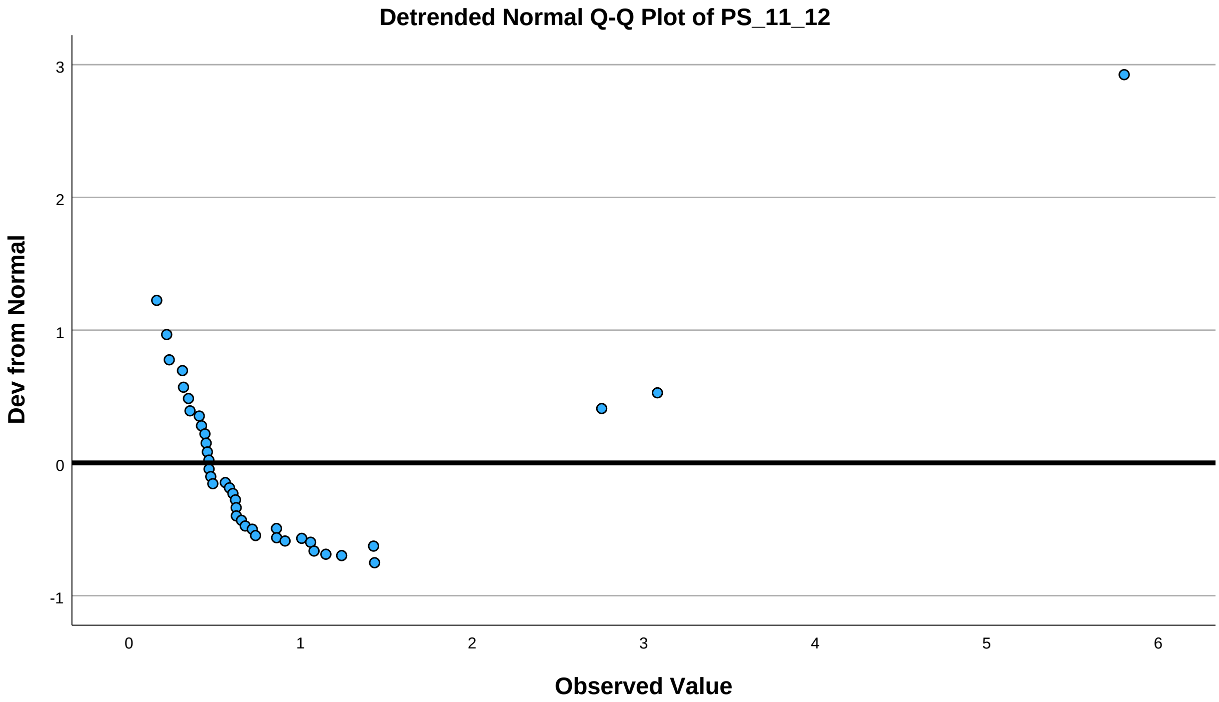
Detrended Normal Q-Q Plot of PS_11_12
Dev from Normal
Observed Value
3
2
1
0
-1
0
1
2
3
4
5
6

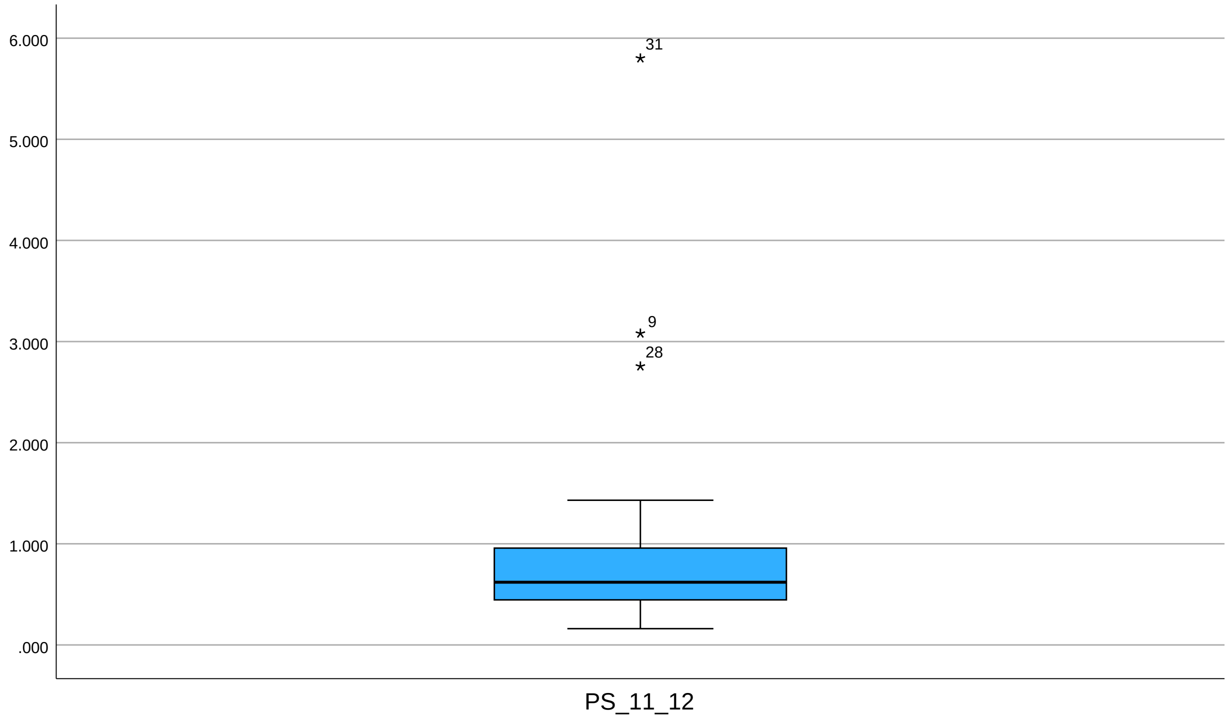
6.000
5.000
4.000
3.000
2.000
1.000
.000
31
9
28
PS_11_12

PS_12_13

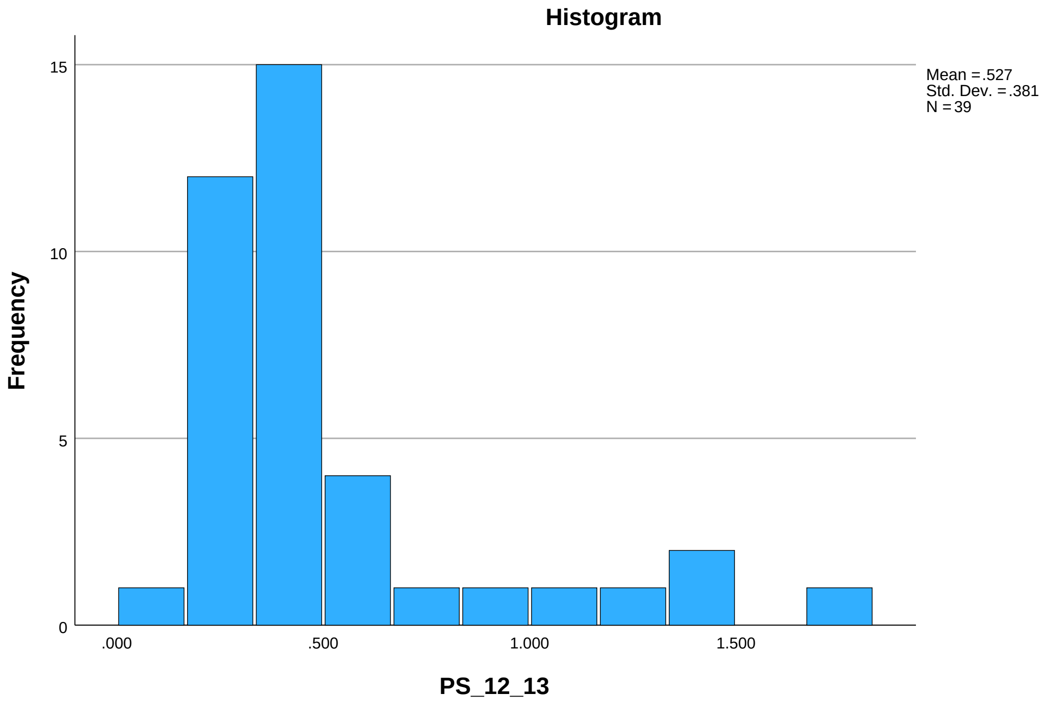


```
PS_12_13 Stem-and-Leaf Plot

 Frequency    Stem &  Leaf

     2.00        1 .  39
     6.00        2 .  004457
    12.00        3 .  122334555677
     8.00        4 .  02356889
     2.00        5 .  34
     2.00        6 .  26
     1.00        7 .  8
     6.00 Extremes    (>=.90)

 Stem width:      .100
 Each leaf:       1 case(s)
```

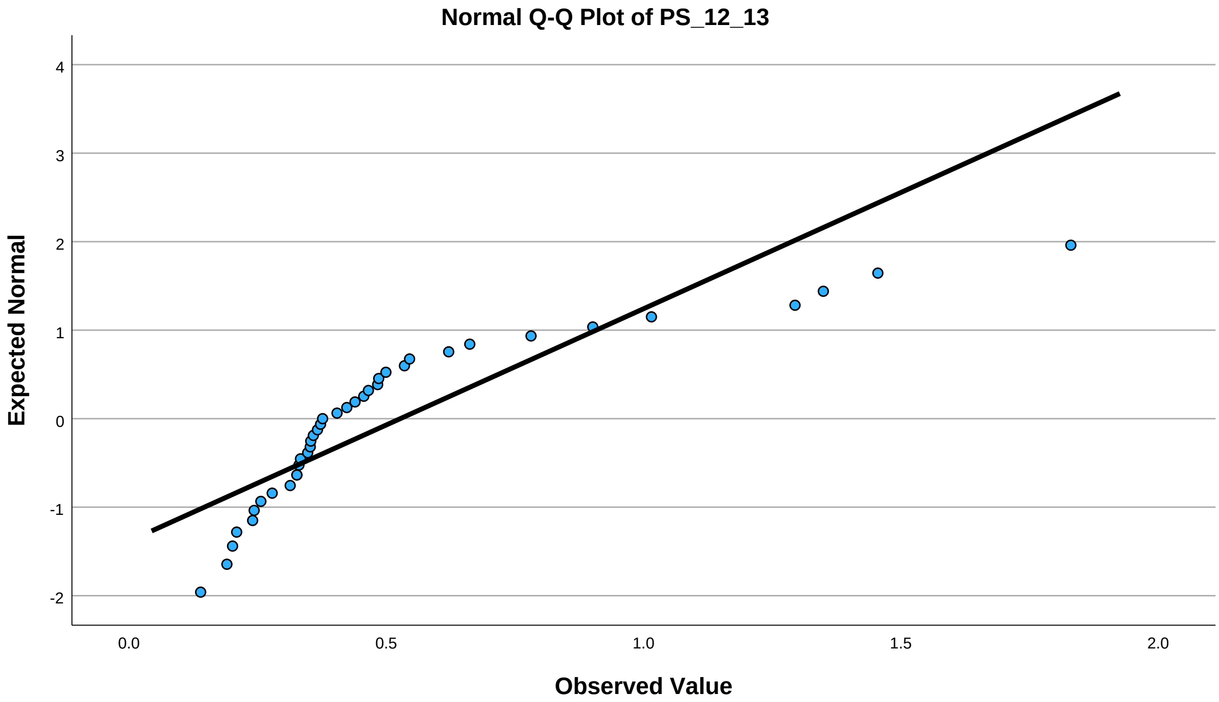
Normal Q-Q Plot of PS_12_13
Expected Normal
4
3
2
1
0
-1
-2
0.0
0.5
1.0
1.5
2.0
Observed Value

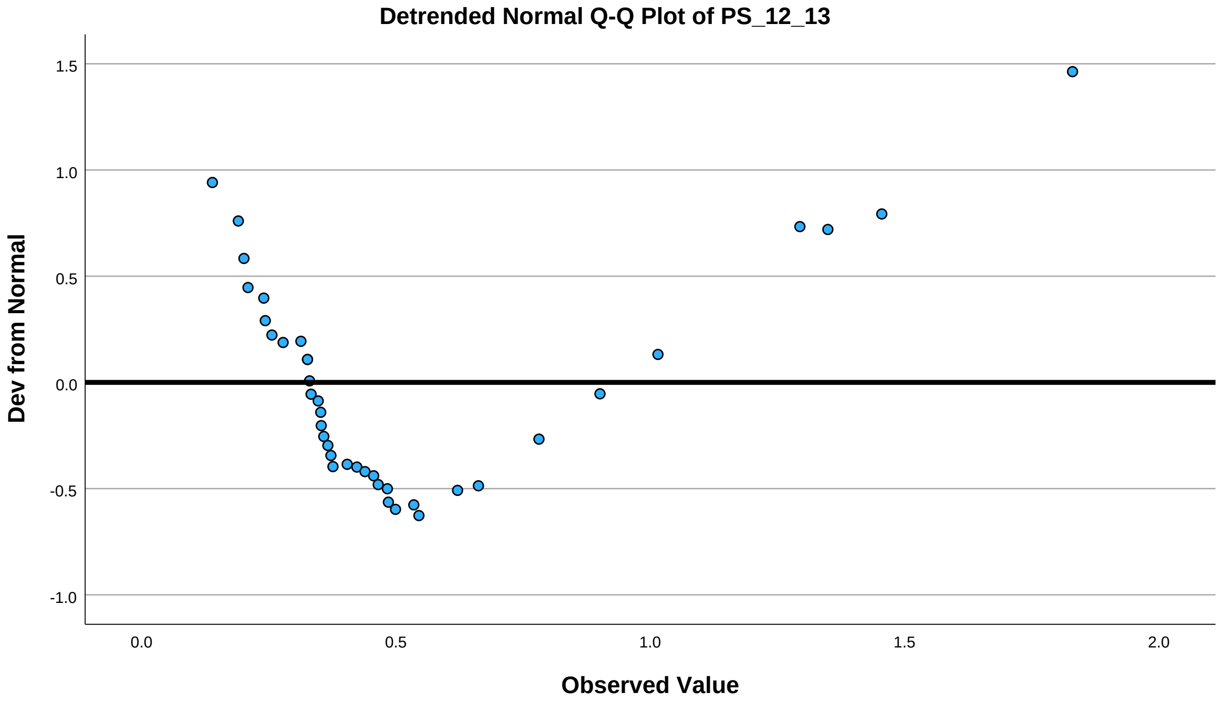
Detrended Normal Q-Q Plot of PS_12_13
Dev from Normal
1.5
1.0
0.5
0.0
-0.5
-1.0
0.0
0.5
1.0
1.5
2.0
Observed Value

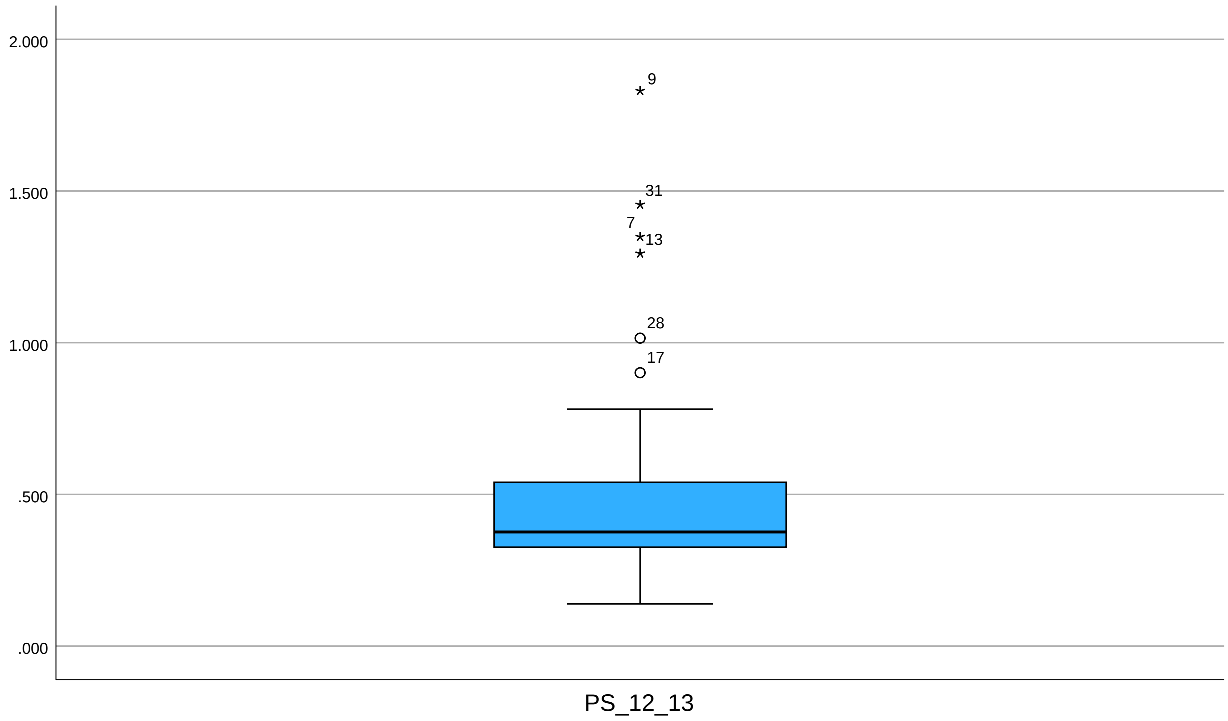

PS_13_14

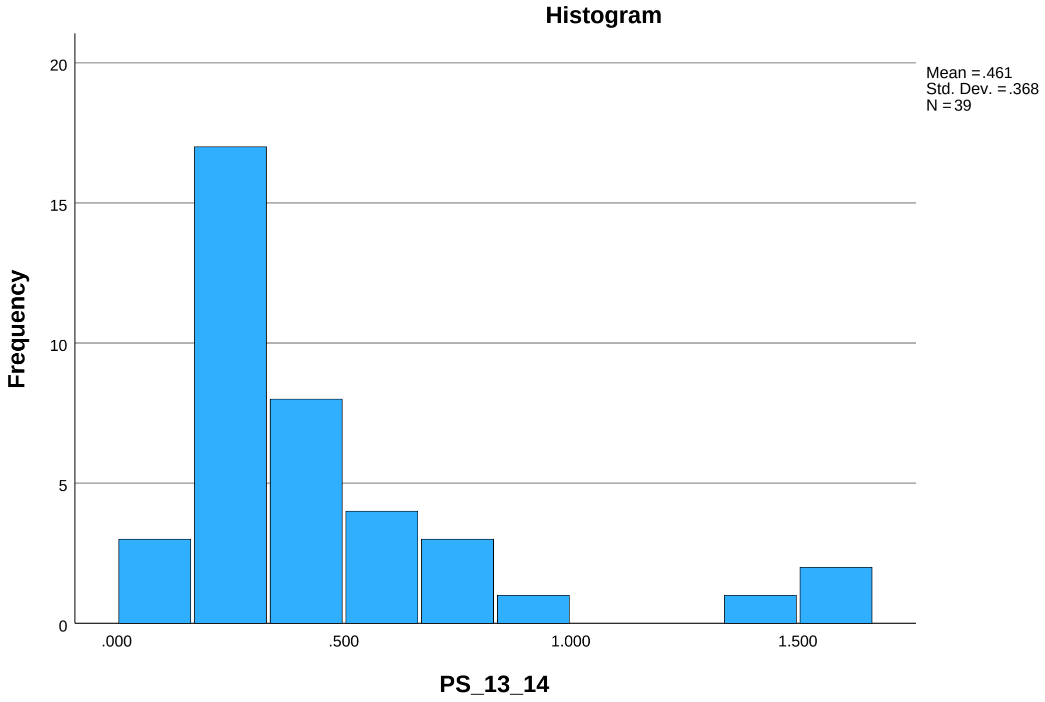

PS_13_14 Stem-and-Leaf Plot

Frequency Stem & Leaf

```
   3.00      1 . 036
  13.00      2 . 0011233345667
   9.00      3 . 112256678
   3.00      4 . 137
   2.00      5 . 05
   3.00      6 . 048
   2.00      7 . 26
    .00      8 .
   1.00      9 . 4
   3.00 Extremes    (>=1.50)

 Stem width:      .100
 Each leaf:       1 case(s)
```

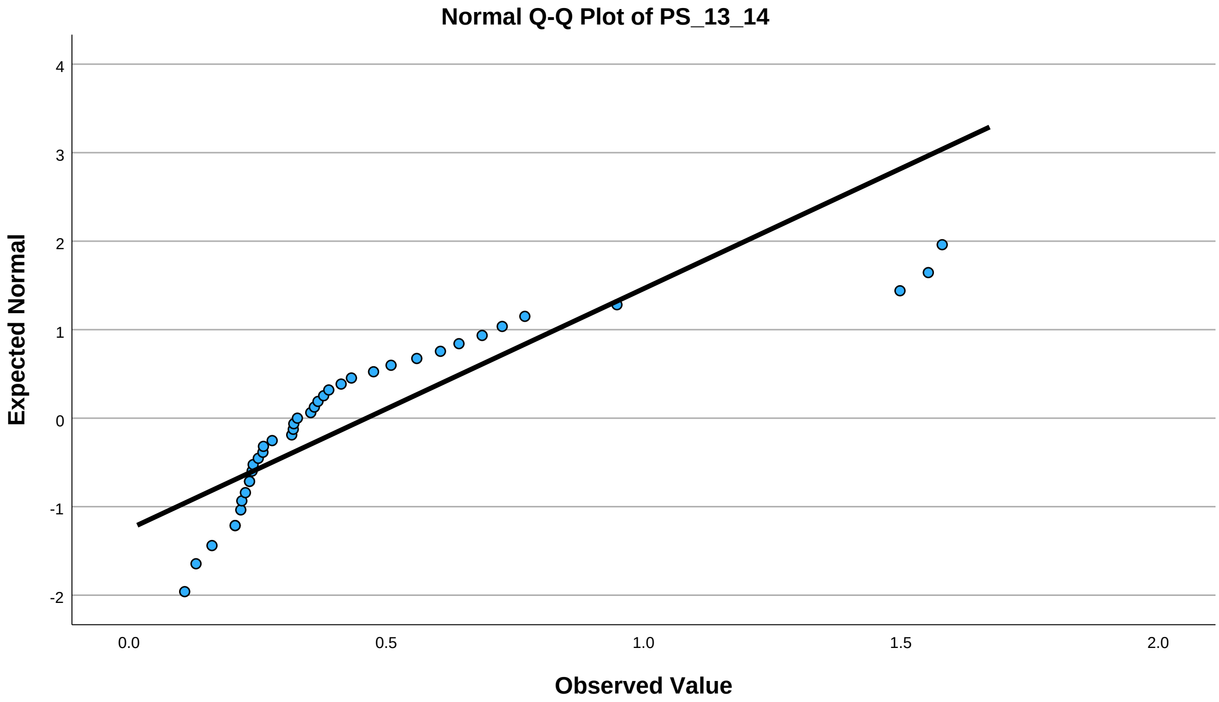

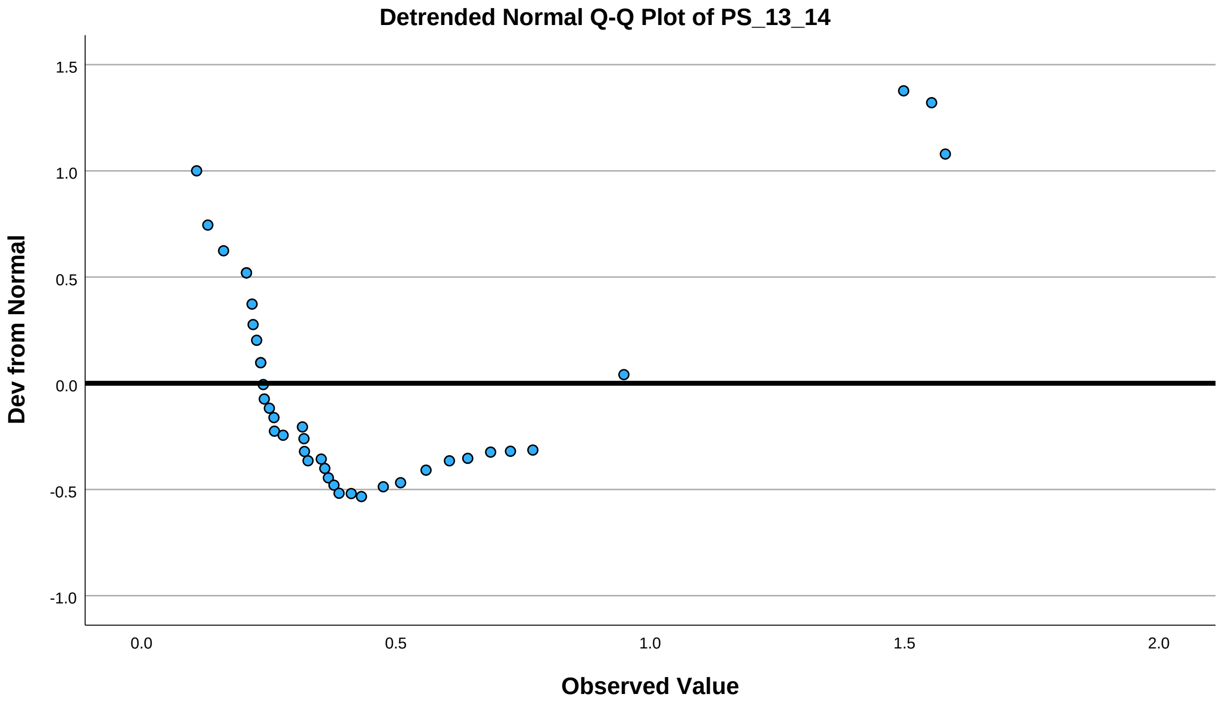
Detrended Normal Q-Q Plot of PS_13_14
Dev from Normal
1.5
1.0
0.5
0.0
-0.5
-1.0
0.0
0.5
1.0
1.5
2.0
Observed Value

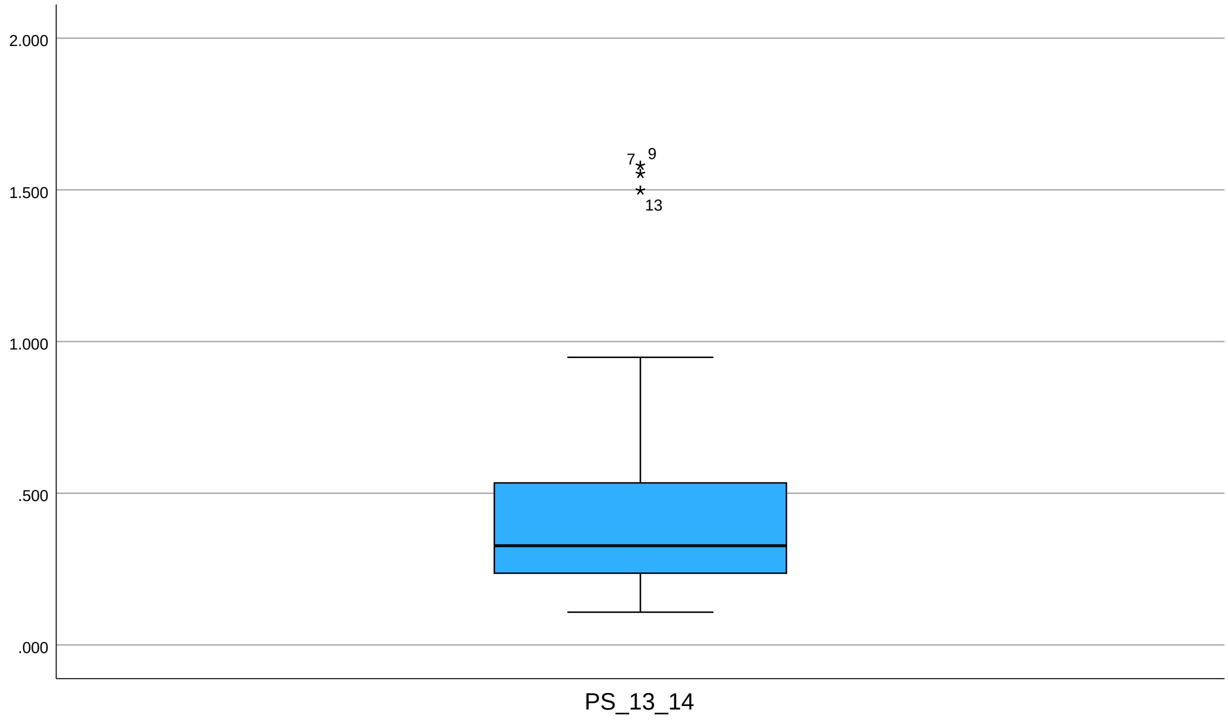
2.000
1.500
1.000
.500
.000
7
9
13
PS_13_14

PS_14_15

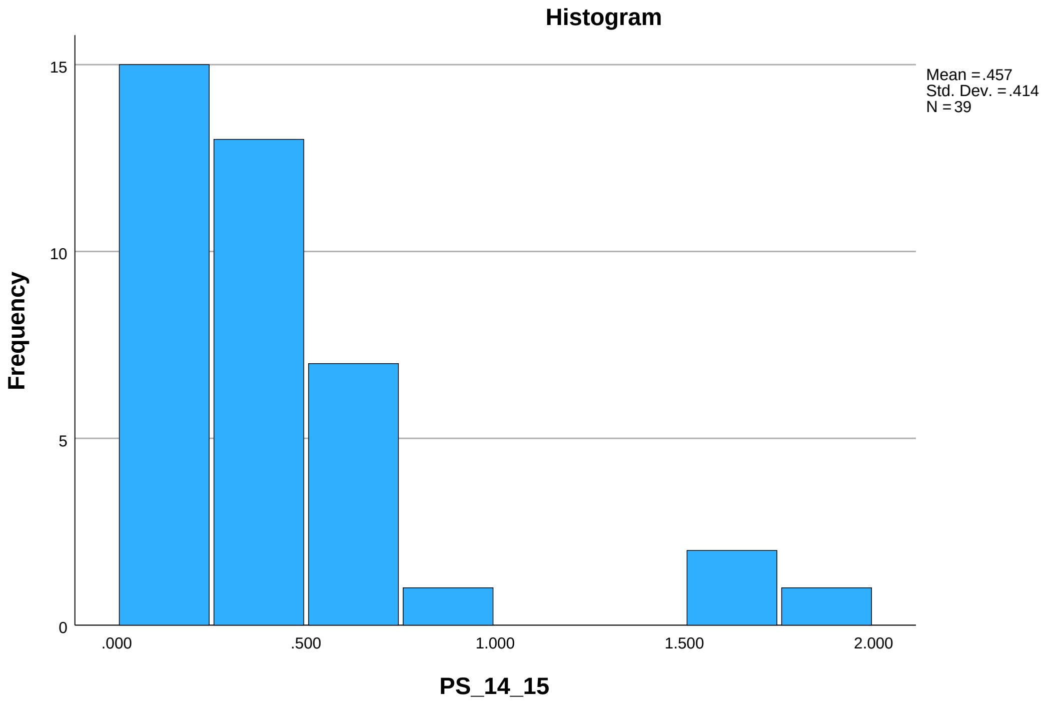


```
PS_14_15 Stem-and-Leaf Plot

 Frequency    Stem &  Leaf

    1.00      0 .  8
     7.00      1 .  2478999
    10.00       2 .  0001234889
     7.00      3 .  0234778
    3.00      4 .  056
    3.00      5 .  227
    3.00      6 .  156
    1.00      7 .  1
     .00     8 .
    1.00      9 .  0
     3.00 Extremes    (>=1.58)

 Stem width:      .100
 Each leaf:       1 case(s)
```

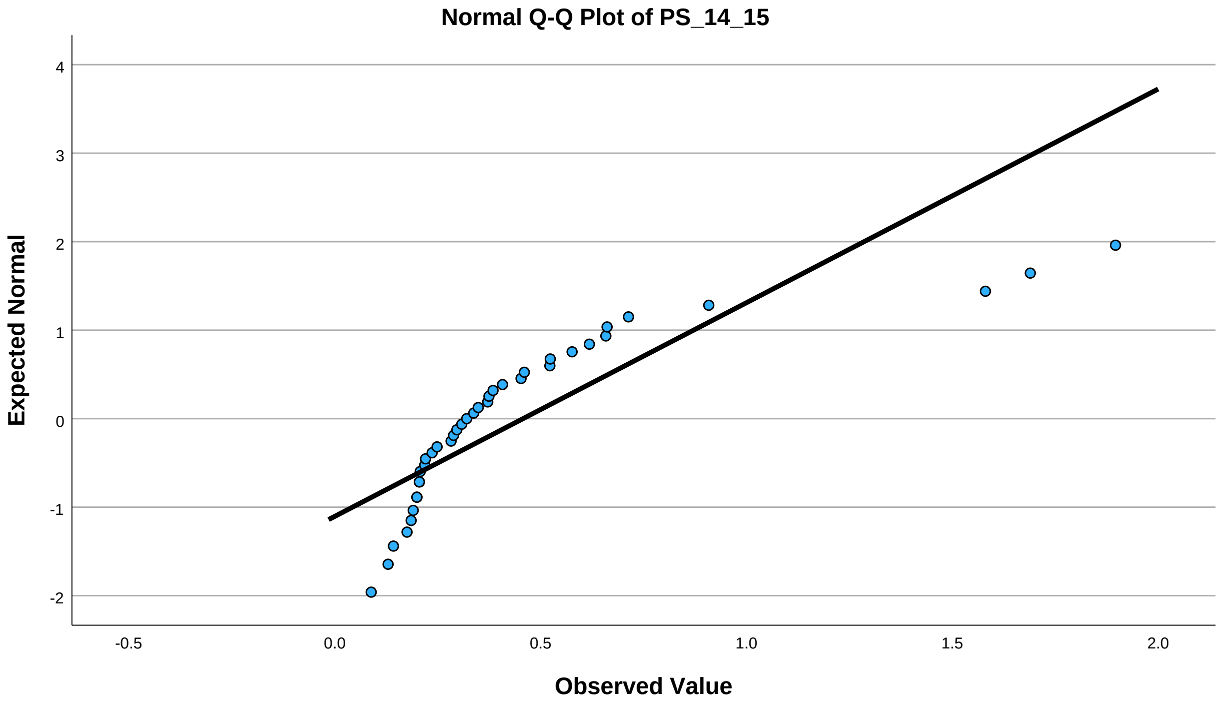
Normal Q-Q Plot of PS_14_15
Expected Normal
4
3
2
1
0
-1
-2
-0.5
0.0
0.5
1.0
1.5
2.0
Observed Value

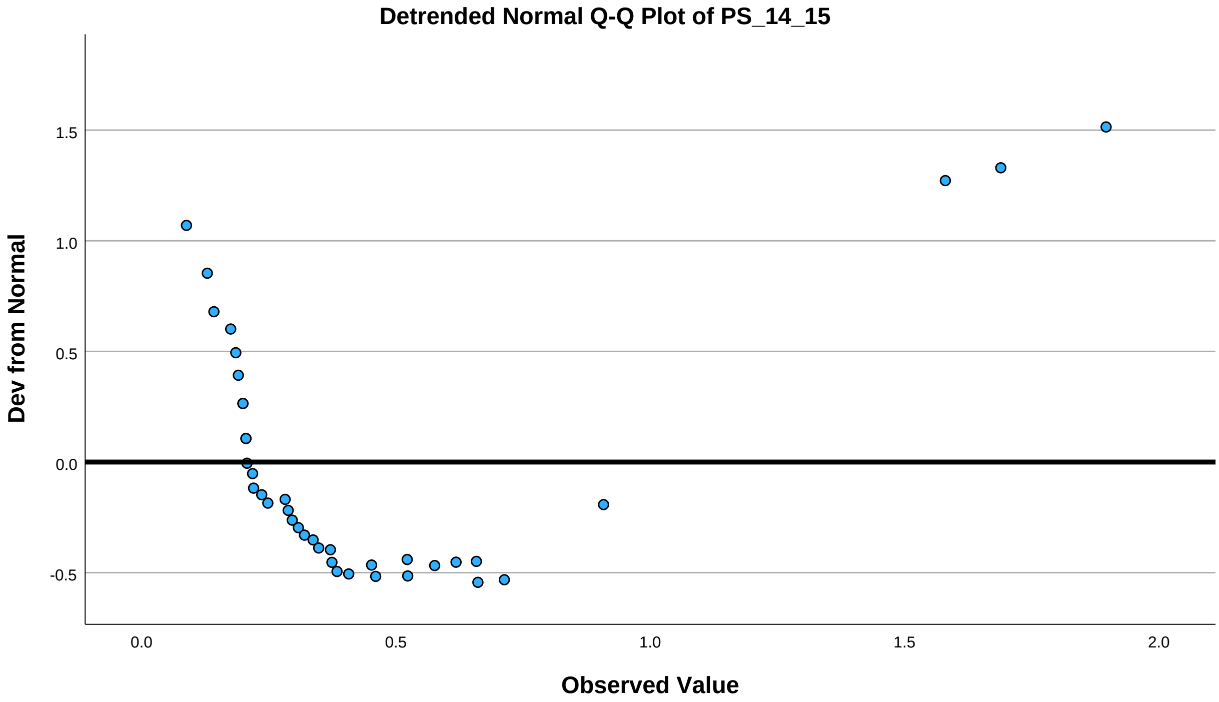
Detrended Normal Q-Q Plot of PS_14_15
Dev from Normal
1.5
1.0
0.5
0.0
-0.5
0.0
0.5
1.0
1.5
2.0
Observed Value

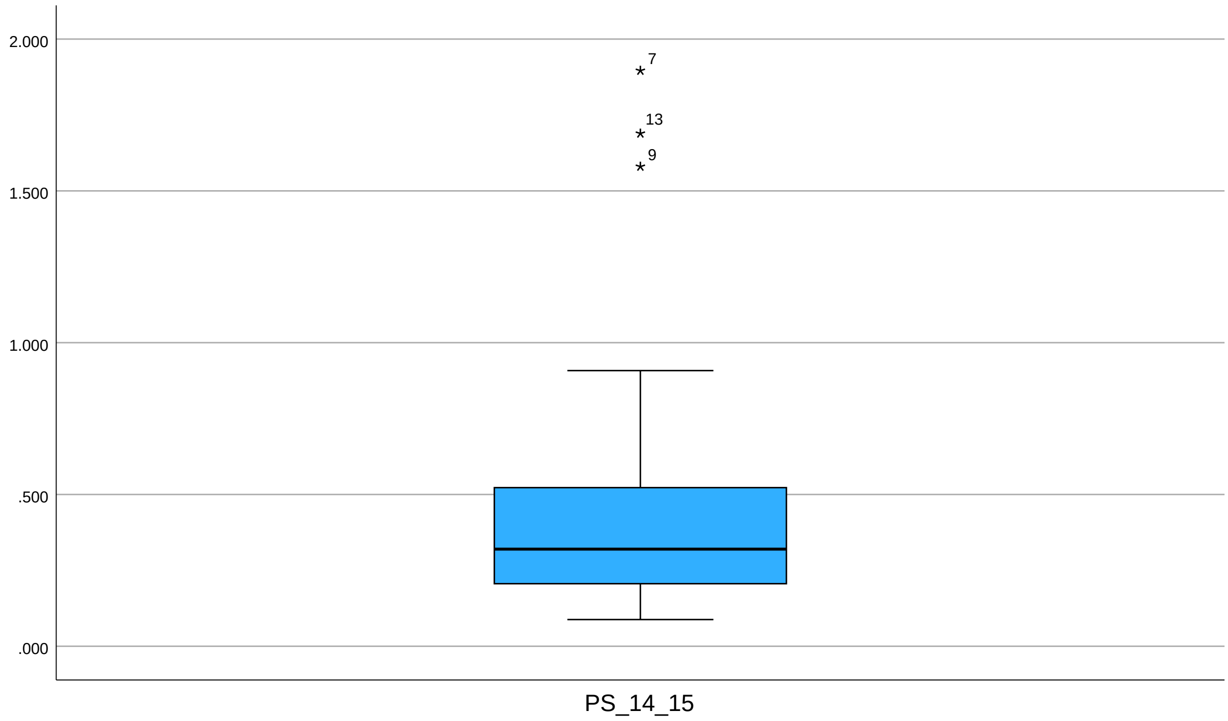

PS_15_16

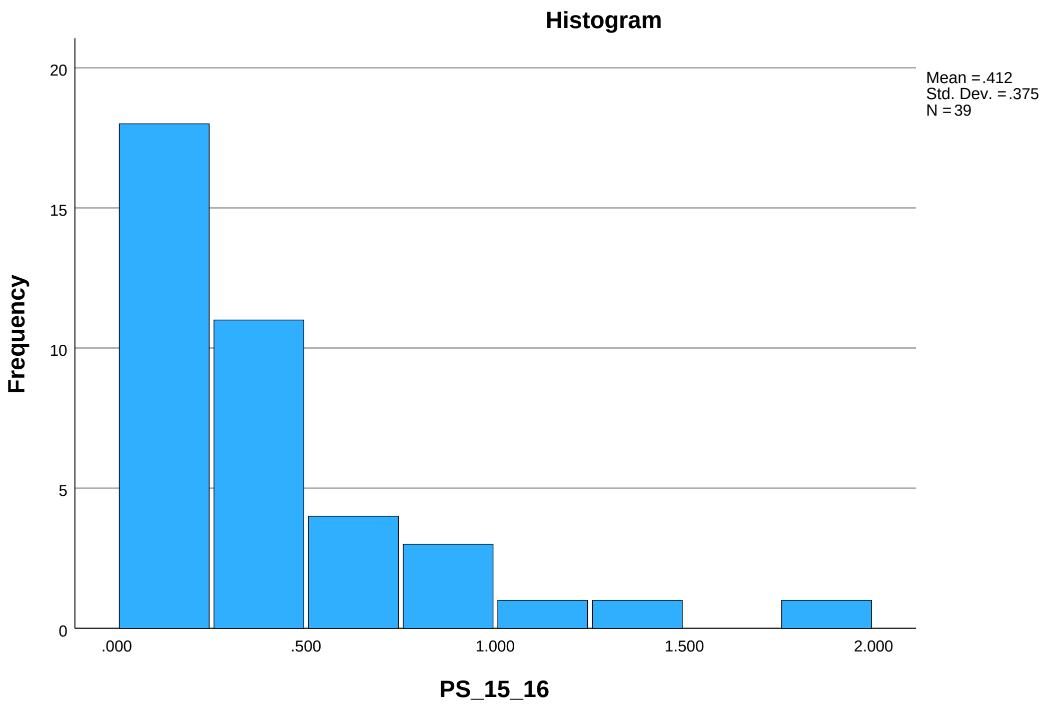

PS_15_16 Stem-and-Leaf Plot

Frequency   Stem & Leaf

```
  2.00      0 . 89
 11.00      1 . 15677788999
 10.00      2 . 1244478899
  4.00      3 . 0057
  2.00      4 . 55
  4.00      5 . 2669
   .00      6 .
  1.00      7 . 9
  1.00      8 . 6
  4.00 Extremes    (>=.96)

Stem width:      .100
Each leaf:       1 case(s)
```

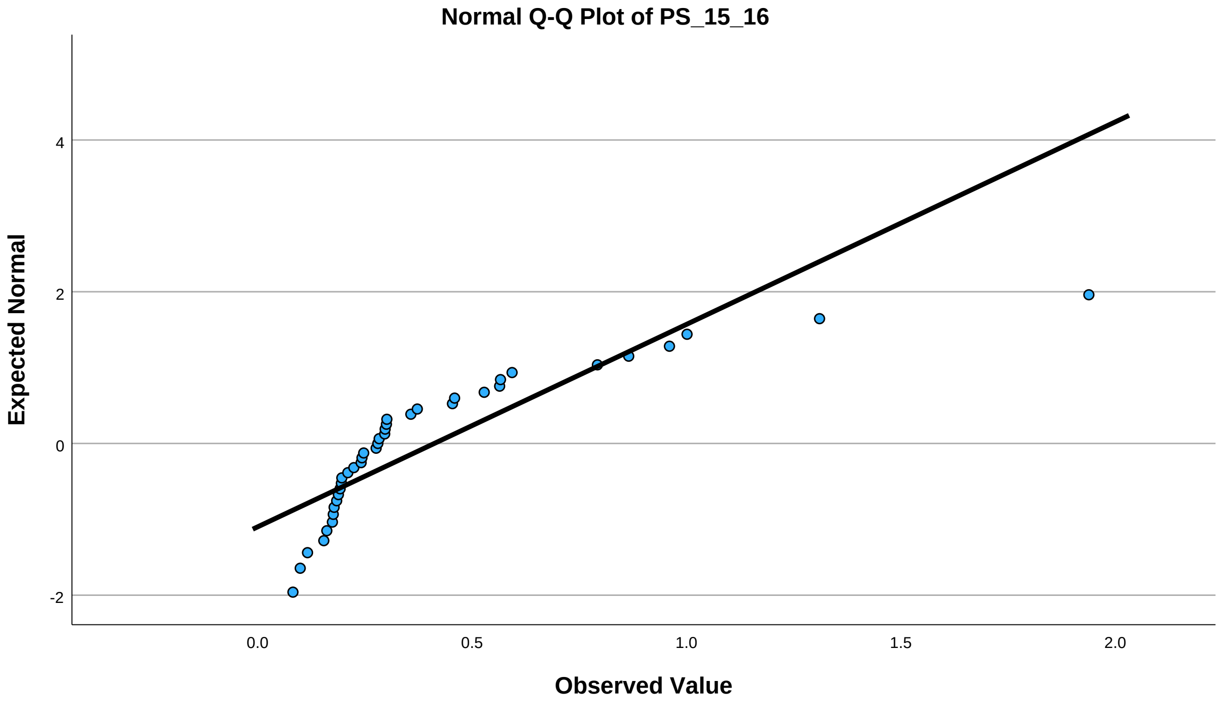

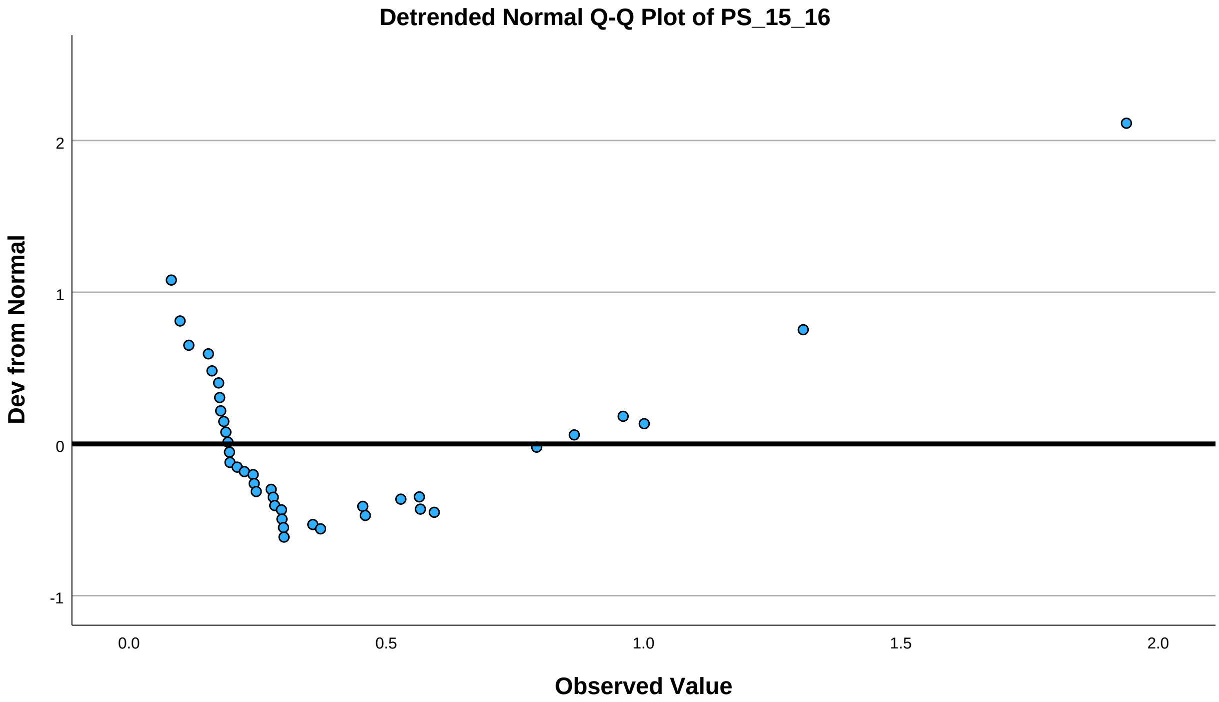
Detrended Normal Q-Q Plot of PS_15_16
Dev from Normal
2
1
0
-1
0.0
0.5
1.0
1.5
2.0
Observed Value

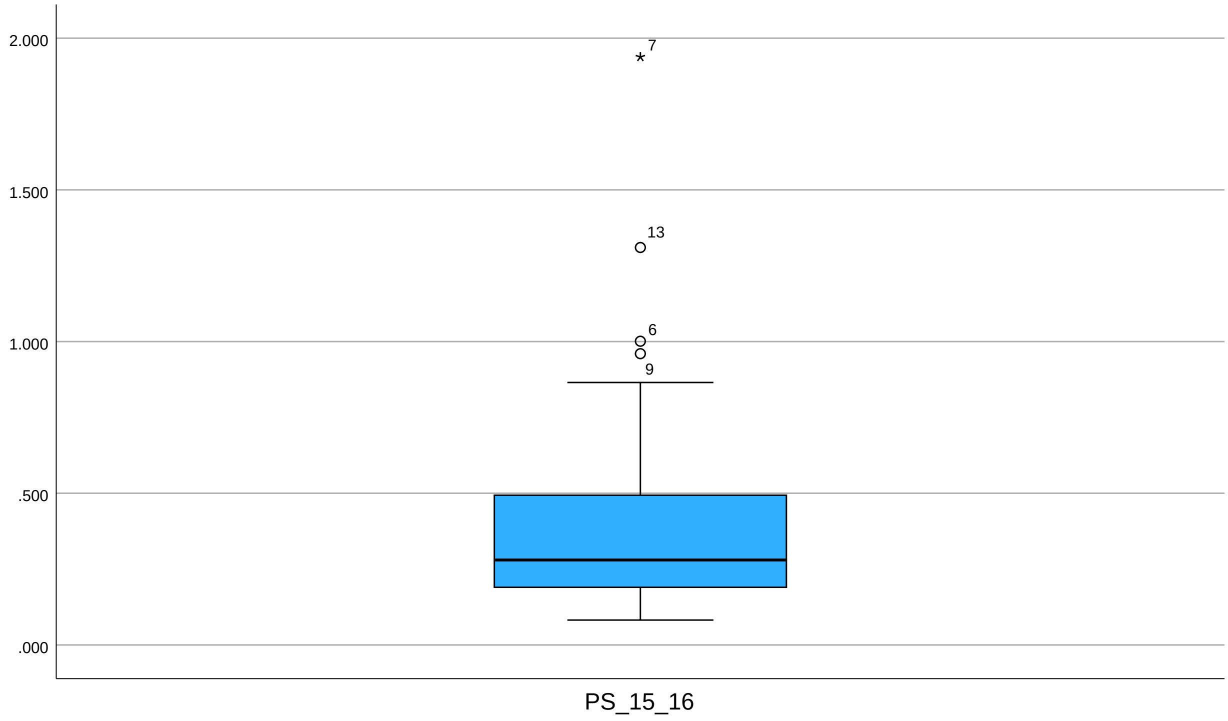
2.000
1.500
1.000
.500
.000
7
13
6
9
PS_15_16

PS_16_17

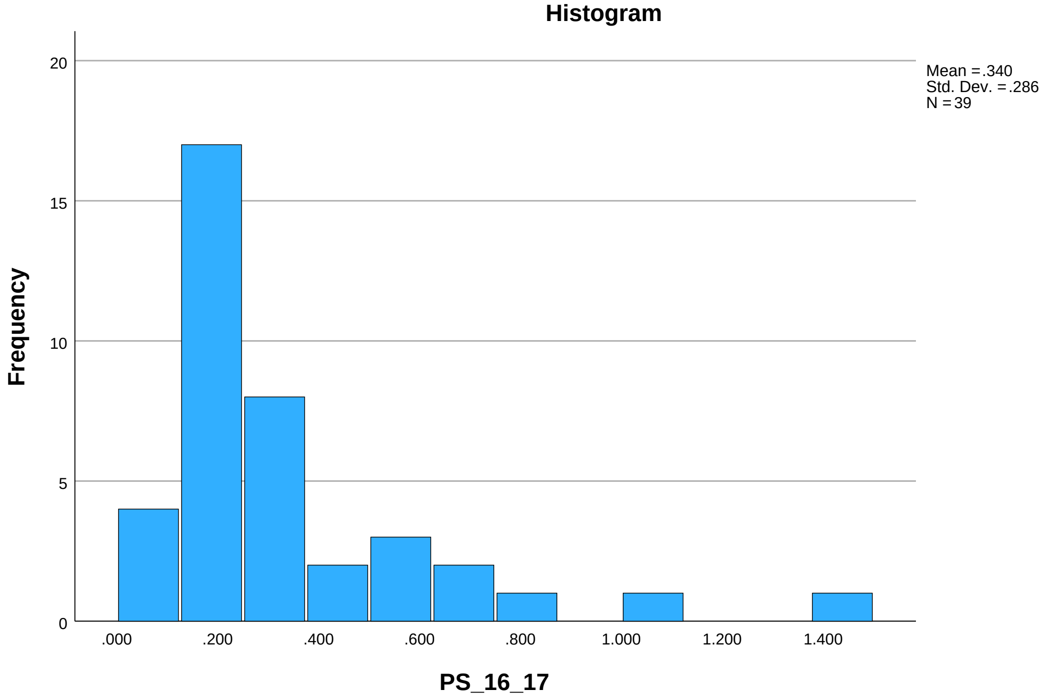


```
PS_16_17 Stem-and-Leaf Plot

 Frequency    Stem &  Leaf

     2.00        0 .  99
    13.00        1 .  1155666777899
    10.00        2 .  0111335568
     6.00        3 .  134578
      .00        4 .
     2.00        5 .  15
     1.00        6 .  0
     5.00 Extremes    (>=.70)

 Stem width:      .100
 Each leaf:       1 case(s)
```

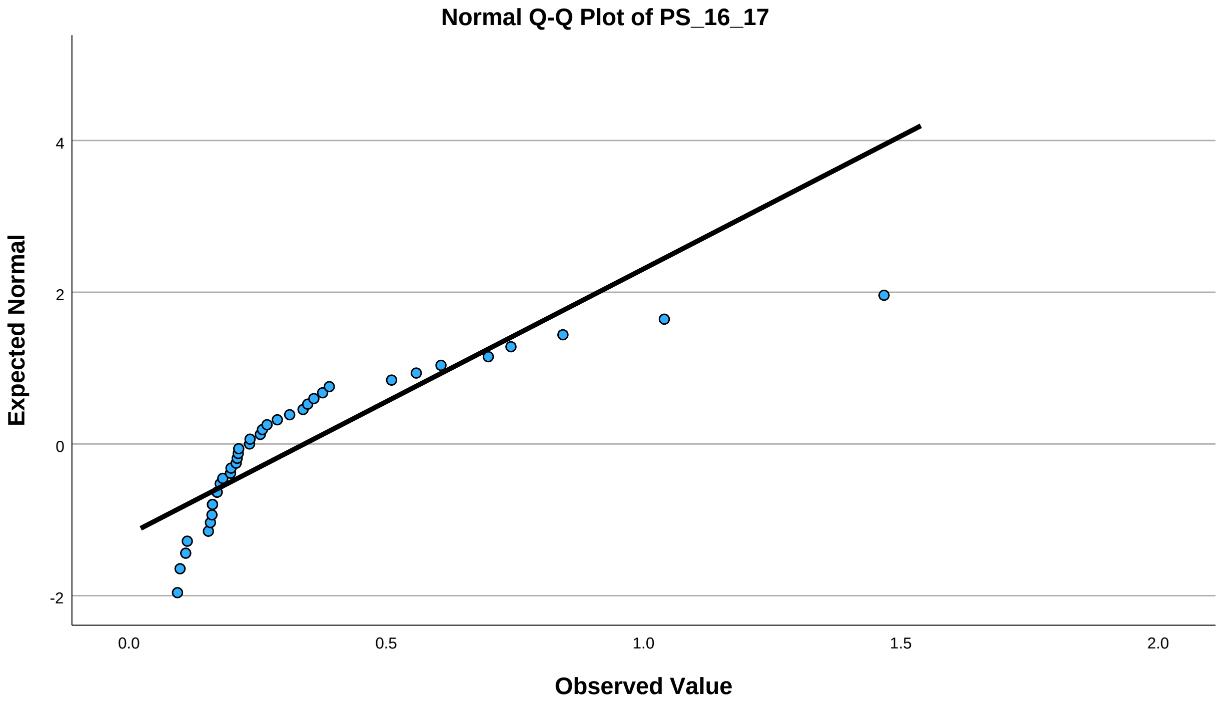
Normal Q-Q Plot of PS_16_17
Expected Normal
4
2
0
-2
0.0
0.5
1.0
1.5
2.0
Observed Value

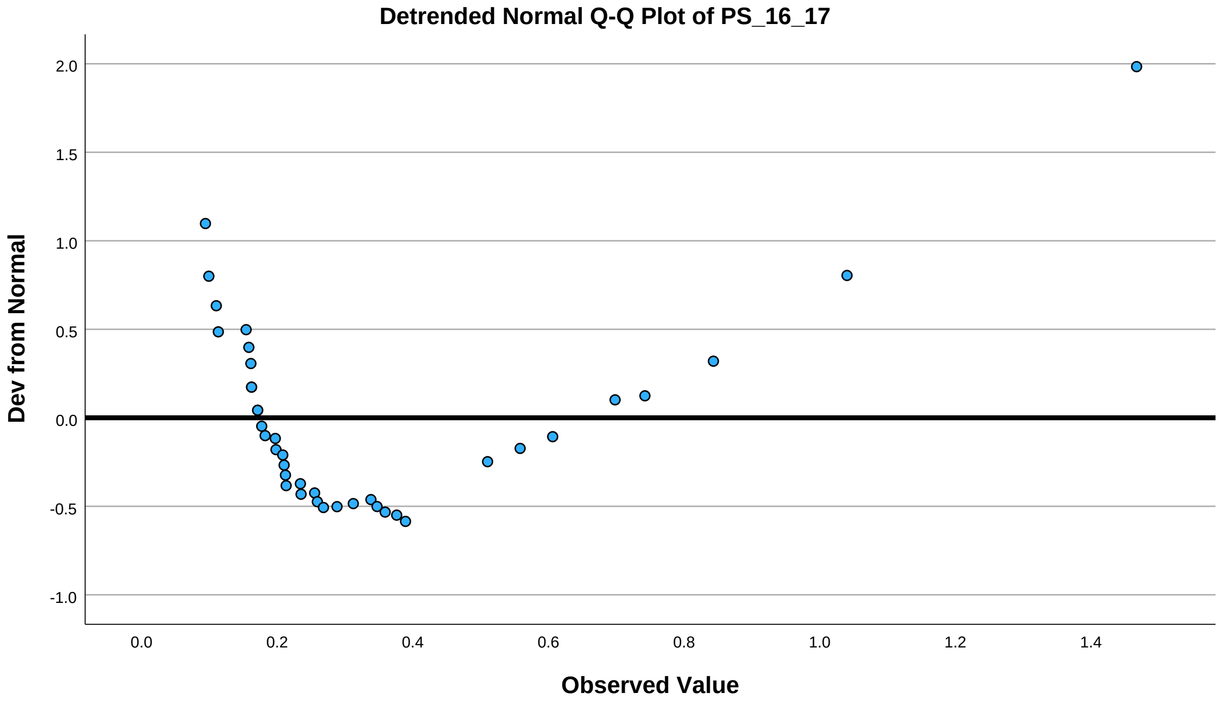
Detrended Normal Q-Q Plot of PS_16_17
Dev from Normal
2.0
1.5
1.0
0.5
0.0
-0.5
-1.0
0.0
0.2
0.4
0.6
0.8
1.0
1.2
1.4
Observed Value

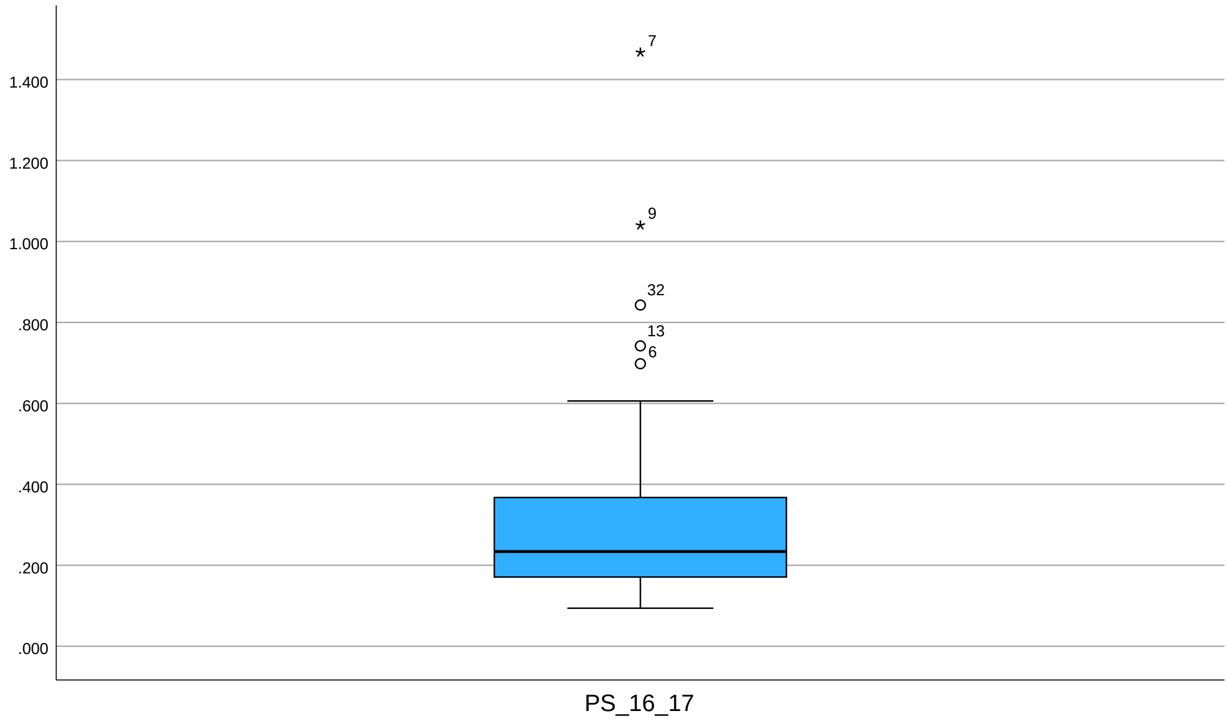

PS_17_18

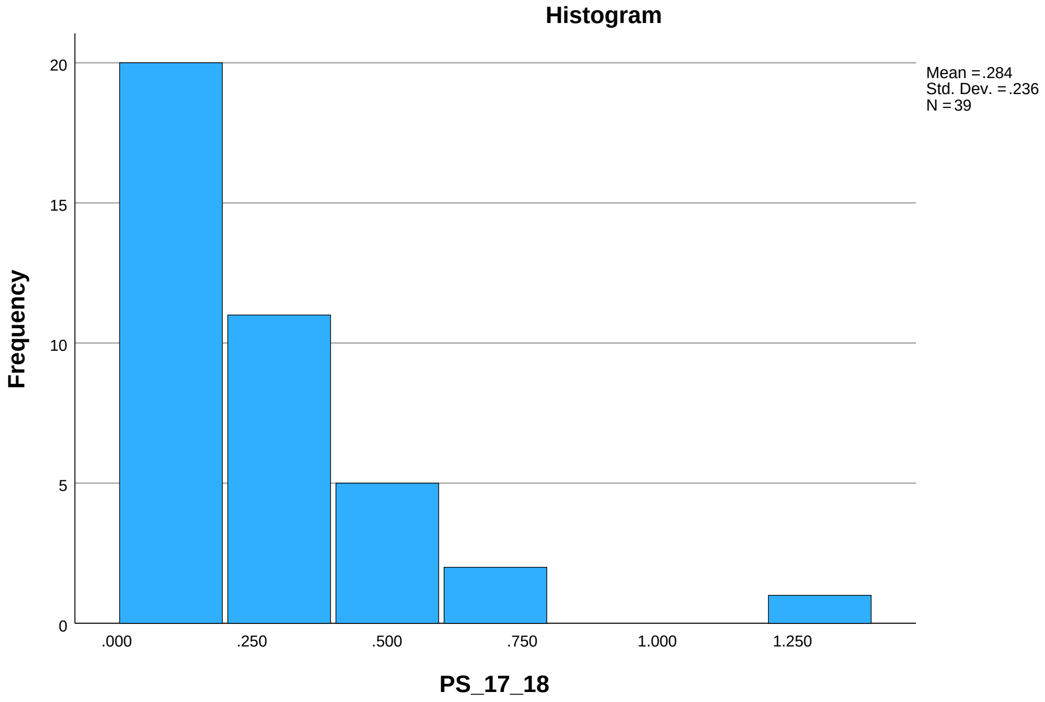

PS_17_18 Stem-and-Leaf Plot

Frequency   Stem & Leaf

```
  1.00      0 . 7
 10.00      1 . 0113344444
  9.00      1 . 556678888
  5.00      2 . 01222
  4.00      2 . 6889
  1.00      3 . 0
  1.00      3 . 9
  2.00      4 . 04
  1.00      4 . 9
  5.00 Extremes    (>=.54)

Stem width:      .100
Each leaf:       1 case(s)
```

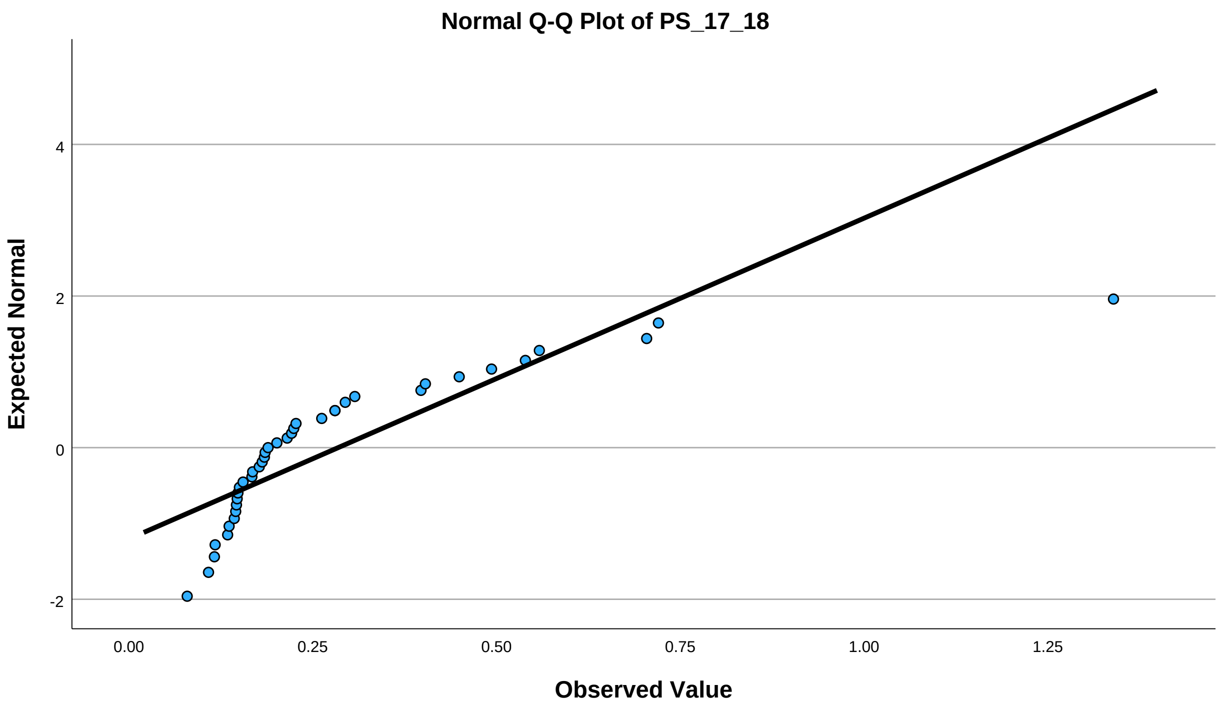

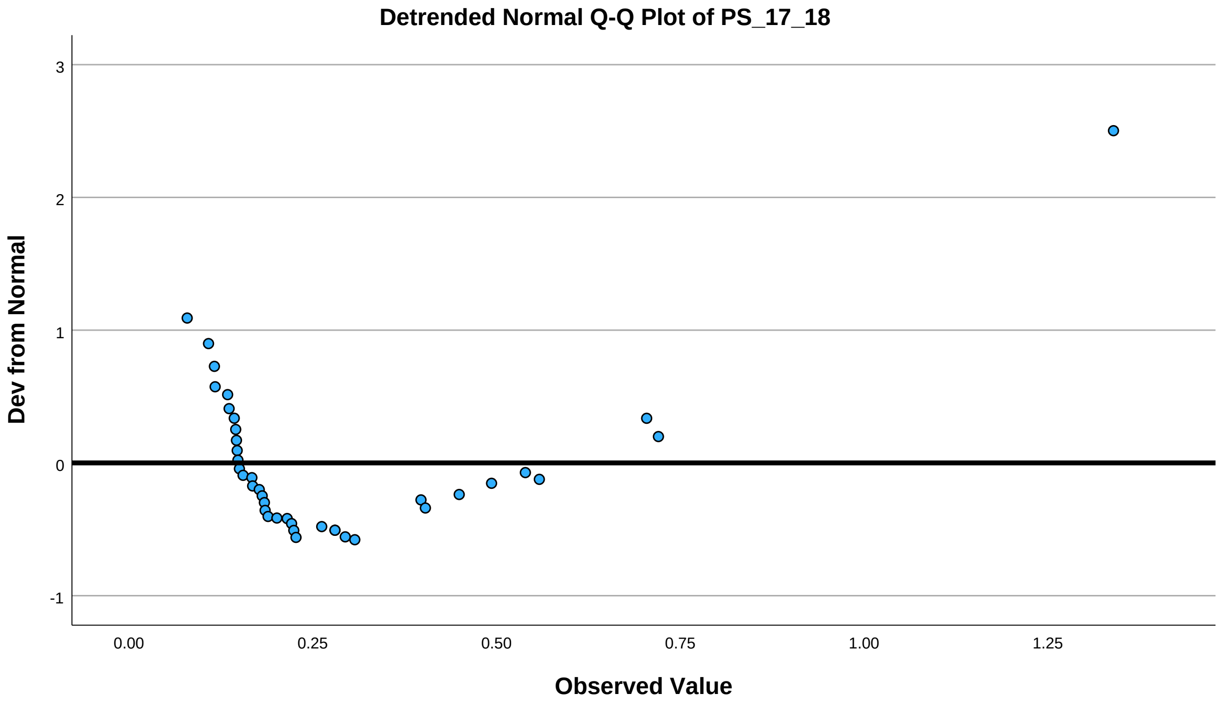
Detrended Normal Q-Q Plot of PS_17_18
Dev from Normal
3
2
1
0
-1
0.00
0.25
0.50
0.75
1.00
1.25
Observed Value

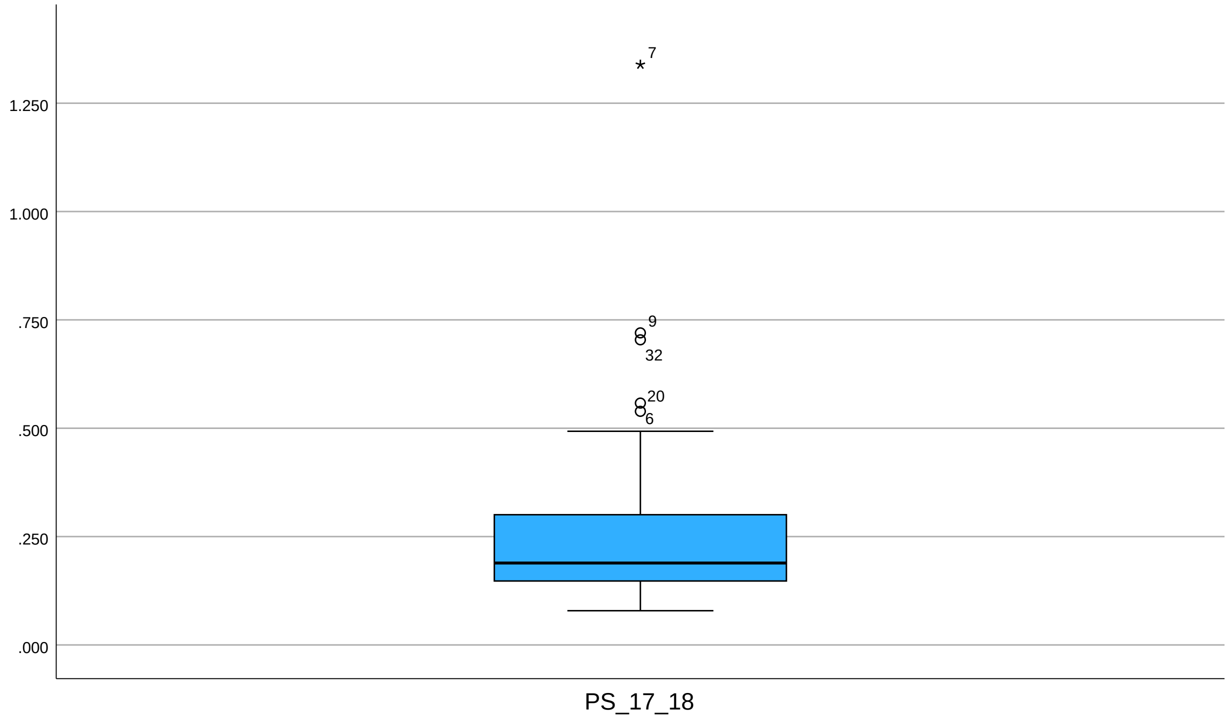
7
1.250
1.000
.750
9
32
20
6
.500
.250
.000
PS_17_18

PS_18_19

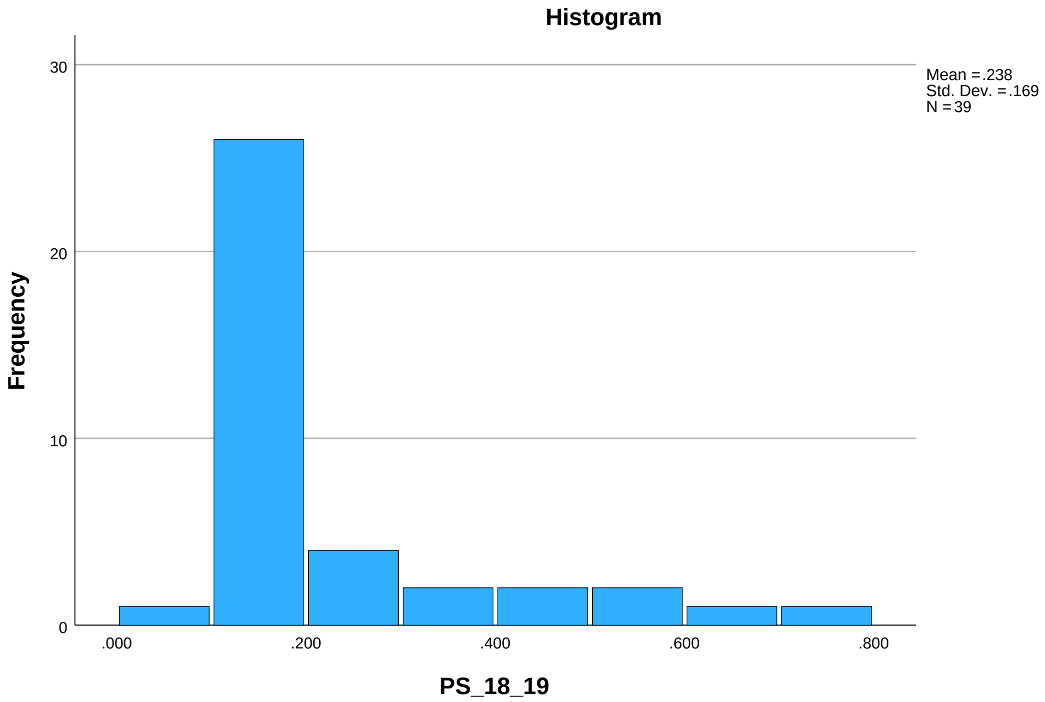


```
PS_18_19 Stem-and-Leaf Plot

 Frequency    Stem &  Leaf

     1.00        0 .  7
    11.00        1 .  00011222344
    15.00        1 .  555666778889999
     2.00        2 .  44
     2.00        2 .  58
     2.00        3 .  04
     6.00 Extremes    (>=.47)

 Stem width:      .100
 Each leaf:        1 case(s)
```

Normal Q-Q Plot of PS_18_19

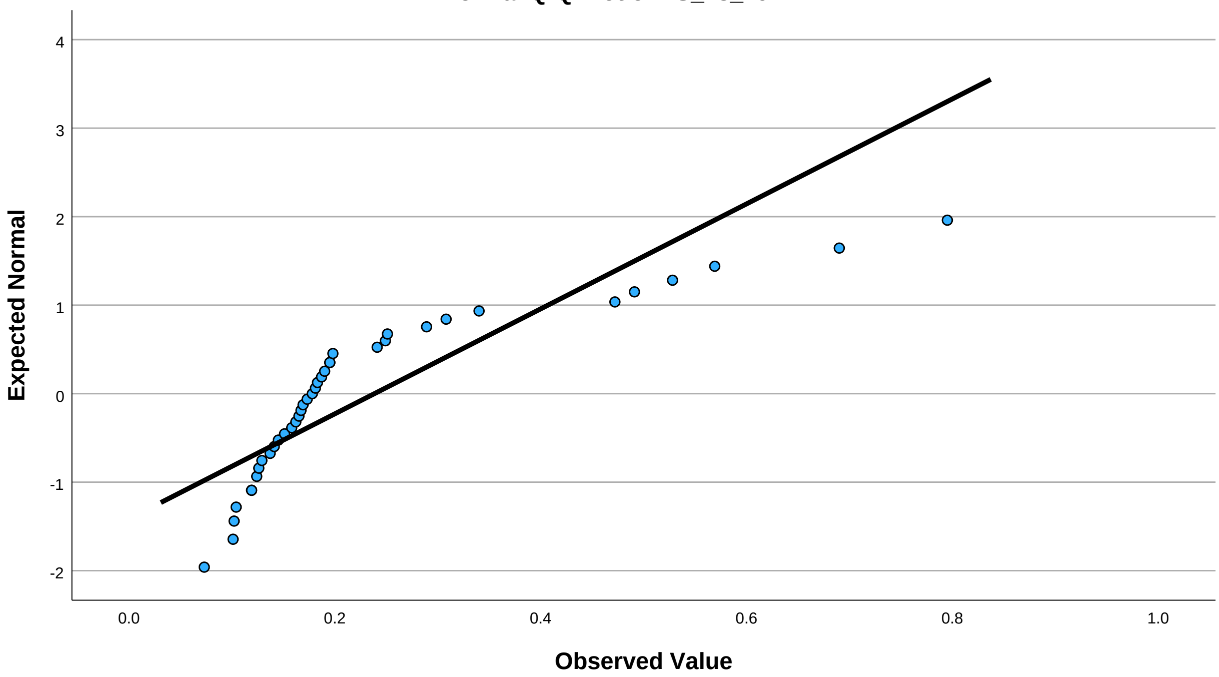

Detrended Normal Q-Q Plot of PS_18_19

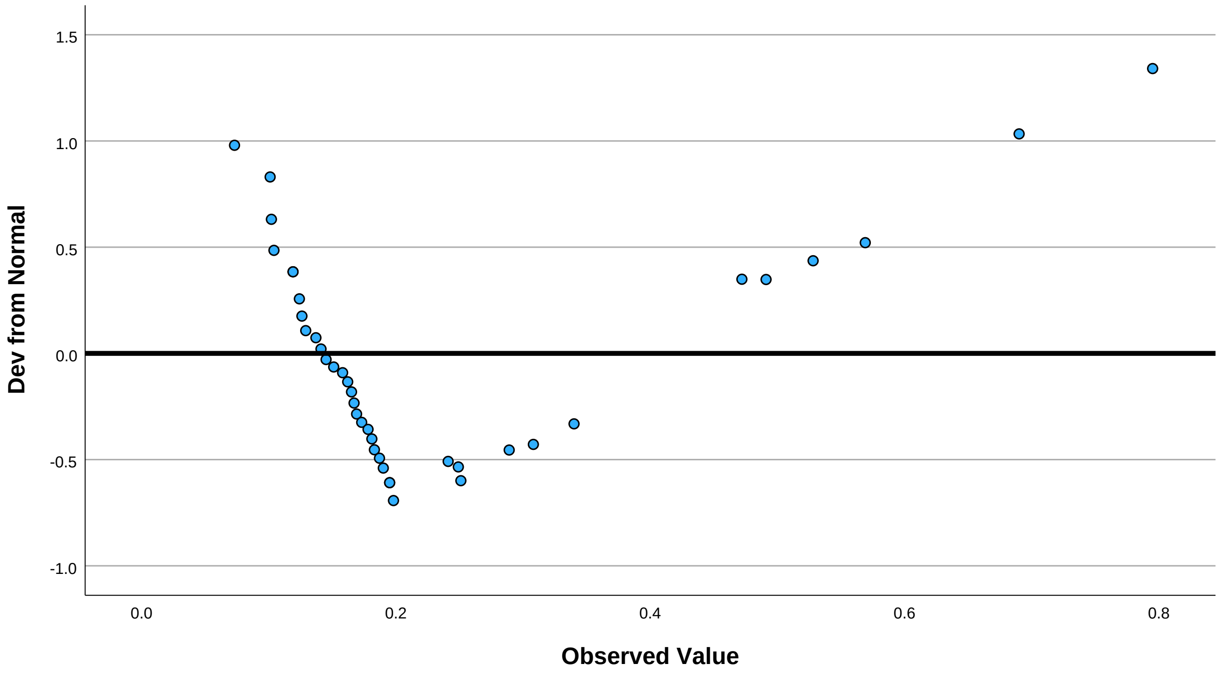

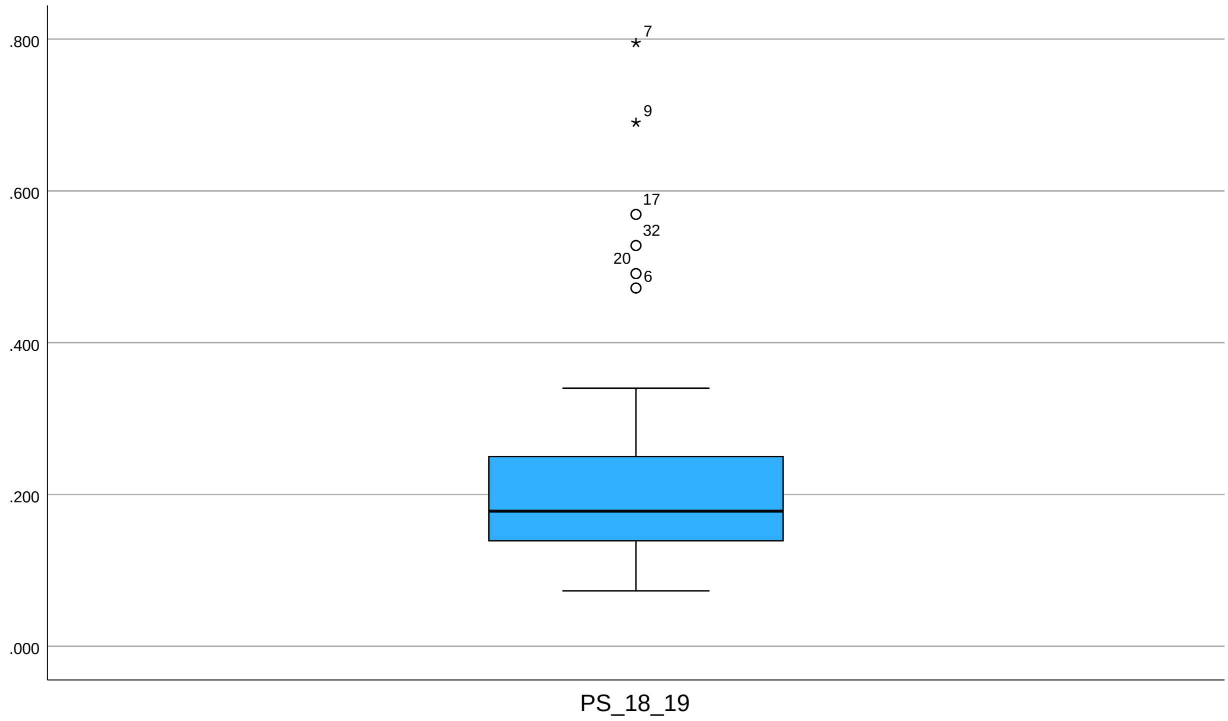

PS_19_20

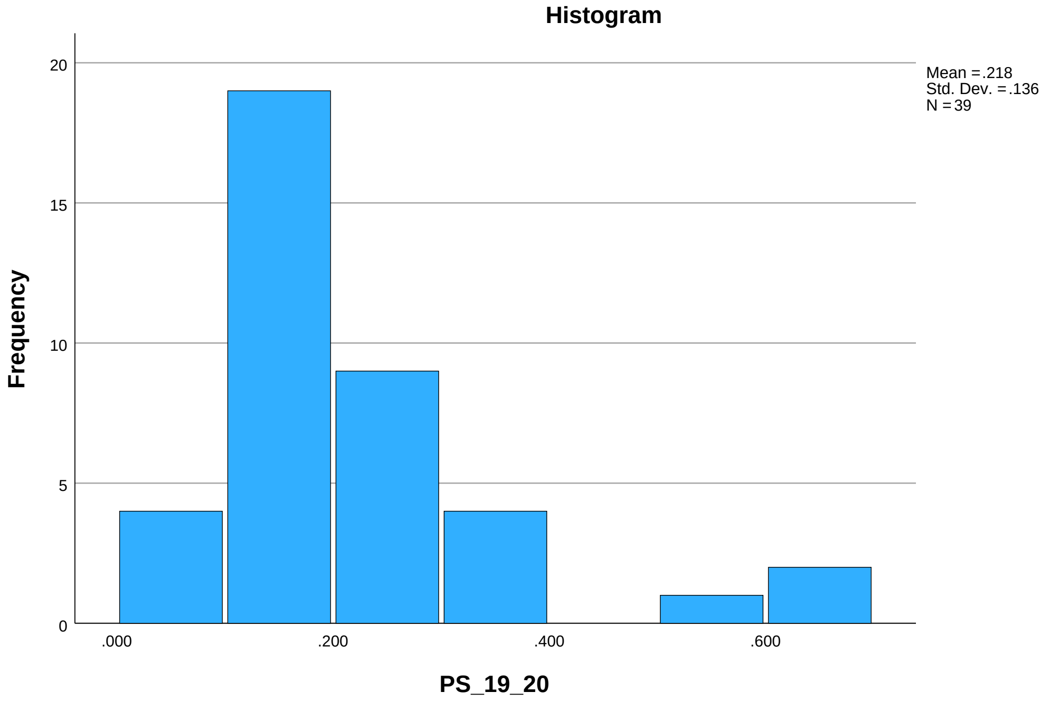


```
PS_19_20 Stem-and-Leaf Plot

 Frequency    Stem &  Leaf
```

```
    4.00       0 .  7899
     9.00        1 .  011222244
    10.00        1 .  5566668899
     8.00        2 .  00112444
    1.00      2 .  6
    2.00      3 .  01
    2.00      3 .  99
     3.00 Extremes    (>=.58)

Stem width:       .100
Each leaf:        1 case(s)
```

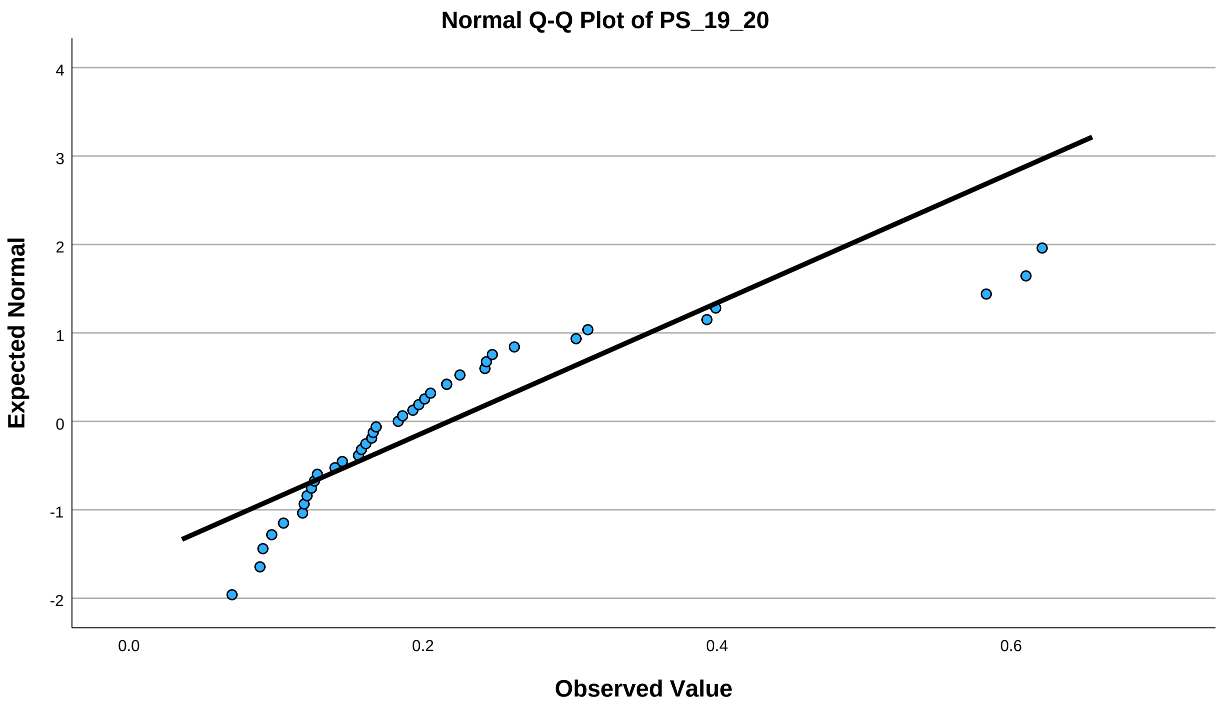

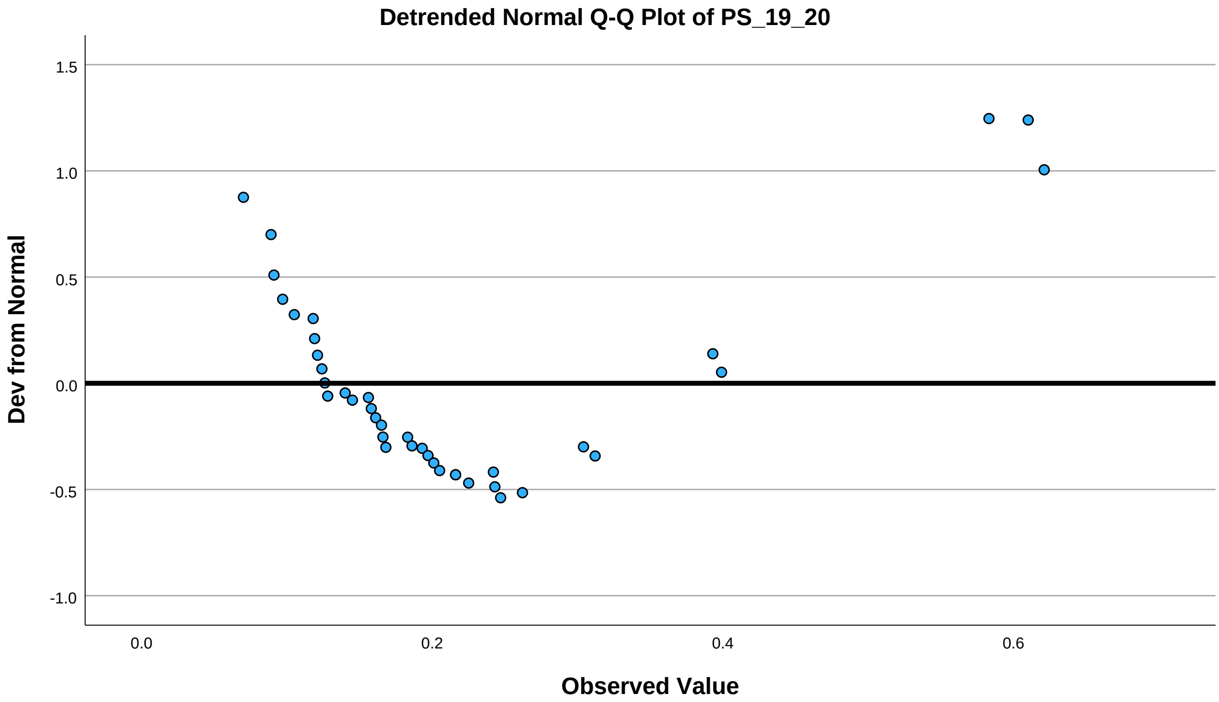
Detrended Normal Q-Q Plot of PS_19_20
Dev from Normal
1.5
1.0
0.5
0.0
-0.5
-1.0
0.0
0.2
0.4
0.6
Observed Value

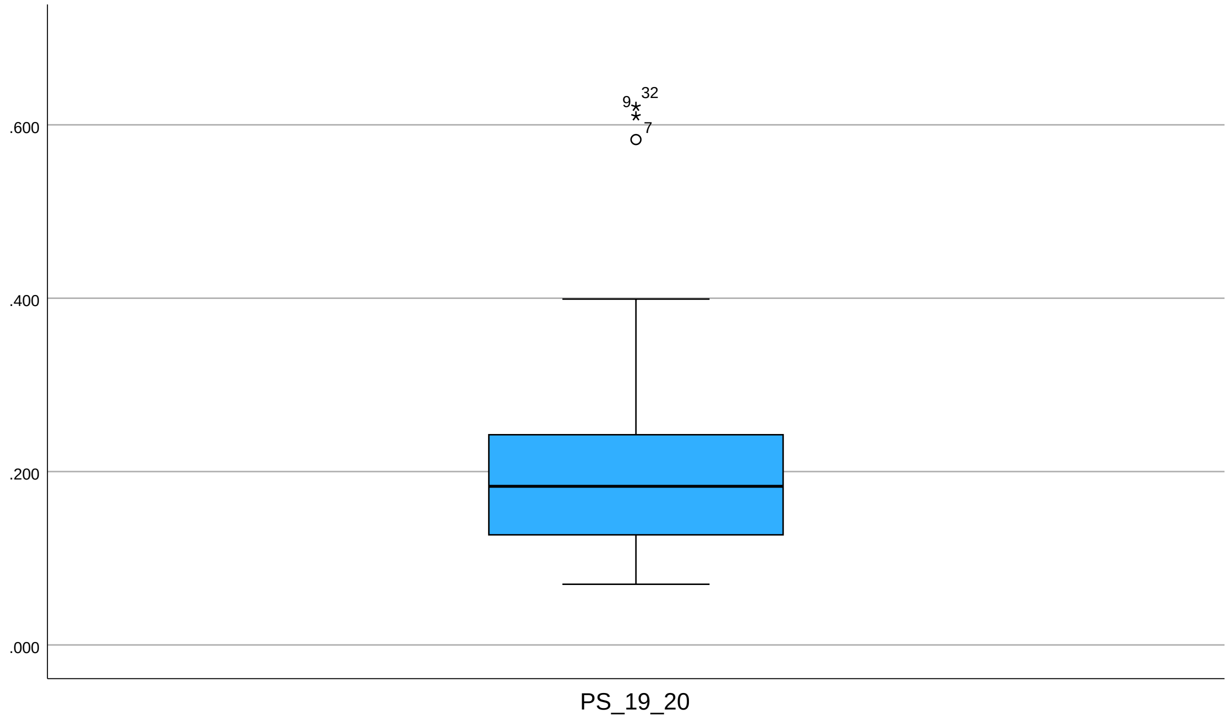
.600
.400
.200
.000
9
32
7
PS_19_20

Explore

Case Processing Summary

	Cases					
	Valid		Missing		Total	
	N	Percent	N	Percent	N	Percent
BrainRate	39	100.0%	0	0.0%	39	100.0%
PermutationEntropy	39	100.0%	0	0.0%	39	100.0%
PermutationEntropy_01	39	100.0%	0	0.0%	39	100.0%
SampleEntropy	39	100.0%	0	0.0%	39	100.0%
SpectralEntropy_bits	39	100.0%	0	0.0%	39	100.0%
SpectralEntropy_01	39	100.0%	0	0.0%	39	100.0%
HiguchiFractalDimension	39	100.0%	0	0.0%	39	100.0%
LempelZivComplexity	39	100.0%	0	0.0%	39	100.0%
RenyiEntropy	39	100.0%	0	0.0%	39	100.0%
TsallisEntropy	39	100.0%	0	0.0%	39	100.0%
PS_4_5	39	100.0%	0	0.0%	39	100.0%
PS_5_6	39	100.0%	0	0.0%	39	100.0%
PS_6_7	39	100.0%	0	0.0%	39	100.0%
PS_7_8	39	100.0%	0	0.0%	39	100.0%
PS_8_9	39	100.0%	0	0.0%	39	100.0%
PS_9_10	39	100.0%	0	0.0%	39	100.0%
PS_10_11	39	100.0%	0	0.0%	39	100.0%
PS_11_12	39	100.0%	0	0.0%	39	100.0%
PS_12_13	39	100.0%	0	0.0%	39	100.0%
PS_13_14	39	100.0%	0	0.0%	39	100.0%
PS_14_15	39	100.0%	0	0.0%	39	100.0%
PS_15_16	39	100.0%	0	0.0%	39	100.0%
PS_16_17	39	100.0%	0	0.0%	39	100.0%
PS_17_18	39	100.0%	0	0.0%	39	100.0%
PS_18_19	39	100.0%	0	0.0%	39	100.0%
PS_19_20	39	100.0%	0	0.0%	39	100.0%

Descriptives

			Statistic	Std. Error
BrainRate	Mean		7.7072	.36621
	95% Confidence Interval for Mean	Lower Bound	6.9658	
		Upper Bound	8.4485	
	5% Trimmed Mean		7.6136	
	Median		7.1100	
	Variance		5.230	
	Std. Deviation		2.28695	
	Minimum		3.33	
	Maximum		14.65	
	Range		11.32	
	Interquartile Range		2.65	
	Skewness		.808	.378
	Kurtosis		.969	.741
PermutationEntropy	Mean		1.4805683908	.00903785000
	95% Confidence Interval for Mean	Lower Bound	1.4622722200	
		Upper Bound	1.4988645616	
	5% Trimmed Mean		1.4812419427	
	Median		1.4742297714	
	Variance		.003	
	Std. Deviation		.05644135517	
	Minimum		1.3534356954	
	Maximum		1.6103614210	
	Range		.25692572562	
	Interquartile Range		.08084887840	
	Skewness		.027	.378
	Kurtosis		-.052	.741
PermutationEntropy_01	Mean		.82632095222	.00504412013
	95% Confidence Interval for Mean	Lower Bound	.81610966487	
		Upper Bound	.83653223957	
	5% Trimmed Mean		.82669686871	
	Median		.82278330138	
	Variance		.001	
	Std. Deviation		.03150052010	
	Minimum		.75536684394	
	Maximum		.89875982164	
	Range		.14339297770	
	Interquartile Range		.04512261818	
	Skewness		.027	.378
	Kurtosis		-.052	.741

Descriptives

			Statistic	Std. Error
SampleEntropy	Mean		.67855731398	.02670435793
	95% Confidence Interval for Mean	Lower Bound	.62449716763	
		Upper Bound	.73261746033	
	5% Trimmed Mean		.68766784031	
	Median		.69254681461	
	Variance		.028	
	Std. Deviation		.16676866184	
	Minimum		.18483819640	
	Maximum		1.0666036481	
	Range		.88176545168	
	Interquartile Range		.17854831245	
	Skewness		-.938	.378
	Kurtosis		2.109	.741
SpectralEntropy_bits	Mean		4.1193801760	.07637191336
	95% Confidence Interval for Mean	Lower Bound	3.9647733203	
		Upper Bound	4.2739870317	
	5% Trimmed Mean		4.1481642719	
	Median		4.1632896256	
	Variance		.227	
	Std. Deviation		.47694244604	
	Minimum		2.5070915800	
	Maximum		5.1043549333	
	Range		2.5972633533	
	Interquartile Range		.47496252255	
	Skewness		-1.087	.378
	Kurtosis		2.855	.741
SpectralEntropy_01	Mean		.58754052977	.01089280244
	95% Confidence Interval for Mean	Lower Bound	.56548920408	
		Upper Bound	.60959185546	
	5% Trimmed Mean		.59164595880	
	Median		.59380326353	
	Variance		.005	
	Std. Deviation		.06802552944	
	Minimum		.35758241585	
	Maximum		.72802588582	
	Range		.37044346998	
	Interquartile Range		.06774313615	
	Skewness		-1.087	.378
	Kurtosis		2.855	.741

Descriptives

			Statistic	Std. Error
HiguchiFractalDimension	Mean		.56187558985	.01540909900
	95% Confidence Interval for Mean	Lower Bound	.53068149976	
		Upper Bound	.59306967994	
	5% Trimmed Mean		.56406283142	
	Median		.59111391886	
	Variance		.009	
	Std. Deviation		.09622979242	
	Minimum		.37534901650	
	Maximum		.70940257323	
	Range		.33405355673	
	Interquartile Range		.18128955657	
	Skewness		-.520	.378
	Kurtosis		-.928	.741
LempelZivComplexity	Mean		.27083017796	.00462525679
	95% Confidence Interval for Mean	Lower Bound	.26146683511	
		Upper Bound	.28019352081	
	5% Trimmed Mean		.26876399748	
	Median		.26332809678	
	Variance		.001	
	Std. Deviation		.02888471939	
	Minimum		.23236927103	
	Maximum		.34905698724	
	Range		.11668771621	
	Interquartile Range		.03488410546	
	Skewness		1.096	.378
	Kurtosis		.696	.741
RenyiEntropy	Mean		7.4096820041	.16299190240
	95% Confidence Interval for Mean	Lower Bound	7.0797221481	
		Upper Bound	7.7396418601	
	5% Trimmed Mean		7.4555522842	
	Median		7.6548842764	
	Variance		1.036	
	Std. Deviation		1.0178841043	
	Minimum		5.2667590973	
	Maximum		8.7231736002	
	Range		3.4564145029	
	Interquartile Range		1.8929640160	
	Skewness		-.623	.378
	Kurtosis		-.679	.741

Descriptives

			Statistic	Std. Error
TsallisEntropy	Mean		1.5777456516	.08338622199
	95% Confidence Interval for Mean	Lower Bound	1.4089390705	
		Upper Bound	1.7465522328	
	5% Trimmed Mean		1.6168819603	
	Median		1.8148924918	
	Variance		.271	
	Std. Deviation		.52074678941	
	Minimum		.38305261510	
	Maximum		1.9867754733	
	Range		1.6037228582	
	Interquartile Range		.85494271399	
	Skewness		-1.096	.378
	Kurtosis		-.257	.741
PS_4_5	Mean		3.35510	.500531
	95% Confidence Interval for Mean	Lower Bound	2.34183	
		Upper Bound	4.36838	
	5% Trimmed Mean		2.91079	
	Median		2.49100	
	Variance		9.771	
	Std. Deviation		3.125817	
	Minimum		.755	
	Maximum		16.890	
	Range		16.135	
	Interquartile Range		1.930	
	Skewness		2.757	.378
	Kurtosis		9.059	.741
PS_5_6	Mean		2.67513	.432849
	95% Confidence Interval for Mean	Lower Bound	1.79887	
		Upper Bound	3.55138	
	5% Trimmed Mean		2.25884	
	Median		1.76000	
	Variance		7.307	
	Std. Deviation		2.703140	
	Minimum		.540	
	Maximum		15.550	
	Range		15.010	
	Interquartile Range		1.750	
	Skewness		3.345	.378
	Kurtosis		13.592	.741

Descriptives

			Statistic	Std. Error
PS_6_7	Mean		1.84131	.218130
	95% Confidence Interval for Mean	Lower Bound	1.39973	
		Upper Bound	2.28289	
	5% Trimmed Mean		1.69362	
	Median		1.37300	
	Variance		1.856	
	Std. Deviation		1.362222	
	Minimum		.371	
	Maximum		7.660	
	Range		7.289	
	Interquartile Range		1.515	
	Skewness		2.358	.378
	Kurtosis		7.770	.741
PS_7_8	Mean		1.60521	.209003
	95% Confidence Interval for Mean	Lower Bound	1.18210	
		Upper Bound	2.02831	
	5% Trimmed Mean		1.46139	
	Median		1.09500	
	Variance		1.704	
	Std. Deviation		1.305226	
	Minimum		.322	
	Maximum		6.392	
	Range		6.070	
	Interquartile Range		1.028	
	Skewness		2.042	.378
	Kurtosis		4.198	.741
PS_8_9	Mean		1.64213	.268976
	95% Confidence Interval for Mean	Lower Bound	1.09762	
		Upper Bound	2.18664	
	5% Trimmed Mean		1.37880	
	Median		1.17600	
	Variance		2.822	
	Std. Deviation		1.679753	
	Minimum		.277	
	Maximum		8.871	
	Range		8.594	
	Interquartile Range		.674	
	Skewness		3.033	.378
	Kurtosis		9.982	.741

Descriptives

			Statistic	Std. Error
PS_9_10	Mean		1.59203	.251414
	95% Confidence Interval for Mean	Lower Bound	1.08306	
		Upper Bound	2.10099	
	5% Trimmed Mean		1.34704	
	Median		1.16400	
	Variance		2.465	
	Std. Deviation		1.570083	
	Minimum		.233	
	Maximum		9.118	
	Range		8.885	
	Interquartile Range		.978	
	Skewness		3.448	.378
	Kurtosis		14.319	.741
PS_10_11	Mean		2.03890	.265420
	95% Confidence Interval for Mean	Lower Bound	1.50158	
		Upper Bound	2.57621	
	5% Trimmed Mean		1.84995	
	Median		1.54100	
	Variance		2.747	
	Std. Deviation		1.657550	
	Minimum		.276	
	Maximum		7.540	
	Range		7.264	
	Interquartile Range		1.532	
	Skewness		1.830	.378
	Kurtosis		3.441	.741
PS_11_12	Mean		1.00249	.118663
	95% Confidence Interval for Mean	Lower Bound	.76227	
		Upper Bound	1.24271	
	5% Trimmed Mean		.92272	
	Median		.71400	
	Variance		.549	
	Std. Deviation		.741050	
	Minimum		.179	
	Maximum		3.460	
	Range		3.281	
	Interquartile Range		.502	
	Skewness		1.803	.378
	Kurtosis		3.114	.741

Descriptives

			Statistic	Std. Error
PS_12_13	Mean		.76731	.080063
	95% Confidence Interval for Mean	Lower Bound	.60523	
		Upper Bound	.92939	
	5% Trimmed Mean		.72106	
	Median		.56900	
	Variance		.250	
	Std. Deviation		.499993	
	Minimum		.155	
	Maximum		2.367	
	Range		2.212	
	Interquartile Range		.570	
	Skewness		1.443	.378
	Kurtosis		1.901	.741
PS_13_14	Mean		.72082	.084265
	95% Confidence Interval for Mean	Lower Bound	.55023	
		Upper Bound	.89141	
	5% Trimmed Mean		.66873	
	Median		.57100	
	Variance		.277	
	Std. Deviation		.526235	
	Minimum		.162	
	Maximum		2.301	
	Range		2.139	
	Interquartile Range		.605	
	Skewness		1.572	.378
	Kurtosis		2.057	.741
PS_14_15	Mean		.66833	.076326
	95% Confidence Interval for Mean	Lower Bound	.51382	
		Upper Bound	.82285	
	5% Trimmed Mean		.62380	
	Median		.51600	
	Variance		.227	
	Std. Deviation		.476657	
	Minimum		.137	
	Maximum		2.032	
	Range		1.895	
	Interquartile Range		.654	
	Skewness		1.382	.378
	Kurtosis		1.475	.741

Descriptives

			Statistic	Std. Error
PS_15_16	Mean		.57515	.068475
	95% Confidence Interval for Mean	Lower Bound	.43653	
		Upper Bound	.71377	
	5% Trimmed Mean		.53278	
	Median		.41900	
	Variance		.183	
	Std. Deviation		.427626	
	Minimum		.120	
	Maximum		1.962	
	Range		1.842	
	Interquartile Range		.505	
	Skewness		1.629	.378
	Kurtosis		2.393	.741
PS_16_17	Mean		.45554	.048824
	95% Confidence Interval for Mean	Lower Bound	.35670	
		Upper Bound	.55438	
	5% Trimmed Mean		.42718	
	Median		.33300	
	Variance		.093	
	Std. Deviation		.304904	
	Minimum		.116	
	Maximum		1.343	
	Range		1.227	
	Interquartile Range		.375	
	Skewness		1.469	.378
	Kurtosis		1.698	.741
PS_17_18	Mean		.39051	.044291
	95% Confidence Interval for Mean	Lower Bound	.30085	
		Upper Bound	.48018	
	5% Trimmed Mean		.35685	
	Median		.29500	
	Variance		.077	
	Std. Deviation		.276600	
	Minimum		.120	
	Maximum		1.534	
	Range		1.414	
	Interquartile Range		.336	
	Skewness		2.245	.378
	Kurtosis		6.847	.741

Descriptives

			Statistic	Std. Error
PS_18_19	Mean		.35192	.046161
	95% Confidence Interval for Mean	Lower Bound	.25847	
		Upper Bound	.44537	
	5% Trimmed Mean		.31049	
	Median		.27200	
	Variance		.083	
	Std. Deviation		.288278	
	Minimum		.103	
	Maximum		1.675	
	Range		1.572	
	Interquartile Range		.254	
	Skewness		2.995	.378
	Kurtosis		11.425	.741
PS_19_20	Mean		.31326	.034819
	95% Confidence Interval for Mean	Lower Bound	.24277	
		Upper Bound	.38374	
	5% Trimmed Mean		.28738	
	Median		.25500	
	Variance		.047	
	Std. Deviation		.217442	
	Minimum		.098	
	Maximum		1.159	
	Range		1.061	
	Interquartile Range		.182	
	Skewness		2.097	.378
	Kurtosis		5.418	.741

Tests of Normality

	Kolmogorov-Smirnov[a]			Shapiro-Wilk		
	Statistic	df	Sig.	Statistic	df	Sig.
BrainRate	.125	39	.131	.958	39	.152
PermutationEntropy	.079	39	.200*	.984	39	.853
PermutationEntropy_01	.079	39	.200*	.984	39	.853
SampleEntropy	.172	39	.005	.919	39	.008
SpectralEntropy_bits	.136	39	.065	.932	39	.021
SpectralEntropy_01	.136	39	.065	.932	39	.021
HiguchiFractalDimension	.140	39	.051	.926	39	.014
LempelZivComplexity	.162	39	.011	.901	39	.002
RenyiEntropy	.126	39	.122	.918	39	.007
TsallisEntropy	.233	39	<.001	.773	39	<.001
PS_4_5	.266	39	<.001	.684	39	<.001
PS_5_6	.269	39	<.001	.620	39	<.001
PS_6_7	.190	39	.001	.777	39	<.001
PS_7_8	.288	39	<.001	.740	39	<.001
PS_8_9	.296	39	<.001	.613	39	<.001
PS_9_10	.241	39	<.001	.624	39	<.001
PS_10_11	.188	39	.001	.808	39	<.001
PS_11_12	.226	39	<.001	.799	39	<.001
PS_12_13	.227	39	<.001	.849	39	<.001
PS_13_14	.215	39	<.001	.825	39	<.001
PS_14_15	.199	39	<.001	.850	39	<.001
PS_15_16	.251	39	<.001	.817	39	<.001
PS_16_17	.231	39	<.001	.837	39	<.001
PS_17_18	.203	39	<.001	.784	39	<.001
PS_18_19	.260	39	<.001	.685	39	<.001
PS_19_20	.226	39	<.001	.792	39	<.001

*. This is a lower bound of the true significance.

a. Lilliefors Significance Correction

BrainRate

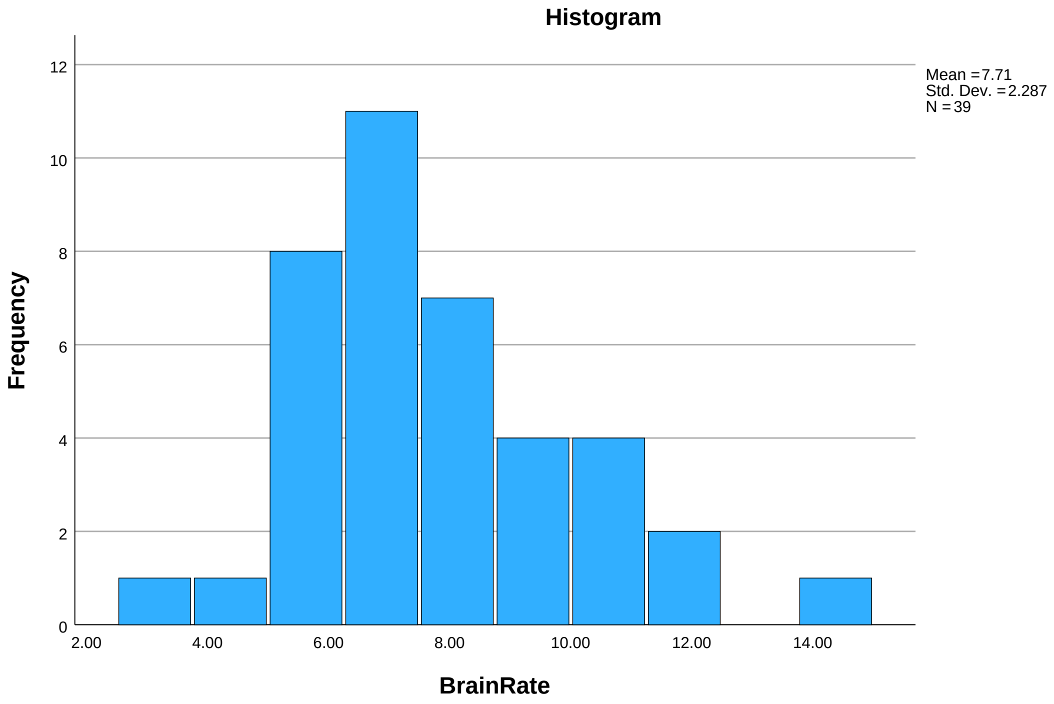


```
BrainRate Stem-and-Leaf Plot

 Frequency    Stem &  Leaf

     1.00        3 .  3
     1.00        4 .  4
     7.00        5 .  1235589
     8.00        6 .  13344558
     6.00        7 .  001289
     7.00        8 .  0045677
     2.00        9 .  56
     3.00       10 .  023
     3.00       11 .  156
     1.00 Extremes    (>=14.7)

 Stem width:      1.00
 Each leaf:       1 case(s)
```

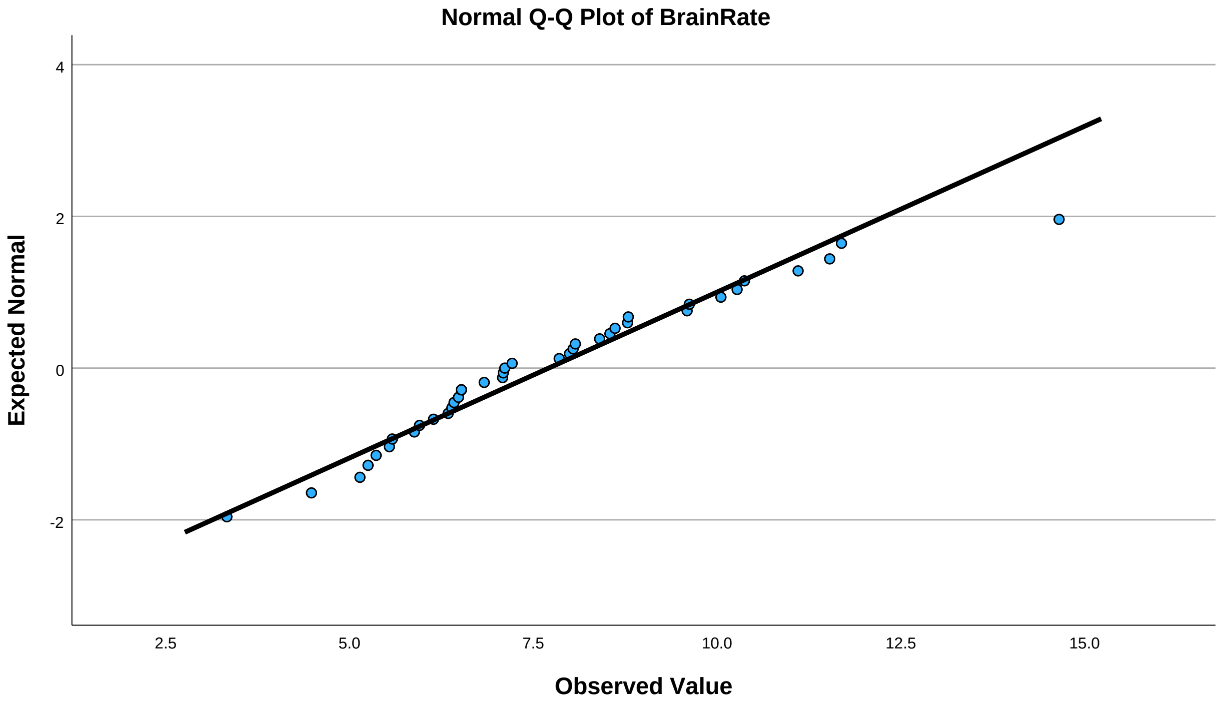
Normal Q-Q Plot of BrainRate
Expected Normal
Observed Value
4
2
0
-2
2.5
5.0
7.5
10.0
12.5
15.0

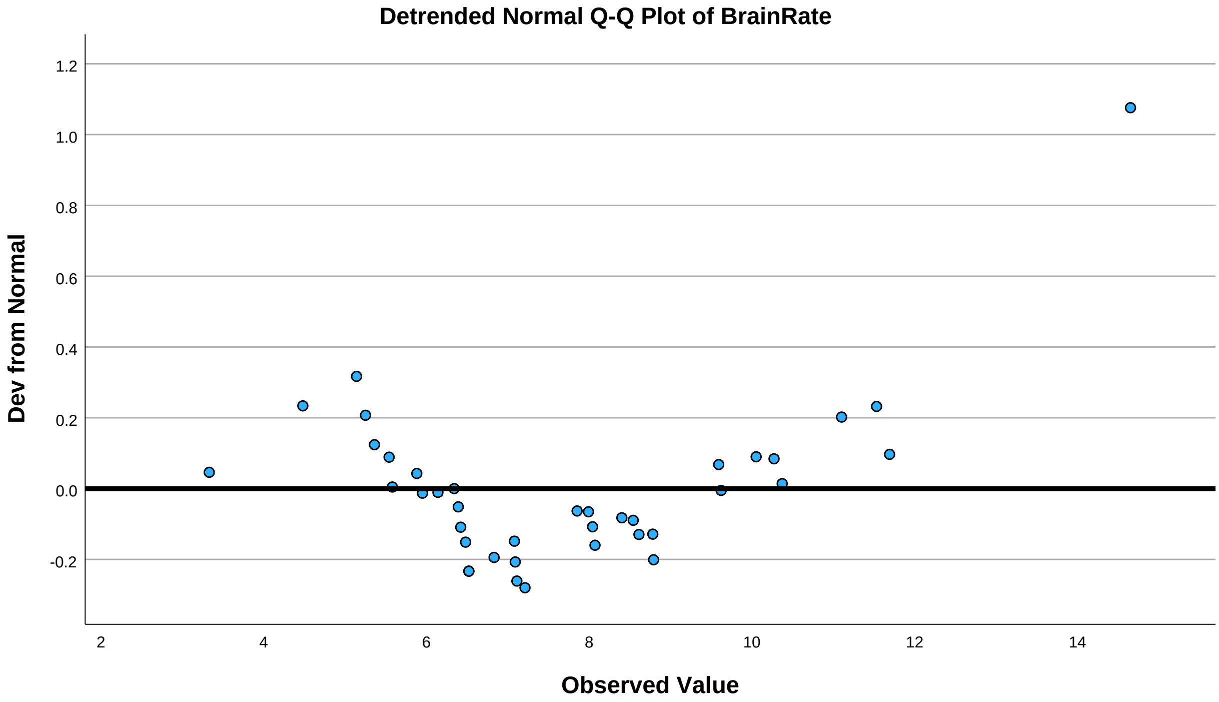
Detrended Normal Q-Q Plot of BrainRate
Dev from Normal
Observed Value
1.2
1.0
0.8
0.6
0.4
0.2
0.0
-0.2
2
4
6
8
10
12
14

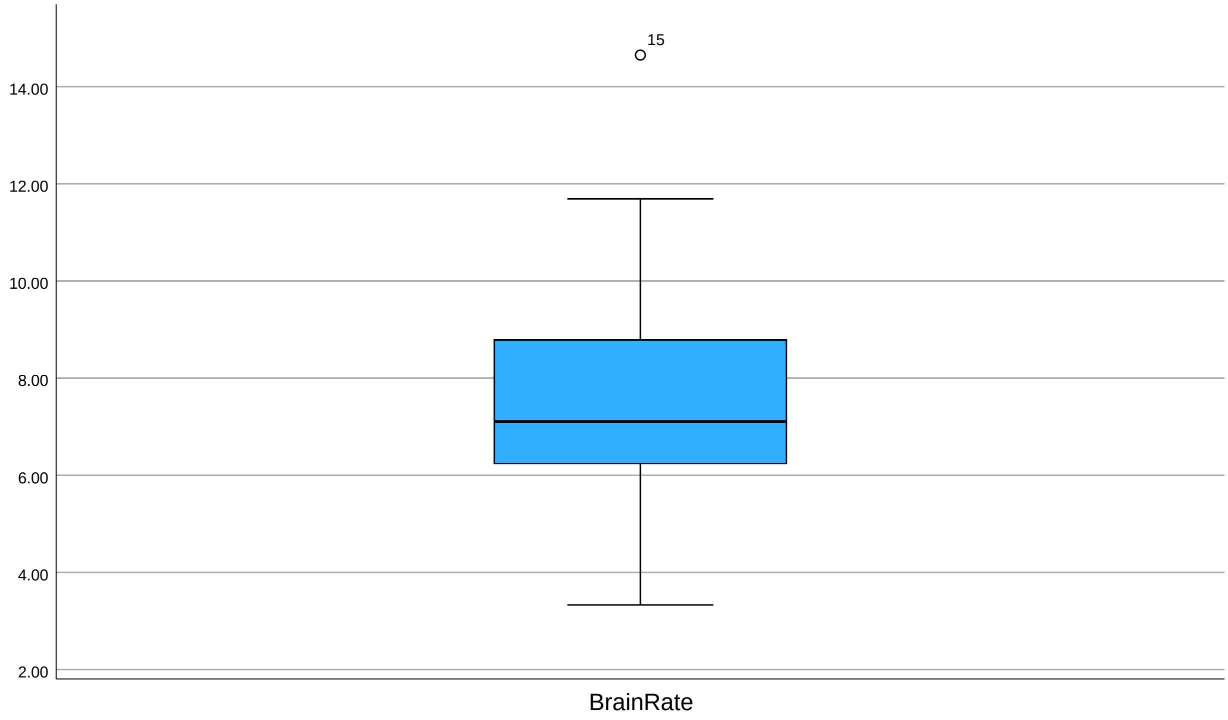

PermutationEntropy

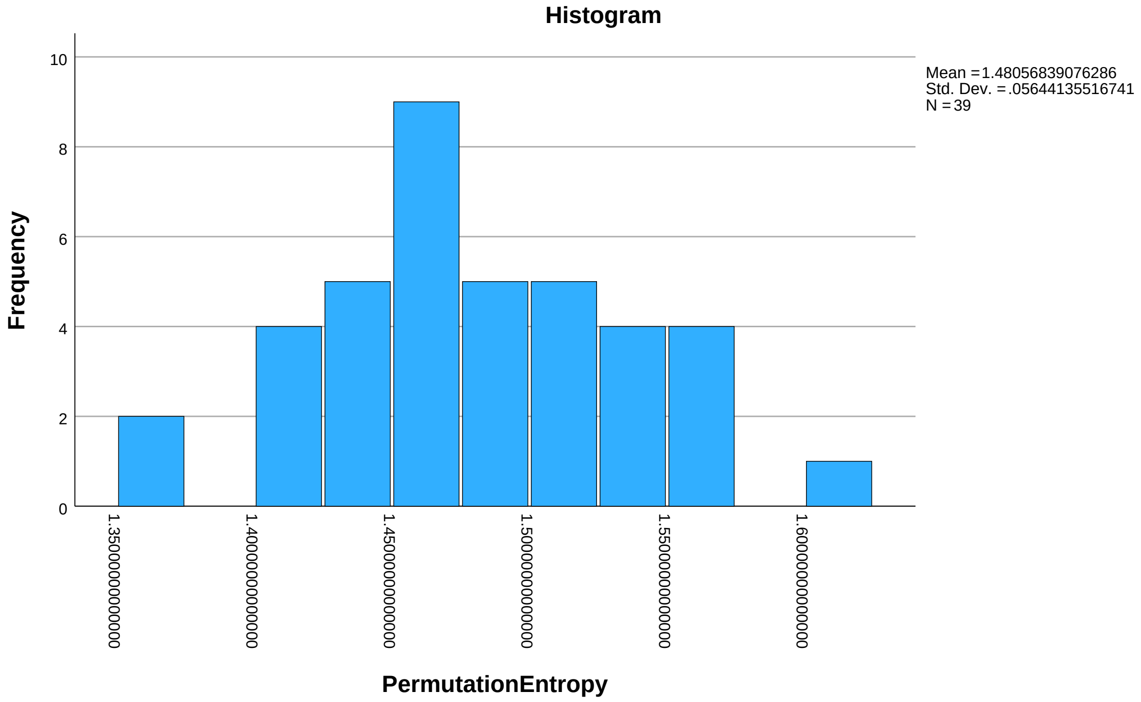

PermutationEntropy Stem-and-Leaf Plot

Frequency   Stem & Leaf

```
  2.00      13 .  56
   9.00      14 .  122223344
  14.00      14 .  55556667777899
   9.00      15 .  011122334
   4.00      15 .  5577
  1.00      16 .  1

Stem width:   .1000000
Each leaf:       1 case(s)
```

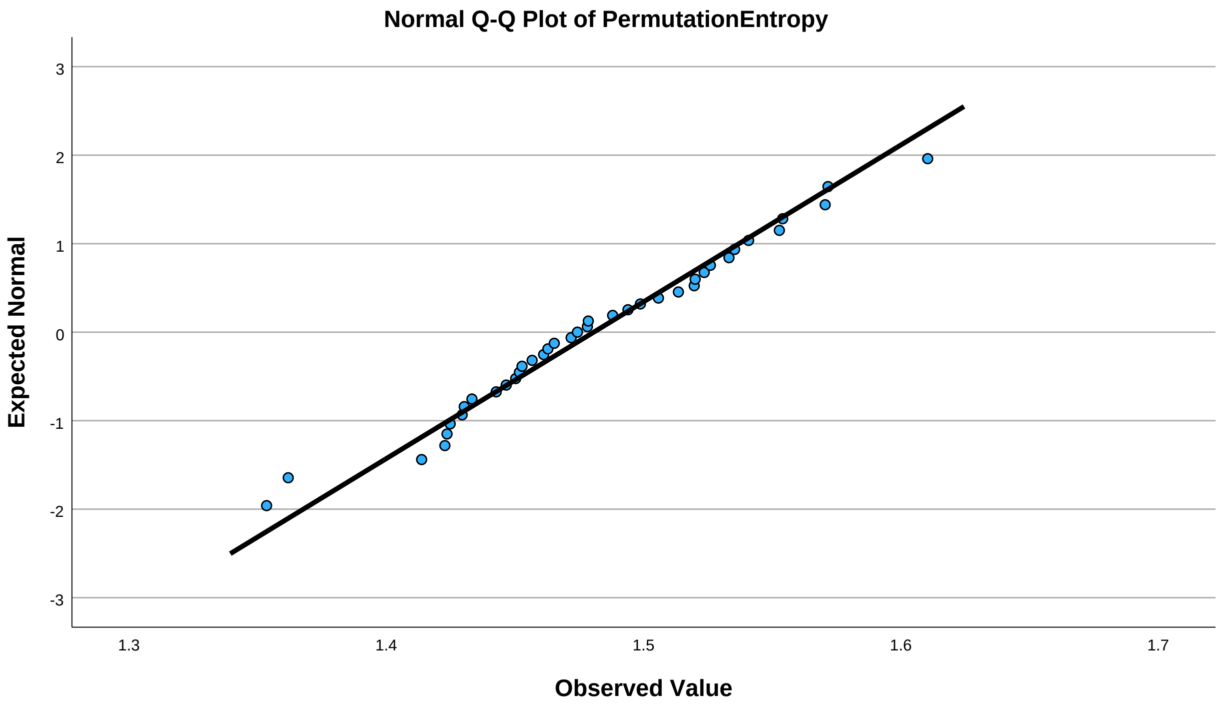

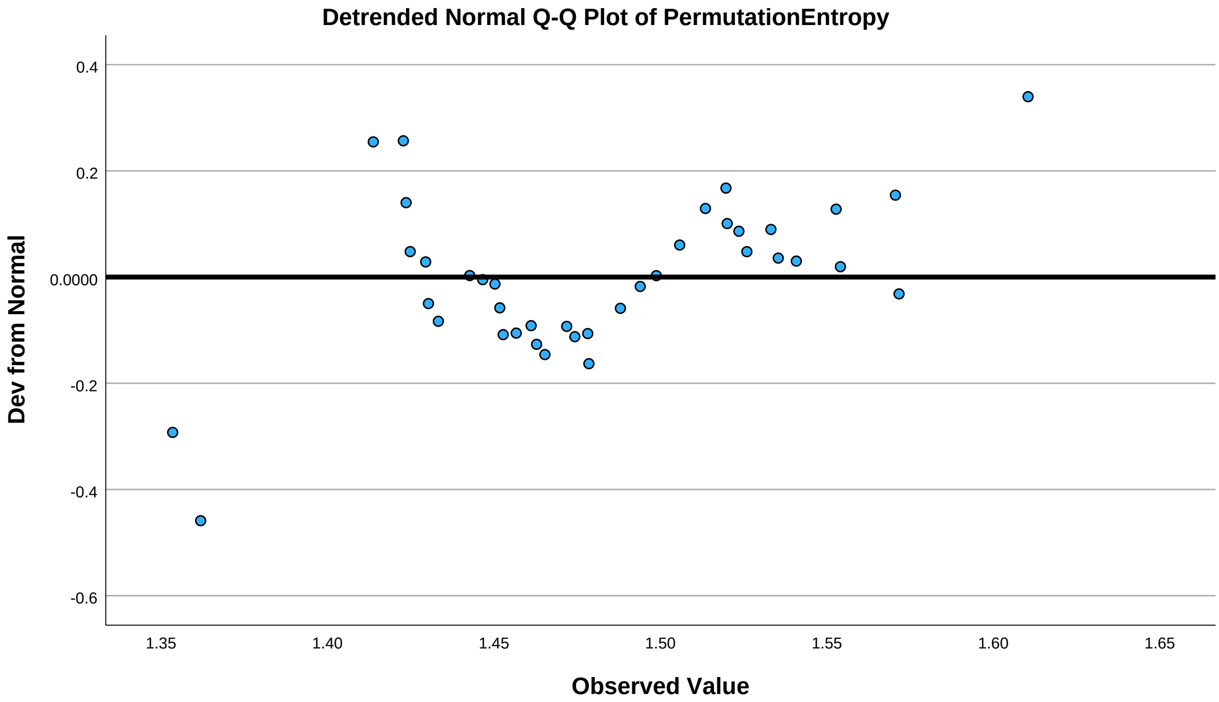

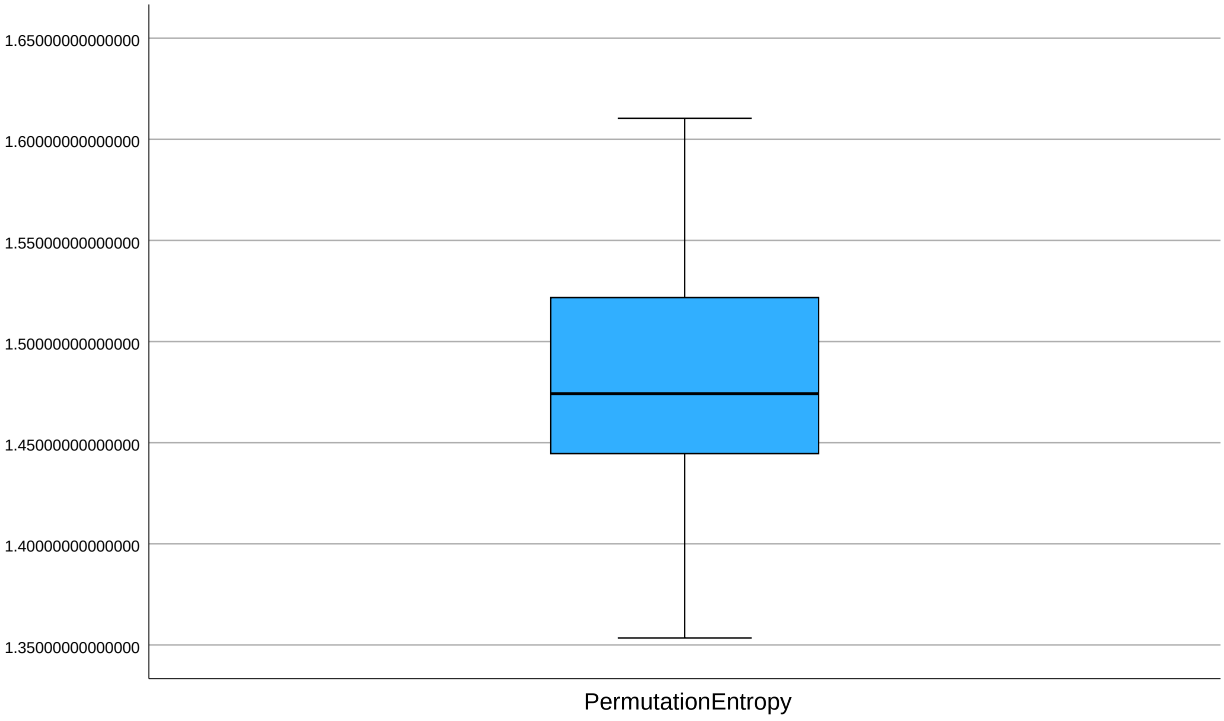

PermutationEntropy_01

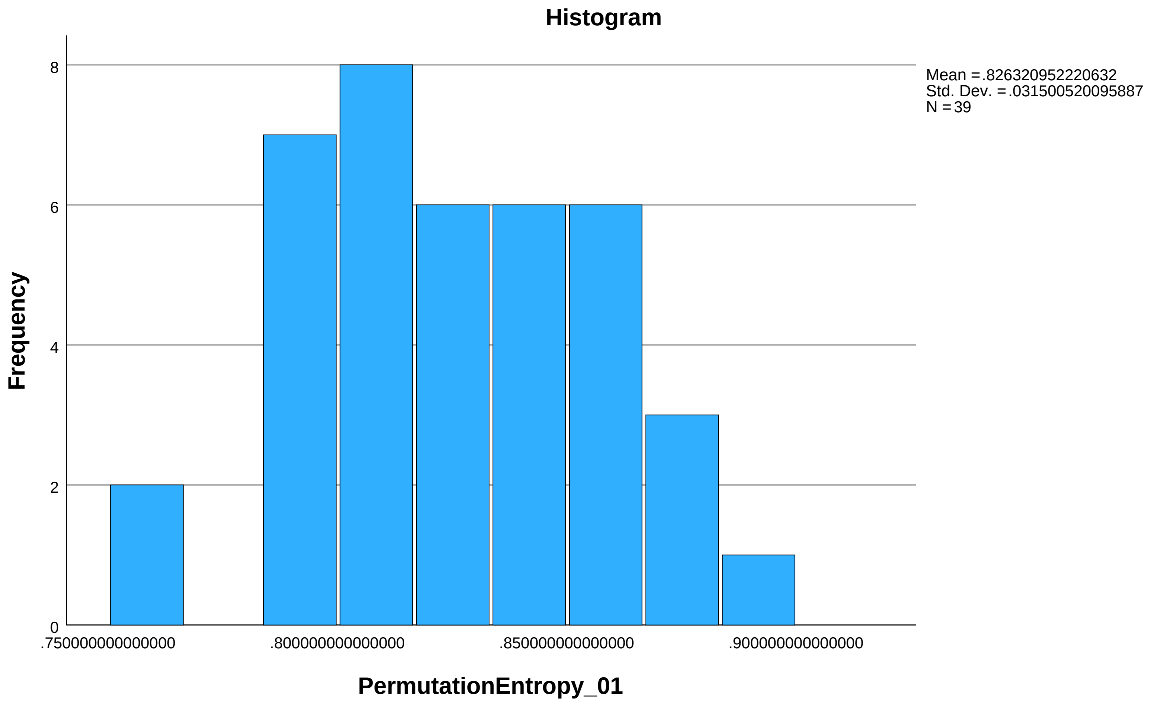


```
PermutationEntropy_01 Stem-and-Leaf Plot

 Frequency    Stem &  Leaf

     1.00        7 .  5
     1.00        7 .  6
     7.00        7 .  8999999
     9.00        8 .  000111111
     7.00        8 .  2222333
     9.00        8 .  444455555
     4.00        8 .  6677
     1.00        8 .  9

 Stem width:   .1000000
 Each leaf:        1 case(s)
```

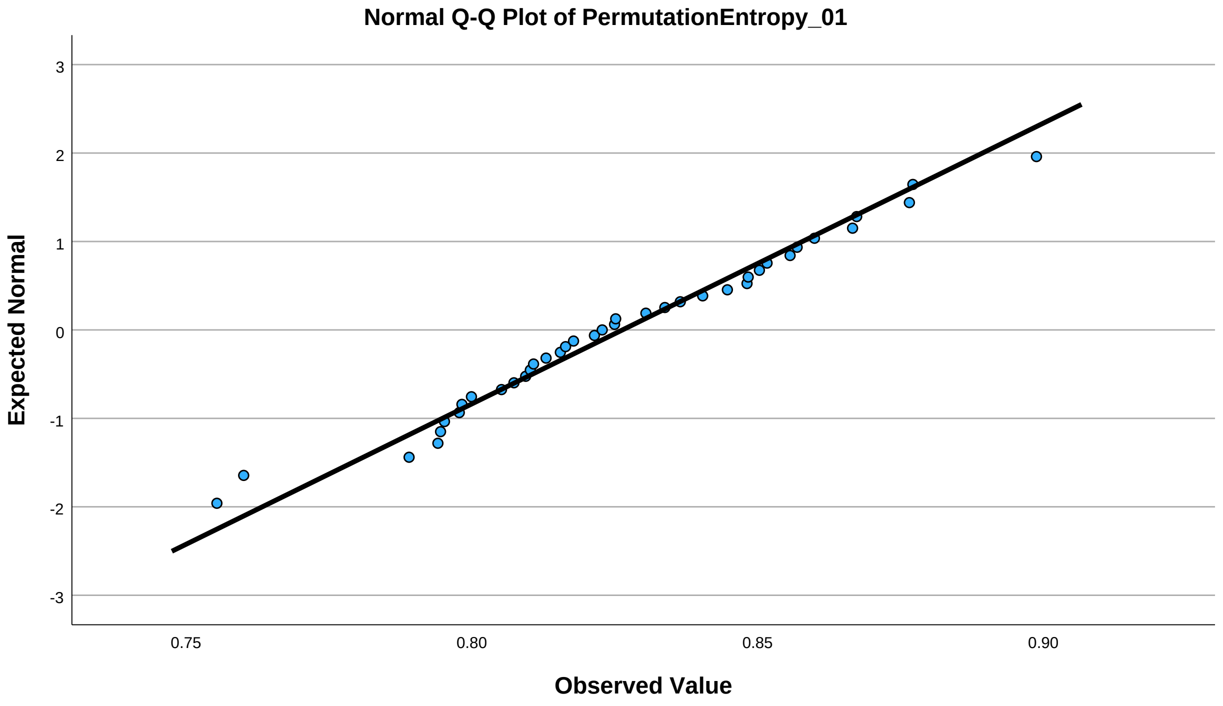
Normal Q-Q Plot of PermutationEntropy_01
Expected Normal
3
2
1
0
-1
-2
-3
0.75
0.80
0.85
0.90
Observed Value

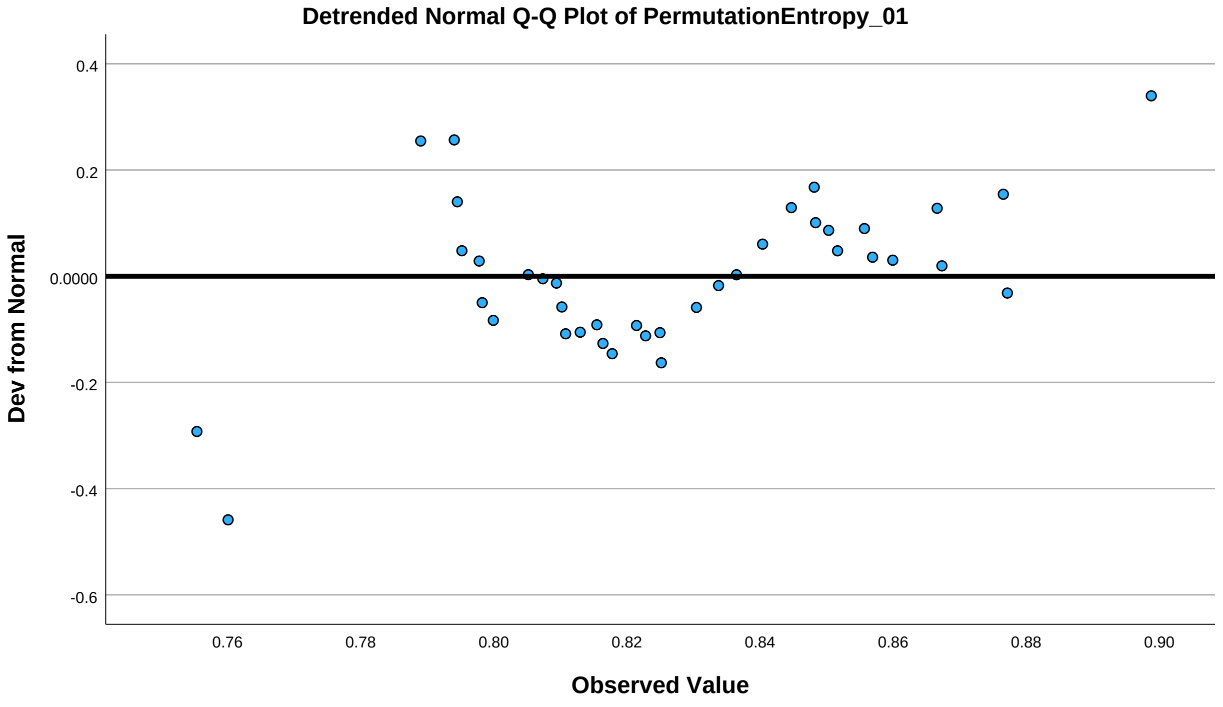
Detrended Normal Q-Q Plot of PermutationEntropy_01
Dev from Normal
0.4
0.2
0.0000
-0.2
-0.4
-0.6
0.76
0.78
0.80
0.82
0.84
0.86
0.88
0.90
Observed Value

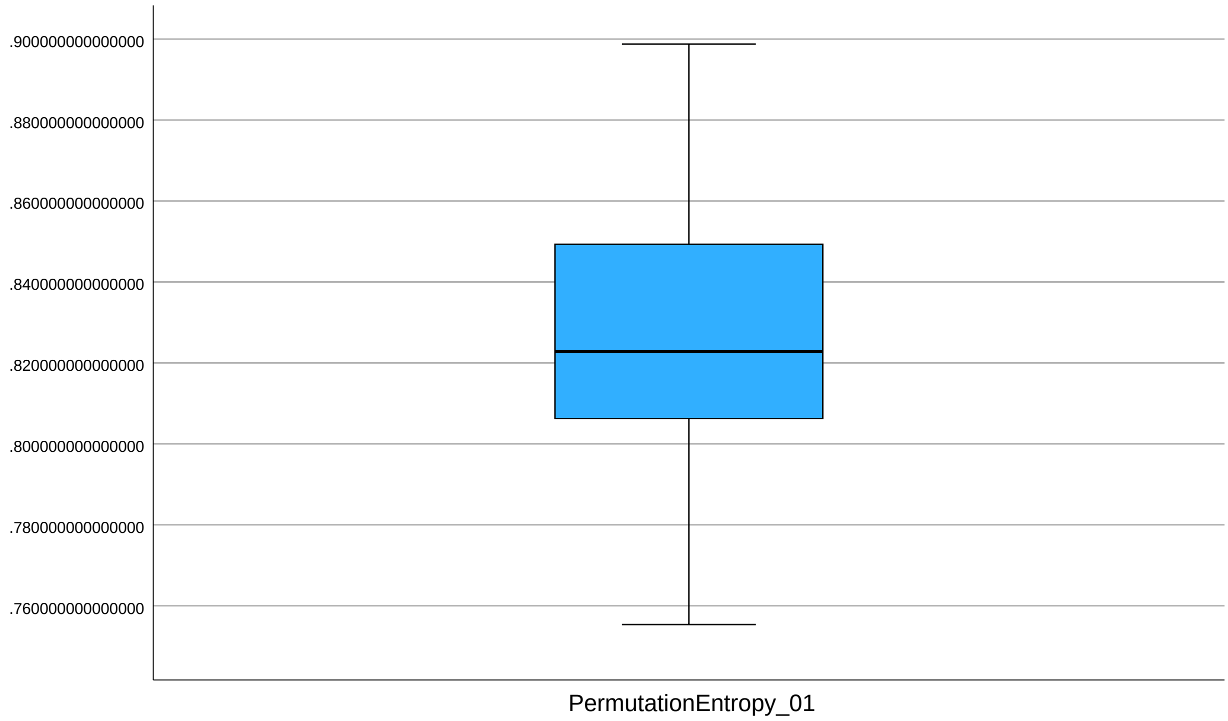

SampleEntropy

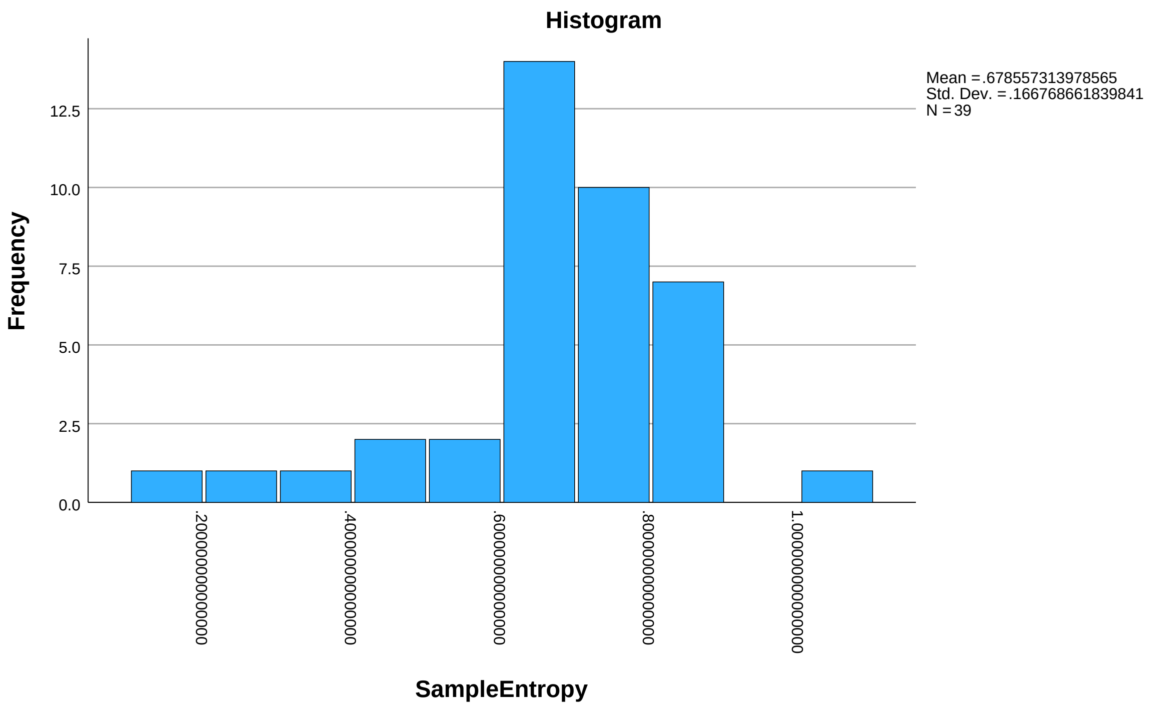

SampleEntropy Stem-and-Leaf Plot

Frequency   Stem & Leaf

```
 3.00 Extremes    (=<.38)
1.00     4 . 1
1.00     4 . 6
 .00     5 .
2.00     5 . 99
5.00     6 . 00034
9.00     6 . 555667899
6.00     7 . 012334
4.00     7 . 6688
5.00     8 . 12234
2.00     8 . 77
 1.00 Extremes    (>=1.07)

Stem width:  .1000000
Each leaf:       1 case(s)
```

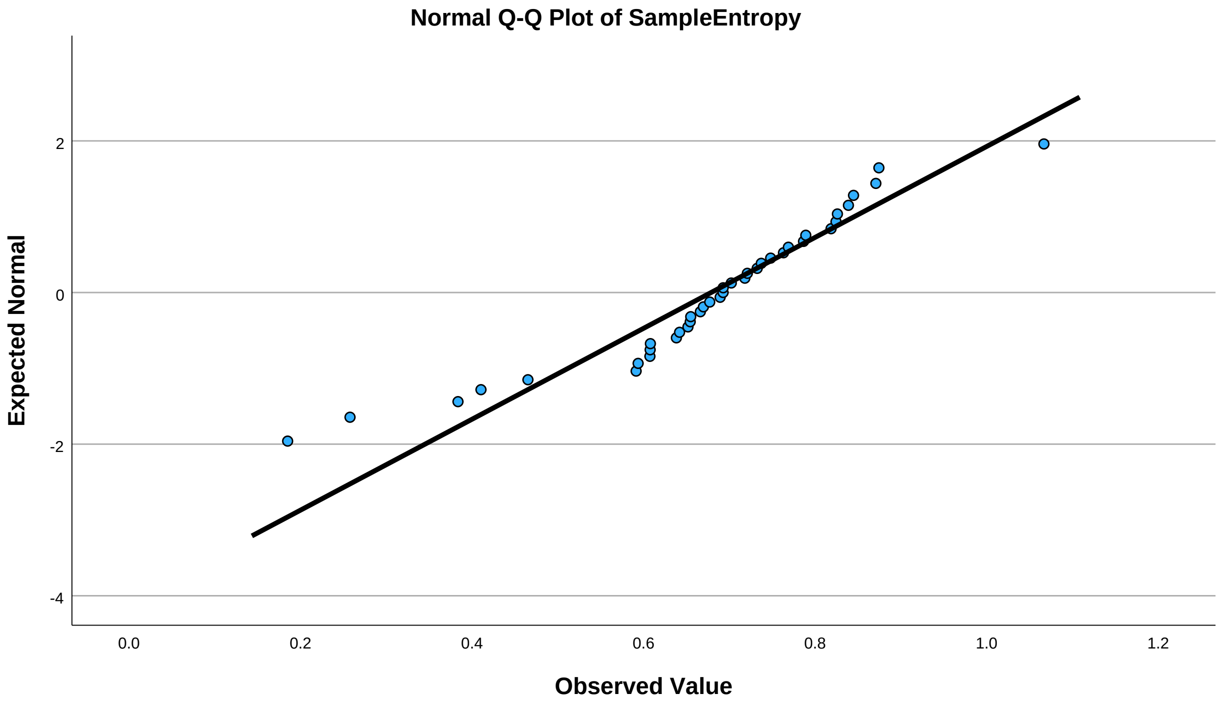

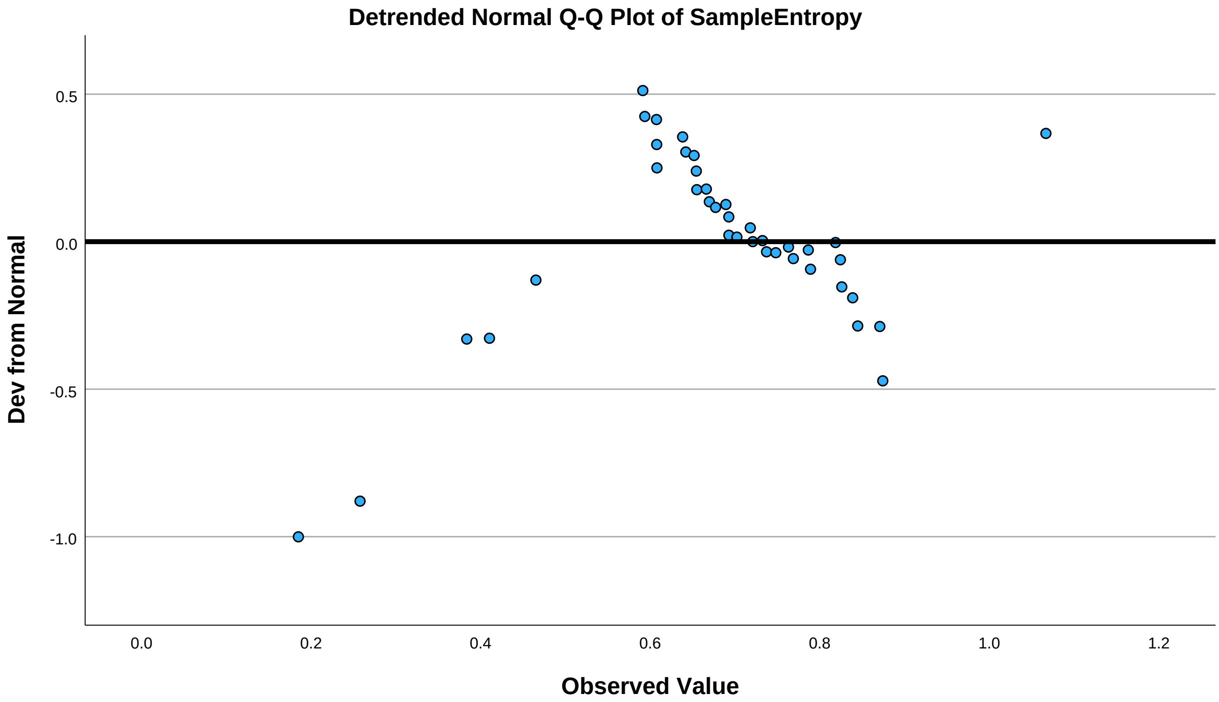

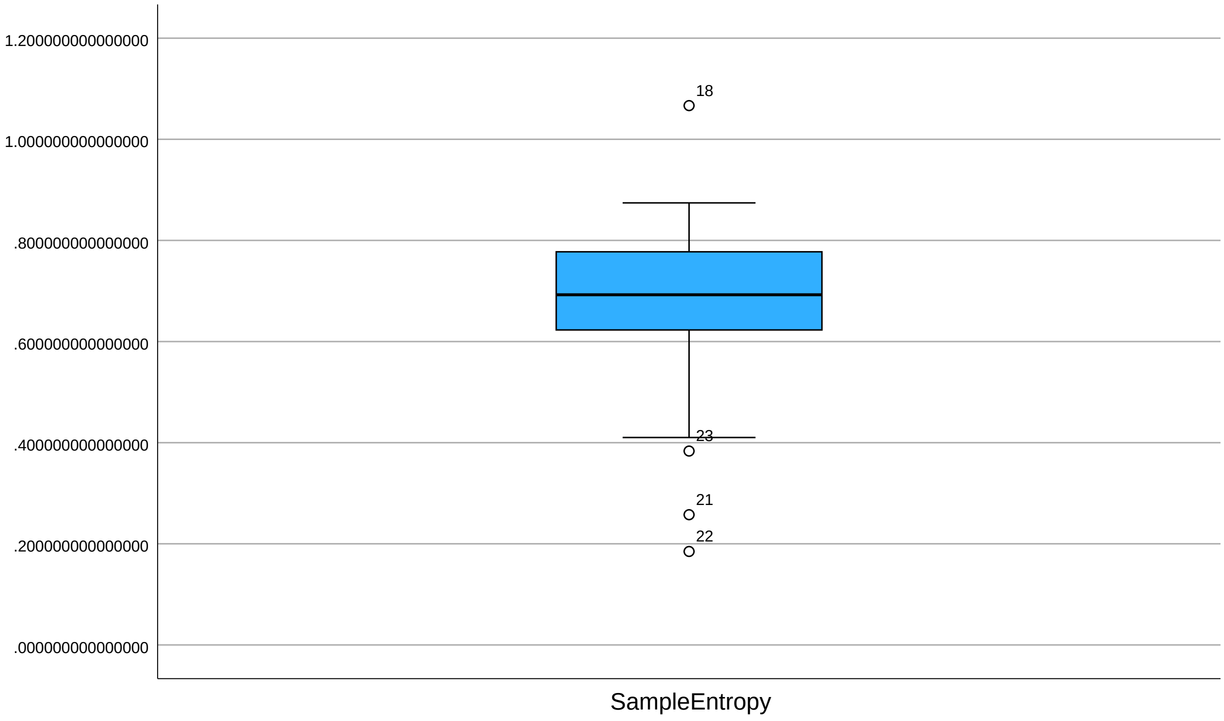

SpectralEntropy_bits

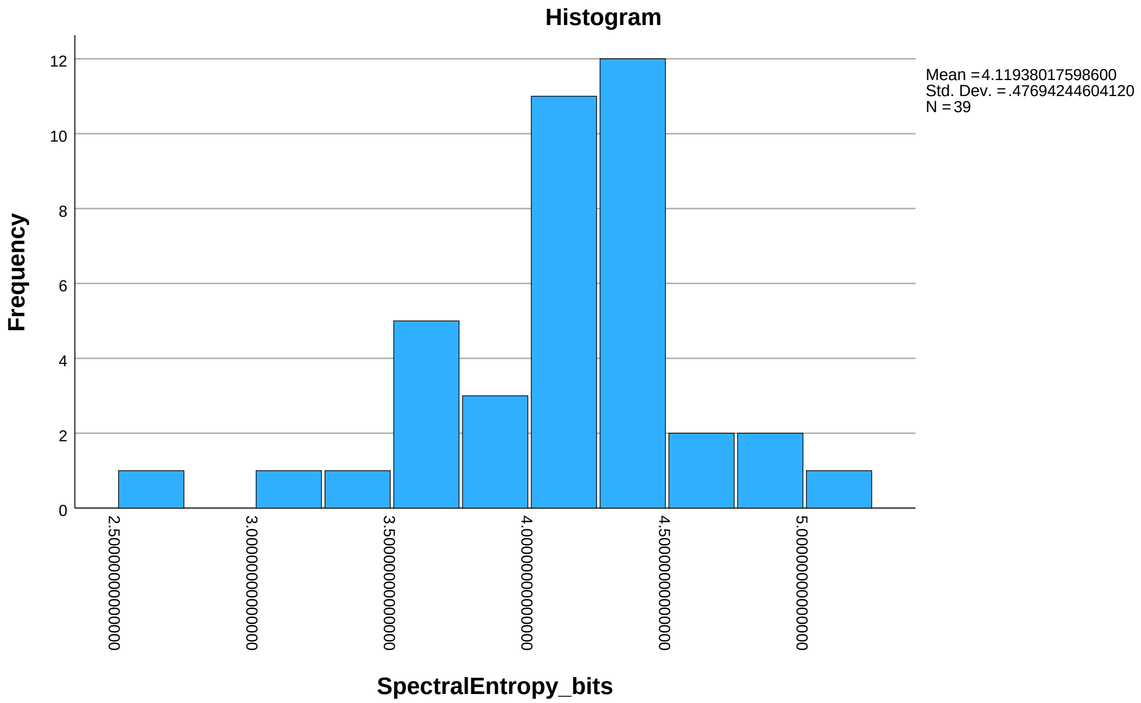


```
SpectralEntropy_bits Stem-and-Leaf Plot

 Frequency    Stem &  Leaf

     2.00 Extremes    (=<3.0)
     1.00        3 .  4
     5.00        3 .  66677
     3.00        3 .  899
    10.00        4 .  0000001111
     9.00        4 .  222333333
     4.00        4 .  4444
     3.00        4 .  667
     1.00        4 .  8
     1.00 Extremes    (>=5.1)

 Stem width:   1.000000
 Each leaf:        1 case(s)
```

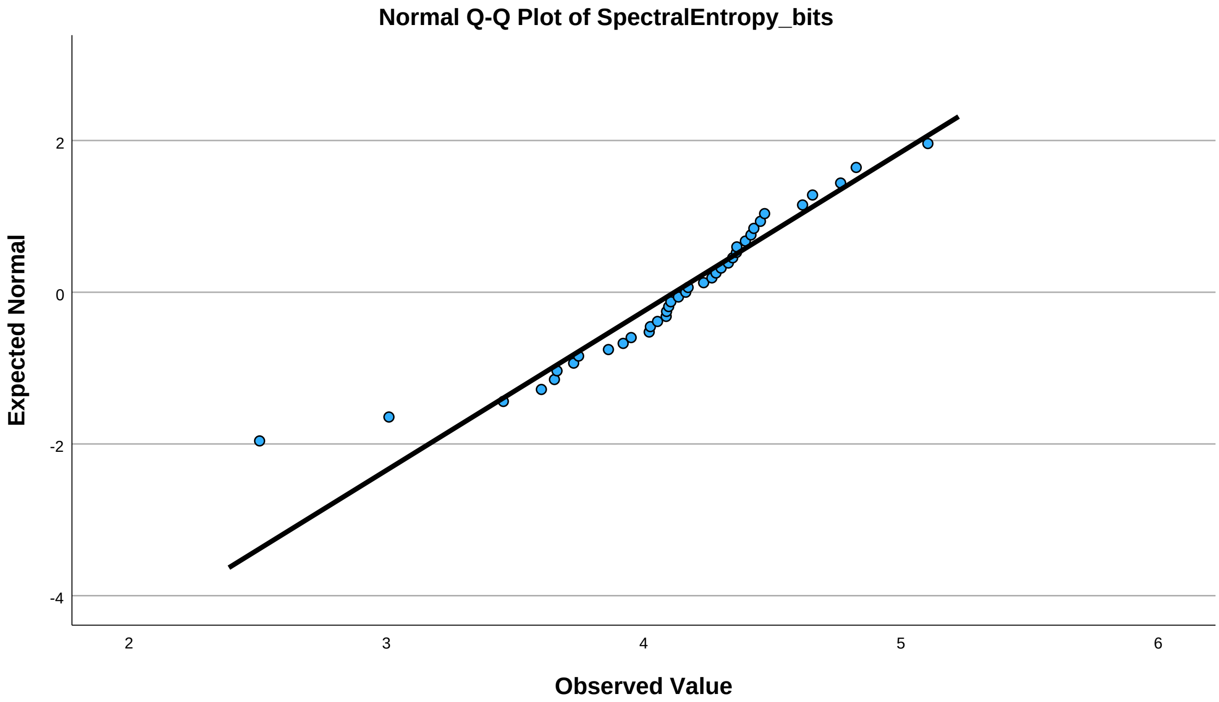
Normal Q-Q Plot of SpectralEntropy_bits
Expected Normal
2
0
-2
-4
2
3
4
5
6
Observed Value

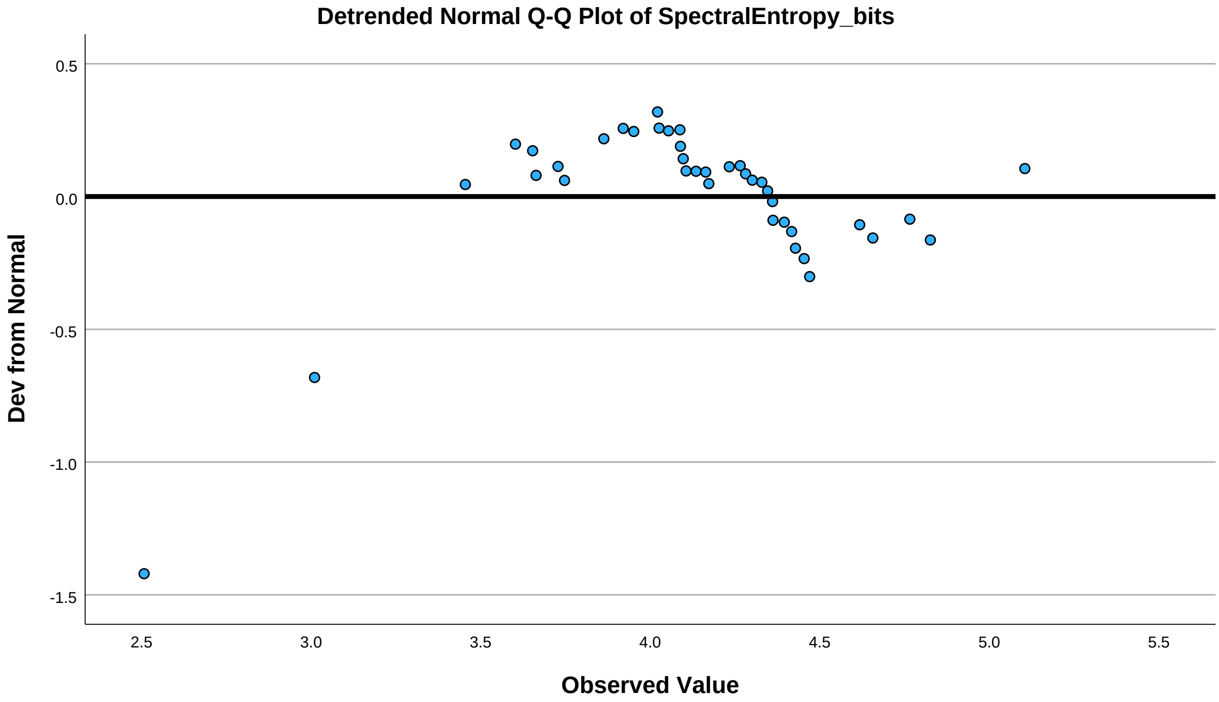
Detrended Normal Q-Q Plot of SpectralEntropy_bits
Dev from Normal
0.5
0.0
-0.5
-1.0
-1.5
2.5
3.0
3.5
4.0
4.5
5.0
5.5
Observed Value

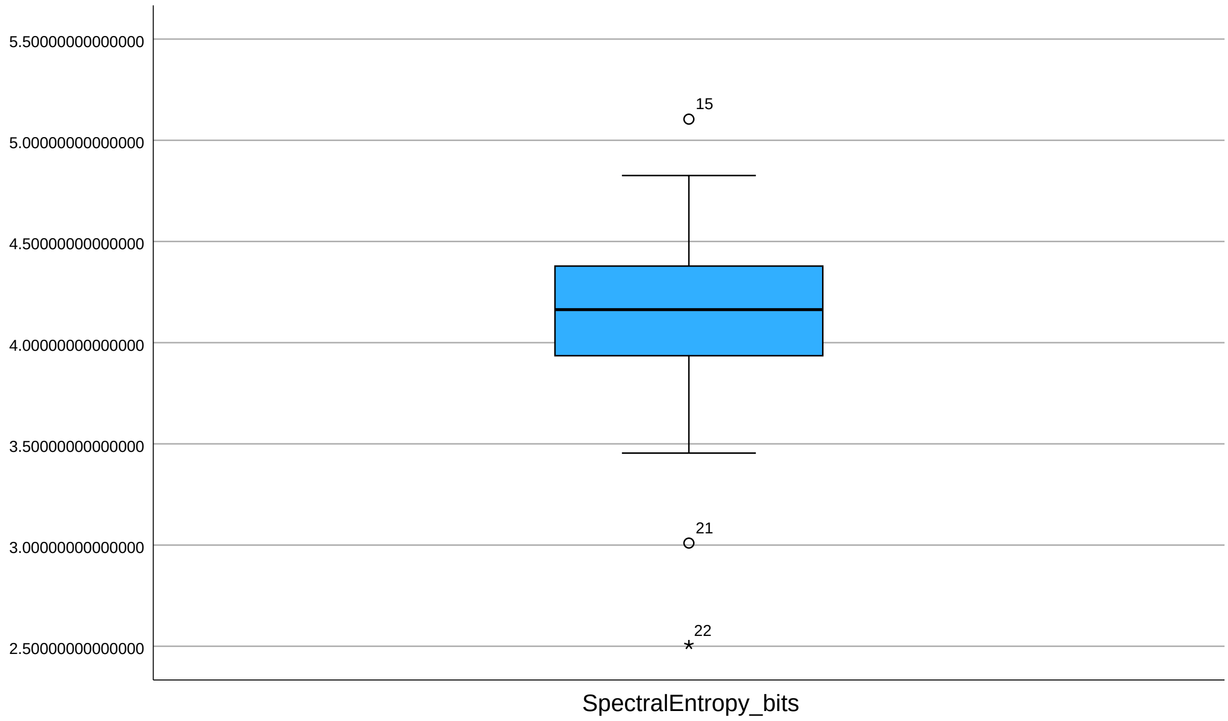

SpectralEntropy_01

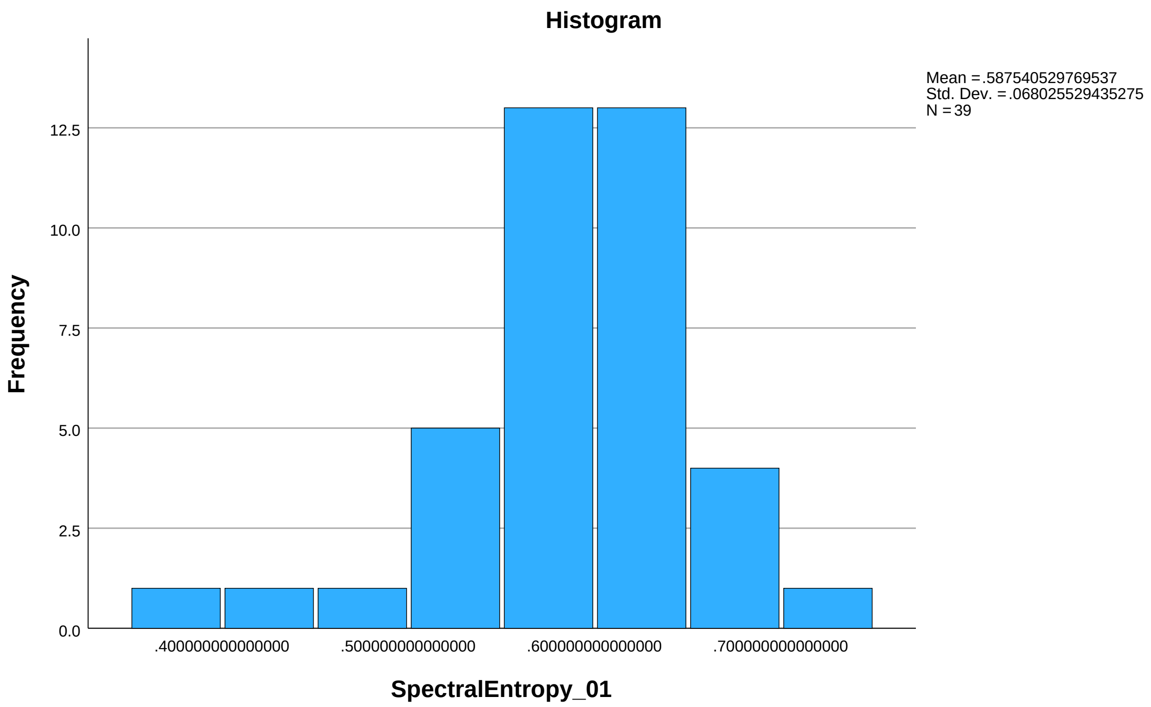

SpectralEntropy_01 Stem-and-Leaf Plot

```
 Frequency    Stem &  Leaf
```

```
 2.00 Extremes    (=<.43)
1.00      4 . 9
 5.00      5 . 12233
13.00      5 . 5567778888899
13.00      6 . 0011112222333
 4.00      6 . 5678
 1.00 Extremes    (>=.73)

Stem width:   .1000000
Each leaf:       1 case(s)
```

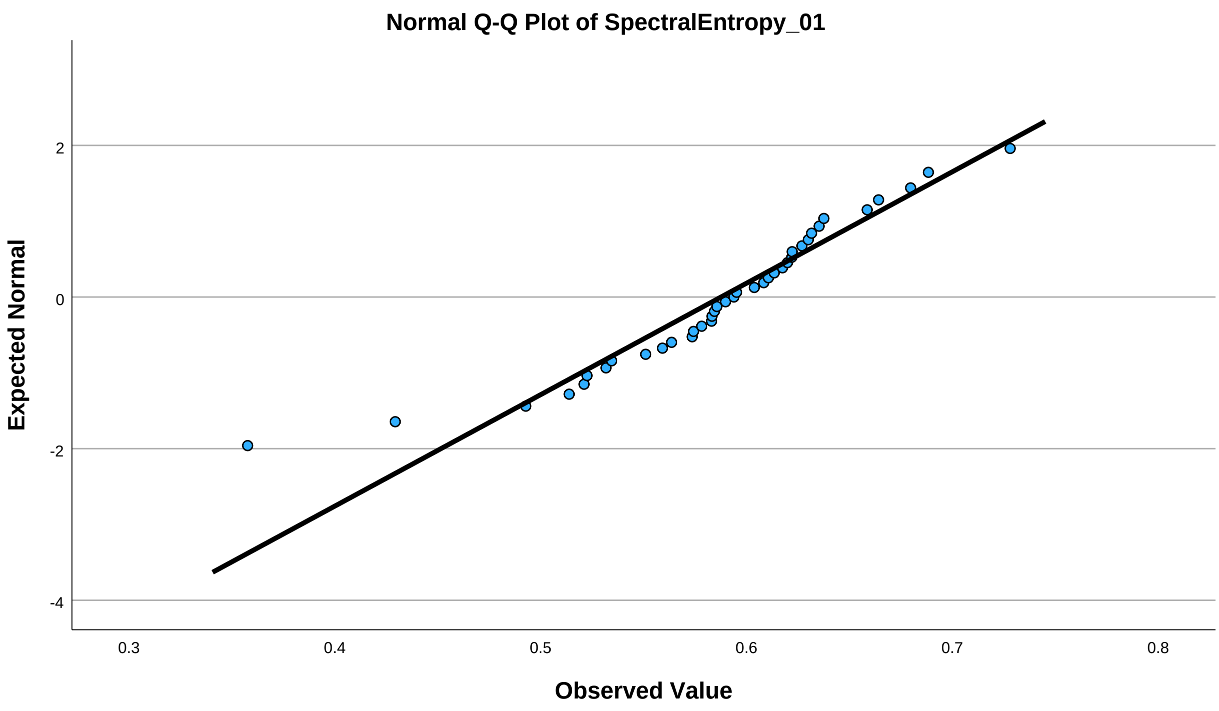

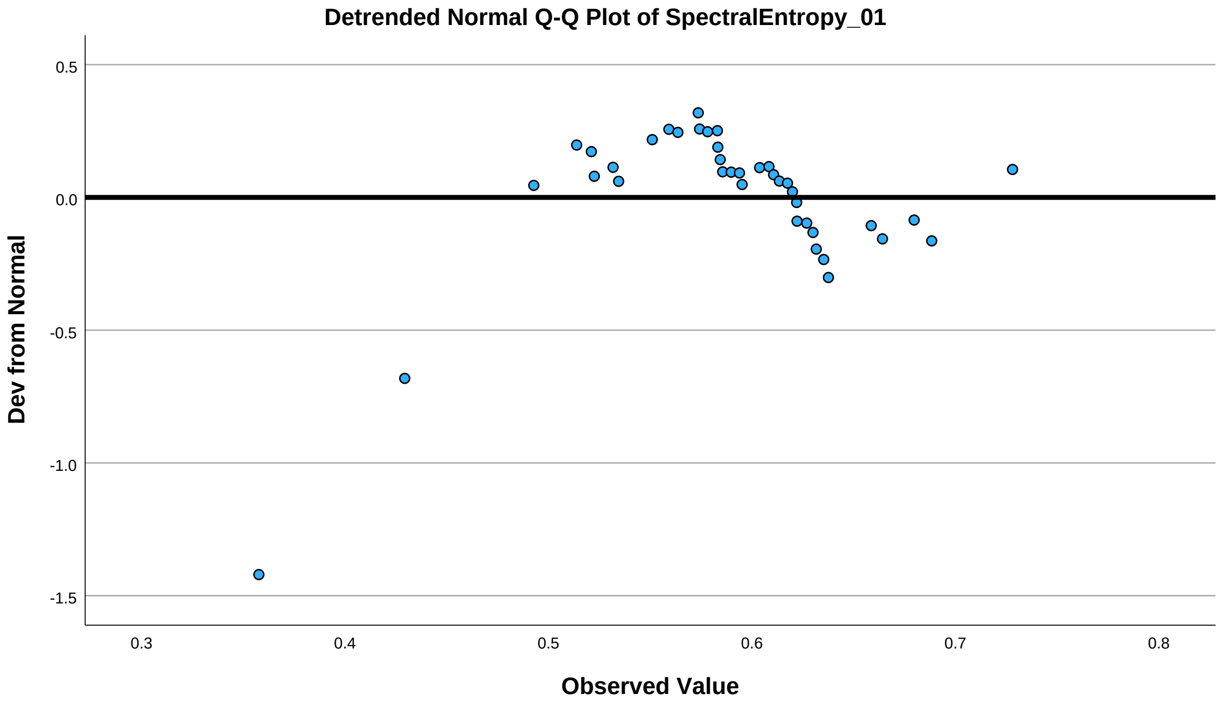

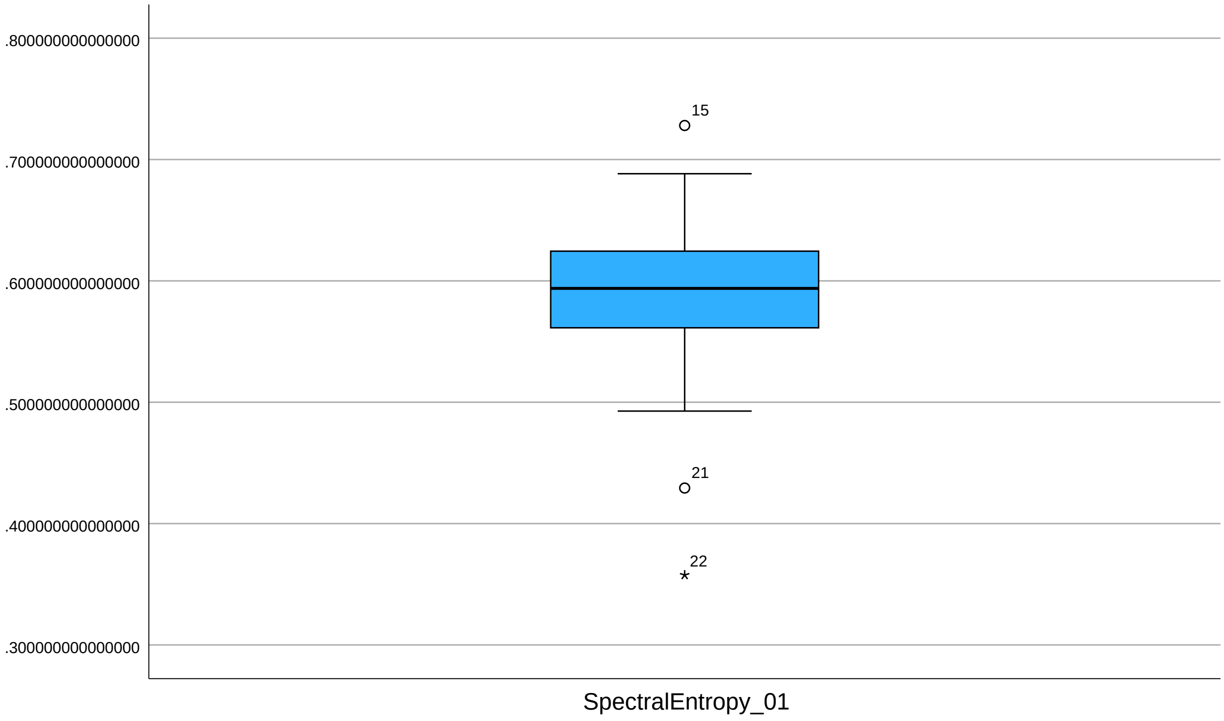

HiguchiFractalDimension

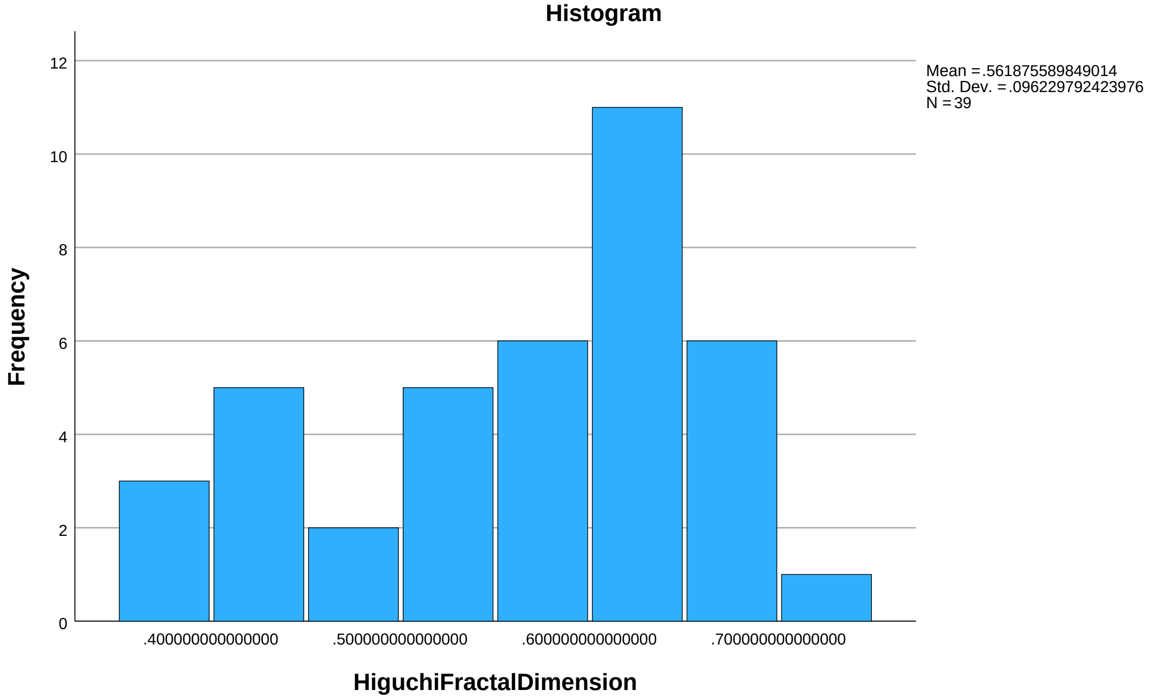


```
HiguchiFractalDimension Stem-and-Leaf Plot

 Frequency    Stem &  Leaf

     3.00        3 .  799
     5.00        4 .  01244
     2.00        4 .  55
     5.00        5 .  11244
     6.00        5 .  567899
    11.00        6 .  00001123344
     6.00        6 .  567789
     1.00        7 .  0

 Stem width:   .1000000
 Each leaf:        1 case(s)
```

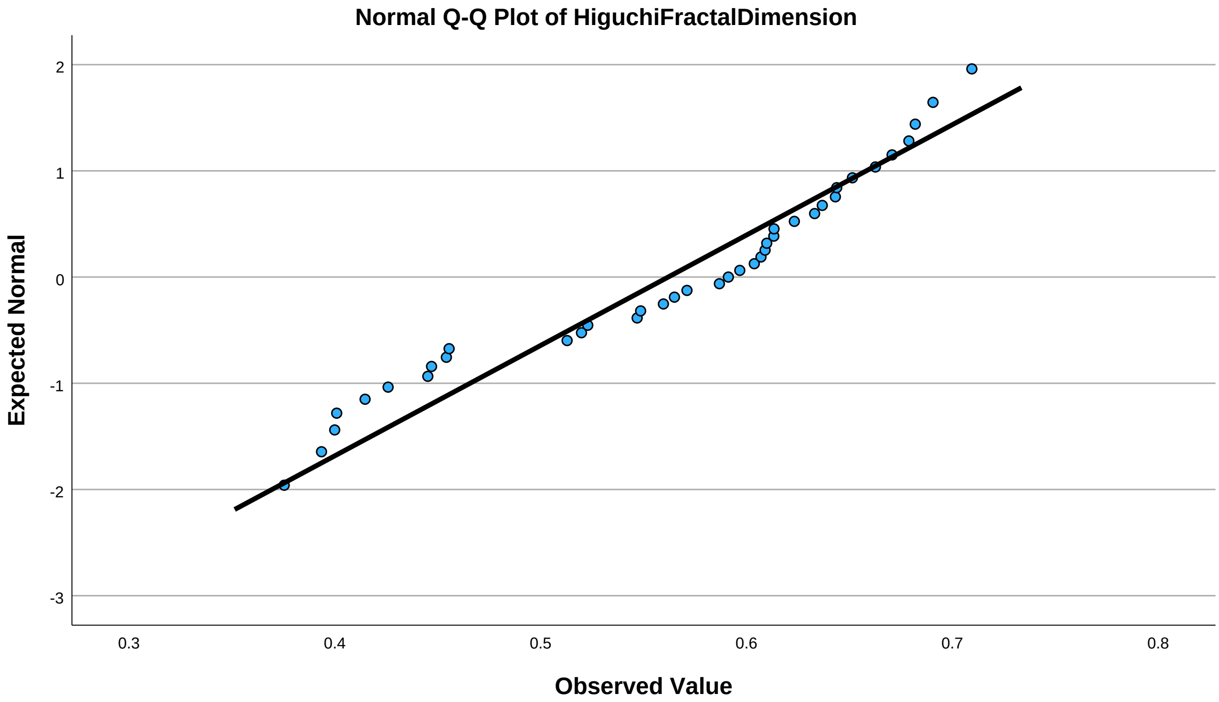

Normal Q-Q Plot of HiguchiFractalDimension
Expected Normal
Observed Value
2
1
0
-1
-2
-3
0.3
0.4
0.5
0.6
0.7
0.8

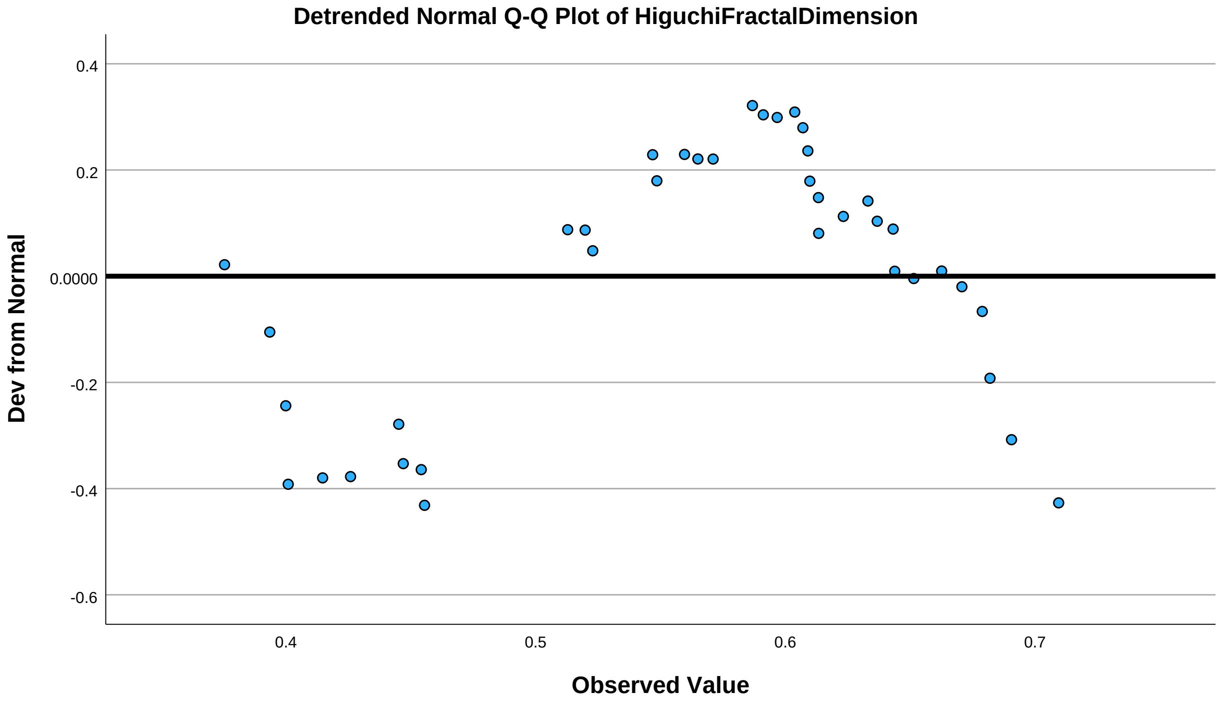

Detrended Normal Q-Q Plot of HiguchiFractalDimension
Dev from Normal
Observed Value
0.4
0.2
0.0000
-0.2
-0.4
-0.6
0.4
0.5
0.6
0.7

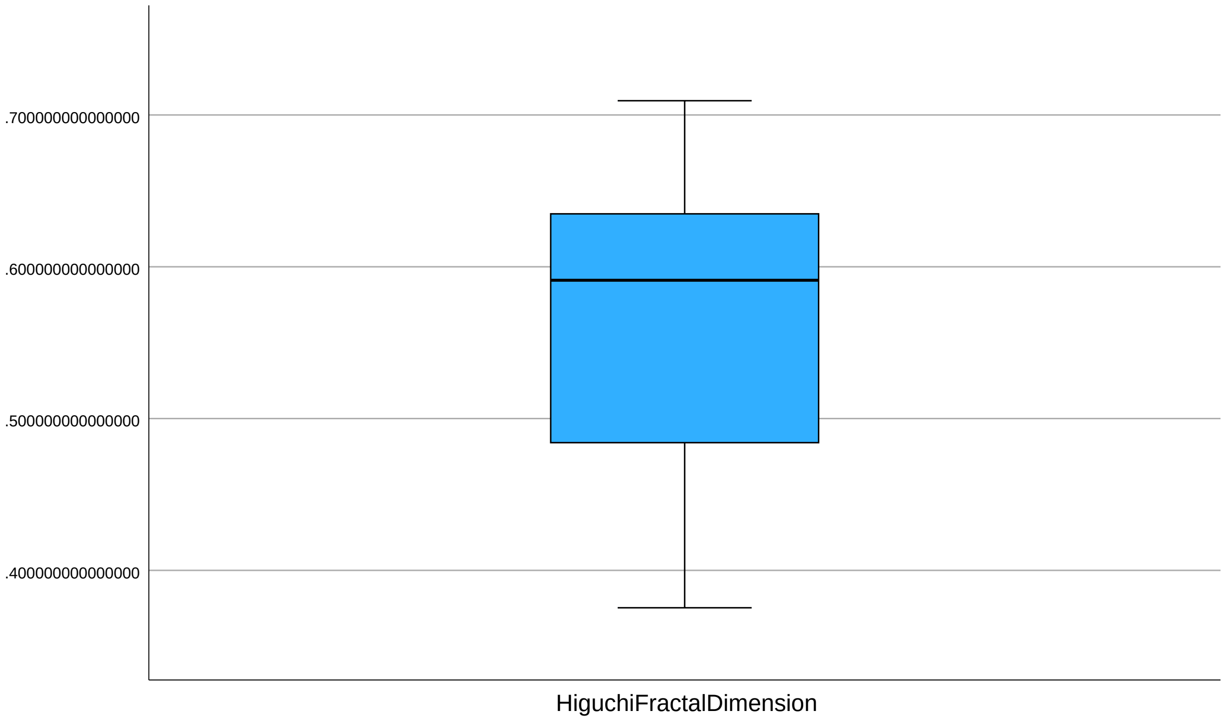

LempelZivComplexity

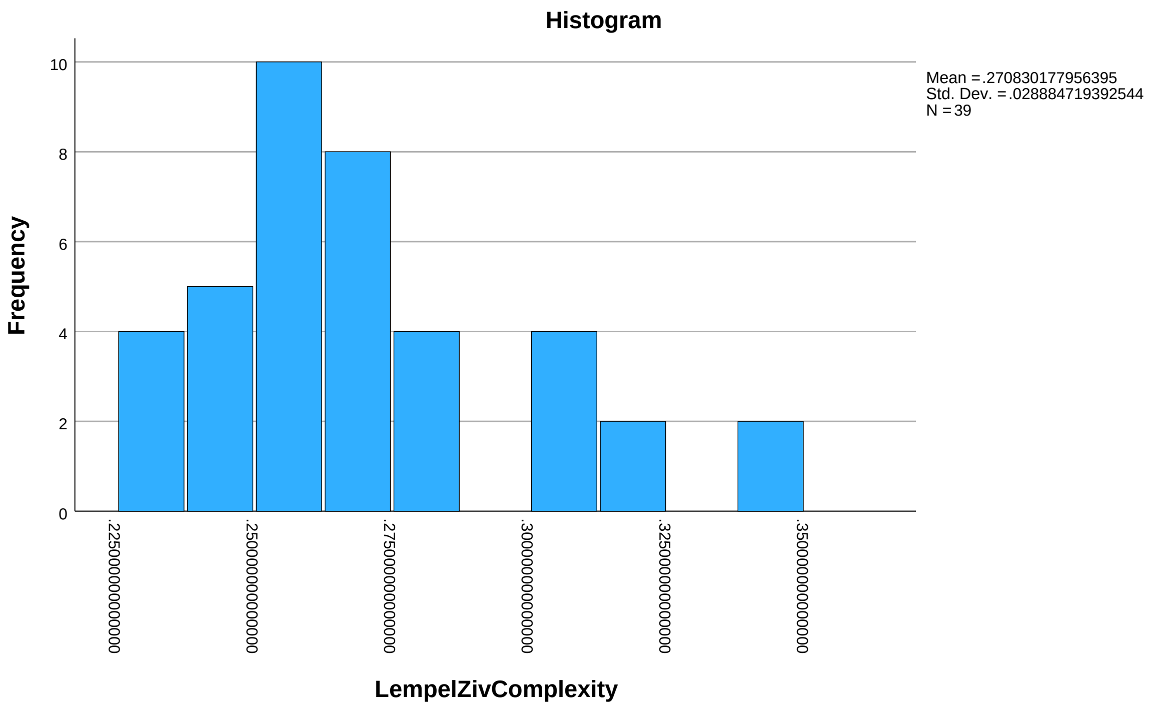


```
LempelZivComplexity Stem-and-Leaf Plot

 Frequency    Stem &  Leaf
```

```
    4.00       23 .  2367
    5.00       24 .  37889
    8.00       25 .  00233688
    7.00       26 .  1134779
    5.00       27 .  12359
    2.00       28 .  55
     .00       29 .
    3.00       30 .  136
    3.00       31 .  179
    2.00 Extremes    (>=.342)

 Stem width:   .0100000
 Each leaf:       1 case(s)
```

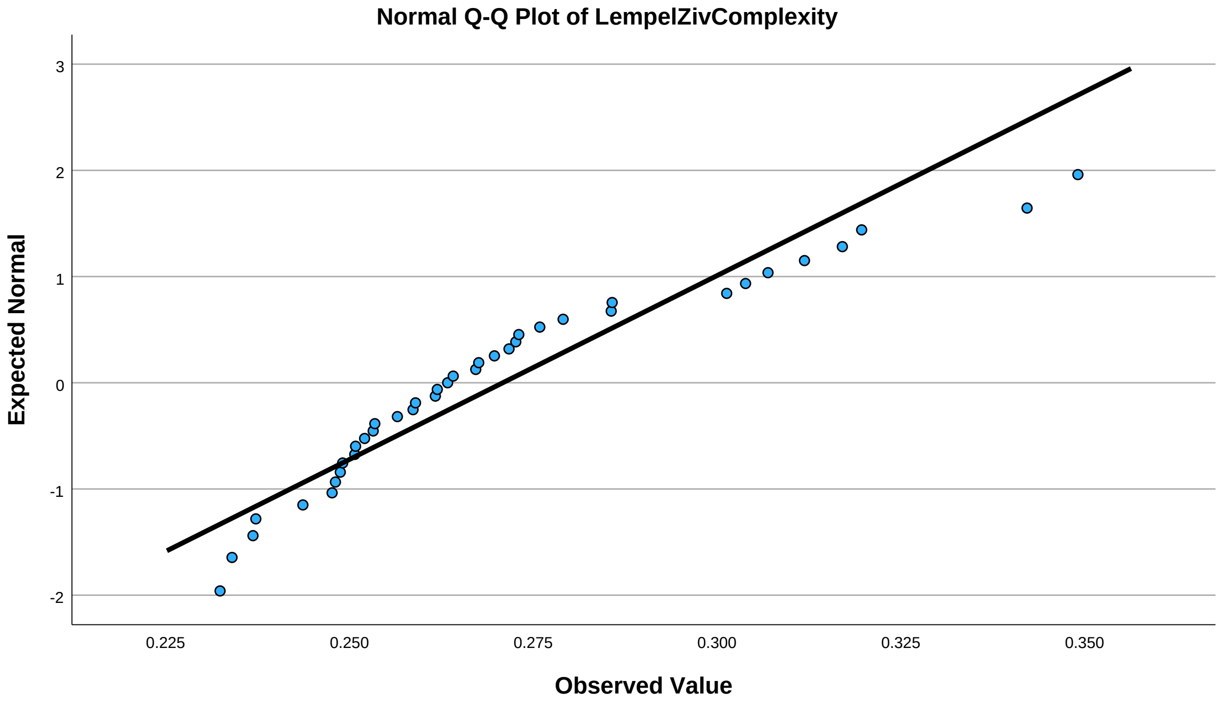

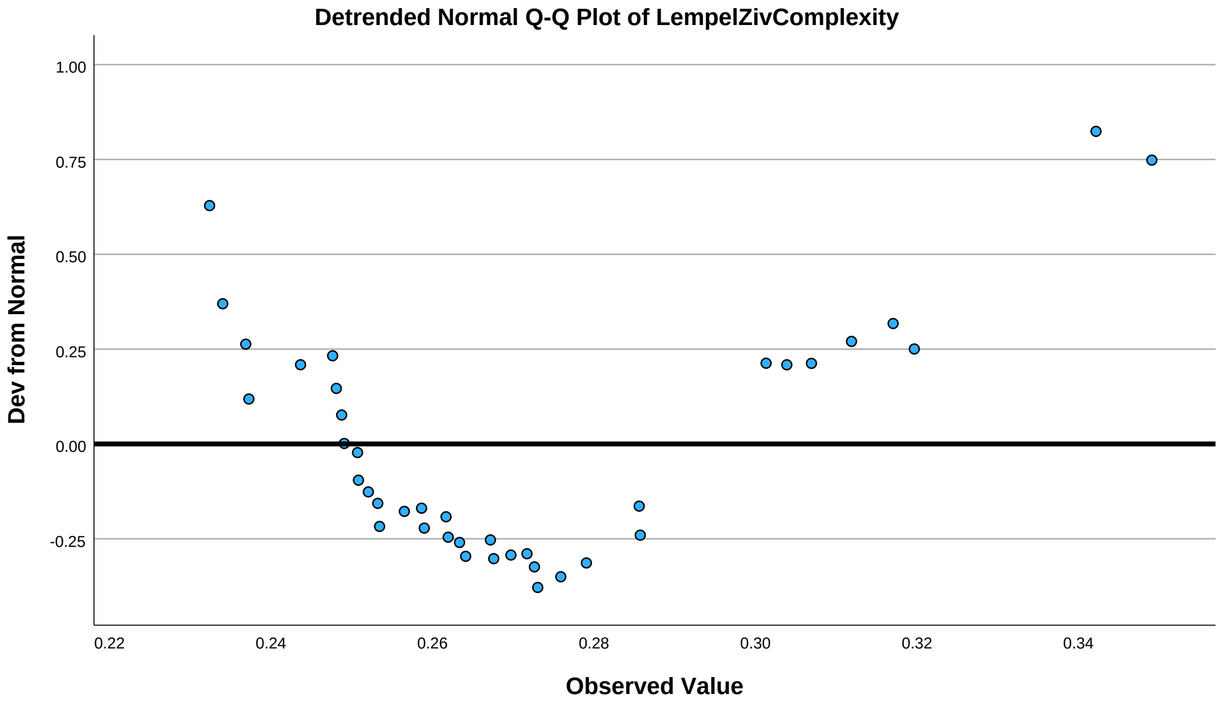

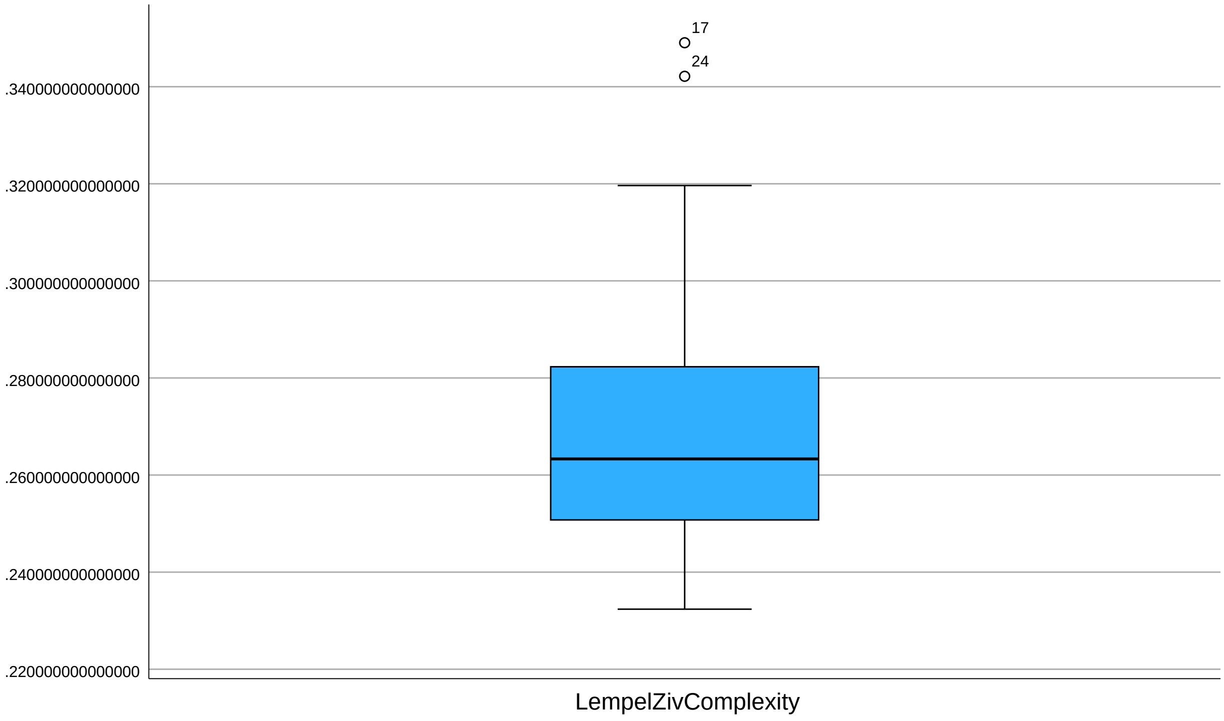

RenyiEntropy

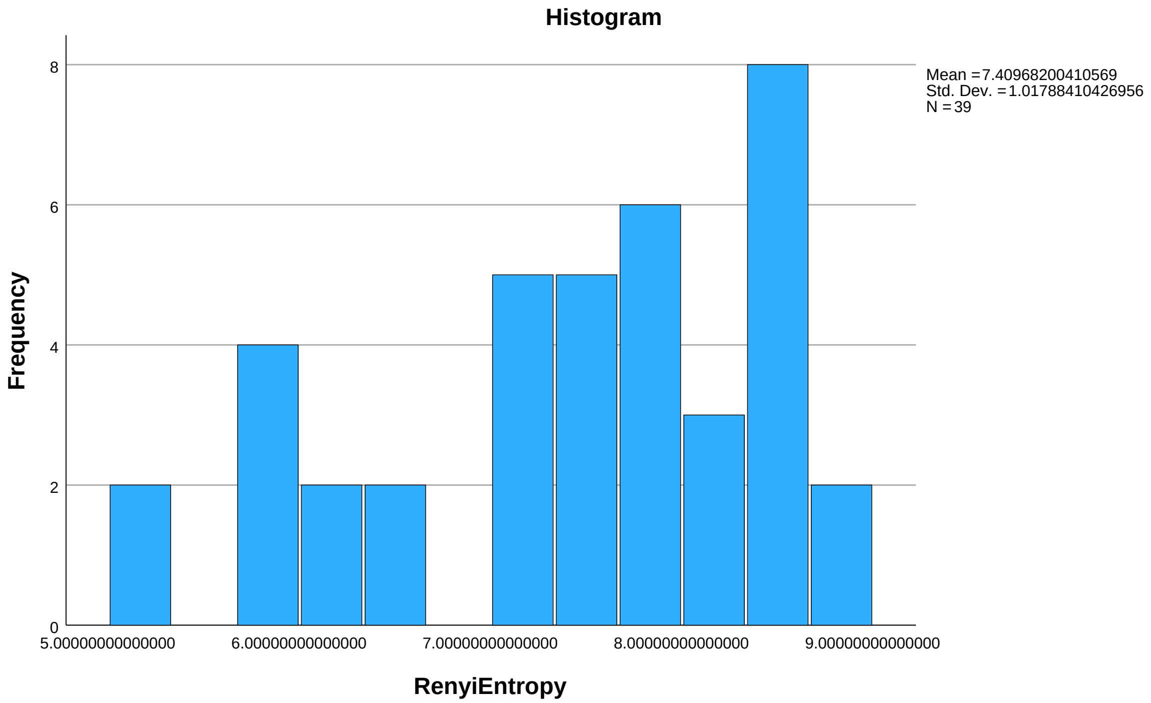


```
RenyiEntropy Stem-and-Leaf Plot

 Frequency    Stem &  Leaf

     2.00        5 .  23
     4.00        5 .  7789
     4.00        6 .  1244
      .00        6 .
     6.00        7 .  012223
    10.00        7 .  5556677779
     6.00        8 .  002344
     7.00        8 .  5556667

 Stem width:   1.000000
 Each leaf:       1 case(s)
```

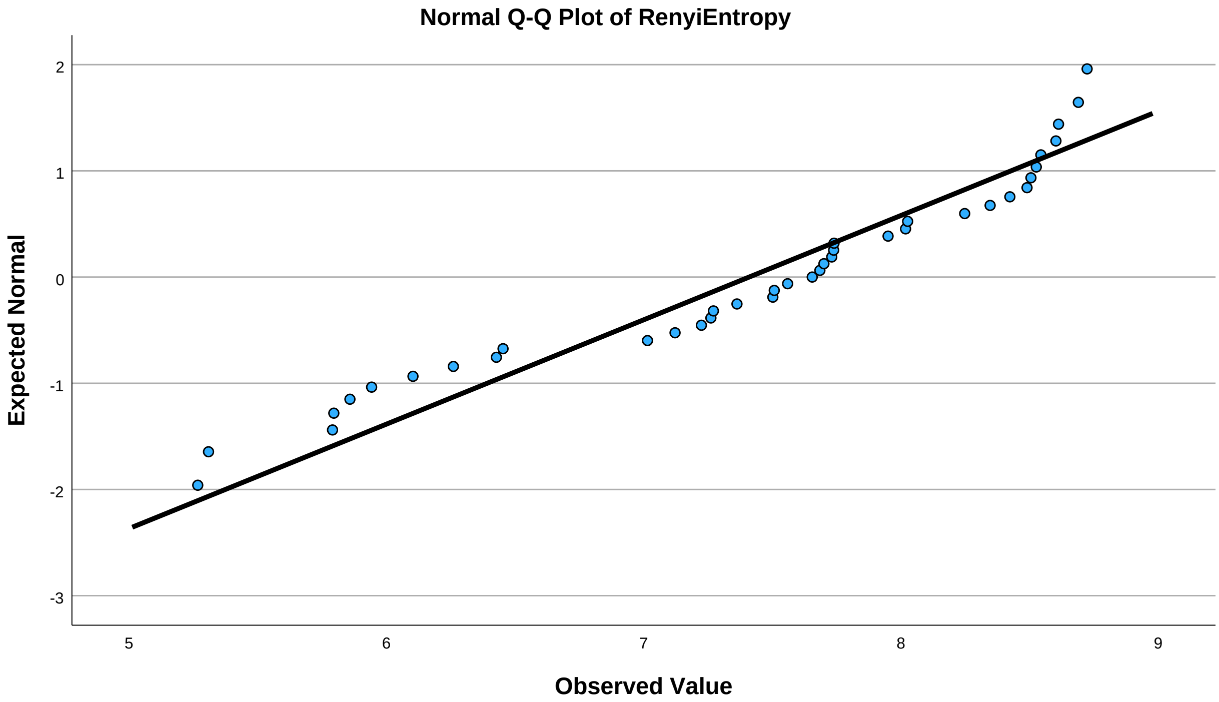
Normal Q-Q Plot of RenyiEntropy
Expected Normal
Observed Value
2
1
0
-1
-2
-3
5
6
7
8
9

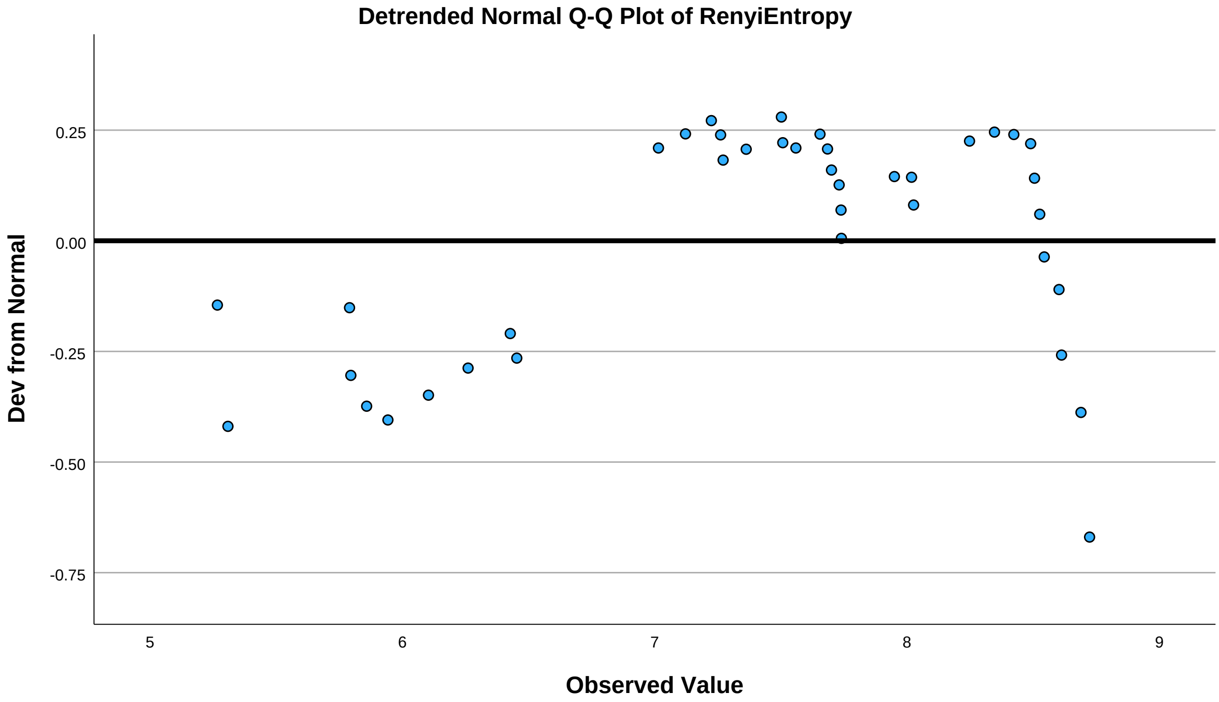
Detrended Normal Q-Q Plot of RenyiEntropy
Dev from Normal
Observed Value
0.25
0.00
-0.25
-0.50
-0.75
5
6
7
8
9

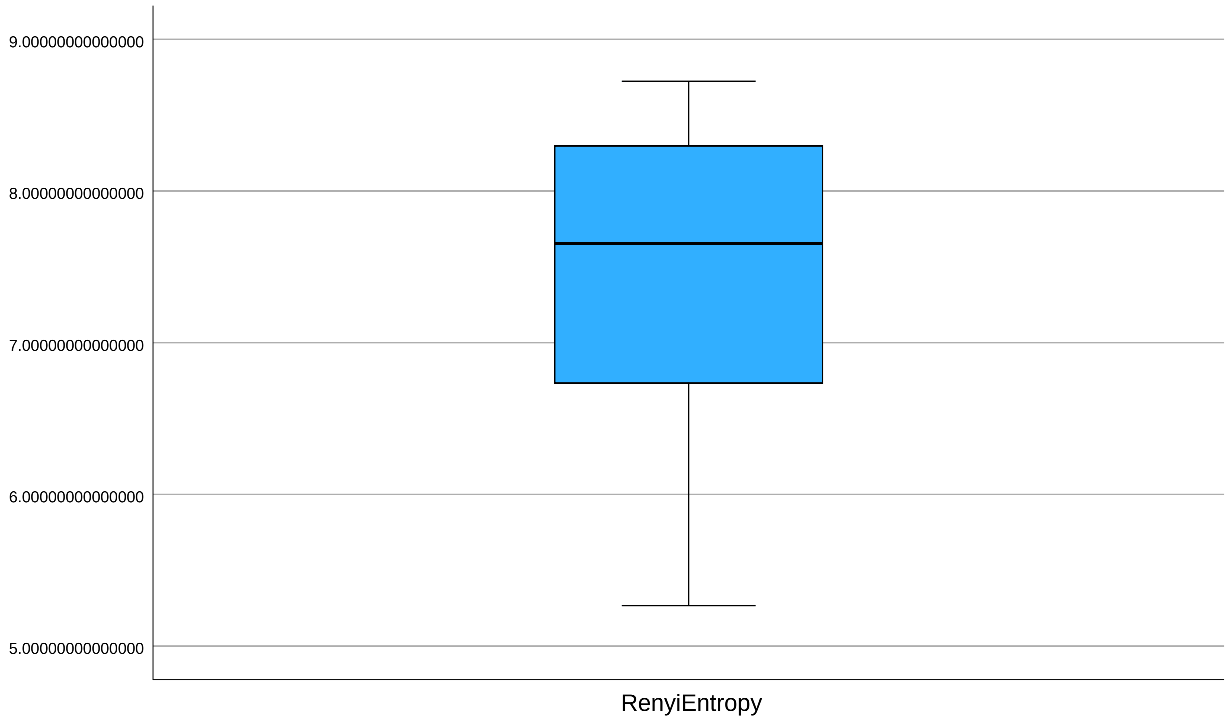

TsallisEntropy

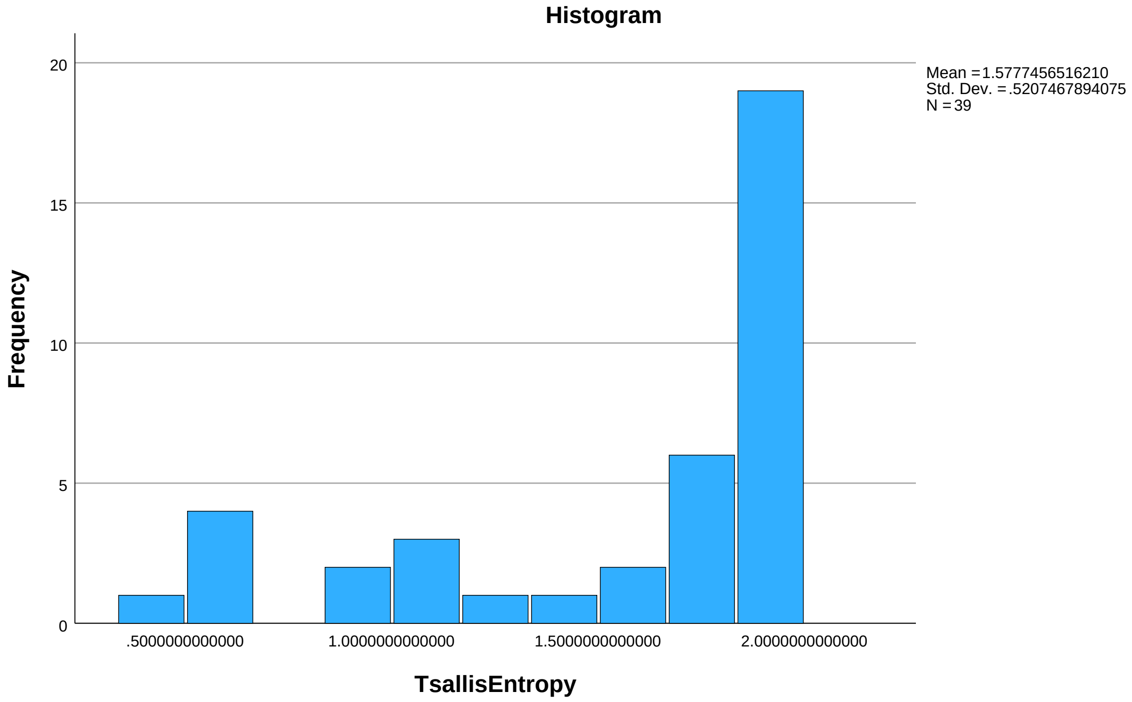

TsallisEntropy Stem-and-Leaf Plot

Frequency   Stem & Leaf

```
 1.00      0 . 3
 3.00      0 . 555
 1.00      0 . 6
 2.00      0 . 89
 3.00      1 . 011
 1.00      1 . 2
 2.00      1 . 45
 6.00      1 . 666777
20.00      1 . 88899999999999999999

Stem width:  1.000000
Each leaf:       1 case(s)
```

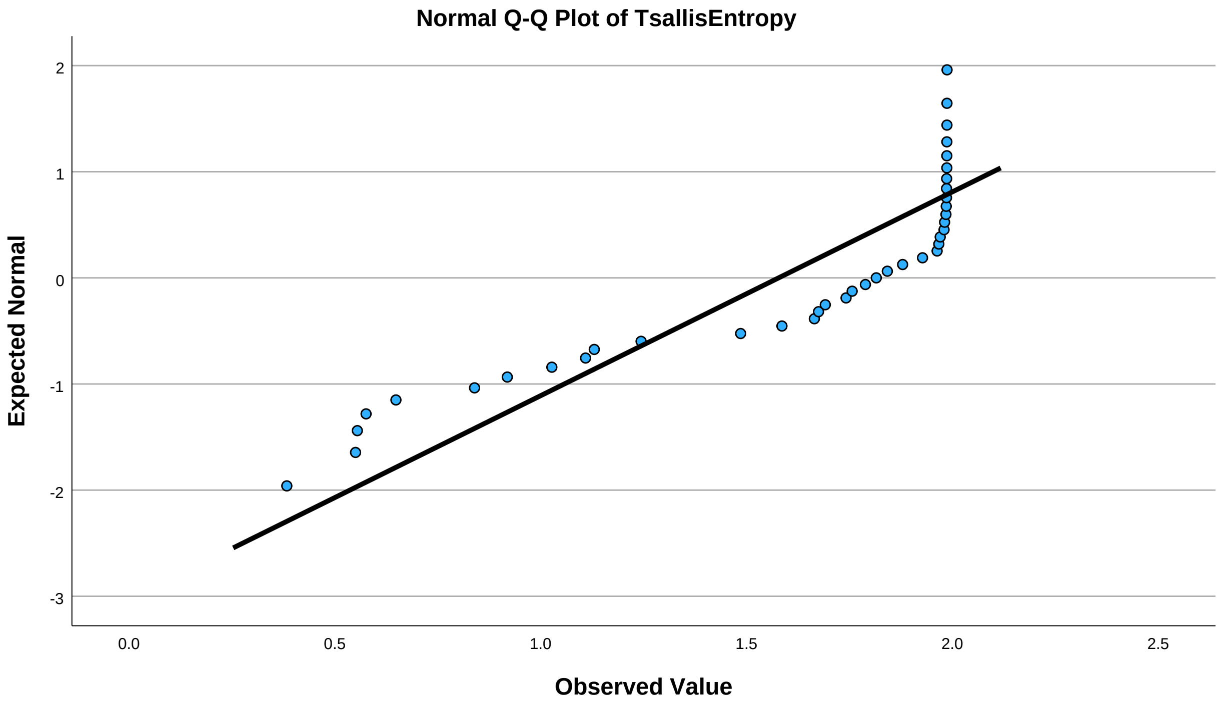

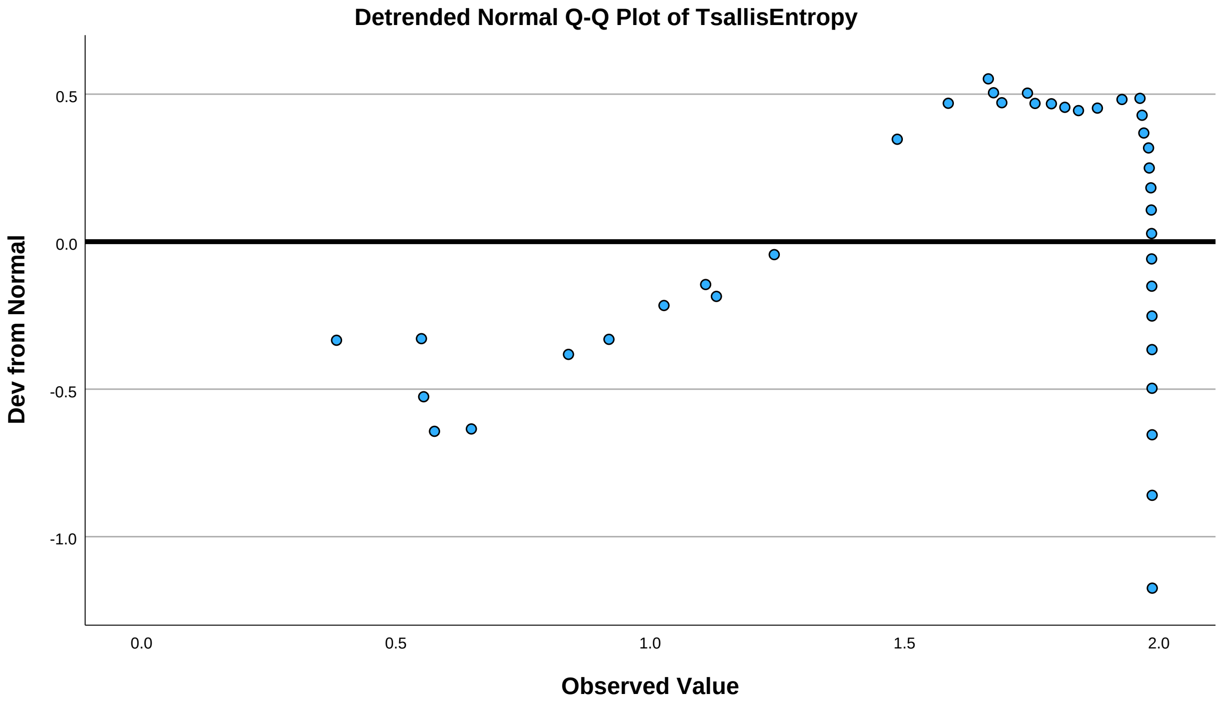
Detrended Normal Q-Q Plot of TsallisEntropy
Dev from Normal
0.5
0.0
-0.5
-1.0
0.0
0.5
1.0
1.5
2.0
Observed Value

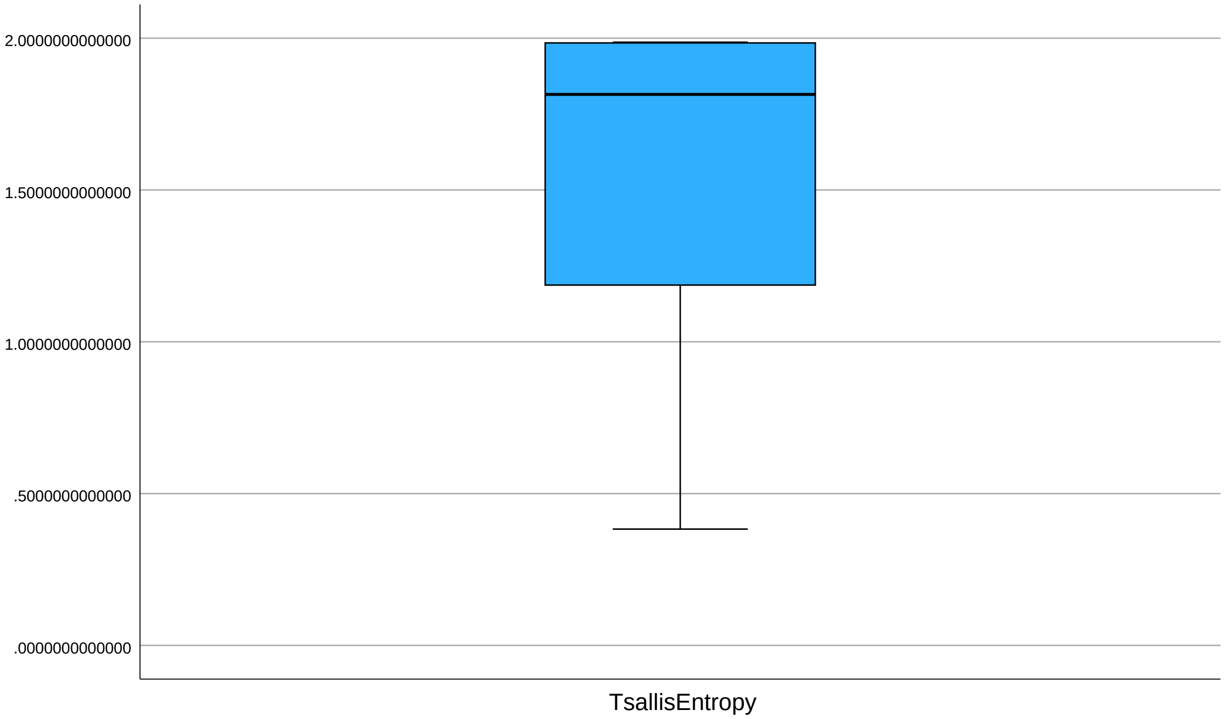
2.0000000000000
1.5000000000000
1.0000000000000
.5000000000000
.0000000000000
TsallisEntropy

PS_4_5

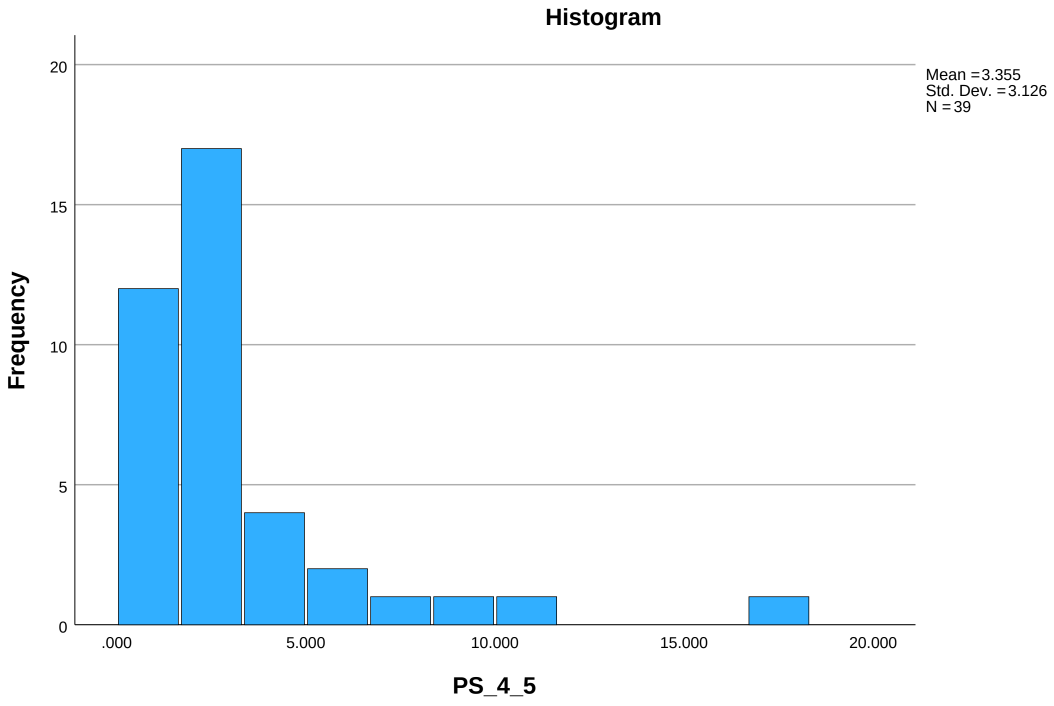

PS_4_5 Stem-and-Leaf Plot

```
 Frequency    Stem &  Leaf

     3.00        0 .  789
     8.00        1 .  23333444
     3.00        1 .  569
     6.00        2 .  223344
     7.00        2 .  5568888
     3.00        3 .  033
     1.00        3 .  6
      .00        4 .
     2.00        4 .  59
      .00        5 .
     1.00        5 .  5
     5.00 Extremes    (>=6.5)

 Stem width:      1.000
 Each leaf:       1 case(s)
```

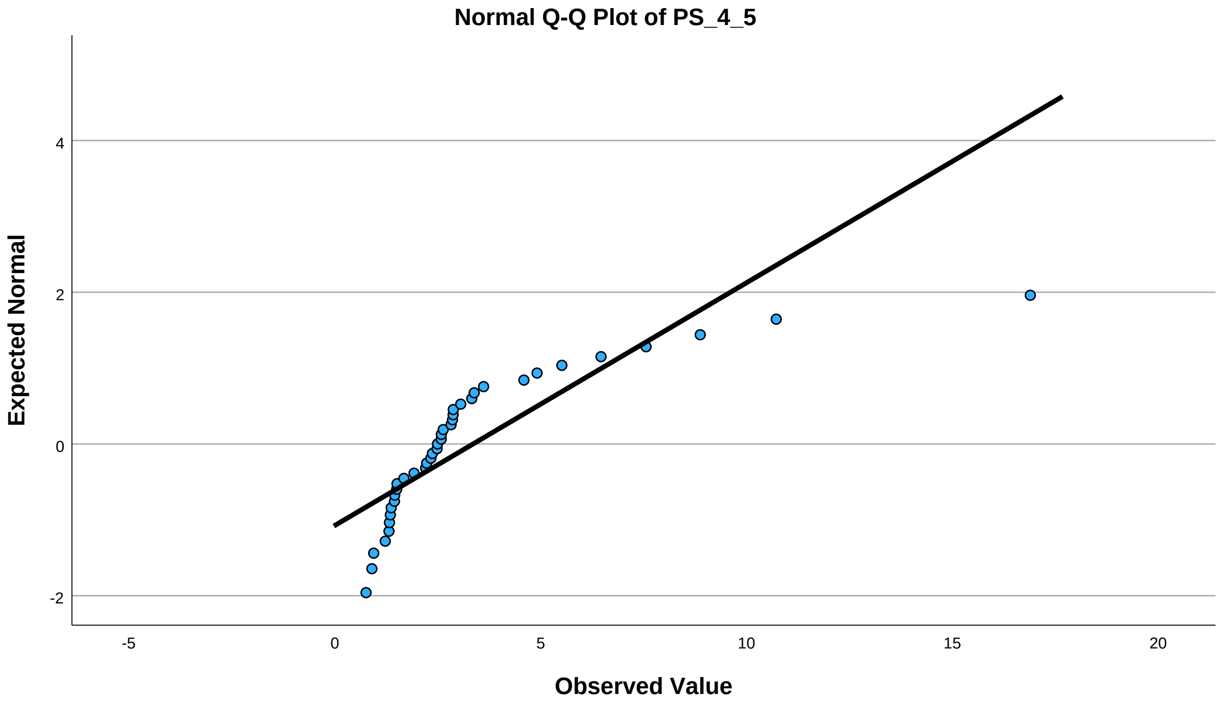
Normal Q-Q Plot of PS_4_5
Expected Normal
Observed Value
4
2
0
-2
-5
0
5
10
15
20

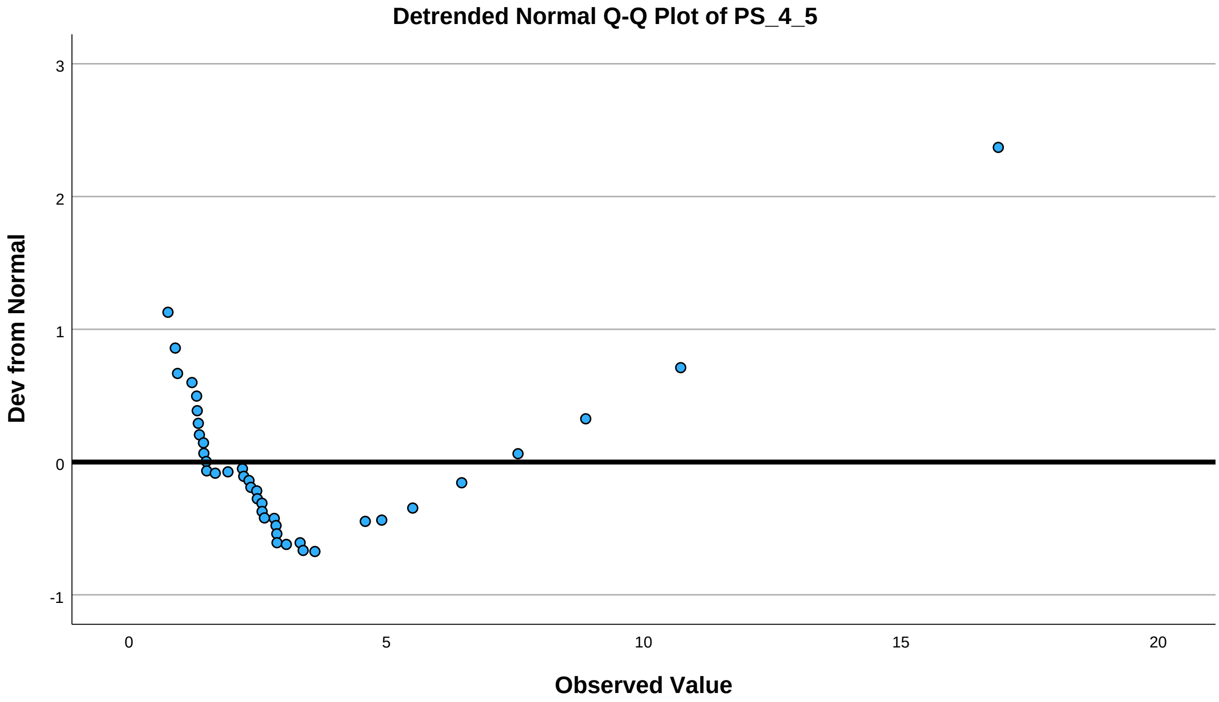
Detrended Normal Q-Q Plot of PS_4_5
Dev from Normal
Observed Value
3
2
1
0
-1
0
5
10
15
20

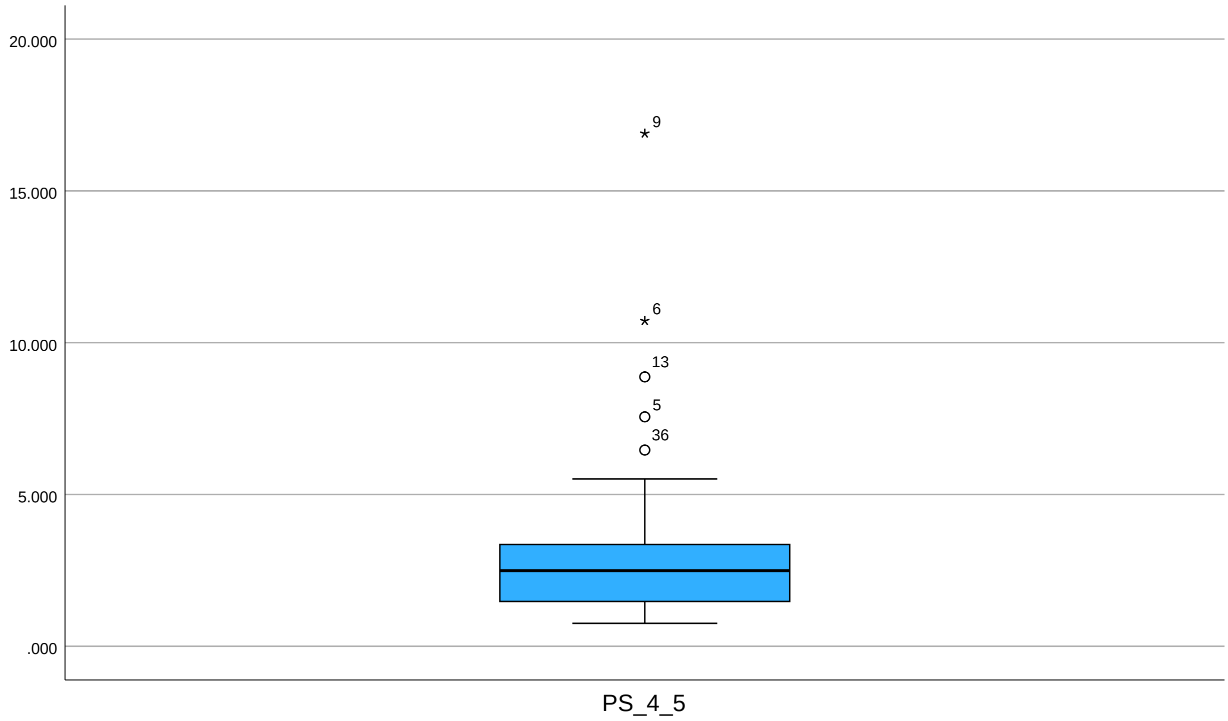

PS_5_6

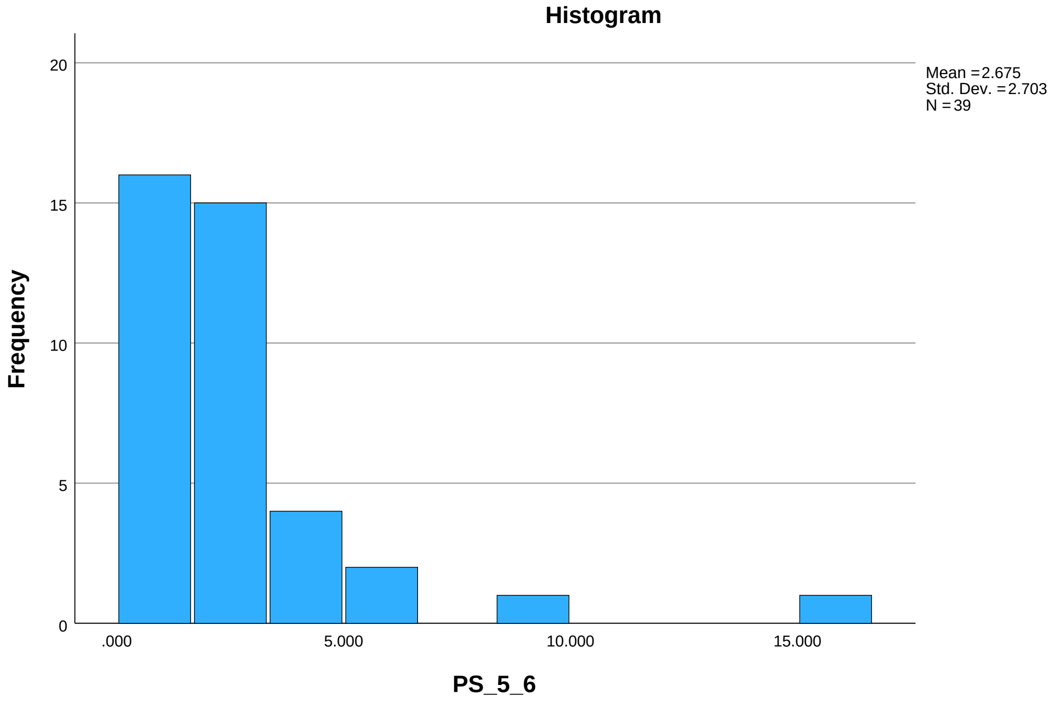

PS_5_6 Stem-and-Leaf Plot

Frequency   Stem & Leaf

```
     5.00        0 .  57889
     6.00        1 .  002334
    14.00        1 .  55566667788889
     3.00        2 .  234
     1.00        2 .  5
     3.00        3 .  133
      .00        3 .
     3.00        4 .  023
     4.00 Extremes    (>=6.1)

 Stem width:      1.000
 Each leaf:        1 case(s)
```

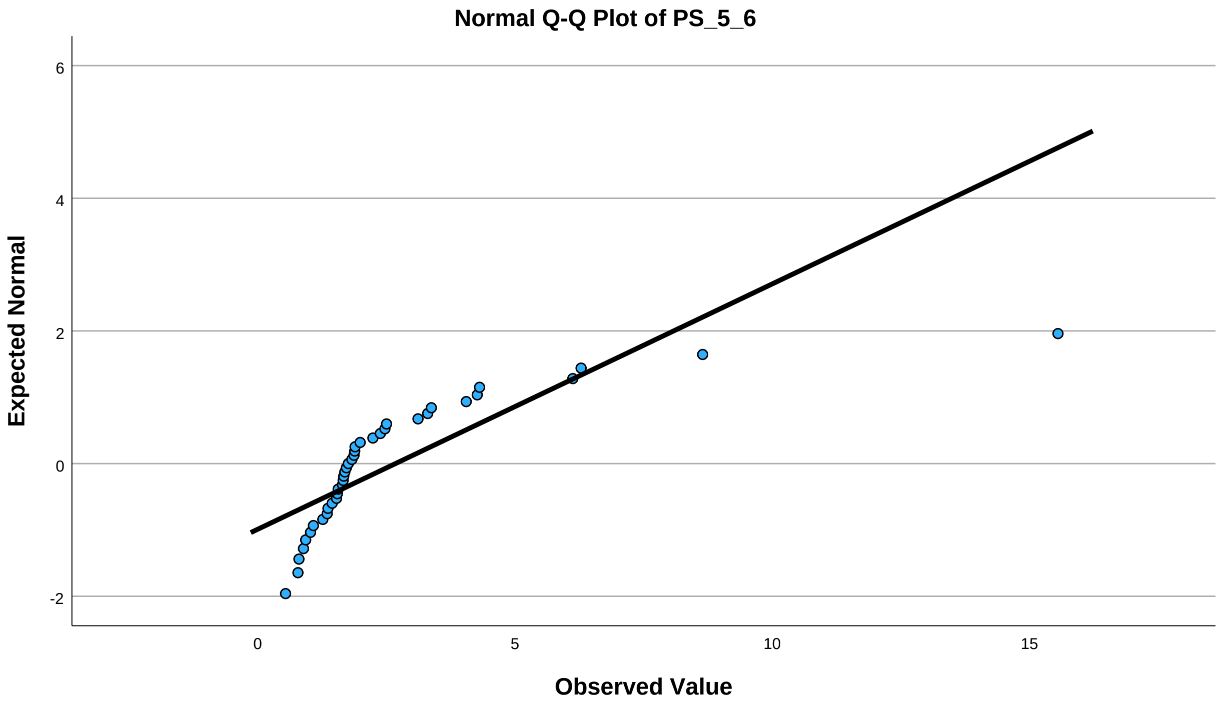

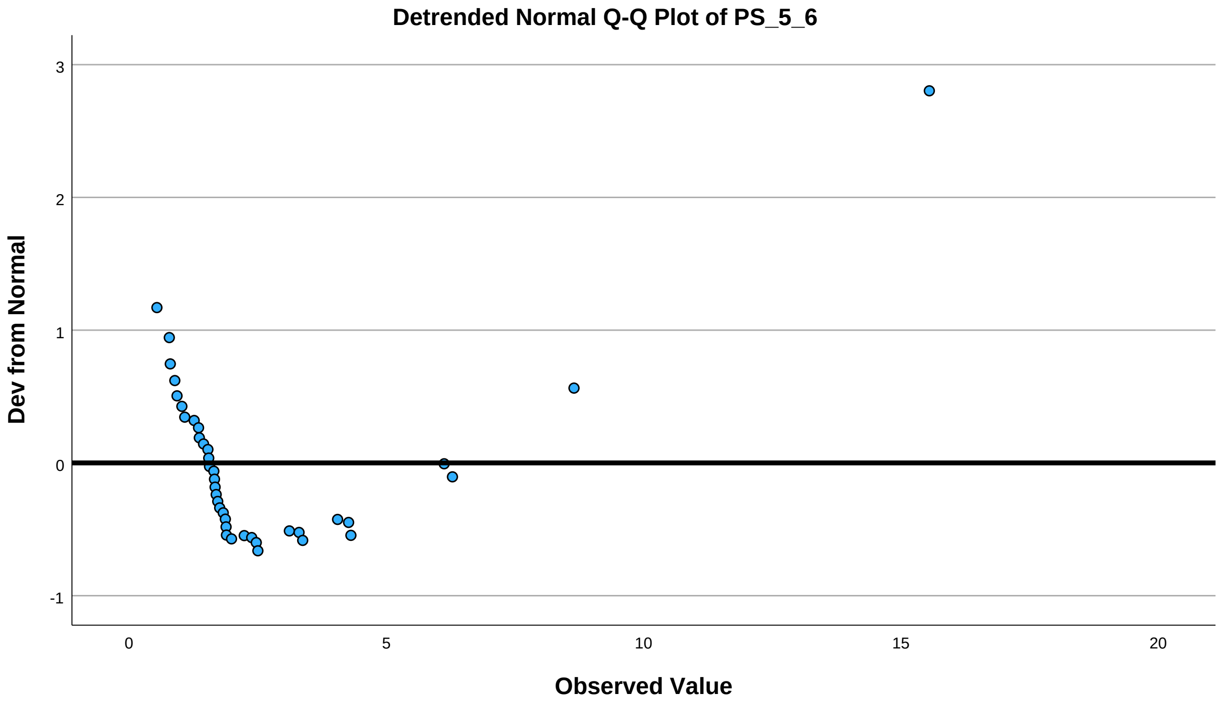
Detrended Normal Q-Q Plot of PS_5_6
Dev from Normal
3
2
1
0
-1
0
5
10
15
20
Observed Value

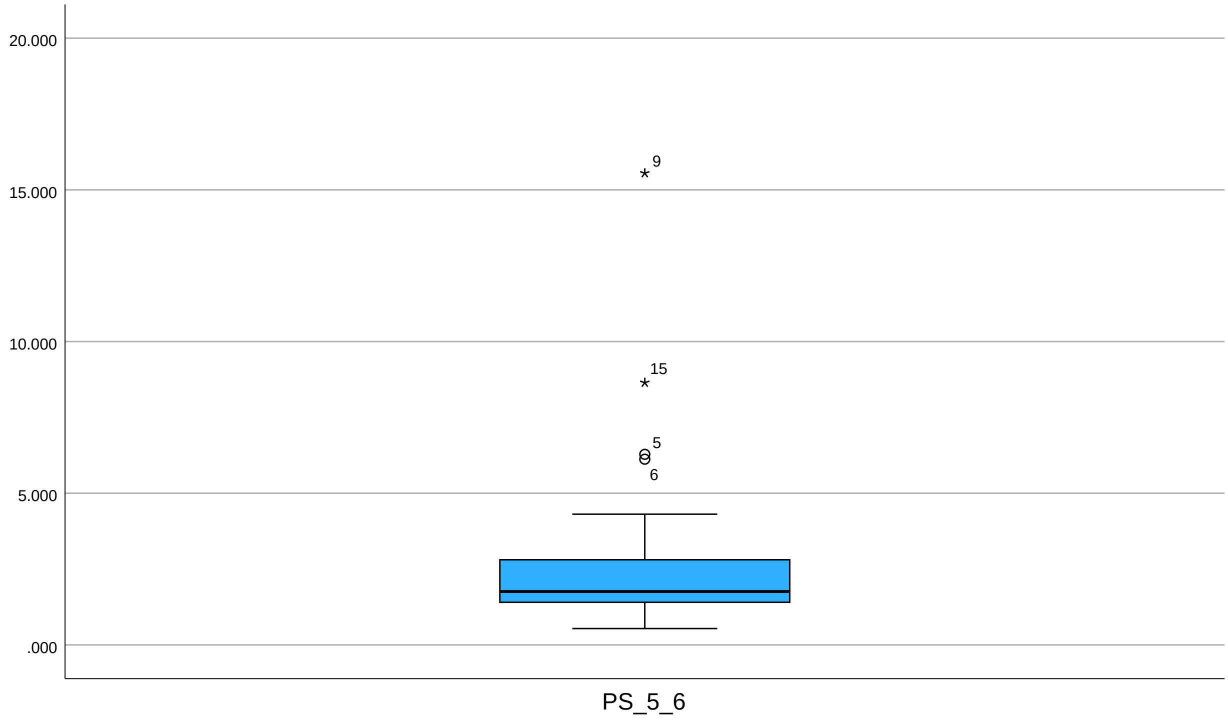
20.000
15.000
10.000
5.000
.000
9
15
5
6
PS_5_6

PS_6_7

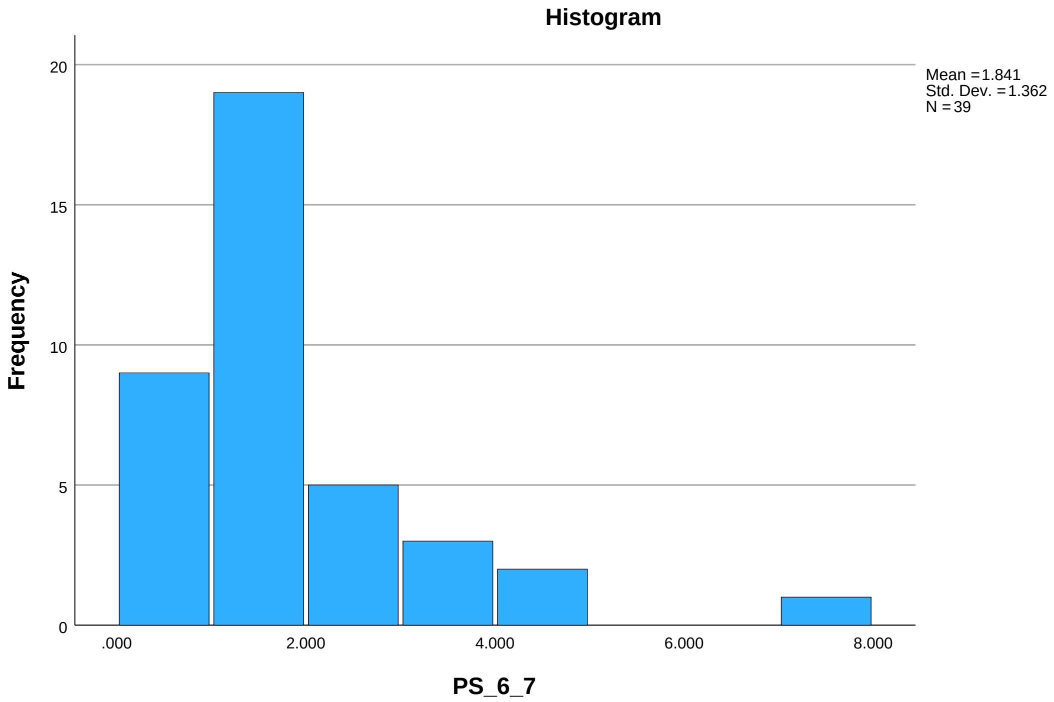


```
PS_6_7 Stem-and-Leaf Plot

 Frequency    Stem &  Leaf

     1.00        0 .  3
     8.00        0 .  56677899
    13.00        1 .  0011112223334
     6.00        1 .  556999
     1.00        2 .  2
     4.00        2 .  5568
     2.00        3 .  33
     1.00        3 .  5
     2.00        4 .  00
     1.00 Extremes    (>=7.7)

 Stem width:      1.000
 Each leaf:       1 case(s)
```

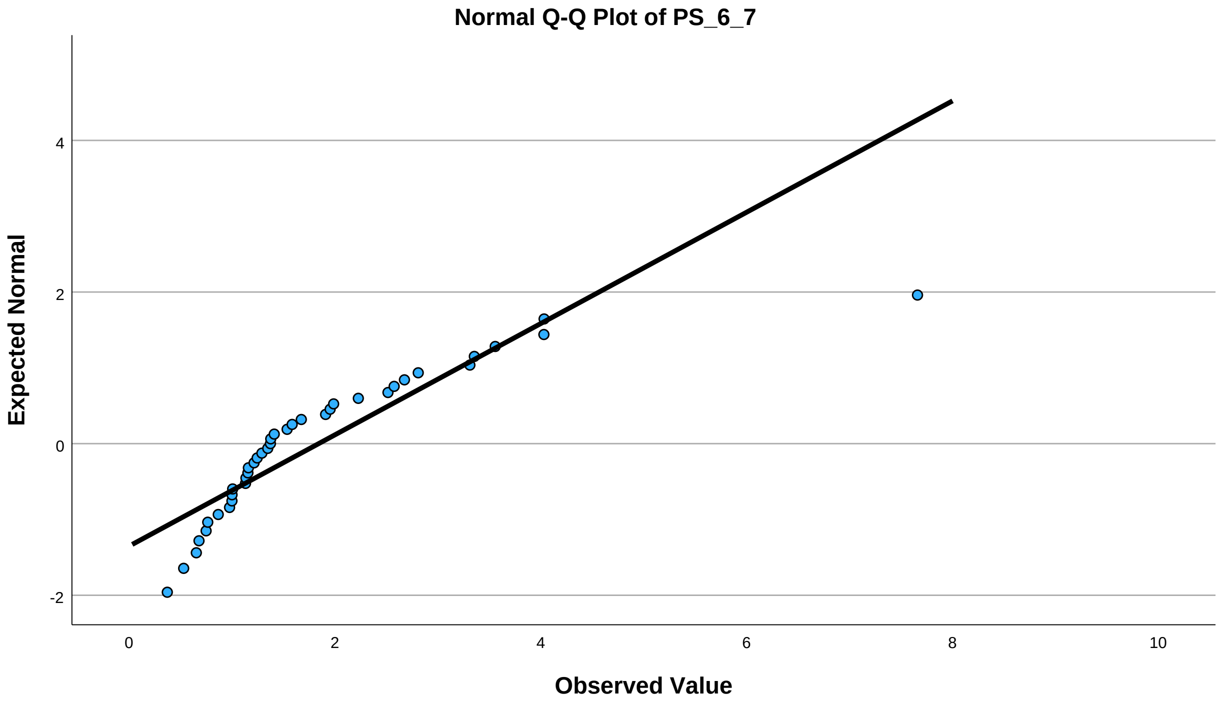
Normal Q-Q Plot of PS_6_7
Expected Normal
Observed Value
4
2
0
-2
0
2
4
6
8
10

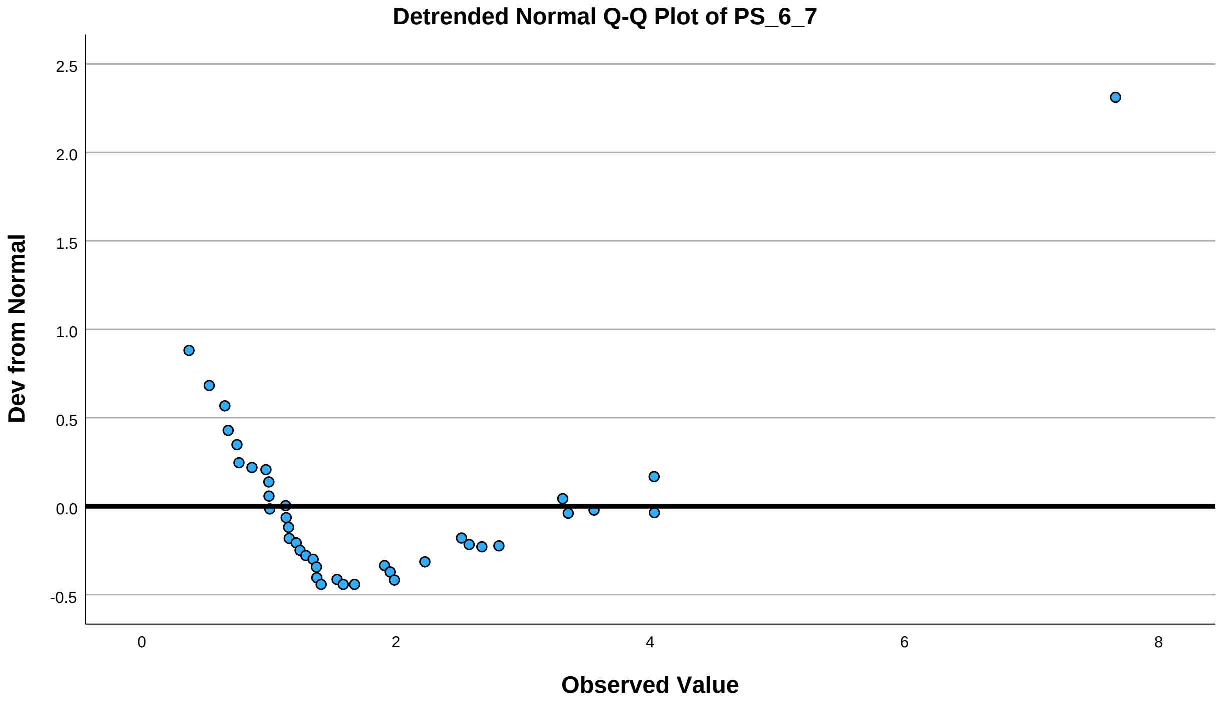
Detrended Normal Q-Q Plot of PS_6_7
Dev from Normal
Observed Value
2.5
2.0
1.5
1.0
0.5
0.0
-0.5
0
2
4
6
8

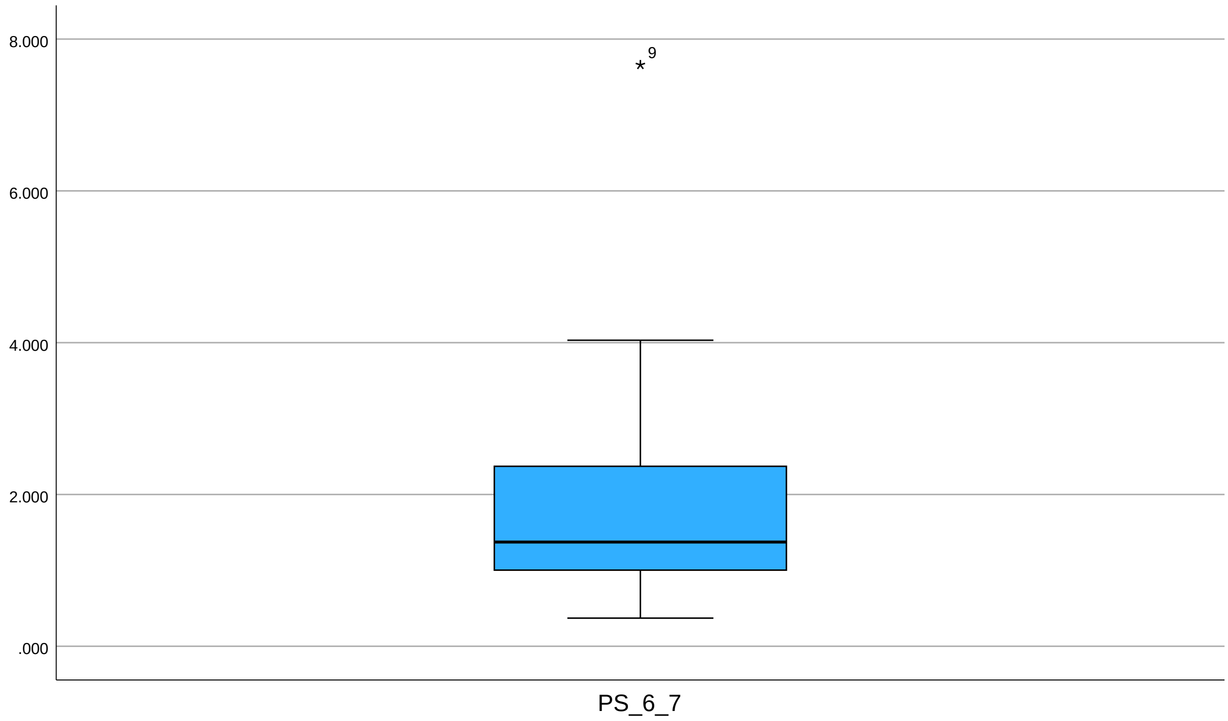

PS_7_8

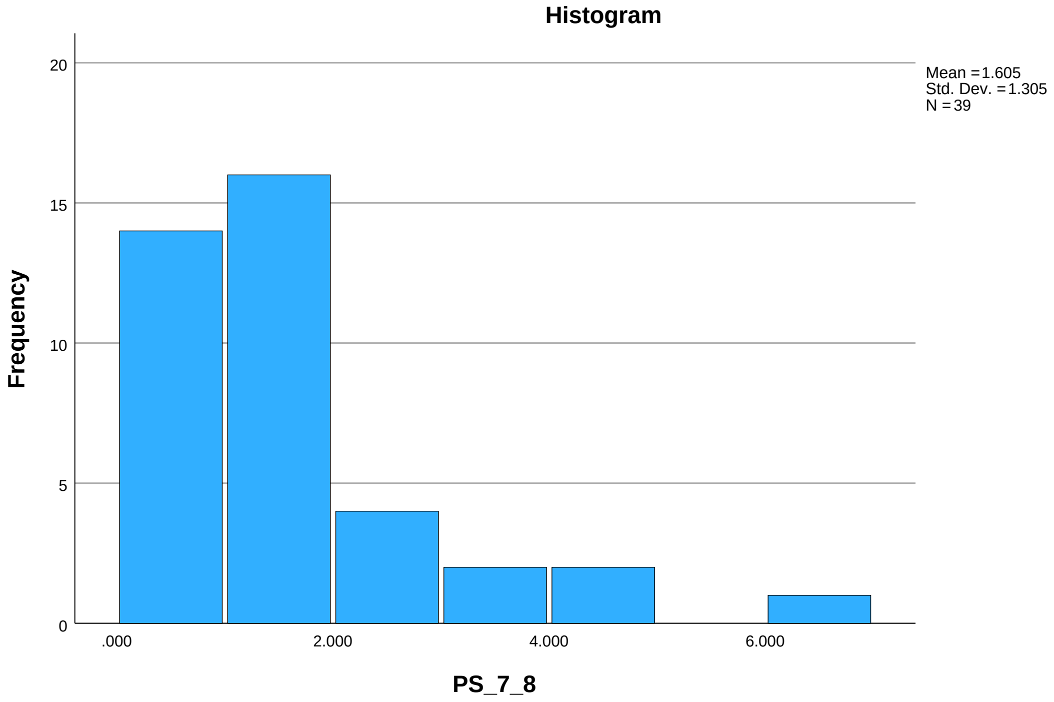


```
PS_7_8 Stem-and-Leaf Plot

 Frequency    Stem &  Leaf
```

```
     2.00        0 .  34
    12.00        0 .  556788888899
    14.00        1 .  00000011122224
     2.00        1 .  68
     2.00        2 .  14
     2.00        2 .  55
     5.00 Extremes    (>=3.7)

 Stem width:      1.000
 Each leaf:        1 case(s)
```

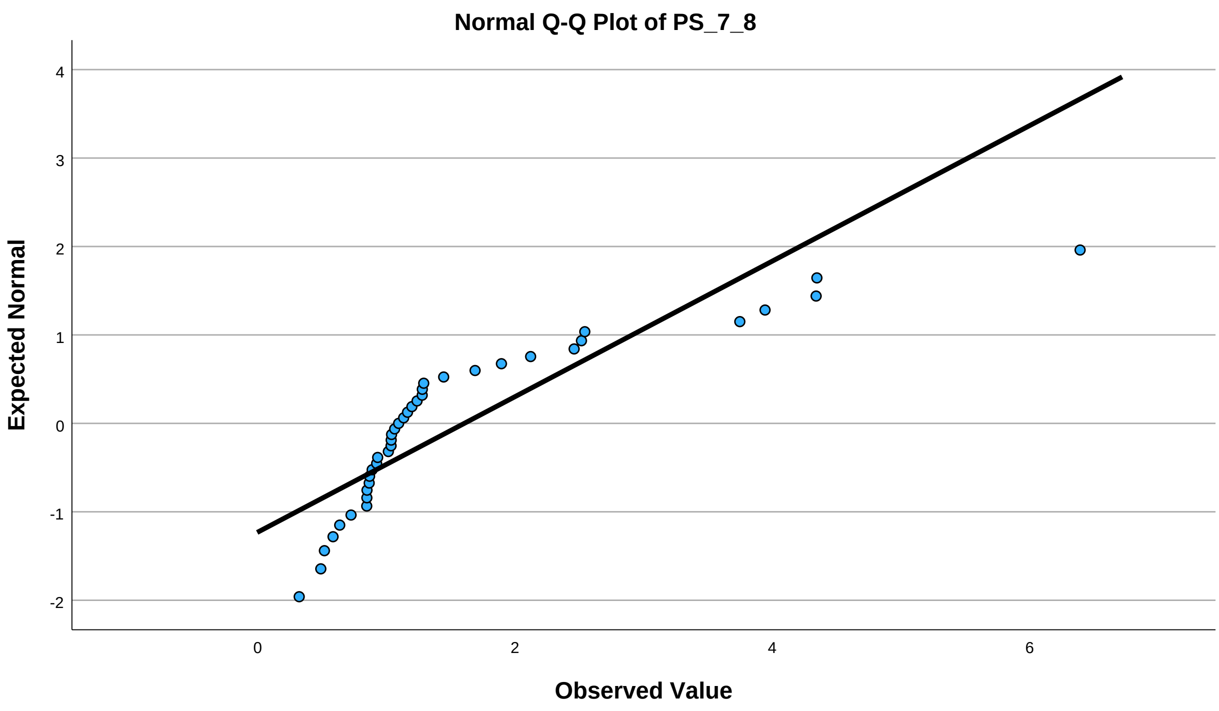

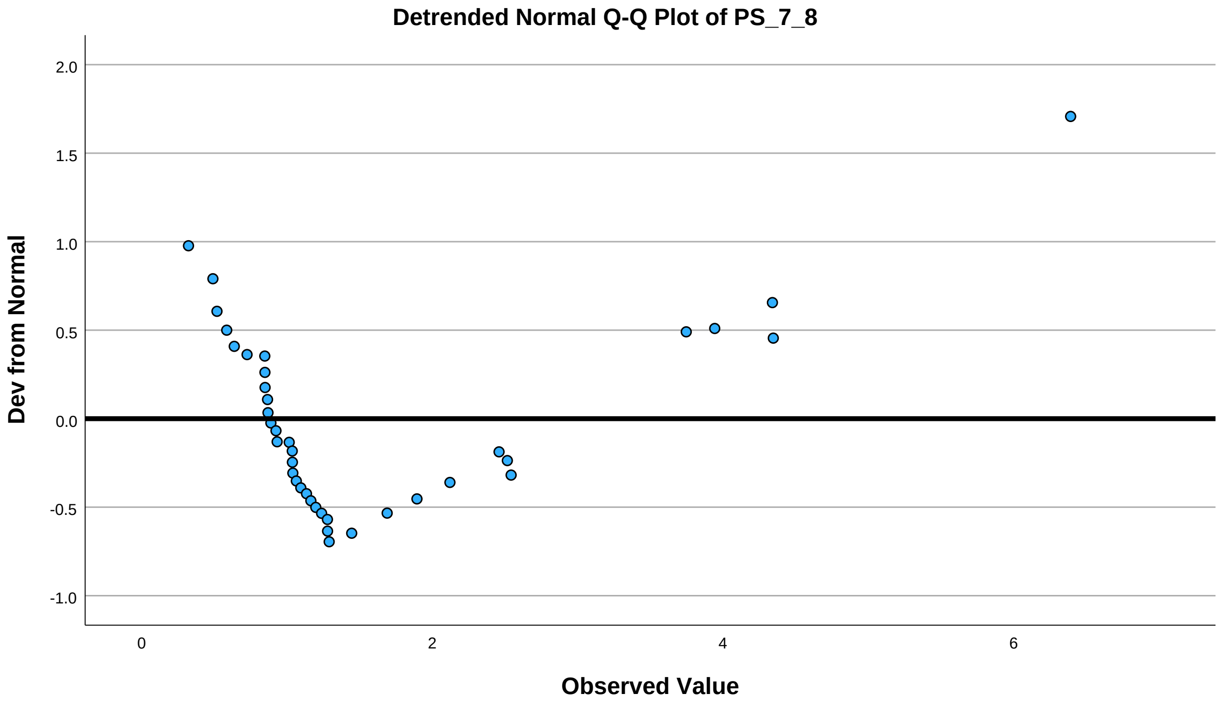
Detrended Normal Q-Q Plot of PS_7_8
Dev from Normal
2.0
1.5
1.0
0.5
0.0
-0.5
-1.0
0
2
4
6
Observed Value

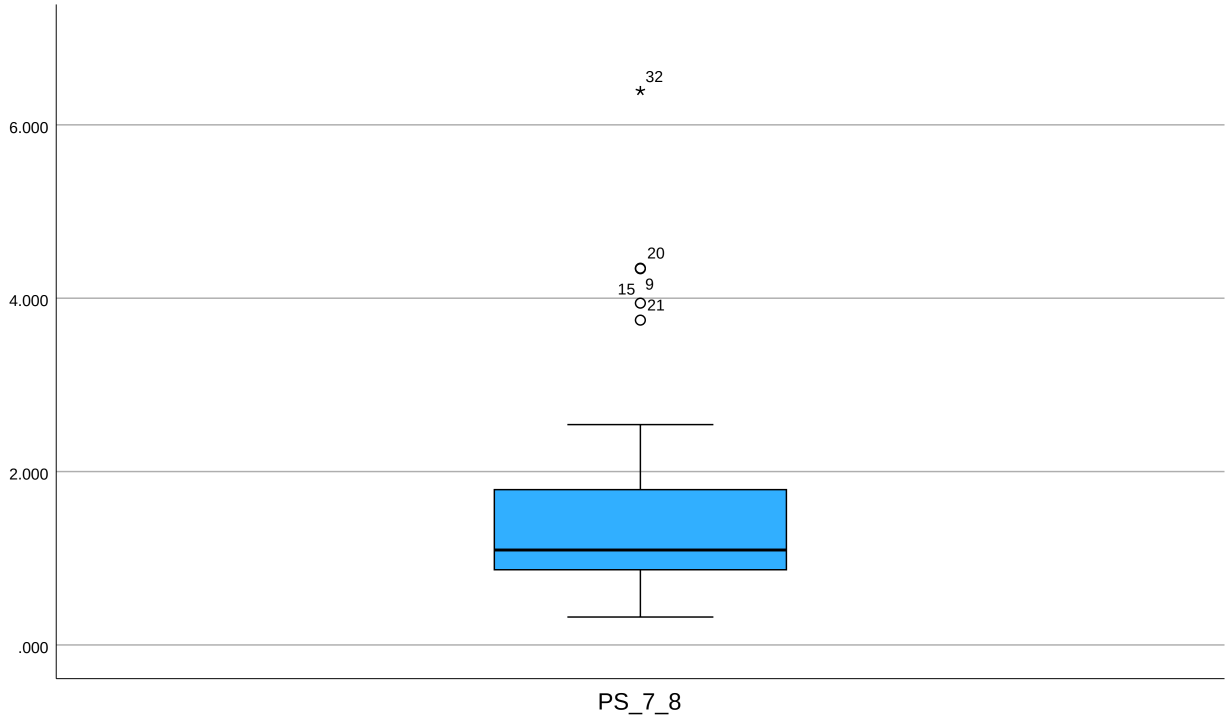
32
20
15
9
21
6.000
4.000
2.000
.000
PS_7_8

PS_8_9

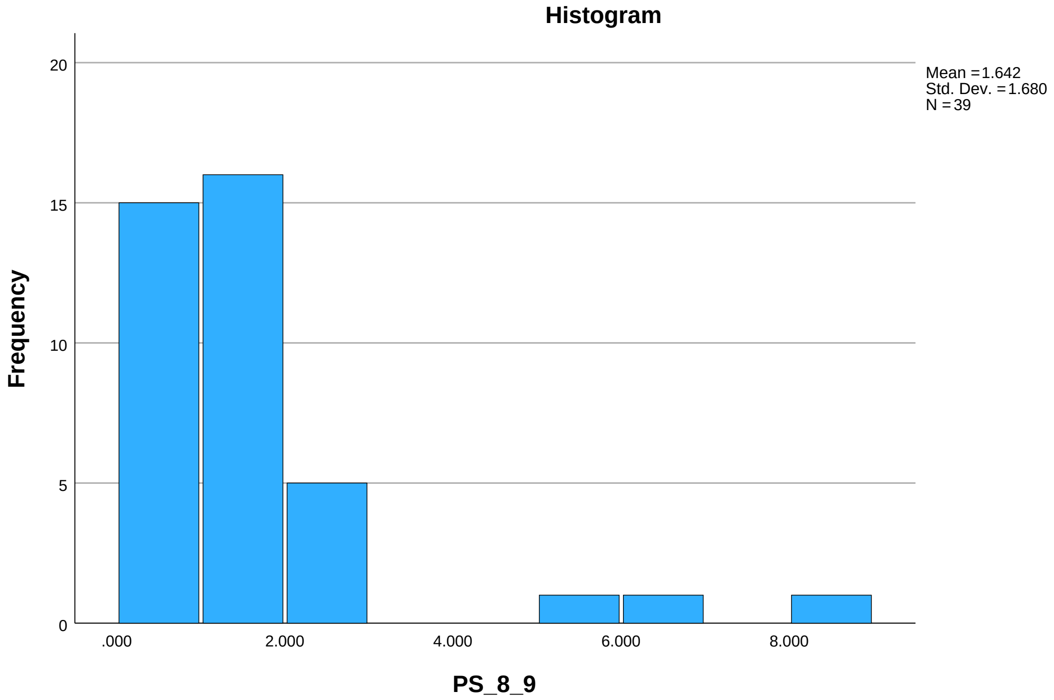


```
PS_8_9 Stem-and-Leaf Plot

 Frequency    Stem &  Leaf

     3.00        0 .  244
    12.00        0 .  556788889999
    14.00        1 .  00001122223344
     2.00        1 .  56
     1.00        2 .  0
     7.00 Extremes    (>=2.4)

 Stem width:      1.000
 Each leaf:       1 case(s)
```

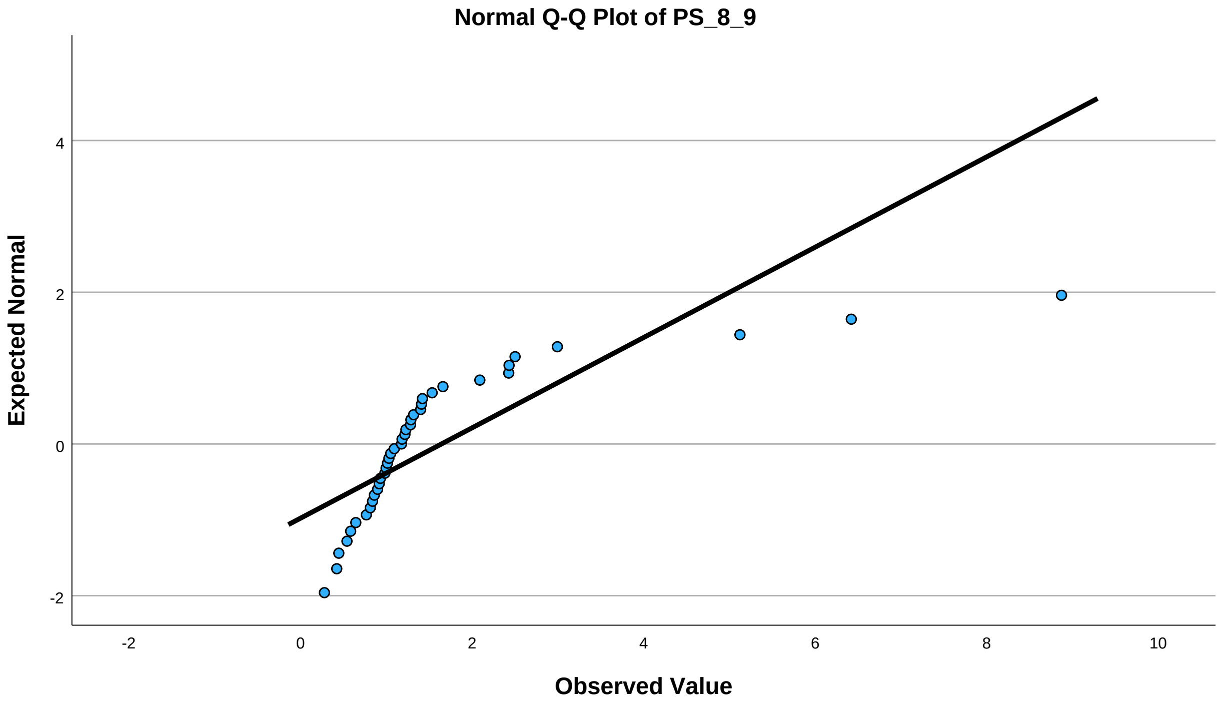
Normal Q-Q Plot of PS_8_9
Expected Normal
4
2
0
-2
-2
0
2
4
6
8
10
Observed Value

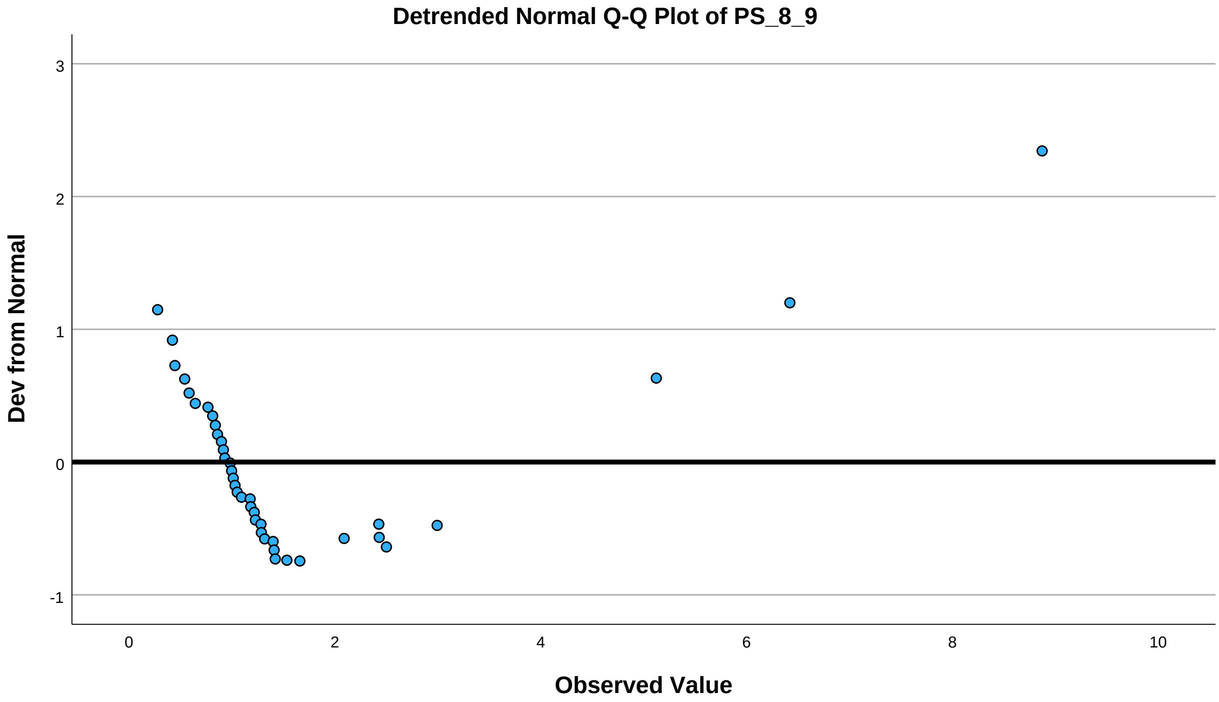
Detrended Normal Q-Q Plot of PS_8_9
Dev from Normal
3
2
1
0
-1
0
2
4
6
8
10
Observed Value

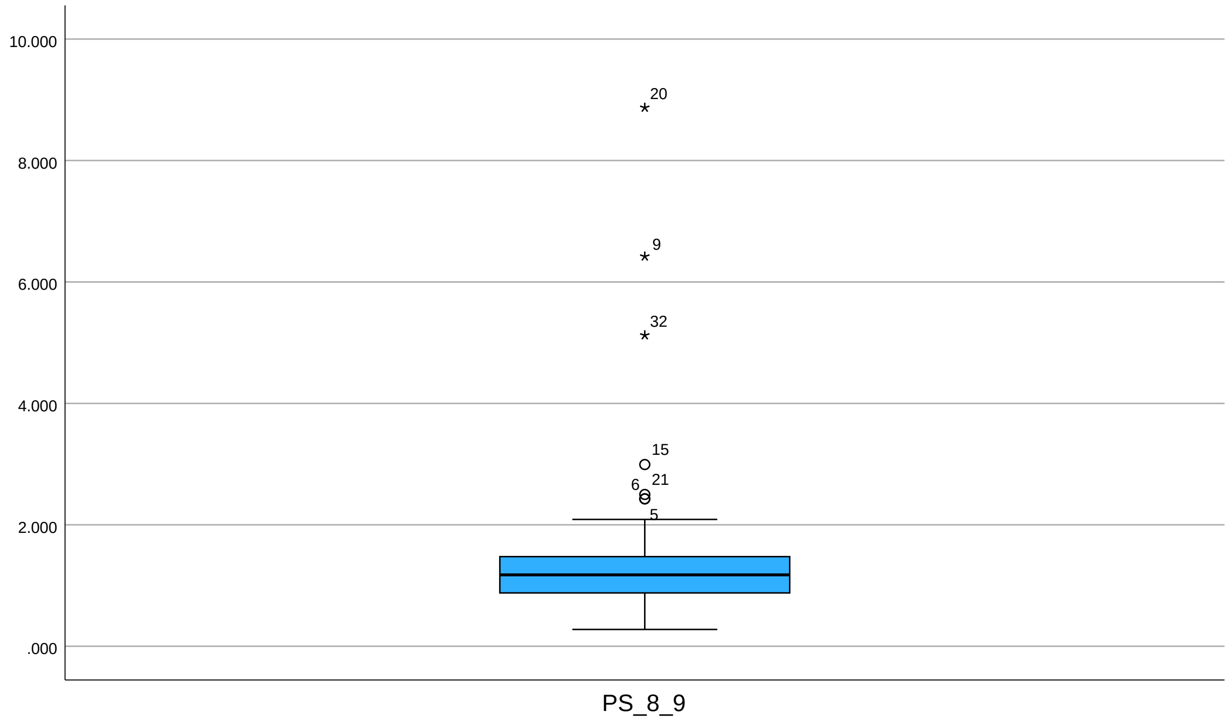

PS_9_10

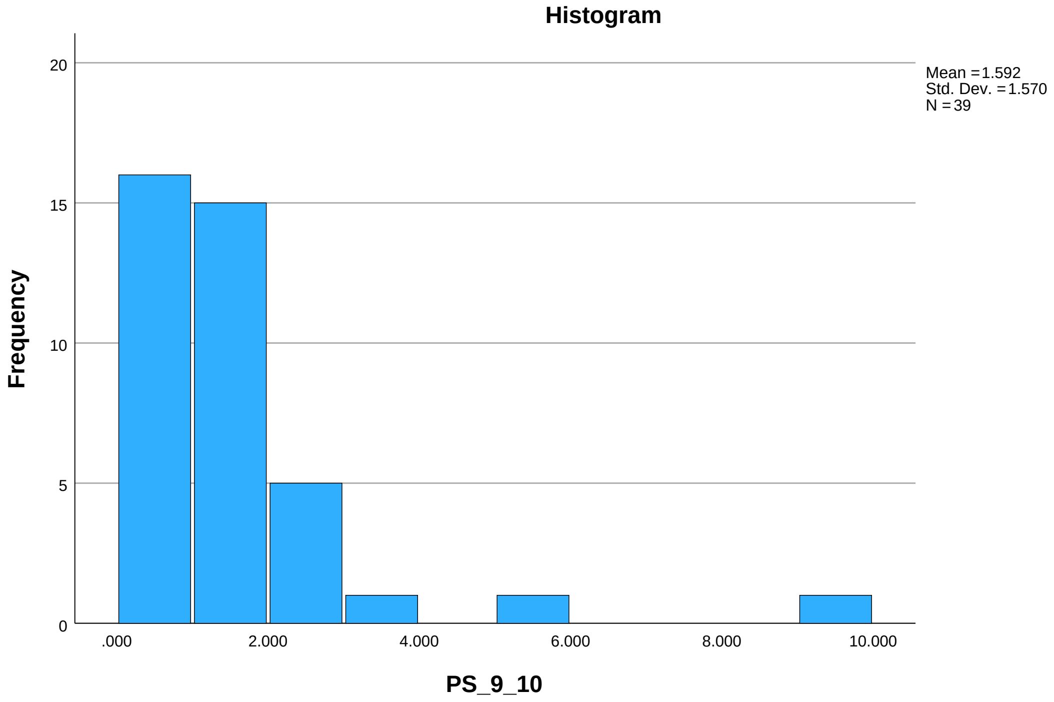


```
PS_9_10 Stem-and-Leaf Plot

 Frequency    Stem &  Leaf
```

```
    4.00        0 .  2334
   12.00        0 .  567778889999
    9.00        1 .  000122334
    6.00        1 .  666788
    5.00        2 .  12224
    3.00 Extremes    (>=3.9)

 Stem width:     1.000
 Each leaf:       1 case(s)
```

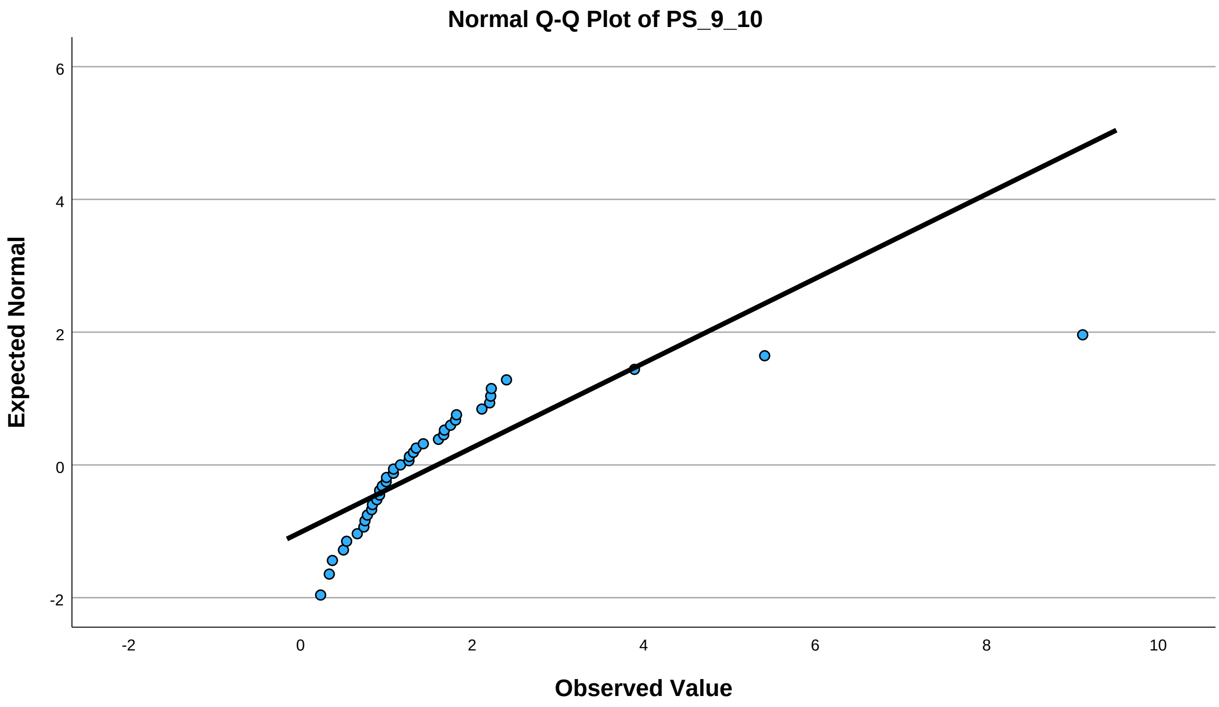

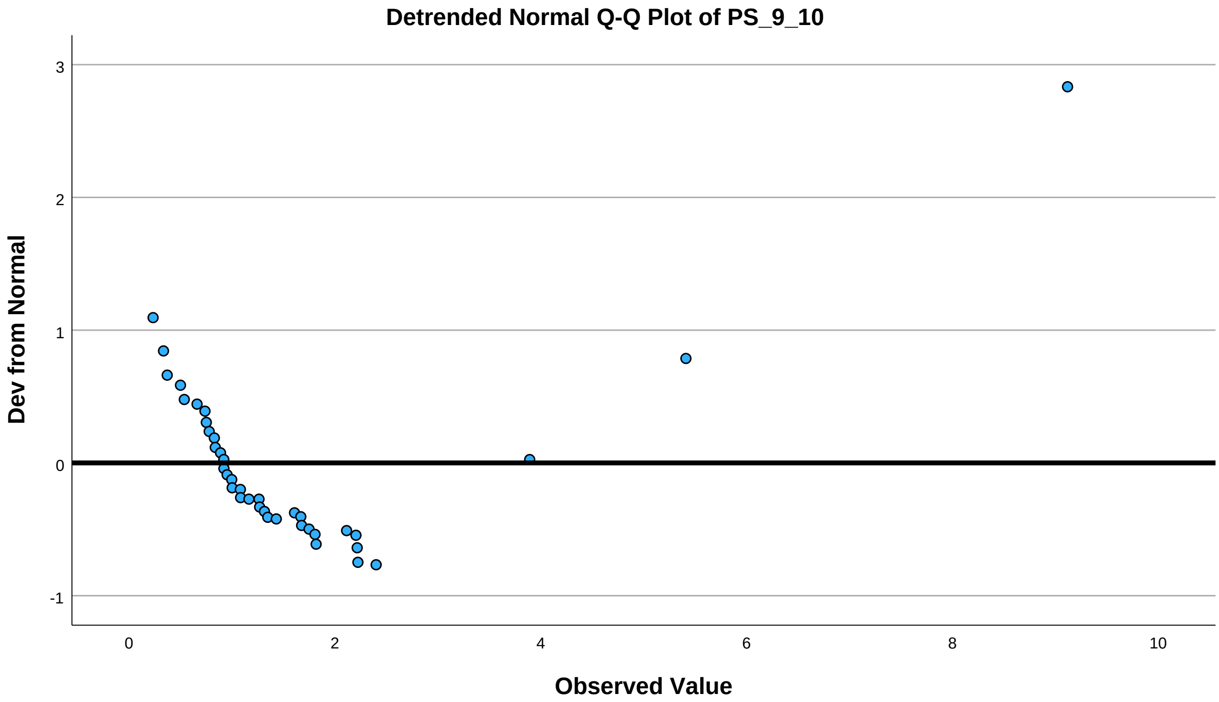

Detrended Normal Q-Q Plot of PS_9_10
Dev from Normal
3
2
1
0
-1
0
2
4
6
8
10
Observed Value

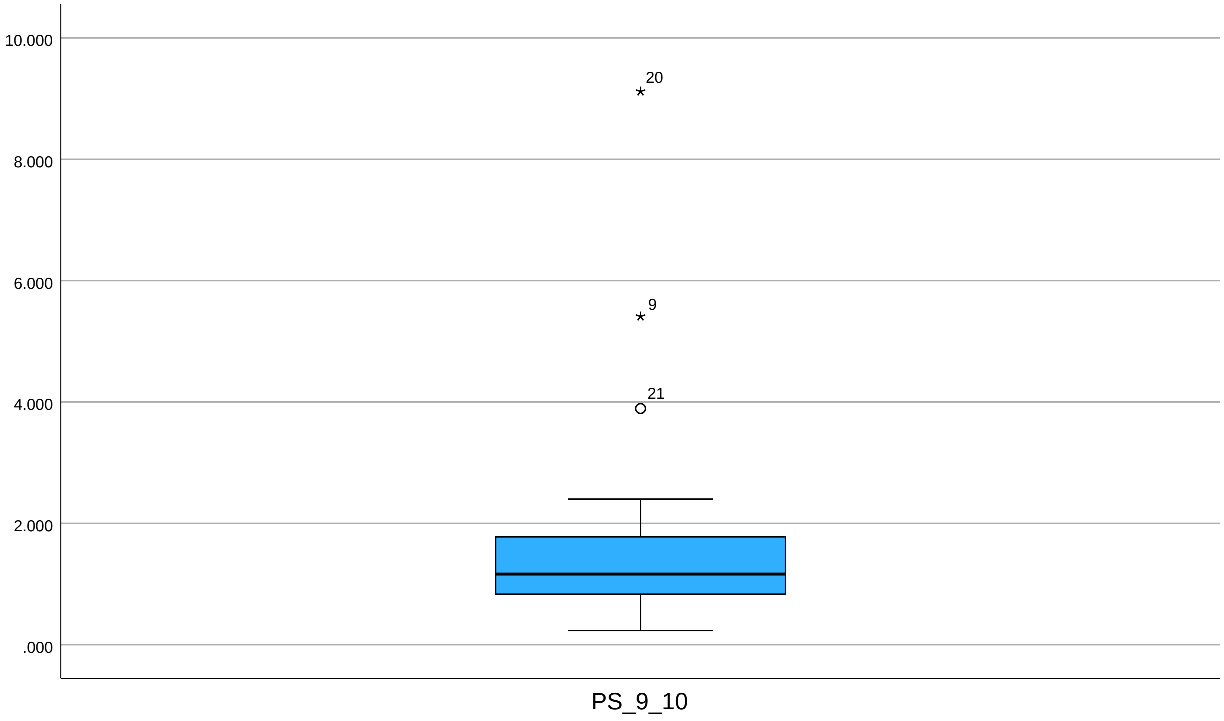

10.000
8.000
6.000
4.000
2.000
.000
20
9
21
PS_9_10

PS_10_11

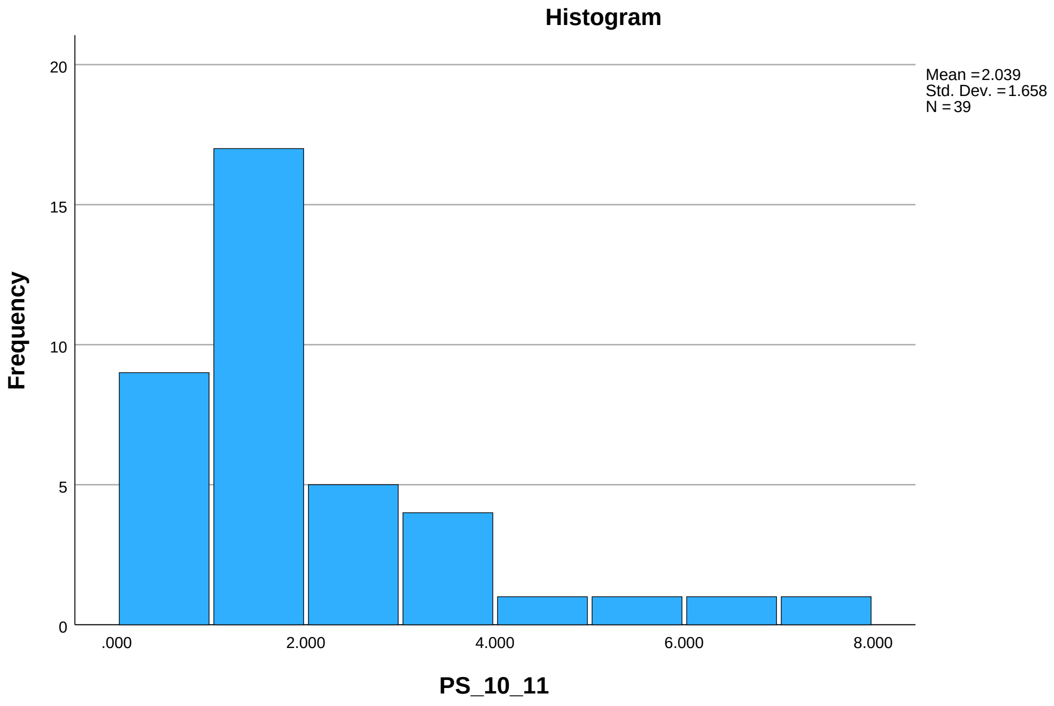


```
PS_10_11 Stem-and-Leaf Plot

 Frequency    Stem &  Leaf

     3.00        0 .  234
     6.00        0 .  677789
     9.00        1 .  000011223
     8.00        1 .  55557799
     2.00        2 .  12
     3.00        2 .  558
     3.00        3 .  134
     1.00        3 .  5
     1.00        4 .  3
     3.00 Extremes    (>=5.4)

 Stem width:      1.000
 Each leaf:       1 case(s)
```

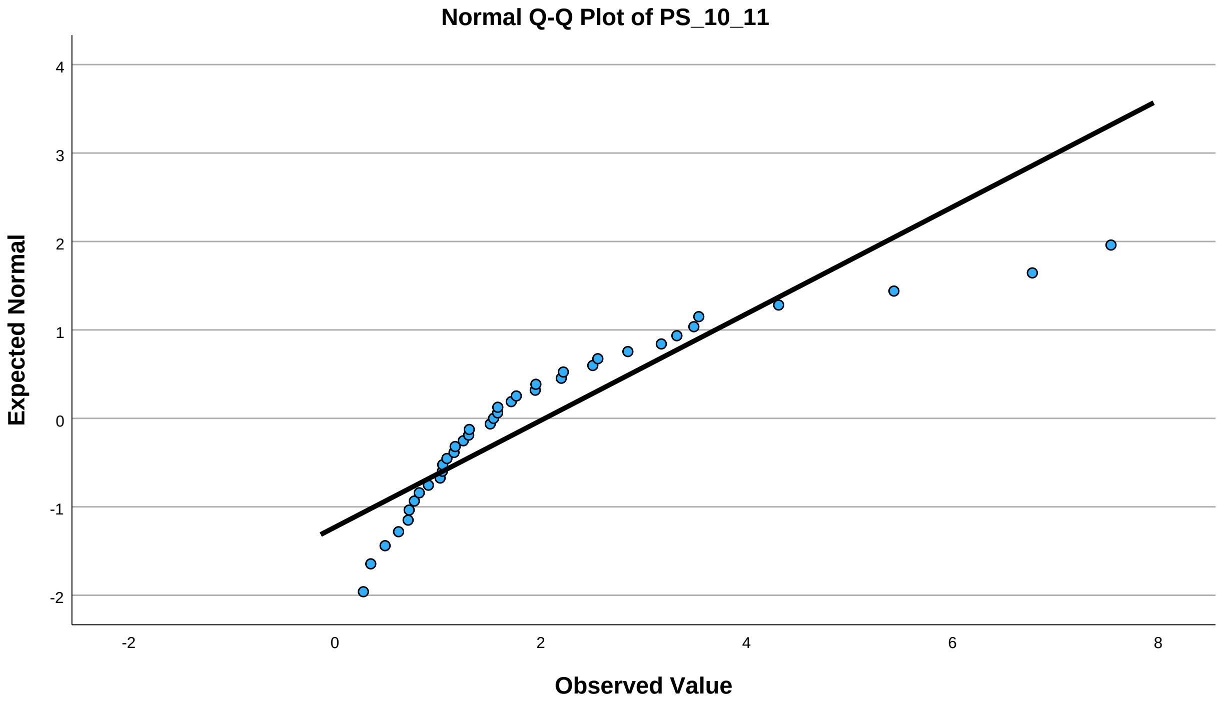
Normal Q-Q Plot of PS_10_11
Expected Normal
4
3
2
1
0
-1
-2
-2
0
2
4
6
8
Observed Value

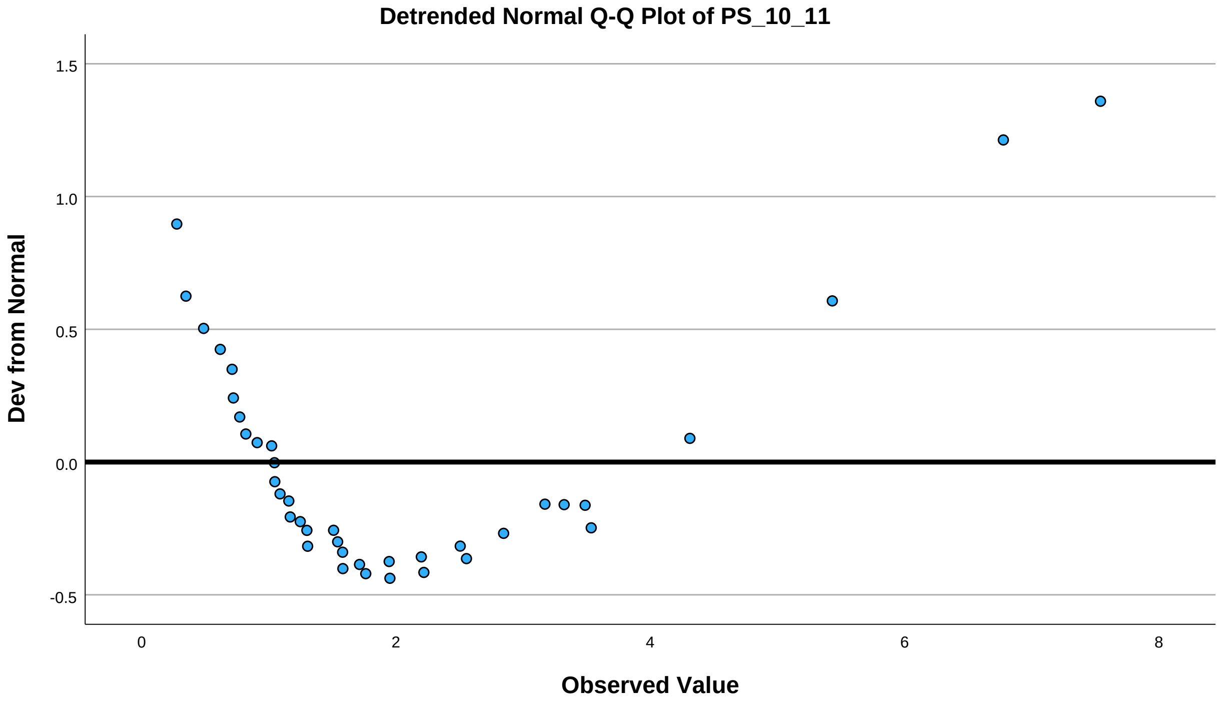
Detrended Normal Q-Q Plot of PS_10_11
Dev from Normal
1.5
1.0
0.5
0.0
-0.5
0
2
4
6
8
Observed Value

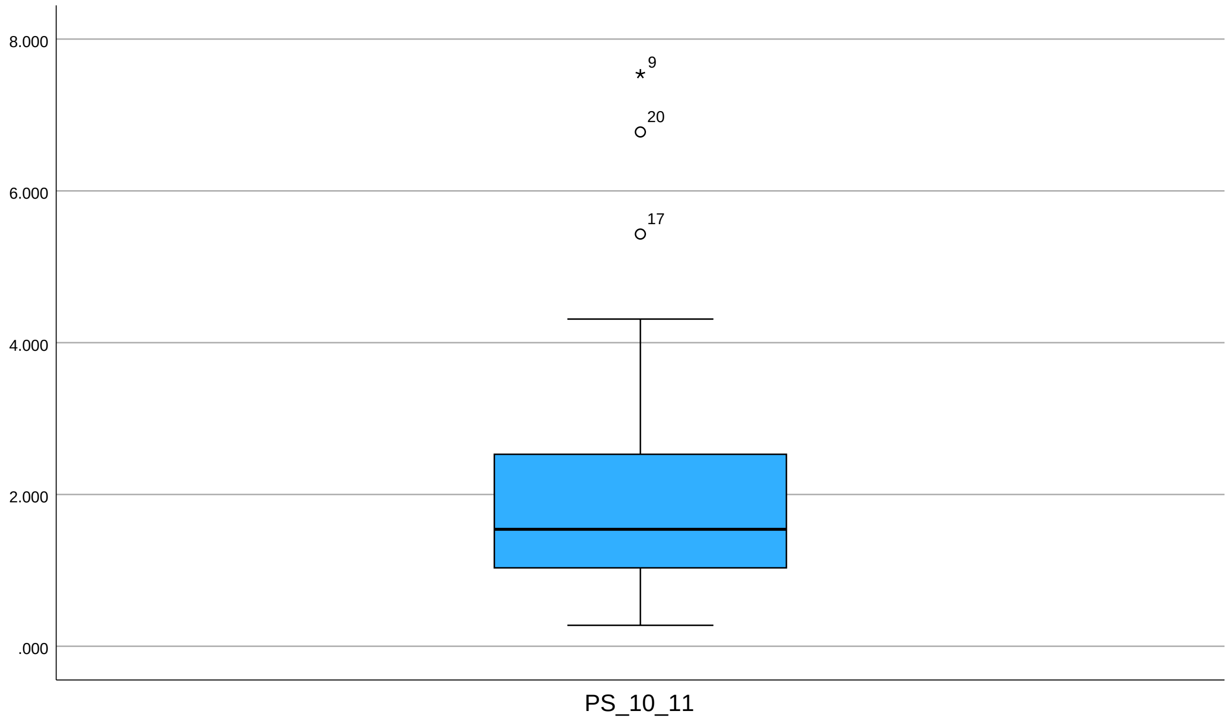

PS_11_12

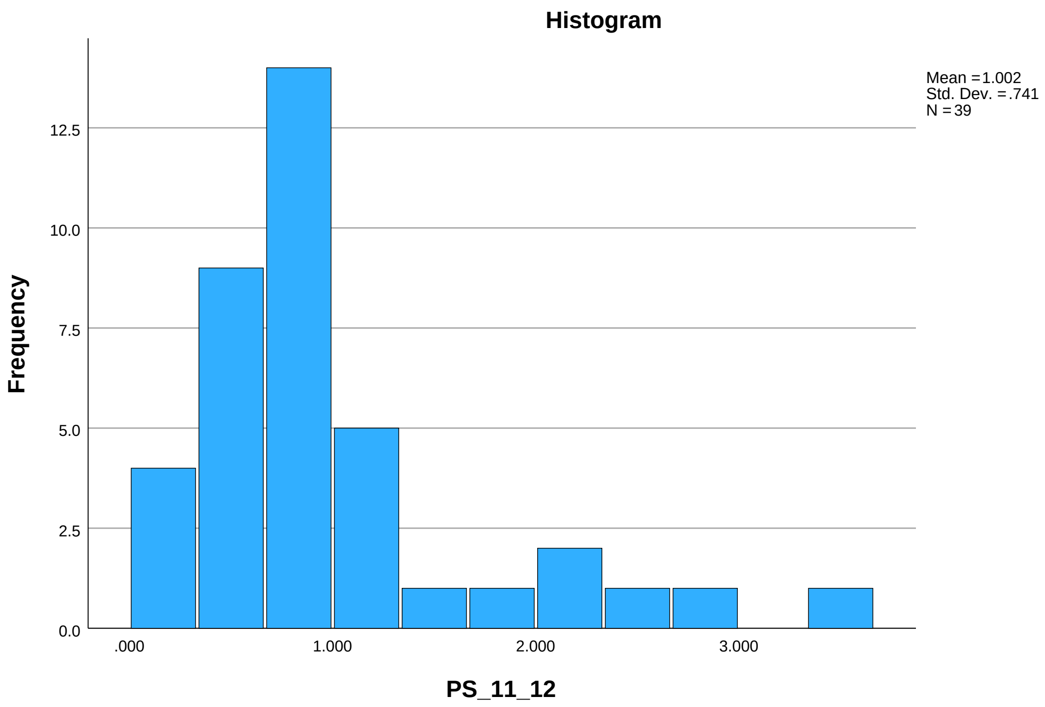

PS_11_12 Stem-and-Leaf Plot

Frequency   Stem & Leaf

```
    1.00        0 .  1
    4.00        0 .  2333
    4.00        0 .  4445
   12.00        0 .  666666666777
    6.00        0 .  889999
    4.00        1 .  0011
    1.00        1 .  3
    1.00        1 .  4
    1.00        1 .  6
    5.00 Extremes    (>=2.2)

 Stem width:      1.000
 Each leaf:        1 case(s)
```

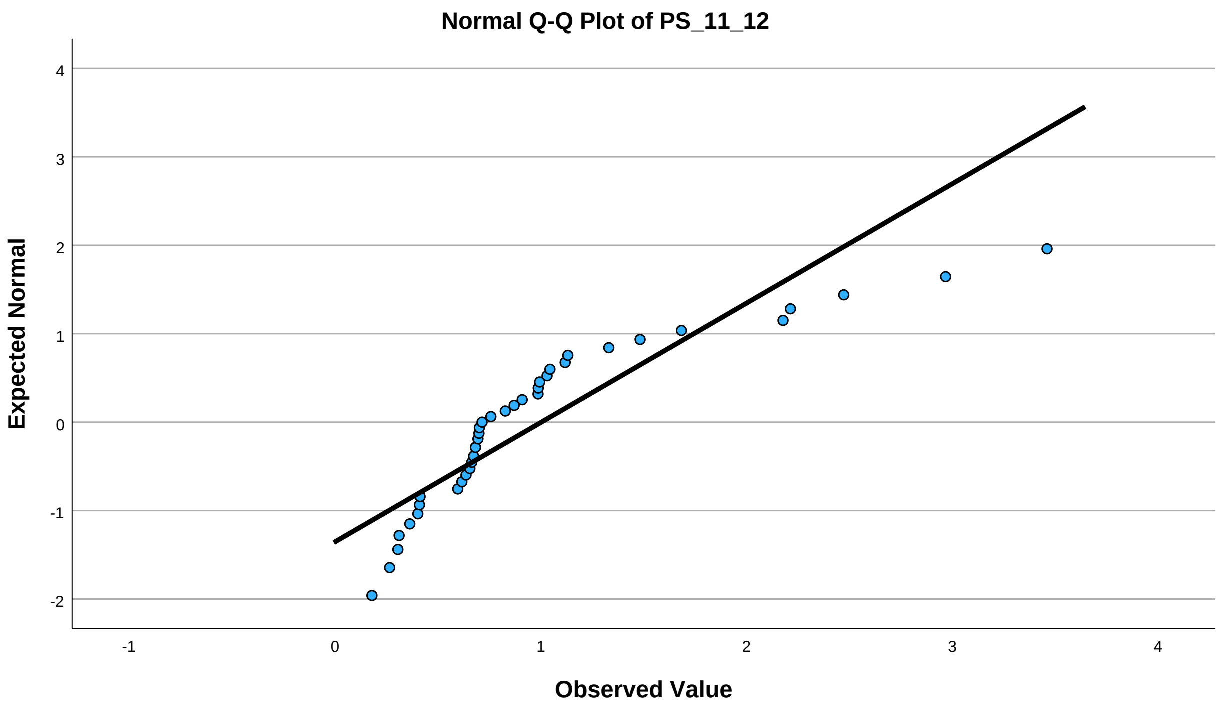

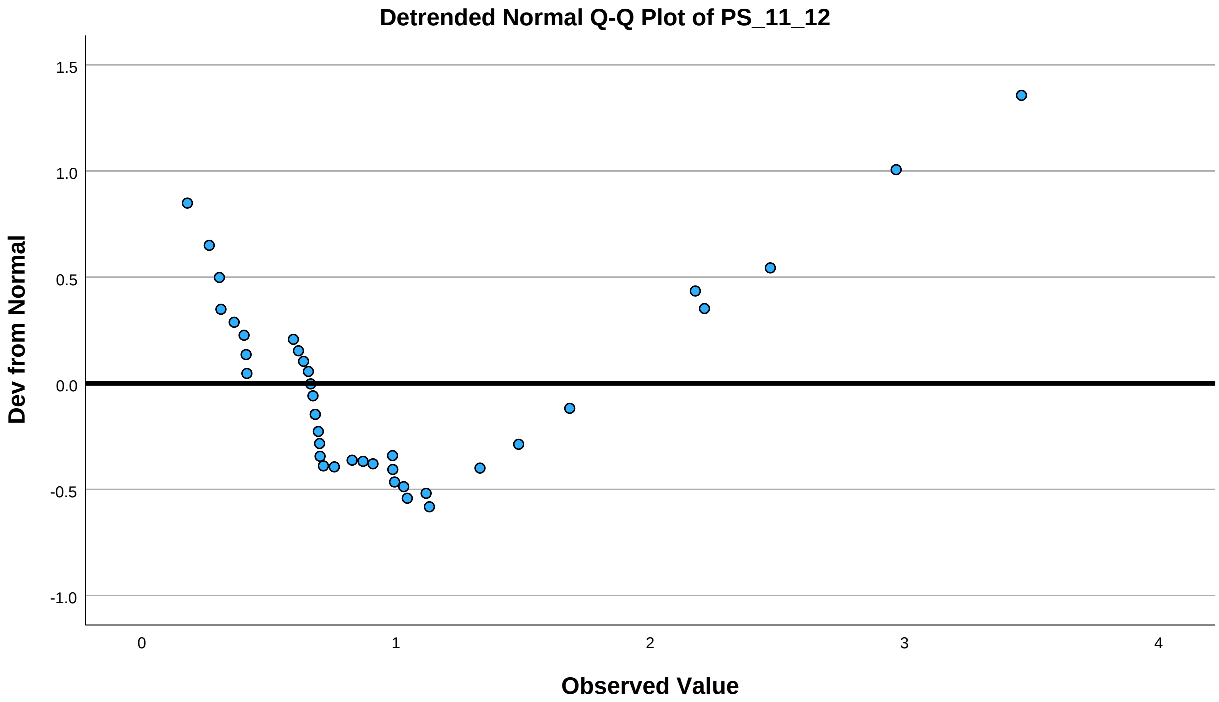
Detrended Normal Q-Q Plot of PS_11_12
Dev from Normal
Observed Value
1.5
1.0
0.5
0.0
-0.5
-1.0
0
1
2
3
4

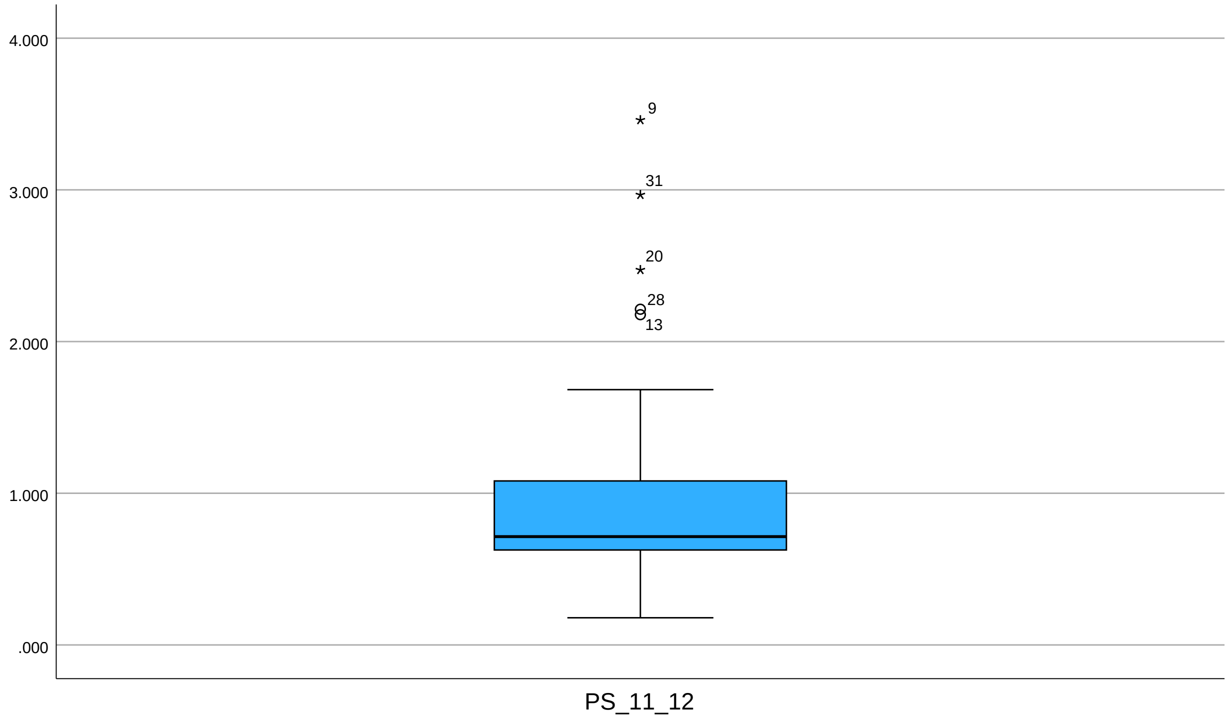
4.000
3.000
2.000
1.000
.000
9
31
20
28
13
PS_11_12

PS_12_13

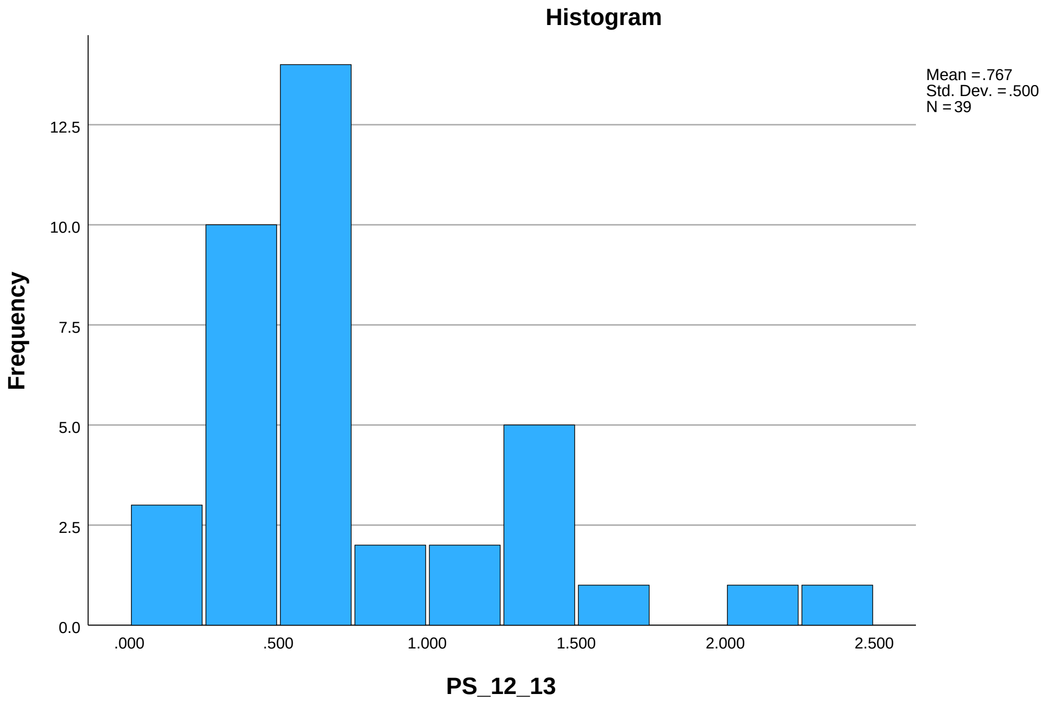


```
PS_12_13 Stem-and-Leaf Plot

 Frequency    Stem &  Leaf

     1.00        0 .  1
     4.00        0 .  2223
    16.00        0 .  4444444455555555
     6.00        0 .  666667
     2.00        0 .  89
     1.00        1 .  0
     5.00        1 .  23333
     2.00        1 .  45
     2.00 Extremes    (>=2.0)

 Stem width:      1.000
 Each leaf:       1 case(s)
```

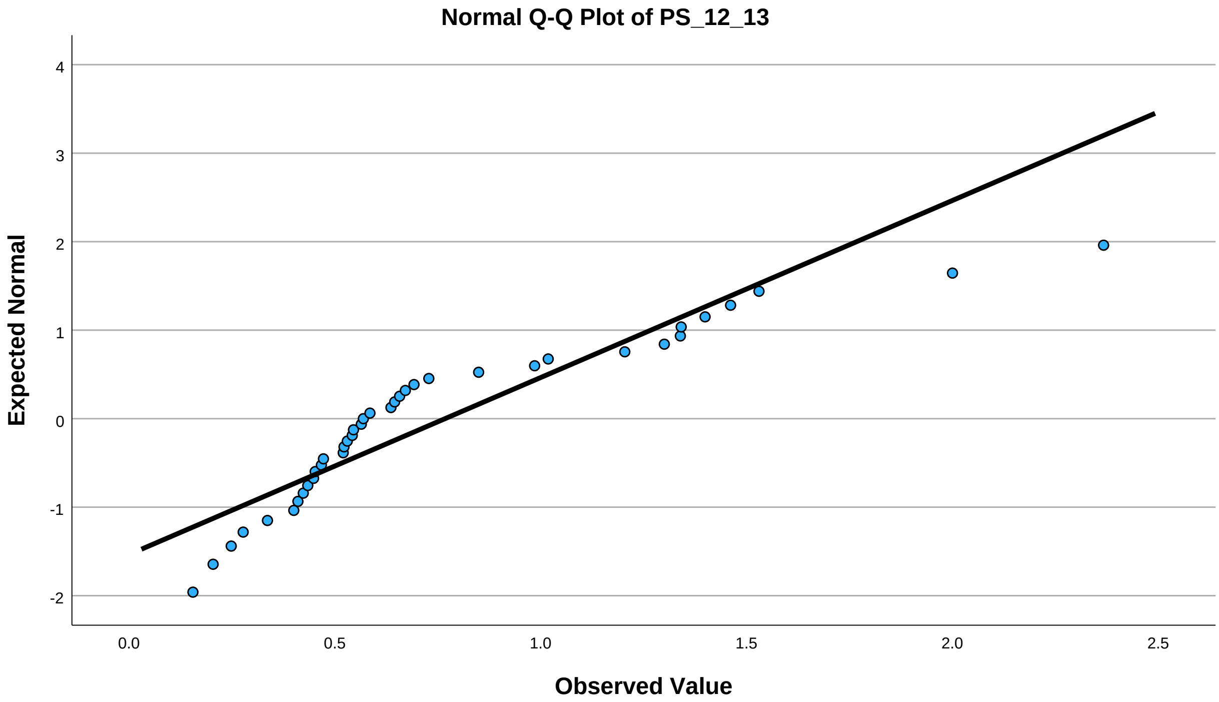
Normal Q-Q Plot of PS_12_13
Expected Normal
4
3
2
1
0
-1
-2
0.0
0.5
1.0
1.5
2.0
2.5
Observed Value

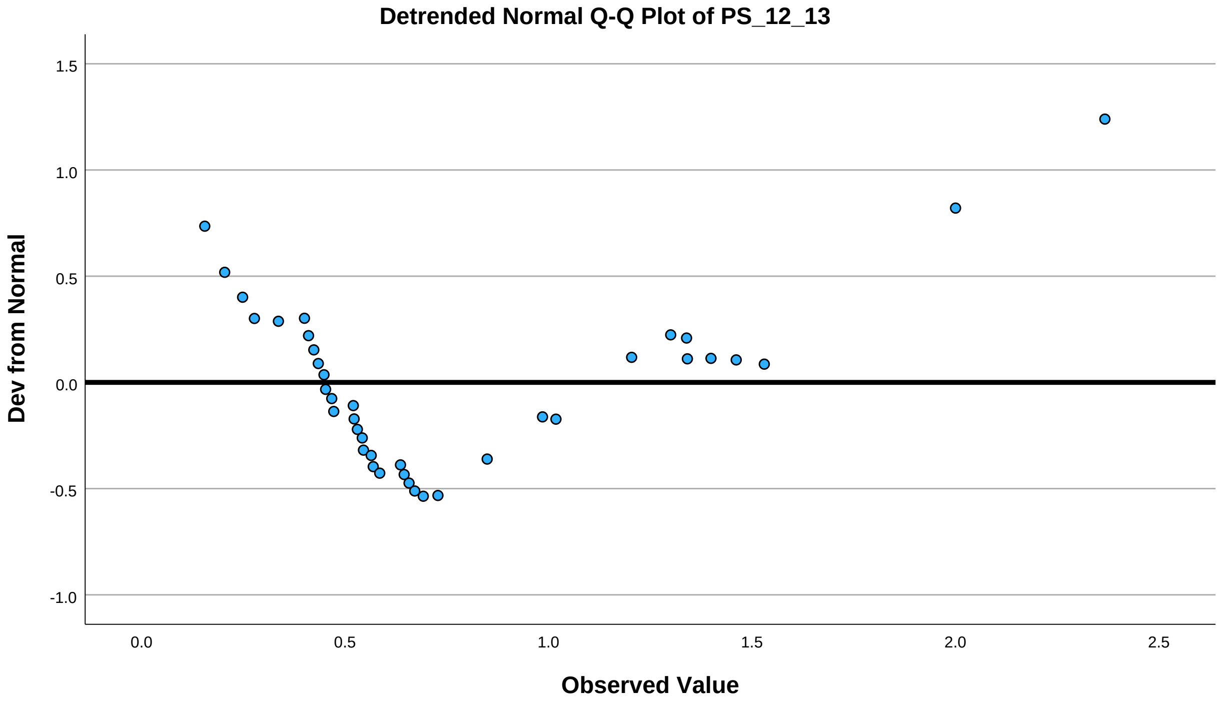
Detrended Normal Q-Q Plot of PS_12_13
Dev from Normal
1.5
1.0
0.5
0.0
-0.5
-1.0
0.0
0.5
1.0
1.5
2.0
2.5
Observed Value

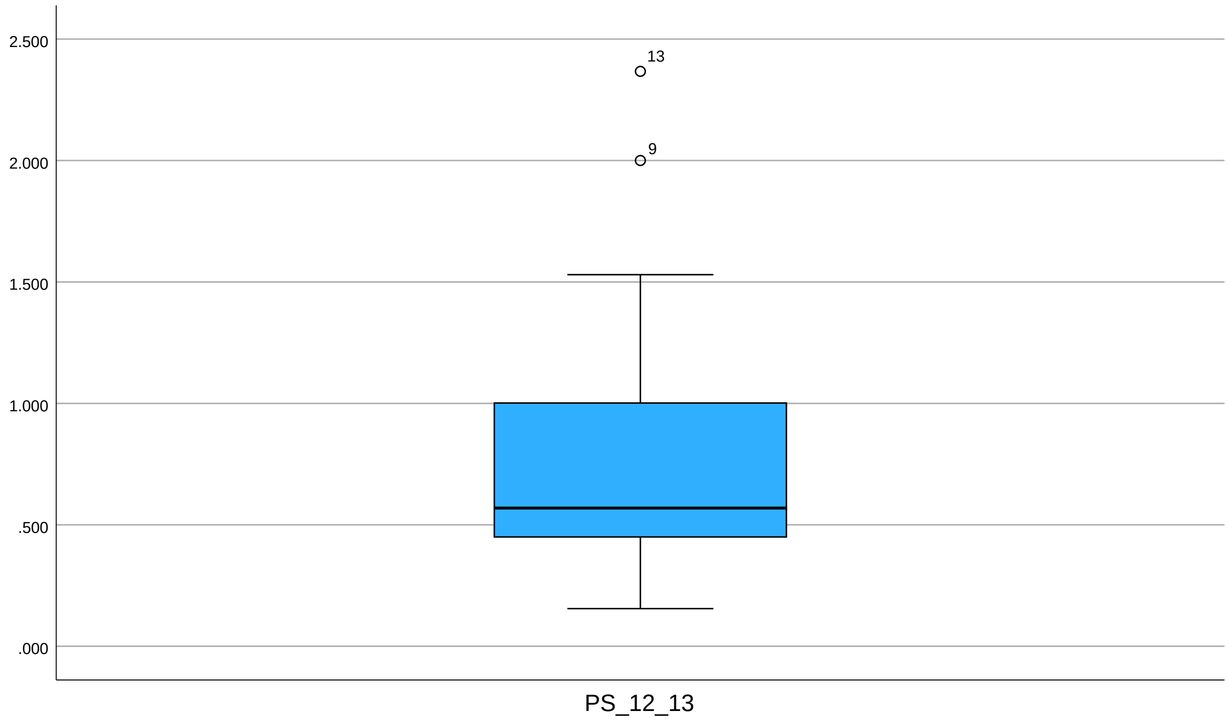

PS_13_14

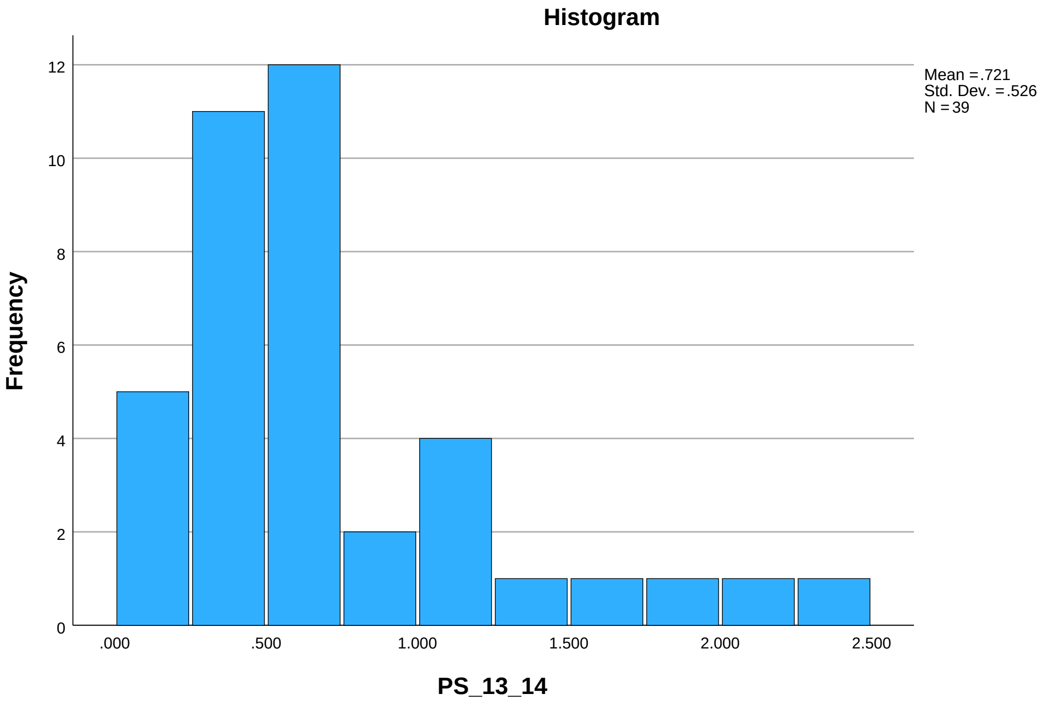


```
PS_13_14 Stem-and-Leaf Plot

 Frequency    Stem &  Leaf
```

```
  2.00       1 .  66
  3.00       2 .  144
  7.00       3 .  0235556
  4.00       4 .  0178
  5.00       5 .  04777
  5.00       6 .  02359
  3.00       7 .  028
   .00       8 .
  1.00       9 .  6
  2.00      10 .  48
  1.00      11 .  5
  2.00      12 .  48
  4.00 Extremes    (>=1.75)

Stem width:      .100
Each leaf:       1 case(s)
```

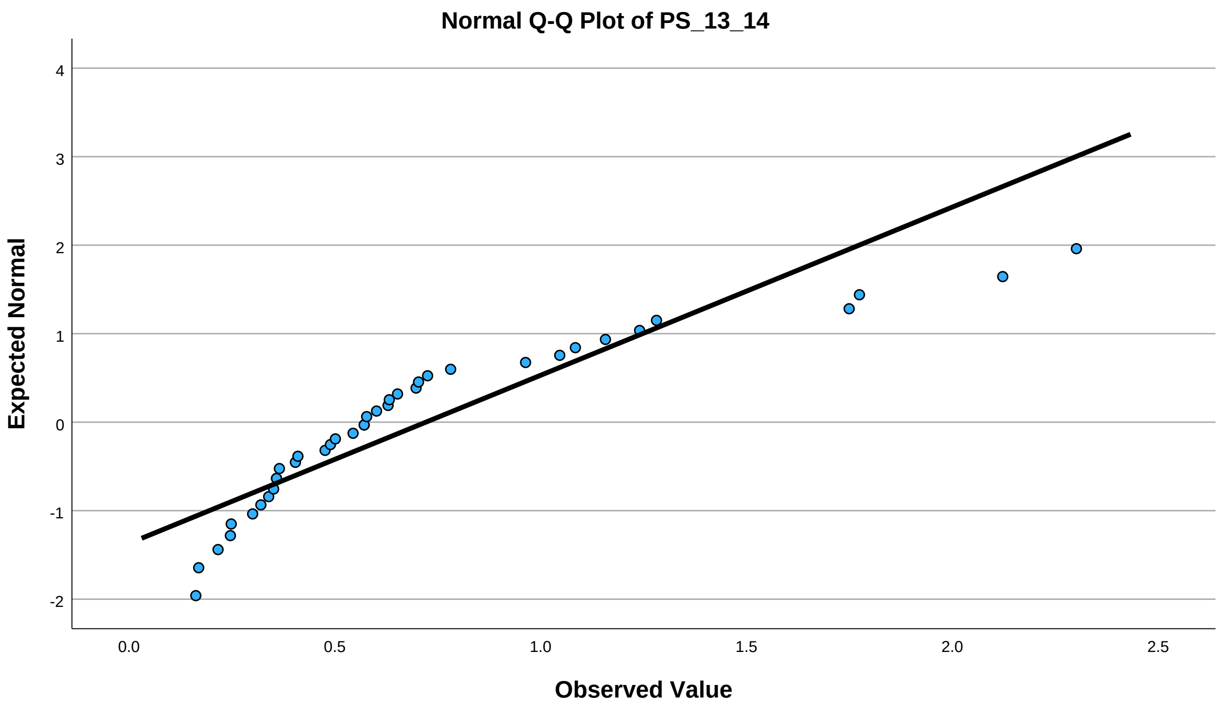

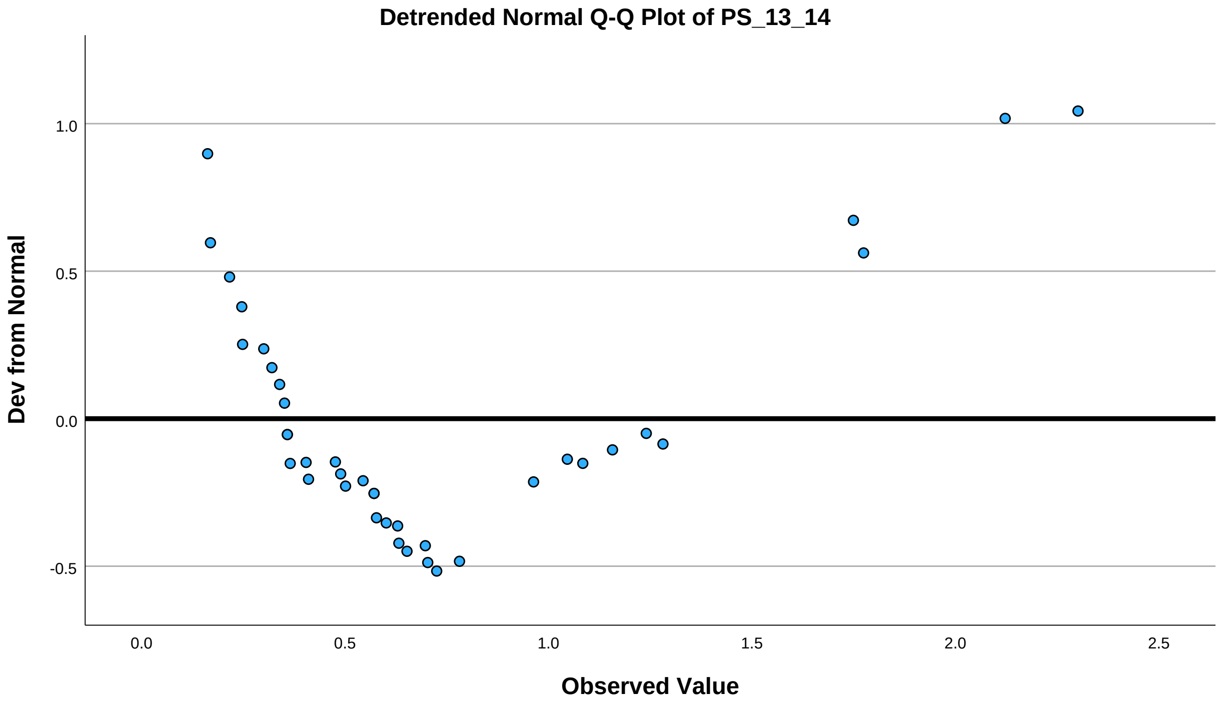
Detrended Normal Q-Q Plot of PS_13_14
Dev from Normal
1.0
0.5
0.0
-0.5
0.0
0.5
1.0
1.5
2.0
2.5
Observed Value

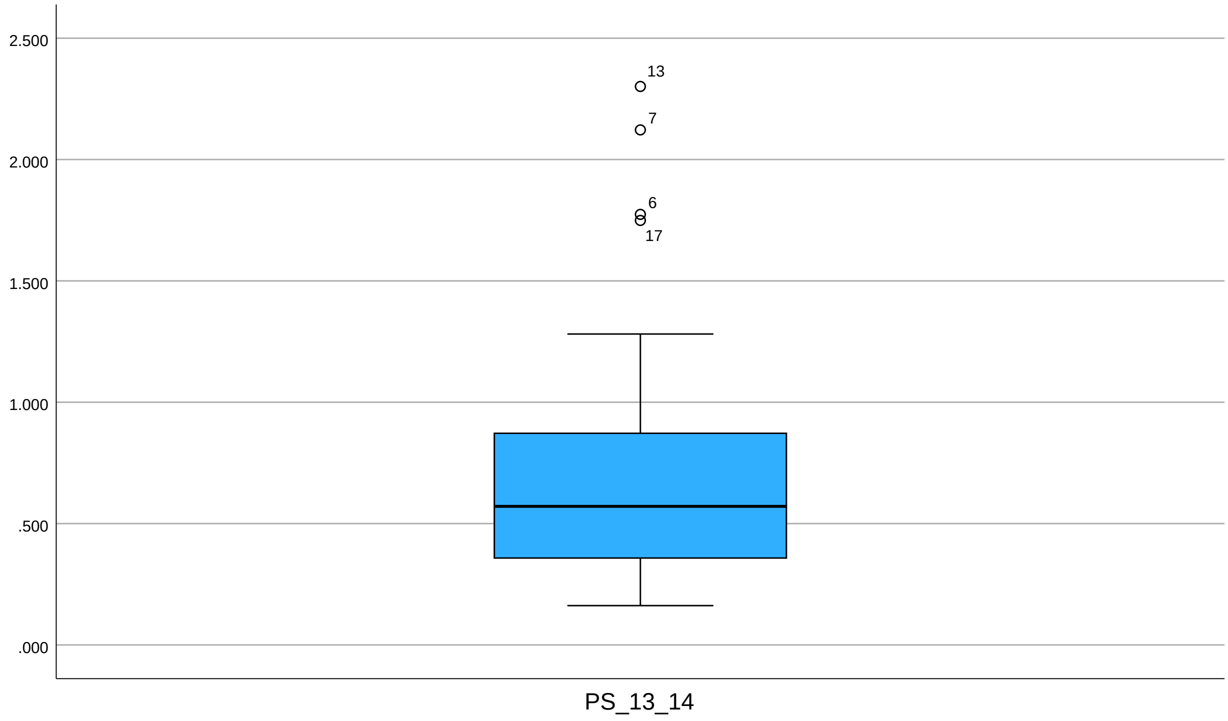
2.500
2.000
1.500
1.000
.500
.000
13
7
6
17
PS_13_14

PS_14_15

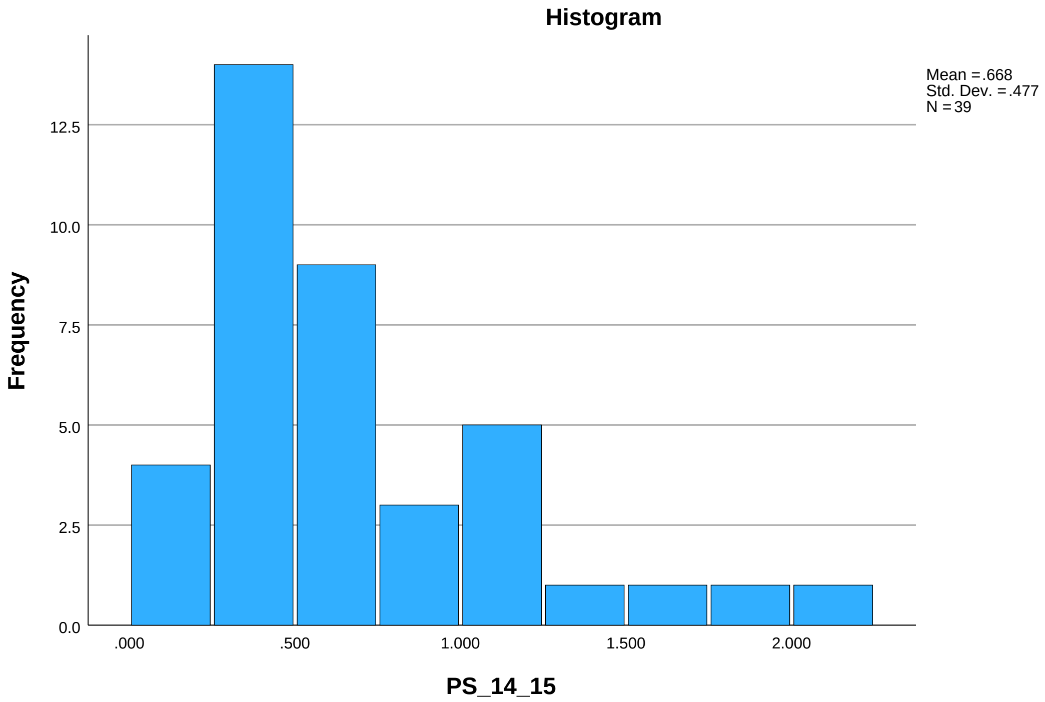


```
PS_14_15 Stem-and-Leaf Plot

 Frequency    Stem &  Leaf

     2.00        0 .  11
    13.00        0 .  2222222333333
    10.00        0 .  4445555555
     2.00        0 .  67
     3.00        0 .  899
     4.00        1 .  0011
     2.00        1 .  23
      .00        1 .
     1.00        1 .  6
     2.00 Extremes    (>=2.0)

 Stem width:      1.000
 Each leaf:       1 case(s)
```

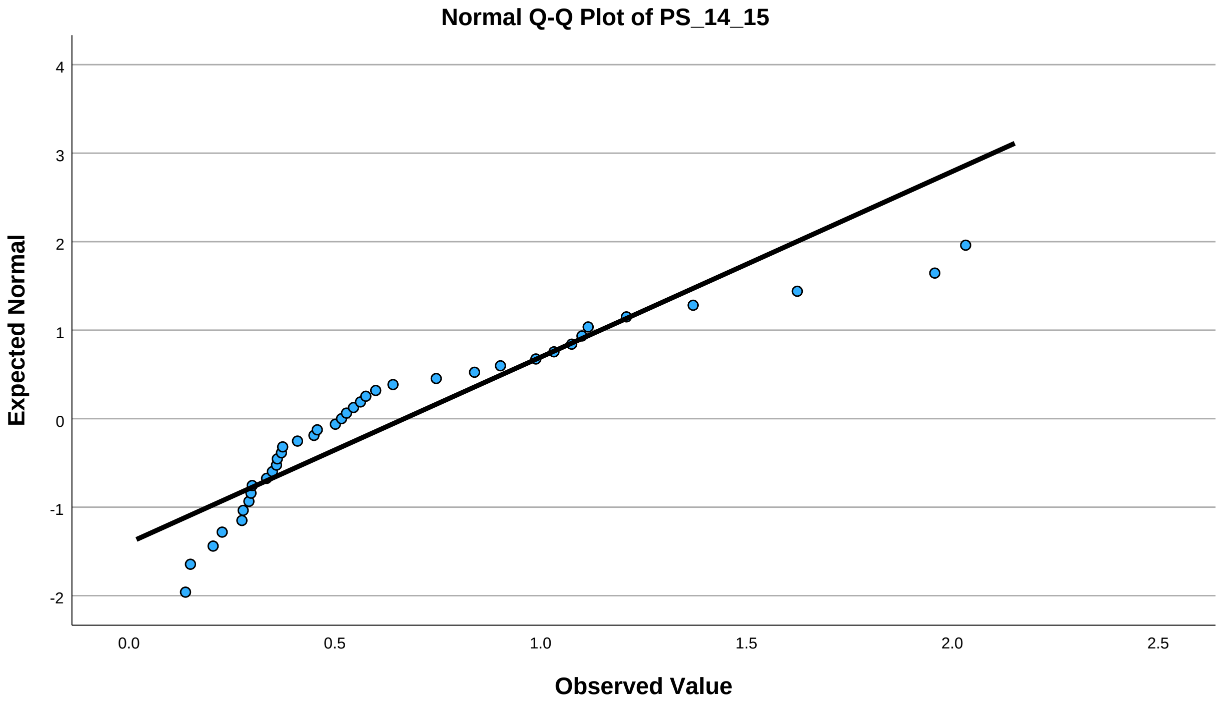
Normal Q-Q Plot of PS_14_15
Expected Normal
4
3
2
1
0
-1
-2
0.0
0.5
1.0
1.5
2.0
2.5
Observed Value

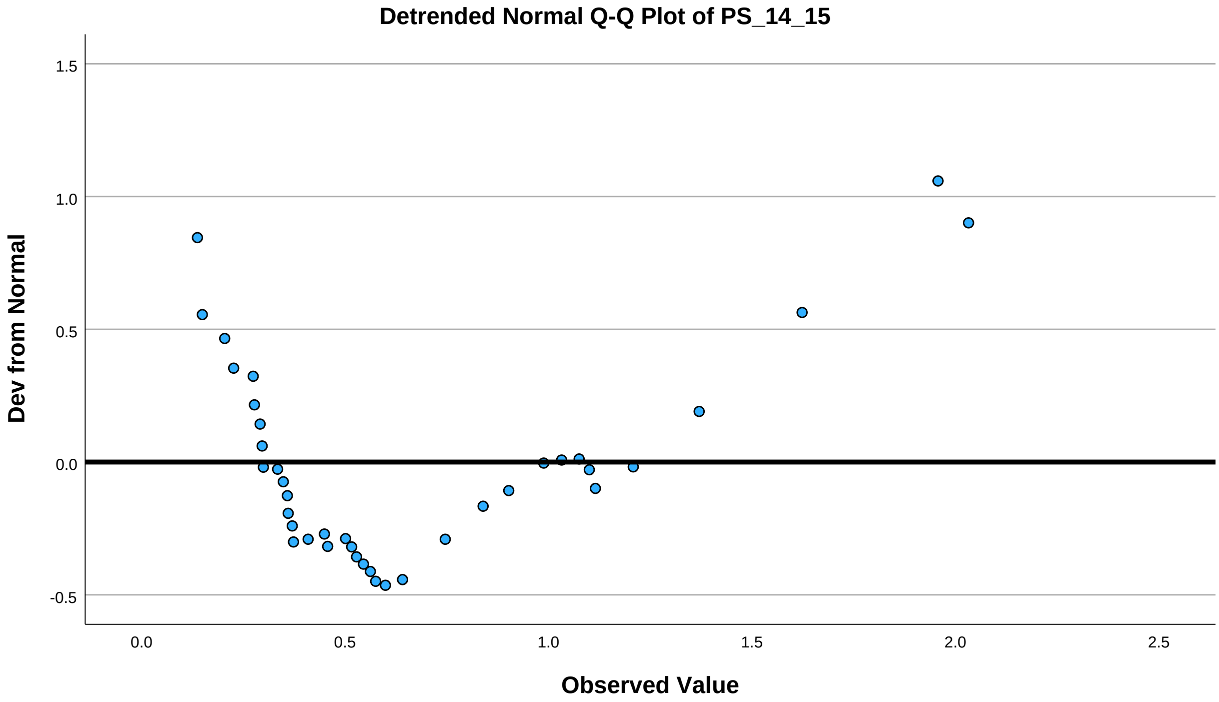
Detrended Normal Q-Q Plot of PS_14_15
Dev from Normal
1.5
1.0
0.5
0.0
-0.5
0.0
0.5
1.0
1.5
2.0
2.5
Observed Value

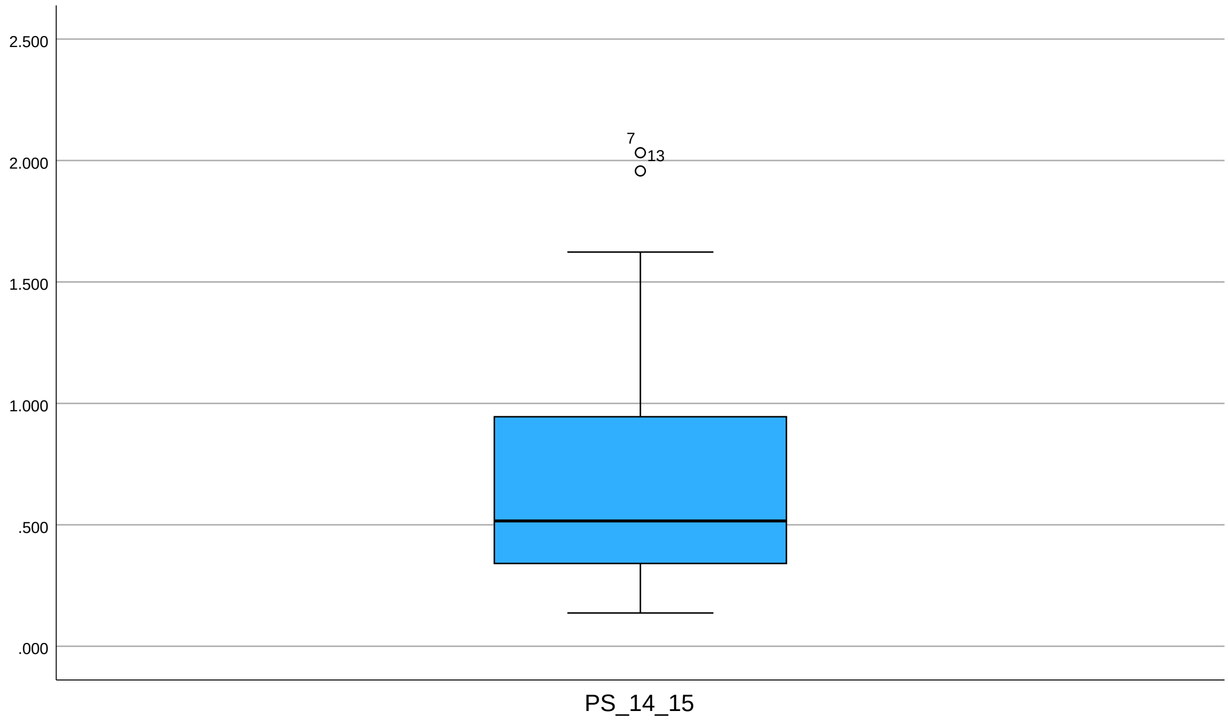

PS_15_16

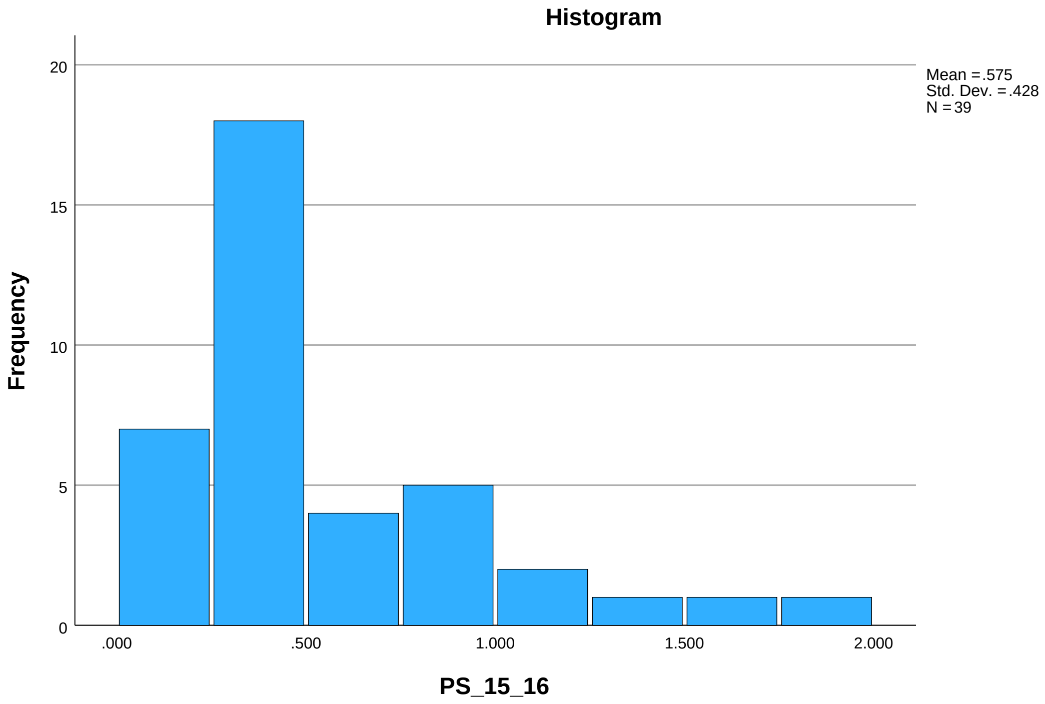

PS_15_16 Stem-and-Leaf Plot

Frequency   Stem & Leaf

```
  3.00      1 .  228
  8.00      2 .  14445688
  4.00      3 .  3345
 10.00      4 .  0011123345
  1.00      5 .  0
  1.00      6 .  9
  4.00      7 .  0088
  1.00      8 .  2
  2.00      9 .  15
   .00     10 .
   .00     11 .
  2.00     12 .  03
  3.00 Extremes    (>=1.49)

Stem width:      .100
Each leaf:       1 case(s)
```

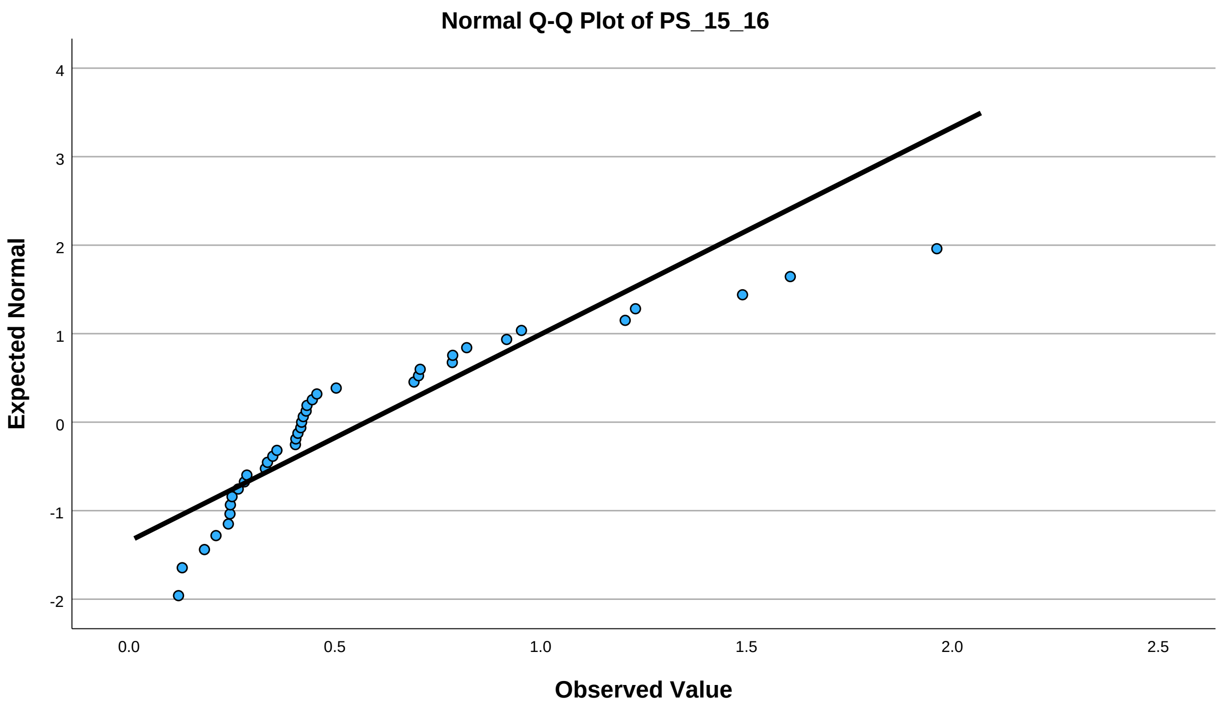

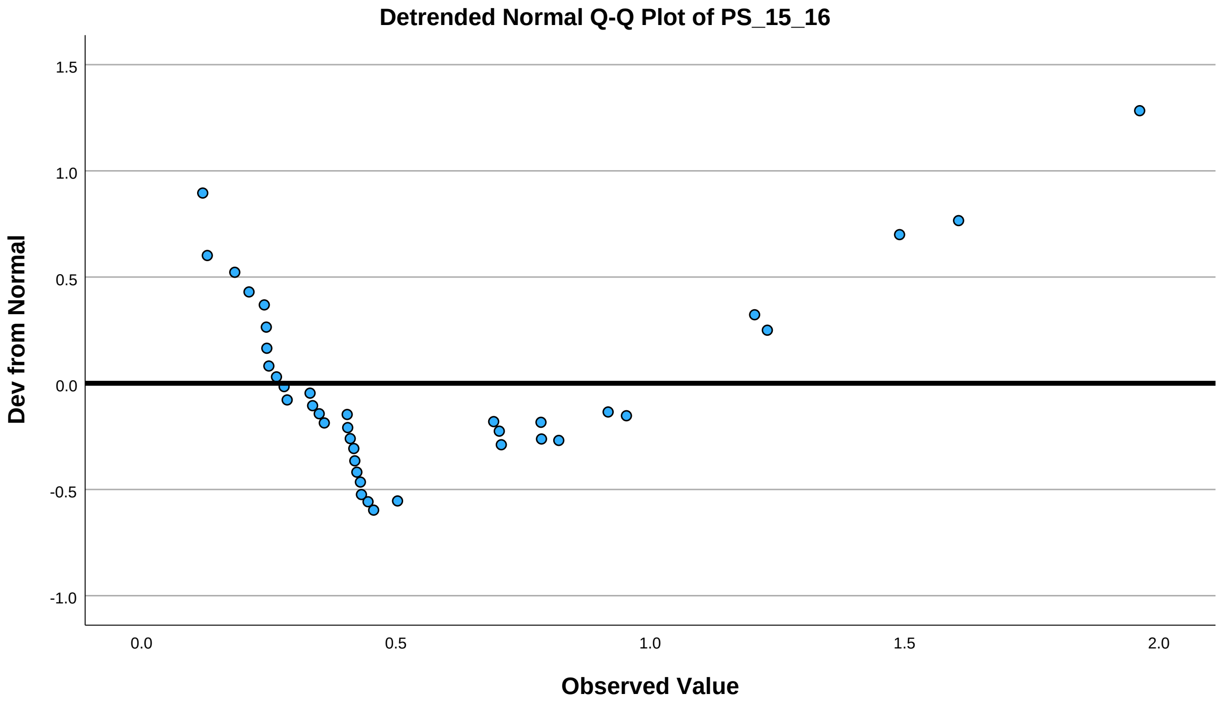
Detrended Normal Q-Q Plot of PS_15_16
Dev from Normal
1.5
1.0
0.5
0.0
-0.5
-1.0
0.0
0.5
1.0
1.5
2.0
Observed Value

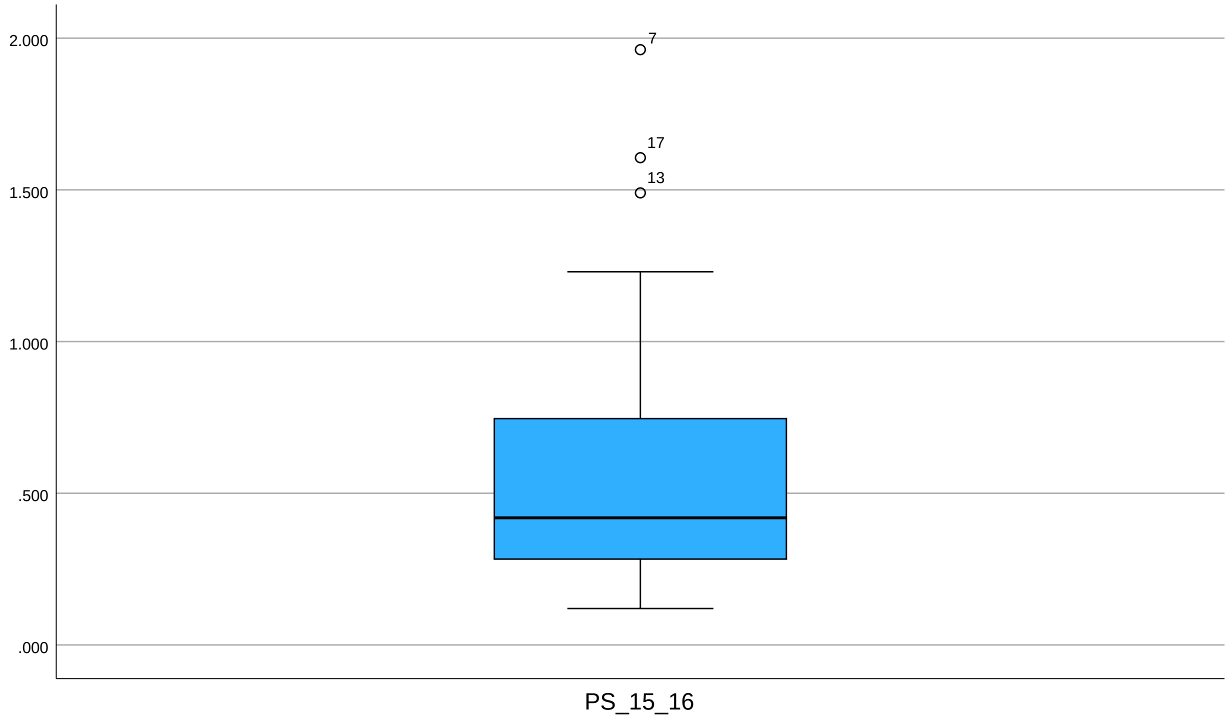
2.000
1.500
1.000
.500
.000
7
17
13
PS_15_16

PS_16_17

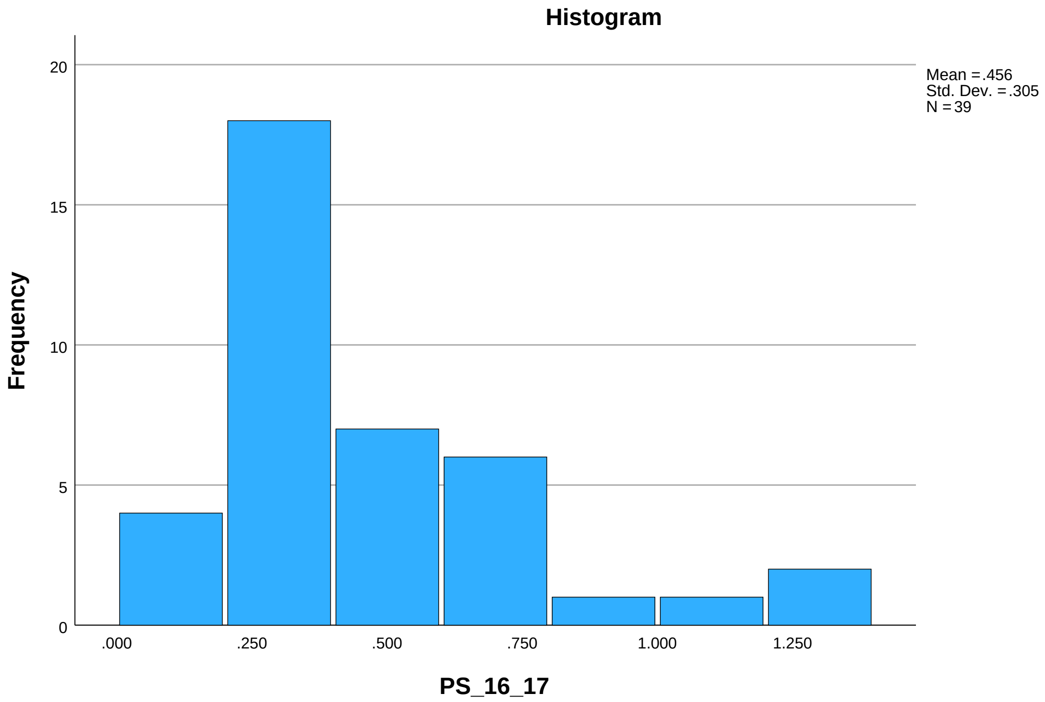


```
PS_16_17 Stem-and-Leaf Plot

 Frequency    Stem &  Leaf

     4.00        1 .  1259
    11.00        2 .  01123457778
     7.00        3 .  0111359
     5.00        4 .  11222
     2.00        5 .  69
     3.00        6 .  239
     3.00        7 .  269
      .00        8 .
     1.00        9 .  1
      .00       10 .
     1.00       11 .  4
     2.00 Extremes    (>=1.25)

 Stem width:      .100
 Each leaf:       1 case(s)
```

Normal Q-Q Plot of PS_16_17

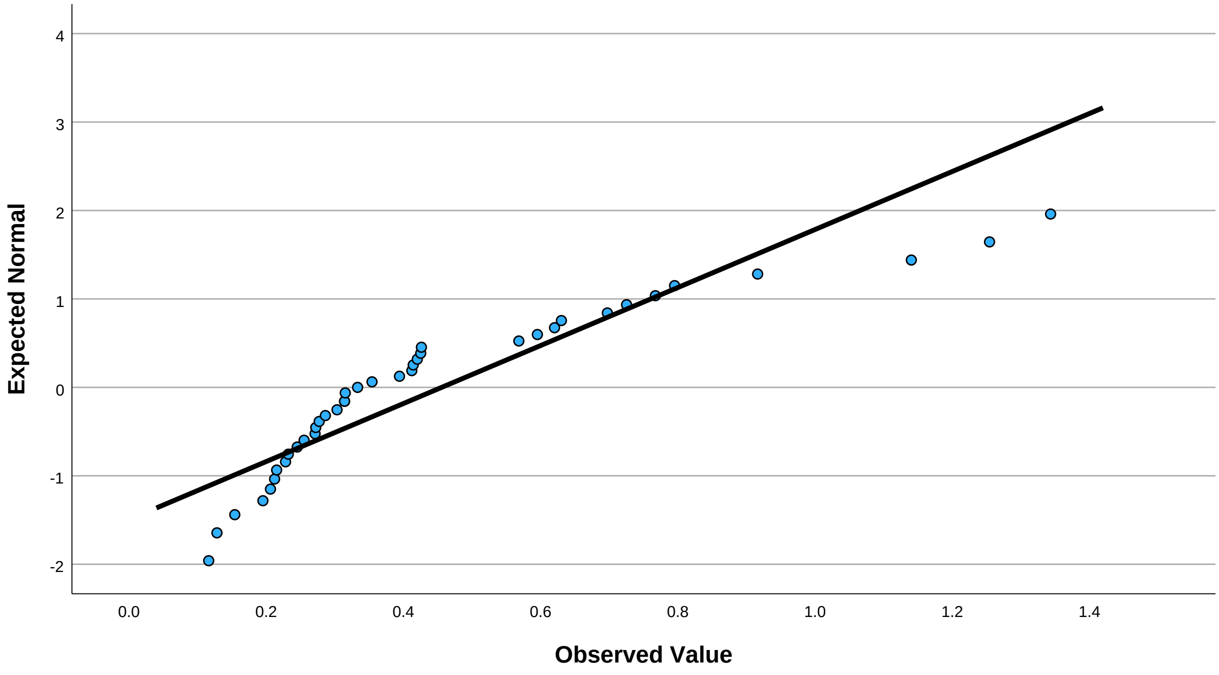

Detrended Normal Q-Q Plot of PS_16_17

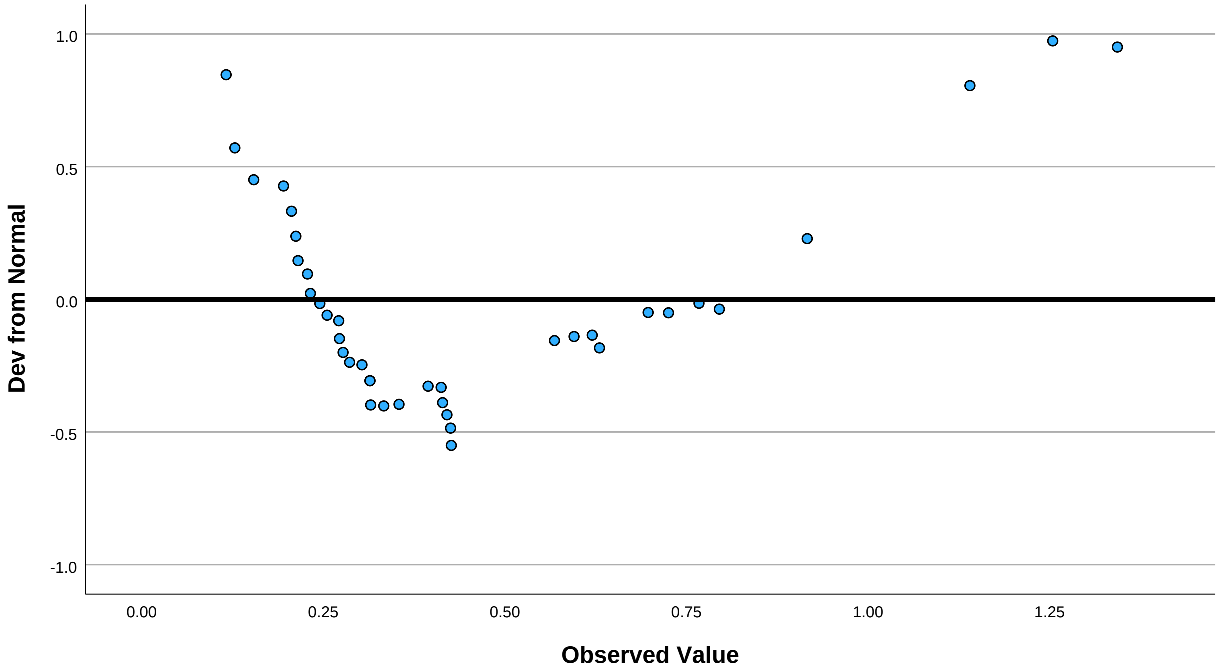

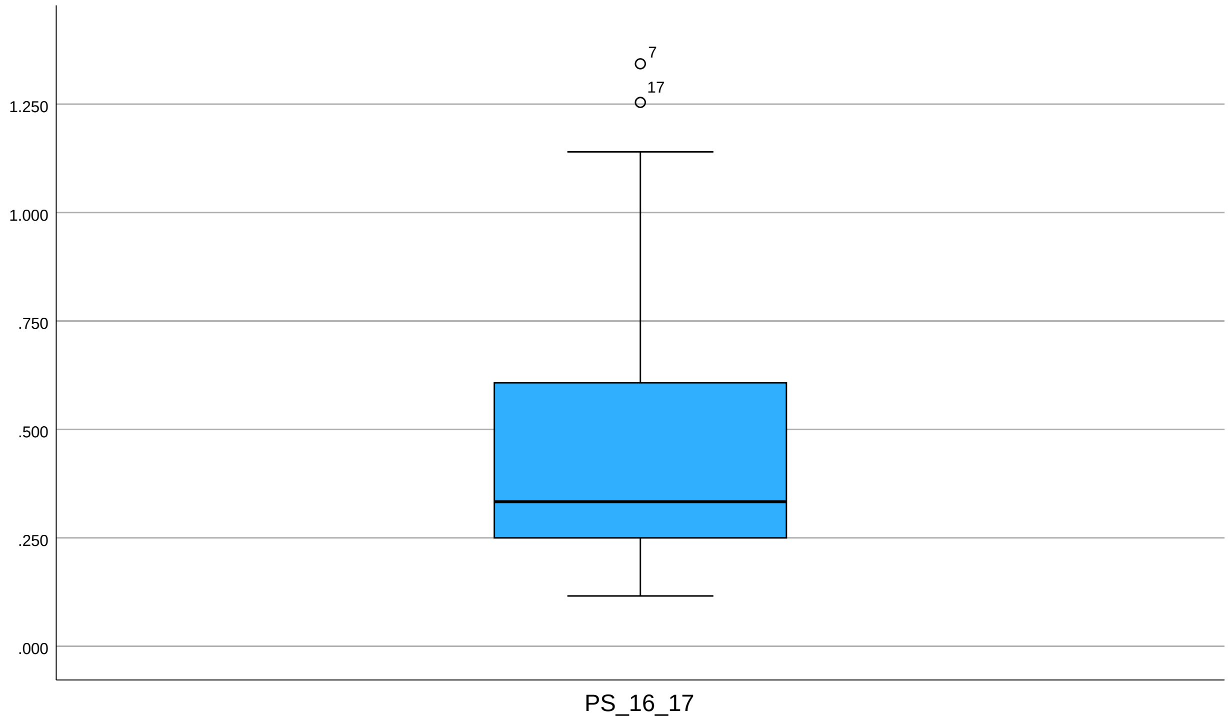

PS_17_18

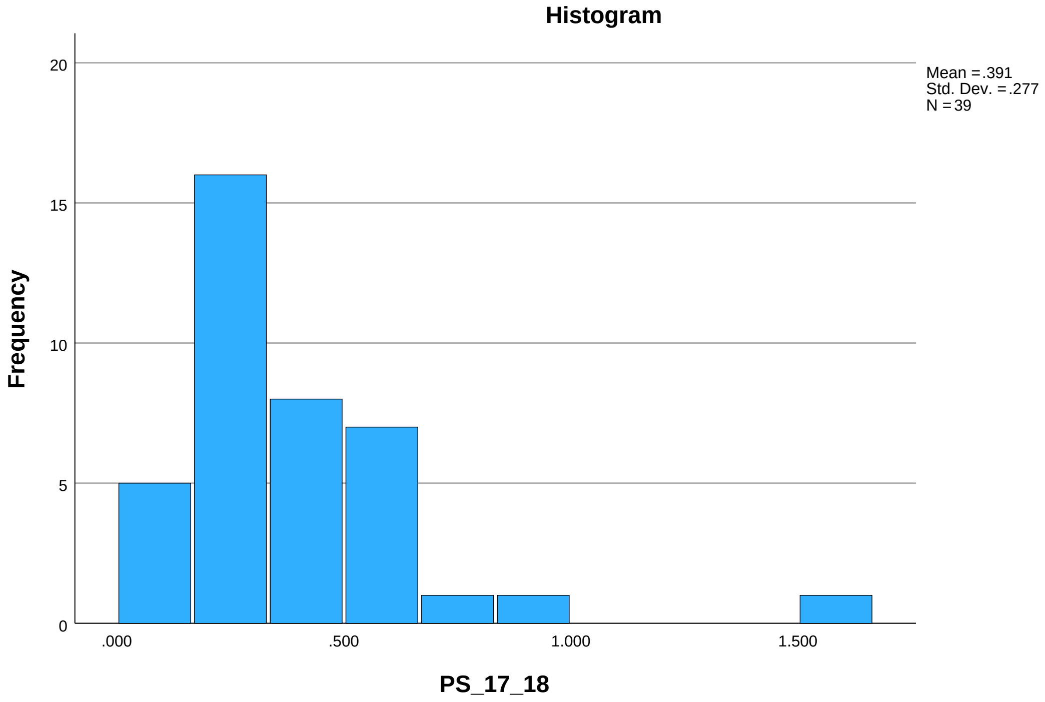


```
PS_17_18 Stem-and-Leaf Plot

 Frequency    Stem &  Leaf
```

```
  10.00        1 .  2245678899
  10.00        2 .  0234577799
   6.00        3 .  233456
   3.00        4 .  279
   2.00        5 .  35
   5.00        6 .  11234
    .00        7 .
   1.00        8 .  2
   1.00        9 .  8
   1.00 Extremes    (>=1.53)

Stem width:       .100
Each leaf:        1 case(s)
```

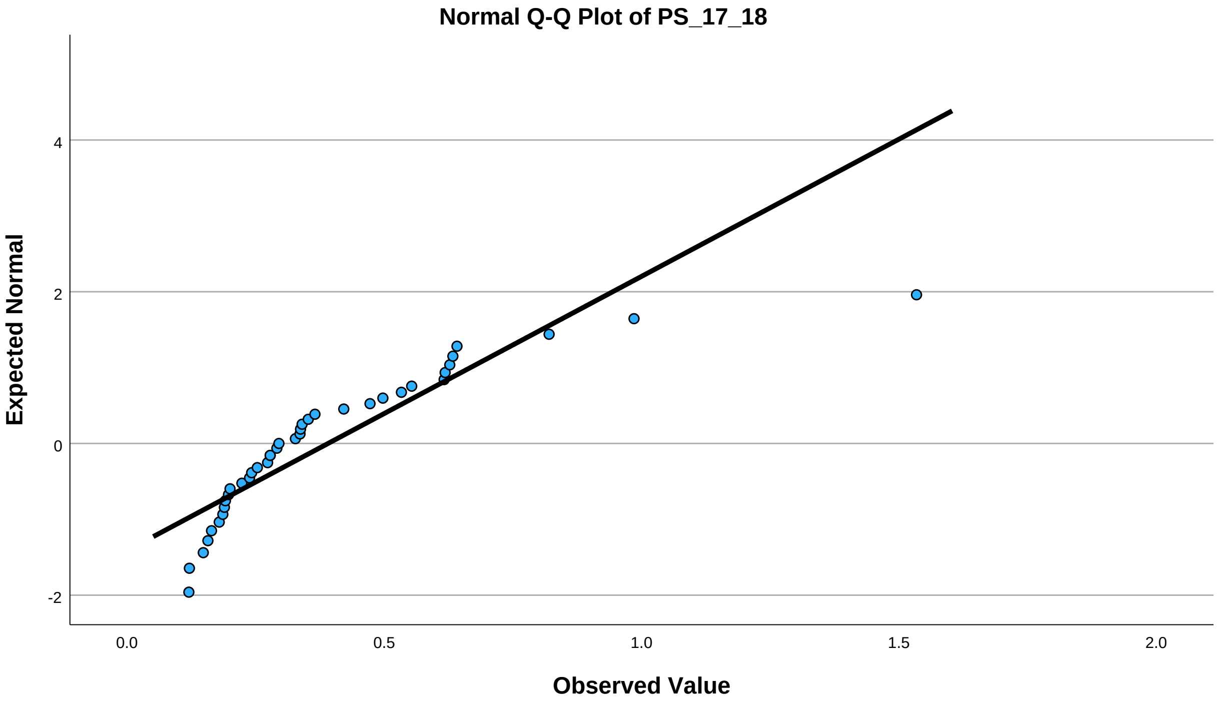

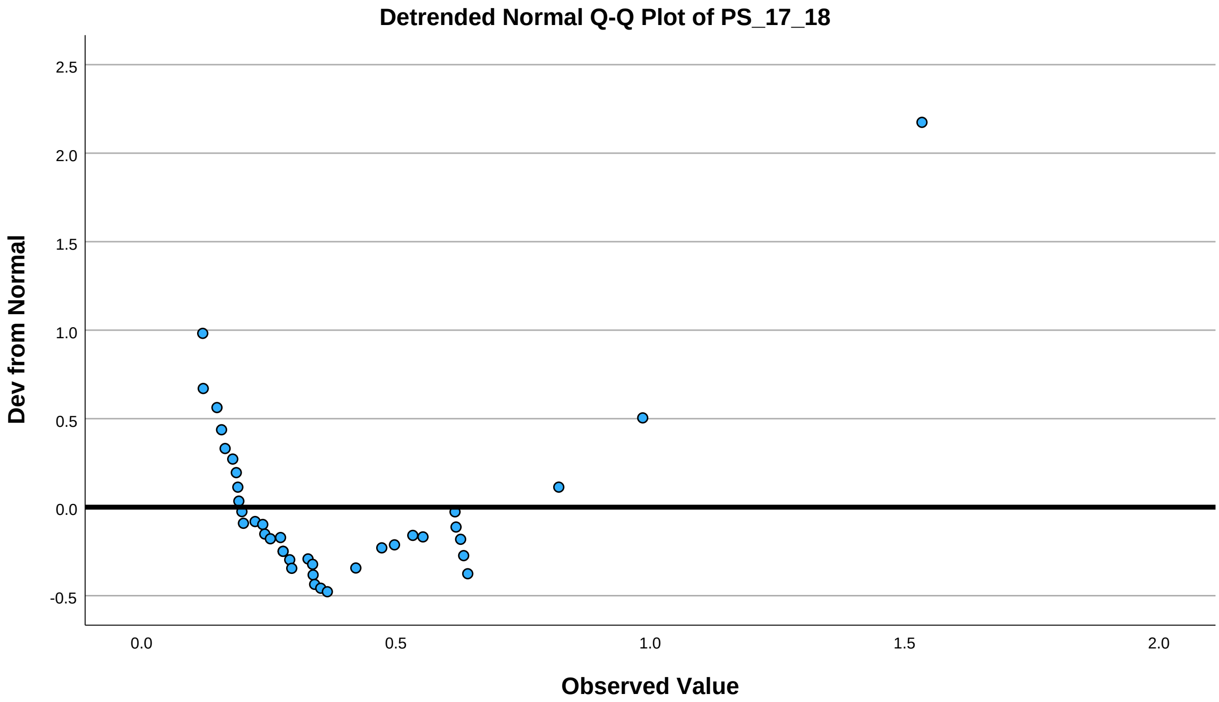
Detrended Normal Q-Q Plot of PS_17_18
Dev from Normal
2.5
2.0
1.5
1.0
0.5
0.0
-0.5
0.0
0.5
1.0
1.5
2.0
Observed Value

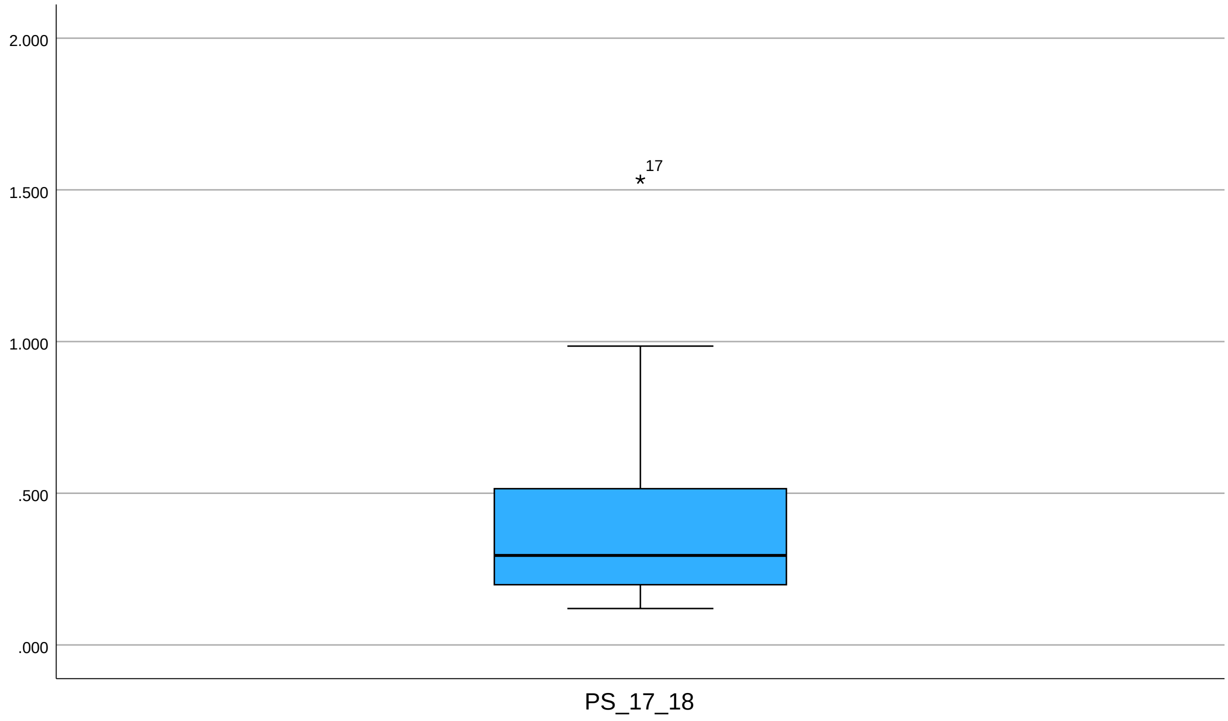
2.000
1.500
1.000
.500
.000
17
PS_17_18

PS_18_19

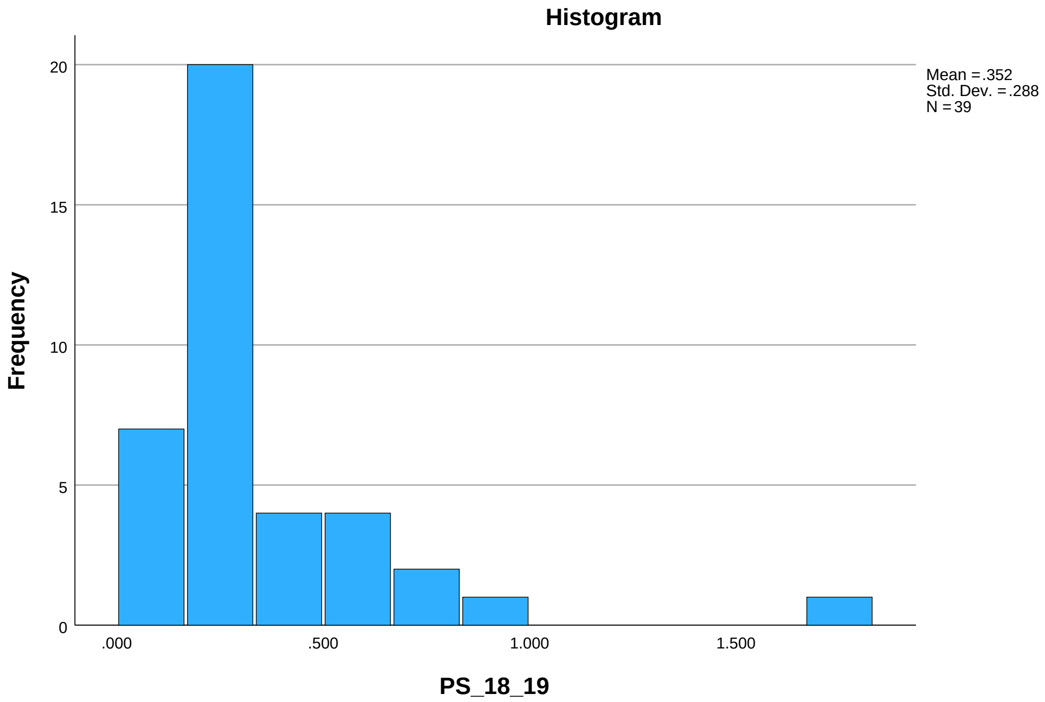


```
PS_18_19 Stem-and-Leaf Plot

 Frequency    Stem &  Leaf

     5.00        1 .  01344
     8.00        1 .  56677999
     2.00        2 .  44
    10.00        2 .  5566777899
     3.00        3 .  003
     1.00        3 .  6
     1.00        4 .  3
     1.00        4 .  9
     3.00        5 .  113
     1.00        5 .  7
      .00        6 .
     1.00        6 .  8
     3.00 Extremes    (>=.73)

 Stem width:      .100
 Each leaf:       1 case(s)
```

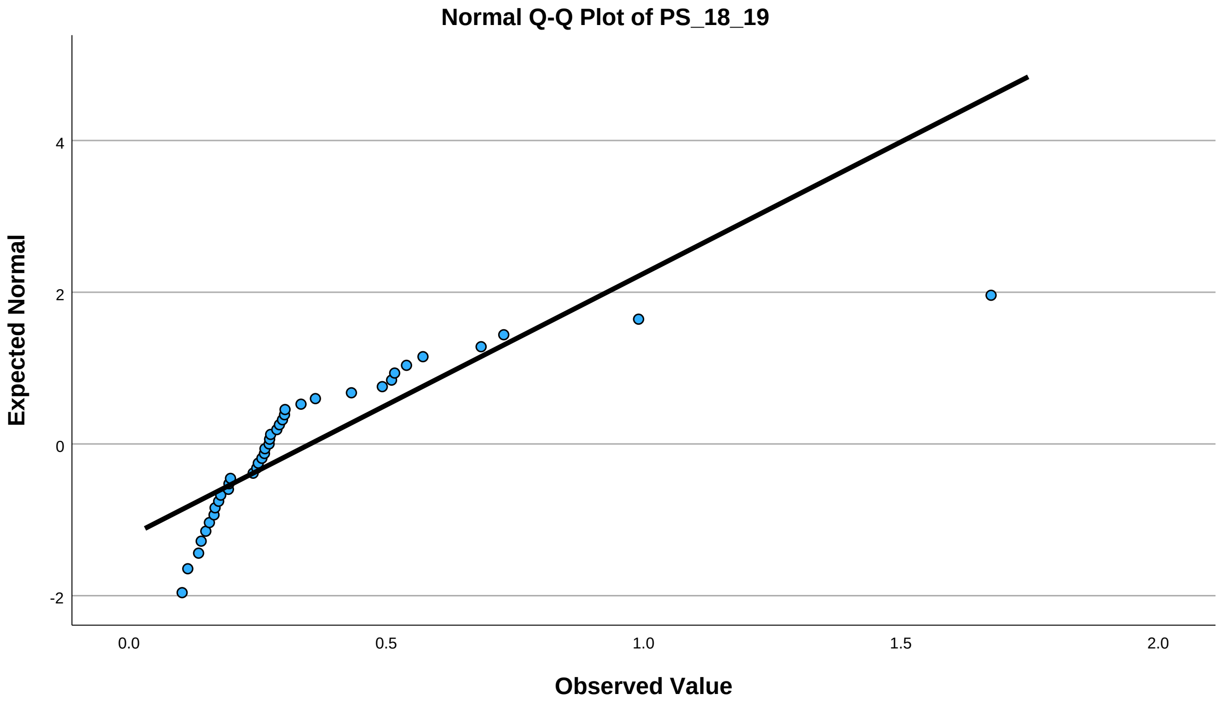
Normal Q-Q Plot of PS_18_19
Expected Normal
4
2
0
-2
0.0
0.5
1.0
1.5
2.0
Observed Value

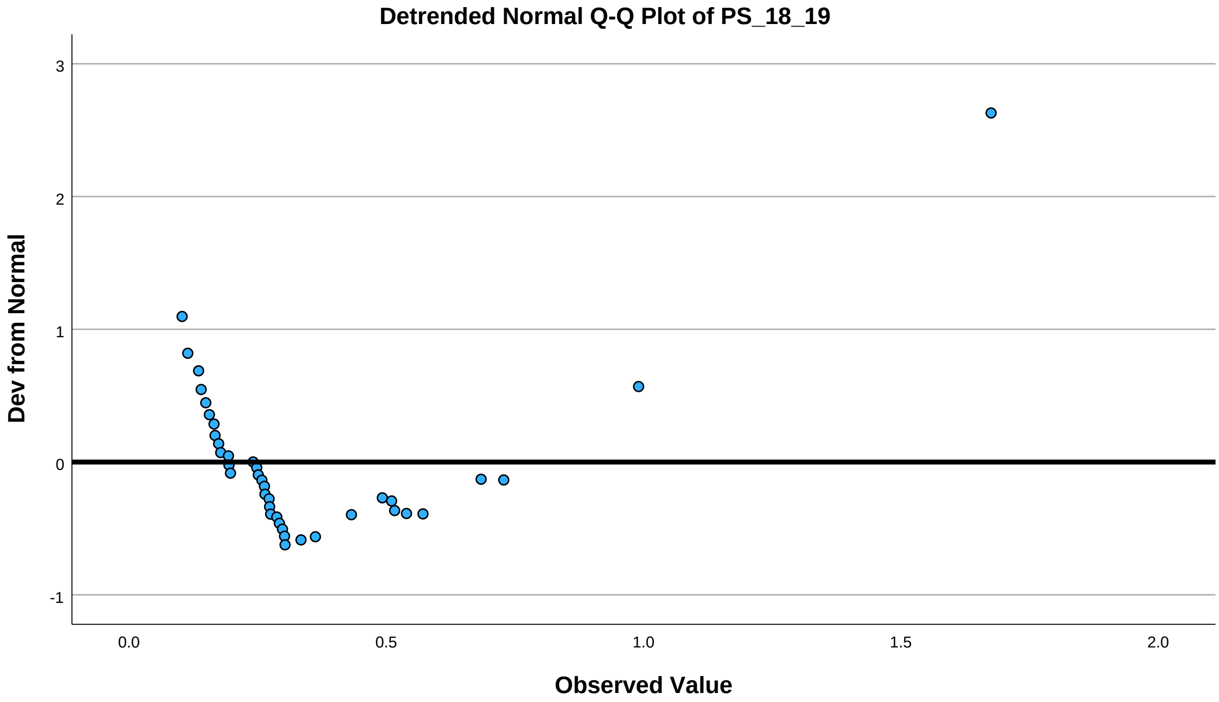
Detrended Normal Q-Q Plot of PS_18_19
Dev from Normal
3
2
1
0
-1
0.0
0.5
1.0
1.5
2.0
Observed Value

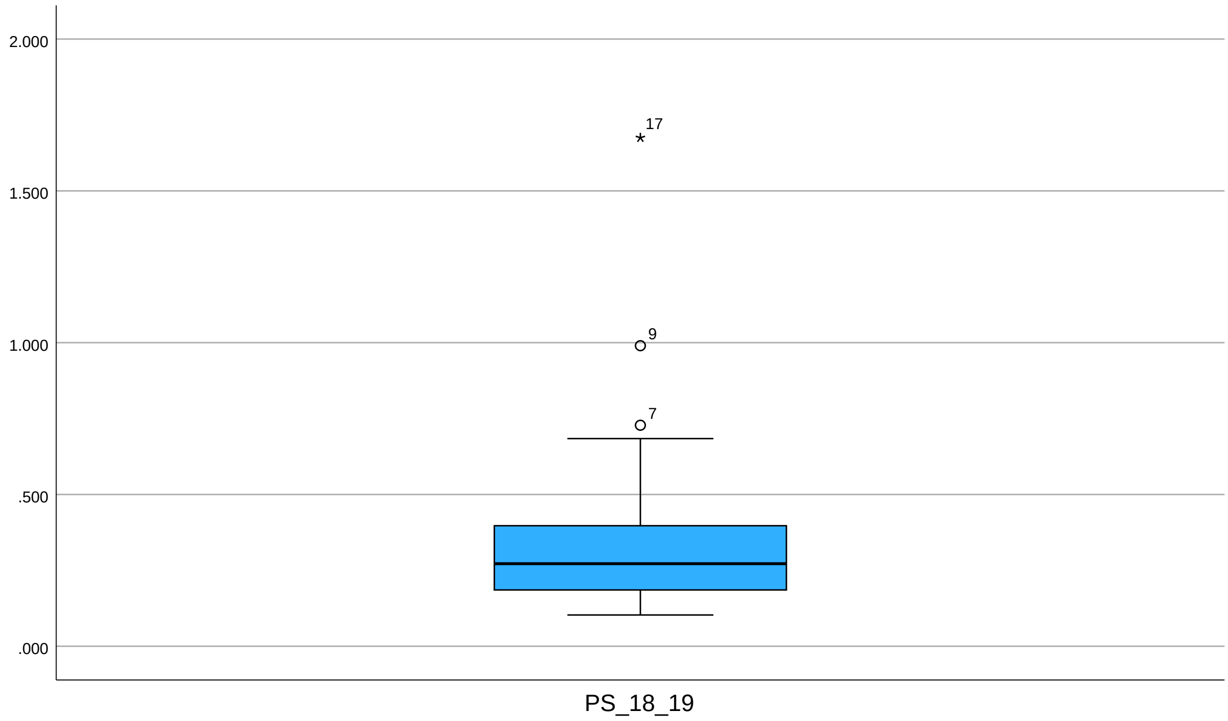

PS_19_20

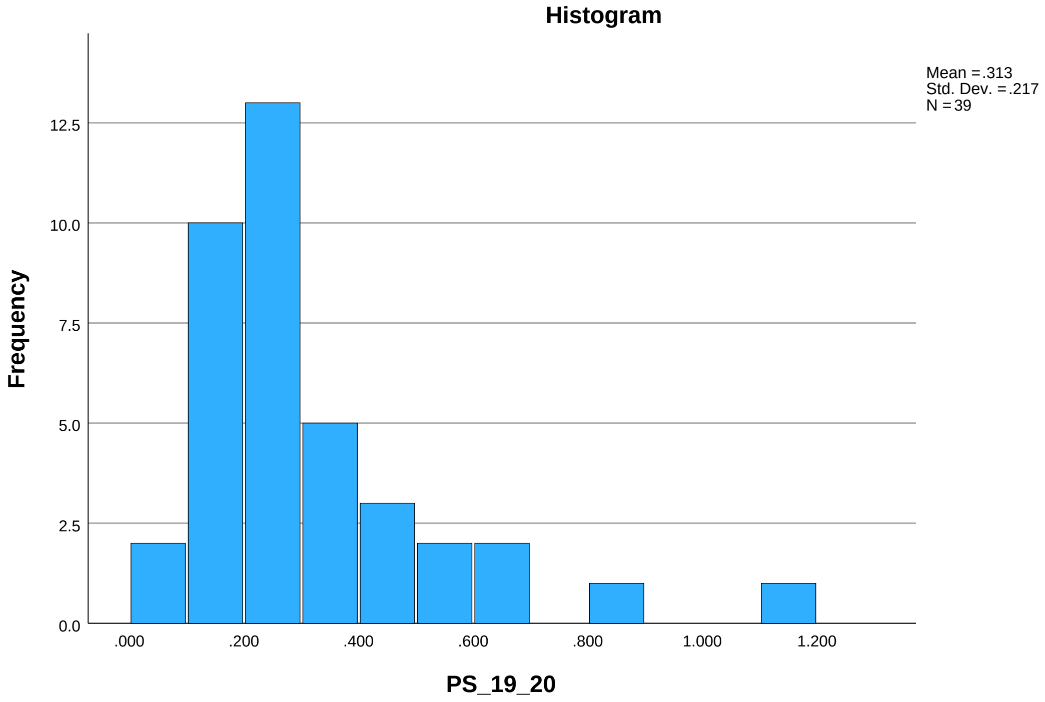

PS_19_20 Stem-and-Leaf Plot

Frequency   Stem & Leaf

```
 2.00      0 .  99
 6.00      1 .  023334
 4.00      1 .  5568
 7.00      2 .  0233444
 6.00      2 .  577799
 5.00      3 .  01123
  .00      3 .
 2.00      4 .  12
 1.00      4 .  5
 2.00      5 .  13
 4.00 Extremes    (>=.66)

 Stem width:      .100
 Each leaf:       1 case(s)
```

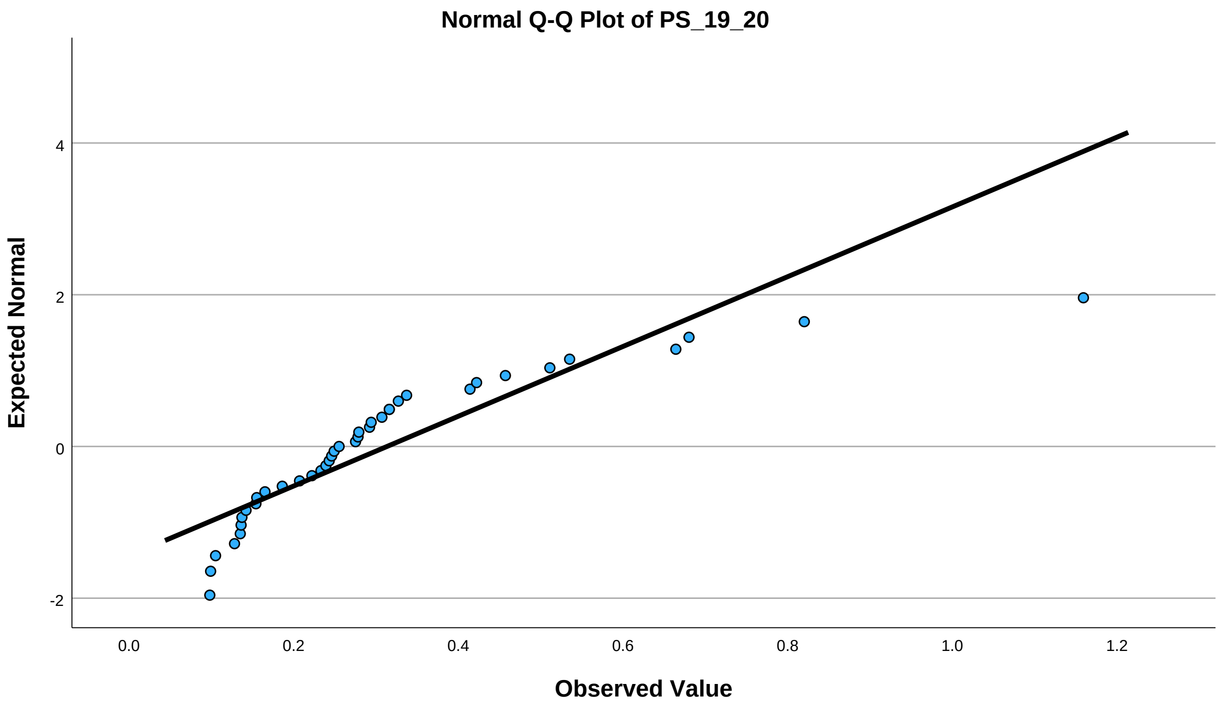

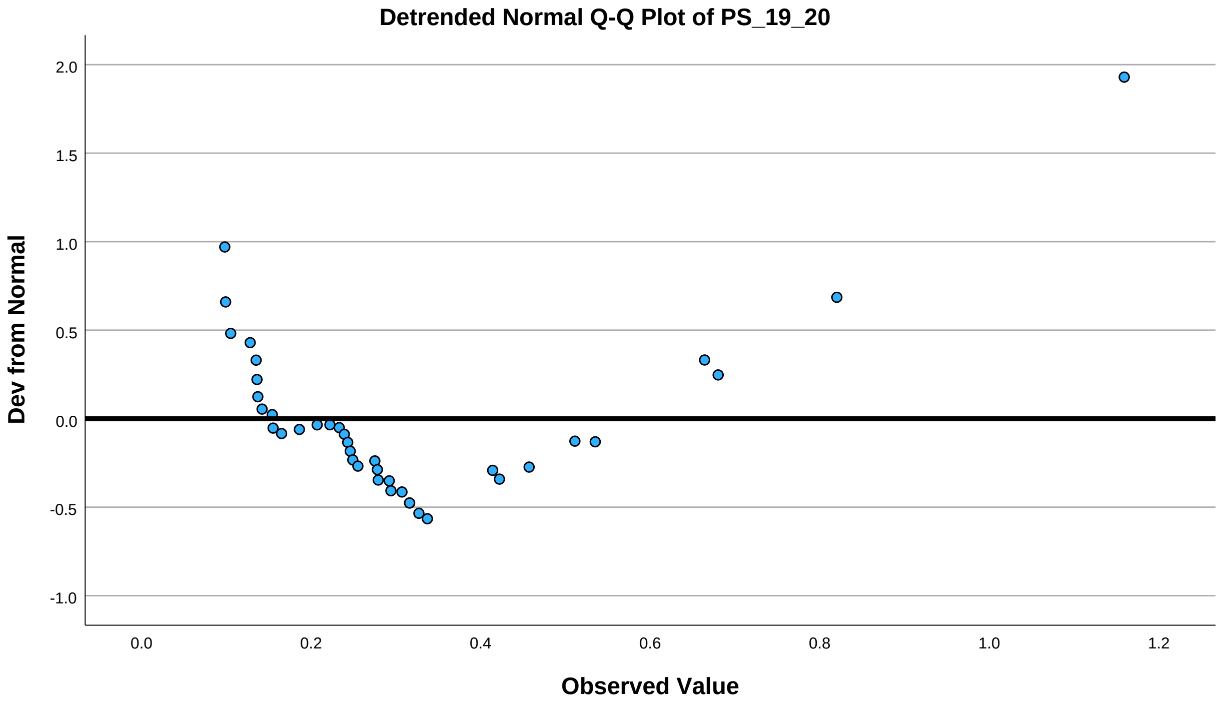

Detrended Normal Q-Q Plot of PS_19_20
Dev from Normal
2.0
1.5
1.0
0.5
0.0
-0.5
-1.0
0.0
0.2
0.4
0.6
0.8
1.0
1.2
Observed Value

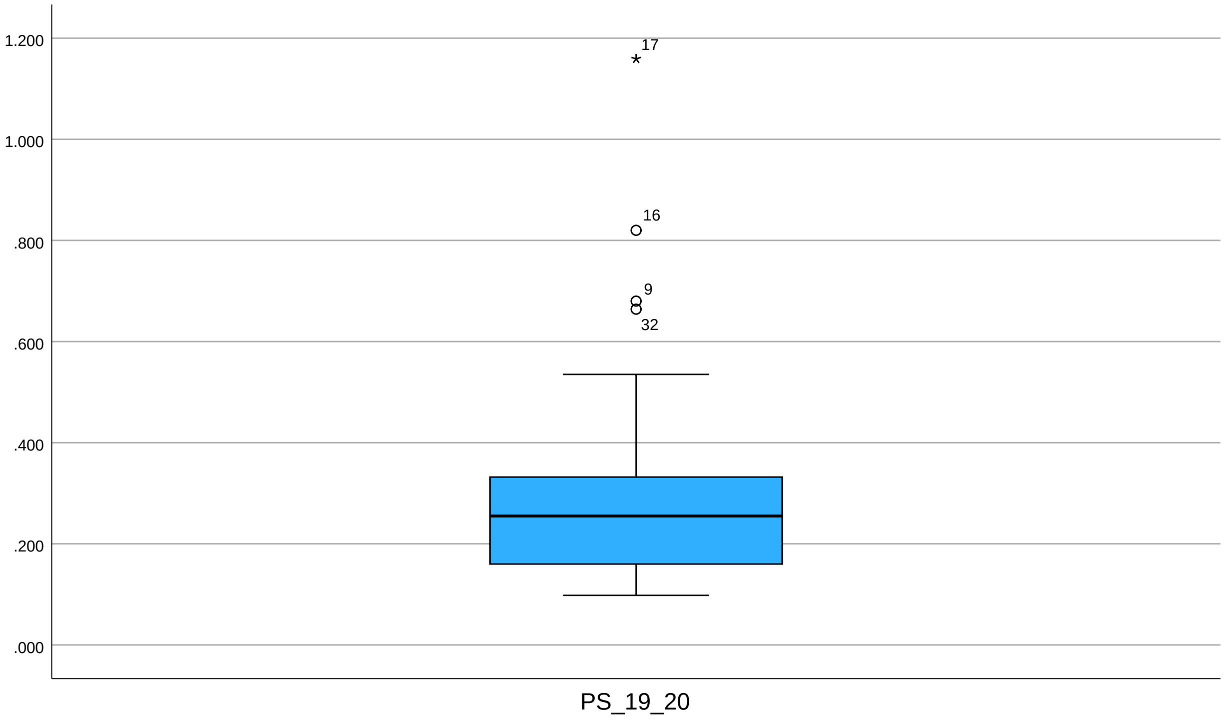

1.200
1.000
.800
.600
.400
.200
.000
17
16
9
32
PS_19_20

Explore

Case Processing Summary

	Cases					
	Valid		Missing		Total	
	N	Percent	N	Percent	N	Percent
BrainRate	39	100.0%	0	0.0%	39	100.0%
PermutationEntropy	39	100.0%	0	0.0%	39	100.0%
PermutationEntropy_01	39	100.0%	0	0.0%	39	100.0%
SampleEntropy	39	100.0%	0	0.0%	39	100.0%
SpectralEntropy_bits	39	100.0%	0	0.0%	39	100.0%
SpectralEntropy_01	39	100.0%	0	0.0%	39	100.0%
HiguchiFractalDimension	39	100.0%	0	0.0%	39	100.0%
LempelZivComplexity	39	100.0%	0	0.0%	39	100.0%
RenyiEntropy	39	100.0%	0	0.0%	39	100.0%
TsallisEntropy	39	100.0%	0	0.0%	39	100.0%
PS_4_5	39	100.0%	0	0.0%	39	100.0%
PS_5_6	39	100.0%	0	0.0%	39	100.0%
PS_6_7	39	100.0%	0	0.0%	39	100.0%
PS_7_8	39	100.0%	0	0.0%	39	100.0%
PS_8_9	39	100.0%	0	0.0%	39	100.0%
PS_9_10	39	100.0%	0	0.0%	39	100.0%
PS_10_11	39	100.0%	0	0.0%	39	100.0%
PS_11_12	39	100.0%	0	0.0%	39	100.0%
PS_12_13	39	100.0%	0	0.0%	39	100.0%
PS_13_14	39	100.0%	0	0.0%	39	100.0%
PS_14_15	39	100.0%	0	0.0%	39	100.0%
PS_15_16	39	100.0%	0	0.0%	39	100.0%
PS_16_17	39	100.0%	0	0.0%	39	100.0%
PS_17_18	39	100.0%	0	0.0%	39	100.0%
PS_18_19	39	100.0%	0	0.0%	39	100.0%
PS_19_20	39	100.0%	0	0.0%	39	100.0%

Descriptives

			Statistic	Std. Error
BrainRate	Mean		6.9856	.46624
	95% Confidence Interval for Mean	Lower Bound	6.0418	
		Upper Bound	7.9295	
	5% Trimmed Mean		6.5949	
	Median		6.3000	
	Variance		8.478	
	Std. Deviation		2.91167	
	Minimum		4.28	
	Maximum		18.03	
	Range		13.75	
	Interquartile Range		2.51	
	Skewness		2.325	.378
	Kurtosis		5.926	.741
PermutationEntropy	Mean		1.4797547301	.01150627178
	95% Confidence Interval for Mean	Lower Bound	1.4564615007	
		Upper Bound	1.5030479596	
	5% Trimmed Mean		1.4803461937	
	Median		1.4736837512	
	Variance		.005	
	Std. Deviation		.07185664424	
	Minimum		1.3494726331	
	Maximum		1.6081808289	
	Range		.25870819581	
	Interquartile Range		.11572290235	
	Skewness		-.093	.378
	Kurtosis		-.754	.741
PermutationEntropy_01	Mean		.82586683958	.00642177255
	95% Confidence Interval for Mean	Lower Bound	.81286664070	
		Upper Bound	.83886703846	
	5% Trimmed Mean		.82619694167	
	Median		.82247856173	
	Variance		.002	
	Std. Deviation		.04010395674	
	Minimum		.75315501678	
	Maximum		.89754281004	
	Range		.14438779326	
	Interquartile Range		.06458618154	
	Skewness		-.093	.378
	Kurtosis		-.754	.741

Descriptives

			Statistic	Std. Error
SampleEntropy	Mean		.70797528927	.02903888674
	95% Confidence Interval for Mean	Lower Bound	.64918913642	
		Upper Bound	.76676144211	
	5% Trimmed Mean		.70024243275	
	Median		.67926924103	
	Variance		.033	
	Std. Deviation		.18134778957	
	Minimum		.38442720274	
	Maximum		1.1687549569	
	Range		.78432775413	
	Interquartile Range		.22641386835	
	Skewness		.826	.378
	Kurtosis		.448	.741
SpectralEntropy_bits	Mean		3.9721837981	.07336296809
	95% Confidence Interval for Mean	Lower Bound	3.8236682336	
		Upper Bound	4.1206993625	
	5% Trimmed Mean		3.9391782855	
	Median		3.9338345114	
	Variance		.210	
	Std. Deviation		.45815158886	
	Minimum		3.3434937729	
	Maximum		5.4261311310	
	Range		2.0826373581	
	Interquartile Range		.46560535307	
	Skewness		1.269	.378
	Kurtosis		1.987	.741
SpectralEntropy_01	Mean		.56654614854	.01046364144
	95% Confidence Interval for Mean	Lower Bound	.54536361386	
		Upper Bound	.58772868321	
	5% Trimmed Mean		.56183862567	
	Median		.56107645182	
	Variance		.004	
	Std. Deviation		.06534541988	
	Minimum		.47687710740	
	Maximum		.77392030430	
	Range		.29704319689	
	Interquartile Range		.06640853821	
	Skewness		1.269	.378
	Kurtosis		1.987	.741

Descriptives

			Statistic	Std. Error
HiguchiFractalDimension	Mean		.58753767059	.01945808147
	95% Confidence Interval for Mean	Lower Bound	.54814684403	
		Upper Bound	.62692849715	
	5% Trimmed Mean		.59473634976	
	Median		.60534161219	
	Variance		.015	
	Std. Deviation		.12151567980	
	Minimum		.25710236439	
	Maximum		.78004889806	
	Range		.52294653368	
	Interquartile Range		.15731838688	
	Skewness		-.919	.378
	Kurtosis		.504	.741
LempelZivComplexity	Mean		.25792348014	.00701002536
	95% Confidence Interval for Mean	Lower Bound	.24373242571	
		Upper Bound	.27211453457	
	5% Trimmed Mean		.25297160375	
	Median		.25175974973	
	Variance		.002	
	Std. Deviation		.04377759435	
	Minimum		.20930903274	
	Maximum		.40243797746	
	Range		.19312894472	
	Interquartile Range		.04241556609	
	Skewness		1.798	.378
	Kurtosis		3.664	.741
RenyiEntropy	Mean		7.9949038303	.12983079574
	95% Confidence Interval for Mean	Lower Bound	7.7320751251	
		Upper Bound	8.2577325355	
	5% Trimmed Mean		8.0625058935	
	Median		8.2917391586	
	Variance		.657	
	Std. Deviation		.81079305953	
	Minimum		5.8970798805	
	Maximum		8.8505852508	
	Range		2.9535053703	
	Interquartile Range		.80199108507	
	Skewness		-1.253	.378
	Kurtosis		.569	.741

Descriptives

			Statistic	Std. Error
TsallisEntropy	Mean		1.9020693031	.04715688099
	95% Confidence Interval for Mean	Lower Bound	1.8066051884	
		Upper Bound	1.9975334178	
	5% Trimmed Mean		1.9623587045	
	Median		1.9857522911	
	Variance		.087	
	Std. Deviation		.29449462739	
	Minimum		.71951741681	
	Maximum		1.9881975968	
	Range		1.2686801800	
	Interquartile Range		.00339942743	
	Skewness		-3.659	.378
	Kurtosis		12.566	.741
PS_4_5	Mean		4.54505	.501275
	95% Confidence Interval for Mean	Lower Bound	3.53027	
		Upper Bound	5.55983	
	5% Trimmed Mean		4.28204	
	Median		3.41100	
	Variance		9.800	
	Std. Deviation		3.130461	
	Minimum		.817	
	Maximum		12.984	
	Range		12.167	
	Interquartile Range		3.496	
	Skewness		1.308	.378
	Kurtosis		1.219	.741
PS_5_6	Mean		3.08615	.362047
	95% Confidence Interval for Mean	Lower Bound	2.35323	
		Upper Bound	3.81908	
	5% Trimmed Mean		2.82504	
	Median		2.53800	
	Variance		5.112	
	Std. Deviation		2.260980	
	Minimum		.423	
	Maximum		11.810	
	Range		11.387	
	Interquartile Range		2.411	
	Skewness		2.144	.378
	Kurtosis		5.600	.741

Descriptives

			Statistic	Std. Error
PS_6_7	Mean		2.42246	.292484
	95% Confidence Interval for Mean	Lower Bound	1.83036	
		Upper Bound	3.01456	
	5% Trimmed Mean		2.20043	
	Median		1.81700	
	Variance		3.336	
	Std. Deviation		1.826561	
	Minimum		.410	
	Maximum		8.601	
	Range		8.191	
	Interquartile Range		1.545	
	Skewness		1.961	.378
	Kurtosis		4.262	.741
PS_7_8	Mean		2.08818	.224161
	95% Confidence Interval for Mean	Lower Bound	1.63439	
		Upper Bound	2.54197	
	5% Trimmed Mean		2.00468	
	Median		1.57100	
	Variance		1.960	
	Std. Deviation		1.399882	
	Minimum		.347	
	Maximum		5.349	
	Range		5.002	
	Interquartile Range		1.422	
	Skewness		1.162	.378
	Kurtosis		.352	.741
PS_8_9	Mean		2.37392	.394571
	95% Confidence Interval for Mean	Lower Bound	1.57516	
		Upper Bound	3.17269	
	5% Trimmed Mean		2.04016	
	Median		1.48000	
	Variance		6.072	
	Std. Deviation		2.464097	
	Minimum		.293	
	Maximum		11.736	
	Range		11.443	
	Interquartile Range		1.354	
	Skewness		2.515	.378
	Kurtosis		6.321	.741

Descriptives

			Statistic	Std. Error
PS_9_10	Mean		2.96210	.655073
	95% Confidence Interval for Mean	Lower Bound	1.63598	
		Upper Bound	4.28823	
	5% Trimmed Mean		2.33447	
	Median		1.71500	
	Variance		16.736	
	Std. Deviation		4.090930	
	Minimum		.230	
	Maximum		25.220	
	Range		24.990	
	Interquartile Range		1.860	
	Skewness		4.527	.378
	Kurtosis		23.929	.741
PS_10_11	Mean		3.67223	.653210
	95% Confidence Interval for Mean	Lower Bound	2.34988	
		Upper Bound	4.99458	
	5% Trimmed Mean		3.11607	
	Median		2.55700	
	Variance		16.641	
	Std. Deviation		4.079293	
	Minimum		.306	
	Maximum		21.601	
	Range		21.295	
	Interquartile Range		2.359	
	Skewness		2.881	.378
	Kurtosis		9.708	.741
PS_11_12	Mean		1.66110	.216464
	95% Confidence Interval for Mean	Lower Bound	1.22289	
		Upper Bound	2.09931	
	5% Trimmed Mean		1.48159	
	Median		1.23300	
	Variance		1.827	
	Std. Deviation		1.351819	
	Minimum		.249	
	Maximum		6.604	
	Range		6.355	
	Interquartile Range		1.382	
	Skewness		2.192	.378
	Kurtosis		5.750	.741

Descriptives

			Statistic	Std. Error
PS_12_13	Mean		1.16397	.123959
	95% Confidence Interval for Mean	Lower Bound	.91303	
		Upper Bound	1.41492	
	5% Trimmed Mean		1.10509	
	Median		.91600	
	Variance		.599	
	Std. Deviation		.774121	
	Minimum		.197	
	Maximum		3.416	
	Range		3.219	
	Interquartile Range		.905	
	Skewness		1.210	.378
	Kurtosis		1.054	.741
PS_13_14	Mean		1.00290	.121441
	95% Confidence Interval for Mean	Lower Bound	.75705	
		Upper Bound	1.24874	
	5% Trimmed Mean		.93183	
	Median		.68600	
	Variance		.575	
	Std. Deviation		.758402	
	Minimum		.188	
	Maximum		3.248	
	Range		3.060	
	Interquartile Range		.848	
	Skewness		1.564	.378
	Kurtosis		1.934	.741
PS_14_15	Mean		.91182	.105621
	95% Confidence Interval for Mean	Lower Bound	.69800	
		Upper Bound	1.12564	
	5% Trimmed Mean		.84973	
	Median		.65800	
	Variance		.435	
	Std. Deviation		.659604	
	Minimum		.206	
	Maximum		2.820	
	Range		2.614	
	Interquartile Range		.782	
	Skewness		1.374	.378
	Kurtosis		1.285	.741

Descriptives

			Statistic	Std. Error
PS_15_16	Mean		.75346	.083345
	95% Confidence Interval for Mean	Lower Bound	.58474	
		Upper Bound	.92218	
	5% Trimmed Mean		.70636	
	Median		.52900	
	Variance		.271	
	Std. Deviation		.520488	
	Minimum		.160	
	Maximum		2.204	
	Range		2.044	
	Interquartile Range		.570	
	Skewness		1.276	.378
	Kurtosis		1.030	.741
PS_16_17	Mean		.61754	.065125
	95% Confidence Interval for Mean	Lower Bound	.48570	
		Upper Bound	.74938	
	5% Trimmed Mean		.58831	
	Median		.42200	
	Variance		.165	
	Std. Deviation		.406703	
	Minimum		.149	
	Maximum		1.619	
	Range		1.470	
	Interquartile Range		.453	
	Skewness		1.177	.378
	Kurtosis		.193	.741
PS_17_18	Mean		.48149	.047901
	95% Confidence Interval for Mean	Lower Bound	.38452	
		Upper Bound	.57846	
	5% Trimmed Mean		.45869	
	Median		.38700	
	Variance		.089	
	Std. Deviation		.299144	
	Minimum		.153	
	Maximum		1.236	
	Range		1.083	
	Interquartile Range		.343	
	Skewness		1.203	.378
	Kurtosis		.345	.741

Descriptives

			Statistic	Std. Error
PS_18_19	Mean		.41064	.048796
	95% Confidence Interval for Mean	Lower Bound	.31186	
		Upper Bound	.50942	
	5% Trimmed Mean		.37052	
	Median		.29900	
	Variance		.093	
	Std. Deviation		.304729	
	Minimum		.117	
	Maximum		1.596	
	Range		1.479	
	Interquartile Range		.251	
	Skewness		2.232	.378
	Kurtosis		5.734	.741
PS_19_20	Mean		.38228	.045326
	95% Confidence Interval for Mean	Lower Bound	.29053	
		Upper Bound	.47404	
	5% Trimmed Mean		.34500	
	Median		.29000	
	Variance		.080	
	Std. Deviation		.283059	
	Minimum		.107	
	Maximum		1.374	
	Range		1.267	
	Interquartile Range		.245	
	Skewness		2.118	.378
	Kurtosis		4.850	.741

Tests of Normality

	Kolmogorov-Smirnov[a]			Shapiro-Wilk		
	Statistic	df	Sig.	Statistic	df	Sig.
BrainRate	.248	39	<.001	.736	39	<.001
PermutationEntropy	.064	39	.200*	.968	39	.318
PermutationEntropy_01	.064	39	.200*	.968	39	.318
SampleEntropy	.137	39	.064	.943	39	.050
SpectralEntropy_bits	.177	39	.004	.893	39	.001
SpectralEntropy_01	.177	39	.004	.893	39	.001
HiguchiFractalDimension	.127	39	.117	.938	39	.033
LempelZivComplexity	.182	39	.002	.824	39	<.001
RenyiEntropy	.218	39	<.001	.835	39	<.001
TsallisEntropy	.487	39	<.001	.314	39	<.001
PS_4_5	.168	39	.007	.864	39	<.001
PS_5_6	.203	39	<.001	.791	39	<.001
PS_6_7	.203	39	<.001	.796	39	<.001
PS_7_8	.212	39	<.001	.855	39	<.001
PS_8_9	.268	39	<.001	.664	39	<.001
PS_9_10	.269	39	<.001	.501	39	<.001
PS_10_11	.279	39	<.001	.658	39	<.001
PS_11_12	.180	39	.003	.778	39	<.001
PS_12_13	.195	39	<.001	.890	39	.001
PS_13_14	.209	39	<.001	.822	39	<.001
PS_14_15	.187	39	.001	.845	39	<.001
PS_15_16	.217	39	<.001	.851	39	<.001
PS_16_17	.235	39	<.001	.830	39	<.001
PS_17_18	.239	39	<.001	.835	39	<.001
PS_18_19	.244	39	<.001	.749	39	<.001
PS_19_20	.220	39	<.001	.759	39	<.001

*. This is a lower bound of the true significance.

a. Lilliefors Significance Correction

BrainRate

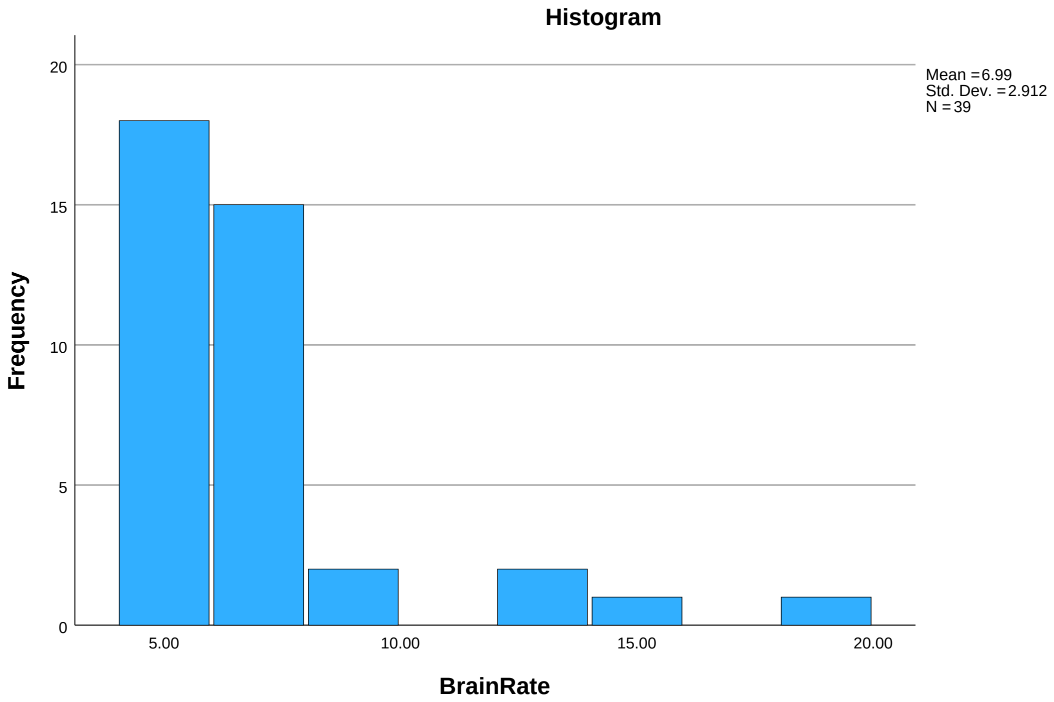


```
BrainRate Stem-and-Leaf Plot

 Frequency    Stem &  Leaf

     2.00        4 .  23
     6.00        4 .  777789
     4.00        5 .  0023
     6.00        5 .  566699
     4.00        6 .  2344
     4.00        6 .  5669
     3.00        7 .  023
     4.00        7 .  5677
      .00        8 .
     1.00        8 .  6
     1.00        9 .  4
     4.00 Extremes    (>=12.1)

 Stem width:      1.00
 Each leaf:       1 case(s)
```

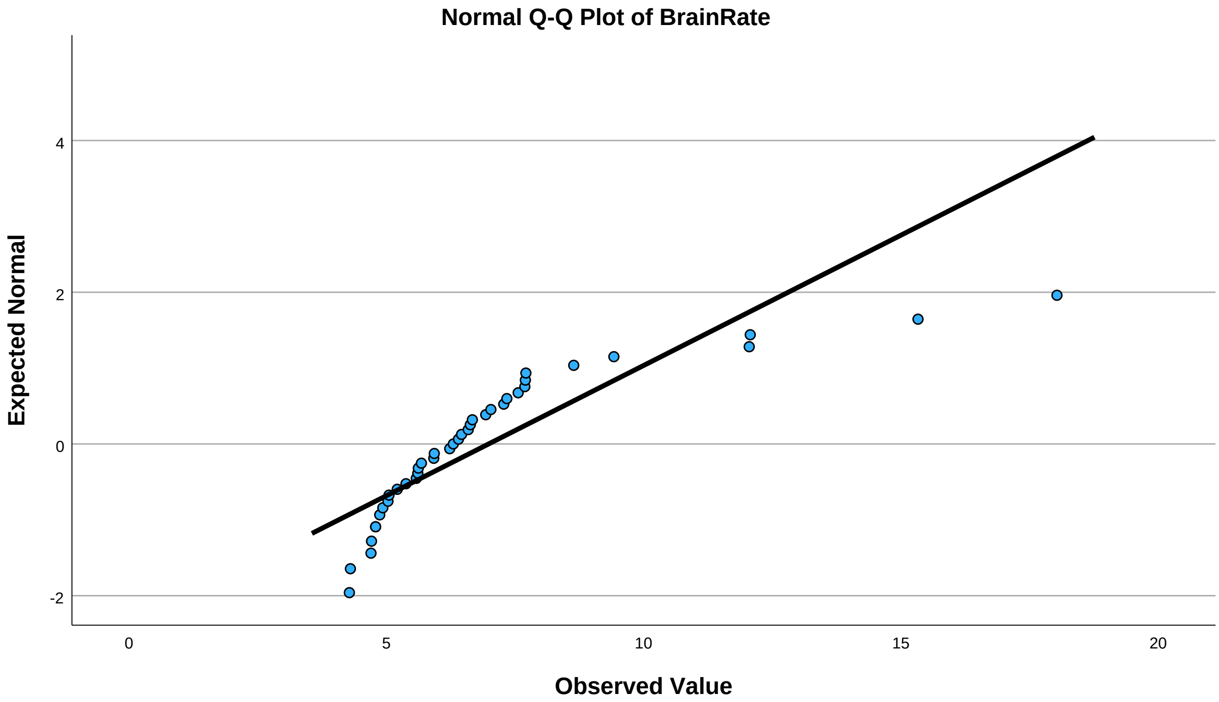
Normal Q-Q Plot of BrainRate
Expected Normal
4
2
0
-2
0
5
10
15
20
Observed Value

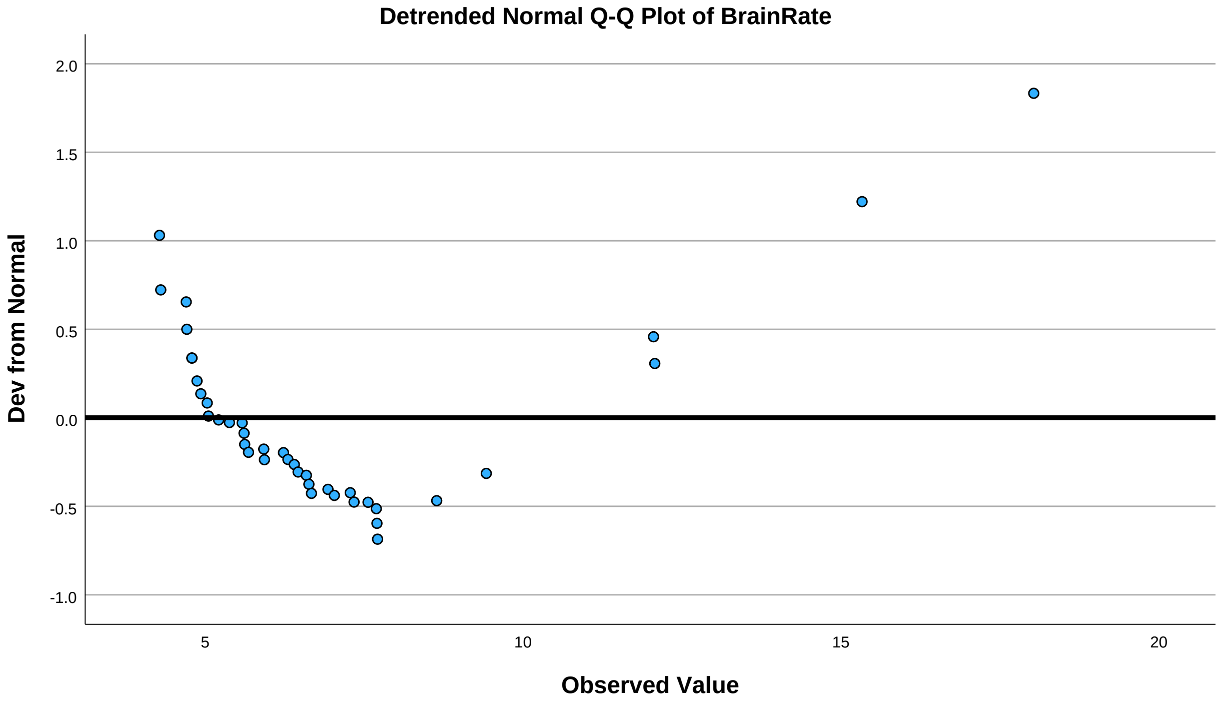
Detrended Normal Q-Q Plot of BrainRate
Dev from Normal
2.0
1.5
1.0
0.5
0.0
-0.5
-1.0
5
10
15
20
Observed Value

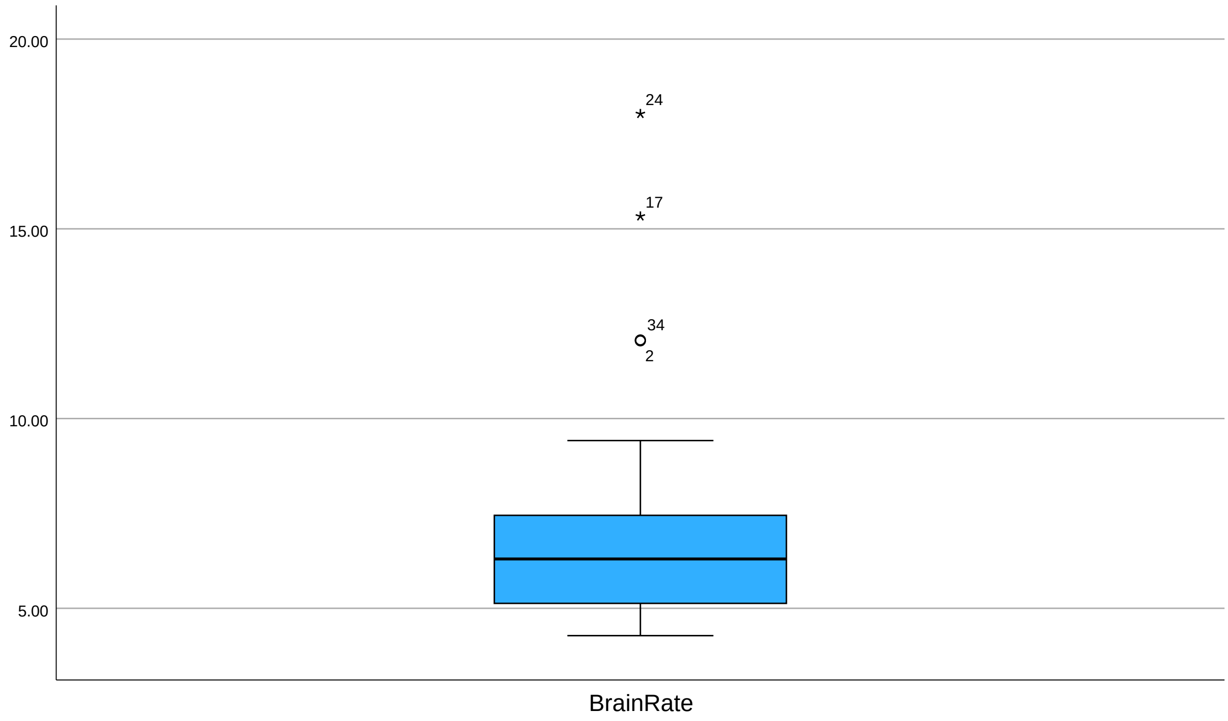

PermutationEntropy

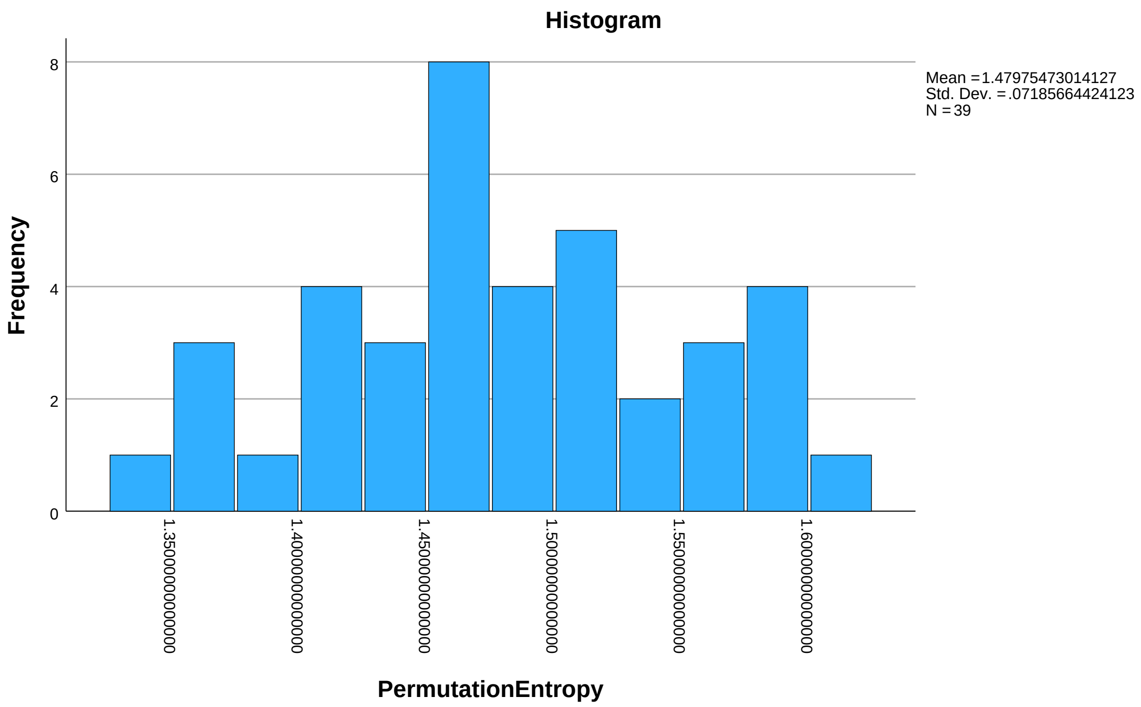

PermutationEntropy Stem-and-Leaf Plot

Frequency   Stem & Leaf

```
 1.00     13 . 4
 4.00     13 . 5559
 7.00     14 . 0011234
12.00     14 . 555556779999
 7.00     15 . 0011144
 7.00     15 . 5668888
 1.00     16 . 0

Stem width:   .1000000
Each leaf:        1 case(s)
```

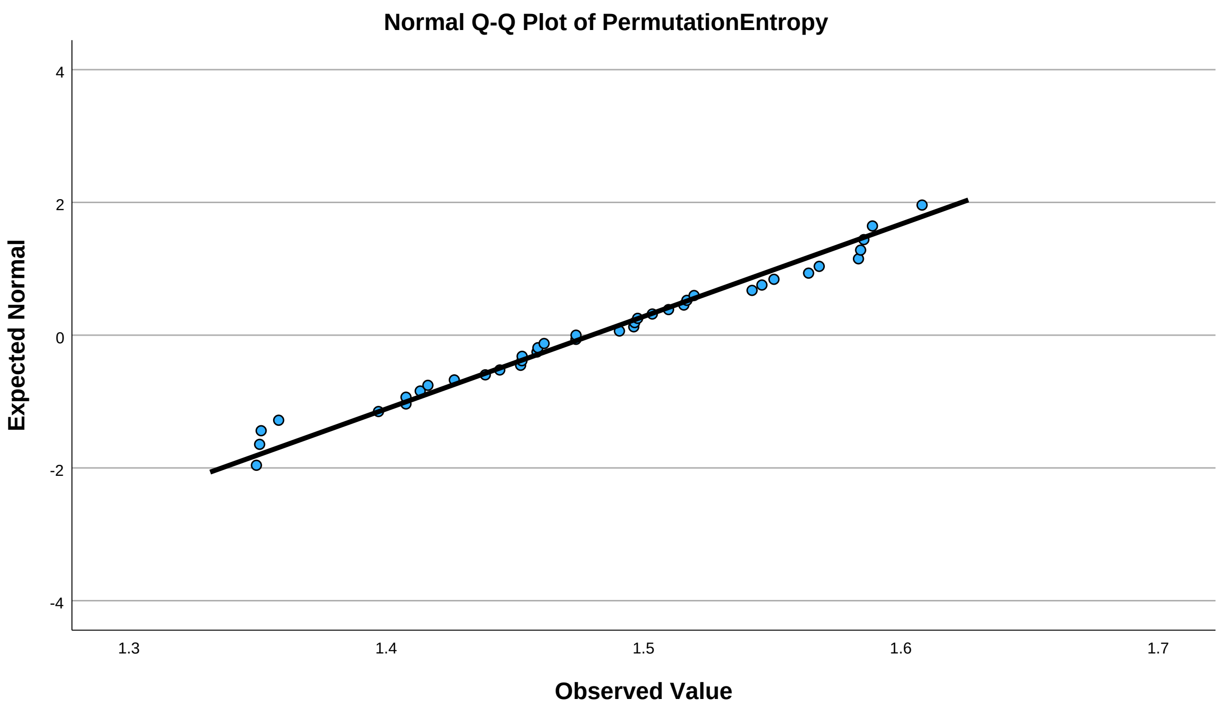

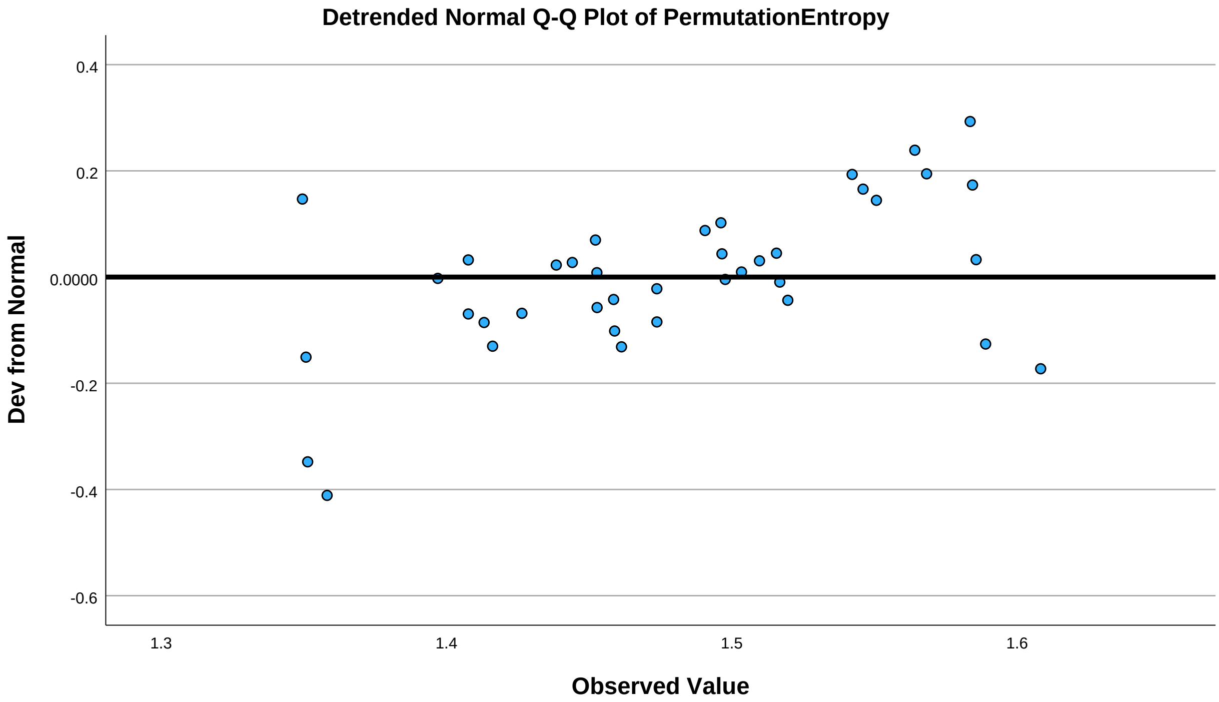

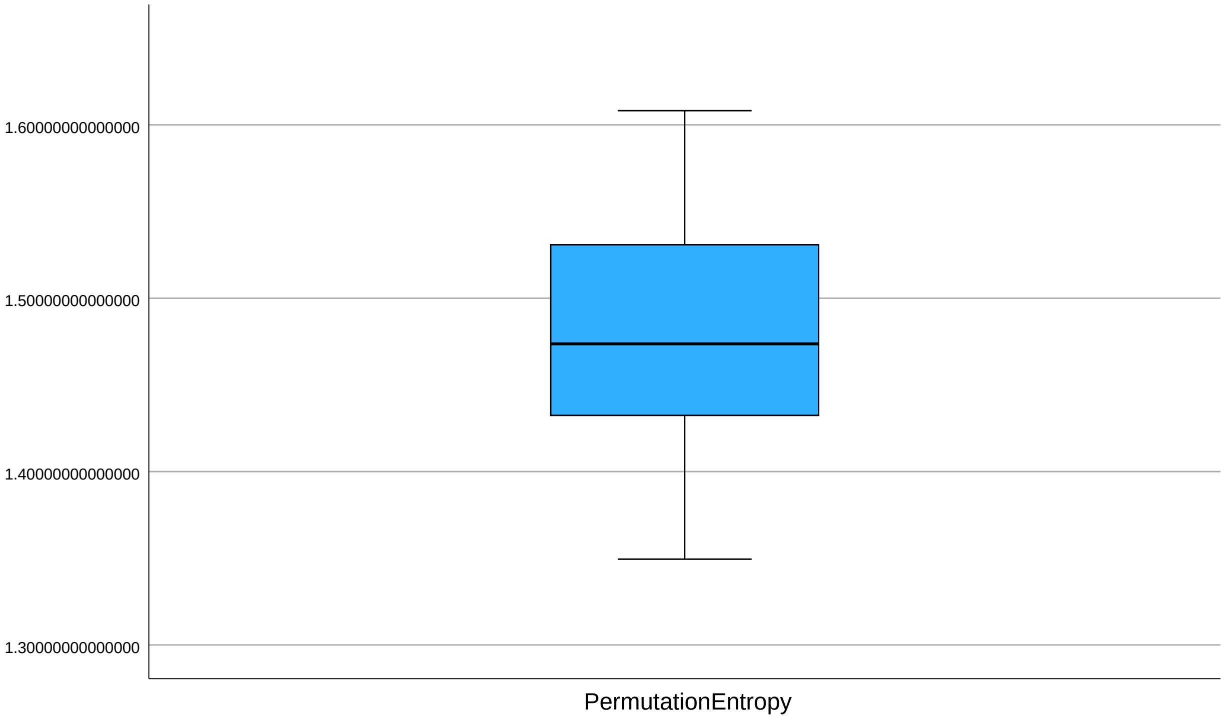

PermutationEntropy_01

Histogram

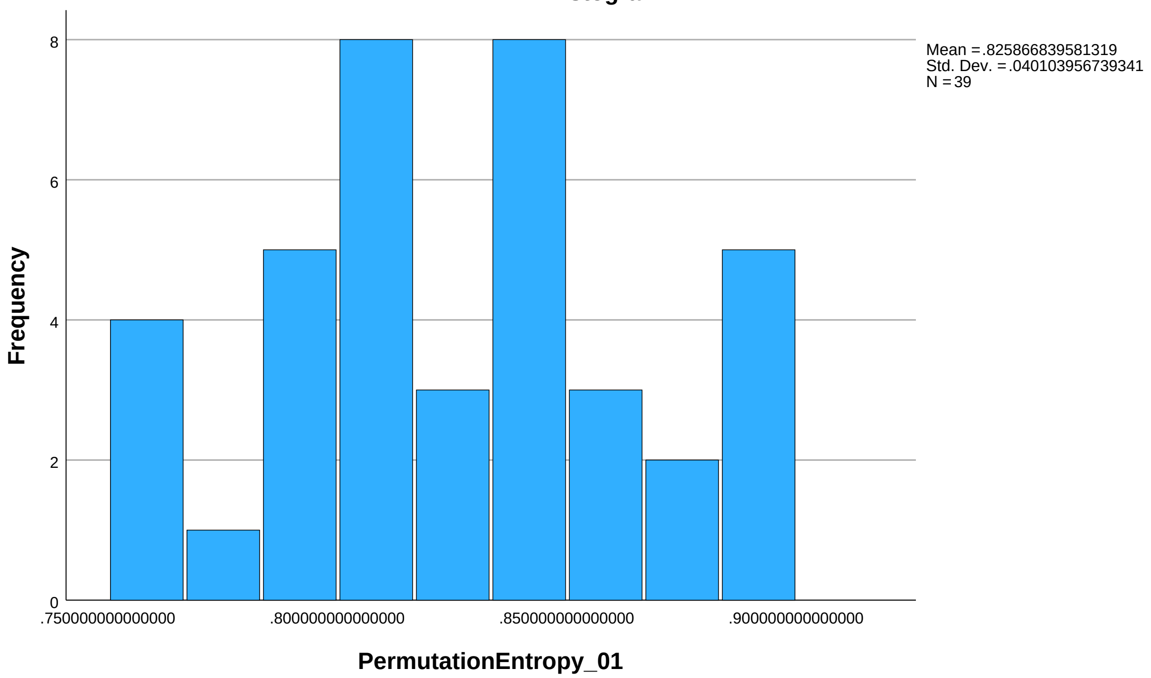


```
PermutationEntropy_01 Stem-and-Leaf Plot

 Frequency    Stem &  Leaf

    4.00       7 .  5555
    1.00       7 .  7
    5.00       7 .  88899
    8.00       8 .  00111111
    7.00       8 .  2233333
    4.00       8 .  4444
    5.00       8 .  66677
    5.00       8 .  88889

 Stem width:   .1000000
 Each leaf:        1 case(s)
```

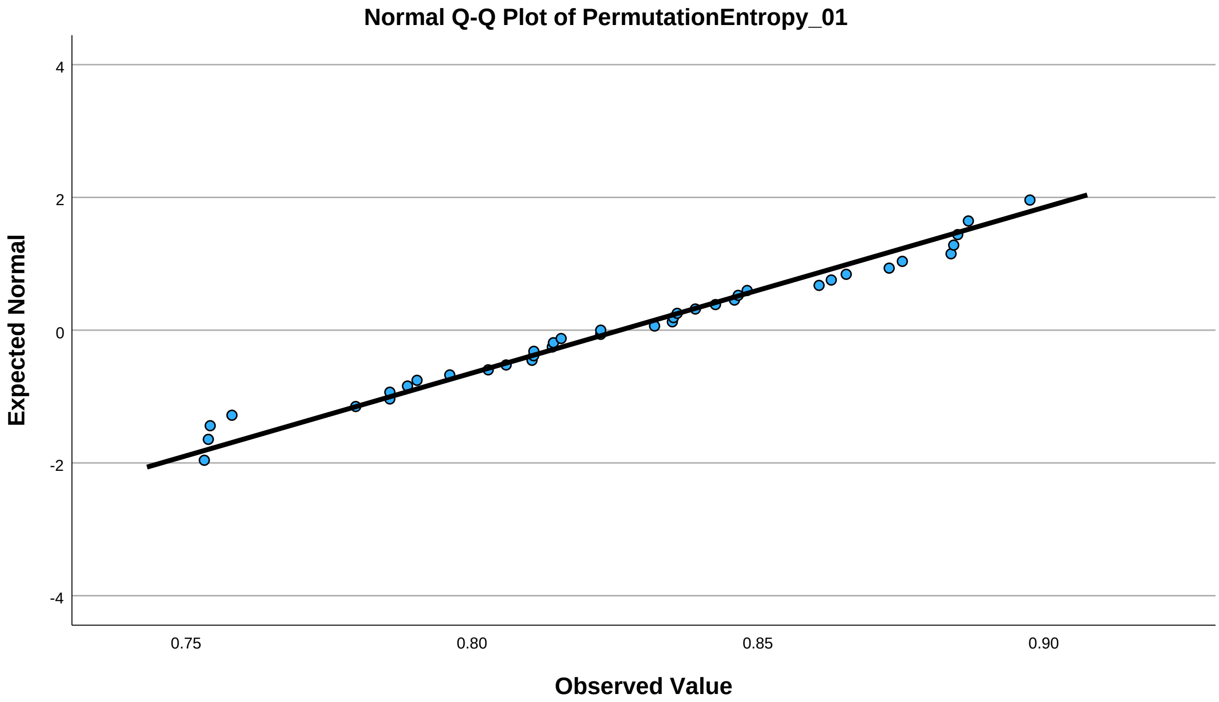
Normal Q-Q Plot of PermutationEntropy_01
Expected Normal
4
2
0
-2
-4
0.75
0.80
0.85
0.90
Observed Value

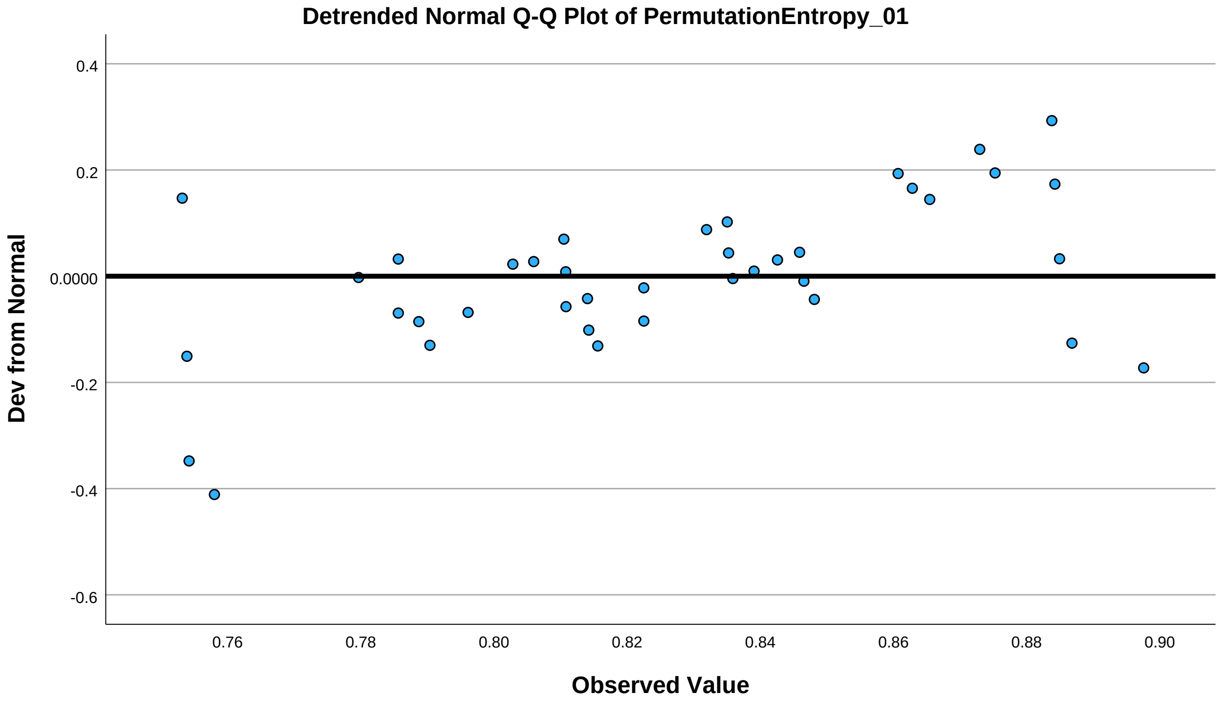
Detrended Normal Q-Q Plot of PermutationEntropy_01
Dev from Normal
0.4
0.2
0.0000
-0.2
-0.4
-0.6
0.76
0.78
0.80
0.82
0.84
0.86
0.88
0.90
Observed Value

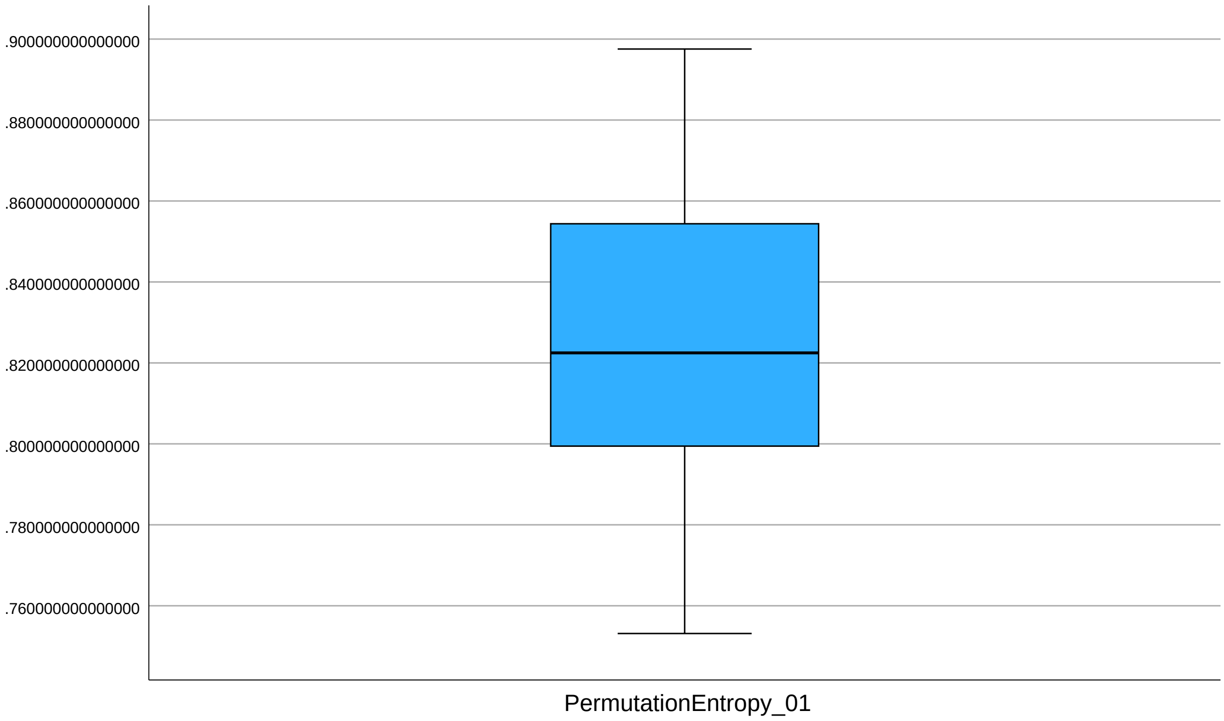

SampleEntropy

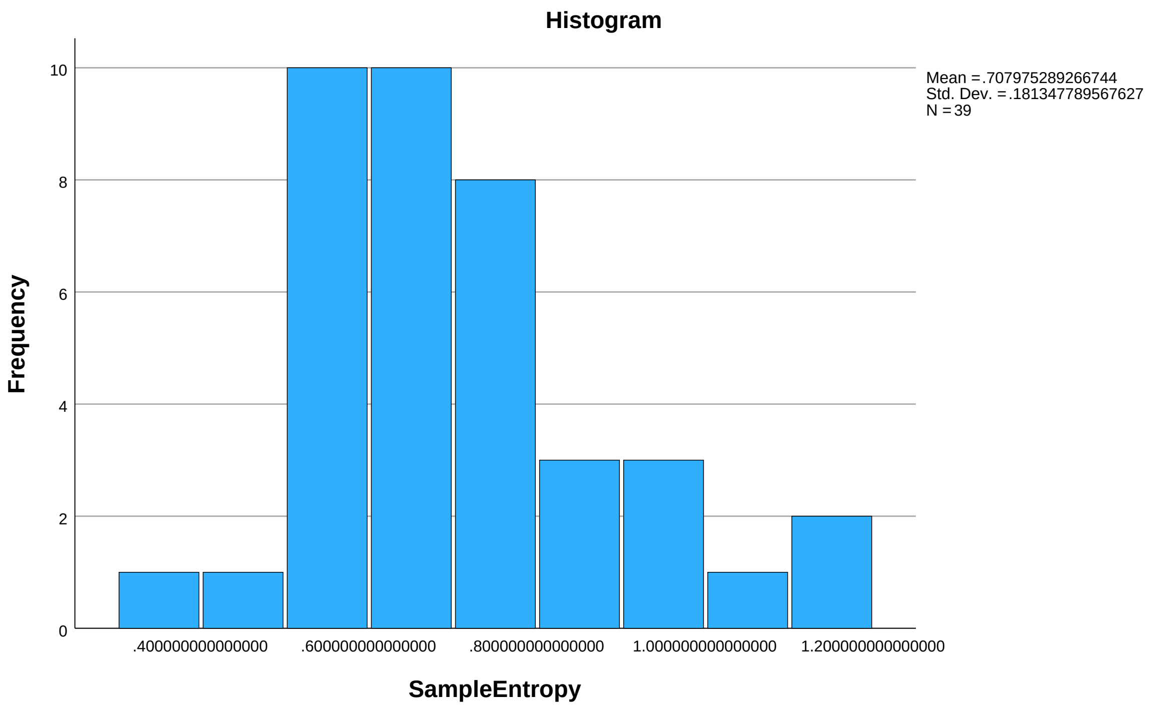

SampleEntropy Stem-and-Leaf Plot

Frequency Stem & Leaf

```
 1.00      3 . 8
 1.00      4 . 2
 10.00       5 . 1123466799
 10.00       6 . 0334457788
  8.00      7 . 00145559
  3.00      8 . 346
  3.00      9 . 289
  1.00     10 . 4
   2.00 Extremes    (>=1.13)

Stem width:  .1000000
Each leaf:       1 case(s)
```

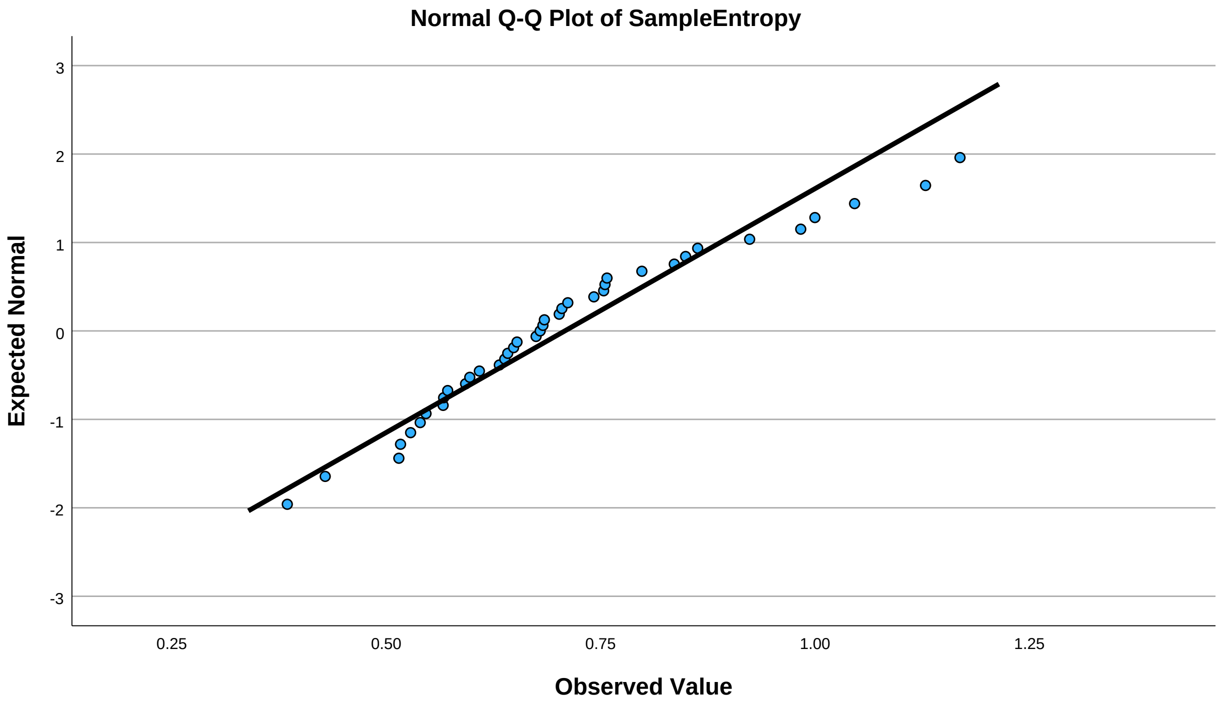

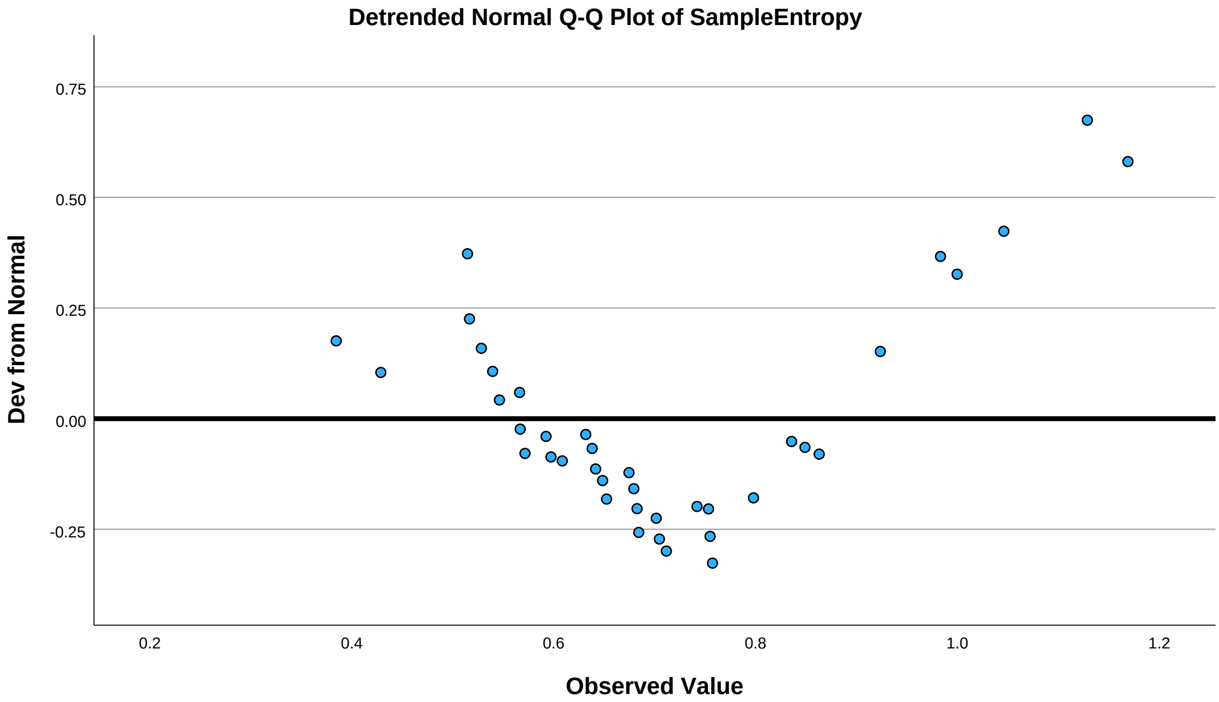

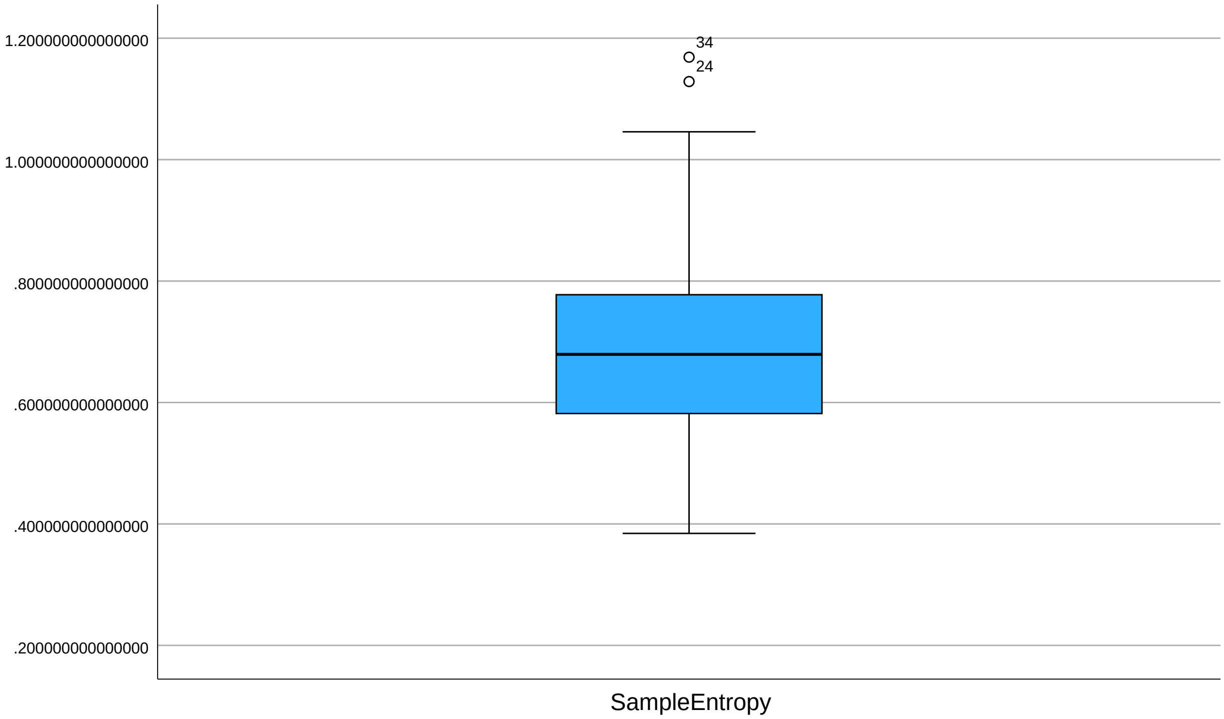

SpectralEntropy_bits

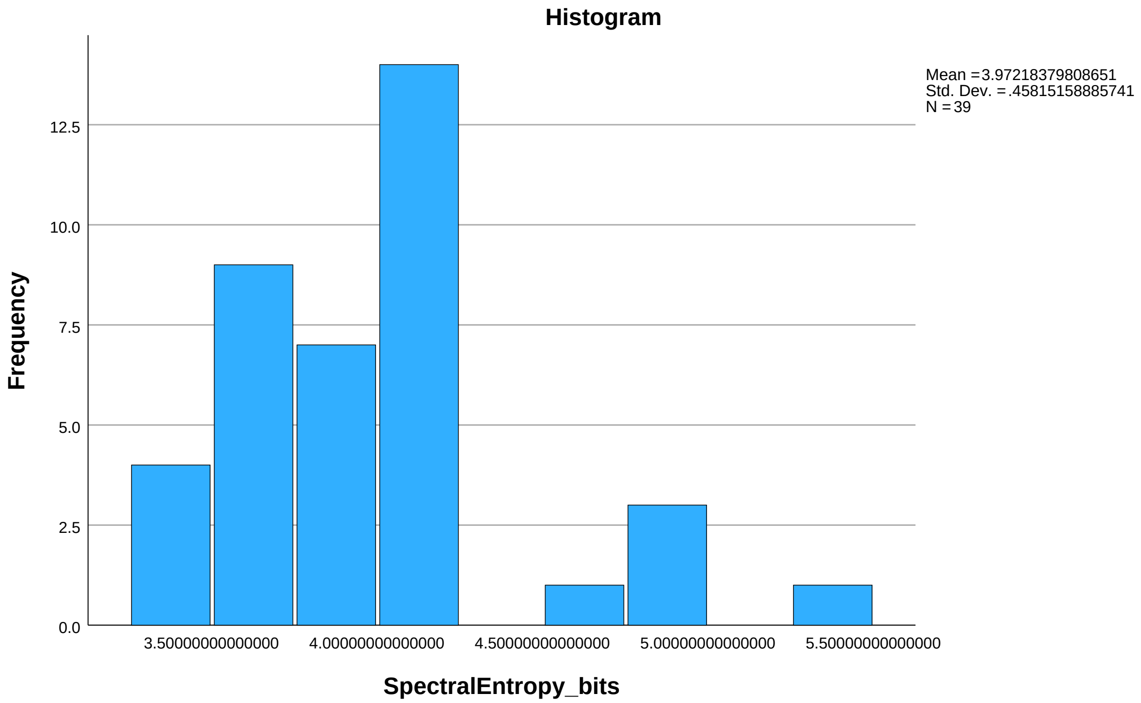


```
SpectralEntropy_bits Stem-and-Leaf Plot

 Frequency    Stem &  Leaf

     2.00       33 .  44
     2.00       34 .  16
     5.00       35 .  24455
     2.00       36 .  48
     3.00       37 .  028
     5.00       38 .  23457
     1.00       39 .  3
     8.00       40 .  22233556
     5.00       41 .  01667
     1.00       42 .  4
      .00       43 .
      .00       44 .
     1.00       45 .  1
     4.00 Extremes    (>=4.86)

 Stem width:   .1000000
 Each leaf:       1 case(s)
```

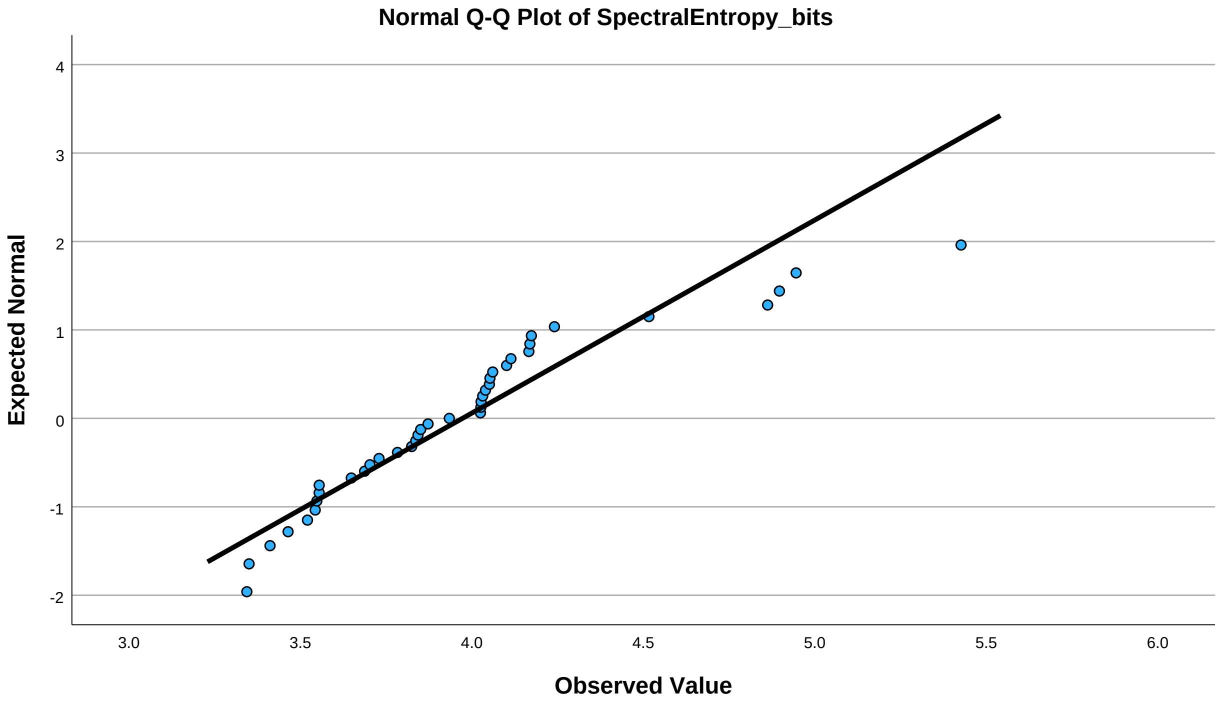
Normal Q-Q Plot of SpectralEntropy_bits
Expected Normal
Observed Value
4
3
2
1
0
-1
-2
3.0
3.5
4.0
4.5
5.0
5.5
6.0

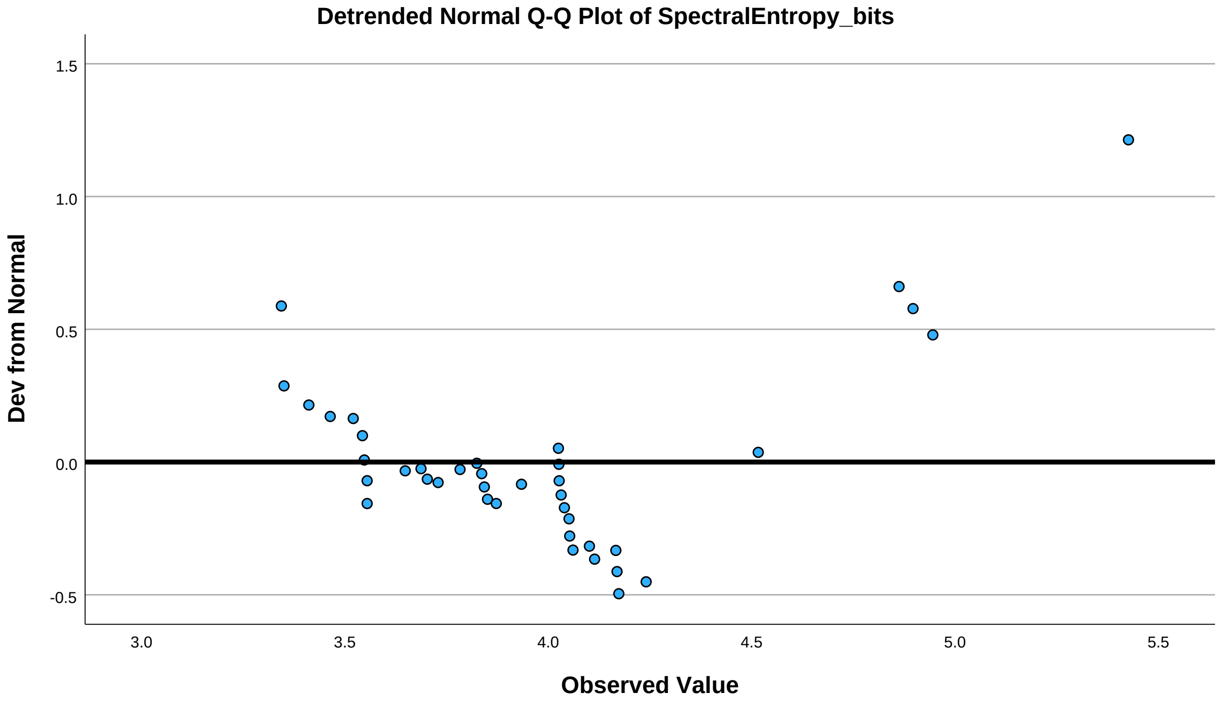
Detrended Normal Q-Q Plot of SpectralEntropy_bits
Dev from Normal
Observed Value
1.5
1.0
0.5
0.0
-0.5
3.0
3.5
4.0
4.5
5.0
5.5

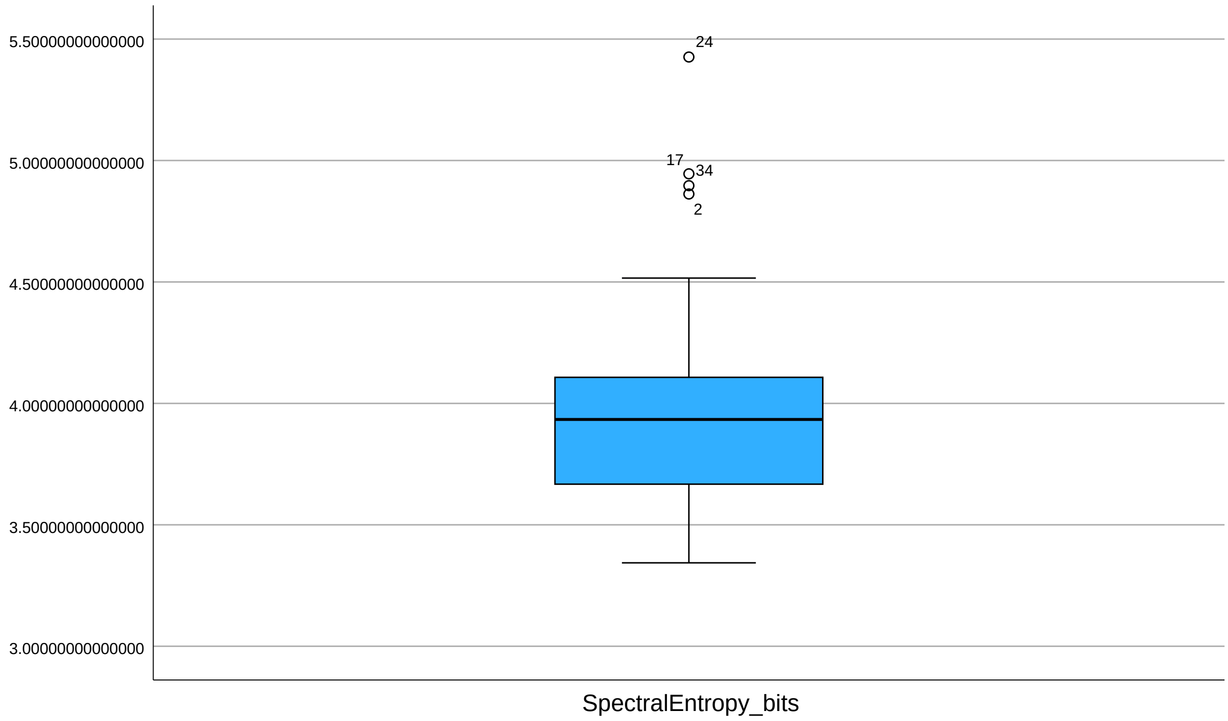

SpectralEntropy_01

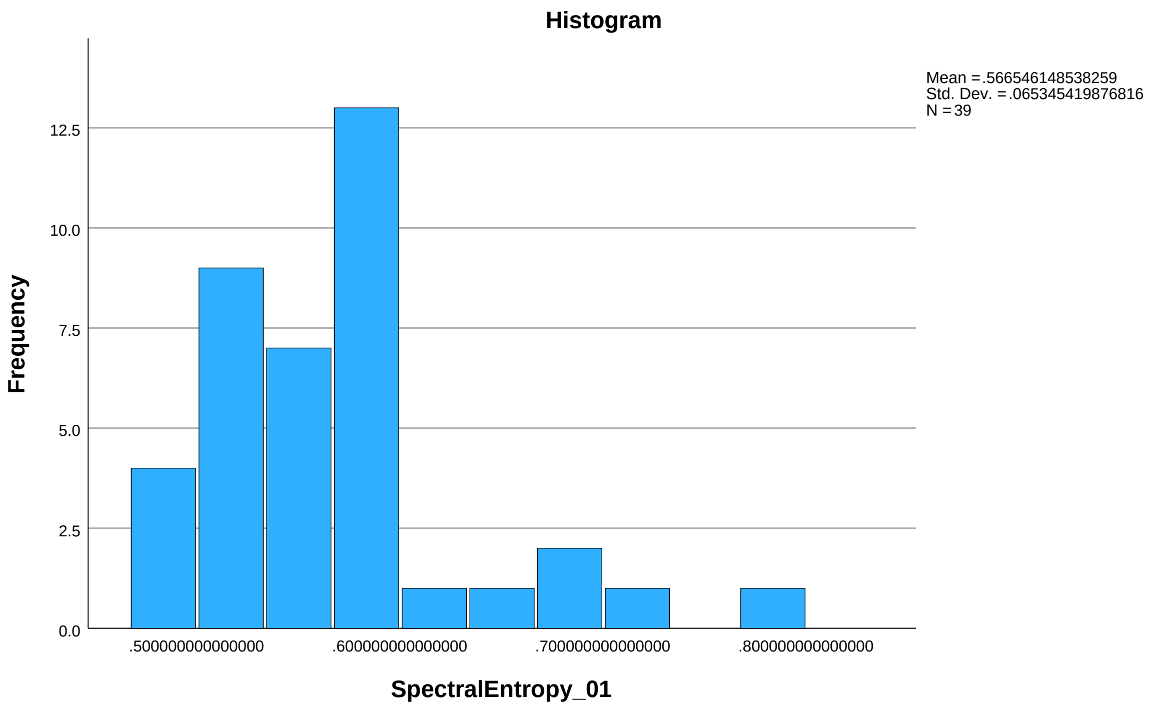

SpectralEntropy_01 Stem-and-Leaf Plot

Frequency   Stem & Leaf

```
  4.00      4 . 7789
 14.00       5 .  00000222334444
 15.00       5 .  567777777788999
  2.00     6 . 04
  4.00 Extremes    (>=.69)

Stem width:   .1000000
Each leaf:       1 case(s)
```

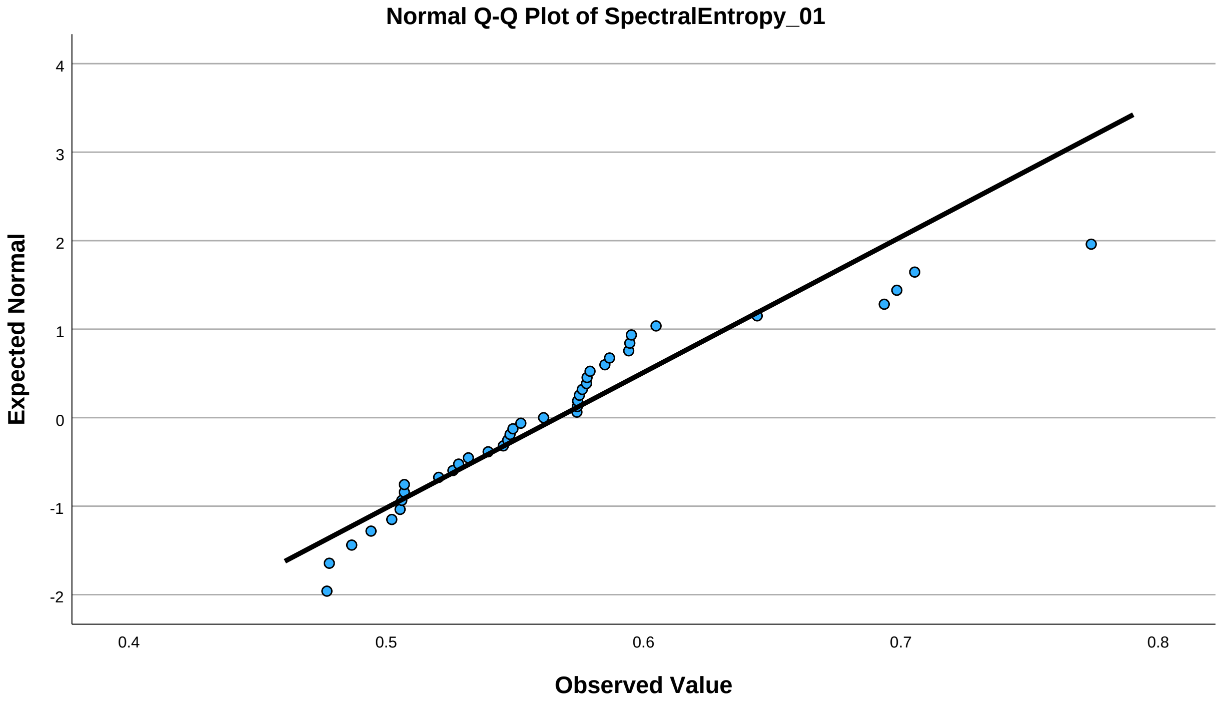

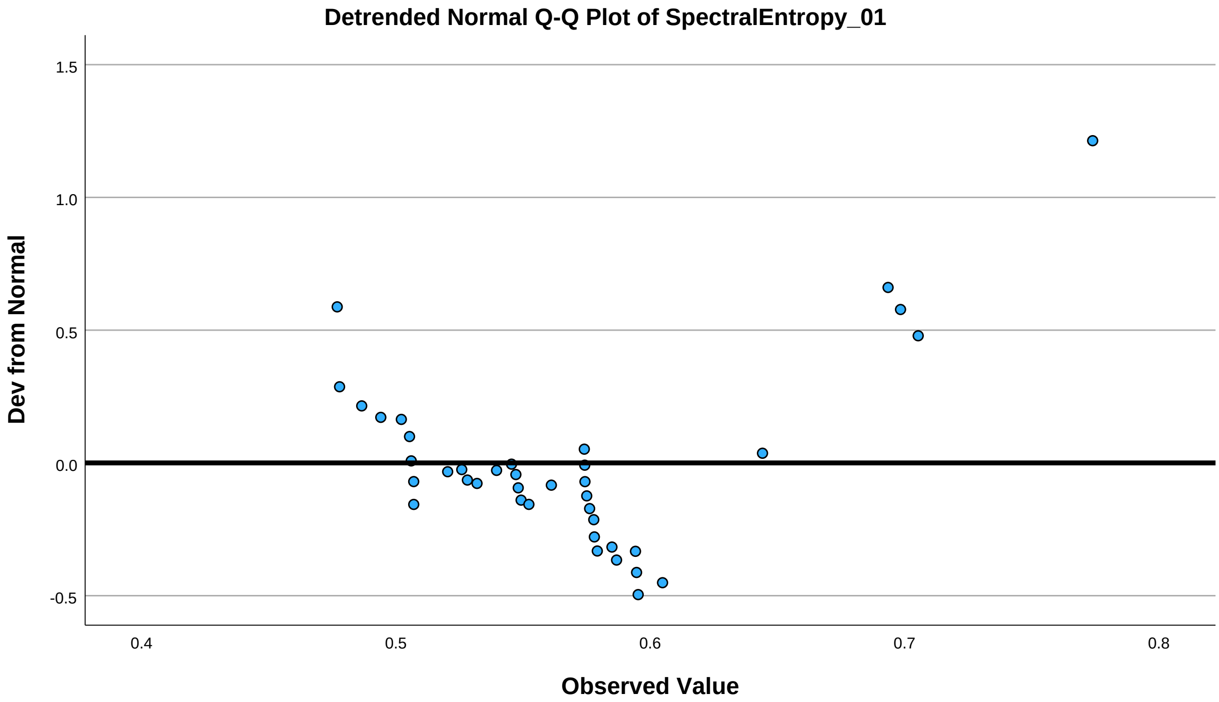

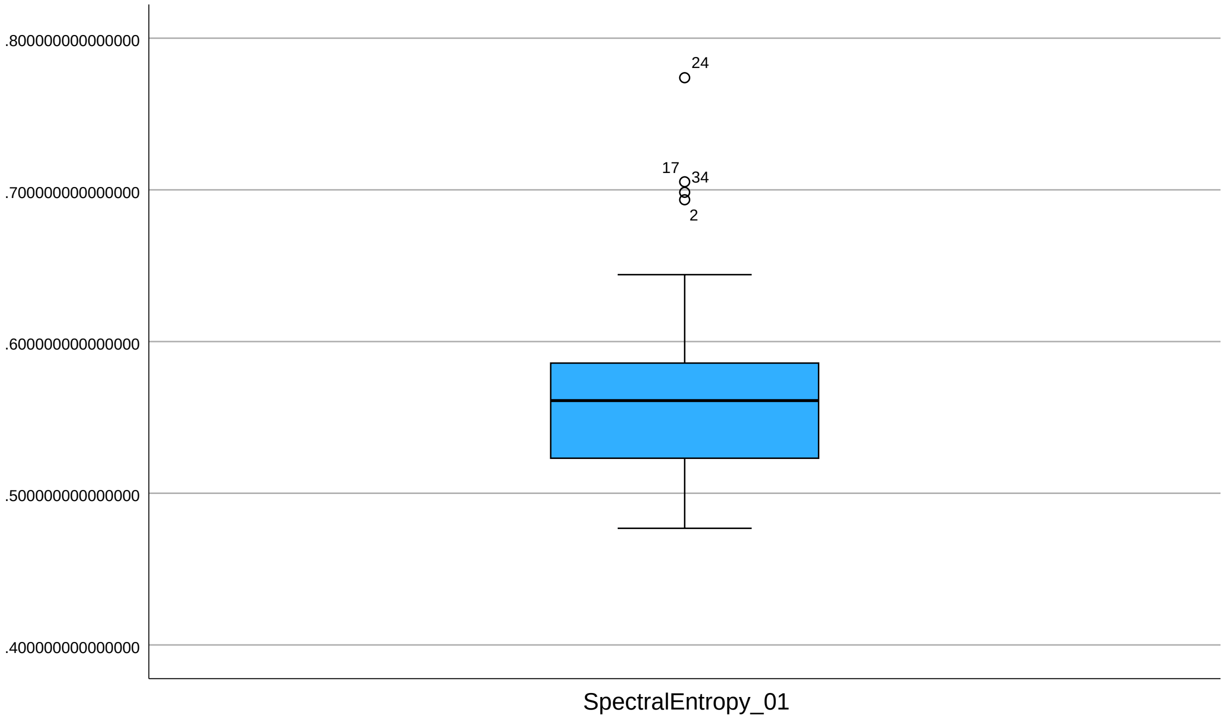

HiguchiFractalDimension

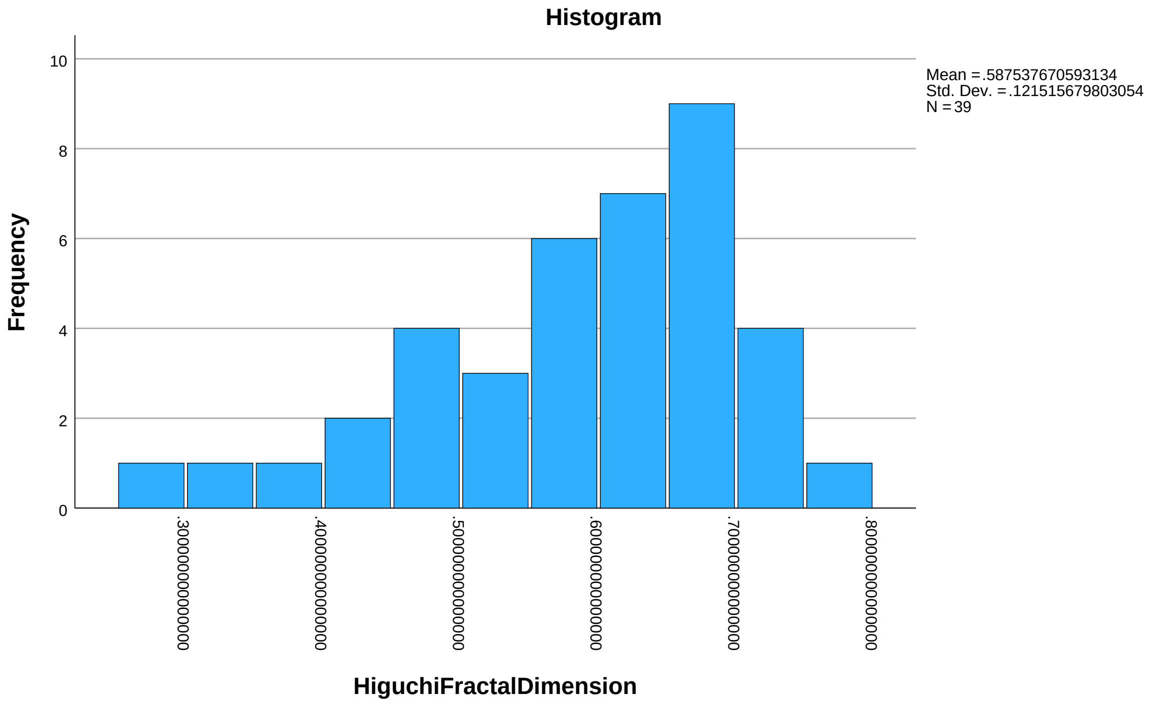


```
HiguchiFractalDimension Stem-and-Leaf Plot

 Frequency    Stem &  Leaf

     1.00 Extremes    (=<.26)
     1.00        3 .  0
     1.00        3 .  7
     2.00        4 .  02
     4.00        4 .  5669
     3.00        5 .  224
     6.00        5 .  678899
     7.00        6 .  0022333
     9.00        6 .  666778999
     4.00        7 .  0134
     1.00        7 .  8

 Stem width:   .1000000
 Each leaf:        1 case(s)
```

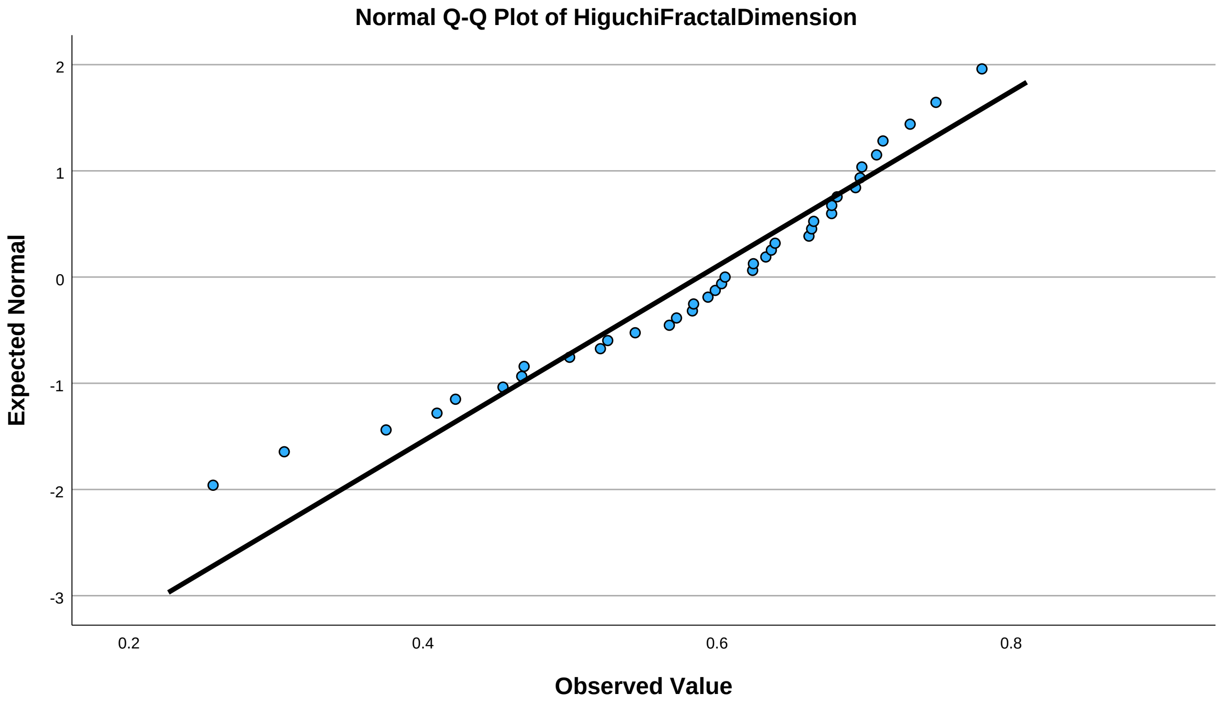
Normal Q-Q Plot of HiguchiFractalDimension
Expected Normal
2
1
0
-1
-2
-3
0.2
0.4
0.6
0.8
Observed Value

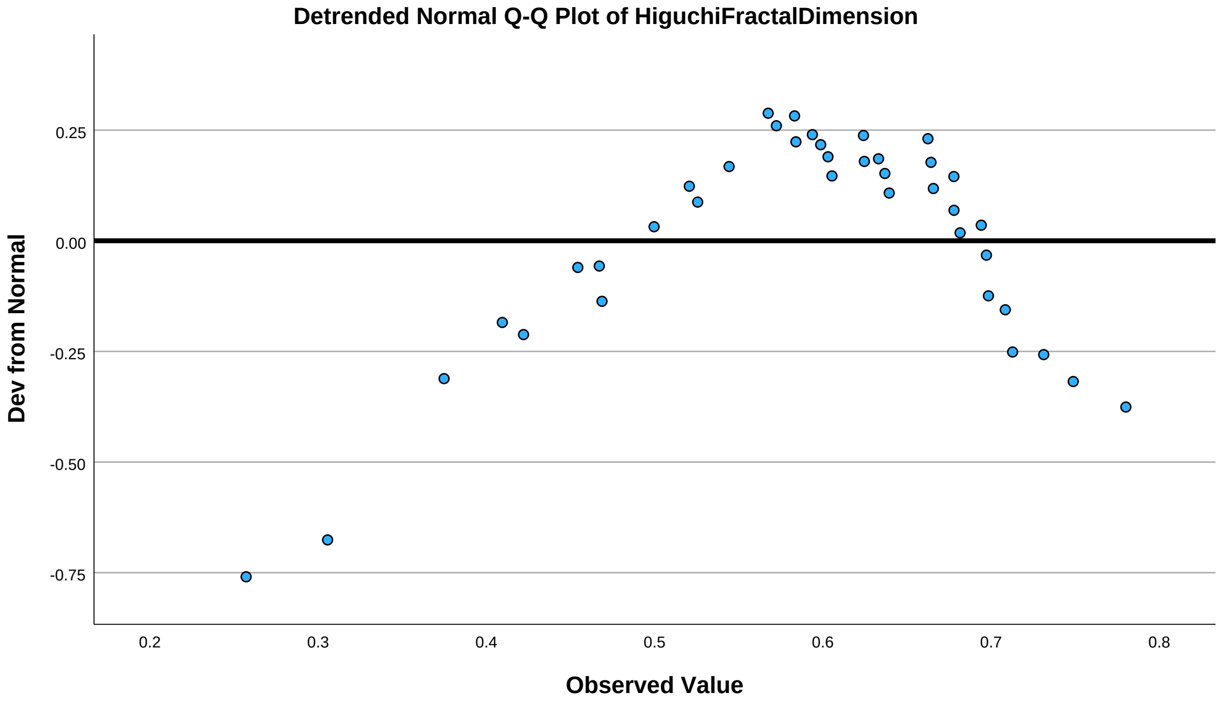
Detrended Normal Q-Q Plot of HiguchiFractalDimension
Dev from Normal
0.25
0.00
-0.25
-0.50
-0.75
0.2
0.3
0.4
0.5
0.6
0.7
0.8
Observed Value

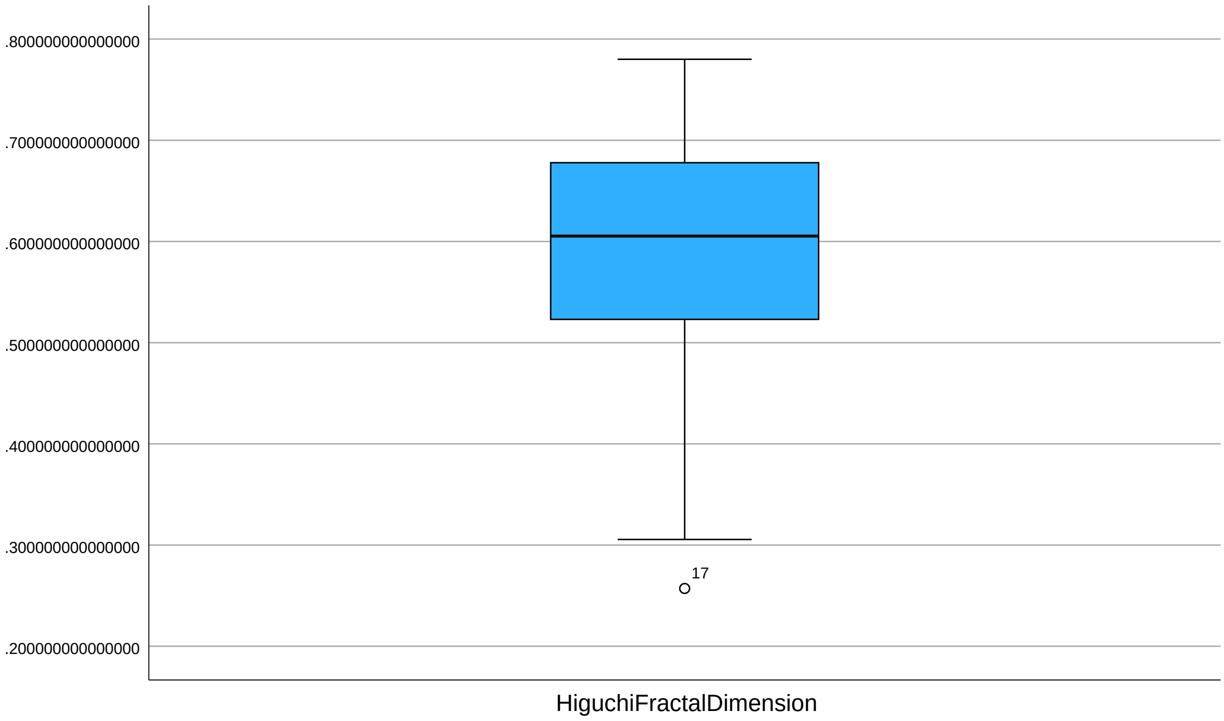

LempelZivComplexity

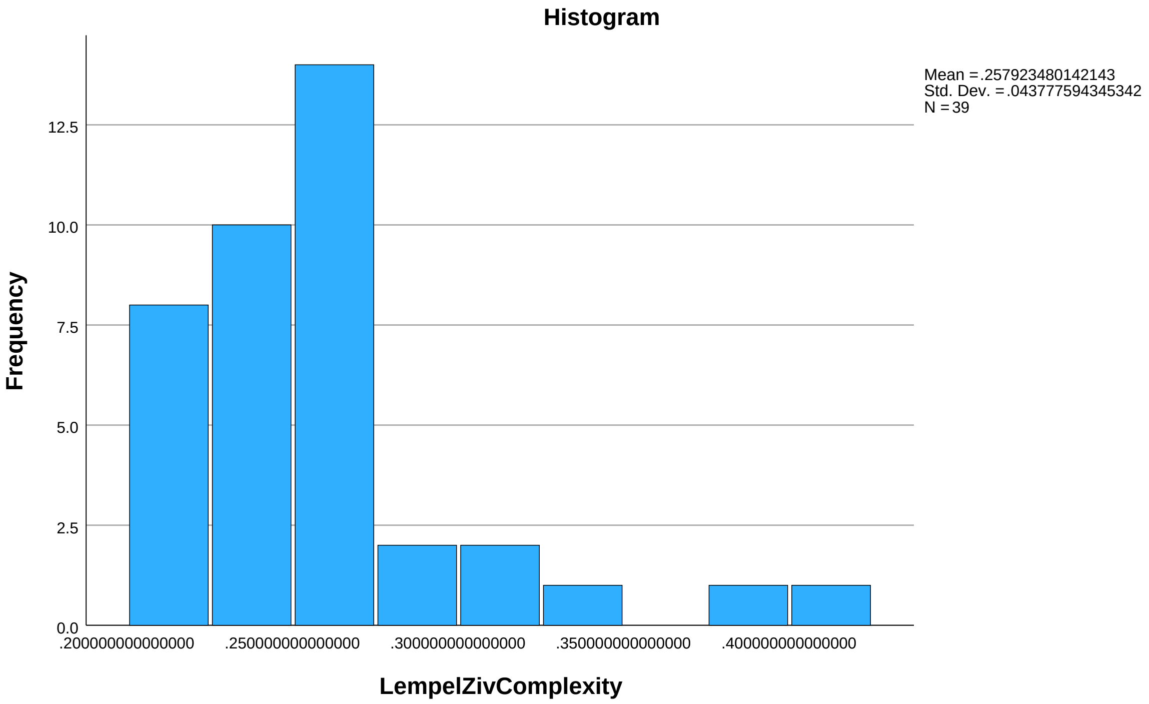


```
LempelZivComplexity Stem-and-Leaf Plot

 Frequency    Stem &  Leaf
```

```
   1.00     20 .  9
   5.00     21 .  03359
   4.00     22 .  1478
   6.00     23 .  111468
   2.00     24 .  07
   7.00     25 .  1167779
   4.00     26 .  1255
   4.00     27 .  0039
   1.00     28 .  6
    .00     29 .
   1.00     30 .  4
   1.00     31 .  2
   3.00 Extremes    (>=.347)

Stem width:   .0100000
Each leaf:       1 case(s)
```

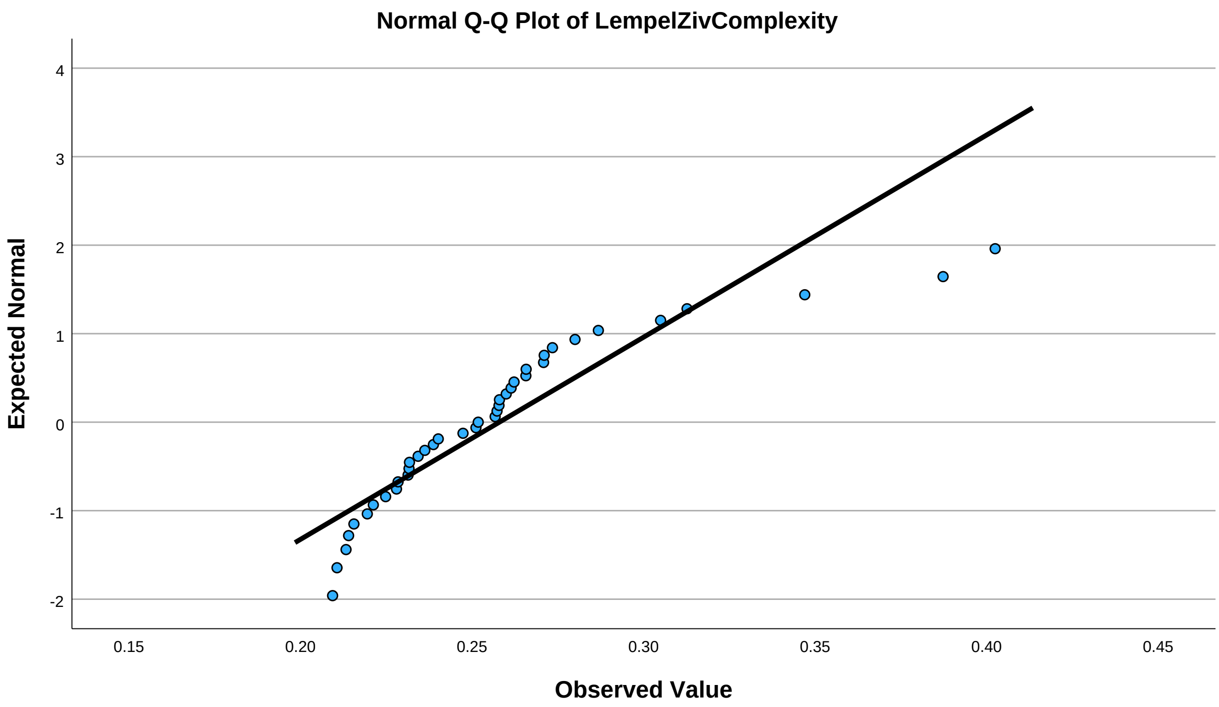

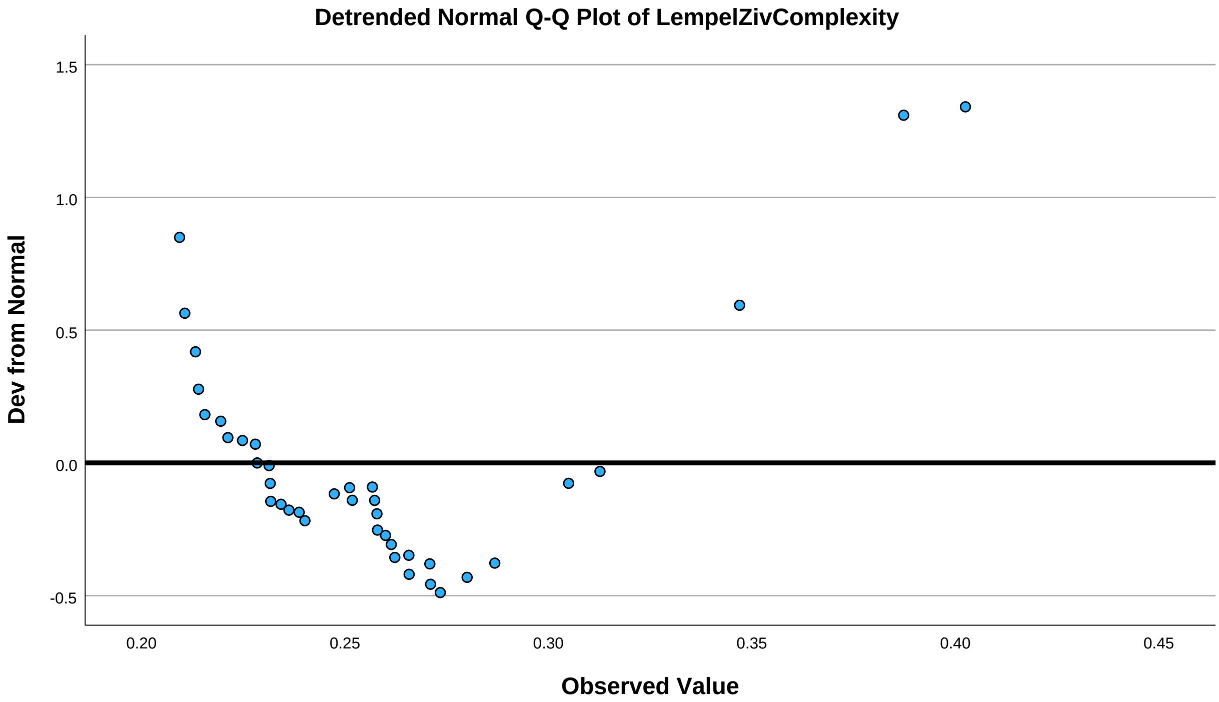

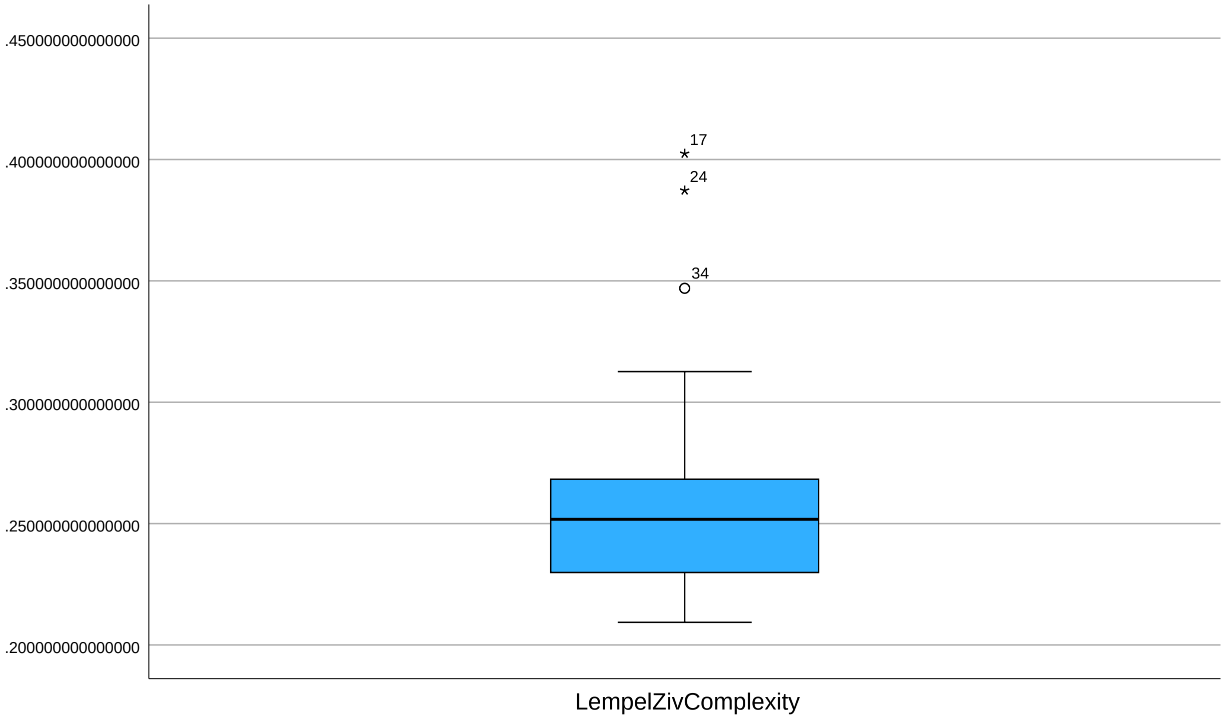

RenyiEntropy

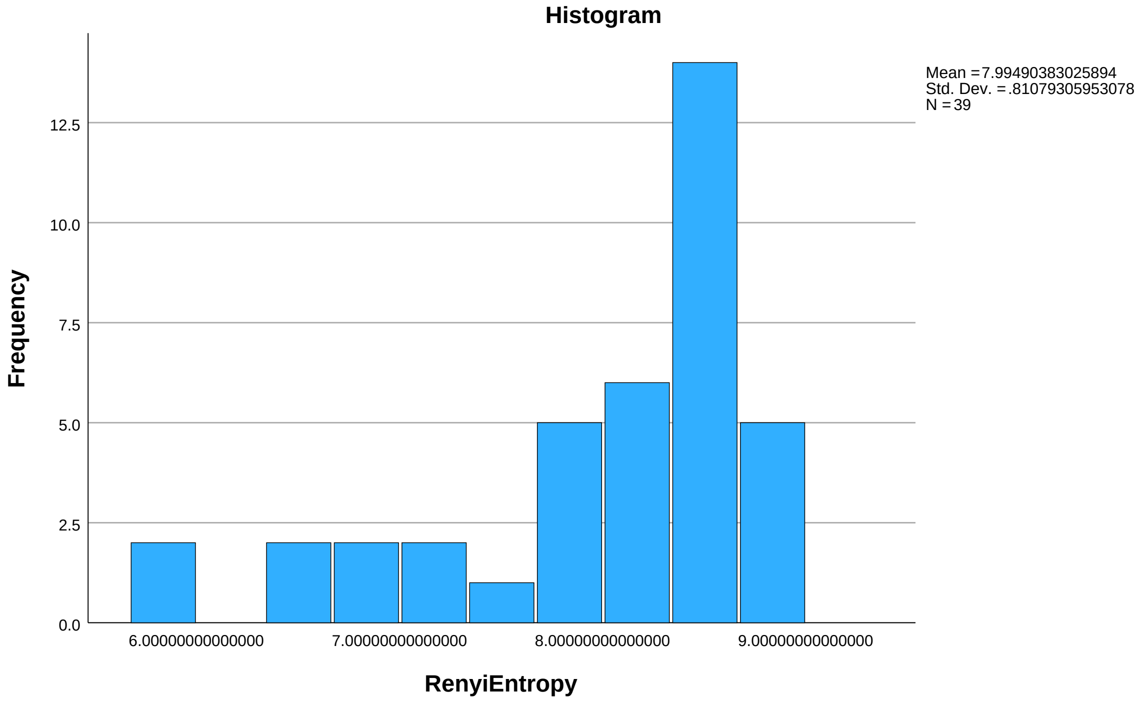


```
RenyiEntropy Stem-and-Leaf Plot

 Frequency    Stem &  Leaf

     3.00 Extremes    (=<6.4)
     3.00       6 .  688
     2.00       7 .  00
     6.00       7 .  577889
    12.00       8 .  022222334444
    13.00       8 .  5555566667788

 Stem width:  1.000000
 Each leaf:       1 case(s)
```

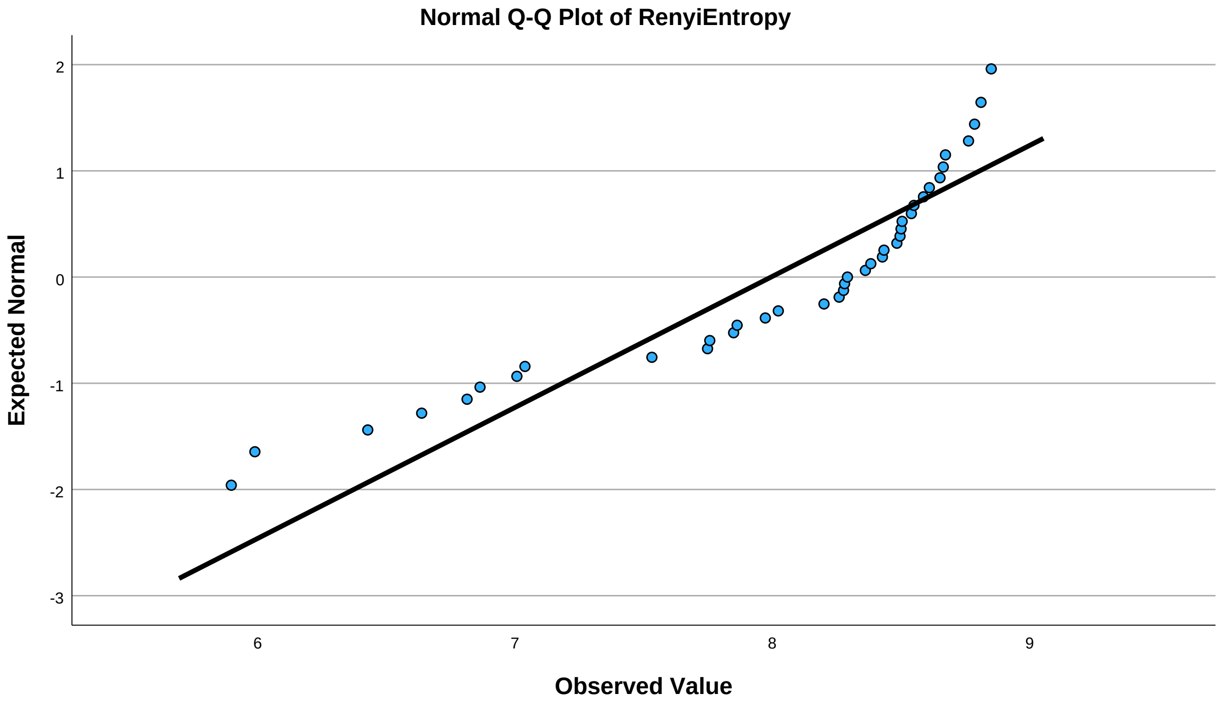
Normal Q-Q Plot of RenyiEntropy
Expected Normal
Observed Value
2
1
0
-1
-2
-3
6
7
8
9

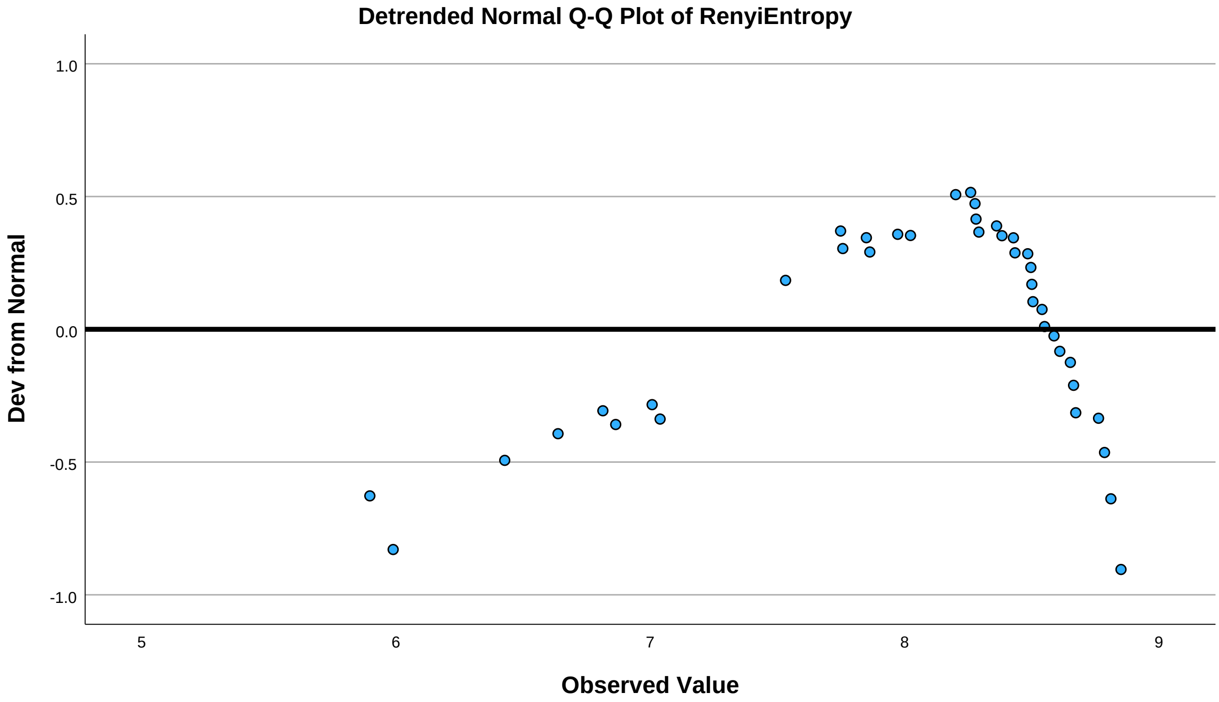
Detrended Normal Q-Q Plot of RenyiEntropy
Dev from Normal
Observed Value
1.0
0.5
0.0
-0.5
-1.0
5
6
7
8
9

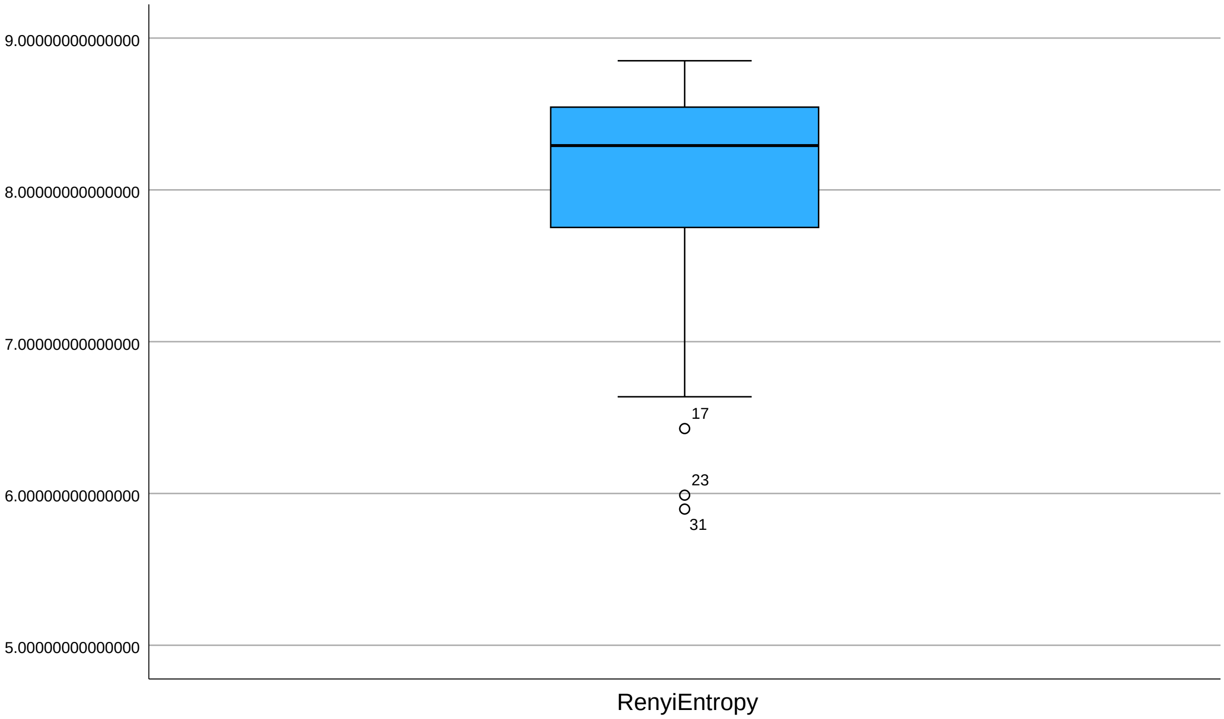

TsallisEntropy

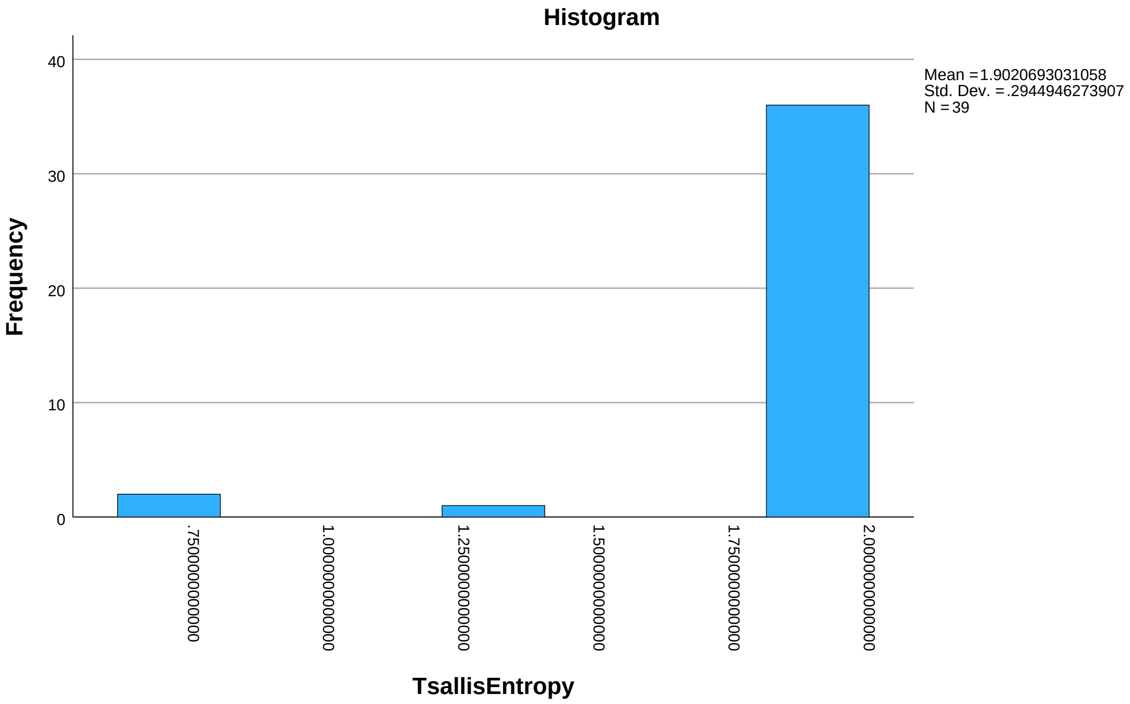


```
TsallisEntropy Stem-and-Leaf Plot

 Frequency    Stem &  Leaf
```

```
 8.00 Extremes    (=<1.9755)
 1.00    1981 .  4
  .00    1982 .
 2.00    1983 .  16
 2.00    1984 .  37
12.00    1985 .  244566778899
10.00    1986 .  0223556666
 3.00    1987 .  122
 1.00    1988 .  1

Stem width:   .0010000
Each leaf:        1 case(s)
```

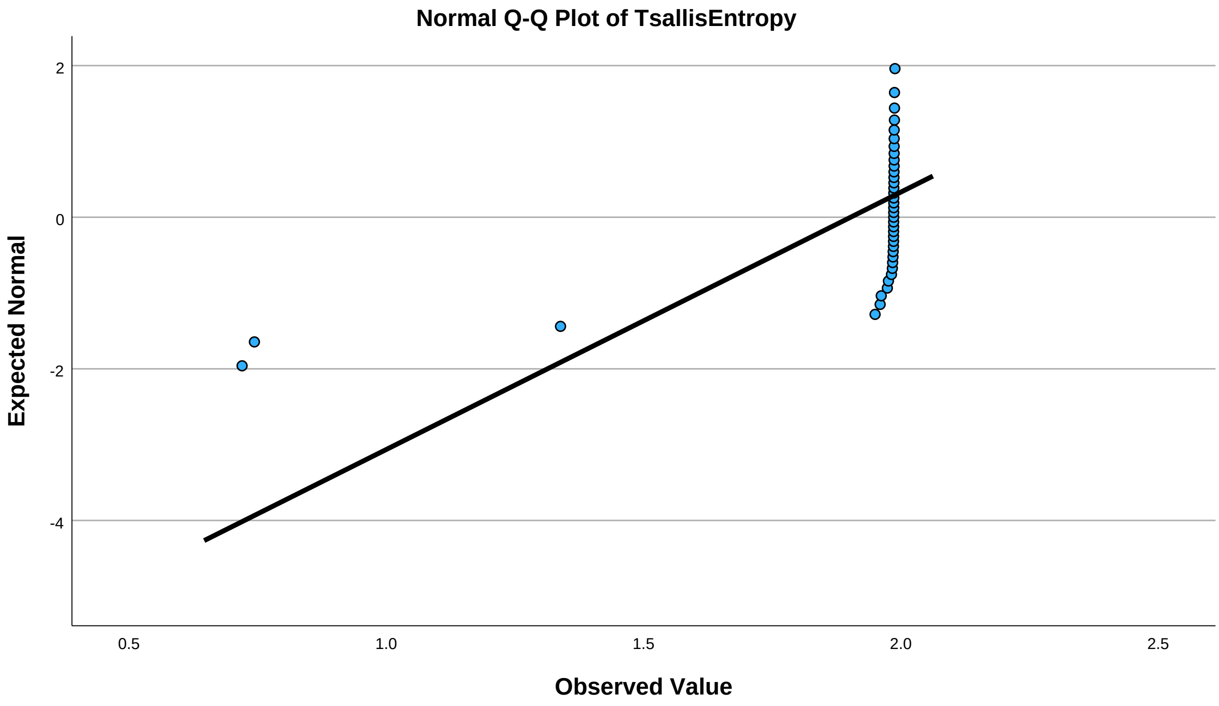

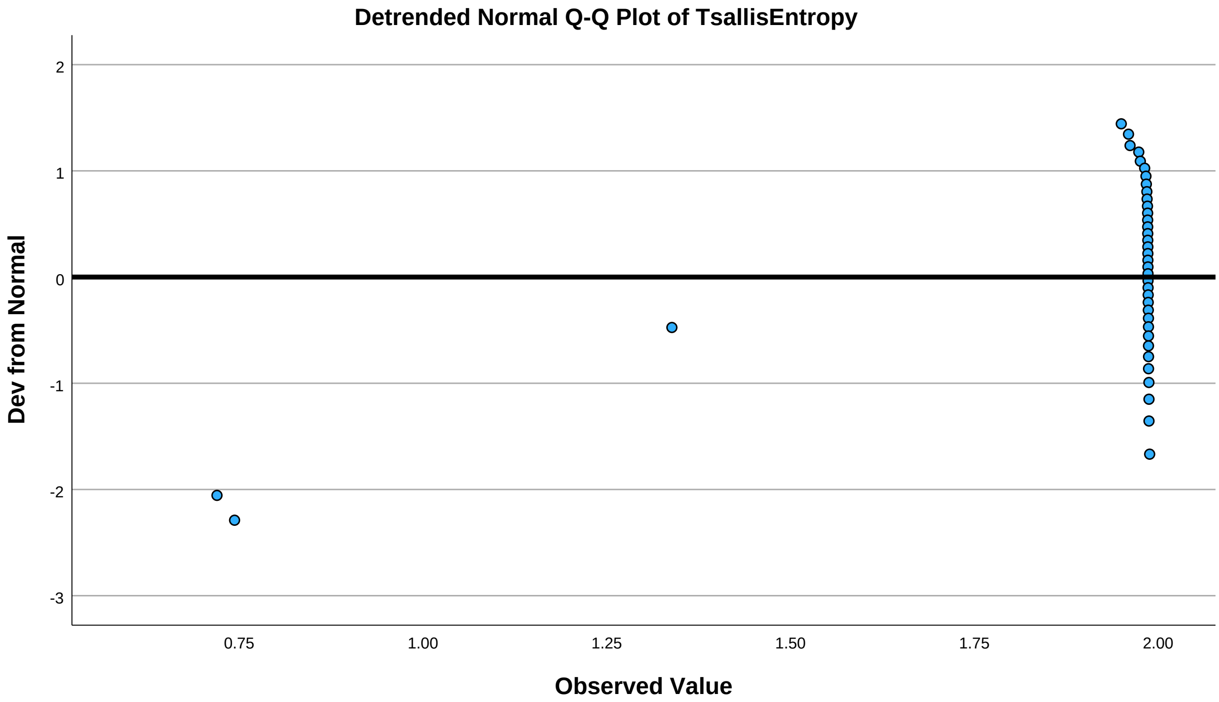
Detrended Normal Q-Q Plot of TsallisEntropy
Dev from Normal
2
1
0
-1
-2
-3
0.75
1.00
1.25
1.50
1.75
2.00
Observed Value

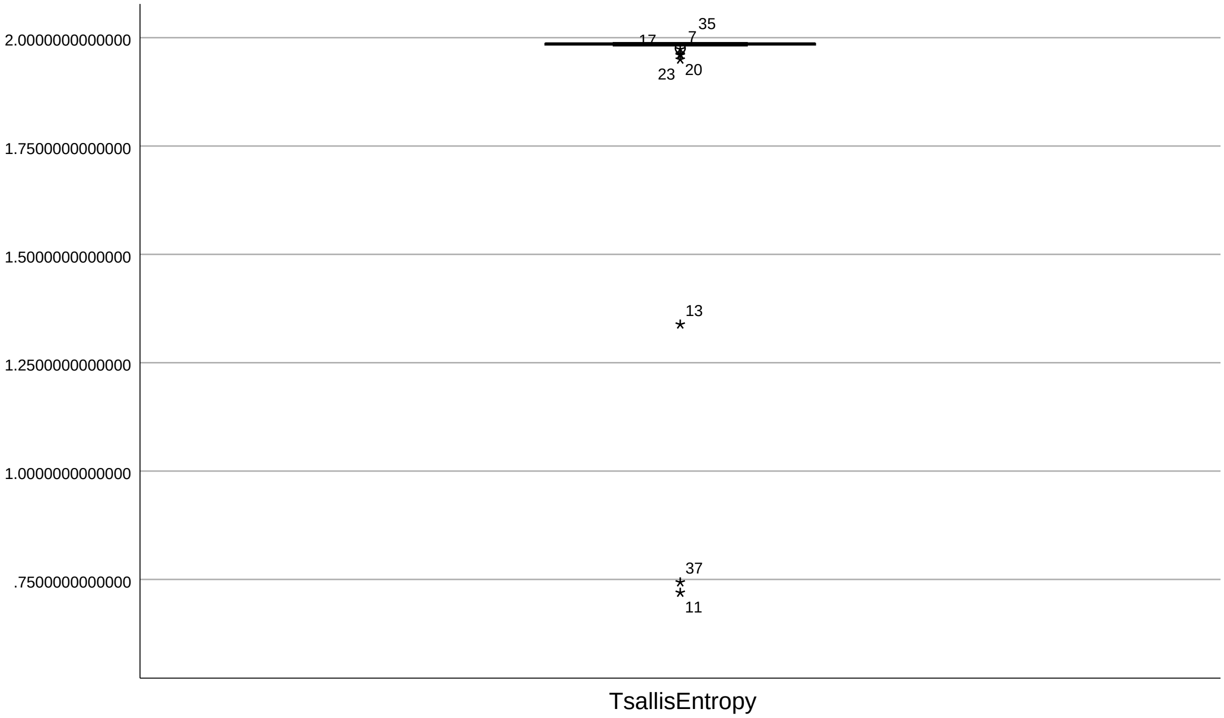
2.0000000000000
1.7500000000000
1.5000000000000
1.2500000000000
1.0000000000000
.7500000000000
35
7
17
23
20
13
37
11
TsallisEntropy

PS_4_5

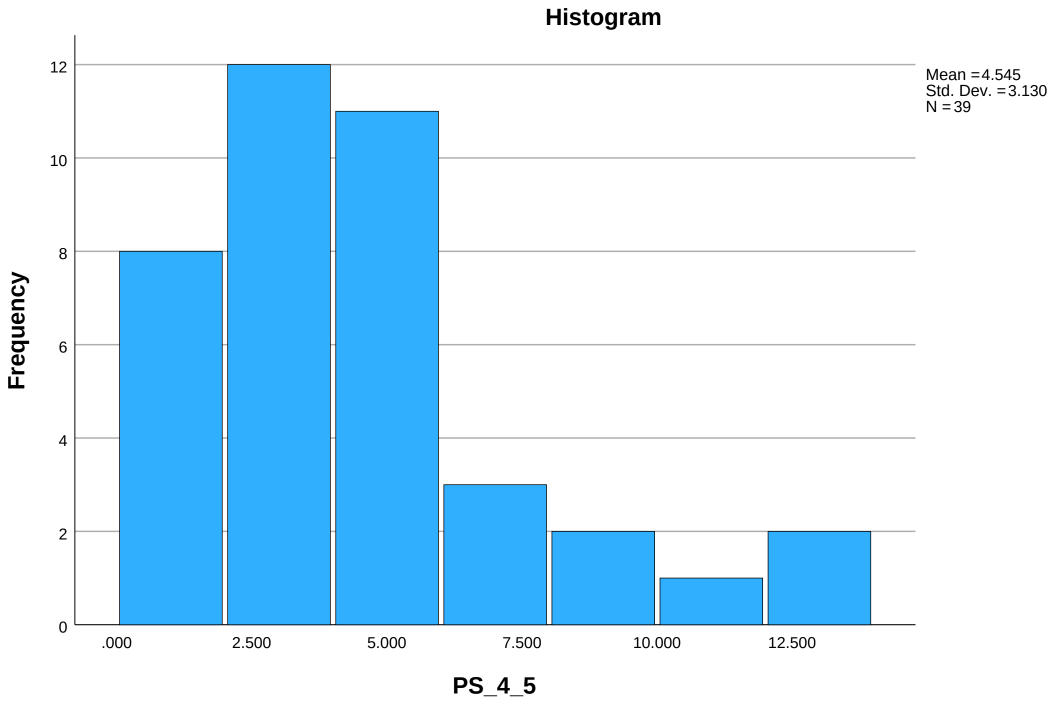


```
PS_4_5 Stem-and-Leaf Plot

 Frequency    Stem &  Leaf

     1.00        0 .  8
     7.00        1 .  1446789
     9.00        2 .  123778999
     3.00        3 .  004
     6.00        4 .  011358
     5.00        5 .  13678
      .00        6 .
     3.00        7 .  388
     2.00        8 .  57
     3.00 Extremes    (>=11.7)

 Stem width:      1.000
 Each leaf:       1 case(s)
```

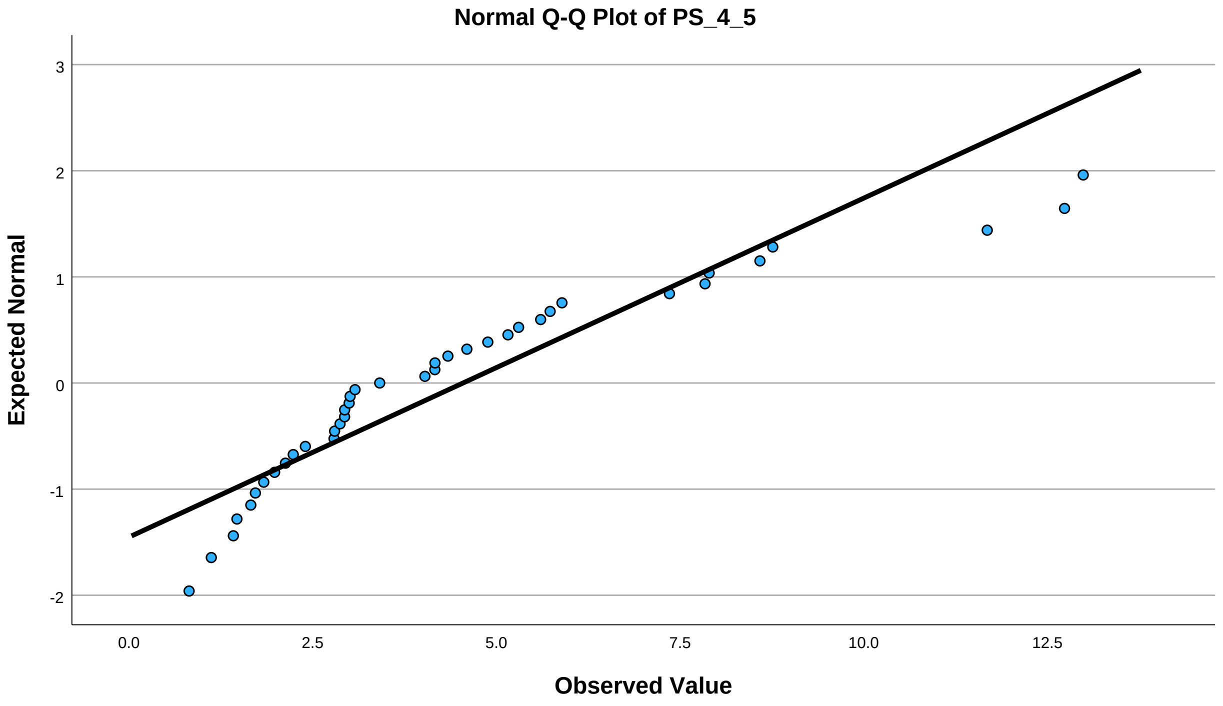
Normal Q-Q Plot of PS_4_5
Expected Normal
Observed Value
3
2
1
0
-1
-2
0.0
2.5
5.0
7.5
10.0
12.5

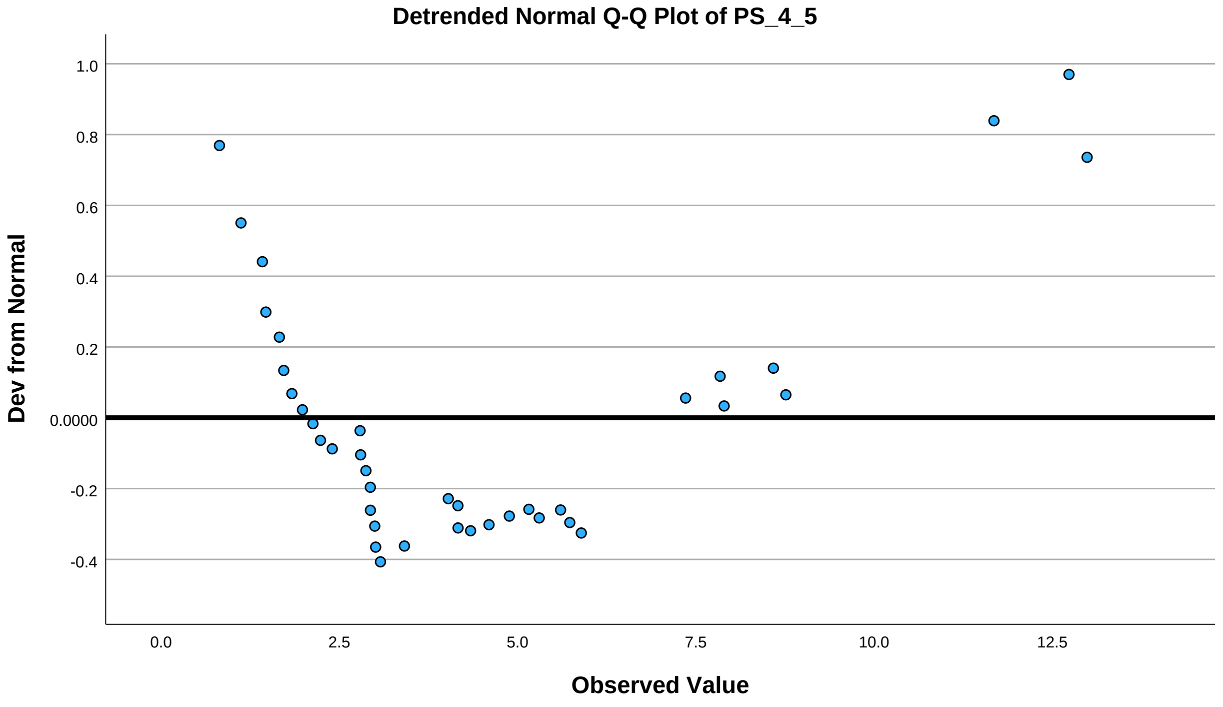
Detrended Normal Q-Q Plot of PS_4_5
Dev from Normal
Observed Value
1.0
0.8
0.6
0.4
0.2
0.0000
-0.2
-0.4
0.0
2.5
5.0
7.5
10.0
12.5

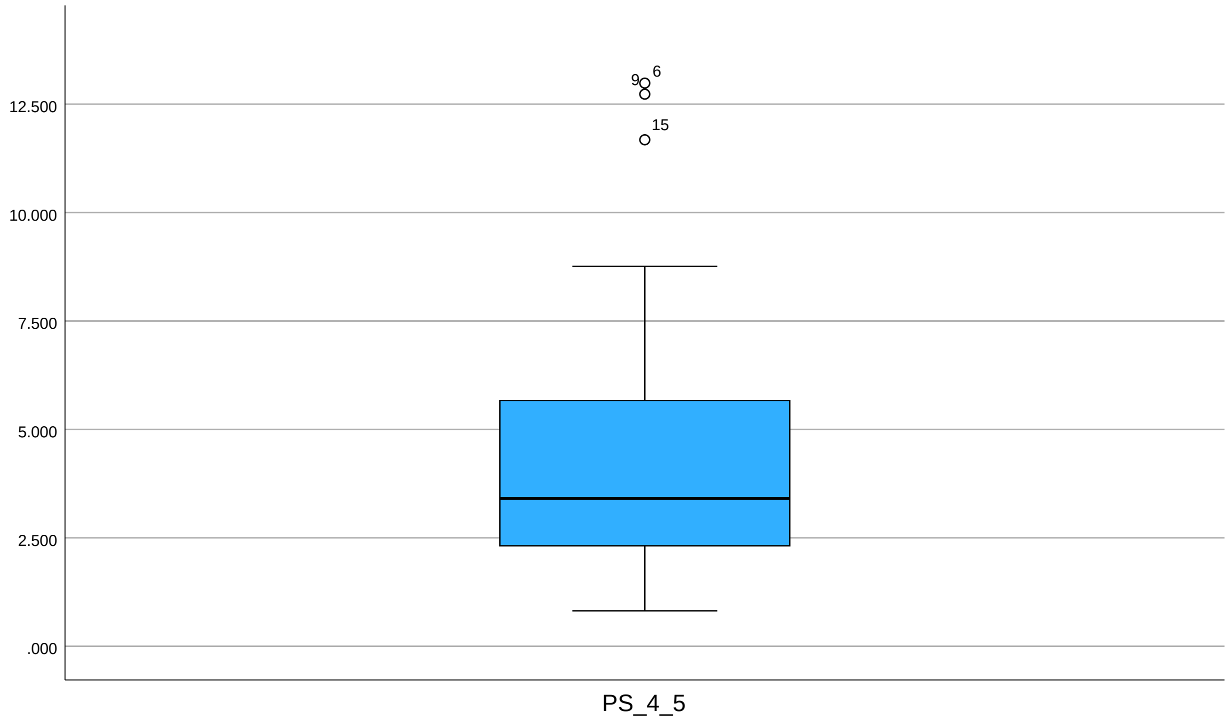

PS_5_6

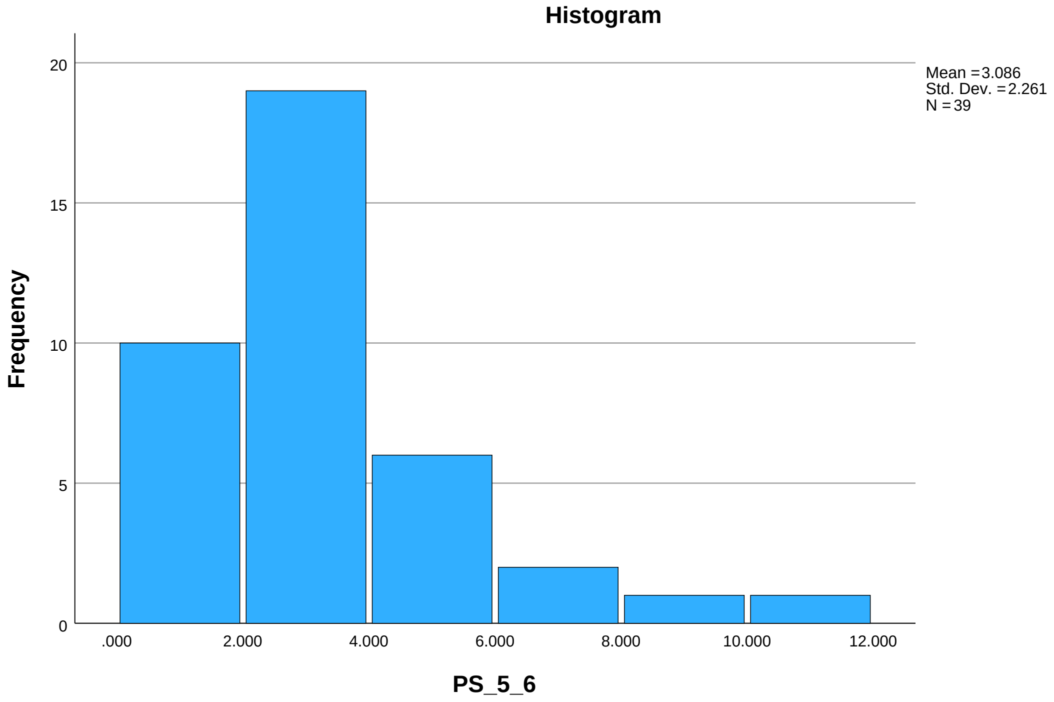

PS_5_6 Stem-and-Leaf Plot

Frequency   Stem & Leaf

```
 1.00      0 . 4
 1.00      0 . 7
 5.00      1 . 00112
 3.00      1 . 556
 8.00      2 . 00012334
 7.00      2 . 5555677
 4.00      3 . 0023
  .00      3 .
 5.00      4 . 00004
 1.00      4 . 7
 4.00 Extremes    (>=6.6)

Stem width:     1.000
Each leaf:       1 case(s)
```

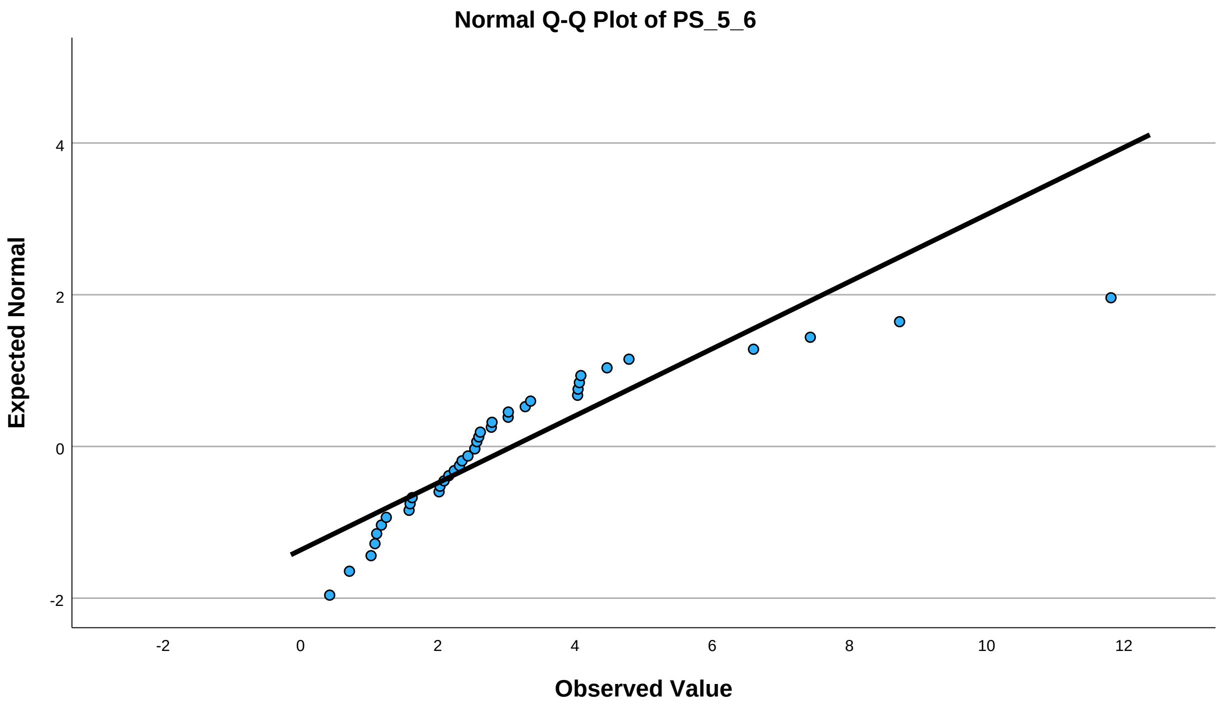

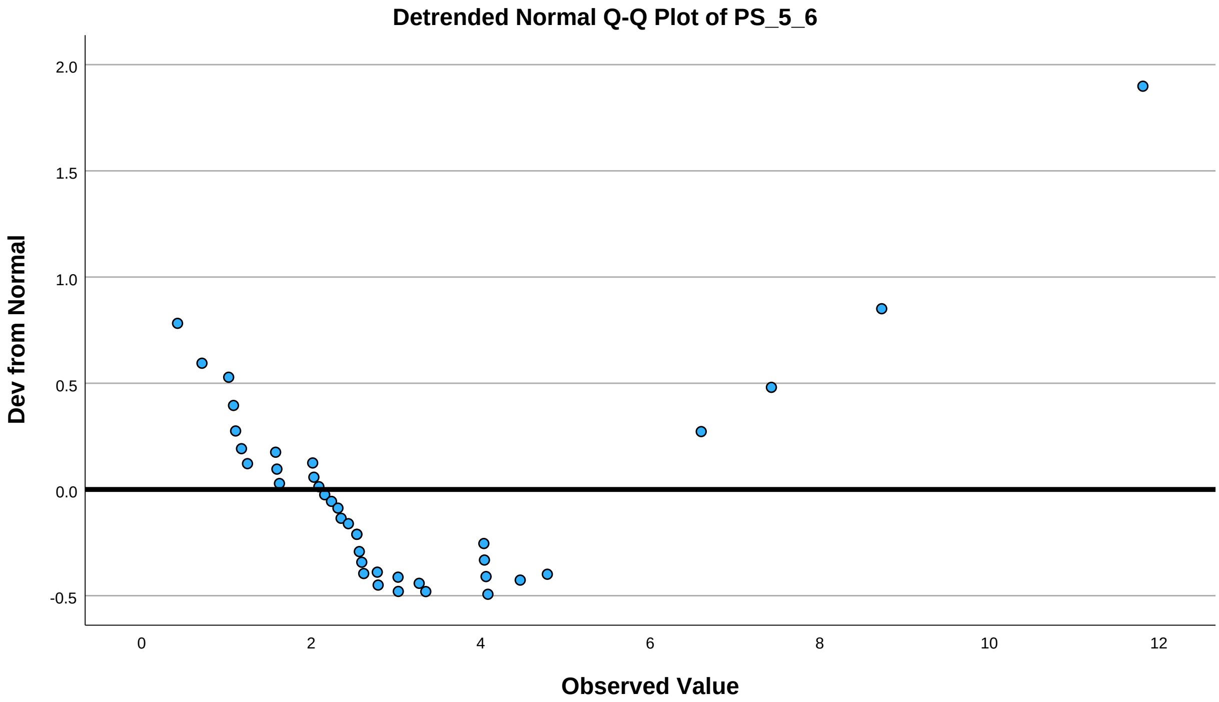
Detrended Normal Q-Q Plot of PS_5_6
Dev from Normal
2.0
1.5
1.0
0.5
0.0
-0.5
0
2
4
6
8
10
12
Observed Value

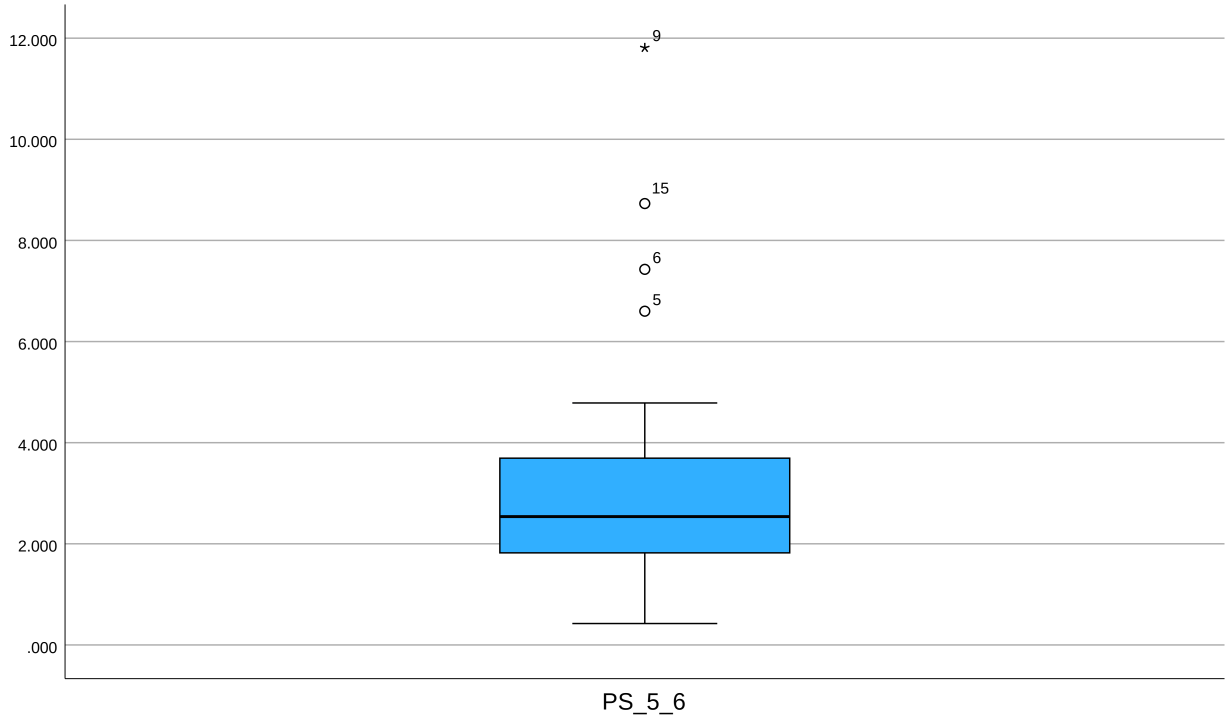
12.000
10.000
8.000
6.000
4.000
2.000
.000
9
15
6
5
PS_5_6

PS_6_7

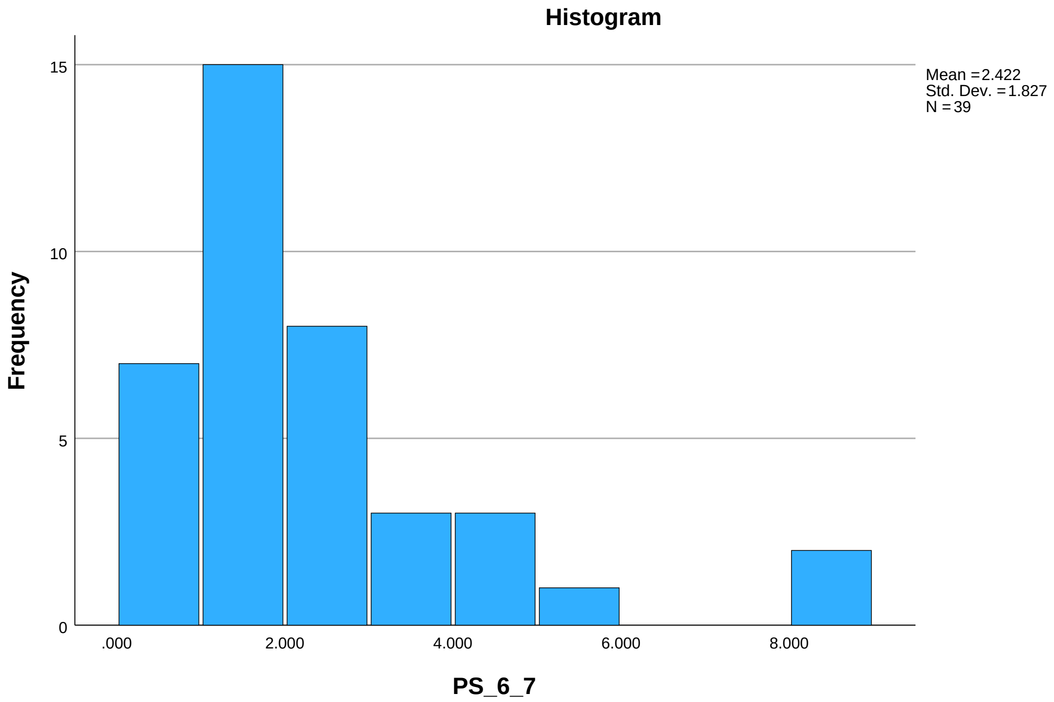

PS_6_7 Stem-and-Leaf Plot

```
 Frequency    Stem &  Leaf

     1.00        0 .  4
     6.00        0 .  568889
     3.00        1 .  112
    12.00        1 .  566666677889
     5.00        2 .  00134
     3.00        2 .  688
     2.00        3 .  04
     1.00        3 .  6
     1.00        4 .  2
     2.00        4 .  57
     3.00 Extremes    (>=5.3)

 Stem width:      1.000
 Each leaf:       1 case(s)
```

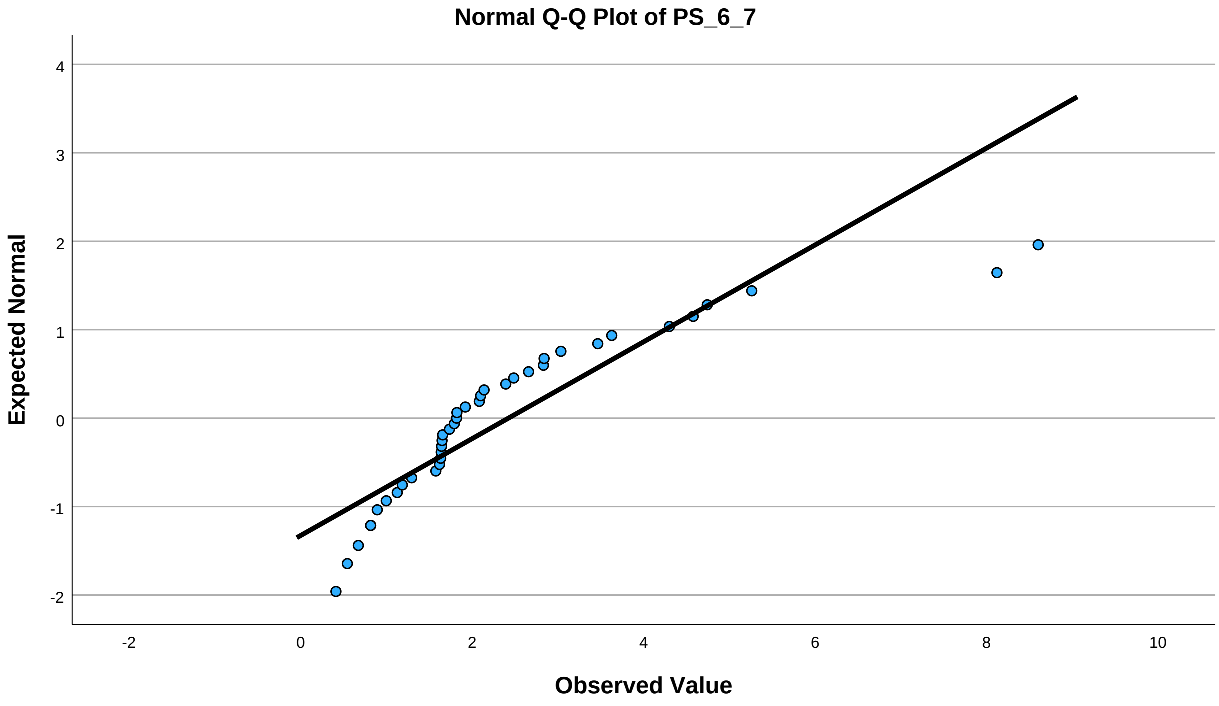
Normal Q-Q Plot of PS_6_7
Expected Normal
Observed Value
4
3
2
1
0
-1
-2
-2
0
2
4
6
8
10

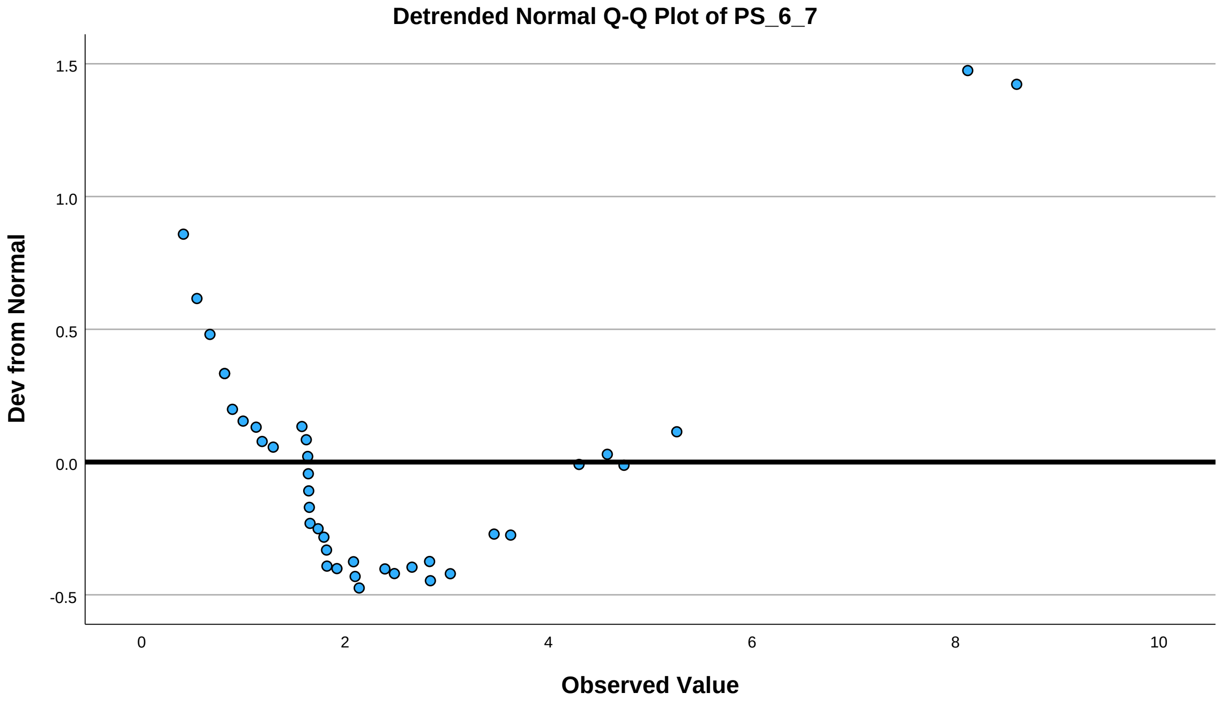
Detrended Normal Q-Q Plot of PS_6_7
Dev from Normal
Observed Value
1.5
1.0
0.5
0.0
-0.5
0
2
4
6
8
10

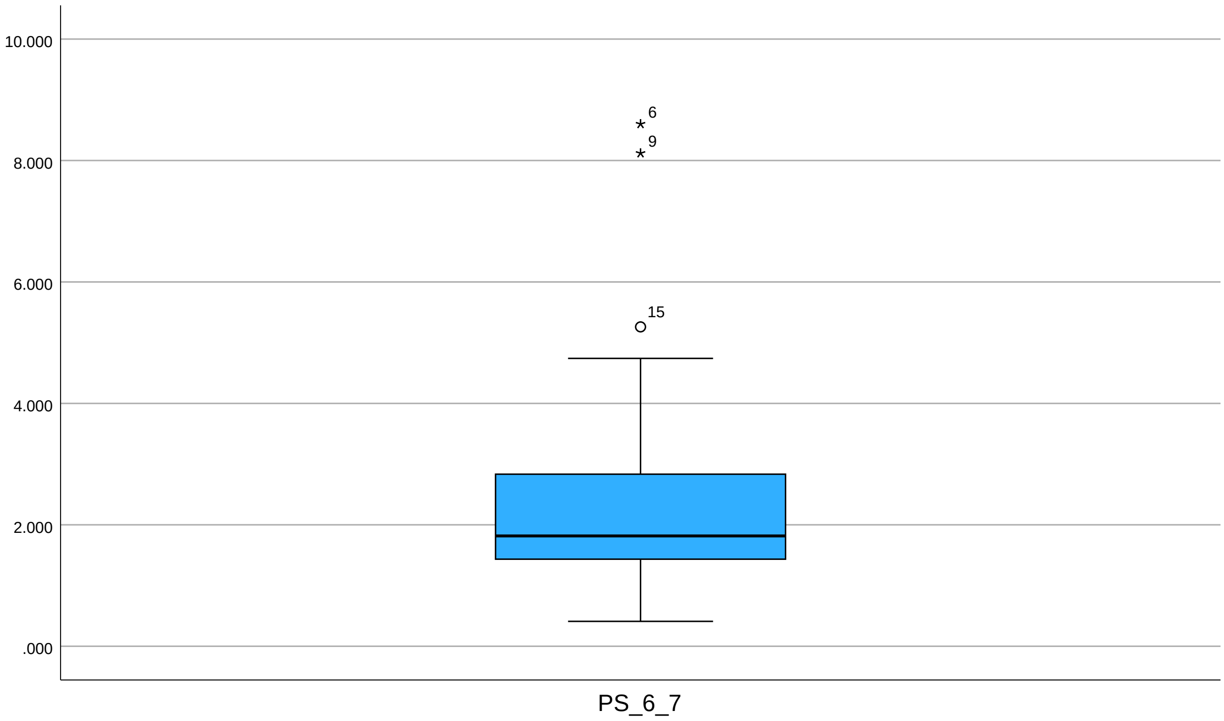

PS_7_8

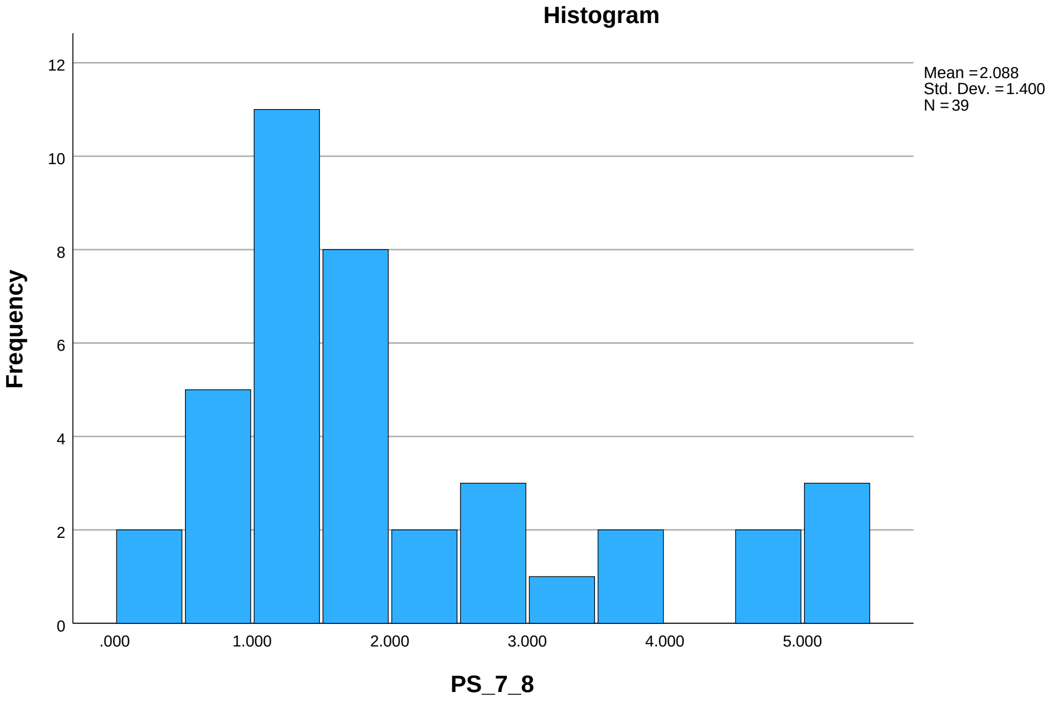

PS_7_8 Stem-and-Leaf Plot

Frequency   Stem & Leaf

```
 2.00     0 . 34
 5.00     0 . 77799
11.00     1 . 01112222344
 8.00     1 . 55678999
 2.00     2 . 04
 3.00     2 . 569
 1.00     3 . 2
 2.00     3 . 58
 5.00 Extremes    (>=4.7)

Stem width:     1.000
Each leaf:       1 case(s)
```

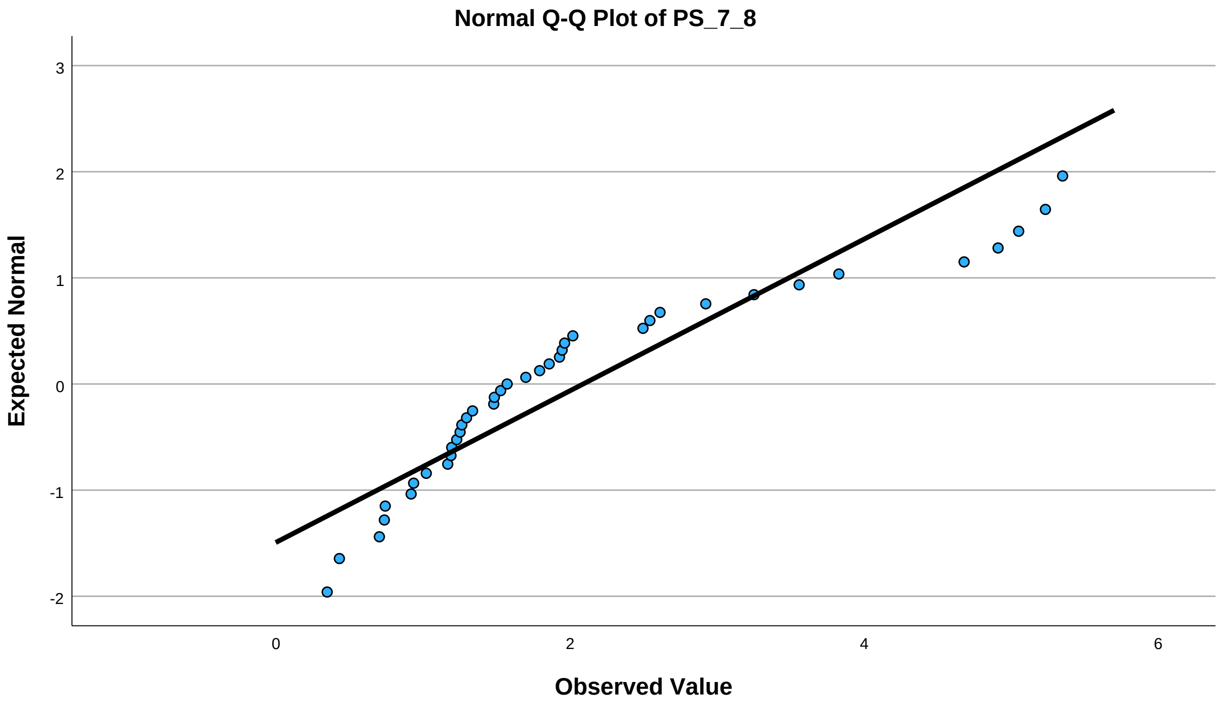

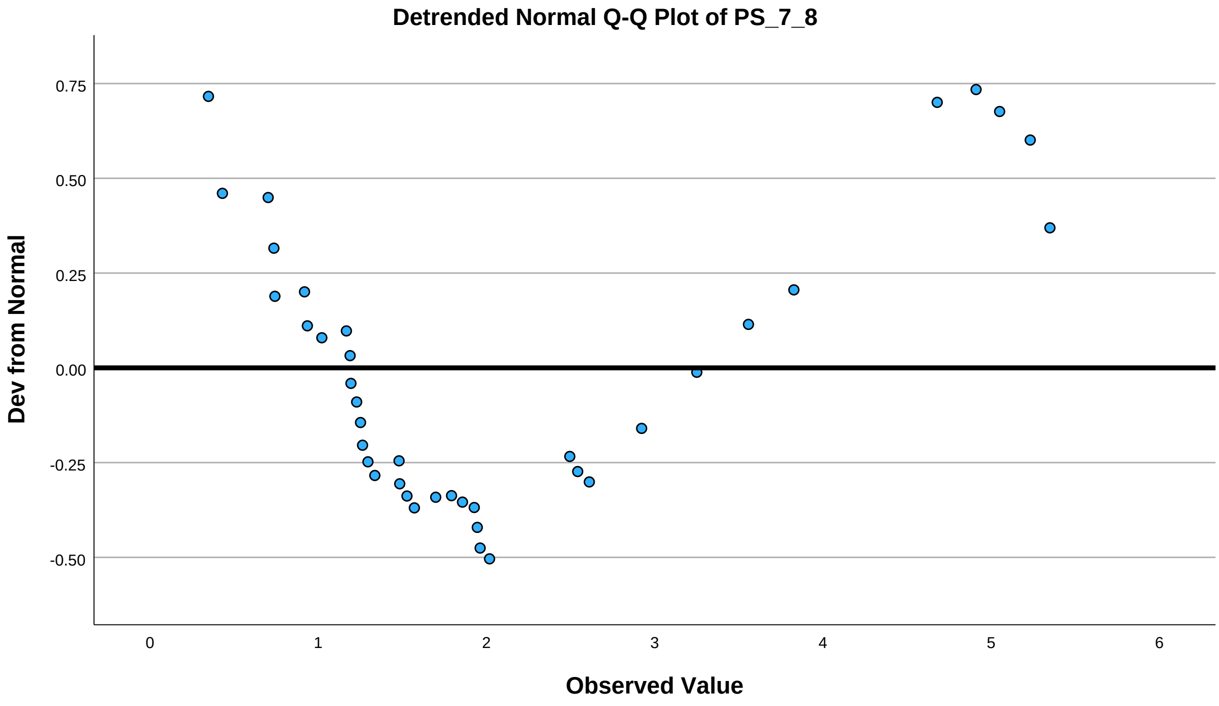
Detrended Normal Q-Q Plot of PS_7_8
Dev from Normal
0.75
0.50
0.25
0.00
-0.25
-0.50
0
1
2
3
4
5
6
Observed Value

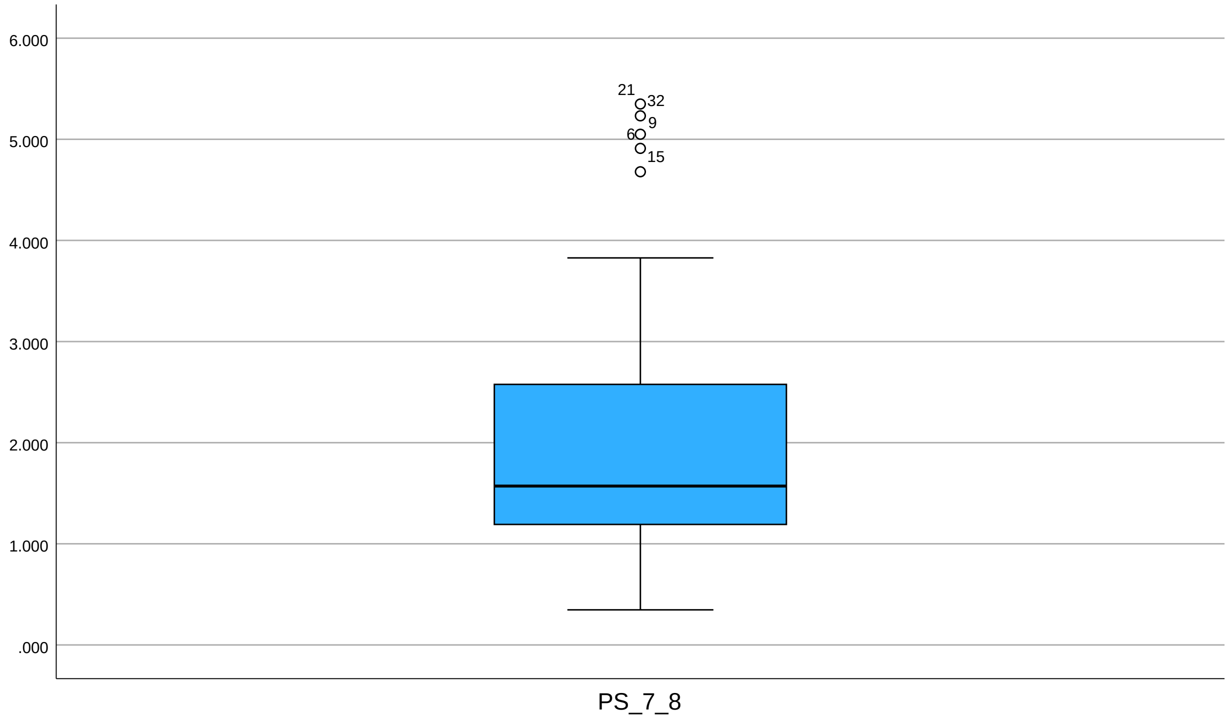
6.000
5.000
4.000
3.000
2.000
1.000
.000
21
32
9
6
15
PS_7_8

PS_8_9

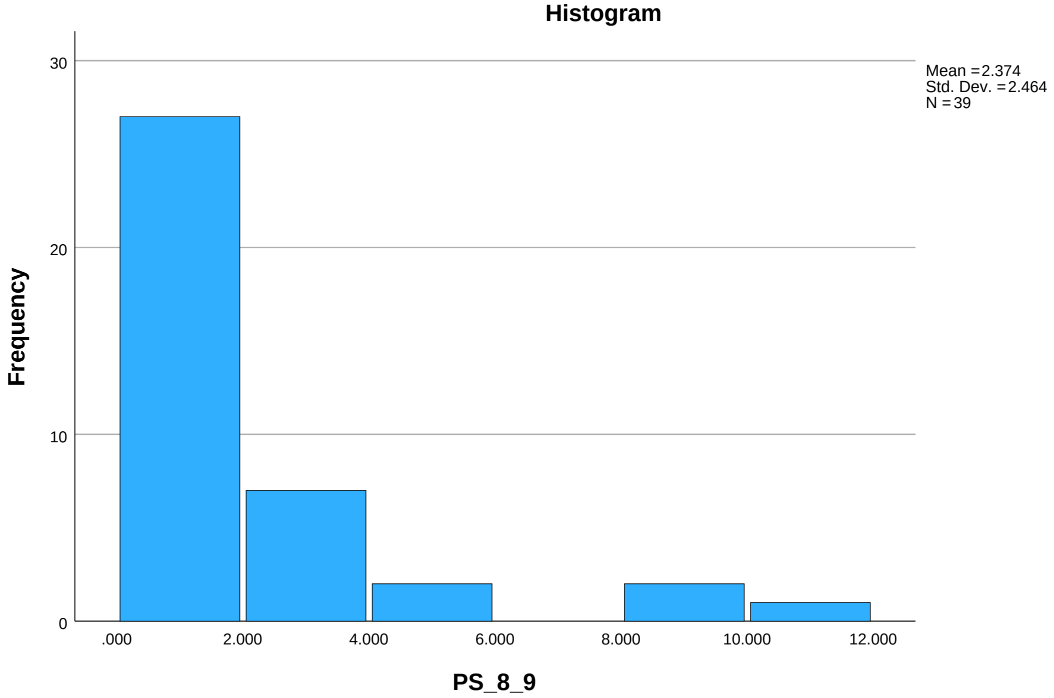


```
PS_8_9 Stem-and-Leaf Plot

 Frequency    Stem &  Leaf

     2.00        0 .  23
     5.00        0 .  57889
    13.00        1 .  0011112223444
     7.00        1 .  5677899
     3.00        2 .  024
     2.00        2 .  89
     1.00        3 .  1
     1.00        3 .  7
     5.00 Extremes    (>=4.8)

 Stem width:      1.000
 Each leaf:       1 case(s)
```

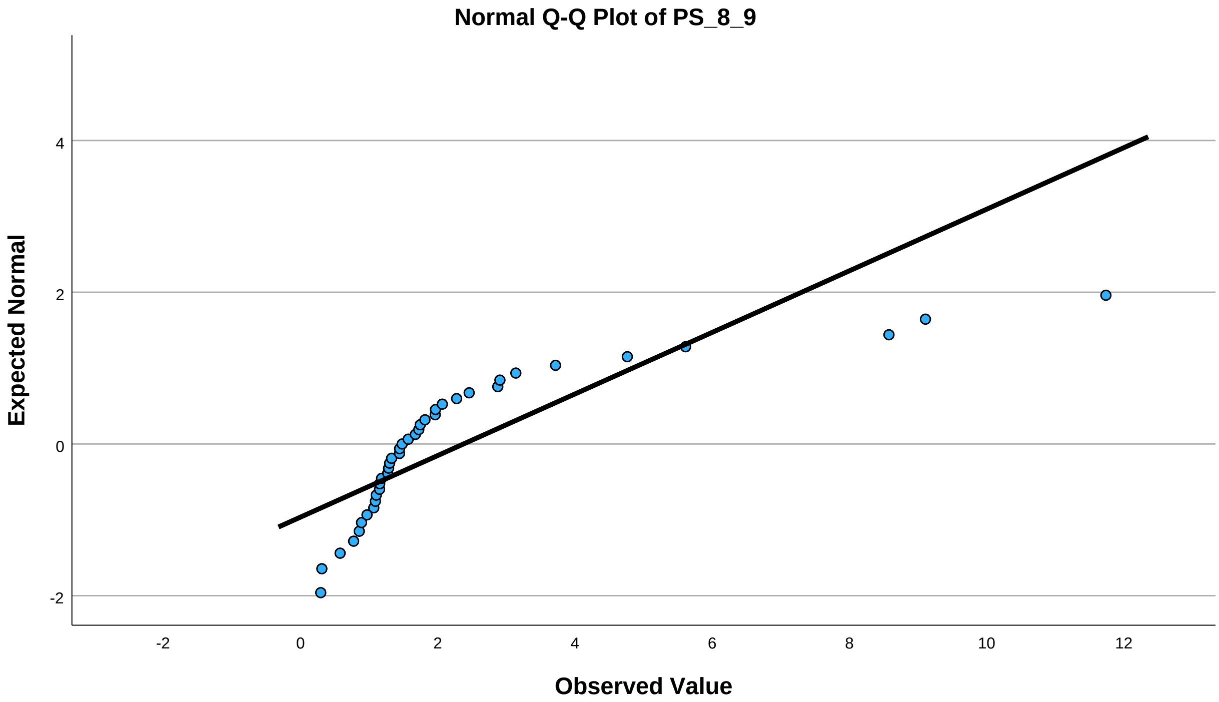
Normal Q-Q Plot of PS_8_9
Expected Normal
4
2
0
-2
-2
0
2
4
6
8
10
12
Observed Value

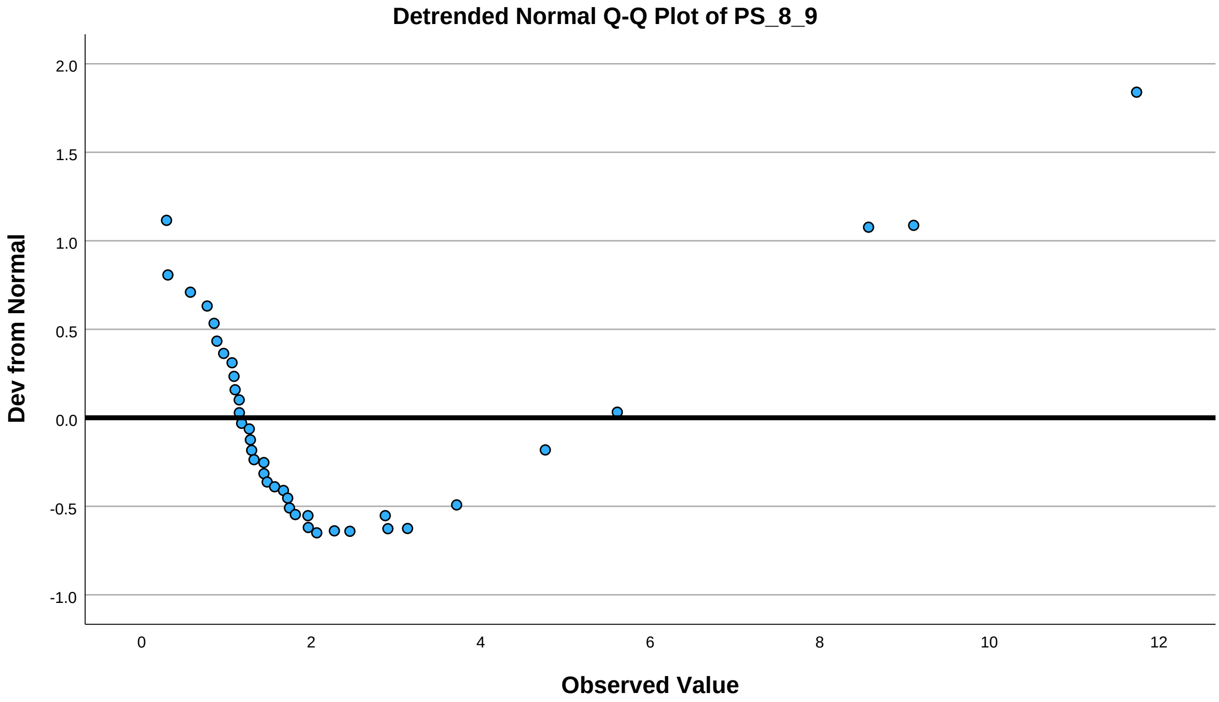
Detrended Normal Q-Q Plot of PS_8_9
Dev from Normal
2.0
1.5
1.0
0.5
0.0
-0.5
-1.0
0
2
4
6
8
10
12
Observed Value

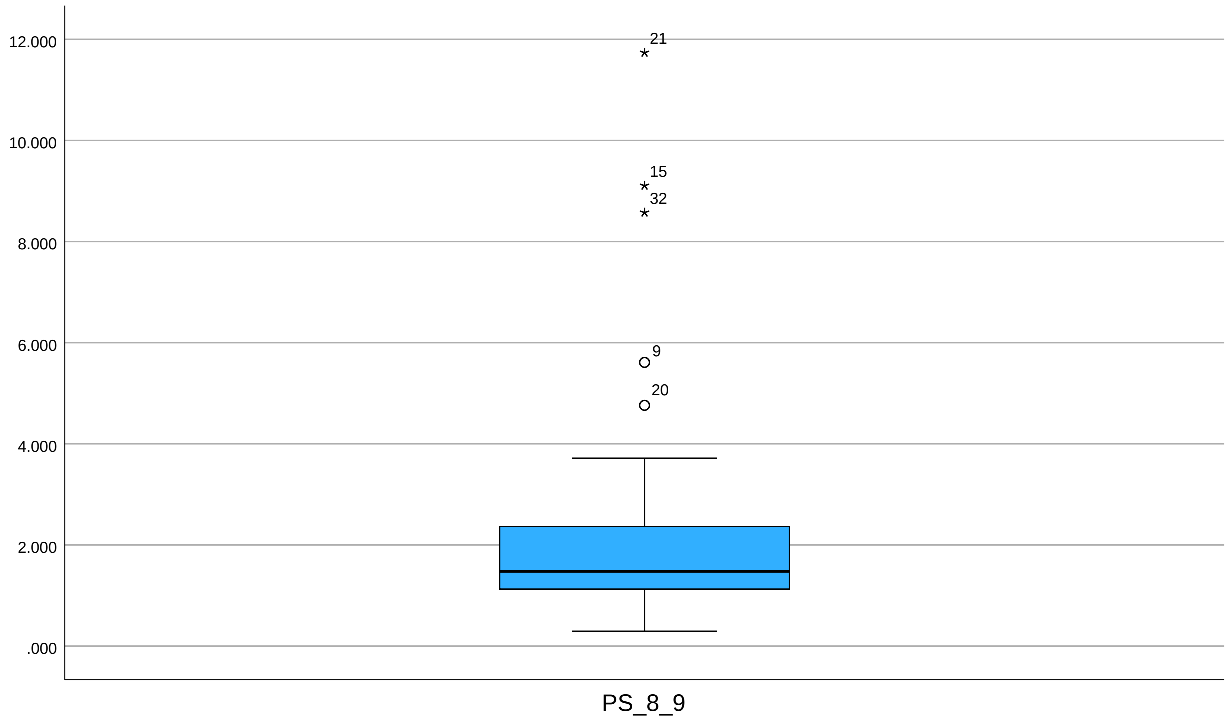

PS_9_10

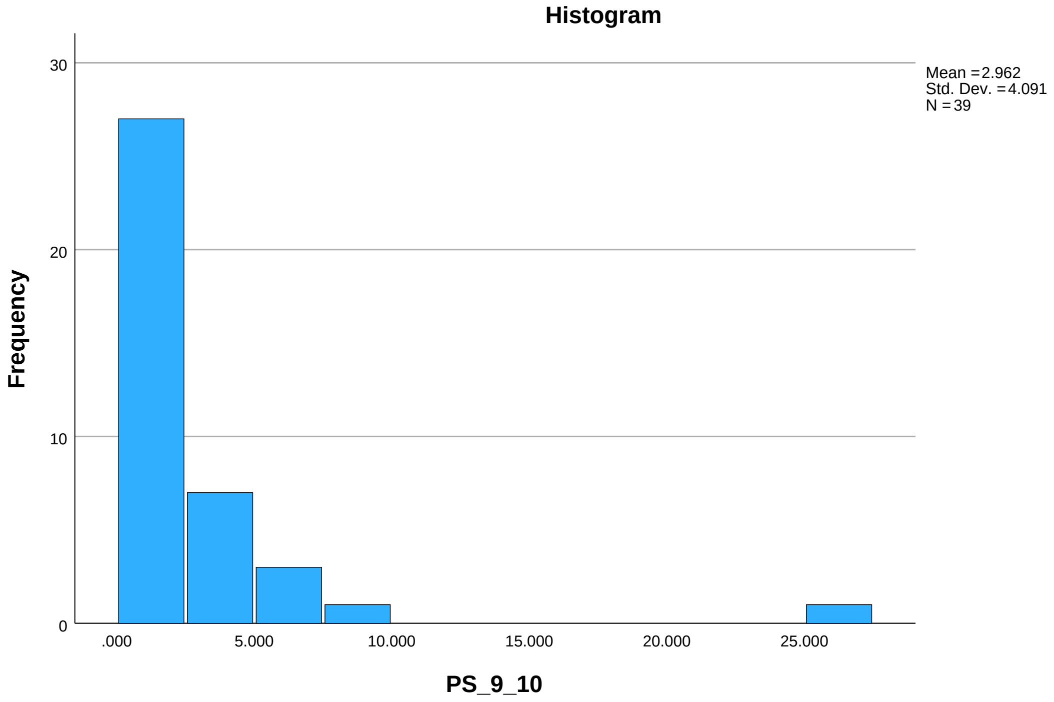

PS_9_10 Stem-and-Leaf Plot

Frequency   Stem & Leaf

```
  3.00      0 . 224
  3.00      0 . 899
  9.00      1 . 012233344
  7.00      1 . 5566799
  5.00      2 . 01233
  1.00      2 . 5
  2.00      3 . 01
  3.00      3 . 557
   .00      4 .
  1.00      4 . 7
   .00      5 .
  1.00      5 . 6
  4.00 Extremes    (>=6.3)

Stem width:      1.000
Each leaf:       1 case(s)
```

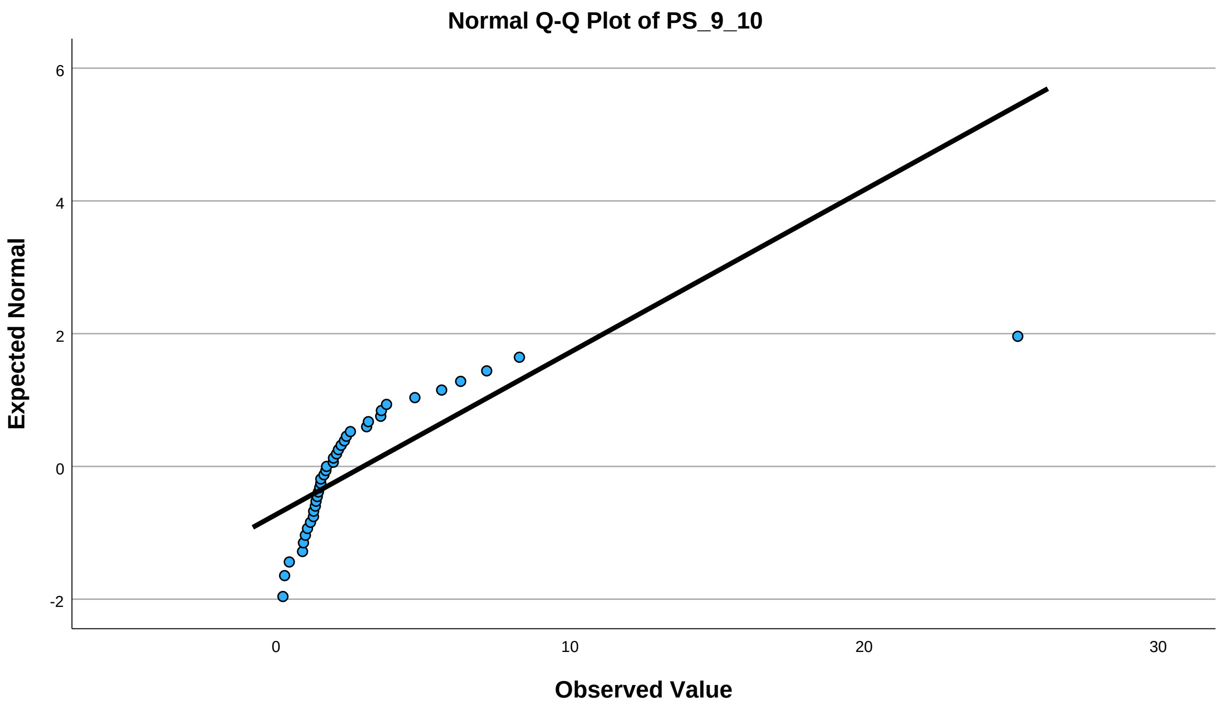

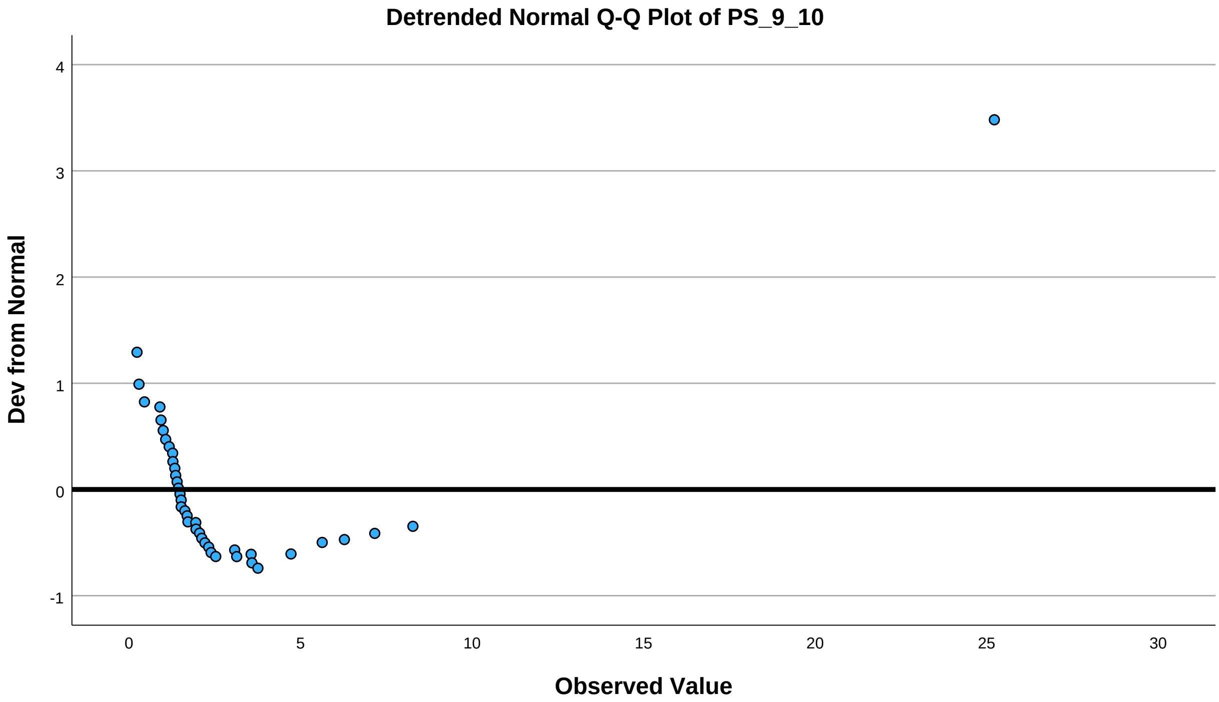
Detrended Normal Q-Q Plot of PS_9_10
Dev from Normal
Observed Value
4
3
2
1
0
-1
0
5
10
15
20
25
30

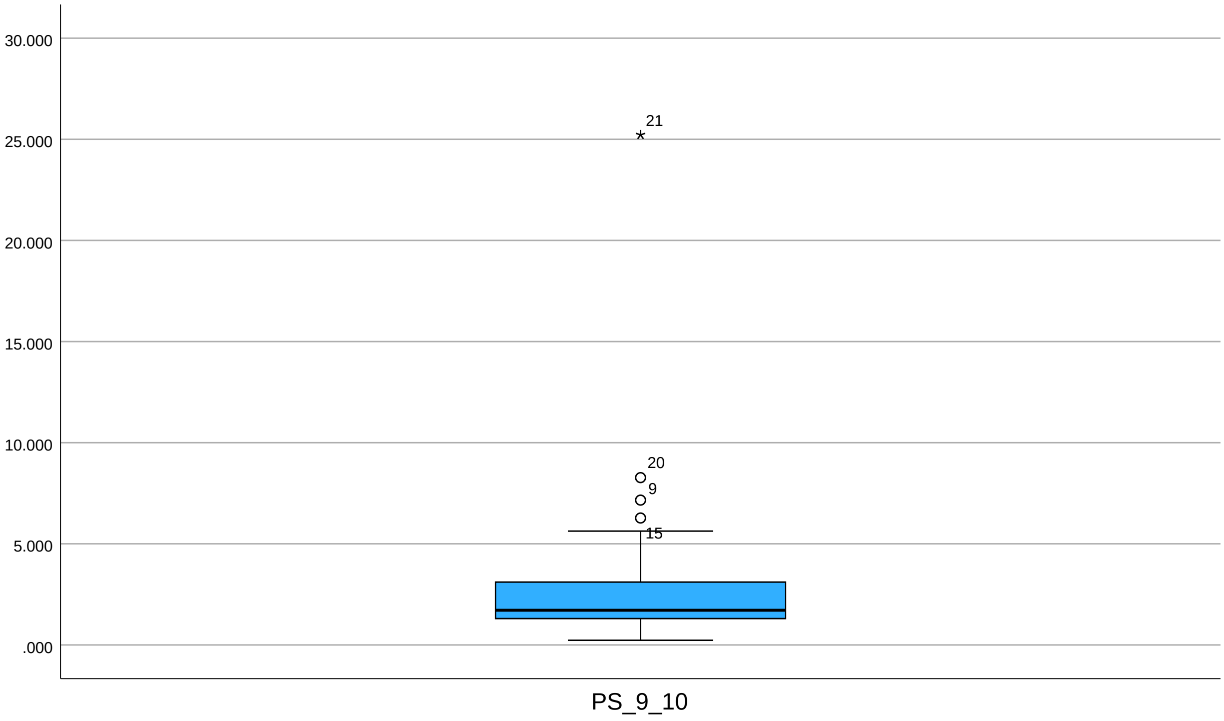
30.000
25.000
20.000
15.000
10.000
5.000
.000
21
20
9
15
PS_9_10

PS_10_11

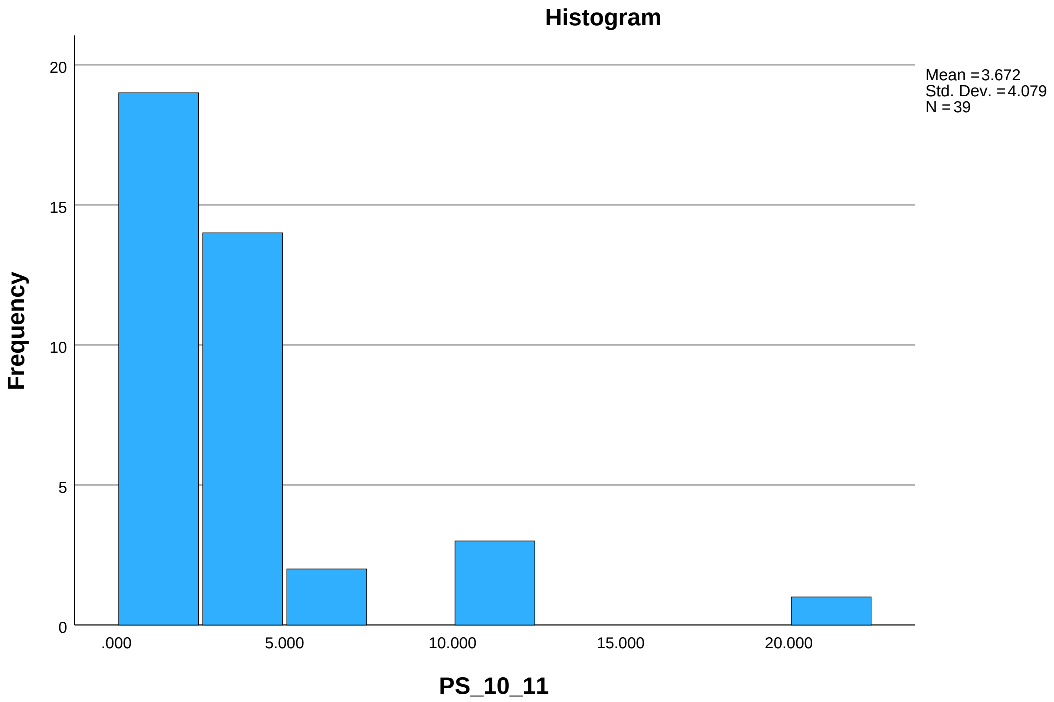


```
PS_10_11 Stem-and-Leaf Plot

 Frequency    Stem &  Leaf

     5.00        0 .  33589
     8.00        1 .  00113667
    11.00        2 .  12233457788
     7.00        3 .  1244568
     2.00        4 .  17
     1.00        5 .  7
     1.00        6 .  3
     4.00 Extremes    (>=11.0)

 Stem width:      1.000
 Each leaf:        1 case(s)
```

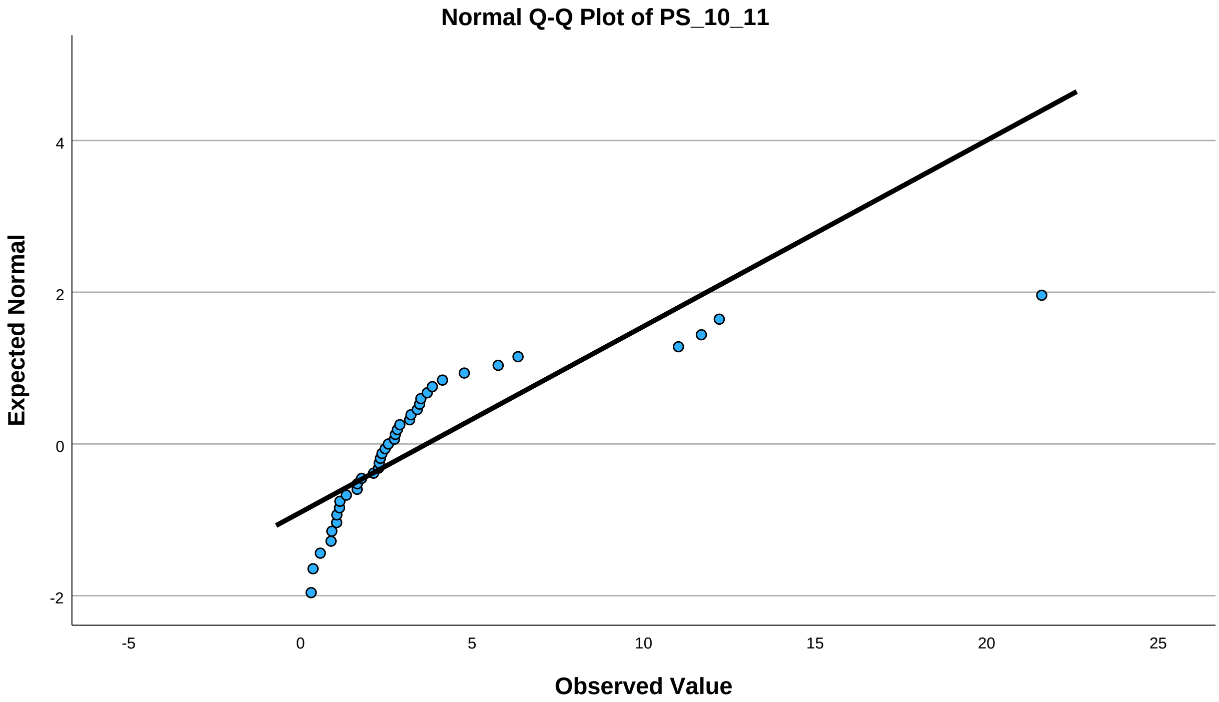
Normal Q-Q Plot of PS_10_11
Expected Normal
Observed Value
4
2
0
-2
-5
0
5
10
15
20
25

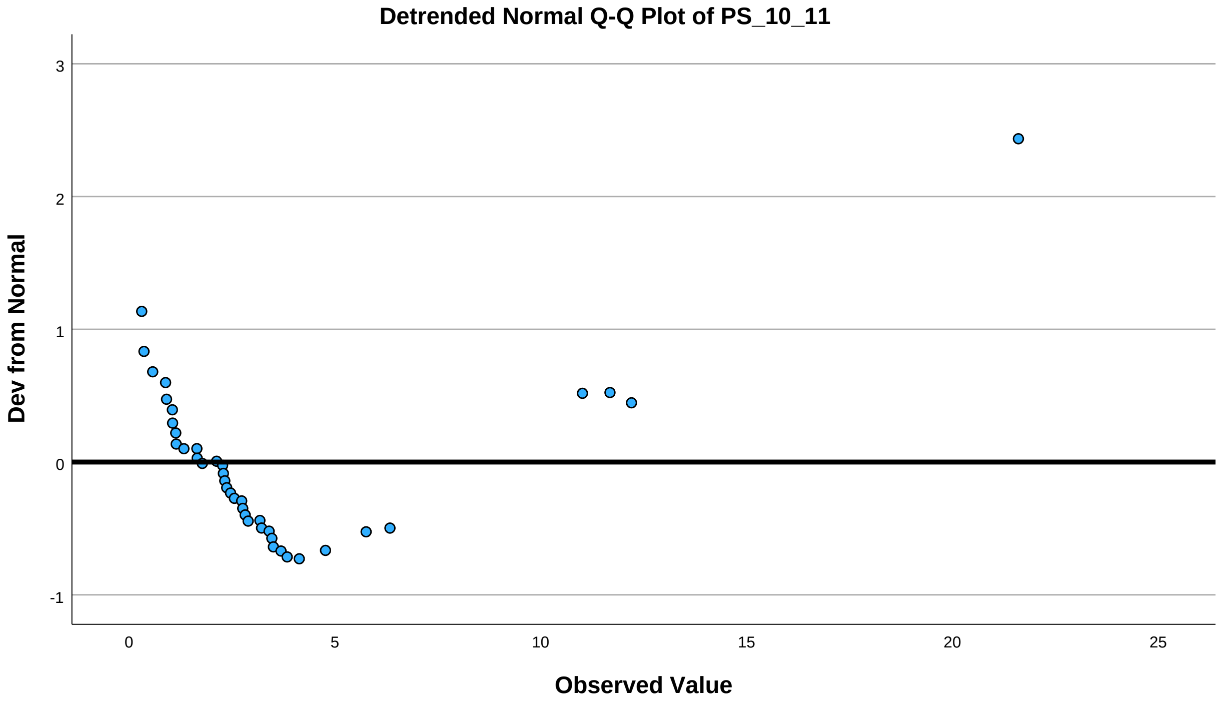
Detrended Normal Q-Q Plot of PS_10_11
Dev from Normal
Observed Value
3
2
1
0
-1
0
5
10
15
20
25

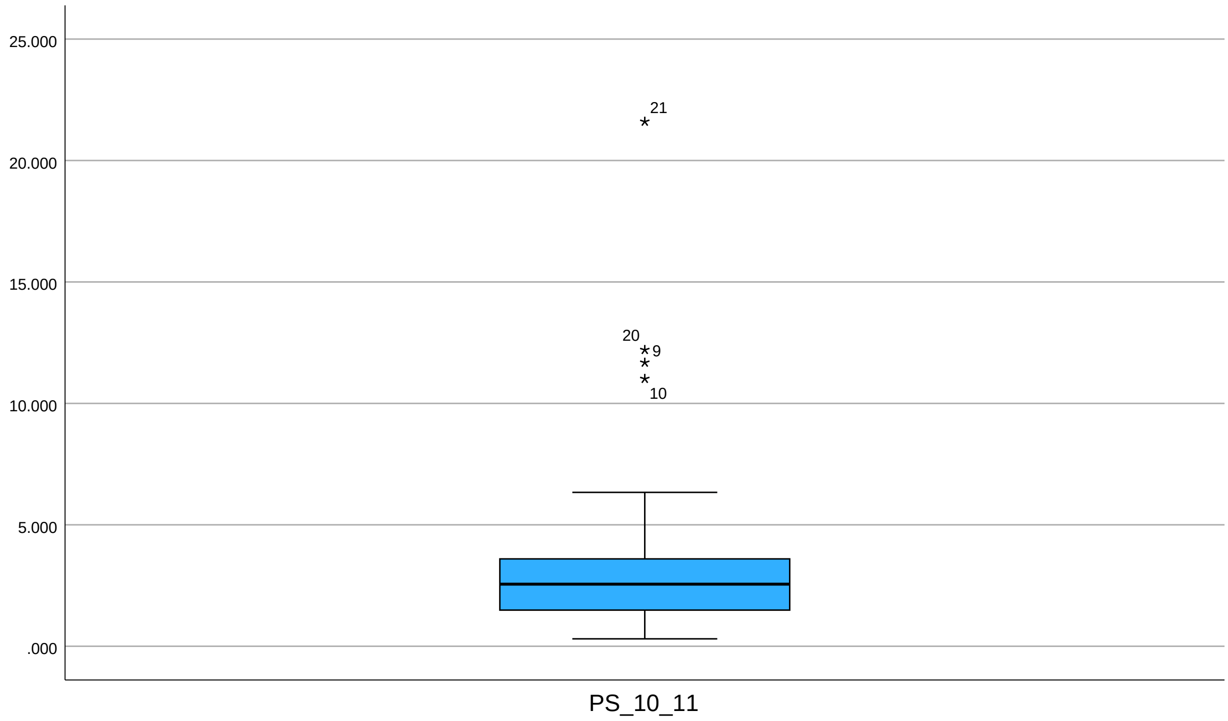

PS_11_12

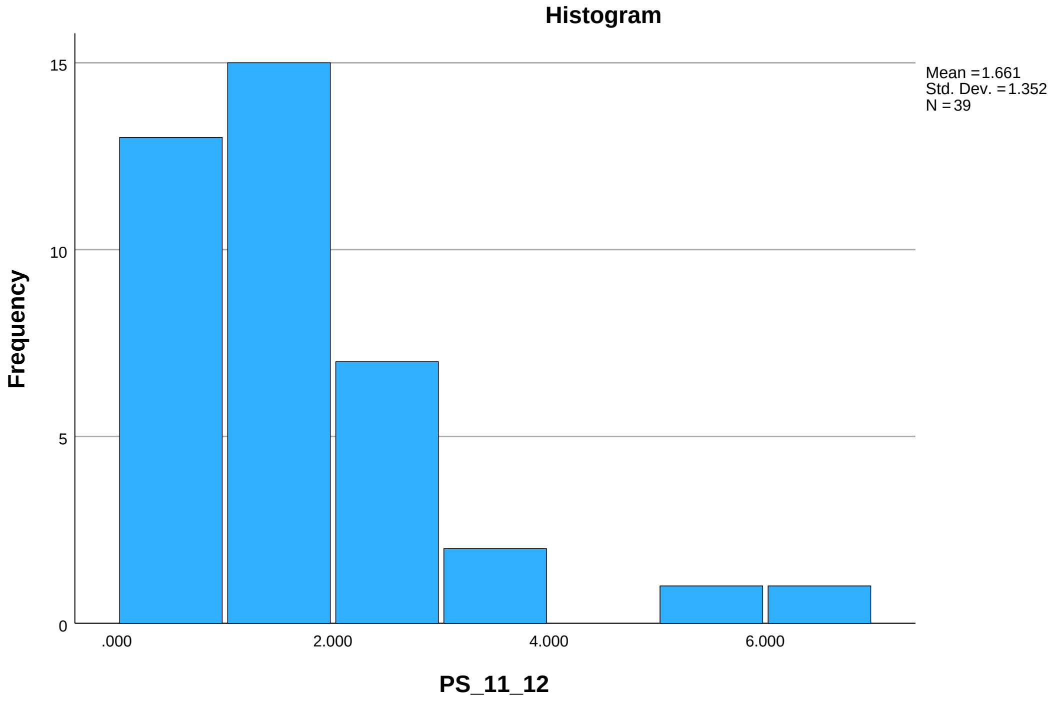

PS_11_12 Stem-and-Leaf Plot

Frequency   Stem & Leaf

```
  4.00      0 .  2244
  9.00      0 .  556678889
 11.00      1 .  01111122344
  4.00      1 .  5688
  3.00      2 .  113
  4.00      2 .  5566
  2.00      3 .  13
  2.00 Extremes    (>=6.0)

Stem width:     1.000
Each leaf:       1 case(s)
```

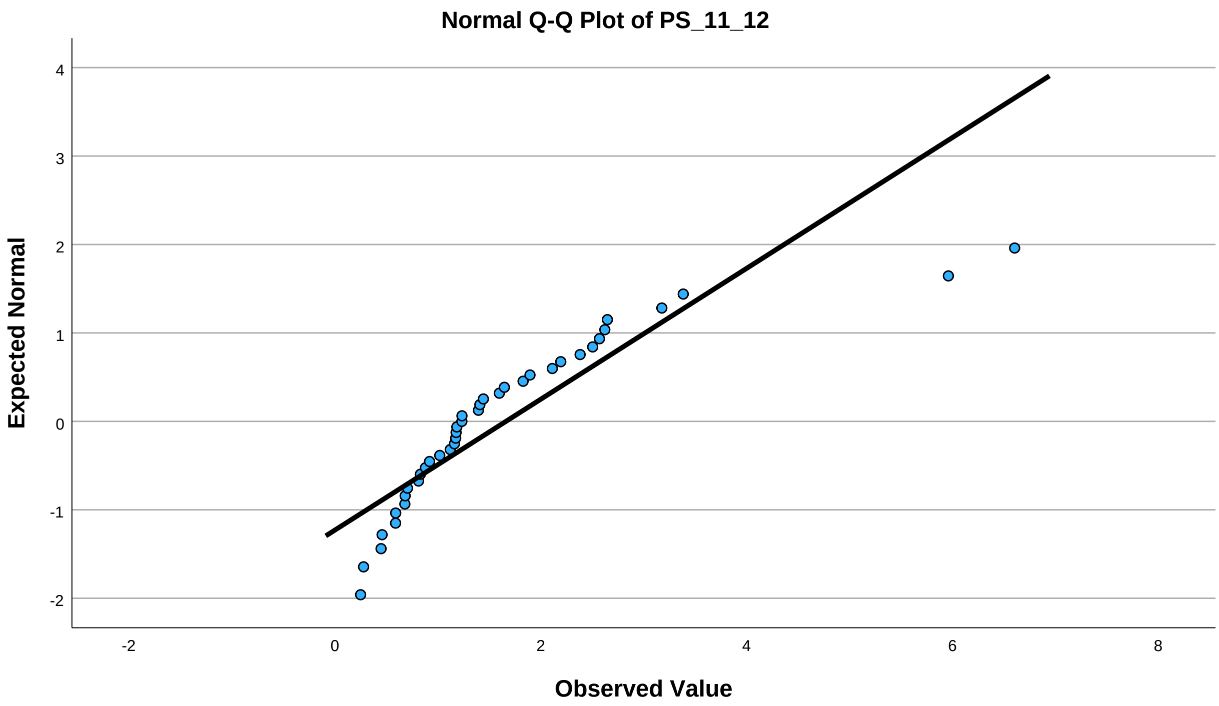

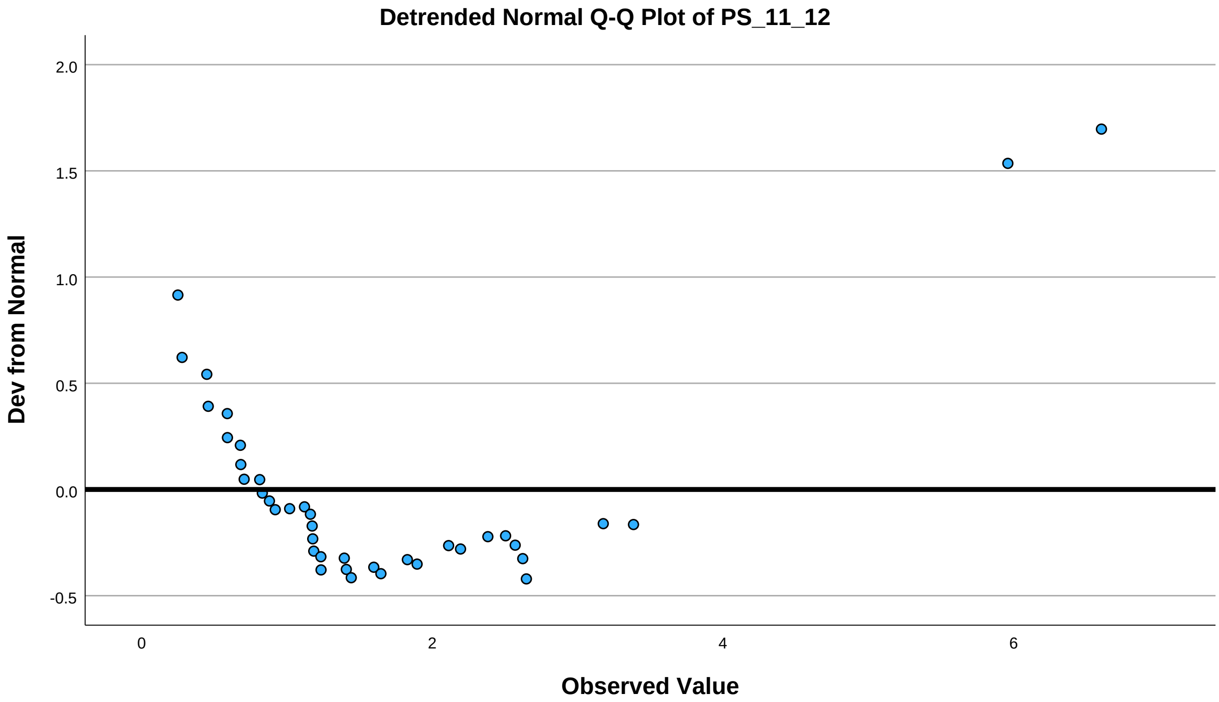
Detrended Normal Q-Q Plot of PS_11_12
Dev from Normal
2.0
1.5
1.0
0.5
0.0
-0.5
0
2
4
6
Observed Value

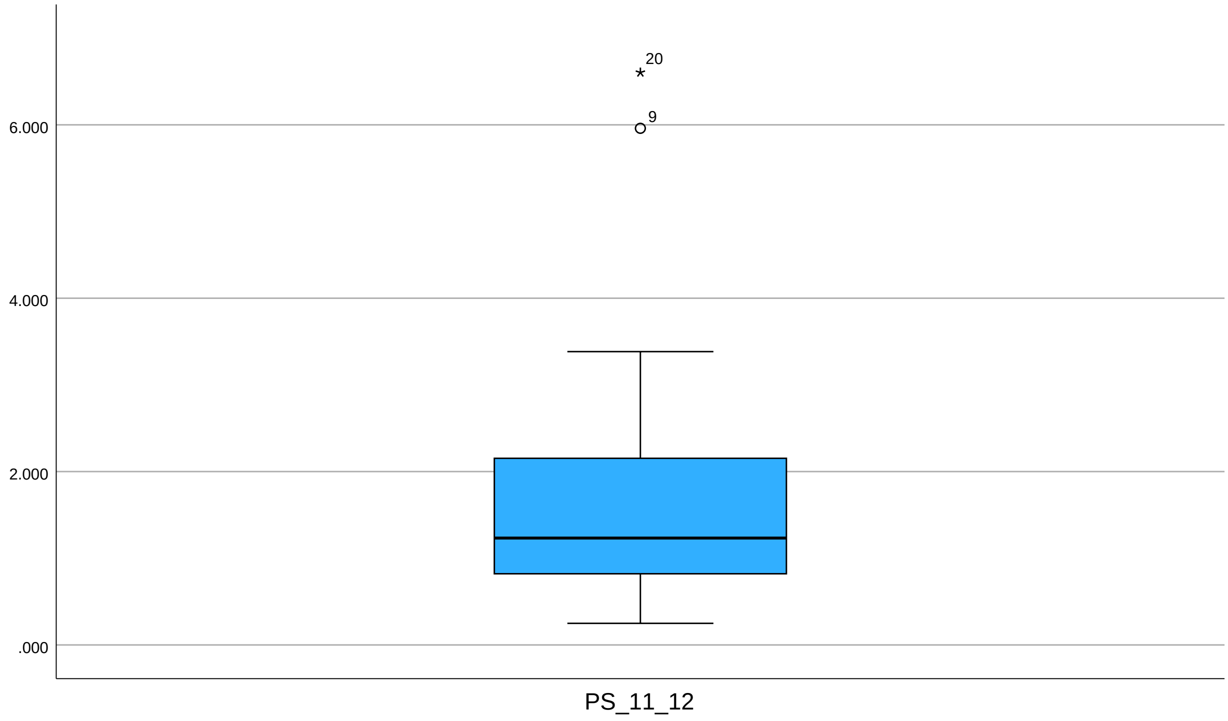
20
9
6.000
4.000
2.000
.000
PS_11_12

PS_12_13

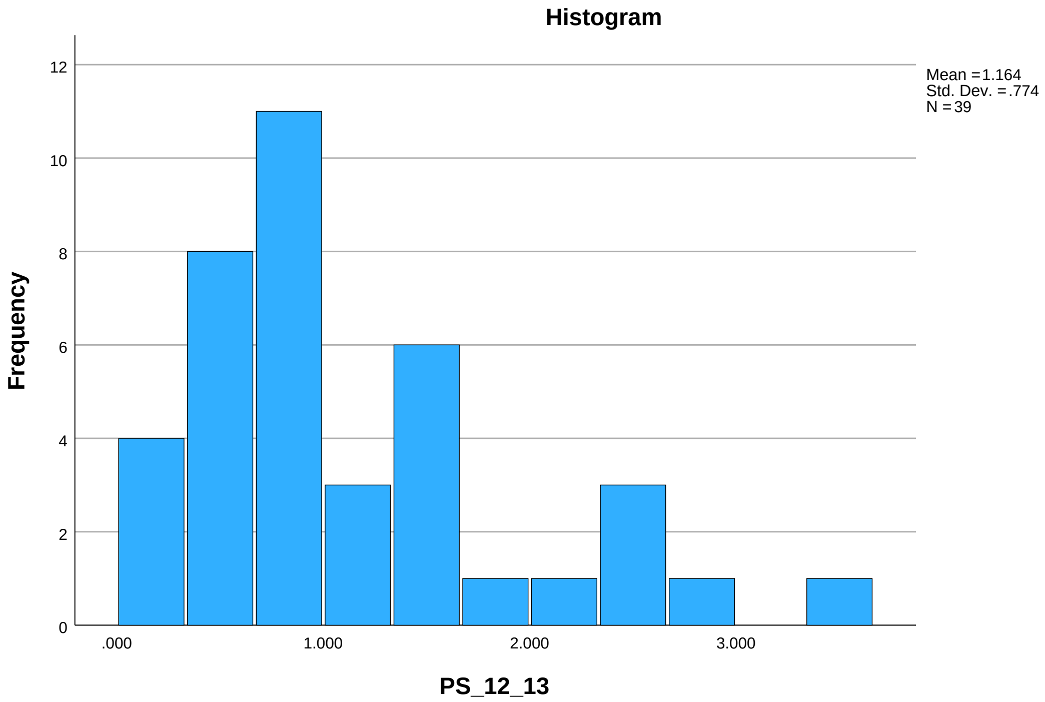


```
PS_12_13 Stem-and-Leaf Plot

 Frequency    Stem &  Leaf

     6.00        0 .  123344
    17.00        0 .  55666677788899999
     6.00        1 .  122344
     4.00        1 .  5557
     3.00        2 .  044
     1.00        2 .  6
     2.00 Extremes    (>=2.9)

 Stem width:      1.000
 Each leaf:       1 case(s)
```

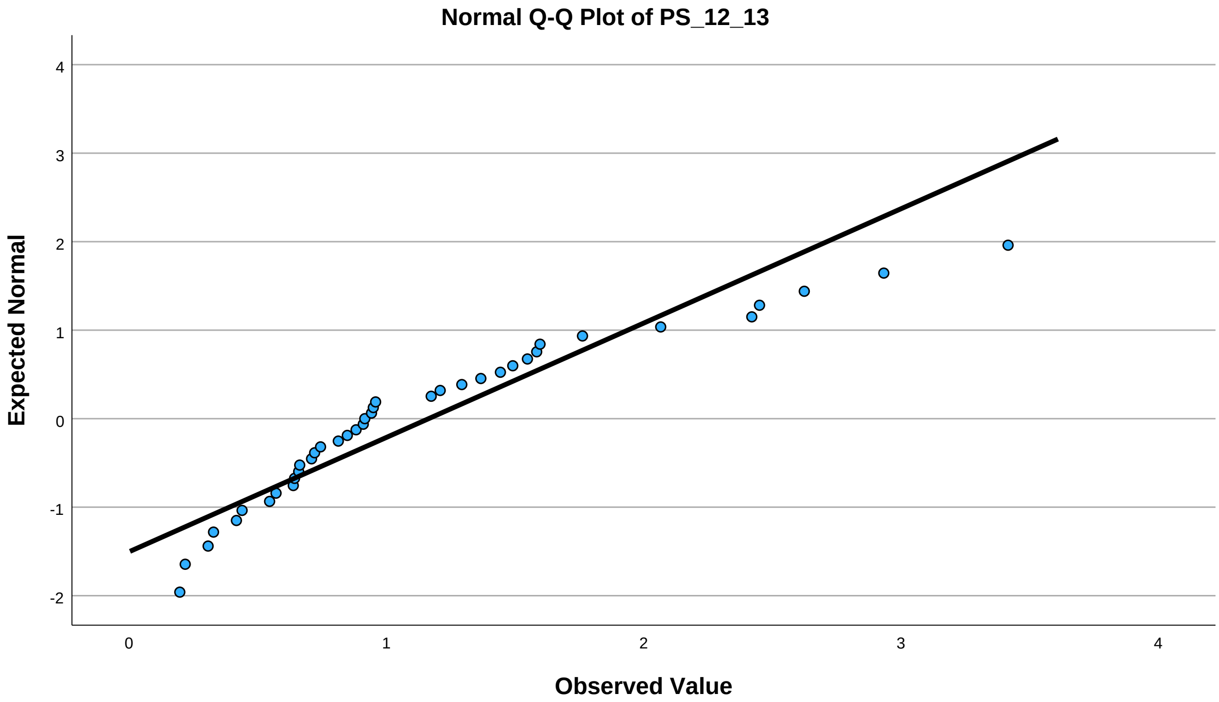
Normal Q-Q Plot of PS_12_13
Expected Normal
Observed Value
4
3
2
1
0
-1
-2
0
1
2
3
4

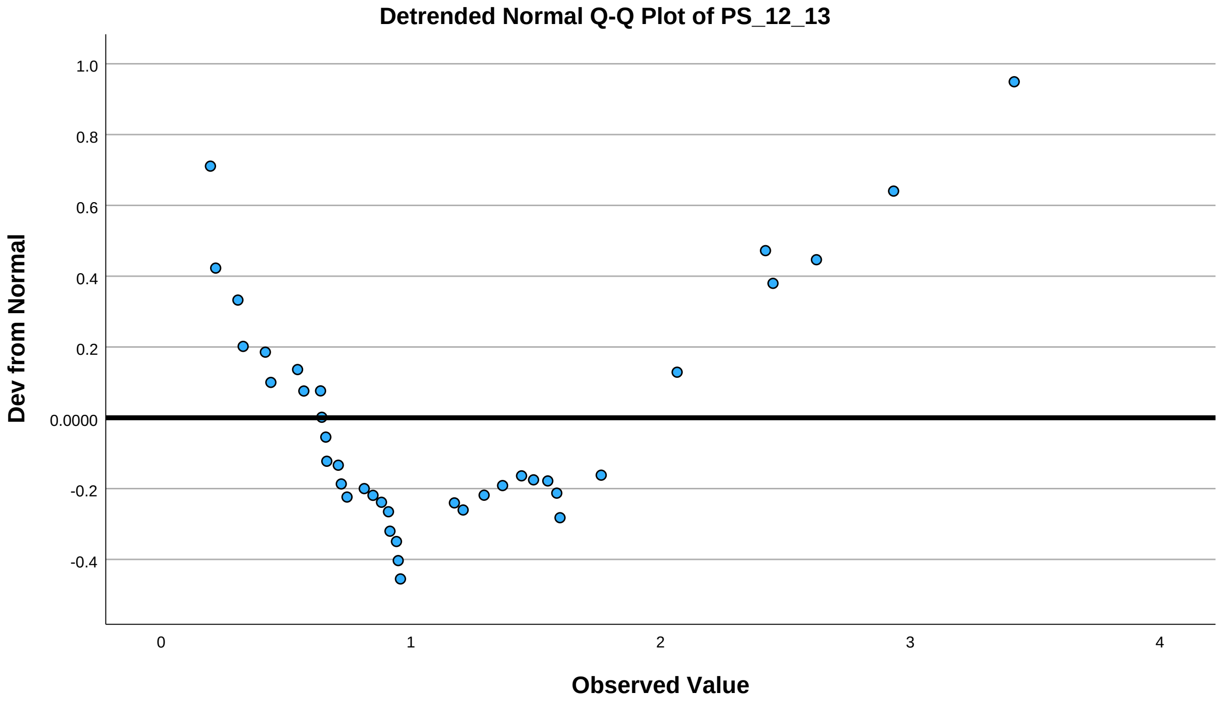
Detrended Normal Q-Q Plot of PS_12_13
Dev from Normal
Observed Value
1.0
0.8
0.6
0.4
0.2
0.0000
-0.2
-0.4
0
1
2
3
4

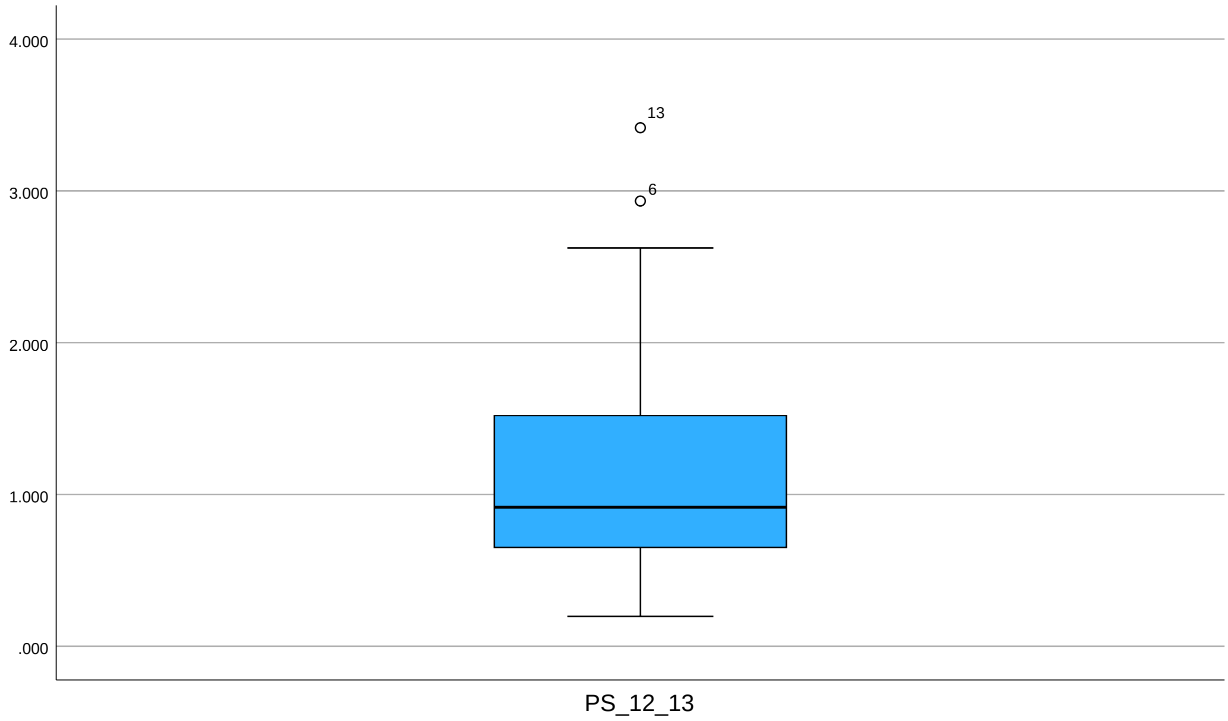

PS_13_14

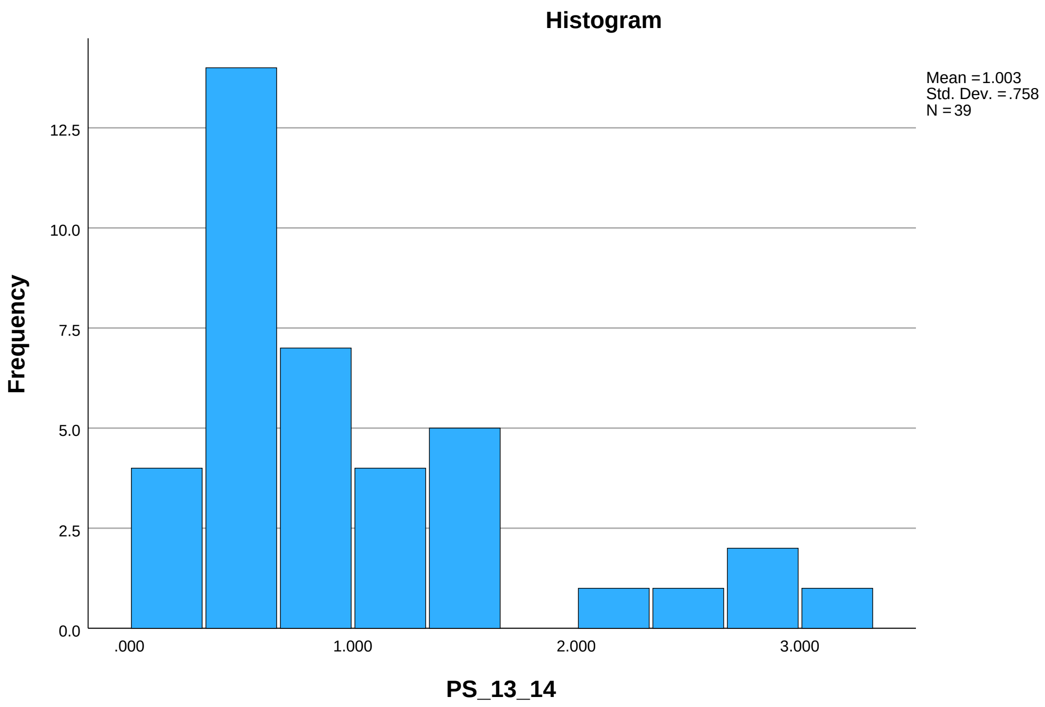


```
PS_13_14 Stem-and-Leaf Plot

 Frequency    Stem &  Leaf
```

```
   9.00        0 .  122334444
  16.00        0 .  5555566666677888
   6.00        1 .  001134
   3.00        1 .  555
   2.00        2 .  03
   3.00 Extremes    (>=2.8)

 Stem width:      1.000
 Each leaf:       1 case(s)
```

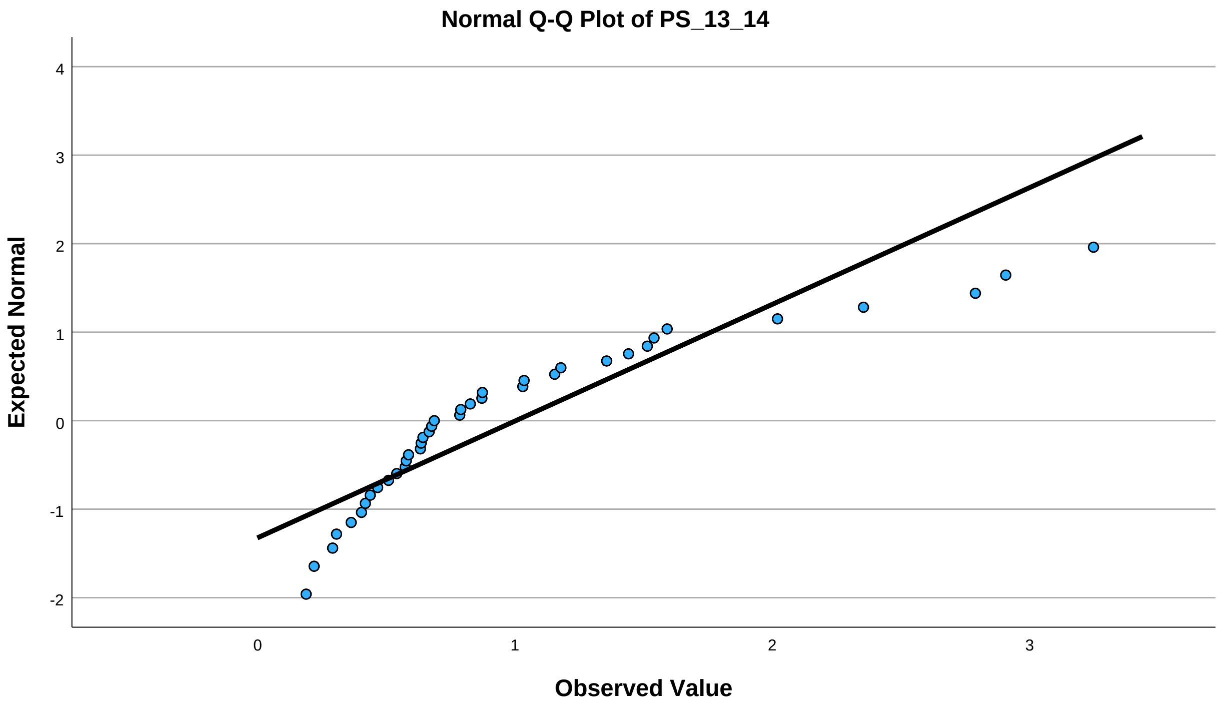

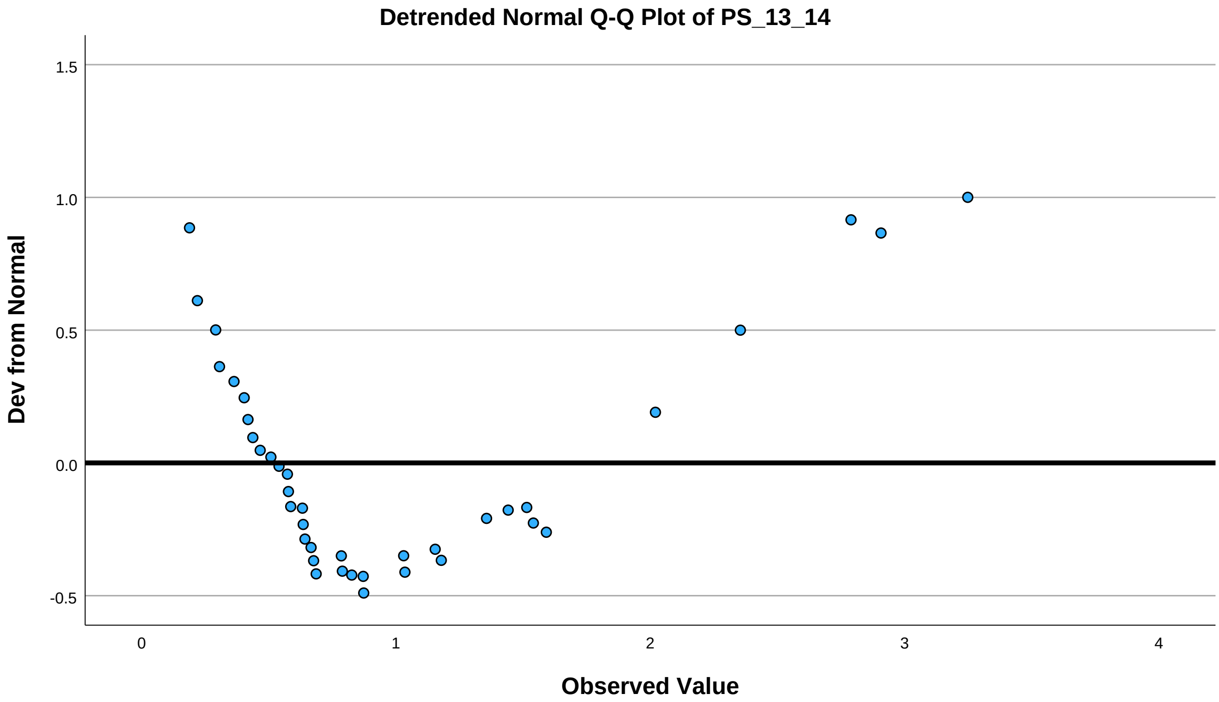
Detrended Normal Q-Q Plot of PS_13_14
Dev from Normal
Observed Value
1.5
1.0
0.5
0.0
-0.5
0
1
2
3
4

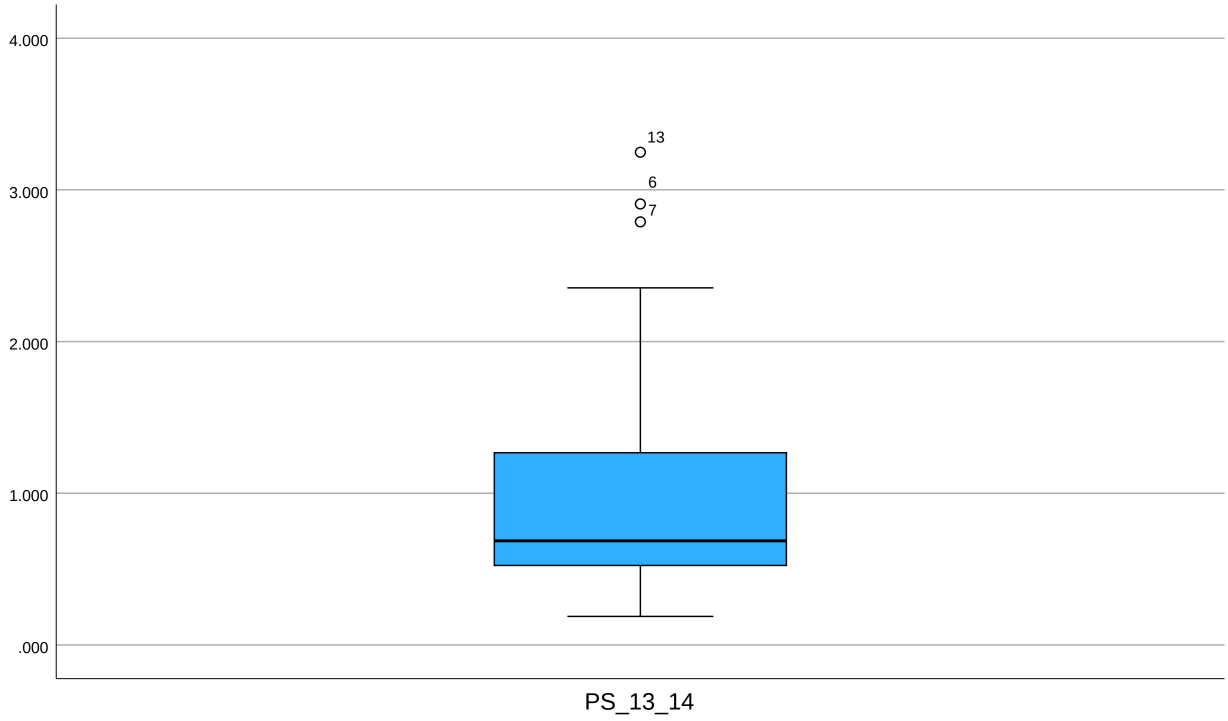
4.000
3.000
2.000
1.000
.000
13
6
7
PS_13_14

PS_14_15

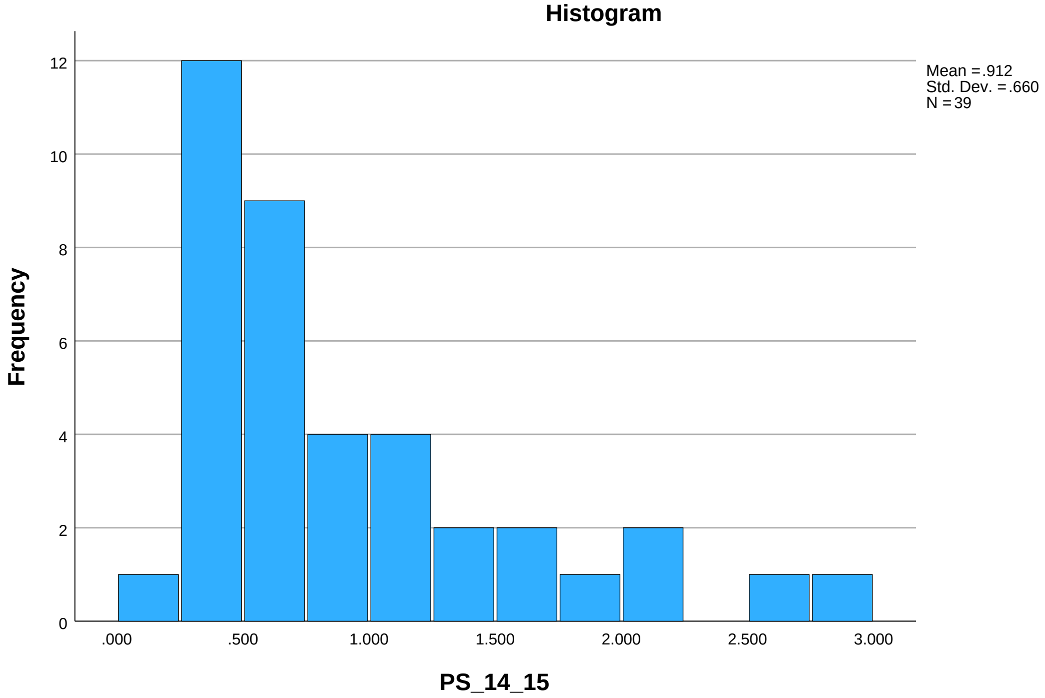


```
PS_14_15 Stem-and-Leaf Plot

 Frequency    Stem &  Leaf

   13.00        0 .  2222334444444
   13.00        0 .  5555666677889
    6.00      1 .  000234
    3.00      1 .  577
    2.00      2 .  01
    2.00 Extremes    (>=2.6)

 Stem width:      1.000
 Each leaf:        1 case(s)
```

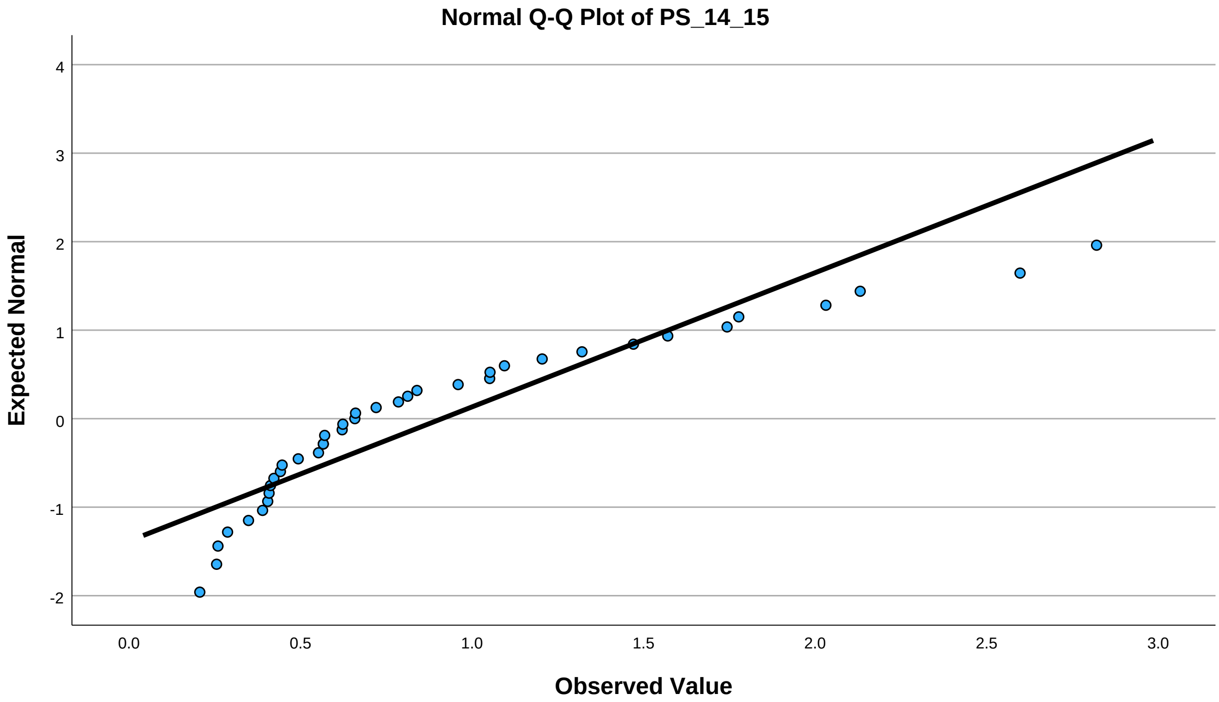
Normal Q-Q Plot of PS_14_15
Expected Normal
4
3
2
1
0
-1
-2
0.0
0.5
1.0
1.5
2.0
2.5
3.0
Observed Value

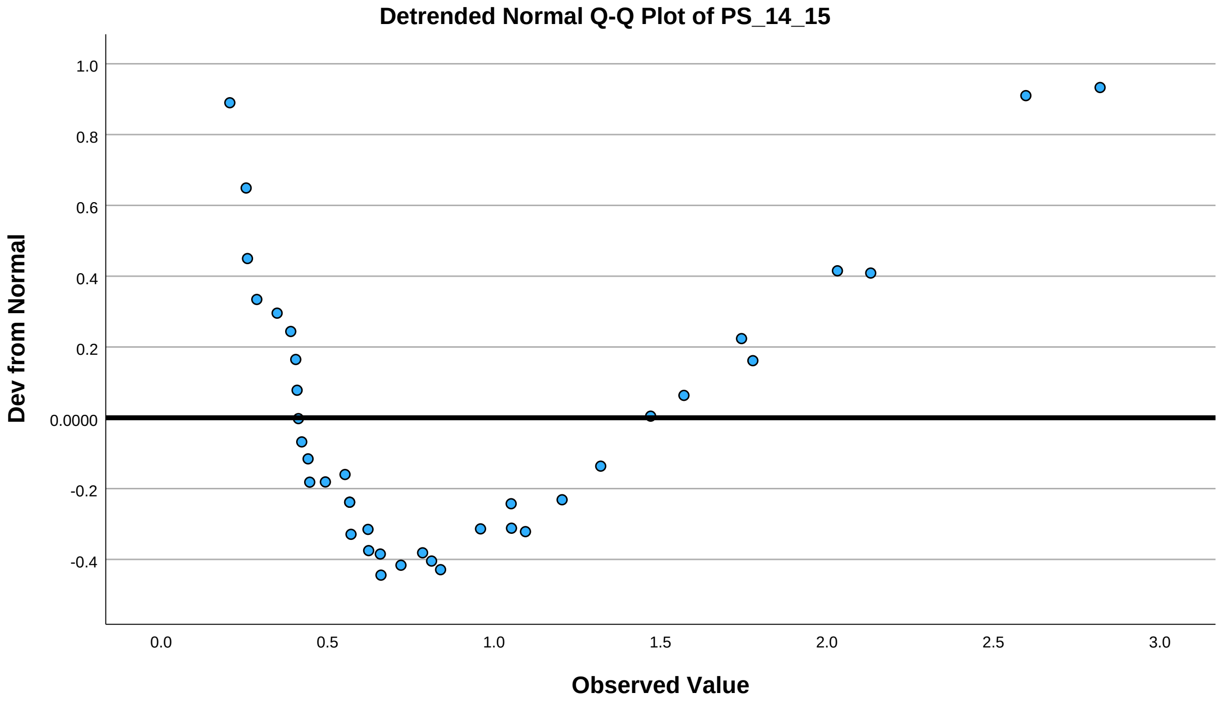
Detrended Normal Q-Q Plot of PS_14_15
Dev from Normal
1.0
0.8
0.6
0.4
0.2
0.0000
-0.2
-0.4
0.0
0.5
1.0
1.5
2.0
2.5
3.0
Observed Value

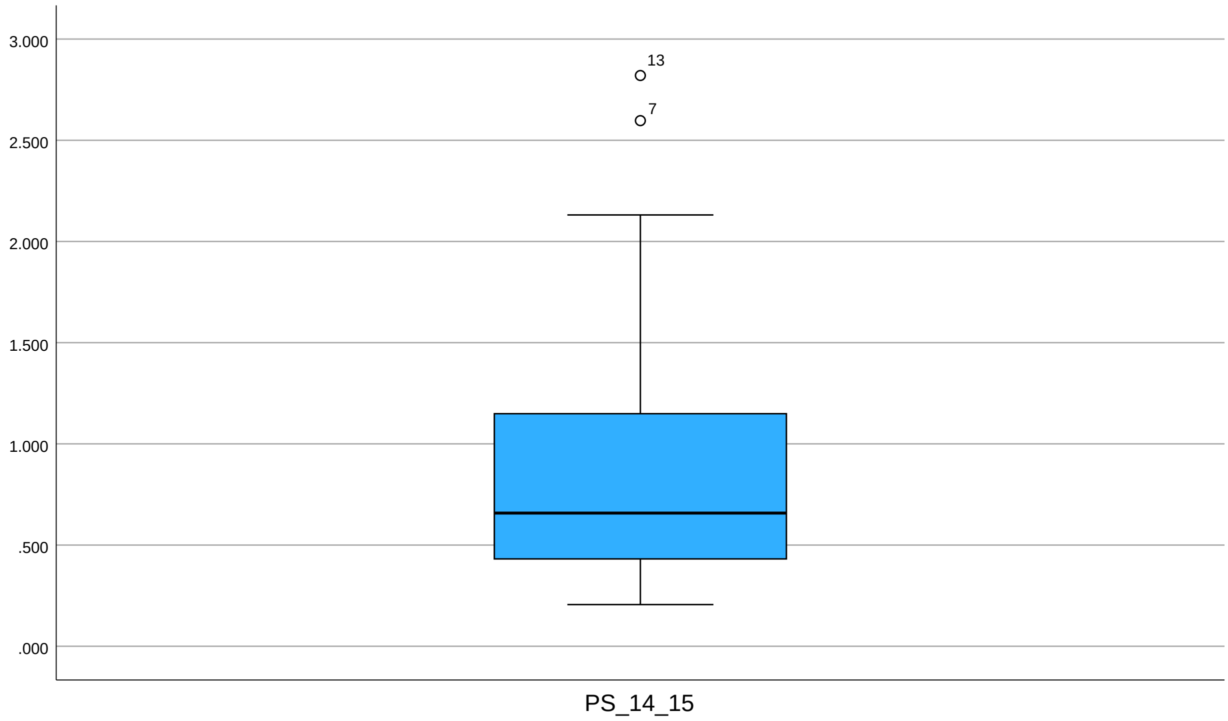

PS_15_16

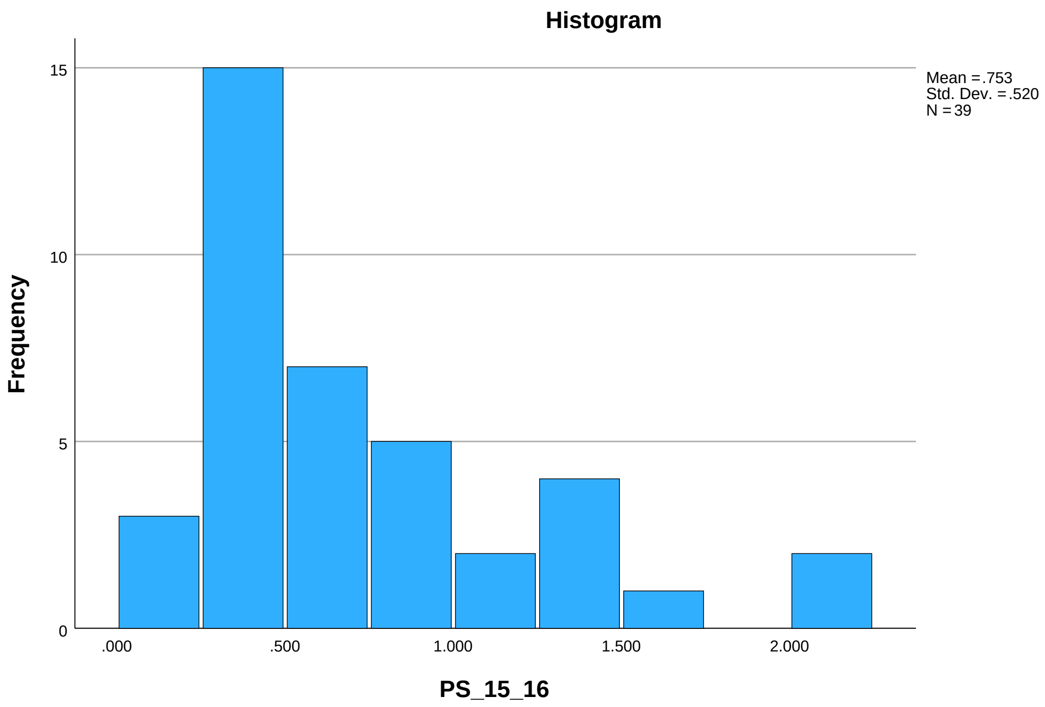

PS_15_16 Stem-and-Leaf Plot

Frequency Stem & Leaf

```
  1.00      0 . 1
  8.00      0 . 22233333
 13.00      0 . 4444444445555
  3.00      0 . 666
  5.00      0 . 89999
  1.00      1 . 1
  1.00      1 . 2
  5.00      1 . 4445
  2.00 Extremes    (>=2.1)

Stem width:     1.000
Each leaf:       1 case(s)
```

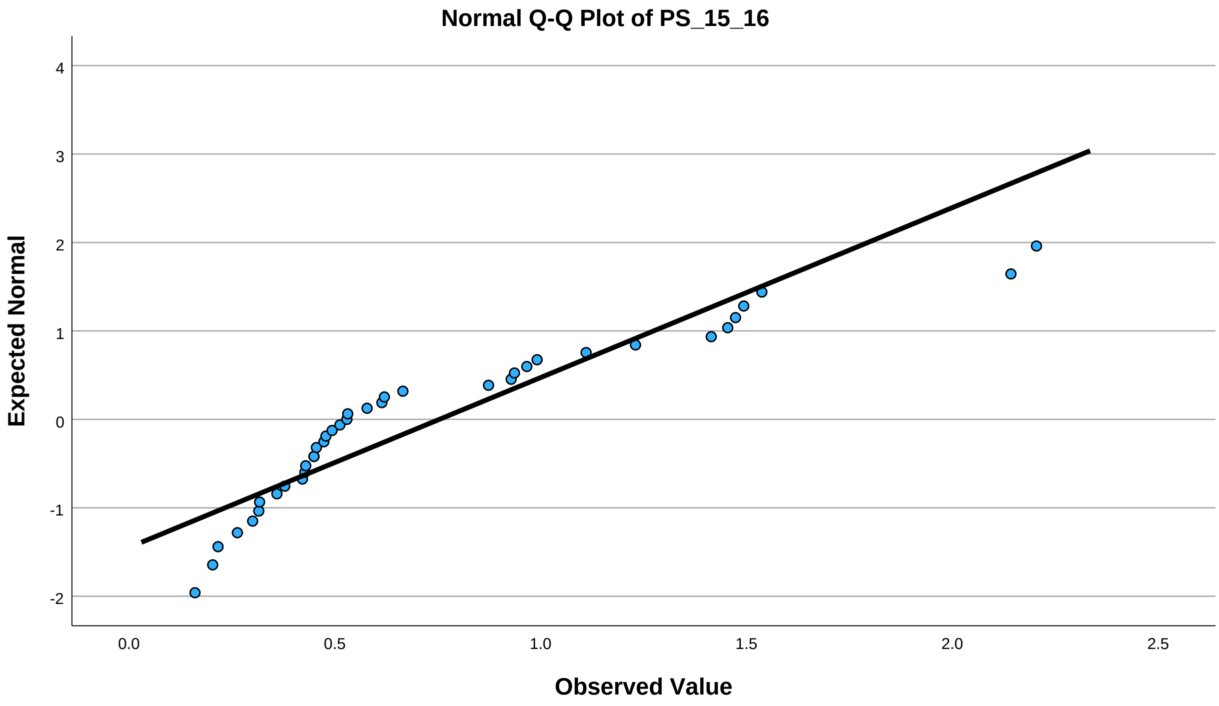

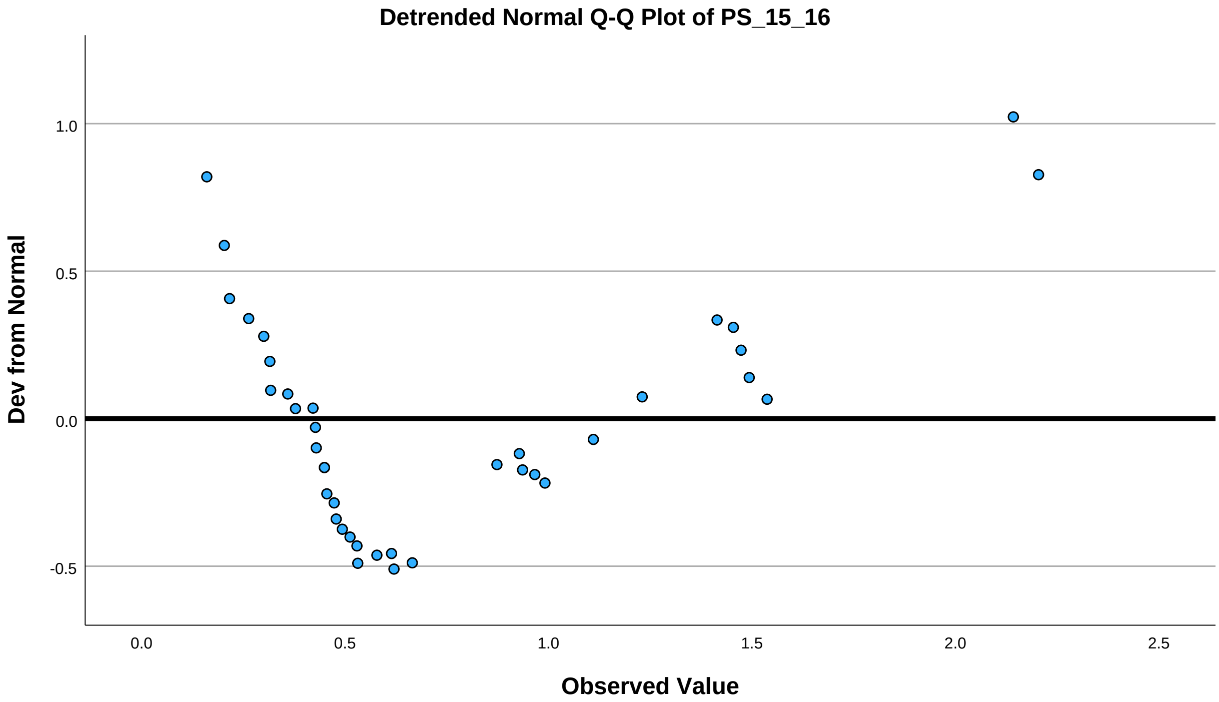
Detrended Normal Q-Q Plot of PS_15_16
Dev from Normal
1.0
0.5
0.0
-0.5
0.0
0.5
1.0
1.5
2.0
2.5
Observed Value

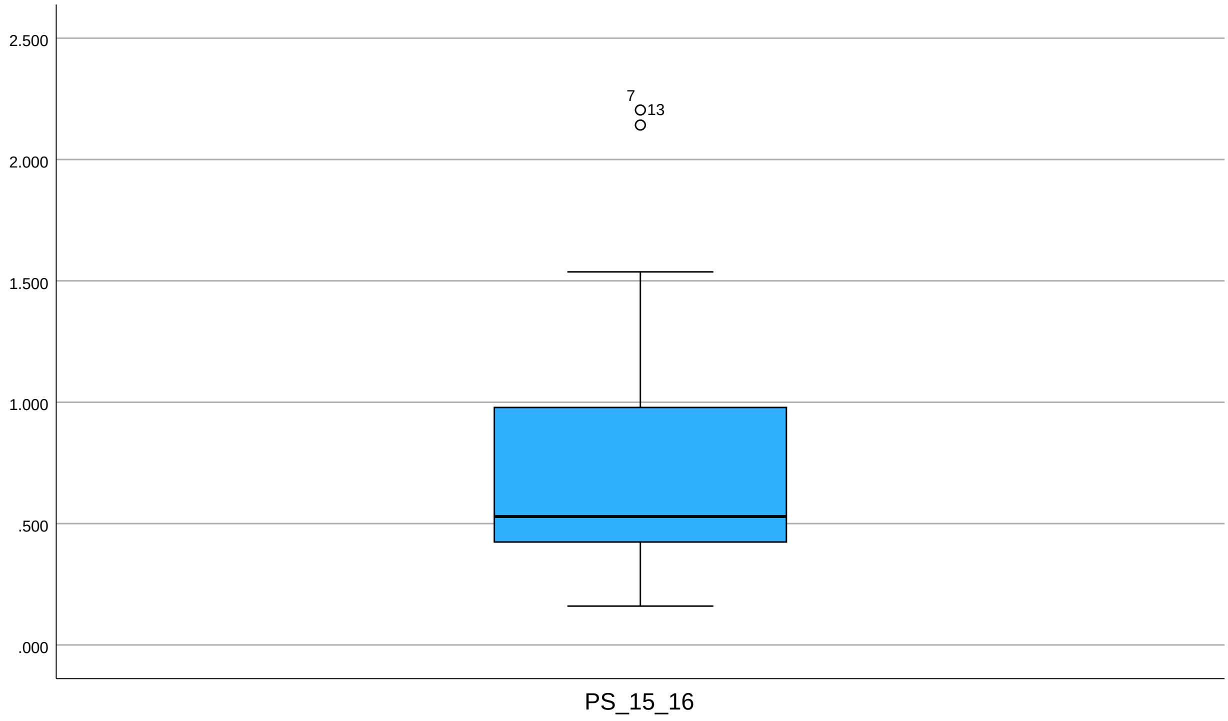
2.500
2.000
1.500
1.000
.500
.000
7
13
PS_15_16

PS_16_17

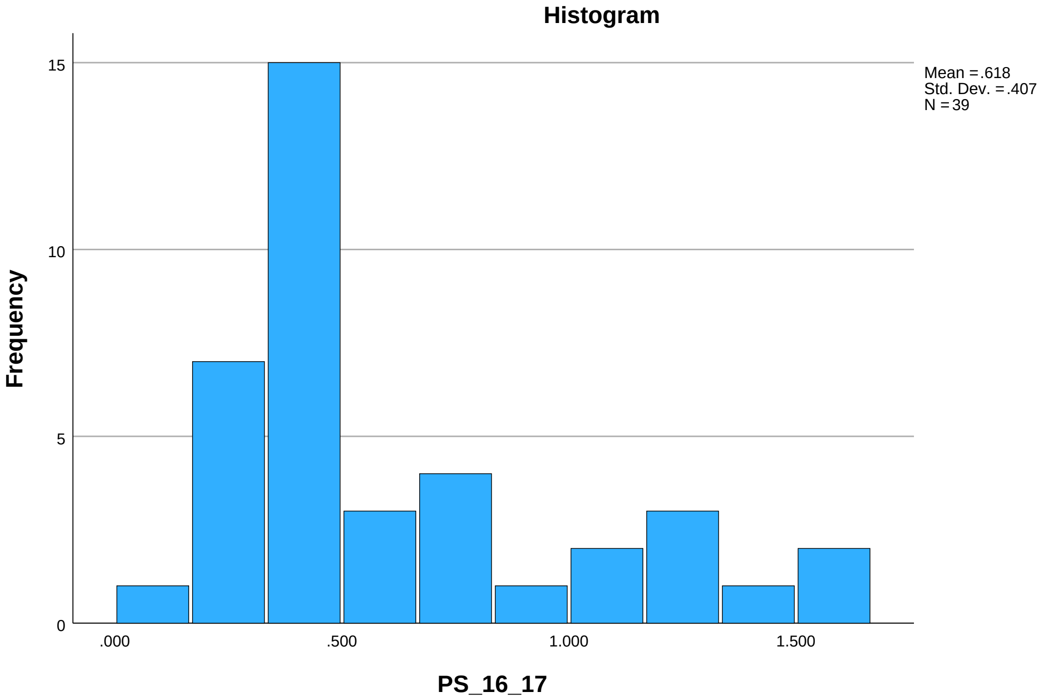


```
PS_16_17 Stem-and-Leaf Plot

 Frequency    Stem &  Leaf

     1.00        0 .  1
    17.00        0 .  22222333333333333
     6.00        0 .  444445
     5.00        0 .  66667
     2.00        0 .  88
     2.00        1 .  01
     3.00        1 .  223
     3.00 Extremes    (>=1.4)

 Stem width:      1.000
 Each leaf:       1 case(s)
```

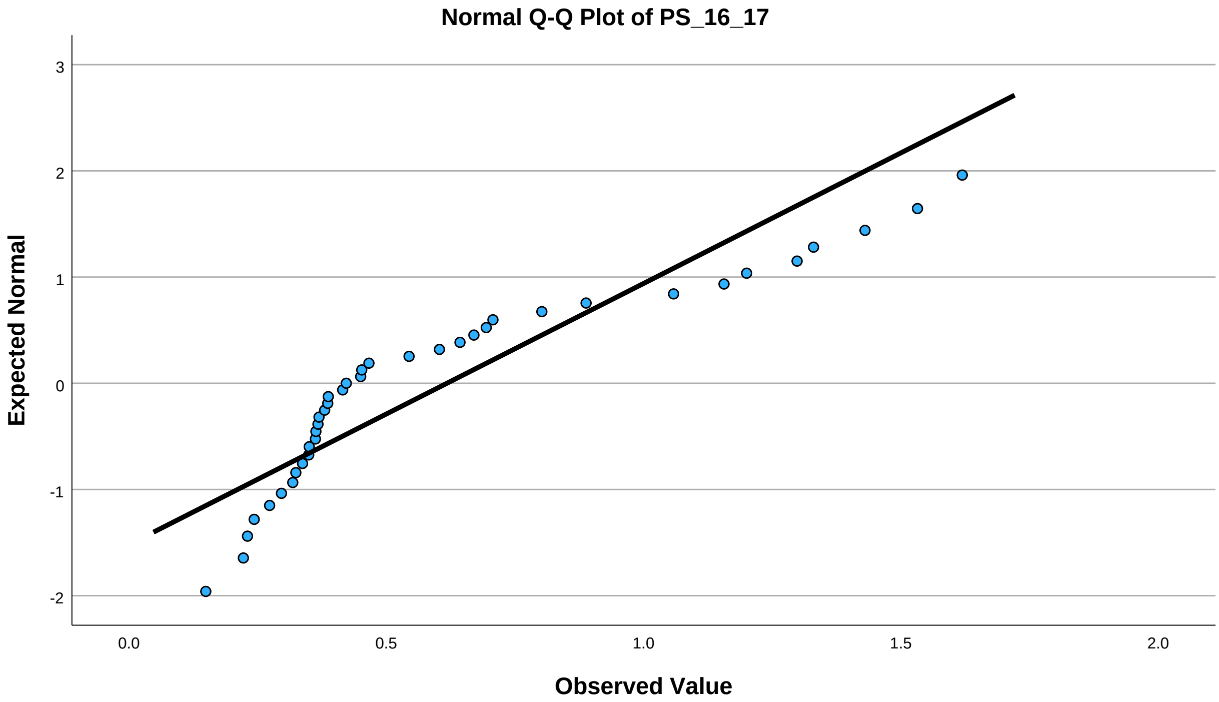
Normal Q-Q Plot of PS_16_17
Expected Normal
3
2
1
0
-1
-2
0.0
0.5
1.0
1.5
2.0
Observed Value

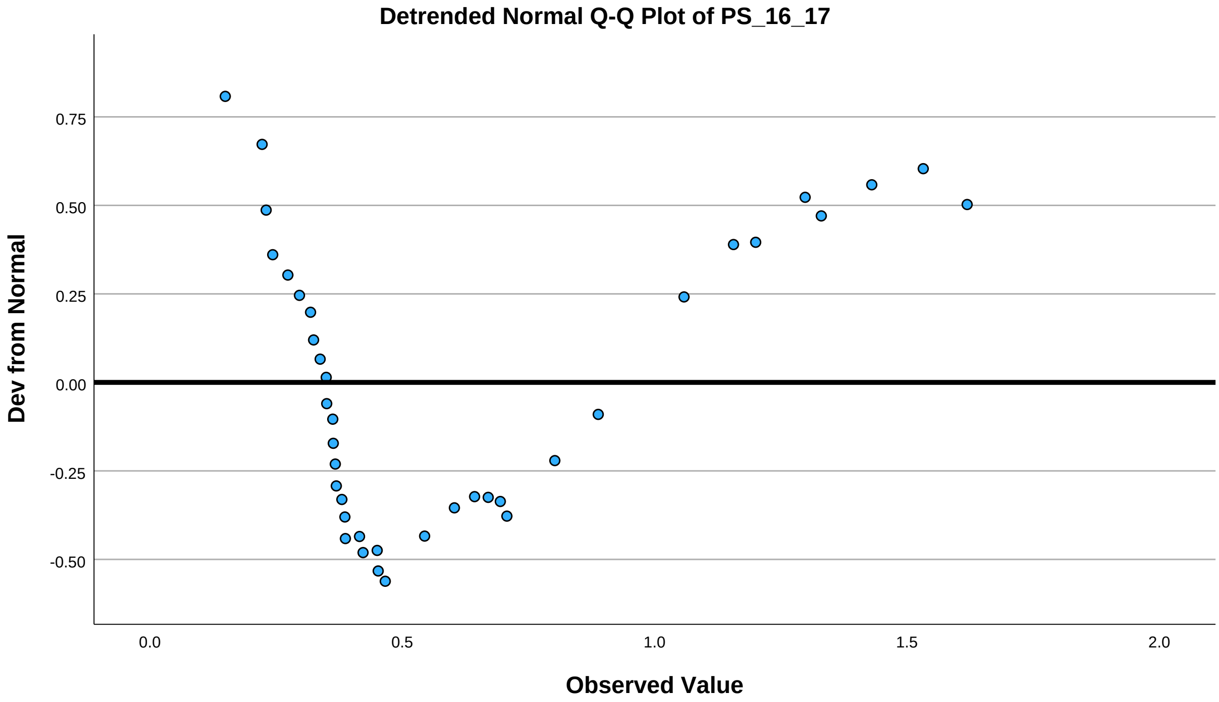
Detrended Normal Q-Q Plot of PS_16_17
Dev from Normal
0.75
0.50
0.25
0.00
-0.25
-0.50
0.0
0.5
1.0
1.5
2.0
Observed Value

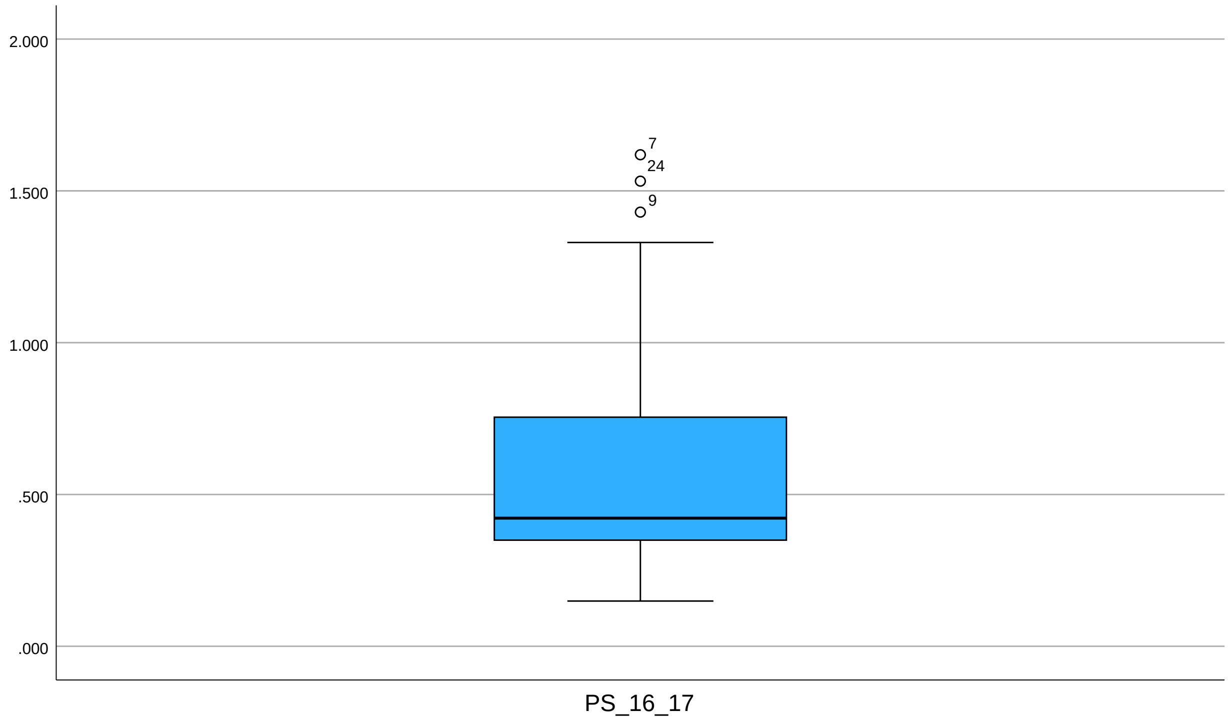

PS_17_18

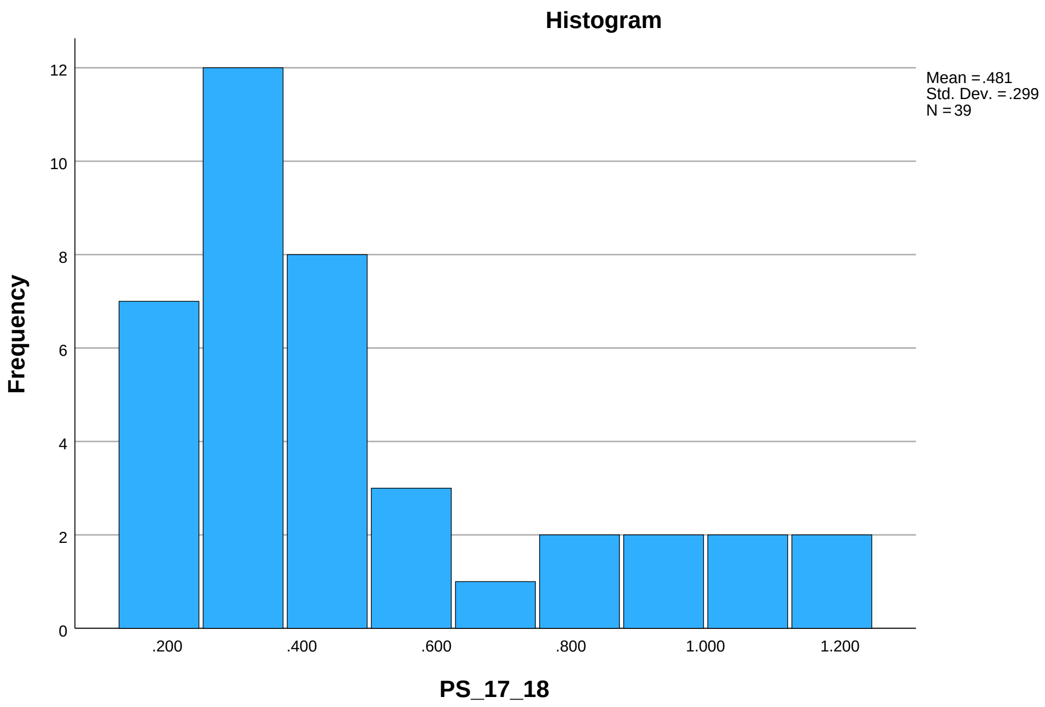

PS_17_18 Stem-and-Leaf Plot

Frequency   Stem & Leaf

```
 4.00      1 . 5789
12.00      2 . 344677888999
 7.00      3 . 2478899
 4.00      4 . 0026
 1.00      5 . 6
 3.00      6 . 118
 1.00      7 . 8
 1.00      8 . 4
 2.00      9 . 07
 2.00     10 . 16
 2.00 Extremes    (>=1.18)

Stem width:      .100
Each leaf:       1 case(s)
```

Normal Q-Q Plot of PS_17_18

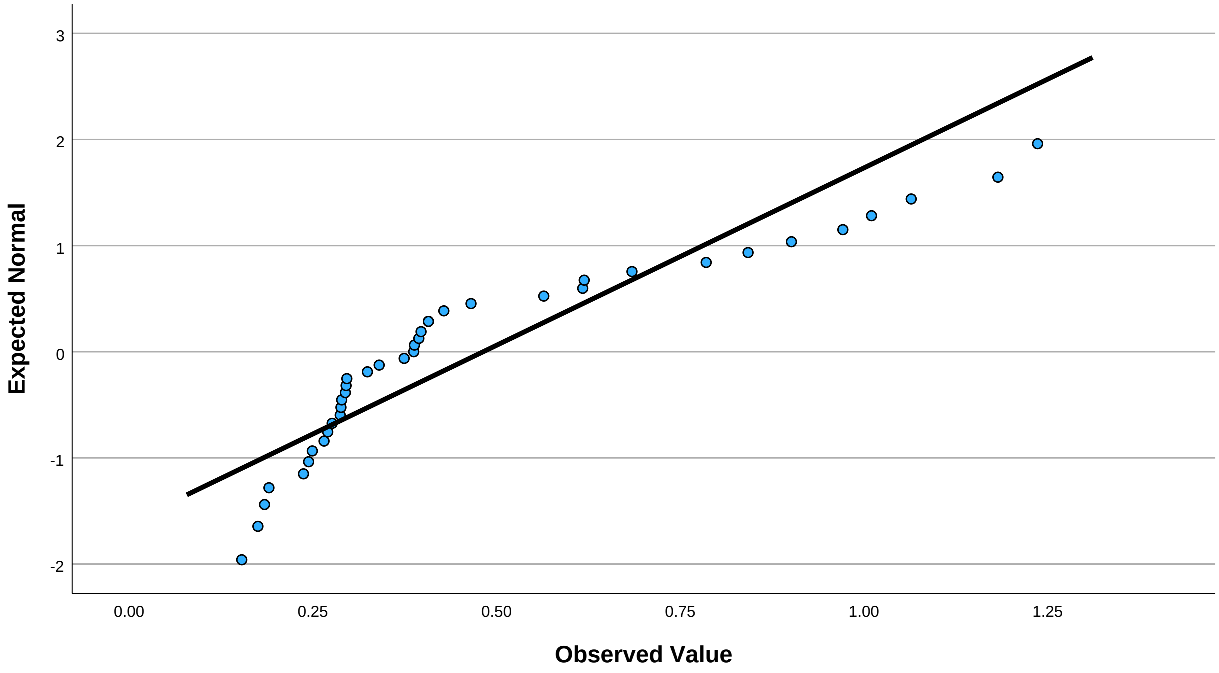

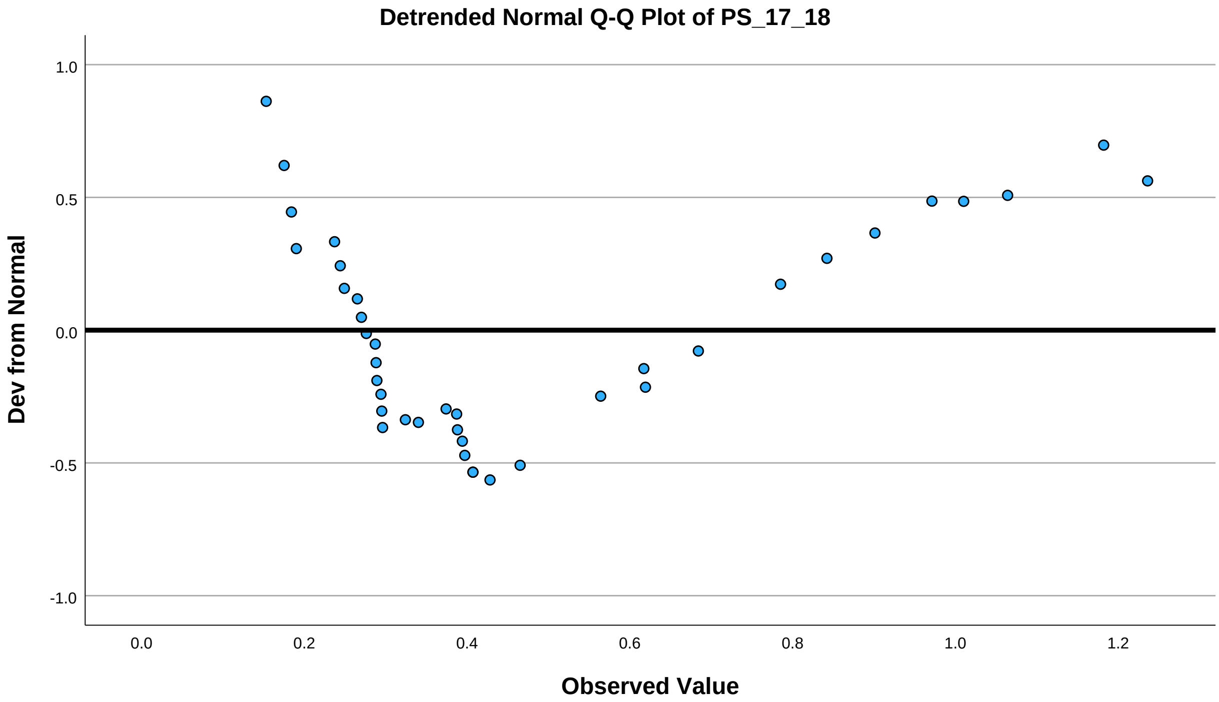
Detrended Normal Q-Q Plot of PS_17_18
1.0
0.5
0.0
-0.5
-1.0
Dev from Normal
0.0
0.2
0.4
0.6
0.8
1.0
1.2
Observed Value

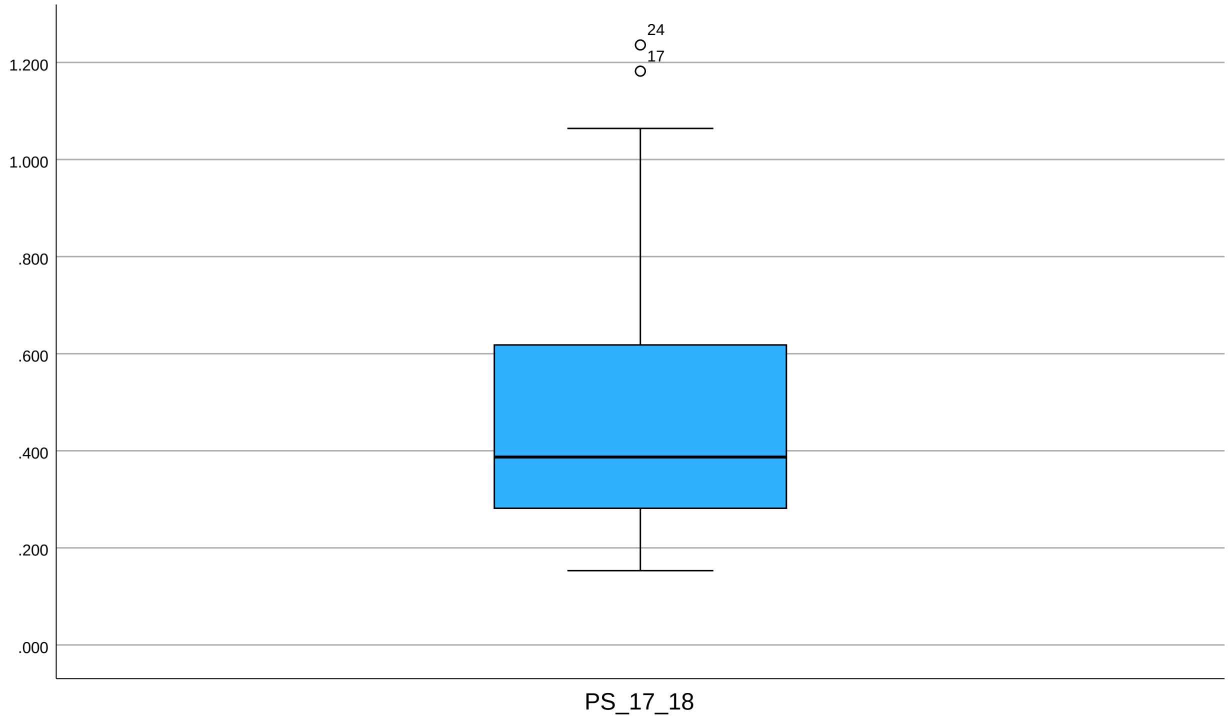
24
17
1.200
1.000
.800
.600
.400
.200
.000
PS_17_18

PS_18_19

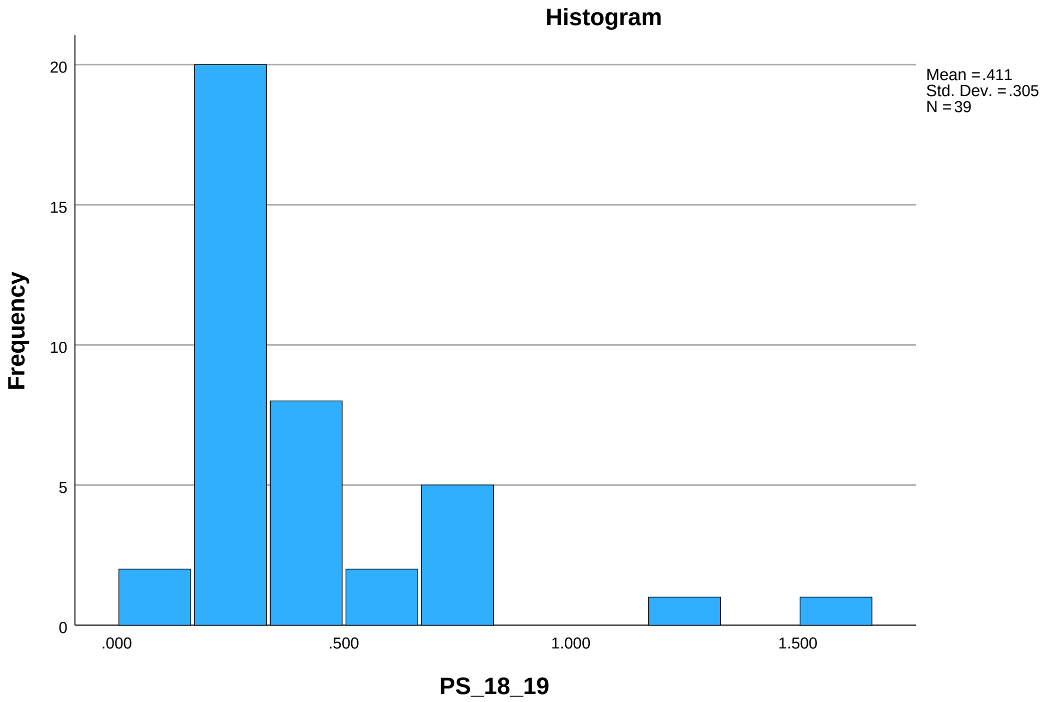


```
PS_18_19 Stem-and-Leaf Plot

 Frequency    Stem &  Leaf

     6.00        1 .  156789
    14.00        2 .  00233444555789
     7.00        3 .  1234789
     3.00        4 .  118
     2.00        5 .  09
     1.00        6 .  9
     1.00        7 .  1
     5.00 Extremes    (>=.80)

 Stem width:      .100
 Each leaf:        1 case(s)
```

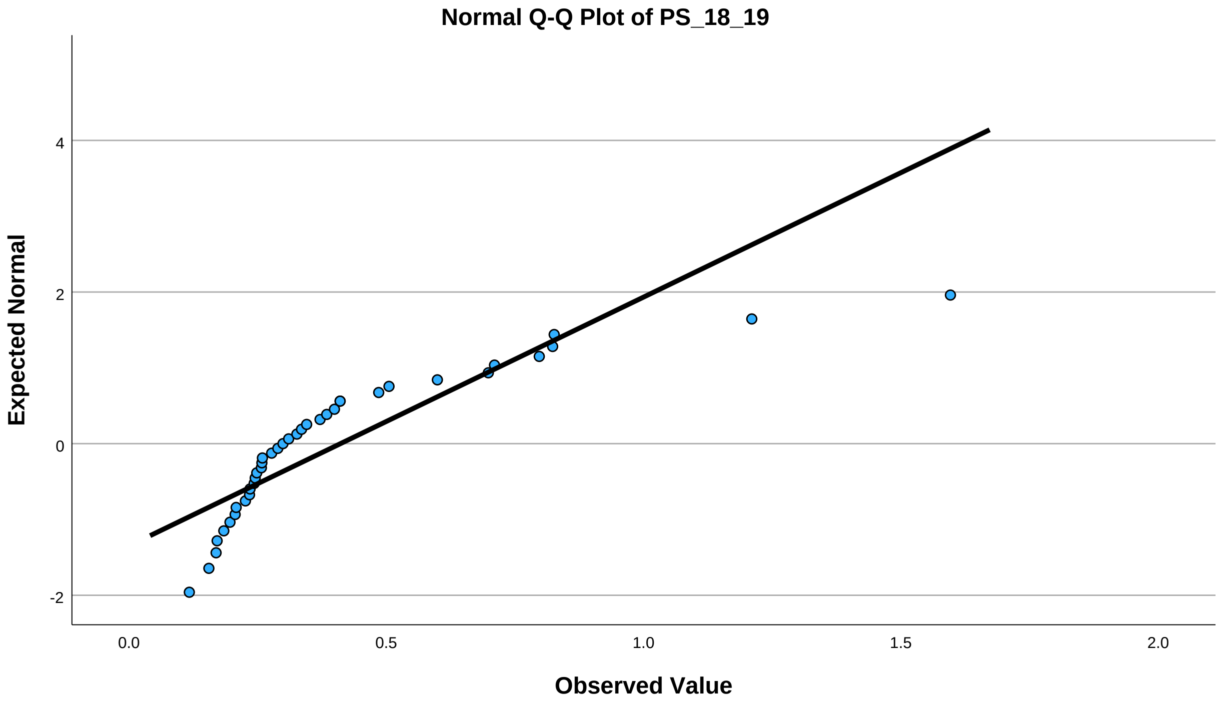
Normal Q-Q Plot of PS_18_19
Expected Normal
4
2
0
-2
0.0
0.5
1.0
1.5
2.0
Observed Value

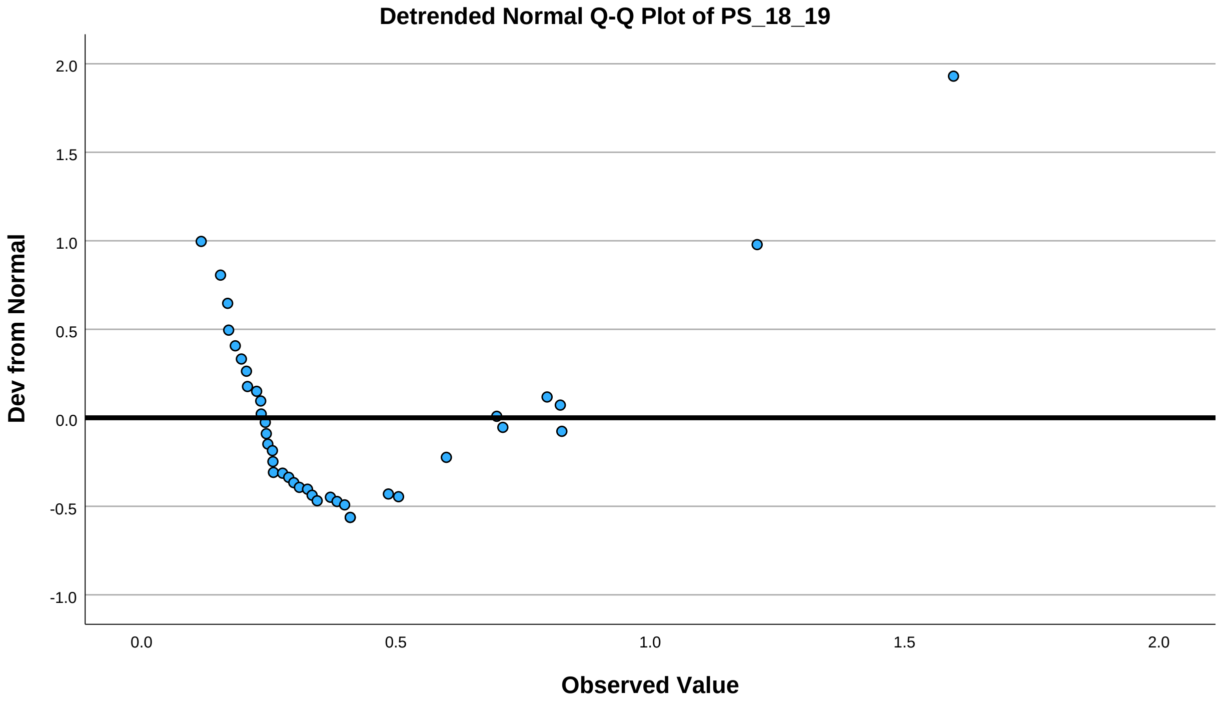
Detrended Normal Q-Q Plot of PS_18_19
Dev from Normal
2.0
1.5
1.0
0.5
0.0
-0.5
-1.0
0.0
0.5
1.0
1.5
2.0
Observed Value

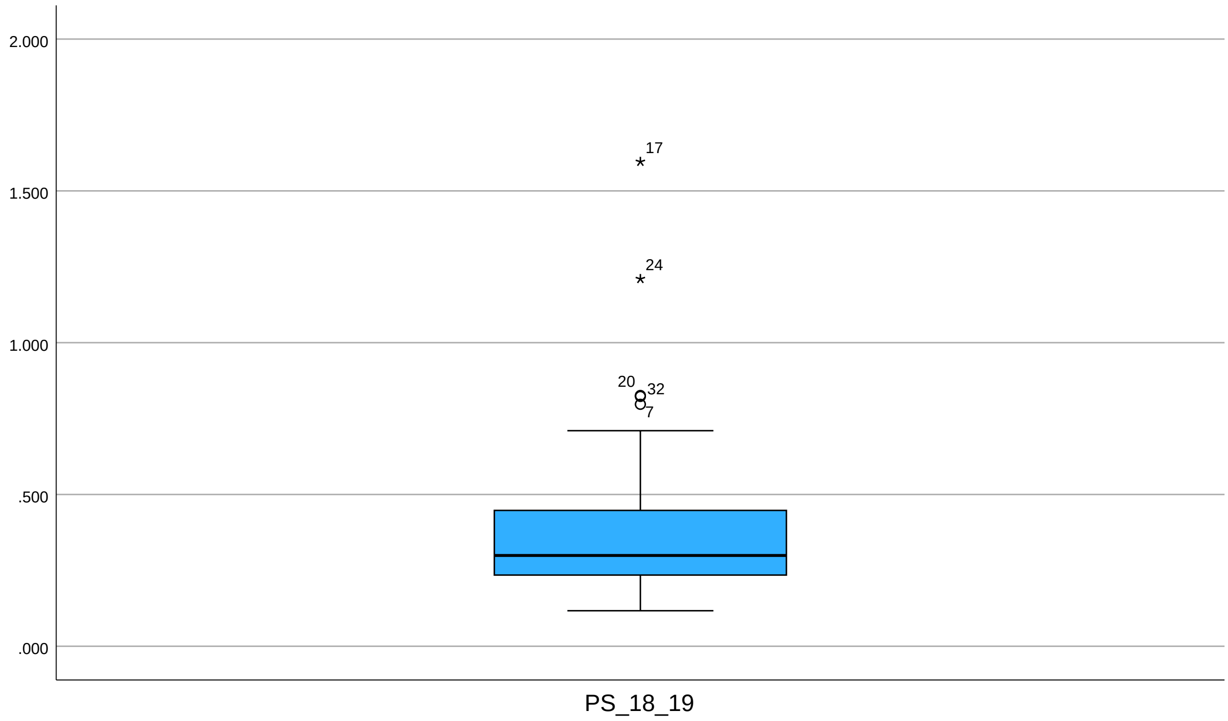

PS_19_20

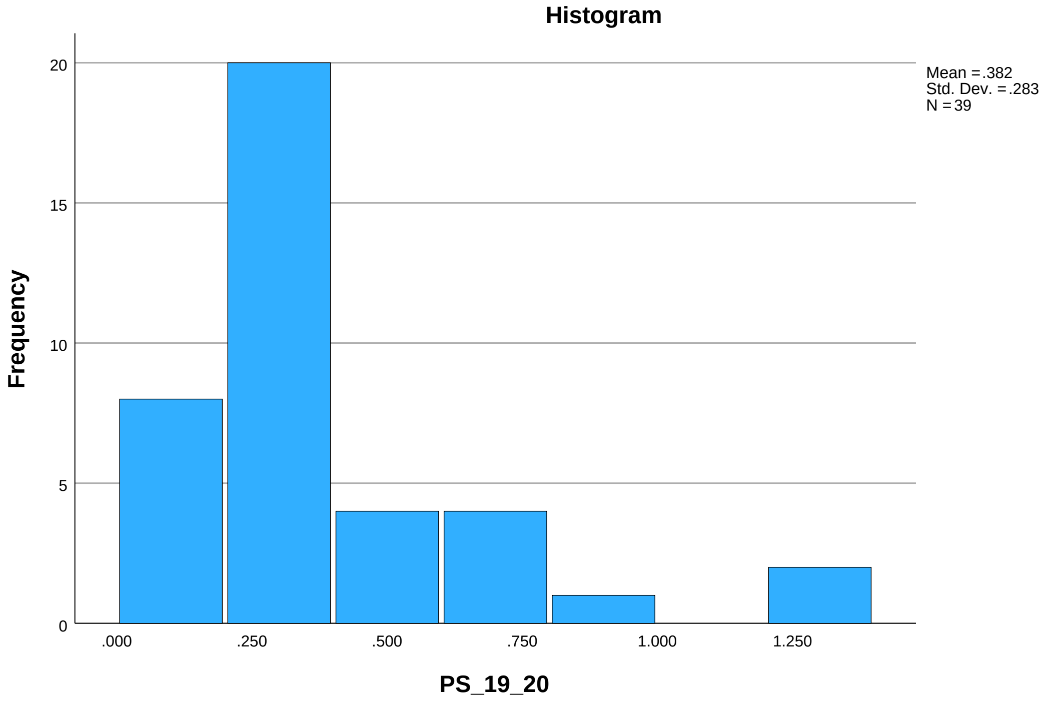

PS_19_20 Stem-and-Leaf Plot

Frequency   Stem & Leaf

```
 8.00      1 .  01377799
14.00       2 .  00111334688999
 6.00      3 .  022678
2.00     4 .  04
2.00     5 .  56
3.00     6 .  029
1.00     7 .  2
 3.00 Extremes    (>=.82)

Stem width:     .100
Each leaf:      1 case(s)
```

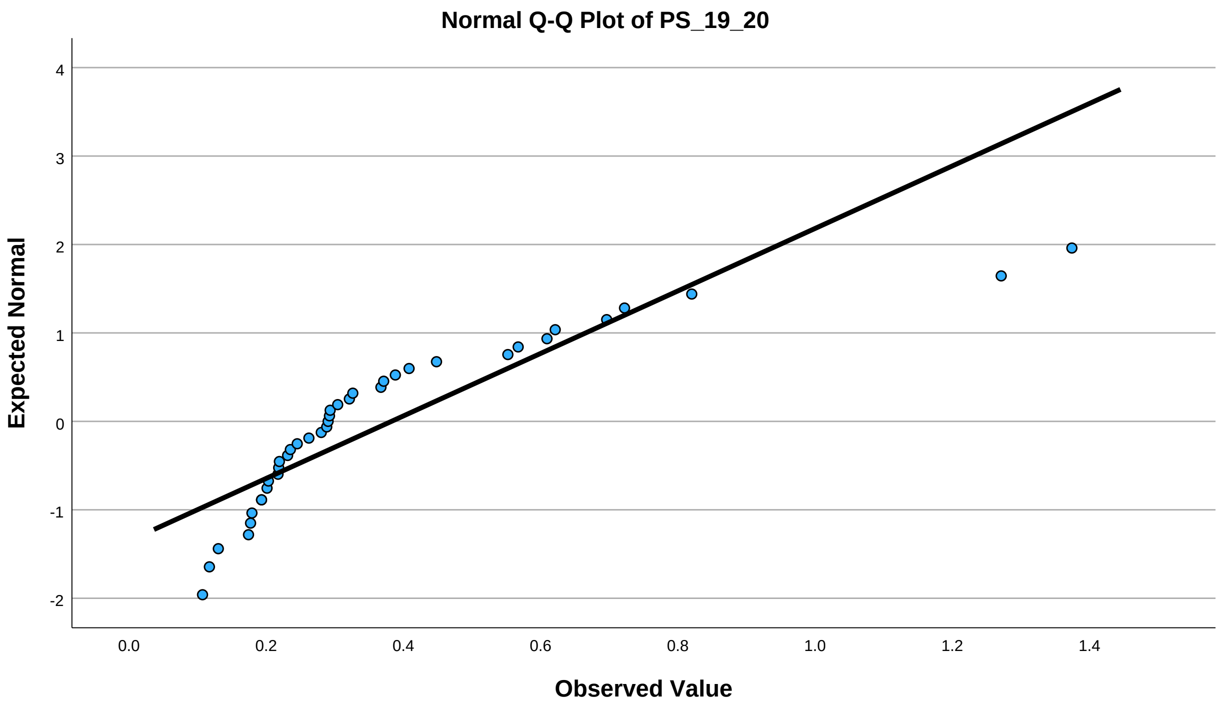

Detrended Normal Q-Q Plot of PS_19_20

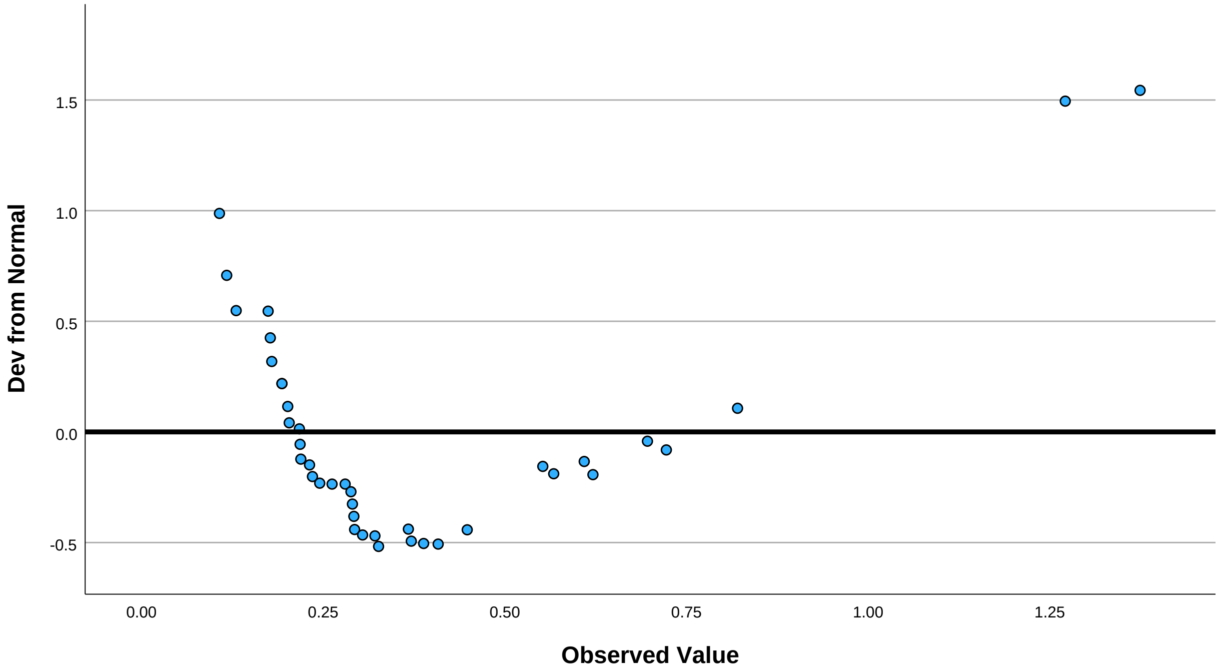

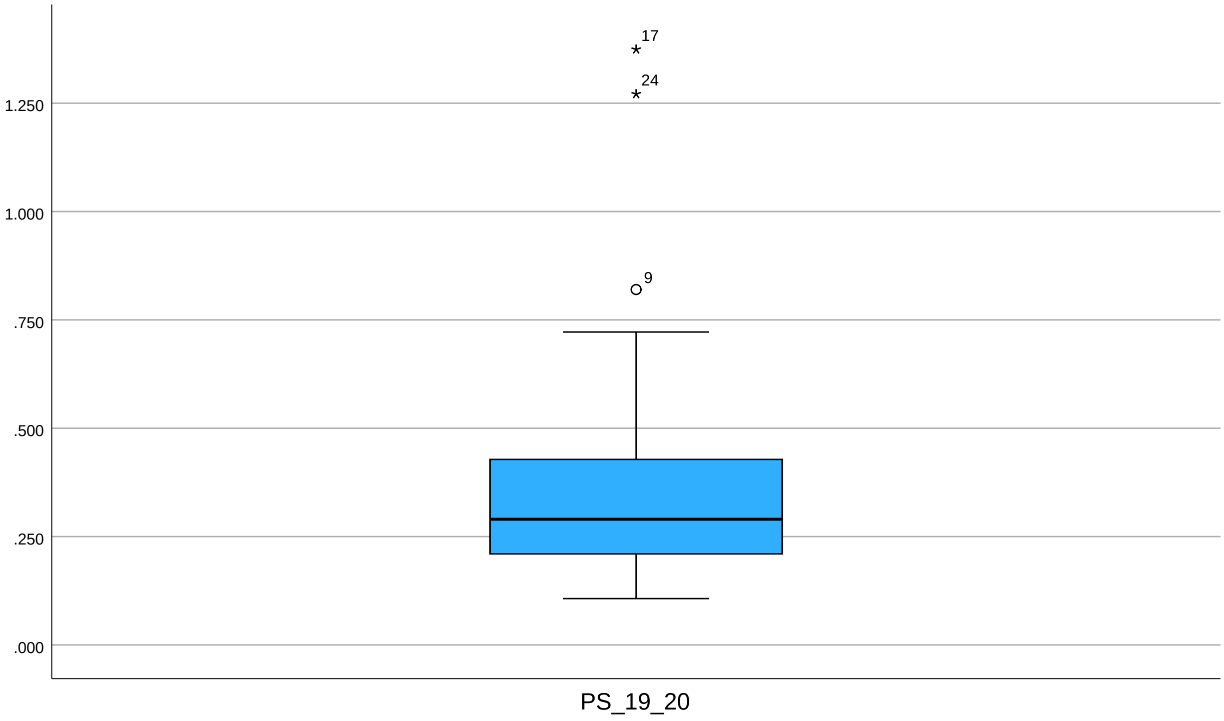

Explore

[DataSet22] C:\Users\aleks\OneDrive\Desktop\Results\SPSS Files\Channel19.sav

Case Processing Summary

	Cases					
	Valid		Missing		Total	
	N	Percent	N	Percent	N	Percent
BrainRate	39	100.0%	0	0.0%	39	100.0%
PermutationEntropy	39	100.0%	0	0.0%	39	100.0%
PermutationEntropy_01	39	100.0%	0	0.0%	39	100.0%
SampleEntropy	39	100.0%	0	0.0%	39	100.0%
SpectralEntropy_bits	39	100.0%	0	0.0%	39	100.0%
SpectralEntropy_01	39	100.0%	0	0.0%	39	100.0%
HiguchiFractalDimension	39	100.0%	0	0.0%	39	100.0%
LempelZivComplexity	39	100.0%	0	0.0%	39	100.0%
RenyiEntropy	39	100.0%	0	0.0%	39	100.0%
TsallisEntropy	39	100.0%	0	0.0%	39	100.0%
PS_4_5	39	100.0%	0	0.0%	39	100.0%
PS_5_6	39	100.0%	0	0.0%	39	100.0%
PS_6_7	39	100.0%	0	0.0%	39	100.0%
PS_7_8	39	100.0%	0	0.0%	39	100.0%
PS_8_9	39	100.0%	0	0.0%	39	100.0%
PS_9_10	39	100.0%	0	0.0%	39	100.0%
PS_10_11	39	100.0%	0	0.0%	39	100.0%
PS_11_12	39	100.0%	0	0.0%	39	100.0%
PS_12_13	39	100.0%	0	0.0%	39	100.0%
PS_13_14	39	100.0%	0	0.0%	39	100.0%
PS_14_15	39	100.0%	0	0.0%	39	100.0%
PS_15_16	39	100.0%	0	0.0%	39	100.0%
PS_16_17	39	100.0%	0	0.0%	39	100.0%
PS_17_18	39	100.0%	0	0.0%	39	100.0%
PS_18_19	39	100.0%	0	0.0%	39	100.0%
PS_19_20	39	100.0%	0	0.0%	39	100.0%

Descriptives

			Statistic	Std. Error
BrainRate	Mean		6.8764	.44304
	95% Confidence Interval for Mean	Lower Bound	5.9795	
		Upper Bound	7.7733	
	5% Trimmed Mean		6.6711	
	Median		6.0200	
	Variance		7.655	
	Std. Deviation		2.76681	
	Minimum		2.38	
	Maximum		14.83	
	Range		12.45	
	Interquartile Range		2.58	
	Skewness		1.403	.378
	Kurtosis		2.003	.741
PermutationEntropy	Mean		1.4870827820	.01168427787
	95% Confidence Interval for Mean	Lower Bound	1.4634291981	
		Upper Bound	1.5107363659	
	5% Trimmed Mean		1.4879196927	
	Median		1.4760051658	
	Variance		.005	
	Std. Deviation		.07296829189	
	Minimum		1.3393810840	
	Maximum		1.6217051196	
	Range		.28232403565	
	Interquartile Range		.10880770945	
	Skewness		-.100	.378
	Kurtosis		-.580	.741
PermutationEntropy_01	Mean		.82995670318	.00652111964
	95% Confidence Interval for Mean	Lower Bound	.81675538664	
		Upper Bound	.84315801972	
	5% Trimmed Mean		.83042379198	
	Median		.82377416788	
	Variance		.002	
	Std. Deviation		.04072437911	
	Minimum		.74752281598	
	Maximum		.90509086040	
	Range		.15756804443	
	Interquartile Range		.06072673889	
	Skewness		-.100	.378
	Kurtosis		-.580	.741

Descriptives

			Statistic	Std. Error
SampleEntropy	Mean		.65677094703	.03068349439
	95% Confidence Interval for Mean	Lower Bound	.59465546006	
		Upper Bound	.71888643400	
	5% Trimmed Mean		.66357024242	
	Median		.64508765415	
	Variance		.037	
	Std. Deviation		.19161836105	
	Minimum		.16242304532	
	Maximum		1.0184283156	
	Range		.85600527031	
	Interquartile Range		.23641608020	
	Skewness		-.626	.378
	Kurtosis		.514	.741
SpectralEntropy_bits	Mean		3.8809798619	.08793741859
	95% Confidence Interval for Mean	Lower Bound	3.7029598649	
		Upper Bound	4.0589998589	
	5% Trimmed Mean		3.8969866006	
	Median		3.8932503338	
	Variance		.302	
	Std. Deviation		.54916900308	
	Minimum		2.1400927267	
	Maximum		5.0366764766	
	Range		2.8965837499	
	Interquartile Range		.56070738488	
	Skewness		-.565	.378
	Kurtosis		2.070	.741
SpectralEntropy_01	Mean		.55353787868	.01254237174
	95% Confidence Interval for Mean	Lower Bound	.52814717453	
		Upper Bound	.57892858283	
	5% Trimmed Mean		.55582089392	
	Median		.55528799624	
	Variance		.006	
	Std. Deviation		.07832708641	
	Minimum		.30523796316	
	Maximum		.71837301704	
	Range		.41313505388	
	Interquartile Range		.07997278714	
	Skewness		-.565	.378
	Kurtosis		2.070	.741

Descriptives

			Statistic	Std. Error
HiguchiFractalDimension	Mean		.58219870890	.01958176151
	95% Confidence Interval for Mean	Lower Bound	.54255750518	
		Upper Bound	.62183991261	
	5% Trimmed Mean		.58791927620	
	Median		.61648085668	
	Variance		.015	
	Std. Deviation		.12228806142	
	Minimum		.25793034453	
	Maximum		.75996456873	
	Range		.50203422420	
	Interquartile Range		.15928261696	
	Skewness		-.827	.378
	Kurtosis		-.039	.741
LempelZivComplexity	Mean		.25858882532	.00719034309
	95% Confidence Interval for Mean	Lower Bound	.24403273673	
		Upper Bound	.27314491391	
	5% Trimmed Mean		.25495798612	
	Median		.25579147647	
	Variance		.002	
	Std. Deviation		.04490367821	
	Minimum		.19280650549	
	Maximum		.39780132129	
	Range		.20499481581	
	Interquartile Range		.04240491990	
	Skewness		1.299	.378
	Kurtosis		1.934	.741
RenyiEntropy	Mean		7.6507998245	.16294405813
	95% Confidence Interval for Mean	Lower Bound	7.3209368242	
		Upper Bound	7.9806628249	
	5% Trimmed Mean		7.6929031990	
	Median		7.9487284530	
	Variance		1.035	
	Std. Deviation		1.0175853169	
	Minimum		5.6105113448	
	Maximum		8.8187870344	
	Range		3.2082756895	
	Interquartile Range		1.8229082603	
	Skewness		-.748	.378
	Kurtosis		-.867	.741

Descriptives

			Statistic	Std. Error
TsallisEntropy	Mean		1.6820950102	.08096114529
	95% Confidence Interval for Mean	Lower Bound	1.5181977402	
		Upper Bound	1.8459922802	
	5% Trimmed Mean		1.7295448546	
	Median		1.9538130769	
	Variance		.256	
	Std. Deviation		.50560219030	
	Minimum		.51276887093	
	Maximum		1.9864854002	
	Range		1.4737165293	
	Interquartile Range		.31267609851	
	Skewness		-1.547	.378
	Kurtosis		.818	.741
PS_4_5	Mean		4.66444	.593429
	95% Confidence Interval for Mean	Lower Bound	3.46310	
		Upper Bound	5.86577	
	5% Trimmed Mean		4.29335	
	Median		3.53100	
	Variance		13.734	
	Std. Deviation		3.705963	
	Minimum		.840	
	Maximum		18.658	
	Range		17.818	
	Interquartile Range		4.806	
	Skewness		1.803	.378
	Kurtosis		4.089	.741
PS_5_6	Mean		3.04621	.328826
	95% Confidence Interval for Mean	Lower Bound	2.38053	
		Upper Bound	3.71188	
	5% Trimmed Mean		2.88286	
	Median		2.34800	
	Variance		4.217	
	Std. Deviation		2.053517	
	Minimum		.448	
	Maximum		8.854	
	Range		8.406	
	Interquartile Range		2.206	
	Skewness		1.315	.378
	Kurtosis		1.248	.741

Descriptives

			Statistic	Std. Error
PS_6_7	Mean		2.33010	.246305
	95% Confidence Interval for Mean	Lower Bound	1.83148	
		Upper Bound	2.82872	
	5% Trimmed Mean		2.19871	
	Median		1.82700	
	Variance		2.366	
	Std. Deviation		1.538173	
	Minimum		.417	
	Maximum		7.130	
	Range		6.713	
	Interquartile Range		1.831	
	Skewness		1.347	.378
	Kurtosis		1.749	.741
PS_7_8	Mean		2.17674	.258072
	95% Confidence Interval for Mean	Lower Bound	1.65430	
		Upper Bound	2.69918	
	5% Trimmed Mean		2.06171	
	Median		1.45600	
	Variance		2.597	
	Std. Deviation		1.611659	
	Minimum		.306	
	Maximum		6.199	
	Range		5.893	
	Interquartile Range		1.636	
	Skewness		1.314	.378
	Kurtosis		.602	.741
PS_8_9	Mean		2.41974	.370975
	95% Confidence Interval for Mean	Lower Bound	1.66874	
		Upper Bound	3.17074	
	5% Trimmed Mean		2.11975	
	Median		1.55500	
	Variance		5.367	
	Std. Deviation		2.316735	
	Minimum		.288	
	Maximum		10.917	
	Range		10.629	
	Interquartile Range		.963	
	Skewness		2.323	.378
	Kurtosis		5.314	.741

Descriptives

			Statistic	Std. Error
PS_9_10	Mean		3.01805	.549702
	95% Confidence Interval for Mean	Lower Bound	1.90524	
		Upper Bound	4.13086	
	5% Trimmed Mean		2.51112	
	Median		2.10400	
	Variance		11.785	
	Std. Deviation		3.432887	
	Minimum		.235	
	Maximum		19.691	
	Range		19.456	
	Interquartile Range		1.394	
	Skewness		3.530	.378
	Kurtosis		14.800	.741
PS_10_11	Mean		4.15523	.786450
	95% Confidence Interval for Mean	Lower Bound	2.56315	
		Upper Bound	5.74732	
	5% Trimmed Mean		3.44191	
	Median		2.65900	
	Variance		24.122	
	Std. Deviation		4.911381	
	Minimum		.253	
	Maximum		22.961	
	Range		22.708	
	Interquartile Range		2.699	
	Skewness		2.693	.378
	Kurtosis		7.161	.741
PS_11_12	Mean		1.75608	.265666
	95% Confidence Interval for Mean	Lower Bound	1.21826	
		Upper Bound	2.29389	
	5% Trimmed Mean		1.54335	
	Median		1.41700	
	Variance		2.753	
	Std. Deviation		1.659085	
	Minimum		.226	
	Maximum		10.048	
	Range		9.822	
	Interquartile Range		1.237	
	Skewness		3.525	.378
	Kurtosis		16.370	.741

Descriptives

			Statistic	Std. Error
PS_12_13	Mean		1.16872	.124525
	95% Confidence Interval for Mean	Lower Bound	.91663	
		Upper Bound	1.42081	
	5% Trimmed Mean		1.08033	
	Median		.94100	
	Variance		.605	
	Std. Deviation		.777661	
	Minimum		.193	
	Maximum		3.770	
	Range		3.577	
	Interquartile Range		.823	
	Skewness		1.864	.378
	Kurtosis		4.341	.741
PS_13_14	Mean		1.03851	.123424
	95% Confidence Interval for Mean	Lower Bound	.78865	
		Upper Bound	1.28837	
	5% Trimmed Mean		.96400	
	Median		.84300	
	Variance		.594	
	Std. Deviation		.770780	
	Minimum		.190	
	Maximum		3.333	
	Range		3.143	
	Interquartile Range		.697	
	Skewness		1.707	.378
	Kurtosis		2.681	.741
PS_14_15	Mean		.92821	.119853
	95% Confidence Interval for Mean	Lower Bound	.68558	
		Upper Bound	1.17083	
	5% Trimmed Mean		.83839	
	Median		.68300	
	Variance		.560	
	Std. Deviation		.748479	
	Minimum		.177	
	Maximum		4.004	
	Range		3.827	
	Interquartile Range		.852	
	Skewness		2.251	.378
	Kurtosis		6.737	.741

Descriptives

			Statistic	Std. Error
PS_15_16	Mean		.78477	.089045
	95% Confidence Interval for Mean	Lower Bound	.60451	
		Upper Bound	.96503	
	5% Trimmed Mean		.72793	
	Median		.54700	
	Variance		.309	
	Std. Deviation		.556087	
	Minimum		.163	
	Maximum		2.589	
	Range		2.426	
	Interquartile Range		.697	
	Skewness		1.536	.378
	Kurtosis		2.337	.741
PS_16_17	Mean		.62387	.066308
	95% Confidence Interval for Mean	Lower Bound	.48964	
		Upper Bound	.75811	
	5% Trimmed Mean		.58782	
	Median		.47300	
	Variance		.171	
	Std. Deviation		.414095	
	Minimum		.162	
	Maximum		1.720	
	Range		1.558	
	Interquartile Range		.377	
	Skewness		1.379	.378
	Kurtosis		1.176	.741
PS_17_18	Mean		.49695	.051069
	95% Confidence Interval for Mean	Lower Bound	.39357	
		Upper Bound	.60033	
	5% Trimmed Mean		.47040	
	Median		.38300	
	Variance		.102	
	Std. Deviation		.318924	
	Minimum		.151	
	Maximum		1.350	
	Range		1.199	
	Interquartile Range		.266	
	Skewness		1.389	.378
	Kurtosis		1.066	.741

Descriptives

			Statistic	Std. Error
PS_18_19	Mean		.40749	.039789
	95% Confidence Interval for Mean	Lower Bound	.32694	
		Upper Bound	.48804	
	5% Trimmed Mean		.38788	
	Median		.32600	
	Variance		.062	
	Std. Deviation		.248483	
	Minimum		.131	
	Maximum		1.068	
	Range		.937	
	Interquartile Range		.286	
	Skewness		1.283	.378
	Kurtosis		.689	.741
PS_19_20	Mean		.36182	.033635
	95% Confidence Interval for Mean	Lower Bound	.29373	
		Upper Bound	.42991	
	5% Trimmed Mean		.34188	
	Median		.29400	
	Variance		.044	
	Std. Deviation		.210048	
	Minimum		.123	
	Maximum		1.020	
	Range		.897	
	Interquartile Range		.253	
	Skewness		1.418	.378
	Kurtosis		1.760	.741

Tests of Normality

	Kolmogorov-Smirnov[a]			Shapiro-Wilk		
	Statistic	df	Sig.	Statistic	df	Sig.
BrainRate	.156	39	.018	.869	39	<.001
PermutationEntropy	.120	39	.171	.972	39	.439
PermutationEntropy_01	.120	39	.171	.972	39	.439
SampleEntropy	.135	39	.072	.962	39	.215
SpectralEntropy_bits	.119	39	.182	.950	39	.084
SpectralEntropy_01	.119	39	.182	.950	39	.084
HiguchiFractalDimension	.161	39	.012	.926	39	.014
LempelZivComplexity	.143	39	.044	.903	39	.003
RenyiEntropy	.176	39	.004	.867	39	<.001
TsallisEntropy	.320	39	<.001	.636	39	<.001
PS_4_5	.191	39	<.001	.819	39	<.001
PS_5_6	.194	39	<.001	.868	39	<.001
PS_6_7	.171	39	.006	.883	39	<.001
PS_7_8	.208	39	<.001	.816	39	<.001
PS_8_9	.307	39	<.001	.692	39	<.001
PS_9_10	.304	39	<.001	.584	39	<.001
PS_10_11	.275	39	<.001	.633	39	<.001
PS_11_12	.234	39	<.001	.657	39	<.001
PS_12_13	.179	39	.003	.828	39	<.001
PS_13_14	.219	39	<.001	.812	39	<.001
PS_14_15	.223	39	<.001	.784	39	<.001
PS_15_16	.206	39	<.001	.843	39	<.001
PS_16_17	.189	39	.001	.840	39	<.001
PS_17_18	.242	39	<.001	.826	39	<.001
PS_18_19	.215	39	<.001	.839	39	<.001
PS_19_20	.180	39	.003	.859	39	<.001

a. Lilliefors Significance Correction

BrainRate

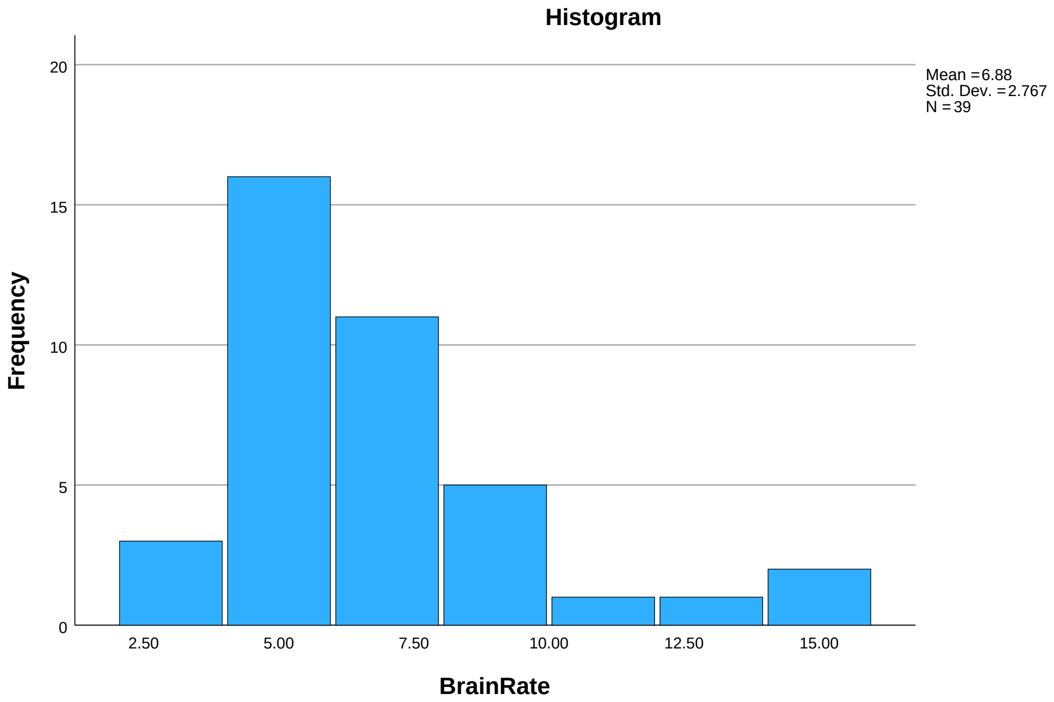


```
BrainRate Stem-and-Leaf Plot

 Frequency    Stem &  Leaf

     1.00        2 .  3
     2.00        3 .  67
     4.00        4 .  1689
    12.00        5 .  012333335688
     6.00        6 .  035789
     5.00        7 .  13447
     4.00        8 .  5688
     1.00        9 .  4
     4.00 Extremes    (>=11.6)

 Stem width:      1.00
 Each leaf:       1 case(s)
```

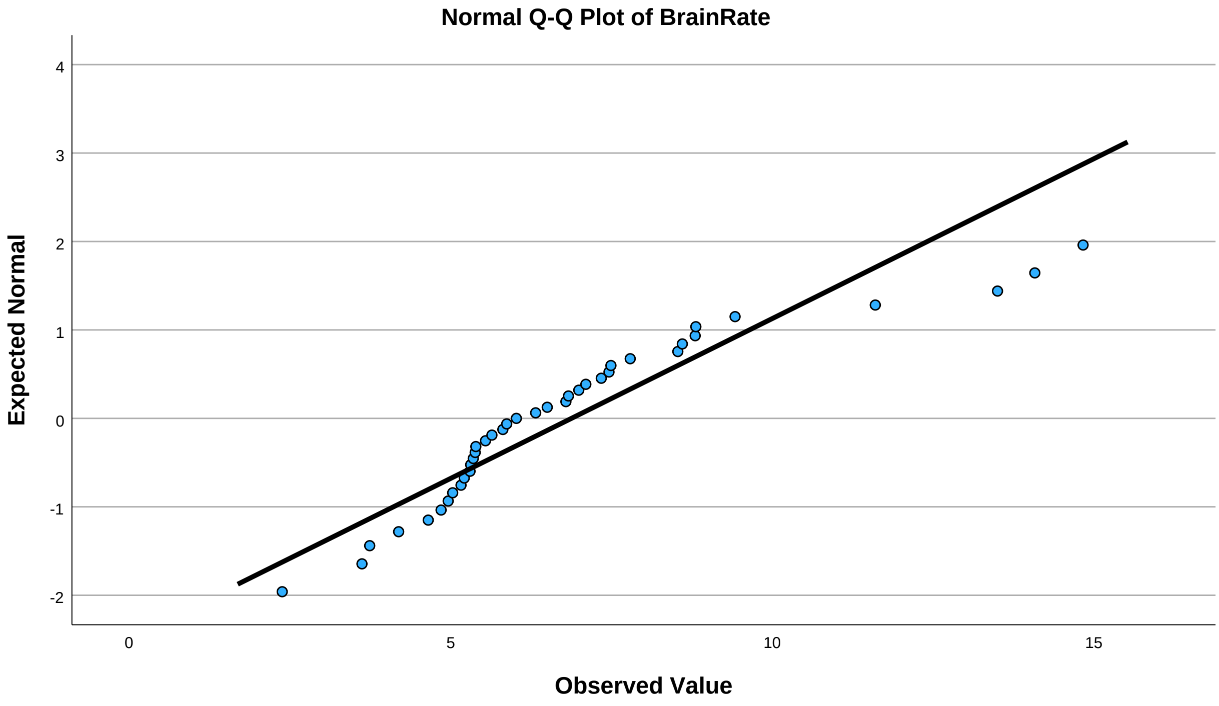
Normal Q-Q Plot of BrainRate
Expected Normal
4
3
2
1
0
-1
-2
0
5
10
15
Observed Value

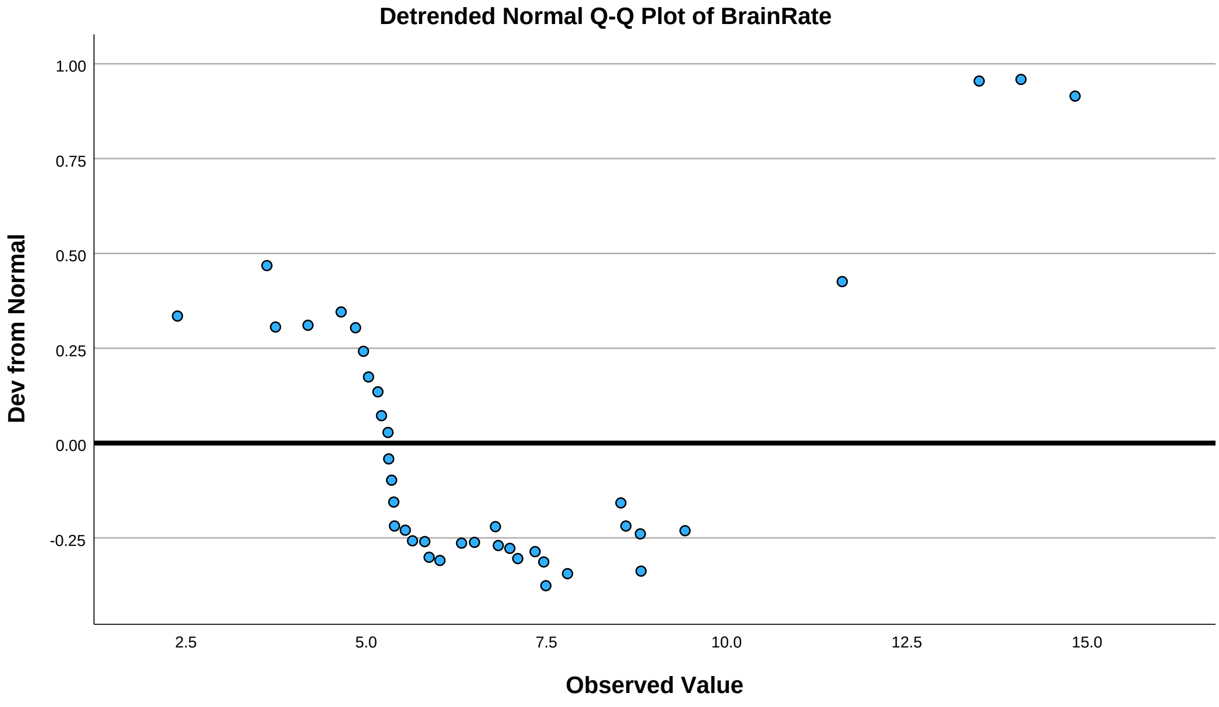
Detrended Normal Q-Q Plot of BrainRate
Dev from Normal
1.00
0.75
0.50
0.25
0.00
-0.25
2.5
5.0
7.5
10.0
12.5
15.0
Observed Value

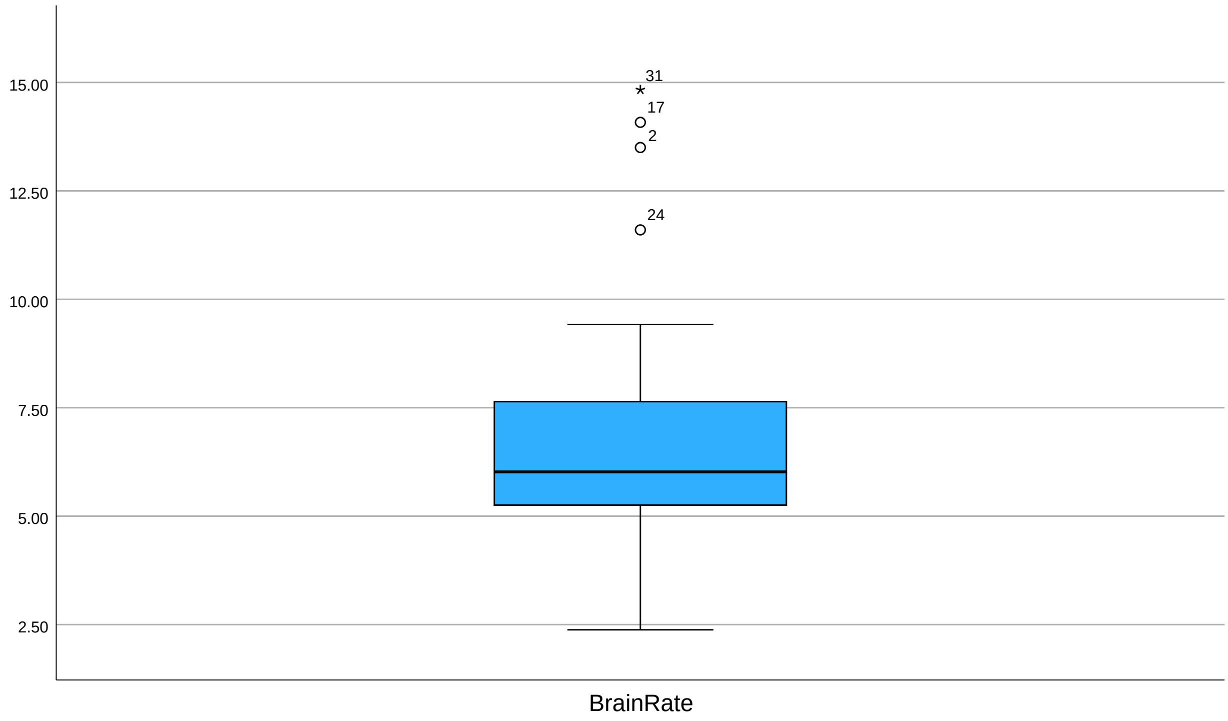

PermutationEntropy

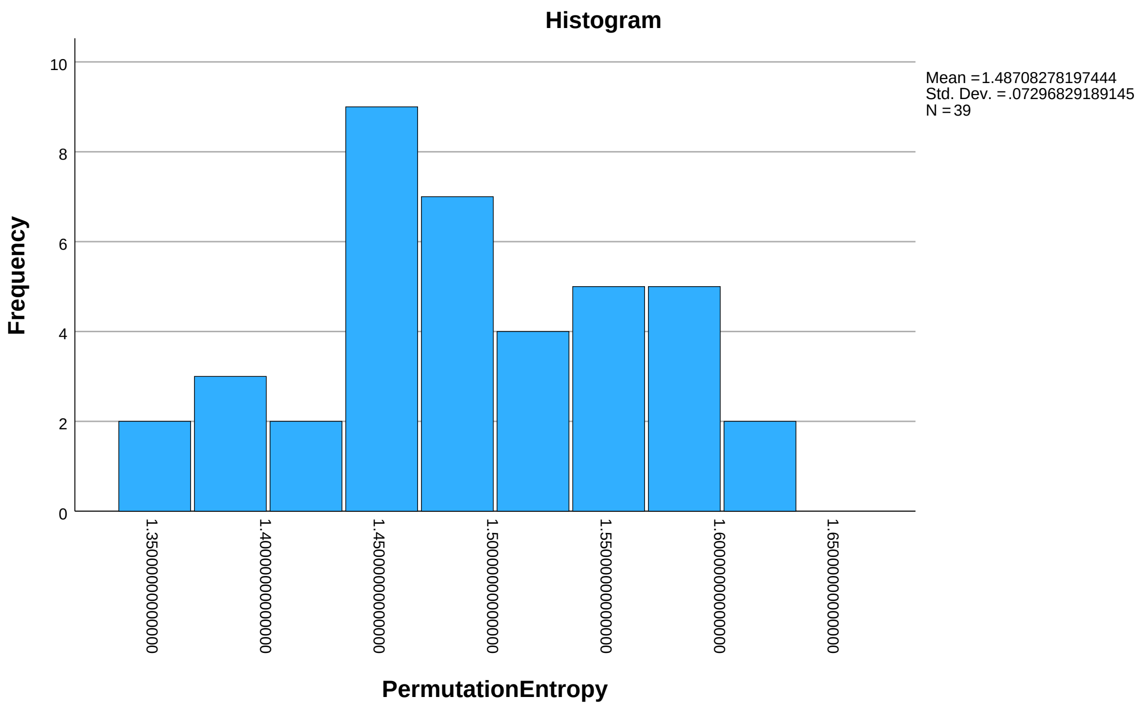

PermutationEntropy Stem-and-Leaf Plot

Frequency   Stem & Leaf

```
     2.00       13 .  33
     3.00       13 .  778
     7.00       14 .  2233444
    11.00       14 .  55566777779
     6.00       15 .  012344
     8.00       15 .  55666779
     2.00       16 .  12

 Stem width:   .1000000
 Each leaf:       1 case(s)
```

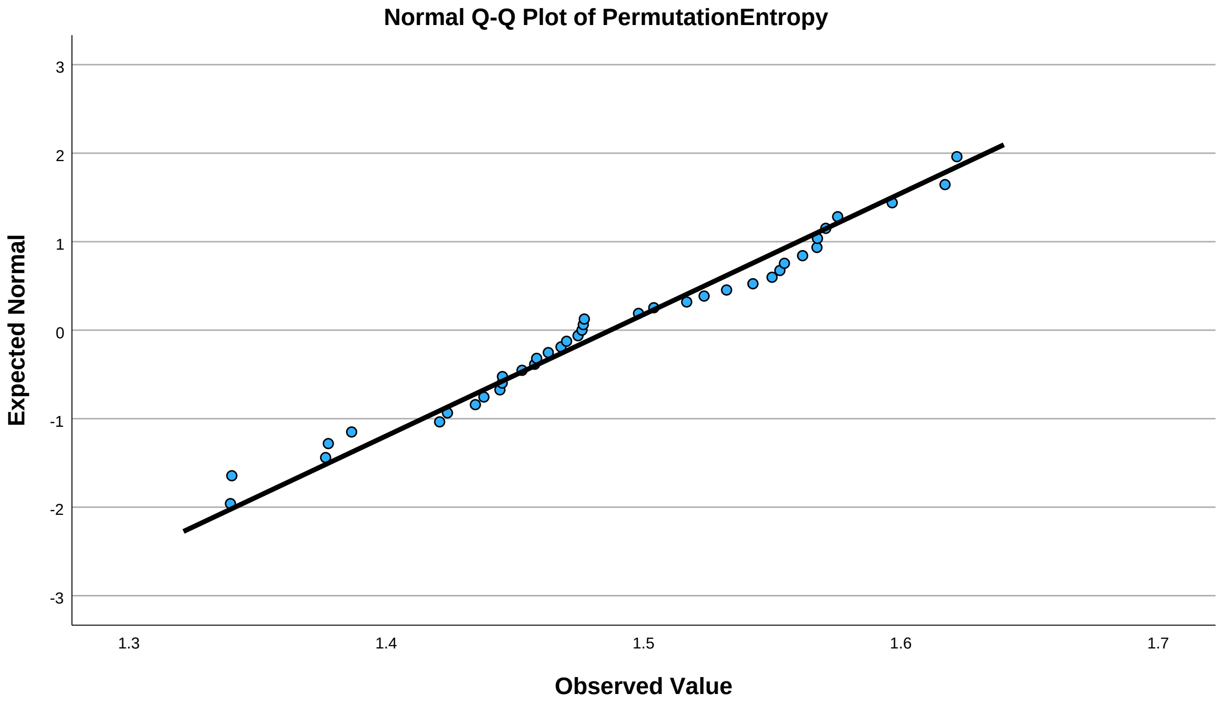

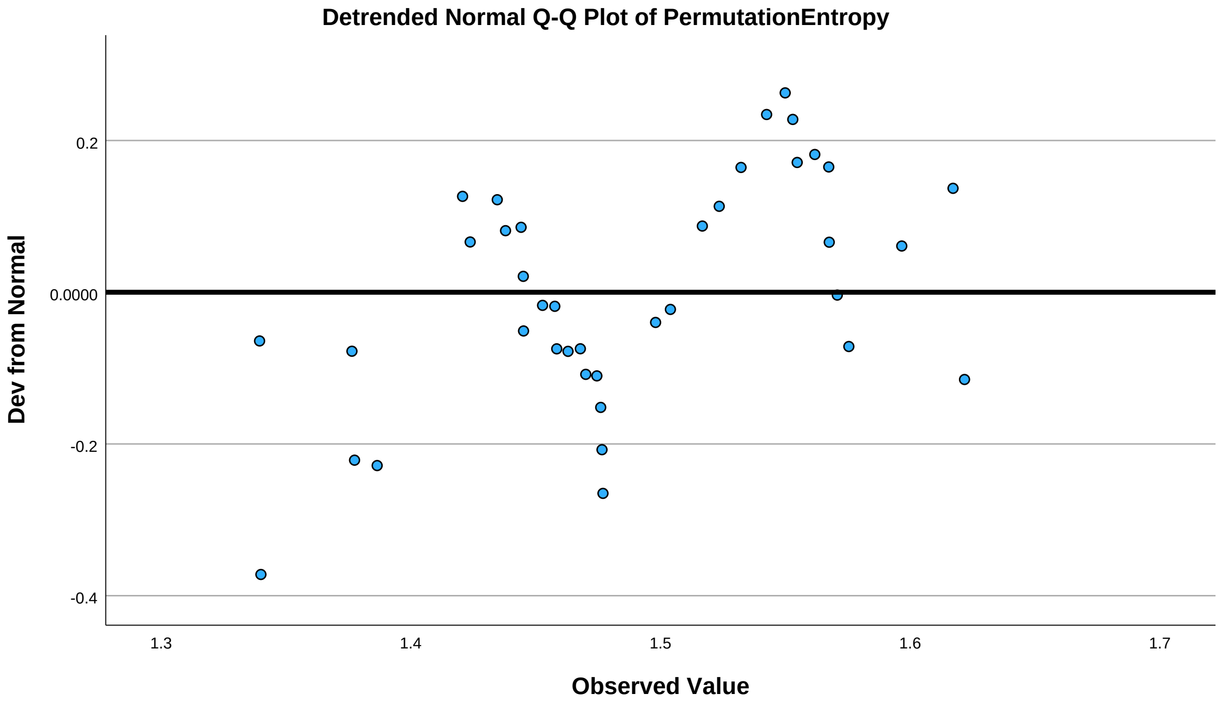

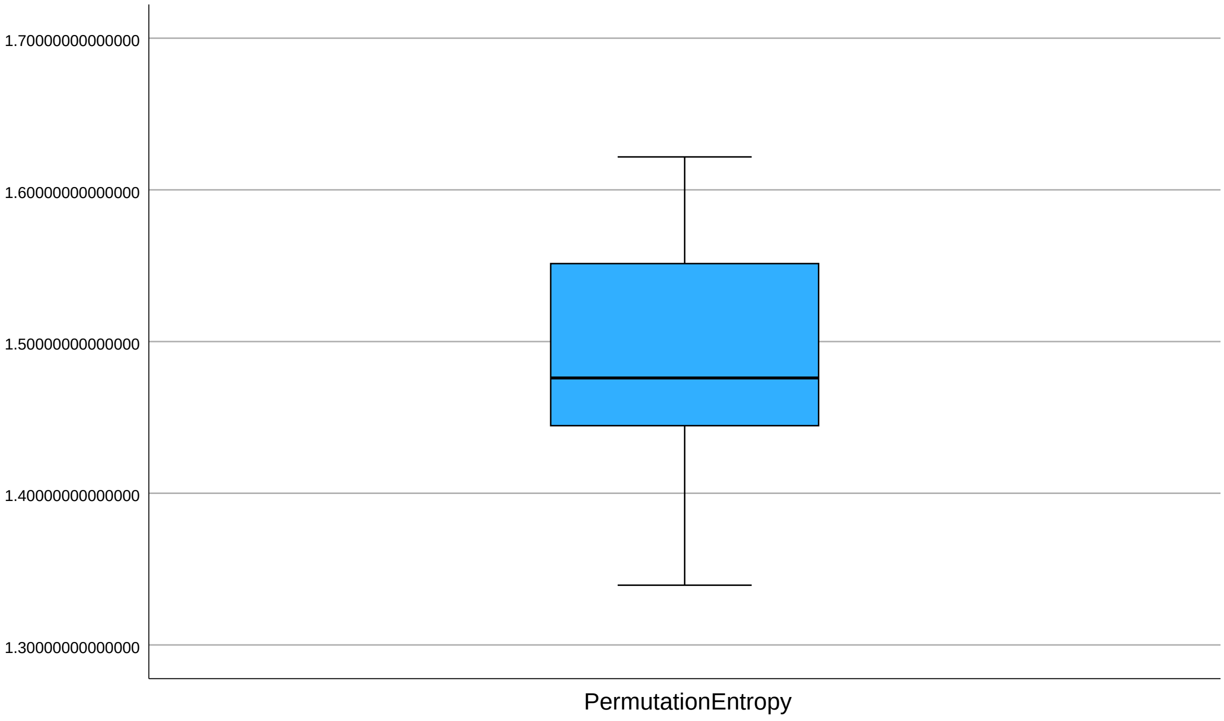

PermutationEntropy_01

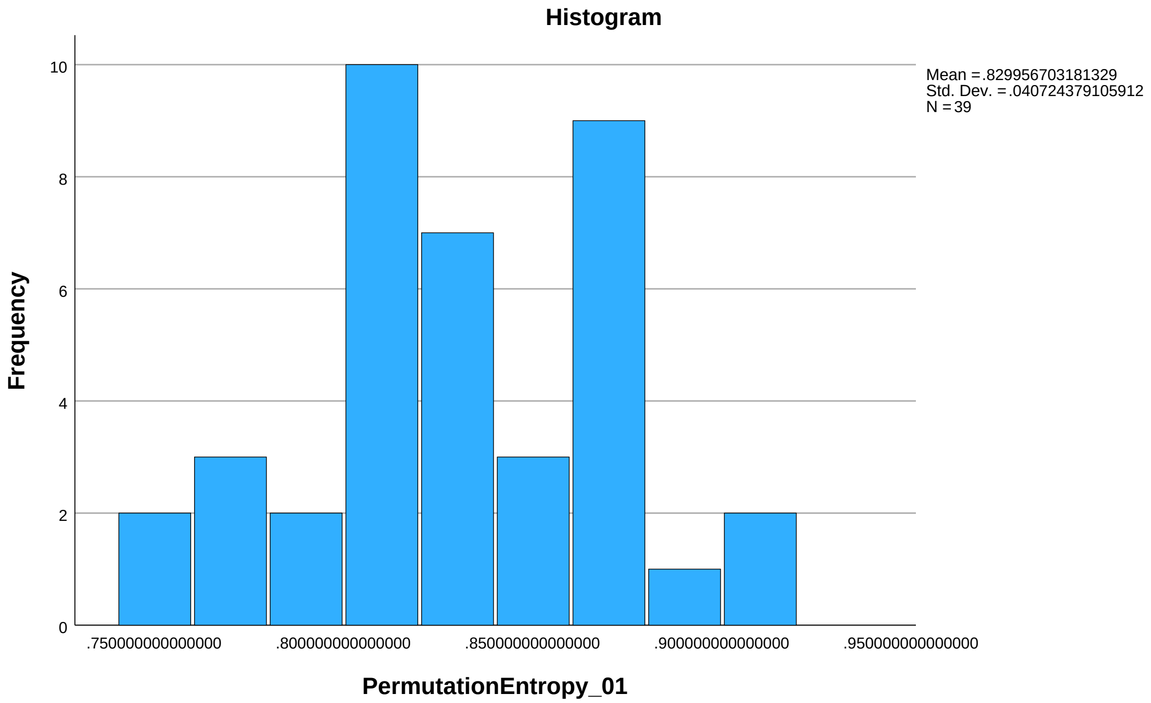


```
PermutationEntropy_01 Stem-and-Leaf Plot

 Frequency    Stem &  Leaf

    2.00       7 .  44
    5.00       7 .  66799
   18.00       8 .  000001111122222334
   12.00       8 .  556666777779
    2.00       9 .  00

 Stem width:   .1000000
 Each leaf:        1 case(s)
```

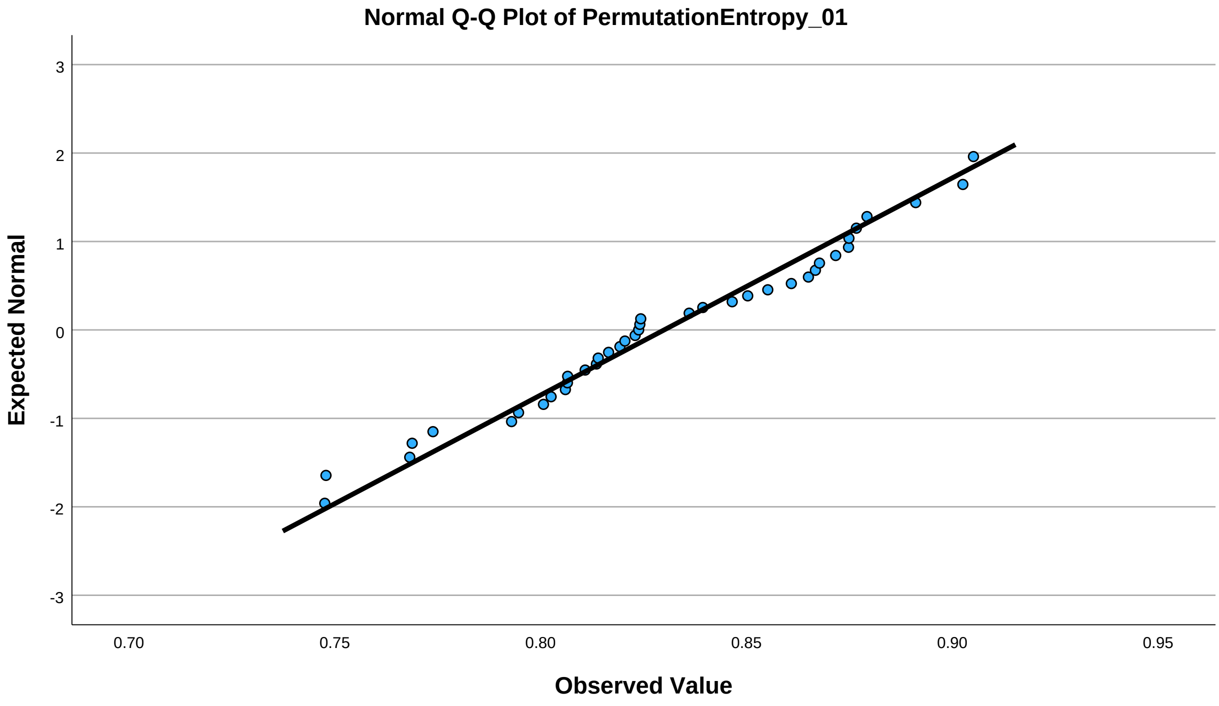
Normal Q-Q Plot of PermutationEntropy_01
Expected Normal
3
2
1
0
-1
-2
-3
0.70
0.75
0.80
0.85
0.90
0.95
Observed Value

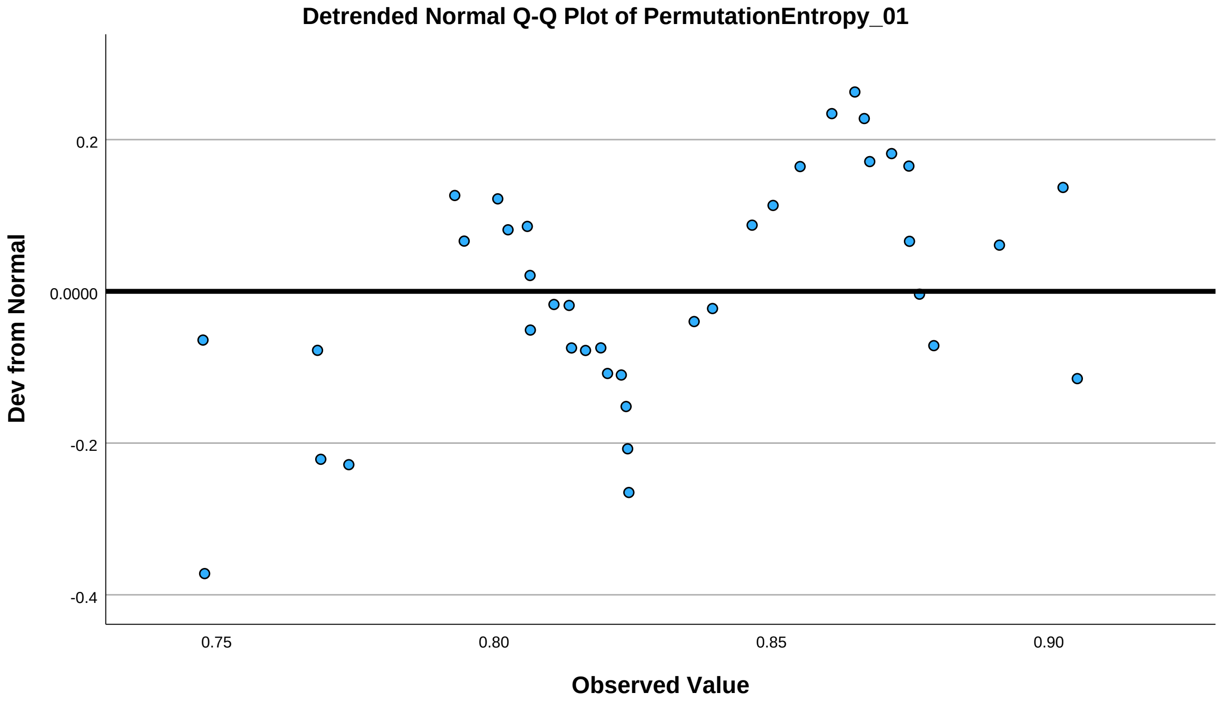
Detrended Normal Q-Q Plot of PermutationEntropy_01
Dev from Normal
0.2
0.0000
-0.2
-0.4
0.75
0.80
0.85
0.90
Observed Value

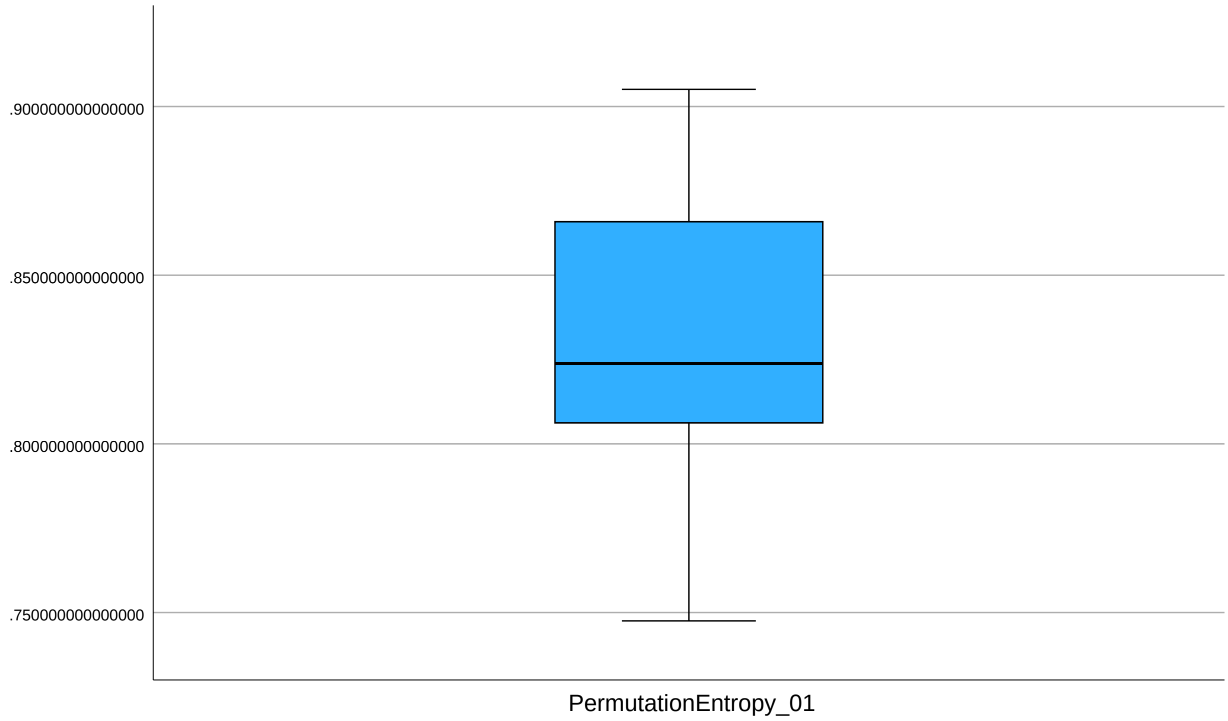

SampleEntropy

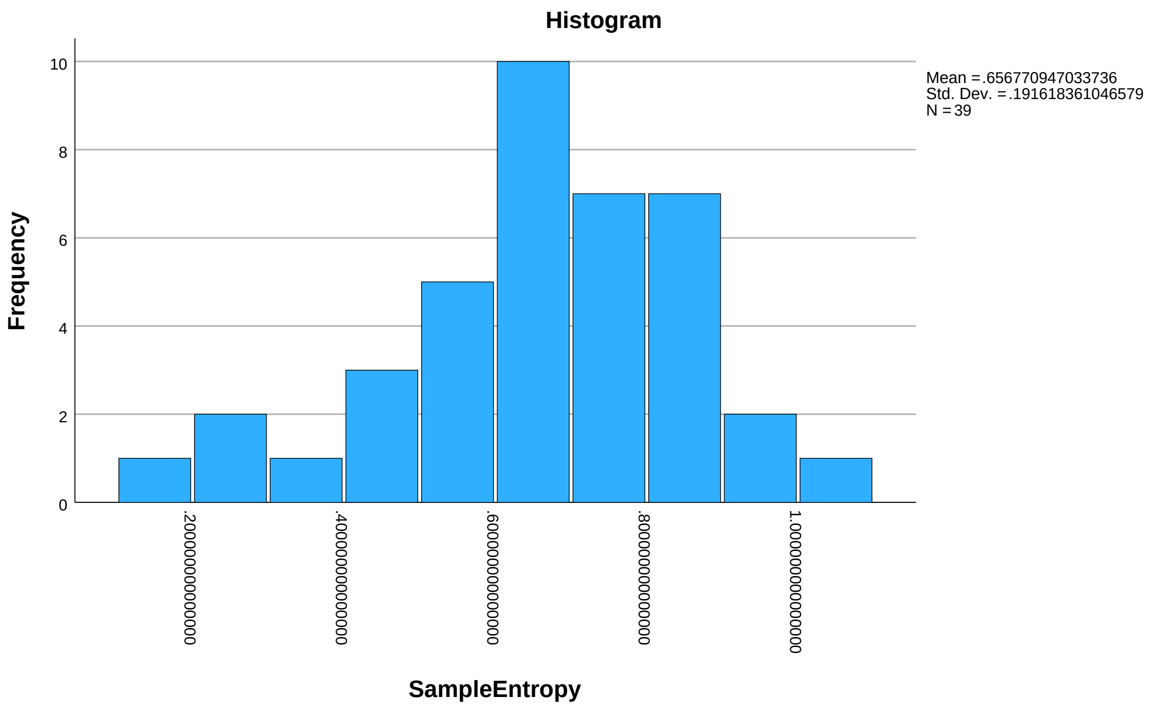


```
SampleEntropy Stem-and-Leaf Plot

 Frequency    Stem &  Leaf
```

```
 3.00 Extremes    (=<.25)
 1.00     3 . 7
 3.00     4 . 477
 5.00     5 . 66789
10.00     6 . 0122224458
 7.00     7 . 0123478
 7.00     8 . 0013669
 2.00     9 . 05
 1.00    10 . 1

Stem width:  .1000000
Each leaf:      1 case(s)
```

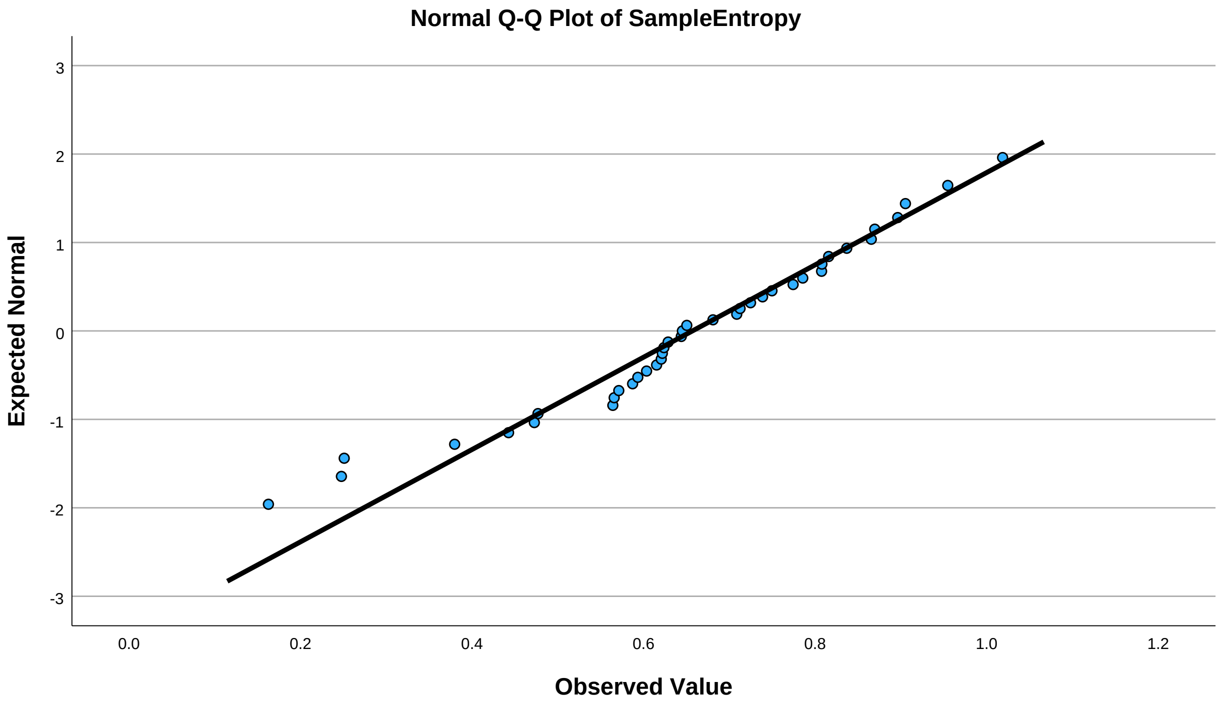

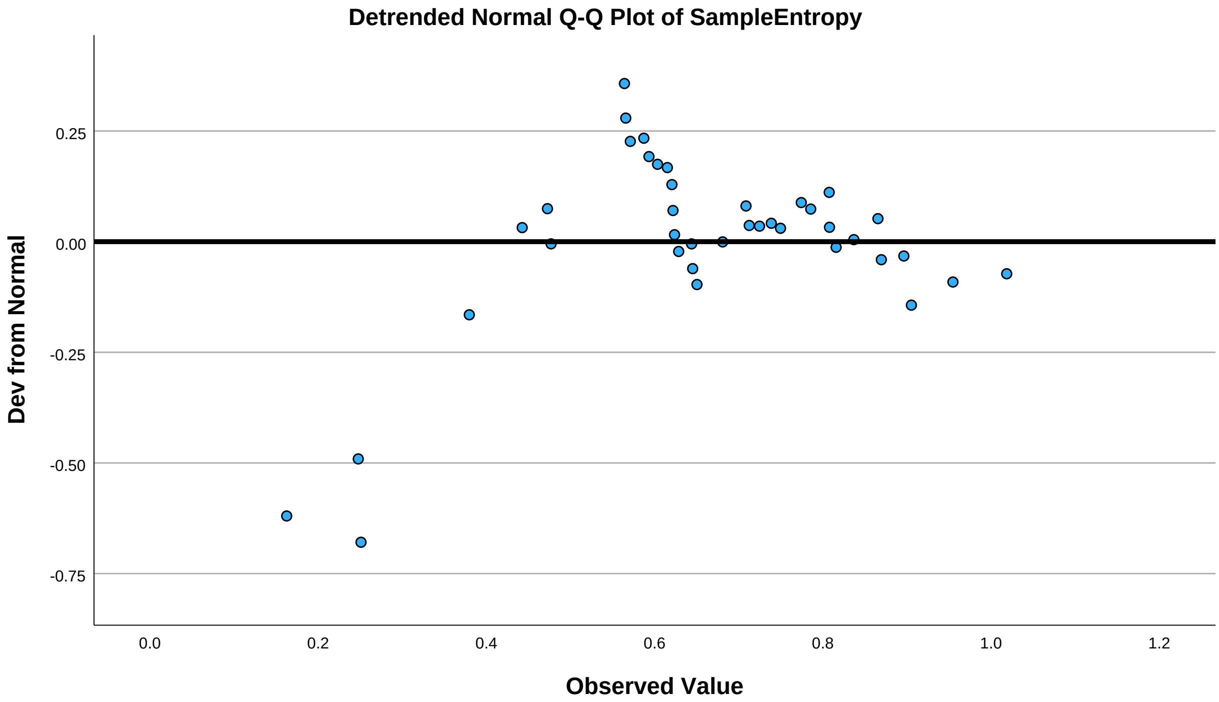

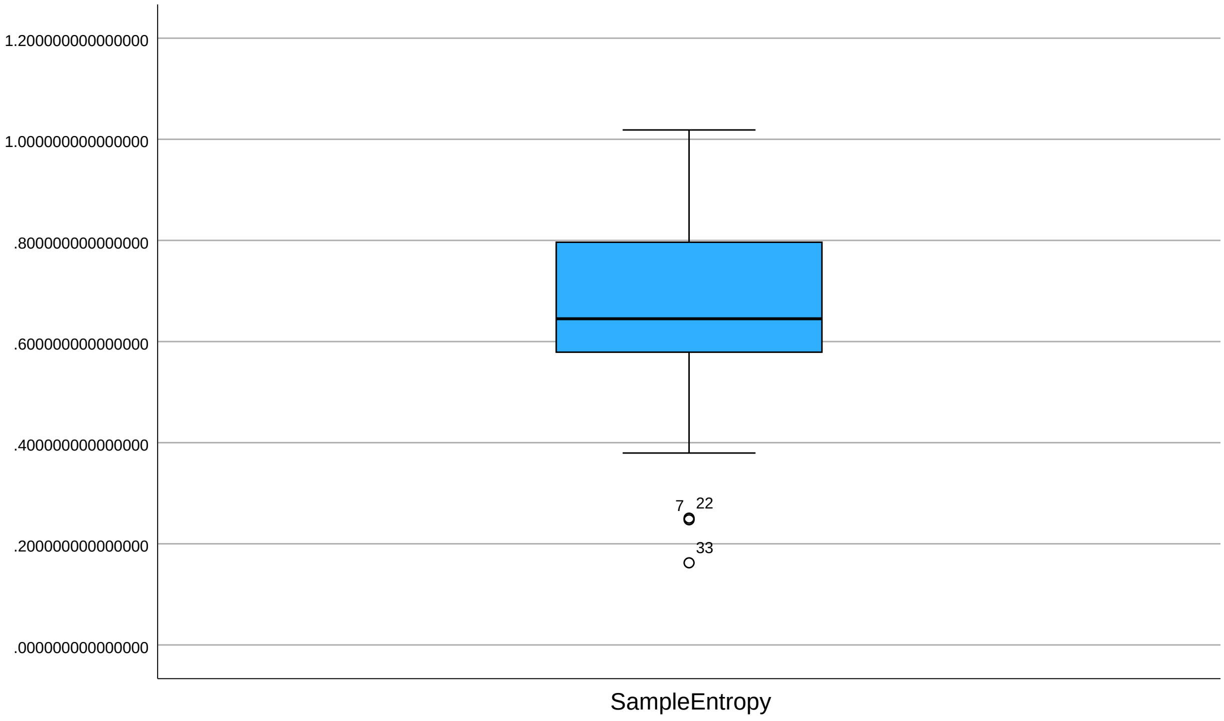

SpectralEntropy_bits

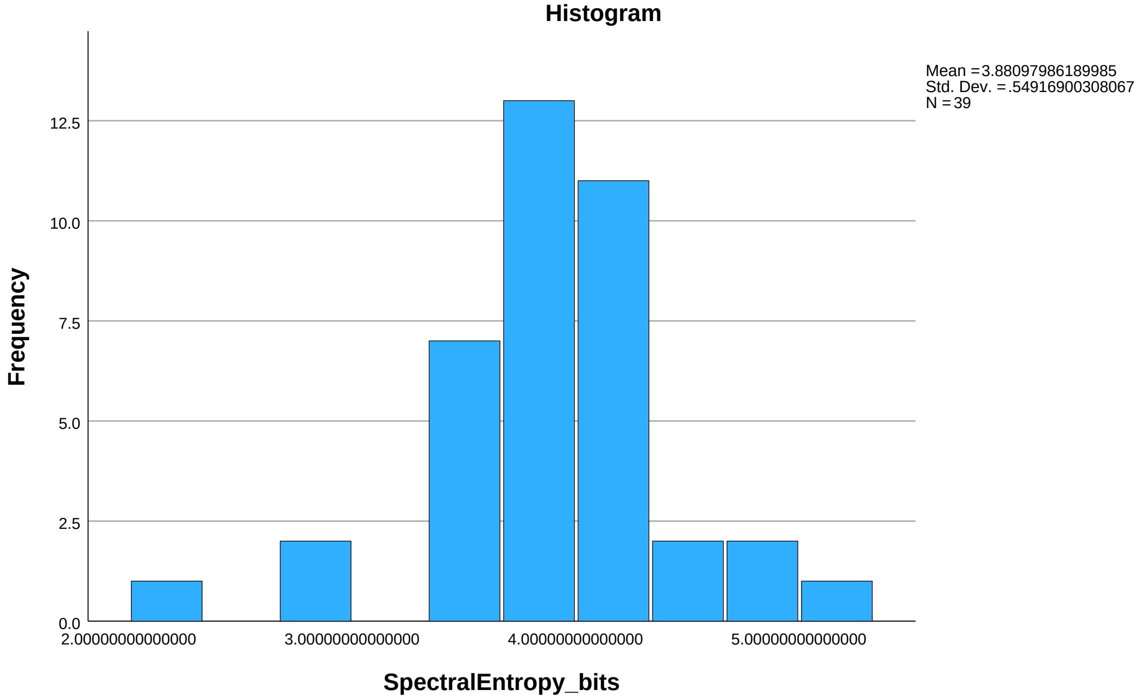


```
SpectralEntropy_bits Stem-and-Leaf Plot

 Frequency    Stem &  Leaf

     2.00 Extremes    (=<2.8)
     1.00        2 .  9
     2.00        3 .  33
    18.00        3 .  555666666777778999
    12.00        4 .  000001122333
     2.00        4 .  67
     2.00 Extremes    (>=5.0)

 Stem width:   1.000000
 Each leaf:        1 case(s)
```

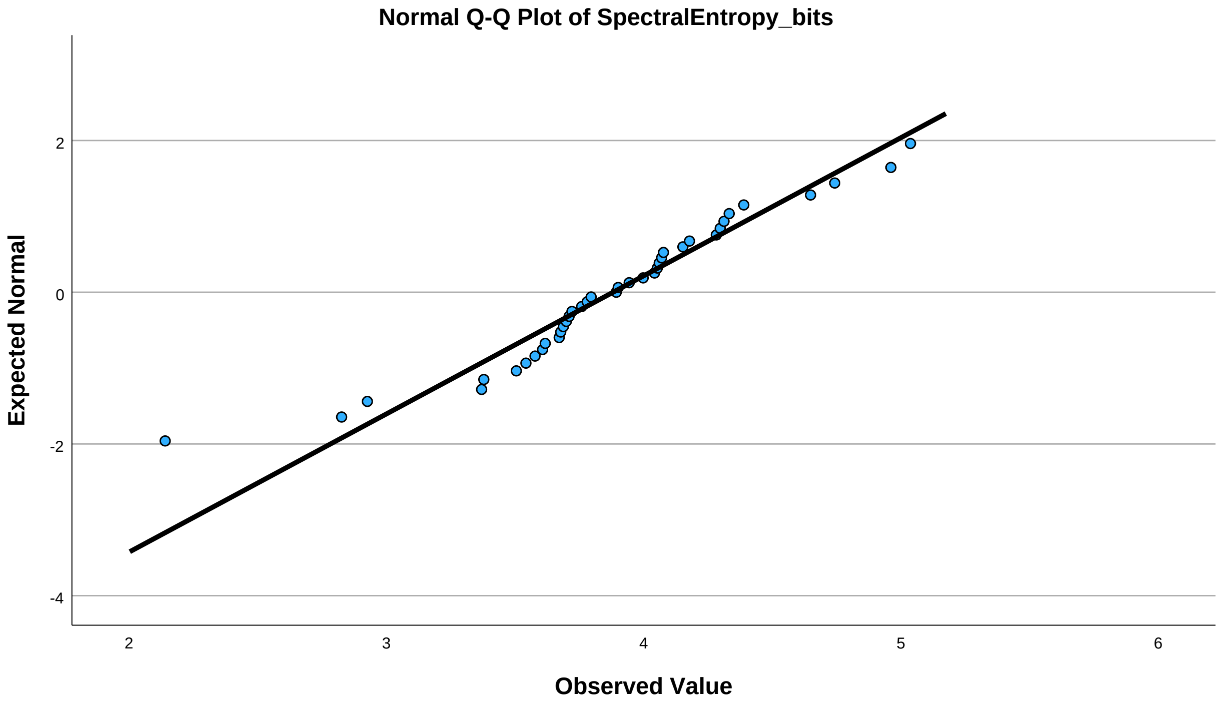
Normal Q-Q Plot of SpectralEntropy_bits
Expected Normal
Observed Value
2
0
-2
-4
2
3
4
5
6

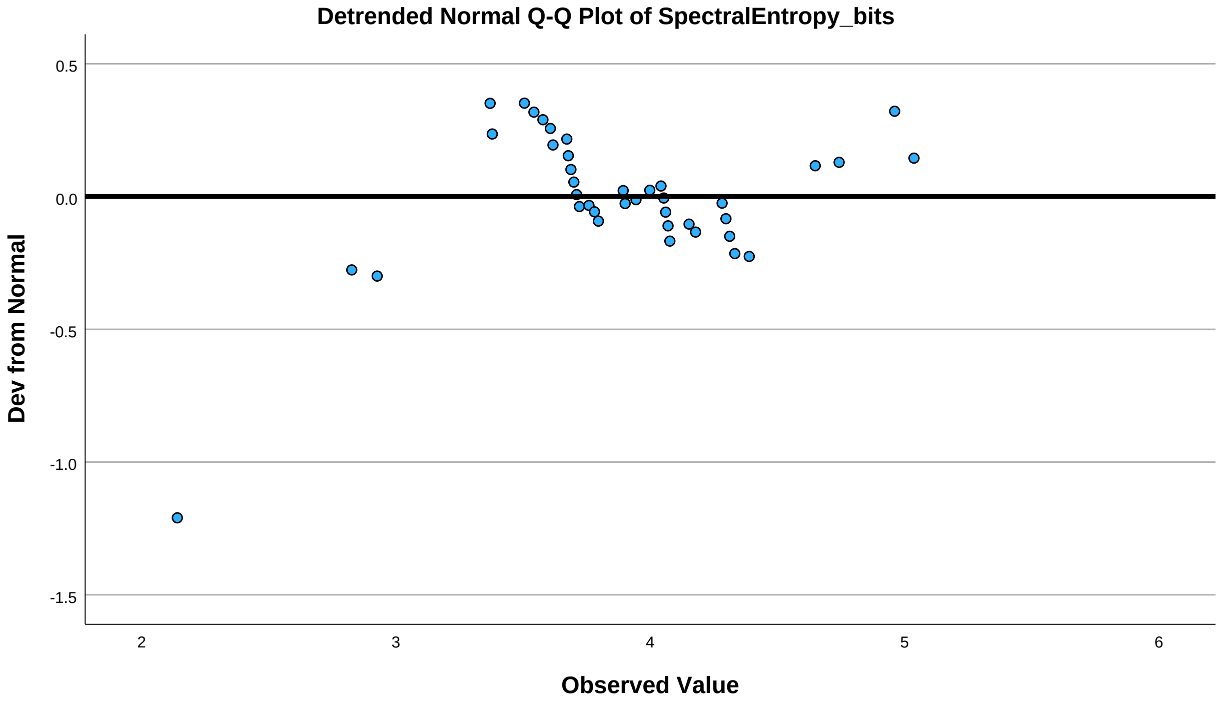
Detrended Normal Q-Q Plot of SpectralEntropy_bits
Dev from Normal
Observed Value
0.5
0.0
-0.5
-1.0
-1.5
2
3
4
5
6

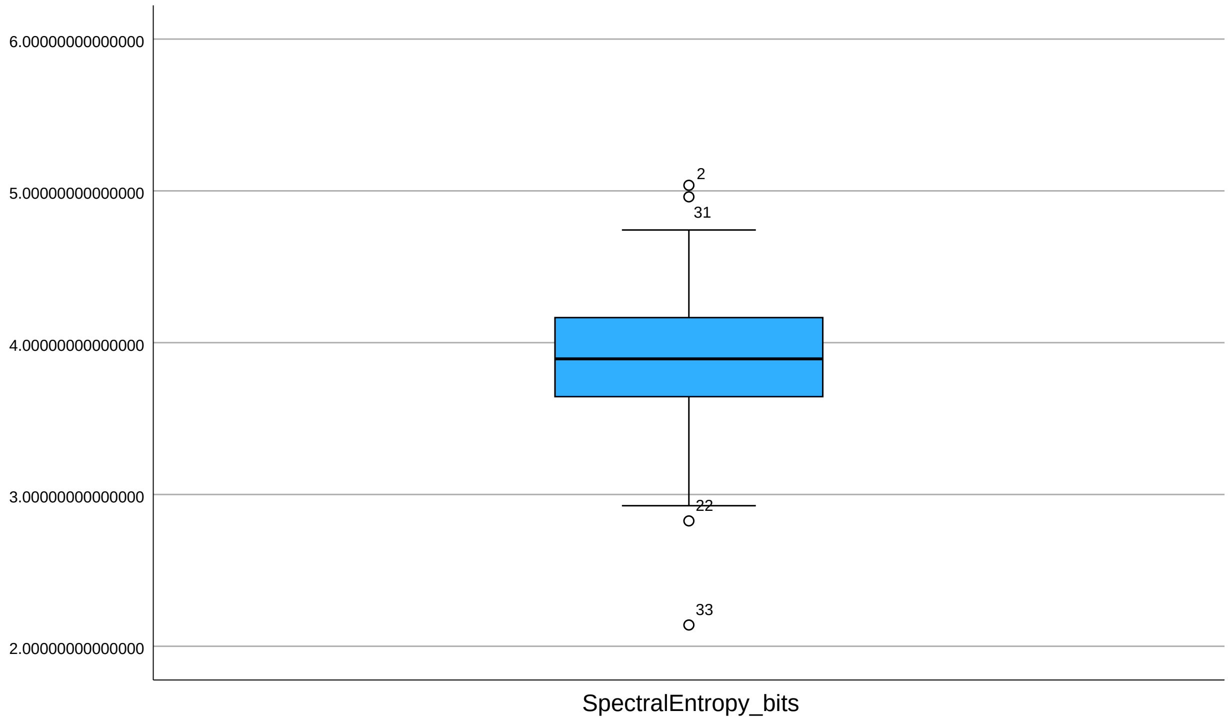

SpectralEntropy_01

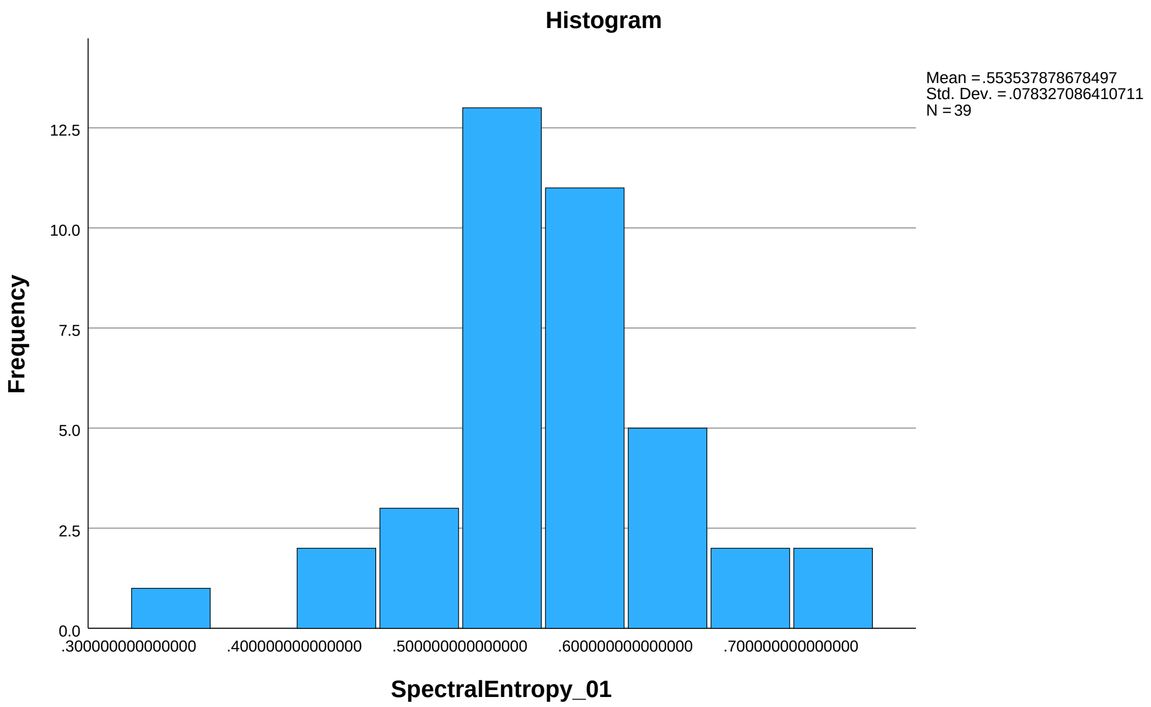


```
SpectralEntropy_01 Stem-and-Leaf Plot

 Frequency    Stem &  Leaf
```

```
 2.00 Extremes    (=<.40)
 1.00      4 .  1
 3.00      4 .  889
13.00      5 .  0111222223334
11.00      5 .  55677778899
 5.00      6 .  11112
 2.00      6 .  67
 2.00 Extremes    (>=.71)

Stem width:   .1000000
Each leaf:       1 case(s)
```

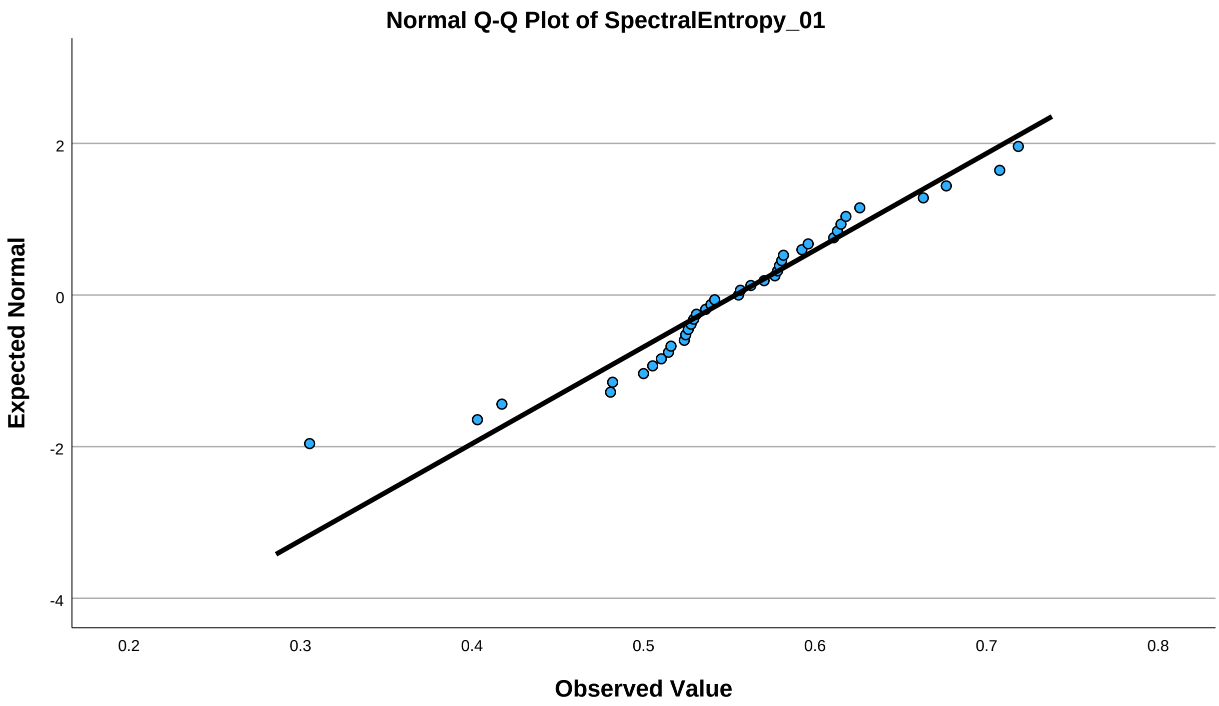

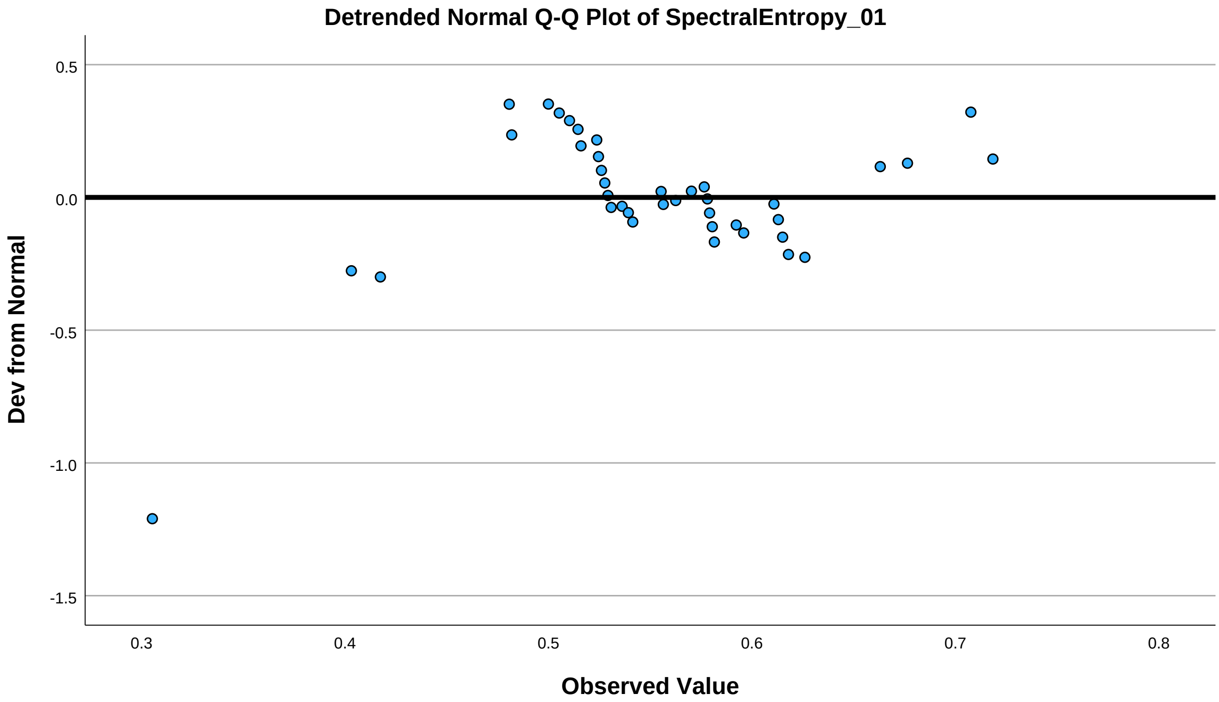

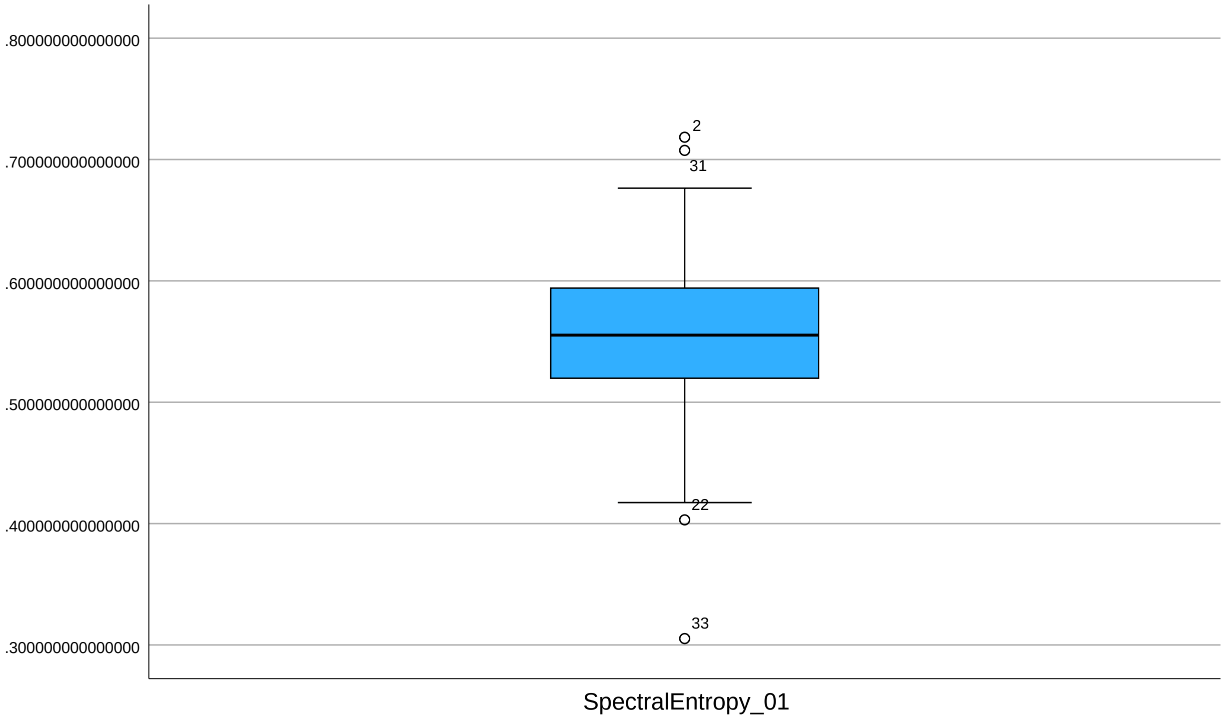

HiguchiFractalDimension

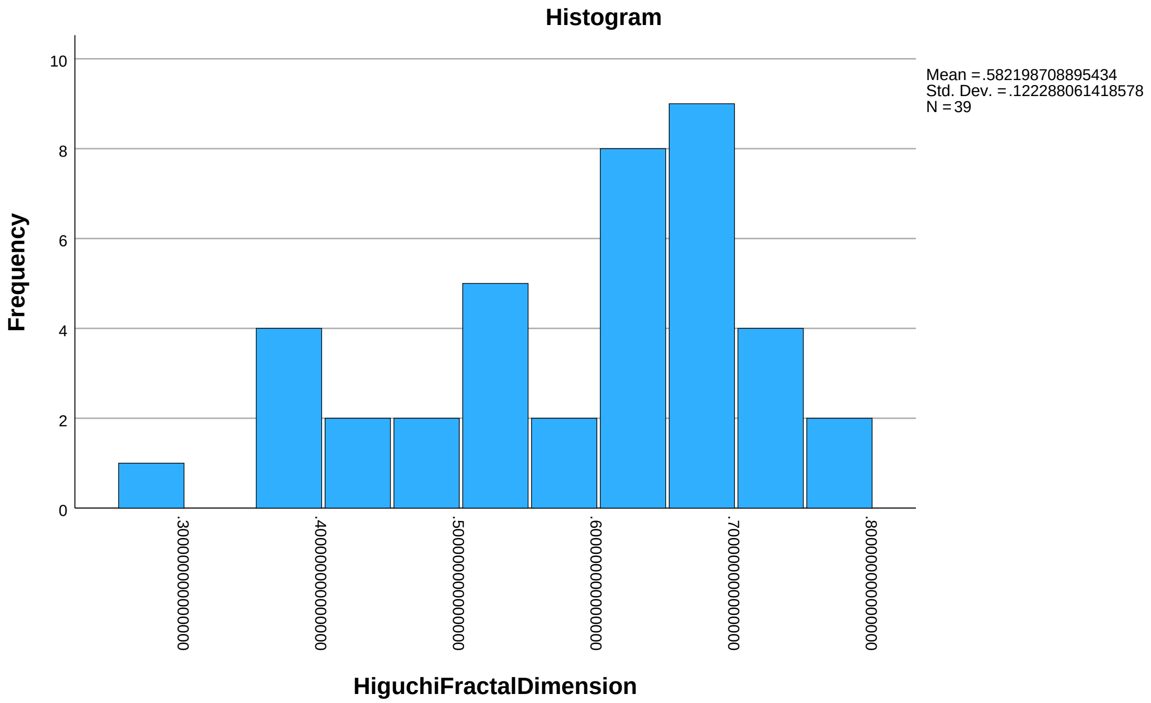


```
HiguchiFractalDimension Stem-and-Leaf Plot

 Frequency    Stem &  Leaf

     1.00 Extremes    (=<.26)
     4.00        3 .  5888
     2.00        4 .  14
     2.00        4 .  78
     5.00        5 .  01144
     2.00        5 .  58
     8.00        6 .  01111344
     9.00        6 .  555666779
     4.00        7 .  0001
     2.00        7 .  55

 Stem width:   .1000000
 Each leaf:       1 case(s)
```

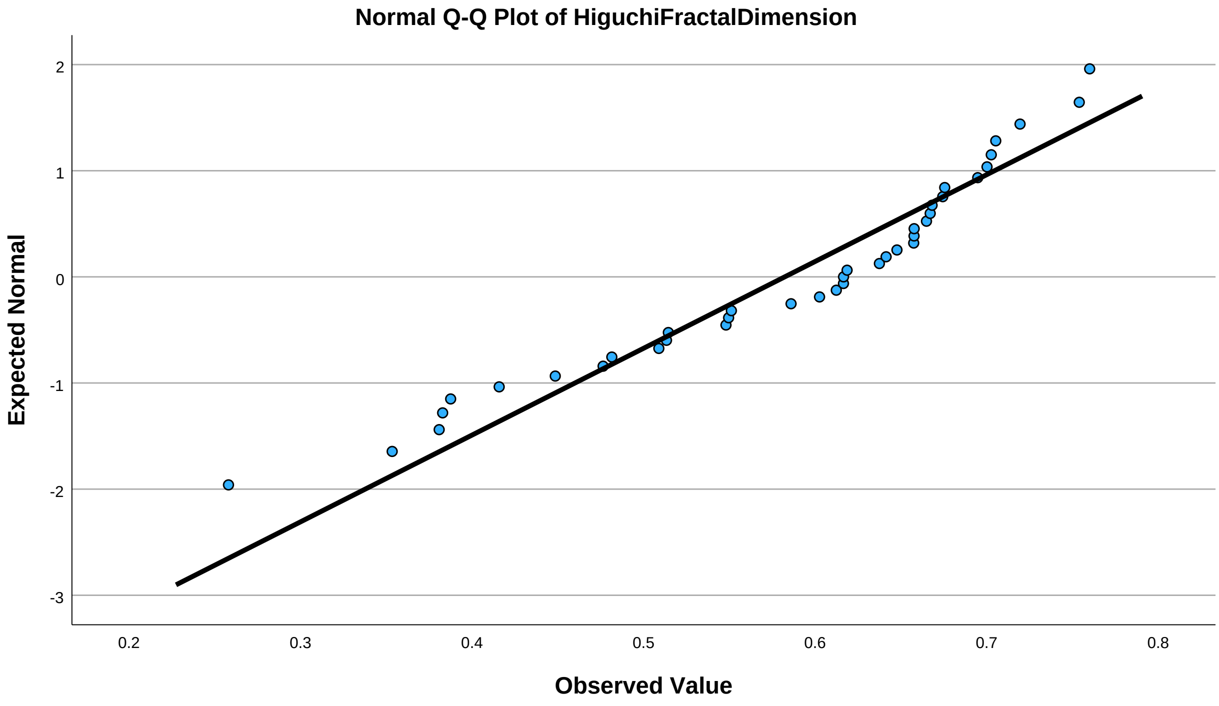
Normal Q-Q Plot of HiguchiFractalDimension
Expected Normal
2
1
0
-1
-2
-3
0.2
0.3
0.4
0.5
0.6
0.7
0.8
Observed Value

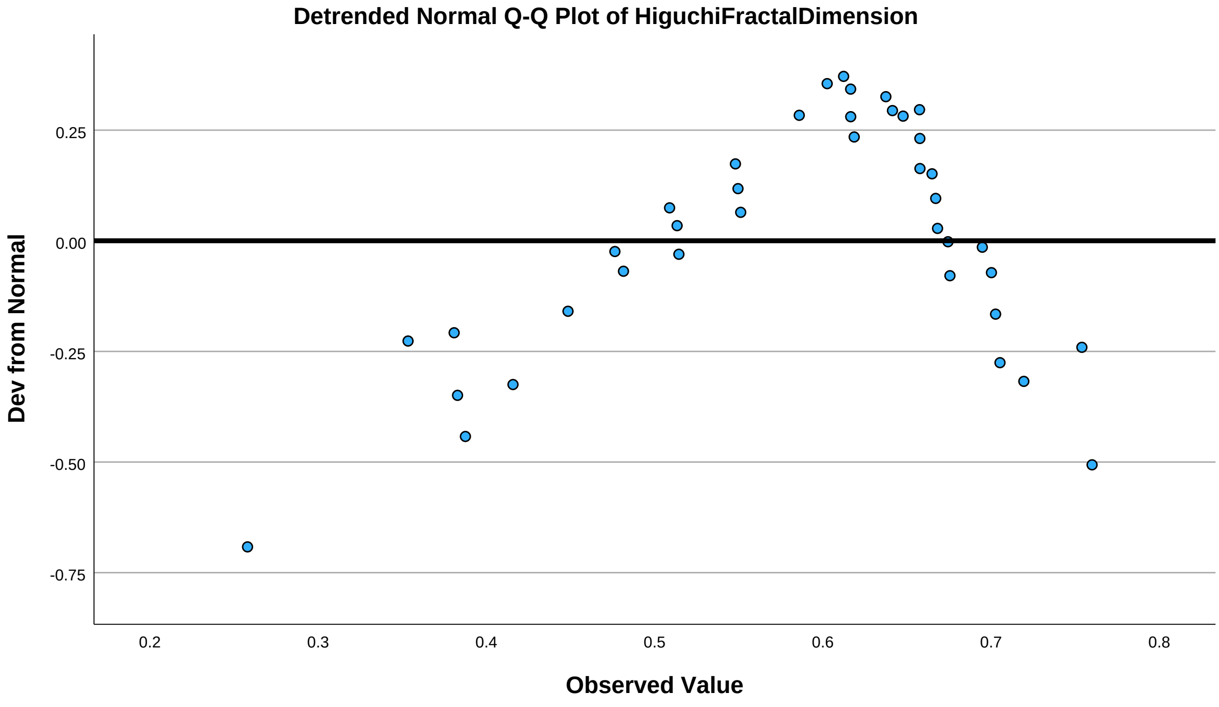
Detrended Normal Q-Q Plot of HiguchiFractalDimension
Dev from Normal
0.25
0.00
-0.25
-0.50
-0.75
0.2
0.3
0.4
0.5
0.6
0.7
0.8
Observed Value

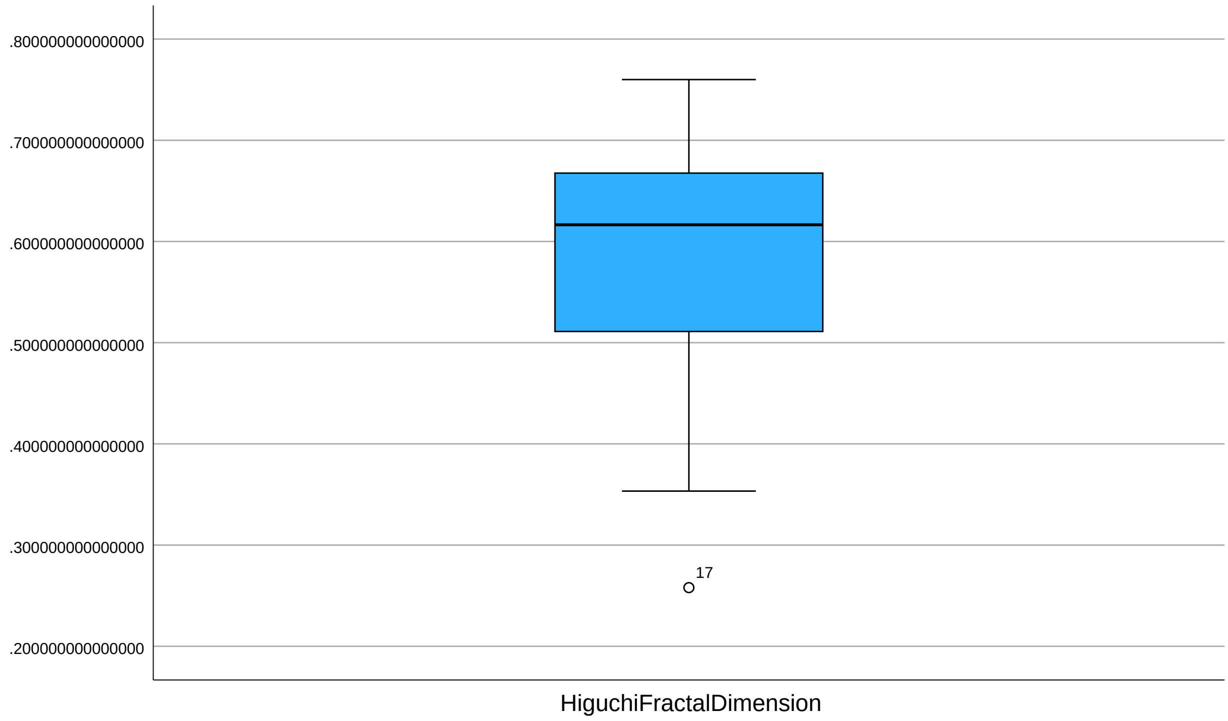

LempelZivComplexity

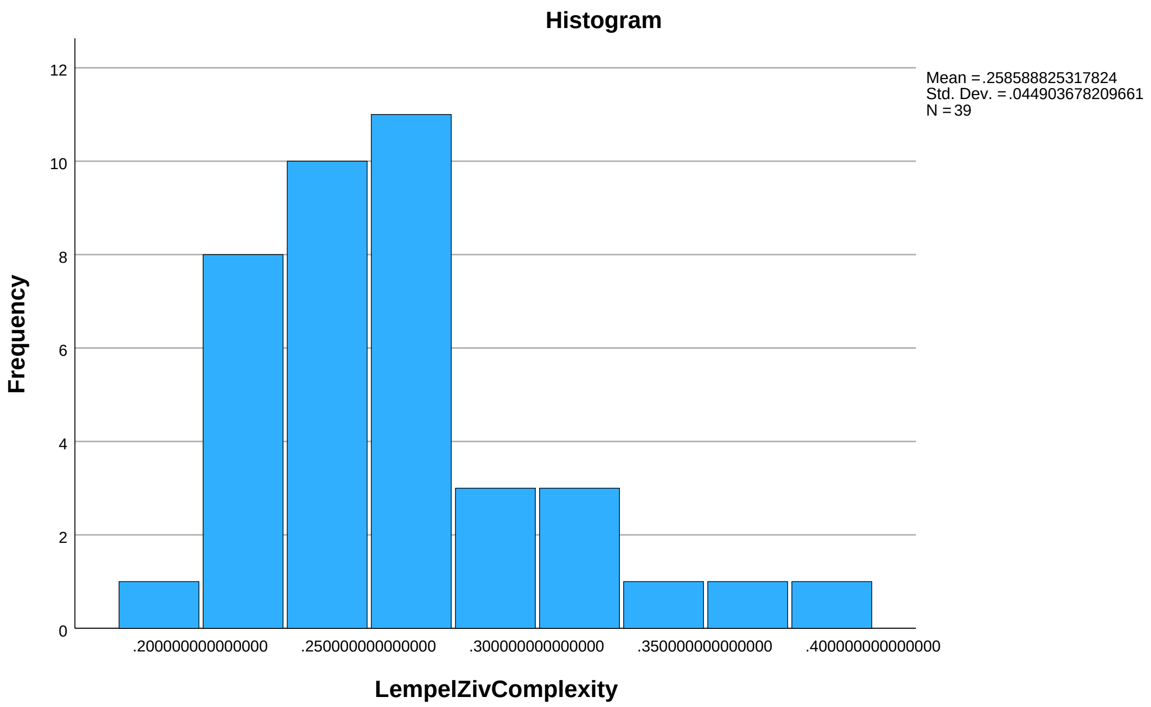

LempelZivComplexity Stem-and-Leaf Plot

Frequency   Stem & Leaf

```
  1.00      1 . 9
 18.00       2 .  000112223333333344
 14.00       2 .  55556666677888
  3.00      3 . 002
  3.00 Extremes    (>=.34)

Stem width:   .1000000
Each leaf:       1 case(s)
```

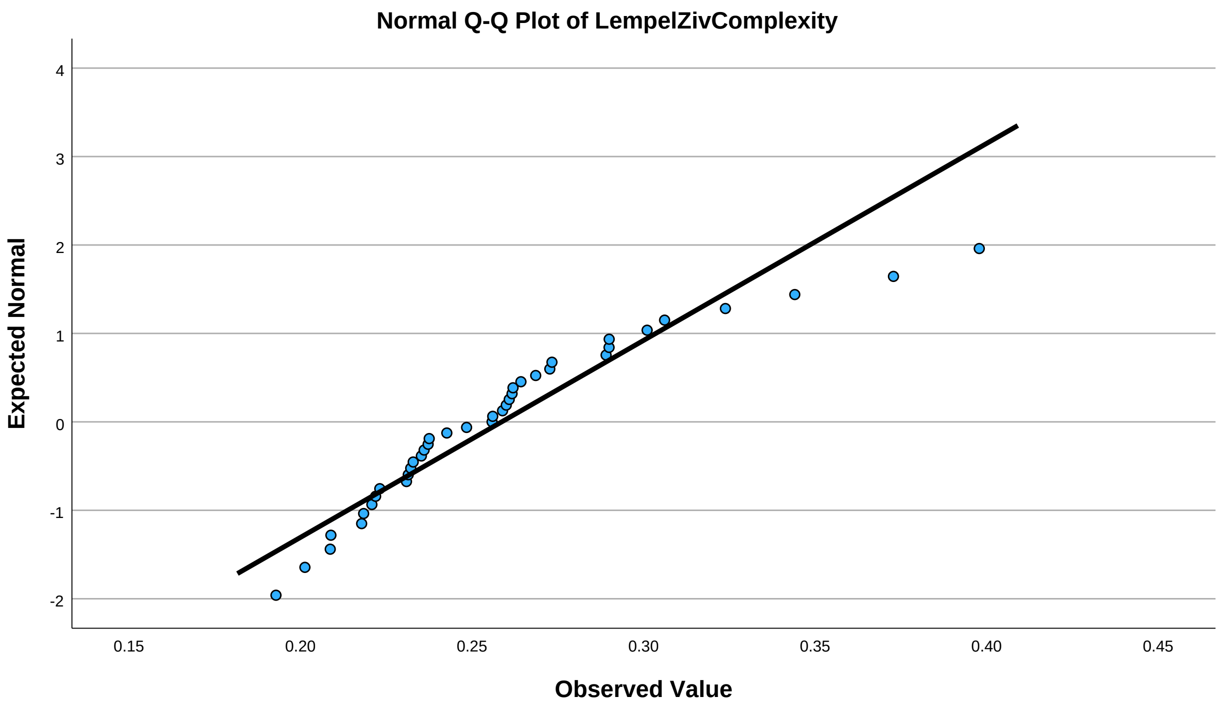

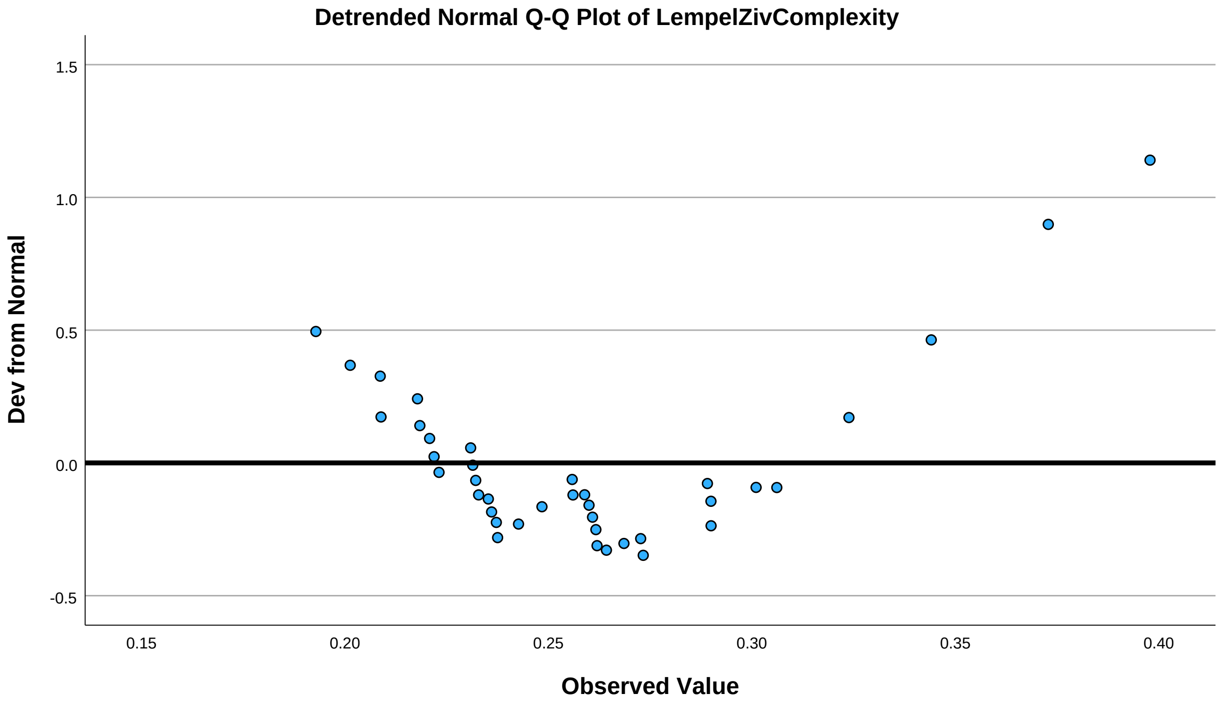

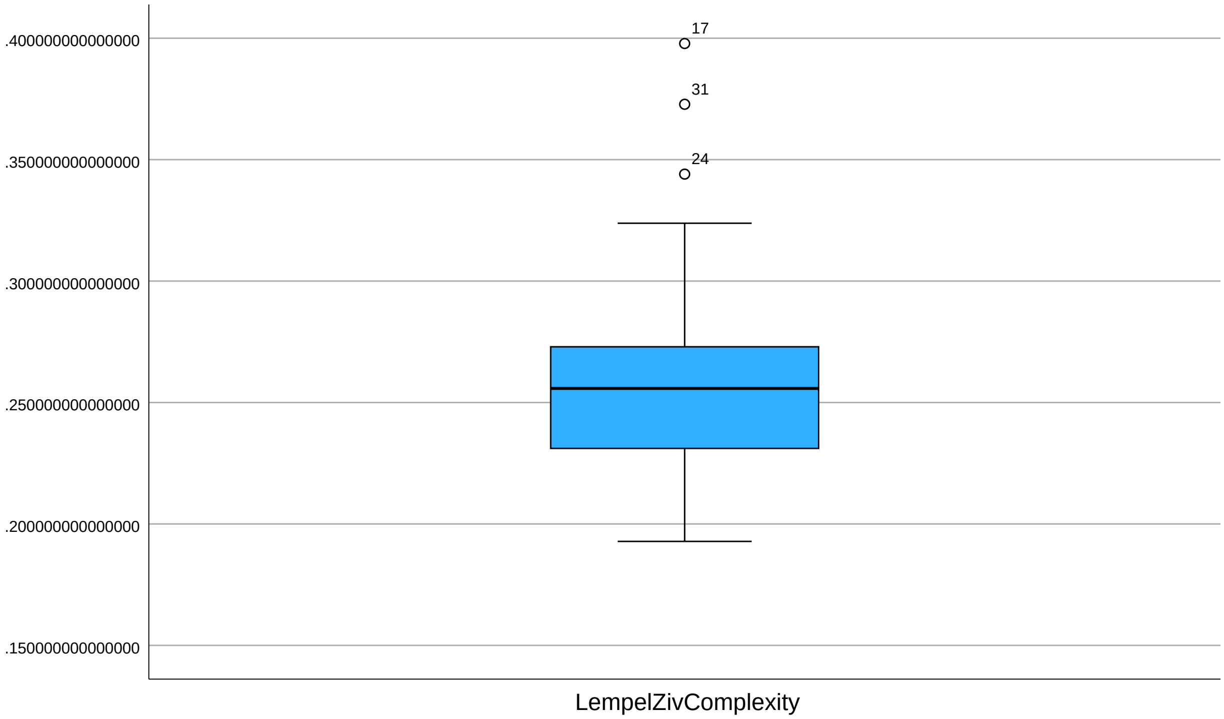

RenyiEntropy

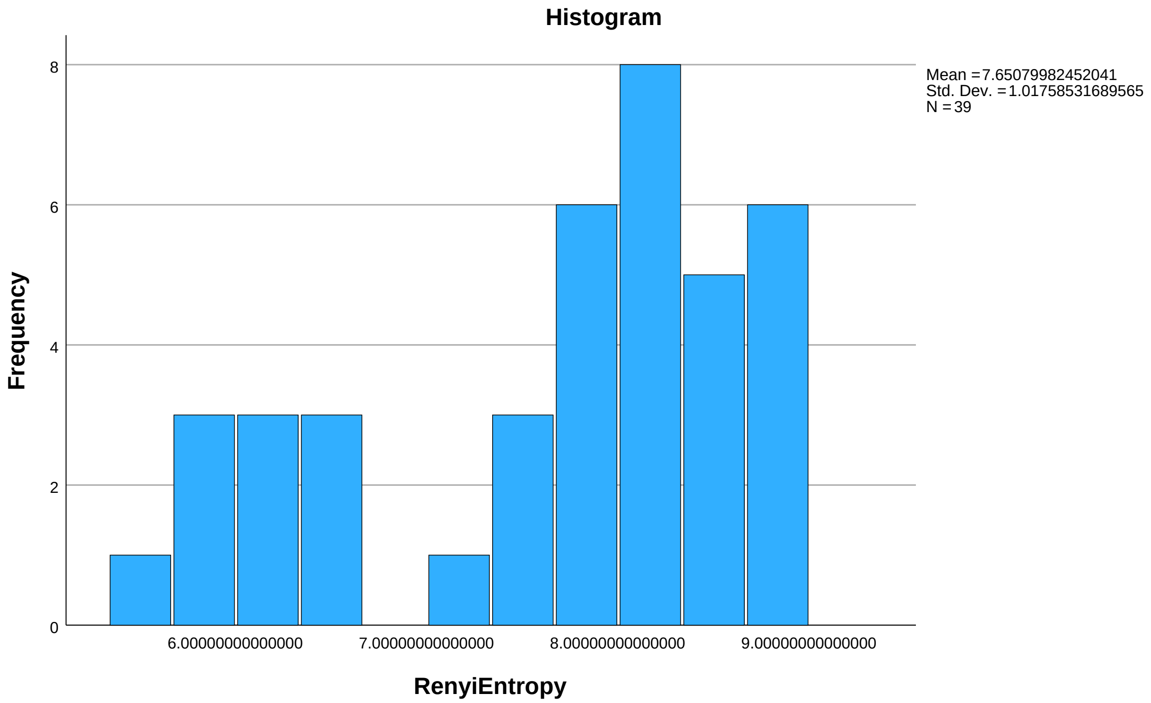


```
RenyiEntropy Stem-and-Leaf Plot

 Frequency    Stem &  Leaf

     4.00        5 .  6899
     5.00        6 .  00133
     1.00        6 .  6
     3.00        7 .  244
     7.00        7 .  5668899
    10.00        8 .  0112233344
     9.00        8 .  566666788

 Stem width:   1.000000
 Each leaf:        1 case(s)
```

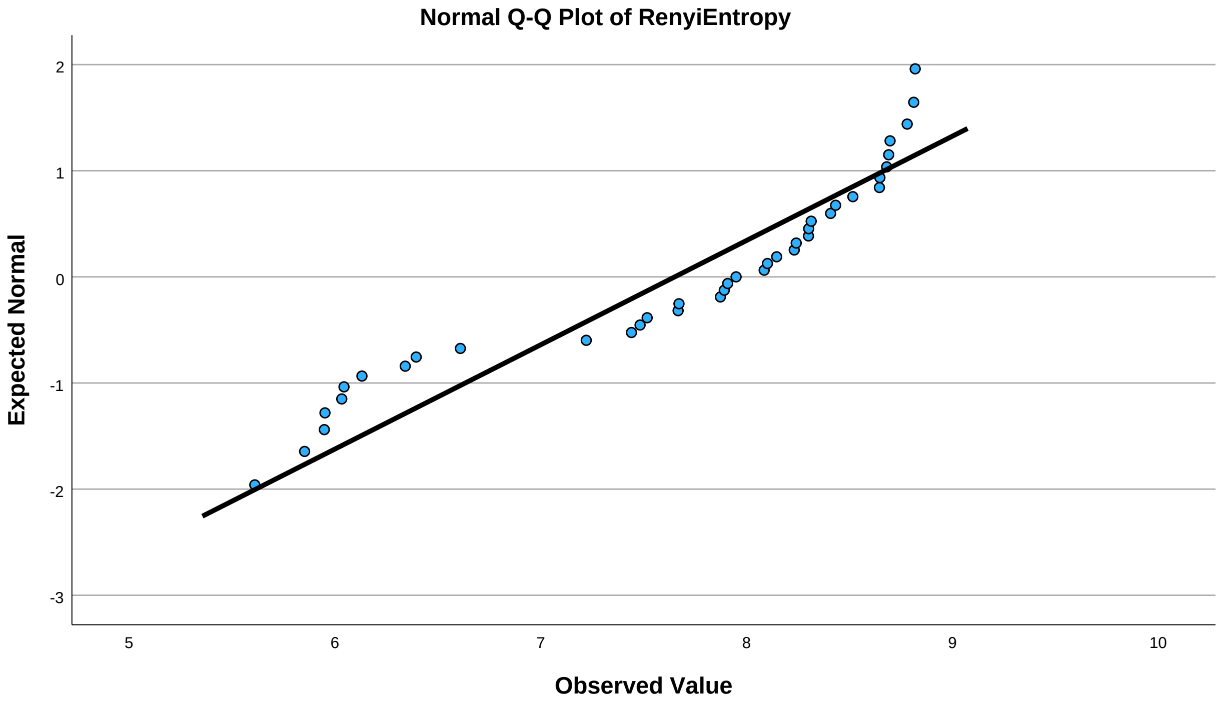
Normal Q-Q Plot of RenyiEntropy
Expected Normal
2
1
0
-1
-2
-3
5
6
7
8
9
10
Observed Value

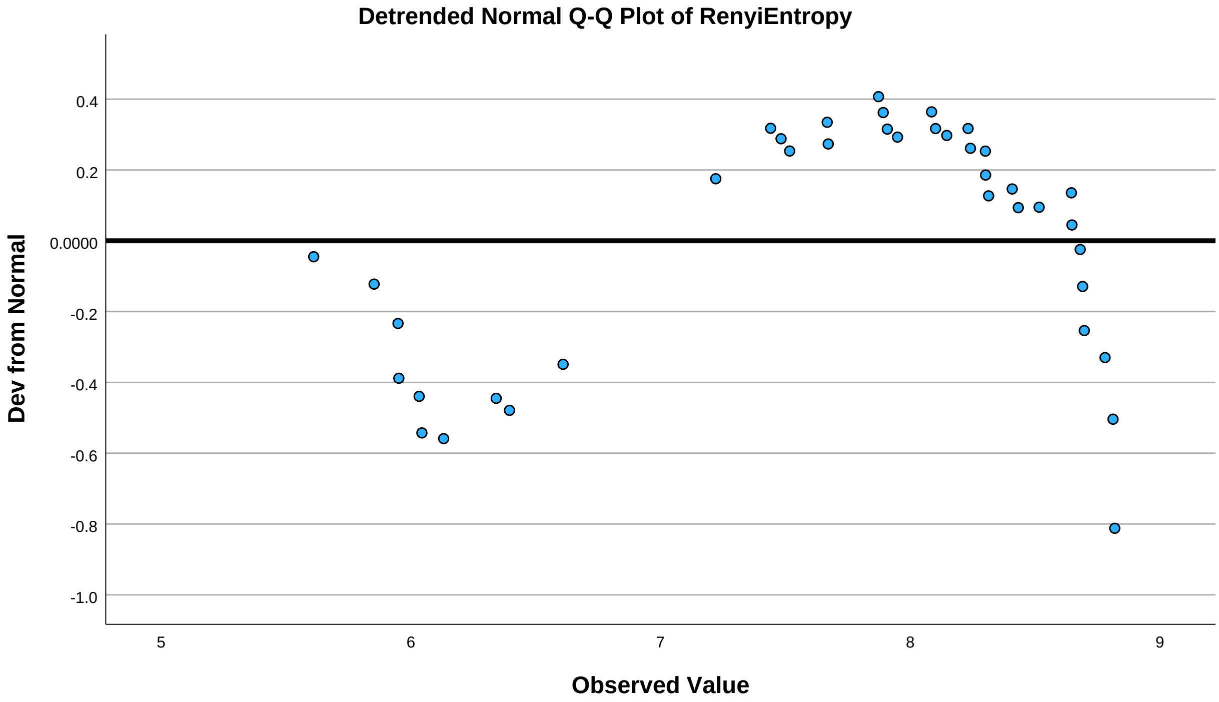
Detrended Normal Q-Q Plot of RenyiEntropy
Dev from Normal
0.4
0.2
0.0000
-0.2
-0.4
-0.6
-0.8
-1.0
5
6
7
8
9
Observed Value

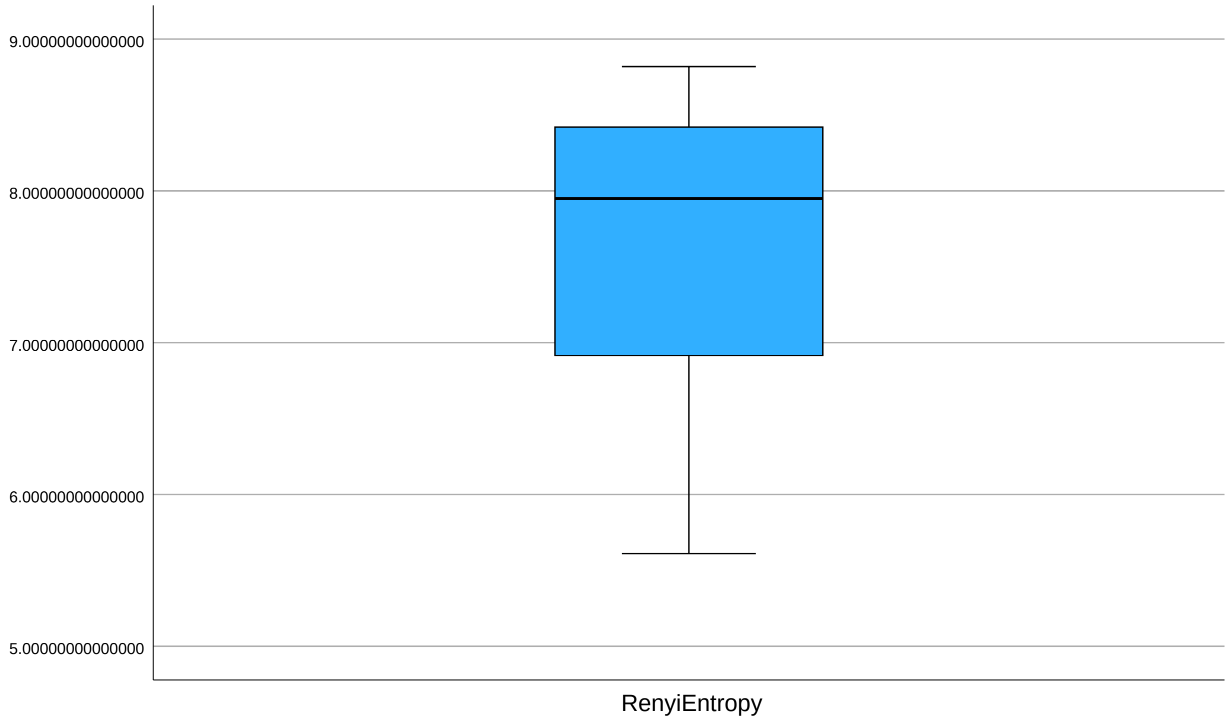

TsallisEntropy

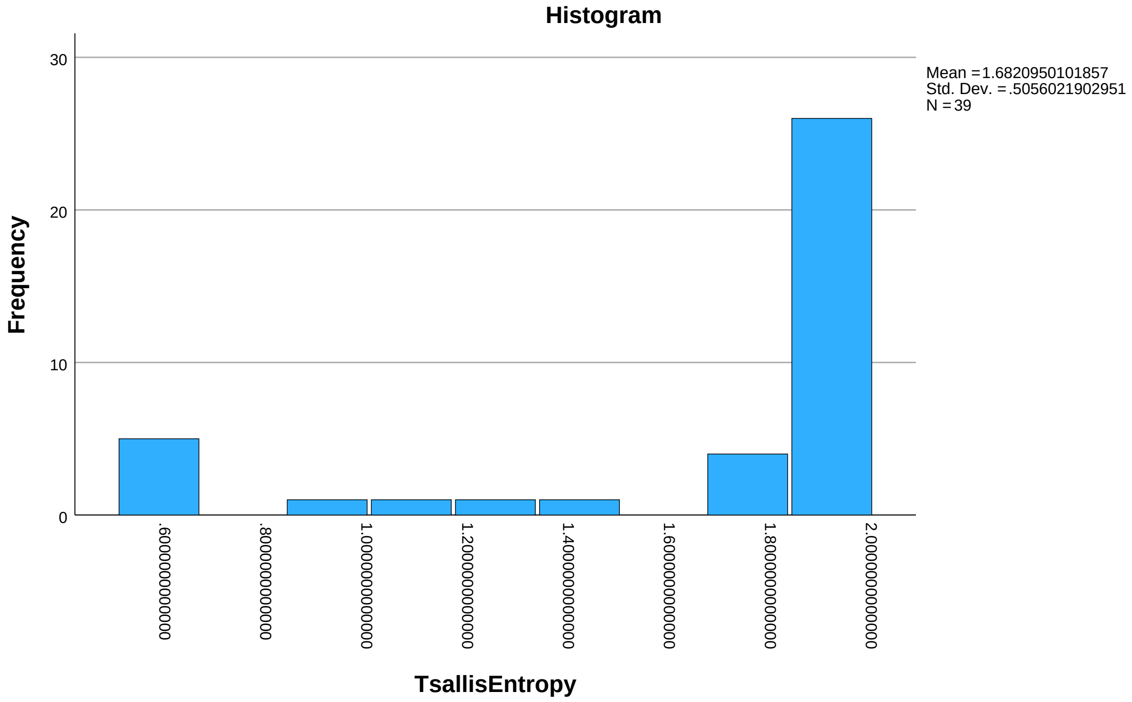

TsallisEntropy Stem-and-Leaf Plot

Frequency   Stem & Leaf

```
    8.00 Extremes    (=<1.24)
    1.00      13 .  6
     .00     14 .
     .00     15 .
    1.00      16 .  7
    1.00      17 .  1
    3.00      18 .  027
   25.00       19 .  1344456677788888888888888

 Stem width:   .1000000
 Each leaf:        1 case(s)
```

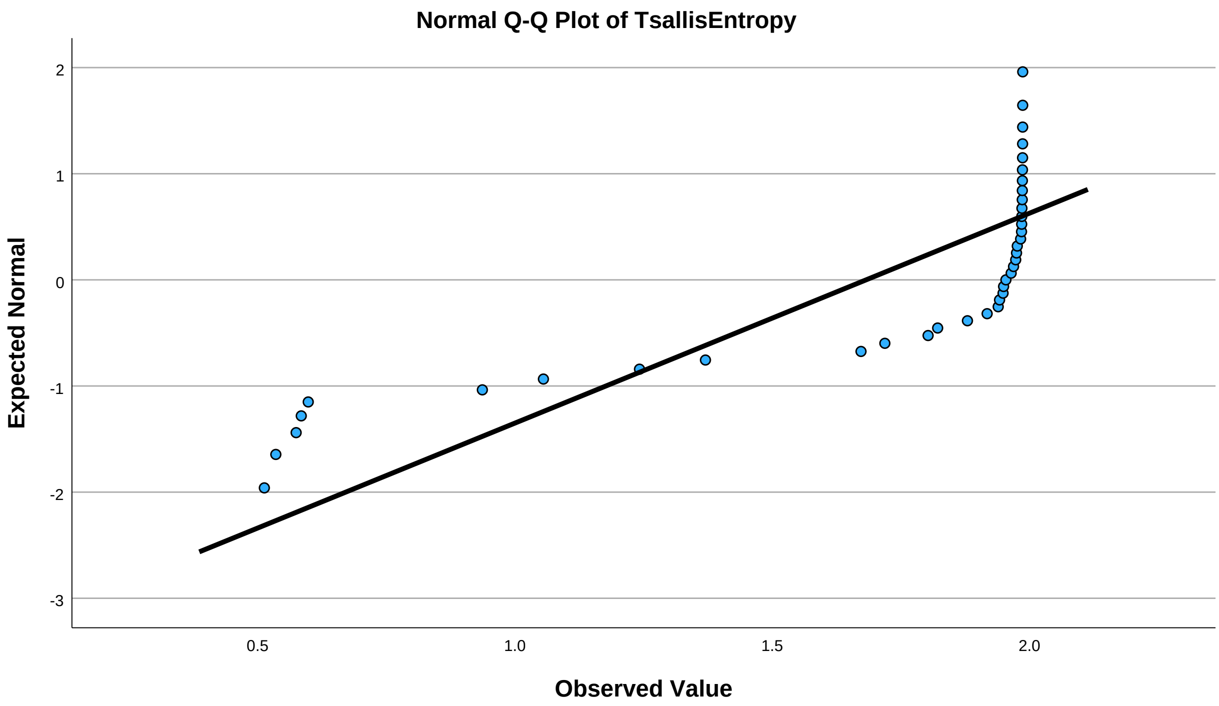

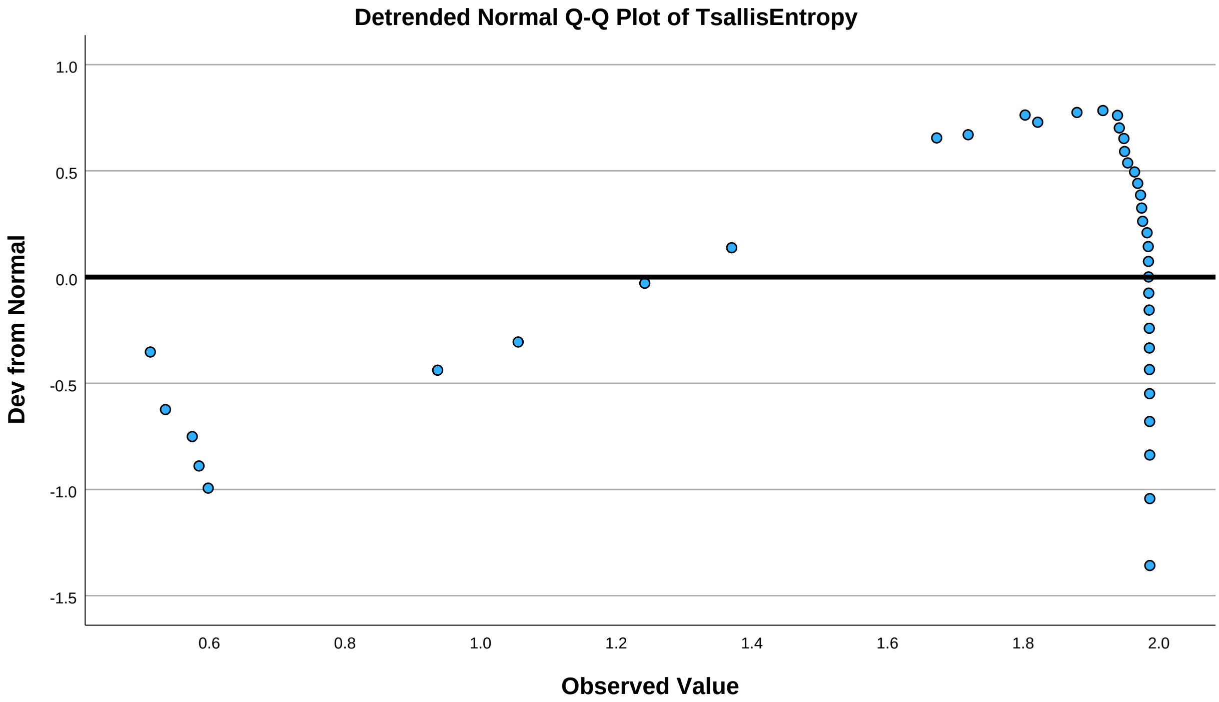
Detrended Normal Q-Q Plot of TsallisEntropy
Dev from Normal
1.0
0.5
0.0
-0.5
-1.0
-1.5
0.6
0.8
1.0
1.2
1.4
1.6
1.8
2.0
Observed Value

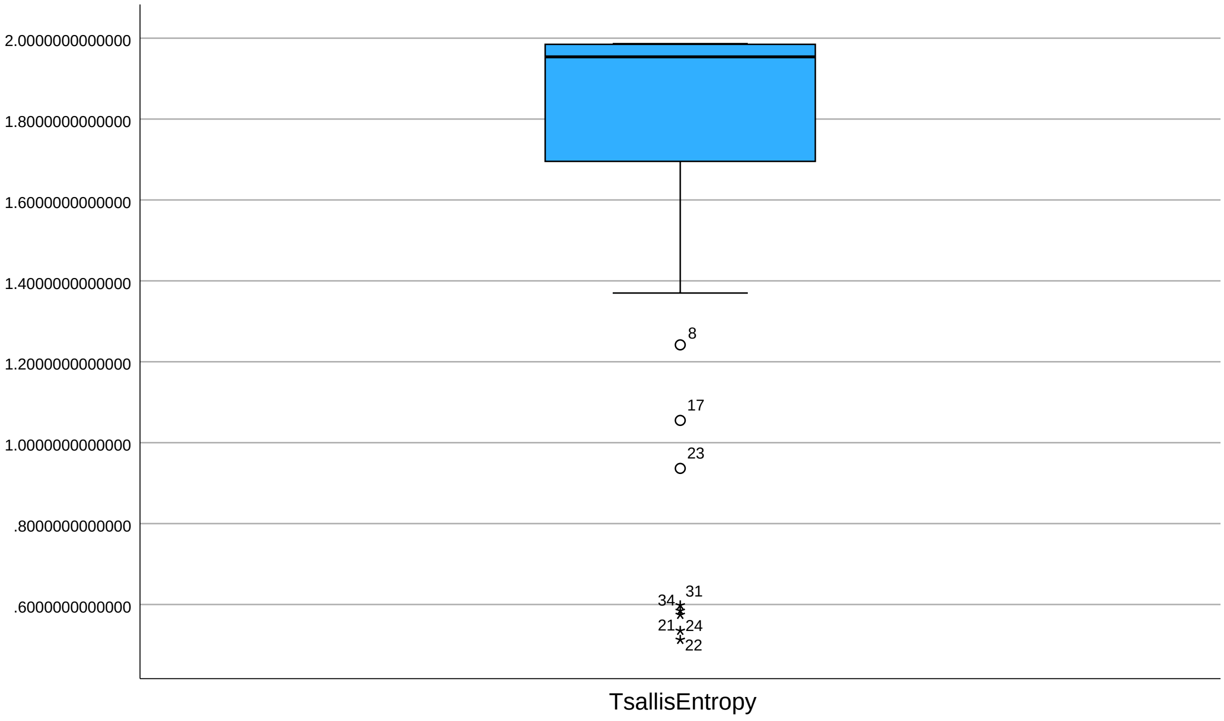
2.0000000000000
1.8000000000000
1.6000000000000
1.4000000000000
1.2000000000000
1.0000000000000
.8000000000000
.6000000000000
8
17
23
34
31
21
24
22
TsallisEntropy

PS_4_5

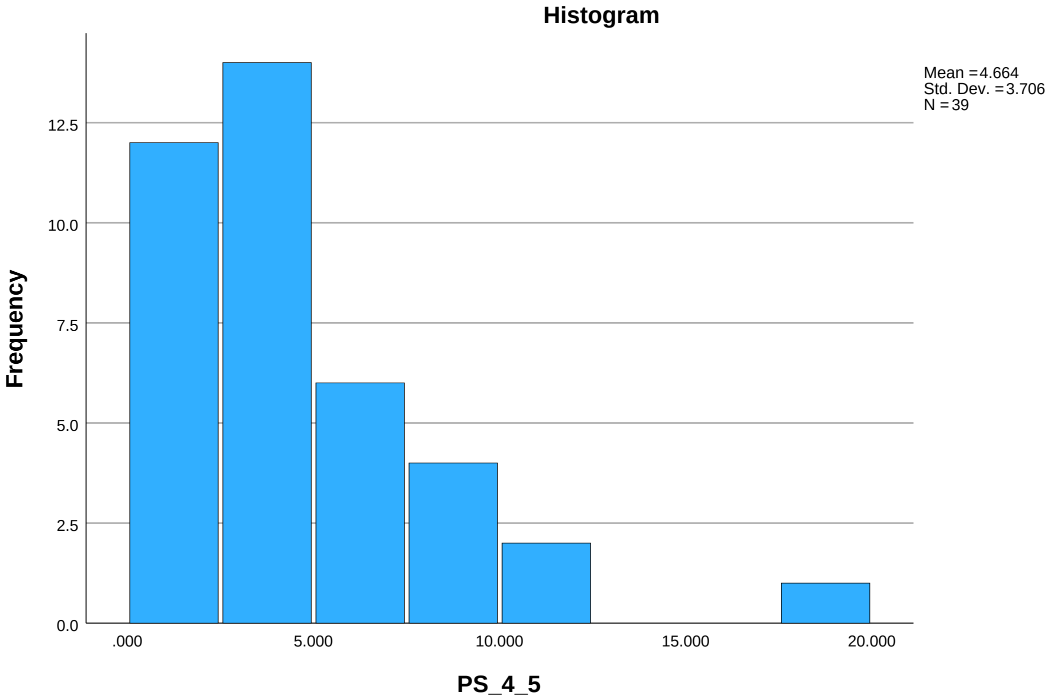

PS_4_5 Stem-and-Leaf Plot

```
 Frequency    Stem &  Leaf

     1.00        0 .  8
     9.00        1 .  034567779
     7.00        2 .  2355569
     5.00        3 .  03567
     4.00        4 .  0034
     3.00        5 .  034
     2.00        6 .  89
     2.00        7 .  48
      .00        8 .
     3.00        9 .  677
     1.00       10 .  6
     1.00       11 .  2
     1.00 Extremes    (>=18.7)

 Stem width:     1.000
 Each leaf:        1 case(s)
```

Normal Q-Q Plot of PS_4_5

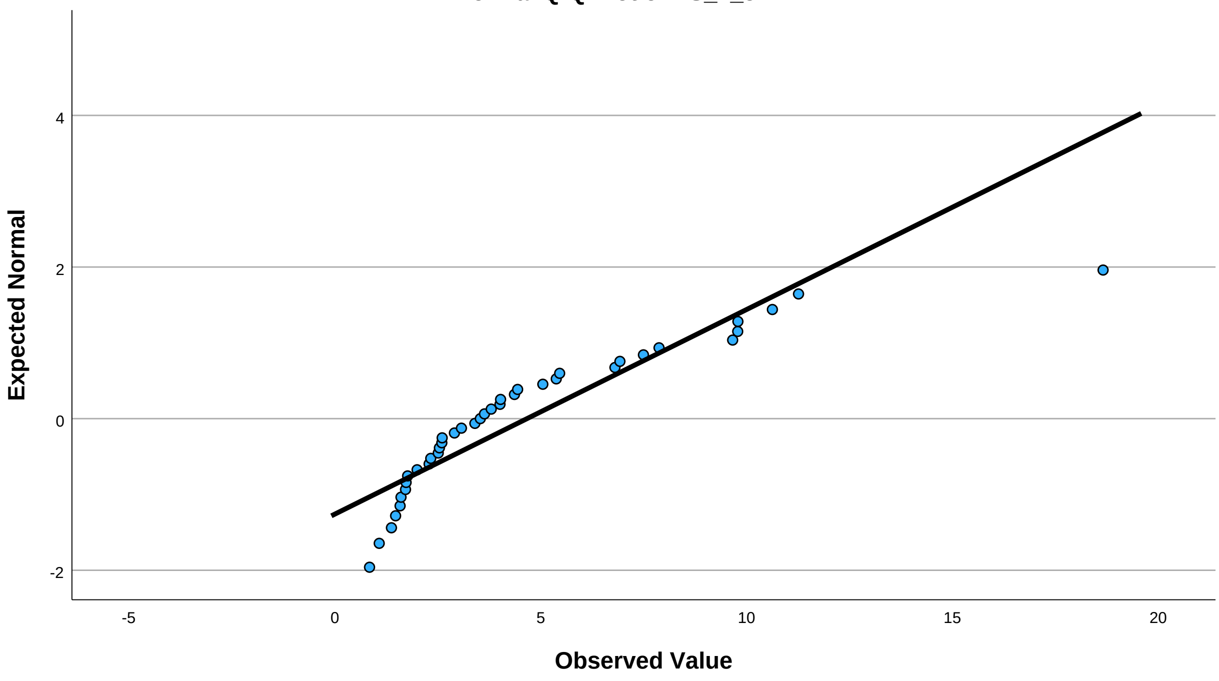

Detrended Normal Q-Q Plot of PS_4_5

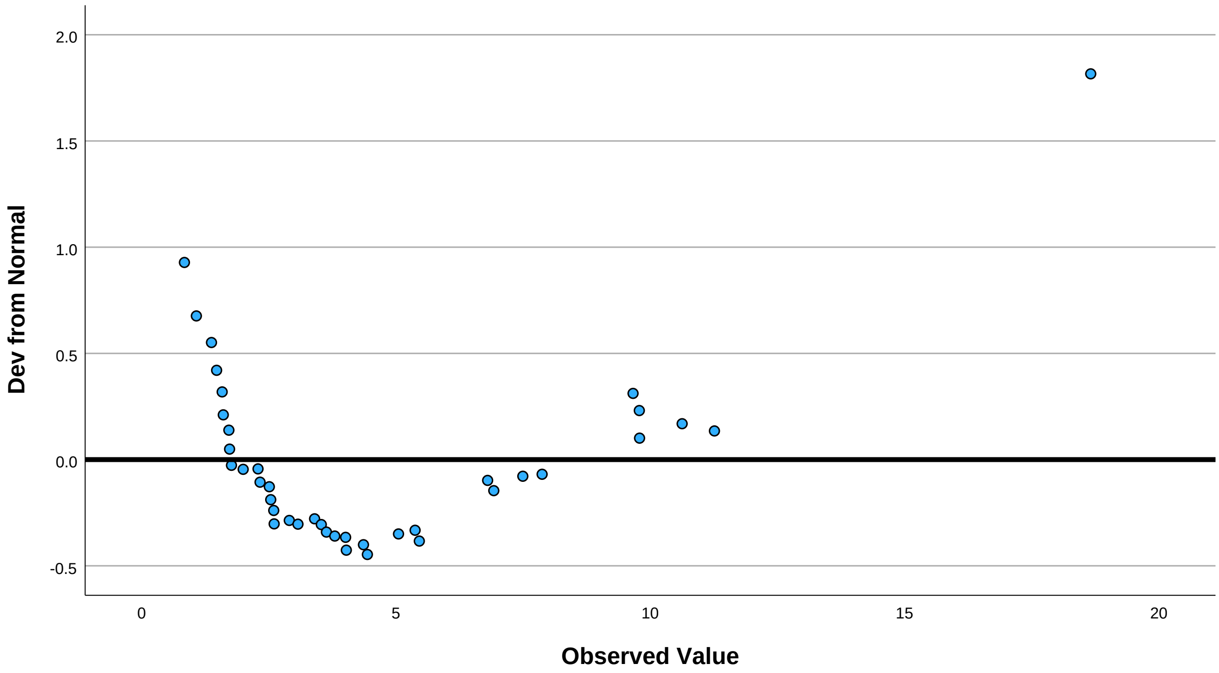

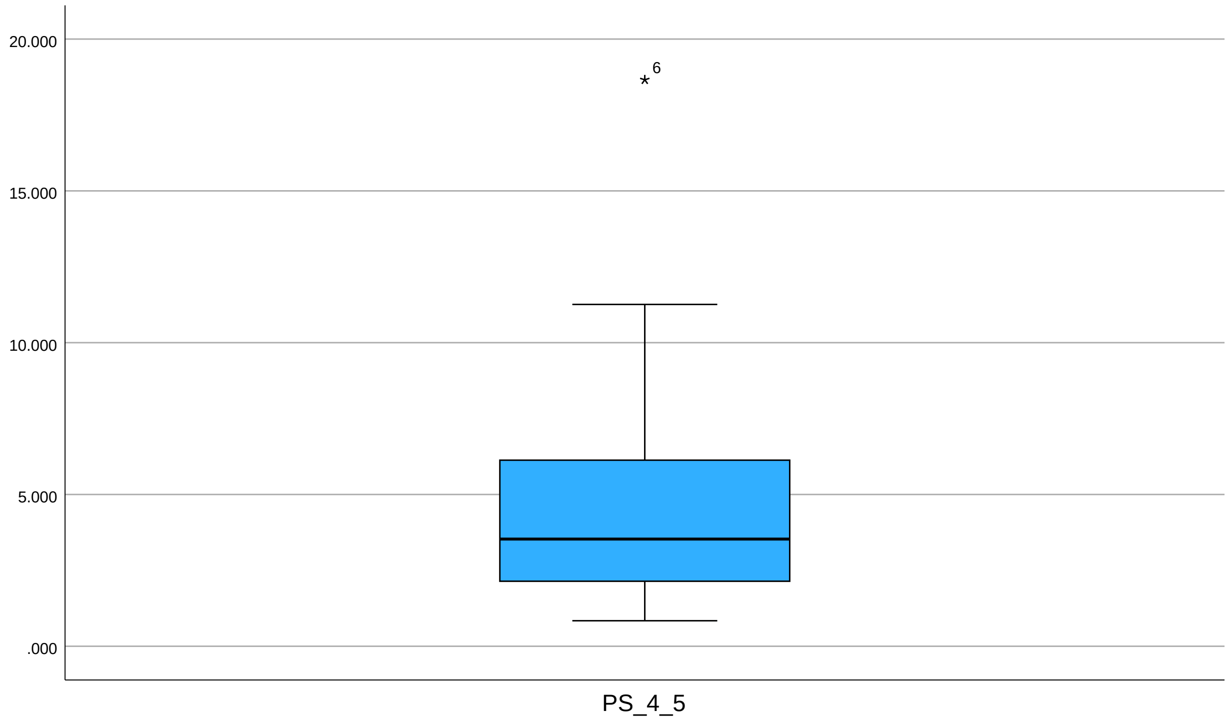

PS_5_6

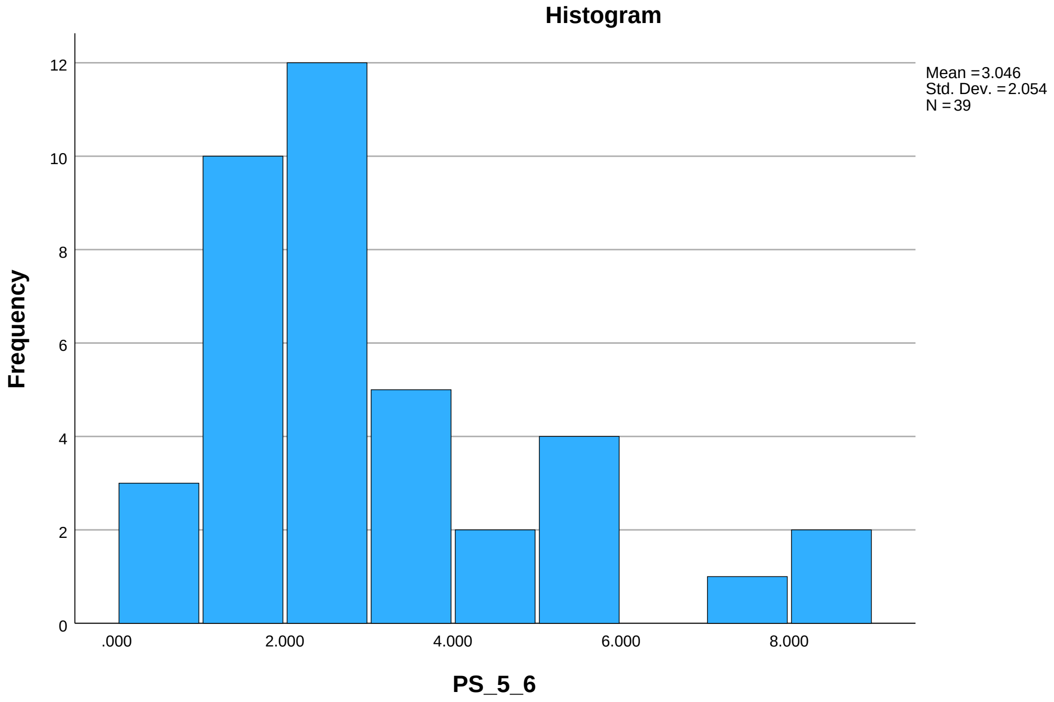


```
PS_5_6 Stem-and-Leaf Plot

 Frequency    Stem &  Leaf
```

```
  1.00      0 .  4
  2.00      0 .  69
  5.00      1 .  11123
  5.00      1 .  56788
  9.00      2 .  111122344
  3.00      2 .  579
  3.00      3 .  124
  2.00      3 .  58
   .00      4 .
  2.00      4 .  66
  1.00      5 .  1
  3.00      5 .  667
  3.00 Extremes    (>=7.7)

Stem width:      1.000
Each leaf:       1 case(s)
```

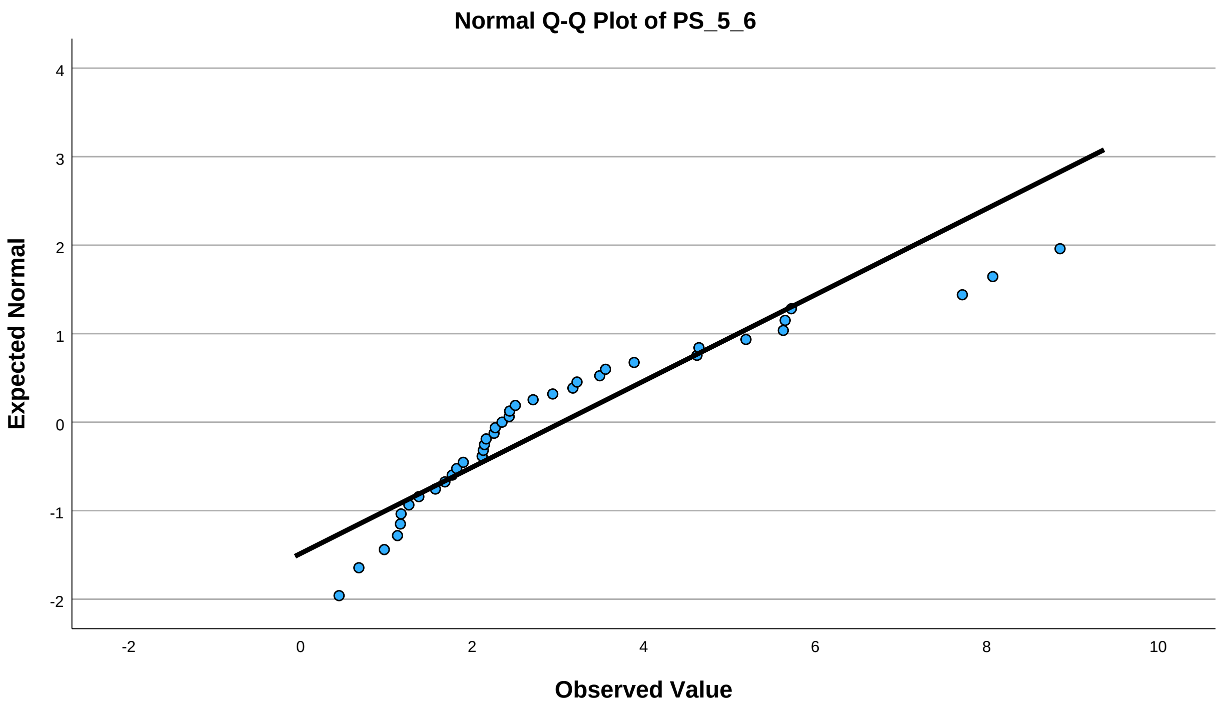

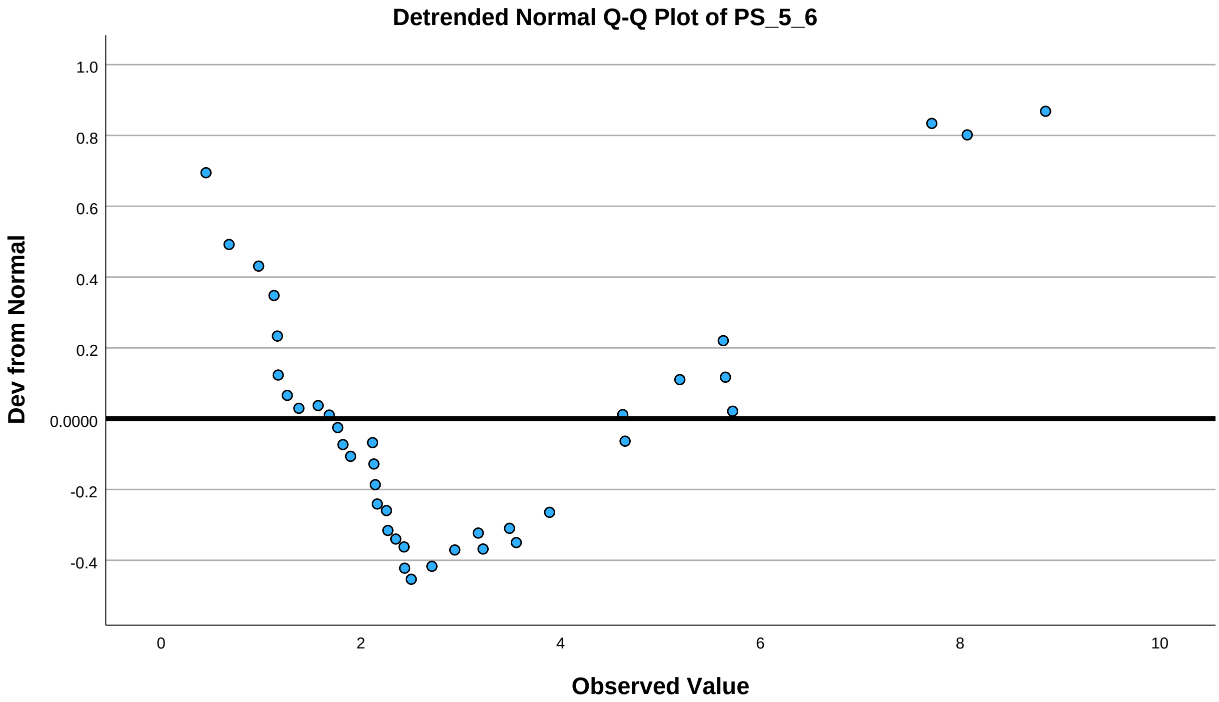
Detrended Normal Q-Q Plot of PS_5_6
Dev from Normal
1.0
0.8
0.6
0.4
0.2
0.0000
-0.2
-0.4
0
2
4
6
8
10
Observed Value

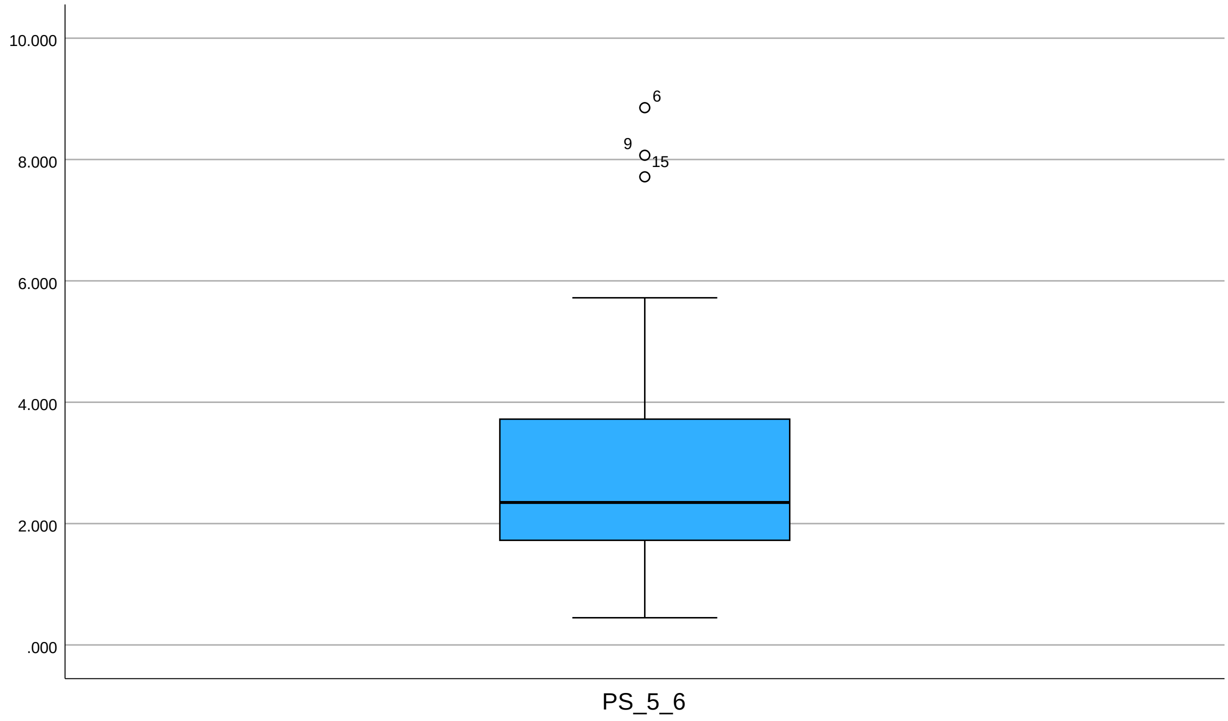
10.000
8.000
6.000
4.000
2.000
.000
6
9
15
PS_5_6

PS_6_7

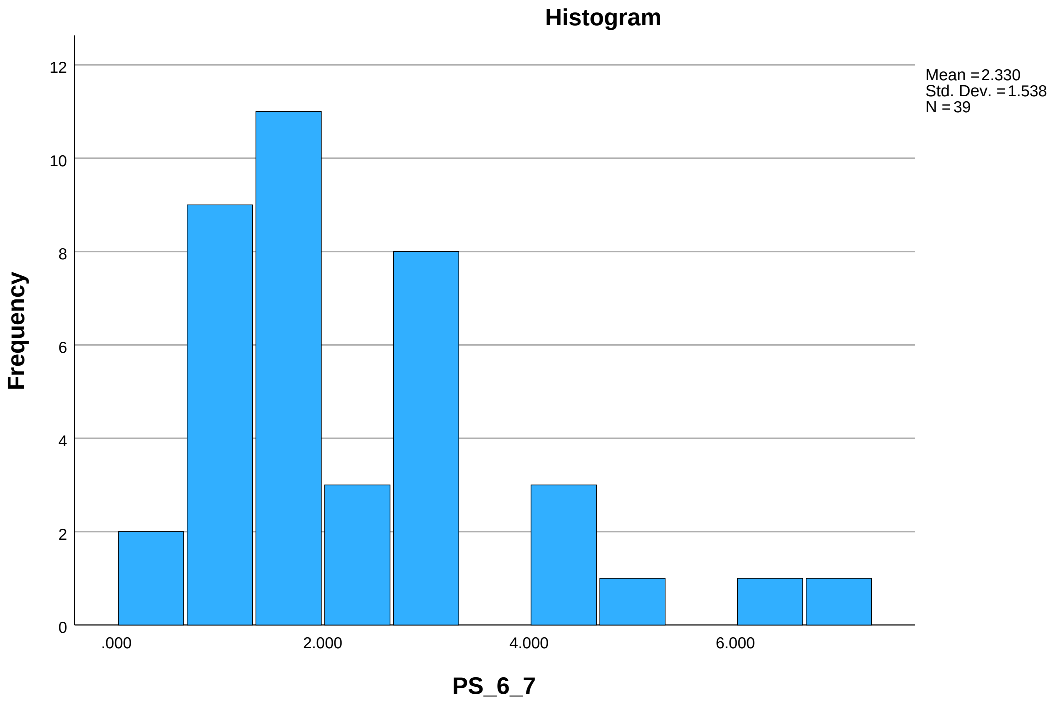


```
PS_6_7 Stem-and-Leaf Plot

 Frequency    Stem &  Leaf

     2.00        0 .  44
     5.00        0 .  78899
     6.00        1 .  122234
     9.00        1 .  556667889
     3.00        2 .  023
     3.00        2 .  779
     5.00        3 .  00123
      .00        3 .
     3.00        4 .  224
      .00        4 .
     1.00        5 .  2
     2.00 Extremes    (>=6.1)

 Stem width:      1.000
 Each leaf:       1 case(s)
```

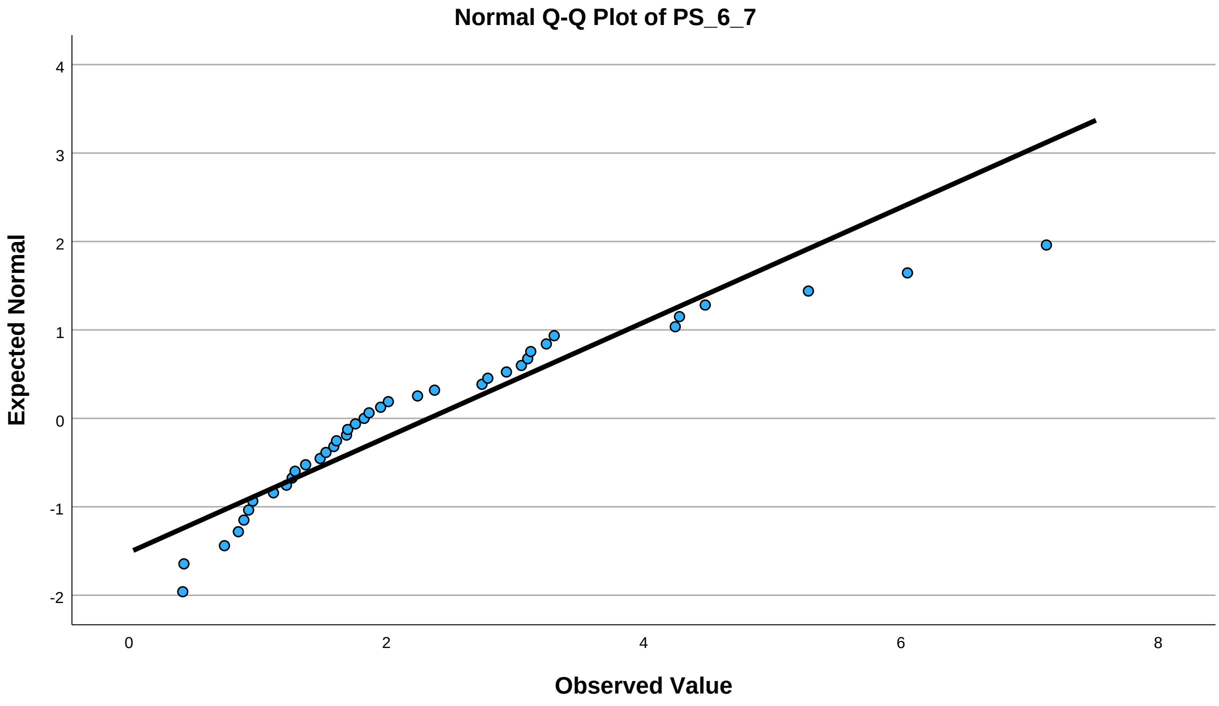
Normal Q-Q Plot of PS_6_7
Expected Normal
Observed Value
4
3
2
1
0
-1
-2
0
2
4
6
8

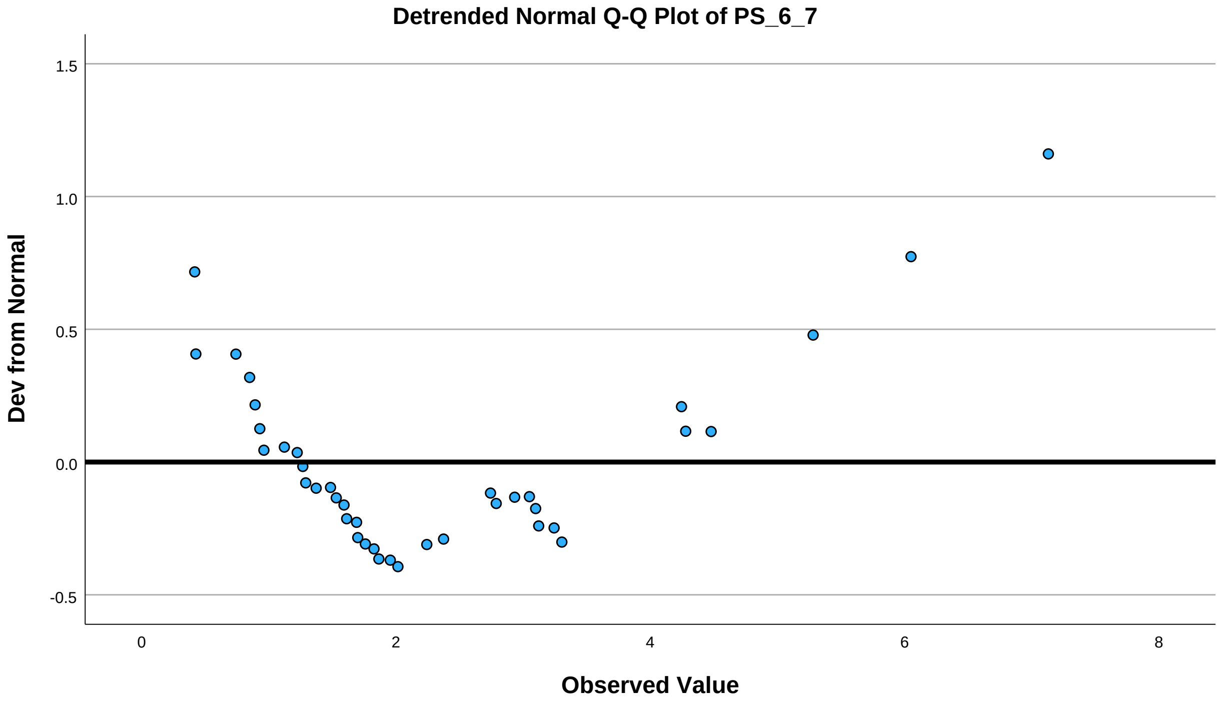
Detrended Normal Q-Q Plot of PS_6_7
Dev from Normal
Observed Value
1.5
1.0
0.5
0.0
-0.5
0
2
4
6
8

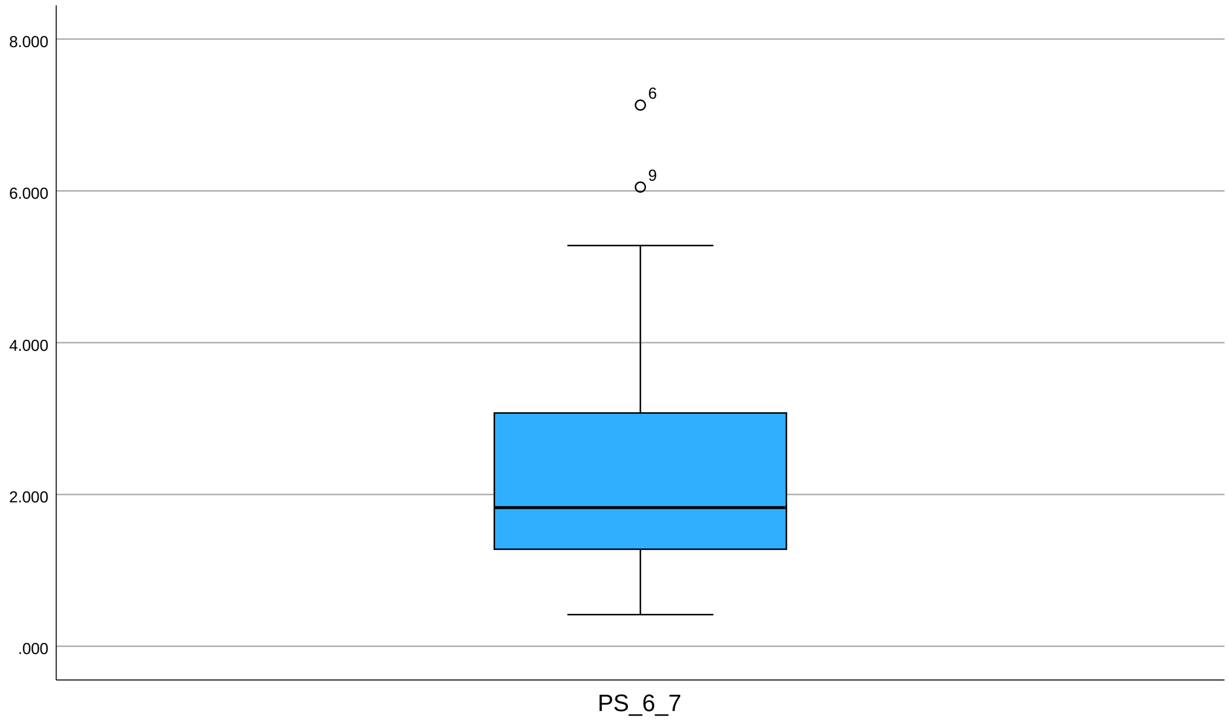

PS_7_8

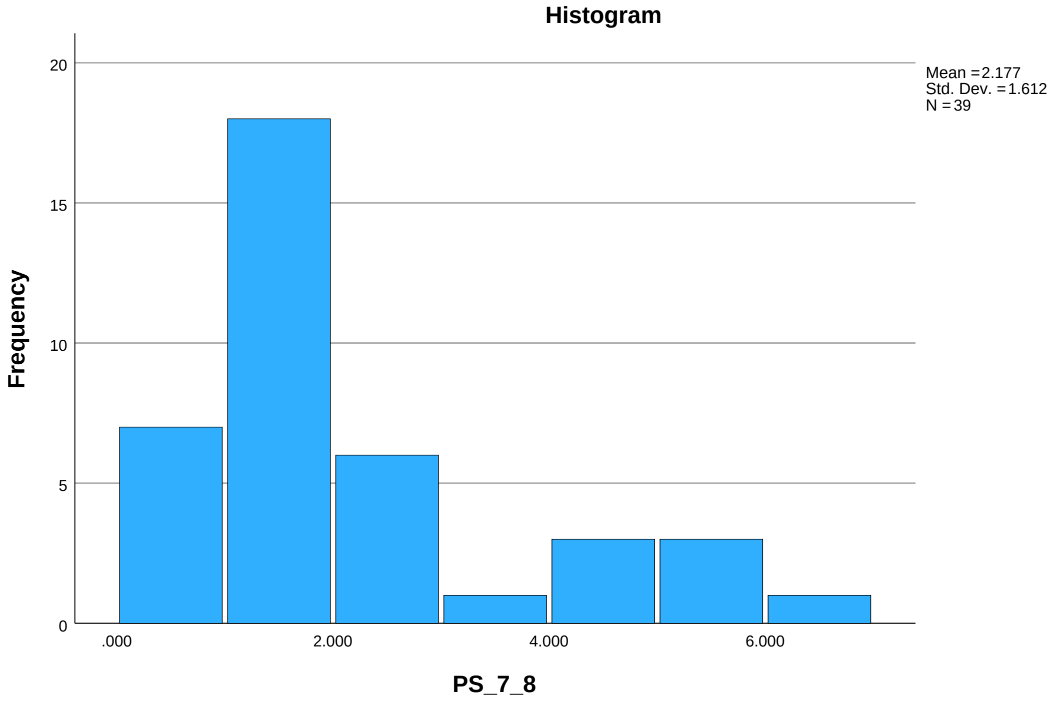


```
PS_7_8 Stem-and-Leaf Plot

 Frequency    Stem &  Leaf
```

```
    2.00      0 . 34
    5.00      0 . 78999
   13.00      1 . 0111112333344
    5.00      1 . 57789
    3.00      2 . 014
    3.00      2 . 579
    1.00      3 . 0
     .00      3 .
    1.00      4 . 1
    1.00      4 . 8
    5.00 Extremes    (>=4.9)

Stem width:     1.000
Each leaf:       1 case(s)
```

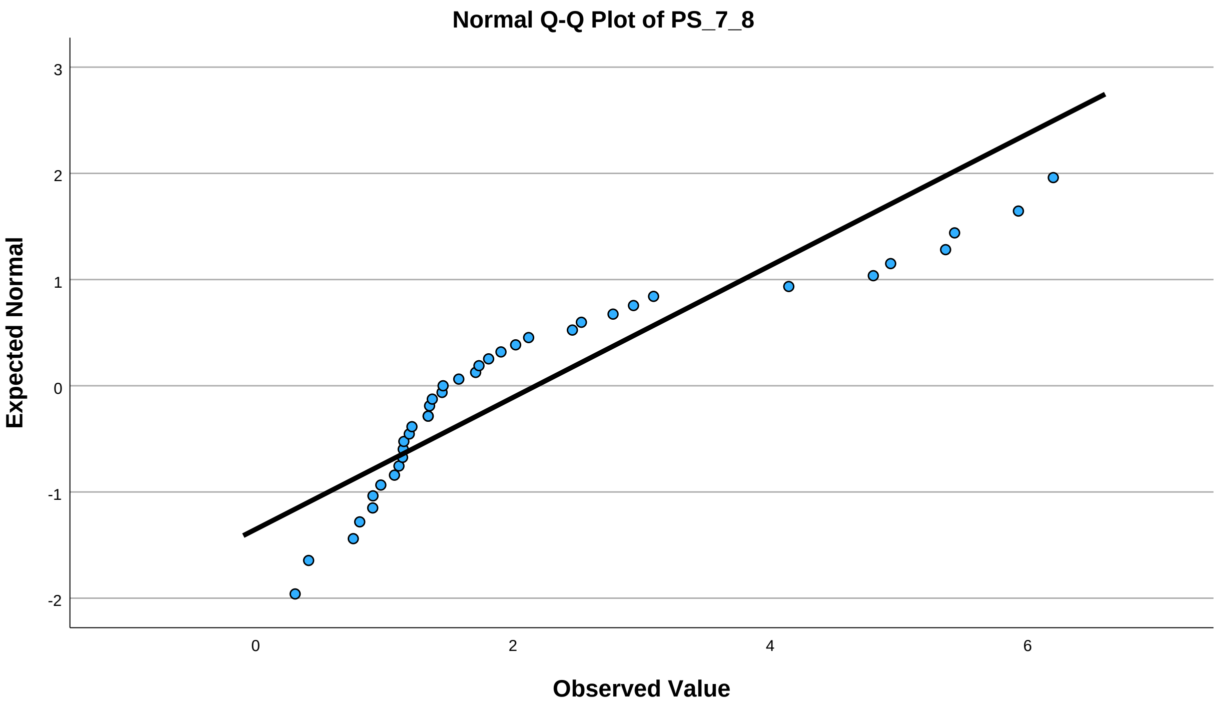

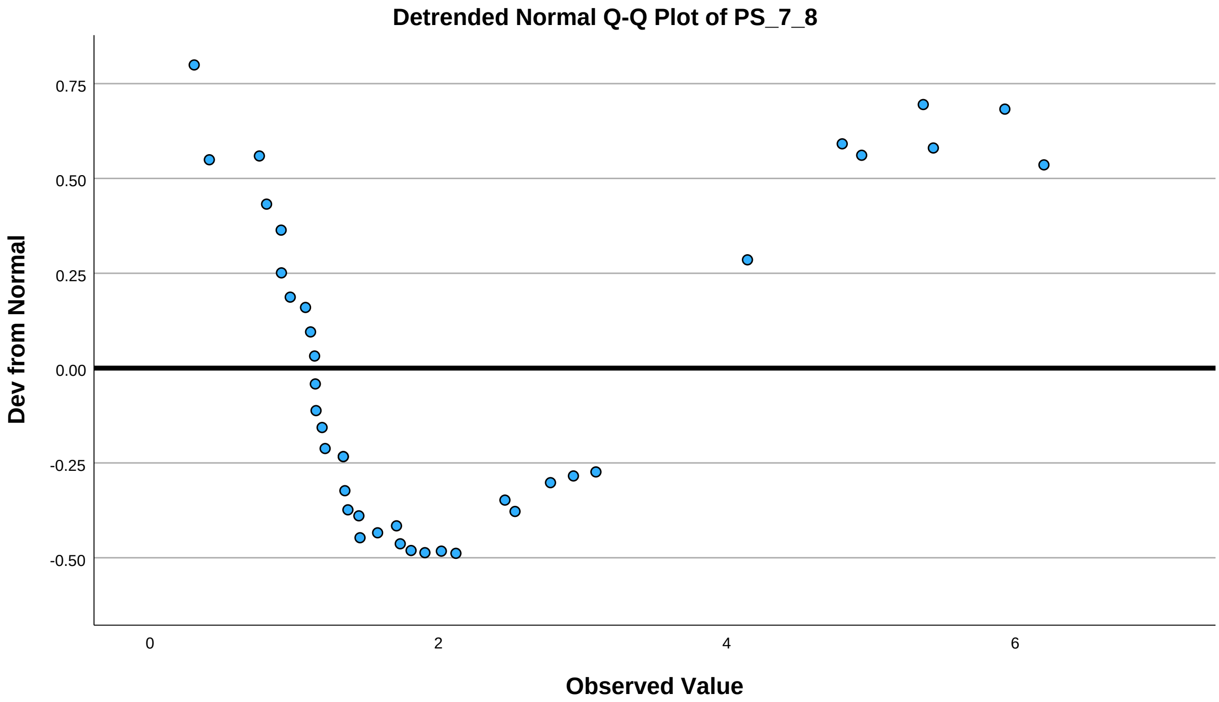
Detrended Normal Q-Q Plot of PS_7_8
Dev from Normal
0.75
0.50
0.25
0.00
-0.25
-0.50
0
2
4
6
Observed Value

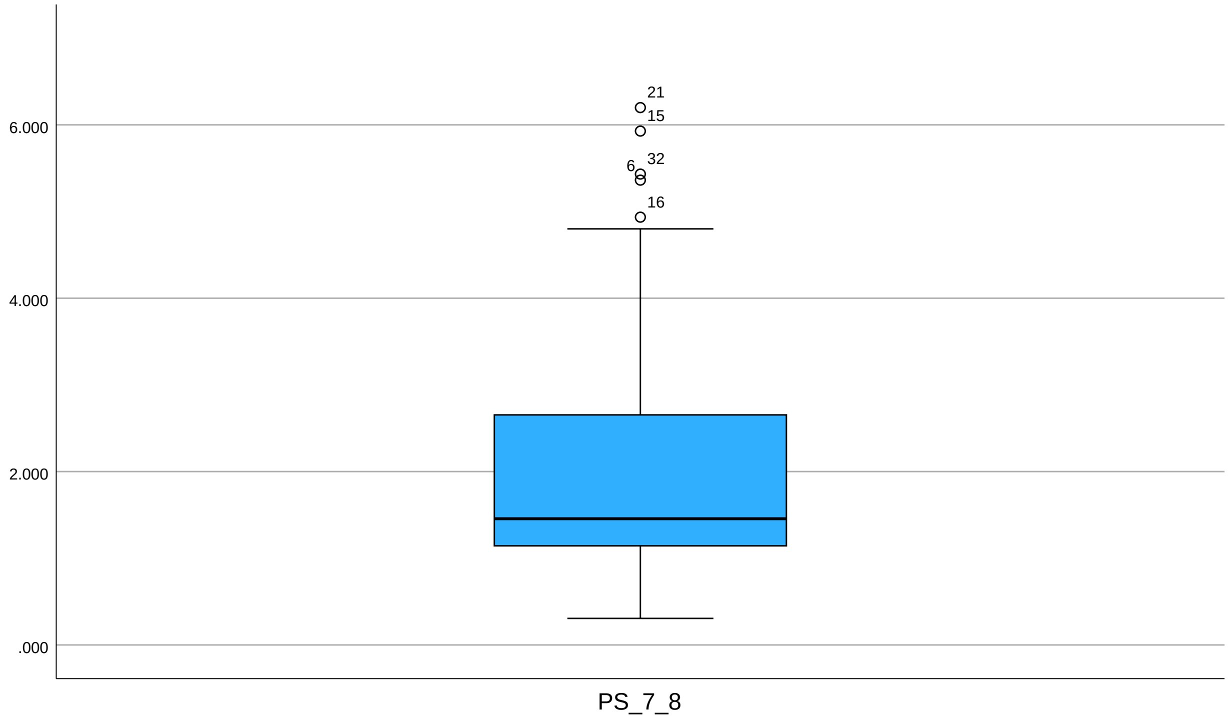
6.000
4.000
2.000
.000
21
15
6
32
16
PS_7_8

PS_8_9

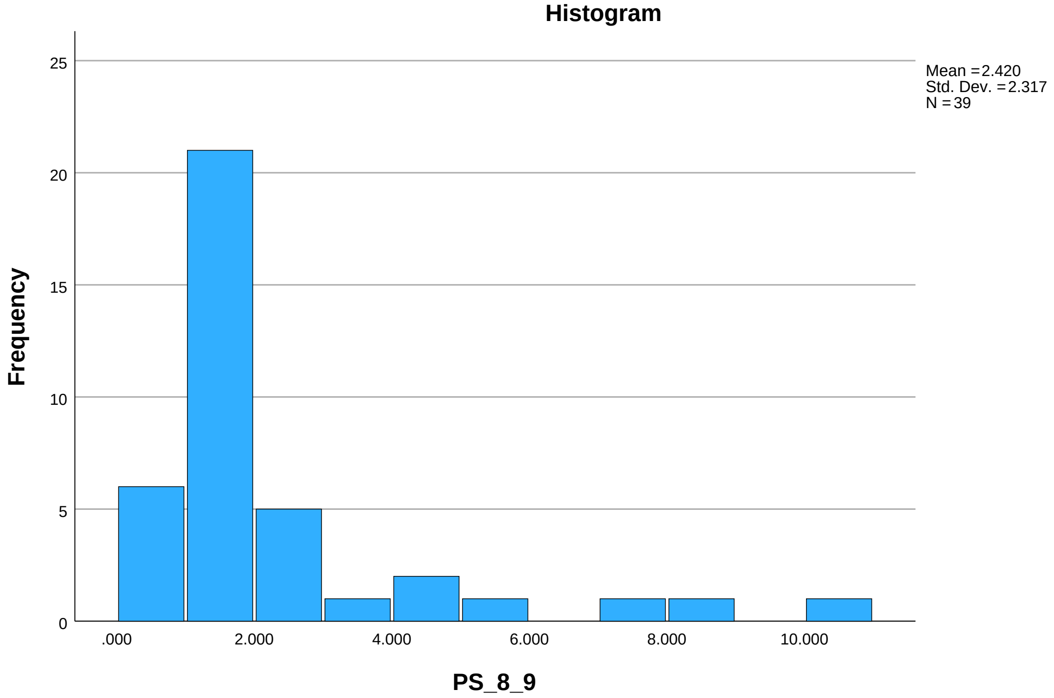


```
PS_8_9 Stem-and-Leaf Plot

 Frequency    Stem &  Leaf

     2.00        0 .  23
     4.00        0 .  6899
    10.00        1 .  0012223344
    11.00        1 .  55555888899
     3.00        2 .  012
     2.00        2 .  89
     7.00 Extremes    (>=3.8)

 Stem width:      1.000
 Each leaf:       1 case(s)
```

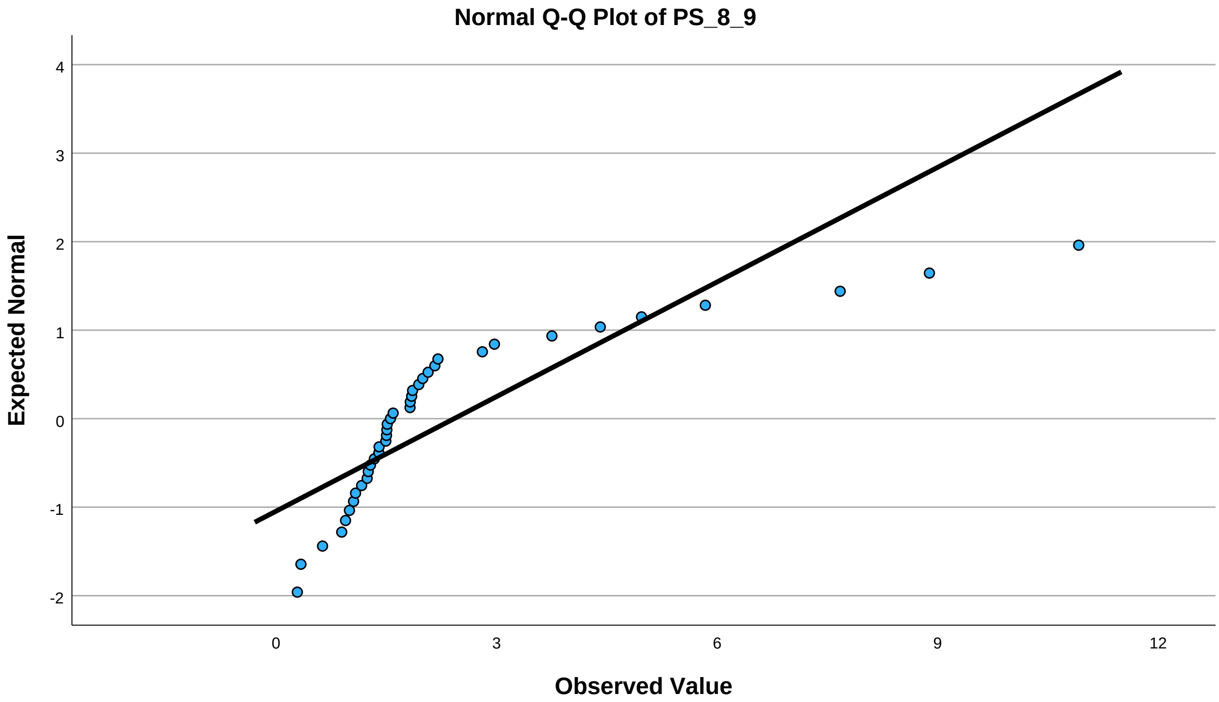
Normal Q-Q Plot of PS_8_9
Expected Normal
4
3
2
1
0
-1
-2
0
3
6
9
12
Observed Value

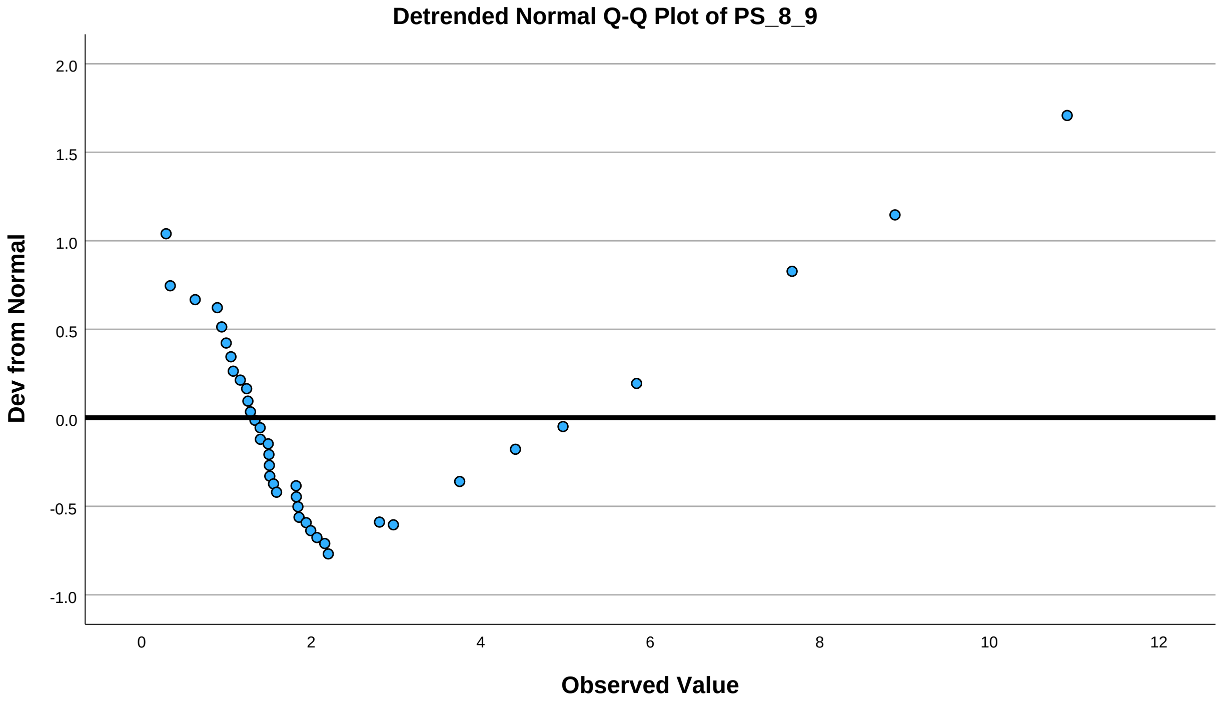
Detrended Normal Q-Q Plot of PS_8_9
Dev from Normal
2.0
1.5
1.0
0.5
0.0
-0.5
-1.0
0
2
4
6
8
10
12
Observed Value

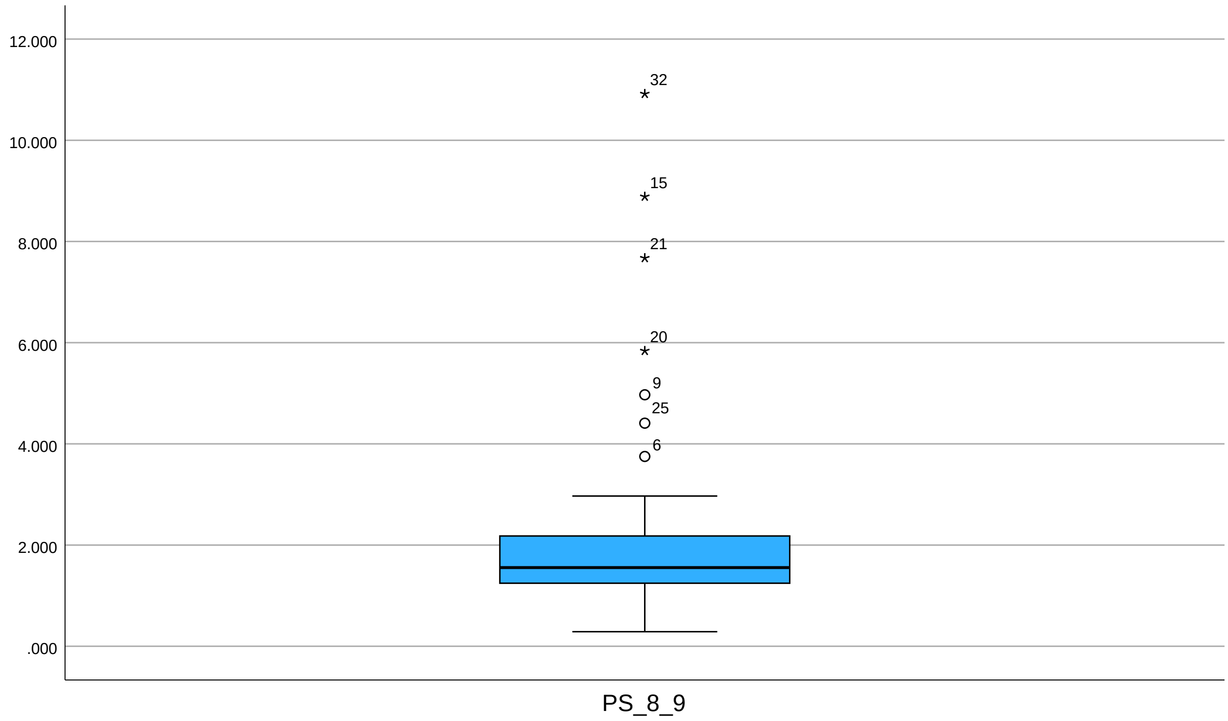

PS_9_10

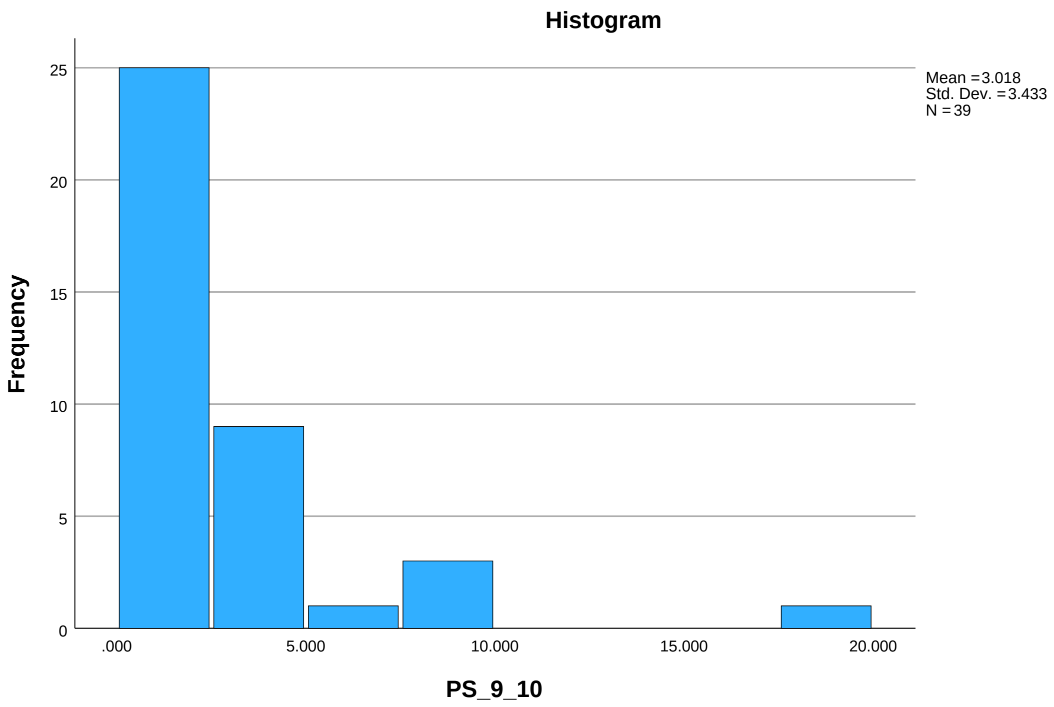

PS_9_10 Stem-and-Leaf Plot

Frequency   Stem & Leaf

```
    2.00     0 .  23
    2.00     0 .  58
    6.00     1 .  122334
    8.00     1 .  66667899
    7.00     2 .  0122244
    6.00     2 .  566789
    1.00     3 .  2
    2.00     3 .  56
    5.00 Extremes    (>=5.2)

Stem width:      1.000
Each leaf:       1 case(s)
```

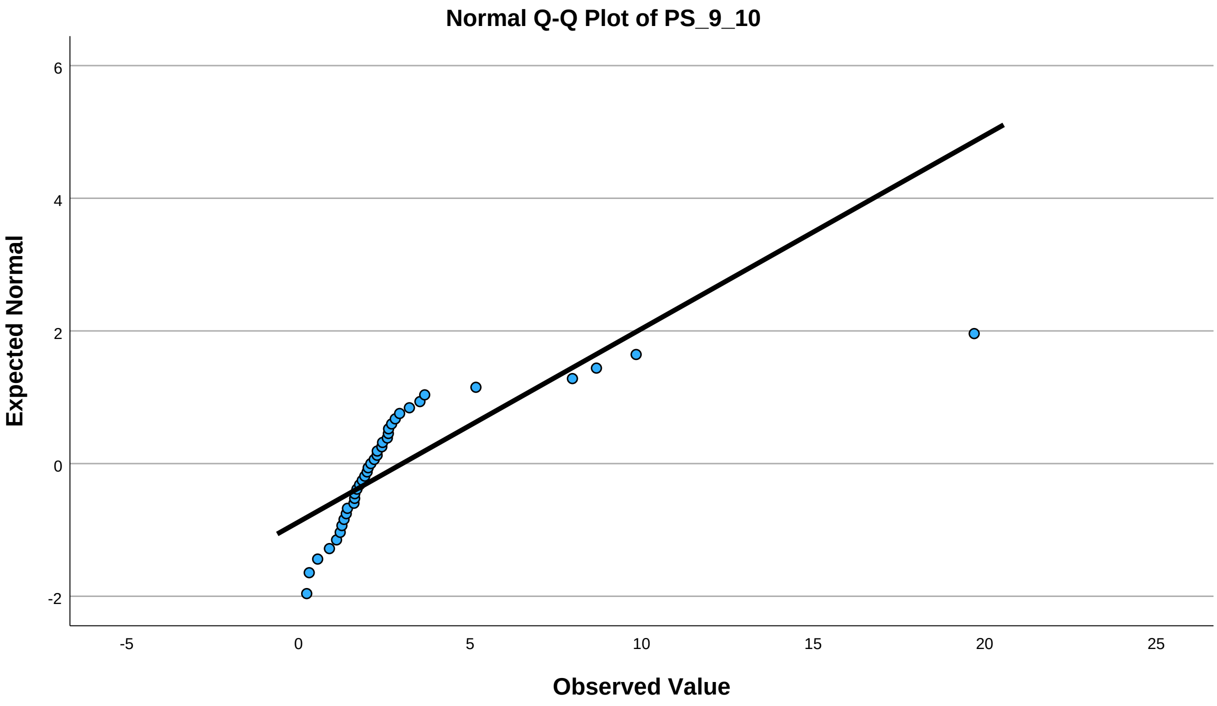

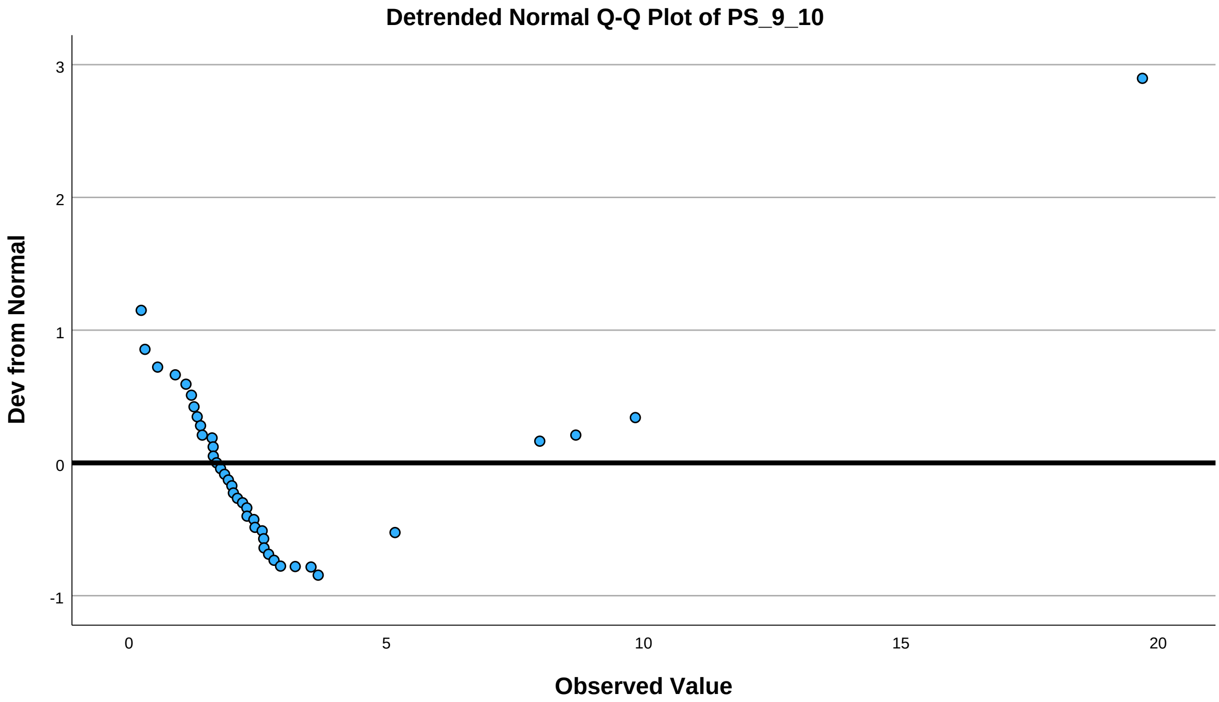
Detrended Normal Q-Q Plot of PS_9_10
Dev from Normal
3
2
1
0
-1
0
5
10
15
20
Observed Value

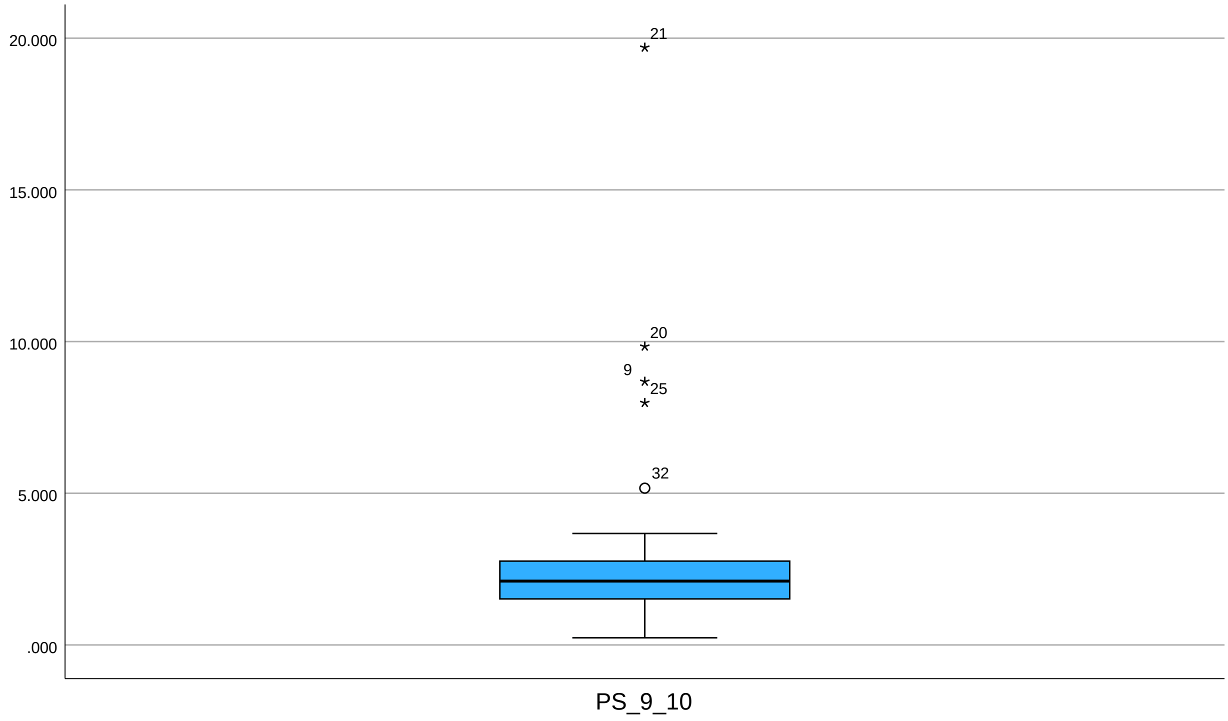
20.000
15.000
10.000
5.000
.000
21
20
9
25
32
PS_9_10

PS_10_11

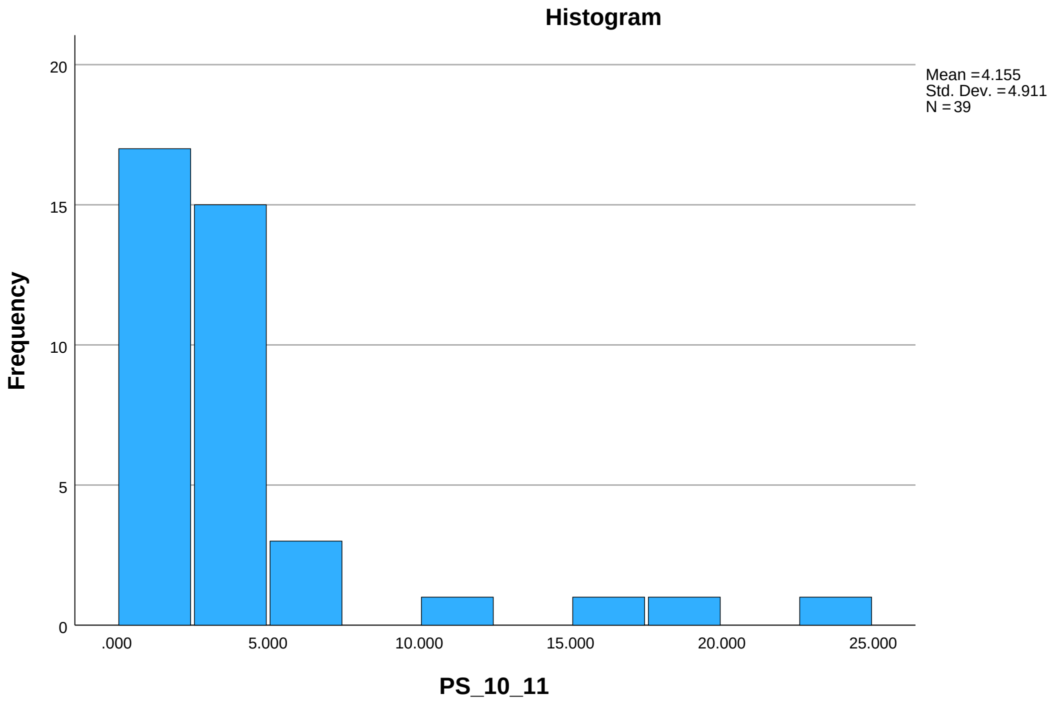


```
PS_10_11 Stem-and-Leaf Plot

 Frequency    Stem &  Leaf

     4.00        0 .  2359
    10.00        1 .  1233566777
     9.00        2 .  124566679
     4.00        3 .  2226
     5.00        4 .  23357
     2.00        5 .  05
     1.00        6 .  7
     4.00 Extremes    (>=10.3)

 Stem width:      1.000
 Each leaf:       1 case(s)
```

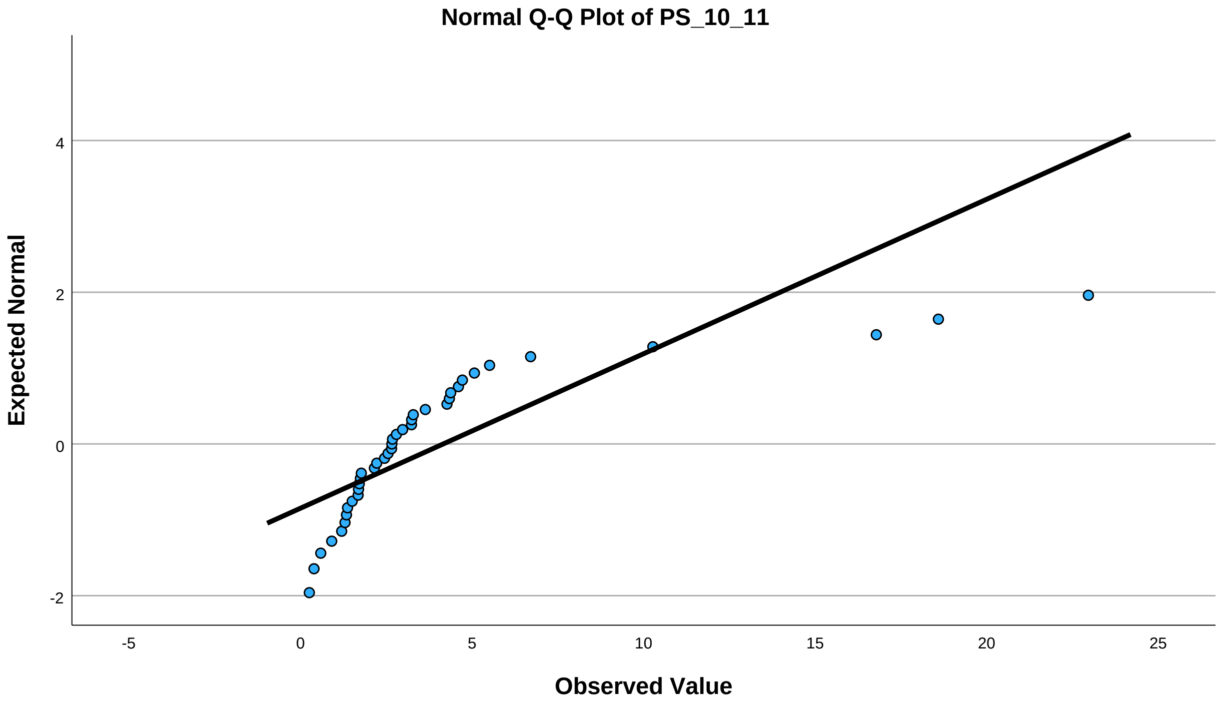
Normal Q-Q Plot of PS_10_11
Expected Normal
4
2
0
-2
-5
0
5
10
15
20
25
Observed Value

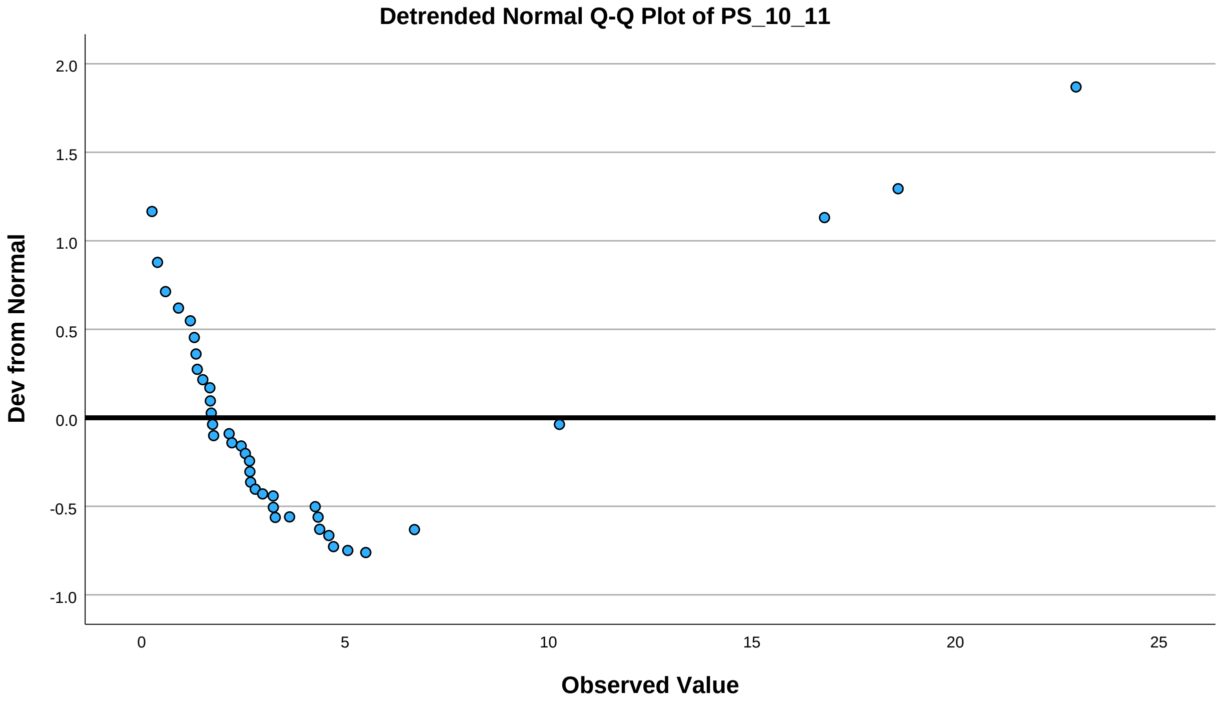
Detrended Normal Q-Q Plot of PS_10_11
Dev from Normal
2.0
1.5
1.0
0.5
0.0
-0.5
-1.0
0
5
10
15
20
25
Observed Value

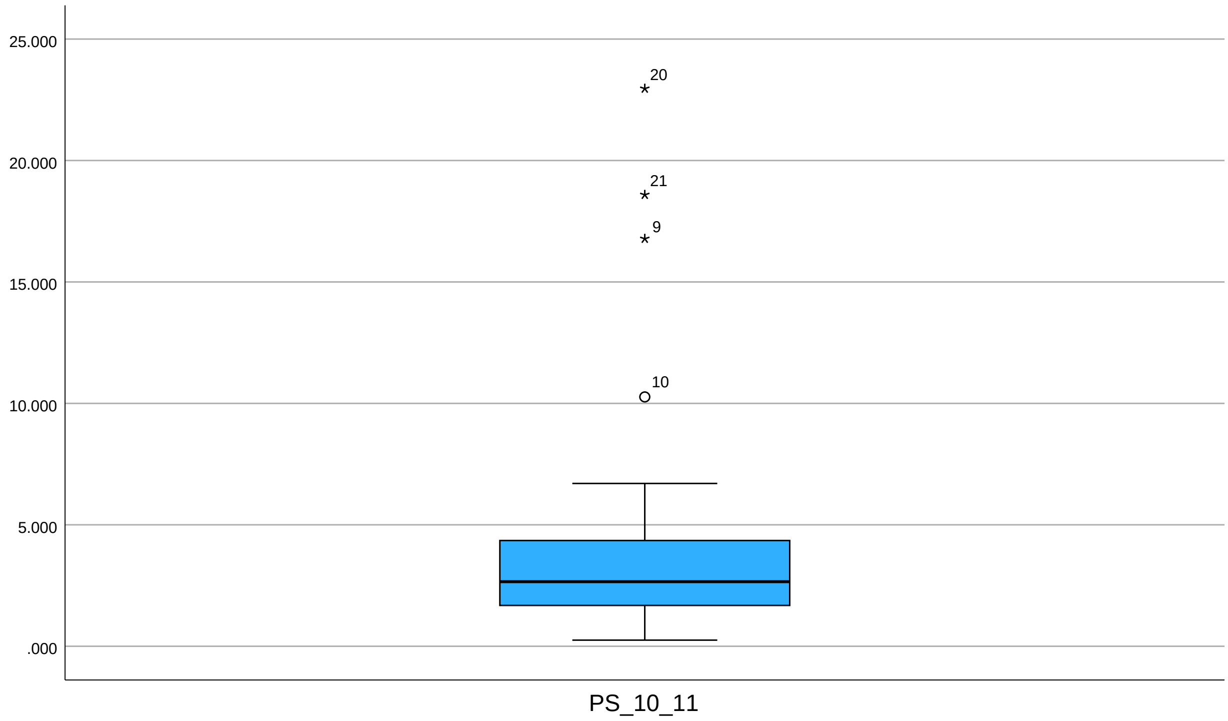

PS_11_12

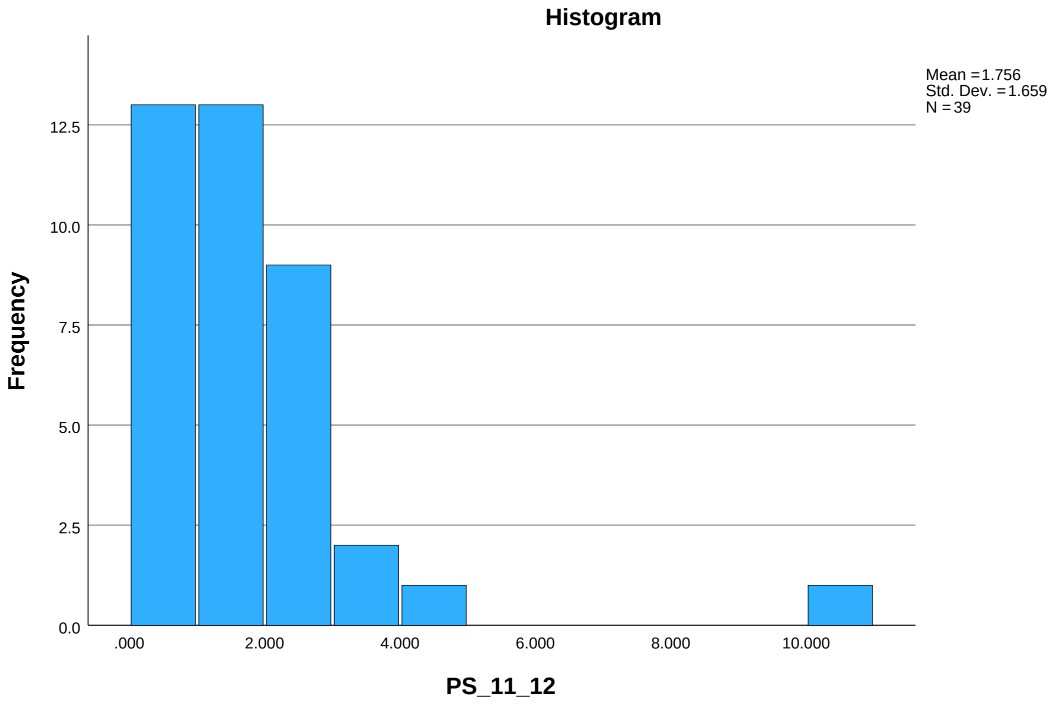

PS_11_12 Stem-and-Leaf Plot

Frequency   Stem & Leaf

```
   4.00        0 .  2244
   9.00        0 .  557788999
   9.00        1 .  001114444
   4.00        1 .  6788
   9.00        2 .  000122334
    .00        2 .
    .00        3 .
   1.00        3 .  8
   3.00 Extremes    (>=4.0)

Stem width:     1.000
Each leaf:        1 case(s)
```

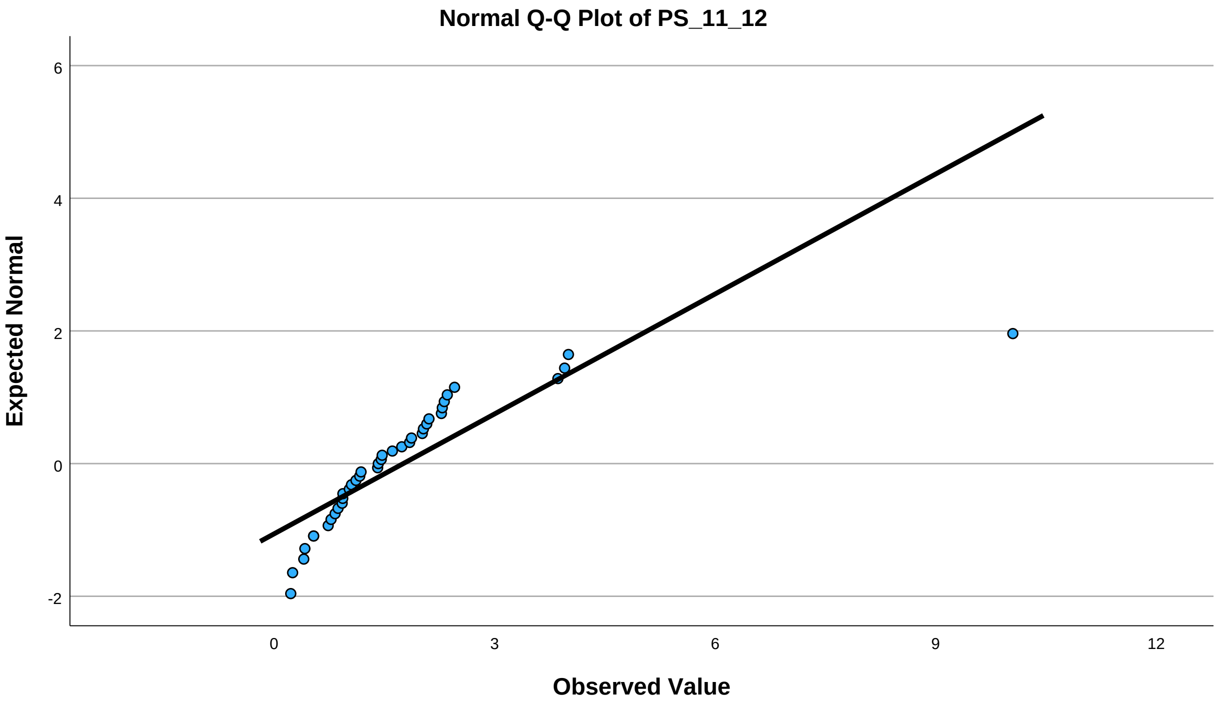

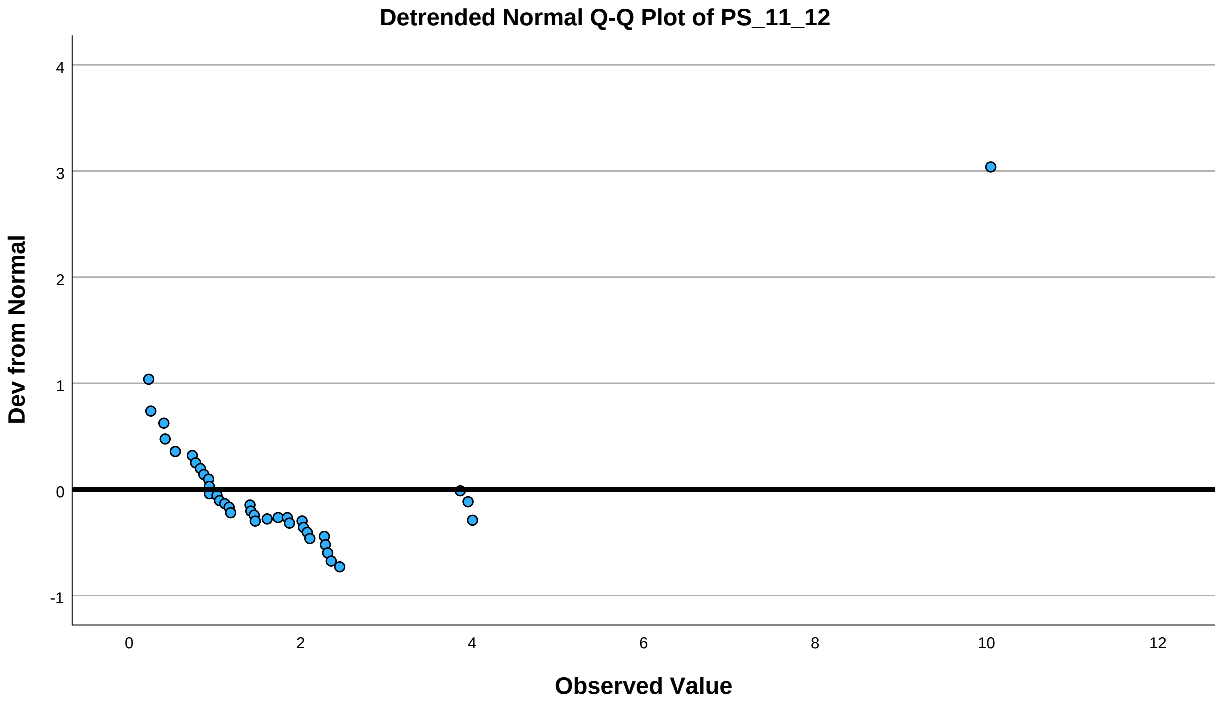
Detrended Normal Q-Q Plot of PS_11_12
Dev from Normal
4
3
2
1
0
-1
0
2
4
6
8
10
12
Observed Value

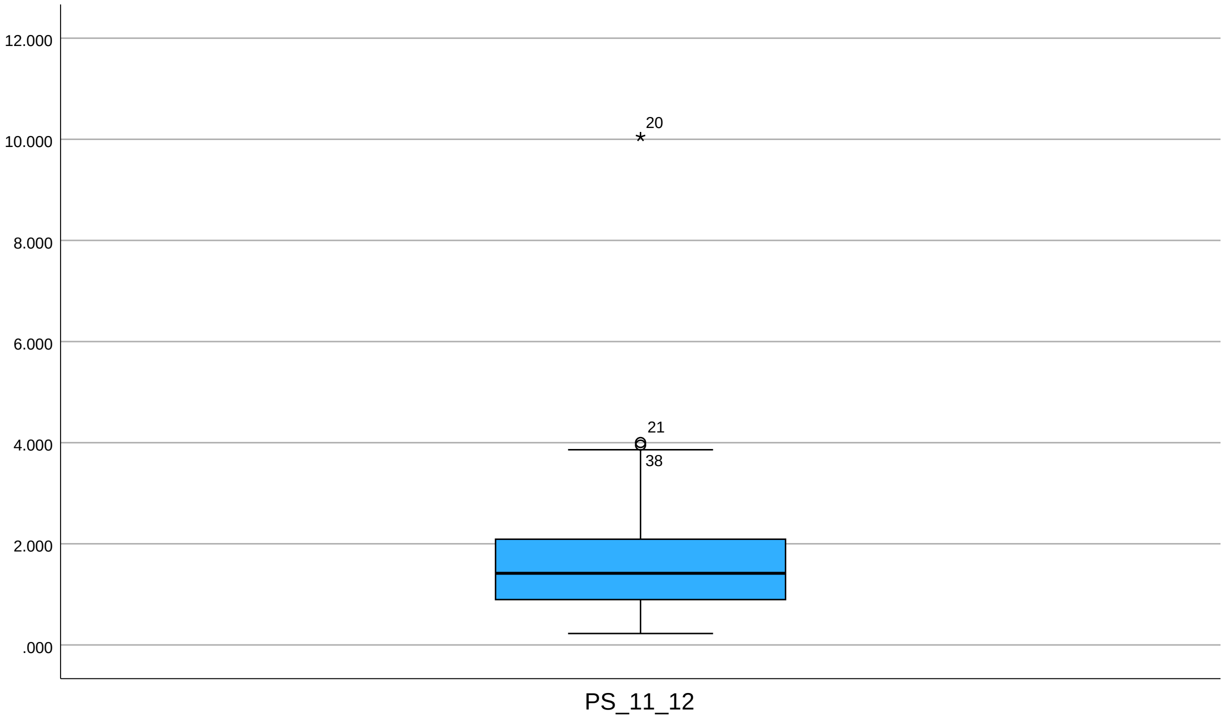
12.000
10.000
8.000
6.000
4.000
2.000
.000
20
21
38
PS_11_12

PS_12_13

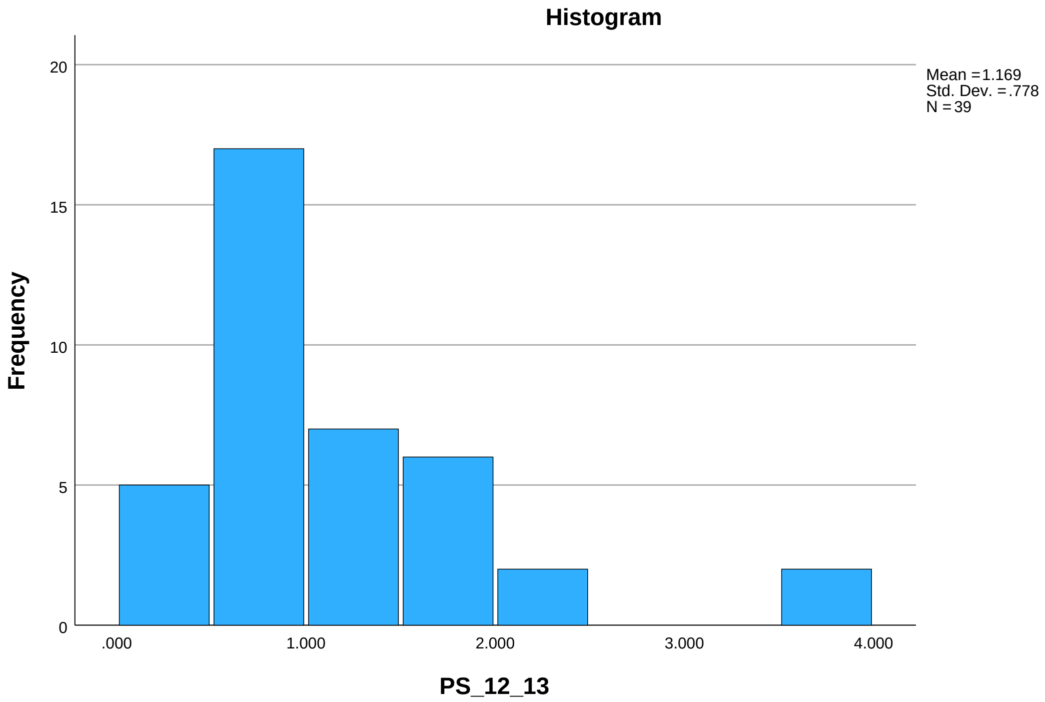

PS_12_13 Stem-and-Leaf Plot

```
 Frequency    Stem &  Leaf

     5.00        0 .  12244
    17.00        0 .  56667777888889999
     7.00        1 .  0022234
     6.00        1 .  555778
     2.00        2 .  02
     2.00 Extremes    (>=3.7)

 Stem width:      1.000
 Each leaf:        1 case(s)
```

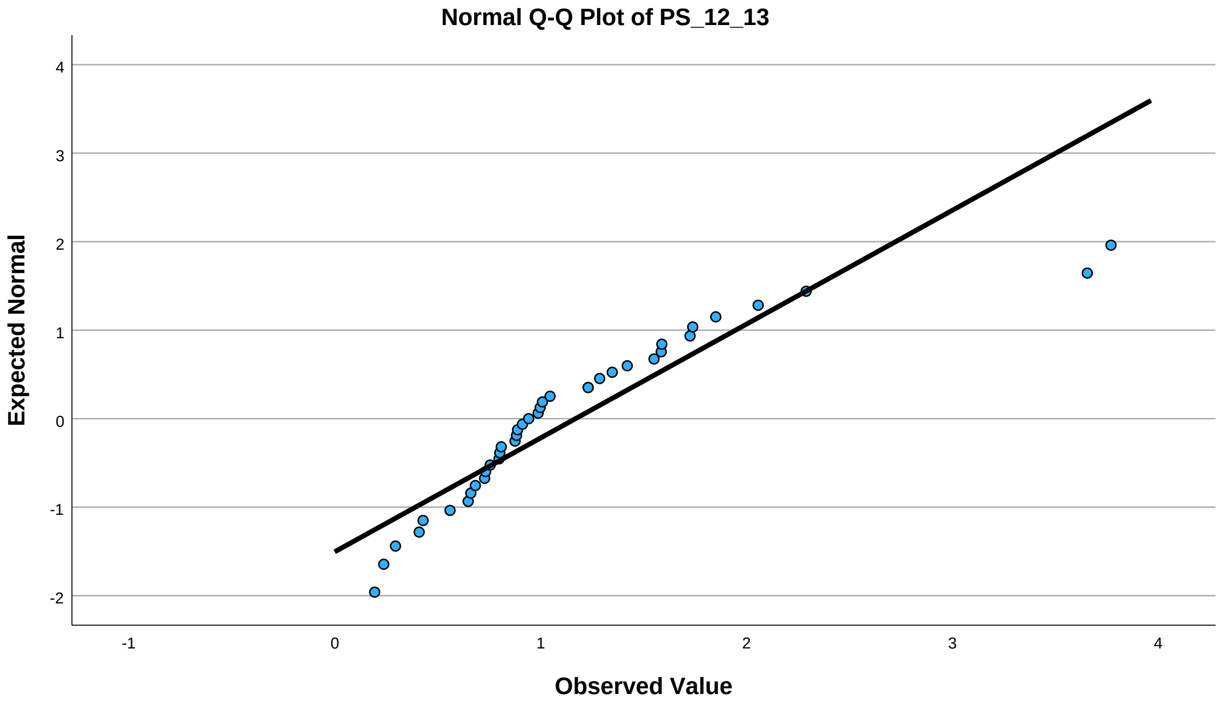
Normal Q-Q Plot of PS_12_13
Expected Normal
Observed Value
4
3
2
1
0
-1
-2
-1
0
1
2
3
4

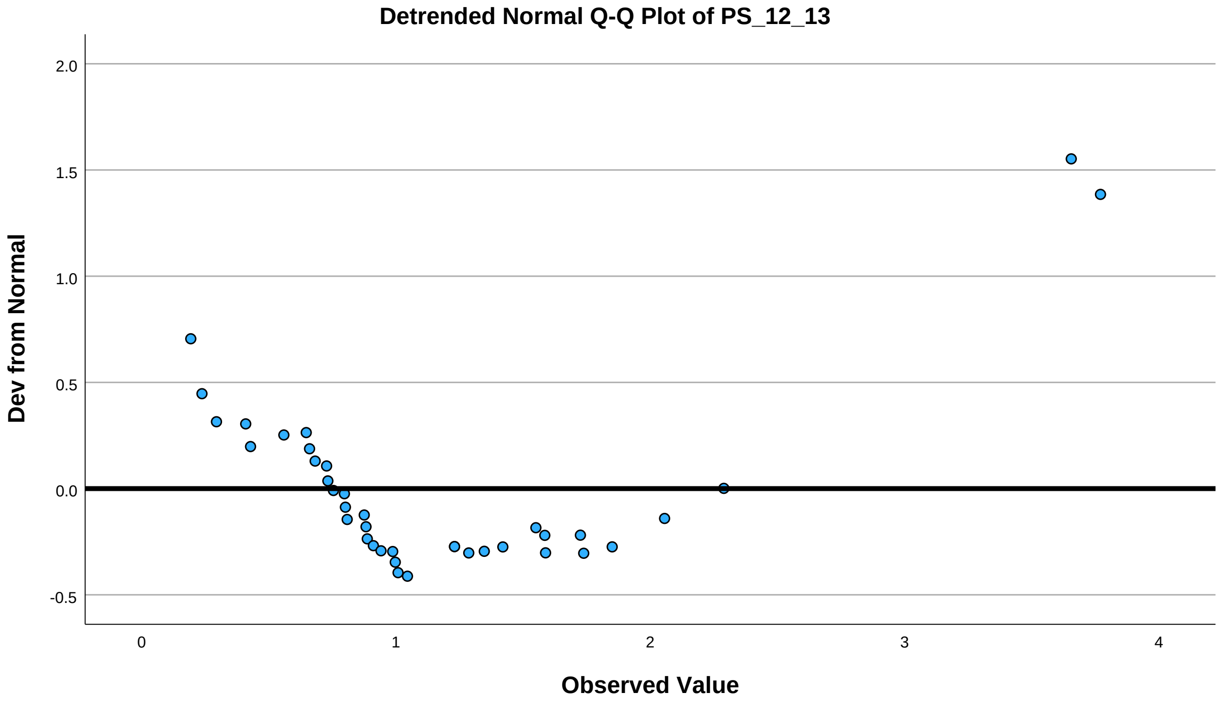
Detrended Normal Q-Q Plot of PS_12_13
Dev from Normal
Observed Value
2.0
1.5
1.0
0.5
0.0
-0.5
0
1
2
3
4

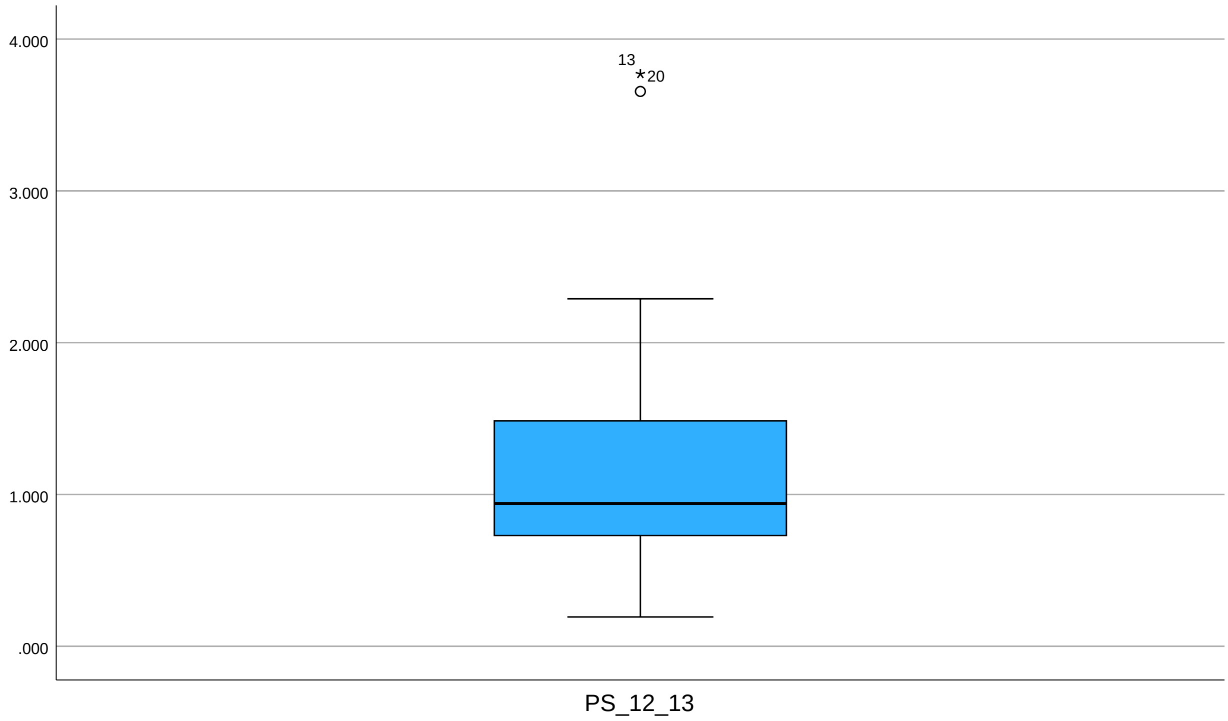

PS_13_14

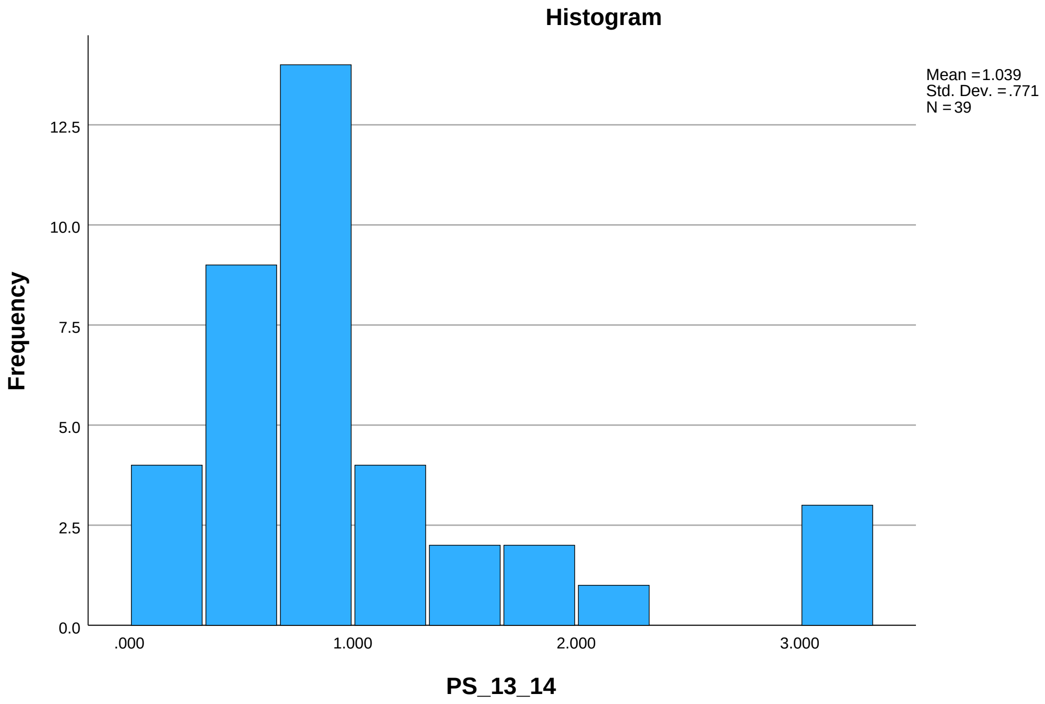

PS_13_14 Stem-and-Leaf Plot

Frequency   Stem & Leaf

```
 6.00      0 . 112333
 21.00      0 .  555555666777788999999
 6.00      1 . 022244
 2.00     1 . 79
 1.00     2 . 1
 3.00 Extremes    (>=3.1)

Stem width:     1.000
Each leaf:       1 case(s)
```

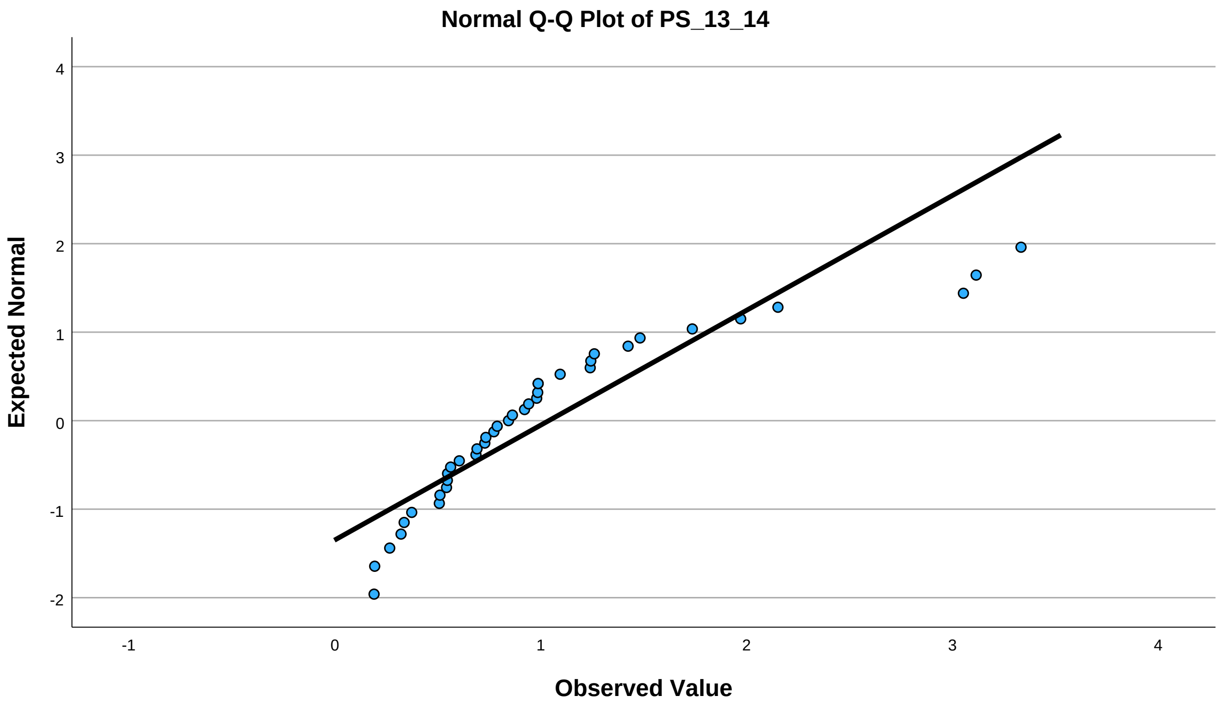

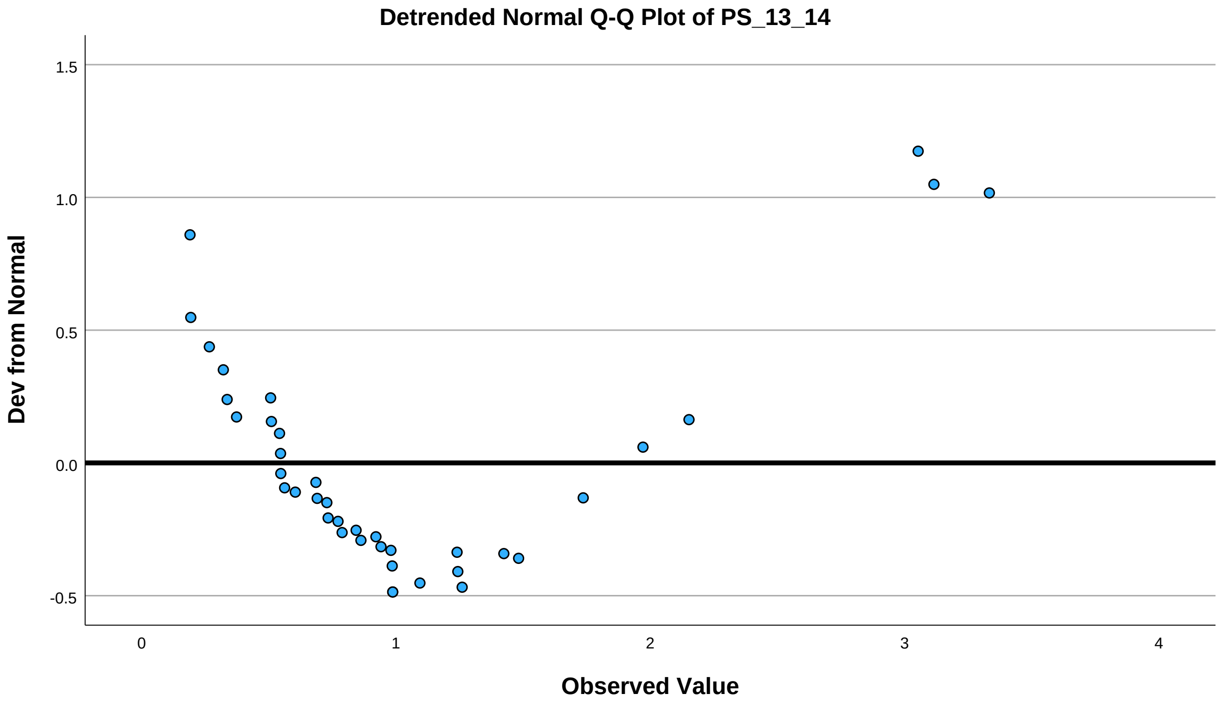
Detrended Normal Q-Q Plot of PS_13_14
Dev from Normal
1.5
1.0
0.5
0.0
-0.5
0
1
2
3
4
Observed Value

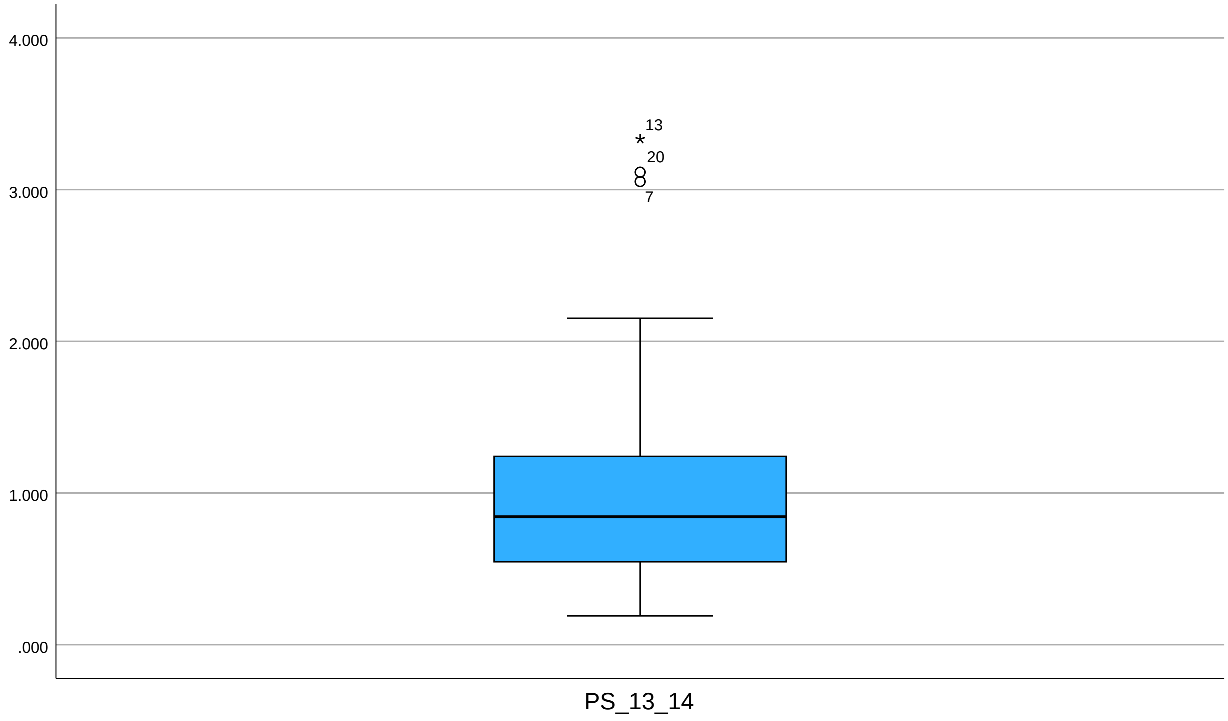
4.000
3.000
2.000
1.000
.000
13
20
7
PS_13_14

PS_14_15

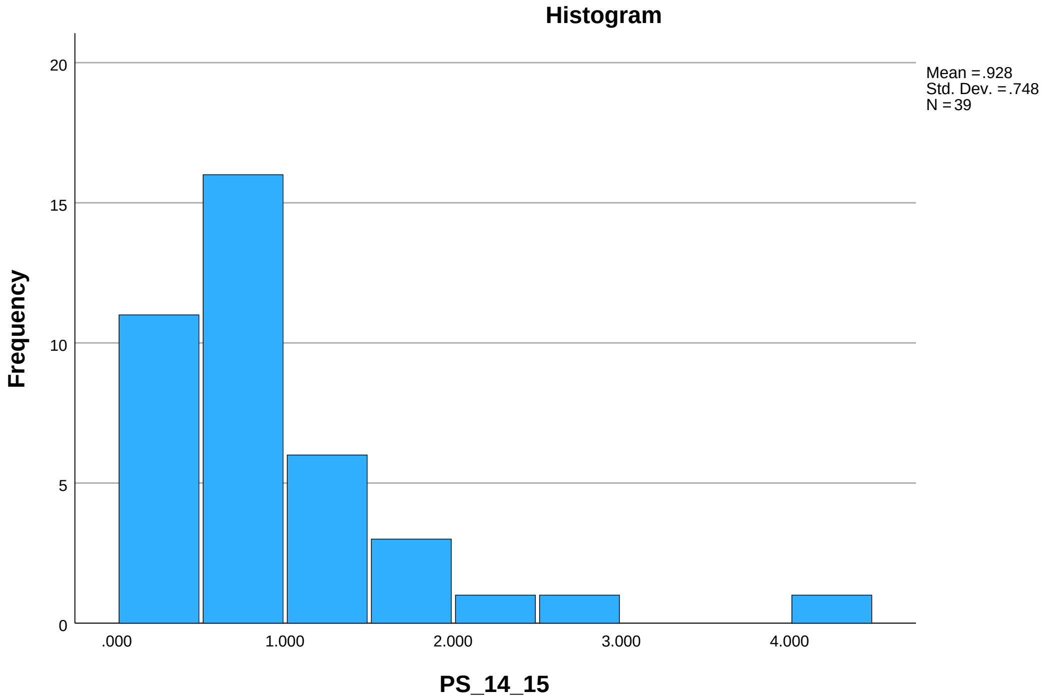


```
PS_14_15 Stem-and-Leaf Plot

 Frequency    Stem &  Leaf

    11.00        0 .  11222334444
    16.00        0 .  5556666667777788
     6.00        1 .  112344
     3.00        1 .  566
     1.00        2 .  2
     2.00 Extremes    (>=2.5)

 Stem width:      1.000
 Each leaf:       1 case(s)
```

Normal Q-Q Plot of PS_14_15

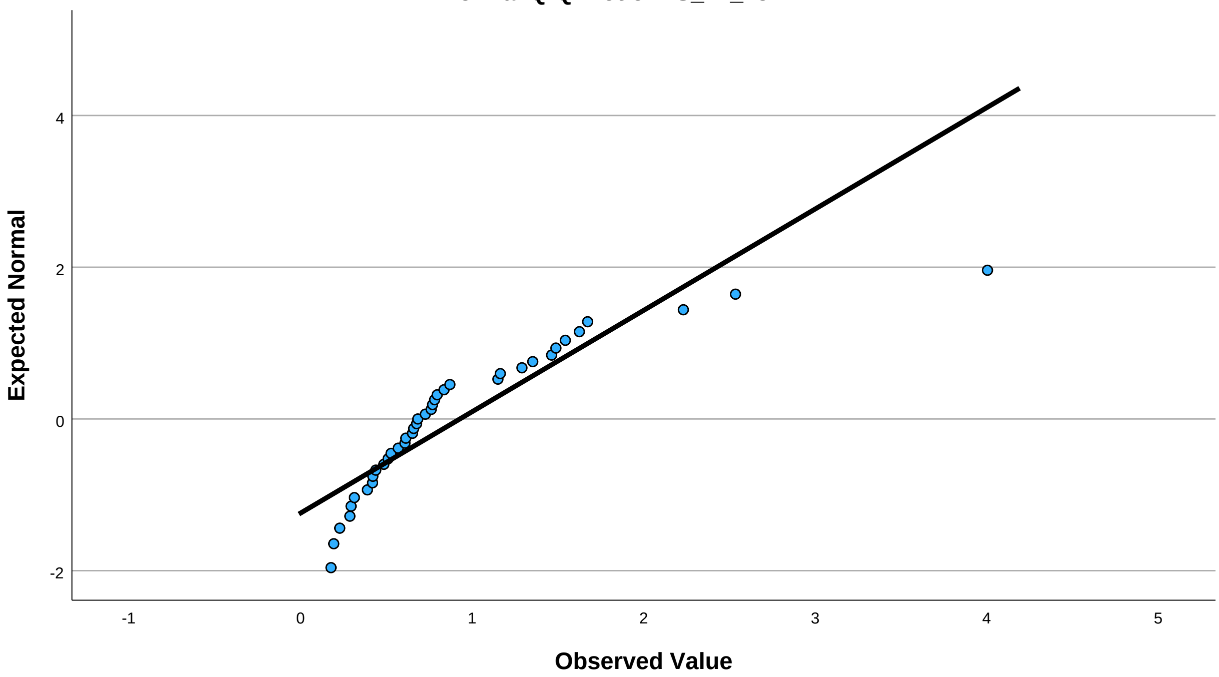

Detrended Normal Q-Q Plot of PS_14_15

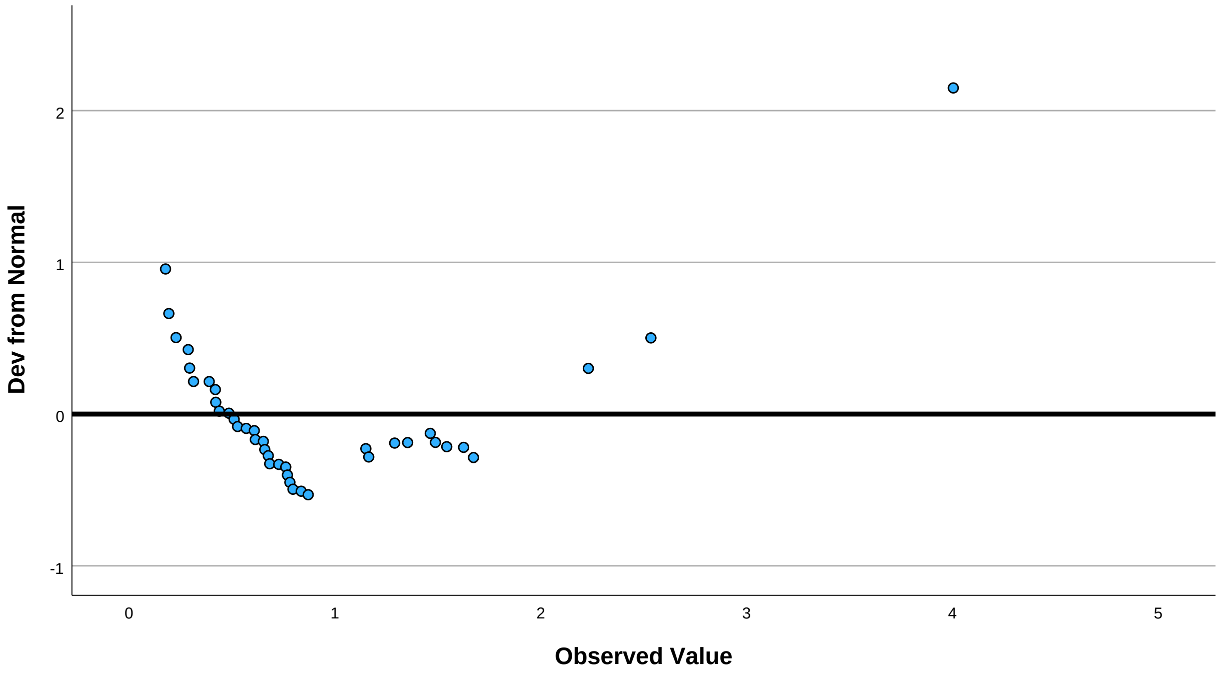

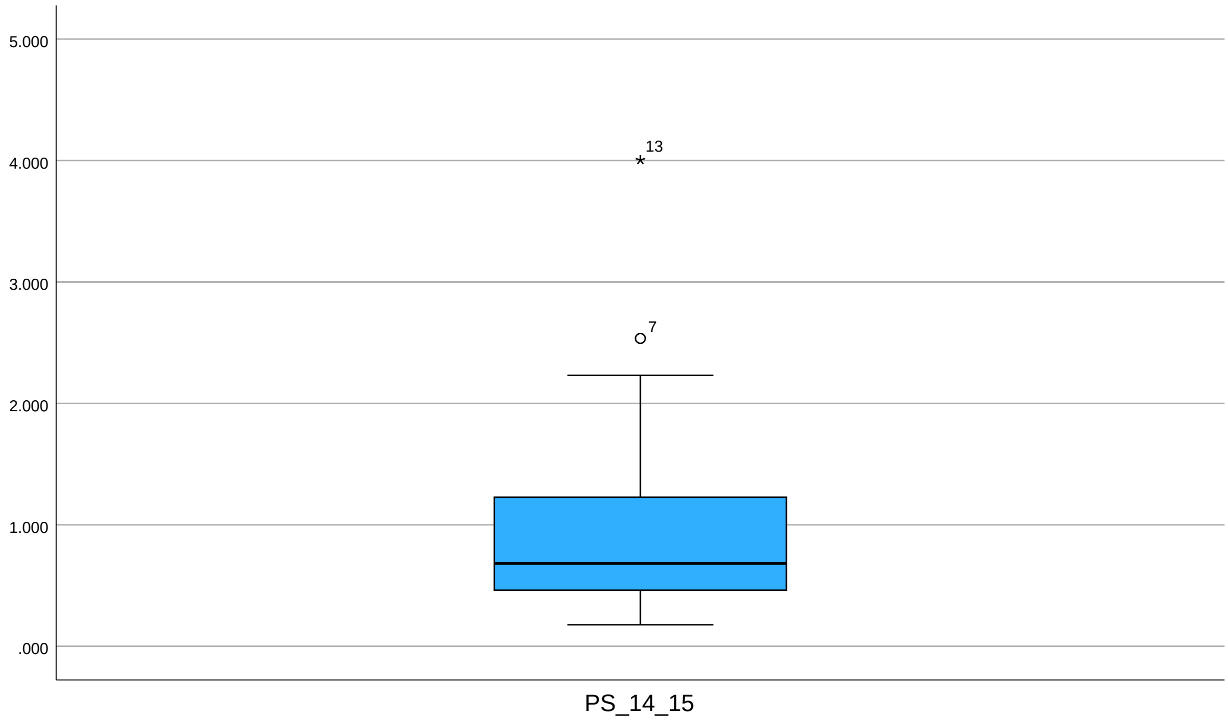

PS_15_16

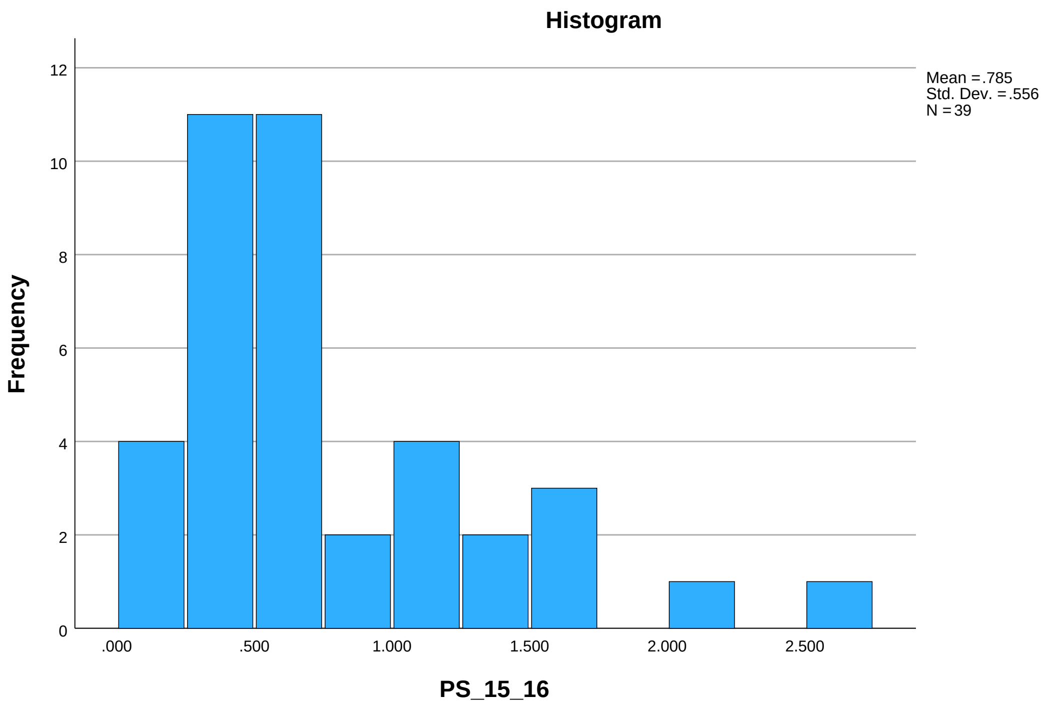

PS_15_16 Stem-and-Leaf Plot

Frequency   Stem & Leaf

```
 2.00      0 . 11
 6.00      0 . 223333
13.00      0 . 4444444555555
 5.00      0 . 66667
 2.00      0 . 89
 3.00      1 . 011
 3.00      1 . 233
 2.00      1 . 55
 1.00      1 . 6
 2.00 Extremes    (>=2.2)

Stem width:     1.000
Each leaf:       1 case(s)
```

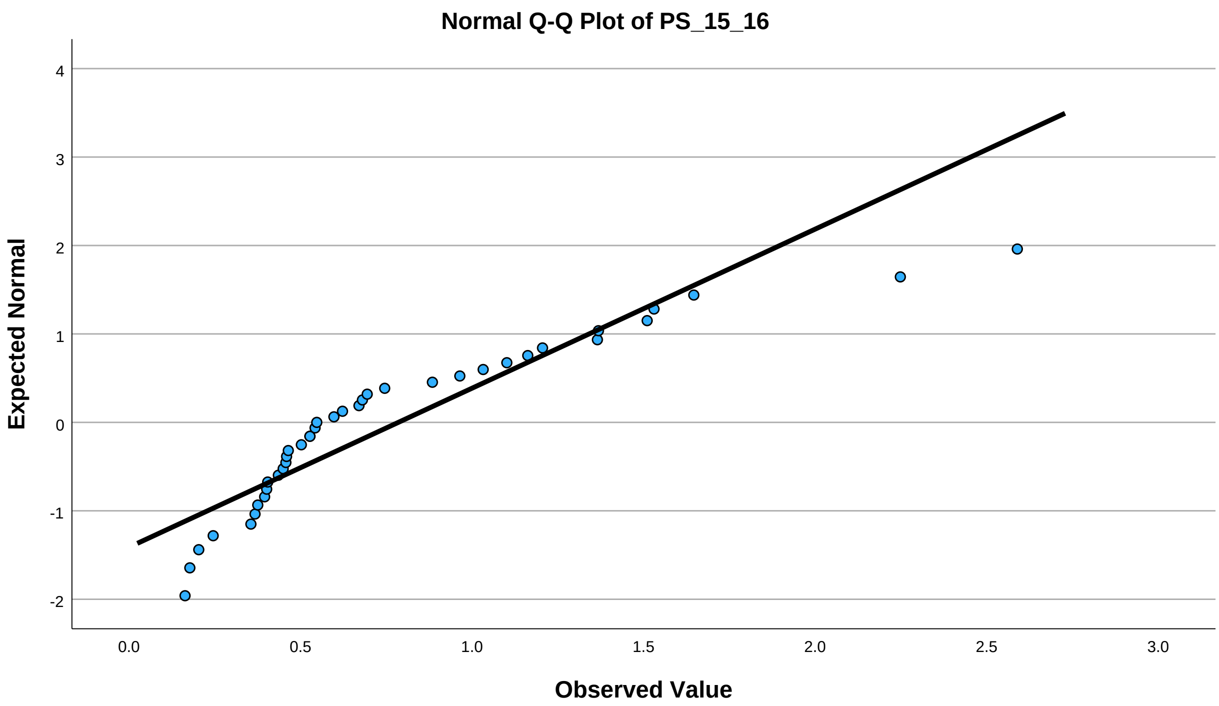

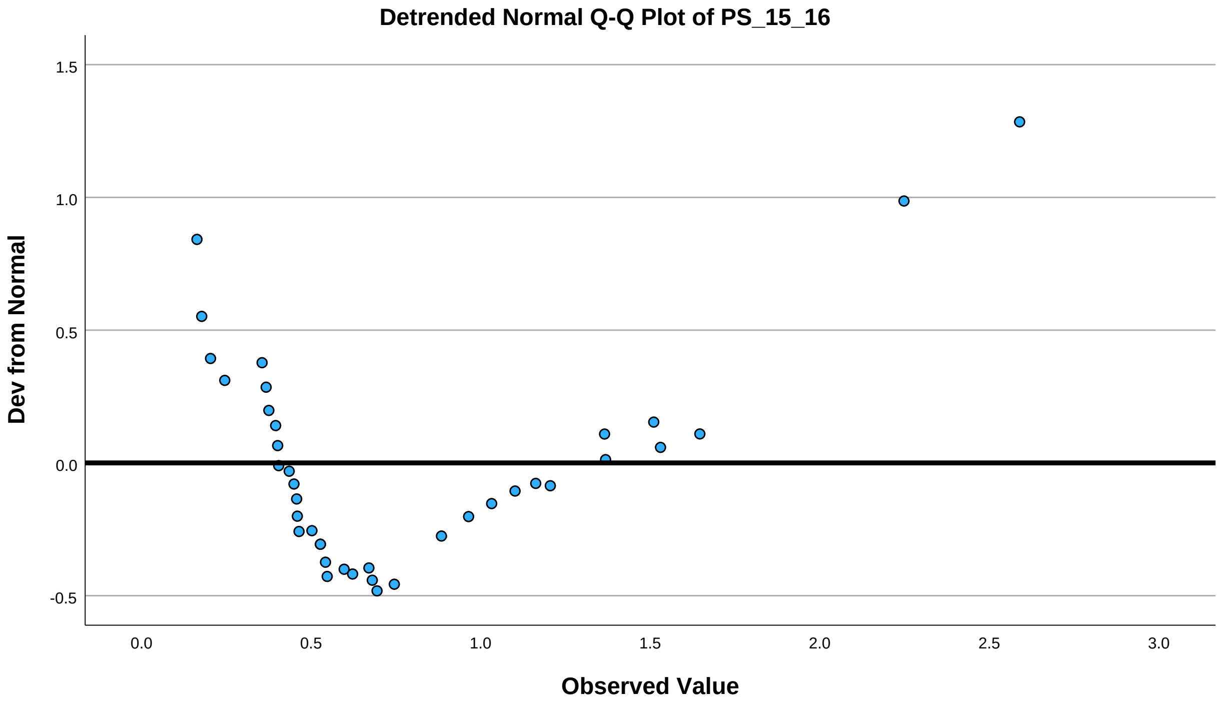

Detrended Normal Q-Q Plot of PS_15_16
Dev from Normal
1.5
1.0
0.5
0.0
-0.5
0.0
0.5
1.0
1.5
2.0
2.5
3.0
Observed Value

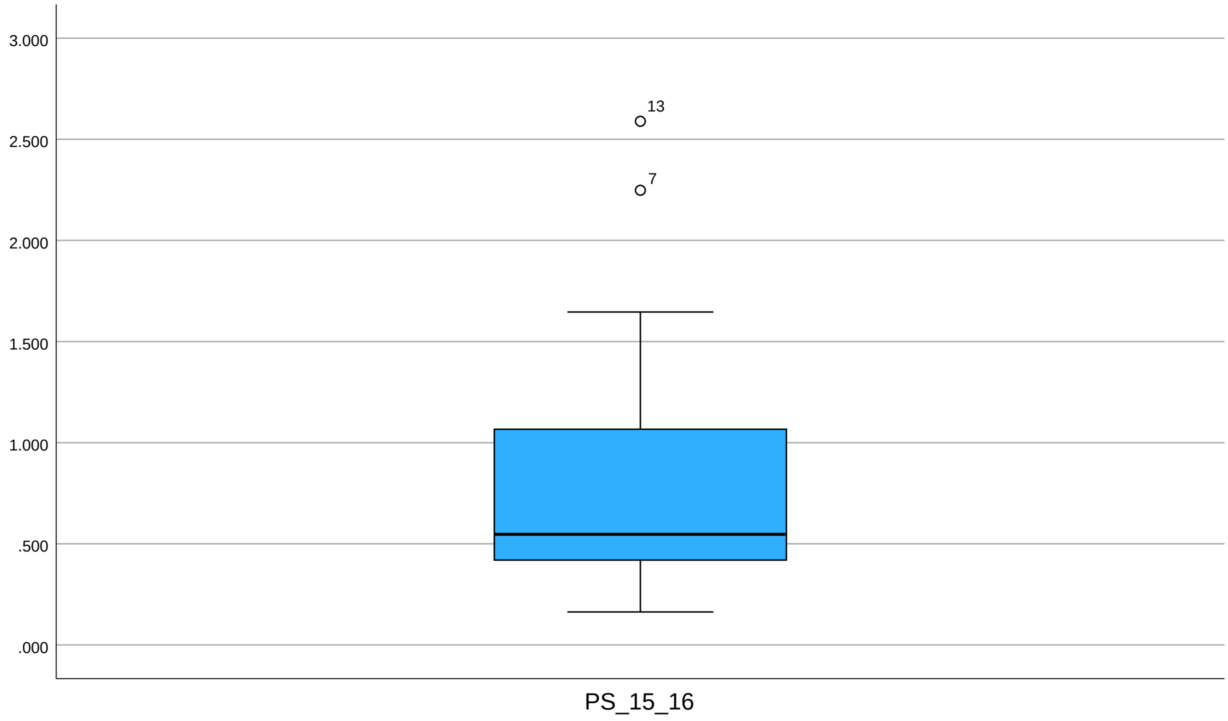

3.000
2.500
2.000
1.500
1.000
.500
.000
13
7
PS_15_16

PS_16_17

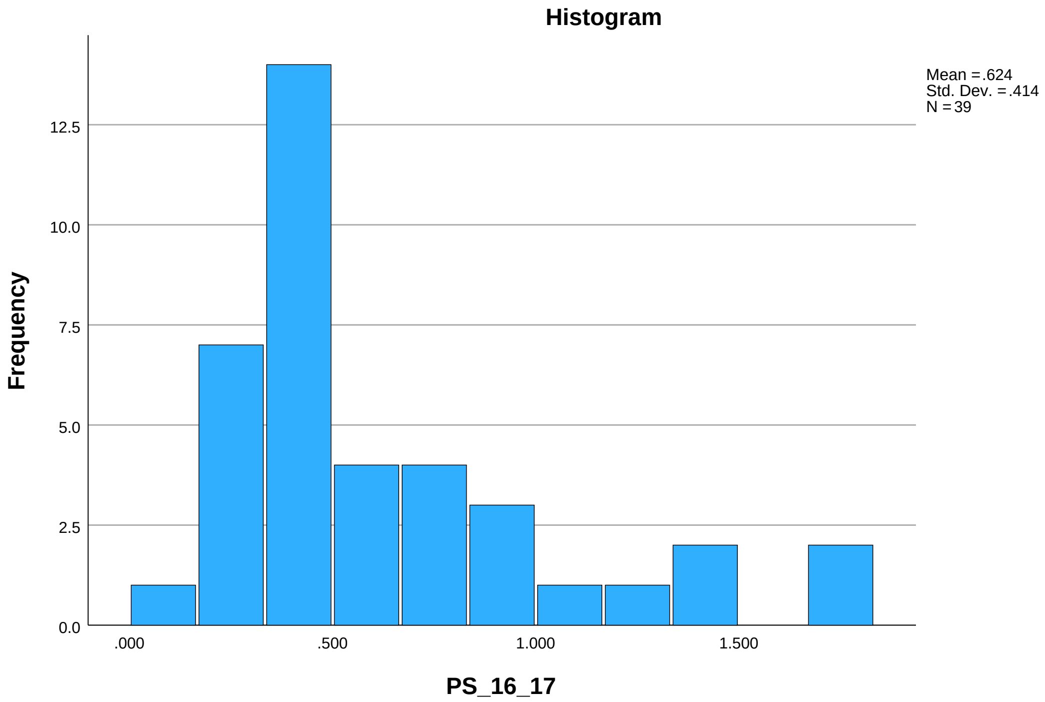


```
PS_16_17 Stem-and-Leaf Plot

 Frequency    Stem &  Leaf

     2.00        1 .  69
     5.00        2 .  23458
     7.00        3 .  0334789
     8.00        4 .  13446779
     1.00        5 .  2
     4.00        6 .  1247
     3.00        7 .  001
     2.00        8 .  59
     1.00        9 .  7
     1.00       10 .  7
     5.00 Extremes    (>=1.32)

 Stem width:      .100
 Each leaf:       1 case(s)
```

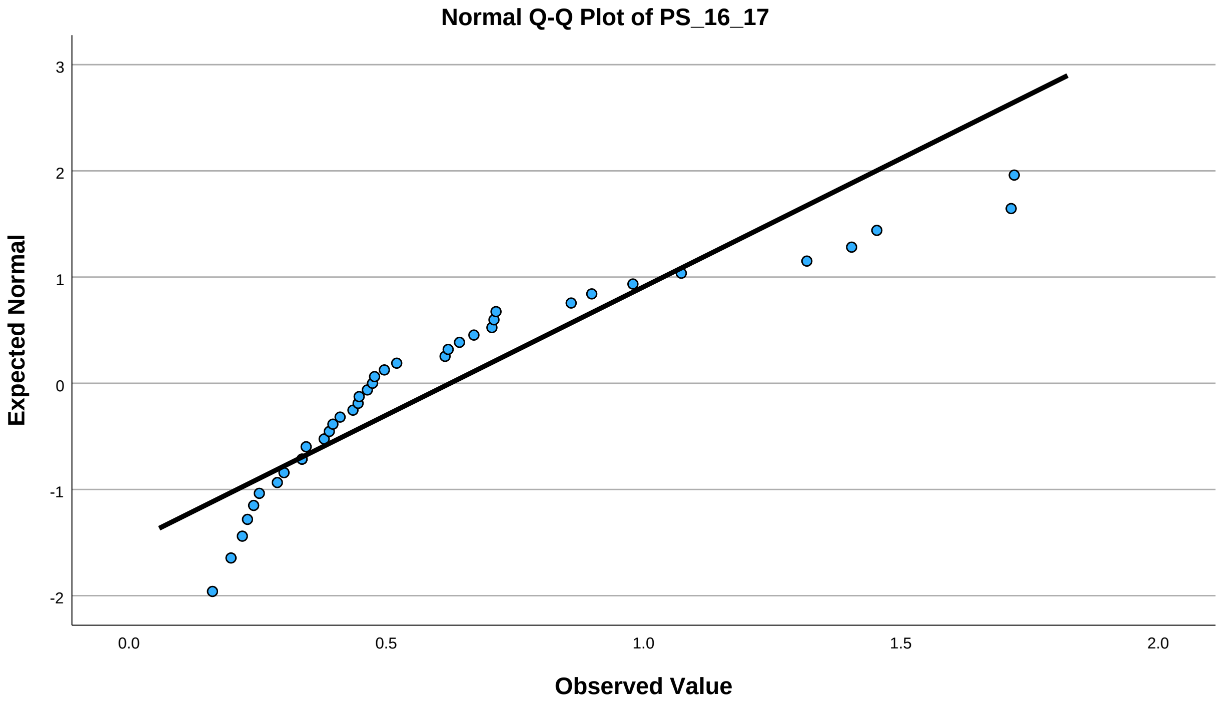
Normal Q-Q Plot of PS_16_17
Expected Normal
3
2
1
0
-1
-2
0.0
0.5
1.0
1.5
2.0
Observed Value

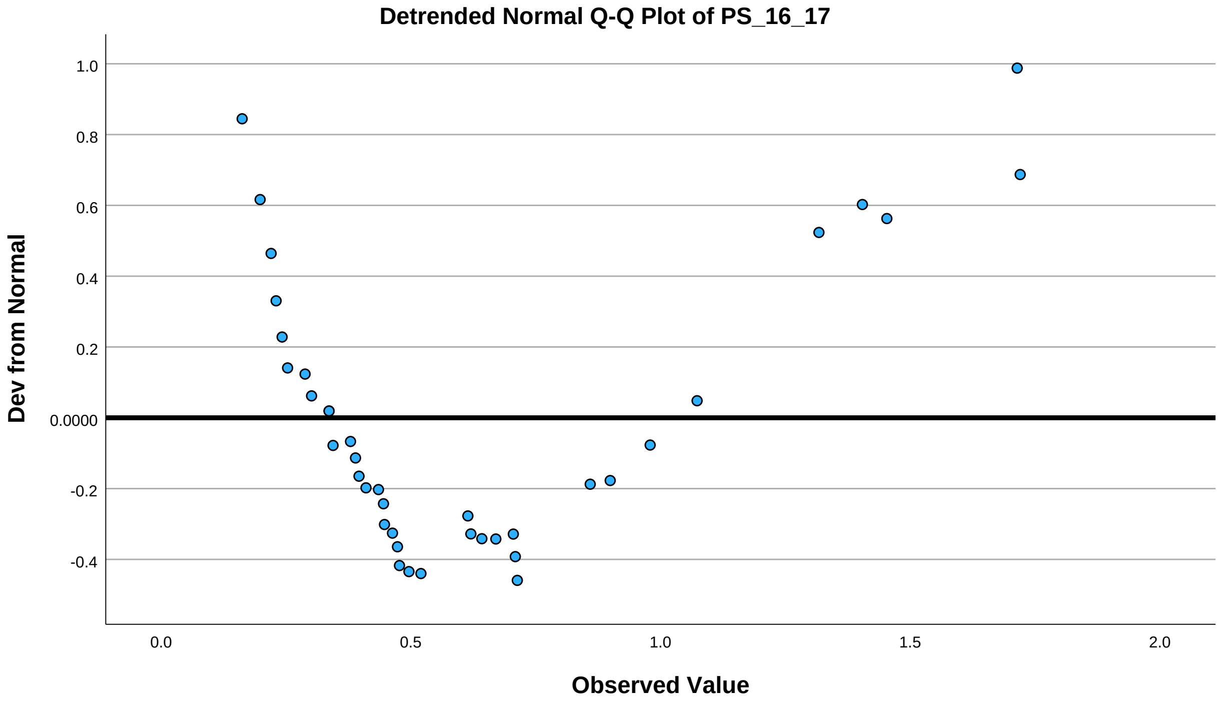
Detrended Normal Q-Q Plot of PS_16_17
Dev from Normal
1.0
0.8
0.6
0.4
0.2
0.0000
-0.2
-0.4
0.0
0.5
1.0
1.5
2.0
Observed Value

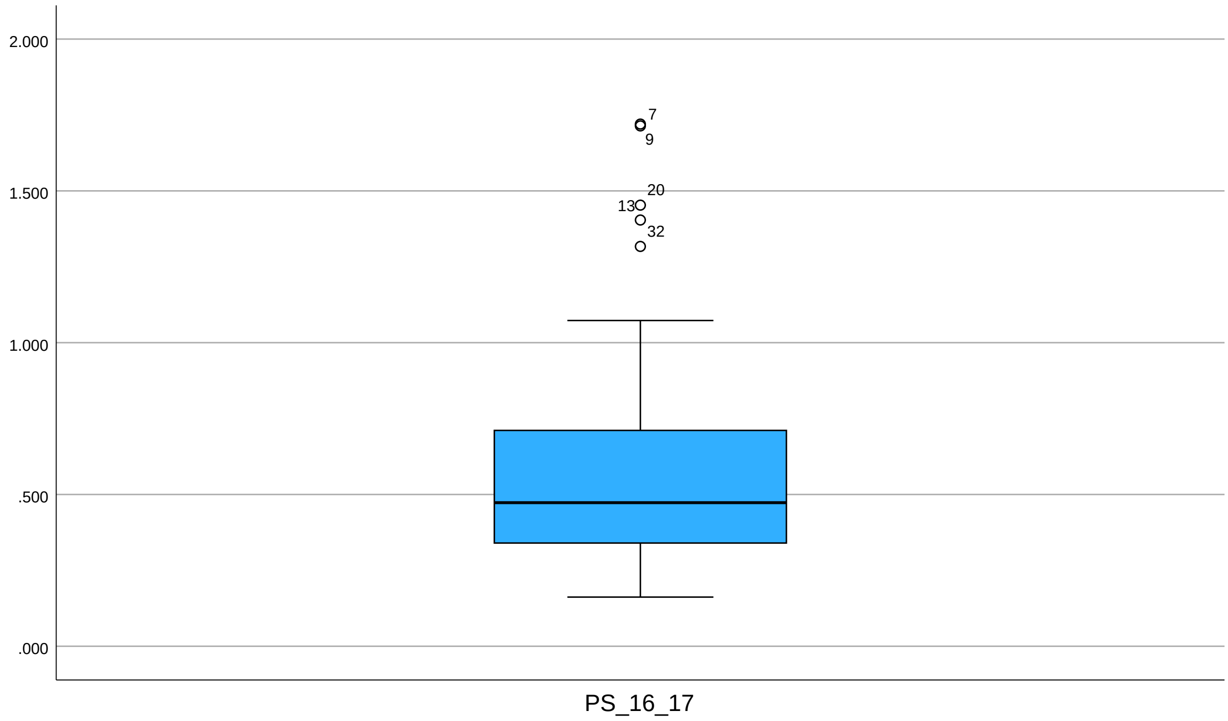

PS_17_18

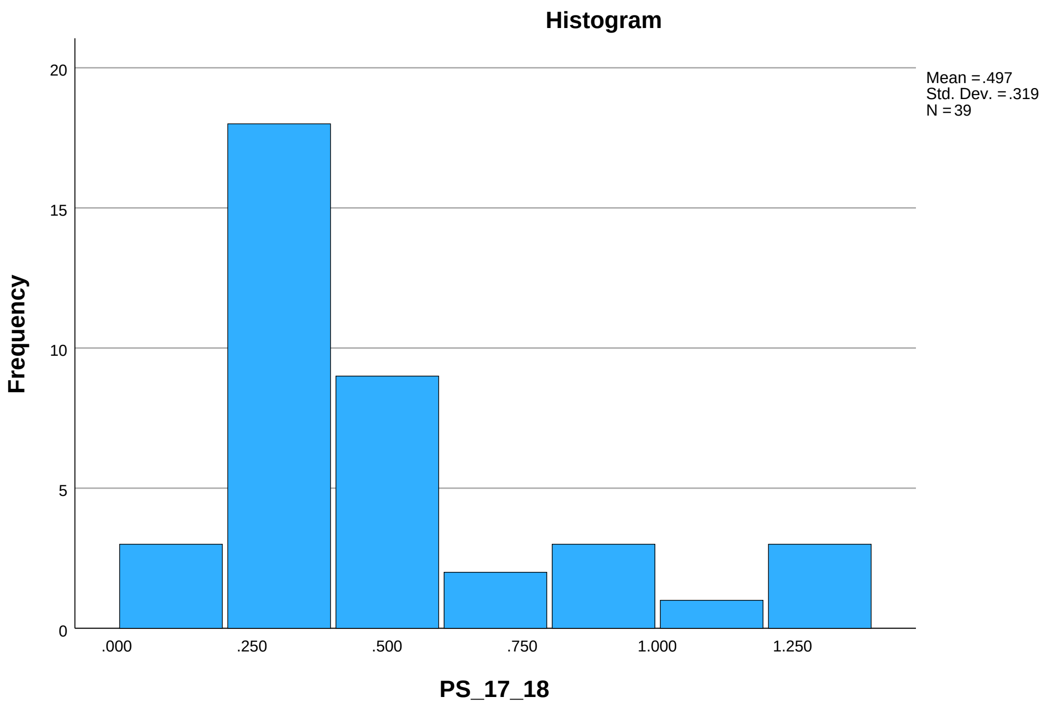

PS_17_18 Stem-and-Leaf Plot

Frequency   Stem & Leaf

```
    3.00        1 .  577
    7.00        2 .  0113579
   11.00        3 .  11244567788
    5.00        4 .  00033
    4.00        5 .  3446
     .00        6 .
    2.00        7 .  08
    2.00        8 .  13
    5.00 Extremes    (>=.98)

 Stem width:      .100
 Each leaf:       1 case(s)
```

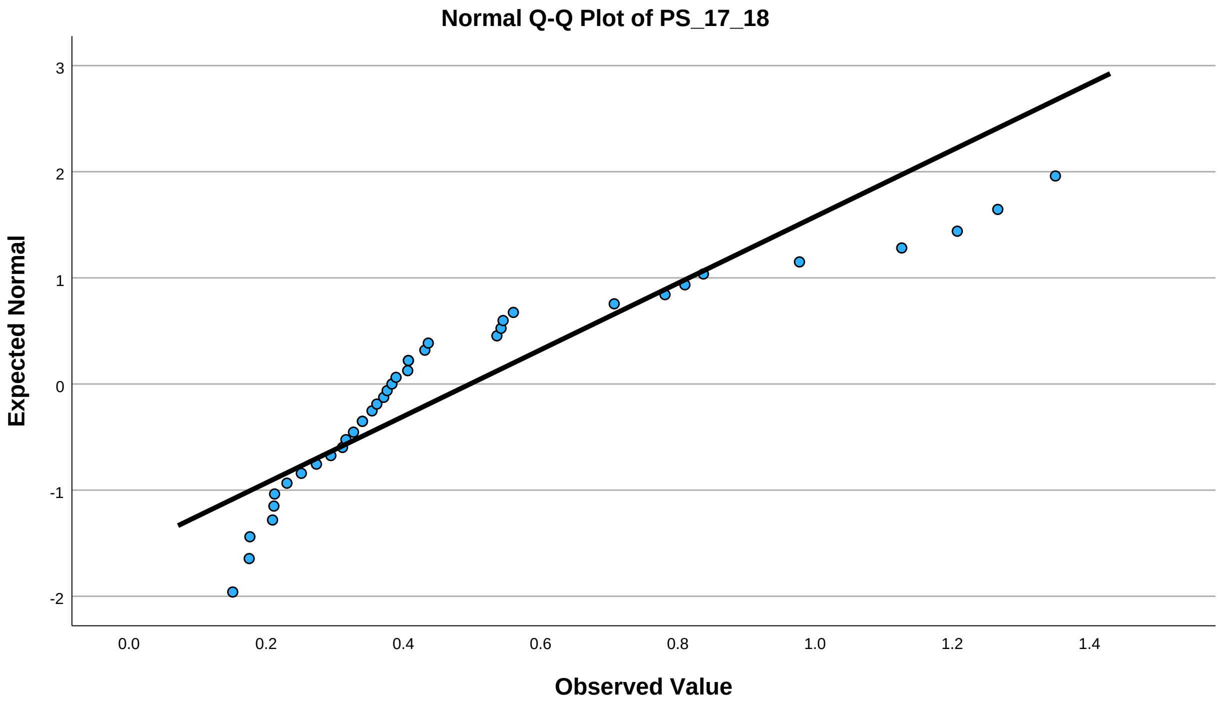

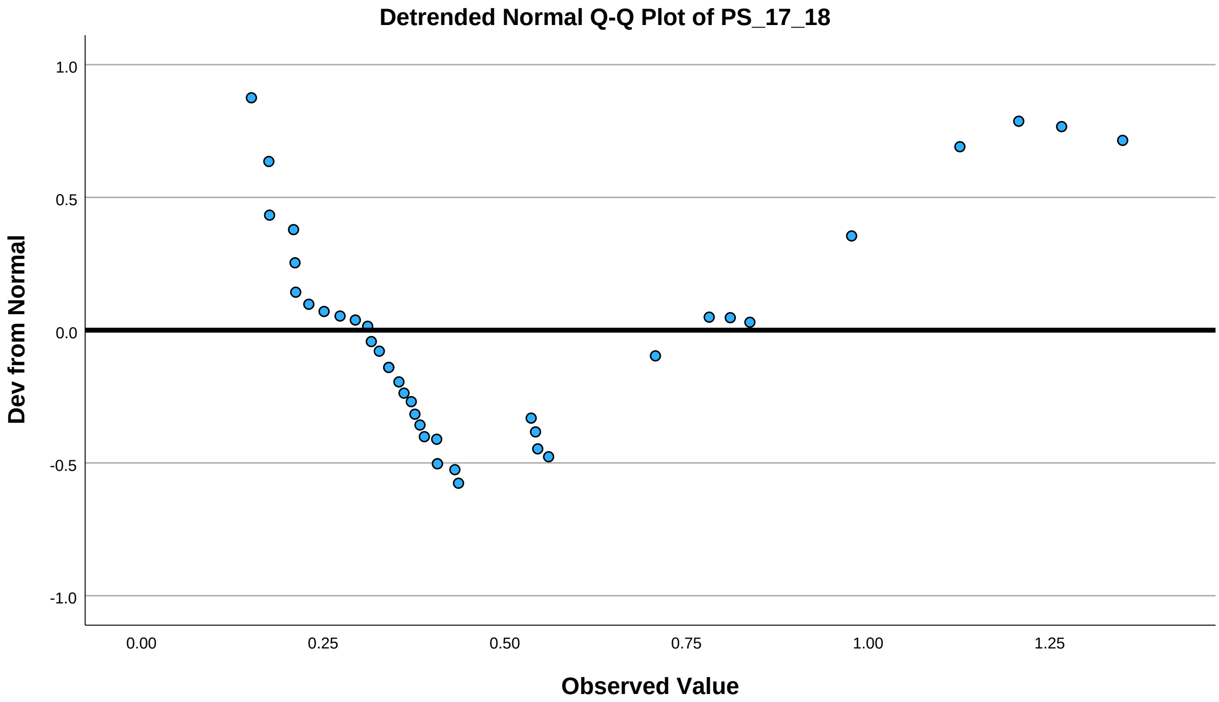
Detrended Normal Q-Q Plot of PS_17_18
Dev from Normal
1.0
0.5
0.0
-0.5
-1.0
0.00
0.25
0.50
0.75
1.00
1.25
Observed Value

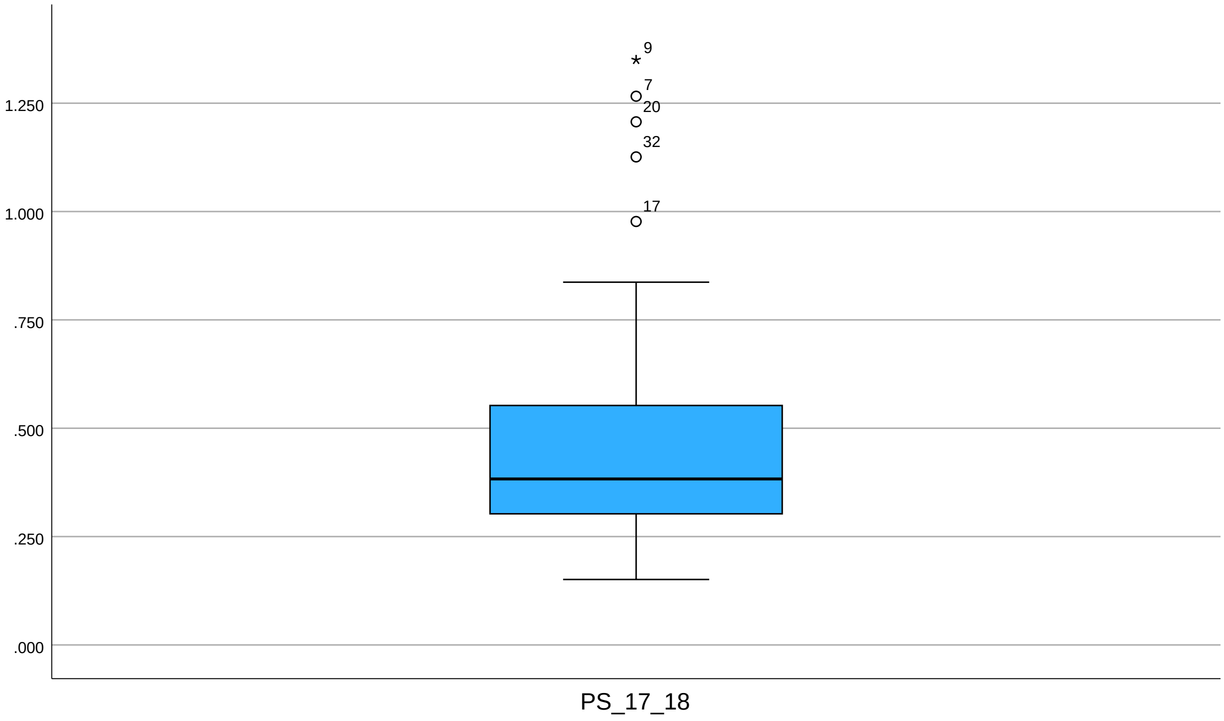
9
7
20
32
17
1.250
1.000
.750
.500
.250
.000
PS_17_18

PS_18_19

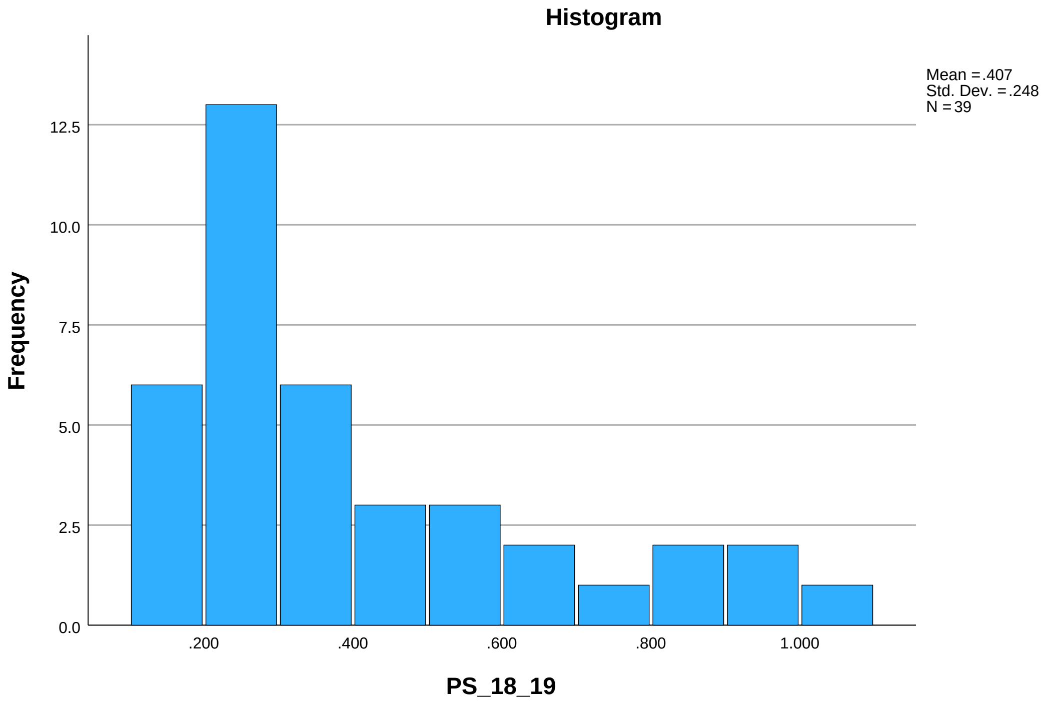


```
PS_18_19 Stem-and-Leaf Plot

 Frequency    Stem &  Leaf

     6.00        1 .  357899
    13.00        2 .  0122345677899
     6.00        3 .  233349
     3.00        4 .  005
     3.00        5 .  012
     2.00        6 .  37
     1.00        7 .  0
     2.00        8 .  46
     1.00        9 .  1
     2.00 Extremes    (>=.98)

 Stem width:      .100
 Each leaf:       1 case(s)
```

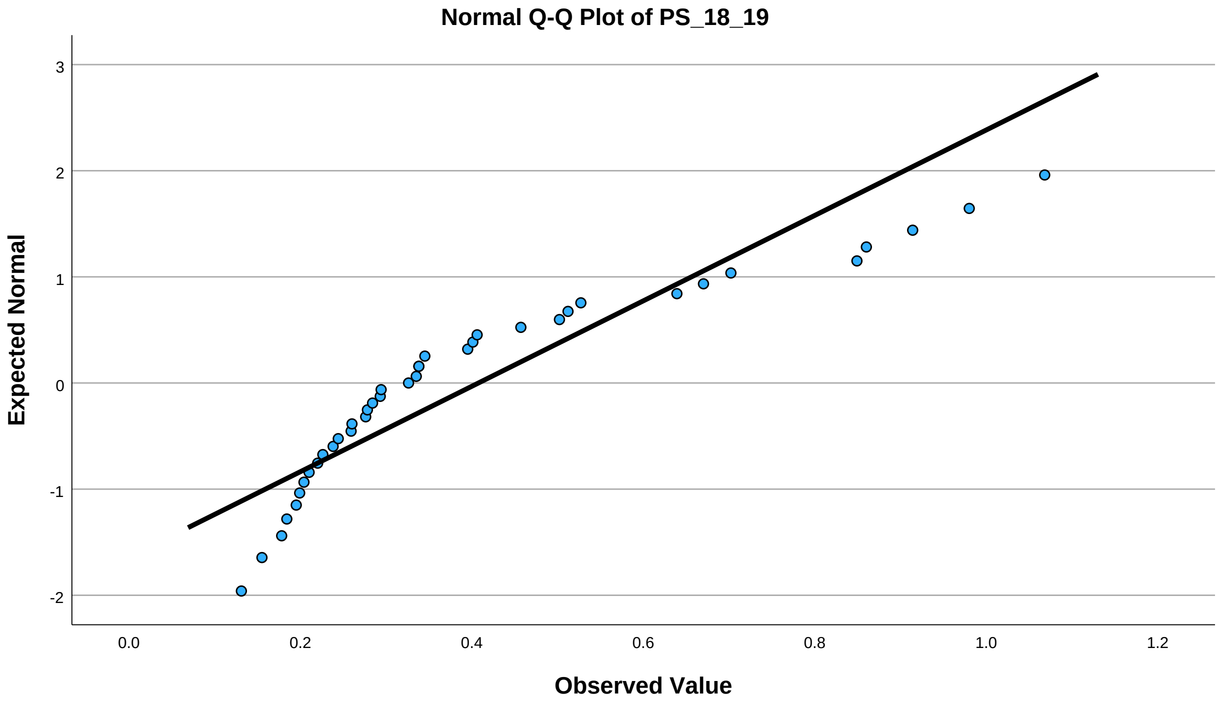
Normal Q-Q Plot of PS_18_19
Expected Normal
3
2
1
0
-1
-2
0.0
0.2
0.4
0.6
0.8
1.0
1.2
Observed Value

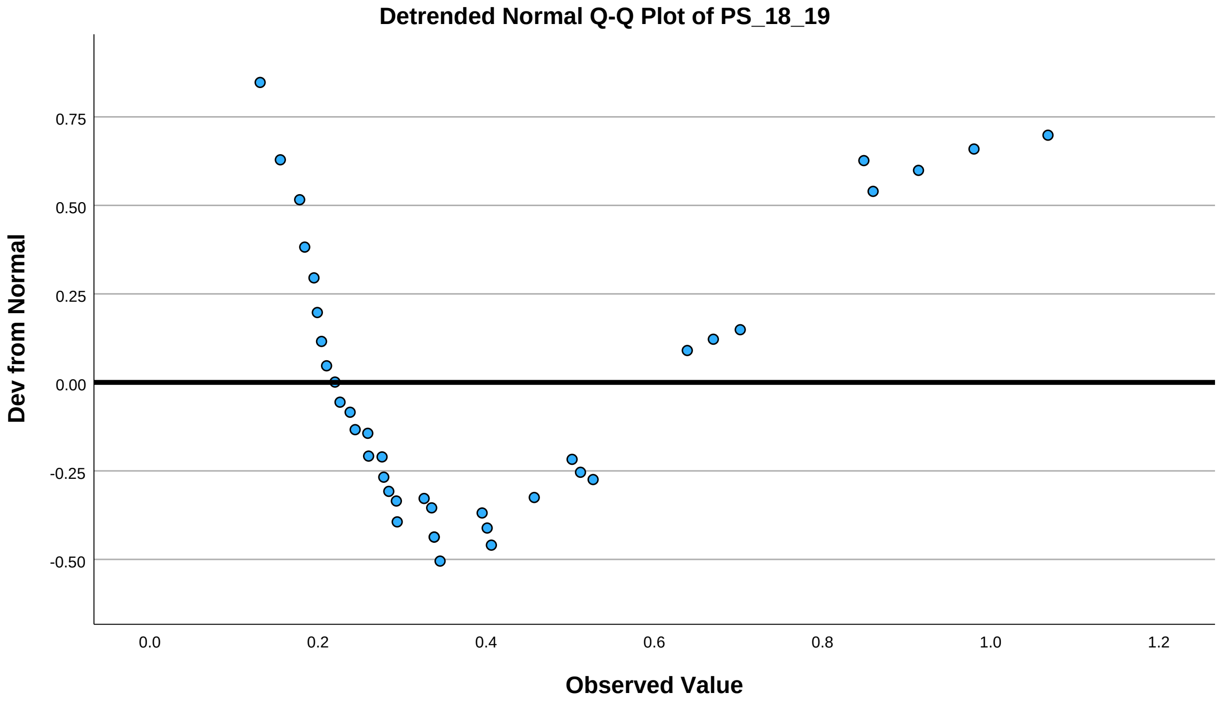
Detrended Normal Q-Q Plot of PS_18_19
Dev from Normal
0.75
0.50
0.25
0.00
-0.25
-0.50
0.0
0.2
0.4
0.6
0.8
1.0
1.2
Observed Value

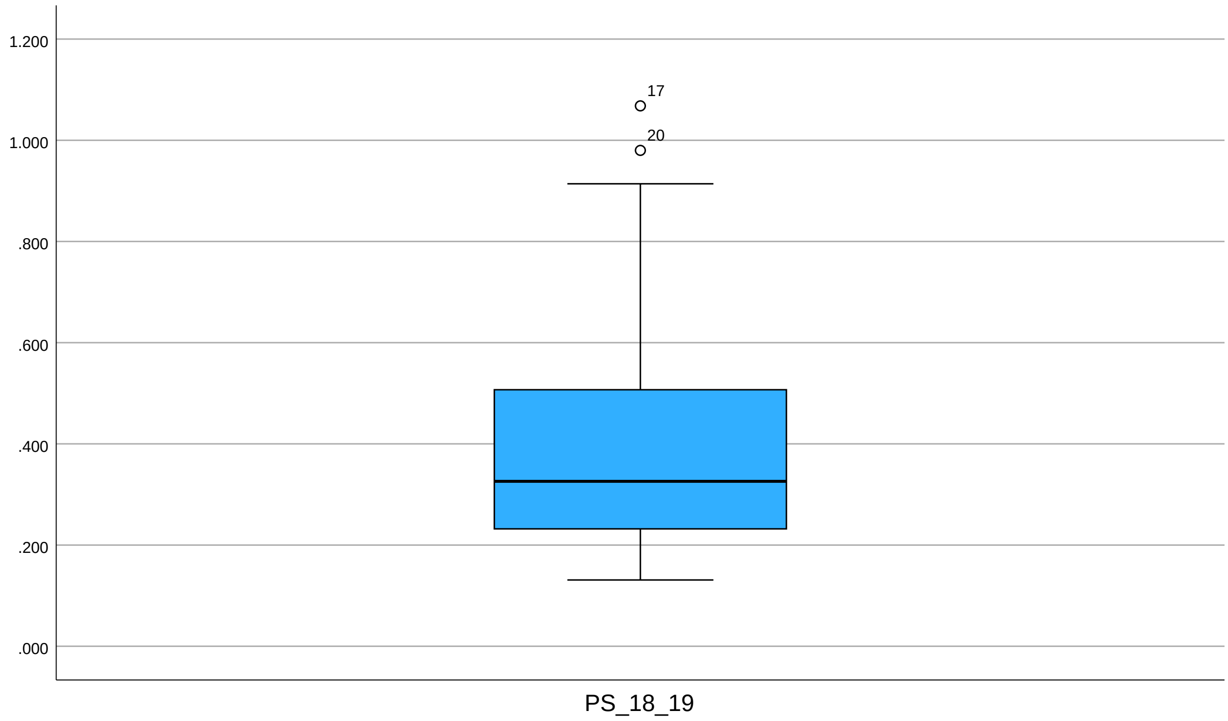

PS_19_20

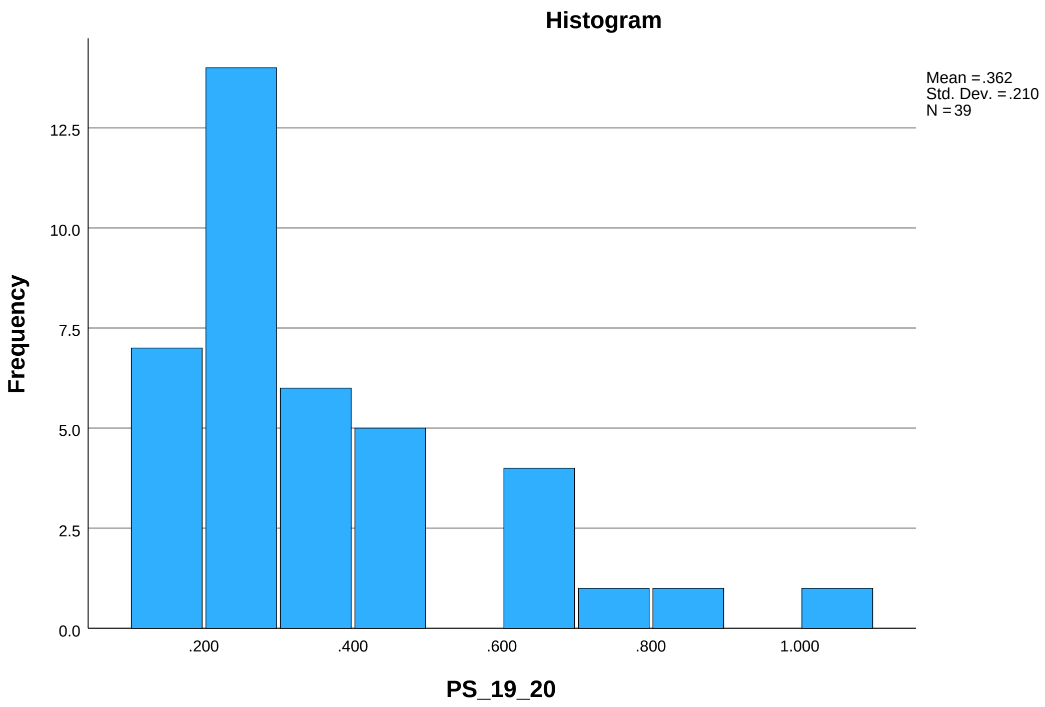

PS_19_20 Stem-and-Leaf Plot

Frequency   Stem & Leaf

```
     7.00        1 .  2446788
    14.00        2 .  01111134677899
     6.00        3 .  034668
     5.00        4 .  23677
      .00        5 .
     4.00        6 .  1227
     1.00        7 .  3
     2.00 Extremes    (>=.88)

 Stem width:      .100
 Each leaf:       1 case(s)
```

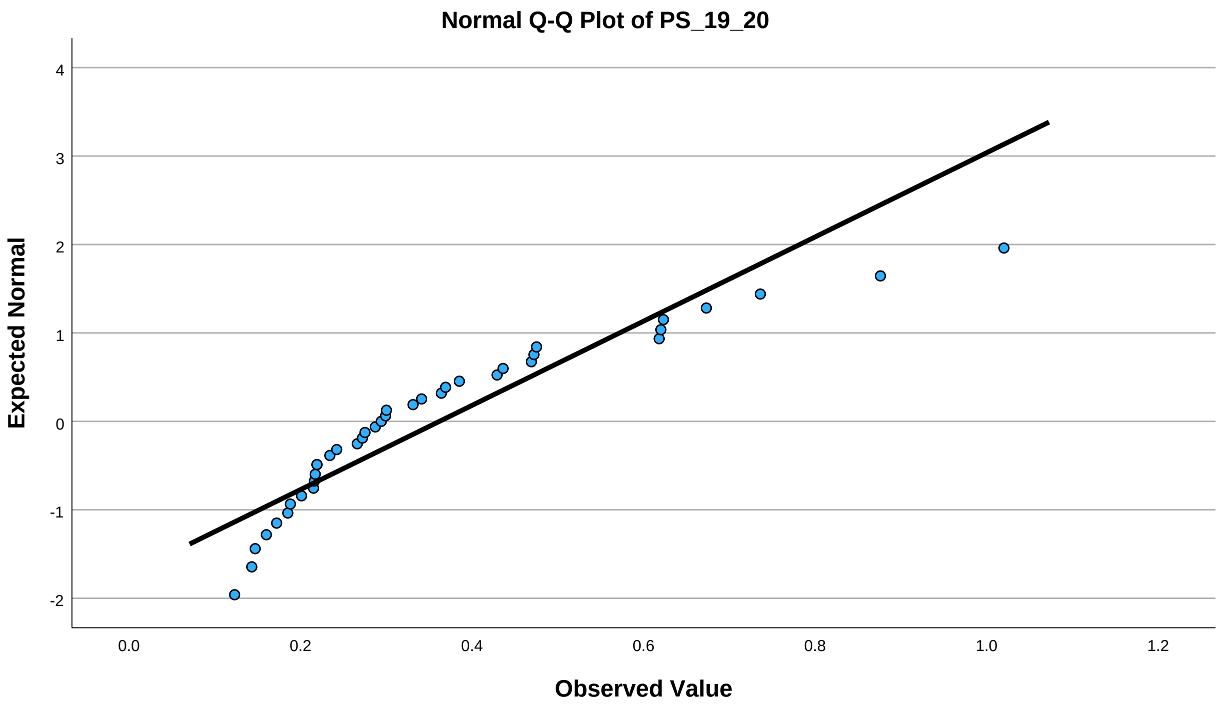

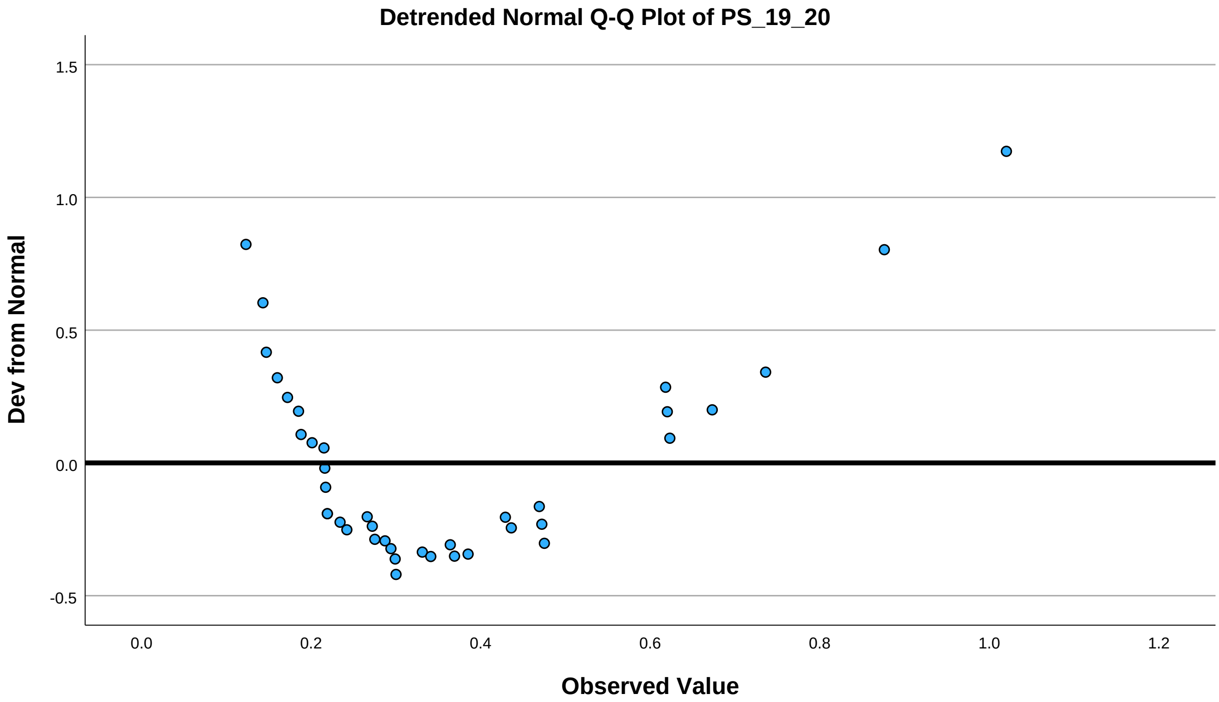
Detrended Normal Q-Q Plot of PS_19_20
Dev from Normal
1.5
1.0
0.5
0.0
-0.5
0.0
0.2
0.4
0.6
0.8
1.0
1.2
Observed Value

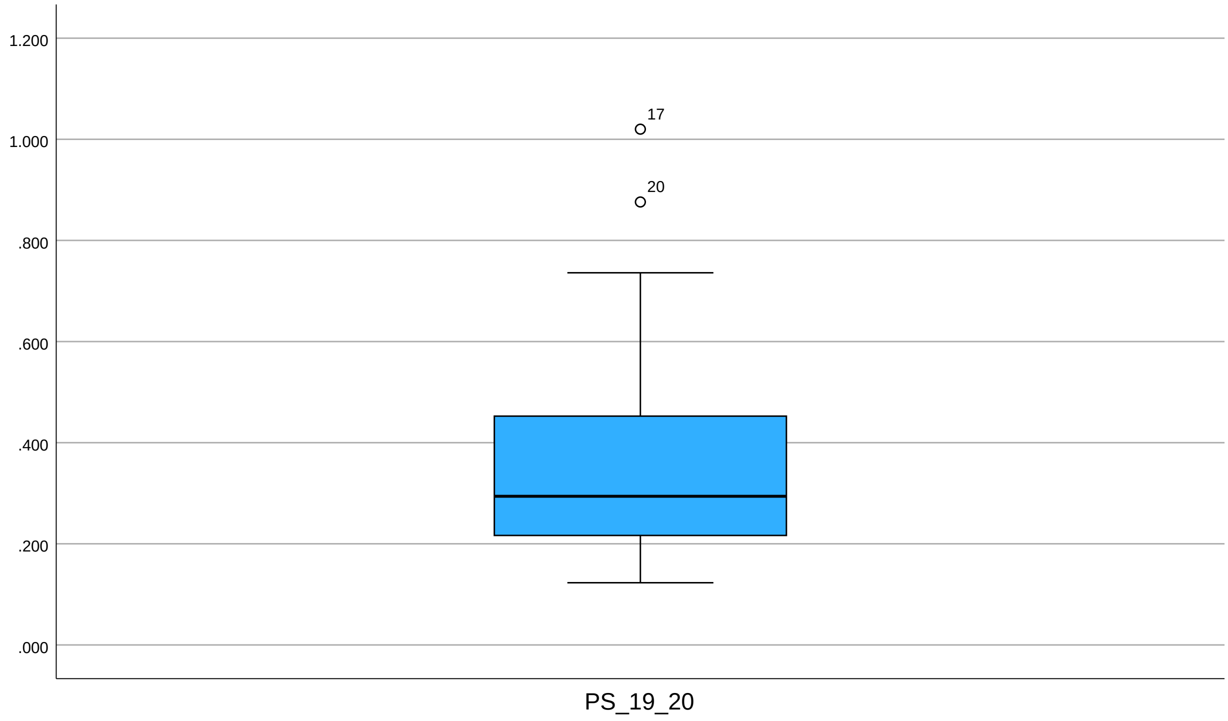
1.200
1.000
.800
.600
.400
.200
.000
17
20
PS_19_20
